# Unveiling the Immune Mechanisms of Hypertension With Single‐Cell Transcriptome‐Wide Mendelian Randomization

**DOI:** 10.1096/fj.202600899R

**Published:** 2026-08-20

**Authors:** Yukang Mao, Peng Li, Chen Cheng, Xiangqing Kong, Wei Sun, Tingting Wu

**Affiliations:** ^1^ Department of Cardiology The First Affiliated Hospital With Nanjing Medical University Nanjing China

**Keywords:** colocalization, expression quantitative trait locus, hypertension, immune cell, Mendelian randomization

## Abstract

Immune dysregulation is closely implicated in hypertension pathogenesis, yet specific therapeutic targets are lacking. We integrated expression quantitative trait loci (eQTLs) for genes expressed in 14 immune cell subsets (OneK1K) with two‐sample Mendelian randomization (MR) and colocalization to provide evidence for cell type‐specific causal effects of eGenes (genes with eQTLs) on hypertension and related complications (FinnGen and UK Biobank). Single cell‐level expression of causal eGenes was compared between individuals with and without hypertension. A tiered framework was built to prioritize crucial eGenes for therapeutic translation. The druggability of the prioritized eGenes was surveyed, with potential on‐target side effects evaluated using phenome‐wide MR. Immune cell‐specific expression of 172 eGenes was causally associated with hypertension, with prioritization of 17 eGenes performed by colocalization. eGenes in naïve and central memory CD4+ T cell (CD4 NC), effector memory CD8+ T cell (CD8 ET), and natural killer cell (NK) were most significantly associated with hypertension. Among these, *FNBP4* and *PRELID1* in multiple cell subsets moonlight as causal drivers of hypertensive heart disease. Higher expression levels of *DDX5* in CD4 NC and CD8 NC, *VIM* in CD8 ET, and *CTSW* in NK were observed in hypertensive patients than normotensive controls. Most eGenes categorized as Tier 1 and 2 targets were druggable, and genetically mimicked therapeutics targeted at Tier 1 targets were predicted to generate limited on‐target side effects. Our findings provided robust genetic insights into the immunological etiology of hypertension, opening up new avenues for the development of immune‐mediated anti‐hypertensive therapies.

## Graphical abstract

This study integrates expression quantitative trait loci data across 14 immune cell subsets with Mendelian randomization and colocalization to uncover 17 eGenes causally associated with hypertension in a cell type‐specific manner, prioritizes three eGenes (*DDX5*, *VIM*, and *CTSW*) with cell type‐specific differential expression levels between hypertensive and normotensive individuals, and evaluates the safety profiles of targeting the prioritized eGenes. Through triangulation of multi‐layered evidence, these findings provide genetic insights into the immunological etiology of hypertension.

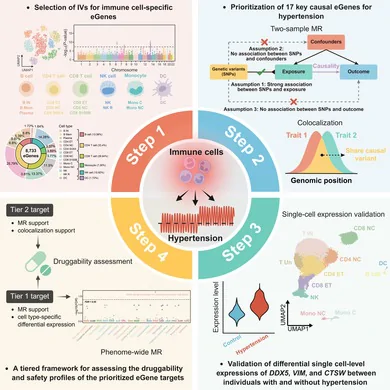

## 1 Introduction

Chronically elevated blood pressure (BP) (termed hypertension) is a public health issue affecting nearly 30% of adults worldwide. As per a NCD Risk Factor Collaboration (NCD‐RisC) report launched in 2021, the number of people aged 30–79 years living with hypertension doubled from 1990 to 2019, reaching up to ~1.3 billion globally [1]. This is quite concerning, as hypertension is a major cardiovascular risk factor and a leading cause of end‐organ damage (e.g., stroke, heart failure, and chronic kidney disease) and cardiovascular disease (CVD) deaths [2]. Despite its high prevalence, the etiology of hypertension remains poorly defined. Most presently used medications merely aim to control BP to a relatively ‘acceptable’ level and curbing severe complications, yet no curative treatment strategy has been achieved by addressing the underlying disease mechanisms [3].

Burgeoning evidence suggests that immune dysregulation may drive hypertension by triggering a systemic, chronic inflammatory response [4, 5]. Hypertension is more prevalent in patients with immune disorders (e.g., rheumatoid arthritis and psoriasis) than those without [6, 7]. Even among individuals who have not undergone classic immune‐related diseases, immune dysregulation mediated by aberrantly increased circulating immune cells and inflammatory cytokines is associated with heightened risk of hypertension [8, 9, 10, 11]. Compelling evidence has also been obtained from Mendelian randomization (MR) for a causal role of immune cells in promoting hypertension [12, 13], consistent with what was found in animal research, in which immunodeficient mice were protected against multiple forms of hypertension and concomitant vascular and renal injury [14, 15, 16]. However, immunomodulation therapy is still under debate for uncertain efficacy in human hypertension, with its clinical use limited by global immunosuppression [17]. As such, more specific targets are warranted for overcoming translational barriers and creating immune‐based therapies tailored to hypertension.

Due to BP's high heritability, genetic lability represents a crucial facet of hypertension pathogenesis. Thus far, genome‐wide association studies (GWASs) have discovered hundreds of risk loci, offering in‐depth interpretation of the inter‐individual variability of BP and the genetic origin of hypertension [18, 19]. This is largely the same case for gene expression traits, as evidenced by large‐scale, consortium‐based GWASs (eQTLGen and GTEx) where genetic variants regulating the expression levels of genes (termed expression quantitative trait loci (eQTLs)) in human blood or across a variety of tissues were identified [20, 21]. While previous MR research has leveraged eQTL data to screen for molecular targets for hypertension [22, 23], there remains a gap; that is, bulk‐level eQTLs cannot capture the transcriptional heterogeneity between distinct cell populations and dissect disease mechanisms in a cell type‐specific manner. In particular, the transcriptional profiles of immune cells are highly variable between individuals [24, 25], with the variations to a large extent explaining the difference in the susceptibility to immune‐related diseases [26]. Recent advances in single‐cell RNA‐seq (scRNA‐seq) technique have enabled a genome‐wide discovery of the complex polygenic architecture that determines cell type‐specific gene expression, which has been employed to delineate the genetically predicted transcriptomic landscape of diseases from a single‐cell resolution [27, 28, 29].

In this study, we sought to systematically investigate the causal roles of genes in 14 immune cell types in hypertension and related complications with MR, which would be integral to enhancing the understanding of the immune mechanisms of hypertension and advancing precision medicine.

## 2 Methods

### 2.1 Study Design

**FIGURE 1:**
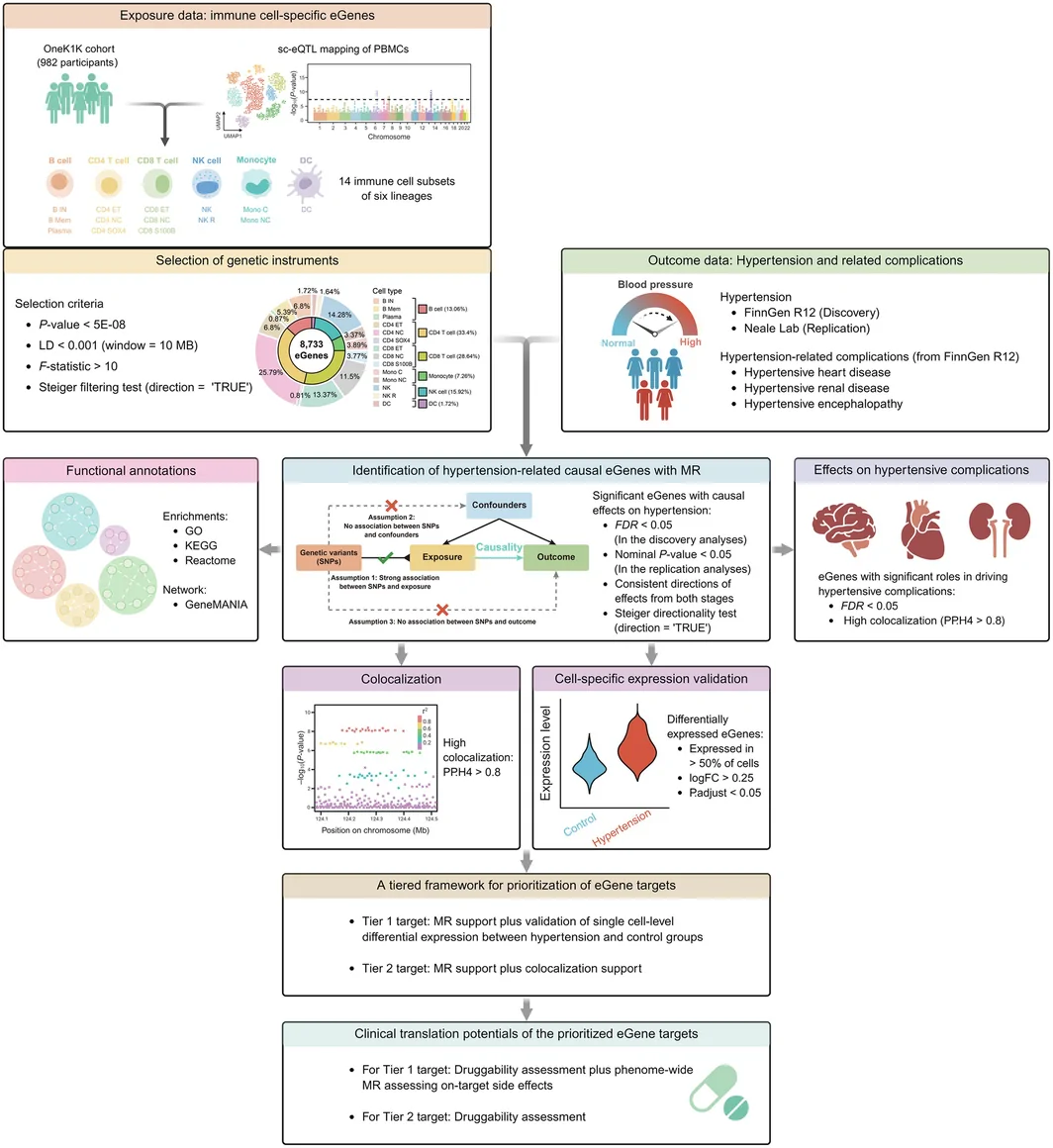
Overview of study design. B IN, immature and naïve B cell; B Mem, memory B cell; CD4 ET, effector memory CD4+ T cell; CD4 NC, naïve and central memory CD4+ T cell; CD4 SOX4, *SOX4*‐expressing CD4+ T cell; CD8 ET, effector memory CD8+ T cell; CD8 NC, naïve and central memory CD8+ T cell; CD8 S100B, *S100B*‐expressing CD8+ T cell; DC, dendritic cell; eGenes, genes with expression quantitative trait loci; *FDR*, false discovery rate; GO: Gene Ontology; KEGG: Kyoto Encyclopedia of Genes and Genomes; LD, linkage disequilibrium; Mono C, classical monocyte; Mono NC, non‐classical monocyte; MR, Mendelian randomization; NK, natural killer cell; NK R, NK‐recruiting cell; PBMC, peripheral blood mononuclear cell; sc‐eQTL, single‐cell expression quantitative trait loci; SNP, single‐nucleotide polymorphism.

The analytical pipeline of our study is illustrated in Figure 1. Briefly, the application of transcriptome‐wide MR requires selection of valid genetic instruments from naturally‐occurring genetic variants in the human genome (usually single‐nucleotide polymorphisms (SNPs)). To fulfill the basic assumptions for MR analyses, instrumental SNPs should (1) be strongly associated with the exposure; (2) not be associated with potential confounders between the exposure and outcome; and (3) only affect the outcome through the exposure but not any other alternative pathway. All the analyses were performed using publicly available GWAS summary statistics that have been approved by respective institutional ethics committees [30]. As such, additional ethical approval was not required in our study. This study adheres to the STROBE‐MR (Strengthening the Reporting of Observational Studies in Epidemiology using Mendelian Randomization) guidelines [31].

### 2.2 Data Source

Single‐cell eQTLs were derived from a large‐scale GWAS of 982 healthy individuals of Northern European ancestry from the OneK1K cohort. By eQTL mapping of over one million peripheral blood mononuclear cells (PBMCs) profiled with scRNA‐seq, a total of 14 distinct immune cell types of different lineages were transcriptionally and phenotypically characterized: three B cell subsets (immature and naïve B cell (B IN), memory B cell (B Mem), and plasma cell), three CD4+ T cell subsets (naïve and central memory CD4+ T cell (CD4 NC), effector memory CD4+ T cell (CD4 ET), and *SOX4*‐expressing CD4+ T cell (CD4 SOX4)), three CD8+ T cell subsets (naïve and central memory CD8+ T cell (CD8 NC), effector memory CD8+ T cell (CD8 ET), and *S100B*‐expressing CD8+ T cell (CD8 S100B)), two natural killer (NK) cell subsets (NK cell (NK), and NK‐recruiting cell (NK R)), two monocyte subsets (classical monocyte (Mono C), and non‐classical monocyte (Mono NC)), and one dendritic cell (DC) subset [32]. We utilized SNPs related to *cis*‐eQTLs (located within ±1 Mb distance of the transcription start site of the corresponding genes), and retained those strongly associated with gene expression at a genome‐wide significance level (*p* < 5 × 10^−8^) as instrumental variables (IVs). Linkage disequilibrium (LD) clumping was implemented to retain SNPs in low LD (*R* ^2^ < 0.001) estimated based on the 1000 Genomes European reference panel [33]. To ensure the statistical power of genetic instruments, we used *F*‐statistic as a major indicator [34]. SNPs with an *F*‐statistic < 10 were considered to have limited statistical power (potentially causing weak instrument bias) and excluded from the analyses.

To ensure the credibility of causal inference, we obtained hypertension GWASs from two European consortiums: the FinnGen study (for discovery) and Neale Lab (for replication). The FinnGen study aims to construct a resource of > 2400 disease‐specific GWASs by combining imputed genotype data generated from samples from Finnish biobanks and digital health record data from Finnish health registries [35]. We included a total of 154 630 cases and 345 634 controls from the R12 release of the FinnGen cohort, with cases identified as the presence of primary hypertension using the International Classification of Diseases, 8–10th Revision (ICD‐8, ICD‐9, and ICD‐10) codes. Neale Lab generates high‐quality disease‐specific GWASs based on the UK Biobank, a nationwide cohort comprising approximately 500 000 community‐dwelling individuals aged 40–69 years from the United Kingdom between 2006 and 2010 [36]. For Neale Lab, hypertension cases were defined based on the ICD‐10 code (1313 cases plus 359 881 controls). We also included three hypertension‐related complications according to the disease category in FinnGen: hypertensive heart disease (HHD), hypertensive renal disease (HRD), and hypertensive encephalopathy (HE). For MR analyses, the exposure and outcome summary statistics were harmonized to ensure consistency of effect alleles and directionality of SNP effects. This included: (1) aligning effect alleles across datasets; (2) removing ambiguous or strand‐incompatible SNPs; and (3) retaining palindromic variants only when allele frequencies allowed for unambiguous alignment, otherwise excluding them. In addition, SNP coordinates established based on the GRCh38/hg38 reference genome were converted to the GRCh37/hg19 reference genome by the ‘liftover’ function deposited in the R package MungeSumstats to ensure genomic consistency across datasets.

### 2.3 Main Analyses

#### 2.3.1 Transcriptome‐Wide MR Analysis

For the MR analyses, we used the Wald ratio method for eGenes with only one IV and used the inverse‐variance weighted (IVW) method for those with two or more IVs [37]. In the scenario that at least three IVs were included, sensitivity analyses based on several alternative MR methods that consider potential biases derived from pleiotropy and heterogeneity were added to boost the robustness of IVW estimates, including MR Egger [38], weighted median [39], simple mode, and weighted mode [40]. The MR estimates for the effects of eGenes on hypertension and related complications were presented as ORs with 95% CIs, with a two‐sided *p*‐value < 0.05 denoting nominal statistical significance. The MR analyses were implemented with the TwoSampleMR package in R (https://mrcieu.github.io/TwoSampleMR/). Regarding multiple hypothesis testing in the MR analyses on the associations between eGenes and hypertension, we applied the Benjamini–Hochberg correction to control for false discovery rate (*FDR*) during the discovery stage while using a nominal *p*‐value threshold of 0.05 when re‐assessing the significant associations during the replication stage. *FDR* correction was also used to adjust for multiple testing when assessing the associations between hypertension‐related eGenes and hypertensive complications.

#### 2.3.2 Steiger Filtering Analysis

To ensure the identified causal associations are in correct directions and rule out reverse causality, we conducted the Steiger filtering tests, which assume that valid IVs should explain more variations in the exposure than in the outcome. If an IV satisfies the criterion (*p*‐value for the Steiger direction (*p*_Steiger_) < 0.05), the results would return ‘TRUE’, suggesting that its direction is correct without reverse causality; otherwise, it would return ‘FALSE’ [41]. IVs failing the Steiger filtering tests were excluded from the MR analyses. In addition, Steiger directionality tests were implemented to validate the correct directions of the associations between the exposure and outcome.

#### 2.3.3 Colocalization Analysis

To delineate whether eGenes and hypertension‐related disease outcomes share the same causal variant within a specific DNA region (usually ±500 kb distance from a gene region for eQTL data), we performed colocalization analyses based on the Bayesian model using the coloc package in R. Under the framework of colocalization, there exist five mutually exclusive hypotheses for genetic variants in a region: (1) genetic variants associate with neither eGenes nor disease outcomes (H0); (2) genetic variants only associate with eGenes (H1); (3) genetic variants only associate with disease outcomes (H2); (4) different genetic variants associate with eGenes and disease outcomes, respectively (H3); and (5) the same genetic variant associates with both eGenes and disease outcomes, indicating that two traits are driven by a shared causal variant (H4) [42]. A posterior probability was assigned to each hypothesis testing, with the posterior probability for H4 (PP.H4) greater than 0.8 deemed high evidence for genetic colocalization. To address the limitation of traditional colocalization method (COLOC) assuming only one causal variant, we applied SuSiE (Sum of Single Effects) colocalization. The fine‐mapping‐based approach enables the identification of multiple causal variants at a single locus, which enhances the accuracy of colocalization when used in combination with COLOC [43]. For SuSiE, colocalization was defined by the maximum PP.H4 from all pairwise comparisons of the identified credible sets. In scenarios where no credible set can be identified by SuSiE, COLOC remained a robust method, as its single‐variant assumption still permits meaningful inference in lower‐powered multiple‐causal cases.

#### 2.3.4 Phenome‐Wide MR

To elucidate potential on‐target side effects associated with genetically mimicked interventions aiming to reduce the risk of hypertension by targeting prioritized eGenes, we performed phenome‐wide MR (Phe‐MR) analyses. A total of > 1400 binary disease phenotypes pertaining to 17 distinct categories defined by the PheCodes of UK Biobank (a unique identifier for phenotype) were assembled in the analyses, which have been explicitly described elsewhere [44]. Summary statistics for phenotypes are available at: https://www.leelabsg.org/resources. Phenotypes with an *FDR* < 0.05 were considered significant side effects.

### 2.4 Single‐Cell RNA‐Seq Data Analysis

We extracted the gene expression matrices of pooled PBMC samples from four patients with hypertension and four normotensive controls from a CITE‐seq (cellular indexing of transcriptomes and epitopes by sequencing) dataset deposited in the NCBI Gene Expression Omnibus (GEO) database (Accession No. GSE212953) [45]. Hypertensive individuals were defined as those with a pre‐existing diagnosis of hypertension (SBP ≥ 140 mmHg or DBP ≥ 90 mmHg) or those taking anti‐hypertensive medications. Data objects for downstream analyses were built following standardized procedures in the Seurat package. We followed a multi‐step pipeline for quality control, including: (1) transforming the raw data to clean data by decontamination of ambient RNA using the decontX package [46]; (2) identifying and removing the doublets with the scDblFinder package [47]; and (3) filtering out low‐quality cells expressing excessively low or high counts of genes (< 200 or > 2500) or with greater than 10% of transcripts of mitochondrial origin (likely to be apoptotic cells). After quality control, data objects from both groups were integrated, normalized, and then scaled by regressing out variables including percentage of mitochondrial genes and difference between G2M and S phase score. The Harmony algorithm was applied to correct for batch effects during data integration [48]. For dimensionality reduction, we executed the principal component analysis incorporating up to 50 principal components (PCs) and determined the optimal cutoff using an elbow plot. In this case, we took the first 20 PCs for cell clustering and projection with UMAP (uniform manifold approximation and projection). Cell clustering was optimized by testing the resolution ranging from 0.1 to 2.0 with clustree [49]. In our analyses, the resolution was set to 0.4, resulting in clustering of major immune cell types almost identical to those identified from the OneK1K data. For each immune cell cluster, annotations were manually performed by referring to the expression of canonical cell markers. Genes differentially expressed (detected in > 50% of cells; log2‐transformed fold change > 0.25; Bonferroni‐adjusted *p*‐value < 0.05) between groups were identified with the FindMarkers function provided by Seurat.

### 2.5 Functional Enrichment and Network Analysis

Functional enrichment analyses based on annotation terms curated in the Gene Ontology (GO), Kyoto Encyclopedia of Genes and Genomes (KEGG), and Reactome pathway databases were carried out using the R packages clusterProfiler and ReactomePA [50, 51]. Enriched functional annotation terms with an *FDR* < 0.05 were deemed significant. To predict and visualize the biological link between eGenes, the protein–protein interaction (PPI) network was constructed with GeneMANIA (https://genemania.org/) [52].

### 2.6 Druggability Analysis

To evaluate whether prioritized eGenes are druggable targets, we interrogated well‐curated repositories: Therapeutic Target Database (https://idrblab.org/ttd/) [53], DrugBank (https://go.drugbank.com) [54], and Open Targets (https://www.targetvalidation.org/) [55]. Detailed information on target druggability including drug name, drug type, development stage, pharmacological action, and approved indications were retrieved.

## 3 Results

### 3.1 Identification of Immune Cell‐Specific Causal eGenes for Hypertension

**FIGURE 2:**
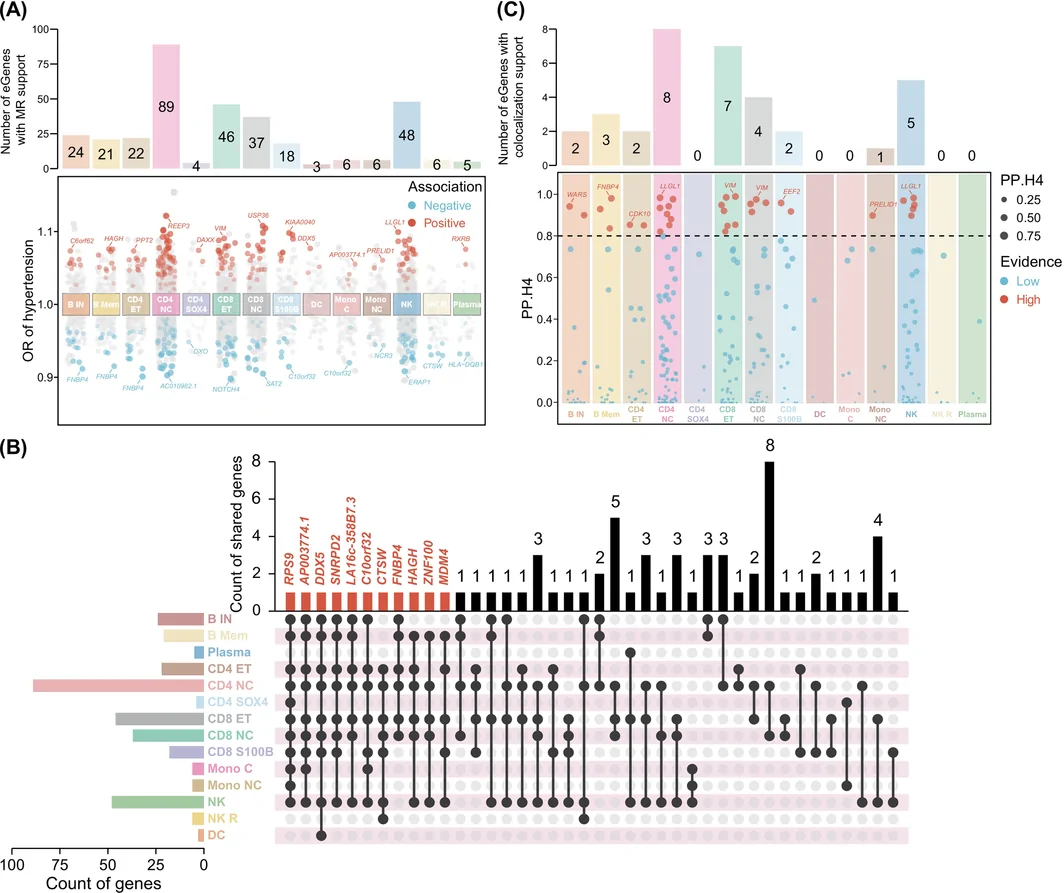
Identification of hypertension‐related eGenes with Mendelian randomization and colocalization. (A) MR results for the associations between eGenes and hypertension, where each dot indicates an eGene and labels of those with the greatest effects for each cell type are displayed (lower panel). eGenes with significantly causal effects on hypertension are highlighted with blue (anti‐hypertensive) and red (pro‐hypertensive) colors. The number of eGenes with evidence support from MR for each cell type (upper panel). (B) The UpSet plot showing eGenes with causal effects on hypertension across multiple cell types. Labels of eGenes with consistent causal effects across five or more cell types are displayed in the plot. (C) Colocalization results for 335 MR‐prioritized causal associations between eGenes and hypertension, where each dot indicates an eGene and the dot size is proportional to PP.H4 (lower panel). eGenes with a PP.H4 > 0.8 are considered to be genetically colocalized with hypertension. Labels of eGenes with the highest PP.H4 for each cell type are displayed in the plot. The number of eGenes with high evidence support from colocalization for each cell type (upper panel). eGenes, genes with expression quantitative trait loci; MR, Mendelian randomization.

To uncover the causality between gene expression profiles in 14 immune cell populations and hypertension, we performed two‐stage MR analyses. Following stringent inclusion criteria, a total of 9123 IVs proxying 8733 cell type‐specific eGenes were obtained, with the majority of eGenes having only one available IV (Table S1). The proportion of eGenes greatly varied across different cell subsets, ranging from 0.81% for CD4 SOX4 to 25.79% for CD4 NC (Figure 1). For the discovery stage (outcome data from FinnGen), 819 IVs were excluded due to absence in the outcome data (*n* = 536), and due to palindromic or ambiguous strands with intermediate allele frequencies (*n* = 283) during data harmonization, with the remaining 8304 IVs mapping onto 8004 cell type‐specific eGenes eligible for subsequent MR analyses. Among the analyzed eGenes, 788 were significantly associated with hypertension after Benjamini–Hochberg correction (Table S2). To strengthen the findings, we re‐assessed the significant associations using outcome data from the Neale Lab with no sample overlap. In the replication stage, nearly half of the eGene–hypertension associations (*n* = 359) retained statistical significance (Table S3). Twenty‐four directionally inconsistent associations between both stages were removed, leaving the remaining 335 (involving 172 eGenes) as highly credible causal associations (Figure 2A). For eGenes with three or more IVs, sensitivity analyses by complementary MR methods supported the robustness of IVW estimates through yielding directionally consistent effect estimates. The Steiger directionality tests demonstrated the correct causal directionality of all the tested associations, ruling out potential bias caused by reverse causation (Tables S2 and S3). The highest number of causal associations was found for CD4 NC (89 associations), followed by NK (48 associations) and CD8 ET (46 associations) (Figure 2A). Significant enrichments for biological processes and pathways associated with immune and inflammatory responses (e.g., MHC class II protein complex assembly and peptide antigen binding) and immune‐mediated diseases (e.g., asthma and viral myocarditis) were observed for the causal eGenes, hinting at immune abnormalities (especially in adaptive immune response) as the fundamental basis for the downstream mechanisms by which eGenes drives hypertension (Figure S1A–C). The PPI network revealed that the eGenes are likely to be biologically connected, with half of them having physical interactions and one third of them being co‐expressed (Figure S1D). Notably, some eGenes ubiquitously expressed in multiple cell subsets appeared to display broad causal link with hypertension, such as *RPS9* (11 subsets), *AP003774.1* (9 subsets), and *DDX5* (9 subsets) (Figure 2B). The cell type‐specific effects of an eGene, albeit of varying magnitude, were overall in consistent directions, hinting at generalized immune‐mediated effects (Tables S2 and S3). However, there were also exceptional cases where directional heterogeneity in cell type‐specific causality exists. Take *MYO16* as an example, its effect on hypertension was in the inverse direction when expressed in CD4 NC, but was in the positive direction when expressed in CD8 NC. Colocalization analyses using the COLOC method suggested that 34 associations with PP.H4 exceeding 0.8 (involving 17 unique eGenes) have high evidence of colocalization (Table S4; Figure S2). Only 9 out of 14 immune cell subsets have causal eGenes receiving additional support from colocalization, with CD4 NC, CD8 ET, and NK taking the top three positions in terms of the number of eGenes (Figure 2C). These results suggested that merely 10.15% (34/335) of the MR–identified associations were attributed to shared causal variants rather than LD–driven confounding. Furthermore, these colocalized signals were largely corroborated by fine‐mapping‐based SuSiE colocalization where credible sets were detectable (Table S5), strengthening the robustness of the colocalization evidence by accounting for potential multiple causal variants.

Altogether, cell‐stratified MR analyses provided compelling evidence for the cell type‐specific causal roles of 172 eGenes in hypertension etiology, with 17 of these prioritized by colocalization analyses. Both analyses supported that CD4 NC, CD8 ET, and NK may be the most pivotal immune cell subsets whose genetically predicted transcriptional profiles significantly associate with the risk of hypertension.

### 3.2 The Causal Roles of Hypertension‐Related eGenes in Hypertension‐Related Complications

We investigated whether hypertension‐related eGenes also contribute to the development of hypertensive end‐organ damage on the heart, kidney, and brain (HHD, HRD, and HE), given that circulating immune cells are responsible not only for initial BP elevation but also for secondary functional impairments upon tissue infiltration [4, 5]. A total of 21 causal associations were observed between genetically predicted expression levels of 11 eGenes in eight cell subsets and HHD after adjusting for multiple testing, with the highest numbers of associations enriched for CD4 NC. The directions for eGene–HHD associations were completely in line with those for eGene–hypertension associations, and no reverse causality was detected by Steiger directionality tests (Table S6). Additionally, high evidence of colocalization was obtained for seven associations of cell type‐specific expression of two eGenes with hypertension, involving *FNBP4* expressed in five lymphocyte subsets (B IN, CD4 NC, CD4 ET, CD8 NC, and CD8 ET) and *PRELID1* expressed in NK and Mono C (Figure S3). By contrast, neither MR nor colocalization suggested that hypertension‐related eGenes were associated with HRD and HE (Tables S7 and S8).

Together, we revealed two hypertension‐related eGenes (*FNBP4* and *PRELID1*) in multiple immune cell subsets that moonlight as potential causal factors for HHD. This demonstrates that immune cell abnormalities may represent shared mechanisms of hypertension and its cardiac complications.

### 3.3 Expression Validation of Hypertension‐Related eGenes in Immune Cells of Hypertensive and Normotensive Individuals

**FIGURE 3:**
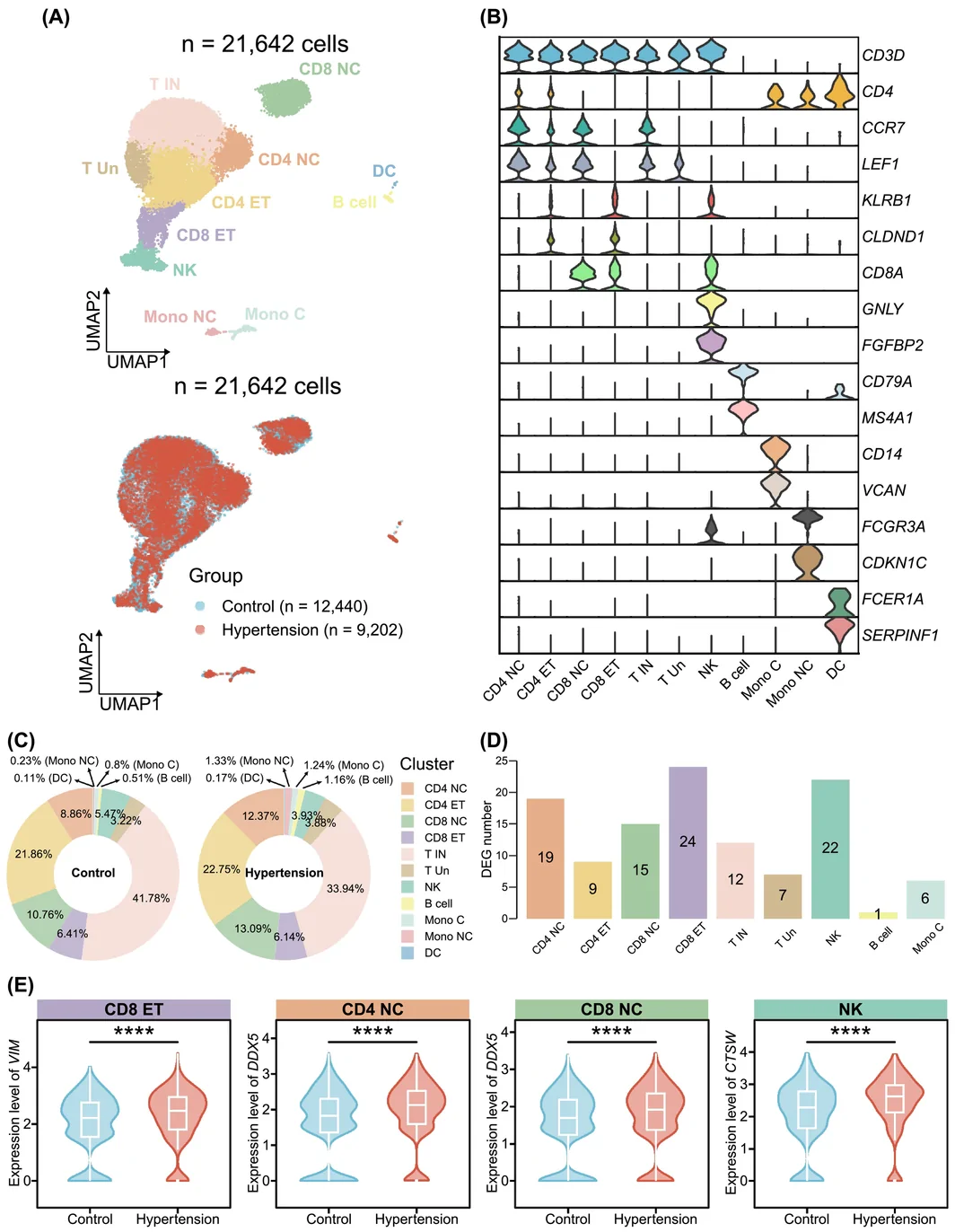
Single‐cell transcriptomic profiling of peripheral immune cells in hypertensive and normotensive individuals. (A) UMAP visualization of 21 642 PBMCs, annotated by cell clusters (top) or grouped by disease status (hypertension vs. control; bottom). (B) Expression of canonical marker genes across the identified immune cell clusters. (C) Proportional abundance of each cell cluster in the hypertension (right) and control (left) groups. (D) Number of DEGs identified in each cell cluster between the hypertension and control groups. (E) Comparison of cell type‐specific expression levels of the prioritized causal eGenes (CD8 ET‐specific *VIM*, CD4 NC‐specific *DDX5*, CD8 NC‐specific *DDX5*, and NK‐specific *CTSW*) between the hypertension and control groups. ****p < 0.0001. DEG, differentially expressed gene; eGenes, genes with expression quantitative trait loci; PBMC, peripheral blood mononuclear cell; UMAP, Unifold Manifold Approximation and Projection.

We conducted single cell‐level expression analyses to compare the expression of causal eGenes in the peripheral immune cells between individuals with and without hypertension. This analysis utilized a previously published CITE‐seq dataset of PBMCs collected from hypertensive patients and normotensive controls. Based on canonical markers (mostly identical to those used in the OneK1K data), 21 642 high‐quality cells (9202 cells from the hypertension group plus 12 440 cells from the control group) were clustered into nine cell types identical to what was identified in the OneK1K cohort (B cell, CD4 NC, CD4 ET, CD8 NC, CD8 ET, NK, Mono C, Mono NC, and DC), as well as two T cell clusters consisting of naïve cells (T IN) and of unknown identity (T Un), respectively (Figure 3A,B). Notably, separation of B IN and B Mem subpopulations could not be achieved because of the limited number of B cells captured in the dataset. This was also confirmed by the clustering results from the clustree method that B cell cluster remained inseparable even at a high resolution value of up to 2.0. Also, three small subpopulations reported in the OneK1K data (CD4 SOX4, CD8 S100B, and NK R) were not present in the analyzed dataset for lacking cell type‐specific expression of characteristic markers. Comparisons in cell composition between the hypertension and control groups revealed that most identified cell clusters have different proportions, albeit being present in both groups. For example, CD4 NC, CD8 NC, and several small clusters (B cell, Mono C, Mono NC, and DC) were more abundant in the hypertension group, whereas T IN and NK were more enriched in the control group (Figure 3C). CD8 ET exhibited the highest number of differentially expressed genes (DEGs) between groups (24 DEGs), followed by NK (22 DEGs) and CD4 NC (19 DEGs) (Figure 3D; Table S9). This suggested that CD4 NC, CD8 ET, and NK may undergo the most prominent transcriptional alternations during hypertension development, coinciding with their identities as key causal contributors to hypertension. Among hypertension‐related causal eGenes, four were determined to be DEGs (including CD4 NC‐specific *DDX5*, CD8 NC‐specific *DDX5*, CD8 ET‐specific *VIM*, and NK‐specific *CTSW*), all of which show higher expression levels in hypertensive patients than normotensive controls (Figure 3E). The expression patterns of *DDX5* and *VIM* were largely in line with their positive causal effects on hypertension, whereas *CTSW* appeared to exhibit a paradoxical expression trend relative to its predicted protective role. Collectively, our observations from scRNA‐seq analyses underscored the key roles of CD4 NC, CD8 ET, and NK in hypertension, and pinpointed three causal eGenes whose expression levels in these cell types are dramatically altered during the disease process (*DDX5*, *VIM*, and *CTSW*).

### 3.4 Therapeutic Potentials of eGenes Prioritized as Crucial Targets

**FIGURE 4:**
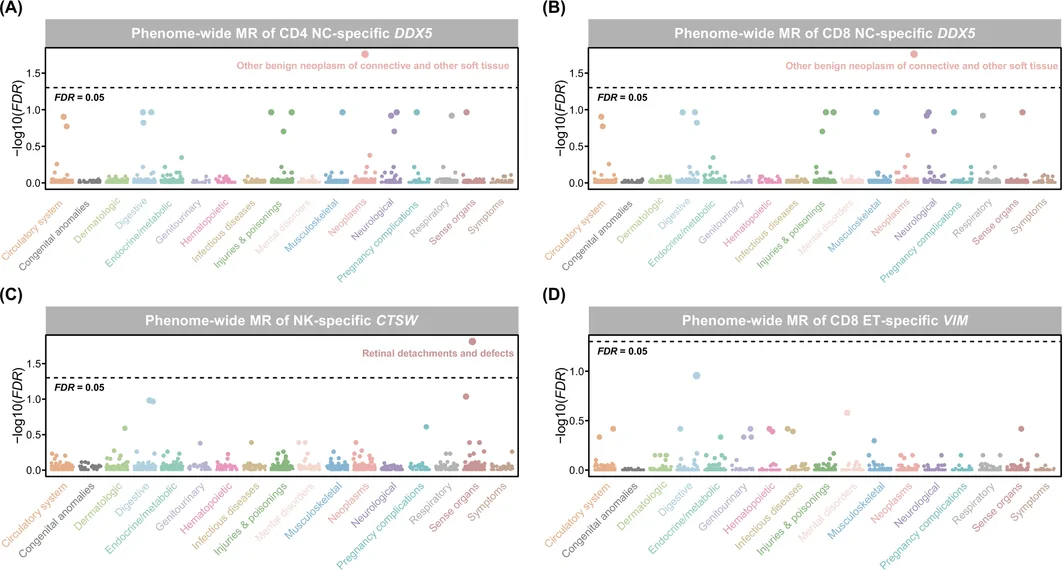
Phenome‐wide Mendelian randomization assessing potential on‐target side effects associated with targeted interventions of eGenes. (A–D) Non‐hypertension phenotypes reaching an *FDR* of < 0.05 are highlighted as significant side effects associated with genetically mimicked targeted interventions of the prioritized eGenes. eGenes, genes with expression quantitative trait loci; *FDR*, false discovery rate.

Based on the abovementioned findings, we built a tiered framework to prioritize two subsets of eGenes as candidate therapeutic targets, including: (1) Tier 1 subset that comprises three eGenes with MR support and with differential expression between immune cells from hypertensive and normotensive individuals (Table 1); and (2) Tier 2 subset that comprises 16 eGenes with MR support plus colocalization support. Druggability analyses suggested that six eGenes—including two Tier 1 targets (*DDX5* and *VIM*) plus four Tier 2 targets (*MDM4*, *WARS*, *EEF2*, and *CDK10*)—are targeted by drugs approved for treating various forms of tumors such as leukemia and infectious diseases such as malaria, albeit not being known targets for hypertension mediations (Tables S10–S12). As such, drug repurposing may represent a major aspect for clinical translation. We also leveraged a Phe‐MR analysis that considers a broad spectrum of disease phenotypes (*n* = 1403) to systematically survey potential on‐target side effects resulting from genetically mimicked targeted interventions of Tier 1 eGene targets. We excluded two phenotypes pertaining to hypertension (“Hypertension” and “Essential hypertension”), retaining 1401 non‐hypertension phenotypes for Phe‐MR. After correcting for multiple comparisons, high genetically proxied *DDX5* expression in both CD4 NC (OR: 1.472, 95% CI: 1.237–1.753) and CD8 NC (OR: 1.586, 95% CI: 1.288–1.952) was causally associated with heightened risk of other benign neoplasm of connective and other soft tissue (Figure 4A,B), suggesting that targeted intervention of *DDX5* might exert dual benefits by not only lowering BP but also potentially reducing the risk of tumor growth. Retinal detachments and defects appeared to be a deleterious side effect related to genetically manipulating *CTSW* expression in NK (OR: 1.141, 95% CI: 1.076–1.211) (Figure 4C), given the association of NK‐specific *CTSW* with hypertension is in the opposite direction (OR: 0.974, 95% CI: 0.961–0.986) (Table S2). Therefore, caution should be taken when developing *CTSW*‐based therapies. In addition, no evidence of beneficial or deleterious side effects was observed for CD8 ET‐specific *VIM* expression (Figure 4D). Summary results of Phe‐MR can be seen in Tables S13–S16. Overall, through druggability assessment and Phe‐MR, we found that six eGenes in immune cells are amenable drug targets that can be repurposed for hypertension treatment, and that genetically mimicked therapies aimed at controlling BP via targeting Tier 1 eGenes present a relatively broad safety profile with limited genetically predicted side effects.

**TABLE 1:** Evidence triangulation for the prioritized Tier 1 eGene targets.

Cell type	eGene	Discovery MR			Replication MR		Single cell‐level validation		
		OR (95% CI)	*p*	*FDR*	OR (95% CI)	*p*	avg_logFC	*p*	*p*.adjust
CD4 NC	*DDX5*	1.04 (1.02, 1.061)	1.06E‐04	0.003	1.008 (1.004, 1.012)	7.78E‐05	0.375	6.82E‐20	1.19E‐15
CD8 NC	*DDX5*	1.048 (1.023, 1.073)	1.06E‐04	0.003	1.01 (1.005, 1.015)	7.78E‐05	0.259	1.81E‐10	3.15E‐06
CD8 ET	*VIM*	1.091 (1.057, 1.125)	6.08E‐08	6.60E‐05	1.012 (1.006, 1.019)	2.26E‐04	0.322	2.05E‐07	0.004
NK	*CTSW*	0.974 (0.961, 0.986)	3.13E‐05	0.001	0.996 (0.994, 0.999)	0.002	0.393	9.37E‐11	1.64E‐06

## 4 Discussion

Accumulating studies on animal models and human individuals with hypertension suggest a pivotal role for immune dysregulation in the pathogenesis of hypertension [4, 5]. In this study, we implemented single cell‐level, transcriptome‐wide MR to systematically look into the causal relationships between immune cell‐specific genes and hypertension. Key findings of our study included: (1) Robust causal associations between 172 eGenes expressed in 14 immune cell subsets and hypertension were identified and validated by two‐stage MR; (2) 17 causal eGenes showed high evidence of genetic colocalization; (3) Two causal eGenes (*FNBP4* and *PRELID1*) in multiple cell types may moonlight as drivers of hypertensive cardiac complications; (4) Significantly differential cell type‐specific expression levels of three hypertension‐related eGenes (*DDX5*, *VIM*, and *CTSW*) between individuals with and without hypertension were validated; (5) We built a tiered framework for therapeutic target prioritization, in which eGenes with MR support and significantly altered expression during hypertension are classified as Tier 1 targets, whereas those with dual evidence support from MR and colocalization are classified as Tier 2 targets; and (6) Six of 19 prioritized Tier 1 and 2 eGenes were known drug targets, and genetically mimicked BP‐reducing treatments by targeting Tier 1 eGenes yielded minimal on‐target side effects. To summarize, our findings provided solid evidence that transcriptional remodeling of immune cells may causally contribute to the development of hypertension, while also outlining eGenes that represent potential therapeutic targets.

On the cellular level, a noticeable finding obtained from triangulation of multiple‐layered evidence (MR, colocalization, and single cell‐level expression) was that CD4 NC, CD8 ET, and NK may represent key immune cell subsets driving hypertension, mediated by their distinct transcriptional alterations. T cells, which are essential functional carriers mediating adaptive immune responses, have been widely implicated in promoting BP elevation and hypertensive end‐organ damage. This was supported by a study by Guzik and colleagues showing that *Rag1(−/−)* mice wholly devoid of T and B cells are resistant to hypertension and vascular dysfunction induced by angiotensin II and deoxycorticosterone acetate salt. Their findings further demonstrated that adoptive transfer of T—but not B—cells can rectify dampened hypertensive responses [56]. Alterations in peripheral lymphocyte composition may have a direct impact on hypertension incidence. Among female individuals who were hypertension‐free at baseline, the proportion of naïve CD4+ T cells was associated with subsequent risk of new‐onset hypertension [57]. Mechanistically, a landmark discovery in the immunological underpinnings of hypertension is the pivotal role of immune memory of particularly T cells in the disease process. Sensitization of hypertensive response (a propensity for becoming hypertensive even when exposed to a mild stimulus) was observed in mice subject to repeated hypertensive stimuli, coinciding with the formation of memory T cells that persist and exhibit greater activation in response to a second stimulus [58]. Human hypertension was also linked with an expansion in the number of both CD4+ and CD8+ memory T cells [59]. Single‐cell transcriptomic profiling of four T cell subsets (among four subsets including CD4+ and CD8+ central memory T cells and effector memory T cell) implied that CD8+ effector memory T cells (CD8 ET) were particularly elevated in individuals with hypertension compared with those without and underwent the most predominant functional remodeling characterized by the enrichment of canonical pathways indicative of mitochondrial oxidative stress, vascular endothelial dysfunction, and inflammatory activation [60]. Further investigations are needed to determine to which extent hypertension is perpetuated by memory T cells formed during initial BP elevation. NK cells are innate immune cells that act as sentinels in classic inflammatory responses, triggering the release of pro‐inflammatory mediators and driving the subsequent potentiation of inflammation cascade through the recruitment of adaptive immune cells. This capacity makes NK cell a critical effector contributing to immune‐related hypertensive response and vascular dysfunction from an early stage, as evidenced by a wealth of observations from animal models of hypertension [61, 62, 63]. Inconsistent with this, lower NK cell activity, measured by IFN‐γ production level during its activation, was reported to be associated with higher BP level in an observational study [64]. Despite the ongoing controversy, it seems that the identity of NK cell cannot be simply recapitulated by a ‘friend‐or‐foe’ concept, given our findings that different NK‐specific eGenes exhibited bidirectional causal effects (both hazardous and protective) on hypertension.

Among MR–prioritized causal eGenes, additional evidence from colocalization and single cell‐level expression validation was obtained for 17 Tier 2 and 3 Tier 1 eGenes, respectively. For *VIM* and *DDX5*, significantly upregulated expressions in specific immune cell subsets in the context of hypertension were validated, aligning with the causal evidence that their genetically predicted cell type‐specific expression levels are associated with heightened risk of hypertension. By contrast, *CTSW* in NK—which played a causal role in protecting against hypertension—also displayed an increased expression level among hypertensive individuals than their normotensive counterparts. This seemingly paradoxical discordance between the directions of causal effect and expression trend may reflect a reactive compensatory response, wherein NK‐specific *CTSW* is upregulated under hypertensive conditions and forms a negative feedback loop. While the secondary reactive increase was captured on the observational level, MR determined the primary, genetically driven protective role of *CTSW*. Therefore, the discordance is likely attributed to the nature of observational design that is intrinsically susceptible to unmeasured confounding or reverse causation (where hypertension causally drives gene expression alternations), underscoring MR's strengths in providing more accurate causal insights into biological plausibility. *VIM* was the only eGene with consistent support from three layers of evidence. While an increase in its expression during hypertension was exclusively captured in CD8 ET, both MR and colocalization results suggested that genetically predicted high expression levels of *VIM* in CD4 NC, CD8 NC, and CD8 ET are comparably correlated with heightened risk of hypertension. Encoded by *VIM*, vimentin is a class of intermediate filament cytoskeletal protein ubiquitously expressed in various mesenchymal and immune cells, regulating cell adhesion, migration, and cell shaping and plasticity [65]. There is compelling evidence that vimentin is broadly implicated in the amplification of immune responses underpinning cancers, injury, autoimmune and infectious diseases. Vimentin in the cell surface has been proven to be effectively targeted by monoclonal antibodies such as Pritumumab, with documentation of its successful application in curbing the growth of vimentin‐positive malignant tumor cells in patients with gliomas [66]. As per our analyses, *VIM* represents a promising candidate for further evaluation in drug repurposing studies, and its genetically proxied expression in CD8 ET displayed a generally favorable safety profile. Given that hypertension is increasingly managed as a disease entity attributable to immune disorder, targeting *VIM* might offer a novel therapeutic avenue for treating inflammation‐driven hypertension. For the remaining Tier 1 eGenes (*DDX5* and *CTSW*), previous literatures predominantly focused on their engagement in modulating the immune microenvironment conducive to oncogenesis and underscored their potential roles as prognostic signatures for solid tumors [67, 68]. Immune cell infiltration into target organs represents a common feature in both carcinogenesis and the inflammatory progression of hypertension; however, to which extent restoring the balance in immune microenvironment via therapeutically targeting *DDX5* and *CTSW* abrogates inflammation and improves hypertension warrants further investigation. In addition, two hypertension‐related eGenes (*FNBP4* and *PRELID1*) were shown to be markers of cardiac damage concomitant with BP elevation. Although development of biomarkers and targeted therapies based on this finding holds potential for concurrently controlling BP and mitigating its downstream organ damage, further mechanistic studies are required.

Our study has the following limitations: (1) despite the inclusion of as many eGenes as possible for the transcriptome‐wide MR analyses, those lacking available genetic instruments cannot be taken into account due to the nature of MR design; (2) the vast majority of GWASs analyzed in the analyses were from European‐only populations. While this is conducive to minimizing biases resulting from racial/ethnic heterogeneity, our findings may not be generalizable to people of other races/ethnicities; (3) the immune‐mediated mechanisms regulating hypertension are inherently pleiotropic and involve intricately linked networks [4, 5]. While our in silico analyses provided causal insights into target prioritization, the extent to which targeting a single eGene may yield adequate therapeutic efficacy and avoid unanticipated on‐target side effects remains uncertain. As such, functional validation and safety assessments with experimental and population‐based research are critical to bridge the gap between genetically inferred causality and therapeutic plausibility; (4) although we restricted the selection of IVs to SNPs within a *cis* region—which are directly involved in regulating the expression levels of the eGenes—the risk of residual pleiotropic bias cannot be entirely dismissed. Specifically, such pleiotropy predominantly exists in scenarios where IVs proxying an eGene influence its expression in cell types other than immune cells, or influence other genes or alternative biological mechanisms unrelated to the corresponding eGenes per se. Moreover, due to the limited number of available IVs that only permitted single‐IV analyses for most eGenes, more rigorous sensitivity analyses (e.g., MR‐Egger) could not be applied to provide a more comprehensive assessment of horizontal pleiotropy; (5) while Steiger filtering was applied to assess potential reverse causality, its efficacy should be interpreted with caution in the context of complex immune–hypertension feedback loops. As Steiger filtering determines correct directionality of causal associations through comparing the variance explained by genetic instruments between the exposure (immune cell‐specific eGenes) and outcome (hypertension), it inherently assumes a unidirectional model and may fail to account for mutual biological feedback loops between two reciprocally related traits. Nevertheless, ensuring correct directionality by Steiger filtering helps to enhance the reliability of our MR analyses and strengthen the robustness of the causal associations through the lens of genetics. Further longitudinal studies are needed to systematically evaluate the temporality and dynamic feedback within the immune–hypertension axis; (6) estimating lifetime effects remains technically challenging when time‐varying exposures (such as BP and gene expression) are measured at a single time point [69]; and (7) it is noteworthy that the identified causal associations were based on genetically predicted expression of genes, but not proteins. This may add an additional layer of uncertainty when assessing the potentials of eGenes as drug targets, given that gene expression at a single‐cell resolution does not always correlate with protein levels of the same target [70].

## 5 Conclusion

In summary, our study provides causal evidence for the molecular mechanisms by which immune cells contribute to the development of hypertension. Through the integration of multiple‐layered evidence, our findings prioritized key immune cell‐specific genes as new targets amenable to therapeutic translation. While these immune‐related targets offer valuable insights for the development of novel anti‐hypertensive strategies, investigations for a deeper mechanistic interpretation of how they directly affect known BP‐regulating components are warranted.

## Author Contributions

**Yukang Mao:** data curation (equal), investigation (equal), writing – original draft (lead), formal analysis (lead), visualization (equal). **Peng Li:** data curation (equal), investigation (equal), methodology (equal), writing – original draft (supporting), validation (lead). **Chen Cheng:** methodology (equal), validation (supporting), visualization (equal). **Xiangqing Kong:** conceptualization (equal), project administration (lead), resources (supporting), supervision (supporting). **Wei Sun:** conceptualization (equal), project administration (supporting), funding acquisition (equal). **Tingting Wu:** conceptualization (equal); funding acquisition (equal), resources (lead), supervision (lead).

## Funding

The study was funded by the Noncommunicable Chronic Diseases‐National Science and Technology Major Project (2024ZD0527300 and 2024ZD0527306) and the National Key Research and Development Program of China (2023YFC2410500).

## Ethics Statement

Ethics approval was not applicable in this study, given all the analyses were performed using publicly available GWAS summary statistics that have been approved by respective institutional ethics committees.

## Consent

All patients provided written informed consent prior to enrollment in the study.

## Conflicts of Interest

The authors declare no conflicts of interest.

## Supporting information


**Table S1:** SNPs used as instrumental variables for 9123 cell type‐specific eGenes.
**Table S2:** Discovery MR analyses on the associations between cell type‐specific eGenes and hypertension.
**Table S3:** Replication MR analyses on the associations between cell type‐specific eGenes and hypertension.
**Table S4:** Colocalization evidence for the associations between cell type‐specific eGenes and hypertension (COLOC method).
**Table S5:** Colocalization evidence for the associations between cell type‐specific eGenes and hypertension (SuSiE method).
**Table S6:** MR and colocalization analyses on the cell type‐specific associations between hypertension‐related eGenes and hypertensive heart disease.
**Table S7:** MR and colocalization analyses on the cell type‐specific associations between hypertension‐related eGenes and hypertensive renal disease.
**Table S8:** MR and colocalization analyses on the cell type‐specific associations between hypertension‐related eGenes and hypertensive encephalopathy.
**Table S9:** Cell type‐specific DEGs between PBMCs of patients with and without hypertension.
**Table S10:** Druggability assessment of Tier 1 and 2 eGene targets with the Therapeutic Target Database.
**Table S11:** Druggability assessment of Tier 1 and 2 eGene targets with the DrugBank.
**Table S12:** Druggability assessment of Tier 1 and 2 eGene targets with the Open Targets Database.
**Table S13:** Phenome‐wide MR analyses on genetically mimicked targeting of CD8 ET‐specific VIM.
**Table S14:** Phenome‐wide MR analyses on genetically mimicked targeting of CD8 NC‐specific DDX5.
**Table S15:** Phenome‐wide MR analyses on genetically mimicked targeting of CD4 NC‐specific DDX5.
**Table S16:** Phenome‐wide MR analyses on genetically mimicked targeting of NK‐specific CTSW.

**Figure S1:** Functional characterization of eGenes causally associated with hypertension.
**Figure S2:** Regional genomic plots showing the colocalization results for the associations between eGenes and hypertension.
**Figure S3:** Regional genomic plots showing the colocalization results for the associations between hypertension‐related eGenes and hypertensive heart disease.

## Data Availability

All supporting data relevant to the study findings are presented in the manuscript or the Supporting Information materials. All the GWAS data analyzed is obtained from publicly available databases or relevant papers. This paper does not generate new data or report original code. Any additional information required to reanalyze the data reported in this paper is available from the lead contact upon reasonable request.
