# Supplementary material for: Unveiling the Immune Mechanisms of Hypertension With Single‐Cell Transcriptome‐Wide Mendelian Randomization
Source: FASEB J. 2026 Aug 20;40(16):e72206. doi: 10.1096/fj.202600899R (PMC13492368; doi:10.1096/fj.202600899R)
Supplement: Supplementary file 1 [file FSB2-40-e72206-s001.pdf]

Unveiling the immune mechanisms of hypertension with single-cell transcriptome-wide Mendelian randomization

Yukang Mao[1†], Peng Li[1†], Chen Cheng[1†], Xiangqing Kong[1], Wei Sun[1*], Tingting Wu[1*]

[1]Department of Cardiology, The First Affiliated Hospital with Nanjing Medical University, Nanjing 210029, China

***Correspondence**

Tingting Wu, M.D., Department of Cardiology, The First Affiliated Hospital with Nanjing Medical University, Nanjing, 210029, China; Email: wutingting@jsph.org.cn

Wei Sun, M.D., Ph.D., Department of Cardiology, The First Affiliated Hospital with Nanjing Medical University, Nanjing, 210029, China; Email: weisun7919@njmu.edu.cn

SUPPLEMENTARY TABLES

Table S1. SNPs used as instrumental variables for 9,123 cell type-specific eGenes.

Table S2. Discovery MR analyses on the associations between cell type-specific eGenes and hypertension.

Table S3. Replication MR analyses on the associations between cell type-specific eGenes and hypertension.

Table S4. Colocalization evidence for the associations between cell type-specific eGenes and hypertension (COLOC method).

Table S5. Colocalization evidence for the associations between cell type-specific eGenes and hypertension (SuSiE method).

Table S6. MR and colocalization analyses on the cell type-specific associations between hypertension-related eGenes and hypertensive heart disease.

Table S7. MR and colocalization analyses on the cell type-specific associations between hypertension-related eGenes and hypertensive renal disease.

Table S8. MR and colocalization analyses on the cell type-specific associations between hypertension-related eGenes and hypertensive encephalopathy.

Table S9. Cell type-specific DEGs between PBMCs of patients with and without hypertension.

Table S10. Druggability assessment of Tier 1 and 2 eGene targets with the Therapeutic Target Database.

Table S11. Druggability assessment of Tier 1 and 2 eGene targets with the DrugBank.

Table S12. Druggability assessment of Tier 1 and 2 eGene targets with the Open Targets Database.

Table S13. Phenome-wide MR analyses on genetically mimicked targeting of CD8 ET-specific *VIM*.

Table S14. Phenome-wide MR analyses on genetically mimicked targeting of CD8 NC-specific *DDX5*.

Table S15. Phenome-wide MR analyses on genetically mimicked targeting of CD4 NC-specific *DDX5*.

Table S16. Phenome-wide MR analyses on genetically mimicked targeting of NK-specific *CTSW*.

Table S1. SNPs used as instrumental variables for 9,123 cell type-specific eGenes.

Cell type	eGene	SNP	CHR	POS	Effect allele	Other allele	EAF	Beta	SE	*P*-value	*F*
B IN	*ABCA1*	rs2437821	9	107686241	C	T	0.2239	-0.3254	0.0560	6.15E-09	33.72
B IN	*ABHD16A*	rs3131786	6	30901872	G	C	0.3666	-0.2872	0.0462	5.02E-10	38.59
B IN	*ABHD16A*	rs9265628	6	31299899	C	T	0.1289	-0.5936	0.0545	1.26E-27	118.39
B IN	*AC005037.3*	rs2136600	2	201742345	T	C	0.2391	-0.6526	0.0521	4.70E-36	156.85
B IN	*AC006129.4*	rs7255742	19	42049821	G	A	0.7898	-0.3741	0.0550	1.02E-11	46.19
B IN	*AC023590.1*	rs2460970	8	119629454	A	G	0.9192	-0.6694	0.0772	4.32E-18	75.02
B IN	*AC079767.4*	rs12397595	2	208526803	G	A	0.4521	0.4379	0.0443	4.71E-23	97.56
B IN	*AC096772.6*	rs13390896	2	208213033	A	G	0.2729	-0.5559	0.0505	3.78E-28	120.78
B IN	*AC104653.1*	rs4849285	2	114587432	T	C	0.5877	0.3263	0.0458	1.02E-12	50.7
B IN	*ACAP1*	rs3809822	17	7234112	G	C	0.2229	-0.3195	0.0516	5.78E-10	38.32
B IN	*ACAT1*	rs4754296	11	108006182	G	T	0.1466	0.3676	0.0637	7.76E-09	33.27
B IN	*ACCS*	rs2074038	11	44087989	T	G	0.1126	0.5156	0.0741	3.50E-12	48.29
B IN	*ACTG1*	rs1139405	17	79478019	A	G	0.8118	0.3147	0.0571	3.56E-08	30.31
B IN	*ACYP2*	rs4671747	2	54229818	T	C	0.4105	-0.4626	0.0447	4.78E-25	106.64
B IN	*ADAM19*	rs113913502	5	156877604	G	C	0.0671	-0.5554	0.0973	1.16E-08	32.48
B IN	*ADPRM*	rs403817	17	10619583	A	T	0.4499	-0.3312	0.0452	2.48E-13	53.47
B IN	*AHI1*	rs58761508	6	135835901	G	A	0.6453	0.6242	0.0452	2.70E-43	189.94
B IN	*AKAP13*	rs11637730	15	85885708	T	A	0.3395	0.3400	0.0466	2.82E-13	53.22
B IN	*AKAP7*	rs76670887	6	131505231	T	G	0.1443	0.3939	0.0615	1.52E-10	40.91
B IN	*ALKBH7*	rs79043335	19	6378610	T	C	0.0780	0.4378	0.0761	8.86E-09	33.01
B IN	*ANK1*	rs999188	8	41435795	G	T	0.4548	-0.4539	0.0443	1.10E-24	104.99
B IN	*ANKDD1A*	rs113935737	15	65123276	A	T	0.3890	-0.3424	0.0457	6.46E-14	56.11
B IN	*ANXA4*	rs6711807	2	69946670	C	T	0.2659	0.5033	0.0512	8.87E-23	96.32
B IN	*ANXA5*	rs6854854	4	122606919	C	T	0.0747	0.4568	0.0782	5.17E-09	34.05
B IN	*ANXA6*	rs2303038	5	150535013	T	C	0.2046	0.4221	0.0544	8.30E-15	60.14
B IN	*AP003774.1*	rs627425	11	64087642	T	C	0.3608	0.4405	0.0456	4.17E-22	93.26
B IN	*AP1S3*	rs1345120	2	224685044	C	T	0.4798	0.4384	0.0442	3.63E-23	98.08
B IN	*ARHGAP24*	rs12506009	4	86563698	G	A	0.3067	-0.9000	0.0453	1.15E-87	393.13
B IN	*ARHGEF3*	rs6804621	3	56764329	T	C	0.1634	0.4913	0.0618	1.85E-15	63.09
B IN	*ARL11*	rs9535274	13	50194394	A	G	0.6485	-0.5013	0.0471	1.95E-26	112.97
B IN	*ARL14EP*	rs7115854	11	30311493	G	A	0.3037	-0.3783	0.0497	2.81E-14	57.75
B IN	*ARL16*	rs7503637	17	79649515	A	G	0.1691	-0.5550	0.0588	3.73E-21	88.93
B IN	*ARL17B*	rs79730878	17	43849415	C	T	0.2110	0.3860	0.0530	3.17E-13	52.99
B IN	*ARSA*	rs2071421	22	51064416	C	T	0.1275	-0.3690	0.0676	4.88E-08	29.7
B IN	*ASPH*	rs59681890	8	62655710	T	C	0.1158	0.4671	0.0783	2.39E-09	35.56
B IN	*ATG10*	rs749401	5	81561369	C	T	0.2282	0.3152	0.0511	6.88E-10	37.98
B IN	*ATP5G3*	rs11894320	2	176022092	G	C	0.1585	0.3716	0.0613	1.33E-09	36.7
B IN	*ATP6V1G2*	rs3130566	6	31102618	G	C	0.1148	-0.6897	0.0612	1.91E-29	126.69
B IN	*ATP6V1G2*	rs4959021	6	31981146	C	T	0.8071	-0.4976	0.0579	8.59E-18	73.66
B IN	*AXIN2*	rs9894067	17	63564778	G	A	0.5285	-0.4059	0.0443	5.62E-20	83.58
B IN	*BCAS4*	rs6096141	20	49490888	C	T	0.3824	-0.2995	0.0464	1.04E-10	41.66
B IN	*BCL2A1*	rs8033549	15	80309299	G	A	0.3329	0.8519	0.0428	3.43E-88	395.55
B IN	*BICD2*	rs10821009	9	95488414	A	C	0.2839	0.4470	0.0497	2.32E-19	80.78
B IN	*BIRC5*	rs16971511	17	76529270	T	C	0.2736	0.6181	0.0477	2.18E-38	167.51
B IN	*BLK*	rs2409780	8	11337587	C	T	0.2580	-0.5156	0.0502	9.94E-25	105.19
B IN	*BRK1*	rs113885243	3	10055671	T	C	0.0877	-0.5275	0.0749	1.92E-12	49.47
B IN	*BTF3*	rs13183753	5	72689328	A	G	0.0792	-0.5440	0.0860	2.51E-10	39.94
B IN	*BTN3A2*	rs67509210	6	26354100	T	G	0.1018	-0.5789	0.0663	2.53E-18	76.07
B IN	*BTNL9*	rs7709346	5	180474080	C	T	0.5457	-0.3604	0.0445	5.76E-16	65.38
B IN	*C10orf32*	rs12775431	10	104611764	C	A	0.2393	-0.3443	0.0526	5.84E-11	42.78
B IN	*C11orf24*	rs312018	11	68066034	A	G	0.3916	0.4814	0.0457	5.34E-26	110.98
B IN	*C12orf57*	rs7138740	12	6983764	A	T	0.2917	0.2930	0.0488	2.00E-09	35.9
B IN	*C17orf97*	rs4567793	17	248856	T	C	0.7616	0.6634	0.0519	2.10E-37	163.02
B IN	*C19orf60*	rs10407212	19	18699445	A	G	0.4756	-0.3337	0.0447	8.07E-14	55.67
B IN	*C1orf86*	rs11587831	1	2110848	G	T	0.2239	-0.3815	0.0544	2.22E-12	49.18
B IN	*C22orf34*	rs6009810	22	50059540	G	A	0.7265	0.2833	0.0513	3.43E-08	30.39
B IN	*C2orf27A*	rs35424057	2	132547620	C	T	0.1963	0.3790	0.0544	3.09E-12	48.53
B IN	*C5orf30*	rs2545662	5	102993771	A	T	0.8147	0.4267	0.0561	2.88E-14	57.7
B IN	*C6orf25*	rs2523856	6	31021630	T	C	0.6243	-0.4101	0.0457	2.74E-19	80.45
B IN	*C6orf25*	rs2844575	6	31334945	C	T	0.4698	0.2455	0.0450	4.80E-08	29.74
B IN	*C6orf48*	rs57165260	6	31836202	C	A	0.0957	-0.5995	0.0801	7.14E-14	55.92
B IN	*C6orf62*	rs3756814	6	24705835	C	T	0.3832	-0.2533	0.0460	3.66E-08	30.26
B IN	*C9orf156*	rs3780419	9	100677134	C	T	0.2565	0.4146	0.0504	1.89E-16	67.58
B IN	*C9orf78*	rs13297609	9	132580686	C	G	0.1853	-0.3334	0.0587	1.32E-08	32.23
B IN	*CAPG*	rs3770102	2	85637837	T	G	0.3911	0.3470	0.0451	1.46E-14	59.03
B IN	*CAPN12*	rs62119115	19	39210593	A	G	0.2446	-0.2929	0.0531	3.55E-08	30.32
B IN	*CARD8*	rs10414394	19	48763008	T	C	0.1432	0.3609	0.0607	2.83E-09	35.22
B IN	*CAST*	rs152005	5	96025847	G	A	0.1633	0.5099	0.0616	1.23E-16	68.43
B IN	*CCDC101*	rs231977	16	28542172	C	T	0.5303	-0.2671	0.0449	2.63E-09	35.37
B IN	*CCDC15*	rs7932003	11	124855162	A	G	0.1113	-0.6347	0.0720	1.21E-18	77.53
B IN	*CCDC163P*	rs945179	1	45992460	T	C	0.6860	-0.3954	0.0468	2.85E-17	71.3
B IN	*CCDC58*	rs3828362	3	122131777	G	C	0.4138	0.4216	0.0449	6.57E-21	87.81
B IN	*CCDC66*	rs17216685	3	56647799	C	T	0.1742	0.5537	0.0603	4.33E-20	84.09
B IN	*CCL4*	rs1719144	17	34431403	A	G	0.2212	0.3391	0.0528	1.39E-10	41.1
B IN	*CCND2-AS1*	rs17770486	12	3771383	C	T	0.1370	0.6100	0.0636	8.68E-22	91.81
B IN	*CCT3*	rs113279830	1	156299392	G	A	0.2804	-0.3234	0.0491	4.39E-11	43.34
B IN	*CCT8*	rs965951	21	30432557	A	G	0.1160	1.0768	0.0644	1.12E-62	278.64

B IN	*CCZ1*	rs7807287	7	6877519	G	A	0.4455	0.4748	0.0442	7.19E-27	114.95
B IN	*CCZ1B*	rs10265409	7	6869304	T	C	0.4393	-0.3477	0.0449	9.75E-15	59.82
B IN	*CD164*	rs35452836	6	109699446	T	A	0.3004	0.2677	0.0487	3.84E-08	30.17
B IN	*CD37*	rs1054770	19	49878115	A	G	0.2640	-0.5557	0.0490	8.07E-30	128.39
B IN	*CD52*	rs11247915	1	26650550	A	C	0.5030	-0.3853	0.0443	3.59E-18	75.38
B IN	*CD81*	rs810225	11	2404602	A	C	0.7693	0.3773	0.0552	8.13E-12	46.64
B IN	*CD83*	rs74405933	6	14095755	A	G	0.0568	-0.6628	0.0984	1.66E-11	45.24
B IN	*CD86*	rs13088702	3	121714019	C	T	0.0739	0.6146	0.0898	7.78E-12	46.73
B IN	*CD86*	rs2681411	3	121785631	A	G	0.6045	0.3051	0.0457	2.46E-11	44.47
B IN	*CD9*	rs797773	12	6307566	T	C	0.4200	-0.2529	0.0459	3.55E-08	30.32
B IN	*CD96*	rs6785519	3	111253069	C	T	0.2612	0.8965	0.0456	3.48E-86	386.35
B IN	*CDC42*	rs2268179	1	22414785	T	G	0.1644	0.4753	0.0602	3.04E-15	62.11
B IN	*CEACAM21*	rs10420992	19	42064462	A	T	0.7206	0.2823	0.0506	2.46E-08	31.03
B IN	*CEBPZ-AS1*	rs72792835	2	37522171	C	T	0.1344	-0.4240	0.0660	1.30E-10	41.23
B IN	*CENPK*	rs2548790	5	65011742	A	G	0.2575	-0.3067	0.0505	1.25E-09	36.82
B IN	*CENPV*	rs11651955	17	16235462	A	G	0.4981	-0.3106	0.0446	3.43E-12	48.33
B IN	*CFLAR*	rs12693930	2	202044232	A	G	0.4685	-0.2845	0.0447	2.01E-10	40.38
B IN	*CHCHD2*	rs2080141	7	56163770	C	T	0.2141	0.5267	0.0533	4.74E-23	97.55
B IN	*CHI3L2*	rs3118207	1	111768576	A	G	0.3631	-0.7992	0.0433	4.18E-76	340.15
B IN	*CHURC1*	rs4902334	14	65385820	C	T	0.3305	0.3183	0.0506	3.28E-10	39.42
B IN	*CIB1*	rs12902511	15	90823039	G	A	0.3768	0.2857	0.0459	5.00E-10	38.6
B IN	*CLK4*	rs1531540	5	178086529	T	C	0.4584	-0.2949	0.0450	5.54E-11	42.89
B IN	*CLLU1*	rs337652	12	92794173	A	T	0.7077	0.3712	0.0485	1.93E-14	58.48
B IN	*CLLU1OS*	rs61935208	12	92813081	G	T	0.2655	0.5805	0.0498	1.95E-31	135.77
B IN	*CLN8*	rs71499040	8	1711918	C	G	0.7034	0.3625	0.0491	1.52E-13	54.44
B IN	*CNDP2*	rs747176	18	72154931	A	G	0.2167	-0.3647	0.0544	2.08E-11	44.8
B IN	*CNOT6L*	rs13144225	4	78717276	C	T	0.8955	-0.4731	0.0693	8.48E-12	46.56
B IN	*CNPPD1*	rs6436124	2	220041433	A	C	0.5814	0.2867	0.0464	6.38E-10	38.12
B IN	*CNTNAP2*	rs1919692	7	148001727	A	G	0.6917	0.6687	0.0470	6.04E-46	202.06
B IN	*COL9A3*	rs66658258	20	61462502	C	G	0.0951	0.5288	0.0895	3.49E-09	34.82
B IN	*COL9A3*	rs6010758	20	61469323	T	C	0.0739	0.5559	0.0835	2.83E-11	44.2
B IN	*COLCA2*	rs12419634	11	111239365	C	G	0.5784	-0.4610	0.0451	1.59E-24	104.26
B IN	*COMMD6*	rs12869081	13	76100090	A	G	0.1381	-0.9442	0.0586	2.50E-58	258.73
B IN	*CORO1B*	rs1638557	11	67215988	G	C	0.4026	-0.2594	0.0456	1.30E-08	32.27
B IN	*CORO1C*	rs10746129	12	109091089	C	T	0.4662	0.5101	0.0439	3.01E-31	134.91
B IN	*COTL1*	rs934166	16	84631300	G	A	0.5577	0.2607	0.0449	6.58E-09	33.59
B IN	*COX6B1*	rs61218414	19	36171716	C	T	0.5113	-0.3453	0.0446	9.19E-15	59.94
B IN	*CPNE1*	rs60646509	20	34303409	G	T	0.1063	-0.5105	0.0770	3.28E-11	43.92
B IN	*CRYBB2*	rs6004662	22	25862124	A	G	0.0960	1.0246	0.0842	4.44E-34	147.83
B IN	*CSTB*	rs35285321	21	45201832	A	G	0.3415	-0.7493	0.0457	1.56E-60	268.82
B IN	*CTA-14H9.5*	rs58616630	6	27474715	G	A	0.0754	-0.5975	0.0753	2.03E-15	62.9
B IN	*CTA-29F11.1*	rs12389	22	47158179	A	G	0.3392	-0.3908	0.0462	2.58E-17	71.49
B IN	*CTC-524C5.2*	rs1050456	17	5336188	G	A	0.2366	0.4959	0.0537	2.64E-20	85.07
B IN	*CTD-2007H13.3*	rs161947	5	98268572	T	C	0.2024	-0.4081	0.0540	4.31E-14	56.9
B IN	*CTD-2017D11.1*	rs57495058	19	24207669	A	G	0.1860	0.3512	0.0576	1.07E-09	37.12
B IN	*CTD-2368P22.1*	rs4801243	19	58513807	C	A	0.2297	0.8388	0.0497	6.75E-64	284.22
B IN	*CTD-2371O3.2*	rs12296039	11	9458828	G	A	0.5033	0.4701	0.0439	9.50E-27	114.39
B IN	*CTD-3110H11.1*	rs11070770	15	50654075	T	C	0.3545	-0.4217	0.0452	1.16E-20	86.7
B IN	*CTH*	rs61778712	1	71222145	A	G	0.2675	-0.6856	0.0470	4.15E-48	211.95
B IN	*CTSH*	rs3825932	15	79235446	C	T	0.6393	0.2948	0.0477	6.19E-10	38.18
B IN	*CYB5A*	rs1790885	18	71908297	C	G	0.7208	-0.8302	0.0459	3.91E-73	326.53
B IN	*DARS*	rs6711493	2	136692553	G	A	0.2109	0.3824	0.0658	6.15E-09	33.72
B IN	*DAZAP2*	rs7314235	12	51631781	G	C	0.6253	-0.2548	0.0467	4.75E-08	29.76
B IN	*DBNDD1*	rs11076653	16	90074001	T	C	0.0574	0.6494	0.0955	1.05E-11	46.13
B IN	*DCLK2*	rs28806288	4	151048211	G	A	0.2005	0.4877	0.0513	2.11E-21	90.06
B IN	*DCXR*	rs111894854	17	79998898	G	A	0.1249	0.9597	0.0646	6.30E-50	220.27
B IN	*DDT*	rs5751777	22	24267047	T	C	0.5906	0.4141	0.0455	9.49E-20	82.54
B IN	*DDX11*	rs4537846	12	31213631	T	C	0.4481	-0.4537	0.0443	1.27E-24	104.71
B IN	*DDX5*	rs1991401	17	62502435	G	A	0.3173	-0.3382	0.0483	2.52E-12	48.93
B IN	*DFNA5*	rs754555	7	24758753	T	C	0.1591	0.4690	0.0608	1.19E-14	59.42
B IN	*DHFR*	rs2115014	5	79899922	G	C	0.6705	-0.2608	0.0475	4.09E-08	30.05
B IN	*DHRS4*	rs147027077	14	24478799	T	A	0.0705	0.4986	0.0896	2.62E-08	30.91
B IN	*DIEXF*	rs598514	1	209994715	T	C	0.5725	-0.2702	0.0455	2.84E-09	35.22
B IN	*DNAJC15*	rs2281778	13	43597977	G	C	0.2269	-0.6278	0.0522	2.63E-33	144.31
B IN	*DNASE1L3*	rs3772985	3	58186719	A	C	0.1169	-0.4809	0.0714	1.69E-11	45.21
B IN	*DOCK8*	rs12156499	9	370199	G	A	0.1449	0.4282	0.0638	1.95E-11	44.93
B IN	*DRAM2*	rs3762374	1	111682118	T	C	0.2783	0.4899	0.0477	8.77E-25	105.44
B IN	*DSE*	rs9488914	6	116690849	T	C	0.4077	-0.2864	0.0463	6.11E-10	38.21
B IN	*DSP*	rs34704640	6	7449465	A	G	0.5602	0.6555	0.0430	1.77E-52	231.95
B IN	*DYNLL2*	rs9900038	17	56169763	G	A	0.7692	-0.3053	0.0515	3.01E-09	35.11
B IN	*EAF2*	rs2331964	3	121542898	C	T	0.6095	-0.8349	0.0427	3.33E-85	381.85
B IN	*EBPL*	rs9562915	13	50283389	C	T	0.2234	-0.4227	0.0534	2.43E-15	62.56
B IN	*ECHDC2*	rs1242331	1	53392908	G	A	0.6574	0.3501	0.0471	1.12E-13	55.03
B IN	*EIF2A*	rs13064363	3	150357070	T	C	0.2391	0.3081	0.0522	3.60E-09	34.76
B IN	*EIF5A*	rs7503161	17	7207964	C	A	0.5615	-1.0407	0.0385	1.20E-160	727.93
B IN	*ELOVL5*	rs2816355	6	53107650	T	C	0.5652	-0.2898	0.0450	1.19E-10	41.4
B IN	*ELP2*	rs681757	18	33744766	G	T	0.3785	0.3683	0.0461	1.39E-15	63.65
B IN	*ELP5*	rs2074218	17	7156186	G	A	0.6135	-0.5875	0.0441	2.08E-40	176.75
B IN	*ENO1*	rs6577536	1	8910110	G	A	0.5116	0.2725	0.0448	1.17E-09	36.95

B IN	*EPSTI1*	rs3825511	13	43566373	C	A	0.7178	-0.4229	0.0502	3.82E-17	70.73
B IN	*ERAP2*	rs27289	5	96346278	T	G	0.5872	-0.6103	0.0434	6.77E-45	197.26
B IN	*ERICH1*	rs6996811	8	606200	C	G	0.5398	0.2547	0.0450	1.53E-08	31.94
B IN	*EVA1C*	rs12627339	21	34279201	T	A	0.2971	-0.4407	0.0490	2.43E-19	80.69
B IN	*EVI2A*	rs2854319	17	29694054	C	G	0.6049	0.4980	0.0456	8.63E-28	119.14
B IN	*FADS2*	rs968567	11	61595564	T	C	0.1535	-0.5058	0.0568	5.61E-19	79.04
B IN	*FAM111A*	rs556507	11	58930836	A	C	0.4751	0.2871	0.0451	1.87E-10	40.51
B IN	*FAM118A*	rs136582	22	45774531	A	T	0.1332	1.4237	0.0603	4.01E-123	555.75
B IN	*FAM167A*	rs2618444	8	11338370	C	A	0.2572	-0.4830	0.0505	1.08E-21	91.37
B IN	*FAM177B*	rs4424514	1	222946310	G	A	0.7586	0.5835	0.0503	4.44E-31	134.14
B IN	*FAM184B*	rs10027755	4	17615954	C	G	0.6081	-0.9345	0.0408	2.29E-116	524.75
B IN	*FAM21A*	rs2338050	10	51804949	A	G	0.3750	0.3160	0.0457	4.83E-12	47.66
B IN	*FAM26F*	rs117361304	6	116783619	G	T	0.0827	-1.3158	0.0748	2.66E-69	308.97
B IN	*FAM26F*	rs483981	6	116763077	C	T	0.7718	0.3097	0.0545	1.36E-08	32.18
B IN	*FAM69B*	rs9411221	9	139622646	A	G	0.5142	0.2823	0.0447	2.80E-10	39.73
B IN	*FANCA*	rs34177108	16	89893375	A	C	0.2263	-0.2842	0.0509	2.29E-08	31.16
B IN	*FAR1*	rs35766662	11	13673313	A	G	0.3313	0.2630	0.0480	4.29E-08	29.95
B IN	*FCER2*	rs12979200	19	7758303	G	A	0.4839	-0.3428	0.0446	1.47E-14	59.02
B IN	*FCRL2*	rs7535596	1	157673356	G	A	0.2401	-0.3469	0.0516	1.82E-11	45.06
B IN	*FCRL3*	rs2210913	1	157668993	T	C	0.4492	0.4876	0.0439	1.09E-28	123.23
B IN	*FCRL5*	rs12741984	1	157534818	A	G	0.2142	1.1269	0.0484	6.22E-120	541.11
B IN	*FCRLA*	rs57678166	1	161679899	C	T	0.1474	0.4877	0.0670	3.51E-13	52.79
B IN	*FIGNL1*	rs6945253	7	50501440	G	T	0.6087	0.3073	0.0454	1.34E-11	45.66
B IN	*FIS1*	rs10260707	7	100863945	G	C	0.0607	-0.7053	0.0918	1.58E-14	58.88
B IN	*FKBPL*	rs1265159	6	31140047	A	G	0.2129	-0.3335	0.0517	1.08E-10	41.58
B IN	*FLVCR1-AS1*	rs7524203	1	213011812	C	G	0.5251	0.3373	0.0446	4.19E-14	56.96
B IN	*FNBP4*	rs10838786	11	47904086	A	G	0.4616	-0.2967	0.0447	3.15E-11	43.99
B IN	*FUT7*	rs7047770	9	139930945	A	G	0.6622	-0.2631	0.0471	2.35E-08	31.12
B IN	*FXYD5*	rs28659908	19	35693120	C	T	0.4020	-0.3121	0.0451	4.44E-12	47.82
B IN	*GAA*	rs8077055	17	78076005	A	C	0.2683	-0.2804	0.0500	1.99E-08	31.44
B IN	*GALNT2*	rs9432148	1	230413313	C	T	0.2381	0.4080	0.0517	3.00E-15	62.14
B IN	*GALT*	rs2812357	9	34655436	C	T	0.6012	0.2489	0.0453	3.97E-08	30.1
B IN	*GATM*	rs12440038	15	45706739	G	A	0.2811	-0.3471	0.0517	1.87E-11	45.01
B IN	*GATS*	rs13232115	7	99670932	C	T	0.2719	-0.4776	0.0494	4.14E-22	93.27
B IN	*GBP7*	rs6659897	1	89527757	T	C	0.5001	-0.4756	0.0439	2.27E-27	117.23
B IN	*GCC2*	rs2139809	2	109045638	C	T	0.5916	0.3853	0.0451	1.25E-17	72.93
B IN	*GCNT2*	rs9295574	6	10531244	A	G	0.4178	-0.2467	0.0452	4.73E-08	29.76
B IN	*GDI2*	rs3736461	10	5855403	C	G	0.1844	-0.4860	0.0547	6.76E-19	78.67
B IN	*GINM1*	rs1125	6	149979416	A	G	0.3414	-0.2821	0.0469	1.75E-09	36.16
B IN	*GM2A*	rs72794132	5	150618993	T	C	0.1649	0.7506	0.0579	1.79E-38	167.9
B IN	*GNG7*	rs10402849	19	2695661	T	C	0.2155	0.3141	0.0554	1.46E-08	32.05
B IN	*GNPTG*	rs57890635	16	1411129	T	C	0.2668	0.3415	0.0533	1.51E-10	40.93
B IN	*GPATCH11*	rs6738282	2	37317457	C	A	0.3552	0.5019	0.0449	5.12E-29	124.73
B IN	*GPM6A*	rs997780	4	176935913	A	C	0.8085	0.4819	0.0599	8.39E-16	64.64
B IN	*GPR155*	rs35155300	2	175255983	A	G	0.2547	0.4539	0.0515	1.15E-18	77.62
B IN	*GRHPR*	rs4878690	9	37422656	T	C	0.3290	0.4508	0.0472	1.18E-21	91.2
B IN	*GS1-124K5.4*	rs12670811	7	65823019	A	C	0.2622	0.2849	0.0509	2.14E-08	31.3
B IN	*GSTM4*	rs2269341	1	110172362	T	C	0.4139	-0.3381	0.0452	7.74E-14	55.76
B IN	*GSTT1*	rs5760147	22	24334948	C	A	0.3844	-0.6058	0.0448	1.39E-41	182.12
B IN	*GTF2H2*	rs28478097	5	70309998	G	C	0.6121	-0.4603	0.0452	2.53E-24	103.35
B IN	*GTF2H4*	rs41546314	6	29910378	T	C	0.2993	0.4984	0.0483	6.07E-25	106.17
B IN	*GTF2H4*	rs9261739	6	30367535	C	T	0.2182	0.3209	0.0550	5.45E-09	33.95
B IN	*GTF2H4*	rs6922051	6	31154820	C	T	0.3517	-0.4173	0.0474	1.21E-18	77.52
B IN	*GTF2H4*	rs3873388	6	31275827	G	A	0.3375	-0.2942	0.0494	2.56E-09	35.42
B IN	*GYPC*	rs10928978	2	127419582	T	G	0.6186	0.3575	0.0456	4.44E-15	61.37
B IN	*HAGHL*	rs1406814	16	778024	C	T	0.2455	0.3224	0.0546	3.45E-09	34.84
B IN	*HCG23*	rs3132472	6	31386131	A	G	0.0992	-0.3947	0.0660	2.27E-09	35.65
B IN	*HCG27*	rs9262619	6	31022266	C	G	0.2796	-0.3469	0.0501	4.22E-12	47.92
B IN	*HEATR5B*	rs6738282	2	37317457	C	A	0.3552	0.2999	0.0458	5.95E-11	42.75
B IN	*HEBP2*	rs9484150	6	138699838	A	G	0.0719	-0.5239	0.0914	9.80E-09	32.81
B IN	*HFE*	rs3804114	6	25602604	T	C	0.2578	0.5083	0.0500	2.78E-24	103.16
B IN	*HIGD2A*	rs13168952	5	175830600	A	G	0.0887	-0.5915	0.0893	3.55E-11	43.76
B IN	*HIST1H1B*	rs9468317	6	28198456	C	T	0.1733	-0.8763	0.0569	1.67E-53	236.63
B IN	*HIST1H2AB*	rs72843784	6	26498758	T	G	0.0715	-0.5477	0.0764	7.43E-13	51.32
B IN	*HIST1H2AH*	rs35111391	6	28103409	T	G	0.0564	0.4794	0.0829	7.23E-09	33.4
B IN	*HIST1H2AM*	rs3118365	6	28839908	A	G	0.0876	0.6644	0.0727	6.18E-20	83.39
B IN	*HIST1H2BF*	rs112510056	6	25505765	A	G	0.1727	-0.5379	0.0592	1.04E-19	82.37
B IN	*HIST1H2BG*	rs13191326	6	27031029	G	A	0.1001	-0.4186	0.0683	8.90E-10	37.48
B IN	*HIST1H2BJ*	rs34158769	6	26336572	A	G	0.0729	-0.6207	0.0755	1.95E-16	67.51
B IN	*HIST1H4I*	rs67662114	6	27932301	A	G	0.0750	-0.5692	0.0749	3.03E-14	57.6
B IN	*HLA-A*	rs2844810	6	29923580	C	G	0.6068	0.4703	0.0446	5.11E-26	111.07
B IN	*HLA-A*	rs1118204	6	30366240	C	T	0.3739	0.2939	0.0464	2.30E-10	40.11
B IN	*HLA-B*	rs2524096	6	31236467	T	G	0.4422	0.7005	0.0427	2.06E-60	268.27
B IN	*HLA-B*	rs3828879	6	31379391	T	C	0.1430	-0.5738	0.0652	1.43E-18	77.19
B IN	*HLA-B*	rs9366777	6	31264715	A	T	0.0923	-0.4656	0.0830	2.02E-08	31.41
B IN	*HLA-C*	rs2249741	6	31240712	C	A	0.5532	0.5116	0.0437	1.15E-31	136.82
B IN	*HLA-C*	rs9264981	6	31280079	T	C	0.5745	0.2625	0.0454	7.52E-09	33.33
B IN	*HLA-DPA1*	rs2163472	6	32983375	T	C	0.4593	0.6053	0.0431	1.05E-44	196.38
B IN	*HLA-DPA1*	rs427809	6	33001066	T	A	0.2798	-0.3237	0.0514	2.92E-10	39.65

B IN	*HLA-DPB1*	rs2163472	6	32983375	T	C	0.4593	0.7081	0.0423	5.61E-63	280.01
B IN	*HLA-DPB1*	rs9277568	6	33057055	C	T	0.2778	0.4192	0.0485	5.44E-18	74.56
B IN	*HLA-DQA1*	rs5002178	6	32611590	A	G	0.6180	0.5346	0.0463	8.19E-31	132.93
B IN	*HLA-DQA2*	rs1391371	6	32603798	T	A	0.2202	1.3727	0.0444	1.00E-200	953.51
B IN	*HLA-DQA2*	rs34241101	6	31936057	T	G	0.0719	0.6733	0.0855	3.38E-15	61.91
B IN	*HLA-DQA2*	rs3134931	6	32190620	C	T	0.3105	-0.4034	0.0474	1.67E-17	72.35
B IN	*HLA-DQB1*	rs1130034	6	32605274	C	T	0.5136	-0.6552	0.0437	7.17E-51	224.59
B IN	*HLA-DQB2*	rs9272625	6	32608014	C	T	0.3507	0.5501	0.0443	2.15E-35	153.84
B IN	*HLA-DRB1*	rs9270589	6	32561390	T	C	0.4134	0.7285	0.0435	6.84E-63	279.61
B IN	*HLA-DRB5*	rs28366319	6	32561495	A	G	0.2843	1.1016	0.0391	8.93E-175	792.78
B IN	*HLA-DRB5*	rs4569	6	31637807	T	C	0.3137	-0.4190	0.0516	4.75E-16	65.76
B IN	*HLA-DRB5*	rs3132948	6	32197219	G	T	0.4645	-0.4305	0.0448	7.29E-22	92.16
B IN	*HLA-F*	rs9258166	6	29688900	C	G	0.1413	0.3911	0.0692	1.61E-08	31.85
B IN	*HMBOX1*	rs10088428	8	28909523	T	C	0.2597	-0.7324	0.0494	8.99E-50	219.56
B IN	*HMGB1*	rs2149851	13	30945921	C	T	0.4668	-0.2553	0.0448	1.23E-08	32.37
B IN	*HOPX*	rs6554379	4	57622222	A	G	0.2726	0.4856	0.0506	8.67E-22	91.81
B IN	*HOXB2*	rs1987164	17	46569052	G	A	0.4537	0.3316	0.0446	1.00E-13	55.25
B IN	*HSBP1L1*	rs9949598	18	77753037	A	G	0.8237	-0.3346	0.0597	2.15E-08	31.29
B IN	*HSD17B11*	rs4443241	4	88288599	C	T	0.7196	0.2853	0.0501	1.21E-08	32.41
B IN	*HSPA1B*	rs536971557	6	32435515	T	C	0.0994	-0.4557	0.0708	1.26E-10	41.29
B IN	*HSPBAP1*	rs12496363	3	122473354	T	C	0.7449	-0.8549	0.0509	2.12E-63	281.95
B IN	*ICA1L*	rs11544338	2	203632510	C	T	0.5593	-0.3556	0.0449	2.37E-15	62.6
B IN	*IFITM3*	rs7942247	11	316349	A	G	0.5502	0.2973	0.0447	2.95E-11	44.12
B IN	*IFNG-AS1*	rs12832728	12	68420403	A	G	0.5290	-0.3488	0.0447	6.37E-15	60.66
B IN	*IGFLR1*	rs3848662	19	36216056	T	G	0.1520	-0.3883	0.0644	1.69E-09	36.23
B IN	*IGLL5*	rs364790	22	23244310	G	A	0.4700	0.3481	0.0448	7.55E-15	60.33
B IN	*IL4R*	rs7195219	16	27277056	A	G	0.3314	-0.2646	0.0471	1.94E-08	31.49
B IN	*INSIG1*	rs28637746	7	155086647	C	T	0.5403	0.2886	0.0452	1.70E-10	40.71
B IN	*IRAK3*	rs2701652	12	66580877	G	C	0.7261	-0.3147	0.0499	2.81E-10	39.72
B IN	*ISCU*	rs10778643	12	108939901	A	T	0.8652	-0.4519	0.0649	3.44E-12	48.32
B IN	*ITGB2*	rs760462	21	46328099	C	T	0.8298	-1.0150	0.0563	1.34E-72	324.09
B IN	*ITGB3BP*	rs6697508	1	63893394	G	A	0.6994	-0.3668	0.0481	2.52E-14	57.96
B IN	*ITM2B*	rs198634	13	48913423	T	C	0.2263	0.3274	0.0518	2.59E-10	39.88
B IN	*ITM2C*	rs62195291	2	231746739	G	C	0.0900	-0.5463	0.0781	2.69E-12	48.8
B IN	*JUP*	rs4606765	17	39917325	C	A	0.7356	-0.3368	0.0519	8.79E-11	41.99
B IN	*JUP*	rs4796703	17	39950674	G	A	0.9164	-0.6988	0.0846	1.49E-16	68.04
B IN	*KANSL1-AS1*	rs199448	17	44809001	G	A	0.1897	1.0116	0.0493	1.01E-93	420.91
B IN	*KB-226F1.1*	rs2097433	22	24296894	G	A	0.5338	-0.4328	0.0445	2.51E-22	94.26
B IN	*KDELR2*	rs6796	7	6502367	C	T	0.2687	-0.4119	0.0489	3.85E-17	70.71
B IN	*KIAA0040*	rs2269650	1	175129691	T	G	0.3691	0.5741	0.0455	1.47E-36	159.15
B IN	*KIAA1841*	rs12713432	2	61265970	G	A	0.2572	-0.3350	0.0542	6.31E-10	38.14
B IN	*KRT10*	rs56261283	17	38961725	A	G	0.1606	0.4599	0.0648	1.22E-12	50.35
B IN	*KTN1*	rs12588172	14	56159354	C	T	0.3476	-0.3006	0.0471	1.70E-10	40.7
B IN	*KYNU*	rs351696	2	143705360	A	G	0.4556	0.3140	0.0448	2.51E-12	48.94
B IN	*L3HYPDH*	rs3742645	14	59931840	T	G	0.3806	-0.2786	0.0466	2.21E-09	35.7
B IN	*L3MBTL3*	rs6569648	6	130349119	T	C	0.7821	-0.4629	0.0509	9.90E-20	82.46
B IN	*LA16c-358B7.3*	rs11647760	16	1367610	A	C	0.6104	-0.3560	0.0453	3.74E-15	61.7
B IN	*LAMB1*	rs1131398	7	107569517	G	A	0.4034	-0.5217	0.0443	4.62E-32	138.62
B IN	*LAMTOR4*	rs3807479	7	99716901	G	C	0.5834	-0.3073	0.0451	9.69E-12	46.3
B IN	*LARGE*	rs5749579	22	33617801	C	T	0.2482	0.8235	0.0498	2.21E-61	272.7
B IN	*LCN8*	rs7041806	9	139648298	A	G	0.1396	1.4642	0.0520	2.08E-174	791.09
B IN	*LGALS9*	rs4239242	17	25974258	C	T	0.3553	-0.2761	0.0468	3.72E-09	34.7
B IN	*LHPP*	rs2124161	10	126242997	C	T	0.4311	0.2491	0.0452	3.59E-08	30.3
B IN	*LIMD1*	rs2673023	3	45711367	C	T	0.7714	-0.6698	0.0493	5.64E-42	183.9
B IN	*LIMS2*	rs10174611	2	128381154	A	G	0.3828	-0.4375	0.0456	8.35E-22	91.89
B IN	*LINC00339*	rs2501281	1	22364958	C	A	0.7057	0.3014	0.0491	8.41E-10	37.59
B IN	*LINC00926*	rs2248534	15	57597961	G	T	0.2669	0.4544	0.0483	5.27E-21	88.25
B IN	*LINC00954*	rs13034115	2	20077653	G	C	0.1396	-0.3870	0.0628	7.08E-10	37.92
B IN	*LINC00969*	rs3827561	3	195674257	G	C	0.4166	0.4255	0.0451	3.96E-21	88.81
B IN	*LONP2*	rs8053839	16	48390512	T	G	0.5150	0.3313	0.0447	1.29E-13	54.76
B IN	*LRRC16A*	rs9461158	6	25401017	G	A	0.4222	-0.4239	0.0452	6.26E-21	87.91
B IN	*LRRC25*	rs1985157	19	18513594	C	T	0.4002	-0.2560	0.0457	2.10E-08	31.34
B IN	*LRRC37A2*	rs2435207	17	44058928	A	G	0.3116	-0.2998	0.0486	7.04E-10	37.93
B IN	*LRRC61*	rs2098053	7	150043205	A	G	0.2477	0.2938	0.0519	1.49E-08	32.01
B IN	*LSM7*	rs12976273	19	2326902	A	G	0.6004	0.4501	0.0452	2.20E-23	99.07
B IN	*LST1*	rs2507960	6	31569704	A	G	0.1848	-0.3683	0.0620	2.79E-09	35.25
B IN	*LTA4H*	rs2300557	12	96427293	T	C	0.0914	-0.6646	0.0812	2.75E-16	66.83
B IN	*LY6G5C*	rs2524075	6	31243859	T	C	0.2469	-0.2982	0.0505	3.47E-09	34.83
B IN	*LY6G5C*	rs1634741	6	31283886	C	T	0.2626	-0.4752	0.0497	1.07E-21	91.4
B IN	*LY86*	rs977785	6	6588881	C	A	0.7413	0.7943	0.0473	3.54E-63	280.93
B IN	*LY9*	rs1412849	1	160835587	A	G	0.1976	-0.3221	0.0561	9.61E-09	32.85
B IN	*LY96*	rs16938755	8	74904136	C	T	0.1463	0.5238	0.0649	7.23E-16	64.94
B IN	*MACROD2*	rs6110879	20	16018957	A	G	0.3778	0.3081	0.0460	2.16E-11	44.73
B IN	*MACROD2*	rs2224735	20	16094380	G	C	0.5905	-0.3288	0.0454	4.30E-13	52.39
B IN	*MARC2*	rs6697965	1	220943801	C	T	0.2308	0.3553	0.0544	6.65E-11	42.53
B IN	*MBOAT1*	rs1327665	6	20210374	A	C	0.4872	0.2860	0.0447	1.63E-10	40.78
B IN	*MBP*	rs34070985	18	74733516	T	C	0.3370	-0.2708	0.0465	5.90E-09	33.8
B IN	*MDGA1*	rs9349050	6	37663624	C	T	0.5194	-0.4430	0.0441	8.88E-24	100.86
B IN	*MDM4*	rs11801289	1	204528969	C	G	0.6747	-0.5234	0.0467	4.20E-29	125.12

B IN	*MED16*	rs1651891	19	868620	A	G	0.2537	0.3428	0.0518	3.66E-11	43.7
B IN	*METTL8*	rs36140780	2	172230563	T	C	0.2361	-0.7818	0.0500	4.16E-55	243.98
B IN	*MFF*	rs11687981	2	228185544	T	C	0.7886	0.3479	0.0562	6.17E-10	38.19
B IN	*MGMT*	rs12254582	10	131279791	G	A	0.2737	-0.3383	0.0486	3.26E-12	48.43
B IN	*MGST3*	rs4147596	1	165601365	C	T	0.4777	-0.5944	0.0431	2.79E-43	189.87
B IN	*MILR1*	rs35528570	17	62580234	G	A	0.4231	0.3211	0.0454	1.44E-12	50.03
B IN	*MLTK*	rs4338932	2	174019112	G	C	0.8139	0.3893	0.0597	6.79E-11	42.49
B IN	*MPHOSPH6*	rs2967375	16	82211418	G	A	0.7478	-0.2824	0.0506	2.37E-08	31.1
B IN	*MRPL21*	rs564849	11	68621440	T	C	0.3006	-0.3362	0.0487	5.15E-12	47.53
B IN	*MRPL34*	rs4430871	19	17366278	C	T	0.6170	-0.2544	0.0456	2.34E-08	31.12
B IN	*MRPL42*	rs7302896	12	93904529	G	A	0.1571	0.3470	0.0597	6.24E-09	33.69
B IN	*MRPL53*	rs6546907	2	74678921	C	T	0.1819	-0.4412	0.0654	1.54E-11	45.39
B IN	*MRPS18C*	rs6535473	4	84364808	C	T	0.4873	0.2600	0.0448	6.61E-09	33.58
B IN	*MSH5*	rs2853915	6	31275289	C	A	0.3846	-0.3149	0.0452	3.37E-12	48.36
B IN	*MSH5*	rs9501374	6	31332332	T	C	0.2709	-0.3052	0.0543	1.85E-08	31.58
B IN	*MSMP*	rs1322045	9	35754500	A	G	0.6501	0.3020	0.0474	1.96E-10	40.42
B IN	*MTSS1*	rs59676492	8	125658126	A	C	0.0782	-0.5108	0.0869	4.18E-09	34.47
B IN	*MTUS1*	rs415226	8	17896138	A	T	0.7521	0.7793	0.0489	2.98E-57	253.79
B IN	*MXRA7*	rs113137369	17	74699440	A	G	0.0828	-0.5469	0.0851	1.31E-10	41.21
B IN	*MYBL2*	rs390076	20	42309916	G	C	0.8309	-0.8646	0.0582	6.77E-50	220.13
B IN	*MYH11*	rs16967494	16	15820863	T	C	0.2539	0.4760	0.0492	3.89E-22	93.39
B IN	*MYLIP*	rs2066399	6	15880540	A	G	0.8119	-0.3622	0.0588	7.26E-10	37.87
B IN	*MYO1E*	rs4775163	15	59695482	T	C	0.4905	-0.2502	0.0449	2.47E-08	31.02
B IN	*MYO7B*	rs2248754	2	128375722	T	C	0.3820	-0.4601	0.0455	4.95E-24	102.02
B IN	*MZT2A*	rs10171879	2	132247549	T	C	0.2176	0.6954	0.0566	1.13E-34	150.55
B IN	*NAA38*	rs11979012	7	117813337	A	C	0.6886	-0.4047	0.0471	8.42E-18	73.7
B IN	*NAAA*	rs11732759	4	76856440	A	T	0.4130	0.2979	0.0450	3.71E-11	43.67
B IN	*NAP1L1*	rs10785228	12	76473721	C	A	0.8115	-0.4959	0.0580	1.29E-17	72.86
B IN	*NAPRT1*	rs9657360	8	144676862	T	C	0.7776	0.6050	0.0539	3.24E-29	125.64
B IN	*NCF4*	rs10854694	22	37256476	A	G	0.4261	0.2554	0.0450	1.36E-08	32.18
B IN	*NDUFA3*	rs76992175	19	54621282	C	T	0.1028	0.4737	0.0722	5.33E-11	42.97
B IN	*NDUFA6*	rs7364180	22	42218856	G	A	0.2855	0.2809	0.0498	1.70E-08	31.74
B IN	*NEK8*	rs2043032	17	27136692	G	A	0.1899	-0.3800	0.0543	2.70E-12	48.8
B IN	*NFKBIZ*	rs635268	3	101602063	A	G	0.8114	-0.3209	0.0575	2.38E-08	31.09
B IN	*NKX6-3*	rs7010327	8	41480509	A	G	0.4582	0.6332	0.0431	5.86E-49	215.84
B IN	*NME1*	rs7207370	17	49231180	G	A	0.3899	0.3704	0.0455	4.19E-16	66.01
B IN	*NME4*	rs77371232	16	447561	T	A	0.4244	0.5449	0.0440	2.68E-35	153.4
B IN	*NMI*	rs55775354	2	152118271	A	G	0.4344	0.3057	0.0448	8.51E-12	46.55
B IN	*NMRAL1*	rs917306	16	4440745	G	C	0.0568	-0.5656	0.1016	2.58E-08	30.93
B IN	*NMRK1*	rs3752955	9	77684690	C	A	0.5423	-0.2779	0.0451	6.95E-10	37.96
B IN	*NOMO1*	rs7405130	16	15147725	C	T	0.8388	0.4547	0.0625	3.57E-13	52.76
B IN	*NQO2*	rs9503373	6	3042606	G	C	0.7858	-0.4040	0.0561	6.20E-13	51.68
B IN	*NSA2*	rs72764687	5	74151336	C	G	0.1340	0.7843	0.0645	5.26E-34	147.49
B IN	*NSUN4*	rs72677602	1	46837487	A	G	0.2819	0.3015	0.0496	1.23E-09	36.84
B IN	*NT5C3A*	rs10951371	7	33092349	G	A	0.6951	-0.3437	0.0492	2.82E-12	48.71
B IN	*NT5C3B*	rs11079016	17	39976024	A	G	0.1493	0.4250	0.0604	2.01E-12	49.38
B IN	*NT5DC3*	rs4964605	12	104224284	C	G	0.1728	0.4730	0.0626	4.01E-14	57.05
B IN	*NT5E*	rs6942065	6	86168265	A	G	0.1375	-0.6143	0.0614	1.41E-23	99.94
B IN	*NTPCR*	rs9424513	1	233074916	C	T	0.2251	0.3130	0.0530	3.54E-09	34.79
B IN	*NUDT2*	rs61573237	9	34346282	T	C	0.1568	0.6242	0.0615	3.25E-24	102.85
B IN	*OPTN*	rs57057378	10	13160035	A	G	0.2929	-0.3974	0.0484	2.34E-16	67.15
B IN	*ORMDL3*	rs8067378	17	38051348	G	A	0.5075	-0.6385	0.0427	1.79E-50	222.77
B IN	*OSTF1*	rs10869496	9	77700084	A	C	0.3854	-0.3851	0.0451	1.31E-17	72.84
B IN	*P2RX5*	rs486474	17	3557495	G	A	0.4640	0.2637	0.0448	4.03E-09	34.54
B IN	*PARK7*	rs6700772	1	8087469	G	C	0.4027	0.2808	0.0458	8.51E-10	37.56
B IN	*PARP14*	rs13071069	3	122440065	T	C	0.1057	-0.7158	0.0862	9.98E-17	68.83
B IN	*PCBP2*	rs10876431	12	53730160	A	G	0.0799	-0.4834	0.0847	1.13E-08	32.53
B IN	*PCSK7*	rs111765459	11	117083373	C	T	0.2126	0.3214	0.0552	5.72E-09	33.86
B IN	*PEX11A*	rs11631930	15	90873264	T	C	0.1874	-0.5199	0.0573	1.23E-19	82.03
B IN	*PEX6*	rs9471972	6	42915021	A	G	0.5240	0.3301	0.0447	1.54E-13	54.41
B IN	*PGBD1*	rs3117425	6	29260431	T	C	0.0841	-0.5863	0.0724	5.72E-16	65.4
B IN	*PHF11*	rs7992294	13	50100756	C	T	0.5079	0.2532	0.0449	1.71E-08	31.73
B IN	*PI4K2B*	rs60527077	4	25161539	A	G	0.0922	-0.4865	0.0755	1.19E-10	41.4
B IN	*PILRB*	rs6953749	7	100000941	T	C	0.1882	0.5330	0.0553	5.11E-22	92.86
B IN	*PINLYP*	rs397706	19	44488085	G	A	0.4147	0.4942	0.0444	8.91E-29	123.64
B IN	*PKHD1L1*	rs1457283	8	110486627	G	A	0.3679	0.6405	0.0438	2.24E-48	213.18
B IN	*PKIG*	rs1061662	20	43243545	T	C	0.1624	-0.6976	0.0603	5.97E-31	133.55
B IN	*PLEK*	rs61142289	2	68580439	C	T	0.1553	-0.4159	0.0650	1.62E-10	40.8
B IN	*PLTP*	rs3091649	20	44537697	A	G	0.5578	-0.3296	0.0447	1.76E-13	54.14
B IN	*PNRC2*	rs6424145	1	24375689	C	G	0.4721	-0.3889	0.0443	1.69E-18	76.86
B IN	*PODXL2*	rs813633	3	127391018	G	A	0.7764	-0.2978	0.0539	3.41E-08	30.4
B IN	*POLM*	rs28382621	7	44122613	G	C	0.0936	0.5614	0.0779	5.78E-13	51.82
B IN	*POLR1D*	rs2232669	13	28195495	T	C	0.1167	0.7478	0.0651	1.51E-30	131.71
B IN	*POLR2J3*	rs9632725	7	102388923	C	T	0.1475	0.3783	0.0612	6.20E-10	38.18
B IN	*POMZP3*	rs60701917	7	76226733	T	G	0.2094	0.4499	0.0542	1.05E-16	68.73
B IN	*PPA1*	rs12355080	10	71963608	A	G	0.3210	0.6426	0.0457	6.31E-45	197.4
B IN	*PPDPF*	rs72629024	20	62152519	G	C	0.1633	-0.9933	0.0585	1.43E-64	287.31
B IN	*PPIA*	rs13240323	7	44816785	A	G	0.3423	-0.4190	0.0454	2.60E-20	85.1
B IN	*PPIL3*	rs7559150	2	201754063	C	T	0.2458	-0.4975	0.0524	2.15E-21	90.02

B IN	*PRKD3*	rs12712528	2	37461139	G	A	0.5255	-0.2838	0.0447	2.23E-10	40.17
B IN	*PRR4*	rs73066852	12	11276541	G	A	0.1426	0.4416	0.0621	1.13E-12	50.5
B IN	*PRR7-AS1*	rs2731670	5	176848019	C	A	0.5365	0.4316	0.0444	2.58E-22	94.21
B IN	*PRRT1*	rs2269711	6	31141612	T	A	0.6608	0.2982	0.0469	1.97E-10	40.41
B IN	*PSMD5-AS1*	rs10760117	9	123586737	G	T	0.5804	-1.0005	0.0393	6.33E-143	646.6
B IN	*PTPLAD2*	rs67375304	9	21013478	G	C	0.1632	0.6654	0.0564	3.78E-32	139.02
B IN	*PTPN4*	rs13015818	2	120523762	T	C	0.6917	0.2973	0.0489	1.22E-09	36.86
B IN	*RABAC1*	rs1965727	19	42452305	C	A	0.6657	0.4438	0.0463	1.01E-21	91.51
B IN	*RAC1*	rs836549	7	6439945	G	A	0.4921	0.3807	0.0444	9.34E-18	73.5
B IN	*RAD51C*	rs62084384	17	57126842	A	G	0.1989	0.3026	0.0542	2.40E-08	31.08
B IN	*RASGRP3*	rs10186362	2	33751664	T	C	0.0965	0.7270	0.0792	4.52E-20	84.01
B IN	*RBM26*	rs4885625	13	79864490	G	T	0.4919	-0.3028	0.0447	1.20E-11	45.88
B IN	*RBM6*	rs7613875	3	49971514	A	C	0.5197	-0.2972	0.0449	3.80E-11	43.63
B IN	*RCSD1*	rs11584355	1	167549747	T	C	0.3867	0.3927	0.0456	6.98E-18	74.07
B IN	*REL*	rs12989427	2	61137506	A	G	0.1595	0.6203	0.0586	3.24E-26	111.97
B IN	*REV3L*	rs240970	6	111636182	T	C	0.7030	0.3186	0.0500	1.80E-10	40.59
B IN	*RGS1*	rs1547624	1	192543837	T	A	0.8230	-0.4022	0.0582	4.83E-12	47.66
B IN	*RHBDF2*	rs3809694	17	74475975	A	C	0.4579	0.3069	0.0448	6.99E-12	46.93
B IN	*RIC3*	rs73409635	11	8122932	A	C	0.0634	-0.5401	0.0879	7.93E-10	37.7
B IN	*RNASE6*	rs8003799	14	21178988	A	T	0.5014	-0.6925	0.0423	3.27E-60	267.35
B IN	*RNGTT*	rs2756370	6	89326244	T	A	0.1640	-0.5976	0.0624	9.75E-22	91.58
B IN	*RP1-29C18.10*	rs5770622	22	50051548	G	T	0.6547	-0.6251	0.0464	1.92E-41	181.47
B IN	*RP1-3J17.3*	rs9450302	6	86289673	T	C	0.6156	-0.6250	0.0443	3.71E-45	198.45
B IN	*RP1-3J17.3*	rs3812139	6	86196990	G	C	0.1043	0.5131	0.0724	1.37E-12	50.12
B IN	*RP11-1070N10.3*	rs179145	14	95983975	A	G	0.3676	-0.2780	0.0467	2.59E-09	35.4
B IN	*RP11-108M9.4*	rs3901404	1	16764703	T	A	0.4603	0.2591	0.0454	1.18E-08	32.46
B IN	*RP11-111A22.1*	rs12913296	15	40844222	A	G	0.4197	0.9216	0.0398	2.34E-118	533.88
B IN	*RP11-138A9.1*	rs205778	7	130644543	G	A	0.9026	0.4680	0.0724	1.01E-10	41.71
B IN	*RP11-139H15.1*	rs11629613	15	55577303	C	T	0.1331	0.4318	0.0719	1.90E-09	36
B IN	*RP11-254F7.2*	rs76958402	2	10180354	A	G	0.1125	0.3919	0.0672	5.62E-09	33.89
B IN	*RP11-298J20.3*	rs2114834	10	126650186	G	A	0.7359	-0.7701	0.0489	8.28E-56	247.19
B IN	*RP11-299J3.8*	rs79493664	3	122109546	A	T	0.1453	-0.3272	0.0595	3.74E-08	30.22
B IN	*RP11-326C3.11*	rs11246065	11	324170	G	A	0.3701	0.2943	0.0469	3.38E-10	39.36
B IN	*RP11-326C3.12*	rs11246068	11	327334	T	C	0.2550	0.3120	0.0517	1.56E-09	36.39
B IN	*RP11-328M4.2*	rs4317403	6	40971173	T	C	0.1980	0.6581	0.0538	2.06E-34	149.35
B IN	*RP11-345J4.5*	rs144208959	16	30323380	A	G	0.4341	-0.3157	0.0446	1.52E-12	49.92
B IN	*RP11-347P5.1*	rs1519601	2	197026769	G	A	0.5905	0.7871	0.0426	2.84E-76	340.92
B IN	*RP11-361F15.2*	rs6899613	6	148553992	A	G	0.7499	-0.3877	0.0515	4.91E-14	56.65
B IN	*RP11-386I14.4*	rs9726525	1	78354585	T	G	0.2537	-0.3412	0.0510	2.28E-11	44.62
B IN	*RP11-428G5.5*	rs1797791	12	32044587	A	T	0.5372	0.4601	0.0440	1.45E-25	109
B IN	*RP11-434C1.1*	rs11054150	12	11156046	G	T	0.1039	0.4128	0.0714	7.22E-09	33.41
B IN	*RP11-446N19.1*	rs4768697	12	46748965	G	C	0.2578	0.3172	0.0506	3.64E-10	39.21
B IN	*RP11-455J20.3*	rs2082020	2	191014587	T	C	0.5753	-0.5941	0.0438	6.30E-42	183.68
B IN	*RP11-457M11.5*	rs72843784	6	26498758	T	G	0.0715	-0.4376	0.0767	1.18E-08	32.45
B IN	*RP11-473I1.5*	rs17567304	16	9187475	G	A	0.1881	-0.5589	0.0572	1.44E-22	95.36
B IN	*RP11-485G7.5*	rs880341	16	11396143	G	C	0.1476	-0.8774	0.0685	1.33E-37	163.93
B IN	*RP11-542K23.7*	rs35918962	9	125168878	T	C	0.1266	0.5308	0.0679	5.48E-15	60.96
B IN	*RP11-596D21.1*	rs2378858	14	31863750	A	G	0.5445	0.4049	0.0447	1.38E-19	81.81
B IN	*RP11-693J15.5*	rs58638543	12	92830683	G	A	0.1719	0.3840	0.0622	6.72E-10	38.02
B IN	*RP11-69E11.4*	rs76571594	1	40205023	A	G	0.0572	0.5616	0.1004	2.23E-08	31.22
B IN	*RP11-755F10.1*	rs11227439	11	66025679	A	G	0.1715	-0.7678	0.0579	3.58E-40	175.66
B IN	*RP11-8L8.2*	rs10133948	14	101393490	G	A	0.2990	-0.5529	0.0472	9.88E-32	137.12
B IN	*RP11-950C14.3*	rs8013780	14	75456449	C	A	0.5265	0.6138	0.0429	2.27E-46	204
B IN	*RP11-960L18.1*	rs12444183	16	81773209	G	A	0.6116	0.2680	0.0459	5.42E-09	33.96
B IN	*RP11-96H19.1*	rs4768697	12	46748965	G	C	0.2578	0.2844	0.0507	2.04E-08	31.39
B IN	*RP13-514E23.2*	rs35832919	4	86866160	C	T	0.2182	-0.6793	0.0534	4.42E-37	161.54
B IN	*RP3-325F22.5*	rs2181096	6	136812315	C	T	0.7959	0.3893	0.0560	3.71E-12	48.17
B IN	*RP5-827C21.4*	rs17576781	1	234537924	C	T	0.0913	0.4484	0.0777	8.04E-09	33.2
B IN	*RPL10A*	rs10947550	6	35428782	A	G	0.5293	-0.6845	0.0428	1.27E-57	255.5
B IN	*RPL11*	rs77766201	1	24006446	A	G	0.1560	-0.7606	0.0611	1.46E-35	154.6
B IN	*RPL12*	rs2247781	9	130208924	C	T	0.5921	0.9868	0.0394	1.58E-138	626.42
B IN	*RPL12*	rs7854003	9	130310834	G	A	0.1464	0.3918	0.0608	1.17E-10	41.42
B IN	*RPL13*	rs12932337	16	89630630	T	C	0.1753	-0.8355	0.0533	1.87E-55	245.57
B IN	*RPL14*	rs75965075	3	40438120	T	C	0.0715	0.6430	0.0850	3.86E-14	57.12
B IN	*RPL22L1*	rs13063927	3	170582888	T	C	0.2425	-0.4177	0.0503	9.47E-17	68.94
B IN	*RPL23A*	rs3809797	17	27065211	C	T	0.0927	-0.8943	0.0766	1.79E-31	135.94
B IN	*RPL27A*	rs61875926	11	8705528	G	C	0.0958	-0.6667	0.0733	9.13E-20	82.62
B IN	*RPL28*	rs17700376	19	55897327	G	A	0.3547	-1.0275	0.0405	4.64E-142	642.63
B IN	*RPL3*	rs6509	22	39714490	T	C	0.4725	0.3424	0.0445	1.48E-14	59.01
B IN	*RPL32*	rs7647881	3	12900430	G	A	0.1773	0.3295	0.0567	6.06E-09	33.75
B IN	*RPL36AL*	rs2147712	14	50091684	A	G	0.7504	0.8580	0.0491	2.56E-68	304.47
B IN	*RPL8*	rs2956171	8	145961954	A	G	0.5129	-1.1178	0.0362	1.00E-200	950.69
B IN	*RPLP2*	rs28694634	11	808423	A	G	0.6588	-0.6029	0.0465	1.96E-38	167.72
B IN	*RPPH1*	rs76311431	14	20786432	G	C	0.1346	0.3725	0.0663	1.91E-08	31.52
B IN	*RPS10*	rs9380433	6	34377626	T	C	0.7750	-0.8883	0.0549	7.15E-59	261.21
B IN	*RPS11*	rs10403394	19	49999345	A	G	0.1225	-0.6947	0.0708	1.02E-22	96.04
B IN	*RPS12*	rs62425109	6	133120782	C	A	0.0580	0.8388	0.0970	5.32E-18	74.61
B IN	*RPS16*	rs640000	19	39926601	T	C	0.3796	0.7089	0.0446	7.18E-57	252.05
B IN	*RPS18*	rs17215231	6	33239869	T	C	0.0640	-1.5873	0.0823	7.54E-83	371.06

B IN	*RPS2*	rs17135718	16	2014834	G	A	0.1151	0.8656	0.0654	5.20E-40	174.92
B IN	*RPS23*	rs9099	5	81571896	G	A	0.1266	0.4082	0.0674	1.40E-09	36.6
B IN	*RPS24*	rs2913134	10	79763775	T	C	0.5775	0.2798	0.0460	1.18E-09	36.93
B IN	*RPS25*	rs61381062	11	118884708	A	G	0.2492	-0.5144	0.0499	6.27E-25	106.11
B IN	*RPS26*	rs773114	12	56379060	T	A	0.3997	1.2035	0.0351	1.00E-200	1173.01
B IN	*RPS3*	rs3094303	11	75114071	G	A	0.0743	-1.1262	0.0880	1.82E-37	163.3
B IN	*RPS3A*	rs1148652	4	151801938	C	T	0.0703	-1.2030	0.0975	5.76E-35	151.88
B IN	*RPS5*	rs731835	19	58898436	C	T	0.3256	-0.9781	0.0423	2.01E-118	534.18
B IN	*RPS6*	rs6475324	9	19361448	A	G	0.0990	-0.8702	0.0807	4.26E-27	115.98
B IN	*RPS7*	rs62106040	2	3627302	T	A	0.0757	-1.1353	0.0861	9.85E-40	173.66
B IN	*RPS8*	rs12120833	1	45241285	G	C	0.1242	-1.1265	0.0575	2.46E-85	382.45
B IN	*RPS9*	rs158366	19	54705317	G	C	0.3901	-0.7450	0.0429	1.23E-67	301.35
B IN	*RPSA*	rs11712093	3	39437665	T	C	0.1555	0.5820	0.0609	1.20E-21	91.17
B IN	*RSRC2*	rs6416298	12	123002143	T	C	0.7480	-0.3981	0.0496	9.89E-16	64.32
B IN	*SAG*	rs2241875	2	234240684	A	G	0.7327	0.5872	0.0482	3.72E-34	148.18
B IN	*SAMD12*	rs2514594	8	119625149	A	G	0.9156	-0.5092	0.0784	8.52E-11	42.05
B IN	*SAR1A*	rs2310991	10	71931646	A	C	0.4271	-0.2995	0.0455	4.46E-11	43.31
B IN	*SCIMP*	rs61182333	17	5133128	C	T	0.8636	-0.5639	0.0665	2.20E-17	71.81
B IN	*SDK2*	rs11655934	17	71587577	A	G	0.1405	-0.3891	0.0647	1.78E-09	36.13
B IN	*SEC61B*	rs75085864	9	101999360	A	G	0.0766	-0.6667	0.0767	3.46E-18	75.46
B IN	*SEL1L3*	rs13147067	4	25877086	T	A	0.4440	0.3693	0.0445	1.05E-16	68.73
B IN	*SELL*	rs4987360	1	169664867	G	A	0.3028	0.3258	0.0487	2.29E-11	44.61
B IN	*SEPSECS*	rs12505146	4	24291220	A	C	0.3213	-0.2986	0.0477	3.84E-10	39.11
B IN	*SEPT2*	rs35533015	2	242191689	A	G	0.0889	0.4860	0.0753	1.06E-10	41.62
B IN	*SERPINE2*	rs1982462	2	224925671	G	A	0.4505	0.3369	0.0448	5.43E-14	56.45
B IN	*SESTD1*	rs340354	2	179946787	A	T	0.7690	-0.3415	0.0548	4.59E-10	38.77
B IN	*SETD9*	rs2662033	5	56213410	G	T	0.8283	0.3618	0.0625	7.14E-09	33.43
B IN	*SF1*	rs533447	11	64561908	C	T	0.8796	-0.5237	0.0780	1.86E-11	45.02
B IN	*SH2B2*	rs803090	7	101950500	G	A	0.7078	0.3132	0.0485	1.04E-10	41.66
B IN	*SH3BGRL3*	rs11247915	1	26650550	A	C	0.5030	0.4609	0.0440	1.01E-25	109.71
B IN	*SH3YL1*	rs7595075	2	264019	A	C	0.3283	-0.5253	0.0458	1.67E-30	131.52
B IN	*SIDT1-AS1*	rs35452897	3	113282075	T	C	0.2149	0.4597	0.0537	1.19E-17	73.02
B IN	*SIDT2*	rs4938343	11	117003528	C	G	0.8041	0.4900	0.0578	2.19E-17	71.82
B IN	*SKAP2*	rs17323934	7	26904330	G	C	0.2183	0.3911	0.0526	9.99E-14	55.26
B IN	*SLC12A7*	rs6895108	5	1113500	A	G	0.5574	0.2848	0.0456	4.40E-10	38.85
B IN	*SLC15A2*	rs7632786	3	121546511	A	G	0.6102	-0.3110	0.0464	2.00E-11	44.87
B IN	*SLC16A4*	rs11102094	1	110921163	T	C	0.4206	0.2904	0.0448	9.30E-11	41.88
B IN	*SLC25A1*	rs807669	22	19154522	C	T	0.6378	-0.2916	0.0462	2.83E-10	39.71
B IN	*SLC2A1-AS1*	rs34964576	1	43409364	C	T	0.1886	0.4692	0.0600	5.11E-15	61.09
B IN	*SLC4A7*	rs2724491	3	27544497	T	C	0.5200	0.2639	0.0448	3.81E-09	34.65
B IN	*SLC7A7*	rs12884337	14	23273114	C	T	0.6181	0.3176	0.0467	1.07E-11	46.09
B IN	*SLC7A7*	rs2073345	14	23292476	C	G	0.3579	-0.2800	0.0478	4.65E-09	34.26
B IN	*SMDT1*	rs2301521	22	42423110	C	G	0.6779	1.2450	0.0379	1.00E-200	1077.01
B IN	*SMIM19*	rs2974366	8	42431768	A	C	0.6247	-0.2860	0.0458	4.14E-10	38.97
B IN	*SNHG7*	rs6874	9	139617836	T	C	0.1153	-1.1376	0.0668	5.83E-65	289.09
B IN	*SNHG8*	rs658642	4	119200274	T	C	0.3810	0.8374	0.0416	5.86E-90	403.65
B IN	*SNHG8*	rs4834708	4	119765039	G	A	0.8840	0.3957	0.0703	1.79E-08	31.64
B IN	*SNRNP25*	rs216598	16	108585	C	T	0.8233	-0.3711	0.0589	2.96E-10	39.62
B IN	*SNRPD2*	rs34399924	19	46200103	A	G	0.2142	-0.4933	0.0552	3.92E-19	79.75
B IN	*SNRPD3*	rs743367	22	24940959	T	C	0.8309	0.3462	0.0624	2.92E-08	30.7
B IN	*SNX29P2*	rs4788162	16	29330963	A	G	0.2822	0.3837	0.0505	2.87E-14	57.7
B IN	*SOCS5*	rs28370327	2	46910064	C	A	0.3758	0.2947	0.0467	2.68E-10	39.81
B IN	*SPATA20*	rs8076632	17	48625928	G	C	0.3650	-0.4285	0.0464	2.50E-20	85.17
B IN	*SPATC1L*	rs9984805	21	47607057	G	A	0.3444	0.2748	0.0477	8.55E-09	33.08
B IN	*SPSB2*	rs7138740	12	6983764	A	T	0.2917	0.2961	0.0488	1.33E-09	36.7
B IN	*SQSTM1*	rs7713219	5	179283170	C	T	0.4522	-0.2912	0.0450	1.01E-10	41.71
B IN	*SRSF10*	rs6424144	1	24375663	A	G	0.4721	-0.3112	0.0446	3.13E-12	48.5
B IN	*SSBP4*	rs4006662	19	18629112	C	T	0.2156	-0.3176	0.0543	5.06E-09	34.1
B IN	*STMN3*	rs879471	20	62269942	T	C	0.5285	0.2832	0.0449	2.74E-10	39.77
B IN	*SULF2*	rs3091426	20	46189755	A	G	0.5891	-0.8489	0.0411	7.46E-95	426.09
B IN	*SULF2*	rs35873170	20	46322502	A	G	0.1071	-0.4501	0.0756	2.59E-09	35.4
B IN	*SURF1*	rs3118667	9	136291063	T	C	0.5241	-0.2747	0.0448	8.64E-10	37.53
B IN	*SVIP*	rs10833819	11	22823282	C	T	0.4498	-0.3421	0.0450	2.83E-14	57.73
B IN	*SYNGR1*	rs5757584	22	39662550	T	C	0.1864	0.3947	0.0628	3.20E-10	39.47
B IN	*SYNJ2*	rs9379316	6	157872377	C	A	0.2060	0.3233	0.0531	1.18E-09	36.92
B IN	*SYS1*	rs2743239	20	44027957	A	T	0.8008	0.3379	0.0560	1.58E-09	36.36
B IN	*TAPBP*	rs3130260	6	33278816	A	G	0.2897	-0.3982	0.0486	2.41E-16	67.1
B IN	*TAPBPL*	rs2243750	12	6567787	C	T	0.3002	0.3549	0.0490	4.54E-13	52.29
B IN	*TBCA*	rs254421	5	77069502	A	G	0.2935	-0.4841	0.0476	3.03E-24	102.99
B IN	*TC2N*	rs2498812	14	92320405	A	G	0.4608	0.2766	0.0449	7.08E-10	37.92
B IN	*TCAP*	rs520054	17	37321538	A	G	0.2153	0.6781	0.0529	1.43E-37	163.78
B IN	*TCL1A*	rs2296310	14	96136775	A	C	0.1314	-0.4714	0.0659	8.78E-13	51
B IN	*TCL1B*	rs2985640	14	96141418	A	G	0.2964	0.2978	0.0481	5.96E-10	38.26
B IN	*TCOF1*	rs11742769	5	149771286	T	C	0.1091	0.4142	0.0701	3.47E-09	34.83
B IN	*TEX35*	rs7527363	1	178492635	C	T	0.5501	-0.4267	0.0442	5.18E-22	92.83
B IN	*THAP7-AS1*	rs1966316	22	21366634	G	A	0.2231	-0.3649	0.0550	3.34E-11	43.88
B IN	*THAP9-AS1*	rs7682657	4	83727406	A	T	0.7859	0.3177	0.0551	8.25E-09	33.15
B IN	*THNSL2*	rs6547757	2	88469770	C	G	0.7416	-0.6224	0.0499	1.09E-35	155.19
B IN	*TIFA*	rs6835890	4	113187346	G	A	0.5126	-0.4907	0.0438	3.73E-29	125.36

B IN	*TIMM10*	rs2848626	11	57283988	T	C	0.7820	-0.6540	0.0502	7.60E-39	169.6
B IN	*TIMM13*	rs6945	19	2426828	C	G	0.4919	0.3284	0.0446	1.74E-13	54.16
B IN	*TLE4*	rs10867416	9	82324152	C	T	0.6410	-0.4172	0.0463	1.93E-19	81.15
B IN	*TLK1*	rs3770433	2	171874024	G	A	0.6329	0.3624	0.0464	6.07E-15	60.75
B IN	*TMEM156*	rs2566097	4	39037336	T	C	0.6608	0.7820	0.0436	7.87E-72	320.56
B IN	*TMEM2*	rs62547055	9	74350747	A	G	0.0778	0.6123	0.0770	1.79E-15	63.15
B IN	*TMEM204*	rs2294624	16	1586255	A	G	0.1329	0.5246	0.0662	2.26E-15	62.7
B IN	*TMEM230*	rs1570031	20	5071680	C	T	0.5294	0.2876	0.0448	1.41E-10	41.06
B IN	*TMEM258*	rs174538	11	61560081	A	G	0.2977	0.2964	0.0487	1.14E-09	37
B IN	*TMEM41B*	rs12363839	11	9250359	T	C	0.2103	-0.3094	0.0556	2.56E-08	30.95
B IN	*TMEM50B*	rs17883129	21	34805197	T	C	0.3467	-0.3061	0.0469	6.42E-11	42.6
B IN	*TMEM80*	rs10902194	11	694257	A	G	0.1323	0.4101	0.0660	5.28E-10	38.49
B IN	*TOMM7*	rs2240728	7	22852499	T	C	0.3422	-0.9473	0.0433	4.92E-106	477.36
B IN	*TPRA1*	rs6439081	3	127408909	T	C	0.7665	0.3124	0.0541	7.76E-09	33.27
B IN	*TRMT112*	rs79423518	11	64105454	A	G	0.0682	-0.7230	0.0976	1.29E-13	54.76
B IN	*TRPM2*	rs2239389	21	45174326	T	C	0.5634	-0.5814	0.0437	2.03E-40	176.79
B IN	*TSGA10*	rs1563108	2	99701153	G	A	0.2934	-0.3121	0.0496	3.18E-10	39.48
B IN	*TSPAN3*	rs17466257	15	77376183	A	C	0.3895	0.4652	0.0448	3.16E-25	107.46
B IN	*TSTD1*	rs2516841	1	161010774	G	A	0.7578	-0.8219	0.0476	1.07E-66	297.05
B IN	*TSTD3*	rs330832	6	100047851	C	G	0.5619	-0.2940	0.0451	7.36E-11	42.33
B IN	*TUBB6*	rs9303766	18	12300849	A	G	0.1269	0.4372	0.0736	2.83E-09	35.22
B IN	*TUFM*	rs8049439	16	28837515	C	T	0.3939	0.7909	0.0427	9.19E-77	343.16
B IN	*U2AF1L4*	rs2239947	19	36244753	C	G	0.1568	0.4547	0.0635	7.84E-13	51.22
B IN	*UBA52*	rs10407212	19	18699445	A	G	0.4756	-0.3366	0.0447	4.90E-14	56.65
B IN	*UBE2J1*	rs12209311	6	90067072	C	T	0.0729	1.2917	0.0766	9.84E-64	283.47
B IN	*UCP2*	rs603950	11	73699136	G	A	0.3879	0.3185	0.0463	5.82E-12	47.29
B IN	*UGT2B17*	rs2603153	4	69365052	T	G	0.5623	-0.4564	0.0445	1.17E-24	104.87
B IN	*UPF3A*	rs3752107	13	115064423	A	G	0.0702	-0.4663	0.0843	3.24E-08	30.5
B IN	*UQCRH*	rs41292543	1	46774783	G	A	0.1009	-0.4963	0.0761	7.11E-11	42.4
B IN	*UROS*	rs2281954	10	127484653	A	G	0.4450	-0.2833	0.0449	2.69E-10	39.81
B IN	*USF1*	rs12116949	1	161042063	A	C	0.2747	0.2832	0.0490	7.30E-09	33.39
B IN	*USP53*	rs11729857	4	120114100	G	C	0.1207	0.8754	0.0678	4.36E-38	166.13
B IN	*VAMP2*	rs4792267	17	8061337	A	G	0.6696	0.3307	0.0490	1.44E-11	45.53
B IN	*VAV3*	rs2125749	1	108484400	A	C	0.2935	-0.3145	0.0498	2.66E-10	39.83
B IN	*VAV3-AS1*	rs12076127	1	108539269	T	C	0.3539	0.3331	0.0462	5.57E-13	51.89
B IN	*VCL*	rs4746177	10	75913033	A	G	0.1369	-0.4333	0.0645	1.90E-11	44.98
B IN	*VDAC2*	rs11543	10	76970613	C	G	0.5682	-0.3111	0.0448	3.89E-12	48.08
B IN	*VPREB3*	rs4822436	22	24093998	G	A	0.1123	0.4755	0.0708	1.82E-11	45.07
B IN	*VPS28*	rs117891721	8	145642886	T	C	0.0569	0.6116	0.0948	1.11E-10	41.54
B IN	*WARS*	rs8015259	14	100795338	C	G	0.2475	0.7696	0.0485	8.33E-57	251.75
B IN	*WASIR2*	rs10266	16	107162	G	C	0.1434	-0.4125	0.0679	1.25E-09	36.81
B IN	*WDR60*	rs28679564	7	158674521	G	C	0.2165	-0.3106	0.0541	9.12E-09	32.95
B IN	*XRRA1*	rs12225387	11	74604767	G	A	0.2742	0.8885	0.0450	6.37E-87	389.73
B IN	*XXbac-BPG252P9.10*	rs2844768	6	30221677	A	G	0.4114	-0.3453	0.0447	1.11E-14	59.57
B IN	*XXbac-BPG252P9.10*	rs115241734	6	31298097	G	A	0.1797	0.2965	0.0513	7.67E-09	33.29
B IN	*XXbac-BPG299F13.17*	rs6922077	6	31163791	C	G	0.2166	0.8844	0.0512	9.82E-67	297.22
B IN	*XXbac-BPG299F13.17*	rs2250287	6	31330251	G	T	0.1615	-0.3352	0.0569	3.76E-09	34.68
B IN	*YBEY*	rs62224180	21	47726332	G	C	0.7751	-0.6392	0.0556	1.36E-30	131.93
B IN	*YEATS4*	rs7136238	12	69752509	T	G	0.4308	-0.3019	0.0448	1.62E-11	45.3
B IN	*YWHAB*	rs6031847	20	43514203	T	C	0.2871	-0.3223	0.0503	1.53E-10	40.91
B IN	*ZBTB9*	rs78633917	6	32501658	A	G	0.1486	-0.4613	0.0581	1.93E-15	63.01
B IN	*ZBTB9*	rs3134996	6	32636866	T	A	0.6557	-0.2846	0.0466	1.05E-09	37.15
B IN	*ZC2HC1A*	rs2953475	8	79638138	A	C	0.2850	-0.4446	0.0507	1.67E-18	76.89
B IN	*ZC3HAV1*	rs10273974	7	138735512	C	A	0.4877	-0.3732	0.0444	4.19E-17	70.54
B IN	*ZDHHC19*	rs6795707	3	195937410	T	G	0.1377	-0.4669	0.0612	2.31E-14	58.13
B IN	*ZFAS1*	rs238164	20	47844341	G	A	0.2042	0.5070	0.0528	8.20E-22	91.92
B IN	*ZFP36L1*	rs12435329	14	69250891	C	T	0.4535	0.2509	0.0449	2.31E-08	31.15
B IN	*ZFP57*	rs9261433	6	30086927	C	T	0.2093	0.6858	0.0524	3.92E-39	170.91
B IN	*ZFP57*	rs3117194	6	29426008	C	T	0.5842	-0.2964	0.0450	4.40E-11	43.34
B IN	*ZKSCAN3*	rs76696782	6	28451829	T	C	0.0452	-0.8514	0.0951	3.51E-19	79.97
B IN	*ZKSCAN4*	rs3117145	6	29046418	T	A	0.0776	-0.4746	0.0752	2.79E-10	39.73
B IN	*ZNF107*	rs112429483	7	64186380	G	A	0.3793	-0.3033	0.0456	2.86E-11	44.18
B IN	*ZNF266*	rs58359623	19	9541406	G	C	0.5767	0.2585	0.0455	1.35E-08	32.2
B IN	*ZNF302*	rs73042013	19	35134619	T	A	0.1260	0.6144	0.0665	2.46E-20	85.21
B IN	*ZNF391*	rs56355079	6	28060398	C	T	0.1097	-0.4353	0.0731	2.55E-09	35.43
B IN	*ZNF506*	rs248944	19	19887174	G	C	0.3979	0.3052	0.0459	2.83E-11	44.2
B IN	*ZNF667-AS1*	rs36147582	19	56991302	T	C	0.2548	0.4129	0.0513	8.53E-16	64.61
B IN	*ZNF677*	rs58934920	19	53773088	G	C	0.2277	-0.3020	0.0537	1.92E-08	31.51
B IN	*ZNF749*	rs1989455	19	57878194	T	G	0.5337	-0.4248	0.0443	8.07E-22	91.95
B IN	*ZNF83*	rs658086	19	53133885	G	A	0.3915	-0.3241	0.0461	2.11E-12	49.27
B IN	*ZNF85*	rs2545225	19	21042460	G	A	0.1318	0.6035	0.0634	1.62E-21	90.57
B IN	*ZNF880*	rs1054612	19	52870052	G	C	0.3964	0.2625	0.0462	1.37E-08	32.16
B IN	*ZNFX1*	rs237706	20	47915390	G	T	0.2203	-0.3250	0.0526	6.68E-10	38.04
B IN	*ZP3*	rs2110465	7	76137284	C	A	0.6728	-0.4978	0.0464	7.66E-27	114.82
B IN	*ZSCAN31*	rs34668037	6	28480023	A	G	0.0855	-0.5751	0.0748	1.49E-14	58.99
B Mem	*AC005253.2*	rs10425018	19	18687175	C	T	0.5896	0.4037	0.0451	3.82E-19	79.8
B Mem	*AC006369.2*	rs111822776	2	37823870	G	A	0.6503	0.3362	0.0462	3.42E-13	52.84
B Mem	*AC021860.1*	rs10019144	4	38686405	A	G	0.1965	-0.3486	0.0535	7.33E-11	42.34
B Mem	*AC023590.1*	rs2460966	8	119624556	G	A	0.9211	-0.7228	0.0778	1.47E-20	86.23

B Mem	*AC079767.4*	rs2663876	2	208519403	C	T	0.3866	-0.3842	0.0457	4.16E-17	70.56
B Mem	*AC104653.1*	rs62170143	2	114572913	A	G	0.5733	-0.3771	0.0454	1.06E-16	68.71
B Mem	*ACCS*	rs2074038	11	44087989	T	G	0.1126	0.4314	0.0744	6.66E-09	33.56
B Mem	*ACYP2*	rs805310	2	54196238	A	G	0.3435	-0.3221	0.0469	6.71E-12	47.01
B Mem	*ADAMTS6*	rs370763	5	64355060	A	T	0.6576	0.2725	0.0475	9.77E-09	32.82
B Mem	*AHI1*	rs13197384	6	135818897	A	C	0.3411	-0.4435	0.0467	2.36E-21	89.83
B Mem	*AKAP13*	rs4407021	15	86000503	T	C	0.5155	0.2760	0.0448	7.05E-10	37.93
B Mem	*AKIP1*	rs11042125	11	8938049	T	A	0.3956	0.2504	0.0451	2.77E-08	30.8
B Mem	*ANK1*	rs13255840	8	41450296	C	T	0.4580	-0.2637	0.0451	4.91E-09	34.15
B Mem	*ANKDD1A*	rs34566328	15	65034289	A	G	0.3949	-0.3902	0.0450	4.28E-18	75.03
B Mem	*ANXA4*	rs6711807	2	69946670	C	T	0.2659	0.5437	0.0510	1.58E-26	113.38
B Mem	*ANXA6*	rs951224	5	150531247	C	T	0.4482	0.3241	0.0450	5.72E-13	51.84
B Mem	*AP003774.1*	rs61886886	11	64104488	T	C	0.3592	0.3711	0.0459	6.29E-16	65.21
B Mem	*AP1S3*	rs6747058	2	224692019	A	G	0.4780	0.2921	0.0449	7.43E-11	42.31
B Mem	*ARHGAP24*	rs12506596	4	86521985	A	G	0.3032	-0.6524	0.0478	2.20E-42	185.77
B Mem	*ARHGEF3*	rs6794215	3	56758203	A	G	0.1612	0.4164	0.0623	2.25E-11	44.65
B Mem	*ARL14EP*	rs10835653	11	30347828	C	T	0.2920	-0.3912	0.0501	5.72E-15	60.87
B Mem	*ARL16*	rs7503637	17	79649515	A	G	0.1691	-0.4921	0.0591	8.15E-17	69.23
B Mem	*ARL17A*	rs62062775	17	44021960	A	G	0.2113	0.3385	0.0521	8.04E-11	42.16
B Mem	*ASCL2*	rs2521269	11	2321095	A	C	0.5657	0.2493	0.0451	3.29E-08	30.47
B Mem	*ATP5G3*	rs2288960	2	176042819	T	C	0.1456	0.3949	0.0623	2.24E-10	40.16
B Mem	*ATP6V1G2*	rs3135365	6	32389255	A	C	0.8158	-0.4508	0.0537	4.70E-17	70.32
B Mem	*ATP6V1G2*	rs45503895	6	30612107	C	A	0.0784	-0.4534	0.0770	3.96E-09	34.57
B Mem	*ATP8A1*	rs9995629	4	42599135	A	C	0.3021	0.3071	0.0469	5.81E-11	42.79
B Mem	*BCAS4*	rs6096205	20	49580970	C	T	0.3590	-0.3009	0.0465	9.33E-11	41.87
B Mem	*BCL2A1*	rs35144820	15	80312144	T	C	0.3283	0.4583	0.0460	2.28E-23	99
B Mem	*BICD2*	rs1552908	9	95373884	G	A	0.2767	0.3256	0.0506	1.26E-10	41.29
B Mem	*BIN1*	rs11682128	2	127839474	A	G	0.3433	0.3118	0.0471	3.60E-11	43.73
B Mem	*BLK*	rs2736337	8	11341880	C	T	0.2548	-0.6476	0.0493	2.07E-39	172.18
B Mem	*BRK1*	rs73117481	3	10160132	T	C	0.1184	-0.4092	0.0685	2.33E-09	35.6
B Mem	*BTF3*	rs10942655	5	72674031	A	G	0.0712	-0.5370	0.0956	1.94E-08	31.49
B Mem	*BTN3A2*	rs67509210	6	26354100	T	G	0.1018	-0.6270	0.0661	2.33E-21	89.86
B Mem	*BTNL9*	rs7706029	5	180461139	G	T	0.4806	-0.3515	0.0446	3.06E-15	62.1
B Mem	*C10orf128*	rs2377997	10	50402624	T	C	0.3494	-0.3193	0.0469	1.04E-11	46.17
B Mem	*C11orf24*	rs10791970	11	68034256	A	G	0.3028	0.4778	0.0484	5.57E-23	97.24
B Mem	*C19orf60*	rs4808823	19	18698019	C	T	0.4762	-0.2845	0.0449	2.27E-10	40.14
B Mem	*C19orf83*	rs2286756	19	46145025	T	G	0.5303	-0.2513	0.0449	2.14E-08	31.3
B Mem	*C1orf220*	rs4650998	1	178531850	A	G	0.5005	0.3368	0.0447	4.62E-14	56.77
B Mem	*C20orf27*	rs45495794	20	3734167	C	T	0.0479	-0.5564	0.0981	1.40E-08	32.12
B Mem	*C6orf48*	rs4947253	6	31820159	T	C	0.1281	-0.3931	0.0690	1.21E-08	32.4
B Mem	*CAMLG*	rs58218430	5	134070027	T	C	0.1121	0.4837	0.0705	6.91E-12	46.96
B Mem	*CAPG*	rs3770102	2	85637837	T	G	0.3911	0.4240	0.0448	2.78E-21	89.51
B Mem	*CAPN12*	rs62119115	19	39210593	A	G	0.2446	-0.3820	0.0529	4.95E-13	52.12
B Mem	*CAST*	rs10039522	5	95990869	A	G	0.1691	0.4120	0.0608	1.28E-11	45.76
B Mem	*CCDC126*	rs71526018	7	23637902	T	A	0.2398	0.4381	0.0499	1.67E-18	76.89
B Mem	*CCDC15*	rs12276686	11	124941520	T	C	0.1551	-0.6381	0.0626	2.22E-24	103.6
B Mem	*CCDC163P*	rs12047498	1	45954632	T	C	0.2230	-0.4996	0.0518	4.83E-22	92.97
B Mem	*CCDC58*	rs3749213	3	122133830	G	T	0.2704	0.4760	0.0498	1.25E-21	91.09
B Mem	*CCDC66*	rs17216685	3	56647799	C	T	0.1742	0.4239	0.0609	3.28E-12	48.42
B Mem	*CCDC85A*	rs10175691	2	56584596	C	A	0.6872	-0.4507	0.0477	3.69E-21	88.95
B Mem	*CCT3*	rs2241106	1	156218910	G	C	0.2702	-0.4230	0.0491	7.44E-18	73.94
B Mem	*CCT8*	rs965951	21	30432557	A	G	0.1160	0.8934	0.0660	8.86E-42	183.01
B Mem	*CCZ1*	rs74688574	7	6855646	G	A	0.4717	0.3395	0.0447	2.99E-14	57.62
B Mem	*CCZ1B*	rs9690561	7	6886751	G	C	0.4431	-0.4287	0.0444	4.74E-22	93.01
B Mem	*CD37*	rs1465697	19	49837246	T	C	0.2637	-0.5973	0.0490	3.08E-34	148.55
B Mem	*CD52*	rs11247915	1	26650550	A	C	0.5030	-0.2631	0.0448	4.27E-09	34.43
B Mem	*CD82*	rs12290127	11	44624480	T	C	0.1693	-0.3342	0.0590	1.50E-08	31.98
B Mem	*CD96*	rs6785519	3	111253069	C	T	0.2612	0.3443	0.0495	3.58E-12	48.24
B Mem	*CDC42*	rs2268179	1	22414785	T	G	0.1644	0.4238	0.0604	2.37E-12	49.05
B Mem	*CEACAM21*	rs3745936	19	42092815	A	T	0.7738	0.3061	0.0549	2.53E-08	30.97
B Mem	*CEBPZ-AS1*	rs11124576	2	37518716	C	T	0.1444	-0.3881	0.0651	2.48E-09	35.48
B Mem	*CENPK*	rs6891955	5	64838758	C	G	0.3836	0.2767	0.0468	3.38E-09	34.88
B Mem	*CFD*	rs2930903	19	887824	C	G	0.4168	0.3422	0.0462	1.36E-13	54.65
B Mem	*CFLAR*	rs6712872	2	202070735	T	A	0.5201	-0.2464	0.0449	4.21E-08	29.99
B Mem	*CHAF1B*	rs9978814	21	37807523	C	A	0.1681	0.7593	0.0606	5.57E-36	156.51
B Mem	*CHCHD10*	rs113586321	22	24099484	A	T	0.0813	-0.4568	0.0801	1.17E-08	32.46
B Mem	*CHCHD2*	rs73142177	7	56302425	G	A	0.1704	-0.4561	0.0559	3.37E-16	66.44
B Mem	*CHI3L2*	rs3118207	1	111768576	A	G	0.3631	-0.6036	0.0450	5.68E-41	179.32
B Mem	*CHMP4B*	rs2626562	20	32409142	A	G	0.5251	-0.2620	0.0449	5.39E-09	33.97
B Mem	*CHPT1*	rs2559851	12	102092989	A	C	0.5836	0.2939	0.0453	8.47E-11	42.06
B Mem	*CIB1*	rs8041338	15	90775913	T	C	0.4822	0.3030	0.0447	1.19E-11	45.9
B Mem	*CITED4*	rs16828219	1	41922249	A	G	0.1240	0.5839	0.0702	9.46E-17	68.94
B Mem	*CLLU1OS*	rs34107810	12	92826680	A	G	0.2643	-0.7699	0.0486	1.29E-56	250.88
B Mem	*CNDP2*	rs8084058	18	72177465	G	A	0.6208	0.2770	0.0456	1.26E-09	36.79
B Mem	*CNPPD1*	rs3795986	2	220033830	G	A	0.6150	0.2637	0.0472	2.28E-08	31.18
B Mem	*CNTNAP2*	rs1919692	7	148001727	A	G	0.6917	0.4258	0.0485	1.60E-18	76.97
B Mem	*COL9A3*	rs751558	20	61463355	A	G	0.2027	-0.3230	0.0563	9.50E-09	32.87
B Mem	*COLCA2*	rs1046872	11	111179390	G	A	0.3668	0.4126	0.0469	1.43E-18	77.2
B Mem	*COMMD6*	rs4885304	13	76088638	A	G	0.1387	-0.7601	0.0601	1.13E-36	159.68

B Mem	*CORO1B*	rs1808279	11	67211569	C	T	0.4238	-0.6583	0.0432	2.39E-52	231.35
B Mem	*CORO1C*	rs961355	12	109065812	C	T	0.4662	0.3085	0.0448	5.99E-12	47.24
B Mem	*COX20*	rs1495946	1	245013807	A	G	0.1307	0.4055	0.0673	1.71E-09	36.2
B Mem	*COX6B1*	rs4806187	19	36149761	G	A	0.5163	-0.3115	0.0447	3.07E-12	48.55
B Mem	*CPNE1*	rs11700299	20	34548865	T	C	0.1054	-0.5869	0.0762	1.34E-14	59.2
B Mem	*CRYBB2*	rs6004662	22	25862124	A	G	0.0960	0.7874	0.0855	3.41E-20	84.56
B Mem	*CSTB*	rs35285321	21	45201832	A	G	0.3415	-0.5569	0.0471	3.28E-32	139.3
B Mem	*CTC-524C5.2*	rs4790264	17	5336791	T	G	0.2353	0.5390	0.0535	6.98E-24	101.34
B Mem	*CTD-2007H13.3*	rs161953	5	98256905	C	T	0.2690	-0.6418	0.0484	4.02E-40	175.43
B Mem	*CTD-2012K14.6*	rs6499166	16	68326917	A	G	0.7223	0.2907	0.0502	7.00E-09	33.47
B Mem	*CTD-2368P22.1*	rs67825948	19	58525067	C	G	0.2268	0.5177	0.0524	5.09E-23	97.41
B Mem	*CTD-2371O3.2*	rs11042350	11	9453209	T	C	0.4988	0.4549	0.0440	4.67E-25	106.69
B Mem	*CTSH*	rs34593439	15	79234957	A	G	0.1119	-0.4870	0.0696	2.53E-12	48.92
B Mem	*CYB5A*	rs9948105	18	71917657	T	G	0.7301	-0.3746	0.0494	3.28E-14	57.44
B Mem	*DAPK2*	rs28656385	15	64338533	G	A	0.8193	-0.3531	0.0598	3.47E-09	34.83
B Mem	*DCXR*	rs9908344	17	79992815	G	A	0.1365	0.6746	0.0639	4.79E-26	111.19
B Mem	*DDR1*	rs2517774	6	29893982	T	C	0.2373	-0.5654	0.0493	1.96E-30	131.19
B Mem	*DDR1*	rs2596573	6	31347930	T	C	0.7687	0.3011	0.0507	2.82E-09	35.23
B Mem	*DDT*	rs5760147	22	24334948	C	A	0.3844	-0.3205	0.0464	4.87E-12	47.64
B Mem	*DDX11*	rs74087920	12	31233053	G	A	0.4488	-0.3745	0.0447	5.08E-17	70.16
B Mem	*DDX5*	rs1991401	17	62502435	G	A	0.3173	-0.2893	0.0485	2.38E-09	35.57
B Mem	*DEGS1*	rs3767732	1	224415509	G	A	0.0802	0.5608	0.0876	1.55E-10	40.89
B Mem	*DHRS4*	rs80326273	14	24461134	A	G	0.0909	0.6049	0.0817	1.32E-13	54.72
B Mem	*DNAJC15*	rs12015	13	43597865	G	A	0.2393	-0.5471	0.0524	1.50E-25	108.93
B Mem	*DOCK8*	rs12156499	9	370199	G	A	0.1449	0.4405	0.0638	4.97E-12	47.6
B Mem	*DRAM2*	rs3762374	1	111682118	T	C	0.2783	0.4816	0.0477	5.83E-24	101.7
B Mem	*DSP*	rs11243176	6	7434286	T	G	0.5645	-0.6971	0.0426	4.12E-60	266.89
B Mem	*EAF2*	rs9834471	3	121487172	G	A	0.6107	-0.7256	0.0438	1.61E-61	273.33
B Mem	*EBPL*	rs3809325	13	50266109	C	T	0.5393	-0.3502	0.0449	5.96E-15	60.79
B Mem	*ECHDC1*	rs9398841	6	127670134	T	G	0.7387	-0.3019	0.0524	8.41E-09	33.11
B Mem	*EIF5A*	rs7503161	17	7207964	C	A	0.5615	-1.0026	0.0392	2.32E-144	653.19
B Mem	*ELP2*	rs592261	18	33752244	A	G	0.3587	0.2840	0.0469	1.36E-09	36.66
B Mem	*ELP5*	rs2074219	17	7156447	G	A	0.6468	-0.5165	0.0448	9.83E-31	132.56
B Mem	*EML6*	rs13033894	2	55010115	C	T	0.2468	0.3646	0.0508	7.13E-13	51.4
B Mem	*ENOSF1*	rs2847157	18	714666	A	G	0.5483	-0.2936	0.0451	7.24E-11	42.37
B Mem	*EPSTI1*	rs3825511	13	43566373	C	A	0.7178	-0.4081	0.0503	4.96E-16	65.68
B Mem	*ERAP2*	rs27298	5	96362800	G	T	0.5903	-0.6532	0.0431	7.02E-52	229.21
B Mem	*ERICH1*	rs11775671	8	589312	A	G	0.3462	-0.2960	0.0472	3.70E-10	39.18
B Mem	*EVI2A*	rs2525570	17	29681245	G	A	0.5970	0.3544	0.0459	1.23E-14	59.37
B Mem	*FADS1*	rs174593	11	61618831	C	T	0.2609	-0.5266	0.0498	4.14E-26	111.48
B Mem	*FAM118A*	rs136582	22	45774531	A	T	0.1332	1.3783	0.0610	4.16E-113	509.8
B Mem	*FAM167A*	rs2736332	8	11339965	C	G	0.2873	0.4039	0.0498	5.13E-16	65.61
B Mem	*FAM177B*	rs12138316	1	222846882	A	G	0.7036	0.3231	0.0493	5.46E-11	42.92
B Mem	*FAM184B*	rs10939740	4	17617490	C	T	0.6230	-0.8302	0.0421	1.55E-86	387.96
B Mem	*FAM26F*	rs117361304	6	116783619	G	T	0.0827	-1.1711	0.0761	2.07E-53	236.21
B Mem	*FAM26F*	rs558144	6	116724291	T	C	0.7610	0.3312	0.0535	6.13E-10	38.2
B Mem	*FAM46A*	rs191786	6	82497480	C	T	0.3759	0.2624	0.0466	1.84E-08	31.6
B Mem	*FCER2*	rs12979200	19	7758303	G	A	0.4839	-0.3029	0.0447	1.26E-11	45.78
B Mem	*FCGR2A*	rs67775399	1	161572353	C	T	0.1844	-0.3515	0.0534	4.74E-11	43.19
B Mem	*FCRL5*	rs11264761	1	157513804	A	G	0.2104	0.5903	0.0536	3.45E-28	120.96
B Mem	*FDFT1*	rs2179011	8	11664895	C	T	0.7331	0.3548	0.0529	1.97E-11	44.91
B Mem	*FIGNL1*	rs11764792	7	50496841	T	C	0.4756	0.2832	0.0447	2.44E-10	40
B Mem	*FLVCR1-AS1*	rs7524203	1	213011812	C	G	0.5251	0.2654	0.0449	3.40E-09	34.87
B Mem	*FNBP4*	rs11039332	11	47695840	A	G	0.2953	-0.3463	0.0481	5.87E-13	51.78
B Mem	*FXYD5*	rs916694	19	35692403	T	C	0.4301	-0.3404	0.0449	3.23E-14	57.47
B Mem	*G3BP2*	rs7679851	4	76553253	T	C	0.3927	-0.2634	0.0459	9.30E-09	32.91
B Mem	*GBP7*	rs521704	1	89680554	A	C	0.4967	-0.7360	0.0419	3.66E-69	308.34
B Mem	*GCC2*	rs2139809	2	109045638	C	T	0.5916	0.3032	0.0454	2.43E-11	44.49
B Mem	*GDI2*	rs11920	10	5805858	A	C	0.2234	-0.2960	0.0529	2.22E-08	31.23
B Mem	*GLTP*	rs9645838	12	110291572	G	A	0.5485	-0.2753	0.0454	1.27E-09	36.78
B Mem	*GM2A*	rs72794132	5	150618993	T	C	0.1649	0.4132	0.0596	4.23E-12	47.92
B Mem	*GNPTG*	rs742460	16	1405537	T	C	0.2936	0.3535	0.0507	3.08E-12	48.54
B Mem	*GPATCH11*	rs6738282	2	37317457	C	A	0.3552	0.3457	0.0457	3.67E-14	57.22
B Mem	*GPM6A*	rs34080115	4	176873913	T	C	0.6128	0.3195	0.0459	3.52E-12	48.28
B Mem	*GRHPR*	rs7856656	9	37421065	T	C	0.3395	0.4138	0.0472	1.74E-18	76.81
B Mem	*GS1-124K5.3*	rs1615892	7	66144596	G	A	0.8821	0.3848	0.0682	1.70E-08	31.74
B Mem	*GS1-124K5.4*	rs3800815	7	66244981	G	A	0.2943	0.2955	0.0489	1.54E-09	36.41
B Mem	*GSTM3*	rs4970736	1	110272645	T	C	0.5889	-0.6240	0.0438	3.94E-46	202.91
B Mem	*GSTM4*	rs11101980	1	110193645	G	T	0.4147	-0.2579	0.0456	1.55E-08	31.92
B Mem	*GSTT1*	rs5760147	22	24334948	C	A	0.3844	-0.5587	0.0452	3.89E-35	152.66
B Mem	*GTF2H2*	rs28478097	5	70309998	G	C	0.6121	-0.4327	0.0454	1.50E-21	90.73
B Mem	*GYPC*	rs893793	2	127416912	G	A	0.6621	0.3290	0.0466	1.69E-12	49.72
B Mem	*HAGH*	rs238680	16	1872160	A	G	0.6943	-0.3312	0.0495	2.16E-11	44.73
B Mem	*HAGHL*	rs12051048	16	783864	A	C	0.2496	0.3335	0.0530	3.20E-10	39.47
B Mem	*HCG25*	rs1612904	6	32669018	A	C	0.6482	-0.4495	0.0456	6.58E-23	96.91
B Mem	*HCG27*	rs3130975	6	31081838	T	C	0.2850	-0.4973	0.0464	8.07E-27	114.72
B Mem	*HCG27*	rs2523627	6	31346445	T	G	0.5915	-0.3850	0.0453	2.02E-17	71.98
B Mem	*HCG27*	rs2894221	6	31461771	A	T	0.3884	-0.3124	0.0457	8.49E-12	46.55
B Mem	*HDHD3*	rs111758952	9	116138460	T	C	0.2792	-0.2795	0.0509	3.98E-08	30.1

B Mem	*HIGD2A*	rs13155545	5	175817499	G	C	0.0942	-0.8074	0.0843	1.02E-21	91.49
B Mem	*HIST1H2AG*	rs78871852	6	26221566	T	A	0.1445	0.5169	0.0687	5.13E-14	56.56
B Mem	*HIST1H2AL*	rs116756717	6	28508095	T	C	0.0516	0.5564	0.0905	8.00E-10	37.68
B Mem	*HIST1H2AM*	rs9393925	6	28682442	C	T	0.8872	-0.3765	0.0681	3.29E-08	30.47
B Mem	*HIST1H2AM*	rs78224691	6	28237110	A	G	0.2239	0.4889	0.0554	1.16E-18	77.61
B Mem	*HIST1H2BG*	rs4634439	6	26598004	G	A	0.1156	0.5153	0.0677	2.73E-14	57.8
B Mem	*HIST1H2BJ*	rs9468168	6	27560236	C	G	0.1064	-0.4763	0.0853	2.34E-08	31.12
B Mem	*HIST1H3D*	rs6912934	6	26459244	T	C	0.8561	0.6267	0.0665	4.44E-21	88.59
B Mem	*HIST1H4E*	rs55706012	6	26266311	C	A	0.0690	-0.7789	0.0755	5.56E-25	106.34
B Mem	*HIST1H4J*	rs9468413	6	28689672	A	C	0.8427	0.3483	0.0607	9.33E-09	32.91
B Mem	*HLA-A*	rs2571389	6	29923503	C	T	0.5408	0.3989	0.0443	2.06E-19	81.02
B Mem	*HLA-B*	rs2524096	6	31236467	T	G	0.4422	0.7316	0.0424	1.23E-66	296.77
B Mem	*HLA-B*	rs1056429	6	31321883	A	G	0.1149	-0.5894	0.0724	4.06E-16	66.07
B Mem	*HLA-C*	rs2249741	6	31240712	C	A	0.5532	0.5371	0.0435	5.59E-35	151.94
B Mem	*HLA-C*	rs9265941	6	31315053	C	T	0.4139	0.2596	0.0450	7.73E-09	33.27
B Mem	*HLA-DPA1*	rs2163472	6	32983375	T	C	0.4593	0.5914	0.0433	1.49E-42	186.55
B Mem	*HLA-DPA1*	rs9469323	6	32996802	T	C	0.1637	-0.3973	0.0623	1.82E-10	40.57
B Mem	*HLA-DPA1*	rs9277568	6	33057055	C	T	0.2778	-0.2848	0.0490	6.22E-09	33.7
B Mem	*HLA-DPB1*	rs2163472	6	32983375	T	C	0.4593	0.7298	0.0421	1.96E-67	300.42
B Mem	*HLA-DPB1*	rs9277384	6	33050463	G	C	0.0643	0.4198	0.0597	2.05E-12	49.34
B Mem	*HLA-DQA1*	rs4713555	6	32575524	T	G	0.2587	0.3562	0.0499	9.11E-13	50.92
B Mem	*HLA-DQA2*	rs909267	6	31746548	C	T	0.0869	-0.6143	0.0912	1.60E-11	45.32
B Mem	*HLA-DQA2*	rs1391371	6	32603798	T	A	0.2202	1.3824	0.0442	1.00E-200	976.79
B Mem	*HLA-DQA2*	rs3134931	6	32190620	C	T	0.3105	-0.4028	0.0474	1.85E-17	72.15
B Mem	*HLA-DQB1*	rs9272553	6	32607008	T	G	0.2882	-0.5034	0.0443	5.74E-30	129.07
B Mem	*HLA-DQB2*	rs9273088	6	32612488	C	A	0.3878	0.4579	0.0445	7.27E-25	105.81
B Mem	*HLA-DRB1*	rs9271492	6	32589122	C	A	0.3824	0.6954	0.0446	1.00E-54	242.23
B Mem	*HLA-DRB5*	rs28366319	6	32561495	A	G	0.2843	1.0706	0.0397	1.70E-160	727.24
B Mem	*HLA-DRB5*	rs707920	6	31629096	C	T	0.3148	-0.3978	0.0517	1.38E-14	59.15
B Mem	*HLA-DRB5*	rs3132948	6	32197219	G	T	0.4645	-0.4054	0.0449	1.83E-19	81.25
B Mem	*HLA-F*	rs9258164	6	29688869	A	G	0.1404	0.4134	0.0694	2.54E-09	35.43
B Mem	*HLA-G*	rs4554349	6	29856279	T	G	0.0638	-0.6128	0.0978	3.70E-10	39.18
B Mem	*HMBOX1*	rs10088428	8	28909523	T	C	0.2597	-0.6122	0.0503	3.93E-34	148.07
B Mem	*HOXB-AS1*	rs4793970	17	46763962	A	G	0.3056	0.3645	0.0494	1.67E-13	54.25
B Mem	*HSBP1L1*	rs3930043	18	77723832	A	G	0.1644	0.3955	0.0606	6.77E-11	42.5
B Mem	*HSPBAP1*	rs12496372	3	122473431	G	C	0.7450	-0.6944	0.0522	2.48E-40	176.39
B Mem	*IER3*	rs1630062	6	29747606	C	T	0.3207	0.2726	0.0471	7.18E-09	33.42
B Mem	*IFITM2*	rs34481144	11	320836	T	C	0.4497	0.2657	0.0448	3.02E-09	35.1
B Mem	*IFITM3*	rs7942247	11	316349	A	G	0.5502	0.2810	0.0448	3.50E-10	39.29
B Mem	*IFNG-AS1*	rs11177007	12	68414744	G	A	0.3776	0.2826	0.0465	1.22E-09	36.86
B Mem	*IGFLR1*	rs2239947	19	36244753	C	G	0.1568	-0.4574	0.0635	5.73E-13	51.83
B Mem	*IGLL5*	rs2856876	22	23249440	A	C	0.1568	0.6289	0.0688	5.92E-20	83.48
B Mem	*ISCU*	rs10778643	12	108939901	A	T	0.8652	-0.4491	0.0650	4.74E-12	47.69
B Mem	*ITGB2*	rs760462	21	46328099	C	T	0.8298	-0.7541	0.0586	5.96E-38	165.51
B Mem	*ITGB3BP*	rs217470	1	64031050	A	C	0.6982	-0.3982	0.0481	1.24E-16	68.41
B Mem	*ITM2B*	rs198604	13	48984063	A	G	0.2252	0.3767	0.0517	3.06E-13	53.06
B Mem	*ITM2C*	rs59481475	2	231750397	A	G	0.1389	-0.4437	0.0655	1.27E-11	45.77
B Mem	*JUP*	rs4606765	17	39917325	C	A	0.7356	-0.4843	0.0513	3.78E-21	88.91
B Mem	*JUP*	rs6503665	17	39953539	C	G	0.9174	-0.4802	0.0856	2.01E-08	31.42
B Mem	*KANSL1-AS1*	rs1378358	17	44787312	T	C	0.1903	0.9608	0.0498	6.47E-83	371.36
B Mem	*KB-226F1.1*	rs5996631	22	24256894	C	T	0.2609	0.7166	0.0492	5.04E-48	211.56
B Mem	*KDELR2*	rs6796	7	6502367	C	T	0.2687	-0.3932	0.0490	1.04E-15	64.23
B Mem	*KDM4B*	rs7250055	19	5133370	T	C	0.2337	0.3249	0.0527	7.11E-10	37.91
B Mem	*KHDC1*	rs7744174	6	73973880	T	C	0.2163	-0.5935	0.0536	1.58E-28	122.51
B Mem	*KIAA0040*	rs2269650	1	175129691	T	G	0.3691	0.4457	0.0462	5.27E-22	92.8
B Mem	*KLK1*	rs3909863	19	51318909	A	G	0.1135	0.4181	0.0743	1.80E-08	31.63
B Mem	*KRTAP5-AS1*	rs61867536	11	1513700	C	T	0.4991	0.5479	0.0437	4.29E-36	157.03
B Mem	*KRTCAP3*	rs1260328	2	27711631	C	T	0.4091	-0.2740	0.0455	1.74E-09	36.17
B Mem	*KYNU*	rs12997823	2	143709091	G	A	0.3387	0.2560	0.0467	4.26E-08	29.97
B Mem	*L3MBTL3*	rs6569648	6	130349119	T	C	0.7821	-0.4524	0.0510	7.09E-19	78.58
B Mem	*LA16c-358B7.3*	rs4984803	16	1349929	A	G	0.6042	-0.4054	0.0449	1.87E-19	81.2
B Mem	*LAMB1*	rs2237683	7	107559137	G	A	0.3469	0.4938	0.0454	1.34E-27	118.27
B Mem	*LARGE*	rs5749579	22	33617801	C	T	0.2482	0.3217	0.0528	1.14E-09	37
B Mem	*LAX1*	rs4951180	1	203738765	C	T	0.6518	0.3161	0.0473	2.42E-11	44.51
B Mem	*LCN8*	rs7041806	9	139648298	A	G	0.1396	1.3562	0.0539	1.13E-139	631.68
B Mem	*LEPROT*	rs10889549	1	65888085	A	C	0.4583	0.2642	0.0449	4.09E-09	34.51
B Mem	*LGALS9*	rs34595284	17	25958791	T	C	0.2365	-0.3177	0.0522	1.12E-09	37.03
B Mem	*LGMN*	rs78693325	14	93165962	C	G	0.1353	0.3893	0.0682	1.16E-08	32.49
B Mem	*LHPP*	rs1869660	10	126227537	G	A	0.8253	0.4051	0.0613	3.81E-11	43.62
B Mem	*LILRB1*	rs6509888	19	55134426	A	G	0.4420	0.3133	0.0451	3.83E-12	48.11
B Mem	*LILRB2*	rs2008238	19	54992671	A	G	0.7898	-0.3271	0.0556	4.06E-09	34.52
B Mem	*LIMD1*	rs13094441	3	45688319	A	T	0.2295	0.6048	0.0498	5.69E-34	147.34
B Mem	*LIMS2*	rs2244880	2	128386644	C	G	0.4319	-0.3960	0.0451	1.51E-18	77.08
B Mem	*LINC00339*	rs4654783	1	22439520	C	T	0.7035	0.3342	0.0494	1.35E-11	45.64
B Mem	*LINC00422*	rs4770135	13	21901874	G	A	0.2742	0.3521	0.0505	3.04E-12	48.57
B Mem	*LINC00926*	rs2248534	15	57597961	G	T	0.2669	0.3938	0.0486	5.42E-16	65.5
B Mem	*LINC00969*	rs3827561	3	195674257	G	C	0.4166	0.3720	0.0453	2.36E-16	67.14
B Mem	*LRRC16A*	rs9467489	6	25397698	G	A	0.4138	-0.3080	0.0456	1.51E-11	45.43
B Mem	*LRRIQ3*	rs2343864	1	74577069	C	G	0.4882	0.4792	0.0439	9.03E-28	119.05

B Mem	*LSM7*	rs11084936	19	2346440	A	G	0.5993	0.3646	0.0455	1.13E-15	64.06
B Mem	*LY6G5C*	rs4947288	6	30696762	G	A	0.2248	0.4513	0.0508	6.68E-19	78.69
B Mem	*LY6G5C*	rs3132486	6	31243170	A	G	0.5435	-0.3009	0.0449	2.06E-11	44.82
B Mem	*LY86*	rs977785	6	6588881	C	A	0.7413	0.7659	0.0476	3.07E-58	258.31
B Mem	*LY96*	rs28435175	8	74908501	A	G	0.1454	0.4673	0.0654	8.99E-13	50.95
B Mem	*LYST*	rs6673237	1	236041129	T	C	0.7574	0.2879	0.0509	1.61E-08	31.86
B Mem	*MANBA*	rs223490	4	103679117	T	G	0.4083	-0.3018	0.0462	6.40E-11	42.61
B Mem	*MARC2*	rs11118600	1	220945713	G	C	0.2390	0.3674	0.0541	1.13E-11	45.99
B Mem	*MCM5*	rs4645771	22	35804752	G	A	0.1480	-0.4843	0.0640	3.68E-14	57.22
B Mem	*MDGA1*	rs6938061	6	37674339	G	A	0.5230	-0.4486	0.0440	2.30E-24	103.54
B Mem	*MDM4*	rs7556371	1	204457336	A	G	0.7044	-0.3723	0.0487	2.17E-14	58.26
B Mem	*MED16*	rs11546307	19	875389	A	G	0.2545	0.3704	0.0518	8.85E-13	50.98
B Mem	*METTL8*	rs12105780	2	172215652	G	A	0.2519	-0.5784	0.0501	7.46E-31	133.11
B Mem	*MGMT*	rs1008982	10	131445731	C	T	0.3802	-0.3329	0.0457	3.43E-13	52.84
B Mem	*MGST3*	rs4147592	1	165600146	A	G	0.4796	-0.4913	0.0438	3.21E-29	125.66
B Mem	*MILR1*	rs35528570	17	62580234	G	A	0.4231	0.2625	0.0455	8.30E-09	33.14
B Mem	*MPHOSPH6*	rs3829549	16	82206353	A	T	0.6399	-0.2631	0.0468	1.95E-08	31.48
B Mem	*MRPL21*	rs646586	11	68681653	G	A	0.3036	-0.3359	0.0482	3.31E-12	48.4
B Mem	*MRPL53*	rs6546907	2	74678921	C	T	0.1819	-0.4436	0.0654	1.18E-11	45.91
B Mem	*MRPS6*	rs2834339	21	35382032	T	C	0.5225	-0.4007	0.0443	1.64E-19	81.47
B Mem	*MRVI1-AS1*	rs2896541	11	10565566	C	T	0.8736	0.3986	0.0655	1.15E-09	36.97
B Mem	*MSH5*	rs3132940	6	32161396	G	T	0.8837	0.5678	0.0655	4.32E-18	75.02
B Mem	*MTDH*	rs2438228	8	98637212	A	G	0.3381	0.4710	0.0479	7.46E-23	96.66
B Mem	*MXRA7*	rs237057	17	74733099	A	G	0.9176	0.7799	0.0872	3.85E-19	79.78
B Mem	*MYBL2*	rs184032	20	42326880	G	A	0.8231	-0.5995	0.0597	9.89E-24	100.65
B Mem	*MYH11*	rs1180588	16	15795048	C	T	0.7230	-0.2962	0.0493	1.88E-09	36.02
B Mem	*MYO7B*	rs2244880	2	128386644	C	G	0.4319	0.4568	0.0448	1.87E-24	103.94
B Mem	*MZT2A*	rs3861757	2	132233317	A	G	0.2083	0.6838	0.0574	1.02E-32	141.61
B Mem	*NAA38*	rs38830	7	117717036	T	A	0.2265	0.3185	0.0508	3.59E-10	39.24
B Mem	*NAGK*	rs6725547	2	71275156	A	G	0.1595	-0.3652	0.0606	1.67E-09	36.25
B Mem	*NAP1L1*	rs1368578	12	76476956	G	A	0.8151	-0.4456	0.0583	2.02E-14	58.39
B Mem	*NAPRT1*	rs9657360	8	144676862	T	C	0.7776	0.5446	0.0543	1.06E-23	100.52
B Mem	*NARFL*	rs4589552	16	767519	C	T	0.1949	0.3357	0.0589	1.18E-08	32.45
B Mem	*NCOA7*	rs61737181	6	126080522	C	G	0.1023	-0.5366	0.0714	5.60E-14	56.39
B Mem	*NDUFA10*	rs7609118	2	240941703	A	C	0.3160	-0.2774	0.0483	9.37E-09	32.9
B Mem	*NDUFA3*	rs254262	19	54612628	G	A	0.7510	-0.2988	0.0511	4.97E-09	34.13
B Mem	*NEK8*	rs12948165	17	27098024	T	A	0.1964	-0.3166	0.0541	4.86E-09	34.17
B Mem	*NME4*	rs77371232	16	447561	T	A	0.4244	0.5316	0.0440	1.52E-33	145.39
B Mem	*NMI*	rs67725431	2	152151259	A	T	0.4622	0.2803	0.0448	3.79E-10	39.14
B Mem	*NMRK1*	rs11144225	9	77707300	T	A	0.3900	-0.2773	0.0453	9.15E-10	37.42
B Mem	*NOMO1*	rs143477575	16	15257639	G	A	0.8394	0.4235	0.0624	1.11E-11	46.04
B Mem	*NQO2*	rs9378352	6	3011149	T	C	0.6054	-0.3150	0.0466	1.36E-11	45.63
B Mem	*NSA2*	rs72764663	5	74082520	A	G	0.1345	0.7434	0.0644	8.65E-31	132.82
B Mem	*NT5C3B*	rs8073219	17	39977734	G	A	0.7569	-0.3913	0.0510	1.66E-14	58.78
B Mem	*NT5E*	rs9362213	6	86136407	A	T	0.1406	-0.5188	0.0617	4.31E-17	70.49
B Mem	*NUDT2*	rs3818830	9	34378999	T	A	0.1539	0.6931	0.0613	1.24E-29	127.54
B Mem	*OPTN*	rs7095146	10	13161409	A	C	0.4919	0.3434	0.0445	1.23E-14	59.37
B Mem	*ORAI2*	rs11444	7	102066873	C	T	0.1678	-0.3516	0.0614	1.00E-08	32.77
B Mem	*ORMDL3*	rs7359623	17	38049589	T	C	0.4963	-0.5491	0.0434	1.11E-36	159.71
B Mem	*OSTF1*	rs34084631	9	77757292	C	G	0.3863	-0.4351	0.0450	4.32E-22	93.19
B Mem	*P2RX1*	rs2074989	17	3808032	G	A	0.4148	-0.3381	0.0454	1.01E-13	55.23
B Mem	*PARP14*	rs13071069	3	122440065	T	C	0.1057	-0.6195	0.0866	8.35E-13	51.09
B Mem	*PILRB*	rs7786844	7	99934008	G	T	0.1844	0.4869	0.0560	3.48E-18	75.44
B Mem	*PIM3*	rs144554206	22	50418996	A	G	0.4777	-0.2634	0.0448	4.09E-09	34.51
B Mem	*PKHD1L1*	rs1783159	8	110514364	A	G	0.3796	0.4903	0.0448	6.16E-28	119.81
B Mem	*PKIG*	rs3091442	20	43174439	G	C	0.1649	-0.4481	0.0610	1.99E-13	53.91
B Mem	*PLA2G4C*	rs8104114	19	48651302	A	G	0.1900	-0.3567	0.0595	2.03E-09	35.87
B Mem	*PLD4*	rs60106058	14	105414252	T	C	0.5414	-0.2562	0.0448	1.10E-08	32.59
B Mem	*PLEK*	rs60061601	2	68622318	C	T	0.1356	-0.4162	0.0659	2.63E-10	39.85
B Mem	*PLTP*	rs3848715	20	44564682	A	G	0.5065	-0.4098	0.0442	1.96E-20	85.66
B Mem	*PNRC2*	rs17184588	1	24365512	C	T	0.4701	-0.3184	0.0446	9.48E-13	50.85
B Mem	*POLM*	rs28382621	7	44122613	G	C	0.0936	0.5540	0.0779	1.18E-12	50.42
B Mem	*POLR1D*	rs12855186	13	28200940	C	T	0.1242	0.5365	0.0655	2.69E-16	66.88
B Mem	*POLR2J*	rs75359550	7	102180473	T	C	0.1492	-0.3584	0.0654	4.21E-08	29.99
B Mem	*POLR2J2*	rs13221979	7	102764723	C	T	0.6564	-0.2727	0.0477	1.10E-08	32.59
B Mem	*POMZP3*	rs11767431	7	76177284	T	C	0.3101	0.2884	0.0482	2.17E-09	35.74
B Mem	*POU5F1*	rs6916685	6	30385184	T	C	0.1814	-0.7329	0.0569	5.82E-38	165.56
B Mem	*POU5F1*	rs4361609	6	31240635	C	G	0.1486	0.6208	0.0650	1.32E-21	90.99
B Mem	*POU5F1*	rs2905708	6	31364603	T	C	0.0841	-0.5888	0.0749	3.88E-15	61.63
B Mem	*PPA1*	rs10740330	10	71970791	G	T	0.3462	0.5127	0.0461	8.79E-29	123.66
B Mem	*PPDPF*	rs72629024	20	62152519	G	C	0.1633	-0.8951	0.0594	2.83E-51	226.44
B Mem	*PPIA*	rs13240323	7	44816785	A	G	0.3423	-0.3612	0.0456	2.50E-15	62.49
B Mem	*PPIL3*	rs7559150	2	201754063	C	T	0.2458	-0.4360	0.0527	1.27E-16	68.36
B Mem	*PPP1R3B*	rs2980757	8	8309097	T	A	0.3858	0.3039	0.0465	6.16E-11	42.68
B Mem	*PRDX2*	rs897804	19	12876964	C	G	0.3850	0.2744	0.0458	2.03E-09	35.87
B Mem	*PRKD3*	rs12622740	2	37466846	A	G	0.5245	-0.2844	0.0447	2.06E-10	40.32
B Mem	*PRR7-AS1*	rs4976695	5	176862963	A	T	0.4999	0.4440	0.0442	8.94E-24	100.85
B Mem	*PRSS16*	rs9368560	6	28159960	C	T	0.2068	-0.3037	0.0549	3.08E-08	30.59
B Mem	*PSMB9*	rs2071480	6	32821859	A	C	0.4101	0.3027	0.0458	3.72E-11	43.67

B Mem	*PSMD5-AS1*	rs10818476	9	123572038	G	A	0.5810	-0.8466	0.0415	1.40E-92	415.67
B Mem	*PSTPIP1*	rs75329354	15	77668065	T	C	0.2710	0.3198	0.0493	8.80E-11	41.99
B Mem	*PTPLAD2*	rs2151001	9	20836862	G	A	0.6261	-0.2843	0.0457	5.15E-10	38.54
B Mem	*PYROXD2*	rs7909131	10	100171471	G	A	0.3418	0.4039	0.0467	4.86E-18	74.78
B Mem	*RABAC1*	rs61007366	19	42436861	A	G	0.6598	0.3647	0.0466	4.76E-15	61.23
B Mem	*RAC1*	rs836516	7	6462203	T	A	0.4416	0.3117	0.0449	3.98E-12	48.04
B Mem	*RASGRP3*	rs57542130	2	33754336	A	C	0.1013	0.4394	0.0780	1.76E-08	31.68
B Mem	*RASSF6*	rs12108404	4	74327001	T	C	0.9346	-0.5188	0.0948	4.46E-08	29.88
B Mem	*RBM26*	rs1324870	13	79872046	T	C	0.5041	0.2509	0.0448	2.17E-08	31.27
B Mem	*RCSD1*	rs28641593	1	167586861	T	C	0.6291	0.2660	0.0472	1.72E-08	31.72
B Mem	*RGS16*	rs584158	1	182603060	A	T	0.1467	0.4783	0.0657	3.27E-13	52.93
B Mem	*RNASE6*	rs8003799	14	21178988	A	T	0.5014	-0.3982	0.0443	2.58E-19	80.57
B Mem	*RNASEH2C*	rs34007146	11	65477954	A	G	0.3457	-0.3029	0.0467	9.11E-11	41.92
B Mem	*RNGTT*	rs2756370	6	89326244	T	A	0.1640	-0.6560	0.0621	4.12E-26	111.49
B Mem	*RP1-29C18.10*	rs5770622	22	50051548	G	T	0.6547	-0.5328	0.0470	7.96E-30	128.42
B Mem	*RP1-3J17.3*	rs12212560	6	86342679	A	G	0.3868	0.5611	0.0448	5.35E-36	156.59
B Mem	*RP1-3J17.3*	rs9444346	6	86165631	G	A	0.1874	0.4509	0.0579	6.66E-15	60.57
B Mem	*RP11-1070N10.3*	rs179145	14	95983975	A	G	0.3676	-0.2774	0.0467	2.79E-09	35.25
B Mem	*RP11-111A22.1*	rs12913296	15	40844222	A	G	0.4197	0.6050	0.0432	1.57E-44	195.59
B Mem	*RP11-119F19.2*	rs1650228	10	81406064	C	T	0.3960	0.2556	0.0465	3.99E-08	30.09
B Mem	*RP11-138A9.1*	rs10808245	7	130632125	T	C	0.5064	0.2819	0.0448	3.11E-10	39.52
B Mem	*RP11-138A9.2*	rs12533527	7	130626948	T	C	0.1594	0.3666	0.0628	5.27E-09	34.02
B Mem	*RP11-138I18.2*	rs11257236	10	11715636	G	A	0.3732	0.2814	0.0466	1.52E-09	36.44
B Mem	*RP11-197K3.1*	rs1533269	3	114214611	A	C	0.3319	0.3184	0.0483	4.22E-11	43.42
B Mem	*RP11-297B17.3*	rs12554596	9	37002142	G	A	0.2084	0.4098	0.0564	3.76E-13	52.66
B Mem	*RP11-298J20.3*	rs10901842	10	126635262	G	A	0.7352	-0.6930	0.0496	2.44E-44	194.71
B Mem	*RP11-326C3.11*	rs10794308	11	324163	T	C	0.3897	-0.2946	0.0468	3.01E-10	39.59
B Mem	*RP11-345J4.5*	rs144208959	16	30323380	A	G	0.4341	-0.3447	0.0445	9.73E-15	59.83
B Mem	*RP11-347P5.1*	rs7571871	2	196963243	A	G	0.3644	-0.7056	0.0441	1.31E-57	255.43
B Mem	*RP11-386I14.4*	rs115525402	1	78443047	C	A	0.0639	-0.4674	0.0844	3.08E-08	30.59
B Mem	*RP11-428G5.5*	rs1259733	12	32030546	T	C	0.6078	-0.3381	0.0456	1.24E-13	54.83
B Mem	*RP11-434C1.1*	rs3911234	12	11349964	A	G	0.6020	-0.2820	0.0455	5.70E-10	38.34
B Mem	*RP11-44F21.5*	rs76512224	4	76070018	A	G	0.1000	-0.5592	0.0757	1.49E-13	54.47
B Mem	*RP11-457M11.5*	rs115810	6	25975883	C	G	0.7090	0.4218	0.0489	6.24E-18	74.29
B Mem	*RP11-473I1.5*	rs34932929	16	9193723	G	T	0.2216	-0.3669	0.0546	1.87E-11	45.01
B Mem	*RP11-514O12.4*	rs6456059	6	166628329	T	C	0.5261	-0.5793	0.0433	7.05E-41	178.89
B Mem	*RP11-542K23.7*	rs35918962	9	125168878	T	C	0.1266	0.6418	0.0674	1.71E-21	90.47
B Mem	*RP11-588H23.3*	rs61616333	12	70887764	C	T	0.1695	0.3883	0.0576	1.58E-11	45.34
B Mem	*RP11-596D21.1*	rs35335466	14	31839771	A	G	0.3623	-0.4123	0.0451	6.62E-20	83.25
B Mem	*RP13-514E23.2*	rs2869408	4	86922721	C	G	0.2174	-0.7597	0.0519	1.62E-48	213.82
B Mem	*RP5-827C21.4*	rs73099916	1	234505024	G	A	0.0926	0.4621	0.0785	3.87E-09	34.62
B Mem	*RPL10A*	rs2076172	6	35436925	T	C	0.5358	-0.4898	0.0443	2.08E-28	121.96
B Mem	*RPL11*	rs77766201	1	24006446	A	G	0.1560	-0.4860	0.0626	7.99E-15	60.21
B Mem	*RPL12*	rs2247781	9	130208924	C	T	0.5921	0.9322	0.0402	7.51E-119	536.15
B Mem	*RPL13*	rs34434037	16	89631008	C	G	0.1664	-0.6688	0.0554	1.33E-33	145.65
B Mem	*RPL14*	rs73080163	3	40533061	T	G	0.2980	0.2839	0.0489	6.44E-09	33.63
B Mem	*RPL22L1*	rs13063927	3	170582888	T	C	0.2425	-0.3267	0.0506	1.08E-10	41.58
B Mem	*RPL23A*	rs9890687	17	26928363	G	A	0.0817	-0.7265	0.0780	1.26E-20	86.53
B Mem	*RPL27A*	rs61875926	11	8705528	G	C	0.0958	-0.6408	0.0734	2.55E-18	76.06
B Mem	*RPL28*	rs17700376	19	55897327	G	A	0.3547	-0.9180	0.0421	2.09E-105	474.48
B Mem	*RPL3*	rs470082	22	39715140	T	C	0.4764	0.2548	0.0448	1.33E-08	32.23
B Mem	*RPL32*	rs2290311	3	12883071	A	T	0.1438	0.3543	0.0619	1.04E-08	32.7
B Mem	*RPL36AL*	rs2147712	14	50091684	A	G	0.7504	0.9124	0.0486	9.23E-79	352.32
B Mem	*RPL8*	rs2958481	8	145983700	G	A	0.4899	1.1061	0.0365	1.00E-200	917.13
B Mem	*RPLP2*	rs28694634	11	808423	A	G	0.6588	-0.6061	0.0465	7.21E-39	169.7
B Mem	*RPS10*	rs7775635	6	34372804	G	A	0.1105	1.2322	0.0788	4.18E-55	243.96
B Mem	*RPS10*	rs7741127	6	34406260	T	G	0.8781	-0.4324	0.0719	1.79E-09	36.12
B Mem	*RPS11*	rs10403394	19	49999345	A	G	0.1225	-0.6121	0.0712	8.25E-18	73.74
B Mem	*RPS12*	rs12204693	6	133140795	T	C	0.0619	0.6661	0.0969	6.14E-12	47.19
B Mem	*RPS14*	rs11167502	5	149833365	A	G	0.2888	-0.2754	0.0487	1.59E-08	31.88
B Mem	*RPS16*	rs640000	19	39926601	T	C	0.3796	0.6864	0.0448	5.58E-53	234.24
B Mem	*RPS18*	rs17215231	6	33239869	T	C	0.0640	-1.5601	0.0826	1.56E-79	355.86
B Mem	*RPS19*	rs10417985	19	42377133	G	T	0.5022	0.2731	0.0448	1.05E-09	37.15
B Mem	*RPS2*	rs17135720	16	2014935	C	T	0.1128	0.8113	0.0659	7.64E-35	151.32
B Mem	*RPS23*	rs226204	5	81610680	A	G	0.7743	0.3360	0.0517	7.99E-11	42.17
B Mem	*RPS24*	rs2913134	10	79763775	T	C	0.5775	0.2683	0.0460	5.58E-09	33.91
B Mem	*RPS25*	rs11217124	11	118889132	G	A	0.2501	-0.5650	0.0496	4.28E-30	129.65
B Mem	*RPS26*	rs773114	12	56379060	T	A	0.3997	1.2089	0.0350	1.00E-200	1193.73
B Mem	*RPS3*	rs11539810	11	75133765	A	G	0.0739	-0.8179	0.0886	2.69E-20	85.03
B Mem	*RPS3A*	rs1201214	4	151815783	C	T	0.0710	-1.2165	0.0981	2.48E-35	153.55
B Mem	*RPS5*	rs731835	19	58898436	C	T	0.3256	-0.9897	0.0421	3.61E-122	551.37
B Mem	*RPS6*	rs10114039	9	19352260	T	A	0.0988	-0.8867	0.0808	5.32E-28	120.1
B Mem	*RPS7*	rs11558965	2	3622912	T	G	0.0770	-1.2543	0.0847	1.29E-49	218.85
B Mem	*RPS8*	rs12120833	1	45241285	G	C	0.1242	-1.0228	0.0586	4.15E-68	303.51
B Mem	*RPS9*	rs3810232	19	54704760	C	A	0.3916	-0.7014	0.0433	4.61E-59	262.09
B Mem	*RPSA*	rs45503293	3	39432785	T	C	0.1594	0.4808	0.0612	3.80E-15	61.68
B Mem	*RSRC2*	rs11058402	12	122981053	G	A	0.7504	-0.3576	0.0505	1.38E-12	50.1
B Mem	*SAR1A*	rs10999158	10	71905695	G	A	0.4420	-0.3007	0.0451	2.58E-11	44.38
B Mem	*SCGB3A1*	rs7730091	5	179690401	T	C	0.3534	0.6092	0.0447	3.24E-42	185

B Mem	*SCIMP*	rs4790752	17	5115877	T	C	0.4301	-0.3353	0.0449	7.79E-14	55.74
B Mem	*SEC61B*	rs75085864	9	101999360	A	G	0.0766	-0.6168	0.0769	1.03E-15	64.23
B Mem	*SEL1L3*	rs13104150	4	25878496	A	G	0.4437	0.3641	0.0445	2.92E-16	66.72
B Mem	*SEPT2*	rs2074771	2	242350944	G	A	0.0944	0.4116	0.0750	4.05E-08	30.06
B Mem	*SERAC1*	rs9457442	6	159124263	T	G	0.1946	0.5916	0.0538	4.39E-28	120.48
B Mem	*SERTAD3*	rs268694	19	40962382	C	T	0.3328	-0.2570	0.0470	4.52E-08	29.85
B Mem	*SESTD1*	rs13382185	2	180059627	A	C	0.2619	0.3364	0.0522	1.19E-10	41.4
B Mem	*SF1*	rs2284301	11	64501991	T	C	0.1032	0.6071	0.0782	8.05E-15	60.2
B Mem	*SH3BGRL3*	rs11247915	1	26650550	A	C	0.5030	0.3352	0.0445	5.31E-14	56.5
B Mem	*SH3YL1*	rs7584915	2	264227	A	G	0.3333	-0.5881	0.0452	1.14E-38	168.8
B Mem	*SIDT1-AS1*	rs34627996	3	113276381	C	G	0.2123	0.4288	0.0539	1.74E-15	63.21
B Mem	*SLC15A2*	rs9834471	3	121487172	G	A	0.6107	-0.2646	0.0466	1.32E-08	32.23
B Mem	*SLC25A1*	rs807466	22	19225650	T	C	0.6640	-0.3048	0.0469	7.96E-11	42.18
B Mem	*SLC25A29*	rs1059264	14	100769163	T	C	0.3348	0.2766	0.0476	6.02E-09	33.76
B Mem	*SLC26A11*	rs1663186	17	77888930	G	A	0.0555	-0.5865	0.0877	2.28E-11	44.62
B Mem	*SMDT1*	rs2301521	22	42423110	C	G	0.6779	1.2218	0.0385	1.00E-200	1007.05
B Mem	*SMIM19*	rs6987853	8	42457450	C	T	0.6238	-0.2869	0.0459	3.95E-10	39.06
B Mem	*SMPD3*	rs73613999	16	68390099	A	C	0.1290	0.4684	0.0701	2.34E-11	44.58
B Mem	*SNHG7*	rs6874	9	139617836	T	C	0.1153	-1.0642	0.0675	5.59E-56	247.97
B Mem	*SNHG8*	rs658642	4	119200274	T	C	0.3810	0.8060	0.0420	4.40E-82	367.54
B Mem	*SNHG8*	rs2389687	4	119734600	G	C	0.8858	0.3875	0.0704	3.74E-08	30.22
B Mem	*SNORD3B-1*	rs12602286	17	19236954	T	G	0.8363	0.7932	0.0666	1.04E-32	141.57
B Mem	*SNORD3D*	rs12953174	17	19375481	T	C	0.7399	0.4589	0.0513	3.46E-19	79.99
B Mem	*SNRPD2*	rs36040835	19	46201213	G	T	0.2051	-0.4944	0.0553	3.86E-19	79.78
B Mem	*SNX24*	rs17477351	5	123000031	A	G	0.1310	-0.4736	0.0652	3.92E-13	52.58
B Mem	*SPATA20*	rs8076470	17	48625828	T	C	0.3932	-0.3956	0.0460	7.61E-18	73.9
B Mem	*SPSB2*	rs7138740	12	6983764	A	T	0.2917	0.2702	0.0489	3.30E-08	30.46
B Mem	*SQSTM1*	rs2241349	5	179260009	A	G	0.3387	-0.3111	0.0476	6.48E-11	42.58
B Mem	*SRSF10*	rs17184588	1	24365512	C	T	0.4701	-0.2658	0.0448	2.92E-09	35.17
B Mem	*STMN3*	rs6062477	20	62287600	T	C	0.5864	0.2848	0.0450	2.46E-10	39.98
B Mem	*SULF2*	rs3091426	20	46189755	A	G	0.5891	-0.8771	0.0407	7.10E-103	462.87
B Mem	*SURF1*	rs3124747	9	136268084	G	A	0.6475	0.3194	0.0478	2.44E-11	44.49
B Mem	*SVIP*	rs11026804	11	22841582	A	G	0.5396	0.2889	0.0454	1.96E-10	40.42
B Mem	*SYNGR1*	rs909685	22	39747671	A	T	0.3216	0.4745	0.0488	2.48E-22	94.29
B Mem	*TAPBP*	rs3130260	6	33278816	A	G	0.2897	-0.3145	0.0489	1.24E-10	41.31
B Mem	*TBCA*	rs254415	5	77071703	T	G	0.2878	-0.3237	0.0488	3.37E-11	43.86
B Mem	*TCEB3*	rs519202	1	24045820	T	C	0.6129	0.2857	0.0455	3.29E-10	39.41
B Mem	*TEN1*	rs2665967	17	74016834	C	T	0.7407	0.3062	0.0511	2.09E-09	35.81
B Mem	*TESC*	rs7978810	12	117498431	G	A	0.3254	0.2838	0.0484	4.47E-09	34.34
B Mem	*THAP7-AS1*	rs370907	22	21361242	A	T	0.3736	-0.2731	0.0471	6.84E-09	33.51
B Mem	*TIMM10*	rs2848626	11	57283988	T	C	0.7820	-0.7149	0.0497	6.82E-47	206.39
B Mem	*TIMM13*	rs7248779	19	2403564	G	T	0.4857	0.2465	0.0449	3.92E-08	30.13
B Mem	*TLE4*	rs2807303	9	82187095	A	G	0.3638	0.3763	0.0465	5.59E-16	65.44
B Mem	*TLK1*	rs10183486	2	171990971	T	C	0.3726	-0.3427	0.0463	1.28E-13	54.77
B Mem	*TMEM156*	rs2711981	4	39039258	T	C	0.6560	0.7193	0.0442	1.35E-59	264.52
B Mem	*TMEM220*	rs376174	17	10618220	C	T	0.4518	-0.3215	0.0453	1.22E-12	50.35
B Mem	*TMEM258*	rs174584	11	61610750	A	G	0.3572	0.3155	0.0472	2.35E-11	44.56
B Mem	*TMEM50B*	rs6517175	21	34857840	C	T	0.4737	-0.2911	0.0448	7.88E-11	42.2
B Mem	*TMEM80*	rs7112962	11	654091	A	G	0.4583	-0.2613	0.0452	7.55E-09	33.32
B Mem	*TOMM7*	rs2240728	7	22852499	T	C	0.3422	-0.9888	0.0427	2.14E-118	534.06
B Mem	*TPGS2*	rs75313810	18	34502171	A	G	0.2623	-0.2931	0.0497	3.61E-09	34.75
B Mem	*TSPAN3*	rs17466257	15	77376183	A	C	0.3895	0.5079	0.0446	4.48E-30	129.56
B Mem	*TSPO*	rs138911	22	43553417	G	T	0.7449	-0.4381	0.0505	4.14E-18	75.1
B Mem	*TSTD1*	rs2516841	1	161010774	G	A	0.7578	-0.5916	0.0495	6.10E-33	142.64
B Mem	*TSTD3*	rs330832	6	100047851	C	G	0.5619	-0.2917	0.0451	1.04E-10	41.66
B Mem	*TTC32*	rs80296077	2	20089000	A	G	0.0661	0.6061	0.0907	2.35E-11	44.56
B Mem	*TUBB6*	rs12965912	18	12303726	T	C	0.1225	0.6761	0.0728	1.64E-20	86.01
B Mem	*TUFM*	rs8049439	16	28837515	C	T	0.3939	0.5918	0.0444	1.70E-40	177.14
B Mem	*TYMP*	rs140522	22	50971266	C	T	0.6583	0.3096	0.0484	1.60E-10	40.82
B Mem	*U2AF1L4*	rs2871921	19	36232420	T	C	0.1557	0.4123	0.0637	9.86E-11	41.76
B Mem	*UBA52*	rs4808823	19	18698019	C	T	0.4762	-0.2515	0.0450	2.24E-08	31.21
B Mem	*UBE2J1*	rs12209311	6	90067072	C	T	0.0729	0.8444	0.0800	4.62E-26	111.26
B Mem	*UCP2*	rs2515087	11	73682872	T	C	0.4077	0.4192	0.0453	2.12E-20	85.5
B Mem	*UPF3A*	rs28539632	13	115064682	A	G	0.0696	-0.5704	0.0847	1.61E-11	45.3
B Mem	*UQCRH*	rs79783387	1	46772031	C	T	0.1272	-0.5453	0.0718	3.02E-14	57.6
B Mem	*USF1*	rs2256633	1	160933467	T	C	0.4547	0.2679	0.0450	2.69E-09	35.33
B Mem	*USP53*	rs34156358	4	120167618	T	C	0.1263	0.5245	0.0664	2.94E-15	62.18
B Mem	*VAMP2*	rs4792267	17	8061337	A	G	0.6696	0.3022	0.0491	7.20E-10	37.89
B Mem	*VAMP5*	rs62165906	2	85801108	T	C	0.0869	-0.4662	0.0735	2.29E-10	40.12
B Mem	*WAC-AS1*	rs7098928	10	28789999	G	A	0.5922	-0.2624	0.0454	7.39E-09	33.36
B Mem	*WARS*	rs8015259	14	100795338	C	G	0.2475	0.6578	0.0494	1.58E-40	177.28
B Mem	*XRCC6BP1*	rs10747788	12	58315338	T	C	0.3126	-0.2954	0.0476	5.55E-10	38.4
B Mem	*XRRA1*	rs12225372	11	74604594	G	A	0.2768	0.7283	0.0465	3.13E-55	244.54
B Mem	*XXbac-BPG181B23.7*	rs9261844	6	30382210	A	G	0.1581	-0.3791	0.0591	1.45E-10	41.01
B Mem	*XXbac-BPG299F13.17*	rs6921663	6	31154620	C	T	0.2178	0.8333	0.0517	2.38E-58	258.82
B Mem	*YBEY*	rs62224180	21	47726332	G	C	0.7751	-0.6757	0.0554	2.90E-34	148.67
B Mem	*YME1L1*	rs10829173	10	27371623	A	G	0.6208	0.2665	0.0475	1.98E-08	31.45
B Mem	*YWHAB*	rs2239535	20	43516156	A	G	0.2479	-0.3374	0.0534	2.66E-10	39.83
B Mem	*ZBTB9*	rs7763411	6	32424992	T	C	0.0913	-0.7350	0.0687	1.05E-26	114.19

B Mem	*ZC2HC1A*	rs6982937	8	79555186	C	T	0.7252	0.4656	0.0516	1.87E-19	81.21
B Mem	*ZC3HAV1*	rs7803490	7	138730361	C	T	0.5046	-0.3423	0.0445	1.50E-14	58.98
B Mem	*ZDHHC19*	rs6788070	3	195932512	T	C	0.2131	0.5154	0.0537	8.70E-22	91.8
B Mem	*ZFAS1*	rs6012617	20	47837126	C	G	0.7966	-0.5696	0.0525	1.94E-27	117.54
B Mem	*ZFP36L1*	rs194762	14	69211893	C	G	0.4325	-0.2579	0.0452	1.12E-08	32.55
B Mem	*ZFP57*	rs1116222	6	30071279	G	T	0.2488	-0.5634	0.0488	7.14E-31	133.2
B Mem	*ZKSCAN3*	rs73403143	6	28726613	T	C	0.0678	0.6430	0.0960	2.08E-11	44.8
B Mem	*ZNF100*	rs6511286	19	21892040	T	C	0.3581	-0.3278	0.0468	2.55E-12	48.91
B Mem	*ZNF302*	rs9789337	19	35166230	C	A	0.1245	0.3966	0.0673	3.84E-09	34.63
B Mem	*ZNF600*	rs4802995	19	53284720	G	C	0.4576	0.2697	0.0448	1.74E-09	36.17
B Mem	*ZNF607*	rs7250800	19	39021754	G	A	0.1110	0.9703	0.0695	3.04E-44	194.27
B Mem	*ZNF667-AS1*	rs7246498	19	57007648	A	C	0.3707	0.2925	0.0474	6.82E-10	37.99
B Mem	*ZNF83*	rs35706759	19	53122472	G	T	0.2834	-0.2949	0.0508	6.29E-09	33.67
B Mem	*ZNF85*	rs73021506	19	21129736	T	C	0.1198	0.6287	0.0662	2.21E-21	89.97
B Mem	*ZP3*	rs11767431	7	76177284	T	C	0.3101	0.4469	0.0476	5.68E-21	88.1
Plasma	*AIF1*	rs17198874	6	31265989	A	G	0.2927	0.3489	0.0547	1.83E-10	40.51
Plasma	*ANXA6*	rs35414841	5	150528928	G	A	0.5995	0.3260	0.0504	9.69E-11	41.75
Plasma	*APOBEC3G*	rs139367	22	39518315	A	G	0.5022	-0.2716	0.0498	4.90E-08	29.66
Plasma	*APOM*	rs9265645	6	31300197	A	G	0.1049	0.6475	0.0718	1.88E-19	81.11
Plasma	*APOM*	rs9267658	6	31845985	C	T	0.8828	0.4015	0.0726	3.20E-08	30.49
Plasma	*BIK*	rs4988388	22	43515108	C	T	0.1559	-0.3868	0.0703	3.82E-08	30.14
Plasma	*C11orf24*	rs2175589	11	68055190	G	A	0.3050	0.4173	0.0541	1.16E-14	59.41
Plasma	*C6orf136*	rs9260078	6	29909033	G	A	0.6225	-0.3176	0.0506	3.37E-10	39.32
Plasma	*CCHCR1*	rs2524225	6	30501464	A	G	0.6366	0.4298	0.0515	7.19E-17	69.4
Plasma	*CCHCR1*	rs9266217	6	31325092	G	A	0.5831	-0.5314	0.0496	9.13E-27	114.35
Plasma	*CCT8*	rs965951	21	30432557	A	G	0.1160	0.4711	0.0759	5.34E-10	38.43
Plasma	*CDKN1A*	rs11759995	6	36116602	T	C	0.3913	-0.3194	0.0509	3.37E-10	39.33
Plasma	*CSTB*	rs35285321	21	45201832	A	G	0.3415	-0.3642	0.0535	9.91E-12	46.2
Plasma	*CTC-524C5.2*	rs3026101	17	5280440	C	T	0.3177	-0.5326	0.0531	1.05E-23	100.42
Plasma	*CTSH*	rs11072817	15	79236815	A	G	0.6581	0.4181	0.0530	2.99E-15	62.08
Plasma	*CTSS*	rs6669855	1	150861229	T	C	0.3613	0.3080	0.0520	3.24E-09	34.92
Plasma	*DDX39B*	rs6940063	6	32428862	C	T	0.1555	0.4668	0.0642	3.49E-13	52.75
Plasma	*DEK*	rs112230930	6	18176718	G	C	0.1328	-0.3943	0.0717	3.74E-08	30.19
Plasma	*DXO*	rs3130477	6	31428920	C	T	0.1014	0.4404	0.0729	1.53E-09	36.38
Plasma	*EHMT2*	rs3130048	6	31613739	C	T	0.2812	-0.3316	0.0563	3.78E-09	34.63
Plasma	*EIF5A*	rs7503161	17	7207964	C	A	0.5615	-0.8696	0.0457	1.07E-80	360.81
Plasma	*ELL2*	rs17085231	5	95222156	T	C	0.2648	0.3279	0.0565	6.68E-09	33.52
Plasma	*ELP5*	rs8067500	17	7172631	T	C	0.6444	-0.4485	0.0503	4.92E-19	79.21
Plasma	*ERAP2*	rs56285155	5	96242857	C	T	0.3646	0.4623	0.0508	9.67E-20	82.42
Plasma	*FAM118A*	rs136582	22	45774531	A	T	0.1332	0.5545	0.0755	2.08E-13	53.76
Plasma	*HIST1H2BN*	rs71557378	6	26903585	G	T	0.0785	-0.5301	0.0830	1.69E-10	40.67
Plasma	*HLA-A*	rs9260408	6	29918917	T	C	0.5798	0.2845	0.0500	1.24E-08	32.32
Plasma	*HLA-B*	rs9264579	6	31235746	A	G	0.5283	0.4626	0.0491	4.24E-21	88.58
Plasma	*HLA-B*	rs28559870	6	31377974	T	C	0.1989	-0.3980	0.0648	8.00E-10	37.64
Plasma	*HLA-C*	rs2249741	6	31240712	C	A	0.5532	0.3259	0.0496	5.06E-11	43.02
Plasma	*HLA-DQA1*	rs560530	6	32577222	A	G	0.1763	0.4577	0.0603	3.22E-14	57.42
Plasma	*HLA-DQA2*	rs1391371	6	32603798	T	A	0.2202	1.0762	0.0550	3.44E-85	381.37
Plasma	*HLA-DQB1*	rs1049225	6	32627747	G	A	0.7761	-0.3871	0.0583	3.25E-11	43.89
Plasma	*HLA-DRB5*	rs35095850	6	32549935	G	C	0.2249	0.7185	0.0493	3.75E-48	211.93
Plasma	*HLA-G*	rs2517585	6	30205201	C	T	0.0614	-0.6812	0.0979	3.47E-12	48.25
Plasma	*IFNG-AS1*	rs2906854	12	68387346	A	G	0.6730	-0.5061	0.0535	3.02E-21	89.25
Plasma	*ITPR3*	rs6935940	6	32683691	C	A	0.2380	-0.3458	0.0586	3.55E-09	34.75
Plasma	*KANSL1-AS1*	rs413519	17	43685826	G	A	0.2040	-0.4436	0.0590	5.55E-14	56.35
Plasma	*LCN8*	rs7041806	9	139648298	A	G	0.1396	0.6125	0.0681	2.32E-19	80.7
Plasma	*LEMD2*	rs9276685	6	32750857	G	A	0.0676	-0.6868	0.0893	1.44E-14	58.99
Plasma	*LIMD1*	rs62242182	3	45683363	A	G	0.2294	0.3749	0.0566	3.54E-11	43.72
Plasma	*LRRC16A*	rs215015	6	25381622	A	C	0.4557	-0.6864	0.0472	5.52E-48	211.15
Plasma	*LST1*	rs885905	6	30890431	G	A	0.7379	-0.3555	0.0556	1.57E-10	40.82
Plasma	*MED23*	rs2181572	6	131666295	C	A	0.0657	-0.6299	0.1012	4.81E-10	38.63
Plasma	*MICB*	rs3132584	6	30688427	T	G	0.2294	-0.4353	0.0605	6.48E-13	51.53
Plasma	*MICB*	rs3130521	6	31196376	C	T	0.5980	-0.4942	0.0505	1.20E-22	95.62
Plasma	*MTHFD1L*	rs6557171	6	152234593	C	T	0.6659	-0.3785	0.0527	6.73E-13	51.46
Plasma	*NFKBIL1*	rs2853944	6	31247758	C	T	0.7074	0.4124	0.0526	4.53E-15	61.27
Plasma	*NT5C3B*	rs35305937	17	39977069	A	G	0.1078	-0.5261	0.0750	2.25E-12	49.1
Plasma	*NUCB2*	rs214068	11	17305650	C	T	0.6047	-0.3592	0.0500	7.10E-13	51.36
Plasma	*PAQR6*	rs9943105	1	156245245	C	T	0.4906	0.4578	0.0489	7.48E-21	87.46
Plasma	*PKHD1L1*	rs1026437	8	110492949	C	G	0.3678	0.3076	0.0508	1.42E-09	36.53
Plasma	*PPDPF*	rs72629024	20	62152519	G	C	0.1633	-0.4157	0.0691	1.82E-09	36.05
Plasma	*PRR3*	rs12665401	6	29717380	C	T	0.3222	0.3188	0.0534	2.37E-09	35.53
Plasma	*PRR3*	rs2516719	6	30223720	T	C	0.0841	-0.7254	0.0933	7.73E-15	60.22
Plasma	*PSMD5-AS1*	rs34025993	9	123516572	G	A	0.5819	-0.3656	0.0503	3.50E-13	52.74
Plasma	*RMND1*	rs6909684	6	152746133	C	T	0.1296	0.5408	0.0749	5.08E-13	52.01
Plasma	*RNF146*	rs553228	6	127829926	C	T	0.8120	-0.8572	0.0639	5.50E-41	179.19
Plasma	*RP1-3J17.3*	rs1885607	6	86394905	G	A	0.6473	0.7029	0.0490	1.07E-46	205.28
Plasma	*RP11-356I2.4*	rs116627788	6	139066945	T	C	0.0653	0.6000	0.1033	6.28E-09	33.64
Plasma	*RPL12*	rs2251551	9	130284652	G	C	0.5934	0.4208	0.0499	3.50E-17	70.82
Plasma	*RPL28*	rs17700376	19	55897327	G	A	0.3547	-0.4038	0.0516	4.99E-15	61.08
Plasma	*RPL36AL*	rs6572589	14	50024344	T	C	0.7153	0.3423	0.0561	1.03E-09	37.14
Plasma	*RPL8*	rs2954658	8	146017782	T	C	0.4990	-0.5032	0.0486	4.41E-25	106.69

Plasma	*RPP21*	rs9261394	6	30064562	G	A	0.5838	0.5851	0.0487	3.49E-33	143.59
Plasma	*RPP21*	rs2517582	6	30808762	T	C	0.4218	-0.2993	0.0512	4.88E-09	34.13
Plasma	*RPS10*	rs4587164	6	34376703	T	G	0.7616	-0.3950	0.0625	2.55E-10	39.87
Plasma	*RPS18*	rs17215231	6	33239869	T	C	0.0640	-0.5863	0.0992	3.46E-09	34.8
Plasma	*RPS26*	rs1131017	12	56435929	G	C	0.5785	-1.1375	0.0405	1.25E-173	786.67
Plasma	*RXRB*	rs9268149	6	32263099	C	T	0.8391	0.3709	0.0668	2.83E-08	30.73
Plasma	*SIRT5*	rs62389221	6	12815161	G	A	0.0780	0.7586	0.0878	5.51E-18	74.46
Plasma	*STK19*	rs4355646	6	32386554	T	C	0.4782	-0.3075	0.0502	8.84E-10	37.45
Plasma	*STK19*	rs184003	6	32150296	A	C	0.1198	0.6833	0.0988	4.70E-12	47.66
Plasma	*SULF2*	rs2425967	20	46185378	C	T	0.5893	-0.4753	0.0493	5.21E-22	92.72
Plasma	*TIMM10*	rs2848626	11	57283988	T	C	0.7820	-0.4671	0.0570	2.50E-16	66.95
Plasma	*TMEM156*	rs2254075	4	39030183	G	A	0.6594	0.3730	0.0517	5.31E-13	51.92
Plasma	*TSPO*	rs138904	22	43544282	C	T	0.6985	-0.3336	0.0547	1.07E-09	37.08
Plasma	*TXNDC11*	rs4780395	16	11809940	G	A	0.5726	-0.3108	0.0504	7.23E-10	37.84
Plasma	*VARS2*	rs1639110	6	31191636	A	G	0.1893	0.5063	0.0575	1.38E-18	77.18
Plasma	*VARS2*	rs9266702	6	31349115	C	T	0.7677	0.5489	0.0551	2.12E-23	99.03
Plasma	*XXbac-BPG299F13.17*	rs1059510	6	30457732	C	T	0.6700	0.3114	0.0526	3.13E-09	34.99
Plasma	*YBEY*	rs914252	21	47719091	A	G	0.7029	0.4478	0.0548	3.06E-16	66.56
Plasma	*ZBTB22*	rs9276757	6	32772856	C	A	0.2266	-0.5154	0.0586	1.37E-18	77.19
Plasma	*ZP3*	rs11975972	7	76028869	T	C	0.4559	0.4602	0.0492	8.09E-21	87.31
CD4 ET	*ABHD16A*	rs9267658	6	31845985	C	T	0.8828	0.5193	0.0649	1.19E-15	63.95
CD4 ET	*ABRACL*	rs7769010	6	139369543	C	T	0.3595	-0.3125	0.0471	3.37E-11	43.86
CD4 ET	*AC006369.2*	rs4670742	2	37835943	G	A	0.5890	-0.6273	0.0432	1.04E-47	210.13
CD4 ET	*AC009961.3*	rs72965351	2	160629133	T	C	0.0847	-0.4565	0.0746	9.65E-10	37.32
CD4 ET	*AC013264.2*	rs10931780	2	198175297	C	G	0.3785	0.7243	0.0434	2.11E-62	277.38
CD4 ET	*AC016757.3*	rs113905150	2	239126774	G	A	0.3156	-0.5592	0.0463	1.50E-33	145.42
CD4 ET	*AC021860.1*	rs1000571	4	38662895	A	G	0.1981	0.3537	0.0538	4.80E-11	43.17
CD4 ET	*AC023590.1*	rs2514611	8	119665997	A	G	0.9210	-0.5383	0.0773	3.26E-12	48.43
CD4 ET	*AC097713.4*	rs10168797	2	235352768	C	A	0.5335	0.2706	0.0448	1.54E-09	36.4
CD4 ET	*AC104653.1*	rs4848353	2	114580423	G	A	0.5510	-0.5172	0.0441	8.53E-32	137.41
CD4 ET	*AC137932.1*	rs4785641	16	89317465	G	A	0.6150	0.2757	0.0461	2.29E-09	35.64
CD4 ET	*ACAT1*	rs2356518	11	107999603	G	A	0.3977	0.2790	0.0454	7.85E-10	37.72
CD4 ET	*ACCS*	rs2074038	11	44087989	T	G	0.1126	0.4159	0.0744	2.30E-08	31.16
CD4 ET	*ACTG1*	rs7503278	17	79479808	T	C	0.8099	0.4193	0.0568	1.50E-13	54.45
CD4 ET	*ACYP2*	rs6742042	2	54218703	C	T	0.4148	-0.4709	0.0447	6.22E-26	110.67
CD4 ET	*ADAMTS6*	rs154940	5	64902091	T	C	0.6113	-0.2651	0.0468	1.43E-08	32.08
CD4 ET	*AGA*	rs13149149	4	178360550	T	C	0.3350	-0.2599	0.0467	2.68E-08	30.87
CD4 ET	*AHI1*	rs13197384	6	135818897	A	C	0.3411	-0.4897	0.0465	6.31E-26	110.65
CD4 ET	*AIF1*	rs9267542	6	31668049	T	C	0.2456	-0.4244	0.0561	3.71E-14	57.2
CD4 ET	*AMICA1*	rs2298831	11	118068752	C	T	0.1596	0.4736	0.0646	2.32E-13	53.6
CD4 ET	*ANAPC13*	rs4435682	3	134344182	T	C	0.3338	-0.2693	0.0478	1.76E-08	31.68
CD4 ET	*ANKDD1A*	rs9920138	15	65164536	C	G	0.6284	-0.4641	0.0457	3.00E-24	103.01
CD4 ET	*ANKRD55*	rs7731626	5	55444683	A	G	0.3526	-0.3242	0.0470	5.26E-12	47.49
CD4 ET	*ANXA5*	rs6854854	4	122606919	C	T	0.0747	0.4560	0.0782	5.53E-09	33.92
CD4 ET	*ANXA6*	rs2303038	5	150535013	T	C	0.2046	0.3575	0.0546	5.93E-11	42.76
CD4 ET	*AP003774.1*	rs479777	11	64107477	C	T	0.3253	0.4386	0.0466	4.96E-21	88.37
CD4 ET	*ARHGAP15*	rs17812607	2	143980969	C	T	0.1490	-0.7112	0.0630	1.61E-29	127.02
CD4 ET	*ARHGEF10*	rs3817692	8	1807855	G	A	0.2947	-0.4717	0.0492	8.40E-22	91.87
CD4 ET	*ARHGEF3*	rs1489948	3	56757717	G	T	0.1634	0.4969	0.0618	9.24E-16	64.45
CD4 ET	*ARL14EP*	rs2065941	11	30363220	C	T	0.3028	-0.3599	0.0498	5.10E-13	52.06
CD4 ET	*ARL16*	rs12945187	17	79642556	C	T	0.2250	-0.5202	0.0521	1.70E-23	99.58
CD4 ET	*ARL17A*	rs557307834	17	44121579	C	T	0.2232	0.3221	0.0514	3.58E-10	39.25
CD4 ET	*ARPC5*	rs2761562	1	183580826	C	T	0.4212	0.3766	0.0450	5.77E-17	69.91
CD4 ET	*ATF7IP2*	rs8057149	16	10461090	C	A	0.5537	-0.2614	0.0457	1.03E-08	32.72
CD4 ET	*ATHL1*	rs12364171	11	296233	C	T	0.3728	0.2551	0.0461	3.03E-08	30.63
CD4 ET	*ATP5G2*	rs916134	12	54069803	G	A	0.2911	0.2806	0.0494	1.30E-08	32.26
CD4 ET	*ATP5G3*	rs268228	2	176046415	A	G	0.8115	-0.3412	0.0572	2.52E-09	35.45
CD4 ET	*ATP6V0E2*	rs4725834	7	149568262	G	A	0.5695	-0.3745	0.0454	1.71E-16	67.77
CD4 ET	*ATP6V1G2*	rs2853992	6	31378301	A	G	0.0715	-0.8009	0.0821	1.73E-22	94.99
CD4 ET	*B3GAT2*	rs495433	6	71606987	T	C	0.1306	-0.3737	0.0684	4.60E-08	29.82
CD4 ET	*B4GALT1*	rs6476398	9	33119241	T	C	0.3350	0.4378	0.0469	1.01E-20	86.96
CD4 ET	*BCAS4*	rs6702	20	49493609	A	G	0.3169	-0.2639	0.0481	4.19E-08	30
CD4 ET	*BIN1*	rs11682128	2	127839474	A	G	0.3433	0.4653	0.0464	1.23E-23	100.22
CD4 ET	*BRK1*	rs73117481	3	10160132	T	C	0.1184	-0.4177	0.0685	1.07E-09	37.12
CD4 ET	*BST2*	rs8102791	19	17505933	C	A	0.8036	0.3778	0.0629	1.86E-09	36.04
CD4 ET	*BTF3*	rs10942655	5	72674031	A	G	0.0712	-0.6709	0.0952	1.78E-12	49.61
CD4 ET	*BTN3A2*	rs67509210	6	26354100	T	G	0.1018	-0.7908	0.0651	6.43E-34	147.09
CD4 ET	*C10orf128*	rs2377997	10	50402624	T	C	0.3494	-0.4115	0.0466	1.00E-18	77.89
CD4 ET	*C10orf32*	rs11191436	10	104637602	T	C	0.2549	-0.3785	0.0517	2.44E-13	53.51
CD4 ET	*C17orf89*	rs2255166	17	79213562	C	T	0.4524	0.3094	0.0450	6.08E-12	47.21
CD4 ET	*C17orf97*	rs7502594	17	260182	C	T	0.2626	-0.7347	0.0499	4.57E-49	216.33
CD4 ET	*C19orf83*	rs2286756	19	46145025	T	G	0.5303	-0.2559	0.0449	1.17E-08	32.47
CD4 ET	*C1orf86*	rs11587831	1	2110848	G	T	0.2239	-0.3775	0.0544	3.84E-12	48.11
CD4 ET	*C1RL-AS1*	rs3782925	12	7262024	T	C	0.3193	0.2576	0.0472	4.66E-08	29.79
CD4 ET	*C22orf34*	rs1109320	22	49989106	A	G	0.5912	0.3373	0.0460	2.35E-13	53.58
CD4 ET	*C2orf27A*	rs7594112	2	132537672	G	T	0.3836	0.4712	0.0452	2.13E-25	108.24
CD4 ET	*C6orf48*	rs12529130	6	31756309	A	C	0.0930	-0.4865	0.0774	3.34E-10	39.38
CD4 ET	*C9orf135*	rs17517599	9	72410323	A	G	0.1191	0.7183	0.0698	7.34E-25	105.79
CD4 ET	*C9orf78*	rs71499491	9	132560991	A	G	0.2328	-0.3648	0.0524	3.40E-12	48.34

CD4 ET	*CA6*	rs4497235	1	8996316	T	A	0.0681	-0.5712	0.0967	3.51E-09	34.81
CD4 ET	*CAMK4*	rs114378220	5	110566360	T	C	0.0635	-0.5224	0.0892	4.66E-09	34.26
CD4 ET	*CAPG*	rs11696093	2	85636195	C	T	0.3028	0.3839	0.0471	3.52E-16	66.35
CD4 ET	*CARD9*	rs13294373	9	139854256	A	T	0.2530	0.6817	0.0488	2.36E-44	194.78
CD4 ET	*CASP7*	rs7067756	10	115516169	G	A	0.7604	0.3125	0.0513	1.09E-09	37.08
CD4 ET	*CASP8*	rs13016963	2	202162811	G	A	0.6165	0.3219	0.0465	4.38E-12	47.85
CD4 ET	*CAST*	rs10039522	5	95990869	A	G	0.1691	0.4271	0.0608	2.13E-12	49.26
CD4 ET	*CCDC122*	rs12874590	13	44433363	A	G	0.4840	0.3147	0.0447	1.99E-12	49.39
CD4 ET	*CCDC146*	rs28807140	7	76767073	C	T	0.1564	-0.3575	0.0584	9.18E-10	37.42
CD4 ET	*CCDC163P*	rs945179	1	45992460	T	C	0.6860	-0.4285	0.0466	3.93E-20	84.29
CD4 ET	*CCDC167*	rs1757178	6	37486310	G	A	0.6345	0.7343	0.0442	6.62E-62	275.1
CD4 ET	*CCDC58*	rs3828362	3	122131777	G	C	0.4138	0.3723	0.0452	1.70E-16	67.78
CD4 ET	*CCDC66*	rs7610922	3	56543750	A	G	0.5240	-0.3560	0.0446	1.40E-15	63.63
CD4 ET	*CCSER2*	rs61866511	10	86251388	A	G	0.1300	-0.6612	0.0684	4.45E-22	93.13
CD4 ET	*CCT3*	rs72710222	1	156285995	C	T	0.2805	-0.4134	0.0487	2.13E-17	71.88
CD4 ET	*CCT8*	rs965951	21	30432557	A	G	0.1160	1.0789	0.0644	5.89E-63	279.91
CD4 ET	*CCZ1*	rs7807287	7	6877519	G	A	0.4455	0.4697	0.0443	2.62E-26	112.38
CD4 ET	*CCZ1B*	rs4265086	7	6885597	T	C	0.4514	-0.4839	0.0441	4.50E-28	120.43
CD4 ET	*CD151*	rs1130276	11	831809	G	T	0.7386	0.4413	0.0524	3.62E-17	70.83
CD4 ET	*CD247*	rs840016	1	167408670	T	C	0.3750	0.2517	0.0458	3.95E-08	30.11
CD4 ET	*CD3G*	rs45508093	11	118224059	G	A	0.1823	-0.3839	0.0568	1.44E-11	45.53
CD4 ET	*CD52*	rs11583258	1	26624270	T	C	0.2918	-0.3284	0.0480	8.12E-12	46.64
CD4 ET	*CD59*	rs4756063	11	33865566	G	A	0.7195	0.2832	0.0495	1.03E-08	32.72
CD4 ET	*CDC42*	rs2268179	1	22414785	T	G	0.1644	0.4711	0.0603	5.43E-15	60.97
CD4 ET	*CDK10*	rs77651727	16	89708267	T	C	0.0591	-0.5637	0.0918	8.37E-10	37.6
CD4 ET	*CEACAM21*	rs3745936	19	42092815	A	T	0.7738	0.4366	0.0545	1.13E-15	64.06
CD4 ET	*CEBPZ-AS1*	rs11689186	2	37446288	G	A	0.1329	-0.4052	0.0658	7.49E-10	37.81
CD4 ET	*CELA1*	rs12829179	12	51719989	G	C	0.2883	0.4550	0.0474	8.87E-22	91.77
CD4 ET	*CENPK*	rs154940	5	64902091	T	C	0.6113	-1.0235	0.0404	7.71E-142	641.62
CD4 ET	*CFD*	rs34828577	19	867872	A	G	0.3375	0.4532	0.0480	3.73E-21	88.93
CD4 ET	*CHCHD2*	rs2908547	7	56176101	T	C	0.7881	-0.4313	0.0539	1.17E-15	63.99
CD4 ET	*CHI3L2*	rs11102223	1	111791134	T	G	0.5042	-0.2500	0.0448	2.46E-08	31.03
CD4 ET	*CHN1*	rs14228	2	175711942	T	C	0.4154	-0.2800	0.0456	8.02E-10	37.68
CD4 ET	*CHPT1*	rs9668831	12	102083778	C	A	0.6328	0.5413	0.0450	2.23E-33	144.63
CD4 ET	*CHURC1*	rs10129479	14	65375180	G	A	0.2134	0.4588	0.0574	1.38E-15	63.67
CD4 ET	*CIB1*	rs28455720	15	90790555	C	G	0.1712	-0.4959	0.0594	6.57E-17	69.66
CD4 ET	*CLEC2B*	rs253148	12	10014936	T	A	0.5510	0.3342	0.0448	8.84E-14	55.5
CD4 ET	*CLK4*	rs530542	5	178022626	G	A	0.4443	0.2458	0.0450	4.56E-08	29.84
CD4 ET	*CMTM7*	rs80258271	3	32501901	C	T	0.0905	-0.4480	0.0784	1.13E-08	32.54
CD4 ET	*CMTM8*	rs7632486	3	32286791	A	G	0.8213	-0.7172	0.0577	1.86E-35	154.12
CD4 ET	*CNN2*	rs4807455	19	1033559	A	G	0.2556	0.3894	0.0500	6.67E-15	60.57
CD4 ET	*CNOT6L*	rs885376	4	78632759	T	A	0.8959	-0.3794	0.0691	3.98E-08	30.1
CD4 ET	*COG5*	rs3779495	7	107250260	A	G	0.7662	-0.3484	0.0535	7.45E-11	42.31
CD4 ET	*COL18A1*	rs7280620	21	46899723	C	T	0.0738	1.1359	0.0852	1.47E-40	177.43
CD4 ET	*COL5A3*	rs9789339	19	10117055	T	C	0.3615	0.4553	0.0463	7.90E-23	96.54
CD4 ET	*COMMD6*	rs9543961	13	76106054	G	T	0.1381	-0.8355	0.0595	9.42E-45	196.6
CD4 ET	*CORO1B*	rs872375	11	67205978	A	G	0.4037	-0.4624	0.0447	4.04E-25	106.97
CD4 ET	*COX14*	rs7138945	12	50539419	G	T	0.3409	0.2543	0.0466	4.68E-08	29.79
CD4 ET	*COX20*	rs10927333	1	245003030	G	T	0.1728	0.3339	0.0608	4.05E-08	30.06
CD4 ET	*COX6B1*	rs4806187	19	36149761	G	A	0.5163	-0.3019	0.0447	1.43E-11	45.54
CD4 ET	*COX7A2L*	rs11895590	2	42406069	C	A	0.4092	0.2853	0.0450	2.30E-10	40.11
CD4 ET	*CPA5*	rs17164864	7	130008015	C	T	0.2126	0.9400	0.0508	1.64E-76	342.01
CD4 ET	*CPNE1*	rs61016611	20	34212151	A	G	0.1104	-0.6498	0.0758	9.81E-18	73.4
CD4 ET	*CRIP2*	rs4983411	14	105916797	A	G	0.6508	0.3341	0.0462	5.06E-13	52.07
CD4 ET	*CRYBB2*	rs6004662	22	25862124	A	G	0.0960	1.0745	0.0839	1.37E-37	163.86
CD4 ET	*CSTB*	rs35285321	21	45201832	A	G	0.3415	-0.3383	0.0482	2.29E-12	49.12
CD4 ET	*CTA-29F11.1*	rs809652	22	47145726	C	T	0.4194	-0.4722	0.0443	1.65E-26	113.31
CD4 ET	*CTB-129P6.4*	rs9797621	19	46166427	A	G	0.3506	-0.5909	0.0449	1.61E-39	172.67
CD4 ET	*CTC-241F20.3*	rs12984612	19	48759091	G	T	0.2207	-0.3261	0.0518	3.04E-10	39.57
CD4 ET	*CTC-524C5.2*	rs1050390	17	5336058	A	G	0.2364	0.5958	0.0533	4.97E-29	124.79
CD4 ET	*CTD-2020K17.4*	rs7212404	17	43317574	A	G	0.1718	-0.4371	0.0618	1.54E-12	49.89
CD4 ET	*CTD-2336O2.1*	rs4876133	8	1710404	C	G	0.7047	0.4026	0.0489	1.92E-16	67.55
CD4 ET	*CTD-2368P22.1*	rs4801243	19	58513807	C	A	0.2297	0.3980	0.0526	3.75E-14	57.18
CD4 ET	*CTD-2371O3.2*	rs1989293	11	9466274	C	T	0.5018	0.3633	0.0444	2.91E-16	66.73
CD4 ET	*CTD-2521M24.9*	rs2303678	19	17533126	G	A	0.7706	0.3673	0.0582	2.69E-10	39.8
CD4 ET	*CTLA4*	rs3087243	2	204738919	A	G	0.4420	0.5416	0.0436	2.32E-35	153.69
CD4 ET	*CTSL*	rs3128507	9	90335288	G	A	0.6371	-0.3635	0.0460	2.83E-15	62.26
CD4 ET	*CTSW*	rs596002	11	65645354	G	A	0.8002	0.7419	0.0544	2.14E-42	185.83
CD4 ET	*DAD1*	rs112795829	14	23044597	A	G	0.0936	0.5364	0.0758	1.52E-12	49.91
CD4 ET	*DARS*	rs60397494	2	136470219	T	C	0.1846	0.4111	0.0699	4.07E-09	34.52
CD4 ET	*DAZAP1*	rs2074897	19	1391235	A	G	0.4470	-0.3073	0.0447	5.88E-12	47.27
CD4 ET	*DAZAP2*	rs7305016	12	51642543	G	A	0.6191	-0.2851	0.0466	9.11E-10	37.43
CD4 ET	*DCP2*	rs2052484	5	112343544	A	G	0.7740	-0.3084	0.0543	1.35E-08	32.19
CD4 ET	*DCXR*	rs8069378	17	80001041	T	C	0.1239	0.4783	0.0671	1.03E-12	50.68
CD4 ET	*DDT*	rs5751777	22	24267047	T	C	0.5906	0.4192	0.0455	3.22E-20	84.68
CD4 ET	*DDX11*	rs4537846	12	31213631	T	C	0.4481	-0.5106	0.0440	3.41E-31	134.66
CD4 ET	*DDX43*	rs3005486	6	74523948	T	C	0.6212	0.5701	0.0448	4.97E-37	161.3
CD4 ET	*DDX5*	rs1991401	17	62502435	G	A	0.3173	-0.4327	0.0479	1.67E-19	81.42
CD4 ET	*DHRS4*	rs80326273	14	24461134	A	G	0.0909	0.6776	0.0814	8.41E-17	69.17

CD4 ET	*DHX32*	rs11244668	10	127531959	T	C	0.4189	0.2982	0.0450	3.44E-11	43.82
CD4 ET	*DNAJC15*	rs2281778	13	43597977	G	C	0.2269	-0.7826	0.0511	4.80E-53	234.54
CD4 ET	*DOCK8*	rs2094462	9	275809	G	A	0.2080	-0.3823	0.0532	6.81E-13	51.49
CD4 ET	*DPYSL4*	rs2814156	10	134011660	G	A	0.0970	0.4885	0.0758	1.13E-10	41.49
CD4 ET	*DRAM2*	rs3795823	1	111726869	A	G	0.2726	0.3726	0.0481	9.61E-15	59.85
CD4 ET	*DYNLL2*	rs9896162	17	56147958	C	T	0.6546	-0.2792	0.0469	2.58E-09	35.4
CD4 ET	*EBPL*	rs7983918	13	50266574	C	A	0.5395	-0.6081	0.0434	1.19E-44	196.14
CD4 ET	*EEF1D*	rs12678314	8	144659608	G	A	0.7490	-0.3104	0.0528	4.24E-09	34.44
CD4 ET	*EIF2A*	rs14434	3	150302010	T	C	0.8405	0.6016	0.0626	7.14E-22	92.19
CD4 ET	*EIF5A*	rs7503161	17	7207964	C	A	0.5615	-1.1152	0.0371	8.50E-199	903.04
CD4 ET	*ELMSAN1*	rs17182544	14	74206855	G	C	0.2930	-0.2683	0.0476	1.74E-08	31.7
CD4 ET	*ELP5*	rs222851	17	7139238	A	G	0.3873	0.4346	0.0451	5.01E-22	92.9
CD4 ET	*EMB*	rs7293370	5	49525181	A	G	0.1137	0.4126	0.0740	2.52E-08	30.98
CD4 ET	*EML4*	rs12467012	2	42605840	C	T	0.4623	-0.2488	0.0450	3.31E-08	30.46
CD4 ET	*EMR1*	rs456320	19	6945141	G	A	0.5217	-0.3343	0.0446	6.57E-14	56.08
CD4 ET	*ENOSF1*	rs7235957	18	717229	C	T	0.5497	-0.2961	0.0451	4.94E-11	43.11
CD4 ET	*ENTPD1*	rs7077566	10	97617943	A	G	0.5211	0.4194	0.0443	2.71E-21	89.56
CD4 ET	*EPHA1-AS1*	rs7794735	7	143104816	A	G	0.0829	-0.5416	0.0792	7.97E-12	46.68
CD4 ET	*EPSTI1*	rs3825511	13	43566373	C	A	0.7178	-0.5437	0.0496	6.16E-28	119.81
CD4 ET	*ERAP2*	rs2927608	5	96252432	A	G	0.4214	0.9745	0.0393	9.57E-136	613.66
CD4 ET	*ERICH1*	rs1703911	8	601043	A	G	0.3541	-0.3453	0.0469	1.73E-13	54.18
CD4 ET	*ESR2*	rs61174206	14	64696200	A	C	0.0741	0.8340	0.0945	1.09E-18	77.73
CD4 ET	*EVI2A*	rs12943365	17	29680526	G	C	0.6267	0.4056	0.0459	1.04E-18	77.83
CD4 ET	*EXOC3*	rs67665078	5	428754	A	G	0.4621	0.3951	0.0443	4.73E-19	79.38
CD4 ET	*FADS1*	rs174548	11	61571348	G	C	0.3094	-0.4023	0.0483	8.72E-17	69.1
CD4 ET	*FADS2*	rs61896141	11	61556039	C	A	0.1516	0.4651	0.0574	5.09E-16	65.63
CD4 ET	*FAM118A*	rs136582	22	45774531	A	T	0.1332	1.5155	0.0589	5.10E-146	660.8
CD4 ET	*FAM153B*	rs62389203	5	175510135	T	C	0.4271	-0.4504	0.0442	2.32E-24	103.52
CD4 ET	*FAM184B*	rs28609167	4	17624887	T	G	0.6072	-0.8524	0.0418	1.80E-92	415.17
CD4 ET	*FAM21A*	rs11003495	10	51806275	C	G	0.6010	-0.2549	0.0458	2.70E-08	30.85
CD4 ET	*FAM26F*	rs9387418	6	116851907	A	C	0.4877	0.4771	0.0439	1.68E-27	117.82
CD4 ET	*FAR1*	rs11022929	11	13678419	G	A	0.3329	0.4213	0.0474	6.30E-19	78.81
CD4 ET	*FAU*	rs544944	11	64862234	T	C	0.4907	0.3437	0.0446	1.29E-14	59.27
CD4 ET	*FBLN5*	rs2267989	14	92339850	A	G	0.4380	-0.5408	0.0436	2.43E-35	153.59
CD4 ET	*FDFT1*	rs1293299	8	11677905	C	A	0.2173	-0.4814	0.0541	5.44E-19	79.1
CD4 ET	*FGF9*	rs7994761	13	22871446	G	A	0.2251	0.3403	0.0541	3.07E-10	39.55
CD4 ET	*FGFRL1*	rs6599390	4	956047	G	A	0.6780	0.4917	0.0474	3.30E-25	107.37
CD4 ET	*FHL3*	rs4360494	1	38455891	C	G	0.5241	-0.3170	0.0447	1.33E-12	50.19
CD4 ET	*FIS1*	rs10230349	7	100897341	C	T	0.0593	-0.5900	0.0929	2.17E-10	40.23
CD4 ET	*FKBP11*	rs10783297	12	49328739	T	C	0.4525	-0.3445	0.0449	1.69E-14	58.74
CD4 ET	*FKBPL*	rs3130696	6	31243884	A	G	0.2467	-0.3323	0.0504	4.17E-11	43.44
CD4 ET	*FLVCR1-AS1*	rs11120049	1	213033642	A	T	0.5074	0.3092	0.0447	4.44E-12	47.82
CD4 ET	*FMN1*	rs345766	15	33403103	C	A	0.2708	0.8211	0.0503	8.26E-60	265.5
CD4 ET	*FN3KRP*	rs3859206	17	80693128	A	G	0.4776	-0.3329	0.0449	1.17E-13	54.95
CD4 ET	*FNBP4*	rs10838786	11	47904086	A	G	0.4616	-0.2633	0.0448	4.18E-09	34.47
CD4 ET	*FTCDNL1*	rs2346585	2	200625156	G	C	0.6585	0.4563	0.0465	1.07E-22	95.95
CD4 ET	*GBP5*	rs55870241	1	89742521	C	T	0.5445	-0.4091	0.0445	3.62E-20	84.44
CD4 ET	*GBP7*	rs495163	1	89648082	A	G	0.4996	0.6291	0.0428	7.62E-49	215.32
CD4 ET	*GCAT*	rs6000899	22	38203796	T	C	0.3177	-0.2707	0.0491	3.60E-08	30.29
CD4 ET	*GCC2*	rs7606621	2	109312728	T	C	0.4010	-0.5226	0.0444	6.04E-32	138.09
CD4 ET	*GDI2*	rs56251728	10	5793444	G	A	0.1973	-0.4194	0.0542	1.03E-14	59.71
CD4 ET	*GDPD5*	rs1783557	11	75219277	C	T	0.8597	-0.5260	0.0658	1.37E-15	63.68
CD4 ET	*GIPC1*	rs116092141	19	14606710	G	C	0.3651	-0.2797	0.0460	1.19E-09	36.9
CD4 ET	*GLTSCR2*	rs3786779	19	48255284	A	G	0.7093	-0.3448	0.0501	5.77E-12	47.31
CD4 ET	*GNG5*	rs6684882	1	84948165	G	A	0.1067	0.4584	0.0750	9.99E-10	37.25
CD4 ET	*GNLY*	rs12151752	2	85934652	C	T	0.2327	0.3179	0.0533	2.48E-09	35.48
CD4 ET	*GNPTG*	rs57890635	16	1411129	T	C	0.2668	0.3216	0.0534	1.71E-09	36.21
CD4 ET	*GPR160*	rs9875531	3	169730124	G	A	0.1806	0.3927	0.0625	3.34E-10	39.38
CD4 ET	*GPR63*	rs6930393	6	98118642	C	G	0.6604	0.9407	0.0445	2.12E-99	446.94
CD4 ET	*GPX4*	rs2074447	19	1095581	G	A	0.2236	-0.4108	0.0533	1.30E-14	59.26
CD4 ET	*GRHPR*	rs4878690	9	37422656	T	C	0.3290	0.4787	0.0470	2.37E-24	103.48
CD4 ET	*GSTM3*	rs4970777	1	110283785	T	C	0.5798	0.5831	0.0438	2.36E-40	176.49
CD4 ET	*GSTM4*	rs1010167	1	110198727	G	C	0.4142	-0.5123	0.0445	1.19E-30	132.18
CD4 ET	*GSTO1*	rs11191967	10	106003861	T	C	0.3084	0.3251	0.0492	3.86E-11	43.59
CD4 ET	*GSTP1*	rs7103713	11	67338242	A	G	0.4054	-0.4987	0.0442	1.76E-29	126.85
CD4 ET	*GSTT1*	rs2097433	22	24296894	G	A	0.5338	0.5806	0.0436	2.01E-40	176.81
CD4 ET	*GTF2H2*	rs28478097	5	70309998	G	C	0.6121	-0.4578	0.0453	4.60E-24	102.16
CD4 ET	*GTF2H4*	rs77580993	6	30059592	G	C	0.2142	0.6313	0.0542	2.53E-31	135.25
CD4 ET	*GTF2H4*	rs2517580	6	30810056	A	G	0.2645	-0.3050	0.0512	2.48E-09	35.48
CD4 ET	*GTF2H4*	rs2524277	6	31407579	A	G	0.0401	0.9722	0.0965	7.05E-24	101.32
CD4 ET	*GTF3A*	rs1475219	13	28037415	T	C	0.7950	0.3249	0.0548	3.04E-09	35.09
CD4 ET	*GYPC*	rs28387094	2	127417987	T	C	0.5613	0.2759	0.0450	9.06E-10	37.44
CD4 ET	*GYPC*	rs74499727	2	127448415	T	C	0.3330	0.2711	0.0474	1.07E-08	32.64
CD4 ET	*GYPC*	rs35651363	2	127519838	A	G	0.2707	0.6020	0.0500	2.03E-33	144.82
CD4 ET	*GZMM*	rs16990714	19	543497	T	C	0.0484	-0.6489	0.1026	2.54E-10	39.92
CD4 ET	*HAGH*	rs238677	16	1883279	C	T	0.3287	0.3535	0.0492	6.99E-13	51.44
CD4 ET	*HCG25*	rs2894251	6	32343236	G	A	0.1010	-0.8823	0.0646	1.87E-42	186.1
CD4 ET	*HCG25*	rs9272553	6	32607008	T	G	0.2882	-0.3127	0.0452	4.50E-12	47.8
CD4 ET	*HCG25*	rs3132136	6	32853987	A	G	0.3927	-0.2741	0.0449	1.03E-09	37.18

CD4 ET	*HEBP2*	rs9495064	6	138709209	G	A	0.0731	-0.5128	0.0915	2.06E-08	31.37
CD4 ET	*HFE*	rs13191659	6	27001055	G	C	0.0592	0.7296	0.0804	1.11E-19	82.24
CD4 ET	*HIGD2A*	rs1065212	5	175811133	C	T	0.0955	-0.7857	0.0840	8.24E-21	87.37
CD4 ET	*HIST1H2BG*	rs13191296	6	25684606	T	C	0.0627	-0.4721	0.0795	2.86E-09	35.2
CD4 ET	*HIST1H2BH*	rs72839445	6	27249686	A	G	0.0719	0.7396	0.0759	1.97E-22	94.74
CD4 ET	*HIST1H4B*	rs66975207	6	26942146	C	A	0.0737	-0.5057	0.0762	3.18E-11	43.97
CD4 ET	*HIVEP3*	rs783629	1	42363275	T	C	0.5567	-0.3720	0.0445	6.74E-17	69.6
CD4 ET	*HLA-A*	rs2571389	6	29923503	C	T	0.5408	0.3587	0.0445	7.00E-16	65
CD4 ET	*HLA-B*	rs2524096	6	31236467	T	G	0.4422	0.7319	0.0424	1.10E-66	296.98
CD4 ET	*HLA-B*	rs9366777	6	31264715	A	T	0.0923	-0.5748	0.0826	3.47E-12	48.3
CD4 ET	*HLA-B*	rs3828879	6	31379391	T	C	0.1430	-0.5687	0.0653	2.95E-18	75.77
CD4 ET	*HLA-C*	rs3931670	6	31243767	C	G	0.2891	0.4800	0.0507	3.11E-21	89.29
CD4 ET	*HLA-DQA2*	rs9272105	6	32599999	A	G	0.5403	0.3103	0.0446	3.61E-12	48.23
CD4 ET	*HLA-DQB1*	rs4455710	6	32608858	T	C	0.3614	-0.2650	0.0456	6.39E-09	33.64
CD4 ET	*HLA-DRB5*	rs9272070	6	32599485	G	T	0.5192	-0.3684	0.0444	1.08E-16	68.68
CD4 ET	*HMBOX1*	rs10088428	8	28909523	T	C	0.2597	-0.5795	0.0505	1.61E-30	131.58
CD4 ET	*HOXB-AS1*	rs6504281	17	46565412	T	C	0.7740	0.4593	0.0551	8.16E-17	69.23
CD4 ET	*HOXB2*	rs11079826	17	46597048	T	C	0.4867	0.5324	0.0435	2.14E-34	149.28
CD4 ET	*HOXB4*	rs2325948	17	46574491	T	C	0.4838	0.3005	0.0447	1.76E-11	45.13
CD4 ET	*HSPA1B*	rs1144709	6	31709349	T	C	0.2765	0.3313	0.0560	3.31E-09	34.92
CD4 ET	*HSPBAP1*	rs12496372	3	122473431	G	C	0.7450	-0.5293	0.0533	2.84E-23	98.57
CD4 ET	*IAH1*	rs10176656	2	9588091	G	A	0.2019	-0.2980	0.0546	4.94E-08	29.68
CD4 ET	*IFI27L2*	rs2273514	14	94595989	C	T	0.2022	-0.6604	0.0537	9.60E-35	150.87
CD4 ET	*IFITM2*	rs10398	11	308180	G	A	0.2135	0.6054	0.0570	2.59E-26	112.41
CD4 ET	*IFITM3*	rs7115703	11	306920	A	T	0.5538	0.6539	0.0427	4.80E-53	234.54
CD4 ET	*IFNG-AS1*	rs3015001	12	68397373	G	C	0.6797	-0.3030	0.0493	7.85E-10	37.72
CD4 ET	*IGFLR1*	rs2239947	19	36244753	C	G	0.1568	-0.3809	0.0637	2.26E-09	35.66
CD4 ET	*IL32*	rs11861531	16	3116325	C	T	0.5537	0.4266	0.0442	4.69E-22	93.02
CD4 ET	*INTS5*	rs11231184	11	62447563	A	C	0.2753	0.2736	0.0490	2.35E-08	31.12
CD4 ET	*ISCA2*	rs10873267	14	74941081	G	A	0.5998	-0.3708	0.0452	2.31E-16	67.18
CD4 ET	*ISCU*	rs10778643	12	108939901	A	T	0.8652	-0.3781	0.0652	6.65E-09	33.57
CD4 ET	*ISG15*	rs1921	1	949608	A	G	0.3869	0.2546	0.0459	2.84E-08	30.75
CD4 ET	*ITGB2*	rs760462	21	46328099	C	T	0.8298	-0.9294	0.0572	1.88E-59	263.87
CD4 ET	*ITGB3BP*	rs7550087	1	63958446	C	A	0.3054	0.4046	0.0478	2.72E-17	71.39
CD4 ET	*ITGB7*	rs12824946	12	53592452	C	T	0.1012	0.5880	0.0807	3.09E-13	53.04
CD4 ET	*JAKMIP1*	rs28380276	4	6173392	C	A	0.5463	-0.2770	0.0451	8.07E-10	37.67
CD4 ET	*JAKMIP2*	rs2436340	5	147162475	A	G	0.3562	0.2947	0.0463	1.90E-10	40.49
CD4 ET	*KANSL1-AS1*	rs55974014	17	43757450	A	C	0.1994	1.2319	0.0459	5.38E-159	720.35
CD4 ET	*KCNAB2*	rs560251	1	6062797	A	G	0.2293	0.3142	0.0523	1.90E-09	36
CD4 ET	*KCTD21-AS1*	rs637149	11	77865961	G	A	0.3608	-0.4958	0.0451	4.13E-28	120.6
CD4 ET	*KDELR2*	rs6796	7	6502367	C	T	0.2687	-0.4491	0.0488	3.28E-20	84.64
CD4 ET	*KIAA0040*	rs760486	1	175137376	T	G	0.3688	0.4025	0.0462	3.19E-18	75.62
CD4 ET	*KIAA1430*	rs2030800	4	186124855	G	A	0.6308	-0.3038	0.0473	1.37E-10	41.12
CD4 ET	*KISS1R*	rs2930891	19	883224	G	A	0.2988	0.2861	0.0498	8.92E-09	33
CD4 ET	*KNOP1*	rs1016971	16	19718725	A	T	0.1759	0.3364	0.0578	5.77E-09	33.84
CD4 ET	*KRT1*	rs2741159	12	53071382	C	A	0.3815	0.6131	0.0436	6.20E-45	197.43
CD4 ET	*KRTCAP3*	rs1647267	2	27651054	T	A	0.3854	0.3129	0.0458	8.14E-12	46.64
CD4 ET	*KTN1*	rs1138345	14	56078739	G	T	0.3035	-0.4505	0.0480	6.79E-21	87.75
CD4 ET	*L3MBTL3*	rs7740107	6	130374461	A	T	0.7512	-0.4252	0.0494	7.68E-18	73.88
CD4 ET	*LA16c-358B7.3*	rs62011260	16	1358561	G	T	0.6087	-0.3658	0.0452	6.30E-16	65.21
CD4 ET	*LAIR2*	rs149731338	19	55020233	T	C	0.1170	-0.3976	0.0673	3.53E-09	34.79
CD4 ET	*LAMB1*	rs13222925	7	107583241	A	G	0.3823	-0.3342	0.0453	1.65E-13	54.27
CD4 ET	*LAMTOR4*	rs11768171	7	99733315	T	C	0.5401	-0.2500	0.0450	2.66E-08	30.88
CD4 ET	*LAX1*	rs7547110	1	203742253	C	G	0.4253	-0.3135	0.0455	5.48E-12	47.41
CD4 ET	*LCLAT1*	rs829636	2	30737801	G	T	0.2274	0.4906	0.0502	1.59E-22	95.16
CD4 ET	*LDLRAP1*	rs61776822	1	25885592	A	G	0.5057	-0.4343	0.0441	7.10E-23	96.76
CD4 ET	*LEPROT*	rs6658922	1	65901208	A	G	0.4061	-0.3411	0.0454	5.39E-14	56.47
CD4 ET	*LGALS3*	rs9919921	14	55695505	G	A	0.3072	0.3835	0.0466	1.84E-16	67.63
CD4 ET	*LIMD1*	rs34355059	3	45682897	T	C	0.2611	0.2708	0.0485	2.33E-08	31.14
CD4 ET	*LINC00176*	rs817343	20	62607160	T	C	0.1914	1.2553	0.0539	3.79E-120	542.09
CD4 ET	*LINC00339*	rs4654783	1	22439520	C	T	0.7035	0.3384	0.0494	7.43E-12	46.81
CD4 ET	*LINC00649*	rs8130507	21	35303960	C	T	0.6025	0.2940	0.0458	1.40E-10	41.08
CD4 ET	*LINC00852*	rs42451	3	10330377	T	C	0.2557	0.4151	0.0492	3.36E-17	70.98
CD4 ET	*LINC00944*	rs1706506	12	127256415	T	C	0.9383	-0.6299	0.1026	8.28E-10	37.62
CD4 ET	*LINC00954*	rs13034115	2	20077653	G	C	0.1396	-0.4954	0.0624	2.01E-15	62.93
CD4 ET	*LINC00969*	rs3827561	3	195674257	G	C	0.4166	0.4551	0.0449	4.28E-24	102.31
CD4 ET	*LMAN1*	rs4108696	18	56980847	T	G	0.2838	0.2814	0.0498	1.58E-08	31.89
CD4 ET	*LPIN1*	rs3762583	2	11924293	A	G	0.1508	-0.4306	0.0651	3.71E-11	43.67
CD4 ET	*LPIN2*	rs9963737	18	3059805	C	T	0.4544	0.2493	0.0448	2.71E-08	30.84
CD4 ET	*LRRC16A*	rs9366619	6	25399005	C	A	0.2715	0.2772	0.0492	1.80E-08	31.63
CD4 ET	*LRRC61*	rs2098053	7	150043205	A	G	0.2477	0.3140	0.0518	1.37E-09	36.63
CD4 ET	*LRRIQ3*	rs524306	1	74682253	T	A	0.4715	0.5583	0.0433	5.85E-38	165.55
CD4 ET	*LSM7*	rs12977961	19	2326897	T	C	0.6020	0.2841	0.0459	5.98E-10	38.25
CD4 ET	*LYRM4*	rs72811688	6	5170707	T	C	0.0814	0.6265	0.0765	2.54E-16	66.99
CD4 ET	*MAF1*	rs56138570	8	145142206	A	G	0.0926	-0.4631	0.0765	1.45E-09	36.53
CD4 ET	*MANBA*	rs10013062	4	103930220	G	A	0.3782	-0.3066	0.0467	5.36E-11	42.95
CD4 ET	*MCC*	rs888844	5	112371641	T	C	0.7470	-0.3646	0.0521	2.53E-12	48.93
CD4 ET	*MDGA1*	rs6458012	6	37661041	A	G	0.6782	0.3338	0.0469	1.14E-12	50.49
CD4 ET	*MDM4*	rs3014606	1	204464150	G	C	0.2631	0.3052	0.0499	9.63E-10	37.32

CD4 ET	*MED16*	rs1651888	19	873743	A	G	0.2929	0.5494	0.0490	3.25E-29	125.63
CD4 ET	*METTL21B*	rs701008	12	58117645	C	T	0.3581	-0.3583	0.0461	7.36E-15	60.38
CD4 ET	*MGMT*	rs11016847	10	131412157	A	G	0.2693	-0.2905	0.0493	3.74E-09	34.69
CD4 ET	*MGST2*	rs4863672	4	140589059	A	T	0.4859	-0.3654	0.0447	2.81E-16	66.8
CD4 ET	*MGST3*	rs10918223	1	165599649	T	C	0.4790	-0.4544	0.0440	5.33E-25	106.43
CD4 ET	*MORN3*	rs28651135	12	122025332	T	C	0.4094	0.2611	0.0457	1.13E-08	32.54
CD4 ET	*MPHOSPH6*	rs2303260	16	82203786	G	C	0.4801	-0.3742	0.0444	3.51E-17	70.89
CD4 ET	*MPZL3*	rs647573	11	118067132	T	C	0.5691	-0.2837	0.0459	6.21E-10	38.18
CD4 ET	*MRPL21*	rs71465408	11	68617022	G	A	0.3006	-0.4121	0.0484	1.62E-17	72.41
CD4 ET	*MRPL34*	rs71338624	19	17414777	G	C	0.3915	0.3324	0.0450	1.45E-13	54.52
CD4 ET	*MRPL54*	rs13960	19	3762698	T	C	0.6148	0.4047	0.0460	1.41E-18	77.22
CD4 ET	*MRPS18C*	rs6535473	4	84364808	C	T	0.4873	0.3380	0.0445	3.28E-14	57.44
CD4 ET	*MSH5*	rs2844632	6	31227901	A	G	0.6289	0.4585	0.0458	1.26E-23	100.17
CD4 ET	*MSH5*	rs9271927	6	32596647	C	T	0.3993	0.3166	0.0448	1.59E-12	49.83
CD4 ET	*MT1E*	rs1610215	16	56659525	G	C	0.5423	0.5293	0.0437	9.34E-34	146.36
CD4 ET	*MT1F*	rs1875231	16	56683745	C	A	0.3943	0.3957	0.0449	1.29E-18	77.41
CD4 ET	*MT1X*	rs1599933	16	56715862	A	C	0.5528	-0.3884	0.0446	2.95E-18	75.77
CD4 ET	*MT2A*	rs1580833	16	56644650	A	C	0.3093	-0.5547	0.0472	7.74E-32	137.6
CD4 ET	*MYH11*	rs881803	16	15802334	T	C	0.6642	-0.3498	0.0473	1.40E-13	54.6
CD4 ET	*MYOM2*	rs7843362	8	2068034	C	T	0.1990	0.7744	0.0580	1.17E-40	177.88
CD4 ET	*MZT2A*	rs1815205	2	132273621	A	G	0.2274	0.7649	0.0542	3.06E-45	198.83
CD4 ET	*NAA38*	rs2896242	7	117824239	C	G	0.6922	-0.3817	0.0472	5.84E-16	65.36
CD4 ET	*NAAA*	rs13138223	4	76859615	T	A	0.3537	0.2889	0.0462	4.01E-10	39.03
CD4 ET	*NAP1L1*	rs1433364	12	76464637	G	A	0.8079	-0.4624	0.0580	1.62E-15	63.35
CD4 ET	*NAPRT1*	rs9657360	8	144676862	T	C	0.7776	0.7240	0.0531	2.71E-42	185.36
CD4 ET	*NBL1*	rs12408663	1	19968058	C	T	0.2431	-0.3246	0.0516	3.09E-10	39.54
CD4 ET	*NCAPG2*	rs73167215	7	158500207	A	G	0.0611	0.5129	0.0846	1.34E-09	36.67
CD4 ET	*NCF2*	rs2182595	1	183661032	G	A	0.6524	-0.5241	0.0453	5.39E-31	133.75
CD4 ET	*NCF4*	rs4821546	22	37268079	G	A	0.7127	-0.3640	0.0500	3.37E-13	52.87
CD4 ET	*NCOA4*	rs10826227	10	51539256	C	T	0.5458	0.2538	0.0450	1.70E-08	31.75
CD4 ET	*NDUFA12*	rs835044	12	95397552	C	T	0.6738	-0.6122	0.0453	1.26E-41	182.31
CD4 ET	*NDUFA3*	rs75622830	19	54611734	G	A	0.0957	0.6167	0.0741	8.66E-17	69.11
CD4 ET	*NDUFS5*	rs4147750	1	39474057	C	A	0.2805	-0.4190	0.0477	1.48E-18	77.13
CD4 ET	*NINJ2*	rs11064141	12	774492	C	T	0.5540	-0.3032	0.0447	1.25E-11	45.8
CD4 ET	*NME1*	rs7207370	17	49231180	G	A	0.3899	0.3439	0.0456	4.96E-14	56.63
CD4 ET	*NME4*	rs77371232	16	447561	T	A	0.4244	0.3824	0.0449	1.51E-17	72.55
CD4 ET	*NMI*	rs55775354	2	152118271	A	G	0.4344	0.3087	0.0447	5.24E-12	47.5
CD4 ET	*NMRK1*	rs3752955	9	77684690	C	A	0.5423	-0.4704	0.0442	1.97E-26	112.94
CD4 ET	*NMT2*	rs10906857	10	15239498	G	A	0.4079	-0.4127	0.0448	3.04E-20	84.79
CD4 ET	*NPC2*	rs4903231	14	74962233	A	G	0.5967	-0.4392	0.0448	1.11E-22	95.87
CD4 ET	*NPDC1*	rs10747042	9	139938606	C	T	0.9026	0.5683	0.0789	6.01E-13	51.74
CD4 ET	*NPHP1*	rs72826803	2	110354383	C	G	0.0486	-0.9552	0.0943	4.16E-24	102.36
CD4 ET	*NQO2*	rs2518581	6	3009944	G	A	0.7344	-0.4500	0.0521	5.37E-18	74.59
CD4 ET	*NR2F6*	rs66606313	19	17345427	C	G	0.5369	-0.2836	0.0450	2.83E-10	39.71
CD4 ET	*NSA2*	rs6877188	5	74031525	A	G	0.1366	0.8338	0.0639	6.30E-39	169.97
CD4 ET	*NSG1*	rs55731118	4	4350915	T	A	0.4050	0.5442	0.0445	2.00E-34	149.42
CD4 ET	*NT5C3A*	rs10951371	7	33092349	G	A	0.6951	-0.2725	0.0494	3.53E-08	30.33
CD4 ET	*NT5C3B*	rs9897034	17	39992584	T	C	0.7555	-0.5230	0.0503	2.40E-25	108.01
CD4 ET	*NTPCR*	rs58790175	1	233061703	T	C	0.1806	0.5716	0.0558	1.35E-24	104.59
CD4 ET	*NUDT2*	rs10814108	9	34374627	C	T	0.1692	0.7448	0.0598	1.38E-35	154.71
CD4 ET	*OCEL1*	rs2288544	19	17337025	T	C	0.2194	-0.3206	0.0550	5.57E-09	33.91
CD4 ET	*ORMDL3*	rs35736272	17	38032680	C	T	0.4591	-0.3978	0.0444	3.36E-19	80.05
CD4 ET	*OSTF1*	rs10869496	9	77700084	A	C	0.3854	-0.2685	0.0455	3.65E-09	34.73
CD4 ET	*OXLD1*	rs11150804	17	79630238	G	A	0.3735	-0.4353	0.0461	3.84E-21	88.87
CD4 ET	*PARK7*	rs225126	1	8090615	G	A	0.6024	0.2832	0.0453	4.22E-10	38.93
CD4 ET	*PASK*	rs3815296	2	242050546	G	A	0.1835	-0.8148	0.0588	1.18E-43	191.59
CD4 ET	*PDIA6*	rs1686482	2	10922516	C	A	0.4782	-0.2551	0.0449	1.38E-08	32.16
CD4 ET	*PDXK*	rs2276528	21	45152500	A	G	0.1480	0.4950	0.0618	1.13E-15	64.06
CD4 ET	*PEX6*	rs3805946	6	42955749	C	T	0.4771	0.2553	0.0448	1.24E-08	32.36
CD4 ET	*PHF11*	rs2274276	13	50095953	G	C	0.5772	0.2502	0.0453	3.29E-08	30.46
CD4 ET	*PHLDB3*	rs34536923	19	44005445	T	C	0.1877	0.3521	0.0579	1.21E-09	36.87
CD4 ET	*PI16*	rs734905	6	36927763	A	C	0.1570	0.4499	0.0588	1.96E-14	58.45
CD4 ET	*PIGP*	rs9975361	21	38450025	C	T	0.5127	-0.2953	0.0447	3.98E-11	43.53
CD4 ET	*PILRB*	rs6955367	7	99961194	G	A	0.1838	0.6867	0.0549	7.49E-36	155.93
CD4 ET	*PKIA*	rs35851166	8	79481806	A	C	0.2057	-0.3404	0.0582	4.92E-09	34.15
CD4 ET	*PLEKHA1*	rs2421017	10	124148167	G	A	0.4972	-0.2826	0.0448	2.77E-10	39.75
CD4 ET	*PLGRKT*	rs9785210	9	5372048	C	A	0.6660	-0.2722	0.0470	6.88E-09	33.5
CD4 ET	*PNRC2*	rs17184588	1	24365512	C	T	0.4701	-0.3234	0.0446	4.08E-13	52.5
CD4 ET	*POLM*	rs77962201	7	44131834	T	C	0.0963	0.4451	0.0768	6.90E-09	33.49
CD4 ET	*POLR1D*	rs2232669	13	28195495	T	C	0.1167	0.7653	0.0650	5.20E-32	138.39
CD4 ET	*POLR2J3*	rs2537466	7	102394415	T	G	0.1468	0.3779	0.0613	6.93E-10	37.96
CD4 ET	*POMZP3*	rs146021635	7	76179719	A	C	0.2083	0.4405	0.0544	5.58E-16	65.45
CD4 ET	*PPA1*	rs7072230	10	71978144	G	A	0.3205	0.8243	0.0440	3.42E-78	349.71
CD4 ET	*PPDPF*	rs72629024	20	62152519	G	C	0.1633	-0.9966	0.0585	4.80E-65	289.48
CD4 ET	*PPFIBP2*	rs7126061	11	7548423	A	G	0.6036	0.4271	0.0456	6.92E-21	87.71
CD4 ET	*PPIA*	rs6850	7	44836314	G	A	0.1572	0.5444	0.0685	2.00E-15	62.94
CD4 ET	*PPIL3*	rs7559150	2	201754063	C	T	0.2458	-0.6077	0.0518	8.02E-32	137.53
CD4 ET	*PPP1R15B*	rs12061474	1	204389314	T	C	0.1101	-0.4043	0.0737	4.05E-08	30.06
CD4 ET	*PPT2*	rs9357120	6	31230717	A	T	0.2862	0.3360	0.0501	2.09E-11	44.8

CD4 ET	*PRDX6*	rs4405161	1	173414992	G	A	0.3102	-0.5401	0.0471	1.96E-30	131.19
CD4 ET	*PRELID1*	rs6879874	5	176730775	T	A	0.7115	0.3286	0.0494	2.75E-11	44.26
CD4 ET	*PRKCB*	rs145719494	16	24044921	G	A	0.0539	1.1144	0.0985	1.12E-29	127.74
CD4 ET	*PRKCQ-AS1*	rs61841332	10	6628224	A	G	0.1784	-0.7499	0.0531	2.39E-45	199.33
CD4 ET	*PRMT2*	rs11910707	21	48042513	T	G	0.3027	0.5141	0.0480	9.93E-27	114.31
CD4 ET	*PRMT2*	rs9975345	21	47843004	C	T	0.5433	0.2522	0.0449	1.99E-08	31.44
CD4 ET	*PRPF6*	rs6062604	20	62633845	C	T	0.8078	-0.3962	0.0604	5.47E-11	42.91
CD4 ET	*PRR4*	rs6488370	12	11445088	T	C	0.8257	-0.3558	0.0582	9.49E-10	37.35
CD4 ET	*PRR7-AS1*	rs2630767	5	176870700	C	T	0.5256	0.5561	0.0437	4.14E-37	161.67
CD4 ET	*PRRT1*	rs2596534	6	31445345	C	T	0.4196	0.2719	0.0456	2.41E-09	35.54
CD4 ET	*PRSS16*	rs35656932	6	28191288	T	G	0.0751	-0.5373	0.0754	1.01E-12	50.72
CD4 ET	*PSD4*	rs2241976	2	113943470	G	A	0.4506	0.2947	0.0453	7.40E-11	42.32
CD4 ET	*PSMB9*	rs28744291	6	32893687	C	A	0.2546	0.2913	0.0510	1.09E-08	32.6
CD4 ET	*PSMD5-AS1*	rs12684934	9	123593159	T	C	0.5794	-1.0688	0.0381	1.70E-173	786.9
CD4 ET	*PSMD5-AS1*	rs306794	9	124152939	T	C	0.0622	0.4863	0.0840	7.02E-09	33.46
CD4 ET	*PSPH*	rs11765904	7	55773037	A	G	0.1973	0.4205	0.0624	1.60E-11	45.32
CD4 ET	*PSTPIP1*	rs11856538	15	77383211	T	G	0.3635	0.3251	0.0452	6.19E-13	51.68
CD4 ET	*PTPN4*	rs13000543	2	120719379	C	T	0.5757	0.2807	0.0454	6.23E-10	38.17
CD4 ET	*PYHIN1*	rs1633280	1	158906555	C	A	0.3120	-0.3514	0.0481	2.65E-13	53.34
CD4 ET	*RABAC1*	rs10416501	19	42456602	G	A	0.6677	0.2716	0.0471	7.80E-09	33.26
CD4 ET	*RAC1*	rs836494	7	6415934	C	G	0.4962	0.3666	0.0444	1.54E-16	67.98
CD4 ET	*RBL2*	rs9931702	16	53526551	T	C	0.4726	-0.2455	0.0450	4.97E-08	29.67
CD4 ET	*RBM43*	rs289904	2	152085643	C	T	0.5281	0.3735	0.0444	4.20E-17	70.53
CD4 ET	*RBP7*	rs4133584	1	11072885	T	C	0.7922	0.4430	0.0571	8.92E-15	60
CD4 ET	*RCSD1*	rs1229403	1	167589943	C	T	0.6284	0.2953	0.0474	4.59E-10	38.77
CD4 ET	*RFWD3*	rs4887925	16	73784416	C	G	0.4099	0.2517	0.0454	2.86E-08	30.74
CD4 ET	*RGS12*	rs3135145	4	3299504	C	T	0.6018	0.3187	0.0453	1.96E-12	49.42
CD4 ET	*RHOC*	rs2999156	1	113247127	C	G	0.5449	0.2497	0.0450	2.85E-08	30.74
CD4 ET	*RIC3*	rs4758295	11	8152080	C	T	0.1306	-0.4483	0.0687	6.60E-11	42.55
CD4 ET	*RILPL2*	rs28404303	12	123905628	C	T	0.6017	-0.2646	0.0452	4.97E-09	34.13
CD4 ET	*RNASEH2C*	rs10896027	11	65420760	G	C	0.3410	-0.2719	0.0470	7.22E-09	33.41
CD4 ET	*RNASET2*	rs453184	6	167387404	C	A	0.5062	-0.7044	0.0423	2.29E-62	277.21
CD4 ET	*RNF166*	rs11076704	16	88777995	T	C	0.3981	-0.2782	0.0450	6.43E-10	38.11
CD4 ET	*RNF168*	rs12490312	3	196231127	A	G	0.6722	-0.3100	0.0497	4.42E-10	38.84
CD4 ET	*ROBO3*	rs11219821	11	124741772	T	C	0.4408	0.2551	0.0451	1.52E-08	31.96
CD4 ET	*RP1-29C18.10*	rs5770622	22	50051548	G	T	0.6547	-0.5901	0.0466	9.50E-37	160.02
CD4 ET	*RP1-3J17.3*	rs9444346	6	86165631	G	A	0.1874	0.3446	0.0583	3.31E-09	34.92
CD4 ET	*RP1-3J17.3*	rs2842603	6	86336337	C	G	0.3789	0.5413	0.0452	4.28E-33	143.34
CD4 ET	*RP11-138A9.1*	rs12533596	7	130627014	T	G	0.2328	0.4091	0.0533	1.73E-14	58.7
CD4 ET	*RP11-138A9.2*	rs12533527	7	130626948	T	C	0.1594	0.4383	0.0626	2.44E-12	48.99
CD4 ET	*RP11-1398P2.1*	rs6811458	4	1606390	C	A	0.5402	0.2884	0.0450	1.52E-10	40.92
CD4 ET	*RP11-1399P15.1*	rs55956548	2	87768679	T	G	0.7418	0.3327	0.0524	2.11E-10	40.28
CD4 ET	*RP11-166B2.5*	rs8052900	16	11940390	C	T	0.6780	-0.3701	0.0473	5.14E-15	61.08
CD4 ET	*RP11-18H21.1*	rs13140099	4	153022375	A	G	0.2594	-0.8120	0.0472	2.84E-66	295.1
CD4 ET	*RP11-245G13.2*	rs7577665	2	11020902	A	G	0.1116	1.0671	0.0698	1.05E-52	232.99
CD4 ET	*RP11-298J20.3*	rs4962711	10	126644482	C	T	0.7354	-0.7953	0.0486	4.30E-60	266.8
CD4 ET	*RP11-299J3.8*	rs6788746	3	122106743	A	G	0.1452	-0.3684	0.0593	5.35E-10	38.47
CD4 ET	*RP11-326C3.11*	rs11246065	11	324170	G	A	0.3701	0.7187	0.0443	4.02E-59	262.36
CD4 ET	*RP11-326C3.12*	rs11246068	11	327334	T	C	0.2550	0.5003	0.0509	8.26E-23	96.46
CD4 ET	*RP11-345J4.5*	rs4471699	16	30320307	T	G	0.4341	-0.3263	0.0446	2.54E-13	53.43
CD4 ET	*RP11-347P5.1*	rs9917251	2	196972941	A	G	0.3546	-0.6567	0.0445	3.51E-49	216.86
CD4 ET	*RP11-382J12.1*	rs441890	8	71564667	C	T	0.4328	0.2494	0.0454	4.00E-08	30.09
CD4 ET	*RP11-403A21.2*	rs12954962	18	21581709	C	T	0.7143	0.5949	0.0499	9.64E-33	141.73
CD4 ET	*RP11-415F23.2*	rs9845427	3	16350642	A	C	0.1984	0.4460	0.0589	3.71E-14	57.2
CD4 ET	*RP11-430H10.1*	rs12795538	11	45361155	T	A	0.1862	-0.3453	0.0569	1.29E-09	36.76
CD4 ET	*RP11-457M11.5*	rs9366698	6	27672035	G	C	0.2807	0.2821	0.0498	1.43E-08	32.08
CD4 ET	*RP11-473I1.5*	rs34932929	16	9193723	G	T	0.2216	-0.3703	0.0546	1.20E-11	45.87
CD4 ET	*RP11-473M20.9*	rs4786384	16	3136919	C	T	0.5202	0.3848	0.0444	4.33E-18	75.01
CD4 ET	*RP11-499P20.2*	rs4335438	10	18796825	C	T	0.4980	-0.2451	0.0449	4.71E-08	29.77
CD4 ET	*RP11-526I2.5*	rs35852295	15	101094174	T	C	0.2753	-0.3196	0.0510	3.75E-10	39.16
CD4 ET	*RP11-539L10.3*	rs36096778	4	6654613	T	A	0.1316	-0.4865	0.0678	7.29E-13	51.36
CD4 ET	*RP11-542K23.7*	rs35918962	9	125168878	T	C	0.1266	0.5951	0.0676	1.39E-18	77.26
CD4 ET	*RP11-545E17.3*	rs55697116	9	131489620	T	C	0.2481	0.3154	0.0509	5.77E-10	38.32
CD4 ET	*RP11-660L16.2*	rs4945008	11	71221248	G	A	0.6878	0.5030	0.0538	9.19E-21	87.15
CD4 ET	*RP11-664D1.1*	rs596856	12	4013581	C	T	0.1248	0.4630	0.0678	8.81E-12	46.48
CD4 ET	*RP11-69E11.4*	rs3820618	1	40223128	C	T	0.1312	0.4212	0.0678	5.27E-10	38.5
CD4 ET	*RP11-841O20.2*	rs75036970	14	53047302	A	G	0.0968	0.6036	0.0799	4.32E-14	56.9
CD4 ET	*RP11-97C16.1*	rs10510238	3	2901647	C	A	0.1214	0.6290	0.0671	7.21E-21	87.63
CD4 ET	*RP13-582O9.5*	rs12681621	8	144343516	A	G	0.2823	0.2719	0.0497	4.41E-08	29.9
CD4 ET	*RP3-325F22.5*	rs6570084	6	136904235	G	A	0.1513	-0.4635	0.0648	8.74E-13	51
CD4 ET	*RP3-510H16.3*	rs2413269	22	35185560	C	G	0.8950	0.5134	0.0733	2.49E-12	48.96
CD4 ET	*RP4-594I10.3*	rs196425	1	24871586	C	T	0.3702	0.3328	0.0461	5.08E-13	52.07
CD4 ET	*RP4-673M15.1*	rs7799332	7	44954786	A	G	0.5255	-0.2546	0.0449	1.38E-08	32.15
CD4 ET	*RP5-1021I20.1*	rs66896407	14	74266771	A	G	0.1398	-0.5080	0.0594	1.17E-17	73.05
CD4 ET	*RP5-1042K10.10*	rs55769635	22	40790101	A	G	0.1809	0.4048	0.0579	2.83E-12	48.71
CD4 ET	*RP5-827C21.4*	rs114190063	1	234538109	G	A	0.0913	0.4950	0.0776	1.76E-10	40.63
CD4 ET	*RPL10A*	rs2076172	6	35436925	T	C	0.5358	-0.6474	0.0432	8.78E-51	224.19
CD4 ET	*RPL11*	rs77766201	1	24006446	A	G	0.1560	-0.5803	0.0622	9.90E-21	87
CD4 ET	*RPL12*	rs2247573	9	130210946	G	A	0.5887	1.0023	0.0391	1.13E-144	654.63

CD4 ET	*RPL13*	rs12932337	16	89630630	T	C	0.1753	-0.7223	0.0542	1.44E-40	177.47
CD4 ET	*RPL14*	rs75965075	3	40438120	T	C	0.0715	0.8958	0.0838	1.09E-26	114.12
CD4 ET	*RPL22L1*	rs34587559	3	170578721	T	C	0.2392	-0.3123	0.0508	7.92E-10	37.7
CD4 ET	*RPL23A*	rs2302205	17	26947046	C	A	0.0821	-0.8758	0.0771	6.40E-30	128.85
CD4 ET	*RPL27A*	rs61875926	11	8705528	G	C	0.0958	-0.5848	0.0736	2.00E-15	62.93
CD4 ET	*RPL28*	rs17700376	19	55897327	G	A	0.3547	-1.0885	0.0394	9.65E-168	760.49
CD4 ET	*RPL3*	rs137620	22	39709776	C	T	0.4729	0.2977	0.0447	2.78E-11	44.24
CD4 ET	*RPL32*	rs2290311	3	12883071	A	T	0.1438	0.4374	0.0616	1.25E-12	50.3
CD4 ET	*RPL36*	rs2485257	19	5655792	T	C	0.0884	-0.4436	0.0789	1.88E-08	31.55
CD4 ET	*RPL36AL*	rs4500635	14	49806704	T	C	0.3950	-0.2614	0.0460	1.36E-08	32.18
CD4 ET	*RPL36AL*	rs2147712	14	50091684	A	G	0.7504	1.0287	0.0472	1.93E-105	474.64
CD4 ET	*RPL8*	rs2954658	8	146017782	T	C	0.4990	-1.0566	0.0374	1.75E-175	796.02
CD4 ET	*RPLP2*	rs28694634	11	808423	A	G	0.6588	-0.7389	0.0454	1.58E-59	264.21
CD4 ET	*RPS10*	rs9380433	6	34377626	T	C	0.7750	-1.0236	0.0535	1.85E-81	364.68
CD4 ET	*RPS11*	rs10403394	19	49999345	A	G	0.1225	-0.7466	0.0705	3.52E-26	111.8
CD4 ET	*RPS12*	rs6917833	6	133099753	C	T	0.4765	0.3453	0.0446	9.83E-15	59.81
CD4 ET	*RPS14*	rs2569106	5	149805130	C	T	0.3138	-0.3007	0.0489	7.66E-10	37.77
CD4 ET	*RPS16*	rs640000	19	39926601	T	C	0.3796	0.4326	0.0465	1.31E-20	86.45
CD4 ET	*RPS18*	rs17215231	6	33239869	T	C	0.0640	-1.5915	0.0823	2.22E-83	373.49
CD4 ET	*RPS19*	rs7259596	19	42369743	C	T	0.5016	0.3348	0.0445	5.60E-14	56.39
CD4 ET	*RPS2*	rs17135718	16	2014834	G	A	0.1151	0.8380	0.0656	2.18E-37	162.94
CD4 ET	*RPS23*	rs226205	5	81593654	T	C	0.7767	0.3869	0.0512	3.97E-14	57.07
CD4 ET	*RPS24*	rs3740253	10	79793688	T	C	0.5917	0.3108	0.0462	1.78E-11	45.11
CD4 ET	*RPS25*	rs4936457	11	118875727	G	A	0.2508	-0.6471	0.0489	6.70E-40	174.42
CD4 ET	*RPS26*	rs773114	12	56379060	T	A	0.3997	1.1972	0.0353	1.00E-200	1149.78
CD4 ET	*RPS3*	rs3094303	11	75114071	G	A	0.0743	-0.8180	0.0898	8.59E-20	82.74
CD4 ET	*RPS3A*	rs6836854	4	151742349	T	C	0.9259	1.2878	0.0961	6.17E-41	179.16
CD4 ET	*RPS5*	rs731835	19	58898436	C	T	0.3256	-0.9942	0.0420	1.19E-123	558.17
CD4 ET	*RPS6*	rs10114039	9	19352260	T	A	0.0988	-0.9941	0.0802	2.56E-35	153.49
CD4 ET	*RPS7*	rs62106040	2	3627302	T	A	0.0757	-1.1062	0.0863	1.22E-37	164.09
CD4 ET	*RPS8*	rs12120833	1	45241285	G	C	0.1242	-0.8094	0.0605	7.43E-41	178.79
CD4 ET	*RPS9*	rs158366	19	54705317	G	C	0.3901	-0.7940	0.0424	2.33E-78	350.47
CD4 ET	*RPSA*	rs45503293	3	39432785	T	C	0.1594	0.7365	0.0598	7.47E-35	151.36
CD4 ET	*RRP7A*	rs136988	22	42946371	T	C	0.1733	0.3977	0.0575	4.68E-12	47.72
CD4 ET	*RSRC2*	rs9988963	12	122991478	G	C	0.7423	-0.3703	0.0496	8.25E-14	55.63
CD4 ET	*SAMD3*	rs7749850	6	130506382	C	A	0.5259	-0.3389	0.0447	3.25E-14	57.46
CD4 ET	*SAR1A*	rs2310991	10	71931646	A	C	0.4271	-0.2950	0.0455	8.81E-11	41.98
CD4 ET	*SCGB3A1*	rs6422334	5	179982151	C	T	0.8922	-1.5227	0.0656	2.64E-119	538.23
CD4 ET	*SEPT2*	rs28446330	2	242392759	C	T	0.0905	0.6153	0.0761	6.31E-16	65.21
CD4 ET	*SERPINB6*	rs3851508	6	2919494	T	G	0.2659	-0.5451	0.0494	2.71E-28	121.43
CD4 ET	*SERPINB9*	rs381854	6	2859686	G	A	0.5386	0.3152	0.0446	1.62E-12	49.79
CD4 ET	*SESTD1*	rs10439300	2	180087991	T	C	0.1880	0.4549	0.0584	6.44E-15	60.64
CD4 ET	*SETD9*	rs978406	5	56259808	T	A	0.1698	-0.4205	0.0625	1.78E-11	45.11
CD4 ET	*SF1*	rs633923	11	64542272	A	C	0.1082	0.5678	0.0788	5.78E-13	51.81
CD4 ET	*SFT2D1*	rs2343590	6	166759622	G	C	0.7466	0.3328	0.0507	5.12E-11	43.04
CD4 ET	*SH3GLB1*	rs263496	1	87215761	C	T	0.1905	0.3209	0.0581	3.41E-08	30.4
CD4 ET	*SH3YL1*	rs7595075	2	264019	A	C	0.3283	-0.9537	0.0416	3.37E-116	523.98
CD4 ET	*SHF*	rs12912809	15	46022647	T	G	0.3317	-0.4465	0.0464	5.79E-22	92.61
CD4 ET	*SIDT1-AS1*	rs16861158	3	113261709	T	G	0.1408	0.5163	0.0627	1.79E-16	67.68
CD4 ET	*SKAP2*	rs73069539	7	26903661	T	C	0.2198	0.3421	0.0523	6.20E-11	42.67
CD4 ET	*SLC12A7*	rs35188965	5	1104938	T	C	0.5560	-0.3473	0.0452	1.56E-14	58.9
CD4 ET	*SLC22A18*	rs450244	11	2940492	C	T	0.9113	-0.6213	0.0780	1.59E-15	63.39
CD4 ET	*SLC25A29*	rs10134767	14	100763954	T	G	0.3371	0.3521	0.0472	8.19E-14	55.64
CD4 ET	*SLC25A3*	rs28372744	12	98987445	A	G	0.0487	0.5530	0.0982	1.80E-08	31.63
CD4 ET	*SLC35F3*	rs516710	1	234447432	C	G	0.3095	-0.7188	0.0473	4.16E-52	230.25
CD4 ET	*SLC44A4*	rs4713417	6	30992734	T	G	0.1609	-0.3624	0.0609	2.62E-09	35.38
CD4 ET	*SLC44A4*	rs11967011	6	31221540	C	T	0.1630	0.4543	0.0583	6.82E-15	60.53
CD4 ET	*SLC4A7*	rs13067781	3	27526605	T	C	0.2349	-0.3815	0.0504	3.86E-14	57.12
CD4 ET	*SLFN5*	rs11656388	17	33531815	A	G	0.3965	0.3405	0.0457	9.39E-14	55.38
CD4 ET	*SMDT1*	rs2301521	22	42423110	C	G	0.6779	1.2724	0.0372	1.00E-200	1168.68
CD4 ET	*SNAP29*	rs1978098	22	21288871	C	T	0.0685	0.5704	0.0818	3.18E-12	48.47
CD4 ET	*SNHG7*	rs6874	9	139617836	T	C	0.1153	-1.0310	0.0678	3.19E-52	230.77
CD4 ET	*SNHG8*	rs658642	4	119200274	T	C	0.3810	1.0724	0.0381	1.98E-174	791.19
CD4 ET	*SNRNP25*	rs1045001	16	107275	G	T	0.8221	-0.3566	0.0591	1.60E-09	36.34
CD4 ET	*SNRPD2*	rs36040835	19	46201213	G	T	0.2051	-0.5017	0.0553	1.10E-19	82.25
CD4 ET	*SOX4*	rs9466028	6	21301005	T	C	0.1477	-0.3782	0.0636	2.80E-09	35.25
CD4 ET	*SPATA20*	rs8076632	17	48625928	G	C	0.3650	-0.3678	0.0467	3.18E-15	62.02
CD4 ET	*SPATC1L*	rs62215189	21	47603995	G	A	0.2446	-0.4878	0.0502	2.75E-22	94.08
CD4 ET	*SPSB2*	rs11064426	12	6953257	C	A	0.3297	0.2951	0.0476	5.69E-10	38.35
CD4 ET	*SRD5A3*	rs36110240	4	56217015	T	C	0.1629	0.4650	0.0605	1.56E-14	58.91
CD4 ET	*SRP14*	rs2250402	15	40322552	T	G	0.9172	-0.5337	0.0839	2.04E-10	40.35
CD4 ET	*SRSF10*	rs6424144	1	24375663	A	G	0.4721	-0.2561	0.0448	1.10E-08	32.59
CD4 ET	*ST6GALNAC1*	rs34835035	17	74697848	A	C	0.5501	0.2958	0.0449	4.26E-11	43.4
CD4 ET	*STMN3*	rs879471	20	62269942	T	C	0.5285	0.4576	0.0441	3.26E-25	107.4
CD4 ET	*SULF2*	rs3091426	20	46189755	A	G	0.5891	-0.7377	0.0423	4.40E-68	303.39
CD4 ET	*SUPT3H*	rs74460828	6	44852136	A	G	0.4158	0.2729	0.0454	1.83E-09	36.08
CD4 ET	*SURF1*	rs567493	9	136185528	G	A	0.7115	0.2876	0.0502	1.03E-08	32.72
CD4 ET	*SYNC*	rs61798931	1	33107443	G	A	0.0985	0.3916	0.0693	1.61E-08	31.85
CD4 ET	*SYNM*	rs260112	15	99686514	T	C	0.3255	0.3158	0.0483	5.95E-11	42.75

CD4 ET	*TAPBPL*	rs758739	12	6626369	C	G	0.7157	-0.2928	0.0503	5.66E-09	33.88
CD4 ET	*TBCA*	rs2662383	5	77008910	A	G	0.2940	-0.4514	0.0478	3.91E-21	88.84
CD4 ET	*THAP7-AS1*	rs28372939	22	21356824	A	G	0.2052	-0.3555	0.0556	1.61E-10	40.81
CD4 ET	*THEM4*	rs2087549	1	151834188	T	C	0.9164	0.6138	0.0849	4.71E-13	52.21
CD4 ET	*THEMIS2*	rs9887847	1	28226317	C	T	0.3803	-0.4791	0.0451	2.49E-26	112.49
CD4 ET	*TIMM10*	rs2848626	11	57283988	T	C	0.7820	-0.7037	0.0498	2.48E-45	199.25
CD4 ET	*TIMM13*	rs7248779	19	2403564	G	T	0.4857	0.2932	0.0447	5.44E-11	42.93
CD4 ET	*TIPARP-AS1*	rs9289971	3	156389947	C	T	0.1529	-0.4114	0.0606	1.14E-11	45.98
CD4 ET	*TLE4*	rs1475676	9	82289153	T	G	0.6177	-0.3248	0.0463	2.32E-12	49.1
CD4 ET	*TMEM120B*	rs11043193	12	122210497	G	C	0.1025	0.5127	0.0789	8.12E-11	42.14
CD4 ET	*TMEM121*	rs35592422	14	105996593	C	G	0.2770	-0.3945	0.0524	5.33E-14	56.49
CD4 ET	*TMEM156*	rs2711970	4	39025844	C	T	0.6591	0.6118	0.0451	6.67E-42	183.57
CD4 ET	*TMEM2*	rs12004614	9	74339777	C	G	0.0797	0.4439	0.0805	3.44E-08	30.38
CD4 ET	*TMEM204*	rs12599859	16	1577184	G	A	0.1364	0.5129	0.0672	2.23E-14	58.2
CD4 ET	*TMEM230*	rs2715	20	5081049	C	T	0.5182	0.3306	0.0447	1.36E-13	54.65
CD4 ET	*TMEM258*	rs174538	11	61560081	A	G	0.2977	0.3532	0.0485	3.16E-13	53
CD4 ET	*TMEM50B*	rs8132535	21	34802946	A	G	0.4703	-0.4015	0.0443	1.35E-19	81.85
CD4 ET	*TMEM8A*	rs11648905	16	425298	T	G	0.4242	-0.4954	0.0438	1.15E-29	127.69
CD4 ET	*TNFRSF14*	rs2495366	1	2488930	A	G	0.5052	0.2485	0.0448	3.00E-08	30.65
CD4 ET	*TNFSF13B*	rs9520848	13	108995449	C	T	0.2650	0.3710	0.0536	4.31E-12	47.88
CD4 ET	*TOMM7*	rs2240728	7	22852499	T	C	0.3422	-1.1999	0.0390	1.00E-200	945.27
CD4 ET	*TPD52*	rs11991941	8	81036178	G	A	0.4419	0.2565	0.0451	1.27E-08	32.31
CD4 ET	*TPGS2*	rs2303508	18	34324376	G	A	0.4676	-0.2788	0.0449	5.11E-10	38.56
CD4 ET	*TRABD2A*	rs1118504	2	84962706	A	T	0.1889	-0.3785	0.0559	1.30E-11	45.73
CD4 ET	*TRAPPC4*	rs4445645	11	118868750	C	G	0.2222	0.2939	0.0531	3.17E-08	30.54
CD4 ET	*TRIM61*	rs6839687	4	165441498	G	A	0.4845	0.2908	0.0450	1.02E-10	41.7
CD4 ET	*TRMT112*	rs79423518	11	64105454	A	G	0.0682	-0.7649	0.0974	4.16E-15	61.5
CD4 ET	*TSHZ2*	rs74963256	20	51588847	C	T	0.1003	0.4113	0.0723	1.28E-08	32.3
CD4 ET	*TSPO*	rs138909	22	43549739	A	T	0.3905	-0.3718	0.0454	2.63E-16	66.93
CD4 ET	*TSTD1*	rs3766383	1	161008572	A	C	0.1552	-0.5641	0.0668	2.97E-17	71.22
CD4 ET	*TSTD3*	rs9402854	6	99958527	C	T	0.5682	-0.3199	0.0451	1.38E-12	50.11
CD4 ET	*TTC32*	rs7594214	2	20106369	C	T	0.0868	0.6143	0.0813	4.06E-14	57.02
CD4 ET	*TUBB2A*	rs3799216	6	3150515	T	C	0.7253	0.2777	0.0504	3.61E-08	30.29
CD4 ET	*TUFM*	rs8049439	16	28837515	C	T	0.3939	0.6425	0.0440	3.18E-48	212.48
CD4 ET	*TXK*	rs12108537	4	48183467	A	G	0.1196	0.4927	0.0728	1.27E-11	45.76
CD4 ET	*TXN*	rs4135183	9	113015602	T	C	0.1295	-0.4328	0.0626	4.91E-12	47.63
CD4 ET	*TYMP*	rs78007348	22	50973817	T	C	0.1028	0.3997	0.0697	9.66E-09	32.84
CD4 ET	*U2AF1L4*	rs112500848	19	36254427	T	C	0.1552	0.6099	0.0630	3.87E-22	93.41
CD4 ET	*U91328.19*	rs80264589	6	26927602	A	G	0.0784	-0.6199	0.0743	7.05E-17	69.52
CD4 ET	*UBA6*	rs11933596	4	68582008	C	A	0.4081	0.2791	0.0457	9.83E-10	37.28
CD4 ET	*UBE2F*	rs72979251	2	238857362	C	T	0.3974	0.2792	0.0462	1.52E-09	36.43
CD4 ET	*UCP2*	rs2515087	11	73682872	T	C	0.4077	0.4932	0.0449	4.37E-28	120.49
CD4 ET	*ULK4*	rs9837273	3	41813104	C	T	0.2003	0.4832	0.0576	4.72E-17	70.31
CD4 ET	*UPF3A*	rs7332374	13	114995733	C	T	0.1634	0.5846	0.0640	6.81E-20	83.2
CD4 ET	*UQCRH*	rs41292543	1	46774783	G	A	0.1009	-0.5587	0.0759	1.83E-13	54.07
CD4 ET	*USP53*	rs11729857	4	120114100	G	C	0.1207	0.5205	0.0698	8.60E-14	55.55
CD4 ET	*UTS2*	rs6689067	1	8164368	C	T	0.4412	-0.3570	0.0447	1.40E-15	63.64
CD4 ET	*VAMP2*	rs8064449	17	8079029	C	G	0.6434	0.3813	0.0478	1.42E-15	63.61
CD4 ET	*VAMP5*	rs28800839	2	85757616	T	C	0.1244	-0.4863	0.0670	3.84E-13	52.62
CD4 ET	*VPS28*	rs117891721	8	145642886	T	C	0.0569	0.6238	0.0948	4.61E-11	43.25
CD4 ET	*WAC-AS1*	rs1034086	10	28934523	C	A	0.5992	-0.2877	0.0451	1.73E-10	40.67
CD4 ET	*WDR86*	rs7782289	7	151091515	G	A	0.6034	0.2557	0.0460	2.63E-08	30.9
CD4 ET	*WIPI2*	rs56112327	7	5174739	T	C	0.3310	0.2966	0.0481	6.92E-10	37.97
CD4 ET	*XBP1*	rs6005866	22	29162940	G	A	0.1211	0.4760	0.0711	2.22E-11	44.67
CD4 ET	*XRCC6BP1*	rs9971803	12	58306097	T	C	0.3134	-0.3455	0.0474	3.13E-13	53.02
CD4 ET	*XRRA1*	rs12225387	11	74604767	G	A	0.2742	0.8293	0.0456	7.34E-74	329.86
CD4 ET	*XXbac-BPG181B23.7*	rs17200249	6	31378654	A	T	0.2368	0.3781	0.0549	5.78E-12	47.31
CD4 ET	*XXbac-BPG252P9.10*	rs9266746	6	31351213	C	T	0.5450	-0.4051	0.0469	6.09E-18	74.34
CD4 ET	*XXbac-BPG299F13.17*	rs6921663	6	31154620	C	T	0.2178	0.9322	0.0508	2.83E-75	336.34
CD4 ET	*YBEY*	rs62224180	21	47726332	G	C	0.7751	-0.6953	0.0552	2.47E-36	158.13
CD4 ET	*YBX3*	rs2607903	12	10876573	A	C	0.9098	-0.4465	0.0818	4.82E-08	29.73
CD4 ET	*YEATS4*	rs10784774	12	69737879	G	A	0.4830	-0.2590	0.0448	7.45E-09	33.35
CD4 ET	*ZC2HC1A*	rs3736169	8	79587855	G	A	0.7129	0.3142	0.0513	8.99E-10	37.46
CD4 ET	*ZC3HAV1*	rs10273974	7	138735512	C	A	0.4877	-0.4527	0.0440	7.84E-25	105.66
CD4 ET	*ZFAS1*	rs237711	20	47926856	G	C	0.2041	0.5794	0.0526	3.17E-28	121.13
CD4 ET	*ZFP57*	rs78960639	6	29763303	A	C	0.3038	-0.4499	0.0472	1.41E-21	90.85
CD4 ET	*ZNF165*	rs172328	6	28790528	T	C	0.1281	-0.3629	0.0614	3.37E-09	34.88
CD4 ET	*ZNF302*	rs113559284	19	35196255	A	G	0.1247	0.4652	0.0670	3.83E-12	48.11
CD4 ET	*ZNF391*	rs67998226	6	28238059	C	T	0.0794	-0.8292	0.0734	1.28E-29	127.48
CD4 ET	*ZNF584*	rs11671005	19	58963530	A	G	0.2602	-0.3849	0.0504	2.17E-14	58.25
CD4 ET	*ZNF593*	rs2490273	1	26489758	T	G	0.7739	-0.3988	0.0529	4.65E-14	56.76
CD4 ET	*ZNF600*	rs12973596	19	53288873	C	T	0.5373	0.3125	0.0449	3.53E-12	48.27
CD4 ET	*ZNF83*	rs73582288	19	53121736	T	C	0.3589	0.3823	0.0463	1.44E-16	68.11
CD4 ET	*ZNF85*	rs2545225	19	21042460	G	A	0.1318	0.6249	0.0632	5.07E-23	97.42
CD4 ET	*ZP3*	rs35613536	7	76143625	A	G	0.3272	0.3906	0.0469	8.26E-17	69.2
CD4 NC	*A1BG*	rs16988668	19	58850756	C	G	0.0597	-0.8289	0.0979	2.56E-17	71.51
CD4 NC	*A1BG*	rs56268519	19	59045904	A	G	0.3295	0.2673	0.0487	4.06E-08	30.06
CD4 NC	*AAK1*	rs2871966	2	69850567	A	G	0.3808	0.3019	0.0463	6.86E-11	42.47
CD4 NC	*ABCA7*	rs2304262	19	1036552	C	T	0.8482	-0.4382	0.0692	2.49E-10	39.96

CD4 NC	*ABCD4*	rs71429048	14	74799143	T	C	0.3300	0.3090	0.0474	7.19E-11	42.38
CD4 NC	*ABCG2*	rs62308057	4	89150178	G	C	0.1394	0.3828	0.0644	2.71E-09	35.31
CD4 NC	*ABHD10*	rs62277638	3	111703315	C	A	0.2132	-0.3324	0.0543	9.17E-10	37.42
CD4 NC	*ABRACL*	rs9389651	6	139366892	T	C	0.3585	-0.3105	0.0472	4.68E-11	43.22
CD4 NC	*AC002331.1*	rs4284645	16	26604035	T	C	0.2434	-0.3784	0.0518	2.72E-13	53.29
CD4 NC	*AC002467.7*	rs13224313	7	107382352	T	G	0.2532	0.3817	0.0525	3.65E-13	52.71
CD4 NC	*AC005037.3*	rs11888171	2	201843257	G	A	0.1932	0.4965	0.0569	2.52E-18	76.08
CD4 NC	*AC005082.12*	rs6967526	7	23159241	A	C	0.4342	0.4314	0.0449	6.98E-22	92.24
CD4 NC	*AC005253.2*	rs2314666	19	18689904	C	T	0.5921	-0.6693	0.0434	1.48E-53	236.88
CD4 NC	*AC006129.2*	rs117151468	19	41992196	C	A	0.0418	-0.5954	0.0997	2.37E-09	35.57
CD4 NC	*AC006369.2*	rs917733	2	37831453	C	T	0.5906	-0.9658	0.0394	1.09E-132	599.62
CD4 NC	*AC007228.11*	rs7258886	19	57068039	T	C	0.2804	0.2868	0.0501	1.06E-08	32.66
CD4 NC	*AC007383.4*	rs6435325	2	207001265	T	C	0.4509	0.3532	0.0450	4.20E-15	61.48
CD4 NC	*AC008746.12*	rs28576683	19	54944607	G	A	0.3261	-0.3277	0.0493	2.96E-11	44.11
CD4 NC	*AC009120.10*	rs4888212	16	74360410	C	G	0.3049	-0.6222	0.0462	2.87E-41	180.67
CD4 NC	*AC009120.4*	rs12446326	16	74405323	G	T	0.1017	0.8175	0.0739	2.02E-28	122.01
CD4 NC	*AC009299.3*	rs11678980	2	162101261	A	G	0.4341	-0.3240	0.0447	4.46E-13	52.32
CD4 NC	*AC009961.3*	rs17813964	2	160599717	G	C	0.0837	0.7488	0.0742	6.34E-24	101.53
CD4 NC	*AC010642.1*	rs3206946	19	58826534	G	A	0.2046	-0.4713	0.0558	2.94E-17	71.24
CD4 NC	*AC010883.5*	rs13399366	2	43461119	T	C	0.1172	0.4930	0.0667	1.45E-13	54.53
CD4 NC	*AC010982.1*	rs1807282	2	114110036	T	A	0.2103	0.3066	0.0543	1.60E-08	31.86
CD4 NC	*AC011484.1*	rs4803966	19	46996928	A	C	0.1460	0.7889	0.0574	5.50E-43	188.52
CD4 NC	*AC013264.2*	rs10199410	2	198155776	T	A	0.3669	1.1485	0.0375	1.00E-200	933.82
CD4 NC	*AC016757.3*	rs6740485	2	239137672	C	T	0.4607	0.4816	0.0439	6.04E-28	119.85
CD4 NC	*AC016757.3*	rs508486	2	239339712	A	C	0.6735	0.2893	0.0483	2.15E-09	35.76
CD4 NC	*AC018816.3*	rs901854	3	4856234	A	G	0.6030	0.4928	0.0448	3.62E-28	120.86
CD4 NC	*AC021860.1*	rs1962053	4	38612357	C	A	0.2004	0.6976	0.0521	7.89E-41	178.67
CD4 NC	*AC022182.3*	rs13278731	8	61862108	T	C	0.1437	0.3438	0.0622	3.28E-08	30.47
CD4 NC	*AC022498.1*	rs2222103	3	187902932	A	G	0.1038	0.6845	0.0731	7.77E-21	87.48
CD4 NC	*AC023590.1*	rs2460970	8	119629454	A	G	0.9192	-0.9394	0.0757	2.33E-35	153.68
CD4 NC	*AC062017.1*	rs843461	2	240319090	T	C	0.6820	0.3240	0.0476	1.01E-11	46.22
CD4 NC	*AC064834.2*	rs59639651	2	196409150	G	A	0.1926	-0.6775	0.0543	8.98E-36	155.57
CD4 NC	*AC068039.4*	rs111357343	2	172616423	A	G	0.2916	0.3293	0.0483	9.59E-12	46.32
CD4 NC	*AC068282.3*	rs6430937	2	128082232	T	C	0.7612	-0.5223	0.0524	2.22E-23	99.05
CD4 NC	*AC068620.1*	rs1401908	4	57285163	T	C	0.4241	-0.2906	0.0450	1.08E-10	41.59
CD4 NC	*AC073115.7*	rs1525831	7	45994409	T	C	0.6958	-0.3294	0.0478	5.27E-12	47.49
CD4 NC	*AC074289.1*	rs2555436	2	64461941	A	G	0.2795	-0.4222	0.0496	1.71E-17	72.31
CD4 NC	*AC084018.1*	rs1168663	12	122234089	A	T	0.8010	0.3274	0.0579	1.60E-08	31.87
CD4 NC	*AC090186.1*	rs6999321	8	52692784	A	G	0.8615	0.8718	0.0747	1.93E-31	135.79
CD4 NC	*AC090186.1*	rs34677273	8	52820813	T	C	0.1901	0.3288	0.0601	4.51E-08	29.86
CD4 NC	*AC091729.9*	rs6953538	7	1196974	G	A	0.6141	-0.3974	0.0465	1.23E-17	72.96
CD4 NC	*AC093157.1*	rs6577222	1	101420502	G	C	0.3000	0.3170	0.0499	2.18E-10	40.21
CD4 NC	*AC093375.1*	rs1528455	2	157169947	T	C	0.2847	-0.6691	0.0484	1.54E-43	191.05
CD4 NC	*AC093901.1*	rs13025020	2	118926955	A	G	0.5195	0.2553	0.0449	1.33E-08	32.22
CD4 NC	*AC097713.4*	rs13428882	2	235354348	T	C	0.6150	-0.4880	0.0446	7.26E-28	119.48
CD4 NC	*AC097724.3*	rs9309664	2	28939626	A	G	0.6143	-0.2554	0.0462	3.15E-08	30.55
CD4 NC	*AC104653.1*	rs11691664	2	114592257	A	G	0.5883	0.7696	0.0428	2.86E-72	322.57
CD4 NC	*AC107622.1*	rs1353998	3	20841094	C	A	0.3070	0.3147	0.0497	2.45E-10	39.99
CD4 NC	*AC116614.1*	rs4854370	2	755008	C	A	0.2514	0.3093	0.0498	5.10E-10	38.56
CD4 NC	*AC137932.1*	rs731136	16	89359421	G	C	0.6537	0.4287	0.0480	3.91E-19	79.75
CD4 NC	*AC144652.1*	rs34021161	7	155086666	T	C	0.5156	0.2682	0.0450	2.62E-09	35.37
CD4 NC	*ACAA2*	rs7227494	18	47269707	A	G	0.2110	0.3616	0.0547	3.88E-11	43.58
CD4 NC	*ACAP1*	rs3809822	17	7234112	G	C	0.2229	-0.3399	0.0515	4.10E-11	43.48
CD4 NC	*ACAT1*	rs72992163	11	108311255	C	G	0.1451	0.7297	0.0626	2.03E-31	135.69
CD4 NC	*ACAT2*	rs2842970	6	160153166	T	C	0.5624	-0.4983	0.0444	3.26E-29	125.63
CD4 NC	*ACBD3*	rs2306120	1	226352498	G	T	0.5941	-0.3191	0.0458	3.36E-12	48.37
CD4 NC	*ACCS*	rs2074038	11	44087989	T	G	0.1126	1.0382	0.0711	3.03E-48	212.57
CD4 NC	*ACER3*	rs6592688	11	76676396	G	A	0.7319	-0.3096	0.0506	9.19E-10	37.41
CD4 NC	*ACOT13*	rs1061925	6	24666007	G	T	0.1129	0.3866	0.0685	1.67E-08	31.78
CD4 NC	*ACOT4*	rs55654418	14	74044019	A	G	0.3900	0.4552	0.0459	3.36E-23	98.23
CD4 NC	*ACP1*	rs300750	2	208794	G	T	0.4984	-0.2746	0.0448	8.79E-10	37.5
CD4 NC	*ACP5*	rs117694210	19	11684412	T	C	0.1230	0.7307	0.0653	4.93E-29	124.81
CD4 NC	*ACPL2*	rs2292404	3	140985697	A	G	0.5778	-0.4332	0.0448	3.89E-22	93.4
CD4 NC	*ACSM1*	rs399499	16	20679530	A	C	0.1063	0.4814	0.0778	6.15E-10	38.2
CD4 NC	*ACTA2*	rs4064	10	90751380	C	G	0.3425	-0.3446	0.0470	2.17E-13	53.74
CD4 NC	*ACTG1*	rs7503278	17	79479808	T	C	0.8099	0.5308	0.0563	3.97E-21	88.81
CD4 NC	*ACTR2*	rs4671651	2	65504311	C	G	0.2806	-0.3177	0.0501	2.35E-10	40.07
CD4 NC	*ACVR1B*	rs11169970	12	52368428	A	G	0.2374	0.3523	0.0531	3.14E-11	44
CD4 NC	*ACY1*	rs729555	3	52478389	T	C	0.1108	-0.3895	0.0651	2.14E-09	35.77
CD4 NC	*ACY3*	rs10791910	11	67467390	T	C	0.0777	0.5252	0.0848	5.99E-10	38.25
CD4 NC	*ACYP2*	rs6742042	2	54218703	C	T	0.4148	-0.8630	0.0413	6.84E-97	435.43
CD4 NC	*ADA*	rs6073507	20	43302719	A	T	0.6962	-0.4248	0.0482	1.29E-18	77.39
CD4 NC	*ADAL*	rs28578454	15	43603032	A	G	0.1285	-0.4707	0.0750	3.46E-10	39.32
CD4 NC	*ADAM10*	rs7161799	15	58770523	T	C	0.0869	0.7696	0.0786	1.22E-22	95.69
CD4 NC	*ADAM17*	rs4278906	2	9661589	T	C	0.6468	-0.4234	0.0473	3.29E-19	80.09
CD4 NC	*ADAMTS6*	rs245541	5	64783403	C	G	0.6103	-0.7067	0.0442	1.88E-57	254.71
CD4 NC	*ADAP1*	rs10807831	7	961221	G	C	0.8522	-0.3472	0.0569	1.02E-09	37.21
CD4 NC	*ADARB2*	rs3793735	10	1282828	C	T	0.3439	0.4980	0.0461	3.43E-27	116.41
CD4 NC	*ADAT1*	rs7189895	16	75665019	G	A	0.3436	0.3182	0.0490	8.66E-11	42.02

CD4 NC	*ADCK1*	rs2364	14	78358457	A	G	0.0480	0.6855	0.1002	7.92E-12	46.69
CD4 NC	*ADCY3*	rs10185143	2	25150758	C	T	0.4786	-0.2759	0.0448	7.04E-10	37.93
CD4 NC	*ADCY7*	rs9934775	16	50383077	T	C	0.1726	0.5069	0.0620	2.77E-16	66.82
CD4 NC	*ADHFE1*	rs2357894	8	67378385	C	T	0.4987	0.2946	0.0448	4.69E-11	43.22
CD4 NC	*ADI1*	rs1130165	2	3502398	T	C	0.1806	0.5168	0.0665	7.79E-15	60.26
CD4 NC	*ADIRF*	rs12783321	10	88733275	A	G	0.6091	-0.3944	0.0454	3.70E-18	75.32
CD4 NC	*ADPGK*	rs12906007	15	72985891	T	C	0.1386	0.5133	0.0625	2.25E-16	67.24
CD4 NC	*ADPRM*	rs2520161	17	10603421	G	A	0.4402	-0.4814	0.0446	4.16E-27	116.03
CD4 NC	*ADTRP*	rs210902	6	11725469	G	A	0.7306	0.4431	0.0555	1.37E-15	63.68
CD4 NC	*AE000662.92*	rs12887841	14	23026438	A	G	0.0951	0.6863	0.0675	2.66E-24	103.25
CD4 NC	*AEBP2*	rs2163224	12	19596266	G	A	0.2216	-0.3307	0.0571	7.12E-09	33.43
CD4 NC	*AF146191.4*	rs11736387	4	190861768	C	G	0.3260	0.2740	0.0477	9.41E-09	32.89
CD4 NC	*AFAP1*	rs56078222	4	7932073	C	T	0.3522	0.6753	0.0441	6.12E-53	234.05
CD4 NC	*AGA*	rs11131799	4	178363378	A	G	0.6460	0.5245	0.0454	8.07E-31	132.96
CD4 NC	*AGAP1*	rs80247210	2	237565426	T	C	0.1337	0.4143	0.0638	8.38E-11	42.08
CD4 NC	*AGAP4*	rs7087633	10	45968987	C	G	0.5198	-0.2495	0.0448	2.63E-08	30.9
CD4 NC	*AGTRAP*	rs6676319	1	11781973	A	G	0.4373	0.2922	0.0453	1.11E-10	41.54
CD4 NC	*AHI1*	rs13197384	6	135818897	A	C	0.3411	-0.8306	0.0437	2.14E-80	359.81
CD4 NC	*AHSA2*	rs12474734	2	61530290	T	C	0.1417	-0.4005	0.0636	3.00E-10	39.59
CD4 NC	*AIF1*	rs2269475	6	31583931	T	C	0.1321	-0.8026	0.0640	3.95E-36	157.19
CD4 NC	*AIG1*	rs1855559	6	143356781	T	C	0.1410	0.4051	0.0743	4.91E-08	29.69
CD4 NC	*AIM1*	rs898893	6	106982732	T	C	0.4718	-0.2927	0.0447	5.83E-11	42.79
CD4 NC	*AK1*	rs10819317	9	130668957	G	A	0.1684	-0.5380	0.0584	3.16E-20	84.71
CD4 NC	*AK3*	rs10123267	9	4757261	T	G	0.0577	-0.5301	0.0943	1.88E-08	31.55
CD4 NC	*AKAP10*	rs385301	17	19806828	T	C	0.2504	0.3432	0.0514	2.49E-11	44.45
CD4 NC	*AKAP12*	rs2011783	6	151625377	A	C	0.0816	0.6926	0.0874	2.33E-15	62.63
CD4 NC	*AKAP8*	rs10419500	19	15486404	G	C	0.7543	0.4236	0.0516	2.16E-16	67.31
CD4 NC	*AKIP1*	rs2742519	11	8933853	G	T	0.5965	0.4528	0.0457	3.72E-23	98.03
CD4 NC	*AKIRIN1*	rs34848020	1	39453785	A	G	0.4368	-0.3830	0.0448	1.17E-17	73.06
CD4 NC	*AKR1C2*	rs67362530	10	5043504	A	G	0.1231	0.5689	0.0706	7.57E-16	64.85
CD4 NC	*AKR1C3*	rs9423597	10	5336050	C	G	0.3648	0.4474	0.0457	1.27E-22	95.61
CD4 NC	*ALDH7A1*	rs79449010	5	125919818	C	T	0.0385	-0.5606	0.1027	4.75E-08	29.76
CD4 NC	*ALDH9A1*	rs12408101	1	165667781	G	T	0.2555	-0.2918	0.0499	5.03E-09	34.11
CD4 NC	*ALG1L*	rs7617162	3	125755450	T	C	0.3523	0.3932	0.0459	1.13E-17	73.12
CD4 NC	*ALKBH7*	rs79751509	19	6372221	T	C	0.0783	0.4978	0.0758	5.26E-11	42.99
CD4 NC	*ALOX5AP*	rs4075473	13	31307172	G	T	0.2356	-0.3117	0.0522	2.41E-09	35.54
CD4 NC	*ALPK1*	rs9995585	4	113224047	A	G	0.2642	0.4769	0.0506	4.29E-21	88.66
CD4 NC	*ALPK2*	rs112957150	18	56540830	A	G	0.1106	-0.7486	0.0738	3.38E-24	102.77
CD4 NC	*AMICA1*	rs10892230	11	118126682	A	C	0.2001	0.7549	0.0551	1.09E-42	187.16
CD4 NC	*ANAPC13*	rs934093	3	134195885	G	A	0.6795	0.5999	0.0469	1.70E-37	163.43
CD4 NC	*ANGEL2*	rs4951475	1	213198068	C	G	0.2791	0.4223	0.0482	1.97E-18	76.56
CD4 NC	*ANK3*	rs7919274	10	62486467	C	T	0.1311	-0.6816	0.0617	2.41E-28	121.66
CD4 NC	*ANKDD1A*	rs1628955	15	65187220	C	T	0.6303	-0.9736	0.0409	4.32E-125	564.78
CD4 NC	*ANKDD1B*	rs10045497	5	74636484	A	C	0.3793	-0.2831	0.0459	6.83E-10	37.99
CD4 NC	*ANKH*	rs2934814	5	14872689	T	C	0.5145	-0.3939	0.0443	6.40E-19	78.78
CD4 NC	*ANKRD55*	rs7731626	5	55444683	A	G	0.3526	-0.7190	0.0446	1.53E-58	259.7
CD4 NC	*ANTXR2*	rs11721624	4	80897253	C	A	0.4435	0.2509	0.0448	2.22E-08	31.22
CD4 NC	*ANXA11*	rs12268619	10	81920660	C	A	0.1283	0.5847	0.0647	1.67E-19	81.43
CD4 NC	*ANXA2*	rs4775266	15	60690788	C	G	0.7144	-0.3030	0.0495	9.04E-10	37.44
CD4 NC	*ANXA2R*	rs550400	5	43054758	C	G	0.3222	0.3503	0.0477	2.04E-13	53.86
CD4 NC	*ANXA5*	rs6534309	4	122609929	C	T	0.0762	1.0045	0.0748	4.03E-41	180
CD4 NC	*ANXA6*	rs2303038	5	150535013	T	C	0.2046	0.6624	0.0531	1.00E-35	155.35
CD4 NC	*AOAH*	rs7785921	7	36762723	G	A	0.7373	-0.5515	0.0495	7.73E-29	123.92
CD4 NC	*AP000442.1*	rs12272999	11	59310017	C	G	0.0538	0.5679	0.1018	2.44E-08	31.05
CD4 NC	*AP000487.5*	rs55905028	11	70127750	A	G	0.1318	0.4456	0.0687	8.68E-11	42.01
CD4 NC	*AP000783.1*	rs55917769	11	123330649	A	C	0.1981	0.3026	0.0536	1.61E-08	31.85
CD4 NC	*AP000936.4*	rs11216277	11	116987046	A	G	0.6274	0.3904	0.0466	5.66E-17	69.95
CD4 NC	*AP001055.6*	rs9985064	21	45589077	G	A	0.8128	0.4490	0.0577	7.23E-15	60.41
CD4 NC	*AP001462.6*	rs12223914	11	64511886	T	G	0.0902	-0.4983	0.0810	7.59E-10	37.78
CD4 NC	*AP001816.1*	rs2044042	4	102236139	T	C	0.2893	-0.2899	0.0489	3.05E-09	35.08
CD4 NC	*AP003774.1*	rs627425	11	64087642	T	C	0.3608	0.8571	0.0421	5.60E-92	412.91
CD4 NC	*AP1M1*	rs448754	19	16358449	C	T	0.7331	0.3506	0.0521	1.74E-11	45.16
CD4 NC	*AP1S1*	rs6970512	7	100798165	G	C	0.7427	-0.3480	0.0519	1.96E-11	44.92
CD4 NC	*AP2A2*	rs4072741	11	976565	C	T	0.5387	0.2638	0.0450	4.58E-09	34.29
CD4 NC	*APOBEC3G*	rs5757467	22	39480259	T	C	0.6436	-0.4527	0.0460	7.57E-23	96.63
CD4 NC	*APOBEC3G*	rs60093835	22	39493356	T	C	0.1280	-0.4844	0.0706	6.88E-12	46.96
CD4 NC	*APOLD1*	rs35071163	12	12883507	C	G	0.4772	-0.4189	0.0442	2.68E-21	89.58
CD4 NC	*AQP8*	rs4787433	16	24323432	C	A	0.3718	-0.2731	0.0463	3.58E-09	34.77
CD4 NC	*ARC*	rs55878729	8	143373005	T	C	0.2648	-0.3252	0.0497	5.88E-11	42.77
CD4 NC	*ARFIP1*	rs11099853	4	153703306	C	T	0.4260	0.3027	0.0451	1.94E-11	44.94
CD4 NC	*ARHGAP11A*	rs34954129	15	32940190	T	A	0.2689	-0.3404	0.0516	4.37E-11	43.35
CD4 NC	*ARHGAP15*	rs13024411	2	144012024	G	A	0.1512	-0.7467	0.0627	1.07E-32	141.51
CD4 NC	*ARHGAP21*	rs4748960	10	24937430	T	C	0.5074	0.2669	0.0449	2.69E-09	35.32
CD4 NC	*ARHGAP44*	rs112616319	17	12925541	C	T	0.1556	0.3609	0.0622	6.46E-09	33.62
CD4 NC	*ARHGAP5*	rs1278926	14	32414145	A	C	0.6181	0.4533	0.0449	5.32E-24	101.88
CD4 NC	*ARHGEF10*	rs60054728	8	1806824	A	G	0.2958	0.7546	0.0470	6.82E-58	256.73
CD4 NC	*ARHGEF26*	rs1848952	3	154088751	T	A	0.0973	0.4300	0.0774	2.72E-08	30.83
CD4 NC	*ARHGEF3*	rs3924228	3	56760744	A	G	0.1635	1.0349	0.0580	3.37E-71	317.67
CD4 NC	*ARHGEF35*	rs1635077	7	144074629	G	A	0.5992	0.2857	0.0459	4.99E-10	38.6

CD4 NC	*ARHGEF5*	rs1635077	7	144074629	G	A	0.5992	-0.2883	0.0459	3.48E-10	39.3
CD4 NC	*ARID5B*	rs6479789	10	63947969	C	T	0.1658	0.3398	0.0589	8.18E-09	33.16
CD4 NC	*ARL14EP*	rs10835653	11	30347828	C	T	0.2920	-0.6945	0.0483	7.24E-47	206.27
CD4 NC	*ARL16*	rs7503637	17	79649515	A	G	0.1691	-1.0822	0.0546	1.64E-87	392.42
CD4 NC	*ARL17A*	rs754593	17	44054696	A	G	0.5787	0.4787	0.0447	9.45E-27	114.4
CD4 NC	*ARL17A*	rs41543512	17	44090778	A	T	0.2276	-0.4275	0.0554	1.20E-14	59.42
CD4 NC	*ARL17B*	rs41543512	17	44090778	A	T	0.2276	-0.5262	0.0550	1.01E-21	91.52
CD4 NC	*ARL17B*	rs117562189	17	44074133	A	G	0.0835	-0.5605	0.0869	1.11E-10	41.53
CD4 NC	*ARL6IP4*	rs4275659	12	123447928	C	T	0.6963	0.3542	0.0498	1.14E-12	50.48
CD4 NC	*ARNTL*	rs9783306	11	13276923	A	C	0.5477	0.2974	0.0452	4.81E-11	43.16
CD4 NC	*ARPC3*	rs6606687	12	110939421	C	T	0.7825	0.3811	0.0515	1.40E-13	54.59
CD4 NC	*ARPC4*	rs293786	3	9829226	C	A	0.6871	0.3231	0.0475	1.04E-11	46.17
CD4 NC	*ARPC5*	rs1174686	1	183606747	A	C	0.3475	0.4676	0.0456	1.10E-24	105
CD4 NC	*ARRDC4*	rs4965124	15	98537670	A	C	0.0677	0.4653	0.0813	1.03E-08	32.72
CD4 NC	*ARRDC5*	rs10404430	19	4892667	G	A	0.3417	0.3549	0.0470	4.48E-14	56.83
CD4 NC	*ARSA*	rs6151429	22	51063477	C	T	0.0698	-0.9637	0.0807	6.63E-33	142.47
CD4 NC	*ARSG*	rs72841868	17	66397740	G	A	0.4596	0.2514	0.0449	2.19E-08	31.26
CD4 NC	*ASB15*	rs7799354	7	123269819	G	A	0.4481	0.3860	0.0446	4.63E-18	74.88
CD4 NC	*ASB16-AS1*	rs730228	17	42239349	C	T	0.3208	-0.4565	0.0485	4.54E-21	88.54
CD4 NC	*ASB6*	rs4837393	9	132387665	C	A	0.5768	0.2591	0.0457	1.42E-08	32.1
CD4 NC	*ASB8*	rs12303741	12	48560820	A	G	0.2894	0.2720	0.0472	8.21E-09	33.16
CD4 NC	*ASL*	rs7788984	7	65915616	G	A	0.4333	-0.3003	0.0453	3.21E-11	43.95
CD4 NC	*ASNSD1*	rs4667299	2	190542687	T	G	0.7280	0.4743	0.0505	6.23E-21	87.92
CD4 NC	*ATF4*	rs1048310	22	39913282	G	T	0.3109	0.2822	0.0488	7.35E-09	33.37
CD4 NC	*ATF5*	rs7255188	19	50420156	T	C	0.3604	-0.3049	0.0469	8.16E-11	42.13
CD4 NC	*ATF6*	rs12029726	1	161787708	T	A	0.0731	0.8794	0.0869	4.53E-24	102.19
CD4 NC	*ATF7IP2*	rs2083953	16	10496828	G	A	0.5261	-0.4058	0.0448	1.27E-19	81.97
CD4 NC	*ATG10*	rs73138787	5	81568934	A	G	0.2898	0.4335	0.0476	8.39E-20	82.79
CD4 NC	*ATHL1*	rs3932433	11	289553	C	G	0.3751	0.6464	0.0437	1.89E-49	218.09
CD4 NC	*ATIC*	rs11651	2	216229692	G	A	0.3219	-0.2765	0.0477	6.72E-09	33.55
CD4 NC	*ATM*	rs2118309	11	108367453	C	A	0.6091	0.4387	0.0445	5.72E-23	97.18
CD4 NC	*ATP10A*	rs1345098	15	26057622	T	G	0.5584	-0.4431	0.0444	1.97E-23	99.29
CD4 NC	*ATP1B2*	rs2075230	17	7487108	G	A	0.1564	-0.5828	0.0616	3.01E-21	89.35
CD4 NC	*ATP1B3*	rs79251184	3	141577497	A	G	0.1370	0.3308	0.0604	4.32E-08	29.94
CD4 NC	*ATP5D*	rs3760987	19	1239881	T	C	0.2017	-0.3041	0.0557	4.68E-08	29.78
CD4 NC	*ATP5G2*	rs916134	12	54069803	G	A	0.2911	0.4837	0.0485	2.11E-23	99.15
CD4 NC	*ATP5G3*	rs268228	2	176046415	A	G	0.8115	-0.5522	0.0564	1.18E-22	95.75
CD4 NC	*ATP5S*	rs111253297	14	50782765	T	A	0.5900	-0.3059	0.0452	1.31E-11	45.71
CD4 NC	*ATP6AP1L*	rs6894520	5	81594748	T	C	0.2925	0.5036	0.0473	1.91E-26	113.01
CD4 NC	*ATP6V0E2*	rs4725834	7	149568262	G	A	0.5695	-0.6859	0.0434	3.91E-56	248.68
CD4 NC	*ATP6V1E2*	rs1868844	2	46768559	A	G	0.3430	0.2849	0.0469	1.19E-09	36.91
CD4 NC	*ATP6V1G3*	rs1384443	1	199125879	A	G	0.2040	-0.6612	0.0580	3.74E-30	129.91
CD4 NC	*ATP8A1*	rs4572908	4	42837764	T	A	0.0680	0.6438	0.0986	6.70E-11	42.52
CD4 NC	*ATP8A2*	rs9578952	13	26591867	A	G	0.4464	0.2992	0.0450	2.97E-11	44.1
CD4 NC	*ATP9B*	rs487395	18	76806843	G	A	0.0986	0.4820	0.0762	2.50E-10	39.95
CD4 NC	*AVL9*	rs10274254	7	32557457	G	T	0.5236	-0.2709	0.0453	2.20E-09	35.72
CD4 NC	*AZI2*	rs1606393	3	28329149	G	A	0.4984	-0.2457	0.0449	4.33E-08	29.93
CD4 NC	*B3GALNT1*	rs6774725	3	160604849	T	C	0.2696	0.3348	0.0516	8.39E-11	42.08
CD4 NC	*B3GAT2*	rs495433	6	71606987	T	C	0.1306	-0.4642	0.0681	9.29E-12	46.38
CD4 NC	*B4GALNT3*	rs7314691	12	661981	A	G	0.2600	0.4877	0.0501	2.26E-22	94.47
CD4 NC	*B4GALT1*	rs6476398	9	33119241	T	C	0.3350	0.6843	0.0453	1.34E-51	227.92
CD4 NC	*B4GALT4*	rs9842789	3	118957370	A	G	0.1995	0.4597	0.0557	1.54E-16	67.98
CD4 NC	*BANF1*	rs649000	11	65742933	C	T	0.4517	-0.3443	0.0446	1.17E-14	59.47
CD4 NC	*BBC3*	rs10424953	19	47705283	A	G	0.2903	-0.2736	0.0489	2.23E-08	31.22
CD4 NC	*BBS2*	rs13337155	16	56504724	C	G	0.2250	-0.3550	0.0508	2.74E-12	48.76
CD4 NC	*BCAS1*	rs290427	20	52673829	A	G	0.4876	-0.3113	0.0448	3.88E-12	48.09
CD4 NC	*BCAS4*	rs2426198	20	49421524	A	G	0.1192	0.8171	0.0676	1.15E-33	145.94
CD4 NC	*BCAS4*	rs12480466	20	49496121	A	G	0.3425	-0.4298	0.0468	3.88E-20	84.31
CD4 NC	*BCL2L14*	rs4763773	12	12225613	G	A	0.2572	0.4864	0.0496	1.04E-22	96
CD4 NC	*BDH2*	rs2623060	4	104047504	A	G	0.4037	0.5105	0.0449	6.84E-30	128.72
CD4 NC	*BEGAIN*	rs11845599	14	101016824	G	A	0.3854	-0.4196	0.0462	9.79E-20	82.48
CD4 NC	*BIK*	rs4988417	22	43520258	C	A	0.8713	0.4552	0.0652	2.83E-12	48.7
CD4 NC	*BIN1*	rs11682128	2	127839474	A	G	0.3433	0.8404	0.0434	1.80E-83	373.91
CD4 NC	*BIN2*	rs4762045	12	51716766	A	G	0.2689	-0.3186	0.0509	3.95E-10	39.06
CD4 NC	*BLK*	rs62489069	8	11338383	G	A	0.2876	-0.4762	0.0488	1.56E-22	95.19
CD4 NC	*BLMH*	rs4429345	17	28458105	G	A	0.4577	0.2565	0.0450	1.23E-08	32.38
CD4 NC	*BLOC1S5*	rs2144432	6	8007178	T	C	0.6334	0.3210	0.0474	1.25E-11	45.8
CD4 NC	*BLVRA*	rs6945433	7	43858926	T	C	0.3909	0.2806	0.0458	8.91E-10	37.47
CD4 NC	*BOLA3-AS1*	rs61239760	2	74412502	T	C	0.1035	0.5529	0.0850	7.63E-11	42.27
CD4 NC	*BPGM*	rs6953140	7	134372275	G	A	0.6221	-0.2665	0.0460	6.76E-09	33.53
CD4 NC	*BRK1*	rs112594161	3	10177860	G	C	0.1089	-0.6193	0.0681	1.01E-19	82.43
CD4 NC	*BST2*	rs8106139	19	17506176	C	T	0.8053	0.7698	0.0615	6.32E-36	156.26
CD4 NC	*BTBD9*	rs9470807	6	38139945	G	A	0.7053	-0.5758	0.0502	1.91E-30	131.24
CD4 NC	*BTF3*	rs10942655	5	72674031	A	G	0.0712	-0.9767	0.0938	2.06E-25	108.31
CD4 NC	*BTN2A1*	rs9379880	6	26496916	A	G	0.1768	0.6736	0.0584	8.24E-31	132.91
CD4 NC	*BTN3A1*	rs3208733	6	26414425	T	C	0.4473	0.3071	0.0456	1.57E-11	45.35
CD4 NC	*BTN3A2*	rs67509210	6	26354100	T	G	0.1018	-1.0559	0.0630	5.64E-63	280
CD4 NC	*C10orf128*	rs7082074	10	50399844	C	A	0.3430	-0.5611	0.0457	1.36E-34	150.18
CD4 NC	*C10orf32*	rs11191421	10	104623053	G	C	0.2357	-0.6871	0.0511	3.81E-41	180.11

CD4 NC	*C10orf54*	rs748113	10	73508791	C	T	0.4424	0.2970	0.0450	4.31E-11	43.38
CD4 NC	*C10orf67*	rs11597935	10	23589787	C	A	0.2427	-0.3086	0.0508	1.27E-09	36.78
CD4 NC	*C10ORF68*	rs11009122	10	33171251	A	G	0.2015	0.3340	0.0553	1.57E-09	36.37
CD4 NC	*C10orf95*	rs72845701	10	104197327	G	A	0.1184	-0.7368	0.0754	1.41E-22	95.4
CD4 NC	*C11orf21*	rs2077112	11	2326451	G	T	0.8654	-0.6358	0.0670	2.34E-21	89.85
CD4 NC	*C11orf24*	rs10896314	11	68054529	T	G	0.3933	0.4132	0.0460	2.51E-19	80.62
CD4 NC	*C11orf63*	rs59230519	11	122736270	G	A	0.1008	0.4686	0.0751	4.32E-10	38.88
CD4 NC	*C11orf71*	rs4938102	11	114291259	G	A	0.4170	-0.3894	0.0448	3.29E-18	75.55
CD4 NC	*C12orf56*	rs34390271	12	64746063	G	A	0.2883	-0.5705	0.0485	5.81E-32	138.17
CD4 NC	*C14orf132*	rs11160298	14	96457297	A	T	0.7929	-0.5609	0.0552	3.03E-24	102.99
CD4 NC	*C14orf178*	rs72685232	14	78133961	T	C	0.1358	-0.5341	0.0657	4.42E-16	65.91
CD4 NC	*C14orf2*	rs11850704	14	104381687	G	A	0.0909	-0.6131	0.0835	2.14E-13	53.76
CD4 NC	*C14orf79*	rs7151377	14	105471886	T	C	0.5113	-0.2459	0.0449	4.27E-08	29.96
CD4 NC	*C15orf40*	rs6603041	15	83670073	C	A	0.5393	0.5117	0.0437	1.01E-31	137.08
CD4 NC	*C15orf61*	rs4776366	15	67882031	G	A	0.8927	0.4370	0.0778	1.96E-08	31.47
CD4 NC	*C16orf13*	rs117667029	16	686337	A	G	0.1175	0.6828	0.0669	1.76E-24	104.07
CD4 NC	*C17orf49*	rs12449315	17	6919636	T	C	0.0734	0.6236	0.0944	4.02E-11	43.51
CD4 NC	*C17orf50*	rs4795090	17	34123597	G	A	0.1858	0.4902	0.0602	3.85E-16	66.18
CD4 NC	*C17orf67*	rs117486106	17	54911028	G	C	0.1121	0.3951	0.0698	1.52E-08	31.96
CD4 NC	*C17orf89*	rs2659008	17	79217002	G	A	0.5311	0.5334	0.0435	1.61E-34	149.84
CD4 NC	*C17orf97*	rs11150882	17	259648	A	G	0.2620	0.8191	0.0491	2.27E-62	277.23
CD4 NC	*C19orf12*	rs62105832	19	30186564	C	T	0.7449	0.3593	0.0521	5.32E-12	47.47
CD4 NC	*C19orf24*	rs12979308	19	1275987	G	C	0.7769	0.4579	0.0510	2.62E-19	80.54
CD4 NC	*C19orf48*	rs55716643	19	51297825	A	G	0.2845	0.4379	0.0493	6.26E-19	78.82
CD4 NC	*C19orf53*	rs8110989	19	13882052	C	G	0.1347	-0.5659	0.0677	6.27E-17	69.75
CD4 NC	*C19orf83*	rs2286756	19	46145025	T	G	0.5303	-0.5295	0.0436	5.82E-34	147.29
CD4 NC	*C1orf174*	rs61770379	1	3827481	T	C	0.3086	0.2696	0.0491	4.00E-08	30.09
CD4 NC	*C1orf228*	rs16832228	1	45224757	A	G	0.3848	-0.3999	0.0456	1.79E-18	76.75
CD4 NC	*C1orf86*	rs11587831	1	2110848	G	T	0.2239	-0.6695	0.0529	9.11E-37	160.1
CD4 NC	*C1QTNF6*	rs4821603	22	37609294	G	A	0.5134	-0.2637	0.0448	3.99E-09	34.56
CD4 NC	*C1QTNF9B-AS1*	rs2119492	13	25110514	A	T	0.2640	-0.4493	0.0492	7.12E-20	83.11
CD4 NC	*C2*	rs2517474	6	31046494	A	G	0.5314	-0.2607	0.0448	6.08E-09	33.74
CD4 NC	*C20orf201*	rs4372964	20	62707527	T	C	0.4502	-0.2576	0.0453	1.28E-08	32.29
CD4 NC	*C20orf27*	rs45495794	20	3734167	C	T	0.0479	-0.8631	0.0969	5.28E-19	79.16
CD4 NC	*C20orf96*	rs6046446	20	258295	T	G	0.4763	0.2964	0.0447	3.40E-11	43.84
CD4 NC	*C21orf91*	rs1352602	21	19160959	C	T	0.2780	-0.3191	0.0502	2.11E-10	40.28
CD4 NC	*C22orf34*	rs5769712	22	50027210	T	C	0.5868	-0.6960	0.0440	1.84E-56	250.18
CD4 NC	*C2ORF15*	rs720229	2	99704273	C	T	0.6102	-0.3990	0.0458	3.20E-18	75.61
CD4 NC	*C2orf27A*	rs6720375	2	132440073	C	G	0.5865	0.7609	0.0421	7.23E-73	325.31
CD4 NC	*C2orf40*	rs13014521	2	106687456	C	G	0.1231	-0.5646	0.0663	1.62E-17	72.41
CD4 NC	*C2orf74*	rs1177307	2	61387388	C	T	0.4492	0.3208	0.0456	1.94E-12	49.45
CD4 NC	*C3orf14*	rs35559789	3	62258871	A	G	0.1639	-0.3827	0.0608	3.04E-10	39.57
CD4 NC	*C4BPB*	rs7542292	1	207268779	G	A	0.4783	0.3169	0.0446	1.24E-12	50.32
CD4 NC	*C4orf32*	rs909033	4	113097758	T	G	0.3790	-0.6137	0.0451	3.80E-42	184.69
CD4 NC	*C4orf33*	rs337274	4	130056680	G	T	0.8159	-0.3937	0.0584	1.51E-11	45.43
CD4 NC	*C5orf17*	rs13155485	5	23977151	G	A	0.1617	-0.4961	0.0559	7.32E-19	78.52
CD4 NC	*C5orf17*	rs16893334	5	24403598	A	G	0.0592	-0.6453	0.0914	1.62E-12	49.79
CD4 NC	*C5orf45*	rs7713219	5	179283170	C	T	0.4522	0.3496	0.0448	6.16E-15	60.73
CD4 NC	*C5orf55*	rs3734141	5	478655	A	G	0.7881	0.3070	0.0562	4.57E-08	29.83
CD4 NC	*C5orf63*	rs28528146	5	126205155	G	A	0.4720	-0.3956	0.0443	4.44E-19	79.5
CD4 NC	*C6orf132*	rs9394866	6	42074106	G	A	0.4718	-0.2480	0.0449	3.39E-08	30.41
CD4 NC	*C6orf25*	rs2249742	6	31240721	T	C	0.4906	0.2600	0.0450	7.49E-09	33.33
CD4 NC	*C6orf48*	rs6921489	6	31804646	T	C	0.1281	-0.6751	0.0680	3.29E-23	98.28
CD4 NC	*C6orf57*	rs7741265	6	71285378	G	A	0.1655	-0.3504	0.0612	1.02E-08	32.73
CD4 NC	*C6orf62*	rs3756814	6	24705835	C	T	0.3832	-0.2677	0.0459	5.71E-09	33.86
CD4 NC	*C7orf13*	rs4716437	7	156442035	C	T	0.2688	-0.3395	0.0508	2.45E-11	44.48
CD4 NC	*C9orf135*	rs7029206	9	72433081	A	G	0.1267	1.4962	0.0597	1.55E-138	626.47
CD4 NC	*C9orf142*	rs925936	9	139845672	T	C	0.2652	-0.3161	0.0507	4.62E-10	38.75
CD4 NC	*C9orf156*	rs3780419	9	100677134	C	T	0.2565	0.5276	0.0498	3.35E-26	111.9
CD4 NC	*C9orf16*	rs4075428	9	130940121	A	G	0.6506	0.2743	0.0470	5.24E-09	34.03
CD4 NC	*C9orf72*	rs4879540	9	27527479	T	A	0.5217	-0.2833	0.0450	2.97E-10	39.62
CD4 NC	*C9orf78*	rs8113	9	132589606	C	T	0.2433	-0.6828	0.0502	3.36E-42	184.93
CD4 NC	*CA6*	rs12091891	1	9000220	G	A	0.0626	1.1969	0.0960	1.10E-35	155.16
CD4 NC	*CA6*	rs10864376	1	9030372	T	C	0.2947	0.4881	0.0497	8.76E-23	96.34
CD4 NC	*CACNA1A*	rs7254351	19	13317980	T	G	0.4403	-0.2702	0.0459	3.94E-09	34.58
CD4 NC	*CALM3*	rs57848713	19	47122671	A	G	0.3495	-0.4761	0.0461	5.62E-25	106.32
CD4 NC	*CALR*	rs2974751	19	13053034	C	A	0.6165	0.3154	0.0458	5.48E-12	47.41
CD4 NC	*CAMK4*	rs114378220	5	110566360	T	C	0.0635	-0.8700	0.0877	3.57E-23	98.11
CD4 NC	*CAMKK2*	rs1169809	12	121688026	A	G	0.2666	-0.3121	0.0498	3.55E-10	39.27
CD4 NC	*CAMLG*	rs79082746	5	134052008	C	A	0.1125	0.6604	0.0698	2.97E-21	89.38
CD4 NC	*CAPG*	rs56352074	2	85584988	C	T	0.3591	0.6365	0.0444	1.33E-46	205.07
CD4 NC	*CAPN10*	rs2953171	2	241536053	T	C	0.1747	-0.3501	0.0598	4.75E-09	34.22
CD4 NC	*CAPN12*	rs35335944	19	39250308	A	G	0.2324	-0.3656	0.0538	1.10E-11	46.05
CD4 NC	*CARD8*	rs9676777	19	48758706	A	G	0.2169	0.5812	0.0508	2.50E-30	130.72
CD4 NC	*CARS*	rs11025044	11	3032753	G	A	0.4078	-0.3623	0.0455	1.62E-15	63.35
CD4 NC	*CARS2*	rs413235	13	111298906	C	T	0.2358	-0.3242	0.0558	6.39E-09	33.64
CD4 NC	*CASP6*	rs726585	4	110569547	C	T	0.6613	-0.2882	0.0477	1.48E-09	36.49
CD4 NC	*CASP7*	rs3124737	10	115497095	G	A	0.3994	0.2542	0.0455	2.27E-08	31.18
CD4 NC	*CASP8*	rs3185378	2	202151439	C	G	0.6057	0.4961	0.0455	1.19E-27	118.5

CD4 NC	*CAST*	rs10075736	5	95990057	T	C	0.1691	0.7117	0.0593	3.90E-33	143.52
CD4 NC	*CBR1*	rs11702262	21	37437223	T	C	0.5068	0.3519	0.0445	2.53E-15	62.47
CD4 NC	*CBR3*	rs11911059	21	37485863	G	A	0.4212	-0.6620	0.0431	3.26E-53	235.31
CD4 NC	*CBR4*	rs17054653	4	169934459	T	C	0.1065	0.4131	0.0717	8.42E-09	33.11
CD4 NC	*CBWD1*	rs573246	9	204014	G	A	0.4325	0.3524	0.0447	3.18E-15	62.03
CD4 NC	*CBWD2*	rs2441607	2	114235478	C	G	0.2032	-0.5278	0.0533	3.89E-23	97.94
CD4 NC	*CC2D1B*	rs72905646	1	53027908	T	C	0.0955	0.4078	0.0742	3.94E-08	30.12
CD4 NC	*CCDC101*	rs151226	16	28549823	C	A	0.5282	-0.5657	0.0434	6.77E-39	169.83
CD4 NC	*CCDC104*	rs2061032	2	55679490	C	T	0.6094	0.3628	0.0454	1.30E-15	63.79
CD4 NC	*CCDC109B*	rs4698748	4	110600928	C	G	0.6537	0.3411	0.0474	6.04E-13	51.73
CD4 NC	*CCDC115*	rs72854907	2	131078454	T	C	0.1923	0.3989	0.0545	2.55E-13	53.42
CD4 NC	*CCDC122*	rs11617551	13	44443977	C	T	0.4855	0.6329	0.0429	3.07E-49	217.13
CD4 NC	*CCDC127*	rs10054902	5	219022	A	G	0.2555	-0.3005	0.0512	4.39E-09	34.37
CD4 NC	*CCDC146*	rs13227255	7	76843912	C	A	0.1408	-0.8125	0.0584	4.58E-44	193.46
CD4 NC	*CCDC152*	rs56046109	5	42744296	T	A	0.2832	0.5358	0.0485	2.01E-28	122.03
CD4 NC	*CCDC163P*	rs6688516	1	45985760	A	G	0.6896	-1.0104	0.0413	6.19E-132	596.17
CD4 NC	*CCDC167*	rs1757178	6	37486310	G	A	0.6345	0.9999	0.0410	3.18E-131	592.91
CD4 NC	*CCDC23*	rs17386017	1	43297082	G	T	0.2755	0.3409	0.0484	1.80E-12	49.59
CD4 NC	*CCDC50*	rs6779820	3	191046793	C	G	0.3165	0.4309	0.0495	3.04E-18	75.71
CD4 NC	*CCDC58*	rs3828362	3	122131777	G	C	0.4138	0.7601	0.0424	9.62E-72	320.16
CD4 NC	*CCDC65*	rs10875885	12	49287450	A	T	0.3968	-0.2702	0.0464	5.93E-09	33.79
CD4 NC	*CCDC66*	rs2317134	3	56648363	C	T	0.4898	0.5849	0.0432	9.12E-42	182.95
CD4 NC	*CCDC77*	rs10774154	12	496695	A	G	0.8047	0.3176	0.0549	7.11E-09	33.43
CD4 NC	*CCDC82*	rs11021552	11	96097932	T	C	0.1531	0.4437	0.0615	5.38E-13	51.96
CD4 NC	*CCDC88B*	rs479552	11	64110683	C	G	0.3664	-0.4223	0.0458	2.83E-20	84.93
CD4 NC	*CCDC88C*	rs7146002	14	91871672	G	T	0.4213	0.2677	0.0454	3.82E-09	34.64
CD4 NC	*CCNB1IP1*	rs12889262	14	20784117	G	A	0.4952	0.2705	0.0448	1.54E-09	36.41
CD4 NC	*CCND1*	rs660963	11	69386962	G	A	0.6387	0.2885	0.0472	9.70E-10	37.31
CD4 NC	*CCND3*	rs113756602	6	41937058	C	T	0.2603	-0.3580	0.0517	4.50E-12	47.8
CD4 NC	*CCS*	rs692892	11	66348503	T	C	0.5106	0.3079	0.0447	5.64E-12	47.35
CD4 NC	*CCSAP*	rs237749	1	229411709	T	G	0.6429	-0.5420	0.0466	3.28E-31	134.74
CD4 NC	*CCSER2*	rs61866492	10	86213924	C	T	0.1297	-1.0786	0.0655	6.86E-61	270.45
CD4 NC	*CCT3*	rs113279830	1	156299392	G	A	0.2804	-0.9195	0.0447	5.71E-94	422.04
CD4 NC	*CCT4*	rs17014577	2	62071338	C	T	0.1564	0.4844	0.0645	5.99E-14	56.26
CD4 NC	*CCT8*	rs965951	21	30432557	A	G	0.1160	1.5120	0.0590	4.66E-145	656.39
CD4 NC	*CCZ1*	rs28558301	7	5937428	C	A	0.5153	-0.4515	0.0442	1.53E-24	104.34
CD4 NC	*CCZ1*	rs7789081	7	6862678	C	T	0.4738	0.7550	0.0418	7.13E-73	325.34
CD4 NC	*CCZ1B*	rs2711224	7	6861880	C	G	0.4738	-0.8528	0.0407	1.83E-97	438.06
CD4 NC	*CD101*	rs1555793	1	117557231	A	G	0.3560	0.2977	0.0468	2.06E-10	40.32
CD4 NC	*CD151*	rs72847212	11	834389	C	T	0.2156	-0.5204	0.0562	1.90E-20	85.72
CD4 NC	*CD164*	rs910730	6	109768295	A	G	0.4283	0.2863	0.0454	2.82E-10	39.72
CD4 NC	*CD247*	rs840012	1	167414872	T	C	0.3734	0.5059	0.0446	8.89E-30	128.2
CD4 NC	*CD37*	rs8107548	19	49870643	C	T	0.2603	-0.3197	0.0503	2.08E-10	40.31
CD4 NC	*CD3E*	rs59138930	11	118173783	T	C	0.3373	0.2918	0.0466	3.75E-10	39.16
CD4 NC	*CD3G*	rs45508093	11	118224059	G	A	0.1823	-0.5087	0.0563	1.68E-19	81.42
CD4 NC	*CD52*	rs11583258	1	26624270	T	C	0.2918	-0.4135	0.0477	4.34E-18	75.01
CD4 NC	*CD53*	rs343807	1	111329066	G	C	0.2339	-0.4525	0.0510	6.97E-19	78.61
CD4 NC	*CD53*	rs12043630	1	111470196	C	G	0.1744	0.4310	0.0603	8.70E-13	51.01
CD4 NC	*CD55*	rs1367067	1	207442364	T	C	0.2730	0.3266	0.0497	4.85E-11	43.15
CD4 NC	*CD59*	rs831625	11	33746436	G	C	0.6211	-0.3345	0.0460	3.63E-13	52.73
CD4 NC	*CD6*	rs4939491	11	60793722	A	G	0.4029	-0.4388	0.0450	1.72E-22	95.01
CD4 NC	*CD7*	rs116065945	17	80269470	T	C	0.1717	-0.3933	0.0572	5.96E-12	47.24
CD4 NC	*CD84*	rs1503846	1	160577620	G	A	0.9071	-0.5084	0.0879	7.34E-09	33.38
CD4 NC	*CDC16*	rs9590507	13	115058408	C	T	0.2384	0.3572	0.0540	3.68E-11	43.69
CD4 NC	*CDC42*	rs2268179	1	22414785	T	G	0.1644	0.6146	0.0596	6.03E-25	106.18
CD4 NC	*CDC42BPA*	rs58496521	1	227582375	C	T	0.1178	0.5029	0.0671	6.47E-14	56.11
CD4 NC	*CDC42EP3*	rs11124593	2	37851468	G	A	0.6509	-0.3289	0.0463	1.18E-12	50.41
CD4 NC	*CDC42SE2*	rs78623258	5	130628103	G	A	0.1843	0.3325	0.0560	2.85E-09	35.21
CD4 NC	*CDC7*	rs6688261	1	91987034	T	C	0.4764	-0.2707	0.0448	1.57E-09	36.37
CD4 NC	*CDCA7L*	rs12700311	7	21891451	G	A	0.7434	0.3484	0.0509	7.31E-12	46.85
CD4 NC	*CDHR3*	rs7800901	7	105571117	T	G	0.5132	0.3090	0.0447	4.82E-12	47.66
CD4 NC	*CDK10*	rs77651727	16	89708267	T	C	0.0591	-0.9091	0.0904	8.46E-24	100.96
CD4 NC	*CDK5R1*	rs7212466	17	30844384	T	C	0.4521	0.4667	0.0440	2.67E-26	112.35
CD4 NC	*CDO1*	rs2650482	5	115122414	G	A	0.8452	-0.6162	0.0611	6.25E-24	101.56
CD4 NC	*CDRT4*	rs8079010	17	16328955	C	T	0.6541	0.2636	0.0477	3.18E-08	30.53
CD4 NC	*CEACAM21*	rs3745936	19	42092815	A	T	0.7738	0.8270	0.0520	5.68E-57	252.51
CD4 NC	*CEBPG*	rs79824755	19	33865323	T	G	0.2282	-0.3429	0.0512	2.20E-11	44.7
CD4 NC	*CEBPZ*	rs10206380	2	37454286	G	A	0.5380	-0.3565	0.0445	1.15E-15	64.02
CD4 NC	*CEBPZ-AS1*	rs11689186	2	37446288	G	A	0.1329	-0.9713	0.0626	2.52E-54	240.4
CD4 NC	*CELA1*	rs12829179	12	51719989	G	C	0.2883	0.9083	0.0437	4.11E-96	431.86
CD4 NC	*CELSR1*	rs35364389	22	46760086	T	C	0.1609	0.6423	0.0621	4.90E-25	106.59
CD4 NC	*CENPC*	rs56073686	4	68303234	A	G	0.0884	-0.4592	0.0718	1.59E-10	40.83
CD4 NC	*CENPK*	rs33394	5	64778944	A	G	0.6081	-1.2762	0.0348	1.00E-200	1338.74
CD4 NC	*CENPM*	rs9620001	22	42330229	C	T	0.2102	0.6339	0.0549	8.25E-31	132.91
CD4 NC	*CENPN*	rs35115578	16	80980289	G	A	0.1398	0.3647	0.0642	1.37E-08	32.17
CD4 NC	*CENPP*	rs10992270	9	94961364	T	C	0.1799	0.3357	0.0592	1.40E-08	32.12
CD4 NC	*CENPU*	rs4862407	4	185654683	C	T	0.6515	0.2633	0.0471	2.28E-08	31.18
CD4 NC	*CENPV*	rs9907546	17	16248083	A	T	0.5217	-0.2828	0.0448	2.71E-10	39.79
CD4 NC	*CENPW*	rs9398804	6	126703390	A	T	0.4714	-0.2519	0.0450	2.21E-08	31.24

CD4 NC	*CEP170B*	rs2841216	14	105322519	G	A	0.6457	0.3694	0.0463	1.50E-15	63.5
CD4 NC	*CEP63*	rs9857242	3	134316378	T	C	0.6497	0.3065	0.0470	6.83E-11	42.48
CD4 NC	*CERS4*	rs62126381	19	8272852	C	T	0.5011	0.3872	0.0444	2.57E-18	76.04
CD4 NC	*CES1*	rs62028648	16	55864517	T	C	0.5947	0.3782	0.0453	6.61E-17	69.64
CD4 NC	*CFD*	rs1683563	19	861593	G	C	0.5805	-0.7406	0.0439	5.59E-64	284.6
CD4 NC	*CFL2*	rs10139365	14	35187323	C	A	0.2237	0.5168	0.0536	5.78E-22	92.61
CD4 NC	*CFLAR*	rs76410935	2	202033322	C	T	0.4989	-0.3888	0.0444	1.91E-18	76.63
CD4 NC	*CHAF1A*	rs760369	19	4449287	C	T	0.2689	-0.3140	0.0511	7.90E-10	37.71
CD4 NC	*CHCHD10*	rs179465	22	24106008	G	C	0.9206	0.6268	0.0815	1.43E-14	59.07
CD4 NC	*CHCHD2*	rs11764629	7	56155035	T	C	0.2157	0.8306	0.0509	8.05E-60	265.55
CD4 NC	*CHCHD5*	rs1437675	2	113298586	C	T	0.5776	0.2860	0.0452	2.48E-10	39.96
CD4 NC	*CHGB*	rs6107697	20	5776083	G	A	0.5834	0.3829	0.0452	2.34E-17	71.69
CD4 NC	*CHI3L2*	rs942697	1	111791503	C	G	0.6352	-0.7957	0.0434	3.42E-75	335.97
CD4 NC	*CHMP2B*	rs12486932	3	87321530	T	C	0.2804	0.3280	0.0497	4.29E-11	43.39
CD4 NC	*CHMP3*	rs4832298	2	86764004	T	C	0.6917	-0.2800	0.0489	1.00E-08	32.77
CD4 NC	*CHMP4A*	rs1242017	14	24675569	G	A	0.2022	-0.6170	0.0561	3.70E-28	120.82
CD4 NC	*CHN1*	rs17198248	2	175703427	G	T	0.4468	-0.5013	0.0441	6.96E-30	128.69
CD4 NC	*CHORDC1*	rs12362112	11	89991692	T	C	0.1960	0.3371	0.0539	4.14E-10	38.97
CD4 NC	*CHPT1*	rs3809260	12	102091017	T	G	0.5805	0.8868	0.0407	4.54E-105	472.93
CD4 NC	*CHRNA1*	rs3816448	2	175613059	C	A	0.3815	-0.2756	0.0459	1.88E-09	36.02
CD4 NC	*CHURC1*	rs10129479	14	65375180	G	A	0.2134	0.8575	0.0549	6.12E-55	243.21
CD4 NC	*CIB1*	rs12904427	15	90789670	T	C	0.1696	-0.7513	0.0582	4.16E-38	166.23
CD4 NC	*CISD1*	rs1867571	10	60038850	G	A	0.2257	-0.3269	0.0567	8.03E-09	33.2
CD4 NC	*CKB*	rs11627052	14	103863476	A	G	0.2119	0.3290	0.0540	1.08E-09	37.1
CD4 NC	*CKMT2-AS1*	rs62365038	5	80597374	C	T	0.0982	-0.6136	0.0751	3.19E-16	66.55
CD4 NC	*CLEC11A*	rs919276	19	51231068	C	T	0.7229	-0.3091	0.0496	4.59E-10	38.76
CD4 NC	*CLEC18A*	rs3192882	16	69975631	T	A	0.3726	-0.2656	0.0467	1.26E-08	32.32
CD4 NC	*CLEC2B*	rs33871	12	10007897	T	C	0.5491	0.4303	0.0444	3.06E-22	93.87
CD4 NC	*CLHC1*	rs17046778	2	55459373	G	T	0.1593	0.4730	0.0607	6.43E-15	60.64
CD4 NC	*CLIC4*	rs2375113	1	25525915	G	A	0.1972	0.3207	0.0550	5.37E-09	33.98
CD4 NC	*CLIP3*	rs62111376	19	36598239	C	T	0.1288	0.4159	0.0706	3.89E-09	34.61
CD4 NC	*CLK4*	rs262029	5	178016218	A	G	0.5839	0.3492	0.0449	7.88E-15	60.24
CD4 NC	*CLN8*	rs71499040	8	1711918	C	G	0.7034	0.2755	0.0494	2.41E-08	31.07
CD4 NC	*CLNS1A*	rs12574999	11	77354007	C	T	0.1123	0.6720	0.0756	5.98E-19	78.91
CD4 NC	*CLUAP1*	rs1859379	16	3608256	C	T	0.5093	0.4031	0.0442	8.24E-20	82.82
CD4 NC	*CMTM3*	rs34213783	16	66729193	C	T	0.4742	-0.3653	0.0445	2.31E-16	67.18
CD4 NC	*CMTM7*	rs149639463	3	32504331	T	C	0.0846	-0.8013	0.0800	1.36E-23	100.03
CD4 NC	*CMTM8*	rs7632486	3	32286791	A	G	0.8213	-1.2476	0.0522	4.60E-126	569.24
CD4 NC	*CMTM8*	rs6803740	3	32344306	G	A	0.1743	0.4811	0.0591	4.03E-16	66.09
CD4 NC	*CNIH4*	rs7517754	1	224544529	G	A	0.7539	0.3306	0.0544	1.24E-09	36.82
CD4 NC	*CNKSR1*	rs6664059	1	26500082	C	T	0.3127	-0.5280	0.0481	5.54E-28	120.02
CD4 NC	*CNN2*	rs2240169	19	1018830	T	C	0.3381	-0.3039	0.0472	1.18E-10	41.41
CD4 NC	*CNN2*	rs4807455	19	1033559	A	G	0.2556	0.5897	0.0489	1.88E-33	144.97
CD4 NC	*CNOT11*	rs2272320	2	101869588	T	G	0.4275	0.2897	0.0458	2.47E-10	39.97
CD4 NC	*CNOT6*	rs62405552	5	180009882	A	C	0.1039	0.4701	0.0757	5.42E-10	38.44
CD4 NC	*CNOT6L*	rs2866689	4	78636187	A	G	0.8956	-0.7691	0.0673	3.15E-30	130.25
CD4 NC	*COA1*	rs1181574	7	43804695	T	C	0.7111	-0.5576	0.0465	3.73E-33	143.61
CD4 NC	*COA6*	rs516710	1	234447432	C	G	0.3095	0.3334	0.0496	1.73E-11	45.16
CD4 NC	*COG5*	rs2520256	7	107162515	T	C	0.2300	0.7835	0.0511	4.57E-53	234.64
CD4 NC	*COL11A2*	rs9278025	6	33338508	A	T	0.3811	-0.2578	0.0468	3.61E-08	30.29
CD4 NC	*COL18A1*	rs2838942	21	46904833	G	A	0.1041	0.5306	0.0707	6.18E-14	56.2
CD4 NC	*COL18A1*	rs2274809	21	46906711	A	G	0.3304	-0.8288	0.0440	4.49E-79	353.76
CD4 NC	*COL18A1*	rs8128707	21	47392733	T	C	0.3561	0.2923	0.0480	1.15E-09	36.97
CD4 NC	*COL6A1*	rs11700724	21	47399969	T	C	0.2274	-0.3502	0.0502	3.10E-12	48.52
CD4 NC	*COL6A2*	rs55783934	21	47529348	A	G	0.1330	-0.5551	0.0647	1.00E-17	73.36
CD4 NC	*COMMD5*	rs59163703	8	146010166	C	T	0.3713	0.2857	0.0467	9.44E-10	37.36
CD4 NC	*COMMD6*	rs9543961	13	76106054	G	T	0.1381	-1.2253	0.0555	5.91E-108	486.17
CD4 NC	*COMMD7*	rs6057633	20	31326776	G	C	0.7410	0.3482	0.0522	2.61E-11	44.36
CD4 NC	*COMP*	rs12611313	19	18886367	C	A	0.1608	0.5263	0.0620	2.01E-17	71.98
CD4 NC	*COMTD1*	rs149901937	10	76929409	T	C	0.0614	1.0433	0.1003	2.44E-25	107.97
CD4 NC	*COPG2*	rs2288081	7	130357296	C	T	0.8403	0.3713	0.0656	1.55E-08	31.92
CD4 NC	*COQ6*	rs10400727	14	74471392	C	T	0.1436	-0.4152	0.0597	3.52E-12	48.28
CD4 NC	*CORO1B*	rs1808279	11	67211569	C	T	0.4238	-0.8475	0.0413	1.49E-93	420.13
CD4 NC	*COX11*	rs12952523	17	53044084	T	G	0.2270	-0.2864	0.0517	3.07E-08	30.6
CD4 NC	*COX14*	rs6580728	12	50541127	G	A	0.3378	0.3964	0.0461	7.80E-18	73.85
CD4 NC	*COX20*	rs10927331	1	244999396	A	G	0.1441	0.4663	0.0638	2.76E-13	53.26
CD4 NC	*COX4I1*	rs11863796	16	85815309	C	T	0.0558	0.7331	0.0974	5.29E-14	56.5
CD4 NC	*COX6B1*	rs4806187	19	36149761	G	A	0.5163	-0.5902	0.0431	1.36E-42	186.73
CD4 NC	*COX7A2L*	rs10204831	2	42623511	T	C	0.5872	-0.5365	0.0438	1.59E-34	149.87
CD4 NC	*CPA5*	rs11761888	7	130007381	C	T	0.2124	1.2915	0.0457	6.87E-176	797.89
CD4 NC	*CPNE1*	rs77120309	20	34218173	T	C	0.1101	-1.1190	0.0727	2.11E-53	236.17
CD4 NC	*CPNE3*	rs7015263	8	87526192	A	C	0.5205	-0.2528	0.0448	1.70E-08	31.74
CD4 NC	*CPSF1*	rs35817095	8	145600697	T	C	0.3667	-0.3925	0.0461	1.56E-17	72.48
CD4 NC	*CRELD2*	rs62234039	22	50338497	G	A	0.1376	0.4003	0.0639	3.71E-10	39.18
CD4 NC	*CRHR1*	rs17688916	17	43778680	A	T	0.1923	0.4543	0.0543	6.34E-17	69.73
CD4 NC	*CRIP2*	rs4983411	14	105916797	A	G	0.6508	0.4824	0.0456	3.29E-26	111.94
CD4 NC	*CRIPAK*	rs57037487	4	1386652	G	A	0.1969	0.3367	0.0575	4.87E-09	34.17
CD4 NC	*CRLF3*	rs11650315	17	29025184	A	C	0.1129	-0.4567	0.0705	9.34E-11	41.87
CD4 NC	*CROCC*	rs761422	1	17301780	G	A	0.4632	0.3205	0.0446	6.82E-13	51.49

CD4 NC	*CROCCP2*	rs11203397	1	17237859	A	G	0.8325	-0.3712	0.0601	6.47E-10	38.1
CD4 NC	*CRYBB2*	rs6004662	22	25862124	A	G	0.0960	1.5873	0.0791	1.77E-89	401.45
CD4 NC	*CRYGN*	rs6965504	7	151138704	C	T	0.3769	0.6217	0.0456	2.83E-42	185.27
CD4 NC	*CRYL1*	rs14236	13	21063524	G	A	0.2470	-0.3296	0.0545	1.48E-09	36.49
CD4 NC	*CRYZ*	rs1465483	1	75195243	C	T	0.6770	-0.6690	0.0455	6.50E-49	215.63
CD4 NC	*CSGALNACT1*	rs28508542	8	19233386	G	T	0.0783	0.6765	0.0874	1.01E-14	59.75
CD4 NC	*CSGALNACT1*	rs28428748	8	19614064	G	C	0.1565	0.4996	0.0586	1.52E-17	72.54
CD4 NC	*CSNK2A2*	rs2550357	16	58213567	C	T	0.1441	-0.4560	0.0632	5.33E-13	51.97
CD4 NC	*CST7*	rs8121732	20	24920726	C	G	0.0834	-0.4074	0.0702	6.60E-09	33.58
CD4 NC	*CSTB*	rs35285321	21	45201832	A	G	0.3415	-0.6021	0.0468	7.98E-38	164.94
CD4 NC	*CTA-211A9.5*	rs5761704	22	27119061	T	C	0.5105	-0.2527	0.0448	1.73E-08	31.71
CD4 NC	*CTA-212D2.2*	rs2327582	6	135383995	A	G	0.3608	-0.6036	0.0449	3.58E-41	180.23
CD4 NC	*CTA-29F11.1*	rs12389	22	47158179	A	G	0.3392	-0.9308	0.0416	7.73E-111	499.39
CD4 NC	*CTA-445C9.15*	rs4275	22	26921725	G	A	0.4260	0.2555	0.0451	1.52E-08	31.96
CD4 NC	*CTB-102L5.4*	rs71354995	19	38791841	G	A	0.2753	0.3130	0.0512	1.00E-09	37.24
CD4 NC	*CTB-179K24.3*	rs10419090	19	45893215	A	C	0.2384	0.5719	0.0522	5.80E-28	119.93
CD4 NC	*CTBP1-AS2*	rs79746330	4	1243329	G	C	0.3664	-0.5191	0.0448	5.20E-31	133.82
CD4 NC	*CTC-241F20.3*	rs9676777	19	48758706	A	G	0.2169	-0.5134	0.0512	1.08E-23	100.48
CD4 NC	*CTC-281F24.1*	rs4796532	17	6556326	A	G	0.1887	0.4438	0.0601	1.46E-13	54.51
CD4 NC	*CTC-425F1.4*	rs2974755	19	13062663	T	C	0.6285	-0.3172	0.0459	4.93E-12	47.62
CD4 NC	*CTC-429P9.1*	rs7259285	19	17153149	A	G	0.4656	-0.4223	0.0444	1.71E-21	90.47
CD4 NC	*CTC-482H14.5*	rs1558138	19	5314996	T	C	0.4874	0.4034	0.0442	7.68E-20	82.96
CD4 NC	*CTC-503J8.4*	rs3787066	19	6223974	C	T	0.2689	0.2899	0.0500	6.81E-09	33.52
CD4 NC	*CTC-507E2.1*	rs1071965	17	33868756	G	A	0.1127	0.4545	0.0767	3.07E-09	35.07
CD4 NC	*CTC-508F8.1*	rs760030	16	21148685	T	C	0.2400	-0.3186	0.0529	1.67E-09	36.26
CD4 NC	*CTC-524C5.2*	rs1050456	17	5336188	G	A	0.2366	1.0873	0.0485	4.24E-111	500.59
CD4 NC	*CTD-2007H13.3*	rs34495	5	98265807	T	G	0.2870	0.6182	0.0471	2.49E-39	171.81
CD4 NC	*CTD-2017D11.1*	rs60068057	19	24220662	T	C	0.1886	0.3899	0.0572	9.61E-12	46.31
CD4 NC	*CTD-2020K17.4*	rs7212404	17	43317574	A	G	0.1718	-1.0153	0.0580	1.50E-68	305.53
CD4 NC	*CTD-2034I4.2*	rs4563005	15	82283954	A	G	0.1663	0.5785	0.0615	4.97E-21	88.36
CD4 NC	*CTD-2116N20.1*	rs11958387	5	64822031	G	A	0.3729	0.5146	0.0459	3.77E-29	125.34
CD4 NC	*CTD-2203K17.1*	rs3756663	5	33440306	A	C	0.2388	0.5176	0.0509	2.89E-24	103.08
CD4 NC	*CTD-2308L22.1*	rs1865506	2	221845006	T	C	0.8329	-0.4317	0.0596	4.21E-13	52.43
CD4 NC	*CTD-2324F15.2*	rs16876253	5	5777625	A	G	0.0684	-1.0733	0.0924	3.50E-31	134.61
CD4 NC	*CTD-2325M2.1*	rs72993911	19	8079591	C	T	0.4489	-0.3675	0.0447	1.93E-16	67.54
CD4 NC	*CTD-2336O2.1*	rs10102731	8	1709020	G	A	0.6983	0.8245	0.0456	3.82E-73	326.58
CD4 NC	*CTD-2357A8.3*	rs74559112	17	76631744	T	C	0.0751	0.8716	0.0927	5.11E-21	88.31
CD4 NC	*CTD-2368P22.1*	rs4801243	19	58513807	C	A	0.2297	0.9490	0.0486	4.39E-85	381.3
CD4 NC	*CTD-2371O3.2*	rs11042350	11	9453209	T	C	0.4988	0.9360	0.0394	1.05E-124	563.01
CD4 NC	*CTD-2515A14.1*	rs12596691	16	24844098	C	T	0.4956	0.4205	0.0442	1.69E-21	90.5
CD4 NC	*CTD-2521M24.9*	rs4808632	19	17518544	T	C	0.1883	-0.6628	0.0607	8.67E-28	119.13
CD4 NC	*CTD-2568A17.5*	rs11671709	19	51310220	A	T	0.2167	-0.6689	0.0520	7.93E-38	164.95
CD4 NC	*CTD-2571L23.8*	rs117556589	19	48398245	C	G	0.0648	0.7785	0.0808	5.59E-22	92.68
CD4 NC	*CTD-2651B20.3*	rs16941238	15	45561904	A	G	0.1218	0.6846	0.0725	3.56E-21	89.02
CD4 NC	*CTD-3037G24.4*	rs11863512	16	12580266	G	C	0.2807	0.5863	0.0495	2.38E-32	139.94
CD4 NC	*CTD-3093M3.1*	rs11881487	19	54904460	A	G	0.4377	-0.3459	0.0448	1.22E-14	59.38
CD4 NC	*CTD-3110H11.1*	rs62022570	15	50607155	C	T	0.3703	0.4736	0.0446	2.74E-26	112.3
CD4 NC	*CTD-3138B18.5*	rs2449635	19	58785227	T	C	0.6665	-0.3640	0.0471	1.07E-14	59.63
CD4 NC	*CTD-3162L10.1*	rs2438514	19	36706633	G	A	0.3956	-0.5241	0.0447	1.05E-31	136.99
CD4 NC	*CTLA4*	rs3087243	2	204738919	A	G	0.4420	0.8715	0.0405	1.04E-102	462.11
CD4 NC	*CTNNA1*	rs10065532	5	138292333	G	A	0.3528	-0.3035	0.0468	9.15E-11	41.91
CD4 NC	*CTNNAL1*	rs7022699	9	111904404	G	A	0.4346	-0.2562	0.0449	1.18E-08	32.46
CD4 NC	*CTSA*	rs3746499	20	44509762	C	T	0.6213	-0.2701	0.0462	4.88E-09	34.17
CD4 NC	*CTSB*	rs1630828	8	11745820	A	G	0.5437	-0.3839	0.0445	6.11E-18	74.33
CD4 NC	*CTSF*	rs2242663	11	66335308	C	T	0.2447	0.5314	0.0514	5.23E-25	106.47
CD4 NC	*CTSH*	rs12148472	15	79231478	C	T	0.1328	0.3938	0.0641	7.99E-10	37.68
CD4 NC	*CTSL*	rs3128499	9	90327095	A	G	0.4661	-0.7789	0.0414	7.80E-79	352.65
CD4 NC	*CTSS*	rs41271951	1	150737220	G	A	0.0680	-0.7321	0.0789	1.67E-20	85.97
CD4 NC	*CTSW*	rs596002	11	65645354	G	A	0.8002	1.2233	0.0490	1.49E-137	621.96
CD4 NC	*CUBN*	rs34434809	10	17187634	C	G	0.2990	0.3888	0.0486	1.22E-15	63.91
CD4 NC	*CYLD*	rs4595801	16	50369888	T	C	0.1729	0.3599	0.0625	8.31E-09	33.13
CD4 NC	*CYP2D6*	rs133376	22	42466905	T	C	0.4395	-0.2549	0.0449	1.35E-08	32.19
CD4 NC	*CYP4V2*	rs7687961	4	187122009	A	C	0.7941	-0.6748	0.0555	5.25E-34	147.5
CD4 NC	*D2HGDH*	rs71351115	2	242677184	A	G	0.2166	-0.3203	0.0552	6.42E-09	33.63
CD4 NC	*DAD1*	rs5742743	14	23056123	A	C	0.1243	0.6813	0.0649	8.28E-26	110.11
CD4 NC	*DAGLB*	rs13235365	7	6456091	T	C	0.2403	0.4159	0.0497	5.98E-17	69.84
CD4 NC	*DAP*	rs5745297	5	10680997	A	G	0.0825	0.5678	0.0845	1.87E-11	45.02
CD4 NC	*DAP3*	rs28582361	1	155753289	C	T	0.3506	-0.4117	0.0461	3.92E-19	79.74
CD4 NC	*DARS*	rs12373779	2	136586840	C	A	0.1983	0.6079	0.0668	8.52E-20	82.76
CD4 NC	*DAZAP1*	rs3848638	19	1383649	T	G	0.4593	-0.4276	0.0441	3.27E-22	93.74
CD4 NC	*DAZAP2*	rs7305016	12	51642543	G	A	0.6191	-0.3926	0.0461	1.77E-17	72.24
CD4 NC	*DBNDD1*	rs75163917	16	90081032	G	T	0.0581	0.9798	0.0932	7.80E-26	110.23
CD4 NC	*DCAF4*	rs12587742	14	73393391	A	G	0.2119	0.4360	0.0566	1.38E-14	59.14
CD4 NC	*DCBLD1*	rs210625	6	117874257	G	A	0.5751	0.2524	0.0452	2.34E-08	31.13
CD4 NC	*DCP2*	rs33563	5	112306279	C	T	0.3011	0.3613	0.0479	4.86E-14	56.67
CD4 NC	*DCT*	rs1891100	13	95203277	T	C	0.3540	-0.4794	0.0471	2.59E-24	103.3
CD4 NC	*DCTN5*	rs62032983	16	23653343	T	C	0.2145	0.3291	0.0544	1.49E-09	36.48
CD4 NC	*DCXR*	rs9903371	17	79993064	G	C	0.1249	1.1390	0.0630	5.04E-73	326.03
CD4 NC	*DDHD2*	rs2293971	8	38284011	T	C	0.2186	0.3162	0.0523	1.52E-09	36.43

CD4 NC	*DDR1-AS1*	rs9264641	6	31238377	C	G	0.8837	-0.5047	0.0636	2.04E-15	62.9
CD4 NC	*DDT*	rs5751775	22	24266726	C	T	0.5903	0.6370	0.0441	2.77E-47	208.18
CD4 NC	*DDTL*	rs5751777	22	24267047	T	C	0.5906	-0.5434	0.0448	7.47E-34	146.8
CD4 NC	*DDX11*	rs11051239	12	31241707	G	C	0.4307	-1.0032	0.0387	2.32E-148	671.54
CD4 NC	*DDX39B-AS1*	rs6911777	6	32409996	C	T	0.0591	-0.6577	0.0827	1.88E-15	63.06
CD4 NC	*DDX39B-AS1*	rs3094101	6	30613182	G	A	0.4008	0.4381	0.0445	7.54E-23	96.64
CD4 NC	*DDX42*	rs1982400	17	61793818	C	T	0.6006	0.3755	0.0455	1.57E-16	67.94
CD4 NC	*DDX5*	rs1991401	17	62502435	G	A	0.3173	-0.5170	0.0475	1.22E-27	118.46
CD4 NC	*DDX55*	rs35064645	12	124065152	T	C	0.2471	0.3313	0.0502	3.98E-11	43.54
CD4 NC	*DDX59*	rs11582610	1	200645101	T	C	0.4091	-0.2734	0.0453	1.66E-09	36.27
CD4 NC	*DDX6*	rs56758835	11	118615340	A	G	0.2150	-0.3225	0.0560	8.56E-09	33.08
CD4 NC	*DDX60L*	rs11728342	4	169361897	T	C	0.2320	0.3397	0.0534	1.96E-10	40.43
CD4 NC	*DEAF1*	rs10902190	11	678561	T	C	0.3000	0.4706	0.0467	6.61E-24	101.45
CD4 NC	*DECR2*	rs1204499	16	465589	G	A	0.5173	0.2711	0.0448	1.44E-09	36.55
CD4 NC	*DGCR6*	rs56333537	22	18888301	A	G	0.0871	-0.6450	0.0787	2.53E-16	67
CD4 NC	*DGCR6L*	rs2904577	22	20293625	A	G	0.4261	-0.3628	0.0448	5.84E-16	65.36
CD4 NC	*DGKQ*	rs11724804	4	965779	A	G	0.4417	0.3575	0.0446	1.07E-15	64.17
CD4 NC	*DHFR*	rs863216	5	79947431	G	A	0.2426	-0.5322	0.0516	6.43E-25	106.06
CD4 NC	*DHRS4*	rs147027077	14	24478799	T	A	0.0705	1.1475	0.0864	2.92E-40	176.07
CD4 NC	*DHRS7*	rs1254028	14	60632246	C	G	0.1879	-0.3917	0.0637	7.98E-10	37.69
CD4 NC	*DHX32*	rs10901473	10	127634125	A	G	0.4136	-0.3767	0.0447	3.82E-17	70.72
CD4 NC	*DHX58*	rs2277621	17	40264661	G	C	0.1940	-0.4395	0.0608	4.80E-13	52.18
CD4 NC	*DIEXF*	rs616702	1	209998053	G	A	0.5472	-0.4659	0.0443	7.11E-26	110.41
CD4 NC	*DIP2A*	rs11701571	21	47963149	G	A	0.2474	0.5401	0.0513	6.52E-26	110.58
CD4 NC	*DIS3L*	rs1978698	15	66666529	T	C	0.2363	-0.4633	0.0494	6.37E-21	87.87
CD4 NC	*DISC1*	rs6541280	1	231754879	C	G	0.6035	0.3676	0.0457	8.20E-16	64.69
CD4 NC	*DIXDC1*	rs11214048	11	111814258	G	C	0.3534	0.3420	0.0469	3.09E-13	53.04
CD4 NC	*DKK3*	rs7395522	11	12000778	C	T	0.8299	-0.5096	0.0600	2.09E-17	71.91
CD4 NC	*DMAP1*	rs67752000	1	44597213	A	G	0.2691	0.2787	0.0499	2.35E-08	31.12
CD4 NC	*DNAH11*	rs1174958	7	22042619	T	G	0.1913	-0.4600	0.0565	3.83E-16	66.18
CD4 NC	*DNAJA1*	rs10971389	9	33081123	C	T	0.3586	-0.4117	0.0462	5.10E-19	79.23
CD4 NC	*DNAJA3*	rs1291699	16	4448496	A	G	0.0759	0.4992	0.0797	3.73E-10	39.17
CD4 NC	*DNAJB1*	rs7253780	19	14643268	C	T	0.4532	-0.3249	0.0449	4.45E-13	52.33
CD4 NC	*DNAJC15*	rs17624230	13	43604332	A	T	0.2260	-1.1887	0.0459	7.83E-148	669.12
CD4 NC	*DNAJC16*	rs1467513	1	15894959	C	T	0.3175	0.4806	0.0474	4.03E-24	102.43
CD4 NC	*DNM1L*	rs11052138	12	32811798	A	C	0.2898	0.2764	0.0499	3.06E-08	30.61
CD4 NC	*DNMT1*	rs12984523	19	10306819	T	C	0.4954	-0.3284	0.0446	1.74E-13	54.17
CD4 NC	*DNPH1*	rs114371775	6	43199730	C	T	0.0828	-0.3858	0.0700	3.61E-08	30.29
CD4 NC	*DOCK8*	rs4741644	9	225834	A	T	0.0519	-0.5102	0.0917	2.63E-08	30.9
CD4 NC	*DOCK8*	rs12156499	9	370199	G	A	0.1449	0.6899	0.0626	3.08E-28	121.18
CD4 NC	*DPYSL4*	rs6560685	10	133967770	G	A	0.7408	-0.7273	0.0482	1.88E-51	227.25
CD4 NC	*DR1*	rs1426318	1	93847420	G	A	0.6352	-0.3788	0.0464	3.49E-16	66.37
CD4 NC	*DRAM2*	rs3762374	1	111682118	T	C	0.2783	0.6105	0.0469	1.00E-38	169.05
CD4 NC	*DRAP1*	rs642293	11	65730217	A	G	0.4467	-0.2858	0.0449	1.94E-10	40.45
CD4 NC	*DST*	rs34733863	6	56761170	C	G	0.2045	-0.6494	0.0535	6.07E-34	147.21
CD4 NC	*DSTN*	rs6075218	20	17485366	G	T	0.3389	0.3342	0.0475	1.90E-12	49.48
CD4 NC	*DSTN*	rs6131985	20	17670429	T	G	0.4306	0.2724	0.0457	2.53E-09	35.45
CD4 NC	*DTNBP1*	rs9476887	6	15662891	A	G	0.1376	0.3515	0.0619	1.33E-08	32.23
CD4 NC	*DUOX1*	rs12899786	15	45469694	G	C	0.3213	0.4109	0.0483	1.70E-17	72.32
CD4 NC	*DUS3L*	rs2260187	19	5799806	G	A	0.2170	-0.5029	0.0577	2.71E-18	75.93
CD4 NC	*DUT*	rs17350938	15	48589981	G	A	0.1448	-0.7353	0.0628	1.21E-31	136.71
CD4 NC	*DYNLL2*	rs8071026	17	56174489	A	G	0.6735	-0.6962	0.0455	8.04E-53	233.51
CD4 NC	*DYNLT1*	rs317792	6	159076766	T	G	0.6303	0.3088	0.0458	1.54E-11	45.39
CD4 NC	*DZIP3*	rs2278911	3	108298260	T	C	0.1156	-0.5876	0.0740	1.93E-15	63.01
CD4 NC	*EBPL*	rs1535470	13	50260029	A	G	0.6296	0.3144	0.0467	1.71E-11	45.18
CD4 NC	*EBPL*	rs3809325	13	50266109	C	T	0.5393	-0.8893	0.0405	7.07E-107	481.23
CD4 NC	*EDN3*	rs259964	20	57824309	G	A	0.5509	0.3720	0.0446	7.83E-17	69.31
CD4 NC	*EEA1*	rs4760498	12	93223366	A	G	0.6319	-0.5918	0.0448	6.96E-40	174.34
CD4 NC	*EEF1D*	rs896955	8	144658370	T	C	0.1390	-0.3755	0.0669	1.97E-08	31.46
CD4 NC	*EEF1D*	rs3812447	8	144671956	A	G	0.0734	0.6703	0.0843	1.79E-15	63.16
CD4 NC	*EEF2*	rs892128	19	3975487	A	G	0.8039	0.3319	0.0552	1.86E-09	36.04
CD4 NC	*EFCAB13*	rs11870252	17	45377825	C	T	0.0756	-0.6722	0.0874	1.47E-14	59.02
CD4 NC	*EFHB*	rs1133328	3	19987539	T	G	0.3696	0.3053	0.0457	2.39E-11	44.53
CD4 NC	*EGFL8*	rs9268851	6	32429582	G	A	0.8774	0.4312	0.0644	2.17E-11	44.72
CD4 NC	*EGLN3*	rs3102134	14	34514918	A	G	0.2482	-0.2963	0.0523	1.50E-08	31.99
CD4 NC	*EI24*	rs519772	11	125528391	G	A	0.3314	-0.5270	0.0452	2.25E-31	135.48
CD4 NC	*EIF2A*	rs6440683	3	150295376	T	C	0.8419	1.0355	0.0593	3.08E-68	304.11
CD4 NC	*EIF2B2*	rs175478	14	75563469	T	C	0.4527	-0.2892	0.0447	1.00E-10	41.73
CD4 NC	*EIF3G*	rs1037686	19	10222835	A	T	0.4408	0.5093	0.0438	3.36E-31	134.69
CD4 NC	*EIF3K*	rs2277941	19	39108723	T	C	0.0866	0.6409	0.0842	2.69E-14	57.83
CD4 NC	*EIF4E3*	rs13067349	3	71761554	C	T	0.6566	0.2823	0.0472	2.27E-09	35.65
CD4 NC	*EIF5A*	rs7503161	17	7207964	C	A	0.5615	-1.1994	0.0350	1.00E-200	1168.92
CD4 NC	*ELMO1*	rs2953535	7	37291877	A	T	0.2955	0.3384	0.0490	4.90E-12	47.63
CD4 NC	*ELMSAN1*	rs11625686	14	74120838	A	G	0.2976	-0.3798	0.0470	6.14E-16	65.26
CD4 NC	*ELOVL5*	rs2294867	6	53153954	A	C	0.3653	-0.2993	0.0469	1.67E-10	40.74
CD4 NC	*ELP2*	rs1970663	18	33749539	T	C	0.3882	0.2927	0.0461	2.24E-10	40.16
CD4 NC	*ELP5*	rs222843	17	7145981	C	T	0.3531	0.9356	0.0407	6.80E-117	527.17
CD4 NC	*EMB*	rs35567312	5	49626919	T	C	0.1147	0.5783	0.0710	3.92E-16	66.14
CD4 NC	*EMC2*	rs16877790	8	109403377	G	T	0.3306	0.3105	0.0472	4.82E-11	43.16

CD4 NC	*EML4*	rs1992259	2	42596520	T	C	0.4615	-0.3685	0.0446	1.44E-16	68.11
CD4 NC	*EMP3*	rs12463018	19	48831777	A	G	0.4138	-0.3135	0.0450	3.34E-12	48.38
CD4 NC	*EMR1*	rs4635445	19	6933754	G	A	0.4493	0.7249	0.0422	3.84E-66	294.5
CD4 NC	*ENC1*	rs77116527	5	73871908	A	G	0.1068	0.4321	0.0649	2.81E-11	44.22
CD4 NC	*ENDOD1*	rs503612	11	94863186	A	C	0.4391	0.4029	0.0450	3.72E-19	79.85
CD4 NC	*ENDOG*	rs3115875	9	131579182	T	C	0.6968	0.3830	0.0493	8.43E-15	60.11
CD4 NC	*ENO1*	rs112653519	1	8959008	T	C	0.2500	0.3294	0.0517	1.88E-10	40.5
CD4 NC	*ENOSF1*	rs2847157	18	714666	A	G	0.5483	-0.5384	0.0439	1.21E-34	150.41
CD4 NC	*ENTPD1*	rs2153913	10	97546426	G	C	0.5305	0.7549	0.0418	7.25E-73	325.31
CD4 NC	*ENTPD3-AS1*	rs9874499	3	40441908	T	C	0.3128	0.3342	0.0483	4.34E-12	47.87
CD4 NC	*ENTPD4*	rs10100628	8	23262008	C	T	0.5609	0.2841	0.0450	2.66E-10	39.83
CD4 NC	*EP400NL*	rs61944633	12	132573740	G	A	0.1323	-0.4647	0.0660	1.85E-12	49.54
CD4 NC	*EPB41L3*	rs7233680	18	5456725	C	T	0.3777	-0.3922	0.0456	7.48E-18	73.94
CD4 NC	*EPC1*	rs2806144	10	32554586	T	C	0.3913	0.2748	0.0458	1.91E-09	35.99
CD4 NC	*EPHA1-AS1*	rs7794735	7	143104816	A	G	0.0829	-1.0077	0.0767	2.16E-39	172.1
CD4 NC	*EPHA1-AS1*	rs68140767	7	143113114	C	T	0.1045	0.5336	0.0773	5.04E-12	47.57
CD4 NC	*EPHA4*	rs7583909	2	222395386	C	G	0.5238	0.2628	0.0448	4.46E-09	34.34
CD4 NC	*EPHB6*	rs10216164	7	142541882	C	T	0.3779	0.3869	0.0469	1.66E-16	67.83
CD4 NC	*EPHX2*	rs891401	8	27381194	C	G	0.1731	0.4935	0.0614	9.31E-16	64.44
CD4 NC	*EPPK1*	rs59265584	8	144950715	C	T	0.1180	-0.3993	0.0658	1.27E-09	36.78
CD4 NC	*EPS8L2*	rs4078520	11	723429	T	C	0.5046	0.4938	0.0439	2.39E-29	126.24
CD4 NC	*EPSTI1*	rs3825511	13	43566373	C	A	0.7178	-0.7780	0.0479	2.40E-59	263.38
CD4 NC	*ERAP1*	rs998509	5	96132795	A	G	0.1254	-0.3867	0.0636	1.17E-09	36.94
CD4 NC	*ERAP2*	rs55770741	5	96220087	T	C	0.5933	-1.2095	0.0344	1.00E-200	1232.88
CD4 NC	*ERCC6L2*	rs591486	9	98650046	G	A	0.4706	-0.2659	0.0449	3.15E-09	35.02
CD4 NC	*ERGIC2*	rs10843390	12	29496991	T	C	0.2943	0.3877	0.0500	8.85E-15	60.01
CD4 NC	*ERICH1*	rs1669681	8	606117	A	G	0.3766	-0.7234	0.0434	2.56E-62	276.99
CD4 NC	*ERO1L*	rs8009184	14	53018758	C	G	0.1355	0.4668	0.0665	2.19E-12	49.2
CD4 NC	*ERP27*	rs1005577	12	15066161	G	C	0.6087	0.2618	0.0456	9.19E-09	32.94
CD4 NC	*ERP29*	rs7114	12	112460749	G	A	0.1231	-0.5526	0.0741	8.76E-14	55.51
CD4 NC	*ESD*	rs1216967	13	47370637	G	A	0.5907	-0.2626	0.0451	5.76E-09	33.85
CD4 NC	*ESR2*	rs1268656	14	64647646	G	T	0.1128	0.6564	0.0694	3.19E-21	89.24
CD4 NC	*ESR2*	rs28418648	14	64698623	C	T	0.0760	1.2681	0.0889	4.02E-46	202.87
CD4 NC	*ESYT2*	rs13244187	7	158512748	G	C	0.6323	-0.2608	0.0466	2.17E-08	31.27
CD4 NC	*ETFB*	rs11881099	19	51865458	T	C	0.2837	-0.2997	0.0485	6.73E-10	38.02
CD4 NC	*ETFDH*	rs11559290	4	159601676	T	C	0.8491	0.3865	0.0635	1.13E-09	37.01
CD4 NC	*ETS1*	rs61907765	11	128391937	T	C	0.2133	0.3270	0.0561	5.54E-09	33.92
CD4 NC	*ETV7*	rs2234071	6	36355372	C	T	0.5322	0.4682	0.0442	3.15E-26	112.02
CD4 NC	*EVI2A*	rs2854319	17	29694054	C	G	0.6049	0.6191	0.0448	1.69E-43	190.86
CD4 NC	*EXOC3*	rs1056465	5	442438	A	T	0.3569	0.8022	0.0417	2.35E-82	368.79
CD4 NC	*EXOSC7*	rs3765173	3	45072769	T	C	0.4150	-0.2810	0.0453	5.43E-10	38.44
CD4 NC	*EYA3*	rs7521893	1	28329729	C	T	0.2593	-0.3748	0.0505	1.15E-13	54.97
CD4 NC	*EZR*	rs923198	6	159246717	T	C	0.4641	0.2820	0.0447	2.90E-10	39.66
CD4 NC	*F5*	rs3917751	1	169576568	A	G	0.3745	-0.3968	0.0467	2.03E-17	71.97
CD4 NC	*FAAH*	rs55707872	1	46680167	C	A	0.3035	0.3184	0.0484	4.73E-11	43.2
CD4 NC	*FADS1*	rs968567	11	61595564	T	C	0.1535	0.7037	0.0557	1.37E-36	159.29
CD4 NC	*FADS2*	rs77053629	11	61519815	T	C	0.0383	-0.5555	0.0981	1.47E-08	32.02
CD4 NC	*FADS2*	rs968567	11	61595564	T	C	0.1535	1.0340	0.0527	1.34E-85	383.66
CD4 NC	*FAHD1*	rs180754	16	1922840	T	C	0.8412	-0.4347	0.0629	4.63E-12	47.74
CD4 NC	*FAIM*	rs773456	3	138325495	C	T	0.5752	0.2529	0.0453	2.37E-08	31.1
CD4 NC	*FAM101B*	rs6598847	17	452403	G	A	0.8357	-0.4696	0.0571	1.91E-16	67.55
CD4 NC	*FAM104A*	rs35330281	17	71217074	T	G	0.5146	0.2634	0.0448	4.18E-09	34.47
CD4 NC	*FAM105A*	rs30610	5	14413568	T	C	0.3459	0.3495	0.0469	9.60E-14	55.33
CD4 NC	*FAM117B*	rs2351117	2	203627412	T	C	0.5528	-0.3748	0.0448	6.08E-17	69.81
CD4 NC	*FAM118A*	rs136582	22	45774531	A	T	0.1332	1.5635	0.0581	1.13E-159	723.46
CD4 NC	*FAM134A*	rs11680709	2	220050707	A	G	0.5685	0.2783	0.0460	1.43E-09	36.55
CD4 NC	*FAM134B*	rs256888	5	16622982	C	T	0.7257	0.5698	0.0492	4.73E-31	134.01
CD4 NC	*FAM13A*	rs2609282	4	89850263	G	A	0.8024	-0.7168	0.0578	2.33E-35	153.68
CD4 NC	*FAM153A*	rs62398510	5	177128865	T	G	0.3147	-0.4091	0.0469	2.66E-18	75.97
CD4 NC	*FAM153B*	rs1643771	5	175546551	A	G	0.4547	-0.8845	0.0402	1.72E-107	484.04
CD4 NC	*FAM153C*	rs2650339	5	177129851	C	A	0.5502	0.3125	0.0447	2.85E-12	48.69
CD4 NC	*FAM154B*	rs114586219	15	82537939	A	C	0.0705	0.9171	0.0803	3.49E-30	130.06
CD4 NC	*FAM184B*	rs12233712	4	17570063	A	T	0.6352	-1.1572	0.0375	1.00E-200	951.33
CD4 NC	*FAM19A1*	rs7428837	3	68106935	T	C	0.4680	-0.5101	0.0437	1.83E-31	135.89
CD4 NC	*FAM207A*	rs928304	21	46403059	T	C	0.1592	-0.4582	0.0612	7.04E-14	55.94
CD4 NC	*FAM211A*	rs55641029	17	16353905	A	G	0.1240	0.4758	0.0657	4.34E-13	52.37
CD4 NC	*FAM21A*	rs7077437	10	51824424	C	T	0.6068	0.4621	0.0450	1.07E-24	105.05
CD4 NC	*FAM26F*	rs1204836	6	116514253	G	A	0.7843	0.3363	0.0541	5.16E-10	38.54
CD4 NC	*FAM26F*	rs2640877	6	116793633	T	C	0.0601	1.4109	0.0921	6.15E-53	234.05
CD4 NC	*FAM43A*	rs813153	3	194407204	C	T	0.6337	-0.5796	0.0445	7.86E-39	169.53
CD4 NC	*FAM45A*	rs11198768	10	120857022	T	C	0.3911	-0.3560	0.0454	4.71E-15	61.25
CD4 NC	*FAM63B*	rs7161799	15	58770523	T	C	0.0869	0.7420	0.0787	4.34E-21	88.63
CD4 NC	*FAM63B*	rs452698	15	59105571	G	A	0.4236	0.2556	0.0457	2.27E-08	31.18
CD4 NC	*FAM66B*	rs2948305	8	8098577	A	G	0.4492	0.3427	0.0449	2.21E-14	58.21
CD4 NC	*FAM85A*	rs2572418	8	11113089	A	G	0.5408	-0.2770	0.0450	7.47E-10	37.82
CD4 NC	*FAM86C1*	rs12285436	11	71548465	G	A	0.5836	-0.2598	0.0455	1.17E-08	32.48
CD4 NC	*FAM92A1*	rs4585714	8	94915411	G	A	0.5822	0.2678	0.0451	2.97E-09	35.13
CD4 NC	*FAM98B*	rs8036878	15	38743892	C	A	0.2525	0.3784	0.0494	1.86E-14	58.55
CD4 NC	*FANCA*	rs72811597	16	89896005	G	C	0.2457	-0.3391	0.0492	5.79E-12	47.3

CD4 NC	*FANCF*	rs12805507	11	22656864	T	C	0.1920	0.3506	0.0565	5.42E-10	38.44
CD4 NC	*FANK1*	rs898325	10	127855523	C	A	0.8236	-0.3763	0.0593	2.18E-10	40.22
CD4 NC	*FAR1*	rs7103760	11	13722680	T	A	0.3322	0.7568	0.0451	3.05E-63	281.22
CD4 NC	*FAS*	rs9658742	10	90764891	C	G	0.5432	0.3585	0.0446	9.34E-16	64.43
CD4 NC	*FAU*	rs544944	11	64862234	T	C	0.4907	0.5389	0.0436	3.77E-35	152.72
CD4 NC	*FBLN5*	rs2267989	14	92339850	A	G	0.4380	-0.8669	0.0405	1.20E-101	457.24
CD4 NC	*FBP1*	rs12553205	9	97401991	A	T	0.4260	0.4219	0.0447	3.98E-21	88.8
CD4 NC	*FBXO10*	rs4345649	9	37487105	T	C	0.0990	0.4178	0.0739	1.53E-08	31.94
CD4 NC	*FBXO2*	rs1883575	1	11713166	G	A	0.5386	-0.3862	0.0444	3.49E-18	75.44
CD4 NC	*FBXO25*	rs10092971	8	406195	A	G	0.3210	0.2658	0.0485	4.22E-08	29.98
CD4 NC	*FBXO3*	rs11032400	11	33839907	A	G	0.3662	0.2759	0.0468	3.72E-09	34.7
CD4 NC	*FBXO7*	rs5754109	22	32872593	A	G	0.2919	-0.3947	0.0499	2.71E-15	62.34
CD4 NC	*FCGRT*	rs2878342	19	50017724	T	C	0.0826	0.4532	0.0810	2.18E-08	31.26
CD4 NC	*FDFT1*	rs1293299	8	11677905	C	A	0.2173	-1.0241	0.0497	2.27E-94	423.87
CD4 NC	*FGD5-AS1*	rs62241835	3	15013348	G	T	0.0913	0.4685	0.0727	1.16E-10	41.44
CD4 NC	*FGFR1*	rs13275043	8	38318337	C	G	0.4763	-0.3390	0.0445	2.76E-14	57.78
CD4 NC	*FGL2*	rs3093273	7	76829275	G	A	0.0905	1.0366	0.0796	9.45E-39	169.17
CD4 NC	*FHIT*	rs2736777	3	60066610	C	T	0.3177	0.4854	0.0479	3.95E-24	102.46
CD4 NC	*FHL3*	rs4360494	1	38455891	C	G	0.5241	-0.6358	0.0428	7.84E-50	219.84
CD4 NC	*FIGNL1*	rs4947641	7	50489038	T	C	0.4833	0.5896	0.0431	1.47E-42	186.58
CD4 NC	*FILIP1L*	rs13079423	3	100028497	A	G	0.2592	-0.4228	0.0514	1.83E-16	67.63
CD4 NC	*FIS1*	rs10260707	7	100863945	G	C	0.0607	-1.0866	0.0898	1.12E-33	146
CD4 NC	*FKBP11*	rs2293449	12	49318546	A	G	0.4680	-0.5838	0.0434	2.95E-41	180.62
CD4 NC	*FKRP*	rs62134831	19	47270181	T	G	0.2451	-0.5143	0.0500	8.03E-25	105.62
CD4 NC	*FLVCR1*	rs12754736	1	213067717	A	G	0.3634	0.2566	0.0463	3.04E-08	30.62
CD4 NC	*FLVCR1-AS1*	rs2279692	1	213030534	T	C	0.5072	0.7218	0.0420	3.55E-66	294.66
CD4 NC	*FMN1*	rs345786	15	33394560	A	T	0.2619	1.3761	0.0419	1.00E-200	1077.5
CD4 NC	*FN3KRP*	rs58867683	17	80679455	A	T	0.4000	-0.6829	0.0437	4.74E-55	243.72
CD4 NC	*FNBP4*	rs7933184	11	47682402	T	C	0.4467	-0.5670	0.0433	4.19E-39	170.78
CD4 NC	*FNIP2*	rs17285611	4	159748216	T	C	0.0738	-0.5425	0.0866	3.71E-10	39.18
CD4 NC	*FNTB*	rs7161378	14	65450780	C	T	0.2390	0.2951	0.0522	1.54E-08	31.93
CD4 NC	*FOXN2*	rs79247094	2	48554955	A	G	0.1181	0.6407	0.0676	2.63E-21	89.62
CD4 NC	*FOXRED1*	rs8177348	11	126153179	C	T	0.7575	0.3119	0.0521	2.22E-09	35.69
CD4 NC	*FRA10AC1*	rs913045	10	95431535	T	C	0.7243	-0.3015	0.0504	2.20E-09	35.71
CD4 NC	*FRG1B*	rs2076376	20	29874829	G	A	0.5146	-0.3297	0.0446	1.42E-13	54.57
CD4 NC	*FRMD4B*	rs9872739	3	69293814	G	C	0.2442	-0.4182	0.0529	2.70E-15	62.35
CD4 NC	*FRMD4B*	rs13059687	3	69246274	G	A	0.9260	0.5649	0.0998	1.50E-08	31.99
CD4 NC	*FRY-AS1*	rs12858463	13	32443378	G	A	0.3158	0.3535	0.0472	6.73E-14	56.03
CD4 NC	*FSBP*	rs16916760	8	95369109	C	T	0.2923	-0.4609	0.0486	2.50E-21	89.72
CD4 NC	*FTCDNL1*	rs13008446	2	200628500	A	G	0.6290	-0.6434	0.0446	3.49E-47	207.72
CD4 NC	*FTSJ3*	rs2727295	17	61887693	T	C	0.5822	0.3689	0.0457	6.43E-16	65.17
CD4 NC	*FUBP1*	rs34517439	1	78450517	A	C	0.1003	-0.5432	0.0683	1.80E-15	63.15
CD4 NC	*FUT10*	rs7841091	8	33299615	G	A	0.5968	-0.4438	0.0451	8.33E-23	96.44
CD4 NC	*FUT2*	rs679574	19	49206108	G	C	0.4651	0.2977	0.0447	2.68E-11	44.31
CD4 NC	*FUT8*	rs1999725	14	65904237	A	G	0.6388	0.3245	0.0469	4.31E-12	47.88
CD4 NC	*FXYD2*	rs7117314	11	117698762	A	G	0.3546	-0.3406	0.0472	5.65E-13	51.86
CD4 NC	*FYB*	rs78666690	5	39201444	T	C	0.3122	-0.3370	0.0478	1.83E-12	49.56
CD4 NC	*G3BP1*	rs1972780	5	151140456	G	A	0.2438	0.3005	0.0513	4.68E-09	34.25
CD4 NC	*G3BP2*	rs13119038	4	76577487	C	G	0.3761	-0.4337	0.0458	2.71E-21	89.56
CD4 NC	*GAA*	rs2304849	17	78075462	C	G	0.2353	-0.3179	0.0519	9.39E-10	37.37
CD4 NC	*GADD45G*	rs1055595	9	92213923	G	T	0.4911	0.2553	0.0448	1.23E-08	32.37
CD4 NC	*GALM*	rs3099952	2	39004215	T	A	0.2920	-0.2698	0.0493	4.51E-08	29.85
CD4 NC	*GALNT10*	rs13154464	5	153454452	C	G	0.3291	0.2958	0.0469	2.97E-10	39.61
CD4 NC	*GALNT2*	rs11122469	1	230355248	G	C	0.2906	0.4467	0.0482	1.92E-20	85.7
CD4 NC	*GALT*	rs2812357	9	34655436	C	T	0.6012	0.5106	0.0442	6.41E-31	133.41
CD4 NC	*GATC*	rs7308053	12	120880133	T	C	0.2984	-0.4289	0.0468	5.13E-20	83.76
CD4 NC	*GBA*	rs370545	1	155175390	G	A	0.5385	-0.2591	0.0450	8.77E-09	33.03
CD4 NC	*GBP3*	rs4655891	1	89484895	A	C	0.4455	0.2926	0.0448	6.76E-11	42.5
CD4 NC	*GBP5*	rs55870241	1	89742521	C	T	0.5445	-0.6383	0.0430	7.52E-50	219.92
CD4 NC	*GBP7*	rs674903	1	89649824	G	A	0.5047	1.0083	0.0383	5.24E-153	692.87
CD4 NC	*GCAT*	rs6000890	22	38187301	T	C	0.6117	-0.5036	0.0458	4.10E-28	120.61
CD4 NC	*GCC2*	rs2139809	2	109045638	C	T	0.5916	0.7500	0.0425	1.05E-69	310.82
CD4 NC	*GDE1*	rs11074400	16	19529203	T	A	0.1450	0.4216	0.0636	3.48E-11	43.8
CD4 NC	*GDI2*	rs3736461	10	5855403	C	G	0.1844	-0.8435	0.0523	1.89E-58	259.28
CD4 NC	*GDPD5*	rs1783557	11	75219277	C	T	0.8597	-0.7782	0.0645	1.66E-33	145.21
CD4 NC	*GFM1*	rs2364274	3	158317365	A	T	0.5314	-0.2843	0.0447	2.09E-10	40.3
CD4 NC	*GFPT1*	rs7422275	2	69528697	T	C	0.3880	0.2636	0.0455	7.07E-09	33.45
CD4 NC	*GGH*	rs13273611	8	63947643	G	A	0.2473	0.2856	0.0508	1.91E-08	31.52
CD4 NC	*GGNBP2*	rs35989249	17	34884149	C	T	0.3997	0.2886	0.0457	2.64E-10	39.84
CD4 NC	*GGT1*	rs2076999	22	25003934	A	G	0.3650	-0.2654	0.0480	3.30E-08	30.46
CD4 NC	*GHITM*	rs76833981	10	85888300	T	C	0.0709	0.5927	0.0947	3.84E-10	39.11
CD4 NC	*GHRL*	rs35681	3	10329377	C	T	0.5345	0.2490	0.0449	2.90E-08	30.71
CD4 NC	*GIMAP2*	rs6952002	7	150389677	C	G	0.3524	-0.4700	0.0463	3.51E-24	102.7
CD4 NC	*GIMAP4*	rs10085554	7	150277387	C	T	0.6178	-0.3182	0.0460	4.60E-12	47.75
CD4 NC	*GINM1*	rs4869923	6	149874899	G	T	0.3911	-0.3014	0.0464	8.41E-11	42.07
CD4 NC	*GINS2*	rs9930110	16	85720780	C	T	0.2924	0.3696	0.0492	5.65E-14	56.37
CD4 NC	*GIPC1*	rs60438220	19	14593176	T	A	0.3699	-0.3469	0.0457	3.11E-14	57.55
CD4 NC	*GJB6*	rs7986974	13	21056301	G	T	0.7055	-0.4789	0.0494	3.51E-22	93.6
CD4 NC	*GLI4*	rs6420186	8	144309119	G	A	0.6382	-0.2741	0.0464	3.36E-09	34.89

CD4 NC	*GLIPR1*	rs1552039	12	75905994	A	G	0.2497	-0.4256	0.0516	1.69E-16	67.8
CD4 NC	*GLIPR1L2*	rs2242435	12	75874913	A	G	0.4118	-0.5310	0.0440	1.43E-33	145.51
CD4 NC	*GLRX5*	rs12431494	14	95993386	C	A	0.4224	0.4216	0.0447	3.95E-21	88.82
CD4 NC	*GLTP*	rs9645838	12	110291572	G	A	0.5485	-0.5829	0.0438	1.91E-40	176.92
CD4 NC	*GLTPD1*	rs307346	1	1260733	C	A	0.9374	-0.5706	0.1021	2.30E-08	31.16
CD4 NC	*GLTSCR2*	rs3786779	19	48255284	A	G	0.7093	-0.5692	0.0490	3.34E-31	134.7
CD4 NC	*GLUL*	rs3856103	1	182050235	G	C	0.4382	-0.3272	0.0450	3.48E-13	52.81
CD4 NC	*GLYCTK*	rs9311474	3	52338852	C	T	0.5092	-0.2860	0.0448	1.66E-10	40.75
CD4 NC	*GM2A*	rs75278160	5	150653612	G	C	0.0874	0.4734	0.0753	3.26E-10	39.43
CD4 NC	*GMPR*	rs66929956	6	16239228	T	C	0.1101	-0.4439	0.0748	2.98E-09	35.12
CD4 NC	*GNAI1*	rs2714450	7	79773935	G	A	0.5774	-0.2550	0.0457	2.41E-08	31.07
CD4 NC	*GNB5*	rs4776011	15	52472713	G	A	0.1520	-0.5076	0.0636	1.52E-15	63.48
CD4 NC	*GNG5*	rs6666572	1	84928736	A	G	0.1078	0.5073	0.0748	1.19E-11	45.89
CD4 NC	*GNG8*	rs12462947	19	47131557	G	A	0.3971	-0.6845	0.0438	3.97E-55	244.07
CD4 NC	*GNMT*	rs3805946	6	42955749	C	T	0.4771	-0.3012	0.0447	1.57E-11	45.35
CD4 NC	*GNPTAB*	rs10860794	12	102217720	A	C	0.2963	0.3782	0.0487	8.36E-15	60.13
CD4 NC	*GNPTG*	rs57890635	16	1411129	T	C	0.2668	0.7442	0.0511	4.63E-48	211.73
CD4 NC	*GOLGA8A*	rs3866558	15	34859970	C	G	0.4062	0.3926	0.0450	2.63E-18	75.99
CD4 NC	*GOLT1B*	rs67187752	12	21648070	T	C	0.1520	0.4293	0.0633	1.21E-11	45.86
CD4 NC	*GOSR2*	rs4968287	17	45024049	C	T	0.5063	0.3957	0.0443	4.07E-19	79.67
CD4 NC	*GPA33*	rs2281963	1	167059462	C	G	0.5028	-0.2467	0.0448	3.76E-08	30.21
CD4 NC	*GPCPD1*	rs6085222	20	5573659	G	A	0.4602	-0.2730	0.0448	1.12E-09	37.02
CD4 NC	*GPR155*	rs11677062	2	175246058	C	G	0.7298	-0.3456	0.0518	2.52E-11	44.43
CD4 NC	*GPR160*	rs9829025	3	169746123	G	A	0.3883	0.7106	0.0442	2.98E-58	258.37
CD4 NC	*GPR18*	rs2230341	13	99947261	T	C	0.1160	0.4550	0.0677	1.77E-11	45.12
CD4 NC	*GPR180*	rs3897473	13	95253920	G	T	0.6346	0.3758	0.0456	1.61E-16	67.89
CD4 NC	*GPR63*	rs6568627	6	97282878	C	T	0.2672	-0.2870	0.0520	3.42E-08	30.39
CD4 NC	*GPX1*	rs9841110	3	49492481	G	C	0.3095	-0.3228	0.0488	3.74E-11	43.66
CD4 NC	*GPX4*	rs4147938	19	1066256	A	G	0.6979	0.2794	0.0499	2.21E-08	31.24
CD4 NC	*GPX4*	rs2074449	19	1095753	A	G	0.2235	-0.6151	0.0523	6.09E-32	138.08
CD4 NC	*GPX7*	rs1047635	1	53074532	A	C	0.5486	-0.2972	0.0448	3.12E-11	44.01
CD4 NC	*GRAMD1A*	rs11084796	19	35500640	G	T	0.1031	0.6647	0.0754	1.24E-18	77.48
CD4 NC	*GRAMD1A*	rs55742440	19	35524824	C	T	0.3917	0.2556	0.0461	2.99E-08	30.65
CD4 NC	*GREM2*	rs4659990	1	240812822	C	A	0.5266	0.2949	0.0449	4.91E-11	43.13
CD4 NC	*GRHPR*	rs4878690	9	37422656	T	C	0.3290	0.9306	0.0430	1.02E-103	466.73
CD4 NC	*GRIN2C*	rs55681270	17	72827323	A	T	0.2876	0.3956	0.0500	2.56E-15	62.45
CD4 NC	*GRK5*	rs10886440	10	121049723	G	T	0.7586	0.3010	0.0527	1.13E-08	32.53
CD4 NC	*GRTP1*	rs9549761	13	114048080	C	T	0.5705	-0.3757	0.0449	5.67E-17	69.95
CD4 NC	*GS1-124K5.3*	rs1144894	7	65542894	G	A	0.5666	-0.4211	0.0448	5.28E-21	88.25
CD4 NC	*GS1-124K5.4*	rs2420821	7	66097333	G	T	0.6446	-0.4821	0.0462	1.92E-25	108.44
CD4 NC	*GS1-251I9.4*	rs7341609	8	92064862	G	A	0.7122	0.3672	0.0490	6.38E-14	56.14
CD4 NC	*GSDMA*	rs3893044	17	38103016	C	T	0.5518	0.3830	0.0448	1.27E-17	72.89
CD4 NC	*GSDMB*	rs17608925	17	38082831	C	T	0.1182	-0.4078	0.0720	1.45E-08	32.05
CD4 NC	*GSDMD*	rs4242487	8	144608634	G	A	0.3646	-0.2918	0.0471	5.66E-10	38.36
CD4 NC	*GSTM1*	rs1056806	1	110233147	T	C	0.0899	0.7389	0.0913	5.85E-16	65.35
CD4 NC	*GSTM1*	rs4970772	1	110250168	A	G	0.3389	-0.2599	0.0469	3.08E-08	30.59
CD4 NC	*GSTM2*	rs1056806	1	110233147	T	C	0.0899	0.5312	0.0921	7.90E-09	33.23
CD4 NC	*GSTM2*	rs12736389	1	110255167	C	G	0.1940	0.3158	0.0575	4.05E-08	30.06
CD4 NC	*GSTM3*	rs1332018	1	110282972	T	G	0.5781	1.0560	0.0382	4.10E-168	762.2
CD4 NC	*GSTM4*	rs1010167	1	110198727	G	C	0.4142	-0.8705	0.0413	9.10E-99	444.04
CD4 NC	*GSTO1*	rs11191967	10	106003861	T	C	0.3084	0.6534	0.0474	3.47E-43	189.44
CD4 NC	*GSTP1*	rs4930444	11	67342580	A	G	0.4200	-0.9629	0.0396	1.20E-130	590.27
CD4 NC	*GSTT1*	rs6003946	22	24249301	T	C	0.0839	-0.5856	0.0879	2.71E-11	44.29
CD4 NC	*GSTT1*	rs5760147	22	24334948	C	A	0.3844	-1.0257	0.0401	2.34E-144	653.17
CD4 NC	*GTF2F1*	rs3088023	19	6393152	A	G	0.1847	-0.3899	0.0614	2.09E-10	40.3
CD4 NC	*GTF2H2*	rs28478097	5	70309998	G	C	0.6121	-0.7319	0.0432	2.92E-64	285.89
CD4 NC	*GTF2H5*	rs62437115	6	158589655	G	C	0.4242	0.2630	0.0451	5.43E-09	33.96
CD4 NC	*GTF3A*	rs1049302	13	27998769	C	T	0.7740	0.3851	0.0558	5.36E-12	47.45
CD4 NC	*GTF3C3*	rs62185601	2	197638979	G	C	0.1305	0.3845	0.0640	1.91E-09	35.99
CD4 NC	*GTPBP8*	rs9866791	3	112709033	C	T	0.7017	0.4188	0.0509	1.91E-16	67.55
CD4 NC	*GTSCR1*	rs11663977	18	68337699	G	T	0.1610	0.5190	0.0631	1.93E-16	67.53
CD4 NC	*GVQW1*	rs57736214	9	32725113	C	T	0.2938	-0.3626	0.0469	1.12E-14	59.56
CD4 NC	*GYG1*	rs3736641	3	148709159	C	G	0.3141	-0.3966	0.0490	5.51E-16	65.47
CD4 NC	*GYPC*	rs13395010	2	127411213	G	A	0.5924	0.3623	0.0451	9.96E-16	64.31
CD4 NC	*GYPC*	rs74499727	2	127448415	T	C	0.3330	0.2615	0.0474	3.53E-08	30.33
CD4 NC	*GYPC*	rs35651363	2	127519838	A	G	0.2707	0.9277	0.0471	2.35E-86	387.13
CD4 NC	*GYPE*	rs12501790	4	144861741	G	T	0.3173	-0.6373	0.0464	6.68E-43	188.14
CD4 NC	*GZMA*	rs58433852	5	54426834	T	C	0.0674	0.5245	0.0943	2.66E-08	30.88
CD4 NC	*GZMM*	rs16990714	19	543497	T	C	0.0484	-0.9081	0.1016	3.86E-19	79.78
CD4 NC	*H1F0*	rs5756825	22	38199950	C	T	0.5411	-0.4139	0.0445	1.46E-20	86.24
CD4 NC	*H2AFY*	rs10072856	5	134601871	T	C	0.0527	0.6401	0.1008	2.18E-10	40.21
CD4 NC	*H2AFZ*	rs2276939	4	100870375	G	T	0.2457	-0.3727	0.0544	7.43E-12	46.82
CD4 NC	*H6PD*	rs9435164	1	9331331	T	A	0.1484	0.5139	0.0641	1.10E-15	64.12
CD4 NC	*HADH*	rs6831530	4	108956423	T	G	0.0775	0.6755	0.0837	6.80E-16	65.06
CD4 NC	*HAGH*	rs238677	16	1883279	C	T	0.3287	0.7941	0.0464	9.61E-66	292.68
CD4 NC	*HAGHL*	rs12597563	16	787738	C	G	0.2149	0.4352	0.0569	2.06E-14	58.35
CD4 NC	*HAR1A*	rs3746746	20	61735112	C	A	0.2612	-0.5240	0.0496	4.11E-26	111.5
CD4 NC	*HAR1B*	rs750699	20	61735660	T	C	0.2522	-0.3298	0.0508	8.82E-11	41.98
CD4 NC	*HAX1*	rs2282227	1	154243097	A	G	0.2882	0.5311	0.0481	2.39E-28	121.68

CD4 NC	*HBS1L*	rs1547247	6	135390836	A	G	0.2850	-0.3993	0.0482	1.21E-16	68.45
CD4 NC	*HCG11*	rs1535274	6	26520747	C	A	0.5994	-0.2970	0.0463	1.38E-10	41.11
CD4 NC	*HCG14*	rs3130248	6	29602936	T	C	0.4990	0.2605	0.0448	6.19E-09	33.71
CD4 NC	*HCG15*	rs3132685	6	29945949	A	G	0.0914	-0.8911	0.0673	4.52E-40	175.2
CD4 NC	*HCG20*	rs1557608	6	30118582	G	T	0.2850	-0.4999	0.0491	2.22E-24	103.6
CD4 NC	*HDDC2*	rs1040803	6	125596957	T	C	0.4843	-0.4449	0.0441	6.27E-24	101.55
CD4 NC	*HDGFRP3*	rs4341707	15	83927857	T	C	0.2908	0.5044	0.0496	2.58E-24	103.31
CD4 NC	*HDHD3*	rs3747816	9	116138013	T	C	0.1818	-0.4402	0.0585	5.44E-14	56.45
CD4 NC	*HEATR3*	rs12918457	16	50088068	A	C	0.7027	0.2835	0.0500	1.46E-08	32.04
CD4 NC	*HEATR6*	rs8066241	17	58157149	C	T	0.1060	0.5067	0.0765	3.42E-11	43.83
CD4 NC	*HEBP1*	rs7956248	12	13177941	A	C	0.5445	-0.4892	0.0441	1.28E-28	122.93
CD4 NC	*HEBP2*	rs9495065	6	138709856	T	C	0.0684	-0.7444	0.0945	3.28E-15	61.97
CD4 NC	*HELLS*	rs11527881	10	96241889	T	C	0.4375	0.5560	0.0437	3.94E-37	161.77
CD4 NC	*HELZ2*	rs6089925	20	62200929	A	G	0.0802	0.5917	0.0911	8.47E-11	42.06
CD4 NC	*HERPUD2*	rs12538255	7	35668920	T	C	0.3745	0.3491	0.0461	3.44E-14	57.35
CD4 NC	*HEXDC*	rs7221247	17	80404958	G	A	0.3750	0.2720	0.0465	4.86E-09	34.18
CD4 NC	*HHLA3*	rs542842	1	70860841	G	A	0.1877	-0.3874	0.0571	1.21E-11	45.86
CD4 NC	*HIGD2A*	rs1065212	5	175811133	C	T	0.0955	-1.3636	0.0798	2.18E-65	291.05
CD4 NC	*HINT1*	rs72801715	5	130151181	G	A	0.0629	-0.5883	0.1002	4.26E-09	34.43
CD4 NC	*HIST1H1D*	rs806786	6	26259748	G	A	0.0782	0.5369	0.0927	7.01E-09	33.46
CD4 NC	*HIST1H2AJ*	rs115709379	6	28518299	A	G	0.1175	-0.3753	0.0686	4.49E-08	29.87
CD4 NC	*HIST1H2BE*	rs57440165	6	26843517	C	A	0.0736	-0.5415	0.0762	1.19E-12	50.4
CD4 NC	*HIST1H3B*	rs13217285	6	26999845	T	C	0.1003	-0.5063	0.0680	9.58E-14	55.34
CD4 NC	*HIST1H4I*	rs13217620	6	27653120	T	C	0.0713	0.5412	0.0770	2.04E-12	49.35
CD4 NC	*HIST1H4K*	rs73403143	6	28726613	T	C	0.0678	0.7105	0.0957	1.15E-13	54.99
CD4 NC	*HIVEP3*	rs783629	1	42363275	T	C	0.5567	-0.6656	0.0427	7.04E-55	242.93
CD4 NC	*HLA-A*	rs7747253	6	29919779	A	C	0.5479	0.4162	0.0442	4.57E-21	88.53
CD4 NC	*HLA-B*	rs2524096	6	31236467	T	G	0.4422	0.7721	0.0420	2.03E-75	337.01
CD4 NC	*HLA-B*	rs9366777	6	31264715	A	T	0.0923	-0.5927	0.0826	7.02E-13	51.43
CD4 NC	*HLA-B*	rs3828879	6	31379391	T	C	0.1430	-0.6060	0.0651	1.28E-20	86.5
CD4 NC	*HLA-C*	rs3931670	6	31243767	C	G	0.2891	0.5442	0.0504	3.55E-27	116.34
CD4 NC	*HLA-DMA*	rs76088152	6	32946322	A	G	0.0639	0.5326	0.0852	4.08E-10	39
CD4 NC	*HLA-DOB*	rs4148876	6	32796793	A	G	0.0646	0.7378	0.0898	2.18E-16	67.29
CD4 NC	*HLA-DPA1*	rs41288893	6	33091605	C	T	0.1228	-0.5460	0.0698	5.35E-15	61
CD4 NC	*HLA-DPB1*	rs1431403	6	33047031	C	T	0.3089	0.3196	0.0488	6.02E-11	42.73
CD4 NC	*HLA-DQA1*	rs1846190	6	32583813	A	G	0.2841	0.3257	0.0492	3.47E-11	43.8
CD4 NC	*HLA-DQA2*	rs35395738	6	32560209	C	T	0.2074	0.8561	0.0501	1.60E-65	291.67
CD4 NC	*HLA-DQA2*	rs9501201	6	32746264	T	G	0.2774	-0.2862	0.0498	9.41E-09	32.89
CD4 NC	*HLA-DQB1*	rs9272557	6	32607048	C	T	0.0740	-0.5461	0.0531	7.89E-25	105.65
CD4 NC	*HLA-DQB1*	rs9275222	6	32659516	T	A	0.4802	-0.2916	0.0448	7.50E-11	42.3
CD4 NC	*HLA-DQB2*	rs419132	6	32210799	G	A	0.3337	-0.3200	0.0539	2.80E-09	35.24
CD4 NC	*HLA-DRB5*	rs28366319	6	32561495	A	G	0.2843	0.6205	0.0450	3.27E-43	189.56
CD4 NC	*HLA-E*	rs1264457	6	30458064	A	G	0.5532	0.2546	0.0456	2.40E-08	31.08
CD4 NC	*HLA-F*	rs1611360	6	29679434	A	G	0.6716	-0.4427	0.0484	6.11E-20	83.41
CD4 NC	*HLA-G*	rs16895223	6	29626949	G	C	0.1439	-0.3729	0.0642	6.23E-09	33.69
CD4 NC	*HLA-G*	rs2248151	6	29914548	T	C	0.1584	0.9452	0.0574	5.02E-61	271.07
CD4 NC	*HLA-G*	rs2523990	6	30077229	G	A	0.5102	0.3063	0.0447	7.08E-12	46.91
CD4 NC	*HMBOX1*	rs10088428	8	28909523	T	C	0.2597	-1.1522	0.0444	9.57E-149	673.31
CD4 NC	*HMBS*	rs4454730	11	118937444	A	G	0.3995	0.4447	0.0454	1.19E-22	95.73
CD4 NC	*HNRNPU*	rs9661182	1	245028509	T	G	0.2415	0.3792	0.0527	6.40E-13	51.61
CD4 NC	*HOPX*	rs4460065	4	57520444	G	A	0.0846	0.5049	0.0855	3.49E-09	34.82
CD4 NC	*HOTAIRM1*	rs2960785	7	26952139	C	T	0.5319	0.3106	0.0447	3.92E-12	48.07
CD4 NC	*HOXB-AS1*	rs16949651	17	46019601	G	A	0.1338	0.4180	0.0701	2.42E-09	35.53
CD4 NC	*HOXB-AS1*	rs11079826	17	46597048	T	C	0.4867	0.7491	0.0417	4.57E-72	321.64
CD4 NC	*HOXB2*	rs12450225	17	46592413	G	A	0.4858	0.8424	0.0407	2.87E-95	427.99
CD4 NC	*HOXB2*	rs7211614	17	46404341	C	T	0.4663	-0.2483	0.0449	3.23E-08	30.5
CD4 NC	*HOXB3*	rs2740760	17	46643389	G	A	0.5446	0.3568	0.0451	2.59E-15	62.43
CD4 NC	*HOXB4*	rs11079826	17	46597048	T	C	0.4867	0.6300	0.0428	5.12E-49	216.11
CD4 NC	*HPCAL4*	rs784600	1	40139553	T	C	0.4692	0.4912	0.0440	5.96E-29	124.43
CD4 NC	*HPS1*	rs11597139	10	100196020	T	A	0.1621	-0.4857	0.0587	1.24E-16	68.41
CD4 NC	*HSBP1L1*	rs11081576	18	77737397	T	C	0.8214	-0.4534	0.0596	2.83E-14	57.73
CD4 NC	*HSD17B11*	rs4443241	4	88288599	C	T	0.7196	0.3805	0.0497	2.02E-14	58.4
CD4 NC	*HSD17B12*	rs57635800	11	43878485	A	G	0.3076	-0.5444	0.0489	8.82E-29	123.66
CD4 NC	*HSD17B13*	rs62305726	4	88255851	G	A	0.1632	0.4526	0.0599	4.14E-14	56.98
CD4 NC	*HSD17B14*	rs8110220	19	49339098	C	T	0.2593	-0.8363	0.0488	8.36E-66	292.96
CD4 NC	*HSD17B8*	rs3117040	6	33164735	A	C	0.7359	-0.3570	0.0488	2.51E-13	53.45
CD4 NC	*HSDL2*	rs3813856	9	115141974	T	G	0.2856	-0.4249	0.0491	5.05E-18	74.71
CD4 NC	*HSP90AA1*	rs3742429	14	102550569	A	G	0.8119	0.4229	0.0610	4.26E-12	47.9
CD4 NC	*HSP90B1*	rs1165685	12	104333866	A	T	0.4221	-0.3065	0.0454	1.42E-11	45.54
CD4 NC	*HSPBAP1*	rs12496363	3	122473354	T	C	0.7449	-1.1270	0.0476	8.51E-124	558.83
CD4 NC	*HTATIP2*	rs10437583	11	20386761	A	T	0.5420	-0.2538	0.0450	1.75E-08	31.69
CD4 NC	*HVCN1*	rs73194023	12	111137940	A	G	0.0795	-0.5637	0.0816	5.05E-12	47.57
CD4 NC	*IAH1*	rs1815892	2	9616728	T	G	0.5608	-0.4221	0.0450	6.26E-21	87.91
CD4 NC	*ICA1L*	rs7597959	2	203637873	T	A	0.5813	-0.5990	0.0440	3.21E-42	185.02
CD4 NC	*ICAM3*	rs281414	19	10450285	A	G	0.1788	0.4262	0.0616	4.51E-12	47.79
CD4 NC	*ID3*	rs2742946	1	23886073	C	T	0.4568	0.3684	0.0447	1.74E-16	67.74
CD4 NC	*IDH2*	rs11638845	15	90642847	G	T	0.5350	-0.2720	0.0452	1.79E-09	36.12
CD4 NC	*IDI1*	rs3793779	10	1079579	T	C	0.3454	-0.2582	0.0471	4.14E-08	30.02
CD4 NC	*IDUA*	rs3796622	4	983060	C	T	0.6617	0.2891	0.0472	9.17E-10	37.42

CD4 NC	*IFI27L1*	rs941478	14	94571518	C	T	0.2256	0.4793	0.0519	2.38E-20	85.28
CD4 NC	*IFI27L2*	rs2273514	14	94595989	C	T	0.2022	-1.1347	0.0490	7.25E-119	536.22
CD4 NC	*IFIT5*	rs303208	10	91165833	T	C	0.2490	-0.3436	0.0529	8.56E-11	42.04
CD4 NC	*IFITM1*	rs28475163	11	327143	A	G	0.2562	0.3399	0.0511	2.90E-11	44.15
CD4 NC	*IFITM2*	rs10751647	11	306884	C	T	0.4259	0.6536	0.0433	2.21E-51	226.93
CD4 NC	*IFITM3*	rs7942247	11	316349	A	G	0.5502	0.9605	0.0391	1.80E-133	603.23
CD4 NC	*IFITM3*	rs7479261	11	351765	C	G	0.3068	-0.2666	0.0480	2.74E-08	30.82
CD4 NC	*IFNG-AS1*	rs12832728	12	68420403	A	G	0.5290	-0.2496	0.0451	3.09E-08	30.59
CD4 NC	*IFT88*	rs34294251	13	21256987	A	G	0.5042	-0.3269	0.0447	2.48E-13	53.47
CD4 NC	*IGFBP3*	rs788765	7	45996985	G	C	0.3043	0.3335	0.0477	2.83E-12	48.7
CD4 NC	*IGFLR1*	rs2871921	19	36232420	T	C	0.1557	-0.9342	0.0607	1.95E-53	236.33
CD4 NC	*IGHMBP2*	rs583182	11	68678634	T	C	0.2951	0.4437	0.0485	6.09E-20	83.42
CD4 NC	*IGLL1*	rs13056126	22	23980968	A	T	0.1917	0.5809	0.0555	1.34E-25	109.16
CD4 NC	*IGSF22*	rs2289965	11	18728650	C	T	0.3625	0.3825	0.0456	4.68E-17	70.32
CD4 NC	*IGSF8*	rs12092798	1	160071819	C	T	0.2134	-0.3871	0.0554	2.92E-12	48.64
CD4 NC	*IKBIP*	rs12368223	12	99090130	A	C	0.1633	0.3099	0.0564	3.84E-08	30.17
CD4 NC	*IKBKE*	rs1059704	1	206648378	A	G	0.6636	-0.3679	0.0483	2.57E-14	57.92
CD4 NC	*IKZF1*	rs10215297	7	50462498	T	A	0.1854	-0.3869	0.0564	6.70E-12	47.02
CD4 NC	*IL18R1*	rs12987977	2	102975336	G	T	0.3833	0.4039	0.0451	3.42E-19	80.02
CD4 NC	*IL24*	rs1150253	1	207071595	A	G	0.4542	-0.5090	0.0439	4.74E-31	134.01
CD4 NC	*IL32*	rs11861531	16	3116325	C	T	0.5537	0.5516	0.0434	6.22E-37	160.86
CD4 NC	*IL6ST*	rs7731626	5	55444683	A	G	0.3526	-0.3589	0.0469	1.87E-14	58.55
CD4 NC	*IMMP2L*	rs40372	7	111057345	C	T	0.5072	0.4123	0.0442	1.10E-20	86.8
CD4 NC	*IMMT*	rs12478097	2	86395961	C	T	0.4891	-0.4105	0.0442	1.73E-20	85.91
CD4 NC	*IMP3*	rs7166281	15	75928192	G	T	0.2661	-0.3244	0.0518	3.83E-10	39.12
CD4 NC	*IMPA1*	rs75812729	8	82543919	G	T	0.1341	-0.3629	0.0654	2.89E-08	30.72
CD4 NC	*IMPA2*	rs1787989	18	11974688	A	T	0.2996	-0.2944	0.0493	2.38E-09	35.57
CD4 NC	*INF2*	rs76386715	14	105171208	T	C	0.1458	-0.7279	0.0618	4.78E-32	138.56
CD4 NC	*ING5*	rs6437277	2	242608984	G	A	0.8010	0.3603	0.0531	1.20E-11	45.88
CD4 NC	*INO80B*	rs2301984	2	74721055	G	A	0.1730	-0.4040	0.0677	2.45E-09	35.51
CD4 NC	*INPP4B*	rs1373036	4	143625262	A	G	0.7682	0.5067	0.0513	5.33E-23	97.32
CD4 NC	*INSR*	rs4640294	19	7297710	A	G	0.7491	-0.4350	0.0563	1.12E-14	59.55
CD4 NC	*INTS5*	rs12291465	11	62437000	T	C	0.2897	0.3273	0.0484	1.36E-11	45.64
CD4 NC	*IP6K2*	rs12491769	3	48768150	A	G	0.0854	0.7754	0.0931	8.11E-17	69.24
CD4 NC	*IPO9*	rs10920259	1	201797696	C	T	0.5219	0.2732	0.0448	1.04E-09	37.18
CD4 NC	*IPP*	rs6697821	1	46216722	G	A	0.3012	-0.3184	0.0487	6.04E-11	42.72
CD4 NC	*IQCB1*	rs80047370	3	121498038	C	T	0.1241	0.4910	0.0620	2.31E-15	62.65
CD4 NC	*IQCH-AS1*	rs4776952	15	67917846	T	C	0.7582	0.3561	0.0547	7.43E-11	42.31
CD4 NC	*IQSEC3*	rs56204927	12	248202	G	C	0.6233	-0.3888	0.0453	8.94E-18	73.58
CD4 NC	*IRAK1BP1*	rs73466232	6	79820534	G	A	0.1086	0.4496	0.0688	6.43E-11	42.6
CD4 NC	*IRF3*	rs2304207	19	50167726	G	C	0.1589	0.4409	0.0614	6.81E-13	51.49
CD4 NC	*IRF7*	rs7932229	11	578759	G	A	0.5596	0.2999	0.0449	2.33E-11	44.58
CD4 NC	*ISCA1*	rs7035034	9	88902663	T	C	0.6802	-0.5347	0.0458	1.70E-31	136.04
CD4 NC	*ISCA2*	rs4903231	14	74962233	A	G	0.5967	-0.8042	0.0419	4.95E-82	367.31
CD4 NC	*ISCU*	rs10778643	12	108939901	A	T	0.8652	-0.6033	0.0643	6.45E-21	87.85
CD4 NC	*ISG15*	rs1891906	1	950243	C	A	0.3814	0.4832	0.0451	8.25E-27	114.67
CD4 NC	*ISG20*	rs3826040	15	89172352	T	C	0.9089	-0.5544	0.0803	5.14E-12	47.54
CD4 NC	*ISM1*	rs1322121	20	13207519	A	T	0.1939	-0.4221	0.0584	4.77E-13	52.19
CD4 NC	*ITCH-AS1*	rs6142147	20	32953344	G	C	0.3105	0.3684	0.0488	4.60E-14	56.78
CD4 NC	*ITGA2*	rs1109526	5	52387323	T	C	0.7256	0.2922	0.0502	5.99E-09	33.77
CD4 NC	*ITGB2*	rs760462	21	46328099	C	T	0.8298	-1.3259	0.0523	6.23E-142	642.05
CD4 NC	*ITGB2-AS1*	rs9983174	21	46349031	C	T	0.4645	0.4075	0.0445	4.86E-20	83.86
CD4 NC	*ITGB3BP*	rs217483	1	64017830	G	A	0.7015	-0.7801	0.0455	7.99E-66	293.05
CD4 NC	*ITGB7*	rs12824946	12	53592452	C	T	0.1012	0.9493	0.0788	2.07E-33	144.78
CD4 NC	*ITIH4*	rs17331178	3	52847544	A	C	0.1130	-0.5415	0.0706	1.74E-14	58.69
CD4 NC	*ITPA*	rs112200085	20	3196390	A	G	0.1148	-0.6894	0.0650	2.70E-26	112.33
CD4 NC	*JAKMIP2*	rs13183927	5	147142688	G	C	0.3308	0.5701	0.0456	8.21E-36	155.74
CD4 NC	*JAZF1*	rs4722758	7	28156606	G	C	0.2341	0.3416	0.0553	6.63E-10	38.05
CD4 NC	*KANK1*	rs72705663	9	515584	T	G	0.0744	-0.4999	0.0833	1.98E-09	35.92
CD4 NC	*KANK1*	rs1976260	9	520211	A	C	0.6622	-0.2990	0.0479	4.25E-10	38.92
CD4 NC	*KANSL1-AS1*	rs56328224	17	43572419	T	C	0.2065	1.3358	0.0429	1.00E-200	969.51
CD4 NC	*KATNBL1*	rs7176526	15	34494756	A	G	0.1424	-0.3567	0.0614	6.13E-09	33.72
CD4 NC	*KB-1507C5.2*	rs1055376	8	103877434	A	G	0.3567	-0.3242	0.0468	4.08E-12	47.99
CD4 NC	*KB-226F1.1*	rs5760147	22	24334948	C	A	0.3844	-0.6956	0.0441	5.29E-56	248.08
CD4 NC	*KB-226F1.2*	rs9608229	22	24399364	G	C	0.5529	0.7812	0.0422	1.50E-76	342.18
CD4 NC	*KBTBD11*	rs7388076	8	1923644	A	G	0.5701	-0.2557	0.0449	1.22E-08	32.39
CD4 NC	*KCNAB2*	rs34522455	1	6060647	T	C	0.1480	0.7482	0.0585	1.99E-37	163.12
CD4 NC	*KCNN1*	rs10411508	19	18110574	A	G	0.3962	0.4012	0.0450	5.26E-19	79.17
CD4 NC	*KCNQ1OT1*	rs7102138	11	2659025	G	C	0.8766	0.4254	0.0696	9.97E-10	37.26
CD4 NC	*KCTD21-AS1*	rs10793289	11	77850517	A	C	0.4647	-0.5938	0.0432	5.40E-43	188.56
CD4 NC	*KDELR2*	rs6796	7	6502367	C	T	0.2687	-0.8685	0.0455	4.23E-81	363.04
CD4 NC	*KIAA0040*	rs2269650	1	175129691	T	G	0.3691	0.7355	0.0442	2.76E-62	276.84
CD4 NC	*KIAA1143*	rs954282	3	44753363	T	C	0.5477	-0.3684	0.0444	1.11E-16	68.62
CD4 NC	*KIAA1328*	rs323331	18	34691672	G	A	0.7125	-0.3769	0.0484	6.97E-15	60.48
CD4 NC	*KIAA1430*	rs2030800	4	186124855	G	A	0.6308	-0.5881	0.0459	1.40E-37	163.82
CD4 NC	*KIAA1551*	rs1259878	12	32047624	T	C	0.3593	0.2539	0.0459	3.13E-08	30.56
CD4 NC	*KIAA1715*	rs1447586	2	176842487	T	C	0.5517	-0.4439	0.0448	3.73E-23	98.03
CD4 NC	*KIF16B*	rs35610860	20	16545368	T	C	0.1349	-0.6529	0.0629	3.02E-25	107.55
CD4 NC	*KIF18B*	rs7220051	17	43459253	T	A	0.4610	-0.7513	0.0417	1.48E-72	323.89

CD4 NC	*KIF2A*	rs1477358	5	61891622	C	T	0.4840	0.3414	0.0445	1.75E-14	58.67
CD4 NC	*KIR3DL2*	rs269919	19	55427348	G	A	0.5980	-0.3027	0.0454	2.56E-11	44.4
CD4 NC	*KISS1R*	rs78047294	19	871987	A	G	0.2410	0.5049	0.0520	2.64E-22	94.16
CD4 NC	*KLF13*	rs28510484	15	31637569	C	G	0.1628	0.5983	0.0590	3.63E-24	102.63
CD4 NC	*KLF5*	rs6562780	13	74289799	T	A	0.7739	-0.4401	0.0526	6.11E-17	69.8
CD4 NC	*KLHDC10*	rs78387641	7	129779264	C	T	0.4263	-0.3038	0.0452	1.76E-11	45.13
CD4 NC	*KLHDC4*	rs7199037	16	87735527	A	G	0.1916	-0.3598	0.0586	8.44E-10	37.58
CD4 NC	*KLHL18*	rs10490798	3	47328744	T	C	0.1545	-0.5459	0.0618	1.07E-18	77.76
CD4 NC	*KLHL20*	rs4472784	1	173396971	T	C	0.3110	-0.3073	0.0481	1.67E-10	40.74
CD4 NC	*KLHL42*	rs10492373	12	27959998	A	G	0.1949	-0.4540	0.0541	4.71E-17	70.31
CD4 NC	*KLHL5*	rs6531685	4	38960263	C	T	0.6079	0.2962	0.0458	9.73E-11	41.79
CD4 NC	*KLHL6*	rs62287198	3	183219656	A	G	0.5186	0.3507	0.0447	4.22E-15	61.47
CD4 NC	*KLHL7-AS1*	rs10250602	7	23303701	C	T	0.3632	-0.3633	0.0463	4.24E-15	61.46
CD4 NC	*KNOP1*	rs4780824	16	19737425	G	A	0.1711	0.4534	0.0578	4.37E-15	61.4
CD4 NC	*KPNA4*	rs2152425	3	160037904	A	G	0.5429	-0.2571	0.0449	1.01E-08	32.76
CD4 NC	*KRT1*	rs2741159	12	53071382	C	A	0.3815	1.0583	0.0381	1.06E-169	769.48
CD4 NC	*KRT10*	rs56261283	17	38961725	A	G	0.1606	0.6361	0.0640	2.68E-23	98.68
CD4 NC	*KRT2*	rs685190	12	53035460	T	C	0.3486	0.2943	0.0466	2.78E-10	39.74
CD4 NC	*KRT72*	rs627826	12	53026125	C	T	0.2685	-0.6910	0.0489	2.61E-45	199.15
CD4 NC	*KRT73*	rs11170205	12	53015371	T	A	0.2528	0.4646	0.0503	2.40E-20	85.25
CD4 NC	*KRTAP5-2*	rs1038723	11	1562713	A	G	0.5646	0.5425	0.0443	1.89E-34	149.52
CD4 NC	*KRTCAP3*	rs1262430	2	27673798	C	T	0.3880	0.7522	0.0428	3.86E-69	308.24
CD4 NC	*KSR2*	rs10775003	12	119341266	G	A	0.0956	0.5510	0.0782	1.84E-12	49.54
CD4 NC	*KTN1*	rs55927221	14	56128866	C	A	0.3400	-0.5699	0.0459	2.19E-35	153.8
CD4 NC	*KYNU*	rs9287356	2	144663667	T	C	0.5170	0.2461	0.0449	4.10E-08	30.04
CD4 NC	*L3MBTL3*	rs7740107	6	130374461	A	T	0.7512	-0.7410	0.0474	3.68E-55	244.22
CD4 NC	*LA16c-358B7.3*	rs11647760	16	1367610	A	C	0.6104	-0.7820	0.0422	1.68E-76	341.97
CD4 NC	*LAIR2*	rs62131569	19	55021542	G	T	0.1168	-0.6564	0.0664	4.98E-23	97.46
CD4 NC	*LAMB1*	rs12672634	7	107582323	C	T	0.3927	-0.9534	0.0399	3.58E-126	569.74
CD4 NC	*LAMC3*	rs10901331	9	133926152	G	C	0.4767	0.2952	0.0448	4.46E-11	43.31
CD4 NC	*LAMTOR4*	rs7341389	7	99735181	G	A	0.6197	-0.3322	0.0456	3.24E-13	52.95
CD4 NC	*LAPTM4B*	rs2449504	8	98792686	C	A	0.1512	-0.4840	0.0648	8.00E-14	55.69
CD4 NC	*LAPTM5*	rs11585511	1	31216969	A	G	0.2702	-0.2814	0.0494	1.26E-08	32.33
CD4 NC	*LAX1*	rs4951181	1	203738781	G	A	0.5623	0.6030	0.0439	5.88E-43	188.39
CD4 NC	*LBH*	rs829636	2	30737801	G	T	0.2274	-0.3340	0.0509	5.33E-11	42.97
CD4 NC	*LCLAT1*	rs1723175	2	30667700	T	C	0.2221	1.0499	0.0456	2.19E-117	529.43
CD4 NC	*LCP1*	rs4942484	13	46697811	T	A	0.9354	-0.6027	0.0887	1.10E-11	46.05
CD4 NC	*LDHA*	rs11024601	11	18301605	C	T	0.5014	0.2684	0.0449	2.26E-09	35.67
CD4 NC	*LDHC*	rs77724297	11	18435458	T	A	0.1209	1.3001	0.0638	2.98E-92	414.16
CD4 NC	*LDLRAP1*	rs6701596	1	25889917	C	T	0.5403	-0.6615	0.0426	2.84E-54	240.16
CD4 NC	*LEKR1*	rs168248	3	156547990	G	A	0.7512	0.3675	0.0538	8.36E-12	46.58
CD4 NC	*LEPROT*	rs6658922	1	65901208	A	G	0.4061	-0.6018	0.0439	8.39E-43	187.69
CD4 NC	*LGALS3*	rs66871576	14	55629050	T	C	0.3836	0.3895	0.0448	3.24E-18	75.58
CD4 NC	*LILRB3*	rs11671310	19	54716663	A	G	0.3208	0.2734	0.0480	1.22E-08	32.38
CD4 NC	*LIMD1*	rs2673023	3	45711367	C	T	0.7714	-0.8061	0.0482	9.07E-63	279.05
CD4 NC	*LIMD2*	rs2665828	17	61961561	G	A	0.2580	0.2906	0.0502	7.03E-09	33.46
CD4 NC	*LIMS2*	rs2255177	2	128393289	C	T	0.3373	-0.3071	0.0479	1.43E-10	41.04
CD4 NC	*LIN52*	rs11622838	14	74669359	G	A	0.6619	-0.3591	0.0481	8.69E-14	55.53
CD4 NC	*LINC00176*	rs817356	20	62614929	C	T	0.1906	1.4972	0.0496	1.79E-200	910.75
CD4 NC	*LINC00240*	rs13195977	6	26759078	A	C	0.1290	-0.6053	0.0631	7.97E-22	91.98
CD4 NC	*LINC00336*	rs3828793	6	32635883	T	C	0.1858	0.4286	0.0624	6.23E-12	47.16
CD4 NC	*LINC00339*	rs12097775	1	22347252	T	C	0.2480	0.7161	0.0492	6.18E-48	211.16
CD4 NC	*LINC00339*	rs7542242	1	22477493	T	C	0.3281	0.2682	0.0474	1.58E-08	31.89
CD4 NC	*LINC00493*	rs11087256	20	18473958	G	T	0.4601	-0.3379	0.0447	3.81E-14	57.15
CD4 NC	*LINC00511*	rs16977301	17	70627018	A	G	0.0625	0.5616	0.0965	5.93E-09	33.79
CD4 NC	*LINC00649*	rs8130507	21	35303960	C	T	0.6025	0.6580	0.0438	3.97E-51	225.76
CD4 NC	*LINC00657*	rs8114927	20	35050323	C	T	0.9559	-0.4986	0.0843	3.27E-09	34.95
CD4 NC	*LINC00662*	rs7259514	19	28291859	A	C	0.8345	-0.3901	0.0603	9.81E-11	41.77
CD4 NC	*LINC00665*	rs2972549	19	36749658	A	G	0.3779	0.3837	0.0461	8.95E-17	69.05
CD4 NC	*LINC00667*	rs7228464	18	5213251	T	C	0.1917	0.4145	0.0554	7.25E-14	55.89
CD4 NC	*LINC00852*	rs582868	3	10323781	A	G	0.3636	-0.4214	0.0456	2.53E-20	85.16
CD4 NC	*LINC00861*	rs16900994	8	126958296	A	G	0.1494	-0.3777	0.0636	2.85E-09	35.21
CD4 NC	*LINC00863*	rs7095182	10	88968166	T	C	0.2935	-0.2865	0.0494	6.58E-09	33.59
CD4 NC	*LINC00886*	rs9820830	3	156542704	T	A	0.5729	-0.2660	0.0453	4.18E-09	34.47
CD4 NC	*LINC00909*	rs12961522	18	72264387	G	T	0.0538	0.7233	0.0945	1.93E-14	58.49
CD4 NC	*LINC00909*	rs7505289	18	72264406	G	A	0.8360	0.4565	0.0627	3.46E-13	52.82
CD4 NC	*LINC00920*	rs11863521	16	66441697	C	T	0.6723	-0.3424	0.0464	1.53E-13	54.42
CD4 NC	*LINC00944*	rs1706506	12	127256415	T	C	0.9383	-0.9458	0.1013	1.01E-20	86.95
CD4 NC	*LINC00951*	rs6458154	6	40354006	G	A	0.5943	0.4091	0.0455	2.25E-19	80.84
CD4 NC	*LINC00954*	rs13034115	2	20077653	G	C	0.1396	-0.9452	0.0595	8.82E-57	251.64
CD4 NC	*LINC00958*	rs12294433	11	13793210	G	C	0.1201	-0.9008	0.0614	1.02E-48	214.75
CD4 NC	*LINC00969*	rs2688446	3	195462025	A	G	0.4311	0.2973	0.0453	5.23E-11	43
CD4 NC	*LINC00969*	rs3827561	3	195674257	G	C	0.4166	0.6799	0.0434	2.39E-55	245.08
CD4 NC	*LINC01137*	rs6681785	1	37939094	T	C	0.3383	-0.3892	0.0476	2.80E-16	66.8
CD4 NC	*LINS*	rs28733387	15	101102708	A	G	0.4623	0.3340	0.0446	6.56E-14	56.08
CD4 NC	*LIPC*	rs7161799	15	58770523	T	C	0.0869	0.7493	0.0787	1.73E-21	90.45
CD4 NC	*LIPT2*	rs640094	11	74218644	C	G	0.6017	-0.3663	0.0459	1.48E-15	63.53
CD4 NC	*LL22NC03-2H8.5*	rs5757641	22	22598882	G	A	0.3591	0.3653	0.0460	2.13E-15	62.81
CD4 NC	*LLGL1*	rs8066387	17	18187621	C	T	0.4085	0.3295	0.0449	2.31E-13	53.61

CD4 NC	*LMAN1*	rs8098998	18	57059665	C	T	0.2892	0.2752	0.0498	3.32E-08	30.45
CD4 NC	*LMOD3*	rs9816032	3	69156421	A	G	0.4419	0.3079	0.0449	7.01E-12	46.93
CD4 NC	*LONP2*	rs8053839	16	48390512	T	G	0.5150	0.5077	0.0438	5.17E-31	133.83
CD4 NC	*LONRF2*	rs764828	2	100908634	C	A	0.4469	-0.6338	0.0428	1.49E-49	218.56
CD4 NC	*LPAR3*	rs12567433	1	85376406	A	G	0.2667	-0.5892	0.0498	2.49E-32	139.85
CD4 NC	*LPIN1*	rs11691509	2	11923179	T	C	0.2947	0.6413	0.0461	4.97E-44	193.3
CD4 NC	*LPIN2*	rs12956008	18	3058706	G	A	0.4538	0.4881	0.0438	7.63E-29	123.94
CD4 NC	*LRRC14*	rs12682297	8	145712860	A	T	0.4794	-0.4646	0.0439	3.81E-26	111.64
CD4 NC	*LRRC16A*	rs17317669	6	25406031	C	T	0.2700	0.6202	0.0476	9.09E-39	169.25
CD4 NC	*LRRC27*	rs3923974	10	134146084	T	C	0.1212	0.6910	0.0702	7.80E-23	96.57
CD4 NC	*LRRC37A*	rs35732828	17	44833217	A	C	0.1348	0.5319	0.0623	1.44E-17	72.65
CD4 NC	*LRRC37A2*	rs1724390	17	43663247	A	C	0.2040	0.5506	0.0525	9.22E-26	109.9
CD4 NC	*LRRC57*	rs62019285	15	42795916	C	G	0.1451	0.3712	0.0637	5.61E-09	33.9
CD4 NC	*LRRC6*	rs7827081	8	133696552	C	T	0.4953	0.2943	0.0447	4.66E-11	43.23
CD4 NC	*LRRC61*	rs3735173	7	150020526	T	C	0.2628	0.6100	0.0503	8.69E-34	146.5
CD4 NC	*LRRC7*	rs1773312	1	70063948	A	G	0.3155	0.2966	0.0476	4.63E-10	38.75
CD4 NC	*LRRIQ3*	rs524306	1	74682253	T	A	0.4715	-0.5091	0.0437	2.10E-31	135.62
CD4 NC	*LSM4*	rs112595944	19	18425758	A	G	0.0646	-0.6547	0.0919	1.06E-12	50.63
CD4 NC	*LSM6*	rs11729547	4	147040848	C	G	0.3060	-0.3657	0.0481	2.89E-14	57.69
CD4 NC	*LSM7*	rs11084936	19	2346440	A	G	0.5993	0.5812	0.0443	2.46E-39	171.84
CD4 NC	*LSMD1*	rs74439044	17	7781019	C	T	0.1040	0.4875	0.0780	4.16E-10	38.96
CD4 NC	*LSR*	rs8107155	19	35705337	T	C	0.6382	0.3197	0.0469	8.98E-12	46.45
CD4 NC	*LSS*	rs62224180	21	47726332	G	C	0.7751	0.3112	0.0570	4.90E-08	29.69
CD4 NC	*LST1*	rs2256974	6	31555392	A	C	0.2026	0.4891	0.0595	2.03E-16	67.44
CD4 NC	*LURAP1*	rs28364741	1	46664249	G	A	0.1712	0.4234	0.0579	2.66E-13	53.33
CD4 NC	*LY75*	rs67073100	2	160675650	A	G	0.3606	0.2863	0.0457	3.71E-10	39.18
CD4 NC	*LY86*	rs41502044	6	6659338	T	G	0.2654	0.3227	0.0520	5.59E-10	38.38
CD4 NC	*LY9*	rs790626	1	160794709	G	A	0.4504	-0.2524	0.0453	2.44E-08	31.05
CD4 NC	*LYRM4*	rs111557465	6	5260694	T	C	0.0736	1.2334	0.0769	7.18E-58	256.62
CD4 NC	*LYRM9*	rs1034895	17	26225558	C	G	0.7169	0.4403	0.0473	1.32E-20	86.43
CD4 NC	*MACF1*	rs16837533	1	39703847	A	G	0.1626	0.5371	0.0612	1.63E-18	76.93
CD4 NC	*MAF1*	rs11136255	8	145159099	A	G	0.8950	0.6128	0.0734	6.55E-17	69.66
CD4 NC	*MAGEF1*	rs2377119	3	184432710	G	A	0.7217	-0.3185	0.0498	1.59E-10	40.84
CD4 NC	*MAL*	rs3105098	2	95698210	C	G	0.7733	-0.4410	0.0516	1.29E-17	72.87
CD4 NC	*MAN1A2*	rs9727433	1	117967602	A	T	0.7613	0.4455	0.0521	1.21E-17	72.99
CD4 NC	*MAN2C1*	rs11634863	15	75766672	A	C	0.2703	0.3549	0.0517	6.46E-12	47.09
CD4 NC	*MANBA*	rs228614	4	103578637	A	G	0.4653	0.6229	0.0429	1.17E-47	209.89
CD4 NC	*MAP2K1*	rs14303	15	66783261	A	C	0.2631	-0.3489	0.0510	7.69E-12	46.75
CD4 NC	*MAP3K7CL*	rs2776233	21	30284575	A	G	0.2599	0.2915	0.0508	9.33E-09	32.91
CD4 NC	*MAP6D1*	rs262969	3	183446783	A	G	0.4226	-0.2608	0.0453	8.76E-09	33.03
CD4 NC	*MAPKAPK5-AS1*	rs688812	12	111962581	T	C	0.2079	0.3736	0.0550	1.10E-11	46.04
CD4 NC	*MARCH10*	rs17683483	17	60802483	A	C	0.1090	0.4549	0.0690	4.38E-11	43.35
CD4 NC	*MARCH2*	rs12150986	19	8483308	T	C	0.7300	-0.3972	0.0497	1.26E-15	63.84
CD4 NC	*MARK3*	rs1951389	14	103898525	C	G	0.3506	-0.3232	0.0462	2.75E-12	48.76
CD4 NC	*MAST3*	rs1566029	19	18258512	C	T	0.5165	-0.4591	0.0440	1.93E-25	108.44
CD4 NC	*MAST4*	rs152630	5	66354787	G	A	0.1681	-0.3376	0.0590	1.04E-08	32.7
CD4 NC	*MAST4*	rs1818068	5	66497850	T	C	0.2825	-0.3134	0.0490	1.54E-10	40.89
CD4 NC	*MATK*	rs11879267	19	3763118	G	A	0.8231	-0.3776	0.0605	4.35E-10	38.87
CD4 NC	*MAX*	rs7150646	14	65540463	T	G	0.3820	0.2758	0.0458	1.75E-09	36.16
CD4 NC	*MBOAT1*	rs2094429	6	20213205	C	T	0.5450	-0.2616	0.0451	6.68E-09	33.56
CD4 NC	*MBP*	rs470929	18	74723459	C	T	0.5925	0.4882	0.0442	2.30E-28	121.76
CD4 NC	*MBP*	rs1667901	18	74849345	C	T	0.7937	-0.3875	0.0542	8.89E-13	50.97
CD4 NC	*MCC*	rs17372511	5	112354898	G	C	0.5141	-0.5489	0.0434	1.18E-36	159.58
CD4 NC	*MCF2L2*	rs6777106	3	183146433	G	C	0.4735	-0.3043	0.0447	1.02E-11	46.19
CD4 NC	*MDGA1*	rs12530135	6	37612100	C	T	0.5867	-0.6674	0.0437	1.12E-52	232.85
CD4 NC	*MDH1*	rs7606045	2	63826528	G	T	0.7132	-0.3509	0.0480	2.58E-13	53.4
CD4 NC	*MDM4*	rs11801289	1	204528969	C	G	0.6747	-0.5305	0.0467	6.67E-30	128.77
CD4 NC	*MDS2*	rs2742946	1	23886073	C	T	0.4568	-0.3688	0.0447	1.61E-16	67.9
CD4 NC	*MEAF6*	rs11264065	1	37984812	T	C	0.5210	0.3908	0.0444	1.24E-18	77.47
CD4 NC	*MED16*	rs6678	19	890165	A	G	0.2610	0.9340	0.0473	8.41E-87	389.17
CD4 NC	*MED19*	rs1510421	11	57741517	A	G	0.2538	0.2943	0.0520	1.52E-08	31.96
CD4 NC	*MED4*	rs1752129	13	48709414	T	C	0.0662	-0.5188	0.0872	2.71E-09	35.31
CD4 NC	*MEF2BNB*	rs4808185	19	19296217	G	A	0.5538	0.2589	0.0451	9.77E-09	32.82
CD4 NC	*MEI1*	rs4822038	22	41958495	A	G	0.2013	0.4197	0.0566	1.25E-13	54.81
CD4 NC	*MEOX1*	rs62078582	17	41725494	A	G	0.4194	0.3650	0.0455	1.03E-15	64.23
CD4 NC	*METTL10*	rs12218394	10	126480591	C	G	0.5563	-0.5044	0.0444	6.45E-30	128.84
CD4 NC	*METTL18*	rs17543611	1	169836175	C	T	0.0898	-0.6593	0.0787	5.48E-17	70.01
CD4 NC	*METTL21B*	rs2069506	12	58142854	A	C	0.3118	0.4537	0.0472	7.69E-22	92.05
CD4 NC	*METTL2B*	rs12539729	7	128142023	T	A	0.6057	-0.3276	0.0465	1.81E-12	49.58
CD4 NC	*MFF*	rs71431034	2	228196571	T	A	0.0870	0.8136	0.0768	3.05E-26	112.08
CD4 NC	*MGMT*	rs1008982	10	131445731	C	T	0.3802	-0.6122	0.0442	1.21E-43	191.54
CD4 NC	*MGRN1*	rs1659503	16	4711791	T	C	0.4485	0.2873	0.0448	1.42E-10	41.05
CD4 NC	*MGST2*	rs795599	4	140588412	C	T	0.4857	-0.6486	0.0429	1.21E-51	228.13
CD4 NC	*MGST3*	rs10800113	1	165571573	G	A	0.5758	-0.6120	0.0434	3.22E-45	198.74
CD4 NC	*MGST3*	rs4314865	1	165621836	C	G	0.0547	0.5519	0.0991	2.56E-08	30.95
CD4 NC	*MIB2*	rs12735861	1	1562895	T	C	0.5750	-0.3494	0.0450	8.72E-15	60.04
CD4 NC	*MICA*	rs3828879	6	31379391	T	C	0.1430	0.3837	0.0660	6.00E-09	33.76
CD4 NC	*MICAL3*	rs9605487	22	18483230	G	A	0.4813	0.3095	0.0447	4.44E-12	47.82
CD4 NC	*MICU3*	rs80067369	8	16968157	T	C	0.0543	0.6078	0.0994	9.73E-10	37.3

CD4 NC	*MICU3*	rs72607371	8	16970354	A	G	0.0951	-0.4301	0.0745	7.81E-09	33.25
CD4 NC	*MINK1*	rs62073635	17	4770197	C	T	0.3331	-0.4260	0.0469	1.11E-19	82.23
CD4 NC	*MKRN1*	rs28563126	7	140170797	A	T	0.1416	-0.6162	0.0661	1.08E-20	86.83
CD4 NC	*MLF2*	rs2302366	12	6862259	C	G	0.3476	-0.3071	0.0466	4.17E-11	43.44
CD4 NC	*MLKL*	rs9932977	16	74735148	A	G	0.5689	0.2612	0.0452	7.60E-09	33.31
CD4 NC	*MLLT4*	rs3800520	6	168280296	G	A	0.3477	0.4709	0.0456	5.42E-25	106.39
CD4 NC	*MMEL1*	rs876938	1	2523212	C	G	0.3392	-0.3889	0.0469	1.10E-16	68.64
CD4 NC	*MORC3*	rs6517342	21	37673381	A	G	0.1579	0.4632	0.0622	9.77E-14	55.3
CD4 NC	*MORN3*	rs3825175	12	122079441	C	T	0.4179	0.7390	0.0426	2.09E-67	300.3
CD4 NC	*MOSPD3*	rs10487157	7	100214487	G	C	0.1889	0.3445	0.0585	3.99E-09	34.56
CD4 NC	*MPHOSPH6*	rs2303260	16	82203786	G	C	0.4801	-0.7352	0.0419	5.08E-69	307.69
CD4 NC	*MPHOSPH9*	rs1463877	12	123473254	C	T	0.7914	-0.5610	0.0564	2.44E-23	98.86
CD4 NC	*MPI*	rs11072511	15	75159433	G	A	0.5182	0.3314	0.0449	1.63E-13	54.29
CD4 NC	*MPPE1*	rs8097892	18	11905953	T	C	0.4593	-0.3170	0.0447	1.37E-12	50.12
CD4 NC	*MPV17L2*	rs10410017	19	18296867	T	C	0.3574	0.2875	0.0466	6.62E-10	38.05
CD4 NC	*MPZL2*	rs3782042	11	118185919	G	A	0.6130	-0.2768	0.0460	1.76E-09	36.15
CD4 NC	*MPZL3*	rs17121997	11	118131049	C	T	0.5030	-0.4359	0.0443	7.04E-23	96.77
CD4 NC	*MRPL14*	rs4711767	6	44081987	C	T	0.5341	-0.3939	0.0443	5.95E-19	78.92
CD4 NC	*MRPL18*	rs9365097	6	160211259	A	G	0.7577	-0.4756	0.0519	4.76E-20	83.91
CD4 NC	*MRPL19*	rs114255554	2	75932627	C	T	0.2465	0.4226	0.0507	7.98E-17	69.27
CD4 NC	*MRPL20*	rs2936661	1	1300946	A	G	0.8196	-0.3548	0.0616	8.39E-09	33.11
CD4 NC	*MRPL21*	rs583182	11	68678634	T	C	0.2951	-0.8613	0.0453	1.73E-80	360.23
CD4 NC	*MRPL28*	rs2363755	16	430292	G	C	0.4125	0.2741	0.0449	1.03E-09	37.18
CD4 NC	*MRPL30*	rs13031919	2	99848932	T	A	0.6056	-0.4119	0.0456	1.59E-19	81.53
CD4 NC	*MRPL32*	rs648956	7	42945367	T	C	0.8581	-0.7282	0.0642	8.04E-30	128.4
CD4 NC	*MRPL34*	rs71338624	19	17414777	G	C	0.3915	0.7128	0.0425	4.60E-63	280.41
CD4 NC	*MRPL4*	rs1059840	19	10370490	A	T	0.1379	0.3960	0.0614	1.12E-10	41.52
CD4 NC	*MRPL41*	rs10867029	9	140442998	A	G	0.2763	-0.3575	0.0488	2.38E-13	53.56
CD4 NC	*MRPL42*	rs12811748	12	93895773	C	T	0.1560	0.8580	0.0572	6.88E-51	224.67
CD4 NC	*MRPL48*	rs1792156	11	73529453	A	G	0.4387	-0.5078	0.0447	5.87E-30	129.02
CD4 NC	*MRPL51*	rs2532513	12	6589171	T	C	0.7091	-0.3079	0.0499	6.99E-10	37.95
CD4 NC	*MRPL53*	rs8469	2	74687550	C	T	0.1667	-0.6536	0.0682	9.86E-22	91.56
CD4 NC	*MRPL54*	rs12459781	19	3762119	C	T	0.6394	0.6712	0.0452	7.54E-50	219.91
CD4 NC	*MRPS10*	rs4424082	6	42194235	A	T	0.6300	0.4523	0.0462	1.15E-22	95.81
CD4 NC	*MRPS18C*	rs1565909	4	84400330	T	C	0.4867	0.4792	0.0439	8.75E-28	119.11
CD4 NC	*MRPS21*	rs844309	1	150366587	T	C	0.3895	-0.4188	0.0450	1.26E-20	86.52
CD4 NC	*MRPS33*	rs9274	7	140706031	A	G	0.0947	-0.5333	0.0807	3.98E-11	43.53
CD4 NC	*MRPS34*	rs2437732	16	1826302	T	G	0.5613	-0.2536	0.0455	2.43E-08	31.05
CD4 NC	*MRPS36*	rs350106	5	68492746	G	A	0.5499	-0.3157	0.0447	1.55E-12	49.88
CD4 NC	*MRPS6*	rs8130507	21	35303960	C	T	0.6025	-0.3459	0.0456	3.42E-14	57.36
CD4 NC	*MSC*	rs3919904	8	72752882	T	C	0.1433	-0.3390	0.0611	2.89E-08	30.71
CD4 NC	*MSH3*	rs420522	5	79902336	A	G	0.2498	0.3965	0.0516	1.53E-14	58.94
CD4 NC	*MSRA*	rs6982308	8	10193772	G	C	0.4969	0.4155	0.0442	5.34E-21	88.22
CD4 NC	*MT1E*	rs1610215	16	56659525	G	C	0.5423	0.7577	0.0418	2.04E-73	327.83
CD4 NC	*MT1F*	rs1875231	16	56683745	C	A	0.3943	0.7347	0.0425	8.25E-67	297.56
CD4 NC	*MT1X*	rs1599933	16	56715862	A	C	0.5528	-0.7133	0.0423	1.09E-63	283.28
CD4 NC	*MT2A*	rs1580833	16	56644650	A	C	0.3093	-0.6735	0.0464	9.62E-48	210.28
CD4 NC	*MT3*	rs1827207	16	56648946	C	T	0.3287	-0.3007	0.0474	2.23E-10	40.17
CD4 NC	*MTERFD2*	rs6437234	2	242019288	T	C	0.1813	-0.4558	0.0609	7.12E-14	55.92
CD4 NC	*MTG2*	rs2184161	20	60776275	G	A	0.5408	0.4307	0.0442	1.78E-22	94.94
CD4 NC	*MTHFR*	rs1476413	1	11852300	T	C	0.2623	0.3136	0.0519	1.47E-09	36.5
CD4 NC	*MTHFS*	rs77462993	15	80186354	A	G	0.0559	0.5264	0.0906	6.27E-09	33.68
CD4 NC	*MTR*	rs10925254	1	237023333	C	T	0.6162	0.4316	0.0458	4.11E-21	88.74
CD4 NC	*MTRF1L*	rs637384	6	153340412	A	C	0.6795	-0.4478	0.0476	5.54E-21	88.15
CD4 NC	*MTSS1*	rs11778063	8	125577110	T	C	0.6697	-0.6671	0.0450	8.78E-50	219.61
CD4 NC	*MTUS1*	rs437548	8	17555029	C	G	0.2177	0.4898	0.0541	1.33E-19	81.88
CD4 NC	*MUC20*	rs712005	3	195473641	A	G	0.8478	-0.5219	0.0613	1.76E-17	72.25
CD4 NC	*MUC20*	rs9881504	3	195656614	C	T	0.6435	0.3038	0.0468	8.83E-11	41.98
CD4 NC	*MX1*	rs464138	21	42798173	C	A	0.4175	-0.3427	0.0452	3.31E-14	57.43
CD4 NC	*MX2*	rs398800	21	42742212	A	C	0.6882	-0.3441	0.0486	1.48E-12	49.98
CD4 NC	*MXRA7*	rs4789349	17	74706341	T	A	0.9117	-0.6460	0.0848	2.65E-14	57.86
CD4 NC	*MYADM*	rs307937	19	54380149	C	G	0.9485	-0.6063	0.0978	5.59E-10	38.38
CD4 NC	*MYCBPAP*	rs6504662	17	48580257	C	T	0.4594	-0.5135	0.0440	1.75E-31	135.99
CD4 NC	*MYH11*	rs16967494	16	15820863	T	C	0.2539	0.7181	0.0476	1.94E-51	227.19
CD4 NC	*MYO15B*	rs3862480	17	73577568	C	T	0.0914	0.8183	0.0831	7.28E-23	96.71
CD4 NC	*MYO16*	rs9520848	13	108995449	C	T	0.2650	0.6367	0.0522	3.42E-34	148.35
CD4 NC	*MYO6*	rs276696	6	76470448	T	C	0.3191	-0.2865	0.0478	2.09E-09	35.81
CD4 NC	*MYO7B*	rs10202094	2	129031590	T	C	0.4196	0.7105	0.0427	4.32E-62	275.95
CD4 NC	*MYOM2*	rs62478456	8	2073189	A	G	0.1883	1.1123	0.0564	1.08E-86	388.67
CD4 NC	*MZT2A*	rs59915006	2	132259374	G	A	0.7817	-1.1815	0.0519	7.84E-115	517.71
CD4 NC	*MZT2A*	rs62176575	2	132343822	G	C	0.2809	-0.2885	0.0501	8.56E-09	33.08
CD4 NC	*N4BP2*	rs7696474	4	40055638	T	C	0.6531	-0.4043	0.0457	9.16E-19	78.07
CD4 NC	*NAA38*	rs1061731	7	117832138	A	G	0.6861	-0.7822	0.0444	1.45E-69	310.18
CD4 NC	*NAAA*	rs111981122	4	76855106	C	A	0.2335	-0.7635	0.0520	1.01E-48	214.76
CD4 NC	*NAP1L1*	rs1433364	12	76464637	G	A	0.8079	-0.7300	0.0566	4.14E-38	166.24
CD4 NC	*NAP1L4*	rs12276060	11	3015572	T	A	0.3858	-0.5840	0.0446	4.04E-39	170.86
CD4 NC	*NAPRT1*	rs9657360	8	144676862	T	C	0.7776	1.1080	0.0492	1.86E-112	506.82
CD4 NC	*NARFL*	rs12443759	16	782132	T	C	0.2107	0.3794	0.0571	3.00E-11	44.09
CD4 NC	*NAV2*	rs11025671	11	20657603	T	C	0.2893	-0.5000	0.0489	1.39E-24	104.52

CD4 NC	*NBL1*	rs10917493	1	19882170	G	T	0.5445	0.5507	0.0436	1.45E-36	159.18
CD4 NC	*NBL1*	rs75061720	1	19892021	T	A	0.1041	0.4918	0.0748	4.85E-11	43.15
CD4 NC	*NBPF1*	rs156917	1	16852865	C	T	0.6114	-0.3014	0.0453	2.92E-11	44.14
CD4 NC	*NBPF3*	rs4079441	1	21761309	T	G	0.7722	-0.3891	0.0521	8.42E-14	55.59
CD4 NC	*NCAPG2*	rs73167215	7	158500207	A	G	0.0611	0.8471	0.0832	2.40E-24	103.45
CD4 NC	*NCBP2*	rs555582	3	196665854	G	A	0.5189	-0.3238	0.0446	3.91E-13	52.58
CD4 NC	*NCF2*	rs1184639	1	183623619	C	G	0.3485	0.9237	0.0415	5.57E-110	495.46
CD4 NC	*NCF4*	rs4821546	22	37268079	G	A	0.7127	-0.4539	0.0496	5.82E-20	83.51
CD4 NC	*NCL*	rs13019380	2	232320581	G	T	0.1067	-0.4763	0.0751	2.25E-10	40.16
CD4 NC	*NCOA7*	rs1544293	6	126175085	G	A	0.6957	-0.4240	0.0480	9.71E-19	77.96
CD4 NC	*NDFIP1*	rs12655443	5	141467856	A	C	0.5060	-0.2655	0.0448	3.07E-09	35.07
CD4 NC	*NDUFA10*	rs7609118	2	240941703	A	C	0.3160	-0.4698	0.0475	4.91E-23	97.48
CD4 NC	*NDUFA12*	rs10859822	12	95396906	A	C	0.3189	0.8481	0.0432	1.05E-85	384.16
CD4 NC	*NDUFA3*	rs62143397	19	54628964	G	C	0.1017	0.9687	0.0697	6.04E-44	192.91
CD4 NC	*NDUFA6*	rs6002719	22	42787282	C	T	0.0488	0.9937	0.0997	2.13E-23	99.13
CD4 NC	*NDUFB1*	rs11845252	14	92605747	C	T	0.4082	-0.3154	0.0460	6.70E-12	47.02
CD4 NC	*NDUFB2*	rs6953568	7	140413459	G	A	0.1745	-0.3919	0.0645	1.25E-09	36.81
CD4 NC	*NDUFS5*	rs6691071	1	39529162	A	C	0.2798	-0.8464	0.0445	9.53E-81	361.42
CD4 NC	*NDUFV3*	rs78926712	21	44318204	A	T	0.0737	-0.6795	0.0860	2.82E-15	62.26
CD4 NC	*NEDD4L*	rs9950709	18	55968327	G	T	0.4925	-0.2743	0.0448	8.96E-10	37.46
CD4 NC	*NEIL2*	rs1293299	8	11677905	C	A	0.2173	-0.3513	0.0546	1.23E-10	41.34
CD4 NC	*NEK1*	rs10520157	4	170533736	T	G	0.1327	0.4456	0.0649	6.42E-12	47.1
CD4 NC	*NEK11*	rs4974477	3	130915439	G	A	0.8088	-0.3137	0.0561	2.26E-08	31.19
CD4 NC	*NELFCD*	rs62205397	20	57597011	T	C	0.1620	-0.4070	0.0609	2.27E-11	44.63
CD4 NC	*NELL2*	rs2658954	12	45241770	A	G	0.8473	0.7035	0.0635	1.68E-28	122.39
CD4 NC	*NEURL1*	rs12253987	10	105330947	A	T	0.1919	-0.3358	0.0589	1.22E-08	32.39
CD4 NC	*NEXN*	rs12732514	1	78640339	T	C	0.4128	0.2776	0.0448	5.97E-10	38.25
CD4 NC	*NFYC-AS1*	rs12409109	1	41192888	T	G	0.2246	0.3292	0.0545	1.54E-09	36.4
CD4 NC	*NINJ2*	rs6489710	12	777008	T	G	0.3842	-0.5392	0.0455	1.88E-32	140.4
CD4 NC	*NINL*	rs11700338	20	25595868	A	G	0.1368	0.4186	0.0705	2.95E-09	35.15
CD4 NC	*NIPSNAP3A*	rs62569025	9	107409559	T	C	0.1153	0.4022	0.0687	4.73E-09	34.23
CD4 NC	*NIPSNAP3B*	rs62565988	9	107526619	A	G	0.1288	0.4656	0.0632	1.81E-13	54.09
CD4 NC	*NIT2*	rs62274146	3	100076980	G	A	0.3209	-0.3091	0.0480	1.23E-10	41.33
CD4 NC	*NKAPL*	rs73391152	6	28689004	T	G	0.0633	0.5701	0.0973	4.71E-09	34.24
CD4 NC	*NLE1*	rs79900800	17	33493071	G	A	0.1086	0.3941	0.0713	3.30E-08	30.46
CD4 NC	*NLRC5*	rs7192652	16	57075180	G	A	0.5176	0.2688	0.0448	2.02E-09	35.88
CD4 NC	*NLRP2*	rs775841	19	55473831	G	A	0.4406	0.3400	0.0448	3.09E-14	57.56
CD4 NC	*NMB*	rs17598603	15	85200211	G	C	0.2636	-0.3074	0.0520	3.30E-09	34.93
CD4 NC	*NME1*	rs7207370	17	49231180	G	A	0.3899	0.6985	0.0434	3.05E-58	258.33
CD4 NC	*NME3*	rs1178437	16	1831912	C	G	0.6900	-0.3358	0.0493	9.33E-12	46.37
CD4 NC	*NME4*	rs77371232	16	447561	T	A	0.4244	0.5162	0.0441	1.37E-31	136.47
CD4 NC	*NME6*	rs35297486	3	48378147	A	T	0.2848	-0.3003	0.0488	7.31E-10	37.86
CD4 NC	*NMI*	rs57201382	2	152131543	T	C	0.4423	0.7558	0.0418	3.83E-73	326.58
CD4 NC	*NMRAL1*	rs9926145	16	4438200	A	G	0.0516	-0.7529	0.1015	1.22E-13	54.86
CD4 NC	*NMRAL1*	rs4786514	16	4576798	C	A	0.7151	-0.3868	0.0491	3.38E-15	61.9
CD4 NC	*NMRK1*	rs11144225	9	77707300	T	A	0.3900	-0.8476	0.0412	4.80E-94	422.38
CD4 NC	*NMT2*	rs12763978	10	15238886	A	C	0.3716	0.5574	0.0452	5.44E-35	151.99
CD4 NC	*NOL9*	rs6692838	1	6610160	T	C	0.8641	0.5373	0.0688	6.02E-15	60.77
CD4 NC	*NOMO1*	rs143477575	16	15257639	G	A	0.8394	-0.5189	0.0620	5.63E-17	69.96
CD4 NC	*NOP10*	rs347818	15	34563598	A	G	0.3243	0.2702	0.0480	1.88E-08	31.55
CD4 NC	*NPAT*	rs227056	11	108262397	T	C	0.5908	0.3285	0.0449	2.55E-13	53.42
CD4 NC	*NPC2*	rs4903231	14	74962233	A	G	0.5967	-0.9094	0.0406	5.02E-111	500.25
CD4 NC	*NPC2*	rs8017691	14	75092905	C	A	0.5668	0.2682	0.0451	2.83E-09	35.22
CD4 NC	*NPCDR1*	rs3772491	3	59969666	A	C	0.4344	-0.3062	0.0450	1.05E-11	46.14
CD4 NC	*NPDC1*	rs28705707	9	139940653	A	C	0.3857	0.3646	0.0457	1.42E-15	63.6
CD4 NC	*NPHP3*	rs12636883	3	132429522	A	C	0.1713	-0.4100	0.0656	4.22E-10	38.93
CD4 NC	*NPIPA5*	rs3198697	16	15129940	T	C	0.3959	0.3169	0.0455	3.39E-12	48.35
CD4 NC	*NPRL3*	rs2562191	16	178543	A	G	0.3891	-0.3321	0.0468	1.22E-12	50.34
CD4 NC	*NQO2*	rs2518581	6	3009944	G	A	0.7344	-0.8715	0.0490	1.22E-70	315.11
CD4 NC	*NR2F6*	rs7253172	19	17332663	C	A	0.3540	0.4324	0.0461	6.49E-21	87.84
CD4 NC	*NRN1*	rs674375	6	6000215	T	C	0.3670	0.2613	0.0469	2.50E-08	31
CD4 NC	*NSA2*	rs72764630	5	73997524	T	A	0.1335	1.2592	0.0598	1.95E-98	442.51
CD4 NC	*NSF*	rs3874943	17	44572068	G	A	0.5235	-0.3451	0.0445	9.20E-15	59.94
CD4 NC	*NSG1*	rs55731118	4	4350915	T	A	0.4050	0.9527	0.0403	2.21E-123	556.94
CD4 NC	*NSG1*	rs7442201	4	4361749	G	A	0.7285	0.2920	0.0494	3.46E-09	34.83
CD4 NC	*NSUN4*	rs5013329	1	46815091	T	C	0.2870	0.3632	0.0493	1.75E-13	54.15
CD4 NC	*NSUN5*	rs10231271	7	72364533	A	G	0.5099	0.3303	0.0446	1.25E-13	54.82
CD4 NC	*NT5C3A*	rs4723240	7	33088926	A	G	0.6955	-0.5859	0.0480	2.90E-34	148.67
CD4 NC	*NT5C3B*	rs9897034	17	39992584	T	C	0.7555	-0.8282	0.0480	9.01E-67	297.39
CD4 NC	*NT5E*	rs12524836	6	86175956	G	A	0.1309	-0.3999	0.0638	3.60E-10	39.24
CD4 NC	*NT5M*	rs41400246	17	17124625	T	C	0.1313	0.4105	0.0652	3.10E-10	39.53
CD4 NC	*NTPCR*	rs56287665	1	233082005	A	T	0.1800	1.2062	0.0498	1.60E-129	585.1
CD4 NC	*NUCB1*	rs117753546	19	49432132	A	G	0.0482	-0.6802	0.1019	2.45E-11	44.48
CD4 NC	*NUCKS1*	rs823095	1	205679239	A	G	0.4161	-0.3053	0.0454	1.86E-11	45.02
CD4 NC	*NUDT12*	rs11750858	5	102896400	C	T	0.1545	-0.7237	0.0595	5.07E-34	147.57
CD4 NC	*NUDT18*	rs7836362	8	21970426	C	T	0.5028	-0.5280	0.0436	8.64E-34	146.51
CD4 NC	*NUDT2*	rs747091	9	34045014	G	T	0.5810	-0.3465	0.0447	9.70E-15	59.83
CD4 NC	*NUDT2*	rs10814102	9	34353879	T	G	0.1522	1.3796	0.0542	6.54E-143	646.54
CD4 NC	*NUP107*	rs2431655	12	69077497	C	T	0.3400	-0.4957	0.0470	4.93E-26	111.13

CD4 NC	*NUTF2*	rs2008173	16	67856240	G	A	0.1859	-0.5296	0.0602	1.44E-18	77.17
CD4 NC	*NXPE3*	rs595230	3	101524761	A	G	0.2178	-0.4550	0.0569	1.26E-15	63.84
CD4 NC	*OCEL1*	rs2288544	19	17337025	T	C	0.2194	-0.6363	0.0535	1.42E-32	140.96
CD4 NC	*OCIAD2*	rs2605254	4	48937377	A	C	0.4686	-0.2471	0.0451	4.30E-08	29.95
CD4 NC	*ODF2L*	rs272514	1	86845563	C	T	0.6265	0.3499	0.0460	2.75E-14	57.79
CD4 NC	*OR2A1-AS1*	rs6949375	7	143773851	G	T	0.1821	-0.3599	0.0536	1.92E-11	44.96
CD4 NC	*ORAI1*	rs12308869	12	122092823	T	C	0.1689	0.5178	0.0589	1.44E-18	77.17
CD4 NC	*ORMDL1*	rs3791767	2	190639915	C	A	0.1939	-0.3722	0.0569	5.96E-11	42.75
CD4 NC	*ORMDL3*	rs17608925	17	38082831	C	T	0.1182	-1.1926	0.0671	9.78E-71	315.55
CD4 NC	*ORMDL3*	rs56301252	17	38109155	G	T	0.6270	0.2608	0.0465	2.11E-08	31.33
CD4 NC	*OSBPL10*	rs4420912	3	31571327	T	A	0.3099	0.4434	0.0495	3.26E-19	80.11
CD4 NC	*OSBPL1A*	rs17797879	18	21973870	T	C	0.5133	-0.3402	0.0445	2.16E-14	58.26
CD4 NC	*OSBPL6*	rs334103	2	179061110	C	T	0.5260	0.4961	0.0437	8.30E-30	128.34
CD4 NC	*OSER1-AS1*	rs6031456	20	42840019	G	A	0.1489	0.5053	0.0654	1.12E-14	59.55
CD4 NC	*OSGIN2*	rs2734823	8	90943728	G	A	0.4008	0.2967	0.0454	6.63E-11	42.54
CD4 NC	*OSTF1*	rs10869496	9	77700084	A	C	0.3854	-0.5844	0.0439	2.12E-40	176.71
CD4 NC	*OXLD1*	rs11150804	17	79630238	G	A	0.3735	-0.7558	0.0438	1.14E-66	296.91
CD4 NC	*OXNAD1*	rs6442592	3	16294425	G	A	0.1542	0.4239	0.0650	7.10E-11	42.4
CD4 NC	*P4HA3*	rs10898954	11	73790243	T	C	0.4575	0.5125	0.0440	2.58E-31	135.21
CD4 NC	*PABPC4*	rs3768321	1	40035928	T	G	0.1819	-0.3241	0.0578	2.06E-08	31.37
CD4 NC	*PADI4*	rs10788663	1	17647045	T	C	0.5992	-0.4505	0.0452	1.97E-23	99.29
CD4 NC	*PADI4*	rs1612843	1	17687161	C	G	0.2723	-0.4494	0.0493	7.42E-20	83.03
CD4 NC	*PAFAH1B2*	rs4938341	11	117002596	A	G	0.1439	0.4460	0.0620	6.48E-13	51.59
CD4 NC	*PAN3*	rs9319424	13	28847135	T	C	0.5928	-0.2770	0.0463	2.25E-09	35.67
CD4 NC	*PAN3-AS1*	rs9512746	13	28128103	A	G	0.3433	0.2742	0.0469	4.96E-09	34.13
CD4 NC	*PANK4*	rs2494620	1	2441358	C	T	0.2476	-0.3913	0.0537	3.24E-13	52.95
CD4 NC	*PANX1*	rs4753126	11	93862020	T	C	0.5080	0.2469	0.0448	3.70E-08	30.24
CD4 NC	*PARK7*	rs379920	1	8028865	C	G	0.5997	0.5184	0.0441	5.80E-32	138.17
CD4 NC	*PARP14*	rs13071069	3	122440065	T	C	0.1057	-0.6301	0.0865	3.31E-13	52.91
CD4 NC	*PARP15*	rs2332240	3	122296834	G	A	0.4499	0.2570	0.0449	1.04E-08	32.71
CD4 NC	*PARVB*	rs2267592	22	44421563	T	G	0.5541	-0.5205	0.0437	9.20E-33	141.82
CD4 NC	*PASK*	rs3815296	2	242050546	G	A	0.1835	-1.0825	0.0563	2.57E-82	368.62
CD4 NC	*PAX8*	rs10175462	2	113988492	A	G	0.3750	-0.5175	0.0457	1.09E-29	127.8
CD4 NC	*PBX3*	rs4837022	9	128475379	C	T	0.6524	0.3126	0.0465	1.79E-11	45.1
CD4 NC	*PCBP2*	rs11170553	12	53822092	T	C	0.0740	-0.6420	0.0826	7.53E-15	60.33
CD4 NC	*PCBP3*	rs3788220	21	47287992	G	A	0.2247	0.3584	0.0522	6.53E-12	47.07
CD4 NC	*PCED1B*	rs215379	12	47754636	T	C	0.4570	0.3622	0.0448	6.18E-16	65.25
CD4 NC	*PCM1*	rs208750	8	17776219	G	T	0.7546	-0.5874	0.0514	2.87E-30	130.44
CD4 NC	*PCMTD1*	rs9298467	8	52806682	T	C	0.7814	0.3563	0.0573	5.18E-10	38.53
CD4 NC	*PCNX*	rs2240341	14	71373580	C	T	0.5444	0.4387	0.0448	1.12E-22	95.85
CD4 NC	*PCSK7*	rs111765459	11	117083373	C	T	0.2126	0.3765	0.0550	7.63E-12	46.76
CD4 NC	*PDCD5*	rs11084660	19	33072118	G	C	0.3634	0.4050	0.0454	5.04E-19	79.25
CD4 NC	*PDE7A*	rs72666512	8	66556843	A	C	0.1633	0.3976	0.0603	4.30E-11	43.38
CD4 NC	*PDE9A*	rs2839581	21	44158405	A	C	0.7004	-0.4713	0.0475	3.63E-23	98.08
CD4 NC	*PDGFB*	rs59744664	22	39647090	G	A	0.3594	-0.2934	0.0466	2.98E-10	39.6
CD4 NC	*PDIA6*	rs1734343	2	10937037	G	T	0.4771	-0.4518	0.0441	1.35E-24	104.59
CD4 NC	*PDK1*	rs12693006	2	173467213	T	C	0.2700	0.3002	0.0529	1.35E-08	32.19
CD4 NC	*PDS5A*	rs17511298	4	39905344	G	C	0.5113	-0.3241	0.0446	3.66E-13	52.71
CD4 NC	*PDXDC1*	rs11861982	16	15369019	T	C	0.1675	0.5250	0.0554	2.75E-21	89.54
CD4 NC	*PDXK*	rs2276528	21	45152500	A	G	0.1480	0.8784	0.0595	2.15E-49	217.83
CD4 NC	*PEBP4*	rs13266950	8	23394940	T	C	0.1422	-0.4856	0.0644	4.82E-14	56.68
CD4 NC	*PERP*	rs1334705	6	138443794	T	C	0.4088	0.2719	0.0456	2.54E-09	35.44
CD4 NC	*PEX5L*	rs11718864	3	179034472	C	G	0.1572	1.0040	0.0546	1.95E-75	337.09
CD4 NC	*PEX6*	rs9296403	6	42925683	T	C	0.4405	0.6470	0.0429	1.69E-51	227.46
CD4 NC	*PFKP*	rs112720077	10	3117988	A	G	0.0522	0.6985	0.1020	7.63E-12	46.76
CD4 NC	*PFN2*	rs11719798	3	149608271	C	T	0.3584	0.5023	0.0456	3.48E-28	120.94
CD4 NC	*PGLS*	rs7251020	19	17629739	T	C	0.5524	0.3792	0.0446	2.01E-17	71.99
CD4 NC	*PGPEP1*	rs12985909	19	18439383	T	C	0.5453	-0.3248	0.0447	3.58E-13	52.75
CD4 NC	*PGS1*	rs72903315	17	76418740	A	G	0.1619	0.4291	0.0595	5.71E-13	51.84
CD4 NC	*PHACTR4*	rs4908411	1	28704978	T	C	0.3223	-0.4372	0.0474	2.85E-20	84.91
CD4 NC	*PHF11*	rs7322908	13	50100090	A	T	0.4796	0.4225	0.0442	1.19E-21	91.18
CD4 NC	*PHGDH*	rs116612731	1	120210231	T	A	0.0753	0.5498	0.0758	4.02E-13	52.53
CD4 NC	*PHLDB3*	rs35681564	19	43959388	A	G	0.1863	0.5277	0.0564	8.01E-21	87.42
CD4 NC	*PHOSPHO2*	rs35489159	2	170472411	A	G	0.2829	-0.3780	0.0484	5.54E-15	60.93
CD4 NC	*PHYKPL*	rs17052412	5	177667225	A	G	0.0788	0.5559	0.0930	2.22E-09	35.69
CD4 NC	*PI16*	rs2177845	6	36915094	T	C	0.1576	1.0059	0.0570	9.02E-70	311.13
CD4 NC	*PI4K2B*	rs60527077	4	25161539	A	G	0.0922	-0.8635	0.0737	1.13E-31	136.85
CD4 NC	*PIGP*	rs7280542	21	38444995	G	A	0.6584	-0.6510	0.0451	3.08E-47	207.97
CD4 NC	*PILRB*	rs75636500	7	99947657	G	C	0.1758	1.0794	0.0515	1.38E-97	438.62
CD4 NC	*PIP4K2A*	rs746203	10	22830541	T	C	0.6149	0.2956	0.0458	1.07E-10	41.6
CD4 NC	*PITRM1*	rs77539019	10	3220134	G	A	0.0810	-0.4564	0.0818	2.46E-08	31.03
CD4 NC	*PKIA*	rs9693457	8	79438103	T	G	0.2018	-0.6504	0.0572	6.41E-30	128.85
CD4 NC	*PKMYT1*	rs224241	16	3317036	G	A	0.2481	0.5586	0.0518	3.85E-27	116.18
CD4 NC	*PLA2G4C*	rs12975454	19	48603755	G	A	0.1612	0.4180	0.0624	2.15E-11	44.74
CD4 NC	*PLA2G6*	rs2235347	22	38522887	G	A	0.4401	0.2465	0.0452	4.85E-08	29.72
CD4 NC	*PLCB2*	rs4244575	15	40596561	G	C	0.2432	0.4778	0.0527	1.18E-19	82.12
CD4 NC	*PLCE1*	rs3740359	10	96100119	T	C	0.1059	-0.4882	0.0805	1.31E-09	36.72
CD4 NC	*PLEC*	rs62523994	8	145026582	A	G	0.4004	-0.5190	0.0445	2.11E-31	135.61
CD4 NC	*PLEK*	rs2120334	2	68616085	T	C	0.2885	0.4422	0.0503	1.56E-18	77.02

CD4 NC	*PLEKHA1*	rs3850765	10	124139910	C	T	0.6133	-0.6055	0.0439	2.40E-43	190.17
CD4 NC	*PLEKHA5*	rs10841164	12	19320243	T	C	0.8036	0.4174	0.0603	4.50E-12	47.8
CD4 NC	*PLEKHG1*	rs6929578	6	151048768	C	T	0.0972	0.5348	0.0785	9.83E-12	46.27
CD4 NC	*PLEKHG5*	rs3986418	1	6621996	C	G	0.8631	0.4338	0.0687	2.76E-10	39.76
CD4 NC	*PLEKHH2*	rs6710830	2	43867946	G	C	0.6123	-0.6638	0.0437	4.78E-52	229.97
CD4 NC	*PLEKHO1*	rs11589922	1	150133323	A	C	0.1587	0.4503	0.0598	5.13E-14	56.56
CD4 NC	*PLGLB1*	rs114731870	2	87260648	T	C	0.3660	0.3190	0.0462	4.86E-12	47.65
CD4 NC	*PLGRKT*	rs2104175	9	5383460	G	C	0.6659	-0.6205	0.0448	1.57E-43	191.02
CD4 NC	*PM20D1*	rs823099	1	205669322	A	C	0.4168	0.4212	0.0449	6.23E-21	87.92
CD4 NC	*PMS1*	rs62185872	2	190554759	A	T	0.1416	0.4377	0.0643	9.79E-12	46.27
CD4 NC	*PNRC2*	rs6424145	1	24375689	C	G	0.4721	-0.7935	0.0412	1.55E-82	369.63
CD4 NC	*POLE2*	rs41345247	14	50108662	C	T	0.3793	-0.2996	0.0456	5.25E-11	43
CD4 NC	*POLI*	rs1380257	18	51799343	A	G	0.4213	-0.3045	0.0458	2.89E-11	44.16
CD4 NC	*POLM*	rs28382621	7	44122613	G	C	0.0936	0.8414	0.0766	4.36E-28	120.49
CD4 NC	*POLR1D*	rs2232669	13	28195495	T	C	0.1167	1.2797	0.0602	3.80E-100	450.36
CD4 NC	*POLR1E*	rs12237351	9	37512808	T	C	0.3164	-0.3487	0.0475	2.05E-13	53.85
CD4 NC	*POLR2I*	rs757223	19	36574798	T	C	0.5733	-0.2932	0.0449	6.79E-11	42.49
CD4 NC	*POLR2J*	rs6961234	7	102100616	G	A	0.6289	0.3289	0.0467	1.90E-12	49.48
CD4 NC	*POLR2J3*	rs62483497	7	102332238	G	A	0.6733	-0.3672	0.0474	9.39E-15	59.9
CD4 NC	*POMC*	rs3754861	2	25393722	C	A	0.0783	0.9186	0.0872	6.10E-26	110.71
CD4 NC	*POMZP3*	rs146021635	7	76179719	A	C	0.2083	0.7795	0.0523	3.44E-50	221.47
CD4 NC	*POP5*	rs787947	12	121006729	G	A	0.6415	-0.2705	0.0472	1.02E-08	32.73
CD4 NC	*POR*	rs59170362	7	75629267	C	G	0.1769	0.3347	0.0601	2.63E-08	30.9
CD4 NC	*PPA1*	rs71480619	10	71674360	T	C	0.1966	0.3011	0.0546	3.56E-08	30.32
CD4 NC	*PPA1*	rs2394668	10	71943951	A	G	0.3192	1.1860	0.0384	1.00E-200	954.14
CD4 NC	*PPAT*	rs57503982	4	57182975	G	A	0.3456	-0.4058	0.0471	6.75E-18	74.14
CD4 NC	*PPDPF*	rs72629024	20	62152519	G	C	0.1633	-1.1423	0.0570	1.80E-89	401.41
CD4 NC	*PPFIBP2*	rs7126061	11	7548423	A	G	0.6036	0.8539	0.0421	1.57E-91	410.85
CD4 NC	*PPIA*	rs10230344	7	44825318	A	G	0.1484	0.8796	0.0707	1.61E-35	154.41
CD4 NC	*PPIE*	rs12094291	1	40226046	A	G	0.3493	-0.3920	0.0462	2.13E-17	71.87
CD4 NC	*PPIL3*	rs7559150	2	201754063	C	T	0.2458	-1.1399	0.0463	5.68E-134	605.52
CD4 NC	*PPM1M*	rs11717383	3	52287468	T	G	0.5085	-0.5004	0.0438	2.81E-30	130.48
CD4 NC	*PPP2R1A*	rs17554825	19	52693298	G	A	0.1404	-0.3839	0.0670	1.01E-08	32.76
CD4 NC	*PPP3R1*	rs6756198	2	68394184	T	C	0.6723	0.2569	0.0466	3.53E-08	30.33
CD4 NC	*PPT1*	rs7524958	1	40565508	G	A	0.8475	0.4839	0.0654	1.37E-13	54.64
CD4 NC	*PPT2-EGFL8*	rs3134755	6	31216300	T	C	0.3858	0.4447	0.0444	1.38E-23	100
CD4 NC	*PPT2-EGFL8*	rs9272278	6	32603656	G	C	0.0513	0.5433	0.0584	1.29E-20	86.48
CD4 NC	*PPT2-EGFL8*	rs28724893	6	32744440	C	T	0.5418	-0.3356	0.0446	5.29E-14	56.5
CD4 NC	*PPWD1*	rs245564	5	64802641	G	T	0.3596	0.5084	0.0463	4.60E-28	120.39
CD4 NC	*PQLC3*	rs11694729	2	11344746	C	A	0.8822	0.6227	0.0666	8.63E-21	87.28
CD4 NC	*PRB3*	rs10772397	12	11138683	T	C	0.6075	0.4109	0.0450	7.25E-20	83.08
CD4 NC	*PRDX2*	rs897804	19	12876964	C	G	0.3850	0.3663	0.0454	7.50E-16	64.87
CD4 NC	*PRDX3*	rs7768	10	120927793	C	G	0.3378	0.3414	0.0474	5.79E-13	51.81
CD4 NC	*PRDX5*	rs79423518	11	64105454	A	G	0.0682	-0.7171	0.0976	2.06E-13	53.84
CD4 NC	*PRDX6*	rs9425411	1	173471890	A	G	0.3050	-1.0245	0.0426	1.58E-127	575.95
CD4 NC	*PRELID1*	rs6879874	5	176730775	T	A	0.7115	0.5928	0.0480	5.41E-35	152
CD4 NC	*PRKAG2-AS1*	rs117728810	7	151574265	A	G	0.0559	-0.7092	0.0975	3.50E-13	52.8
CD4 NC	*PRKAR1B*	rs4072186	7	756483	C	G	0.1785	0.4891	0.0553	9.65E-19	77.97
CD4 NC	*PRKCB*	rs145719494	16	24044921	G	A	0.0539	1.6331	0.0944	5.40E-67	298.41
CD4 NC	*PRKCQ-AS1*	rs41284471	10	6626214	A	G	0.1785	-1.0427	0.0500	1.58E-96	433.76
CD4 NC	*PRKD3*	rs12712528	2	37461139	G	A	0.5255	-0.3309	0.0446	1.14E-13	54.99
CD4 NC	*PRMT2*	rs56411654	21	47872880	A	G	0.0737	0.5938	0.0851	3.00E-12	48.59
CD4 NC	*PRMT2*	rs11910707	21	48042513	T	G	0.3027	0.7945	0.0459	3.90E-67	299.05
CD4 NC	*PROSC*	rs7463174	8	37635649	G	A	0.1539	0.4809	0.0599	9.95E-16	64.31
CD4 NC	*PROSER2*	rs74358513	10	11905544	C	A	0.1186	0.5505	0.0681	6.52E-16	65.14
CD4 NC	*PRPF40A*	rs7596408	2	153473435	A	G	0.3520	0.3130	0.0471	2.99E-11	44.09
CD4 NC	*PRPF4B*	rs1969836	6	4020658	A	G	0.6092	-0.2852	0.0462	6.45E-10	38.1
CD4 NC	*PRPF6*	rs817356	20	62614929	C	T	0.1906	0.8847	0.0580	1.39E-52	232.43
CD4 NC	*PRR24*	rs4804036	19	47767643	A	G	0.3258	0.4104	0.0476	6.14E-18	74.32
CD4 NC	*PRR4*	rs61928603	12	11311787	T	C	0.8175	-0.5301	0.0587	1.64E-19	81.46
CD4 NC	*PRR7-AS1*	rs466256	5	176875956	A	C	0.5216	1.0551	0.0378	9.90E-172	778.8
CD4 NC	*PSAT1*	rs7019885	9	80967717	G	A	0.2583	0.3297	0.0523	2.87E-10	39.68
CD4 NC	*PSD4*	rs2241976	2	113943470	G	A	0.4506	0.4991	0.0443	1.91E-29	126.68
CD4 NC	*PSMB11*	rs941718	14	23495048	C	T	0.3076	-0.7462	0.0464	3.45E-58	258.08
CD4 NC	*PSMB3*	rs2338115	17	36929578	T	C	0.5309	-0.3108	0.0450	4.82E-12	47.66
CD4 NC	*PSMB9*	rs2071480	6	32821859	A	C	0.4101	0.3214	0.0457	2.01E-12	49.37
CD4 NC	*PSMD2*	rs6845	3	184026703	T	C	0.4571	-0.3949	0.0443	5.25E-19	79.17
CD4 NC	*PSMD5-AS1*	rs34025993	9	123516572	G	A	0.5819	-1.2833	0.0346	1.00E-200	1371.07
CD4 NC	*PSMD5-AS1*	rs306794	9	124152939	T	C	0.0622	0.4810	0.0840	1.02E-08	32.73
CD4 NC	*PSME4*	rs805345	2	54194071	A	G	0.5065	-0.3046	0.0447	9.00E-12	46.44
CD4 NC	*PSMF1*	rs6074148	20	1098486	T	C	0.7003	0.2686	0.0490	4.16E-08	30.01
CD4 NC	*PSMG1*	rs79688076	21	40623567	G	C	0.1299	-0.3746	0.0680	3.66E-08	30.26
CD4 NC	*PSMG4*	rs11242829	6	3254984	C	T	0.8055	0.3305	0.0594	2.57E-08	30.94
CD4 NC	*PSPH*	rs6593279	7	55803970	A	G	0.2274	0.7294	0.0574	5.40E-37	161.14
CD4 NC	*PSTPIP1*	rs3784788	15	77759896	T	C	0.2697	0.4718	0.0486	2.60E-22	94.19
CD4 NC	*PSTPIP2*	rs79643869	18	43568320	A	G	0.1427	-0.5377	0.0664	5.37E-16	65.52
CD4 NC	*PTCH1*	rs28377268	9	98225056	T	G	0.1202	0.4475	0.0719	4.75E-10	38.7
CD4 NC	*PTEN*	rs10887759	10	89603424	A	G	0.1325	0.3568	0.0625	1.16E-08	32.49
CD4 NC	*PTGER2*	rs17125362	14	52796829	G	A	0.2180	-0.3264	0.0547	2.46E-09	35.49

CD4 NC	*PTGIR*	rs11083840	19	47119910	G	T	0.4095	-0.7591	0.0428	2.75E-70	313.49
CD4 NC	*PTPLA*	rs10218862	10	17707668	T	C	0.5540	-0.2702	0.0449	1.83E-09	36.07
CD4 NC	*PTPLAD2*	rs1113401	9	21012170	A	G	0.5340	-0.4672	0.0439	2.14E-26	112.79
CD4 NC	*PTPN4*	rs10196708	2	120682788	A	G	0.2994	-0.7394	0.0466	9.09E-57	251.58
CD4 NC	*PTPRN2*	rs75990842	7	157271704	C	G	0.0500	0.5465	0.0999	4.56E-08	29.84
CD4 NC	*PTTG1IP*	rs84191	21	46269496	G	A	0.5191	0.3021	0.0447	1.39E-11	45.59
CD4 NC	*PWWP2A*	rs1106055	5	159491520	G	A	0.5964	-0.5085	0.0448	7.47E-30	128.55
CD4 NC	*PYGB*	rs1044573	20	25206654	G	A	0.4940	0.3222	0.0446	5.00E-13	52.1
CD4 NC	*PYHIN1*	rs2325704	1	158909164	A	C	0.7626	0.5986	0.0506	2.63E-32	139.74
CD4 NC	*PYROXD1*	rs10770810	12	21594835	T	G	0.4370	0.4188	0.0448	8.60E-21	87.28
CD4 NC	*QDPR*	rs10020740	4	17515688	C	T	0.2526	-0.3713	0.0515	5.57E-13	51.89
CD4 NC	*R3HDM4*	rs1683565	19	880602	G	A	0.2970	0.4235	0.0493	9.43E-18	73.48
CD4 NC	*RAB27B*	rs28572052	18	52391388	C	T	0.1132	0.5930	0.0718	1.50E-16	68.03
CD4 NC	*RAB2A*	rs2981277	8	61488824	C	T	0.3931	0.2714	0.0465	5.22E-09	34.04
CD4 NC	*RAB34*	rs3809797	17	27065211	C	T	0.0927	-0.4360	0.0787	3.01E-08	30.64
CD4 NC	*RAB4A*	rs237747	1	229410325	A	G	0.6401	-0.2672	0.0477	2.11E-08	31.33
CD4 NC	*RABAC1*	rs9304600	19	42467973	A	G	0.6643	0.6511	0.0449	1.32E-47	209.66
CD4 NC	*RABL2B*	rs187225588	22	51197602	A	T	0.0510	0.5484	0.0918	2.33E-09	35.6
CD4 NC	*RAC1*	rs836549	7	6439945	G	A	0.4921	0.7027	0.0422	2.39E-62	277.13
CD4 NC	*RAC2*	rs9610680	22	37621951	A	G	0.1885	0.3825	0.0589	8.37E-11	42.08
CD4 NC	*RAD51C*	rs66480139	17	57101538	A	G	0.2097	0.4416	0.0533	1.24E-16	68.4
CD4 NC	*RAD54B*	rs12676373	8	95362118	G	C	0.2925	0.3311	0.0492	1.64E-11	45.27
CD4 NC	*RAMP1*	rs11686156	2	238814823	C	A	0.5434	0.3024	0.0450	1.87E-11	45.01
CD4 NC	*RAMP3*	rs6957463	7	45204780	C	T	0.2113	0.4057	0.0547	1.15E-13	54.98
CD4 NC	*RAPGEF6*	rs11956351	5	131137224	A	G	0.7993	-0.4328	0.0569	2.93E-14	57.66
CD4 NC	*RASA3*	rs7490427	13	114917931	G	C	0.4980	0.2600	0.0449	6.87E-09	33.5
CD4 NC	*RASA4*	rs60209301	7	102111176	T	C	0.0669	0.6004	0.0997	1.73E-09	36.18
CD4 NC	*RASGRF2*	rs113185115	5	80290046	A	G	0.2554	0.2798	0.0491	1.22E-08	32.38
CD4 NC	*RBAK-RBAKDN*	rs9689983	7	5024540	A	G	0.0550	-0.9975	0.0864	7.45E-31	133.11
CD4 NC	*RBL1*	rs77938357	20	35872251	C	G	0.1840	0.3372	0.0581	6.49E-09	33.61
CD4 NC	*RBL2*	rs6499617	16	53467722	G	A	0.4814	-0.5435	0.0435	9.39E-36	155.48
CD4 NC	*RBM11*	rs9978229	21	15613760	T	C	0.4263	0.3561	0.0446	1.34E-15	63.72
CD4 NC	*RBM26-AS1*	rs9574421	13	79980340	T	C	0.0867	0.6105	0.0758	8.04E-16	64.73
CD4 NC	*RBM43*	rs67303849	2	152096613	T	C	0.4333	-0.7531	0.0418	1.74E-72	323.57
CD4 NC	*RBM6*	rs11130227	3	49993319	G	A	0.4840	-0.4517	0.0441	1.13E-24	104.93
CD4 NC	*RBMS1*	rs28468464	2	161305497	G	A	0.1965	-0.6799	0.0554	1.34E-34	150.2
CD4 NC	*RCHY1*	rs112882054	4	76300596	A	T	0.2082	-0.3463	0.0549	2.83E-10	39.71
CD4 NC	*RCSD1*	rs4657680	1	167575104	T	C	0.6404	0.5009	0.0467	7.66E-27	114.82
CD4 NC	*RCSD1*	rs7541226	1	167671442	A	G	0.2899	0.5415	0.0470	1.01E-30	132.51
CD4 NC	*RDH13*	rs1671216	19	55554076	A	G	0.6152	0.4060	0.0456	5.42E-19	79.11
CD4 NC	*RDH5*	rs3138142	12	56115585	T	C	0.2120	-0.3362	0.0532	2.52E-10	39.94
CD4 NC	*REEP3*	rs67044923	10	65377538	T	C	0.2523	0.2721	0.0493	3.48E-08	30.36
CD4 NC	*REEP4*	rs56401147	8	21996617	A	T	0.1972	0.3449	0.0571	1.57E-09	36.37
CD4 NC	*REV3L*	rs240972	6	111638335	T	C	0.6610	0.4470	0.0476	6.38E-21	87.87
CD4 NC	*RFC4*	rs62294446	3	186498536	C	G	0.2570	-0.3177	0.0521	1.05E-09	37.16
CD4 NC	*RFTN1*	rs9845427	3	16350642	A	C	0.1984	0.4263	0.0590	4.92E-13	52.13
CD4 NC	*RGCC*	rs421706	13	42087771	C	T	0.5584	0.2768	0.0450	7.69E-10	37.76
CD4 NC	*RGMB*	rs10056188	5	97817143	T	A	0.5190	-0.3345	0.0446	6.38E-14	56.14
CD4 NC	*RGS10*	rs2917929	10	121300515	A	T	0.0841	-0.4388	0.0778	1.66E-08	31.79
CD4 NC	*RGS12*	rs2749782	4	3339761	C	T	0.5959	-0.4454	0.0447	2.23E-23	99.04
CD4 NC	*RGS14*	rs10051765	5	176799992	C	T	0.3470	-0.3367	0.0482	2.96E-12	48.62
CD4 NC	*RGS19*	rs8121509	20	62712053	C	T	0.4618	0.2590	0.0450	8.85E-09	33.01
CD4 NC	*RHBDL1*	rs12933682	16	721456	C	T	0.3814	-0.3218	0.0465	4.44E-12	47.82
CD4 NC	*RHOB*	rs2602160	2	20645506	T	C	0.7266	0.2837	0.0507	2.23E-08	31.22
CD4 NC	*RHOC*	rs2999158	1	113239478	C	T	0.6802	0.4230	0.0469	1.85E-19	81.23
CD4 NC	*RIBC2*	rs5765371	22	45838887	C	T	0.5201	0.5354	0.0435	8.77E-35	151.05
CD4 NC	*RIC3*	rs4758295	11	8152080	C	T	0.1306	-0.5734	0.0682	4.03E-17	70.62
CD4 NC	*RICTOR*	rs1239259	5	38985269	A	C	0.3662	-0.2646	0.0463	1.11E-08	32.57
CD4 NC	*RINL*	rs8110393	19	39360720	A	G	0.5021	0.2703	0.0448	1.63E-09	36.3
CD4 NC	*RLN1*	rs1853218	9	5322310	C	T	0.2475	0.3855	0.0536	6.64E-13	51.54
CD4 NC	*RMI2*	rs10163410	16	11430615	C	G	0.3009	0.3836	0.0472	4.65E-16	65.8
CD4 NC	*RNASEH1-AS1*	rs12629	2	3609064	A	G	0.3129	-0.4229	0.0486	3.19E-18	75.61
CD4 NC	*RNASEH2C*	rs11227250	11	65438345	T	C	0.3441	-0.5454	0.0455	4.80E-33	143.11
CD4 NC	*RNASEK-C17orf49*	rs311747	17	6836880	T	A	0.5187	-0.3927	0.0443	7.82E-19	78.39
CD4 NC	*RNASET2*	rs1044059	6	167369897	A	C	0.4957	1.1053	0.0365	1.00E-200	915.46
CD4 NC	*RNF13*	rs3911491	3	149708906	T	C	0.7029	0.3169	0.0487	7.55E-11	42.29
CD4 NC	*RNF145*	rs17718834	5	158529996	T	C	0.0933	0.5142	0.0738	3.15E-12	48.5
CD4 NC	*RNF157*	rs12942176	17	74206117	G	A	0.4909	0.2814	0.0448	3.36E-10	39.37
CD4 NC	*RNF166*	rs57350670	16	88761231	A	C	0.2723	-0.4828	0.0497	2.54E-22	94.24
CD4 NC	*RNF168*	rs9681103	3	196211219	C	T	0.1991	-0.5789	0.0515	2.45E-29	126.19
CD4 NC	*RNF168*	rs9837962	3	196287618	A	C	0.7388	-0.2859	0.0503	1.29E-08	32.28
CD4 NC	*RNF175*	rs12644640	4	154677610	C	T	0.4348	-0.4593	0.0443	3.38E-25	107.32
CD4 NC	*RNF207*	rs10864433	1	6261871	A	G	0.2754	-0.2861	0.0504	1.34E-08	32.2
CD4 NC	*RNF212*	rs13132174	4	1133382	C	G	0.6813	0.3421	0.0488	2.45E-12	48.98
CD4 NC	*RNF24*	rs241623	20	3921964	C	T	0.4374	-0.4387	0.0443	4.16E-23	97.81
CD4 NC	*RNF8*	rs2284923	6	37348920	T	C	0.4598	-0.2548	0.0450	1.44E-08	32.07
CD4 NC	*ROBO3*	rs11219822	11	124746644	C	A	0.4301	0.5603	0.0437	1.34E-37	163.9
CD4 NC	*ROBO4*	rs12281805	11	124771763	G	A	0.3648	-0.5593	0.0445	2.82E-36	157.86
CD4 NC	*RP1-102H19.8*	rs410683	6	88008920	C	T	0.4312	0.3140	0.0451	3.24E-12	48.44

CD4 NC	*RP1-130H16.16*	rs5753111	22	30779211	C	T	0.7518	0.3951	0.0500	2.86E-15	62.23
CD4 NC	*RP1-140A9.1*	rs2272908	1	1721479	T	C	0.4815	0.3697	0.0444	8.34E-17	69.19
CD4 NC	*RP1-153P14.5*	rs12212090	6	37431808	G	A	0.6415	-0.3581	0.0466	1.63E-14	58.82
CD4 NC	*RP1-178F15.4*	rs4351684	1	153636860	G	A	0.5481	-0.2518	0.0449	2.05E-08	31.38
CD4 NC	*RP1-187B23.1*	rs2982304	1	25965178	A	G	0.2773	0.3155	0.0545	7.25E-09	33.4
CD4 NC	*RP1-197B17.3*	rs73104155	12	48094573	C	T	0.0791	0.5050	0.0855	3.56E-09	34.78
CD4 NC	*RP1-28O10.1*	rs12034758	1	209920545	A	G	0.2107	0.3932	0.0540	3.26E-13	52.94
CD4 NC	*RP1-29C18.10*	rs5770622	22	50051548	G	T	0.6547	-1.0657	0.0413	1.65E-146	663.05
CD4 NC	*RP1-39G22.7*	rs9326050	1	40721908	C	T	0.4895	-0.2878	0.0447	1.22E-10	41.35
CD4 NC	*RP1-3J17.3*	rs12212560	6	86342679	A	G	0.3868	0.7806	0.0429	6.88E-74	329.99
CD4 NC	*RP1-3J17.3*	rs220410	6	86869791	T	C	0.7561	-0.3446	0.0531	8.77E-11	41.99
CD4 NC	*RP1-40E16.12*	rs12196777	6	3064523	T	C	0.5465	-0.5934	0.0437	4.81E-42	184.22
CD4 NC	*RP1-55C23.7*	rs3756974	6	133052283	G	A	0.3243	0.6765	0.0455	4.89E-50	220.77
CD4 NC	*RP1-90J20.8*	rs4959756	6	3022511	C	T	0.6275	-0.5760	0.0455	8.95E-37	160.14
CD4 NC	*RP11-1026M7.2*	rs76027440	5	177114332	T	C	0.0964	0.5200	0.0781	2.74E-11	44.27
CD4 NC	*RP11-103G8.2*	rs13065451	3	150422721	A	C	0.4748	-0.3467	0.0446	7.62E-15	60.31
CD4 NC	*RP11-104L21.3*	rs2995091	1	167434277	C	T	0.3575	0.2893	0.0461	3.39E-10	39.36
CD4 NC	*RP11-104N10.1*	rs62070320	16	89573689	G	A	0.4606	0.2667	0.0452	3.61E-09	34.75
CD4 NC	*RP11-1060J15.4*	rs76851022	12	27851984	T	C	0.0563	1.5676	0.0836	1.62E-78	351.2
CD4 NC	*RP11-108M9.4*	rs279028	1	16790901	T	C	0.6340	-0.3750	0.0468	1.05E-15	64.21
CD4 NC	*RP11-108M9.4*	rs7517838	1	17276627	T	C	0.4041	0.2612	0.0451	7.01E-09	33.46
CD4 NC	*RP11-108M9.6*	rs696095	1	16802890	A	G	0.5362	-0.3058	0.0453	1.45E-11	45.5
CD4 NC	*RP11-110I1.11*	rs75067211	11	118854372	T	C	0.2386	0.3050	0.0508	1.95E-09	35.95
CD4 NC	*RP11-111M22.3*	rs111819496	11	76150395	T	C	0.0416	-0.7124	0.0975	2.81E-13	53.23
CD4 NC	*RP11-1191J2.5*	rs113529517	4	668353	A	C	0.1271	0.5323	0.0680	5.16E-15	61.07
CD4 NC	*RP11-119D9.1*	rs11605408	11	67686720	C	T	0.1759	-0.4269	0.0560	2.49E-14	57.98
CD4 NC	*RP11-119F19.2*	rs4933803	10	81579625	C	T	0.5923	0.4270	0.0455	6.76E-21	87.76
CD4 NC	*RP11-12J10.3*	rs7076785	10	125943420	G	A	0.0518	0.9549	0.1013	4.04E-21	88.77
CD4 NC	*RP11-131N11.4*	rs9415614	10	62514136	G	A	0.4115	-0.3221	0.0451	8.99E-13	50.95
CD4 NC	*RP11-134O21.1*	rs6997239	8	2606290	G	C	0.4787	-0.3275	0.0446	2.04E-13	53.86
CD4 NC	*RP11-137H2.4*	rs11185932	10	82295398	A	G	0.3372	-0.5264	0.0463	5.42E-30	129.18
CD4 NC	*RP11-138A9.1*	rs2293750	7	130629772	T	A	0.5186	0.4115	0.0443	1.56E-20	86.11
CD4 NC	*RP11-138A9.2*	rs205724	7	130623257	C	A	0.8419	0.4430	0.0601	1.63E-13	54.3
CD4 NC	*RP11-1398P2.1*	rs12501769	4	1608224	A	G	0.5268	0.6191	0.0432	1.38E-46	204.99
CD4 NC	*RP11-1399P15.1*	rs55956548	2	87768679	T	G	0.7418	0.6639	0.0507	3.26E-39	171.28
CD4 NC	*RP11-139H15.1*	rs7162777	15	55590228	A	G	0.2149	0.3801	0.0549	4.48E-12	47.8
CD4 NC	*RP11-1407O15.2*	rs12949161	17	36245490	A	G	0.0700	0.5403	0.0940	9.09E-09	32.96
CD4 NC	*RP11-140A10.3*	rs2814166	10	133993559	G	A	0.8498	-0.6830	0.0588	3.79E-31	134.45
CD4 NC	*RP11-140I16.3*	rs2622567	8	56416733	T	G	0.2216	-0.5183	0.0515	8.24E-24	101.01
CD4 NC	*RP11-143J12.2*	rs56317320	18	9173892	T	G	0.6142	0.2964	0.0457	8.61E-11	42.03
CD4 NC	*RP11-147L13.2*	rs11077450	17	66249979	T	C	0.3181	0.2751	0.0476	7.70E-09	33.28
CD4 NC	*RP11-157J24.2*	rs7758049	6	1547591	T	C	0.5075	-0.3522	0.0445	2.41E-15	62.57
CD4 NC	*RP11-160H22.5*	rs55698100	1	174227433	C	T	0.2667	0.2948	0.0490	1.73E-09	36.18
CD4 NC	*RP11-161M6.3*	rs11640890	16	1046630	A	G	0.4796	-0.3860	0.0443	3.17E-18	75.62
CD4 NC	*RP11-166B2.1*	rs393329	16	12070422	C	T	0.5888	-0.5326	0.0450	2.27E-32	140.03
CD4 NC	*RP11-166B2.5*	rs33643	16	11986149	G	A	0.3618	-0.6395	0.0452	1.78E-45	199.92
CD4 NC	*RP11-166B2.5*	rs71385125	16	12027781	C	A	0.0806	0.5037	0.0890	1.50E-08	31.99
CD4 NC	*RP11-166P13.3*	rs55638944	17	55174147	G	T	0.3728	-0.2805	0.0466	1.71E-09	36.21
CD4 NC	*RP11-169K16.9*	rs848218	1	16275506	C	T	0.2006	0.3185	0.0581	4.22E-08	29.98
CD4 NC	*RP11-173B14.5*	rs9600517	13	76237724	G	A	0.5370	-0.4186	0.0442	2.96E-21	89.39
CD4 NC	*RP11-176H8.1*	rs34024670	14	31896969	A	G	0.3862	0.2523	0.0462	4.84E-08	29.72
CD4 NC	*RP11-179B2.2*	rs3802725	10	102673063	T	G	0.4297	0.2754	0.0451	1.06E-09	37.13
CD4 NC	*RP11-17M16.2*	rs12961878	18	74567762	T	A	0.2917	0.5295	0.0467	9.16E-30	128.14
CD4 NC	*RP11-187E13.1*	rs1278926	14	32414145	A	C	0.6181	-0.2763	0.0456	1.37E-09	36.63
CD4 NC	*RP11-18H21.1*	rs13140099	4	153022375	A	G	0.2594	-1.1052	0.0435	2.06E-142	644.25
CD4 NC	*RP11-18H21.1*	rs34961631	4	152990636	G	A	0.4441	0.3314	0.0450	1.79E-13	54.11
CD4 NC	*RP11-190A12.8*	rs4656858	1	159867866	G	A	0.4020	-0.5764	0.0439	2.50E-39	171.8
CD4 NC	*RP11-218M22.1*	rs55800929	12	752360	A	G	0.1477	0.5273	0.0597	1.06E-18	77.78
CD4 NC	*RP11-223C24.1*	rs1373036	4	143625262	A	G	0.7682	0.4476	0.0516	4.10E-18	75.12
CD4 NC	*RP11-23P13.6*	rs1704389	15	42201339	G	A	0.4250	-0.3141	0.0454	4.71E-12	47.71
CD4 NC	*RP11-245G13.2*	rs7604967	2	11020995	T	C	0.1179	1.5393	0.0626	2.36E-133	602.69
CD4 NC	*RP11-254F7.2*	rs11886661	2	10239944	G	C	0.1270	0.4125	0.0632	6.69E-11	42.52
CD4 NC	*RP11-273G15.2*	rs4270938	8	144093864	T	C	0.5628	-0.4042	0.0449	2.24E-19	80.85
CD4 NC	*RP11-274B21.10*	rs12706858	7	128347584	G	C	0.1479	0.4951	0.0656	4.41E-14	56.86
CD4 NC	*RP11-278H7.4*	rs57252948	1	244824681	G	A	0.0632	0.7247	0.0948	2.10E-14	58.31
CD4 NC	*RP11-292F22.7*	rs10906938	10	47641131	C	T	0.6581	-0.2841	0.0490	6.54E-09	33.6
CD4 NC	*RP11-293A21.1*	rs13138942	4	26866763	T	C	0.5200	-0.3005	0.0449	2.25E-11	44.65
CD4 NC	*RP11-293M10.2*	rs1045430	14	75130235	G	T	0.4979	-0.4194	0.0442	2.44E-21	89.77
CD4 NC	*RP11-294J22.6*	rs17563146	17	48769329	T	C	0.1201	-0.5120	0.0644	1.88E-15	63.05
CD4 NC	*RP11-296O14.3*	rs9425411	1	173471890	A	G	0.3050	-0.2892	0.0486	2.69E-09	35.32
CD4 NC	*RP11-297N6.4*	rs2645435	8	11657716	G	A	0.8150	-0.4035	0.0581	3.70E-12	48.18
CD4 NC	*RP11-298J20.3*	rs10901835	10	126589712	T	G	0.7337	-1.2802	0.0418	1.00E-200	936.2
CD4 NC	*RP11-299J3.8*	rs12636106	3	122164156	T	C	0.1515	-0.6688	0.0574	2.05E-31	135.67
CD4 NC	*RP11-303E16.5*	rs56286107	16	81117832	A	C	0.0733	-0.8364	0.0915	6.35E-20	83.34
CD4 NC	*RP11-305K5.1*	rs907060	3	156255087	G	T	0.8181	0.3468	0.0582	2.58E-09	35.4
CD4 NC	*RP11-306I1.2*	rs59033716	1	165561136	G	C	0.0775	0.6832	0.0777	1.39E-18	77.25
CD4 NC	*RP11-314C16.1*	rs6923272	6	8498639	G	T	0.6167	0.5483	0.0440	1.31E-35	154.82
CD4 NC	*RP11-315D16.4*	rs116529867	15	68570158	G	T	0.3316	-0.6773	0.0449	1.69E-51	227.46
CD4 NC	*RP11-324I22.4*	rs10764048	10	35857000	G	A	0.3435	0.6170	0.0461	6.40E-41	179.08

CD4 NC	*RP11-325F22.2*	rs2003506	7	104761741	G	A	0.3588	0.2709	0.0460	3.78E-09	34.66
CD4 NC	*RP11-326C3.11*	rs11246065	11	324170	G	A	0.3701	1.0975	0.0393	1.26E-171	778.31
CD4 NC	*RP11-326C3.11*	rs9666761	11	330061	G	A	0.6599	-0.3122	0.0487	1.49E-10	40.96
CD4 NC	*RP11-326C3.12*	rs11246068	11	327334	T	C	0.2550	1.2110	0.0433	2.26E-172	781.75
CD4 NC	*RP11-326C3.15*	rs61876250	11	325418	C	G	0.2514	0.6156	0.0505	3.35E-34	148.39
CD4 NC	*RP11-334C17.5*	rs2304856	17	78175483	C	T	0.5261	-0.4643	0.0442	9.18E-26	109.91
CD4 NC	*RP11-345J4.5*	rs144208959	16	30323380	A	G	0.4341	-0.6766	0.0424	2.99E-57	253.79
CD4 NC	*RP11-347P5.1*	rs10804069	2	197014692	A	G	0.6118	0.8667	0.0417	4.61E-96	431.63
CD4 NC	*RP11-367G6.3*	rs9461090	6	25016224	C	T	0.1425	-0.4075	0.0699	5.53E-09	33.93
CD4 NC	*RP11-377D9.3*	rs10845696	12	13180332	G	A	0.1781	-0.8228	0.0572	5.80E-47	206.71
CD4 NC	*RP11-379F4.9*	rs2244996	3	158519446	C	T	0.6911	-0.3744	0.0480	5.84E-15	60.83
CD4 NC	*RP11-382J12.1*	rs13252584	8	71689031	G	A	0.4957	-0.6260	0.0429	2.46E-48	212.99
CD4 NC	*RP11-385D13.3*	rs2938158	17	15447364	C	T	0.3405	-0.3517	0.0474	1.16E-13	54.97
CD4 NC	*RP11-392P7.6*	rs741432	12	13087790	C	G	0.3266	0.2771	0.0484	1.06E-08	32.67
CD4 NC	*RP11-395B7.4*	rs11766024	7	100647819	T	C	0.4314	0.3108	0.0448	4.07E-12	47.99
CD4 NC	*RP11-403A21.2*	rs12954962	18	21581709	C	T	0.7143	0.5735	0.0501	2.14E-30	131.02
CD4 NC	*RP11-415F23.2*	rs9845427	3	16350642	A	C	0.1984	0.9257	0.0558	8.71E-62	274.56
CD4 NC	*RP11-415F23.4*	rs6442593	3	16296927	A	G	0.1582	0.4681	0.0650	6.02E-13	51.73
CD4 NC	*RP11-420G6.4*	rs9392441	6	2885930	C	T	0.3599	-0.3378	0.0469	5.65E-13	51.86
CD4 NC	*RP11-423H2.3*	rs56083597	5	177322056	A	C	0.5611	-0.2523	0.0452	2.44E-08	31.04
CD4 NC	*RP11-425D10.10*	rs1476387	6	109764535	T	G	0.4288	0.3224	0.0452	1.03E-12	50.68
CD4 NC	*RP11-429E11.3*	rs2150130	20	60294793	G	A	0.6185	-0.2958	0.0457	9.45E-11	41.85
CD4 NC	*RP11-430H10.1*	rs12418562	11	45373480	C	G	0.1739	0.9508	0.0546	6.05E-68	302.76
CD4 NC	*RP11-434C1.1*	rs10772397	12	11138683	T	C	0.6075	-0.4430	0.0449	5.43E-23	97.28
CD4 NC	*RP11-445H22.3*	rs11696392	20	43300331	G	C	0.5943	0.4220	0.0448	4.52E-21	88.55
CD4 NC	*RP11-446N19.1*	rs4768697	12	46748965	G	C	0.2578	0.6723	0.0487	2.68E-43	189.95
CD4 NC	*RP11-448A19.1*	rs36083190	7	129222963	T	C	0.3887	0.3136	0.0457	7.10E-12	46.9
CD4 NC	*RP11-44F14.2*	rs1362428	16	53423883	T	A	0.7641	-0.3409	0.0520	5.36E-11	42.95
CD4 NC	*RP11-44F14.8*	rs2908795	16	53417443	G	A	0.8021	-0.3068	0.0557	3.59E-08	30.3
CD4 NC	*RP11-44F21.5*	rs61508612	4	76034601	A	G	0.1446	0.6896	0.0669	6.35E-25	106.08
CD4 NC	*RP11-457M11.5*	rs569545490	6	26701786	T	C	0.1431	-0.4503	0.0673	2.19E-11	44.7
CD4 NC	*RP11-459F6.3*	rs16960143	16	58404877	A	G	0.0858	-0.8827	0.0956	2.70E-20	85.02
CD4 NC	*RP11-462G2.1*	rs56131495	6	71275276	G	A	0.1345	-0.3963	0.0635	4.42E-10	38.84
CD4 NC	*RP11-473I1.5*	rs12918271	16	9200547	C	T	0.2190	-0.9275	0.0509	3.72E-74	331.22
CD4 NC	*RP11-473M20.14*	rs1053871	16	3181963	C	T	0.3808	0.2523	0.0456	3.13E-08	30.57
CD4 NC	*RP11-473M20.7*	rs1043298	16	3110522	A	T	0.4815	-0.2462	0.0451	4.68E-08	29.78
CD4 NC	*RP11-473M20.9*	rs4786384	16	3136919	C	T	0.5202	0.6099	0.0430	1.26E-45	200.6
CD4 NC	*RP11-474N24.6*	rs29204	18	9927719	C	G	0.1228	0.7885	0.0658	4.64E-33	143.18
CD4 NC	*RP11-47L3.1*	rs28881186	17	33631801	G	C	0.0528	0.7880	0.1005	4.60E-15	61.3
CD4 NC	*RP11-483I13.5*	rs547364	1	108742123	G	C	0.4778	0.3284	0.0446	1.76E-13	54.15
CD4 NC	*RP11-492I21.1*	rs4650921	1	175513270	C	G	0.5063	-0.2599	0.0448	6.62E-09	33.57
CD4 NC	*RP11-493L12.3*	rs855176	12	47651781	C	T	0.2906	0.3793	0.0471	8.05E-16	64.73
CD4 NC	*RP11-493L12.4*	rs1101796	12	47647013	T	G	0.2991	0.5013	0.0462	1.80E-27	117.68
CD4 NC	*RP11-497H16.9*	rs28478097	5	70309998	G	C	0.6121	-0.2965	0.0459	1.09E-10	41.57
CD4 NC	*RP11-498P14.5*	rs3747506	9	100071989	G	T	0.1843	-0.4666	0.0551	2.46E-17	71.59
CD4 NC	*RP11-499F3.2*	rs35650972	15	82295601	A	C	0.1562	0.4397	0.0627	2.33E-12	49.08
CD4 NC	*RP11-499P20.2*	rs6482597	10	18964840	G	A	0.3776	0.4606	0.0463	2.34E-23	98.95
CD4 NC	*RP11-502I4.3*	rs4261476	15	67920465	G	A	0.7576	-0.2999	0.0547	4.12E-08	30.03
CD4 NC	*RP11-509J21.2*	rs1334063	9	3652155	A	T	0.6795	-0.2976	0.0477	4.39E-10	38.85
CD4 NC	*RP11-513M16.7*	rs10114093	9	19359457	C	G	0.1102	0.4640	0.0782	3.00E-09	35.11
CD4 NC	*RP11-517P14.2*	rs10899795	10	43869097	A	C	0.2395	-0.3090	0.0551	2.05E-08	31.39
CD4 NC	*RP11-522I20.3*	rs12235741	9	86310303	G	T	0.1721	0.3913	0.0629	5.01E-10	38.6
CD4 NC	*RP11-526I2.5*	rs36053285	15	101114640	C	T	0.4253	-0.6702	0.0433	6.23E-54	238.6
CD4 NC	*RP11-527L4.2*	rs231458	17	41994794	T	C	0.3595	0.2925	0.0466	3.56E-10	39.26
CD4 NC	*RP11-531F16.4*	rs872425	3	183203545	G	C	0.0731	-0.5980	0.0844	1.35E-12	50.15
CD4 NC	*RP11-532F6.3*	rs2244443	6	711150	C	A	0.7103	0.4857	0.0512	2.31E-21	89.88
CD4 NC	*RP11-536K7.3*	rs34847630	10	6794051	T	C	0.0515	0.5201	0.0930	2.25E-08	31.21
CD4 NC	*RP11-538P18.2*	rs1714505	3	158237051	T	C	0.0735	-0.7261	0.0878	1.32E-16	68.29
CD4 NC	*RP11-539L10.3*	rs7662433	4	6676485	A	G	0.1633	-0.9871	0.0582	1.95E-64	286.69
CD4 NC	*RP11-542K23.7*	rs35918962	9	125168878	T	C	0.1266	1.3046	0.0618	8.27E-99	444.23
CD4 NC	*RP11-542M13.3*	rs305060	16	85975916	C	T	0.4678	0.2485	0.0450	3.46E-08	30.37
CD4 NC	*RP11-543C4.1*	rs7161519	14	100088980	T	C	0.5520	-0.2610	0.0454	9.16E-09	32.95
CD4 NC	*RP11-545E17.3*	rs55697116	9	131489620	T	C	0.2481	1.0527	0.0450	3.71E-121	546.73
CD4 NC	*RP11-545M17.1*	rs12881405	14	38992066	A	G	0.1240	-0.6404	0.0672	1.65E-21	90.54
CD4 NC	*RP11-572M11.4*	rs3889123	3	112725442	C	G	0.7022	0.3645	0.0512	1.13E-12	50.5
CD4 NC	*RP11-589P10.5*	rs10852889	17	6900845	T	C	0.5804	0.3268	0.0450	4.01E-13	52.53
CD4 NC	*RP11-58B17.2*	rs2648580	3	9495385	G	A	0.7345	-0.3529	0.0506	3.19E-12	48.47
CD4 NC	*RP11-596D21.1*	rs12883273	14	31743604	C	T	0.3933	-0.6111	0.0441	1.36E-43	191.3
CD4 NC	*RP11-617D20.1*	rs73232890	4	38685041	A	G	0.1917	0.3273	0.0536	1.02E-09	37.2
CD4 NC	*RP11-629O1.2*	rs58201941	8	134585018	C	G	0.2174	0.3468	0.0533	7.94E-11	42.19
CD4 NC	*RP11-641A6.2*	rs34155853	12	52974306	A	G	0.1217	0.7274	0.0659	2.68E-28	121.46
CD4 NC	*RP11-656D10.3*	rs11208254	1	40959506	T	G	0.3269	-0.4374	0.0478	5.27E-20	83.7
CD4 NC	*RP11-65I12.1*	rs7578633	2	113978650	T	C	0.3727	-0.3411	0.0466	2.47E-13	53.48
CD4 NC	*RP11-65L3.4*	rs67350921	2	179859205	C	T	0.1159	0.4132	0.0705	4.51E-09	34.32
CD4 NC	*RP11-660L16.2*	rs12807827	11	71203790	C	A	0.6914	0.8645	0.0513	1.19E-63	283.09
CD4 NC	*RP11-661A12.5*	rs2980269	8	144585511	C	T	0.4968	-0.3435	0.0445	1.22E-14	59.39
CD4 NC	*RP11-661P17.1*	rs7169129	15	92024587	C	T	0.4478	-0.3383	0.0449	5.09E-14	56.58
CD4 NC	*RP11-664D1.1*	rs2336757	12	4014358	G	A	0.1452	0.4957	0.0630	3.48E-15	61.85
CD4 NC	*RP11-664D1.1*	rs609018	12	4071901	G	T	0.4284	-0.3899	0.0446	2.22E-18	76.33

CD4 NC	*RP11-667K14.4*	rs8065937	17	1732977	T	G	0.7434	0.5724	0.0545	8.03E-26	110.17
CD4 NC	*RP11-669C19.1*	rs56060128	3	20321346	T	C	0.1942	-0.3534	0.0548	1.13E-10	41.5
CD4 NC	*RP11-669E14.6*	rs199441	17	44773783	G	A	0.7937	-0.6354	0.0525	9.37E-34	146.35
CD4 NC	*RP11-673E1.1*	rs35677072	4	144515896	C	A	0.1311	-0.6314	0.0626	6.37E-24	101.52
CD4 NC	*RP11-692C24.1*	rs4396513	15	92217321	G	A	0.2809	-0.6499	0.0476	1.63E-42	186.37
CD4 NC	*RP11-69E11.4*	rs3820618	1	40223128	C	T	0.1312	1.0060	0.0644	6.00E-55	243.25
CD4 NC	*RP11-69E11.4*	rs1180348	1	40001276	G	A	0.6314	-0.2666	0.0465	9.95E-09	32.78
CD4 NC	*RP11-730K11.1*	rs297495	11	121290293	A	G	0.4672	-0.2518	0.0448	1.94E-08	31.49
CD4 NC	*RP11-755F10.1*	rs11227439	11	66025679	A	G	0.1715	0.7559	0.0580	6.98E-39	169.77
CD4 NC	*RP11-773D16.1*	rs7921725	10	81588553	C	T	0.7183	-0.3278	0.0500	5.30E-11	42.97
CD4 NC	*RP11-792A8.4*	rs78996796	7	66301193	T	C	0.0468	1.1790	0.0944	8.56E-36	155.66
CD4 NC	*RP11-796G6.2*	rs11848014	14	102145928	G	A	0.0803	-0.5153	0.0868	2.93E-09	35.16
CD4 NC	*RP11-797A18.4*	rs11632999	15	77436995	G	T	0.2235	-0.4547	0.0549	1.19E-16	68.49
CD4 NC	*RP11-798G7.5*	rs62063853	17	44074613	G	A	0.2030	-0.6485	0.0519	6.97E-36	156.07
CD4 NC	*RP11-798G7.6*	rs3874943	17	44572068	G	A	0.5235	-0.3311	0.0446	1.10E-13	55.07
CD4 NC	*RP11-817O13.8*	rs58341256	15	75594175	T	C	0.4027	0.2545	0.0455	2.19E-08	31.25
CD4 NC	*RP11-81A22.5*	rs9889517	17	4704175	A	G	0.4583	0.2551	0.0448	1.26E-08	32.33
CD4 NC	*RP11-83C7.1*	rs986507	4	38334509	G	T	0.5517	-0.4387	0.0445	5.96E-23	97.1
CD4 NC	*RP11-841O20.2*	rs72684254	14	53058992	A	G	0.0963	0.7507	0.0787	1.47E-21	90.77
CD4 NC	*RP11-85G18.6*	rs7477200	10	27508125	G	C	0.7460	-0.5328	0.0515	4.69E-25	106.68
CD4 NC	*RP11-867G23.8*	rs1785639	11	66100430	A	G	0.6751	0.2720	0.0486	2.19E-08	31.25
CD4 NC	*RP11-894P9.1*	rs35140615	14	104150352	A	G	0.2069	-0.4034	0.0544	1.18E-13	54.92
CD4 NC	*RP11-89K11.1*	rs58819255	15	102249817	T	C	0.1966	-0.4257	0.0543	4.63E-15	61.29
CD4 NC	*RP11-92G12.3*	rs10919993	1	200627312	A	C	0.4076	-0.6161	0.0436	2.05E-45	199.63
CD4 NC	*RP11-936I5.1*	rs312825	17	75371822	C	T	0.6921	-0.5572	0.0472	4.06E-32	138.88
CD4 NC	*RP11-93B14.6*	rs910927	20	61696208	C	A	0.5943	0.4999	0.0445	2.92E-29	125.85
CD4 NC	*RP11-944C7.1*	rs2401841	14	90123909	C	G	0.2827	-0.3158	0.0498	2.30E-10	40.11
CD4 NC	*RP11-950C14.3*	rs12882664	14	75446778	T	C	0.5290	-0.6865	0.0423	3.36E-59	262.71
CD4 NC	*RP11-950C14.7*	rs7303	14	75520065	C	T	0.4790	-0.3462	0.0446	8.90E-15	60
CD4 NC	*RP11-96H19.1*	rs11613912	12	46739740	T	C	0.2693	0.6899	0.0484	4.19E-46	202.78
CD4 NC	*RP11-973H7.1*	rs888262	18	12750218	A	C	0.4132	0.3713	0.0447	9.95E-17	68.84
CD4 NC	*RP11-973H7.4*	rs2542153	18	12783898	C	T	0.5607	-0.3076	0.0451	8.69E-12	46.51
CD4 NC	*RP11-981P6.1*	rs73437338	12	90054619	C	T	0.1582	-0.4748	0.0598	2.10E-15	62.84
CD4 NC	*RP13-270P17.3*	rs1373000	18	3260834	C	T	0.8415	0.4178	0.0578	5.08E-13	52.07
CD4 NC	*RP13-578N3.3*	rs35601522	4	144438595	T	C	0.1650	-0.5475	0.0582	5.11E-21	88.31
CD4 NC	*RP13-582O9.5*	rs4614011	8	144370473	C	T	0.5364	-0.3074	0.0448	6.81E-12	46.99
CD4 NC	*RP3-325F22.5*	rs9389405	6	136891074	T	C	0.1473	-0.7955	0.0633	2.91E-36	157.8
CD4 NC	*RP4-539M6.22*	rs77539689	22	30690904	A	C	0.0922	-0.5166	0.0798	9.42E-11	41.85
CD4 NC	*RP4-539M6.22*	rs5997646	22	30830312	T	A	0.1911	-0.4185	0.0571	2.26E-13	53.66
CD4 NC	*RP4-564F22.5*	rs707578	20	37055875	C	T	0.7849	0.7572	0.0553	1.18E-42	187.01
CD4 NC	*RP4-594I10.3*	rs196425	1	24871586	C	T	0.3702	0.5154	0.0452	3.83E-30	129.87
CD4 NC	*RP4-673M15.1*	rs7799332	7	44954786	A	G	0.5255	-0.3838	0.0444	5.33E-18	74.6
CD4 NC	*RP4-798A10.2*	rs279781	1	16777812	C	G	0.3328	-0.5326	0.0450	2.36E-32	139.96
CD4 NC	*RP5-1021I20.1*	rs66896407	14	74266771	A	G	0.1398	0.7330	0.0581	1.76E-36	158.79
CD4 NC	*RP5-1042K10.10*	rs73167004	22	40732953	A	G	0.1731	-0.7993	0.0557	1.11E-46	205.41
CD4 NC	*RP5-1065J22.8*	rs180029	1	109633973	T	C	0.1776	0.3729	0.0588	2.24E-10	40.17
CD4 NC	*RP5-1068E13.7*	rs7262320	20	18775235	T	G	0.1754	0.4133	0.0575	6.86E-13	51.48
CD4 NC	*RP5-1092A3.4*	rs2078348	1	28563084	T	C	0.2321	-0.4057	0.0514	2.97E-15	62.16
CD4 NC	*RP5-1159O4.2*	rs1859605	7	7687840	A	G	0.3739	0.4103	0.0465	1.12E-18	77.68
CD4 NC	*RP5-821D11.7*	rs5758487	22	42226395	T	C	0.5283	0.3219	0.0447	5.80E-13	51.81
CD4 NC	*RP5-827C21.4*	rs10429821	1	234512471	C	T	0.4570	0.5975	0.0433	3.07E-43	189.68
CD4 NC	*RP5-827C21.4*	rs538237	1	234554599	G	A	0.0717	0.5488	0.0934	4.22E-09	34.45
CD4 NC	*RP5-882C2.2*	rs10208	17	42300278	T	C	0.3309	-0.3053	0.0476	1.37E-10	41.12
CD4 NC	*RP5-903G2.2*	rs7104257	11	67544442	C	G	0.6423	0.3394	0.0469	4.68E-13	52.23
CD4 NC	*RP6-109B7.3*	rs7410685	22	46451813	T	C	0.1001	0.4537	0.0761	2.52E-09	35.45
CD4 NC	*RPA1*	rs34589281	17	1691953	T	C	0.3545	-0.2666	0.0457	5.47E-09	33.95
CD4 NC	*RPA2*	rs2236074	1	28211384	A	G	0.3691	-0.3348	0.0458	2.61E-13	53.37
CD4 NC	*RPH3AL*	rs12951485	17	185083	T	C	0.7915	-0.3697	0.0547	1.40E-11	45.57
CD4 NC	*RPL10A*	rs2076172	6	35436925	T	C	0.5358	-0.9048	0.0404	5.79E-111	499.97
CD4 NC	*RPL11*	rs7521548	1	24010230	A	G	0.8374	0.8252	0.0606	3.68E-42	184.75
CD4 NC	*RPL12*	rs2247781	9	130208924	C	T	0.5921	1.1118	0.0371	1.66E-197	897.12
CD4 NC	*RPL12*	rs7854003	9	130310834	G	A	0.1464	0.4137	0.0607	9.70E-12	46.29
CD4 NC	*RPL13*	rs12932337	16	89630630	T	C	0.1753	-0.8370	0.0532	1.10E-55	246.62
CD4 NC	*RPL14*	rs75965075	3	40438120	T	C	0.0715	1.1482	0.0821	1.89E-44	195.21
CD4 NC	*RPL14*	rs11916725	3	40320627	A	G	0.0478	0.6714	0.1000	1.88E-11	45
CD4 NC	*RPL22L1*	rs13063927	3	170582888	T	C	0.2425	-0.4622	0.0501	2.62E-20	85.08
CD4 NC	*RPL23A*	rs2302205	17	26947046	C	A	0.0821	-1.1959	0.0747	1.07E-57	255.83
CD4 NC	*RPL27A*	rs61875926	11	8705528	G	C	0.0958	-0.8773	0.0721	4.49E-34	147.81
CD4 NC	*RPL28*	rs17700376	19	55897327	G	A	0.3547	-1.1543	0.0381	1.00E-200	915.54
CD4 NC	*RPL3*	rs6509	22	39714490	T	C	0.4725	0.2693	0.0448	1.83E-09	36.07
CD4 NC	*RPL32*	rs2290311	3	12883071	A	T	0.1438	0.5466	0.0612	3.99E-19	79.71
CD4 NC	*RPL36AL*	rs7160151	14	49803164	G	A	0.3045	-0.3039	0.0497	9.36E-10	37.38
CD4 NC	*RPL36AL*	rs1952013	14	50087357	G	C	0.7531	1.2828	0.0430	6.69E-196	889.75
CD4 NC	*RPL8*	rs2722495	8	145971666	T	C	0.5147	-1.1105	0.0364	1.00E-200	928.5
CD4 NC	*RPLP2*	rs28694634	11	808423	A	G	0.6588	-0.8421	0.0444	2.66E-80	359.38
CD4 NC	*RPRD2*	rs12136332	1	150305992	T	C	0.2084	0.3311	0.0546	1.28E-09	36.77
CD4 NC	*RPRM*	rs6727183	2	154378628	A	G	0.4303	0.4919	0.0446	3.15E-28	121.14
CD4 NC	*RPS10*	rs9380433	6	34377626	T	C	0.7750	-1.1344	0.0522	1.11E-104	471.15
CD4 NC	*RPS11*	rs17555056	19	49999647	G	C	0.1165	-0.8432	0.0701	2.49E-33	144.41

CD4 NC	*RPS12*	rs7357079	6	133148461	T	C	0.4426	0.4210	0.0446	3.81E-21	88.89
CD4 NC	*RPS14*	rs2245384	5	149817268	C	G	0.3033	-0.3077	0.0487	2.73E-10	39.78
CD4 NC	*RPS16*	rs480595	19	39925482	T	A	0.3758	0.4654	0.0465	1.49E-23	99.85
CD4 NC	*RPS17*	rs783538	15	83253143	G	C	0.3232	-0.3929	0.0476	1.48E-16	68.06
CD4 NC	*RPS18*	rs17215231	6	33239869	T	C	0.0640	-1.5894	0.0823	4.07E-83	372.29
CD4 NC	*RPS19*	rs12984383	19	42371522	C	G	0.4967	0.3637	0.0444	2.67E-16	66.9
CD4 NC	*RPS2*	rs17135718	16	2014834	G	A	0.1151	0.9550	0.0647	2.61E-49	217.44
CD4 NC	*RPS23*	rs226205	5	81593654	T	C	0.7767	0.4796	0.0508	3.44E-21	89.09
CD4 NC	*RPS24*	rs2913134	10	79763775	T	C	0.5775	0.3790	0.0456	9.72E-17	68.88
CD4 NC	*RPS25*	rs11602764	11	118888619	G	A	0.2652	-0.9113	0.0457	2.48E-88	396.19
CD4 NC	*RPS26*	rs773114	12	56379060	T	A	0.3997	1.2071	0.0350	1.00E-200	1186.58
CD4 NC	*RPS27L*	rs1126308	15	63429086	C	A	0.4824	0.2486	0.0449	3.03E-08	30.63
CD4 NC	*RPS3*	rs3094303	11	75114071	G	A	0.0743	-1.1263	0.0880	1.78E-37	163.34
CD4 NC	*RPS3A*	rs6836854	4	151742349	T	C	0.9259	1.2862	0.0961	7.91E-41	178.66
CD4 NC	*RPS5*	rs731835	19	58898436	C	T	0.3256	-1.1909	0.0385	1.00E-200	956.81
CD4 NC	*RPS6*	rs6475324	9	19361448	A	G	0.0990	-1.2758	0.0777	1.54E-60	268.85
CD4 NC	*RPS6KA2*	rs9459780	6	167271632	G	C	0.1796	0.4006	0.0591	1.19E-11	45.89
CD4 NC	*RPS6KL1*	rs11159109	14	75389074	A	G	0.3563	0.2646	0.0469	1.67E-08	31.78
CD4 NC	*RPS7*	rs62106040	2	3627302	T	A	0.0757	-1.4359	0.0835	3.29E-66	294.81
CD4 NC	*RPS7*	rs4850068	2	3655743	T	C	0.6690	-0.3080	0.0480	1.38E-10	41.11
CD4 NC	*RPS8*	rs12120833	1	45241285	G	C	0.1242	-0.9184	0.0596	1.47E-53	236.89
CD4 NC	*RPS9*	rs158366	19	54705317	G	C	0.3901	-0.9258	0.0407	2.74E-114	515.22
CD4 NC	*RPSA*	rs60217375	3	39440578	A	T	0.1555	0.9571	0.0580	3.44E-61	271.83
CD4 NC	*RQCD1*	rs4674319	2	219510320	T	C	0.5607	0.2527	0.0453	2.48E-08	31.01
CD4 NC	*RRP1B*	rs11089093	21	45121161	T	C	0.6104	-0.3024	0.0452	2.23E-11	44.67
CD4 NC	*RRP7A*	rs136988	22	42946371	T	C	0.1733	0.5648	0.0568	2.56E-23	98.77
CD4 NC	*RSL24D1*	rs28544512	15	55405966	T	C	0.1763	0.3767	0.0591	1.90E-10	40.49
CD4 NC	*RSPH9*	rs62401765	6	44098266	T	C	0.0565	0.5733	0.0904	2.22E-10	40.18
CD4 NC	*RSRC2*	rs6416298	12	123002143	T	C	0.7480	-0.4984	0.0491	3.33E-24	102.8
CD4 NC	*RSU1*	rs2248852	10	16859400	C	G	0.1365	0.4012	0.0637	3.08E-10	39.54
CD4 NC	*RTCA*	rs56215925	1	100707489	G	A	0.8302	-0.3930	0.0618	2.00E-10	40.39
CD4 NC	*RTKN2*	rs6479794	10	63983394	T	C	0.8731	-0.6618	0.0654	4.39E-24	102.26
CD4 NC	*RTN4*	rs2588512	2	55192082	G	A	0.2018	0.4142	0.0566	2.62E-13	53.37
CD4 NC	*RUNX2*	rs3799984	6	44893640	G	C	0.5068	0.3429	0.0445	1.33E-14	59.22
CD4 NC	*RWDD1*	rs72951241	6	116819150	A	G	0.0527	0.6614	0.0986	1.95E-11	44.92
CD4 NC	*S100B*	rs11701571	21	47963149	G	A	0.2474	0.3007	0.0523	9.07E-09	32.96
CD4 NC	*S100B*	rs11089085	21	48039367	G	A	0.6194	-0.5595	0.0451	2.28E-35	153.72
CD4 NC	*SAAL1*	rs10832882	11	18117821	G	A	0.2437	-0.4706	0.0506	1.53E-20	86.15
CD4 NC	*SAMD10*	rs817352	20	62611631	T	C	0.1853	0.5383	0.0604	5.05E-19	79.25
CD4 NC	*SAMD12*	rs2460970	8	119629454	A	G	0.9192	-0.8884	0.0760	1.54E-31	136.24
CD4 NC	*SAMD3*	rs41285310	6	130505374	C	A	0.5086	-0.7963	0.0412	4.79E-83	371.96
CD4 NC	*SAMD9L*	rs10255997	7	92798514	G	A	0.0518	-0.5802	0.0987	4.14E-09	34.49
CD4 NC	*SAMM50*	rs2294920	22	44351351	C	T	0.1283	-0.7723	0.0714	2.78E-27	116.83
CD4 NC	*SAPCD1-AS1*	rs9468936	6	31269884	G	T	0.3778	0.5369	0.0457	6.58E-32	137.92
CD4 NC	*SAPCD1-AS1*	rs530559160	6	31353792	G	T	0.0766	0.5937	0.0801	1.22E-13	54.86
CD4 NC	*SAPCD1-AS1*	rs2734574	6	31493888	A	T	0.3822	-0.3215	0.0460	2.63E-12	48.85
CD4 NC	*SAR1A*	rs15801	10	71910663	T	C	0.3974	-0.5136	0.0447	1.44E-30	131.8
CD4 NC	*SARDH*	rs10993769	9	136564488	T	C	0.3348	0.3171	0.0465	9.51E-12	46.33
CD4 NC	*SARS2*	rs7508539	19	39417440	A	G	0.6516	-0.3185	0.0455	2.57E-12	48.89
CD4 NC	*SAT2*	rs858521	17	7530147	C	G	0.3948	0.4658	0.0454	1.13E-24	104.95
CD4 NC	*SAYSD1*	rs7349859	6	39109161	C	G	0.5235	-0.3367	0.0445	4.08E-14	57.01
CD4 NC	*SC5D*	rs12275106	11	121170025	C	A	0.0740	0.4660	0.0833	2.22E-08	31.23
CD4 NC	*SCAMP3*	rs1076556	1	155234821	G	A	0.2668	0.5512	0.0484	4.35E-30	129.62
CD4 NC	*SCAND3*	rs2023463	6	29413003	T	C	0.0839	-0.7749	0.0711	1.20E-27	118.49
CD4 NC	*SCAPER*	rs2461871	15	76822418	G	C	0.3444	0.2924	0.0467	3.87E-10	39.1
CD4 NC	*SCCPDH*	rs35628008	1	246885067	C	T	0.3614	-0.3112	0.0466	2.36E-11	44.55
CD4 NC	*SCFD1*	rs61976741	14	31197813	T	C	0.2536	-0.3011	0.0530	1.32E-08	32.24
CD4 NC	*SCGB3A1*	rs7730055	5	180007386	G	A	0.8901	-1.5648	0.0650	3.18E-128	579.15
CD4 NC	*SCO2*	rs1138404	22	50964446	T	A	0.0998	0.4230	0.0736	9.21E-09	32.93
CD4 NC	*SCRN2*	rs2010952	17	45892859	C	A	0.1251	-0.4913	0.0640	1.64E-14	58.8
CD4 NC	*SDCBP2-AS1*	rs34019978	20	1306103	T	C	0.1246	-0.4439	0.0650	8.53E-12	46.54
CD4 NC	*SDK2*	rs11655934	17	71587577	A	G	0.1405	-0.5365	0.0641	5.95E-17	69.85
CD4 NC	*SDR39U1*	rs111636420	14	24918722	G	A	0.8891	0.5064	0.0756	2.09E-11	44.79
CD4 NC	*SDSL*	rs12820985	12	113796856	C	A	0.0744	0.7098	0.0872	4.00E-16	66.1
CD4 NC	*SELM*	rs11703403	22	31507338	G	A	0.5262	0.4404	0.0442	1.95E-23	99.31
CD4 NC	*SEMA3A*	rs2272221	7	83643687	A	G	0.3230	-0.3940	0.0476	1.27E-16	68.36
CD4 NC	*SEPT2*	rs3732276	2	242343404	T	C	0.0938	1.2287	0.0702	1.30E-68	305.82
CD4 NC	*SEPW1*	rs2334298	19	48280978	A	G	0.6775	0.3490	0.0489	9.63E-13	50.81
CD4 NC	*SERP1*	rs7374917	3	150311463	G	A	0.8637	0.4982	0.0675	1.60E-13	54.33
CD4 NC	*SERPINB1*	rs11242809	6	2937295	T	C	0.2565	-0.3346	0.0506	3.85E-11	43.6
CD4 NC	*SERPINB6*	rs11242809	6	2937295	T	C	0.2565	-0.9181	0.0465	8.70E-87	389.11
CD4 NC	*SERPINB9*	rs389260	6	2857442	C	T	0.4666	0.4781	0.0440	1.59E-27	117.93
CD4 NC	*SERPINE2*	rs865749	2	224900602	C	T	0.6572	-0.5992	0.0453	5.58E-40	174.78
CD4 NC	*SERPINF1*	rs12450371	17	1667674	T	C	0.3372	0.4900	0.0462	2.98E-26	112.13
CD4 NC	*SESN1*	rs6916819	6	109371129	C	T	0.1236	0.9795	0.0629	1.23E-54	241.82
CD4 NC	*SESN3*	rs4409785	11	95311422	C	T	0.1722	-0.5441	0.0575	2.96E-21	89.39
CD4 NC	*SESTD1*	rs10205477	2	180020847	G	A	0.1872	0.7226	0.0563	1.19E-37	164.14
CD4 NC	*SETD5-AS1*	rs2596916	3	9422210	C	T	0.5958	0.3565	0.0456	5.16E-15	61.07
CD4 NC	*SETD9*	rs185220	5	56205357	G	A	0.8359	0.8888	0.0601	1.49E-49	218.56

CD4 NC	*SF1*	rs533447	11	64561908	C	T	0.8796	-0.6059	0.0777	6.11E-15	60.74
CD4 NC	*SFI1*	rs5753691	22	31952045	G	A	0.2178	0.4502	0.0540	7.52E-17	69.39
CD4 NC	*SFT2D1*	rs10862	6	166755975	C	T	0.7497	0.3669	0.0508	5.00E-13	52.1
CD4 NC	*SFXN4*	rs10749291	10	120920588	C	T	0.4801	0.4422	0.0441	1.03E-23	100.58
CD4 NC	*SH2D2A*	rs11579761	1	156693018	G	C	0.0475	0.5200	0.0926	1.97E-08	31.46
CD4 NC	*SH2D3A*	rs12611400	19	6751707	A	G	0.0866	0.4520	0.0773	4.89E-09	34.16
CD4 NC	*SH3BP5*	rs7610033	3	15344254	G	A	0.3398	0.3185	0.0471	1.43E-11	45.54
CD4 NC	*SH3GLB1*	rs111571748	1	87273083	A	G	0.0751	0.6715	0.0783	1.01E-17	73.35
CD4 NC	*SH3YL1*	rs62114494	2	211317	T	C	0.3273	-1.1884	0.0373	1.00E-200	1011.61
CD4 NC	*SHFM1*	rs12671864	7	96269575	G	C	0.1705	0.4191	0.0611	6.85E-12	46.97
CD4 NC	*SHMT1*	rs669340	17	18263957	C	G	0.6337	0.2789	0.0463	1.66E-09	36.26
CD4 NC	*SIDT1-AS1*	rs35452897	3	113282075	T	C	0.2149	0.9738	0.0498	4.63E-85	381.19
CD4 NC	*SIRPB1*	rs2318043	20	1596473	G	A	0.2164	0.3837	0.0528	3.52E-13	52.79
CD4 NC	*SIRPG*	rs2277760	20	1616892	G	A	0.2544	-0.3698	0.0541	8.24E-12	46.61
CD4 NC	*SIVA1*	rs2498796	14	105243220	A	G	0.3189	-0.2997	0.0496	1.51E-09	36.45
CD4 NC	*SKA3*	rs73167443	13	21370652	C	T	0.1216	0.5665	0.0650	2.97E-18	75.76
CD4 NC	*SKAP2*	rs17323934	7	26904330	G	C	0.2183	0.5558	0.0518	7.06E-27	114.98
CD4 NC	*SKIV2L*	rs444921	6	31932177	T	C	0.1290	0.3975	0.0653	1.16E-09	36.95
CD4 NC	*SLC12A5*	rs2274755	20	44639692	T	G	0.1493	0.5681	0.0603	4.70E-21	88.47
CD4 NC	*SLC12A7*	rs35188965	5	1104938	T	C	0.5560	-0.6033	0.0437	2.93E-43	189.78
CD4 NC	*SLC16A4*	rs12120793	1	110911766	A	G	0.4352	0.4193	0.0442	2.55E-21	89.68
CD4 NC	*SLC18A2*	rs61865312	10	119126662	T	C	0.2779	0.3046	0.0509	2.23E-09	35.69
CD4 NC	*SLC18B1*	rs12191058	6	133123332	C	G	0.0728	-0.6150	0.0877	2.30E-12	49.11
CD4 NC	*SLC22A18*	rs1661052	11	2942593	A	G	0.9084	-1.1592	0.0742	4.76E-55	243.71
CD4 NC	*SLC22A31*	rs8052423	16	89269178	T	G	0.1419	-0.6534	0.0609	8.00E-27	114.73
CD4 NC	*SLC22A5*	rs274555	5	131722951	T	C	0.4205	0.2647	0.0455	6.07E-09	33.74
CD4 NC	*SLC25A1*	rs1780640	22	19174597	T	C	0.6542	-0.6588	0.0447	2.91E-49	217.23
CD4 NC	*SLC25A24*	rs663045	1	108743059	C	G	0.5428	0.3938	0.0446	9.84E-19	77.93
CD4 NC	*SLC25A26*	rs13078983	3	66356902	C	T	0.2178	-0.5063	0.0515	8.57E-23	96.38
CD4 NC	*SLC25A29*	rs1059264	14	100769163	T	C	0.3348	0.8986	0.0431	1.68E-96	433.64
CD4 NC	*SLC25A3*	rs28372744	12	98987445	A	G	0.0487	1.0055	0.0963	1.65E-25	108.74
CD4 NC	*SLC25A37*	rs2942219	8	23439308	G	A	0.2573	-0.3132	0.0497	2.94E-10	39.63
CD4 NC	*SLC25A51*	rs7852991	9	37876559	T	C	0.1447	0.5422	0.0632	1.00E-17	73.36
CD4 NC	*SLC26A5*	rs17149018	7	103111953	T	G	0.0591	0.6637	0.0953	3.33E-12	48.38
CD4 NC	*SLC2A1-AS1*	rs900835	1	43412455	T	C	0.1884	0.5104	0.0595	1.03E-17	73.3
CD4 NC	*SLC2A11*	rs113638588	22	24193999	G	C	0.0852	-0.4817	0.0767	3.39E-10	39.35
CD4 NC	*SLC2A4RG*	rs2315007	20	62343845	C	T	0.6998	-0.3632	0.0471	1.22E-14	59.38
CD4 NC	*SLC35F3*	rs611676	1	234451146	G	A	0.3248	0.8501	0.0447	1.09E-80	361.16
CD4 NC	*SLC38A1*	rs11183389	12	46588626	C	T	0.2648	-0.4669	0.0501	1.25E-20	86.55
CD4 NC	*SLC40A1*	rs71434664	2	190452068	T	G	0.2407	0.4228	0.0504	4.55E-17	70.38
CD4 NC	*SLC41A3*	rs56379527	3	125756207	A	G	0.3523	0.3339	0.0462	4.80E-13	52.18
CD4 NC	*SLC44A5*	rs1249827	1	75855910	A	G	0.5253	-0.3778	0.0444	1.72E-17	72.29
CD4 NC	*SLC4A7*	rs13100751	3	27454964	G	A	0.2136	-0.5839	0.0510	2.48E-30	130.73
CD4 NC	*SLC7A5*	rs184446886	16	87887496	G	A	0.0860	0.5278	0.0938	1.83E-08	31.61
CD4 NC	*SLC7A8*	rs59358210	14	23631372	A	G	0.1073	1.1155	0.0687	2.45E-59	263.34
CD4 NC	*SLCO3A1*	rs73523611	15	92013324	G	A	0.2142	0.3223	0.0546	3.62E-09	34.75
CD4 NC	*SLFN5*	rs11656388	17	33531815	A	G	0.3965	0.4677	0.0451	3.55E-25	107.23
CD4 NC	*SMAD5*	rs7717420	5	135542246	T	C	0.3239	0.5269	0.0481	6.55E-28	119.69
CD4 NC	*SMAGP*	rs7311832	12	51618856	A	G	0.7754	0.4452	0.0523	1.71E-17	72.31
CD4 NC	*SMARCE1*	rs8066541	17	38779942	A	G	0.5046	-0.3286	0.0446	1.70E-13	54.21
CD4 NC	*SMCR5*	rs9900803	17	17650978	T	C	0.6227	-0.4055	0.0479	2.42E-17	71.62
CD4 NC	*SMDT1*	rs2301521	22	42423110	C	G	0.6779	1.3470	0.0363	1.00E-200	1371.07
CD4 NC	*SMIM19*	rs2974366	8	42431768	A	C	0.6247	-0.5508	0.0445	3.17E-35	153.07
CD4 NC	*SMIM2-AS1*	rs9595066	13	44706268	C	T	0.8254	0.8647	0.0599	3.09E-47	207.96
CD4 NC	*SMIM8*	rs7746545	6	88090225	G	A	0.4656	-0.3581	0.0446	9.38E-16	64.43
CD4 NC	*SMYD2*	rs6687629	1	214495139	A	G	0.4217	-0.2929	0.0449	6.87E-11	42.47
CD4 NC	*SNAI3*	rs9932972	16	88757341	G	A	0.2441	-0.3354	0.0520	1.10E-10	41.55
CD4 NC	*SNAP29*	rs1978098	22	21288871	C	T	0.0685	0.9889	0.0797	2.40E-35	153.61
CD4 NC	*SNHG18*	rs6882340	5	9539960	C	G	0.0815	0.4370	0.0742	3.82E-09	34.64
CD4 NC	*SNHG7*	rs11145844	9	139613998	T	C	0.1148	-1.4005	0.0640	3.31E-106	478.15
CD4 NC	*SNHG8*	rs55747202	4	119083152	T	C	0.3719	1.1588	0.0365	1.00E-200	1003.9
CD4 NC	*SNHG8*	rs76533601	4	119777070	T	C	0.0809	-0.4786	0.0831	8.31E-09	33.13
CD4 NC	*SNORD3B-1*	rs959071	17	19142226	T	C	0.1827	0.5592	0.0676	1.37E-16	68.21
CD4 NC	*SNORD3B-2*	rs77232220	17	19262534	C	G	0.1953	0.4641	0.0547	2.32E-17	71.7
CD4 NC	*SNORD3D*	rs11869950	17	19375429	C	T	0.7399	-0.3515	0.0517	1.06E-11	46.13
CD4 NC	*SNPH*	rs6041150	20	1270094	G	A	0.8249	0.3840	0.0599	1.46E-10	40.99
CD4 NC	*SNRNP25*	rs216598	16	108585	C	T	0.8233	-0.6102	0.0578	5.05E-26	111.09
CD4 NC	*SNRNP35*	rs13303107	12	123939487	T	C	0.2065	0.3659	0.0550	2.80E-11	44.22
CD4 NC	*SNRPC*	rs9462026	6	34797033	G	C	0.3481	-0.5140	0.0475	2.47E-27	117.06
CD4 NC	*SNRPD2*	rs36040835	19	46201213	G	T	0.2051	-0.9961	0.0514	1.45E-83	374.34
CD4 NC	*SNRPF*	rs75081992	12	96244337	C	T	0.1754	0.3648	0.0567	1.24E-10	41.32
CD4 NC	*SNTG2*	rs4241330	2	830942	A	G	0.1332	-0.4330	0.0674	1.28E-10	41.25
CD4 NC	*SNX16*	rs10504741	8	82755353	A	C	0.6070	-0.3241	0.0454	9.33E-13	50.88
CD4 NC	*SNX25*	rs12501185	4	186203264	G	A	0.4227	0.3596	0.0449	1.11E-15	64.1
CD4 NC	*SNX9*	rs9347773	6	158349250	T	C	0.2217	0.3563	0.0532	2.07E-11	44.81
CD4 NC	*SOCS2*	rs7953280	12	94136009	C	G	0.5127	-0.3305	0.0446	1.22E-13	54.86
CD4 NC	*SOD2*	rs78550031	6	160149179	C	T	0.0474	0.6387	0.1021	3.89E-10	39.09
CD4 NC	*SOX6*	rs36101996	11	17757417	A	C	0.0504	0.8053	0.0939	9.91E-18	73.38
CD4 NC	*SP110*	rs6436915	2	231034524	T	G	0.4305	-0.3740	0.0445	4.51E-17	70.39

CD4 NC	*SP140*	rs10205568	2	231131021	G	A	0.0600	-0.6094	0.1011	1.68E-09	36.24
CD4 NC	*SP140L*	rs6707578	2	231174420	A	G	0.3993	-0.3310	0.0452	2.38E-13	53.55
CD4 NC	*SP140L*	rs4349338	2	231268834	G	A	0.4908	-0.2510	0.0448	2.16E-08	31.29
CD4 NC	*SPAG7*	rs72828872	17	4953756	A	G	0.1310	0.4701	0.0709	3.27E-11	43.92
CD4 NC	*SPATA20*	rs9890200	17	48624523	C	A	0.3676	-0.6806	0.0447	2.97E-52	230.92
CD4 NC	*SPATA21*	rs673126	1	16732518	C	T	0.5191	-0.3246	0.0447	4.00E-13	52.53
CD4 NC	*SPATA24*	rs4074031	5	138738096	T	A	0.5413	-0.2471	0.0450	4.13E-08	30.03
CD4 NC	*SPATA33*	rs11647958	16	89720909	A	T	0.4295	-0.2976	0.0448	3.04E-11	44.06
CD4 NC	*SPATA9*	rs213888	5	94981993	A	G	0.3183	0.5382	0.0469	1.79E-30	131.38
CD4 NC	*SPATC1L*	rs62215189	21	47603995	G	A	0.2446	-1.0043	0.0457	5.90E-107	481.58
CD4 NC	*SPATC1L*	rs2070426	21	47850484	C	G	0.5456	0.2783	0.0449	5.46E-10	38.43
CD4 NC	*SPCS1*	rs11130310	3	52674667	T	C	0.4272	0.3379	0.0456	1.33E-13	54.69
CD4 NC	*SPECC1*	rs8067545	17	19912710	C	T	0.5032	0.3265	0.0446	2.41E-13	53.53
CD4 NC	*SPG21*	rs11637902	15	65278805	T	C	0.5659	0.3648	0.0446	2.98E-16	66.68
CD4 NC	*SPINK2*	rs4864602	4	57624491	C	A	0.2192	-0.5450	0.0536	2.70E-24	103.22
CD4 NC	*SPINK2*	rs781539	4	57685913	C	G	0.6523	-0.5491	0.0453	9.30E-34	146.36
CD4 NC	*SPNS1*	rs11643297	16	28973268	G	A	0.7029	-0.3887	0.0492	2.75E-15	62.31
CD4 NC	*SPOCK2*	rs3088251	10	73822343	C	T	0.1164	-0.5137	0.0755	9.98E-12	46.24
CD4 NC	*SPPL3*	rs508595	12	121198891	G	C	0.5006	0.2630	0.0448	4.33E-09	34.4
CD4 NC	*SPRYD4*	rs73114872	12	56861034	C	T	0.1775	0.3521	0.0561	3.34E-10	39.38
CD4 NC	*SPSB2*	rs2269359	12	6993653	A	G	0.2882	0.5402	0.0478	1.23E-29	127.56
CD4 NC	*SRA1*	rs801458	5	139953189	C	T	0.5393	-0.4219	0.0443	1.59E-21	90.62
CD4 NC	*SRD5A3*	rs36110240	4	56217015	T	C	0.1629	0.8318	0.0584	4.75E-46	202.54
CD4 NC	*SRGN*	rs7915745	10	70870380	T	C	0.5688	-0.2890	0.0453	1.71E-10	40.69
CD4 NC	*SRI*	rs1063964	7	87835469	A	G	0.3146	0.4230	0.0476	6.06E-19	78.89
CD4 NC	*SRP14*	rs16924528	15	40328292	G	A	0.3179	-0.4837	0.0458	4.82E-26	111.18
CD4 NC	*SRP14-AS1*	rs16924528	15	40328292	G	A	0.3179	0.2678	0.0467	1.01E-08	32.76
CD4 NC	*SRR*	rs72819572	17	2180771	C	T	0.3395	-0.3100	0.0468	3.42E-11	43.83
CD4 NC	*SRSF10*	rs6424144	1	24375663	A	G	0.4721	-0.5889	0.0431	1.83E-42	186.14
CD4 NC	*SSBP4*	rs10423802	19	18567901	T	C	0.2060	-0.4843	0.0544	5.52E-19	79.07
CD4 NC	*ST3GAL5*	rs12990400	2	86081014	A	T	0.2631	-0.4618	0.0506	6.55E-20	83.28
CD4 NC	*ST6GALNAC1*	rs34503528	17	74703015	C	A	0.4491	-0.4888	0.0439	9.54E-29	123.5
CD4 NC	*ST6GALNAC3*	rs1251525	1	76487624	A	G	0.8167	-0.3277	0.0598	4.28E-08	29.96
CD4 NC	*ST6GALNAC6*	rs10819317	9	130668957	G	A	0.1684	-0.6596	0.0577	3.13E-30	130.27
CD4 NC	*STIM2*	rs35499018	4	27006111	A	C	0.6339	-0.2765	0.0470	3.96E-09	34.57
CD4 NC	*STK32C*	rs2698779	10	134157875	A	G	0.2273	0.6010	0.0507	1.78E-32	140.51
CD4 NC	*STMN1*	rs11581608	1	26338183	G	A	0.1998	-0.3658	0.0545	1.97E-11	44.91
CD4 NC	*STMN3*	rs879471	20	62269942	T	C	0.5285	0.6850	0.0425	1.61E-58	259.6
CD4 NC	*STOX1*	rs55746511	10	70603360	A	G	0.1421	0.7284	0.0676	4.23E-27	115.99
CD4 NC	*STRC*	rs478104	15	43594900	G	A	0.1506	-0.5756	0.0732	3.77E-15	61.69
CD4 NC	*STX16-NPEPL1*	rs11086654	20	57263390	C	T	0.3701	0.2704	0.0457	3.27E-09	34.94
CD4 NC	*STX2*	rs10848205	12	131280714	C	T	0.5684	0.2922	0.0454	1.22E-10	41.35
CD4 NC	*STX8*	rs35666827	17	9327820	G	A	0.2341	0.2786	0.0509	4.49E-08	29.86
CD4 NC	*STX8*	rs7219198	17	9149392	C	G	0.2799	0.2807	0.0492	1.18E-08	32.45
CD4 NC	*STXBP2*	rs2303115	19	7708214	A	G	0.3932	-0.3933	0.0456	6.66E-18	74.16
CD4 NC	*STYX*	rs12435056	14	53254224	A	G	0.3927	-0.3434	0.0460	8.35E-14	55.61
CD4 NC	*STYXL1*	rs6467960	7	75627458	A	G	0.9043	-0.5947	0.0734	5.54E-16	65.46
CD4 NC	*SUCLG2*	rs9828283	3	67402841	C	T	0.4393	0.3244	0.0448	4.58E-13	52.27
CD4 NC	*SULF2*	rs3091426	20	46189755	A	G	0.5891	-0.9890	0.0391	3.68E-141	638.51
CD4 NC	*SULF2*	rs4810653	20	46317485	C	T	0.6594	-0.2634	0.0475	2.85E-08	30.74
CD4 NC	*SUMF2*	rs58981281	7	56047951	A	G	0.2199	0.3765	0.0538	2.63E-12	48.84
CD4 NC	*SUPT3H*	rs12524323	6	45303853	A	C	0.4300	0.6833	0.0427	1.28E-57	255.47
CD4 NC	*SURF1*	rs34423753	9	136185960	A	G	0.4048	0.2771	0.0453	9.76E-10	37.3
CD4 NC	*SURF1*	rs3124747	9	136268084	G	A	0.6475	0.5321	0.0468	6.31E-30	128.88
CD4 NC	*SUSD3*	rs74513746	9	95826011	A	C	0.1182	0.4279	0.0706	1.37E-09	36.63
CD4 NC	*SUSD4*	rs34766468	1	223685201	T	A	0.0721	-0.7205	0.0860	5.28E-17	70.08
CD4 NC	*SUSD5*	rs4245887	3	32326748	T	C	0.7823	-0.3239	0.0553	4.69E-09	34.25
CD4 NC	*SVIP*	rs10833824	11	22847485	T	C	0.5443	0.4081	0.0449	1.07E-19	82.31
CD4 NC	*SYCE1L*	rs9926976	16	77251394	T	C	0.4063	0.2596	0.0458	1.50E-08	31.99
CD4 NC	*SYNC*	rs61798931	1	33107443	G	A	0.0985	0.6622	0.0682	2.92E-22	93.96
CD4 NC	*SYNE3*	rs28483795	14	95916931	C	T	0.2386	-0.3112	0.0524	2.95E-09	35.14
CD4 NC	*SYNJ2*	rs2502601	6	158517308	G	A	0.5180	0.3051	0.0447	8.30E-12	46.6
CD4 NC	*SYNM*	rs34878632	15	99645262	T	C	0.3903	0.4834	0.0447	2.73E-27	116.86
CD4 NC	*SYS1*	rs1008953	20	43980726	C	T	0.7724	0.3979	0.0533	8.41E-14	55.59
CD4 NC	*SYTL2*	rs1564550	11	85535361	T	A	0.2355	-0.2956	0.0539	4.12E-08	30.03
CD4 NC	*TAF6*	rs1050542	7	99704827	G	A	0.5835	-0.2581	0.0454	1.31E-08	32.25
CD4 NC	*TAMM41*	rs2454145	3	11887044	T	A	0.4412	-0.4446	0.0444	1.33E-23	100.07
CD4 NC	*TAPBP*	rs3130260	6	33278816	A	G	0.2897	-0.6135	0.0474	2.19E-38	167.5
CD4 NC	*TAPBPL*	rs2532496	12	6564448	G	A	0.3063	0.4996	0.0483	4.20E-25	106.9
CD4 NC	*TAS2R31*	rs6488331	12	11119119	C	T	0.8151	0.5157	0.0585	1.17E-18	77.59
CD4 NC	*TBC1D2B*	rs2044099	15	78332022	C	T	0.2614	0.3064	0.0497	6.92E-10	37.97
CD4 NC	*TBC1D4*	rs9593060	13	75875380	A	G	0.5325	0.3050	0.0449	1.13E-11	45.99
CD4 NC	*TBC1D4*	rs2274758	13	75894015	C	T	0.4719	-0.3515	0.0445	2.74E-15	62.32
CD4 NC	*TBC1D9B*	rs152234	5	179335060	G	A	0.9134	0.5742	0.0737	6.84E-15	60.52
CD4 NC	*TBCA*	rs254415	5	77071703	T	G	0.2878	-0.7977	0.0458	5.83E-68	302.83
CD4 NC	*TBCD*	rs9303014	17	80824369	T	G	0.5610	-0.6974	0.0426	4.12E-60	266.89
CD4 NC	*TC2N*	rs61990134	14	92334242	T	C	0.3506	-0.3100	0.0465	2.51E-11	44.43
CD4 NC	*TCEA3*	rs504145	1	23788310	T	C	0.8067	0.3449	0.0599	8.76E-09	33.03
CD4 NC	*TCN2*	rs5753231	22	31003069	T	C	0.1842	0.3009	0.0532	1.58E-08	31.89

CD4 NC	*TCTEX1D2*	rs2270769	3	196044894	C	G	0.3187	0.2901	0.0488	2.74E-09	35.29
CD4 NC	*TECPR1*	rs4727388	7	97865458	C	T	0.3999	0.3369	0.0453	1.07E-13	55.12
CD4 NC	*TECR*	rs8108187	19	14618483	T	G	0.1332	-0.3791	0.0645	4.19E-09	34.46
CD4 NC	*TEN1*	rs3760131	17	73996887	C	T	0.7540	0.6185	0.0509	5.36E-34	147.46
CD4 NC	*TEPP*	rs2405128	16	58020167	A	G	0.8359	0.5576	0.0607	4.11E-20	84.2
CD4 NC	*TERT*	rs10064734	5	860423	A	G	0.0763	-0.6933	0.0882	3.82E-15	61.67
CD4 NC	*TESC*	rs3736417	12	117537379	T	G	0.5565	0.3013	0.0447	1.58E-11	45.34
CD4 NC	*TEX29*	rs34832639	13	112540737	A	G	0.3705	0.2978	0.0462	1.11E-10	41.53
CD4 NC	*TFDP1*	rs4076410	13	114305732	A	G	0.1119	0.4253	0.0732	6.21E-09	33.7
CD4 NC	*TFPT*	rs79584852	19	54614627	A	G	0.1227	-0.5339	0.0639	6.70E-17	69.62
CD4 NC	*TGM4*	rs3749189	3	44986836	C	T	0.1840	0.4371	0.0554	2.95E-15	62.17
CD4 NC	*THAP5*	rs10258344	7	108208587	C	G	0.2287	0.3812	0.0558	8.48E-12	46.56
CD4 NC	*THAP7-AS1*	rs28372939	22	21356824	A	G	0.2052	-0.7140	0.0537	2.69E-40	176.23
CD4 NC	*THAP9-AS1*	rs7682657	4	83727406	A	T	0.7859	0.5840	0.0540	2.73E-27	116.86
CD4 NC	*THEM4*	rs7542137	1	151868892	T	C	0.6000	0.5083	0.0446	4.35E-30	129.62
CD4 NC	*THEMIS2*	rs3766396	1	28221554	G	A	0.3801	-0.9017	0.0413	1.23E-105	475.53
CD4 NC	*THNSL2*	rs56027847	2	88453438	G	A	0.0686	0.6401	0.0866	1.44E-13	54.54
CD4 NC	*THOC3*	rs62389203	5	175510135	T	C	0.4271	-0.2819	0.0449	3.54E-10	39.27
CD4 NC	*THRA*	rs883871	17	38252660	A	G	0.1425	0.6332	0.0665	1.66E-21	90.53
CD4 NC	*TIAM2*	rs9767124	6	155556164	G	C	0.6773	0.3165	0.0479	3.85E-11	43.6
CD4 NC	*TIFA*	rs6855562	4	113230540	A	G	0.2660	0.3764	0.0507	1.17E-13	54.95
CD4 NC	*TIMM10*	rs2848626	11	57283988	T	C	0.7820	-1.2527	0.0427	4.51E-189	858.4
CD4 NC	*TIMM13*	rs6945	19	2426828	C	G	0.4919	0.6115	0.0429	5.33E-46	202.31
CD4 NC	*TIPARP-AS1*	rs9289971	3	156389947	C	T	0.1529	-0.8934	0.0578	5.76E-54	238.75
CD4 NC	*TIPRL*	rs12760223	1	168132898	T	C	0.2492	-0.3704	0.0527	2.17E-12	49.23
CD4 NC	*TLE4*	rs4877500	9	82302854	G	A	0.6372	-0.5624	0.0454	3.27E-35	153
CD4 NC	*TLK1*	rs34004615	2	171843623	A	G	0.3521	-0.6004	0.0451	2.03E-40	176.8
CD4 NC	*TMED4*	rs217368	7	44615527	A	G	0.4303	-0.2627	0.0450	5.16E-09	34.06
CD4 NC	*TMED9*	rs28496282	5	177032372	C	G	0.5624	0.4724	0.0443	1.33E-26	113.72
CD4 NC	*TMEM101*	rs77536093	17	42092328	G	A	0.0441	-1.0574	0.1001	4.23E-26	111.44
CD4 NC	*TMEM106C*	rs11168332	12	48353477	G	A	0.3424	-0.3478	0.0469	1.15E-13	54.99
CD4 NC	*TMEM120B*	rs73413708	12	122213144	C	G	0.0964	1.0560	0.0756	2.37E-44	194.77
CD4 NC	*TMEM121*	rs35240373	14	105969396	G	A	0.2796	-0.5586	0.0517	3.26E-27	116.51
CD4 NC	*TMEM128*	rs67635842	4	4244078	C	T	0.3550	-0.2937	0.0468	3.37E-10	39.37
CD4 NC	*TMEM14C*	rs1045911	6	10723449	C	A	0.1387	0.5214	0.0656	1.97E-15	62.97
CD4 NC	*TMEM156*	rs6819988	4	39038665	T	C	0.5743	0.7404	0.0423	1.25E-68	305.89
CD4 NC	*TMEM159*	rs34976834	16	21150905	T	C	0.4129	0.2973	0.0452	4.76E-11	43.19
CD4 NC	*TMEM161B-AS1*	rs2305104	5	87620670	C	T	0.2604	0.3336	0.0510	6.17E-11	42.68
CD4 NC	*TMEM18*	rs12990777	2	674963	A	G	0.7414	-0.5617	0.0495	8.19E-30	128.36
CD4 NC	*TMEM199*	rs4795433	17	26716821	T	C	0.5258	0.2888	0.0448	1.16E-10	41.44
CD4 NC	*TMEM2*	rs4745123	9	74393843	G	A	0.7815	-0.3596	0.0558	1.13E-10	41.49
CD4 NC	*TMEM204*	rs2294624	16	1586255	A	G	0.1329	0.7925	0.0648	1.96E-34	149.45
CD4 NC	*TMEM230*	rs2326588	20	5058061	C	T	0.5212	0.6614	0.0427	3.54E-54	239.72
CD4 NC	*TMEM244*	rs113871222	6	130159481	G	C	0.4529	0.2870	0.0447	1.37E-10	41.12
CD4 NC	*TMEM258*	rs174538	11	61560081	A	G	0.2977	0.6333	0.0469	1.74E-41	181.66
CD4 NC	*TMEM41B*	rs7939704	11	9304702	C	T	0.2032	-0.4768	0.0558	1.33E-17	72.79
CD4 NC	*TMEM45B*	rs7948511	11	129756694	C	A	0.5711	0.6440	0.0430	1.15E-50	223.64
CD4 NC	*TMEM50A*	rs55794721	1	25561667	A	G	0.3493	-0.2709	0.0458	3.33E-09	34.91
CD4 NC	*TMEM50B*	rs928739	21	34824203	T	C	0.4731	-0.8673	0.0404	3.62E-102	459.63
CD4 NC	*TMEM71*	rs13250526	8	133721028	T	A	0.1478	0.4814	0.0680	1.48E-12	49.98
CD4 NC	*TMEM80*	rs28392994	11	679915	A	G	0.4778	-0.4766	0.0441	3.57E-27	116.33
CD4 NC	*TMEM87B*	rs4848979	2	112816236	T	G	0.3264	0.3255	0.0480	1.16E-11	45.95
CD4 NC	*TMEM8A*	rs28533314	16	339082	T	C	0.1832	-0.3131	0.0571	4.20E-08	29.99
CD4 NC	*TMEM8A*	rs2363755	16	430292	G	C	0.4125	-0.9320	0.0396	2.74E-122	551.92
CD4 NC	*TMEM97*	rs614877	17	26612996	T	C	0.4531	-0.3024	0.0449	1.60E-11	45.31
CD4 NC	*TMEM9B-AS1*	rs2316521	11	9006883	T	A	0.2009	0.3253	0.0566	9.12E-09	32.95
CD4 NC	*TMOD1*	rs2805803	9	100337953	C	T	0.4672	-0.4570	0.0440	3.17E-25	107.45
CD4 NC	*TMTC4*	rs9582406	13	101280900	C	G	0.3456	0.4396	0.0465	3.11E-21	89.29
CD4 NC	*TNFRSF10A*	rs6982233	8	23061792	C	T	0.2383	-0.3492	0.0521	2.01E-11	44.87
CD4 NC	*TNFRSF14*	rs28734787	1	2504363	C	T	0.1458	0.5860	0.0637	3.49E-20	84.52
CD4 NC	*TNFRSF18*	rs57260149	1	1140290	C	T	0.0589	-0.5295	0.0965	4.07E-08	30.05
CD4 NC	*TNFRSF1B*	rs1201171	1	12192270	G	A	0.7055	-0.3218	0.0497	9.30E-11	41.88
CD4 NC	*TNFSF10*	rs3136589	3	172237826	G	T	0.1789	-0.3882	0.0586	3.58E-11	43.74
CD4 NC	*TNFSF13B*	rs9514831	13	108973576	A	G	0.2718	0.5178	0.0524	4.90E-23	97.49
CD4 NC	*TNFSF13B*	rs78679073	13	109027657	T	A	0.1830	0.3454	0.0580	2.64E-09	35.36
CD4 NC	*TNFSF8*	rs5003740	9	117660601	C	G	0.1875	-0.5233	0.0584	3.16E-19	80.17
CD4 NC	*TNFSF9*	rs12984568	19	5999483	A	G	0.1912	0.7347	0.0573	1.17E-37	164.17
CD4 NC	*TNNT3*	rs3781962	11	1907609	G	C	0.3696	-0.2840	0.0470	1.51E-09	36.44
CD4 NC	*TNRC6A*	rs79080004	16	24842933	A	G	0.1355	-0.4230	0.0712	2.81E-09	35.24
CD4 NC	*TOMM20*	rs74868495	1	235313921	A	G	0.1452	-0.3719	0.0622	2.30E-09	35.63
CD4 NC	*TOMM7*	rs2240728	7	22852499	T	C	0.3422	-1.3296	0.0364	1.00E-200	1330.65
CD4 NC	*TOR3A*	rs1128952	1	179065125	T	C	0.3083	-0.2757	0.0491	2.01E-08	31.42
CD4 NC	*TP53I11*	rs73464507	11	45123931	C	G	0.1567	0.3406	0.0610	2.36E-08	31.11
CD4 NC	*TP53TG1*	rs802035	7	86969559	T	C	0.7363	0.3693	0.0495	8.85E-14	55.49
CD4 NC	*TPD52*	rs7009579	8	81064734	G	A	0.4496	0.5368	0.0438	1.38E-34	150.15
CD4 NC	*TPGS1*	rs12610275	19	521407	G	A	0.5039	0.4448	0.0441	5.76E-24	101.72
CD4 NC	*TPGS2*	rs28794232	18	34578888	A	G	0.2585	-0.7832	0.0469	1.61E-62	277.91
CD4 NC	*TPI1*	rs9737890	12	6990453	T	A	0.2868	-0.3928	0.0487	6.85E-16	65.04
CD4 NC	*TPK1*	rs10261806	7	144532104	A	G	0.4353	-0.2821	0.0453	4.87E-10	38.65

CD4 NC	*TPR*	rs76317001	1	186272489	A	G	0.1533	-0.4261	0.0611	3.16E-12	48.49
CD4 NC	*TPRA1*	rs62264113	3	127292333	A	G	0.1061	-0.5248	0.0737	1.09E-12	50.58
CD4 NC	*TPST2*	rs4275	22	26921725	G	A	0.4260	-0.2603	0.0451	8.06E-09	33.19
CD4 NC	*TRABD2A*	rs11674022	2	85050488	A	G	0.0675	-0.6212	0.0876	1.34E-12	50.17
CD4 NC	*TRAPPC12-AS1*	rs4641971	2	3481857	T	G	0.3846	-0.5767	0.0452	3.07E-37	162.26
CD4 NC	*TRAPPC2L*	rs3826061	16	88927221	C	T	0.3581	-0.5844	0.0468	1.02E-35	155.31
CD4 NC	*TRAPPC4*	rs8192696	11	118895686	T	C	0.2133	0.4157	0.0532	5.60E-15	60.91
CD4 NC	*TRAT1*	rs2715718	3	108568416	T	C	0.5241	0.2564	0.0448	1.06E-08	32.66
CD4 NC	*TRIB2*	rs2288192	2	12864766	T	C	0.2270	-0.4106	0.0555	1.40E-13	54.59
CD4 NC	*TRIB3*	rs7263207	20	363733	T	C	0.0830	-0.4734	0.0824	9.38E-09	32.9
CD4 NC	*TRIM16*	rs10221188	17	15587575	A	G	0.5095	0.2785	0.0448	4.96E-10	38.62
CD4 NC	*TRIM23*	rs463911	5	64988425	A	G	0.6382	-0.4360	0.0467	9.49E-21	87.09
CD4 NC	*TRIM31*	rs2517653	6	30121185	A	G	0.2499	-0.3816	0.0509	6.49E-14	56.1
CD4 NC	*TRIM35*	rs2322605	8	27164449	A	G	0.4432	-0.2977	0.0447	2.82E-11	44.21
CD4 NC	*TRIM4*	rs503115	7	99459144	C	T	0.5928	0.3062	0.0453	1.43E-11	45.53
CD4 NC	*TRIM66*	rs10840105	11	8699662	G	A	0.3774	0.3265	0.0453	5.97E-13	51.75
CD4 NC	*TRMT112*	rs79423518	11	64105454	A	G	0.0682	-0.9897	0.0964	9.69E-25	105.24
CD4 NC	*TRMT12*	rs4132855	8	125462121	G	A	0.4126	0.2607	0.0454	9.63E-09	32.85
CD4 NC	*TRMT6*	rs4813779	20	5776624	T	C	0.5835	-0.2639	0.0456	7.23E-09	33.4
CD4 NC	*TSEN54*	rs7225469	17	73516934	C	T	0.9005	-0.4284	0.0751	1.15E-08	32.5
CD4 NC	*TSGA10*	rs11692669	2	99750346	A	C	0.6085	0.6010	0.0446	1.82E-41	181.58
CD4 NC	*TSHZ2*	rs719721	20	50975497	G	A	0.2765	-0.3550	0.0497	9.49E-13	50.84
CD4 NC	*TSHZ2*	rs74963256	20	51588847	C	T	0.1003	0.5605	0.0718	5.72E-15	60.87
CD4 NC	*TSKS*	rs3745477	19	50265549	G	T	0.7036	-0.6346	0.0495	1.10E-37	164.31
CD4 NC	*TSPAN15*	rs61848436	10	71247714	T	C	0.2259	0.3086	0.0526	4.51E-09	34.32
CD4 NC	*TSPAN32*	rs2244891	11	2319355	T	C	0.7435	-0.3918	0.0529	1.30E-13	54.73
CD4 NC	*TSPO*	rs138909	22	43549739	A	T	0.3905	-0.5758	0.0443	1.04E-38	168.98
CD4 NC	*TSTD1*	rs3766383	1	161008572	A	C	0.1552	-1.0205	0.0638	1.32E-57	255.42
CD4 NC	*TSTD1*	rs4559524	1	161045128	A	G	0.1202	0.5851	0.0693	2.94E-17	71.24
CD4 NC	*TSTD3*	rs9402863	6	99971429	G	A	0.5634	-0.6854	0.0429	1.74E-57	254.87
CD4 NC	*TTC12*	rs1893695	11	113206085	C	A	0.4692	-0.2928	0.0449	6.81E-11	42.49
CD4 NC	*TTC16*	rs12554049	9	130442993	T	C	0.4799	0.3758	0.0445	3.14E-17	71.11
CD4 NC	*TTC24*	rs12405992	1	156594364	C	T	0.2411	-0.3749	0.0535	2.44E-12	49
CD4 NC	*TTC3*	rs56356911	21	38583628	G	A	0.3196	0.3674	0.0480	1.89E-14	58.52
CD4 NC	*TTC32*	rs7563410	2	20096936	A	C	0.0885	1.0611	0.0786	1.46E-41	182.01
CD4 NC	*TTC39B*	rs17327373	9	15294331	T	C	0.2889	-0.4424	0.0480	3.15E-20	84.72
CD4 NC	*TUBA1B*	rs7976890	12	49519185	G	A	0.3635	0.4014	0.0465	6.04E-18	74.36
CD4 NC	*TUFM*	rs8049439	16	28837515	C	T	0.3939	1.0906	0.0383	2.61E-178	809
CD4 NC	*TWSG1*	rs9956536	18	9360863	A	G	0.5997	-0.2813	0.0455	6.44E-10	38.11
CD4 NC	*TXK*	rs58924403	4	48131879	G	C	0.1156	0.6083	0.0731	8.37E-17	69.18
CD4 NC	*TXN2*	rs2011106	22	36890342	C	T	0.5575	-0.4085	0.0452	1.68E-19	81.42
CD4 NC	*TXNDC15*	rs9327697	5	134279940	A	G	0.1397	0.4582	0.0639	7.73E-13	51.24
CD4 NC	*TXNL4A*	rs11081576	18	77737397	T	C	0.8214	-0.3541	0.0600	3.52E-09	34.8
CD4 NC	*TYMP*	rs9616864	22	50976152	C	T	0.1038	0.7121	0.0689	4.89E-25	106.6
CD4 NC	*U2AF1L4*	rs112500848	19	36254427	T	C	0.1552	1.0887	0.0594	4.60E-75	335.38
CD4 NC	*UACA*	rs56739943	15	71049952	A	G	0.1046	-0.4832	0.0791	1.01E-09	37.23
CD4 NC	*UBA52*	rs8110371	19	18667272	C	A	0.5875	-0.3024	0.0455	3.15E-11	43.99
CD4 NC	*UBA6*	rs4860262	4	68480525	T	C	0.4045	0.5585	0.0445	3.46E-36	157.46
CD4 NC	*UBA6-AS1*	rs12646549	4	68553737	C	G	0.1467	0.5307	0.0637	7.63E-17	69.36
CD4 NC	*UBASH3A*	rs1893592	21	43855067	C	A	0.2651	0.4511	0.0476	2.57E-21	89.67
CD4 NC	*UBE2B*	rs244657	5	133448850	G	A	0.9146	-0.5304	0.0852	4.91E-10	38.64
CD4 NC	*UBE2E2*	rs6808513	3	23481113	A	G	0.5585	-0.3106	0.0448	4.31E-12	47.88
CD4 NC	*UBE2F*	rs11682046	2	239006650	C	T	0.4943	0.5045	0.0438	1.03E-30	132.48
CD4 NC	*UBE2I*	rs6600135	16	1362199	A	G	0.8884	-0.4374	0.0659	3.21E-11	43.95
CD4 NC	*UBE2L3*	rs5754422	22	21980257	G	A	0.2197	-0.4511	0.0572	3.23E-15	61.99
CD4 NC	*UBE2Q2*	rs78911138	15	76275641	T	G	0.0477	-0.7567	0.0970	6.28E-15	60.69
CD4 NC	*UBE3B*	rs142089760	12	109872015	A	G	0.0754	0.5396	0.0843	1.56E-10	40.87
CD4 NC	*UBE3C*	rs62491932	7	157021274	C	A	0.0979	0.5755	0.0761	3.86E-14	57.12
CD4 NC	*UBE4B*	rs7528979	1	10093457	T	C	0.1920	0.4310	0.0593	3.77E-13	52.65
CD4 NC	*UBR7*	rs1263397	14	93682589	G	A	0.6791	-0.3322	0.0477	3.23E-12	48.45
CD4 NC	*UBXN10-AS1*	rs7535938	1	20513040	A	T	0.2342	-0.2977	0.0535	2.60E-08	30.92
CD4 NC	*UBXN11*	rs6598942	1	26531034	T	C	0.6268	-0.2713	0.0469	7.11E-09	33.44
CD4 NC	*UBXN6*	rs732716	19	4366219	G	A	0.2853	-0.4678	0.0492	2.02E-21	90.14
CD4 NC	*UCP2*	rs2515087	11	73682872	T	C	0.4077	0.6684	0.0436	5.96E-53	234.11
CD4 NC	*UFL1*	rs1127175	6	96999725	T	C	0.3543	0.2639	0.0465	1.39E-08	32.13
CD4 NC	*UFSP2*	rs10000869	4	186339449	G	A	0.7494	0.3254	0.0524	5.30E-10	38.48
CD4 NC	*ULK4*	rs112265441	3	41836068	A	G	0.1568	0.8668	0.0584	9.12E-50	219.54
CD4 NC	*UNC45B*	rs77591182	17	33578330	C	T	0.1041	0.4327	0.0756	1.07E-08	32.65
CD4 NC	*UPF3A*	rs7331680	13	115000650	T	G	0.1614	1.0625	0.0605	3.92E-69	308.2
CD4 NC	*UPF3A*	rs7998623	13	114973772	T	C	0.1738	0.3571	0.0619	7.76E-09	33.27
CD4 NC	*UPK3BL*	rs59924467	7	102342723	C	T	0.1106	0.9119	0.0705	2.83E-38	166.99
CD4 NC	*UQCC2*	rs2281830	6	33695130	A	G	0.2867	-0.3637	0.0500	3.63E-13	52.72
CD4 NC	*UQCRH*	rs41292543	1	46774783	G	A	0.1009	-1.0046	0.0734	1.30E-42	186.82
CD4 NC	*URGCP*	rs2595722	7	44010922	G	A	0.5194	0.3011	0.0447	1.61E-11	45.3
CD4 NC	*UROS*	rs947582	10	127495354	G	C	0.4453	-0.4374	0.0442	4.34E-23	97.73
CD4 NC	*USF1*	rs12116949	1	161042063	A	C	0.2747	0.4740	0.0482	7.91E-23	96.54
CD4 NC	*USP10*	rs8050719	16	84780468	A	G	0.3013	-0.3683	0.0490	5.35E-14	56.48
CD4 NC	*USP36*	rs11658134	17	76739141	A	G	0.5132	-0.3038	0.0447	1.03E-11	46.17
CD4 NC	*USP44*	rs6538656	12	95964031	A	C	0.6199	-0.2860	0.0459	4.66E-10	38.74

CD4 NC	*USP53*	rs34478210	4	120135901	G	A	0.1222	1.2741	0.0639	2.50E-88	396.17
CD4 NC	*USP7*	rs2447918	16	8996377	T	C	0.3730	0.2560	0.0464	3.52E-08	30.33
CD4 NC	*UTS2*	rs161804	1	7975638	G	A	0.6789	0.5909	0.0474	1.02E-35	155.31
CD4 NC	*VAMP2*	rs4792267	17	8061337	A	G	0.6696	0.5680	0.0478	1.42E-32	140.96
CD4 NC	*VAMP4*	rs6679354	1	171668002	C	T	0.1986	0.6816	0.0541	2.29E-36	158.28
CD4 NC	*VAMP5*	rs13421434	2	85807610	A	C	0.1162	-0.9207	0.0651	1.85E-45	199.84
CD4 NC	*VCL*	rs11000888	10	75901929	C	G	0.1332	-0.7116	0.0634	3.01E-29	125.79
CD4 NC	*VDAC2*	rs1259498	10	76986741	C	T	0.4687	-0.4006	0.0444	1.92E-19	81.15
CD4 NC	*VIM*	rs57465155	10	17276066	T	C	0.3195	0.2761	0.0488	1.48E-08	32.01
CD4 NC	*VMAC*	rs146033453	19	5908163	C	T	0.1323	-0.4421	0.0633	2.95E-12	48.62
CD4 NC	*VOPP1*	rs6976519	7	55519649	G	A	0.5487	0.3049	0.0450	1.23E-11	45.84
CD4 NC	*VPS13C*	rs12916800	15	62346685	G	A	0.5488	-0.2486	0.0451	3.53E-08	30.33
CD4 NC	*VPS28*	rs117891721	8	145642886	T	C	0.0569	1.2081	0.0917	1.27E-39	173.15
CD4 NC	*VPS53*	rs4968139	17	449447	G	A	0.4184	-0.3071	0.0449	8.13E-12	46.64
CD4 NC	*VRK3*	rs4802643	19	50477230	T	C	0.1933	-0.4808	0.0586	2.21E-16	67.27
CD4 NC	*VWA7*	rs622871	6	31878495	G	A	0.7499	-0.4451	0.0486	4.96E-20	83.82
CD4 NC	*VWDE*	rs847912	7	12457164	G	T	0.2178	0.6137	0.0533	1.08E-30	132.37
CD4 NC	*WAC-AS1*	rs2807741	10	28783350	T	C	0.5259	-0.3019	0.0447	1.41E-11	45.56
CD4 NC	*WAC-AS1*	rs7098928	10	28789999	G	A	0.5922	-0.5303	0.0441	2.96E-33	144.07
CD4 NC	*WASF2*	rs9438522	1	27809584	A	G	0.0963	0.5321	0.0799	2.77E-11	44.24
CD4 NC	*WBP1L*	rs284854	10	104574562	G	T	0.5709	0.3551	0.0451	3.36E-15	61.91
CD4 NC	*WBSCR16*	rs6955671	7	74489486	C	T	0.4577	-0.2690	0.0450	2.19E-09	35.73
CD4 NC	*WBSCR27*	rs6974182	7	73242977	C	T	0.7255	-0.6934	0.0503	2.95E-43	189.76
CD4 NC	*WDR17*	rs35772136	4	177569252	G	A	0.2880	-0.4754	0.0488	2.19E-22	94.53
CD4 NC	*WDR18*	rs2240150	19	991063	C	A	0.1309	0.4935	0.0680	3.87E-13	52.6
CD4 NC	*WDR60*	rs57298399	7	158659884	C	G	0.0459	-0.5981	0.1027	5.83E-09	33.82
CD4 NC	*WDR73*	rs7181035	15	85162429	A	G	0.8306	-0.3265	0.0591	3.38E-08	30.41
CD4 NC	*WDR86*	rs12703130	7	151095131	A	G	0.5202	0.3441	0.0446	1.27E-14	59.31
CD4 NC	*WDR86*	rs2075000	7	151133792	A	G	0.1200	0.4717	0.0758	4.95E-10	38.62
CD4 NC	*WDSUB1*	rs2159876	2	160099278	A	G	0.3320	-0.4428	0.0471	5.29E-21	88.24
CD4 NC	*WDYHV1*	rs6982267	8	124472489	A	G	0.3581	-0.3823	0.0471	4.76E-16	65.76
CD4 NC	*WIPI2*	rs6972792	7	5169532	T	C	0.3347	0.4844	0.0475	1.86E-24	103.96
CD4 NC	*WRB*	rs9974828	21	40772045	T	C	0.6885	-0.2613	0.0474	3.47E-08	30.36
CD4 NC	*WWP1*	rs72690442	8	87530791	T	G	0.2549	-0.3347	0.0526	2.01E-10	40.38
CD4 NC	*XAB2*	rs35020672	19	7697956	C	T	0.3631	-0.2727	0.0462	3.45E-09	34.84
CD4 NC	*XBP1*	rs2881496	22	28921265	G	C	0.8731	-0.9022	0.0641	5.49E-45	197.67
CD4 NC	*XKR9*	rs2639901	8	71917751	G	A	0.4959	0.3867	0.0444	2.80E-18	75.87
CD4 NC	*XPC*	rs2305843	3	14242148	C	T	0.1393	0.4155	0.0712	5.45E-09	33.95
CD4 NC	*XPNPEP1*	rs3862006	10	111750764	A	G	0.1662	0.3686	0.0622	3.09E-09	35.06
CD4 NC	*XRCC2*	rs17813864	7	152337646	C	T	0.2145	0.3593	0.0524	6.89E-12	46.96
CD4 NC	*XRCC6BP1*	rs10431506	12	58311524	A	G	0.3136	-0.6973	0.0453	1.83E-53	236.46
CD4 NC	*XRRA1*	rs61734374	11	74457056	G	A	0.2737	1.2518	0.0387	1.00E-200	1042.2
CD4 NC	*XXbac-BPG181B23.7*	rs28530646	6	31364769	A	G	0.2731	-0.5472	0.0523	1.21E-25	109.35
CD4 NC	*XXbac-BPG181M17.6*	rs9277919	6	33125484	T	C	0.3253	0.3444	0.0479	6.53E-13	51.57
CD4 NC	*XXbac-BPG249D20.9*	rs6936943	6	30352986	A	G	0.4496	0.2922	0.0457	1.58E-10	40.84
CD4 NC	*XXbac-BPG249D20.9*	rs9266702	6	31349115	C	T	0.7677	0.5121	0.0497	7.56E-25	105.73
CD4 NC	*XXbac-BPG299F13.17*	rs6921663	6	31154620	C	T	0.2178	1.2155	0.0470	1.56E-147	667.75
CD4 NC	*XXbac-BPG299F13.17*	rs9261742	6	30368663	A	G	0.2468	0.4827	0.0536	2.14E-19	80.94
CD4 NC	*XXbac-BPG299F13.17*	rs2250287	6	31330251	G	T	0.1615	-0.4044	0.0566	9.22E-13	50.9
CD4 NC	*XXbac-BPG308K3.5*	rs113510833	6	29671067	T	C	0.0925	-0.4564	0.0695	5.09E-11	43.06
CD4 NC	*XYLB*	rs7649153	3	38467753	A	T	0.6646	-0.3760	0.0475	2.54E-15	62.47
CD4 NC	*YBEY*	rs62224180	21	47726332	G	C	0.7751	-1.1487	0.0507	1.38E-113	512
CD4 NC	*YBX3*	rs12317203	12	10876059	T	G	0.3863	-0.3830	0.0450	1.69E-17	72.33
CD4 NC	*YEATS4*	rs11177615	12	69754189	A	G	0.4260	-0.5628	0.0435	2.41E-38	167.31
CD4 NC	*YIPF5*	rs6864309	5	143550217	T	G	0.1801	-0.4262	0.0603	1.50E-12	49.94
CD4 NC	*YME1L1*	rs7919316	10	27467486	A	G	0.6352	0.3754	0.0470	1.41E-15	63.62
CD4 NC	*YWHAB*	rs4931	20	43530234	C	A	0.2911	-0.3645	0.0501	3.34E-13	52.89
CD4 NC	*YWHAH*	rs9609396	22	32363761	T	C	0.1525	0.4249	0.0600	1.41E-12	50.07
CD4 NC	*ZADH2*	rs9240	18	72910687	C	T	0.4306	-0.3780	0.0446	2.50E-17	71.56
CD4 NC	*ZBTB1*	rs3783726	14	64904340	T	C	0.2260	-0.2917	0.0531	3.99E-08	30.1
CD4 NC	*ZBTB32*	rs73590559	19	36194251	T	A	0.1042	0.4912	0.0812	1.45E-09	36.52
CD4 NC	*ZC2HC1A*	rs1466526	8	79555474	G	C	0.7233	0.4701	0.0509	2.58E-20	85.12
CD4 NC	*ZC3H3*	rs10089146	8	144526269	T	C	0.2897	0.3892	0.0482	6.46E-16	65.16
CD4 NC	*ZC3H8*	rs6759283	2	112967145	G	A	0.1417	0.4724	0.0632	7.67E-14	55.78
CD4 NC	*ZC3HAV1*	rs7803490	7	138730361	C	T	0.5046	-0.8303	0.0408	5.60E-92	412.91
CD4 NC	*ZDHHC2*	rs7460815	8	17083856	T	G	0.5163	-0.2765	0.0448	6.86E-10	37.98
CD4 NC	*ZDHHC24*	rs17496586	11	66315512	C	G	0.2372	0.3451	0.0525	5.07E-11	43.06
CD4 NC	*ZDHHC4*	rs11559147	7	6617247	G	C	0.7006	0.4837	0.0488	3.93E-23	97.93
CD4 NC	*ZDHHC7*	rs75271179	16	85005716	T	C	0.1896	0.3321	0.0558	2.73E-09	35.29
CD4 NC	*ZFAS1*	rs237714	20	47922900	T	C	0.2041	0.8231	0.0508	4.04E-59	262.35
CD4 NC	*ZFP57*	rs416568	6	29647628	A	T	0.2687	0.5601	0.0477	7.07E-32	137.78
CD4 NC	*ZFP57*	rs2394186	6	29816421	G	A	0.1592	0.4030	0.0593	1.12E-11	46.02
CD4 NC	*ZFP90*	rs1170439	16	68608511	C	T	0.7759	0.3639	0.0547	2.94E-11	44.13
CD4 NC	*ZMAT3*	rs79440597	3	178727181	T	C	0.0619	-0.5448	0.0927	4.18E-09	34.47
CD4 NC	*ZNF100*	rs35717293	19	21912755	G	A	0.5539	-0.6251	0.0435	7.37E-47	206.23
CD4 NC	*ZNF107*	rs6970764	7	64103954	G	C	0.3895	0.2552	0.0456	2.20E-08	31.25
CD4 NC	*ZNF138*	rs7804455	7	64304481	A	C	0.9059	0.4320	0.0785	3.74E-08	30.22
CD4 NC	*ZNF154*	rs9676736	19	58234915	C	T	0.1603	0.3834	0.0617	5.05E-10	38.58
CD4 NC	*ZNF160*	rs4801950	19	53582131	T	G	0.8865	-0.5839	0.0673	4.20E-18	75.07

CD4 NC	*ZNF208*	rs12327638	19	21753035	G	C	0.3425	-0.2743	0.0480	1.12E-08	32.55
CD4 NC	*ZNF257*	rs62110984	19	22252332	A	G	0.1515	0.6334	0.0613	4.60E-25	106.72
CD4 NC	*ZNF264*	rs4801440	19	57706378	C	G	0.0530	-0.7793	0.0988	3.13E-15	62.06
CD4 NC	*ZNF266*	rs8108483	19	9545030	T	C	0.5729	0.4626	0.0446	3.32E-25	107.36
CD4 NC	*ZNF30*	rs28693537	19	35436707	C	T	0.1875	0.5073	0.0592	1.09E-17	73.19
CD4 NC	*ZNF302*	rs151181991	19	35150352	G	A	0.1236	0.8107	0.0654	2.74E-35	153.35
CD4 NC	*ZNF302*	rs73040297	19	35127398	G	A	0.1791	-0.4032	0.0573	2.00E-12	49.38
CD4 NC	*ZNF324*	rs4801601	19	59008566	A	G	0.7644	0.3493	0.0534	6.03E-11	42.72
CD4 NC	*ZNF396*	rs4291985	18	32957283	T	C	0.5153	-0.2657	0.0448	3.00E-09	35.12
CD4 NC	*ZNF404*	rs4802201	19	44358042	T	C	0.4876	-0.2543	0.0448	1.41E-08	32.11
CD4 NC	*ZNF419*	rs2074206	19	58116143	G	T	0.8038	-0.3157	0.0562	1.90E-08	31.53
CD4 NC	*ZNF429*	rs1113577	19	21749035	C	T	0.6045	-0.4278	0.0448	1.36E-21	90.92
CD4 NC	*ZNF480*	rs13343641	19	52825032	T	C	0.1952	-0.5259	0.0546	5.94E-22	92.56
CD4 NC	*ZNF502*	rs56084453	3	44762830	G	A	0.2115	0.6699	0.0527	5.19E-37	161.22
CD4 NC	*ZNF503-AS1*	rs4746260	10	76842244	G	T	0.0603	0.7141	0.0997	8.04E-13	51.17
CD4 NC	*ZNF506*	rs7255725	19	19936913	G	C	0.4640	0.3980	0.0443	2.84E-19	80.38
CD4 NC	*ZNF516*	rs117983097	18	74072636	A	G	0.4931	0.4322	0.0441	1.24E-22	95.66
CD4 NC	*ZNF544*	rs260503	19	58790095	C	T	0.6680	-0.3494	0.0472	1.33E-13	54.7
CD4 NC	*ZNF552*	rs2802	19	58315273	A	G	0.3320	0.5471	0.0473	5.76E-31	133.62
CD4 NC	*ZNF557*	rs8101341	19	7080316	G	T	0.3893	0.3539	0.0457	9.11E-15	59.96
CD4 NC	*ZNF559*	rs28384491	19	9434872	T	C	0.0862	-0.6337	0.0736	7.00E-18	74.06
CD4 NC	*ZNF566*	rs8110282	19	36938398	T	C	0.3021	-0.2853	0.0480	2.72E-09	35.3
CD4 NC	*ZNF568*	rs496730	19	37451439	T	C	0.5217	0.3498	0.0448	6.09E-15	60.75
CD4 NC	*ZNF582-AS1*	rs55874363	19	56907158	T	C	0.5011	-0.3433	0.0445	1.25E-14	59.33
CD4 NC	*ZNF584*	rs34023391	19	58913368	A	G	0.2018	-0.6753	0.0534	1.18E-36	159.58
CD4 NC	*ZNF586*	rs58754895	19	58361549	G	A	0.3780	-0.3095	0.0468	3.70E-11	43.68
CD4 NC	*ZNF587*	rs28374851	19	58361328	C	G	0.3054	0.3591	0.0497	5.10E-13	52.06
CD4 NC	*ZNF593*	rs8768	1	26497340	C	A	0.7703	-0.6000	0.0515	2.29E-31	135.45
CD4 NC	*ZNF595*	rs6839041	4	72048	G	T	0.2721	0.3577	0.0506	1.54E-12	49.9
CD4 NC	*ZNF600*	rs4802995	19	53284720	G	C	0.4576	0.5476	0.0434	1.89E-36	158.66
CD4 NC	*ZNF638*	rs7590738	2	71525698	A	G	0.5572	-0.4855	0.0441	3.61E-28	120.87
CD4 NC	*ZNF652*	rs17637241	17	47447375	C	T	0.1133	0.4893	0.0740	3.70E-11	43.68
CD4 NC	*ZNF667-AS1*	rs145011	19	57065189	C	T	0.2519	-0.3600	0.0507	1.19E-12	50.4
CD4 NC	*ZNF677*	rs61576844	19	53761591	G	T	0.2247	-0.7657	0.0512	1.75E-50	222.81
CD4 NC	*ZNF680*	rs67005038	7	64099391	A	G	0.2316	-0.3127	0.0528	3.19E-09	34.99
CD4 NC	*ZNF684*	rs55818767	1	40986535	A	G	0.1729	-0.3267	0.0577	1.46E-08	32.04
CD4 NC	*ZNF708*	rs62107531	19	21509065	T	C	0.2063	0.3253	0.0538	1.45E-09	36.53
CD4 NC	*ZNF717*	rs11706909	3	75887744	G	C	0.4048	-0.2623	0.0452	6.32E-09	33.66
CD4 NC	*ZNF718*	rs6841277	4	190053	G	A	0.4057	-0.2962	0.0457	9.52E-11	41.83
CD4 NC	*ZNF737*	rs1987880	19	21518961	C	G	0.3345	0.3115	0.0486	1.40E-10	41.07
CD4 NC	*ZNF749*	rs11665675	19	57939619	T	C	0.5072	0.6513	0.0427	1.46E-52	232.32
CD4 NC	*ZNF772*	rs8113039	19	57999182	G	A	0.2809	-0.3260	0.0490	2.90E-11	44.15
CD4 NC	*ZNF781*	rs2058852	19	38164602	G	C	0.4272	-0.3393	0.0448	3.46E-14	57.34
CD4 NC	*ZNF783*	rs56007083	7	149004018	G	A	0.1006	0.4960	0.0797	4.79E-10	38.68
CD4 NC	*ZNF786*	rs12530946	7	148887942	G	A	0.6026	0.3970	0.0451	1.36E-18	77.3
CD4 NC	*ZNF793*	rs11083426	19	38017173	G	C	0.1903	0.3667	0.0573	1.60E-10	40.82
CD4 NC	*ZNF83*	rs7256557	19	53119166	A	G	0.3634	0.7107	0.0441	1.53E-58	259.7
CD4 NC	*ZNF846*	rs10415549	19	9853957	C	T	0.2073	0.3111	0.0567	4.09E-08	30.05
CD4 NC	*ZNF85*	rs149764210	19	21139850	A	G	0.1238	0.9430	0.0628	5.76E-51	225.02
CD4 NC	*ZNF879*	rs7733067	5	178425714	C	T	0.7598	-0.3276	0.0553	3.16E-09	35.01
CD4 NC	*ZNF880*	rs4802926	19	52880265	T	C	0.3936	0.3361	0.0461	3.14E-13	53.01
CD4 NC	*ZNFX1*	rs1567865	20	47881967	T	C	0.1948	-0.3334	0.0536	4.87E-10	38.65
CD4 NC	*ZNHIT1*	rs1056678	7	100860636	C	T	0.2342	0.4289	0.0536	1.25E-15	63.86
CD4 NC	*ZNHIT6*	rs11161664	1	86185379	T	C	0.4257	0.3166	0.0451	2.14E-12	49.25
CD4 NC	*ZNRD1*	rs56052612	6	29945802	C	G	0.0672	0.7052	0.0965	2.67E-13	53.33
CD4 NC	*ZP3*	rs11772694	7	76195020	A	G	0.3120	0.8941	0.0438	1.68E-92	415.3
CD4 NC	*ZSCAN18*	rs157376	19	58663457	A	T	0.8398	0.4109	0.0653	3.15E-10	39.5
CD4 NC	*ZSCAN23*	rs9257190	6	28764974	G	A	0.1358	-0.3675	0.0605	1.27E-09	36.78
CD4 NC	*ZSCAN23*	rs12663002	6	28441634	T	C	0.1349	-0.8020	0.0653	1.14E-34	150.52
CD4 NC	*ZSWIM7*	rs6416868	17	15924370	G	A	0.5948	-0.3897	0.0448	3.35E-18	75.52
CD4 NC	*ZYX*	rs10266621	7	143086832	C	T	0.4532	0.2579	0.0454	1.38E-08	32.15
CD4 SOX4	*AC013264.2*	rs2564389	2	198260098	T	A	0.6893	0.3454	0.0504	7.41E-12	46.81
CD4 SOX4	*AGPAT1*	rs2524079	6	31242174	A	G	0.4519	0.4130	0.0479	7.05E-18	74.03
CD4 SOX4	*AKAP7*	rs6939185	6	132139187	G	A	0.6168	0.3094	0.0488	2.31E-10	40.09
CD4 SOX4	*ATG5*	rs9399944	6	106234161	C	T	0.1179	-0.5193	0.0722	6.55E-13	51.55
CD4 SOX4	*B3GALT4*	rs3891175	6	32634467	T	C	0.1389	0.3996	0.0562	1.18E-12	50.39
CD4 SOX4	*B3GALT4*	rs241408	6	32862711	G	A	0.5512	0.4120	0.0473	3.23E-18	75.57
CD4 SOX4	*BTN2A1*	rs13196692	6	27379119	T	C	0.0746	-0.4786	0.0820	5.22E-09	34.02
CD4 SOX4	*C6orf136*	rs2246856	6	29732775	G	C	0.0451	-0.7573	0.1049	5.11E-13	52.04
CD4 SOX4	*C6orf136*	rs416568	6	29647628	A	T	0.2687	0.2916	0.0524	2.57E-08	30.94
CD4 SOX4	*C6orf226*	rs4711722	6	42505551	G	A	0.5899	0.3048	0.0484	2.94E-10	39.62
CD4 SOX4	*CENPK*	rs155061	5	64851146	G	A	0.6436	-0.3899	0.0502	7.84E-15	60.23
CD4 SOX4	*DAXX*	rs7383144	6	32312353	T	C	0.7837	0.3196	0.0559	1.09E-08	32.61
CD4 SOX4	*DDAH2*	rs2844520	6	31407758	T	G	0.0589	0.6734	0.0924	3.15E-13	52.99
CD4 SOX4	*DHX16*	rs2523966	6	29939240	A	G	0.6029	0.4425	0.0477	1.85E-20	85.74
CD4 SOX4	*DHX16*	rs9391682	6	30357291	T	G	0.3450	0.2995	0.0519	7.79E-09	33.25
CD4 SOX4	*DXO*	rs4713589	6	32659435	A	G	0.6123	-0.3894	0.0484	9.10E-16	64.47
CD4 SOX4	*EIF5A*	rs7503161	17	7207964	C	A	0.5615	-0.5407	0.0473	2.98E-30	130.33
CD4 SOX4	*ERAP2*	rs27436	5	96330966	C	T	0.5879	-0.2859	0.0484	3.40E-09	34.86

CD4 SOX4	*FAM118A*	rs738171	22	45721284	C	T	0.8812	-0.8134	0.0759	8.57E-27	114.56
CD4 SOX4	*GNL1*	rs2524123	6	31265314	C	T	0.3618	0.2969	0.0511	6.05E-09	33.74
CD4 SOX4	*HIST1H1D*	rs7752550	6	25331455	G	T	0.1428	0.4957	0.0657	4.38E-14	56.86
CD4 SOX4	*HLA-B*	rs2524096	6	31236467	T	G	0.4422	0.4868	0.0474	1.06E-24	105.03
CD4 SOX4	*HLA-B*	rs2267637	6	30578335	T	C	0.1189	-0.4602	0.0803	1.02E-08	32.73
CD4 SOX4	*HLA-DMA*	rs3129877	6	32408597	A	G	0.2743	0.4031	0.0524	1.48E-14	58.99
CD4 SOX4	*HLA-DQB1*	rs17840121	6	32577693	C	G	0.1225	-0.4261	0.0664	1.35E-10	41.15
CD4 SOX4	*HLA-DRB5*	rs9270664	6	32566149	A	G	0.6060	-0.2806	0.0496	1.58E-08	31.88
CD4 SOX4	*IER3*	rs2844786	6	30144420	A	C	0.2822	0.4258	0.0516	1.49E-16	68.02
CD4 SOX4	*IER3*	rs9266702	6	31349115	C	T	0.7677	-0.4819	0.0534	1.91E-19	81.14
CD4 SOX4	*IFNGR1*	rs6570167	6	137793391	C	G	0.1554	0.7506	0.0620	9.32E-34	146.32
CD4 SOX4	*IL32*	rs11861531	16	3116325	C	T	0.5537	0.3387	0.0477	1.30E-12	50.21
CD4 SOX4	*KANSL1-AS1*	rs576904891	17	44252468	A	G	0.1759	0.4289	0.0608	1.80E-12	49.58
CD4 SOX4	*LTA*	rs12110481	6	30998312	T	G	0.3725	0.4193	0.0493	1.72E-17	72.27
CD4 SOX4	*LTA*	rs3130977	6	31081989	C	T	0.3318	-0.5341	0.0488	7.63E-28	119.35
CD4 SOX4	*LTA*	rs2922996	6	31337207	C	G	0.4229	-0.3499	0.0477	2.16E-13	53.73
CD4 SOX4	*MICA*	rs12174870	6	30436681	A	G	0.3865	-0.4043	0.0488	1.14E-16	68.55
CD4 SOX4	*NEU1*	rs17213337	6	32713888	C	T	0.5240	-0.3188	0.0478	2.58E-11	44.37
CD4 SOX4	*NFKBIL1*	rs12665339	6	30601232	G	A	0.1490	0.5613	0.0661	2.01E-17	71.97
CD4 SOX4	*PBX2*	rs7775759	6	31352446	A	G	0.3345	0.3804	0.0527	5.06E-13	52.06
CD4 SOX4	*PRR3*	rs2524054	6	31252396	C	A	0.7710	0.5087	0.0523	2.12E-22	94.57
CD4 SOX4	*PSMD5-AS1*	rs12684934	9	123593159	T	C	0.5794	-0.4506	0.0479	5.39E-21	88.18
CD4 SOX4	*RNASET2*	rs239936	6	167411616	T	C	0.5518	-0.3501	0.0484	4.58E-13	52.26
CD4 SOX4	*RNGTT*	rs4519980	6	88592819	G	A	0.5886	-0.6284	0.0469	5.48E-41	179.34
CD4 SOX4	*RP1-3J17.3*	rs60618624	6	85592175	A	G	0.0637	-0.8567	0.0956	3.26E-19	80.09
CD4 SOX4	*RP11-347P5.1*	rs6741307	2	196998315	T	C	0.6378	-0.2720	0.0497	4.28E-08	29.95
CD4 SOX4	*RPL12*	rs2243898	9	130219990	C	G	0.5970	0.3663	0.0483	3.50E-14	57.3
CD4 SOX4	*RPL28*	rs17700376	19	55897327	G	A	0.3547	-0.5999	0.0485	3.35E-35	152.91
CD4 SOX4	*RPL36AL*	rs1952013	14	50087357	G	C	0.7531	0.3614	0.0561	1.17E-10	41.42
CD4 SOX4	*RPL4*	rs4776733	15	66408407	A	G	0.2750	0.3123	0.0555	1.87E-08	31.55
CD4 SOX4	*RPL8*	rs2954658	8	146017782	T	C	0.4990	-0.4401	0.0472	1.17E-20	86.65
CD4 SOX4	*RPP21*	rs9261893	6	30388936	C	T	0.1957	-0.4376	0.0614	1.04E-12	50.65
CD4 SOX4	*RPP21*	rs9468874	6	31102241	A	G	0.2652	-0.3493	0.0558	3.91E-10	39.07
CD4 SOX4	*RPP21*	rs28380862	6	31311795	A	C	0.1598	-0.4524	0.0613	1.52E-13	54.42
CD4 SOX4	*RPS10*	rs9380433	6	34377626	T	C	0.7750	-0.5326	0.0614	4.13E-18	75.08
CD4 SOX4	*RPS18*	rs17215231	6	33239869	T	C	0.0640	-1.0344	0.0932	1.34E-28	122.78
CD4 SOX4	*RPS2*	rs72764891	16	2013588	C	T	0.1386	0.4184	0.0670	4.17E-10	38.94
CD4 SOX4	*RPS26*	rs10876864	12	56401085	A	G	0.5810	-1.1962	0.0376	1.00E-200	1010.8
CD4 SOX4	*RPS3A*	rs61442068	4	152104206	T	C	0.0618	-0.7627	0.0979	6.56E-15	60.59
CD4 SOX4	*RPS5*	rs4584938	19	58899303	G	C	0.3318	-0.3059	0.0508	1.70E-09	36.2
CD4 SOX4	*RPS8*	rs11589147	1	45319121	T	A	0.1178	-0.3782	0.0685	3.37E-08	30.41
CD4 SOX4	*RPS9*	rs34629529	19	54701846	A	G	0.3950	-0.3411	0.0487	2.45E-12	48.97
CD4 SOX4	*RXRB*	rs1548306	6	32427179	T	A	0.6721	-0.4277	0.0502	1.58E-17	72.44
CD4 SOX4	*SGK1*	rs7755228	6	135017476	G	T	0.4702	-0.4762	0.0475	1.14E-23	100.34
CD4 SOX4	*SH3YL1*	rs55936726	2	258210	C	T	0.3311	-0.3149	0.0500	2.89E-10	39.66
CD4 SOX4	*SKIV2L*	rs9271348	6	32583742	A	G	0.7413	0.3827	0.0522	2.33E-13	53.58
CD4 SOX4	*SKIV2L*	rs17196137	6	30933397	A	C	0.2492	0.2893	0.0530	4.78E-08	29.73
CD4 SOX4	*SMDT1*	rs5996108	22	42490967	T	C	0.3267	-0.5797	0.0500	4.48E-31	134.08
CD4 SOX4	*SMIM8*	rs6918408	6	89028389	C	T	0.6316	-0.3199	0.0499	1.43E-10	41.03
CD4 SOX4	*SNHG8*	rs788666	4	119219168	T	C	0.3793	0.4973	0.0477	2.10E-25	108.23
CD4 SOX4	*STK19*	rs2905708	6	31364603	T	C	0.0841	0.5725	0.0803	9.98E-13	50.73
CD4 SOX4	*STK19*	rs17885557	6	31237087	A	C	0.1347	0.4783	0.0718	2.68E-11	44.29
CD4 SOX4	*SUPT3H*	rs12196818	6	44911414	A	G	0.4328	-0.4146	0.0477	3.46E-18	75.43
CD4 SOX4	*SYNCRIP*	rs12211894	6	85333759	G	A	0.4664	-0.3482	0.0477	2.83E-13	53.2
CD4 SOX4	*SYNGAP1*	rs2187668	6	32605884	T	C	0.1371	-0.4984	0.0703	1.35E-12	50.14
CD4 SOX4	*SYTL3*	rs2451979	6	159559273	T	C	0.5908	-0.5286	0.0479	2.83E-28	121.31
CD4 SOX4	*TMEM181*	rs1561680	6	158936352	T	A	0.4145	0.5122	0.0471	1.39E-27	118.16
CD4 SOX4	*TNF*	rs685031	6	31881731	A	G	0.6908	-0.3693	0.0493	6.84E-14	55.98
CD4 SOX4	*TOMM7*	rs2240728	7	22852499	T	C	0.3422	-0.4722	0.0509	1.61E-20	86.02
CD4 SOX4	*TRIM26*	rs111696429	6	29908309	A	G	0.0423	-0.6087	0.1067	1.16E-08	32.47
CD4 SOX4	*TRIM39*	rs77580993	6	30059592	G	C	0.2142	0.5154	0.0587	1.74E-18	76.79
CD4 SOX4	*TRIM39*	rs62400982	6	30805446	A	G	0.0431	-0.5846	0.1026	1.22E-08	32.37
CD4 SOX4	*TULP4*	rs117760808	6	158775018	G	C	0.0895	-0.6240	0.0861	4.32E-13	52.37
CD4 SOX4	*UQCC2*	rs1776880	6	34155988	T	C	0.4252	0.3407	0.0480	1.31E-12	50.19
CD4 SOX4	*XXbac-BPG299F13.17*	rs3130140	6	30357780	C	T	0.4626	0.2739	0.0487	1.85E-08	31.57
CD8 ET	*ABRACL*	rs62441852	6	139356848	C	G	0.4110	-0.2853	0.0460	5.71E-10	38.34
CD8 ET	*AC005253.2*	rs2058077	19	18671055	T	C	0.5894	-0.4478	0.0449	2.10E-23	99.16
CD8 ET	*AC006369.2*	rs917732	2	37831553	G	A	0.5902	-0.9587	0.0395	4.14E-130	587.8
CD8 ET	*AC007383.4*	rs6435330	2	207010558	T	G	0.4597	-0.4692	0.0443	3.58E-26	111.77
CD8 ET	*AC010642.1*	rs3206946	19	58826534	G	A	0.2046	-0.4155	0.0560	1.18E-13	54.93
CD8 ET	*AC013264.2*	rs1366836	2	198172148	G	T	0.3781	0.5334	0.0449	1.61E-32	140.72
CD8 ET	*AC016757.3*	rs11694250	2	239138259	C	T	0.3233	0.3833	0.0468	2.71E-16	66.87
CD8 ET	*AC018816.3*	rs3805025	3	4869590	G	A	0.2043	-0.3317	0.0566	4.60E-09	34.28
CD8 ET	*AC020571.3*	rs17231419	2	197127249	T	C	0.2482	-0.2954	0.0520	1.39E-08	32.14
CD8 ET	*AC021860.1*	rs9998440	4	38607801	C	A	0.1999	0.4031	0.0538	6.57E-14	56.08
CD8 ET	*AC022498.1*	rs13082889	3	187898523	C	T	0.1118	0.4744	0.0722	5.03E-11	43.08
CD8 ET	*AC023590.1*	rs2514610	8	119659371	A	G	0.9210	-0.6021	0.0771	5.53E-15	60.94
CD8 ET	*AC090186.1*	rs13267846	8	52786158	A	G	0.8614	0.5154	0.0767	1.77E-11	45.12
CD8 ET	*AC091729.9*	rs2960835	7	1202963	G	A	0.6139	-0.2559	0.0468	4.53E-08	29.85

CD8 ET	*AC104653.1*	rs4848353	2	114580423	G	A	0.5510	0.4471	0.0445	9.01E-24	100.83
CD8 ET	*AC131056.3*	rs8064426	17	34819750	A	G	0.1685	-0.4844	0.0652	1.10E-13	55.06
CD8 ET	*ACAT1*	rs10749914	11	108012077	A	G	0.5973	-0.2971	0.0453	5.45E-11	42.92
CD8 ET	*ACCS*	rs2074038	11	44087989	T	G	0.1126	0.7204	0.0732	7.65E-23	96.61
CD8 ET	*ACOT4*	rs3742819	14	74058832	T	C	0.3857	0.2890	0.0465	4.92E-10	38.63
CD8 ET	*ACP5*	rs35541942	19	11702322	T	A	0.3536	0.3111	0.0477	6.65E-11	42.53
CD8 ET	*ACTA2*	rs1324551	10	90751516	T	C	0.5074	-0.4928	0.0438	2.06E-29	126.53
CD8 ET	*ACYP2*	rs10183655	2	54210781	T	C	0.4133	-0.6215	0.0437	6.84E-46	201.81
CD8 ET	*ADAM10*	rs7161799	15	58770523	T	C	0.0869	0.6409	0.0792	5.90E-16	65.34
CD8 ET	*ADAMTS1*	rs402007	21	28217320	G	C	0.7618	-0.3710	0.0540	6.32E-12	47.13
CD8 ET	*ADCY7*	rs4785412	16	50385468	C	T	0.1672	0.4564	0.0623	2.34E-13	53.59
CD8 ET	*ADCY9*	rs2532004	16	4161793	C	T	0.6575	-0.4007	0.0482	9.12E-17	69.01
CD8 ET	*ADRB1*	rs68122733	10	115831533	G	A	0.1723	0.4836	0.0600	8.04E-16	64.73
CD8 ET	*AGA*	rs11131799	4	178363378	A	G	0.6460	0.3173	0.0464	8.35E-12	46.59
CD8 ET	*AGTRAP*	rs17875970	1	11811886	T	C	0.1465	-0.5152	0.0627	2.07E-16	67.4
CD8 ET	*AHI1*	rs13197384	6	135818897	A	C	0.3411	-0.6424	0.0455	2.84E-45	198.98
CD8 ET	*AIF1*	rs2844475	6	31583155	A	G	0.3901	0.3559	0.0460	1.02E-14	59.73
CD8 ET	*AK5*	rs4949655	1	77897688	C	T	0.1594	0.6812	0.0574	1.59E-32	140.73
CD8 ET	*AKAP13*	rs11630755	15	86108737	G	A	0.3517	-0.3366	0.0475	1.36E-12	50.14
CD8 ET	*AKIP1*	rs10840160	11	9053536	C	T	0.4004	0.2580	0.0450	9.60E-09	32.85
CD8 ET	*AKIRIN1*	rs12043492	1	39457006	T	C	0.4366	-0.2521	0.0452	2.52E-08	30.98
CD8 ET	*AKR1C3*	rs10904452	10	5347916	G	A	0.2423	-0.3502	0.0533	4.83E-11	43.16
CD8 ET	*AL589743.1*	rs28593759	14	20680230	C	G	0.2608	0.7179	0.0491	2.08E-48	213.32
CD8 ET	*ALG1L*	rs11710518	3	125783844	T	G	0.3435	-0.3895	0.0462	3.35E-17	70.98
CD8 ET	*AMICA1*	rs12286604	11	118084728	A	G	0.1949	0.3959	0.0579	7.98E-12	46.68
CD8 ET	*ANAPC13*	rs9857242	3	134316378	T	C	0.6497	0.4860	0.0462	6.69E-26	110.53
CD8 ET	*ANK3*	rs7071548	10	62490361	G	A	0.3109	-0.4373	0.0472	2.00E-20	85.62
CD8 ET	*ANKDD1A*	rs1628955	15	65187220	C	T	0.6303	-0.5474	0.0453	1.15E-33	145.95
CD8 ET	*ANXA11*	rs56122289	10	81746027	A	G	0.0801	0.6713	0.0783	1.01E-17	73.33
CD8 ET	*ANXA2R*	rs35137321	5	42987922	A	G	0.1642	0.3254	0.0587	3.03E-08	30.63
CD8 ET	*ANXA5*	rs6534309	4	122609929	C	T	0.0762	0.5206	0.0774	1.71E-11	45.19
CD8 ET	*ANXA6*	rs2303038	5	150535013	T	C	0.2046	0.4575	0.0542	3.27E-17	71.03
CD8 ET	*AOAH*	rs2228410	7	36763672	T	C	0.3411	-0.5441	0.0460	3.15E-32	139.38
CD8 ET	*AOAH*	rs7806624	7	36824655	T	C	0.2085	0.4844	0.0537	1.86E-19	81.22
CD8 ET	*AP000783.1*	rs56383769	11	123329867	A	T	0.1982	0.3053	0.0536	1.24E-08	32.36
CD8 ET	*AP001055.6*	rs62229740	21	45445374	A	G	0.0897	-0.4518	0.0755	2.17E-09	35.74
CD8 ET	*AP003774.1*	rs479777	11	64107477	C	T	0.3253	0.7306	0.0446	2.19E-60	268.15
CD8 ET	*AP2A2*	rs7395773	11	970503	T	C	0.5164	0.2978	0.0447	2.85E-11	44.19
CD8 ET	*APMAP*	rs56183883	20	24897942	A	G	0.3346	-0.3851	0.0459	4.89E-17	70.24
CD8 ET	*APOBEC3G*	rs17537581	22	39482809	A	G	0.1077	-0.6088	0.0749	4.51E-16	65.86
CD8 ET	*APOBEC3H*	rs60093835	22	39493356	T	C	0.1280	-0.6335	0.0700	1.40E-19	81.77
CD8 ET	*ARHGAP11A*	rs34954129	15	32940190	T	A	0.2689	-0.2981	0.0518	8.55E-09	33.08
CD8 ET	*ARHGAP15*	rs13024411	2	144012024	G	A	0.1512	-0.4318	0.0642	1.80E-11	45.08
CD8 ET	*ARHGEF26*	rs13085039	3	153930006	C	A	0.1015	0.4347	0.0767	1.44E-08	32.06
CD8 ET	*ARHGEF3*	rs6978	3	56761715	A	G	0.1613	0.6351	0.0612	3.11E-25	107.49
CD8 ET	*ARL14EP*	rs7927974	11	30351223	G	A	0.3057	-0.3226	0.0497	8.23E-11	42.12
CD8 ET	*ARL16*	rs61065803	17	79638049	C	A	0.1648	-0.7911	0.0577	9.68E-43	187.4
CD8 ET	*ARL17A*	rs557307834	17	44121579	C	T	0.2232	0.5052	0.0506	1.74E-23	99.53
CD8 ET	*ARL17B*	rs41543512	17	44090778	A	T	0.2276	-0.3068	0.0558	3.86E-08	30.16
CD8 ET	*ARPC3*	rs10082809	12	110515411	T	C	0.2269	-0.2883	0.0514	2.05E-08	31.39
CD8 ET	*ARPC4*	rs6780456	3	9865239	A	G	0.6550	0.2904	0.0469	5.74E-10	38.33
CD8 ET	*ARPC5*	rs75435975	1	183583993	G	A	0.3698	0.3263	0.0463	1.75E-12	49.64
CD8 ET	*ARSA*	rs2071421	22	51064416	C	T	0.1275	-0.6785	0.0664	1.57E-24	104.3
CD8 ET	*ASAH1*	rs34288580	8	17931603	G	T	0.0879	-0.4671	0.0836	2.29E-08	31.16
CD8 ET	*ASCL2*	rs2651838	11	2319531	T	A	0.5700	0.4210	0.0445	3.17E-21	89.25
CD8 ET	*ASNSD1*	rs1132360	2	190526224	T	C	0.7251	0.4068	0.0511	1.70E-15	63.25
CD8 ET	*ATG10*	rs75458562	5	81503241	G	C	0.0828	-0.4719	0.0753	3.62E-10	39.23
CD8 ET	*ATHL1*	rs3932433	11	289553	C	G	0.3751	0.5469	0.0445	8.95E-35	151.01
CD8 ET	*ATP13A4*	rs4686670	3	193798401	C	T	0.3875	-0.2754	0.0458	1.79E-09	36.11
CD8 ET	*ATP5G1*	rs318095	17	46974734	C	T	0.5271	-0.3732	0.0445	4.76E-17	70.29
CD8 ET	*ATP5G3*	rs268172	2	176071906	G	A	0.8166	-0.5188	0.0575	1.78E-19	81.3
CD8 ET	*ATP6V0E2*	rs6949457	7	149584953	A	T	0.4485	0.4102	0.0448	5.23E-20	83.72
CD8 ET	*ATP8A1*	rs62303561	4	42632114	G	C	0.2909	0.2909	0.0475	9.15E-10	37.42
CD8 ET	*ATP9A*	rs6512745	20	50382325	G	C	0.4485	-0.3542	0.0449	3.03E-15	62.12
CD8 ET	*B3GAT1*	rs77954628	11	134262154	G	A	0.1852	-0.3638	0.0556	5.95E-11	42.75
CD8 ET	*B3GAT2*	rs651984	6	71599887	G	A	0.0632	-0.6170	0.0990	4.64E-10	38.74
CD8 ET	*B4GALNT3*	rs7314691	12	661981	A	G	0.2600	0.5749	0.0496	5.02E-31	133.89
CD8 ET	*B4GALT1*	rs6476398	9	33119241	T	C	0.3350	0.4744	0.0467	3.09E-24	102.95
CD8 ET	*B4GALT4*	rs13065867	3	118959210	T	C	0.2010	0.3574	0.0561	1.84E-10	40.55
CD8 ET	*BCAS4*	rs6122981	20	49495326	A	G	0.3389	-0.3145	0.0473	2.90E-11	44.15
CD8 ET	*BCR*	rs10775764	22	23503569	C	T	0.4955	0.3060	0.0447	7.20E-12	46.88
CD8 ET	*BIN1*	rs10194375	2	127839781	A	C	0.3384	0.7520	0.0446	8.79E-64	283.7
CD8 ET	*BOK*	rs34031668	2	242533379	C	T	0.2921	-0.3442	0.0496	3.89E-12	48.08
CD8 ET	*BRD7*	rs9935454	16	50377831	A	C	0.1671	0.4291	0.0624	5.98E-12	47.24
CD8 ET	*BRK1*	rs112937420	3	10001851	A	C	0.0893	-0.5069	0.0747	1.15E-11	45.96
CD8 ET	*BTBD9*	rs9470811	6	38148584	G	A	0.7023	-0.3015	0.0516	4.94E-09	34.14
CD8 ET	*BTN2A1*	rs3734544	6	26468660	A	G	0.1771	0.3633	0.0598	1.21E-09	36.88
CD8 ET	*BTN3A1*	rs56190518	6	26413975	T	C	0.1725	0.4038	0.0638	2.45E-10	39.99
CD8 ET	*BTN3A2*	rs67509210	6	26354100	T	G	0.1018	-0.9693	0.0638	4.06E-52	230.3

CD8 ET	*C10orf128*	rs2377991	10	50425252	G	A	0.7342	-0.6141	0.0485	8.65E-37	160.21
CD8 ET	*C10orf32*	rs11191421	10	104623053	G	C	0.2357	-0.4836	0.0524	2.61E-20	85.09
CD8 ET	*C10orf54*	rs748113	10	73508791	C	T	0.4424	0.2921	0.0451	9.03E-11	41.94
CD8 ET	*C11orf21*	rs2074022	11	2324041	G	A	0.9112	-0.7712	0.0872	9.02E-19	78.1
CD8 ET	*C11orf24*	rs2450908	11	68022006	T	C	0.4271	-0.4495	0.0443	3.68E-24	102.6
CD8 ET	*C11orf73*	rs10898447	11	85935753	T	G	0.3262	0.2668	0.0486	4.09E-08	30.05
CD8 ET	*C14orf2*	rs11850704	14	104381687	G	A	0.0909	-0.6347	0.0834	2.82E-14	57.74
CD8 ET	*C15orf40*	rs11259963	15	83641353	C	G	0.5436	0.2680	0.0448	2.21E-09	35.71
CD8 ET	*C16orf13*	rs117667029	16	686337	A	G	0.1175	0.6720	0.0669	1.01E-23	100.62
CD8 ET	*C17orf50*	rs8080959	17	34140454	A	G	0.1918	-0.7937	0.0575	2.29E-43	190.26
CD8 ET	*C17orf89*	rs11655759	17	79205736	A	G	0.3681	-0.4457	0.0447	1.98E-23	99.28
CD8 ET	*C17orf97*	rs7503725	17	260142	T	G	0.2626	0.5585	0.0511	8.71E-28	119.12
CD8 ET	*C19orf24*	rs12979308	19	1275987	G	C	0.7769	0.4430	0.0510	4.02E-18	75.16
CD8 ET	*C19orf83*	rs2286756	19	46145025	T	G	0.5303	-0.5733	0.0433	4.77E-40	175.09
CD8 ET	*C1orf63*	rs609320	1	25717365	G	C	0.1505	0.4171	0.0602	4.24E-12	47.91
CD8 ET	*C1orf86*	rs11587831	1	2110848	G	T	0.2239	-0.4439	0.0541	2.33E-16	67.16
CD8 ET	*C2*	rs12524667	6	32389334	T	C	0.0975	-0.4758	0.0726	5.70E-11	42.83
CD8 ET	*C20orf27*	rs45495794	20	3734167	C	T	0.0479	-0.5990	0.0979	9.63E-10	37.32
CD8 ET	*C21orf119*	rs79309054	21	33772536	A	G	0.0887	0.4780	0.0800	2.33E-09	35.61
CD8 ET	*C22orf34*	rs9616333	22	50050847	A	G	0.5837	-0.4543	0.0455	1.95E-23	99.31
CD8 ET	*C2orf27A*	rs6720375	2	132440073	C	G	0.5865	0.5508	0.0439	4.55E-36	156.92
CD8 ET	*C2orf74*	rs1729673	2	61389799	A	G	0.4225	-0.3103	0.0462	1.78E-11	45.11
CD8 ET	*C5orf17*	rs7701333	5	23725743	T	C	0.7567	0.7630	0.0485	7.14E-56	247.48
CD8 ET	*C5orf56*	rs35377719	5	131789456	A	G	0.1204	-0.3884	0.0685	1.43E-08	32.08
CD8 ET	*C6orf25*	rs4947288	6	30696762	G	A	0.2248	0.4033	0.0510	2.71E-15	62.34
CD8 ET	*C6orf25*	rs9271344	6	32583328	C	T	0.3832	0.2959	0.0468	2.51E-10	39.94
CD8 ET	*C6orf47-AS1*	rs2857600	6	31582287	C	T	0.8821	-0.6277	0.0635	4.77E-23	97.54
CD8 ET	*C6orf47-AS1*	rs9267123	6	31427395	C	G	0.1136	-0.5015	0.0624	8.87E-16	64.54
CD8 ET	*C6orf62*	rs3756814	6	24705835	C	T	0.3832	-0.2526	0.0460	3.97E-08	30.1
CD8 ET	*C9orf156*	rs7864655	9	100679836	G	T	0.2566	0.5157	0.0499	4.41E-25	106.8
CD8 ET	*C9orf78*	rs13295391	9	132555302	A	G	0.2335	-0.5506	0.0515	1.06E-26	114.17
CD8 ET	*CALHM2*	rs114011803	10	105265089	G	C	0.0401	-0.5919	0.1021	6.79E-09	33.53
CD8 ET	*CAMLG*	rs12654944	5	134036784	A	G	0.1122	0.5009	0.0705	1.18E-12	50.41
CD8 ET	*CAPG*	rs11696093	2	85636195	C	T	0.3028	0.4460	0.0468	1.55E-21	90.66
CD8 ET	*CAPN12*	rs62119115	19	39210593	A	G	0.2446	-0.4234	0.0527	9.25E-16	64.45
CD8 ET	*CARD8*	rs9676777	19	48758706	A	G	0.2169	0.3828	0.0518	1.41E-13	54.58
CD8 ET	*CARHSP1*	rs9928800	16	8936910	G	A	0.2594	0.5593	0.0496	1.60E-29	127.04
CD8 ET	*CASP8*	rs7560328	2	202164837	C	A	0.6056	0.3979	0.0460	5.55E-18	74.52
CD8 ET	*CAST*	rs152005	5	96025847	G	A	0.1633	0.7596	0.0602	1.66E-36	158.91
CD8 ET	*CCDC101*	rs240707	16	28524612	G	C	0.4565	-0.3651	0.0448	3.68E-16	66.26
CD8 ET	*CCDC115*	rs12232961	2	131089343	T	G	0.1969	0.3381	0.0545	5.44E-10	38.43
CD8 ET	*CCDC122*	rs7318147	13	44445153	C	G	0.4792	-0.3621	0.0447	5.81E-16	65.37
CD8 ET	*CCDC146*	rs12704823	7	76822127	T	C	0.1494	-0.5717	0.0582	9.53E-23	96.17
CD8 ET	*CCDC152*	rs56046109	5	42744296	T	A	0.2832	0.2886	0.0495	5.70E-09	33.86
CD8 ET	*CCDC163P*	rs6688516	1	45985760	A	G	0.6896	-0.4535	0.0466	2.41E-22	94.34
CD8 ET	*CCDC167*	rs1757178	6	37486310	G	A	0.6345	0.7190	0.0444	4.68E-59	262.05
CD8 ET	*CCDC25*	rs9797	8	27591849	A	G	0.4804	0.2683	0.0449	2.33E-09	35.6
CD8 ET	*CCDC50*	rs4687210	3	191046770	A	G	0.2155	-0.4364	0.0530	1.92E-16	67.54
CD8 ET	*CCDC58*	rs3828362	3	122131777	G	C	0.4138	0.6123	0.0438	1.71E-44	195.42
CD8 ET	*CCDC66*	rs282534	3	56634544	A	G	0.7746	-0.4534	0.0545	8.53E-17	69.14
CD8 ET	*CCDC82*	rs11021562	11	96108105	G	A	0.1522	0.3624	0.0617	4.20E-09	34.46
CD8 ET	*CCDC88B*	rs1199045	11	64117878	T	C	0.3602	-0.2704	0.0463	5.02E-09	34.11
CD8 ET	*CCL3*	rs1719123	17	34413390	T	A	0.2373	-0.4857	0.0519	7.89E-21	87.45
CD8 ET	*CCL3L1*	rs4796217	17	34819191	T	C	0.6647	0.3322	0.0479	4.20E-12	47.93
CD8 ET	*CCL3L3*	rs7208788	17	34823204	A	G	0.1610	0.5868	0.0573	1.19E-24	104.85
CD8 ET	*CCL3L3*	rs17138295	17	34981225	T	C	0.6366	-0.3089	0.0463	2.60E-11	44.36
CD8 ET	*CCL4L1*	rs7208788	17	34823204	A	G	0.1610	0.4857	0.0578	4.12E-17	70.58
CD8 ET	*CCL4L2*	rs11651338	17	34816489	T	C	0.5666	0.2613	0.0452	7.46E-09	33.34
CD8 ET	*CCSER2*	rs11598450	10	86126945	G	A	0.1304	-0.6777	0.0679	1.81E-23	99.45
CD8 ET	*CCT3*	rs12132919	1	156318141	A	C	0.2794	-0.4588	0.0485	3.30E-21	89.17
CD8 ET	*CCT8*	rs965951	21	30432557	A	G	0.1160	1.0990	0.0642	1.23E-65	292.18
CD8 ET	*CCZ1*	rs7807287	7	6877519	G	A	0.4455	0.6912	0.0427	4.70E-59	262.05
CD8 ET	*CCZ1*	rs6979676	7	6054987	A	G	0.2512	-0.2851	0.0515	3.16E-08	30.54
CD8 ET	*CCZ1B*	rs2711224	7	6861880	C	G	0.4738	-0.7737	0.0416	3.90E-77	344.87
CD8 ET	*CD101*	rs9332416	1	117551984	A	G	0.3557	0.4452	0.0461	4.99E-22	92.9
CD8 ET	*CD151*	rs74915614	11	857789	T	C	0.0901	0.6014	0.0783	1.59E-14	58.87
CD8 ET	*CD160*	rs3753436	1	145718113	G	A	0.4908	-0.3241	0.0449	5.56E-13	51.89
CD8 ET	*CD27-AS1*	rs1059501	12	6560573	T	G	0.5426	0.2482	0.0452	4.11E-08	30.04
CD8 ET	*CD300A*	rs72844326	17	72459091	G	T	0.0582	-1.0303	0.1007	1.42E-24	104.49
CD8 ET	*CD300A*	rs4788836	17	72465238	G	A	0.3875	-0.2803	0.0462	1.27E-09	36.78
CD8 ET	*CD37*	rs8107548	19	49870643	C	T	0.2603	-0.3562	0.0502	1.27E-12	50.28
CD8 ET	*CD3E*	rs17540708	11	118150127	G	T	0.3083	0.3184	0.0480	3.35E-11	43.87
CD8 ET	*CD48*	rs1126644	1	160648549	T	C	0.7658	-0.4056	0.0518	4.88E-15	61.18
CD8 ET	*CD55*	rs1588143	1	207519523	G	T	0.6942	-0.2994	0.0484	6.37E-10	38.13
CD8 ET	*CD8B*	rs56153774	2	87052746	G	T	0.1834	-0.4039	0.0535	4.39E-14	56.87
CD8 ET	*CDC14A*	rs12568038	1	100879914	T	C	0.3834	-0.4271	0.0450	2.24E-21	89.93
CD8 ET	*CDC42*	rs2268179	1	22414785	T	G	0.1644	0.5179	0.0601	6.56E-18	74.19
CD8 ET	*CDC42SE2*	rs78623258	5	130628103	G	A	0.1843	0.5241	0.0552	2.21E-21	89.96
CD8 ET	*CDC7*	rs61798047	1	92016934	T	C	0.4774	-0.2469	0.0448	3.67E-08	30.25

CD8 ET	*CDK10*	rs77651727	16	89708267	T	C	0.0591	-0.7076	0.0913	9.33E-15	59.91
CD8 ET	*CDK5R1*	rs118092144	17	30778945	A	G	0.4588	-0.2456	0.0449	4.57E-08	29.83
CD8 ET	*CEACAM21*	rs3745936	19	42092815	A	T	0.7738	0.6494	0.0534	4.42E-34	147.84
CD8 ET	*CEBPZ-AS1*	rs61208496	2	37464230	T	C	0.1277	-0.6207	0.0674	3.34E-20	84.6
CD8 ET	*CENPK*	rs154940	5	64902091	T	C	0.6113	-0.9965	0.0408	9.03E-132	595.42
CD8 ET	*CENPM*	rs4822064	22	42279506	C	G	0.1344	0.4517	0.0659	7.38E-12	46.83
CD8 ET	*CENPP*	rs10820982	9	95280906	T	C	0.1801	0.4293	0.0609	1.84E-12	49.55
CD8 ET	*CEP78*	rs10867175	9	80905745	G	T	0.0828	-0.8238	0.0896	3.78E-20	84.36
CD8 ET	*CERS4*	rs62126381	19	8272852	C	T	0.5011	0.2857	0.0448	1.73E-10	40.67
CD8 ET	*CES1*	rs62028648	16	55864517	T	C	0.5947	0.4707	0.0448	8.44E-26	110.07
CD8 ET	*CFD*	rs10424998	19	874647	C	G	0.2509	0.7017	0.0500	1.05E-44	196.38
CD8 ET	*CFLAR*	rs10190751	2	202006096	A	G	0.2094	-0.4746	0.0553	8.88E-18	73.6
CD8 ET	*CHCHD2*	rs68051560	7	56208694	A	G	0.1690	-0.7191	0.0546	1.24E-39	173.2
CD8 ET	*CHI3L2*	rs2252482	1	111759762	T	C	0.9009	-1.2431	0.0688	4.95E-73	326.07
CD8 ET	*CHMP4A*	rs1242029	14	24668567	C	A	0.1973	-0.3444	0.0582	3.23E-09	34.97
CD8 ET	*CHMP4B*	rs6059520	20	32470133	G	A	0.5118	-0.2602	0.0451	7.79E-09	33.26
CD8 ET	*CHPF*	rs13010015	2	220421479	G	A	0.2507	0.3101	0.0517	1.92E-09	35.98
CD8 ET	*CHPT1*	rs2559850	12	102093459	A	G	0.5861	0.5207	0.0442	4.69E-32	138.59
CD8 ET	*CIB1*	rs8025610	15	90776984	C	G	0.1658	-0.6670	0.0586	5.22E-30	129.26
CD8 ET	*CKB*	rs11625044	14	104009760	T	A	0.2259	0.3632	0.0529	6.55E-12	47.06
CD8 ET	*CKMT2-AS1*	rs62365867	5	80625353	T	C	0.0951	-0.4616	0.0787	4.39E-09	34.37
CD8 ET	*CLEC2B*	rs2908700	12	10004648	T	C	0.5465	0.4348	0.0443	9.63E-23	96.15
CD8 ET	*CLHC1*	rs75442422	2	55405037	A	T	0.1013	0.4354	0.0748	5.73E-09	33.86
CD8 ET	*CLIC3*	rs7862602	9	139840471	T	G	0.5429	0.2767	0.0448	6.59E-10	38.06
CD8 ET	*CLK4*	rs628857	5	178014668	C	G	0.4662	0.3502	0.0445	3.74E-15	61.71
CD8 ET	*CMC1*	rs7638881	3	28569680	A	C	0.4207	0.3129	0.0451	4.14E-12	47.96
CD8 ET	*CMPK1*	rs9436430	1	47783966	G	C	0.1932	0.3490	0.0577	1.43E-09	36.55
CD8 ET	*CMSS1*	rs4244712	3	99679461	A	G	0.3986	-0.2706	0.0457	3.30E-09	34.93
CD8 ET	*CMTM3*	rs37177	16	66566239	A	G	0.4757	-0.2874	0.0448	1.34E-10	41.17
CD8 ET	*CMTM7*	rs149639463	3	32504331	T	C	0.0846	-0.6050	0.0809	7.78E-14	55.75
CD8 ET	*CMTM8*	rs7632486	3	32286791	A	G	0.8213	-0.3441	0.0595	7.29E-09	33.39
CD8 ET	*CNKSR3*	rs12212325	6	154087432	G	T	0.3325	0.4117	0.0468	1.47E-18	77.14
CD8 ET	*CNN2*	rs4807455	19	1033559	A	G	0.2556	0.3441	0.0501	6.82E-12	46.98
CD8 ET	*CNOT6L*	rs4859871	4	78628215	C	T	0.8962	-0.4751	0.0688	4.90E-12	47.63
CD8 ET	*COA1*	rs2240516	7	43687296	T	C	0.5885	-0.3497	0.0448	5.66E-15	60.89
CD8 ET	*COG5*	rs2520256	7	107162515	T	C	0.2300	0.6990	0.0518	1.53E-41	181.93
CD8 ET	*COL18A1*	rs13339980	21	46902435	A	C	0.0597	1.0755	0.0927	3.88E-31	134.4
CD8 ET	*COL5A3*	rs12985798	19	10124048	G	A	0.1672	-0.4508	0.0575	4.35E-15	61.41
CD8 ET	*COL6A2*	rs9680145	21	47517598	G	C	0.8581	0.3551	0.0632	1.95E-08	31.48
CD8 ET	*COLGALT2*	rs4233115	1	183936470	A	G	0.5994	0.4942	0.0442	5.12E-29	124.73
CD8 ET	*COMMD5*	rs2958517	8	146019378	A	G	0.5102	-0.2742	0.0449	9.89E-10	37.27
CD8 ET	*COMMD6*	rs4885304	13	76088638	A	G	0.1387	-0.7867	0.0599	2.14E-39	172.12
CD8 ET	*COQ5*	rs651627	12	121009198	T	C	0.2994	0.3610	0.0471	1.84E-14	58.57
CD8 ET	*CORO1B*	rs7131041	11	67230184	T	A	0.4107	-0.8836	0.0409	2.16E-103	465.24
CD8 ET	*COX20*	rs1053700	1	245006827	G	A	0.1427	0.3718	0.0641	6.66E-09	33.56
CD8 ET	*COX6B1*	rs2285421	19	36168914	C	T	0.5080	-0.4778	0.0439	1.43E-27	118.14
CD8 ET	*COX7A2L*	rs1981664	2	42577170	G	A	0.5905	-0.3255	0.0449	3.99E-13	52.54
CD8 ET	*CPA5*	rs11761888	7	130007381	C	T	0.2124	0.7370	0.0526	1.29E-44	195.98
CD8 ET	*CPNE1*	rs7261284	20	34244104	A	T	0.1068	-1.0132	0.0736	3.72E-43	189.3
CD8 ET	*CRELD2*	rs9616205	22	50309915	T	A	0.1982	0.3998	0.0580	5.45E-12	47.42
CD8 ET	*CRIP2*	rs35203032	14	105951319	C	T	0.0839	-0.4313	0.0762	1.53E-08	31.95
CD8 ET	*CROCC*	rs7513616	1	17298496	G	A	0.4696	0.3453	0.0445	8.98E-15	59.99
CD8 ET	*CRYBB2*	rs6004662	22	25862124	A	G	0.0960	1.4029	0.0811	5.25E-67	298.46
CD8 ET	*CRYZ*	rs1465483	1	75195243	C	T	0.6770	-0.3444	0.0474	3.73E-13	52.67
CD8 ET	*CSGALNACT1*	rs62496184	8	19319423	A	G	0.5222	-0.4285	0.0442	3.16E-22	93.81
CD8 ET	*CSTB*	rs35285321	21	45201832	A	G	0.3415	-0.5073	0.0474	1.06E-26	114.17
CD8 ET	*CTA-14H9.5*	rs9467736	6	26380032	G	A	0.3116	-0.2809	0.0496	1.49E-08	32
CD8 ET	*CTA-29F11.1*	rs12389	22	47158179	A	G	0.3392	-0.6158	0.0448	6.55E-43	188.18
CD8 ET	*CTA-445C9.15*	rs4275	22	26921725	G	A	0.4260	0.3382	0.0449	4.79E-14	56.7
CD8 ET	*CTBP1-AS2*	rs2878539	4	1243877	T	C	0.4296	0.3189	0.0452	1.76E-12	49.64
CD8 ET	*CTC-241F20.3*	rs12984612	19	48759091	G	T	0.2207	-0.2985	0.0519	8.74E-09	33.04
CD8 ET	*CTC-425F1.4*	rs2900660	19	13022859	A	C	0.6129	-0.2606	0.0459	1.34E-08	32.2
CD8 ET	*CTC-507E2.1*	rs2889311	17	33907649	A	G	0.1919	-0.5784	0.0582	2.81E-23	98.59
CD8 ET	*CTC-524C5.2*	rs1050390	17	5336058	A	G	0.2364	0.6066	0.0532	4.24E-30	129.67
CD8 ET	*CTD-2007H13.3*	rs4448016	5	98373608	C	T	0.3156	-0.2837	0.0475	2.25E-09	35.67
CD8 ET	*CTD-2020K17.4*	rs8065345	17	43332015	G	A	0.1642	-0.3398	0.0620	4.23E-08	29.98
CD8 ET	*CTD-2196E14.4*	rs35635	16	23693770	G	T	0.2722	-0.5260	0.0493	1.39E-26	113.64
CD8 ET	*CTD-2325M2.1*	rs10413053	19	8104555	C	G	0.4286	0.4004	0.0448	4.31E-19	79.56
CD8 ET	*CTD-2368P22.1*	rs4801243	19	58513807	C	A	0.2297	0.5190	0.0520	1.96E-23	99.3
CD8 ET	*CTD-2371O3.2*	rs12296039	11	9458828	G	A	0.5033	0.7264	0.0419	3.52E-67	299.26
CD8 ET	*CTD-2515A14.1*	rs200540	16	24733849	A	G	0.4154	-0.2606	0.0454	9.67E-09	32.84
CD8 ET	*CTLA4*	rs3087243	2	204738919	A	G	0.4420	0.3435	0.0447	1.53E-14	58.94
CD8 ET	*CTSF*	rs2242663	11	66335308	C	T	0.2447	0.3553	0.0522	1.04E-11	46.16
CD8 ET	*CTSG*	rs2273843	14	25103409	C	T	0.1235	-0.5336	0.0697	1.90E-14	58.51
CD8 ET	*CTSH*	rs75564875	15	79230389	G	T	0.2038	-0.3859	0.0574	1.86E-11	45.03
CD8 ET	*CTSW*	rs596002	11	65645354	G	A	0.8002	1.1955	0.0494	3.41E-129	583.6
CD8 ET	*DAD1*	rs5742783	14	23045995	G	A	0.0986	0.6134	0.0745	1.80E-16	67.67
CD8 ET	*DAP*	rs5745297	5	10680997	A	G	0.0825	0.4657	0.0849	4.08E-08	30.05
CD8 ET	*DARS*	rs141928185	2	135991896	T	C	0.1290	0.4287	0.0780	3.83E-08	30.17

CD8 ET	*DAZAP1*	rs71337011	19	1354195	G	C	0.4541	-0.2798	0.0448	4.08E-10	39
CD8 ET	*DAZAP2*	rs10876154	12	51607890	T	C	0.6416	-0.4225	0.0469	1.93E-19	81.15
CD8 ET	*DBNDD1*	rs79690969	16	90076227	A	T	0.0561	-0.7258	0.0961	4.23E-14	56.94
CD8 ET	*DCXR*	rs111894854	17	79998898	G	A	0.1249	1.0733	0.0636	6.68E-64	284.24
CD8 ET	*DDR1*	rs2187977	6	30367751	A	C	0.3475	-0.3111	0.0469	3.19E-11	43.97
CD8 ET	*DDR1*	rs545914110	6	31091992	G	A	0.5478	0.4375	0.0441	3.20E-23	98.33
CD8 ET	*DDRGK1*	rs2295553	20	3184134	T	C	0.5238	-0.2975	0.0447	2.82E-11	44.21
CD8 ET	*DDT*	rs9624364	22	24311474	A	G	0.0711	-0.8888	0.0917	3.11E-22	93.84
CD8 ET	*DDTL*	rs4822451	22	24248166	G	T	0.6207	0.5604	0.0454	5.09E-35	152.13
CD8 ET	*DDX11*	rs4931424	12	31229317	C	T	0.4487	-0.6983	0.0425	1.37E-60	269.08
CD8 ET	*DDX5*	rs1991401	17	62502435	G	A	0.3173	-0.5105	0.0475	6.05E-27	115.29
CD8 ET	*DDX6*	rs10892251	11	118543563	T	C	0.2462	-0.3286	0.0537	9.63E-10	37.32
CD8 ET	*DENND3*	rs7462379	8	142134478	T	C	0.3262	0.3424	0.0481	1.09E-12	50.57
CD8 ET	*DGCR6*	rs56333537	22	18888301	A	G	0.0871	-0.4592	0.0794	7.26E-09	33.4
CD8 ET	*DGCR6L*	rs2904577	22	20293625	A	G	0.4261	-0.2821	0.0451	4.09E-10	38.99
CD8 ET	*DGCR8*	rs4589855	22	20106069	A	C	0.0559	0.6110	0.0978	4.16E-10	38.96
CD8 ET	*DGKD*	rs1441093	2	234272484	G	A	0.7455	-0.2900	0.0523	2.91E-08	30.7
CD8 ET	*DGKQ*	rs11724804	4	965779	A	G	0.4417	0.2506	0.0450	2.49E-08	31.01
CD8 ET	*DHRS4*	rs77491846	14	24458752	A	G	0.0909	0.9840	0.0797	4.64E-35	152.31
CD8 ET	*DHRS7*	rs7401258	14	60361153	A	C	0.8856	0.5136	0.0677	3.42E-14	57.36
CD8 ET	*DHX32*	rs12359733	10	127620582	A	G	0.4114	-0.2730	0.0451	1.47E-09	36.5
CD8 ET	*DIP2A*	rs2070435	21	47961711	A	G	0.3259	1.0296	0.0413	3.89E-137	620.04
CD8 ET	*DIS3L*	rs12101389	15	66657048	C	T	0.2917	-0.3284	0.0473	3.94E-12	48.06
CD8 ET	*DMKN*	rs8111522	19	35974202	A	G	0.0867	-0.4327	0.0738	4.43E-09	34.35
CD8 ET	*DNAI2*	rs3186520	17	72257713	T	C	0.1409	-0.6254	0.0594	6.54E-26	110.58
CD8 ET	*DNAJA1*	rs10971358	9	33038049	C	G	0.3605	-0.2628	0.0467	1.84E-08	31.6
CD8 ET	*DNAJC15*	rs17624230	13	43604332	A	T	0.2260	-0.9921	0.0487	3.31E-92	413.95
CD8 ET	*DOCK8*	rs34755173	9	324752	T	C	0.1491	-0.5935	0.0583	2.44E-24	103.42
CD8 ET	*DPY19L2*	rs11175143	12	64128976	T	C	0.5168	0.6136	0.0430	4.20E-46	202.78
CD8 ET	*DRAM2*	rs3762374	1	111682118	T	C	0.2783	0.3880	0.0482	7.79E-16	64.79
CD8 ET	*DTNBP1*	rs9476864	6	15611827	C	T	0.5410	0.3656	0.0447	2.82E-16	66.79
CD8 ET	*DUOX1*	rs74011388	15	45493122	T	C	0.1109	-0.4297	0.0762	1.73E-08	31.71
CD8 ET	*DUS3L*	rs11878476	19	5798285	C	T	0.2130	-0.3534	0.0583	1.33E-09	36.69
CD8 ET	*DYNLL2*	rs12950704	17	56168713	C	G	0.6730	-0.4201	0.0473	6.24E-19	78.83
CD8 ET	*DZIP3*	rs13084166	3	108395273	C	A	0.2943	0.5487	0.0479	2.50E-30	130.71
CD8 ET	*EBPL*	rs3809325	13	50266109	C	T	0.5393	-0.6490	0.0431	2.42E-51	226.75
CD8 ET	*ECHDC2*	rs1242331	1	53392908	G	A	0.6574	0.2983	0.0473	2.91E-10	39.65
CD8 ET	*EFHB*	rs11128927	3	19988437	C	G	0.3711	-0.4699	0.0450	1.41E-25	109.06
CD8 ET	*EGFL8*	rs9468925	6	31258837	A	G	0.3700	0.3207	0.0471	9.37E-12	46.36
CD8 ET	*EGFL8*	rs9277752	6	33094600	A	G	0.3669	0.5466	0.0455	2.66E-33	144.28
CD8 ET	*EIF2A*	rs6440683	3	150295376	T	C	0.8419	0.5239	0.0629	7.95E-17	69.28
CD8 ET	*EIF3G*	rs2305792	19	10221151	G	C	0.4399	0.4211	0.0443	2.14E-21	90.03
CD8 ET	*EIF4G3*	rs4654735	1	21432001	C	T	0.4158	0.2499	0.0457	4.59E-08	29.82
CD8 ET	*EIF5A*	rs7503161	17	7207964	C	A	0.5615	-1.2116	0.0347	1.00E-200	1216.18
CD8 ET	*EIF6*	rs8122819	20	33881586	T	C	0.2227	0.3045	0.0554	3.97E-08	30.1
CD8 ET	*ELP5*	rs2074219	17	7156447	G	A	0.6468	-0.9164	0.0410	1.48E-110	498.1
CD8 ET	*EMB*	rs8188217	5	49478029	T	C	0.5478	-0.4636	0.0443	1.12E-25	109.51
CD8 ET	*EML4*	rs7597228	2	42329711	G	C	0.3745	-0.3869	0.0455	1.75E-17	72.26
CD8 ET	*EML4*	rs11884524	2	42381860	G	T	0.4071	0.3058	0.0449	1.00E-11	46.23
CD8 ET	*EMP3*	rs17295412	19	48841185	A	G	0.4395	-0.2511	0.0450	2.43E-08	31.05
CD8 ET	*EMR2*	rs888875	19	14491673	C	G	0.3195	0.4001	0.0479	6.23E-17	69.76
CD8 ET	*ENDOG*	rs10988134	9	131595407	T	C	0.2542	-0.3430	0.0515	2.71E-11	44.29
CD8 ET	*ENTPD1*	rs10882663	10	97536612	C	T	0.5305	0.2522	0.0450	2.05E-08	31.38
CD8 ET	*ENTPD3-AS1*	rs7644643	3	40350209	G	A	0.3809	0.2622	0.0463	1.54E-08	31.93
CD8 ET	*EPB41L4A*	rs6863537	5	111932280	G	T	0.8600	-0.4178	0.0637	5.43E-11	42.93
CD8 ET	*EPC1*	rs1475436	10	32610703	C	T	0.4219	-0.2773	0.0452	8.65E-10	37.53
CD8 ET	*EPHA4*	rs4613266	2	222393635	C	T	0.5242	0.2567	0.0448	1.02E-08	32.74
CD8 ET	*EPS8L2*	rs3934992	11	771716	C	A	0.4410	0.3431	0.0445	1.31E-14	59.24
CD8 ET	*EPSTI1*	rs3825511	13	43566373	C	A	0.7178	-0.6351	0.0490	2.29E-38	167.42
CD8 ET	*ERAP2*	rs2927608	5	96252432	A	G	0.4214	1.0591	0.0378	1.91E-172	782.08
CD8 ET	*ERN1*	rs2077041	17	62153231	C	G	0.1610	-0.5830	0.0613	1.94E-21	90.22
CD8 ET	*ESR2*	rs1268656	14	64647646	G	T	0.1128	0.4880	0.0701	3.46E-12	48.31
CD8 ET	*ESR2*	rs944045	14	64701446	C	T	0.0757	1.2390	0.0890	4.33E-44	193.57
CD8 ET	*ETV7*	rs6457910	6	36372201	C	T	0.5262	0.4455	0.0443	7.68E-24	101.15
CD8 ET	*EVA1C*	rs9305503	21	33776646	A	T	0.2991	-0.3367	0.0496	1.09E-11	46.07
CD8 ET	*EVI2A*	rs2854334	17	29715500	G	A	0.6079	0.4296	0.0460	1.05E-20	86.88
CD8 ET	*EXOC3*	rs12188321	5	428418	G	C	0.4608	0.4932	0.0438	1.86E-29	126.73
CD8 ET	*FADS1*	rs174529	11	61543961	C	T	0.3647	0.3947	0.0465	1.97E-17	72.03
CD8 ET	*FADS2*	rs968567	11	61595564	T	C	0.1535	0.9890	0.0532	4.74E-77	344.48
CD8 ET	*FAH*	rs4778583	15	80455521	T	A	0.5987	-0.4189	0.0452	2.06E-20	85.56
CD8 ET	*FAM110B*	rs28848865	8	59253540	G	T	0.2845	-0.7424	0.0458	5.78E-59	261.63
CD8 ET	*FAM118A*	rs136564	22	45764874	C	T	0.1341	1.5315	0.0584	1.13E-151	686.75
CD8 ET	*FAM129A*	rs234108	1	184942671	A	G	0.4667	-0.3180	0.0447	1.08E-12	50.59
CD8 ET	*FAM134A*	rs3795986	2	220033830	G	A	0.6150	0.3325	0.0470	1.42E-12	50.05
CD8 ET	*FAM154B*	rs28883606	15	82509054	G	A	0.3661	-0.4039	0.0465	3.82E-18	75.26
CD8 ET	*FAM179A*	rs13409097	2	29240005	C	T	0.5134	0.2726	0.0449	1.32E-09	36.71
CD8 ET	*FAM184B*	rs6854733	4	17640526	T	C	0.6032	-1.0499	0.0389	1.65E-160	727.3
CD8 ET	*FAM21A*	rs10762956	10	51806645	C	G	0.6077	0.3402	0.0456	8.41E-14	55.59
CD8 ET	*FAM26F*	rs1057192	6	116783330	A	G	0.2439	-0.6252	0.0512	2.86E-34	148.7

CD8 ET	*FAM26F*	rs1741819	6	116849485	A	G	0.7359	0.3949	0.0503	3.98E-15	61.58
CD8 ET	*FAM43A*	rs1773225	3	194403578	G	A	0.4763	-0.4631	0.0442	1.01E-25	109.73
CD8 ET	*FAM83D*	rs220546	20	37246038	T	C	0.1338	-0.3722	0.0662	1.90E-08	31.53
CD8 ET	*FAR1*	rs12275525	11	13742804	C	G	0.3311	0.5752	0.0465	4.04E-35	152.58
CD8 ET	*FAU*	rs1196043	11	64887560	G	T	0.4919	0.3870	0.0444	2.92E-18	75.79
CD8 ET	*FBLN5*	rs2267989	14	92339850	A	G	0.4380	-0.4275	0.0443	4.47E-22	93.12
CD8 ET	*FBXO2*	rs56052340	1	11716755	A	G	0.0748	0.5469	0.0857	1.76E-10	40.63
CD8 ET	*FCGBP*	rs34797395	19	40353576	C	A	0.3545	0.4818	0.0462	1.67E-25	108.72
CD8 ET	*FCRL3*	rs2210913	1	157668993	T	C	0.4492	0.4188	0.0443	3.02E-21	89.35
CD8 ET	*FDFT1*	rs1293299	8	11677905	C	A	0.2173	-0.4085	0.0544	5.83E-14	56.31
CD8 ET	*FGD5-AS1*	rs11717398	3	15102441	C	G	0.2947	0.3338	0.0483	4.83E-12	47.66
CD8 ET	*FGFBP2*	rs55927777	4	15961010	T	C	0.3775	-0.2816	0.0461	9.91E-10	37.27
CD8 ET	*FGFR1*	rs2468964	8	38333062	G	T	0.6141	-0.3156	0.0454	3.67E-12	48.19
CD8 ET	*FGL2*	rs3093273	7	76829275	G	A	0.0905	0.9350	0.0803	2.43E-31	135.33
CD8 ET	*FHAD1*	rs763821	1	15668276	A	G	0.3848	0.4307	0.0450	1.14E-21	91.28
CD8 ET	*FHL3*	rs67631072	1	38461821	T	C	0.5175	-0.6118	0.0430	7.22E-46	201.7
CD8 ET	*FIBP*	rs588114	11	65662752	A	G	0.1996	0.3542	0.0566	3.87E-10	39.1
CD8 ET	*FIGNL1*	rs4947641	7	50489038	T	C	0.4833	0.3985	0.0443	2.26E-19	80.84
CD8 ET	*FIS1*	rs74662330	7	100865866	T	C	0.0582	-0.8628	0.0915	4.29E-21	88.66
CD8 ET	*FKBP11*	rs11168779	12	49288629	A	G	0.3919	-0.4863	0.0455	1.22E-26	113.89
CD8 ET	*FLVCR1-AS1*	rs12125982	1	213056892	G	A	0.5078	0.5357	0.0435	8.09E-35	151.21
CD8 ET	*FMN1*	rs345781	15	33395917	G	A	0.2708	0.7366	0.0509	2.15E-47	208.69
CD8 ET	*FN3KRP*	rs2246577	17	80680394	C	T	0.5992	0.5140	0.0450	2.87E-30	130.44
CD8 ET	*FNBP4*	rs1471712	11	47912150	A	G	0.5506	0.3676	0.0445	1.46E-16	68.08
CD8 ET	*FOXN2*	rs138659652	2	48655341	T	C	0.1373	0.4448	0.0659	1.45E-11	45.5
CD8 ET	*FRG1B*	rs2376538	20	29834368	A	G	0.5069	-0.2602	0.0448	6.37E-09	33.65
CD8 ET	*FTCDNL1*	rs11687699	2	200626678	A	G	0.6303	-0.4156	0.0460	1.71E-19	81.38
CD8 ET	*FZD6*	rs827537	8	104322247	T	G	0.2547	0.6450	0.0502	7.79E-38	164.98
CD8 ET	*G3BP2*	rs34659736	4	76593967	G	C	0.3743	-0.3542	0.0462	1.73E-14	58.7
CD8 ET	*GALM*	rs72795179	2	38931153	A	G	0.4442	0.2461	0.0450	4.44E-08	29.89
CD8 ET	*GALT*	rs2812357	9	34655436	C	T	0.6012	0.2832	0.0452	3.77E-10	39.15
CD8 ET	*GAPDH*	rs3741915	12	6643927	G	T	0.2623	0.3781	0.0496	2.55E-14	57.94
CD8 ET	*GAS1*	rs4878050	9	89888014	C	A	0.8105	-0.3235	0.0582	2.71E-08	30.84
CD8 ET	*GBP5*	rs56069047	1	89742517	T	C	0.5456	-0.4920	0.0440	5.56E-29	124.57
CD8 ET	*GBP7*	rs683100	1	89653720	T	C	0.4965	0.7933	0.0413	2.27E-82	368.86
CD8 ET	*GCC2*	rs2139809	2	109045638	C	T	0.5916	0.6641	0.0433	4.10E-53	234.85
CD8 ET	*GCHFR*	rs72733486	15	40858382	T	C	0.1297	-0.4541	0.0633	7.53E-13	51.3
CD8 ET	*GDE1*	rs8053672	16	19524766	C	T	0.2775	0.3205	0.0515	4.89E-10	38.64
CD8 ET	*GDI2*	rs74116316	10	5845061	G	A	0.2072	-0.5778	0.0526	4.39E-28	120.48
CD8 ET	*GEMIN7*	rs1054470	19	45653234	C	T	0.6434	-0.2643	0.0475	2.56E-08	30.96
CD8 ET	*GIMAP2*	rs60774275	7	150342420	T	C	0.1095	-0.4997	0.0706	1.41E-12	50.06
CD8 ET	*GIPC1*	rs116092141	19	14606710	G	C	0.3651	-0.2832	0.0460	7.43E-10	37.83
CD8 ET	*GLIPR1*	rs35619460	12	75886695	C	A	0.2532	-0.3573	0.0519	5.98E-12	47.24
CD8 ET	*GLIPR1L2*	rs7297580	12	76038674	C	T	0.3858	0.4024	0.0450	3.69E-19	79.87
CD8 ET	*GLTP*	rs11832113	12	110303124	A	C	0.4577	-0.3025	0.0447	1.27E-11	45.76
CD8 ET	*GLTSCR2*	rs3786779	19	48255284	A	G	0.7093	-0.4980	0.0494	6.76E-24	101.4
CD8 ET	*GMIP*	rs7248885	19	19753292	A	G	0.3996	0.2666	0.0462	7.82E-09	33.25
CD8 ET	*GNAQ*	rs4744855	9	80575017	C	T	0.4560	-0.3029	0.0448	1.30E-11	45.73
CD8 ET	*GNG5*	rs6666572	1	84928736	A	G	0.1078	0.4294	0.0751	1.06E-08	32.67
CD8 ET	*GNLY*	rs12151742	2	85933003	T	C	0.2260	0.8977	0.0500	5.78E-72	321.18
CD8 ET	*GNPTG*	rs57890635	16	1411129	T	C	0.2668	0.5165	0.0526	8.94E-23	96.3
CD8 ET	*GOLT1B*	rs16923862	12	21659973	C	T	0.1562	0.3617	0.0628	8.23E-09	33.15
CD8 ET	*GPR135*	rs60675976	14	60018584	G	C	0.5252	0.5632	0.0436	4.15E-38	166.23
CD8 ET	*GPR56*	rs8045257	16	57700469	A	G	0.5098	-0.2478	0.0449	3.28E-08	30.47
CD8 ET	*GPR63*	rs454859	6	97211800	G	A	0.6948	-0.3881	0.0485	1.28E-15	63.81
CD8 ET	*GREM2*	rs17698283	1	241050375	A	G	0.1047	0.4964	0.0702	1.56E-12	49.87
CD8 ET	*GRHPR*	rs4878690	9	37422656	T	C	0.3290	0.6061	0.0462	2.68E-39	171.67
CD8 ET	*GRIN2C*	rs6501735	17	72775340	G	C	0.5655	0.3262	0.0458	1.12E-12	50.52
CD8 ET	*GS1-124K5.4*	rs2465121	7	65621004	G	A	0.7450	-0.3512	0.0509	5.39E-12	47.44
CD8 ET	*GS1-251I9.4*	rs7341609	8	92064862	G	A	0.7122	0.2988	0.0492	1.26E-09	36.8
CD8 ET	*GSC*	rs61574875	14	95403353	C	G	0.2187	-0.3207	0.0543	3.44E-09	34.85
CD8 ET	*GSDMA*	rs72832958	17	38093881	A	C	0.1001	0.9608	0.0686	1.46E-44	195.73
CD8 ET	*GSE1*	rs11649034	16	85718480	G	A	0.6819	-0.2839	0.0484	4.57E-09	34.29
CD8 ET	*GSTM2*	rs1056806	1	110233147	T	C	0.0899	0.5109	0.0921	2.92E-08	30.7
CD8 ET	*GSTM3*	rs1332018	1	110282972	T	G	0.5781	0.6955	0.0430	5.73E-59	261.65
CD8 ET	*GSTM4*	rs1010167	1	110198727	G	C	0.4142	-0.6703	0.0434	6.72E-54	238.45
CD8 ET	*GSTO1*	rs7896536	10	106005958	A	G	0.2960	0.4493	0.0494	1.02E-19	82.41
CD8 ET	*GSTP1*	rs4930444	11	67342580	A	G	0.4200	-0.4953	0.0443	4.72E-29	124.89
CD8 ET	*GSTT1*	rs5760147	22	24334948	C	A	0.3844	-0.6470	0.0445	7.73E-48	210.71
CD8 ET	*GTF2H2*	rs28478097	5	70309998	G	C	0.6121	-0.5861	0.0445	1.06E-39	173.5
CD8 ET	*GTPBP4*	rs3763720	10	1045099	T	C	0.0975	0.4480	0.0752	2.57E-09	35.41
CD8 ET	*GYG1*	rs34578445	3	148723023	C	A	0.3134	-0.2855	0.0484	3.67E-09	34.72
CD8 ET	*GYPC*	rs2119151	2	127408871	T	G	0.5928	0.2929	0.0455	1.18E-10	41.42
CD8 ET	*GYPC*	rs35651363	2	127519838	A	G	0.2707	0.4671	0.0507	3.38E-20	84.58
CD8 ET	*H2AFZ*	rs6836304	4	100995816	C	T	0.2524	-0.3146	0.0549	9.83E-09	32.81
CD8 ET	*HAGH*	rs8044343	16	1881145	C	G	0.3129	0.4833	0.0491	7.58E-23	96.63
CD8 ET	*HAR1A*	rs111435192	20	61734190	A	G	0.2461	-0.4115	0.0507	4.62E-16	65.82
CD8 ET	*HAX1*	rs2282227	1	154243097	A	G	0.2882	0.3181	0.0491	9.08E-11	41.92
CD8 ET	*HBA1*	rs78360966	16	151262	T	C	0.0818	0.6919	0.0887	6.39E-15	60.65

CD8 ET	*HCG14*	rs9295778	6	28684183	G	T	0.8428	0.6182	0.0595	2.99E-25	107.57
CD8 ET	*HCG14*	rs2394661	6	29672941	C	T	0.4749	0.2943	0.0447	4.63E-11	43.24
CD8 ET	*HCG25*	rs111625658	6	32339988	T	C	0.0960	0.6259	0.0723	4.91E-18	74.76
CD8 ET	*HDDC2*	rs2275623	6	125574696	G	A	0.4680	-0.3188	0.0447	1.03E-12	50.68
CD8 ET	*HDGFRP3*	rs28567531	15	83834324	G	C	0.0708	0.6112	0.0850	6.61E-13	51.55
CD8 ET	*HEBP2*	rs9495063	6	138708184	A	C	0.0680	-0.9679	0.0937	4.98E-25	106.56
CD8 ET	*HELLS*	rs11187985	10	96257048	C	A	0.4280	0.4051	0.0446	9.93E-20	82.45
CD8 ET	*HEXDC*	rs4072532	17	80394080	A	G	0.3204	0.3232	0.0489	3.78E-11	43.64
CD8 ET	*HHEX*	rs9420589	10	94450233	T	G	0.4130	-0.2585	0.0452	1.07E-08	32.64
CD8 ET	*HIGD2A*	rs1065212	5	175811133	C	T	0.0955	-0.8633	0.0836	5.14E-25	106.5
CD8 ET	*HIP1*	rs111778729	7	75368110	T	C	0.0618	0.5824	0.0974	2.27E-09	35.66
CD8 ET	*HIST1H1B*	rs17709145	6	28004556	G	T	0.0472	0.5637	0.0925	1.11E-09	37.04
CD8 ET	*HIST1H2AH*	rs34788973	6	27879200	A	C	0.0756	0.4873	0.0752	9.39E-11	41.86
CD8 ET	*HIST1H2AI*	rs35546888	6	28558890	A	G	0.2203	0.3585	0.0527	1.08E-11	46.09
CD8 ET	*HIST1H2AJ*	rs13193532	6	28622914	G	A	0.1195	0.4860	0.0612	1.96E-15	62.97
CD8 ET	*HIST1H2BI*	rs72839445	6	27249686	A	G	0.0719	0.6113	0.0765	1.35E-15	63.71
CD8 ET	*HIST1H2BL*	rs9257184	6	28756088	T	C	0.0826	0.4636	0.0742	4.20E-10	38.94
CD8 ET	*HIST1H2BM*	rs35982103	6	27243134	T	C	0.1838	-0.4699	0.0569	1.47E-16	68.07
CD8 ET	*HIST1H2BO*	rs76542212	6	28198122	T	C	0.0584	-0.5902	0.0850	3.81E-12	48.12
CD8 ET	*HIST1H3H*	rs9393925	6	28682442	C	T	0.8872	0.4179	0.0680	8.03E-10	37.68
CD8 ET	*HIST1H3J*	rs3118365	6	28839908	A	G	0.0876	-0.4173	0.0736	1.45E-08	32.05
CD8 ET	*HIST1H4B*	rs9379945	6	26907831	C	T	0.1259	-0.4311	0.0645	2.32E-11	44.59
CD8 ET	*HIST1H4F*	rs57440165	6	26843517	C	A	0.0736	0.8354	0.0748	5.61E-29	124.55
CD8 ET	*HIST1H4I*	rs67662114	6	27932301	A	G	0.0750	-0.9223	0.0730	1.51E-36	159.11
CD8 ET	*HIST1H4K*	rs7741443	6	28241263	T	A	0.1747	-0.3486	0.0607	9.26E-09	32.92
CD8 ET	*HIVEP3*	rs1004870	1	42370787	T	C	0.5896	-0.2786	0.0452	7.19E-10	37.89
CD8 ET	*HLA-A*	rs9260043	6	29907740	G	A	0.3897	0.4087	0.0457	3.62E-19	79.91
CD8 ET	*HLA-B*	rs2524096	6	31236467	T	G	0.4422	0.8038	0.0417	6.63E-83	371.31
CD8 ET	*HLA-B*	rs9366777	6	31264715	A	T	0.0923	-0.5581	0.0827	1.48E-11	45.47
CD8 ET	*HLA-B*	rs2244020	6	31347451	G	A	0.4150	-0.4268	0.0464	3.79E-20	84.35
CD8 ET	*HLA-C*	rs2395471	6	31240692	A	G	0.4050	0.4766	0.0455	1.01E-25	109.71
CD8 ET	*HLA-C*	rs2442752	6	31351764	C	T	0.3908	0.2640	0.0455	6.67E-09	33.56
CD8 ET	*HLA-DOB*	rs4148876	6	32796793	A	G	0.0646	0.5597	0.0905	6.27E-10	38.16
CD8 ET	*HLA-DPA1*	rs6934289	6	33044956	C	T	0.1214	-0.4996	0.0735	1.06E-11	46.11
CD8 ET	*HLA-DPB1*	rs928976	6	33049211	T	C	0.3313	0.4535	0.0473	8.26E-22	91.91
CD8 ET	*HLA-DQA1*	rs9272785	6	32610401	A	G	0.1783	0.9053	0.0526	2.51E-66	295.35
CD8 ET	*HLA-DQA2*	rs9272461	6	32605609	A	G	0.1774	1.3324	0.0460	2.34E-184	836.76
CD8 ET	*HLA-DQA2*	rs77000541	6	32635605	A	G	0.2974	0.5285	0.0439	2.48E-33	144.42
CD8 ET	*HLA-DQB1*	rs9272426	6	32605189	G	A	0.3067	-0.5479	0.0437	5.45E-36	156.56
CD8 ET	*HLA-DQB1*	rs9275522	6	32674970	T	C	0.2350	-0.3209	0.0541	2.99E-09	35.12
CD8 ET	*HLA-DQB2*	rs2227956	6	31778272	A	G	0.8407	-0.5072	0.0570	5.72E-19	79
CD8 ET	*HLA-DRB5*	rs9267546	6	31673436	A	G	0.1391	-0.5048	0.0833	1.36E-09	36.65
CD8 ET	*HLA-DRB5*	rs28366319	6	32561495	A	G	0.2843	0.9554	0.0415	2.82E-117	528.93
CD8 ET	*HLA-F*	rs577795245	6	29682542	C	T	0.6721	-0.3940	0.0487	5.56E-16	65.45
CD8 ET	*HLA-G*	rs2248151	6	29914548	T	C	0.1584	0.8260	0.0584	1.79E-45	199.9
CD8 ET	*HLA-G*	rs28454792	6	30061010	T	C	0.1611	-0.3924	0.0618	2.22E-10	40.18
CD8 ET	*HMBOX1*	rs10088428	8	28909523	T	C	0.2597	-0.8511	0.0483	1.72E-69	309.84
CD8 ET	*HMGN1*	rs73364034	21	40744651	C	A	0.3113	0.3037	0.0478	2.18E-10	40.22
CD8 ET	*HNRNPU*	rs9661182	1	245028509	T	G	0.2415	0.3132	0.0530	3.33E-09	34.91
CD8 ET	*HOPX*	rs4864602	4	57624491	C	A	0.2192	-0.3218	0.0545	3.60E-09	34.76
CD8 ET	*HOXB-AS1*	rs12450225	17	46592413	G	A	0.4858	0.5243	0.0436	2.45E-33	144.44
CD8 ET	*HOXB2*	rs62066684	17	46597488	G	A	0.4867	0.6413	0.0427	6.31E-51	224.84
CD8 ET	*HOXB4*	rs8077407	17	46558036	A	G	0.4837	0.2843	0.0447	2.06E-10	40.33
CD8 ET	*HPGD*	rs45545934	4	175442061	C	T	0.5198	0.4031	0.0442	8.25E-20	82.82
CD8 ET	*HSBP1L1*	rs1430597	18	77725699	G	A	0.1727	0.4245	0.0597	1.18E-12	50.42
CD8 ET	*HSD17B11*	rs10028441	4	88290265	A	G	0.2986	0.3506	0.0489	7.24E-13	51.37
CD8 ET	*HSD17B14*	rs10407029	19	49340858	T	C	0.2779	0.2934	0.0513	1.11E-08	32.58
CD8 ET	*HSP90B1*	rs1165673	12	104320510	C	A	0.6058	-0.2728	0.0454	1.90E-09	36
CD8 ET	*HSPBAP1*	rs13093464	3	122507436	A	G	0.7480	-0.6004	0.0530	9.28E-30	128.12
CD8 ET	*IAH1*	rs13392064	2	9626365	G	C	0.5673	-0.2850	0.0456	4.15E-10	38.96
CD8 ET	*ICA1L*	rs62194159	2	203646897	T	C	0.4446	-0.2614	0.0449	5.95E-09	33.78
CD8 ET	*IDI1*	rs7094331	10	1022816	C	T	0.7004	-0.5126	0.0487	6.26E-26	110.66
CD8 ET	*IFI27L1*	rs941477	14	94571353	T	A	0.4042	0.2928	0.0458	1.57E-10	40.85
CD8 ET	*IFITM2*	rs1059091	11	309127	G	A	0.3507	0.5193	0.0470	1.98E-28	122.05
CD8 ET	*IFITM2*	rs7117996	11	349122	C	T	0.2915	0.3617	0.0487	1.09E-13	55.08
CD8 ET	*IFITM3*	rs7942247	11	316349	A	G	0.5502	0.8208	0.0410	2.57E-89	400.7
CD8 ET	*IFITM3*	rs7117996	11	349122	C	T	0.2915	0.3393	0.0488	3.48E-12	48.3
CD8 ET	*IFNG-AS1*	rs4141135	12	68418830	G	T	0.5591	-0.3000	0.0453	3.68E-11	43.69
CD8 ET	*IGFLR1*	rs12459634	19	36230174	C	T	0.1502	-0.4890	0.0637	1.68E-14	58.76
CD8 ET	*IGHMBP2*	rs7131462	11	68610143	A	G	0.5149	-0.2610	0.0450	6.82E-09	33.52
CD8 ET	*IKBKE*	rs11117909	1	206649952	A	G	0.1344	0.4342	0.0693	3.74E-10	39.16
CD8 ET	*IL18R1*	rs55927292	2	102964861	T	C	0.2722	0.2945	0.0492	2.14E-09	35.76
CD8 ET	*IL32*	rs11861531	16	3116325	C	T	0.5537	0.3424	0.0446	1.57E-14	58.88
CD8 ET	*INSR*	rs10414152	19	7300731	T	C	0.1412	-0.5486	0.0720	2.51E-14	57.97
CD8 ET	*INTS5*	rs11231184	11	62447563	A	C	0.2753	0.3009	0.0489	7.65E-10	37.77
CD8 ET	*IQCB1*	rs10934559	3	121480038	A	G	0.1271	0.3713	0.0621	2.30E-09	35.63
CD8 ET	*IRF1*	rs35426692	5	131798403	G	C	0.1254	-0.4293	0.0680	2.82E-10	39.72
CD8 ET	*ISCA1*	rs7035034	9	88902663	T	C	0.6802	-0.3522	0.0467	4.72E-14	56.73
CD8 ET	*ISCA2*	rs10873267	14	74941081	G	A	0.5998	-0.3716	0.0452	2.02E-16	67.45

CD8 ET	*ISG15*	rs1921	1	949608	A	G	0.3869	0.3416	0.0456	6.60E-14	56.07
CD8 ET	*ITCH*	rs1205340	20	32923871	A	G	0.4899	-0.4498	0.0440	1.59E-24	104.26
CD8 ET	*ITCH-AS1*	rs6088525	20	33141272	C	T	0.3509	0.4072	0.0476	1.22E-17	72.98
CD8 ET	*ITGAD*	rs17708876	16	31398466	C	T	0.1681	0.3684	0.0598	7.41E-10	37.83
CD8 ET	*ITGB2*	rs760462	21	46328099	C	T	0.8298	-1.2249	0.0538	9.56E-115	517.32
CD8 ET	*ITGB2-AS1*	rs4818995	21	46370591	C	T	0.8559	-0.3647	0.0649	1.91E-08	31.52
CD8 ET	*ITGB3BP*	rs217483	1	64017830	G	A	0.7015	-0.3743	0.0482	8.45E-15	60.11
CD8 ET	*ITPA*	rs9101	20	3204084	A	G	0.3038	0.3387	0.0484	2.53E-12	48.92
CD8 ET	*IVNS1ABP*	rs10732976	1	185409061	A	G	0.7012	0.3621	0.0483	6.45E-14	56.12
CD8 ET	*JAKMIP1*	rs60046962	4	6179711	T	C	0.5199	-0.4340	0.0442	9.20E-23	96.24
CD8 ET	*KANSL1-AS1*	rs199533	17	44828931	A	G	0.1904	1.3100	0.0445	1.83E-190	864.79
CD8 ET	*KB-1980E6.3*	rs17186084	8	103541166	A	G	0.0898	0.5653	0.0851	3.12E-11	44.01
CD8 ET	*KB-226F1.1*	rs5760147	22	24334948	C	A	0.3844	-0.3248	0.0464	2.44E-12	48.99
CD8 ET	*KCNAB2*	rs61760766	1	6064811	A	G	0.1363	0.6056	0.0620	1.52E-22	95.25
CD8 ET	*KCNK12*	rs3771273	2	47658474	T	A	0.4022	0.2607	0.0463	1.75E-08	31.69
CD8 ET	*KCTD10*	rs56406401	12	109900538	C	T	0.0609	0.7036	0.0814	5.29E-18	74.62
CD8 ET	*KCTD21-AS1*	rs10793282	11	77805236	A	G	0.4710	-0.3653	0.0446	2.48E-16	67.04
CD8 ET	*KDELR2*	rs6796	7	6502367	C	T	0.2687	-0.6541	0.0475	3.87E-43	189.22
CD8 ET	*KIAA0040*	rs2269650	1	175129691	T	G	0.3691	0.4377	0.0463	3.03E-21	89.34
CD8 ET	*KIAA0930*	rs5765231	22	45641411	G	T	0.4408	0.3795	0.0445	1.50E-17	72.56
CD8 ET	*KIF16B*	rs6044134	20	16555032	A	G	0.4477	0.3595	0.0449	1.25E-15	63.87
CD8 ET	*KIF19*	rs4788840	17	72476020	A	G	0.4586	-0.2837	0.0450	2.98E-10	39.61
CD8 ET	*KIR3DL1*	rs56162245	19	55379326	T	A	0.3111	-0.3033	0.0487	4.70E-10	38.72
CD8 ET	*KLF13*	rs4779867	15	31652783	C	G	0.1624	0.3532	0.0629	1.94E-08	31.49
CD8 ET	*KLHDC4*	rs2290019	16	87741662	A	C	0.2032	-0.4615	0.0563	2.49E-16	67.04
CD8 ET	*KLRC1*	rs78547237	12	10594848	A	C	0.8225	-0.3992	0.0609	5.50E-11	42.9
CD8 ET	*KLRC2*	rs60110638	12	10597311	T	A	0.2278	-0.6510	0.0546	9.44E-33	141.77
CD8 ET	*KLRC4*	rs2460740	12	10581446	G	T	0.6476	0.5572	0.0463	2.63E-33	144.3
CD8 ET	*KLRD1*	rs17808267	12	10418779	G	T	0.2262	0.4459	0.0522	1.27E-17	72.89
CD8 ET	*KLRK1*	rs60110638	12	10597311	T	A	0.2278	-0.3907	0.0559	2.80E-12	48.73
CD8 ET	*KRT73*	rs34899403	12	53037114	A	G	0.2864	0.3950	0.0490	7.40E-16	64.89
CD8 ET	*KTN1*	rs8007183	14	56081149	A	G	0.3499	-0.3635	0.0468	8.49E-15	60.1
CD8 ET	*KYNU*	rs7420443	2	143596902	A	T	0.4020	-0.3868	0.0450	7.95E-18	73.81
CD8 ET	*L3HYPDH*	rs1253176	14	59894956	T	C	0.5319	0.2728	0.0449	1.28E-09	36.76
CD8 ET	*L3MBTL3*	rs7740107	6	130374461	A	T	0.7512	-0.4900	0.0491	1.88E-23	99.38
CD8 ET	*LA16c-358B7.3*	rs11647760	16	1367610	A	C	0.6104	-0.5194	0.0444	1.43E-31	136.39
CD8 ET	*LAIR1*	rs74463408	19	54871664	C	G	0.1326	-0.7970	0.0686	3.19E-31	134.79
CD8 ET	*LAIR2*	rs73070113	19	55014172	T	A	0.1204	-0.7726	0.0658	7.06E-32	137.78
CD8 ET	*LAMB1*	rs2237685	7	107574784	A	G	0.3829	-0.6536	0.0434	2.34E-51	226.81
CD8 ET	*LAMTOR4*	rs7341389	7	99735181	G	A	0.6197	-0.2991	0.0457	6.14E-11	42.69
CD8 ET	*LAPTM5*	rs11585511	1	31216969	A	G	0.2702	-0.2822	0.0494	1.15E-08	32.51
CD8 ET	*LATS2*	rs9552341	13	21637808	G	C	0.1892	0.3678	0.0584	3.07E-10	39.55
CD8 ET	*LAX1*	rs4951181	1	203738781	G	A	0.5623	0.4472	0.0449	2.16E-23	99.11
CD8 ET	*LCLAT1*	rs72853154	2	30666530	G	C	0.2265	0.3088	0.0514	1.87E-09	36.03
CD8 ET	*LCP1*	rs1409430	13	46701233	T	A	0.9368	-0.5558	0.0883	3.04E-10	39.57
CD8 ET	*LDHC*	rs11599997	11	18431563	G	A	0.1223	0.7059	0.0688	1.06E-24	105.07
CD8 ET	*LEPROT*	rs55889533	1	65900357	T	G	0.3901	-0.3567	0.0456	5.06E-15	61.11
CD8 ET	*LIMD1*	rs2673023	3	45711367	C	T	0.7714	-0.4218	0.0508	9.89E-17	68.85
CD8 ET	*LIN52*	rs2358628	14	74631757	C	A	0.6609	-0.3256	0.0482	1.37E-11	45.61
CD8 ET	*LINC00152*	rs55956548	2	87768679	T	G	0.7418	0.2983	0.0525	1.32E-08	32.24
CD8 ET	*LINC00176*	rs816933	20	62662515	G	T	0.1906	0.5852	0.0596	1.01E-22	96.06
CD8 ET	*LINC00240*	rs66868086	6	27865902	T	A	0.0743	-0.5870	0.0752	5.95E-15	60.79
CD8 ET	*LINC00339*	rs11801382	1	22352108	T	C	0.2534	0.5319	0.0502	2.84E-26	112.23
CD8 ET	*LINC00426*	rs7983230	13	30942955	A	G	0.3075	0.3459	0.0484	9.00E-13	50.95
CD8 ET	*LINC00493*	rs3748450	20	18470664	G	A	0.8600	-0.3914	0.0658	2.73E-09	35.29
CD8 ET	*LINC00649*	rs9977699	21	35350902	T	C	0.4237	-0.4602	0.0446	5.94E-25	106.21
CD8 ET	*LINC00909*	rs28708663	18	72258352	T	C	0.1546	-0.3484	0.0635	4.10E-08	30.04
CD8 ET	*LINC00920*	rs2289149	16	66442406	T	C	0.6726	-0.6673	0.0442	2.11E-51	227.02
CD8 ET	*LINC00944*	rs1706506	12	127256415	T	C	0.9383	-0.9942	0.1011	7.91E-23	96.54
CD8 ET	*LINC00969*	rs3827561	3	195674257	G	C	0.4166	0.5437	0.0444	1.91E-34	149.5
CD8 ET	*LINC00969*	rs2251330	3	195460550	A	G	0.4306	0.2505	0.0454	3.48E-08	30.36
CD8 ET	*LINC01137*	rs2492301	1	37939173	C	T	0.5262	0.2731	0.0450	1.31E-09	36.72
CD8 ET	*LIPC*	rs113194763	15	58773939	A	G	0.0587	-0.5524	0.0890	5.30E-10	38.48
CD8 ET	*LL22NC03-2H8.5*	rs5757641	22	22598882	G	A	0.3591	0.3070	0.0463	3.23E-11	43.94
CD8 ET	*LLGL1*	rs8076336	17	18212614	T	G	0.4069	0.3052	0.0450	1.25E-11	45.8
CD8 ET	*LONP2*	rs8053839	16	48390512	T	G	0.5150	0.2706	0.0449	1.73E-09	36.19
CD8 ET	*LPIN1*	rs3762583	2	11924293	A	G	0.1508	-0.6388	0.0642	2.46E-23	98.85
CD8 ET	*LPIN2*	rs11665199	18	3057978	T	C	0.3719	0.2930	0.0454	1.06E-10	41.63
CD8 ET	*LRRC16B*	rs2877590	14	24512726	T	C	0.4792	0.3663	0.0444	1.63E-16	67.87
CD8 ET	*LRRC37A*	rs6503454	17	44118269	G	A	0.1501	-0.6124	0.0585	1.30E-25	109.21
CD8 ET	*LRRC37A2*	rs531934295	17	43670696	C	G	0.1833	0.4771	0.0559	1.37E-17	72.74
CD8 ET	*LRRC61*	rs3735167	7	150039555	T	C	0.2485	0.3020	0.0517	5.18E-09	34.05
CD8 ET	*LRRIQ3*	rs11806946	1	74663709	A	G	0.4704	-0.3868	0.0443	2.68E-18	75.96
CD8 ET	*LSM7*	rs11084936	19	2346440	A	G	0.5993	0.3878	0.0454	1.33E-17	72.8
CD8 ET	*LY6G5B*	rs1005598	6	31276760	C	T	0.6087	-0.3129	0.0449	3.23E-12	48.45
CD8 ET	*LYAR*	rs2980088	4	4268315	C	G	0.7218	-0.4874	0.0481	4.20E-24	102.35
CD8 ET	*LYRM4*	rs111557465	6	5260694	T	C	0.0736	1.0272	0.0786	4.59E-39	170.6
CD8 ET	*LYST*	rs2011390	1	235945502	G	A	0.7619	-0.3206	0.0507	2.56E-10	39.9
CD8 ET	*MAD1L1*	rs6967442	7	1926090	G	A	0.4437	-0.2956	0.0449	4.72E-11	43.2

CD8 ET	*MAF1*	rs56138570	8	145142206	A	G	0.0926	-0.7590	0.0753	6.86E-24	101.38
CD8 ET	*MAP3K5*	rs9402850	6	137164622	T	G	0.5967	0.3076	0.0453	1.10E-11	46.04
CD8 ET	*MARCH2*	rs12150986	19	8483308	T	C	0.7300	-0.3255	0.0499	7.10E-11	42.4
CD8 ET	*MAST3*	rs10409167	19	18275532	A	G	0.5211	-0.4042	0.0443	7.84E-20	82.92
CD8 ET	*MBP*	rs7241461	18	74778515	T	G	0.4675	-0.3841	0.0444	4.91E-18	74.76
CD8 ET	*MC1R*	rs2270460	16	89972416	A	C	0.0957	0.5213	0.0752	4.11E-12	47.98
CD8 ET	*MCC*	rs33554	5	112312736	C	G	0.4997	0.3029	0.0447	1.19E-11	45.89
CD8 ET	*MCF2L2*	rs6777106	3	183146433	G	C	0.4735	-0.3349	0.0446	6.18E-14	56.2
CD8 ET	*MDGA1*	rs9349050	6	37663624	C	T	0.5194	-0.6793	0.0424	8.88E-58	256.2
CD8 ET	*MDM4*	rs6681905	1	204535789	G	T	0.6799	-0.3526	0.0478	1.68E-13	54.24
CD8 ET	*MEAF6*	rs114745536	1	37958339	C	T	0.0676	-0.5148	0.0823	4.03E-10	39.02
CD8 ET	*MED16*	rs11546307	19	875389	A	G	0.2545	0.7465	0.0496	3.62E-51	225.95
CD8 ET	*METTL10*	rs2363340	10	126461368	A	C	0.5566	-0.2765	0.0454	1.17E-09	36.95
CD8 ET	*METTL18*	rs74122419	1	169861438	T	C	0.0959	-0.4860	0.0780	4.68E-10	38.73
CD8 ET	*MFF*	rs6436679	2	228183507	G	A	0.7905	0.4199	0.0564	9.98E-14	55.26
CD8 ET	*MGMT*	rs1008982	10	131445731	C	T	0.3802	-0.5126	0.0449	3.11E-30	130.28
CD8 ET	*MGST2*	rs3822045	4	140587414	C	A	0.2832	-0.3663	0.0502	2.87E-13	53.19
CD8 ET	*MGST3*	rs10800113	1	165571573	G	A	0.5758	-0.4061	0.0446	9.31E-20	82.58
CD8 ET	*MIAT*	rs6005127	22	27069557	T	C	0.2598	0.5412	0.0516	1.07E-25	109.6
CD8 ET	*MICB*	rs3828905	6	31464902	G	A	0.3350	0.2698	0.0482	2.11E-08	31.32
CD8 ET	*MIF*	rs2000466	22	24237862	G	T	0.1725	-0.5219	0.0581	2.76E-19	80.44
CD8 ET	*MINK1*	rs9895848	17	4738958	T	C	0.3298	-0.3070	0.0472	8.00E-11	42.17
CD8 ET	*MIR4435-1HG*	rs143087699	2	112187041	G	A	0.7228	-0.5336	0.0491	1.72E-27	117.78
CD8 ET	*MKRN1*	rs28563126	7	140170797	A	T	0.1416	-0.4481	0.0668	1.91E-11	44.97
CD8 ET	*MLF1*	rs10936149	3	158293893	G	T	0.1701	0.4662	0.0620	5.60E-14	56.39
CD8 ET	*MLF2*	rs1997510	12	6871891	T	C	0.3253	-0.2673	0.0477	2.03E-08	31.4
CD8 ET	*MLLT4*	rs3800531	6	168336052	A	G	0.3514	0.3122	0.0461	1.32E-11	45.69
CD8 ET	*MLTK*	rs3754747	2	173965296	A	G	0.1589	-0.4019	0.0633	2.16E-10	40.23
CD8 ET	*MPHOSPH6*	rs2303260	16	82203786	G	C	0.4801	-0.5025	0.0437	1.42E-30	131.83
CD8 ET	*MPHOSPH9*	rs2049114	12	123657338	C	T	0.7793	-0.3218	0.0575	2.22E-08	31.22
CD8 ET	*MPZL3*	rs12275546	11	118109097	A	G	0.7036	-0.3093	0.0507	1.07E-09	37.11
CD8 ET	*MRPL10*	rs17774272	17	45910616	C	T	0.3880	-0.3128	0.0455	6.49E-12	47.08
CD8 ET	*MRPL21*	rs482172	11	68668437	A	C	0.2904	-0.4260	0.0487	2.06E-18	76.48
CD8 ET	*MRPL32*	rs643599	7	42984190	T	C	0.8580	-0.5446	0.0651	5.70E-17	69.94
CD8 ET	*MRPL34*	rs35116124	19	17413160	T	C	0.3919	0.4110	0.0447	3.50E-20	84.51
CD8 ET	*MRPL41*	rs2254149	9	140441177	G	A	0.4605	0.3522	0.0445	2.53E-15	62.47
CD8 ET	*MRPL42*	rs12811748	12	93895773	C	T	0.1560	0.3571	0.0600	2.58E-09	35.41
CD8 ET	*MRPL53*	rs2270016	2	74686329	T	C	0.1735	-0.3798	0.0680	2.38E-08	31.09
CD8 ET	*MRPL54*	rs13960	19	3762698	T	C	0.6148	0.4560	0.0458	2.15E-23	99.11
CD8 ET	*MRPS18C*	rs1565909	4	84400330	T	C	0.4867	0.3458	0.0445	7.96E-15	60.22
CD8 ET	*MRPS21*	rs834233	1	150354276	A	G	0.3675	-0.2724	0.0463	4.05E-09	34.53
CD8 ET	*MRPS6*	rs2154568	21	35325805	G	C	0.4974	-0.3233	0.0447	4.58E-13	52.27
CD8 ET	*MSC*	rs3779760	8	72754639	G	C	0.2975	0.4323	0.0485	4.65E-19	79.41
CD8 ET	*MSH5*	rs2524080	6	31242089	T	A	0.7256	-0.3151	0.0484	7.20E-11	42.38
CD8 ET	*MT1E*	rs1580833	16	56644650	A	C	0.3093	-0.7321	0.0459	3.00E-57	253.78
CD8 ET	*MT1E*	rs11640851	16	56673227	A	C	0.6435	-0.3145	0.0466	1.43E-11	45.53
CD8 ET	*MT1F*	rs62038694	16	56690912	A	G	0.3937	0.4469	0.0447	1.43E-23	99.92
CD8 ET	*MT1X*	rs1599933	16	56715862	A	C	0.5528	-0.5142	0.0439	1.08E-31	136.93
CD8 ET	*MT2A*	rs1580833	16	56644650	A	C	0.3093	-1.1104	0.0411	4.17E-161	730.04
CD8 ET	*MTFP1*	rs5994294	22	30758236	T	C	0.1805	-0.3317	0.0580	1.05E-08	32.68
CD8 ET	*MTG2*	rs1627191	20	60784512	C	G	0.7302	0.3285	0.0502	6.02E-11	42.73
CD8 ET	*MTSS1*	rs55822443	8	125771210	T	C	0.2757	0.3278	0.0493	3.04E-11	44.06
CD8 ET	*MTSS1*	rs11778063	8	125577110	T	C	0.6697	-0.2719	0.0471	7.90E-09	33.23
CD8 ET	*MX1*	rs464138	21	42798173	C	A	0.4175	-0.3764	0.0450	6.47E-17	69.69
CD8 ET	*MXRA7*	rs2240773	17	74710783	A	C	0.0787	0.5336	0.0874	1.02E-09	37.22
CD8 ET	*MYCBPAP*	rs73351675	17	48585554	C	T	0.4566	0.4572	0.0443	6.38E-25	106.07
CD8 ET	*MYH11*	rs16967494	16	15820863	T	C	0.2539	0.4843	0.0492	6.75E-23	96.86
CD8 ET	*MYL12A*	rs3819221	18	3246391	G	C	0.2132	-0.3700	0.0580	1.83E-10	40.56
CD8 ET	*MYO15B*	rs76124218	17	73584441	C	G	0.0623	0.8604	0.0980	1.63E-18	76.94
CD8 ET	*MYO1F*	rs4804311	19	8615589	G	A	0.0908	0.8174	0.0830	6.92E-23	96.81
CD8 ET	*MYOM2*	rs13272507	8	2100135	G	C	0.2753	-0.8525	0.0474	3.10E-72	322.42
CD8 ET	*MZT2A*	rs12691980	2	132251685	A	G	0.2151	1.0605	0.0534	1.26E-87	392.96
CD8 ET	*NAA38*	rs7796145	7	117834763	T	G	0.6872	-0.4829	0.0467	4.24E-25	106.88
CD8 ET	*NAAA*	rs2292533	4	76861859	T	C	0.4012	0.4695	0.0444	3.83E-26	111.63
CD8 ET	*NAP1L1*	rs1368578	12	76476956	G	A	0.8151	-0.3614	0.0586	6.72E-10	38.02
CD8 ET	*NAPRT1*	rs9657360	8	144676862	T	C	0.7776	0.9085	0.0515	1.40E-69	310.25
CD8 ET	*NARG2*	rs28578662	15	60772674	C	T	0.2020	-0.3581	0.0557	1.28E-10	41.26
CD8 ET	*NBEAL2*	rs7640397	3	46989828	T	C	0.5694	0.3367	0.0453	1.07E-13	55.12
CD8 ET	*NBPF1*	rs10907231	1	16851440	T	C	0.3223	0.2968	0.0468	2.22E-10	40.18
CD8 ET	*NBPF3*	rs12566538	1	21814187	A	G	0.7816	-0.3988	0.0522	2.27E-14	58.16
CD8 ET	*NCAPG2*	rs73165322	7	158453165	A	G	0.0610	0.6858	0.0857	1.22E-15	63.91
CD8 ET	*NCF4*	rs2284027	22	37265079	C	G	0.7841	-0.3374	0.0557	1.39E-09	36.6
CD8 ET	*NCL*	rs13019380	2	232320581	G	T	0.1067	-0.4796	0.0751	1.68E-10	40.72
CD8 ET	*NCOA7*	rs11963811	6	126215101	C	T	0.1903	-0.3416	0.0569	1.90E-09	36
CD8 ET	*NDUFA12*	rs844333	12	95453279	G	C	0.6826	-0.5319	0.0461	9.06E-31	132.72
CD8 ET	*NDUFA3*	rs75622830	19	54611734	G	A	0.0957	0.7722	0.0733	6.34E-26	110.64
CD8 ET	*NDUFA6*	rs7292416	22	42807216	C	G	0.0459	0.7369	0.1011	3.07E-13	53.06
CD8 ET	*NDUFB2*	rs2364397	7	140405706	G	A	0.1697	-0.4431	0.0654	1.28E-11	45.75
CD8 ET	*NDUFC2*	rs689195	11	77781173	C	T	0.8429	0.3467	0.0627	3.22E-08	30.51

CD8 ET	*NDUFS5*	rs12043492	1	39457006	T	C	0.4366	-0.7361	0.0423	7.74E-68	302.27
CD8 ET	*NDUFS8*	rs4147779	11	67804807	A	G	0.7515	0.2969	0.0518	9.78E-09	32.82
CD8 ET	*NEAT1*	rs1144924	11	65203561	T	C	0.1978	0.3331	0.0563	3.35E-09	34.9
CD8 ET	*NELFCD*	rs17851253	20	57564695	T	C	0.1031	-0.5517	0.0735	6.01E-14	56.25
CD8 ET	*NFAM1*	rs2144413	22	43772658	T	C	0.5494	-0.4325	0.0442	1.19E-22	95.74
CD8 ET	*NINJ1*	rs12235416	9	95888667	C	T	0.1996	-0.6296	0.0564	6.27E-29	124.33
CD8 ET	*NIT2*	rs6771481	3	100135624	C	T	0.3801	-0.2656	0.0469	1.44E-08	32.06
CD8 ET	*NME1*	rs7207370	17	49231180	G	A	0.3899	0.4386	0.0452	3.07E-22	93.86
CD8 ET	*NME4*	rs77371232	16	447561	T	A	0.4244	0.7148	0.0426	3.23E-63	281.11
CD8 ET	*NMI*	rs17798290	2	152126473	G	T	0.4378	0.4803	0.0440	9.02E-28	119.05
CD8 ET	*NMRAL1*	rs934929	16	4587789	G	A	0.2743	0.3013	0.0500	1.72E-09	36.19
CD8 ET	*NMRK1*	rs11144225	9	77707300	T	A	0.3900	-0.5801	0.0437	3.89E-40	175.5
CD8 ET	*NOP10*	rs6495660	15	34612472	A	G	0.1473	0.3622	0.0629	8.42E-09	33.11
CD8 ET	*NOTCH4*	rs9271348	6	32583742	A	G	0.7413	-0.2936	0.0491	2.23E-09	35.69
CD8 ET	*NPC2*	rs61980863	14	74951808	A	G	0.4190	-0.3936	0.0447	1.31E-18	77.36
CD8 ET	*NPDC1*	rs28705707	9	139940653	A	C	0.3857	0.3626	0.0457	2.04E-15	62.89
CD8 ET	*NPIPA5*	rs153797	16	15538931	A	G	0.4873	0.4161	0.0442	4.84E-21	88.42
CD8 ET	*NQO2*	rs10223369	6	3018174	C	T	0.6782	-0.6413	0.0475	1.34E-41	182.18
CD8 ET	*NR2F6*	rs66606313	19	17345427	C	G	0.5369	-0.2803	0.0450	4.55E-10	38.78
CD8 ET	*NSA2*	rs72764630	5	73997524	T	A	0.1335	0.8644	0.0637	5.31E-42	184.02
CD8 ET	*NSG1*	rs2369105	4	4353086	G	A	0.7724	0.9326	0.0492	5.07E-80	358.1
CD8 ET	*NSG1*	rs4689412	4	4388017	G	A	0.5112	0.2518	0.0448	1.98E-08	31.45
CD8 ET	*NSUN4*	rs17413701	1	46818452	G	A	0.2594	0.3764	0.0510	1.66E-13	54.26
CD8 ET	*NSUN5*	rs10263853	7	72368344	T	G	0.5080	0.3140	0.0446	1.97E-12	49.41
CD8 ET	*NT5C3A*	rs2022230	7	33119907	A	G	0.7001	-0.3699	0.0498	1.17E-13	54.94
CD8 ET	*NT5C3B*	rs9897034	17	39992584	T	C	0.7555	-0.7608	0.0486	3.07E-55	244.58
CD8 ET	*NTPCR*	rs9424513	1	233074916	C	T	0.2251	0.6804	0.0512	2.30E-40	176.55
CD8 ET	*NUB1*	rs10256728	7	151067589	T	A	0.6885	0.3134	0.0485	1.07E-10	41.61
CD8 ET	*NUCB2*	rs214094	11	17293714	G	A	0.6043	-0.7496	0.0423	3.79E-70	312.85
CD8 ET	*NUDT14*	rs10873554	14	105648606	A	G	0.6217	0.2883	0.0461	3.98E-10	39.04
CD8 ET	*NUDT2*	rs4879776	9	34337967	A	G	0.1673	0.8413	0.0592	8.44E-46	201.39
CD8 ET	*NUP107*	rs2431655	12	69077497	C	T	0.3400	-0.3303	0.0477	4.50E-12	47.8
CD8 ET	*NUTF2*	rs59600980	16	67972257	C	T	0.1929	-0.4317	0.0599	5.55E-13	51.9
CD8 ET	*NXPE3*	rs4377474	3	101419903	A	G	0.0660	-0.5583	0.0907	7.62E-10	37.78
CD8 ET	*OASL*	rs7965349	12	121471931	T	C	0.1966	-0.3321	0.0574	7.37E-09	33.37
CD8 ET	*ORAI1*	rs7398511	12	122072829	A	C	0.1691	0.5193	0.0587	9.67E-19	77.97
CD8 ET	*ORAI2*	rs12669274	7	102076562	A	G	0.5236	0.3542	0.0445	1.69E-15	63.27
CD8 ET	*ORMDL3*	rs4065275	17	38080865	G	A	0.5069	0.8601	0.0404	2.24E-100	451.41
CD8 ET	*OSBPL5*	rs61503147	11	3171861	C	G	0.5642	0.2799	0.0454	6.92E-10	37.96
CD8 ET	*OSTF1*	rs11144219	9	77698483	A	G	0.3845	-0.3826	0.0451	2.14E-17	71.86
CD8 ET	*OSTF1*	rs10869515	9	77767481	T	C	0.2863	-0.3317	0.0508	6.84E-11	42.48
CD8 ET	*OXLD1*	rs11150804	17	79630238	G	A	0.3735	-0.3272	0.0466	2.18E-12	49.21
CD8 ET	*OXNAD1*	rs842268	3	16325182	C	T	0.4143	-0.3681	0.0448	2.17E-16	67.3
CD8 ET	*P4HA3*	rs2848557	11	73898409	C	T	0.7042	0.3891	0.0496	4.45E-15	61.36
CD8 ET	*PAPD4*	rs3829808	5	78909324	G	T	0.1105	0.4463	0.0777	9.43E-09	32.89
CD8 ET	*PARK7*	rs400736	1	8078309	C	T	0.5952	0.3744	0.0448	6.44E-17	69.7
CD8 ET	*PARVB*	rs6006622	22	44424108	T	C	0.6099	-0.2862	0.0455	3.34E-10	39.39
CD8 ET	*PASK*	rs7599243	2	242065306	A	G	0.1667	-0.3802	0.0628	1.37E-09	36.64
CD8 ET	*PAX8*	rs7589901	2	113993344	C	A	0.3680	-0.3395	0.0467	3.48E-13	52.81
CD8 ET	*PCBP2*	rs11170553	12	53822092	T	C	0.0740	-0.5023	0.0831	1.48E-09	36.48
CD8 ET	*PCM1*	rs409217	8	17816597	G	C	0.7858	-0.4267	0.0547	5.81E-15	60.84
CD8 ET	*PCNX*	rs2240533	14	71541026	C	T	0.3294	-0.3459	0.0483	7.93E-13	51.19
CD8 ET	*PCSK7*	rs74830	11	117090558	T	C	0.4053	0.3090	0.0459	1.68E-11	45.22
CD8 ET	*PDCD5*	rs34040260	19	33074614	C	A	0.3641	0.2740	0.0459	2.38E-09	35.56
CD8 ET	*PDCD6*	rs2244029	5	311478	C	T	0.6910	-0.3023	0.0495	1.05E-09	37.15
CD8 ET	*PDE4B*	rs571146	1	66753955	T	C	0.3446	0.2772	0.0466	2.78E-09	35.26
CD8 ET	*PDE7A*	rs72666512	8	66556843	A	C	0.1633	0.3526	0.0605	5.44E-09	33.96
CD8 ET	*PDIA6*	rs1734435	2	10928275	G	A	0.2774	-0.3769	0.0506	9.32E-14	55.39
CD8 ET	*PDK2*	rs1063647	17	48187884	T	C	0.5420	-0.2576	0.0450	1.02E-08	32.73
CD8 ET	*PDS5B*	rs7983065	13	33380786	T	C	0.4512	-0.2709	0.0449	1.65E-09	36.28
CD8 ET	*PDXDC1*	rs35811052	16	15128416	G	A	0.2479	0.3840	0.0505	2.77E-14	57.77
CD8 ET	*PDXK*	rs2276528	21	45152500	A	G	0.1480	0.5065	0.0617	2.31E-16	67.18
CD8 ET	*PDZD8*	rs61865312	10	119126662	T	C	0.2779	0.3039	0.0509	2.44E-09	35.51
CD8 ET	*PEX11G*	rs2287916	19	7528497	G	A	0.5195	-0.2536	0.0448	1.54E-08	31.94
CD8 ET	*PEX6*	rs10948059	6	42928461	T	C	0.4686	0.3031	0.0447	1.18E-11	45.91
CD8 ET	*PFKP*	rs872398	10	3135699	T	C	0.3231	0.3463	0.0464	8.47E-14	55.58
CD8 ET	*PGBD1*	rs3117145	6	29046418	T	A	0.0776	0.4664	0.0752	5.70E-10	38.34
CD8 ET	*PGLS*	rs10416082	19	17630461	A	G	0.5440	0.3171	0.0449	1.60E-12	49.82
CD8 ET	*PHACTR4*	rs9660549	1	28688657	G	A	0.3146	-0.2917	0.0481	1.28E-09	36.77
CD8 ET	*PHPT1*	rs2784075	9	139792926	C	T	0.7819	-0.3506	0.0535	5.43E-11	42.93
CD8 ET	*PI4K2B*	rs62411567	4	25164735	C	T	0.0897	-0.5428	0.0755	6.71E-13	51.52
CD8 ET	*PIGP*	rs7280542	21	38444995	G	A	0.6584	-0.4163	0.0466	3.87E-19	79.77
CD8 ET	*PILRB*	rs6955367	7	99961194	G	A	0.1838	1.1189	0.0507	8.16E-108	485.53
CD8 ET	*PIP4K2A*	rs4747443	10	22844326	C	A	0.6207	0.2571	0.0459	2.15E-08	31.29
CD8 ET	*PIWIL4*	rs73528744	11	94833768	C	T	0.0885	0.7471	0.0849	1.35E-18	77.31
CD8 ET	*PLA2G16*	rs4471424	11	63318725	C	T	0.4406	-0.2855	0.0453	2.89E-10	39.66
CD8 ET	*PLAGL1*	rs34047741	6	144368546	A	G	0.2094	0.3282	0.0554	3.13E-09	35.03
CD8 ET	*PLCB2*	rs2229691	15	40589002	G	A	0.2466	0.3197	0.0529	1.50E-09	36.46
CD8 ET	*PLCH2*	rs3935660	1	2351879	T	C	0.1957	0.3318	0.0569	5.38E-09	33.98

CD8 ET	*PLEK*	rs10496164	2	68610120	G	A	0.2875	0.5660	0.0498	5.65E-30	129.1
CD8 ET	*PLGLB1*	rs62144417	2	87303060	G	C	0.3640	0.2695	0.0463	5.91E-09	33.8
CD8 ET	*PLGRKT*	rs2381277	9	5381870	G	A	0.6665	-0.3319	0.0465	9.11E-13	50.92
CD8 ET	*PLOD1*	rs2273290	1	12018378	G	C	0.3332	-0.3801	0.0471	7.48E-16	64.87
CD8 ET	*PLS1*	rs6801610	3	142848367	G	A	0.4798	-0.6304	0.0428	4.35E-49	216.43
CD8 ET	*PLTP*	rs3091649	20	44537697	A	G	0.5578	-0.3457	0.0447	1.02E-14	59.73
CD8 ET	*PLXDC1*	rs62076619	17	37248261	G	A	0.2857	0.4465	0.0478	1.05E-20	86.89
CD8 ET	*PMEPA1*	rs2426713	20	55963389	C	T	0.5378	-0.3897	0.0444	1.72E-18	76.83
CD8 ET	*PNMT*	rs12453217	17	37741982	C	T	0.7800	0.6221	0.0545	3.37E-30	130.12
CD8 ET	*PNRC2*	rs17184588	1	24365512	C	T	0.4701	-0.5821	0.0432	1.94E-41	181.45
CD8 ET	*POLM*	rs28382621	7	44122613	G	C	0.0936	0.6691	0.0775	5.77E-18	74.45
CD8 ET	*POLR1D*	rs2232669	13	28195495	T	C	0.1167	0.8052	0.0647	1.58E-35	154.45
CD8 ET	*POLR2I*	rs2227260	19	36605647	C	T	0.6241	-0.2703	0.0457	3.38E-09	34.88
CD8 ET	*POLR2J*	rs6961234	7	102100616	G	A	0.6289	0.2730	0.0469	5.81E-09	33.83
CD8 ET	*POLR2J3*	rs12537647	7	102358917	C	T	0.6427	-0.2953	0.0468	2.88E-10	39.68
CD8 ET	*POMC*	rs3754861	2	25393722	C	A	0.0783	0.7526	0.0880	1.26E-17	72.91
CD8 ET	*POMZP3*	rs146021635	7	76179719	A	C	0.2083	0.6086	0.0535	6.05E-30	128.96
CD8 ET	*POU5F1*	rs2905708	6	31364603	T	C	0.0841	0.6971	0.0744	7.51E-21	87.55
CD8 ET	*POU5F1*	rs3094639	6	30206753	C	T	0.4314	-0.4462	0.0441	4.81E-24	102.08
CD8 ET	*PPA1*	rs7077538	10	71993094	G	T	0.3217	0.7775	0.0445	2.04E-68	304.92
CD8 ET	*PPDPF*	rs72629024	20	62152519	G	C	0.1633	-1.1696	0.0566	9.29E-95	425.65
CD8 ET	*PPFIBP2*	rs7126061	11	7548423	A	G	0.6036	0.3216	0.0460	2.73E-12	48.77
CD8 ET	*PPIA*	rs4724322	7	44822490	T	C	0.1484	0.6909	0.0718	6.57E-22	92.36
CD8 ET	*PPIE*	rs6670841	1	40220284	G	C	0.3493	-0.2693	0.0466	7.80E-09	33.26
CD8 ET	*PPIL3*	rs2136600	2	201742345	T	C	0.2391	-0.6085	0.0523	3.08E-31	134.86
CD8 ET	*PPIL6*	rs184918019	6	110202478	A	G	0.0312	0.7150	0.0999	8.33E-13	51.1
CD8 ET	*PPM1M*	rs2276834	3	52325759	G	A	0.5089	-0.3475	0.0445	6.05E-15	60.76
CD8 ET	*PPP1R14C*	rs111671759	6	150632231	C	T	0.1379	0.3649	0.0650	1.93E-08	31.5
CD8 ET	*PPP5C*	rs8106249	19	46862470	T	A	0.3075	-0.2911	0.0473	7.67E-10	37.77
CD8 ET	*PPT1*	rs4660382	1	40559207	T	C	0.8833	-0.4301	0.0724	2.85E-09	35.21
CD8 ET	*PPWD1*	rs79871714	5	64826562	G	C	0.3550	0.3632	0.0470	1.10E-14	59.58
CD8 ET	*PQLC3*	rs6706126	2	11434748	A	G	0.8820	0.4629	0.0670	4.91E-12	47.62
CD8 ET	*PRDX2*	rs897804	19	12876964	C	G	0.3850	0.5219	0.0446	1.32E-31	136.53
CD8 ET	*PRDX5*	rs11231746	11	64070940	C	T	0.3003	-0.6340	0.0458	1.48E-43	191.13
CD8 ET	*PRDX6*	rs4472784	1	173396971	T	C	0.3110	-0.4422	0.0476	1.43E-20	86.28
CD8 ET	*PRELID1*	rs6879874	5	176730775	T	A	0.7115	0.3850	0.0491	4.71E-15	61.25
CD8 ET	*PRKCB*	rs114827361	16	24045354	G	A	0.0539	0.9669	0.0993	2.20E-22	94.52
CD8 ET	*PRMT2*	rs7278089	21	47955501	A	T	0.7488	-0.3151	0.0524	1.76E-09	36.15
CD8 ET	*PRMT2*	rs2070435	21	47961711	A	G	0.3259	1.0733	0.0406	3.78E-154	698.11
CD8 ET	*PROSC*	rs7463174	8	37635649	G	A	0.1539	0.3529	0.0604	5.04E-09	34.11
CD8 ET	*PRR4*	rs61928603	12	11311787	T	C	0.8175	-0.3675	0.0593	5.81E-10	38.3
CD8 ET	*PRR7-AS1*	rs466256	5	176875956	A	C	0.5216	0.7014	0.0425	3.57E-61	271.75
CD8 ET	*PRSS57*	rs10412005	19	690639	T	C	0.5855	0.3269	0.0456	7.53E-13	51.3
CD8 ET	*PSD4*	rs2241976	2	113943470	G	A	0.4506	0.3535	0.0450	4.13E-15	61.51
CD8 ET	*PSMB3*	rs2338115	17	36929578	T	C	0.5309	-0.3635	0.0448	4.60E-16	65.83
CD8 ET	*PSMB9*	rs17587	6	32825090	A	G	0.2619	0.3021	0.0506	2.40E-09	35.55
CD8 ET	*PSMD5-AS1*	rs34025993	9	123516572	G	A	0.5819	-1.1951	0.0353	1.00E-200	1145.02
CD8 ET	*PSPH*	rs6593279	7	55803970	A	G	0.2274	0.4910	0.0587	6.27E-17	69.75
CD8 ET	*PSTPIP1*	rs35093280	15	77386853	T	C	0.3583	0.3712	0.0450	1.63E-16	67.87
CD8 ET	*PTAR1*	rs1330333	9	72313038	A	C	0.1293	0.3846	0.0676	1.27E-08	32.31
CD8 ET	*PTGDR*	rs8022207	14	52619837	A	G	0.3067	-0.4599	0.0489	5.03E-21	88.34
CD8 ET	*PTGDS*	rs4880082	9	139878828	C	T	0.7109	0.4152	0.0489	2.11E-17	71.9
CD8 ET	*PTP4A3*	rs34642025	8	142415650	G	A	0.3135	0.5338	0.0485	3.37E-28	121
CD8 ET	*PTPLAD2*	rs1857649	9	21008725	G	A	0.5340	-0.2774	0.0448	5.81E-10	38.31
CD8 ET	*PTPN2*	rs559406	18	12857002	T	G	0.5296	-0.2492	0.0451	3.25E-08	30.49
CD8 ET	*PTPN4*	rs3101946	2	120580542	A	C	0.3079	-0.2939	0.0489	1.88E-09	36.02
CD8 ET	*PTPRA*	rs677344	20	2846289	A	G	0.5352	-0.2697	0.0448	1.72E-09	36.19
CD8 ET	*PWWP2A*	rs1106055	5	159491520	G	A	0.5964	-0.2828	0.0458	6.82E-10	37.99
CD8 ET	*PXT1*	rs3734339	6	36562982	C	T	0.1175	0.3689	0.0668	3.28E-08	30.47
CD8 ET	*PYHIN1*	rs2325704	1	158909164	A	C	0.7626	0.6517	0.0502	1.73E-38	167.97
CD8 ET	*QDPR*	rs10020773	4	17526682	C	A	0.2377	-0.3279	0.0526	4.63E-10	38.75
CD8 ET	*R3HDM4*	rs11546307	19	875389	A	G	0.2545	0.5852	0.0508	9.80E-31	132.57
CD8 ET	*RAB11A*	rs75567160	15	66135748	A	G	0.0847	0.6037	0.0734	1.92E-16	67.54
CD8 ET	*RAB34*	rs67590677	17	26887271	A	G	0.0812	-0.4312	0.0790	4.85E-08	29.72
CD8 ET	*RAB6B*	rs1880667	3	133602268	A	C	0.2707	0.3188	0.0511	4.43E-10	38.83
CD8 ET	*RABAC1*	rs11665965	19	42429833	A	G	0.6630	0.2856	0.0468	1.08E-09	37.1
CD8 ET	*RAC1*	rs836516	7	6462203	T	A	0.4416	0.6591	0.0429	2.45E-53	235.88
CD8 ET	*RAC2*	rs4821611	22	37622475	A	G	0.1520	0.3618	0.0649	2.48E-08	31.02
CD8 ET	*RAD51C*	rs35797125	17	56736547	A	G	0.1986	0.3819	0.0540	1.57E-12	49.86
CD8 ET	*RALGDS*	rs560297	9	136005637	G	A	0.6000	-0.2702	0.0453	2.37E-09	35.57
CD8 ET	*RAMP1*	rs6741923	2	238799206	G	C	0.7239	-0.3413	0.0500	8.70E-12	46.51
CD8 ET	*RASA3*	rs3118896	13	114931769	G	C	0.4162	0.2887	0.0453	1.83E-10	40.56
CD8 ET	*RBAK-RBAKDN*	rs9689983	7	5024540	A	G	0.0550	-0.5790	0.0884	5.66E-11	42.85
CD8 ET	*RBL2*	rs9927982	16	53474108	T	C	0.4714	-0.3739	0.0445	4.43E-17	70.43
CD8 ET	*RBM43*	rs55775354	2	152118271	A	G	0.4344	-0.6121	0.0431	7.21E-46	201.7
CD8 ET	*RBMS2*	rs10735863	12	56921966	T	C	0.5022	0.4237	0.0441	8.19E-22	91.92
CD8 ET	*RCN1*	rs4922580	11	32143936	C	G	0.5242	-0.5187	0.0437	1.54E-32	140.8
CD8 ET	*RCSD1*	rs2862865	1	167673555	G	A	0.2864	0.3959	0.0477	1.13E-16	68.59
CD8 ET	*RDH13*	rs1654410	19	55524813	T	C	0.5070	0.2873	0.0447	1.35E-10	41.16

CD8 ET	*RDH5*	rs3138142	12	56115585	T	C	0.2120	-0.3028	0.0533	1.30E-08	32.26
CD8 ET	*REEP4*	rs12678092	8	21994951	T	C	0.1734	0.5396	0.0587	4.07E-20	84.22
CD8 ET	*REEP5*	rs153569	5	112274827	C	T	0.4735	0.2780	0.0449	5.87E-10	38.29
CD8 ET	*RGS10*	rs11198977	10	121256622	G	T	0.0450	-0.6630	0.0997	2.98E-11	44.1
CD8 ET	*RGS17*	rs1281973	6	153471311	T	C	0.5184	-0.3369	0.0446	4.23E-14	56.94
CD8 ET	*RHOC*	rs937053	1	113246229	A	T	0.5401	0.3923	0.0445	1.12E-18	77.68
CD8 ET	*RHOH*	rs13151567	4	40250140	A	G	0.5830	-0.2727	0.0456	2.27E-09	35.65
CD8 ET	*RNASEH1-AS1*	rs4583476	2	3608756	C	T	0.3128	-0.2751	0.0491	2.18E-08	31.26
CD8 ET	*RNASEH2C*	rs34007146	11	65477954	A	G	0.3457	-0.3800	0.0464	2.82E-16	66.79
CD8 ET	*RNASET2*	rs2149084	6	167370988	C	G	0.4944	0.5642	0.0434	1.23E-38	168.64
CD8 ET	*RNF166*	rs11649075	16	88766740	T	C	0.1936	-0.4248	0.0558	2.66E-14	57.86
CD8 ET	*RNF168*	rs28625638	3	196197104	C	G	0.1624	-0.3739	0.0561	2.58E-11	44.38
CD8 ET	*ROBO3*	rs11219821	11	124741772	T	C	0.4408	0.2683	0.0450	2.56E-09	35.42
CD8 ET	*RP1-111D6.3*	rs7744115	6	150529051	C	T	0.1275	-0.6499	0.0648	1.14E-23	100.37
CD8 ET	*RP1-244F24.1*	rs11751172	6	45552731	T	C	0.2598	0.2877	0.0518	2.77E-08	30.8
CD8 ET	*RP1-29C18.10*	rs8138399	22	50044824	T	C	0.6367	-0.8299	0.0441	7.56E-79	352.72
CD8 ET	*RP1-3J17.3*	rs7771612	6	86264374	G	T	0.6109	-0.7032	0.0437	2.86E-58	258.45
CD8 ET	*RP1-3J17.3*	rs9359656	6	86179835	C	T	0.1044	0.4978	0.0723	5.79E-12	47.3
CD8 ET	*RP1-40E16.12*	rs12665605	6	3067003	A	G	0.5491	-0.2690	0.0454	3.05E-09	35.08
CD8 ET	*RP1-55C23.7*	rs1883617	6	133072650	G	A	0.3480	0.8210	0.0434	8.21E-80	357.14
CD8 ET	*RP1-8B1.4*	rs9357500	6	46097580	G	T	0.5142	0.5081	0.0438	3.72E-31	134.49
CD8 ET	*RP11-108M9.4*	rs3901404	1	16764703	T	A	0.4603	0.2616	0.0454	8.56E-09	33.08
CD8 ET	*RP11-108M9.4*	rs28711566	1	17215933	T	C	0.0493	-0.5402	0.0909	2.82E-09	35.23
CD8 ET	*RP11-119F19.2*	rs4933802	10	81578891	T	C	0.5923	0.3431	0.0459	7.80E-14	55.74
CD8 ET	*RP11-137H2.4*	rs11186029	10	82315290	C	T	0.2942	0.4362	0.0485	2.53E-19	80.61
CD8 ET	*RP11-138A9.1*	rs205725	7	130623437	T	C	0.9081	0.6162	0.0741	9.13E-17	69.01
CD8 ET	*RP11-138A9.2*	rs205724	7	130623257	C	A	0.8419	0.4898	0.0599	2.82E-16	66.79
CD8 ET	*RP11-166B2.5*	rs435108	16	12009614	A	G	0.9304	-0.8197	0.0953	8.09E-18	73.78
CD8 ET	*RP11-18H21.1*	rs13151283	4	153027177	C	T	0.2641	-0.5273	0.0489	4.18E-27	116.02
CD8 ET	*RP11-218M22.1*	rs758792	12	676900	G	A	0.8461	-0.3663	0.0619	3.25E-09	34.95
CD8 ET	*RP11-222K16.2*	rs72902589	3	27754726	C	G	0.2329	0.3792	0.0514	1.65E-13	54.27
CD8 ET	*RP11-23P13.6*	rs77996527	15	42177085	A	G	0.0506	0.5833	0.0949	7.94E-10	37.7
CD8 ET	*RP11-245G13.2*	rs7604815	2	11020809	T	C	0.1127	0.6464	0.0720	2.85E-19	80.38
CD8 ET	*RP11-274B21.10*	rs34960471	7	128293294	A	G	0.1467	-0.3746	0.0664	1.66E-08	31.8
CD8 ET	*RP11-279F6.3*	rs71393466	15	70034953	G	A	0.0861	0.4677	0.0748	4.07E-10	39
CD8 ET	*RP11-294J22.6*	rs17563146	17	48769329	T	C	0.1201	-0.3767	0.0649	6.42E-09	33.64
CD8 ET	*RP11-298J20.3*	rs2114834	10	126650186	G	A	0.7359	-1.0602	0.0456	2.54E-119	538.31
CD8 ET	*RP11-299J3.8*	rs12636106	3	122164156	T	C	0.1515	-0.3332	0.0589	1.52E-08	31.97
CD8 ET	*RP11-305L7.1*	rs7046632	9	93919341	T	C	0.2002	-0.5819	0.0539	3.75E-27	116.23
CD8 ET	*RP11-305L7.3*	rs10820831	9	93903636	A	G	0.5937	0.4653	0.0447	2.30E-25	108.09
CD8 ET	*RP11-326C3.11*	rs11246065	11	324170	G	A	0.3701	0.8904	0.0425	1.66E-97	438.26
CD8 ET	*RP11-326C3.11*	rs6598043	11	324630	C	G	0.6761	-0.3118	0.0491	2.19E-10	40.21
CD8 ET	*RP11-334C17.5*	rs2304856	17	78175483	C	T	0.5261	-0.3458	0.0448	1.19E-14	59.44
CD8 ET	*RP11-345J4.5*	rs4471699	16	30320307	T	G	0.4341	-0.4764	0.0439	1.87E-27	117.61
CD8 ET	*RP11-347P5.1*	rs4850675	2	197030087	A	G	0.5880	0.7392	0.0430	2.83E-66	295.11
CD8 ET	*RP11-367G6.3*	rs4711076	6	25091714	C	T	0.2543	-0.3896	0.0521	7.60E-14	55.79
CD8 ET	*RP11-369E15.3*	rs6989851	8	20897084	T	C	0.8362	-0.4361	0.0620	1.99E-12	49.39
CD8 ET	*RP11-382J12.1*	rs7814274	8	71570411	C	T	0.5000	-0.2920	0.0447	6.80E-11	42.49
CD8 ET	*RP11-383H13.1*	rs13257737	8	72752975	A	C	0.1418	-0.3492	0.0611	1.10E-08	32.6
CD8 ET	*RP11-395B7.4*	rs3924886	7	100656003	C	G	0.4971	0.2738	0.0448	9.97E-10	37.26
CD8 ET	*RP11-430H10.1*	rs12418562	11	45373480	C	G	0.1739	0.4829	0.0578	6.87E-17	69.57
CD8 ET	*RP11-434C1.1*	rs7976950	12	11349454	C	T	0.6020	-0.3018	0.0454	3.04E-11	44.06
CD8 ET	*RP11-439L18.1*	rs12193417	6	142357391	T	C	0.2112	-0.3452	0.0552	3.89E-10	39.09
CD8 ET	*RP11-445H22.3*	rs11696392	20	43300331	G	C	0.5943	0.2599	0.0454	1.06E-08	32.65
CD8 ET	*RP11-448A19.1*	rs10954245	7	129229787	A	G	0.3928	0.3397	0.0456	9.13E-14	55.43
CD8 ET	*RP11-473I1.5*	rs12918271	16	9200547	C	T	0.2190	-0.6998	0.0529	5.99E-40	174.64
CD8 ET	*RP11-473M20.9*	rs4786384	16	3136919	C	T	0.5202	0.5258	0.0436	1.80E-33	145.06
CD8 ET	*RP11-47L3.1*	rs7221768	17	33562776	T	C	0.4045	-0.2754	0.0461	2.38E-09	35.56
CD8 ET	*RP11-485G4.2*	rs73236059	3	141877407	G	T	0.1916	0.3139	0.0557	1.75E-08	31.69
CD8 ET	*RP11-493L12.4*	rs1101796	12	47647013	T	G	0.2991	0.3538	0.0469	4.46E-14	56.84
CD8 ET	*RP11-498D10.6*	rs35593117	16	71377311	A	G	0.5929	0.2954	0.0460	1.33E-10	41.18
CD8 ET	*RP11-499P20.2*	rs7904263	10	18880525	A	C	0.3683	0.2872	0.0473	1.28E-09	36.76
CD8 ET	*RP11-509J21.2*	rs10814395	9	3660648	C	G	0.6857	-0.3281	0.0479	7.28E-12	46.86
CD8 ET	*RP11-526I2.5*	rs36053285	15	101114640	C	T	0.4253	-0.3662	0.0453	5.86E-16	65.35
CD8 ET	*RP11-532F6.3*	rs2804744	6	733163	T	C	0.7252	0.3294	0.0524	3.14E-10	39.5
CD8 ET	*RP11-538P18.2*	rs76182200	3	158180219	T	C	0.0750	-0.6056	0.0896	1.35E-11	45.65
CD8 ET	*RP11-539L10.2*	rs3822263	4	6697673	C	T	0.7466	-0.4045	0.0524	1.21E-14	59.4
CD8 ET	*RP11-539L10.3*	rs2301787	4	6677063	A	G	0.1652	-0.4774	0.0616	9.09E-15	59.96
CD8 ET	*RP11-571L19.8*	rs28730602	4	100001682	T	G	0.1362	-0.4931	0.0634	7.43E-15	60.36
CD8 ET	*RP11-586D19.2*	rs73075449	4	6158873	G	A	0.0768	0.6551	0.0921	1.11E-12	50.54
CD8 ET	*RP11-596D21.1*	rs12883273	14	31743604	C	T	0.3933	-0.4895	0.0449	1.29E-27	118.35
CD8 ET	*RP11-611D20.2*	rs3013301	9	139431719	A	G	0.3581	-0.3820	0.0466	2.44E-16	67.08
CD8 ET	*RP11-629O1.2*	rs7837708	8	134591109	A	G	0.4290	-0.3707	0.0452	2.32E-16	67.18
CD8 ET	*RP11-656D10.3*	rs10789144	1	40955156	T	A	0.5066	0.2988	0.0449	2.77E-11	44.24
CD8 ET	*RP11-65I12.1*	rs10175462	2	113988492	A	G	0.3750	0.7106	0.0443	6.26E-58	256.9
CD8 ET	*RP11-660L16.2*	rs11606611	11	71164046	G	C	0.6900	0.6828	0.0530	5.96E-38	165.52
CD8 ET	*RP11-669E14.6*	rs2435204	17	43988205	G	A	0.2099	0.4148	0.0527	3.33E-15	61.94
CD8 ET	*RP11-69E11.4*	rs3820618	1	40223128	C	T	0.1312	0.6055	0.0671	1.77E-19	81.32
CD8 ET	*RP11-755F10.1*	rs10896076	11	65840668	G	A	0.2217	-0.3607	0.0534	1.44E-11	45.52

CD8 ET	*RP11-77H9.2*	rs620126	16	9399509	T	C	0.1989	0.6203	0.0533	2.79E-31	135.06
CD8 ET	*RP11-792A8.4*	rs78996796	7	66301193	T	C	0.0468	0.6769	0.0970	2.94E-12	48.63
CD8 ET	*RP11-798G7.6*	rs3874943	17	44572068	G	A	0.5235	-0.2504	0.0449	2.37E-08	31.1
CD8 ET	*RP11-81H14.2*	rs731938	12	68843191	G	A	0.6401	0.8768	0.0411	5.86E-101	454.08
CD8 ET	*RP11-841O20.2*	rs8009184	14	53018758	C	G	0.1355	0.3985	0.0667	2.32E-09	35.62
CD8 ET	*RP11-950C14.3*	rs175016	14	75459633	T	C	0.4662	0.3863	0.0443	2.93E-18	75.78
CD8 ET	*RP11-950C14.7*	rs7147118	14	75610170	C	G	0.4838	0.5717	0.0434	1.17E-39	173.31
CD8 ET	*RP11-96H19.1*	rs11613912	12	46739740	T	C	0.2693	-0.5597	0.0493	7.05E-30	128.66
CD8 ET	*RP11-973H7.1*	rs2542152	18	12782761	C	T	0.4054	-0.3936	0.0454	4.06E-18	75.14
CD8 ET	*RP11-973H7.4*	rs592390	18	12822314	C	T	0.4453	-0.3054	0.0449	1.07E-11	46.09
CD8 ET	*RP11-98G7.1*	rs4127047	1	156794822	G	C	0.6464	-0.3007	0.0472	1.93E-10	40.45
CD8 ET	*RP3-325F22.5*	rs3757163	6	136874451	C	T	0.1539	-0.5627	0.0639	1.25E-18	77.46
CD8 ET	*RP3-466P17.1*	rs11756359	6	145639105	C	T	0.4727	-0.3443	0.0445	1.02E-14	59.73
CD8 ET	*RP3-510H16.3*	rs45519932	22	36007464	A	C	0.0808	0.4355	0.0791	3.73E-08	30.23
CD8 ET	*RP4-539M6.22*	rs5997646	22	30830312	T	A	0.1911	-0.4218	0.0571	1.46E-13	54.51
CD8 ET	*RP4-745E8.2*	rs17354986	1	20585773	T	C	0.2062	0.4710	0.0520	1.27E-19	81.97
CD8 ET	*RP4-758J18.13*	rs3766169	1	1497201	C	A	0.3233	-0.4525	0.0488	1.77E-20	85.85
CD8 ET	*RP4-799D16.1*	rs311452	1	25392364	A	G	0.2847	0.3094	0.0499	5.60E-10	38.38
CD8 ET	*RP5-1021I20.1*	rs12890967	14	74296119	G	T	0.1339	0.5516	0.0600	3.70E-20	84.4
CD8 ET	*RP5-1042K10.10*	rs139919	22	40726183	C	T	0.1767	-0.3884	0.0581	2.32E-11	44.59
CD8 ET	*RP5-827C21.4*	rs6695813	1	234515608	C	A	0.0910	0.6232	0.0777	1.04E-15	64.22
CD8 ET	*RP6-109B7.3*	rs73177006	22	46454712	A	G	0.1024	0.5364	0.0756	1.30E-12	50.22
CD8 ET	*RPA2*	rs2236074	1	28211384	A	G	0.3691	-0.3935	0.0455	5.60E-18	74.51
CD8 ET	*RPL10A*	rs2076172	6	35436925	T	C	0.5358	-0.7916	0.0418	6.11E-80	357.72
CD8 ET	*RPL11*	rs77766201	1	24006446	A	G	0.1560	-0.9442	0.0597	2.33E-56	249.71
CD8 ET	*RPL12*	rs2247781	9	130208924	C	T	0.5921	1.0073	0.0391	1.11E-146	663.83
CD8 ET	*RPL13*	rs3211568	16	89629780	A	G	0.1858	-0.6899	0.0543	5.21E-37	161.21
CD8 ET	*RPL14*	rs75965075	3	40438120	T	C	0.0715	0.6346	0.0850	8.34E-14	55.61
CD8 ET	*RPL22L1*	rs34934154	3	170574875	C	T	0.2506	-0.4287	0.0497	6.13E-18	74.33
CD8 ET	*RPL23A*	rs2302205	17	26947046	C	A	0.0821	-0.9339	0.0767	4.25E-34	147.92
CD8 ET	*RPL27A*	rs61875926	11	8705528	G	C	0.0958	-0.5315	0.0739	6.16E-13	51.69
CD8 ET	*RPL28*	rs17700376	19	55897327	G	A	0.3547	-1.2241	0.0365	1.00E-200	1125.42
CD8 ET	*RPL3*	rs470082	22	39715140	T	C	0.4764	0.2987	0.0447	2.34E-11	44.57
CD8 ET	*RPL32*	rs2290311	3	12883071	A	T	0.1438	0.5345	0.0612	2.52E-18	76.08
CD8 ET	*RPL36AL*	rs1952013	14	50087357	G	C	0.7531	1.0954	0.0463	6.52E-124	559.37
CD8 ET	*RPL8*	rs2954658	8	146017782	T	C	0.4990	-1.0482	0.0376	2.94E-171	776.63
CD8 ET	*RPL9*	rs13103023	4	39457617	A	G	0.3195	0.4017	0.0473	1.98E-17	72.02
CD8 ET	*RPLP2*	rs12277141	11	798865	C	G	0.6564	-0.3731	0.0476	4.87E-15	61.19
CD8 ET	*RPPH1*	rs76311431	14	20786432	G	C	0.1346	0.3701	0.0663	2.36E-08	31.11
CD8 ET	*RPS10*	rs9380433	6	34377626	T	C	0.7750	-1.1225	0.0524	5.93E-102	458.65
CD8 ET	*RPS11*	rs10403394	19	49999345	A	G	0.1225	-0.6016	0.0713	3.10E-17	71.14
CD8 ET	*RPS12*	rs12203738	6	133188449	G	A	0.0637	0.6019	0.0925	7.49E-11	42.3
CD8 ET	*RPS14*	rs2245384	5	149817268	C	G	0.3033	-0.3406	0.0486	2.50E-12	48.95
CD8 ET	*RPS16*	rs480595	19	39925482	T	A	0.3758	0.4650	0.0465	1.61E-23	99.69
CD8 ET	*RPS18*	rs17215231	6	33239869	T	C	0.0640	-1.5902	0.0823	3.30E-83	372.7
CD8 ET	*RPS19*	rs7257666	19	42360356	A	G	0.4966	0.3384	0.0445	2.99E-14	57.62
CD8 ET	*RPS2*	rs17135720	16	2014935	C	T	0.1128	0.7816	0.0661	2.81E-32	139.61
CD8 ET	*RPS23*	rs226198	5	81573992	T	C	0.7755	0.4948	0.0507	1.65E-22	95.09
CD8 ET	*RPS24*	rs2913134	10	79763775	T	C	0.5775	0.3804	0.0456	7.43E-17	69.41
CD8 ET	*RPS25*	rs11602764	11	118888619	G	A	0.2652	-0.7156	0.0477	6.18E-51	224.88
CD8 ET	*RPS26*	rs773114	12	56379060	T	A	0.3997	1.2091	0.0350	1.00E-200	1194.3
CD8 ET	*RPS3*	rs3094304	11	75118158	G	A	0.0727	-0.8224	0.0900	6.35E-20	83.34
CD8 ET	*RPS3A*	rs1148652	4	151801938	C	T	0.0703	-1.2771	0.0970	1.39E-39	172.98
CD8 ET	*RPS5*	rs731835	19	58898436	C	T	0.3256	-1.0875	0.0405	1.40E-158	718.45
CD8 ET	*RPS6*	rs13298176	9	19363521	C	T	0.0908	-0.6447	0.0821	3.96E-15	61.59
CD8 ET	*RPS7*	rs62106040	2	3627302	T	A	0.0757	-1.2504	0.0852	8.95E-49	215
CD8 ET	*RPS8*	rs12120833	1	45241285	G	C	0.1242	-1.0301	0.0586	3.10E-69	308.67
CD8 ET	*RPS9*	rs158366	19	54705317	G	C	0.3901	-0.9234	0.0408	1.57E-113	511.74
CD8 ET	*RPSA*	rs28576532	3	39447788	G	A	0.1555	0.8635	0.0587	6.02E-49	215.78
CD8 ET	*RRAS2*	rs2915407	11	14330260	T	C	0.7547	0.4906	0.0518	2.77E-21	89.52
CD8 ET	*RRP7A*	rs137092	22	42984131	G	A	0.1378	0.5001	0.0614	3.70E-16	66.25
CD8 ET	*RSRC2*	rs6416298	12	123002143	T	C	0.7480	-0.4376	0.0494	8.18E-19	78.3
CD8 ET	*RTKN2*	rs4979775	10	63981847	A	G	0.7616	-0.4072	0.0511	1.58E-15	63.4
CD8 ET	*RUNX2*	rs6938214	6	45544364	A	G	0.2433	-0.3362	0.0526	1.58E-10	40.84
CD8 ET	*S100B*	rs9306156	21	47960493	C	T	0.3298	0.6817	0.0453	2.96E-51	226.34
CD8 ET	*SACM1L*	rs6765732	3	45685386	A	G	0.6315	-0.4957	0.0464	1.25E-26	113.85
CD8 ET	*SAMD12*	rs2514610	8	119659371	A	G	0.9210	-0.4328	0.0776	2.49E-08	31.01
CD8 ET	*SAMD3*	rs41285310	6	130505374	C	A	0.5086	-0.3236	0.0446	4.14E-13	52.47
CD8 ET	*SAMHD1*	rs6513797	20	35567722	C	G	0.1742	0.3485	0.0607	9.33E-09	32.91
CD8 ET	*SAMM50*	rs62228002	22	44356480	T	G	0.1273	-0.5053	0.0730	4.49E-12	47.8
CD8 ET	*SAMSN1*	rs2223092	21	15927837	C	A	0.4886	0.3462	0.0446	8.42E-15	60.11
CD8 ET	*SAMSN1*	rs467140	21	15965927	G	T	0.5506	-0.2962	0.0450	4.43E-11	43.32
CD8 ET	*SAR1A*	rs9971029	10	71916461	A	G	0.3972	-0.4669	0.0450	2.91E-25	107.62
CD8 ET	*SAT2*	rs1642771	17	7504060	C	G	0.4008	0.3438	0.0457	5.59E-14	56.39
CD8 ET	*SC5D*	rs12275106	11	121170025	C	A	0.0740	0.5181	0.0831	4.61E-10	38.76
CD8 ET	*SCAMP3*	rs1076556	1	155234821	G	A	0.2668	0.4208	0.0490	9.58E-18	73.45
CD8 ET	*SCCPDH*	rs10924809	1	246862588	T	C	0.3099	-0.3507	0.0487	6.11E-13	51.71
CD8 ET	*SCD5*	rs4572871	4	83760887	G	A	0.7766	-0.4988	0.0537	1.63E-20	86.03
CD8 ET	*SCGB3A1*	rs7729771	5	179993813	C	T	0.8901	-1.4188	0.0669	6.45E-100	449.31

CD8 ET	*SCRN2*	rs2325752	17	45921906	C	T	0.5233	-0.3571	0.0446	1.26E-15	63.84
CD8 ET	*SEC61B*	rs79754161	9	101985668	T	C	0.0730	-0.6793	0.0787	6.23E-18	74.3
CD8 ET	*SEPT2*	rs2074771	2	242350944	G	A	0.0944	0.8375	0.0731	2.23E-30	130.94
CD8 ET	*SERAC1*	rs12662156	6	158589039	T	G	0.1271	0.3459	0.0631	4.15E-08	30.02
CD8 ET	*SERPINB6*	rs62391543	6	2940740	T	C	0.2038	-0.4697	0.0538	2.69E-18	75.95
CD8 ET	*SESTD1*	rs13389956	2	180112641	G	C	0.1818	0.4474	0.0587	2.50E-14	57.97
CD8 ET	*SETD5-AS1*	rs2648535	3	9408443	C	A	0.5954	0.2536	0.0460	3.44E-08	30.38
CD8 ET	*SETD9*	rs185220	5	56205357	G	A	0.8359	0.5153	0.0624	1.40E-16	68.16
CD8 ET	*SF1*	rs533447	11	64561908	C	T	0.8796	-0.6873	0.0773	6.12E-19	78.87
CD8 ET	*SFT2D1*	rs4598048	6	166732624	G	C	0.7415	0.3736	0.0506	1.51E-13	54.44
CD8 ET	*SFXN4*	rs10749291	10	120920588	C	T	0.4801	0.2884	0.0447	1.12E-10	41.51
CD8 ET	*SGCD*	rs1545773	5	155239884	G	A	0.3630	0.3133	0.0468	2.07E-11	44.82
CD8 ET	*SH3GLB1*	rs263433	1	87180889	G	A	0.2315	0.3126	0.0538	6.43E-09	33.63
CD8 ET	*SH3TC1*	rs11729860	4	8230945	G	A	0.3130	0.4546	0.0471	4.71E-22	93.02
CD8 ET	*SH3YL1*	rs55936726	2	258210	C	T	0.3311	-0.3967	0.0463	1.10E-17	73.18
CD8 ET	*SHFM1*	rs12671864	7	96269575	G	C	0.1705	0.4468	0.0610	2.36E-13	53.57
CD8 ET	*SHMT1*	rs669340	17	18263957	C	G	0.6337	0.2583	0.0463	2.48E-08	31.01
CD8 ET	*SIDT1-AS1*	rs35452897	3	113282075	T	C	0.2149	0.7126	0.0523	2.42E-42	185.58
CD8 ET	*SIRPA*	rs1049901	20	1147775	A	G	0.0730	-0.7192	0.0805	4.09E-19	79.66
CD8 ET	*SIRPB1*	rs2252155	20	1508001	G	A	0.8553	0.4471	0.0597	7.28E-14	55.88
CD8 ET	*SKAP2*	rs17323934	7	26904330	G	C	0.2183	0.5441	0.0518	9.19E-26	109.9
CD8 ET	*SLC12A7*	rs35188965	5	1104938	T	C	0.5560	-0.5345	0.0442	1.27E-33	145.74
CD8 ET	*SLC14A1*	rs7228161	18	43266885	A	C	0.5472	-0.3091	0.0448	5.18E-12	47.52
CD8 ET	*SLC15A4*	rs11059919	12	129289190	A	G	0.5998	-0.2802	0.0460	1.14E-09	36.99
CD8 ET	*SLC16A4*	rs12120793	1	110911766	A	G	0.4352	0.3140	0.0447	2.10E-12	49.29
CD8 ET	*SLC22A18*	rs386111	11	2933605	G	A	0.7712	-0.3767	0.0519	4.05E-13	52.51
CD8 ET	*SLC25A1*	rs807668	22	19162686	G	A	0.6545	-0.5059	0.0457	1.82E-28	122.23
CD8 ET	*SLC25A24*	rs12118280	1	108738780	C	T	0.2365	0.2848	0.0517	3.71E-08	30.23
CD8 ET	*SLC25A26*	rs13078983	3	66356902	C	T	0.2178	-0.2949	0.0524	1.80E-08	31.64
CD8 ET	*SLC25A29*	rs1059264	14	100769163	T	C	0.3348	0.9757	0.0421	6.21E-119	536.52
CD8 ET	*SLC25A51*	rs7852991	9	37876559	T	C	0.1447	0.3614	0.0639	1.55E-08	31.92
CD8 ET	*SLC38A1*	rs7308375	12	46585291	G	A	0.3089	-0.2842	0.0487	5.31E-09	34
CD8 ET	*SLC44A2*	rs3087969	19	10738639	T	C	0.7830	0.3702	0.0538	5.71E-12	47.33
CD8 ET	*SLC44A4*	rs3131383	6	31704294	T	G	0.0935	-0.4196	0.0686	9.84E-10	37.28
CD8 ET	*SLC4A7*	rs4973776	3	27530208	A	G	0.2167	-0.4746	0.0515	3.12E-20	84.74
CD8 ET	*SLFN5*	rs11656388	17	33531815	A	G	0.3965	0.3807	0.0455	6.31E-17	69.74
CD8 ET	*SMDT1*	rs2301521	22	42423110	C	G	0.6779	1.2135	0.0387	1.00E-200	983.41
CD8 ET	*SMIM19*	rs2923429	8	42389743	G	A	0.6251	-0.4702	0.0450	1.43E-25	109.03
CD8 ET	*SMIM20*	rs2305670	4	25915858	A	C	0.2457	-0.3470	0.0503	5.17E-12	47.52
CD8 ET	*SMPD3*	rs12927428	16	68425493	G	C	0.5121	0.2518	0.0450	2.18E-08	31.26
CD8 ET	*SNAP29*	rs73162844	22	21211220	C	T	0.0680	0.6758	0.0817	1.35E-16	68.24
CD8 ET	*SNHG7*	rs11145844	9	139613998	T	C	0.1148	-0.9162	0.0688	1.90E-40	176.92
CD8 ET	*SNHG8*	rs705837	4	119225940	T	C	0.3810	0.9772	0.0397	1.16E-133	604.09
CD8 ET	*SNORD3B-2*	rs77232220	17	19262534	C	G	0.1953	0.4729	0.0547	5.40E-18	74.58
CD8 ET	*SNRNP25*	rs216594	16	103009	T	A	0.8277	-0.3833	0.0591	9.14E-11	41.91
CD8 ET	*SNRPC*	rs2764208	6	34714322	G	A	0.3488	-0.2688	0.0485	2.99E-08	30.65
CD8 ET	*SNRPD2*	rs36040835	19	46201213	G	T	0.2051	-0.7249	0.0539	3.34E-41	180.37
CD8 ET	*SNRPF*	rs80247972	12	96270298	T	C	0.1753	0.3239	0.0568	1.17E-08	32.47
CD8 ET	*SNX11*	rs2240122	17	46152559	A	C	0.2193	-0.3472	0.0561	6.22E-10	38.17
CD8 ET	*SOCS6*	rs1942172	18	68774209	A	T	0.4798	0.3586	0.0446	9.06E-16	64.49
CD8 ET	*SOD2*	rs41267779	6	160147058	C	G	0.0495	0.5563	0.1014	4.06E-08	30.06
CD8 ET	*SP110*	rs2288328	2	231106244	C	G	0.3247	0.2840	0.0469	1.42E-09	36.57
CD8 ET	*SPATA20*	rs989128	17	48636534	A	G	0.3595	-0.4653	0.0462	7.70E-24	101.15
CD8 ET	*SPATC1L*	rs62215189	21	47603995	G	A	0.2446	-0.6814	0.0490	6.08E-44	192.9
CD8 ET	*SPECC1*	rs10459969	17	19935901	C	T	0.1985	0.4095	0.0550	9.59E-14	55.34
CD8 ET	*SPG21*	rs2414867	15	65298775	T	C	0.5528	0.2596	0.0449	7.52E-09	33.33
CD8 ET	*SPNS1*	rs8045689	16	28988269	T	C	0.7030	-0.3372	0.0494	8.99E-12	46.44
CD8 ET	*SPON2*	rs6836335	4	1165748	C	T	0.1148	0.4281	0.0729	4.34E-09	34.39
CD8 ET	*SPRYD4*	rs2657910	12	56891649	A	G	0.1288	0.3527	0.0628	1.99E-08	31.44
CD8 ET	*SPSB1*	rs4465231	1	9347278	A	G	0.2585	0.3090	0.0503	7.83E-10	37.73
CD8 ET	*SPSB2*	rs2269359	12	6993653	A	G	0.2882	0.5590	0.0477	9.18E-32	137.26
CD8 ET	*SPTBN1*	rs13016942	2	54779234	C	T	0.7524	-0.3562	0.0511	3.15E-12	48.5
CD8 ET	*SQSTM1*	rs475716	5	179249433	C	T	0.5242	-0.2770	0.0447	5.98E-10	38.25
CD8 ET	*SRA1*	rs2563253	5	139962167	G	A	0.5395	-0.2473	0.0450	3.75E-08	30.22
CD8 ET	*SRD5A3*	rs36110240	4	56217015	T	C	0.1629	0.5633	0.0601	6.88E-21	87.72
CD8 ET	*SRP14*	rs16924528	15	40328292	G	A	0.3179	-0.3650	0.0464	3.62E-15	61.77
CD8 ET	*SRSF10*	rs6424144	1	24375663	A	G	0.4721	-0.5183	0.0436	1.41E-32	140.98
CD8 ET	*ST6GALNAC6*	rs10819317	9	130668957	G	A	0.1684	-0.4371	0.0588	1.09E-13	55.08
CD8 ET	*STARD3NL*	rs9639788	7	38220597	G	A	0.2537	0.3229	0.0509	2.15E-10	40.24
CD8 ET	*STK32C*	rs7085852	10	134144005	C	T	0.1782	-0.3542	0.0593	2.35E-09	35.59
CD8 ET	*STK4*	rs6031924	20	43627183	G	A	0.1737	0.3970	0.0601	3.84E-11	43.61
CD8 ET	*STMN3*	rs879471	20	62269942	T	C	0.5285	0.4871	0.0439	1.46E-28	122.65
CD8 ET	*STX8*	rs7207541	17	9344134	G	A	0.2376	0.3016	0.0500	1.64E-09	36.29
CD8 ET	*STX8*	rs72814104	17	9127852	T	C	0.2427	0.2906	0.0524	2.94E-08	30.69
CD8 ET	*STXBP2*	rs2303115	19	7708214	A	G	0.3932	-0.3190	0.0459	3.77E-12	48.14
CD8 ET	*SULF2*	rs3091426	20	46189755	A	G	0.5891	-0.8081	0.0416	3.34E-84	377.26
CD8 ET	*SURF1*	rs3118667	9	136291063	T	C	0.5241	-0.4394	0.0441	2.18E-23	99.09
CD8 ET	*SVIP*	rs6483880	11	22852740	A	G	0.5468	0.2880	0.0454	2.23E-10	40.17
CD8 ET	*SYNE1*	rs9478342	6	152916571	T	C	0.1307	0.5346	0.0687	7.24E-15	60.41

CD8 ET	*SYNGR1*	rs909685	22	39747671	A	T	0.3216	0.8464	0.0460	1.08E-75	338.26
CD8 ET	*SYNM*	rs1044356	15	99645813	A	G	0.3406	-0.4189	0.0471	6.09E-19	78.88
CD8 ET	*TAGLN2*	rs6427499	1	159891917	T	C	0.4829	0.3868	0.0444	2.95E-18	75.77
CD8 ET	*TAMM41*	rs1498090	3	11890647	T	C	0.4272	-0.2560	0.0454	1.71E-08	31.73
CD8 ET	*TAPBP*	rs2239841	6	33281842	T	G	0.2873	-0.3045	0.0490	5.07E-10	38.57
CD8 ET	*TAPBPL*	rs2534711	12	6570821	G	T	0.3045	0.6349	0.0475	9.75E-41	178.25
CD8 ET	*TBCA*	rs254415	5	77071703	T	G	0.2878	-0.7065	0.0466	7.23E-52	229.15
CD8 ET	*TC2N*	rs2498851	14	92332548	A	C	0.1782	0.6155	0.0581	3.19E-26	111.99
CD8 ET	*TCF25*	rs60862594	16	89978338	A	G	0.0941	0.6341	0.0757	5.28E-17	70.08
CD8 ET	*TECR*	rs891089	19	14674235	T	C	0.5505	-0.3518	0.0447	3.63E-15	61.76
CD8 ET	*TEN1*	rs12945114	17	73993546	T	C	0.7534	0.4454	0.0517	6.71E-18	74.15
CD8 ET	*TEPP*	rs4784020	16	58010708	C	T	0.8341	0.4552	0.0604	4.92E-14	56.64
CD8 ET	*TESC*	rs3736417	12	117537379	T	G	0.5565	0.2658	0.0448	3.01E-09	35.11
CD8 ET	*TESPA1*	rs266673	12	55141026	C	A	0.6678	-0.3094	0.0462	2.21E-11	44.68
CD8 ET	*TEX12*	rs17114972	11	113009703	A	G	0.1244	0.4433	0.0770	8.54E-09	33.08
CD8 ET	*TFPT*	rs80269846	19	54614095	G	A	0.1310	-0.4920	0.0628	4.72E-15	61.25
CD8 ET	*THAP7-AS1*	rs7292605	22	21365271	A	G	0.2086	-0.4668	0.0553	2.94E-17	71.24
CD8 ET	*THAP9-AS1*	rs4484290	4	83755424	T	C	0.7772	0.5018	0.0539	1.26E-20	86.52
CD8 ET	*THEM4*	rs10788814	1	151853754	C	T	0.9155	0.5473	0.0831	4.52E-11	43.29
CD8 ET	*THEMIS2*	rs1884469	1	28239434	A	G	0.3803	-0.9350	0.0409	7.89E-116	522.29
CD8 ET	*TIFA*	rs4834247	4	113179077	T	C	0.5244	-0.2906	0.0447	8.07E-11	42.15
CD8 ET	*TIMM10*	rs2848626	11	57283988	T	C	0.7820	-0.8827	0.0482	5.40E-75	335.06
CD8 ET	*TIMM13*	rs6945	19	2426828	C	G	0.4919	0.3808	0.0444	9.04E-18	73.56
CD8 ET	*TIPARP-AS1*	rs9822906	3	156384295	G	A	0.1423	-0.4294	0.0621	4.76E-12	47.68
CD8 ET	*TLE4*	rs4877500	9	82302854	G	A	0.6372	-0.4214	0.0462	7.76E-20	82.94
CD8 ET	*TLK1*	rs55996750	2	171878189	G	A	0.3583	-0.3769	0.0464	4.55E-16	65.85
CD8 ET	*TMA7*	rs9881491	3	48487911	A	G	0.4359	0.2590	0.0450	8.77E-09	33.03
CD8 ET	*TMC4*	rs36653	19	54668487	T	C	0.2599	-0.5235	0.0482	1.75E-27	117.74
CD8 ET	*TMCO3*	rs2257460	13	114131954	T	C	0.2056	-0.3761	0.0567	3.18E-11	43.97
CD8 ET	*TMED9*	rs186803	5	177016095	C	T	0.5554	0.3223	0.0449	6.90E-13	51.47
CD8 ET	*TMEM101*	rs77536093	17	42092328	G	A	0.0441	-0.5854	0.1021	9.73E-09	32.83
CD8 ET	*TMEM120B*	rs73413708	12	122213144	C	G	0.0964	0.9520	0.0763	1.08E-35	155.2
CD8 ET	*TMEM121*	rs35592422	14	105996593	C	G	0.2770	-0.4457	0.0522	1.42E-17	72.67
CD8 ET	*TMEM156*	rs6819988	4	39038665	T	C	0.5743	0.5818	0.0436	1.52E-40	177.37
CD8 ET	*TMEM18*	rs2293083	2	676177	G	C	0.7439	-0.3394	0.0508	2.36E-11	44.56
CD8 ET	*TMEM2*	rs62547055	9	74350747	A	G	0.0778	0.5298	0.0773	7.15E-12	46.89
CD8 ET	*TMEM204*	rs2294624	16	1586255	A	G	0.1329	0.9262	0.0638	9.24E-48	210.36
CD8 ET	*TMEM230*	rs4315602	20	5061017	C	T	0.5174	0.4591	0.0441	2.09E-25	108.28
CD8 ET	*TMEM244*	rs113781346	6	130158232	T	C	0.4466	0.3485	0.0445	4.73E-15	61.24
CD8 ET	*TMEM258*	rs102274	11	61557826	C	T	0.3344	0.3362	0.0476	1.55E-12	49.88
CD8 ET	*TMEM50A*	rs55794721	1	25561667	A	G	0.3493	-0.3965	0.0453	2.17E-18	76.38
CD8 ET	*TMEM50B*	rs13049439	21	34846997	A	G	0.5332	0.4650	0.0440	4.15E-26	111.48
CD8 ET	*TMEM55A*	rs7016498	8	92050604	G	T	0.7055	-0.2948	0.0491	1.91E-09	35.99
CD8 ET	*TMEM80*	rs7112962	11	654091	A	G	0.4583	-0.3740	0.0448	7.17E-17	69.48
CD8 ET	*TMEM8A*	rs2363755	16	430292	G	C	0.4125	-0.4326	0.0443	1.43E-22	95.37
CD8 ET	*TMOD1*	rs2480721	9	100337311	A	G	0.4641	-0.2551	0.0449	1.30E-08	32.26
CD8 ET	*TMTC4*	rs7987438	13	101326992	A	C	0.3103	0.3012	0.0485	5.35E-10	38.47
CD8 ET	*TNFRSF14*	rs3748825	1	2483270	C	T	0.5115	0.3034	0.0447	1.09E-11	46.06
CD8 ET	*TNRC18*	rs67800908	7	5429212	A	G	0.5048	0.2673	0.0448	2.42E-09	35.53
CD8 ET	*TOMM7*	rs2240728	7	22852499	T	C	0.3422	-1.2433	0.0380	1.00E-200	1070.44
CD8 ET	*TP53I11*	rs747614	11	45119181	C	A	0.2737	0.6264	0.0492	3.73E-37	161.87
CD8 ET	*TPD52*	rs2033066	8	81050423	C	T	0.4469	0.6120	0.0432	1.71E-45	199.99
CD8 ET	*TPGS1*	rs62130840	19	512359	G	C	0.2458	0.3158	0.0495	1.78E-10	40.61
CD8 ET	*TPGS2*	rs948636	18	34361762	C	G	0.2855	-0.5570	0.0480	4.46E-31	134.13
CD8 ET	*TPM1*	rs73431508	15	63340416	G	A	0.1364	-0.4091	0.0655	4.10E-10	38.99
CD8 ET	*TPM2*	rs1538535	9	35681299	T	C	0.2588	-0.3268	0.0504	8.65E-11	42.02
CD8 ET	*TPRA1*	rs920233	3	127295084	T	C	0.2195	-0.3382	0.0541	3.95E-10	39.06
CD8 ET	*TPST2*	rs4275	22	26921725	G	A	0.4260	-0.3175	0.0449	1.61E-12	49.81
CD8 ET	*TRAPPC12-AS1*	rs12993215	2	3473727	C	T	0.3980	-0.3785	0.0460	1.90E-16	67.56
CD8 ET	*TRAPPC2L*	rs3826061	16	88927221	C	T	0.3581	-0.4108	0.0478	8.55E-18	73.67
CD8 ET	*TRAPPC4*	rs60065241	11	118883491	A	C	0.2219	0.4517	0.0525	8.21E-18	73.75
CD8 ET	*TRIM23*	rs42884	5	64952870	C	T	0.6339	-0.3337	0.0471	1.38E-12	50.11
CD8 ET	*TRIM47*	rs9908862	17	73873394	G	A	0.1537	0.4086	0.0613	2.65E-11	44.33
CD8 ET	*TRMT112*	rs616563	11	63877501	C	G	0.3934	0.3249	0.0454	8.53E-13	51.05
CD8 ET	*TRMT112*	rs79423518	11	64105454	A	G	0.0682	-1.0125	0.0962	6.98E-26	110.45
CD8 ET	*TSGA10*	rs4340581	2	99725025	T	C	0.6316	0.3669	0.0464	2.55E-15	62.46
CD8 ET	*TSPAN15*	rs61848397	10	71225584	A	C	0.2261	0.3473	0.0525	3.71E-11	43.67
CD8 ET	*TSPAN32*	rs12800998	11	2317951	T	C	0.3391	-0.5426	0.0451	2.88E-33	144.12
CD8 ET	*TSPO*	rs138910	22	43550055	C	T	0.7417	-0.5247	0.0502	1.28E-25	109.25
CD8 ET	*TSTD1*	rs3766383	1	161008572	A	C	0.1552	-0.8007	0.0655	2.21E-34	149.21
CD8 ET	*TSTD3*	rs4618535	6	99935669	T	C	0.7378	-0.4230	0.0498	2.05E-17	71.95
CD8 ET	*TTC16*	rs565073	9	130510673	G	A	0.6321	0.3666	0.0457	1.02E-15	64.26
CD8 ET	*TTC32*	rs35957485	2	20090677	A	G	0.0880	0.7450	0.0801	1.41E-20	86.31
CD8 ET	*TTC38*	rs9615347	22	46728153	G	A	0.1060	-0.8710	0.0720	9.94E-34	146.23
CD8 ET	*TUBA1B*	rs7958241	12	49509262	G	A	0.3538	0.3653	0.0469	6.52E-15	60.61
CD8 ET	*TUBB2A*	rs9392464	6	3145127	G	A	0.7429	0.2918	0.0504	6.90E-09	33.49
CD8 ET	*TUFM*	rs8049439	16	28837515	C	T	0.3939	0.7791	0.0428	4.01E-74	331.07
CD8 ET	*TXN*	rs1049927	9	113018755	C	T	0.1259	-0.3963	0.0634	3.95E-10	39.06
CD8 ET	*TYMP*	rs9616864	22	50976152	C	T	0.1038	0.4734	0.0700	1.33E-11	45.68

CD8 ET	*U2AF1L4*	rs2871921	19	36232420	T	C	0.1557	0.6296	0.0628	1.16E-23	100.33
CD8 ET	*U91328.19*	rs73730705	6	25383843	C	T	0.1480	0.5341	0.0605	1.00E-18	77.9
CD8 ET	*UAP1*	rs6700298	1	162511529	A	C	0.5228	-0.2498	0.0449	2.62E-08	30.91
CD8 ET	*UBA52*	rs8110371	19	18667272	C	A	0.5875	-0.5922	0.0440	2.90E-41	180.66
CD8 ET	*UBA6*	rs2253064	4	68478067	C	T	0.5160	-0.3256	0.0447	3.13E-13	53.01
CD8 ET	*UBE2L3*	rs5998509	22	21917479	T	C	0.2076	-0.4798	0.0578	1.01E-16	68.81
CD8 ET	*UBE2Q2*	rs35880865	15	76073665	A	G	0.3196	0.3213	0.0483	3.02E-11	44.08
CD8 ET	*UBXN6*	rs7769	19	4360543	A	G	0.2393	-0.3960	0.0525	4.79E-14	56.7
CD8 ET	*UCP2*	rs2515087	11	73682872	T	C	0.4077	0.4596	0.0451	2.07E-24	103.74
CD8 ET	*UFSP2*	rs13107209	4	186348817	A	G	0.7371	0.3147	0.0515	9.76E-10	37.3
CD8 ET	*ULK4*	rs73073235	3	42012693	A	C	0.1576	0.5622	0.0602	9.48E-21	87.09
CD8 ET	*UNC5B-AS1*	rs10999732	10	72981204	G	A	0.1430	-0.3964	0.0631	3.27E-10	39.43
CD8 ET	*UPF3A*	rs3752107	13	115064423	A	G	0.0702	-0.7453	0.0833	3.61E-19	79.91
CD8 ET	*UPK3BL*	rs11981219	7	102339168	T	C	0.8859	-0.6426	0.0720	4.65E-19	79.41
CD8 ET	*UQCRH*	rs41292543	1	46774783	G	A	0.1009	-0.7612	0.0750	3.25E-24	102.85
CD8 ET	*UROS*	rs1571278	10	127485254	G	A	0.4424	-0.2911	0.0449	8.70E-11	42.01
CD8 ET	*USF1*	rs12116949	1	161042063	A	C	0.2747	0.3205	0.0488	5.36E-11	42.95
CD8 ET	*USP44*	rs10859898	12	95957901	T	C	0.1405	0.4293	0.0646	2.96E-11	44.11
CD8 ET	*VAMP2*	rs4792267	17	8061337	A	G	0.6696	0.4007	0.0487	1.85E-16	67.61
CD8 ET	*VAMP4*	rs12119232	1	171711152	C	A	0.1951	0.4419	0.0555	1.71E-15	63.24
CD8 ET	*VAMP5*	rs17508727	2	85799776	A	G	0.1165	-0.9856	0.0642	3.40E-53	235.22
CD8 ET	*VCL*	rs12355188	10	75993245	A	C	0.6024	0.4042	0.0451	2.93E-19	80.32
CD8 ET	*VDAC2*	rs11543	10	76970613	C	G	0.5682	-0.2821	0.0449	3.39E-10	39.35
CD8 ET	*VIM*	rs3758411	10	17271110	C	G	0.2870	0.3332	0.0491	1.11E-11	46.03
CD8 ET	*VIT*	rs12618630	2	37025076	G	A	0.7255	-0.3299	0.0498	3.33E-11	43.89
CD8 ET	*VOPP1*	rs10230195	7	55636629	G	A	0.3757	0.2730	0.0456	2.15E-09	35.76
CD8 ET	*VPS28*	rs117891721	8	145642886	T	C	0.0569	1.0220	0.0929	3.86E-28	120.73
CD8 ET	*WAC-AS1*	rs2993986	10	28800023	T	C	0.7960	-0.5954	0.0522	3.87E-30	129.85
CD8 ET	*WARS*	rs1570305	14	100808155	A	G	0.7850	-0.3118	0.0538	6.91E-09	33.49
CD8 ET	*WBP1L*	rs2265309	10	104487871	C	T	0.5179	0.2496	0.0449	2.71E-08	30.85
CD8 ET	*WBSCR22*	rs143598667	7	73091122	T	C	0.0467	0.5775	0.0972	2.88E-09	35.19
CD8 ET	*WDR37*	rs4880758	10	1085572	G	C	0.1033	0.4722	0.0760	5.15E-10	38.54
CD8 ET	*WDR63*	rs72720544	1	85497262	C	T	0.2425	-0.3002	0.0520	7.89E-09	33.24
CD8 ET	*WIPI1*	rs28449485	17	66449844	G	T	0.1229	-0.3824	0.0680	1.91E-08	31.52
CD8 ET	*WNK1*	rs34038797	12	740009	G	C	0.4812	-0.2535	0.0449	1.62E-08	31.84
CD8 ET	*XBP1*	rs6005892	22	29199258	A	C	0.3336	0.3087	0.0484	1.76E-10	40.63
CD8 ET	*XCL1*	rs1040508	1	168500750	T	C	0.3842	0.8829	0.0422	3.66E-97	436.68
CD8 ET	*XCL2*	rs141673522	1	168517508	T	C	0.1712	-0.9426	0.0558	6.18E-64	284.4
CD8 ET	*XRCC6BP1*	rs10431506	12	58311524	A	G	0.3136	-0.3854	0.0473	3.48E-16	66.38
CD8 ET	*XRRA1*	rs61894839	11	74626366	A	G	0.2756	0.9878	0.0437	3.91E-113	509.92
CD8 ET	*XXbac-BPG181B23.7*	rs66609536	6	31362120	A	G	0.2726	-0.2962	0.0534	2.92E-08	30.69
CD8 ET	*XXbac-BPG252P9.10*	rs1264372	6	30769726	T	C	0.2185	0.3010	0.0509	3.40E-09	34.87
CD8 ET	*XXbac-BPG252P9.9*	rs1150772	6	30384671	C	G	0.1489	-0.3953	0.0616	1.36E-10	41.14
CD8 ET	*XXbac-BPG299F13.17*	rs6921663	6	31154620	C	T	0.2178	1.0599	0.0493	1.41E-102	461.51
CD8 ET	*XXbac-BPG299F13.17*	rs9261742	6	30368663	A	G	0.2468	0.4070	0.0539	4.45E-14	56.84
CD8 ET	*XXbac-BPG299F13.17*	rs2250287	6	31330251	G	T	0.1615	-0.3351	0.0569	3.79E-09	34.66
CD8 ET	*YBEY*	rs62224180	21	47726332	G	C	0.7751	-0.7706	0.0547	4.36E-45	198.13
CD8 ET	*YBX3*	rs12317203	12	10876059	T	G	0.3863	-0.3307	0.0452	2.55E-13	53.42
CD8 ET	*YEATS4*	rs11561378	12	69767949	A	G	0.4326	-0.5227	0.0437	6.06E-33	142.65
CD8 ET	*YME1L1*	rs4749235	10	27481667	A	G	0.5879	0.2723	0.0456	2.30E-09	35.62
CD8 ET	*YWHAB*	rs6017442	20	43527337	T	C	0.2901	-0.5327	0.0493	3.37E-27	116.45
CD8 ET	*YWHAH*	rs7290696	22	32341684	T	C	0.2316	0.3070	0.0530	6.83E-09	33.51
CD8 ET	*ZBTB1*	rs28827477	14	64946510	C	T	0.2940	-0.3115	0.0487	1.57E-10	40.86
CD8 ET	*ZC3HAV1*	rs10270221	7	138734838	G	A	0.5050	-0.5749	0.0432	2.35E-40	176.5
CD8 ET	*ZDHHC4*	rs11559147	7	6617247	G	C	0.7006	0.3493	0.0494	1.58E-12	49.85
CD8 ET	*ZFAS1*	rs237714	20	47922900	T	C	0.2041	0.6401	0.0522	1.40E-34	150.11
CD8 ET	*ZFP57*	rs3131875	6	29666111	C	T	0.4611	0.4091	0.0442	2.23E-20	85.4
CD8 ET	*ZFP90*	rs11865339	16	68565296	T	C	0.7620	0.4168	0.0530	3.65E-15	61.76
CD8 ET	*ZNF100*	rs6511291	19	21950402	C	T	0.5555	-0.3729	0.0450	1.19E-16	68.49
CD8 ET	*ZNF138*	rs56063644	7	64364866	C	T	0.4472	0.2574	0.0452	1.23E-08	32.37
CD8 ET	*ZNF165*	rs3131337	6	28796071	C	G	0.0902	-0.4793	0.0728	4.48E-11	43.3
CD8 ET	*ZNF165*	rs61173069	6	28725704	G	T	0.1621	-0.7072	0.0560	1.49E-36	159.12
CD8 ET	*ZNF205-AS1*	rs56037566	16	3114026	A	T	0.1011	-0.6656	0.0744	3.62E-19	79.9
CD8 ET	*ZNF257*	rs7248898	19	22245354	A	G	0.2955	0.3021	0.0498	1.26E-09	36.79
CD8 ET	*ZNF266*	rs6512138	19	9560304	A	G	0.6311	0.2894	0.0465	4.67E-10	38.73
CD8 ET	*ZNF302*	rs73042013	19	35134619	T	A	0.1260	0.5436	0.0668	4.10E-16	66.05
CD8 ET	*ZNF391*	rs9468121	6	27382381	G	A	0.7594	-0.5853	0.0514	4.76E-30	129.44
CD8 ET	*ZNF44*	rs1399699	19	12373005	T	G	0.3111	-0.3391	0.0483	2.31E-12	49.1
CD8 ET	*ZNF480*	rs12975893	19	52865649	G	C	0.5110	0.2453	0.0448	4.48E-08	29.87
CD8 ET	*ZNF502*	rs56084453	3	44762830	G	A	0.2115	0.3328	0.0544	9.30E-10	37.39
CD8 ET	*ZNF506*	rs4808992	19	19920912	C	T	0.1808	0.3635	0.0591	7.50E-10	37.81
CD8 ET	*ZNF519*	rs12607953	18	14125085	C	T	0.5360	-0.2500	0.0449	2.50E-08	31
CD8 ET	*ZNF559*	rs28384491	19	9434872	T	C	0.0862	-0.4738	0.0742	1.70E-10	40.71
CD8 ET	*ZNF584*	rs36090113	19	58912384	C	G	0.2097	-0.4180	0.0538	7.64E-15	60.3
CD8 ET	*ZNF593*	rs12117236	1	26523507	C	T	0.2432	0.4527	0.0520	3.00E-18	75.73
CD8 ET	*ZNF600*	rs4802995	19	53284720	G	C	0.4576	0.6160	0.0429	1.08E-46	205.47
CD8 ET	*ZNF677*	rs61576844	19	53761591	G	T	0.2247	-0.3252	0.0537	1.39E-09	36.61
CD8 ET	*ZNF749*	rs7255035	19	57943697	G	A	0.5087	0.4365	0.0441	4.67E-23	97.58
CD8 ET	*ZNF786*	rs62507489	7	148781070	C	G	0.6174	0.2767	0.0461	1.93E-09	35.97

CD8 ET	*ZNF793*	rs35540940	19	38008235	A	G	0.1762	0.3480	0.0587	3.03E-09	35.09
CD8 ET	*ZNF83*	rs7256557	19	53119166	A	G	0.3634	0.4354	0.0460	2.71E-21	89.56
CD8 ET	*ZNF846*	rs10414720	19	9780361	C	A	0.1410	0.3729	0.0648	8.90E-09	33
CD8 ET	*ZNF85*	rs2545225	19	21042460	G	A	0.1318	0.7076	0.0628	1.84E-29	126.77
CD8 ET	*ZNRD1*	rs35144883	6	29952482	T	C	0.0678	0.7022	0.0962	2.82E-13	53.22
CD8 ET	*ZP3*	rs11772694	7	76195020	A	G	0.3120	0.7773	0.0451	1.43E-66	296.47
CD8 ET	*ZSWIM7*	rs6416868	17	15924370	G	A	0.5948	-0.3414	0.0450	3.30E-14	57.43
CD8 NC	*ABCG2*	rs7688567	4	88426498	A	G	0.4006	-0.6263	0.0452	1.10E-43	191.72
CD8 NC	*ABHD16A*	rs2763981	6	31840021	A	T	0.7096	0.3545	0.0478	1.25E-13	54.82
CD8 NC	*AC002331.1*	rs11642354	16	26604708	T	C	0.2577	0.3167	0.0510	5.17E-10	38.53
CD8 NC	*AC004854.4*	rs4724368	7	45187733	T	C	0.8126	-0.4937	0.0579	1.55E-17	72.51
CD8 NC	*AC005253.2*	rs59798925	19	18671740	T	C	0.5478	-0.3232	0.0449	6.21E-13	51.67
CD8 NC	*AC006369.2*	rs917733	2	37831453	C	T	0.5906	-0.7633	0.0420	7.47E-74	329.83
CD8 NC	*AC007383.4*	rs4147713	2	207015772	C	A	0.4843	-0.6591	0.0428	1.37E-53	237.03
CD8 NC	*AC009120.10*	rs889641	16	74333380	C	T	0.3044	-0.2907	0.0479	1.31E-09	36.73
CD8 NC	*AC010642.1*	rs3206946	19	58826534	G	A	0.2046	-0.3193	0.0563	1.44E-08	32.07
CD8 NC	*AC011484.1*	rs11670534	19	47003906	T	C	0.1454	0.3939	0.0594	3.46E-11	43.81
CD8 NC	*AC013264.2*	rs10931780	2	198175297	C	G	0.3785	0.9406	0.0409	8.19E-117	526.8
CD8 NC	*AC016757.3*	rs6740485	2	239137672	C	T	0.4607	0.3189	0.0447	1.00E-12	50.74
CD8 NC	*AC018816.3*	rs901854	3	4856234	A	G	0.6030	0.2637	0.0458	8.41E-09	33.11
CD8 NC	*AC021860.1*	rs10032639	4	38674728	T	A	0.1976	0.4208	0.0534	3.47E-15	61.86
CD8 NC	*AC022498.1*	rs13096994	3	187897283	A	G	0.1122	-0.5848	0.0718	3.70E-16	66.25
CD8 NC	*AC023590.1*	rs2514610	8	119659371	A	G	0.9210	-0.5594	0.0772	4.37E-13	52.36
CD8 NC	*AC024592.12*	rs56094126	19	5170922	T	G	0.1805	0.4545	0.0584	7.11E-15	60.44
CD8 NC	*AC062017.1*	rs908258	2	240309087	A	G	0.4263	0.2752	0.0453	1.27E-09	36.78
CD8 NC	*AC064834.2*	rs10497761	2	196442199	C	A	0.2188	0.3717	0.0548	1.13E-11	46
CD8 NC	*AC084018.1*	rs1168668	12	122227451	G	C	0.8073	0.3481	0.0584	2.55E-09	35.43
CD8 NC	*AC090186.1*	rs13267846	8	52786158	A	G	0.8614	0.5875	0.0764	1.46E-14	59.04
CD8 NC	*AC091729.9*	rs6953538	7	1196974	G	A	0.6141	-0.3342	0.0467	8.67E-13	51.02
CD8 NC	*AC092301.3*	rs4452060	19	45347911	A	C	0.4294	0.4420	0.0446	4.16E-23	97.81
CD8 NC	*AC104653.1*	rs10185135	2	114586483	A	G	0.5968	0.4085	0.0454	2.50E-19	80.63
CD8 NC	*AC144652.1*	rs28694084	7	155086502	G	A	0.5345	0.2766	0.0451	8.83E-10	37.49
CD8 NC	*ACAT1*	rs1519073	11	107986418	G	C	0.2483	-0.3681	0.0507	4.03E-13	52.52
CD8 NC	*ACAT2*	rs2758353	6	160125467	T	C	0.4734	-0.2812	0.0447	3.27E-10	39.43
CD8 NC	*ACCS*	rs2074038	11	44087989	T	G	0.1126	0.6358	0.0736	5.84E-18	74.42
CD8 NC	*ACOT4*	rs4903128	14	74075935	C	T	0.5356	0.2927	0.0447	5.90E-11	42.77
CD8 NC	*ACTA2*	rs1324551	10	90751516	T	C	0.5074	-0.2566	0.0448	1.03E-08	32.73
CD8 NC	*ACTG1*	rs7503278	17	79479808	T	C	0.8099	0.3595	0.0570	2.80E-10	39.73
CD8 NC	*ACYP2*	rs6742042	2	54218703	C	T	0.4148	-0.6077	0.0438	1.02E-43	191.87
CD8 NC	*ADA*	rs6103817	20	43302409	C	T	0.7169	-0.2886	0.0492	4.51E-09	34.32
CD8 NC	*ADAM10*	rs7161799	15	58770523	T	C	0.0869	0.4479	0.0799	2.06E-08	31.37
CD8 NC	*ADAMTS6*	rs245531	5	64780736	A	T	0.6103	-0.4080	0.0462	1.09E-18	77.72
CD8 NC	*ADIRF*	rs10887680	10	88710510	G	A	0.1207	0.4179	0.0667	3.75E-10	39.16
CD8 NC	*ADPRM*	rs421541	17	10632499	A	G	0.4496	-0.3189	0.0453	1.94E-12	49.44
CD8 NC	*ADTRP*	rs849013	6	11732787	A	G	0.7615	0.3392	0.0568	2.30E-09	35.63
CD8 NC	*AE000662.92*	rs12888408	14	23026044	G	C	0.0929	0.3776	0.0690	4.37E-08	29.92
CD8 NC	*AFAP1*	rs62290601	4	7939008	A	T	0.3496	0.3254	0.0462	1.94E-12	49.45
CD8 NC	*AGA*	rs13149149	4	178360550	T	C	0.3350	-0.2968	0.0466	1.93E-10	40.45
CD8 NC	*AGAP4*	rs11239505	10	45882741	G	A	0.2160	0.8175	0.0530	9.75E-54	237.71
CD8 NC	*AGTRAP*	rs61773930	1	11790386	C	A	0.2666	-0.2859	0.0495	7.58E-09	33.31
CD8 NC	*AHI1*	rs13197384	6	135818897	A	C	0.3411	-0.5793	0.0459	1.91E-36	158.63
CD8 NC	*AHSA2*	rs62150966	2	61199056	G	A	0.1602	-0.3229	0.0585	3.48E-08	30.36
CD8 NC	*AIF1*	rs2844475	6	31583155	A	G	0.3901	0.5039	0.0453	8.98E-29	123.62
CD8 NC	*AKAP10*	rs4925055	17	19803450	T	C	0.2198	0.2979	0.0536	2.75E-08	30.82
CD8 NC	*AKAP12*	rs7742470	6	151629019	C	T	0.8394	0.6646	0.0612	1.69E-27	117.81
CD8 NC	*AKAP8*	rs10419500	19	15486404	G	C	0.7543	0.3470	0.0519	2.24E-11	44.66
CD8 NC	*AKIRIN1*	rs34848020	1	39453785	A	G	0.4368	-0.2821	0.0452	4.17E-10	38.95
CD8 NC	*AMICA1*	rs12289120	11	118066609	T	C	0.1595	0.5423	0.0643	3.28E-17	71.02
CD8 NC	*ANAPC13*	rs4074521	3	134325536	A	G	0.3456	-0.3117	0.0470	3.21E-11	43.95
CD8 NC	*ANK3*	rs12217839	10	62496212	T	C	0.3069	-0.3307	0.0478	4.75E-12	47.69
CD8 NC	*ANKDD1A*	rs1719264	15	65176535	T	C	0.6288	-0.6688	0.0443	1.89E-51	227.24
CD8 NC	*ANKRD55*	rs7731626	5	55444683	A	G	0.3526	-0.2906	0.0471	6.88E-10	37.98
CD8 NC	*ANXA5*	rs6534309	4	122609929	C	T	0.0762	0.6948	0.0767	1.26E-19	81.99
CD8 NC	*ANXA6*	rs2303038	5	150535013	T	C	0.2046	0.4752	0.0541	1.70E-18	76.85
CD8 NC	*AOAH*	rs7785921	7	36762723	G	A	0.7373	-0.3458	0.0505	7.23E-12	46.87
CD8 NC	*AP000936.4*	rs640621	11	116726769	G	C	0.6039	-0.3889	0.0459	2.29E-17	71.73
CD8 NC	*AP003774.1*	rs627425	11	64087642	T	C	0.3608	0.5856	0.0447	3.02E-39	171.43
CD8 NC	*APOLD1*	rs2024385	12	12888438	A	T	0.4227	-0.2611	0.0452	7.70E-09	33.28
CD8 NC	*ARHGAP15*	rs62171742	2	143995246	C	T	0.1515	-0.6315	0.0634	2.36E-23	98.93
CD8 NC	*ARHGEF10*	rs4875950	8	1806229	C	A	0.2792	0.3133	0.0509	7.61E-10	37.78
CD8 NC	*ARHGEF3*	rs6765444	3	56763619	G	A	0.1634	0.7515	0.0604	1.49E-35	154.57
CD8 NC	*ARL14EP*	rs7102836	11	30315627	T	C	0.2929	-0.3603	0.0502	7.03E-13	51.43
CD8 NC	*ARL16*	rs113250157	17	79634541	C	T	0.1649	-0.6254	0.0588	1.86E-26	113.07
CD8 NC	*ARL17A*	rs557307834	17	44121579	C	T	0.2232	0.3871	0.0511	3.67E-14	57.22
CD8 NC	*ARL17B*	rs41543512	17	44090778	A	T	0.2276	-0.3185	0.0558	1.13E-08	32.53
CD8 NC	*ARPC5*	rs1926825	1	183690371	A	G	0.6556	-0.3632	0.0462	3.82E-15	61.66
CD8 NC	*ARSA*	rs6151419	22	51064915	A	G	0.1475	-0.4447	0.0605	2.03E-13	53.86
CD8 NC	*ASL*	rs801215	7	66011938	T	A	0.6264	0.2600	0.0466	2.45E-08	31.03
CD8 NC	*ATF7IP2*	rs2083953	16	10496828	G	A	0.5261	-0.3205	0.0451	1.24E-12	50.32

CD8 NC	*ATG10*	rs150934	5	81600339	A	G	0.7230	-0.2897	0.0489	3.22E-09	34.98
CD8 NC	*ATHL1*	rs3932433	11	289553	C	G	0.3751	0.4456	0.0451	4.70E-23	97.57
CD8 NC	*ATM*	rs227056	11	108262397	T	C	0.5908	0.2926	0.0450	8.05E-11	42.16
CD8 NC	*ATP5D*	rs35369365	19	1220004	G	A	0.2032	-0.3090	0.0555	2.62E-08	30.9
CD8 NC	*ATP5G2*	rs7314664	12	53957586	C	T	0.1262	0.4112	0.0662	5.36E-10	38.46
CD8 NC	*ATP5G3*	rs21818	2	176035845	A	G	0.8107	-0.3689	0.0572	1.17E-10	41.44
CD8 NC	*ATP6AP1L*	rs6894520	5	81594748	T	C	0.2925	-0.2766	0.0483	1.03E-08	32.72
CD8 NC	*ATP6V0E2*	rs4348397	7	149579787	T	G	0.5686	-0.4682	0.0450	2.11E-25	108.26
CD8 NC	*B3GALNT1*	rs11713441	3	160906855	C	T	0.2929	0.7835	0.0468	6.93E-63	279.59
CD8 NC	*B3GAT2*	rs651984	6	71599887	G	A	0.0632	-0.5924	0.0991	2.28E-09	35.65
CD8 NC	*B4GALT1*	rs6476400	9	33123106	A	T	0.3352	0.5021	0.0465	3.63E-27	116.3
CD8 NC	*B4GALT4*	rs9842789	3	118957370	A	G	0.1995	0.3407	0.0561	1.29E-09	36.75
CD8 NC	*BCAS4*	rs1124952	20	49434526	G	A	0.1268	0.6812	0.0677	8.33E-24	100.99
CD8 NC	*BCAS4*	rs4811098	20	49496411	C	T	0.3650	-0.3216	0.0465	4.90E-12	47.63
CD8 NC	*BIN1*	rs11682128	2	127839474	A	G	0.3433	0.5714	0.0458	9.92E-36	155.37
CD8 NC	*BIRC7*	rs1047822	20	61873039	C	T	0.5230	-0.4804	0.0439	8.05E-28	119.28
CD8 NC	*BNIP1*	rs112356729	5	172544816	T	C	0.1228	-0.3886	0.0671	7.04E-09	33.46
CD8 NC	*BRK1*	rs73117481	3	10160132	T	C	0.1184	-0.5706	0.0679	4.37E-17	70.46
CD8 NC	*BST2*	rs8106139	19	17506176	C	T	0.8053	0.4360	0.0632	5.36E-12	47.45
CD8 NC	*BTBD9*	rs737171	6	38140505	G	A	0.6992	-0.3745	0.0511	2.25E-13	53.66
CD8 NC	*BTF3*	rs10942655	5	72674031	A	G	0.0712	-0.8576	0.0944	1.01E-19	82.43
CD8 NC	*BTN2A1*	rs3734544	6	26468660	A	G	0.1771	0.3972	0.0596	2.74E-11	44.26
CD8 NC	*BTN3A1*	rs4712994	6	26429655	A	C	0.1712	0.4284	0.0642	2.49E-11	44.45
CD8 NC	*BTN3A2*	rs67509210	6	26354100	T	G	0.1018	-0.8932	0.0644	1.01E-43	191.89
CD8 NC	*C10orf128*	rs17009325	10	50406022	G	A	0.3426	-0.4885	0.0462	3.72E-26	111.69
CD8 NC	*C10orf32*	rs10786716	10	104625178	C	T	0.3627	-0.4050	0.0468	4.94E-18	74.75
CD8 NC	*C11orf21*	rs2521272	11	2327151	C	T	0.8655	-0.3895	0.0678	8.97E-09	32.99
CD8 NC	*C14orf2*	rs11850704	14	104381687	G	A	0.0909	-0.4806	0.0840	1.04E-08	32.69
CD8 NC	*C15orf40*	rs1037403	15	83664191	G	C	0.5398	0.3229	0.0446	4.48E-13	52.31
CD8 NC	*C16orf13*	rs117667029	16	686337	A	G	0.1175	0.4437	0.0679	6.43E-11	42.6
CD8 NC	*C17orf50*	rs35517765	17	34107165	C	T	0.1922	-0.6531	0.0592	2.64E-28	121.49
CD8 NC	*C17orf89*	rs2659008	17	79217002	G	A	0.5311	0.3845	0.0444	4.33E-18	75.01
CD8 NC	*C17orf97*	rs11150881	17	259304	G	A	0.2620	0.6088	0.0508	3.78E-33	143.59
CD8 NC	*C19orf83*	rs2286756	19	46145025	T	G	0.5303	-0.3711	0.0444	6.87E-17	69.57
CD8 NC	*C1orf228*	rs11577974	1	45376232	A	C	0.3797	-0.3292	0.0459	7.63E-13	51.27
CD8 NC	*C1orf86*	rs11587831	1	2110848	G	T	0.2239	-0.4503	0.0541	8.26E-17	69.2
CD8 NC	*C2*	rs7452226	6	31303394	G	A	0.6144	-0.4385	0.0442	3.56E-23	98.12
CD8 NC	*C2*	rs28383456	6	32609453	T	C	0.1069	-0.2825	0.0486	6.04E-09	33.75
CD8 NC	*C20orf27*	rs45495794	20	3734167	C	T	0.0479	-0.6455	0.0978	4.10E-11	43.48
CD8 NC	*C22orf34*	rs8141219	22	50044546	G	A	0.6739	-0.3706	0.0488	3.01E-14	57.61
CD8 NC	*C2orf27A*	rs6720375	2	132440073	C	G	0.5865	0.3992	0.0448	4.91E-19	79.3
CD8 NC	*C4orf32*	rs1040278	4	113079391	C	T	0.4601	-0.3457	0.0448	1.22E-14	59.39
CD8 NC	*C5orf17*	rs77446464	5	23491428	T	G	0.0494	0.5726	0.0979	5.03E-09	34.11
CD8 NC	*C6orf25*	rs2245822	6	31230800	A	G	0.2246	0.2996	0.0514	5.47E-09	33.94
CD8 NC	*C6orf48*	rs4947253	6	31820159	T	C	0.1281	-0.5374	0.0685	4.28E-15	61.44
CD8 NC	*C6orf57*	rs74733138	6	71271450	T	C	0.1188	-0.4251	0.0701	1.30E-09	36.74
CD8 NC	*C9orf135*	rs7029206	9	72433081	A	G	0.1267	1.3314	0.0621	5.10E-102	458.94
CD8 NC	*C9orf156*	rs12235434	9	100671500	T	C	0.2571	0.3950	0.0504	4.73E-15	61.24
CD8 NC	*C9orf78*	rs1590705	9	132588228	G	C	0.2530	-0.5099	0.0506	6.32E-24	101.54
CD8 NC	*CA6*	rs4497235	1	8996316	T	A	0.0681	0.7418	0.0961	1.18E-14	59.45
CD8 NC	*CA6*	rs4908522	1	9028172	C	G	0.2616	0.3147	0.0518	1.25E-09	36.81
CD8 NC	*CALM3*	rs1124638	19	47135222	G	A	0.4571	-0.2688	0.0452	2.81E-09	35.24
CD8 NC	*CAMK4*	rs114378220	5	110566360	T	C	0.0635	-0.5830	0.0890	5.67E-11	42.85
CD8 NC	*CAMLG*	rs56308774	5	133907554	G	C	0.1241	0.4481	0.0686	6.58E-11	42.55
CD8 NC	*CAPG*	rs56352074	2	85584988	C	T	0.3591	0.3923	0.0459	1.23E-17	72.95
CD8 NC	*CAPN12*	rs62119115	19	39210593	A	G	0.2446	-0.3644	0.0529	5.74E-12	47.32
CD8 NC	*CARD8*	rs10409934	19	48762572	G	T	0.1556	0.4453	0.0585	2.60E-14	57.9
CD8 NC	*CARHSP1*	rs2437704	16	8950273	C	T	0.7204	-0.3086	0.0494	4.10E-10	38.98
CD8 NC	*CARS*	rs437087	11	3083133	G	A	0.4097	-0.7580	0.0427	2.18E-70	313.96
CD8 NC	*CASP7*	rs2227309	10	115489167	A	G	0.2497	-0.2919	0.0506	8.26E-09	33.15
CD8 NC	*CASP8*	rs3185378	2	202151439	C	G	0.6057	0.3299	0.0463	1.05E-12	50.64
CD8 NC	*CAST*	rs10075736	5	95990057	T	C	0.1691	0.5272	0.0604	2.44E-18	76.14
CD8 NC	*CBR1*	rs2835268	21	37449136	G	C	0.4993	0.2515	0.0448	2.03E-08	31.4
CD8 NC	*CBR3*	rs11911059	21	37485863	G	A	0.4212	-0.4168	0.0447	1.19E-20	86.64
CD8 NC	*CCDC101*	rs497523	16	28577931	C	T	0.3561	0.3458	0.0463	7.94E-14	55.71
CD8 NC	*CCDC109B*	rs11724306	4	110583165	G	A	0.6672	0.2991	0.0478	3.84E-10	39.11
CD8 NC	*CCDC122*	rs11617551	13	44443977	C	T	0.4855	0.4268	0.0443	5.27E-22	92.8
CD8 NC	*CCDC146*	rs10488499	7	76834066	G	A	0.1408	-0.5035	0.0601	5.59E-17	69.97
CD8 NC	*CCDC152*	rs28919892	5	42809308	G	A	0.2843	0.3238	0.0494	5.54E-11	42.89
CD8 NC	*CCDC163P*	rs4660306	1	45978675	C	T	0.6856	-0.6063	0.0456	2.68E-40	176.24
CD8 NC	*CCDC167*	rs2797803	6	37487340	C	T	0.6482	0.8203	0.0437	1.82E-78	350.97
CD8 NC	*CCDC58*	rs3828362	3	122131777	G	C	0.4138	0.5034	0.0445	1.12E-29	127.74
CD8 NC	*CCDC66*	rs282534	3	56634544	A	G	0.7746	-0.5845	0.0538	1.73E-27	117.77
CD8 NC	*CCSER2*	rs57190292	10	86269259	T	C	0.1297	-0.7915	0.0676	1.24E-31	136.66
CD8 NC	*CCT3*	rs12141236	1	156302575	C	A	0.2804	-0.6312	0.0475	2.33E-40	176.51
CD8 NC	*CCT8*	rs965951	21	30432557	A	G	0.1160	1.3368	0.0615	1.25E-104	470.92
CD8 NC	*CCZ1*	rs58793660	7	5936956	G	T	0.6739	-0.2790	0.0479	5.77E-09	33.84
CD8 NC	*CCZ1*	rs4720686	7	6868354	A	C	0.4380	0.5487	0.0438	5.84E-36	156.42
CD8 NC	*CCZ1B*	rs2711224	7	6861880	C	G	0.4738	-0.5792	0.0434	1.01E-40	178.18

CD8 NC	*CD151*	rs78744412	11	895596	T	C	0.0892	0.5413	0.0790	7.46E-12	46.81
CD8 NC	*CD164*	rs1322124	6	110062873	G	T	0.3796	0.2585	0.0462	2.26E-08	31.19
CD8 NC	*CD247*	rs840012	1	167414872	T	C	0.3734	0.3614	0.0454	1.62E-15	63.35
CD8 NC	*CD300A*	rs12939435	17	72463215	A	G	0.4215	-0.3321	0.0455	2.73E-13	53.28
CD8 NC	*CD52*	rs72649360	1	26662904	T	C	0.2993	-0.2951	0.0479	6.93E-10	37.96
CD8 NC	*CD53*	rs343808	1	111330007	T	C	0.2318	-0.3010	0.0517	5.88E-09	33.8
CD8 NC	*CD6*	rs12791045	11	60767964	T	C	0.2323	-0.2874	0.0523	4.00E-08	30.09
CD8 NC	*CD7*	rs11658034	17	80272873	T	C	0.1703	-0.3175	0.0578	4.00E-08	30.09
CD8 NC	*CDC42*	rs2268179	1	22414785	T	G	0.1644	0.4790	0.0602	1.83E-15	63.11
CD8 NC	*CDC42SE2*	rs78623258	5	130628103	G	A	0.1843	0.3276	0.0560	4.90E-09	34.16
CD8 NC	*CDK10*	rs2010623	16	89729025	G	A	0.4369	-0.2728	0.0448	1.17E-09	36.94
CD8 NC	*CDK5R1*	rs62065492	17	30822607	T	C	0.4449	0.2479	0.0449	3.34E-08	30.44
CD8 NC	*CDO1*	rs249490	5	115131150	C	T	0.7838	0.5366	0.0547	9.77E-23	96.13
CD8 NC	*CEACAM21*	rs3745936	19	42092815	A	T	0.7738	0.5336	0.0540	5.33E-23	97.32
CD8 NC	*CEBPZ*	rs7560904	2	37465309	G	A	0.4801	-0.2521	0.0450	2.07E-08	31.36
CD8 NC	*CEBPZ-AS1*	rs10198835	2	37434380	T	C	0.1318	-0.5866	0.0654	2.96E-19	80.3
CD8 NC	*CELA1*	rs12829179	12	51719989	G	C	0.2883	0.5146	0.0471	9.16E-28	119.02
CD8 NC	*CENPK*	rs404781	5	64794829	T	G	0.6135	-1.1498	0.0380	1.00E-200	913.51
CD8 NC	*CERS4*	rs62126381	19	8272852	C	T	0.5011	0.3108	0.0447	3.46E-12	48.31
CD8 NC	*CES1*	rs2244614	16	55844617	A	G	0.6039	0.3156	0.0457	5.10E-12	47.55
CD8 NC	*CFD*	rs2930903	19	887824	C	G	0.4168	0.6659	0.0443	4.82E-51	225.38
CD8 NC	*CFLAR*	rs12693929	2	202038249	G	A	0.5008	-0.2774	0.0448	5.95E-10	38.26
CD8 NC	*CHCHD10*	rs179465	22	24106008	G	C	0.9206	0.4518	0.0821	3.69E-08	30.24
CD8 NC	*CHCHD2*	rs2080141	7	56163770	C	T	0.2141	0.6477	0.0526	6.72E-35	151.57
CD8 NC	*CHI3L2*	rs11102222	1	111791078	C	T	0.6365	-0.3990	0.0463	6.89E-18	74.1
CD8 NC	*CHPT1*	rs2695295	12	102086805	T	A	0.5814	0.6506	0.0432	3.66E-51	225.92
CD8 NC	*CHURC1*	rs10129479	14	65375180	G	A	0.2134	0.5595	0.0570	9.17E-23	96.25
CD8 NC	*CIB1*	rs8025610	15	90776984	C	G	0.1658	-0.5027	0.0595	2.83E-17	71.32
CD8 NC	*CLEC2B*	rs2594072	12	10005778	G	A	0.5521	0.3228	0.0449	6.22E-13	51.67
CD8 NC	*CLIC3*	rs7862602	9	139840471	T	G	0.5429	0.2537	0.0449	1.58E-08	31.89
CD8 NC	*CLK4*	rs576097	5	178037216	A	G	0.4453	0.2879	0.0448	1.33E-10	41.18
CD8 NC	*CLNS1A*	rs17135969	11	77626963	T	A	0.1102	0.4468	0.0755	3.26E-09	34.95
CD8 NC	*CLUAP1*	rs9925749	16	3617503	G	T	0.5225	0.2685	0.0448	2.03E-09	35.87
CD8 NC	*CMTM7*	rs80258271	3	32501901	C	T	0.0905	-0.6621	0.0777	1.52E-17	72.53
CD8 NC	*CMTM8*	rs7632486	3	32286791	A	G	0.8213	-0.9628	0.0557	6.11E-67	298.16
CD8 NC	*CNKSR1*	rs12565756	1	26498196	T	G	0.3126	0.2785	0.0492	1.52E-08	31.96
CD8 NC	*CNN2*	rs4807455	19	1033559	A	G	0.2556	0.3534	0.0501	1.77E-12	49.62
CD8 NC	*CNOT6L*	rs2866689	4	78636187	A	G	0.8956	-0.5554	0.0684	4.76E-16	65.76
CD8 NC	*COA1*	rs10264159	7	43655836	C	T	0.6140	0.3233	0.0464	3.12E-12	48.51
CD8 NC	*COG5*	rs10264412	7	107183877	A	G	0.2315	0.5660	0.0527	5.95E-27	115.32
CD8 NC	*COL18A1*	rs7280620	21	46899723	C	T	0.0738	1.3497	0.0835	7.85E-59	261.02
CD8 NC	*COL18A1*	rs406179	21	47357236	A	G	0.3526	-0.2736	0.0462	3.15E-09	35.02
CD8 NC	*COL6A2*	rs7282491	21	47503880	C	T	0.2909	-0.3207	0.0503	1.80E-10	40.59
CD8 NC	*COMMD6*	rs4885304	13	76088638	A	G	0.1387	-0.8584	0.0594	2.19E-47	208.64
CD8 NC	*COMTD1*	rs139930031	10	76875676	A	G	0.0615	0.5931	0.1025	7.19E-09	33.42
CD8 NC	*CORO1B*	rs7110021	11	67253564	C	T	0.4096	-0.6353	0.0435	2.09E-48	213.31
CD8 NC	*COX14*	rs7307230	12	50512632	C	T	0.3414	0.2865	0.0464	6.85E-10	37.98
CD8 NC	*COX6B1*	rs4806187	19	36149761	G	A	0.5163	-0.4026	0.0443	9.83E-20	82.48
CD8 NC	*COX7A2L*	rs10183278	2	42571486	T	C	0.5843	-0.3903	0.0445	1.91E-18	76.63
CD8 NC	*CPA5*	rs17164864	7	130008015	C	T	0.2126	1.2769	0.0460	1.29E-169	769.09
CD8 NC	*CPNE1*	rs77120309	20	34218173	T	C	0.1101	-0.8409	0.0747	2.33E-29	126.29
CD8 NC	*CRELD2*	rs117800401	22	50343738	G	T	0.1409	0.3506	0.0638	3.94E-08	30.11
CD8 NC	*CRHR1*	rs7220206	17	43507403	A	G	0.1736	-0.4285	0.0578	1.26E-13	54.81
CD8 NC	*CRIP2*	rs4983411	14	105916797	A	G	0.6508	0.3837	0.0460	7.87E-17	69.3
CD8 NC	*CRYBB2*	rs6004662	22	25862124	A	G	0.0960	1.2869	0.0822	2.98E-55	244.64
CD8 NC	*CRYGN*	rs12539860	7	151135503	T	C	0.3752	-0.5877	0.0460	2.31E-37	162.83
CD8 NC	*CRYZ*	rs1465483	1	75195243	C	T	0.6770	-0.3985	0.0472	2.99E-17	71.21
CD8 NC	*CSGALNACT1*	rs73204703	8	19622801	T	C	0.1544	0.3538	0.0596	2.88E-09	35.19
CD8 NC	*CSTB*	rs35285321	21	45201832	A	G	0.3415	-0.4539	0.0477	1.86E-21	90.3
CD8 NC	*CTA-29F11.1*	rs12389	22	47158179	A	G	0.3392	-0.7482	0.0437	1.13E-65	292.37
CD8 NC	*CTBP1-AS2*	rs1250101	4	1236111	A	G	0.3754	-0.3084	0.0456	1.33E-11	45.67
CD8 NC	*CTC-241F20.3*	rs16981882	19	48755267	C	T	0.2168	-0.3209	0.0521	7.07E-10	37.92
CD8 NC	*CTC-425F1.4*	rs2242517	19	13002563	G	T	0.3957	0.3004	0.0454	3.85E-11	43.6
CD8 NC	*CTC-524C5.2*	rs980406	17	5338783	C	G	0.2545	0.7317	0.0506	2.27E-47	208.57
CD8 NC	*CTD-2007H13.3*	rs327811	5	98271175	G	A	0.2908	0.3189	0.0485	4.72E-11	43.2
CD8 NC	*CTD-2020K17.4*	rs7212404	17	43317574	A	G	0.1718	-0.5863	0.0612	9.24E-22	91.69
CD8 NC	*CTD-2116N20.1*	rs245541	5	64783403	C	G	0.6103	0.6694	0.0446	5.09E-51	225.27
CD8 NC	*CTD-2336O2.1*	rs6558535	8	1710632	C	T	0.7078	0.5650	0.0481	7.42E-32	137.68
CD8 NC	*CTD-2368P22.1*	rs4801243	19	58513807	C	A	0.2297	0.6540	0.0512	2.37E-37	162.78
CD8 NC	*CTD-2371O3.2*	rs9667574	11	9475540	C	A	0.4987	-0.6433	0.0427	2.70E-51	226.53
CD8 NC	*CTD-2547L24.3*	rs1961541	14	91894819	T	C	0.4443	-0.3794	0.0446	1.82E-17	72.19
CD8 NC	*CTD-2651B20.3*	rs34378704	15	45441410	A	C	0.1608	0.6253	0.0586	1.32E-26	113.74
CD8 NC	*CTD-3110H11.1*	rs111954657	15	50672855	A	G	0.3627	-0.3323	0.0455	2.78E-13	53.25
CD8 NC	*CTLA4*	rs3087243	2	204738919	A	G	0.4420	0.5505	0.0436	1.42E-36	159.23
CD8 NC	*CTNNAL1*	rs10979605	9	111663793	T	C	0.0867	0.4842	0.0863	2.03E-08	31.41
CD8 NC	*CTSF*	rs2242663	11	66335308	C	T	0.2447	0.3056	0.0524	5.49E-09	33.94
CD8 NC	*CTSL*	rs3118874	9	90352845	G	T	0.4326	-0.3467	0.0447	9.11E-15	59.96
CD8 NC	*CTSS*	rs41271951	1	150737220	G	A	0.0680	-0.5825	0.0795	2.38E-13	53.55
CD8 NC	*CTSW*	rs596002	11	65645354	G	A	0.8002	1.1956	0.0494	3.14E-129	583.76

CD8 NC	*CYLD*	rs11649621	16	50343193	A	G	0.1906	0.3371	0.0585	8.46E-09	33.1
CD8 NC	*CYP2D6*	rs9607871	22	42436322	A	G	0.1877	-0.5476	0.0560	1.39E-22	95.43
CD8 NC	*CYP4V2*	rs7687961	4	187122009	A	C	0.7941	-0.4556	0.0567	9.06E-16	64.49
CD8 NC	*DAD1*	rs5742783	14	23045995	G	A	0.0986	0.5387	0.0748	5.90E-13	51.77
CD8 NC	*DARS*	rs6743537	2	136702601	A	G	0.1953	0.4457	0.0685	7.61E-11	42.27
CD8 NC	*DAZAP1*	rs266807	19	1400679	G	A	0.4639	-0.3188	0.0446	8.84E-13	50.98
CD8 NC	*DAZAP2*	rs7305016	12	51642543	G	A	0.6191	-0.2706	0.0466	6.41E-09	33.64
CD8 NC	*DBNDD1*	rs79690969	16	90076227	A	T	0.0561	0.5664	0.0966	4.60E-09	34.28
CD8 NC	*DCP2*	rs7721666	5	112347710	C	T	0.7184	-0.3170	0.0507	3.92E-10	39.07
CD8 NC	*DCXR*	rs111894854	17	79998898	G	A	0.1249	0.8274	0.0656	1.74E-36	158.83
CD8 NC	*DDR1*	rs9263986	6	31191070	G	A	0.5885	-0.4174	0.0444	5.55E-21	88.15
CD8 NC	*DDR1*	rs2394893	6	31206719	A	G	0.5834	-0.2745	0.0460	2.40E-09	35.55
CD8 NC	*DDRGK1*	rs1056881	20	3169729	A	G	0.3935	-0.2761	0.0462	2.19E-09	35.73
CD8 NC	*DDT*	rs5751777	22	24267047	T	C	0.5906	0.4602	0.0453	3.00E-24	103.01
CD8 NC	*DDTL*	rs2000469	22	24245292	G	A	0.6506	0.6034	0.0460	2.23E-39	172.03
CD8 NC	*DDX11*	rs3881296	12	31237700	A	G	0.4487	-0.6553	0.0429	1.06E-52	232.96
CD8 NC	*DDX5*	rs1991401	17	62502435	G	A	0.3173	-0.4338	0.0479	1.33E-19	81.88
CD8 NC	*DHRS4*	rs77491846	14	24458752	A	G	0.0909	0.7871	0.0808	2.04E-22	94.67
CD8 NC	*DHX32*	rs10794057	10	127729734	C	T	0.5476	-0.2558	0.0452	1.56E-08	31.92
CD8 NC	*DIP2A*	rs2070435	21	47961711	A	G	0.3259	0.4493	0.0469	9.66E-22	91.6
CD8 NC	*DISC1*	rs12042938	1	231816842	T	C	0.4058	-0.2734	0.0456	2.06E-09	35.84
CD8 NC	*DIXDC1*	rs11214048	11	111814258	G	C	0.3534	0.4227	0.0466	1.13E-19	82.21
CD8 NC	*DNAJA1*	rs10813927	9	33047910	T	C	0.3609	-0.2885	0.0466	6.06E-10	38.22
CD8 NC	*DNAJB1*	rs7253780	19	14643268	C	T	0.4532	-0.2675	0.0451	2.93E-09	35.16
CD8 NC	*DNAJC15*	rs17553284	13	43597706	T	C	0.2267	-0.9222	0.0495	1.47E-77	346.81
CD8 NC	*DOCK8*	rs10973952	9	396293	A	G	0.2418	-0.4131	0.0517	1.41E-15	63.63
CD8 NC	*DPYSL4*	rs881347	10	134000130	T	C	0.5430	-0.3510	0.0447	4.05E-15	61.55
CD8 NC	*DR1*	rs1426318	1	93847420	G	A	0.6352	-0.3064	0.0467	5.45E-11	42.92
CD8 NC	*DRAM2*	rs6680418	1	111638612	G	A	0.2678	0.3839	0.0490	4.95E-15	61.16
CD8 NC	*DUS3L*	rs2446210	19	5783634	C	T	0.2133	-0.3430	0.0588	5.56E-09	33.91
CD8 NC	*DUT*	rs28536045	15	48606649	T	G	0.7868	0.3751	0.0573	5.85E-11	42.78
CD8 NC	*DYNLL2*	rs12950704	17	56168713	C	G	0.6730	-0.4517	0.0471	8.97E-22	91.74
CD8 NC	*EBPL*	rs1535470	13	50260029	A	G	0.6296	0.2771	0.0468	3.32E-09	34.91
CD8 NC	*EBPL*	rs7983918	13	50266574	C	A	0.5395	-0.6879	0.0427	2.38E-58	258.82
CD8 NC	*EDN3*	rs259965	20	57821556	G	A	0.5165	-0.4478	0.0440	2.67E-24	103.24
CD8 NC	*EEA1*	rs73209563	12	93325586	G	A	0.4226	0.2603	0.0453	9.26E-09	32.92
CD8 NC	*EEF1D*	rs12678314	8	144659608	G	A	0.7490	-0.3186	0.0528	1.60E-09	36.33
CD8 NC	*EGFL8*	rs12665166	6	31208242	T	C	0.2042	-0.3316	0.0577	9.25E-09	32.92
CD8 NC	*EI24*	rs12804009	11	125438802	A	G	0.3064	-0.2663	0.0472	1.66E-08	31.79
CD8 NC	*EIF2A*	rs14434	3	150302010	T	C	0.8405	0.7760	0.0616	2.00E-36	158.55
CD8 NC	*EIF3G*	rs7710	19	10226256	G	A	0.4494	0.3039	0.0448	1.18E-11	45.91
CD8 NC	*EIF5A*	rs7503161	17	7207964	C	A	0.5615	-1.1356	0.0366	1.00E-200	959.66
CD8 NC	*ELP2*	rs592261	18	33752244	A	G	0.3587	0.3099	0.0468	3.45E-11	43.81
CD8 NC	*ELP5*	rs2074219	17	7156447	G	A	0.6468	-0.6317	0.0440	1.01E-46	205.61
CD8 NC	*EMB*	rs34928038	5	49488127	T	C	0.1076	0.4642	0.0749	5.67E-10	38.35
CD8 NC	*EML4*	rs6544527	2	42494273	T	C	0.4309	-0.3408	0.0452	4.99E-14	56.62
CD8 NC	*EMP3*	rs17295412	19	48841185	A	G	0.4395	-0.3109	0.0448	4.02E-12	48.02
CD8 NC	*EMR1*	rs3895916	19	6927996	G	A	0.4523	0.3355	0.0448	6.91E-14	55.98
CD8 NC	*ENOSF1*	rs6506511	18	750937	C	T	0.5265	-0.3600	0.0445	6.08E-16	65.28
CD8 NC	*ENTPD1*	rs2226163	10	97628994	G	A	0.5200	0.5574	0.0434	1.07E-37	164.35
CD8 NC	*EPHA1-AS1*	rs7794735	7	143104816	A	G	0.0829	-0.6630	0.0787	3.64E-17	70.82
CD8 NC	*EPHX2*	rs6990733	8	27404137	A	G	0.1677	0.4475	0.0624	7.42E-13	51.32
CD8 NC	*EPSTI1*	rs3825511	13	43566373	C	A	0.7178	-0.6254	0.0491	3.77E-37	161.86
CD8 NC	*ERAP2*	rs2927608	5	96252432	A	G	0.4214	1.0624	0.0378	4.21E-174	789.69
CD8 NC	*ERC1*	rs10774481	12	1100637	C	G	0.5133	0.2727	0.0449	1.21E-09	36.89
CD8 NC	*ERICH1*	rs1703910	8	601226	T	G	0.3588	-0.3780	0.0465	4.17E-16	66.02
CD8 NC	*ESR1*	rs115429675	6	151819857	T	C	0.0639	-0.8703	0.1010	6.71E-18	74.15
CD8 NC	*ESR2*	rs28418648	14	64698623	C	T	0.0760	0.9952	0.0908	5.90E-28	119.89
CD8 NC	*ETS1*	rs61907765	11	128391937	T	C	0.2133	0.3183	0.0561	1.41E-08	32.11
CD8 NC	*ETV7*	rs2234071	6	36355372	C	T	0.5322	0.2606	0.0451	7.39E-09	33.36
CD8 NC	*EVI2A*	rs2854308	17	29666539	G	A	0.6133	0.4059	0.0462	1.47E-18	77.13
CD8 NC	*EXOC3*	rs56182240	5	423169	T	C	0.3559	0.5021	0.0443	8.33E-30	128.33
CD8 NC	*FADS1*	rs174591	11	61617676	A	T	0.2568	0.3314	0.0516	1.29E-10	41.23
CD8 NC	*FADS2*	rs968567	11	61595564	T	C	0.1535	0.5964	0.0564	3.66E-26	111.73
CD8 NC	*FAM101B*	rs12450578	17	455383	G	C	0.2069	0.3345	0.0537	4.75E-10	38.7
CD8 NC	*FAM117B*	rs11680951	2	203631359	A	G	0.5584	-0.3089	0.0451	7.34E-12	46.84
CD8 NC	*FAM118A*	rs136582	22	45774531	A	T	0.1332	1.5498	0.0583	1.21E-155	704.96
CD8 NC	*FAM134A*	rs2385392	2	220049511	C	T	0.6012	0.3138	0.0466	1.58E-11	45.34
CD8 NC	*FAM134B*	rs256888	5	16622982	C	T	0.7257	0.3886	0.0501	8.68E-15	60.05
CD8 NC	*FAM13A*	rs56221166	4	89835762	G	T	0.0915	0.5611	0.0753	8.97E-14	55.47
CD8 NC	*FAM153B*	rs62389203	5	175510135	T	C	0.4271	-0.6528	0.0428	1.89E-52	231.81
CD8 NC	*FAM154B*	rs113311244	15	82421868	A	G	0.0507	0.6866	0.0975	1.94E-12	49.44
CD8 NC	*FAM157B*	rs76975097	9	140741439	T	C	0.1211	-0.5994	0.0654	4.71E-20	83.93
CD8 NC	*FAM184B*	rs10939740	4	17617490	C	T	0.6230	-1.0786	0.0384	1.69E-173	786.92
CD8 NC	*FAM211A*	rs12951804	17	16358661	A	T	0.1187	0.4116	0.0669	7.58E-10	37.79
CD8 NC	*FAM21A*	rs2338050	10	51804949	A	G	0.3750	-0.2861	0.0458	4.31E-10	38.89
CD8 NC	*FAM26F*	rs1204836	6	116514253	G	A	0.7843	0.3270	0.0541	1.55E-09	36.4
CD8 NC	*FAM26F*	rs2640866	6	116806899	C	T	0.0532	1.1478	0.0961	6.81E-33	142.42
CD8 NC	*FAM43A*	rs9834693	3	194383487	A	G	0.3667	-0.2963	0.0464	1.75E-10	40.64

CD8 NC	*FAM63B*	rs12910580	15	59272053	C	T	0.2119	0.3140	0.0554	1.44E-08	32.07
CD8 NC	*FAR1*	rs11022929	11	13678419	G	A	0.3329	0.4582	0.0472	3.00E-22	93.91
CD8 NC	*FAU*	rs1196043	11	64887560	G	T	0.4919	0.3801	0.0444	1.21E-17	72.98
CD8 NC	*FBLN5*	rs2267989	14	92339850	A	G	0.4380	-0.6756	0.0426	9.17E-57	251.56
CD8 NC	*FBP1*	rs28369693	9	97384111	T	G	0.4257	0.2614	0.0453	8.12E-09	33.18
CD8 NC	*FBXO2*	rs1883575	1	11713166	G	A	0.5386	-0.2534	0.0449	1.69E-08	31.76
CD8 NC	*FCGBP*	rs4803311	19	40409821	T	C	0.7472	-0.2908	0.0514	1.52E-08	31.96
CD8 NC	*FCRL3*	rs58517866	1	157669580	A	T	0.2051	0.3450	0.0548	3.17E-10	39.49
CD8 NC	*FDFT1*	rs13276352	8	11657838	C	T	0.2074	-0.5990	0.0560	1.01E-26	114.27
CD8 NC	*FGFR1*	rs17182134	8	38324281	A	C	0.0789	-0.4807	0.0832	7.42E-09	33.35
CD8 NC	*FGL2*	rs2075761	7	76828953	T	C	0.0923	0.5731	0.0823	3.37E-12	48.36
CD8 NC	*FHIT*	rs2736777	3	60066610	C	T	0.3177	0.2884	0.0487	3.26E-09	34.95
CD8 NC	*FHL3*	rs11583126	1	38419218	T	C	0.4209	-0.3492	0.0450	8.12E-15	60.18
CD8 NC	*FIGNL1*	rs7789635	7	50473610	C	T	0.4780	0.3757	0.0444	2.56E-17	71.51
CD8 NC	*FIS1*	rs10487887	7	100862209	G	C	0.0657	-0.6677	0.0858	7.31E-15	60.39
CD8 NC	*FKBP11*	rs4760662	12	49370857	A	G	0.3940	-0.4816	0.0454	2.69E-26	112.33
CD8 NC	*FLVCR1-AS1*	rs12125982	1	213056892	G	A	0.5078	0.4851	0.0438	1.82E-28	122.22
CD8 NC	*FMN1*	rs345786	15	33394560	A	T	0.2619	1.0271	0.0481	4.13E-101	454.78
CD8 NC	*FN3KRP*	rs58867683	17	80679455	A	T	0.4000	-0.3794	0.0456	9.39E-17	68.95
CD8 NC	*FNBP4*	rs7933184	11	47682402	T	C	0.4467	-0.3236	0.0446	4.22E-13	52.43
CD8 NC	*FNTB*	rs7144259	14	65537559	A	T	0.2986	0.2705	0.0483	2.09E-08	31.35
CD8 NC	*FOXN2*	rs79073127	2	48573895	C	G	0.1195	0.4013	0.0683	4.22E-09	34.45
CD8 NC	*FRG1B*	rs6142639	20	29813865	G	T	0.5004	-0.3186	0.0446	9.15E-13	50.92
CD8 NC	*FTCDNL1*	rs13008446	2	200628500	A	G	0.6290	-0.3329	0.0464	7.05E-13	51.42
CD8 NC	*FUT10*	rs1113663	8	33323127	T	C	0.5864	-0.2626	0.0457	9.49E-09	32.88
CD8 NC	*FUT2*	rs281378	19	49214066	A	G	0.3582	-0.5808	0.0460	1.69E-36	158.87
CD8 NC	*FXYD2*	rs11216569	11	117699377	T	C	0.4427	-0.2921	0.0452	1.00E-10	41.73
CD8 NC	*FXYD2*	rs7118414	11	117811548	C	G	0.4119	0.2827	0.0458	6.68E-10	38.04
CD8 NC	*G3BP2*	rs1553985	4	76554604	G	A	0.3907	-0.3379	0.0458	1.51E-13	54.45
CD8 NC	*GALNT2*	rs12120720	1	230352681	G	A	0.2910	0.3784	0.0484	5.61E-15	60.91
CD8 NC	*GALT*	rs2812357	9	34655436	C	T	0.6012	0.2701	0.0453	2.41E-09	35.54
CD8 NC	*GATM*	rs1617984	15	45622662	G	A	0.4143	0.4067	0.0464	1.95E-18	76.59
CD8 NC	*GBP3*	rs4656078	1	89479074	T	C	0.4456	0.2503	0.0450	2.61E-08	30.91
CD8 NC	*GBP5*	rs55870241	1	89742521	C	T	0.5445	-0.4477	0.0443	4.96E-24	102.01
CD8 NC	*GBP7*	rs674903	1	89649824	G	A	0.5047	0.6648	0.0425	4.65E-55	243.75
CD8 NC	*GCC2*	rs2139809	2	109045638	C	T	0.5916	0.6011	0.0438	7.18E-43	188
CD8 NC	*GDI2*	rs72772354	10	5813361	G	A	0.1908	-0.5983	0.0539	1.13E-28	123.16
CD8 NC	*GIMAP2*	rs79807098	7	150386627	T	C	0.1072	-0.4211	0.0694	1.28E-09	36.76
CD8 NC	*GIMAP4*	rs1879871	7	150300393	G	A	0.6168	-0.2664	0.0462	7.80E-09	33.26
CD8 NC	*GJB6*	rs9552198	13	21058433	A	G	0.2740	-0.4835	0.0516	7.88E-21	87.45
CD8 NC	*GLIPR1*	rs1552039	12	75905994	A	G	0.2497	-0.3213	0.0520	6.59E-10	38.06
CD8 NC	*GLIPR1L2*	rs73191008	12	75770752	G	T	0.3719	-0.2904	0.0457	2.20E-10	40.2
CD8 NC	*GLIS3*	rs56795713	9	4297036	C	A	0.0684	-0.4783	0.0814	4.17E-09	34.47
CD8 NC	*GLRX5*	rs12431440	14	95993103	G	C	0.4081	0.3276	0.0453	4.83E-13	52.17
CD8 NC	*GLTP*	rs11832113	12	110303124	A	C	0.4577	-0.2652	0.0448	3.21E-09	34.98
CD8 NC	*GLTSCR2*	rs3786779	19	48255284	A	G	0.7093	-0.4263	0.0498	1.06E-17	73.25
CD8 NC	*GNG8*	rs12463013	19	47131688	A	C	0.3968	0.3607	0.0458	3.52E-15	61.83
CD8 NC	*GNLY*	rs12151621	2	85934499	A	C	0.2263	0.3162	0.0538	4.05E-09	34.53
CD8 NC	*GNPTG*	rs57890635	16	1411129	T	C	0.2668	0.4184	0.0530	3.05E-15	62.11
CD8 NC	*GOLGA8A*	rs3866558	15	34859970	C	G	0.4062	0.3322	0.0452	2.11E-13	53.79
CD8 NC	*GOLT1B*	rs11046090	12	21658531	C	A	0.1578	0.3759	0.0632	2.76E-09	35.27
CD8 NC	*GPR160*	rs9829025	3	169746123	G	A	0.3883	0.5216	0.0456	2.53E-30	130.69
CD8 NC	*GPR18*	rs7332358	13	100023529	A	G	0.0980	0.4254	0.0711	2.18E-09	35.73
CD8 NC	*GPX4*	rs2074449	19	1095753	A	G	0.2235	-0.3657	0.0535	8.27E-12	46.61
CD8 NC	*GRAMD1A*	rs786503	19	35478034	G	T	0.5933	0.2875	0.0454	2.42E-10	40.01
CD8 NC	*GRHPR*	rs4878690	9	37422656	T	C	0.3290	0.6788	0.0457	5.32E-50	220.61
CD8 NC	*GS1-124K5.4*	rs2946580	7	65531842	A	G	0.7418	-0.3049	0.0510	2.27E-09	35.65
CD8 NC	*GSTM3*	rs1332018	1	110282972	T	G	0.5781	0.7961	0.0419	2.59E-80	359.43
CD8 NC	*GSTM4*	rs1010167	1	110198727	G	C	0.4142	-0.5827	0.0441	6.13E-40	174.6
CD8 NC	*GSTO1*	rs2164624	10	106013445	A	G	0.3301	0.3920	0.0475	1.50E-16	68.03
CD8 NC	*GSTP1*	rs614080	11	67347287	A	G	0.4951	0.6059	0.0430	4.11E-45	198.25
CD8 NC	*GSTT1*	rs5760147	22	24334948	C	A	0.3844	-0.6894	0.0442	6.52E-55	243.08
CD8 NC	*GTF2H2*	rs28478097	5	70309998	G	C	0.6121	-0.5520	0.0447	4.70E-35	152.28
CD8 NC	*GTF2H4*	rs4947302	6	31074128	T	C	0.0797	0.9874	0.0814	7.06E-34	146.91
CD8 NC	*GTPBP8*	rs3889123	3	112725442	C	G	0.7022	0.2839	0.0515	3.53E-08	30.33
CD8 NC	*GTSCR1*	rs76731088	18	68358823	G	A	0.1579	0.4086	0.0636	1.34E-10	41.17
CD8 NC	*GYPC*	rs35651363	2	127519838	A	G	0.2707	0.5669	0.0502	1.41E-29	127.28
CD8 NC	*GZMM*	rs16990714	19	543497	T	C	0.0484	-0.6600	0.1026	1.23E-10	41.33
CD8 NC	*H6PD*	rs72641821	1	9333514	A	G	0.1711	0.3741	0.0607	7.24E-10	37.88
CD8 NC	*HAGH*	rs8044343	16	1881145	C	G	0.3129	0.5171	0.0489	4.20E-26	111.45
CD8 NC	*HAGHL*	rs1054788	16	779689	C	G	0.2219	-0.2852	0.0514	2.90E-08	30.71
CD8 NC	*HAX1*	rs12750321	1	154251626	G	A	0.2882	0.3574	0.0489	2.77E-13	53.26
CD8 NC	*HCG25*	rs9269076	6	32440805	A	C	0.5519	-0.2934	0.0455	1.18E-10	41.41
CD8 NC	*HCG25*	rs213203	6	33238404	C	A	0.5561	-0.4523	0.0440	8.82E-25	105.43
CD8 NC	*HDGFRP3*	rs72755947	15	83638985	A	G	0.0628	0.5281	0.0857	7.19E-10	37.89
CD8 NC	*HEBP1*	rs67498669	12	13154077	T	C	0.5276	-0.2660	0.0450	3.44E-09	34.84
CD8 NC	*HEBP2*	rs9495065	6	138709856	T	C	0.0684	-0.6814	0.0947	6.26E-13	51.66
CD8 NC	*HELLS*	rs2265364	10	96145246	C	T	0.4419	0.2638	0.0451	4.76E-09	34.22
CD8 NC	*HFE*	rs35608615	6	27040402	C	G	0.0997	-0.3774	0.0685	3.57E-08	30.31

CD8 NC	*HIGD2A*	rs3172921	5	175815782	C	G	0.0953	-1.0236	0.0827	3.49E-35	152.87
CD8 NC	*HIST1H2AD*	rs4712972	6	25772047	G	A	0.8634	0.3861	0.0654	3.57E-09	34.77
CD8 NC	*HIST1H2AI*	rs35656932	6	28191288	T	G	0.0751	0.4937	0.0755	6.24E-11	42.66
CD8 NC	*HIST1H2BB*	rs9393718	6	26407482	A	G	0.1023	-0.4604	0.0680	1.30E-11	45.72
CD8 NC	*HIST1H2BF*	rs4711112	6	26470862	G	A	0.7722	0.3361	0.0557	1.56E-09	36.39
CD8 NC	*HIST1H2BI*	rs72839445	6	27249686	A	G	0.0719	-0.5293	0.0768	5.59E-12	47.37
CD8 NC	*HIST1H2BJ*	rs76909894	6	27840715	C	T	0.0659	0.4776	0.0852	2.09E-08	31.35
CD8 NC	*HIST1H2BL*	rs9295778	6	28684183	G	T	0.8428	-0.8434	0.0580	7.46E-48	210.79
CD8 NC	*HIST1H2BO*	rs13215804	6	28415572	G	A	0.3245	-0.6692	0.0449	3.40E-50	221.5
CD8 NC	*HIST1H3H*	rs7775835	6	28678357	C	T	0.8747	0.4018	0.0676	2.79E-09	35.25
CD8 NC	*HIST1H3I*	rs3131337	6	28796071	C	G	0.0902	-0.6211	0.0722	7.83E-18	73.85
CD8 NC	*HIVEP3*	rs1004870	1	42370787	T	C	0.5896	-0.3633	0.0449	6.00E-16	65.31
CD8 NC	*HLA-A*	rs2571389	6	29923503	C	T	0.5408	0.3270	0.0446	2.22E-13	53.69
CD8 NC	*HLA-B*	rs2524096	6	31236467	T	G	0.4422	0.7593	0.0422	1.52E-72	323.84
CD8 NC	*HLA-B*	rs9366777	6	31264715	A	T	0.0923	-0.5784	0.0826	2.54E-12	48.91
CD8 NC	*HLA-B*	rs3828879	6	31379391	T	C	0.1430	-0.6012	0.0651	2.65E-20	85.06
CD8 NC	*HLA-C*	rs3931670	6	31243767	C	G	0.2891	0.5300	0.0505	8.93E-26	109.96
CD8 NC	*HLA-DQA1*	rs9272785	6	32610401	A	G	0.1783	0.3627	0.0560	9.76E-11	41.78
CD8 NC	*HLA-DQA2*	rs9272785	6	32610401	A	G	0.1783	0.8944	0.0527	1.59E-64	287.1
CD8 NC	*HLA-DQA2*	rs77000541	6	32635605	A	G	0.2974	0.3034	0.0450	1.64E-11	45.27
CD8 NC	*HLA-DQB1*	rs9272557	6	32607048	C	T	0.0740	-0.5390	0.0531	3.40E-24	102.76
CD8 NC	*HLA-DQB2*	rs6935940	6	32683691	C	A	0.2380	-0.3835	0.0525	2.88E-13	53.18
CD8 NC	*HLA-DRB5*	rs9271853	6	32595117	T	C	0.4427	0.6156	0.0451	1.90E-42	186.06
CD8 NC	*HLA-F*	rs9258164	6	29688869	A	G	0.1404	0.5263	0.0690	2.35E-14	58.1
CD8 NC	*HLA-G*	rs1061156	6	29913266	T	G	0.1564	0.5783	0.0603	9.12E-22	91.71
CD8 NC	*HMBOX1*	rs10088428	8	28909523	T	C	0.2597	-0.7501	0.0492	2.08E-52	231.62
CD8 NC	*HNRNPU*	rs9728823	1	245030141	A	G	0.2626	0.3073	0.0513	2.17E-09	35.74
CD8 NC	*HOXB-AS1*	rs1987164	17	46569052	G	A	0.4537	0.4568	0.0440	2.89E-25	107.64
CD8 NC	*HOXB2*	rs4793870	17	46581184	C	T	0.6285	0.6015	0.0452	1.89E-40	176.94
CD8 NC	*HOXB4*	rs11869101	17	46568013	G	T	0.4863	0.3481	0.0445	5.26E-15	61.04
CD8 NC	*HPCAL4*	rs784600	1	40139553	T	C	0.4692	0.3071	0.0449	7.64E-12	46.76
CD8 NC	*HSD17B12*	rs10734518	11	43861748	A	G	0.6710	0.2890	0.0486	2.78E-09	35.26
CD8 NC	*HSD17B14*	rs8110220	19	49339098	C	T	0.2593	-0.4336	0.0516	4.25E-17	70.51
CD8 NC	*HSPBAP1*	rs13093464	3	122507436	A	G	0.7480	-0.7510	0.0519	2.22E-47	208.62
CD8 NC	*IAH1*	rs6747020	2	9644304	A	G	0.5357	-0.2598	0.0452	9.12E-09	32.95
CD8 NC	*ICA1L*	rs62194159	2	203646897	T	C	0.4446	-0.3571	0.0446	1.16E-15	64.02
CD8 NC	*ICAM3*	rs281414	19	10450285	A	G	0.1788	0.3951	0.0617	1.51E-10	40.93
CD8 NC	*IFI27L2*	rs2273514	14	94595989	C	T	0.2022	-0.5184	0.0545	1.99E-21	90.17
CD8 NC	*IFITM2*	rs10398	11	308180	G	A	0.2135	0.6770	0.0566	5.75E-33	142.75
CD8 NC	*IFITM3*	rs7115703	11	306920	A	T	0.5538	0.6805	0.0424	6.34E-58	256.87
CD8 NC	*IFNG-AS1*	rs11177006	12	68413097	G	A	0.3766	0.3717	0.0462	8.58E-16	64.6
CD8 NC	*IGFLR1*	rs112500848	19	36254427	T	C	0.1552	-0.5501	0.0633	3.70E-18	75.32
CD8 NC	*IKZF1*	rs7806674	7	50423963	G	C	0.5832	0.2694	0.0451	2.38E-09	35.56
CD8 NC	*IL24*	rs291083	1	207086969	G	A	0.4456	-0.2992	0.0449	2.79E-11	44.23
CD8 NC	*IL32*	rs28372698	16	3115111	A	T	0.4056	0.2792	0.0458	1.07E-09	37.11
CD8 NC	*IL32*	rs9928554	16	3126735	A	G	0.0771	0.8009	0.0916	2.28E-18	76.27
CD8 NC	*IMMT*	rs1124686	2	86376474	C	T	0.5036	-0.2542	0.0450	1.58E-08	31.88
CD8 NC	*INF2*	rs113514625	14	105174598	T	C	0.1455	-0.4884	0.0631	9.96E-15	59.78
CD8 NC	*INPP4B*	rs1373036	4	143625262	A	G	0.7682	0.3064	0.0521	4.17E-09	34.47
CD8 NC	*INTS5*	rs11231184	11	62447563	A	C	0.2753	0.3031	0.0489	5.70E-10	38.34
CD8 NC	*IQSEC3*	rs11064628	12	240863	A	G	0.2516	-0.2819	0.0503	2.12E-08	31.31
CD8 NC	*IRF7*	rs4963128	11	589564	C	T	0.6605	0.2867	0.0466	7.64E-10	37.77
CD8 NC	*ISCA1*	rs9314750	9	88896956	G	A	0.6802	-0.3853	0.0466	1.38E-16	68.2
CD8 NC	*ISCA2*	rs4903231	14	74962233	A	G	0.5967	-0.5196	0.0443	1.04E-31	137.02
CD8 NC	*ISG15*	rs1921	1	949608	A	G	0.3869	0.3150	0.0457	5.37E-12	47.45
CD8 NC	*ITGB2*	rs760462	21	46328099	C	T	0.8298	-1.1092	0.0553	1.59E-89	401.66
CD8 NC	*ITGB2-AS1*	rs12626759	21	46351134	G	T	0.6364	-0.2734	0.0467	4.82E-09	34.19
CD8 NC	*ITGB3BP*	rs855316	1	64069495	A	G	0.7016	-0.4159	0.0481	5.19E-18	74.66
CD8 NC	*ITGB7*	rs2272300	12	53587468	G	T	0.1020	0.6043	0.0806	6.47E-14	56.11
CD8 NC	*ITM2C*	rs60237566	2	231742711	G	T	0.2267	0.5541	0.0535	4.24E-25	106.88
CD8 NC	*JAKMIP2*	rs2436340	5	147162475	A	G	0.3562	0.3013	0.0462	7.22E-11	42.37
CD8 NC	*KANSL1-AS1*	rs62056877	17	43778828	G	T	0.2070	1.2951	0.0436	1.10E-193	879.58
CD8 NC	*KB-226F1.1*	rs5760147	22	24334948	C	A	0.3844	-0.4152	0.0460	1.73E-19	81.36
CD8 NC	*KCNAB2*	rs61762129	1	6021704	C	T	0.1508	0.5386	0.0616	2.38E-18	76.19
CD8 NC	*KCNN1*	rs10411508	19	18110574	A	G	0.3962	0.2629	0.0456	8.03E-09	33.2
CD8 NC	*KCTD21-AS1*	rs10793289	11	77850517	A	C	0.4647	-0.3245	0.0447	3.88E-13	52.6
CD8 NC	*KDELR2*	rs6796	7	6502367	C	T	0.2687	-0.5188	0.0484	8.27E-27	114.67
CD8 NC	*KIAA0040*	rs2269650	1	175129691	T	G	0.3691	0.4963	0.0459	3.41E-27	116.42
CD8 NC	*KIAA1430*	rs3112884	4	186129622	G	A	0.6329	-0.3552	0.0473	5.84E-14	56.31
CD8 NC	*KIF16B*	rs6111195	20	16565025	G	A	0.1419	-0.4379	0.0634	4.98E-12	47.6
CD8 NC	*KISS1R*	rs2930894	19	881161	A	G	0.2958	0.3467	0.0497	3.12E-12	48.51
CD8 NC	*KLF13*	rs28510484	15	31637569	C	G	0.1628	0.3324	0.0601	3.16E-08	30.54
CD8 NC	*KLRC4*	rs11053802	12	10597207	T	C	0.6503	0.5508	0.0464	1.77E-32	140.52
CD8 NC	*KLRD1*	rs17808345	12	10433531	G	A	0.2297	0.3888	0.0521	8.38E-14	55.6
CD8 NC	*KRT1*	rs2741159	12	53071382	C	A	0.3815	0.8520	0.0412	8.73E-95	425.78
CD8 NC	*KRT10*	rs56261283	17	38961725	A	G	0.1606	0.3760	0.0650	7.40E-09	33.36
CD8 NC	*KRT72*	rs627826	12	53026125	C	T	0.2685	-0.4524	0.0504	2.75E-19	80.44
CD8 NC	*KRT73*	rs609307	12	53027400	A	C	0.2408	0.3105	0.0526	3.51E-09	34.81
CD8 NC	*KRTCAP3*	rs780088	2	27716004	C	T	0.3863	0.5270	0.0447	3.91E-32	138.95

CD8 NC	*KTN1*	rs6573051	14	56120600	G	A	0.3558	-0.4570	0.0461	3.88E-23	97.95
CD8 NC	*L3MBTL3*	rs6569648	6	130349119	T	C	0.7821	-0.5100	0.0507	8.21E-24	101.02
CD8 NC	*LA16c-358B7.3*	rs11647760	16	1367610	A	C	0.6104	-0.5125	0.0445	9.91E-31	132.55
CD8 NC	*LAIR2*	rs73070113	19	55014172	T	A	0.1204	-0.3809	0.0675	1.69E-08	31.75
CD8 NC	*LAMB1*	rs66999558	7	107544531	A	G	0.3873	-0.6592	0.0433	2.10E-52	231.61
CD8 NC	*LAMTOR4*	rs12878	7	99747130	A	G	0.6115	-0.3510	0.0455	1.23E-14	59.37
CD8 NC	*LAX1*	rs7547110	1	203742253	C	G	0.4253	-0.3870	0.0452	1.08E-17	73.21
CD8 NC	*LCLAT1*	rs4952148	2	30777908	G	T	0.2222	0.6797	0.0495	5.48E-43	188.53
CD8 NC	*LCNL1*	rs11145948	9	139857912	A	G	0.5066	-0.2491	0.0449	2.84E-08	30.75
CD8 NC	*LDHC*	rs11606494	11	18445800	A	G	0.1179	0.7627	0.0687	1.15E-28	123.13
CD8 NC	*LDLRAP1*	rs6701596	1	25889917	C	T	0.5403	-0.5015	0.0438	2.46E-30	130.75
CD8 NC	*LEPROT*	rs6658922	1	65901208	A	G	0.4061	-0.4153	0.0450	2.85E-20	84.92
CD8 NC	*LGALS3*	rs10873087	14	55565875	C	A	0.2648	0.3128	0.0505	5.64E-10	38.36
CD8 NC	*LIMD1*	rs2673023	3	45711367	C	T	0.7714	-0.5674	0.0500	8.17E-30	128.37
CD8 NC	*LINC00176*	rs817356	20	62614929	C	T	0.1906	1.4071	0.0514	2.74E-165	749.24
CD8 NC	*LINC00339*	rs11801382	1	22352108	T	C	0.2534	0.4437	0.0506	1.81E-18	76.73
CD8 NC	*LINC00649*	rs734746	21	35346897	T	G	0.2581	0.5413	0.0494	5.95E-28	119.88
CD8 NC	*LINC00884*	rs4687456	3	193501914	C	A	0.4712	-0.5382	0.0436	5.21E-35	152.08
CD8 NC	*LINC00954*	rs13034115	2	20077653	G	C	0.1396	-0.6657	0.0615	2.88E-27	116.75
CD8 NC	*LINC00969*	rs6804822	3	195709662	A	G	0.2393	0.5806	0.0523	1.37E-28	122.78
CD8 NC	*LINC01137*	rs2783726	1	37931095	C	G	0.5217	0.2651	0.0450	3.80E-09	34.65
CD8 NC	*LIPC*	rs7161799	15	58770523	T	C	0.0869	0.5411	0.0796	1.05E-11	46.13
CD8 NC	*LL22NC03-2H8.5*	rs5757699	22	22613265	A	G	0.1674	-0.3349	0.0602	2.62E-08	30.91
CD8 NC	*LONP2*	rs8053839	16	48390512	T	G	0.5150	0.3720	0.0446	6.84E-17	69.58
CD8 NC	*LPIN1*	rs11695702	2	11922054	G	A	0.2964	0.4178	0.0474	1.23E-18	77.49
CD8 NC	*LPIN2*	rs11659454	18	3058316	T	C	0.3516	0.3832	0.0454	3.29E-17	71.02
CD8 NC	*LRRC14*	rs13254911	8	145728735	T	C	0.4808	-0.3082	0.0446	5.03E-12	47.58
CD8 NC	*LRRC16A*	rs9348676	6	25398426	C	T	0.2717	0.3883	0.0489	1.95E-15	62.98
CD8 NC	*LRRC37A*	rs79475968	17	44326895	C	T	0.1521	-0.5197	0.0582	4.25E-19	79.59
CD8 NC	*LRRC37A2*	rs2106785	17	43919105	T	C	0.2037	0.3591	0.0535	1.90E-11	44.98
CD8 NC	*LRRC61*	rs1464751	7	150021282	T	C	0.2552	0.3213	0.0521	6.97E-10	37.95
CD8 NC	*LRRIQ3*	rs6702487	1	74641019	T	C	0.5186	-0.2745	0.0448	9.11E-10	37.43
CD8 NC	*LSM7*	rs12977961	19	2326897	T	C	0.6020	0.2972	0.0458	8.98E-11	41.95
CD8 NC	*LYAR*	rs2916450	4	4271623	G	C	0.7233	-0.2778	0.0490	1.47E-08	32.03
CD8 NC	*LYRM4*	rs111557465	6	5260694	T	C	0.0736	0.7882	0.0800	6.90E-23	96.81
CD8 NC	*MACF1*	rs115236337	1	39662602	T	C	0.0543	0.5467	0.0935	5.08E-09	34.09
CD8 NC	*MANBA*	rs223492	4	103675108	C	G	0.3798	-0.2971	0.0470	2.51E-10	39.94
CD8 NC	*MAST3*	rs8112290	19	18270491	A	G	0.5153	-0.2780	0.0448	5.56E-10	38.39
CD8 NC	*MBP*	rs7232502	18	74727618	T	C	0.3821	0.2953	0.0467	2.56E-10	39.9
CD8 NC	*MCC*	rs3733969	5	112328377	G	A	0.7542	-0.4005	0.0519	1.15E-14	59.5
CD8 NC	*MDGA1*	rs9349050	6	37663624	C	T	0.5194	-0.3844	0.0443	4.39E-18	74.98
CD8 NC	*MDH1*	rs1023820	2	63764963	C	G	0.7290	-0.2735	0.0487	1.89E-08	31.54
CD8 NC	*MDM4*	rs11801289	1	204528969	C	G	0.6747	-0.4547	0.0471	4.96E-22	92.92
CD8 NC	*MDS2*	rs2742946	1	23886073	C	T	0.4568	-0.2485	0.0451	3.68E-08	30.25
CD8 NC	*MEAF6*	rs11264065	1	37984812	T	C	0.5210	0.3173	0.0447	1.20E-12	50.38
CD8 NC	*MED16*	rs11546307	19	875389	A	G	0.2545	0.6526	0.0503	1.89E-38	167.79
CD8 NC	*METTL10*	rs2559509	10	126551900	A	C	0.5470	-0.3009	0.0452	2.78E-11	44.23
CD8 NC	*METTL21B*	rs1021469	12	58208164	A	G	0.3103	0.3155	0.0479	4.49E-11	43.3
CD8 NC	*MFF*	rs71431033	2	228183471	C	T	0.0853	0.4914	0.0817	1.82E-09	36.08
CD8 NC	*MGMT*	rs1008982	10	131445731	C	T	0.3802	-0.4362	0.0453	5.87E-22	92.58
CD8 NC	*MGST2*	rs3822045	4	140587414	C	A	0.2832	-0.3586	0.0502	9.10E-13	50.93
CD8 NC	*MGST3*	rs6686129	1	165591948	C	T	0.6836	-0.4171	0.0471	7.70E-19	78.42
CD8 NC	*MICU3*	rs74614084	8	16885542	G	A	0.0517	0.6034	0.1011	2.41E-09	35.54
CD8 NC	*MORN3*	rs10743188	12	122098914	C	G	0.4107	0.3718	0.0452	1.98E-16	67.48
CD8 NC	*MPHOSPH6*	rs2303260	16	82203786	G	C	0.4801	-0.4517	0.0440	1.06E-24	105.07
CD8 NC	*MPHOSPH9*	rs1790126	12	123683563	T	C	0.7739	-0.3962	0.0568	3.02E-12	48.58
CD8 NC	*MPZL3*	rs1573559	11	118104627	A	G	0.5023	-0.2858	0.0449	1.96E-10	40.43
CD8 NC	*MRPL14*	rs4714751	6	44101544	T	C	0.5150	-0.2807	0.0448	3.62E-10	39.23
CD8 NC	*MRPL18*	rs9347340	6	160188891	C	T	0.7563	-0.3573	0.0523	8.51E-12	46.55
CD8 NC	*MRPL21*	rs583182	11	68678634	T	C	0.2951	-0.5436	0.0480	9.59E-30	128.05
CD8 NC	*MRPL32*	rs11770172	7	42915143	T	A	0.1251	0.4700	0.0670	2.25E-12	49.15
CD8 NC	*MRPL34*	rs71338624	19	17414777	G	C	0.3915	0.4628	0.0444	1.77E-25	108.6
CD8 NC	*MRPL42*	rs11107039	12	93850902	A	G	0.1583	0.4331	0.0593	2.78E-13	53.25
CD8 NC	*MRPL48*	rs9705336	11	73421287	G	A	0.5547	0.2681	0.0456	4.27E-09	34.43
CD8 NC	*MRPL53*	rs1859735	2	74738322	G	A	0.1729	-0.4452	0.0676	4.47E-11	43.31
CD8 NC	*MRPL54*	rs2302307	19	3761652	A	G	0.6117	0.4801	0.0456	6.82E-26	110.49
CD8 NC	*MRPS18C*	rs4235062	4	84351153	A	G	0.5221	0.3012	0.0447	1.62E-11	45.29
CD8 NC	*MSH5*	rs3815087	6	31093587	A	G	0.2332	0.3202	0.0586	4.57E-08	29.83
CD8 NC	*MT1E*	rs1610215	16	56659525	G	C	0.5423	0.5110	0.0438	2.03E-31	135.69
CD8 NC	*MT1F*	rs1875231	16	56683745	C	A	0.3943	0.4382	0.0447	1.14E-22	95.82
CD8 NC	*MT1X*	rs1599933	16	56715862	A	C	0.5528	-0.5197	0.0439	2.19E-32	140.11
CD8 NC	*MT2A*	rs12600038	16	56648626	A	G	0.3288	-0.5888	0.0459	1.26E-37	164.03
CD8 NC	*MTERFD2*	rs6708265	2	242047750	G	A	0.1835	-0.3900	0.0611	1.72E-10	40.68
CD8 NC	*MTG2*	rs4925355	20	60773684	T	C	0.5673	0.2491	0.0451	3.26E-08	30.49
CD8 NC	*MTSS1*	rs11779706	8	125585578	C	T	0.7038	-0.2846	0.0485	4.33E-09	34.4
CD8 NC	*MTUS1*	rs673842	8	17562803	A	G	0.1763	0.3463	0.0575	1.77E-09	36.13
CD8 NC	*MUC20*	rs3107766	3	195449618	G	A	0.7894	-0.3339	0.0555	1.76E-09	36.15
CD8 NC	*MUC20*	rs4927680	3	195659169	G	A	0.3613	0.3361	0.0467	6.33E-13	51.64
CD8 NC	*MX1*	rs464138	21	42798173	C	A	0.4175	-0.2846	0.0454	3.58E-10	39.25

CD8 NC	*MYCBPAP*	rs73351675	17	48585554	C	T	0.4566	-0.3023	0.0450	1.93E-11	44.95
CD8 NC	*MYH11*	rs55871015	16	15810211	A	G	0.2572	0.4345	0.0496	2.01E-18	76.53
CD8 NC	*MYO15B*	rs76124218	17	73584441	C	G	0.0623	0.6623	0.0988	2.02E-11	44.86
CD8 NC	*MYO16*	rs9559298	13	108995713	G	A	0.2759	-0.6165	0.0510	1.07E-33	146.09
CD8 NC	*MYOM2*	rs10112042	8	2119753	T	G	0.1768	0.6846	0.0612	5.05E-29	124.76
CD8 NC	*MZT2A*	rs7605741	2	132276412	G	A	0.2151	0.9113	0.0550	1.02E-61	274.25
CD8 NC	*NAA38*	rs6964911	7	117859904	A	G	0.6809	-0.5187	0.0462	2.82E-29	125.91
CD8 NC	*NAAA*	rs6816425	4	76983992	C	T	0.3955	-0.3482	0.0463	5.16E-14	56.55
CD8 NC	*NABP1*	rs34957901	2	192545643	G	A	0.0925	-0.4820	0.0755	1.71E-10	40.69
CD8 NC	*NAP1L1*	rs1433364	12	76464637	G	A	0.8079	-0.5992	0.0574	1.57E-25	108.84
CD8 NC	*NAP1L4*	rs12276060	11	3015572	T	A	0.3858	-0.3524	0.0459	1.62E-14	58.82
CD8 NC	*NAPRT1*	rs9657360	8	144676862	T	C	0.7776	0.8019	0.0525	1.17E-52	232.77
CD8 NC	*NBL1*	rs12736909	1	19889272	G	A	0.4134	0.3919	0.0448	2.05E-18	76.48
CD8 NC	*NBL1*	rs2792036	1	19911802	C	T	0.2230	0.4113	0.0554	1.10E-13	55.06
CD8 NC	*NBPF3*	rs61775933	1	21765690	C	T	0.2063	-0.3028	0.0529	1.04E-08	32.7
CD8 NC	*NCAPG2*	rs73165344	7	158478691	T	C	0.0618	0.5132	0.0844	1.21E-09	36.88
CD8 NC	*NCF2*	rs1174657	1	183616926	C	T	0.3851	0.4193	0.0451	1.34E-20	86.41
CD8 NC	*NCF4*	rs5756376	22	37271013	C	G	0.7362	-0.3301	0.0502	4.91E-11	43.12
CD8 NC	*NCL*	rs12992177	2	232312968	G	C	0.1063	-0.4371	0.0756	7.51E-09	33.33
CD8 NC	*NDUFA10*	rs10166776	2	240967312	A	G	0.3472	-0.3034	0.0470	1.03E-10	41.68
CD8 NC	*NDUFA12*	rs10859822	12	95396906	A	C	0.3189	0.5524	0.0458	1.89E-33	144.96
CD8 NC	*NDUFA3*	rs79307734	19	54629209	T	C	0.1018	0.6590	0.0716	3.36E-20	84.59
CD8 NC	*NDUFA6*	rs6002719	22	42787282	C	T	0.0488	0.6721	0.1011	2.97E-11	44.11
CD8 NC	*NDUFS5*	rs4147750	1	39474057	C	A	0.2805	-0.5700	0.0468	4.32E-34	147.88
CD8 NC	*NEXN*	rs1328452	1	78775182	A	G	0.1522	0.4331	0.0651	2.84E-11	44.19
CD8 NC	*NINJ2*	rs11064103	12	766741	T	C	0.2727	-0.3909	0.0503	7.79E-15	60.27
CD8 NC	*NME1*	rs7207370	17	49231180	G	A	0.3899	0.3805	0.0455	6.04E-17	69.82
CD8 NC	*NME4*	rs77371232	16	447561	T	A	0.4244	0.3954	0.0448	1.07E-18	77.77
CD8 NC	*NMI*	rs11683487	2	152142549	T	G	0.4357	0.4463	0.0442	5.19E-24	101.93
CD8 NC	*NMRK1*	rs11144225	9	77707300	T	A	0.3900	-0.5884	0.0437	2.37E-41	181.05
CD8 NC	*NMT2*	rs10906857	10	15239498	G	A	0.4079	-0.4560	0.0446	1.39E-24	104.53
CD8 NC	*NOMO1*	rs6498523	16	15048643	T	C	0.8581	-0.3746	0.0652	9.32E-09	32.91
CD8 NC	*NOTCH4*	rs2524073	6	31244235	A	G	0.1283	-0.7337	0.0622	3.77E-32	139.03
CD8 NC	*NPC2*	rs4903231	14	74962233	A	G	0.5967	-0.5853	0.0439	1.51E-40	177.38
CD8 NC	*NPDC1*	rs28705707	9	139940653	A	C	0.3857	0.3164	0.0459	5.20E-12	47.51
CD8 NC	*NQO2*	rs138616686	6	3003970	G	A	0.2788	-0.5081	0.0490	3.77E-25	107.11
CD8 NC	*NQO2*	rs57826671	6	3025499	T	C	0.5556	-0.2869	0.0456	3.29E-10	39.41
CD8 NC	*NR2F6*	rs66606313	19	17345427	C	G	0.5369	-0.2548	0.0450	1.55E-08	31.92
CD8 NC	*NSA2*	rs112820313	5	74167947	A	G	0.1345	1.0376	0.0624	4.73E-62	275.77
CD8 NC	*NSG1*	rs2369105	4	4353086	G	A	0.7724	0.8995	0.0496	1.49E-73	328.45
CD8 NC	*NT5C3A*	rs10244292	7	33115943	G	A	0.7144	-0.4509	0.0504	3.87E-19	79.77
CD8 NC	*NT5C3B*	rs9897034	17	39992584	T	C	0.7555	-0.5903	0.0499	2.55E-32	139.8
CD8 NC	*NTPCR*	rs6673130	1	233081593	T	C	0.2235	0.7760	0.0505	2.58E-53	235.77
CD8 NC	*NUCKS1*	rs823094	1	205689807	G	T	0.4146	-0.2557	0.0457	2.15E-08	31.29
CD8 NC	*NUDT18*	rs6557791	8	21949663	C	T	0.6061	0.3054	0.0452	1.39E-11	45.59
CD8 NC	*NUDT2*	rs747091	9	34045014	G	T	0.5810	-0.2784	0.0450	6.10E-10	38.21
CD8 NC	*NUDT2*	rs61573237	9	34346282	T	C	0.1568	1.0516	0.0582	5.54E-73	325.84
CD8 NC	*NUP107*	rs2468418	12	69079909	C	T	0.3394	-0.2869	0.0479	2.09E-09	35.82
CD8 NC	*NUTF2*	rs79327462	16	67958058	A	G	0.0421	-0.6903	0.1022	1.42E-11	45.55
CD8 NC	*ORAI1*	rs6486787	12	122066988	C	G	0.1986	0.3494	0.0569	8.13E-10	37.65
CD8 NC	*ORMDL3*	rs17608925	17	38082831	C	T	0.1182	-0.8626	0.0698	4.52E-35	152.36
CD8 NC	*ORMDL3*	rs56301252	17	38109155	G	T	0.6270	0.2574	0.0466	3.21E-08	30.51
CD8 NC	*OSBPL10*	rs12639135	3	31561796	C	T	0.3124	0.3472	0.0497	2.92E-12	48.65
CD8 NC	*OSGIN1*	rs3826129	16	83935283	A	G	0.0506	-0.6386	0.0986	9.26E-11	41.89
CD8 NC	*OSTF1*	rs10869496	9	77700084	A	C	0.3854	-0.3785	0.0451	4.78E-17	70.28
CD8 NC	*OXLD1*	rs11150804	17	79630238	G	A	0.3735	-0.5881	0.0452	1.15E-38	168.77
CD8 NC	*OXNAD1*	rs62233919	3	16357348	C	A	0.1811	0.3677	0.0597	7.46E-10	37.82
CD8 NC	*P4HA3*	rs1726744	11	73865782	A	C	0.4856	-0.6466	0.0429	2.65E-51	226.57
CD8 NC	*PADI4*	rs1612843	1	17687161	C	G	0.2723	-0.2882	0.0499	7.67E-09	33.29
CD8 NC	*PARK7*	rs225093	1	8040177	C	T	0.6419	0.2920	0.0460	2.20E-10	40.2
CD8 NC	*PARVB*	rs2267592	22	44421563	T	G	0.5541	-0.3357	0.0446	5.24E-14	56.52
CD8 NC	*PASK*	rs6708265	2	242047750	G	A	0.1835	-0.8421	0.0586	8.26E-47	206.01
CD8 NC	*PAX8*	rs1049137	2	113975110	G	A	0.2516	0.4414	0.0510	5.10E-18	74.69
CD8 NC	*PCBP2*	rs12828029	12	53864012	G	A	0.1506	-0.3512	0.0615	1.10E-08	32.58
CD8 NC	*PCM1*	rs208058	8	17820433	A	G	0.7828	-0.3733	0.0548	9.53E-12	46.33
CD8 NC	*PCSK7*	rs4936368	11	117093486	T	C	0.2099	0.3106	0.0552	1.87E-08	31.56
CD8 NC	*PDCD5*	rs11084660	19	33072118	G	C	0.3634	0.2933	0.0459	1.65E-10	40.76
CD8 NC	*PDE6G*	rs11150804	17	79630238	G	A	0.3735	-0.3025	0.0467	9.19E-11	41.9
CD8 NC	*PDE9A*	rs2839581	21	44158405	A	C	0.7004	-0.3526	0.0481	2.24E-13	53.68
CD8 NC	*PDIA6*	rs6432141	2	10913170	G	A	0.5741	-0.2723	0.0449	1.33E-09	36.69
CD8 NC	*PDXDC1*	rs151160356	16	15198457	T	C	0.1096	0.4038	0.0648	4.49E-10	38.81
CD8 NC	*PDXK*	rs2276528	21	45152500	A	G	0.1480	0.5153	0.0617	6.72E-17	69.61
CD8 NC	*PEX6*	rs9471974	6	42918984	A	T	0.4376	0.3806	0.0446	1.42E-17	72.67
CD8 NC	*PFN2*	rs13065732	3	149685233	G	C	0.4179	0.2731	0.0455	1.91E-09	35.99
CD8 NC	*PGLS*	rs11086074	19	17619767	T	C	0.4209	-0.2602	0.0452	8.28E-09	33.14
CD8 NC	*PHACTR4*	rs10902704	1	28735707	C	A	0.3489	-0.2923	0.0468	4.36E-10	38.87
CD8 NC	*PHF11*	rs7322908	13	50100090	A	T	0.4796	0.2620	0.0448	5.15E-09	34.06
CD8 NC	*PHLDB3*	rs2599438	19	43981272	C	A	0.2432	0.3742	0.0530	1.69E-12	49.71
CD8 NC	*PI16*	rs753635	6	36934762	A	G	0.1530	0.5781	0.0595	2.70E-22	94.12

CD8 NC	*PI4K2B*	rs62411567	4	25164735	C	T	0.0897	-0.4524	0.0758	2.46E-09	35.5
CD8 NC	*PIGP*	rs2835637	21	38533467	T	A	0.3234	0.4017	0.0477	3.87E-17	70.7
CD8 NC	*PILRB*	rs75636500	7	99947657	G	C	0.1758	0.8386	0.0540	2.21E-54	240.66
CD8 NC	*PKIA*	rs7844056	8	79473033	T	C	0.2011	-0.5237	0.0578	1.24E-19	82.02
CD8 NC	*PLEC*	rs10107388	8	145004944	C	T	0.3842	-0.2913	0.0464	3.52E-10	39.28
CD8 NC	*PLEK*	rs7355584	2	68598289	G	A	0.3177	0.3054	0.0492	5.53E-10	38.4
CD8 NC	*PLEKHA1*	rs3850765	10	124139910	C	T	0.6133	-0.3829	0.0452	2.36E-17	71.68
CD8 NC	*PLEKHH2*	rs13408620	2	43870368	A	G	0.6245	-0.3500	0.0462	3.45E-14	57.34
CD8 NC	*PLGRKT*	rs2381277	9	5381870	G	A	0.6665	-0.3386	0.0464	3.06E-13	53.06
CD8 NC	*PNRC2*	rs6424145	1	24375689	C	G	0.4721	-0.4982	0.0437	4.62E-30	129.5
CD8 NC	*POLM*	rs79603064	7	44101696	A	G	0.0906	0.5511	0.0785	2.19E-12	49.2
CD8 NC	*POLR1D*	rs2232669	13	28195495	T	C	0.1167	0.9683	0.0635	1.65E-52	232.08
CD8 NC	*POLR2J3*	rs62483497	7	102332238	G	A	0.6733	-0.3191	0.0476	2.00E-11	44.88
CD8 NC	*POMC*	rs3754861	2	25393722	C	A	0.0783	0.7353	0.0881	7.14E-17	69.49
CD8 NC	*POMZP3*	rs150542665	7	76184086	G	A	0.2057	0.4721	0.0545	4.38E-18	74.99
CD8 NC	*POU5F1*	rs2516719	6	30223720	T	C	0.0841	0.6133	0.0844	3.74E-13	52.67
CD8 NC	*PPA1*	rs7077538	10	71993094	G	T	0.3217	1.0040	0.0417	3.73E-128	578.83
CD8 NC	*PPDPF*	rs72629024	20	62152519	G	C	0.1633	-1.0251	0.0582	2.35E-69	309.23
CD8 NC	*PPFIA3*	rs112378716	19	50122758	C	G	0.3442	0.6559	0.0451	6.22E-48	211.15
CD8 NC	*PPFIBP2*	rs7126061	11	7548423	A	G	0.6036	0.5475	0.0449	2.98E-34	148.62
CD8 NC	*PPIA*	rs59952188	7	44853106	T	A	0.1379	0.7186	0.0745	5.11E-22	92.86
CD8 NC	*PPIE*	rs2463260	1	40229368	G	A	0.4258	-0.2861	0.0451	2.33E-10	40.09
CD8 NC	*PPIL3*	rs2136600	2	201742345	T	C	0.2391	-0.8441	0.0505	1.16E-62	278.57
CD8 NC	*PPIL6*	rs736275	6	109031487	G	A	0.1895	0.3627	0.0611	2.86E-09	35.21
CD8 NC	*PPM1M*	rs6445358	3	52308845	G	C	0.5086	-0.2981	0.0447	2.65E-11	44.33
CD8 NC	*PPWD1*	rs154629	5	64977299	G	A	0.6323	-0.3593	0.0470	2.04E-14	58.37
CD8 NC	*PQLC3*	rs6759490	2	11401165	C	T	0.8194	0.3191	0.0575	2.92E-08	30.7
CD8 NC	*PRDX2*	rs897804	19	12876964	C	G	0.3850	0.2903	0.0457	2.15E-10	40.24
CD8 NC	*PRDX6*	rs9425411	1	173471890	A	G	0.3050	-0.7970	0.0454	7.63E-69	306.88
CD8 NC	*PRELID1*	rs6879874	5	176730775	T	A	0.7115	0.3966	0.0491	6.54E-16	65.13
CD8 NC	*PRKAR1B*	rs6945181	7	758196	A	G	0.4922	-0.2494	0.0449	2.69E-08	30.86
CD8 NC	*PRKCB*	rs11643225	16	24046655	T	G	0.0532	1.3006	0.0978	2.22E-40	176.61
CD8 NC	*PRKCQ-AS1*	rs61841332	10	6628224	A	G	0.1784	-0.9076	0.0517	4.56E-69	307.9
CD8 NC	*PRMT2*	rs59977673	21	47922091	T	C	0.4090	-0.5120	0.0442	4.71E-31	134.02
CD8 NC	*PRMT2*	rs60526571	21	48049518	C	A	0.2065	0.4306	0.0541	1.82E-15	63.12
CD8 NC	*PRPF6*	rs817352	20	62611631	T	C	0.1853	0.5183	0.0605	1.07E-17	73.24
CD8 NC	*PRR24*	rs3826791	19	47760632	A	C	0.3258	0.2957	0.0480	7.26E-10	37.87
CD8 NC	*PRR4*	rs2600337	12	11293130	C	G	0.8444	-0.4483	0.0608	1.60E-13	54.34
CD8 NC	*PRR7-AS1*	rs466256	5	176875956	A	C	0.5216	0.7334	0.0422	1.18E-67	301.43
CD8 NC	*PRSS16*	rs66975207	6	26942146	C	A	0.0737	-0.4366	0.0764	1.10E-08	32.58
CD8 NC	*PSD4*	rs2241976	2	113943470	G	A	0.4506	0.3369	0.0451	8.00E-14	55.69
CD8 NC	*PSMB9*	rs2071480	6	32821859	A	C	0.4101	0.2746	0.0459	2.12E-09	35.79
CD8 NC	*PSMD5-AS1*	rs34025993	9	123516572	G	A	0.5819	-1.1833	0.0356	1.00E-200	1103
CD8 NC	*PSPH*	rs62456603	7	55794322	T	C	0.0584	0.6858	0.0980	2.55E-12	48.9
CD8 NC	*PSTPIP1*	rs74666689	15	77786731	T	C	0.2679	0.4277	0.0488	1.95E-18	76.58
CD8 NC	*PTGER2*	rs708508	14	52801487	A	C	0.7254	0.2998	0.0516	6.17E-09	33.71
CD8 NC	*PTGIR*	rs4267438	19	47123402	G	T	0.3559	-0.3914	0.0465	3.99E-17	70.64
CD8 NC	*PTPLAD2*	rs1113401	9	21012170	A	G	0.5340	-0.2792	0.0448	4.46E-10	38.82
CD8 NC	*PTPN4*	rs3101946	2	120580542	A	C	0.3079	-0.4601	0.0483	1.50E-21	90.73
CD8 NC	*PVRIG*	rs1048303	7	100804140	T	C	0.5818	0.3438	0.0450	2.21E-14	58.21
CD8 NC	*PWWP2A*	rs4540217	5	159494252	C	T	0.6486	-0.3095	0.0472	5.35E-11	42.96
CD8 NC	*PYHIN1*	rs856080	1	158910194	A	G	0.2318	-0.3215	0.0523	7.99E-10	37.69
CD8 NC	*RABAC1*	rs11665965	19	42429833	A	G	0.6630	0.5611	0.0455	6.29E-35	151.7
CD8 NC	*RAC1*	rs2742122	7	6413687	C	T	0.4945	0.5333	0.0435	1.53E-34	149.94
CD8 NC	*RBAK-RBAKDN*	rs141183220	7	5027296	G	A	0.0485	-0.6343	0.0889	9.52E-13	50.84
CD8 NC	*RBL2*	rs4281707	16	53501946	A	G	0.4652	-0.3794	0.0446	1.68E-17	72.34
CD8 NC	*RBM43*	rs55775354	2	152118271	A	G	0.4344	-0.5142	0.0437	6.70E-32	137.89
CD8 NC	*RBM6*	rs7428430	3	50174184	T	C	0.5080	0.2803	0.0448	3.80E-10	39.13
CD8 NC	*RCSD1*	rs4657680	1	167575104	T	C	0.6404	0.3908	0.0473	1.33E-16	68.27
CD8 NC	*RCSD1*	rs4656550	1	167670439	T	C	0.2853	0.4271	0.0476	3.09E-19	80.21
CD8 NC	*RDH13*	rs1671216	19	55554076	A	G	0.6152	0.2808	0.0461	1.11E-09	37.04
CD8 NC	*RDH5*	rs3138144	12	56114769	C	G	0.4678	-0.2557	0.0448	1.19E-08	32.45
CD8 NC	*RGS12*	rs3129308	4	3309272	A	G	0.5913	-0.2631	0.0454	6.94E-09	33.48
CD8 NC	*RGS14*	rs10051765	5	176799992	C	T	0.3470	-0.2786	0.0484	8.80E-09	33.02
CD8 NC	*RIC3*	rs59123180	11	8177369	C	G	0.1332	-0.3807	0.0682	2.37E-08	31.1
CD8 NC	*RNASEH2C*	rs11227250	11	65438345	T	C	0.3441	-0.3162	0.0467	1.25E-11	45.8
CD8 NC	*RNASET2*	rs376097	6	167410878	G	A	0.5062	-0.8334	0.0409	2.37E-92	414.62
CD8 NC	*RNF145*	rs17718834	5	158529996	T	C	0.0933	0.4331	0.0740	4.92E-09	34.15
CD8 NC	*RNF166*	rs12148926	16	88779323	T	C	0.2769	0.3505	0.0507	4.70E-12	47.71
CD8 NC	*RNF168*	rs9865308	3	196231072	G	T	0.7031	-0.3522	0.0503	2.43E-12	49
CD8 NC	*RNF175*	rs2445617	4	154659028	T	C	0.4882	-0.2453	0.0448	4.53E-08	29.85
CD8 NC	*RNF24*	rs241602	20	3912359	T	C	0.4937	-0.2676	0.0448	2.32E-09	35.61
CD8 NC	*ROBO3*	rs11219822	11	124746644	C	A	0.4301	0.3315	0.0449	1.61E-13	54.32
CD8 NC	*RP1-257A7.5*	rs59178279	6	13927510	G	A	0.2135	0.3924	0.0547	7.41E-13	51.33
CD8 NC	*RP1-265C24.8*	rs3118362	6	28785085	T	C	0.9328	0.5117	0.0788	8.18E-11	42.13
CD8 NC	*RP1-29C18.10*	rs8138399	22	50044824	T	C	0.6367	-0.7253	0.0452	5.40E-58	257.19
CD8 NC	*RP1-30M3.5*	rs9969023	6	25211551	T	C	0.0706	-0.5554	0.0771	5.92E-13	51.77
CD8 NC	*RP1-3J17.3*	rs6903114	6	86406671	C	T	0.7040	0.7015	0.0465	1.60E-51	227.56
CD8 NC	*RP1-40E16.12*	rs12663589	6	3069057	T	C	0.5524	-0.3480	0.0451	1.16E-14	59.49

CD8 NC	*RP11-103G8.2*	rs9814796	3	150422469	A	G	0.3440	0.2810	0.0466	1.68E-09	36.23
CD8 NC	*RP11-1060J15.4*	rs144049521	12	27848343	G	A	0.0568	1.1324	0.0867	5.98E-39	170.08
CD8 NC	*RP11-108M9.4*	rs157252	1	16796721	C	A	0.5375	-0.3145	0.0453	3.80E-12	48.13
CD8 NC	*RP11-1191J2.5*	rs28586910	4	656604	T	G	0.1647	0.3853	0.0621	5.34E-10	38.47
CD8 NC	*RP11-137H2.4*	rs11185932	10	82295398	A	G	0.3372	-0.2922	0.0473	6.73E-10	38.02
CD8 NC	*RP11-138A9.1*	rs17165295	7	130622564	G	A	0.1919	0.4064	0.0571	1.06E-12	50.63
CD8 NC	*RP11-138A9.2*	rs205724	7	130623257	C	A	0.8419	0.4605	0.0600	1.64E-14	58.8
CD8 NC	*RP11-1398P2.1*	rs4865439	4	1598368	C	T	0.5282	0.3688	0.0447	1.59E-16	67.92
CD8 NC	*RP11-1399P15.1*	rs55956548	2	87768679	T	G	0.7418	0.4284	0.0520	1.76E-16	67.72
CD8 NC	*RP11-158I9.7*	rs594603	11	117923079	T	C	0.1284	0.8450	0.0660	1.71E-37	163.42
CD8 NC	*RP11-166B2.1*	rs12930612	16	12072203	A	G	0.3194	-0.7228	0.0461	1.95E-55	245.48
CD8 NC	*RP11-166B2.5*	rs33643	16	11986149	G	A	0.3618	-0.3871	0.0467	1.12E-16	68.6
CD8 NC	*RP11-186N15.3*	rs2768465	10	12574113	T	C	0.6066	-0.4146	0.0454	7.05E-20	83.13
CD8 NC	*RP11-18H21.1*	rs13140099	4	153022375	A	G	0.2594	-0.9185	0.0461	2.19E-88	396.44
CD8 NC	*RP11-18H21.1*	rs7668777	4	152932663	T	C	0.4090	0.2583	0.0460	2.00E-08	31.43
CD8 NC	*RP11-245G13.2*	rs7577665	2	11020902	A	G	0.1116	1.2657	0.0679	1.84E-77	346.37
CD8 NC	*RP11-273G15.2*	rs13279735	8	144097453	G	A	0.3606	-0.2682	0.0467	9.59E-09	32.86
CD8 NC	*RP11-291B21.2*	rs61913114	12	10709389	G	A	0.4123	-0.3302	0.0459	6.55E-13	51.57
CD8 NC	*RP11-294J22.6*	rs12946653	17	48748185	G	A	0.7354	0.2834	0.0506	2.13E-08	31.31
CD8 NC	*RP11-298J20.3*	rs7099073	10	126609647	T	G	0.7315	-1.0394	0.0461	1.02E-112	508.01
CD8 NC	*RP11-299J3.8*	rs9289198	3	122277160	T	G	0.1459	-0.4337	0.0586	1.41E-13	54.58
CD8 NC	*RP11-326C3.11*	rs11246065	11	324170	G	A	0.3701	0.7863	0.0437	1.79E-72	323.51
CD8 NC	*RP11-326C3.11*	rs10794309	11	325948	T	C	0.6765	-0.2789	0.0493	1.50E-08	31.99
CD8 NC	*RP11-326C3.12*	rs11246068	11	327334	T	C	0.2550	0.8176	0.0486	1.62E-63	282.48
CD8 NC	*RP11-326C3.15*	rs7395116	11	324880	C	G	0.2517	-0.4461	0.0513	3.19E-18	75.61
CD8 NC	*RP11-334C17.5*	rs6565648	17	78188256	T	C	0.3456	0.2666	0.0469	1.31E-08	32.25
CD8 NC	*RP11-345J4.5*	rs144208959	16	30323380	A	G	0.4341	-0.4674	0.0439	1.96E-26	112.96
CD8 NC	*RP11-347P5.1*	rs34830320	2	196962919	G	A	0.4054	-0.7159	0.0433	2.08E-61	272.83
CD8 NC	*RP11-382J12.1*	rs62530785	8	71612304	T	A	0.4967	-0.3672	0.0444	1.41E-16	68.15
CD8 NC	*RP11-395B7.4*	rs13246386	7	100660639	G	C	0.1448	-0.3609	0.0613	4.02E-09	34.54
CD8 NC	*RP11-403A21.2*	rs17259569	18	21583692	C	G	0.1597	-0.4698	0.0614	1.99E-14	58.42
CD8 NC	*RP11-415F23.2*	rs2280247	3	16358902	G	A	0.1746	0.7729	0.0619	9.31E-36	155.49
CD8 NC	*RP11-430H10.1*	rs12418562	11	45373480	C	G	0.1739	0.5364	0.0576	1.23E-20	86.57
CD8 NC	*RP11-434C1.1*	rs10772397	12	11138683	T	C	0.6075	-0.2630	0.0456	8.09E-09	33.19
CD8 NC	*RP11-457M11.5*	rs806977	6	26189615	G	A	0.5334	-0.2657	0.0448	3.12E-09	35.04
CD8 NC	*RP11-473I1.5*	rs12918271	16	9200547	C	T	0.2190	-0.4834	0.0542	4.54E-19	79.46
CD8 NC	*RP11-473M20.9*	rs4786384	16	3136919	C	T	0.5202	0.4256	0.0442	5.90E-22	92.57
CD8 NC	*RP11-474G23.2*	rs11678521	2	68533154	T	A	0.5174	0.5848	0.0432	7.61E-42	183.31
CD8 NC	*RP11-499P20.2*	rs4748483	10	18822667	C	G	0.4696	0.2853	0.0451	2.51E-10	39.94
CD8 NC	*RP11-526I2.5*	rs36053285	15	101114640	C	T	0.4253	-0.3572	0.0453	3.10E-15	62.08
CD8 NC	*RP11-536K7.3*	rs17555802	10	6583622	G	A	0.0908	-0.5139	0.0761	1.41E-11	45.56
CD8 NC	*RP11-539L10.3*	rs36102500	4	6669749	A	G	0.1447	-0.6437	0.0636	4.20E-24	102.35
CD8 NC	*RP11-53O19.3*	rs10447145	5	44689796	G	T	0.3819	0.6357	0.0445	3.30E-46	203.26
CD8 NC	*RP11-542K23.7*	rs35918962	9	125168878	T	C	0.1266	0.8600	0.0661	1.07E-38	168.93
CD8 NC	*RP11-545E17.3*	rs55697116	9	131489620	T	C	0.2481	0.6268	0.0493	5.77E-37	161.01
CD8 NC	*RP11-589P10.5*	rs312466	17	6899221	A	G	0.4097	-0.2544	0.0453	1.96E-08	31.47
CD8 NC	*RP11-596D21.1*	rs4981092	14	31790565	C	T	0.4129	-0.3941	0.0452	2.68E-18	75.96
CD8 NC	*RP11-641A6.2*	rs34155853	12	52974306	A	G	0.1217	0.5931	0.0666	5.57E-19	79.05
CD8 NC	*RP11-660L16.2*	rs12807827	11	71203790	C	A	0.6914	0.5556	0.0536	3.58E-25	107.21
CD8 NC	*RP11-664D1.1*	rs588616	12	4008755	G	A	0.1491	0.6835	0.0619	2.49E-28	121.6
CD8 NC	*RP11-664D1.1*	rs609018	12	4071901	G	T	0.4284	-0.3679	0.0447	1.80E-16	67.68
CD8 NC	*RP11-669E14.6*	rs4327090	17	43902541	G	A	0.1778	-0.5389	0.0546	5.76E-23	97.17
CD8 NC	*RP11-677M14.3*	rs12281805	11	124771763	G	A	0.3648	-0.5999	0.0442	5.52E-42	183.94
CD8 NC	*RP11-69E11.4*	rs3820618	1	40223128	C	T	0.1312	0.4680	0.0677	4.61E-12	47.75
CD8 NC	*RP11-712L6.5*	rs627911	11	126072688	T	C	0.7610	-0.3833	0.0521	1.90E-13	54
CD8 NC	*RP11-774O3.3*	rs4689718	4	7390159	C	T	0.3034	0.2727	0.0496	3.92E-08	30.13
CD8 NC	*RP11-77P16.4*	rs56218598	3	129823899	A	G	0.4027	0.3725	0.0450	1.19E-16	68.49
CD8 NC	*RP11-792A8.4*	rs76949143	7	66009851	A	T	0.0634	0.8111	0.0931	2.97E-18	75.76
CD8 NC	*RP11-841O20.2*	rs11626437	14	53154620	T	C	0.2710	0.3391	0.0502	1.48E-11	45.47
CD8 NC	*RP11-936I5.1*	rs93075	17	75370468	G	T	0.6892	-0.4371	0.0477	4.83E-20	83.88
CD8 NC	*RP11-950C14.3*	rs449131	14	75589878	T	C	0.4527	-0.3314	0.0446	1.04E-13	55.17
CD8 NC	*RP11-96H19.1*	rs4768697	12	46748965	G	C	0.2578	0.3243	0.0506	1.44E-10	41.02
CD8 NC	*RP3-325F22.5*	rs9389398	6	136831056	C	T	0.1758	-0.3825	0.0604	2.35E-10	40.07
CD8 NC	*RP4-539M6.22*	rs5994305	22	30820707	G	A	0.1901	-0.3219	0.0572	1.87E-08	31.56
CD8 NC	*RP4-594I10.3*	rs196425	1	24871586	C	T	0.3702	0.4489	0.0456	6.59E-23	96.9
CD8 NC	*RP4-798A10.2*	rs279781	1	16777812	C	G	0.3328	-0.2905	0.0461	3.04E-10	39.57
CD8 NC	*RP5-1021I20.1*	rs72723709	14	74273973	T	C	0.1400	0.3830	0.0597	1.42E-10	41.05
CD8 NC	*RP5-1042K10.10*	rs6001894	22	40779197	C	T	0.1788	-0.5462	0.0572	1.31E-21	90.99
CD8 NC	*RP5-827C21.4*	rs17576781	1	234537924	C	T	0.0913	0.7139	0.0767	1.29E-20	86.47
CD8 NC	*RPL10A*	rs2076172	6	35436925	T	C	0.5358	-0.7290	0.0425	4.49E-66	294.19
CD8 NC	*RPL11*	rs77766201	1	24006446	A	G	0.1560	-0.8351	0.0606	3.09E-43	189.67
CD8 NC	*RPL12*	rs2247781	9	130208924	C	T	0.5921	0.9660	0.0397	1.08E-130	590.48
CD8 NC	*RPL12*	rs7854003	9	130310834	G	A	0.1464	0.3536	0.0609	6.48E-09	33.62
CD8 NC	*RPL13*	rs12932337	16	89630630	T	C	0.1753	-0.7494	0.0540	7.47E-44	192.49
CD8 NC	*RPL14*	rs75965075	3	40438120	T	C	0.0715	1.0188	0.0830	1.28E-34	150.29
CD8 NC	*RPL14*	rs4973906	3	40310349	C	T	0.9381	-0.5715	0.0946	1.53E-09	36.42
CD8 NC	*RPL22L1*	rs34587559	3	170578721	T	C	0.2392	-0.3272	0.0508	1.14E-10	41.48
CD8 NC	*RPL23A*	rs2302205	17	26947046	C	A	0.0821	-0.8847	0.0770	1.53E-30	131.69
CD8 NC	*RPL27A*	rs61875926	11	8705528	G	C	0.0958	-0.6455	0.0734	1.40E-18	77.24

CD8 NC	*RPL28*	rs17700376	19	55897327	G	A	0.3547	-1.1214	0.0388	1.06E-183	833.75
CD8 NC	*RPL3*	rs470082	22	39715140	T	C	0.4764	0.2825	0.0448	2.73E-10	39.78
CD8 NC	*RPL32*	rs2290311	3	12883071	A	T	0.1438	0.5592	0.0611	5.55E-20	83.6
CD8 NC	*RPL36AL*	rs2147712	14	50091684	A	G	0.7504	1.0837	0.0464	1.35E-120	544.16
CD8 NC	*RPL8*	rs2955194	8	145988907	T	G	0.4950	1.1195	0.0361	1.00E-200	960.03
CD8 NC	*RPLP2*	rs28694634	11	808423	A	G	0.6588	-0.7912	0.0449	1.81E-69	309.75
CD8 NC	*RPS10*	rs9380433	6	34377626	T	C	0.7750	-0.9381	0.0544	1.48E-66	296.39
CD8 NC	*RPS11*	rs10403394	19	49999345	A	G	0.1225	-0.7683	0.0704	1.02E-27	118.82
CD8 NC	*RPS12*	rs138246297	6	133167642	C	G	0.0608	0.7047	0.0963	2.53E-13	53.43
CD8 NC	*RPS14*	rs2245384	5	149817268	C	G	0.3033	-0.3056	0.0487	3.61E-10	39.23
CD8 NC	*RPS16*	rs480595	19	39925482	T	A	0.3758	0.4102	0.0468	1.87E-18	76.66
CD8 NC	*RPS17*	rs12916980	15	83306447	G	C	0.4319	-0.2500	0.0453	3.39E-08	30.41
CD8 NC	*RPS18*	rs17215231	6	33239869	T	C	0.0640	-1.5912	0.0823	2.46E-83	373.29
CD8 NC	*RPS19*	rs12984383	19	42371522	C	G	0.4967	0.3172	0.0446	1.15E-12	50.47
CD8 NC	*RPS2*	rs17135718	16	2014834	G	A	0.1151	0.8698	0.0654	2.05E-40	176.78
CD8 NC	*RPS23*	rs226205	5	81593654	T	C	0.7767	0.4076	0.0511	1.48E-15	63.53
CD8 NC	*RPS24*	rs2913134	10	79763775	T	C	0.5775	0.3106	0.0459	1.31E-11	45.71
CD8 NC	*RPS25*	rs11602764	11	118888619	G	A	0.2652	-0.7517	0.0474	1.03E-56	251.34
CD8 NC	*RPS26*	rs773114	12	56379060	T	A	0.3997	1.2010	0.0352	1.00E-200	1163.62
CD8 NC	*RPS3*	rs3094303	11	75114071	G	A	0.0743	-0.9416	0.0892	4.70E-26	111.23
CD8 NC	*RPS3A*	rs6836854	4	151742349	T	C	0.9259	1.2678	0.0963	1.30E-39	173.1
CD8 NC	*RPS5*	rs731835	19	58898436	C	T	0.3256	-1.0332	0.0414	3.33E-137	620.35
CD8 NC	*RPS6*	rs6475324	9	19361448	A	G	0.0990	-1.1448	0.0789	9.30E-48	210.35
CD8 NC	*RPS6KA2*	rs12216502	6	167267072	A	G	0.1820	-0.3305	0.0595	2.72E-08	30.83
CD8 NC	*RPS7*	rs11558965	2	3622912	T	G	0.0770	-1.2572	0.0847	7.22E-50	220
CD8 NC	*RPS8*	rs12120833	1	45241285	G	C	0.1242	-0.7952	0.0606	2.24E-39	172.03
CD8 NC	*RPS9*	rs34629529	19	54701846	A	G	0.3950	-0.8719	0.0414	1.18E-98	443.52
CD8 NC	*RPSA*	rs28720962	3	39426441	T	C	0.1558	0.7272	0.0599	7.10E-34	146.9
CD8 NC	*RRP7A*	rs136988	22	42946371	T	C	0.1733	0.4575	0.0573	1.38E-15	63.67
CD8 NC	*RSRC2*	rs2062506	12	123084836	G	A	0.7422	-0.3847	0.0496	8.67E-15	60.05
CD8 NC	*RTKN2*	rs2172070	10	64052889	A	G	0.8690	-0.4612	0.0654	1.78E-12	49.61
CD8 NC	*S100B*	rs11701571	21	47963149	G	A	0.2474	0.3057	0.0523	5.10E-09	34.08
CD8 NC	*S100B*	rs11089085	21	48039367	G	A	0.6194	-0.3636	0.0461	3.20E-15	62.01
CD8 NC	*SAMD10*	rs817356	20	62614929	C	T	0.1906	0.3760	0.0609	6.44E-10	38.11
CD8 NC	*SAMD3*	rs41285310	6	130505374	C	A	0.5086	-0.6272	0.0429	1.78E-48	213.63
CD8 NC	*SAMM50*	rs6006595	22	44368039	C	T	0.1292	-0.4106	0.0733	2.15E-08	31.29
CD8 NC	*SAR1A*	rs2310991	10	71931646	A	C	0.4271	-0.4060	0.0450	1.93E-19	81.14
CD8 NC	*SAT2*	rs858520	17	7530271	T	C	0.3938	0.2755	0.0462	2.50E-09	35.47
CD8 NC	*SCAMP3*	rs2242576	1	155231545	G	T	0.2676	0.4000	0.0492	4.19E-16	66.01
CD8 NC	*SCD5*	rs4461514	4	83752924	G	T	0.2078	0.3432	0.0551	4.69E-10	38.72
CD8 NC	*SCGB3A1*	rs7730055	5	180007386	G	A	0.8901	-1.5419	0.0653	2.78E-123	556.47
CD8 NC	*SDK2*	rs11655934	17	71587577	A	G	0.1405	-0.3838	0.0647	2.98E-09	35.12
CD8 NC	*SELM*	rs11703403	22	31507338	G	A	0.5262	0.3040	0.0447	1.10E-11	46.05
CD8 NC	*SEPT2*	rs35533015	2	242191689	A	G	0.0889	0.9889	0.0726	2.93E-42	185.21
CD8 NC	*SERPINB6*	rs62391542	6	2940666	T	C	0.2533	-0.7579	0.0482	9.57E-56	246.9
CD8 NC	*SERPINB9*	rs12529647	6	2885595	C	T	0.2354	-0.3827	0.0526	3.46E-13	52.82
CD8 NC	*SERPINE2*	rs282254	2	224907347	T	C	0.5483	-0.2522	0.0452	2.32E-08	31.14
CD8 NC	*SERPINF1*	rs7220762	17	1668248	T	C	0.3379	0.3854	0.0467	1.62E-16	67.89
CD8 NC	*SESN1*	rs2798646	6	109310596	A	C	0.8789	-0.3913	0.0669	4.91E-09	34.15
CD8 NC	*SESN3*	rs4409785	11	95311422	C	T	0.1722	-0.4068	0.0581	2.49E-12	48.95
CD8 NC	*SESTD1*	rs10181291	2	180098404	C	A	0.2621	0.3255	0.0523	4.75E-10	38.7
CD8 NC	*SETD5-AS1*	rs2255558	3	9416832	T	C	0.5922	0.2908	0.0458	2.18E-10	40.21
CD8 NC	*SETD9*	rs2662022	5	56261491	A	G	0.1638	-0.4998	0.0623	1.00E-15	64.3
CD8 NC	*SF1*	rs533447	11	64561908	C	T	0.8796	-0.4742	0.0781	1.29E-09	36.76
CD8 NC	*SFT2D1*	rs2343590	6	166759622	G	C	0.7466	0.3804	0.0505	4.98E-14	56.62
CD8 NC	*SH3YL1*	rs7595075	2	264019	A	C	0.3283	-1.0530	0.0401	3.66E-152	688.99
CD8 NC	*SIDT1-AS1*	rs3773693	3	113293438	A	T	0.1691	0.5721	0.0572	1.46E-23	99.89
CD8 NC	*SIRPG*	rs6043403	20	1615228	T	A	0.3142	-0.3090	0.0504	8.97E-10	37.46
CD8 NC	*SKAP2*	rs17323934	7	26904330	G	C	0.2183	0.3842	0.0526	2.76E-13	53.27
CD8 NC	*SLC12A5*	rs3918241	20	44635735	A	T	0.1436	-0.5792	0.0603	7.24E-22	92.17
CD8 NC	*SLC12A7*	rs35188965	5	1104938	T	C	0.5560	-0.4810	0.0446	3.56E-27	116.34
CD8 NC	*SLC22A18*	rs440150	11	2936657	A	G	0.9083	-0.5891	0.0761	1.02E-14	59.73
CD8 NC	*SLC25A1*	rs2854643	22	19189706	C	T	0.6537	-0.3123	0.0466	2.05E-11	44.83
CD8 NC	*SLC25A24*	rs547364	1	108742123	G	C	0.4778	0.3196	0.0446	7.79E-13	51.23
CD8 NC	*SLC25A26*	rs13091146	3	66451522	A	G	0.1884	-0.3936	0.0535	1.97E-13	53.92
CD8 NC	*SLC25A29*	rs10134767	14	100763954	T	G	0.3371	0.5317	0.0463	1.44E-30	131.81
CD8 NC	*SLC25A3*	rs28372744	12	98987445	A	G	0.0487	0.7785	0.0974	1.34E-15	63.72
CD8 NC	*SLC25A51*	rs73646116	9	37873464	A	G	0.1483	0.3732	0.0640	5.56E-09	33.91
CD8 NC	*SLC2A1-AS1*	rs7534555	1	43413319	C	G	0.1854	0.3465	0.0604	9.59E-09	32.86
CD8 NC	*SLC35F3*	rs595595	1	234449908	A	G	0.3927	-0.3464	0.0464	8.65E-14	55.54
CD8 NC	*SLC38A1*	rs7311868	12	46585492	C	T	0.2651	-0.2823	0.0508	2.80E-08	30.78
CD8 NC	*SLC40A1*	rs71434664	2	190452068	T	G	0.2407	0.2777	0.0509	4.79E-08	29.74
CD8 NC	*SLC4A7*	rs4973776	3	27530208	A	G	0.2167	-0.4239	0.0517	2.53E-16	67
CD8 NC	*SLC7A8*	rs59358210	14	23631372	A	G	0.1073	0.6108	0.0720	2.23E-17	71.79
CD8 NC	*SLFN5*	rs11656388	17	33531815	A	G	0.3965	0.3720	0.0456	3.31E-16	66.47
CD8 NC	*SMAD5*	rs1990198	5	135459494	C	G	0.3212	0.3127	0.0495	2.57E-10	39.9
CD8 NC	*SMAGP*	rs7308873	12	51643242	G	A	0.7720	0.2998	0.0530	1.51E-08	31.98
CD8 NC	*SMDT1*	rs2301521	22	42423110	C	G	0.6779	1.3193	0.0361	1.00E-200	1335.88
CD8 NC	*SMIM19*	rs6987853	8	42457450	C	T	0.6238	-0.2894	0.0459	2.76E-10	39.76

CD8 NC	*SNAP29*	rs5761464	22	21298018	G	C	0.0664	0.6345	0.0829	1.98E-14	58.43
CD8 NC	*SNHG7*	rs11145844	9	139613998	T	C	0.1148	-1.1604	0.0667	1.02E-67	301.72
CD8 NC	*SNHG8*	rs658642	4	119200274	T	C	0.3810	1.1030	0.0375	2.29E-190	864.34
CD8 NC	*SNORD3B-2*	rs77232220	17	19262534	C	G	0.1953	0.3659	0.0551	3.22E-11	43.95
CD8 NC	*SNRNP25*	rs11866537	16	90538	C	T	0.1765	0.3320	0.0589	1.70E-08	31.75
CD8 NC	*SNRPC*	rs9469861	6	34726136	A	T	0.3490	-0.3243	0.0483	1.92E-11	44.95
CD8 NC	*SNRPD2*	rs34399924	19	46200103	A	G	0.2142	-0.7778	0.0534	4.68E-48	211.71
CD8 NC	*SNTG2*	rs4241334	2	860229	C	T	0.6767	-0.5329	0.0470	7.71E-30	128.48
CD8 NC	*SPATA20*	rs8076632	17	48625928	G	C	0.3650	-0.4056	0.0465	2.67E-18	75.96
CD8 NC	*SPATC1L*	rs62215189	21	47603995	G	A	0.2446	-0.6926	0.0489	1.72E-45	199.98
CD8 NC	*SPCS1*	rs4687629	3	52615227	C	G	0.4270	0.2534	0.0459	3.49E-08	30.35
CD8 NC	*SPINK2*	rs35867715	4	57627999	C	T	0.2331	-0.4532	0.0521	3.38E-18	75.5
CD8 NC	*SPNS1*	rs7140	16	28995757	A	C	0.7024	-0.2813	0.0497	1.47E-08	32.02
CD8 NC	*SPRYD4*	rs73114872	12	56861034	C	T	0.1775	0.3180	0.0562	1.49E-08	32
CD8 NC	*SPSB2*	rs66621175	12	6965805	C	T	0.3162	0.3389	0.0480	1.75E-12	49.64
CD8 NC	*SRA1*	rs73269505	5	139973781	T	C	0.2993	-0.2935	0.0487	1.68E-09	36.24
CD8 NC	*SRD5A3*	rs11935857	4	56204865	A	C	0.1899	0.4191	0.0587	9.60E-13	50.82
CD8 NC	*SRI*	rs1063964	7	87835469	A	G	0.3146	0.3188	0.0480	3.09E-11	44.03
CD8 NC	*SRP14*	rs75578790	15	40332440	G	A	0.0814	0.5923	0.0863	6.87E-12	46.97
CD8 NC	*SRR*	rs77530532	17	2208874	A	G	0.3371	-0.2774	0.0470	3.50E-09	34.81
CD8 NC	*SRSF10*	rs6424145	1	24375689	C	G	0.4721	-0.4542	0.0440	5.39E-25	106.4
CD8 NC	*SSBP4*	rs10402247	19	18522805	C	A	0.2067	-0.3204	0.0557	9.05E-09	32.97
CD8 NC	*ST6GALNAC6*	rs10819317	9	130668957	G	A	0.1684	-0.3905	0.0590	3.63E-11	43.71
CD8 NC	*STK32C*	rs72863829	10	134189128	T	C	0.1667	0.3243	0.0564	8.88E-09	33
CD8 NC	*STK4*	rs6130724	20	43640738	A	G	0.1636	0.3337	0.0610	4.41E-08	29.9
CD8 NC	*STMN3*	rs879471	20	62269942	T	C	0.5285	0.5440	0.0436	9.36E-36	155.48
CD8 NC	*STOX1*	rs35233764	10	70580477	C	T	0.1299	-0.7840	0.0711	2.68E-28	121.46
CD8 NC	*STYX*	rs11846010	14	53172922	C	T	0.6264	0.2631	0.0466	1.64E-08	31.81
CD8 NC	*SULF2*	rs3091426	20	46189755	A	G	0.5891	-0.8580	0.0410	2.15E-97	437.74
CD8 NC	*SUPT3H*	rs4711805	6	44870102	C	T	0.4143	0.3729	0.0450	1.21E-16	68.45
CD8 NC	*SURF1*	rs3118667	9	136291063	T	C	0.5241	-0.3722	0.0444	5.35E-17	70.06
CD8 NC	*SVIP*	rs10833823	11	22832056	C	T	0.3039	-0.3084	0.0488	2.62E-10	39.86
CD8 NC	*SYNGR1*	rs909685	22	39747671	A	T	0.3216	0.5117	0.0486	6.63E-26	110.55
CD8 NC	*SYNJ2*	rs1744164	6	158513415	A	G	0.6095	0.2718	0.0460	3.49E-09	34.82
CD8 NC	*TAGLN2*	rs2252815	1	159889686	A	G	0.5075	-0.2457	0.0449	4.56E-08	29.83
CD8 NC	*TAPBP*	rs3130260	6	33278816	A	G	0.2897	-0.4052	0.0485	6.93E-17	69.55
CD8 NC	*TAPBPL*	rs2532496	12	6564448	G	A	0.3063	0.3841	0.0488	3.59E-15	61.79
CD8 NC	*TBCA*	rs254415	5	77071703	T	G	0.2878	-0.5774	0.0476	6.96E-34	146.94
CD8 NC	*TBCD*	rs8075894	17	80853155	G	T	0.4679	0.3460	0.0446	8.21E-15	60.16
CD8 NC	*TBX6*	rs7698	16	30125800	A	G	0.0851	-0.5926	0.0827	7.54E-13	51.29
CD8 NC	*TC2N*	rs2402076	14	92322416	C	T	0.4973	-0.2630	0.0448	4.36E-09	34.38
CD8 NC	*TCTEX1D2*	rs55996879	3	196046300	T	C	0.2583	0.3107	0.0528	3.96E-09	34.57
CD8 NC	*TEN1*	rs12945114	17	73993546	T	C	0.7534	0.3507	0.0521	1.61E-11	45.3
CD8 NC	*THAP5*	rs2227272	7	108209623	C	T	0.2327	0.3065	0.0555	3.39E-08	30.41
CD8 NC	*THAP7-AS1*	rs2239961	22	21363960	A	G	0.2024	-0.4165	0.0555	6.20E-14	56.19
CD8 NC	*THAP9-AS1*	rs4560402	4	83762954	G	A	0.7856	0.4020	0.0547	1.96E-13	53.93
CD8 NC	*THEM4*	rs7542137	1	151868892	T	C	0.6000	0.2831	0.0456	5.55E-10	38.4
CD8 NC	*THEMIS2*	rs6691656	1	28221130	G	C	0.3801	-0.6566	0.0439	1.36E-50	223.31
CD8 NC	*TIMM10*	rs2848626	11	57283988	T	C	0.7820	-1.0156	0.0466	2.78E-105	473.91
CD8 NC	*TIMM13*	rs6945	19	2426828	C	G	0.4919	0.3869	0.0443	2.57E-18	76.04
CD8 NC	*TIPARP-AS1*	rs9289971	3	156389947	C	T	0.1529	-0.3938	0.0607	8.54E-11	42.04
CD8 NC	*TIPRL*	rs4540637	1	168103545	G	A	0.4981	-0.2603	0.0450	7.54E-09	33.32
CD8 NC	*TLE4*	rs2807303	9	82187095	A	G	0.3638	0.4355	0.0462	4.25E-21	88.67
CD8 NC	*TLK1*	rs34004615	2	171843623	A	G	0.3521	-0.3624	0.0464	6.03E-15	60.77
CD8 NC	*TMEM101*	rs77536093	17	42092328	G	A	0.0441	-0.7134	0.1016	2.25E-12	49.15
CD8 NC	*TMEM120B*	rs73413716	12	122215128	G	A	0.0993	0.7749	0.0785	5.42E-23	97.29
CD8 NC	*TMEM121*	rs11622603	14	105967942	G	A	0.2761	-0.4632	0.0522	7.43E-19	78.49
CD8 NC	*TMEM156*	rs6819988	4	39038665	T	C	0.5743	0.5453	0.0439	1.96E-35	154.01
CD8 NC	*TMEM18*	rs2293083	2	676177	G	C	0.7439	-0.3803	0.0506	5.96E-14	56.27
CD8 NC	*TMEM2*	rs117975395	9	74377843	C	T	0.0877	0.4025	0.0725	2.83E-08	30.76
CD8 NC	*TMEM204*	rs2745183	16	1581770	G	A	0.1397	0.5363	0.0670	1.24E-15	63.88
CD8 NC	*TMEM230*	rs1570031	20	5071680	C	T	0.5294	0.5030	0.0438	1.74E-30	131.43
CD8 NC	*TMEM244*	rs9492393	6	130154667	C	T	0.3324	0.4587	0.0464	4.42E-23	97.69
CD8 NC	*TMEM254-AS1*	rs2342607	10	81821489	T	C	0.0964	-0.5098	0.0808	2.85E-10	39.69
CD8 NC	*TMEM258*	rs174589	11	61615803	G	C	0.1903	0.4306	0.0546	3.01E-15	62.13
CD8 NC	*TMEM41B*	rs10840188	11	9241219	A	G	0.2161	-0.3552	0.0552	1.20E-10	41.38
CD8 NC	*TMEM45B*	rs2241573	11	129793305	G	A	0.3167	0.3277	0.0479	7.54E-12	46.79
CD8 NC	*TMEM50A*	rs111941366	1	25736369	T	C	0.3868	-0.2505	0.0451	2.82E-08	30.76
CD8 NC	*TMEM50B*	rs2834222	21	34824771	A	G	0.4717	-0.5227	0.0436	4.70E-33	143.15
CD8 NC	*TMEM80*	rs10794330	11	686714	A	G	0.4585	-0.4222	0.0446	2.82E-21	89.48
CD8 NC	*TMEM8A*	rs2363755	16	430292	G	C	0.4125	-0.5433	0.0436	1.22E-35	154.96
CD8 NC	*TMPRSS11E*	rs1972333	4	69302719	C	G	0.4616	-0.6200	0.0432	1.00E-46	205.63
CD8 NC	*TNFRSF14*	rs12129508	1	2511388	A	G	0.1479	0.4089	0.0644	2.18E-10	40.21
CD8 NC	*TNFSF13B*	rs9301290	13	109004653	G	C	0.2718	0.2763	0.0506	4.80E-08	29.74
CD8 NC	*TNFSF8*	rs5003740	9	117660601	C	G	0.1875	-0.3390	0.0591	9.59E-09	32.86
CD8 NC	*TNNI2*	rs234861	11	2853821	A	G	0.8194	0.6347	0.0560	8.47E-30	128.3
CD8 NC	*TOMM7*	rs2240728	7	22852499	T	C	0.3422	-1.2119	0.0387	1.00E-200	977.86
CD8 NC	*TP53TG1*	rs802035	7	86969559	T	C	0.7363	0.2886	0.0498	6.81E-09	33.52
CD8 NC	*TPD52*	rs7011637	8	81025007	T	C	0.4397	0.4116	0.0445	2.31E-20	85.33

CD8 NC	*TPGS1*	rs55714911	19	522145	C	T	0.2790	0.3323	0.0487	9.33E-12	46.37
CD8 NC	*TPGS2*	rs948636	18	34361762	C	G	0.2855	-0.5056	0.0484	1.37E-25	109.11
CD8 NC	*TPRA1*	rs6776442	3	127166771	G	A	0.9220	0.5104	0.0901	1.47E-08	32.03
CD8 NC	*TRAPPC12-AS1*	rs4641971	2	3481857	T	G	0.3846	-0.3389	0.0465	3.11E-13	53.03
CD8 NC	*TRAPPC2L*	rs28656031	16	88927044	G	A	0.3321	-0.4198	0.0489	9.77E-18	73.41
CD8 NC	*TRAPPC4*	rs4445645	11	118868750	C	G	0.2222	0.3500	0.0530	3.84E-11	43.6
CD8 NC	*TRIM23*	rs463911	5	64988425	A	G	0.6382	-0.2690	0.0473	1.32E-08	32.24
CD8 NC	*TRMT112*	rs79423518	11	64105454	A	G	0.0682	-0.8936	0.0969	2.82E-20	84.94
CD8 NC	*TSGA10*	rs11692669	2	99750346	A	C	0.6085	0.3181	0.0461	5.05E-12	47.57
CD8 NC	*TSHZ2*	rs74963256	20	51588847	C	T	0.1003	0.4676	0.0721	8.97E-11	41.95
CD8 NC	*TSPAN32*	rs2651838	11	2319531	T	A	0.5700	0.3061	0.0450	1.03E-11	46.18
CD8 NC	*TSPO*	rs138909	22	43549739	A	T	0.3905	-0.3768	0.0454	1.00E-16	68.82
CD8 NC	*TSTD1*	rs57839241	1	160996971	G	C	0.1596	-0.7792	0.0649	3.38E-33	143.8
CD8 NC	*TSTD1*	rs4559524	1	161045128	A	G	0.1202	0.5274	0.0695	3.23E-14	57.47
CD8 NC	*TSTD3*	rs330873	6	100017156	C	T	0.7279	-0.3870	0.0491	3.12E-15	62.06
CD8 NC	*TTC16*	rs538927	9	130488494	T	C	0.5908	0.2526	0.0452	2.31E-08	31.15
CD8 NC	*TTC32*	rs35957485	2	20090677	A	G	0.0880	0.7603	0.0800	2.11E-21	90.06
CD8 NC	*TTC39B*	rs7023154	9	15291834	C	G	0.2876	-0.2745	0.0487	1.69E-08	31.75
CD8 NC	*TUFM*	rs8049439	16	28837515	C	T	0.3939	0.7691	0.0429	6.15E-72	321.05
CD8 NC	*TXK*	rs12108537	4	48183467	A	G	0.1196	0.4331	0.0729	2.89E-09	35.18
CD8 NC	*TXLNB*	rs2273509	6	139094355	C	T	0.1592	0.4441	0.0594	7.36E-14	55.86
CD8 NC	*TXN2*	rs4821495	22	36893251	A	G	0.5562	-0.3002	0.0457	4.88E-11	43.14
CD8 NC	*TYMP*	rs9616864	22	50976152	C	T	0.1038	0.5496	0.0697	3.11E-15	62.07
CD8 NC	*U2AF1L4*	rs112500848	19	36254427	T	C	0.1552	0.6685	0.0627	1.58E-26	113.38
CD8 NC	*UBA52*	rs8110371	19	18667272	C	A	0.5875	-0.3154	0.0455	4.16E-12	47.95
CD8 NC	*UBA6*	rs3860688	4	68486467	G	A	0.4049	0.3116	0.0456	8.49E-12	46.55
CD8 NC	*UBASH3A*	rs1893592	21	43855067	C	A	0.2651	0.3259	0.0481	1.26E-11	45.78
CD8 NC	*UBE2E3*	rs78376944	2	181851997	A	T	0.2658	0.2739	0.0493	2.67E-08	30.87
CD8 NC	*UBE2F*	rs13025510	2	239017140	A	G	0.1480	-0.3130	0.0566	3.21E-08	30.52
CD8 NC	*UBE2L3*	rs5754100	22	21916166	C	T	0.2047	-0.3245	0.0586	3.07E-08	30.6
CD8 NC	*UBXN6*	rs732716	19	4366219	G	A	0.2853	-0.3501	0.0497	1.92E-12	49.46
CD8 NC	*UCP2*	rs2515087	11	73682872	T	C	0.4077	0.5584	0.0445	3.82E-36	157.26
CD8 NC	*ULK4*	rs6771928	3	41750973	A	G	0.1774	0.4463	0.0603	1.29E-13	54.76
CD8 NC	*UPF3A*	rs7332374	13	114995733	C	T	0.1634	0.6279	0.0638	7.58E-23	96.63
CD8 NC	*UPK3BL*	rs62483499	7	102339195	G	A	0.6283	0.5401	0.0453	7.73E-33	142.17
CD8 NC	*UQCRH*	rs41292543	1	46774783	G	A	0.1009	-0.8070	0.0747	3.43E-27	116.41
CD8 NC	*USF1*	rs12116949	1	161042063	A	C	0.2747	0.3088	0.0489	2.69E-10	39.81
CD8 NC	*USP36*	rs7218899	17	76739850	T	C	0.5206	-0.2466	0.0448	3.84E-08	30.17
CD8 NC	*USP53*	rs11729857	4	120114100	G	C	0.1207	0.9965	0.0669	3.89E-50	221.23
CD8 NC	*UTS2*	rs9434555	1	7981453	T	C	0.6262	-0.3102	0.0478	8.51E-11	42.05
CD8 NC	*VAMP2*	rs4792267	17	8061337	A	G	0.6696	0.4556	0.0484	5.07E-21	88.33
CD8 NC	*VAMP4*	rs17649050	1	171713808	C	G	0.1963	0.3512	0.0558	3.17E-10	39.48
CD8 NC	*VAMP5*	rs28479378	2	85750407	T	C	0.1223	-0.7216	0.0651	1.54E-28	122.55
CD8 NC	*VCL*	rs12414280	10	75934400	T	C	0.5821	0.3697	0.0448	1.64E-16	67.86
CD8 NC	*VIM*	rs4747300	10	17280869	C	G	0.3046	0.3292	0.0486	1.32E-11	45.69
CD8 NC	*VPS28*	rs117891721	8	145642886	T	C	0.0569	0.7938	0.0941	3.27E-17	71.03
CD8 NC	*VRK3*	rs8111526	19	50475114	C	G	0.2949	-0.2901	0.0521	2.57E-08	30.95
CD8 NC	*VWDE*	rs4721111	7	12544057	G	A	0.9318	0.5689	0.0882	1.11E-10	41.53
CD8 NC	*WAC-AS1*	rs2807740	10	28784483	T	C	0.7939	-0.4303	0.0524	2.29E-16	67.2
CD8 NC	*WBP1L*	rs284862	10	104572081	T	C	0.5709	0.2675	0.0454	3.83E-09	34.64
CD8 NC	*WBSCR27*	rs4507641	7	73196890	T	C	0.2785	-0.6874	0.0499	3.03E-43	189.71
CD8 NC	*WDYHV1*	rs13273416	8	124436414	T	G	0.3583	-0.2829	0.0475	2.50E-09	35.47
CD8 NC	*WIPI2*	rs62441996	7	5173155	C	T	0.3287	0.3138	0.0482	7.71E-11	42.24
CD8 NC	*XBP1*	rs2881496	22	28921265	G	C	0.8731	-0.6443	0.0658	1.15E-22	95.81
CD8 NC	*XCL1*	rs141673522	1	168517508	T	C	0.1712	0.4565	0.0590	1.05E-14	59.67
CD8 NC	*XKR9*	rs7825815	8	71986405	A	T	0.4170	-0.3972	0.0445	4.12E-19	79.65
CD8 NC	*XRCC6BP1*	rs10747788	12	58315338	T	C	0.3126	-0.3500	0.0474	1.59E-13	54.34
CD8 NC	*XRRA1*	rs12225372	11	74604594	G	A	0.2768	1.0605	0.0426	5.63E-137	619.3
CD8 NC	*XXbac-BPG181B23.7*	rs2905709	6	31364569	C	T	0.3750	-0.2592	0.0475	4.80E-08	29.73
CD8 NC	*XXbac-BPG252P9.9*	rs1264377	6	30764907	A	G	0.1456	-0.5955	0.0594	1.09E-23	100.45
CD8 NC	*XXbac-BPG252P9.9*	rs5006724	6	31337850	C	T	0.6071	-0.2756	0.0463	2.59E-09	35.4
CD8 NC	*XXbac-BPG299F13.17*	rs6921663	6	31154620	C	T	0.2178	1.0736	0.0491	5.86E-106	477.01
CD8 NC	*XXbac-BPG299F13.17*	rs367364	6	32019946	T	C	0.1252	0.3981	0.0614	9.12E-11	41.92
CD8 NC	*YBEY*	rs62224180	21	47726332	G	C	0.7751	-0.8224	0.0543	7.23E-52	229.15
CD8 NC	*YBX3*	rs3816566	12	10870398	T	C	0.2087	0.3917	0.0549	9.30E-13	50.88
CD8 NC	*YEATS4*	rs10784774	12	69737879	G	A	0.4830	-0.3204	0.0446	6.76E-13	51.51
CD8 NC	*YME1L1*	rs11015586	10	27467495	T	C	0.6157	0.2774	0.0472	4.09E-09	34.51
CD8 NC	*YWHAH*	rs2246704	22	32349063	C	T	0.4054	0.2497	0.0453	3.63E-08	30.28
CD8 NC	*ZC3HAV1*	rs7803490	7	138730361	C	T	0.5046	-0.5892	0.0431	1.70E-42	186.28
CD8 NC	*ZFAS1*	rs238200	20	47899652	T	C	0.2056	0.6820	0.0517	1.16E-39	173.34
CD8 NC	*ZFP57*	rs1611283	6	29730128	A	G	0.2287	-0.3555	0.0536	3.44E-11	43.82
CD8 NC	*ZFP90*	rs698719	16	68560125	T	C	0.7574	0.3153	0.0531	2.88E-09	35.19
CD8 NC	*ZNF100*	rs7252798	19	21908053	T	C	0.4439	0.3517	0.0451	6.39E-15	60.65
CD8 NC	*ZNF160*	rs329706	19	53584202	G	C	0.8835	-0.3733	0.0673	2.86E-08	30.74
CD8 NC	*ZNF266*	rs10406143	19	9548422	A	C	0.4964	0.2741	0.0448	9.07E-10	37.44
CD8 NC	*ZNF302*	rs9789337	19	35166230	C	A	0.1245	0.6560	0.0663	4.14E-23	97.82
CD8 NC	*ZNF429*	rs1976385	19	21672022	C	T	0.3747	0.2560	0.0457	2.10E-08	31.34
CD8 NC	*ZNF584*	rs11668420	19	58937836	A	G	0.2117	-0.4570	0.0534	1.19E-17	73.01
CD8 NC	*ZNF593*	rs2490273	1	26489758	T	G	0.7739	-0.3441	0.0531	9.03E-11	41.94

CD8 NC	*ZNF600*	rs4802995	19	53284720	G	C	0.4576	0.4174	0.0442	3.56E-21	89.03
CD8 NC	*ZNF638*	rs7578166	2	71630041	C	A	0.6154	-0.2627	0.0457	8.74E-09	33.03
CD8 NC	*ZNF670*	rs6674691	1	246537683	T	C	0.1779	-0.4795	0.0574	6.54E-17	69.67
CD8 NC	*ZNF677*	rs61576844	19	53761591	G	T	0.2247	-0.4950	0.0530	9.79E-21	87.03
CD8 NC	*ZNF749*	rs3746224	19	57949536	C	T	0.5114	0.3751	0.0444	3.13E-17	71.11
CD8 NC	*ZNF786*	rs7788925	7	148831621	G	A	0.6271	0.2563	0.0465	3.48E-08	30.36
CD8 NC	*ZNF83*	rs73582288	19	53121736	T	C	0.3589	0.5108	0.0456	4.27E-29	125.09
CD8 NC	*ZNF85*	rs73005122	19	21114806	T	C	0.1227	0.6483	0.0649	1.81E-23	99.46
CD8 NC	*ZNF880*	rs1054612	19	52870052	G	C	0.3964	0.2579	0.0463	2.49E-08	31.01
CD8 NC	*ZP3*	rs144021789	7	76184774	A	G	0.3103	0.5548	0.0469	3.20E-32	139.35
CD8 NC	*ZSCAN18*	rs8105716	19	58622262	G	A	0.8034	0.3484	0.0593	4.12E-09	34.49
CD8 NC	*ZSCAN31*	rs9257566	6	29144532	T	C	0.1140	-0.4722	0.0688	6.78E-12	47
CD8 NC	*ZSCAN9*	rs16901847	6	28375851	A	G	0.2094	0.4706	0.0528	4.99E-19	79.27
CD8 NC	*ZSWIM7*	rs6416868	17	15924370	G	A	0.5948	-0.2680	0.0453	3.21E-09	34.98
CD8 S100B	*ABHD16A*	rs7742179	6	31159020	A	T	0.3846	0.5272	0.0451	1.53E-31	136.25
CD8 S100B	*ABHD16A*	rs9271775	6	32594328	T	C	0.7508	0.5580	0.0588	2.47E-21	89.74
CD8 S100B	*AC006369.2*	rs9752750	2	37824224	C	A	0.5972	-0.5055	0.0441	2.05E-30	131.11
CD8 S100B	*AC013264.2*	rs1366836	2	198172148	G	T	0.3781	0.4949	0.0452	6.35E-28	119.75
CD8 S100B	*AC090186.1*	rs9298467	8	52806682	T	C	0.7814	0.3300	0.0575	9.24E-09	32.93
CD8 S100B	*ACYP2*	rs7559380	2	54356797	T	A	0.4611	-0.3139	0.0447	2.21E-12	49.19
CD8 S100B	*AGER*	rs77032877	6	32501811	C	T	0.0978	-0.4775	0.0664	6.47E-13	51.59
CD8 S100B	*AGER*	rs2454138	6	32570401	A	G	0.0574	0.2909	0.0502	6.83E-09	33.51
CD8 S100B	*AHI1*	rs13197384	6	135818897	A	C	0.3411	-0.2968	0.0474	3.73E-10	39.17
CD8 S100B	*AKAP5*	rs8020475	14	65654008	C	T	0.0985	0.7737	0.0767	6.66E-24	101.43
CD8 S100B	*ANKDD1A*	rs1719264	15	65176535	T	C	0.6288	0.5427	0.0453	4.15E-33	143.4
CD8 S100B	*ANXA6*	rs2303038	5	150535013	T	C	0.2046	0.3396	0.0547	5.35E-10	38.47
CD8 S100B	*AOAH*	rs2228410	7	36763672	T	C	0.3411	-0.3164	0.0472	1.99E-11	44.89
CD8 S100B	*AOAH*	rs7806624	7	36824655	T	C	0.2085	0.3699	0.0542	8.82E-12	46.48
CD8 S100B	*AP003774.1*	rs627425	11	64087642	T	C	0.3608	0.3316	0.0461	6.15E-13	51.69
CD8 S100B	*APOM*	rs3132514	6	31142758	C	T	0.1591	0.4851	0.0545	5.15E-19	79.21
CD8 S100B	*ARHGAP15*	rs13024411	2	144012024	G	A	0.1512	-0.5402	0.0638	2.66E-17	71.44
CD8 S100B	*ARHGEF3*	rs1489948	3	56757717	C	T	0.1634	0.3520	0.0624	1.68E-08	31.77
CD8 S100B	*ARL16*	rs7503637	17	79649515	A	G	0.1691	-0.3987	0.0595	2.04E-11	44.84
CD8 S100B	*ATHL1*	rs3932433	11	289553	C	G	0.3751	0.3097	0.0457	1.19E-11	45.9
CD8 S100B	*ATP6V1G2*	rs1264377	6	30764907	A	G	0.1456	-0.6836	0.0589	3.63E-31	134.54
CD8 S100B	*ATP6V1G2*	rs3130625	6	31597915	G	A	0.8715	-0.4601	0.0632	3.21E-13	52.96
CD8 S100B	*BIN1*	rs11682128	2	127839474	A	G	0.3433	0.3372	0.0470	7.57E-13	51.29
CD8 S100B	*BTF3*	rs10942655	5	72674031	A	G	0.0712	-0.6495	0.0953	9.29E-12	46.38
CD8 S100B	*BTN3A2*	rs67509210	6	26354100	T	G	0.1018	-0.6814	0.0658	4.12E-25	106.94
CD8 S100B	*C10orf128*	rs10857439	10	50417799	C	T	0.3586	-0.4351	0.0457	1.86E-21	90.31
CD8 S100B	*C10orf32*	rs11191436	10	104637602	T	C	0.2549	-0.3281	0.0519	2.59E-10	39.88
CD8 S100B	*C17orf97*	rs11150882	17	259648	A	G	0.2620	-0.7237	0.0500	1.75E-47	209.09
CD8 S100B	*C22orf34*	rs8141219	22	50044546	G	A	0.6739	0.5217	0.0481	1.91E-27	117.57
CD8 S100B	*C9orf135*	rs17518030	9	72427646	C	G	0.1251	0.5847	0.0686	1.62E-17	72.41
CD8 S100B	*CAMLG*	rs12654944	5	134036784	A	G	0.1122	0.4245	0.0708	2.00E-09	35.9
CD8 S100B	*CARHSP1*	rs9936117	16	8937087	G	A	0.2650	0.3399	0.0506	1.81E-11	45.07
CD8 S100B	*CARS*	rs3782069	11	3033275	A	G	0.3993	-0.4219	0.0453	1.23E-20	86.57
CD8 S100B	*CASP8*	rs13016963	2	202162811	G	A	0.6165	0.3318	0.0465	9.47E-13	50.85
CD8 S100B	*CAST*	rs10039522	5	95990869	A	G	0.1691	0.3455	0.0611	1.56E-08	31.91
CD8 S100B	*CCDC122*	rs12873099	13	44396247	A	G	0.4775	-0.3601	0.0446	6.48E-16	65.15
CD8 S100B	*CCDC146*	rs17230010	7	76870998	C	T	0.1424	-0.3609	0.0606	2.58E-09	35.41
CD8 S100B	*CCDC163P*	rs882803	1	45976147	A	G	0.6770	0.4589	0.0465	5.85E-23	97.14
CD8 S100B	*CCDC167*	rs1757178	6	37486310	G	A	0.6345	0.4994	0.0460	1.88E-27	117.6
CD8 S100B	*CCDC58*	rs6438726	3	122110921	T	C	0.2689	0.3617	0.0504	6.95E-13	51.45
CD8 S100B	*CCL3L3*	rs7208788	17	34823204	A	G	0.1610	0.3760	0.0582	1.05E-10	41.64
CD8 S100B	*CCL4*	rs1719141	17	34430450	T	G	0.2237	0.2900	0.0529	4.23E-08	29.98
CD8 S100B	*CCNG2*	rs729240	4	78063137	A	G	0.4176	0.2987	0.0455	5.01E-11	43.09
CD8 S100B	*CCT3*	rs12563629	1	156303961	T	C	0.2793	-0.2935	0.0492	2.44E-09	35.51
CD8 S100B	*CCT8*	rs965951	21	30432557	A	G	0.1160	0.8487	0.0663	1.73E-37	163.39
CD8 S100B	*CCZ1*	rs74688574	7	6855646	G	A	0.4717	0.2943	0.0449	5.42E-11	42.93
CD8 S100B	*CCZ1B*	rs7798575	7	6885171	T	C	0.4439	-0.4262	0.0444	8.77E-22	91.79
CD8 S100B	*CD151*	rs61876748	11	833262	G	T	0.7330	0.3051	0.0531	8.87E-09	33.01
CD8 S100B	*CD300A*	rs2293191	17	72470423	G	T	0.4198	-0.2697	0.0457	3.55E-09	34.78
CD8 S100B	*CDC42*	rs4654783	1	22439520	C	T	0.7035	-0.3175	0.0495	1.42E-10	41.05
CD8 S100B	*CELA1*	rs7306746	12	51709282	C	T	0.2787	-0.2909	0.0485	2.07E-09	35.83
CD8 S100B	*CENPK*	rs154940	5	64902091	T	C	0.6113	-0.7286	0.0441	1.91E-61	272.99
CD8 S100B	*CES1*	rs2244614	16	55844617	A	G	0.6039	0.3125	0.0458	8.50E-12	46.55
CD8 S100B	*CFD*	rs2930901	19	886945	G	A	0.2656	0.3431	0.0515	2.64E-11	44.33
CD8 S100B	*CHCHD2*	rs2908547	7	56176101	T	C	0.7881	-0.4109	0.0540	2.67E-14	57.85
CD8 S100B	*CHPT1*	rs2114918	12	102084518	T	C	0.6327	0.3429	0.0460	9.14E-14	55.43
CD8 S100B	*CLEC2B*	rs33871	12	10007897	T	C	0.5491	0.2491	0.0451	3.37E-08	30.42
CD8 S100B	*CMC1*	rs10510607	3	28286261	T	C	0.1753	0.3736	0.0583	1.46E-10	40.99
CD8 S100B	*CMTM8*	rs7632486	3	32286791	A	G	0.8213	-0.4681	0.0591	2.31E-15	62.65
CD8 S100B	*COG5*	rs2222845	7	107281829	T	C	0.7623	-0.4038	0.0536	4.75E-14	56.71
CD8 S100B	*COL18A1*	rs73386859	21	46886524	T	G	0.0609	0.7265	0.0932	6.60E-15	60.59
CD8 S100B	*COL5A3*	rs34729515	19	10119450	A	C	0.1938	0.4063	0.0543	7.56E-14	55.8
CD8 S100B	*COMMD6*	rs9543961	13	76106054	G	T	0.1381	-0.4556	0.0617	1.56E-13	54.39
CD8 S100B	*CORO1B*	rs1638564	11	67223920	G	C	0.4238	-0.3580	0.0451	2.17E-15	62.77
CD8 S100B	*CPA5*	rs11761888	7	130007381	C	T	0.2124	0.7860	0.0522	3.49E-51	226.02

CD8 S100B	*CPNE1*	rs7272916	20	34562914	A	T	0.1044	-0.4613	0.0775	2.66E-09	35.34
CD8 S100B	*CSTB*	rs11089098	21	45234744	G	T	0.3824	-0.3456	0.0467	1.43E-13	54.56
CD8 S100B	*CTA-29F11.1*	rs12389	22	47158179	A	G	0.3392	-0.3831	0.0462	1.17E-16	68.52
CD8 S100B	*CTC-524C5.2*	rs57745425	17	5327528	T	C	0.2227	0.4511	0.0550	2.33E-16	67.17
CD8 S100B	*CTD-2368P22.1*	rs4801542	19	58522974	G	A	0.2257	-0.4284	0.0528	4.80E-16	65.74
CD8 S100B	*CTD-2371O3.2*	rs10840244	11	9486063	G	T	0.5097	-0.5981	0.0431	7.68E-44	192.43
CD8 S100B	*CTLA4*	rs1427680	2	204729795	A	G	0.5962	-0.5128	0.0444	7.47E-31	133.11
CD8 S100B	*CTSW*	rs596002	11	65645354	G	A	0.8002	0.9425	0.0526	1.07E-71	319.95
CD8 S100B	*DCXR*	rs9903371	17	79993064	G	C	0.1249	0.3726	0.0679	3.98E-08	30.1
CD8 S100B	*DDX11*	rs3881296	12	31237700	A	G	0.4487	-0.3083	0.0449	6.97E-12	46.94
CD8 S100B	*DDX5*	rs1991401	17	62502435	G	A	0.3173	-0.3497	0.0483	4.38E-13	52.36
CD8 S100B	*DIP2A*	rs2070435	21	47961711	A	G	0.3259	0.4897	0.0467	1.01E-25	109.71
CD8 S100B	*DNAJC15*	rs2281779	13	43597980	A	G	0.2358	-0.5521	0.0526	8.20E-26	110.13
CD8 S100B	*DTX2*	rs1179610	7	75246126	C	T	0.1802	0.4140	0.0586	1.67E-12	49.74
CD8 S100B	*DYNLL2*	rs8071026	17	56174489	A	G	0.6735	-0.2917	0.0478	1.06E-09	37.13
CD8 S100B	*EBPL*	rs3809325	13	50266109	C	T	0.5393	-0.3473	0.0449	1.05E-14	59.68
CD8 S100B	*EEF2*	rs892128	19	3975487	A	G	0.8039	0.3227	0.0553	5.28E-09	34.01
CD8 S100B	*EIF2A*	rs14434	3	150302010	T	C	0.8405	0.4471	0.0633	1.63E-12	49.78
CD8 S100B	*EIF5A*	rs7503161	17	7207964	C	A	0.5615	-0.9438	0.0401	2.36E-122	552.21
CD8 S100B	*ELP5*	rs222851	17	7139238	A	G	0.3873	0.4071	0.0452	2.18E-19	80.91
CD8 S100B	*EPSTI1*	rs2014439	13	43563136	C	G	0.7204	-0.3530	0.0505	2.85E-12	48.69
CD8 S100B	*ERAP2*	rs27436	5	96330966	C	T	0.5879	-0.6421	0.0432	5.72E-50	220.46
CD8 S100B	*ERN1*	rs196904	17	62119555	T	C	0.8272	0.3762	0.0618	1.14E-09	37
CD8 S100B	*ESR2*	rs28418648	14	64698623	C	T	0.0760	0.5774	0.0927	4.77E-10	38.69
CD8 S100B	*EXOC3*	rs13356700	5	477719	G	A	0.4837	0.2725	0.0449	1.28E-09	36.77
CD8 S100B	*FADS1*	rs174622	11	61631927	A	G	0.2230	-0.6022	0.0531	8.49E-30	128.29
CD8 S100B	*FAM118A*	rs136582	22	45774531	A	T	0.1332	1.3205	0.0618	2.36E-101	455.89
CD8 S100B	*FAM153B*	rs10042710	5	175579380	G	A	0.4979	-0.2926	0.0448	6.23E-11	42.66
CD8 S100B	*FAM184B*	rs10011865	4	17645710	A	G	0.6030	-0.5793	0.0444	6.34E-39	169.96
CD8 S100B	*FAM26F*	rs1057192	6	116783330	A	G	0.2439	-0.4235	0.0523	5.82E-16	65.36
CD8 S100B	*FAM85A*	rs35697449	8	11822903	C	T	0.7147	0.5736	0.0490	1.28E-31	136.61
CD8 S100B	*FAM86B1*	rs1293326	8	11694765	C	G	0.2485	0.6120	0.0500	2.07E-34	149.34
CD8 S100B	*FAU*	rs1196043	11	64887560	G	T	0.4919	0.2588	0.0449	8.29E-09	33.14
CD8 S100B	*FBLN5*	rs2267989	14	92339850	A	G	0.4380	-0.3705	0.0446	9.13E-17	69.01
CD8 S100B	*FDFT1*	rs1293299	8	11677905	C	A	0.2173	-0.3299	0.0547	1.61E-09	36.32
CD8 S100B	*FHL3*	rs67631072	1	38461821	T	C	0.5175	-0.2568	0.0449	1.08E-08	32.63
CD8 S100B	*FKBP11*	rs11168769	12	49269999	A	C	0.4012	-0.2694	0.0461	5.05E-09	34.1
CD8 S100B	*FKBPL*	rs2894255	6	32390332	T	C	0.1264	0.5041	0.0633	1.73E-15	63.22
CD8 S100B	*FMN1*	rs2619174	15	33402084	G	C	0.2710	-0.7320	0.0511	1.49E-46	204.83
CD8 S100B	*GBP5*	rs4656109	1	89740474	G	A	0.5719	-0.2536	0.0455	2.41E-08	31.07
CD8 S100B	*GBP7*	rs523772	1	89698147	G	A	0.4852	0.4198	0.0442	2.15E-21	90.02
CD8 S100B	*GCC2*	rs7606621	2	109312728	T	C	0.4010	-0.3874	0.0452	9.86E-18	73.39
CD8 S100B	*GLTSCR2*	rs3786779	19	48255284	A	G	0.7093	-0.3943	0.0499	2.81E-15	62.27
CD8 S100B	*GNLY*	rs12151752	2	85934652	C	T	0.2327	0.5752	0.0522	3.10E-28	121.17
CD8 S100B	*GOLGA6L4*	rs9989259	15	84667242	G	C	0.3990	-0.3774	0.0453	7.48E-17	69.4
CD8 S100B	*GPR160*	rs6787266	3	169777572	G	A	0.4348	-0.2838	0.0455	4.55E-10	38.78
CD8 S100B	*GRHPR*	rs4878690	9	37422656	T	C	0.3290	0.2832	0.0479	3.25E-09	34.96
CD8 S100B	*GSTM3*	rs4540683	1	110270908	G	T	0.5919	0.3773	0.0453	8.37E-17	69.18
CD8 S100B	*GSTM4*	rs1010167	1	110198727	G	C	0.4142	-0.3031	0.0455	2.81E-11	44.22
CD8 S100B	*GSTP1*	rs4320958	11	67310334	A	G	0.3917	-0.3483	0.0452	1.26E-14	59.32
CD8 S100B	*GSTT1*	rs5760147	22	24334948	C	A	0.3844	-0.3858	0.0461	6.19E-17	69.77
CD8 S100B	*GTF2H2*	rs28478097	5	70309998	G	C	0.6121	-0.2518	0.0461	4.75E-08	29.76
CD8 S100B	*GYPC*	rs34999508	2	127523332	G	C	0.2721	0.3361	0.0513	5.80E-11	42.8
CD8 S100B	*HCG27*	rs2524129	6	31265135	G	C	0.7708	-0.7107	0.0475	1.30E-50	223.41
CD8 S100B	*HCG27*	rs204999	6	32109979	G	A	0.2741	0.3760	0.0485	8.53E-15	60.09
CD8 S100B	*HIGD2A*	rs13155545	5	175817499	G	C	0.0942	-0.5349	0.0855	3.92E-10	39.07
CD8 S100B	*HIST1H2AB*	rs57440165	6	26843517	C	A	0.0736	-0.6218	0.0759	2.58E-16	66.97
CD8 S100B	*HIST1H2AG*	rs67092078	6	27054772	A	G	0.0727	-0.4877	0.0763	1.61E-10	40.81
CD8 S100B	*HIST1H2AK*	rs7766356	6	28400538	C	T	0.1648	-0.5333	0.0551	3.81E-22	93.44
CD8 S100B	*HIST1H2BF*	rs67092078	6	27054772	A	G	0.0727	-0.5247	0.0761	5.54E-12	47.39
CD8 S100B	*HIST1H2BG*	rs35942569	6	26339131	G	A	0.0750	0.4252	0.0760	2.23E-08	31.22
CD8 S100B	*HIST1H3D*	rs13200888	6	26100198	A	G	0.0936	-0.5893	0.0708	8.49E-17	69.15
CD8 S100B	*HIST1H4H*	rs35144506	6	26739487	G	A	0.0915	0.5686	0.0719	2.56E-15	62.45
CD8 S100B	*HLA-A*	rs7747253	6	29919779	A	C	0.5479	0.2545	0.0448	1.39E-08	32.13
CD8 S100B	*HLA-B*	rs2524096	6	31236467	T	G	0.4422	0.7092	0.0427	4.70E-62	275.78
CD8 S100B	*HLA-B*	rs9366777	6	31264715	A	T	0.0923	-0.5424	0.0828	5.69E-11	42.84
CD8 S100B	*HLA-B*	rs3828879	6	31379391	T	C	0.1430	-0.5690	0.0653	2.96E-18	75.76
CD8 S100B	*HLA-C*	rs3931670	6	31243767	C	G	0.2891	0.3976	0.0511	7.62E-15	60.31
CD8 S100B	*HLA-DOA*	rs3117106	6	32343369	C	T	0.1435	-0.5332	0.0621	8.58E-18	73.66
CD8 S100B	*HLA-DOB*	rs35214850	6	32018573	C	A	0.0856	-0.4654	0.0757	8.04E-10	37.67
CD8 S100B	*HLA-DQA2*	rs9272226	6	32602396	T	C	0.6594	-0.4088	0.0464	1.14E-18	77.64
CD8 S100B	*HLA-DQA2*	rs6927022	6	32612397	G	A	0.3361	0.2471	0.0449	3.65E-08	30.27
CD8 S100B	*HLA-DQB1*	rs28383322	6	32592796	T	C	0.1617	0.3605	0.0537	1.89E-11	44.99
CD8 S100B	*HLA-DRB5*	rs9272105	6	32599999	A	G	0.5403	-0.2744	0.0448	8.93E-10	37.47
CD8 S100B	*HMBOX1*	rs10088428	8	28909523	T	C	0.2597	-0.4353	0.0513	2.02E-17	71.98
CD8 S100B	*HOXB2*	rs4793870	17	46581184	C	T	0.6285	0.4491	0.0461	2.16E-22	94.56
CD8 S100B	*HSPA1L*	rs3129907	6	32323745	A	G	0.7951	-0.5203	0.0519	1.17E-23	100.32
CD8 S100B	*HSPA1L*	rs77032877	6	32501811	C	T	0.0978	-0.3760	0.0667	1.77E-08	31.67
CD8 S100B	*HSPBAP1*	rs1877374	3	122409504	A	G	0.2367	0.4024	0.0559	5.84E-13	51.8

CD8 S100B	*IFITM2*	rs10398	11	308180	G	A	0.2135	0.5747	0.0572	1.00E-23	100.62
CD8 S100B	*IFITM3*	rs7942247	11	316349	A	G	0.5502	0.5476	0.0435	2.21E-36	158.35
CD8 S100B	*IFNG-AS1*	rs12832728	12	68420403	A	G	0.5290	-0.2606	0.0451	7.46E-09	33.34
CD8 S100B	*IL32*	rs9928554	16	3126735	A	G	0.0771	0.5257	0.0927	1.41E-08	32.11
CD8 S100B	*ISCA2*	rs10873267	14	74941081	G	A	0.5998	-0.2650	0.0456	6.26E-09	33.68
CD8 S100B	*ITGB2*	rs760462	21	46328099	C	T	0.8298	-0.7342	0.0587	7.28E-36	155.98
CD8 S100B	*KANSL1-AS1*	rs55974014	17	43757450	A	C	0.1994	0.9652	0.0497	4.43E-84	376.7
CD8 S100B	*KCNAB2*	rs17432446	1	5952726	T	C	0.1515	0.3518	0.0613	9.64E-09	32.85
CD8 S100B	*KDELR2*	rs6796	7	6502367	C	T	0.2687	-0.3774	0.0491	1.51E-14	58.96
CD8 S100B	*KIAA0040*	rs2285216	1	175133012	T	C	0.5302	0.2600	0.0448	6.68E-09	33.56
CD8 S100B	*KIAA1430*	rs3108296	4	186123731	T	G	0.6356	-0.2696	0.0477	1.60E-08	31.86
CD8 S100B	*KLRC4*	rs2460740	12	10581446	G	T	0.6476	0.3139	0.0476	4.06E-11	43.5
CD8 S100B	*KRTCAP3*	rs2010087	2	27637235	T	C	0.3790	-0.3485	0.0456	2.16E-14	58.26
CD8 S100B	*KTN1*	rs12588935	14	55996897	C	A	0.5508	0.2667	0.0453	4.02E-09	34.54
CD8 S100B	*LAMB1*	rs6961243	7	107521404	A	G	0.3866	0.5350	0.0442	1.06E-33	146.11
CD8 S100B	*LDLRAP1*	rs6701596	1	25889917	C	T	0.5403	-0.2794	0.0449	4.74E-10	38.7
CD8 S100B	*LGALS3*	rs58553156	14	55582694	A	T	0.3198	0.2991	0.0468	1.72E-10	40.67
CD8 S100B	*LINC00176*	rs817356	20	62614929	C	T	0.1906	0.8184	0.0585	1.97E-44	195.13
CD8 S100B	*LINC00339*	rs12097775	1	22347252	T	C	0.2480	0.3289	0.0514	1.56E-10	40.87
CD8 S100B	*LINC00649*	rs8130507	21	35303960	C	T	0.6025	0.2642	0.0459	8.80E-09	33.02
CD8 S100B	*LINC00969*	rs9886966	3	195317047	A	G	0.4732	0.2960	0.0450	4.70E-11	43.21
CD8 S100B	*LRRC16A*	rs716797	6	25393306	A	G	0.5571	0.3393	0.0447	3.18E-14	57.5
CD8 S100B	*LYRM4*	rs111557465	6	5260694	T	C	0.0736	0.4924	0.0813	1.38E-09	36.62
CD8 S100B	*MDC1*	rs2523938	6	29926976	G	A	0.1619	0.3612	0.0662	4.86E-08	29.71
CD8 S100B	*MDM4*	rs12119098	1	204521577	A	G	0.6730	-0.3064	0.0478	1.40E-10	41.08
CD8 S100B	*MED16*	rs2930893	19	882724	C	G	0.2624	0.3258	0.0516	2.64E-10	39.84
CD8 S100B	*MGST3*	rs6686129	1	165591948	C	T	0.6836	-0.2803	0.0476	3.88E-09	34.61
CD8 S100B	*MPHOSPH6*	rs2303260	16	82203786	G	C	0.4801	-0.3533	0.0445	2.05E-15	62.89
CD8 S100B	*MRPL21*	rs602805	11	68662076	T	A	0.2901	-0.2836	0.0492	8.21E-09	33.16
CD8 S100B	*MRPL34*	rs8100463	19	17423144	A	G	0.2917	0.3017	0.0483	4.24E-10	38.92
CD8 S100B	*MT1E*	rs1610215	16	56659525	G	C	0.5423	0.3533	0.0447	2.55E-15	62.46
CD8 S100B	*MT1F*	rs11647190	16	56684241	G	A	0.5724	-0.2507	0.0453	3.02E-08	30.63
CD8 S100B	*MT1X*	rs1599933	16	56715862	A	C	0.5528	-0.2559	0.0451	1.38E-08	32.14
CD8 S100B	*MT2A*	rs1580833	16	56644650	A	C	0.3093	-0.6367	0.0467	2.58E-42	185.46
CD8 S100B	*MYOM2*	rs79200728	8	2082753	G	T	0.0859	-0.7710	0.0869	7.43E-19	78.49
CD8 S100B	*MZT2A*	rs34295369	2	132288853	A	G	0.2023	0.5765	0.0586	8.01E-23	96.52
CD8 S100B	*NAA38*	rs6964911	7	117859904	A	G	0.6809	-0.3577	0.0470	2.71E-14	57.82
CD8 S100B	*NAP1L1*	rs1059143	12	76449900	C	T	0.8157	-0.3308	0.0587	1.77E-08	31.67
CD8 S100B	*NAPRT1*	rs896955	8	144658370	T	C	0.1390	-0.6385	0.0659	3.17E-22	93.8
CD8 S100B	*NCAPG2*	rs73167215	7	158500207	A	G	0.0611	0.5424	0.0846	1.41E-10	41.06
CD8 S100B	*NDUFA12*	rs12820518	12	95592212	T	C	0.3004	0.2949	0.0477	6.10E-10	38.21
CD8 S100B	*NDUFA3*	rs2576453	19	54632253	A	G	0.6129	-0.2740	0.0461	2.71E-09	35.31
CD8 S100B	*NDUFS5*	rs4147750	1	39474057	C	A	0.2805	-0.3330	0.0480	4.18E-12	47.94
CD8 S100B	*NME4*	rs77371232	16	447561	T	A	0.4244	0.2602	0.0453	9.45E-09	32.88
CD8 S100B	*NMI*	rs3854012	2	152137106	T	C	0.4433	0.2606	0.0450	6.75E-09	33.54
CD8 S100B	*NMRK1*	rs3752955	9	77684690	C	A	0.5423	-0.2936	0.0450	7.05E-11	42.42
CD8 S100B	*NPC2*	rs10873267	14	74941081	G	A	0.5998	-0.2610	0.0456	1.06E-08	32.67
CD8 S100B	*NPDC1*	rs28705707	9	139940653	A	C	0.3857	0.2592	0.0461	1.83E-08	31.61
CD8 S100B	*NQO2*	rs62392501	6	3024583	G	T	0.7974	-0.3220	0.0582	3.21E-08	30.51
CD8 S100B	*NSA2*	rs72764675	5	74122399	C	T	0.1338	0.5458	0.0658	1.09E-16	68.65
CD8 S100B	*NSG1*	rs2369105	4	4353086	G	A	0.7724	0.7519	0.0510	3.03E-49	217.15
CD8 S100B	*NT5C3B*	rs8073219	17	39977734	G	A	0.7569	-0.4021	0.0510	3.03E-15	62.12
CD8 S100B	*NTPCR*	rs57413238	1	233074848	A	G	0.1814	0.3455	0.0569	1.27E-09	36.79
CD8 S100B	*NUDT2*	rs3818830	9	34378999	T	A	0.1539	0.7695	0.0608	1.16E-36	159.62
CD8 S100B	*ORMDL3*	rs12944882	17	37983492	C	T	0.5308	-0.4006	0.0444	1.80E-19	81.28
CD8 S100B	*OXLD1*	rs11150804	17	79630238	G	A	0.3735	-0.2684	0.0468	9.82E-09	32.81
CD8 S100B	*PASK*	rs6437234	2	242019288	T	C	0.1813	-0.4495	0.0609	1.63E-13	54.3
CD8 S100B	*PEX6*	rs9471972	6	42915021	A	G	0.5240	0.2742	0.0449	1.03E-09	37.19
CD8 S100B	*PIGP*	rs738862	21	38442136	C	T	0.6279	-0.2931	0.0466	3.19E-10	39.48
CD8 S100B	*PILRB*	rs6955367	7	99961194	G	A	0.1838	0.5981	0.0555	4.54E-27	115.85
CD8 S100B	*PLEK*	rs12622659	2	68646279	A	G	0.3320	0.2800	0.0485	7.98E-09	33.21
CD8 S100B	*PNRC2*	rs6424144	1	24375663	A	G	0.4721	-0.2804	0.0448	3.76E-10	39.15
CD8 S100B	*POLR1D*	rs7317084	13	28198098	T	C	0.1171	0.4836	0.0660	2.40E-13	53.54
CD8 S100B	*PPA1*	rs7077538	10	71993094	G	T	0.3217	0.6599	0.0455	1.47E-47	209.43
CD8 S100B	*PPAP2C*	rs3813154	19	292082	T	C	0.5039	0.4693	0.0439	1.21E-26	113.92
CD8 S100B	*PPDPF*	rs72629024	20	62152519	G	C	0.1633	-0.7797	0.0603	3.35E-38	166.65
CD8 S100B	*PPIA*	rs7804671	7	44846055	C	T	0.1399	0.5554	0.0743	7.69E-14	55.77
CD8 S100B	*PPIL3*	rs7559150	2	201754063	C	T	0.2458	-0.4277	0.0527	5.04E-16	65.65
CD8 S100B	*PPP4R1L*	rs6026241	20	56980705	A	T	0.3394	0.3204	0.0471	1.05E-11	46.14
CD8 S100B	*PPT2*	rs9267954	6	32213052	T	A	0.2261	0.3738	0.0501	8.14E-14	55.66
CD8 S100B	*PRDX6*	rs9425411	1	173471890	A	G	0.3050	-0.3586	0.0484	1.27E-13	54.78
CD8 S100B	*PRKCB*	rs114827361	16	24045354	G	A	0.0539	0.7253	0.1005	5.23E-13	52.01
CD8 S100B	*PRKCQ-AS1*	rs41284471	10	6626214	A	G	0.1785	-0.4987	0.0545	5.80E-20	83.51
CD8 S100B	*PRMT2*	rs2070435	21	47961711	A	G	0.3259	0.4973	0.0467	1.61E-26	113.34
CD8 S100B	*PRMT2*	rs7278089	21	47955501	A	T	0.7488	-0.3154	0.0524	1.74E-09	36.18
CD8 S100B	*PRR7-AS1*	rs2545794	5	176894756	T	C	0.5086	0.3515	0.0446	3.43E-15	61.88
CD8 S100B	*PRSS16*	rs112863641	6	28217931	T	C	0.0796	0.4748	0.0771	7.46E-10	37.82
CD8 S100B	*PSMD5-AS1*	rs12343516	9	123603469	C	A	0.5777	-0.8479	0.0414	4.97E-93	417.73
CD8 S100B	*PSPH*	rs6593279	7	55803970	A	G	0.2274	-0.6422	0.0580	1.67E-28	122.39

CD8 S100B	*RAB6B*	rs1949881	3	133602195	T	C	0.3172	0.4929	0.0481	1.25E-24	104.73
CD8 S100B	*RAC1*	rs7805985	7	6397141	A	C	0.4869	0.2844	0.0448	2.10E-10	40.29
CD8 S100B	*RBM43*	rs289924	2	152093290	G	A	0.5645	0.3726	0.0446	6.19E-17	69.77
CD8 S100B	*RNASET2*	rs12212247	6	167413539	C	T	0.4368	-0.4717	0.0441	1.06E-26	114.18
CD8 S100B	*RP1-29C18.10*	rs5770622	22	50051548	G	T	0.6547	-0.3756	0.0478	3.87E-15	61.64
CD8 S100B	*RP1-3J17.3*	rs12212560	6	86342679	A	G	0.3868	0.3627	0.0459	2.70E-15	62.34
CD8 S100B	*RP1-3J17.3*	rs9444346	6	86165631	G	A	0.1874	0.3825	0.0582	4.86E-11	43.15
CD8 S100B	*RP11-18H21.1*	rs1991223	4	153019169	A	G	0.2910	-0.5579	0.0482	5.88E-31	133.58
CD8 S100B	*RP11-291B21.2*	rs2417745	12	10715012	T	G	0.4150	0.2476	0.0453	4.62E-08	29.81
CD8 S100B	*RP11-298J20.3*	rs2114834	10	126650186	G	A	0.7359	-0.4755	0.0509	1.03E-20	86.93
CD8 S100B	*RP11-326C3.11*	rs11246065	11	324170	G	A	0.3701	0.5339	0.0458	1.81E-31	135.91
CD8 S100B	*RP11-326C3.12*	rs7395116	11	324880	C	G	0.2517	0.3757	0.0516	3.24E-13	52.95
CD8 S100B	*RP11-347P5.1*	rs1568368	2	197008529	T	C	0.6434	0.5683	0.0452	3.03E-36	157.72
CD8 S100B	*RP11-403A21.2*	rs1429437	18	21573386	C	A	0.7092	0.2872	0.0509	1.72E-08	31.72
CD8 S100B	*RP11-457M11.5*	rs34332556	6	27357414	G	C	0.0780	-0.6240	0.0759	2.03E-16	67.44
CD8 S100B	*RP11-473I1.5*	rs9935095	16	9224535	C	G	0.2051	-0.3225	0.0575	2.06E-08	31.37
CD8 S100B	*RP11-473M20.9*	rs4786384	16	3136919	C	T	0.5202	0.3482	0.0446	5.56E-15	60.93
CD8 S100B	*RP11-664D1.1*	rs2336757	12	4014358	G	A	0.1452	0.3976	0.0634	3.49E-10	39.3
CD8 S100B	*RP11-664D1.1*	rs609018	12	4071901	G	T	0.4284	-0.2569	0.0451	1.23E-08	32.38
CD8 S100B	*RP11-81H14.2*	rs731938	12	68843191	G	A	0.6401	0.2969	0.0455	6.76E-11	42.5
CD8 S100B	*RP5-1042K10.10*	rs11913132	22	40778778	G	A	0.1891	0.6969	0.0559	1.16E-35	155.05
CD8 S100B	*RPL10A*	rs2076172	6	35436925	T	C	0.5358	-0.5547	0.0439	1.40E-36	159.25
CD8 S100B	*RPL11*	rs7521548	1	24010230	A	G	0.8374	0.6620	0.0617	8.02E-27	114.73
CD8 S100B	*RPL12*	rs2247573	9	130210946	G	A	0.5887	0.7992	0.0419	4.78E-81	362.8
CD8 S100B	*RPL13*	rs364343	16	89612638	A	G	0.2099	-0.6130	0.0524	1.15E-31	136.82
CD8 S100B	*RPL14*	rs75965075	3	40438120	T	C	0.0715	0.6208	0.0851	3.01E-13	53.09
CD8 S100B	*RPL23A*	rs2302205	17	26947046	C	A	0.0821	-0.6343	0.0784	5.68E-16	65.41
CD8 S100B	*RPL28*	rs17700376	19	55897327	G	A	0.3547	-1.0243	0.0406	1.16E-140	636.22
CD8 S100B	*RPL3*	rs137685	22	39739628	T	C	0.4163	0.2651	0.0449	3.48E-09	34.83
CD8 S100B	*RPL32*	rs2290311	3	12883071	A	T	0.1438	0.3383	0.0620	4.76E-08	29.75
CD8 S100B	*RPL36AL*	rs1952013	14	50087357	G	C	0.7531	0.7299	0.0503	1.11E-47	209.99
CD8 S100B	*RPL8*	rs2954658	8	146017782	T	C	0.4990	-0.9162	0.0398	1.62E-117	530.03
CD8 S100B	*RPLP2*	rs12277141	11	798865	C	G	0.6564	-0.5117	0.0470	1.26E-27	118.39
CD8 S100B	*RPS10*	rs9380433	6	34377626	T	C	0.7750	-0.9131	0.0547	1.48E-62	278.08
CD8 S100B	*RPS11*	rs10403394	19	49999345	A	G	0.1225	-0.5705	0.0714	1.38E-15	63.66
CD8 S100B	*RPS12*	rs60412119	6	133210388	G	A	0.0719	0.5861	0.0898	6.88E-11	42.47
CD8 S100B	*RPS18*	rs17215231	6	33239869	T	C	0.0640	-1.4932	0.0834	9.75E-72	320.14
CD8 S100B	*RPS19*	rs10415452	19	42382745	G	A	0.4580	0.2661	0.0450	3.38E-09	34.88
CD8 S100B	*RPS2*	rs338787	16	2008095	A	G	0.1299	0.6113	0.0658	1.52E-20	86.16
CD8 S100B	*RPS23*	rs7707921	5	81538046	A	T	0.7729	0.4276	0.0506	2.88E-17	71.28
CD8 S100B	*RPS24*	rs2913134	10	79763775	T	C	0.5775	0.2717	0.0460	3.59E-09	34.76
CD8 S100B	*RPS25*	rs11602764	11	118888619	G	A	0.2652	-0.4996	0.0492	2.89E-24	103.08
CD8 S100B	*RPS26*	rs773114	12	56379060	T	A	0.3997	1.1963	0.0353	1.00E-200	1145.08
CD8 S100B	*RPS3A*	rs1148652	4	151801938	C	T	0.0703	-1.1645	0.0978	1.12E-32	141.43
CD8 S100B	*RPS5*	rs731835	19	58898436	C	T	0.3256	-0.8041	0.0444	3.12E-73	326.98
CD8 S100B	*RPS6*	rs6475324	9	19361448	A	G	0.0990	-0.8092	0.0811	1.91E-23	99.35
CD8 S100B	*RPS7*	rs62106040	2	3627302	T	A	0.0757	-0.9447	0.0873	2.87E-27	116.76
CD8 S100B	*RPS8*	rs12120833	1	45241285	G	C	0.1242	-0.7029	0.0612	1.62E-30	131.58
CD8 S100B	*RPS9*	rs158366	19	54705317	G	C	0.3901	-0.6865	0.0434	2.97E-56	249.23
CD8 S100B	*RPSA*	rs6804150	3	39475433	C	T	0.1427	0.6161	0.0633	2.33E-22	94.41
CD8 S100B	*S100B*	rs9306156	21	47960493	C	T	0.3298	0.8648	0.0435	4.74E-88	394.9
CD8 S100B	*S100B*	rs13048683	21	48035163	A	T	0.1980	-0.3883	0.0538	5.05E-13	52.08
CD8 S100B	*SCD5*	rs4572871	4	83760887	G	A	0.7766	0.4404	0.0540	3.56E-16	66.33
CD8 S100B	*SCGB3A1*	rs76820754	5	179936737	A	G	0.1030	1.3132	0.0692	2.30E-80	359.67
CD8 S100B	*SEPT2*	rs75590598	2	242185654	A	G	0.0887	0.4529	0.0754	1.89E-09	36.01
CD8 S100B	*SERPINB6*	rs1885294	6	2932996	C	G	0.2042	-0.4027	0.0543	1.19E-13	54.92
CD8 S100B	*SERPINF1*	rs6502953	17	1670809	A	G	0.3364	-0.2962	0.0471	3.29E-10	39.42
CD8 S100B	*SETD9*	rs702686	5	56201350	G	A	0.7091	-0.3294	0.0514	1.46E-10	41
CD8 S100B	*SH3YL1*	rs7601944	2	229456	G	A	0.3333	-0.6033	0.0452	1.19E-40	177.85
CD8 S100B	*SLC12A7*	rs4458624	5	1109314	C	G	0.5658	0.4389	0.0450	1.65E-22	95.08
CD8 S100B	*SLC25A29*	rs8007427	14	100771572	T	C	0.2430	-0.4413	0.0523	3.36E-17	70.98
CD8 S100B	*SLC25A3*	rs28372744	12	98987445	A	G	0.0487	0.5399	0.0983	3.98E-08	30.1
CD8 S100B	*SLFN5*	rs7221768	17	33562776	T	C	0.4045	0.2955	0.0461	1.43E-10	41.03
CD8 S100B	*SMDT1*	rs8143153	22	42515637	C	G	0.3381	-1.0570	0.0415	4.55E-143	647.26
CD8 S100B	*SMIM19*	rs55881007	8	42367290	G	A	0.3727	0.2543	0.0460	3.14E-08	30.55
CD8 S100B	*SNHG7*	rs11145844	9	139613998	T	C	0.1148	-0.8793	0.0691	4.35E-37	161.57
CD8 S100B	*SNHG8*	rs788666	4	119219168	T	C	0.3793	0.8367	0.0417	1.31E-89	402.04
CD8 S100B	*SNORD3B-1*	rs59415593	17	19223224	G	T	0.1431	-0.5726	0.0697	2.04E-16	67.42
CD8 S100B	*SNORD3B-2*	rs12937053	17	19348126	A	G	0.5794	0.2648	0.0457	6.82E-09	33.52
CD8 S100B	*SNRPD2*	rs34399924	19	46200103	A	G	0.2142	-0.4006	0.0556	5.81E-13	51.8
CD8 S100B	*SPATC1L*	rs62215189	21	47603995	G	A	0.2446	-0.3277	0.0509	1.24E-10	41.31
CD8 S100B	*SRP14*	rs2898984	15	40324075	C	G	0.9173	-0.5432	0.0856	2.22E-10	40.18
CD8 S100B	*SRSF10*	rs6424144	1	24375663	A	G	0.4721	-0.2502	0.0449	2.44E-08	31.05
CD8 S100B	*STMN3*	rs879471	20	62269942	T	C	0.5285	0.2873	0.0449	1.53E-10	40.91
CD8 S100B	*STXBP5*	rs1575374	6	146927319	G	A	0.5727	-0.2597	0.0452	9.35E-09	32.9
CD8 S100B	*SULF2*	rs6125037	20	46143709	G	A	0.4029	-0.5220	0.0446	1.39E-31	136.44
CD8 S100B	*SURF1*	rs567493	9	136185528	G	A	0.7115	0.2984	0.0502	2.81E-09	35.24
CD8 S100B	*SYNGR1*	rs909685	22	39747671	A	T	0.3216	0.5545	0.0484	2.13E-30	131.03
CD8 S100B	*TBCA*	rs374745	5	76983163	T	C	0.2939	-0.3914	0.0481	4.21E-16	66

CD8 S100B	*THEMIS2*	rs9887847	1	28226317	C	T	0.3803	-0.4796	0.0451	2.32E-26	112.63
CD8 S100B	*TIMM10*	rs2649652	11	57324428	G	A	0.7810	-0.5946	0.0506	6.85E-32	137.84
CD8 S100B	*TIMM13*	rs12459733	19	2419249	C	T	0.4708	0.2609	0.0449	6.11E-09	33.73
CD8 S100B	*TLE4*	rs2807301	9	82200253	C	T	0.3838	0.3553	0.0462	1.43E-14	59.08
CD8 S100B	*TMEM120B*	rs73413708	12	122213144	C	G	0.0964	0.4605	0.0787	4.97E-09	34.13
CD8 S100B	*TMEM156*	rs6819988	4	39038665	T	C	0.5743	0.3088	0.0451	7.65E-12	46.76
CD8 S100B	*TMEM204*	rs12599859	16	1577184	G	A	0.1364	0.3722	0.0677	3.80E-08	30.19
CD8 S100B	*TMEM50B*	rs1044218	21	34821570	A	G	0.4718	-0.3009	0.0448	1.79E-11	45.1
CD8 S100B	*TNFSF13B*	rs9514831	13	108973576	A	G	0.2718	0.2975	0.0533	2.37E-08	31.1
CD8 S100B	*TOMM7*	rs2240728	7	22852499	T	C	0.3422	-0.9900	0.0427	1.20E-118	535.21
CD8 S100B	*TPGS2*	rs28794232	18	34578888	A	G	0.2585	-0.2910	0.0499	5.47E-09	33.94
CD8 S100B	*TPP2*	rs35722492	13	103330038	A	T	0.3609	-0.2543	0.0461	3.59E-08	30.3
CD8 S100B	*TRMT112*	rs28395880	11	64033748	C	T	0.0710	-0.5723	0.0940	1.13E-09	37.01
CD8 S100B	*TSTD1*	rs12042437	1	161023296	T	C	0.1248	-0.4912	0.0767	1.49E-10	40.96
CD8 S100B	*TTC12*	rs11214589	11	113245048	A	G	0.4943	0.4575	0.0440	2.56E-25	107.88
CD8 S100B	*TUFM*	rs12928404	16	28847246	C	T	0.4054	0.4750	0.0450	5.29E-26	111
CD8 S100B	*U2AF1L4*	rs3848662	19	36216056	T	G	0.1520	0.3565	0.0646	3.37E-08	30.42
CD8 S100B	*UBA52*	rs2314664	19	18691942	G	A	0.5931	-0.2999	0.0456	4.96E-11	43.11
CD8 S100B	*UCP2*	rs72982971	11	73680776	T	C	0.2512	0.3599	0.0527	8.27E-12	46.61
CD8 S100B	*USP53*	rs11729857	4	120114100	G	C	0.1207	0.5569	0.0696	1.29E-15	63.8
CD8 S100B	*VAMP2*	rs12944041	17	8073097	A	G	0.5159	0.2607	0.0455	1.00E-08	32.76
CD8 S100B	*VAMP5*	rs2121397	2	85798858	A	G	0.1164	-0.3985	0.0676	3.83E-09	34.64
CD8 S100B	*VARS2*	rs916570	6	30066031	G	A	0.2732	0.3981	0.0497	1.13E-15	64.06
CD8 S100B	*XBP1*	rs5762815	22	29205076	G	A	0.3318	0.2704	0.0485	2.53E-08	30.98
CD8 S100B	*XCL1*	rs1040508	1	168500750	T	C	0.3842	0.3064	0.0465	4.46E-11	43.31
CD8 S100B	*XRRA1*	rs61734374	11	74457056	G	A	0.2737	0.5197	0.0480	2.43E-27	117.09
CD8 S100B	*XXbac-BPG181B23.7*	rs9261737	6	30366014	T	C	0.1136	-0.5179	0.0646	1.09E-15	64.13
CD8 S100B	*XXbac-BPG181B23.7*	rs3905495	6	31265539	A	G	0.3395	0.3557	0.0474	6.40E-14	56.13
CD8 S100B	*XXbac-BPG252P9.10*	rs2077314	6	30428436	C	A	0.6536	0.3636	0.0456	1.61E-15	63.36
CD8 S100B	*XXbac-BPG252P9.10*	rs2853915	6	31275289	C	A	0.3846	0.5094	0.0443	1.39E-30	131.88
CD8 S100B	*XXbac-BPG252P9.10*	rs2074511	6	30889389	C	T	0.1727	-0.3576	0.0615	6.02E-09	33.76
CD8 S100B	*XXbac-BPG299F13.17*	rs6921663	6	31154620	C	T	0.2178	0.6929	0.0529	3.37E-39	171.21
CD8 S100B	*YBEY*	rs62224180	21	47726332	G	C	0.7751	-0.4530	0.0566	1.19E-15	63.96
CD8 S100B	*ZBTB9*	rs9268851	6	32429582	G	A	0.8774	-0.5371	0.0640	4.98E-17	70.2
CD8 S100B	*ZBTB9*	rs35144104	6	33463961	T	C	0.3039	0.6059	0.0489	3.15E-35	153.08
CD8 S100B	*ZC3HAV1*	rs10273974	7	138735512	C	A	0.4877	-0.3762	0.0444	2.37E-17	71.66
CD8 S100B	*ZFAS1*	rs237711	20	47926856	G	C	0.2041	0.5127	0.0530	3.74E-22	93.47
CD8 S100B	*ZFP57*	rs739780	6	29780621	G	C	0.3441	-0.6440	0.0449	1.16E-46	205.33
CD8 S100B	*ZFP57*	rs3132681	6	30060041	A	C	0.3712	-0.3308	0.0459	5.65E-13	51.86
CD8 S100B	*ZKSCAN3*	rs9257793	6	29335154	C	T	0.1003	0.7762	0.0682	5.04E-30	129.32
CD8 S100B	*ZKSCAN4*	rs7775835	6	28678357	C	T	0.8747	0.5261	0.0672	4.85E-15	61.2
CD8 S100B	*ZNF677*	rs6509740	19	53738985	C	T	0.2524	-0.3020	0.0520	6.35E-09	33.66
CD8 S100B	*ZNF85*	rs10402582	19	21036569	A	G	0.1346	-0.4630	0.0637	3.61E-13	52.74
CD8 S100B	*ZP3*	rs35613536	7	76143625	A	G	0.3272	-0.4490	0.0467	6.35E-22	92.43
CD8 S100B	*ZSCAN12*	rs7742957	6	28535685	T	C	0.2219	0.4020	0.0527	2.33E-14	58.11
Mono C	*ABCC3*	rs4793665	17	48712087	T	C	0.5769	-0.3557	0.0451	2.95E-15	62.17
Mono C	*ABHD16A*	rs9262617	6	31022206	A	G	0.2796	-0.5502	0.0495	9.35E-29	123.54
Mono C	*ABHD16A*	rs2853982	6	31378751	G	A	0.1540	0.4297	0.0627	7.19E-12	46.88
Mono C	*ABHD16A*	rs9265579	6	31298890	T	C	0.3390	0.2465	0.0452	4.82E-08	29.73
Mono C	*ABRACL*	rs12196247	6	139350182	A	G	0.2977	-0.3082	0.0501	7.88E-10	37.71
Mono C	*ABTB1*	rs113761591	3	127305355	T	C	0.2014	0.4492	0.0549	2.84E-16	66.78
Mono C	*AC000003.2*	rs7208708	17	10049841	C	T	0.3144	0.3470	0.0484	7.39E-13	51.33
Mono C	*AC002451.3*	rs7776702	7	95238041	C	G	0.4607	-0.5029	0.0444	8.56E-30	128.27
Mono C	*AC016757.3*	rs8835	2	239147036	G	C	0.3236	-0.6400	0.0457	1.42E-44	195.78
Mono C	*AC091814.2*	rs74737544	12	10086812	T	G	0.0575	-0.7080	0.1011	2.55E-12	48.91
Mono C	*ACP5*	rs78934187	19	11695690	G	A	0.3703	0.3486	0.0476	2.49E-13	53.46
Mono C	*ACTA2*	rs7901656	10	90766213	C	T	0.3355	-0.3395	0.0478	1.24E-12	50.32
Mono C	*ADK*	rs67594352	10	75928933	C	T	0.1452	0.6186	0.0631	1.14E-22	95.81
Mono C	*AFAP1*	rs55938116	4	7933940	A	C	0.3479	-0.6934	0.0443	4.08E-55	244.01
Mono C	*ANAPC13*	rs7374325	3	134275601	C	G	0.6796	0.3312	0.0485	8.49E-12	46.55
Mono C	*ANP32E*	rs72696855	1	150364873	A	G	0.1051	0.4069	0.0706	8.36E-09	33.12
Mono C	*ANPEP*	rs11073891	15	90363995	C	A	0.4325	-0.5767	0.0441	4.12E-39	170.81
Mono C	*ANXA6*	rs3811988	5	150515042	A	C	0.2481	0.3287	0.0516	1.93E-10	40.45
Mono C	*AOAH*	rs4723559	7	36746017	A	G	0.5607	-0.2669	0.0453	3.82E-09	34.64
Mono C	*AP003774.1*	rs627425	11	64087642	T	C	0.3608	-0.3255	0.0464	2.24E-12	49.16
Mono C	*APBB3*	rs6550	5	139948616	A	T	0.3057	0.3336	0.0489	8.72E-12	46.5
Mono C	*APOBEC3A*	rs12628403	22	39358037	C	A	0.0772	-0.6278	0.0834	5.14E-14	56.56
Mono C	*APOBEC3B*	rs12628403	22	39358037	C	A	0.0772	0.5990	0.0835	7.30E-13	51.36
Mono C	*ARPC5*	rs1926825	1	183690371	A	G	0.6556	-0.3400	0.0466	2.99E-13	53.1
Mono C	*ARRDC4*	rs1491180	15	98505857	G	A	0.7862	-0.3407	0.0566	1.70E-09	36.21
Mono C	*B3GNT5*	rs1813856	3	182944134	A	G	0.5577	0.2695	0.0457	3.66E-09	34.73
Mono C	*BCAT1*	rs11047696	12	25051883	C	T	0.2014	-0.3809	0.0558	9.05E-12	46.43
Mono C	*BRI3*	rs7790229	7	97919338	A	G	0.4926	-0.2488	0.0451	3.56E-08	30.31
Mono C	*BRK1*	rs7645759	3	10157368	T	C	0.1182	-0.5675	0.0684	1.03E-16	68.78
Mono C	*BSG*	rs11085113	19	563613	A	G	0.2504	-0.3530	0.0551	1.47E-10	40.98
Mono C	*BTBD9*	rs9349116	6	39333977	C	T	0.6711	-0.4001	0.0473	2.59E-17	71.49
Mono C	*C10orf32*	rs12775431	10	104611764	C	A	0.2393	-0.3345	0.0530	2.70E-10	39.8
Mono C	*C10orf88*	rs77020305	10	124809718	C	A	0.1100	0.5976	0.0707	2.74E-17	71.38
Mono C	*C11orf21*	rs12224967	11	2321284	A	G	0.4112	-0.2706	0.0457	3.07E-09	35.07

Mono C	*C17orf97*	rs4567793	17	248856	T	C	0.7616	0.6812	0.0521	5.02E-39	170.42
Mono C	*C19orf60*	rs10407212	19	18699445	A	G	0.4756	-0.3141	0.0451	3.13E-12	48.5
Mono C	*C1orf85*	rs759329	1	156230936	G	A	0.4958	-0.4486	0.0443	4.36E-24	102.27
Mono C	*C20orf27*	rs6139202	20	3746070	T	A	0.0776	0.9382	0.0840	5.77E-29	124.49
Mono C	*CAPG*	rs35666320	2	85626524	T	C	0.1341	-0.4839	0.0634	2.23E-14	58.2
Mono C	*CASC8*	rs9297756	8	128440167	A	C	0.1573	0.8281	0.0638	1.49E-38	168.26
Mono C	*CAST*	rs6860934	5	96001842	A	G	0.2252	0.3995	0.0534	7.39E-14	55.85
Mono C	*CAT*	rs11032682	11	34443753	T	G	0.3956	0.2841	0.0459	6.02E-10	38.24
Mono C	*CCHCR1*	rs2516647	6	30526845	G	T	0.6246	-0.3561	0.0468	2.79E-14	57.76
Mono C	*CCHCR1*	rs530559160	6	31353792	G	T	0.0766	0.5658	0.0807	2.37E-12	49.05
Mono C	*CCL3*	rs1634520	17	34436610	G	A	0.2374	0.4221	0.0518	3.53E-16	66.35
Mono C	*CCL3L1*	rs11651338	17	34816489	T	C	0.5666	0.2803	0.0455	7.00E-10	37.94
Mono C	*CCL3L3*	rs7208788	17	34823204	A	G	0.1610	0.5976	0.0576	3.10E-25	107.49
Mono C	*CCT8*	rs965951	21	30432557	A	G	0.1160	0.6003	0.0682	1.30E-18	77.38
Mono C	*CCZ1B*	rs74688574	7	6855646	G	A	0.4717	-0.4351	0.0445	1.50E-22	95.28
Mono C	*CD151*	rs8672	11	838634	G	C	0.6810	0.4721	0.0493	1.04E-21	91.46
Mono C	*CD52*	rs10902717	1	26486092	C	T	0.6124	0.2567	0.0464	3.22E-08	30.51
Mono C	*CD52*	rs11589222	1	26645806	T	C	0.7402	1.0434	0.0462	6.53E-113	508.89
Mono C	*CD55*	rs2914937	1	207488768	A	G	0.6978	0.5251	0.0478	4.05E-28	120.64
Mono C	*CD9*	rs7956847	12	6245619	T	C	0.6196	-0.3570	0.0466	1.95E-14	58.46
Mono C	*CDA*	rs72650813	1	20935986	T	A	0.1165	0.6941	0.0689	7.27E-24	101.26
Mono C	*CDC42EP1*	rs2076087	22	37960365	A	G	0.4350	0.3231	0.0457	1.47E-12	49.98
Mono C	*CEACAM21*	rs10420992	19	42064462	A	T	0.7206	-0.4593	0.0503	6.54E-20	83.28
Mono C	*CES1*	rs8192935	16	55861794	G	A	0.6607	0.4544	0.0467	2.35E-22	94.39
Mono C	*CFD*	rs71335276	19	859368	T	G	0.2211	0.8497	0.0517	9.15E-61	269.87
Mono C	*CHCHD2*	rs6951202	7	56156156	T	C	0.2141	0.3619	0.0544	2.82E-11	44.21
Mono C	*CHMP4B*	rs2747536	20	32404128	G	C	0.5240	-0.3244	0.0451	6.59E-13	51.56
Mono C	*CHN2*	rs245900	7	29194429	T	C	0.4040	0.3718	0.0455	3.24E-16	66.51
Mono C	*CHST13*	rs869464	3	126268953	G	T	0.5833	0.3612	0.0458	3.21E-15	62.01
Mono C	*CLEC12A*	rs7313235	12	10132283	T	C	0.2673	0.9347	0.0467	6.04E-89	398.99
Mono C	*CNN2*	rs62131196	19	1032474	T	G	0.1310	-0.4153	0.0643	1.05E-10	41.63
Mono C	*COA6*	rs10910420	1	234509259	C	G	0.5027	0.2937	0.0450	6.73E-11	42.51
Mono C	*COMMD6*	rs4885304	13	76088638	A	G	0.1387	-0.3757	0.0624	1.73E-09	36.18
Mono C	*COX6B1*	rs61218414	19	36171716	C	T	0.5113	-0.3055	0.0450	1.14E-11	45.98
Mono C	*CPVL*	rs11971828	7	29188870	A	G	0.3760	-0.3137	0.0464	1.37E-11	45.61
Mono C	*CRYZ*	rs1327090	1	75266479	A	G	0.6288	0.3131	0.0461	1.13E-11	46
Mono C	*CSGALNACT1*	rs4922089	8	19568628	T	C	0.7414	0.3492	0.0510	7.61E-12	46.77
Mono C	*CST3*	rs2424582	20	23622550	A	G	0.7864	0.7153	0.0531	2.39E-41	181.03
Mono C	*CTC-524C5.2*	rs8072363	17	5335752	T	C	0.2350	-0.4021	0.0547	2.01E-13	53.88
Mono C	*CTSB*	rs709822	8	11702313	G	C	0.2769	0.3906	0.0501	6.11E-15	60.74
Mono C	*CTSH*	rs2289702	15	79237293	T	C	0.1100	-0.5852	0.0696	4.00E-17	70.63
Mono C	*CTSS*	rs41271951	1	150737220	G	A	0.0680	-0.5722	0.0801	9.08E-13	50.93
Mono C	*CYP27A1*	rs12623740	2	219665715	A	T	0.5034	0.3231	0.0449	6.39E-13	51.62
Mono C	*DARS2*	rs4504924	1	173051346	T	G	0.5042	0.4535	0.0443	1.51E-24	104.37
Mono C	*DBI*	rs4411704	2	120120065	A	C	0.1908	-0.4531	0.0558	4.66E-16	65.8
Mono C	*DDT*	rs5760147	22	24334948	C	A	0.3844	-0.5178	0.0457	1.02E-29	127.93
Mono C	*DEGS1*	rs3767732	1	224415509	G	A	0.0802	0.5242	0.0883	2.94E-09	35.15
Mono C	*DNAJC15*	rs17624230	13	43604332	A	T	0.2260	-0.5874	0.0526	5.99E-29	124.42
Mono C	*DRAM2*	rs3762374	1	111682118	T	C	0.2783	0.4760	0.0481	3.94E-23	97.92
Mono C	*DYSF*	rs77690539	2	71648742	T	A	0.0624	0.4987	0.0824	1.45E-09	36.52
Mono C	*EBPL*	rs9562915	13	50283389	C	T	0.2234	-0.3139	0.0541	6.66E-09	33.56
Mono C	*EIF5A*	rs7503161	17	7207964	C	A	0.5615	-0.5853	0.0442	4.56E-40	175.18
Mono C	*EIF6*	rs6120889	20	33870053	A	G	0.2207	0.3451	0.0552	4.19E-10	38.94
Mono C	*ELP5*	rs2074217	17	7150552	G	A	0.6295	-0.4427	0.0454	1.86E-22	94.85
Mono C	*EPHA1-AS1*	rs11771145	7	143110762	A	G	0.3567	0.5610	0.0461	3.87E-34	148.1
Mono C	*ERAP2*	rs2927608	5	96252432	A	G	0.4214	0.4837	0.0445	1.78E-27	117.71
Mono C	*F13A1*	rs12204494	6	6333084	G	T	0.3704	0.3754	0.0457	2.27E-16	67.22
Mono C	*FADS2*	rs61897793	11	61599347	A	G	0.1538	-0.4300	0.0578	9.85E-14	55.28
Mono C	*FAM118A*	rs136582	22	45774531	A	T	0.1332	0.9975	0.0656	3.04E-52	230.86
Mono C	*FAM184B*	rs6819132	4	17689752	T	G	0.3722	-0.5117	0.0452	9.96E-30	127.97
Mono C	*FAM26F*	rs2637665	6	116822449	T	C	0.5182	-0.5312	0.0439	9.55E-34	146.31
Mono C	*FAR1*	rs12417546	11	13671322	T	C	0.3214	0.3077	0.0485	2.20E-10	40.19
Mono C	*FCER1A*	rs2251746	1	159272060	C	T	0.2691	0.4843	0.0487	2.87E-23	98.54
Mono C	*FCER1G*	rs4233366	1	161159147	T	C	0.2757	0.6694	0.0501	1.10E-40	177.99
Mono C	*FCER2*	rs1990975	19	7766274	C	T	0.7160	-0.2856	0.0503	1.38E-08	32.15
Mono C	*FCGR1A*	rs72692851	1	149997588	G	C	0.0757	0.5227	0.0770	1.13E-11	46
Mono C	*FCN1*	rs12377780	9	137808961	A	C	0.3285	0.6232	0.0460	8.05E-42	183.19
Mono C	*FHL3*	rs4634868	1	38465315	A	G	0.2201	0.3388	0.0539	3.31E-10	39.4
Mono C	*FKBPL*	rs2524080	6	31242089	T	A	0.7256	-0.4722	0.0480	7.79E-23	96.57
Mono C	*FOLR3*	rs80225022	11	71869707	T	C	0.0858	1.1405	0.0791	3.83E-47	207.53
Mono C	*FPR1*	rs3764542	19	52257673	G	C	0.7000	0.4185	0.0469	4.76E-19	79.36
Mono C	*GAA*	rs12450199	17	78076592	C	A	0.3781	-0.2680	0.0467	9.59E-09	32.85
Mono C	*GBP7*	rs4233337	1	89636947	T	C	0.4778	-0.6524	0.0429	3.21E-52	230.76
Mono C	*GPR27*	rs7634474	3	71829467	G	A	0.8036	-0.3934	0.0590	2.59E-11	44.37
Mono C	*GPX4*	rs17526264	19	1103768	T	A	0.0544	-0.5843	0.0980	2.45E-09	35.5
Mono C	*GRHPR*	rs4878690	9	37422656	T	C	0.3290	0.3917	0.0477	2.34E-16	67.15
Mono C	*GSN*	rs10818519	9	124012935	C	G	0.4803	0.3485	0.0448	7.76E-15	60.27
Mono C	*GSTK1*	rs2140904	7	143009487	T	C	0.0559	-0.5442	0.0950	1.01E-08	32.76
Mono C	*GSTM4*	rs11101981	1	110204650	C	T	0.3642	-0.2565	0.0467	3.91E-08	30.13

Mono C	*GSTP1*	rs4891	11	67353970	C	T	0.3435	0.3496	0.0471	1.09E-13	55.08
Mono C	*GSTT1*	rs5760147	22	24334948	C	A	0.3844	-0.4379	0.0462	2.47E-21	89.74
Mono C	*H1F0*	rs2285179	22	38211954	A	G	0.5383	-0.2884	0.0453	1.86E-10	40.52
Mono C	*HCG27*	rs9380159	6	30186681	T	C	0.2155	-0.7777	0.0507	4.62E-53	234.61
Mono C	*HCG27*	rs9267659	6	31846234	G	A	0.8103	-0.3563	0.0545	6.35E-11	42.62
Mono C	*HIGD2A*	rs3172921	5	175815782	C	G	0.0953	-0.6080	0.0854	1.09E-12	50.57
Mono C	*HIST1H2AE*	rs11751646	6	26304801	G	A	0.1747	0.4654	0.0608	1.95E-14	58.46
Mono C	*HIST1H3D*	rs66975207	6	26942146	C	A	0.0737	0.4806	0.0768	3.87E-10	39.1
Mono C	*HIST1H4A*	rs72843784	6	26498758	T	G	0.0715	-0.7408	0.0760	1.96E-22	94.75
Mono C	*HIST1H4J*	rs3118362	6	28785085	T	C	0.9328	0.4398	0.0795	3.16E-08	30.54
Mono C	*HLA-A*	rs7767668	6	29923023	T	C	0.5336	0.3672	0.0447	2.13E-16	67.34
Mono C	*HLA-B*	rs915660	6	31143835	G	C	0.1834	-0.3574	0.0578	6.31E-10	38.14
Mono C	*HLA-B*	rs2524096	6	31236467	T	G	0.4422	0.7136	0.0429	3.68E-62	276.26
Mono C	*HLA-B*	rs9366777	6	31264715	A	T	0.0923	-0.5313	0.0833	1.82E-10	40.57
Mono C	*HLA-C*	rs2249741	6	31240712	C	A	0.5532	0.4884	0.0441	1.81E-28	122.23
Mono C	*HLA-DOB*	rs2849016	6	32209262	C	T	0.2750	0.5442	0.0588	2.28E-20	85.35
Mono C	*HLA-DPB1*	rs13197513	6	32990121	C	T	0.1186	-0.3896	0.0708	3.82E-08	30.18
Mono C	*HLA-DQA1*	rs661330	6	32577472	C	T	0.0602	0.3333	0.0573	5.96E-09	33.78
Mono C	*HLA-DQA1*	rs9272535	6	32606756	A	G	0.0891	-0.4957	0.0509	2.22E-22	94.5
Mono C	*HLA-DQA2*	rs909267	6	31746548	C	T	0.0869	-0.5124	0.0921	2.64E-08	30.89
Mono C	*HLA-DQA2*	rs3134931	6	32190620	C	T	0.3105	-0.2943	0.0481	9.60E-10	37.33
Mono C	*HLA-DQA2*	rs1391371	6	32603798	T	A	0.2202	1.2108	0.0478	2.22E-141	639.49
Mono C	*HLA-DQB1*	rs9271377	6	32587165	G	T	0.4033	-0.2791	0.0468	2.38E-09	35.56
Mono C	*HLA-DQB1*	rs9272553	6	32607008	T	G	0.2882	-0.7324	0.0428	9.12E-66	292.77
Mono C	*HLA-DQB2*	rs35656734	6	32600153	T	C	0.2865	-0.4045	0.0500	6.20E-16	65.24
Mono C	*HLA-DRB1*	rs9272492	6	32606052	G	T	0.3637	-0.3384	0.0465	3.38E-13	52.86
Mono C	*HLA-DRB1*	rs28383456	6	32609453	T	C	0.1069	-0.3291	0.0487	1.46E-11	45.49
Mono C	*HLA-DRB5*	rs9272105	6	32599999	A	G	0.5403	-0.8987	0.0402	9.71E-111	498.93
Mono C	*HLA-DRB5*	rs6457598	6	32444475	C	T	0.6962	0.3535	0.0503	2.20E-12	49.19
Mono C	*HLA-G*	rs2517587	6	30205151	T	C	0.0613	-0.6278	0.0889	1.63E-12	49.79
Mono C	*HMBOX1*	rs10088428	8	28909523	T	C	0.2597	-0.2876	0.0521	3.41E-08	30.4
Mono C	*HMGN1*	rs4818029	21	40731931	G	T	0.4842	0.2532	0.0451	2.02E-08	31.41
Mono C	*HP*	rs9941087	16	72118324	A	G	0.3683	-0.5321	0.0465	2.47E-30	130.74
Mono C	*HSBP1L1*	rs8099311	18	77917062	G	A	0.3715	0.2909	0.0468	4.89E-10	38.64
Mono C	*HSD17B8*	rs3129966	6	32381280	C	G	0.0905	0.5084	0.0703	4.81E-13	52.17
Mono C	*HSPA1B*	rs2395004	6	31412513	C	G	0.4368	0.2640	0.0451	4.83E-09	34.18
Mono C	*HSPA1L*	rs2596573	6	31347930	T	C	0.7687	-0.4692	0.0503	1.15E-20	86.7
Mono C	*ICAM4*	rs281437	19	10397238	T	C	0.2936	0.4500	0.0480	6.73E-21	87.76
Mono C	*IFITM3*	rs34481144	11	320836	T	C	0.4497	-0.5451	0.0437	1.18E-35	155.02
Mono C	*IFNGR2*	rs9974603	21	34776300	A	C	0.1489	0.4216	0.0622	1.22E-11	45.85
Mono C	*IGFLR1*	rs62112163	19	36275074	A	G	0.1885	-0.3423	0.0575	2.58E-09	35.41
Mono C	*ITGB2*	rs760462	21	46328099	C	T	0.8298	-1.2826	0.0533	6.80E-128	577.62
Mono C	*ITPR3*	rs9271788	6	32594734	G	A	0.2907	0.3966	0.0447	6.50E-19	78.75
Mono C	*JUP*	rs9911745	17	39942449	T	C	0.9159	-0.7309	0.0847	6.20E-18	74.3
Mono C	*KANSL1-AS1*	rs56328224	17	43572419	T	C	0.2065	0.6635	0.0520	2.47E-37	162.69
Mono C	*KCNMA1*	rs816828	10	79291868	T	C	0.5360	0.3022	0.0450	1.81E-11	45.07
Mono C	*KDELR2*	rs6796	7	6502367	C	T	0.2687	-0.3597	0.0495	3.59E-13	52.74
Mono C	*KIAA1598*	rs72833408	10	118723484	T	G	0.2283	0.5381	0.0519	3.68E-25	107.15
Mono C	*LAP3*	rs6449310	4	17575530	C	T	0.6443	-0.4524	0.0460	8.33E-23	96.44
Mono C	*LGALS1*	rs78684352	22	38059043	G	C	0.0860	0.7432	0.0741	1.10E-23	100.43
Mono C	*LGALS12*	rs1404504	11	63274768	G	T	0.6128	-0.2602	0.0460	1.50E-08	31.99
Mono C	*LGALS2*	rs7291467	22	37972628	A	G	0.5718	-1.0725	0.0384	1.71E-171	777.69
Mono C	*LGALS9*	rs28425473	17	25970421	T	G	0.2417	-0.4414	0.0519	1.76E-17	72.25
Mono C	*LILRA3*	rs367070	19	54800500	G	A	0.1991	-0.7474	0.0515	1.11E-47	209.98
Mono C	*LILRA5*	rs12980744	19	54811680	G	A	0.2079	0.5011	0.0572	2.06E-18	76.48
Mono C	*LINC00969*	rs4247595	3	195672574	C	G	0.2256	0.3701	0.0559	3.70E-11	43.67
Mono C	*LIPA*	rs1412445	10	91002804	T	C	0.3383	0.5263	0.0466	1.30E-29	127.44
Mono C	*LITAF*	rs11075003	16	11700163	G	T	0.1867	-0.6394	0.0545	9.15E-32	137.26
Mono C	*LMNA*	rs78261031	1	156112470	A	G	0.0797	0.6286	0.0904	3.54E-12	48.26
Mono C	*LPAR1*	rs1610009	9	113999400	A	G	0.2353	-0.4265	0.0509	5.19E-17	70.12
Mono C	*LRRC8B*	rs1380416	1	89949213	C	T	0.5075	-0.2662	0.0451	3.52E-09	34.8
Mono C	*LTA4H*	rs1561091	12	96197201	T	G	0.2456	0.2987	0.0541	3.42E-08	30.39
Mono C	*LTA4H*	rs6538697	12	96403610	C	T	0.0943	-0.7019	0.0810	4.54E-18	74.92
Mono C	*LY96*	rs6984696	8	74855616	C	A	0.1466	0.3697	0.0665	2.73E-08	30.83
Mono C	*LYZ*	rs4761234	12	69732105	C	T	0.4502	-0.9110	0.0401	2.58E-114	515.32
Mono C	*MAK*	rs474669	6	10765180	C	T	0.7868	0.7008	0.0521	2.69E-41	180.8
Mono C	*MANBA*	rs223490	4	103679117	T	G	0.4083	-0.2990	0.0465	1.28E-10	41.26
Mono C	*MANEA*	rs9488321	6	96016318	T	C	0.6278	0.4225	0.0460	4.13E-20	84.18
Mono C	*MAP3K7CL*	rs2471946	21	30533397	G	C	0.3689	0.4568	0.0457	1.44E-23	99.91
Mono C	*MARCO*	rs13425622	2	119707051	G	T	0.2698	-0.4977	0.0529	5.04E-21	88.33
Mono C	*MDC1*	rs7773677	6	30336424	G	C	0.2878	-0.2866	0.0514	2.42E-08	31.06
Mono C	*MDC1*	rs7768179	6	29973978	G	A	0.1105	-0.6045	0.0752	8.95E-16	64.51
Mono C	*METTL22*	rs62018925	16	8701727	A	G	0.1367	0.3774	0.0641	3.88E-09	34.61
Mono C	*MGMT*	rs77900915	10	131364237	T	C	0.0817	-0.5173	0.0833	5.38E-10	38.45
Mono C	*MGST3*	rs4147596	1	165601365	C	T	0.4777	-0.4165	0.0445	7.88E-21	87.45
Mono C	*MICALL1*	rs881144	22	37471290	A	G	0.1119	0.4524	0.0708	1.69E-10	40.71
Mono C	*MIF*	rs5751775	22	24266726	C	T	0.5903	-0.3420	0.0461	1.21E-13	54.88
Mono C	*MRPL21*	rs71465408	11	68617022	G	A	0.3006	-0.3067	0.0491	4.22E-10	38.93
Mono C	*MRPL54*	rs12459781	19	3762119	C	T	0.6394	0.3737	0.0473	2.92E-15	62.19

Mono C	*MX1*	rs56003111	21	42792768	T	C	0.1043	0.4223	0.0737	9.80E-09	32.81
Mono C	*MYO1G*	rs59388988	7	45017202	C	G	0.2235	0.3125	0.0565	3.20E-08	30.52
Mono C	*NAAA*	rs17287042	4	76825139	G	A	0.2697	0.3433	0.0505	1.08E-11	46.09
Mono C	*NAAA*	rs28759823	4	76844849	T	C	0.3055	-0.2773	0.0505	3.95E-08	30.11
Mono C	*NAGK*	rs2287327	2	71297982	T	C	0.3307	-0.3324	0.0472	1.81E-12	49.58
Mono C	*NAP1L1*	rs1433364	12	76464637	G	A	0.8079	-0.3829	0.0587	7.09E-11	42.41
Mono C	*NAPRT1*	rs9657360	8	144676862	T	C	0.7776	0.5344	0.0547	1.45E-22	95.34
Mono C	*NDUFA12*	rs835044	12	95397552	C	T	0.6738	-0.3731	0.0470	1.98E-15	62.95
Mono C	*NDUFS5*	rs11205522	1	39465139	C	A	0.2802	-0.5865	0.0470	1.16E-35	155.06
Mono C	*NDUFV3*	rs4148974	21	44323720	T	C	0.0607	-0.5567	0.0989	1.80E-08	31.64
Mono C	*NEU4*	rs80304737	2	242764651	T	G	0.0760	-0.7263	0.0911	1.53E-15	63.46
Mono C	*NFXL1*	rs10008818	4	47930450	T	C	0.3667	0.3731	0.0467	1.36E-15	63.69
Mono C	*NKAPL*	rs172328	6	28790528	T	C	0.1281	-0.4638	0.0614	4.37E-14	56.88
Mono C	*NME1*	rs7207370	17	49231180	G	A	0.3899	0.2792	0.0462	1.50E-09	36.46
Mono C	*NME4*	rs77371232	16	447561	T	A	0.4244	0.3920	0.0451	3.60E-18	75.38
Mono C	*NOMO3*	rs7184653	16	16294465	C	T	0.7614	-0.3457	0.0535	1.07E-10	41.61
Mono C	*NOP10*	rs12593384	15	34634626	G	A	0.1581	0.8602	0.0586	8.53E-49	215.09
Mono C	*NQO2*	rs2518581	6	3009944	G	A	0.7344	-0.6636	0.0512	1.81E-38	167.87
Mono C	*NRG1*	rs2466077	8	32432753	T	G	0.5386	0.5144	0.0440	1.59E-31	136.17
Mono C	*NT5C3B*	rs7208062	17	39988601	T	C	0.7477	-0.5014	0.0508	5.99E-23	97.09
Mono C	*NT5DC3*	rs11111797	12	104208972	T	A	0.1602	-0.6565	0.0630	1.96E-25	108.4
Mono C	*NUDT14*	rs2242636	14	105614032	G	C	0.6501	0.2629	0.0472	2.63E-08	30.9
Mono C	*NUDT2*	rs10814102	9	34353879	T	G	0.1522	0.5101	0.0627	4.15E-16	66.03
Mono C	*OLIG1*	rs2834081	21	34432792	T	A	0.7643	-0.3041	0.0519	4.57E-09	34.29
Mono C	*OSCAR*	rs4442925	19	54554950	T	C	0.4637	0.3501	0.0450	7.55E-15	60.32
Mono C	*OSTF1*	rs11144249	9	77754494	A	G	0.3827	-0.2604	0.0460	1.52E-08	31.96
Mono C	*PADI2*	rs2746510	1	17424302	C	T	0.6051	0.3222	0.0463	3.61E-12	48.23
Mono C	*PLA2G16*	rs7482863	11	63382002	C	G	0.8656	-0.4411	0.0629	2.40E-12	49.02
Mono C	*PNRC2*	rs6424144	1	24375663	A	G	0.4721	-0.2754	0.0451	9.82E-10	37.28
Mono C	*POLR1D*	rs2232669	13	28195495	T	C	0.1167	0.5279	0.0667	2.40E-15	62.57
Mono C	*POLR2L*	rs28404903	11	841763	C	G	0.6825	-0.4371	0.0495	9.91E-19	77.92
Mono C	*POMZP3*	rs1799122	7	76275659	G	A	0.2138	-0.3280	0.0551	2.64E-09	35.36
Mono C	*PPA1*	rs10823493	10	71949471	T	A	0.3217	0.2883	0.0479	1.69E-09	36.22
Mono C	*PPDPF*	rs72629024	20	62152519	G	C	0.1633	-0.6939	0.0613	9.95E-30	127.97
Mono C	*PPIL3*	rs2136600	2	201742345	T	C	0.2391	-0.4389	0.0536	2.63E-16	66.92
Mono C	*PPT1*	rs4660382	1	40559207	T	C	0.8833	0.6591	0.0720	5.34E-20	83.68
Mono C	*PRDX2*	rs897804	19	12876964	C	G	0.3850	0.3336	0.0459	3.50E-13	52.8
Mono C	*PRELID1*	rs10051822	5	176735187	T	G	0.7774	0.5218	0.0551	2.76E-21	89.52
Mono C	*PRKCB*	rs9806836	16	23862213	T	A	0.5811	0.4467	0.0449	2.31E-23	98.97
Mono C	*PROK2*	rs7634474	3	71829467	G	A	0.8036	-0.4247	0.0589	5.46E-13	51.92
Mono C	*PRR7-AS1*	rs2545797	5	176864202	C	T	0.5264	-0.3858	0.0450	9.39E-18	73.48
Mono C	*PSAP*	rs4747199	10	73569318	T	C	0.2854	0.3163	0.0498	2.18E-10	40.22
Mono C	*PSMD5-AS1*	rs13299616	9	123594803	C	T	0.5817	-0.4801	0.0449	1.21E-26	113.92
Mono C	*PVRL2*	rs60389450	19	45372184	C	A	0.2557	0.3493	0.0505	4.75E-12	47.69
Mono C	*RAC1*	rs836551	7	6441799	C	G	0.4869	0.6030	0.0433	4.58E-44	193.45
Mono C	*RAD51C*	rs35828828	17	56718205	T	C	0.1991	0.3037	0.0547	2.83E-08	30.76
Mono C	*RGS1*	rs1547624	1	192543837	T	A	0.8230	-0.6636	0.0573	5.21E-31	133.82
Mono C	*RNASE2*	rs2233859	14	21359808	A	C	0.4106	0.3721	0.0449	1.13E-16	68.58
Mono C	*RNF144B*	rs1886248	6	18399394	T	C	0.6443	-0.4023	0.0471	1.41E-17	72.68
Mono C	*RNF166*	rs8048125	16	88751207	C	T	0.7048	0.2715	0.0487	2.47E-08	31.02
Mono C	*RNPEP*	rs12091788	1	201954429	A	G	0.5893	0.3924	0.0458	1.07E-17	73.23
Mono C	*ROGDI*	rs2290562	16	4853816	A	G	0.2822	0.5994	0.0480	7.57E-36	155.9
Mono C	*RP1-3J17.3*	rs4579322	6	86165540	A	T	0.6900	0.6151	0.0471	4.83E-39	170.5
Mono C	*RP11-1143G9.4*	rs1670737	12	69774877	A	G	0.5389	0.9974	0.0389	2.50E-145	657.61
Mono C	*RP11-245G13.2*	rs61179776	2	11017287	T	G	0.0640	0.6042	0.0932	8.87E-11	41.97
Mono C	*RP11-290F20.3*	rs6125968	20	48909078	A	G	0.3317	-0.2620	0.0478	4.10E-08	30.04
Mono C	*RP11-347P5.1*	rs1401723	2	197035827	T	C	0.5852	0.5159	0.0449	1.71E-30	131.46
Mono C	*RP11-362F19.1*	rs6842402	4	142215923	C	T	0.4700	-0.3458	0.0449	1.35E-14	59.18
Mono C	*RP11-661A12.5*	rs391615	8	144565680	G	A	0.4882	0.4992	0.0441	9.52E-30	128.06
Mono C	*RP3-460G2.2*	rs12525110	6	141219538	G	A	0.2318	0.4684	0.0505	1.67E-20	85.97
Mono C	*RPH3A*	rs7966149	12	113327018	T	C	0.4116	0.3953	0.0456	4.56E-18	74.91
Mono C	*RPL10A*	rs2076172	6	35436925	T	C	0.5358	-0.3245	0.0454	8.75E-13	51
Mono C	*RPL12*	rs2243784	9	130220956	A	G	0.5914	0.5447	0.0444	1.38E-34	150.14
Mono C	*RPL13*	rs35878298	16	89609694	G	A	0.1765	-0.3458	0.0566	9.98E-10	37.25
Mono C	*RPL14*	rs11718621	3	40362122	C	T	0.5880	0.2636	0.0460	1.01E-08	32.75
Mono C	*RPL28*	rs17700376	19	55897327	G	A	0.3547	-0.4202	0.0466	2.04E-19	81.03
Mono C	*RPL36AL*	rs11157696	14	50105746	G	C	0.7481	0.5059	0.0520	2.26E-22	94.46
Mono C	*RPL8*	rs2954658	8	146017782	T	C	0.4990	-0.7754	0.0418	5.92E-77	344.03
Mono C	*RPLP2*	rs12277141	11	798865	C	G	0.6564	-0.2669	0.0483	3.33E-08	30.44
Mono C	*RPPH1*	rs79030532	14	20814865	G	A	0.1336	0.6832	0.0649	6.13E-26	110.7
Mono C	*RPS10*	rs4587164	6	34376703	T	G	0.7616	-0.5658	0.0558	3.53E-24	102.68
Mono C	*RPS16*	rs640000	19	39926601	T	C	0.3796	0.5849	0.0459	3.35E-37	162.08
Mono C	*RPS18*	rs17215231	6	33239869	T	C	0.0640	-0.9607	0.0881	1.13E-27	118.61
Mono C	*RPS19*	rs73549152	19	42361054	G	T	0.5020	0.2504	0.0451	2.88E-08	30.72
Mono C	*RPS2*	rs17135716	16	2014778	G	A	0.1130	0.6603	0.0672	8.99E-23	96.29
Mono C	*RPS25*	rs111804650	11	118878960	T	C	0.0870	-0.5005	0.0787	2.02E-10	40.36
Mono C	*RPS26*	rs773114	12	56379060	T	A	0.3997	1.1259	0.0372	1.00E-200	912.05
Mono C	*RPS3*	rs3094303	11	75114071	G	A	0.0743	-0.5397	0.0915	3.71E-09	34.7
Mono C	*RPS3A*	rs6836854	4	151742349	T	C	0.9259	0.8575	0.0993	5.90E-18	74.4

Mono C	*RPS5*	rs731835	19	58898436	C	T	0.3256	-0.3016	0.0480	3.30E-10	39.41
Mono C	*RPS8*	rs11589147	1	45319121	T	A	0.1178	-0.3716	0.0644	8.06E-09	33.19
Mono C	*RPS9*	rs158366	19	54705317	G	C	0.3901	-0.5905	0.0445	3.24E-40	175.86
Mono C	*S100A12*	rs3014874	1	153337943	A	G	0.2677	-0.4355	0.0514	2.25E-17	71.76
Mono C	*S100A12*	rs61803119	1	153390202	T	G	0.1071	0.4142	0.0694	2.34E-09	35.59
Mono C	*S100A8*	rs61803338	1	153353031	G	T	0.1028	0.4291	0.0704	1.12E-09	37.02
Mono C	*S100A9*	rs61803338	1	153353031	G	T	0.1028	0.4691	0.0703	2.54E-11	44.41
Mono C	*S100P*	rs3822263	4	6697673	C	T	0.7466	0.3886	0.0529	1.94E-13	53.95
Mono C	*SCML4*	rs62422961	6	107130589	A	T	0.0563	0.6998	0.0953	2.14E-13	53.76
Mono C	*SELL*	rs4987360	1	169664867	G	A	0.3028	-0.5454	0.0480	6.43E-30	128.84
Mono C	*SERPINB10*	rs8099238	18	61586637	C	A	0.2438	-0.4564	0.0551	1.25E-16	68.39
Mono C	*SETD9*	rs166169	5	56194768	C	T	0.8418	0.3575	0.0651	4.02E-08	30.08
Mono C	*SH3BGRL3*	rs4471263	1	26622700	T	C	0.7389	-0.6113	0.0505	1.06E-33	146.1
Mono C	*SH3BP5*	rs1287469	3	15312276	T	C	0.5979	-0.2903	0.0461	3.16E-10	39.49
Mono C	*SIRPB1*	rs2253427	20	1552430	C	T	0.7868	-0.6564	0.0517	5.44E-37	161.12
Mono C	*SLC12A7*	rs35188965	5	1104938	T	C	0.5560	-0.2901	0.0457	2.23E-10	40.17
Mono C	*SLC22A18AS*	rs438263	11	2925574	A	C	0.4204	0.3476	0.0456	2.40E-14	58.05
Mono C	*SLC24A4*	rs61977309	14	92927402	A	G	0.0930	-0.4219	0.0773	4.83E-08	29.72
Mono C	*SMDT1*	rs2301521	22	42423110	C	G	0.6779	0.7701	0.0456	5.82E-64	284.51
Mono C	*SNHG7*	rs6874	9	139617836	T	C	0.1153	-0.5513	0.0712	9.79E-15	59.81
Mono C	*SNHG8*	rs658642	4	119200274	T	C	0.3810	0.6033	0.0442	1.75E-42	186.22
Mono C	*SNORD3B-1*	rs959071	17	19142226	T	C	0.1827	0.5735	0.0680	3.48E-17	70.91
Mono C	*SNORD3B-2*	rs77232220	17	19262534	C	G	0.1953	0.4525	0.0552	2.37E-16	67.13
Mono C	*SNORD3D*	rs7214134	17	19375856	A	T	0.7400	-0.3474	0.0521	2.50E-11	44.44
Mono C	*SPATA20*	rs9890200	17	48624523	C	A	0.3676	-0.3909	0.0469	7.52E-17	69.39
Mono C	*SRD5A3*	rs36110240	4	56217015	T	C	0.1629	0.3692	0.0613	1.69E-09	36.22
Mono C	*STEAP4*	rs10266002	7	87968189	A	G	0.7513	0.3139	0.0504	4.90E-10	38.64
Mono C	*STXBP2*	rs710951	19	7710269	G	A	0.6340	0.6105	0.0448	2.71E-42	185.36
Mono C	*SULF2*	rs78055051	20	46356316	A	G	0.1154	-0.4380	0.0651	1.69E-11	45.21
Mono C	*SURF1*	rs739469	9	136298729	C	G	0.4377	-0.2974	0.0456	6.82E-11	42.48
Mono C	*SUSD3*	rs4073460	9	95797999	T	C	0.5451	-0.2998	0.0453	3.53E-11	43.77
Mono C	*TACSTD2*	rs11207240	1	58950155	G	C	0.2877	-0.4639	0.0487	1.67E-21	90.52
Mono C	*TBKBP1*	rs12948299	17	45403057	C	T	0.6755	0.4393	0.0473	1.59E-20	86.07
Mono C	*TCF19*	rs1029237	6	30138662	T	C	0.2168	-0.3666	0.0531	5.05E-12	47.57
Mono C	*TEN1*	rs12945114	17	73993546	T	C	0.7534	0.3157	0.0525	1.83E-09	36.07
Mono C	*THBS1*	rs28790585	15	39668870	T	C	0.3034	0.4052	0.0490	1.37E-16	68.21
Mono C	*THEMIS*	rs72969618	6	128136515	T	C	0.0993	0.7250	0.0686	3.83E-26	111.63
Mono C	*THEMIS2*	rs34341922	1	28243696	A	G	0.6128	0.3981	0.0459	3.89E-18	75.22
Mono C	*TIMM10*	rs2848626	11	57283988	T	C	0.7820	-0.5526	0.0512	3.38E-27	116.43
Mono C	*TKT*	rs141078441	3	53286932	T	C	0.1806	-0.4008	0.0569	1.84E-12	49.55
Mono C	*TLE4*	rs10780319	9	82277217	C	A	0.6357	-0.3541	0.0468	3.67E-14	57.22
Mono C	*TMC4*	rs2241790	19	54655647	T	C	0.3689	-0.3134	0.0469	2.26E-11	44.64
Mono C	*TMEM150B*	rs1172820	19	55815894	C	T	0.5455	-0.2631	0.0453	6.12E-09	33.73
Mono C	*TMEM176A*	rs6464100	7	150477909	A	G	0.6357	-1.0937	0.0391	2.35E-172	781.65
Mono C	*TMEM176B*	rs7806458	7	150476888	A	G	0.6363	-1.1554	0.0378	1.00E-200	933.81
Mono C	*TMEM220*	rs367351	17	10624389	C	T	0.4499	-0.4108	0.0452	9.70E-20	82.5
Mono C	*TMEM50A*	rs909832	1	25754025	G	C	0.3849	-0.3161	0.0452	2.66E-12	48.82
Mono C	*TMEM91*	rs111867059	19	41900809	T	C	0.1266	-0.3562	0.0643	2.99E-08	30.65
Mono C	*TMTC1*	rs186216	12	29936914	G	C	0.2991	0.6284	0.0469	5.62E-41	179.33
Mono C	*TNNT1*	rs891187	19	55671374	T	C	0.1931	0.6597	0.0568	3.12E-31	134.83
Mono C	*TOMM7*	rs2240728	7	22852499	T	C	0.3422	-0.8254	0.0451	6.18E-75	334.78
Mono C	*TPTEP1*	rs8139954	22	17238335	A	C	0.3903	0.2526	0.0462	4.41E-08	29.9
Mono C	*TREML1*	rs60617111	6	41355505	A	G	0.1550	-0.8631	0.0622	8.84E-44	192.15
Mono C	*TSPAN33*	rs12667978	7	128777461	T	C	0.5526	-0.2931	0.0454	1.04E-10	41.67
Mono C	*TSPAN4*	rs7947690	11	833589	G	T	0.6881	-0.3700	0.0501	1.50E-13	54.46
Mono C	*TSPO*	rs113515	22	43556922	G	C	0.3875	0.3259	0.0459	1.22E-12	50.34
Mono C	*TSTD1*	rs10908821	1	161008535	G	C	0.1307	0.3737	0.0675	3.05E-08	30.61
Mono C	*TTC39C*	rs4800173	18	21619931	G	A	0.7600	-0.4355	0.0565	1.22E-14	59.38
Mono C	*TUFM*	rs8049439	16	28837515	C	T	0.3939	0.4257	0.0457	1.27E-20	86.51
Mono C	*TYMP*	rs361725	22	50971047	C	T	0.6078	0.4434	0.0471	4.66E-21	88.49
Mono C	*UAP1L1*	rs72763269	9	140005916	A	G	0.3647	0.2998	0.0464	1.06E-10	41.63
Mono C	*UBE2D1*	rs4390300	10	60144207	A	G	0.4827	-0.2777	0.0451	7.38E-10	37.84
Mono C	*UCP2*	rs603950	11	73699136	G	A	0.3879	0.4076	0.0462	1.15E-18	77.62
Mono C	*UPK3A*	rs2472787	22	45673477	C	A	0.7066	0.2861	0.0496	8.28E-09	33.14
Mono C	*UTP20*	rs12310954	12	101111183	A	G	0.1778	-0.6229	0.0586	2.22E-26	112.71
Mono C	*VARS2*	rs1264448	6	30542999	A	G	0.7139	-0.4432	0.0484	5.49E-20	83.62
Mono C	*VASH1*	rs1861425	14	77191198	T	C	0.7272	-0.3905	0.0521	6.67E-14	56.05
Mono C	*VNN1*	rs4897612	6	133035325	C	A	0.3679	0.3590	0.0472	2.86E-14	57.71
Mono C	*VPS28*	rs117891721	8	145642886	T	C	0.0569	0.8870	0.0943	5.06E-21	88.33
Mono C	*VSTM1*	rs10500316	19	54553697	C	T	0.5198	-0.5030	0.0441	4.04E-30	129.76
Mono C	*WAC-AS1*	rs2993987	10	28800170	T	C	0.7969	-0.3338	0.0538	5.48E-10	38.42
Mono C	*XRCC6BP1*	rs10431506	12	58311524	A	G	0.3136	-0.2855	0.0479	2.60E-09	35.39
Mono C	*XRRA1*	rs11236306	11	74757597	A	G	0.2665	0.3568	0.0494	5.02E-13	52.09
Mono C	*XXbac-BPG181B23.7*	rs553108	6	31840455	G	A	0.6087	-0.3366	0.0457	1.69E-13	54.23
Mono C	*XXbac-BPG252P9.10*	rs2523722	6	30165273	T	C	0.1860	0.3065	0.0553	2.92E-08	30.69
Mono C	*XXbac-BPG299F13.17*	rs2844773	6	30207495	A	C	0.1336	0.3561	0.0627	1.35E-08	32.19
Mono C	*YBEY*	rs66928986	21	47714607	A	G	0.8203	-0.5516	0.0628	1.66E-18	76.9
Mono C	*ZFAS1*	rs6648	20	47895242	G	A	0.2034	0.5193	0.0531	1.49E-22	95.28
Mono C	*ZKSCAN3*	rs3130750	6	29159970	T	C	0.2061	0.3860	0.0539	8.12E-13	51.15

Mono C	*ZKSCAN4*	rs7739915	6	28770683	T	G	0.1279	0.5747	0.0609	3.57E-21	89.02
Mono C	*ZNF184*	rs34481509	6	28261417	G	A	0.0594	0.5163	0.0808	1.70E-10	40.7
Mono C	*ZNF506*	rs7260393	19	19886410	G	C	0.1812	0.3346	0.0596	1.96E-08	31.46
Mono C	*ZNF593*	rs11577318	1	26601570	G	A	0.1811	0.3441	0.0573	1.96E-09	35.94
Mono C	*ZNRD1-AS1*	rs113135194	6	30990055	C	T	0.8946	0.4782	0.0687	3.42E-12	48.33
Mono C	*ZP3*	rs4728722	7	76052091	G	A	0.5836	0.2914	0.0457	1.84E-10	40.54
Mono C	*ZSCAN12*	rs442694	6	29356687	T	G	0.0800	0.6555	0.0731	3.20E-19	80.14
Mono NC	*ABCC3*	rs12604031	17	48712705	A	G	0.6157	-0.2610	0.0467	2.28E-08	31.17
Mono NC	*ABHD16A*	rs630379	6	31922254	C	A	0.7439	0.4466	0.0493	1.29E-19	81.93
Mono NC	*ABRACL*	rs12196247	6	139350182	A	G	0.2977	-0.3029	0.0511	3.01E-09	35.1
Mono NC	*AC000003.2*	rs7208708	17	10049841	C	T	0.3144	0.4538	0.0488	1.44E-20	86.26
Mono NC	*AC002451.3*	rs10085780	7	95226249	G	A	0.3541	-0.7019	0.0461	2.02E-52	231.65
Mono NC	*ACP5*	rs149147675	19	11690676	C	T	0.1268	0.8127	0.0669	6.34E-34	147.11
Mono NC	*ACTA2*	rs6586163	10	90752018	C	A	0.5075	0.4669	0.0450	3.44E-25	107.28
Mono NC	*ADK*	rs67594352	10	75928933	C	T	0.1452	0.6636	0.0641	3.78E-25	107.09
Mono NC	*AFMID*	rs111582188	17	76188292	G	A	0.2827	0.3163	0.0518	1.03E-09	37.19
Mono NC	*AGER*	rs7748141	6	31288877	C	T	0.1003	0.6419	0.0666	5.81E-22	92.59
Mono NC	*AHI1*	rs72980251	6	135781159	A	G	0.0797	0.4762	0.0830	9.48E-09	32.87
Mono NC	*ANPEP*	rs12442778	15	90360038	G	A	0.4321	-0.4125	0.0459	2.73E-19	80.45
Mono NC	*ANXA1*	rs10124476	9	75889773	C	T	0.7134	0.3159	0.0520	1.22E-09	36.85
Mono NC	*ANXA4*	rs35999343	2	69966731	A	G	0.2655	-0.4526	0.0506	3.71E-19	79.84
Mono NC	*AP1AR*	rs4834266	4	113278172	C	A	0.8350	-0.4350	0.0593	2.21E-13	53.69
Mono NC	*APOBEC3A*	rs12628403	22	39358037	C	A	0.0772	-0.5849	0.0851	6.33E-12	47.12
Mono NC	*BATF3*	rs9308424	1	212877776	A	G	0.3259	-0.2833	0.0489	6.93E-09	33.48
Mono NC	*BCAT1*	rs11047695	12	25051596	C	T	0.2020	0.4119	0.0568	3.97E-13	52.55
Mono NC	*BCR*	rs111397379	22	23661565	C	A	0.2294	0.3727	0.0542	5.91E-12	47.26
Mono NC	*BEGAIN*	rs11623078	14	100964549	T	C	0.3950	0.6120	0.0455	3.58E-41	180.21
Mono NC	*BIRC7*	rs11908424	20	61863955	T	C	0.0850	0.9102	0.0762	7.18E-33	142.3
Mono NC	*BLVRA*	rs1181597	7	43789148	T	C	0.5688	0.3839	0.0458	4.97E-17	70.2
Mono NC	*BRK1*	rs6783705	3	10164783	G	A	0.1184	-0.4766	0.0699	9.06E-12	46.42
Mono NC	*C1QA*	rs12033074	1	22966609	G	C	0.3085	0.3365	0.0505	2.67E-11	44.31
Mono NC	*C1QB*	rs6691043	1	22967713	A	G	0.3114	0.3954	0.0500	2.70E-15	62.34
Mono NC	*C20orf27*	rs6139202	20	3746070	T	A	0.0776	0.8309	0.0862	5.30E-22	92.78
Mono NC	*CAT*	rs2284369	11	34468936	G	A	0.2281	-0.3058	0.0557	4.07E-08	30.05
Mono NC	*CCDC170*	rs73783018	6	151762344	G	A	0.1810	-0.4947	0.0611	5.64E-16	65.42
Mono NC	*CCT8*	rs60260312	21	30404396	A	G	0.1025	0.5061	0.0724	2.71E-12	48.78
Mono NC	*CCZ1B*	rs2711224	7	6861880	C	G	0.4738	-0.4295	0.0454	3.06E-21	89.32
Mono NC	*CD151*	rs28681202	11	834098	T	C	0.6896	0.6642	0.0493	2.08E-41	181.29
Mono NC	*CD300C*	rs1171210	17	72552036	C	G	0.5096	-0.3311	0.0457	4.27E-13	52.4
Mono NC	*CD52*	rs4471263	1	26622700	T	C	0.7389	1.1164	0.0460	3.25E-130	588.21
Mono NC	*CD55*	rs2914937	1	207488768	A	G	0.6978	0.5954	0.0482	4.55E-35	152.33
Mono NC	*CDA*	rs6704070	1	20910770	T	C	0.4876	-0.5624	0.0444	9.74E-37	159.95
Mono NC	*CDH23*	rs6480555	10	73538896	A	T	0.5457	-0.2753	0.0460	2.15E-09	35.76
Mono NC	*CEACAM3*	rs10426020	19	42308031	A	G	0.3973	0.8962	0.0427	6.26E-98	440.15
Mono NC	*CFD*	rs71335276	19	859368	T	G	0.2211	0.9009	0.0521	6.23E-67	298.09
Mono NC	*CHMP4B*	rs2626562	20	32409142	A	G	0.5251	-0.2887	0.0460	3.34E-10	39.38
Mono NC	*CHPT1*	rs2695295	12	102086805	T	A	0.5814	0.2572	0.0465	3.27E-08	30.47
Mono NC	*CHST13*	rs744233	3	126249265	A	G	0.2994	-0.4204	0.0504	7.50E-17	69.39
Mono NC	*CHST2*	rs924577	3	143094363	A	C	0.8224	0.3316	0.0598	2.90E-08	30.71
Mono NC	*CKB*	rs911554	14	103999371	T	G	0.3688	-0.3080	0.0469	5.28E-11	42.98
Mono NC	*CLCN1*	rs62472680	7	143048248	A	G	0.1251	-0.4741	0.0697	1.06E-11	46.12
Mono NC	*CLEC12A*	rs999185	12	10113258	T	C	0.2653	0.9969	0.0467	3.33E-101	455.16
Mono NC	*CLEC4F*	rs12991771	2	71053379	G	T	0.6611	-0.4096	0.0473	4.72E-18	74.83
Mono NC	*CNN2*	rs62131196	19	1032474	T	G	0.1310	-0.4548	0.0654	3.44E-12	48.32
Mono NC	*COPG2*	rs4067229	7	130412296	T	C	0.4759	-0.2757	0.0459	1.89E-09	36.01
Mono NC	*COTL1*	rs2018540	16	84631268	C	T	0.5492	0.2571	0.0461	2.47E-08	31.02
Mono NC	*CPNE1*	rs12479546	20	34479277	T	C	0.1046	-0.5049	0.0793	1.89E-10	40.49
Mono NC	*CRYZ*	rs1409785	1	75248122	A	G	0.6302	0.4711	0.0463	2.47E-24	103.38
Mono NC	*CST3*	rs13039536	20	23612305	G	A	0.2199	-0.7383	0.0527	1.29E-44	195.96
Mono NC	*CTB-61M7.2*	rs4806601	19	55388875	C	T	0.2758	0.7520	0.0497	8.48E-52	228.8
Mono NC	*CTD-2006K23.1*	rs905710	17	72563060	T	G	0.3268	-0.4414	0.0481	4.38E-20	84.06
Mono NC	*CTSC*	rs11019518	11	88091814	G	A	0.3390	0.4180	0.0491	1.62E-17	72.41
Mono NC	*CTSH*	rs12148472	15	79231478	C	T	0.1328	-0.5781	0.0650	5.60E-19	79.04
Mono NC	*CYBRD1*	rs6704600	2	172384763	G	T	0.1728	0.4798	0.0608	3.09E-15	62.07
Mono NC	*CYP1B1*	rs232568	2	38394651	A	G	0.3463	0.2667	0.0478	2.33E-08	31.13
Mono NC	*DDT*	rs2739344	22	24298293	C	A	0.5724	0.3330	0.0468	1.09E-12	50.56
Mono NC	*DNAJC15*	rs11618553	13	43607650	G	A	0.2265	-0.4734	0.0541	2.20E-18	76.34
Mono NC	*DOK2*	rs1479056	8	21763393	G	A	0.5836	0.4260	0.0460	1.96E-20	85.65
Mono NC	*EIF5A*	rs7503161	17	7207964	C	A	0.5615	-0.7714	0.0433	6.30E-71	316.39
Mono NC	*ELP2*	rs592261	18	33752244	A	G	0.3587	0.2834	0.0481	3.70E-09	34.7
Mono NC	*ELP5*	rs929229	17	7105399	G	A	0.6587	-0.3242	0.0474	8.12E-12	46.64
Mono NC	*EPHA1-AS1*	rs10228407	7	143127771	G	T	0.4946	0.3953	0.0454	3.26E-18	75.56
Mono NC	*ERAP2*	rs2927608	5	96252432	A	G	0.4214	0.5800	0.0447	1.87E-38	167.8
Mono NC	*FADS2*	rs7943728	11	61547068	A	G	0.1529	-0.6737	0.0574	7.83E-32	137.56
Mono NC	*FAM118A*	rs4605479	22	45797954	A	G	0.1275	1.0427	0.0663	8.48E-56	247.11
Mono NC	*FAM184B*	rs10015354	4	17659526	G	C	0.6121	-0.3718	0.0467	1.76E-15	63.18
Mono NC	*FAM26F*	rs117361304	6	116783619	G	T	0.0827	-1.1076	0.0786	4.20E-45	198.18
Mono NC	*FBP1*	rs10761350	9	97407889	A	G	0.4567	0.2535	0.0464	4.62E-08	29.81
Mono NC	*FCAR*	rs12972637	19	55398101	G	A	0.6751	0.4685	0.0488	7.53E-22	92.08

Mono NC	*FCER1G*	rs2070902	1	161187665	T	C	0.2663	0.4921	0.0528	1.14E-20	86.71
Mono NC	*FCN1*	rs7388879	9	137845625	A	C	0.2708	0.6026	0.0505	8.02E-33	142.08
Mono NC	*FHL3*	rs4634868	1	38465315	A	G	0.2201	0.4181	0.0546	1.93E-14	58.48
Mono NC	*FIBCD1*	rs12683313	9	133868398	T	C	0.3689	0.3159	0.0480	4.80E-11	43.17
Mono NC	*FKBPL*	rs2763981	6	31840021	A	T	0.7096	-0.3900	0.0489	1.53E-15	63.46
Mono NC	*FLVCR1-AS1*	rs12744746	1	213055505	T	G	0.5394	-0.3571	0.0458	6.36E-15	60.66
Mono NC	*FPR1*	rs4801892	19	52256501	G	A	0.7011	0.5671	0.0470	1.39E-33	145.55
Mono NC	*GAPDH*	rs7967416	12	6660997	G	A	0.2679	-0.3914	0.0508	1.29E-14	59.27
Mono NC	*GAPT*	rs62366403	5	57768350	A	G	0.3148	-0.2998	0.0485	6.16E-10	38.19
Mono NC	*GBP7*	rs674903	1	89649824	G	A	0.5047	0.3379	0.0457	1.41E-13	54.57
Mono NC	*GDI2*	rs11255662	10	5914303	G	A	0.4224	-0.2637	0.0465	1.41E-08	32.11
Mono NC	*GFI1B*	rs1633768	9	135879138	T	C	0.2701	0.4778	0.0510	7.07E-21	87.66
Mono NC	*GLIPR1*	rs1824980	12	75911318	T	C	0.6161	-0.2900	0.0476	1.15E-09	36.97
Mono NC	*GLUL*	rs1281340	1	182115787	G	A	0.3872	-0.3111	0.0479	8.66E-11	42.01
Mono NC	*GM2A*	rs153449	5	150649153	T	C	0.2841	-0.4770	0.0516	2.28E-20	85.35
Mono NC	*GMPR*	rs6912988	6	16100654	C	T	0.4995	-0.4433	0.0452	9.72E-23	96.12
Mono NC	*GPRC5C*	rs2706528	17	72442416	C	A	0.9205	-0.8738	0.0812	5.29E-27	115.54
Mono NC	*GRAMD1A*	rs7247980	19	35482114	T	C	0.2945	-0.4093	0.0502	3.46E-16	66.38
Mono NC	*GSTP1*	rs7940813	11	67346866	T	C	0.4027	-0.7788	0.0431	4.27E-73	326.33
Mono NC	*GTF2H2*	rs4704274	5	70681475	A	G	0.6329	-0.2717	0.0476	1.18E-08	32.45
Mono NC	*HCAR2*	rs600501	12	123195014	G	A	0.5551	-0.7002	0.0437	1.00E-57	255.94
Mono NC	*HCG18*	rs1264543	6	30382328	G	A	0.8941	-0.3899	0.0685	1.29E-08	32.28
Mono NC	*HIGD2A*	rs13155545	5	175817499	G	C	0.0942	-0.6520	0.0872	7.53E-14	55.81
Mono NC	*HIST1H2BN*	rs76542212	6	28198122	T	C	0.0584	0.5623	0.0873	1.16E-10	41.44
Mono NC	*HIST1H3D*	rs116418977	6	25365888	T	C	0.0481	-0.5645	0.0895	2.87E-10	39.67
Mono NC	*HIST1H4J*	rs13193532	6	28622914	G	A	0.1195	0.3636	0.0632	8.69E-09	33.04
Mono NC	*HLA-A*	rs7767668	6	29923023	T	C	0.5336	0.4052	0.0454	4.18E-19	79.61
Mono NC	*HLA-B*	rs2524096	6	31236467	T	G	0.4422	0.7901	0.0429	8.46E-76	338.71
Mono NC	*HLA-B*	rs2844532	6	31351175	C	G	0.1970	-0.4241	0.0600	1.63E-12	49.77
Mono NC	*HLA-C*	rs2249741	6	31240712	C	A	0.5532	0.4353	0.0453	6.59E-22	92.34
Mono NC	*HLA-DOA*	rs204988	6	32167253	A	G	0.0350	0.6527	0.0995	5.46E-11	42.91
Mono NC	*HLA-DOB*	rs204992	6	32156908	A	G	0.2018	0.3851	0.0532	4.62E-13	52.25
Mono NC	*HLA-DOB*	rs13212435	6	32422348	T	C	0.1540	-0.6209	0.0603	7.92E-25	105.63
Mono NC	*HLA-DPA1*	rs6457711	6	33045272	A	C	0.2766	-0.3333	0.0543	8.12E-10	37.65
Mono NC	*HLA-DQA1*	rs1391371	6	32603798	T	A	0.2202	0.6752	0.0547	5.75E-35	151.87
Mono NC	*HLA-DQA2*	rs1391371	6	32603798	T	A	0.2202	1.2410	0.0482	3.39E-146	661.54
Mono NC	*HLA-DQA2*	rs204993	6	32155581	G	A	0.2513	0.3380	0.0500	1.35E-11	45.65
Mono NC	*HLA-DQB1*	rs1130034	6	32605274	C	T	0.5136	-0.6184	0.0451	8.09E-43	187.74
Mono NC	*HLA-DRB1*	rs9270505	6	32559216	G	A	0.5223	0.2905	0.0463	3.39E-10	39.35
Mono NC	*HLA-DRB5*	rs9272105	6	32599999	A	G	0.5403	-0.9194	0.0406	2.71E-113	510.6
Mono NC	*HLA-DRB5*	rs7889	6	31605448	G	C	0.5911	0.3830	0.0474	6.35E-16	65.19
Mono NC	*HLA-G*	rs3130380	6	30279130	A	G	0.0895	0.5817	0.0724	9.71E-16	64.35
Mono NC	*HMGN1*	rs8128901	21	40710419	A	G	0.4354	0.4135	0.0454	8.24E-20	82.81
Mono NC	*HSPA1B*	rs3130967	6	31063413	C	T	0.9380	-0.5133	0.0876	4.55E-09	34.3
Mono NC	*HSPA1B*	rs2507997	6	31314781	G	A	0.2677	-0.5236	0.0491	1.45E-26	113.54
Mono NC	*HSPBAP1*	rs13093464	3	122507436	A	G	0.7480	-0.4192	0.0553	3.34E-14	57.4
Mono NC	*ICAM3*	rs7254041	19	10448636	G	T	0.2231	-0.3598	0.0551	6.45E-11	42.59
Mono NC	*ICAM4*	rs281438	19	10399375	G	T	0.2939	0.3305	0.0495	2.45E-11	44.48
Mono NC	*IFI30*	rs2921	19	18288810	G	A	0.2385	-0.3306	0.0559	3.43E-09	34.85
Mono NC	*IFITM2*	rs12801980	11	293188	C	T	0.3586	0.4265	0.0469	1.04E-19	82.35
Mono NC	*IFITM3*	rs34481144	11	320836	T	C	0.4497	-0.6336	0.0439	2.82E-47	208.12
Mono NC	*IQGAP2*	rs11747990	5	75705617	C	T	0.4843	0.3280	0.0457	7.21E-13	51.38
Mono NC	*ITGB2*	rs760462	21	46328099	C	T	0.8298	-0.7739	0.0599	3.52E-38	166.54
Mono NC	*JUP*	rs9910080	17	39949608	C	T	0.9162	-0.9900	0.0849	2.04E-31	135.66
Mono NC	*KANSL1-AS1*	rs413519	17	43685826	G	A	0.2040	0.7652	0.0524	2.42E-48	212.99
Mono NC	*KCNMA1*	rs2670107	10	79318838	C	T	0.4644	0.6049	0.0441	7.96E-43	187.77
Mono NC	*KCNN4*	rs56681946	19	44283031	C	T	0.3520	0.3718	0.0470	2.72E-15	62.33
Mono NC	*KIAA1586*	rs2397215	6	56197920	C	G	0.3370	0.3560	0.0486	2.28E-13	53.63
Mono NC	*KNDC1*	rs11595788	10	134999200	T	A	0.8409	-0.3843	0.0653	4.04E-09	34.53
Mono NC	*L1TD1*	rs11207933	1	62676091	A	C	0.1366	-0.4004	0.0716	2.23E-08	31.22
Mono NC	*L3MBTL3*	rs6569648	6	130349119	T	C	0.7821	-0.2929	0.0529	3.05E-08	30.61
Mono NC	*LAIR2*	rs59342471	19	55014031	T	C	0.0904	0.5921	0.0791	7.39E-14	55.84
Mono NC	*LAP3*	rs7670500	4	17586957	G	A	0.6435	-0.4111	0.0470	2.27E-18	76.27
Mono NC	*LGALS2*	rs2076088	22	37960725	T	G	0.7267	0.4015	0.0506	2.19E-15	62.75
Mono NC	*LGALS9*	rs3751092	17	25958091	G	A	0.2668	-0.5435	0.0506	6.23E-27	115.21
Mono NC	*LILRA2*	rs7249811	19	55087313	G	T	0.0942	-0.6230	0.0773	7.65E-16	64.82
Mono NC	*LILRA3*	rs416867	19	54796630	A	G	0.1946	-0.7457	0.0528	2.52E-45	199.2
Mono NC	*LILRA5*	rs12979675	19	54824872	C	T	0.2201	0.4952	0.0570	3.58E-18	75.38
Mono NC	*LIMD1*	rs267233	3	45642223	G	C	0.2384	0.3138	0.0523	1.99E-09	35.91
Mono NC	*LINC00969*	rs6804822	3	195709662	A	G	0.2393	0.3128	0.0549	1.21E-08	32.4
Mono NC	*LIPA*	rs2246949	10	91004668	T	C	0.5326	0.3994	0.0455	1.75E-18	76.79
Mono NC	*LITAF*	rs11075003	16	11700163	G	T	0.1867	-0.4282	0.0566	4.07E-14	57.01
Mono NC	*LPL*	rs78299715	8	19905904	G	C	0.0851	0.6311	0.0813	8.27E-15	60.14
Mono NC	*LST1*	rs2256974	6	31555392	A	C	0.2026	0.4762	0.0611	6.32E-15	60.67
Mono NC	*LTA*	rs3873304	6	30586031	A	G	0.0787	0.4641	0.0788	3.82E-09	34.64
Mono NC	*LTA4H*	rs4441106	12	96419455	G	A	0.0943	-0.6476	0.0831	6.33E-15	60.67
Mono NC	*LY6G5C*	rs1264372	6	30769726	T	C	0.2185	-0.5141	0.0513	1.22E-23	100.22
Mono NC	*LY86*	rs3765285	6	6589277	G	C	0.7407	0.4418	0.0510	4.77E-18	74.81
Mono NC	*LY96*	rs10957691	8	74906729	G	A	0.1462	0.5095	0.0667	2.18E-14	58.24

Mono NC	*LYPD2*	rs13279795	8	143838669	A	G	0.3876	0.3409	0.0466	2.46E-13	53.48
Mono NC	*LYZ*	rs4761234	12	69732105	C	T	0.4502	-0.8849	0.0412	2.57E-102	460.26
Mono NC	*MAD1L1*	rs7801421	7	1989888	G	C	0.3435	0.3026	0.0478	2.47E-10	39.97
Mono NC	*MAP3K7CL*	rs2776252	21	30531705	A	G	0.3710	0.3583	0.0470	2.48E-14	57.98
Mono NC	*MCM9*	rs11752928	6	118977768	G	C	0.4391	0.3054	0.0460	3.03E-11	44.06
Mono NC	*MDC1*	rs9261373	6	30059137	G	T	0.1596	-0.6205	0.0680	7.29E-20	83.06
Mono NC	*MDC1*	rs9261739	6	30367535	C	T	0.2182	-0.3250	0.0564	8.30E-09	33.13
Mono NC	*MDC1*	rs2844495	6	31483136	G	A	0.3277	-0.3501	0.0483	4.04E-13	52.51
Mono NC	*MEG3*	rs12881545	14	101176212	C	G	0.6387	0.4035	0.0489	1.59E-16	67.91
Mono NC	*MGLL*	rs664910	3	127474030	A	G	0.7023	0.3544	0.0495	7.79E-13	51.22
Mono NC	*MGME1*	rs62209763	20	17965814	T	C	0.1227	-0.3936	0.0696	1.55E-08	31.93
Mono NC	*MGST3*	rs4147596	1	165601365	C	T	0.4777	-0.3573	0.0456	4.63E-15	61.28
Mono NC	*MIF*	rs1006771	22	24314006	T	G	0.6577	-0.2764	0.0492	1.92E-08	31.51
Mono NC	*MLLT4*	rs4709164	6	167724546	G	A	0.3070	-0.5231	0.0490	1.30E-26	113.76
Mono NC	*MMEL1*	rs2843401	1	2528133	C	T	0.6600	-0.2859	0.0484	3.48E-09	34.82
Mono NC	*MROH6*	rs2242095	8	144678874	T	C	0.1707	0.3882	0.0634	8.97E-10	37.46
Mono NC	*MRPL54*	rs2302307	19	3761652	A	G	0.6117	0.2966	0.0476	4.70E-10	38.72
Mono NC	*MX1*	rs56003111	21	42792768	T	C	0.1043	0.4924	0.0748	4.60E-11	43.25
Mono NC	*NAAA*	rs9993150	4	76816881	T	C	0.4431	0.3861	0.0457	2.95E-17	71.23
Mono NC	*NAGK*	rs11680831	2	71292522	C	T	0.3666	-0.3638	0.0475	1.86E-14	58.55
Mono NC	*NAP1L1*	rs1368579	12	76428534	G	A	0.8166	-0.3416	0.0610	2.14E-08	31.29
Mono NC	*NAPRT1*	rs9657360	8	144676862	T	C	0.7776	0.5079	0.0558	9.24E-20	82.59
Mono NC	*NCR3*	rs2853928	6	31257511	C	A	0.7705	-0.4576	0.0502	8.29E-20	82.8
Mono NC	*NDUFA3*	rs7253859	19	54604319	G	A	0.1043	0.4344	0.0736	3.65E-09	34.73
Mono NC	*NDUFS5*	rs11205522	1	39465139	C	A	0.2802	-0.3852	0.0490	4.07E-15	61.53
Mono NC	*NME4*	rs77371232	16	447561	T	A	0.4244	0.3451	0.0462	7.62E-14	55.78
Mono NC	*NOP10*	rs2219668	15	34632857	C	T	0.1565	0.7976	0.0605	1.08E-39	173.46
Mono NC	*NQO2*	rs2756078	6	3010103	A	G	0.7852	-0.4298	0.0569	4.08E-14	57.01
Mono NC	*NRM*	rs2285795	6	30120912	T	C	0.2369	-0.4177	0.0532	4.22E-15	61.46
Mono NC	*NRM*	rs12110481	6	30998312	T	G	0.3725	-0.2694	0.0478	1.73E-08	31.71
Mono NC	*NUDT14*	rs11160838	14	105648901	C	T	0.6260	0.2624	0.0474	3.05E-08	30.61
Mono NC	*NUDT2*	rs10972031	9	34268702	T	C	0.1707	0.4874	0.0619	3.46E-15	61.86
Mono NC	*OAS1*	rs11066451	12	113361443	A	G	0.6622	-0.4632	0.0480	4.81E-22	92.96
Mono NC	*OLIG1*	rs928736	21	34439889	T	C	0.7089	-0.2896	0.0498	6.14E-09	33.72
Mono NC	*OSBPL8*	rs75084335	12	76981753	G	C	0.0609	0.5403	0.0889	1.20E-09	36.89
Mono NC	*OSCAR*	rs10500316	19	54553697	C	T	0.5198	-0.3635	0.0457	1.74E-15	63.2
Mono NC	*OSTF1*	rs13289799	9	77762831	G	A	0.2946	-0.3150	0.0490	1.27E-10	41.27
Mono NC	*P2RX1*	rs12600392	17	3820698	G	A	0.6982	0.4226	0.0483	2.13E-18	76.4
Mono NC	*PDGFC*	rs2042637	4	157745198	G	A	0.3522	0.3133	0.0488	1.31E-10	41.2
Mono NC	*PHF11*	rs9526567	13	50093452	A	G	0.5325	0.2561	0.0461	2.68E-08	30.86
Mono NC	*PLEK*	rs62143899	2	68604553	T	C	0.2980	-0.3095	0.0502	7.02E-10	37.93
Mono NC	*POLR1D*	rs7317084	13	28198098	T	C	0.1171	0.4463	0.0678	4.65E-11	43.23
Mono NC	*POLR2L*	rs9704919	11	842682	G	A	0.6834	-0.3136	0.0511	8.11E-10	37.65
Mono NC	*POMZP3*	rs6956929	7	76002921	C	T	0.6047	0.2709	0.0468	6.92E-09	33.48
Mono NC	*PPA1*	rs10823508	10	72038464	G	T	0.3733	0.3111	0.0478	7.63E-11	42.26
Mono NC	*PPDPF*	rs72629024	20	62152519	G	C	0.1633	-0.8659	0.0612	1.78E-45	199.89
Mono NC	*PPP1R17*	rs12666349	7	31728180	C	T	0.1987	-0.5389	0.0578	1.05E-20	86.88
Mono NC	*PPT1*	rs11584362	1	40631460	T	C	0.1715	0.4114	0.0568	4.40E-13	52.34
Mono NC	*PRDX2*	rs897804	19	12876964	C	G	0.3850	0.3378	0.0467	4.71E-13	52.21
Mono NC	*PRELID1*	rs17078781	5	176544228	A	G	0.1291	-0.5446	0.0679	1.04E-15	64.22
Mono NC	*PRR7-AS1*	rs2545796	5	176864269	C	T	0.4992	-0.6293	0.0440	2.41E-46	203.86
Mono NC	*PSAP*	rs727415	10	73582752	A	T	0.2893	0.3225	0.0505	1.76E-10	40.63
Mono NC	*PSMB9*	rs9276915	6	32865911	A	G	0.2548	0.3263	0.0520	3.45E-10	39.32
Mono NC	*PSMD5-AS1*	rs10760117	9	123586737	G	T	0.5804	-0.4738	0.0458	4.50E-25	106.75
Mono NC	*RAC1*	rs836550	7	6440437	G	A	0.4049	0.4788	0.0457	1.08E-25	109.58
Mono NC	*RIN1*	rs1190621	11	66090307	C	G	0.8972	0.5832	0.0817	9.77E-13	50.78
Mono NC	*RIN3*	rs61975764	14	93014929	A	G	0.4503	-0.2606	0.0461	1.59E-08	31.88
Mono NC	*RNF13*	rs9814695	3	149725801	T	C	0.2443	0.3700	0.0520	1.09E-12	50.56
Mono NC	*RNF144B*	rs1886248	6	18399394	T	C	0.6443	-0.4985	0.0475	9.43E-26	109.84
Mono NC	*RNPEP*	rs4950805	1	201950291	T	G	0.5931	0.3938	0.0467	3.27E-17	71.02
Mono NC	*ROGDI*	rs2290562	16	4853816	A	G	0.2822	0.4247	0.0499	1.63E-17	72.39
Mono NC	*RP1-102H19.8*	rs7707	6	88174102	G	C	0.2744	-0.2959	0.0503	3.96E-09	34.57
Mono NC	*RP1-197B17.3*	rs12426347	12	48043868	C	A	0.0808	0.4811	0.0875	3.80E-08	30.18
Mono NC	*RP1-29C18.10*	rs12628438	22	50003305	C	G	0.1800	-0.5353	0.0588	8.58E-20	82.73
Mono NC	*RP1-3J17.3*	rs12212560	6	86342679	A	G	0.3868	0.3904	0.0469	8.67E-17	69.1
Mono NC	*RP1-3J17.3*	rs9444346	6	86165631	G	A	0.1874	0.3642	0.0597	1.04E-09	37.16
Mono NC	*RP11-1143G9.4*	rs1670737	12	69774877	A	G	0.5389	0.5972	0.0443	2.06E-41	181.31
Mono NC	*RP11-298J20.3*	rs1896530	10	126581925	T	A	0.7826	0.6807	0.0563	1.30E-33	145.68
Mono NC	*RP11-347P5.1*	rs1519611	2	196944333	A	G	0.5861	0.5906	0.0454	1.04E-38	168.95
Mono NC	*RP11-362F19.1*	rs2321966	4	142228292	T	C	0.4724	-0.3101	0.0459	1.40E-11	45.57
Mono NC	*RP11-367G6.3*	rs13202592	6	25935591	T	C	0.0664	0.6495	0.0797	3.82E-16	66.18
Mono NC	*RP11-386I14.4*	rs115525402	1	78443047	C	A	0.0639	-0.4750	0.0865	4.06E-08	30.06
Mono NC	*RP11-514O12.4*	rs13204302	6	167287772	A	G	0.6681	0.3050	0.0493	6.32E-10	38.14
Mono NC	*RP3-460G2.2*	rs620746	6	141130072	C	G	0.7642	0.6818	0.0500	2.76E-42	185.3
Mono NC	*RP3-525N10.2*	rs639338	6	69463872	A	G	0.3420	0.4668	0.0478	1.46E-22	95.32
Mono NC	*RPH3A*	rs10850089	12	113323067	T	C	0.4091	0.3436	0.0468	2.04E-13	53.85
Mono NC	*RPL10A*	rs2076172	6	35436925	T	C	0.5358	-0.3250	0.0462	2.08E-12	49.3
Mono NC	*RPL12*	rs10987645	9	130181223	G	C	0.5913	0.4493	0.0458	1.04E-22	95.99
Mono NC	*RPL13A*	rs61141826	19	49978447	A	G	0.1051	-0.5182	0.0783	3.72E-11	43.66

Mono NC	*RPL28*	rs17700376	19	55897327	G	A	0.3547	-0.5283	0.0469	1.94E-29	126.64
Mono NC	*RPL36AL*	rs3007030	14	50086563	T	C	0.7494	0.4823	0.0528	6.39E-20	83.31
Mono NC	*RPL8*	rs2954654	8	146008168	G	A	0.5017	0.7363	0.0430	7.41E-66	293.16
Mono NC	*RPS10*	rs9380433	6	34377626	T	C	0.7750	-0.5929	0.0585	3.72E-24	102.57
Mono NC	*RPS16*	rs640000	19	39926601	T	C	0.3796	0.8323	0.0445	4.18E-78	349.28
Mono NC	*RPS18*	rs112199989	6	33308380	A	G	0.0656	-0.9305	0.0886	8.62E-26	110.02
Mono NC	*RPS19*	rs7257666	19	42360356	A	G	0.4966	0.6320	0.0439	4.80E-47	207.06
Mono NC	*RPS2*	rs113892113	16	2013488	T	G	0.1127	0.3968	0.0697	1.27E-08	32.31
Mono NC	*RPS23*	rs4703879	5	81553815	G	A	0.7726	0.2870	0.0524	4.39E-08	29.91
Mono NC	*RPS25*	rs45573242	11	118853995	C	A	0.0882	-0.6188	0.0790	4.61E-15	61.29
Mono NC	*RPS26*	rs10876864	12	56401085	A	G	0.5810	-1.2028	0.0358	1.00E-200	1125.99
Mono NC	*RPS3*	rs11539810	11	75133765	A	G	0.0739	-0.5796	0.0919	2.79E-10	39.73
Mono NC	*RPS3A*	rs9884963	4	151626244	G	C	0.9257	0.9532	0.0998	1.34E-21	90.95
Mono NC	*RPS5*	rs731835	19	58898436	C	T	0.3256	-0.3780	0.0486	7.32E-15	60.38
Mono NC	*RPS7*	rs62106040	2	3627302	T	A	0.0757	-0.5788	0.0912	2.21E-10	40.19
Mono NC	*RPS8*	rs12120833	1	45241285	G	C	0.1242	-0.3685	0.0643	9.93E-09	32.79
Mono NC	*RPS9*	rs158366	19	54705317	G	C	0.3901	-0.4019	0.0465	5.14E-18	74.67
Mono NC	*S100A10*	rs2338210	1	151904864	T	A	0.9203	-0.4828	0.0880	4.06E-08	30.06
Mono NC	*SECTM1*	rs8079688	17	80290581	A	G	0.4790	0.2609	0.0459	1.36E-08	32.18
Mono NC	*SH3BGRL3*	rs11589222	1	26645806	T	C	0.7402	-0.6573	0.0511	7.49E-38	165.04
Mono NC	*SIDT2*	rs4938343	11	117003528	C	G	0.8041	0.5147	0.0591	3.14E-18	75.63
Mono NC	*SIRPB1*	rs2253427	20	1552430	C	T	0.7868	-0.6607	0.0526	3.36E-36	157.5
Mono NC	*SLC11A1*	rs62182117	2	219228608	T	C	0.1060	-0.4059	0.0733	3.10E-08	30.58
Mono NC	*SLC17A5*	rs3003182	6	74678611	C	A	0.3698	0.3914	0.0471	9.54E-17	68.91
Mono NC	*SLC22A18*	rs28730759	11	2797566	T	C	0.1340	-0.3643	0.0647	1.77E-08	31.66
Mono NC	*SLC22A18*	rs367035	11	2923826	G	A	0.4319	-0.3267	0.0463	1.65E-12	49.76
Mono NC	*SLC24A4*	rs61977309	14	92927402	A	G	0.0930	-0.4704	0.0786	2.17E-09	35.74
Mono NC	*SMDT1*	rs133377	22	42466950	T	C	0.6612	0.4849	0.0484	1.20E-23	100.26
Mono NC	*SNAP29*	rs5760036	22	21045651	T	C	0.0763	0.5034	0.0842	2.25E-09	35.67
Mono NC	*SNHG8*	rs658642	4	119200274	T	C	0.3810	0.6506	0.0446	3.36E-48	212.35
Mono NC	*SOD1*	rs9976258	21	32975998	C	T	0.4049	-0.2931	0.0470	4.51E-10	38.8
Mono NC	*SOD2*	rs78550031	6	160149179	C	T	0.0474	0.6207	0.1047	3.07E-09	35.06
Mono NC	*SQRDL*	rs16946621	15	45950481	T	C	0.1049	0.4831	0.0825	4.86E-09	34.17
Mono NC	*SSBP3*	rs11206344	1	54852994	A	C	0.4911	-0.2550	0.0461	3.10E-08	30.58
Mono NC	*ST3GAL3*	rs57259875	1	45063912	A	C	0.2525	-0.4837	0.0515	5.53E-21	88.14
Mono NC	*SULF2*	rs78055051	20	46356316	A	G	0.1154	-0.4077	0.0664	8.21E-10	37.63
Mono NC	*SULF2*	rs4530426	20	46103128	A	C	0.2872	-0.2836	0.0505	1.93E-08	31.5
Mono NC	*SULT1A1*	rs11648192	16	28618708	T	C	0.4363	0.3118	0.0460	1.24E-11	45.81
Mono NC	*TCF19*	rs9261535	6	30127323	A	G	0.2164	-0.6723	0.0526	1.82E-37	163.28
Mono NC	*TCF19*	rs6910297	6	31472115	T	C	0.2716	-0.3123	0.0522	2.15E-09	35.75
Mono NC	*THEMIS2*	rs1467465	1	28211403	G	A	0.6584	-0.4072	0.0482	2.89E-17	71.27
Mono NC	*TIMM10*	rs2581921	11	57222607	G	C	0.7639	-0.3262	0.0529	7.10E-10	37.91
Mono NC	*TLE4*	rs1475674	9	82286286	G	C	0.6144	-0.3150	0.0475	3.22E-11	43.94
Mono NC	*TMEM176A*	rs7806458	7	150476888	A	G	0.6363	-0.9133	0.0427	2.47E-101	455.75
Mono NC	*TMEM176B*	rs4725361	7	150460891	T	G	0.6377	-1.0574	0.0405	4.53E-150	679.32
Mono NC	*TMEM50A*	rs72660908	1	25583610	G	C	0.3476	-0.3582	0.0467	1.66E-14	58.77
Mono NC	*TMPRSS3*	rs1109352	21	43799723	A	G	0.2793	0.7087	0.0485	2.66E-48	212.81
Mono NC	*TMTC1*	rs4931216	12	29921735	G	A	0.4069	0.2714	0.0471	8.39E-09	33.11
Mono NC	*TNFSF12*	rs80067372	17	7452752	A	G	0.2537	0.3092	0.0513	1.65E-09	36.27
Mono NC	*TNNI2*	rs907612	11	1874221	T	C	0.3817	-0.2993	0.0473	2.53E-10	39.92
Mono NC	*TNNT1*	rs891187	19	55671374	T	C	0.1931	0.3939	0.0591	2.74E-11	44.26
Mono NC	*TOMM7*	rs2240728	7	22852499	T	C	0.3422	-0.7651	0.0465	8.94E-61	269.9
Mono NC	*TTC39C*	rs4239439	18	21620065	A	G	0.7536	-0.3777	0.0570	3.30E-11	43.89
Mono NC	*TUFM*	rs12928404	16	28847246	C	T	0.4054	0.4434	0.0463	1.09E-21	91.35
Mono NC	*TYMP*	rs361725	22	50971047	C	T	0.6078	0.3499	0.0484	4.76E-13	52.19
Mono NC	*UCP2*	rs653529	11	73702071	C	T	0.3875	0.3862	0.0472	2.62E-16	66.93
Mono NC	*USP49*	rs9369298	6	41576640	G	A	0.2926	0.2990	0.0515	6.48E-09	33.61
Mono NC	*UTF1*	rs11595788	10	134999200	T	A	0.8409	-0.4710	0.0650	4.33E-13	52.37
Mono NC	*VARS2*	rs113565451	6	31190175	T	C	0.1547	0.5227	0.0635	1.83E-16	67.63
Mono NC	*VASH1*	rs10483877	14	77182302	T	C	0.7277	-0.3421	0.0533	1.36E-10	41.13
Mono NC	*VMO1*	rs9901226	17	4669747	A	T	0.8256	0.4869	0.0567	8.80E-18	73.61
Mono NC	*VNN1*	rs2050153	6	133035775	T	C	0.3662	0.3607	0.0481	6.71E-14	56.03
Mono NC	*VPS52*	rs7763262	6	32424882	C	T	0.6859	-0.3417	0.0490	2.95E-12	48.61
Mono NC	*VPS53*	rs4968070	17	446162	G	A	0.4857	-0.4384	0.0452	3.01E-22	93.89
Mono NC	*VSTM1*	rs8111849	19	54566176	C	T	0.2219	0.7173	0.0537	1.08E-40	178.03
Mono NC	*WAC-AS1*	rs2993985	10	28799240	T	C	0.7915	-0.3759	0.0539	3.17E-12	48.48
Mono NC	*XRRA1*	rs11236306	11	74757597	A	G	0.2665	0.3368	0.0504	2.31E-11	44.6
Mono NC	*XXbac-BPG181B23.7*	rs12665339	6	30601232	G	A	0.1490	-0.4891	0.0636	1.51E-14	58.96
Mono NC	*XXbac-BPG181B23.7*	rs9267654	6	31841009	G	A	0.8424	-0.3253	0.0595	4.52E-08	29.85
Mono NC	*XXbac-BPG299F13.17*	rs74618856	6	31325745	C	T	0.5634	-0.2684	0.0461	5.86E-09	33.81
Mono NC	*YBEY*	rs35210219	21	47769134	T	A	0.1482	0.4051	0.0700	7.26E-09	33.39
Mono NC	*ZFAS1*	rs238164	20	47844341	G	A	0.2042	0.5539	0.0539	9.34E-25	105.3
Mono NC	*ZFP36L1*	rs382666	14	69182036	T	C	0.4736	0.3255	0.0457	1.11E-12	50.54
Mono NC	*ZKSCAN8*	rs9295778	6	28684183	G	T	0.8428	-0.3929	0.0621	2.48E-10	39.96
Mono NC	*ZNF593*	rs12117236	1	26523507	C	T	0.2432	0.5191	0.0530	1.10E-22	95.88
Mono NC	*ZNRD1-AS1*	rs9261742	6	30368663	A	G	0.2468	0.4092	0.0553	1.35E-13	54.66
Mono NC	*ZP3*	rs4728775	7	76131695	G	A	0.6410	0.5993	0.0461	1.05E-38	168.93
NK	*ABR*	rs12948620	17	1023292	T	C	0.2026	0.3118	0.0569	4.16E-08	30.01
NK	*ABRACL*	rs2498639	6	139345136	T	C	0.9506	-0.5467	0.0936	5.24E-09	34.02

NK	*AC000003.2*	rs7208708	17	10049841	C	T	0.3144	0.5224	0.0472	1.88E-28	122.14
NK	*AC005037.3*	rs3851973	2	201732878	G	A	0.1258	-0.4817	0.0680	1.37E-12	50.11
NK	*AC005082.12*	rs858300	7	23244631	G	A	0.4046	-0.5788	0.0445	9.93E-39	169.05
NK	*AC005253.2*	rs2314664	19	18691942	G	A	0.5931	-0.3989	0.0452	1.09E-18	77.72
NK	*AC006369.2*	rs917733	2	37831453	C	T	0.5906	-0.8749	0.0407	1.54E-102	461.27
NK	*AC007383.4*	rs6758398	2	207138479	G	A	0.5301	0.6450	0.0429	4.44E-51	225.52
NK	*AC009120.10*	rs4889487	16	74279778	C	G	0.2862	0.5666	0.0472	3.29E-33	143.84
NK	*AC010642.1*	rs3206946	19	58826534	G	A	0.2046	-0.3920	0.0561	2.79E-12	48.72
NK	*AC016757.3*	rs881933	2	239151041	C	G	0.2995	0.4318	0.0471	5.27E-20	83.69
NK	*AC021860.1*	rs73232881	4	38664131	C	T	0.1966	0.4506	0.0534	3.42E-17	70.93
NK	*AC022498.1*	rs13096994	3	187897283	A	G	0.1122	0.5324	0.0720	1.41E-13	54.58
NK	*AC023590.1*	rs2514611	8	119665997	A	G	0.9210	-0.6188	0.0770	8.91E-16	64.52
NK	*AC026202.1*	rs9811522	3	5879366	A	G	0.1851	1.0202	0.0529	6.40E-83	371.34
NK	*AC067959.1*	rs11902050	2	22575507	A	G	0.0872	-1.0320	0.0824	5.16E-36	156.64
NK	*AC069277.2*	rs28590359	3	6543136	A	G	0.1760	-0.8124	0.0568	1.77E-46	204.47
NK	*AC090186.1*	rs6999321	8	52692784	A	G	0.8615	0.6313	0.0760	9.85E-17	68.85
NK	*AC091729.9*	rs7784043	7	1210554	T	G	0.5580	-0.2895	0.0457	2.37E-10	40.05
NK	*AC093157.1*	rs7554702	1	101246098	T	C	0.4139	0.3068	0.0451	1.08E-11	46.07
NK	*AC100830.3*	rs525182	15	64780293	A	G	0.9374	-0.7254	0.1003	4.78E-13	52.18
NK	*AC104653.1*	rs57106417	2	114583321	A	G	0.5884	0.5282	0.0448	4.10E-32	138.84
NK	*AC131056.3*	rs9911791	17	34820996	G	A	0.1966	-0.3437	0.0605	1.32E-08	32.24
NK	*AC132186.1*	rs7191006	16	66126856	T	C	0.3305	0.5334	0.0468	4.07E-30	129.74
NK	*ACAA2*	rs621101	18	47363009	T	C	0.4874	-0.3603	0.0444	5.22E-16	65.57
NK	*ACAP1*	rs3809822	17	7234112	G	C	0.2229	-0.2904	0.0517	1.89E-08	31.54
NK	*ACAT1*	rs10749914	11	108012077	A	G	0.5973	-0.3519	0.0451	6.08E-15	60.74
NK	*ACCS*	rs2074038	11	44087989	T	G	0.1126	0.4950	0.0742	2.53E-11	44.42
NK	*ACOT4*	rs10143135	14	74049986	C	G	0.3806	0.3343	0.0465	6.51E-13	51.58
NK	*ACTA2*	rs1324551	10	90751516	T	C	0.5074	-0.4358	0.0441	4.84E-23	97.5
NK	*ACTN4*	rs62121821	19	39175617	T	C	0.0579	-0.5705	0.0993	9.23E-09	32.93
NK	*ACYP2*	rs11694711	2	54199061	A	C	0.4147	-0.6920	0.0431	5.14E-58	257.26
NK	*ADAM10*	rs7161799	15	58770523	T	C	0.0869	0.6762	0.0790	1.18E-17	73.03
NK	*ADARB2*	rs4880803	10	1294025	A	G	0.3079	-0.6493	0.0461	5.17E-45	197.77
NK	*ADCY7*	rs4595801	16	50369888	T	C	0.1729	0.4596	0.0621	1.38E-13	54.62
NK	*ADCY9*	rs7204629	16	4110036	T	C	0.6199	-0.4565	0.0464	7.86E-23	96.54
NK	*ADHFE1*	rs2357894	8	67378385	C	T	0.4987	0.3302	0.0446	1.41E-13	54.58
NK	*ADRB1*	rs68122733	10	115831533	G	A	0.1723	0.5209	0.0599	3.32E-18	75.52
NK	*AE000662.92*	rs12888861	14	23025932	T	A	0.6332	-0.2602	0.0467	2.59E-08	30.93
NK	*AGA*	rs11131799	4	178363378	A	G	0.6460	0.2953	0.0465	2.17E-10	40.22
NK	*AGAP1*	rs2138497	2	236619055	G	C	0.3990	0.3353	0.0450	9.53E-14	55.34
NK	*AGPAT2*	rs9411217	9	139591685	A	G	0.7739	-0.4885	0.0533	5.19E-20	83.72
NK	*AGTRAP*	rs17875934	1	11804288	T	C	0.1457	-0.6026	0.0625	5.22E-22	92.8
NK	*AHI1*	rs13197384	6	135818897	A	C	0.3411	-0.5933	0.0459	2.73E-38	167.05
NK	*AHNAK*	rs74679312	11	62194434	G	A	0.0524	0.5160	0.0940	4.05E-08	30.06
NK	*AK5*	rs12093881	1	77899640	T	C	0.1595	1.3063	0.0506	5.92E-147	665.01
NK	*AKAP8*	rs4809192	19	15541091	C	A	0.7509	0.3144	0.0521	1.59E-09	36.34
NK	*AKR1C3*	rs1344355	10	5338562	C	A	0.2306	-0.4352	0.0535	4.15E-16	66.02
NK	*AL359091.2*	rs1005362	9	130638467	T	C	0.5179	-0.2503	0.0448	2.37E-08	31.1
NK	*ALDH16A1*	rs2914668	19	49969310	G	A	0.7590	-0.3072	0.0533	8.02E-09	33.2
NK	*ALG1L*	rs12630001	3	125685806	T	C	0.2635	0.4795	0.0515	1.19E-20	86.63
NK	*AMICA1*	rs11216824	11	118096818	A	G	0.1957	0.4550	0.0570	1.49E-15	63.51
NK	*ANAPC13*	rs9834981	3	134282279	G	T	0.6789	0.3537	0.0480	1.75E-13	54.15
NK	*ANKDD1A*	rs1719264	15	65176535	T	C	0.6288	-0.3047	0.0464	5.19E-11	43.01
NK	*ANXA11*	rs12268619	10	81920660	C	A	0.1283	0.5473	0.0649	3.37E-17	70.96
NK	*ANXA2*	rs67255518	15	60760828	T	C	0.1338	0.4943	0.0633	5.51E-15	60.94
NK	*ANXA2R*	rs550400	5	43054758	C	G	0.3222	0.3412	0.0477	8.70E-13	51.01
NK	*ANXA5*	rs6534309	4	122609929	C	T	0.0762	0.8114	0.0760	1.41E-26	113.6
NK	*ANXA6*	rs2303038	5	150535013	T	C	0.2046	0.5106	0.0540	3.09E-21	89.29
NK	*AOAH*	rs2228410	7	36763672	T	C	0.3411	-0.5773	0.0458	2.14E-36	158.39
NK	*AOAH*	rs7806624	7	36824655	T	C	0.2085	0.3452	0.0543	1.99E-10	40.39
NK	*AP001055.6*	rs35985715	21	45490684	A	G	0.1701	-0.3422	0.0584	4.67E-09	34.25
NK	*AP003774.1*	rs2510066	11	64052447	T	C	0.3605	0.7678	0.0431	6.26E-71	316.4
NK	*AP1G2*	rs12431716	14	24021526	A	G	0.0934	-0.4484	0.0761	3.80E-09	34.65
NK	*AP2A1*	rs79349312	19	50275178	A	G	0.1030	-0.6058	0.0857	1.55E-12	49.88
NK	*AP2A2*	rs10751665	11	941300	A	G	0.5360	0.4453	0.0443	8.43E-24	100.95
NK	*APC2*	rs1631848	19	868551	C	T	0.2983	0.6369	0.0482	7.31E-40	174.23
NK	*APMAP*	rs11550623	20	24973247	A	C	0.0807	-0.5189	0.0724	7.57E-13	51.28
NK	*APOBEC3G*	rs17537581	22	39482809	A	G	0.1077	-0.7289	0.0744	1.11E-22	95.86
NK	*APOBEC3H*	rs59728386	22	39506202	A	G	0.1339	-0.4873	0.0687	1.30E-12	50.23
NK	*ARHGAP15*	rs16858573	2	143875725	C	T	0.1120	-0.4134	0.0687	1.78E-09	36.12
NK	*ARHGEF10*	rs6980781	8	1765371	A	G	0.1168	0.5087	0.0761	2.30E-11	44.6
NK	*ARHGEF3*	rs1489948	3	56757717	C	T	0.1634	0.6053	0.0613	5.61E-23	97.21
NK	*ARL16*	rs7503637	17	79649515	A	G	0.1691	-0.7036	0.0579	6.18E-34	147.16
NK	*ARL17A*	rs557307834	17	44121579	C	T	0.2232	0.3944	0.0511	1.17E-14	59.45
NK	*ARPC4*	rs7613920	3	9871984	T	G	0.6948	0.4283	0.0477	2.75E-19	80.44
NK	*ARPC5*	rs75435975	1	183583993	G	A	0.3698	0.3438	0.0462	1.00E-13	55.25
NK	*ARSA*	rs2071421	22	51064416	C	T	0.1275	-0.6122	0.0667	4.38E-20	84.06
NK	*ASCL2*	rs2521269	11	2321095	A	C	0.5657	0.4402	0.0444	3.29E-23	98.26
NK	*ASNSD1*	rs1132360	2	190526224	T	C	0.7251	0.3429	0.0513	2.43E-11	44.49
NK	*ATG10*	rs12108681	5	81278375	C	T	0.2259	0.3677	0.0508	4.73E-13	52.2

NK	*ATHL1*	rs10902119	11	288115	C	T	0.3851	0.6018	0.0439	9.87E-43	187.34
NK	*ATIC*	rs1546778	2	216143705	T	A	0.7252	-0.3063	0.0512	2.21E-09	35.7
NK	*ATM*	rs11212527	11	108021205	C	T	0.5840	-0.3069	0.0449	8.07E-12	46.65
NK	*ATP5D*	rs3760987	19	1239881	T	C	0.2017	-0.3181	0.0556	1.07E-08	32.64
NK	*ATP5G1*	rs12602746	17	47031741	C	A	0.5221	-0.3392	0.0446	2.84E-14	57.72
NK	*ATP5G3*	rs21818	2	176035845	A	G	0.8107	-0.3519	0.0573	8.22E-10	37.63
NK	*ATP6AP1L*	rs226202	5	81571669	T	C	0.7072	-0.3385	0.0480	1.76E-12	49.62
NK	*ATP6V1G2*	rs2524129	6	31265135	G	C	0.7708	-0.5494	0.0486	1.31E-29	127.41
NK	*ATP9A*	rs6091367	20	50388229	G	A	0.4408	-0.5242	0.0441	1.25E-32	141.19
NK	*ATP9B*	rs499548	18	76817189	A	G	0.0842	0.4834	0.0811	2.52E-09	35.45
NK	*B3GAT1*	rs78760579	11	134281332	G	T	0.1221	-0.4020	0.0622	1.05E-10	41.63
NK	*B3GAT2*	rs495433	6	71606987	T	C	0.1306	-0.4048	0.0683	3.05E-09	35.08
NK	*B3GNT7*	rs16825944	2	232291866	T	C	0.2821	-0.3619	0.0529	8.07E-12	46.65
NK	*B4GALNT3*	rs7314691	12	661981	A	G	0.2600	0.6926	0.0488	1.08E-45	200.88
NK	*B4GALT1*	rs12352863	9	33116941	C	G	0.1858	0.4795	0.0556	6.89E-18	74.09
NK	*B4GALT4*	rs9842789	3	118957370	A	G	0.1995	0.3906	0.0560	2.97E-12	48.6
NK	*BIN1*	rs10194375	2	127839781	A	C	0.3384	0.9088	0.0429	1.03E-99	448.32
NK	*BNC2*	rs10810655	9	16877138	C	G	0.5530	0.3604	0.0460	4.50E-15	61.33
NK	*BOK*	rs35717482	2	242510982	A	G	0.1471	-0.7193	0.0635	1.06E-29	127.84
NK	*BPGM*	rs2113449	7	134309840	T	A	0.5086	-0.3281	0.0446	1.83E-13	54.07
NK	*BRD7*	rs4595801	16	50369888	T	C	0.1729	0.5171	0.0619	6.51E-17	69.67
NK	*BRK1*	rs112594161	3	10177860	G	C	0.1089	-0.4993	0.0687	3.52E-13	52.78
NK	*BSPRY*	rs7873862	9	116113396	T	C	0.2109	-0.4120	0.0572	6.06E-13	51.72
NK	*BST1*	rs4698415	4	15743391	C	T	0.7924	-0.4082	0.0543	5.72E-14	56.34
NK	*BTBD9*	rs9470806	6	38139938	A	G	0.6956	-0.4196	0.0509	1.66E-16	67.82
NK	*BTN2A1*	rs2273558	6	26466035	C	A	0.3391	0.3659	0.0493	1.13E-13	55.01
NK	*BTN3A1*	rs56190518	6	26413975	T	C	0.1725	0.3603	0.0639	1.73E-08	31.71
NK	*BTN3A2*	rs67509210	6	26354100	T	G	0.1018	-1.0193	0.0634	3.30E-58	258.14
NK	*C10orf128*	rs17774010	10	50391910	G	A	0.2156	0.8268	0.0493	3.11E-63	281.15
NK	*C10orf128*	rs2292201	10	50506613	T	G	0.1401	-0.3812	0.0646	3.59E-09	34.76
NK	*C10orf32*	rs11191421	10	104623053	G	C	0.2357	-0.4572	0.0525	3.08E-18	75.68
NK	*C10orf54*	rs748113	10	73508791	C	T	0.4424	0.4000	0.0446	3.12E-19	80.19
NK	*C10orf95*	rs72845701	10	104197327	G	A	0.1184	-0.4241	0.0766	3.09E-08	30.59
NK	*C11orf21*	rs11022157	11	2322829	A	C	0.3383	-0.4377	0.0458	1.28E-21	91.03
NK	*C11orf24*	rs2512620	11	68025180	A	G	0.4388	-0.4452	0.0443	8.52E-24	100.93
NK	*C14orf2*	rs11850704	14	104381687	G	A	0.0909	-0.5252	0.0838	3.73E-10	39.16
NK	*C16orf13*	rs117667029	16	686337	A	G	0.1175	0.5372	0.0676	1.84E-15	63.09
NK	*C17orf49*	rs312460	17	6926130	C	A	0.9046	-0.6678	0.0881	3.51E-14	57.3
NK	*C17orf89*	rs2659008	17	79217002	G	A	0.5311	0.4303	0.0441	1.85E-22	94.85
NK	*C17orf97*	rs11150882	17	259648	A	G	0.2620	0.6128	0.0508	1.52E-33	145.37
NK	*C19orf24*	rs12609287	19	1271274	T	C	0.7729	0.3149	0.0515	9.43E-10	37.36
NK	*C19orf66*	rs12460842	19	10222195	G	A	0.5674	0.2525	0.0452	2.29E-08	31.16
NK	*C19orf83*	rs2286756	19	46145025	T	G	0.5303	-0.6386	0.0428	2.17E-50	222.36
NK	*C1orf173*	rs1146400	1	75479182	A	G	0.7290	0.3650	0.0516	1.53E-12	49.9
NK	*C1orf86*	rs11587831	1	2110848	G	T	0.2239	-0.3890	0.0543	8.00E-13	51.17
NK	*C2*	rs3132541	6	31098734	C	A	0.1048	0.8442	0.0610	1.57E-43	190.99
NK	*C2*	rs35395738	6	32560209	C	T	0.2074	-0.3009	0.0534	1.77E-08	31.67
NK	*C22orf34*	rs4524218	22	50018639	A	G	0.5846	-0.4464	0.0456	1.34E-22	95.49
NK	*C2ORF15*	rs13018891	2	99755099	G	C	0.6451	0.2730	0.0472	7.19E-09	33.41
NK	*C2orf27A*	rs6720375	2	132440073	C	G	0.5865	0.4639	0.0445	1.73E-25	108.63
NK	*C4orf33*	rs318513	4	129816821	T	C	0.1680	0.3614	0.0602	1.94E-09	35.96
NK	*C5orf17*	rs13155485	5	23977151	G	A	0.1617	-0.6052	0.0554	7.93E-28	119.29
NK	*C5orf34*	rs10042992	5	43561759	T	C	0.4467	-0.3274	0.0448	2.77E-13	53.25
NK	*C5orf56*	rs2522051	5	131797578	C	T	0.4649	0.3237	0.0447	4.61E-13	52.25
NK	*C6orf62*	rs3756814	6	24705835	C	T	0.3832	-0.2697	0.0459	4.33E-09	34.4
NK	*C9orf156*	rs3780419	9	100677134	C	T	0.2565	0.4293	0.0503	1.44E-17	72.64
NK	*C9orf78*	rs8113	9	132589606	C	T	0.2433	-0.4824	0.0514	6.15E-21	87.93
NK	*CACNA1A*	rs7254351	19	13317980	T	G	0.4403	-0.2925	0.0458	1.75E-10	40.64
NK	*CALR*	rs10419627	19	13036677	A	G	0.6134	0.2558	0.0459	2.48E-08	31.01
NK	*CAMLG*	rs12655433	5	133979933	G	A	0.1170	0.4981	0.0690	5.27E-13	51.99
NK	*CAP1*	rs7556253	1	40612199	A	G	0.1639	0.3227	0.0583	3.04E-08	30.61
NK	*CAPN12*	rs35335944	19	39250308	A	G	0.2324	-0.6009	0.0527	3.99E-30	129.77
NK	*CARD16*	rs539595	11	104905047	A	G	0.1689	-0.4177	0.0608	6.34E-12	47.12
NK	*CARHSP1*	rs7190732	16	8930563	C	T	0.2567	0.5926	0.0496	6.39E-33	142.53
NK	*CASP8*	rs10804111	2	202145612	T	C	0.6051	0.4094	0.0459	4.90E-19	79.3
NK	*CAST*	rs10039522	5	95990869	A	G	0.1691	0.8097	0.0587	2.64E-43	189.96
NK	*CAT*	rs1157669	11	34417870	C	T	0.5311	0.2918	0.0447	6.89E-11	42.46
NK	*CCDC101*	rs3743963	16	28604686	G	A	0.5283	-0.2505	0.0449	2.44E-08	31.04
NK	*CCDC122*	rs1822969	13	44418164	A	G	0.4805	0.2669	0.0451	3.15E-09	35.01
NK	*CCDC146*	rs35446630	7	76868115	C	T	0.1425	-0.6150	0.0594	4.35E-25	106.82
NK	*CCDC152*	rs62373000	5	42730620	A	T	0.2874	0.3761	0.0490	1.60E-14	58.84
NK	*CCDC163P*	rs781066	1	45943156	C	T	0.3103	0.4610	0.0466	4.14E-23	97.81
NK	*CCDC25*	rs9797	8	27591849	A	G	0.4804	0.2705	0.0449	1.72E-09	36.19
NK	*CCDC50*	rs4687210	3	191046770	A	G	0.2155	-0.5381	0.0526	1.33E-24	104.6
NK	*CCDC58*	rs3828362	3	122131777	G	C	0.4138	0.5693	0.0441	3.49E-38	166.56
NK	*CCDC66*	rs17216685	3	56647799	C	T	0.1742	0.5696	0.0602	3.19E-21	89.23
NK	*CCDC69*	rs919727	5	150597053	C	T	0.1098	0.4006	0.0729	3.86E-08	30.15
NK	*CCDC74A*	rs143759515	2	132278118	T	C	0.0950	0.5195	0.0748	3.87E-12	48.08
NK	*CCDC88B*	rs61886926	11	64133552	T	C	0.3672	-0.2875	0.0462	4.85E-10	38.65

NK	*CCL3L1*	rs11651338	17	34816489	T	C	0.5666	0.2832	0.0451	3.53E-10	39.27
NK	*CCL3L3*	rs7208788	17	34823204	A	G	0.1610	0.6905	0.0566	3.42E-34	148.33
NK	*CCL4L1*	rs7208788	17	34823204	A	G	0.1610	0.5287	0.0576	4.05E-20	84.21
NK	*CCL4L2*	rs11651338	17	34816489	T	C	0.5666	0.2504	0.0452	3.12E-08	30.56
NK	*CCS*	rs4930387	11	66449905	G	A	0.5197	0.3628	0.0445	3.54E-16	66.33
NK	*CCSAP*	rs237819	1	229452387	T	C	0.6421	-0.2876	0.0477	1.64E-09	36.28
NK	*CCSER2*	rs17663697	10	86276294	A	G	0.1301	-0.5731	0.0686	6.87E-17	69.56
NK	*CCT8*	rs971281	21	30420279	G	A	0.1314	0.7690	0.0641	3.73E-33	143.59
NK	*CCZ1*	rs58793660	7	5936956	G	T	0.6739	-0.4036	0.0475	1.81E-17	72.19
NK	*CCZ1*	rs7807287	7	6877519	G	A	0.4455	0.6037	0.0434	5.10E-44	193.22
NK	*CCZ1B*	rs2711224	7	6861880	C	G	0.4738	-0.7378	0.0420	4.08E-69	308.09
NK	*CD151*	rs72847212	11	834389	C	T	0.2156	-0.6604	0.0554	8.45E-33	141.97
NK	*CD27-AS1*	rs1059501	12	6560573	T	G	0.5426	0.2516	0.0452	2.64E-08	30.89
NK	*CD300A*	rs72844326	17	72459091	G	T	0.0582	-1.0415	0.1006	4.18E-25	106.89
NK	*CD300A*	rs11868856	17	72463603	A	C	0.3870	0.3781	0.0458	1.53E-16	67.99
NK	*CD300C*	rs7209406	17	72473553	C	T	0.4538	-0.2519	0.0452	2.55E-08	30.95
NK	*CD300LB*	rs12939435	17	72463215	A	G	0.4215	0.4732	0.0448	4.31E-26	111.39
NK	*CD37*	rs10405465	19	49869298	T	C	0.2717	-0.4589	0.0494	1.43E-20	86.27
NK	*CD3E*	rs3759009	11	118173850	A	G	0.3079	1.1441	0.0400	2.90E-180	817.87
NK	*CD47*	rs326361	3	107819041	A	G	0.4481	0.4227	0.0444	1.68E-21	90.49
NK	*CD48*	rs7515941	1	160646561	A	G	0.7647	-0.4234	0.0518	3.20E-16	66.53
NK	*CD52*	rs11577318	1	26601570	G	A	0.1811	-0.6641	0.0554	4.70E-33	143.14
NK	*CD55*	rs12021745	1	207466599	T	C	0.2733	0.3082	0.0498	5.83E-10	38.29
NK	*CD79B*	rs73326393	17	62071159	C	T	0.1638	0.3848	0.0599	1.37E-10	41.11
NK	*CD81*	rs731909	11	2408624	C	G	0.8413	-0.3958	0.0628	2.98E-10	39.6
NK	*CD8A*	rs6547705	2	87044316	A	G	0.7872	-0.6033	0.0554	1.18E-27	118.51
NK	*CDC14A*	rs12568038	1	100879914	T	C	0.3834	-0.3256	0.0454	7.60E-13	51.27
NK	*CDC42*	rs2473290	1	22358457	C	T	0.7720	-0.3517	0.0532	3.84E-11	43.6
NK	*CDC42SE2*	rs78623258	5	130628103	G	A	0.1843	0.5144	0.0552	1.27E-20	86.5
NK	*CDK10*	rs77651727	16	89708267	T	C	0.0591	-0.7800	0.0910	1.03E-17	73.29
NK	*CEACAM21*	rs3745936	19	42092815	A	T	0.7738	0.5493	0.0539	2.39E-24	103.45
NK	*CEBPZ-AS1*	rs10198835	2	37434380	T	C	0.1318	-0.6149	0.0653	4.39E-21	88.6
NK	*CELA1*	rs9651999	12	51742948	T	G	0.2852	-0.3179	0.0481	3.97E-11	43.54
NK	*CENPK*	rs245561	5	64804698	T	C	0.3865	0.5768	0.0453	3.36E-37	162.06
NK	*CENPU*	rs11132254	4	185611592	T	G	0.3248	-0.2916	0.0480	1.28E-09	36.77
NK	*CEP63*	rs7645295	3	134307734	G	T	0.6749	0.2655	0.0484	4.01E-08	30.08
NK	*CEP78*	rs10867165	9	80849929	G	C	0.0811	-0.9824	0.0869	1.18E-29	127.63
NK	*CERS4*	rs12982350	19	8272146	G	C	0.4845	0.2819	0.0448	2.99E-10	39.59
NK	*CES1*	rs62028648	16	55864517	T	C	0.5947	0.2653	0.0457	6.40E-09	33.64
NK	*CFD*	rs10424998	19	874647	C	G	0.2509	0.5577	0.0510	7.62E-28	119.37
NK	*CFLAR*	rs10190751	2	202006096	A	G	0.2094	-0.4314	0.0555	7.22E-15	60.41
NK	*CHCHD2*	rs2016580	7	56184642	T	C	0.1721	-0.7263	0.0542	6.72E-41	178.96
NK	*CHI3L2*	rs2252482	1	111759762	T	C	0.9009	-1.4621	0.0662	3.90E-108	486.94
NK	*CHMP4A*	rs2855098	14	24719540	C	G	0.8700	0.3994	0.0647	6.48E-10	38.09
NK	*CHMP4B*	rs13041078	20	32435497	T	G	0.5057	-0.3261	0.0449	3.63E-13	52.72
NK	*CHPT1*	rs2559850	12	102093459	A	G	0.5861	0.4938	0.0444	8.53E-29	123.71
NK	*CHURC1*	rs1957426	14	65355575	G	A	0.7345	-0.3194	0.0555	8.48E-09	33.09
NK	*CIAO1*	rs3171927	2	96952833	G	A	0.3021	-0.2882	0.0488	3.60E-09	34.75
NK	*CIB1*	rs1104971	15	90795138	A	G	0.4014	-0.3674	0.0455	6.34E-16	65.19
NK	*CISD1*	rs1111821	10	59935221	G	C	0.2244	-0.3372	0.0569	3.09E-09	35.05
NK	*CLEC11A*	rs11084024	19	51228634	A	G	0.2329	-0.2999	0.0545	3.66E-08	30.26
NK	*CLEC12A*	rs584856	12	10150069	A	G	0.5577	0.3357	0.0449	7.21E-14	55.89
NK	*CLEC2B*	rs2594072	12	10005778	G	A	0.5521	0.4707	0.0442	1.58E-26	113.38
NK	*CLHC1*	rs17046778	2	55459373	G	T	0.1593	0.3823	0.0610	3.70E-10	39.18
NK	*CLIC3*	rs7040970	9	139859013	C	T	0.5087	-0.4048	0.0443	6.20E-20	83.38
NK	*CLK4*	rs11249554	5	178053487	T	G	0.4312	0.2974	0.0448	3.33E-11	43.88
NK	*CMC1*	rs1982560	3	28520197	T	C	0.4245	0.3331	0.0450	1.42E-13	54.56
NK	*CMTM3*	rs34213783	16	66729193	C	T	0.4742	-0.3471	0.0446	7.15E-15	60.42
NK	*CNN2*	rs4807440	19	1026477	T	G	0.6175	-0.3917	0.0460	1.76E-17	72.24
NK	*CNOT6L*	rs57868780	4	78797333	A	G	0.1121	0.5494	0.0668	1.98E-16	67.47
NK	*COA1*	rs1181574	7	43804695	T	C	0.7111	-0.3913	0.0474	1.45E-16	68.09
NK	*COG5*	rs2520256	7	107162515	T	C	0.2300	0.6181	0.0523	3.32E-32	139.26
NK	*COL18A1*	rs9977482	21	46899119	T	C	0.2188	-0.3387	0.0519	6.93E-11	42.45
NK	*COL5A3*	rs34480538	19	10117242	C	T	0.1926	0.6716	0.0529	5.80E-37	160.98
NK	*COL6A1*	rs2839029	21	47291826	A	G	0.5824	-0.6469	0.0429	2.37E-51	226.76
NK	*COLGALT2*	rs4233115	1	183936470	A	G	0.5994	0.6371	0.0432	3.18E-49	217.03
NK	*COMMD5*	rs2722472	8	146047236	T	C	0.3989	0.3738	0.0460	4.45E-16	65.89
NK	*COMMD6*	rs4885304	13	76088638	A	G	0.1387	-0.6830	0.0606	1.76E-29	126.84
NK	*CORO1B*	rs10274	11	67202826	A	G	0.4070	-0.5979	0.0437	1.56E-42	186.43
NK	*COX20*	rs12139064	1	245000918	C	A	0.1454	0.4237	0.0640	3.51E-11	43.78
NK	*COX6B1*	rs4806187	19	36149761	G	A	0.5163	-0.3104	0.0447	3.70E-12	48.17
NK	*COX7A2L*	rs1981664	2	42577170	G	A	0.5905	-0.2691	0.0451	2.34E-09	35.59
NK	*CPNE1*	rs7261284	20	34244104	A	T	0.1068	-0.9291	0.0742	5.29E-36	156.6
NK	*CPSF1*	rs10111654	8	145602304	C	T	0.4470	-0.2629	0.0451	5.63E-09	33.88
NK	*CRIP1*	rs59732071	14	105954949	G	A	0.1631	0.4401	0.0603	2.84E-13	53.2
NK	*CRIPAK*	rs11538062	4	1165764	T	C	0.1326	0.3480	0.0637	4.69E-08	29.78
NK	*CRLF3*	rs11657985	17	29094775	A	G	0.1369	-0.5743	0.0676	1.88E-17	72.11
NK	*CROCC*	rs12745538	1	17299983	G	A	0.0572	0.4800	0.0830	7.35E-09	33.37
NK	*CRTAM*	rs4369419	11	122716041	A	G	0.3645	0.4581	0.0466	8.33E-23	96.43

NK	*CRYBB2*	rs6004658	22	25856231	T	C	0.0961	1.4289	0.0808	4.74E-70	312.37
NK	*CRYZ*	rs1465483	1	75195243	C	T	0.6770	-0.2856	0.0476	1.99E-09	35.9
NK	*CSGALNACT1*	rs62496184	8	19319423	A	G	0.5222	-0.6665	0.0426	3.08E-55	244.54
NK	*CST3*	rs13039536	20	23612305	G	A	0.2199	-0.4129	0.0533	9.41E-15	59.89
NK	*CST7*	rs6114991	20	24980580	G	A	0.0873	0.5177	0.0689	5.66E-14	56.37
NK	*CSTB*	rs35285321	21	45201832	A	G	0.3415	-0.6113	0.0468	4.94E-39	170.44
NK	*CTA-29F11.1*	rs12389	22	47158179	A	G	0.3392	-0.4915	0.0457	5.10E-27	115.61
NK	*CTA-445C9.15*	rs4275	22	26921725	G	A	0.4260	0.5417	0.0438	3.88E-35	152.65
NK	*CTBP1-AS2*	rs2878539	4	1243877	T	C	0.4296	0.3269	0.0452	4.73E-13	52.2
NK	*CTC-425F1.4*	rs2974755	19	13062663	T	C	0.6285	-0.2999	0.0460	6.94E-11	42.44
NK	*CTC-503J8.4*	rs10414026	19	6246479	A	G	0.2978	0.4170	0.0484	7.08E-18	74.03
NK	*CTC-524C5.2*	rs1050390	17	5336058	A	G	0.2364	0.5660	0.0535	3.35E-26	111.89
NK	*CTD-2007H13.3*	rs327811	5	98271175	G	A	0.2908	0.2673	0.0486	3.89E-08	30.14
NK	*CTD-2325M2.1*	rs4804056	19	8027762	G	A	0.5254	0.3388	0.0446	3.02E-14	57.6
NK	*CTD-2353F22.1*	rs6887423	5	36696501	C	T	0.5490	0.4411	0.0444	3.23E-23	98.3
NK	*CTD-2357A8.3*	rs56121534	17	76621949	T	C	0.4253	0.3931	0.0450	2.45E-18	76.12
NK	*CTD-2368P22.1*	rs4801243	19	58513807	C	A	0.2297	0.8573	0.0495	3.85E-67	299.05
NK	*CTD-2371O3.2*	rs2290423	11	9450377	G	T	0.5094	0.7068	0.0421	3.80E-63	280.75
NK	*CTD-3138B18.5*	rs437229	19	58790675	C	T	0.6727	-0.3003	0.0474	2.45E-10	39.98
NK	*CTD-3162L10.1*	rs2972525	19	36747279	C	T	0.4477	-0.5019	0.0442	7.20E-30	128.6
NK	*CTSF*	rs2242663	11	66335308	C	T	0.2447	0.4302	0.0519	1.21E-16	68.45
NK	*CTSG*	rs6573880	14	25048853	C	T	0.1016	-0.8009	0.0738	1.81E-27	117.66
NK	*CTSS*	rs66958685	1	150675007	A	C	0.2586	-0.3168	0.0503	3.11E-10	39.52
NK	*CTSW*	rs677931	11	65654665	C	G	0.1996	-1.0607	0.0515	4.30E-94	422.55
NK	*CXCR4*	rs16832995	2	136893864	T	C	0.1079	-0.4700	0.0840	2.17E-08	31.26
NK	*CYTL1*	rs4689195	4	5022616	A	G	0.8478	-0.9459	0.0577	1.98E-60	268.31
NK	*DAD1*	rs74036418	14	23025946	T	C	0.0922	0.4506	0.0773	5.52E-09	33.92
NK	*DAP*	rs5745297	5	10680997	A	G	0.0825	0.5772	0.0845	8.50E-12	46.55
NK	*DAPK1*	rs12554893	9	90047023	T	C	0.0445	0.8283	0.1017	3.92E-16	66.14
NK	*DAPK1*	rs888334	9	90097435	A	G	0.4352	0.2793	0.0453	6.97E-10	37.95
NK	*DAZAP1*	rs2668420	19	1367014	C	T	0.4715	-0.2604	0.0448	6.13E-09	33.72
NK	*DAZAP2*	rs7314235	12	51631781	G	C	0.6253	-0.3231	0.0464	3.45E-12	48.31
NK	*DBI*	rs6711245	2	120046905	G	T	0.6362	0.3390	0.0464	2.85E-13	53.19
NK	*DBNL*	rs140776140	7	44094935	T	C	0.0584	-0.5631	0.0984	1.07E-08	32.65
NK	*DCTN5*	rs152449	16	23710160	C	T	0.2823	0.3044	0.0508	2.10E-09	35.8
NK	*DCXR*	rs9903371	17	79993064	G	C	0.1249	1.0705	0.0637	2.17E-63	281.86
NK	*DDR1*	rs12201084	6	30382904	T	A	0.1932	-0.3701	0.0570	8.57E-11	42.03
NK	*DDR1*	rs750332	6	31607050	T	C	0.8338	-0.4803	0.0584	1.93E-16	67.53
NK	*DDRGK1*	rs6037501	20	3191205	T	G	0.2280	-0.3161	0.0548	7.97E-09	33.21
NK	*DDT*	rs5760147	22	24334948	C	A	0.3844	-0.3449	0.0463	9.25E-14	55.4
NK	*DDTL*	rs4822452	22	24254363	T	C	0.6202	0.5059	0.0457	1.89E-28	122.13
NK	*DDX11*	rs3881296	12	31237700	A	G	0.4487	-0.5848	0.0434	2.61E-41	180.84
NK	*DDX42*	rs2665826	17	61944924	G	A	0.2692	0.3074	0.0495	5.21E-10	38.51
NK	*DDX5*	rs1991401	17	62502435	G	A	0.3173	-0.5326	0.0474	2.47E-29	126.16
NK	*DDX6*	rs112561833	11	118603736	C	A	0.2301	-0.3174	0.0539	3.94E-09	34.58
NK	*DECR2*	rs3743894	16	460543	C	A	0.5119	-0.2793	0.0447	4.32E-10	38.88
NK	*DFNB31*	rs2031875	9	117306807	G	T	0.0926	0.4258	0.0781	4.95E-08	29.67
NK	*DGKQ*	rs13101828	4	965720	G	A	0.4441	0.2508	0.0449	2.39E-08	31.09
NK	*DHRS4*	rs1811890	14	24470138	T	C	0.0783	0.8037	0.0884	1.01E-19	82.4
NK	*DHRS7*	rs17834340	14	60657126	T	G	0.1155	-0.3962	0.0684	7.05E-09	33.45
NK	*DHX32*	rs11244669	10	127532782	C	A	0.3710	-0.2658	0.0456	5.57E-09	33.91
NK	*DIP2A*	rs2839301	21	47948427	G	C	0.3110	1.1791	0.0390	1.00E-200	913.63
NK	*DLG5*	rs2579139	10	79646712	T	C	0.2943	-0.2801	0.0494	1.41E-08	32.1
NK	*DLL1*	rs7763693	6	170725187	C	A	0.6058	0.2658	0.0458	6.59E-09	33.58
NK	*DMKN*	rs8111522	19	35974202	A	G	0.0867	-0.7230	0.0726	2.19E-23	99.07
NK	*DNAJA1*	rs7469374	9	33035114	G	A	0.3606	-0.3238	0.0465	3.27E-12	48.42
NK	*DNAJC15*	rs17064045	13	43598322	G	C	0.2357	-0.8731	0.0499	1.75E-68	305.2
NK	*DOCK8*	rs17720878	9	281845	G	A	0.1649	-0.4512	0.0565	1.38E-15	63.66
NK	*DOK7*	rs10805009	4	3501802	C	G	0.5180	0.2604	0.0448	6.37E-09	33.65
NK	*DPYSL4*	rs2818405	10	134031876	T	G	0.0998	-0.4756	0.0748	2.05E-10	40.33
NK	*DRAM2*	rs10857846	1	111550437	A	G	0.2639	0.3634	0.0497	2.62E-13	53.36
NK	*DRAXIN*	rs55706902	1	11724708	C	T	0.1880	-0.3971	0.0561	1.45E-12	50.01
NK	*DST*	rs16888222	6	56767158	A	G	0.2594	0.2887	0.0525	3.92E-08	30.12
NK	*DSTN*	rs13037434	20	17484736	C	G	0.1941	0.3772	0.0573	4.47E-11	43.3
NK	*DSTN*	rs6034875	20	17627727	A	G	0.5725	-0.3093	0.0456	1.22E-11	45.84
NK	*DTD1*	rs6136420	20	18562842	T	C	0.1465	0.3932	0.0649	1.39E-09	36.6
NK	*DTNBP1*	rs2619539	6	15620855	G	C	0.5411	0.7470	0.0420	1.02E-70	315.43
NK	*DUOX1*	rs1706816	15	45460422	G	A	0.8406	0.4895	0.0683	7.51E-13	51.3
NK	*DYNLL2*	rs9900038	17	56169763	G	A	0.7692	-0.4208	0.0511	1.70E-16	67.78
NK	*DYNLRB1*	rs2378134	20	32938085	T	C	0.4986	0.3151	0.0446	1.64E-12	49.77
NK	*DZIP3*	rs13059124	3	108408614	T	C	0.2937	0.5448	0.0480	7.09E-30	128.63
NK	*EBPL*	rs1535470	13	50260029	A	G	0.6296	0.3075	0.0467	4.76E-11	43.18
NK	*EBPL*	rs3809325	13	50266109	C	T	0.5393	-0.5670	0.0437	1.57E-38	168.14
NK	*ECHDC2*	rs11206043	1	53402552	T	C	0.6243	0.3067	0.0467	4.95E-11	43.1
NK	*EFCAB4B*	rs242024	12	3787094	A	T	0.0941	0.5088	0.0738	5.32E-12	47.46
NK	*EFHB*	rs1133328	3	19987539	T	G	0.3696	0.3091	0.0457	1.32E-11	45.68
NK	*EGFL8*	rs2524080	6	31242089	T	A	0.7256	0.4812	0.0476	5.37E-24	101.84
NK	*EIF2A*	rs1568585	3	150332011	T	G	0.2645	0.3091	0.0508	1.15E-09	36.97
NK	*EIF3G*	rs2305795	19	10226052	A	G	0.5697	0.8235	0.0413	1.38E-88	397.31

NK	*EIF3K*	rs115347874	19	39072205	G	C	0.0751	0.5585	0.0980	1.22E-08	32.38
NK	*EIF4G3*	rs9426652	1	21458151	A	G	0.4669	0.4218	0.0445	2.35E-21	89.83
NK	*EIF5A*	rs7503161	17	7207964	C	A	0.5615	-1.2367	0.0340	1.00E-200	1322.59
NK	*ELP5*	rs2654186	17	7151111	C	A	0.6470	-1.0757	0.0384	2.59E-172	781.38
NK	*EMB*	rs7704686	5	49844829	A	T	0.5519	-0.4796	0.0442	2.22E-27	117.26
NK	*EMILIN1*	rs898031	2	28203294	T	C	0.3091	-0.2835	0.0483	4.21E-09	34.45
NK	*EML4*	rs4953236	2	42333345	A	G	0.3743	-0.3674	0.0455	6.74E-16	65.07
NK	*EMP3*	rs11665	19	48833806	G	C	0.4408	-0.2479	0.0450	3.67E-08	30.25
NK	*ENC1*	rs10070279	5	73898568	G	A	0.1142	0.5691	0.0634	2.87E-19	80.35
NK	*ENDOG*	rs3115875	9	131579182	T	C	0.6968	0.2942	0.0497	3.15E-09	35.01
NK	*ENO1*	rs11121242	1	8906301	G	A	0.5098	0.4353	0.0441	5.87E-23	97.12
NK	*ENPP4*	rs7754582	6	46067273	A	G	0.5124	0.3521	0.0445	2.69E-15	62.35
NK	*ENPP5*	rs7769738	6	46110726	G	A	0.5140	0.3199	0.0447	8.43E-13	51.07
NK	*ENTPD3-AS1*	rs13100795	3	40513974	A	C	0.6677	-0.3294	0.0479	5.85E-12	47.28
NK	*EPDR1*	rs3807194	7	37952391	T	C	0.2454	-0.3263	0.0517	2.70E-10	39.79
NK	*EPHX4*	rs6656473	1	92416449	A	G	0.0613	0.5523	0.0903	9.79E-10	37.29
NK	*EPSTI1*	rs3825511	13	43566373	C	A	0.7178	-0.6041	0.0492	1.38E-34	150.13
NK	*ERAP1*	rs27524	5	96101944	G	A	0.6179	-0.2713	0.0467	6.38E-09	33.64
NK	*ERAP2*	rs2927608	5	96252432	A	G	0.4214	1.0916	0.0372	2.33E-189	859.62
NK	*ERI1*	rs13252797	8	8861430	A	G	0.4273	-0.3127	0.0448	3.03E-12	48.57
NK	*ERMP1*	rs10815290	9	5841963	A	G	0.1313	-0.4225	0.0637	3.32E-11	43.88
NK	*ERN1*	rs74787440	17	62121787	T	G	0.1591	-0.7247	0.0604	3.51E-33	143.72
NK	*ESR2*	rs61174206	14	64696200	A	C	0.0741	1.1611	0.0926	4.94E-36	156.73
NK	*ESR2*	rs1256121	14	64802779	C	G	0.1410	0.4901	0.0628	6.19E-15	60.71
NK	*ETV7*	rs11757823	6	36356322	T	C	0.5287	0.4304	0.0444	3.11E-22	93.82
NK	*EVA1C*	rs9305503	21	33776646	A	T	0.2991	-0.4374	0.0491	5.52E-19	79.06
NK	*EVI2A*	rs2525570	17	29681245	G	A	0.5970	0.4685	0.0454	5.77E-25	106.26
NK	*EXOC3*	rs957791	5	430217	T	C	0.4419	0.4050	0.0442	5.46E-20	83.63
NK	*F2R*	rs250753	5	76037551	G	A	0.7397	0.5636	0.0491	1.87E-30	131.27
NK	*FADS1*	rs174555	11	61579760	C	T	0.2998	0.2835	0.0492	8.32E-09	33.13
NK	*FADS2*	rs968567	11	61595564	T	C	0.1535	0.9098	0.0540	1.30E-63	282.88
NK	*FAH*	rs1370274	15	80452075	C	T	0.5993	-0.5715	0.0444	5.55E-38	165.64
NK	*FAM101B*	rs6598847	17	452403	G	A	0.8357	-0.3474	0.0575	1.56E-09	36.38
NK	*FAM118A*	rs2142552	22	45719497	G	C	0.8669	-1.3846	0.0610	6.37E-114	513.48
NK	*FAM129A*	rs234108	1	184942671	A	G	0.4667	-0.3175	0.0447	1.17E-12	50.42
NK	*FAM132B*	rs3820817	2	239075935	G	A	0.5902	0.2673	0.0460	6.09E-09	33.73
NK	*FAM154B*	rs114586219	15	82537939	A	C	0.0705	0.6375	0.0818	6.32E-15	60.67
NK	*FAM184B*	rs10939740	4	17617490	C	T	0.6230	-1.0408	0.0391	4.39E-156	706.9
NK	*FAM26F*	rs1057192	6	116783330	A	G	0.2439	-0.8651	0.0492	4.42E-69	307.93
NK	*FAM26F*	rs1741819	6	116849485	A	G	0.7359	0.4283	0.0501	1.30E-17	72.83
NK	*FAM3C*	rs11771477	7	121004803	C	T	0.1816	0.3069	0.0557	3.60E-08	30.29
NK	*FAM43A*	rs1773218	3	194407020	G	A	0.6320	-0.3238	0.0458	1.52E-12	49.91
NK	*FAM53B*	rs112140735	10	126464649	A	G	0.1156	-0.4401	0.0700	3.22E-10	39.45
NK	*FAM63B*	rs7161799	15	58770523	T	C	0.0869	0.4818	0.0798	1.56E-09	36.38
NK	*FAM64A*	rs56202638	17	6361942	T	C	0.3253	0.3995	0.0474	3.61E-17	70.83
NK	*FAR1*	rs7130044	11	13715649	C	T	0.3326	0.5148	0.0469	4.57E-28	120.39
NK	*FAU*	rs544944	11	64862234	T	C	0.4907	0.3084	0.0447	5.38E-12	47.44
NK	*FBLN5*	rs2284339	14	92340901	G	A	0.4576	-0.3152	0.0446	1.67E-12	49.73
NK	*FBP1*	rs10993269	9	97388953	G	T	0.4970	0.2726	0.0448	1.14E-09	36.99
NK	*FBXO2*	rs1883575	1	11713166	G	A	0.5386	-0.3556	0.0446	1.46E-15	63.55
NK	*FBXO6*	rs3117066	1	11725908	G	A	0.4880	-0.3455	0.0445	8.56E-15	60.07
NK	*FCER1G*	rs11421	1	161188936	C	T	0.1523	-0.3532	0.0626	1.65E-08	31.8
NK	*FCGBP*	rs28540820	19	40407728	T	C	0.7513	-0.3164	0.0516	8.97E-10	37.46
NK	*FCGR2A*	rs71632979	1	161526397	G	A	0.1352	0.6292	0.0697	1.79E-19	81.28
NK	*FCRL3*	rs2210912	1	157668701	C	A	0.4700	0.3979	0.0443	2.55E-19	80.58
NK	*FES*	rs146845689	15	91317368	G	A	0.0487	0.6203	0.1026	1.47E-09	36.49
NK	*FES*	rs2071410	15	91420940	G	C	0.3129	-0.7383	0.0461	1.20E-57	255.58
NK	*FGFBP2*	rs4698432	4	15964763	T	C	0.3763	-0.5037	0.0451	5.69E-29	124.51
NK	*FGFR1*	rs17182002	8	38327630	A	C	0.3897	0.5903	0.0440	5.81E-41	179.25
NK	*FGL2*	rs2075761	7	76828953	T	C	0.0923	0.8540	0.0810	5.75E-26	110.82
NK	*FHAD1*	rs763821	1	15668276	A	G	0.3848	-0.5386	0.0444	7.53E-34	146.77
NK	*FHL3*	rs4360494	1	38455891	C	G	0.5241	-0.4166	0.0443	4.94E-21	88.37
NK	*FIBP*	rs596002	11	65645354	G	A	0.8002	-0.5410	0.0556	2.34E-22	94.39
NK	*FIG4*	rs12190372	6	110084534	A	G	0.3604	-0.2851	0.0466	9.66E-10	37.31
NK	*FIS1*	rs10255195	7	100897812	A	G	0.0602	-0.7379	0.0916	8.15E-16	64.69
NK	*FKBP11*	rs11168790	12	49311528	C	A	0.3919	-0.5417	0.0451	3.54E-33	143.7
NK	*FKRP*	rs62134782	19	47205419	G	C	0.2222	-0.2989	0.0523	1.11E-08	32.58
NK	*FLVCR1-AS1*	rs61832055	1	213015222	G	A	0.5073	0.4366	0.0441	4.22E-23	97.77
NK	*FMN1*	rs61999983	15	33352167	C	T	0.1757	-0.4681	0.0609	1.51E-14	58.96
NK	*FN3KRP*	rs2280251	17	80675105	C	G	0.3963	-0.5415	0.0448	1.18E-33	145.87
NK	*FNBP4*	rs28498823	11	47914941	G	C	0.5521	0.3067	0.0447	7.08E-12	46.9
NK	*FOXN2*	rs77981979	2	48209839	C	T	0.1192	0.4732	0.0668	1.35E-12	50.15
NK	*FOXRED1*	rs587891	11	126081336	C	T	0.7785	0.2943	0.0533	3.37E-08	30.42
NK	*FRG1B*	rs2379649	20	29649222	C	T	0.4962	0.2680	0.0448	2.15E-09	35.76
NK	*FUT7*	rs13301354	9	139924637	C	T	0.6439	-0.4826	0.0454	2.30E-26	112.63
NK	*FXYD5*	rs56050577	19	35660138	T	G	0.0735	-0.6513	0.0831	4.69E-15	61.25
NK	*FXYD5*	rs11670649	19	35665515	C	A	0.2848	-0.4153	0.0477	3.42E-18	75.47
NK	*G3BP2*	rs1553985	4	76554604	G	A	0.3907	-0.3794	0.0456	8.56E-17	69.13
NK	*GADD45G*	rs7357754	9	92207308	G	A	0.4871	0.3137	0.0446	2.06E-12	49.32

NK	*GALNT2*	rs641038	1	230324801	A	G	0.2258	-0.3262	0.0552	3.40E-09	34.87
NK	*GALT*	rs2812357	9	34655436	C	T	0.6012	0.2931	0.0452	8.80E-11	41.98
NK	*GAPDH*	rs11064213	12	6580376	G	A	0.2081	0.4014	0.0557	5.57E-13	51.88
NK	*GBP3*	rs4656083	1	89503665	A	G	0.4371	0.2807	0.0450	4.24E-10	38.91
NK	*GBP5*	rs55870241	1	89742521	C	T	0.5445	-0.3691	0.0447	1.39E-16	68.18
NK	*GBP7*	rs674903	1	89649824	G	A	0.5047	0.8200	0.0410	3.78E-89	399.89
NK	*GCC2*	rs2139809	2	109045638	C	T	0.5916	0.5046	0.0444	7.23E-30	128.6
NK	*GCHFR*	rs72735627	15	41057507	T	C	0.0849	-1.0201	0.0717	5.91E-46	202.08
NK	*GDE1*	rs4780810	16	19497948	C	T	0.8460	-0.3524	0.0634	2.73E-08	30.83
NK	*GDI2*	rs3736461	10	5855403	C	G	0.1844	-0.4839	0.0547	9.65E-19	77.96
NK	*GEMIN7*	rs6509193	19	45600291	G	A	0.6391	-0.3564	0.0474	5.31E-14	56.49
NK	*GGA1*	rs35662099	22	38053909	T	C	0.4335	0.2519	0.0453	2.74E-08	30.82
NK	*GIMAP2*	rs60774275	7	150342420	T	C	0.1095	-0.4857	0.0706	6.02E-12	47.22
NK	*GIPC1*	rs116092141	19	14606710	G	C	0.3651	-0.3645	0.0457	1.51E-15	63.48
NK	*GLRX*	rs28926210	5	95153223	A	G	0.3703	-0.2613	0.0468	2.32E-08	31.14
NK	*GLTP*	rs4766642	12	110313754	C	T	0.5874	-0.2855	0.0459	4.77E-10	38.69
NK	*GLTPD1*	rs1810745	1	1257593	G	A	0.9375	-0.6201	0.1020	1.21E-09	36.87
NK	*GLTSCR2*	rs11083892	19	48256428	T	C	0.5591	-0.3735	0.0452	1.51E-16	68.01
NK	*GMIP*	rs45458192	19	19750822	T	C	0.3873	0.2789	0.0463	1.75E-09	36.16
NK	*GNB5*	rs7163597	15	52490075	C	T	0.8969	-0.4765	0.0837	1.22E-08	32.38
NK	*GNG5*	rs11163986	1	84973275	T	G	0.1230	0.3942	0.0707	2.50E-08	31
NK	*GNGT2*	rs4794029	17	47280301	C	T	0.6793	-0.3052	0.0481	2.22E-10	40.18
NK	*GNLY*	rs3755007	2	85920249	G	T	0.3609	-0.9391	0.0421	2.46E-110	497.03
NK	*GNMT*	rs6940814	6	42924932	G	A	0.5791	0.3616	0.0455	1.99E-15	62.94
NK	*GNPTG*	rs742460	16	1405537	T	C	0.2936	0.5255	0.0499	6.24E-26	110.66
NK	*GOLGA8A*	rs3866558	15	34859970	C	G	0.4062	0.2980	0.0454	5.13E-11	43.03
NK	*GOLGA8Q*	rs11857533	15	31472389	T	C	0.0629	-0.4995	0.0885	1.69E-08	31.75
NK	*GOLM1*	rs4333693	9	88692049	A	C	0.2471	0.3769	0.0525	7.02E-13	51.43
NK	*GPLD1*	rs6903070	6	25153166	T	A	0.9456	-0.9436	0.0988	1.33E-21	90.95
NK	*GPR56*	rs8054093	16	57687714	C	A	0.5890	0.3248	0.0450	5.54E-13	51.89
NK	*GRAMD1C*	rs7632377	3	113550915	T	C	0.7347	-0.5587	0.0508	4.06E-28	120.62
NK	*GRHPR*	rs4878690	9	37422656	T	C	0.3290	0.5250	0.0467	2.82E-29	125.9
NK	*GRIK4*	rs949055	11	120779520	T	C	0.6768	-0.9938	0.0412	7.73E-129	581.9
NK	*GS1-124K5.4*	rs1860471	7	66082946	C	T	0.7420	-0.3731	0.0509	2.24E-13	53.66
NK	*GS1-251I9.4*	rs1138489	8	92080575	A	G	0.6310	0.2601	0.0465	2.20E-08	31.24
NK	*GSAP*	rs1181898	7	76993577	T	C	0.5310	-0.6399	0.0431	8.47E-50	219.66
NK	*GSC*	rs10135136	14	95405708	A	G	0.2932	-0.3584	0.0496	5.21E-13	52.01
NK	*GSDMA*	rs72832957	17	38093085	G	A	0.1001	0.4360	0.0714	1.03E-09	37.19
NK	*GSTM1*	rs12046107	1	110164657	G	A	0.0705	-0.5658	0.1003	1.68E-08	31.76
NK	*GSTM3*	rs1332018	1	110282972	T	G	0.5781	0.4254	0.0448	2.31E-21	89.86
NK	*GSTM4*	rs1010167	1	110198727	G	C	0.4142	-0.6424	0.0436	3.73E-49	216.71
NK	*GSTO1*	rs10883990	10	106018558	G	A	0.3164	0.3186	0.0483	4.20E-11	43.43
NK	*GSTP1*	rs614080	11	67347287	A	G	0.4951	0.4410	0.0441	1.37E-23	99.99
NK	*GSTT1*	rs5760147	22	24334948	C	A	0.3844	-0.8159	0.0429	1.58E-80	360.38
NK	*GTF2H2*	rs28478097	5	70309998	G	C	0.6121	-0.6219	0.0442	5.37E-45	197.7
NK	*GTF2H4*	rs2517614	6	30163955	A	G	0.1860	-0.5205	0.0541	6.09E-22	92.5
NK	*GTPBP4*	rs3763720	10	1045099	T	C	0.0975	0.7242	0.0741	1.42E-22	95.37
NK	*GYPC*	rs12692112	2	127399116	T	C	0.0570	-0.5546	0.1008	3.71E-08	30.23
NK	*GZMH*	rs11158812	14	25083383	A	G	0.5178	0.3364	0.0445	4.25E-14	56.92
NK	*H2AFV*	rs12702091	7	44860763	A	C	0.4028	0.2644	0.0453	5.48E-09	33.94
NK	*H6PD*	rs61783460	1	9333128	A	C	0.1714	0.3839	0.0607	2.54E-10	39.91
NK	*HAGH*	rs238677	16	1883279	C	T	0.3287	0.4547	0.0488	1.18E-20	86.64
NK	*HBA1*	rs78360966	16	151262	T	C	0.0818	1.4752	0.0834	4.90E-70	312.3
NK	*HBA2*	rs78360966	16	151262	T	C	0.0818	1.3845	0.0843	1.15E-60	269.39
NK	*HBQ1*	rs111682761	16	178735	G	C	0.0904	0.8897	0.0839	2.88E-26	112.19
NK	*HCG17*	rs1264538	6	30389258	T	C	0.4307	-0.5687	0.0441	4.75E-38	165.94
NK	*HCG17*	rs545040429	6	31175667	G	A	0.2373	-0.3323	0.0548	1.29E-09	36.74
NK	*HCG27*	rs9357094	6	30167476	C	T	0.2158	-0.4129	0.0526	4.13E-15	61.5
NK	*HDDC2*	rs7752672	6	125587423	C	G	0.4843	-0.4439	0.0441	7.89E-24	101.09
NK	*HDHD3*	rs3750525	9	116139260	A	C	0.3309	-0.3672	0.0483	2.87E-14	57.7
NK	*HEBP2*	rs9484150	6	138699838	A	G	0.0719	-0.9988	0.0893	4.65E-29	124.91
NK	*HEG1*	rs4368548	3	124727778	T	C	0.6202	-0.4334	0.0458	3.17E-21	89.24
NK	*HELLS*	rs945123	10	96199921	T	C	0.3995	0.3223	0.0453	1.17E-12	50.42
NK	*HELZ2*	rs34830600	20	62197370	A	G	0.0819	0.5544	0.0904	8.46E-10	37.57
NK	*HES6*	rs12731	2	239087912	A	G	0.4488	-0.4476	0.0443	5.45E-24	101.82
NK	*HEXDC*	rs4074069	17	80377406	C	T	0.3134	0.4347	0.0476	6.49E-20	83.28
NK	*HHEX*	rs7918084	10	94429467	T	C	0.5614	0.4291	0.0443	3.74E-22	93.46
NK	*HIF1A-AS2*	rs10873150	14	62783552	T	C	0.1885	-0.4553	0.0562	5.41E-16	65.5
NK	*HIGD2A*	rs13182101	5	175828347	G	A	0.0905	-0.7386	0.0867	1.67E-17	72.34
NK	*HIP1*	rs112641413	7	75315474	T	C	0.0613	0.8669	0.0965	2.57E-19	80.57
NK	*HIST1H1B*	rs73403143	6	28726613	T	C	0.0678	0.5470	0.0963	1.33E-08	32.21
NK	*HIST1H2AD*	rs71559014	6	27122444	G	A	0.0589	-0.8783	0.0792	1.47E-28	122.64
NK	*HIST1H2AD*	rs198823	6	26122933	T	G	0.6326	-0.2591	0.0474	4.44E-08	29.88
NK	*HIST1H2AJ*	rs7775835	6	28678357	C	T	0.8747	-0.5825	0.0669	3.18E-18	75.61
NK	*HIST1H2BB*	rs13220495	6	26441640	T	C	0.0717	0.4247	0.0769	3.40E-08	30.4
NK	*HIST1H2BI*	rs67092078	6	27054772	A	G	0.0727	0.5408	0.0760	1.14E-12	50.47
NK	*HIST1H2BL*	rs1233579	6	28712663	G	A	0.0824	-0.6444	0.0737	2.19E-18	76.35
NK	*HIST1H2BO*	rs1233582	6	28714044	G	C	0.1443	0.6039	0.0578	1.59E-25	108.8
NK	*HIST1H3H*	rs11752919	6	28403603	C	T	0.3837	0.4301	0.0451	1.40E-21	90.85

NK	*HIST1H3I*	rs73403143	6	28726613	T	C	0.0678	-0.9371	0.0947	4.32E-23	97.73
NK	*HIST1H4B*	rs66757203	6	26454956	T	C	0.0829	0.5159	0.0720	7.79E-13	51.22
NK	*HIST1H4B*	rs11757088	6	25948458	C	T	0.1732	0.6111	0.0555	3.51E-28	120.91
NK	*HIST1H4I*	rs139757732	6	28084616	C	T	0.0339	0.7231	0.1021	1.41E-12	50.07
NK	*HIST1H4K*	rs7742957	6	28535685	T	C	0.2219	-0.3012	0.0530	1.33E-08	32.22
NK	*HLA-A*	rs9260043	6	29907740	G	A	0.3897	0.4665	0.0454	8.68E-25	105.45
NK	*HLA-B*	rs2524096	6	31236467	T	G	0.4422	0.8052	0.0417	2.93E-83	372.89
NK	*HLA-B*	rs9366777	6	31264715	A	T	0.0923	-0.5376	0.0828	8.21E-11	42.12
NK	*HLA-B*	rs2244020	6	31347451	G	A	0.4150	-0.4086	0.0465	1.56E-18	77.01
NK	*HLA-C*	rs17191797	6	31228540	G	T	0.1109	0.7117	0.0746	1.35E-21	90.93
NK	*HLA-DOA*	rs3129903	6	32310211	G	A	0.7837	0.3097	0.0522	3.06E-09	35.07
NK	*HLA-DPA1*	rs34159555	6	33078707	A	G	0.1177	-0.4655	0.0732	2.00E-10	40.38
NK	*HLA-DQA1*	rs41269945	6	32607853	T	A	0.0976	0.8237	0.0532	4.56E-54	239.19
NK	*HLA-DQA1*	rs77000541	6	32635605	A	G	0.2974	0.3228	0.0450	7.02E-13	51.43
NK	*HLA-DQA2*	rs9272461	6	32605609	A	G	0.1774	1.1114	0.0497	7.20E-111	499.48
NK	*HLA-DQA2*	rs77000541	6	32635605	A	G	0.2974	0.4730	0.0443	1.18E-26	113.94
NK	*HLA-DQB1*	rs9272426	6	32605189	G	A	0.3067	-0.4750	0.0442	6.14E-27	115.24
NK	*HLA-DRB1*	rs9270824	6	32570055	G	T	0.4762	-0.3195	0.0482	3.42E-11	43.83
NK	*HLA-DRB5*	rs9272070	6	32599485	G	T	0.5192	-0.4978	0.0437	5.06E-30	129.3
NK	*HLA-F*	rs7759272	6	29684994	C	T	0.1463	0.7310	0.0662	2.32E-28	121.73
NK	*HLA-G*	rs16895223	6	29626949	G	C	0.1439	-0.3590	0.0642	2.26E-08	31.19
NK	*HLA-G*	rs1061156	6	29913266	T	G	0.1564	0.7944	0.0590	2.49E-41	180.94
NK	*HLA-G*	rs2523990	6	30077229	G	A	0.5102	0.2462	0.0449	4.11E-08	30.03
NK	*HMBOX1*	rs10088428	8	28909523	T	C	0.2597	-0.8286	0.0485	2.22E-65	290.98
NK	*HMGN2*	rs1055323	1	26797654	T	C	0.2552	0.3002	0.0497	1.54E-09	36.41
NK	*HNRNPU*	rs9661182	1	245028509	T	G	0.2415	0.3016	0.0530	1.26E-08	32.33
NK	*HOPX*	rs4865137	4	57525669	C	T	0.4675	0.2971	0.0450	3.94E-11	43.55
NK	*HOXB-AS1*	rs2326013	17	46619102	A	C	0.4565	0.3224	0.0446	4.83E-13	52.16
NK	*HOXB2*	rs4793870	17	46581184	C	T	0.6285	0.5153	0.0457	1.91E-29	126.67
NK	*HPGD*	rs11133039	4	175397646	A	G	0.6200	-0.3138	0.0462	1.10E-11	46.05
NK	*HPGD*	rs45545934	4	175442061	C	T	0.5198	0.4819	0.0438	4.03E-28	120.63
NK	*HSBP1L1*	rs1864529	18	77748975	A	G	0.8400	-0.4054	0.0630	1.25E-10	41.29
NK	*HSD17B11*	rs57154655	4	88287167	T	C	0.1228	0.3878	0.0659	3.92E-09	34.59
NK	*HSD17B12*	rs1914773	11	43866294	A	T	0.6709	0.2727	0.0487	2.13E-08	31.31
NK	*HSP90B1*	rs1866070	12	104310556	T	C	0.3942	0.2524	0.0455	2.87E-08	30.73
NK	*HSPBAP1*	rs4678214	3	122502093	T	C	0.7460	-0.6306	0.0526	4.63E-33	143.17
NK	*IAH1*	rs12692386	2	9695906	G	A	0.6605	-0.2801	0.0476	3.83E-09	34.63
NK	*ICA1L*	rs10439267	2	203923447	T	C	0.4087	0.2762	0.0459	1.75E-09	36.16
NK	*IDI1*	rs11599238	10	1012487	G	A	0.0980	0.6643	0.0745	4.68E-19	79.39
NK	*IFI27L1*	rs941480	14	94571793	C	T	0.2307	0.3682	0.0519	1.33E-12	50.18
NK	*IFITM1*	rs10794314	11	350567	T	A	0.2924	0.3593	0.0487	1.62E-13	54.31
NK	*IFITM2*	rs1059091	11	309127	G	A	0.3507	0.6438	0.0461	2.77E-44	194.44
NK	*IFITM2*	rs11246108	11	350917	A	G	0.2912	0.6482	0.0471	3.56E-43	189.36
NK	*IFITM3*	rs34481144	11	320836	T	C	0.4497	-0.9138	0.0397	4.41E-117	527.97
NK	*IFITM3*	rs67463956	11	302619	C	G	0.1156	0.4354	0.0718	1.33E-09	36.7
NK	*IFITM3*	rs58474296	11	346381	T	C	0.4243	-0.3326	0.0450	1.41E-13	54.57
NK	*IGF2BP2*	rs6789741	3	185479733	T	G	0.2115	0.3203	0.0564	1.32E-08	32.23
NK	*IGFBP7*	rs781666	4	57830981	G	A	0.3559	-0.2614	0.0469	2.52E-08	30.98
NK	*IGFBP7*	rs4865182	4	57963611	A	G	0.1938	0.3941	0.0590	2.41E-11	44.51
NK	*IGFLR1*	rs112500848	19	36254427	T	C	0.1552	-0.5609	0.0633	7.67E-19	78.41
NK	*IGLL1*	rs6519462	22	23999138	T	C	0.1847	-0.9200	0.0555	8.12E-62	274.66
NK	*IKBKE*	rs28584674	1	206652524	C	T	0.0855	0.5569	0.0833	2.30E-11	44.6
NK	*IL10RB*	rs2834167	21	34640788	G	A	0.2649	0.2862	0.0511	2.12E-08	31.32
NK	*IL12RB1*	rs845381	19	18181779	A	G	0.1955	-0.3339	0.0551	1.33E-09	36.69
NK	*IL12RB2*	rs17129778	1	67787691	T	A	0.1646	0.4133	0.0592	2.97E-12	48.6
NK	*IL18R1*	rs4851566	2	102972799	C	G	0.3949	-0.3169	0.0463	7.58E-12	46.77
NK	*IL18RAP*	rs1420105	2	103035119	C	T	0.5085	-0.2860	0.0448	1.66E-10	40.74
NK	*IL27RA*	rs6511905	19	14142616	G	C	0.2530	-0.3491	0.0528	3.64E-11	43.7
NK	*IL32*	rs12448657	16	3114081	C	A	0.6476	0.4001	0.0461	3.89E-18	75.21
NK	*IL32*	rs2179016	16	3128428	G	C	0.6553	0.3016	0.0468	1.15E-10	41.45
NK	*ILKAP*	rs6760421	2	239073046	T	C	0.3182	0.2784	0.0470	3.13E-09	35.02
NK	*IMPDH1*	rs76089614	7	128091315	A	G	0.0589	0.6126	0.0973	3.09E-10	39.53
NK	*IP6K2*	rs12496747	3	48819376	T	G	0.0802	0.5747	0.0942	1.04E-09	37.17
NK	*IQCB1*	rs6438652	3	121480375	G	A	0.6099	0.2647	0.0466	1.30E-08	32.26
NK	*IQSEC3*	rs56204927	12	248202	G	C	0.6233	-0.5001	0.0447	4.57E-29	124.94
NK	*IRF1*	rs2269821	5	131758314	G	A	0.8737	0.4412	0.0671	4.85E-11	43.14
NK	*IRF6*	rs7545542	1	209979635	T	C	0.3847	0.4099	0.0453	1.40E-19	81.77
NK	*IRF7*	rs1807012	11	546377	G	A	0.3756	-0.2512	0.0458	4.10E-08	30.04
NK	*ISCA1*	rs791321	9	88953554	T	C	0.3169	0.2777	0.0470	3.51E-09	34.8
NK	*ITCH*	rs7271970	20	32987666	G	A	0.4921	-0.2983	0.0447	2.43E-11	44.49
NK	*ITCH-AS1*	rs72497006	20	32909056	A	G	0.3117	-0.3166	0.0489	9.86E-11	41.76
NK	*ITGB2*	rs760462	21	46328099	C	T	0.8298	-1.3735	0.0515	6.72E-157	710.64
NK	*ITGB2-AS1*	rs9983174	21	46349031	C	T	0.4645	0.2609	0.0450	6.90E-09	33.49
NK	*ITPA*	rs8362	20	3193978	A	G	0.3117	0.4555	0.0481	3.09E-21	89.29
NK	*ITPR3*	rs3916279	6	33574950	C	A	0.2472	-0.2993	0.0531	1.75E-08	31.68
NK	*IVNS1ABP*	rs10732976	1	185409061	A	G	0.7012	0.7871	0.0455	3.83E-67	299.06
NK	*JAKMIP1*	rs60046962	4	6179711	T	C	0.5199	-0.5376	0.0436	5.75E-35	151.87
NK	*JAKMIP2*	rs2436340	5	147162475	A	G	0.3562	0.2553	0.0464	3.71E-08	30.23
NK	*KANSL1-AS1*	rs1378358	17	44787312	T	C	0.1903	1.3105	0.0445	6.22E-191	866.84

NK	*KB-226F1.1*	rs5760147	22	24334948	C	A	0.3844	-0.3627	0.0462	4.23E-15	61.45
NK	*KCNAB2*	rs61760766	1	6064811	A	G	0.1363	0.5851	0.0621	4.44E-21	88.58
NK	*KCNK12*	rs879722	2	47741016	C	G	0.3843	0.2861	0.0465	7.85E-10	37.72
NK	*KCTD10*	rs56406401	12	109900538	C	T	0.0609	1.0732	0.0792	7.67E-42	183.27
NK	*KDELR2*	rs6796	7	6502367	C	T	0.2687	-0.7303	0.0469	1.07E-54	242.06
NK	*KDSR*	rs402348	18	61034420	G	T	0.2202	0.3411	0.0555	7.67E-10	37.76
NK	*KHK*	rs4665943	2	27295885	T	C	0.3518	0.2577	0.0468	3.59E-08	30.29
NK	*KIAA0040*	rs2269650	1	175129691	T	G	0.3691	0.3522	0.0466	4.27E-14	56.92
NK	*KIAA1377*	rs1441928	11	101790671	C	T	0.4986	-0.2450	0.0449	4.81E-08	29.73
NK	*KIAA1671*	rs6004417	22	25464310	G	A	0.2211	0.3472	0.0534	7.74E-11	42.23
NK	*KIAA1683*	rs2384989	19	18379549	A	G	0.2969	0.2913	0.0502	6.69E-09	33.55
NK	*KIF16B*	rs6135798	20	16545359	A	T	0.4442	0.3655	0.0450	4.40E-16	65.9
NK	*KIR2DL3*	rs2296369	19	55224544	A	C	0.1171	0.4671	0.0755	6.20E-10	38.17
NK	*KIR3DL1*	rs56162245	19	55379326	T	A	0.3111	-0.5209	0.0477	9.35E-28	118.97
NK	*KIR3DL2*	rs4487030	19	55376569	A	G	0.5771	0.3823	0.0447	1.15E-17	73.08
NK	*KIR3DL2*	rs1433097	19	55418458	C	T	0.1831	0.3504	0.0614	1.14E-08	32.52
NK	*KIR3DX1*	rs8106451	19	55040648	A	G	0.4113	0.2502	0.0457	4.27E-08	29.96
NK	*KLB*	rs80150520	4	39539245	T	G	0.1974	-0.5248	0.0541	3.05E-22	93.87
NK	*KLF7*	rs2037893	2	208031796	G	C	0.6536	0.2722	0.0468	6.21E-09	33.69
NK	*KLHDC4*	rs2290019	16	87741662	A	C	0.2032	-0.5453	0.0559	1.83E-22	94.88
NK	*KLHL25*	rs11637216	15	86205957	G	T	0.2118	-0.3621	0.0562	1.21E-10	41.36
NK	*KLRC1*	rs78547237	12	10594848	A	C	0.8225	-0.8280	0.0585	2.03E-45	199.63
NK	*KLRC2*	rs12318583	12	10579084	G	A	0.1994	-0.7028	0.0549	1.66E-37	163.46
NK	*KLRC2*	rs10845128	12	10585083	T	C	0.8144	-0.5143	0.0596	5.95E-18	74.38
NK	*KLRC3*	rs11053802	12	10597207	T	C	0.6503	-0.3591	0.0474	3.58E-14	57.27
NK	*KLRC4*	rs10772275	12	10563592	C	T	0.4399	-0.3164	0.0448	1.68E-12	49.71
NK	*KLRD1*	rs12813197	12	10471317	G	C	0.6551	-0.5298	0.0467	8.25E-30	128.33
NK	*KLRK1*	rs17549004	12	10562623	A	G	0.1824	-0.3662	0.0574	1.82E-10	40.57
NK	*KRT10*	rs3169914	17	39032395	T	C	0.1731	-0.3950	0.0632	4.20E-10	38.93
NK	*KRT72*	rs672275	12	53025733	G	A	0.2685	-0.4072	0.0506	8.71E-16	64.56
NK	*KRT73*	rs11170181	12	52978365	A	T	0.1210	0.3807	0.0685	2.72E-08	30.83
NK	*KTN1*	rs17684916	14	56113917	C	A	0.3441	-0.3734	0.0468	1.57E-15	63.41
NK	*KYNU*	rs2381356	2	143686832	C	A	0.0727	-0.6106	0.0944	1.01E-10	41.71
NK	*L3MBTL3*	rs7740107	6	130374461	A	T	0.7512	-0.3752	0.0496	4.01E-14	57.04
NK	*LA16c-358B7.3*	rs11647760	16	1367610	A	C	0.6104	-0.5963	0.0439	5.18E-42	184.05
NK	*LAIR1*	rs74463408	19	54871664	C	G	0.1326	-1.0555	0.0666	1.69E-56	250.31
NK	*LAIR2*	rs73070113	19	55014172	T	A	0.1204	-0.8179	0.0655	7.96E-36	155.79
NK	*LAIR2*	rs8106451	19	55040648	A	G	0.4113	0.2754	0.0456	1.54E-09	36.41
NK	*LAMB1*	rs7786987	7	107562657	G	A	0.3905	-0.5677	0.0439	3.37E-38	166.63
NK	*LAPTM5*	rs35351292	1	31208042	T	C	0.2608	-0.2796	0.0511	4.48E-08	29.86
NK	*LAT*	rs11645302	16	28951457	T	C	0.1794	-0.4195	0.0564	1.02E-13	55.21
NK	*LAX1*	rs7547110	1	203742253	C	G	0.4253	-0.3660	0.0453	6.30E-16	65.2
NK	*LBX2*	rs1721245	2	74130806	C	T	0.7967	0.3430	0.0535	1.50E-10	40.94
NK	*LCNL1*	rs7040970	9	139859013	C	T	0.5087	-0.2607	0.0448	6.17E-09	33.71
NK	*LCP1*	rs7323698	13	46702602	A	G	0.9323	-0.5331	0.0875	1.12E-09	37.02
NK	*LDHC*	rs56151802	11	18439567	G	A	0.1178	0.7266	0.0689	4.94E-26	111.12
NK	*LEPROT*	rs10493377	1	65879252	G	A	0.4570	0.4055	0.0444	6.81E-20	83.19
NK	*LGALS9B*	rs4470200	17	20403618	A	T	0.4494	-0.6203	0.0431	5.46E-47	206.81
NK	*LGR6*	rs10920352	1	202148042	C	T	0.4324	-0.2639	0.0456	6.90E-09	33.49
NK	*LILRB4*	rs62123425	19	55400292	A	G	0.1748	0.3861	0.0571	1.32E-11	45.68
NK	*LIMD1*	rs2673023	3	45711367	C	T	0.7714	-0.4506	0.0507	5.86E-19	78.94
NK	*LIN52*	rs11622838	14	74669359	G	A	0.6619	-0.2676	0.0484	3.34E-08	30.43
NK	*LINC00035*	rs4717114	7	73285093	G	C	0.5548	0.2726	0.0454	1.86E-09	36.04
NK	*LINC00299*	rs13412757	2	8458080	A	G	0.3290	-0.2952	0.0471	3.61E-10	39.23
NK	*LINC00339*	rs4654783	1	22439520	C	T	0.7035	0.5532	0.0484	2.75E-30	130.51
NK	*LINC00565*	rs6560935	13	114945867	A	G	0.5439	-0.4405	0.0442	2.13E-23	99.12
NK	*LINC00665*	rs2972527	19	36746225	A	C	0.4761	0.2628	0.0450	5.15E-09	34.06
NK	*LINC00861*	rs16900994	8	126958296	A	G	0.1494	-0.3506	0.0637	3.65E-08	30.26
NK	*LINC00886*	rs9818684	3	156524220	T	G	0.5631	0.3377	0.0451	6.55E-14	56.08
NK	*LINC00920*	rs2289149	16	66442406	T	C	0.6726	-0.6988	0.0440	7.07E-57	252.05
NK	*LINC00944*	rs1706506	12	127256415	T	C	0.9383	-0.5733	0.1028	2.42E-08	31.05
NK	*LINC00954*	rs13034115	2	20077653	G	C	0.1396	-0.4644	0.0625	1.10E-13	55.06
NK	*LINC00969*	rs3827561	3	195674257	G	C	0.4166	0.5431	0.0444	2.32E-34	149.1
NK	*LINC01137*	rs11264060	1	37944506	A	G	0.3371	-0.2725	0.0481	1.44E-08	32.06
NK	*LINGO2*	rs7026138	9	29295342	C	T	0.2619	0.3115	0.0506	7.53E-10	37.8
NK	*LIPC*	rs7161799	15	58770523	T	C	0.0869	0.5725	0.0795	5.85E-13	51.78
NK	*LL22NC03-2H8.5*	rs5757641	22	22598882	G	A	0.3591	0.3179	0.0462	6.11E-12	47.19
NK	*LLGL1*	rs8076336	17	18212614	T	G	0.4069	0.2613	0.0452	7.36E-09	33.37
NK	*LONP2*	rs8053839	16	48390512	T	G	0.5150	0.2836	0.0449	2.64E-10	39.84
NK	*LPIN1*	rs3762583	2	11924293	A	G	0.1508	-0.6331	0.0642	6.34E-23	96.97
NK	*LRRC37A2*	rs77875796	17	44051612	G	A	0.2016	0.3419	0.0536	1.83E-10	40.55
NK	*LRRC6*	rs4081781	8	133694084	A	G	0.4955	0.5372	0.0435	5.17E-35	152.07
NK	*LRRC61*	rs1962004	7	150020381	A	G	0.2428	0.3148	0.0519	1.28E-09	36.76
NK	*LRRFIP1*	rs6730431	2	238608399	A	G	0.2219	-0.3649	0.0530	5.95E-12	47.24
NK	*LRRIQ3*	rs11806946	1	74663709	A	G	0.4704	-0.3297	0.0446	1.40E-13	54.59
NK	*LSM7*	rs62119754	19	2356277	G	A	0.5987	0.4038	0.0453	5.18E-19	79.19
NK	*LSS*	rs2839183	21	47687144	A	G	0.3937	0.2603	0.0458	1.35E-08	32.19
NK	*LTA4H*	rs6538697	12	96403610	C	T	0.0943	0.4495	0.0814	3.34E-08	30.43
NK	*LTBP1*	rs12617290	2	32341408	G	A	0.4144	-0.5335	0.0444	2.74E-33	144.2

NK	*LY6E*	rs12548954	8	144077243	T	A	0.4908	-0.3588	0.0445	7.49E-16	64.86
NK	*LY6G5B*	rs4959027	6	32383050	G	A	0.8145	-0.3235	0.0535	1.43E-09	36.55
NK	*LY6G6F*	rs2253051	6	31462002	C	G	0.1345	0.5873	0.0623	4.21E-21	88.68
NK	*LYAR*	rs11723339	4	4271886	T	C	0.6169	-0.7028	0.0439	9.69E-58	256
NK	*LYRM4*	rs111557465	6	5260694	T	C	0.0736	0.6336	0.0807	4.27E-15	61.44
NK	*LYST*	rs2891663	1	236034274	C	T	0.7649	-0.2862	0.0509	1.91E-08	31.51
NK	*MAD1L1*	rs34021847	7	2037761	A	G	0.3445	0.3581	0.0467	1.79E-14	58.63
NK	*MAF1*	rs7830598	8	145180327	G	A	0.0941	-0.4249	0.0756	1.91E-08	31.52
NK	*MAFF*	rs132972	22	38562056	C	T	0.5445	0.2474	0.0449	3.56E-08	30.31
NK	*MAGEF1*	rs13068715	3	184424614	G	A	0.7367	-0.3022	0.0511	3.34E-09	34.9
NK	*MAP3K5*	rs9321570	6	137095679	C	G	0.6144	0.3020	0.0457	4.02E-11	43.51
NK	*MAP6D1*	rs166398	3	183446977	A	G	0.4363	0.3078	0.0450	7.87E-12	46.7
NK	*MARCH10*	rs17683483	17	60802483	A	C	0.1090	0.4201	0.0691	1.24E-09	36.83
NK	*MARCH2*	rs890849	19	8478250	C	G	0.1809	0.3569	0.0593	1.74E-09	36.16
NK	*MAST1*	rs62109295	19	13708257	T	C	0.6522	-0.3853	0.0473	3.91E-16	66.14
NK	*MAST3*	rs10409167	19	18275532	A	G	0.5211	-0.4417	0.0442	1.46E-23	99.87
NK	*MAST4*	rs1697142	5	66495847	C	T	0.4091	-0.3058	0.0456	1.91E-11	44.96
NK	*MBP*	rs4890887	18	74779614	A	C	0.3929	0.5407	0.0446	8.41E-34	146.55
NK	*MBP*	rs470640	18	74720775	G	A	0.3978	0.2712	0.0458	3.26E-09	34.95
NK	*MC1R*	rs11645970	16	89958118	A	G	0.0905	0.9487	0.0737	6.88E-38	165.21
NK	*MCF2L2*	rs6777106	3	183146433	G	C	0.4735	-0.3600	0.0445	6.17E-16	65.24
NK	*MDGA1*	rs9349050	6	37663624	C	T	0.5194	-0.6306	0.0428	4.17E-49	216.49
NK	*MDM4*	rs6681905	1	204535789	G	T	0.6799	-0.2977	0.0480	5.68E-10	38.35
NK	*MEAF6*	rs11586555	1	38031798	G	A	0.0676	-0.5342	0.0824	8.87E-11	41.97
NK	*MED16*	rs2930893	19	882724	C	G	0.2624	0.8232	0.0485	9.83E-65	288.02
NK	*MEI1*	rs739129	22	42118249	A	G	0.8076	-0.3205	0.0573	2.16E-08	31.27
NK	*MESP1*	rs2165072	15	90302629	G	A	0.2484	0.3376	0.0508	3.12E-11	44.01
NK	*METTL18*	rs12144122	1	169851365	G	A	0.0894	-0.4580	0.0796	8.86E-09	33
NK	*MFF*	rs13416290	2	228231295	A	C	0.2122	-0.3317	0.0569	5.43E-09	33.95
NK	*MGME1*	rs11551768	20	17950545	T	A	0.1239	-0.5114	0.0674	3.24E-14	57.46
NK	*MGMT*	rs10741191	10	131307276	G	C	0.5810	0.5598	0.0439	3.39E-37	162.04
NK	*MGST2*	rs8192024	4	140592641	A	C	0.3241	-0.3429	0.0486	1.69E-12	49.71
NK	*MGST3*	rs4147596	1	165601365	C	T	0.4777	-0.5735	0.0432	3.86E-40	175.5
NK	*MIAT*	rs6005127	22	27069557	T	C	0.2598	0.6573	0.0509	4.00E-38	166.29
NK	*MICAL3*	rs58845524	22	18484060	G	A	0.5273	-0.2859	0.0447	1.63E-10	40.78
NK	*MICB*	rs13214865	6	30995158	T	C	0.0497	-0.5369	0.0928	7.23E-09	33.4
NK	*MIF*	rs5751775	22	24266726	C	T	0.5903	-0.5631	0.0446	1.80E-36	158.74
NK	*MIF*	rs1007889	22	24239243	G	T	0.3315	-0.2786	0.0478	5.64E-09	33.88
NK	*MIR181A2HG*	rs4076333	9	127385609	C	T	0.5288	-0.3181	0.0446	1.00E-12	50.73
NK	*MIR4435-1HG*	rs143087699	2	112187041	G	A	0.7228	-0.4538	0.0495	5.21E-20	83.72
NK	*MKRN1*	rs7777768	7	140182268	T	C	0.1572	-0.3837	0.0638	1.82E-09	36.08
NK	*MLC1*	rs137919	22	50499964	C	T	0.2909	-0.2924	0.0484	1.51E-09	36.44
NK	*MLF1*	rs12633687	3	158338168	T	G	0.1668	0.4763	0.0622	1.89E-14	58.52
NK	*MLF2*	rs2302366	12	6862259	C	G	0.3476	-0.2955	0.0466	2.25E-10	40.15
NK	*MLKL*	rs9932977	16	74735148	A	G	0.5689	0.2532	0.0452	2.19E-08	31.25
NK	*MMAB*	rs7974321	12	109990369	C	T	0.1912	-0.3321	0.0570	5.62E-09	33.89
NK	*MMEL1*	rs4648663	1	2573727	C	A	0.8592	-0.4402	0.0599	2.02E-13	53.87
NK	*MPHOSPH6*	rs2303260	16	82203786	G	C	0.4801	-0.5149	0.0436	4.00E-32	138.89
NK	*MPHOSPH9*	rs28472704	12	123887369	T	C	0.2109	0.4214	0.0575	2.42E-13	53.51
NK	*MRPL10*	rs17774171	17	45907599	G	A	0.3879	-0.5652	0.0442	2.18E-37	162.92
NK	*MRPL14*	rs4714751	6	44101544	T	C	0.5150	-0.2593	0.0448	7.37E-09	33.36
NK	*MRPL18*	rs9365097	6	160211259	A	G	0.7577	-0.2919	0.0526	2.80E-08	30.77
NK	*MRPL21*	rs1272609	11	68618182	C	T	0.2913	-0.4103	0.0486	3.07E-17	71.14
NK	*MRPL32*	rs612464	7	42956347	A	C	0.8580	-0.5397	0.0649	9.26E-17	68.97
NK	*MRPL34*	rs35116124	19	17413160	T	C	0.3919	0.4290	0.0446	6.29E-22	92.43
NK	*MRPL41*	rs2254149	9	140441177	G	A	0.4605	0.5469	0.0435	2.64E-36	157.97
NK	*MRPL54*	rs12459781	19	3762119	C	T	0.6394	0.5458	0.0461	2.59E-32	139.75
NK	*MRPS10*	rs10948028	6	42183867	T	G	0.6244	0.3147	0.0467	1.60E-11	45.31
NK	*MRPS18C*	rs6535473	4	84364808	C	T	0.4873	0.2864	0.0447	1.53E-10	40.91
NK	*MRPS6*	rs11088269	21	35383937	G	A	0.1199	0.4498	0.0721	4.41E-10	38.84
NK	*MSH3*	rs2431221	5	79934230	T	G	0.3764	-0.2928	0.0462	2.43E-10	40
NK	*MT1E*	rs12600038	16	56648626	A	G	0.3288	-0.3354	0.0473	1.28E-12	50.25
NK	*MT1F*	rs1875231	16	56683745	C	A	0.3943	0.3677	0.0451	3.33E-16	66.46
NK	*MT1X*	rs1599933	16	56715862	A	C	0.5528	-0.2536	0.0451	1.85E-08	31.58
NK	*MT2A*	rs1580833	16	56644650	A	C	0.3093	-0.9514	0.0435	5.40E-106	477.12
NK	*MTSS1*	rs34281960	8	125766752	C	G	0.2997	0.3604	0.0477	4.01E-14	57.04
NK	*MTSS1*	rs13282164	8	125649900	T	C	0.1308	-0.4497	0.0696	1.02E-10	41.69
NK	*MTUS1*	rs11785687	8	17687842	A	G	0.0762	-0.6491	0.0826	4.04E-15	61.55
NK	*MUC20*	rs2688493	3	195431255	G	A	0.8571	-0.3573	0.0628	1.30E-08	32.26
NK	*MVP*	rs11863734	16	29848946	G	A	0.1427	-0.3644	0.0627	6.34E-09	33.66
NK	*MX1*	rs456018	21	42793724	G	A	0.4144	-0.3316	0.0452	2.29E-13	53.62
NK	*MX2*	rs398800	21	42742212	A	C	0.6882	-0.3751	0.0485	1.06E-14	59.65
NK	*MXRA7*	rs9303003	17	74705876	C	A	0.9133	-0.5216	0.0853	9.47E-10	37.35
NK	*MYCBPAP*	rs73351675	17	48585554	C	T	0.4566	0.3607	0.0448	8.31E-16	64.66
NK	*MYH11*	rs13330959	16	15846698	T	C	0.3780	0.3741	0.0455	2.12E-16	67.35
NK	*MYL12A*	rs12606063	18	3249866	G	C	0.1420	-0.7807	0.0652	5.24E-33	142.92
NK	*MYO15B*	rs76124218	17	73584441	C	G	0.0623	0.5930	0.0990	2.10E-09	35.8
NK	*MYO1F*	rs4804311	19	8615589	G	A	0.0908	0.7536	0.0833	1.49E-19	81.64
NK	*MYO1G*	rs6946581	7	44998714	A	G	0.6454	-0.2952	0.0477	6.15E-10	38.19

NK	*MYOM2*	rs62480001	8	2082015	C	G	0.3417	1.2262	0.0381	1.00E-200	1034.23
NK	*MYRF*	rs520987	11	61521446	A	C	0.4986	0.3298	0.0446	1.42E-13	54.56
NK	*MZT2A*	rs3817572	2	132256505	C	A	0.3209	0.8700	0.0461	2.15E-79	355.18
NK	*NAA38*	rs7796145	7	117834763	T	G	0.6872	-0.4931	0.0466	3.69E-26	111.7
NK	*NAA40*	rs2508584	11	63757446	C	T	0.4251	0.2471	0.0450	4.09E-08	30.04
NK	*NAAA*	rs7661762	4	76829939	T	A	0.3083	0.2748	0.0477	8.46E-09	33.09
NK	*NAAA*	rs6532084	4	76929593	G	A	0.0817	-0.9106	0.0838	1.64E-27	117.86
NK	*NAPG*	rs649777	18	10525518	C	G	0.4604	-0.2732	0.0449	1.19E-09	36.91
NK	*NAPRT1*	rs9657360	8	144676862	T	C	0.7776	0.8469	0.0521	2.18E-59	263.54
NK	*NBEAL2*	rs6763236	3	47094611	T	C	0.5716	0.4338	0.0448	3.40E-22	93.65
NK	*NBPF1*	rs10492988	1	16826296	G	C	0.3053	0.2658	0.0476	2.31E-08	31.15
NK	*NBPF3*	rs113548640	1	21766776	C	G	0.2133	0.4462	0.0513	3.40E-18	75.48
NK	*NCAPG2*	rs73165322	7	158453165	A	G	0.0610	0.6286	0.0859	2.57E-13	53.39
NK	*NCL*	rs4973396	2	232291320	G	A	0.2661	-0.4857	0.0534	9.21E-20	82.59
NK	*NCOA7*	rs490361	6	126153253	C	T	0.3009	0.3619	0.0487	1.02E-13	55.22
NK	*NCR3*	rs986475	6	31556709	G	A	0.0891	-0.8811	0.0856	7.22E-25	105.81
NK	*NDUFA10*	rs10166776	2	240967312	A	G	0.3472	-0.2991	0.0470	1.92E-10	40.46
NK	*NDUFA12*	rs835044	12	95397552	C	T	0.6738	-0.7645	0.0440	1.21E-67	301.35
NK	*NDUFA3*	rs254262	19	54612628	G	A	0.7510	-0.4950	0.0503	7.53E-23	96.63
NK	*NDUFA6*	rs7292416	22	42807216	C	G	0.0459	0.5998	0.1015	3.46E-09	34.83
NK	*NDUFB2*	rs2968555	7	140379261	G	A	0.1666	-0.4317	0.0645	2.21E-11	44.68
NK	*NDUFC2*	rs7115272	11	77829178	C	T	0.1647	-0.3707	0.0628	3.67E-09	34.72
NK	*NDUFS5*	rs12043492	1	39457006	T	C	0.4366	-0.6849	0.0428	1.11E-57	255.73
NK	*NDUFS8*	rs4147779	11	67804807	A	G	0.7515	0.5006	0.0509	8.56E-23	96.37
NK	*NEAT1*	rs512715	11	65191208	G	C	0.7296	0.3340	0.0505	3.65E-11	43.7
NK	*NELFCD*	rs236729	20	57579975	C	T	0.1832	-0.3250	0.0578	1.87E-08	31.56
NK	*NEXN*	rs7536433	1	78023173	T	C	0.2169	-0.3724	0.0549	1.14E-11	45.98
NK	*NFE2L3*	rs10270428	7	25238174	G	A	0.4378	-0.5464	0.0436	5.41E-36	156.55
NK	*NGRN*	rs112396280	15	91713611	A	G	0.3125	-0.2723	0.0492	3.05E-08	30.61
NK	*NINJ1*	rs13299270	9	95887108	T	C	0.1572	-0.5565	0.0629	8.60E-19	78.19
NK	*NIPAL2*	rs10105510	8	99259641	A	C	0.3002	0.2688	0.0491	4.47E-08	29.87
NK	*NKAPL*	rs6933672	6	28941688	A	G	0.1154	-0.4132	0.0707	5.11E-09	34.07
NK	*NLRP2*	rs269913	19	55481669	C	T	0.4883	0.3349	0.0446	5.59E-14	56.39
NK	*NME1*	rs16949649	17	49230308	C	T	0.3855	0.3899	0.0455	9.77E-18	73.4
NK	*NME4*	rs77371232	16	447561	T	A	0.4244	0.7799	0.0419	3.32E-77	345.15
NK	*NMI*	rs3854012	2	152137106	T	C	0.4433	0.4038	0.0444	9.24E-20	82.59
NK	*NMRAL1*	rs2918601	16	4441032	G	T	0.0476	-0.5829	0.1012	8.36E-09	33.12
NK	*NMRAL1*	rs11639998	16	4527109	C	T	0.7379	-0.2953	0.0502	4.04E-09	34.53
NK	*NMRK1*	rs11144225	9	77707300	T	A	0.3900	-0.6307	0.0434	6.37E-48	211.07
NK	*NMUR1*	rs4973442	2	232389301	C	T	0.3000	-0.3882	0.0491	2.80E-15	62.27
NK	*NOP10*	rs12593384	15	34634626	G	A	0.1581	0.3531	0.0610	6.92E-09	33.49
NK	*NPRL3*	rs111682761	16	178735	G	C	0.0904	0.6799	0.0849	1.19E-15	63.95
NK	*NQO2*	rs138616686	6	3003970	G	A	0.2788	-0.6636	0.0480	2.12E-43	190.4
NK	*NQO2*	rs1040861	6	3023956	A	C	0.5547	-0.2737	0.0456	1.96E-09	35.94
NK	*NRROS*	rs939874	3	196366964	A	G	0.3954	-0.4224	0.0462	6.14E-20	83.39
NK	*NSA2*	rs6883278	5	74070053	A	T	0.1393	0.8539	0.0632	1.24E-41	182.32
NK	*NSG1*	rs55731118	4	4350915	T	A	0.4050	0.7848	0.0424	2.32E-76	341.28
NK	*NSG1*	rs7442201	4	4361749	G	A	0.7285	0.3061	0.0494	5.67E-10	38.35
NK	*NSUN4*	rs6662982	1	46877180	A	G	0.2858	0.3545	0.0489	4.24E-13	52.42
NK	*NSUN5*	rs10231271	7	72364533	A	G	0.5099	0.2718	0.0448	1.27E-09	36.78
NK	*NT5C*	rs729405	17	73185657	G	A	0.6754	-0.2990	0.0494	1.42E-09	36.56
NK	*NT5C3B*	rs8073219	17	39977734	G	A	0.7569	-0.9100	0.0472	9.10E-83	370.64
NK	*NTPCR*	rs9424513	1	233074916	C	T	0.2251	0.4761	0.0524	9.99E-20	82.43
NK	*NUB1*	rs406624	7	151058623	T	A	0.7168	0.2893	0.0504	9.32E-09	32.91
NK	*NUCB2*	rs214068	11	17305650	C	T	0.6047	-0.6831	0.0430	6.00E-57	252.37
NK	*NUDT14*	rs11625862	14	105644382	A	G	0.6039	0.4606	0.0450	1.45E-24	104.44
NK	*NUDT2*	rs3892022	9	34378177	T	C	0.1492	0.7217	0.0614	6.62E-32	137.89
NK	*NUP107*	rs2259592	12	69082669	G	A	0.3509	-0.3016	0.0471	1.49E-10	40.95
NK	*NUTF2*	rs7184978	16	67736840	T	A	0.0494	-0.7593	0.1008	5.05E-14	56.59
NK	*NXPE3*	rs3796278	3	101498077	G	A	0.0695	-0.5666	0.0887	1.72E-10	40.68
NK	*OAT*	rs4144117	10	126107506	C	A	0.8231	0.3401	0.0572	2.77E-09	35.27
NK	*ORAI1*	rs7398511	12	122072829	A	C	0.1691	0.3336	0.0594	2.00E-08	31.42
NK	*ORAI2*	rs12669274	7	102076562	A	G	0.5236	0.3914	0.0443	1.03E-18	77.84
NK	*ORMDL3*	rs8074437	17	38076137	G	T	0.4865	0.8021	0.0412	1.39E-84	378.96
NK	*OSBPL5*	rs1562355	11	3163396	G	T	0.6150	0.3951	0.0456	4.27E-18	75.03
NK	*OSTF1*	rs10869496	9	77700084	A	C	0.3854	-0.4199	0.0449	8.71E-21	87.25
NK	*OSTF1*	rs773023	9	77768894	A	G	0.2882	-0.4160	0.0505	1.65E-16	67.83
NK	*OVCH1-AS1*	rs10492377	12	29620447	G	A	0.1207	-0.5795	0.0691	4.98E-17	70.19
NK	*OXNAD1*	rs842255	3	16301564	A	G	0.5743	-0.4513	0.0445	3.43E-24	102.73
NK	*P2RY11*	rs2305790	19	10218408	A	G	0.2467	0.3227	0.0501	1.20E-10	41.37
NK	*P4HA3*	rs1783538	11	73969750	G	A	0.4794	-0.7449	0.0420	2.73E-70	313.47
NK	*PABPC1*	rs73282499	8	101822215	A	G	0.1612	0.5756	0.0593	2.70E-22	94.11
NK	*PADI4*	rs6695214	1	17719843	C	T	0.6275	-0.4457	0.0470	2.26E-21	89.91
NK	*PAK1*	rs2154754	11	77178698	A	G	0.3232	0.2653	0.0476	2.53E-08	30.97
NK	*PAPD4*	rs62365175	5	78926076	A	G	0.0641	0.4886	0.0885	3.36E-08	30.42
NK	*PARK7*	rs400736	1	8078309	C	T	0.5952	0.3637	0.0448	5.05E-16	65.64
NK	*PARP14*	rs4678206	3	122396567	G	C	0.1042	-0.5699	0.0856	2.76E-11	44.24
NK	*PARP8*	rs11960066	5	49921295	G	A	0.3636	-0.2872	0.0456	2.99E-10	39.59
NK	*PARVB*	rs6006622	22	44424108	T	C	0.6099	-0.3816	0.0452	3.04E-17	71.16

NK	*PAX8*	rs35129549	2	114002779	T	A	0.2900	0.3875	0.0505	1.73E-14	58.7
NK	*PCBP2*	rs11170517	12	53753909	T	C	0.1317	-0.3933	0.0660	2.50E-09	35.46
NK	*PCNT*	rs9306156	21	47960493	C	T	0.3298	0.2941	0.0474	5.71E-10	38.34
NK	*PCNX*	rs36567	14	71350617	G	C	0.6941	0.3612	0.0495	2.87E-13	53.18
NK	*PCSK5*	rs12347453	9	78724005	T	G	0.4866	0.4173	0.0442	3.55E-21	89.02
NK	*PCSK6*	rs3784481	15	101932164	A	G	0.6188	0.2757	0.0463	2.55E-09	35.42
NK	*PCSK7*	rs2277287	11	117096652	A	G	0.2154	0.3579	0.0542	3.99E-11	43.52
NK	*PDCD5*	rs34040260	19	33074614	C	A	0.3641	0.3027	0.0458	3.91E-11	43.56
NK	*PDE4B*	rs571146	1	66753955	T	C	0.3446	0.4578	0.0459	1.92E-23	99.33
NK	*PDLIM1*	rs35540768	10	97054903	T	G	0.0487	0.6334	0.0932	1.05E-11	46.13
NK	*PDS5B*	rs9315172	13	33208172	T	C	0.4053	-0.2620	0.0453	7.36E-09	33.37
NK	*PDXDC1*	rs4985124	16	15125441	G	T	0.2984	0.3092	0.0486	2.03E-10	40.35
NK	*PDXK*	rs2276528	21	45152500	A	G	0.1480	0.5083	0.0617	1.80E-16	67.66
NK	*PEX6*	rs9462858	6	42946490	A	C	0.4402	0.3458	0.0447	1.02E-14	59.72
NK	*PGLS*	rs10416082	19	17630461	A	G	0.5440	0.4025	0.0445	1.55E-19	81.56
NK	*PHPT1*	rs872463	9	139744807	G	A	0.7418	-0.3853	0.0507	2.96E-14	57.64
NK	*PI4K2B*	rs60527077	4	25161539	A	G	0.0922	-0.5260	0.0754	3.04E-12	48.56
NK	*PIF1*	rs832881	15	65149682	T	C	0.4624	0.3569	0.0445	9.90E-16	64.31
NK	*PIGC*	rs1023479	1	172355132	A	G	0.8165	0.3239	0.0593	4.78E-08	29.74
NK	*PIGP*	rs7280542	21	38444995	G	A	0.6584	-0.3270	0.0469	3.24E-12	48.43
NK	*PILRB*	rs11771936	7	99943283	G	A	0.1794	1.1976	0.0496	7.63E-129	581.92
NK	*PIP4K2A*	rs8341	10	22825133	C	T	0.6507	0.2849	0.0462	7.01E-10	37.94
NK	*PLCL1*	rs1541953	2	198898344	T	A	0.4991	0.3299	0.0446	1.34E-13	54.67
NK	*PLEK*	rs10496164	2	68610120	G	A	0.2875	0.4158	0.0505	1.92E-16	67.54
NK	*PLGRKT*	rs2381277	9	5381870	G	A	0.6665	-0.3094	0.0465	2.95E-11	44.11
NK	*PLOD1*	rs58495963	1	12012980	A	G	0.3336	-0.6452	0.0456	1.58E-45	200.12
NK	*PLTP*	rs441346	20	44539492	C	G	0.5569	-0.5932	0.0433	9.17E-43	187.49
NK	*PLXDC1*	rs36179135	17	37249939	A	G	0.2889	0.8564	0.0446	4.45E-82	367.48
NK	*PLXND1*	rs6786987	3	129311568	A	T	0.7492	0.3213	0.0528	1.12E-09	37.03
NK	*PNPLA2*	rs1138714	11	825110	G	A	0.5328	0.2523	0.0454	2.69E-08	30.85
NK	*PNRC2*	rs6424144	1	24375663	A	G	0.4721	-0.5703	0.0433	1.07E-39	173.47
NK	*POLM*	rs28745596	7	44111189	C	T	0.0912	0.4402	0.0785	2.08E-08	31.35
NK	*POLR1D*	rs10492487	13	28199368	A	T	0.1219	0.5078	0.0657	1.05E-14	59.68
NK	*POLR2I*	rs757223	19	36574798	T	C	0.5733	-0.3590	0.0447	9.39E-16	64.41
NK	*POLR2J3*	rs2537466	7	102394415	T	G	0.1468	0.4099	0.0612	2.06E-11	44.81
NK	*POMC*	rs3754861	2	25393722	C	A	0.0783	0.6528	0.0885	1.59E-13	54.34
NK	*POMZP3*	rs146568439	7	76214017	C	A	0.2111	0.4468	0.0542	1.59E-16	67.91
NK	*POU5F1*	rs2844747	6	30339593	C	T	0.6047	0.2522	0.0452	2.34E-08	31.12
NK	*POU5F1*	rs2524054	6	31252396	C	A	0.7710	-0.6250	0.0481	1.21E-38	168.65
NK	*POU5F1*	rs9265745	6	31302718	G	A	0.3927	0.3736	0.0451	1.18E-16	68.5
NK	*PPA1*	rs7077538	10	71993094	G	T	0.3217	0.6441	0.0456	3.29E-45	198.67
NK	*PPDPF*	rs72629024	20	62152519	G	C	0.1633	-1.0057	0.0584	2.08E-66	295.68
NK	*PPFIBP2*	rs4758197	11	7561609	A	G	0.6092	0.3415	0.0461	1.32E-13	54.7
NK	*PPIA*	rs4724322	7	44822490	T	C	0.1484	0.5042	0.0726	3.87E-12	48.08
NK	*PPIE*	rs12094291	1	40226046	A	G	0.3493	-0.4059	0.0461	1.39E-18	77.24
NK	*PPIL3*	rs2136600	2	201742345	T	C	0.2391	-0.6490	0.0521	1.22E-35	154.94
NK	*PPM1M*	rs610060	3	52273421	A	G	0.4932	0.3071	0.0447	6.36E-12	47.11
NK	*PPP1R9A*	rs4727302	7	94523663	A	G	0.6091	-0.4727	0.0448	4.55E-26	111.28
NK	*PPP5C*	rs62136099	19	46847901	T	C	0.2759	-0.5760	0.0471	2.37E-34	149.06
NK	*PQLC3*	rs3911110	2	11318477	T	C	0.8816	0.5635	0.0670	4.19E-17	70.53
NK	*PRDM11*	rs11601593	11	44505619	T	C	0.4854	0.5920	0.0431	7.37E-43	187.92
NK	*PRDX2*	rs897804	19	12876964	C	G	0.3850	0.5996	0.0441	4.01E-42	184.56
NK	*PRDX3*	rs11198811	10	120937283	C	G	0.3330	0.2737	0.0482	1.32E-08	32.24
NK	*PRDX5*	rs11231746	11	64070940	C	T	0.3003	-0.7286	0.0450	7.02E-59	261.22
NK	*PRELID1*	rs7707346	5	176544299	G	C	0.1951	-0.5066	0.0571	7.53E-19	78.45
NK	*PRKCB*	rs114827361	16	24045354	G	A	0.0539	0.7941	0.1001	2.20E-15	62.74
NK	*PRKCB*	rs780169	16	23754561	G	A	0.2582	0.2957	0.0505	4.80E-09	34.19
NK	*PRMT2*	rs2839301	21	47948427	G	C	0.3110	1.1832	0.0389	1.00E-200	924.37
NK	*PRMT2*	rs7278089	21	47955501	A	T	0.7488	-0.4621	0.0518	4.53E-19	79.45
NK	*PRR7-AS1*	rs335466	5	176889530	A	C	0.5187	0.6610	0.0429	1.16E-53	237.33
NK	*PRSS57*	rs10412005	19	690639	T	C	0.5855	0.6671	0.0436	6.35E-53	233.95
NK	*PRX*	rs79521384	19	40876864	T	C	0.0662	0.7481	0.0845	8.41E-19	78.23
NK	*PSD4*	rs2241976	2	113943470	G	A	0.4506	0.3451	0.0451	1.91E-14	58.5
NK	*PSMB3*	rs11653487	17	36942631	T	C	0.5262	-0.3303	0.0449	1.93E-13	53.95
NK	*PSMB9*	rs17587	6	32825090	A	G	0.2619	0.3837	0.0503	2.46E-14	58
NK	*PSMD5-AS1*	rs34025993	9	123516572	G	A	0.5819	-1.1429	0.0366	1.00E-200	974.59
NK	*PSPH*	rs10899768	7	55824205	G	A	0.2112	0.4120	0.0601	7.03E-12	46.92
NK	*PSTPIP1*	rs33931897	15	77357312	C	G	0.3861	-0.4057	0.0458	7.87E-19	78.36
NK	*PTGDR*	rs10149899	14	52624207	T	C	0.3065	-0.5870	0.0481	3.24E-34	148.43
NK	*PTGDS*	rs2271869	9	139848273	G	A	0.4508	0.5744	0.0436	1.23E-39	173.19
NK	*PTGER4*	rs9292776	5	40437845	A	G	0.4579	-0.3307	0.0446	1.21E-13	54.88
NK	*PWWP2A*	rs1106055	5	159491520	G	A	0.5964	-0.3182	0.0457	3.42E-12	48.33
NK	*PXK*	rs11714515	3	58410431	G	A	0.4480	-0.2685	0.0449	2.26E-09	35.66
NK	*PYCR2*	rs77157663	1	226111945	C	T	0.0538	0.5696	0.0954	2.35E-09	35.58
NK	*PYHIN1*	rs2325704	1	158909164	A	C	0.7626	0.6086	0.0505	2.04E-33	144.79
NK	*PYROXD2*	rs10883090	10	100167236	C	G	0.2603	-0.2949	0.0504	4.92E-09	34.15
NK	*QDPR*	rs10020773	4	17526682	C	A	0.2377	-0.4545	0.0521	2.79E-18	75.87
NK	*R3HDM4*	rs1683588	19	870489	G	A	0.2519	0.5910	0.0508	2.71E-31	135.1
NK	*RAB11A*	rs12911521	15	66148594	C	G	0.0860	0.4836	0.0738	5.61E-11	42.86

NK	RAB24	rs72813176	5	176709333	A	G	0.0799	0.4629	0.0749	6.48E-10	38.09
NK	RAB38	rs302644	11	87913471	T	G	0.3121	0.3317	0.0488	1.05E-11	46.13
NK	RAC1	rs836549	7	6439945	G	A	0.4921	0.6670	0.0425	1.59E-55	245.86
NK	RAD51C	rs34426768	17	56683385	T	C	0.1961	0.5477	0.0535	1.36E-24	104.57
NK	RAI14	rs10060556	5	34531099	T	C	0.1423	-0.5432	0.0643	2.92E-17	71.25
NK	RAMP1	rs7578855	2	238801297	T	C	0.6222	-0.6059	0.0444	1.73E-42	186.23
NK	RAMP1	rs34934971	2	238811515	T	A	0.0919	-0.4825	0.0802	1.78E-09	36.12
NK	RAP1GAP2	rs35193075	17	2938715	G	C	0.1281	0.4777	0.0684	2.91E-12	48.64
NK	RASA3	rs56781357	13	114922114	A	G	0.2037	0.4799	0.0548	1.88E-18	76.65
NK	RBAK-RBAKDN	rs77684954	7	5101858	A	G	0.0555	-0.5030	0.0866	6.36E-09	33.65
NK	RBL2	rs9932007	16	53456661	A	G	0.4740	-0.3202	0.0447	7.89E-13	51.2
NK	RBM43	rs55775354	2	152118271	A	G	0.4344	-0.5739	0.0433	5.12E-40	174.93
NK	RBM6	rs7642934	3	50174848	A	G	0.5011	0.3527	0.0445	2.43E-15	62.54
NK	RBPMS2	rs584422	15	64768156	A	G	0.9356	-1.0891	0.1002	1.54E-27	117.97
NK	RCN1	rs4922580	11	32143936	C	G	0.5242	-0.4119	0.0443	1.33E-20	86.41
NK	RCSD1	rs4656550	1	167670439	T	C	0.2853	0.3623	0.0479	4.00E-14	57.05
NK	REEP4	rs12678092	8	21994951	T	C	0.1734	0.5237	0.0588	5.39E-19	79.11
NK	RFC4	rs62294446	3	186498536	C	G	0.2570	-0.3113	0.0521	2.29E-09	35.63
NK	RFTN1	rs76204094	3	16635851	G	A	0.1398	0.3882	0.0652	2.61E-09	35.38
NK	RGS10	rs1556591	10	121275903	T	C	0.7811	0.4600	0.0538	1.15E-17	73.07
NK	RGS17	rs596359	6	153457053	T	C	0.5188	-0.4960	0.0438	1.05E-29	127.86
NK	RHOC	rs139444463	1	112980131	A	G	0.0405	0.9045	0.0992	7.73E-20	82.94
NK	RIOK3	rs36091929	18	21003267	G	C	0.5046	0.3390	0.0445	2.69E-14	57.83
NK	RMDN3	rs10744948	15	40961189	C	T	0.8270	0.4908	0.0615	1.47E-15	63.53
NK	RNASEH2C	rs11227250	11	65438345	T	C	0.3441	-0.4779	0.0459	2.44E-25	107.96
NK	RNASET2	rs453184	6	167387404	C	A	0.5062	-0.5866	0.0432	6.68E-42	183.55
NK	RNF166	rs12148926	16	88779323	T	C	0.2769	0.3636	0.0506	7.04E-13	51.42
NK	RNF167	rs11654312	17	4817602	G	C	0.0596	-0.5333	0.0923	7.56E-09	33.31
NK	RNF168	rs111360550	3	196201495	G	T	0.1627	-0.3505	0.0561	4.20E-10	38.93
NK	RNF19A	rs10107200	8	101452935	T	C	0.7404	-0.3055	0.0537	1.29E-08	32.28
NK	RORB	rs10781209	9	76878353	G	A	0.6481	0.3639	0.0471	1.17E-14	59.46
NK	RP1-168L15.5	rs2179177	6	166806793	T	C	0.6915	0.2722	0.0487	2.33E-08	31.13
NK	RP1-265C24.8	rs9257182	6	28754817	A	C	0.1587	0.3540	0.0568	4.60E-10	38.76
NK	RP1-29C18.10	rs5770622	22	50051548	G	T	0.6547	-0.8540	0.0443	5.63E-83	371.59
NK	RP1-30M3.5	rs34991172	6	25480328	G	T	0.0543	-0.6488	0.0818	2.20E-15	62.74
NK	RP1-3J17.3	rs2842614	6	86306069	T	G	0.3868	0.7595	0.0432	2.52E-69	309.04
NK	RP1-3J17.3	rs3812139	6	86196990	G	C	0.1043	0.5359	0.0723	1.26E-13	54.8
NK	RP1-40E16.12	rs7739320	6	3054146	T	C	0.5459	-0.2615	0.0453	8.13E-09	33.17
NK	RP1-8B1.4	rs10807334	6	46071116	A	G	0.5150	0.7841	0.0414	6.40E-80	357.59
NK	RP11-1000B6.3	rs4779556	15	32173245	C	T	0.9495	0.8017	0.1006	1.60E-15	63.37
NK	RP11-1055B8.4	rs8079005	17	79342071	T	C	0.3558	-0.3156	0.0466	1.25E-11	45.79
NK	RP11-1055B8.7	rs112895628	17	79356092	C	G	0.1892	-0.6173	0.0545	8.77E-30	128.21
NK	RP11-108M9.4	rs279028	1	16790901	T	C	0.6340	-0.3508	0.0469	7.05E-14	55.93
NK	RP11-108M9.4	rs28711566	1	17215933	T	C	0.0493	-0.5032	0.0910	3.24E-08	30.49
NK	RP11-119F19.2	rs2983793	10	81445802	G	A	0.4028	-0.3236	0.0461	2.21E-12	49.19
NK	RP11-121A8.1	rs2735161	7	38374309	C	T	0.3133	-0.3338	0.0483	4.86E-12	47.64
NK	RP11-137H2.4	rs873257	10	82280140	T	C	0.4706	0.6816	0.0425	5.26E-58	257.21
NK	RP11-138A9.2	rs205724	7	130623257	C	A	0.8419	0.3539	0.0604	4.54E-09	34.3
NK	RP11-157J24.2	rs4959141	6	1543080	T	A	0.4042	-0.3302	0.0449	1.86E-13	54.03
NK	RP11-166B2.5	rs33621	16	12008513	A	G	0.3222	-0.3162	0.0475	2.70E-11	44.29
NK	RP11-245G13.2	rs7604967	2	11020995	T	C	0.1179	1.3010	0.0658	6.83E-87	389.54
NK	RP11-254F7.2	rs75817661	2	10181354	C	T	0.1130	0.3915	0.0671	5.38E-09	33.97
NK	RP11-261C10.3	rs10803122	1	243348854	T	A	0.4735	-0.2928	0.0447	5.97E-11	42.74
NK	RP11-266L9.2	rs12930390	16	25854365	A	C	0.1469	-0.5285	0.0602	1.72E-18	76.82
NK	RP11-273G15.2	rs1130985	8	144099971	C	G	0.3306	-0.5880	0.0461	3.51E-37	161.98
NK	RP11-294J22.6	rs72839214	17	48751721	T	C	0.1214	-0.4051	0.0657	7.15E-10	37.9
NK	RP11-298J20.3	rs10901842	10	126635262	G	A	0.7352	-1.0452	0.0459	1.00E-114	517.17
NK	RP11-299J3.8	rs4585137	3	122226160	G	C	0.1423	0.3685	0.0644	1.07E-08	32.65
NK	RP11-305L7.1	rs7046632	9	93919341	T	C	0.2002	-0.4794	0.0544	1.31E-18	77.36
NK	RP11-305L7.3	rs10991784	9	93917094	C	T	0.2013	-0.5133	0.0544	3.70E-21	88.94
NK	RP11-306I1.2	rs72693852	1	165539843	T	C	0.0926	-0.5631	0.0730	1.25E-14	59.33
NK	RP11-308N19.1	rs34575265	9	108943801	T	C	0.2912	-0.4041	0.0511	2.63E-15	62.39
NK	RP11-324I22.4	rs10827503	10	35724454	A	G	0.4894	0.2595	0.0448	7.09E-09	33.44
NK	RP11-326C3.11	rs11246065	11	324170	G	A	0.3701	0.8618	0.0428	5.11E-90	403.87
NK	RP11-326C3.11	rs9666761	11	330061	G	A	0.6599	-0.3245	0.0487	2.65E-11	44.32
NK	RP11-326C3.15	rs6421983	11	320394	T	C	0.4462	0.4114	0.0442	1.35E-20	86.38
NK	RP11-326C3.15	rs12281495	11	348460	C	T	0.4394	-0.2493	0.0453	3.67E-08	30.25
NK	RP11-345J4.5	rs144208959	16	30323380	A	G	0.4341	-0.5476	0.0434	1.92E-36	158.61
NK	RP11-347P5.1	rs1519600	2	197026716	C	T	0.6361	0.6901	0.0442	7.54E-55	242.77
NK	RP11-369E15.3	rs12681667	8	20894827	G	C	0.1394	0.5455	0.0641	1.79E-17	72.2
NK	RP11-395B7.4	rs7778998	7	100718747	C	T	0.5371	0.2723	0.0449	1.36E-09	36.65
NK	RP11-403P17.3	rs61066285	16	66631542	G	A	0.1266	0.4207	0.0762	3.35E-08	30.43
NK	RP11-413H22.2	rs4784664	16	56447286	C	T	0.5275	0.3036	0.0449	1.33E-11	45.67
NK	RP11-426C22.5	rs12932306	16	29148307	T	G	0.3029	-0.2745	0.0480	1.09E-08	32.6
NK	RP11-428G5.5	rs1797791	12	32044587	A	T	0.5372	0.2476	0.0449	3.49E-08	30.35
NK	RP11-430H10.1	rs12418562	11	45373480	C	G	0.1739	0.3900	0.0582	2.06E-11	44.81
NK	RP11-434C1.1	rs3911234	12	11349964	A	G	0.6020	-0.3761	0.0451	7.85E-17	69.3
NK	RP11-448A19.1	rs34498715	7	129246036	A	T	0.3952	0.3640	0.0455	1.20E-15	63.93
NK	RP11-473I1.5	rs12918271	16	9200547	C	T	0.2190	-0.6682	0.0531	2.78E-36	157.87

NK	*RP11-473M20.7*	rs9924885	16	3107921	T	G	0.1573	0.3605	0.0576	3.86E-10	39.1
NK	*RP11-473M20.9*	rs4786384	16	3136919	C	T	0.5202	0.5153	0.0437	4.08E-32	138.85
NK	*RP11-483I13.5*	rs663045	1	108743059	C	G	0.5428	0.2462	0.0451	4.83E-08	29.72
NK	*RP11-485G4.2*	rs73236059	3	141877407	G	T	0.1916	0.3873	0.0555	2.88E-12	48.66
NK	*RP11-485G4.2*	rs4386518	3	141991267	C	G	0.5519	0.2680	0.0449	2.32E-09	35.61
NK	*RP11-48B3.3*	rs78808634	8	81970067	A	G	0.1067	-0.6318	0.0762	1.15E-16	68.54
NK	*RP11-493L12.4*	rs855162	12	47643682	T	C	0.3004	0.3292	0.0470	2.39E-12	49.03
NK	*RP11-509J21.2*	rs1548299	9	3650174	C	A	0.6580	0.3661	0.0471	7.48E-15	60.34
NK	*RP11-514O12.4*	rs1044059	6	167369897	A	C	0.4957	-0.3409	0.0446	2.20E-14	58.22
NK	*RP11-539G18.2*	rs1396952	4	40056859	C	A	0.6712	-0.7332	0.0437	4.60E-63	280.37
NK	*RP11-539L10.2*	rs3822262	4	6697766	G	T	0.2437	0.4715	0.0522	1.66E-19	81.43
NK	*RP11-539L10.3*	rs4689500	4	6665938	A	G	0.1454	-0.5044	0.0640	3.32E-15	61.94
NK	*RP11-596D21.1*	rs10872851	14	31781575	C	T	0.3934	-0.5112	0.0448	3.88E-30	129.83
NK	*RP11-611D20.2*	rs3013306	9	139440845	A	G	0.6188	-0.3075	0.0467	4.44E-11	43.32
NK	*RP11-63P12.6*	rs34965542	9	74920447	A	G	0.0796	-0.4573	0.0819	2.30E-08	31.15
NK	*RP11-656D10.3*	rs11208254	1	40959506	T	G	0.3269	-0.3784	0.0480	3.30E-15	61.94
NK	*RP11-660L16.2*	rs11606612	11	71164047	A	C	0.6901	0.5963	0.0536	9.80E-29	123.43
NK	*RP11-669E14.6*	rs2957297	17	44368212	A	C	0.1694	-0.4468	0.0567	3.26E-15	61.97
NK	*RP11-69E11.4*	rs3820618	1	40223128	C	T	0.1312	0.7801	0.0661	4.01E-32	138.89
NK	*RP11-791G15.2*	rs1198843	2	10907647	A	G	0.3792	0.3048	0.0468	7.45E-11	42.31
NK	*RP11-792A8.4*	rs62468711	7	65836474	T	C	0.0465	0.8123	0.0981	1.23E-16	68.42
NK	*RP11-81H14.2*	rs731938	12	68843191	G	A	0.6401	1.1136	0.0372	7.63E-197	893.97
NK	*RP11-841O20.2*	rs72684280	14	53155585	A	C	0.1157	0.4275	0.0667	1.47E-10	40.98
NK	*RP11-894P9.1*	rs35140615	14	104150352	A	G	0.2069	-0.3296	0.0546	1.61E-09	36.31
NK	*RP11-932O9.9*	rs8028128	15	30973176	T	C	0.0671	0.5155	0.0857	1.82E-09	36.08
NK	*RP11-950C14.3*	rs449131	14	75589878	T	C	0.4527	0.4766	0.0439	1.72E-27	117.76
NK	*RP11-973H7.1*	rs2542157	18	12787247	G	T	0.4055	-0.4607	0.0450	1.36E-24	104.56
NK	*RP11-973H7.4*	rs2542152	18	12782761	C	T	0.4054	-0.3705	0.0455	3.64E-16	66.28
NK	*RP13-582O9.5*	rs113864337	8	144344261	T	G	0.2868	0.2852	0.0496	8.91E-09	32.99
NK	*RP3-325F22.5*	rs9376205	6	136899087	A	T	0.1247	-0.4903	0.0678	4.84E-13	52.16
NK	*RP5-1021I20.1*	rs12890741	14	74261657	C	G	0.1401	0.5167	0.0592	2.53E-18	76.06
NK	*RP5-821D11.7*	rs2413660	22	42247360	A	G	0.4269	-0.2901	0.0451	1.30E-10	41.22
NK	*RP5-827C21.4*	rs10910423	1	234517594	G	A	0.4593	0.3546	0.0447	2.22E-15	62.72
NK	*RP6-109B7.3*	rs6519999	22	46444452	A	T	0.0971	0.4512	0.0820	3.71E-08	30.23
NK	*RPA2*	rs9701457	1	28216736	T	C	0.3744	-0.3524	0.0459	1.66E-14	58.78
NK	*RPL10A*	rs2076172	6	35436925	T	C	0.5358	-0.6129	0.0435	3.75E-45	198.4
NK	*RPL11*	rs77766201	1	24006446	A	G	0.1560	-0.8238	0.0607	5.23E-42	184.03
NK	*RPL12*	rs2247781	9	130208924	C	T	0.5921	0.9091	0.0405	2.25E-111	501.79
NK	*RPL13*	rs12932337	16	89630630	T	C	0.1753	-0.6122	0.0549	6.79E-29	124.16
NK	*RPL13A*	rs60365978	19	49987811	T	C	0.1154	-0.6183	0.0753	2.22E-16	67.25
NK	*RPL14*	rs2371183	3	40334247	C	T	0.3380	-0.4026	0.0471	1.29E-17	72.86
NK	*RPL22L1*	rs35192046	3	170584064	C	A	0.2423	-0.4992	0.0499	1.36E-23	100
NK	*RPL23A*	rs2286518	17	26889996	C	G	0.0812	-0.6212	0.0782	1.97E-15	62.96
NK	*RPL27A*	rs61875926	11	8705528	G	C	0.0958	-0.4491	0.0741	1.38E-09	36.62
NK	*RPL28*	rs17700376	19	55897327	G	A	0.3547	-1.2245	0.0364	1.00E-200	1126.53
NK	*RPL3*	rs5750772	22	39602968	T	C	0.6105	-0.2863	0.0455	3.13E-10	39.51
NK	*RPL32*	rs2290311	3	12883071	A	T	0.1438	0.4247	0.0617	5.67E-12	47.34
NK	*RPL36AL*	rs2147712	14	50091684	A	G	0.7504	0.9328	0.0483	5.45E-83	371.66
NK	*RPL8*	rs2954658	8	146017782	T	C	0.4990	-0.8904	0.0401	2.81E-109	492.18
NK	*RPL9*	rs13103023	4	39457617	A	G	0.3195	0.4152	0.0472	1.47E-18	77.14
NK	*RPPH1*	rs56343177	14	20836173	G	C	0.1344	0.4111	0.0652	2.96E-10	39.61
NK	*RPS10*	rs9380433	6	34377626	T	C	0.7750	-1.0133	0.0537	1.56E-79	355.82
NK	*RPS11*	rs10403394	19	49999345	A	G	0.1225	-0.4315	0.0719	1.96E-09	35.93
NK	*RPS12*	rs78340398	6	133210387	T	C	0.0588	0.5560	0.0952	5.17E-09	34.05
NK	*RPS14*	rs2245384	5	149817268	C	G	0.3033	-0.2910	0.0488	2.46E-09	35.49
NK	*RPS16*	rs484379	19	39925054	G	T	0.3787	0.5558	0.0459	1.02E-33	146.17
NK	*RPS18*	rs17215231	6	33239869	T	C	0.0640	-1.5600	0.0826	1.62E-79	355.74
NK	*RPS2*	rs17135718	16	2014834	G	A	0.1151	0.6282	0.0668	5.39E-21	88.2
NK	*RPS23*	rs226205	5	81593654	T	C	0.7767	0.4260	0.0510	6.69E-17	69.61
NK	*RPS24*	rs2493568	10	79772854	T	C	0.5769	0.3712	0.0457	4.29E-16	65.96
NK	*RPS25*	rs111804650	11	118878960	T	C	0.0870	-0.9900	0.0757	4.00E-39	170.85
NK	*RPS26*	rs773114	12	56379060	T	A	0.3997	1.2083	0.0350	1.00E-200	1191.11
NK	*RPS3*	rs3094304	11	75118158	G	A	0.0727	-0.8476	0.0899	4.07E-21	88.75
NK	*RPS3A*	rs61442068	4	152104206	T	C	0.0618	-1.0745	0.0897	4.85E-33	143.07
NK	*RPS5*	rs731835	19	58898436	C	T	0.3256	-0.9648	0.0425	2.65E-114	515.22
NK	*RPS6KL1*	rs7146041	14	75395429	A	G	0.3591	0.3341	0.0465	6.88E-13	51.47
NK	*RPS7*	rs62106040	2	3627302	T	A	0.0757	-0.8756	0.0877	1.72E-23	99.54
NK	*RPS8*	rs12120833	1	45241285	G	C	0.1242	-0.9477	0.0594	2.14E-57	254.42
NK	*RPS9*	rs34629529	19	54701846	A	G	0.3950	-0.9047	0.0409	3.02E-108	487.45
NK	*RPSA*	rs60217375	3	39440578	A	T	0.1555	0.6544	0.0602	1.74E-27	117.74
NK	*RPTOR*	rs9319610	17	78653614	A	C	0.4626	0.2862	0.0449	1.90E-10	40.48
NK	*RRAS2*	rs7952194	11	14429088	T	A	0.2630	-0.4084	0.0518	3.02E-15	62.12
NK	*RRP7A*	rs136988	22	42946371	T	C	0.1733	0.4288	0.0574	7.96E-14	55.7
NK	*RSRC2*	rs7314638	12	122983460	G	A	0.7506	-0.5012	0.0498	7.99E-24	101.06
NK	*RTDR1*	rs73152631	22	23542099	A	G	0.2577	-0.7717	0.0477	9.35E-59	260.65
NK	*RTN4*	rs2588512	2	55192082	G	A	0.2018	0.3593	0.0568	2.59E-10	39.87
NK	*RXRA*	rs1536474	9	137298096	G	T	0.7362	-0.3329	0.0515	1.00E-10	41.73
NK	*S100A10*	rs2999528	1	151952885	T	C	0.5753	-0.3588	0.0448	1.23E-15	63.88
NK	*S100A13*	rs999418	1	153600124	C	T	0.5289	-0.2832	0.0448	2.49E-10	39.96

NK	*S100B*	rs9306156	21	47960493	C	T	0.3298	1.1730	0.0385	1.00E-200	925.39
NK	*S1PR5*	rs28580742	19	10635196	C	T	0.3591	0.3194	0.0473	1.49E-11	45.45
NK	*SACM1L*	rs2742433	3	45738107	T	C	0.3692	0.7720	0.0444	1.07E-67	301.6
NK	*SAMD12*	rs2514594	8	119625149	A	G	0.9156	-0.6938	0.0777	4.27E-19	79.57
NK	*SAMD3*	rs41285310	6	130505374	C	A	0.5086	-0.2597	0.0448	7.01E-09	33.46
NK	*SAMHD1*	rs6513797	20	35567722	C	G	0.1742	0.5335	0.0600	5.92E-19	78.93
NK	*SAMSN1*	rs1882917	21	15930957	G	T	0.5232	0.3214	0.0446	5.77E-13	51.81
NK	*SAR1A*	rs2310991	10	71931646	A	C	0.4271	-0.5399	0.0443	3.36E-34	148.37
NK	*SAT2*	rs858527	17	7498191	T	C	0.4006	0.3372	0.0458	1.76E-13	54.14
NK	*SBF1*	rs76275199	22	50883660	A	G	0.0744	0.4738	0.0819	7.24E-09	33.4
NK	*SCAMP3*	rs11264352	1	155255837	C	T	0.2693	0.2864	0.0493	6.16E-09	33.71
NK	*SCAND1*	rs6060699	20	34523833	T	C	0.1124	-0.4910	0.0772	1.98E-10	40.4
NK	*SCD5*	rs4484290	4	83755424	T	C	0.7772	-0.5067	0.0539	5.08E-21	88.31
NK	*SCRN2*	rs2325752	17	45921906	C	T	0.5233	-0.3451	0.0447	1.16E-14	59.47
NK	*SDSL*	rs12820985	12	113796856	C	A	0.0744	0.7675	0.0870	1.09E-18	77.73
NK	*SEC61B*	rs79754161	9	101985668	T	C	0.0730	-0.8557	0.0778	3.94E-28	120.68
NK	*SELL*	rs7553	1	169660002	T	G	0.3967	-0.2559	0.0460	2.61E-08	30.91
NK	*SEMA4D*	rs62557302	9	92135501	T	C	0.1657	-0.3465	0.0544	1.89E-10	40.49
NK	*SEPT2*	rs2074771	2	242350944	G	A	0.0944	0.7461	0.0736	3.98E-24	102.44
NK	*SERAC1*	rs13206168	6	158599688	A	G	0.4240	0.2965	0.0450	4.37E-11	43.35
NK	*SERPINB6*	rs72838699	6	2937490	G	A	0.2043	-0.4430	0.0541	2.65E-16	66.91
NK	*SERPINB6*	rs13210939	6	2942519	A	G	0.3376	0.4294	0.0458	6.97E-21	87.69
NK	*SERPINB9*	rs318448	6	2931354	A	C	0.6751	-0.3239	0.0473	7.49E-12	46.8
NK	*SERPINE2*	rs12476888	2	224893921	T	C	0.5488	-0.4358	0.0444	1.02E-22	96.03
NK	*SETD5-AS1*	rs2596913	3	9436472	C	T	0.5965	0.3307	0.0457	4.69E-13	52.22
NK	*SETD9*	rs7702800	5	56271610	T	C	0.7821	0.4096	0.0563	3.49E-13	52.8
NK	*SF1*	rs684720	11	64548429	G	C	0.1072	0.5030	0.0771	6.94E-11	42.44
NK	*SH3BP5*	rs9831807	3	15342850	C	T	0.3460	0.3002	0.0471	1.78E-10	40.61
NK	*SH3YL1*	rs7595075	2	264019	A	C	0.3283	-0.3133	0.0468	2.12E-11	44.76
NK	*SHFM1*	rs12671864	7	96269575	G	C	0.1705	0.3506	0.0613	1.08E-08	32.63
NK	*SHMT1*	rs4925179	17	18253029	T	A	0.6504	0.2883	0.0467	6.73E-10	38.01
NK	*SIDT1-AS1*	rs35452897	3	113282075	T	C	0.2149	0.7172	0.0522	6.31E-43	188.23
NK	*SKAP2*	rs73069539	7	26903661	T	C	0.2198	0.5308	0.0515	6.40E-25	106.05
NK	*SLC12A5*	rs2274755	20	44639692	T	G	0.1493	-0.4531	0.0608	9.58E-14	55.33
NK	*SLC12A7*	rs35188965	5	1104938	T	C	0.5560	-0.7068	0.0429	4.77E-61	271.14
NK	*SLC14A1*	rs16978452	18	43267071	A	G	0.1767	-0.4585	0.0577	2.04E-15	62.89
NK	*SLC15A4*	rs36076444	12	129237297	G	A	0.0835	-0.4175	0.0765	4.82E-08	29.72
NK	*SLC15A4*	rs4760588	12	129280712	A	G	0.6968	-0.4635	0.0491	3.56E-21	89.01
NK	*SLC16A4*	rs2279722	1	110919903	C	T	0.4361	0.3334	0.0446	7.77E-14	55.74
NK	*SLC1A7*	rs58140496	1	53479298	A	G	0.0794	-0.5612	0.0901	4.75E-10	38.69
NK	*SLC1A7*	rs899976	1	53593813	A	G	0.2350	0.2967	0.0518	9.92E-09	32.79
NK	*SLC22A18*	rs450244	11	2940492	C	T	0.9113	-0.5148	0.0784	5.04E-11	43.07
NK	*SLC22A5*	rs4705938	5	131694077	C	T	0.4554	0.4598	0.0440	1.59E-25	108.81
NK	*SLC25A1*	rs2854643	22	19189706	C	T	0.6537	-0.5383	0.0455	2.69E-32	139.68
NK	*SLC25A24*	rs61799377	1	108741774	A	T	0.2365	0.3700	0.0515	6.51E-13	51.58
NK	*SLC25A26*	rs142408865	3	66287334	C	A	0.3156	-0.3323	0.0486	7.99E-12	46.67
NK	*SLC25A29*	rs10134767	14	100763954	T	G	0.3371	0.9667	0.0420	6.01E-117	527.35
NK	*SLC25A3*	rs28372744	12	98987445	A	G	0.0487	0.6469	0.0979	3.95E-11	43.54
NK	*SLC25A51*	rs2005084	9	37884237	G	A	0.1507	0.3818	0.0637	2.11E-09	35.79
NK	*SLC27A1*	rs4808656	19	17604731	A	G	0.8640	0.5202	0.0710	2.39E-13	53.54
NK	*SLC44A2*	rs78671428	19	10707400	T	G	0.0789	-0.5427	0.0844	1.28E-10	41.24
NK	*SLC44A4*	rs2253487	6	31249127	A	G	0.4049	-0.2834	0.0457	5.40E-10	38.44
NK	*SLC4A7*	rs13100751	3	27454964	G	A	0.2136	-0.2997	0.0523	9.99E-09	32.77
NK	*SLC7A7*	rs1242735	14	23196040	T	A	0.8474	0.3798	0.0613	5.88E-10	38.28
NK	*SLFN12*	rs1838150	17	33760211	T	C	0.9009	-0.4880	0.0837	5.49E-09	33.94
NK	*SLFN5*	rs11656388	17	33531815	A	G	0.3965	0.4235	0.0453	9.80E-21	87.01
NK	*SMAP1*	rs781977	6	71373224	G	A	0.4816	0.2848	0.0448	1.98E-10	40.4
NK	*SMDT1*	rs133377	22	42466950	T	C	0.6612	0.9296	0.0433	2.18E-102	460.59
NK	*SMIM19*	rs6474401	8	42367068	C	T	0.3727	0.4234	0.0453	8.66E-21	87.26
NK	*SMIM20*	rs2305670	4	25915858	A	C	0.2457	-0.2932	0.0505	6.20E-09	33.7
NK	*SMOX*	rs6139427	20	4432150	A	G	0.0576	-0.5079	0.0864	4.18E-09	34.47
NK	*SMPD3*	rs7190307	16	68218336	A	G	0.4954	-0.2604	0.0450	7.00E-09	33.46
NK	*SMPDL3B*	rs6564	1	28212975	T	C	0.3803	0.5878	0.0445	6.83E-40	174.36
NK	*SNAI3*	rs11645983	16	88742585	T	G	0.2209	-0.3334	0.0534	4.23E-10	38.92
NK	*SNAP29*	rs165616	22	21173308	T	C	0.4390	0.5665	0.0436	1.19E-38	168.68
NK	*SNHG7*	rs4880135	9	139615162	A	C	0.6291	-0.5078	0.0447	7.33E-30	128.57
NK	*SNHG8*	rs788649	4	119231736	C	T	0.3811	0.8193	0.0418	1.90E-85	382.92
NK	*SNORD3B-1*	rs4924986	17	19242467	G	A	0.1430	0.5773	0.0698	1.29E-16	68.32
NK	*SNORD3B-2*	rs12937053	17	19348126	A	G	0.5794	0.3709	0.0453	2.62E-16	66.92
NK	*SNRNP25*	rs216599	16	109671	G	A	0.8421	-0.3578	0.0606	3.61E-09	34.75
NK	*SNRPD2*	rs36040835	19	46201213	G	T	0.2051	-0.4703	0.0554	2.11E-17	71.89
NK	*SNX18*	rs2121131	5	53942843	T	C	0.6564	0.3387	0.0473	8.14E-13	51.14
NK	*SNX9*	rs9347751	6	158326704	T	G	0.2246	-0.3080	0.0530	6.36E-09	33.65
NK	*SPATA20*	rs9890200	17	48624523	C	A	0.3676	-0.4743	0.0462	9.02E-25	105.37
NK	*SPATC1L*	rs62215189	21	47603995	G	A	0.2446	-0.5718	0.0498	1.47E-30	131.74
NK	*SPATC1L*	rs62224180	21	47726332	G	C	0.7751	-0.3754	0.0568	4.00E-11	43.52
NK	*SPECC1*	rs2008758	17	19893590	T	C	0.1163	0.5921	0.0657	2.07E-19	81
NK	*SPNS1*	rs11643297	16	28973268	G	A	0.7029	-0.3199	0.0495	9.89E-11	41.75
NK	*SPOCK2*	rs1245546	10	73846826	T	C	0.4440	-0.3206	0.0448	8.35E-13	51.09

NK	*SPON2*	rs6836158	4	1111594	G	A	0.1151	0.5845	0.0724	6.82E-16	65.04
NK	*SPRYD4*	rs2657907	12	56913639	A	C	0.1552	0.3585	0.0585	9.21E-10	37.41
NK	*SPSB1*	rs11803236	1	9344415	G	C	0.3432	0.4191	0.0471	5.93E-19	78.92
NK	*SPSB2*	rs9737890	12	6990453	T	A	0.2868	0.5688	0.0477	9.41E-33	141.76
NK	*SPTBN1*	rs7564719	2	54829731	C	A	0.2320	-0.5200	0.0505	8.09E-25	105.59
NK	*SPTLC3*	rs13045508	20	12905468	A	G	0.5312	0.2924	0.0451	9.03E-11	41.93
NK	*SRD5A3*	rs11935857	4	56204865	A	C	0.1899	0.5245	0.0583	2.28E-19	80.81
NK	*SRI*	rs1063964	7	87835469	A	G	0.3146	0.2711	0.0481	1.79E-08	31.64
NK	*SRP14*	rs2250402	15	40322552	T	G	0.9172	-0.6103	0.0837	2.99E-13	53.1
NK	*SRR*	rs17834627	17	2198892	C	T	0.3386	-0.2818	0.0469	1.91E-09	35.99
NK	*SRSF10*	rs6424145	1	24375689	C	G	0.4721	-0.4642	0.0439	4.26E-26	111.41
NK	*ST3GAL5*	rs72842060	2	86064849	A	T	0.2084	-0.3998	0.0564	1.36E-12	50.13
NK	*ST6GAL1*	rs9820270	3	186609682	C	T	0.2645	-0.2813	0.0505	2.54E-08	30.97
NK	*STK32C*	rs7085852	10	134144005	C	T	0.1782	-0.3273	0.0594	3.56E-08	30.31
NK	*STXBP2*	rs11260005	19	7699804	G	C	0.4023	-0.2551	0.0459	2.72E-08	30.83
NK	*STYX*	rs1952094	14	53279367	G	A	0.5131	-0.3157	0.0446	1.48E-12	49.97
NK	*SULF2*	rs2425967	20	46185378	C	T	0.5893	-0.7228	0.0425	5.69E-65	289.11
NK	*SURF1*	rs3118667	9	136291063	T	C	0.5241	-0.4343	0.0441	7.46E-23	96.65
NK	*SVIP*	rs10833823	11	22832056	C	T	0.3039	-0.3905	0.0485	8.09E-16	64.71
NK	*SYK*	rs290243	9	93571337	A	G	0.2371	-0.4055	0.0539	5.50E-14	56.42
NK	*SYNC*	rs61798768	1	33179866	A	G	0.1669	0.3804	0.0568	2.05E-11	44.82
NK	*SYNGR1*	rs909685	22	39747671	A	T	0.3216	1.0513	0.0434	7.07E-130	586.66
NK	*SYTL2*	rs290200	11	85421392	G	A	0.6559	0.2635	0.0470	2.03E-08	31.4
NK	*TAGLN2*	rs6427499	1	159891917	T	C	0.4829	0.3520	0.0445	2.72E-15	62.33
NK	*TAMM41*	rs392621	3	11886365	C	T	0.4395	-0.2485	0.0452	3.78E-08	30.19
NK	*TAPBP*	rs1800837	6	33272626	T	C	0.2005	0.3258	0.0561	6.23E-09	33.69
NK	*TAPBPL*	rs2534711	12	6570821	G	T	0.3045	0.6291	0.0475	5.94E-40	174.64
NK	*TBC1D12*	rs7922956	10	96318359	G	A	0.3494	0.3273	0.0460	1.08E-12	50.59
NK	*TBCA*	rs254405	5	77080789	A	G	0.4546	-0.2786	0.0449	5.27E-10	38.49
NK	*TC2N*	rs2267989	14	92339850	A	G	0.4380	-0.5469	0.0436	3.59E-36	157.37
NK	*TCF25*	rs2270460	16	89972416	A	C	0.0957	1.3617	0.0692	2.92E-86	386.65
NK	*TCIRG1*	rs3133269	11	67804156	T	C	0.7402	0.4302	0.0502	1.02E-17	73.32
NK	*TECR*	rs930090	19	14667417	G	A	0.5553	-0.6112	0.0433	2.43E-45	199.27
NK	*TEN1*	rs2069528	17	73997474	A	G	0.7529	0.4218	0.0517	3.51E-16	66.35
NK	*TESC*	rs73203865	12	117501150	T	C	0.0363	-0.6749	0.1005	1.91E-11	44.97
NK	*TFPT*	rs80269846	19	54614095	G	A	0.1310	-0.5646	0.0625	1.63E-19	81.46
NK	*THAP7-AS1*	rs2239961	22	21363960	A	G	0.2024	-0.4372	0.0554	3.06E-15	62.09
NK	*THAP9-AS1*	rs12506410	4	83721656	G	T	0.7874	0.5091	0.0544	8.18E-21	87.37
NK	*THEM6*	rs2717600	8	143813988	G	A	0.5442	-0.2451	0.0449	4.82E-08	29.72
NK	*THEMIS2*	rs6564	1	28212975	T	C	0.3803	-0.9797	0.0402	7.45E-131	591.14
NK	*TIAM1*	rs9980047	21	32724413	T	C	0.7349	0.3259	0.0509	1.46E-10	41
NK	*TIGIT*	rs62265703	3	114013479	G	A	0.2279	0.2963	0.0532	2.60E-08	30.92
NK	*TIMM10*	rs2848626	11	57283988	T	C	0.7820	-0.7154	0.0497	5.85E-47	206.67
NK	*TIPARP-AS1*	rs9822906	3	156384295	G	A	0.1423	-0.4178	0.0622	1.80E-11	45.08
NK	*TLE4*	rs2807303	9	82187095	A	G	0.3638	0.5344	0.0456	1.15E-31	136.8
NK	*TLK1*	rs2557794	2	171868342	T	G	0.3580	-0.3617	0.0465	7.07E-15	60.45
NK	*TLR6*	rs17429273	4	38868900	G	A	0.2436	0.3528	0.0497	1.28E-12	50.24
NK	*TM7SF3*	rs146970669	12	27103449	A	G	0.1053	0.4881	0.0782	4.43E-10	38.83
NK	*TMA7*	rs9881491	3	48487911	A	G	0.4359	0.2587	0.0450	9.25E-09	32.92
NK	*TMC4*	rs36653	19	54668487	T	C	0.2599	-0.5775	0.0479	1.62E-33	145.24
NK	*TMC6*	rs34712518	17	76121031	T	C	0.0652	0.4942	0.0877	1.74E-08	31.7
NK	*TMED9*	rs6634	5	177023098	T	A	0.4354	-0.3516	0.0449	4.60E-15	61.29
NK	*TMEM101*	rs77536093	17	42092328	G	A	0.0441	-0.5930	0.1020	6.20E-09	33.7
NK	*TMEM120B*	rs11043192	12	122209613	A	G	0.0974	1.0464	0.0760	4.46E-43	188.92
NK	*TMEM156*	rs6819988	4	39038665	T	C	0.5743	0.6912	0.0428	8.53E-59	260.83
NK	*TMEM163*	rs7558554	2	135357203	C	G	0.6013	0.3194	0.0467	8.09E-12	46.64
NK	*TMEM171*	rs7706869	5	72242669	T	C	0.1401	0.3907	0.0633	6.69E-10	38.03
NK	*TMEM2*	rs4745123	9	74393843	G	A	0.7815	-0.4420	0.0555	1.59E-15	63.38
NK	*TMEM204*	rs2294624	16	1586255	A	G	0.1329	0.9751	0.0634	2.12E-53	236.13
NK	*TMEM220*	rs3826448	17	10633181	G	A	0.4587	0.2773	0.0452	8.80E-10	37.49
NK	*TMEM230*	rs4315602	20	5061017	C	T	0.5174	0.4425	0.0442	1.25E-23	100.18
NK	*TMEM241*	rs8098618	18	21069269	T	C	0.4937	-0.2678	0.0448	2.23E-09	35.68
NK	*TMEM50B*	rs4816452	21	34810330	A	G	0.4717	-0.5272	0.0436	1.25E-33	145.76
NK	*TMEM55A*	rs2740792	8	91997679	G	A	0.7111	-0.3416	0.0502	1.06E-11	46.11
NK	*TMEM71*	rs2467958	8	133785881	G	A	0.4181	-0.3074	0.0449	8.00E-12	46.66
NK	*TMEM80*	rs11246262	11	680647	C	A	0.4796	-0.3823	0.0446	1.05E-17	73.26
NK	*TMEM8A*	rs11248930	16	425785	T	C	0.4445	-0.2928	0.0447	5.83E-11	42.78
NK	*TMOD1*	rs2480721	9	100337311	A	G	0.4641	-0.3256	0.0446	2.94E-13	53.13
NK	*TMPPE*	rs6799644	3	32714877	A	G	0.1896	0.5615	0.0586	9.52E-22	91.62
NK	*TMSB10*	rs13411337	2	85114700	C	G	0.1741	0.3275	0.0588	2.58E-08	30.93
NK	*TMTC4*	rs1283196	13	101362258	G	A	0.3121	0.3096	0.0484	1.54E-10	40.88
NK	*TNFRSF14*	rs2227312	1	2487663	A	C	0.4936	0.3099	0.0446	3.83E-12	48.11
NK	*TNFRSF18*	rs12036216	1	1153113	A	G	0.1101	-0.6950	0.0691	8.67E-24	100.9
NK	*TNFRSF4*	rs78107697	1	1161866	T	C	0.1076	-0.5472	0.0700	5.45E-15	60.96
NK	*TNIP1*	rs4958890	5	150477087	T	C	0.5147	0.2552	0.0448	1.24E-08	32.35
NK	*TNRC18*	rs10233187	7	5430375	C	T	0.5538	0.2884	0.0451	1.56E-10	40.87
NK	*TOMM34*	rs2234197	20	43589041	A	G	0.2132	-0.2920	0.0532	4.12E-08	30.03
NK	*TOMM7*	rs2240728	7	22852499	T	C	0.3422	-1.1494	0.0400	3.53E-181	822.07
NK	*TOX*	rs78302344	8	60063767	A	C	0.1636	0.4397	0.0634	4.04E-12	48

NK	*TP53I11*	rs12363092	11	45121892	T	G	0.2620	0.3222	0.0508	2.29E-10	40.12
NK	*TP53TG1*	rs802035	7	86969559	T	C	0.7363	0.3249	0.0497	6.17E-11	42.67
NK	*TPD52*	rs1346146	8	81047278	T	C	0.4449	0.6382	0.0430	7.98E-50	219.77
NK	*TPGS1*	rs12610275	19	521407	G	A	0.5039	0.3181	0.0446	1.02E-12	50.69
NK	*TPGS2*	rs948636	18	34361762	C	G	0.2855	-0.4489	0.0486	2.76E-20	84.97
NK	*TPM1*	rs11071710	15	63309193	G	A	0.1724	-0.3515	0.0598	4.15E-09	34.48
NK	*TPRA1*	rs2099589	3	127320529	A	C	0.7794	0.3774	0.0539	2.57E-12	48.89
NK	*TPST2*	rs4275	22	26921725	G	A	0.4260	-0.3197	0.0449	1.11E-12	50.53
NK	*TRAM1*	rs13252584	8	71689031	G	A	0.4957	0.2496	0.0449	2.61E-08	30.91
NK	*TRAPPC12-AS1*	rs12993215	2	3473727	C	T	0.3980	-0.3581	0.0461	7.82E-15	60.25
NK	*TRAPPC2L*	rs34150867	16	88921634	G	C	0.3103	-0.3290	0.0507	8.67E-11	42.01
NK	*TRAPPC4*	rs11607569	11	118880668	C	T	0.2148	0.4861	0.0524	1.87E-20	85.74
NK	*TRIM47*	rs9908862	17	73873394	G	A	0.1537	-0.3709	0.0614	1.56E-09	36.38
NK	*TRMT112*	rs11231746	11	64070940	C	T	0.3003	-0.5450	0.0464	7.98E-32	137.52
NK	*TRMT12*	rs4132855	8	125462121	G	A	0.4126	0.2747	0.0454	1.44E-09	36.53
NK	*TRPC3*	rs6838639	4	122899165	A	G	0.2469	-0.8553	0.0488	6.78E-69	307.08
NK	*TSGA10*	rs11123770	2	99825102	G	A	0.6033	0.3656	0.0457	1.27E-15	63.83
NK	*TSPAN15*	rs61848397	10	71225584	A	C	0.2261	0.4817	0.0519	1.80E-20	85.82
NK	*TSPAN32*	rs2651838	11	2319531	T	A	0.5700	0.6263	0.0432	1.23E-47	209.76
NK	*TSPO*	rs138910	22	43550055	C	T	0.7417	-0.5501	0.0500	3.79E-28	120.75
NK	*TSTD1*	rs3766383	1	161008572	A	C	0.1552	-0.8825	0.0649	4.27E-42	184.43
NK	*TSTD3*	rs9402863	6	99971429	G	A	0.5634	-0.4579	0.0445	7.87E-25	105.64
NK	*TTC16*	rs3824523	9	130426944	G	T	0.6069	0.3151	0.0452	3.01E-12	48.58
NK	*TTC32*	rs35957485	2	20090677	A	G	0.0880	0.6607	0.0805	2.25E-16	67.22
NK	*TTC38*	rs739165	22	46684528	T	C	0.1045	-1.0227	0.0709	3.30E-47	207.81
NK	*TUBB2A*	rs909963	6	3157442	G	C	0.6671	0.2613	0.0474	3.59E-08	30.29
NK	*TUFM*	rs8049439	16	28837515	C	T	0.3939	0.6269	0.0442	9.39E-46	201.16
NK	*TXK*	rs13136882	4	48172798	T	C	0.1152	0.7374	0.0725	2.68E-24	103.22
NK	*TYROBP*	rs7251879	19	36385928	G	A	0.0605	-0.9863	0.1004	9.18E-23	96.24
NK	*U2AF1L4*	rs2239947	19	36244753	C	G	0.1568	0.6447	0.0626	7.16E-25	105.83
NK	*UBA52*	rs2058077	19	18671055	T	C	0.5894	-0.3994	0.0452	9.11E-19	78.07
NK	*UBA6*	rs9994257	4	68577083	C	G	0.4081	0.4066	0.0452	2.21E-19	80.87
NK	*UBA6-AS1*	rs10518042	4	68602903	C	T	0.1479	0.3844	0.0640	1.88E-09	36.02
NK	*UBE2L3*	rs131656	22	21917450	A	G	0.2172	-0.4401	0.0572	1.44E-14	59.05
NK	*UBE3B*	rs67342900	12	110003399	A	G	0.0757	0.4623	0.0842	4.08E-08	30.04
NK	*UCP2*	rs34408426	11	73697519	G	A	0.3845	0.5469	0.0452	1.23E-33	145.78
NK	*UFL1*	rs9386528	6	96928514	A	G	0.3149	0.2672	0.0475	1.79E-08	31.64
NK	*UFSP2*	rs10000869	4	186339449	G	A	0.7494	0.2887	0.0525	3.83E-08	30.17
NK	*ULK4*	rs12634670	3	41875299	C	G	0.1527	0.6343	0.0609	2.03E-25	108.33
NK	*UNC5B-AS1*	rs59587808	10	72977060	C	G	0.1462	0.3886	0.0636	9.72E-10	37.3
NK	*UPF3A*	rs7999630	13	115064482	A	G	0.1269	0.4814	0.0696	4.62E-12	47.74
NK	*UPK3BL*	rs10277684	7	102365475	G	A	0.8822	-0.5166	0.0718	6.02E-13	51.73
NK	*UQCRH*	rs41292543	1	46774783	G	A	0.1009	-0.6009	0.0757	2.13E-15	62.8
NK	*UROS*	rs11244646	10	127475151	T	A	0.4516	-0.2965	0.0448	3.72E-11	43.66
NK	*USF1*	rs12116949	1	161042063	A	C	0.2747	0.3904	0.0486	9.37E-16	64.42
NK	*UVSSA*	rs10024529	4	1363886	A	G	0.1118	0.3823	0.0698	4.23E-08	29.98
NK	*VAMP2*	rs1476161	17	8090908	G	A	0.5760	0.2899	0.0464	4.29E-10	38.89
NK	*VAMP4*	rs7543682	1	171656678	A	G	0.1933	0.3362	0.0562	2.22E-09	35.7
NK	*VAMP5*	rs28479378	2	85750407	T	C	0.1223	-0.8016	0.0646	2.40E-35	153.6
NK	*VAV3*	rs2125749	1	108484400	A	C	0.2935	-0.3311	0.0497	2.79E-11	44.23
NK	*VCL*	rs1874151	10	75867614	T	C	0.5965	0.4178	0.0449	1.42E-20	86.28
NK	*VEGFA*	rs9462921	6	43694634	G	C	0.7158	0.4795	0.0504	1.99E-21	90.16
NK	*VIM*	rs3758418	10	17245638	C	G	0.3150	0.2668	0.0478	2.35E-08	31.12
NK	*VIPR2*	rs4143341	7	159054952	G	A	0.5904	-0.3123	0.0451	4.15E-12	47.95
NK	*VIT*	rs1558701	2	37027819	T	C	0.7256	0.3587	0.0501	7.81E-13	51.22
NK	*VOPP1*	rs12718970	7	55572141	T	G	0.3470	0.2584	0.0465	2.67E-08	30.86
NK	*VPS28*	rs117891721	8	145642886	T	C	0.0569	1.0803	0.0926	1.81E-31	135.89
NK	*WAC-AS1*	rs3121059	10	28797312	C	G	0.7951	-0.5479	0.0519	4.37E-26	111.36
NK	*WAC-AS1*	rs2800737	10	28943072	C	T	0.3358	-0.3295	0.0484	9.92E-12	46.24
NK	*WARS*	rs34040257	14	100810099	T	C	0.2050	0.3863	0.0538	7.21E-13	51.37
NK	*WDR1*	rs887725	4	10183895	T	C	0.4705	0.2770	0.0447	6.03E-10	38.23
NK	*WDR37*	rs34407608	10	1066710	T	C	0.0968	0.4581	0.0750	1.01E-09	37.23
NK	*WDR60*	rs28679564	7	158674521	G	C	0.2165	-0.2953	0.0541	4.78E-08	29.74
NK	*WDR93*	rs72630480	15	89649883	T	A	0.1291	-1.0749	0.0599	4.69E-72	321.55
NK	*WIPI1*	rs78095701	17	66443609	C	A	0.1216	-0.4514	0.0678	2.78E-11	44.23
NK	*XCL1*	rs1040508	1	168500750	T	C	0.3842	0.8053	0.0431	6.99E-78	348.25
NK	*XCL2*	rs141673522	1	168517508	T	C	0.1712	-1.0743	0.0545	1.24E-86	388.36
NK	*XRCC6BP1*	rs9971803	12	58306097	T	C	0.3134	-0.3270	0.0475	5.61E-12	47.36
NK	*XRRA1*	rs12225387	11	74604767	G	A	0.2742	0.9979	0.0436	3.88E-116	523.64
NK	*XXbac-BPG252P9.9*	rs9468922	6	31255286	A	G	0.3384	0.4068	0.0476	1.18E-17	73.02
NK	*XXbac-BPG299F13.17*	rs6921663	6	31154620	C	T	0.2178	1.0716	0.0491	1.92E-105	474.6
NK	*XXbac-BPG299F13.17*	rs2596461	6	31416993	G	A	0.6007	-0.2861	0.0461	5.40E-10	38.44
NK	*XXbac-BPG299F13.17*	rs2853982	6	31378751	G	A	0.1540	-0.5771	0.0617	7.98E-21	87.42
NK	*XXYLT1-AS2*	rs950256	3	194847752	T	C	0.1819	0.5408	0.0594	8.36E-20	82.78
NK	*YBEY*	rs62224180	21	47726332	G	C	0.7751	-0.7011	0.0552	5.66E-37	161.03
NK	*YEATS4*	rs57954211	12	69748397	A	C	0.4286	-0.3516	0.0446	3.25E-15	61.97
NK	*YWHAB*	rs8356	20	43536455	T	C	0.2794	-0.4572	0.0498	4.43E-20	84.04
NK	*ZAP70*	rs6543040	2	98343254	T	C	0.4694	0.2477	0.0450	3.83E-08	30.17
NK	*ZC3H7A*	rs2072498	16	11853238	C	T	0.1961	0.3250	0.0548	3.07E-09	35.06

NK	*ZC3HAV1*	rs10270221	7	138734838	G	A	0.5050	-0.5325	0.0435	2.00E-34	149.4
NK	*ZDHHC4*	rs11559147	7	6617247	G	C	0.7006	0.3037	0.0496	9.01E-10	37.45
NK	*ZFAND3*	rs10807188	6	37789028	G	A	0.3437	-0.2559	0.0456	2.03E-08	31.4
NK	*ZFAS1*	rs237714	20	47922900	T	C	0.2041	0.4786	0.0531	2.00E-19	81.06
NK	*ZFP57*	rs2982836	6	29649609	A	G	0.2527	-0.4245	0.0515	1.65E-16	67.84
NK	*ZFP57*	rs362529	6	29530868	T	C	0.1489	0.3503	0.0629	2.62E-08	30.91
NK	*ZFP90*	rs698718	16	68560185	G	A	0.7622	0.5245	0.0525	1.65E-23	99.63
NK	*ZMAT4*	rs4282554	8	40627587	G	T	0.9094	-1.1356	0.0954	1.08E-32	141.48
NK	*ZNF100*	rs7250085	19	21891497	A	C	0.3580	0.4127	0.0465	6.73E-19	78.67
NK	*ZNF165*	rs1233582	6	28714044	G	C	0.1443	0.4373	0.0586	8.67E-14	55.53
NK	*ZNF264*	rs4801440	19	57706378	C	G	0.0530	-0.6165	0.0994	5.63E-10	38.36
NK	*ZNF266*	rs10406056	19	9536418	T	C	0.5698	0.3846	0.0449	1.06E-17	73.24
NK	*ZNF302*	rs9789337	19	35166230	C	A	0.1245	0.6819	0.0661	6.11E-25	106.14
NK	*ZNF324*	rs10415547	19	59006510	C	T	0.7430	0.3078	0.0514	2.08E-09	35.82
NK	*ZNF391*	rs9461355	6	27295506	T	A	0.1306	-0.8219	0.0631	8.25E-39	169.42
NK	*ZNF429*	rs11085487	19	21818466	A	T	0.5949	-0.2560	0.0455	1.84E-08	31.59
NK	*ZNF44*	rs2438531	19	12384988	C	T	0.3148	-0.4459	0.0479	1.34E-20	86.39
NK	*ZNF492*	rs62120195	19	23832535	G	T	0.1197	-0.7618	0.0707	4.63E-27	115.8
NK	*ZNF506*	rs7260393	19	19886410	G	C	0.1812	0.4297	0.0589	2.88E-13	53.17
NK	*ZNF516*	rs117983097	18	74072636	A	G	0.4931	0.3149	0.0447	1.77E-12	49.62
NK	*ZNF584*	rs62117595	19	58950383	C	T	0.0794	0.6355	0.0832	2.18E-14	58.24
NK	*ZNF587*	rs3745134	19	58373635	G	C	0.3840	0.2677	0.0469	1.11E-08	32.56
NK	*ZNF593*	rs2232646	1	26496370	T	C	0.2962	0.3205	0.0489	5.42E-11	42.93
NK	*ZNF600*	rs4802995	19	53284720	G	C	0.4576	0.7496	0.0417	3.65E-72	322.05
NK	*ZNF677*	rs8107040	19	53757622	G	C	0.2477	-0.3297	0.0522	2.66E-10	39.82
NK	*ZNF718*	rs10006358	4	125490	G	A	0.4428	-0.3273	0.0451	3.83E-13	52.61
NK	*ZNF749*	rs12610163	19	57892729	C	T	0.5240	0.4003	0.0444	1.81E-19	81.26
NK	*ZNF781*	rs2891657	19	38197084	C	G	0.5927	-0.2989	0.0460	8.19E-11	42.12
NK	*ZNF791*	rs1529472	19	12732258	T	C	0.6356	0.2808	0.0472	2.70E-09	35.31
NK	*ZNF823*	rs799193	19	11891003	A	G	0.7957	0.4248	0.0551	1.30E-14	59.25
NK	*ZNF83*	rs1015304	19	53121171	G	A	0.3661	0.4036	0.0461	1.94E-18	76.59
NK	*ZNF85*	rs2545225	19	21042460	G	A	0.1318	0.8296	0.0620	7.12E-41	178.85
NK	*ZNF880*	rs324126	19	52886137	C	T	0.5445	-0.2660	0.0451	3.70E-09	34.7
NK	*ZNHIT1*	rs2072168	7	100861870	T	G	0.2329	0.3839	0.0540	1.22E-12	50.34
NK	*ZNHIT6*	rs11161664	1	86185379	T	C	0.4257	0.2533	0.0453	2.20E-08	31.24
NK	*ZNRD1*	rs11753957	6	29980190	T	C	0.0672	0.6839	0.0962	1.15E-12	50.47
NK	*ZP3*	rs35613536	7	76143625	A	G	0.3272	0.7935	0.0440	1.49E-72	323.83
NK	*ZSWIM7*	rs2779207	17	15870401	A	T	0.5839	-0.4143	0.0447	1.83E-20	85.78
NK R	*AC000003.2*	rs78624212	17	10061385	T	C	0.2696	-0.3802	0.0515	1.49E-13	54.47
NK R	*AC006369.2*	rs10205753	2	37823509	C	T	0.5385	0.5013	0.0440	4.60E-30	129.5
NK R	*AC016831.7*	rs12533406	7	129958612	T	G	0.0684	0.9717	0.0781	1.68E-35	154.31
NK R	*AC021860.1*	rs10032014	4	38625778	A	G	0.1988	-0.3076	0.0545	1.71E-08	31.74
NK R	*APOM*	rs6415128	6	32330894	A	G	0.6330	-0.3143	0.0455	5.03E-12	47.58
NK R	*ATXN1*	rs140208219	6	17329010	A	G	0.2439	0.3813	0.0531	7.12E-13	51.41
NK R	*BIN1*	rs11682128	2	127839474	A	G	0.3433	0.3520	0.0473	9.59E-14	55.34
NK R	*BTN3A2*	rs2072806	6	26385093	G	C	0.1049	-0.4383	0.0675	8.63E-11	42.02
NK R	*C11orf21*	rs11022157	11	2322829	A	C	0.3383	-0.2837	0.0468	1.31E-09	36.73
NK R	*C19orf83*	rs2286756	19	46145025	T	G	0.5303	-0.2707	0.0451	1.98E-09	35.92
NK R	*C6orf136*	rs2517685	6	29931035	T	G	0.3606	0.3188	0.0465	7.12E-12	46.9
NK R	*CAPG*	rs6741368	2	85603064	A	G	0.2497	-0.3461	0.0519	2.69E-11	44.3
NK R	*CCHCR1*	rs9266669	6	31348077	A	G	0.1261	0.6514	0.0605	5.06E-27	115.63
NK R	*CCT8*	rs965951	21	30432557	A	G	0.1160	0.4829	0.0687	2.03E-12	49.35
NK R	*CCZ1B*	rs2711224	7	6861880	C	G	0.4738	-0.3354	0.0450	9.11E-14	55.44
NK R	*CD3E*	rs3759009	11	118173850	A	G	0.3079	0.8690	0.0444	3.02E-85	382.03
NK R	*CD52*	rs1071849	1	26646726	G	A	0.7403	0.3196	0.0519	7.44E-10	37.82
NK R	*CDC42*	rs7544210	1	22455142	A	G	0.5587	-0.2554	0.0455	2.01E-08	31.42
NK R	*CHI3L2*	rs2252482	1	111759762	T	C	0.9009	-0.5404	0.0741	2.92E-13	53.15
NK R	*CLIC3*	rs7040970	9	139859013	C	T	0.5087	-0.2721	0.0451	1.62E-09	36.3
NK R	*COMMD6*	rs4885306	13	76098486	T	C	0.2272	-0.3663	0.0525	3.10E-12	48.52
NK R	*CST3*	rs3761279	20	23620075	G	C	0.2097	-0.3017	0.0549	4.00E-08	30.09
NK R	*CSTB*	rs35285321	21	45201832	A	G	0.3415	-0.3430	0.0485	1.56E-12	49.87
NK R	*CTD-2368P22.1*	rs3810128	19	58483709	G	A	0.2400	-0.4099	0.0526	6.66E-15	60.57
NK R	*CTSW*	rs622614	11	65663239	C	T	0.7945	0.3750	0.0569	4.25E-11	43.41
NK R	*CYTL1*	rs6446312	4	5019932	G	C	0.8147	-0.5185	0.0561	2.22E-20	85.41
NK R	*DCBLD1*	rs7757149	6	116939485	A	G	0.1970	-0.5562	0.0553	8.15E-24	101.03
NK R	*DCXR*	rs12940462	17	80004161	T	C	0.1635	0.3563	0.0583	9.99E-10	37.25
NK R	*DHX16*	rs9391683	6	30361358	A	G	0.3526	0.2941	0.0487	1.55E-09	36.39
NK R	*DIP2A*	rs9306156	21	47960493	C	T	0.3298	0.4832	0.0469	7.40E-25	105.77
NK R	*DNAJC15*	rs2281778	13	43597977	G	C	0.2269	-0.3074	0.0541	1.30E-08	32.27
NK R	*EIF3G*	rs2305795	19	10226052	A	G	0.5697	0.4399	0.0447	7.56E-23	96.63
NK R	*EIF5A*	rs7503161	17	7207964	C	A	0.5615	-0.8388	0.0418	1.05E-89	402.47
NK R	*ELP5*	rs2074218	17	7156186	G	A	0.6135	-0.2980	0.0460	9.03E-11	41.93
NK R	*ERAP2*	rs2548226	5	96275186	T	C	0.3882	0.4592	0.0450	1.68E-24	104.15
NK R	*FAM118A*	rs104664	22	45711854	A	G	0.8660	-0.7112	0.0676	6.78E-26	110.5
NK R	*FAM184B*	rs10029790	4	17559213	C	T	0.3965	-0.4696	0.0456	6.32E-25	106.09
NK R	*FAM26F*	rs9387418	6	116851907	A	C	0.4877	0.2520	0.0452	2.43E-08	31.05
NK R	*FCER1G*	rs3813627	1	161195148	T	G	0.3407	-0.2913	0.0468	4.94E-10	38.62
NK R	*FCRL3*	rs2485066	1	157632501	T	C	0.5247	-0.2897	0.0453	1.62E-10	40.79
NK R	*FES*	rs6227	15	91425232	T	C	0.3153	-0.3378	0.0489	5.10E-12	47.55

NK R	*FGFR1OP*	rs2235283	6	166884848	G	T	0.3282	0.4547	0.0478	2.01E-21	90.15
NK R	*GCC2*	rs13022595	2	109038026	T	C	0.5991	0.3234	0.0457	1.54E-12	49.9
NK R	*GNLY*	rs3755007	2	85920249	G	T	0.3609	-0.5792	0.0459	1.92E-36	158.62
NK R	*GSTT1*	rs2097433	22	24296894	G	A	0.5338	-0.5399	0.0442	2.57E-34	148.91
NK R	*HIST1H2BC*	rs67092078	6	27054772	A	G	0.0727	0.6649	0.0760	2.25E-18	76.3
NK R	*HIST1H4E*	rs3799380	6	26467182	C	T	0.2053	-0.6133	0.0582	5.32E-26	110.98
NK R	*HLA-A*	rs1143146	6	29910358	G	C	0.4939	0.2505	0.0452	3.07E-08	30.6
NK R	*HLA-B*	rs2524096	6	31236467	T	G	0.4422	0.6207	0.0437	8.47E-46	201.38
NK R	*HLA-B*	rs9394058	6	31279815	T	C	0.1075	-0.5669	0.0836	1.16E-11	45.94
NK R	*HLA-B*	rs1056429	6	31321883	A	G	0.1149	-0.5782	0.0730	2.32E-15	62.64
NK R	*HLA-C*	rs58019823	6	31241215	G	A	0.1090	0.5521	0.0764	5.00E-13	52.1
NK R	*HLA-DQA1*	rs6927022	6	32612397	G	A	0.3361	0.3238	0.0449	5.43E-13	51.94
NK R	*HLA-DQA2*	rs204993	6	32155581	G	A	0.2513	0.3475	0.0490	1.37E-12	50.12
NK R	*HLA-DQA2*	rs6913471	6	32339925	A	T	0.3370	-0.2694	0.0470	9.92E-09	32.79
NK R	*HLA-DQA2*	rs34763586	6	32608998	C	T	0.1322	0.5578	0.0555	8.54E-24	100.94
NK R	*HLA-DRB1*	rs9271573	6	32590501	C	A	0.5476	-0.2597	0.0462	1.84E-08	31.59
NK R	*HLA-DRB5*	rs9272070	6	32599485	G	T	0.5192	-0.3429	0.0448	1.98E-14	58.43
NK R	*HLA-G*	rs8347	6	30037686	C	T	0.1623	-0.7539	0.0657	1.71E-30	131.46
NK R	*HLA-G*	rs2074504	6	30530245	C	T	0.6895	-0.3472	0.0486	9.51E-13	50.84
NK R	*HMGN4*	rs35848276	6	27521096	T	C	0.0742	-0.7981	0.0751	2.26E-26	112.67
NK R	*HSF2*	rs9490547	6	123088139	C	G	0.7862	0.7076	0.0584	9.22E-34	146.38
NK R	*HSP90AA1*	rs13749	14	102514227	C	T	0.1871	-0.3487	0.0620	1.90E-08	31.53
NK R	*HSPA1B*	rs1625792	6	31306420	A	G	0.1062	0.7803	0.0640	3.49E-34	148.31
NK R	*HSPA1B*	rs116029711	6	32603564	C	T	0.1435	-0.5476	0.0691	2.22E-15	62.73
NK R	*IFITM2*	rs10398	11	308180	G	A	0.2135	0.4210	0.0583	5.13E-13	52.05
NK R	*IFITM3*	rs34481144	11	320836	T	C	0.4497	-0.5786	0.0435	2.22E-40	176.6
NK R	*IL32*	rs45499297	16	3115272	C	T	0.1074	0.5874	0.0763	1.33E-14	59.22
NK R	*ITGB2*	rs760462	21	46328099	C	T	0.8298	-0.8155	0.0585	3.64E-44	193.91
NK R	*ITPR3*	rs1794282	6	32666526	T	C	0.0896	-0.4492	0.0705	1.90E-10	40.49
NK R	*KANSL1-AS1*	rs56328224	17	43572419	T	C	0.2065	0.5402	0.0527	1.25E-24	104.74
NK R	*KLRC2*	rs77926410	12	10568525	G	A	0.1203	0.6069	0.0663	5.59E-20	83.58
NK R	*KLRD1*	rs11608254	12	10480733	C	T	0.3159	0.2853	0.0488	5.04E-09	34.1
NK R	*LTA*	rs9267890	6	32202086	G	T	0.8874	0.5468	0.0659	1.03E-16	68.77
NK R	*LY6G5C*	rs2844690	6	30940032	T	C	0.1547	0.5248	0.0646	4.44E-16	65.9
NK R	*LYAR*	rs11723339	4	4271886	T	C	0.6169	-0.4739	0.0458	4.52E-25	106.75
NK R	*MAP3K5*	rs9389437	6	137081542	T	G	0.4653	0.5732	0.0435	1.33E-39	173.06
NK R	*MBOAT1*	rs73376681	6	19808175	G	A	0.0927	0.8826	0.0782	1.51E-29	127.15
NK R	*MICA*	rs2524222	6	30511170	T	C	0.2039	-0.3653	0.0530	5.24E-12	47.5
NK R	*MICA*	rs9265745	6	31302718	G	A	0.3927	0.3677	0.0454	5.62E-16	65.43
NK R	*MIF*	rs2253552	22	24297287	C	T	0.5714	-0.2648	0.0462	9.68E-09	32.84
NK R	*MORC2*	rs1049583	22	32352791	A	G	0.3035	0.8714	0.0448	2.10E-84	378.18
NK R	*MYOM2*	rs79200728	8	2082753	G	T	0.0859	0.7494	0.0876	1.16E-17	73.07
NK R	*NSA2*	rs112820313	5	74167947	A	G	0.1345	0.3936	0.0667	3.66E-09	34.72
NK R	*NT5C3B*	rs9906048	17	40032683	T	C	0.8069	0.3759	0.0556	1.38E-11	45.61
NK R	*OSTF1*	rs3780180	9	77727884	T	C	0.3228	-0.2689	0.0470	1.07E-08	32.64
NK R	*PABPC1*	rs73282499	8	101822215	A	G	0.1612	0.3920	0.0605	8.96E-11	41.95
NK R	*PILRB*	rs11771936	7	99943283	G	A	0.1794	0.3908	0.0568	6.07E-12	47.21
NK R	*PPDPF*	rs72629024	20	62152519	G	C	0.1633	-0.5510	0.0620	6.62E-19	78.71
NK R	*PRMT2*	rs2070435	21	47961711	A	G	0.3259	0.4430	0.0472	6.80E-21	87.74
NK R	*PRR7-AS1*	rs2545794	5	176894756	T	C	0.5086	-0.5405	0.0439	7.57E-35	151.33
NK R	*PSMD5-AS1*	rs12343516	9	123603469	C	A	0.5777	-0.5417	0.0445	4.56E-34	147.77
NK R	*PTGDR*	rs4391970	14	52635729	G	A	0.3048	-0.3652	0.0497	2.06E-13	53.83
NK R	*QRSL1*	rs7761376	6	106488970	G	T	0.5221	0.2809	0.0450	4.49E-10	38.81
NK R	*RAC1*	rs836472	7	6434904	A	G	0.2623	0.2907	0.0508	1.05E-08	32.69
NK R	*RAMP1*	rs6431562	2	238804162	G	T	0.8707	-0.3631	0.0660	3.80E-08	30.19
NK R	*RAMP1*	rs11686156	2	238814823	C	A	0.5434	0.3471	0.0452	1.51E-14	58.97
NK R	*RP1-3J17.3*	rs76488172	6	86532530	C	T	0.0425	0.7157	0.1030	3.65E-12	48.2
NK R	*RP11-291B21.2*	rs4492899	12	10598426	G	A	0.6556	0.4855	0.0475	1.46E-24	104.42
NK R	*RP11-326C3.11*	rs10794308	11	324163	T	C	0.3897	0.3170	0.0470	1.56E-11	45.36
NK R	*RP11-347P5.1*	rs34830320	2	196962919	G	A	0.4054	-0.3409	0.0460	1.27E-13	54.78
NK R	*RP11-81H14.2*	rs11177237	12	68850086	C	T	0.4121	0.3421	0.0454	4.75E-14	56.71
NK R	*RPL10A*	rs10947550	6	35428782	A	G	0.5293	-0.2716	0.0455	2.37E-09	35.57
NK R	*RPL12*	rs2253669	9	130230514	G	A	0.5944	0.4705	0.0450	1.25E-25	109.29
NK R	*RPL13*	rs3211568	16	89629780	A	G	0.1858	-0.3507	0.0563	4.84E-10	38.66
NK R	*RPL28*	rs17700376	19	55897327	G	A	0.3547	-0.8155	0.0436	5.33E-78	348.82
NK R	*RPL36AL*	rs2147712	14	50091684	A	G	0.7504	0.4373	0.0524	7.12E-17	69.49
NK R	*RPL8*	rs2954658	8	146017782	T	C	0.4990	-0.6280	0.0432	5.55E-48	211.37
NK R	*RPLP2*	rs7479101	11	802115	G	A	0.6480	-0.2638	0.0481	4.19E-08	30
NK R	*RPS10*	rs6924982	6	34269856	T	A	0.2272	0.6935	0.0565	1.35E-34	150.19
NK R	*RPS18*	rs17215231	6	33239869	T	C	0.0640	-1.3458	0.0853	4.39E-56	248.44
NK R	*RPS2*	rs338787	16	2008095	A	G	0.1299	0.3992	0.0670	2.62E-09	35.38
NK R	*RPS26*	rs773114	12	56379060	T	A	0.3997	1.1802	0.0360	1.00E-200	1075.39
NK R	*RPS3A*	rs1148652	4	151801938	C	T	0.0703	-0.9198	0.0998	3.10E-20	84.75
NK R	*RPS5*	rs731835	19	58898436	C	T	0.3256	-0.5284	0.0469	2.15E-29	126.45
NK R	*RPS7*	rs62106047	2	3630883	G	T	0.0899	-0.5423	0.0836	8.77E-11	41.99
NK R	*RPS8*	rs12126557	1	45257201	T	C	0.1238	-0.4813	0.0627	1.69E-14	58.75
NK R	*RPS9*	rs12980600	19	54707248	T	A	0.2684	0.4873	0.0495	6.86E-23	96.82
NK R	*RPSA*	rs73058289	3	39437714	T	A	0.1547	0.3389	0.0620	4.64E-08	29.8
NK R	*S100A13*	rs2274739	1	153612133	A	G	0.5530	-0.3408	0.0450	3.42E-14	57.36

NK R	*S100A4*	rs2480696	1	153506765	C	T	0.0965	-0.4847	0.0771	3.17E-10	39.48
NK R	*S100B*	rs9306153	21	47900279	G	C	0.3686	-0.4086	0.0464	1.36E-18	77.3
NK R	*SMDT1*	rs28515621	22	42534126	T	G	0.6587	0.5320	0.0473	2.28E-29	126.33
NK R	*SNCA*	rs1871399	4	90783545	G	C	0.3724	0.4420	0.0456	3.09E-22	93.85
NK R	*SNHG8*	rs658642	4	119200274	T	C	0.3810	0.4966	0.0449	1.86E-28	122.18
NK R	*SPINT2*	rs7249688	19	38753676	C	A	0.2340	0.2851	0.0515	3.09E-08	30.58
NK R	*SULF2*	rs59954210	20	46098043	C	T	0.2887	0.4699	0.0488	6.42E-22	92.4
NK R	*SYNGAP1*	rs9271147	6	32577385	C	T	0.8463	-0.5362	0.0632	2.22E-17	71.79
NK R	*SYNGR1*	rs11704615	22	39696834	G	A	0.2130	-0.5343	0.0591	1.51E-19	81.63
NK R	*TCF19*	rs2285801	6	30209493	G	A	0.4267	0.4378	0.0445	7.25E-23	96.71
NK R	*TCF19*	rs2250264	6	30821187	A	G	0.2484	-0.3296	0.0526	3.77E-10	39.15
NK R	*TCIRG1*	rs4147778	11	67804605	A	C	0.7401	0.3054	0.0510	2.12E-09	35.79
NK R	*THEMIS2*	rs7356	1	28218100	C	T	0.3802	-0.4190	0.0457	5.03E-20	83.79
NK R	*TIMM10*	rs2848626	11	57283988	T	C	0.7820	-0.3268	0.0522	3.75E-10	39.16
NK R	*TJAP1*	rs507964	6	44186390	G	T	0.6639	-0.2635	0.0473	2.55E-08	30.96
NK R	*TOMM7*	rs2240728	7	22852499	T	C	0.3422	-0.6195	0.0469	7.55E-40	174.18
NK R	*TSPO*	rs25245	22	43561385	G	A	0.6952	-0.2756	0.0493	2.30E-08	31.16
NK R	*UCP2*	rs2515087	11	73682872	T	C	0.4077	0.3135	0.0460	9.76E-12	46.28
NK R	*VARS2*	rs3130513	6	31178084	G	C	0.4934	0.2623	0.0451	6.10E-09	33.73
NK R	*VPS52*	rs9268219	6	32284108	G	T	0.0862	0.7269	0.0715	2.97E-24	103.03
NK R	*WRNIP1*	rs3800122	6	1899671	C	T	0.5798	-0.2560	0.0462	3.02E-08	30.63
NK R	*XCL1*	rs61801307	1	168496949	G	C	0.1731	0.3687	0.0589	3.88E-10	39.09
NK R	*XCL2*	rs141673522	1	168517508	T	C	0.1712	-0.4384	0.0595	1.75E-13	54.16
NK R	*XRRA1*	rs61894839	11	74626366	A	G	0.2756	0.3112	0.0493	2.81E-10	39.72
NK R	*XXbac-BPG299F13.17*	rs3132479	6	31274752	G	A	0.5077	-0.2861	0.0452	2.39E-10	40.03
NK R	*YBEY*	rs12626184	21	47715296	C	T	0.6944	-0.3008	0.0497	1.46E-09	36.51
NK R	*ZBTB22*	rs55890913	6	32385596	C	T	0.1017	0.6396	0.0721	7.67E-19	78.42
NK R	*ZKSCAN8*	rs276362	6	27938235	C	A	0.3214	-0.3438	0.0510	1.54E-11	45.38
NK R	*ZNF600*	rs4802995	19	53284720	G	C	0.4576	0.3108	0.0450	4.71E-12	47.71
NK R	*ZNF667-AS1*	rs862701	19	57863937	C	T	0.7160	-0.3001	0.0512	4.50E-09	34.32
NK R	*ZNRD1-AS1*	rs3094639	6	30206753	C	T	0.4314	0.3874	0.0447	4.52E-18	74.92
NK R	*ZNRD1-AS1*	rs3129987	6	30766204	C	T	0.8747	0.8428	0.0606	6.49E-44	192.76
NK R	*ZSCAN16*	rs3118365	6	28839908	A	G	0.0876	-0.4161	0.0741	1.98E-08	31.45
DC	*AC000003.2*	rs7208708	17	10049841	C	T	0.3144	0.3161	0.0485	7.29E-11	42.35
DC	*AGER*	rs9264185	6	31219982	A	G	0.2656	-0.5269	0.0476	1.72E-28	122.33
DC	*AGER*	rs9271720	6	32593507	A	G	0.2394	-0.2794	0.0467	2.26E-09	35.66
DC	*ANPEP*	rs11073890	15	90352611	G	A	0.5242	0.3494	0.0448	6.27E-15	60.69
DC	*ANXA1*	rs2795114	9	75771314	C	G	0.2085	0.3286	0.0588	2.27E-08	31.18
DC	*ANXA5*	rs6857766	4	122611285	A	G	0.1968	0.3336	0.0563	3.12E-09	35.03
DC	*APOM*	rs2394886	6	31194677	A	G	0.2236	0.4218	0.0513	1.89E-16	67.57
DC	*ATAT1*	rs9468882	6	31151039	A	T	0.3022	0.3739	0.0490	2.44E-14	58.02
DC	*B3GALT4*	rs2894251	6	32343236	G	A	0.1010	-0.4558	0.0674	1.36E-11	45.63
DC	*BAG2*	rs56003634	6	57019459	T	C	0.0440	-0.6155	0.0984	3.98E-10	39.04
DC	*BLOC1S1*	rs1081732	12	56148291	G	C	0.0925	0.4791	0.0853	1.92E-08	31.51
DC	*BRK1*	rs73017232	3	10059664	C	T	0.0963	-0.5055	0.0722	2.45E-12	48.98
DC	*C20orf27*	rs45495794	20	3734167	C	T	0.0479	-0.5843	0.0987	3.23E-09	34.97
DC	*C20orf27*	rs6139202	20	3746070	T	A	0.0776	0.8130	0.0847	8.43E-22	91.87
DC	*C6orf136*	rs3130458	6	31154434	T	C	0.2602	-0.4459	0.0493	1.39E-19	81.79
DC	*C6orf136*	rs8347	6	30037686	C	T	0.1623	-0.3678	0.0675	4.95E-08	29.67
DC	*CCHCR1*	rs9261739	6	30367535	C	T	0.2182	-0.3437	0.0553	5.25E-10	38.5
DC	*CCHCR1*	rs2853926	6	31263051	G	A	0.2837	-0.4119	0.0499	1.54E-16	67.98
DC	*CCT8*	rs965951	21	30432557	A	G	0.1160	0.7753	0.0673	9.50E-31	132.63
DC	*CCZ1B*	rs7789081	7	6862678	C	T	0.4738	-0.2906	0.0452	1.26E-10	41.28
DC	*CD300C*	rs1171210	17	72552036	C	G	0.5096	-0.2792	0.0451	5.84E-10	38.3
DC	*CD52*	rs7537477	1	26618829	T	C	0.7385	0.5682	0.0508	4.76E-29	124.88
DC	*CFD*	rs71335276	19	859368	T	G	0.2211	0.4632	0.0543	1.52E-17	72.53
DC	*CLEC12A*	rs7304783	12	10115391	C	T	0.2709	0.5788	0.0498	2.82E-31	135.04
DC	*COMMD6*	rs9543962	13	76107167	T	C	0.1412	-0.4435	0.0621	9.26E-13	50.89
DC	*CPNE1*	rs7261284	20	34244104	A	T	0.1068	-0.5259	0.0768	7.39E-12	46.82
DC	*CTA-445C9.15*	rs5761654	22	27037688	C	G	0.4178	0.3505	0.0461	3.03E-14	57.59
DC	*CTC-524C5.2*	rs980407	17	5338818	C	G	0.2545	-0.4482	0.0527	1.89E-17	72.1
DC	*CTSH*	rs2289702	15	79237293	T	C	0.1100	-0.4783	0.0700	8.46E-12	46.56
DC	*DBI*	rs77901185	2	120089882	G	A	0.2087	-0.5348	0.0536	1.97E-23	99.28
DC	*DCXR*	rs9903371	17	79993064	G	C	0.1249	0.3864	0.0683	1.52E-08	31.96
DC	*DDT*	rs5751775	22	24266726	C	T	0.5903	0.2895	0.0463	4.13E-10	38.97
DC	*DDX5*	rs1991401	17	62502435	G	A	0.3173	-0.2736	0.0489	2.15E-08	31.29
DC	*DNAJA1*	rs10971316	9	33013962	A	G	0.4026	-0.2588	0.0461	1.98E-08	31.45
DC	*DNAJC15*	rs17624339	13	43612074	G	A	0.2249	-0.4369	0.0535	3.01E-16	66.66
DC	*DOPEY2*	rs33999808	21	37587798	A	G	0.7911	0.3111	0.0556	2.24E-08	31.21
DC	*DRAM2*	rs657801	1	111736389	T	C	0.6829	0.2726	0.0482	1.56E-08	31.91
DC	*EIF5A*	rs7503161	17	7207964	C	A	0.5615	-0.8458	0.0417	1.87E-91	410.49
DC	*ELP5*	rs222835	17	7134129	G	A	0.5937	-0.2733	0.0455	1.86E-09	36.05
DC	*ENPP4*	rs538242	6	46828874	C	T	0.9233	0.8385	0.0874	8.71E-22	91.8
DC	*ERAP2*	rs2549798	5	96245903	C	A	0.5153	-0.3405	0.0449	3.15E-14	57.52
DC	*F13A1*	rs927332	6	6331873	C	T	0.4808	0.2509	0.0452	2.75E-08	30.81
DC	*FAM118A*	rs136567	22	45766485	A	G	0.1359	1.1707	0.0639	4.41E-75	335.45
DC	*FAM184B*	rs13145480	4	17565143	C	T	0.3248	-0.5482	0.0470	1.87E-31	135.85
DC	*FAM26F*	rs2637678	6	116787378	C	T	0.4086	0.4639	0.0452	9.63E-25	105.25
DC	*FCER1G*	rs2070901	1	161185058	T	G	0.2764	0.3587	0.0518	4.38E-12	47.85

DC	*FCGR2B*	rs67295569	1	161597821	G	T	0.5010	-0.3869	0.0447	4.58E-18	74.9
DC	*FCN1*	rs10858293	9	137809685	A	C	0.3344	0.2730	0.0477	1.07E-08	32.63
DC	*FPR1*	rs11882804	19	52251157	T	C	0.7141	0.3625	0.0478	3.41E-14	57.36
DC	*GAA*	rs12950270	17	78146429	C	G	0.7123	-0.3175	0.0500	2.24E-10	40.17
DC	*GDI2*	rs3736461	10	5855403	C	G	0.1844	-0.3820	0.0556	6.17E-12	47.18
DC	*GNPDA2*	rs10938310	4	43815025	A	G	0.8899	-0.8444	0.0797	2.93E-26	112.16
DC	*GRHPR*	rs10973326	9	37419497	A	G	0.3364	0.3354	0.0478	2.34E-12	49.07
DC	*GSTM4*	rs2269341	1	110172362	T	C	0.4139	-0.2603	0.0458	1.35E-08	32.19
DC	*GSTT1*	rs113413	22	24292264	C	T	0.3267	0.7187	0.0465	6.55E-54	238.49
DC	*HCG11*	rs34332556	6	27357414	G	C	0.0780	0.6241	0.0764	3.14E-16	66.57
DC	*HIST1H2BN*	rs9295778	6	28684183	G	T	0.8428	-0.4358	0.0608	7.74E-13	51.24
DC	*HIVEP2*	rs7771884	6	143899055	C	T	0.3816	-0.4167	0.0453	3.91E-20	84.29
DC	*HLA-A*	rs5027914	6	29922215	A	G	0.5211	0.3695	0.0447	1.44E-16	68.12
DC	*HLA-B*	rs2524096	6	31236467	T	G	0.4422	0.5865	0.0440	1.43E-40	177.48
DC	*HLA-B*	rs1056429	6	31321883	A	G	0.1149	-0.4488	0.0735	1.02E-09	37.21
DC	*HLA-C*	rs17408553	6	31239407	T	G	0.3872	0.3665	0.0470	5.98E-15	60.78
DC	*HLA-DPA1*	rs2213309	6	33045255	C	T	0.4712	-0.2844	0.0457	4.79E-10	38.68
DC	*HLA-DQA1*	rs73730372	6	32584581	T	C	0.1258	0.7229	0.0732	5.09E-23	97.41
DC	*HLA-DQA2*	rs1391371	6	32603798	T	A	0.2202	1.3106	0.0460	3.49E-178	808.4
DC	*HLA-DQA2*	rs443198	6	32190406	G	A	0.3676	0.3337	0.0460	3.87E-13	52.6
DC	*HLA-DQB1*	rs7751376	6	32605437	G	C	0.4462	-0.3582	0.0454	2.90E-15	62.2
DC	*HLA-DQB2*	rs11751024	6	32586236	A	C	0.3684	-0.3553	0.0456	6.25E-15	60.7
DC	*HLA-DRB1*	rs9271720	6	32593507	A	G	0.2394	0.4424	0.0461	7.51E-22	92.09
DC	*HLA-DRB5*	rs9272105	6	32599999	A	G	0.5403	-0.8986	0.0402	1.36E-110	498.26
DC	*HLA-DRB5*	rs3819720	6	32804570	A	G	0.3230	0.3697	0.0473	5.53E-15	60.94
DC	*HSPA1B*	rs2442714	6	31373260	C	G	0.5194	-0.4769	0.0444	6.47E-27	115.15
DC	*HSPA1B*	rs3021302	6	32623150	C	T	0.1616	0.5478	0.0601	7.93E-20	82.9
DC	*HYLS1*	rs689051	11	125292490	A	G	0.1488	-0.6615	0.0626	4.38E-26	111.37
DC	*IFITM3*	rs34481144	11	320836	T	C	0.4497	-0.4517	0.0443	2.19E-24	103.63
DC	*IGFLR1*	rs62112162	19	36264030	A	G	0.1659	-0.3972	0.0622	1.75E-10	40.64
DC	*IGLL1*	rs13056126	22	23980968	A	T	0.1917	0.3174	0.0571	2.64E-08	30.89
DC	*ITGB2*	rs760462	21	46328099	C	T	0.8298	-0.5556	0.0602	2.58E-20	85.11
DC	*KANSL1-AS1*	rs62061733	17	44018399	G	A	0.2103	0.4706	0.0527	4.31E-19	79.56
DC	*KCNK17*	rs4535541	6	39292984	T	C	0.5771	0.3128	0.0450	3.80E-12	48.13
DC	*LGALS1*	rs7291867	22	38062112	G	C	0.3433	-0.3198	0.0480	2.61E-11	44.36
DC	*LGALS2*	rs5756736	22	37970189	A	G	0.5727	-0.6334	0.0440	6.24E-47	206.56
DC	*LY6G5C*	rs112905979	6	30665559	T	C	0.0721	-0.7911	0.0772	1.25E-24	104.75
DC	*LY6G5C*	rs3104412	6	32585967	G	A	0.4863	-0.2869	0.0453	2.37E-10	40.05
DC	*LYZ*	rs4761234	12	69732105	C	T	0.4502	-0.6580	0.0429	5.27E-53	234.35
DC	*MDC1*	rs3130387	6	30187887	T	C	0.4160	-0.4112	0.0447	3.70E-20	84.4
DC	*MICA*	rs36057735	6	31319923	G	C	0.1564	-0.5008	0.0576	3.47E-18	75.44
DC	*MICB*	rs17192526	6	31266041	C	T	0.4090	0.3832	0.0458	5.65E-17	69.95
DC	*MZT2A*	rs2103256	2	132270136	T	C	0.2185	0.3203	0.0582	3.79E-08	30.19
DC	*NAPRT1*	rs9657360	8	144676862	T	C	0.7776	0.4383	0.0552	1.93E-15	63
DC	*NDUFS5*	rs11205621	1	39530005	A	T	0.2785	-0.3184	0.0485	5.00E-11	43.09
DC	*NQO2*	rs10223369	6	3018174	C	T	0.6782	-0.2814	0.0496	1.45E-08	32.06
DC	*NSA2*	rs72764630	5	73997524	T	A	0.1335	0.4043	0.0665	1.22E-09	36.86
DC	*NT5C3B*	rs8073987	17	40090520	T	C	0.7553	-0.3154	0.0523	1.60E-09	36.34
DC	*PPA1*	rs7901747	10	71984752	A	G	0.3213	0.4272	0.0474	1.87E-19	81.21
DC	*PPARA*	rs8139990	22	46501355	T	G	0.3625	0.4938	0.0465	2.52E-26	112.46
DC	*PPDPF*	rs72629024	20	62152519	G	C	0.1633	-0.5807	0.0619	6.89E-21	87.72
DC	*PPIL3*	rs2136600	2	201742345	T	C	0.2391	-0.3197	0.0541	3.33E-09	34.91
DC	*PSMD5-AS1*	rs4837796	9	123610288	T	C	0.5730	-0.4589	0.0450	2.04E-24	103.77
DC	*RAC1*	rs10256583	7	6408218	C	T	0.4673	0.5765	0.0435	5.27E-40	174.89
DC	*RNASET2*	rs239934	6	167412048	A	G	0.4987	-0.2673	0.0452	3.33E-09	34.91
DC	*ROGDI*	rs2290562	16	4853816	A	G	0.2822	0.2826	0.0495	1.14E-08	32.52
DC	*RP1-3J17.3*	rs4441928	6	86241894	C	T	0.6528	0.4566	0.0465	8.96E-23	96.29
DC	*RP11-1143G9.4*	rs1670737	12	69774877	A	G	0.5389	0.7844	0.0418	1.36E-78	351.54
DC	*RP11-347P5.1*	rs7581626	2	197000907	A	G	0.6430	0.4393	0.0463	2.14E-21	90.02
DC	*RP11-385F7.1*	rs10948386	6	47829141	G	T	0.6467	-0.3864	0.0464	7.94E-17	69.28
DC	*RP11-47L3.1*	rs73279289	17	33735298	T	G	0.0920	-0.4882	0.0814	2.01E-09	35.89
DC	*RP6-91H8.3*	rs17109053	14	71609698	C	G	0.2878	0.3756	0.0510	1.74E-13	54.16
DC	*RPL10A*	rs10947550	6	35428782	A	G	0.5293	-0.3044	0.0454	2.03E-11	44.85
DC	*RPL12*	rs2247781	9	130208924	C	T	0.5921	0.5901	0.0441	8.17E-41	178.59
DC	*RPL13*	rs12932337	16	89630630	T	C	0.1753	-0.4489	0.0561	1.24E-15	63.88
DC	*RPL22L1*	rs7649979	3	170653423	T	C	0.3618	-0.2636	0.0473	2.53E-08	30.97
DC	*RPL28*	rs17700376	19	55897327	G	A	0.3547	-0.7254	0.0446	1.32E-59	264.56
DC	*RPL36AL*	rs4581611	14	50043520	T	A	0.7274	0.3874	0.0510	3.03E-14	57.59
DC	*RPL8*	rs2954658	8	146017782	T	C	0.4990	-0.6660	0.0429	1.81E-54	241.05
DC	*RPP21*	rs9918306	6	30419771	T	C	0.1812	0.3485	0.0594	4.49E-09	34.33
DC	*RPPH1*	rs6575124	14	20786827	C	T	0.2559	-0.2876	0.0523	3.87E-08	30.15
DC	*RPS10*	rs4587164	6	34376703	T	G	0.7616	-0.5584	0.0558	1.54E-23	99.77
DC	*RPS11*	rs73056062	19	50004499	T	C	0.1126	-0.4712	0.0722	6.69E-11	42.52
DC	*RPS16*	rs484379	19	39925054	G	T	0.3787	0.3432	0.0473	4.21E-13	52.44
DC	*RPS18*	rs17215231	6	33239869	T	C	0.0640	-1.3197	0.0856	1.17E-53	237.34
DC	*RPS19*	rs7259596	19	42369743	C	T	0.5016	0.2470	0.0452	4.52E-08	29.85
DC	*RPS2*	rs17135716	16	2014778	G	A	0.1130	0.6741	0.0672	1.09E-23	100.46
DC	*RPS26*	rs773114	12	56379060	T	A	0.3997	1.1681	0.0363	1.00E-200	1034.62
DC	*RPS3A*	rs12508270	4	152114234	C	T	0.0617	-0.7772	0.0924	3.95E-17	70.66

DC	*RPS5*	rs731835	19	58898436	C	T	0.3256	-0.5308	0.0469	1.22E-29	127.57
DC	*RPS7*	rs62106040	2	3627302	T	A	0.0757	-0.5526	0.0897	7.17E-10	37.9
DC	*RPS8*	rs12120833	1	45241285	G	C	0.1242	-0.4522	0.0629	6.34E-13	51.63
DC	*RPS9*	rs10048513	19	54704720	C	G	0.2755	0.4883	0.0492	3.48E-23	98.16
DC	*SESN1*	rs2768558	6	109258284	C	T	0.2491	0.4568	0.0529	5.64E-18	74.49
DC	*SH3BGRL3*	rs11589222	1	26645806	T	C	0.7402	-0.3281	0.0519	2.58E-10	39.88
DC	*SIRPB1*	rs34600126	20	1597433	T	G	0.2103	0.3103	0.0537	7.31E-09	33.38
DC	*SMDT1*	rs133377	22	42466950	T	C	0.6612	0.7454	0.0456	5.17E-60	266.43
DC	*SNAP29*	rs5761520	22	21312889	T	C	0.0678	0.4636	0.0849	4.72E-08	29.77
DC	*SNHG7*	rs11145844	9	139613998	T	C	0.1148	-0.5733	0.0713	8.65E-16	64.58
DC	*SNHG8*	rs658642	4	119200274	T	C	0.3810	0.5238	0.0447	1.15E-31	136.81
DC	*SULF2*	rs2425978	20	46226478	T	C	0.2851	-0.3314	0.0498	2.80E-11	44.22
DC	*SYNGAP1*	rs984778	6	32400088	T	C	0.6901	0.5926	0.0461	6.85E-38	165.23
DC	*SYNGAP1*	rs9272559	6	32607124	C	T	0.0734	0.3986	0.0543	2.21E-13	53.69
DC	*SYNGR1*	rs5757599	22	39670181	C	A	0.2831	0.3191	0.0538	3.02E-09	35.1
DC	*THEMIS2*	rs3766398	1	28209305	G	A	0.6190	-0.4062	0.0468	4.04E-18	75.15
DC	*TIMM10*	rs2649662	11	57352562	C	A	0.7817	-0.4166	0.0525	2.04E-15	62.89
DC	*TMEM176A*	rs7806458	7	150476888	A	G	0.6363	0.6379	0.0448	6.37E-46	201.94
DC	*TMEM176B*	rs9691673	7	150479586	G	C	0.7020	0.4567	0.0485	4.63E-21	88.5
DC	*TNF*	rs143015185	6	31397689	T	A	0.0639	0.5360	0.0969	3.21E-08	30.51
DC	*TNF*	rs9267549	6	31677391	A	G	0.0931	-0.5490	0.0685	1.14E-15	64.04
DC	*TOMM7*	rs2240728	7	22852499	T	C	0.3422	-0.6078	0.0470	2.95E-38	166.9
DC	*TRAM2*	rs13192439	6	52366808	C	G	0.1653	0.5899	0.0633	1.23E-20	86.57
DC	*TRIM39*	rs2517614	6	30163955	A	G	0.1860	-0.6466	0.0537	2.23E-33	144.62
DC	*TRIM39*	rs1611637	6	29836741	G	A	0.5781	-0.2595	0.0457	1.36E-08	32.18
DC	*TSTD1*	rs10908821	1	161008535	G	C	0.1307	0.4289	0.0673	1.88E-10	40.5
DC	*TUFM*	rs56404918	16	28846840	G	T	0.3812	0.4587	0.0457	1.00E-23	100.62
DC	*TYMP*	rs140522	22	50971266	C	T	0.6583	0.3819	0.0485	3.36E-15	61.91
DC	*VARS2*	rs3823374	6	29944148	A	C	0.3310	-0.5972	0.0466	1.15E-37	164.2
DC	*VARS2*	rs9391682	6	30357291	T	G	0.3450	-0.4219	0.0483	2.59E-18	76.02
DC	*VARS2*	rs6899875	6	31154897	A	G	0.1739	0.3749	0.0586	1.53E-10	40.91
DC	*VPS28*	rs117891721	8	145642886	T	C	0.0569	0.5582	0.0957	5.34E-09	33.99
DC	*WASF1*	rs117975843	6	111327888	T	G	0.1200	0.4703	0.0713	4.36E-11	43.36
DC	*XRRA1*	rs10899059	11	74746553	G	C	0.2864	-0.3796	0.0485	4.74E-15	61.24
DC	*XXbac-BPG299F13.17*	rs3131113	6	30366642	T	C	0.3449	0.2827	0.0474	2.53E-09	35.44
DC	*ZBTB22*	rs9268219	6	32284108	G	T	0.0862	-0.5844	0.0723	6.20E-16	65.24
DC	*ZNF322*	rs13217620	6	27653120	T	C	0.0713	-0.5859	0.0773	3.56E-14	57.28
DC	*ZNRD1-AS1*	rs35480831	6	29954719	T	C	0.0644	-0.5908	0.0980	1.64E-09	36.29
DC	*ZNRD1-AS1*	rs2517521	6	31027336	G	A	0.8877	-0.6504	0.0648	1.10E-23	100.44
DC	*ZSCAN16*	rs73403143	6	28726613	T	C	0.0678	0.6299	0.0967	7.36E-11	42.33

Table S2. Discovery MR analyses on the associations between cell type-specific eGenes and hypertension.

Cell type	eGene	Method	nSNP	β	SE	OR (95% CI)	*P*-value	*FDR*	Steiger pval	Steiger direction
B IN	*ABCA1*	Wald ratio	1	0.0584	0.0175	1.06 (1.024, 1.097)	8.32E-04	0.015	1.84E-08	TRUE
B IN	*ABHD16A*	IVW	2	0.0112	0.0087	1.011 (0.994, 1.029)	0.201	0.485	6.68E-36	TRUE
B IN	*AC005037.3*	Wald ratio	1	0.0078	0.0091	1.008 (0.99, 1.026)	0.393	0.672	4.57E-34	TRUE
B IN	*AC006129.4*	Wald ratio	1	0.0294	0.0153	1.03 (0.999, 1.061)	0.054	0.233	2.79E-11	TRUE
B IN	*AC023590.1*	Wald ratio	1	-0.0049	0.0149	0.995 (0.967, 1.025)	0.743	0.881	1.39E-17	TRUE
B IN	*AC079767.4*	Wald ratio	1	0.0294	0.0115	1.03 (1.007, 1.053)	0.011	0.085	7.59E-22	TRUE
B IN	*AC096772.6*	Wald ratio	1	0.0204	0.0099	1.021 (1.001, 1.041)	0.04	0.195	1.24E-26	TRUE
B IN	*AC104653.1*	Wald ratio	1	0.0198	0.0161	1.02 (0.988, 1.053)	0.219	0.51	2.49E-12	TRUE
B IN	*ACAP1*	Wald ratio	1	0.0135	0.0193	1.014 (0.976, 1.053)	0.485	0.736	9.53E-10	TRUE
B IN	*ACAT1*	Wald ratio	1	-0.0489	0.0165	0.952 (0.922, 0.984)	0.003	0.035	2.10E-08	TRUE
B IN	*ACCS*	Wald ratio	1	-0.0034	0.0176	0.997 (0.963, 1.032)	0.847	0.935	5.93E-12	TRUE
B IN	*ACTG1*	Wald ratio	1	-0.0465	0.0215	0.955 (0.915, 0.996)	0.031	0.166	7.39E-08	TRUE
B IN	*ACYP2*	Wald ratio	1	0.0058	0.0109	1.006 (0.984, 1.028)	0.598	0.803	4.50E-24	TRUE
B IN	*ADAM19*	Wald ratio	1	0.0032	0.0159	1.003 (0.972, 1.035)	0.84	0.933	1.54E-08	TRUE
B IN	*AHI1*	Wald ratio	1	-0.0132	0.0086	0.987 (0.97, 1.004)	0.124	0.379	2.39E-40	TRUE
B IN	*AKAP13*	Wald ratio	1	0.0042	0.0159	1.004 (0.973, 1.036)	0.793	0.909	5.35E-13	TRUE
B IN	*AKAP7*	Wald ratio	1	0.0084	0.0154	1.008 (0.978, 1.039)	0.589	0.796	2.51E-10	TRUE
B IN	*ALKBH7*	Wald ratio	1	0.0071	0.0214	1.007 (0.966, 1.05)	0.739	0.881	1.22E-08	TRUE
B IN	*ANK1*	Wald ratio	1	0.0137	0.0116	1.014 (0.991, 1.037)	0.236	0.53	1.30E-23	TRUE
B IN	*ANXA4*	Wald ratio	1	-0.0332	0.0104	0.967 (0.948, 0.987)	0.001	0.02	1.80E-21	TRUE
B IN	*ANXA5*	Wald ratio	1	-0.0091	0.0247	0.991 (0.944, 1.04)	0.712	0.872	7.31E-09	TRUE
B IN	*ANXA6*	Wald ratio	1	0.0007	0.0132	1.001 (0.975, 1.027)	0.958	0.985	1.70E-14	TRUE
B IN	*AP003774.1*	Wald ratio	1	-0.0398	0.0116	0.961 (0.939, 0.983)	5.86E-04	0.011	8.28E-21	TRUE
B IN	*AP1S3*	Wald ratio	1	-0.0115	0.0115	0.989 (0.966, 1.011)	0.318	0.614	3.10E-22	TRUE
B IN	*ARHGAP24*	Wald ratio	1	0.0058	0.0061	1.006 (0.994, 1.018)	0.338	0.631	1.42E-77	TRUE
B IN	*ARHGEF3*	Wald ratio	1	-0.0022	0.0134	0.998 (0.972, 1.024)	0.872	0.947	4.18E-15	TRUE
B IN	*ARL11*	Wald ratio	1	-0.0229	0.0103	0.977 (0.958, 0.997)	0.026	0.149	5.03E-25	TRUE
B IN	*ARL14EP*	Wald ratio	1	-0.0272	0.0139	0.973 (0.947, 1)	0.052	0.225	1.02E-13	TRUE
B IN	*ARL16*	Wald ratio	1	0.0144	0.0118	1.014 (0.991, 1.038)	0.222	0.514	2.56E-20	TRUE
B IN	*ARL17B*	Wald ratio	1	-0.0553	0.0238	0.946 (0.903, 0.991)	0.02	0.128	1.15E-12	TRUE
B IN	*ARSA*	Wald ratio	1	0.0282	0.0280	1.029 (0.974, 1.087)	0.314	0.608	7.60E-08	TRUE
B IN	*ASPH*	Wald ratio	1	0.0002	0.0146	1 (0.972, 1.029)	0.991	0.996	3.14E-09	TRUE
B IN	*ATG10*	Wald ratio	1	0.0245	0.0196	1.025 (0.986, 1.065)	0.212	0.5	1.31E-09	TRUE
B IN	*ATP5G3*	Wald ratio	1	-0.0049	0.0228	0.995 (0.952, 1.041)	0.829	0.929	1.87E-09	TRUE
B IN	*ATP6V1G2*	Wald ratio	1	-0.0045	0.0109	0.996 (0.974, 1.017)	0.681	0.854	3.71E-28	TRUE
B IN	*AXIN2*	Wald ratio	1	-0.0067	0.0125	0.993 (0.969, 1.018)	0.592	0.797	2.50E-19	TRUE
B IN	*BCAS4*	Wald ratio	1	0.0185	0.0170	1.019 (0.985, 1.053)	0.277	0.571	2.02E-10	TRUE
B IN	*BCL2A1*	Wald ratio	1	-0.0202	0.0061	0.98 (0.968, 0.992)	9.00E-04	0.016	3.67E-77	TRUE
B IN	*BICD2*	Wald ratio	1	0.0177	0.0130	1.018 (0.992, 1.044)	0.174	0.452	1.31E-18	TRUE
B IN	*BIRC5*	Wald ratio	1	0.0280	0.0098	1.028 (1.009, 1.048)	0.004	0.044	1.10E-35	TRUE
B IN	*BLK*	Wald ratio	1	-0.0523	0.0112	0.949 (0.929, 0.97)	2.75E-06	2.37E-04	5.48E-23	TRUE
B IN	*BRK1*	Wald ratio	1	-0.0239	0.0174	0.976 (0.944, 1.01)	0.17	0.447	4.76E-12	TRUE
B IN	*BTF3*	Wald ratio	1	0.0318	0.0174	1.032 (0.998, 1.068)	0.068	0.267	5.81E-10	TRUE
B IN	*BTN3A2*	Wald ratio	1	0.0220	0.0154	1.022 (0.992, 1.054)	0.153	0.423	1.27E-17	TRUE
B IN	*BTNL9*	Wald ratio	1	-0.0006	0.0140	0.999 (0.972, 1.027)	0.967	0.989	1.32E-15	TRUE
B IN	*C10orf32*	Wald ratio	1	-0.0811	0.0175	0.922 (0.891, 0.954)	3.45E-06	2.63E-04	3.17E-10	TRUE
B IN	*C11orf24*	Wald ratio	1	-0.0005	0.0107	1 (0.979, 1.021)	0.966	0.988	4.73E-25	TRUE
B IN	*C12orf57*	Wald ratio	1	-0.0022	0.0198	0.998 (0.96, 1.037)	0.91	0.962	2.71E-09	TRUE
B IN	*C17orf97*	Wald ratio	1	0.0121	0.0090	1.012 (0.994, 1.03)	0.179	0.459	3.65E-35	TRUE
B IN	*C19orf60*	Wald ratio	1	0.0052	0.0152	1.005 (0.976, 1.036)	0.734	0.88	1.65E-13	TRUE
B IN	*C1orf86*	Wald ratio	1	0.0032	0.0160	1.003 (0.972, 1.035)	0.84	0.932	3.83E-12	TRUE
B IN	*C22orf34*	Wald ratio	1	0.0518	0.0201	1.053 (1.012, 1.096)	0.01	0.082	7.86E-08	TRUE
B IN	*C2orf27A*	Wald ratio	1	-0.0171	0.0189	0.983 (0.947, 1.02)	0.365	0.653	6.53E-12	TRUE
B IN	*C5orf30*	Wald ratio	1	-0.0068	0.0154	0.993 (0.964, 1.024)	0.661	0.844	6.35E-14	TRUE
B IN	*C6orf25*	IVW	2	0.0164	0.0397	1.017 (0.94, 1.099)	0.68	0.853	3.77E-25	TRUE
B IN	*C6orf48*	Wald ratio	1	0.0531	0.0105	1.054 (1.033, 1.076)	4.37E-07	9.75E-05	6.70E-13	TRUE
B IN	*C6orf62*	Wald ratio	1	0.0709	0.0206	1.073 (1.031, 1.118)	5.87E-04	0.011	1.03E-07	TRUE
B IN	*C9orf156*	Wald ratio	1	0.0076	0.0143	1.008 (0.98, 1.036)	0.594	0.799	5.41E-16	TRUE
B IN	*C9orf78*	Wald ratio	1	-0.0117	0.0186	0.988 (0.953, 1.025)	0.529	0.757	1.95E-08	TRUE
B IN	*CAPG*	Wald ratio	1	-0.0103	0.0147	0.99 (0.962, 1.019)	0.484	0.736	3.62E-14	TRUE
B IN	*CAPN12*	Wald ratio	1	-0.0043	0.0218	0.996 (0.954, 1.039)	0.844	0.935	4.58E-08	TRUE
B IN	*CARD8*	Wald ratio	1	0.0365	0.0233	1.037 (0.991, 1.086)	0.116	0.363	5.58E-09	TRUE
B IN	*CAST*	Wald ratio	1	-0.0181	0.0137	0.982 (0.956, 1.009)	0.185	0.465	4.77E-16	TRUE
B IN	*CCDC101*	Wald ratio	1	-0.0196	0.0190	0.981 (0.945, 1.018)	0.303	0.598	4.50E-09	TRUE
B IN	*CCDC15*	Wald ratio	1	-0.0066	0.0158	0.993 (0.963, 1.025)	0.676	0.853	4.30E-18	TRUE
B IN	*CCDC163P*	Wald ratio	1	-0.0111	0.0135	0.989 (0.963, 1.015)	0.412	0.686	9.96E-17	TRUE
B IN	*CCDC66*	Wald ratio	1	0.0152	0.0130	1.015 (0.99, 1.042)	0.241	0.534	2.50E-19	TRUE
B IN	*CCND2-AS1*	Wald ratio	1	-0.0036	0.0122	0.996 (0.973, 1.021)	0.766	0.896	4.49E-21	TRUE
B IN	*CCT3*	Wald ratio	1	0.0290	0.0170	1.029 (0.996, 1.064)	0.088	0.313	1.05E-10	TRUE
B IN	*CCT8*	Wald ratio	1	-0.0009	0.0091	0.999 (0.981, 1.017)	0.922	0.965	1.90E-57	TRUE
B IN	*CCZ1*	Wald ratio	1	0.0149	0.0109	1.015 (0.994, 1.037)	0.171	0.448	1.36E-25	TRUE
B IN	*CCZ1B*	Wald ratio	1	-0.0174	0.0149	0.983 (0.954, 1.012)	0.242	0.534	2.88E-14	TRUE
B IN	*CD164*	Wald ratio	1	-0.0611	0.0207	0.941 (0.903, 0.98)	0.003	0.036	9.64E-08	TRUE
B IN	*CD37*	Wald ratio	1	0.0009	0.0121	1.001 (0.978, 1.025)	0.938	0.975	1.43E-28	TRUE
B IN	*CD52*	Wald ratio	1	0.0069	0.0130	1.007 (0.981, 1.033)	0.598	0.803	1.26E-17	TRUE
B IN	*CD81*	Wald ratio	1	0.0364	0.0142	1.037 (1.009, 1.066)	0.01	0.084	2.71E-11	TRUE

B IN	*CD83*	Wald ratio	1	-0.0099	0.0142	0.99 (0.963, 1.018)	0.484	0.736	3.09E-11	TRUE
B IN	*CD86*	IVW	2	0.0050	0.0108	1.005 (0.984, 1.026)	0.645	0.834	8.50E-22	TRUE
B IN	*CD9*	Wald ratio	1	-0.0134	0.0206	0.987 (0.948, 1.027)	0.514	0.75	5.12E-08	TRUE
B IN	*CD96*	Wald ratio	1	0.0060	0.0062	1.006 (0.994, 1.018)	0.333	0.626	2.14E-76	TRUE
B IN	*CDC42*	Wald ratio	1	0.0093	0.0134	1.009 (0.983, 1.036)	0.49	0.738	8.07E-15	TRUE
B IN	*CEACAM21*	Wald ratio	1	-0.0257	0.0190	0.975 (0.939, 1.012)	0.177	0.456	4.25E-08	TRUE
B IN	*CEBPZ-AS1*	Wald ratio	1	0.0129	0.0198	1.013 (0.974, 1.053)	0.515	0.75	2.22E-10	TRUE
B IN	*CENPK*	Wald ratio	1	0.0381	0.0179	1.039 (1.003, 1.076)	0.033	0.172	2.95E-09	TRUE
B IN	*CENPV*	Wald ratio	1	0.0529	0.0162	1.054 (1.021, 1.088)	0.001	0.018	1.47E-11	TRUE
B IN	*CFLAR*	Wald ratio	1	0.0016	0.0177	1.002 (0.967, 1.037)	0.93	0.97	2.89E-10	TRUE
B IN	*CHCHD2*	Wald ratio	1	0.0053	0.0121	1.005 (0.982, 1.029)	0.659	0.843	3.14E-22	TRUE
B IN	*CHI3L2*	Wald ratio	1	-0.0007	0.0064	0.999 (0.987, 1.012)	0.909	0.961	1.43E-68	TRUE
B IN	*CHURC1*	Wald ratio	1	0.0122	0.0166	1.012 (0.98, 1.046)	0.462	0.722	5.56E-10	TRUE
B IN	*CIB1*	Wald ratio	1	-0.0041	0.0177	0.996 (0.962, 1.031)	0.816	0.921	7.29E-10	TRUE
B IN	*CLK4*	Wald ratio	1	0.0171	0.0172	1.017 (0.984, 1.052)	0.319	0.614	1.08E-10	TRUE
B IN	*CLLU1*	Wald ratio	1	-0.0492	0.0149	0.952 (0.924, 0.98)	9.84E-04	0.017	1.11E-13	TRUE
B IN	*CLLU1OS*	Wald ratio	1	0.0310	0.0096	1.031 (1.012, 1.051)	0.001	0.02	2.23E-29	TRUE
B IN	*CLN8*	Wald ratio	1	0.0564	0.0164	1.058 (1.025, 1.093)	5.76E-04	0.011	8.16E-13	TRUE
B IN	*CNDP2*	Wald ratio	1	0.0315	0.0198	1.032 (0.993, 1.073)	0.111	0.354	5.01E-11	TRUE
B IN	*CNOT6L*	Wald ratio	1	0.0315	0.0166	1.032 (0.999, 1.066)	0.058	0.243	2.31E-11	TRUE
B IN	*CNPPD1*	Wald ratio	1	-0.0572	0.0180	0.944 (0.912, 0.978)	0.001	0.021	2.04E-09	TRUE
B IN	*CNTNAP2*	Wald ratio	1	-0.0072	0.0082	0.993 (0.977, 1.009)	0.381	0.666	7.56E-43	TRUE
B IN	*COL9A3*	IVW	2	-0.0053	0.0133	0.995 (0.969, 1.021)	0.687	0.858	4.36E-19	TRUE
B IN	*COMMD6*	Wald ratio	1	0.0121	0.0074	1.012 (0.998, 1.027)	0.101	0.337	2.58E-53	TRUE
B IN	*CORO1B*	Wald ratio	1	-0.0061	0.0209	0.994 (0.954, 1.035)	0.77	0.898	1.75E-08	TRUE
B IN	*CORO1C*	Wald ratio	1	-0.0087	0.0099	0.991 (0.972, 1.011)	0.38	0.666	1.05E-29	TRUE
B IN	*COTL1*	Wald ratio	1	-0.0168	0.0197	0.983 (0.946, 1.022)	0.394	0.673	1.05E-08	TRUE
B IN	*COX6B1*	Wald ratio	1	-0.0325	0.0146	0.968 (0.941, 0.996)	0.026	0.149	3.88E-14	TRUE
B IN	*CPNE1*	Wald ratio	1	-0.0115	0.0185	0.989 (0.953, 1.025)	0.537	0.762	5.82E-11	TRUE
B IN	*CRYBB2*	Wald ratio	1	0.0119	0.0095	1.012 (0.993, 1.031)	0.208	0.494	3.43E-32	TRUE
B IN	*CSTB*	Wald ratio	1	0.0024	0.0069	1.002 (0.989, 1.016)	0.73	0.88	1.46E-55	TRUE
B IN	*CTA-14H9.5*	Wald ratio	1	0.0222	0.0196	1.022 (0.984, 1.062)	0.256	0.551	6.41E-15	TRUE
B IN	*CTA-29F11.1*	Wald ratio	1	-0.0086	0.0136	0.991 (0.965, 1.018)	0.527	0.756	8.50E-17	TRUE
B IN	*CTC-524C5.2*	Wald ratio	1	0.0195	0.0111	1.02 (0.998, 1.042)	0.078	0.292	1.99E-19	TRUE
B IN	*CTD-2007H13.3*	Wald ratio	1	0.0355	0.0195	1.036 (0.997, 1.077)	0.068	0.268	1.47E-13	TRUE
B IN	*CTD-2017D11.1*	Wald ratio	1	0.0139	0.0183	1.014 (0.978, 1.051)	0.448	0.713	1.75E-09	TRUE
B IN	*CTD-2368P22.1*	Wald ratio	1	0.0025	0.0074	1.003 (0.988, 1.017)	0.734	0.88	2.12E-58	TRUE
B IN	*CTD-2371O3.2*	Wald ratio	1	-0.0018	0.0110	0.998 (0.977, 1.02)	0.873	0.947	1.01E-25	TRUE
B IN	*CTD-3110H11.1*	Wald ratio	1	-0.0403	0.0142	0.96 (0.934, 0.988)	0.005	0.047	1.41E-19	TRUE
B IN	*CTH*	Wald ratio	1	0.0015	0.0081	1.002 (0.986, 1.018)	0.853	0.938	6.36E-45	TRUE
B IN	*CTSH*	Wald ratio	1	-0.0192	0.0175	0.981 (0.948, 1.015)	0.272	0.566	1.14E-09	TRUE
B IN	*CYB5A*	Wald ratio	1	-0.0037	0.0067	0.996 (0.983, 1.009)	0.579	0.789	5.38E-66	TRUE
B IN	*DARS*	Wald ratio	1	-0.0460	0.0183	0.955 (0.921, 0.99)	0.012	0.092	1.49E-08	TRUE
B IN	*DBNDD1*	Wald ratio	1	0.0018	0.0148	1.002 (0.973, 1.031)	0.904	0.959	1.68E-11	TRUE
B IN	*DCLK2*	Wald ratio	1	-0.0159	0.0140	0.984 (0.957, 1.012)	0.256	0.551	1.46E-20	TRUE
B IN	*DCXR*	Wald ratio	1	-0.0064	0.0074	0.994 (0.979, 1.008)	0.392	0.672	2.51E-46	TRUE
B IN	*DDT*	Wald ratio	1	-0.0138	0.0126	0.986 (0.962, 1.011)	0.273	0.567	5.08E-19	TRUE
B IN	*DDX11*	Wald ratio	1	-0.0151	0.0112	0.985 (0.964, 1.007)	0.179	0.459	1.59E-23	TRUE
B IN	*DDX5*	Wald ratio	1	0.0599	0.0155	1.062 (1.03, 1.094)	1.06E-04	0.003	1.32E-11	TRUE
B IN	*DFNA5*	Wald ratio	1	-0.0190	0.0140	0.981 (0.955, 1.008)	0.174	0.452	3.73E-14	TRUE
B IN	*DHFR*	Wald ratio	1	0.0316	0.0201	1.032 (0.992, 1.074)	0.116	0.363	7.33E-08	TRUE
B IN	*DHRS4*	Wald ratio	1	0.0209	0.0329	1.021 (0.957, 1.089)	0.526	0.755	3.81E-08	TRUE
B IN	*DIEXF*	Wald ratio	1	-0.0307	0.0186	0.97 (0.935, 1.006)	0.1	0.334	5.70E-09	TRUE
B IN	*DNAJC15*	Wald ratio	1	0.0098	0.0095	1.01 (0.991, 1.029)	0.303	0.598	1.52E-31	TRUE
B IN	*DNASE1L3*	Wald ratio	1	-0.0033	0.0144	0.997 (0.969, 1.025)	0.816	0.921	2.74E-11	TRUE
B IN	*DOCK8*	Wald ratio	1	0.0206	0.0185	1.021 (0.985, 1.059)	0.265	0.559	4.09E-11	TRUE
B IN	*DRAM2*	Wald ratio	1	-0.0037	0.0110	0.996 (0.975, 1.018)	0.734	0.88	7.28E-24	TRUE
B IN	*DSE*	Wald ratio	1	-0.0126	0.0176	0.988 (0.954, 1.022)	0.476	0.732	1.01E-09	TRUE
B IN	*DSP*	Wald ratio	1	0.0032	0.0077	1.003 (0.988, 1.019)	0.679	0.853	1.17E-48	TRUE
B IN	*DYNLL2*	Wald ratio	1	-0.0590	0.0207	0.943 (0.905, 0.982)	0.004	0.046	8.23E-09	TRUE
B IN	*EAF2*	Wald ratio	1	0.0047	0.0064	1.005 (0.992, 1.017)	0.461	0.721	1.07E-75	TRUE
B IN	*EBPL*	Wald ratio	1	0.0224	0.0145	1.023 (0.994, 1.052)	0.122	0.375	8.73E-15	TRUE
B IN	*EIF2A*	Wald ratio	1	-0.0032	0.0194	0.997 (0.959, 1.035)	0.868	0.945	4.88E-09	TRUE
B IN	*EIF5A*	Wald ratio	1	-0.0078	0.0052	0.992 (0.982, 1.002)	0.134	0.394	6.57E-131	TRUE
B IN	*ELOVL5*	Wald ratio	1	-0.0108	0.0175	0.989 (0.956, 1.024)	0.538	0.762	2.02E-10	TRUE
B IN	*ELP2*	Wald ratio	1	0.0214	0.0142	1.022 (0.994, 1.05)	0.132	0.393	5.08E-15	TRUE
B IN	*ENO1*	Wald ratio	1	-0.0069	0.0185	0.993 (0.958, 1.03)	0.709	0.87	1.73E-09	TRUE
B IN	*EPSTI1*	Wald ratio	1	0.0086	0.0141	1.009 (0.981, 1.037)	0.54	0.762	1.22E-16	TRUE
B IN	*ERAP2*	Wald ratio	1	0.0386	0.0084	1.039 (1.022, 1.057)	4.46E-06	3.05E-04	5.62E-41	TRUE
B IN	*EVA1C*	Wald ratio	1	-0.0033	0.0129	0.997 (0.972, 1.022)	0.799	0.913	8.87E-19	TRUE
B IN	*EVI2A*	Wald ratio	1	-0.0153	0.0103	0.985 (0.965, 1.005)	0.136	0.399	2.04E-26	TRUE
B IN	*FADS2*	Wald ratio	1	-0.0229	0.0160	0.977 (0.947, 1.009)	0.153	0.423	3.07E-18	TRUE
B IN	*FAM111A*	Wald ratio	1	-0.0076	0.0175	0.992 (0.959, 1.027)	0.663	0.845	2.98E-10	TRUE
B IN	*FAM118A*	Wald ratio	1	0.0006	0.0047	1.001 (0.992, 1.01)	0.895	0.957	6.00E-105	TRUE
B IN	*FAM167A*	Wald ratio	1	-0.0561	0.0119	0.945 (0.924, 0.968)	2.39E-06	2.13E-04	3.36E-20	TRUE
B IN	*FAM177B*	Wald ratio	1	0.0115	0.0110	1.012 (0.99, 1.034)	0.297	0.592	1.61E-29	TRUE
B IN	*FAM184B*	Wald ratio	1	0.0025	0.0058	1.003 (0.991, 1.014)	0.663	0.845	8.00E-100	TRUE
B IN	*FAM21A*	Wald ratio	1	0.0497	0.0169	1.051 (1.017, 1.086)	0.003	0.037	1.85E-11	TRUE
B IN	*FAM26F*	IVW	2	-0.0027	0.0166	0.997 (0.965, 1.03)	0.872	0.947	4.85E-72	TRUE

B IN	*FAM69B*	Wald ratio	1	-0.0410	0.0181	0.96 (0.926, 0.995)	0.024	0.141	7.29E-10	TRUE
B IN	*FANCA*	Wald ratio	1	-0.0827	0.0222	0.921 (0.882, 0.962)	1.94E-04	0.005	7.08E-08	TRUE
B IN	*FAR1*	Wald ratio	1	-0.0042	0.0212	0.996 (0.955, 1.038)	0.844	0.935	5.50E-08	TRUE
B IN	*FCER2*	Wald ratio	1	-0.0006	0.0148	0.999 (0.971, 1.029)	0.966	0.988	2.93E-14	TRUE
B IN	*FCRL2*	Wald ratio	1	0.0007	0.0170	1.001 (0.968, 1.035)	0.965	0.988	2.79E-11	TRUE
B IN	*FCRL3*	Wald ratio	1	0.0067	0.0104	1.007 (0.986, 1.028)	0.522	0.754	2.05E-27	TRUE
B IN	*FCRL5*	Wald ratio	1	0.0005	0.0057	1 (0.989, 1.012)	0.93	0.97	1.30E-102	TRUE
B IN	*FCRLA*	Wald ratio	1	0.0118	0.0143	1.012 (0.984, 1.041)	0.411	0.686	7.89E-13	TRUE
B IN	*FIGNL1*	Wald ratio	1	0.0034	0.0166	1.003 (0.971, 1.037)	0.838	0.932	2.18E-11	TRUE
B IN	*FIS1*	Wald ratio	1	0.0069	0.0151	1.007 (0.978, 1.037)	0.649	0.835	3.59E-14	TRUE
B IN	*FKBPL*	Wald ratio	1	0.0127	0.0211	1.013 (0.972, 1.056)	0.549	0.769	1.84E-10	TRUE
B IN	*FNBP4*	Wald ratio	1	-0.0925	0.0174	0.912 (0.881, 0.943)	1.08E-07	6.60E-05	2.15E-10	TRUE
B IN	*FUT7*	Wald ratio	1	-0.0066	0.0198	0.993 (0.956, 1.033)	0.738	0.881	3.17E-08	TRUE
B IN	*FXYD5*	Wald ratio	1	0.0075	0.0168	1.008 (0.975, 1.041)	0.656	0.84	8.05E-12	TRUE
B IN	*GAA*	Wald ratio	1	-0.0069	0.0212	0.993 (0.953, 1.035)	0.743	0.881	2.69E-08	TRUE
B IN	*GALNT2*	Wald ratio	1	0.0369	0.0159	1.038 (1.006, 1.07)	0.02	0.129	1.39E-14	TRUE
B IN	*GALT*	Wald ratio	1	0.1049	0.0212	1.111 (1.065, 1.158)	7.32E-07	9.75E-05	1.59E-07	TRUE
B IN	*GATM*	Wald ratio	1	0.0391	0.0157	1.04 (1.008, 1.072)	0.013	0.094	5.88E-11	TRUE
B IN	*GATS*	Wald ratio	1	-0.0273	0.0116	0.973 (0.951, 0.995)	0.019	0.122	5.23E-21	TRUE
B IN	*GBP7*	Wald ratio	1	-0.0015	0.0107	0.999 (0.978, 1.02)	0.891	0.957	2.65E-26	TRUE
B IN	*GCC2*	Wald ratio	1	0.0114	0.0140	1.012 (0.984, 1.04)	0.414	0.686	4.56E-17	TRUE
B IN	*GCNT2*	Wald ratio	1	-0.0022	0.0206	0.998 (0.958, 1.039)	0.914	0.962	5.92E-08	TRUE
B IN	*GDI2*	Wald ratio	1	-0.0078	0.0132	0.992 (0.967, 1.018)	0.555	0.774	2.66E-18	TRUE
B IN	*GINM1*	Wald ratio	1	-0.0372	0.0184	0.964 (0.929, 0.999)	0.044	0.207	3.93E-09	TRUE
B IN	*GM2A*	Wald ratio	1	-0.0026	0.0096	0.997 (0.979, 1.016)	0.788	0.909	2.24E-36	TRUE
B IN	*GNG7*	Wald ratio	1	0.0504	0.0178	1.052 (1.016, 1.089)	0.005	0.048	3.69E-08	TRUE
B IN	*GNPTG*	Wald ratio	1	-0.0199	0.0158	0.98 (0.951, 1.011)	0.208	0.494	3.05E-10	TRUE
B IN	*GPATCH11*	Wald ratio	1	0.0125	0.0104	1.013 (0.992, 1.033)	0.227	0.52	1.34E-27	TRUE
B IN	*GPM6A*	Wald ratio	1	0.0023	0.0133	1.002 (0.976, 1.029)	0.866	0.944	1.97E-15	TRUE
B IN	*GPR155*	Wald ratio	1	0.0304	0.0134	1.031 (1.004, 1.058)	0.024	0.142	8.30E-18	TRUE
B IN	*GRHPR*	Wald ratio	1	-0.0139	0.0115	0.986 (0.964, 1.009)	0.227	0.52	8.71E-21	TRUE
B IN	*GS1-124K5.4*	Wald ratio	1	0.0049	0.0206	1.005 (0.965, 1.046)	0.811	0.919	2.82E-08	TRUE
B IN	*GSTM4*	Wald ratio	1	-0.0282	0.0150	0.972 (0.944, 1.001)	0.061	0.25	2.62E-13	TRUE
B IN	*GTF2H4*	IVW	3	-0.0526	0.0089	0.949 (0.932, 0.965)	3.22E-09	2.58E-05	7.81E-34	TRUE
B IN	*GTF2H4*	MR Egger	3	-0.0176	0.0639	0.983 (0.867, 1.114)	0.829	NA	NA	NA
B IN	*GTF2H4*	Weighted median	3	-0.0489	0.0115	0.952 (0.931, 0.974)	2.24E-05	NA	NA	NA
B IN	*GTF2H4*	Simple mode	3	-0.0455	0.0141	0.955 (0.929, 0.982)	0.084	NA	NA	NA
B IN	*GTF2H4*	Weighted mode	3	-0.0474	0.0135	0.954 (0.929, 0.979)	0.072	NA	NA	NA
B IN	*GYPC*	Wald ratio	1	0.0148	0.0146	1.015 (0.986, 1.044)	0.309	0.603	1.29E-14	TRUE
B IN	*HAGHL*	Wald ratio	1	0.0477	0.0166	1.049 (1.015, 1.083)	0.004	0.043	9.45E-09	TRUE
B IN	*HCG23*	Wald ratio	1	-0.0261	0.0222	0.974 (0.933, 1.018)	0.24	0.534	4.07E-09	TRUE
B IN	*HCG27*	Wald ratio	1	-0.0101	0.0155	0.99 (0.96, 1.02)	0.514	0.75	8.15E-12	TRUE
B IN	*HEATR5B*	Wald ratio	1	0.0210	0.0174	1.021 (0.987, 1.057)	0.227	0.52	1.23E-10	TRUE
B IN	*HEBP2*	Wald ratio	1	0.0254	0.0230	1.026 (0.981, 1.073)	0.269	0.563	1.64E-08	TRUE
B IN	*HFE*	Wald ratio	1	0.0043	0.0114	1.004 (0.982, 1.027)	0.709	0.87	2.16E-23	TRUE
B IN	*HIGD2A*	Wald ratio	1	-0.0139	0.0167	0.986 (0.954, 1.019)	0.405	0.681	6.69E-11	TRUE
B IN	*HIST1H1B*	Wald ratio	1	0.0088	0.0090	1.009 (0.991, 1.027)	0.329	0.624	2.20E-49	TRUE
B IN	*HIST1H2AB*	Wald ratio	1	0.0110	0.0214	1.011 (0.969, 1.054)	0.607	0.808	1.47E-12	TRUE
B IN	*HIST1H2AH*	Wald ratio	1	0.0142	0.0250	1.014 (0.966, 1.065)	0.568	0.782	1.07E-08	TRUE
B IN	*HIST1H2AM*	Wald ratio	1	-0.0143	0.0171	0.986 (0.953, 1.019)	0.404	0.681	3.07E-19	TRUE
B IN	*HIST1H2BF*	Wald ratio	1	0.0078	0.0127	1.008 (0.983, 1.033)	0.538	0.762	4.58E-19	TRUE
B IN	*HIST1H2BG*	Wald ratio	1	0.0040	0.0230	1.004 (0.96, 1.05)	0.863	0.943	1.26E-09	TRUE
B IN	*HIST1H2BJ*	Wald ratio	1	0.0160	0.0185	1.016 (0.98, 1.054)	0.386	0.669	6.30E-16	TRUE
B IN	*HIST1H4I*	Wald ratio	1	0.0264	0.0203	1.027 (0.987, 1.069)	0.194	0.474	8.88E-14	TRUE
B IN	*HLA-A*	IVW	2	0.0271	0.0093	1.028 (1.009, 1.046)	0.004	0.039	2.10E-34	TRUE
B IN	*HLA-B*	IVW	3	0.0039	0.0225	1.004 (0.961, 1.049)	0.861	0.942	3.50E-87	TRUE
B IN	*HLA-B*	MR Egger	3	0.2043	0.0574	1.227 (1.096, 1.373)	0.174	NA	NA	NA
B IN	*HLA-B*	Weighted median	3	0.0055	0.0070	1.006 (0.992, 1.019)	0.433	NA	NA	NA
B IN	*HLA-B*	Simple mode	3	-0.0387	0.0138	0.962 (0.936, 0.988)	0.107	NA	NA	NA
B IN	*HLA-B*	Weighted mode	3	0.0256	0.0090	1.026 (1.008, 1.044)	0.105	NA	NA	NA
B IN	*HLA-C*	Wald ratio	1	0.0134	0.0098	1.013 (0.994, 1.033)	0.173	0.451	5.51E-30	TRUE
B IN	*HLA-DPA1*	IVW	2	-0.0021	0.0170	0.998 (0.965, 1.032)	0.904	0.959	2.94E-52	TRUE
B IN	*HLA-DPB1*	Wald ratio	1	0.0050	0.0071	1.005 (0.991, 1.019)	0.483	0.736	1.62E-57	TRUE
B IN	*HLA-DQA1*	Wald ratio	1	0.0478	0.0096	1.049 (1.029, 1.069)	6.92E-07	9.75E-05	1.90E-28	TRUE
B IN	*HLA-DQA2*	IVW	2	-0.0167	0.0111	0.983 (0.962, 1.005)	0.132	0.393	2.14E-31	TRUE
B IN	*HLA-DRB1*	Wald ratio	1	0.0077	0.0070	1.008 (0.994, 1.022)	0.268	0.562	2.54E-57	TRUE
B IN	*HLA-DRB5*	IVW	3	0.0207	0.0160	1.021 (0.989, 1.053)	0.196	0.477	6.61E-222	TRUE
B IN	*HLA-DRB5*	MR Egger	3	0.0399	0.0463	1.041 (0.95, 1.139)	0.547	NA	NA	NA
B IN	*HLA-DRB5*	Weighted median	3	0.0263	0.0054	1.027 (1.016, 1.037)	8.98E-07	NA	NA	NA
B IN	*HLA-DRB5*	Simple mode	3	0.0349	0.0065	1.036 (1.022, 1.049)	0.033	NA	NA	NA
B IN	*HLA-DRB5*	Weighted mode	3	0.0283	0.0053	1.029 (1.018, 1.039)	0.034	NA	NA	NA
B IN	*HLA-F*	Wald ratio	1	-0.0180	0.0204	0.982 (0.944, 1.022)	0.379	0.665	2.51E-08	TRUE
B IN	*HMBOX1*	Wald ratio	1	-0.0139	0.0076	0.986 (0.972, 1.001)	0.066	0.263	6.29E-46	TRUE
B IN	*HMGB1*	Wald ratio	1	-0.0179	0.0205	0.982 (0.944, 1.022)	0.382	0.666	1.94E-08	TRUE
B IN	*HOPX*	Wald ratio	1	0.0135	0.0122	1.014 (0.99, 1.038)	0.267	0.561	6.26E-21	TRUE
B IN	*HOXB2*	Wald ratio	1	-0.0158	0.0152	0.984 (0.956, 1.014)	0.299	0.594	2.55E-13	TRUE
B IN	*HSBP1L1*	Wald ratio	1	-0.0781	0.0230	0.925 (0.884, 0.967)	6.67E-04	0.013	6.17E-08	TRUE
B IN	*HSD17B11*	Wald ratio	1	0.0548	0.0187	1.056 (1.018, 1.096)	0.003	0.038	3.18E-08	TRUE
B IN	*HSPBAP1*	Wald ratio	1	0.0056	0.0064	1.006 (0.993, 1.018)	0.382	0.666	8.05E-58	TRUE

B IN	*ICA1L*	Wald ratio	1	-0.0223	0.0142	0.978 (0.951, 1.006)	0.117	0.365	8.61E-15	TRUE
B IN	*IFITM3*	Wald ratio	1	0.0005	0.0171	1 (0.967, 1.035)	0.978	0.991	4.43E-11	TRUE
B IN	*IFNG-AS1*	Wald ratio	1	-0.0105	0.0145	0.99 (0.962, 1.018)	0.471	0.729	1.65E-14	TRUE
B IN	*IGFLR1*	Wald ratio	1	-0.0142	0.0253	0.986 (0.938, 1.036)	0.574	0.786	2.59E-09	TRUE
B IN	*IGLL5*	Wald ratio	1	0.0072	0.0148	1.007 (0.978, 1.037)	0.625	0.822	1.80E-14	TRUE
B IN	*IL4R*	Wald ratio	1	-0.0019	0.0203	0.998 (0.959, 1.039)	0.926	0.968	2.48E-08	TRUE
B IN	*INSIG1*	Wald ratio	1	0.0078	0.0175	1.008 (0.974, 1.043)	0.656	0.84	2.71E-10	TRUE
B IN	*IRAK3*	Wald ratio	1	0.0072	0.0170	1.007 (0.974, 1.041)	0.672	0.85	4.39E-10	TRUE
B IN	*ISCU*	Wald ratio	1	0.0003	0.0157	1 (0.97, 1.032)	0.984	0.994	5.52E-12	TRUE
B IN	*ITGB2*	Wald ratio	1	-0.0008	0.0062	0.999 (0.987, 1.011)	0.897	0.957	1.07E-65	TRUE
B IN	*ITGB3BP*	Wald ratio	1	0.0068	0.0146	1.007 (0.978, 1.036)	0.641	0.831	5.65E-14	TRUE
B IN	*ITM2B*	Wald ratio	1	-0.0025	0.0175	0.998 (0.964, 1.032)	0.888	0.955	3.75E-10	TRUE
B IN	*ITM2C*	Wald ratio	1	0.0031	0.0127	1.003 (0.978, 1.028)	0.808	0.918	4.67E-12	TRUE
B IN	*JUP*	IVW	2	-0.0375	0.0177	0.963 (0.93, 0.997)	0.034	0.175	2.73E-25	TRUE
B IN	*KANSL1-AS1*	Wald ratio	1	-0.0205	0.0089	0.98 (0.963, 0.997)	0.021	0.131	7.56E-82	TRUE
B IN	*KDELR2*	Wald ratio	1	0.0424	0.0131	1.043 (1.017, 1.07)	0.001	0.02	3.18E-16	TRUE
B IN	*KIAA1841*	Wald ratio	1	0.0144	0.0166	1.014 (0.982, 1.048)	0.388	0.669	1.09E-09	TRUE
B IN	*KRT10*	Wald ratio	1	0.0138	0.0157	1.014 (0.983, 1.046)	0.379	0.665	2.66E-12	TRUE
B IN	*KTN1*	Wald ratio	1	0.0519	0.0173	1.053 (1.018, 1.09)	0.003	0.033	5.53E-10	TRUE
B IN	*KYNU*	Wald ratio	1	-0.0418	0.0160	0.959 (0.929, 0.99)	0.009	0.076	8.97E-12	TRUE
B IN	*L3HYPDH*	Wald ratio	1	0.0191	0.0202	1.019 (0.98, 1.06)	0.345	0.638	3.73E-09	TRUE
B IN	*L3MBTL3*	Wald ratio	1	-0.0046	0.0133	0.995 (0.97, 1.022)	0.726	0.878	3.95E-19	TRUE
B IN	*LA16c-358B7.3*	Wald ratio	1	0.0600	0.0150	1.062 (1.031, 1.094)	6.58E-05	0.002	3.00E-14	TRUE
B IN	*LAMB1*	Wald ratio	1	0.0087	0.0098	1.009 (0.989, 1.028)	0.379	0.666	1.90E-30	TRUE
B IN	*LARGE*	Wald ratio	1	0.0014	0.0073	1.001 (0.987, 1.016)	0.847	0.935	2.52E-56	TRUE
B IN	*LCN8*	Wald ratio	1	-0.0088	0.0056	0.991 (0.98, 1.002)	0.119	0.369	3.83E-140	TRUE
B IN	*LGALS9*	Wald ratio	1	0.0223	0.0195	1.023 (0.984, 1.062)	0.251	0.545	6.49E-09	TRUE
B IN	*LHPP*	Wald ratio	1	-0.0365	0.0212	0.964 (0.925, 1.005)	0.086	0.311	6.70E-08	TRUE
B IN	*LIMD1*	Wald ratio	1	0.0078	0.0087	1.008 (0.991, 1.025)	0.368	0.656	2.42E-39	TRUE
B IN	*LIMS2*	Wald ratio	1	0.0200	0.0119	1.02 (0.997, 1.044)	0.093	0.323	7.66E-21	TRUE
B IN	*LINC00339*	Wald ratio	1	-0.0325	0.0178	0.968 (0.935, 1.002)	0.067	0.265	1.85E-09	TRUE
B IN	*LINC00926*	Wald ratio	1	-0.0174	0.0126	0.983 (0.959, 1.007)	0.168	0.443	3.79E-20	TRUE
B IN	*LINC00954*	Wald ratio	1	-0.0176	0.0203	0.983 (0.944, 1.022)	0.385	0.668	1.22E-09	TRUE
B IN	*LINC00969*	Wald ratio	1	-0.0105	0.0120	0.99 (0.966, 1.013)	0.384	0.668	2.36E-20	TRUE
B IN	*LRRC16A*	Wald ratio	1	-0.0053	0.0118	0.995 (0.972, 1.018)	0.652	0.836	3.05E-20	TRUE
B IN	*LRRC25*	Wald ratio	1	-0.0494	0.0198	0.952 (0.916, 0.989)	0.013	0.095	4.83E-08	TRUE
B IN	*LRRC37A2*	Wald ratio	1	-0.0018	0.0171	0.998 (0.965, 1.032)	0.916	0.963	9.82E-10	TRUE
B IN	*LRRC61*	Wald ratio	1	-0.0527	0.0194	0.949 (0.913, 0.985)	0.007	0.06	3.66E-08	TRUE
B IN	*LSM7*	Wald ratio	1	0.0364	0.0114	1.037 (1.014, 1.06)	0.001	0.02	4.95E-22	TRUE
B IN	*LST1*	Wald ratio	1	0.0421	0.0168	1.043 (1.009, 1.078)	0.012	0.093	7.02E-09	TRUE
B IN	*LTA4H*	Wald ratio	1	0.0115	0.0096	1.012 (0.993, 1.031)	0.231	0.524	9.82E-16	TRUE
B IN	*LY6G5C*	Wald ratio	1	-0.0733	0.0205	0.929 (0.893, 0.967)	3.48E-04	0.008	1.13E-08	TRUE
B IN	*LY86*	Wald ratio	1	-0.0046	0.0072	0.995 (0.982, 1.009)	0.52	0.753	1.06E-57	TRUE
B IN	*LY9*	Wald ratio	1	-0.0215	0.0194	0.979 (0.942, 1.017)	0.267	0.561	1.61E-08	TRUE
B IN	*LY96*	Wald ratio	1	0.0153	0.0167	1.015 (0.983, 1.049)	0.361	0.65	2.22E-15	TRUE
B IN	*MACROD2*	IVW	2	-0.0044	0.0116	0.996 (0.973, 1.018)	0.704	0.867	3.68E-23	TRUE
B IN	*MARC2*	Wald ratio	1	-0.0005	0.0164	1 (0.968, 1.032)	0.977	0.991	9.73E-11	TRUE
B IN	*MBOAT1*	Wald ratio	1	0.0260	0.0177	1.026 (0.991, 1.063)	0.142	0.409	3.49E-10	TRUE
B IN	*MBP*	Wald ratio	1	-0.0239	0.0193	0.976 (0.94, 1.014)	0.215	0.505	1.04E-08	TRUE
B IN	*MDGA1*	Wald ratio	1	-0.0090	0.0115	0.991 (0.969, 1.014)	0.435	0.702	7.61E-23	TRUE
B IN	*MDM4*	Wald ratio	1	-0.0437	0.0116	0.957 (0.936, 0.979)	1.71E-04	0.004	3.76E-27	TRUE
B IN	*MED16*	Wald ratio	1	0.0015	0.0186	1.001 (0.966, 1.039)	0.938	0.975	5.54E-11	TRUE
B IN	*METTL8*	Wald ratio	1	-0.0106	0.0073	0.989 (0.975, 1.004)	0.147	0.416	1.26E-50	TRUE
B IN	*MFF*	Wald ratio	1	-0.0012	0.0163	0.999 (0.967, 1.031)	0.943	0.978	8.56E-10	TRUE
B IN	*MGMT*	Wald ratio	1	-0.0237	0.0164	0.977 (0.946, 1.009)	0.149	0.418	8.08E-12	TRUE
B IN	*MGST3*	Wald ratio	1	0.0003	0.0084	1 (0.984, 1.017)	0.97	0.989	1.03E-40	TRUE
B IN	*MILR1*	Wald ratio	1	-0.0412	0.0158	0.96 (0.93, 0.99)	0.009	0.076	5.31E-12	TRUE
B IN	*MLTK*	Wald ratio	1	-0.0117	0.0170	0.988 (0.956, 1.022)	0.49	0.738	1.20E-10	TRUE
B IN	*MPHOSPH6*	Wald ratio	1	0.0546	0.0217	1.056 (1.012, 1.102)	0.012	0.092	5.44E-08	TRUE
B IN	*MRPL21*	Wald ratio	1	0.0133	0.0154	1.013 (0.983, 1.044)	0.388	0.669	1.05E-11	TRUE
B IN	*MRPL34*	Wald ratio	1	0.0229	0.0203	1.023 (0.983, 1.065)	0.26	0.555	3.84E-08	TRUE
B IN	*MRPL42*	Wald ratio	1	0.0337	0.0191	1.034 (0.996, 1.074)	0.078	0.292	1.25E-08	TRUE
B IN	*MRPL53*	Wald ratio	1	-0.0077	0.0161	0.992 (0.961, 1.024)	0.631	0.825	2.70E-11	TRUE
B IN	*MRPS18C*	Wald ratio	1	-0.0024	0.0193	0.998 (0.96, 1.036)	0.899	0.958	8.73E-09	TRUE
B IN	*MSH5*	IVW	2	-0.0538	0.0368	0.948 (0.882, 1.018)	0.144	0.411	2.03E-18	TRUE
B IN	*MSMP*	Wald ratio	1	-0.0616	0.0174	0.94 (0.909, 0.973)	3.89E-04	0.009	7.39E-10	TRUE
B IN	*MTSS1*	Wald ratio	1	-0.0171	0.0181	0.983 (0.949, 1.018)	0.343	0.636	6.91E-09	TRUE
B IN	*MTUS1*	Wald ratio	1	0.0110	0.0080	1.011 (0.995, 1.027)	0.169	0.444	1.78E-52	TRUE
B IN	*MXRA7*	Wald ratio	1	-0.0054	0.0150	0.995 (0.966, 1.024)	0.717	0.874	2.06E-10	TRUE
B IN	*MYBL2*	Wald ratio	1	-0.0175	0.0081	0.983 (0.967, 0.998)	0.031	0.167	5.99E-46	TRUE
B IN	*MYH11*	Wald ratio	1	-0.0212	0.0121	0.979 (0.956, 1.002)	0.079	0.294	3.88E-21	TRUE
B IN	*MYLIP*	Wald ratio	1	0.0224	0.0187	1.023 (0.986, 1.061)	0.231	0.524	1.36E-09	TRUE
B IN	*MYO1E*	Wald ratio	1	0.0353	0.0201	1.036 (0.996, 1.078)	0.08	0.296	4.70E-08	TRUE
B IN	*MYO7B*	Wald ratio	1	0.0191	0.0113	1.019 (0.997, 1.042)	0.09	0.318	6.56E-23	TRUE
B IN	*MZT2A*	Wald ratio	1	-0.0139	0.0082	0.986 (0.97, 1.002)	0.091	0.319	1.24E-32	TRUE
B IN	*NAA38*	Wald ratio	1	-0.0099	0.0155	0.99 (0.961, 1.021)	0.523	0.754	2.94E-17	TRUE
B IN	*NAP1L1*	Wald ratio	1	-0.0115	0.0133	0.989 (0.963, 1.015)	0.386	0.669	4.78E-17	TRUE
B IN	*NAPRT1*	Wald ratio	1	-0.0167	0.0101	0.983 (0.964, 1.003)	0.099	0.333	1.09E-27	TRUE
B IN	*NCF4*	Wald ratio	1	0.0109	0.0201	1.011 (0.972, 1.052)	0.587	0.794	1.96E-08	TRUE

B IN	*NDUFA3*	Wald ratio	1	0.0063	0.0177	1.006 (0.972, 1.042)	0.722	0.877	8.63E-11	TRUE
B IN	*NDUFA6*	Wald ratio	1	0.0034	0.0209	1.003 (0.963, 1.045)	0.871	0.947	2.22E-08	TRUE
B IN	*NEK8*	Wald ratio	1	0.0085	0.0169	1.008 (0.976, 1.042)	0.617	0.817	5.06E-12	TRUE
B IN	*NFKBIZ*	Wald ratio	1	0.0060	0.0183	1.006 (0.971, 1.043)	0.742	0.881	3.20E-08	TRUE
B IN	*NKX6-3*	Wald ratio	1	-0.0094	0.0083	0.991 (0.975, 1.007)	0.258	0.552	2.07E-45	TRUE
B IN	*NME1*	Wald ratio	1	0.0047	0.0140	1.005 (0.978, 1.033)	0.737	0.88	1.08E-15	TRUE
B IN	*NMI*	Wald ratio	1	0.0387	0.0165	1.039 (1.006, 1.074)	0.019	0.122	2.65E-11	TRUE
B IN	*NMRAL1*	Wald ratio	1	0.0126	0.0215	1.013 (0.971, 1.056)	0.558	0.776	3.69E-08	TRUE
B IN	*NMRK1*	Wald ratio	1	-0.0037	0.0184	0.996 (0.961, 1.033)	0.84	0.932	9.96E-10	TRUE
B IN	*NOMO1*	Wald ratio	1	-0.0502	0.0166	0.951 (0.92, 0.983)	0.003	0.031	1.60E-12	TRUE
B IN	*NQO2*	Wald ratio	1	-0.0243	0.0172	0.976 (0.944, 1.009)	0.157	0.428	1.64E-12	TRUE
B IN	*NSA2*	Wald ratio	1	0.0226	0.0100	1.023 (1.003, 1.043)	0.024	0.142	6.66E-32	TRUE
B IN	*NSUN4*	Wald ratio	1	-0.0029	0.0182	0.997 (0.962, 1.033)	0.875	0.948	1.72E-09	TRUE
B IN	*NT5C3A*	Wald ratio	1	-0.0260	0.0170	0.974 (0.942, 1.007)	0.127	0.382	7.23E-12	TRUE
B IN	*NT5C3B*	Wald ratio	1	0.0303	0.0141	1.031 (1.003, 1.06)	0.031	0.167	6.32E-12	TRUE
B IN	*NT5DC3*	Wald ratio	1	0.0146	0.0158	1.015 (0.984, 1.047)	0.355	0.646	1.02E-13	TRUE
B IN	*NT5E*	Wald ratio	1	-0.0026	0.0131	0.997 (0.972, 1.023)	0.842	0.934	9.14E-23	TRUE
B IN	*NTPCR*	Wald ratio	1	0.0282	0.0205	1.029 (0.988, 1.071)	0.17	0.446	6.56E-09	TRUE
B IN	*OPTN*	Wald ratio	1	0.0084	0.0160	1.008 (0.977, 1.041)	0.601	0.805	6.62E-16	TRUE
B IN	*ORMDL3*	Wald ratio	1	0.0025	0.0079	1.002 (0.987, 1.018)	0.754	0.888	6.00E-47	TRUE
B IN	*OSTF1*	Wald ratio	1	-0.0101	0.0142	0.99 (0.963, 1.018)	0.477	0.732	4.57E-17	TRUE
B IN	*P2RX5*	Wald ratio	1	0.0082	0.0191	1.008 (0.971, 1.047)	0.669	0.848	5.82E-09	TRUE
B IN	*PARK7*	Wald ratio	1	0.0406	0.0189	1.041 (1.004, 1.081)	0.032	0.168	2.04E-09	TRUE
B IN	*PARP14*	Wald ratio	1	0.0012	0.0097	1.001 (0.982, 1.02)	0.904	0.959	2.55E-16	TRUE
B IN	*PCBP2*	Wald ratio	1	-0.0846	0.0218	0.919 (0.881, 0.959)	1.01E-04	0.003	3.79E-08	TRUE
B IN	*PCSK7*	Wald ratio	1	-0.0255	0.0179	0.975 (0.941, 1.01)	0.156	0.427	1.06E-08	TRUE
B IN	*PEX11A*	Wald ratio	1	0.0088	0.0125	1.009 (0.984, 1.034)	0.481	0.734	5.56E-19	TRUE
B IN	*PEX6*	Wald ratio	1	0.0110	0.0155	1.011 (0.981, 1.042)	0.478	0.733	3.46E-13	TRUE
B IN	*PGBD1*	Wald ratio	1	0.0171	0.0193	1.017 (0.979, 1.056)	0.376	0.664	1.76E-15	TRUE
B IN	*PI4K2B*	Wald ratio	1	0.0052	0.0250	1.005 (0.957, 1.056)	0.833	0.93	1.80E-10	TRUE
B IN	*PILRB*	Wald ratio	1	0.0130	0.0113	1.013 (0.991, 1.036)	0.25	0.543	3.89E-21	TRUE
B IN	*PINLYP*	Wald ratio	1	0.0071	0.0103	1.007 (0.987, 1.028)	0.49	0.738	1.74E-27	TRUE
B IN	*PKHD1L1*	Wald ratio	1	0.0036	0.0081	1.004 (0.988, 1.02)	0.652	0.837	4.37E-45	TRUE
B IN	*PKIG*	Wald ratio	1	0.0025	0.0112	1.002 (0.981, 1.025)	0.825	0.926	1.41E-29	TRUE
B IN	*PLEK*	Wald ratio	1	0.0218	0.0165	1.022 (0.99, 1.056)	0.186	0.466	3.31E-10	TRUE
B IN	*PLTP*	Wald ratio	1	0.0374	0.0157	1.038 (1.007, 1.071)	0.017	0.116	6.71E-13	TRUE
B IN	*PODXL2*	Wald ratio	1	-0.0084	0.0195	0.992 (0.954, 1.03)	0.665	0.846	4.66E-08	TRUE
B IN	*POLM*	Wald ratio	1	0.0095	0.0143	1.01 (0.982, 1.038)	0.507	0.745	1.21E-12	TRUE
B IN	*POLR1D*	Wald ratio	1	0.0016	0.0114	1.002 (0.979, 1.024)	0.888	0.955	3.18E-29	TRUE
B IN	*POLR2J3*	Wald ratio	1	0.0126	0.0190	1.013 (0.976, 1.051)	0.509	0.747	1.01E-09	TRUE
B IN	*POMZP3*	Wald ratio	1	0.0216	0.0153	1.022 (0.992, 1.053)	0.158	0.429	4.25E-16	TRUE
B IN	*PPA1*	Wald ratio	1	0.0038	0.0080	1.004 (0.988, 1.02)	0.632	0.826	4.69E-42	TRUE
B IN	*PPDPF*	Wald ratio	1	-0.0072	0.0067	0.993 (0.98, 1.006)	0.283	0.575	9.77E-59	TRUE
B IN	*PPIA*	Wald ratio	1	-0.0091	0.0125	0.991 (0.967, 1.016)	0.47	0.727	1.30E-19	TRUE
B IN	*PPIL3*	Wald ratio	1	0.0086	0.0118	1.009 (0.986, 1.032)	0.467	0.725	1.25E-20	TRUE
B IN	*PRKD3*	Wald ratio	1	0.0546	0.0181	1.056 (1.019, 1.094)	0.002	0.031	7.20E-10	TRUE
B IN	*PRR4*	Wald ratio	1	-0.0073	0.0206	0.993 (0.953, 1.034)	0.725	0.878	2.10E-12	TRUE
B IN	*PRR7-AS1*	Wald ratio	1	0.0074	0.0118	1.007 (0.984, 1.031)	0.529	0.757	1.66E-21	TRUE
B IN	*PSMD5-AS1*	Wald ratio	1	0.0041	0.0051	1.004 (0.994, 1.014)	0.418	0.689	6.32E-119	TRUE
B IN	*PTPLAD2*	Wald ratio	1	-0.0508	0.0117	0.95 (0.929, 0.972)	1.29E-05	6.87E-04	9.09E-30	TRUE
B IN	*PTPN4*	Wald ratio	1	-0.0059	0.0177	0.994 (0.96, 1.029)	0.738	0.881	1.78E-09	TRUE
B IN	*RABAC1*	Wald ratio	1	0.0196	0.0126	1.02 (0.995, 1.045)	0.119	0.369	8.68E-21	TRUE
B IN	*RAC1*	Wald ratio	1	-0.0380	0.0133	0.963 (0.938, 0.988)	0.004	0.044	7.37E-17	TRUE
B IN	*RAD51C*	Wald ratio	1	0.0114	0.0189	1.011 (0.975, 1.05)	0.546	0.767	3.45E-08	TRUE
B IN	*RASGRP3*	Wald ratio	1	0.0036	0.0129	1.004 (0.978, 1.029)	0.78	0.905	1.83E-19	TRUE
B IN	*RBM26*	Wald ratio	1	-0.0105	0.0168	0.99 (0.958, 1.023)	0.533	0.76	2.21E-11	TRUE
B IN	*RBM6*	Wald ratio	1	-0.0724	0.0170	0.93 (0.9, 0.962)	1.96E-05	9.43E-04	1.90E-10	TRUE
B IN	*RCSD1*	Wald ratio	1	-0.0195	0.0128	0.981 (0.956, 1.006)	0.129	0.388	3.41E-17	TRUE
B IN	*REL*	Wald ratio	1	-0.0188	0.0099	0.981 (0.963, 1.001)	0.057	0.24	6.91E-25	TRUE
B IN	*REV3L*	Wald ratio	1	0.0139	0.0177	1.014 (0.98, 1.05)	0.43	0.7	3.17E-10	TRUE
B IN	*RGS1*	Wald ratio	1	-0.0197	0.0177	0.981 (0.947, 1.015)	0.267	0.561	1.07E-11	TRUE
B IN	*RHBDF2*	Wald ratio	1	0.0170	0.0164	1.017 (0.985, 1.05)	0.3	0.596	1.49E-11	TRUE
B IN	*RIC3*	Wald ratio	1	-0.0020	0.0185	0.998 (0.962, 1.035)	0.914	0.962	1.10E-09	TRUE
B IN	*RNGTT*	Wald ratio	1	-0.0033	0.0117	0.997 (0.974, 1.02)	0.781	0.905	4.97E-21	TRUE
B IN	*RP1-29C18.10*	Wald ratio	1	-0.0181	0.0091	0.982 (0.965, 1)	0.046	0.21	1.34E-38	TRUE
B IN	*RP1-3J17.3*	IVW	2	0.0021	0.0073	1.002 (0.988, 1.017)	0.772	0.899	4.05E-56	TRUE
B IN	*RP11-1070N10.3*	Wald ratio	1	-0.0273	0.0191	0.973 (0.937, 1.01)	0.152	0.422	4.94E-09	TRUE
B IN	*RP11-111A22.1*	Wald ratio	1	-0.0054	0.0056	0.995 (0.984, 1.006)	0.335	0.628	4.32E-101	TRUE
B IN	*RP11-138A9.1*	Wald ratio	1	0.0190	0.0278	1.019 (0.965, 1.076)	0.495	0.74	1.77E-10	TRUE
B IN	*RP11-139H15.1*	Wald ratio	1	0.0082	0.0180	1.008 (0.973, 1.044)	0.649	0.835	2.83E-09	TRUE
B IN	*RP11-254F7.2*	Wald ratio	1	0.0191	0.0246	1.019 (0.971, 1.07)	0.437	0.703	8.81E-09	TRUE
B IN	*RP11-298J20.3*	Wald ratio	1	0.0194	0.0073	1.02 (1.005, 1.034)	0.008	0.069	6.94E-51	TRUE
B IN	*RP11-299J3.8*	Wald ratio	1	-0.0107	0.0220	0.989 (0.948, 1.033)	0.627	0.822	5.17E-08	TRUE
B IN	*RP11-326C3.11*	Wald ratio	1	-0.0271	0.0197	0.973 (0.936, 1.012)	0.169	0.444	6.84E-10	TRUE
B IN	*RP11-326C3.12*	Wald ratio	1	-0.0181	0.0185	0.982 (0.947, 1.018)	0.325	0.62	2.68E-09	TRUE
B IN	*RP11-328M4.2*	Wald ratio	1	-0.0104	0.0088	0.99 (0.973, 1.007)	0.236	0.53	1.64E-32	TRUE
B IN	*RP11-345J4.5*	Wald ratio	1	-0.0361	0.0163	0.965 (0.934, 0.996)	0.027	0.154	4.93E-12	TRUE
B IN	*RP11-347P5.1*	Wald ratio	1	0.0024	0.0066	1.002 (0.99, 1.015)	0.718	0.874	1.26E-68	TRUE
B IN	*RP11-361F15.2*	Wald ratio	1	-0.0082	0.0146	0.992 (0.964, 1.021)	0.574	0.786	1.10E-13	TRUE

B IN	*RP11-386I14.4*	Wald ratio	1	-0.0115	0.0182	0.989 (0.954, 1.024)	0.528	0.757	4.11E-11	TRUE
B IN	*RP11-434C1.1*	Wald ratio	1	-0.0153	0.0181	0.985 (0.95, 1.02)	0.399	0.678	1.14E-08	TRUE
B IN	*RP11-446N19.1*	Wald ratio	1	-0.0140	0.0179	0.986 (0.952, 1.021)	0.434	0.702	6.24E-10	TRUE
B IN	*RP11-455J20.3*	Wald ratio	1	-0.0120	0.0085	0.988 (0.972, 1.005)	0.158	0.429	3.58E-39	TRUE
B IN	*RP11-457M11.5*	Wald ratio	1	0.0138	0.0268	1.014 (0.962, 1.069)	0.607	0.808	1.69E-08	TRUE
B IN	*RP11-473I1.5*	Wald ratio	1	-0.0276	0.0106	0.973 (0.953, 0.993)	0.009	0.076	2.19E-21	TRUE
B IN	*RP11-485G7.5*	Wald ratio	1	0.0092	0.0080	1.009 (0.994, 1.025)	0.249	0.542	2.19E-35	TRUE
B IN	*RP11-542K23.7*	Wald ratio	1	0.0151	0.0201	1.015 (0.976, 1.056)	0.453	0.717	1.45E-14	TRUE
B IN	*RP11-596D21.1*	Wald ratio	1	0.0303	0.0125	1.031 (1.006, 1.056)	0.015	0.106	1.21E-18	TRUE
B IN	*RP11-693J15.5*	Wald ratio	1	-0.0081	0.0190	0.992 (0.956, 1.03)	0.669	0.848	1.02E-09	TRUE
B IN	*RP11-69E11.4*	Wald ratio	1	0.0700	0.0219	1.073 (1.027, 1.12)	0.001	0.021	6.04E-08	TRUE
B IN	*RP11-755F10.1*	Wald ratio	1	-0.0198	0.0086	0.98 (0.964, 0.997)	0.021	0.131	2.12E-37	TRUE
B IN	*RP11-8L8.2*	Wald ratio	1	0.0101	0.0098	1.01 (0.991, 1.03)	0.3	0.596	4.10E-30	TRUE
B IN	*RP11-950C14.3*	Wald ratio	1	0.0143	0.0082	1.014 (0.998, 1.031)	0.081	0.299	5.40E-43	TRUE
B IN	*RP11-960L18.1*	Wald ratio	1	-0.0310	0.0201	0.969 (0.932, 1.008)	0.123	0.377	1.04E-08	TRUE
B IN	*RP11-96H19.1*	Wald ratio	1	-0.0156	0.0199	0.985 (0.947, 1.024)	0.434	0.702	3.08E-08	TRUE
B IN	*RP13-514E23.2*	Wald ratio	1	-0.0330	0.0090	0.967 (0.951, 0.985)	2.45E-04	0.006	2.45E-34	TRUE
B IN	*RP3-325F22.5*	Wald ratio	1	0.0005	0.0174	1 (0.967, 1.035)	0.978	0.991	5.97E-12	TRUE
B IN	*RP5-827C21.4*	Wald ratio	1	-0.0099	0.0241	0.99 (0.944, 1.038)	0.68	0.853	1.14E-08	TRUE
B IN	*RPL10A*	Wald ratio	1	-0.0067	0.0075	0.993 (0.979, 1.008)	0.372	0.661	6.23E-53	TRUE
B IN	*RPL11*	Wald ratio	1	-0.0144	0.0092	0.986 (0.968, 1.004)	0.119	0.369	1.84E-33	TRUE
B IN	*RPL12*	IVW	2	-0.0128	0.0195	0.987 (0.95, 1.026)	0.511	0.748	1.79E-131	TRUE
B IN	*RPL13*	Wald ratio	1	-0.0206	0.0102	0.98 (0.96, 0.999)	0.043	0.202	9.29E-51	TRUE
B IN	*RPL14*	Wald ratio	1	0.0186	0.0166	1.019 (0.986, 1.052)	0.264	0.558	1.05E-13	TRUE
B IN	*RPL22L1*	Wald ratio	1	0.0033	0.0155	1.003 (0.973, 1.034)	0.833	0.93	2.50E-16	TRUE
B IN	*RPL23A*	Wald ratio	1	-0.0219	0.0100	0.978 (0.959, 0.998)	0.028	0.157	1.24E-29	TRUE
B IN	*RPL27A*	Wald ratio	1	0.0101	0.0119	1.01 (0.987, 1.034)	0.4	0.678	4.45E-19	TRUE
B IN	*RPL28*	Wald ratio	1	0.0039	0.0051	1.004 (0.994, 1.014)	0.438	0.703	2.50E-118	TRUE
B IN	*RPL3*	Wald ratio	1	0.0139	0.0148	1.014 (0.985, 1.044)	0.347	0.64	3.96E-14	TRUE
B IN	*RPL32*	Wald ratio	1	0.0257	0.0196	1.026 (0.987, 1.066)	0.19	0.471	1.09E-08	TRUE
B IN	*RPL36AL*	Wald ratio	1	-0.0045	0.0070	0.996 (0.982, 1.009)	0.524	0.754	5.38E-62	TRUE
B IN	*RPL8*	Wald ratio	1	-0.0081	0.0046	0.992 (0.983, 1.001)	0.074	0.286	1.90E-162	TRUE
B IN	*RPLP2*	Wald ratio	1	-0.0235	0.0090	0.977 (0.96, 0.994)	0.009	0.076	8.74E-36	TRUE
B IN	*RPPH1*	Wald ratio	1	0.0140	0.0177	1.014 (0.979, 1.05)	0.43	0.7	2.90E-08	TRUE
B IN	*RPS10*	Wald ratio	1	-0.0069	0.0068	0.993 (0.98, 1.006)	0.305	0.599	5.92E-54	TRUE
B IN	*RPS11*	Wald ratio	1	0.0292	0.0123	1.03 (1.005, 1.055)	0.017	0.115	1.45E-21	TRUE
B IN	*RPS12*	Wald ratio	1	0.0162	0.0102	1.016 (0.996, 1.037)	0.114	0.361	2.70E-17	TRUE
B IN	*RPS16*	Wald ratio	1	-0.0066	0.0073	0.993 (0.979, 1.008)	0.363	0.651	2.76E-52	TRUE
B IN	*RPS18*	Wald ratio	1	-0.0121	0.0071	0.988 (0.974, 1.002)	0.087	0.312	1.78E-73	TRUE
B IN	*RPS2*	Wald ratio	1	-0.0242	0.0090	0.976 (0.959, 0.994)	0.007	0.066	3.63E-37	TRUE
B IN	*RPS23*	Wald ratio	1	0.0176	0.0205	1.018 (0.978, 1.06)	0.391	0.672	2.34E-09	TRUE
B IN	*RPS24*	Wald ratio	1	-0.0034	0.0186	0.997 (0.961, 1.034)	0.855	0.938	1.65E-09	TRUE
B IN	*RPS25*	Wald ratio	1	-0.0088	0.0111	0.991 (0.97, 1.013)	0.428	0.699	6.50E-24	TRUE
B IN	*RPS26*	Wald ratio	1	-0.0099	0.0043	0.99 (0.982, 0.999)	0.022	0.132	2.51E-191	TRUE
B IN	*RPS3*	Wald ratio	1	-0.0124	0.0070	0.988 (0.974, 1.001)	0.075	0.287	4.06E-35	TRUE
B IN	*RPS3A*	Wald ratio	1	-0.0045	0.0065	0.996 (0.983, 1.008)	0.492	0.738	4.01E-33	TRUE
B IN	*RPS5*	Wald ratio	1	0.0077	0.0057	1.008 (0.997, 1.019)	0.177	0.456	5.55E-101	TRUE
B IN	*RPS6*	Wald ratio	1	-0.0145	0.0124	0.986 (0.962, 1.01)	0.242	0.534	7.64E-26	TRUE
B IN	*RPS7*	Wald ratio	1	0.0034	0.0110	1.003 (0.982, 1.025)	0.754	0.888	1.71E-37	TRUE
B IN	*RPS8*	Wald ratio	1	0.0017	0.0063	1.002 (0.989, 1.014)	0.791	0.909	5.76E-76	TRUE
B IN	*RPS9*	Wald ratio	1	0.0264	0.0069	1.027 (1.013, 1.041)	1.26E-04	0.003	1.97E-60	TRUE
B IN	*RPSA*	Wald ratio	1	-0.0280	0.0136	0.972 (0.947, 0.999)	0.039	0.193	1.25E-20	TRUE
B IN	*RSRC2*	Wald ratio	1	-0.0090	0.0156	0.991 (0.961, 1.022)	0.564	0.78	2.66E-15	TRUE
B IN	*SAG*	Wald ratio	1	0.0277	0.0097	1.028 (1.009, 1.048)	0.004	0.044	6.64E-32	TRUE
B IN	*SAMD12*	Wald ratio	1	-0.0128	0.0207	0.987 (0.948, 1.028)	0.537	0.762	1.46E-10	TRUE
B IN	*SAR1A*	Wald ratio	1	0.0126	0.0168	1.013 (0.98, 1.047)	0.453	0.717	8.15E-11	TRUE
B IN	*SDK2*	Wald ratio	1	0.0305	0.0185	1.031 (0.994, 1.069)	0.099	0.333	3.64E-09	TRUE
B IN	*SEC61B*	Wald ratio	1	-0.0135	0.0152	0.987 (0.958, 1.016)	0.373	0.661	1.39E-17	TRUE
B IN	*SELL*	Wald ratio	1	-0.0020	0.0176	0.998 (0.964, 1.033)	0.908	0.961	3.56E-11	TRUE
B IN	*SEPSECS*	Wald ratio	1	0.0138	0.0181	1.014 (0.979, 1.05)	0.443	0.708	6.55E-10	TRUE
B IN	*SEPT2*	Wald ratio	1	0.0121	0.0184	1.012 (0.976, 1.049)	0.512	0.748	1.83E-10	TRUE
B IN	*SERPINE2*	Wald ratio	1	-0.0265	0.0150	0.974 (0.946, 1.003)	0.077	0.289	1.80E-13	TRUE
B IN	*SESTD1*	Wald ratio	1	-0.0216	0.0191	0.979 (0.943, 1.016)	0.258	0.552	8.57E-10	TRUE
B IN	*SETD9*	Wald ratio	1	-0.0510	0.0184	0.95 (0.917, 0.985)	0.006	0.054	1.84E-08	TRUE
B IN	*SF1*	Wald ratio	1	-0.0316	0.0148	0.969 (0.941, 0.997)	0.032	0.17	5.28E-11	TRUE
B IN	*SH2B2*	Wald ratio	1	0.0195	0.0181	1.02 (0.984, 1.056)	0.281	0.575	2.02E-10	TRUE
B IN	*SH3BGRL3*	Wald ratio	1	-0.0057	0.0109	0.994 (0.973, 1.016)	0.598	0.803	1.07E-24	TRUE
B IN	*SH3YL1*	Wald ratio	1	0.0407	0.0101	1.042 (1.021, 1.062)	5.45E-05	0.002	2.31E-28	TRUE
B IN	*SIDT1-AS1*	Wald ratio	1	-0.0134	0.0145	0.987 (0.959, 1.015)	0.356	0.647	4.54E-17	TRUE
B IN	*SIDT2*	Wald ratio	1	-0.0473	0.0125	0.954 (0.931, 0.977)	1.53E-04	0.004	2.29E-16	TRUE
B IN	*SKAP2*	Wald ratio	1	-0.0136	0.0166	0.986 (0.955, 1.019)	0.412	0.686	2.37E-13	TRUE
B IN	*SLC12A7*	Wald ratio	1	0.0091	0.0181	1.009 (0.974, 1.046)	0.615	0.814	6.94E-10	TRUE
B IN	*SLC15A2*	Wald ratio	1	0.0138	0.0171	1.014 (0.981, 1.048)	0.419	0.69	3.83E-11	TRUE
B IN	*SLC16A4*	Wald ratio	1	0.0334	0.0173	1.034 (1, 1.07)	0.053	0.231	2.31E-10	TRUE
B IN	*SLC25A1*	Wald ratio	1	-0.0054	0.0182	0.995 (0.96, 1.031)	0.767	0.897	4.27E-10	TRUE
B IN	*SLC2A1-AS1*	Wald ratio	1	0.0162	0.0125	1.016 (0.992, 1.042)	0.195	0.476	1.62E-14	TRUE
B IN	*SLC7A7*	IVW	2	0.0072	0.0197	1.007 (0.969, 1.047)	0.715	0.873	3.75E-19	TRUE
B IN	*SMDT1*	Wald ratio	1	0.0079	0.0042	1.008 (1, 1.016)	0.057	0.239	3.13E-179	TRUE
B IN	*SMIM19*	Wald ratio	1	0.0141	0.0183	1.014 (0.979, 1.051)	0.439	0.703	7.04E-10	TRUE

B IN	*SNHG7*	Wald ratio	1	-0.0147	0.0087	0.985 (0.969, 1.002)	0.091	0.318	7.09E-59	TRUE
B IN	*SNHG8*	IVW	2	-0.0047	0.0061	0.995 (0.983, 1.007)	0.441	0.706	3.53E-90	TRUE
B IN	*SNRNP25*	Wald ratio	1	0.0303	0.0168	1.031 (0.997, 1.065)	0.071	0.277	6.77E-10	TRUE
B IN	*SNRPD2*	Wald ratio	1	-0.0390	0.0125	0.962 (0.939, 0.986)	0.002	0.025	4.18E-18	TRUE
B IN	*SNRPD3*	Wald ratio	1	0.0096	0.0202	1.01 (0.97, 1.05)	0.635	0.827	4.05E-08	TRUE
B IN	*SNX29P2*	Wald ratio	1	0.0028	0.0138	1.003 (0.976, 1.03)	0.84	0.932	5.86E-14	TRUE
B IN	*SOCS5*	Wald ratio	1	0.0012	0.0181	1.001 (0.966, 1.037)	0.946	0.979	3.80E-10	TRUE
B IN	*SPATA20*	Wald ratio	1	0.0205	0.0125	1.021 (0.996, 1.046)	0.101	0.337	1.80E-19	TRUE
B IN	*SPATC1L*	Wald ratio	1	0.0295	0.0186	1.03 (0.993, 1.068)	0.113	0.359	1.62E-08	TRUE
B IN	*SPSB2*	Wald ratio	1	-0.0022	0.0196	0.998 (0.96, 1.037)	0.91	0.962	1.82E-09	TRUE
B IN	*SQSTM1*	Wald ratio	1	-0.0368	0.0174	0.964 (0.932, 0.997)	0.034	0.175	2.65E-10	TRUE
B IN	*SRSF10*	Wald ratio	1	-0.0187	0.0162	0.981 (0.951, 1.013)	0.248	0.541	7.14E-12	TRUE
B IN	*SSBP4*	Wald ratio	1	-0.0058	0.0189	0.994 (0.958, 1.032)	0.758	0.889	7.05E-09	TRUE
B IN	*STMN3*	Wald ratio	1	0.0349	0.0178	1.036 (1, 1.072)	0.05	0.222	6.57E-10	TRUE
B IN	*SULF2*	IVW	2	0.0069	0.0057	1.007 (0.996, 1.018)	0.225	0.519	1.91E-95	TRUE
B IN	*SURF1*	Wald ratio	1	-0.0373	0.0186	0.963 (0.929, 0.999)	0.045	0.208	2.00E-09	TRUE
B IN	*SVIP*	Wald ratio	1	-0.0006	0.0150	0.999 (0.97, 1.029)	0.97	0.989	5.47E-14	TRUE
B IN	*SYNGR1*	Wald ratio	1	-0.0272	0.0147	0.973 (0.945, 1.002)	0.065	0.262	7.36E-10	TRUE
B IN	*SYNJ2*	Wald ratio	1	-0.0034	0.0196	0.997 (0.959, 1.036)	0.864	0.943	1.66E-09	TRUE
B IN	*TAPBP*	Wald ratio	1	0.0086	0.0131	1.009 (0.983, 1.035)	0.511	0.748	7.14E-16	TRUE
B IN	*TAPBPL*	Wald ratio	1	0.0058	0.0156	1.006 (0.975, 1.037)	0.711	0.871	8.76E-13	TRUE
B IN	*TBCA*	Wald ratio	1	0.0099	0.0114	1.01 (0.988, 1.033)	0.385	0.668	2.90E-23	TRUE
B IN	*TC2N*	Wald ratio	1	-0.0033	0.0182	0.997 (0.962, 1.033)	0.855	0.938	1.01E-09	TRUE
B IN	*TCAP*	Wald ratio	1	-0.0199	0.0093	0.98 (0.963, 0.998)	0.031	0.167	4.02E-35	TRUE
B IN	*TCL1A*	Wald ratio	1	0.0191	0.0140	1.019 (0.992, 1.048)	0.172	0.45	2.25E-12	TRUE
B IN	*TCL1B*	Wald ratio	1	-0.0301	0.0187	0.97 (0.935, 1.007)	0.107	0.348	1.26E-09	TRUE
B IN	*TCOF1*	Wald ratio	1	-0.0160	0.0202	0.984 (0.946, 1.024)	0.428	0.699	5.55E-09	TRUE
B IN	*TEX35*	Wald ratio	1	0.0267	0.0118	1.027 (1.004, 1.051)	0.024	0.142	6.23E-21	TRUE
B IN	*THAP7-AS1*	Wald ratio	1	0.0370	0.0160	1.038 (1.006, 1.071)	0.021	0.131	9.66E-11	TRUE
B IN	*THAP9-AS1*	Wald ratio	1	0.0984	0.0212	1.103 (1.059, 1.15)	3.31E-06	2.62E-04	3.38E-08	TRUE
B IN	*TIFA*	Wald ratio	1	0.0117	0.0103	1.012 (0.992, 1.032)	0.255	0.549	9.72E-28	TRUE
B IN	*TIMM10*	Wald ratio	1	-0.0015	0.0098	0.998 (0.979, 1.018)	0.875	0.948	9.77E-37	TRUE
B IN	*TLE4*	Wald ratio	1	-0.0067	0.0129	0.993 (0.969, 1.019)	0.602	0.805	7.91E-19	TRUE
B IN	*TLK1*	Wald ratio	1	0.0060	0.0151	1.006 (0.977, 1.036)	0.692	0.861	1.41E-14	TRUE
B IN	*TMEM156*	Wald ratio	1	0.0053	0.0065	1.005 (0.993, 1.018)	0.419	0.69	7.62E-65	TRUE
B IN	*TMEM2*	Wald ratio	1	0.0100	0.0140	1.01 (0.983, 1.038)	0.477	0.732	4.91E-15	TRUE
B IN	*TMEM204*	Wald ratio	1	0.0137	0.0195	1.014 (0.976, 1.053)	0.481	0.734	6.12E-15	TRUE
B IN	*TMEM230*	Wald ratio	1	0.0105	0.0177	1.011 (0.976, 1.046)	0.551	0.769	2.37E-10	TRUE
B IN	*TMEM258*	Wald ratio	1	0.0166	0.0178	1.017 (0.982, 1.053)	0.353	0.644	1.95E-09	TRUE
B IN	*TMEM41B*	Wald ratio	1	-0.0135	0.0198	0.987 (0.949, 1.026)	0.495	0.74	3.75E-08	TRUE
B IN	*TMEM50B*	Wald ratio	1	-0.0077	0.0172	0.992 (0.96, 1.026)	0.653	0.837	1.06E-10	TRUE
B IN	*TMEM80*	Wald ratio	1	-0.0035	0.0197	0.996 (0.959, 1.036)	0.857	0.939	7.57E-10	TRUE
B IN	*TOMM7*	Wald ratio	1	0.0036	0.0054	1.004 (0.993, 1.014)	0.503	0.743	6.00E-92	TRUE
B IN	*TPRA1*	Wald ratio	1	0.0225	0.0177	1.023 (0.988, 1.059)	0.203	0.489	1.36E-08	TRUE
B IN	*TRMT112*	Wald ratio	1	0.0207	0.0178	1.021 (0.986, 1.057)	0.247	0.54	3.38E-13	TRUE
B IN	*TRPM2*	Wald ratio	1	-0.0075	0.0087	0.992 (0.976, 1.01)	0.386	0.669	5.74E-38	TRUE
B IN	*TSGA10*	Wald ratio	1	-0.0248	0.0186	0.975 (0.941, 1.012)	0.182	0.462	6.36E-10	TRUE
B IN	*TSPAN3*	Wald ratio	1	0.0041	0.0113	1.004 (0.982, 1.027)	0.713	0.872	2.86E-24	TRUE
B IN	*TSTD1*	Wald ratio	1	0.0110	0.0071	1.011 (0.997, 1.025)	0.123	0.377	2.28E-60	TRUE
B IN	*TUBB6*	Wald ratio	1	0.0006	0.0196	1.001 (0.963, 1.04)	0.975	0.99	3.73E-09	TRUE
B IN	*TUFM*	Wald ratio	1	0.0128	0.0064	1.013 (1, 1.026)	0.046	0.21	1.75E-68	TRUE
B IN	*U2AF1L4*	Wald ratio	1	-0.0129	0.0201	0.987 (0.949, 1.027)	0.522	0.754	1.61E-12	TRUE
B IN	*UBA52*	Wald ratio	1	0.0051	0.0151	1.005 (0.976, 1.035)	0.734	0.88	1.02E-13	TRUE
B IN	*UBE2J1*	Wald ratio	1	0.0023	0.0078	1.002 (0.987, 1.018)	0.771	0.899	2.82E-58	TRUE
B IN	*UCP2*	Wald ratio	1	0.0060	0.0161	1.006 (0.975, 1.038)	0.711	0.871	1.02E-11	TRUE
B IN	*UGT2B17*	Wald ratio	1	0.0137	0.0119	1.014 (0.99, 1.038)	0.249	0.542	1.36E-23	TRUE
B IN	*UPF3A*	Wald ratio	1	-0.0372	0.0210	0.963 (0.925, 1.004)	0.076	0.288	6.15E-08	TRUE
B IN	*UQCRH*	Wald ratio	1	-0.0407	0.0193	0.96 (0.924, 0.997)	0.035	0.179	1.88E-10	TRUE
B IN	*UROS*	Wald ratio	1	0.0211	0.0179	1.021 (0.986, 1.058)	0.239	0.534	5.19E-10	TRUE
B IN	*USF1*	Wald ratio	1	-0.0223	0.0209	0.978 (0.939, 1.019)	0.285	0.577	1.22E-08	TRUE
B IN	*USP53*	Wald ratio	1	-0.0041	0.0074	0.996 (0.982, 1.011)	0.584	0.793	5.80E-36	TRUE
B IN	*VAMP2*	Wald ratio	1	0.0312	0.0181	1.032 (0.996, 1.069)	0.086	0.311	3.63E-11	TRUE
B IN	*VAV3*	Wald ratio	1	0.0000	0.0171	1 (0.967, 1.034)	0.998	0.999	3.70E-10	TRUE
B IN	*VAV3-AS1*	Wald ratio	1	0.0008	0.0172	1.001 (0.968, 1.035)	0.963	0.988	9.63E-13	TRUE
B IN	*VCL*	Wald ratio	1	-0.0167	0.0193	0.983 (0.947, 1.021)	0.388	0.669	3.70E-11	TRUE
B IN	*VPREB3*	Wald ratio	1	0.0134	0.0174	1.014 (0.979, 1.049)	0.441	0.706	3.45E-11	TRUE
B IN	*VPS28*	Wald ratio	1	0.0264	0.0162	1.027 (0.995, 1.06)	0.102	0.338	2.52E-10	TRUE
B IN	*WARS*	Wald ratio	1	-0.0405	0.0081	0.96 (0.945, 0.976)	5.67E-07	9.75E-05	4.68E-51	TRUE
B IN	*WASIR2*	Wald ratio	1	-0.0127	0.0262	0.987 (0.938, 1.039)	0.627	0.822	1.90E-09	TRUE
B IN	*WDR60*	Wald ratio	1	-0.0247	0.0186	0.976 (0.941, 1.012)	0.185	0.464	1.62E-08	TRUE
B IN	*XRRA1*	Wald ratio	1	-0.0072	0.0060	0.993 (0.981, 1.005)	0.232	0.525	6.65E-77	TRUE
B IN	*XXbac-BPG252P9.10*	Wald ratio	1	-0.0077	0.0147	0.992 (0.964, 1.021)	0.6	0.804	2.63E-14	TRUE
B IN	*XXbac-BPG299F13.17*	IVW	2	0.0028	0.0248	1.003 (0.955, 1.053)	0.91	0.962	3.36E-70	TRUE
B IN	*YBEY*	Wald ratio	1	-0.0012	0.0093	0.999 (0.981, 1.017)	0.898	0.957	2.86E-29	TRUE
B IN	*YEATS4*	Wald ratio	1	-0.0007	0.0172	0.999 (0.966, 1.034)	0.966	0.988	2.48E-11	TRUE
B IN	*YWHAB*	Wald ratio	1	0.0317	0.0185	1.032 (0.995, 1.07)	0.086	0.312	3.49E-10	TRUE
B IN	*ZC2HC1A*	Wald ratio	1	-0.0026	0.0128	0.997 (0.973, 1.023)	0.836	0.93	5.41E-18	TRUE
B IN	*ZC3HAV1*	Wald ratio	1	0.0081	0.0135	1.008 (0.982, 1.035)	0.548	0.769	1.33E-16	TRUE
B IN	*ZDHHC19*	Wald ratio	1	-0.0061	0.0157	0.994 (0.964, 1.025)	0.7	0.866	5.05E-14	TRUE

B IN	*ZFAS1*	Wald ratio	1	0.0091	0.0123	1.009 (0.985, 1.034)	0.458	0.719	5.11E-21	TRUE
B IN	*ZFP36L1*	Wald ratio	1	-0.0385	0.0200	0.962 (0.925, 1.001)	0.055	0.235	4.60E-08	TRUE
B IN	*ZFP57*	IVW	2	0.0062	0.0073	1.006 (0.992, 1.021)	0.401	0.679	2.87E-48	TRUE
B IN	*ZKSCAN3*	Wald ratio	1	0.0101	0.0149	1.01 (0.981, 1.04)	0.499	0.741	1.48E-18	TRUE
B IN	*ZKSCAN4*	Wald ratio	1	0.0213	0.0239	1.021 (0.975, 1.071)	0.375	0.663	4.99E-10	TRUE
B IN	*ZNF107*	Wald ratio	1	0.0192	0.0166	1.019 (0.987, 1.053)	0.249	0.541	5.98E-11	TRUE
B IN	*ZNF302*	Wald ratio	1	-0.0126	0.0120	0.988 (0.965, 1.011)	0.294	0.587	1.40E-19	TRUE
B IN	*ZNF391*	Wald ratio	1	-0.0346	0.0273	0.966 (0.916, 1.019)	0.206	0.493	4.65E-09	TRUE
B IN	*ZNF667-AS1*	Wald ratio	1	-0.0316	0.0147	0.969 (0.941, 0.997)	0.031	0.167	3.99E-15	TRUE
B IN	*ZNF677*	Wald ratio	1	-0.0325	0.0204	0.968 (0.93, 1.007)	0.111	0.354	3.55E-08	TRUE
B IN	*ZNF749*	Wald ratio	1	0.0214	0.0119	1.022 (0.998, 1.046)	0.072	0.278	7.79E-21	TRUE
B IN	*ZNF83*	Wald ratio	1	-0.0254	0.0160	0.975 (0.945, 1.006)	0.113	0.359	5.59E-12	TRUE
B IN	*ZNF85*	Wald ratio	1	0.0194	0.0128	1.02 (0.994, 1.046)	0.13	0.39	1.33E-20	TRUE
B IN	*ZNF880*	Wald ratio	1	-0.0263	0.0198	0.974 (0.937, 1.013)	0.183	0.463	2.41E-08	TRUE
B IN	*ZNFX1*	Wald ratio	1	-0.0156	0.0188	0.985 (0.949, 1.022)	0.407	0.682	1.14E-09	TRUE
B IN	*ZP3*	Wald ratio	1	0.0287	0.0118	1.029 (1.006, 1.053)	0.015	0.106	2.33E-25	TRUE
B IN	*ZSCAN31*	Wald ratio	1	-0.0266	0.0139	0.974 (0.948, 1.001)	0.056	0.238	5.51E-14	TRUE
B Mem	*AC005253.2*	Wald ratio	1	-0.0069	0.0129	0.993 (0.968, 1.019)	0.592	0.798	1.52E-18	TRUE
B Mem	*AC006369.2*	Wald ratio	1	-0.0064	0.0163	0.994 (0.962, 1.026)	0.695	0.863	6.72E-13	TRUE
B Mem	*AC021860.1*	Wald ratio	1	-0.0405	0.0188	0.96 (0.926, 0.996)	0.031	0.167	1.96E-10	TRUE
B Mem	*AC023590.1*	Wald ratio	1	-0.0091	0.0146	0.991 (0.963, 1.02)	0.534	0.761	7.29E-20	TRUE
B Mem	*AC079767.4*	Wald ratio	1	0.0005	0.0135	1 (0.974, 1.027)	0.971	0.989	1.07E-16	TRUE
B Mem	*AC104653.1*	Wald ratio	1	-0.0087	0.0135	0.991 (0.965, 1.018)	0.518	0.752	3.26E-16	TRUE
B Mem	*ACCS*	Wald ratio	1	-0.0040	0.0210	0.996 (0.956, 1.038)	0.847	0.935	8.95E-09	TRUE
B Mem	*ACYP2*	Wald ratio	1	0.0141	0.0160	1.014 (0.983, 1.046)	0.375	0.664	1.37E-11	TRUE
B Mem	*ADAMTS6*	Wald ratio	1	-0.0563	0.0198	0.945 (0.909, 0.983)	0.004	0.046	2.53E-08	TRUE
B Mem	*AHI1*	Wald ratio	1	-0.0191	0.0121	0.981 (0.958, 1.005)	0.116	0.362	1.94E-20	TRUE
B Mem	*AKAP13*	Wald ratio	1	-0.0205	0.0182	0.98 (0.945, 1.015)	0.262	0.557	1.29E-09	TRUE
B Mem	*ANK1*	Wald ratio	1	0.0225	0.0199	1.023 (0.984, 1.064)	0.258	0.552	8.47E-09	TRUE
B Mem	*ANKDD1A*	Wald ratio	1	-0.0175	0.0131	0.983 (0.958, 1.008)	0.179	0.459	2.02E-17	TRUE
B Mem	*ANXA4*	Wald ratio	1	-0.0308	0.0096	0.97 (0.952, 0.988)	0.001	0.02	6.47E-25	TRUE
B Mem	*ANXA6*	Wald ratio	1	0.0047	0.0155	1.005 (0.975, 1.036)	0.764	0.894	1.07E-12	TRUE
B Mem	*AP003774.1*	Wald ratio	1	-0.0472	0.0137	0.954 (0.929, 0.98)	5.99E-04	0.012	4.67E-15	TRUE
B Mem	*AP1S3*	Wald ratio	1	-0.0171	0.0173	0.983 (0.95, 1.017)	0.324	0.618	1.42E-10	TRUE
B Mem	*ARHGAP24*	Wald ratio	1	0.0081	0.0084	1.008 (0.992, 1.025)	0.337	0.629	1.09E-39	TRUE
B Mem	*ARHGEF3*	Wald ratio	1	-0.0030	0.0158	0.997 (0.967, 1.028)	0.85	0.936	3.57E-11	TRUE
B Mem	*ARL14EP*	Wald ratio	1	-0.0371	0.0140	0.964 (0.938, 0.99)	0.008	0.068	2.86E-14	TRUE
B Mem	*ARL16*	Wald ratio	1	0.0162	0.0133	1.016 (0.99, 1.043)	0.222	0.514	3.12E-16	TRUE
B Mem	*ARL17A*	Wald ratio	1	-0.0278	0.0193	0.973 (0.937, 1.01)	0.149	0.418	1.75E-10	TRUE
B Mem	*ASCL2*	Wald ratio	1	-0.0265	0.0204	0.974 (0.936, 1.014)	0.193	0.474	5.56E-08	TRUE
B Mem	*ATP5G3*	Wald ratio	1	-0.0013	0.0219	0.999 (0.957, 1.043)	0.953	0.983	3.19E-10	TRUE
B Mem	*ATP6V1G2*	IVW	2	-0.0547	0.0339	0.947 (0.886, 1.012)	0.106	0.346	6.42E-24	TRUE
B Mem	*ATP8A1*	Wald ratio	1	0.0217	0.0173	1.022 (0.988, 1.057)	0.21	0.497	1.21E-10	TRUE
B Mem	*BCAS4*	Wald ratio	1	0.0291	0.0173	1.03 (0.995, 1.065)	0.092	0.321	2.16E-10	TRUE
B Mem	*BCL2A1*	Wald ratio	1	-0.0378	0.0113	0.963 (0.942, 0.985)	8.49E-04	0.015	5.41E-22	TRUE
B Mem	*BICD2*	Wald ratio	1	0.0254	0.0181	1.026 (0.99, 1.063)	0.162	0.436	2.66E-10	TRUE
B Mem	*BIN1*	Wald ratio	1	0.0313	0.0165	1.032 (0.999, 1.066)	0.058	0.243	9.23E-11	TRUE
B Mem	*BLK*	Wald ratio	1	-0.0417	0.0089	0.959 (0.943, 0.976)	2.63E-06	2.31E-04	3.75E-36	TRUE
B Mem	*BRK1*	Wald ratio	1	-0.0304	0.0215	0.97 (0.93, 1.012)	0.157	0.428	4.44E-09	TRUE
B Mem	*BTF3*	Wald ratio	1	0.0560	0.0188	1.058 (1.019, 1.097)	0.003	0.034	5.03E-08	TRUE
B Mem	*BTN3A2*	Wald ratio	1	0.0203	0.0142	1.02 (0.992, 1.049)	0.153	0.423	1.80E-20	TRUE
B Mem	*C10orf128*	Wald ratio	1	-0.0083	0.0162	0.992 (0.961, 1.024)	0.609	0.809	1.86E-11	TRUE
B Mem	*C11orf24*	Wald ratio	1	-0.0044	0.0115	0.996 (0.973, 1.018)	0.703	0.867	3.55E-22	TRUE
B Mem	*C19orf60*	Wald ratio	1	0.0061	0.0179	1.006 (0.971, 1.042)	0.733	0.88	3.50E-10	TRUE
B Mem	*C19orf83*	Wald ratio	1	-0.0438	0.0201	0.957 (0.92, 0.996)	0.029	0.159	4.56E-08	TRUE
B Mem	*C1orf220*	Wald ratio	1	0.0152	0.0150	1.015 (0.986, 1.046)	0.31	0.604	1.21E-13	TRUE
B Mem	*C20orf27*	Wald ratio	1	-0.0058	0.0261	0.994 (0.945, 1.046)	0.824	0.926	1.86E-08	TRUE
B Mem	*C6orf48*	Wald ratio	1	0.0486	0.0141	1.05 (1.021, 1.079)	5.43E-04	0.011	3.62E-08	TRUE
B Mem	*CAMLG*	Wald ratio	1	-0.0008	0.0133	0.999 (0.974, 1.026)	0.95	0.981	1.10E-11	TRUE
B Mem	*CAPG*	Wald ratio	1	-0.0084	0.0120	0.992 (0.968, 1.015)	0.484	0.736	1.57E-20	TRUE
B Mem	*CAPN12*	Wald ratio	1	-0.0033	0.0167	0.997 (0.965, 1.03)	0.844	0.935	9.01E-13	TRUE
B Mem	*CAST*	Wald ratio	1	-0.0060	0.0178	0.994 (0.96, 1.029)	0.737	0.88	2.16E-11	TRUE
B Mem	*CCDC126*	Wald ratio	1	-0.0077	0.0145	0.992 (0.965, 1.021)	0.596	0.801	6.10E-18	TRUE
B Mem	*CCDC15*	Wald ratio	1	0.0007	0.0120	1.001 (0.978, 1.024)	0.951	0.981	1.53E-23	TRUE
B Mem	*CCDC163P*	Wald ratio	1	0.0362	0.0113	1.037 (1.014, 1.06)	0.001	0.02	8.56E-21	TRUE
B Mem	*CCDC58*	Wald ratio	1	0.0269	0.0111	1.027 (1.005, 1.05)	0.015	0.106	1.51E-20	TRUE
B Mem	*CCDC66*	Wald ratio	1	0.0199	0.0170	1.02 (0.987, 1.055)	0.241	0.534	7.48E-12	TRUE
B Mem	*CCDC85A*	Wald ratio	1	0.0030	0.0117	1.003 (0.98, 1.026)	0.799	0.913	1.72E-20	TRUE
B Mem	*CCT3*	Wald ratio	1	0.0259	0.0130	1.026 (1, 1.053)	0.047	0.215	4.31E-17	TRUE
B Mem	*CCT8*	Wald ratio	1	-0.0011	0.0110	0.999 (0.978, 1.021)	0.922	0.965	2.28E-39	TRUE
B Mem	*CD37*	Wald ratio	1	0.0024	0.0112	1.002 (0.981, 1.025)	0.832	0.93	1.43E-32	TRUE
B Mem	*CD52*	Wald ratio	1	0.0101	0.0191	1.01 (0.973, 1.049)	0.598	0.803	6.32E-09	TRUE
B Mem	*CD82*	Wald ratio	1	-0.0024	0.0252	0.998 (0.95, 1.048)	0.925	0.967	1.93E-08	TRUE
B Mem	*CD96*	Wald ratio	1	0.0157	0.0162	1.016 (0.984, 1.049)	0.333	0.626	7.68E-12	TRUE
B Mem	*CDC42*	Wald ratio	1	0.0104	0.0151	1.01 (0.981, 1.041)	0.49	0.738	4.74E-12	TRUE
B Mem	*CEACAM21*	Wald ratio	1	-0.0228	0.0188	0.977 (0.942, 1.014)	0.227	0.52	4.22E-08	TRUE
B Mem	*CEBPZ-AS1*	Wald ratio	1	-0.0111	0.0199	0.989 (0.951, 1.028)	0.577	0.788	3.76E-09	TRUE
B Mem	*CENPK*	Wald ratio	1	0.0277	0.0189	1.028 (0.991, 1.067)	0.144	0.412	6.41E-09	TRUE
B Mem	*CFD*	Wald ratio	1	0.0054	0.0153	1.005 (0.976, 1.036)	0.725	0.878	2.74E-13	TRUE

B Mem	*CHAF1B*	Wald ratio	1	0.0203	0.0092	1.021 (1.002, 1.039)	0.027	0.152	1.10E-33	TRUE
B Mem	*CHCHD10*	Wald ratio	1	-0.0079	0.0218	0.992 (0.951, 1.035)	0.715	0.873	1.62E-08	TRUE
B Mem	*CHCHD2*	Wald ratio	1	0.0084	0.0144	1.008 (0.98, 1.037)	0.56	0.778	9.55E-16	TRUE
B Mem	*CHI3L2*	Wald ratio	1	-0.0010	0.0085	0.999 (0.982, 1.016)	0.909	0.961	1.20E-38	TRUE
B Mem	*CHMP4B*	Wald ratio	1	-0.0443	0.0195	0.957 (0.921, 0.994)	0.023	0.138	1.24E-08	TRUE
B Mem	*CHPT1*	Wald ratio	1	0.0339	0.0174	1.035 (1, 1.07)	0.051	0.225	2.13E-10	TRUE
B Mem	*CIB1*	Wald ratio	1	-0.0172	0.0166	0.983 (0.951, 1.016)	0.301	0.596	2.47E-11	TRUE
B Mem	*CITED4*	Wald ratio	1	0.0202	0.0117	1.02 (0.997, 1.044)	0.084	0.308	4.30E-16	TRUE
B Mem	*CLLU1OS*	Wald ratio	1	-0.0234	0.0073	0.977 (0.963, 0.991)	0.001	0.02	2.07E-51	TRUE
B Mem	*CNDP2*	Wald ratio	1	0.0049	0.0195	1.005 (0.967, 1.044)	0.8	0.914	1.81E-09	TRUE
B Mem	*CNPPD1*	Wald ratio	1	-0.0337	0.0202	0.967 (0.929, 1.006)	0.095	0.327	4.26E-08	TRUE
B Mem	*CNTNAP2*	Wald ratio	1	-0.0113	0.0129	0.989 (0.964, 1.014)	0.381	0.666	6.69E-18	TRUE
B Mem	*COL9A3*	Wald ratio	1	0.0575	0.0197	1.059 (1.019, 1.101)	0.004	0.039	2.51E-08	TRUE
B Mem	*COLCA2*	Wald ratio	1	-0.0281	0.0123	0.972 (0.949, 0.996)	0.023	0.138	1.03E-17	TRUE
B Mem	*COMMD6*	Wald ratio	1	0.0149	0.0092	1.015 (0.997, 1.034)	0.105	0.343	1.91E-34	TRUE
B Mem	*CORO1B*	Wald ratio	1	0.0010	0.0079	1.001 (0.986, 1.017)	0.898	0.957	1.27E-48	TRUE
B Mem	*CORO1C*	Wald ratio	1	-0.0140	0.0164	0.986 (0.955, 1.018)	0.393	0.672	1.21E-11	TRUE
B Mem	*COX6B1*	Wald ratio	1	-0.0357	0.0162	0.965 (0.935, 0.996)	0.027	0.155	9.61E-12	TRUE
B Mem	*CPNE1*	Wald ratio	1	-0.0074	0.0164	0.993 (0.961, 1.025)	0.652	0.836	3.07E-14	TRUE
B Mem	*CRYBB2*	Wald ratio	1	0.0155	0.0123	1.016 (0.991, 1.04)	0.208	0.494	2.08E-19	TRUE
B Mem	*CSTB*	Wald ratio	1	0.0032	0.0093	1.003 (0.985, 1.022)	0.73	0.88	1.06E-30	TRUE
B Mem	*CTC-524C5.2*	Wald ratio	1	0.0180	0.0102	1.018 (0.998, 1.039)	0.078	0.292	9.30E-23	TRUE
B Mem	*CTD-2007H13.3*	Wald ratio	1	0.0038	0.0108	1.004 (0.983, 1.025)	0.727	0.878	7.86E-38	TRUE
B Mem	*CTD-2012K14.6*	Wald ratio	1	-0.0169	0.0190	0.983 (0.947, 1.021)	0.374	0.662	1.12E-08	TRUE
B Mem	*CTD-2368P22.1*	Wald ratio	1	0.0030	0.0120	1.003 (0.98, 1.027)	0.806	0.916	3.08E-22	TRUE
B Mem	*CTD-2371O3.2*	Wald ratio	1	0.0003	0.0114	1 (0.978, 1.023)	0.982	0.993	3.52E-24	TRUE
B Mem	*CTSH*	Wald ratio	1	-0.0382	0.0155	0.963 (0.934, 0.992)	0.014	0.101	8.64E-12	TRUE
B Mem	*CYB5A*	Wald ratio	1	-0.0044	0.0148	0.996 (0.967, 1.025)	0.768	0.897	6.87E-14	TRUE
B Mem	*DCXR*	Wald ratio	1	-0.0120	0.0101	0.988 (0.969, 1.008)	0.238	0.532	7.17E-25	TRUE
B Mem	*DDX5*	Wald ratio	1	0.0700	0.0181	1.073 (1.035, 1.111)	1.06E-04	0.003	8.59E-09	TRUE
B Mem	*DEGS1*	Wald ratio	1	0.0101	0.0195	1.01 (0.972, 1.049)	0.603	0.806	2.53E-10	TRUE
B Mem	*DNAJC15*	Wald ratio	1	0.0106	0.0109	1.011 (0.989, 1.032)	0.333	0.626	1.88E-24	TRUE
B Mem	*DOCK8*	Wald ratio	1	0.0201	0.0180	1.02 (0.985, 1.057)	0.265	0.559	1.10E-11	TRUE
B Mem	*DRAM2*	Wald ratio	1	-0.0038	0.0112	0.996 (0.975, 1.018)	0.734	0.88	4.25E-23	TRUE
B Mem	*DSP*	Wald ratio	1	-0.0030	0.0073	0.997 (0.983, 1.011)	0.683	0.856	3.47E-55	TRUE
B Mem	*EAF2*	Wald ratio	1	0.0054	0.0073	1.005 (0.991, 1.02)	0.465	0.725	2.79E-56	TRUE
B Mem	*EBPL*	Wald ratio	1	0.0085	0.0145	1.009 (0.98, 1.038)	0.556	0.774	1.48E-14	TRUE
B Mem	*ECHDC1*	Wald ratio	1	-0.0131	0.0189	0.987 (0.951, 1.024)	0.488	0.738	1.27E-08	TRUE
B Mem	*EIF5A*	Wald ratio	1	-0.0081	0.0054	0.992 (0.981, 1.002)	0.134	0.394	1.24E-119	TRUE
B Mem	*ELP2*	Wald ratio	1	0.0146	0.0187	1.015 (0.978, 1.053)	0.435	0.702	2.23E-09	TRUE
B Mem	*EML6*	Wald ratio	1	-0.0056	0.0172	0.994 (0.961, 1.029)	0.746	0.883	1.33E-12	TRUE
B Mem	*ENOSF1*	Wald ratio	1	0.0280	0.0172	1.028 (0.994, 1.064)	0.103	0.34	1.67E-10	TRUE
B Mem	*EPSTI1*	Wald ratio	1	0.0089	0.0146	1.009 (0.981, 1.038)	0.54	0.762	1.40E-15	TRUE
B Mem	*ERAP2*	Wald ratio	1	0.0359	0.0079	1.037 (1.021, 1.053)	5.04E-06	3.39E-04	5.32E-47	TRUE
B Mem	*ERICH1*	Wald ratio	1	-0.0112	0.0179	0.989 (0.955, 1.024)	0.532	0.76	6.06E-10	TRUE
B Mem	*EVI2A*	Wald ratio	1	-0.0195	0.0144	0.981 (0.953, 1.009)	0.177	0.456	3.82E-14	TRUE
B Mem	*FADS1*	Wald ratio	1	-0.0123	0.0113	0.988 (0.966, 1.01)	0.277	0.571	6.01E-25	TRUE
B Mem	*FAM118A*	Wald ratio	1	0.0006	0.0048	1.001 (0.991, 1.01)	0.895	0.957	1.66E-97	TRUE
B Mem	*FAM167A*	Wald ratio	1	0.0655	0.0142	1.068 (1.038, 1.098)	3.84E-06	2.79E-04	5.82E-15	TRUE
B Mem	*FAM177B*	Wald ratio	1	0.0147	0.0178	1.015 (0.98, 1.051)	0.408	0.684	1.01E-10	TRUE
B Mem	*FAM26F*	IVW	2	-0.0046	0.0199	0.995 (0.957, 1.035)	0.817	0.922	1.40E-59	TRUE
B Mem	*FAM46A*	Wald ratio	1	-0.0018	0.0204	0.998 (0.959, 1.039)	0.93	0.97	2.34E-08	TRUE
B Mem	*FCER2*	Wald ratio	1	-0.0007	0.0167	0.999 (0.967, 1.033)	0.966	0.988	1.95E-11	TRUE
B Mem	*FCGR2A*	Wald ratio	1	0.0227	0.0182	1.023 (0.987, 1.06)	0.213	0.502	1.00E-10	TRUE
B Mem	*FCRL5*	Wald ratio	1	0.0010	0.0108	1.001 (0.98, 1.022)	0.928	0.969	4.56E-27	TRUE
B Mem	*FDFT1*	Wald ratio	1	0.0069	0.0157	1.007 (0.976, 1.038)	0.659	0.842	3.39E-11	TRUE
B Mem	*FIGNL1*	Wald ratio	1	0.0075	0.0181	1.008 (0.972, 1.044)	0.679	0.853	3.82E-10	TRUE
B Mem	*FNBP4*	Wald ratio	1	-0.0886	0.0176	0.915 (0.884, 0.947)	4.85E-07	9.75E-05	4.74E-12	TRUE
B Mem	*FXYD5*	Wald ratio	1	0.0056	0.0152	1.006 (0.976, 1.036)	0.712	0.872	6.93E-14	TRUE
B Mem	*G3BP2*	Wald ratio	1	0.0021	0.0196	1.002 (0.964, 1.041)	0.914	0.962	1.21E-08	TRUE
B Mem	*GBP7*	Wald ratio	1	-0.0002	0.0069	1 (0.986, 1.013)	0.981	0.993	6.86E-63	TRUE
B Mem	*GCC2*	Wald ratio	1	0.0145	0.0178	1.015 (0.98, 1.051)	0.414	0.686	4.63E-11	TRUE
B Mem	*GDI2*	Wald ratio	1	-0.0061	0.0201	0.994 (0.955, 1.034)	0.761	0.893	2.97E-08	TRUE
B Mem	*GLTP*	Wald ratio	1	-0.0223	0.0187	0.978 (0.943, 1.015)	0.235	0.529	2.34E-09	TRUE
B Mem	*GM2A*	Wald ratio	1	-0.0047	0.0175	0.995 (0.962, 1.03)	0.788	0.909	7.29E-12	TRUE
B Mem	*GNPTG*	Wald ratio	1	-0.0149	0.0150	0.985 (0.957, 1.015)	0.32	0.615	6.69E-12	TRUE
B Mem	*GPATCH11*	Wald ratio	1	0.0182	0.0151	1.018 (0.989, 1.049)	0.227	0.52	1.03E-13	TRUE
B Mem	*GPM6A*	Wald ratio	1	0.0115	0.0166	1.012 (0.979, 1.045)	0.488	0.738	6.93E-12	TRUE
B Mem	*GRHPR*	Wald ratio	1	-0.0163	0.0125	0.984 (0.96, 1.008)	0.193	0.474	8.49E-18	TRUE
B Mem	*GS1-124K5.3*	Wald ratio	1	0.0037	0.0191	1.004 (0.967, 1.042)	0.847	0.935	2.23E-08	TRUE
B Mem	*GS1-124K5.4*	Wald ratio	1	0.0050	0.0194	1.005 (0.967, 1.044)	0.798	0.913	2.18E-09	TRUE
B Mem	*GSTM3*	Wald ratio	1	0.0047	0.0082	1.005 (0.989, 1.021)	0.563	0.78	4.35E-43	TRUE
B Mem	*GSTM4*	Wald ratio	1	-0.0336	0.0196	0.967 (0.931, 1.005)	0.086	0.312	2.98E-08	TRUE
B Mem	*GYPC*	Wald ratio	1	0.0176	0.0160	1.018 (0.986, 1.05)	0.27	0.563	3.88E-12	TRUE
B Mem	*HAGH*	Wald ratio	1	0.0735	0.0155	1.076 (1.044, 1.109)	2.05E-06	1.89E-04	1.28E-10	TRUE
B Mem	*HAGHL*	Wald ratio	1	0.0452	0.0160	1.046 (1.014, 1.079)	0.005	0.048	9.66E-10	TRUE
B Mem	*HCG25*	Wald ratio	1	-0.0431	0.0117	0.958 (0.936, 0.98)	2.27E-04	0.006	1.67E-21	TRUE
B Mem	*HCG27*	IVW	2	-0.0012	0.0112	0.999 (0.977, 1.021)	0.911	0.962	3.55E-37	TRUE
B Mem	*HDHD3*	Wald ratio	1	-0.0295	0.0207	0.971 (0.932, 1.011)	0.153	0.423	6.89E-08	TRUE

B Mem	*HIGD2A*	Wald ratio	1	-0.0096	0.0122	0.99 (0.967, 1.014)	0.429	0.699	6.39E-21	TRUE
B Mem	*HIST1H2AG*	Wald ratio	1	0.0478	0.0137	1.049 (1.021, 1.077)	4.64E-04	0.01	2.98E-13	TRUE
B Mem	*HIST1H2AL*	Wald ratio	1	-0.0167	0.0209	0.983 (0.944, 1.024)	0.422	0.693	1.34E-09	TRUE
B Mem	*HIST1H2AM*	IVW	2	0.0249	0.0364	1.025 (0.955, 1.101)	0.493	0.739	7.47E-25	TRUE
B Mem	*HIST1H2BG*	Wald ratio	1	-0.0262	0.0168	0.974 (0.943, 1.007)	0.119	0.369	8.75E-14	TRUE
B Mem	*HIST1H2BJ*	Wald ratio	1	-0.0690	0.0239	0.933 (0.891, 0.978)	0.004	0.042	5.90E-08	TRUE
B Mem	*HIST1H3D*	Wald ratio	1	-0.0434	0.0110	0.958 (0.937, 0.978)	8.09E-05	0.003	8.95E-20	TRUE
B Mem	*HIST1H4E*	Wald ratio	1	0.0147	0.0148	1.015 (0.986, 1.045)	0.321	0.616	6.37E-24	TRUE
B Mem	*HIST1H4J*	Wald ratio	1	0.0148	0.0304	1.015 (0.956, 1.077)	0.625	0.822	1.34E-08	TRUE
B Mem	*HLA-B*	IVW	2	0.0181	0.0137	1.018 (0.991, 1.046)	0.186	0.465	9.88E-80	TRUE
B Mem	*HLA-C*	IVW	2	0.0208	0.0193	1.021 (0.983, 1.06)	0.279	0.572	3.50E-41	TRUE
B Mem	*HLA-DPA1*	Wald ratio	1	0.0060	0.0085	1.006 (0.989, 1.023)	0.483	0.736	6.63E-40	TRUE
B Mem	*HLA-DPB1*	IVW	2	0.0106	0.0136	1.011 (0.984, 1.038)	0.437	0.703	9.71E-76	TRUE
B Mem	*HLA-DQA1*	Wald ratio	1	-0.0013	0.0156	0.999 (0.969, 1.03)	0.935	0.973	1.56E-12	TRUE
B Mem	*HLA-DQA2*	IVW	2	-0.0082	0.0116	0.992 (0.97, 1.015)	0.48	0.734	1.34E-27	TRUE
B Mem	*HLA-DRB1*	Wald ratio	1	-0.0006	0.0078	0.999 (0.984, 1.015)	0.943	0.978	1.09E-50	TRUE
B Mem	*HLA-DRB5*	IVW	3	0.0214	0.0163	1.022 (0.989, 1.055)	0.189	0.47	6.12E-198	TRUE
B Mem	*HLA-DRB5*	MR Egger	3	0.0410	0.0459	1.042 (0.952, 1.14)	0.536	NA	NA	NA
B Mem	*HLA-DRB5*	Weighted median	3	0.0273	0.0056	1.028 (1.016, 1.039)	9.87E-07	NA	NA	NA
B Mem	*HLA-DRB5*	Simple mode	3	0.0355	0.0069	1.036 (1.022, 1.05)	0.036	NA	NA	NA
B Mem	*HLA-DRB5*	Weighted mode	3	0.0293	0.0057	1.03 (1.018, 1.041)	0.036	NA	NA	NA
B Mem	*HLA-F*	Wald ratio	1	-0.0170	0.0193	0.983 (0.947, 1.021)	0.379	0.665	4.19E-09	TRUE
B Mem	*HMBOX1*	Wald ratio	1	-0.0166	0.0090	0.984 (0.966, 1.001)	0.066	0.263	4.12E-32	TRUE
B Mem	*HOXB-AS1*	Wald ratio	1	0.0421	0.0144	1.043 (1.014, 1.073)	0.003	0.038	7.59E-13	TRUE
B Mem	*HSBP1L1*	Wald ratio	1	-0.0661	0.0194	0.936 (0.901, 0.972)	6.74E-04	0.013	2.59E-10	TRUE
B Mem	*HSPBAP1*	Wald ratio	1	0.0069	0.0079	1.007 (0.991, 1.023)	0.383	0.667	6.85E-38	TRUE
B Mem	*IER3*	Wald ratio	1	0.0166	0.0218	1.017 (0.974, 1.061)	0.445	0.709	1.11E-08	TRUE
B Mem	*IFITM2*	Wald ratio	1	-0.0108	0.0191	0.989 (0.953, 1.027)	0.573	0.786	4.57E-09	TRUE
B Mem	*IFITM3*	Wald ratio	1	0.0005	0.0181	1.001 (0.966, 1.037)	0.978	0.991	4.88E-10	TRUE
B Mem	*IFNG-AS1*	Wald ratio	1	-0.0064	0.0185	0.994 (0.958, 1.03)	0.729	0.88	1.79E-09	TRUE
B Mem	*IGFLR1*	Wald ratio	1	0.0128	0.0200	1.013 (0.974, 1.053)	0.522	0.754	1.19E-12	TRUE
B Mem	*IGLL5*	Wald ratio	1	0.0083	0.0104	1.008 (0.988, 1.029)	0.426	0.696	2.90E-19	TRUE
B Mem	*ISCU*	Wald ratio	1	0.0003	0.0159	1 (0.97, 1.032)	0.984	0.994	7.54E-12	TRUE
B Mem	*ITGB2*	Wald ratio	1	-0.0011	0.0083	0.999 (0.983, 1.015)	0.897	0.957	6.10E-36	TRUE
B Mem	*ITGB3BP*	Wald ratio	1	0.0069	0.0134	1.007 (0.981, 1.034)	0.608	0.809	3.60E-16	TRUE
B Mem	*ITM2B*	Wald ratio	1	-0.0019	0.0152	0.998 (0.969, 1.028)	0.898	0.957	5.55E-13	TRUE
B Mem	*ITM2C*	Wald ratio	1	0.0037	0.0148	1.004 (0.975, 1.033)	0.803	0.915	2.09E-11	TRUE
B Mem	*JUP*	IVW	2	-0.0280	0.0289	0.972 (0.919, 1.029)	0.331	0.625	2.54E-27	TRUE
B Mem	*KANSL1-AS1*	Wald ratio	1	-0.0220	0.0094	0.978 (0.96, 0.996)	0.02	0.125	2.58E-73	TRUE
B Mem	*KB-226F1.1*	Wald ratio	1	0.0035	0.0084	1.004 (0.987, 1.02)	0.677	0.853	8.69E-45	TRUE
B Mem	*KDELR2*	Wald ratio	1	0.0444	0.0138	1.045 (1.018, 1.074)	0.001	0.02	6.99E-15	TRUE
B Mem	*KDM4B*	Wald ratio	1	-0.0125	0.0170	0.988 (0.955, 1.021)	0.461	0.721	1.18E-09	TRUE
B Mem	*KHDC1*	Wald ratio	1	-0.0053	0.0098	0.995 (0.976, 1.014)	0.587	0.794	2.75E-27	TRUE
B Mem	*KLK1*	Wald ratio	1	0.0130	0.0162	1.013 (0.981, 1.046)	0.421	0.692	2.75E-08	TRUE
B Mem	*KRTAP5-AS1*	Wald ratio	1	-0.0459	0.0092	0.955 (0.938, 0.972)	5.68E-07	9.75E-05	3.76E-33	TRUE
B Mem	*KRTCAP3*	Wald ratio	1	0.0466	0.0185	1.048 (1.01, 1.086)	0.012	0.09	4.47E-09	TRUE
B Mem	*KYNU*	Wald ratio	1	-0.0321	0.0203	0.968 (0.931, 1.008)	0.114	0.361	7.63E-08	TRUE
B Mem	*L3MBTL3*	Wald ratio	1	-0.0047	0.0136	0.995 (0.969, 1.022)	0.726	0.878	2.53E-18	TRUE
B Mem	*LA16c-358B7.3*	Wald ratio	1	0.0499	0.0132	1.051 (1.024, 1.079)	1.52E-04	0.004	2.72E-18	TRUE
B Mem	*LAMB1*	Wald ratio	1	-0.0090	0.0104	0.991 (0.971, 1.012)	0.39	0.671	2.28E-26	TRUE
B Mem	*LARGE*	Wald ratio	1	0.0036	0.0187	1.004 (0.967, 1.041)	0.847	0.935	1.60E-09	TRUE
B Mem	*LAX1*	Wald ratio	1	0.0139	0.0166	1.014 (0.982, 1.047)	0.402	0.679	4.64E-11	TRUE
B Mem	*LCN8*	Wald ratio	1	-0.0095	0.0061	0.991 (0.979, 1.002)	0.119	0.369	2.68E-116	TRUE
B Mem	*LEPROT*	Wald ratio	1	0.0272	0.0191	1.028 (0.99, 1.067)	0.154	0.424	7.65E-09	TRUE
B Mem	*LGALS9*	Wald ratio	1	0.0598	0.0197	1.062 (1.022, 1.103)	0.002	0.029	3.38E-09	TRUE
B Mem	*LGMN*	Wald ratio	1	-0.0221	0.0187	0.978 (0.943, 1.015)	0.238	0.532	1.96E-08	TRUE
B Mem	*LHPP*	Wald ratio	1	-0.0240	0.0177	0.976 (0.943, 1.011)	0.176	0.456	8.33E-11	TRUE
B Mem	*LILRB1*	Wald ratio	1	0.0066	0.0161	1.007 (0.975, 1.039)	0.682	0.855	6.90E-12	TRUE
B Mem	*LILRB2*	Wald ratio	1	0.0268	0.0203	1.027 (0.987, 1.069)	0.187	0.466	7.39E-09	TRUE
B Mem	*LIMD1*	Wald ratio	1	0.0088	0.0096	1.009 (0.99, 1.028)	0.361	0.651	3.57E-32	TRUE
B Mem	*LINC00339*	Wald ratio	1	-0.0263	0.0160	0.974 (0.944, 1.005)	0.1	0.334	3.36E-11	TRUE
B Mem	*LINC00422*	Wald ratio	1	-0.0045	0.0150	0.995 (0.967, 1.025)	0.763	0.893	5.34E-12	TRUE
B Mem	*LINC00926*	Wald ratio	1	-0.0201	0.0146	0.98 (0.952, 1.008)	0.168	0.443	1.99E-15	TRUE
B Mem	*LINC00969*	Wald ratio	1	-0.0120	0.0138	0.988 (0.962, 1.015)	0.384	0.668	7.55E-16	TRUE
B Mem	*LRRC16A*	Wald ratio	1	-0.0196	0.0166	0.981 (0.949, 1.013)	0.237	0.531	3.24E-11	TRUE
B Mem	*LSM7*	Wald ratio	1	0.0494	0.0141	1.051 (1.022, 1.08)	4.56E-04	0.01	8.27E-15	TRUE
B Mem	*LY6G5C*	Wald ratio	1	0.0369	0.0134	1.038 (1.011, 1.065)	0.006	0.055	6.00E-18	TRUE
B Mem	*LY86*	Wald ratio	1	-0.0048	0.0074	0.995 (0.981, 1.01)	0.52	0.753	1.58E-53	TRUE
B Mem	*LY96*	Wald ratio	1	0.0171	0.0188	1.017 (0.981, 1.055)	0.362	0.651	2.00E-12	TRUE
B Mem	*LYST*	Wald ratio	1	0.0216	0.0216	1.022 (0.979, 1.066)	0.317	0.613	2.58E-08	TRUE
B Mem	*MANBA*	Wald ratio	1	0.0729	0.0171	1.076 (1.04, 1.112)	2.04E-05	9.61E-04	3.12E-10	TRUE
B Mem	*MARC2*	Wald ratio	1	-0.0004	0.0159	1 (0.969, 1.031)	0.982	0.993	1.75E-11	TRUE
B Mem	*MCM5*	Wald ratio	1	-0.0018	0.0137	0.998 (0.972, 1.025)	0.895	0.957	7.26E-14	TRUE
B Mem	*MDGA1*	Wald ratio	1	-0.0182	0.0114	0.982 (0.96, 1.004)	0.11	0.353	3.08E-23	TRUE
B Mem	*MDM4*	Wald ratio	1	-0.0678	0.0170	0.934 (0.904, 0.966)	6.77E-05	0.002	1.56E-13	TRUE
B Mem	*MED16*	Wald ratio	1	0.0006	0.0172	1.001 (0.967, 1.035)	0.973	0.989	1.50E-12	TRUE
B Mem	*METTL8*	Wald ratio	1	-0.0140	0.0098	0.986 (0.967, 1.005)	0.153	0.423	3.13E-29	TRUE
B Mem	*MGMT*	Wald ratio	1	-0.0135	0.0154	0.987 (0.957, 1.017)	0.381	0.666	7.85E-13	TRUE
B Mem	*MGST3*	Wald ratio	1	-0.0007	0.0102	0.999 (0.979, 1.02)	0.946	0.979	5.07E-28	TRUE

B Mem	*MILR1*	Wald ratio	1	-0.0504	0.0193	0.951 (0.916, 0.988)	0.009	0.076	2.05E-08	TRUE
B Mem	*MPHOSPH6*	Wald ratio	1	0.0407	0.0205	1.042 (1, 1.084)	0.048	0.215	3.97E-08	TRUE
B Mem	*MRPL21*	Wald ratio	1	0.0254	0.0154	1.026 (0.995, 1.057)	0.098	0.333	8.76E-12	TRUE
B Mem	*MRPL53*	Wald ratio	1	-0.0077	0.0160	0.992 (0.962, 1.024)	0.631	0.825	2.09E-11	TRUE
B Mem	*MRPS6*	Wald ratio	1	0.0111	0.0126	1.011 (0.986, 1.037)	0.38	0.666	7.79E-19	TRUE
B Mem	*MRVI1-AS1*	Wald ratio	1	0.0241	0.0194	1.024 (0.986, 1.064)	0.212	0.5	2.15E-09	TRUE
B Mem	*MTDH*	Wald ratio	1	0.0097	0.0113	1.01 (0.988, 1.032)	0.392	0.672	5.71E-22	TRUE
B Mem	*MXRA7*	Wald ratio	1	-0.0026	0.0104	0.997 (0.977, 1.018)	0.801	0.915	1.37E-18	TRUE
B Mem	*MYBL2*	Wald ratio	1	-0.0234	0.0116	0.977 (0.955, 0.999)	0.044	0.207	1.43E-22	TRUE
B Mem	*MYH11*	Wald ratio	1	-0.0267	0.0186	0.974 (0.939, 1.01)	0.152	0.422	3.62E-09	TRUE
B Mem	*MZT2A*	Wald ratio	1	-0.0139	0.0084	0.986 (0.97, 1.003)	0.097	0.332	7.14E-31	TRUE
B Mem	*NAA38*	Wald ratio	1	0.0044	0.0207	1.004 (0.965, 1.046)	0.832	0.93	5.26E-10	TRUE
B Mem	*NAGK*	Wald ratio	1	-0.0221	0.0216	0.978 (0.938, 1.02)	0.307	0.6	2.89E-09	TRUE
B Mem	*NAP1L1*	Wald ratio	1	-0.0120	0.0148	0.988 (0.96, 1.017)	0.417	0.688	5.12E-14	TRUE
B Mem	*NAPRT1*	Wald ratio	1	-0.0185	0.0112	0.982 (0.96, 1.003)	0.099	0.333	1.30E-22	TRUE
B Mem	*NARFL*	Wald ratio	1	0.0437	0.0163	1.045 (1.012, 1.079)	0.007	0.066	2.91E-08	TRUE
B Mem	*NCOA7*	Wald ratio	1	-0.0368	0.0160	0.964 (0.934, 0.995)	0.022	0.133	2.20E-13	TRUE
B Mem	*NDUFA3*	Wald ratio	1	0.0024	0.0191	1.002 (0.966, 1.041)	0.9	0.958	6.61E-09	TRUE
B Mem	*NEK8*	Wald ratio	1	0.0116	0.0201	1.012 (0.973, 1.052)	0.565	0.781	7.27E-09	TRUE
B Mem	*NMRK1*	Wald ratio	1	-0.0136	0.0200	0.986 (0.949, 1.026)	0.495	0.74	1.48E-09	TRUE
B Mem	*NQO2*	Wald ratio	1	-0.0022	0.0168	0.998 (0.965, 1.031)	0.896	0.957	2.16E-11	TRUE
B Mem	*NSA2*	Wald ratio	1	0.0243	0.0106	1.025 (1.004, 1.046)	0.021	0.132	5.50E-29	TRUE
B Mem	*NT5C3B*	Wald ratio	1	-0.0002	0.0136	1 (0.973, 1.027)	0.986	0.995	3.26E-14	TRUE
B Mem	*NT5E*	Wald ratio	1	-0.0027	0.0153	0.997 (0.968, 1.028)	0.861	0.941	1.17E-16	TRUE
B Mem	*NUDT2*	Wald ratio	1	-0.0056	0.0098	0.994 (0.976, 1.014)	0.569	0.782	2.69E-28	TRUE
B Mem	*OPTN*	Wald ratio	1	-0.0130	0.0147	0.987 (0.959, 1.016)	0.376	0.665	3.26E-14	TRUE
B Mem	*ORAI2*	Wald ratio	1	0.0280	0.0187	1.028 (0.991, 1.067)	0.135	0.396	1.85E-08	TRUE
B Mem	*ORMDL3*	Wald ratio	1	0.0026	0.0092	1.003 (0.985, 1.021)	0.779	0.904	9.21E-35	TRUE
B Mem	*OSTF1*	Wald ratio	1	-0.0054	0.0125	0.995 (0.971, 1.019)	0.664	0.846	2.47E-21	TRUE
B Mem	*P2RX1*	Wald ratio	1	0.0114	0.0150	1.011 (0.982, 1.042)	0.446	0.71	2.35E-13	TRUE
B Mem	*PARP14*	Wald ratio	1	0.0014	0.0112	1.001 (0.98, 1.024)	0.904	0.959	1.46E-12	TRUE
B Mem	*PILRB*	Wald ratio	1	0.0093	0.0127	1.009 (0.985, 1.035)	0.463	0.723	1.32E-17	TRUE
B Mem	*PIM3*	Wald ratio	1	0.0637	0.0193	1.066 (1.026, 1.107)	9.63E-04	0.016	1.24E-08	TRUE
B Mem	*PKHD1L1*	Wald ratio	1	0.0062	0.0105	1.006 (0.986, 1.027)	0.558	0.776	9.82E-27	TRUE
B Mem	*PKIG*	Wald ratio	1	0.0059	0.0175	1.006 (0.972, 1.041)	0.736	0.88	3.92E-13	TRUE
B Mem	*PLD4*	Wald ratio	1	0.0330	0.0201	1.034 (0.994, 1.075)	0.1	0.336	2.09E-08	TRUE
B Mem	*PLEK*	Wald ratio	1	0.0458	0.0180	1.047 (1.011, 1.085)	0.011	0.086	7.39E-10	TRUE
B Mem	*PLTP*	Wald ratio	1	-0.0327	0.0123	0.968 (0.945, 0.991)	0.008	0.068	2.15E-19	TRUE
B Mem	*PNRC2*	Wald ratio	1	-0.0211	0.0158	0.979 (0.949, 1.01)	0.182	0.462	2.40E-12	TRUE
B Mem	*POLM*	Wald ratio	1	0.0096	0.0145	1.01 (0.981, 1.039)	0.507	0.745	2.40E-12	TRUE
B Mem	*POLR1D*	Wald ratio	1	-0.0087	0.0147	0.991 (0.963, 1.02)	0.556	0.774	7.76E-16	TRUE
B Mem	*POLR2J2*	Wald ratio	1	-0.0219	0.0196	0.978 (0.941, 1.017)	0.264	0.558	1.84E-08	TRUE
B Mem	*POMZP3*	Wald ratio	1	0.0473	0.0206	1.048 (1.007, 1.092)	0.022	0.134	5.21E-09	TRUE
B Mem	*POU5F1*	IVW	3	-0.0017	0.0072	0.998 (0.984, 1.013)	0.814	0.921	3.00E-78	TRUE
B Mem	*POU5F1*	MR Egger	3	0.0954	0.0887	1.1 (0.925, 1.309)	0.477	NA	NA	NA
B Mem	*POU5F1*	Weighted median	3	-0.0007	0.0077	0.999 (0.984, 1.015)	0.927	NA	NA	NA
B Mem	*POU5F1*	Simple mode	3	0.0006	0.0097	1.001 (0.982, 1.02)	0.958	NA	NA	NA
B Mem	*POU5F1*	Weighted mode	3	-0.0004	0.0087	1 (0.983, 1.017)	0.964	NA	NA	NA
B Mem	*PPA1*	Wald ratio	1	0.0052	0.0099	1.005 (0.986, 1.025)	0.602	0.805	1.59E-27	TRUE
B Mem	*PPDPF*	Wald ratio	1	-0.0080	0.0074	0.992 (0.978, 1.007)	0.283	0.575	1.97E-47	TRUE
B Mem	*PPIA*	Wald ratio	1	-0.0105	0.0146	0.99 (0.962, 1.018)	0.47	0.727	6.78E-15	TRUE
B Mem	*PPIL3*	Wald ratio	1	0.0098	0.0134	1.01 (0.984, 1.037)	0.467	0.725	3.97E-16	TRUE
B Mem	*PRKD3*	Wald ratio	1	0.0547	0.0180	1.056 (1.02, 1.094)	0.002	0.03	6.71E-10	TRUE
B Mem	*PRSS16*	Wald ratio	1	0.0088	0.0232	1.009 (0.964, 1.056)	0.705	0.867	4.17E-08	TRUE
B Mem	*PSMB9*	Wald ratio	1	-0.0080	0.0170	0.992 (0.959, 1.026)	0.638	0.829	6.30E-11	TRUE
B Mem	*PSMD5-AS1*	Wald ratio	1	0.0052	0.0060	1.005 (0.993, 1.017)	0.391	0.672	1.74E-81	TRUE
B Mem	*PSTPIP1*	Wald ratio	1	0.0017	0.0176	1.002 (0.968, 1.037)	0.921	0.965	1.30E-10	TRUE
B Mem	*PTPLAD2*	Wald ratio	1	-0.0617	0.0189	0.94 (0.906, 0.976)	0.001	0.018	1.70E-09	TRUE
B Mem	*PYROXD2*	Wald ratio	1	0.0209	0.0134	1.021 (0.995, 1.048)	0.12	0.37	2.46E-17	TRUE
B Mem	*RABAC1*	Wald ratio	1	0.0273	0.0147	1.028 (0.998, 1.058)	0.064	0.259	1.84E-14	TRUE
B Mem	*RASGRP3*	Wald ratio	1	0.0004	0.0207	1 (0.961, 1.042)	0.985	0.994	2.20E-08	TRUE
B Mem	*RASSF6*	Wald ratio	1	0.0032	0.0201	1.003 (0.965, 1.043)	0.872	0.947	5.66E-08	TRUE
B Mem	*RBM26*	Wald ratio	1	-0.0127	0.0203	0.987 (0.949, 1.027)	0.53	0.758	3.15E-08	TRUE
B Mem	*RCSD1*	Wald ratio	1	-0.0293	0.0202	0.971 (0.933, 1.01)	0.148	0.417	3.08E-08	TRUE
B Mem	*RGS16*	Wald ratio	1	-0.0086	0.0137	0.991 (0.965, 1.018)	0.528	0.756	6.94E-13	TRUE
B Mem	*RNASEH2C*	Wald ratio	1	-0.0010	0.0184	0.999 (0.964, 1.036)	0.957	0.984	1.33E-10	TRUE
B Mem	*RNGTT*	Wald ratio	1	-0.0030	0.0107	0.997 (0.976, 1.018)	0.781	0.905	4.14E-25	TRUE
B Mem	*RP1-29C18.10*	Wald ratio	1	-0.0212	0.0106	0.979 (0.959, 1)	0.046	0.21	3.57E-28	TRUE
B Mem	*RP1-3J17.3*	IVW	2	0.0021	0.0079	1.002 (0.987, 1.018)	0.792	0.909	5.39E-50	TRUE
B Mem	*RP11-1070N10.3*	Wald ratio	1	-0.0274	0.0191	0.973 (0.937, 1.01)	0.152	0.422	5.31E-09	TRUE
B Mem	*RP11-111A22.1*	Wald ratio	1	-0.0083	0.0086	0.992 (0.975, 1.009)	0.335	0.628	1.39E-41	TRUE
B Mem	*RP11-119F19.2*	Wald ratio	1	0.0496	0.0199	1.051 (1.011, 1.093)	0.013	0.096	8.91E-08	TRUE
B Mem	*RP11-138A9.1*	Wald ratio	1	0.0152	0.0185	1.015 (0.979, 1.053)	0.412	0.686	5.41E-10	TRUE
B Mem	*RP11-138A9.2*	Wald ratio	1	0.0092	0.0159	1.009 (0.978, 1.041)	0.561	0.778	7.88E-09	TRUE
B Mem	*RP11-138I18.2*	Wald ratio	1	-0.0031	0.0186	0.997 (0.961, 1.034)	0.866	0.944	2.11E-09	TRUE
B Mem	*RP11-197K3.1*	Wald ratio	1	-0.0276	0.0165	0.973 (0.942, 1.005)	0.096	0.329	1.00E-10	TRUE
B Mem	*RP11-297B17.3*	Wald ratio	1	-0.0098	0.0187	0.99 (0.955, 1.027)	0.6	0.804	7.68E-13	TRUE
B Mem	*RP11-298J20.3*	Wald ratio	1	0.0214	0.0081	1.022 (1.006, 1.038)	0.008	0.071	5.47E-41	TRUE
B Mem	*RP11-326C3.11*	Wald ratio	1	0.0132	0.0185	1.013 (0.977, 1.051)	0.474	0.73	5.10E-10	TRUE

B Mem	*RP11-345J4.5*	Wald ratio	1	-0.0330	0.0150	0.968 (0.94, 0.996)	0.027	0.154	4.07E-14	TRUE
B Mem	*RP11-347P5.1*	Wald ratio	1	0.0007	0.0075	1.001 (0.986, 1.016)	0.929	0.97	3.74E-53	TRUE
B Mem	*RP11-386I14.4*	Wald ratio	1	-0.0211	0.0193	0.979 (0.943, 1.017)	0.273	0.567	4.97E-08	TRUE
B Mem	*RP11-428G5.5*	Wald ratio	1	-0.0011	0.0156	0.999 (0.969, 1.03)	0.942	0.977	2.29E-13	TRUE
B Mem	*RP11-434C1.1*	Wald ratio	1	-0.0007	0.0188	0.999 (0.963, 1.037)	0.971	0.989	7.85E-10	TRUE
B Mem	*RP11-44F21.5*	Wald ratio	1	0.0268	0.0137	1.027 (1, 1.055)	0.051	0.224	4.98E-13	TRUE
B Mem	*RP11-457M11.5*	Wald ratio	1	0.0176	0.0132	1.018 (0.992, 1.044)	0.183	0.463	2.86E-17	TRUE
B Mem	*RP11-473I1.5*	Wald ratio	1	-0.0329	0.0154	0.968 (0.939, 0.997)	0.033	0.172	5.27E-11	TRUE
B Mem	*RP11-514O12.4*	Wald ratio	1	0.0063	0.0088	1.006 (0.989, 1.024)	0.469	0.727	2.06E-38	TRUE
B Mem	*RP11-542K23.7*	Wald ratio	1	0.0125	0.0166	1.013 (0.98, 1.046)	0.453	0.717	1.03E-20	TRUE
B Mem	*RP11-588H23.3*	Wald ratio	1	-0.0179	0.0162	0.982 (0.952, 1.014)	0.271	0.564	3.32E-11	TRUE
B Mem	*RP11-596D21.1*	Wald ratio	1	-0.0192	0.0128	0.981 (0.957, 1.006)	0.133	0.394	4.25E-19	TRUE
B Mem	*RP13-514E23.2*	Wald ratio	1	-0.0297	0.0078	0.971 (0.956, 0.986)	1.49E-04	0.004	2.52E-44	TRUE
B Mem	*RP5-827C21.4*	Wald ratio	1	-0.0063	0.0235	0.994 (0.949, 1.041)	0.787	0.909	5.37E-09	TRUE
B Mem	*RPL10A*	Wald ratio	1	-0.0140	0.0106	0.986 (0.966, 1.007)	0.187	0.466	5.11E-27	TRUE
B Mem	*RPL11*	Wald ratio	1	-0.0225	0.0144	0.978 (0.95, 1.006)	0.119	0.369	2.72E-14	TRUE
B Mem	*RPL12*	Wald ratio	1	-0.0194	0.0057	0.981 (0.97, 0.992)	7.26E-04	0.013	1.74E-100	TRUE
B Mem	*RPL13*	Wald ratio	1	-0.0250	0.0128	0.975 (0.951, 1)	0.051	0.224	1.32E-31	TRUE
B Mem	*RPL14*	Wald ratio	1	-0.0609	0.0187	0.941 (0.907, 0.976)	0.001	0.018	1.89E-08	TRUE
B Mem	*RPL22L1*	Wald ratio	1	0.0042	0.0198	1.004 (0.966, 1.044)	0.833	0.93	1.65E-10	TRUE
B Mem	*RPL23A*	Wald ratio	1	-0.0235	0.0125	0.977 (0.953, 1.001)	0.06	0.247	1.04E-19	TRUE
B Mem	*RPL27A*	Wald ratio	1	0.0105	0.0124	1.011 (0.986, 1.035)	0.4	0.678	1.03E-17	TRUE
B Mem	*RPL28*	Wald ratio	1	0.0044	0.0057	1.004 (0.993, 1.016)	0.438	0.703	1.99E-91	TRUE
B Mem	*RPL3*	Wald ratio	1	0.0186	0.0199	1.019 (0.98, 1.059)	0.35	0.643	2.11E-08	TRUE
B Mem	*RPL32*	Wald ratio	1	0.0343	0.0204	1.035 (0.994, 1.077)	0.093	0.323	2.01E-08	TRUE
B Mem	*RPL36AL*	Wald ratio	1	-0.0042	0.0066	0.996 (0.983, 1.009)	0.524	0.754	1.49E-70	TRUE
B Mem	*RPL8*	Wald ratio	1	-0.0042	0.0046	0.996 (0.987, 1.005)	0.356	0.647	2.77E-158	TRUE
B Mem	*RPLP2*	Wald ratio	1	-0.0234	0.0090	0.977 (0.96, 0.994)	0.009	0.076	3.59E-36	TRUE
B Mem	*RPS10*	IVW	2	-0.0163	0.0203	0.984 (0.945, 1.024)	0.421	0.692	2.91E-60	TRUE
B Mem	*RPS11*	Wald ratio	1	0.0331	0.0139	1.034 (1.006, 1.062)	0.017	0.115	5.51E-17	TRUE
B Mem	*RPS12*	Wald ratio	1	0.0203	0.0127	1.021 (0.995, 1.046)	0.111	0.354	1.55E-11	TRUE
B Mem	*RPS14*	Wald ratio	1	-0.0217	0.0196	0.979 (0.942, 1.017)	0.268	0.562	2.62E-08	TRUE
B Mem	*RPS16*	Wald ratio	1	-0.0068	0.0075	0.993 (0.979, 1.008)	0.363	0.651	5.96E-49	TRUE
B Mem	*RPS18*	Wald ratio	1	-0.0123	0.0072	0.988 (0.974, 1.002)	0.087	0.312	8.16E-71	TRUE
B Mem	*RPS19*	Wald ratio	1	0.0181	0.0185	1.018 (0.982, 1.056)	0.326	0.62	1.83E-09	TRUE
B Mem	*RPS2*	Wald ratio	1	-0.0258	0.0097	0.975 (0.956, 0.993)	0.008	0.067	1.46E-32	TRUE
B Mem	*RPS23*	Wald ratio	1	-0.0284	0.0185	0.972 (0.937, 1.008)	0.125	0.38	1.79E-10	TRUE
B Mem	*RPS24*	Wald ratio	1	-0.0035	0.0194	0.996 (0.959, 1.035)	0.855	0.938	7.51E-09	TRUE
B Mem	*RPS25*	Wald ratio	1	-0.0071	0.0101	0.993 (0.973, 1.013)	0.485	0.736	1.08E-28	TRUE
B Mem	*RPS26*	Wald ratio	1	-0.0099	0.0043	0.99 (0.982, 0.999)	0.022	0.132	6.21E-194	TRUE
B Mem	*RPS3*	Wald ratio	1	-0.0169	0.0096	0.983 (0.965, 1.002)	0.079	0.295	2.01E-19	TRUE
B Mem	*RPS3A*	Wald ratio	1	-0.0045	0.0065	0.996 (0.983, 1.008)	0.487	0.738	1.88E-33	TRUE
B Mem	*RPS5*	Wald ratio	1	0.0076	0.0056	1.008 (0.997, 1.019)	0.177	0.456	9.55E-104	TRUE
B Mem	*RPS6*	Wald ratio	1	-0.0144	0.0122	0.986 (0.962, 1.009)	0.236	0.53	1.14E-26	TRUE
B Mem	*RPS7*	Wald ratio	1	0.0030	0.0100	1.003 (0.984, 1.023)	0.761	0.892	3.31E-46	TRUE
B Mem	*RPS8*	Wald ratio	1	0.0018	0.0069	1.002 (0.988, 1.016)	0.791	0.909	6.12E-62	TRUE
B Mem	*RPS9*	Wald ratio	1	0.0280	0.0073	1.028 (1.014, 1.043)	1.27E-04	0.003	2.70E-53	TRUE
B Mem	*RPSA*	Wald ratio	1	-0.0339	0.0165	0.967 (0.936, 0.998)	0.039	0.192	1.59E-14	TRUE
B Mem	*RSRC2*	Wald ratio	1	-0.0098	0.0176	0.99 (0.957, 1.025)	0.576	0.787	2.71E-12	TRUE
B Mem	*SAR1A*	Wald ratio	1	0.0124	0.0168	1.012 (0.98, 1.046)	0.459	0.719	4.78E-11	TRUE
B Mem	*SCGB3A1*	Wald ratio	1	0.0176	0.0085	1.018 (1.001, 1.035)	0.039	0.191	2.89E-39	TRUE
B Mem	*SCIMP*	Wald ratio	1	-0.0182	0.0159	0.982 (0.952, 1.013)	0.251	0.545	2.08E-13	TRUE
B Mem	*SEC61B*	Wald ratio	1	-0.0146	0.0164	0.985 (0.954, 1.018)	0.373	0.661	3.10E-15	TRUE
B Mem	*SEL1L3*	Wald ratio	1	0.0036	0.0143	1.004 (0.976, 1.032)	0.803	0.915	7.41E-16	TRUE
B Mem	*SEPT2*	Wald ratio	1	0.0187	0.0226	1.019 (0.975, 1.065)	0.409	0.685	6.06E-08	TRUE
B Mem	*SERAC1*	Wald ratio	1	0.0354	0.0119	1.036 (1.012, 1.06)	0.003	0.034	2.20E-26	TRUE
B Mem	*SERTAD3*	Wald ratio	1	-0.0331	0.0222	0.967 (0.926, 1.011)	0.137	0.399	7.92E-08	TRUE
B Mem	*SESTD1*	Wald ratio	1	-0.0046	0.0179	0.995 (0.961, 1.031)	0.796	0.911	1.82E-10	TRUE
B Mem	*SF1*	Wald ratio	1	-0.0004	0.0135	1 (0.973, 1.026)	0.976	0.991	1.63E-14	TRUE
B Mem	*SH3BGRL3*	Wald ratio	1	-0.0079	0.0150	0.992 (0.963, 1.022)	0.598	0.803	1.17E-13	TRUE
B Mem	*SH3YL1*	Wald ratio	1	0.0392	0.0089	1.04 (1.022, 1.058)	1.04E-05	5.93E-04	1.42E-35	TRUE
B Mem	*SIDT1-AS1*	Wald ratio	1	-0.0145	0.0155	0.986 (0.956, 1.016)	0.35	0.643	5.16E-15	TRUE
B Mem	*SLC15A2*	Wald ratio	1	0.0147	0.0201	1.015 (0.976, 1.056)	0.465	0.725	2.00E-08	TRUE
B Mem	*SLC25A1*	Wald ratio	1	-0.0179	0.0175	0.982 (0.949, 1.017)	0.309	0.603	1.54E-10	TRUE
B Mem	*SLC25A29*	Wald ratio	1	-0.0137	0.0191	0.986 (0.95, 1.024)	0.473	0.73	9.28E-09	TRUE
B Mem	*SLC26A11*	Wald ratio	1	-0.0121	0.0178	0.988 (0.954, 1.023)	0.496	0.74	4.18E-11	TRUE
B Mem	*SMDT1*	Wald ratio	1	0.0081	0.0042	1.008 (1, 1.017)	0.057	0.239	5.36E-170	TRUE
B Mem	*SMIM19*	Wald ratio	1	0.0130	0.0184	1.013 (0.977, 1.05)	0.481	0.734	6.61E-10	TRUE
B Mem	*SMPD3*	Wald ratio	1	-0.0203	0.0147	0.98 (0.952, 1.009)	0.167	0.442	5.25E-11	TRUE
B Mem	*SNHG7*	Wald ratio	1	-0.0157	0.0093	0.984 (0.967, 1.002)	0.091	0.318	2.67E-51	TRUE
B Mem	*SNHG8*	IVW	2	-0.0037	0.0064	0.996 (0.984, 1.009)	0.563	0.78	3.13E-83	TRUE
B Mem	*SNORD3B-1*	Wald ratio	1	0.0024	0.0084	1.002 (0.986, 1.019)	0.772	0.899	3.64E-31	TRUE
B Mem	*SNORD3D*	Wald ratio	1	-0.0086	0.0117	0.991 (0.969, 1.014)	0.461	0.721	1.49E-18	TRUE
B Mem	*SNRPD2*	Wald ratio	1	-0.0405	0.0125	0.96 (0.937, 0.984)	0.001	0.02	4.30E-18	TRUE
B Mem	*SNX24*	Wald ratio	1	0.0345	0.0150	1.035 (1.005, 1.066)	0.022	0.134	1.39E-12	TRUE
B Mem	*SPATA20*	Wald ratio	1	0.0105	0.0132	1.011 (0.985, 1.037)	0.429	0.699	2.83E-17	TRUE
B Mem	*SPSB2*	Wald ratio	1	-0.0024	0.0215	0.998 (0.956, 1.04)	0.91	0.962	4.17E-08	TRUE
B Mem	*SQSTM1*	Wald ratio	1	-0.0095	0.0165	0.991 (0.959, 1.023)	0.564	0.78	1.11E-10	TRUE
B Mem	*SRSF10*	Wald ratio	1	-0.0253	0.0190	0.975 (0.939, 1.012)	0.182	0.462	5.39E-09	TRUE

B Mem	*STMN3*	Wald ratio	1	0.0200	0.0187	1.02 (0.984, 1.058)	0.284	0.576	4.63E-10	TRUE
B Mem	*SULF2*	Wald ratio	1	0.0057	0.0057	1.006 (0.994, 1.017)	0.324	0.618	2.05E-89	TRUE
B Mem	*SURF1*	Wald ratio	1	0.0068	0.0186	1.007 (0.971, 1.044)	0.715	0.873	4.07E-11	TRUE
B Mem	*SVIP*	Wald ratio	1	-0.0164	0.0178	0.984 (0.95, 1.019)	0.356	0.647	3.57E-10	TRUE
B Mem	*SYNGR1*	Wald ratio	1	-0.0217	0.0113	0.979 (0.957, 1)	0.054	0.233	2.73E-21	TRUE
B Mem	*TAPBP*	Wald ratio	1	0.0109	0.0166	1.011 (0.979, 1.044)	0.511	0.748	2.13E-10	TRUE
B Mem	*TBCA*	Wald ratio	1	0.0145	0.0172	1.015 (0.981, 1.049)	0.399	0.678	6.37E-11	TRUE
B Mem	*TCEB3*	Wald ratio	1	0.0098	0.0180	1.01 (0.975, 1.046)	0.584	0.793	5.30E-10	TRUE
B Mem	*TEN1*	Wald ratio	1	-0.0247	0.0204	0.976 (0.937, 1.015)	0.227	0.52	3.79E-09	TRUE
B Mem	*TESC*	Wald ratio	1	0.0579	0.0202	1.06 (1.019, 1.102)	0.004	0.044	1.20E-08	TRUE
B Mem	*TIMM10*	Wald ratio	1	-0.0014	0.0090	0.999 (0.981, 1.016)	0.875	0.948	7.25E-44	TRUE
B Mem	*TIMM13*	Wald ratio	1	0.0656	0.0206	1.068 (1.026, 1.112)	0.001	0.021	1.04E-07	TRUE
B Mem	*TLE4*	Wald ratio	1	-0.0116	0.0144	0.988 (0.961, 1.017)	0.419	0.69	1.67E-15	TRUE
B Mem	*TLK1*	Wald ratio	1	0.0162	0.0160	1.016 (0.985, 1.049)	0.309	0.603	3.20E-13	TRUE
B Mem	*TMEM156*	Wald ratio	1	0.0049	0.0071	1.005 (0.991, 1.019)	0.488	0.738	1.15E-54	TRUE
B Mem	*TMEM220*	Wald ratio	1	0.0136	0.0157	1.014 (0.983, 1.045)	0.388	0.669	2.64E-12	TRUE
B Mem	*TMEM258*	Wald ratio	1	0.0192	0.0162	1.019 (0.988, 1.052)	0.235	0.529	4.98E-11	TRUE
B Mem	*TMEM50B*	Wald ratio	1	-0.0553	0.0173	0.946 (0.915, 0.979)	0.001	0.02	2.81E-10	TRUE
B Mem	*TMEM80*	Wald ratio	1	-0.0490	0.0193	0.952 (0.917, 0.989)	0.011	0.086	1.83E-08	TRUE
B Mem	*TOMM7*	Wald ratio	1	0.0035	0.0052	1.003 (0.993, 1.014)	0.503	0.743	3.09E-101	TRUE
B Mem	*TPGS2*	Wald ratio	1	-0.0208	0.0197	0.979 (0.942, 1.018)	0.292	0.585	6.16E-09	TRUE
B Mem	*TSPAN3*	Wald ratio	1	0.0038	0.0103	1.004 (0.984, 1.024)	0.713	0.872	9.62E-29	TRUE
B Mem	*TSPO*	Wald ratio	1	0.0021	0.0133	1.002 (0.976, 1.028)	0.877	0.949	1.25E-17	TRUE
B Mem	*TSTD1*	Wald ratio	1	0.0152	0.0099	1.015 (0.996, 1.035)	0.123	0.377	4.23E-31	TRUE
B Mem	*TTC32*	Wald ratio	1	0.0243	0.0205	1.025 (0.984, 1.067)	0.236	0.53	4.98E-11	TRUE
B Mem	*TUBB6*	Wald ratio	1	0.0003	0.0126	1 (0.976, 1.025)	0.978	0.991	6.36E-20	TRUE
B Mem	*TUFM*	Wald ratio	1	0.0172	0.0086	1.017 (1, 1.035)	0.046	0.21	9.23E-38	TRUE
B Mem	*TYMP*	Wald ratio	1	0.0356	0.0175	1.036 (1.001, 1.072)	0.042	0.201	4.00E-10	TRUE
B Mem	*UBA52*	Wald ratio	1	0.0069	0.0202	1.007 (0.968, 1.048)	0.733	0.88	3.02E-08	TRUE
B Mem	*UBE2J1*	Wald ratio	1	0.0035	0.0119	1.003 (0.98, 1.027)	0.771	0.899	4.64E-25	TRUE
B Mem	*UCP2*	Wald ratio	1	0.0056	0.0120	1.006 (0.982, 1.03)	0.64	0.83	9.68E-20	TRUE
B Mem	*UPF3A*	Wald ratio	1	-0.0301	0.0171	0.97 (0.938, 1.004)	0.079	0.295	4.10E-11	TRUE
B Mem	*UQCRH*	Wald ratio	1	-0.0258	0.0159	0.975 (0.945, 1.005)	0.104	0.342	9.85E-14	TRUE
B Mem	*USF1*	Wald ratio	1	-0.0088	0.0194	0.991 (0.954, 1.03)	0.648	0.834	3.96E-09	TRUE
B Mem	*USP53*	Wald ratio	1	-0.0333	0.0129	0.967 (0.943, 0.992)	0.01	0.08	1.49E-14	TRUE
B Mem	*VAMP2*	Wald ratio	1	0.0341	0.0198	1.035 (0.995, 1.076)	0.086	0.311	1.55E-09	TRUE
B Mem	*VAMP5*	Wald ratio	1	0.0273	0.0253	1.028 (0.978, 1.08)	0.281	0.575	4.32E-10	TRUE
B Mem	*WAC-AS1*	Wald ratio	1	0.0078	0.0201	1.008 (0.969, 1.048)	0.696	0.864	1.04E-08	TRUE
B Mem	*WARS*	Wald ratio	1	-0.0474	0.0095	0.954 (0.936, 0.972)	5.67E-07	9.75E-05	4.66E-37	TRUE
B Mem	*XRCC6BP1*	Wald ratio	1	-0.0358	0.0181	0.965 (0.931, 1)	0.048	0.215	1.30E-09	TRUE
B Mem	*XRRA1*	Wald ratio	1	-0.0088	0.0073	0.991 (0.977, 1.006)	0.228	0.521	8.41E-51	TRUE
B Mem	*XXbac-BPG181B23.7*	Wald ratio	1	0.0165	0.0225	1.017 (0.973, 1.062)	0.464	0.725	2.52E-10	TRUE
B Mem	*XXbac-BPG299F13.17*	Wald ratio	1	0.0094	0.0067	1.009 (0.996, 1.023)	0.162	0.436	2.10E-53	TRUE
B Mem	*YBEY*	Wald ratio	1	-0.0011	0.0088	0.999 (0.982, 1.016)	0.898	0.957	1.30E-32	TRUE
B Mem	*YME1L1*	Wald ratio	1	0.0135	0.0200	1.014 (0.975, 1.054)	0.498	0.741	2.92E-08	TRUE
B Mem	*YWHAB*	Wald ratio	1	0.0427	0.0182	1.044 (1.007, 1.082)	0.019	0.123	7.07E-10	TRUE
B Mem	*ZBTB9*	Wald ratio	1	-0.0076	0.0117	0.992 (0.97, 1.015)	0.513	0.75	1.39E-25	TRUE
B Mem	*ZC2HC1A*	Wald ratio	1	-0.0040	0.0123	0.996 (0.972, 1.02)	0.742	0.881	7.13E-19	TRUE
B Mem	*ZC3HAV1*	Wald ratio	1	0.0109	0.0147	1.011 (0.982, 1.04)	0.459	0.72	3.76E-14	TRUE
B Mem	*ZDHHC19*	Wald ratio	1	0.0022	0.0123	1.002 (0.978, 1.027)	0.86	0.941	4.28E-21	TRUE
B Mem	*ZFAS1*	Wald ratio	1	0.0079	0.0109	1.008 (0.987, 1.03)	0.468	0.726	3.02E-26	TRUE
B Mem	*ZFP57*	Wald ratio	1	-0.0202	0.0110	0.98 (0.959, 1.001)	0.066	0.263	3.68E-29	TRUE
B Mem	*ZKSCAN3*	Wald ratio	1	0.0391	0.0406	1.04 (0.96, 1.126)	0.335	0.628	4.15E-11	TRUE
B Mem	*ZNF100*	Wald ratio	1	0.0730	0.0159	1.076 (1.043, 1.11)	4.36E-06	3.02E-04	1.66E-11	TRUE
B Mem	*ZNF302*	Wald ratio	1	-0.0189	0.0185	0.981 (0.946, 1.018)	0.307	0.6	6.48E-09	TRUE
B Mem	*ZNF607*	Wald ratio	1	0.0123	0.0088	1.012 (0.995, 1.03)	0.164	0.438	3.21E-41	TRUE
B Mem	*ZNF667-AS1*	Wald ratio	1	-0.0461	0.0180	0.955 (0.922, 0.989)	0.01	0.083	1.85E-09	TRUE
B Mem	*ZNF83*	Wald ratio	1	-0.0270	0.0186	0.973 (0.939, 1.01)	0.148	0.417	1.17E-08	TRUE
B Mem	*ZNF85*	Wald ratio	1	0.0204	0.0123	1.021 (0.996, 1.046)	0.097	0.332	1.88E-20	TRUE
B Mem	*ZP3*	Wald ratio	1	0.0305	0.0133	1.031 (1.004, 1.058)	0.022	0.134	5.86E-20	TRUE
CD4 ET	*ABRACL*	Wald ratio	1	0.0582	0.0170	1.06 (1.025, 1.096)	6.07E-04	0.012	1.34E-10	TRUE
CD4 ET	*AC006369.2*	Wald ratio	1	0.0091	0.0083	1.009 (0.993, 1.026)	0.275	0.568	2.46E-44	TRUE
CD4 ET	*AC009961.3*	Wald ratio	1	-0.0184	0.0175	0.982 (0.949, 1.016)	0.293	0.587	1.72E-09	TRUE
CD4 ET	*AC016757.3*	Wald ratio	1	0.0090	0.0098	1.009 (0.99, 1.029)	0.358	0.649	8.63E-32	TRUE
CD4 ET	*AC021860.1*	Wald ratio	1	0.0420	0.0187	1.043 (1.005, 1.082)	0.025	0.146	1.34E-10	TRUE
CD4 ET	*AC023590.1*	Wald ratio	1	-0.0095	0.0198	0.991 (0.953, 1.03)	0.633	0.826	6.04E-12	TRUE
CD4 ET	*AC097713.4*	Wald ratio	1	-0.0075	0.0188	0.993 (0.957, 1.03)	0.69	0.86	2.28E-09	TRUE
CD4 ET	*AC104653.1*	Wald ratio	1	-0.0068	0.0098	0.993 (0.974, 1.013)	0.487	0.738	3.03E-30	TRUE
CD4 ET	*AC137932.1*	Wald ratio	1	0.0375	0.0185	1.038 (1.001, 1.076)	0.042	0.202	5.12E-09	TRUE
CD4 ET	*ACAT1*	Wald ratio	1	-0.0495	0.0182	0.952 (0.918, 0.986)	0.007	0.06	2.21E-09	TRUE
CD4 ET	*ACCS*	Wald ratio	1	-0.0042	0.0218	0.996 (0.954, 1.039)	0.847	0.935	3.00E-08	TRUE
CD4 ET	*ACTG1*	Wald ratio	1	-0.0343	0.0162	0.966 (0.936, 0.997)	0.034	0.175	5.32E-13	TRUE
CD4 ET	*ACYP2*	Wald ratio	1	0.0044	0.0107	1.004 (0.984, 1.026)	0.68	0.853	6.45E-25	TRUE
CD4 ET	*ADAMTS6*	Wald ratio	1	0.0274	0.0197	1.028 (0.989, 1.068)	0.165	0.439	2.54E-08	TRUE
CD4 ET	*AGA*	Wald ratio	1	-0.0151	0.0200	0.985 (0.947, 1.024)	0.449	0.714	3.99E-08	TRUE
CD4 ET	*AHI1*	Wald ratio	1	-0.0173	0.0110	0.983 (0.962, 1.004)	0.116	0.362	1.10E-24	TRUE
CD4 ET	*AIF1*	Wald ratio	1	0.0457	0.0123	1.047 (1.022, 1.072)	1.97E-04	0.005	2.37E-13	TRUE
CD4 ET	*AMICA1*	Wald ratio	1	0.0308	0.0137	1.031 (1.004, 1.059)	0.024	0.144	8.37E-13	TRUE
CD4 ET	*ANAPC13*	Wald ratio	1	-0.0208	0.0209	0.979 (0.94, 1.02)	0.321	0.616	2.81E-08	TRUE

CD4 ET	*ANKDD1A*	Wald ratio	1	0.0026	0.0111	1.003 (0.981, 1.025)	0.815	0.921	2.18E-23	TRUE
CD4 ET	*ANKRD55*	Wald ratio	1	-0.0026	0.0173	0.997 (0.964, 1.032)	0.881	0.951	8.67E-12	TRUE
CD4 ET	*ANXA5*	Wald ratio	1	-0.0091	0.0247	0.991 (0.944, 1.04)	0.712	0.872	7.81E-09	TRUE
CD4 ET	*ANXA6*	Wald ratio	1	0.0008	0.0156	1.001 (0.971, 1.032)	0.958	0.985	8.76E-11	TRUE
CD4 ET	*AP003774.1*	Wald ratio	1	-0.0380	0.0118	0.963 (0.941, 0.985)	0.001	0.02	7.48E-20	TRUE
CD4 ET	*ARHGAP15*	Wald ratio	1	0.0078	0.0115	1.008 (0.985, 1.031)	0.496	0.741	3.61E-28	TRUE
CD4 ET	*ARHGEF10*	Wald ratio	1	-0.0058	0.0118	0.994 (0.971, 1.017)	0.621	0.82	4.72E-21	TRUE
CD4 ET	*ARHGEF3*	Wald ratio	1	-0.0025	0.0132	0.998 (0.972, 1.024)	0.85	0.936	2.18E-15	TRUE
CD4 ET	*ARL14EP*	Wald ratio	1	-0.0284	0.0147	0.972 (0.944, 1)	0.053	0.231	1.60E-12	TRUE
CD4 ET	*ARL16*	Wald ratio	1	0.0273	0.0120	1.028 (1.004, 1.052)	0.023	0.137	2.64E-22	TRUE
CD4 ET	*ARPC5*	Wald ratio	1	-0.0057	0.0134	0.994 (0.968, 1.021)	0.67	0.848	1.69E-16	TRUE
CD4 ET	*ATF7IP2*	Wald ratio	1	0.0497	0.0194	1.051 (1.012, 1.092)	0.01	0.083	2.48E-08	TRUE
CD4 ET	*ATP5G2*	Wald ratio	1	0.0157	0.0210	1.016 (0.975, 1.059)	0.454	0.717	1.97E-08	TRUE
CD4 ET	*ATP5G3*	Wald ratio	1	-0.0068	0.0217	0.993 (0.952, 1.036)	0.753	0.888	3.59E-09	TRUE
CD4 ET	*ATP6V0E2*	Wald ratio	1	0.0216	0.0134	1.022 (0.995, 1.049)	0.108	0.35	7.23E-16	TRUE
CD4 ET	*ATP6V1G2*	Wald ratio	1	-0.0315	0.0263	0.969 (0.92, 1.02)	0.231	0.524	1.45E-21	TRUE
CD4 ET	*B3GAT2*	Wald ratio	1	0.0334	0.0256	1.034 (0.983, 1.087)	0.191	0.471	7.73E-08	TRUE
CD4 ET	*B4GALT1*	Wald ratio	1	0.0113	0.0131	1.011 (0.986, 1.038)	0.387	0.669	5.66E-20	TRUE
CD4 ET	*BCAS4*	Wald ratio	1	0.0329	0.0193	1.033 (0.995, 1.073)	0.088	0.315	7.75E-08	TRUE
CD4 ET	*BIN1*	Wald ratio	1	0.0210	0.0111	1.021 (0.999, 1.044)	0.058	0.243	1.66E-22	TRUE
CD4 ET	*BRK1*	Wald ratio	1	-0.0298	0.0210	0.971 (0.931, 1.012)	0.157	0.428	2.09E-09	TRUE
CD4 ET	*BST2*	Wald ratio	1	0.0451	0.0153	1.046 (1.015, 1.078)	0.003	0.037	5.33E-09	TRUE
CD4 ET	*BTF3*	Wald ratio	1	0.0448	0.0151	1.046 (1.015, 1.077)	0.003	0.034	7.25E-12	TRUE
CD4 ET	*BTN3A2*	Wald ratio	1	0.0161	0.0113	1.016 (0.994, 1.039)	0.153	0.423	5.22E-32	TRUE
CD4 ET	*C10orf128*	Wald ratio	1	-0.0064	0.0126	0.994 (0.969, 1.018)	0.609	0.809	3.74E-18	TRUE
CD4 ET	*C10orf32*	Wald ratio	1	-0.0774	0.0157	0.925 (0.897, 0.954)	8.67E-07	1.03E-04	2.02E-12	TRUE
CD4 ET	*C17orf89*	Wald ratio	1	0.0506	0.0163	1.052 (1.019, 1.086)	0.002	0.026	2.41E-11	TRUE
CD4 ET	*C17orf97*	Wald ratio	1	0.0093	0.0079	1.009 (0.994, 1.025)	0.241	0.534	1.70E-45	TRUE
CD4 ET	*C19orf83*	Wald ratio	1	-0.0430	0.0197	0.958 (0.922, 0.996)	0.029	0.159	2.55E-08	TRUE
CD4 ET	*C1orf86*	Wald ratio	1	0.0033	0.0162	1.003 (0.972, 1.036)	0.84	0.932	6.50E-12	TRUE
CD4 ET	*C1RL-AS1*	Wald ratio	1	-0.0207	0.0213	0.979 (0.939, 1.021)	0.33	0.624	7.21E-08	TRUE
CD4 ET	*C22orf34*	Wald ratio	1	0.0272	0.0156	1.028 (0.997, 1.06)	0.081	0.298	7.22E-13	TRUE
CD4 ET	*C2orf27A*	Wald ratio	1	-0.0062	0.0118	0.994 (0.971, 1.017)	0.597	0.802	2.12E-24	TRUE
CD4 ET	*C6orf48*	Wald ratio	1	0.0171	0.0137	1.017 (0.99, 1.045)	0.212	0.5	6.52E-10	TRUE
CD4 ET	*C9orf135*	Wald ratio	1	-0.0149	0.0102	0.985 (0.966, 1.005)	0.144	0.411	1.01E-23	TRUE
CD4 ET	*CA6*	Wald ratio	1	0.0156	0.0189	1.016 (0.979, 1.054)	0.408	0.684	5.65E-09	TRUE
CD4 ET	*CAMK4*	Wald ratio	1	0.0222	0.0207	1.022 (0.982, 1.065)	0.286	0.577	7.93E-09	TRUE
CD4 ET	*CAPG*	Wald ratio	1	-0.0185	0.0140	0.982 (0.955, 1.009)	0.187	0.466	1.29E-15	TRUE
CD4 ET	*CARD9*	Wald ratio	1	0.0074	0.0082	1.007 (0.991, 1.024)	0.366	0.654	1.92E-41	TRUE
CD4 ET	*CASP7*	Wald ratio	1	0.0109	0.0183	1.011 (0.975, 1.048)	0.553	0.772	1.71E-09	TRUE
CD4 ET	*CASP8*	Wald ratio	1	-0.0451	0.0161	0.956 (0.926, 0.987)	0.005	0.051	1.61E-11	TRUE
CD4 ET	*CAST*	Wald ratio	1	-0.0058	0.0171	0.994 (0.961, 1.028)	0.737	0.88	3.84E-12	TRUE
CD4 ET	*CCDC122*	Wald ratio	1	-0.0131	0.0160	0.987 (0.957, 1.018)	0.414	0.686	4.16E-12	TRUE
CD4 ET	*CCDC146*	Wald ratio	1	0.0399	0.0261	1.041 (0.989, 1.095)	0.126	0.381	1.87E-09	TRUE
CD4 ET	*CCDC163P*	Wald ratio	1	-0.0102	0.0124	0.99 (0.966, 1.014)	0.412	0.686	1.99E-19	TRUE
CD4 ET	*CCDC167*	Wald ratio	1	-0.0008	0.0069	0.999 (0.986, 1.013)	0.913	0.962	8.58E-57	TRUE
CD4 ET	*CCDC66*	Wald ratio	1	-0.0080	0.0142	0.992 (0.965, 1.02)	0.574	0.786	3.69E-15	TRUE
CD4 ET	*CCSER2*	Wald ratio	1	-0.0248	0.0131	0.976 (0.951, 1.001)	0.059	0.244	4.65E-21	TRUE
CD4 ET	*CCT3*	Wald ratio	1	0.0225	0.0133	1.023 (0.996, 1.05)	0.09	0.318	1.04E-16	TRUE
CD4 ET	*CCT8*	Wald ratio	1	-0.0009	0.0091	0.999 (0.982, 1.017)	0.922	0.965	1.11E-57	TRUE
CD4 ET	*CCZ1*	Wald ratio	1	0.0150	0.0110	1.015 (0.994, 1.037)	0.171	0.448	4.48E-25	TRUE
CD4 ET	*CCZ1B*	Wald ratio	1	-0.0165	0.0108	0.984 (0.963, 1.005)	0.126	0.382	1.15E-26	TRUE
CD4 ET	*CD151*	Wald ratio	1	0.0534	0.0144	1.055 (1.025, 1.085)	2.14E-04	0.005	3.57E-16	TRUE
CD4 ET	*CD247*	Wald ratio	1	-0.0062	0.0227	0.994 (0.951, 1.039)	0.784	0.907	5.18E-08	TRUE
CD4 ET	*CD3G*	Wald ratio	1	-0.0129	0.0206	0.987 (0.948, 1.028)	0.533	0.76	2.63E-11	TRUE
CD4 ET	*CD52*	Wald ratio	1	-0.0197	0.0168	0.98 (0.949, 1.013)	0.24	0.534	1.79E-11	TRUE
CD4 ET	*CD59*	Wald ratio	1	-0.0115	0.0189	0.989 (0.953, 1.026)	0.543	0.765	1.52E-08	TRUE
CD4 ET	*CDC42*	Wald ratio	1	0.0094	0.0136	1.009 (0.983, 1.037)	0.49	0.738	1.40E-14	TRUE
CD4 ET	*CDK10*	Wald ratio	1	-0.0590	0.0179	0.943 (0.91, 0.976)	9.77E-04	0.017	2.72E-09	TRUE
CD4 ET	*CEACAM21*	Wald ratio	1	-0.0160	0.0132	0.984 (0.959, 1.01)	0.227	0.52	3.75E-15	TRUE
CD4 ET	*CEBPZ-AS1*	Wald ratio	1	0.0273	0.0222	1.028 (0.984, 1.073)	0.219	0.511	1.41E-09	TRUE
CD4 ET	*CELA1*	Wald ratio	1	0.0042	0.0132	1.004 (0.979, 1.031)	0.75	0.886	4.62E-21	TRUE
CD4 ET	*CENPK*	Wald ratio	1	0.0071	0.0051	1.007 (0.997, 1.017)	0.165	0.439	6.59E-118	TRUE
CD4 ET	*CFD*	Wald ratio	1	-0.0027	0.0128	0.997 (0.973, 1.023)	0.835	0.93	1.70E-20	TRUE
CD4 ET	*CHCHD2*	Wald ratio	1	0.0079	0.0148	1.008 (0.979, 1.038)	0.591	0.797	3.08E-15	TRUE
CD4 ET	*CHI3L2*	Wald ratio	1	0.0076	0.0201	1.008 (0.969, 1.048)	0.704	0.867	3.35E-08	TRUE
CD4 ET	*CHN1*	Wald ratio	1	-0.0067	0.0180	0.993 (0.959, 1.029)	0.709	0.87	1.20E-09	TRUE
CD4 ET	*CHPT1*	Wald ratio	1	0.0004	0.0098	1 (0.981, 1.02)	0.964	0.988	7.91E-32	TRUE
CD4 ET	*CHURC1*	Wald ratio	1	0.0252	0.0131	1.026 (1, 1.052)	0.054	0.232	5.80E-15	TRUE
CD4 ET	*CIB1*	Wald ratio	1	-0.0438	0.0158	0.957 (0.928, 0.987)	0.005	0.053	4.46E-16	TRUE
CD4 ET	*CLK4*	Wald ratio	1	0.0039	0.0214	1.004 (0.963, 1.047)	0.856	0.939	5.81E-08	TRUE
CD4 ET	*CMTM7*	Wald ratio	1	0.0332	0.0194	1.034 (0.995, 1.074)	0.087	0.312	2.19E-08	TRUE
CD4 ET	*CMTM8*	Wald ratio	1	0.0017	0.0101	1.002 (0.982, 1.022)	0.865	0.943	1.10E-33	TRUE
CD4 ET	*CNN2*	Wald ratio	1	0.0196	0.0147	1.02 (0.991, 1.05)	0.181	0.462	2.13E-14	TRUE
CD4 ET	*CNOT6L*	Wald ratio	1	0.0392	0.0207	1.04 (0.999, 1.083)	0.059	0.244	7.72E-08	TRUE
CD4 ET	*COG5*	Wald ratio	1	0.0854	0.0168	1.089 (1.054, 1.125)	3.53E-07	8.32E-05	4.52E-10	TRUE
CD4 ET	*COL18A1*	Wald ratio	1	-0.0279	0.0142	0.973 (0.946, 1)	0.049	0.22	8.03E-38	TRUE
CD4 ET	*COL5A3*	Wald ratio	1	0.0517	0.0113	1.053 (1.03, 1.077)	4.38E-06	3.02E-04	2.89E-21	TRUE
CD4 ET	*COMMD6*	Wald ratio	1	0.0136	0.0084	1.014 (0.997, 1.03)	0.104	0.343	1.31E-41	TRUE

CD4 ET	*CORO1B*	Wald ratio	1	0.0017	0.0113	1.002 (0.98, 1.024)	0.884	0.953	3.25E-24	TRUE
CD4 ET	*COX14*	Wald ratio	1	-0.0684	0.0216	0.934 (0.895, 0.974)	0.001	0.021	1.22E-07	TRUE
CD4 ET	*COX20*	Wald ratio	1	-0.0072	0.0242	0.993 (0.947, 1.041)	0.766	0.896	5.34E-08	TRUE
CD4 ET	*COX6B1*	Wald ratio	1	-0.0368	0.0167	0.964 (0.933, 0.996)	0.027	0.155	4.17E-11	TRUE
CD4 ET	*COX7A2L*	Wald ratio	1	-0.0205	0.0185	0.98 (0.945, 1.016)	0.266	0.561	4.38E-10	TRUE
CD4 ET	*CPA5*	Wald ratio	1	-0.0057	0.0066	0.994 (0.982, 1.007)	0.386	0.669	1.18E-68	TRUE
CD4 ET	*CPNE1*	Wald ratio	1	-0.0061	0.0146	0.994 (0.966, 1.023)	0.677	0.853	3.13E-17	TRUE
CD4 ET	*CRIP2*	Wald ratio	1	-0.0227	0.0166	0.978 (0.946, 1.01)	0.173	0.451	1.34E-12	TRUE
CD4 ET	*CRYBB2*	Wald ratio	1	0.0114	0.0090	1.011 (0.994, 1.03)	0.208	0.494	2.39E-35	TRUE
CD4 ET	*CSTB*	Wald ratio	1	0.0053	0.0153	1.005 (0.976, 1.036)	0.73	0.88	4.12E-12	TRUE
CD4 ET	*CTA-29F11.1*	Wald ratio	1	-0.0080	0.0108	0.992 (0.971, 1.013)	0.456	0.719	2.19E-25	TRUE
CD4 ET	*CTB-129P6.4*	Wald ratio	1	0.0081	0.0089	1.008 (0.991, 1.026)	0.362	0.651	3.73E-37	TRUE
CD4 ET	*CTC-241F20.3*	Wald ratio	1	-0.0343	0.0218	0.966 (0.926, 1.008)	0.115	0.362	6.50E-10	TRUE
CD4 ET	*CTC-524C5.2*	Wald ratio	1	0.0162	0.0091	1.016 (0.998, 1.035)	0.076	0.289	1.71E-27	TRUE
CD4 ET	*CTD-2020K17.4*	Wald ratio	1	-0.0157	0.0141	0.984 (0.958, 1.012)	0.263	0.558	3.58E-12	TRUE
CD4 ET	*CTD-2336O2.1*	Wald ratio	1	0.0469	0.0143	1.048 (1.019, 1.078)	9.95E-04	0.017	1.46E-15	TRUE
CD4 ET	*CTD-2368P22.1*	Wald ratio	1	0.0053	0.0156	1.005 (0.975, 1.037)	0.734	0.88	7.91E-14	TRUE
CD4 ET	*CTD-2371O3.2*	Wald ratio	1	0.0011	0.0143	1.001 (0.973, 1.03)	0.94	0.976	6.95E-16	TRUE
CD4 ET	*CTD-2521M24.9*	Wald ratio	1	0.0398	0.0152	1.041 (1.01, 1.072)	0.009	0.073	7.76E-10	TRUE
CD4 ET	*CTLA4*	Wald ratio	1	-0.0026	0.0099	0.997 (0.978, 1.017)	0.788	0.909	1.41E-33	TRUE
CD4 ET	*CTSL*	Wald ratio	1	-0.0103	0.0145	0.99 (0.962, 1.018)	0.479	0.733	7.58E-15	TRUE
CD4 ET	*CTSW*	Wald ratio	1	-0.0390	0.0092	0.962 (0.945, 0.979)	2.34E-05	0.001	6.86E-39	TRUE
CD4 ET	*DAD1*	Wald ratio	1	-0.0059	0.0154	0.994 (0.965, 1.025)	0.702	0.866	2.82E-12	TRUE
CD4 ET	*DARS*	Wald ratio	1	-0.0304	0.0172	0.97 (0.938, 1.003)	0.078	0.292	8.32E-09	TRUE
CD4 ET	*DAZAP1*	Wald ratio	1	0.0429	0.0167	1.044 (1.01, 1.079)	0.01	0.083	1.98E-11	TRUE
CD4 ET	*DAZAP2*	Wald ratio	1	-0.0619	0.0178	0.94 (0.908, 0.973)	5.23E-04	0.011	3.10E-09	TRUE
CD4 ET	*DCP2*	Wald ratio	1	0.0456	0.0182	1.047 (1.01, 1.085)	0.012	0.093	3.18E-08	TRUE
CD4 ET	*DCXR*	Wald ratio	1	-0.0095	0.0149	0.991 (0.962, 1.02)	0.525	0.755	2.10E-12	TRUE
CD4 ET	*DDT*	Wald ratio	1	-0.0136	0.0125	0.986 (0.963, 1.011)	0.273	0.567	1.84E-19	TRUE
CD4 ET	*DDX11*	Wald ratio	1	-0.0134	0.0100	0.987 (0.968, 1.006)	0.179	0.459	1.47E-29	TRUE
CD4 ET	*DDX43*	Wald ratio	1	-0.0067	0.0090	0.993 (0.976, 1.011)	0.452	0.717	5.75E-35	TRUE
CD4 ET	*DDX5*	Wald ratio	1	0.0468	0.0121	1.048 (1.023, 1.073)	1.06E-04	0.003	2.55E-18	TRUE
CD4 ET	*DHX32*	Wald ratio	1	-0.0017	0.0171	0.998 (0.965, 1.032)	0.92	0.965	5.25E-11	TRUE
CD4 ET	*DNAJC15*	Wald ratio	1	0.0079	0.0076	1.008 (0.993, 1.023)	0.303	0.598	5.66E-49	TRUE
CD4 ET	*DOCK8*	Wald ratio	1	0.0329	0.0191	1.033 (0.995, 1.073)	0.085	0.311	1.98E-12	TRUE
CD4 ET	*DPYSL4*	Wald ratio	1	-0.0266	0.0190	0.974 (0.938, 1.011)	0.161	0.434	2.41E-10	TRUE
CD4 ET	*DRAM2*	Wald ratio	1	-0.0004	0.0143	1 (0.972, 1.028)	0.979	0.992	1.93E-14	TRUE
CD4 ET	*DYNLL2*	Wald ratio	1	-0.0323	0.0193	0.968 (0.932, 1.006)	0.095	0.327	5.23E-09	TRUE
CD4 ET	*EBPL*	Wald ratio	1	0.0048	0.0083	1.005 (0.989, 1.021)	0.564	0.78	8.68E-42	TRUE
CD4 ET	*EEF1D*	Wald ratio	1	0.0344	0.0179	1.035 (0.999, 1.072)	0.055	0.234	8.98E-09	TRUE
CD4 ET	*EIF2A*	Wald ratio	1	-0.0159	0.0114	0.984 (0.962, 1.006)	0.163	0.437	5.88E-21	TRUE
CD4 ET	*EIF5A*	Wald ratio	1	-0.0073	0.0049	0.993 (0.983, 1.002)	0.134	0.394	4.81E-156	TRUE
CD4 ET	*ELMSAN1*	Wald ratio	1	0.0166	0.0198	1.017 (0.978, 1.057)	0.401	0.679	2.67E-08	TRUE
CD4 ET	*EML4*	Wald ratio	1	-0.0563	0.0202	0.945 (0.908, 0.984)	0.005	0.053	7.99E-08	TRUE
CD4 ET	*EMR1*	Wald ratio	1	0.0209	0.0152	1.021 (0.991, 1.052)	0.168	0.444	1.90E-13	TRUE
CD4 ET	*ENOSF1*	Wald ratio	1	0.0153	0.0170	1.015 (0.982, 1.05)	0.369	0.657	9.37E-11	TRUE
CD4 ET	*ENTPD1*	Wald ratio	1	0.0231	0.0120	1.023 (1, 1.048)	0.054	0.233	2.53E-20	TRUE
CD4 ET	*EPHA1-AS1*	Wald ratio	1	0.0424	0.0216	1.043 (1, 1.088)	0.05	0.222	2.24E-11	TRUE
CD4 ET	*EPSTI1*	Wald ratio	1	0.0067	0.0109	1.007 (0.985, 1.029)	0.54	0.762	9.96E-27	TRUE
CD4 ET	*ERICH1*	Wald ratio	1	-0.0058	0.0154	0.994 (0.965, 1.025)	0.704	0.867	3.48E-13	TRUE
CD4 ET	*ESR2*	Wald ratio	1	-0.0415	0.0170	0.959 (0.928, 0.992)	0.014	0.103	8.43E-18	TRUE
CD4 ET	*EVI2A*	Wald ratio	1	-0.0214	0.0127	0.979 (0.955, 1.003)	0.091	0.32	6.03E-18	TRUE
CD4 ET	*EXOC3*	Wald ratio	1	-0.0032	0.0128	0.997 (0.972, 1.022)	0.803	0.915	1.66E-18	TRUE
CD4 ET	*FADS1*	Wald ratio	1	-0.0246	0.0128	0.976 (0.952, 1)	0.054	0.233	4.27E-16	TRUE
CD4 ET	*FADS2*	Wald ratio	1	0.0274	0.0174	1.028 (0.993, 1.063)	0.115	0.362	2.01E-15	TRUE
CD4 ET	*FAM118A*	Wald ratio	1	0.0006	0.0044	1.001 (0.992, 1.009)	0.895	0.957	2.11E-121	TRUE
CD4 ET	*FAM153B*	Wald ratio	1	0.0278	0.0114	1.028 (1.006, 1.051)	0.014	0.102	4.56E-23	TRUE
CD4 ET	*FAM184B*	Wald ratio	1	0.0028	0.0064	1.003 (0.99, 1.015)	0.66	0.843	1.50E-81	TRUE
CD4 ET	*FAM21A*	Wald ratio	1	0.0614	0.0209	1.063 (1.021, 1.108)	0.003	0.038	6.83E-08	TRUE
CD4 ET	*FAM26F*	Wald ratio	1	-0.0067	0.0106	0.993 (0.973, 1.014)	0.525	0.755	2.54E-26	TRUE
CD4 ET	*FAR1*	Wald ratio	1	-0.0025	0.0132	0.998 (0.972, 1.024)	0.851	0.936	2.13E-18	TRUE
CD4 ET	*FAU*	Wald ratio	1	-0.0018	0.0146	0.998 (0.97, 1.027)	0.901	0.958	2.65E-14	TRUE
CD4 ET	*FBLN5*	Wald ratio	1	0.0026	0.0093	1.003 (0.984, 1.021)	0.779	0.904	1.49E-33	TRUE
CD4 ET	*FDFT1*	Wald ratio	1	0.0455	0.0142	1.047 (1.018, 1.076)	0.001	0.02	5.92E-18	TRUE
CD4 ET	*FGF9*	Wald ratio	1	-0.0771	0.0182	0.926 (0.893, 0.959)	2.18E-05	9.97E-04	1.36E-09	TRUE
CD4 ET	*FGFRL1*	Wald ratio	1	-0.0130	0.0117	0.987 (0.965, 1.01)	0.269	0.563	4.13E-24	TRUE
CD4 ET	*FIS1*	Wald ratio	1	0.0080	0.0181	1.008 (0.973, 1.044)	0.659	0.842	3.44E-10	TRUE
CD4 ET	*FKBPL*	Wald ratio	1	-0.0661	0.0184	0.936 (0.903, 0.97)	3.29E-04	0.008	1.72E-10	TRUE
CD4 ET	*FMN1*	Wald ratio	1	-0.0088	0.0067	0.991 (0.978, 1.004)	0.192	0.473	1.15E-54	TRUE
CD4 ET	*FN3KRP*	Wald ratio	1	0.0150	0.0155	1.015 (0.985, 1.046)	0.334	0.627	2.88E-13	TRUE
CD4 ET	*FNBP4*	Wald ratio	1	-0.1042	0.0196	0.901 (0.867, 0.936)	1.08E-07	6.60E-05	2.10E-08	TRUE
CD4 ET	*FTCDNL1*	Wald ratio	1	-0.0034	0.0134	0.997 (0.971, 1.023)	0.801	0.915	6.19E-22	TRUE
CD4 ET	*GBP5*	Wald ratio	1	-0.0316	0.0124	0.969 (0.946, 0.993)	0.011	0.085	3.67E-19	TRUE
CD4 ET	*GBP7*	Wald ratio	1	0.0006	0.0081	1.001 (0.985, 1.017)	0.943	0.978	1.35E-45	TRUE
CD4 ET	*GCAT*	Wald ratio	1	-0.0386	0.0199	0.962 (0.925, 1)	0.052	0.227	7.09E-08	TRUE
CD4 ET	*GCC2*	Wald ratio	1	0.0078	0.0104	1.008 (0.988, 1.029)	0.451	0.716	2.28E-30	TRUE
CD4 ET	*GDI2*	Wald ratio	1	-0.0106	0.0147	0.989 (0.961, 1.018)	0.47	0.727	2.62E-14	TRUE
CD4 ET	*GDPD5*	Wald ratio	1	-0.0016	0.0124	0.998 (0.975, 1.023)	0.899	0.958	3.10E-15	TRUE
CD4 ET	*GIPC1*	Wald ratio	1	-0.0507	0.0190	0.951 (0.916, 0.987)	0.008	0.067	3.25E-09	TRUE

CD4 ET	*GLTSCR2*	Wald ratio	1	-0.0098	0.0158	0.99 (0.96, 1.021)	0.537	0.762	1.09E-11	TRUE
CD4 ET	*GNG5*	Wald ratio	1	0.0082	0.0227	1.008 (0.964, 1.054)	0.719	0.875	1.48E-09	TRUE
CD4 ET	*GNLY*	Wald ratio	1	-0.0251	0.0178	0.975 (0.942, 1.01)	0.157	0.428	4.71E-09	TRUE
CD4 ET	*GNPTG*	Wald ratio	1	-0.0211	0.0167	0.979 (0.948, 1.012)	0.208	0.494	3.15E-09	TRUE
CD4 ET	*GPR63*	Wald ratio	1	0.0021	0.0058	1.002 (0.991, 1.014)	0.716	0.874	5.64E-87	TRUE
CD4 ET	*GPX4*	Wald ratio	1	-0.0153	0.0158	0.985 (0.955, 1.016)	0.333	0.626	3.53E-14	TRUE
CD4 ET	*GRHPR*	Wald ratio	1	-0.0131	0.0108	0.987 (0.966, 1.008)	0.227	0.52	2.68E-23	TRUE
CD4 ET	*GSTM3*	Wald ratio	1	-0.0137	0.0087	0.986 (0.97, 1.003)	0.114	0.361	9.76E-38	TRUE
CD4 ET	*GSTO1*	Wald ratio	1	0.0304	0.0175	1.031 (0.996, 1.067)	0.083	0.303	9.42E-11	TRUE
CD4 ET	*GSTP1*	Wald ratio	1	0.0173	0.0106	1.017 (0.997, 1.039)	0.101	0.337	6.21E-28	TRUE
CD4 ET	*GTF2H4*	IVW	2	0.0376	0.0172	1.038 (1.004, 1.074)	0.029	0.16	2.97E-31	TRUE
CD4 ET	*GTF3A*	Wald ratio	1	0.0270	0.0180	1.027 (0.992, 1.064)	0.134	0.396	5.85E-09	TRUE
CD4 ET	*GYPC*	IVW	3	-0.0064	0.0076	0.994 (0.979, 1.008)	0.399	0.678	2.64E-50	TRUE
CD4 ET	*GYPC*	MR Egger	3	-0.0152	0.0201	0.985 (0.947, 1.025)	0.589	NA	NA	NA
CD4 ET	*GYPC*	Weighted median	3	-0.0081	0.0085	0.992 (0.975, 1.009)	0.342	NA	NA	NA
CD4 ET	*GYPC*	Simple mode	3	-0.0079	0.0110	0.992 (0.971, 1.014)	0.547	NA	NA	NA
CD4 ET	*GYPC*	Weighted mode	3	-0.0086	0.0096	0.991 (0.973, 1.01)	0.465	NA	NA	NA
CD4 ET	*GZMM*	Wald ratio	1	0.0152	0.0225	1.015 (0.972, 1.061)	0.498	0.741	4.28E-10	TRUE
CD4 ET	*HAGH*	Wald ratio	1	0.0597	0.0144	1.062 (1.032, 1.092)	3.48E-05	0.001	4.26E-12	TRUE
CD4 ET	*HCG25*	IVW	2	-0.0137	0.0202	0.986 (0.948, 1.026)	0.499	0.742	2.59E-49	TRUE
CD4 ET	*HEBP2*	Wald ratio	1	0.0258	0.0235	1.026 (0.98, 1.075)	0.272	0.566	3.36E-08	TRUE
CD4 ET	*HFE*	Wald ratio	1	0.0036	0.0155	1.004 (0.973, 1.035)	0.819	0.923	4.19E-19	TRUE
CD4 ET	*HIGD2A*	Wald ratio	1	-0.0108	0.0125	0.989 (0.965, 1.014)	0.387	0.669	4.68E-20	TRUE
CD4 ET	*HIST1H2BG*	Wald ratio	1	0.0185	0.0258	1.019 (0.968, 1.072)	0.475	0.731	4.52E-09	TRUE
CD4 ET	*HIST1H2BH*	Wald ratio	1	-0.0145	0.0160	0.986 (0.955, 1.017)	0.364	0.653	1.45E-21	TRUE
CD4 ET	*HIST1H4B*	Wald ratio	1	0.0150	0.0231	1.015 (0.97, 1.062)	0.516	0.751	5.72E-11	TRUE
CD4 ET	*HIVEP3*	Wald ratio	1	0.0286	0.0136	1.029 (1.002, 1.057)	0.035	0.179	3.58E-16	TRUE
CD4 ET	*HLA-B*	IVW	3	0.0030	0.0213	1.003 (0.962, 1.046)	0.889	0.955	6.90E-99	TRUE
CD4 ET	*HLA-B*	MR Egger	3	0.2431	0.0464	1.275 (1.164, 1.397)	0.12	NA	NA	NA
CD4 ET	*HLA-B*	Weighted median	3	0.0025	0.0066	1.003 (0.99, 1.016)	0.7	NA	NA	NA
CD4 ET	*HLA-B*	Simple mode	3	-0.0353	0.0112	0.965 (0.944, 0.987)	0.088	NA	NA	NA
CD4 ET	*HLA-B*	Weighted mode	3	0.0271	0.0081	1.027 (1.011, 1.044)	0.079	NA	NA	NA
CD4 ET	*HLA-C*	Wald ratio	1	-0.0164	0.0113	0.984 (0.962, 1.006)	0.146	0.415	2.38E-20	TRUE
CD4 ET	*HLA-DQA2*	Wald ratio	1	-0.0188	0.0166	0.981 (0.95, 1.014)	0.257	0.551	8.13E-12	TRUE
CD4 ET	*HMBOX1*	Wald ratio	1	-0.0175	0.0095	0.983 (0.964, 1.001)	0.066	0.263	7.73E-29	TRUE
CD4 ET	*HOXB-AS1*	Wald ratio	1	-0.0070	0.0134	0.993 (0.967, 1.019)	0.599	0.804	2.43E-16	TRUE
CD4 ET	*HOXB2*	Wald ratio	1	-0.0105	0.0095	0.99 (0.971, 1.008)	0.27	0.563	1.63E-32	TRUE
CD4 ET	*HOXB4*	Wald ratio	1	-0.0224	0.0168	0.978 (0.946, 1.011)	0.184	0.463	3.94E-11	TRUE
CD4 ET	*HSPA1B*	Wald ratio	1	-0.0144	0.0175	0.986 (0.952, 1.02)	0.412	0.686	5.33E-09	TRUE
CD4 ET	*HSPBAP1*	Wald ratio	1	0.0091	0.0104	1.009 (0.989, 1.03)	0.383	0.667	2.33E-22	TRUE
CD4 ET	*IAH1*	Wald ratio	1	0.0078	0.0230	1.008 (0.963, 1.054)	0.735	0.88	6.54E-08	TRUE
CD4 ET	*IFI27L2*	Wald ratio	1	0.0053	0.0092	1.005 (0.987, 1.024)	0.568	0.782	5.99E-33	TRUE
CD4 ET	*IFITM2*	Wald ratio	1	-0.0039	0.0106	0.996 (0.976, 1.017)	0.713	0.872	2.80E-25	TRUE
CD4 ET	*IFNG-AS1*	Wald ratio	1	-0.0076	0.0176	0.992 (0.959, 1.027)	0.666	0.847	1.19E-09	TRUE
CD4 ET	*IGFLR1*	Wald ratio	1	0.0154	0.0240	1.015 (0.969, 1.064)	0.522	0.754	3.52E-09	TRUE
CD4 ET	*IL32*	Wald ratio	1	-0.0105	0.0118	0.99 (0.967, 1.013)	0.376	0.664	3.22E-21	TRUE
CD4 ET	*INTS5*	Wald ratio	1	-0.0290	0.0223	0.971 (0.93, 1.015)	0.193	0.474	4.01E-08	TRUE
CD4 ET	*ISCA2*	Wald ratio	1	0.0110	0.0137	1.011 (0.984, 1.039)	0.424	0.694	7.23E-16	TRUE
CD4 ET	*ISCU*	Wald ratio	1	0.0004	0.0188	1 (0.964, 1.038)	0.984	0.994	8.54E-09	TRUE
CD4 ET	*ISG15*	Wald ratio	1	-0.0464	0.0198	0.955 (0.918, 0.992)	0.019	0.123	6.21E-08	TRUE
CD4 ET	*ITGB2*	Wald ratio	1	-0.0009	0.0067	0.999 (0.986, 1.012)	0.897	0.957	1.04E-54	TRUE
CD4 ET	*ITGB3BP*	Wald ratio	1	0.0065	0.0132	1.007 (0.981, 1.033)	0.623	0.822	8.45E-17	TRUE
CD4 ET	*JAKMIP1*	Wald ratio	1	0.0220	0.0183	1.022 (0.986, 1.06)	0.229	0.523	1.51E-09	TRUE
CD4 ET	*JAKMIP2*	Wald ratio	1	0.0577	0.0185	1.059 (1.022, 1.099)	0.002	0.025	6.36E-10	TRUE
CD4 ET	*KANSL1-AS1*	Wald ratio	1	-0.0185	0.0075	0.982 (0.967, 0.996)	0.014	0.103	2.43E-129	TRUE
CD4 ET	*KCNAB2*	Wald ratio	1	0.0094	0.0202	1.009 (0.97, 1.05)	0.641	0.831	2.84E-09	TRUE
CD4 ET	*KCTD21-AS1*	Wald ratio	1	-0.0175	0.0106	0.983 (0.962, 1.003)	0.099	0.334	1.12E-26	TRUE
CD4 ET	*KDELR2*	Wald ratio	1	0.0389	0.0121	1.04 (1.015, 1.064)	0.001	0.02	4.36E-19	TRUE
CD4 ET	*KIAA1430*	Wald ratio	1	0.0105	0.0171	1.011 (0.977, 1.045)	0.538	0.762	2.32E-10	TRUE
CD4 ET	*KISS1R*	Wald ratio	1	0.0020	0.0213	1.002 (0.961, 1.045)	0.926	0.968	1.16E-08	TRUE
CD4 ET	*KNOP1*	Wald ratio	1	-0.0076	0.0210	0.992 (0.952, 1.034)	0.717	0.874	8.12E-09	TRUE
CD4 ET	*KRT1*	Wald ratio	1	-0.0043	0.0083	0.996 (0.98, 1.012)	0.604	0.806	4.73E-42	TRUE
CD4 ET	*KTN1*	Wald ratio	1	0.0232	0.0116	1.024 (1.001, 1.047)	0.044	0.208	6.19E-20	TRUE
CD4 ET	*L3MBTL3*	Wald ratio	1	-0.0114	0.0134	0.989 (0.963, 1.015)	0.393	0.672	2.92E-17	TRUE
CD4 ET	*LA16c-358B7.3*	Wald ratio	1	0.0594	0.0147	1.061 (1.031, 1.092)	5.05E-05	0.002	5.79E-15	TRUE
CD4 ET	*LAMB1*	Wald ratio	1	0.0163	0.0154	1.016 (0.986, 1.048)	0.289	0.582	4.14E-13	TRUE
CD4 ET	*LAMTOR4*	Wald ratio	1	-0.0347	0.0202	0.966 (0.928, 1.005)	0.085	0.309	5.02E-08	TRUE
CD4 ET	*LCLAT1*	Wald ratio	1	-0.0014	0.0124	0.999 (0.975, 1.023)	0.912	0.962	8.45E-22	TRUE
CD4 ET	*LDLRAP1*	Wald ratio	1	0.0135	0.0119	1.014 (0.99, 1.037)	0.257	0.551	6.14E-22	TRUE
CD4 ET	*LEPROT*	Wald ratio	1	0.0006	0.0156	1.001 (0.97, 1.032)	0.969	0.989	1.02E-13	TRUE
CD4 ET	*LGALS3*	Wald ratio	1	-0.0115	0.0132	0.989 (0.963, 1.014)	0.382	0.666	5.96E-16	TRUE
CD4 ET	*LIMD1*	Wald ratio	1	0.0228	0.0206	1.023 (0.982, 1.065)	0.27	0.563	3.79E-08	TRUE
CD4 ET	*LINC00176*	Wald ratio	1	0.0041	0.0053	1.004 (0.994, 1.015)	0.444	0.709	1.70E-102	TRUE
CD4 ET	*LINC00339*	Wald ratio	1	-0.0260	0.0158	0.974 (0.945, 1.005)	0.1	0.334	1.89E-11	TRUE
CD4 ET	*LINC00649*	Wald ratio	1	-0.0072	0.0174	0.993 (0.959, 1.027)	0.679	0.853	2.23E-10	TRUE
CD4 ET	*LINC00852*	Wald ratio	1	0.0273	0.0147	1.028 (0.999, 1.058)	0.062	0.255	1.71E-16	TRUE
CD4 ET	*LINC00944*	Wald ratio	1	0.0076	0.0141	1.008 (0.98, 1.036)	0.59	0.797	1.29E-09	TRUE
CD4 ET	*LINC00954*	Wald ratio	1	-0.0138	0.0159	0.986 (0.956, 1.017)	0.385	0.668	5.80E-15	TRUE
CD4 ET	*LINC00969*	Wald ratio	1	-0.0098	0.0113	0.99 (0.969, 1.012)	0.384	0.668	4.01E-23	TRUE

CD4 ET	*LMAN1*	Wald ratio	1	0.0123	0.0191	1.012 (0.975, 1.051)	0.52	0.753	2.32E-08	TRUE
CD4 ET	*LPIN1*	Wald ratio	1	0.0373	0.0155	1.038 (1.007, 1.07)	0.016	0.111	1.10E-10	TRUE
CD4 ET	*LPIN2*	Wald ratio	1	-0.0250	0.0204	0.975 (0.937, 1.015)	0.22	0.511	4.53E-08	TRUE
CD4 ET	*LRRC16A*	Wald ratio	1	-0.0271	0.0205	0.973 (0.935, 1.013)	0.186	0.466	3.12E-08	TRUE
CD4 ET	*LRRC61*	Wald ratio	1	-0.0493	0.0181	0.952 (0.919, 0.986)	0.007	0.06	3.76E-09	TRUE
CD4 ET	*LSM7*	Wald ratio	1	0.0577	0.0180	1.059 (1.023, 1.097)	0.001	0.02	1.93E-09	TRUE
CD4 ET	*MAF1*	Wald ratio	1	0.0214	0.0209	1.022 (0.981, 1.064)	0.306	0.599	2.53E-09	TRUE
CD4 ET	*MANBA*	Wald ratio	1	0.0614	0.0170	1.063 (1.029, 1.099)	3.00E-04	0.007	2.20E-10	TRUE
CD4 ET	*MDGA1*	Wald ratio	1	0.0031	0.0179	1.003 (0.969, 1.039)	0.861	0.941	2.00E-12	TRUE
CD4 ET	*MDM4*	Wald ratio	1	-0.0922	0.0220	0.912 (0.873, 0.952)	2.70E-05	0.001	3.98E-09	TRUE
CD4 ET	*MED16*	Wald ratio	1	0.0019	0.0112	1.002 (0.98, 1.024)	0.866	0.944	5.38E-28	TRUE
CD4 ET	*METTL21B*	Wald ratio	1	0.0226	0.0143	1.023 (0.995, 1.052)	0.115	0.362	2.53E-14	TRUE
CD4 ET	*MGMT*	Wald ratio	1	-0.0302	0.0190	0.97 (0.935, 1.007)	0.113	0.359	7.31E-09	TRUE
CD4 ET	*MGST3*	Wald ratio	1	-0.0007	0.0111	0.999 (0.978, 1.021)	0.951	0.981	4.05E-24	TRUE
CD4 ET	*MORN3*	Wald ratio	1	-0.0350	0.0196	0.966 (0.929, 1.004)	0.075	0.287	2.24E-08	TRUE
CD4 ET	*MPZL3*	Wald ratio	1	0.0261	0.0178	1.026 (0.991, 1.063)	0.143	0.41	1.26E-09	TRUE
CD4 ET	*MRPL21*	Wald ratio	1	0.0110	0.0126	1.011 (0.986, 1.036)	0.382	0.667	5.97E-17	TRUE
CD4 ET	*MRPL54*	Wald ratio	1	0.0102	0.0129	1.01 (0.985, 1.036)	0.429	0.699	5.74E-18	TRUE
CD4 ET	*MRPS18C*	Wald ratio	1	-0.0019	0.0149	0.998 (0.969, 1.028)	0.899	0.958	6.48E-14	TRUE
CD4 ET	*MSH5*	IVW	2	0.0046	0.0290	1.005 (0.949, 1.063)	0.873	0.947	2.17E-34	TRUE
CD4 ET	*MT1X*	Wald ratio	1	0.0051	0.0134	1.005 (0.979, 1.032)	0.702	0.866	9.88E-18	TRUE
CD4 ET	*MT2A*	Wald ratio	1	-0.0170	0.0106	0.983 (0.963, 1.004)	0.11	0.353	4.36E-30	TRUE
CD4 ET	*MYH11*	Wald ratio	1	-0.0102	0.0150	0.99 (0.961, 1.019)	0.498	0.741	3.12E-13	TRUE
CD4 ET	*MYOM2*	Wald ratio	1	-0.0081	0.0078	0.992 (0.977, 1.007)	0.294	0.587	3.90E-38	TRUE
CD4 ET	*MZT2A*	Wald ratio	1	-0.0129	0.0074	0.987 (0.973, 1.002)	0.081	0.299	5.26E-42	TRUE
CD4 ET	*NAA38*	Wald ratio	1	-0.0100	0.0162	0.99 (0.959, 1.022)	0.535	0.761	1.64E-15	TRUE
CD4 ET	*NAAA*	Wald ratio	1	0.0341	0.0177	1.035 (0.999, 1.071)	0.054	0.233	9.36E-10	TRUE
CD4 ET	*NAP1L1*	Wald ratio	1	-0.0112	0.0143	0.989 (0.962, 1.017)	0.432	0.701	4.57E-15	TRUE
CD4 ET	*NAPRT1*	Wald ratio	1	-0.0139	0.0084	0.986 (0.97, 1.003)	0.099	0.333	1.95E-39	TRUE
CD4 ET	*NBL1*	Wald ratio	1	0.0522	0.0192	1.054 (1.015, 1.094)	0.006	0.06	9.07E-10	TRUE
CD4 ET	*NCAPG2*	Wald ratio	1	-0.0200	0.0218	0.98 (0.939, 1.023)	0.36	0.65	2.28E-09	TRUE
CD4 ET	*NCF2*	Wald ratio	1	-0.0103	0.0101	0.99 (0.97, 1.01)	0.309	0.603	1.90E-29	TRUE
CD4 ET	*NCF4*	Wald ratio	1	-0.0203	0.0160	0.98 (0.95, 1.011)	0.203	0.487	8.76E-13	TRUE
CD4 ET	*NDUFA12*	Wald ratio	1	0.0045	0.0093	1.005 (0.986, 1.023)	0.626	0.822	3.89E-39	TRUE
CD4 ET	*NDUFA3*	Wald ratio	1	0.0034	0.0137	1.003 (0.977, 1.031)	0.802	0.915	2.33E-16	TRUE
CD4 ET	*NDUFS5*	Wald ratio	1	-0.0085	0.0139	0.992 (0.965, 1.019)	0.54	0.763	5.61E-18	TRUE
CD4 ET	*NINJ2*	Wald ratio	1	-0.0190	0.0174	0.981 (0.948, 1.015)	0.273	0.567	2.64E-11	TRUE
CD4 ET	*NME1*	Wald ratio	1	0.0051	0.0151	1.005 (0.976, 1.035)	0.737	0.88	1.03E-13	TRUE
CD4 ET	*NMI*	Wald ratio	1	0.0383	0.0163	1.039 (1.006, 1.073)	0.019	0.122	1.67E-11	TRUE
CD4 ET	*NMRK1*	Wald ratio	1	-0.0022	0.0109	0.998 (0.977, 1.019)	0.84	0.932	2.02E-25	TRUE
CD4 ET	*NMT2*	Wald ratio	1	-0.0231	0.0124	0.977 (0.954, 1.001)	0.062	0.253	2.37E-19	TRUE
CD4 ET	*NPC2*	Wald ratio	1	0.0075	0.0116	1.007 (0.985, 1.031)	0.519	0.753	7.57E-22	TRUE
CD4 ET	*NPDC1*	Wald ratio	1	-0.0040	0.0157	0.996 (0.966, 1.027)	0.799	0.913	1.11E-12	TRUE
CD4 ET	*NPHP1*	Wald ratio	1	-0.0062	0.0121	0.994 (0.971, 1.018)	0.609	0.809	3.35E-23	TRUE
CD4 ET	*NQO2*	Wald ratio	1	-0.0014	0.0134	0.999 (0.973, 1.025)	0.914	0.962	1.57E-17	TRUE
CD4 ET	*NSA2*	Wald ratio	1	0.0218	0.0094	1.022 (1.003, 1.041)	0.02	0.13	2.71E-36	TRUE
CD4 ET	*NT5C3A*	Wald ratio	1	-0.0328	0.0215	0.968 (0.928, 1.009)	0.127	0.382	6.28E-08	TRUE
CD4 ET	*NT5C3B*	Wald ratio	1	0.0001	0.0102	1 (0.98, 1.02)	0.991	0.996	1.88E-24	TRUE
CD4 ET	*NTPCR*	Wald ratio	1	0.0185	0.0117	1.019 (0.996, 1.042)	0.114	0.36	1.87E-23	TRUE
CD4 ET	*NUDT2*	Wald ratio	1	-0.0135	0.0089	0.987 (0.97, 1.004)	0.129	0.389	1.72E-33	TRUE
CD4 ET	*OCEL1*	Wald ratio	1	-0.0084	0.0184	0.992 (0.957, 1.028)	0.647	0.834	8.04E-09	TRUE
CD4 ET	*ORMDL3*	Wald ratio	1	-0.0072	0.0127	0.993 (0.969, 1.018)	0.571	0.785	1.36E-18	TRUE
CD4 ET	*OSTF1*	Wald ratio	1	-0.0144	0.0203	0.986 (0.947, 1.026)	0.477	0.732	5.70E-09	TRUE
CD4 ET	*OXLD1*	Wald ratio	1	0.0051	0.0118	1.005 (0.982, 1.029)	0.668	0.848	1.91E-20	TRUE
CD4 ET	*PARK7*	Wald ratio	1	-0.0110	0.0183	0.989 (0.954, 1.025)	0.548	0.769	6.84E-10	TRUE
CD4 ET	*PASK*	Wald ratio	1	-0.0035	0.0082	0.997 (0.981, 1.013)	0.669	0.848	6.01E-41	TRUE
CD4 ET	*PDIA6*	Wald ratio	1	-0.0377	0.0199	0.963 (0.926, 1.001)	0.059	0.244	2.77E-08	TRUE
CD4 ET	*PDXK*	Wald ratio	1	0.0044	0.0134	1.004 (0.978, 1.031)	0.741	0.881	2.77E-15	TRUE
CD4 ET	*PEX6*	Wald ratio	1	0.0133	0.0201	1.013 (0.974, 1.054)	0.507	0.745	1.84E-08	TRUE
CD4 ET	*PHLDB3*	Wald ratio	1	-0.0085	0.0177	0.992 (0.958, 1.027)	0.633	0.827	1.84E-09	TRUE
CD4 ET	*PI16*	Wald ratio	1	-0.0106	0.0159	0.989 (0.959, 1.021)	0.505	0.745	4.75E-14	TRUE
CD4 ET	*PIGP*	Wald ratio	1	-0.0129	0.0171	0.987 (0.955, 1.021)	0.453	0.717	7.30E-11	TRUE
CD4 ET	*PILRB*	Wald ratio	1	0.0065	0.0090	1.007 (0.989, 1.024)	0.465	0.725	6.51E-34	TRUE
CD4 ET	*PKIA*	Wald ratio	1	-0.0194	0.0183	0.981 (0.946, 1.017)	0.288	0.58	8.32E-09	TRUE
CD4 ET	*PLEKHA1*	Wald ratio	1	0.0589	0.0184	1.061 (1.023, 1.1)	0.001	0.02	9.31E-10	TRUE
CD4 ET	*PLGRKT*	Wald ratio	1	0.0022	0.0191	1.002 (0.965, 1.04)	0.909	0.961	9.05E-09	TRUE
CD4 ET	*PNRC2*	Wald ratio	1	-0.0208	0.0156	0.979 (0.95, 1.01)	0.182	0.462	1.07E-12	TRUE
CD4 ET	*POLM*	Wald ratio	1	0.0110	0.0178	1.011 (0.976, 1.047)	0.538	0.762	1.03E-08	TRUE
CD4 ET	*POLR1D*	Wald ratio	1	0.0016	0.0112	1.002 (0.98, 1.024)	0.888	0.955	1.46E-30	TRUE
CD4 ET	*POLR2J3*	Wald ratio	1	0.0121	0.0190	1.012 (0.975, 1.051)	0.524	0.754	1.12E-09	TRUE
CD4 ET	*POMZP3*	Wald ratio	1	0.0213	0.0157	1.022 (0.991, 1.053)	0.173	0.451	2.03E-15	TRUE
CD4 ET	*PPA1*	Wald ratio	1	0.0035	0.0062	1.003 (0.991, 1.016)	0.578	0.789	4.03E-70	TRUE
CD4 ET	*PPDPF*	Wald ratio	1	-0.0071	0.0067	0.993 (0.98, 1.006)	0.283	0.575	3.92E-59	TRUE
CD4 ET	*PPFIBP2*	Wald ratio	1	-0.0079	0.0122	0.992 (0.969, 1.016)	0.517	0.752	3.63E-20	TRUE
CD4 ET	*PPIA*	Wald ratio	1	0.0169	0.0144	1.017 (0.989, 1.046)	0.241	0.534	6.39E-15	TRUE
CD4 ET	*PPIL3*	Wald ratio	1	0.0070	0.0096	1.007 (0.988, 1.026)	0.467	0.725	2.91E-30	TRUE
CD4 ET	*PPP1R15B*	Wald ratio	1	0.0278	0.0163	1.028 (0.996, 1.062)	0.088	0.314	7.49E-08	TRUE
CD4 ET	*PPT2*	Wald ratio	1	0.0709	0.0154	1.073 (1.042, 1.106)	4.02E-06	2.85E-04	1.19E-10	TRUE
CD4 ET	*PRDX6*	Wald ratio	1	-0.0123	0.0097	0.988 (0.969, 1.007)	0.206	0.493	7.01E-29	TRUE

CD4 ET	*PRELID1*	Wald ratio	1	0.0847	0.0166	1.088 (1.053, 1.124)	3.52E-07	8.32E-05	1.77E-10	TRUE
CD4 ET	*PRKCB*	Wald ratio	1	0.0142	0.0116	1.014 (0.991, 1.038)	0.221	0.513	3.37E-28	TRUE
CD4 ET	*PRKCQ-AS1*	Wald ratio	1	-0.0052	0.0082	0.995 (0.979, 1.011)	0.53	0.758	2.18E-42	TRUE
CD4 ET	*PRMT2*	IVW	2	-0.0039	0.0091	0.996 (0.978, 1.014)	0.666	0.847	1.59E-33	TRUE
CD4 ET	*PRPF6*	Wald ratio	1	0.0151	0.0168	1.015 (0.982, 1.049)	0.368	0.656	1.03E-10	TRUE
CD4 ET	*PRR4*	Wald ratio	1	0.0033	0.0200	1.003 (0.965, 1.044)	0.869	0.946	1.33E-09	TRUE
CD4 ET	*PRR7-AS1*	Wald ratio	1	0.0119	0.0091	1.012 (0.994, 1.03)	0.191	0.471	6.58E-35	TRUE
CD4 ET	*PRRT1*	Wald ratio	1	-0.0247	0.0185	0.976 (0.941, 1.012)	0.182	0.462	4.48E-09	TRUE
CD4 ET	*PRSS16*	Wald ratio	1	0.0290	0.0215	1.029 (0.987, 1.074)	0.177	0.456	2.57E-12	TRUE
CD4 ET	*PSD4*	Wald ratio	1	-0.0160	0.0173	0.984 (0.951, 1.018)	0.353	0.644	1.40E-10	TRUE
CD4 ET	*PSMB9*	Wald ratio	1	-0.0336	0.0205	0.967 (0.929, 1.007)	0.102	0.338	2.09E-08	TRUE
CD4 ET	*PSMD5-AS1*	IVW	2	0.0030	0.0048	1.003 (0.994, 1.012)	0.524	0.754	1.27E-155	TRUE
CD4 ET	*PSPH*	Wald ratio	1	-0.0086	0.0140	0.991 (0.965, 1.019)	0.537	0.762	2.91E-11	TRUE
CD4 ET	*PSTPIP1*	Wald ratio	1	-0.0042	0.0160	0.996 (0.965, 1.028)	0.791	0.909	1.14E-12	TRUE
CD4 ET	*PTPN4*	Wald ratio	1	-0.0052	0.0179	0.995 (0.96, 1.03)	0.772	0.899	9.17E-10	TRUE
CD4 ET	*PYHIN1*	Wald ratio	1	0.0032	0.0149	1.003 (0.974, 1.033)	0.831	0.929	4.97E-13	TRUE
CD4 ET	*RABAC1*	Wald ratio	1	0.0316	0.0205	1.032 (0.991, 1.075)	0.124	0.378	1.47E-08	TRUE
CD4 ET	*RBL2*	Wald ratio	1	-0.0190	0.0216	0.981 (0.94, 1.024)	0.377	0.665	7.50E-08	TRUE
CD4 ET	*RBM43*	Wald ratio	1	-0.0276	0.0136	0.973 (0.947, 0.999)	0.042	0.201	2.24E-16	TRUE
CD4 ET	*RBP7*	Wald ratio	1	-0.0307	0.0147	0.97 (0.942, 0.998)	0.037	0.185	3.60E-14	TRUE
CD4 ET	*RCSD1*	Wald ratio	1	-0.0210	0.0182	0.979 (0.945, 1.015)	0.249	0.542	8.63E-10	TRUE
CD4 ET	*RGS12*	Wald ratio	1	0.0303	0.0159	1.031 (0.999, 1.063)	0.056	0.238	5.74E-12	TRUE
CD4 ET	*RIC3*	Wald ratio	1	-0.0066	0.0172	0.993 (0.96, 1.027)	0.701	0.866	1.07E-10	TRUE
CD4 ET	*RILPL2*	Wald ratio	1	0.0251	0.0193	1.025 (0.987, 1.065)	0.193	0.474	8.96E-09	TRUE
CD4 ET	*RNASEH2C*	Wald ratio	1	0.0188	0.0208	1.019 (0.978, 1.061)	0.365	0.653	1.16E-08	TRUE
CD4 ET	*RNASET2*	Wald ratio	1	-0.0203	0.0071	0.98 (0.966, 0.994)	0.004	0.046	2.33E-56	TRUE
CD4 ET	*RNF166*	Wald ratio	1	-0.0670	0.0185	0.935 (0.902, 0.97)	2.83E-04	0.007	2.33E-09	TRUE
CD4 ET	*RNF168*	Wald ratio	1	-0.0132	0.0178	0.987 (0.953, 1.022)	0.459	0.719	7.44E-10	TRUE
CD4 ET	*ROBO3*	Wald ratio	1	0.0038	0.0199	1.004 (0.965, 1.044)	0.849	0.936	2.00E-08	TRUE
CD4 ET	*RP1-29C18.10*	Wald ratio	1	-0.0192	0.0096	0.981 (0.963, 1)	0.046	0.21	2.00E-34	TRUE
CD4 ET	*RP1-3J17.3*	IVW	2	0.0018	0.0086	1.002 (0.985, 1.019)	0.832	0.93	1.39E-40	TRUE
CD4 ET	*RP11-138A9.1*	Wald ratio	1	0.0118	0.0131	1.012 (0.986, 1.038)	0.367	0.655	4.55E-14	TRUE
CD4 ET	*RP11-138A9.2*	Wald ratio	1	0.0077	0.0133	1.008 (0.982, 1.034)	0.561	0.778	4.72E-12	TRUE
CD4 ET	*RP11-1398P2.1*	Wald ratio	1	-0.0388	0.0175	0.962 (0.93, 0.995)	0.026	0.15	4.01E-10	TRUE
CD4 ET	*RP11-166B2.5*	Wald ratio	1	-0.0030	0.0143	0.997 (0.97, 1.025)	0.834	0.93	1.13E-14	TRUE
CD4 ET	*RP11-18H21.1*	Wald ratio	1	-0.0030	0.0074	0.997 (0.983, 1.012)	0.689	0.86	2.28E-60	TRUE
CD4 ET	*RP11-245G13.2*	Wald ratio	1	-0.0126	0.0091	0.988 (0.97, 1.005)	0.168	0.444	1.38E-48	TRUE
CD4 ET	*RP11-298J20.3*	Wald ratio	1	0.0174	0.0071	1.018 (1.004, 1.032)	0.014	0.101	1.44E-54	TRUE
CD4 ET	*RP11-299J3.8*	Wald ratio	1	-0.0093	0.0195	0.991 (0.954, 1.029)	0.636	0.827	8.32E-10	TRUE
CD4 ET	*RP11-326C3.11*	Wald ratio	1	-0.0111	0.0081	0.989 (0.973, 1.005)	0.169	0.444	4.62E-54	TRUE
CD4 ET	*RP11-326C3.12*	Wald ratio	1	-0.0113	0.0115	0.989 (0.967, 1.011)	0.325	0.62	6.63E-22	TRUE
CD4 ET	*RP11-345J4.5*	Wald ratio	1	-0.0353	0.0158	0.965 (0.936, 0.996)	0.025	0.146	9.05E-13	TRUE
CD4 ET	*RP11-347P5.1*	Wald ratio	1	0.0010	0.0081	1.001 (0.985, 1.017)	0.899	0.958	7.10E-46	TRUE
CD4 ET	*RP11-382J12.1*	Wald ratio	1	0.0049	0.0203	1.005 (0.966, 1.046)	0.808	0.918	5.20E-08	TRUE
CD4 ET	*RP11-403A21.2*	Wald ratio	1	-0.0150	0.0093	0.985 (0.967, 1.003)	0.108	0.348	6.63E-31	TRUE
CD4 ET	*RP11-415F23.2*	Wald ratio	1	-0.0018	0.0162	0.998 (0.967, 1.03)	0.913	0.962	7.26E-14	TRUE
CD4 ET	*RP11-430H10.1*	Wald ratio	1	0.0425	0.0185	1.043 (1.006, 1.082)	0.021	0.132	3.16E-09	TRUE
CD4 ET	*RP11-457M11.5*	Wald ratio	1	0.0514	0.0196	1.053 (1.013, 1.094)	0.009	0.073	3.45E-08	TRUE
CD4 ET	*RP11-473I1.5*	Wald ratio	1	-0.0326	0.0153	0.968 (0.939, 0.997)	0.033	0.172	3.46E-11	TRUE
CD4 ET	*RP11-473M20.9*	Wald ratio	1	-0.0130	0.0132	0.987 (0.962, 1.013)	0.326	0.62	1.78E-17	TRUE
CD4 ET	*RP11-499P20.2*	Wald ratio	1	-0.0645	0.0206	0.938 (0.9, 0.976)	0.002	0.024	1.22E-07	TRUE
CD4 ET	*RP11-526I2.5*	Wald ratio	1	0.0068	0.0175	1.007 (0.973, 1.042)	0.697	0.864	5.76E-10	TRUE
CD4 ET	*RP11-539L10.3*	Wald ratio	1	-0.0139	0.0146	0.986 (0.958, 1.015)	0.339	0.632	1.66E-12	TRUE
CD4 ET	*RP11-542K23.7*	Wald ratio	1	0.0134	0.0179	1.014 (0.979, 1.05)	0.453	0.717	5.58E-18	TRUE
CD4 ET	*RP11-545E17.3*	Wald ratio	1	-0.0260	0.0179	0.974 (0.941, 1.009)	0.146	0.415	1.17E-09	TRUE
CD4 ET	*RP11-660L16.2*	Wald ratio	1	-0.0105	0.0102	0.99 (0.97, 1.01)	0.304	0.599	5.52E-20	TRUE
CD4 ET	*RP11-664D1.1*	Wald ratio	1	-0.0026	0.0189	0.997 (0.961, 1.035)	0.89	0.956	1.42E-11	TRUE
CD4 ET	*RP11-69E11.4*	Wald ratio	1	0.0367	0.0173	1.037 (1.003, 1.073)	0.034	0.175	1.28E-09	TRUE
CD4 ET	*RP11-841O20.2*	Wald ratio	1	0.0048	0.0131	1.005 (0.979, 1.031)	0.717	0.874	9.14E-14	TRUE
CD4 ET	*RP11-97C16.1*	Wald ratio	1	-0.0059	0.0128	0.994 (0.97, 1.019)	0.647	0.834	3.50E-20	TRUE
CD4 ET	*RP13-582O9.5*	Wald ratio	1	0.0017	0.0205	1.002 (0.962, 1.043)	0.933	0.972	5.49E-08	TRUE
CD4 ET	*RP3-325F22.5*	Wald ratio	1	0.0022	0.0175	1.002 (0.969, 1.037)	0.897	0.957	1.53E-12	TRUE
CD4 ET	*RP3-510H16.3*	Wald ratio	1	0.0307	0.0188	1.031 (0.994, 1.07)	0.102	0.338	6.62E-12	TRUE
CD4 ET	*RP4-594I10.3*	Wald ratio	1	-0.0136	0.0155	0.987 (0.957, 1.017)	0.381	0.666	1.14E-12	TRUE
CD4 ET	*RP4-673M15.1*	Wald ratio	1	-0.0350	0.0209	0.966 (0.927, 1.006)	0.093	0.323	2.64E-08	TRUE
CD4 ET	*RP5-1021I20.1*	Wald ratio	1	0.0405	0.0131	1.041 (1.015, 1.068)	0.002	0.026	9.92E-17	TRUE
CD4 ET	*RP5-1042K10.10*	Wald ratio	1	0.0218	0.0149	1.022 (0.993, 1.052)	0.143	0.41	7.10E-12	TRUE
CD4 ET	*RP5-827C21.4*	Wald ratio	1	-0.0081	0.0218	0.992 (0.95, 1.035)	0.711	0.871	2.76E-10	TRUE
CD4 ET	*RPL10A*	Wald ratio	1	-0.0106	0.0080	0.989 (0.974, 1.005)	0.187	0.466	6.09E-47	TRUE
CD4 ET	*RPL11*	Wald ratio	1	-0.0188	0.0121	0.981 (0.958, 1.005)	0.119	0.369	7.33E-20	TRUE
CD4 ET	*RPL12*	Wald ratio	1	-0.0178	0.0053	0.982 (0.972, 0.993)	8.72E-04	0.015	4.67E-119	TRUE
CD4 ET	*RPL13*	Wald ratio	1	-0.0239	0.0118	0.976 (0.954, 0.999)	0.043	0.202	8.12E-38	TRUE
CD4 ET	*RPL14*	Wald ratio	1	0.0133	0.0119	1.013 (0.99, 1.037)	0.264	0.558	1.79E-25	TRUE
CD4 ET	*RPL22L1*	Wald ratio	1	0.0044	0.0209	1.004 (0.964, 1.046)	0.835	0.93	1.13E-09	TRUE
CD4 ET	*RPL23A*	Wald ratio	1	-0.0192	0.0104	0.981 (0.961, 1.001)	0.064	0.258	2.72E-28	TRUE
CD4 ET	*RPL27A*	Wald ratio	1	0.0115	0.0136	1.012 (0.985, 1.039)	0.4	0.678	5.72E-15	TRUE
CD4 ET	*RPL28*	Wald ratio	1	0.0037	0.0048	1.004 (0.994, 1.013)	0.438	0.703	4.72E-136	TRUE
CD4 ET	*RPL3*	Wald ratio	1	0.0171	0.0171	1.017 (0.984, 1.052)	0.316	0.611	5.55E-11	TRUE
CD4 ET	*RPL32*	Wald ratio	1	0.0278	0.0165	1.028 (0.995, 1.062)	0.093	0.323	3.49E-12	TRUE

CD4 ET	*RPL36*	Wald ratio	1	-0.0353	0.0208	0.965 (0.927, 1.006)	0.09	0.318	3.56E-08	TRUE
CD4 ET	*RPL36AL*	IVW	2	-0.0055	0.0062	0.994 (0.982, 1.007)	0.372	0.661	5.86E-103	TRUE
CD4 ET	*RPL8*	Wald ratio	1	-0.0049	0.0048	0.995 (0.986, 1.004)	0.303	0.598	4.14E-141	TRUE
CD4 ET	*RPLP2*	Wald ratio	1	-0.0192	0.0074	0.981 (0.967, 0.995)	0.009	0.076	4.79E-54	TRUE
CD4 ET	*RPS10*	Wald ratio	1	-0.0060	0.0059	0.994 (0.983, 1.006)	0.305	0.599	1.34E-72	TRUE
CD4 ET	*RPS11*	Wald ratio	1	0.0272	0.0114	1.028 (1.005, 1.051)	0.017	0.115	9.28E-25	TRUE
CD4 ET	*RPS12*	Wald ratio	1	0.0257	0.0148	1.026 (0.997, 1.056)	0.082	0.3	3.52E-14	TRUE
CD4 ET	*RPS14*	Wald ratio	1	-0.0178	0.0177	0.982 (0.949, 1.017)	0.313	0.608	1.36E-09	TRUE
CD4 ET	*RPS16*	Wald ratio	1	-0.0108	0.0119	0.989 (0.966, 1.013)	0.363	0.651	7.35E-20	TRUE
CD4 ET	*RPS18*	Wald ratio	1	-0.0120	0.0070	0.988 (0.975, 1.002)	0.087	0.312	6.69E-74	TRUE
CD4 ET	*RPS19*	Wald ratio	1	0.0166	0.0150	1.017 (0.987, 1.047)	0.269	0.563	1.49E-13	TRUE
CD4 ET	*RPS2*	Wald ratio	1	-0.0250	0.0093	0.975 (0.958, 0.993)	0.007	0.066	7.75E-35	TRUE
CD4 ET	*RPS23*	Wald ratio	1	-0.0228	0.0159	0.977 (0.947, 1.008)	0.151	0.422	1.20E-13	TRUE
CD4 ET	*RPS24*	Wald ratio	1	-0.0053	0.0169	0.995 (0.962, 1.028)	0.754	0.888	2.95E-11	TRUE
CD4 ET	*RPS25*	Wald ratio	1	-0.0086	0.0088	0.991 (0.974, 1.009)	0.331	0.624	1.76E-37	TRUE
CD4 ET	*RPS26*	Wald ratio	1	-0.0100	0.0043	0.99 (0.982, 0.999)	0.022	0.132	2.20E-188	TRUE
CD4 ET	*RPS3*	Wald ratio	1	-0.0171	0.0096	0.983 (0.965, 1.002)	0.075	0.287	6.04E-19	TRUE
CD4 ET	*RPS3A*	Wald ratio	1	-0.0040	0.0061	0.996 (0.984, 1.008)	0.514	0.75	1.76E-38	TRUE
CD4 ET	*RPS5*	Wald ratio	1	0.0076	0.0056	1.008 (0.997, 1.019)	0.177	0.456	7.80E-105	TRUE
CD4 ET	*RPS6*	Wald ratio	1	-0.0129	0.0108	0.987 (0.966, 1.008)	0.236	0.53	2.50E-33	TRUE
CD4 ET	*RPS7*	Wald ratio	1	0.0035	0.0113	1.004 (0.982, 1.026)	0.754	0.888	1.28E-35	TRUE
CD4 ET	*RPS8*	Wald ratio	1	0.0023	0.0088	1.002 (0.985, 1.02)	0.791	0.909	1.67E-38	TRUE
CD4 ET	*RPS9*	Wald ratio	1	0.0248	0.0065	1.025 (1.012, 1.038)	1.26E-04	0.003	3.69E-69	TRUE
CD4 ET	*RPSA*	Wald ratio	1	-0.0222	0.0107	0.978 (0.958, 0.999)	0.039	0.192	1.04E-32	TRUE
CD4 ET	*RSRC2*	Wald ratio	1	-0.0095	0.0168	0.991 (0.958, 1.024)	0.57	0.784	1.82E-13	TRUE
CD4 ET	*SAMD3*	Wald ratio	1	-0.0032	0.0150	0.997 (0.968, 1.027)	0.83	0.929	6.62E-14	TRUE
CD4 ET	*SAR1A*	Wald ratio	1	0.0128	0.0171	1.013 (0.98, 1.047)	0.453	0.717	1.57E-10	TRUE
CD4 ET	*SCGB3A1*	Wald ratio	1	0.0062	0.0056	1.006 (0.995, 1.017)	0.263	0.558	9.93E-102	TRUE
CD4 ET	*SEPT2*	Wald ratio	1	0.0096	0.0157	1.01 (0.979, 1.041)	0.542	0.764	1.75E-15	TRUE
CD4 ET	*SERPINB6*	Wald ratio	1	0.0075	0.0106	1.008 (0.987, 1.029)	0.476	0.731	4.92E-27	TRUE
CD4 ET	*SERPINB9*	Wald ratio	1	0.0038	0.0162	1.004 (0.973, 1.036)	0.813	0.92	2.87E-12	TRUE
CD4 ET	*SESTD1*	Wald ratio	1	0.0095	0.0148	1.01 (0.981, 1.039)	0.522	0.754	1.62E-14	TRUE
CD4 ET	*SETD9*	Wald ratio	1	-0.0405	0.0157	0.96 (0.931, 0.99)	0.01	0.08	5.73E-11	TRUE
CD4 ET	*SF1*	Wald ratio	1	0.0017	0.0145	1.002 (0.974, 1.031)	0.906	0.96	1.02E-12	TRUE
CD4 ET	*SFT2D1*	Wald ratio	1	0.0359	0.0164	1.037 (1.004, 1.07)	0.029	0.158	1.41E-10	TRUE
CD4 ET	*SH3GLB1*	Wald ratio	1	-0.0190	0.0210	0.981 (0.942, 1.022)	0.367	0.655	5.22E-08	TRUE
CD4 ET	*SH3YL1*	Wald ratio	1	0.0224	0.0056	1.023 (1.012, 1.034)	5.45E-05	0.002	2.90E-98	TRUE
CD4 ET	*SHF*	Wald ratio	1	0.0142	0.0122	1.014 (0.99, 1.039)	0.242	0.535	4.41E-21	TRUE
CD4 ET	*SIDT1-AS1*	Wald ratio	1	-0.0232	0.0167	0.977 (0.946, 1.01)	0.166	0.441	7.00E-16	TRUE
CD4 ET	*SKAP2*	Wald ratio	1	-0.0144	0.0189	0.986 (0.95, 1.023)	0.447	0.712	1.12E-10	TRUE
CD4 ET	*SLC12A7*	Wald ratio	1	-0.0163	0.0150	0.984 (0.955, 1.013)	0.275	0.568	4.39E-14	TRUE
CD4 ET	*SLC22A18*	Wald ratio	1	-0.0078	0.0157	0.992 (0.962, 1.023)	0.617	0.817	4.06E-15	TRUE
CD4 ET	*SLC25A29*	Wald ratio	1	-0.0108	0.0151	0.989 (0.961, 1.019)	0.475	0.73	1.89E-13	TRUE
CD4 ET	*SLC25A3*	Wald ratio	1	0.0096	0.0231	1.01 (0.965, 1.057)	0.678	0.853	2.49E-08	TRUE
CD4 ET	*SLC35F3*	Wald ratio	1	0.0113	0.0077	1.011 (0.996, 1.027)	0.142	0.409	4.81E-48	TRUE
CD4 ET	*SLC44A4*	IVW	2	0.0139	0.0108	1.014 (0.993, 1.036)	0.197	0.48	1.12E-22	TRUE
CD4 ET	*SLFN5*	Wald ratio	1	-0.0091	0.0152	0.991 (0.962, 1.021)	0.55	0.769	2.08E-13	TRUE
CD4 ET	*SMDT1*	Wald ratio	1	0.0078	0.0041	1.008 (1, 1.016)	0.057	0.239	5.29E-191	TRUE
CD4 ET	*SNAP29*	Wald ratio	1	0.0425	0.0186	1.043 (1.006, 1.082)	0.023	0.137	1.02E-11	TRUE
CD4 ET	*SNHG7*	Wald ratio	1	-0.0162	0.0096	0.984 (0.966, 1.003)	0.091	0.318	4.41E-48	TRUE
CD4 ET	*SNHG8*	Wald ratio	1	-0.0023	0.0051	0.998 (0.988, 1.008)	0.647	0.834	1.11E-140	TRUE
CD4 ET	*SNRNP25*	Wald ratio	1	0.0312	0.0175	1.032 (0.997, 1.068)	0.074	0.286	3.40E-09	TRUE
CD4 ET	*SNRPD2*	Wald ratio	1	-0.0399	0.0123	0.961 (0.938, 0.984)	0.001	0.02	1.34E-18	TRUE
CD4 ET	*SOX4*	Wald ratio	1	0.0333	0.0209	1.034 (0.992, 1.077)	0.111	0.355	5.54E-09	TRUE
CD4 ET	*SPATA20*	Wald ratio	1	0.0239	0.0145	1.024 (0.995, 1.054)	0.101	0.337	1.17E-14	TRUE
CD4 ET	*SPATC1L*	Wald ratio	1	0.0024	0.0138	1.002 (0.976, 1.03)	0.864	0.943	1.45E-21	TRUE
CD4 ET	*SRD5A3*	Wald ratio	1	-0.0338	0.0154	0.967 (0.938, 0.996)	0.028	0.157	6.34E-14	TRUE
CD4 ET	*SRP14*	Wald ratio	1	0.0676	0.0159	1.07 (1.037, 1.104)	2.14E-05	9.86E-04	9.25E-10	TRUE
CD4 ET	*SRSF10*	Wald ratio	1	-0.0227	0.0197	0.978 (0.941, 1.016)	0.248	0.541	1.86E-08	TRUE
CD4 ET	*ST6GALNAC1*	Wald ratio	1	-0.0316	0.0171	0.969 (0.937, 1.002)	0.064	0.258	1.07E-10	TRUE
CD4 ET	*STMN3*	Wald ratio	1	0.0216	0.0110	1.022 (1, 1.044)	0.05	0.222	5.97E-24	TRUE
CD4 ET	*SULF2*	Wald ratio	1	0.0067	0.0068	1.007 (0.993, 1.02)	0.324	0.618	1.08E-61	TRUE
CD4 ET	*SUPT3H*	Wald ratio	1	-0.0298	0.0187	0.971 (0.936, 1.007)	0.111	0.354	3.67E-09	TRUE
CD4 ET	*SURF1*	Wald ratio	1	-0.0253	0.0216	0.975 (0.935, 1.017)	0.242	0.535	1.75E-08	TRUE
CD4 ET	*SYNC*	Wald ratio	1	0.0048	0.0172	1.005 (0.971, 1.039)	0.782	0.905	2.16E-08	TRUE
CD4 ET	*SYNM*	Wald ratio	1	0.0002	0.0174	1 (0.967, 1.035)	0.992	0.996	8.69E-11	TRUE
CD4 ET	*TAPBPL*	Wald ratio	1	-0.0038	0.0200	0.996 (0.958, 1.036)	0.848	0.935	7.63E-09	TRUE
CD4 ET	*TBCA*	Wald ratio	1	0.0104	0.0123	1.01 (0.986, 1.035)	0.396	0.675	2.31E-20	TRUE
CD4 ET	*THAP7-AS1*	Wald ratio	1	0.0351	0.0167	1.036 (1.002, 1.07)	0.036	0.181	4.09E-10	TRUE
CD4 ET	*THEM4*	Wald ratio	1	0.0521	0.0155	1.053 (1.022, 1.086)	7.94E-04	0.014	2.31E-12	TRUE
CD4 ET	*THEMIS2*	Wald ratio	1	0.0099	0.0108	1.01 (0.989, 1.032)	0.362	0.651	3.46E-25	TRUE
CD4 ET	*TIMM10*	Wald ratio	1	-0.0014	0.0091	0.999 (0.981, 1.017)	0.875	0.948	1.70E-42	TRUE
CD4 ET	*TIMM13*	Wald ratio	1	0.0551	0.0173	1.057 (1.022, 1.093)	0.001	0.021	1.98E-10	TRUE
CD4 ET	*TIPARP-AS1*	Wald ratio	1	0.0447	0.0175	1.046 (1.01, 1.082)	0.011	0.085	3.71E-11	TRUE
CD4 ET	*TLE4*	Wald ratio	1	-0.0003	0.0161	1 (0.969, 1.032)	0.987	0.995	3.77E-12	TRUE
CD4 ET	*TMEM120B*	Wald ratio	1	-0.0288	0.0220	0.972 (0.931, 1.014)	0.19	0.47	1.70E-10	TRUE
CD4 ET	*TMEM121*	Wald ratio	1	0.0042	0.0142	1.004 (0.977, 1.033)	0.766	0.896	1.09E-13	TRUE
CD4 ET	*TMEM156*	Wald ratio	1	0.0065	0.0083	1.007 (0.99, 1.023)	0.434	0.702	2.63E-39	TRUE
CD4 ET	*TMEM2*	Wald ratio	1	0.0142	0.0199	1.014 (0.976, 1.055)	0.476	0.732	5.03E-08	TRUE

CD4 ET	*TMEM204*	Wald ratio	1	-0.0017	0.0191	0.998 (0.962, 1.036)	0.928	0.969	4.44E-14	TRUE
CD4 ET	*TMEM230*	Wald ratio	1	0.0085	0.0154	1.009 (0.979, 1.039)	0.58	0.79	2.92E-13	TRUE
CD4 ET	*TMEM258*	Wald ratio	1	0.0139	0.0150	1.014 (0.985, 1.044)	0.353	0.644	7.39E-13	TRUE
CD4 ET	*TMEM50B*	Wald ratio	1	-0.0415	0.0125	0.959 (0.936, 0.983)	9.15E-04	0.016	1.67E-18	TRUE
CD4 ET	*TMEM8A*	Wald ratio	1	0.0020	0.0103	1.002 (0.982, 1.022)	0.845	0.935	2.10E-28	TRUE
CD4 ET	*TNFRSF14*	Wald ratio	1	0.0256	0.0203	1.026 (0.986, 1.067)	0.208	0.494	5.04E-08	TRUE
CD4 ET	*TNFSF13B*	Wald ratio	1	-0.0568	0.0168	0.945 (0.914, 0.976)	6.99E-04	0.013	1.90E-11	TRUE
CD4 ET	*TOMM7*	Wald ratio	1	0.0029	0.0043	1.003 (0.994, 1.011)	0.503	0.743	2.77E-162	TRUE
CD4 ET	*TPD52*	Wald ratio	1	-0.0351	0.0196	0.966 (0.929, 1.003)	0.073	0.283	2.50E-08	TRUE
CD4 ET	*TPGS2*	Wald ratio	1	-0.0184	0.0182	0.982 (0.947, 1.017)	0.312	0.606	9.21E-10	TRUE
CD4 ET	*TRABD2A*	Wald ratio	1	0.0198	0.0175	1.02 (0.986, 1.056)	0.258	0.552	2.77E-11	TRUE
CD4 ET	*TRAPPC4*	Wald ratio	1	0.0320	0.0202	1.033 (0.992, 1.074)	0.113	0.359	5.75E-08	TRUE
CD4 ET	*TRIM61*	Wald ratio	1	-0.0111	0.0173	0.989 (0.956, 1.023)	0.522	0.754	1.75E-10	TRUE
CD4 ET	*TRMT112*	Wald ratio	1	0.0195	0.0169	1.02 (0.987, 1.054)	0.247	0.54	1.28E-14	TRUE
CD4 ET	*TSHZ2*	Wald ratio	1	-0.0015	0.0193	0.999 (0.962, 1.037)	0.94	0.976	1.64E-08	TRUE
CD4 ET	*TSPO*	Wald ratio	1	0.0156	0.0140	1.016 (0.988, 1.044)	0.264	0.558	9.13E-16	TRUE
CD4 ET	*TSTD1*	Wald ratio	1	0.0046	0.0127	1.005 (0.98, 1.03)	0.718	0.874	8.77E-17	TRUE
CD4 ET	*TSTD3*	Wald ratio	1	-0.0156	0.0158	0.985 (0.954, 1.016)	0.325	0.62	3.09E-12	TRUE
CD4 ET	*TTC32*	Wald ratio	1	0.0334	0.0154	1.034 (1.003, 1.066)	0.029	0.16	1.56E-13	TRUE
CD4 ET	*TUBB2A*	Wald ratio	1	0.0276	0.0211	1.028 (0.986, 1.071)	0.189	0.47	6.10E-08	TRUE
CD4 ET	*TUFM*	Wald ratio	1	0.0158	0.0079	1.016 (1, 1.032)	0.046	0.21	1.53E-44	TRUE
CD4 ET	*TXK*	Wald ratio	1	0.0050	0.0141	1.005 (0.978, 1.033)	0.722	0.877	2.16E-11	TRUE
CD4 ET	*TXN*	Wald ratio	1	0.0344	0.0169	1.035 (1.001, 1.07)	0.042	0.201	1.43E-11	TRUE
CD4 ET	*TYMP*	Wald ratio	1	-0.0024	0.0276	0.998 (0.945, 1.053)	0.93	0.97	1.25E-08	TRUE
CD4 ET	*U91328.19*	Wald ratio	1	0.0120	0.0187	1.012 (0.976, 1.05)	0.521	0.753	2.21E-16	TRUE
CD4 ET	*UBA6*	Wald ratio	1	0.0153	0.0185	1.015 (0.979, 1.053)	0.407	0.682	1.65E-09	TRUE
CD4 ET	*UBE2F*	Wald ratio	1	-0.0213	0.0180	0.979 (0.945, 1.014)	0.237	0.531	2.76E-09	TRUE
CD4 ET	*UCP2*	Wald ratio	1	0.0048	0.0102	1.005 (0.985, 1.025)	0.64	0.83	6.78E-27	TRUE
CD4 ET	*ULK4*	Wald ratio	1	0.0247	0.0130	1.025 (0.999, 1.051)	0.057	0.239	2.38E-16	TRUE
CD4 ET	*UQCRH*	Wald ratio	1	-0.0361	0.0172	0.965 (0.933, 0.998)	0.035	0.179	6.36E-13	TRUE
CD4 ET	*USP53*	Wald ratio	1	-0.0068	0.0125	0.993 (0.969, 1.018)	0.584	0.793	1.88E-13	TRUE
CD4 ET	*UTS2*	Wald ratio	1	-0.0326	0.0145	0.968 (0.941, 0.996)	0.025	0.145	6.57E-15	TRUE
CD4 ET	*VAMP2*	Wald ratio	1	0.0370	0.0152	1.038 (1.007, 1.069)	0.015	0.104	7.14E-15	TRUE
CD4 ET	*VAMP5*	Wald ratio	1	0.0262	0.0184	1.027 (0.99, 1.064)	0.154	0.425	1.04E-12	TRUE
CD4 ET	*VPS28*	Wald ratio	1	0.0259	0.0158	1.026 (0.995, 1.059)	0.102	0.338	1.09E-10	TRUE
CD4 ET	*WAC-AS1*	Wald ratio	1	0.0189	0.0180	1.019 (0.984, 1.056)	0.293	0.587	3.27E-10	TRUE
CD4 ET	*WDR86*	Wald ratio	1	-0.0492	0.0204	0.952 (0.915, 0.991)	0.016	0.11	5.91E-08	TRUE
CD4 ET	*WIPI2*	Wald ratio	1	-0.0220	0.0176	0.978 (0.945, 1.013)	0.21	0.497	1.32E-09	TRUE
CD4 ET	*XBP1*	Wald ratio	1	0.0249	0.0132	1.025 (0.999, 1.052)	0.059	0.244	5.80E-11	TRUE
CD4 ET	*XRCC6BP1*	Wald ratio	1	-0.0311	0.0155	0.969 (0.94, 0.999)	0.044	0.208	1.03E-12	TRUE
CD4 ET	*XRRA1*	Wald ratio	1	-0.0077	0.0064	0.992 (0.98, 1.005)	0.232	0.525	2.20E-66	TRUE
CD4 ET	*XXbac-BPG181B23.7*	Wald ratio	1	0.0089	0.0148	1.009 (0.98, 1.039)	0.549	0.769	1.09E-11	TRUE
CD4 ET	*XXbac-BPG252P9.10*	Wald ratio	1	-0.0601	0.0134	0.942 (0.917, 0.967)	7.64E-06	4.74E-04	8.91E-17	TRUE
CD4 ET	*XXbac-BPG299F13.17*	Wald ratio	1	0.0084	0.0060	1.008 (0.997, 1.02)	0.162	0.436	1.79E-67	TRUE
CD4 ET	*YBEY*	Wald ratio	1	-0.0011	0.0085	0.999 (0.982, 1.016)	0.898	0.957	1.74E-34	TRUE
CD4 ET	*YBX3*	Wald ratio	1	0.0357	0.0209	1.036 (0.995, 1.08)	0.087	0.312	8.89E-08	TRUE
CD4 ET	*YEATS4*	Wald ratio	1	-0.0022	0.0196	0.998 (0.96, 1.037)	0.911	0.962	9.77E-09	TRUE
CD4 ET	*ZC2HC1A*	Wald ratio	1	-0.0043	0.0181	0.996 (0.961, 1.032)	0.814	0.921	1.29E-09	TRUE
CD4 ET	*ZC3HAV1*	Wald ratio	1	0.0067	0.0111	1.007 (0.985, 1.029)	0.548	0.769	7.35E-24	TRUE
CD4 ET	*ZFAS1*	Wald ratio	1	0.0087	0.0107	1.009 (0.988, 1.03)	0.421	0.691	5.90E-27	TRUE
CD4 ET	*ZNF165*	Wald ratio	1	0.0061	0.0246	1.006 (0.959, 1.056)	0.805	0.916	4.68E-09	TRUE
CD4 ET	*ZNF302*	Wald ratio	1	-0.0166	0.0158	0.983 (0.953, 1.014)	0.292	0.586	8.38E-12	TRUE
CD4 ET	*ZNF391*	Wald ratio	1	0.0190	0.0139	1.019 (0.992, 1.047)	0.172	0.449	4.08E-28	TRUE
CD4 ET	*ZNF584*	Wald ratio	1	0.0303	0.0148	1.031 (1.001, 1.061)	0.04	0.196	8.30E-14	TRUE
CD4 ET	*ZNF593*	Wald ratio	1	0.0304	0.0154	1.031 (1, 1.063)	0.049	0.221	1.65E-13	TRUE
CD4 ET	*ZNF600*	Wald ratio	1	-0.0003	0.0163	1 (0.968, 1.032)	0.985	0.994	5.66E-12	TRUE
CD4 ET	*ZNF83*	Wald ratio	1	-0.0231	0.0136	0.977 (0.951, 1.004)	0.09	0.318	6.34E-16	TRUE
CD4 ET	*ZNF85*	Wald ratio	1	0.0188	0.0124	1.019 (0.994, 1.044)	0.13	0.39	5.26E-22	TRUE
CD4 ET	*ZP3*	Wald ratio	1	0.0375	0.0151	1.038 (1.008, 1.069)	0.013	0.096	4.97E-16	TRUE
CD4 NC	*A1BG*	IVW	2	0.0255	0.0205	1.026 (0.985, 1.068)	0.214	0.503	1.21E-23	TRUE
CD4 NC	*AAK1*	Wald ratio	1	0.0294	0.0171	1.03 (0.996, 1.065)	0.086	0.312	1.62E-10	TRUE
CD4 NC	*ABCA7*	Wald ratio	1	0.0127	0.0137	1.013 (0.986, 1.04)	0.352	0.644	4.50E-10	TRUE
CD4 NC	*ABCG2*	Wald ratio	1	0.0164	0.0233	1.017 (0.971, 1.064)	0.481	0.735	4.26E-09	TRUE
CD4 NC	*ABHD10*	Wald ratio	1	0.0443	0.0193	1.045 (1.007, 1.086)	0.022	0.133	2.28E-09	TRUE
CD4 NC	*ABRACL*	Wald ratio	1	0.0569	0.0171	1.059 (1.024, 1.095)	9.00E-04	0.016	1.78E-10	TRUE
CD4 NC	*AC002331.1*	Wald ratio	1	-0.0325	0.0158	0.968 (0.939, 0.998)	0.039	0.193	9.16E-13	TRUE
CD4 NC	*AC002467.7*	Wald ratio	1	0.0175	0.0141	1.018 (0.99, 1.046)	0.214	0.503	9.38E-13	TRUE
CD4 NC	*AC005082.12*	Wald ratio	1	-0.0024	0.0119	0.998 (0.975, 1.021)	0.841	0.933	3.52E-21	TRUE
CD4 NC	*AC005253.2*	Wald ratio	1	0.0042	0.0078	1.004 (0.989, 1.02)	0.589	0.796	1.50E-49	TRUE
CD4 NC	*AC006129.2*	Wald ratio	1	-0.0199	0.0367	0.98 (0.912, 1.053)	0.588	0.794	3.59E-09	TRUE
CD4 NC	*AC006369.2*	Wald ratio	1	0.0058	0.0054	1.006 (0.995, 1.017)	0.283	0.575	1.63E-111	TRUE
CD4 NC	*AC007228.11*	Wald ratio	1	0.0202	0.0197	1.02 (0.982, 1.061)	0.303	0.598	1.74E-08	TRUE
CD4 NC	*AC007383.4*	Wald ratio	1	0.0046	0.0142	1.005 (0.977, 1.033)	0.745	0.883	9.70E-15	TRUE
CD4 NC	*AC008746.12*	Wald ratio	1	0.0122	0.0178	1.012 (0.978, 1.048)	0.492	0.738	5.39E-11	TRUE
CD4 NC	*AC009120.10*	Wald ratio	1	-0.0219	0.0083	0.978 (0.963, 0.994)	0.008	0.07	2.76E-38	TRUE
CD4 NC	*AC009120.4*	Wald ratio	1	0.0249	0.0094	1.025 (1.006, 1.044)	0.008	0.07	9.31E-27	TRUE
CD4 NC	*AC009299.3*	Wald ratio	1	-0.0348	0.0160	0.966 (0.936, 0.997)	0.029	0.16	1.53E-12	TRUE
CD4 NC	*AC009961.3*	Wald ratio	1	0.0113	0.0106	1.011 (0.99, 1.033)	0.289	0.582	6.29E-23	TRUE
CD4 NC	*AC010642.1*	Wald ratio	1	0.0166	0.0172	1.017 (0.983, 1.052)	0.336	0.628	1.08E-16	TRUE

CD4 NC	*AC010883.5*	Wald ratio	1	0.0286	0.0190	1.029 (0.991, 1.068)	0.132	0.394	4.25E-13	TRUE
CD4 NC	*AC010982.1*	Wald ratio	1	-0.1024	0.0200	0.903 (0.868, 0.939)	3.02E-07	8.32E-05	7.05E-08	TRUE
CD4 NC	*AC011484.1*	Wald ratio	1	-0.0145	0.0095	0.986 (0.967, 1.004)	0.128	0.385	4.44E-40	TRUE
CD4 NC	*AC016757.3*	IVW	2	-0.0148	0.0114	0.985 (0.964, 1.008)	0.194	0.475	2.09E-35	TRUE
CD4 NC	*AC018816.3*	Wald ratio	1	0.0139	0.0106	1.014 (0.993, 1.035)	0.19	0.47	8.46E-27	TRUE
CD4 NC	*AC021860.1*	Wald ratio	1	0.0217	0.0095	1.022 (1.003, 1.041)	0.023	0.137	5.47E-38	TRUE
CD4 NC	*AC022182.3*	Wald ratio	1	0.0113	0.0216	1.011 (0.969, 1.055)	0.6	0.805	4.59E-08	TRUE
CD4 NC	*AC022498.1*	Wald ratio	1	-0.0035	0.0148	0.997 (0.968, 1.026)	0.814	0.921	3.43E-20	TRUE
CD4 NC	*AC023590.1*	Wald ratio	1	-0.0035	0.0106	0.997 (0.976, 1.017)	0.743	0.881	1.47E-33	TRUE
CD4 NC	*AC062017.1*	Wald ratio	1	0.0036	0.0166	1.004 (0.971, 1.037)	0.83	0.929	1.66E-11	TRUE
CD4 NC	*AC064834.2*	Wald ratio	1	-0.0250	0.0091	0.975 (0.958, 0.993)	0.006	0.056	2.22E-33	TRUE
CD4 NC	*AC068282.3*	Wald ratio	1	-0.0376	0.0108	0.963 (0.943, 0.984)	5.33E-04	0.011	5.57E-22	TRUE
CD4 NC	*AC068620.1*	Wald ratio	1	0.0329	0.0176	1.033 (0.998, 1.07)	0.062	0.253	2.61E-10	TRUE
CD4 NC	*AC073115.7*	Wald ratio	1	0.0228	0.0172	1.023 (0.989, 1.058)	0.184	0.463	1.24E-11	TRUE
CD4 NC	*AC074289.1*	Wald ratio	1	0.0134	0.0134	1.013 (0.987, 1.04)	0.318	0.614	6.54E-17	TRUE
CD4 NC	*AC084018.1*	Wald ratio	1	-0.0096	0.0188	0.99 (0.955, 1.028)	0.609	0.809	2.27E-08	TRUE
CD4 NC	*AC090186.1*	IVW	2	-0.0251	0.0080	0.975 (0.96, 0.991)	0.002	0.025	5.29E-37	TRUE
CD4 NC	*AC091729.9*	Wald ratio	1	-0.0248	0.0132	0.975 (0.951, 1.001)	0.059	0.245	6.65E-17	TRUE
CD4 NC	*AC093157.1*	Wald ratio	1	-0.0019	0.0192	0.998 (0.961, 1.036)	0.921	0.965	3.14E-10	TRUE
CD4 NC	*AC093375.1*	Wald ratio	1	-0.0033	0.0090	0.997 (0.979, 1.015)	0.717	0.874	7.36E-41	TRUE
CD4 NC	*AC093901.1*	Wald ratio	1	0.0354	0.0198	1.036 (0.997, 1.077)	0.073	0.283	2.62E-08	TRUE
CD4 NC	*AC097713.4*	Wald ratio	1	0.0254	0.0107	1.026 (1.004, 1.047)	0.018	0.118	2.62E-26	TRUE
CD4 NC	*AC097724.3*	Wald ratio	1	0.0230	0.0199	1.023 (0.984, 1.064)	0.249	0.542	5.14E-08	TRUE
CD4 NC	*AC104653.1*	Wald ratio	1	0.0084	0.0068	1.008 (0.995, 1.022)	0.218	0.51	4.54E-65	TRUE
CD4 NC	*AC107622.1*	Wald ratio	1	0.0029	0.0169	1.003 (0.97, 1.037)	0.863	0.943	3.59E-10	TRUE
CD4 NC	*AC116614.1*	Wald ratio	1	-0.0139	0.0190	0.986 (0.95, 1.024)	0.465	0.725	8.52E-10	TRUE
CD4 NC	*AC137932.1*	Wald ratio	1	0.0497	0.0120	1.051 (1.027, 1.076)	3.21E-05	0.001	6.22E-18	TRUE
CD4 NC	*AC144652.1*	Wald ratio	1	0.0142	0.0188	1.014 (0.978, 1.052)	0.449	0.714	4.19E-09	TRUE
CD4 NC	*ACAA2*	Wald ratio	1	0.0174	0.0160	1.018 (0.986, 1.05)	0.274	0.568	7.86E-11	TRUE
CD4 NC	*ACAP1*	Wald ratio	1	0.0127	0.0181	1.013 (0.977, 1.049)	0.485	0.736	7.40E-11	TRUE
CD4 NC	*ACAT1*	Wald ratio	1	-0.0257	0.0082	0.975 (0.959, 0.99)	0.002	0.024	2.22E-29	TRUE
CD4 NC	*ACAT2*	Wald ratio	1	0.0005	0.0102	1.001 (0.981, 1.021)	0.96	0.986	5.10E-28	TRUE
CD4 NC	*ACBD3*	Wald ratio	1	-0.0203	0.0162	0.98 (0.949, 1.012)	0.211	0.498	7.86E-12	TRUE
CD4 NC	*ACCS*	Wald ratio	1	-0.0017	0.0087	0.998 (0.981, 1.016)	0.847	0.935	4.86E-45	TRUE
CD4 NC	*ACER3*	Wald ratio	1	0.0318	0.0179	1.032 (0.997, 1.069)	0.076	0.288	1.99E-09	TRUE
CD4 NC	*ACOT13*	Wald ratio	1	-0.0885	0.0222	0.915 (0.876, 0.956)	6.57E-05	0.002	5.58E-08	TRUE
CD4 NC	*ACP1*	Wald ratio	1	0.0619	0.0184	1.064 (1.026, 1.103)	7.69E-04	0.014	2.91E-09	TRUE
CD4 NC	*ACP5*	Wald ratio	1	0.0016	0.0103	1.002 (0.982, 1.022)	0.875	0.948	7.85E-28	TRUE
CD4 NC	*ACPL2*	Wald ratio	1	-0.0229	0.0118	0.977 (0.955, 1)	0.053	0.231	4.17E-21	TRUE
CD4 NC	*ACSM1*	Wald ratio	1	-0.0129	0.0178	0.987 (0.953, 1.022)	0.47	0.727	1.02E-09	TRUE
CD4 NC	*ACTA2*	Wald ratio	1	0.0327	0.0154	1.033 (1.002, 1.065)	0.034	0.175	7.52E-13	TRUE
CD4 NC	*ACTG1*	Wald ratio	1	-0.0271	0.0128	0.973 (0.949, 0.998)	0.034	0.175	3.92E-20	TRUE
CD4 NC	*ACTR2*	Wald ratio	1	0.0263	0.0197	1.027 (0.988, 1.067)	0.18	0.461	4.78E-10	TRUE
CD4 NC	*ACVR1B*	Wald ratio	1	-0.0074	0.0173	0.993 (0.959, 1.027)	0.669	0.848	5.29E-11	TRUE
CD4 NC	*ACY1*	Wald ratio	1	0.0802	0.0269	1.083 (1.028, 1.142)	0.003	0.034	6.14E-09	TRUE
CD4 NC	*ACY3*	Wald ratio	1	0.0240	0.0179	1.024 (0.989, 1.061)	0.18	0.46	1.17E-09	TRUE
CD4 NC	*ACYP2*	Wald ratio	1	0.0024	0.0059	1.002 (0.991, 1.014)	0.68	0.853	5.19E-85	TRUE
CD4 NC	*ADA*	Wald ratio	1	0.0097	0.0123	1.01 (0.986, 1.034)	0.428	0.699	5.30E-18	TRUE
CD4 NC	*ADAL*	Wald ratio	1	0.0160	0.0243	1.016 (0.969, 1.066)	0.508	0.747	5.74E-10	TRUE
CD4 NC	*ADAM10*	Wald ratio	1	-0.0454	0.0162	0.956 (0.926, 0.986)	0.005	0.051	2.05E-21	TRUE
CD4 NC	*ADAM17*	Wald ratio	1	0.0187	0.0120	1.019 (0.995, 1.043)	0.118	0.368	1.96E-18	TRUE
CD4 NC	*ADAMTS6*	Wald ratio	1	0.0111	0.0074	1.011 (0.997, 1.026)	0.134	0.395	1.30E-52	TRUE
CD4 NC	*ADAP1*	Wald ratio	1	0.0002	0.0210	1 (0.96, 1.042)	0.993	0.997	1.37E-09	TRUE
CD4 NC	*ADARB2*	Wald ratio	1	0.0166	0.0110	1.017 (0.995, 1.039)	0.131	0.392	7.32E-26	TRUE
CD4 NC	*ADAT1*	Wald ratio	1	0.0260	0.0166	1.026 (0.993, 1.06)	0.118	0.367	1.95E-10	TRUE
CD4 NC	*ADCK1*	Wald ratio	1	-0.0176	0.0167	0.983 (0.951, 1.015)	0.294	0.587	1.68E-11	TRUE
CD4 NC	*ADCY3*	Wald ratio	1	0.0224	0.0185	1.023 (0.986, 1.06)	0.226	0.52	1.32E-09	TRUE
CD4 NC	*ADCY7*	Wald ratio	1	-0.0348	0.0143	0.966 (0.939, 0.993)	0.015	0.105	1.52E-15	TRUE
CD4 NC	*ADHFE1*	Wald ratio	1	-0.0447	0.0171	0.956 (0.925, 0.989)	0.009	0.076	1.45E-10	TRUE
CD4 NC	*ADI1*	Wald ratio	1	-0.0217	0.0116	0.979 (0.956, 1.001)	0.062	0.253	2.95E-14	TRUE
CD4 NC	*ADIRF*	Wald ratio	1	0.0340	0.0137	1.035 (1.007, 1.063)	0.013	0.096	2.69E-17	TRUE
CD4 NC	*ADPGK*	Wald ratio	1	-0.0266	0.0151	0.974 (0.945, 1.003)	0.079	0.293	9.87E-16	TRUE
CD4 NC	*ADPRM*	Wald ratio	1	0.0099	0.0106	1.01 (0.989, 1.031)	0.35	0.643	6.71E-26	TRUE
CD4 NC	*ADTRP*	Wald ratio	1	-0.0365	0.0122	0.964 (0.941, 0.988)	0.003	0.034	8.28E-15	TRUE
CD4 NC	*AE000662.92*	Wald ratio	1	-0.0149	0.0146	0.985 (0.957, 1.014)	0.307	0.6	2.75E-23	TRUE
CD4 NC	*AEBP2*	Wald ratio	1	-0.0413	0.0168	0.96 (0.928, 0.992)	0.014	0.1	1.70E-08	TRUE
CD4 NC	*AF146191.4*	Wald ratio	1	0.0111	0.0199	1.011 (0.972, 1.051)	0.579	0.789	1.37E-08	TRUE
CD4 NC	*AFAP1*	Wald ratio	1	-0.0013	0.0078	0.999 (0.984, 1.014)	0.871	0.947	4.00E-49	TRUE
CD4 NC	*AGA*	Wald ratio	1	-0.0158	0.0098	0.984 (0.966, 1.003)	0.106	0.346	3.69E-29	TRUE
CD4 NC	*AGAP1*	Wald ratio	1	0.0278	0.0204	1.028 (0.988, 1.07)	0.173	0.451	1.78E-10	TRUE
CD4 NC	*AGTRAP*	Wald ratio	1	0.0681	0.0173	1.071 (1.035, 1.107)	7.84E-05	0.003	4.78E-10	TRUE
CD4 NC	*AHI1*	Wald ratio	1	-0.0102	0.0065	0.99 (0.977, 1.003)	0.116	0.362	1.48E-71	TRUE
CD4 NC	*AHSA2*	Wald ratio	1	0.0142	0.0176	1.014 (0.98, 1.05)	0.42	0.691	5.21E-10	TRUE
CD4 NC	*AIF1*	Wald ratio	1	0.0280	0.0069	1.028 (1.015, 1.042)	4.62E-05	0.002	2.15E-33	TRUE
CD4 NC	*AIG1*	Wald ratio	1	0.0216	0.0176	1.022 (0.987, 1.058)	0.22	0.512	8.05E-08	TRUE
CD4 NC	*AIM1*	Wald ratio	1	-0.0312	0.0175	0.969 (0.937, 1.003)	0.075	0.288	1.41E-10	TRUE
CD4 NC	*AK1*	Wald ratio	1	0.0081	0.0120	1.008 (0.985, 1.032)	0.498	0.741	1.53E-19	TRUE
CD4 NC	*AK3*	Wald ratio	1	-0.0020	0.0153	0.998 (0.969, 1.028)	0.898	0.957	2.42E-08	TRUE
CD4 NC	*AKAP10*	Wald ratio	1	0.0045	0.0175	1.005 (0.971, 1.04)	0.796	0.912	4.01E-11	TRUE

CD4 NC	*AKAP12*	Wald ratio	1	-0.0071	0.0128	0.993 (0.968, 1.018)	0.578	0.789	5.99E-15	TRUE
CD4 NC	*AKAP8*	Wald ratio	1	0.0312	0.0148	1.032 (1.002, 1.062)	0.035	0.176	1.08E-15	TRUE
CD4 NC	*AKIP1*	Wald ratio	1	-0.0073	0.0119	0.993 (0.97, 1.016)	0.538	0.762	2.69E-22	TRUE
CD4 NC	*AKIRIN1*	Wald ratio	1	-0.0053	0.0135	0.995 (0.969, 1.021)	0.695	0.863	3.66E-17	TRUE
CD4 NC	*AKR1C2*	Wald ratio	1	-0.0072	0.0131	0.993 (0.968, 1.019)	0.585	0.794	2.04E-15	TRUE
CD4 NC	*AKR1C3*	Wald ratio	1	-0.0222	0.0118	0.978 (0.956, 1.001)	0.06	0.247	1.44E-21	TRUE
CD4 NC	*ALDH7A1*	Wald ratio	1	-0.0007	0.0176	0.999 (0.965, 1.034)	0.969	0.989	5.85E-08	TRUE
CD4 NC	*ALDH9A1*	Wald ratio	1	-0.0055	0.0186	0.995 (0.959, 1.032)	0.769	0.897	6.99E-09	TRUE
CD4 NC	*ALG1L*	Wald ratio	1	0.0213	0.0149	1.021 (0.992, 1.052)	0.155	0.426	5.19E-17	TRUE
CD4 NC	*ALKBH7*	Wald ratio	1	0.0087	0.0188	1.009 (0.972, 1.047)	0.646	0.834	8.78E-11	TRUE
CD4 NC	*ALOX5AP*	Wald ratio	1	0.0357	0.0192	1.036 (0.998, 1.076)	0.063	0.255	5.16E-09	TRUE
CD4 NC	*ALPK1*	Wald ratio	1	0.0162	0.0121	1.016 (0.992, 1.041)	0.181	0.462	3.07E-20	TRUE
CD4 NC	*ALPK2*	Wald ratio	1	0.0220	0.0096	1.022 (1.003, 1.042)	0.022	0.137	5.94E-23	TRUE
CD4 NC	*AMICA1*	Wald ratio	1	0.0024	0.0081	1.002 (0.987, 1.018)	0.77	0.898	3.99E-40	TRUE
CD4 NC	*ANAPC13*	Wald ratio	1	-0.0076	0.0094	0.992 (0.974, 1.011)	0.417	0.688	2.27E-35	TRUE
CD4 NC	*ANGEL2*	Wald ratio	1	0.0289	0.0129	1.029 (1.004, 1.056)	0.024	0.144	1.36E-17	TRUE
CD4 NC	*ANK3*	Wald ratio	1	0.0314	0.0112	1.032 (1.009, 1.055)	0.005	0.051	1.18E-26	TRUE
CD4 NC	*ANKDD1A*	Wald ratio	1	0.0011	0.0053	1.001 (0.991, 1.012)	0.835	0.93	2.32E-106	TRUE
CD4 NC	*ANKDD1B*	Wald ratio	1	0.0118	0.0179	1.012 (0.977, 1.048)	0.511	0.748	1.11E-09	TRUE
CD4 NC	*ANKH*	Wald ratio	1	0.0094	0.0129	1.009 (0.984, 1.035)	0.469	0.727	2.66E-18	TRUE
CD4 NC	*ANKRD55*	Wald ratio	1	-0.0012	0.0078	0.999 (0.984, 1.014)	0.881	0.951	6.27E-54	TRUE
CD4 NC	*ANTXR2*	Wald ratio	1	0.0059	0.0201	1.006 (0.967, 1.046)	0.767	0.897	2.97E-08	TRUE
CD4 NC	*ANXA11*	Wald ratio	1	-0.0116	0.0140	0.988 (0.962, 1.016)	0.407	0.682	7.80E-19	TRUE
CD4 NC	*ANXA2*	Wald ratio	1	0.0859	0.0202	1.09 (1.047, 1.134)	2.07E-05	9.71E-04	3.78E-09	TRUE
CD4 NC	*ANXA2R*	Wald ratio	1	0.0039	0.0173	1.004 (0.97, 1.039)	0.823	0.926	3.87E-13	TRUE
CD4 NC	*ANXA5*	Wald ratio	1	-0.0041	0.0112	0.996 (0.974, 1.018)	0.714	0.872	1.02E-38	TRUE
CD4 NC	*ANXA6*	Wald ratio	1	0.0004	0.0084	1 (0.984, 1.017)	0.958	0.985	5.92E-34	TRUE
CD4 NC	*AOAH*	Wald ratio	1	-0.0354	0.0116	0.965 (0.944, 0.987)	0.002	0.029	4.69E-27	TRUE
CD4 NC	*AP000442.1*	Wald ratio	1	0.0373	0.0159	1.038 (1.006, 1.071)	0.019	0.122	5.37E-08	TRUE
CD4 NC	*AP000487.5*	Wald ratio	1	0.0080	0.0171	1.008 (0.975, 1.043)	0.639	0.83	1.43E-10	TRUE
CD4 NC	*AP000783.1*	Wald ratio	1	-0.0318	0.0235	0.969 (0.925, 1.014)	0.176	0.456	2.82E-08	TRUE
CD4 NC	*AP001462.6*	Wald ratio	1	-0.0019	0.0166	0.998 (0.966, 1.031)	0.908	0.961	1.06E-09	TRUE
CD4 NC	*AP001816.1*	Wald ratio	1	0.0009	0.0178	1.001 (0.967, 1.036)	0.96	0.986	4.03E-09	TRUE
CD4 NC	*AP003774.1*	Wald ratio	1	-0.0205	0.0060	0.98 (0.968, 0.991)	5.86E-04	0.011	4.37E-80	TRUE
CD4 NC	*AP1M1*	Wald ratio	1	0.0110	0.0171	1.011 (0.978, 1.046)	0.519	0.753	3.18E-11	TRUE
CD4 NC	*AP1S1*	Wald ratio	1	0.0184	0.0174	1.019 (0.984, 1.054)	0.291	0.583	4.03E-11	TRUE
CD4 NC	*AP2A2*	Wald ratio	1	0.0741	0.0190	1.077 (1.037, 1.118)	9.86E-05	0.003	1.59E-08	TRUE
CD4 NC	*APOBEC3G*	IVW	2	0.0060	0.0092	1.006 (0.988, 1.024)	0.516	0.751	2.28E-33	TRUE
CD4 NC	*AQP8*	Wald ratio	1	0.0399	0.0195	1.041 (1.002, 1.081)	0.041	0.197	7.90E-09	TRUE
CD4 NC	*ARC*	Wald ratio	1	-0.0165	0.0172	0.984 (0.951, 1.017)	0.337	0.629	1.13E-10	TRUE
CD4 NC	*ARFIP1*	Wald ratio	1	-0.0084	0.0166	0.992 (0.96, 1.024)	0.612	0.812	3.40E-11	TRUE
CD4 NC	*ARHGAP11A*	Wald ratio	1	-0.0088	0.0164	0.991 (0.96, 1.024)	0.591	0.797	7.51E-11	TRUE
CD4 NC	*ARHGAP15*	Wald ratio	1	0.0073	0.0109	1.007 (0.986, 1.029)	0.505	0.745	4.53E-31	TRUE
CD4 NC	*ARHGAP21*	Wald ratio	1	-0.0333	0.0189	0.967 (0.932, 1.004)	0.078	0.292	5.58E-09	TRUE
CD4 NC	*ARHGAP44*	Wald ratio	1	0.0163	0.0171	1.016 (0.983, 1.051)	0.341	0.635	1.05E-08	TRUE
CD4 NC	*ARHGAP5*	Wald ratio	1	-0.0015	0.0113	0.999 (0.977, 1.021)	0.896	0.957	3.56E-23	TRUE
CD4 NC	*ARHGEF10*	Wald ratio	1	0.0040	0.0074	1.004 (0.99, 1.019)	0.589	0.795	2.90E-53	TRUE
CD4 NC	*ARHGEF26*	Wald ratio	1	0.0586	0.0255	1.06 (1.009, 1.115)	0.022	0.134	5.89E-08	TRUE
CD4 NC	*ARHGEF3*	Wald ratio	1	-0.0013	0.0064	0.999 (0.986, 1.011)	0.841	0.933	1.61E-64	TRUE
CD4 NC	*ARHGEF35*	Wald ratio	1	0.0174	0.0176	1.018 (0.983, 1.053)	0.323	0.618	8.95E-10	TRUE
CD4 NC	*ARHGEF5*	Wald ratio	1	-0.0173	0.0175	0.983 (0.95, 1.017)	0.323	0.618	6.32E-10	TRUE
CD4 NC	*ARID5B*	Wald ratio	1	-0.0497	0.0213	0.951 (0.913, 0.992)	0.019	0.124	1.88E-08	TRUE
CD4 NC	*ARL14EP*	Wald ratio	1	-0.0209	0.0079	0.979 (0.964, 0.995)	0.008	0.068	3.45E-43	TRUE
CD4 NC	*ARL16*	Wald ratio	1	0.0074	0.0060	1.007 (0.996, 1.019)	0.222	0.514	2.32E-77	TRUE
CD4 NC	*ARL17A*	IVW	2	0.0184	0.0222	1.019 (0.975, 1.064)	0.408	0.683	1.03E-39	TRUE
CD4 NC	*ARL17B*	IVW	2	0.0013	0.0102	1.001 (0.981, 1.022)	0.899	0.958	6.30E-31	TRUE
CD4 NC	*ARL6IP4*	Wald ratio	1	0.0328	0.0161	1.033 (1.001, 1.066)	0.041	0.198	3.57E-12	TRUE
CD4 NC	*ARNTL*	Wald ratio	1	0.0366	0.0170	1.037 (1.003, 1.072)	0.031	0.166	1.31E-10	TRUE
CD4 NC	*ARPC3*	Wald ratio	1	0.0275	0.0148	1.028 (0.998, 1.058)	0.064	0.258	4.56E-13	TRUE
CD4 NC	*ARPC4*	Wald ratio	1	0.0269	0.0188	1.027 (0.99, 1.066)	0.151	0.422	2.44E-11	TRUE
CD4 NC	*ARPC5*	Wald ratio	1	-0.0150	0.0113	0.985 (0.964, 1.007)	0.186	0.466	1.38E-23	TRUE
CD4 NC	*ARRDC4*	Wald ratio	1	-0.0241	0.0203	0.976 (0.938, 1.016)	0.235	0.529	1.76E-08	TRUE
CD4 NC	*ARRDC5*	Wald ratio	1	0.0072	0.0156	1.007 (0.977, 1.039)	0.643	0.832	9.78E-14	TRUE
CD4 NC	*ARSA*	Wald ratio	1	0.0259	0.0140	1.026 (0.998, 1.055)	0.065	0.262	5.33E-31	TRUE
CD4 NC	*ARSG*	Wald ratio	1	0.0202	0.0201	1.02 (0.981, 1.061)	0.313	0.608	3.49E-08	TRUE
CD4 NC	*ASB15*	Wald ratio	1	-0.0176	0.0131	0.983 (0.958, 1.008)	0.179	0.459	2.17E-17	TRUE
CD4 NC	*ASB16-AS1*	Wald ratio	1	0.0221	0.0119	1.022 (0.999, 1.046)	0.062	0.254	4.00E-20	TRUE
CD4 NC	*ASB6*	Wald ratio	1	0.0206	0.0194	1.021 (0.983, 1.06)	0.289	0.582	2.32E-08	TRUE
CD4 NC	*ASB8*	Wald ratio	1	0.0074	0.0223	1.007 (0.964, 1.052)	0.738	0.881	1.14E-08	TRUE
CD4 NC	*ASL*	Wald ratio	1	-0.0104	0.0168	0.99 (0.958, 1.023)	0.537	0.762	5.71E-11	TRUE
CD4 NC	*ASNSD1*	Wald ratio	1	-0.0100	0.0116	0.99 (0.968, 1.013)	0.389	0.67	3.59E-20	TRUE
CD4 NC	*ATF4*	Wald ratio	1	0.0534	0.0191	1.055 (1.016, 1.095)	0.005	0.052	1.90E-08	TRUE
CD4 NC	*ATF5*	Wald ratio	1	-0.0823	0.0166	0.921 (0.891, 0.951)	6.92E-07	9.75E-05	4.76E-10	TRUE
CD4 NC	*ATF6*	Wald ratio	1	0.0068	0.0118	1.007 (0.984, 1.03)	0.561	0.778	3.73E-23	TRUE
CD4 NC	*ATF7IP2*	Wald ratio	1	0.0342	0.0124	1.035 (1.01, 1.06)	0.006	0.057	1.27E-18	TRUE
CD4 NC	*ATG10*	Wald ratio	1	0.0198	0.0126	1.02 (0.995, 1.045)	0.116	0.363	5.46E-19	TRUE
CD4 NC	*ATHL1*	Wald ratio	1	-0.0027	0.0087	0.997 (0.98, 1.014)	0.752	0.887	4.67E-46	TRUE
CD4 NC	*ATIC*	Wald ratio	1	-0.0445	0.0202	0.956 (0.919, 0.995)	0.027	0.155	1.50E-08	TRUE
CD4 NC	*ATM*	Wald ratio	1	-0.0176	0.0121	0.983 (0.959, 1.006)	0.147	0.416	5.73E-22	TRUE

CD4 NC	*ATP10A*	Wald ratio	1	0.0152	0.0117	1.015 (0.992, 1.039)	0.191	0.472	2.00E-22	TRUE
CD4 NC	*ATP1B2*	Wald ratio	1	-0.0269	0.0108	0.973 (0.953, 0.994)	0.013	0.096	3.51E-20	TRUE
CD4 NC	*ATP1B3*	Wald ratio	1	0.0099	0.0243	1.01 (0.963, 1.059)	0.684	0.856	5.83E-08	TRUE
CD4 NC	*ATP5D*	Wald ratio	1	-0.0560	0.0202	0.946 (0.909, 0.984)	0.005	0.053	1.12E-07	TRUE
CD4 NC	*ATP5G2*	Wald ratio	1	0.0091	0.0122	1.009 (0.985, 1.034)	0.454	0.717	1.68E-22	TRUE
CD4 NC	*ATP5G3*	Wald ratio	1	-0.0042	0.0134	0.996 (0.97, 1.022)	0.753	0.888	6.99E-22	TRUE
CD4 NC	*ATP6AP1L*	Wald ratio	1	0.0174	0.0108	1.018 (0.996, 1.039)	0.108	0.35	3.72E-25	TRUE
CD4 NC	*ATP6V0E2*	Wald ratio	1	0.0118	0.0073	1.012 (0.997, 1.027)	0.108	0.35	1.86E-51	TRUE
CD4 NC	*ATP6V1E2*	Wald ratio	1	0.0302	0.0188	1.031 (0.993, 1.069)	0.108	0.349	2.44E-09	TRUE
CD4 NC	*ATP6V1G3*	Wald ratio	1	0.0152	0.0095	1.015 (0.997, 1.034)	0.109	0.352	1.49E-28	TRUE
CD4 NC	*ATP8A1*	Wald ratio	1	-0.0104	0.0180	0.99 (0.955, 1.025)	0.563	0.78	1.15E-10	TRUE
CD4 NC	*ATP8A2*	Wald ratio	1	-0.0006	0.0169	0.999 (0.967, 1.033)	0.971	0.989	4.47E-11	TRUE
CD4 NC	*ATP9B*	Wald ratio	1	-0.0249	0.0177	0.975 (0.942, 1.01)	0.159	0.431	5.16E-10	TRUE
CD4 NC	*AVL9*	Wald ratio	1	0.0330	0.0187	1.034 (0.996, 1.072)	0.077	0.291	4.59E-09	TRUE
CD4 NC	*AZI2*	Wald ratio	1	0.0133	0.0206	1.013 (0.973, 1.055)	0.519	0.753	6.19E-08	TRUE
CD4 NC	*B3GALNT1*	Wald ratio	1	-0.0889	0.0171	0.915 (0.885, 0.946)	1.98E-07	6.60E-05	5.26E-10	TRUE
CD4 NC	*B3GAT2*	Wald ratio	1	0.0269	0.0206	1.027 (0.987, 1.07)	0.191	0.471	2.13E-11	TRUE
CD4 NC	*B4GALNT3*	Wald ratio	1	0.0095	0.0120	1.01 (0.986, 1.034)	0.432	0.701	1.56E-21	TRUE
CD4 NC	*B4GALT1*	Wald ratio	1	0.0072	0.0084	1.007 (0.991, 1.024)	0.387	0.669	8.99E-48	TRUE
CD4 NC	*B4GALT4*	Wald ratio	1	-0.0252	0.0155	0.975 (0.946, 1.005)	0.104	0.342	6.59E-16	TRUE
CD4 NC	*BANF1*	Wald ratio	1	-0.0042	0.0149	0.996 (0.967, 1.025)	0.779	0.904	2.54E-14	TRUE
CD4 NC	*BBC3*	Wald ratio	1	0.0491	0.0201	1.05 (1.01, 1.093)	0.014	0.103	5.04E-08	TRUE
CD4 NC	*BBS2*	Wald ratio	1	0.0143	0.0187	1.014 (0.978, 1.052)	0.444	0.709	5.58E-12	TRUE
CD4 NC	*BCAS1*	Wald ratio	1	-0.0083	0.0163	0.992 (0.961, 1.024)	0.611	0.812	7.21E-12	TRUE
CD4 NC	*BCAS4*	IVW	2	0.0080	0.0088	1.008 (0.991, 1.026)	0.368	0.656	2.61E-53	TRUE
CD4 NC	*BCL2L14*	Wald ratio	1	0.0315	0.0119	1.032 (1.008, 1.056)	0.008	0.07	1.64E-21	TRUE
CD4 NC	*BDH2*	Wald ratio	1	-0.0211	0.0100	0.979 (0.96, 0.998)	0.035	0.176	3.29E-28	TRUE
CD4 NC	*BEGAIN*	Wald ratio	1	-0.0082	0.0122	0.992 (0.968, 1.016)	0.5	0.742	4.44E-19	TRUE
CD4 NC	*BIK*	Wald ratio	1	-0.0542	0.0188	0.947 (0.913, 0.983)	0.004	0.042	1.09E-11	TRUE
CD4 NC	*BIN1*	Wald ratio	1	0.0116	0.0061	1.012 (1, 1.024)	0.058	0.243	6.49E-74	TRUE
CD4 NC	*BIN2*	Wald ratio	1	0.0750	0.0189	1.078 (1.039, 1.119)	7.49E-05	0.003	1.60E-09	TRUE
CD4 NC	*BLK*	Wald ratio	1	-0.0510	0.0117	0.95 (0.929, 0.972)	1.35E-05	7.04E-04	4.89E-21	TRUE
CD4 NC	*BLMH*	Wald ratio	1	-0.0874	0.0197	0.916 (0.882, 0.952)	9.32E-06	5.39E-04	4.64E-08	TRUE
CD4 NC	*BLOC1S5*	Wald ratio	1	0.0164	0.0165	1.017 (0.984, 1.05)	0.321	0.616	2.56E-11	TRUE
CD4 NC	*BLVRA*	Wald ratio	1	0.0180	0.0183	1.018 (0.982, 1.055)	0.325	0.619	1.57E-09	TRUE
CD4 NC	*BOLA3-AS1*	Wald ratio	1	0.0124	0.0183	1.012 (0.977, 1.049)	0.496	0.741	1.34E-10	TRUE
CD4 NC	*BPGM*	Wald ratio	1	0.0221	0.0203	1.022 (0.983, 1.064)	0.275	0.568	1.14E-08	TRUE
CD4 NC	*BRK1*	Wald ratio	1	-0.0206	0.0142	0.98 (0.953, 1.007)	0.148	0.417	6.17E-19	TRUE
CD4 NC	*BST2*	Wald ratio	1	0.0217	0.0075	1.022 (1.007, 1.037)	0.004	0.043	1.74E-33	TRUE
CD4 NC	*BTBD9*	Wald ratio	1	0.0065	0.0091	1.006 (0.989, 1.025)	0.48	0.734	5.20E-29	TRUE
CD4 NC	*BTF3*	Wald ratio	1	0.0308	0.0103	1.031 (1.011, 1.052)	0.003	0.034	6.12E-24	TRUE
CD4 NC	*BTN2A1*	Wald ratio	1	0.0008	0.0110	1.001 (0.979, 1.023)	0.944	0.978	1.76E-29	TRUE
CD4 NC	*BTN3A1*	Wald ratio	1	-0.0132	0.0164	0.987 (0.956, 1.019)	0.422	0.693	3.02E-11	TRUE
CD4 NC	*BTN3A2*	Wald ratio	1	0.0120	0.0084	1.012 (0.996, 1.029)	0.153	0.423	2.71E-57	TRUE
CD4 NC	*C10orf128*	Wald ratio	1	-0.0046	0.0092	0.995 (0.978, 1.014)	0.62	0.819	7.88E-33	TRUE
CD4 NC	*C10orf32*	Wald ratio	1	-0.0387	0.0089	0.962 (0.945, 0.979)	1.40E-05	7.21E-04	9.15E-38	TRUE
CD4 NC	*C10orf54*	Wald ratio	1	0.0162	0.0175	1.016 (0.982, 1.052)	0.353	0.644	8.32E-11	TRUE
CD4 NC	*C10orf67*	Wald ratio	1	-0.0108	0.0192	0.989 (0.953, 1.027)	0.575	0.786	1.97E-09	TRUE
CD4 NC	*C10ORF68*	Wald ratio	1	-0.0413	0.0188	0.96 (0.925, 0.996)	0.028	0.156	3.73E-09	TRUE
CD4 NC	*C10orf95*	Wald ratio	1	-0.0277	0.0134	0.973 (0.947, 0.999)	0.039	0.19	1.72E-21	TRUE
CD4 NC	*C11orf21*	Wald ratio	1	-0.0076	0.0100	0.992 (0.973, 1.012)	0.448	0.713	1.38E-20	TRUE
CD4 NC	*C11orf24*	Wald ratio	1	0.0037	0.0124	1.004 (0.98, 1.028)	0.762	0.893	9.33E-19	TRUE
CD4 NC	*C11orf63*	Wald ratio	1	0.0064	0.0204	1.006 (0.967, 1.047)	0.754	0.888	6.47E-10	TRUE
CD4 NC	*C11orf71*	Wald ratio	1	-0.0037	0.0136	0.996 (0.97, 1.023)	0.787	0.909	1.05E-17	TRUE
CD4 NC	*C12orf56*	Wald ratio	1	-0.0032	0.0101	0.997 (0.977, 1.017)	0.75	0.886	1.77E-30	TRUE
CD4 NC	*C14orf132*	Wald ratio	1	0.0091	0.0127	1.009 (0.984, 1.035)	0.476	0.731	2.72E-23	TRUE
CD4 NC	*C14orf178*	Wald ratio	1	0.0182	0.0131	1.018 (0.992, 1.045)	0.166	0.442	1.64E-15	TRUE
CD4 NC	*C14orf2*	Wald ratio	1	0.0276	0.0155	1.028 (0.997, 1.06)	0.075	0.287	6.67E-13	TRUE
CD4 NC	*C14orf79*	Wald ratio	1	-0.0218	0.0207	0.978 (0.94, 1.019)	0.293	0.587	6.75E-08	TRUE
CD4 NC	*C15orf40*	Wald ratio	1	0.0002	0.0098	1 (0.981, 1.02)	0.985	0.994	2.51E-30	TRUE
CD4 NC	*C15orf61*	Wald ratio	1	-0.0069	0.0160	0.993 (0.962, 1.025)	0.666	0.847	2.71E-08	TRUE
CD4 NC	*C16orf13*	Wald ratio	1	0.0000	0.0093	1 (0.982, 1.018)	0.997	0.999	1.20E-23	TRUE
CD4 NC	*C17orf49*	Wald ratio	1	-0.0142	0.0119	0.986 (0.963, 1.009)	0.232	0.525	8.38E-11	TRUE
CD4 NC	*C17orf67*	Wald ratio	1	-0.0259	0.0195	0.974 (0.938, 1.012)	0.185	0.465	2.66E-08	TRUE
CD4 NC	*C17orf89*	Wald ratio	1	0.0274	0.0094	1.028 (1.009, 1.047)	0.004	0.041	3.20E-32	TRUE
CD4 NC	*C17orf97*	Wald ratio	1	-0.0085	0.0071	0.992 (0.978, 1.005)	0.23	0.524	7.40E-57	TRUE
CD4 NC	*C19orf24*	Wald ratio	1	0.0345	0.0137	1.035 (1.008, 1.063)	0.012	0.092	2.29E-18	TRUE
CD4 NC	*C19orf48*	Wald ratio	1	0.0166	0.0118	1.017 (0.993, 1.041)	0.159	0.432	3.38E-18	TRUE
CD4 NC	*C19orf53*	Wald ratio	1	-0.0086	0.0118	0.991 (0.969, 1.015)	0.47	0.728	2.03E-16	TRUE
CD4 NC	*C19orf83*	Wald ratio	1	-0.0208	0.0095	0.979 (0.961, 0.998)	0.029	0.159	7.01E-32	TRUE
CD4 NC	*C1orf174*	Wald ratio	1	0.0459	0.0197	1.047 (1.007, 1.088)	0.02	0.126	8.61E-08	TRUE
CD4 NC	*C1orf228*	Wald ratio	1	-0.0034	0.0127	0.997 (0.972, 1.022)	0.789	0.909	5.90E-18	TRUE
CD4 NC	*C1orf86*	Wald ratio	1	0.0018	0.0091	1.002 (0.984, 1.02)	0.84	0.932	7.37E-35	TRUE
CD4 NC	*C1QTNF6*	Wald ratio	1	-0.0103	0.0192	0.99 (0.953, 1.028)	0.59	0.797	5.94E-09	TRUE
CD4 NC	*C1QTNF9B-AS1*	Wald ratio	1	-0.0054	0.0124	0.995 (0.971, 1.019)	0.661	0.844	3.00E-19	TRUE
CD4 NC	*C2*	Wald ratio	1	-0.0067	0.0193	0.993 (0.956, 1.032)	0.726	0.878	8.52E-09	TRUE
CD4 NC	*C20orf27*	Wald ratio	1	-0.0038	0.0168	0.996 (0.964, 1.03)	0.824	0.926	1.83E-18	TRUE
CD4 NC	*C20orf96*	Wald ratio	1	-0.0199	0.0169	0.98 (0.948, 1.013)	0.241	0.534	7.07E-11	TRUE
CD4 NC	*C21orf91*	Wald ratio	1	-0.0009	0.0188	0.999 (0.963, 1.037)	0.962	0.988	3.00E-10	TRUE

CD4 NC	*C22orf34*	Wald ratio	1	-0.0138	0.0075	0.986 (0.972, 1.001)	0.067	0.265	1.13E-51	TRUE
CD4 NC	*C2ORF15*	Wald ratio	1	0.0033	0.0127	1.003 (0.979, 1.029)	0.797	0.913	1.02E-17	TRUE
CD4 NC	*C2orf27A*	Wald ratio	1	0.0104	0.0070	1.01 (0.997, 1.024)	0.137	0.399	1.78E-65	TRUE
CD4 NC	*C2orf40*	Wald ratio	1	-0.0113	0.0115	0.989 (0.967, 1.011)	0.324	0.618	6.22E-17	TRUE
CD4 NC	*C2orf74*	Wald ratio	1	-0.0572	0.0158	0.944 (0.916, 0.974)	2.79E-04	0.007	9.58E-12	TRUE
CD4 NC	*C3orf14*	Wald ratio	1	0.0116	0.0176	1.012 (0.977, 1.047)	0.508	0.747	5.06E-10	TRUE
CD4 NC	*C4BPB*	Wald ratio	1	0.0351	0.0160	1.036 (1.004, 1.069)	0.028	0.156	4.06E-12	TRUE
CD4 NC	*C4orf32*	Wald ratio	1	0.0123	0.0085	1.012 (0.996, 1.029)	0.146	0.415	2.35E-39	TRUE
CD4 NC	*C4orf33*	Wald ratio	1	0.0035	0.0179	1.003 (0.969, 1.039)	0.846	0.935	2.43E-11	TRUE
CD4 NC	*C5orf17*	IVW	2	0.0150	0.0368	1.015 (0.944, 1.091)	0.683	0.856	1.74E-29	TRUE
CD4 NC	*C5orf45*	Wald ratio	1	0.0307	0.0145	1.031 (1.002, 1.061)	0.034	0.175	2.57E-14	TRUE
CD4 NC	*C5orf55*	Wald ratio	1	0.0223	0.0225	1.023 (0.978, 1.069)	0.321	0.616	7.10E-08	TRUE
CD4 NC	*C5orf63*	Wald ratio	1	-0.0082	0.0132	0.992 (0.966, 1.018)	0.533	0.76	1.81E-18	TRUE
CD4 NC	*C6orf132*	Wald ratio	1	-0.0620	0.0207	0.94 (0.903, 0.979)	0.003	0.032	8.65E-08	TRUE
CD4 NC	*C6orf25*	Wald ratio	1	0.0326	0.0194	1.033 (0.995, 1.073)	0.092	0.323	1.46E-08	TRUE
CD4 NC	*C6orf48*	Wald ratio	1	0.0287	0.0082	1.029 (1.013, 1.046)	4.60E-04	0.01	8.16E-22	TRUE
CD4 NC	*C6orf57*	Wald ratio	1	-0.0671	0.0174	0.935 (0.904, 0.967)	1.12E-04	0.003	3.38E-08	TRUE
CD4 NC	*C6orf62*	Wald ratio	1	0.0671	0.0195	1.069 (1.029, 1.111)	5.87E-04	0.011	1.76E-08	TRUE
CD4 NC	*C7orf13*	Wald ratio	1	-0.0072	0.0163	0.993 (0.962, 1.025)	0.659	0.843	4.18E-11	TRUE
CD4 NC	*C9orf135*	Wald ratio	1	-0.0072	0.0048	0.993 (0.984, 1.002)	0.135	0.396	1.61E-115	TRUE
CD4 NC	*C9orf142*	Wald ratio	1	0.0286	0.0169	1.029 (0.996, 1.064)	0.09	0.318	1.01E-09	TRUE
CD4 NC	*C9orf156*	Wald ratio	1	0.0060	0.0112	1.006 (0.984, 1.028)	0.594	0.799	3.84E-25	TRUE
CD4 NC	*C9orf16*	Wald ratio	1	0.0244	0.0190	1.025 (0.987, 1.064)	0.201	0.484	9.36E-09	TRUE
CD4 NC	*C9orf78*	Wald ratio	1	-0.0010	0.0087	0.999 (0.982, 1.016)	0.906	0.96	9.76E-40	TRUE
CD4 NC	*CA6*	IVW	2	-0.0012	0.0096	0.999 (0.98, 1.018)	0.897	0.957	1.58E-58	TRUE
CD4 NC	*CACNA1A*	Wald ratio	1	0.0087	0.0190	1.009 (0.972, 1.047)	0.648	0.834	5.75E-09	TRUE
CD4 NC	*CALM3*	Wald ratio	1	0.0047	0.0119	1.005 (0.982, 1.028)	0.69	0.86	4.94E-24	TRUE
CD4 NC	*CALR*	Wald ratio	1	0.0015	0.0171	1.002 (0.968, 1.036)	0.929	0.97	8.86E-12	TRUE
CD4 NC	*CAMK4*	Wald ratio	1	0.0133	0.0125	1.013 (0.989, 1.038)	0.286	0.577	3.15E-22	TRUE
CD4 NC	*CAMKK2*	Wald ratio	1	0.0123	0.0171	1.012 (0.979, 1.047)	0.473	0.73	5.97E-10	TRUE
CD4 NC	*CAMLG*	Wald ratio	1	0.0000	0.0097	1 (0.981, 1.019)	0.999	1	1.26E-20	TRUE
CD4 NC	*CAPG*	Wald ratio	1	-0.0076	0.0081	0.992 (0.977, 1.008)	0.347	0.64	2.08E-43	TRUE
CD4 NC	*CAPN10*	Wald ratio	1	0.0182	0.0174	1.018 (0.984, 1.054)	0.293	0.587	8.02E-09	TRUE
CD4 NC	*CAPN12*	Wald ratio	1	-0.0179	0.0188	0.982 (0.947, 1.019)	0.34	0.634	2.24E-11	TRUE
CD4 NC	*CARD8*	Wald ratio	1	0.0195	0.0123	1.02 (0.995, 1.045)	0.113	0.359	1.02E-28	TRUE
CD4 NC	*CARS*	Wald ratio	1	0.0221	0.0139	1.022 (0.995, 1.051)	0.111	0.355	6.03E-15	TRUE
CD4 NC	*CARS2*	Wald ratio	1	0.0214	0.0178	1.022 (0.987, 1.058)	0.228	0.521	1.11E-08	TRUE
CD4 NC	*CASP6*	Wald ratio	1	-0.0115	0.0188	0.989 (0.953, 1.026)	0.542	0.764	2.31E-09	TRUE
CD4 NC	*CASP7*	Wald ratio	1	0.0502	0.0210	1.051 (1.009, 1.096)	0.017	0.114	5.09E-08	TRUE
CD4 NC	*CASP8*	Wald ratio	1	-0.0317	0.0104	0.969 (0.949, 0.989)	0.002	0.029	5.62E-26	TRUE
CD4 NC	*CAST*	Wald ratio	1	-0.0035	0.0103	0.997 (0.977, 1.017)	0.737	0.88	1.52E-31	TRUE
CD4 NC	*CBR1*	Wald ratio	1	-0.0259	0.0143	0.974 (0.947, 1.002)	0.071	0.275	9.95E-15	TRUE
CD4 NC	*CBR3*	Wald ratio	1	0.0040	0.0079	1.004 (0.989, 1.02)	0.615	0.814	2.89E-49	TRUE
CD4 NC	*CBR4*	Wald ratio	1	-0.0090	0.0227	0.991 (0.948, 1.036)	0.69	0.86	1.18E-08	TRUE
CD4 NC	*CBWD1*	Wald ratio	1	-0.0089	0.0144	0.991 (0.964, 1.019)	0.534	0.761	8.22E-15	TRUE
CD4 NC	*CBWD2*	Wald ratio	1	-0.0187	0.0116	0.982 (0.959, 1.004)	0.107	0.348	4.30E-22	TRUE
CD4 NC	*CC2D1B*	Wald ratio	1	-0.0111	0.0212	0.989 (0.949, 1.031)	0.599	0.804	5.49E-08	TRUE
CD4 NC	*CCDC101*	Wald ratio	1	-0.0087	0.0090	0.991 (0.974, 1.009)	0.331	0.625	1.38E-36	TRUE
CD4 NC	*CCDC104*	Wald ratio	1	0.0727	0.0152	1.075 (1.044, 1.108)	1.60E-06	1.62E-04	1.46E-14	TRUE
CD4 NC	*CCDC109B*	Wald ratio	1	-0.0045	0.0154	0.995 (0.966, 1.026)	0.77	0.897	1.13E-12	TRUE
CD4 NC	*CCDC115*	Wald ratio	1	-0.0032	0.0177	0.997 (0.963, 1.032)	0.857	0.939	4.74E-13	TRUE
CD4 NC	*CCDC122*	Wald ratio	1	-0.0065	0.0080	0.994 (0.978, 1.009)	0.415	0.687	9.67E-46	TRUE
CD4 NC	*CCDC127*	Wald ratio	1	-0.0519	0.0187	0.949 (0.915, 0.985)	0.006	0.054	1.15E-08	TRUE
CD4 NC	*CCDC146*	Wald ratio	1	0.0077	0.0114	1.008 (0.985, 1.031)	0.501	0.743	3.01E-41	TRUE
CD4 NC	*CCDC152*	Wald ratio	1	0.0106	0.0099	1.011 (0.991, 1.03)	0.284	0.576	4.40E-27	TRUE
CD4 NC	*CCDC163P*	Wald ratio	1	-0.0043	0.0053	0.996 (0.985, 1.006)	0.411	0.686	4.45E-111	TRUE
CD4 NC	*CCDC167*	Wald ratio	1	-0.0006	0.0051	0.999 (0.99, 1.009)	0.913	0.962	7.26E-111	TRUE
CD4 NC	*CCDC23*	Wald ratio	1	0.0341	0.0153	1.035 (1.004, 1.066)	0.026	0.148	5.82E-12	TRUE
CD4 NC	*CCDC50*	Wald ratio	1	-0.0234	0.0130	0.977 (0.952, 1.002)	0.072	0.278	1.74E-17	TRUE
CD4 NC	*CCDC65*	Wald ratio	1	0.0104	0.0190	1.01 (0.974, 1.049)	0.585	0.794	8.74E-09	TRUE
CD4 NC	*CCDC66*	Wald ratio	1	-0.0059	0.0086	0.994 (0.978, 1.011)	0.49	0.738	3.28E-39	TRUE
CD4 NC	*CCDC77*	Wald ratio	1	-0.0265	0.0178	0.974 (0.941, 1.008)	0.136	0.399	1.33E-08	TRUE
CD4 NC	*CCDC82*	Wald ratio	1	0.0008	0.0171	1.001 (0.968, 1.035)	0.963	0.988	9.31E-13	TRUE
CD4 NC	*CCDC88B*	Wald ratio	1	0.0392	0.0121	1.04 (1.016, 1.065)	0.001	0.019	3.80E-19	TRUE
CD4 NC	*CCDC88C*	Wald ratio	1	-0.0134	0.0192	0.987 (0.95, 1.025)	0.487	0.738	5.93E-09	TRUE
CD4 NC	*CCNB1IP1*	Wald ratio	1	0.0121	0.0187	1.012 (0.976, 1.05)	0.519	0.753	2.43E-09	TRUE
CD4 NC	*CCND1*	Wald ratio	1	0.0397	0.0197	1.04 (1.001, 1.081)	0.044	0.207	2.24E-09	TRUE
CD4 NC	*CCND3*	Wald ratio	1	0.0020	0.0169	1.002 (0.969, 1.036)	0.904	0.96	7.39E-12	TRUE
CD4 NC	*CCS*	Wald ratio	1	0.0094	0.0164	1.009 (0.977, 1.043)	0.567	0.782	1.05E-11	TRUE
CD4 NC	*CCSAP*	Wald ratio	1	-0.0239	0.0097	0.976 (0.958, 0.995)	0.013	0.098	2.48E-29	TRUE
CD4 NC	*CCSER2*	Wald ratio	1	-0.0154	0.0080	0.985 (0.969, 1)	0.055	0.236	2.13E-55	TRUE
CD4 NC	*CCT3*	Wald ratio	1	0.0102	0.0060	1.01 (0.998, 1.022)	0.088	0.313	2.89E-82	TRUE
CD4 NC	*CCT4*	Wald ratio	1	-0.0379	0.0130	0.963 (0.939, 0.988)	0.004	0.039	2.87E-13	TRUE
CD4 NC	*CCT8*	Wald ratio	1	-0.0006	0.0065	0.999 (0.987, 1.012)	0.922	0.965	9.66E-121	TRUE
CD4 NC	*CCZ1*	Wald ratio	1	-0.0022	0.0112	0.998 (0.976, 1.02)	0.848	0.935	1.15E-23	TRUE
CD4 NC	*CD101*	Wald ratio	1	0.0045	0.0170	1.005 (0.972, 1.039)	0.79	0.909	3.12E-10	TRUE
CD4 NC	*CD151*	Wald ratio	1	0.0408	0.0129	1.042 (1.016, 1.068)	0.002	0.022	2.55E-19	TRUE
CD4 NC	*CD164*	Wald ratio	1	-0.0216	0.0176	0.979 (0.945, 1.013)	0.22	0.512	5.50E-10	TRUE
CD4 NC	*CD247*	Wald ratio	1	0.0041	0.0114	1.004 (0.982, 1.027)	0.715	0.873	1.80E-28	TRUE

CD4 NC	*CD37*	Wald ratio	1	0.0034	0.0210	1.003 (0.963, 1.046)	0.87	0.946	3.05E-10	TRUE
CD4 NC	*CD3E*	Wald ratio	1	0.0457	0.0185	1.047 (1.01, 1.085)	0.013	0.098	1.02E-09	TRUE
CD4 NC	*CD3G*	Wald ratio	1	-0.0097	0.0155	0.99 (0.961, 1.021)	0.533	0.76	7.25E-19	TRUE
CD4 NC	*CD52*	Wald ratio	1	-0.0157	0.0133	0.984 (0.959, 1.011)	0.24	0.534	1.92E-17	TRUE
CD4 NC	*CD53*	IVW	2	0.0303	0.0096	1.031 (1.012, 1.05)	0.002	0.022	6.57E-30	TRUE
CD4 NC	*CD55*	Wald ratio	1	-0.0412	0.0183	0.96 (0.926, 0.995)	0.024	0.142	1.36E-10	TRUE
CD4 NC	*CD59*	Wald ratio	1	-0.0077	0.0157	0.992 (0.962, 1.023)	0.623	0.822	7.34E-13	TRUE
CD4 NC	*CD6*	Wald ratio	1	-0.0008	0.0127	0.999 (0.975, 1.024)	0.95	0.981	8.92E-22	TRUE
CD4 NC	*CD7*	Wald ratio	1	0.0082	0.0152	1.008 (0.979, 1.039)	0.592	0.797	1.10E-11	TRUE
CD4 NC	*CD84*	Wald ratio	1	-0.0095	0.0195	0.991 (0.954, 1.029)	0.627	0.822	1.06E-08	TRUE
CD4 NC	*CDC42*	Wald ratio	1	0.0072	0.0104	1.007 (0.987, 1.028)	0.49	0.738	6.00E-24	TRUE
CD4 NC	*CDC42BPA*	Wald ratio	1	0.0171	0.0189	1.017 (0.98, 1.056)	0.365	0.654	1.61E-13	TRUE
CD4 NC	*CDC42EP3*	Wald ratio	1	0.0107	0.0165	1.011 (0.979, 1.044)	0.516	0.751	2.40E-12	TRUE
CD4 NC	*CDC42SE2*	Wald ratio	1	0.0195	0.0193	1.02 (0.982, 1.059)	0.311	0.605	4.86E-09	TRUE
CD4 NC	*CDC7*	Wald ratio	1	-0.0009	0.0187	0.999 (0.963, 1.036)	0.962	0.987	2.11E-09	TRUE
CD4 NC	*CDCA7L*	Wald ratio	1	-0.0150	0.0158	0.985 (0.955, 1.016)	0.342	0.635	1.51E-11	TRUE
CD4 NC	*CDHR3*	Wald ratio	1	-0.0082	0.0164	0.992 (0.96, 1.024)	0.616	0.815	8.87E-12	TRUE
CD4 NC	*CDK10*	Wald ratio	1	-0.0366	0.0111	0.964 (0.943, 0.985)	9.77E-04	0.017	2.13E-22	TRUE
CD4 NC	*CDK5R1*	Wald ratio	1	0.0247	0.0108	1.025 (1.004, 1.047)	0.022	0.135	6.87E-25	TRUE
CD4 NC	*CDO1*	Wald ratio	1	-0.0047	0.0103	0.995 (0.975, 1.016)	0.65	0.835	4.77E-23	TRUE
CD4 NC	*CDRT4*	Wald ratio	1	-0.0237	0.0200	0.977 (0.939, 1.016)	0.235	0.529	5.23E-08	TRUE
CD4 NC	*CEACAM21*	Wald ratio	1	-0.0084	0.0070	0.992 (0.978, 1.005)	0.227	0.52	2.76E-52	TRUE
CD4 NC	*CEBPG*	Wald ratio	1	0.0070	0.0174	1.007 (0.973, 1.042)	0.69	0.86	3.71E-11	TRUE
CD4 NC	*CEBPZ*	Wald ratio	1	0.0106	0.0141	1.011 (0.983, 1.039)	0.453	0.717	3.27E-15	TRUE
CD4 NC	*CEBPZ-AS1*	Wald ratio	1	0.0114	0.0093	1.011 (0.993, 1.03)	0.219	0.511	5.12E-50	TRUE
CD4 NC	*CELA1*	Wald ratio	1	0.0021	0.0066	1.002 (0.989, 1.015)	0.75	0.886	1.93E-84	TRUE
CD4 NC	*CELSR1*	Wald ratio	1	-0.0030	0.0103	0.997 (0.977, 1.017)	0.772	0.899	4.14E-24	TRUE
CD4 NC	*CENPC*	Wald ratio	1	0.0033	0.0224	1.003 (0.96, 1.048)	0.882	0.952	2.35E-10	TRUE
CD4 NC	*CENPK*	Wald ratio	1	0.0066	0.0041	1.007 (0.999, 1.015)	0.105	0.344	4.17E-212	TRUE
CD4 NC	*CENPM*	Wald ratio	1	-0.0046	0.0100	0.995 (0.976, 1.015)	0.65	0.835	2.13E-29	TRUE
CD4 NC	*CENPP*	Wald ratio	1	0.0055	0.0218	1.005 (0.963, 1.049)	0.802	0.915	1.88E-08	TRUE
CD4 NC	*CENPU*	Wald ratio	1	0.0263	0.0205	1.027 (0.986, 1.069)	0.2	0.484	3.88E-08	TRUE
CD4 NC	*CEP170B*	Wald ratio	1	-0.0239	0.0141	0.976 (0.95, 1.004)	0.09	0.318	5.82E-15	TRUE
CD4 NC	*CEP63*	Wald ratio	1	-0.0062	0.0182	0.994 (0.959, 1.03)	0.735	0.88	1.09E-10	TRUE
CD4 NC	*CERS4*	Wald ratio	1	-0.0079	0.0132	0.992 (0.967, 1.018)	0.55	0.769	9.41E-18	TRUE
CD4 NC	*CES1*	Wald ratio	1	-0.0147	0.0140	0.985 (0.959, 1.013)	0.293	0.587	2.41E-16	TRUE
CD4 NC	*CFD*	Wald ratio	1	-0.0015	0.0073	0.998 (0.984, 1.013)	0.835	0.93	1.66E-58	TRUE
CD4 NC	*CFL2*	Wald ratio	1	0.0033	0.0118	1.003 (0.98, 1.027)	0.778	0.904	3.05E-21	TRUE
CD4 NC	*CFLAR*	Wald ratio	1	0.0240	0.0129	1.024 (0.999, 1.051)	0.063	0.256	1.14E-17	TRUE
CD4 NC	*CHAF1A*	Wald ratio	1	-0.0375	0.0201	0.963 (0.926, 1.002)	0.062	0.254	1.77E-09	TRUE
CD4 NC	*CHCHD10*	Wald ratio	1	0.0119	0.0173	1.012 (0.978, 1.047)	0.489	0.738	3.55E-14	TRUE
CD4 NC	*CHCHD2*	Wald ratio	1	0.0033	0.0077	1.003 (0.988, 1.018)	0.668	0.848	6.21E-55	TRUE
CD4 NC	*CHCHD5*	Wald ratio	1	0.0378	0.0176	1.039 (1.003, 1.075)	0.032	0.169	6.27E-10	TRUE
CD4 NC	*CHGB*	Wald ratio	1	-0.0046	0.0133	0.995 (0.97, 1.022)	0.73	0.88	6.95E-17	TRUE
CD4 NC	*CHI3L2*	Wald ratio	1	0.0016	0.0064	1.002 (0.989, 1.014)	0.807	0.917	8.77E-68	TRUE
CD4 NC	*CHMP2B*	Wald ratio	1	-0.0236	0.0166	0.977 (0.945, 1.009)	0.155	0.426	9.51E-11	TRUE
CD4 NC	*CHMP3*	Wald ratio	1	0.0162	0.0195	1.016 (0.978, 1.056)	0.405	0.681	1.56E-08	TRUE
CD4 NC	*CHMP4A*	Wald ratio	1	0.0257	0.0097	1.026 (1.007, 1.046)	0.008	0.068	1.62E-26	TRUE
CD4 NC	*CHN1*	Wald ratio	1	-0.0074	0.0100	0.993 (0.973, 1.012)	0.46	0.721	1.72E-28	TRUE
CD4 NC	*CHORDC1*	Wald ratio	1	-0.0021	0.0180	0.998 (0.963, 1.034)	0.905	0.96	5.88E-10	TRUE
CD4 NC	*CHPT1*	Wald ratio	1	0.0109	0.0058	1.011 (1, 1.022)	0.058	0.243	9.61E-91	TRUE
CD4 NC	*CHRNA1*	Wald ratio	1	0.0609	0.0183	1.063 (1.025, 1.102)	8.66E-04	0.015	5.95E-09	TRUE
CD4 NC	*CHURC1*	Wald ratio	1	0.0135	0.0070	1.014 (1, 1.028)	0.054	0.232	2.39E-50	TRUE
CD4 NC	*CIB1*	Wald ratio	1	-0.0276	0.0106	0.973 (0.953, 0.993)	0.009	0.077	1.70E-35	TRUE
CD4 NC	*CISD1*	Wald ratio	1	-0.0062	0.0181	0.994 (0.959, 1.03)	0.734	0.88	1.11E-08	TRUE
CD4 NC	*CKB*	Wald ratio	1	-0.0846	0.0201	0.919 (0.883, 0.956)	2.49E-05	0.001	4.43E-09	TRUE
CD4 NC	*CKMT2-AS1*	Wald ratio	1	-0.0165	0.0190	0.984 (0.948, 1.021)	0.385	0.668	1.00E-15	TRUE
CD4 NC	*CLEC11A*	Wald ratio	1	0.0069	0.0173	1.007 (0.973, 1.042)	0.69	0.86	7.03E-10	TRUE
CD4 NC	*CLEC2B*	Wald ratio	1	0.0037	0.0117	1.004 (0.981, 1.027)	0.755	0.888	1.70E-21	TRUE
CD4 NC	*CLHC1*	Wald ratio	1	-0.0779	0.0188	0.925 (0.892, 0.96)	3.38E-05	0.001	5.29E-14	TRUE
CD4 NC	*CLIC4*	Wald ratio	1	0.0117	0.0204	1.012 (0.972, 1.053)	0.567	0.782	7.99E-09	TRUE
CD4 NC	*CLIP3*	Wald ratio	1	-0.0108	0.0193	0.989 (0.953, 1.027)	0.575	0.787	5.83E-09	TRUE
CD4 NC	*CLK4*	Wald ratio	1	0.0213	0.0144	1.021 (0.993, 1.051)	0.14	0.405	2.61E-14	TRUE
CD4 NC	*CLN8*	Wald ratio	1	0.0743	0.0216	1.077 (1.033, 1.124)	5.76E-04	0.011	6.94E-08	TRUE
CD4 NC	*CLNS1A*	Wald ratio	1	0.0375	0.0095	1.038 (1.019, 1.058)	8.21E-05	0.003	8.53E-18	TRUE
CD4 NC	*CLUAP1*	Wald ratio	1	0.0557	0.0125	1.057 (1.032, 1.084)	8.55E-06	5.07E-04	1.64E-18	TRUE
CD4 NC	*CMTM3*	Wald ratio	1	0.0139	0.0138	1.014 (0.987, 1.042)	0.315	0.609	7.75E-16	TRUE
CD4 NC	*CMTM7*	Wald ratio	1	0.0182	0.0109	1.018 (0.997, 1.04)	0.096	0.329	1.65E-22	TRUE
CD4 NC	*CMTM8*	IVW	2	0.0037	0.0062	1.004 (0.992, 1.016)	0.551	0.77	5.64E-133	TRUE
CD4 NC	*CNIH4*	Wald ratio	1	0.0073	0.0181	1.007 (0.972, 1.044)	0.686	0.857	1.85E-09	TRUE
CD4 NC	*CNKSR1*	Wald ratio	1	0.0196	0.0112	1.02 (0.998, 1.042)	0.079	0.295	1.54E-26	TRUE
CD4 NC	*CNN2*	IVW	2	0.0134	0.0084	1.014 (0.997, 1.03)	0.111	0.355	3.01E-42	TRUE
CD4 NC	*CNOT11*	Wald ratio	1	0.0038	0.0174	1.004 (0.97, 1.039)	0.826	0.926	3.67E-10	TRUE
CD4 NC	*CNOT6*	Wald ratio	1	0.0080	0.0193	1.008 (0.971, 1.047)	0.678	0.853	8.29E-10	TRUE
CD4 NC	*CNOT6L*	Wald ratio	1	0.0193	0.0102	1.02 (0.999, 1.04)	0.059	0.244	1.46E-28	TRUE
CD4 NC	*COA1*	Wald ratio	1	0.0029	0.0102	1.003 (0.983, 1.023)	0.775	0.901	1.43E-31	TRUE
CD4 NC	*COA6*	Wald ratio	1	-0.0243	0.0166	0.976 (0.945, 1.008)	0.142	0.409	4.04E-11	TRUE
CD4 NC	*COG5*	Wald ratio	1	0.0372	0.0075	1.038 (1.023, 1.053)	6.43E-07	9.75E-05	6.66E-48	TRUE
CD4 NC	*COL18A1*	IVW	3	0.0082	0.0125	1.008 (0.984, 1.033)	0.515	0.751	2.26E-101	TRUE

CD4 NC	*COL18A1*	MR Egger	3	0.0201	0.0398	1.02 (0.944, 1.103)	0.702	NA	NA	NA
CD4 NC	*COL18A1*	Weighted median	3	0.0127	0.0064	1.013 (1, 1.026)	0.047	NA	NA	NA
CD4 NC	*COL18A1*	Simple mode	3	0.0101	0.0113	1.01 (0.988, 1.033)	0.466	NA	NA	NA
CD4 NC	*COL18A1*	Weighted mode	3	0.0139	0.0066	1.014 (1.001, 1.027)	0.17	NA	NA	NA
CD4 NC	*COL6A1*	Wald ratio	1	-0.0037	0.0164	0.996 (0.965, 1.029)	0.822	0.926	5.33E-12	TRUE
CD4 NC	*COL6A2*	Wald ratio	1	0.0118	0.0179	1.012 (0.977, 1.048)	0.509	0.747	3.50E-17	TRUE
CD4 NC	*COMMD5*	Wald ratio	1	-0.0030	0.0186	0.997 (0.961, 1.034)	0.87	0.946	1.33E-09	TRUE
CD4 NC	*COMMD6*	Wald ratio	1	0.0093	0.0057	1.009 (0.998, 1.021)	0.104	0.343	4.89E-93	TRUE
CD4 NC	*COMMD7*	Wald ratio	1	-0.0609	0.0156	0.941 (0.912, 0.97)	9.66E-05	0.003	1.21E-10	TRUE
CD4 NC	*COMP*	Wald ratio	1	-0.0200	0.0157	0.98 (0.95, 1.011)	0.204	0.491	8.47E-17	TRUE
CD4 NC	*COMTD1*	Wald ratio	1	-0.0015	0.0124	0.999 (0.975, 1.023)	0.904	0.959	2.01E-24	TRUE
CD4 NC	*COPG2*	Wald ratio	1	-0.0025	0.0172	0.998 (0.964, 1.032)	0.886	0.954	2.01E-08	TRUE
CD4 NC	*COQ6*	Wald ratio	1	0.0411	0.0169	1.042 (1.008, 1.077)	0.015	0.105	1.17E-11	TRUE
CD4 NC	*CORO1B*	Wald ratio	1	0.0008	0.0061	1.001 (0.989, 1.013)	0.898	0.957	1.64E-82	TRUE
CD4 NC	*COX11*	Wald ratio	1	0.0169	0.0202	1.017 (0.978, 1.058)	0.402	0.679	4.64E-08	TRUE
CD4 NC	*COX14*	Wald ratio	1	-0.0425	0.0139	0.958 (0.933, 0.985)	0.002	0.028	6.70E-17	TRUE
CD4 NC	*COX20*	Wald ratio	1	0.0059	0.0182	1.006 (0.971, 1.043)	0.746	0.883	5.36E-13	TRUE
CD4 NC	*COX6B1*	Wald ratio	1	-0.0188	0.0085	0.981 (0.965, 0.998)	0.027	0.155	1.45E-39	TRUE
CD4 NC	*COX7A2L*	Wald ratio	1	-0.0307	0.0096	0.97 (0.952, 0.988)	0.001	0.02	3.67E-32	TRUE
CD4 NC	*CPA5*	Wald ratio	1	-0.0042	0.0048	0.996 (0.986, 1.005)	0.378	0.665	1.89E-141	TRUE
CD4 NC	*CPNE1*	Wald ratio	1	-0.0033	0.0085	0.997 (0.98, 1.013)	0.7	0.866	1.84E-49	TRUE
CD4 NC	*CPNE3*	Wald ratio	1	-0.0519	0.0200	0.949 (0.913, 0.987)	0.01	0.079	4.04E-08	TRUE
CD4 NC	*CPSF1*	Wald ratio	1	0.0099	0.0132	1.01 (0.984, 1.036)	0.452	0.717	5.51E-17	TRUE
CD4 NC	*CRHR1*	Wald ratio	1	-0.0687	0.0235	0.934 (0.892, 0.978)	0.003	0.039	4.61E-16	TRUE
CD4 NC	*CRIP2*	Wald ratio	1	-0.0157	0.0115	0.984 (0.962, 1.007)	0.173	0.451	5.54E-25	TRUE
CD4 NC	*CRIPAK*	Wald ratio	1	-0.0392	0.0199	0.962 (0.925, 1)	0.049	0.219	1.04E-08	TRUE
CD4 NC	*CRLF3*	Wald ratio	1	0.0304	0.0207	1.031 (0.99, 1.074)	0.143	0.41	2.04E-10	TRUE
CD4 NC	*CROCC*	Wald ratio	1	0.0088	0.0161	1.009 (0.978, 1.041)	0.584	0.793	1.37E-12	TRUE
CD4 NC	*CRYBB2*	Wald ratio	1	0.0077	0.0061	1.008 (0.996, 1.02)	0.208	0.494	6.71E-79	TRUE
CD4 NC	*CRYGN*	Wald ratio	1	-0.0184	0.0083	0.982 (0.966, 0.998)	0.026	0.151	2.81E-39	TRUE
CD4 NC	*CRYL1*	Wald ratio	1	0.0272	0.0183	1.028 (0.991, 1.065)	0.137	0.399	2.92E-09	TRUE
CD4 NC	*CRYZ*	Wald ratio	1	-0.0095	0.0080	0.991 (0.975, 1.006)	0.231	0.524	2.35E-45	TRUE
CD4 NC	*CSGALNACT1*	IVW	2	0.0022	0.0109	1.002 (0.981, 1.024)	0.842	0.934	4.36E-31	TRUE
CD4 NC	*CSNK2A2*	Wald ratio	1	-0.0194	0.0128	0.981 (0.956, 1.006)	0.132	0.393	1.46E-12	TRUE
CD4 NC	*CSTB*	Wald ratio	1	0.0030	0.0086	1.003 (0.986, 1.02)	0.73	0.88	8.91E-36	TRUE
CD4 NC	*CTA-211A9.5*	Wald ratio	1	-0.0111	0.0200	0.989 (0.951, 1.028)	0.579	0.789	2.48E-08	TRUE
CD4 NC	*CTA-212D2.2*	Wald ratio	1	-0.0106	0.0092	0.989 (0.972, 1.008)	0.252	0.545	1.44E-38	TRUE
CD4 NC	*CTA-29F11.1*	Wald ratio	1	-0.0036	0.0057	0.996 (0.985, 1.008)	0.527	0.756	1.34E-95	TRUE
CD4 NC	*CTA-445C9.15*	Wald ratio	1	0.0249	0.0197	1.025 (0.986, 1.066)	0.207	0.494	2.61E-08	TRUE
CD4 NC	*CTB-102L5.4*	Wald ratio	1	-0.0095	0.0166	0.991 (0.959, 1.023)	0.567	0.782	1.57E-09	TRUE
CD4 NC	*CTB-179K24.3*	Wald ratio	1	-0.0193	0.0105	0.981 (0.961, 1.001)	0.068	0.267	1.67E-26	TRUE
CD4 NC	*CTBP1-AS2*	Wald ratio	1	-0.0445	0.0104	0.956 (0.937, 0.976)	1.93E-05	9.38E-04	9.11E-29	TRUE
CD4 NC	*CTC-241F20.3*	Wald ratio	1	-0.0221	0.0139	0.978 (0.952, 1.005)	0.113	0.359	1.29E-22	TRUE
CD4 NC	*CTC-281F24.1*	Wald ratio	1	0.0259	0.0147	1.026 (0.997, 1.056)	0.079	0.293	4.61E-13	TRUE
CD4 NC	*CTC-425F1.4*	Wald ratio	1	-0.0024	0.0171	0.998 (0.965, 1.032)	0.889	0.955	8.11E-12	TRUE
CD4 NC	*CTC-429P9.1*	Wald ratio	1	-0.0286	0.0119	0.972 (0.949, 0.995)	0.016	0.112	2.01E-20	TRUE
CD4 NC	*CTC-482H14.5*	Wald ratio	1	-0.0309	0.0125	0.97 (0.946, 0.994)	0.013	0.098	7.14E-19	TRUE
CD4 NC	*CTC-503J8.4*	Wald ratio	1	-0.0257	0.0231	0.975 (0.932, 1.02)	0.266	0.561	1.16E-08	TRUE
CD4 NC	*CTC-507E2.1*	Wald ratio	1	0.0211	0.0157	1.021 (0.99, 1.053)	0.18	0.46	5.68E-09	TRUE
CD4 NC	*CTC-508F8.1*	Wald ratio	1	-0.0189	0.0178	0.981 (0.948, 1.016)	0.289	0.582	2.92E-09	TRUE
CD4 NC	*CTC-524C5.2*	Wald ratio	1	0.0089	0.0050	1.009 (0.999, 1.019)	0.078	0.292	2.35E-95	TRUE
CD4 NC	*CTD-2007H13.3*	Wald ratio	1	-0.0118	0.0109	0.988 (0.967, 1.01)	0.278	0.571	6.04E-37	TRUE
CD4 NC	*CTD-2017D11.1*	Wald ratio	1	0.0135	0.0160	1.014 (0.982, 1.046)	0.399	0.678	1.91E-11	TRUE
CD4 NC	*CTD-2020K17.4*	Wald ratio	1	-0.0068	0.0061	0.993 (0.982, 1.005)	0.263	0.558	4.88E-62	TRUE
CD4 NC	*CTD-2034I4.2*	Wald ratio	1	-0.0071	0.0106	0.993 (0.972, 1.014)	0.507	0.745	2.68E-20	TRUE
CD4 NC	*CTD-2116N20.1*	Wald ratio	1	0.0152	0.0102	1.015 (0.995, 1.036)	0.135	0.396	1.16E-27	TRUE
CD4 NC	*CTD-2203K17.1*	Wald ratio	1	-0.0183	0.0117	0.982 (0.96, 1.005)	0.116	0.363	3.77E-23	TRUE
CD4 NC	*CTD-2308L22.1*	Wald ratio	1	0.0141	0.0183	1.014 (0.978, 1.051)	0.443	0.708	9.25E-13	TRUE
CD4 NC	*CTD-2324F15.2*	Wald ratio	1	-0.0042	0.0092	0.996 (0.978, 1.014)	0.646	0.834	9.76E-30	TRUE
CD4 NC	*CTD-2325M2.1*	Wald ratio	1	-0.0013	0.0137	0.999 (0.972, 1.026)	0.926	0.968	4.72E-16	TRUE
CD4 NC	*CTD-2336O2.1*	Wald ratio	1	0.0198	0.0069	1.02 (1.006, 1.034)	0.004	0.044	2.94E-65	TRUE
CD4 NC	*CTD-2357A8.3*	Wald ratio	1	0.0028	0.0123	1.003 (0.979, 1.027)	0.822	0.926	2.30E-20	TRUE
CD4 NC	*CTD-2368P22.1*	Wald ratio	1	0.0022	0.0065	1.002 (0.989, 1.015)	0.734	0.88	9.72E-76	TRUE
CD4 NC	*CTD-2371O3.2*	Wald ratio	1	0.0001	0.0056	1 (0.989, 1.011)	0.982	0.993	3.73E-106	TRUE
CD4 NC	*CTD-2515A14.1*	Wald ratio	1	-0.0262	0.0122	0.974 (0.951, 0.998)	0.032	0.168	1.79E-20	TRUE
CD4 NC	*CTD-2521M24.9*	Wald ratio	1	0.0208	0.0094	1.021 (1.002, 1.04)	0.027	0.153	2.87E-26	TRUE
CD4 NC	*CTD-2568A17.5*	Wald ratio	1	0.0032	0.0087	1.003 (0.986, 1.02)	0.71	0.871	9.00E-36	TRUE
CD4 NC	*CTD-2571L23.8*	Wald ratio	1	0.0176	0.0115	1.018 (0.995, 1.041)	0.127	0.383	4.95E-21	TRUE
CD4 NC	*CTD-2651B20.3*	Wald ratio	1	-0.0110	0.0119	0.989 (0.966, 1.012)	0.357	0.648	2.18E-20	TRUE
CD4 NC	*CTD-3037G24.4*	Wald ratio	1	0.0135	0.0093	1.014 (0.995, 1.032)	0.149	0.418	1.38E-30	TRUE
CD4 NC	*CTD-3093M3.1*	Wald ratio	1	0.0058	0.0152	1.006 (0.976, 1.036)	0.701	0.866	2.74E-14	TRUE
CD4 NC	*CTD-3110H11.1*	Wald ratio	1	0.0239	0.0122	1.024 (1, 1.049)	0.05	0.222	6.08E-25	TRUE
CD4 NC	*CTD-3138B18.5*	Wald ratio	1	0.0124	0.0146	1.012 (0.984, 1.042)	0.397	0.677	2.84E-14	TRUE
CD4 NC	*CTD-3162L10.1*	Wald ratio	1	0.0154	0.0100	1.015 (0.996, 1.036)	0.126	0.381	5.56E-30	TRUE
CD4 NC	*CTLA4*	Wald ratio	1	-0.0016	0.0061	0.998 (0.986, 1.01)	0.788	0.909	1.47E-89	TRUE
CD4 NC	*CTNNA1*	Wald ratio	1	-0.0075	0.0171	0.993 (0.96, 1.026)	0.659	0.843	1.49E-10	TRUE
CD4 NC	*CTNNAL1*	Wald ratio	1	-0.0088	0.0198	0.991 (0.954, 1.03)	0.655	0.839	1.66E-08	TRUE
CD4 NC	*CTSA*	Wald ratio	1	0.0430	0.0200	1.044 (1.004, 1.086)	0.032	0.168	1.09E-08	TRUE
CD4 NC	*CTSB*	Wald ratio	1	0.0063	0.0132	1.006 (0.981, 1.033)	0.63	0.824	2.05E-17	TRUE

CD4 NC	*CTSF*	Wald ratio	1	-0.0132	0.0116	0.987 (0.965, 1.01)	0.254	0.548	6.41E-24	TRUE
CD4 NC	*CTSH*	Wald ratio	1	0.0485	0.0181	1.05 (1.013, 1.088)	0.007	0.066	2.21E-09	TRUE
CD4 NC	*CTSL*	Wald ratio	1	0.0100	0.0065	1.01 (0.997, 1.023)	0.124	0.378	2.61E-70	TRUE
CD4 NC	*CTSS*	Wald ratio	1	0.0233	0.0139	1.024 (0.996, 1.052)	0.094	0.326	1.25E-19	TRUE
CD4 NC	*CTSW*	Wald ratio	1	-0.0237	0.0056	0.977 (0.966, 0.987)	2.34E-05	0.001	1.19E-113	TRUE
CD4 NC	*CUBN*	Wald ratio	1	0.0605	0.0149	1.062 (1.032, 1.094)	5.08E-05	0.002	1.07E-14	TRUE
CD4 NC	*CYLD*	Wald ratio	1	-0.0491	0.0201	0.952 (0.915, 0.99)	0.015	0.104	1.96E-08	TRUE
CD4 NC	*CYP2D6*	Wald ratio	1	-0.0692	0.0197	0.933 (0.898, 0.97)	4.40E-04	0.01	4.06E-08	TRUE
CD4 NC	*CYP4V2*	Wald ratio	1	0.0025	0.0092	1.003 (0.985, 1.021)	0.784	0.906	2.39E-32	TRUE
CD4 NC	*D2HGDH*	Wald ratio	1	-0.0282	0.0179	0.972 (0.939, 1.007)	0.115	0.362	1.23E-08	TRUE
CD4 NC	*DAD1*	Wald ratio	1	-0.0021	0.0109	0.998 (0.977, 1.019)	0.85	0.936	7.60E-25	TRUE
CD4 NC	*DAGLB*	Wald ratio	1	-0.0314	0.0141	0.969 (0.943, 0.996)	0.027	0.151	3.34E-16	TRUE
CD4 NC	*DAP*	Wald ratio	1	-0.0206	0.0164	0.98 (0.949, 1.012)	0.21	0.497	4.08E-11	TRUE
CD4 NC	*DAP3*	Wald ratio	1	0.0222	0.0125	1.022 (0.998, 1.048)	0.077	0.29	2.50E-18	TRUE
CD4 NC	*DARS*	Wald ratio	1	-0.0247	0.0117	0.976 (0.953, 0.998)	0.035	0.179	6.81E-19	TRUE
CD4 NC	*DAZAP1*	Wald ratio	1	0.0316	0.0120	1.032 (1.008, 1.057)	0.009	0.073	4.72E-21	TRUE
CD4 NC	*DAZAP2*	Wald ratio	1	-0.0449	0.0130	0.956 (0.932, 0.981)	5.23E-04	0.011	1.67E-16	TRUE
CD4 NC	*DBNDD1*	Wald ratio	1	0.0020	0.0098	1.002 (0.983, 1.021)	0.838	0.932	7.25E-25	TRUE
CD4 NC	*DCAF4*	Wald ratio	1	0.0116	0.0134	1.012 (0.985, 1.039)	0.389	0.67	3.62E-14	TRUE
CD4 NC	*DCBLD1*	Wald ratio	1	-0.0084	0.0211	0.992 (0.951, 1.034)	0.69	0.86	3.20E-08	TRUE
CD4 NC	*DCP2*	Wald ratio	1	0.0322	0.0149	1.033 (1.003, 1.063)	0.031	0.164	1.84E-13	TRUE
CD4 NC	*DCT*	Wald ratio	1	0.0149	0.0108	1.015 (0.994, 1.037)	0.166	0.442	3.15E-23	TRUE
CD4 NC	*DCTN5*	Wald ratio	1	0.0247	0.0174	1.025 (0.991, 1.061)	0.155	0.426	2.88E-09	TRUE
CD4 NC	*DCXR*	Wald ratio	1	-0.0056	0.0062	0.994 (0.982, 1.007)	0.365	0.653	8.59E-66	TRUE
CD4 NC	*DDHD2*	Wald ratio	1	0.0586	0.0209	1.06 (1.018, 1.105)	0.005	0.051	4.24E-09	TRUE
CD4 NC	*DDT*	Wald ratio	1	-0.0101	0.0082	0.99 (0.974, 1.006)	0.219	0.511	6.29E-44	TRUE
CD4 NC	*DDTL*	Wald ratio	1	0.0105	0.0096	1.011 (0.992, 1.03)	0.273	0.567	5.02E-32	TRUE
CD4 NC	*DDX39B-AS1*	IVW	2	0.0326	0.0195	1.033 (0.994, 1.073)	0.094	0.325	9.87E-37	TRUE
CD4 NC	*DDX42*	Wald ratio	1	-0.0339	0.0140	0.967 (0.94, 0.994)	0.015	0.106	8.91E-16	TRUE
CD4 NC	*DDX5*	Wald ratio	1	0.0392	0.0101	1.04 (1.02, 1.061)	1.06E-04	0.003	8.51E-26	TRUE
CD4 NC	*DDX55*	Wald ratio	1	-0.0186	0.0195	0.982 (0.945, 1.02)	0.339	0.632	7.74E-11	TRUE
CD4 NC	*DDX59*	Wald ratio	1	-0.0580	0.0184	0.944 (0.91, 0.978)	0.002	0.023	5.03E-09	TRUE
CD4 NC	*DDX6*	Wald ratio	1	-0.0257	0.0188	0.975 (0.939, 1.011)	0.172	0.45	1.54E-08	TRUE
CD4 NC	*DDX60L*	Wald ratio	1	-0.0141	0.0167	0.986 (0.954, 1.019)	0.399	0.678	3.48E-10	TRUE
CD4 NC	*DEAF1*	Wald ratio	1	-0.0193	0.0111	0.981 (0.96, 1.002)	0.083	0.302	8.71E-23	TRUE
CD4 NC	*DECR2*	Wald ratio	1	0.0564	0.0188	1.058 (1.02, 1.098)	0.003	0.033	4.22E-09	TRUE
CD4 NC	*DGCR6L*	Wald ratio	1	0.0059	0.0139	1.006 (0.979, 1.034)	0.67	0.848	1.53E-15	TRUE
CD4 NC	*DGKQ*	Wald ratio	1	-0.0119	0.0142	0.988 (0.961, 1.016)	0.4	0.679	3.14E-15	TRUE
CD4 NC	*DHFR*	Wald ratio	1	0.0058	0.0107	1.006 (0.985, 1.027)	0.587	0.794	5.96E-24	TRUE
CD4 NC	*DHRS4*	Wald ratio	1	0.0091	0.0143	1.009 (0.981, 1.038)	0.526	0.755	7.03E-38	TRUE
CD4 NC	*DHRS7*	Wald ratio	1	-0.0275	0.0204	0.973 (0.935, 1.013)	0.178	0.457	1.55E-09	TRUE
CD4 NC	*DHX32*	Wald ratio	1	0.0022	0.0135	1.002 (0.976, 1.029)	0.869	0.946	1.04E-16	TRUE
CD4 NC	*DHX58*	Wald ratio	1	0.0181	0.0154	1.018 (0.988, 1.049)	0.24	0.534	1.19E-12	TRUE
CD4 NC	*DIEXF*	Wald ratio	1	-0.0219	0.0108	0.978 (0.958, 0.999)	0.042	0.202	1.51E-24	TRUE
CD4 NC	*DIP2A*	Wald ratio	1	0.0146	0.0103	1.015 (0.994, 1.035)	0.156	0.427	1.06E-24	TRUE
CD4 NC	*DIS3L*	Wald ratio	1	0.0006	0.0127	1.001 (0.976, 1.026)	0.964	0.988	2.64E-20	TRUE
CD4 NC	*DISC1*	Wald ratio	1	-0.0118	0.0139	0.988 (0.962, 1.015)	0.394	0.673	2.45E-15	TRUE
CD4 NC	*DKK3*	Wald ratio	1	0.0166	0.0116	1.017 (0.994, 1.04)	0.152	0.423	9.30E-17	TRUE
CD4 NC	*DMAP1*	Wald ratio	1	0.0024	0.0205	1.002 (0.963, 1.044)	0.906	0.96	3.00E-08	TRUE
CD4 NC	*DNAH11*	Wald ratio	1	-0.0103	0.0132	0.99 (0.964, 1.016)	0.435	0.702	1.16E-15	TRUE
CD4 NC	*DNAJA1*	Wald ratio	1	-0.0452	0.0131	0.956 (0.932, 0.981)	5.53E-04	0.011	6.08E-18	TRUE
CD4 NC	*DNAJA3*	Wald ratio	1	0.0568	0.0185	1.058 (1.021, 1.098)	0.002	0.027	1.19E-09	TRUE
CD4 NC	*DNAJC15*	Wald ratio	1	0.0043	0.0049	1.004 (0.995, 1.014)	0.382	0.666	2.42E-122	TRUE
CD4 NC	*DNAJC16*	Wald ratio	1	0.0114	0.0110	1.011 (0.99, 1.033)	0.301	0.596	4.07E-23	TRUE
CD4 NC	*DNM1L*	Wald ratio	1	0.0555	0.0240	1.057 (1.009, 1.108)	0.021	0.13	6.63E-08	TRUE
CD4 NC	*DNMT1*	Wald ratio	1	0.0201	0.0153	1.02 (0.99, 1.051)	0.191	0.472	4.70E-13	TRUE
CD4 NC	*DNPH1*	Wald ratio	1	-0.0073	0.0233	0.993 (0.948, 1.039)	0.756	0.888	4.78E-08	TRUE
CD4 NC	*DOCK8*	IVW	2	0.0140	0.0102	1.014 (0.994, 1.035)	0.171	0.449	1.24E-34	TRUE
CD4 NC	*DPYSL4*	Wald ratio	1	-0.0079	0.0080	0.992 (0.977, 1.008)	0.323	0.618	1.30E-47	TRUE
CD4 NC	*DR1*	Wald ratio	1	-0.0248	0.0140	0.976 (0.949, 1.003)	0.076	0.289	1.50E-15	TRUE
CD4 NC	*DRAM2*	Wald ratio	1	-0.0030	0.0088	0.997 (0.98, 1.014)	0.734	0.88	1.39E-36	TRUE
CD4 NC	*DRAP1*	Wald ratio	1	-0.0073	0.0180	0.993 (0.958, 1.028)	0.686	0.857	3.05E-10	TRUE
CD4 NC	*DST*	Wald ratio	1	-0.0088	0.0090	0.991 (0.974, 1.009)	0.33	0.624	3.91E-32	TRUE
CD4 NC	*DSTN*	IVW	2	-0.0462	0.0121	0.955 (0.932, 0.978)	1.41E-04	0.004	8.25E-20	TRUE
CD4 NC	*DTNBP1*	Wald ratio	1	-0.0104	0.0261	0.99 (0.94, 1.042)	0.689	0.86	1.84E-08	TRUE
CD4 NC	*DUOX1*	Wald ratio	1	-0.0081	0.0128	0.992 (0.967, 1.017)	0.527	0.756	5.70E-17	TRUE
CD4 NC	*DUS3L*	Wald ratio	1	-0.0044	0.0120	0.996 (0.973, 1.019)	0.716	0.873	9.07E-18	TRUE
CD4 NC	*DUT*	Wald ratio	1	0.0107	0.0104	1.011 (0.99, 1.032)	0.302	0.598	4.93E-30	TRUE
CD4 NC	*DYNLL2*	Wald ratio	1	-0.0176	0.0081	0.983 (0.967, 0.998)	0.03	0.161	1.83E-48	TRUE
CD4 NC	*DYNLT1*	Wald ratio	1	-0.0115	0.0165	0.989 (0.957, 1.021)	0.486	0.737	2.88E-11	TRUE
CD4 NC	*DZIP3*	Wald ratio	1	0.0063	0.0124	1.006 (0.982, 1.031)	0.608	0.809	4.91E-15	TRUE
CD4 NC	*EBPL*	IVW	2	0.0078	0.0127	1.008 (0.983, 1.033)	0.539	0.762	2.29E-108	TRUE
CD4 NC	*EEA1*	Wald ratio	1	-0.0106	0.0086	0.989 (0.973, 1.006)	0.215	0.505	2.11E-37	TRUE
CD4 NC	*EEF1D*	IVW	2	0.0038	0.0207	1.004 (0.964, 1.045)	0.854	0.938	2.96E-22	TRUE
CD4 NC	*EEF2*	Wald ratio	1	0.0837	0.0188	1.087 (1.048, 1.128)	8.31E-06	4.97E-04	7.90E-09	TRUE
CD4 NC	*EFCAB13*	Wald ratio	1	-0.0236	0.0184	0.977 (0.942, 1.013)	0.201	0.484	4.42E-14	TRUE
CD4 NC	*EFHB*	Wald ratio	1	0.0055	0.0172	1.005 (0.972, 1.04)	0.751	0.886	3.93E-11	TRUE
CD4 NC	*EGFL8*	Wald ratio	1	0.0886	0.0169	1.093 (1.057, 1.129)	1.53E-07	6.60E-05	1.49E-10	TRUE
CD4 NC	*EGLN3*	Wald ratio	1	0.0060	0.0184	1.006 (0.97, 1.043)	0.746	0.883	2.04E-08	TRUE

CD4 NC	*EI24*	Wald ratio	1	0.0184	0.0103	1.019 (0.998, 1.039)	0.075	0.287	1.26E-29	TRUE
CD4 NC	*EIF2A*	Wald ratio	1	-0.0092	0.0066	0.991 (0.978, 1.004)	0.163	0.437	1.08E-61	TRUE
CD4 NC	*EIF2B2*	Wald ratio	1	0.0289	0.0175	1.029 (0.995, 1.065)	0.098	0.333	2.30E-10	TRUE
CD4 NC	*EIF3K*	Wald ratio	1	0.0109	0.0172	1.011 (0.977, 1.046)	0.525	0.755	6.36E-14	TRUE
CD4 NC	*EIF4E3*	Wald ratio	1	-0.0098	0.0200	0.99 (0.952, 1.03)	0.624	0.822	3.39E-09	TRUE
CD4 NC	*EIF5A*	Wald ratio	1	-0.0068	0.0045	0.993 (0.984, 1.002)	0.134	0.394	2.94E-191	TRUE
CD4 NC	*ELMO1*	Wald ratio	1	-0.0304	0.0156	0.97 (0.941, 1)	0.051	0.224	1.39E-11	TRUE
CD4 NC	*ELMSAN1*	Wald ratio	1	0.0114	0.0141	1.011 (0.984, 1.04)	0.418	0.689	1.84E-15	TRUE
CD4 NC	*ELOVL5*	Wald ratio	1	-0.0126	0.0191	0.988 (0.951, 1.025)	0.511	0.748	2.84E-10	TRUE
CD4 NC	*ELP2*	Wald ratio	1	0.0203	0.0179	1.021 (0.985, 1.057)	0.257	0.552	4.30E-10	TRUE
CD4 NC	*EMB*	Wald ratio	1	0.0071	0.0169	1.007 (0.974, 1.041)	0.676	0.853	1.04E-15	TRUE
CD4 NC	*EMC2*	Wald ratio	1	-0.0016	0.0176	0.998 (0.965, 1.033)	0.927	0.969	7.26E-11	TRUE
CD4 NC	*EML4*	Wald ratio	1	-0.0380	0.0137	0.963 (0.937, 0.989)	0.005	0.053	9.32E-16	TRUE
CD4 NC	*EMP3*	Wald ratio	1	-0.0014	0.0170	0.999 (0.966, 1.032)	0.933	0.972	5.48E-12	TRUE
CD4 NC	*EMR1*	Wald ratio	1	-0.0018	0.0071	0.998 (0.984, 1.012)	0.804	0.915	2.63E-60	TRUE
CD4 NC	*ENC1*	Wald ratio	1	-0.0006	0.0197	0.999 (0.961, 1.039)	0.977	0.991	4.22E-11	TRUE
CD4 NC	*ENDOD1*	Wald ratio	1	-0.0267	0.0125	0.974 (0.95, 0.998)	0.033	0.173	2.73E-18	TRUE
CD4 NC	*ENDOG*	Wald ratio	1	0.0283	0.0157	1.029 (0.998, 1.061)	0.072	0.278	3.10E-14	TRUE
CD4 NC	*ENO1*	Wald ratio	1	-0.0205	0.0177	0.98 (0.946, 1.014)	0.247	0.54	3.67E-10	TRUE
CD4 NC	*ENOSF1*	Wald ratio	1	0.0153	0.0094	1.015 (0.997, 1.034)	0.103	0.34	1.28E-32	TRUE
CD4 NC	*ENTPD3-AS1*	Wald ratio	1	-0.0492	0.0156	0.952 (0.923, 0.982)	0.002	0.023	1.77E-11	TRUE
CD4 NC	*ENTPD4*	Wald ratio	1	-0.0169	0.0179	0.983 (0.949, 1.018)	0.345	0.638	4.82E-10	TRUE
CD4 NC	*EP400NL*	Wald ratio	1	0.0229	0.0164	1.023 (0.991, 1.057)	0.163	0.436	4.62E-12	TRUE
CD4 NC	*EPB41L3*	Wald ratio	1	0.0064	0.0131	1.006 (0.981, 1.033)	0.627	0.822	2.48E-17	TRUE
CD4 NC	*EPC1*	Wald ratio	1	-0.0718	0.0193	0.931 (0.896, 0.967)	2.07E-04	0.005	6.66E-09	TRUE
CD4 NC	*EPHA1-AS1*	IVW	2	0.0081	0.0213	1.008 (0.967, 1.051)	0.702	0.866	2.51E-49	TRUE
CD4 NC	*EPHB6*	Wald ratio	1	0.0134	0.0138	1.013 (0.986, 1.041)	0.332	0.626	5.60E-16	TRUE
CD4 NC	*EPHX2*	Wald ratio	1	-0.0172	0.0152	0.983 (0.954, 1.013)	0.259	0.553	3.05E-15	TRUE
CD4 NC	*EPPK1*	Wald ratio	1	-0.0043	0.0178	0.996 (0.962, 1.031)	0.811	0.919	1.81E-09	TRUE
CD4 NC	*EPS8L2*	Wald ratio	1	0.0137	0.0103	1.014 (0.994, 1.034)	0.183	0.462	7.12E-28	TRUE
CD4 NC	*EPSTI1*	Wald ratio	1	0.0047	0.0076	1.005 (0.99, 1.02)	0.54	0.762	1.77E-54	TRUE
CD4 NC	*ERAP1*	Wald ratio	1	-0.0439	0.0175	0.957 (0.925, 0.99)	0.012	0.093	3.05E-09	TRUE
CD4 NC	*ERAP2*	Wald ratio	1	0.0212	0.0042	1.021 (1.013, 1.03)	5.57E-07	9.75E-05	2.80E-197	TRUE
CD4 NC	*ERCC6L2*	Wald ratio	1	0.0015	0.0190	1.002 (0.965, 1.04)	0.936	0.974	4.19E-09	TRUE
CD4 NC	*ERGIC2*	Wald ratio	1	-0.0043	0.0143	0.996 (0.968, 1.024)	0.762	0.893	1.96E-14	TRUE
CD4 NC	*ERICH1*	Wald ratio	1	-0.0024	0.0073	0.998 (0.983, 1.012)	0.74	0.881	4.49E-57	TRUE
CD4 NC	*ERO1L*	Wald ratio	1	-0.0075	0.0142	0.993 (0.965, 1.021)	0.6	0.804	4.18E-12	TRUE
CD4 NC	*ERP27*	Wald ratio	1	-0.0031	0.0204	0.997 (0.958, 1.038)	0.878	0.95	1.21E-08	TRUE
CD4 NC	*ERP29*	Wald ratio	1	0.0745	0.0137	1.077 (1.049, 1.107)	5.95E-08	6.60E-05	9.08E-13	TRUE
CD4 NC	*ESD*	Wald ratio	1	0.0032	0.0194	1.003 (0.966, 1.042)	0.868	0.945	7.71E-09	TRUE
CD4 NC	*ESR2*	IVW	2	0.0052	0.0271	1.005 (0.953, 1.06)	0.847	0.935	1.50E-66	TRUE
CD4 NC	*ESYT2*	Wald ratio	1	0.0279	0.0216	1.028 (0.986, 1.073)	0.196	0.478	3.72E-08	TRUE
CD4 NC	*ETFDH*	Wald ratio	1	0.0544	0.0244	1.056 (1.007, 1.108)	0.025	0.147	2.75E-09	TRUE
CD4 NC	*ETS1*	Wald ratio	1	0.0061	0.0182	1.006 (0.971, 1.043)	0.737	0.88	7.76E-09	TRUE
CD4 NC	*ETV7*	Wald ratio	1	-0.0157	0.0108	0.984 (0.964, 1.005)	0.144	0.411	5.52E-25	TRUE
CD4 NC	*EVI2A*	Wald ratio	1	-0.0123	0.0083	0.988 (0.972, 1.004)	0.136	0.399	1.54E-40	TRUE
CD4 NC	*EXOC3*	Wald ratio	1	0.0054	0.0065	1.005 (0.993, 1.018)	0.406	0.682	2.20E-73	TRUE
CD4 NC	*EXOSC7*	Wald ratio	1	-0.0309	0.0181	0.97 (0.936, 1.005)	0.088	0.314	1.18E-09	TRUE
CD4 NC	*EYA3*	Wald ratio	1	0.0323	0.0154	1.033 (1.002, 1.064)	0.036	0.18	4.10E-13	TRUE
CD4 NC	*EZR*	Wald ratio	1	-0.0354	0.0178	0.965 (0.932, 1)	0.047	0.214	6.96E-10	TRUE
CD4 NC	*F5*	Wald ratio	1	-0.0134	0.0127	0.987 (0.962, 1.012)	0.291	0.583	7.90E-17	TRUE
CD4 NC	*FAAH*	Wald ratio	1	-0.0093	0.0170	0.991 (0.958, 1.024)	0.585	0.794	8.12E-11	TRUE
CD4 NC	*FADS1*	Wald ratio	1	0.0165	0.0115	1.017 (0.994, 1.04)	0.153	0.423	2.05E-34	TRUE
CD4 NC	*FADS2*	IVW	2	0.0129	0.0075	1.013 (0.998, 1.028)	0.083	0.304	2.40E-86	TRUE
CD4 NC	*FAHD1*	Wald ratio	1	0.0375	0.0134	1.038 (1.011, 1.066)	0.005	0.051	1.70E-11	TRUE
CD4 NC	*FAIM*	Wald ratio	1	-0.0565	0.0215	0.945 (0.906, 0.986)	0.009	0.073	5.59E-08	TRUE
CD4 NC	*FAM101B*	Wald ratio	1	0.0071	0.0160	1.007 (0.976, 1.039)	0.659	0.842	5.31E-16	TRUE
CD4 NC	*FAM105A*	Wald ratio	1	0.0243	0.0165	1.025 (0.992, 1.058)	0.142	0.41	2.81E-13	TRUE
CD4 NC	*FAM117B*	Wald ratio	1	-0.0204	0.0135	0.98 (0.954, 1.006)	0.132	0.393	2.62E-16	TRUE
CD4 NC	*FAM118A*	Wald ratio	1	0.0006	0.0042	1.001 (0.992, 1.009)	0.895	0.957	7.16E-131	TRUE
CD4 NC	*FAM134A*	Wald ratio	1	-0.0606	0.0185	0.941 (0.908, 0.976)	0.001	0.018	4.52E-09	TRUE
CD4 NC	*FAM134B*	Wald ratio	1	0.0128	0.0100	1.013 (0.993, 1.033)	0.198	0.481	1.93E-29	TRUE
CD4 NC	*FAM13A*	Wald ratio	1	-0.0175	0.0081	0.983 (0.967, 0.998)	0.031	0.167	3.81E-33	TRUE
CD4 NC	*FAM153A*	Wald ratio	1	0.0233	0.0132	1.024 (0.997, 1.05)	0.077	0.291	1.51E-17	TRUE
CD4 NC	*FAM153B*	Wald ratio	1	0.0163	0.0057	1.016 (1.005, 1.028)	0.005	0.047	3.26E-92	TRUE
CD4 NC	*FAM153C*	Wald ratio	1	0.0000	0.0161	1 (0.969, 1.032)	0.998	0.999	4.59E-12	TRUE
CD4 NC	*FAM154B*	Wald ratio	1	-0.0058	0.0094	0.994 (0.976, 1.013)	0.537	0.762	8.62E-29	TRUE
CD4 NC	*FAM184B*	Wald ratio	1	0.0011	0.0047	1.001 (0.992, 1.01)	0.814	0.921	2.46E-163	TRUE
CD4 NC	*FAM19A1*	Wald ratio	1	0.0066	0.0099	1.007 (0.987, 1.026)	0.506	0.745	5.97E-30	TRUE
CD4 NC	*FAM207A*	Wald ratio	1	-0.0099	0.0151	0.99 (0.961, 1.02)	0.513	0.749	1.60E-13	TRUE
CD4 NC	*FAM211A*	Wald ratio	1	-0.0330	0.0194	0.968 (0.931, 1.005)	0.089	0.315	1.28E-12	TRUE
CD4 NC	*FAM21A*	Wald ratio	1	-0.0335	0.0116	0.967 (0.945, 0.989)	0.004	0.041	2.69E-23	TRUE
CD4 NC	*FAM26F*	IVW	2	-0.0024	0.0108	0.998 (0.977, 1.019)	0.824	0.926	9.06E-60	TRUE
CD4 NC	*FAM43A*	Wald ratio	1	-0.0102	0.0094	0.99 (0.972, 1.008)	0.279	0.572	1.68E-36	TRUE
CD4 NC	*FAM45A*	Wald ratio	1	-0.0030	0.0143	0.997 (0.969, 1.025)	0.834	0.93	1.04E-14	TRUE
CD4 NC	*FAM63B*	IVW	2	-0.0151	0.0375	0.985 (0.915, 1.06)	0.687	0.858	2.06E-27	TRUE
CD4 NC	*FAM86C1*	Wald ratio	1	0.0486	0.0204	1.05 (1.009, 1.093)	0.017	0.115	2.68E-08	TRUE
CD4 NC	*FAM92A1*	Wald ratio	1	0.0580	0.0190	1.06 (1.021, 1.1)	0.002	0.028	8.58E-09	TRUE
CD4 NC	*FAM98B*	Wald ratio	1	0.0030	0.0158	1.003 (0.972, 1.034)	0.851	0.936	3.85E-14	TRUE

CD4 NC	*FANCA*	Wald ratio	1	-0.0689	0.0179	0.933 (0.901, 0.967)	1.14E-04	0.003	2.87E-11	TRUE
CD4 NC	*FANCF*	Wald ratio	1	-0.0077	0.0161	0.992 (0.962, 1.024)	0.631	0.824	8.44E-10	TRUE
CD4 NC	*FANK1*	Wald ratio	1	-0.0042	0.0181	0.996 (0.961, 1.032)	0.815	0.921	3.26E-10	TRUE
CD4 NC	*FAR1*	Wald ratio	1	-0.0031	0.0074	0.997 (0.983, 1.011)	0.676	0.853	7.95E-58	TRUE
CD4 NC	*FAU*	Wald ratio	1	-0.0012	0.0093	0.999 (0.981, 1.017)	0.901	0.958	2.03E-33	TRUE
CD4 NC	*FBLN5*	Wald ratio	1	0.0016	0.0058	1.002 (0.99, 1.013)	0.779	0.904	9.74E-89	TRUE
CD4 NC	*FBXO10*	Wald ratio	1	0.0346	0.0172	1.035 (1.001, 1.071)	0.045	0.208	3.17E-08	TRUE
CD4 NC	*FBXO2*	Wald ratio	1	-0.0326	0.0134	0.968 (0.943, 0.994)	0.015	0.104	2.52E-17	TRUE
CD4 NC	*FBXO25*	Wald ratio	1	0.0281	0.0218	1.028 (0.985, 1.073)	0.199	0.482	7.05E-08	TRUE
CD4 NC	*FBXO3*	Wald ratio	1	0.0187	0.0199	1.019 (0.98, 1.059)	0.347	0.64	6.15E-09	TRUE
CD4 NC	*FBXO7*	Wald ratio	1	-0.0059	0.0134	0.994 (0.968, 1.021)	0.661	0.844	6.64E-15	TRUE
CD4 NC	*FCGRT*	Wald ratio	1	-0.0460	0.0278	0.955 (0.904, 1.009)	0.099	0.333	4.09E-08	TRUE
CD4 NC	*FDFT1*	Wald ratio	1	0.0214	0.0067	1.022 (1.008, 1.035)	0.001	0.02	5.05E-82	TRUE
CD4 NC	*FGD5-AS1*	Wald ratio	1	0.0653	0.0381	1.067 (0.991, 1.15)	0.087	0.312	2.70E-10	TRUE
CD4 NC	*FGL2*	Wald ratio	1	-0.0079	0.0082	0.992 (0.976, 1.008)	0.335	0.628	1.86E-36	TRUE
CD4 NC	*FHIT*	Wald ratio	1	-0.0297	0.0109	0.971 (0.95, 0.992)	0.007	0.06	8.29E-23	TRUE
CD4 NC	*FIGNL1*	Wald ratio	1	0.0050	0.0087	1.005 (0.988, 1.022)	0.562	0.779	6.12E-40	TRUE
CD4 NC	*FIS1*	Wald ratio	1	0.0045	0.0098	1.004 (0.985, 1.024)	0.649	0.835	5.21E-32	TRUE
CD4 NC	*FKBP11*	Wald ratio	1	0.0120	0.0087	1.012 (0.995, 1.029)	0.166	0.441	1.39E-38	TRUE
CD4 NC	*FKRP*	Wald ratio	1	-0.0025	0.0118	0.998 (0.975, 1.021)	0.835	0.93	6.33E-24	TRUE
CD4 NC	*FLVCR1*	Wald ratio	1	0.0112	0.0196	1.011 (0.973, 1.051)	0.568	0.782	4.31E-08	TRUE
CD4 NC	*FLVCR1-AS1*	Wald ratio	1	-0.0079	0.0071	0.992 (0.978, 1.006)	0.264	0.558	4.57E-60	TRUE
CD4 NC	*FMN1*	Wald ratio	1	-0.0050	0.0040	0.995 (0.987, 1.003)	0.217	0.508	1.17E-179	TRUE
CD4 NC	*FN3KRP*	Wald ratio	1	0.0145	0.0078	1.015 (0.999, 1.03)	0.063	0.255	1.86E-50	TRUE
CD4 NC	*FNBP4*	Wald ratio	1	-0.0433	0.0093	0.958 (0.94, 0.975)	2.84E-06	2.39E-04	6.92E-36	TRUE
CD4 NC	*FNIP2*	Wald ratio	1	-0.0024	0.0157	0.998 (0.967, 1.029)	0.876	0.949	5.34E-10	TRUE
CD4 NC	*FNTB*	Wald ratio	1	0.0281	0.0208	1.029 (0.988, 1.071)	0.176	0.456	2.71E-08	TRUE
CD4 NC	*FOXN2*	Wald ratio	1	0.0622	0.0144	1.064 (1.035, 1.095)	1.56E-05	7.86E-04	6.43E-20	TRUE
CD4 NC	*FOXRED1*	Wald ratio	1	-0.0271	0.0194	0.973 (0.937, 1.011)	0.163	0.437	4.22E-09	TRUE
CD4 NC	*FRA10AC1*	Wald ratio	1	-0.0472	0.0184	0.954 (0.92, 0.989)	0.01	0.083	5.67E-09	TRUE
CD4 NC	*FRG1B*	Wald ratio	1	-0.0585	0.0153	0.943 (0.915, 0.972)	1.29E-04	0.004	8.65E-13	TRUE
CD4 NC	*FRMD4B*	IVW	2	-0.0022	0.0118	0.998 (0.975, 1.021)	0.855	0.938	2.23E-22	TRUE
CD4 NC	*FRY-AS1*	Wald ratio	1	0.0058	0.0152	1.006 (0.976, 1.036)	0.701	0.866	1.41E-13	TRUE
CD4 NC	*FSBP*	Wald ratio	1	0.0022	0.0118	1.002 (0.979, 1.026)	0.853	0.938	1.16E-20	TRUE
CD4 NC	*FTCDNL1*	Wald ratio	1	-0.0006	0.0092	0.999 (0.982, 1.017)	0.945	0.979	3.82E-44	TRUE
CD4 NC	*FTSJ3*	Wald ratio	1	-0.0348	0.0142	0.966 (0.939, 0.993)	0.015	0.104	3.37E-15	TRUE
CD4 NC	*FUBP1*	Wald ratio	1	0.0258	0.0139	1.026 (0.999, 1.054)	0.062	0.255	7.31E-15	TRUE
CD4 NC	*FUT10*	Wald ratio	1	0.0021	0.0116	1.002 (0.98, 1.025)	0.853	0.938	4.76E-22	TRUE
CD4 NC	*FUT8*	Wald ratio	1	-0.0289	0.0158	0.972 (0.942, 1.002)	0.068	0.266	1.18E-11	TRUE
CD4 NC	*FXYD2*	Wald ratio	1	0.0139	0.0149	1.014 (0.985, 1.044)	0.351	0.643	1.29E-12	TRUE
CD4 NC	*FYB*	Wald ratio	1	-0.0114	0.0157	0.989 (0.959, 1.02)	0.467	0.725	3.74E-12	TRUE
CD4 NC	*G3BP1*	Wald ratio	1	0.0001	0.0180	1 (0.965, 1.036)	0.994	0.998	6.04E-09	TRUE
CD4 NC	*G3BP2*	Wald ratio	1	0.0005	0.0121	1 (0.977, 1.024)	0.967	0.989	1.18E-20	TRUE
CD4 NC	*GAA*	Wald ratio	1	-0.0084	0.0200	0.992 (0.954, 1.031)	0.675	0.852	1.41E-09	TRUE
CD4 NC	*GADD45G*	Wald ratio	1	0.0253	0.0197	1.026 (0.987, 1.066)	0.199	0.482	2.14E-08	TRUE
CD4 NC	*GALNT10*	Wald ratio	1	-0.0310	0.0182	0.969 (0.936, 1.005)	0.088	0.313	6.60E-10	TRUE
CD4 NC	*GALNT2*	Wald ratio	1	-0.0117	0.0123	0.988 (0.965, 1.012)	0.339	0.632	1.07E-19	TRUE
CD4 NC	*GALT*	Wald ratio	1	0.0511	0.0103	1.052 (1.031, 1.074)	7.32E-07	9.75E-05	1.52E-28	TRUE
CD4 NC	*GATC*	Wald ratio	1	0.0163	0.0126	1.016 (0.992, 1.042)	0.195	0.477	3.08E-19	TRUE
CD4 NC	*GBA*	Wald ratio	1	0.0348	0.0195	1.035 (0.996, 1.076)	0.075	0.288	1.75E-08	TRUE
CD4 NC	*GBP3*	Wald ratio	1	0.0186	0.0172	1.019 (0.985, 1.054)	0.279	0.572	1.34E-10	TRUE
CD4 NC	*GBP5*	Wald ratio	1	-0.0203	0.0079	0.98 (0.965, 0.995)	0.011	0.085	8.45E-46	TRUE
CD4 NC	*GBP7*	Wald ratio	1	0.0003	0.0050	1 (0.991, 1.01)	0.946	0.979	2.62E-126	TRUE
CD4 NC	*GCAT*	Wald ratio	1	0.0210	0.0103	1.021 (1.001, 1.042)	0.041	0.199	1.33E-26	TRUE
CD4 NC	*GCC2*	Wald ratio	1	0.0059	0.0072	1.006 (0.992, 1.02)	0.414	0.686	4.35E-63	TRUE
CD4 NC	*GDE1*	Wald ratio	1	0.0001	0.0192	1 (0.963, 1.038)	0.995	0.999	5.17E-11	TRUE
CD4 NC	*GDI2*	Wald ratio	1	-0.0045	0.0076	0.996 (0.981, 1.01)	0.555	0.774	1.01E-53	TRUE
CD4 NC	*GDPD5*	Wald ratio	1	-0.0011	0.0084	0.999 (0.983, 1.015)	0.899	0.958	6.30E-32	TRUE
CD4 NC	*GFPT1*	Wald ratio	1	0.0436	0.0196	1.045 (1.005, 1.086)	0.027	0.151	1.59E-08	TRUE
CD4 NC	*GGH*	Wald ratio	1	0.0276	0.0224	1.028 (0.984, 1.074)	0.217	0.508	3.23E-08	TRUE
CD4 NC	*GGT1*	Wald ratio	1	-0.0194	0.0205	0.981 (0.942, 1.021)	0.344	0.637	5.12E-08	TRUE
CD4 NC	*GHITM*	Wald ratio	1	0.0068	0.0204	1.007 (0.967, 1.048)	0.739	0.881	5.80E-10	TRUE
CD4 NC	*GHRL*	Wald ratio	1	0.0215	0.0203	1.022 (0.982, 1.063)	0.291	0.584	4.64E-08	TRUE
CD4 NC	*GIMAP2*	Wald ratio	1	-0.0003	0.0112	1 (0.978, 1.022)	0.98	0.993	2.31E-23	TRUE
CD4 NC	*GIMAP4*	Wald ratio	1	0.0113	0.0176	1.011 (0.977, 1.047)	0.519	0.753	8.86E-12	TRUE
CD4 NC	*GINM1*	Wald ratio	1	-0.0221	0.0170	0.978 (0.946, 1.011)	0.194	0.475	1.75E-10	TRUE
CD4 NC	*GINS2*	Wald ratio	1	-0.0090	0.0156	0.991 (0.961, 1.022)	0.562	0.779	1.27E-13	TRUE
CD4 NC	*GIPC1*	Wald ratio	1	-0.0422	0.0154	0.959 (0.93, 0.988)	0.006	0.057	1.46E-13	TRUE
CD4 NC	*GJB6*	Wald ratio	1	-0.0025	0.0111	0.997 (0.976, 1.019)	0.822	0.926	1.86E-21	TRUE
CD4 NC	*GLI4*	Wald ratio	1	-0.0030	0.0195	0.997 (0.96, 1.036)	0.878	0.95	4.55E-09	TRUE
CD4 NC	*GLIPR1*	Wald ratio	1	0.0090	0.0144	1.009 (0.981, 1.038)	0.531	0.758	5.03E-16	TRUE
CD4 NC	*GLIPR1L2*	Wald ratio	1	0.0141	0.0097	1.014 (0.995, 1.034)	0.143	0.411	1.09E-31	TRUE
CD4 NC	*GLTP*	Wald ratio	1	-0.0105	0.0088	0.99 (0.973, 1.007)	0.235	0.529	6.51E-38	TRUE
CD4 NC	*GLTPD1*	Wald ratio	1	-0.0399	0.0152	0.961 (0.933, 0.99)	0.009	0.075	5.44E-08	TRUE
CD4 NC	*GLTSCR2*	Wald ratio	1	-0.0059	0.0096	0.994 (0.976, 1.013)	0.537	0.762	1.01E-29	TRUE
CD4 NC	*GLYCTK*	Wald ratio	1	-0.0442	0.0176	0.957 (0.924, 0.99)	0.012	0.094	4.72E-10	TRUE
CD4 NC	*GM2A*	Wald ratio	1	-0.0132	0.0243	0.987 (0.941, 1.035)	0.587	0.794	5.25E-10	TRUE
CD4 NC	*GMPR*	Wald ratio	1	-0.0265	0.0219	0.974 (0.933, 1.017)	0.227	0.52	5.34E-09	TRUE
CD4 NC	*GNAI1*	Wald ratio	1	0.0131	0.0208	1.013 (0.973, 1.055)	0.529	0.757	3.50E-08	TRUE

CD4 NC	*GNB5*	Wald ratio	1	-0.0036	0.0150	0.996 (0.968, 1.026)	0.808	0.918	3.57E-15	TRUE
CD4 NC	*GNG5*	Wald ratio	1	0.0071	0.0206	1.007 (0.967, 1.049)	0.731	0.88	2.02E-11	TRUE
CD4 NC	*GNG8*	Wald ratio	1	0.0014	0.0078	1.001 (0.986, 1.017)	0.857	0.939	5.29E-51	TRUE
CD4 NC	*GNMT*	Wald ratio	1	-0.0113	0.0170	0.989 (0.956, 1.022)	0.507	0.745	2.91E-11	TRUE
CD4 NC	*GNPTAB*	Wald ratio	1	-0.0369	0.0148	0.964 (0.936, 0.992)	0.013	0.094	3.88E-14	TRUE
CD4 NC	*GNPTG*	Wald ratio	1	-0.0091	0.0072	0.991 (0.977, 1.005)	0.208	0.494	1.35E-44	TRUE
CD4 NC	*GOLT1B*	Wald ratio	1	0.0462	0.0199	1.047 (1.007, 1.089)	0.02	0.126	3.69E-11	TRUE
CD4 NC	*GOSR2*	Wald ratio	1	0.0633	0.0127	1.065 (1.039, 1.092)	6.19E-07	9.75E-05	8.76E-18	TRUE
CD4 NC	*GPCPD1*	Wald ratio	1	0.0163	0.0186	1.016 (0.98, 1.054)	0.383	0.667	1.90E-09	TRUE
CD4 NC	*GPR155*	Wald ratio	1	0.0240	0.0170	1.024 (0.991, 1.059)	0.158	0.429	5.69E-11	TRUE
CD4 NC	*GPR160*	Wald ratio	1	0.0224	0.0072	1.023 (1.008, 1.037)	0.002	0.026	7.91E-53	TRUE
CD4 NC	*GPR18*	Wald ratio	1	0.0228	0.0163	1.023 (0.991, 1.056)	0.162	0.436	4.03E-11	TRUE
CD4 NC	*GPR180*	Wald ratio	1	-0.0017	0.0140	0.998 (0.971, 1.026)	0.903	0.959	4.01E-16	TRUE
CD4 NC	*GPR63*	Wald ratio	1	-0.0197	0.0194	0.98 (0.944, 1.019)	0.31	0.604	5.38E-08	TRUE
CD4 NC	*GPX4*	IVW	2	-0.0103	0.0093	0.99 (0.972, 1.008)	0.27	0.563	2.50E-38	TRUE
CD4 NC	*GPX7*	Wald ratio	1	0.0005	0.0171	1.001 (0.968, 1.035)	0.975	0.99	4.68E-11	TRUE
CD4 NC	*GRAMD1A*	IVW	2	-0.0154	0.0239	0.985 (0.94, 1.032)	0.517	0.752	4.99E-25	TRUE
CD4 NC	*GREM2*	Wald ratio	1	-0.0090	0.0171	0.991 (0.958, 1.025)	0.598	0.803	8.37E-11	TRUE
CD4 NC	*GRHPR*	Wald ratio	1	-0.0067	0.0056	0.993 (0.982, 1.004)	0.227	0.52	5.65E-90	TRUE
CD4 NC	*GRIN2C*	Wald ratio	1	0.0275	0.0141	1.028 (1, 1.057)	0.051	0.225	1.05E-14	TRUE
CD4 NC	*GRK5*	Wald ratio	1	0.0302	0.0206	1.031 (0.99, 1.073)	0.143	0.41	2.07E-08	TRUE
CD4 NC	*GRTP1*	Wald ratio	1	0.0160	0.0138	1.016 (0.989, 1.044)	0.249	0.542	2.17E-16	TRUE
CD4 NC	*GS1-124K5.3*	Wald ratio	1	0.0077	0.0120	1.008 (0.984, 1.032)	0.52	0.753	2.81E-20	TRUE
CD4 NC	*GS1-124K5.4*	Wald ratio	1	0.0033	0.0115	1.003 (0.981, 1.026)	0.777	0.903	1.73E-24	TRUE
CD4 NC	*GS1-251I9.4*	Wald ratio	1	0.0011	0.0145	1.001 (0.973, 1.03)	0.939	0.976	1.21E-13	TRUE
CD4 NC	*GSDMA*	Wald ratio	1	-0.0105	0.0132	0.99 (0.964, 1.016)	0.429	0.699	4.60E-17	TRUE
CD4 NC	*GSDMB*	Wald ratio	1	-0.0061	0.0174	0.994 (0.961, 1.028)	0.724	0.878	1.99E-08	TRUE
CD4 NC	*GSDMD*	Wald ratio	1	-0.0008	0.0174	0.999 (0.966, 1.034)	0.965	0.988	7.81E-10	TRUE
CD4 NC	*GSTM1*	Wald ratio	1	-0.0494	0.0199	0.952 (0.915, 0.99)	0.013	0.098	6.96E-08	TRUE
CD4 NC	*GSTM2*	Wald ratio	1	-0.0328	0.0223	0.968 (0.926, 1.011)	0.143	0.41	7.09E-08	TRUE
CD4 NC	*GSTM3*	Wald ratio	1	-0.0073	0.0048	0.993 (0.983, 1.002)	0.125	0.38	6.00E-136	TRUE
CD4 NC	*GSTO1*	Wald ratio	1	0.0151	0.0087	1.015 (0.998, 1.033)	0.083	0.303	3.34E-40	TRUE
CD4 NC	*GSTP1*	Wald ratio	1	0.0099	0.0055	1.01 (0.999, 1.021)	0.071	0.276	9.85E-110	TRUE
CD4 NC	*GSTT1*	Wald ratio	1	-0.0143	0.0251	0.986 (0.938, 1.035)	0.567	0.782	4.78E-11	TRUE
CD4 NC	*GTF2F1*	Wald ratio	1	-0.0213	0.0174	0.979 (0.946, 1.013)	0.223	0.516	4.12E-10	TRUE
CD4 NC	*GTF3A*	Wald ratio	1	0.0157	0.0146	1.016 (0.987, 1.045)	0.284	0.576	1.17E-11	TRUE
CD4 NC	*GTF3C3*	Wald ratio	1	-0.0462	0.0231	0.955 (0.913, 0.999)	0.045	0.21	4.28E-09	TRUE
CD4 NC	*GTPBP8*	Wald ratio	1	-0.0009	0.0127	0.999 (0.975, 1.024)	0.943	0.978	4.65E-16	TRUE
CD4 NC	*GTSCR1*	Wald ratio	1	0.0016	0.0130	1.002 (0.977, 1.027)	0.899	0.958	4.78E-16	TRUE
CD4 NC	*GVQW1*	Wald ratio	1	0.0412	0.0166	1.042 (1.009, 1.076)	0.013	0.096	5.09E-14	TRUE
CD4 NC	*GYG1*	Wald ratio	1	-0.0054	0.0130	0.995 (0.97, 1.02)	0.678	0.853	1.44E-15	TRUE
CD4 NC	*GYPC*	IVW	3	-0.0025	0.0059	0.997 (0.986, 1.009)	0.67	0.848	2.15E-109	TRUE
CD4 NC	*GYPC*	MR Egger	3	-0.0106	0.0141	0.989 (0.963, 1.017)	0.59	NA	NA	NA
CD4 NC	*GYPC*	Weighted median	3	-0.0041	0.0056	0.996 (0.985, 1.007)	0.472	NA	NA	NA
CD4 NC	*GYPC*	Simple mode	3	-0.0065	0.0095	0.994 (0.975, 1.012)	0.567	NA	NA	NA
CD4 NC	*GYPC*	Weighted mode	3	-0.0058	0.0059	0.994 (0.983, 1.006)	0.429	NA	NA	NA
CD4 NC	*GYPE*	Wald ratio	1	-0.0104	0.0084	0.99 (0.974, 1.006)	0.217	0.509	4.45E-40	TRUE
CD4 NC	*GZMA*	Wald ratio	1	0.0087	0.0205	1.009 (0.969, 1.05)	0.671	0.849	3.66E-08	TRUE
CD4 NC	*GZMM*	Wald ratio	1	0.0109	0.0160	1.011 (0.98, 1.043)	0.498	0.741	1.62E-18	TRUE
CD4 NC	*H1F0*	Wald ratio	1	0.0236	0.0123	1.024 (0.999, 1.049)	0.055	0.235	1.22E-19	TRUE
CD4 NC	*H2AFY*	Wald ratio	1	-0.0054	0.0180	0.995 (0.96, 1.03)	0.766	0.896	3.33E-10	TRUE
CD4 NC	*H2AFZ*	Wald ratio	1	0.0289	0.0156	1.029 (0.998, 1.061)	0.063	0.257	2.01E-11	TRUE
CD4 NC	*H6PD*	Wald ratio	1	-0.0297	0.0164	0.971 (0.94, 1.002)	0.069	0.272	4.51E-15	TRUE
CD4 NC	*HADH*	Wald ratio	1	0.0281	0.0189	1.028 (0.991, 1.067)	0.137	0.399	2.56E-15	TRUE
CD4 NC	*HAGH*	Wald ratio	1	0.0266	0.0064	1.027 (1.014, 1.04)	3.48E-05	0.001	8.85E-59	TRUE
CD4 NC	*HAGHL*	Wald ratio	1	0.0356	0.0125	1.036 (1.011, 1.062)	0.004	0.045	1.03E-13	TRUE
CD4 NC	*HAR1A*	Wald ratio	1	0.0187	0.0112	1.019 (0.997, 1.042)	0.095	0.327	7.75E-25	TRUE
CD4 NC	*HAR1B*	Wald ratio	1	0.0339	0.0179	1.034 (0.999, 1.071)	0.058	0.244	2.17E-10	TRUE
CD4 NC	*HAX1*	Wald ratio	1	-0.0090	0.0098	0.991 (0.972, 1.01)	0.357	0.648	4.82E-27	TRUE
CD4 NC	*HBS1L*	Wald ratio	1	0.0213	0.0133	1.022 (0.995, 1.049)	0.11	0.353	5.20E-16	TRUE
CD4 NC	*HCG11*	Wald ratio	1	0.0207	0.0170	1.021 (0.987, 1.055)	0.224	0.517	2.76E-10	TRUE
CD4 NC	*HCG14*	Wald ratio	1	0.0074	0.0194	1.007 (0.97, 1.047)	0.702	0.866	8.74E-09	TRUE
CD4 NC	*HCG20*	Wald ratio	1	-0.0093	0.0107	0.991 (0.97, 1.012)	0.386	0.669	2.18E-23	TRUE
CD4 NC	*HDDC2*	Wald ratio	1	0.0194	0.0115	1.02 (0.997, 1.043)	0.091	0.319	8.15E-23	TRUE
CD4 NC	*HDGFRP3*	Wald ratio	1	-0.0064	0.0112	0.994 (0.972, 1.016)	0.564	0.78	2.20E-23	TRUE
CD4 NC	*HDHD3*	Wald ratio	1	-0.0125	0.0169	0.988 (0.955, 1.021)	0.458	0.719	1.29E-13	TRUE
CD4 NC	*HEATR3*	Wald ratio	1	-0.0059	0.0210	0.994 (0.954, 1.036)	0.778	0.904	1.96E-08	TRUE
CD4 NC	*HEATR6*	Wald ratio	1	-0.0485	0.0166	0.953 (0.922, 0.984)	0.003	0.038	1.18E-10	TRUE
CD4 NC	*HEBP2*	Wald ratio	1	0.0184	0.0162	1.019 (0.987, 1.051)	0.258	0.552	1.01E-14	TRUE
CD4 NC	*HELLS*	Wald ratio	1	-0.0138	0.0092	0.986 (0.969, 1.004)	0.137	0.399	6.94E-35	TRUE
CD4 NC	*HELZ2*	Wald ratio	1	0.0054	0.0281	1.005 (0.952, 1.062)	0.847	0.935	1.29E-10	TRUE
CD4 NC	*HERPUD2*	Wald ratio	1	-0.0389	0.0153	0.962 (0.933, 0.991)	0.011	0.086	1.50E-13	TRUE
CD4 NC	*HEXDC*	Wald ratio	1	-0.0194	0.0195	0.981 (0.944, 1.019)	0.322	0.618	8.08E-09	TRUE
CD4 NC	*HHLA3*	Wald ratio	1	-0.0120	0.0178	0.988 (0.954, 1.023)	0.502	0.743	2.26E-11	TRUE
CD4 NC	*HIGD2A*	Wald ratio	1	-0.0062	0.0072	0.994 (0.98, 1.008)	0.387	0.669	1.74E-59	TRUE
CD4 NC	*HINT1*	Wald ratio	1	0.0839	0.0160	1.087 (1.054, 1.122)	1.70E-07	6.60E-05	2.09E-08	TRUE
CD4 NC	*HIST1H1D*	Wald ratio	1	0.0071	0.0205	1.007 (0.967, 1.048)	0.731	0.88	9.77E-09	TRUE
CD4 NC	*HIST1H2AJ*	Wald ratio	1	-0.0361	0.0299	0.965 (0.91, 1.023)	0.227	0.52	7.34E-08	TRUE
CD4 NC	*HIST1H2BE*	Wald ratio	1	0.0158	0.0217	1.016 (0.974, 1.06)	0.467	0.725	2.47E-12	TRUE

CD4 NC	*HIST1H3B*	Wald ratio	1	0.0004	0.0189	1 (0.964, 1.038)	0.985	0.994	1.76E-13	TRUE
CD4 NC	*HIST1H4I*	Wald ratio	1	-0.0235	0.0216	0.977 (0.936, 1.019)	0.275	0.568	4.64E-12	TRUE
CD4 NC	*HIST1H4K*	Wald ratio	1	0.0354	0.0367	1.036 (0.964, 1.113)	0.335	0.628	2.83E-13	TRUE
CD4 NC	*HIVEP3*	Wald ratio	1	0.0160	0.0076	1.016 (1.001, 1.031)	0.035	0.179	3.02E-50	TRUE
CD4 NC	*HLA-A*	Wald ratio	1	0.0286	0.0122	1.029 (1.005, 1.054)	0.019	0.124	4.87E-20	TRUE
CD4 NC	*HLA-B*	IVW	3	0.0029	0.0202	1.003 (0.964, 1.044)	0.885	0.954	1.10E-112	TRUE
CD4 NC	*HLA-B*	MR Egger	3	0.2300	0.0434	1.259 (1.156, 1.37)	0.119	NA	NA	NA
CD4 NC	*HLA-B*	Weighted median	3	0.0027	0.0063	1.003 (0.99, 1.015)	0.668	NA	NA	NA
CD4 NC	*HLA-B*	Simple mode	3	-0.0339	0.0108	0.967 (0.946, 0.987)	0.088	NA	NA	NA
CD4 NC	*HLA-B*	Weighted mode	3	0.0256	0.0077	1.026 (1.011, 1.041)	0.08	NA	NA	NA
CD4 NC	*HLA-C*	Wald ratio	1	-0.0145	0.0100	0.986 (0.967, 1.005)	0.146	0.415	7.37E-26	TRUE
CD4 NC	*HLA-DMA*	Wald ratio	1	-0.0293	0.0172	0.971 (0.939, 1.004)	0.089	0.317	8.95E-10	TRUE
CD4 NC	*HLA-DOB*	Wald ratio	1	-0.0024	0.0131	0.998 (0.972, 1.024)	0.852	0.937	5.49E-16	TRUE
CD4 NC	*HLA-DPA1*	Wald ratio	1	0.0305	0.0118	1.031 (1.007, 1.055)	0.01	0.082	2.60E-14	TRUE
CD4 NC	*HLA-DPB1*	Wald ratio	1	0.0378	0.0183	1.039 (1.002, 1.076)	0.039	0.191	1.58E-10	TRUE
CD4 NC	*HLA-DQA1*	Wald ratio	1	0.0890	0.0171	1.093 (1.057, 1.13)	1.97E-07	6.60E-05	2.27E-10	TRUE
CD4 NC	*HLA-DQA2*	Wald ratio	1	-0.0109	0.0182	0.989 (0.955, 1.025)	0.548	0.769	1.39E-08	TRUE
CD4 NC	*HLA-DQB2*	Wald ratio	1	-0.0513	0.0211	0.95 (0.912, 0.99)	0.015	0.104	6.90E-09	TRUE
CD4 NC	*HLA-DRB5*	Wald ratio	1	0.0476	0.0096	1.049 (1.029, 1.069)	6.24E-07	9.75E-05	2.09E-39	TRUE
CD4 NC	*HLA-E*	Wald ratio	1	0.0171	0.0197	1.017 (0.979, 1.057)	0.386	0.669	3.68E-08	TRUE
CD4 NC	*HLA-F*	Wald ratio	1	-0.0107	0.0117	0.989 (0.967, 1.012)	0.357	0.648	3.13E-19	TRUE
CD4 NC	*HLA-G*	IVW	3	0.0112	0.0077	1.011 (0.996, 1.027)	0.145	0.415	2.97E-80	TRUE
CD4 NC	*HLA-G*	MR Egger	3	-0.0053	0.0143	0.995 (0.967, 1.023)	0.775	NA	NA	NA
CD4 NC	*HLA-G*	Weighted median	3	0.0064	0.0077	1.006 (0.991, 1.022)	0.402	NA	NA	NA
CD4 NC	*HLA-G*	Simple mode	3	0.0066	0.0120	1.007 (0.983, 1.031)	0.639	NA	NA	NA
CD4 NC	*HLA-G*	Weighted mode	3	0.0062	0.0089	1.006 (0.989, 1.024)	0.56	NA	NA	NA
CD4 NC	*HMBOX1*	Wald ratio	1	-0.0088	0.0048	0.991 (0.982, 1.001)	0.066	0.263	1.49E-122	TRUE
CD4 NC	*HMBS*	Wald ratio	1	0.0363	0.0124	1.037 (1.012, 1.062)	0.003	0.039	2.10E-21	TRUE
CD4 NC	*HNRNPU*	Wald ratio	1	-0.0447	0.0144	0.956 (0.93, 0.984)	0.002	0.025	2.89E-12	TRUE
CD4 NC	*HOPX*	Wald ratio	1	-0.0052	0.0219	0.995 (0.953, 1.038)	0.813	0.92	4.82E-09	TRUE
CD4 NC	*HOTAIRM1*	Wald ratio	1	-0.0696	0.0163	0.933 (0.903, 0.963)	1.92E-05	9.36E-04	2.26E-11	TRUE
CD4 NC	*HOXB-AS1*	IVW	2	-0.0046	0.0069	0.995 (0.982, 1.009)	0.503	0.743	4.61E-76	TRUE
CD4 NC	*HOXB2*	IVW	2	-0.0065	0.0058	0.994 (0.982, 1.005)	0.26	0.554	3.57E-94	TRUE
CD4 NC	*HOXB3*	Wald ratio	1	-0.0057	0.0145	0.994 (0.966, 1.023)	0.695	0.863	6.24E-15	TRUE
CD4 NC	*HOXB4*	Wald ratio	1	-0.0089	0.0080	0.991 (0.976, 1.007)	0.27	0.563	1.80E-45	TRUE
CD4 NC	*HPCAL4*	Wald ratio	1	-0.0117	0.0103	0.988 (0.969, 1.008)	0.255	0.549	1.49E-27	TRUE
CD4 NC	*HPS1*	Wald ratio	1	0.0046	0.0134	1.005 (0.979, 1.031)	0.732	0.88	3.39E-16	TRUE
CD4 NC	*HSBP1L1*	Wald ratio	1	-0.0575	0.0169	0.944 (0.913, 0.976)	6.80E-04	0.013	1.66E-13	TRUE
CD4 NC	*HSD17B11*	Wald ratio	1	0.0411	0.0140	1.042 (1.014, 1.071)	0.003	0.038	1.03E-13	TRUE
CD4 NC	*HSD17B12*	Wald ratio	1	-0.0351	0.0101	0.965 (0.947, 0.985)	4.98E-04	0.01	6.44E-27	TRUE
CD4 NC	*HSD17B13*	Wald ratio	1	-0.0280	0.0160	0.972 (0.942, 1.003)	0.081	0.3	1.38E-13	TRUE
CD4 NC	*HSD17B14*	Wald ratio	1	-0.0080	0.0069	0.992 (0.979, 1.005)	0.241	0.534	9.71E-60	TRUE
CD4 NC	*HSD17B8*	Wald ratio	1	-0.0055	0.0153	0.995 (0.965, 1.025)	0.722	0.877	4.93E-13	TRUE
CD4 NC	*HSDL2*	Wald ratio	1	-0.0026	0.0140	0.997 (0.97, 1.025)	0.851	0.936	1.53E-17	TRUE
CD4 NC	*HSP90AA1*	Wald ratio	1	-0.0133	0.0150	0.987 (0.958, 1.016)	0.374	0.663	8.84E-12	TRUE
CD4 NC	*HSPBAP1*	Wald ratio	1	0.0043	0.0049	1.004 (0.995, 1.014)	0.382	0.666	3.88E-105	TRUE
CD4 NC	*HVCN1*	Wald ratio	1	-0.0063	0.0199	0.994 (0.956, 1.033)	0.751	0.887	8.77E-12	TRUE
CD4 NC	*IAH1*	Wald ratio	1	0.0211	0.0119	1.021 (0.998, 1.045)	0.076	0.288	5.20E-20	TRUE
CD4 NC	*ICAM3*	Wald ratio	1	0.0669	0.0141	1.069 (1.04, 1.099)	2.05E-06	1.89E-04	2.96E-11	TRUE
CD4 NC	*ID3*	Wald ratio	1	0.0104	0.0139	1.01 (0.983, 1.038)	0.454	0.717	5.41E-16	TRUE
CD4 NC	*IDH2*	Wald ratio	1	0.0507	0.0188	1.052 (1.014, 1.091)	0.007	0.063	4.81E-09	TRUE
CD4 NC	*IDI1*	Wald ratio	1	0.0421	0.0210	1.043 (1.001, 1.087)	0.045	0.208	8.24E-08	TRUE
CD4 NC	*IDUA*	Wald ratio	1	-0.0235	0.0196	0.977 (0.94, 1.015)	0.23	0.524	1.70E-09	TRUE
CD4 NC	*IFI27L1*	Wald ratio	1	-0.0259	0.0117	0.974 (0.952, 0.997)	0.027	0.151	2.18E-19	TRUE
CD4 NC	*IFI27L2*	Wald ratio	1	0.0031	0.0054	1.003 (0.993, 1.014)	0.568	0.782	1.26E-101	TRUE
CD4 NC	*IFIT5*	Wald ratio	1	-0.0081	0.0163	0.992 (0.961, 1.024)	0.618	0.817	1.42E-10	TRUE
CD4 NC	*IFITM1*	Wald ratio	1	-0.0148	0.0170	0.985 (0.953, 1.019)	0.385	0.668	5.57E-11	TRUE
CD4 NC	*IFITM2*	Wald ratio	1	-0.0026	0.0082	0.997 (0.982, 1.014)	0.756	0.888	9.76E-48	TRUE
CD4 NC	*IFITM3*	IVW	2	-0.0019	0.0085	0.998 (0.982, 1.015)	0.82	0.925	1.92E-124	TRUE
CD4 NC	*IFNG-AS1*	Wald ratio	1	-0.0146	0.0203	0.985 (0.947, 1.025)	0.471	0.729	4.54E-08	TRUE
CD4 NC	*IFT88*	Wald ratio	1	0.0124	0.0154	1.013 (0.982, 1.044)	0.419	0.69	5.63E-13	TRUE
CD4 NC	*IGFBP3*	Wald ratio	1	0.0226	0.0170	1.023 (0.989, 1.057)	0.182	0.462	6.85E-12	TRUE
CD4 NC	*IGHMBP2*	Wald ratio	1	-0.0125	0.0117	0.988 (0.965, 1.01)	0.284	0.577	3.31E-19	TRUE
CD4 NC	*IGLL1*	Wald ratio	1	0.0194	0.0105	1.02 (0.999, 1.041)	0.065	0.262	2.49E-24	TRUE
CD4 NC	*IGSF22*	Wald ratio	1	0.0030	0.0132	1.003 (0.977, 1.029)	0.823	0.926	1.29E-16	TRUE
CD4 NC	*IGSF8*	Wald ratio	1	-0.0037	0.0146	0.996 (0.968, 1.025)	0.8	0.914	5.08E-12	TRUE
CD4 NC	*IKBIP*	Wald ratio	1	0.0199	0.0218	1.02 (0.977, 1.065)	0.361	0.651	5.88E-08	TRUE
CD4 NC	*IKBKE*	Wald ratio	1	0.0019	0.0142	1.002 (0.974, 1.03)	0.892	0.957	5.16E-14	TRUE
CD4 NC	*IKZF1*	Wald ratio	1	-0.0169	0.0181	0.983 (0.949, 1.019)	0.35	0.643	1.39E-11	TRUE
CD4 NC	*IL18R1*	Wald ratio	1	-0.0042	0.0127	0.996 (0.971, 1.021)	0.742	0.881	1.26E-18	TRUE
CD4 NC	*IL24*	Wald ratio	1	0.0130	0.0101	1.013 (0.993, 1.033)	0.199	0.482	1.93E-29	TRUE
CD4 NC	*IL32*	Wald ratio	1	-0.0081	0.0092	0.992 (0.974, 1.01)	0.376	0.664	7.56E-35	TRUE
CD4 NC	*IL6ST*	Wald ratio	1	-0.0023	0.0156	0.998 (0.968, 1.029)	0.881	0.951	3.81E-14	TRUE
CD4 NC	*IMMP2L*	Wald ratio	1	0.0170	0.0122	1.017 (0.993, 1.042)	0.164	0.438	7.54E-20	TRUE
CD4 NC	*IMMT*	Wald ratio	1	-0.0131	0.0122	0.987 (0.964, 1.011)	0.285	0.577	1.01E-19	TRUE
CD4 NC	*IMP3*	Wald ratio	1	0.0101	0.0204	1.01 (0.971, 1.051)	0.62	0.819	6.05E-10	TRUE
CD4 NC	*IMPA1*	Wald ratio	1	0.0470	0.0187	1.048 (1.01, 1.087)	0.012	0.093	6.58E-08	TRUE
CD4 NC	*IMPA2*	Wald ratio	1	0.0337	0.0188	1.034 (0.997, 1.073)	0.073	0.283	4.98E-09	TRUE
CD4 NC	*INF2*	Wald ratio	1	0.0107	0.0089	1.011 (0.993, 1.029)	0.23	0.523	2.30E-30	TRUE

CD4 NC	*ING5*	Wald ratio	1	-0.0356	0.0168	0.965 (0.934, 0.997)	0.034	0.175	3.45E-11	TRUE
CD4 NC	*INO80B*	Wald ratio	1	-0.0118	0.0177	0.988 (0.955, 1.023)	0.506	0.745	3.82E-09	TRUE
CD4 NC	*INPP4B*	Wald ratio	1	-0.0417	0.0137	0.959 (0.934, 0.985)	0.002	0.029	1.05E-21	TRUE
CD4 NC	*INTS5*	Wald ratio	1	-0.0282	0.0183	0.972 (0.938, 1.008)	0.124	0.379	3.26E-11	TRUE
CD4 NC	*IP6K2*	Wald ratio	1	-0.0092	0.0133	0.991 (0.965, 1.017)	0.491	0.738	2.57E-16	TRUE
CD4 NC	*IPO9*	Wald ratio	1	-0.0199	0.0184	0.98 (0.945, 1.016)	0.28	0.574	1.86E-09	TRUE
CD4 NC	*IPP*	Wald ratio	1	-0.0009	0.0177	0.999 (0.965, 1.034)	0.96	0.986	8.92E-11	TRUE
CD4 NC	*IQCB1*	Wald ratio	1	0.0026	0.0159	1.003 (0.972, 1.034)	0.871	0.947	5.19E-15	TRUE
CD4 NC	*IQCH-AS1*	Wald ratio	1	0.0483	0.0152	1.049 (1.019, 1.081)	0.002	0.022	2.64E-10	TRUE
CD4 NC	*IQSEC3*	Wald ratio	1	0.0056	0.0133	1.006 (0.98, 1.032)	0.675	0.852	2.87E-17	TRUE
CD4 NC	*IRAK1BP1*	Wald ratio	1	-0.0277	0.0193	0.973 (0.936, 1.01)	0.151	0.422	1.41E-10	TRUE
CD4 NC	*IRF3*	Wald ratio	1	-0.0054	0.0156	0.995 (0.965, 1.025)	0.728	0.879	1.28E-12	TRUE
CD4 NC	*IRF7*	Wald ratio	1	0.0080	0.0174	1.008 (0.974, 1.043)	0.647	0.834	3.99E-11	TRUE
CD4 NC	*ISCA1*	Wald ratio	1	0.0187	0.0095	1.019 (1, 1.038)	0.05	0.222	1.06E-29	TRUE
CD4 NC	*ISCA2*	Wald ratio	1	0.0041	0.0063	1.004 (0.992, 1.017)	0.519	0.753	3.44E-73	TRUE
CD4 NC	*ISCU*	Wald ratio	1	0.0002	0.0118	1 (0.977, 1.024)	0.984	0.994	2.64E-20	TRUE
CD4 NC	*ISG15*	Wald ratio	1	-0.0246	0.0104	0.976 (0.956, 0.996)	0.018	0.12	2.42E-25	TRUE
CD4 NC	*ISG20*	Wald ratio	1	0.0081	0.0182	1.008 (0.973, 1.045)	0.654	0.839	9.27E-12	TRUE
CD4 NC	*ISM1*	Wald ratio	1	0.0181	0.0156	1.018 (0.988, 1.05)	0.244	0.537	1.18E-12	TRUE
CD4 NC	*ITCH-AS1*	Wald ratio	1	0.0171	0.0150	1.017 (0.988, 1.047)	0.254	0.548	1.25E-13	TRUE
CD4 NC	*ITGA2*	Wald ratio	1	-0.0105	0.0192	0.99 (0.953, 1.028)	0.585	0.794	8.82E-09	TRUE
CD4 NC	*ITGB2*	Wald ratio	1	-0.0006	0.0047	0.999 (0.99, 1.009)	0.897	0.957	1.61E-118	TRUE
CD4 NC	*ITGB2-AS1*	Wald ratio	1	-0.0058	0.0127	0.994 (0.97, 1.019)	0.645	0.834	2.11E-19	TRUE
CD4 NC	*ITGB3BP*	Wald ratio	1	0.0032	0.0068	1.003 (0.99, 1.017)	0.636	0.827	5.69E-60	TRUE
CD4 NC	*ITIH4*	Wald ratio	1	0.0172	0.0163	1.017 (0.985, 1.05)	0.292	0.586	4.81E-14	TRUE
CD4 NC	*ITPA*	Wald ratio	1	0.0054	0.0135	1.005 (0.979, 1.032)	0.689	0.859	2.96E-25	TRUE
CD4 NC	*JAKMIP2*	Wald ratio	1	0.0264	0.0101	1.027 (1.007, 1.047)	0.009	0.074	1.93E-33	TRUE
CD4 NC	*JAZF1*	Wald ratio	1	0.0137	0.0203	1.014 (0.974, 1.055)	0.5	0.743	1.08E-09	TRUE
CD4 NC	*KANK1*	IVW	2	0.0098	0.0170	1.01 (0.977, 1.044)	0.564	0.78	5.50E-18	TRUE
CD4 NC	*KANSL1-AS1*	Wald ratio	1	-0.0162	0.0069	0.984 (0.971, 0.997)	0.018	0.12	1.05E-164	TRUE
CD4 NC	*KATNBL1*	Wald ratio	1	-0.0047	0.0211	0.995 (0.955, 1.037)	0.825	0.926	8.31E-09	TRUE
CD4 NC	*KB-1507C5.2*	Wald ratio	1	0.0687	0.0157	1.071 (1.039, 1.105)	1.18E-05	6.52E-04	2.42E-11	TRUE
CD4 NC	*KBTBD11*	Wald ratio	1	0.0250	0.0200	1.025 (0.986, 1.066)	0.212	0.499	2.10E-08	TRUE
CD4 NC	*KCNAB2*	Wald ratio	1	0.0137	0.0103	1.014 (0.993, 1.035)	0.187	0.466	3.44E-35	TRUE
CD4 NC	*KCNN1*	Wald ratio	1	0.0139	0.0131	1.014 (0.988, 1.04)	0.288	0.581	2.51E-18	TRUE
CD4 NC	*KCNQ1OT1*	Wald ratio	1	-0.0564	0.0186	0.945 (0.911, 0.98)	0.002	0.03	3.01E-09	TRUE
CD4 NC	*KCTD21-AS1*	Wald ratio	1	0.0019	0.0085	1.002 (0.985, 1.019)	0.821	0.925	2.06E-40	TRUE
CD4 NC	*KDELR2*	Wald ratio	1	0.0201	0.0062	1.02 (1.008, 1.033)	0.001	0.02	1.46E-71	TRUE
CD4 NC	*KIAA1143*	Wald ratio	1	0.0076	0.0137	1.008 (0.981, 1.035)	0.578	0.789	3.30E-16	TRUE
CD4 NC	*KIAA1328*	Wald ratio	1	-0.0138	0.0141	0.986 (0.959, 1.014)	0.329	0.624	1.96E-14	TRUE
CD4 NC	*KIAA1430*	Wald ratio	1	0.0054	0.0088	1.005 (0.988, 1.023)	0.538	0.762	1.71E-35	TRUE
CD4 NC	*KIAA1551*	Wald ratio	1	-0.0095	0.0201	0.991 (0.952, 1.03)	0.635	0.827	4.34E-08	TRUE
CD4 NC	*KIAA1715*	Wald ratio	1	-0.0230	0.0114	0.977 (0.956, 0.999)	0.044	0.207	4.89E-22	TRUE
CD4 NC	*KIF16B*	Wald ratio	1	0.0004	0.0108	1 (0.98, 1.022)	0.972	0.989	2.36E-24	TRUE
CD4 NC	*KIF2A*	Wald ratio	1	0.0131	0.0148	1.013 (0.984, 1.043)	0.376	0.664	4.59E-14	TRUE
CD4 NC	*KIR3DL2*	Wald ratio	1	0.0096	0.0168	1.01 (0.977, 1.044)	0.568	0.782	4.52E-11	TRUE
CD4 NC	*KISS1R*	Wald ratio	1	0.0040	0.0132	1.004 (0.978, 1.03)	0.763	0.894	1.47E-21	TRUE
CD4 NC	*KLF13*	Wald ratio	1	0.0123	0.0112	1.012 (0.99, 1.035)	0.271	0.564	3.81E-23	TRUE
CD4 NC	*KLF5*	Wald ratio	1	-0.0002	0.0124	1 (0.976, 1.024)	0.985	0.994	1.54E-16	TRUE
CD4 NC	*KLHDC10*	Wald ratio	1	0.0432	0.0169	1.044 (1.01, 1.079)	0.01	0.084	5.64E-11	TRUE
CD4 NC	*KLHDC4*	Wald ratio	1	0.0097	0.0164	1.01 (0.978, 1.043)	0.553	0.772	1.34E-09	TRUE
CD4 NC	*KLHL18*	Wald ratio	1	-0.0082	0.0137	0.992 (0.966, 1.019)	0.55	0.769	4.12E-18	TRUE
CD4 NC	*KLHL20*	Wald ratio	1	-0.0168	0.0170	0.983 (0.951, 1.017)	0.324	0.618	3.11E-10	TRUE
CD4 NC	*KLHL42*	Wald ratio	1	0.0135	0.0146	1.014 (0.985, 1.043)	0.356	0.647	1.67E-16	TRUE
CD4 NC	*KLHL5*	Wald ratio	1	0.0342	0.0173	1.035 (1, 1.07)	0.048	0.217	2.45E-10	TRUE
CD4 NC	*KLHL6*	Wald ratio	1	-0.0069	0.0147	0.993 (0.965, 1.022)	0.64	0.83	1.02E-14	TRUE
CD4 NC	*KLHL7-AS1*	Wald ratio	1	0.0276	0.0154	1.028 (0.997, 1.06)	0.074	0.285	1.61E-14	TRUE
CD4 NC	*KNOP1*	Wald ratio	1	-0.0079	0.0160	0.992 (0.961, 1.024)	0.62	0.819	1.07E-14	TRUE
CD4 NC	*KPNA4*	Wald ratio	1	0.0298	0.0200	1.03 (0.991, 1.071)	0.137	0.399	1.86E-08	TRUE
CD4 NC	*KRT1*	Wald ratio	1	-0.0025	0.0048	0.998 (0.988, 1.007)	0.604	0.806	1.72E-137	TRUE
CD4 NC	*KRT10*	Wald ratio	1	0.0100	0.0113	1.01 (0.988, 1.033)	0.379	0.665	2.22E-22	TRUE
CD4 NC	*KRT2*	Wald ratio	1	-0.0385	0.0186	0.962 (0.928, 0.998)	0.039	0.191	6.85E-10	TRUE
CD4 NC	*KRT72*	Wald ratio	1	-0.0219	0.0080	0.978 (0.963, 0.994)	0.006	0.058	8.22E-42	TRUE
CD4 NC	*KRT73*	Wald ratio	1	-0.0046	0.0131	0.995 (0.97, 1.021)	0.728	0.879	1.04E-19	TRUE
CD4 NC	*KRTAP5-2*	Wald ratio	1	-0.0465	0.0096	0.955 (0.937, 0.973)	1.46E-06	1.50E-04	9.91E-32	TRUE
CD4 NC	*KSR2*	Wald ratio	1	-0.0274	0.0157	0.973 (0.943, 1.003)	0.08	0.297	5.15E-12	TRUE
CD4 NC	*KTN1*	Wald ratio	1	0.0274	0.0092	1.028 (1.009, 1.047)	0.003	0.035	5.55E-33	TRUE
CD4 NC	*KYNU*	Wald ratio	1	0.0406	0.0206	1.041 (1, 1.084)	0.049	0.22	8.08E-08	TRUE
CD4 NC	*L3MBTL3*	Wald ratio	1	-0.0066	0.0077	0.993 (0.979, 1.009)	0.393	0.672	7.69E-51	TRUE
CD4 NC	*LA16c-358B7.3*	Wald ratio	1	0.0273	0.0068	1.028 (1.014, 1.042)	6.58E-05	0.002	1.28E-67	TRUE
CD4 NC	*LAIR2*	Wald ratio	1	0.0400	0.0137	1.041 (1.013, 1.069)	0.003	0.038	9.45E-22	TRUE
CD4 NC	*LAMTOR4*	Wald ratio	1	-0.0132	0.0155	0.987 (0.957, 1.017)	0.393	0.672	7.40E-13	TRUE
CD4 NC	*LAPTM4B*	Wald ratio	1	0.0243	0.0165	1.025 (0.992, 1.058)	0.141	0.408	2.37E-13	TRUE
CD4 NC	*LAPTM5*	Wald ratio	1	-0.0600	0.0196	0.942 (0.906, 0.979)	0.002	0.028	3.42E-08	TRUE
CD4 NC	*LAX1*	Wald ratio	1	0.0000	0.0084	1 (0.984, 1.017)	0.996	0.999	1.95E-40	TRUE
CD4 NC	*LBH*	Wald ratio	1	0.0020	0.0181	1.002 (0.967, 1.038)	0.912	0.962	8.04E-11	TRUE
CD4 NC	*LCLAT1*	Wald ratio	1	-0.0007	0.0058	0.999 (0.988, 1.011)	0.897	0.957	1.05E-100	TRUE
CD4 NC	*LCP1*	Wald ratio	1	-0.0209	0.0196	0.979 (0.942, 1.018)	0.287	0.579	2.32E-11	TRUE
CD4 NC	*LDHA*	Wald ratio	1	0.0188	0.0188	1.019 (0.982, 1.057)	0.319	0.614	3.85E-09	TRUE

CD4 NC	*LDHC*	Wald ratio	1	-0.0093	0.0066	0.991 (0.978, 1.004)	0.157	0.428	5.01E-81	TRUE
CD4 NC	*LDLRAP1*	Wald ratio	1	0.0057	0.0076	1.006 (0.991, 1.021)	0.458	0.719	4.13E-50	TRUE
CD4 NC	*LEKR1*	Wald ratio	1	-0.0090	0.0150	0.991 (0.962, 1.021)	0.548	0.769	1.55E-11	TRUE
CD4 NC	*LEPROT*	Wald ratio	1	0.0003	0.0089	1 (0.983, 1.018)	0.969	0.989	2.73E-40	TRUE
CD4 NC	*LGALS3*	Wald ratio	1	0.0050	0.0129	1.005 (0.98, 1.031)	0.701	0.866	1.08E-17	TRUE
CD4 NC	*LILRB3*	Wald ratio	1	0.0596	0.0193	1.061 (1.022, 1.102)	0.002	0.026	3.35E-08	TRUE
CD4 NC	*LIMD1*	Wald ratio	1	0.0065	0.0072	1.007 (0.992, 1.021)	0.368	0.656	2.78E-57	TRUE
CD4 NC	*LIMD2*	Wald ratio	1	-0.0909	0.0207	0.913 (0.877, 0.951)	1.16E-05	6.46E-04	2.72E-08	TRUE
CD4 NC	*LIMS2*	Wald ratio	1	0.0454	0.0183	1.046 (1.01, 1.085)	0.013	0.098	4.06E-10	TRUE
CD4 NC	*LIN52*	Wald ratio	1	-0.0017	0.0156	0.998 (0.968, 1.029)	0.912	0.962	1.65E-13	TRUE
CD4 NC	*LINC00240*	Wald ratio	1	0.0143	0.0133	1.014 (0.988, 1.041)	0.282	0.575	5.72E-21	TRUE
CD4 NC	*LINC00336*	Wald ratio	1	-0.0372	0.0142	0.963 (0.937, 0.991)	0.009	0.075	2.13E-11	TRUE
CD4 NC	*LINC00339*	IVW	2	0.0029	0.0127	1.003 (0.978, 1.028)	0.816	0.921	2.87E-53	TRUE
CD4 NC	*LINC00493*	Wald ratio	1	0.0059	0.0149	1.006 (0.977, 1.036)	0.693	0.862	8.19E-14	TRUE
CD4 NC	*LINC00511*	Wald ratio	1	-0.0031	0.0189	0.997 (0.961, 1.035)	0.872	0.947	7.92E-09	TRUE
CD4 NC	*LINC00649*	Wald ratio	1	-0.0032	0.0078	0.997 (0.982, 1.012)	0.679	0.853	1.73E-47	TRUE
CD4 NC	*LINC00657*	Wald ratio	1	-0.0027	0.0473	0.997 (0.909, 1.094)	0.955	0.983	4.31E-09	TRUE
CD4 NC	*LINC00662*	Wald ratio	1	0.0046	0.0147	1.005 (0.976, 1.034)	0.756	0.888	1.54E-10	TRUE
CD4 NC	*LINC00665*	Wald ratio	1	-0.0266	0.0137	0.974 (0.948, 1)	0.051	0.225	4.43E-16	TRUE
CD4 NC	*LINC00667*	Wald ratio	1	-0.0006	0.0170	0.999 (0.967, 1.033)	0.971	0.989	1.35E-13	TRUE
CD4 NC	*LINC00852*	Wald ratio	1	-0.0138	0.0125	0.986 (0.962, 1.011)	0.271	0.565	1.47E-19	TRUE
CD4 NC	*LINC00861*	Wald ratio	1	-0.0285	0.0186	0.972 (0.937, 1.008)	0.125	0.38	5.58E-09	TRUE
CD4 NC	*LINC00863*	Wald ratio	1	0.0507	0.0192	1.052 (1.013, 1.092)	0.008	0.07	1.65E-08	TRUE
CD4 NC	*LINC00909*	IVW	2	-0.0153	0.0118	0.985 (0.962, 1.008)	0.195	0.477	3.48E-26	TRUE
CD4 NC	*LINC00920*	Wald ratio	1	0.0049	0.0154	1.005 (0.975, 1.036)	0.75	0.886	3.03E-13	TRUE
CD4 NC	*LINC00944*	Wald ratio	1	0.0051	0.0094	1.005 (0.987, 1.024)	0.59	0.797	4.98E-20	TRUE
CD4 NC	*LINC00951*	Wald ratio	1	0.0231	0.0123	1.023 (0.999, 1.048)	0.06	0.247	1.55E-18	TRUE
CD4 NC	*LINC00954*	Wald ratio	1	-0.0072	0.0083	0.993 (0.977, 1.009)	0.385	0.668	3.22E-52	TRUE
CD4 NC	*LINC00958*	Wald ratio	1	0.0092	0.0105	1.009 (0.989, 1.03)	0.379	0.666	2.86E-45	TRUE
CD4 NC	*LINC00969*	Wald ratio	1	-0.0066	0.0075	0.993 (0.979, 1.008)	0.384	0.668	5.39E-51	TRUE
CD4 NC	*LINS*	Wald ratio	1	0.0074	0.0151	1.007 (0.978, 1.038)	0.625	0.822	1.42E-13	TRUE
CD4 NC	*LIPC*	Wald ratio	1	-0.0466	0.0166	0.954 (0.924, 0.986)	0.005	0.051	2.39E-20	TRUE
CD4 NC	*LIPT2*	Wald ratio	1	0.0006	0.0140	1.001 (0.974, 1.028)	0.964	0.988	3.25E-15	TRUE
CD4 NC	*LL22NC03-2H8.5*	Wald ratio	1	0.0015	0.0145	1.002 (0.973, 1.03)	0.916	0.963	4.71E-15	TRUE
CD4 NC	*LLGL1*	Wald ratio	1	0.0745	0.0153	1.077 (1.046, 1.11)	1.13E-06	1.22E-04	1.88E-12	TRUE
CD4 NC	*LMAN1*	Wald ratio	1	0.0140	0.0192	1.014 (0.977, 1.053)	0.467	0.725	4.89E-08	TRUE
CD4 NC	*LMOD3*	Wald ratio	1	0.0131	0.0172	1.013 (0.98, 1.048)	0.444	0.709	1.38E-11	TRUE
CD4 NC	*LONRF2*	Wald ratio	1	-0.0022	0.0079	0.998 (0.982, 1.013)	0.785	0.907	3.69E-46	TRUE
CD4 NC	*LPAR3*	Wald ratio	1	-0.0104	0.0089	0.99 (0.973, 1.007)	0.241	0.534	1.25E-30	TRUE
CD4 NC	*LPIN1*	Wald ratio	1	-0.0043	0.0082	0.996 (0.98, 1.012)	0.598	0.803	2.98E-41	TRUE
CD4 NC	*LPIN2*	Wald ratio	1	-0.0125	0.0104	0.988 (0.968, 1.008)	0.23	0.524	1.93E-27	TRUE
CD4 NC	*LRRC16A*	Wald ratio	1	-0.0120	0.0092	0.988 (0.97, 1.006)	0.188	0.469	2.17E-36	TRUE
CD4 NC	*LRRC27*	Wald ratio	1	0.0010	0.0104	1.001 (0.981, 1.022)	0.925	0.968	4.31E-22	TRUE
CD4 NC	*LRRC37A2*	Wald ratio	1	-0.0413	0.0167	0.96 (0.929, 0.991)	0.013	0.098	2.36E-24	TRUE
CD4 NC	*LRRC57*	Wald ratio	1	0.0357	0.0225	1.036 (0.992, 1.083)	0.112	0.356	1.08E-08	TRUE
CD4 NC	*LRRC6*	Wald ratio	1	0.0118	0.0172	1.012 (0.978, 1.047)	0.493	0.738	8.33E-11	TRUE
CD4 NC	*LRRC61*	Wald ratio	1	-0.0421	0.0089	0.959 (0.942, 0.976)	2.26E-06	2.06E-04	3.67E-31	TRUE
CD4 NC	*LRRC7*	Wald ratio	1	-0.0050	0.0195	0.995 (0.958, 1.034)	0.797	0.913	6.82E-10	TRUE
CD4 NC	*LSM4*	Wald ratio	1	-0.0492	0.0130	0.952 (0.928, 0.977)	1.55E-04	0.004	5.64E-12	TRUE
CD4 NC	*LSM6*	Wald ratio	1	-0.0519	0.0148	0.949 (0.922, 0.977)	4.39E-04	0.01	1.76E-13	TRUE
CD4 NC	*LSM7*	Wald ratio	1	0.0310	0.0088	1.031 (1.014, 1.05)	4.56E-04	0.01	2.26E-36	TRUE
CD4 NC	*LSR*	Wald ratio	1	0.0307	0.0164	1.031 (0.999, 1.065)	0.061	0.25	2.43E-11	TRUE
CD4 NC	*LSS*	Wald ratio	1	0.0024	0.0190	1.002 (0.966, 1.04)	0.898	0.957	6.17E-08	TRUE
CD4 NC	*LST1*	Wald ratio	1	0.0629	0.0132	1.065 (1.038, 1.093)	1.75E-06	1.71E-04	2.57E-15	TRUE
CD4 NC	*LURAP1*	Wald ratio	1	0.0135	0.0165	1.014 (0.981, 1.047)	0.412	0.686	6.06E-13	TRUE
CD4 NC	*LY75*	Wald ratio	1	0.0461	0.0180	1.047 (1.011, 1.085)	0.01	0.082	1.04E-09	TRUE
CD4 NC	*LY9*	Wald ratio	1	-0.0092	0.0199	0.991 (0.953, 1.03)	0.645	0.834	3.39E-08	TRUE
CD4 NC	*LYRM4*	Wald ratio	1	0.0087	0.0067	1.009 (0.995, 1.022)	0.197	0.48	5.00E-53	TRUE
CD4 NC	*LYRM9*	Wald ratio	1	0.0130	0.0134	1.013 (0.987, 1.04)	0.332	0.626	7.60E-20	TRUE
CD4 NC	*MACF1*	Wald ratio	1	0.0507	0.0124	1.052 (1.027, 1.078)	4.37E-05	0.002	2.27E-17	TRUE
CD4 NC	*MAF1*	Wald ratio	1	0.0202	0.0152	1.02 (0.991, 1.051)	0.183	0.462	2.64E-16	TRUE
CD4 NC	*MAGEF1*	Wald ratio	1	0.0118	0.0175	1.012 (0.978, 1.047)	0.5	0.742	2.71E-10	TRUE
CD4 NC	*MAL*	Wald ratio	1	-0.0137	0.0149	0.986 (0.958, 1.016)	0.358	0.648	4.87E-17	TRUE
CD4 NC	*MAN1A2*	Wald ratio	1	0.0344	0.0127	1.035 (1.01, 1.061)	0.007	0.062	8.86E-17	TRUE
CD4 NC	*MAN2C1*	Wald ratio	1	-0.0195	0.0174	0.981 (0.948, 1.015)	0.262	0.557	1.41E-11	TRUE
CD4 NC	*MANBA*	Wald ratio	1	0.0096	0.0081	1.01 (0.994, 1.026)	0.232	0.525	2.91E-44	TRUE
CD4 NC	*MAP2K1*	Wald ratio	1	-0.0516	0.0163	0.95 (0.92, 0.981)	0.002	0.022	3.06E-11	TRUE
CD4 NC	*MAP3K7CL*	Wald ratio	1	-0.0137	0.0198	0.986 (0.949, 1.025)	0.49	0.738	1.41E-08	TRUE
CD4 NC	*MAP6D1*	Wald ratio	1	0.0131	0.0204	1.013 (0.973, 1.055)	0.521	0.753	1.31E-08	TRUE
CD4 NC	*MARCH10*	Wald ratio	1	0.0276	0.0162	1.028 (0.996, 1.061)	0.088	0.313	1.05E-10	TRUE
CD4 NC	*MARCH2*	Wald ratio	1	0.0385	0.0160	1.039 (1.007, 1.072)	0.016	0.111	6.34E-15	TRUE
CD4 NC	*MARK3*	Wald ratio	1	-0.0717	0.0161	0.931 (0.902, 0.961)	8.96E-06	5.27E-04	1.70E-11	TRUE
CD4 NC	*MAST3*	Wald ratio	1	0.0327	0.0110	1.033 (1.011, 1.056)	0.003	0.035	5.75E-24	TRUE
CD4 NC	*MAST4*	IVW	2	-0.0316	0.0349	0.969 (0.905, 1.037)	0.365	0.654	2.32E-17	TRUE
CD4 NC	*MATK*	Wald ratio	1	-0.0006	0.0160	0.999 (0.969, 1.031)	0.97	0.989	6.03E-10	TRUE
CD4 NC	*MAX*	Wald ratio	1	0.0072	0.0185	1.007 (0.971, 1.044)	0.698	0.865	2.57E-09	TRUE
CD4 NC	*MBOAT1*	Wald ratio	1	-0.0235	0.0198	0.977 (0.94, 1.015)	0.234	0.528	1.16E-08	TRUE
CD4 NC	*MBP*	IVW	2	-0.0121	0.0089	0.988 (0.971, 1.005)	0.173	0.451	9.52E-40	TRUE
CD4 NC	*MDGA1*	Wald ratio	1	0.0019	0.0080	1.002 (0.986, 1.018)	0.807	0.917	7.12E-49	TRUE

CD4 NC	*MDH1*	Wald ratio	1	0.0200	0.0171	1.02 (0.987, 1.055)	0.243	0.535	6.55E-13	TRUE
CD4 NC	*MDM4*	Wald ratio	1	-0.0431	0.0115	0.958 (0.936, 0.98)	1.71E-04	0.004	7.09E-28	TRUE
CD4 NC	*MDS2*	Wald ratio	1	-0.0104	0.0139	0.99 (0.963, 1.017)	0.454	0.717	5.02E-16	TRUE
CD4 NC	*MEAF6*	Wald ratio	1	-0.0224	0.0133	0.978 (0.953, 1.004)	0.092	0.323	7.15E-18	TRUE
CD4 NC	*MED16*	Wald ratio	1	-0.0003	0.0067	1 (0.987, 1.013)	0.959	0.986	3.30E-77	TRUE
CD4 NC	*MED19*	Wald ratio	1	-0.0201	0.0185	0.98 (0.945, 1.016)	0.278	0.571	2.50E-08	TRUE
CD4 NC	*MED4*	Wald ratio	1	0.0261	0.0182	1.026 (0.991, 1.064)	0.151	0.422	5.17E-09	TRUE
CD4 NC	*MEF2BNB*	Wald ratio	1	0.0227	0.0200	1.023 (0.984, 1.064)	0.257	0.551	1.65E-08	TRUE
CD4 NC	*MEI1*	Wald ratio	1	0.0419	0.0141	1.043 (1.014, 1.072)	0.003	0.035	5.84E-13	TRUE
CD4 NC	*MEOX1*	Wald ratio	1	-0.0147	0.0142	0.985 (0.958, 1.013)	0.299	0.594	3.26E-15	TRUE
CD4 NC	*METTL18*	Wald ratio	1	-0.0011	0.0151	0.999 (0.97, 1.029)	0.942	0.977	1.41E-16	TRUE
CD4 NC	*METTL21B*	Wald ratio	1	-0.0167	0.0117	0.983 (0.961, 1.006)	0.155	0.426	6.38E-21	TRUE
CD4 NC	*METTL2B*	Wald ratio	1	0.0144	0.0157	1.015 (0.984, 1.046)	0.359	0.65	3.92E-12	TRUE
CD4 NC	*MFF*	Wald ratio	1	-0.0028	0.0127	0.997 (0.973, 1.022)	0.828	0.928	3.05E-25	TRUE
CD4 NC	*MGMT*	Wald ratio	1	-0.0073	0.0084	0.993 (0.977, 1.009)	0.381	0.666	7.99E-41	TRUE
CD4 NC	*MGRN1*	Wald ratio	1	0.0395	0.0176	1.04 (1.005, 1.077)	0.025	0.145	3.78E-10	TRUE
CD4 NC	*MGST2*	Wald ratio	1	-0.0051	0.0078	0.995 (0.98, 1.01)	0.512	0.748	7.21E-48	TRUE
CD4 NC	*MGST3*	IVW	2	-0.0004	0.0075	1 (0.985, 1.014)	0.962	0.988	6.74E-51	TRUE
CD4 NC	*MIB2*	Wald ratio	1	-0.0414	0.0150	0.959 (0.932, 0.988)	0.006	0.055	4.48E-14	TRUE
CD4 NC	*MICA*	Wald ratio	1	0.0421	0.0176	1.043 (1.008, 1.079)	0.017	0.113	1.42E-08	TRUE
CD4 NC	*MICAL3*	Wald ratio	1	0.0117	0.0164	1.012 (0.98, 1.045)	0.477	0.732	8.72E-12	TRUE
CD4 NC	*MICU3*	IVW	2	0.0258	0.0140	1.026 (0.998, 1.055)	0.066	0.263	5.90E-17	TRUE
CD4 NC	*MINK1*	Wald ratio	1	0.0306	0.0128	1.031 (1.006, 1.057)	0.016	0.112	9.86E-19	TRUE
CD4 NC	*MKRN1*	Wald ratio	1	-0.0380	0.0129	0.963 (0.939, 0.987)	0.003	0.037	1.39E-19	TRUE
CD4 NC	*MLF2*	Wald ratio	1	-0.0166	0.0170	0.984 (0.951, 1.017)	0.329	0.623	8.15E-11	TRUE
CD4 NC	*MLKL*	Wald ratio	1	-0.0700	0.0194	0.932 (0.898, 0.968)	2.98E-04	0.007	2.42E-08	TRUE
CD4 NC	*MLLT4*	Wald ratio	1	0.0025	0.0109	1.003 (0.981, 1.024)	0.817	0.922	4.43E-24	TRUE
CD4 NC	*MMEL1*	Wald ratio	1	-0.0115	0.0136	0.989 (0.963, 1.015)	0.396	0.676	3.63E-16	TRUE
CD4 NC	*MORC3*	Wald ratio	1	-0.0411	0.0153	0.96 (0.931, 0.989)	0.007	0.066	4.27E-13	TRUE
CD4 NC	*MORN3*	Wald ratio	1	-0.0083	0.0069	0.992 (0.978, 1.005)	0.231	0.524	4.61E-61	TRUE
CD4 NC	*MOSPD3*	Wald ratio	1	0.0236	0.0177	1.024 (0.989, 1.06)	0.182	0.462	7.29E-09	TRUE
CD4 NC	*MPHOSPH9*	Wald ratio	1	-0.0315	0.0117	0.969 (0.947, 0.991)	0.007	0.063	4.42E-22	TRUE
CD4 NC	*MPI*	Wald ratio	1	-0.0506	0.0153	0.951 (0.923, 0.98)	9.28E-04	0.016	8.39E-13	TRUE
CD4 NC	*MPPE1*	Wald ratio	1	-0.0038	0.0165	0.996 (0.965, 1.029)	0.816	0.921	2.44E-12	TRUE
CD4 NC	*MPV17L2*	Wald ratio	1	0.0437	0.0189	1.045 (1.007, 1.084)	0.021	0.132	1.68E-09	TRUE
CD4 NC	*MPZL2*	Wald ratio	1	0.0488	0.0189	1.05 (1.012, 1.09)	0.01	0.08	4.59E-09	TRUE
CD4 NC	*MPZL3*	Wald ratio	1	0.0080	0.0115	1.008 (0.985, 1.031)	0.49	0.738	5.04E-22	TRUE
CD4 NC	*MRPL14*	Wald ratio	1	-0.0049	0.0128	0.995 (0.97, 1.02)	0.7	0.866	2.18E-18	TRUE
CD4 NC	*MRPL18*	Wald ratio	1	-0.0014	0.0137	0.999 (0.972, 1.026)	0.92	0.965	1.79E-19	TRUE
CD4 NC	*MRPL19*	Wald ratio	1	-0.0338	0.0131	0.967 (0.942, 0.992)	0.01	0.082	4.96E-16	TRUE
CD4 NC	*MRPL20*	Wald ratio	1	-0.0204	0.0178	0.98 (0.946, 1.015)	0.251	0.544	1.43E-08	TRUE
CD4 NC	*MRPL21*	Wald ratio	1	0.0065	0.0060	1.006 (0.995, 1.018)	0.284	0.577	8.38E-72	TRUE
CD4 NC	*MRPL32*	Wald ratio	1	0.0092	0.0132	1.009 (0.983, 1.036)	0.485	0.736	1.93E-28	TRUE
CD4 NC	*MRPL4*	Wald ratio	1	-0.0247	0.0217	0.976 (0.935, 1.018)	0.253	0.547	2.20E-10	TRUE
CD4 NC	*MRPL41*	Wald ratio	1	0.0650	0.0180	1.067 (1.03, 1.105)	2.99E-04	0.007	1.33E-12	TRUE
CD4 NC	*MRPL42*	Wald ratio	1	0.0139	0.0077	1.014 (0.999, 1.029)	0.071	0.276	6.66E-47	TRUE
CD4 NC	*MRPL48*	Wald ratio	1	-0.0074	0.0100	0.993 (0.973, 1.012)	0.456	0.719	1.48E-28	TRUE
CD4 NC	*MRPL51*	Wald ratio	1	0.0177	0.0186	1.018 (0.981, 1.056)	0.341	0.635	1.23E-09	TRUE
CD4 NC	*MRPL53*	Wald ratio	1	-0.0063	0.0111	0.994 (0.972, 1.015)	0.569	0.782	5.66E-21	TRUE
CD4 NC	*MRPL54*	Wald ratio	1	0.0069	0.0078	1.007 (0.992, 1.023)	0.377	0.665	2.98E-46	TRUE
CD4 NC	*MRPS10*	Wald ratio	1	-0.0035	0.0123	0.997 (0.973, 1.021)	0.777	0.904	6.70E-22	TRUE
CD4 NC	*MRPS18C*	Wald ratio	1	-0.0010	0.0105	0.999 (0.979, 1.02)	0.922	0.965	1.08E-26	TRUE
CD4 NC	*MRPS21*	Wald ratio	1	-0.0545	0.0122	0.947 (0.925, 0.97)	7.90E-06	4.83E-04	2.93E-19	TRUE
CD4 NC	*MRPS33*	Wald ratio	1	-0.0232	0.0192	0.977 (0.941, 1.014)	0.226	0.52	8.33E-11	TRUE
CD4 NC	*MRPS34*	Wald ratio	1	0.0964	0.0199	1.101 (1.059, 1.145)	1.34E-06	1.42E-04	9.80E-08	TRUE
CD4 NC	*MRPS36*	Wald ratio	1	0.0522	0.0161	1.054 (1.021, 1.087)	0.001	0.019	6.87E-12	TRUE
CD4 NC	*MRPS6*	Wald ratio	1	0.0061	0.0148	1.006 (0.977, 1.036)	0.679	0.853	7.43E-14	TRUE
CD4 NC	*MSC*	Wald ratio	1	-0.0040	0.0238	0.996 (0.951, 1.044)	0.868	0.945	3.72E-08	TRUE
CD4 NC	*MSH3*	Wald ratio	1	-0.0088	0.0143	0.991 (0.964, 1.019)	0.538	0.762	3.68E-14	TRUE
CD4 NC	*MT1X*	Wald ratio	1	0.0028	0.0073	1.003 (0.989, 1.017)	0.702	0.866	3.26E-58	TRUE
CD4 NC	*MT2A*	Wald ratio	1	-0.0140	0.0088	0.986 (0.969, 1.003)	0.11	0.353	3.15E-44	TRUE
CD4 NC	*MT3*	Wald ratio	1	-0.0352	0.0192	0.965 (0.93, 1.002)	0.067	0.265	5.20E-10	TRUE
CD4 NC	*MTERFD2*	Wald ratio	1	-0.0049	0.0147	0.995 (0.967, 1.024)	0.742	0.881	1.46E-13	TRUE
CD4 NC	*MTG2*	Wald ratio	1	0.0261	0.0118	1.026 (1.003, 1.051)	0.028	0.155	2.25E-21	TRUE
CD4 NC	*MTHFS*	Wald ratio	1	0.0284	0.0213	1.029 (0.987, 1.073)	0.183	0.462	1.13E-08	TRUE
CD4 NC	*MTRF1L*	Wald ratio	1	0.0181	0.0125	1.018 (0.994, 1.044)	0.148	0.417	4.07E-20	TRUE
CD4 NC	*MTSS1*	Wald ratio	1	0.0120	0.0085	1.012 (0.995, 1.029)	0.159	0.43	4.72E-46	TRUE
CD4 NC	*MTUS1*	Wald ratio	1	0.0034	0.0116	1.003 (0.981, 1.026)	0.766	0.896	5.10E-19	TRUE
CD4 NC	*MUC20*	Wald ratio	1	-0.0055	0.0169	0.995 (0.962, 1.028)	0.747	0.884	1.39E-10	TRUE
CD4 NC	*MX1*	Wald ratio	1	0.0194	0.0148	1.02 (0.99, 1.05)	0.189	0.47	9.68E-14	TRUE
CD4 NC	*MX2*	Wald ratio	1	0.0103	0.0158	1.01 (0.98, 1.042)	0.514	0.75	2.97E-12	TRUE
CD4 NC	*MXRA7*	Wald ratio	1	0.0045	0.0127	1.005 (0.98, 1.03)	0.72	0.876	5.71E-14	TRUE
CD4 NC	*MYADM*	Wald ratio	1	-0.0062	0.0218	0.994 (0.952, 1.037)	0.777	0.903	8.24E-10	TRUE
CD4 NC	*MYCBPAP*	Wald ratio	1	-0.0006	0.0099	0.999 (0.98, 1.019)	0.952	0.982	4.24E-30	TRUE
CD4 NC	*MYH11*	Wald ratio	1	-0.0140	0.0080	0.986 (0.971, 1.002)	0.079	0.294	2.17E-47	TRUE
CD4 NC	*MYO15B*	Wald ratio	1	0.0099	0.0142	1.01 (0.982, 1.038)	0.486	0.737	5.23E-22	TRUE
CD4 NC	*MYO16*	Wald ratio	1	-0.0331	0.0098	0.967 (0.949, 0.986)	6.99E-04	0.013	8.13E-32	TRUE
CD4 NC	*MYO6*	Wald ratio	1	-0.0028	0.0184	0.997 (0.962, 1.034)	0.881	0.951	2.87E-09	TRUE
CD4 NC	*MYO7B*	Wald ratio	1	-0.0088	0.0072	0.991 (0.977, 1.005)	0.224	0.518	1.29E-56	TRUE

CD4 NC	*MYOM2*	Wald ratio	1	-0.0047	0.0055	0.995 (0.985, 1.006)	0.392	0.672	7.73E-77	TRUE
CD4 NC	*MZT2A*	Wald ratio	1	-0.0086	0.0196	0.991 (0.954, 1.03)	0.663	0.845	1.21E-08	TRUE
CD4 NC	*N4BP2*	Wald ratio	1	0.0086	0.0132	1.009 (0.983, 1.035)	0.512	0.748	3.63E-18	TRUE
CD4 NC	*NAA38*	Wald ratio	1	-0.0054	0.0079	0.995 (0.979, 1.01)	0.495	0.74	5.15E-63	TRUE
CD4 NC	*NAAA*	Wald ratio	1	0.0147	0.0075	1.015 (1, 1.03)	0.048	0.217	5.58E-45	TRUE
CD4 NC	*NAP1L1*	Wald ratio	1	-0.0071	0.0090	0.993 (0.976, 1.011)	0.432	0.701	6.30E-36	TRUE
CD4 NC	*NAPRT1*	Wald ratio	1	-0.0091	0.0055	0.991 (0.98, 1.002)	0.099	0.333	2.04E-96	TRUE
CD4 NC	*NARFL*	Wald ratio	1	0.0482	0.0143	1.049 (1.02, 1.079)	7.67E-04	0.014	1.18E-10	TRUE
CD4 NC	*NAV2*	Wald ratio	1	-0.0198	0.0108	0.98 (0.96, 1.001)	0.067	0.265	2.15E-23	TRUE
CD4 NC	*NBL1*	IVW	2	0.0083	0.0151	1.008 (0.979, 1.039)	0.584	0.793	1.53E-45	TRUE
CD4 NC	*NBPF1*	Wald ratio	1	-0.0400	0.0176	0.961 (0.928, 0.995)	0.023	0.14	8.41E-11	TRUE
CD4 NC	*NBPF3*	Wald ratio	1	0.0004	0.0151	1 (0.971, 1.03)	0.981	0.993	1.55E-13	TRUE
CD4 NC	*NCAPG2*	Wald ratio	1	-0.0121	0.0132	0.988 (0.963, 1.014)	0.36	0.65	2.38E-23	TRUE
CD4 NC	*NCBP2*	Wald ratio	1	0.0346	0.0161	1.035 (1.003, 1.068)	0.032	0.168	1.33E-12	TRUE
CD4 NC	*NCF2*	Wald ratio	1	-0.0058	0.0057	0.994 (0.983, 1.005)	0.309	0.603	8.33E-95	TRUE
CD4 NC	*NCF4*	Wald ratio	1	-0.0163	0.0128	0.984 (0.959, 1.009)	0.203	0.487	3.45E-19	TRUE
CD4 NC	*NCL*	Wald ratio	1	-0.0415	0.0190	0.959 (0.924, 0.996)	0.029	0.16	5.77E-10	TRUE
CD4 NC	*NDFIP1*	Wald ratio	1	0.0233	0.0190	1.024 (0.986, 1.062)	0.219	0.51	5.52E-09	TRUE
CD4 NC	*NDUFA12*	Wald ratio	1	0.0025	0.0069	1.003 (0.989, 1.016)	0.712	0.872	3.17E-76	TRUE
CD4 NC	*NDUFA3*	Wald ratio	1	0.0030	0.0086	1.003 (0.986, 1.02)	0.726	0.878	3.19E-41	TRUE
CD4 NC	*NDUFB1*	Wald ratio	1	-0.0059	0.0163	0.994 (0.963, 1.026)	0.719	0.875	1.17E-11	TRUE
CD4 NC	*NDUFB2*	Wald ratio	1	-0.0311	0.0175	0.969 (0.937, 1.003)	0.075	0.287	2.68E-09	TRUE
CD4 NC	*NDUFS5*	Wald ratio	1	-0.0061	0.0069	0.994 (0.981, 1.007)	0.378	0.665	4.47E-72	TRUE
CD4 NC	*NDUFV3*	Wald ratio	1	0.0162	0.0129	1.016 (0.991, 1.042)	0.206	0.494	9.16E-15	TRUE
CD4 NC	*NEDD4L*	Wald ratio	1	-0.0067	0.0184	0.993 (0.958, 1.03)	0.715	0.873	1.33E-09	TRUE
CD4 NC	*NEIL2*	Wald ratio	1	0.0623	0.0195	1.064 (1.024, 1.106)	0.001	0.02	4.32E-10	TRUE
CD4 NC	*NEK1*	Wald ratio	1	0.0035	0.0189	1.004 (0.967, 1.041)	0.853	0.938	1.06E-11	TRUE
CD4 NC	*NEK11*	Wald ratio	1	-0.0045	0.0232	0.996 (0.951, 1.042)	0.846	0.935	2.95E-08	TRUE
CD4 NC	*NEURL1*	Wald ratio	1	0.0369	0.0230	1.038 (0.992, 1.085)	0.109	0.352	2.29E-08	TRUE
CD4 NC	*NEXN*	Wald ratio	1	-0.0267	0.0182	0.974 (0.94, 1.009)	0.142	0.41	1.21E-09	TRUE
CD4 NC	*NFYC-AS1*	Wald ratio	1	0.0151	0.0184	1.015 (0.979, 1.053)	0.411	0.686	2.55E-09	TRUE
CD4 NC	*NINJ2*	Wald ratio	1	-0.0031	0.0094	0.997 (0.979, 1.015)	0.739	0.881	6.37E-31	TRUE
CD4 NC	*NINL*	Wald ratio	1	0.0336	0.0182	1.034 (0.998, 1.072)	0.064	0.259	6.23E-09	TRUE
CD4 NC	*NIPSNAP3A*	Wald ratio	1	-0.0001	0.0233	1 (0.955, 1.047)	0.995	0.999	6.11E-09	TRUE
CD4 NC	*NIPSNAP3B*	Wald ratio	1	-0.0041	0.0194	0.996 (0.959, 1.034)	0.831	0.93	3.45E-13	TRUE
CD4 NC	*NIT2*	Wald ratio	1	-0.0410	0.0174	0.96 (0.928, 0.993)	0.019	0.121	3.41E-10	TRUE
CD4 NC	*NKAPL*	Wald ratio	1	0.0355	0.0464	1.036 (0.946, 1.135)	0.444	0.709	7.40E-09	TRUE
CD4 NC	*NLRC5*	Wald ratio	1	0.0020	0.0189	1.002 (0.966, 1.04)	0.915	0.963	2.74E-09	TRUE
CD4 NC	*NLRP2*	Wald ratio	1	-0.0036	0.0151	0.996 (0.967, 1.026)	0.812	0.92	6.36E-14	TRUE
CD4 NC	*NMB*	Wald ratio	1	-0.0786	0.0195	0.924 (0.89, 0.96)	5.50E-05	0.002	1.21E-08	TRUE
CD4 NC	*NME1*	Wald ratio	1	0.0025	0.0074	1.002 (0.988, 1.017)	0.737	0.88	1.28E-53	TRUE
CD4 NC	*NME3*	Wald ratio	1	0.0794	0.0152	1.083 (1.051, 1.115)	1.87E-07	6.60E-05	6.70E-11	TRUE
CD4 NC	*NMI*	Wald ratio	1	0.0134	0.0067	1.013 (1, 1.027)	0.045	0.208	1.56E-65	TRUE
CD4 NC	*NMRAL1*	IVW	2	0.0168	0.0109	1.017 (0.995, 1.039)	0.125	0.38	2.16E-27	TRUE
CD4 NC	*NMRK1*	Wald ratio	1	-0.0045	0.0065	0.996 (0.983, 1.008)	0.495	0.74	1.08E-82	TRUE
CD4 NC	*NOL9*	Wald ratio	1	-0.0046	0.0160	0.995 (0.965, 1.027)	0.774	0.901	1.35E-14	TRUE
CD4 NC	*NOP10*	Wald ratio	1	0.0255	0.0212	1.026 (0.984, 1.069)	0.23	0.523	3.16E-08	TRUE
CD4 NC	*NPAT*	Wald ratio	1	-0.0327	0.0158	0.968 (0.938, 0.998)	0.039	0.19	8.63E-13	TRUE
CD4 NC	*NPC2*	IVW	2	0.0081	0.0151	1.008 (0.979, 1.038)	0.592	0.798	6.95E-108	TRUE
CD4 NC	*NPCDR1*	Wald ratio	1	0.0214	0.0164	1.022 (0.989, 1.055)	0.193	0.474	2.38E-11	TRUE
CD4 NC	*NPDC1*	Wald ratio	1	0.0131	0.0143	1.013 (0.985, 1.042)	0.359	0.65	4.24E-15	TRUE
CD4 NC	*NPHP3*	Wald ratio	1	-0.0106	0.0154	0.989 (0.96, 1.02)	0.489	0.738	7.02E-10	TRUE
CD4 NC	*NPIPA5*	Wald ratio	1	-0.0229	0.0161	0.977 (0.947, 1.009)	0.156	0.428	8.34E-12	TRUE
CD4 NC	*NPRL3*	Wald ratio	1	-0.0019	0.0154	0.998 (0.968, 1.029)	0.901	0.958	2.11E-12	TRUE
CD4 NC	*NQO2*	Wald ratio	1	-0.0007	0.0069	0.999 (0.986, 1.013)	0.914	0.962	4.36E-64	TRUE
CD4 NC	*NR2F6*	Wald ratio	1	0.0089	0.0129	1.009 (0.984, 1.035)	0.491	0.738	3.48E-20	TRUE
CD4 NC	*NRN1*	Wald ratio	1	0.0328	0.0200	1.033 (0.994, 1.075)	0.1	0.335	4.64E-08	TRUE
CD4 NC	*NSA2*	Wald ratio	1	0.0141	0.0062	1.014 (1.002, 1.027)	0.024	0.143	1.58E-85	TRUE
CD4 NC	*NSUN4*	Wald ratio	1	0.0018	0.0149	1.002 (0.973, 1.031)	0.902	0.959	3.24E-13	TRUE
CD4 NC	*NSUN5*	Wald ratio	1	-0.0337	0.0154	0.967 (0.938, 0.996)	0.029	0.158	4.55E-13	TRUE
CD4 NC	*NT5C3A*	Wald ratio	1	-0.0153	0.0100	0.985 (0.966, 1.004)	0.125	0.38	2.68E-32	TRUE
CD4 NC	*NT5C3B*	Wald ratio	1	0.0001	0.0064	1 (0.988, 1.013)	0.991	0.996	6.62E-61	TRUE
CD4 NC	*NT5E*	Wald ratio	1	-0.0077	0.0202	0.992 (0.954, 1.032)	0.701	0.866	5.52E-10	TRUE
CD4 NC	*NT5M*	Wald ratio	1	0.0143	0.0189	1.014 (0.978, 1.053)	0.448	0.713	5.30E-10	TRUE
CD4 NC	*NTPCR*	Wald ratio	1	0.0098	0.0055	1.01 (0.999, 1.021)	0.078	0.292	6.09E-109	TRUE
CD4 NC	*NUCB1*	Wald ratio	1	-0.0367	0.0248	0.964 (0.918, 1.012)	0.139	0.402	5.69E-11	TRUE
CD4 NC	*NUCKS1*	Wald ratio	1	0.0201	0.0166	1.02 (0.988, 1.054)	0.225	0.519	4.01E-11	TRUE
CD4 NC	*NUDT12*	Wald ratio	1	0.0181	0.0106	1.018 (0.997, 1.04)	0.088	0.313	4.86E-32	TRUE
CD4 NC	*NUDT18*	Wald ratio	1	-0.0076	0.0096	0.992 (0.974, 1.011)	0.431	0.7	4.90E-32	TRUE
CD4 NC	*NUDT2*	IVW	2	-0.0062	0.0046	0.994 (0.985, 1.003)	0.174	0.453	3.91E-144	TRUE
CD4 NC	*NUP107*	Wald ratio	1	-0.0192	0.0104	0.981 (0.961, 1.001)	0.066	0.263	9.91E-25	TRUE
CD4 NC	*NUTF2*	Wald ratio	1	0.0136	0.0123	1.014 (0.99, 1.038)	0.268	0.562	6.62E-18	TRUE
CD4 NC	*NXPE3*	Wald ratio	1	-0.0265	0.0142	0.974 (0.947, 1.001)	0.062	0.253	5.24E-15	TRUE
CD4 NC	*OCEL1*	Wald ratio	1	-0.0042	0.0093	0.996 (0.978, 1.014)	0.647	0.834	5.25E-31	TRUE
CD4 NC	*OCIAD2*	Wald ratio	1	0.0563	0.0204	1.058 (1.016, 1.101)	0.006	0.055	1.02E-07	TRUE
CD4 NC	*ODF2L*	Wald ratio	1	-0.0034	0.0153	0.997 (0.967, 1.027)	0.822	0.926	5.67E-14	TRUE
CD4 NC	*OR2A1-AS1*	Wald ratio	1	0.0192	0.0185	1.019 (0.983, 1.057)	0.301	0.596	3.92E-11	TRUE
CD4 NC	*ORAI1*	Wald ratio	1	-0.0227	0.0147	0.978 (0.95, 1.006)	0.123	0.378	7.81E-18	TRUE
CD4 NC	*ORMDL1*	Wald ratio	1	-0.0128	0.0168	0.987 (0.955, 1.02)	0.449	0.714	1.08E-10	TRUE

CD4 NC	*ORMDL3*	Wald ratio	1	-0.0021	0.0059	0.998 (0.986, 1.01)	0.724	0.878	4.35E-64	TRUE
CD4 NC	*OSBPL10*	Wald ratio	1	0.0075	0.0129	1.008 (0.982, 1.033)	0.561	0.778	1.33E-18	TRUE
CD4 NC	*OSBPL1A*	Wald ratio	1	-0.0237	0.0149	0.977 (0.948, 1.005)	0.111	0.354	7.09E-14	TRUE
CD4 NC	*OSBPL6*	Wald ratio	1	-0.0034	0.0106	0.997 (0.976, 1.017)	0.748	0.885	1.65E-28	TRUE
CD4 NC	*OSER1-AS1*	Wald ratio	1	0.0442	0.0137	1.045 (1.017, 1.074)	0.001	0.02	6.51E-14	TRUE
CD4 NC	*OSGIN2*	Wald ratio	1	-0.0136	0.0175	0.986 (0.953, 1.021)	0.435	0.702	1.20E-10	TRUE
CD4 NC	*OSTF1*	Wald ratio	1	-0.0066	0.0093	0.993 (0.975, 1.012)	0.477	0.732	5.44E-38	TRUE
CD4 NC	*OXLD1*	Wald ratio	1	0.0029	0.0068	1.003 (0.99, 1.016)	0.668	0.848	1.09E-60	TRUE
CD4 NC	*OXNAD1*	Wald ratio	1	0.0060	0.0170	1.006 (0.973, 1.04)	0.724	0.878	1.14E-10	TRUE
CD4 NC	*P4HA3*	Wald ratio	1	-0.0025	0.0099	0.998 (0.978, 1.017)	0.804	0.915	6.66E-30	TRUE
CD4 NC	*PABPC4*	Wald ratio	1	-0.0984	0.0210	0.906 (0.87, 0.944)	2.68E-06	2.33E-04	8.09E-08	TRUE
CD4 NC	*PADI4*	IVW	2	0.0081	0.0088	1.008 (0.991, 1.026)	0.36	0.65	1.51E-42	TRUE
CD4 NC	*PAFAH1B2*	Wald ratio	1	0.0142	0.0145	1.014 (0.986, 1.044)	0.326	0.62	1.49E-12	TRUE
CD4 NC	*PAN3*	Wald ratio	1	0.0193	0.0182	1.02 (0.984, 1.057)	0.288	0.58	3.91E-09	TRUE
CD4 NC	*PAN3-AS1*	Wald ratio	1	-0.0064	0.0191	0.994 (0.957, 1.031)	0.739	0.881	6.96E-09	TRUE
CD4 NC	*PANK4*	Wald ratio	1	-0.0320	0.0151	0.969 (0.94, 0.998)	0.034	0.175	1.10E-12	TRUE
CD4 NC	*PANX1*	Wald ratio	1	-0.0498	0.0206	0.951 (0.914, 0.991)	0.015	0.107	8.17E-08	TRUE
CD4 NC	*PARK7*	Wald ratio	1	0.0103	0.0101	1.01 (0.991, 1.031)	0.305	0.599	2.51E-30	TRUE
CD4 NC	*PARP14*	Wald ratio	1	0.0013	0.0110	1.001 (0.98, 1.023)	0.904	0.959	5.97E-13	TRUE
CD4 NC	*PARP15*	Wald ratio	1	-0.0598	0.0196	0.942 (0.907, 0.979)	0.002	0.029	2.81E-08	TRUE
CD4 NC	*PARVB*	Wald ratio	1	0.0055	0.0099	1.006 (0.986, 1.025)	0.578	0.789	3.72E-31	TRUE
CD4 NC	*PASK*	Wald ratio	1	-0.0026	0.0061	0.997 (0.985, 1.009)	0.669	0.848	1.71E-73	TRUE
CD4 NC	*PAX8*	Wald ratio	1	-0.0180	0.0098	0.982 (0.963, 1.001)	0.067	0.265	4.38E-28	TRUE
CD4 NC	*PBX3*	Wald ratio	1	-0.0452	0.0183	0.956 (0.922, 0.991)	0.014	0.099	5.58E-11	TRUE
CD4 NC	*PCBP2*	Wald ratio	1	-0.0779	0.0198	0.925 (0.89, 0.962)	8.45E-05	0.003	5.71E-14	TRUE
CD4 NC	*PCBP3*	Wald ratio	1	-0.0155	0.0193	0.985 (0.948, 1.023)	0.422	0.692	1.30E-11	TRUE
CD4 NC	*PCED1B*	Wald ratio	1	0.0147	0.0139	1.015 (0.988, 1.043)	0.289	0.582	2.01E-15	TRUE
CD4 NC	*PCM1*	Wald ratio	1	-0.0139	0.0103	0.986 (0.966, 1.006)	0.179	0.458	1.03E-28	TRUE
CD4 NC	*PCMTD1*	Wald ratio	1	-0.0263	0.0187	0.974 (0.939, 1.01)	0.16	0.433	1.04E-09	TRUE
CD4 NC	*PCNX*	Wald ratio	1	0.0410	0.0115	1.042 (1.019, 1.066)	3.80E-04	0.009	2.59E-21	TRUE
CD4 NC	*PCSK7*	Wald ratio	1	-0.0217	0.0153	0.979 (0.95, 1.008)	0.156	0.427	1.82E-11	TRUE
CD4 NC	*PDE7A*	Wald ratio	1	0.0219	0.0179	1.022 (0.987, 1.059)	0.221	0.513	9.00E-11	TRUE
CD4 NC	*PDE9A*	Wald ratio	1	0.0049	0.0119	1.005 (0.982, 1.029)	0.682	0.855	2.41E-22	TRUE
CD4 NC	*PDGFB*	Wald ratio	1	0.0546	0.0178	1.056 (1.02, 1.094)	0.002	0.027	9.66E-10	TRUE
CD4 NC	*PDIA6*	Wald ratio	1	-0.0224	0.0113	0.978 (0.956, 1)	0.046	0.212	2.24E-23	TRUE
CD4 NC	*PDK1*	Wald ratio	1	-0.0247	0.0180	0.976 (0.942, 1.011)	0.172	0.45	2.39E-08	TRUE
CD4 NC	*PDXK*	Wald ratio	1	0.0025	0.0075	1.002 (0.988, 1.017)	0.741	0.881	5.26E-46	TRUE
CD4 NC	*PEBP4*	Wald ratio	1	-0.0358	0.0145	0.965 (0.938, 0.993)	0.013	0.098	2.03E-13	TRUE
CD4 NC	*PERP*	Wald ratio	1	-0.0129	0.0187	0.987 (0.952, 1.024)	0.491	0.738	3.98E-09	TRUE
CD4 NC	*PEX5L*	Wald ratio	1	-0.0064	0.0078	0.994 (0.979, 1.009)	0.416	0.688	8.51E-68	TRUE
CD4 NC	*PFKP*	Wald ratio	1	0.0069	0.0140	1.007 (0.98, 1.035)	0.623	0.822	1.37E-11	TRUE
CD4 NC	*PFN2*	Wald ratio	1	-0.0121	0.0103	0.988 (0.968, 1.008)	0.242	0.534	7.64E-27	TRUE
CD4 NC	*PGLS*	Wald ratio	1	0.0076	0.0135	1.008 (0.981, 1.035)	0.573	0.786	6.52E-17	TRUE
CD4 NC	*PGPEP1*	Wald ratio	1	0.0564	0.0163	1.058 (1.025, 1.092)	5.52E-04	0.011	1.84E-12	TRUE
CD4 NC	*PGS1*	Wald ratio	1	0.0195	0.0184	1.02 (0.984, 1.057)	0.289	0.582	1.35E-12	TRUE
CD4 NC	*PHACTR4*	Wald ratio	1	0.0435	0.0119	1.045 (1.02, 1.069)	2.64E-04	0.006	4.49E-19	TRUE
CD4 NC	*PHGDH*	Wald ratio	1	0.0089	0.0214	1.009 (0.968, 1.052)	0.676	0.853	7.91E-13	TRUE
CD4 NC	*PHLDB3*	Wald ratio	1	-0.0075	0.0119	0.992 (0.97, 1.016)	0.528	0.756	4.15E-20	TRUE
CD4 NC	*PHOSPHO2*	Wald ratio	1	-0.0420	0.0157	0.959 (0.93, 0.989)	0.007	0.066	2.80E-14	TRUE
CD4 NC	*PHYKPL*	Wald ratio	1	0.0236	0.0194	1.024 (0.986, 1.063)	0.224	0.517	4.02E-09	TRUE
CD4 NC	*PI16*	Wald ratio	1	-0.0057	0.0069	0.994 (0.981, 1.008)	0.407	0.682	3.87E-63	TRUE
CD4 NC	*PI4K2B*	Wald ratio	1	0.0030	0.0141	1.003 (0.976, 1.031)	0.833	0.93	3.08E-30	TRUE
CD4 NC	*PIGP*	Wald ratio	1	0.0032	0.0081	1.003 (0.987, 1.019)	0.692	0.861	4.17E-44	TRUE
CD4 NC	*PILRB*	Wald ratio	1	0.0042	0.0057	1.004 (0.993, 1.015)	0.467	0.725	1.95E-85	TRUE
CD4 NC	*PIP4K2A*	Wald ratio	1	-0.0165	0.0176	0.984 (0.95, 1.018)	0.347	0.64	2.00E-10	TRUE
CD4 NC	*PITRM1*	Wald ratio	1	-0.0050	0.0192	0.995 (0.958, 1.033)	0.794	0.911	3.25E-08	TRUE
CD4 NC	*PKIA*	Wald ratio	1	-0.0100	0.0097	0.99 (0.971, 1.009)	0.301	0.597	1.84E-28	TRUE
CD4 NC	*PKMYT1*	Wald ratio	1	-0.0337	0.0099	0.967 (0.948, 0.986)	6.77E-04	0.013	1.94E-25	TRUE
CD4 NC	*PLA2G4C*	Wald ratio	1	-0.0258	0.0193	0.975 (0.938, 1.012)	0.181	0.462	4.79E-11	TRUE
CD4 NC	*PLA2G6*	Wald ratio	1	0.0004	0.0207	1 (0.961, 1.042)	0.985	0.994	5.94E-08	TRUE
CD4 NC	*PLCB2*	Wald ratio	1	-0.0174	0.0119	0.983 (0.96, 1.006)	0.145	0.413	7.20E-19	TRUE
CD4 NC	*PLCE1*	Wald ratio	1	0.1431	0.0275	1.154 (1.093, 1.218)	1.95E-07	6.60E-05	6.90E-09	TRUE
CD4 NC	*PLEC*	Wald ratio	1	0.0173	0.0099	1.017 (0.998, 1.037)	0.082	0.3	1.16E-29	TRUE
CD4 NC	*PLEK*	Wald ratio	1	0.0120	0.0128	1.012 (0.987, 1.038)	0.352	0.644	6.67E-18	TRUE
CD4 NC	*PLEKHA1*	Wald ratio	1	0.0051	0.0091	1.005 (0.987, 1.023)	0.577	0.788	1.22E-40	TRUE
CD4 NC	*PLEKHA5*	Wald ratio	1	-0.0107	0.0154	0.989 (0.96, 1.02)	0.487	0.738	8.79E-12	TRUE
CD4 NC	*PLEKHG1*	Wald ratio	1	0.0491	0.0136	1.05 (1.023, 1.079)	2.92E-04	0.007	4.42E-11	TRUE
CD4 NC	*PLEKHG5*	Wald ratio	1	-0.0085	0.0198	0.992 (0.954, 1.031)	0.666	0.847	4.33E-10	TRUE
CD4 NC	*PLEKHH2*	Wald ratio	1	0.0140	0.0077	1.014 (0.999, 1.03)	0.07	0.274	6.71E-48	TRUE
CD4 NC	*PLEKHO1*	Wald ratio	1	-0.0482	0.0159	0.953 (0.924, 0.983)	0.002	0.03	2.57E-13	TRUE
CD4 NC	*PLGRKT*	Wald ratio	1	-0.0058	0.0083	0.994 (0.978, 1.011)	0.485	0.737	9.06E-41	TRUE
CD4 NC	*PM20D1*	Wald ratio	1	-0.0246	0.0120	0.976 (0.953, 0.999)	0.041	0.196	5.78E-20	TRUE
CD4 NC	*PMS1*	Wald ratio	1	-0.0112	0.0176	0.989 (0.955, 1.024)	0.526	0.755	1.83E-11	TRUE
CD4 NC	*POLE2*	Wald ratio	1	-0.0022	0.0169	0.998 (0.965, 1.032)	0.898	0.957	7.96E-11	TRUE
CD4 NC	*POLI*	Wald ratio	1	-0.0361	0.0166	0.964 (0.934, 0.996)	0.029	0.16	8.12E-11	TRUE
CD4 NC	*POLM*	Wald ratio	1	0.0063	0.0096	1.006 (0.988, 1.025)	0.507	0.745	7.41E-27	TRUE
CD4 NC	*POLR1D*	Wald ratio	1	0.0009	0.0067	1.001 (0.988, 1.014)	0.888	0.955	1.24E-87	TRUE
CD4 NC	*POLR1E*	Wald ratio	1	-0.0326	0.0148	0.968 (0.94, 0.996)	0.028	0.156	7.32E-13	TRUE
CD4 NC	*POLR2I*	Wald ratio	1	0.0227	0.0175	1.023 (0.988, 1.059)	0.195	0.477	1.43E-10	TRUE

CD4 NC	*POLR2J*	Wald ratio	1	-0.0372	0.0154	0.963 (0.935, 0.993)	0.015	0.106	6.53E-12	TRUE
CD4 NC	*POLR2J3*	Wald ratio	1	0.0353	0.0142	1.036 (1.007, 1.065)	0.013	0.097	4.31E-14	TRUE
CD4 NC	*POMC*	Wald ratio	1	-0.0202	0.0102	0.98 (0.961, 1)	0.047	0.213	1.28E-24	TRUE
CD4 NC	*POMZP3*	Wald ratio	1	0.0121	0.0088	1.012 (0.995, 1.03)	0.173	0.451	2.04E-46	TRUE
CD4 NC	*POP5*	Wald ratio	1	0.0077	0.0191	1.008 (0.971, 1.046)	0.689	0.86	1.43E-08	TRUE
CD4 NC	*POR*	Wald ratio	1	0.0122	0.0161	1.012 (0.981, 1.045)	0.452	0.717	3.91E-08	TRUE
CD4 NC	*PPA1*	IVW	2	0.0006	0.0054	1.001 (0.99, 1.011)	0.918	0.965	1.91E-179	TRUE
CD4 NC	*PPAT*	Wald ratio	1	0.0248	0.0140	1.025 (0.997, 1.054)	0.076	0.288	3.64E-17	TRUE
CD4 NC	*PPDPF*	Wald ratio	1	-0.0062	0.0058	0.994 (0.983, 1.005)	0.283	0.575	5.86E-79	TRUE
CD4 NC	*PPFIBP2*	Wald ratio	1	-0.0040	0.0061	0.996 (0.984, 1.008)	0.517	0.752	9.79E-81	TRUE
CD4 NC	*PPIA*	Wald ratio	1	0.0090	0.0093	1.009 (0.991, 1.028)	0.333	0.626	1.47E-33	TRUE
CD4 NC	*PPIE*	Wald ratio	1	0.0223	0.0133	1.023 (0.996, 1.05)	0.094	0.326	1.03E-16	TRUE
CD4 NC	*PPIL3*	Wald ratio	1	0.0037	0.0051	1.004 (0.994, 1.014)	0.467	0.725	1.38E-112	TRUE
CD4 NC	*PPM1M*	Wald ratio	1	-0.0257	0.0101	0.975 (0.956, 0.994)	0.011	0.085	1.81E-28	TRUE
CD4 NC	*PPP3R1*	Wald ratio	1	0.0074	0.0214	1.007 (0.966, 1.051)	0.729	0.88	4.71E-08	TRUE
CD4 NC	*PPT1*	Wald ratio	1	0.0528	0.0124	1.054 (1.029, 1.08)	1.90E-05	9.34E-04	9.64E-13	TRUE
CD4 NC	*PPT2-EGFL8*	IVW	3	0.0098	0.0126	1.01 (0.985, 1.035)	0.437	0.703	1.24E-58	TRUE
CD4 NC	*PPT2-EGFL8*	MR Egger	3	0.1027	0.0368	1.108 (1.031, 1.191)	0.219	NA	NA	NA
CD4 NC	*PPT2-EGFL8*	Weighted median	3	0.0143	0.0084	1.014 (0.998, 1.031)	0.09	NA	NA	NA
CD4 NC	*PPT2-EGFL8*	Simple mode	3	0.0163	0.0118	1.016 (0.993, 1.04)	0.3	NA	NA	NA
CD4 NC	*PPT2-EGFL8*	Weighted mode	3	0.0177	0.0094	1.018 (0.999, 1.037)	0.202	NA	NA	NA
CD4 NC	*PPWD1*	Wald ratio	1	0.0169	0.0103	1.017 (0.997, 1.038)	0.101	0.337	1.23E-26	TRUE
CD4 NC	*PQLC3*	Wald ratio	1	0.0515	0.0186	1.053 (1.015, 1.092)	0.006	0.054	1.05E-19	TRUE
CD4 NC	*PRB3*	Wald ratio	1	0.0032	0.0128	1.003 (0.978, 1.029)	0.799	0.913	2.83E-19	TRUE
CD4 NC	*PRDX3*	Wald ratio	1	-0.0245	0.0160	0.976 (0.946, 1.007)	0.124	0.379	1.59E-12	TRUE
CD4 NC	*PRDX5*	Wald ratio	1	0.0208	0.0180	1.021 (0.986, 1.058)	0.247	0.54	5.29E-13	TRUE
CD4 NC	*PRDX6*	Wald ratio	1	-0.0064	0.0051	0.994 (0.984, 1.004)	0.207	0.494	1.06E-107	TRUE
CD4 NC	*PRELID1*	Wald ratio	1	0.0469	0.0092	1.048 (1.029, 1.067)	3.52E-07	8.32E-05	3.73E-32	TRUE
CD4 NC	*PRKAG2-AS1*	Wald ratio	1	0.0540	0.0165	1.056 (1.022, 1.09)	0.001	0.018	1.71E-12	TRUE
CD4 NC	*PRKAR1B*	Wald ratio	1	0.0252	0.0164	1.026 (0.993, 1.059)	0.125	0.38	5.33E-18	TRUE
CD4 NC	*PRKCB*	Wald ratio	1	0.0097	0.0079	1.01 (0.994, 1.026)	0.221	0.513	1.03E-60	TRUE
CD4 NC	*PRKCQ-AS1*	Wald ratio	1	-0.0039	0.0059	0.996 (0.985, 1.008)	0.511	0.748	1.22E-84	TRUE
CD4 NC	*PRKD3*	Wald ratio	1	0.0468	0.0155	1.048 (1.017, 1.08)	0.002	0.031	5.45E-13	TRUE
CD4 NC	*PRMT2*	IVW	2	0.0009	0.0058	1.001 (0.989, 1.012)	0.883	0.952	7.16E-76	TRUE
CD4 NC	*PROSC*	Wald ratio	1	-0.0140	0.0134	0.986 (0.96, 1.012)	0.298	0.593	3.15E-15	TRUE
CD4 NC	*PROSER2*	Wald ratio	1	-0.0129	0.0139	0.987 (0.961, 1.015)	0.355	0.646	2.02E-15	TRUE
CD4 NC	*PRPF40A*	Wald ratio	1	-0.0506	0.0188	0.951 (0.916, 0.986)	0.007	0.064	9.71E-11	TRUE
CD4 NC	*PRPF4B*	Wald ratio	1	0.0078	0.0189	1.008 (0.971, 1.046)	0.678	0.853	9.80E-10	TRUE
CD4 NC	*PRR24*	Wald ratio	1	-0.0077	0.0126	0.992 (0.968, 1.017)	0.543	0.765	2.15E-17	TRUE
CD4 NC	*PRR4*	Wald ratio	1	-0.0053	0.0167	0.995 (0.963, 1.028)	0.75	0.886	6.28E-19	TRUE
CD4 NC	*PRR7-AS1*	Wald ratio	1	0.0054	0.0048	1.005 (0.996, 1.015)	0.258	0.552	1.46E-138	TRUE
CD4 NC	*PSAT1*	Wald ratio	1	-0.0043	0.0181	0.996 (0.961, 1.032)	0.811	0.919	4.26E-10	TRUE
CD4 NC	*PSD4*	Wald ratio	1	-0.0095	0.0102	0.991 (0.971, 1.011)	0.353	0.644	4.77E-28	TRUE
CD4 NC	*PSMB11*	Wald ratio	1	-0.0179	0.0073	0.982 (0.968, 0.996)	0.015	0.104	5.80E-53	TRUE
CD4 NC	*PSMB9*	Wald ratio	1	-0.0075	0.0160	0.992 (0.962, 1.024)	0.638	0.829	3.78E-12	TRUE
CD4 NC	*PSMD2*	Wald ratio	1	-0.0045	0.0128	0.996 (0.971, 1.021)	0.728	0.879	1.91E-18	TRUE
CD4 NC	*PSMD5-AS1*	IVW	2	0.0030	0.0039	1.003 (0.995, 1.011)	0.452	0.717	4.83E-239	TRUE
CD4 NC	*PSME4*	Wald ratio	1	-0.0040	0.0167	0.996 (0.964, 1.029)	0.81	0.919	1.50E-11	TRUE
CD4 NC	*PSMF1*	Wald ratio	1	0.0251	0.0205	1.025 (0.985, 1.067)	0.22	0.511	6.86E-08	TRUE
CD4 NC	*PSMG1*	Wald ratio	1	-0.0428	0.0200	0.958 (0.921, 0.996)	0.032	0.17	7.54E-08	TRUE
CD4 NC	*PSMG4*	Wald ratio	1	-0.0065	0.0221	0.993 (0.951, 1.038)	0.768	0.897	3.42E-08	TRUE
CD4 NC	*PSPH*	Wald ratio	1	-0.0005	0.0079	1 (0.984, 1.015)	0.95	0.981	4.27E-35	TRUE
CD4 NC	*PSTPIP1*	Wald ratio	1	0.0076	0.0122	1.008 (0.984, 1.032)	0.533	0.76	1.66E-21	TRUE
CD4 NC	*PSTPIP2*	Wald ratio	1	0.0010	0.0143	1.001 (0.973, 1.029)	0.944	0.978	1.24E-15	TRUE
CD4 NC	*PTCH1*	Wald ratio	1	0.0404	0.0179	1.041 (1.005, 1.078)	0.024	0.143	1.21E-09	TRUE
CD4 NC	*PTEN*	Wald ratio	1	0.0480	0.0224	1.049 (1.004, 1.096)	0.032	0.169	2.50E-08	TRUE
CD4 NC	*PTGER2*	Wald ratio	1	0.0312	0.0188	1.032 (0.994, 1.071)	0.097	0.332	4.99E-09	TRUE
CD4 NC	*PTGIR*	Wald ratio	1	0.0019	0.0070	1.002 (0.988, 1.016)	0.789	0.909	9.59E-64	TRUE
CD4 NC	*PTPLA*	Wald ratio	1	-0.0073	0.0186	0.993 (0.957, 1.03)	0.695	0.863	2.68E-09	TRUE
CD4 NC	*PTPLAD2*	Wald ratio	1	-0.0150	0.0109	0.985 (0.964, 1.006)	0.169	0.444	3.72E-25	TRUE
CD4 NC	*PTPN4*	Wald ratio	1	-0.0023	0.0072	0.998 (0.984, 1.012)	0.745	0.883	2.29E-52	TRUE
CD4 NC	*PTPRN2*	Wald ratio	1	-0.0229	0.0238	0.977 (0.933, 1.024)	0.335	0.628	7.05E-08	TRUE
CD4 NC	*PTTG1IP*	Wald ratio	1	0.0013	0.0168	1.001 (0.969, 1.035)	0.938	0.975	2.17E-11	TRUE
CD4 NC	*PWWP2A*	Wald ratio	1	0.0024	0.0106	1.002 (0.982, 1.023)	0.825	0.926	1.43E-28	TRUE
CD4 NC	*PYGB*	Wald ratio	1	0.0107	0.0157	1.011 (0.98, 1.042)	0.493	0.738	1.06E-12	TRUE
CD4 NC	*PYHIN1*	Wald ratio	1	0.0042	0.0093	1.004 (0.986, 1.023)	0.65	0.835	9.20E-31	TRUE
CD4 NC	*PYROXD1*	Wald ratio	1	-0.0394	0.0120	0.961 (0.939, 0.984)	0.001	0.017	1.27E-19	TRUE
CD4 NC	*QDPR*	Wald ratio	1	0.0244	0.0164	1.025 (0.992, 1.058)	0.137	0.399	1.51E-12	TRUE
CD4 NC	*R3HDM4*	Wald ratio	1	-0.0020	0.0146	0.998 (0.97, 1.027)	0.888	0.955	2.72E-17	TRUE
CD4 NC	*RAB27B*	Wald ratio	1	-0.0009	0.0150	0.999 (0.97, 1.029)	0.954	0.983	3.66E-16	TRUE
CD4 NC	*RAB2A*	Wald ratio	1	0.0520	0.0189	1.053 (1.015, 1.093)	0.006	0.056	1.36E-08	TRUE
CD4 NC	*RAB34*	Wald ratio	1	-0.0450	0.0205	0.956 (0.918, 0.995)	0.028	0.157	6.33E-08	TRUE
CD4 NC	*RAB4A*	Wald ratio	1	-0.0505	0.0196	0.951 (0.915, 0.988)	0.01	0.082	4.93E-08	TRUE
CD4 NC	*RABAC1*	Wald ratio	1	0.0132	0.0086	1.013 (0.996, 1.03)	0.126	0.381	3.98E-44	TRUE
CD4 NC	*RABL2B*	Wald ratio	1	0.0287	0.0174	1.029 (0.995, 1.065)	0.1	0.334	4.71E-09	TRUE
CD4 NC	*RAC1*	Wald ratio	1	-0.0206	0.0072	0.98 (0.966, 0.993)	0.004	0.044	2.44E-56	TRUE
CD4 NC	*RAC2*	Wald ratio	1	0.0126	0.0178	1.013 (0.978, 1.049)	0.478	0.733	1.48E-10	TRUE
CD4 NC	*RAD54B*	Wald ratio	1	-0.0030	0.0164	0.997 (0.965, 1.03)	0.853	0.938	2.63E-11	TRUE
CD4 NC	*RAMP1*	Wald ratio	1	0.0123	0.0167	1.012 (0.98, 1.046)	0.461	0.721	3.51E-11	TRUE

CD4 NC	*RAMP3*	Wald ratio	1	-0.0333	0.0174	0.967 (0.935, 1.001)	0.056	0.238	3.85E-13	TRUE
CD4 NC	*RAPGEF6*	Wald ratio	1	-0.0176	0.0128	0.983 (0.958, 1.008)	0.171	0.448	8.79E-14	TRUE
CD4 NC	*RASA4*	Wald ratio	1	0.0367	0.0122	1.037 (1.013, 1.062)	0.003	0.031	5.10E-09	TRUE
CD4 NC	*RASGRF2*	Wald ratio	1	0.0092	0.0227	1.009 (0.965, 1.055)	0.686	0.857	1.71E-08	TRUE
CD4 NC	*RBAK-RBAKDN*	Wald ratio	1	-0.0069	0.0127	0.993 (0.969, 1.018)	0.589	0.796	2.02E-29	TRUE
CD4 NC	*RBL1*	Wald ratio	1	-0.0381	0.0193	0.963 (0.927, 1)	0.048	0.218	1.37E-08	TRUE
CD4 NC	*RBL2*	Wald ratio	1	0.0006	0.0096	1.001 (0.982, 1.02)	0.953	0.983	5.59E-34	TRUE
CD4 NC	*RBM11*	Wald ratio	1	-0.0108	0.0143	0.989 (0.962, 1.017)	0.452	0.717	3.79E-15	TRUE
CD4 NC	*RBM26-AS1*	Wald ratio	1	0.0047	0.0107	1.005 (0.984, 1.026)	0.66	0.843	2.08E-15	TRUE
CD4 NC	*RBM43*	Wald ratio	1	-0.0159	0.0067	0.984 (0.971, 0.997)	0.017	0.116	7.07E-65	TRUE
CD4 NC	*RBM6*	Wald ratio	1	-0.0475	0.0112	0.954 (0.933, 0.975)	2.14E-05	9.86E-04	5.09E-23	TRUE
CD4 NC	*RBMS1*	Wald ratio	1	0.0362	0.0092	1.037 (1.018, 1.056)	9.15E-05	0.003	4.58E-32	TRUE
CD4 NC	*RCHY1*	Wald ratio	1	-0.0027	0.0186	0.997 (0.962, 1.034)	0.885	0.954	4.09E-10	TRUE
CD4 NC	*RCSD1*	IVW	2	-0.0054	0.0074	0.995 (0.98, 1.009)	0.469	0.727	6.11E-58	TRUE
CD4 NC	*RDH13*	Wald ratio	1	-0.0168	0.0131	0.983 (0.958, 1.009)	0.201	0.484	2.81E-18	TRUE
CD4 NC	*RDH5*	Wald ratio	1	0.0102	0.0201	1.01 (0.971, 1.051)	0.614	0.814	4.05E-10	TRUE
CD4 NC	*REEP3*	Wald ratio	1	0.1148	0.0217	1.122 (1.075, 1.17)	1.21E-07	6.60E-05	1.52E-07	TRUE
CD4 NC	*REEP4*	Wald ratio	1	0.0331	0.0197	1.034 (0.995, 1.074)	0.093	0.323	3.25E-09	TRUE
CD4 NC	*REV3L*	Wald ratio	1	0.0053	0.0122	1.005 (0.981, 1.03)	0.665	0.847	3.09E-20	TRUE
CD4 NC	*RFC4*	Wald ratio	1	0.0160	0.0163	1.016 (0.984, 1.049)	0.325	0.62	1.83E-09	TRUE
CD4 NC	*RFTN1*	Wald ratio	1	-0.0018	0.0169	0.998 (0.966, 1.032)	0.913	0.962	8.71E-13	TRUE
CD4 NC	*RGCC*	Wald ratio	1	-0.0034	0.0183	0.997 (0.962, 1.033)	0.854	0.938	1.09E-09	TRUE
CD4 NC	*RGS10*	Wald ratio	1	0.0017	0.0195	1.002 (0.964, 1.041)	0.929	0.97	2.13E-08	TRUE
CD4 NC	*RGS12*	Wald ratio	1	-0.0214	0.0113	0.979 (0.957, 1.001)	0.059	0.244	2.89E-22	TRUE
CD4 NC	*RGS14*	Wald ratio	1	-0.0166	0.0152	0.984 (0.955, 1.013)	0.277	0.57	6.62E-12	TRUE
CD4 NC	*RHBDL1*	Wald ratio	1	-0.0355	0.0157	0.965 (0.936, 0.995)	0.023	0.14	1.39E-11	TRUE
CD4 NC	*RHOB*	Wald ratio	1	0.0214	0.0205	1.022 (0.981, 1.064)	0.296	0.591	3.59E-08	TRUE
CD4 NC	*RIBC2*	Wald ratio	1	-0.0047	0.0094	0.995 (0.977, 1.014)	0.616	0.816	5.33E-33	TRUE
CD4 NC	*RIC3*	Wald ratio	1	-0.0052	0.0134	0.995 (0.969, 1.021)	0.701	0.866	1.18E-16	TRUE
CD4 NC	*RICTOR*	Wald ratio	1	-0.0196	0.0201	0.981 (0.943, 1.02)	0.33	0.624	1.79E-08	TRUE
CD4 NC	*RINL*	Wald ratio	1	0.0247	0.0186	1.025 (0.988, 1.063)	0.186	0.465	3.07E-09	TRUE
CD4 NC	*RLN1*	Wald ratio	1	-0.0158	0.0168	0.984 (0.952, 1.017)	0.347	0.64	1.51E-12	TRUE
CD4 NC	*RMI2*	Wald ratio	1	-0.0003	0.0149	1 (0.971, 1.029)	0.985	0.994	1.07E-15	TRUE
CD4 NC	*RNASEH1-AS1*	Wald ratio	1	0.0158	0.0124	1.016 (0.992, 1.041)	0.202	0.487	1.49E-17	TRUE
CD4 NC	*RNASEH2C*	Wald ratio	1	0.0022	0.0102	1.002 (0.982, 1.022)	0.831	0.929	1.73E-31	TRUE
CD4 NC	*RNASET2*	Wald ratio	1	-0.0129	0.0046	0.987 (0.978, 0.996)	0.005	0.047	4.32E-157	TRUE
CD4 NC	*RNF13*	Wald ratio	1	-0.0258	0.0182	0.974 (0.94, 1.01)	0.156	0.427	1.63E-10	TRUE
CD4 NC	*RNF145*	Wald ratio	1	0.0448	0.0157	1.046 (1.014, 1.079)	0.004	0.046	1.20E-11	TRUE
CD4 NC	*RNF157*	Wald ratio	1	-0.0005	0.0179	1 (0.965, 1.035)	0.978	0.991	4.68E-10	TRUE
CD4 NC	*RNF166*	Wald ratio	1	-0.0264	0.0130	0.974 (0.949, 0.999)	0.043	0.202	2.92E-21	TRUE
CD4 NC	*RNF168*	IVW	2	-0.0074	0.0096	0.993 (0.974, 1.011)	0.436	0.703	4.69E-36	TRUE
CD4 NC	*RNF175*	Wald ratio	1	0.0216	0.0110	1.022 (1, 1.044)	0.049	0.221	6.16E-24	TRUE
CD4 NC	*RNF207*	Wald ratio	1	-0.0033	0.0214	0.997 (0.956, 1.039)	0.876	0.948	1.75E-08	TRUE
CD4 NC	*RNF212*	Wald ratio	1	0.0333	0.0157	1.034 (1.002, 1.066)	0.034	0.175	7.55E-12	TRUE
CD4 NC	*RNF24*	Wald ratio	1	-0.0338	0.0118	0.967 (0.945, 0.989)	0.004	0.045	7.71E-22	TRUE
CD4 NC	*RNF8*	Wald ratio	1	-0.0634	0.0203	0.939 (0.902, 0.977)	0.002	0.024	3.92E-08	TRUE
CD4 NC	*ROBO4*	Wald ratio	1	0.0063	0.0093	1.006 (0.988, 1.025)	0.501	0.743	2.62E-34	TRUE
CD4 NC	*RP1-102H19.8*	Wald ratio	1	-0.0077	0.0164	0.992 (0.961, 1.025)	0.64	0.83	5.98E-12	TRUE
CD4 NC	*RP1-130H16.16*	Wald ratio	1	0.0270	0.0157	1.027 (0.996, 1.059)	0.086	0.311	1.09E-14	TRUE
CD4 NC	*RP1-140A9.1*	Wald ratio	1	-0.0283	0.0136	0.972 (0.947, 0.998)	0.038	0.187	4.33E-16	TRUE
CD4 NC	*RP1-153P14.5*	Wald ratio	1	0.0050	0.0143	1.005 (0.977, 1.034)	0.727	0.878	3.57E-14	TRUE
CD4 NC	*RP1-178F15.4*	Wald ratio	1	-0.0475	0.0200	0.954 (0.917, 0.992)	0.017	0.116	4.59E-08	TRUE
CD4 NC	*RP1-187B23.1*	Wald ratio	1	0.0062	0.0180	1.006 (0.971, 1.042)	0.731	0.88	1.01E-08	TRUE
CD4 NC	*RP1-197B17.3*	Wald ratio	1	-0.0356	0.0162	0.965 (0.935, 0.996)	0.028	0.156	8.18E-09	TRUE
CD4 NC	*RP1-28O10.1*	Wald ratio	1	-0.0393	0.0140	0.961 (0.935, 0.988)	0.005	0.051	1.37E-12	TRUE
CD4 NC	*RP1-29C18.10*	Wald ratio	1	-0.0106	0.0053	0.989 (0.979, 1)	0.046	0.21	6.37E-121	TRUE
CD4 NC	*RP1-39G22.7*	Wald ratio	1	0.0717	0.0175	1.074 (1.038, 1.112)	3.98E-05	0.002	5.46E-10	TRUE
CD4 NC	*RP1-3J17.3*	IVW	2	0.0035	0.0062	1.004 (0.991, 1.016)	0.571	0.785	7.61E-80	TRUE
CD4 NC	*RP1-40E16.12*	Wald ratio	1	-0.0069	0.0087	0.993 (0.976, 1.01)	0.426	0.697	1.98E-39	TRUE
CD4 NC	*RP1-55C23.7*	Wald ratio	1	0.0017	0.0078	1.002 (0.987, 1.017)	0.827	0.928	1.35E-46	TRUE
CD4 NC	*RP1-90J20.8*	Wald ratio	1	-0.0121	0.0097	0.988 (0.969, 1.007)	0.208	0.495	1.28E-34	TRUE
CD4 NC	*RP11-1026M7.2*	Wald ratio	1	0.0171	0.0229	1.017 (0.973, 1.064)	0.455	0.718	5.08E-11	TRUE
CD4 NC	*RP11-103G8.2*	Wald ratio	1	-0.0020	0.0145	0.998 (0.97, 1.027)	0.891	0.957	1.61E-14	TRUE
CD4 NC	*RP11-104L21.3*	Wald ratio	1	0.0081	0.0200	1.008 (0.969, 1.048)	0.683	0.856	5.25E-10	TRUE
CD4 NC	*RP11-104N10.1*	Wald ratio	1	-0.0310	0.0189	0.969 (0.934, 1.006)	0.102	0.338	7.16E-09	TRUE
CD4 NC	*RP11-1060J15.4*	Wald ratio	1	-0.0083	0.0084	0.992 (0.976, 1.008)	0.32	0.615	3.09E-70	TRUE
CD4 NC	*RP11-108M9.4*	IVW	2	0.0181	0.0114	1.018 (0.996, 1.041)	0.111	0.355	5.72E-23	TRUE
CD4 NC	*RP11-108M9.6*	Wald ratio	1	0.0144	0.0165	1.014 (0.982, 1.048)	0.383	0.667	2.87E-11	TRUE
CD4 NC	*RP11-110I1.11*	Wald ratio	1	0.0064	0.0194	1.006 (0.969, 1.045)	0.742	0.881	2.80E-09	TRUE
CD4 NC	*RP11-111M22.3*	Wald ratio	1	-0.0042	0.0156	0.996 (0.966, 1.027)	0.789	0.909	5.35E-13	TRUE
CD4 NC	*RP11-1191J2.5*	Wald ratio	1	0.0048	0.0130	1.005 (0.98, 1.031)	0.713	0.872	1.20E-14	TRUE
CD4 NC	*RP11-119D9.1*	Wald ratio	1	0.0130	0.0188	1.013 (0.976, 1.051)	0.491	0.738	6.02E-14	TRUE
CD4 NC	*RP11-119F19.2*	Wald ratio	1	-0.0264	0.0120	0.974 (0.951, 0.997)	0.028	0.158	6.68E-20	TRUE
CD4 NC	*RP11-12J10.3*	Wald ratio	1	0.0136	0.0085	1.014 (0.997, 1.031)	0.11	0.353	3.22E-20	TRUE
CD4 NC	*RP11-131N11.4*	Wald ratio	1	0.0316	0.0158	1.032 (1.001, 1.065)	0.046	0.211	2.80E-12	TRUE
CD4 NC	*RP11-137H2.4*	Wald ratio	1	0.0089	0.0104	1.009 (0.989, 1.03)	0.391	0.672	1.45E-28	TRUE
CD4 NC	*RP11-138A9.2*	Wald ratio	1	0.0026	0.0175	1.003 (0.969, 1.038)	0.882	0.952	3.05E-13	TRUE
CD4 NC	*RP11-1398P2.1*	Wald ratio	1	-0.0145	0.0081	0.986 (0.97, 1.001)	0.075	0.287	3.56E-43	TRUE
CD4 NC	*RP11-139H15.1*	Wald ratio	1	-0.0176	0.0176	0.983 (0.949, 1.017)	0.317	0.612	9.61E-12	TRUE

CD4 NC	*RP11-140A10.3*	Wald ratio	1	-0.0087	0.0100	0.991 (0.972, 1.011)	0.384	0.668	1.29E-29	TRUE
CD4 NC	*RP11-143J12.2*	Wald ratio	1	0.0349	0.0177	1.035 (1, 1.072)	0.049	0.22	2.17E-10	TRUE
CD4 NC	*RP11-147L13.2*	Wald ratio	1	0.0083	0.0192	1.008 (0.971, 1.047)	0.666	0.847	1.09E-08	TRUE
CD4 NC	*RP11-157J24.2*	Wald ratio	1	-0.0144	0.0143	0.986 (0.959, 1.014)	0.313	0.607	7.22E-15	TRUE
CD4 NC	*RP11-160H22.5*	Wald ratio	1	0.0291	0.0189	1.03 (0.992, 1.068)	0.124	0.379	3.44E-09	TRUE
CD4 NC	*RP11-161M6.3*	Wald ratio	1	0.0076	0.0132	1.008 (0.982, 1.034)	0.567	0.782	1.14E-17	TRUE
CD4 NC	*RP11-166B2.1*	Wald ratio	1	0.0173	0.0095	1.017 (0.999, 1.036)	0.068	0.267	1.60E-30	TRUE
CD4 NC	*RP11-166B2.5*	IVW	2	0.0019	0.0098	1.002 (0.983, 1.021)	0.846	0.935	3.92E-51	TRUE
CD4 NC	*RP11-166P13.3*	Wald ratio	1	-0.0026	0.0180	0.997 (0.963, 1.033)	0.883	0.952	2.35E-09	TRUE
CD4 NC	*RP11-169K16.9*	Wald ratio	1	0.0234	0.0176	1.024 (0.989, 1.06)	0.185	0.464	7.12E-08	TRUE
CD4 NC	*RP11-173B14.5*	Wald ratio	1	-0.0107	0.0121	0.989 (0.966, 1.013)	0.377	0.665	1.80E-20	TRUE
CD4 NC	*RP11-176H8.1*	Wald ratio	1	-0.0471	0.0200	0.954 (0.917, 0.992)	0.018	0.12	1.04E-07	TRUE
CD4 NC	*RP11-179B2.2*	Wald ratio	1	-0.0641	0.0183	0.938 (0.905, 0.972)	4.61E-04	0.01	3.61E-09	TRUE
CD4 NC	*RP11-17M16.2*	Wald ratio	1	0.0176	0.0101	1.018 (0.998, 1.038)	0.08	0.297	3.60E-28	TRUE
CD4 NC	*RP11-187E13.1*	Wald ratio	1	0.0024	0.0186	1.002 (0.967, 1.04)	0.896	0.957	1.89E-09	TRUE
CD4 NC	*RP11-18H21.1*	IVW	2	0.0034	0.0154	1.003 (0.973, 1.034)	0.828	0.928	1.56E-140	TRUE
CD4 NC	*RP11-190A12.8*	Wald ratio	1	0.0109	0.0089	1.011 (0.993, 1.029)	0.223	0.516	6.52E-37	TRUE
CD4 NC	*RP11-218M22.1*	Wald ratio	1	0.0215	0.0125	1.022 (0.997, 1.047)	0.086	0.312	6.25E-18	TRUE
CD4 NC	*RP11-223C24.1*	Wald ratio	1	-0.0472	0.0155	0.954 (0.925, 0.983)	0.002	0.029	3.65E-17	TRUE
CD4 NC	*RP11-23P13.6*	Wald ratio	1	0.0387	0.0163	1.039 (1.007, 1.073)	0.018	0.117	1.52E-11	TRUE
CD4 NC	*RP11-245G13.2*	Wald ratio	1	-0.0086	0.0062	0.991 (0.979, 1.004)	0.167	0.442	7.26E-112	TRUE
CD4 NC	*RP11-254F7.2*	Wald ratio	1	-0.0006	0.0212	0.999 (0.959, 1.042)	0.976	0.991	9.80E-11	TRUE
CD4 NC	*RP11-273G15.2*	Wald ratio	1	0.0579	0.0125	1.06 (1.034, 1.086)	3.34E-06	2.62E-04	4.45E-18	TRUE
CD4 NC	*RP11-274B21.10*	Wald ratio	1	-0.0025	0.0135	0.997 (0.971, 1.024)	0.85	0.936	8.81E-14	TRUE
CD4 NC	*RP11-278H7.4*	Wald ratio	1	-0.0182	0.0193	0.982 (0.945, 1.02)	0.347	0.64	5.55E-14	TRUE
CD4 NC	*RP11-293A21.1*	Wald ratio	1	-0.0042	0.0170	0.996 (0.963, 1.03)	0.806	0.916	3.63E-11	TRUE
CD4 NC	*RP11-293M10.2*	Wald ratio	1	-0.0388	0.0120	0.962 (0.94, 0.985)	0.001	0.02	3.90E-20	TRUE
CD4 NC	*RP11-294J22.6*	Wald ratio	1	-0.0375	0.0221	0.963 (0.922, 1.006)	0.09	0.318	7.22E-15	TRUE
CD4 NC	*RP11-296O14.3*	Wald ratio	1	-0.0228	0.0181	0.977 (0.943, 1.013)	0.207	0.494	4.90E-09	TRUE
CD4 NC	*RP11-298J20.3*	Wald ratio	1	0.0127	0.0044	1.013 (1.004, 1.021)	0.004	0.041	6.53E-160	TRUE
CD4 NC	*RP11-299J3.8*	Wald ratio	1	0.0023	0.0109	1.002 (0.981, 1.024)	0.834	0.93	5.28E-30	TRUE
CD4 NC	*RP11-303E16.5*	Wald ratio	1	-0.0113	0.0099	0.989 (0.97, 1.008)	0.253	0.547	3.55E-19	TRUE
CD4 NC	*RP11-305K5.1*	Wald ratio	1	0.0115	0.0194	1.012 (0.974, 1.051)	0.552	0.771	3.95E-09	TRUE
CD4 NC	*RP11-306I1.2*	Wald ratio	1	0.0170	0.0132	1.017 (0.991, 1.044)	0.198	0.481	6.84E-18	TRUE
CD4 NC	*RP11-314C16.1*	Wald ratio	1	0.0040	0.0101	1.004 (0.984, 1.024)	0.695	0.863	9.00E-34	TRUE
CD4 NC	*RP11-315D16.4*	Wald ratio	1	-0.0228	0.0083	0.977 (0.962, 0.994)	0.006	0.057	3.60E-47	TRUE
CD4 NC	*RP11-324I22.4*	Wald ratio	1	-0.0108	0.0090	0.989 (0.972, 1.007)	0.23	0.524	2.48E-38	TRUE
CD4 NC	*RP11-325F22.2*	Wald ratio	1	0.0486	0.0200	1.05 (1.009, 1.092)	0.015	0.106	9.17E-09	TRUE
CD4 NC	*RP11-326C3.11*	IVW	2	-0.0043	0.0106	0.996 (0.975, 1.017)	0.687	0.858	2.60E-157	TRUE
CD4 NC	*RP11-326C3.12*	Wald ratio	1	-0.0047	0.0048	0.995 (0.986, 1.005)	0.325	0.62	4.66E-139	TRUE
CD4 NC	*RP11-326C3.15*	Wald ratio	1	-0.0085	0.0094	0.992 (0.973, 1.01)	0.364	0.653	2.22E-32	TRUE
CD4 NC	*RP11-334C17.5*	Wald ratio	1	0.0072	0.0109	1.007 (0.986, 1.029)	0.508	0.747	1.03E-24	TRUE
CD4 NC	*RP11-345J4.5*	Wald ratio	1	-0.0168	0.0076	0.983 (0.969, 0.998)	0.027	0.154	3.09E-52	TRUE
CD4 NC	*RP11-347P5.1*	Wald ratio	1	0.0042	0.0060	1.004 (0.992, 1.016)	0.489	0.738	2.89E-84	TRUE
CD4 NC	*RP11-377D9.3*	Wald ratio	1	-0.0001	0.0080	1 (0.984, 1.016)	0.986	0.994	5.78E-44	TRUE
CD4 NC	*RP11-379F4.9*	Wald ratio	1	0.0435	0.0140	1.044 (1.016, 1.074)	0.002	0.026	3.39E-14	TRUE
CD4 NC	*RP11-382J12.1*	Wald ratio	1	-0.0002	0.0080	1 (0.984, 1.016)	0.977	0.991	3.65E-45	TRUE
CD4 NC	*RP11-385D13.3*	Wald ratio	1	-0.0281	0.0149	0.972 (0.944, 1.001)	0.059	0.244	3.84E-13	TRUE
CD4 NC	*RP11-392P7.6*	Wald ratio	1	0.0116	0.0200	1.012 (0.973, 1.052)	0.564	0.78	1.54E-08	TRUE
CD4 NC	*RP11-395B7.4*	Wald ratio	1	0.0133	0.0166	1.013 (0.981, 1.047)	0.422	0.692	8.26E-12	TRUE
CD4 NC	*RP11-403A21.2*	Wald ratio	1	-0.0156	0.0097	0.985 (0.966, 1.003)	0.108	0.348	8.98E-29	TRUE
CD4 NC	*RP11-415F23.2*	Wald ratio	1	-0.0008	0.0078	0.999 (0.984, 1.015)	0.913	0.962	1.08E-56	TRUE
CD4 NC	*RP11-415F23.4*	Wald ratio	1	0.0130	0.0161	1.013 (0.982, 1.045)	0.42	0.69	1.32E-12	TRUE
CD4 NC	*RP11-420G6.4*	Wald ratio	1	-0.0025	0.0149	0.997 (0.969, 1.027)	0.866	0.944	1.02E-12	TRUE
CD4 NC	*RP11-425D10.10*	Wald ratio	1	-0.0197	0.0156	0.98 (0.951, 1.011)	0.208	0.494	2.55E-12	TRUE
CD4 NC	*RP11-429E11.3*	Wald ratio	1	0.0224	0.0180	1.023 (0.987, 1.059)	0.215	0.505	1.93E-10	TRUE
CD4 NC	*RP11-430H10.1*	Wald ratio	1	-0.0141	0.0071	0.986 (0.972, 1)	0.047	0.214	2.90E-61	TRUE
CD4 NC	*RP11-434C1.1*	Wald ratio	1	-0.0030	0.0118	0.997 (0.974, 1.02)	0.799	0.913	3.29E-22	TRUE
CD4 NC	*RP11-446N19.1*	Wald ratio	1	-0.0066	0.0084	0.993 (0.977, 1.01)	0.434	0.702	1.53E-40	TRUE
CD4 NC	*RP11-448A19.1*	Wald ratio	1	-0.0040	0.0163	0.996 (0.965, 1.028)	0.805	0.916	1.19E-11	TRUE
CD4 NC	*RP11-44F14.2*	Wald ratio	1	-0.0106	0.0161	0.989 (0.959, 1.021)	0.511	0.748	9.47E-11	TRUE
CD4 NC	*RP11-44F14.8*	Wald ratio	1	0.0236	0.0199	1.024 (0.985, 1.065)	0.235	0.529	5.88E-08	TRUE
CD4 NC	*RP11-44F21.5*	Wald ratio	1	-0.0138	0.0098	0.986 (0.968, 1.005)	0.159	0.432	8.63E-24	TRUE
CD4 NC	*RP11-459F6.3*	Wald ratio	1	0.0082	0.0104	1.008 (0.988, 1.029)	0.431	0.701	1.38E-19	TRUE
CD4 NC	*RP11-462G2.1*	Wald ratio	1	-0.0305	0.0184	0.97 (0.936, 1.006)	0.098	0.333	9.54E-10	TRUE
CD4 NC	*RP11-473I1.5*	Wald ratio	1	-0.0132	0.0061	0.987 (0.975, 0.999)	0.032	0.168	2.58E-66	TRUE
CD4 NC	*RP11-473M20.14*	Wald ratio	1	0.0074	0.0211	1.007 (0.967, 1.05)	0.726	0.878	4.19E-08	TRUE
CD4 NC	*RP11-473M20.9*	Wald ratio	1	-0.0082	0.0083	0.992 (0.976, 1.008)	0.326	0.62	1.53E-42	TRUE
CD4 NC	*RP11-474N24.6*	Wald ratio	1	-0.0070	0.0108	0.993 (0.972, 1.014)	0.518	0.752	2.09E-31	TRUE
CD4 NC	*RP11-47L3.1*	Wald ratio	1	-0.0508	0.0180	0.951 (0.918, 0.985)	0.005	0.049	2.52E-14	TRUE
CD4 NC	*RP11-493L12.3*	Wald ratio	1	-0.0188	0.0153	0.981 (0.952, 1.011)	0.218	0.509	2.74E-15	TRUE
CD4 NC	*RP11-493L12.4*	Wald ratio	1	-0.0110	0.0116	0.989 (0.967, 1.012)	0.344	0.637	3.12E-26	TRUE
CD4 NC	*RP11-498P14.5*	Wald ratio	1	0.0188	0.0141	1.019 (0.991, 1.047)	0.182	0.462	1.05E-16	TRUE
CD4 NC	*RP11-499F3.2*	Wald ratio	1	-0.0099	0.0141	0.99 (0.963, 1.018)	0.482	0.735	4.68E-12	TRUE
CD4 NC	*RP11-499P20.2*	Wald ratio	1	-0.0208	0.0115	0.979 (0.958, 1.002)	0.07	0.274	2.91E-22	TRUE
CD4 NC	*RP11-502I4.3*	Wald ratio	1	-0.0570	0.0181	0.945 (0.912, 0.979)	0.002	0.023	1.08E-07	TRUE
CD4 NC	*RP11-509J21.2*	Wald ratio	1	0.0247	0.0196	1.025 (0.986, 1.065)	0.208	0.494	8.52E-10	TRUE
CD4 NC	*RP11-513M16.7*	Wald ratio	1	0.0333	0.0196	1.034 (0.995, 1.074)	0.09	0.317	6.08E-09	TRUE
CD4 NC	*RP11-517P14.2*	Wald ratio	1	-0.0145	0.0183	0.986 (0.951, 1.022)	0.43	0.7	3.10E-08	TRUE

CD4 NC	*RP11-522I20.3*	Wald ratio	1	0.0057	0.0200	1.006 (0.967, 1.046)	0.775	0.901	7.41E-10	TRUE
CD4 NC	*RP11-526I2.5*	Wald ratio	1	0.0095	0.0076	1.01 (0.995, 1.025)	0.212	0.499	1.13E-49	TRUE
CD4 NC	*RP11-527L4.2*	Wald ratio	1	-0.0093	0.0180	0.991 (0.956, 1.026)	0.604	0.806	5.68E-10	TRUE
CD4 NC	*RP11-531F16.4*	Wald ratio	1	0.0129	0.0190	1.013 (0.976, 1.051)	0.496	0.74	2.76E-12	TRUE
CD4 NC	*RP11-532F6.3*	Wald ratio	1	-0.0131	0.0117	0.987 (0.965, 1.01)	0.265	0.559	1.57E-20	TRUE
CD4 NC	*RP11-536K7.3*	Wald ratio	1	-0.0270	0.0255	0.973 (0.926, 1.023)	0.29	0.583	3.62E-08	TRUE
CD4 NC	*RP11-538P18.2*	Wald ratio	1	0.0185	0.0119	1.019 (0.995, 1.043)	0.12	0.37	5.53E-16	TRUE
CD4 NC	*RP11-539L10.3*	Wald ratio	1	-0.0084	0.0070	0.992 (0.978, 1.005)	0.228	0.521	1.38E-58	TRUE
CD4 NC	*RP11-542K23.7*	Wald ratio	1	0.0061	0.0082	1.006 (0.99, 1.022)	0.453	0.717	2.27E-86	TRUE
CD4 NC	*RP11-542M13.3*	Wald ratio	1	0.0260	0.0203	1.026 (0.986, 1.068)	0.2	0.484	5.81E-08	TRUE
CD4 NC	*RP11-543C4.1*	Wald ratio	1	0.0149	0.0193	1.015 (0.977, 1.054)	0.44	0.705	1.41E-08	TRUE
CD4 NC	*RP11-545E17.3*	Wald ratio	1	-0.0078	0.0054	0.992 (0.982, 1.003)	0.146	0.415	5.89E-103	TRUE
CD4 NC	*RP11-545M17.1*	Wald ratio	1	-0.0284	0.0129	0.972 (0.948, 0.997)	0.027	0.154	1.79E-20	TRUE
CD4 NC	*RP11-572M11.4*	Wald ratio	1	-0.0012	0.0146	0.999 (0.971, 1.028)	0.933	0.972	1.92E-12	TRUE
CD4 NC	*RP11-589P10.5*	Wald ratio	1	-0.0244	0.0162	0.976 (0.945, 1.007)	0.132	0.394	1.11E-12	TRUE
CD4 NC	*RP11-58B17.2*	Wald ratio	1	0.0167	0.0178	1.017 (0.982, 1.053)	0.351	0.643	6.78E-12	TRUE
CD4 NC	*RP11-596D21.1*	Wald ratio	1	0.0204	0.0083	1.021 (1.004, 1.037)	0.013	0.098	2.25E-40	TRUE
CD4 NC	*RP11-617D20.1*	Wald ratio	1	0.0424	0.0200	1.043 (1.003, 1.085)	0.034	0.175	2.42E-09	TRUE
CD4 NC	*RP11-629O1.2*	Wald ratio	1	-0.0185	0.0185	0.982 (0.947, 1.018)	0.316	0.61	1.53E-10	TRUE
CD4 NC	*RP11-641A6.2*	Wald ratio	1	-0.0088	0.0133	0.991 (0.966, 1.017)	0.51	0.748	4.73E-27	TRUE
CD4 NC	*RP11-656D10.3*	Wald ratio	1	0.0258	0.0126	1.026 (1.001, 1.052)	0.04	0.195	4.25E-19	TRUE
CD4 NC	*RP11-65I12.1*	Wald ratio	1	-0.0271	0.0149	0.973 (0.945, 1.002)	0.068	0.269	7.74E-13	TRUE
CD4 NC	*RP11-65L3.4*	Wald ratio	1	0.0008	0.0250	1.001 (0.953, 1.051)	0.975	0.99	5.87E-09	TRUE
CD4 NC	*RP11-660L16.2*	Wald ratio	1	-0.0043	0.0060	0.996 (0.984, 1.007)	0.47	0.727	4.48E-58	TRUE
CD4 NC	*RP11-661A12.5*	Wald ratio	1	-0.0176	0.0149	0.983 (0.954, 1.012)	0.236	0.53	3.58E-14	TRUE
CD4 NC	*RP11-661P17.1*	Wald ratio	1	-0.0094	0.0154	0.991 (0.961, 1.021)	0.544	0.765	1.16E-13	TRUE
CD4 NC	*RP11-664D1.1*	IVW	2	0.0136	0.0101	1.014 (0.994, 1.034)	0.178	0.458	2.76E-32	TRUE
CD4 NC	*RP11-667K14.4*	Wald ratio	1	-0.0104	0.0095	0.99 (0.971, 1.008)	0.273	0.567	1.11E-24	TRUE
CD4 NC	*RP11-669C19.1*	Wald ratio	1	0.0449	0.0176	1.046 (1.01, 1.083)	0.011	0.085	3.30E-10	TRUE
CD4 NC	*RP11-673E1.1*	Wald ratio	1	-0.0264	0.0121	0.974 (0.951, 0.997)	0.029	0.16	1.02E-22	TRUE
CD4 NC	*RP11-692C24.1*	Wald ratio	1	-0.0017	0.0084	0.998 (0.982, 1.015)	0.842	0.934	5.37E-40	TRUE
CD4 NC	*RP11-69E11.4*	IVW	2	0.0179	0.0068	1.018 (1.005, 1.032)	0.009	0.073	1.44E-59	TRUE
CD4 NC	*RP11-730K11.1*	Wald ratio	1	-0.0358	0.0201	0.965 (0.928, 1.004)	0.075	0.288	3.76E-08	TRUE
CD4 NC	*RP11-755F10.1*	Wald ratio	1	0.0201	0.0087	1.02 (1.003, 1.038)	0.021	0.131	2.96E-36	TRUE
CD4 NC	*RP11-773D16.1*	Wald ratio	1	0.0284	0.0163	1.029 (0.996, 1.062)	0.082	0.302	1.28E-10	TRUE
CD4 NC	*RP11-792A8.4*	Wald ratio	1	0.0055	0.0080	1.006 (0.99, 1.021)	0.487	0.738	7.20E-34	TRUE
CD4 NC	*RP11-796G6.2*	Wald ratio	1	0.0923	0.0283	1.097 (1.038, 1.159)	0.001	0.018	8.97E-09	TRUE
CD4 NC	*RP11-797A18.4*	Wald ratio	1	-0.0386	0.0136	0.962 (0.937, 0.988)	0.004	0.046	7.93E-16	TRUE
CD4 NC	*RP11-798G7.5*	Wald ratio	1	0.0338	0.0142	1.034 (1.006, 1.064)	0.017	0.116	1.47E-33	TRUE
CD4 NC	*RP11-817O13.8*	Wald ratio	1	0.0639	0.0205	1.066 (1.024, 1.11)	0.002	0.025	6.01E-08	TRUE
CD4 NC	*RP11-81A22.5*	Wald ratio	1	-0.0292	0.0202	0.971 (0.933, 1.01)	0.148	0.417	2.28E-08	TRUE
CD4 NC	*RP11-841O20.2*	Wald ratio	1	0.0039	0.0106	1.004 (0.983, 1.025)	0.711	0.871	7.58E-21	TRUE
CD4 NC	*RP11-85G18.6*	Wald ratio	1	-0.0090	0.0114	0.991 (0.969, 1.013)	0.432	0.701	4.95E-24	TRUE
CD4 NC	*RP11-867G23.8*	Wald ratio	1	-0.0243	0.0203	0.976 (0.938, 1.016)	0.231	0.524	3.66E-08	TRUE
CD4 NC	*RP11-894P9.1*	Wald ratio	1	-0.0251	0.0149	0.975 (0.947, 1.004)	0.093	0.323	3.67E-13	TRUE
CD4 NC	*RP11-89K11.1*	Wald ratio	1	0.0018	0.0159	1.002 (0.971, 1.034)	0.911	0.962	9.90E-15	TRUE
CD4 NC	*RP11-92G12.3*	Wald ratio	1	-0.0266	0.0082	0.974 (0.958, 0.989)	0.001	0.018	9.03E-42	TRUE
CD4 NC	*RP11-936I5.1*	Wald ratio	1	0.0090	0.0094	1.009 (0.991, 1.028)	0.339	0.632	1.76E-30	TRUE
CD4 NC	*RP11-93B14.6*	Wald ratio	1	0.0210	0.0102	1.021 (1.001, 1.042)	0.04	0.193	1.20E-27	TRUE
CD4 NC	*RP11-944C7.1*	Wald ratio	1	0.0286	0.0174	1.029 (0.995, 1.065)	0.1	0.334	5.09E-10	TRUE
CD4 NC	*RP11-950C14.3*	Wald ratio	1	-0.0123	0.0073	0.988 (0.974, 1.002)	0.095	0.326	4.82E-54	TRUE
CD4 NC	*RP11-950C14.7*	Wald ratio	1	-0.0279	0.0145	0.972 (0.945, 1.001)	0.055	0.234	3.40E-14	TRUE
CD4 NC	*RP11-96H19.1*	Wald ratio	1	-0.0067	0.0082	0.993 (0.977, 1.009)	0.416	0.688	5.28E-43	TRUE
CD4 NC	*RP11-973H7.1*	Wald ratio	1	0.0090	0.0142	1.009 (0.981, 1.038)	0.525	0.755	3.06E-16	TRUE
CD4 NC	*RP11-973H7.4*	Wald ratio	1	0.0296	0.0167	1.03 (0.997, 1.064)	0.076	0.288	2.29E-11	TRUE
CD4 NC	*RP13-270P17.3*	Wald ratio	1	0.0017	0.0171	1.002 (0.969, 1.036)	0.921	0.965	8.96E-13	TRUE
CD4 NC	*RP13-578N3.3*	Wald ratio	1	-0.0244	0.0136	0.976 (0.95, 1.002)	0.072	0.28	4.36E-20	TRUE
CD4 NC	*RP13-582O9.5*	Wald ratio	1	-0.0032	0.0168	0.997 (0.965, 1.03)	0.849	0.936	1.12E-11	TRUE
CD4 NC	*RP3-325F22.5*	Wald ratio	1	0.0071	0.0103	1.007 (0.987, 1.028)	0.492	0.738	2.72E-34	TRUE
CD4 NC	*RP4-539M6.22*	IVW	2	-0.0108	0.0116	0.989 (0.967, 1.012)	0.35	0.643	1.08E-22	TRUE
CD4 NC	*RP4-564F22.5*	Wald ratio	1	-0.0099	0.0087	0.99 (0.973, 1.007)	0.254	0.548	6.98E-40	TRUE
CD4 NC	*RP4-594I10.3*	Wald ratio	1	-0.0088	0.0100	0.991 (0.972, 1.011)	0.381	0.666	1.07E-28	TRUE
CD4 NC	*RP4-673M15.1*	Wald ratio	1	-0.0233	0.0139	0.977 (0.951, 1.004)	0.093	0.323	2.81E-17	TRUE
CD4 NC	*RP4-798A10.2*	Wald ratio	1	-0.0021	0.0098	0.998 (0.979, 1.017)	0.833	0.93	7.36E-31	TRUE
CD4 NC	*RP5-1021I20.1*	Wald ratio	1	-0.0280	0.0091	0.972 (0.955, 0.99)	0.002	0.026	6.25E-34	TRUE
CD4 NC	*RP5-1042K10.10*	Wald ratio	1	-0.0115	0.0076	0.989 (0.974, 1.003)	0.131	0.392	2.52E-43	TRUE
CD4 NC	*RP5-1065J22.8*	Wald ratio	1	0.0054	0.0159	1.005 (0.975, 1.037)	0.735	0.88	3.44E-10	TRUE
CD4 NC	*RP5-1068E13.7*	Wald ratio	1	0.0320	0.0155	1.033 (1.002, 1.064)	0.039	0.192	2.21E-12	TRUE
CD4 NC	*RP5-1092A3.4*	Wald ratio	1	0.0506	0.0144	1.052 (1.023, 1.082)	4.52E-04	0.01	2.05E-14	TRUE
CD4 NC	*RP5-1159O4.2*	Wald ratio	1	0.0018	0.0123	1.002 (0.978, 1.026)	0.883	0.952	3.61E-18	TRUE
CD4 NC	*RP5-821D11.7*	Wald ratio	1	-0.0263	0.0156	0.974 (0.945, 1.004)	0.093	0.323	1.67E-12	TRUE
CD4 NC	*RP5-827C21.4*	IVW	2	0.0007	0.0075	1.001 (0.986, 1.016)	0.925	0.968	5.45E-50	TRUE
CD4 NC	*RP5-882C2.2*	Wald ratio	1	0.0361	0.0182	1.037 (1, 1.074)	0.047	0.215	3.40E-10	TRUE
CD4 NC	*RP5-903G2.2*	Wald ratio	1	0.0066	0.0155	1.007 (0.976, 1.038)	0.672	0.849	9.17E-13	TRUE
CD4 NC	*RP6-109B7.3*	Wald ratio	1	-0.0177	0.0235	0.982 (0.938, 1.029)	0.45	0.715	4.03E-09	TRUE
CD4 NC	*RPA1*	Wald ratio	1	0.0323	0.0200	1.033 (0.993, 1.074)	0.106	0.346	1.06E-08	TRUE
CD4 NC	*RPA2*	Wald ratio	1	0.0207	0.0154	1.021 (0.99, 1.052)	0.18	0.461	7.00E-13	TRUE
CD4 NC	*RPH3AL*	Wald ratio	1	0.0088	0.0180	1.009 (0.974, 1.045)	0.626	0.822	2.47E-11	TRUE
CD4 NC	*RPL10A*	Wald ratio	1	-0.0076	0.0057	0.992 (0.981, 1.004)	0.187	0.466	1.99E-95	TRUE

CD4 NC	*RPL12*	IVW	2	-0.0117	0.0172	0.988 (0.956, 1.022)	0.496	0.74	1.37E-177	TRUE
CD4 NC	*RPL13*	Wald ratio	1	-0.0206	0.0102	0.98 (0.96, 0.999)	0.043	0.202	5.92E-51	TRUE
CD4 NC	*RPL14*	IVW	2	0.0102	0.0089	1.01 (0.993, 1.028)	0.25	0.543	8.53E-54	TRUE
CD4 NC	*RPL22L1*	Wald ratio	1	0.0030	0.0140	1.003 (0.976, 1.031)	0.833	0.93	1.07E-19	TRUE
CD4 NC	*RPL23A*	Wald ratio	1	-0.0141	0.0076	0.986 (0.971, 1.001)	0.064	0.258	1.02E-52	TRUE
CD4 NC	*RPL27A*	Wald ratio	1	0.0076	0.0091	1.008 (0.99, 1.026)	0.4	0.678	2.79E-32	TRUE
CD4 NC	*RPL28*	Wald ratio	1	0.0035	0.0045	1.004 (0.995, 1.012)	0.438	0.703	3.87E-158	TRUE
CD4 NC	*RPL3*	Wald ratio	1	0.0177	0.0189	1.018 (0.981, 1.056)	0.347	0.64	3.11E-09	TRUE
CD4 NC	*RPL32*	Wald ratio	1	0.0222	0.0132	1.022 (0.996, 1.049)	0.093	0.323	2.46E-18	TRUE
CD4 NC	*RPL36AL*	IVW	2	-0.0049	0.0046	0.995 (0.986, 1.004)	0.283	0.575	1.81E-173	TRUE
CD4 NC	*RPL8*	Wald ratio	1	-0.0079	0.0046	0.992 (0.983, 1.001)	0.084	0.307	1.93E-159	TRUE
CD4 NC	*RPLP2*	Wald ratio	1	-0.0169	0.0065	0.983 (0.971, 0.996)	0.009	0.076	3.91E-71	TRUE
CD4 NC	*RPRD2*	Wald ratio	1	-0.0702	0.0183	0.932 (0.899, 0.966)	1.22E-04	0.003	4.74E-09	TRUE
CD4 NC	*RPRM*	Wald ratio	1	-0.0253	0.0107	0.975 (0.955, 0.996)	0.018	0.117	1.22E-26	TRUE
CD4 NC	*RPS10*	Wald ratio	1	-0.0054	0.0053	0.995 (0.984, 1.005)	0.305	0.599	8.85E-91	TRUE
CD4 NC	*RPS11*	Wald ratio	1	0.0241	0.0101	1.024 (1.004, 1.045)	0.017	0.115	2.88E-31	TRUE
CD4 NC	*RPS12*	Wald ratio	1	0.0254	0.0121	1.026 (1.002, 1.05)	0.036	0.182	3.73E-20	TRUE
CD4 NC	*RPS14*	Wald ratio	1	-0.0194	0.0175	0.981 (0.948, 1.015)	0.267	0.561	5.17E-10	TRUE
CD4 NC	*RPS16*	Wald ratio	1	-0.0114	0.0111	0.989 (0.967, 1.01)	0.304	0.598	1.37E-22	TRUE
CD4 NC	*RPS17*	Wald ratio	1	0.0037	0.0133	1.004 (0.978, 1.03)	0.779	0.904	3.92E-16	TRUE
CD4 NC	*RPS18*	Wald ratio	1	-0.0120	0.0070	0.988 (0.974, 1.002)	0.087	0.312	1.09E-73	TRUE
CD4 NC	*RPS2*	Wald ratio	1	-0.0219	0.0082	0.978 (0.963, 0.994)	0.007	0.066	2.66E-45	TRUE
CD4 NC	*RPS23*	Wald ratio	1	-0.0184	0.0128	0.982 (0.957, 1.007)	0.151	0.422	2.59E-20	TRUE
CD4 NC	*RPS24*	Wald ratio	1	-0.0025	0.0137	0.997 (0.971, 1.025)	0.855	0.938	2.54E-16	TRUE
CD4 NC	*RPS25*	Wald ratio	1	-0.0007	0.0060	0.999 (0.988, 1.011)	0.908	0.961	2.11E-78	TRUE
CD4 NC	*RPS26*	Wald ratio	1	-0.0099	0.0043	0.99 (0.982, 0.999)	0.022	0.132	4.90E-193	TRUE
CD4 NC	*RPS27L*	Wald ratio	1	-0.0599	0.0203	0.942 (0.905, 0.98)	0.003	0.036	7.66E-08	TRUE
CD4 NC	*RPS3*	Wald ratio	1	-0.0124	0.0070	0.988 (0.974, 1.001)	0.075	0.287	3.99E-35	TRUE
CD4 NC	*RPS3A*	Wald ratio	1	-0.0040	0.0061	0.996 (0.984, 1.008)	0.514	0.75	2.19E-38	TRUE
CD4 NC	*RPS5*	Wald ratio	1	0.0063	0.0047	1.006 (0.997, 1.016)	0.177	0.456	1.65E-163	TRUE
CD4 NC	*RPS6*	Wald ratio	1	-0.0099	0.0084	0.99 (0.974, 1.007)	0.242	0.534	2.53E-55	TRUE
CD4 NC	*RPS6KA2*	Wald ratio	1	-0.0158	0.0149	0.984 (0.956, 1.014)	0.29	0.583	2.50E-11	TRUE
CD4 NC	*RPS6KL1*	Wald ratio	1	0.0705	0.0212	1.073 (1.029, 1.119)	9.05E-04	0.016	4.73E-08	TRUE
CD4 NC	*RPS7*	IVW	2	0.0031	0.0079	1.003 (0.988, 1.019)	0.697	0.864	6.31E-73	TRUE
CD4 NC	*RPS8*	Wald ratio	1	0.0020	0.0077	1.002 (0.987, 1.017)	0.791	0.909	1.25E-49	TRUE
CD4 NC	*RPS9*	Wald ratio	1	0.0213	0.0055	1.021 (1.01, 1.033)	1.26E-04	0.003	6.36E-97	TRUE
CD4 NC	*RPSA*	Wald ratio	1	-0.0166	0.0083	0.984 (0.968, 1)	0.045	0.209	1.26E-55	TRUE
CD4 NC	*RQCD1*	Wald ratio	1	-0.0011	0.0201	0.999 (0.96, 1.039)	0.954	0.983	3.12E-08	TRUE
CD4 NC	*RSL24D1*	Wald ratio	1	-0.0396	0.0190	0.961 (0.926, 0.998)	0.037	0.186	4.75E-10	TRUE
CD4 NC	*RSPH9*	Wald ratio	1	-0.0509	0.0204	0.95 (0.913, 0.989)	0.013	0.095	6.26E-10	TRUE
CD4 NC	*RSRC2*	Wald ratio	1	-0.0072	0.0125	0.993 (0.969, 1.017)	0.564	0.78	2.80E-23	TRUE
CD4 NC	*RSU1*	Wald ratio	1	0.0567	0.0153	1.058 (1.027, 1.091)	2.14E-04	0.005	1.18E-09	TRUE
CD4 NC	*RTCA*	Wald ratio	1	0.0088	0.0192	1.009 (0.972, 1.047)	0.648	0.834	3.19E-10	TRUE
CD4 NC	*RTKN2*	Wald ratio	1	0.0188	0.0134	1.019 (0.993, 1.046)	0.16	0.432	5.18E-23	TRUE
CD4 NC	*RTN4*	Wald ratio	1	-0.0324	0.0161	0.968 (0.938, 0.999)	0.044	0.206	8.73E-13	TRUE
CD4 NC	*RWDD1*	Wald ratio	1	0.0063	0.0164	1.006 (0.975, 1.039)	0.701	0.866	3.30E-11	TRUE
CD4 NC	*S100B*	IVW	2	0.0043	0.0109	1.004 (0.983, 1.026)	0.691	0.86	6.03E-42	TRUE
CD4 NC	*SAAL1*	Wald ratio	1	-0.0137	0.0118	0.986 (0.964, 1.01)	0.246	0.54	9.37E-20	TRUE
CD4 NC	*SAMD10*	Wald ratio	1	0.0102	0.0124	1.01 (0.986, 1.035)	0.41	0.685	2.21E-18	TRUE
CD4 NC	*SAMD12*	Wald ratio	1	-0.0037	0.0112	0.996 (0.975, 1.018)	0.743	0.881	4.32E-30	TRUE
CD4 NC	*SAMD3*	Wald ratio	1	-0.0021	0.0064	0.998 (0.985, 1.01)	0.739	0.881	4.14E-74	TRUE
CD4 NC	*SAMD9L*	Wald ratio	1	-0.0279	0.0247	0.972 (0.927, 1.021)	0.258	0.552	7.18E-09	TRUE
CD4 NC	*SAMM50*	Wald ratio	1	-0.0035	0.0087	0.997 (0.98, 1.014)	0.69	0.86	3.61E-26	TRUE
CD4 NC	*SAR1A*	Wald ratio	1	0.0066	0.0098	1.007 (0.987, 1.026)	0.504	0.744	3.94E-29	TRUE
CD4 NC	*SARDH*	Wald ratio	1	-0.0574	0.0163	0.944 (0.914, 0.975)	4.49E-04	0.01	4.17E-11	TRUE
CD4 NC	*SARS2*	Wald ratio	1	-0.0217	0.0169	0.979 (0.947, 1.011)	0.199	0.482	6.15E-12	TRUE
CD4 NC	*SAT2*	Wald ratio	1	-0.0590	0.0112	0.943 (0.922, 0.964)	1.44E-07	6.60E-05	7.84E-23	TRUE
CD4 NC	*SC5D*	Wald ratio	1	-0.0536	0.0256	0.948 (0.901, 0.997)	0.037	0.182	4.61E-08	TRUE
CD4 NC	*SCAMP3*	Wald ratio	1	0.0032	0.0102	1.003 (0.983, 1.024)	0.755	0.888	9.11E-29	TRUE
CD4 NC	*SCAND3*	Wald ratio	1	0.0055	0.0145	1.005 (0.977, 1.034)	0.706	0.868	1.65E-26	TRUE
CD4 NC	*SCAPER*	Wald ratio	1	-0.0455	0.0181	0.956 (0.922, 0.99)	0.012	0.093	1.06E-09	TRUE
CD4 NC	*SCFD1*	Wald ratio	1	-0.0171	0.0200	0.983 (0.945, 1.022)	0.391	0.672	2.05E-08	TRUE
CD4 NC	*SCGB3A1*	Wald ratio	1	0.0059	0.0054	1.006 (0.995, 1.017)	0.277	0.571	2.79E-108	TRUE
CD4 NC	*SCO2*	Wald ratio	1	0.0122	0.0269	1.012 (0.96, 1.067)	0.65	0.835	1.31E-08	TRUE
CD4 NC	*SCRN2*	Wald ratio	1	-0.0291	0.0168	0.971 (0.94, 1.004)	0.083	0.304	5.71E-14	TRUE
CD4 NC	*SDCBP2-AS1*	Wald ratio	1	-0.0004	0.0164	1 (0.968, 1.032)	0.979	0.992	1.33E-11	TRUE
CD4 NC	*SDK2*	Wald ratio	1	0.0222	0.0134	1.022 (0.996, 1.05)	0.099	0.333	2.71E-16	TRUE
CD4 NC	*SDR39U1*	Wald ratio	1	-0.0246	0.0198	0.976 (0.938, 1.014)	0.214	0.502	4.53E-11	TRUE
CD4 NC	*SDSL*	Wald ratio	1	-0.0308	0.0131	0.97 (0.945, 0.995)	0.019	0.123	2.09E-15	TRUE
CD4 NC	*SELM*	Wald ratio	1	0.0270	0.0115	1.027 (1.005, 1.051)	0.018	0.12	3.10E-22	TRUE
CD4 NC	*SEMA3A*	Wald ratio	1	0.0095	0.0134	1.01 (0.983, 1.036)	0.479	0.733	3.96E-16	TRUE
CD4 NC	*SEPT2*	Wald ratio	1	0.0060	0.0076	1.006 (0.991, 1.021)	0.431	0.701	3.40E-62	TRUE
CD4 NC	*SEPW1*	Wald ratio	1	0.0121	0.0152	1.012 (0.982, 1.043)	0.426	0.696	2.06E-12	TRUE
CD4 NC	*SERP1*	Wald ratio	1	-0.0203	0.0158	0.98 (0.95, 1.011)	0.199	0.481	4.33E-13	TRUE
CD4 NC	*SERPINB9*	Wald ratio	1	-0.0052	0.0113	0.995 (0.973, 1.017)	0.642	0.831	2.23E-26	TRUE
CD4 NC	*SERPINE2*	Wald ratio	1	-0.0137	0.0093	0.986 (0.969, 1.005)	0.142	0.409	1.97E-37	TRUE
CD4 NC	*SERPINF1*	Wald ratio	1	0.0087	0.0104	1.009 (0.988, 1.029)	0.401	0.679	3.96E-25	TRUE
CD4 NC	*SESN1*	Wald ratio	1	-0.0101	0.0072	0.99 (0.976, 1.004)	0.161	0.435	3.09E-50	TRUE
CD4 NC	*SESN3*	Wald ratio	1	-0.0099	0.0124	0.99 (0.966, 1.015)	0.425	0.695	1.74E-20	TRUE

CD4 NC	*SESTD1*	Wald ratio	1	0.0060	0.0093	1.006 (0.988, 1.025)	0.519	0.753	1.50E-35	TRUE
CD4 NC	*SETD5-AS1*	Wald ratio	1	-0.0040	0.0143	0.996 (0.969, 1.024)	0.78	0.905	1.16E-14	TRUE
CD4 NC	*SETD9*	Wald ratio	1	-0.0208	0.0075	0.979 (0.965, 0.994)	0.005	0.053	1.73E-45	TRUE
CD4 NC	*SF1*	Wald ratio	1	-0.0273	0.0128	0.973 (0.949, 0.998)	0.032	0.17	2.56E-14	TRUE
CD4 NC	*SFI1*	Wald ratio	1	-0.0070	0.0145	0.993 (0.965, 1.022)	0.632	0.825	2.21E-16	TRUE
CD4 NC	*SFT2D1*	Wald ratio	1	0.0335	0.0148	1.034 (1.004, 1.065)	0.024	0.142	1.74E-12	TRUE
CD4 NC	*SFXN4*	Wald ratio	1	-0.0204	0.0115	0.98 (0.958, 1.002)	0.076	0.288	1.33E-22	TRUE
CD4 NC	*SH2D2A*	Wald ratio	1	-0.0033	0.0174	0.997 (0.963, 1.031)	0.849	0.936	2.57E-08	TRUE
CD4 NC	*SH2D3A*	Wald ratio	1	-0.0275	0.0191	0.973 (0.937, 1.01)	0.15	0.419	9.13E-09	TRUE
CD4 NC	*SH3BP5*	Wald ratio	1	-0.0137	0.0168	0.986 (0.954, 1.02)	0.417	0.688	2.76E-11	TRUE
CD4 NC	*SH3GLB1*	Wald ratio	1	0.0075	0.0166	1.008 (0.975, 1.041)	0.651	0.836	3.25E-17	TRUE
CD4 NC	*SH3YL1*	Wald ratio	1	0.0166	0.0044	1.017 (1.008, 1.026)	1.54E-04	0.004	1.27E-169	TRUE
CD4 NC	*SHFM1*	Wald ratio	1	0.0006	0.0145	1.001 (0.973, 1.029)	0.969	0.989	1.08E-11	TRUE
CD4 NC	*SHMT1*	Wald ratio	1	0.0394	0.0190	1.04 (1.002, 1.08)	0.038	0.187	3.81E-09	TRUE
CD4 NC	*SIDT1-AS1*	Wald ratio	1	-0.0063	0.0068	0.994 (0.98, 1.007)	0.356	0.647	1.62E-75	TRUE
CD4 NC	*SIRPB1*	Wald ratio	1	-0.0135	0.0165	0.987 (0.955, 1.019)	0.413	0.686	7.92E-13	TRUE
CD4 NC	*SIRPG*	Wald ratio	1	0.0099	0.0149	1.01 (0.981, 1.04)	0.505	0.745	1.56E-11	TRUE
CD4 NC	*SIVA1*	Wald ratio	1	0.0216	0.0179	1.022 (0.987, 1.058)	0.227	0.52	2.77E-09	TRUE
CD4 NC	*SKA3*	Wald ratio	1	0.0036	0.0129	1.004 (0.979, 1.029)	0.779	0.904	9.56E-18	TRUE
CD4 NC	*SKAP2*	Wald ratio	1	-0.0096	0.0117	0.99 (0.968, 1.013)	0.412	0.686	1.04E-25	TRUE
CD4 NC	*SKIV2L*	Wald ratio	1	-0.0329	0.0237	0.968 (0.924, 1.014)	0.165	0.439	2.25E-09	TRUE
CD4 NC	*SLC12A5*	Wald ratio	1	0.0261	0.0120	1.026 (1.003, 1.051)	0.03	0.162	4.67E-20	TRUE
CD4 NC	*SLC12A7*	Wald ratio	1	-0.0094	0.0086	0.991 (0.974, 1.008)	0.275	0.568	1.98E-40	TRUE
CD4 NC	*SLC16A4*	Wald ratio	1	0.0235	0.0120	1.024 (1, 1.048)	0.05	0.222	2.42E-20	TRUE
CD4 NC	*SLC18A2*	Wald ratio	1	0.0298	0.0176	1.03 (0.995, 1.066)	0.09	0.318	4.57E-09	TRUE
CD4 NC	*SLC18B1*	Wald ratio	1	-0.0248	0.0137	0.975 (0.95, 1.002)	0.071	0.275	6.46E-12	TRUE
CD4 NC	*SLC22A18*	Wald ratio	1	-0.0039	0.0084	0.996 (0.98, 1.013)	0.641	0.83	7.45E-51	TRUE
CD4 NC	*SLC22A31*	Wald ratio	1	0.0011	0.0118	1.001 (0.978, 1.025)	0.925	0.968	8.35E-26	TRUE
CD4 NC	*SLC22A5*	Wald ratio	1	0.0814	0.0191	1.085 (1.045, 1.126)	1.98E-05	9.50E-04	2.30E-08	TRUE
CD4 NC	*SLC25A1*	Wald ratio	1	-0.0077	0.0081	0.992 (0.977, 1.008)	0.342	0.635	1.00E-45	TRUE
CD4 NC	*SLC25A26*	Wald ratio	1	0.0006	0.0118	1.001 (0.978, 1.024)	0.956	0.984	4.63E-22	TRUE
CD4 NC	*SLC25A29*	Wald ratio	1	-0.0042	0.0059	0.996 (0.984, 1.007)	0.473	0.73	1.35E-84	TRUE
CD4 NC	*SLC25A3*	Wald ratio	1	0.0053	0.0127	1.005 (0.981, 1.031)	0.678	0.853	1.60E-24	TRUE
CD4 NC	*SLC25A37*	Wald ratio	1	-0.0012	0.0181	0.999 (0.964, 1.035)	0.946	0.979	4.17E-10	TRUE
CD4 NC	*SLC25A51*	Wald ratio	1	-0.0288	0.0120	0.972 (0.949, 0.995)	0.016	0.112	6.63E-17	TRUE
CD4 NC	*SLC26A5*	Wald ratio	1	0.0243	0.0134	1.025 (0.998, 1.052)	0.068	0.269	9.30E-12	TRUE
CD4 NC	*SLC2A1-AS1*	Wald ratio	1	0.0135	0.0115	1.014 (0.991, 1.037)	0.241	0.534	4.34E-17	TRUE
CD4 NC	*SLC2A11*	Wald ratio	1	-0.0136	0.0283	0.986 (0.933, 1.043)	0.63	0.824	5.38E-10	TRUE
CD4 NC	*SLC2A4RG*	Wald ratio	1	-0.0286	0.0158	0.972 (0.942, 1.002)	0.07	0.274	4.41E-14	TRUE
CD4 NC	*SLC35F3*	Wald ratio	1	-0.0094	0.0063	0.991 (0.978, 1.003)	0.137	0.399	7.98E-72	TRUE
CD4 NC	*SLC38A1*	Wald ratio	1	-0.0107	0.0121	0.989 (0.966, 1.013)	0.376	0.664	6.96E-20	TRUE
CD4 NC	*SLC40A1*	Wald ratio	1	0.0329	0.0134	1.033 (1.007, 1.061)	0.014	0.101	2.80E-16	TRUE
CD4 NC	*SLC41A3*	Wald ratio	1	0.0249	0.0176	1.025 (0.99, 1.061)	0.157	0.428	1.29E-12	TRUE
CD4 NC	*SLC44A5*	Wald ratio	1	-0.0130	0.0136	0.987 (0.961, 1.014)	0.338	0.632	6.51E-17	TRUE
CD4 NC	*SLC7A5*	Wald ratio	1	-0.0122	0.0196	0.988 (0.951, 1.027)	0.536	0.762	2.66E-08	TRUE
CD4 NC	*SLC7A8*	Wald ratio	1	-0.0025	0.0074	0.997 (0.983, 1.012)	0.73	0.88	1.50E-54	TRUE
CD4 NC	*SLCO3A1*	Wald ratio	1	0.0437	0.0175	1.045 (1.01, 1.081)	0.012	0.094	9.03E-09	TRUE
CD4 NC	*SLFN5*	Wald ratio	1	-0.0066	0.0111	0.993 (0.972, 1.015)	0.55	0.769	3.52E-24	TRUE
CD4 NC	*SMAD5*	Wald ratio	1	0.0110	0.0105	1.011 (0.991, 1.032)	0.294	0.587	1.29E-26	TRUE
CD4 NC	*SMAGP*	Wald ratio	1	0.0110	0.0128	1.011 (0.986, 1.037)	0.391	0.672	6.22E-17	TRUE
CD4 NC	*SMARCE1*	Wald ratio	1	0.0276	0.0153	1.028 (0.998, 1.059)	0.072	0.279	5.40E-13	TRUE
CD4 NC	*SMCR5*	Wald ratio	1	0.0251	0.0131	1.025 (0.999, 1.052)	0.055	0.236	1.28E-16	TRUE
CD4 NC	*SMDT1*	Wald ratio	1	0.0073	0.0038	1.007 (1, 1.015)	0.057	0.239	8.53E-216	TRUE
CD4 NC	*SMIM19*	Wald ratio	1	0.0073	0.0095	1.007 (0.989, 1.026)	0.439	0.703	2.45E-33	TRUE
CD4 NC	*SMIM2-AS1*	Wald ratio	1	-0.0053	0.0087	0.995 (0.978, 1.012)	0.539	0.762	4.79E-44	TRUE
CD4 NC	*SMIM8*	Wald ratio	1	-0.0142	0.0140	0.986 (0.959, 1.013)	0.313	0.608	2.94E-15	TRUE
CD4 NC	*SMYD2*	Wald ratio	1	-0.0467	0.0173	0.954 (0.922, 0.987)	0.007	0.064	2.14E-10	TRUE
CD4 NC	*SNAI3*	Wald ratio	1	-0.0492	0.0179	0.952 (0.919, 0.986)	0.006	0.057	3.40E-10	TRUE
CD4 NC	*SNAP29*	Wald ratio	1	0.0245	0.0107	1.025 (1.003, 1.047)	0.023	0.137	4.19E-33	TRUE
CD4 NC	*SNHG18*	Wald ratio	1	0.0213	0.0260	1.022 (0.971, 1.075)	0.412	0.686	6.13E-09	TRUE
CD4 NC	*SNHG7*	Wald ratio	1	-0.0114	0.0071	0.989 (0.975, 1.002)	0.107	0.348	1.02E-91	TRUE
CD4 NC	*SNHG8*	IVW	2	-0.0018	0.0045	0.998 (0.989, 1.007)	0.686	0.857	9.03E-189	TRUE
CD4 NC	*SNORD3B-1*	Wald ratio	1	-0.0040	0.0119	0.996 (0.973, 1.02)	0.736	0.88	3.72E-16	TRUE
CD4 NC	*SNORD3B-2*	Wald ratio	1	-0.0118	0.0165	0.988 (0.957, 1.021)	0.473	0.73	7.94E-17	TRUE
CD4 NC	*SNORD3D*	Wald ratio	1	0.0113	0.0153	1.011 (0.981, 1.042)	0.461	0.721	2.02E-11	TRUE
CD4 NC	*SNPH*	Wald ratio	1	0.0216	0.0144	1.022 (0.993, 1.051)	0.135	0.396	3.16E-10	TRUE
CD4 NC	*SNRNP25*	Wald ratio	1	0.0184	0.0102	1.019 (0.998, 1.039)	0.071	0.277	9.93E-25	TRUE
CD4 NC	*SNRNP35*	Wald ratio	1	-0.0325	0.0173	0.968 (0.936, 1.001)	0.06	0.249	7.26E-11	TRUE
CD4 NC	*SNRPC*	Wald ratio	1	-0.0355	0.0104	0.965 (0.946, 0.985)	6.72E-04	0.013	1.29E-25	TRUE
CD4 NC	*SNRPD2*	Wald ratio	1	-0.0201	0.0062	0.98 (0.968, 0.992)	0.001	0.02	1.56E-73	TRUE
CD4 NC	*SNRPF*	Wald ratio	1	-0.0223	0.0174	0.978 (0.945, 1.012)	0.198	0.481	2.53E-10	TRUE
CD4 NC	*SNTG2*	Wald ratio	1	0.0098	0.0147	1.01 (0.981, 1.039)	0.505	0.745	2.21E-10	TRUE
CD4 NC	*SNX16*	Wald ratio	1	0.0620	0.0169	1.064 (1.029, 1.1)	2.39E-04	0.006	4.86E-12	TRUE
CD4 NC	*SNX25*	Wald ratio	1	-0.0093	0.0144	0.991 (0.963, 1.019)	0.518	0.752	3.03E-15	TRUE
CD4 NC	*SNX9*	Wald ratio	1	-0.0057	0.0174	0.994 (0.961, 1.029)	0.741	0.881	3.44E-11	TRUE
CD4 NC	*SOD2*	Wald ratio	1	0.0082	0.0192	1.008 (0.971, 1.047)	0.67	0.848	6.03E-10	TRUE
CD4 NC	*SOX6*	Wald ratio	1	0.0062	0.0128	1.006 (0.981, 1.032)	0.628	0.822	3.25E-17	TRUE
CD4 NC	*SP110*	Wald ratio	1	0.0101	0.0136	1.01 (0.984, 1.037)	0.459	0.72	1.50E-16	TRUE
CD4 NC	*SP140*	Wald ratio	1	-0.0067	0.0213	0.993 (0.953, 1.036)	0.753	0.888	2.41E-09	TRUE

CD4 NC	*SP140L*	IVW	2	0.0091	0.0122	1.009 (0.985, 1.034)	0.456	0.719	2.74E-20	TRUE
CD4 NC	*SPAG7*	Wald ratio	1	-0.0295	0.0143	0.971 (0.944, 0.999)	0.039	0.192	8.82E-11	TRUE
CD4 NC	*SPATA20*	Wald ratio	1	0.0127	0.0079	1.013 (0.997, 1.029)	0.106	0.346	3.94E-48	TRUE
CD4 NC	*SPATA21*	Wald ratio	1	-0.0220	0.0158	0.978 (0.949, 1.009)	0.163	0.437	1.07E-12	TRUE
CD4 NC	*SPATA9*	Wald ratio	1	0.0069	0.0096	1.007 (0.988, 1.026)	0.472	0.729	4.92E-29	TRUE
CD4 NC	*SPCS1*	Wald ratio	1	0.0499	0.0151	1.051 (1.021, 1.083)	9.20E-04	0.016	6.92E-13	TRUE
CD4 NC	*SPECC1*	Wald ratio	1	0.0346	0.0155	1.035 (1.004, 1.067)	0.025	0.146	8.62E-13	TRUE
CD4 NC	*SPG21*	Wald ratio	1	-0.0184	0.0142	0.982 (0.955, 1.009)	0.195	0.477	1.09E-15	TRUE
CD4 NC	*SPINK2*	IVW	2	-0.0028	0.0073	0.997 (0.983, 1.012)	0.696	0.864	1.62E-58	TRUE
CD4 NC	*SPNS1*	Wald ratio	1	0.0251	0.0137	1.025 (0.998, 1.053)	0.068	0.266	1.08E-14	TRUE
CD4 NC	*SPOCK2*	Wald ratio	1	-0.0064	0.0149	0.994 (0.965, 1.023)	0.667	0.848	1.75E-11	TRUE
CD4 NC	*SPRYD4*	Wald ratio	1	0.0306	0.0185	1.031 (0.994, 1.069)	0.098	0.333	7.29E-10	TRUE
CD4 NC	*SPSB2*	Wald ratio	1	-0.0016	0.0108	0.998 (0.978, 1.02)	0.885	0.954	2.18E-28	TRUE
CD4 NC	*SRA1*	Wald ratio	1	-0.0344	0.0124	0.966 (0.943, 0.99)	0.005	0.053	2.18E-20	TRUE
CD4 NC	*SRD5A3*	Wald ratio	1	-0.0189	0.0086	0.981 (0.965, 0.998)	0.028	0.157	1.35E-42	TRUE
CD4 NC	*SRGN*	Wald ratio	1	0.0161	0.0175	1.016 (0.982, 1.052)	0.358	0.649	3.12E-10	TRUE
CD4 NC	*SRI*	Wald ratio	1	-0.0148	0.0125	0.985 (0.961, 1.01)	0.239	0.533	3.01E-18	TRUE
CD4 NC	*SRR*	Wald ratio	1	0.0216	0.0168	1.022 (0.989, 1.056)	0.199	0.483	7.34E-11	TRUE
CD4 NC	*SRSF10*	Wald ratio	1	-0.0099	0.0086	0.99 (0.974, 1.007)	0.248	0.541	1.03E-39	TRUE
CD4 NC	*SSBP4*	Wald ratio	1	0.0062	0.0127	1.006 (0.982, 1.031)	0.624	0.822	2.11E-18	TRUE
CD4 NC	*ST3GAL5*	Wald ratio	1	-0.0264	0.0116	0.974 (0.952, 0.996)	0.023	0.137	5.69E-19	TRUE
CD4 NC	*ST6GALNAC1*	Wald ratio	1	-0.0191	0.0103	0.981 (0.961, 1.001)	0.064	0.258	3.22E-27	TRUE
CD4 NC	*ST6GALNAC3*	Wald ratio	1	-0.0077	0.0199	0.992 (0.954, 1.032)	0.699	0.865	5.75E-08	TRUE
CD4 NC	*ST6GALNAC6*	Wald ratio	1	0.0066	0.0098	1.007 (0.988, 1.026)	0.498	0.741	8.04E-29	TRUE
CD4 NC	*STIM2*	Wald ratio	1	0.0144	0.0192	1.015 (0.977, 1.053)	0.452	0.717	6.23E-09	TRUE
CD4 NC	*STK32C*	Wald ratio	1	0.0301	0.0122	1.031 (1.006, 1.055)	0.013	0.098	1.79E-30	TRUE
CD4 NC	*STMN1*	Wald ratio	1	0.0253	0.0193	1.026 (0.988, 1.065)	0.19	0.47	4.37E-11	TRUE
CD4 NC	*STMN3*	Wald ratio	1	0.0144	0.0074	1.015 (1, 1.029)	0.05	0.222	2.21E-53	TRUE
CD4 NC	*STOX1*	Wald ratio	1	0.0221	0.0088	1.022 (1.005, 1.04)	0.012	0.094	1.40E-25	TRUE
CD4 NC	*STRC*	Wald ratio	1	-0.0301	0.0152	0.97 (0.942, 1)	0.048	0.217	1.53E-14	TRUE
CD4 NC	*STX16-NPEPL1*	Wald ratio	1	0.0119	0.0192	1.012 (0.975, 1.051)	0.536	0.762	5.00E-09	TRUE
CD4 NC	*STX2*	Wald ratio	1	-0.0035	0.0176	0.997 (0.963, 1.031)	0.842	0.934	1.84E-10	TRUE
CD4 NC	*STX8*	IVW	2	0.0325	0.0159	1.033 (1.001, 1.066)	0.041	0.197	4.95E-15	TRUE
CD4 NC	*STXBP2*	Wald ratio	1	-0.0149	0.0128	0.985 (0.961, 1.01)	0.246	0.54	2.86E-17	TRUE
CD4 NC	*STYX*	Wald ratio	1	0.0337	0.0147	1.034 (1.005, 1.064)	0.022	0.132	3.22E-13	TRUE
CD4 NC	*STYXL1*	Wald ratio	1	-0.0262	0.0164	0.974 (0.943, 1.006)	0.11	0.353	2.19E-15	TRUE
CD4 NC	*SUCLG2*	Wald ratio	1	0.0144	0.0157	1.015 (0.984, 1.046)	0.36	0.65	1.05E-12	TRUE
CD4 NC	*SULF2*	IVW	2	0.0052	0.0049	1.005 (0.996, 1.015)	0.295	0.589	1.44E-130	TRUE
CD4 NC	*SUMF2*	Wald ratio	1	-0.0271	0.0148	0.973 (0.945, 1.002)	0.068	0.268	7.41E-12	TRUE
CD4 NC	*SUPT3H*	Wald ratio	1	-0.0112	0.0075	0.989 (0.975, 1.003)	0.135	0.396	9.41E-53	TRUE
CD4 NC	*SURF1*	IVW	2	0.0074	0.0096	1.007 (0.989, 1.026)	0.439	0.703	5.78E-38	TRUE
CD4 NC	*SUSD3*	Wald ratio	1	-0.0053	0.0191	0.995 (0.958, 1.033)	0.782	0.905	1.97E-09	TRUE
CD4 NC	*SUSD4*	Wald ratio	1	-0.0133	0.0149	0.987 (0.958, 1.016)	0.372	0.661	1.84E-16	TRUE
CD4 NC	*SUSD5*	Wald ratio	1	0.0006	0.0185	1.001 (0.965, 1.038)	0.976	0.991	6.09E-09	TRUE
CD4 NC	*SVIP*	Wald ratio	1	-0.0114	0.0126	0.989 (0.965, 1.013)	0.367	0.655	5.26E-19	TRUE
CD4 NC	*SYCE1L*	Wald ratio	1	0.0182	0.0213	1.018 (0.977, 1.062)	0.394	0.673	2.33E-08	TRUE
CD4 NC	*SYNC*	Wald ratio	1	0.0028	0.0102	1.003 (0.983, 1.023)	0.782	0.905	1.60E-21	TRUE
CD4 NC	*SYNE3*	Wald ratio	1	-0.0024	0.0198	0.998 (0.96, 1.037)	0.903	0.959	3.97E-09	TRUE
CD4 NC	*SYNJ2*	Wald ratio	1	-0.0122	0.0165	0.988 (0.956, 1.02)	0.458	0.719	1.61E-11	TRUE
CD4 NC	*SYNM*	Wald ratio	1	-0.0019	0.0107	0.998 (0.977, 1.019)	0.858	0.939	3.21E-26	TRUE
CD4 NC	*SYS1*	Wald ratio	1	-0.0085	0.0147	0.992 (0.963, 1.02)	0.562	0.779	1.86E-13	TRUE
CD4 NC	*SYTL2*	Wald ratio	1	0.0375	0.0174	1.038 (1.003, 1.074)	0.031	0.167	8.53E-08	TRUE
CD4 NC	*TAF6*	Wald ratio	1	-0.0345	0.0197	0.966 (0.93, 1.004)	0.08	0.296	2.55E-08	TRUE
CD4 NC	*TAPBP*	Wald ratio	1	0.0056	0.0085	1.006 (0.989, 1.023)	0.511	0.748	3.32E-36	TRUE
CD4 NC	*TAPBPL*	Wald ratio	1	0.0042	0.0111	1.004 (0.983, 1.026)	0.706	0.868	3.73E-24	TRUE
CD4 NC	*TAS2R31*	Wald ratio	1	0.0070	0.0172	1.007 (0.974, 1.042)	0.687	0.858	4.16E-18	TRUE
CD4 NC	*TBC1D2B*	Wald ratio	1	0.0087	0.0185	1.009 (0.973, 1.046)	0.636	0.827	1.07E-09	TRUE
CD4 NC	*TBC1D4*	IVW	2	0.0109	0.0111	1.011 (0.989, 1.033)	0.329	0.624	1.71E-25	TRUE
CD4 NC	*TBC1D9B*	Wald ratio	1	0.0174	0.0160	1.018 (0.986, 1.05)	0.275	0.568	2.00E-14	TRUE
CD4 NC	*TBCA*	Wald ratio	1	0.0059	0.0070	1.006 (0.992, 1.02)	0.399	0.678	1.23E-61	TRUE
CD4 NC	*TBCD*	Wald ratio	1	0.0015	0.0073	1.001 (0.987, 1.016)	0.839	0.932	3.01E-55	TRUE
CD4 NC	*TC2N*	Wald ratio	1	-0.0078	0.0169	0.992 (0.96, 1.026)	0.642	0.831	4.31E-11	TRUE
CD4 NC	*TCEA3*	Wald ratio	1	-0.0305	0.0200	0.97 (0.933, 1.009)	0.127	0.384	1.63E-08	TRUE
CD4 NC	*TCN2*	Wald ratio	1	-0.0008	0.0242	0.999 (0.953, 1.048)	0.973	0.989	1.99E-08	TRUE
CD4 NC	*TCTEX1D2*	Wald ratio	1	-0.0260	0.0193	0.974 (0.938, 1.012)	0.179	0.458	5.09E-09	TRUE
CD4 NC	*TECPR1*	Wald ratio	1	-0.0036	0.0149	0.996 (0.968, 1.026)	0.808	0.917	2.10E-13	TRUE
CD4 NC	*TECR*	Wald ratio	1	0.0481	0.0215	1.049 (1.006, 1.094)	0.025	0.146	9.66E-09	TRUE
CD4 NC	*TEN1*	Wald ratio	1	-0.0081	0.0108	0.992 (0.971, 1.013)	0.452	0.717	3.12E-32	TRUE
CD4 NC	*TEPP*	Wald ratio	1	-0.0305	0.0121	0.97 (0.947, 0.993)	0.012	0.093	4.04E-19	TRUE
CD4 NC	*TERT*	Wald ratio	1	-0.0066	0.0129	0.993 (0.969, 1.019)	0.606	0.807	9.45E-15	TRUE
CD4 NC	*TESC*	Wald ratio	1	-0.0550	0.0177	0.946 (0.914, 0.98)	0.002	0.025	5.95E-11	TRUE
CD4 NC	*TEX29*	Wald ratio	1	-0.0236	0.0173	0.977 (0.944, 1.01)	0.174	0.452	2.33E-10	TRUE
CD4 NC	*TFDP1*	Wald ratio	1	0.0247	0.0223	1.025 (0.981, 1.071)	0.269	0.563	1.06E-08	TRUE
CD4 NC	*TFPT*	Wald ratio	1	0.0035	0.0150	1.004 (0.974, 1.033)	0.813	0.921	1.82E-16	TRUE
CD4 NC	*TGM4*	Wald ratio	1	0.0124	0.0162	1.012 (0.981, 1.045)	0.445	0.709	8.05E-15	TRUE
CD4 NC	*THAP5*	Wald ratio	1	0.0296	0.0150	1.03 (1, 1.061)	0.049	0.22	2.36E-11	TRUE
CD4 NC	*THAP7-AS1*	Wald ratio	1	0.0175	0.0083	1.018 (1.001, 1.034)	0.036	0.181	1.47E-37	TRUE
CD4 NC	*THAP9-AS1*	Wald ratio	1	0.0535	0.0115	1.055 (1.031, 1.079)	3.31E-06	2.62E-04	2.52E-25	TRUE
CD4 NC	*THEM4*	Wald ratio	1	0.0218	0.0103	1.022 (1.002, 1.043)	0.035	0.176	2.17E-28	TRUE

CD4 NC	*THEMIS2*	Wald ratio	1	0.0054	0.0057	1.005 (0.994, 1.017)	0.345	0.637	1.54E-91	TRUE
CD4 NC	*THNSL2*	Wald ratio	1	-0.0310	0.0163	0.97 (0.939, 1.001)	0.058	0.242	4.75E-13	TRUE
CD4 NC	*THOC3*	Wald ratio	1	0.0445	0.0181	1.045 (1.009, 1.083)	0.014	0.102	9.65E-10	TRUE
CD4 NC	*THRA*	Wald ratio	1	-0.0080	0.0104	0.992 (0.972, 1.012)	0.439	0.703	1.00E-20	TRUE
CD4 NC	*TIAM2*	Wald ratio	1	0.0147	0.0168	1.015 (0.982, 1.049)	0.383	0.667	7.32E-11	TRUE
CD4 NC	*TIFA*	Wald ratio	1	0.0218	0.0153	1.022 (0.992, 1.053)	0.154	0.425	3.35E-13	TRUE
CD4 NC	*TIMM10*	Wald ratio	1	-0.0008	0.0051	0.999 (0.989, 1.009)	0.875	0.948	1.86E-150	TRUE
CD4 NC	*TIPARP-AS1*	Wald ratio	1	0.0206	0.0081	1.021 (1.005, 1.037)	0.011	0.085	2.44E-49	TRUE
CD4 NC	*TIPRL*	Wald ratio	1	-0.0032	0.0174	0.997 (0.963, 1.031)	0.856	0.939	3.72E-12	TRUE
CD4 NC	*TLE4*	Wald ratio	1	-0.0067	0.0096	0.993 (0.975, 1.012)	0.486	0.737	2.41E-33	TRUE
CD4 NC	*TLK1*	Wald ratio	1	0.0068	0.0092	1.007 (0.989, 1.025)	0.459	0.719	5.32E-38	TRUE
CD4 NC	*TMED4*	Wald ratio	1	0.0499	0.0204	1.051 (1.01, 1.094)	0.015	0.104	1.24E-08	TRUE
CD4 NC	*TMEM101*	Wald ratio	1	-0.0257	0.0177	0.975 (0.941, 1.009)	0.146	0.415	7.25E-25	TRUE
CD4 NC	*TMEM106C*	Wald ratio	1	0.0026	0.0150	1.003 (0.974, 1.033)	0.862	0.942	2.19E-13	TRUE
CD4 NC	*TMEM120B*	Wald ratio	1	-0.0108	0.0099	0.989 (0.97, 1.009)	0.275	0.568	2.16E-41	TRUE
CD4 NC	*TMEM121*	Wald ratio	1	0.0067	0.0101	1.007 (0.987, 1.027)	0.51	0.748	4.73E-26	TRUE
CD4 NC	*TMEM128*	Wald ratio	1	0.0481	0.0185	1.049 (1.012, 1.088)	0.009	0.077	9.49E-10	TRUE
CD4 NC	*TMEM14C*	Wald ratio	1	-0.0101	0.0169	0.99 (0.958, 1.023)	0.551	0.769	5.17E-15	TRUE
CD4 NC	*TMEM156*	Wald ratio	1	0.0028	0.0068	1.003 (0.989, 1.016)	0.684	0.856	2.51E-62	TRUE
CD4 NC	*TMEM159*	Wald ratio	1	0.0163	0.0172	1.016 (0.983, 1.051)	0.343	0.635	9.18E-11	TRUE
CD4 NC	*TMEM161B-AS1*	Wald ratio	1	0.0313	0.0168	1.032 (0.998, 1.066)	0.062	0.253	1.53E-10	TRUE
CD4 NC	*TMEM18*	Wald ratio	1	0.0022	0.0103	1.002 (0.982, 1.023)	0.83	0.929	1.55E-28	TRUE
CD4 NC	*TMEM199*	Wald ratio	1	-0.0698	0.0175	0.933 (0.901, 0.965)	7.04E-05	0.002	5.05E-10	TRUE
CD4 NC	*TMEM2*	Wald ratio	1	-0.0144	0.0160	0.986 (0.955, 1.017)	0.369	0.657	2.09E-10	TRUE
CD4 NC	*TMEM204*	Wald ratio	1	0.0091	0.0129	1.009 (0.984, 1.035)	0.481	0.734	1.23E-32	TRUE
CD4 NC	*TMEM230*	Wald ratio	1	0.0045	0.0077	1.005 (0.99, 1.02)	0.557	0.775	4.51E-50	TRUE
CD4 NC	*TMEM258*	Wald ratio	1	0.0078	0.0083	1.008 (0.991, 1.024)	0.353	0.644	6.69E-39	TRUE
CD4 NC	*TMEM41B*	Wald ratio	1	-0.0114	0.0127	0.989 (0.964, 1.014)	0.371	0.659	5.00E-17	TRUE
CD4 NC	*TMEM45B*	Wald ratio	1	-0.0095	0.0080	0.991 (0.975, 1.006)	0.232	0.525	7.11E-47	TRUE
CD4 NC	*TMEM50A*	Wald ratio	1	0.0522	0.0196	1.054 (1.014, 1.095)	0.008	0.067	8.65E-09	TRUE
CD4 NC	*TMEM50B*	Wald ratio	1	-0.0185	0.0058	0.982 (0.971, 0.993)	0.001	0.02	4.85E-88	TRUE
CD4 NC	*TMEM71*	Wald ratio	1	0.0270	0.0179	1.027 (0.992, 1.064)	0.131	0.393	3.87E-12	TRUE
CD4 NC	*TMEM80*	Wald ratio	1	-0.0288	0.0106	0.972 (0.952, 0.992)	0.006	0.059	1.33E-25	TRUE
CD4 NC	*TMEM87B*	Wald ratio	1	0.0525	0.0174	1.054 (1.019, 1.09)	0.003	0.031	4.32E-11	TRUE
CD4 NC	*TMEM97*	Wald ratio	1	-0.0542	0.0166	0.947 (0.917, 0.979)	0.001	0.018	6.31E-11	TRUE
CD4 NC	*TMEM9B-AS1*	Wald ratio	1	-0.0174	0.0198	0.983 (0.945, 1.022)	0.382	0.666	1.44E-08	TRUE
CD4 NC	*TMOD1*	Wald ratio	1	0.0068	0.0111	1.007 (0.985, 1.029)	0.543	0.765	3.19E-24	TRUE
CD4 NC	*TNFRSF10A*	Wald ratio	1	-0.0099	0.0176	0.99 (0.957, 1.025)	0.573	0.786	3.57E-11	TRUE
CD4 NC	*TNFRSF14*	Wald ratio	1	0.0191	0.0132	1.019 (0.993, 1.046)	0.148	0.417	2.28E-19	TRUE
CD4 NC	*TNFRSF18*	Wald ratio	1	0.0579	0.0191	1.06 (1.021, 1.1)	0.003	0.031	1.04E-07	TRUE
CD4 NC	*TNFRSF1B*	Wald ratio	1	-0.0169	0.0162	0.983 (0.953, 1.015)	0.298	0.593	1.80E-10	TRUE
CD4 NC	*TNFSF10*	Wald ratio	1	-0.0153	0.0155	0.985 (0.955, 1.015)	0.321	0.616	7.06E-11	TRUE
CD4 NC	*TNFSF13B*	IVW	2	-0.0173	0.0368	0.983 (0.914, 1.056)	0.638	0.829	4.05E-30	TRUE
CD4 NC	*TNFSF8*	Wald ratio	1	-0.0083	0.0116	0.992 (0.969, 1.014)	0.472	0.729	1.36E-18	TRUE
CD4 NC	*TNFSF9*	Wald ratio	1	-0.0067	0.0082	0.993 (0.978, 1.009)	0.414	0.686	1.63E-35	TRUE
CD4 NC	*TNRC6A*	Wald ratio	1	-0.0516	0.0146	0.95 (0.923, 0.977)	4.19E-04	0.009	9.15E-09	TRUE
CD4 NC	*TOMM20*	Wald ratio	1	0.0107	0.0164	1.011 (0.979, 1.044)	0.512	0.748	3.59E-09	TRUE
CD4 NC	*TOMM7*	Wald ratio	1	0.0026	0.0039	1.003 (0.995, 1.01)	0.503	0.743	1.09E-211	TRUE
CD4 NC	*TOR3A*	Wald ratio	1	-0.0081	0.0191	0.992 (0.955, 1.03)	0.672	0.849	2.78E-08	TRUE
CD4 NC	*TP53I11*	Wald ratio	1	-0.0282	0.0202	0.972 (0.934, 1.011)	0.163	0.437	4.12E-08	TRUE
CD4 NC	*TP53TG1*	Wald ratio	1	-0.0024	0.0151	0.998 (0.968, 1.027)	0.871	0.947	1.70E-13	TRUE
CD4 NC	*TPD52*	Wald ratio	1	-0.0157	0.0094	0.984 (0.967, 1.003)	0.093	0.323	1.47E-32	TRUE
CD4 NC	*TPGS1*	Wald ratio	1	-0.0037	0.0116	0.996 (0.974, 1.019)	0.749	0.886	4.17E-23	TRUE
CD4 NC	*TPGS2*	Wald ratio	1	-0.0073	0.0074	0.993 (0.978, 1.007)	0.318	0.614	4.83E-57	TRUE
CD4 NC	*TPI1*	Wald ratio	1	0.0005	0.0148	1.001 (0.972, 1.03)	0.973	0.989	1.55E-15	TRUE
CD4 NC	*TPK1*	Wald ratio	1	0.0049	0.0189	1.005 (0.968, 1.043)	0.795	0.911	7.16E-10	TRUE
CD4 NC	*TPR*	Wald ratio	1	0.0328	0.0151	1.033 (1.003, 1.064)	0.031	0.164	9.75E-12	TRUE
CD4 NC	*TPRA1*	Wald ratio	1	-0.0172	0.0148	0.983 (0.955, 1.012)	0.245	0.539	2.59E-12	TRUE
CD4 NC	*TPST2*	Wald ratio	1	-0.0244	0.0194	0.976 (0.94, 1.014)	0.207	0.494	1.41E-08	TRUE
CD4 NC	*TRABD2A*	Wald ratio	1	0.0472	0.0237	1.048 (1.001, 1.098)	0.046	0.212	4.10E-12	TRUE
CD4 NC	*TRAPPC12-AS1*	Wald ratio	1	0.0277	0.0089	1.028 (1.01, 1.046)	0.002	0.025	1.32E-34	TRUE
CD4 NC	*TRAPPC2L*	Wald ratio	1	0.0230	0.0092	1.023 (1.005, 1.042)	0.013	0.095	2.18E-33	TRUE
CD4 NC	*TRAPPC4*	Wald ratio	1	0.0338	0.0148	1.034 (1.005, 1.065)	0.022	0.134	2.48E-14	TRUE
CD4 NC	*TRAT1*	Wald ratio	1	-0.0601	0.0197	0.942 (0.906, 0.979)	0.002	0.029	2.88E-08	TRUE
CD4 NC	*TRIB2*	Wald ratio	1	-0.0315	0.0140	0.969 (0.943, 0.996)	0.024	0.143	5.21E-13	TRUE
CD4 NC	*TRIB3*	Wald ratio	1	0.0287	0.0150	1.029 (0.999, 1.06)	0.055	0.234	1.94E-08	TRUE
CD4 NC	*TRIM16*	Wald ratio	1	0.0193	0.0182	1.02 (0.984, 1.056)	0.287	0.579	9.08E-10	TRUE
CD4 NC	*TRIM23*	Wald ratio	1	0.0192	0.0122	1.019 (0.995, 1.044)	0.114	0.361	7.11E-20	TRUE
CD4 NC	*TRIM31*	Wald ratio	1	-0.0066	0.0139	0.993 (0.967, 1.021)	0.636	0.828	1.40E-13	TRUE
CD4 NC	*TRIM35*	Wald ratio	1	0.0137	0.0169	1.014 (0.981, 1.048)	0.416	0.688	5.33E-11	TRUE
CD4 NC	*TRIM4*	Wald ratio	1	-0.0148	0.0169	0.985 (0.953, 1.018)	0.381	0.666	2.83E-11	TRUE
CD4 NC	*TRIM66*	Wald ratio	1	-0.0125	0.0157	0.988 (0.958, 1.018)	0.427	0.697	1.30E-12	TRUE
CD4 NC	*TRMT112*	Wald ratio	1	0.0151	0.0130	1.015 (0.99, 1.041)	0.247	0.54	1.15E-23	TRUE
CD4 NC	*TRMT12*	Wald ratio	1	-0.0162	0.0198	0.984 (0.947, 1.023)	0.412	0.686	1.50E-08	TRUE
CD4 NC	*TRMT6*	Wald ratio	1	0.0065	0.0193	1.006 (0.969, 1.045)	0.737	0.88	1.01E-08	TRUE
CD4 NC	*TSEN54*	Wald ratio	1	-0.0428	0.0184	0.958 (0.924, 0.993)	0.02	0.125	2.60E-08	TRUE
CD4 NC	*TSGA10*	Wald ratio	1	-0.0014	0.0084	0.999 (0.982, 1.015)	0.871	0.947	4.48E-39	TRUE
CD4 NC	*TSHZ2*	IVW	2	0.0348	0.0428	1.035 (0.952, 1.126)	0.417	0.688	1.50E-25	TRUE
CD4 NC	*TSKS*	Wald ratio	1	0.0020	0.0084	1.002 (0.986, 1.019)	0.81	0.919	1.12E-35	TRUE

CD4 NC	*TSPAN15*	Wald ratio	1	-0.0127	0.0205	0.987 (0.949, 1.028)	0.535	0.761	6.84E-09	TRUE
CD4 NC	*TSPAN32*	Wald ratio	1	-0.0646	0.0135	0.937 (0.913, 0.963)	1.64E-06	1.65E-04	1.09E-12	TRUE
CD4 NC	*TSPO*	Wald ratio	1	0.0101	0.0090	1.01 (0.992, 1.028)	0.264	0.558	2.19E-36	TRUE
CD4 NC	*TSTD1*	IVW	2	0.0056	0.0060	1.006 (0.994, 1.017)	0.351	0.644	1.30E-73	TRUE
CD4 NC	*TSTD3*	Wald ratio	1	-0.0031	0.0074	0.997 (0.982, 1.012)	0.675	0.852	5.95E-53	TRUE
CD4 NC	*TTC12*	Wald ratio	1	0.0520	0.0173	1.053 (1.018, 1.09)	0.003	0.032	2.33E-10	TRUE
CD4 NC	*TTC16*	Wald ratio	1	-0.0361	0.0135	0.965 (0.939, 0.99)	0.008	0.067	2.14E-16	TRUE
CD4 NC	*TTC24*	Wald ratio	1	0.0448	0.0161	1.046 (1.013, 1.079)	0.005	0.052	9.25E-12	TRUE
CD4 NC	*TTC3*	Wald ratio	1	0.0009	0.0145	1.001 (0.973, 1.03)	0.948	0.98	3.75E-14	TRUE
CD4 NC	*TTC32*	Wald ratio	1	0.0185	0.0089	1.019 (1.001, 1.037)	0.037	0.183	1.11E-38	TRUE
CD4 NC	*TTC39B*	Wald ratio	1	0.0203	0.0120	1.021 (0.997, 1.045)	0.089	0.317	2.29E-19	TRUE
CD4 NC	*TUBA1B*	Wald ratio	1	0.0269	0.0128	1.027 (1.002, 1.053)	0.036	0.182	3.68E-17	TRUE
CD4 NC	*TUFM*	Wald ratio	1	0.0093	0.0047	1.009 (1, 1.019)	0.046	0.21	1.62E-142	TRUE
CD4 NC	*TWSG1*	Wald ratio	1	-0.0369	0.0188	0.964 (0.929, 1)	0.05	0.222	1.49E-09	TRUE
CD4 NC	*TXK*	Wald ratio	1	0.0033	0.0114	1.003 (0.981, 1.026)	0.775	0.901	2.29E-16	TRUE
CD4 NC	*TXN2*	Wald ratio	1	0.0053	0.0124	1.005 (0.981, 1.03)	0.665	0.847	6.72E-19	TRUE
CD4 NC	*TXNDC15*	Wald ratio	1	0.0049	0.0137	1.005 (0.978, 1.032)	0.721	0.876	1.46E-12	TRUE
CD4 NC	*TXNL4A*	Wald ratio	1	-0.0737	0.0217	0.929 (0.89, 0.969)	6.80E-04	0.013	1.10E-08	TRUE
CD4 NC	*TYMP*	Wald ratio	1	-0.0033	0.0153	0.997 (0.967, 1.027)	0.829	0.929	4.01E-24	TRUE
CD4 NC	*UACA*	Wald ratio	1	0.0558	0.0168	1.057 (1.023, 1.093)	8.80E-04	0.015	3.29E-09	TRUE
CD4 NC	*UBA52*	Wald ratio	1	0.0094	0.0172	1.009 (0.976, 1.044)	0.586	0.794	5.49E-11	TRUE
CD4 NC	*UBA6*	Wald ratio	1	0.0078	0.0093	1.008 (0.99, 1.026)	0.397	0.677	3.45E-34	TRUE
CD4 NC	*UBA6-AS1*	Wald ratio	1	-0.0230	0.0140	0.977 (0.951, 1.004)	0.101	0.336	3.41E-16	TRUE
CD4 NC	*UBASH3A*	Wald ratio	1	-0.0154	0.0120	0.985 (0.962, 1.008)	0.2	0.484	1.86E-20	TRUE
CD4 NC	*UBE2B*	Wald ratio	1	-0.0065	0.0164	0.993 (0.962, 1.026)	0.69	0.86	7.49E-10	TRUE
CD4 NC	*UBE2E2*	Wald ratio	1	-0.0060	0.0168	0.994 (0.962, 1.027)	0.723	0.877	7.61E-12	TRUE
CD4 NC	*UBE2F*	Wald ratio	1	-0.0166	0.0100	0.984 (0.964, 1.003)	0.099	0.333	4.68E-29	TRUE
CD4 NC	*UBE2I*	Wald ratio	1	0.0760	0.0200	1.079 (1.037, 1.122)	1.47E-04	0.004	1.42E-10	TRUE
CD4 NC	*UBE2L3*	Wald ratio	1	-0.0010	0.0120	0.999 (0.976, 1.023)	0.937	0.974	6.94E-15	TRUE
CD4 NC	*UBE2Q2*	Wald ratio	1	0.0509	0.0116	1.052 (1.028, 1.077)	1.22E-05	6.70E-04	5.53E-14	TRUE
CD4 NC	*UBE3B*	Wald ratio	1	0.0626	0.0192	1.065 (1.025, 1.105)	0.001	0.018	5.49E-10	TRUE
CD4 NC	*UBE3C*	Wald ratio	1	0.0190	0.0188	1.019 (0.982, 1.058)	0.312	0.606	1.02E-13	TRUE
CD4 NC	*UBE4B*	Wald ratio	1	-0.0197	0.0138	0.981 (0.954, 1.007)	0.153	0.423	1.02E-12	TRUE
CD4 NC	*UBR7*	Wald ratio	1	0.0067	0.0162	1.007 (0.975, 1.039)	0.68	0.854	5.86E-12	TRUE
CD4 NC	*UBXN10-AS1*	Wald ratio	1	-0.0314	0.0193	0.969 (0.933, 1.006)	0.104	0.343	4.79E-08	TRUE
CD4 NC	*UBXN11*	Wald ratio	1	-0.0093	0.0189	0.991 (0.955, 1.028)	0.624	0.822	1.03E-08	TRUE
CD4 NC	*UBXN6*	Wald ratio	1	-0.0236	0.0135	0.977 (0.951, 1.003)	0.08	0.297	1.80E-20	TRUE
CD4 NC	*UCP2*	Wald ratio	1	0.0035	0.0075	1.004 (0.989, 1.018)	0.64	0.83	4.75E-49	TRUE
CD4 NC	*UFL1*	Wald ratio	1	-0.0096	0.0202	0.99 (0.952, 1.031)	0.636	0.828	1.97E-08	TRUE
CD4 NC	*UFSP2*	Wald ratio	1	-0.0169	0.0185	0.983 (0.948, 1.02)	0.36	0.65	9.29E-10	TRUE
CD4 NC	*UNC45B*	Wald ratio	1	0.0099	0.0204	1.01 (0.97, 1.051)	0.627	0.822	1.53E-08	TRUE
CD4 NC	*UPF3A*	Wald ratio	1	0.0298	0.0186	1.03 (0.993, 1.068)	0.109	0.352	1.48E-08	TRUE
CD4 NC	*UPK3BL*	Wald ratio	1	0.0071	0.0096	1.007 (0.988, 1.026)	0.459	0.719	4.38E-36	TRUE
CD4 NC	*UQCC2*	Wald ratio	1	-0.0017	0.0148	0.998 (0.97, 1.028)	0.911	0.962	6.52E-13	TRUE
CD4 NC	*UQCRH*	Wald ratio	1	-0.0201	0.0095	0.98 (0.962, 0.999)	0.035	0.179	1.32E-39	TRUE
CD4 NC	*URGCP*	Wald ratio	1	-0.0040	0.0168	0.996 (0.964, 1.029)	0.811	0.919	2.63E-11	TRUE
CD4 NC	*USF1*	Wald ratio	1	-0.0134	0.0125	0.987 (0.963, 1.011)	0.285	0.577	6.60E-22	TRUE
CD4 NC	*USP10*	Wald ratio	1	-0.0142	0.0168	0.986 (0.954, 1.019)	0.398	0.678	1.31E-13	TRUE
CD4 NC	*USP36*	Wald ratio	1	0.0830	0.0169	1.087 (1.051, 1.123)	8.76E-07	1.03E-04	6.74E-11	TRUE
CD4 NC	*USP44*	Wald ratio	1	-0.0089	0.0177	0.991 (0.957, 1.026)	0.614	0.814	7.33E-10	TRUE
CD4 NC	*USP53*	Wald ratio	1	-0.0031	0.0051	0.997 (0.987, 1.007)	0.545	0.766	3.18E-78	TRUE
CD4 NC	*USP7*	Wald ratio	1	0.0577	0.0211	1.059 (1.017, 1.104)	0.006	0.058	8.41E-08	TRUE
CD4 NC	*UTS2*	Wald ratio	1	-0.0221	0.0095	0.978 (0.96, 0.996)	0.019	0.124	2.01E-33	TRUE
CD4 NC	*VAMP2*	Wald ratio	1	0.0181	0.0106	1.018 (0.997, 1.04)	0.086	0.311	9.94E-31	TRUE
CD4 NC	*VAMP4*	Wald ratio	1	0.0064	0.0099	1.006 (0.987, 1.026)	0.522	0.754	2.13E-34	TRUE
CD4 NC	*VAMP5*	Wald ratio	1	0.0137	0.0098	1.014 (0.994, 1.033)	0.164	0.439	2.73E-42	TRUE
CD4 NC	*VCL*	Wald ratio	1	-0.0102	0.0118	0.99 (0.967, 1.013)	0.385	0.668	7.01E-28	TRUE
CD4 NC	*VDAC2*	Wald ratio	1	-0.0328	0.0126	0.968 (0.944, 0.992)	0.009	0.076	1.77E-18	TRUE
CD4 NC	*VIM*	Wald ratio	1	0.0973	0.0191	1.102 (1.062, 1.144)	3.34E-07	8.32E-05	6.55E-08	TRUE
CD4 NC	*VMAC*	Wald ratio	1	-0.0110	0.0189	0.989 (0.953, 1.026)	0.559	0.777	5.66E-12	TRUE
CD4 NC	*VOPP1*	Wald ratio	1	-0.0070	0.0165	0.993 (0.961, 1.026)	0.671	0.849	2.13E-11	TRUE
CD4 NC	*VPS13C*	Wald ratio	1	0.0089	0.0224	1.009 (0.966, 1.054)	0.692	0.861	4.78E-08	TRUE
CD4 NC	*VPS28*	Wald ratio	1	0.0134	0.0082	1.013 (0.997, 1.03)	0.102	0.338	4.51E-37	TRUE
CD4 NC	*VPS53*	Wald ratio	1	-0.0720	0.0168	0.931 (0.9, 0.962)	1.83E-05	9.06E-04	4.47E-11	TRUE
CD4 NC	*VRK3*	Wald ratio	1	-0.0182	0.0147	0.982 (0.954, 1.011)	0.215	0.505	8.07E-16	TRUE
CD4 NC	*VWA7*	Wald ratio	1	-0.0119	0.0122	0.988 (0.965, 1.012)	0.329	0.624	2.64E-19	TRUE
CD4 NC	*VWDE*	Wald ratio	1	-0.0101	0.0095	0.99 (0.972, 1.009)	0.289	0.582	3.67E-29	TRUE
CD4 NC	*WAC-AS1*	IVW	2	0.0131	0.0156	1.013 (0.983, 1.045)	0.4	0.679	6.51E-43	TRUE
CD4 NC	*WBSCR16*	Wald ratio	1	-0.0920	0.0194	0.912 (0.878, 0.947)	2.05E-06	1.89E-04	9.90E-09	TRUE
CD4 NC	*WBSCR27*	Wald ratio	1	-0.0102	0.0078	0.99 (0.975, 1.005)	0.192	0.473	2.25E-40	TRUE
CD4 NC	*WDR17*	Wald ratio	1	0.0084	0.0126	1.008 (0.984, 1.034)	0.504	0.744	1.44E-21	TRUE
CD4 NC	*WDR18*	Wald ratio	1	-0.0100	0.0152	0.99 (0.961, 1.02)	0.512	0.748	8.23E-13	TRUE
CD4 NC	*WDR60*	Wald ratio	1	-0.0134	0.0175	0.987 (0.953, 1.021)	0.445	0.709	9.09E-09	TRUE
CD4 NC	*WDR73*	Wald ratio	1	-0.0396	0.0194	0.961 (0.925, 0.998)	0.041	0.198	6.83E-08	TRUE
CD4 NC	*WDR86*	IVW	2	-0.0301	0.0282	0.97 (0.918, 1.025)	0.285	0.577	1.17E-22	TRUE
CD4 NC	*WDYHV1*	Wald ratio	1	0.0076	0.0136	1.008 (0.981, 1.035)	0.578	0.789	1.32E-15	TRUE
CD4 NC	*WIPI2*	Wald ratio	1	-0.0125	0.0108	0.988 (0.967, 1.009)	0.246	0.539	2.10E-23	TRUE
CD4 NC	*WRB*	Wald ratio	1	0.0701	0.0230	1.073 (1.025, 1.122)	0.002	0.029	8.92E-08	TRUE
CD4 NC	*WWP1*	Wald ratio	1	-0.0311	0.0164	0.969 (0.939, 1.001)	0.058	0.242	4.78E-10	TRUE

CD4 NC	*XAB2*	Wald ratio	1	-0.0178	0.0188	0.982 (0.947, 1.019)	0.343	0.636	5.74E-09	TRUE
CD4 NC	*XBP1*	Wald ratio	1	0.0059	0.0072	1.006 (0.992, 1.02)	0.418	0.689	5.04E-42	TRUE
CD4 NC	*XKR9*	Wald ratio	1	-0.0005	0.0130	0.999 (0.974, 1.025)	0.969	0.989	8.26E-18	TRUE
CD4 NC	*XPC*	Wald ratio	1	-0.0090	0.0145	0.991 (0.963, 1.02)	0.538	0.762	8.20E-09	TRUE
CD4 NC	*XPNPEP1*	Wald ratio	1	-0.0219	0.0195	0.978 (0.942, 1.016)	0.261	0.555	5.40E-09	TRUE
CD4 NC	*XRCC2*	Wald ratio	1	0.0195	0.0166	1.02 (0.987, 1.053)	0.24	0.534	1.53E-11	TRUE
CD4 NC	*XRCC6BP1*	Wald ratio	1	-0.0153	0.0077	0.985 (0.97, 1)	0.046	0.21	4.59E-49	TRUE
CD4 NC	*XRRA1*	Wald ratio	1	-0.0063	0.0043	0.994 (0.985, 1.002)	0.143	0.41	6.75E-175	TRUE
CD4 NC	*XXbac-BPG181B23.7*	Wald ratio	1	-0.0117	0.0100	0.988 (0.969, 1.008)	0.24	0.534	1.68E-24	TRUE
CD4 NC	*XXbac-BPG181M17.6*	Wald ratio	1	0.0117	0.0165	1.012 (0.98, 1.045)	0.48	0.734	1.38E-12	TRUE
CD4 NC	*XXbac-BPG299F13.17*	IVW	3	-0.0005	0.0135	1 (0.973, 1.026)	0.972	0.989	4.12E-182	TRUE
CD4 NC	*XXbac-BPG299F13.17*	MR Egger	3	0.0359	0.0188	1.037 (0.999, 1.075)	0.307	NA	NA	NA
CD4 NC	*XXbac-BPG299F13.17*	Weighted median	3	0.0019	0.0044	1.002 (0.993, 1.011)	0.662	NA	NA	NA
CD4 NC	*XXbac-BPG299F13.17*	Simple mode	3	-0.0128	0.0119	0.987 (0.965, 1.011)	0.396	NA	NA	NA
CD4 NC	*XXbac-BPG299F13.17*	Weighted mode	3	0.0035	0.0046	1.004 (0.994, 1.013)	0.526	NA	NA	NA
CD4 NC	*XXbac-BPG308K3.5*	Wald ratio	1	0.0111	0.0202	1.011 (0.972, 1.052)	0.583	0.793	8.72E-11	TRUE
CD4 NC	*XYLB*	Wald ratio	1	-0.0171	0.0137	0.983 (0.957, 1.01)	0.213	0.502	8.23E-15	TRUE
CD4 NC	*YBEY*	Wald ratio	1	-0.0007	0.0051	0.999 (0.989, 1.009)	0.898	0.957	7.25E-98	TRUE
CD4 NC	*YBX3*	Wald ratio	1	0.0066	0.0136	1.007 (0.98, 1.034)	0.627	0.822	5.39E-17	TRUE
CD4 NC	*YEATS4*	Wald ratio	1	-0.0007	0.0092	0.999 (0.981, 1.018)	0.942	0.977	2.63E-36	TRUE
CD4 NC	*YIPF5*	Wald ratio	1	-0.0094	0.0190	0.991 (0.954, 1.028)	0.622	0.821	2.88E-12	TRUE
CD4 NC	*YME1L1*	Wald ratio	1	0.0109	0.0141	1.011 (0.983, 1.039)	0.439	0.703	4.00E-15	TRUE
CD4 NC	*YWHAB*	Wald ratio	1	0.0315	0.0160	1.032 (1, 1.065)	0.049	0.22	1.08E-12	TRUE
CD4 NC	*YWHAH*	Wald ratio	1	-0.0345	0.0155	0.966 (0.937, 0.996)	0.026	0.151	4.59E-12	TRUE
CD4 NC	*ZADH2*	Wald ratio	1	0.0029	0.0134	1.003 (0.977, 1.03)	0.831	0.929	7.05E-17	TRUE
CD4 NC	*ZBTB1*	Wald ratio	1	-0.0192	0.0194	0.981 (0.944, 1.019)	0.322	0.618	6.21E-08	TRUE
CD4 NC	*ZBTB32*	Wald ratio	1	0.0169	0.0133	1.017 (0.991, 1.044)	0.203	0.489	2.71E-09	TRUE
CD4 NC	*ZC2HC1A*	Wald ratio	1	-0.0032	0.0121	0.997 (0.973, 1.021)	0.793	0.909	1.07E-19	TRUE
CD4 NC	*ZC3H3*	Wald ratio	1	-0.0031	0.0141	0.997 (0.97, 1.025)	0.829	0.929	1.56E-15	TRUE
CD4 NC	*ZC3H8*	Wald ratio	1	0.0081	0.0149	1.008 (0.979, 1.038)	0.587	0.794	1.68E-13	TRUE
CD4 NC	*ZC3HAV1*	Wald ratio	1	0.0045	0.0061	1.004 (0.993, 1.016)	0.459	0.72	4.69E-81	TRUE
CD4 NC	*ZDHHC2*	Wald ratio	1	-0.0187	0.0183	0.981 (0.947, 1.017)	0.306	0.6	1.23E-09	TRUE
CD4 NC	*ZDHHC24*	Wald ratio	1	0.0000	0.0151	1 (0.971, 1.03)	0.998	0.999	7.42E-11	TRUE
CD4 NC	*ZDHHC4*	Wald ratio	1	-0.0447	0.0107	0.956 (0.936, 0.976)	2.72E-05	0.001	1.28E-21	TRUE
CD4 NC	*ZDHHC7*	Wald ratio	1	-0.0402	0.0207	0.961 (0.923, 1)	0.052	0.226	5.94E-09	TRUE
CD4 NC	*ZFAS1*	Wald ratio	1	0.0059	0.0076	1.006 (0.991, 1.021)	0.436	0.703	3.09E-54	TRUE
CD4 NC	*ZFP57*	IVW	2	-0.0253	0.0098	0.975 (0.956, 0.994)	0.01	0.08	1.26E-41	TRUE
CD4 NC	*ZFP90*	Wald ratio	1	0.0251	0.0182	1.025 (0.989, 1.063)	0.168	0.444	6.53E-11	TRUE
CD4 NC	*ZMAT3*	Wald ratio	1	-0.0113	0.0168	0.989 (0.957, 1.022)	0.501	0.743	6.43E-09	TRUE
CD4 NC	*ZNF100*	Wald ratio	1	-0.0435	0.0081	0.957 (0.942, 0.973)	7.16E-08	6.60E-05	1.80E-42	TRUE
CD4 NC	*ZNF138*	Wald ratio	1	-0.0191	0.0206	0.981 (0.942, 1.022)	0.354	0.645	5.76E-08	TRUE
CD4 NC	*ZNF154*	Wald ratio	1	-0.0071	0.0161	0.993 (0.962, 1.025)	0.659	0.842	7.80E-10	TRUE
CD4 NC	*ZNF160*	Wald ratio	1	0.0310	0.0154	1.032 (1.001, 1.063)	0.044	0.206	2.54E-17	TRUE
CD4 NC	*ZNF257*	Wald ratio	1	0.0256	0.0101	1.026 (1.006, 1.046)	0.011	0.086	1.06E-23	TRUE
CD4 NC	*ZNF264*	Wald ratio	1	0.0057	0.0137	1.006 (0.979, 1.033)	0.678	0.853	7.54E-15	TRUE
CD4 NC	*ZNF266*	Wald ratio	1	0.0277	0.0109	1.028 (1.006, 1.05)	0.011	0.088	7.80E-24	TRUE
CD4 NC	*ZNF30*	Wald ratio	1	0.0039	0.0143	1.004 (0.976, 1.032)	0.785	0.907	3.28E-17	TRUE
CD4 NC	*ZNF302*	IVW	2	-0.0058	0.0079	0.994 (0.979, 1.01)	0.464	0.724	3.34E-46	TRUE
CD4 NC	*ZNF324*	Wald ratio	1	0.0215	0.0166	1.022 (0.989, 1.055)	0.194	0.475	1.27E-10	TRUE
CD4 NC	*ZNF396*	Wald ratio	1	-0.0107	0.0196	0.989 (0.952, 1.028)	0.586	0.794	4.51E-09	TRUE
CD4 NC	*ZNF404*	Wald ratio	1	-0.0068	0.0198	0.993 (0.955, 1.033)	0.731	0.88	1.93E-08	TRUE
CD4 NC	*ZNF419*	Wald ratio	1	-0.0152	0.0176	0.985 (0.952, 1.019)	0.388	0.669	2.93E-08	TRUE
CD4 NC	*ZNF429*	Wald ratio	1	-0.0098	0.0120	0.99 (0.967, 1.014)	0.414	0.686	8.47E-21	TRUE
CD4 NC	*ZNF480*	Wald ratio	1	0.0092	0.0133	1.009 (0.983, 1.036)	0.49	0.738	3.71E-21	TRUE
CD4 NC	*ZNF502*	Wald ratio	1	-0.0285	0.0090	0.972 (0.955, 0.989)	0.002	0.022	2.17E-34	TRUE
CD4 NC	*ZNF503-AS1*	Wald ratio	1	-0.0022	0.0183	0.998 (0.963, 1.034)	0.905	0.96	1.40E-12	TRUE
CD4 NC	*ZNF544*	Wald ratio	1	0.0096	0.0152	1.01 (0.98, 1.04)	0.525	0.755	2.93E-13	TRUE
CD4 NC	*ZNF552*	Wald ratio	1	-0.0004	0.0096	1 (0.981, 1.019)	0.964	0.988	1.25E-29	TRUE
CD4 NC	*ZNF557*	Wald ratio	1	0.0307	0.0144	1.031 (1.002, 1.061)	0.033	0.172	3.73E-14	TRUE
CD4 NC	*ZNF559*	Wald ratio	1	0.0083	0.0170	1.008 (0.975, 1.043)	0.627	0.822	2.33E-17	TRUE
CD4 NC	*ZNF566*	Wald ratio	1	0.0072	0.0193	1.007 (0.97, 1.046)	0.71	0.871	3.92E-09	TRUE
CD4 NC	*ZNF568*	Wald ratio	1	-0.0220	0.0144	0.978 (0.951, 1.006)	0.126	0.382	2.08E-14	TRUE
CD4 NC	*ZNF584*	Wald ratio	1	0.0146	0.0089	1.015 (0.997, 1.033)	0.101	0.337	2.02E-34	TRUE
CD4 NC	*ZNF586*	Wald ratio	1	0.0090	0.0164	1.009 (0.977, 1.042)	0.585	0.794	6.41E-11	TRUE
CD4 NC	*ZNF587*	Wald ratio	1	-0.0033	0.0145	0.997 (0.969, 1.025)	0.817	0.922	9.37E-13	TRUE
CD4 NC	*ZNF593*	Wald ratio	1	0.0200	0.0103	1.02 (1, 1.041)	0.051	0.224	1.38E-29	TRUE
CD4 NC	*ZNF595*	Wald ratio	1	0.0108	0.0149	1.011 (0.982, 1.041)	0.469	0.727	3.16E-12	TRUE
CD4 NC	*ZNF638*	Wald ratio	1	0.0187	0.0104	1.019 (0.998, 1.04)	0.072	0.278	1.06E-26	TRUE
CD4 NC	*ZNF652*	Wald ratio	1	-0.0025	0.0153	0.998 (0.968, 1.028)	0.873	0.947	5.73E-11	TRUE
CD4 NC	*ZNF667-AS1*	Wald ratio	1	0.0216	0.0149	1.022 (0.992, 1.052)	0.147	0.416	3.10E-12	TRUE
CD4 NC	*ZNF677*	Wald ratio	1	-0.0108	0.0082	0.989 (0.974, 1.005)	0.184	0.464	1.11E-46	TRUE
CD4 NC	*ZNF680*	Wald ratio	1	0.0361	0.0186	1.037 (1, 1.075)	0.052	0.228	6.88E-09	TRUE
CD4 NC	*ZNF684*	Wald ratio	1	0.0265	0.0213	1.027 (0.985, 1.071)	0.215	0.504	2.50E-08	TRUE
CD4 NC	*ZNF708*	Wald ratio	1	-0.0391	0.0200	0.962 (0.925, 1)	0.051	0.224	3.23E-09	TRUE
CD4 NC	*ZNF718*	Wald ratio	1	-0.0061	0.0170	0.994 (0.961, 1.028)	0.72	0.876	1.51E-10	TRUE
CD4 NC	*ZNF737*	Wald ratio	1	0.0463	0.0171	1.047 (1.013, 1.083)	0.007	0.062	4.25E-10	TRUE
CD4 NC	*ZNF749*	Wald ratio	1	-0.0097	0.0078	0.99 (0.975, 1.005)	0.209	0.496	1.71E-48	TRUE
CD4 NC	*ZNF772*	Wald ratio	1	-0.0135	0.0162	0.987 (0.956, 1.018)	0.403	0.681	5.52E-11	TRUE
CD4 NC	*ZNF783*	Wald ratio	1	0.0037	0.0144	1.004 (0.976, 1.032)	0.798	0.913	7.04E-10	TRUE

CD4 NC	*ZNF786*	Wald ratio	1	0.0222	0.0136	1.022 (0.996, 1.05)	0.101	0.337	7.64E-18	TRUE
CD4 NC	*ZNF793*	Wald ratio	1	-0.0469	0.0180	0.954 (0.921, 0.988)	0.009	0.076	4.70E-10	TRUE
CD4 NC	*ZNF83*	Wald ratio	1	-0.0137	0.0073	0.986 (0.972, 1.001)	0.062	0.253	1.98E-53	TRUE
CD4 NC	*ZNF846*	Wald ratio	1	-0.0142	0.0191	0.986 (0.95, 1.024)	0.457	0.719	6.00E-08	TRUE
CD4 NC	*ZNF85*	Wald ratio	1	0.0135	0.0082	1.014 (0.997, 1.03)	0.099	0.333	5.21E-47	TRUE
CD4 NC	*ZNF879*	Wald ratio	1	-0.0001	0.0174	1 (0.966, 1.034)	0.994	0.998	4.12E-09	TRUE
CD4 NC	*ZNF880*	Wald ratio	1	-0.0231	0.0154	0.977 (0.948, 1.007)	0.135	0.396	8.76E-13	TRUE
CD4 NC	*ZNFX1*	Wald ratio	1	-0.0135	0.0187	0.987 (0.951, 1.023)	0.471	0.728	8.12E-10	TRUE
CD4 NC	*ZNHIT1*	Wald ratio	1	0.0185	0.0158	1.019 (0.988, 1.051)	0.241	0.534	4.09E-15	TRUE
CD4 NC	*ZNHIT6*	Wald ratio	1	-0.0214	0.0161	0.979 (0.948, 1.01)	0.184	0.464	5.22E-12	TRUE
CD4 NC	*ZP3*	Wald ratio	1	0.0153	0.0066	1.015 (1.002, 1.029)	0.021	0.132	6.71E-81	TRUE
CD4 NC	*ZSCAN18*	Wald ratio	1	-0.0299	0.0158	0.971 (0.941, 1.001)	0.058	0.243	7.36E-10	TRUE
CD4 NC	*ZSCAN23*	IVW	2	-0.0039	0.0082	0.996 (0.98, 1.012)	0.633	0.826	1.84E-42	TRUE
CD4 NC	*ZSWIM7*	Wald ratio	1	0.0421	0.0132	1.043 (1.016, 1.07)	0.001	0.02	3.20E-17	TRUE
CD4 NC	*ZYX*	Wald ratio	1	0.0117	0.0195	1.012 (0.974, 1.051)	0.548	0.769	2.02E-08	TRUE
CD4 SOX4	*AC013264.2*	Wald ratio	1	-0.0005	0.0148	1 (0.971, 1.029)	0.975	0.99	1.22E-11	TRUE
CD4 SOX4	*AGPAT1*	Wald ratio	1	0.0132	0.0125	1.013 (0.989, 1.038)	0.292	0.585	3.20E-17	TRUE
CD4 SOX4	*AKAP7*	Wald ratio	1	-0.0332	0.0164	0.967 (0.937, 0.999)	0.043	0.204	5.65E-10	TRUE
CD4 SOX4	*ATG5*	Wald ratio	1	-0.0106	0.0182	0.989 (0.955, 1.025)	0.561	0.778	1.40E-12	TRUE
CD4 SOX4	*B3GALT4*	IVW	2	0.0312	0.0195	1.032 (0.993, 1.072)	0.11	0.353	3.77E-29	TRUE
CD4 SOX4	*BTN2A1*	Wald ratio	1	0.0264	0.0245	1.027 (0.979, 1.077)	0.281	0.575	8.95E-09	TRUE
CD4 SOX4	*C6orf136*	IVW	2	-0.0427	0.0259	0.958 (0.911, 1.008)	0.1	0.334	1.21E-19	TRUE
CD4 SOX4	*C6orf226*	Wald ratio	1	-0.0030	0.0169	0.997 (0.964, 1.031)	0.857	0.939	4.43E-10	TRUE
CD4 SOX4	*CENPK*	Wald ratio	1	0.0210	0.0135	1.021 (0.995, 1.048)	0.119	0.369	2.80E-14	TRUE
CD4 SOX4	*DAXX*	Wald ratio	1	0.0716	0.0209	1.074 (1.031, 1.119)	6.10E-04	0.012	3.12E-08	TRUE
CD4 SOX4	*DDAH2*	Wald ratio	1	0.0402	0.0362	1.041 (0.97, 1.118)	0.266	0.561	8.13E-13	TRUE
CD4 SOX4	*DHX16*	IVW	2	0.0008	0.0207	1.001 (0.961, 1.042)	0.969	0.989	1.40E-27	TRUE
CD4 SOX4	*DXO*	Wald ratio	1	-0.0529	0.0134	0.948 (0.924, 0.974)	8.24E-05	0.003	7.99E-15	TRUE
CD4 SOX4	*EIF5A*	Wald ratio	1	-0.0150	0.0100	0.985 (0.966, 1.005)	0.134	0.394	1.57E-28	TRUE
CD4 SOX4	*ERAP2*	Wald ratio	1	0.0811	0.0180	1.084 (1.047, 1.123)	6.43E-06	4.08E-04	1.34E-08	TRUE
CD4 SOX4	*FAM118A*	Wald ratio	1	-0.0030	0.0087	0.997 (0.98, 1.014)	0.727	0.878	1.32E-25	TRUE
CD4 SOX4	*GNL1*	Wald ratio	1	0.0193	0.0204	1.019 (0.98, 1.061)	0.344	0.637	9.98E-09	TRUE
CD4 SOX4	*HIST1H1D*	Wald ratio	1	0.0295	0.0188	1.03 (0.993, 1.069)	0.117	0.365	1.43E-13	TRUE
CD4 SOX4	*HLA-B*	IVW	2	0.0378	0.0098	1.039 (1.019, 1.059)	1.08E-04	0.003	3.23E-31	TRUE
CD4 SOX4	*HLA-DMA*	Wald ratio	1	-0.0301	0.0127	0.97 (0.946, 0.995)	0.018	0.118	6.54E-14	TRUE
CD4 SOX4	*HLA-DQB1*	Wald ratio	1	0.0156	0.0247	1.016 (0.968, 1.066)	0.529	0.757	2.35E-10	TRUE
CD4 SOX4	*HLA-DRB5*	Wald ratio	1	-0.0708	0.0179	0.932 (0.899, 0.965)	7.80E-05	0.003	5.03E-08	TRUE
CD4 SOX4	*IER3*	Wald ratio	1	-0.0068	0.0128	0.993 (0.969, 1.018)	0.596	0.801	4.73E-16	TRUE
CD4 SOX4	*IFNGR1*	Wald ratio	1	-0.0004	0.0103	1 (0.98, 1.02)	0.965	0.988	5.60E-32	TRUE
CD4 SOX4	*IL32*	Wald ratio	1	-0.0132	0.0149	0.987 (0.958, 1.016)	0.376	0.664	2.94E-12	TRUE
CD4 SOX4	*LTA*	IVW	2	0.0200	0.0208	1.02 (0.979, 1.063)	0.338	0.631	3.87E-44	TRUE
CD4 SOX4	*MICA*	Wald ratio	1	0.0107	0.0125	1.011 (0.986, 1.036)	0.391	0.672	4.10E-16	TRUE
CD4 SOX4	*NFKBIL1*	Wald ratio	1	0.0047	0.0109	1.005 (0.983, 1.026)	0.665	0.846	6.90E-17	TRUE
CD4 SOX4	*PBX2*	Wald ratio	1	0.0080	0.0137	1.008 (0.981, 1.035)	0.561	0.778	1.09E-12	TRUE
CD4 SOX4	*PRR3*	Wald ratio	1	0.0529	0.0116	1.054 (1.031, 1.079)	5.26E-06	3.48E-04	7.49E-21	TRUE
CD4 SOX4	*PSMD5-AS1*	Wald ratio	1	0.0095	0.0113	1.01 (0.987, 1.032)	0.402	0.679	3.61E-20	TRUE
CD4 SOX4	*RNASET2*	Wald ratio	1	-0.0266	0.0144	0.974 (0.947, 1.002)	0.064	0.259	1.44E-12	TRUE
CD4 SOX4	*RNGTT*	Wald ratio	1	0.0049	0.0080	1.005 (0.989, 1.021)	0.542	0.764	2.90E-38	TRUE
CD4 SOX4	*RP1-3J17.3*	Wald ratio	1	0.0173	0.0170	1.017 (0.984, 1.052)	0.307	0.601	1.77E-18	TRUE
CD4 SOX4	*RP11-347P5.1*	Wald ratio	1	-0.0028	0.0196	0.997 (0.96, 1.036)	0.885	0.954	5.53E-08	TRUE
CD4 SOX4	*RPL12*	Wald ratio	1	-0.0493	0.0146	0.952 (0.925, 0.98)	7.61E-04	0.014	2.00E-13	TRUE
CD4 SOX4	*RPL28*	Wald ratio	1	0.0068	0.0087	1.007 (0.99, 1.024)	0.438	0.703	4.08E-33	TRUE
CD4 SOX4	*RPL36AL*	Wald ratio	1	-0.0139	0.0167	0.986 (0.954, 1.019)	0.405	0.681	2.16E-10	TRUE
CD4 SOX4	*RPL4*	Wald ratio	1	-0.0108	0.0204	0.989 (0.95, 1.03)	0.596	0.801	2.70E-08	TRUE
CD4 SOX4	*RPL8*	Wald ratio	1	-0.0118	0.0115	0.988 (0.966, 1.011)	0.303	0.598	7.97E-20	TRUE
CD4 SOX4	*RPP21*	IVW	2	0.0177	0.0125	1.018 (0.993, 1.043)	0.156	0.427	7.64E-25	TRUE
CD4 SOX4	*RPS10*	Wald ratio	1	-0.0116	0.0113	0.988 (0.967, 1.011)	0.305	0.599	1.92E-17	TRUE
CD4 SOX4	*RPS18*	Wald ratio	1	-0.0185	0.0108	0.982 (0.961, 1.003)	0.087	0.312	5.42E-27	TRUE
CD4 SOX4	*RPS2*	Wald ratio	1	-0.0436	0.0177	0.957 (0.925, 0.991)	0.014	0.101	1.11E-09	TRUE
CD4 SOX4	*RPS26*	Wald ratio	1	-0.0125	0.0043	0.988 (0.979, 0.996)	0.003	0.038	2.40E-165	TRUE
CD4 SOX4	*RPS3A*	Wald ratio	1	0.0197	0.0209	1.02 (0.979, 1.063)	0.348	0.641	1.94E-14	TRUE
CD4 SOX4	*RPS5*	Wald ratio	1	0.0274	0.0181	1.028 (0.992, 1.065)	0.129	0.389	3.38E-09	TRUE
CD4 SOX4	*RPS8*	Wald ratio	1	0.0093	0.0189	1.009 (0.973, 1.047)	0.622	0.821	4.74E-08	TRUE
CD4 SOX4	*RPS9*	Wald ratio	1	0.0575	0.0153	1.059 (1.028, 1.091)	1.64E-04	0.004	1.24E-11	TRUE
CD4 SOX4	*RXRB*	Wald ratio	1	-0.0377	0.0123	0.963 (0.94, 0.986)	0.002	0.028	1.36E-16	TRUE
CD4 SOX4	*SGK1*	Wald ratio	1	-0.0199	0.0109	0.98 (0.96, 1.001)	0.067	0.265	1.79E-22	TRUE
CD4 SOX4	*SH3YL1*	Wald ratio	1	0.0729	0.0166	1.076 (1.041, 1.111)	1.11E-05	6.26E-04	1.28E-09	TRUE
CD4 SOX4	*SKIV2L*	IVW	2	0.0562	0.0319	1.058 (0.994, 1.126)	0.079	0.293	2.74E-19	TRUE
CD4 SOX4	*SMDT1*	Wald ratio	1	0.0175	0.0089	1.018 (1, 1.036)	0.05	0.222	3.51E-29	TRUE
CD4 SOX4	*SMIM8*	Wald ratio	1	-0.0141	0.0164	0.986 (0.955, 1.018)	0.39	0.671	2.64E-10	TRUE
CD4 SOX4	*SNHG8*	Wald ratio	1	-0.0048	0.0110	0.995 (0.974, 1.017)	0.663	0.845	2.57E-24	TRUE
CD4 SOX4	*STK19*	Wald ratio	1	0.0428	0.0398	1.044 (0.965, 1.128)	0.282	0.575	2.41E-12	TRUE
CD4 SOX4	*SUPT3H*	Wald ratio	1	0.0200	0.0123	1.02 (0.996, 1.045)	0.102	0.338	2.01E-17	TRUE
CD4 SOX4	*SYNCRIP*	Wald ratio	1	0.0527	0.0145	1.054 (1.025, 1.084)	2.69E-04	0.007	1.54E-12	TRUE
CD4 SOX4	*SYTL3*	Wald ratio	1	-0.0042	0.0099	0.996 (0.977, 1.015)	0.671	0.849	6.03E-27	TRUE
CD4 SOX4	*TNF*	Wald ratio	1	-0.0206	0.0142	0.98 (0.953, 1.007)	0.148	0.417	2.10E-13	TRUE
CD4 SOX4	*TOMM7*	Wald ratio	1	0.0073	0.0109	1.007 (0.986, 1.029)	0.503	0.743	9.38E-20	TRUE
CD4 SOX4	*TRIM26*	Wald ratio	1	0.0002	0.0451	1 (0.916, 1.093)	0.997	0.999	1.50E-08	TRUE
CD4 SOX4	*TRIM39*	Wald ratio	1	-0.0666	0.0178	0.936 (0.904, 0.969)	1.78E-04	0.005	3.78E-08	TRUE

CD4 SOX4	*TULP4*	Wald ratio	1	0.0087	0.0206	1.009 (0.969, 1.05)	0.672	0.85	8.98E-13	TRUE
CD4 SOX4	*UQCC2*	Wald ratio	1	-0.0333	0.0149	0.967 (0.939, 0.996)	0.025	0.146	4.39E-12	TRUE
CD4 SOX4	*XXbac-BPG299F13.17*	Wald ratio	1	-0.0247	0.0184	0.976 (0.941, 1.012)	0.181	0.462	3.22E-08	TRUE
CD8 ET	*ABRACL*	Wald ratio	1	0.0691	0.0185	1.072 (1.033, 1.111)	1.90E-04	0.005	2.13E-09	TRUE
CD8 ET	*AC005253.2*	Wald ratio	1	0.0064	0.0116	1.006 (0.984, 1.03)	0.585	0.794	1.54E-22	TRUE
CD8 ET	*AC006369.2*	Wald ratio	1	0.0051	0.0055	1.005 (0.994, 1.016)	0.355	0.646	1.03E-109	TRUE
CD8 ET	*AC007383.4*	Wald ratio	1	-0.0030	0.0107	0.997 (0.976, 1.018)	0.779	0.904	3.64E-25	TRUE
CD8 ET	*AC010642.1*	Wald ratio	1	0.0188	0.0195	1.019 (0.981, 1.059)	0.336	0.628	2.92E-13	TRUE
CD8 ET	*AC013264.2*	Wald ratio	1	0.0115	0.0094	1.012 (0.993, 1.03)	0.223	0.516	8.61E-31	TRUE
CD8 ET	*AC016757.3*	Wald ratio	1	-0.0131	0.0141	0.987 (0.96, 1.015)	0.353	0.644	8.77E-16	TRUE
CD8 ET	*AC018816.3*	Wald ratio	1	0.0081	0.0175	1.008 (0.974, 1.043)	0.643	0.832	6.69E-09	TRUE
CD8 ET	*AC020571.3*	Wald ratio	1	-0.0278	0.0196	0.973 (0.936, 1.011)	0.156	0.428	2.48E-08	TRUE
CD8 ET	*AC021860.1*	Wald ratio	1	0.0379	0.0165	1.039 (1.006, 1.073)	0.022	0.132	2.56E-13	TRUE
CD8 ET	*AC022498.1*	Wald ratio	1	-0.0112	0.0209	0.989 (0.949, 1.03)	0.591	0.797	8.60E-11	TRUE
CD8 ET	*AC023590.1*	Wald ratio	1	-0.0092	0.0178	0.991 (0.957, 1.026)	0.603	0.806	1.35E-14	TRUE
CD8 ET	*AC090186.1*	Wald ratio	1	-0.0386	0.0154	0.962 (0.934, 0.992)	0.012	0.093	5.57E-11	TRUE
CD8 ET	*AC091729.9*	Wald ratio	1	-0.0468	0.0206	0.954 (0.917, 0.994)	0.023	0.138	9.57E-08	TRUE
CD8 ET	*AC104653.1*	Wald ratio	1	0.0079	0.0114	1.008 (0.986, 1.031)	0.487	0.738	7.43E-23	TRUE
CD8 ET	*ACCS*	Wald ratio	1	-0.0024	0.0126	0.998 (0.973, 1.022)	0.847	0.935	4.41E-22	TRUE
CD8 ET	*ACOT4*	Wald ratio	1	0.0269	0.0182	1.027 (0.991, 1.065)	0.14	0.404	1.01E-09	TRUE
CD8 ET	*ACP5*	Wald ratio	1	-0.0406	0.0181	0.96 (0.927, 0.995)	0.025	0.146	1.83E-10	TRUE
CD8 ET	*ACTA2*	Wald ratio	1	0.0080	0.0102	1.008 (0.988, 1.028)	0.433	0.701	4.76E-28	TRUE
CD8 ET	*ACYP2*	Wald ratio	1	0.0068	0.0081	1.007 (0.991, 1.023)	0.403	0.68	8.25E-43	TRUE
CD8 ET	*ADAM10*	Wald ratio	1	-0.0545	0.0195	0.947 (0.912, 0.984)	0.005	0.051	3.55E-15	TRUE
CD8 ET	*ADAMTS1*	Wald ratio	1	-0.0038	0.0166	0.996 (0.964, 1.029)	0.817	0.921	1.06E-11	TRUE
CD8 ET	*ADCY7*	Wald ratio	1	-0.0374	0.0163	0.963 (0.933, 0.995)	0.022	0.134	8.53E-13	TRUE
CD8 ET	*ADCY9*	Wald ratio	1	0.0123	0.0130	1.012 (0.987, 1.038)	0.342	0.635	3.15E-16	TRUE
CD8 ET	*ADRB1*	Wald ratio	1	-0.0483	0.0123	0.953 (0.93, 0.976)	9.15E-05	0.003	6.95E-15	TRUE
CD8 ET	*AGA*	Wald ratio	1	-0.0262	0.0162	0.974 (0.944, 1.006)	0.106	0.346	2.10E-11	TRUE
CD8 ET	*AHI1*	Wald ratio	1	-0.0132	0.0084	0.987 (0.971, 1.003)	0.116	0.362	4.47E-42	TRUE
CD8 ET	*AK5*	Wald ratio	1	0.0039	0.0100	1.004 (0.984, 1.024)	0.699	0.866	5.63E-31	TRUE
CD8 ET	*AKAP13*	Wald ratio	1	-0.0282	0.0164	0.972 (0.941, 1.004)	0.086	0.311	3.82E-12	TRUE
CD8 ET	*AKIP1*	Wald ratio	1	-0.0136	0.0196	0.987 (0.949, 1.025)	0.488	0.738	1.45E-08	TRUE
CD8 ET	*AKIRIN1*	Wald ratio	1	-0.0089	0.0205	0.991 (0.952, 1.032)	0.663	0.845	3.47E-08	TRUE
CD8 ET	*AKR1C3*	Wald ratio	1	-0.0246	0.0168	0.976 (0.944, 1.008)	0.143	0.41	1.08E-10	TRUE
CD8 ET	*AL589743.1*	Wald ratio	1	-0.0045	0.0077	0.996 (0.981, 1.011)	0.562	0.779	4.44E-45	TRUE
CD8 ET	*ALG1L*	Wald ratio	1	-0.0210	0.0151	0.979 (0.951, 1.009)	0.165	0.44	1.43E-16	TRUE
CD8 ET	*AMICA1*	Wald ratio	1	0.0058	0.0156	1.006 (0.975, 1.037)	0.711	0.871	1.38E-11	TRUE
CD8 ET	*ANAPC13*	Wald ratio	1	-0.0039	0.0115	0.996 (0.974, 1.019)	0.735	0.88	6.67E-25	TRUE
CD8 ET	*ANK3*	Wald ratio	1	-0.0273	0.0122	0.973 (0.95, 0.997)	0.025	0.146	1.86E-19	TRUE
CD8 ET	*ANKDD1A*	Wald ratio	1	0.0020	0.0094	1.002 (0.984, 1.021)	0.835	0.93	4.69E-32	TRUE
CD8 ET	*ANXA11*	Wald ratio	1	0.0254	0.0146	1.026 (0.997, 1.055)	0.082	0.301	5.28E-17	TRUE
CD8 ET	*ANXA2R*	Wald ratio	1	-0.0358	0.0297	0.965 (0.91, 1.023)	0.228	0.521	5.02E-08	TRUE
CD8 ET	*ANXA5*	Wald ratio	1	-0.0079	0.0216	0.992 (0.951, 1.035)	0.714	0.872	2.88E-11	TRUE
CD8 ET	*ANXA6*	Wald ratio	1	0.0006	0.0122	1.001 (0.977, 1.025)	0.958	0.985	8.57E-17	TRUE
CD8 ET	*AOAH*	IVW	2	-0.0249	0.0112	0.975 (0.954, 0.997)	0.026	0.15	1.50E-50	TRUE
CD8 ET	*AP000783.1*	Wald ratio	1	-0.0315	0.0233	0.969 (0.926, 1.014)	0.176	0.456	2.19E-08	TRUE
CD8 ET	*AP001055.6*	Wald ratio	1	0.0207	0.0175	1.021 (0.986, 1.057)	0.237	0.532	3.91E-09	TRUE
CD8 ET	*AP003774.1*	Wald ratio	1	-0.0228	0.0071	0.977 (0.964, 0.991)	0.001	0.02	1.37E-54	TRUE
CD8 ET	*AP2A2*	Wald ratio	1	0.0646	0.0169	1.067 (1.032, 1.103)	1.28E-04	0.003	1.28E-10	TRUE
CD8 ET	*APMAP*	Wald ratio	1	0.0100	0.0142	1.01 (0.982, 1.039)	0.481	0.735	1.60E-16	TRUE
CD8 ET	*APOBEC3G*	Wald ratio	1	0.0117	0.0141	1.012 (0.984, 1.04)	0.407	0.682	1.38E-15	TRUE
CD8 ET	*APOBEC3H*	Wald ratio	1	0.0135	0.0122	1.014 (0.99, 1.038)	0.269	0.563	7.37E-19	TRUE
CD8 ET	*ARHGAP11A*	Wald ratio	1	-0.0101	0.0187	0.99 (0.954, 1.027)	0.591	0.797	1.24E-08	TRUE
CD8 ET	*ARHGAP15*	Wald ratio	1	0.0126	0.0189	1.013 (0.976, 1.051)	0.505	0.745	3.31E-11	TRUE
CD8 ET	*ARHGEF26*	Wald ratio	1	0.0282	0.0208	1.029 (0.987, 1.071)	0.176	0.456	2.54E-08	TRUE
CD8 ET	*ARHGEF3*	Wald ratio	1	-0.0017	0.0104	0.998 (0.978, 1.019)	0.867	0.944	2.57E-24	TRUE
CD8 ET	*ARL14EP*	Wald ratio	1	-0.0322	0.0164	0.968 (0.938, 1)	0.049	0.219	2.08E-10	TRUE
CD8 ET	*ARL16*	Wald ratio	1	0.0162	0.0087	1.016 (0.999, 1.034)	0.065	0.26	8.78E-40	TRUE
CD8 ET	*ARL17B*	Wald ratio	1	-0.0100	0.0179	0.99 (0.956, 1.025)	0.575	0.787	5.43E-08	TRUE
CD8 ET	*ARPC3*	Wald ratio	1	0.0243	0.0196	1.025 (0.986, 1.065)	0.215	0.505	3.46E-08	TRUE
CD8 ET	*ARPC4*	Wald ratio	1	0.0287	0.0198	1.029 (0.99, 1.07)	0.146	0.415	1.16E-09	TRUE
CD8 ET	*ARPC5*	Wald ratio	1	-0.0068	0.0155	0.993 (0.964, 1.024)	0.663	0.845	3.28E-12	TRUE
CD8 ET	*ARSA*	Wald ratio	1	0.0153	0.0152	1.015 (0.986, 1.046)	0.314	0.608	1.68E-23	TRUE
CD8 ET	*ASAH1*	Wald ratio	1	0.0032	0.0188	1.003 (0.967, 1.041)	0.867	0.945	2.97E-08	TRUE
CD8 ET	*ASNSD1*	Wald ratio	1	-0.0101	0.0135	0.99 (0.964, 1.016)	0.454	0.717	4.74E-15	TRUE
CD8 ET	*ATG10*	Wald ratio	1	-0.0258	0.0173	0.975 (0.942, 1.008)	0.135	0.397	7.53E-10	TRUE
CD8 ET	*ATHL1*	Wald ratio	1	-0.0032	0.0103	0.997 (0.977, 1.017)	0.752	0.887	4.92E-33	TRUE
CD8 ET	*ATP13A4*	Wald ratio	1	0.0119	0.0183	1.012 (0.976, 1.049)	0.516	0.751	2.81E-09	TRUE
CD8 ET	*ATP5G3*	Wald ratio	1	-0.0035	0.0143	0.996 (0.969, 1.025)	0.805	0.916	6.60E-19	TRUE
CD8 ET	*ATP8A1*	Wald ratio	1	0.0129	0.0184	1.013 (0.977, 1.05)	0.483	0.735	1.49E-09	TRUE
CD8 ET	*B3GAT1*	Wald ratio	1	-0.0495	0.0189	0.952 (0.917, 0.988)	0.009	0.074	1.83E-10	TRUE
CD8 ET	*B3GAT2*	Wald ratio	1	0.0135	0.0215	1.014 (0.972, 1.057)	0.531	0.758	7.56E-10	TRUE
CD8 ET	*B4GALNT3*	Wald ratio	1	0.0080	0.0102	1.008 (0.988, 1.028)	0.432	0.701	1.59E-29	TRUE
CD8 ET	*B4GALT1*	Wald ratio	1	0.0104	0.0121	1.011 (0.987, 1.035)	0.387	0.669	2.96E-23	TRUE
CD8 ET	*B4GALT4*	Wald ratio	1	-0.0322	0.0199	0.968 (0.931, 1.007)	0.106	0.345	4.06E-10	TRUE
CD8 ET	*BCAS4*	Wald ratio	1	0.0279	0.0163	1.028 (0.996, 1.062)	0.087	0.312	7.10E-11	TRUE
CD8 ET	*BCR*	Wald ratio	1	0.0114	0.0165	1.011 (0.979, 1.045)	0.49	0.738	1.38E-11	TRUE
CD8 ET	*BIN1*	Wald ratio	1	0.0133	0.0068	1.013 (1, 1.027)	0.052	0.226	8.13E-58	TRUE

CD8 ET	*BOK*	Wald ratio	1	-0.0002	0.0175	1 (0.966, 1.035)	0.991	0.996	6.21E-12	TRUE
CD8 ET	*BRD7*	Wald ratio	1	-0.0399	0.0174	0.961 (0.929, 0.994)	0.022	0.133	1.86E-11	TRUE
CD8 ET	*BRK1*	Wald ratio	1	-0.0212	0.0180	0.979 (0.945, 1.014)	0.238	0.532	2.51E-11	TRUE
CD8 ET	*BTBD9*	Wald ratio	1	0.0144	0.0175	1.015 (0.98, 1.05)	0.409	0.684	7.87E-09	TRUE
CD8 ET	*BTN2A1*	Wald ratio	1	0.0011	0.0204	1.001 (0.962, 1.042)	0.959	0.985	1.63E-09	TRUE
CD8 ET	*BTN3A1*	Wald ratio	1	0.0744	0.0168	1.077 (1.042, 1.113)	9.36E-06	5.39E-04	1.15E-09	TRUE
CD8 ET	*BTN3A2*	Wald ratio	1	0.0131	0.0092	1.013 (0.995, 1.032)	0.153	0.423	4.60E-48	TRUE
CD8 ET	*C10orf128*	Wald ratio	1	-0.0092	0.0092	0.991 (0.973, 1.009)	0.318	0.614	1.08E-34	TRUE
CD8 ET	*C10orf32*	Wald ratio	1	-0.0550	0.0127	0.946 (0.923, 0.97)	1.40E-05	7.21E-04	5.42E-19	TRUE
CD8 ET	*C10orf54*	Wald ratio	1	0.0165	0.0178	1.017 (0.982, 1.053)	0.353	0.644	1.70E-10	TRUE
CD8 ET	*C11orf21*	Wald ratio	1	-0.0020	0.0088	0.998 (0.981, 1.015)	0.824	0.926	3.03E-18	TRUE
CD8 ET	*C11orf24*	Wald ratio	1	0.0001	0.0112	1 (0.978, 1.022)	0.99	0.996	2.40E-23	TRUE
CD8 ET	*C11orf73*	Wald ratio	1	0.0129	0.0193	1.013 (0.975, 1.052)	0.503	0.744	5.89E-08	TRUE
CD8 ET	*C14orf2*	Wald ratio	1	0.0266	0.0149	1.027 (0.997, 1.058)	0.075	0.287	9.73E-14	TRUE
CD8 ET	*C16orf13*	Wald ratio	1	0.0000	0.0094	1 (0.982, 1.019)	0.997	0.999	6.11E-23	TRUE
CD8 ET	*C17orf89*	Wald ratio	1	0.0385	0.0117	1.039 (1.016, 1.063)	9.92E-04	0.017	4.70E-22	TRUE
CD8 ET	*C17orf97*	Wald ratio	1	-0.0122	0.0104	0.988 (0.968, 1.008)	0.243	0.535	1.77E-26	TRUE
CD8 ET	*C19orf24*	Wald ratio	1	0.0357	0.0142	1.036 (1.008, 1.066)	0.012	0.092	2.95E-17	TRUE
CD8 ET	*C19orf83*	Wald ratio	1	-0.0192	0.0088	0.981 (0.964, 0.998)	0.029	0.159	2.55E-37	TRUE
CD8 ET	*C1orf63*	Wald ratio	1	0.0319	0.0155	1.032 (1.002, 1.064)	0.039	0.191	1.25E-11	TRUE
CD8 ET	*C1orf86*	Wald ratio	1	0.0028	0.0137	1.003 (0.976, 1.03)	0.84	0.932	5.88E-16	TRUE
CD8 ET	*C2*	Wald ratio	1	0.0450	0.0137	1.046 (1.018, 1.074)	9.84E-04	0.017	2.12E-10	TRUE
CD8 ET	*C20orf27*	Wald ratio	1	-0.0054	0.0243	0.995 (0.948, 1.043)	0.824	0.926	1.37E-09	TRUE
CD8 ET	*C21orf119*	Wald ratio	1	-0.0238	0.0216	0.976 (0.936, 1.019)	0.27	0.563	4.08E-09	TRUE
CD8 ET	*C22orf34*	Wald ratio	1	-0.0173	0.0114	0.983 (0.961, 1.005)	0.131	0.392	2.16E-22	TRUE
CD8 ET	*C2orf27A*	Wald ratio	1	0.0144	0.0097	1.014 (0.995, 1.034)	0.137	0.399	6.22E-34	TRUE
CD8 ET	*C2orf74*	Wald ratio	1	0.0481	0.0163	1.049 (1.016, 1.083)	0.003	0.036	6.41E-11	TRUE
CD8 ET	*C5orf17*	Wald ratio	1	-0.0066	0.0075	0.993 (0.979, 1.008)	0.384	0.668	1.92E-51	TRUE
CD8 ET	*C5orf56*	Wald ratio	1	0.0151	0.0213	1.015 (0.974, 1.058)	0.478	0.733	2.15E-08	TRUE
CD8 ET	*C6orf25*	IVW	2	0.0132	0.0325	1.013 (0.951, 1.08)	0.685	0.856	8.74E-24	TRUE
CD8 ET	*C6orf47-AS1*	Wald ratio	1	-0.0070	0.0168	0.993 (0.961, 1.026)	0.678	0.853	2.27E-15	TRUE
CD8 ET	*C6orf62*	Wald ratio	1	0.0711	0.0207	1.074 (1.031, 1.118)	5.87E-04	0.011	1.11E-07	TRUE
CD8 ET	*C9orf156*	Wald ratio	1	0.0074	0.0115	1.007 (0.985, 1.03)	0.521	0.753	4.40E-24	TRUE
CD8 ET	*C9orf78*	Wald ratio	1	-0.0050	0.0108	0.995 (0.974, 1.016)	0.642	0.831	1.29E-25	TRUE
CD8 ET	*CALHM2*	Wald ratio	1	0.0462	0.0210	1.047 (1.005, 1.091)	0.028	0.157	1.51E-08	TRUE
CD8 ET	*CAMLG*	Wald ratio	1	0.0000	0.0128	1 (0.975, 1.025)	0.999	0.999	1.96E-12	TRUE
CD8 ET	*CAPG*	Wald ratio	1	-0.0159	0.0120	0.984 (0.961, 1.008)	0.187	0.466	1.18E-20	TRUE
CD8 ET	*CAPN12*	Wald ratio	1	-0.0030	0.0151	0.997 (0.968, 1.027)	0.844	0.935	2.19E-15	TRUE
CD8 ET	*CARD8*	Wald ratio	1	0.0296	0.0187	1.03 (0.993, 1.068)	0.113	0.359	4.20E-13	TRUE
CD8 ET	*CARHSP1*	Wald ratio	1	0.0140	0.0099	1.014 (0.995, 1.034)	0.159	0.43	5.08E-28	TRUE
CD8 ET	*CASP8*	Wald ratio	1	-0.0395	0.0130	0.961 (0.937, 0.986)	0.002	0.029	4.83E-17	TRUE
CD8 ET	*CAST*	Wald ratio	1	-0.0121	0.0092	0.988 (0.97, 1.006)	0.185	0.465	2.31E-34	TRUE
CD8 ET	*CCDC115*	Wald ratio	1	-0.0027	0.0209	0.997 (0.957, 1.039)	0.895	0.957	7.70E-10	TRUE
CD8 ET	*CCDC146*	Wald ratio	1	0.0103	0.0162	1.01 (0.979, 1.043)	0.526	0.756	6.53E-22	TRUE
CD8 ET	*CCDC152*	Wald ratio	1	0.0197	0.0183	1.02 (0.984, 1.057)	0.284	0.576	9.64E-09	TRUE
CD8 ET	*CCDC163P*	Wald ratio	1	-0.0097	0.0118	0.99 (0.968, 1.013)	0.411	0.686	1.68E-21	TRUE
CD8 ET	*CCDC167*	Wald ratio	1	-0.0008	0.0071	0.999 (0.985, 1.013)	0.913	0.962	2.22E-54	TRUE
CD8 ET	*CCDC25*	Wald ratio	1	-0.0283	0.0187	0.972 (0.937, 1.008)	0.131	0.392	4.54E-09	TRUE
CD8 ET	*CCDC50*	Wald ratio	1	0.0199	0.0134	1.02 (0.994, 1.047)	0.137	0.399	7.74E-16	TRUE
CD8 ET	*CCDC66*	Wald ratio	1	0.0164	0.0142	1.017 (0.989, 1.045)	0.247	0.541	3.18E-16	TRUE
CD8 ET	*CCDC82*	Wald ratio	1	0.0004	0.0209	1 (0.96, 1.042)	0.983	0.994	5.46E-09	TRUE
CD8 ET	*CCDC88B*	Wald ratio	1	0.0601	0.0189	1.062 (1.023, 1.102)	0.001	0.021	1.46E-08	TRUE
CD8 ET	*CCSER2*	Wald ratio	1	-0.0249	0.0128	0.975 (0.951, 1)	0.052	0.226	2.44E-22	TRUE
CD8 ET	*CCT3*	Wald ratio	1	0.0204	0.0120	1.021 (0.997, 1.045)	0.088	0.314	2.78E-20	TRUE
CD8 ET	*CCT8*	Wald ratio	1	-0.0009	0.0089	0.999 (0.982, 1.017)	0.922	0.965	6.26E-60	TRUE
CD8 ET	*CCZ1*	IVW	2	0.0061	0.0108	1.006 (0.985, 1.028)	0.572	0.786	6.48E-63	TRUE
CD8 ET	*CD101*	Wald ratio	1	0.0034	0.0114	1.003 (0.981, 1.026)	0.765	0.896	2.67E-21	TRUE
CD8 ET	*CD151*	Wald ratio	1	-0.0185	0.0149	0.982 (0.953, 1.011)	0.215	0.505	4.69E-14	TRUE
CD8 ET	*CD160*	Wald ratio	1	-0.0211	0.0158	0.979 (0.949, 1.01)	0.182	0.462	1.45E-12	TRUE
CD8 ET	*CD27-AS1*	Wald ratio	1	0.0061	0.0206	1.006 (0.966, 1.047)	0.768	0.897	5.40E-08	TRUE
CD8 ET	*CD300A*	IVW	2	0.0075	0.0135	1.008 (0.981, 1.034)	0.579	0.789	1.52E-32	TRUE
CD8 ET	*CD37*	Wald ratio	1	0.0031	0.0188	1.003 (0.967, 1.041)	0.87	0.946	2.21E-12	TRUE
CD8 ET	*CD3E*	Wald ratio	1	-0.0389	0.0168	0.962 (0.931, 0.994)	0.021	0.132	9.66E-11	TRUE
CD8 ET	*CD48*	Wald ratio	1	0.0158	0.0146	1.016 (0.987, 1.045)	0.281	0.575	1.44E-14	TRUE
CD8 ET	*CD55*	Wald ratio	1	-0.0482	0.0193	0.953 (0.918, 0.99)	0.013	0.094	1.70E-09	TRUE
CD8 ET	*CD8B*	Wald ratio	1	0.0089	0.0156	1.009 (0.979, 1.04)	0.568	0.782	9.94E-14	TRUE
CD8 ET	*CDC14A*	Wald ratio	1	-0.0082	0.0119	0.992 (0.969, 1.015)	0.491	0.738	1.28E-20	TRUE
CD8 ET	*CDC42*	Wald ratio	1	0.0085	0.0123	1.009 (0.984, 1.033)	0.49	0.738	2.37E-17	TRUE
CD8 ET	*CDC42SE2*	Wald ratio	1	0.0124	0.0122	1.012 (0.988, 1.037)	0.311	0.605	1.45E-20	TRUE
CD8 ET	*CDC7*	Wald ratio	1	0.0202	0.0204	1.02 (0.98, 1.062)	0.321	0.616	5.74E-08	TRUE
CD8 ET	*CDK10*	Wald ratio	1	-0.0470	0.0143	0.954 (0.928, 0.981)	9.77E-04	0.017	5.59E-14	TRUE
CD8 ET	*CDK5R1*	Wald ratio	1	-0.0281	0.0206	0.972 (0.934, 1.012)	0.172	0.45	7.76E-08	TRUE
CD8 ET	*CEACAM21*	Wald ratio	1	-0.0107	0.0089	0.989 (0.972, 1.007)	0.227	0.52	3.32E-32	TRUE
CD8 ET	*CEBPZ-AS1*	Wald ratio	1	0.0117	0.0145	1.012 (0.983, 1.041)	0.42	0.69	1.70E-19	TRUE
CD8 ET	*CENPK*	Wald ratio	1	0.0073	0.0052	1.007 (0.997, 1.018)	0.165	0.439	1.01E-110	TRUE
CD8 ET	*CENPM*	Wald ratio	1	0.0014	0.0176	1.001 (0.967, 1.037)	0.938	0.975	1.18E-11	TRUE
CD8 ET	*CENPP*	Wald ratio	1	0.0018	0.0172	1.002 (0.969, 1.036)	0.916	0.963	3.09E-12	TRUE
CD8 ET	*CEP78*	Wald ratio	1	-0.0018	0.0093	0.998 (0.98, 1.017)	0.851	0.936	1.49E-19	TRUE
CD8 ET	*CERS4*	Wald ratio	1	-0.0107	0.0179	0.989 (0.955, 1.025)	0.55	0.769	2.88E-10	TRUE

CD8 ET	*CES1*	Wald ratio	1	-0.0118	0.0113	0.988 (0.967, 1.01)	0.293	0.587	1.14E-24	TRUE
CD8 ET	*CFD*	Wald ratio	1	0.0004	0.0091	1 (0.983, 1.018)	0.963	0.988	5.70E-42	TRUE
CD8 ET	*CFLAR*	Wald ratio	1	0.0306	0.0144	1.031 (1.002, 1.061)	0.034	0.175	5.34E-17	TRUE
CD8 ET	*CHCHD2*	Wald ratio	1	0.0051	0.0091	1.005 (0.987, 1.023)	0.576	0.788	2.41E-37	TRUE
CD8 ET	*CHI3L2*	Wald ratio	1	-0.0052	0.0068	0.995 (0.982, 1.008)	0.444	0.709	7.63E-66	TRUE
CD8 ET	*CHMP4A*	Wald ratio	1	0.0436	0.0169	1.045 (1.011, 1.08)	0.01	0.081	8.21E-09	TRUE
CD8 ET	*CHMP4B*	Wald ratio	1	-0.0323	0.0194	0.968 (0.932, 1.006)	0.096	0.329	1.51E-08	TRUE
CD8 ET	*CHPF*	Wald ratio	1	-0.0096	0.0181	0.99 (0.956, 1.026)	0.597	0.802	2.92E-09	TRUE
CD8 ET	*CHPT1*	Wald ratio	1	0.0189	0.0098	1.019 (1, 1.039)	0.054	0.233	3.26E-30	TRUE
CD8 ET	*CIB1*	Wald ratio	1	-0.0381	0.0135	0.963 (0.938, 0.988)	0.005	0.048	3.64E-28	TRUE
CD8 ET	*CKB*	Wald ratio	1	-0.0872	0.0180	0.916 (0.885, 0.949)	1.26E-06	1.34E-04	4.34E-11	TRUE
CD8 ET	*CKMT2-AS1*	Wald ratio	1	-0.0249	0.0260	0.975 (0.927, 1.026)	0.338	0.631	7.30E-09	TRUE
CD8 ET	*CLEC2B*	Wald ratio	1	0.0028	0.0116	1.003 (0.98, 1.026)	0.813	0.92	5.58E-22	TRUE
CD8 ET	*CLHC1*	Wald ratio	1	-0.0795	0.0244	0.924 (0.88, 0.969)	0.001	0.018	1.69E-08	TRUE
CD8 ET	*CLIC3*	Wald ratio	1	0.0579	0.0182	1.06 (1.022, 1.098)	0.001	0.021	2.11E-09	TRUE
CD8 ET	*CMC1*	Wald ratio	1	0.0277	0.0165	1.028 (0.995, 1.062)	0.094	0.324	1.09E-11	TRUE
CD8 ET	*CMPK1*	Wald ratio	1	0.0240	0.0216	1.024 (0.982, 1.069)	0.267	0.561	2.56E-09	TRUE
CD8 ET	*CMSS1*	Wald ratio	1	-0.0214	0.0191	0.979 (0.943, 1.016)	0.263	0.558	5.73E-09	TRUE
CD8 ET	*CMTM3*	Wald ratio	1	-0.0118	0.0175	0.988 (0.955, 1.023)	0.501	0.743	2.30E-10	TRUE
CD8 ET	*CMTM7*	Wald ratio	1	0.0240	0.0144	1.024 (0.996, 1.054)	0.096	0.329	2.46E-13	TRUE
CD8 ET	*CMTM8*	Wald ratio	1	0.0036	0.0210	1.004 (0.963, 1.046)	0.865	0.943	9.70E-09	TRUE
CD8 ET	*CNKSR3*	Wald ratio	1	-0.0175	0.0127	0.983 (0.959, 1.007)	0.169	0.444	7.45E-18	TRUE
CD8 ET	*CNN2*	Wald ratio	1	0.0222	0.0166	1.022 (0.99, 1.056)	0.181	0.462	1.59E-11	TRUE
CD8 ET	*CNOT6L*	Wald ratio	1	0.0313	0.0166	1.032 (0.999, 1.066)	0.059	0.244	1.37E-11	TRUE
CD8 ET	*COA1*	Wald ratio	1	-0.0007	0.0147	0.999 (0.971, 1.028)	0.96	0.986	1.18E-14	TRUE
CD8 ET	*COG5*	Wald ratio	1	0.0417	0.0084	1.043 (1.026, 1.06)	6.43E-07	9.75E-05	5.84E-38	TRUE
CD8 ET	*COL18A1*	Wald ratio	1	-0.0480	0.0164	0.953 (0.923, 0.984)	0.003	0.039	3.64E-29	TRUE
CD8 ET	*COL5A3*	Wald ratio	1	0.0155	0.0158	1.016 (0.985, 1.048)	0.326	0.62	1.25E-14	TRUE
CD8 ET	*COL6A2*	Wald ratio	1	0.0281	0.0277	1.029 (0.974, 1.086)	0.309	0.603	3.13E-08	TRUE
CD8 ET	*COLGALT2*	Wald ratio	1	0.0006	0.0103	1.001 (0.981, 1.021)	0.951	0.981	7.77E-28	TRUE
CD8 ET	*COMMD5*	Wald ratio	1	-0.0222	0.0184	0.978 (0.943, 1.014)	0.229	0.523	1.84E-09	TRUE
CD8 ET	*COMMD6*	Wald ratio	1	0.0144	0.0089	1.015 (0.997, 1.032)	0.105	0.343	7.08E-37	TRUE
CD8 ET	*COQ5*	Wald ratio	1	-0.0193	0.0149	0.981 (0.953, 1.01)	0.197	0.48	5.50E-14	TRUE
CD8 ET	*CORO1B*	Wald ratio	1	0.0011	0.0059	1.001 (0.99, 1.013)	0.845	0.935	4.13E-90	TRUE
CD8 ET	*COX20*	Wald ratio	1	0.0078	0.0229	1.008 (0.964, 1.054)	0.734	0.88	9.29E-09	TRUE
CD8 ET	*COX6B1*	Wald ratio	1	-0.0241	0.0105	0.976 (0.956, 0.997)	0.022	0.135	4.70E-26	TRUE
CD8 ET	*COX7A2L*	Wald ratio	1	-0.0464	0.0158	0.955 (0.925, 0.985)	0.003	0.038	1.73E-12	TRUE
CD8 ET	*CPA5*	Wald ratio	1	-0.0074	0.0084	0.993 (0.976, 1.009)	0.378	0.665	1.11E-41	TRUE
CD8 ET	*CPNE1*	Wald ratio	1	-0.0055	0.0093	0.995 (0.977, 1.013)	0.557	0.775	1.82E-40	TRUE
CD8 ET	*CRELD2*	Wald ratio	1	-0.0445	0.0170	0.957 (0.925, 0.989)	0.009	0.075	1.88E-11	TRUE
CD8 ET	*CRIP2*	Wald ratio	1	0.0518	0.0262	1.053 (1, 1.109)	0.048	0.217	3.27E-08	TRUE
CD8 ET	*CROCC*	Wald ratio	1	0.0053	0.0149	1.005 (0.976, 1.035)	0.721	0.876	2.03E-14	TRUE
CD8 ET	*CRYBB2*	Wald ratio	1	0.0087	0.0069	1.009 (0.995, 1.023)	0.208	0.494	1.04E-60	TRUE
CD8 ET	*CRYZ*	Wald ratio	1	-0.0185	0.0155	0.982 (0.952, 1.012)	0.231	0.524	9.42E-13	TRUE
CD8 ET	*CSTB*	Wald ratio	1	0.0035	0.0102	1.004 (0.984, 1.024)	0.73	0.88	1.22E-25	TRUE
CD8 ET	*CTA-14H9.5*	Wald ratio	1	0.0764	0.0195	1.079 (1.039, 1.122)	9.06E-05	0.003	4.91E-08	TRUE
CD8 ET	*CTA-29F11.1*	Wald ratio	1	-0.0054	0.0086	0.995 (0.978, 1.012)	0.527	0.756	3.09E-40	TRUE
CD8 ET	*CTA-445C9.15*	Wald ratio	1	0.0188	0.0149	1.019 (0.99, 1.049)	0.207	0.494	1.35E-13	TRUE
CD8 ET	*CTBP1-AS2*	Wald ratio	1	-0.0209	0.0159	0.979 (0.949, 1.01)	0.187	0.466	4.32E-12	TRUE
CD8 ET	*CTC-241F20.3*	Wald ratio	1	-0.0375	0.0238	0.963 (0.919, 1.009)	0.115	0.362	1.65E-08	TRUE
CD8 ET	*CTC-425F1.4*	Wald ratio	1	-0.0017	0.0207	0.998 (0.959, 1.04)	0.936	0.974	1.72E-08	TRUE
CD8 ET	*CTC-507E2.1*	Wald ratio	1	-0.0138	0.0115	0.986 (0.964, 1.009)	0.231	0.524	2.65E-22	TRUE
CD8 ET	*CTC-524C5.2*	Wald ratio	1	0.0159	0.0090	1.016 (0.998, 1.034)	0.076	0.289	1.81E-28	TRUE
CD8 ET	*CTD-2007H13.3*	Wald ratio	1	0.0199	0.0238	1.02 (0.974, 1.069)	0.404	0.681	3.69E-09	TRUE
CD8 ET	*CTD-2020K17.4*	Wald ratio	1	0.0041	0.0195	1.004 (0.967, 1.043)	0.834	0.93	5.44E-08	TRUE
CD8 ET	*CTD-2196E14.4*	Wald ratio	1	-0.0119	0.0104	0.988 (0.968, 1.009)	0.254	0.548	2.25E-25	TRUE
CD8 ET	*CTD-2368P22.1*	Wald ratio	1	0.0041	0.0120	1.004 (0.981, 1.028)	0.734	0.88	1.32E-22	TRUE
CD8 ET	*CTD-2371O3.2*	Wald ratio	1	-0.0011	0.0071	0.999 (0.985, 1.013)	0.873	0.947	3.36E-61	TRUE
CD8 ET	*CTD-2515A14.1*	Wald ratio	1	-0.0622	0.0193	0.94 (0.905, 0.976)	0.001	0.02	2.75E-08	TRUE
CD8 ET	*CTLA4*	Wald ratio	1	-0.0042	0.0155	0.996 (0.966, 1.027)	0.788	0.909	3.28E-14	TRUE
CD8 ET	*CTSF*	Wald ratio	1	-0.0198	0.0173	0.98 (0.948, 1.014)	0.254	0.548	2.24E-11	TRUE
CD8 ET	*CTSG*	Wald ratio	1	0.0164	0.0141	1.017 (0.989, 1.045)	0.244	0.537	5.44E-14	TRUE
CD8 ET	*CTSH*	Wald ratio	1	-0.0091	0.0160	0.991 (0.96, 1.022)	0.569	0.783	3.32E-11	TRUE
CD8 ET	*CTSW*	Wald ratio	1	-0.0242	0.0057	0.976 (0.965, 0.987)	2.34E-05	0.001	1.12E-107	TRUE
CD8 ET	*DAD1*	Wald ratio	1	-0.0043	0.0134	0.996 (0.97, 1.022)	0.749	0.886	4.80E-16	TRUE
CD8 ET	*DAP*	Wald ratio	1	-0.0251	0.0200	0.975 (0.938, 1.014)	0.21	0.497	6.78E-08	TRUE
CD8 ET	*DARS*	Wald ratio	1	-0.0395	0.0189	0.961 (0.926, 0.997)	0.036	0.182	7.84E-08	TRUE
CD8 ET	*DAZAP2*	Wald ratio	1	-0.0330	0.0121	0.968 (0.945, 0.991)	0.006	0.059	1.86E-18	TRUE
CD8 ET	*DBNDD1*	Wald ratio	1	-0.0075	0.0134	0.993 (0.967, 1.019)	0.578	0.789	9.54E-14	TRUE
CD8 ET	*DCXR*	Wald ratio	1	-0.0057	0.0067	0.994 (0.981, 1.007)	0.392	0.672	3.02E-58	TRUE
CD8 ET	*DDRGK1*	Wald ratio	1	-0.0201	0.0172	0.98 (0.948, 1.014)	0.242	0.535	5.91E-11	TRUE
CD8 ET	*DDT*	Wald ratio	1	-0.0163	0.0116	0.984 (0.962, 1.007)	0.162	0.436	2.72E-21	TRUE
CD8 ET	*DDTL*	Wald ratio	1	-0.0103	0.0094	0.99 (0.972, 1.008)	0.273	0.567	4.43E-33	TRUE
CD8 ET	*DDX11*	Wald ratio	1	-0.0098	0.0073	0.99 (0.976, 1.004)	0.177	0.456	2.59E-55	TRUE
CD8 ET	*DDX5*	Wald ratio	1	0.0397	0.0102	1.04 (1.02, 1.062)	1.06E-04	0.003	3.66E-25	TRUE
CD8 ET	*DDX6*	Wald ratio	1	-0.0374	0.0179	0.963 (0.93, 0.998)	0.037	0.184	2.26E-09	TRUE
CD8 ET	*DENND3*	Wald ratio	1	-0.0020	0.0163	0.998 (0.966, 1.03)	0.9	0.958	1.88E-12	TRUE
CD8 ET	*DGCR6L*	Wald ratio	1	0.0076	0.0179	1.008 (0.973, 1.044)	0.67	0.848	6.33E-10	TRUE
CD8 ET	*DGCR8*	Wald ratio	1	0.0258	0.0177	1.026 (0.991, 1.062)	0.145	0.413	8.52E-10	TRUE

CD8 ET	*DGKD*	Wald ratio	1	0.0714	0.0200	1.074 (1.033, 1.117)	3.66E-04	0.008	8.57E-08	TRUE
CD8 ET	*DGKQ*	Wald ratio	1	-0.0170	0.0202	0.983 (0.945, 1.023)	0.4	0.679	3.79E-08	TRUE
CD8 ET	*DHRS7*	Wald ratio	1	-0.0172	0.0117	0.983 (0.961, 1.006)	0.142	0.409	1.05E-13	TRUE
CD8 ET	*DHX32*	Wald ratio	1	0.0007	0.0187	1.001 (0.965, 1.038)	0.971	0.989	1.98E-09	TRUE
CD8 ET	*DIP2A*	Wald ratio	1	0.0063	0.0053	1.006 (0.996, 1.017)	0.236	0.53	1.18E-114	TRUE
CD8 ET	*DIS3L*	Wald ratio	1	-0.0010	0.0170	0.999 (0.966, 1.033)	0.952	0.982	6.38E-12	TRUE
CD8 ET	*DMKN*	Wald ratio	1	-0.0197	0.0237	0.98 (0.936, 1.027)	0.405	0.681	7.10E-09	TRUE
CD8 ET	*DNAI2*	Wald ratio	1	-0.0059	0.0103	0.994 (0.974, 1.014)	0.566	0.782	7.26E-25	TRUE
CD8 ET	*DNAJA1*	Wald ratio	1	-0.0733	0.0205	0.929 (0.893, 0.967)	3.39E-04	0.008	5.53E-08	TRUE
CD8 ET	*DNAJC15*	Wald ratio	1	0.0051	0.0059	1.005 (0.994, 1.017)	0.382	0.666	3.48E-81	TRUE
CD8 ET	*DOCK8*	Wald ratio	1	0.0305	0.0131	1.031 (1.005, 1.058)	0.02	0.125	4.49E-23	TRUE
CD8 ET	*DPY19L2*	Wald ratio	1	0.0190	0.0083	1.019 (1.003, 1.036)	0.022	0.137	1.28E-42	TRUE
CD8 ET	*DRAM2*	Wald ratio	1	-0.0047	0.0139	0.995 (0.969, 1.023)	0.734	0.88	1.95E-15	TRUE
CD8 ET	*DTNBP1*	Wald ratio	1	0.0336	0.0140	1.034 (1.006, 1.063)	0.016	0.111	1.54E-15	TRUE
CD8 ET	*DUOX1*	Wald ratio	1	0.0246	0.0186	1.025 (0.988, 1.063)	0.187	0.466	3.00E-08	TRUE
CD8 ET	*DUS3L*	Wald ratio	1	-0.0062	0.0170	0.994 (0.961, 1.028)	0.717	0.874	1.96E-09	TRUE
CD8 ET	*DYNLL2*	Wald ratio	1	-0.0286	0.0134	0.972 (0.947, 0.998)	0.033	0.171	4.46E-18	TRUE
CD8 ET	*DZIP3*	Wald ratio	1	0.0164	0.0100	1.017 (0.997, 1.037)	0.099	0.333	1.05E-28	TRUE
CD8 ET	*EBPL*	Wald ratio	1	0.0046	0.0078	1.005 (0.989, 1.02)	0.556	0.774	1.26E-47	TRUE
CD8 ET	*EFHB*	Wald ratio	1	0.0038	0.0111	1.004 (0.982, 1.026)	0.734	0.88	1.33E-24	TRUE
CD8 ET	*EGFL8*	IVW	2	0.0109	0.0085	1.011 (0.994, 1.028)	0.198	0.481	1.89E-43	TRUE
CD8 ET	*EIF2A*	Wald ratio	1	-0.0183	0.0131	0.982 (0.957, 1.007)	0.163	0.437	3.24E-16	TRUE
CD8 ET	*EIF4G3*	Wald ratio	1	-0.0573	0.0204	0.944 (0.907, 0.983)	0.005	0.051	1.10E-07	TRUE
CD8 ET	*EIF5A*	Wald ratio	1	-0.0067	0.0045	0.993 (0.985, 1.002)	0.134	0.394	3.41E-197	TRUE
CD8 ET	*EIF6*	Wald ratio	1	-0.0558	0.0199	0.946 (0.91, 0.983)	0.005	0.051	9.58E-08	TRUE
CD8 ET	*EMB*	Wald ratio	1	0.0210	0.0113	1.021 (0.999, 1.044)	0.063	0.257	2.12E-24	TRUE
CD8 ET	*EML4*	IVW	2	-0.0030	0.0123	0.997 (0.973, 1.021)	0.809	0.918	8.04E-28	TRUE
CD8 ET	*EMP3*	Wald ratio	1	0.0053	0.0210	1.005 (0.965, 1.047)	0.801	0.915	3.20E-08	TRUE
CD8 ET	*EMR2*	Wald ratio	1	-0.0219	0.0133	0.978 (0.953, 1.004)	0.098	0.333	2.84E-16	TRUE
CD8 ET	*ENDOG*	Wald ratio	1	0.0285	0.0193	1.029 (0.991, 1.069)	0.139	0.403	6.23E-11	TRUE
CD8 ET	*ENTPD1*	Wald ratio	1	0.0541	0.0200	1.056 (1.015, 1.098)	0.007	0.062	4.97E-08	TRUE
CD8 ET	*ENTPD3-AS1*	Wald ratio	1	-0.0567	0.0196	0.945 (0.909, 0.982)	0.004	0.042	3.95E-08	TRUE
CD8 ET	*EPB41L4A*	Wald ratio	1	-0.0261	0.0160	0.974 (0.944, 1.005)	0.102	0.338	1.27E-10	TRUE
CD8 ET	*EPC1*	Wald ratio	1	-0.0194	0.0182	0.981 (0.946, 1.016)	0.286	0.577	1.55E-09	TRUE
CD8 ET	*EPHA4*	Wald ratio	1	0.0257	0.0196	1.026 (0.987, 1.066)	0.191	0.471	1.79E-08	TRUE
CD8 ET	*EPS8L2*	Wald ratio	1	0.0154	0.0147	1.016 (0.987, 1.045)	0.294	0.587	3.67E-14	TRUE
CD8 ET	*EPSTI1*	Wald ratio	1	0.0057	0.0094	1.006 (0.987, 1.024)	0.54	0.762	3.37E-36	TRUE
CD8 ET	*ERN1*	Wald ratio	1	-0.0320	0.0122	0.969 (0.946, 0.992)	0.009	0.075	2.45E-20	TRUE
CD8 ET	*ESR2*	IVW	2	0.0018	0.0319	1.002 (0.941, 1.066)	0.955	0.983	1.34E-53	TRUE
CD8 ET	*ETV7*	Wald ratio	1	-0.0163	0.0113	0.984 (0.962, 1.006)	0.15	0.42	8.83E-23	TRUE
CD8 ET	*EVA1C*	Wald ratio	1	-0.0427	0.0166	0.958 (0.928, 0.99)	0.01	0.081	3.59E-11	TRUE
CD8 ET	*EVI2A*	Wald ratio	1	-0.0173	0.0120	0.983 (0.96, 1.006)	0.148	0.417	7.43E-20	TRUE
CD8 ET	*FADS1*	Wald ratio	1	0.0139	0.0129	1.014 (0.989, 1.04)	0.282	0.575	7.70E-17	TRUE
CD8 ET	*FADS2*	Wald ratio	1	0.0117	0.0082	1.012 (0.996, 1.028)	0.153	0.423	6.62E-69	TRUE
CD8 ET	*FAM110B*	Wald ratio	1	0.0094	0.0073	1.009 (0.995, 1.024)	0.199	0.483	5.87E-54	TRUE
CD8 ET	*FAM118A*	Wald ratio	1	0.0003	0.0043	1 (0.992, 1.009)	0.94	0.976	2.22E-125	TRUE
CD8 ET	*FAM129A*	Wald ratio	1	-0.0113	0.0161	0.989 (0.958, 1.021)	0.484	0.736	2.23E-12	TRUE
CD8 ET	*FAM134A*	Wald ratio	1	-0.0267	0.0160	0.974 (0.944, 1.005)	0.095	0.327	3.91E-12	TRUE
CD8 ET	*FAM154B*	Wald ratio	1	0.0318	0.0133	1.032 (1.006, 1.06)	0.017	0.113	2.68E-17	TRUE
CD8 ET	*FAM179A*	Wald ratio	1	-0.0763	0.0186	0.927 (0.893, 0.961)	3.95E-05	0.002	5.19E-09	TRUE
CD8 ET	*FAM184B*	Wald ratio	1	0.0010	0.0052	1.001 (0.991, 1.011)	0.847	0.935	2.05E-131	TRUE
CD8 ET	*FAM21A*	Wald ratio	1	-0.0460	0.0157	0.955 (0.926, 0.985)	0.003	0.038	3.97E-13	TRUE
CD8 ET	*FAM26F*	IVW	2	-0.0166	0.0223	0.984 (0.941, 1.027)	0.456	0.719	1.07E-47	TRUE
CD8 ET	*FAM43A*	Wald ratio	1	-0.0076	0.0110	0.992 (0.971, 1.014)	0.492	0.738	1.14E-24	TRUE
CD8 ET	*FAM83D*	Wald ratio	1	-0.0129	0.0235	0.987 (0.943, 1.034)	0.583	0.792	2.71E-08	TRUE
CD8 ET	*FAR1*	Wald ratio	1	-0.0040	0.0097	0.996 (0.977, 1.015)	0.683	0.856	2.52E-33	TRUE
CD8 ET	*FAU*	Wald ratio	1	-0.0004	0.0130	1 (0.974, 1.025)	0.973	0.989	8.59E-18	TRUE
CD8 ET	*FBLN5*	Wald ratio	1	0.0033	0.0118	1.003 (0.98, 1.027)	0.779	0.904	2.39E-21	TRUE
CD8 ET	*FBXO2*	Wald ratio	1	0.0087	0.0167	1.009 (0.976, 1.042)	0.602	0.805	2.87E-10	TRUE
CD8 ET	*FCGBP*	Wald ratio	1	0.0065	0.0112	1.007 (0.985, 1.029)	0.559	0.778	1.74E-24	TRUE
CD8 ET	*FCRL3*	Wald ratio	1	0.0078	0.0122	1.008 (0.984, 1.032)	0.522	0.754	1.66E-20	TRUE
CD8 ET	*FDFT1*	Wald ratio	1	0.0536	0.0167	1.055 (1.021, 1.09)	0.001	0.02	3.09E-13	TRUE
CD8 ET	*FGD5-AS1*	Wald ratio	1	-0.0001	0.0198	1 (0.962, 1.039)	0.998	0.999	7.63E-12	TRUE
CD8 ET	*FGFBP2*	Wald ratio	1	0.0296	0.0187	1.03 (0.993, 1.069)	0.113	0.359	2.04E-09	TRUE
CD8 ET	*FGFR1*	Wald ratio	1	0.0399	0.0162	1.041 (1.008, 1.074)	0.014	0.101	1.23E-11	TRUE
CD8 ET	*FGL2*	Wald ratio	1	-0.0087	0.0090	0.991 (0.974, 1.009)	0.335	0.628	8.99E-30	TRUE
CD8 ET	*FHAD1*	Wald ratio	1	-0.0159	0.0120	0.984 (0.961, 1.008)	0.187	0.466	8.80E-21	TRUE
CD8 ET	*FHL3*	Wald ratio	1	0.0057	0.0085	1.006 (0.989, 1.023)	0.499	0.741	7.90E-43	TRUE
CD8 ET	*FIBP*	Wald ratio	1	0.0770	0.0197	1.08 (1.039, 1.123)	8.99E-05	0.003	1.55E-09	TRUE
CD8 ET	*FIGNL1*	Wald ratio	1	0.0074	0.0128	1.007 (0.982, 1.033)	0.562	0.779	9.39E-19	TRUE
CD8 ET	*FIS1*	Wald ratio	1	0.0054	0.0124	1.005 (0.981, 1.03)	0.66	0.843	2.13E-20	TRUE
CD8 ET	*FKBP11*	Wald ratio	1	0.0028	0.0106	1.003 (0.982, 1.024)	0.791	0.909	1.34E-25	TRUE
CD8 ET	*FLVCR1-AS1*	Wald ratio	1	-0.0107	0.0095	0.989 (0.971, 1.008)	0.264	0.558	6.82E-33	TRUE
CD8 ET	*FMN1*	Wald ratio	1	-0.0100	0.0075	0.99 (0.976, 1.005)	0.182	0.462	5.37E-44	TRUE
CD8 ET	*FN3KRP*	Wald ratio	1	0.0194	0.0103	1.02 (0.999, 1.04)	0.059	0.246	1.34E-28	TRUE
CD8 ET	*FNBP4*	Wald ratio	1	-0.0703	0.0143	0.932 (0.906, 0.959)	8.54E-07	1.03E-04	2.01E-15	TRUE
CD8 ET	*FOXN2*	Wald ratio	1	0.0773	0.0192	1.08 (1.04, 1.122)	5.66E-05	0.002	7.18E-11	TRUE
CD8 ET	*FRG1B*	Wald ratio	1	-0.0767	0.0194	0.926 (0.892, 0.962)	7.36E-05	0.002	2.23E-08	TRUE
CD8 ET	*FTCDNL1*	Wald ratio	1	-0.0014	0.0142	0.999 (0.971, 1.027)	0.919	0.965	6.01E-19	TRUE

CD8 ET	*FZD6*	Wald ratio	1	0.0017	0.0091	1.002 (0.984, 1.02)	0.849	0.936	8.03E-36	TRUE
CD8 ET	*G3BP2*	Wald ratio	1	0.0015	0.0148	1.001 (0.973, 1.031)	0.919	0.965	3.48E-14	TRUE
CD8 ET	*GALM*	Wald ratio	1	-0.0315	0.0205	0.969 (0.931, 1.009)	0.124	0.378	7.87E-08	TRUE
CD8 ET	*GALT*	Wald ratio	1	0.0922	0.0186	1.097 (1.057, 1.137)	7.32E-07	9.75E-05	2.00E-09	TRUE
CD8 ET	*GAPDH*	Wald ratio	1	-0.0160	0.0144	0.984 (0.957, 1.012)	0.266	0.561	7.08E-14	TRUE
CD8 ET	*GAS1*	Wald ratio	1	0.0197	0.0191	1.02 (0.982, 1.059)	0.302	0.597	4.32E-08	TRUE
CD8 ET	*GBP5*	Wald ratio	1	-0.0263	0.0103	0.974 (0.955, 0.994)	0.011	0.084	2.76E-27	TRUE
CD8 ET	*GBP7*	Wald ratio	1	0.0005	0.0064	1 (0.988, 1.013)	0.94	0.976	1.17E-73	TRUE
CD8 ET	*GCC2*	Wald ratio	1	0.0066	0.0081	1.007 (0.991, 1.023)	0.414	0.686	4.31E-49	TRUE
CD8 ET	*GCHFR*	Wald ratio	1	0.0195	0.0173	1.02 (0.986, 1.055)	0.259	0.553	1.81E-12	TRUE
CD8 ET	*GDE1*	Wald ratio	1	-0.0038	0.0193	0.996 (0.959, 1.035)	0.844	0.935	7.06E-10	TRUE
CD8 ET	*GDI2*	Wald ratio	1	-0.0096	0.0108	0.99 (0.97, 1.012)	0.376	0.664	8.29E-27	TRUE
CD8 ET	*GEMIN7*	Wald ratio	1	-0.0231	0.0213	0.977 (0.937, 1.019)	0.277	0.57	4.14E-08	TRUE
CD8 ET	*GIMAP2*	Wald ratio	1	-0.0279	0.0158	0.973 (0.943, 1.003)	0.078	0.292	4.01E-12	TRUE
CD8 ET	*GIPC1*	Wald ratio	1	-0.0501	0.0188	0.951 (0.917, 0.987)	0.008	0.067	2.06E-09	TRUE
CD8 ET	*GLIPR1*	Wald ratio	1	0.0112	0.0172	1.011 (0.978, 1.046)	0.515	0.751	1.14E-11	TRUE
CD8 ET	*GLIPR1L2*	Wald ratio	1	-0.0119	0.0133	0.988 (0.963, 1.014)	0.373	0.662	1.68E-18	TRUE
CD8 ET	*GLTP*	Wald ratio	1	-0.0279	0.0168	0.972 (0.941, 1.005)	0.096	0.329	3.20E-11	TRUE
CD8 ET	*GLTSCR2*	Wald ratio	1	-0.0068	0.0109	0.993 (0.972, 1.015)	0.537	0.762	5.50E-23	TRUE
CD8 ET	*GMIP*	Wald ratio	1	-0.0065	0.0190	0.994 (0.957, 1.031)	0.732	0.88	1.09E-08	TRUE
CD8 ET	*GNAQ*	Wald ratio	1	-0.0067	0.0166	0.993 (0.961, 1.026)	0.686	0.857	2.24E-11	TRUE
CD8 ET	*GNG5*	Wald ratio	1	0.0084	0.0243	1.008 (0.961, 1.058)	0.731	0.88	1.46E-08	TRUE
CD8 ET	*GNLY*	Wald ratio	1	-0.0083	0.0063	0.992 (0.98, 1.004)	0.186	0.465	8.65E-65	TRUE
CD8 ET	*GNPTG*	Wald ratio	1	-0.0131	0.0104	0.987 (0.967, 1.007)	0.208	0.494	8.01E-22	TRUE
CD8 ET	*GOLT1B*	Wald ratio	1	0.0531	0.0236	1.055 (1.007, 1.104)	0.024	0.143	1.85E-08	TRUE
CD8 ET	*GPR56*	Wald ratio	1	-0.0164	0.0205	0.984 (0.945, 1.024)	0.423	0.693	4.91E-08	TRUE
CD8 ET	*GPR63*	Wald ratio	1	0.0123	0.0139	1.012 (0.985, 1.04)	0.376	0.664	3.78E-15	TRUE
CD8 ET	*GREM2*	Wald ratio	1	0.0042	0.0160	1.004 (0.973, 1.036)	0.795	0.911	2.78E-12	TRUE
CD8 ET	*GRHPR*	Wald ratio	1	-0.0103	0.0086	0.99 (0.973, 1.006)	0.227	0.52	6.88E-37	TRUE
CD8 ET	*GS1-124K5.4*	Wald ratio	1	0.0024	0.0168	1.002 (0.97, 1.036)	0.885	0.953	8.85E-12	TRUE
CD8 ET	*GS1-251I9.4*	Wald ratio	1	0.0014	0.0178	1.001 (0.967, 1.037)	0.939	0.976	1.71E-09	TRUE
CD8 ET	*GSC*	Wald ratio	1	0.0142	0.0222	1.014 (0.971, 1.059)	0.523	0.754	5.29E-09	TRUE
CD8 ET	*GSDMA*	Wald ratio	1	0.0001	0.0071	1 (0.986, 1.014)	0.989	0.996	7.45E-42	TRUE
CD8 ET	*GSE1*	Wald ratio	1	0.0091	0.0195	1.009 (0.971, 1.048)	0.642	0.831	6.66E-09	TRUE
CD8 ET	*GSTM3*	Wald ratio	1	-0.0112	0.0073	0.989 (0.975, 1.003)	0.125	0.38	6.90E-54	TRUE
CD8 ET	*GSTO1*	Wald ratio	1	0.0288	0.0127	1.029 (1.004, 1.055)	0.024	0.142	8.55E-19	TRUE
CD8 ET	*GSTP1*	Wald ratio	1	0.0193	0.0107	1.019 (0.998, 1.041)	0.071	0.276	1.66E-27	TRUE
CD8 ET	*GTPBP4*	Wald ratio	1	-0.0446	0.0419	0.956 (0.881, 1.038)	0.287	0.579	4.45E-09	TRUE
CD8 ET	*GYG1*	Wald ratio	1	-0.0024	0.0181	0.998 (0.963, 1.034)	0.893	0.957	4.93E-09	TRUE
CD8 ET	*GYPC*	IVW	2	0.0002	0.0173	1 (0.967, 1.035)	0.993	0.997	2.88E-29	TRUE
CD8 ET	*H2AFZ*	Wald ratio	1	0.0257	0.0184	1.026 (0.99, 1.064)	0.164	0.438	1.77E-08	TRUE
CD8 ET	*HAGH*	Wald ratio	1	0.0492	0.0106	1.05 (1.029, 1.073)	3.61E-06	2.70E-04	2.81E-21	TRUE
CD8 ET	*HAR1A*	Wald ratio	1	0.0249	0.0145	1.025 (0.996, 1.055)	0.086	0.312	1.92E-15	TRUE
CD8 ET	*HAX1*	Wald ratio	1	-0.0151	0.0164	0.985 (0.954, 1.017)	0.357	0.648	1.70E-10	TRUE
CD8 ET	*HBA1*	Wald ratio	1	-0.0167	0.0112	0.983 (0.962, 1.005)	0.137	0.399	2.15E-14	TRUE
CD8 ET	*HCG14*	IVW	2	0.0182	0.0121	1.018 (0.995, 1.043)	0.132	0.393	1.05E-34	TRUE
CD8 ET	*HCG25*	Wald ratio	1	-0.0348	0.0104	0.966 (0.946, 0.986)	8.42E-04	0.015	4.81E-17	TRUE
CD8 ET	*HDDC2*	Wald ratio	1	0.0310	0.0161	1.031 (0.999, 1.065)	0.055	0.234	3.12E-12	TRUE
CD8 ET	*HDGFRP3*	Wald ratio	1	0.0050	0.0153	1.005 (0.975, 1.036)	0.744	0.882	1.24E-12	TRUE
CD8 ET	*HEBP2*	Wald ratio	1	0.0143	0.0125	1.014 (0.99, 1.04)	0.252	0.545	6.15E-24	TRUE
CD8 ET	*HELLS*	Wald ratio	1	-0.0119	0.0128	0.988 (0.964, 1.013)	0.353	0.644	4.97E-19	TRUE
CD8 ET	*HEXDC*	Wald ratio	1	-0.0101	0.0171	0.99 (0.957, 1.024)	0.555	0.774	6.62E-11	TRUE
CD8 ET	*HHEX*	Wald ratio	1	0.0169	0.0196	1.017 (0.979, 1.057)	0.387	0.669	1.68E-08	TRUE
CD8 ET	*HIGD2A*	Wald ratio	1	-0.0098	0.0114	0.99 (0.968, 1.013)	0.387	0.669	5.59E-24	TRUE
CD8 ET	*HIP1*	Wald ratio	1	-0.0058	0.0197	0.994 (0.957, 1.033)	0.768	0.897	3.22E-09	TRUE
CD8 ET	*HIST1H1B*	Wald ratio	1	-0.0060	0.0241	0.994 (0.948, 1.042)	0.801	0.915	1.60E-09	TRUE
CD8 ET	*HIST1H2AH*	Wald ratio	1	-0.0354	0.0237	0.965 (0.921, 1.011)	0.134	0.396	2.06E-10	TRUE
CD8 ET	*HIST1H2AI*	Wald ratio	1	0.0156	0.0227	1.016 (0.972, 1.062)	0.492	0.738	2.03E-11	TRUE
CD8 ET	*HIST1H2BI*	Wald ratio	1	-0.0176	0.0194	0.983 (0.946, 1.021)	0.364	0.653	4.02E-15	TRUE
CD8 ET	*HIST1H2BL*	Wald ratio	1	-0.0248	0.0246	0.976 (0.93, 1.024)	0.315	0.609	7.62E-10	TRUE
CD8 ET	*HIST1H2BO*	Wald ratio	1	0.0221	0.0176	1.022 (0.988, 1.058)	0.211	0.498	8.91E-12	TRUE
CD8 ET	*HIST1H3H*	Wald ratio	1	0.0307	0.0226	1.031 (0.986, 1.078)	0.176	0.456	1.56E-09	TRUE
CD8 ET	*HIST1H3J*	Wald ratio	1	0.0228	0.0273	1.023 (0.97, 1.079)	0.404	0.681	2.24E-08	TRUE
CD8 ET	*HIST1H4B*	Wald ratio	1	0.0223	0.0193	1.023 (0.985, 1.062)	0.248	0.541	4.88E-11	TRUE
CD8 ET	*HIST1H4F*	Wald ratio	1	-0.0102	0.0141	0.99 (0.963, 1.017)	0.467	0.725	1.16E-27	TRUE
CD8 ET	*HIST1H4I*	Wald ratio	1	0.0163	0.0125	1.016 (0.992, 1.042)	0.194	0.474	2.09E-34	TRUE
CD8 ET	*HIST1H4K*	Wald ratio	1	-0.1103	0.0259	0.896 (0.851, 0.942)	2.04E-05	9.61E-04	3.40E-08	TRUE
CD8 ET	*HIVEP3*	Wald ratio	1	0.0133	0.0185	1.013 (0.977, 1.051)	0.474	0.73	1.18E-09	TRUE
CD8 ET	*HLA-B*	IVW	2	0.0128	0.0258	1.013 (0.963, 1.065)	0.619	0.818	2.01E-87	TRUE
CD8 ET	*HLA-C*	IVW	2	0.0072	0.0094	1.007 (0.989, 1.026)	0.443	0.708	6.22E-33	TRUE
CD8 ET	*HLA-DOB*	Wald ratio	1	-0.0032	0.0173	0.997 (0.964, 1.031)	0.852	0.937	8.97E-10	TRUE
CD8 ET	*HLA-DPA1*	Wald ratio	1	0.0346	0.0127	1.035 (1.01, 1.061)	0.006	0.059	3.67E-11	TRUE
CD8 ET	*HLA-DPB1*	Wald ratio	1	0.0273	0.0127	1.028 (1.002, 1.054)	0.032	0.169	9.15E-21	TRUE
CD8 ET	*HLA-DQB1*	Wald ratio	1	-0.0344	0.0166	0.966 (0.935, 0.998)	0.038	0.189	6.70E-09	TRUE
CD8 ET	*HLA-DQB2*	Wald ratio	1	-0.0526	0.0133	0.949 (0.924, 0.974)	7.78E-05	0.003	8.15E-18	TRUE
CD8 ET	*HLA-DRB5*	IVW	2	0.0265	0.0129	1.027 (1.001, 1.053)	0.039	0.192	1.20E-112	TRUE
CD8 ET	*HLA-G*	IVW	2	0.0092	0.0089	1.009 (0.992, 1.027)	0.302	0.598	2.05E-53	TRUE
CD8 ET	*HMBOX1*	Wald ratio	1	-0.0119	0.0065	0.988 (0.976, 1.001)	0.066	0.263	1.37E-62	TRUE
CD8 ET	*HMGN1*	Wald ratio	1	0.0567	0.0196	1.058 (1.019, 1.1)	0.004	0.041	6.83E-10	TRUE

CD8 ET	*HNRNPU*	Wald ratio	1	-0.0542	0.0174	0.947 (0.916, 0.98)	0.002	0.025	9.75E-09	TRUE
CD8 ET	*HOPX*	Wald ratio	1	-0.0066	0.0185	0.993 (0.958, 1.03)	0.723	0.877	5.12E-09	TRUE
CD8 ET	*HOXB-AS1*	Wald ratio	1	-0.0108	0.0097	0.989 (0.971, 1.008)	0.265	0.559	1.49E-31	TRUE
CD8 ET	*HOXB2*	Wald ratio	1	-0.0092	0.0079	0.991 (0.976, 1.006)	0.242	0.534	4.16E-47	TRUE
CD8 ET	*HOXB4*	Wald ratio	1	-0.0255	0.0178	0.975 (0.941, 1.009)	0.152	0.423	4.30E-10	TRUE
CD8 ET	*HPGD*	Wald ratio	1	-0.0070	0.0127	0.993 (0.969, 1.018)	0.579	0.789	3.61E-19	TRUE
CD8 ET	*HSBP1L1*	Wald ratio	1	-0.0614	0.0181	0.94 (0.908, 0.974)	6.94E-04	0.013	5.59E-12	TRUE
CD8 ET	*HSD17B11*	Wald ratio	1	-0.0190	0.0164	0.981 (0.95, 1.013)	0.247	0.541	1.75E-12	TRUE
CD8 ET	*HSD17B14*	Wald ratio	1	0.0174	0.0195	1.018 (0.979, 1.057)	0.373	0.661	1.75E-08	TRUE
CD8 ET	*HSP90B1*	Wald ratio	1	-0.0250	0.0188	0.975 (0.94, 1.012)	0.184	0.463	3.55E-09	TRUE
CD8 ET	*HSPBAP1*	Wald ratio	1	0.0090	0.0092	1.009 (0.991, 1.027)	0.327	0.62	2.52E-28	TRUE
CD8 ET	*ICA1L*	Wald ratio	1	-0.0482	0.0204	0.953 (0.916, 0.992)	0.018	0.118	1.40E-08	TRUE
CD8 ET	*IDI1*	Wald ratio	1	0.0168	0.0122	1.017 (0.993, 1.042)	0.168	0.443	1.00E-24	TRUE
CD8 ET	*IFITM2*	IVW	2	-0.0081	0.0088	0.992 (0.975, 1.009)	0.359	0.649	7.13E-41	TRUE
CD8 ET	*IFITM3*	IVW	2	-0.0020	0.0064	0.998 (0.985, 1.011)	0.751	0.886	2.80E-95	TRUE
CD8 ET	*IFNG-AS1*	Wald ratio	1	-0.0138	0.0170	0.986 (0.954, 1.02)	0.418	0.689	6.88E-11	TRUE
CD8 ET	*IGFLR1*	Wald ratio	1	0.0128	0.0187	1.013 (0.976, 1.051)	0.495	0.74	4.12E-14	TRUE
CD8 ET	*IGHMBP2*	Wald ratio	1	-0.0730	0.0199	0.93 (0.894, 0.967)	2.43E-04	0.006	2.20E-08	TRUE
CD8 ET	*IKBKE*	Wald ratio	1	0.0111	0.0166	1.011 (0.979, 1.045)	0.505	0.745	6.20E-10	TRUE
CD8 ET	*IL18R1*	Wald ratio	1	0.0080	0.0190	1.008 (0.971, 1.046)	0.675	0.852	3.15E-09	TRUE
CD8 ET	*IL32*	Wald ratio	1	-0.0131	0.0148	0.987 (0.959, 1.016)	0.376	0.664	4.14E-14	TRUE
CD8 ET	*INSR*	Wald ratio	1	-0.0551	0.0112	0.946 (0.926, 0.967)	9.45E-07	1.08E-04	2.41E-13	TRUE
CD8 ET	*INTS5*	Wald ratio	1	-0.0264	0.0203	0.974 (0.936, 1.013)	0.193	0.474	1.47E-09	TRUE
CD8 ET	*IQCB1*	Wald ratio	1	0.0022	0.0214	1.002 (0.961, 1.045)	0.919	0.965	3.11E-09	TRUE
CD8 ET	*IRF1*	Wald ratio	1	0.0135	0.0193	1.014 (0.976, 1.053)	0.483	0.735	4.76E-10	TRUE
CD8 ET	*ISCA1*	Wald ratio	1	0.0284	0.0145	1.029 (1, 1.058)	0.05	0.222	1.67E-13	TRUE
CD8 ET	*ISCA2*	Wald ratio	1	0.0109	0.0137	1.011 (0.984, 1.038)	0.424	0.694	6.34E-16	TRUE
CD8 ET	*ISG15*	Wald ratio	1	-0.0346	0.0148	0.966 (0.938, 0.994)	0.019	0.123	2.61E-13	TRUE
CD8 ET	*ITCH*	Wald ratio	1	-0.0059	0.0114	0.994 (0.972, 1.017)	0.606	0.808	1.37E-23	TRUE
CD8 ET	*ITCH-AS1*	Wald ratio	1	0.0104	0.0133	1.01 (0.985, 1.037)	0.434	0.702	4.39E-17	TRUE
CD8 ET	*ITGAD*	Wald ratio	1	-0.0042	0.0172	0.996 (0.963, 1.03)	0.806	0.916	1.07E-09	TRUE
CD8 ET	*ITGB2*	Wald ratio	1	-0.0007	0.0051	0.999 (0.989, 1.009)	0.897	0.957	9.81E-99	TRUE
CD8 ET	*ITGB2-AS1*	Wald ratio	1	-0.0221	0.0219	0.978 (0.937, 1.021)	0.314	0.608	3.06E-08	TRUE
CD8 ET	*ITGB3BP*	Wald ratio	1	0.0068	0.0143	1.007 (0.979, 1.035)	0.636	0.827	1.99E-14	TRUE
CD8 ET	*ITPA*	Wald ratio	1	0.0313	0.0178	1.032 (0.996, 1.068)	0.079	0.295	6.98E-12	TRUE
CD8 ET	*IVNS1ABP*	Wald ratio	1	0.0150	0.0154	1.015 (0.985, 1.046)	0.331	0.624	1.64E-13	TRUE
CD8 ET	*JAKMIP1*	Wald ratio	1	0.0071	0.0117	1.007 (0.984, 1.03)	0.546	0.767	6.25E-22	TRUE
CD8 ET	*KANSL1-AS1*	Wald ratio	1	-0.0162	0.0069	0.984 (0.971, 0.997)	0.018	0.119	2.89E-150	TRUE
CD8 ET	*KB-1980E6.3*	Wald ratio	1	-0.0102	0.0132	0.99 (0.965, 1.016)	0.438	0.703	5.81E-11	TRUE
CD8 ET	*KCNAB2*	Wald ratio	1	0.0116	0.0130	1.012 (0.986, 1.038)	0.374	0.662	1.13E-21	TRUE
CD8 ET	*KCTD10*	Wald ratio	1	0.0090	0.0124	1.009 (0.985, 1.034)	0.466	0.725	1.95E-17	TRUE
CD8 ET	*KCTD21-AS1*	Wald ratio	1	0.0016	0.0138	1.002 (0.975, 1.029)	0.908	0.961	6.04E-16	TRUE
CD8 ET	*KDELR2*	Wald ratio	1	0.0267	0.0083	1.027 (1.011, 1.044)	0.001	0.02	8.75E-40	TRUE
CD8 ET	*KIAA0930*	Wald ratio	1	0.0184	0.0133	1.019 (0.992, 1.045)	0.166	0.441	6.69E-17	TRUE
CD8 ET	*KIF16B*	Wald ratio	1	0.0286	0.0140	1.029 (1.001, 1.058)	0.042	0.199	5.50E-15	TRUE
CD8 ET	*KIF19*	Wald ratio	1	-0.0327	0.0179	0.968 (0.934, 1.002)	0.067	0.266	6.86E-10	TRUE
CD8 ET	*KIR3DL1*	Wald ratio	1	0.0095	0.0174	1.009 (0.976, 1.044)	0.586	0.794	7.48E-10	TRUE
CD8 ET	*KLHDC4*	Wald ratio	1	-0.0028	0.0119	0.997 (0.974, 1.021)	0.816	0.921	6.33E-16	TRUE
CD8 ET	*KLRC2*	Wald ratio	1	-0.0166	0.0093	0.984 (0.966, 1.002)	0.075	0.287	7.09E-31	TRUE
CD8 ET	*KLRC4*	Wald ratio	1	-0.0108	0.0095	0.989 (0.971, 1.008)	0.257	0.551	1.61E-31	TRUE
CD8 ET	*KLRD1*	Wald ratio	1	-0.0214	0.0128	0.979 (0.955, 1.004)	0.093	0.324	6.36E-17	TRUE
CD8 ET	*KLRK1*	Wald ratio	1	-0.0277	0.0155	0.973 (0.944, 1.003)	0.075	0.287	7.75E-12	TRUE
CD8 ET	*KRT73*	Wald ratio	1	0.0650	0.0142	1.067 (1.038, 1.097)	4.86E-06	3.29E-04	8.12E-15	TRUE
CD8 ET	*KTN1*	Wald ratio	1	0.0444	0.0143	1.045 (1.016, 1.075)	0.002	0.026	4.82E-14	TRUE
CD8 ET	*L3HYPDH*	Wald ratio	1	0.0206	0.0197	1.021 (0.982, 1.061)	0.297	0.592	2.26E-09	TRUE
CD8 ET	*L3MBTL3*	Wald ratio	1	-0.0099	0.0116	0.99 (0.968, 1.013)	0.393	0.672	1.58E-22	TRUE
CD8 ET	*LA16c-358B7.3*	Wald ratio	1	0.0411	0.0103	1.042 (1.021, 1.063)	6.58E-05	0.002	2.46E-29	TRUE
CD8 ET	*LAIR2*	Wald ratio	1	0.0333	0.0114	1.034 (1.011, 1.057)	0.003	0.039	7.86E-30	TRUE
CD8 ET	*LAMB1*	Wald ratio	1	0.0080	0.0079	1.008 (0.993, 1.024)	0.306	0.6	1.61E-47	TRUE
CD8 ET	*LAMTOR4*	Wald ratio	1	-0.0147	0.0172	0.985 (0.953, 1.019)	0.393	0.672	1.14E-10	TRUE
CD8 ET	*LAPTM5*	Wald ratio	1	-0.0598	0.0195	0.942 (0.907, 0.979)	0.002	0.028	3.15E-08	TRUE
CD8 ET	*LATS2*	Wald ratio	1	-0.0510	0.0161	0.95 (0.921, 0.981)	0.002	0.022	1.02E-09	TRUE
CD8 ET	*LAX1*	Wald ratio	1	0.0001	0.0114	1 (0.978, 1.023)	0.996	0.999	1.25E-22	TRUE
CD8 ET	*LCLAT1*	Wald ratio	1	-0.0023	0.0197	0.998 (0.96, 1.037)	0.909	0.961	2.55E-09	TRUE
CD8 ET	*LCP1*	Wald ratio	1	-0.0193	0.0211	0.981 (0.941, 1.022)	0.362	0.651	5.43E-10	TRUE
CD8 ET	*LDHC*	Wald ratio	1	-0.0190	0.0120	0.981 (0.958, 1.005)	0.113	0.359	1.50E-23	TRUE
CD8 ET	*LEPROT*	Wald ratio	1	-0.0004	0.0150	1 (0.971, 1.029)	0.977	0.991	1.05E-14	TRUE
CD8 ET	*LIMD1*	Wald ratio	1	0.0124	0.0138	1.012 (0.986, 1.04)	0.368	0.656	3.34E-16	TRUE
CD8 ET	*LIN52*	Wald ratio	1	0.0146	0.0173	1.015 (0.981, 1.05)	0.4	0.679	2.69E-11	TRUE
CD8 ET	*LINC00176*	Wald ratio	1	0.0099	0.0114	1.01 (0.988, 1.033)	0.388	0.669	7.60E-22	TRUE
CD8 ET	*LINC00240*	Wald ratio	1	0.0238	0.0198	1.024 (0.985, 1.065)	0.23	0.524	1.82E-14	TRUE
CD8 ET	*LINC00339*	Wald ratio	1	0.0121	0.0108	1.012 (0.991, 1.034)	0.261	0.556	4.30E-25	TRUE
CD8 ET	*LINC00426*	Wald ratio	1	0.0059	0.0151	1.006 (0.977, 1.036)	0.697	0.864	1.70E-12	TRUE
CD8 ET	*LINC00493*	Wald ratio	1	-0.0324	0.0190	0.968 (0.933, 1.005)	0.089	0.317	5.57E-09	TRUE
CD8 ET	*LINC00649*	Wald ratio	1	-0.0180	0.0110	0.982 (0.961, 1.004)	0.101	0.337	8.99E-24	TRUE
CD8 ET	*LINC00909*	Wald ratio	1	-0.0081	0.0211	0.992 (0.952, 1.034)	0.699	0.866	5.51E-08	TRUE
CD8 ET	*LINC00920*	Wald ratio	1	0.0037	0.0078	1.004 (0.988, 1.019)	0.636	0.827	1.04E-47	TRUE
CD8 ET	*LINC00944*	Wald ratio	1	0.0048	0.0090	1.005 (0.987, 1.023)	0.59	0.797	5.27E-22	TRUE
CD8 ET	*LINC00969*	Wald ratio	1	-0.0082	0.0094	0.992 (0.974, 1.01)	0.384	0.668	1.30E-32	TRUE

CD8 ET	*LINC01137*	Wald ratio	1	-0.0025	0.0188	0.998 (0.962, 1.035)	0.895	0.957	1.81E-09	TRUE
CD8 ET	*LIPC*	Wald ratio	1	0.0526	0.0240	1.054 (1.006, 1.105)	0.028	0.157	1.32E-09	TRUE
CD8 ET	*LL22NC03-2H8.5*	Wald ratio	1	0.0018	0.0172	1.002 (0.969, 1.036)	0.916	0.963	4.95E-11	TRUE
CD8 ET	*LLGL1*	Wald ratio	1	0.0809	0.0166	1.084 (1.05, 1.12)	1.07E-06	1.17E-04	7.95E-11	TRUE
CD8 ET	*LPIN1*	Wald ratio	1	0.0252	0.0105	1.025 (1.005, 1.047)	0.016	0.111	3.93E-22	TRUE
CD8 ET	*LPIN2*	Wald ratio	1	-0.0113	0.0180	0.989 (0.955, 1.024)	0.53	0.758	1.81E-10	TRUE
CD8 ET	*LRRC16B*	Wald ratio	1	0.0289	0.0138	1.029 (1.002, 1.058)	0.037	0.182	8.23E-16	TRUE
CD8 ET	*LRRC37A*	Wald ratio	1	-0.0457	0.0099	0.955 (0.937, 0.974)	3.95E-06	2.83E-04	8.26E-24	TRUE
CD8 ET	*LRRC61*	Wald ratio	1	-0.0541	0.0188	0.947 (0.913, 0.983)	0.004	0.043	1.39E-08	TRUE
CD8 ET	*LRRIQ3*	Wald ratio	1	-0.0013	0.0130	0.999 (0.974, 1.025)	0.922	0.965	8.10E-18	TRUE
CD8 ET	*LSM7*	Wald ratio	1	0.0464	0.0133	1.048 (1.021, 1.075)	4.56E-04	0.01	1.29E-16	TRUE
CD8 ET	*LYAR*	Wald ratio	1	-0.0145	0.0112	0.986 (0.964, 1.007)	0.195	0.476	4.74E-23	TRUE
CD8 ET	*LYRM4*	Wald ratio	1	0.0104	0.0081	1.01 (0.995, 1.027)	0.197	0.48	1.16E-36	TRUE
CD8 ET	*LYST*	Wald ratio	1	-0.0179	0.0192	0.982 (0.946, 1.02)	0.352	0.644	4.62E-10	TRUE
CD8 ET	*MAD1L1*	Wald ratio	1	-0.0514	0.0176	0.95 (0.918, 0.983)	0.004	0.039	1.59E-10	TRUE
CD8 ET	*MAF1*	Wald ratio	1	0.0130	0.0127	1.013 (0.988, 1.039)	0.306	0.599	6.64E-23	TRUE
CD8 ET	*MAP3K5*	Wald ratio	1	0.0267	0.0165	1.027 (0.994, 1.061)	0.107	0.347	2.74E-11	TRUE
CD8 ET	*MARCH2*	Wald ratio	1	0.0470	0.0195	1.048 (1.009, 1.089)	0.016	0.111	2.05E-10	TRUE
CD8 ET	*MAST3*	Wald ratio	1	0.0340	0.0125	1.035 (1.01, 1.06)	0.007	0.06	8.03E-19	TRUE
CD8 ET	*MBP*	Wald ratio	1	0.0154	0.0132	1.016 (0.99, 1.042)	0.24	0.534	2.16E-17	TRUE
CD8 ET	*MC1R*	Wald ratio	1	0.0228	0.0182	1.023 (0.987, 1.06)	0.211	0.498	9.52E-12	TRUE
CD8 ET	*MDGA1*	Wald ratio	1	-0.0059	0.0075	0.994 (0.98, 1.009)	0.435	0.702	4.27E-53	TRUE
CD8 ET	*MDM4*	Wald ratio	1	-0.0673	0.0173	0.935 (0.904, 0.967)	1.02E-04	0.003	1.03E-12	TRUE
CD8 ET	*MEAF6*	Wald ratio	1	-0.0409	0.0169	0.96 (0.929, 0.992)	0.015	0.107	1.08E-09	TRUE
CD8 ET	*MED16*	Wald ratio	1	0.0003	0.0085	1 (0.984, 1.017)	0.973	0.989	1.26E-47	TRUE
CD8 ET	*METTL10*	Wald ratio	1	0.0024	0.0185	1.002 (0.967, 1.039)	0.896	0.957	1.62E-09	TRUE
CD8 ET	*METTL18*	Wald ratio	1	-0.0014	0.0203	0.999 (0.96, 1.039)	0.945	0.978	6.54E-10	TRUE
CD8 ET	*MFF*	Wald ratio	1	-0.0008	0.0136	0.999 (0.973, 1.026)	0.955	0.983	1.85E-13	TRUE
CD8 ET	*MGMT*	Wald ratio	1	-0.0087	0.0100	0.991 (0.972, 1.011)	0.381	0.666	8.82E-29	TRUE
CD8 ET	*MGST2*	Wald ratio	1	-0.0073	0.0150	0.993 (0.964, 1.022)	0.627	0.822	5.84E-13	TRUE
CD8 ET	*MGST3*	Wald ratio	1	-0.0029	0.0129	0.997 (0.972, 1.023)	0.819	0.924	3.55E-19	TRUE
CD8 ET	*MIAT*	Wald ratio	1	-0.0030	0.0101	0.997 (0.977, 1.017)	0.768	0.897	1.01E-24	TRUE
CD8 ET	*MIF*	Wald ratio	1	0.0044	0.0116	1.004 (0.982, 1.027)	0.708	0.869	1.05E-18	TRUE
CD8 ET	*MINK1*	Wald ratio	1	0.0538	0.0179	1.055 (1.019, 1.093)	0.003	0.032	2.71E-10	TRUE
CD8 ET	*MKRN1*	Wald ratio	1	-0.0522	0.0177	0.949 (0.917, 0.983)	0.003	0.037	6.82E-11	TRUE
CD8 ET	*MLF1*	Wald ratio	1	0.0693	0.0133	1.072 (1.044, 1.1)	1.76E-07	6.60E-05	5.61E-13	TRUE
CD8 ET	*MLF2*	Wald ratio	1	-0.0122	0.0197	0.988 (0.951, 1.027)	0.537	0.762	2.94E-08	TRUE
CD8 ET	*MLLT4*	Wald ratio	1	0.0014	0.0164	1.001 (0.97, 1.034)	0.933	0.972	2.07E-11	TRUE
CD8 ET	*MLTK*	Wald ratio	1	-0.0008	0.0187	0.999 (0.963, 1.037)	0.965	0.988	3.07E-10	TRUE
CD8 ET	*MPHOSPH9*	Wald ratio	1	-0.0594	0.0205	0.942 (0.905, 0.981)	0.004	0.041	5.63E-08	TRUE
CD8 ET	*MPZL3*	Wald ratio	1	0.0076	0.0178	1.008 (0.973, 1.043)	0.669	0.848	1.61E-09	TRUE
CD8 ET	*MRPL10*	Wald ratio	1	-0.0304	0.0162	0.97 (0.94, 1.001)	0.061	0.252	1.78E-11	TRUE
CD8 ET	*MRPL21*	Wald ratio	1	0.0130	0.0122	1.013 (0.989, 1.038)	0.288	0.58	9.09E-18	TRUE
CD8 ET	*MRPL32*	Wald ratio	1	0.0221	0.0186	1.022 (0.986, 1.06)	0.236	0.53	2.19E-16	TRUE
CD8 ET	*MRPL41*	Wald ratio	1	-0.0171	0.0148	0.983 (0.955, 1.012)	0.248	0.541	8.01E-15	TRUE
CD8 ET	*MRPL42*	Wald ratio	1	0.0335	0.0185	1.034 (0.997, 1.072)	0.071	0.276	5.41E-09	TRUE
CD8 ET	*MRPL53*	Wald ratio	1	-0.0122	0.0191	0.988 (0.952, 1.026)	0.523	0.754	3.46E-08	TRUE
CD8 ET	*MRPL54*	Wald ratio	1	0.0091	0.0115	1.009 (0.987, 1.032)	0.429	0.699	1.74E-22	TRUE
CD8 ET	*MRPS18C*	Wald ratio	1	-0.0014	0.0145	0.999 (0.971, 1.027)	0.922	0.965	1.65E-14	TRUE
CD8 ET	*MRPS21*	Wald ratio	1	-0.0751	0.0188	0.928 (0.894, 0.963)	6.64E-05	0.002	1.46E-08	TRUE
CD8 ET	*MSC*	Wald ratio	1	0.0068	0.0126	1.007 (0.982, 1.032)	0.59	0.797	1.83E-18	TRUE
CD8 ET	*MSH5*	Wald ratio	1	-0.0711	0.0179	0.931 (0.899, 0.965)	7.11E-05	0.002	3.21E-10	TRUE
CD8 ET	*MT1E*	IVW	2	-0.0102	0.0073	0.99 (0.976, 1.004)	0.166	0.441	1.02E-65	TRUE
CD8 ET	*MT1F*	Wald ratio	1	0.0056	0.0115	1.006 (0.983, 1.029)	0.627	0.822	1.04E-22	TRUE
CD8 ET	*MT1X*	Wald ratio	1	0.0039	0.0102	1.004 (0.984, 1.024)	0.702	0.866	3.22E-30	TRUE
CD8 ET	*MT2A*	Wald ratio	1	-0.0085	0.0053	0.992 (0.981, 1.002)	0.11	0.353	3.56E-131	TRUE
CD8 ET	*MTFP1*	Wald ratio	1	-0.0129	0.0196	0.987 (0.95, 1.026)	0.511	0.748	1.56E-08	TRUE
CD8 ET	*MTG2*	Wald ratio	1	0.0469	0.0192	1.048 (1.009, 1.088)	0.014	0.103	1.77E-10	TRUE
CD8 ET	*MTSS1*	IVW	2	0.0368	0.0134	1.037 (1.011, 1.065)	0.006	0.057	2.69E-18	TRUE
CD8 ET	*MX1*	Wald ratio	1	0.0177	0.0135	1.018 (0.991, 1.045)	0.189	0.47	2.59E-16	TRUE
CD8 ET	*MXRA7*	Wald ratio	1	0.0057	0.0154	1.006 (0.976, 1.036)	0.712	0.872	1.51E-09	TRUE
CD8 ET	*MYCBPAP*	Wald ratio	1	0.0030	0.0111	1.003 (0.981, 1.025)	0.789	0.909	5.24E-24	TRUE
CD8 ET	*MYH11*	Wald ratio	1	-0.0208	0.0119	0.979 (0.957, 1.002)	0.079	0.294	7.60E-22	TRUE
CD8 ET	*MYL12A*	Wald ratio	1	-0.0100	0.0151	0.99 (0.961, 1.02)	0.509	0.748	3.10E-10	TRUE
CD8 ET	*MYO15B*	Wald ratio	1	-0.0115	0.0157	0.989 (0.959, 1.019)	0.462	0.721	6.44E-18	TRUE
CD8 ET	*MYO1F*	Wald ratio	1	-0.0197	0.0119	0.98 (0.958, 1.004)	0.097	0.331	7.47E-22	TRUE
CD8 ET	*MYOM2*	Wald ratio	1	-0.0050	0.0068	0.995 (0.982, 1.008)	0.457	0.719	3.38E-65	TRUE
CD8 ET	*MZT2A*	Wald ratio	1	-0.0089	0.0054	0.991 (0.981, 1.002)	0.099	0.333	2.65E-77	TRUE
CD8 ET	*NAA38*	Wald ratio	1	-0.0088	0.0128	0.991 (0.967, 1.016)	0.494	0.739	4.32E-24	TRUE
CD8 ET	*NAAA*	Wald ratio	1	0.0242	0.0108	1.025 (1.003, 1.046)	0.025	0.145	9.42E-25	TRUE
CD8 ET	*NAP1L1*	Wald ratio	1	-0.0148	0.0182	0.985 (0.951, 1.021)	0.417	0.688	1.14E-09	TRUE
CD8 ET	*NAPRT1*	Wald ratio	1	-0.0111	0.0067	0.989 (0.976, 1.002)	0.099	0.333	1.02E-62	TRUE
CD8 ET	*NARG2*	Wald ratio	1	-0.0545	0.0215	0.947 (0.908, 0.988)	0.011	0.089	3.70E-10	TRUE
CD8 ET	*NBEAL2*	Wald ratio	1	0.0107	0.0152	1.011 (0.981, 1.041)	0.481	0.734	2.43E-13	TRUE
CD8 ET	*NBPF1*	Wald ratio	1	-0.0383	0.0188	0.962 (0.928, 0.998)	0.041	0.198	5.50E-10	TRUE
CD8 ET	*NBPF3*	Wald ratio	1	0.0042	0.0148	1.004 (0.976, 1.034)	0.775	0.901	4.82E-14	TRUE
CD8 ET	*NCF4*	Wald ratio	1	-0.0097	0.0202	0.99 (0.952, 1.03)	0.631	0.825	2.11E-09	TRUE
CD8 ET	*NCL*	Wald ratio	1	-0.0412	0.0189	0.96 (0.925, 0.996)	0.029	0.16	4.37E-10	TRUE
CD8 ET	*NCOA7*	Wald ratio	1	-0.0581	0.0188	0.944 (0.909, 0.979)	0.002	0.027	5.64E-09	TRUE

CD8 ET	*NDUFA12*	Wald ratio	1	0.0062	0.0109	1.006 (0.985, 1.028)	0.568	0.782	2.46E-29	TRUE
CD8 ET	*NDUFA3*	Wald ratio	1	0.0027	0.0109	1.003 (0.981, 1.024)	0.802	0.915	6.10E-25	TRUE
CD8 ET	*NDUFA6*	Wald ratio	1	0.0013	0.0202	1.001 (0.962, 1.042)	0.948	0.98	5.46E-13	TRUE
CD8 ET	*NDUFB2*	Wald ratio	1	-0.0278	0.0155	0.973 (0.943, 1.003)	0.073	0.282	3.33E-11	TRUE
CD8 ET	*NDUFC2*	Wald ratio	1	0.0447	0.0221	1.046 (1.001, 1.092)	0.043	0.203	6.49E-08	TRUE
CD8 ET	*NDUFS5*	Wald ratio	1	-0.0031	0.0070	0.997 (0.983, 1.011)	0.663	0.845	1.16E-61	TRUE
CD8 ET	*NDUFS8*	Wald ratio	1	-0.0481	0.0178	0.953 (0.92, 0.987)	0.007	0.063	2.45E-08	TRUE
CD8 ET	*NEAT1*	Wald ratio	1	-0.0184	0.0197	0.982 (0.944, 1.02)	0.351	0.643	5.56E-09	TRUE
CD8 ET	*NFAM1*	Wald ratio	1	-0.0166	0.0117	0.984 (0.961, 1.006)	0.154	0.425	1.12E-21	TRUE
CD8 ET	*NINJ1*	Wald ratio	1	0.0105	0.0099	1.011 (0.991, 1.03)	0.288	0.58	1.51E-27	TRUE
CD8 ET	*NIT2*	Wald ratio	1	-0.0446	0.0199	0.956 (0.92, 0.994)	0.025	0.146	3.16E-08	TRUE
CD8 ET	*NME1*	Wald ratio	1	0.0040	0.0118	1.004 (0.981, 1.028)	0.737	0.88	1.72E-21	TRUE
CD8 ET	*NMI*	Wald ratio	1	0.0207	0.0105	1.021 (1, 1.042)	0.048	0.217	2.66E-26	TRUE
CD8 ET	*NMRAL1*	Wald ratio	1	-0.0198	0.0200	0.98 (0.943, 1.02)	0.323	0.618	2.96E-09	TRUE
CD8 ET	*NMRK1*	Wald ratio	1	-0.0065	0.0096	0.993 (0.975, 1.012)	0.495	0.74	9.21E-38	TRUE
CD8 ET	*NOP10*	Wald ratio	1	-0.0056	0.0206	0.994 (0.955, 1.035)	0.786	0.908	1.15E-08	TRUE
CD8 ET	*NOTCH4*	Wald ratio	1	-0.1076	0.0214	0.898 (0.861, 0.937)	5.07E-07	9.75E-05	1.08E-08	TRUE
CD8 ET	*NPC2*	Wald ratio	1	0.0119	0.0130	1.012 (0.986, 1.038)	0.36	0.65	5.63E-18	TRUE
CD8 ET	*NPDC1*	Wald ratio	1	0.0132	0.0144	1.013 (0.985, 1.042)	0.359	0.65	5.98E-15	TRUE
CD8 ET	*NPIPA5*	Wald ratio	1	0.0295	0.0121	1.03 (1.006, 1.055)	0.015	0.105	5.33E-20	TRUE
CD8 ET	*NQO2*	Wald ratio	1	-0.0018	0.0087	0.998 (0.981, 1.015)	0.836	0.93	3.52E-39	TRUE
CD8 ET	*NSA2*	Wald ratio	1	0.0205	0.0091	1.021 (1.003, 1.039)	0.024	0.143	4.98E-39	TRUE
CD8 ET	*NSG1*	IVW	2	0.0062	0.0090	1.006 (0.989, 1.024)	0.494	0.74	1.59E-81	TRUE
CD8 ET	*NSUN4*	Wald ratio	1	-0.0026	0.0146	0.997 (0.969, 1.026)	0.859	0.94	3.13E-13	TRUE
CD8 ET	*NSUN5*	Wald ratio	1	-0.0353	0.0162	0.965 (0.935, 0.996)	0.029	0.16	6.27E-12	TRUE
CD8 ET	*NT5C3A*	Wald ratio	1	-0.0309	0.0158	0.97 (0.94, 1)	0.051	0.223	3.98E-13	TRUE
CD8 ET	*NT5C3B*	Wald ratio	1	0.0001	0.0070	1 (0.986, 1.014)	0.991	0.996	3.80E-51	TRUE
CD8 ET	*NTPCR*	Wald ratio	1	0.0130	0.0095	1.013 (0.994, 1.032)	0.17	0.446	8.47E-38	TRUE
CD8 ET	*NUB1*	Wald ratio	1	-0.0068	0.0181	0.993 (0.959, 1.029)	0.706	0.868	1.70E-10	TRUE
CD8 ET	*NUCB2*	Wald ratio	1	-0.0336	0.0068	0.967 (0.954, 0.98)	6.73E-07	9.75E-05	3.88E-62	TRUE
CD8 ET	*NUDT14*	Wald ratio	1	0.0006	0.0181	1.001 (0.966, 1.037)	0.972	0.989	5.54E-10	TRUE
CD8 ET	*NUDT2*	Wald ratio	1	-0.0119	0.0079	0.988 (0.973, 1.004)	0.132	0.393	1.48E-42	TRUE
CD8 ET	*NUP107*	Wald ratio	1	-0.0288	0.0156	0.972 (0.942, 1.002)	0.066	0.263	1.24E-11	TRUE
CD8 ET	*NUTF2*	Wald ratio	1	0.0108	0.0153	1.011 (0.981, 1.042)	0.478	0.733	1.18E-12	TRUE
CD8 ET	*NXPE3*	Wald ratio	1	-0.0166	0.0176	0.984 (0.95, 1.018)	0.344	0.637	1.33E-09	TRUE
CD8 ET	*OASL*	Wald ratio	1	0.0160	0.0181	1.016 (0.981, 1.053)	0.377	0.665	1.18E-08	TRUE
CD8 ET	*ORAI1*	Wald ratio	1	-0.0225	0.0147	0.978 (0.95, 1.006)	0.125	0.38	5.33E-18	TRUE
CD8 ET	*ORAI2*	Wald ratio	1	-0.0039	0.0143	0.996 (0.969, 1.024)	0.783	0.906	3.99E-15	TRUE
CD8 ET	*ORMDL3*	Wald ratio	1	-0.0012	0.0059	0.999 (0.987, 1.01)	0.835	0.93	8.70E-88	TRUE
CD8 ET	*OSTF1*	IVW	2	-0.0013	0.0106	0.999 (0.978, 1.02)	0.905	0.96	6.76E-27	TRUE
CD8 ET	*OXLD1*	Wald ratio	1	0.0067	0.0157	1.007 (0.976, 1.038)	0.668	0.848	4.04E-12	TRUE
CD8 ET	*OXNAD1*	Wald ratio	1	-0.0592	0.0140	0.943 (0.917, 0.969)	2.22E-05	0.001	2.28E-15	TRUE
CD8 ET	*P4HA3*	Wald ratio	1	0.0273	0.0145	1.028 (0.999, 1.057)	0.059	0.245	1.74E-14	TRUE
CD8 ET	*PAPD4*	Wald ratio	1	0.0089	0.0183	1.009 (0.973, 1.046)	0.626	0.822	1.35E-08	TRUE
CD8 ET	*PARK7*	Wald ratio	1	-0.0054	0.0139	0.995 (0.968, 1.022)	0.699	0.865	1.85E-16	TRUE
CD8 ET	*PARVB*	Wald ratio	1	0.0160	0.0183	1.016 (0.98, 1.053)	0.38	0.666	5.89E-10	TRUE
CD8 ET	*PASK*	Wald ratio	1	0.0018	0.0177	1.002 (0.968, 1.037)	0.919	0.965	1.87E-09	TRUE
CD8 ET	*PAX8*	Wald ratio	1	-0.0352	0.0150	0.965 (0.937, 0.994)	0.019	0.123	1.26E-12	TRUE
CD8 ET	*PCBP2*	Wald ratio	1	-0.0995	0.0253	0.905 (0.861, 0.951)	8.45E-05	0.003	5.58E-09	TRUE
CD8 ET	*PCM1*	Wald ratio	1	-0.0144	0.0153	0.986 (0.957, 1.016)	0.346	0.639	1.63E-14	TRUE
CD8 ET	*PCNX*	Wald ratio	1	0.0356	0.0160	1.036 (1.004, 1.069)	0.026	0.147	2.68E-12	TRUE
CD8 ET	*PCSK7*	Wald ratio	1	0.0245	0.0164	1.025 (0.992, 1.058)	0.135	0.396	3.96E-11	TRUE
CD8 ET	*PDCD5*	Wald ratio	1	-0.0179	0.0184	0.982 (0.947, 1.018)	0.33	0.624	4.03E-09	TRUE
CD8 ET	*PDCD6*	Wald ratio	1	0.0563	0.0182	1.058 (1.021, 1.096)	0.002	0.026	3.23E-09	TRUE
CD8 ET	*PDE4B*	Wald ratio	1	-0.0482	0.0191	0.953 (0.918, 0.989)	0.012	0.09	7.03E-09	TRUE
CD8 ET	*PDE7A*	Wald ratio	1	0.0247	0.0202	1.025 (0.985, 1.066)	0.221	0.513	9.56E-09	TRUE
CD8 ET	*PDIA6*	Wald ratio	1	-0.0369	0.0156	0.964 (0.935, 0.994)	0.018	0.118	3.65E-13	TRUE
CD8 ET	*PDK2*	Wald ratio	1	-0.0059	0.0196	0.994 (0.957, 1.033)	0.762	0.893	1.39E-08	TRUE
CD8 ET	*PDS5B*	Wald ratio	1	-0.0095	0.0188	0.991 (0.955, 1.028)	0.612	0.812	2.50E-09	TRUE
CD8 ET	*PDXDC1*	Wald ratio	1	-0.0168	0.0152	0.983 (0.955, 1.013)	0.269	0.563	7.63E-14	TRUE
CD8 ET	*PDXK*	Wald ratio	1	0.0043	0.0131	1.004 (0.979, 1.03)	0.741	0.881	6.10E-16	TRUE
CD8 ET	*PDZD8*	Wald ratio	1	0.0299	0.0176	1.03 (0.995, 1.067)	0.09	0.318	4.99E-09	TRUE
CD8 ET	*PEX11G*	Wald ratio	1	-0.0405	0.0199	0.96 (0.924, 0.999)	0.042	0.201	3.19E-08	TRUE
CD8 ET	*PEX6*	Wald ratio	1	0.0138	0.0170	1.014 (0.981, 1.048)	0.417	0.688	2.31E-11	TRUE
CD8 ET	*PFKP*	Wald ratio	1	0.0098	0.0153	1.01 (0.98, 1.041)	0.522	0.754	1.91E-13	TRUE
CD8 ET	*PGBD1*	Wald ratio	1	-0.0216	0.0244	0.979 (0.933, 1.026)	0.375	0.663	9.93E-10	TRUE
CD8 ET	*PGLS*	Wald ratio	1	0.0089	0.0161	1.009 (0.978, 1.041)	0.58	0.789	3.12E-12	TRUE
CD8 ET	*PHACTR4*	Wald ratio	1	0.0679	0.0180	1.07 (1.033, 1.109)	1.56E-04	0.004	4.64E-09	TRUE
CD8 ET	*PHPT1*	Wald ratio	1	-0.0569	0.0183	0.945 (0.911, 0.979)	0.002	0.025	1.93E-10	TRUE
CD8 ET	*PI4K2B*	Wald ratio	1	0.0047	0.0224	1.005 (0.962, 1.05)	0.833	0.93	1.21E-12	TRUE
CD8 ET	*PIGP*	Wald ratio	1	0.0050	0.0126	1.005 (0.98, 1.03)	0.692	0.861	1.46E-18	TRUE
CD8 ET	*PILRB*	Wald ratio	1	0.0040	0.0055	1.004 (0.993, 1.015)	0.465	0.725	2.81E-93	TRUE
CD8 ET	*PIP4K2A*	Wald ratio	1	-0.0358	0.0203	0.965 (0.927, 1.004)	0.078	0.292	4.13E-08	TRUE
CD8 ET	*PIWIL4*	Wald ratio	1	0.0401	0.0107	1.041 (1.019, 1.063)	1.87E-04	0.005	1.68E-17	TRUE
CD8 ET	*PLA2G16*	Wald ratio	1	0.0156	0.0178	1.016 (0.981, 1.052)	0.381	0.666	5.13E-10	TRUE
CD8 ET	*PLAGL1*	Wald ratio	1	-0.0275	0.0174	0.973 (0.94, 1.007)	0.115	0.362	6.14E-09	TRUE
CD8 ET	*PLCB2*	Wald ratio	1	-0.0525	0.0184	0.949 (0.915, 0.984)	0.004	0.044	4.28E-09	TRUE
CD8 ET	*PLCH2*	Wald ratio	1	-0.0154	0.0163	0.985 (0.954, 1.017)	0.345	0.638	8.80E-09	TRUE
CD8 ET	*PLEK*	Wald ratio	1	0.0094	0.0100	1.009 (0.99, 1.029)	0.351	0.644	1.56E-28	TRUE

CD8 ET	*PLGRKT*	Wald ratio	1	-0.0107	0.0156	0.989 (0.96, 1.02)	0.492	0.738	1.89E-12	TRUE
CD8 ET	*PLS1*	Wald ratio	1	-0.0003	0.0080	1 (0.984, 1.015)	0.967	0.989	8.14E-46	TRUE
CD8 ET	*PLTP*	Wald ratio	1	0.0356	0.0150	1.036 (1.006, 1.067)	0.017	0.116	4.51E-14	TRUE
CD8 ET	*PLXDC1*	Wald ratio	1	0.0067	0.0137	1.007 (0.98, 1.034)	0.624	0.822	5.05E-20	TRUE
CD8 ET	*PMEPA1*	Wald ratio	1	-0.0072	0.0130	0.993 (0.968, 1.018)	0.579	0.789	6.34E-18	TRUE
CD8 ET	*PNMT*	Wald ratio	1	0.0103	0.0101	1.01 (0.991, 1.031)	0.306	0.6	1.02E-28	TRUE
CD8 ET	*PNRC2*	Wald ratio	1	-0.0115	0.0087	0.989 (0.972, 1.005)	0.182	0.462	9.26E-39	TRUE
CD8 ET	*POLM*	Wald ratio	1	0.0080	0.0120	1.008 (0.985, 1.032)	0.507	0.745	2.07E-17	TRUE
CD8 ET	*POLR1D*	Wald ratio	1	0.0015	0.0106	1.002 (0.981, 1.023)	0.888	0.955	9.33E-34	TRUE
CD8 ET	*POLR2I*	Wald ratio	1	0.0284	0.0194	1.029 (0.99, 1.069)	0.144	0.411	6.43E-09	TRUE
CD8 ET	*POLR2J*	Wald ratio	1	-0.0449	0.0185	0.956 (0.922, 0.991)	0.015	0.106	1.39E-08	TRUE
CD8 ET	*POLR2J3*	Wald ratio	1	0.0284	0.0173	1.029 (0.994, 1.064)	0.101	0.337	6.31E-10	TRUE
CD8 ET	*POMC*	Wald ratio	1	-0.0246	0.0124	0.976 (0.952, 1)	0.047	0.213	7.07E-17	TRUE
CD8 ET	*POMZP3*	Wald ratio	1	0.0154	0.0113	1.016 (0.993, 1.038)	0.173	0.451	2.05E-28	TRUE
CD8 ET	*POU5F1*	IVW	2	-0.0011	0.0126	0.999 (0.975, 1.024)	0.93	0.97	2.92E-44	TRUE
CD8 ET	*PPA1*	Wald ratio	1	0.0034	0.0066	1.003 (0.991, 1.016)	0.603	0.806	4.08E-62	TRUE
CD8 ET	*PPDPF*	Wald ratio	1	-0.0061	0.0057	0.994 (0.983, 1.005)	0.283	0.575	4.18E-83	TRUE
CD8 ET	*PPFIBP2*	Wald ratio	1	-0.0105	0.0162	0.99 (0.959, 1.022)	0.517	0.752	5.37E-12	TRUE
CD8 ET	*PPIA*	Wald ratio	1	0.0116	0.0119	1.012 (0.988, 1.035)	0.33	0.624	4.57E-21	TRUE
CD8 ET	*PPIE*	Wald ratio	1	0.0320	0.0194	1.033 (0.994, 1.072)	0.098	0.333	1.51E-08	TRUE
CD8 ET	*PPIL3*	Wald ratio	1	0.0083	0.0097	1.008 (0.989, 1.028)	0.393	0.672	1.05E-29	TRUE
CD8 ET	*PPIL6*	Wald ratio	1	-0.0359	0.0247	0.965 (0.919, 1.013)	0.146	0.415	2.22E-12	TRUE
CD8 ET	*PPM1M*	Wald ratio	1	-0.0365	0.0145	0.964 (0.937, 0.992)	0.012	0.093	2.88E-14	TRUE
CD8 ET	*PPP1R14C*	Wald ratio	1	-0.0456	0.0205	0.955 (0.918, 0.995)	0.026	0.15	4.16E-08	TRUE
CD8 ET	*PPP5C*	Wald ratio	1	0.0143	0.0202	1.014 (0.975, 1.055)	0.479	0.733	1.26E-09	TRUE
CD8 ET	*PPT1*	Wald ratio	1	-0.0537	0.0184	0.948 (0.914, 0.982)	0.003	0.039	7.96E-09	TRUE
CD8 ET	*PPWD1*	Wald ratio	1	0.0236	0.0144	1.024 (0.995, 1.053)	0.102	0.338	3.78E-14	TRUE
CD8 ET	*PQLC3*	Wald ratio	1	0.0687	0.0250	1.071 (1.02, 1.125)	0.006	0.056	1.78E-11	TRUE
CD8 ET	*PRDX5*	Wald ratio	1	-0.0074	0.0090	0.993 (0.975, 1.01)	0.407	0.682	9.29E-41	TRUE
CD8 ET	*PRDX6*	Wald ratio	1	-0.0117	0.0118	0.988 (0.966, 1.012)	0.324	0.618	8.22E-20	TRUE
CD8 ET	*PRELID1*	Wald ratio	1	0.0723	0.0142	1.075 (1.045, 1.105)	3.52E-07	8.32E-05	5.36E-14	TRUE
CD8 ET	*PRKCB*	Wald ratio	1	0.0164	0.0134	1.017 (0.99, 1.044)	0.221	0.513	1.82E-21	TRUE
CD8 ET	*PRMT2*	IVW	2	0.0071	0.0048	1.007 (0.998, 1.017)	0.142	0.41	2.02E-142	TRUE
CD8 ET	*PROSC*	Wald ratio	1	-0.0191	0.0183	0.981 (0.947, 1.017)	0.298	0.593	8.49E-09	TRUE
CD8 ET	*PRR4*	Wald ratio	1	-0.0077	0.0241	0.992 (0.947, 1.04)	0.75	0.886	8.64E-10	TRUE
CD8 ET	*PRR7-AS1*	Wald ratio	1	0.0081	0.0072	1.008 (0.994, 1.022)	0.258	0.552	7.18E-56	TRUE
CD8 ET	*PRSS57*	Wald ratio	1	0.0035	0.0155	1.003 (0.973, 1.034)	0.823	0.926	1.36E-12	TRUE
CD8 ET	*PSD4*	Wald ratio	1	-0.0134	0.0144	0.987 (0.959, 1.015)	0.353	0.644	1.17E-14	TRUE
CD8 ET	*PSMB9*	Wald ratio	1	-0.0297	0.0200	0.971 (0.934, 1.009)	0.137	0.399	4.65E-09	TRUE
CD8 ET	*PSMD5-AS1*	Wald ratio	1	0.0039	0.0043	1.004 (0.996, 1.012)	0.361	0.65	1.52E-188	TRUE
CD8 ET	*PSPH*	Wald ratio	1	-0.0007	0.0118	0.999 (0.976, 1.023)	0.95	0.981	1.60E-16	TRUE
CD8 ET	*PSTPIP1*	Wald ratio	1	0.0039	0.0142	1.004 (0.976, 1.032)	0.786	0.907	4.28E-16	TRUE
CD8 ET	*PTAR1*	Wald ratio	1	0.0218	0.0201	1.022 (0.983, 1.063)	0.278	0.571	2.10E-08	TRUE
CD8 ET	*PTGDR*	Wald ratio	1	0.0247	0.0119	1.025 (1.001, 1.049)	0.038	0.188	4.80E-20	TRUE
CD8 ET	*PTGDS*	Wald ratio	1	0.0077	0.0130	1.008 (0.982, 1.034)	0.552	0.771	6.90E-17	TRUE
CD8 ET	*PTP4A3*	Wald ratio	1	0.0190	0.0106	1.019 (0.998, 1.041)	0.075	0.287	9.88E-27	TRUE
CD8 ET	*PTPLAD2*	Wald ratio	1	-0.0260	0.0183	0.974 (0.94, 1.01)	0.156	0.427	1.16E-09	TRUE
CD8 ET	*PTPN4*	Wald ratio	1	-0.0060	0.0179	0.994 (0.96, 1.029)	0.737	0.88	2.72E-09	TRUE
CD8 ET	*PTPRA*	Wald ratio	1	0.0175	0.0188	1.018 (0.981, 1.056)	0.352	0.644	2.92E-09	TRUE
CD8 ET	*PWWP2A*	Wald ratio	1	0.0042	0.0191	1.004 (0.967, 1.042)	0.825	0.926	9.83E-10	TRUE
CD8 ET	*PXT1*	Wald ratio	1	0.0432	0.0207	1.044 (1.003, 1.088)	0.037	0.185	6.74E-08	TRUE
CD8 ET	*PYHIN1*	Wald ratio	1	0.0039	0.0085	1.004 (0.987, 1.021)	0.65	0.835	2.40E-36	TRUE
CD8 ET	*QDPR*	Wald ratio	1	0.0408	0.0193	1.042 (1.003, 1.082)	0.034	0.175	1.13E-09	TRUE
CD8 ET	*R3HDM4*	Wald ratio	1	0.0004	0.0109	1 (0.979, 1.022)	0.973	0.989	2.03E-29	TRUE
CD8 ET	*RAB11A*	Wald ratio	1	-0.0077	0.0135	0.992 (0.967, 1.019)	0.569	0.782	5.58E-16	TRUE
CD8 ET	*RAB34*	Wald ratio	1	-0.0406	0.0211	0.96 (0.921, 1.001)	0.054	0.232	9.39E-08	TRUE
CD8 ET	*RAB6B*	Wald ratio	1	-0.0027	0.0167	0.997 (0.965, 1.03)	0.871	0.947	6.37E-10	TRUE
CD8 ET	*RABAC1*	Wald ratio	1	0.0294	0.0195	1.03 (0.991, 1.07)	0.132	0.393	2.16E-09	TRUE
CD8 ET	*RAC2*	Wald ratio	1	0.0101	0.0198	1.01 (0.972, 1.05)	0.61	0.81	3.48E-08	TRUE
CD8 ET	*RAD51C*	Wald ratio	1	0.0039	0.0150	1.004 (0.975, 1.034)	0.795	0.911	2.79E-12	TRUE
CD8 ET	*RALGDS*	Wald ratio	1	-0.0090	0.0189	0.991 (0.955, 1.028)	0.634	0.827	3.52E-09	TRUE
CD8 ET	*RAMP1*	Wald ratio	1	0.0119	0.0174	1.012 (0.978, 1.047)	0.495	0.74	1.65E-11	TRUE
CD8 ET	*RASA3*	Wald ratio	1	0.0269	0.0178	1.027 (0.992, 1.064)	0.131	0.392	3.93E-10	TRUE
CD8 ET	*RBAK-RBAKDN*	Wald ratio	1	-0.0118	0.0219	0.988 (0.947, 1.032)	0.589	0.796	9.64E-11	TRUE
CD8 ET	*RBL2*	Wald ratio	1	0.0005	0.0140	1.001 (0.973, 1.028)	0.971	0.989	1.14E-16	TRUE
CD8 ET	*RBM43*	Wald ratio	1	-0.0193	0.0082	0.981 (0.965, 0.997)	0.019	0.122	2.12E-42	TRUE
CD8 ET	*RBMS2*	Wald ratio	1	-0.0100	0.0119	0.99 (0.967, 1.013)	0.399	0.678	5.33E-21	TRUE
CD8 ET	*RCSD1*	Wald ratio	1	-0.0002	0.0139	1 (0.973, 1.027)	0.991	0.996	2.75E-16	TRUE
CD8 ET	*RDH13*	Wald ratio	1	-0.0256	0.0178	0.975 (0.941, 1.009)	0.149	0.418	2.87E-10	TRUE
CD8 ET	*RDH5*	Wald ratio	1	0.0113	0.0224	1.011 (0.968, 1.057)	0.614	0.814	1.86E-08	TRUE
CD8 ET	*REEP4*	Wald ratio	1	0.0216	0.0136	1.022 (0.995, 1.05)	0.113	0.359	2.79E-19	TRUE
CD8 ET	*REEP5*	Wald ratio	1	0.0605	0.0183	1.062 (1.025, 1.101)	9.24E-04	0.016	1.96E-09	TRUE
CD8 ET	*RGS10*	Wald ratio	1	0.0108	0.0326	1.011 (0.948, 1.077)	0.741	0.881	4.88E-11	TRUE
CD8 ET	*RGS17*	Wald ratio	1	0.0049	0.0150	1.005 (0.976, 1.035)	0.742	0.881	8.86E-14	TRUE
CD8 ET	*RHOH*	Wald ratio	1	0.0325	0.0188	1.033 (0.996, 1.072)	0.083	0.303	4.70E-09	TRUE
CD8 ET	*RNASEH1-AS1*	Wald ratio	1	0.0236	0.0190	1.024 (0.986, 1.063)	0.215	0.505	3.68E-08	TRUE
CD8 ET	*RNASEH2C*	Wald ratio	1	-0.0008	0.0147	0.999 (0.971, 1.028)	0.957	0.984	6.69E-16	TRUE
CD8 ET	*RNF168*	Wald ratio	1	-0.0206	0.0172	0.98 (0.947, 1.013)	0.23	0.524	5.48E-11	TRUE
CD8 ET	*ROBO3*	Wald ratio	1	0.0036	0.0189	1.004 (0.967, 1.041)	0.849	0.936	3.52E-09	TRUE

CD8 ET	*RP1-111D6.3*	Wald ratio	1	0.0097	0.0117	1.01 (0.987, 1.033)	0.407	0.682	9.81E-23	TRUE
CD8 ET	*RP1-244F24.1*	Wald ratio	1	-0.0437	0.0206	0.957 (0.919, 0.997)	0.034	0.175	5.75E-08	TRUE
CD8 ET	*RP1-29C18.10*	Wald ratio	1	-0.0140	0.0065	0.986 (0.974, 0.999)	0.03	0.164	4.11E-70	TRUE
CD8 ET	*RP1-3J17.3*	IVW	2	0.0021	0.0067	1.002 (0.989, 1.015)	0.748	0.885	1.39E-67	TRUE
CD8 ET	*RP1-40E16.12*	Wald ratio	1	-0.0163	0.0191	0.984 (0.948, 1.021)	0.396	0.675	4.97E-09	TRUE
CD8 ET	*RP1-55C23.7*	Wald ratio	1	0.0023	0.0064	1.002 (0.99, 1.015)	0.72	0.876	1.69E-71	TRUE
CD8 ET	*RP1-8B1.4*	Wald ratio	1	0.0025	0.0099	1.002 (0.983, 1.022)	0.803	0.915	9.31E-30	TRUE
CD8 ET	*RP11-119F19.2*	Wald ratio	1	-0.0335	0.0150	0.967 (0.939, 0.996)	0.025	0.146	2.98E-13	TRUE
CD8 ET	*RP11-137H2.4*	Wald ratio	1	-0.0303	0.0131	0.97 (0.946, 0.995)	0.021	0.13	2.05E-18	TRUE
CD8 ET	*RP11-138A9.1*	Wald ratio	1	0.0097	0.0214	1.01 (0.968, 1.053)	0.65	0.835	2.64E-16	TRUE
CD8 ET	*RP11-138A9.2*	Wald ratio	1	0.0023	0.0158	1.002 (0.972, 1.034)	0.882	0.952	6.92E-16	TRUE
CD8 ET	*RP11-166B2.5*	Wald ratio	1	0.0337	0.0122	1.034 (1.01, 1.059)	0.006	0.054	6.23E-17	TRUE
CD8 ET	*RP11-18H21.1*	Wald ratio	1	-0.0040	0.0114	0.996 (0.974, 1.018)	0.725	0.878	5.16E-26	TRUE
CD8 ET	*RP11-218M22.1*	Wald ratio	1	0.0305	0.0198	1.031 (0.992, 1.072)	0.124	0.379	6.33E-09	TRUE
CD8 ET	*RP11-222K16.2*	Wald ratio	1	-0.0269	0.0155	0.974 (0.944, 1.004)	0.083	0.305	5.14E-13	TRUE
CD8 ET	*RP11-23P13.6*	Wald ratio	1	-0.0169	0.0273	0.983 (0.932, 1.037)	0.535	0.761	1.27E-09	TRUE
CD8 ET	*RP11-245G13.2*	Wald ratio	1	-0.0181	0.0150	0.982 (0.954, 1.011)	0.228	0.521	1.49E-18	TRUE
CD8 ET	*RP11-279F6.3*	Wald ratio	1	-0.0192	0.0222	0.981 (0.939, 1.025)	0.388	0.669	7.11E-10	TRUE
CD8 ET	*RP11-294J22.6*	Wald ratio	1	-0.0510	0.0301	0.95 (0.896, 1.008)	0.09	0.318	1.27E-08	TRUE
CD8 ET	*RP11-298J20.3*	Wald ratio	1	0.0141	0.0053	1.014 (1.004, 1.025)	0.008	0.069	3.98E-101	TRUE
CD8 ET	*RP11-299J3.8*	Wald ratio	1	0.0046	0.0219	1.005 (0.962, 1.049)	0.834	0.93	2.00E-08	TRUE
CD8 ET	*RP11-305L7.1*	Wald ratio	1	-0.0159	0.0113	0.984 (0.963, 1.006)	0.16	0.433	7.57E-26	TRUE
CD8 ET	*RP11-305L7.3*	Wald ratio	1	0.0093	0.0111	1.009 (0.988, 1.031)	0.401	0.679	2.62E-24	TRUE
CD8 ET	*RP11-326C3.11*	IVW	2	-0.0052	0.0108	0.995 (0.974, 1.016)	0.63	0.824	4.08E-99	TRUE
CD8 ET	*RP11-334C17.5*	Wald ratio	1	0.0097	0.0146	1.01 (0.981, 1.039)	0.508	0.747	2.93E-14	TRUE
CD8 ET	*RP11-345J4.5*	Wald ratio	1	-0.0242	0.0108	0.976 (0.956, 0.997)	0.025	0.146	5.85E-26	TRUE
CD8 ET	*RP11-347P5.1*	Wald ratio	1	0.0033	0.0070	1.003 (0.99, 1.017)	0.631	0.825	2.40E-60	TRUE
CD8 ET	*RP11-367G6.3*	Wald ratio	1	0.0012	0.0147	1.001 (0.973, 1.03)	0.937	0.974	1.43E-13	TRUE
CD8 ET	*RP11-369E15.3*	Wald ratio	1	0.0050	0.0147	1.005 (0.976, 1.034)	0.735	0.88	3.60E-12	TRUE
CD8 ET	*RP11-382J12.1*	Wald ratio	1	-0.0008	0.0172	0.999 (0.966, 1.033)	0.961	0.987	9.99E-11	TRUE
CD8 ET	*RP11-383H13.1*	Wald ratio	1	-0.0041	0.0231	0.996 (0.952, 1.042)	0.859	0.94	1.45E-08	TRUE
CD8 ET	*RP11-430H10.1*	Wald ratio	1	-0.0278	0.0140	0.973 (0.946, 1)	0.047	0.214	3.50E-16	TRUE
CD8 ET	*RP11-434C1.1*	Wald ratio	1	-0.0008	0.0176	0.999 (0.965, 1.034)	0.964	0.988	4.58E-11	TRUE
CD8 ET	*RP11-439L18.1*	Wald ratio	1	0.0485	0.0206	1.05 (1.008, 1.093)	0.018	0.12	1.02E-09	TRUE
CD8 ET	*RP11-448A19.1*	Wald ratio	1	-0.0043	0.0151	0.996 (0.967, 1.026)	0.773	0.901	1.83E-13	TRUE
CD8 ET	*RP11-473I1.5*	Wald ratio	1	-0.0175	0.0081	0.983 (0.967, 0.998)	0.032	0.168	3.06E-37	TRUE
CD8 ET	*RP11-473M20.9*	Wald ratio	1	-0.0095	0.0097	0.991 (0.972, 1.01)	0.326	0.62	1.05E-31	TRUE
CD8 ET	*RP11-47L3.1*	Wald ratio	1	0.0034	0.0189	1.003 (0.967, 1.041)	0.856	0.939	3.27E-09	TRUE
CD8 ET	*RP11-485G4.2*	Wald ratio	1	0.0223	0.0221	1.023 (0.979, 1.068)	0.314	0.608	2.80E-08	TRUE
CD8 ET	*RP11-493L12.4*	Wald ratio	1	-0.0155	0.0164	0.985 (0.953, 1.017)	0.344	0.637	1.14E-13	TRUE
CD8 ET	*RP11-498D10.6*	Wald ratio	1	0.0356	0.0174	1.036 (1.002, 1.072)	0.041	0.196	3.37E-10	TRUE
CD8 ET	*RP11-499P20.2*	Wald ratio	1	-0.0360	0.0185	0.965 (0.93, 1)	0.052	0.226	2.87E-09	TRUE
CD8 ET	*RP11-509J21.2*	Wald ratio	1	0.0145	0.0178	1.015 (0.98, 1.051)	0.414	0.686	1.45E-11	TRUE
CD8 ET	*RP11-526I2.5*	Wald ratio	1	0.0173	0.0139	1.017 (0.99, 1.045)	0.212	0.499	2.04E-15	TRUE
CD8 ET	*RP11-532F6.3*	Wald ratio	1	-0.0357	0.0179	0.965 (0.932, 0.999)	0.046	0.211	7.54E-10	TRUE
CD8 ET	*RP11-538P18.2*	Wald ratio	1	0.0210	0.0143	1.021 (0.993, 1.05)	0.143	0.41	3.18E-11	TRUE
CD8 ET	*RP11-539L10.2*	Wald ratio	1	0.0060	0.0153	1.006 (0.976, 1.037)	0.695	0.863	2.73E-14	TRUE
CD8 ET	*RP11-539L10.3*	Wald ratio	1	-0.0172	0.0144	0.983 (0.956, 1.011)	0.233	0.527	2.71E-14	TRUE
CD8 ET	*RP11-571L19.8*	Wald ratio	1	-0.0260	0.0180	0.974 (0.941, 1.009)	0.148	0.417	2.45E-14	TRUE
CD8 ET	*RP11-586D19.2*	Wald ratio	1	0.0285	0.0192	1.029 (0.991, 1.068)	0.137	0.399	2.93E-12	TRUE
CD8 ET	*RP11-596D21.1*	Wald ratio	1	0.0255	0.0103	1.026 (1.005, 1.047)	0.013	0.098	4.65E-26	TRUE
CD8 ET	*RP11-611D20.2*	Wald ratio	1	-0.0005	0.0134	0.999 (0.974, 1.026)	0.97	0.989	5.79E-16	TRUE
CD8 ET	*RP11-629O1.2*	Wald ratio	1	0.0006	0.0140	1.001 (0.974, 1.028)	0.966	0.988	5.52E-16	TRUE
CD8 ET	*RP11-65I12.1*	Wald ratio	1	0.0131	0.0071	1.013 (0.999, 1.027)	0.067	0.265	6.39E-53	TRUE
CD8 ET	*RP11-660L16.2*	Wald ratio	1	-0.0072	0.0075	0.993 (0.978, 1.008)	0.342	0.635	9.53E-36	TRUE
CD8 ET	*RP11-669E14.6*	Wald ratio	1	-0.0535	0.0223	0.948 (0.907, 0.99)	0.016	0.111	1.59E-14	TRUE
CD8 ET	*RP11-69E11.4*	Wald ratio	1	0.0255	0.0121	1.026 (1.002, 1.05)	0.034	0.175	1.35E-18	TRUE
CD8 ET	*RP11-755F10.1*	Wald ratio	1	-0.0437	0.0177	0.957 (0.925, 0.991)	0.014	0.099	4.53E-11	TRUE
CD8 ET	*RP11-77H9.2*	Wald ratio	1	-0.0018	0.0099	0.998 (0.979, 1.018)	0.857	0.939	6.90E-30	TRUE
CD8 ET	*RP11-792A8.4*	Wald ratio	1	0.0096	0.0139	1.01 (0.983, 1.037)	0.487	0.738	5.84E-12	TRUE
CD8 ET	*RP11-81H14.2*	Wald ratio	1	-0.0023	0.0061	0.998 (0.986, 1.01)	0.705	0.867	3.59E-88	TRUE
CD8 ET	*RP11-841O20.2*	Wald ratio	1	-0.0087	0.0167	0.991 (0.959, 1.024)	0.6	0.804	3.49E-09	TRUE
CD8 ET	*RP11-950C14.3*	Wald ratio	1	-0.0224	0.0131	0.978 (0.953, 1.003)	0.086	0.312	1.62E-17	TRUE
CD8 ET	*RP11-96H19.1*	Wald ratio	1	0.0082	0.0101	1.008 (0.988, 1.028)	0.416	0.688	1.80E-28	TRUE
CD8 ET	*RP11-973H7.1*	Wald ratio	1	0.0229	0.0129	1.023 (0.998, 1.049)	0.076	0.289	2.25E-17	TRUE
CD8 ET	*RP11-973H7.4*	Wald ratio	1	0.0136	0.0165	1.014 (0.981, 1.047)	0.41	0.685	2.11E-11	TRUE
CD8 ET	*RP11-98G7.1*	Wald ratio	1	0.0300	0.0173	1.03 (0.996, 1.066)	0.082	0.301	4.41E-10	TRUE
CD8 ET	*RP3-325F22.5*	Wald ratio	1	0.0040	0.0141	1.004 (0.977, 1.032)	0.774	0.901	4.23E-18	TRUE
CD8 ET	*RP3-466P17.1*	Wald ratio	1	0.0121	0.0148	1.012 (0.983, 1.042)	0.414	0.686	2.68E-14	TRUE
CD8 ET	*RP3-510H16.3*	Wald ratio	1	-0.0204	0.0248	0.98 (0.933, 1.029)	0.412	0.686	5.58E-08	TRUE
CD8 ET	*RP4-539M6.22*	Wald ratio	1	-0.0064	0.0153	0.994 (0.964, 1.024)	0.677	0.853	3.00E-13	TRUE
CD8 ET	*RP4-745E8.2*	Wald ratio	1	0.0114	0.0127	1.011 (0.987, 1.037)	0.37	0.659	6.18E-19	TRUE
CD8 ET	*RP4-758J18.13*	Wald ratio	1	0.0087	0.0119	1.009 (0.986, 1.032)	0.463	0.723	9.10E-20	TRUE
CD8 ET	*RP4-799D16.1*	Wald ratio	1	0.0623	0.0176	1.064 (1.028, 1.102)	3.88E-04	0.009	1.99E-09	TRUE
CD8 ET	*RP5-1021I20.1*	Wald ratio	1	-0.0401	0.0128	0.961 (0.937, 0.985)	0.002	0.024	4.69E-19	TRUE
CD8 ET	*RP5-1042K10.10*	Wald ratio	1	-0.0265	0.0158	0.974 (0.944, 1.005)	0.094	0.326	5.69E-11	TRUE
CD8 ET	*RP5-827C21.4*	Wald ratio	1	-0.0049	0.0174	0.995 (0.962, 1.03)	0.78	0.904	2.52E-15	TRUE
CD8 ET	*RP6-109B7.3*	Wald ratio	1	-0.0097	0.0201	0.99 (0.952, 1.03)	0.63	0.824	2.50E-12	TRUE
CD8 ET	*RPA2*	Wald ratio	1	0.0176	0.0131	1.018 (0.992, 1.044)	0.18	0.461	2.59E-17	TRUE

CD8 ET	*RPL10A*	Wald ratio	1	-0.0087	0.0066	0.991 (0.979, 1.004)	0.187	0.466	2.82E-71	TRUE
CD8 ET	*RPL11*	Wald ratio	1	-0.0116	0.0074	0.988 (0.974, 1.003)	0.119	0.369	1.16E-51	TRUE
CD8 ET	*RPL12*	Wald ratio	1	-0.0180	0.0053	0.982 (0.972, 0.992)	7.26E-04	0.013	1.95E-120	TRUE
CD8 ET	*RPL13*	Wald ratio	1	-0.0254	0.0124	0.975 (0.951, 0.999)	0.04	0.195	1.21E-34	TRUE
CD8 ET	*RPL14*	Wald ratio	1	0.0188	0.0168	1.019 (0.986, 1.053)	0.264	0.558	2.20E-13	TRUE
CD8 ET	*RPL22L1*	Wald ratio	1	-0.0067	0.0146	0.993 (0.965, 1.022)	0.646	0.834	2.04E-17	TRUE
CD8 ET	*RPL23A*	Wald ratio	1	-0.0180	0.0097	0.982 (0.964, 1.001)	0.064	0.258	4.44E-32	TRUE
CD8 ET	*RPL27A*	Wald ratio	1	0.0126	0.0150	1.013 (0.983, 1.043)	0.4	0.678	1.37E-12	TRUE
CD8 ET	*RPL28*	Wald ratio	1	0.0033	0.0043	1.003 (0.995, 1.012)	0.438	0.703	4.14E-186	TRUE
CD8 ET	*RPL3*	Wald ratio	1	0.0159	0.0170	1.016 (0.983, 1.05)	0.35	0.643	4.61E-11	TRUE
CD8 ET	*RPL32*	Wald ratio	1	0.0227	0.0135	1.023 (0.996, 1.05)	0.093	0.323	1.39E-17	TRUE
CD8 ET	*RPL36AL*	Wald ratio	1	-0.0046	0.0055	0.995 (0.985, 1.006)	0.405	0.681	3.08E-105	TRUE
CD8 ET	*RPL8*	Wald ratio	1	-0.0050	0.0048	0.995 (0.986, 1.004)	0.303	0.598	2.71E-138	TRUE
CD8 ET	*RPL9*	Wald ratio	1	-0.0203	0.0129	0.98 (0.955, 1.005)	0.117	0.365	9.27E-17	TRUE
CD8 ET	*RPLP2*	Wald ratio	1	-0.0396	0.0146	0.961 (0.934, 0.989)	0.007	0.062	2.50E-14	TRUE
CD8 ET	*RPPH1*	Wald ratio	1	0.0141	0.0178	1.014 (0.979, 1.05)	0.43	0.7	3.56E-08	TRUE
CD8 ET	*RPS10*	Wald ratio	1	-0.0055	0.0054	0.995 (0.984, 1.005)	0.305	0.599	1.08E-88	TRUE
CD8 ET	*RPS11*	Wald ratio	1	0.0337	0.0141	1.034 (1.006, 1.063)	0.017	0.115	1.91E-16	TRUE
CD8 ET	*RPS12*	Wald ratio	1	0.0201	0.0139	1.02 (0.993, 1.049)	0.148	0.417	1.64E-10	TRUE
CD8 ET	*RPS14*	Wald ratio	1	-0.0175	0.0158	0.983 (0.953, 1.014)	0.267	0.561	5.66E-12	TRUE
CD8 ET	*RPS16*	Wald ratio	1	-0.0114	0.0111	0.989 (0.967, 1.01)	0.304	0.598	1.47E-22	TRUE
CD8 ET	*RPS18*	Wald ratio	1	-0.0120	0.0070	0.988 (0.974, 1.002)	0.087	0.312	9.19E-74	TRUE
CD8 ET	*RPS19*	Wald ratio	1	0.0165	0.0149	1.017 (0.987, 1.047)	0.269	0.563	8.22E-14	TRUE
CD8 ET	*RPS2*	Wald ratio	1	-0.0268	0.0100	0.974 (0.955, 0.993)	0.008	0.067	2.97E-30	TRUE
CD8 ET	*RPS23*	Wald ratio	1	-0.0175	0.0124	0.983 (0.959, 1.007)	0.158	0.43	1.51E-21	TRUE
CD8 ET	*RPS24*	Wald ratio	1	-0.0025	0.0137	0.998 (0.971, 1.025)	0.855	0.938	1.97E-16	TRUE
CD8 ET	*RPS25*	Wald ratio	1	-0.0009	0.0077	0.999 (0.984, 1.014)	0.908	0.961	2.10E-47	TRUE
CD8 ET	*RPS26*	Wald ratio	1	-0.0099	0.0043	0.99 (0.982, 0.999)	0.022	0.132	5.27E-194	TRUE
CD8 ET	*RPS3*	Wald ratio	1	-0.0171	0.0096	0.983 (0.965, 1.002)	0.074	0.286	4.56E-19	TRUE
CD8 ET	*RPS3A*	Wald ratio	1	-0.0042	0.0062	0.996 (0.984, 1.008)	0.492	0.738	2.86E-37	TRUE
CD8 ET	*RPS5*	Wald ratio	1	0.0069	0.0051	1.007 (0.997, 1.017)	0.177	0.456	1.45E-129	TRUE
CD8 ET	*RPS6*	Wald ratio	1	-0.0195	0.0167	0.981 (0.949, 1.013)	0.242	0.535	1.22E-14	TRUE
CD8 ET	*RPS7*	Wald ratio	1	0.0031	0.0100	1.003 (0.984, 1.023)	0.754	0.888	1.80E-45	TRUE
CD8 ET	*RPS8*	Wald ratio	1	0.0018	0.0069	1.002 (0.988, 1.015)	0.791	0.909	7.12E-63	TRUE
CD8 ET	*RPS9*	Wald ratio	1	0.0213	0.0056	1.022 (1.01, 1.033)	1.26E-04	0.003	2.34E-96	TRUE
CD8 ET	*RPSA*	Wald ratio	1	-0.0189	0.0092	0.981 (0.964, 0.999)	0.039	0.192	3.74E-45	TRUE
CD8 ET	*RRAS2*	Wald ratio	1	-0.0180	0.0110	0.982 (0.961, 1.004)	0.102	0.338	2.30E-20	TRUE
CD8 ET	*RRP7A*	Wald ratio	1	-0.0158	0.0170	0.984 (0.952, 1.018)	0.354	0.645	1.18E-15	TRUE
CD8 ET	*RSRC2*	Wald ratio	1	-0.0082	0.0142	0.992 (0.965, 1.02)	0.564	0.78	3.16E-18	TRUE
CD8 ET	*RTKN2*	Wald ratio	1	0.0238	0.0161	1.024 (0.992, 1.057)	0.138	0.4	5.68E-15	TRUE
CD8 ET	*RUNX2*	Wald ratio	1	0.0442	0.0180	1.045 (1.009, 1.083)	0.014	0.102	4.45E-10	TRUE
CD8 ET	*S100B*	Wald ratio	1	0.0094	0.0080	1.009 (0.994, 1.025)	0.239	0.533	2.17E-47	TRUE
CD8 ET	*SACM1L*	Wald ratio	1	-0.0097	0.0103	0.99 (0.971, 1.011)	0.348	0.641	1.86E-25	TRUE
CD8 ET	*SAMD12*	Wald ratio	1	-0.0129	0.0247	0.987 (0.941, 1.036)	0.603	0.806	3.51E-08	TRUE
CD8 ET	*SAMD3*	Wald ratio	1	-0.0052	0.0157	0.995 (0.965, 1.026)	0.739	0.881	7.92E-13	TRUE
CD8 ET	*SAMHD1*	Wald ratio	1	-0.0206	0.0185	0.98 (0.945, 1.016)	0.267	0.561	1.57E-08	TRUE
CD8 ET	*SAMM50*	Wald ratio	1	-0.0053	0.0133	0.995 (0.969, 1.021)	0.687	0.858	8.03E-12	TRUE
CD8 ET	*SAMSN1*	IVW	2	0.0006	0.0111	1.001 (0.979, 1.023)	0.959	0.985	1.61E-24	TRUE
CD8 ET	*SAR1A*	Wald ratio	1	0.0075	0.0108	1.007 (0.986, 1.029)	0.49	0.738	3.05E-24	TRUE
CD8 ET	*SAT2*	Wald ratio	1	-0.0805	0.0152	0.923 (0.896, 0.95)	1.12E-07	6.60E-05	5.78E-13	TRUE
CD8 ET	*SC5D*	Wald ratio	1	-0.0482	0.0231	0.953 (0.911, 0.997)	0.037	0.182	1.12E-09	TRUE
CD8 ET	*SCAMP3*	Wald ratio	1	0.0042	0.0134	1.004 (0.978, 1.031)	0.755	0.888	2.94E-17	TRUE
CD8 ET	*SCCPDH*	Wald ratio	1	0.0030	0.0153	1.003 (0.973, 1.034)	0.844	0.935	1.10E-12	TRUE
CD8 ET	*SCD5*	Wald ratio	1	-0.0541	0.0124	0.947 (0.925, 0.971)	1.30E-05	6.87E-04	3.55E-19	TRUE
CD8 ET	*SCGB3A1*	Wald ratio	1	0.0067	0.0060	1.007 (0.995, 1.019)	0.261	0.556	4.33E-87	TRUE
CD8 ET	*SCRN2*	Wald ratio	1	-0.0433	0.0141	0.958 (0.931, 0.984)	0.002	0.028	7.91E-15	TRUE
CD8 ET	*SEC61B*	Wald ratio	1	-0.0193	0.0160	0.981 (0.951, 1.012)	0.228	0.521	2.73E-17	TRUE
CD8 ET	*SEPT2*	Wald ratio	1	0.0092	0.0111	1.009 (0.987, 1.031)	0.409	0.685	6.33E-29	TRUE
CD8 ET	*SERAC1*	Wald ratio	1	-0.0275	0.0210	0.973 (0.934, 1.014)	0.189	0.47	6.97E-08	TRUE
CD8 ET	*SERPINB6*	Wald ratio	1	0.0138	0.0139	1.014 (0.987, 1.042)	0.323	0.618	1.14E-17	TRUE
CD8 ET	*SESTD1*	Wald ratio	1	0.0056	0.0149	1.006 (0.977, 1.035)	0.706	0.868	5.44E-14	TRUE
CD8 ET	*SETD5-AS1*	Wald ratio	1	-0.0061	0.0200	0.994 (0.956, 1.034)	0.76	0.892	4.55E-08	TRUE
CD8 ET	*SETD9*	Wald ratio	1	-0.0359	0.0129	0.965 (0.941, 0.989)	0.005	0.053	9.05E-16	TRUE
CD8 ET	*SF1*	Wald ratio	1	-0.0240	0.0112	0.976 (0.955, 0.998)	0.032	0.17	4.39E-18	TRUE
CD8 ET	*SFT2D1*	Wald ratio	1	0.0359	0.0147	1.037 (1.007, 1.067)	0.014	0.103	5.92E-13	TRUE
CD8 ET	*SFXN4*	Wald ratio	1	-0.0313	0.0176	0.969 (0.936, 1.003)	0.076	0.288	2.65E-10	TRUE
CD8 ET	*SGCD*	Wald ratio	1	0.0094	0.0162	1.009 (0.978, 1.042)	0.565	0.78	3.68E-11	TRUE
CD8 ET	*SH3GLB1*	Wald ratio	1	-0.0008	0.0208	0.999 (0.959, 1.041)	0.97	0.989	8.30E-09	TRUE
CD8 ET	*SH3TC1*	Wald ratio	1	-0.0058	0.0119	0.994 (0.971, 1.018)	0.626	0.822	2.74E-21	TRUE
CD8 ET	*SH3YL1*	Wald ratio	1	0.0578	0.0132	1.06 (1.033, 1.087)	1.11E-05	6.26E-04	1.49E-16	TRUE
CD8 ET	*SHFM1*	Wald ratio	1	0.0005	0.0136	1.001 (0.974, 1.028)	0.969	0.989	4.21E-13	TRUE
CD8 ET	*SHMT1*	Wald ratio	1	0.0426	0.0205	1.043 (1.002, 1.086)	0.038	0.187	5.11E-08	TRUE
CD8 ET	*SIDT1-AS1*	Wald ratio	1	-0.0086	0.0093	0.991 (0.973, 1.01)	0.356	0.647	1.16E-39	TRUE
CD8 ET	*SIRPA*	Wald ratio	1	0.0076	0.0143	1.008 (0.98, 1.036)	0.593	0.798	1.62E-18	TRUE
CD8 ET	*SIRPB1*	Wald ratio	1	0.0044	0.0165	1.004 (0.973, 1.037)	0.788	0.909	1.46E-13	TRUE
CD8 ET	*SKAP2*	Wald ratio	1	-0.0098	0.0119	0.99 (0.967, 1.014)	0.412	0.686	1.11E-24	TRUE
CD8 ET	*SLC12A7*	Wald ratio	1	-0.0106	0.0097	0.989 (0.971, 1.008)	0.275	0.568	8.13E-32	TRUE
CD8 ET	*SLC14A1*	Wald ratio	1	-0.0064	0.0164	0.994 (0.962, 1.026)	0.697	0.864	9.18E-12	TRUE
CD8 ET	*SLC15A4*	Wald ratio	1	0.0261	0.0182	1.026 (0.99, 1.064)	0.153	0.423	2.23E-09	TRUE

CD8 ET	*SLC16A4*	Wald ratio	1	0.0313	0.0160	1.032 (1, 1.065)	0.05	0.222	6.20E-12	TRUE
CD8 ET	*SLC22A18*	Wald ratio	1	0.0365	0.0167	1.037 (1.004, 1.072)	0.029	0.159	1.39E-12	TRUE
CD8 ET	*SLC25A1*	Wald ratio	1	-0.0100	0.0106	0.99 (0.97, 1.011)	0.342	0.635	3.79E-27	TRUE
CD8 ET	*SLC25A24*	Wald ratio	1	-0.0326	0.0197	0.968 (0.931, 1.006)	0.098	0.333	6.81E-08	TRUE
CD8 ET	*SLC25A26*	Wald ratio	1	0.0011	0.0203	1.001 (0.962, 1.042)	0.956	0.984	2.27E-08	TRUE
CD8 ET	*SLC25A29*	Wald ratio	1	-0.0039	0.0054	0.996 (0.986, 1.007)	0.473	0.73	1.29E-101	TRUE
CD8 ET	*SLC25A51*	Wald ratio	1	-0.0432	0.0180	0.958 (0.924, 0.992)	0.016	0.112	3.52E-08	TRUE
CD8 ET	*SLC38A1*	Wald ratio	1	-0.0275	0.0195	0.973 (0.936, 1.011)	0.158	0.429	9.81E-09	TRUE
CD8 ET	*SLC44A2*	Wald ratio	1	0.0133	0.0178	1.013 (0.979, 1.049)	0.454	0.717	1.12E-11	TRUE
CD8 ET	*SLC44A4*	Wald ratio	1	-0.0296	0.0209	0.971 (0.932, 1.011)	0.156	0.427	1.94E-09	TRUE
CD8 ET	*SLFN5*	Wald ratio	1	-0.0081	0.0136	0.992 (0.966, 1.019)	0.55	0.769	1.95E-16	TRUE
CD8 ET	*SMDT1*	Wald ratio	1	0.0081	0.0043	1.008 (1, 1.017)	0.057	0.239	7.95E-167	TRUE
CD8 ET	*SMIM19*	Wald ratio	1	0.0082	0.0111	1.008 (0.987, 1.03)	0.46	0.721	1.62E-24	TRUE
CD8 ET	*SMIM20*	Wald ratio	1	0.0244	0.0158	1.025 (0.993, 1.057)	0.123	0.377	1.30E-11	TRUE
CD8 ET	*SNAP29*	Wald ratio	1	0.0357	0.0157	1.036 (1.005, 1.069)	0.023	0.138	7.29E-16	TRUE
CD8 ET	*SNHG7*	Wald ratio	1	-0.0174	0.0108	0.983 (0.962, 1.004)	0.107	0.348	8.19E-38	TRUE
CD8 ET	*SNHG8*	Wald ratio	1	-0.0019	0.0056	0.998 (0.987, 1.009)	0.738	0.88	1.58E-112	TRUE
CD8 ET	*SNORD3B-2*	Wald ratio	1	-0.0116	0.0162	0.988 (0.958, 1.02)	0.473	0.73	1.99E-17	TRUE
CD8 ET	*SNRNP25*	Wald ratio	1	0.0295	0.0162	1.03 (0.998, 1.063)	0.069	0.271	2.20E-10	TRUE
CD8 ET	*SNRPC*	Wald ratio	1	-0.0696	0.0200	0.933 (0.897, 0.97)	4.93E-04	0.01	8.59E-08	TRUE
CD8 ET	*SNRPD2*	Wald ratio	1	-0.0276	0.0085	0.973 (0.957, 0.989)	0.001	0.02	4.36E-38	TRUE
CD8 ET	*SNRPF*	Wald ratio	1	-0.0222	0.0196	0.978 (0.941, 1.016)	0.259	0.553	1.95E-08	TRUE
CD8 ET	*SNX11*	Wald ratio	1	0.0156	0.0230	1.016 (0.971, 1.062)	0.498	0.741	1.02E-09	TRUE
CD8 ET	*SOD2*	Wald ratio	1	0.0088	0.0202	1.009 (0.97, 1.049)	0.664	0.846	5.53E-08	TRUE
CD8 ET	*SP110*	Wald ratio	1	-0.0240	0.0180	0.976 (0.942, 1.011)	0.182	0.462	2.69E-09	TRUE
CD8 ET	*SPATA20*	Wald ratio	1	0.0211	0.0116	1.021 (0.998, 1.045)	0.069	0.27	1.04E-22	TRUE
CD8 ET	*SPATC1L*	Wald ratio	1	0.0017	0.0099	1.002 (0.982, 1.021)	0.864	0.943	2.88E-41	TRUE
CD8 ET	*SPECC1*	Wald ratio	1	0.0239	0.0158	1.024 (0.993, 1.056)	0.131	0.392	2.85E-13	TRUE
CD8 ET	*SPG21*	Wald ratio	1	-0.0280	0.0199	0.972 (0.935, 1.011)	0.16	0.433	1.37E-08	TRUE
CD8 ET	*SPNS1*	Wald ratio	1	0.0296	0.0158	1.03 (0.999, 1.063)	0.062	0.253	2.43E-11	TRUE
CD8 ET	*SPON2*	Wald ratio	1	-0.0051	0.0186	0.995 (0.959, 1.032)	0.782	0.905	6.03E-09	TRUE
CD8 ET	*SPRYD4*	Wald ratio	1	0.0473	0.0209	1.048 (1.006, 1.092)	0.024	0.142	4.32E-08	TRUE
CD8 ET	*SPSB1*	Wald ratio	1	0.0291	0.0179	1.03 (0.994, 1.066)	0.104	0.342	1.64E-09	TRUE
CD8 ET	*SPSB2*	Wald ratio	1	-0.0015	0.0104	0.998 (0.978, 1.019)	0.885	0.954	2.46E-30	TRUE
CD8 ET	*SPTBN1*	Wald ratio	1	-0.0197	0.0182	0.98 (0.946, 1.016)	0.278	0.571	7.02E-12	TRUE
CD8 ET	*SQSTM1*	Wald ratio	1	-0.0338	0.0183	0.967 (0.933, 1.002)	0.065	0.262	1.34E-09	TRUE
CD8 ET	*SRA1*	Wald ratio	1	-0.0595	0.0211	0.942 (0.904, 0.982)	0.005	0.049	9.09E-08	TRUE
CD8 ET	*SRD5A3*	Wald ratio	1	-0.0279	0.0127	0.973 (0.949, 0.997)	0.028	0.157	6.76E-20	TRUE
CD8 ET	*SRSF10*	Wald ratio	1	-0.0112	0.0097	0.989 (0.97, 1.008)	0.248	0.541	7.41E-31	TRUE
CD8 ET	*ST6GALNAC6*	Wald ratio	1	0.0100	0.0147	1.01 (0.981, 1.04)	0.498	0.741	2.47E-13	TRUE
CD8 ET	*STARD3NL*	Wald ratio	1	0.0049	0.0199	1.005 (0.967, 1.045)	0.804	0.916	3.23E-10	TRUE
CD8 ET	*STK32C*	Wald ratio	1	-0.0063	0.0178	0.994 (0.96, 1.029)	0.723	0.877	3.38E-09	TRUE
CD8 ET	*STK4*	Wald ratio	1	-0.0043	0.0166	0.996 (0.964, 1.029)	0.793	0.91	6.11E-11	TRUE
CD8 ET	*STMN3*	Wald ratio	1	0.0203	0.0104	1.021 (1, 1.041)	0.05	0.222	5.02E-27	TRUE
CD8 ET	*STX8*	IVW	2	0.0409	0.0187	1.042 (1.004, 1.081)	0.029	0.158	6.06E-16	TRUE
CD8 ET	*STXBP2*	Wald ratio	1	-0.0183	0.0158	0.982 (0.952, 1.013)	0.246	0.54	8.54E-12	TRUE
CD8 ET	*SULF2*	Wald ratio	1	0.0061	0.0062	1.006 (0.994, 1.019)	0.324	0.618	8.23E-75	TRUE
CD8 ET	*SURF1*	Wald ratio	1	-0.0233	0.0116	0.977 (0.955, 0.999)	0.045	0.208	2.97E-22	TRUE
CD8 ET	*SVIP*	Wald ratio	1	-0.0169	0.0178	0.983 (0.95, 1.018)	0.343	0.636	4.06E-10	TRUE
CD8 ET	*SYNE1*	Wald ratio	1	-0.0141	0.0137	0.986 (0.96, 1.013)	0.302	0.597	2.07E-14	TRUE
CD8 ET	*SYNGR1*	Wald ratio	1	-0.0122	0.0063	0.988 (0.976, 1)	0.054	0.233	1.22E-67	TRUE
CD8 ET	*SYNM*	Wald ratio	1	0.0061	0.0130	1.006 (0.981, 1.032)	0.637	0.829	2.30E-18	TRUE
CD8 ET	*TAGLN2*	Wald ratio	1	0.0032	0.0130	1.003 (0.978, 1.029)	0.804	0.915	9.39E-18	TRUE
CD8 ET	*TAMM41*	Wald ratio	1	0.0140	0.0199	1.014 (0.975, 1.054)	0.483	0.736	2.54E-08	TRUE
CD8 ET	*TAPBP*	Wald ratio	1	0.0121	0.0172	1.012 (0.979, 1.047)	0.482	0.735	8.40E-10	TRUE
CD8 ET	*TAPBPL*	Wald ratio	1	0.0023	0.0087	1.002 (0.985, 1.02)	0.791	0.909	2.12E-38	TRUE
CD8 ET	*TBCA*	Wald ratio	1	0.0066	0.0079	1.007 (0.991, 1.022)	0.399	0.678	5.19E-48	TRUE
CD8 ET	*TC2N*	Wald ratio	1	0.0009	0.0117	1.001 (0.978, 1.024)	0.938	0.975	2.99E-25	TRUE
CD8 ET	*TCF25*	Wald ratio	1	0.0199	0.0178	1.02 (0.985, 1.056)	0.263	0.558	2.03E-16	TRUE
CD8 ET	*TECR*	Wald ratio	1	0.0122	0.0144	1.012 (0.984, 1.041)	0.398	0.678	1.01E-14	TRUE
CD8 ET	*TEN1*	Wald ratio	1	-0.0111	0.0150	0.989 (0.96, 1.018)	0.458	0.719	2.46E-17	TRUE
CD8 ET	*TEPP*	Wald ratio	1	-0.0348	0.0148	0.966 (0.938, 0.994)	0.019	0.122	1.98E-13	TRUE
CD8 ET	*TESC*	Wald ratio	1	-0.0623	0.0201	0.94 (0.903, 0.977)	0.002	0.025	8.78E-09	TRUE
CD8 ET	*TESPA1*	Wald ratio	1	0.0313	0.0183	1.032 (0.996, 1.069)	0.086	0.312	5.49E-11	TRUE
CD8 ET	*TEX12*	Wald ratio	1	0.0161	0.0127	1.016 (0.991, 1.042)	0.205	0.493	1.50E-08	TRUE
CD8 ET	*TFPT*	Wald ratio	1	-0.0080	0.0154	0.992 (0.962, 1.022)	0.604	0.806	1.16E-14	TRUE
CD8 ET	*THAP7-AS1*	Wald ratio	1	0.0272	0.0127	1.028 (1.002, 1.054)	0.033	0.171	1.66E-16	TRUE
CD8 ET	*THAP9-AS1*	Wald ratio	1	0.0538	0.0123	1.055 (1.03, 1.081)	1.30E-05	6.87E-04	2.82E-19	TRUE
CD8 ET	*THEM4*	Wald ratio	1	0.0538	0.0164	1.055 (1.022, 1.09)	0.001	0.017	1.70E-10	TRUE
CD8 ET	*THEMIS2*	Wald ratio	1	0.0053	0.0055	1.005 (0.994, 1.016)	0.337	0.629	3.26E-99	TRUE
CD8 ET	*TIFA*	Wald ratio	1	0.0197	0.0174	1.02 (0.986, 1.055)	0.256	0.549	1.61E-10	TRUE
CD8 ET	*TIMM10*	Wald ratio	1	-0.0011	0.0073	0.999 (0.985, 1.013)	0.875	0.948	1.19E-67	TRUE
CD8 ET	*TIPARP-AS1*	Wald ratio	1	0.0401	0.0165	1.041 (1.008, 1.075)	0.015	0.105	1.56E-11	TRUE
CD8 ET	*TLE4*	Wald ratio	1	-0.0089	0.0128	0.991 (0.967, 1.016)	0.486	0.737	3.59E-19	TRUE
CD8 ET	*TLK1*	Wald ratio	1	0.0113	0.0147	1.011 (0.983, 1.041)	0.443	0.708	1.36E-15	TRUE
CD8 ET	*TMA7*	Wald ratio	1	-0.0065	0.0198	0.993 (0.956, 1.033)	0.741	0.881	1.21E-08	TRUE
CD8 ET	*TMC4*	Wald ratio	1	-0.0130	0.0110	0.987 (0.966, 1.009)	0.238	0.532	3.40E-26	TRUE
CD8 ET	*TMCO3*	Wald ratio	1	-0.0306	0.0182	0.97 (0.936, 1.005)	0.093	0.323	7.70E-11	TRUE
CD8 ET	*TMED9*	Wald ratio	1	0.0020	0.0157	1.002 (0.972, 1.033)	0.9	0.958	1.21E-12	TRUE

CD8 ET	*TMEM101*	Wald ratio	1	-0.0465	0.0320	0.955 (0.897, 1.016)	0.146	0.415	1.78E-08	TRUE
CD8 ET	*TMEM120B*	Wald ratio	1	-0.0119	0.0109	0.988 (0.967, 1.01)	0.275	0.568	1.09E-33	TRUE
CD8 ET	*TMEM121*	Wald ratio	1	0.0037	0.0126	1.004 (0.979, 1.029)	0.766	0.896	4.25E-17	TRUE
CD8 ET	*TMEM156*	Wald ratio	1	0.0035	0.0087	1.004 (0.987, 1.021)	0.684	0.856	3.41E-38	TRUE
CD8 ET	*TMEM18*	Wald ratio	1	0.0038	0.0171	1.004 (0.971, 1.038)	0.824	0.926	3.78E-11	TRUE
CD8 ET	*TMEM2*	Wald ratio	1	0.0115	0.0162	1.012 (0.98, 1.044)	0.477	0.732	1.38E-11	TRUE
CD8 ET	*TMEM204*	Wald ratio	1	0.0078	0.0110	1.008 (0.986, 1.03)	0.481	0.734	1.76E-44	TRUE
CD8 ET	*TMEM230*	Wald ratio	1	0.0062	0.0111	1.006 (0.985, 1.028)	0.575	0.786	2.12E-24	TRUE
CD8 ET	*TMEM258*	Wald ratio	1	0.0156	0.0152	1.016 (0.986, 1.046)	0.305	0.599	3.50E-12	TRUE
CD8 ET	*TMEM50A*	Wald ratio	1	0.0356	0.0134	1.036 (1.009, 1.064)	0.008	0.067	1.74E-17	TRUE
CD8 ET	*TMEM55A*	Wald ratio	1	0.0015	0.0182	1.002 (0.966, 1.038)	0.934	0.972	2.58E-09	TRUE
CD8 ET	*TMEM80*	Wald ratio	1	-0.0343	0.0135	0.966 (0.941, 0.992)	0.011	0.086	4.44E-16	TRUE
CD8 ET	*TMOD1*	Wald ratio	1	0.0114	0.0199	1.011 (0.973, 1.052)	0.567	0.782	1.89E-08	TRUE
CD8 ET	*TMTC4*	Wald ratio	1	-0.0238	0.0170	0.977 (0.944, 1.01)	0.163	0.437	1.07E-09	TRUE
CD8 ET	*TNFRSF14*	Wald ratio	1	0.0208	0.0166	1.021 (0.988, 1.055)	0.21	0.497	2.44E-11	TRUE
CD8 ET	*TNRC18*	Wald ratio	1	0.0155	0.0194	1.016 (0.978, 1.055)	0.425	0.695	3.92E-09	TRUE
CD8 ET	*TOMM7*	Wald ratio	1	0.0028	0.0041	1.003 (0.995, 1.011)	0.503	0.743	4.90E-179	TRUE
CD8 ET	*TP53I11*	Wald ratio	1	-0.0239	0.0094	0.976 (0.959, 0.995)	0.011	0.086	1.16E-34	TRUE
CD8 ET	*TPD52*	Wald ratio	1	-0.0147	0.0082	0.985 (0.97, 1.001)	0.074	0.284	3.24E-42	TRUE
CD8 ET	*TPGS1*	Wald ratio	1	0.0019	0.0203	1.002 (0.963, 1.043)	0.925	0.968	2.58E-10	TRUE
CD8 ET	*TPGS2*	Wald ratio	1	-0.0030	0.0103	0.997 (0.977, 1.017)	0.769	0.897	1.12E-29	TRUE
CD8 ET	*TPM1*	Wald ratio	1	-0.0123	0.0170	0.988 (0.955, 1.021)	0.471	0.729	6.87E-10	TRUE
CD8 ET	*TPM2*	Wald ratio	1	0.0481	0.0186	1.049 (1.012, 1.088)	0.01	0.079	2.59E-10	TRUE
CD8 ET	*TPRA1*	Wald ratio	1	0.0052	0.0173	1.005 (0.972, 1.04)	0.765	0.895	5.91E-10	TRUE
CD8 ET	*TPST2*	Wald ratio	1	-0.0200	0.0159	0.98 (0.95, 1.011)	0.207	0.494	3.89E-12	TRUE
CD8 ET	*TRAPPC12-AS1*	Wald ratio	1	0.0418	0.0136	1.043 (1.015, 1.071)	0.002	0.026	1.35E-15	TRUE
CD8 ET	*TRAPPC2L*	Wald ratio	1	0.0327	0.0131	1.033 (1.007, 1.06)	0.013	0.095	5.91E-17	TRUE
CD8 ET	*TRAPPC4*	Wald ratio	1	0.0177	0.0132	1.018 (0.992, 1.044)	0.18	0.46	3.72E-17	TRUE
CD8 ET	*TRIM23*	Wald ratio	1	0.0197	0.0157	1.02 (0.989, 1.052)	0.21	0.497	3.35E-12	TRUE
CD8 ET	*TRIM47*	Wald ratio	1	-0.0491	0.0217	0.952 (0.912, 0.993)	0.023	0.14	7.66E-11	TRUE
CD8 ET	*TRMT112*	IVW	2	0.0111	0.0100	1.011 (0.992, 1.031)	0.266	0.561	2.37E-37	TRUE
CD8 ET	*TSGA10*	Wald ratio	1	0.0029	0.0139	1.003 (0.976, 1.031)	0.836	0.93	5.78E-15	TRUE
CD8 ET	*TSPAN15*	Wald ratio	1	-0.0113	0.0182	0.989 (0.954, 1.025)	0.535	0.761	6.57E-11	TRUE
CD8 ET	*TSPAN32*	Wald ratio	1	-0.0241	0.0108	0.976 (0.956, 0.997)	0.026	0.148	3.04E-31	TRUE
CD8 ET	*TSPO*	Wald ratio	1	0.0058	0.0112	1.006 (0.984, 1.028)	0.606	0.807	1.32E-24	TRUE
CD8 ET	*TSTD1*	Wald ratio	1	0.0032	0.0089	1.003 (0.986, 1.021)	0.718	0.874	1.14E-32	TRUE
CD8 ET	*TSTD3*	Wald ratio	1	-0.0140	0.0125	0.986 (0.962, 1.011)	0.262	0.557	8.15E-17	TRUE
CD8 ET	*TTC16*	Wald ratio	1	-0.0171	0.0150	0.983 (0.955, 1.012)	0.255	0.549	3.33E-15	TRUE
CD8 ET	*TTC32*	Wald ratio	1	0.0268	0.0126	1.027 (1.002, 1.053)	0.034	0.175	1.27E-19	TRUE
CD8 ET	*TTC38*	Wald ratio	1	0.0011	0.0099	1.001 (0.982, 1.021)	0.909	0.961	3.93E-32	TRUE
CD8 ET	*TUBA1B*	Wald ratio	1	0.0371	0.0142	1.038 (1.009, 1.067)	0.009	0.076	3.19E-14	TRUE
CD8 ET	*TUBB2A*	Wald ratio	1	0.0217	0.0202	1.022 (0.982, 1.063)	0.284	0.576	1.16E-08	TRUE
CD8 ET	*TUFM*	Wald ratio	1	0.0130	0.0065	1.013 (1, 1.026)	0.046	0.21	2.45E-66	TRUE
CD8 ET	*TXN*	Wald ratio	1	0.0354	0.0185	1.036 (0.999, 1.074)	0.056	0.237	9.20E-10	TRUE
CD8 ET	*TYMP*	Wald ratio	1	-0.0050	0.0230	0.995 (0.951, 1.041)	0.829	0.929	2.17E-11	TRUE
CD8 ET	*U91328.19*	Wald ratio	1	0.0140	0.0186	1.014 (0.978, 1.052)	0.452	0.717	4.09E-18	TRUE
CD8 ET	*UAP1*	Wald ratio	1	-0.0716	0.0202	0.931 (0.895, 0.968)	3.87E-04	0.009	7.70E-08	TRUE
CD8 ET	*UBA52*	Wald ratio	1	0.0048	0.0088	1.005 (0.988, 1.022)	0.586	0.794	8.45E-39	TRUE
CD8 ET	*UBA6*	Wald ratio	1	0.0108	0.0155	1.011 (0.981, 1.042)	0.485	0.736	6.81E-13	TRUE
CD8 ET	*UBE2L3*	Wald ratio	1	-0.0092	0.0116	0.991 (0.969, 1.014)	0.428	0.699	3.28E-16	TRUE
CD8 ET	*UBE2Q2*	Wald ratio	1	-0.0284	0.0162	0.972 (0.942, 1.003)	0.079	0.293	7.48E-11	TRUE
CD8 ET	*UBXN6*	Wald ratio	1	-0.0243	0.0171	0.976 (0.944, 1.009)	0.156	0.427	1.43E-13	TRUE
CD8 ET	*UCP2*	Wald ratio	1	0.0051	0.0110	1.005 (0.984, 1.027)	0.64	0.83	1.72E-23	TRUE
CD8 ET	*UFSP2*	Wald ratio	1	-0.0145	0.0195	0.986 (0.949, 1.024)	0.456	0.719	1.60E-09	TRUE
CD8 ET	*ULK4*	Wald ratio	1	0.0255	0.0118	1.026 (1.002, 1.05)	0.03	0.163	9.00E-20	TRUE
CD8 ET	*UNC5B-AS1*	Wald ratio	1	-0.0094	0.0164	0.991 (0.959, 1.023)	0.567	0.782	5.30E-10	TRUE
CD8 ET	*UPF3A*	Wald ratio	1	-0.0233	0.0131	0.977 (0.952, 1.002)	0.076	0.288	2.32E-18	TRUE
CD8 ET	*UPK3BL*	Wald ratio	1	0.0101	0.0136	1.01 (0.984, 1.037)	0.457	0.719	1.98E-18	TRUE
CD8 ET	*UQCRH*	Wald ratio	1	-0.0265	0.0126	0.974 (0.95, 0.998)	0.035	0.179	5.31E-23	TRUE
CD8 ET	*UROS*	Wald ratio	1	0.0183	0.0175	1.018 (0.984, 1.054)	0.296	0.59	1.69E-10	TRUE
CD8 ET	*USF1*	Wald ratio	1	-0.0198	0.0185	0.98 (0.946, 1.017)	0.285	0.577	1.07E-10	TRUE
CD8 ET	*USP44*	Wald ratio	1	0.0060	0.0202	1.006 (0.967, 1.047)	0.767	0.897	4.80E-11	TRUE
CD8 ET	*VAMP2*	Wald ratio	1	0.0257	0.0150	1.026 (0.996, 1.057)	0.086	0.311	8.11E-16	TRUE
CD8 ET	*VAMP4*	Wald ratio	1	0.0127	0.0155	1.013 (0.982, 1.044)	0.412	0.686	4.87E-15	TRUE
CD8 ET	*VAMP5*	Wald ratio	1	0.0133	0.0092	1.013 (0.995, 1.032)	0.146	0.415	5.50E-49	TRUE
CD8 ET	*VCL*	Wald ratio	1	-0.0168	0.0126	0.983 (0.959, 1.008)	0.183	0.462	1.61E-18	TRUE
CD8 ET	*VIM*	Wald ratio	1	0.0867	0.0160	1.091 (1.057, 1.125)	6.08E-08	6.60E-05	8.33E-11	TRUE
CD8 ET	*VIT*	Wald ratio	1	0.0292	0.0160	1.03 (0.998, 1.063)	0.069	0.27	8.35E-11	TRUE
CD8 ET	*VOPP1*	Wald ratio	1	0.0069	0.0191	1.007 (0.97, 1.045)	0.719	0.874	3.10E-09	TRUE
CD8 ET	*VPS28*	Wald ratio	1	0.0158	0.0097	1.016 (0.997, 1.035)	0.102	0.338	1.05E-26	TRUE
CD8 ET	*WAC-AS1*	Wald ratio	1	0.0121	0.0108	1.012 (0.991, 1.034)	0.261	0.555	1.22E-28	TRUE
CD8 ET	*WARS*	Wald ratio	1	-0.0862	0.0208	0.917 (0.881, 0.956)	3.35E-05	0.001	2.52E-08	TRUE
CD8 ET	*WBP1L*	Wald ratio	1	0.0210	0.0202	1.021 (0.982, 1.063)	0.299	0.595	4.32E-08	TRUE
CD8 ET	*WBSCR22*	Wald ratio	1	-0.0009	0.0220	0.999 (0.957, 1.043)	0.967	0.989	3.79E-09	TRUE
CD8 ET	*WDR37*	Wald ratio	1	-0.0377	0.0399	0.963 (0.891, 1.041)	0.344	0.637	9.15E-10	TRUE
CD8 ET	*WDR63*	Wald ratio	1	0.0135	0.0202	1.014 (0.974, 1.054)	0.505	0.745	1.19E-08	TRUE
CD8 ET	*WIPI1*	Wald ratio	1	-0.0124	0.0247	0.988 (0.941, 1.037)	0.617	0.816	2.70E-08	TRUE
CD8 ET	*XBP1*	Wald ratio	1	0.0842	0.0168	1.088 (1.053, 1.124)	5.79E-07	9.75E-05	9.89E-10	TRUE
CD8 ET	*XCL1*	Wald ratio	1	0.0105	0.0057	1.011 (0.999, 1.022)	0.066	0.263	1.07E-84	TRUE

CD8 ET	*XRCC6BP1*	Wald ratio	1	-0.0277	0.0139	0.973 (0.947, 0.999)	0.046	0.21	1.62E-15	TRUE
CD8 ET	*XRRA1*	Wald ratio	1	-0.0062	0.0054	0.994 (0.983, 1.004)	0.247	0.541	4.03E-97	TRUE
CD8 ET	*XXbac-BPG181B23.7*	Wald ratio	1	-0.0223	0.0184	0.978 (0.943, 1.014)	0.225	0.519	4.85E-08	TRUE
CD8 ET	*XXbac-BPG252P9.10*	Wald ratio	1	0.0262	0.0182	1.027 (0.991, 1.064)	0.149	0.418	6.43E-09	TRUE
CD8 ET	*XXbac-BPG252P9.9*	Wald ratio	1	-0.0497	0.0176	0.952 (0.919, 0.985)	0.005	0.048	4.26E-10	TRUE
CD8 ET	*XXbac-BPG299F13.17*	IVW	3	-0.0003	0.0156	1 (0.97, 1.031)	0.986	0.995	3.22E-122	TRUE
CD8 ET	*XXbac-BPG299F13.17*	MR Egger	3	0.0403	0.0210	1.041 (0.999, 1.085)	0.305	NA	NA	NA
CD8 ET	*XXbac-BPG299F13.17*	Weighted median	3	0.0026	0.0050	1.003 (0.993, 1.012)	0.611	NA	NA	NA
CD8 ET	*XXbac-BPG299F13.17*	Simple mode	3	-0.0149	0.0151	0.985 (0.956, 1.015)	0.43	NA	NA	NA
CD8 ET	*XXbac-BPG299F13.17*	Weighted mode	3	0.0045	0.0050	1.004 (0.995, 1.014)	0.467	NA	NA	NA
CD8 ET	*YBEY*	Wald ratio	1	-0.0010	0.0077	0.999 (0.984, 1.014)	0.898	0.957	2.75E-42	TRUE
CD8 ET	*YBX3*	Wald ratio	1	0.0077	0.0158	1.008 (0.977, 1.039)	0.627	0.822	5.23E-13	TRUE
CD8 ET	*YEATS4*	Wald ratio	1	-0.0034	0.0099	0.997 (0.977, 1.016)	0.728	0.879	2.29E-31	TRUE
CD8 ET	*YME1L1*	Wald ratio	1	0.0194	0.0192	1.02 (0.982, 1.059)	0.312	0.606	3.95E-09	TRUE
CD8 ET	*YWHAB*	Wald ratio	1	0.0212	0.0110	1.021 (1, 1.044)	0.053	0.232	8.74E-26	TRUE
CD8 ET	*YWHAH*	Wald ratio	1	-0.0483	0.0185	0.953 (0.919, 0.988)	0.009	0.075	1.70E-08	TRUE
CD8 ET	*ZBTB1*	Wald ratio	1	-0.0432	0.0180	0.958 (0.925, 0.992)	0.016	0.111	4.36E-10	TRUE
CD8 ET	*ZC3HAV1*	Wald ratio	1	0.0054	0.0088	1.005 (0.988, 1.023)	0.538	0.762	5.66E-38	TRUE
CD8 ET	*ZDHHC4*	Wald ratio	1	-0.0620	0.0148	0.94 (0.913, 0.968)	2.72E-05	0.001	9.34E-12	TRUE
CD8 ET	*ZFAS1*	Wald ratio	1	0.0076	0.0097	1.008 (0.989, 1.027)	0.436	0.703	9.42E-33	TRUE
CD8 ET	*ZFP57*	Wald ratio	1	0.0086	0.0123	1.009 (0.985, 1.033)	0.483	0.736	1.11E-19	TRUE
CD8 ET	*ZFP90*	Wald ratio	1	0.0226	0.0156	1.023 (0.992, 1.055)	0.148	0.417	1.24E-14	TRUE
CD8 ET	*ZNF100*	Wald ratio	1	-0.0726	0.0135	0.93 (0.906, 0.955)	8.14E-08	6.60E-05	1.94E-15	TRUE
CD8 ET	*ZNF138*	Wald ratio	1	-0.0098	0.0197	0.99 (0.953, 1.029)	0.619	0.819	1.76E-08	TRUE
CD8 ET	*ZNF165*	IVW	2	-0.0017	0.0093	0.998 (0.98, 1.017)	0.858	0.939	7.55E-46	TRUE
CD8 ET	*ZNF205-AS1*	Wald ratio	1	0.0196	0.0177	1.02 (0.985, 1.056)	0.267	0.561	1.81E-18	TRUE
CD8 ET	*ZNF257*	Wald ratio	1	0.0630	0.0184	1.065 (1.027, 1.104)	6.20E-04	0.012	4.16E-09	TRUE
CD8 ET	*ZNF266*	Wald ratio	1	0.0548	0.0174	1.056 (1.021, 1.093)	0.002	0.023	1.51E-09	TRUE
CD8 ET	*ZNF302*	Wald ratio	1	-0.0142	0.0135	0.986 (0.96, 1.012)	0.294	0.587	1.36E-15	TRUE
CD8 ET	*ZNF391*	Wald ratio	1	0.0305	0.0089	1.031 (1.013, 1.049)	5.83E-04	0.011	4.47E-28	TRUE
CD8 ET	*ZNF44*	Wald ratio	1	-0.0300	0.0163	0.97 (0.94, 1.002)	0.066	0.263	6.56E-12	TRUE
CD8 ET	*ZNF502*	Wald ratio	1	-0.0573	0.0181	0.944 (0.911, 0.978)	0.002	0.022	2.91E-09	TRUE
CD8 ET	*ZNF506*	Wald ratio	1	0.0273	0.0169	1.028 (0.994, 1.062)	0.106	0.346	1.57E-09	TRUE
CD8 ET	*ZNF519*	Wald ratio	1	-0.0098	0.0207	0.99 (0.951, 1.031)	0.635	0.827	3.48E-08	TRUE
CD8 ET	*ZNF559*	Wald ratio	1	0.0111	0.0228	1.011 (0.967, 1.057)	0.627	0.822	2.74E-10	TRUE
CD8 ET	*ZNF584*	Wald ratio	1	0.0233	0.0143	1.024 (0.995, 1.053)	0.103	0.341	2.67E-14	TRUE
CD8 ET	*ZNF593*	Wald ratio	1	0.0330	0.0130	1.034 (1.008, 1.06)	0.011	0.086	2.26E-17	TRUE
CD8 ET	*ZNF677*	Wald ratio	1	-0.0255	0.0192	0.975 (0.939, 1.012)	0.184	0.464	2.63E-09	TRUE
CD8 ET	*ZNF749*	Wald ratio	1	-0.0147	0.0116	0.985 (0.963, 1.008)	0.205	0.491	4.40E-22	TRUE
CD8 ET	*ZNF786*	Wald ratio	1	0.0130	0.0197	1.013 (0.975, 1.053)	0.51	0.748	3.03E-09	TRUE
CD8 ET	*ZNF793*	Wald ratio	1	-0.0522	0.0191	0.949 (0.914, 0.985)	0.006	0.058	8.05E-09	TRUE
CD8 ET	*ZNF83*	Wald ratio	1	-0.0223	0.0120	0.978 (0.955, 1.001)	0.062	0.253	2.48E-20	TRUE
CD8 ET	*ZNF846*	Wald ratio	1	-0.0357	0.0175	0.965 (0.932, 0.999)	0.041	0.198	1.89E-08	TRUE
CD8 ET	*ZNF85*	Wald ratio	1	0.0166	0.0109	1.017 (0.995, 1.039)	0.13	0.39	6.07E-28	TRUE
CD8 ET	*ZNRD1*	Wald ratio	1	-0.0127	0.0215	0.987 (0.947, 1.03)	0.556	0.774	5.94E-13	TRUE
CD8 ET	*ZP3*	Wald ratio	1	0.0176	0.0076	1.018 (1.003, 1.033)	0.021	0.132	5.00E-60	TRUE
CD8 ET	*ZSWIM7*	Wald ratio	1	0.0481	0.0150	1.049 (1.019, 1.081)	0.001	0.02	1.79E-13	TRUE
CD8 NC	*ABCG2*	Wald ratio	1	0.0282	0.0081	1.029 (1.012, 1.045)	4.75E-04	0.01	3.38E-40	TRUE
CD8 NC	*ABHD16A*	Wald ratio	1	0.0342	0.0162	1.035 (1.003, 1.068)	0.034	0.175	4.45E-13	TRUE
CD8 NC	*AC002331.1*	Wald ratio	1	0.0342	0.0187	1.035 (0.998, 1.073)	0.067	0.265	1.16E-09	TRUE
CD8 NC	*AC004854.4*	Wald ratio	1	0.0281	0.0171	1.029 (0.995, 1.063)	0.099	0.333	7.59E-17	TRUE
CD8 NC	*AC005253.2*	Wald ratio	1	0.0099	0.0161	1.01 (0.979, 1.042)	0.538	0.762	1.28E-12	TRUE
CD8 NC	*AC006369.2*	Wald ratio	1	0.0074	0.0069	1.007 (0.994, 1.021)	0.283	0.575	2.04E-66	TRUE
CD8 NC	*AC007383.4*	Wald ratio	1	-0.0009	0.0076	0.999 (0.984, 1.014)	0.904	0.96	1.07E-49	TRUE
CD8 NC	*AC009120.10*	Wald ratio	1	-0.0430	0.0177	0.958 (0.925, 0.992)	0.015	0.105	3.32E-09	TRUE
CD8 NC	*AC010642.1*	Wald ratio	1	0.0244	0.0254	1.025 (0.975, 1.077)	0.336	0.628	2.30E-08	TRUE
CD8 NC	*AC011484.1*	Wald ratio	1	-0.0270	0.0190	0.973 (0.938, 1.01)	0.156	0.427	7.73E-11	TRUE
CD8 NC	*AC016757.3*	Wald ratio	1	-0.0323	0.0158	0.968 (0.939, 0.999)	0.041	0.197	3.15E-12	TRUE
CD8 NC	*AC018816.3*	Wald ratio	1	0.0260	0.0198	1.026 (0.987, 1.067)	0.19	0.47	1.49E-08	TRUE
CD8 NC	*AC021860.1*	Wald ratio	1	0.0334	0.0158	1.034 (1.002, 1.066)	0.034	0.175	1.49E-14	TRUE
CD8 NC	*AC022498.1*	Wald ratio	1	0.0046	0.0170	1.005 (0.972, 1.039)	0.789	0.909	9.35E-16	TRUE
CD8 NC	*AC023590.1*	Wald ratio	1	-0.0099	0.0191	0.99 (0.954, 1.028)	0.603	0.806	8.86E-13	TRUE
CD8 NC	*AC024592.12*	Wald ratio	1	-0.0062	0.0134	0.994 (0.968, 1.02)	0.642	0.831	1.68E-14	TRUE
CD8 NC	*AC062017.1*	Wald ratio	1	0.0082	0.0183	1.008 (0.973, 1.045)	0.654	0.838	1.91E-09	TRUE
CD8 NC	*AC064834.2*	Wald ratio	1	0.0561	0.0158	1.058 (1.025, 1.091)	3.86E-04	0.009	4.92E-11	TRUE
CD8 NC	*AC084018.1*	Wald ratio	1	0.0086	0.0181	1.009 (0.973, 1.045)	0.636	0.827	3.78E-09	TRUE
CD8 NC	*AC090186.1*	Wald ratio	1	-0.0338	0.0135	0.967 (0.942, 0.993)	0.012	0.093	6.58E-14	TRUE
CD8 NC	*AC091729.9*	Wald ratio	1	-0.0295	0.0156	0.971 (0.942, 1.001)	0.059	0.245	2.62E-12	TRUE
CD8 NC	*AC092301.3*	Wald ratio	1	0.0405	0.0115	1.041 (1.018, 1.065)	4.08E-04	0.009	1.03E-21	TRUE
CD8 NC	*AC104653.1*	Wald ratio	1	0.0139	0.0129	1.014 (0.989, 1.04)	0.284	0.576	1.25E-18	TRUE
CD8 NC	*AC144652.1*	Wald ratio	1	0.0131	0.0182	1.013 (0.978, 1.05)	0.473	0.73	1.44E-09	TRUE
CD8 NC	*ACAT1*	Wald ratio	1	-0.0563	0.0161	0.945 (0.916, 0.976)	4.86E-04	0.01	2.08E-12	TRUE
CD8 NC	*ACAT2*	Wald ratio	1	0.0109	0.0179	1.011 (0.976, 1.047)	0.543	0.765	5.36E-10	TRUE
CD8 NC	*ACCS*	Wald ratio	1	-0.0027	0.0142	0.997 (0.97, 1.026)	0.847	0.935	1.76E-17	TRUE
CD8 NC	*ACOT4*	Wald ratio	1	0.0376	0.0174	1.038 (1.004, 1.074)	0.03	0.163	1.60E-10	TRUE
CD8 NC	*ACTA2*	Wald ratio	1	0.0154	0.0197	1.016 (0.977, 1.055)	0.433	0.701	1.58E-08	TRUE
CD8 NC	*ACTG1*	Wald ratio	1	-0.0400	0.0189	0.961 (0.926, 0.997)	0.034	0.175	6.99E-10	TRUE
CD8 NC	*ACYP2*	Wald ratio	1	0.0034	0.0083	1.003 (0.987, 1.02)	0.68	0.853	5.24E-41	TRUE
CD8 NC	*ADA*	Wald ratio	1	0.0135	0.0182	1.014 (0.978, 1.05)	0.458	0.719	7.06E-09	TRUE

CD8 NC	*ADAM10*	Wald ratio	1	-0.0780	0.0278	0.925 (0.876, 0.977)	0.005	0.051	5.13E-08	TRUE
CD8 NC	*ADAMTS6*	Wald ratio	1	0.0193	0.0128	1.02 (0.994, 1.045)	0.132	0.393	5.92E-18	TRUE
CD8 NC	*ADIRF*	Wald ratio	1	-0.0097	0.0207	0.99 (0.951, 1.031)	0.639	0.83	5.89E-10	TRUE
CD8 NC	*ADPRM*	Wald ratio	1	0.0133	0.0159	1.013 (0.982, 1.045)	0.402	0.68	4.09E-12	TRUE
CD8 NC	*ADTRP*	Wald ratio	1	-0.0408	0.0168	0.96 (0.929, 0.992)	0.015	0.104	5.71E-09	TRUE
CD8 NC	*AE000662.92*	Wald ratio	1	-0.0258	0.0267	0.975 (0.925, 1.027)	0.334	0.627	6.75E-08	TRUE
CD8 NC	*AFAP1*	Wald ratio	1	-0.0033	0.0163	0.997 (0.965, 1.029)	0.841	0.933	3.36E-12	TRUE
CD8 NC	*AGA*	Wald ratio	1	-0.0132	0.0175	0.987 (0.954, 1.021)	0.449	0.714	3.35E-10	TRUE
CD8 NC	*AGAP4*	Wald ratio	1	0.0096	0.0070	1.01 (0.996, 1.024)	0.167	0.443	1.80E-49	TRUE
CD8 NC	*AHI1*	Wald ratio	1	-0.0146	0.0093	0.985 (0.968, 1.004)	0.116	0.362	3.00E-34	TRUE
CD8 NC	*AKAP10*	Wald ratio	1	0.0136	0.0207	1.014 (0.973, 1.056)	0.51	0.748	3.99E-08	TRUE
CD8 NC	*AKAP12*	Wald ratio	1	0.0017	0.0111	1.002 (0.98, 1.024)	0.875	0.948	2.04E-26	TRUE
CD8 NC	*AKAP8*	Wald ratio	1	0.0380	0.0180	1.039 (1.003, 1.076)	0.035	0.176	6.22E-11	TRUE
CD8 NC	*AKIRIN1*	Wald ratio	1	-0.0072	0.0184	0.993 (0.958, 1.029)	0.695	0.863	6.40E-10	TRUE
CD8 NC	*AMICA1*	Wald ratio	1	0.0269	0.0120	1.027 (1.003, 1.052)	0.025	0.145	1.92E-16	TRUE
CD8 NC	*ANAPC13*	Wald ratio	1	-0.0049	0.0179	0.995 (0.961, 1.031)	0.784	0.907	5.16E-11	TRUE
CD8 NC	*ANK3*	Wald ratio	1	-0.0399	0.0162	0.961 (0.931, 0.992)	0.014	0.099	1.58E-11	TRUE
CD8 NC	*ANKDD1A*	Wald ratio	1	0.0030	0.0077	1.003 (0.988, 1.018)	0.696	0.864	8.95E-48	TRUE
CD8 NC	*ANKRD55*	Wald ratio	1	-0.0029	0.0193	0.997 (0.96, 1.036)	0.881	0.951	9.72E-10	TRUE
CD8 NC	*ANXA5*	Wald ratio	1	-0.0059	0.0162	0.994 (0.963, 1.026)	0.714	0.872	4.98E-19	TRUE
CD8 NC	*ANXA6*	Wald ratio	1	0.0006	0.0118	1.001 (0.978, 1.024)	0.958	0.985	5.19E-18	TRUE
CD8 NC	*AOAH*	Wald ratio	1	-0.0565	0.0185	0.945 (0.911, 0.98)	0.002	0.029	2.80E-11	TRUE
CD8 NC	*AP003774.1*	Wald ratio	1	-0.0299	0.0087	0.971 (0.954, 0.987)	5.86E-04	0.011	2.62E-36	TRUE
CD8 NC	*ARHGAP15*	Wald ratio	1	0.0089	0.0129	1.009 (0.984, 1.035)	0.492	0.738	1.81E-22	TRUE
CD8 NC	*ARHGEF10*	Wald ratio	1	0.0278	0.0181	1.028 (0.992, 1.065)	0.125	0.38	1.56E-09	TRUE
CD8 NC	*ARHGEF3*	Wald ratio	1	-0.0013	0.0087	0.999 (0.982, 1.016)	0.881	0.952	8.89E-34	TRUE
CD8 NC	*ARL14EP*	Wald ratio	1	-0.0401	0.0152	0.961 (0.933, 0.99)	0.008	0.07	2.71E-12	TRUE
CD8 NC	*ARL16*	Wald ratio	1	0.0204	0.0111	1.021 (0.999, 1.043)	0.065	0.26	4.04E-25	TRUE
CD8 NC	*ARL17B*	Wald ratio	1	-0.0097	0.0173	0.99 (0.957, 1.024)	0.575	0.787	1.64E-08	TRUE
CD8 NC	*ARPC5*	Wald ratio	1	-0.0208	0.0146	0.979 (0.952, 1.008)	0.154	0.425	1.29E-14	TRUE
CD8 NC	*ARSA*	Wald ratio	1	0.0537	0.0199	1.055 (1.015, 1.097)	0.007	0.064	8.52E-13	TRUE
CD8 NC	*ASL*	Wald ratio	1	-0.0037	0.0211	0.996 (0.956, 1.038)	0.861	0.941	3.17E-08	TRUE
CD8 NC	*ATF7IP2*	Wald ratio	1	0.0432	0.0158	1.044 (1.012, 1.077)	0.006	0.057	4.79E-12	TRUE
CD8 NC	*ATG10*	Wald ratio	1	0.0229	0.0200	1.023 (0.984, 1.064)	0.253	0.546	5.64E-09	TRUE
CD8 NC	*ATHL1*	Wald ratio	1	-0.0040	0.0126	0.996 (0.972, 1.021)	0.752	0.887	2.95E-22	TRUE
CD8 NC	*ATM*	Wald ratio	1	-0.0367	0.0178	0.964 (0.931, 0.998)	0.039	0.19	2.09E-10	TRUE
CD8 NC	*ATP5D*	Wald ratio	1	-0.0529	0.0198	0.948 (0.912, 0.986)	0.007	0.066	6.25E-08	TRUE
CD8 NC	*ATP5G2*	Wald ratio	1	0.0900	0.0208	1.094 (1.05, 1.14)	1.59E-05	7.94E-04	2.34E-09	TRUE
CD8 NC	*ATP5G3*	Wald ratio	1	-0.0050	0.0201	0.995 (0.957, 1.035)	0.804	0.916	1.79E-10	TRUE
CD8 NC	*ATP6AP1L*	Wald ratio	1	-0.0317	0.0197	0.969 (0.932, 1.007)	0.108	0.35	1.95E-08	TRUE
CD8 NC	*ATP6V0E2*	Wald ratio	1	0.0178	0.0107	1.018 (0.997, 1.04)	0.098	0.333	3.48E-24	TRUE
CD8 NC	*B3GALNT1*	Wald ratio	1	-0.0269	0.0069	0.973 (0.96, 0.987)	1.05E-04	0.003	1.76E-56	TRUE
CD8 NC	*B3GAT2*	Wald ratio	1	0.0140	0.0224	1.014 (0.971, 1.06)	0.531	0.758	3.53E-09	TRUE
CD8 NC	*B4GALT1*	Wald ratio	1	0.0100	0.0114	1.01 (0.988, 1.033)	0.378	0.665	5.77E-26	TRUE
CD8 NC	*B4GALT4*	Wald ratio	1	-0.0340	0.0209	0.967 (0.928, 1.007)	0.104	0.342	2.66E-09	TRUE
CD8 NC	*BCAS4*	IVW	2	0.0094	0.0131	1.009 (0.984, 1.036)	0.475	0.731	2.58E-34	TRUE
CD8 NC	*BIN1*	Wald ratio	1	0.0171	0.0090	1.017 (0.999, 1.035)	0.058	0.243	1.55E-33	TRUE
CD8 NC	*BIRC7*	Wald ratio	1	0.0235	0.0105	1.024 (1.003, 1.045)	0.025	0.145	2.72E-26	TRUE
CD8 NC	*BNIP1*	Wald ratio	1	0.0202	0.0210	1.02 (0.979, 1.063)	0.334	0.627	1.15E-08	TRUE
CD8 NC	*BRK1*	Wald ratio	1	-0.0218	0.0154	0.978 (0.949, 1.008)	0.157	0.428	1.86E-16	TRUE
CD8 NC	*BST2*	Wald ratio	1	0.0382	0.0133	1.039 (1.012, 1.066)	0.004	0.043	2.00E-11	TRUE
CD8 NC	*BTBD9*	Wald ratio	1	0.0097	0.0141	1.01 (0.982, 1.038)	0.49	0.738	4.95E-13	TRUE
CD8 NC	*BTF3*	Wald ratio	1	0.0350	0.0118	1.036 (1.012, 1.06)	0.003	0.034	1.12E-18	TRUE
CD8 NC	*BTN2A1*	Wald ratio	1	0.0010	0.0186	1.001 (0.965, 1.038)	0.959	0.985	4.15E-11	TRUE
CD8 NC	*BTN3A1*	Wald ratio	1	0.0707	0.0158	1.073 (1.041, 1.107)	7.74E-06	4.76E-04	1.35E-10	TRUE
CD8 NC	*BTN3A2*	Wald ratio	1	0.0142	0.0100	1.014 (0.995, 1.034)	0.153	0.423	9.44E-41	TRUE
CD8 NC	*C10orf128*	Wald ratio	1	-0.0055	0.0106	0.995 (0.974, 1.015)	0.604	0.806	4.20E-25	TRUE
CD8 NC	*C10orf32*	Wald ratio	1	0.0200	0.0131	1.02 (0.994, 1.047)	0.127	0.384	2.47E-17	TRUE
CD8 NC	*C11orf21*	Wald ratio	1	-0.0128	0.0163	0.987 (0.956, 1.019)	0.429	0.699	1.39E-08	TRUE
CD8 NC	*C14orf2*	Wald ratio	1	0.0352	0.0197	1.036 (0.996, 1.077)	0.075	0.287	2.07E-08	TRUE
CD8 NC	*C16orf13*	Wald ratio	1	0.0001	0.0143	1 (0.972, 1.028)	0.997	0.999	9.35E-11	TRUE
CD8 NC	*C17orf89*	Wald ratio	1	0.0380	0.0131	1.039 (1.012, 1.066)	0.004	0.041	3.64E-17	TRUE
CD8 NC	*C17orf97*	Wald ratio	1	-0.0109	0.0096	0.989 (0.971, 1.008)	0.253	0.546	2.23E-31	TRUE
CD8 NC	*C19orf83*	Wald ratio	1	-0.0297	0.0136	0.971 (0.945, 0.997)	0.029	0.159	3.75E-16	TRUE
CD8 NC	*C1orf228*	Wald ratio	1	-0.0059	0.0156	0.994 (0.964, 1.025)	0.704	0.867	1.45E-12	TRUE
CD8 NC	*C1orf86*	Wald ratio	1	0.0027	0.0135	1.003 (0.976, 1.03)	0.84	0.932	2.19E-16	TRUE
CD8 NC	*C2*	Wald ratio	1	-0.0412	0.0118	0.96 (0.938, 0.982)	4.94E-04	0.01	8.72E-22	TRUE
CD8 NC	*C20orf27*	Wald ratio	1	-0.0050	0.0225	0.995 (0.952, 1.04)	0.824	0.926	6.44E-11	TRUE
CD8 NC	*C22orf34*	Wald ratio	1	-0.0330	0.0146	0.967 (0.94, 0.996)	0.024	0.142	1.21E-13	TRUE
CD8 NC	*C2orf27A*	Wald ratio	1	0.0198	0.0133	1.02 (0.994, 1.047)	0.137	0.399	2.77E-18	TRUE
CD8 NC	*C4orf32*	Wald ratio	1	0.0196	0.0148	1.02 (0.991, 1.05)	0.186	0.466	3.75E-14	TRUE
CD8 NC	*C5orf17*	Wald ratio	1	0.0035	0.0169	1.004 (0.971, 1.037)	0.835	0.93	6.84E-09	TRUE
CD8 NC	*C6orf25*	Wald ratio	1	-0.0169	0.0218	0.983 (0.942, 1.026)	0.438	0.703	8.58E-09	TRUE
CD8 NC	*C6orf48*	Wald ratio	1	0.0356	0.0103	1.036 (1.016, 1.057)	5.43E-04	0.011	2.85E-14	TRUE
CD8 NC	*C6orf57*	Wald ratio	1	-0.0636	0.0165	0.938 (0.909, 0.969)	1.16E-04	0.003	4.79E-09	TRUE
CD8 NC	*C9orf135*	Wald ratio	1	-0.0081	0.0054	0.992 (0.982, 1.003)	0.135	0.396	1.44E-88	TRUE
CD8 NC	*C9orf156*	Wald ratio	1	0.0101	0.0150	1.01 (0.981, 1.04)	0.501	0.743	1.22E-14	TRUE
CD8 NC	*C9orf78*	Wald ratio	1	-0.0050	0.0115	0.995 (0.973, 1.018)	0.664	0.845	4.77E-23	TRUE
CD8 NC	*CA6*	IVW	2	-0.0064	0.0119	0.994 (0.971, 1.017)	0.592	0.797	7.49E-23	TRUE

CD8 NC	*CALM3*	Wald ratio	1	-0.0016	0.0191	0.998 (0.962, 1.037)	0.934	0.972	3.75E-09	TRUE
CD8 NC	*CAMK4*	Wald ratio	1	0.0199	0.0186	1.02 (0.984, 1.058)	0.286	0.577	1.13E-10	TRUE
CD8 NC	*CAMLG*	Wald ratio	1	0.0003	0.0143	1 (0.973, 1.029)	0.982	0.993	9.61E-11	TRUE
CD8 NC	*CAPG*	Wald ratio	1	-0.0123	0.0131	0.988 (0.963, 1.013)	0.347	0.64	4.71E-17	TRUE
CD8 NC	*CAPN12*	Wald ratio	1	-0.0034	0.0175	0.997 (0.963, 1.031)	0.844	0.935	9.56E-12	TRUE
CD8 NC	*CARD8*	Wald ratio	1	0.0352	0.0176	1.036 (1.001, 1.072)	0.045	0.209	9.67E-14	TRUE
CD8 NC	*CARHSP1*	Wald ratio	1	0.0073	0.0180	1.007 (0.972, 1.044)	0.685	0.857	6.31E-10	TRUE
CD8 NC	*CARS*	Wald ratio	1	0.0092	0.0067	1.009 (0.996, 1.023)	0.165	0.439	1.80E-63	TRUE
CD8 NC	*CASP7*	Wald ratio	1	0.0170	0.0191	1.017 (0.98, 1.056)	0.374	0.662	1.32E-08	TRUE
CD8 NC	*CASP8*	Wald ratio	1	-0.0477	0.0157	0.953 (0.925, 0.983)	0.002	0.029	4.50E-12	TRUE
CD8 NC	*CAST*	Wald ratio	1	-0.0047	0.0139	0.995 (0.969, 1.023)	0.737	0.88	8.12E-18	TRUE
CD8 NC	*CBR3*	Wald ratio	1	0.0063	0.0125	1.006 (0.982, 1.031)	0.615	0.814	5.71E-20	TRUE
CD8 NC	*CCDC101*	Wald ratio	1	-0.0210	0.0153	0.979 (0.95, 1.009)	0.17	0.447	2.27E-13	TRUE
CD8 NC	*CCDC109B*	Wald ratio	1	0.0136	0.0182	1.014 (0.978, 1.05)	0.453	0.717	6.50E-10	TRUE
CD8 NC	*CCDC122*	Wald ratio	1	-0.0096	0.0118	0.99 (0.968, 1.014)	0.415	0.687	3.48E-21	TRUE
CD8 NC	*CCDC146*	Wald ratio	1	0.0124	0.0184	1.013 (0.977, 1.05)	0.5	0.743	1.79E-16	TRUE
CD8 NC	*CCDC152*	Wald ratio	1	0.0176	0.0163	1.018 (0.986, 1.051)	0.281	0.575	1.10E-10	TRUE
CD8 NC	*CCDC163P*	Wald ratio	1	-0.0097	0.0087	0.99 (0.974, 1.007)	0.266	0.561	8.41E-38	TRUE
CD8 NC	*CCDC167*	Wald ratio	1	0.0015	0.0062	1.002 (0.989, 1.014)	0.809	0.918	1.89E-70	TRUE
CD8 NC	*CCDC66*	Wald ratio	1	0.0127	0.0110	1.013 (0.991, 1.035)	0.247	0.541	3.31E-26	TRUE
CD8 NC	*CCSER2*	Wald ratio	1	-0.0209	0.0109	0.979 (0.959, 1.001)	0.056	0.239	7.81E-30	TRUE
CD8 NC	*CCT3*	Wald ratio	1	0.0148	0.0087	1.015 (0.998, 1.032)	0.089	0.316	1.03E-37	TRUE
CD8 NC	*CCT8*	Wald ratio	1	-0.0007	0.0073	0.999 (0.985, 1.014)	0.922	0.965	4.27E-91	TRUE
CD8 NC	*CCZ1*	IVW	2	0.0132	0.0084	1.013 (0.997, 1.03)	0.117	0.365	1.10E-42	TRUE
CD8 NC	*CD151*	Wald ratio	1	-0.0185	0.0166	0.982 (0.95, 1.014)	0.267	0.561	1.62E-11	TRUE
CD8 NC	*CD164*	Wald ratio	1	-0.0430	0.0197	0.958 (0.922, 0.996)	0.03	0.161	4.79E-08	TRUE
CD8 NC	*CD247*	Wald ratio	1	0.0058	0.0159	1.006 (0.975, 1.038)	0.715	0.873	3.96E-15	TRUE
CD8 NC	*CD300A*	Wald ratio	1	-0.0093	0.0154	0.991 (0.961, 1.021)	0.545	0.767	5.80E-13	TRUE
CD8 NC	*CD52*	Wald ratio	1	-0.0131	0.0187	0.987 (0.951, 1.024)	0.485	0.736	1.14E-09	TRUE
CD8 NC	*CD53*	Wald ratio	1	0.0447	0.0191	1.046 (1.007, 1.086)	0.019	0.123	1.37E-08	TRUE
CD8 NC	*CD6*	Wald ratio	1	-0.0049	0.0232	0.995 (0.951, 1.041)	0.832	0.93	5.16E-08	TRUE
CD8 NC	*CD7*	Wald ratio	1	0.0048	0.0187	1.005 (0.969, 1.042)	0.799	0.913	5.21E-08	TRUE
CD8 NC	*CDC42*	Wald ratio	1	0.0092	0.0133	1.009 (0.983, 1.036)	0.49	0.738	4.98E-15	TRUE
CD8 NC	*CDC42SE2*	Wald ratio	1	0.0198	0.0196	1.02 (0.982, 1.06)	0.311	0.605	8.19E-09	TRUE
CD8 NC	*CDK5R1*	Wald ratio	1	0.0242	0.0203	1.025 (0.984, 1.066)	0.234	0.527	5.50E-08	TRUE
CD8 NC	*CDO1*	Wald ratio	1	0.0114	0.0111	1.011 (0.99, 1.034)	0.306	0.599	7.89E-22	TRUE
CD8 NC	*CEACAM21*	Wald ratio	1	-0.0131	0.0108	0.987 (0.966, 1.008)	0.227	0.52	4.84E-22	TRUE
CD8 NC	*CEBPZ*	Wald ratio	1	0.0722	0.0201	1.075 (1.033, 1.118)	3.22E-04	0.007	6.23E-08	TRUE
CD8 NC	*CEBPZ-AS1*	Wald ratio	1	0.0194	0.0153	1.02 (0.989, 1.051)	0.205	0.492	1.58E-18	TRUE
CD8 NC	*CELA1*	Wald ratio	1	0.0037	0.0117	1.004 (0.981, 1.027)	0.75	0.886	1.25E-26	TRUE
CD8 NC	*CENPK*	Wald ratio	1	0.0068	0.0045	1.007 (0.998, 1.016)	0.134	0.394	1.70E-157	TRUE
CD8 NC	*CERS4*	Wald ratio	1	-0.0098	0.0165	0.99 (0.959, 1.023)	0.55	0.769	6.63E-12	TRUE
CD8 NC	*CFD*	Wald ratio	1	0.0028	0.0079	1.003 (0.987, 1.018)	0.725	0.878	1.97E-47	TRUE
CD8 NC	*CFLAR*	Wald ratio	1	0.0340	0.0181	1.035 (0.998, 1.072)	0.061	0.25	1.35E-09	TRUE
CD8 NC	*CHCHD10*	Wald ratio	1	0.0166	0.0240	1.017 (0.97, 1.066)	0.489	0.738	5.37E-08	TRUE
CD8 NC	*CHCHD2*	Wald ratio	1	0.0043	0.0098	1.004 (0.985, 1.024)	0.659	0.843	4.06E-33	TRUE
CD8 NC	*CHI3L2*	Wald ratio	1	0.0041	0.0129	1.004 (0.979, 1.03)	0.752	0.887	2.16E-17	TRUE
CD8 NC	*CHURC1*	Wald ratio	1	0.0207	0.0107	1.021 (1, 1.043)	0.054	0.232	1.08E-21	TRUE
CD8 NC	*CIB1*	Wald ratio	1	-0.0506	0.0179	0.951 (0.918, 0.985)	0.005	0.048	2.06E-16	TRUE
CD8 NC	*CLEC2B*	Wald ratio	1	0.0052	0.0157	1.005 (0.975, 1.037)	0.741	0.881	1.17E-12	TRUE
CD8 NC	*CLIC3*	Wald ratio	1	0.0631	0.0198	1.065 (1.025, 1.107)	0.001	0.021	4.34E-08	TRUE
CD8 NC	*CLK4*	Wald ratio	1	0.0139	0.0181	1.014 (0.979, 1.051)	0.444	0.709	2.34E-10	TRUE
CD8 NC	*CLNS1A*	Wald ratio	1	0.0587	0.0143	1.06 (1.031, 1.091)	4.14E-05	0.002	1.23E-08	TRUE
CD8 NC	*CLUAP1*	Wald ratio	1	0.0741	0.0187	1.077 (1.038, 1.117)	7.82E-05	0.003	7.51E-09	TRUE
CD8 NC	*CMTM7*	Wald ratio	1	0.0225	0.0131	1.023 (0.997, 1.049)	0.087	0.312	7.68E-17	TRUE
CD8 NC	*CMTM8*	Wald ratio	1	0.0013	0.0075	1.001 (0.987, 1.016)	0.865	0.943	5.36E-61	TRUE
CD8 NC	*CNKSR1*	Wald ratio	1	-0.0373	0.0212	0.963 (0.924, 1.004)	0.078	0.292	2.96E-08	TRUE
CD8 NC	*CNN2*	Wald ratio	1	0.0216	0.0162	1.022 (0.99, 1.055)	0.181	0.462	4.38E-12	TRUE
CD8 NC	*CNOT6L*	Wald ratio	1	0.0268	0.0142	1.027 (0.999, 1.056)	0.059	0.244	2.10E-15	TRUE
CD8 NC	*COA1*	Wald ratio	1	0.0103	0.0160	1.01 (0.979, 1.043)	0.517	0.752	6.10E-12	TRUE
CD8 NC	*COG5*	Wald ratio	1	0.0508	0.0103	1.052 (1.031, 1.074)	8.92E-07	1.03E-04	5.70E-25	TRUE
CD8 NC	*COL18A1*	IVW	2	-0.0151	0.0133	0.985 (0.96, 1.011)	0.257	0.551	5.83E-64	TRUE
CD8 NC	*COL6A2*	Wald ratio	1	0.0205	0.0185	1.021 (0.984, 1.058)	0.267	0.561	3.47E-10	TRUE
CD8 NC	*COMMD6*	Wald ratio	1	0.0132	0.0081	1.013 (0.997, 1.03)	0.105	0.343	6.53E-44	TRUE
CD8 NC	*CORO1B*	Wald ratio	1	0.0020	0.0082	1.002 (0.986, 1.018)	0.806	0.916	3.62E-45	TRUE
CD8 NC	*COX14*	Wald ratio	1	-0.0595	0.0192	0.942 (0.907, 0.978)	0.002	0.025	2.15E-09	TRUE
CD8 NC	*COX6B1*	Wald ratio	1	-0.0276	0.0125	0.973 (0.949, 0.997)	0.027	0.155	8.10E-19	TRUE
CD8 NC	*COX7A2L*	Wald ratio	1	-0.0378	0.0132	0.963 (0.938, 0.988)	0.004	0.045	1.66E-17	TRUE
CD8 NC	*CPA5*	Wald ratio	1	-0.0042	0.0049	0.996 (0.986, 1.005)	0.386	0.669	2.85E-137	TRUE
CD8 NC	*CPNE1*	Wald ratio	1	-0.0043	0.0113	0.996 (0.974, 1.018)	0.7	0.866	4.40E-28	TRUE
CD8 NC	*CRELD2*	Wald ratio	1	-0.0412	0.0202	0.96 (0.922, 0.998)	0.041	0.199	7.95E-08	TRUE
CD8 NC	*CRHR1*	Wald ratio	1	0.0934	0.0265	1.098 (1.042, 1.156)	4.20E-04	0.009	7.09E-13	TRUE
CD8 NC	*CRIP2*	Wald ratio	1	-0.0197	0.0145	0.98 (0.953, 1.009)	0.173	0.451	3.20E-16	TRUE
CD8 NC	*CRYBB2*	Wald ratio	1	0.0095	0.0075	1.01 (0.995, 1.025)	0.208	0.494	8.41E-51	TRUE
CD8 NC	*CRYGN*	Wald ratio	1	0.0155	0.0088	1.016 (0.998, 1.033)	0.078	0.292	4.99E-35	TRUE
CD8 NC	*CRYZ*	Wald ratio	1	-0.0160	0.0134	0.984 (0.959, 1.01)	0.231	0.524	1.20E-16	TRUE
CD8 NC	*CSGALNACT1*	Wald ratio	1	0.0164	0.0204	1.017 (0.977, 1.058)	0.422	0.693	4.64E-09	TRUE
CD8 NC	*CSTB*	Wald ratio	1	0.0039	0.0114	1.004 (0.982, 1.027)	0.73	0.88	9.38E-21	TRUE
CD8 NC	*CTA-29F11.1*	Wald ratio	1	-0.0045	0.0071	0.996 (0.982, 1.009)	0.527	0.756	8.49E-60	TRUE

CD8 NC	*CTBP1-AS2*	Wald ratio	1	-0.0732	0.0174	0.929 (0.898, 0.962)	2.64E-05	0.001	7.00E-11	TRUE
CD8 NC	*CTC-425F1.4*	Wald ratio	1	0.0078	0.0179	1.008 (0.973, 1.044)	0.663	0.845	6.45E-11	TRUE
CD8 NC	*CTC-524C5.2*	Wald ratio	1	0.0103	0.0073	1.01 (0.996, 1.025)	0.159	0.43	5.94E-44	TRUE
CD8 NC	*CTD-2007H13.3*	Wald ratio	1	-0.0192	0.0211	0.981 (0.941, 1.023)	0.364	0.653	9.00E-11	TRUE
CD8 NC	*CTD-2020K17.4*	Wald ratio	1	-0.0117	0.0105	0.988 (0.968, 1.009)	0.263	0.558	6.67E-21	TRUE
CD8 NC	*CTD-2116N20.1*	Wald ratio	1	-0.0117	0.0078	0.988 (0.973, 1.004)	0.134	0.395	4.23E-47	TRUE
CD8 NC	*CTD-2336O2.1*	Wald ratio	1	0.0363	0.0105	1.037 (1.016, 1.059)	5.57E-04	0.011	1.05E-29	TRUE
CD8 NC	*CTD-2368P22.1*	Wald ratio	1	0.0032	0.0095	1.003 (0.985, 1.022)	0.734	0.88	2.36E-35	TRUE
CD8 NC	*CTD-2371O3.2*	Wald ratio	1	0.0002	0.0081	1 (0.984, 1.016)	0.982	0.993	9.66E-48	TRUE
CD8 NC	*CTD-2547L24.3*	Wald ratio	1	0.0047	0.0136	1.005 (0.978, 1.032)	0.727	0.878	5.47E-17	TRUE
CD8 NC	*CTD-2651B20.3*	Wald ratio	1	-0.0124	0.0108	0.988 (0.967, 1.009)	0.251	0.545	2.15E-25	TRUE
CD8 NC	*CTD-3110H11.1*	Wald ratio	1	-0.0522	0.0180	0.949 (0.916, 0.983)	0.004	0.041	1.22E-12	TRUE
CD8 NC	*CTLA4*	Wald ratio	1	-0.0026	0.0097	0.997 (0.979, 1.017)	0.788	0.909	1.14E-34	TRUE
CD8 NC	*CTNNAL1*	Wald ratio	1	-0.0445	0.0183	0.956 (0.923, 0.991)	0.015	0.104	4.60E-08	TRUE
CD8 NC	*CTSF*	Wald ratio	1	-0.0230	0.0202	0.977 (0.939, 1.017)	0.254	0.548	9.45E-09	TRUE
CD8 NC	*CTSL*	Wald ratio	1	0.0219	0.0147	1.022 (0.993, 1.052)	0.136	0.397	3.01E-14	TRUE
CD8 NC	*CTSS*	Wald ratio	1	0.0293	0.0175	1.03 (0.995, 1.066)	0.094	0.326	7.15E-13	TRUE
CD8 NC	*CTSW*	Wald ratio	1	-0.0242	0.0057	0.976 (0.965, 0.987)	2.34E-05	0.001	1.06E-107	TRUE
CD8 NC	*CYLD*	Wald ratio	1	-0.0453	0.0199	0.956 (0.919, 0.994)	0.023	0.138	1.91E-08	TRUE
CD8 NC	*CYP2D6*	Wald ratio	1	0.0412	0.0147	1.042 (1.012, 1.073)	0.005	0.051	2.30E-21	TRUE
CD8 NC	*CYP4V2*	Wald ratio	1	0.0037	0.0136	1.004 (0.977, 1.031)	0.784	0.906	2.20E-15	TRUE
CD8 NC	*DAD1*	Wald ratio	1	-0.0049	0.0153	0.995 (0.966, 1.025)	0.749	0.886	1.11E-12	TRUE
CD8 NC	*DARS*	Wald ratio	1	-0.0437	0.0159	0.957 (0.928, 0.987)	0.006	0.056	2.41E-10	TRUE
CD8 NC	*DAZAP1*	Wald ratio	1	0.0438	0.0161	1.045 (1.012, 1.078)	0.007	0.062	3.44E-12	TRUE
CD8 NC	*DAZAP2*	Wald ratio	1	-0.0652	0.0188	0.937 (0.903, 0.972)	5.23E-04	0.011	1.98E-08	TRUE
CD8 NC	*DBNDD1*	Wald ratio	1	0.0096	0.0172	1.01 (0.976, 1.044)	0.578	0.789	6.85E-09	TRUE
CD8 NC	*DCP2*	Wald ratio	1	0.0477	0.0165	1.049 (1.015, 1.083)	0.004	0.042	1.19E-09	TRUE
CD8 NC	*DCXR*	Wald ratio	1	-0.0074	0.0086	0.993 (0.976, 1.01)	0.392	0.672	1.87E-34	TRUE
CD8 NC	*DDR1*	IVW	2	-0.0502	0.0124	0.951 (0.928, 0.974)	5.27E-05	0.002	5.44E-28	TRUE
CD8 NC	*DDRGK1*	Wald ratio	1	0.0076	0.0193	1.008 (0.97, 1.047)	0.693	0.862	3.19E-09	TRUE
CD8 NC	*DDT*	Wald ratio	1	-0.0124	0.0113	0.988 (0.966, 1.01)	0.273	0.567	3.17E-23	TRUE
CD8 NC	*DDTL*	Wald ratio	1	-0.0106	0.0089	0.989 (0.972, 1.007)	0.234	0.529	5.78E-37	TRUE
CD8 NC	*DDX11*	Wald ratio	1	-0.0103	0.0078	0.99 (0.975, 1.005)	0.183	0.462	1.35E-48	TRUE
CD8 NC	*DDX5*	Wald ratio	1	0.0467	0.0120	1.048 (1.023, 1.073)	1.06E-04	0.003	2.06E-18	TRUE
CD8 NC	*DHX32*	Wald ratio	1	0.0209	0.0206	1.021 (0.981, 1.063)	0.31	0.604	2.51E-08	TRUE
CD8 NC	*DIP2A*	Wald ratio	1	0.0143	0.0121	1.014 (0.991, 1.039)	0.236	0.53	7.15E-21	TRUE
CD8 NC	*DISC1*	Wald ratio	1	-0.0133	0.0187	0.987 (0.951, 1.024)	0.478	0.733	3.27E-09	TRUE
CD8 NC	*DNAJA1*	Wald ratio	1	-0.0674	0.0186	0.935 (0.901, 0.97)	3.01E-04	0.007	2.19E-09	TRUE
CD8 NC	*DNAJC15*	Wald ratio	1	0.0065	0.0065	1.006 (0.994, 1.019)	0.318	0.614	1.85E-69	TRUE
CD8 NC	*DOCK8*	Wald ratio	1	0.0345	0.0157	1.035 (1.004, 1.067)	0.029	0.158	6.50E-15	TRUE
CD8 NC	*DPYSL4*	Wald ratio	1	0.0193	0.0143	1.02 (0.991, 1.049)	0.177	0.456	1.33E-14	TRUE
CD8 NC	*DR1*	Wald ratio	1	-0.0306	0.0173	0.97 (0.938, 1.003)	0.076	0.289	1.32E-10	TRUE
CD8 NC	*DRAM2*	Wald ratio	1	-0.0069	0.0142	0.993 (0.966, 1.021)	0.629	0.824	1.20E-14	TRUE
CD8 NC	*DUT*	Wald ratio	1	0.0142	0.0167	1.014 (0.982, 1.048)	0.395	0.674	1.09E-10	TRUE
CD8 NC	*DYNLL2*	Wald ratio	1	-0.0266	0.0124	0.974 (0.95, 0.998)	0.033	0.171	9.88E-21	TRUE
CD8 NC	*EBPL*	IVW	2	0.0105	0.0158	1.011 (0.98, 1.042)	0.504	0.744	2.96E-63	TRUE
CD8 NC	*EEA1*	Wald ratio	1	-0.0418	0.0194	0.959 (0.923, 0.996)	0.032	0.168	2.02E-08	TRUE
CD8 NC	*EEF1D*	Wald ratio	1	0.0335	0.0174	1.034 (0.999, 1.07)	0.055	0.234	3.53E-09	TRUE
CD8 NC	*EGFL8*	Wald ratio	1	-0.0411	0.0166	0.96 (0.929, 0.991)	0.013	0.097	2.19E-08	TRUE
CD8 NC	*EI24*	Wald ratio	1	0.0191	0.0206	1.019 (0.979, 1.061)	0.354	0.645	2.61E-08	TRUE
CD8 NC	*EIF2A*	Wald ratio	1	-0.0123	0.0088	0.988 (0.971, 1.005)	0.163	0.437	2.83E-34	TRUE
CD8 NC	*EIF3G*	Wald ratio	1	-0.0404	0.0166	0.96 (0.93, 0.992)	0.015	0.106	3.71E-11	TRUE
CD8 NC	*EIF5A*	Wald ratio	1	-0.0072	0.0048	0.993 (0.984, 1.002)	0.134	0.394	8.05E-164	TRUE
CD8 NC	*ELP2*	Wald ratio	1	0.0134	0.0172	1.013 (0.98, 1.048)	0.435	0.702	6.41E-11	TRUE
CD8 NC	*EMB*	Wald ratio	1	0.0087	0.0214	1.009 (0.967, 1.052)	0.684	0.856	8.64E-10	TRUE
CD8 NC	*EML4*	Wald ratio	1	-0.0310	0.0149	0.969 (0.942, 0.998)	0.038	0.188	1.84E-13	TRUE
CD8 NC	*EMP3*	Wald ratio	1	0.0043	0.0169	1.004 (0.971, 1.038)	0.801	0.915	6.90E-12	TRUE
CD8 NC	*EMR1*	Wald ratio	1	-0.0072	0.0154	0.993 (0.963, 1.023)	0.64	0.83	1.49E-13	TRUE
CD8 NC	*ENOSF1*	Wald ratio	1	0.0096	0.0140	1.01 (0.982, 1.038)	0.491	0.738	1.74E-15	TRUE
CD8 NC	*EPHA1-AS1*	Wald ratio	1	0.0346	0.0176	1.035 (1, 1.072)	0.05	0.222	1.92E-16	TRUE
CD8 NC	*EPHX2*	Wald ratio	1	-0.0186	0.0169	0.982 (0.95, 1.015)	0.271	0.564	1.77E-12	TRUE
CD8 NC	*EPSTI1*	Wald ratio	1	0.0058	0.0095	1.006 (0.987, 1.025)	0.54	0.762	4.16E-35	TRUE
CD8 NC	*ERICH1*	Wald ratio	1	-0.0079	0.0140	0.992 (0.965, 1.02)	0.572	0.786	1.16E-15	TRUE
CD8 NC	*ESR1*	Wald ratio	1	0.0134	0.0116	1.013 (0.991, 1.037)	0.251	0.544	2.87E-17	TRUE
CD8 NC	*ESR2*	Wald ratio	1	-0.0294	0.0136	0.971 (0.945, 0.997)	0.03	0.162	1.98E-26	TRUE
CD8 NC	*ETS1*	Wald ratio	1	0.0063	0.0187	1.006 (0.97, 1.044)	0.737	0.88	1.93E-08	TRUE
CD8 NC	*ETV7*	Wald ratio	1	-0.0283	0.0193	0.972 (0.936, 1.01)	0.144	0.411	1.37E-08	TRUE
CD8 NC	*EVI2A*	Wald ratio	1	-0.0199	0.0126	0.98 (0.956, 1.005)	0.114	0.36	8.07E-18	TRUE
CD8 NC	*EXOC3*	Wald ratio	1	0.0106	0.0103	1.011 (0.991, 1.031)	0.301	0.596	2.35E-28	TRUE
CD8 NC	*FADS1*	Wald ratio	1	0.0103	0.0186	1.01 (0.974, 1.048)	0.578	0.789	2.15E-10	TRUE
CD8 NC	*FADS2*	Wald ratio	1	0.0194	0.0136	1.02 (0.993, 1.047)	0.153	0.423	6.24E-25	TRUE
CD8 NC	*FAM101B*	Wald ratio	1	0.0144	0.0190	1.014 (0.977, 1.053)	0.45	0.714	8.02E-10	TRUE
CD8 NC	*FAM117B*	Wald ratio	1	-0.0259	0.0164	0.974 (0.944, 1.006)	0.114	0.361	1.83E-11	TRUE
CD8 NC	*FAM118A*	Wald ratio	1	0.0006	0.0043	1.001 (0.992, 1.009)	0.895	0.957	4.22E-128	TRUE
CD8 NC	*FAM134B*	Wald ratio	1	0.0188	0.0146	1.019 (0.99, 1.049)	0.198	0.481	2.69E-14	TRUE
CD8 NC	*FAM13A*	Wald ratio	1	-0.0215	0.0174	0.979 (0.946, 1.013)	0.218	0.509	2.44E-13	TRUE
CD8 NC	*FAM153B*	Wald ratio	1	0.0192	0.0078	1.019 (1.004, 1.035)	0.014	0.102	4.62E-48	TRUE
CD8 NC	*FAM154B*	Wald ratio	1	-0.0191	0.0142	0.981 (0.954, 1.009)	0.179	0.459	4.78E-12	TRUE
CD8 NC	*FAM157B*	Wald ratio	1	0.0204	0.0168	1.021 (0.988, 1.055)	0.224	0.517	2.77E-19	TRUE

CD8 NC	*FAM211A*	Wald ratio	1	-0.0353	0.0225	0.965 (0.924, 1.009)	0.116	0.363	1.57E-09	TRUE
CD8 NC	*FAM21A*	Wald ratio	1	-0.0549	0.0187	0.947 (0.913, 0.982)	0.003	0.037	1.32E-09	TRUE
CD8 NC	*FAM26F*	IVW	2	-0.0031	0.0129	0.997 (0.972, 1.023)	0.81	0.919	1.96E-40	TRUE
CD8 NC	*FAM43A*	Wald ratio	1	-0.0093	0.0180	0.991 (0.956, 1.026)	0.604	0.806	2.86E-10	TRUE
CD8 NC	*FAM63B*	Wald ratio	1	0.0402	0.0168	1.041 (1.007, 1.076)	0.017	0.115	3.28E-08	TRUE
CD8 NC	*FAR1*	Wald ratio	1	-0.0023	0.0121	0.998 (0.974, 1.022)	0.851	0.936	1.58E-21	TRUE
CD8 NC	*FAU*	Wald ratio	1	-0.0004	0.0132	1 (0.974, 1.026)	0.973	0.989	3.32E-17	TRUE
CD8 NC	*FBLN5*	Wald ratio	1	0.0021	0.0075	1.002 (0.988, 1.017)	0.779	0.904	2.24E-52	TRUE
CD8 NC	*FBP1*	Wald ratio	1	0.0057	0.0194	1.006 (0.968, 1.045)	0.767	0.897	1.11E-08	TRUE
CD8 NC	*FBXO2*	Wald ratio	1	-0.0498	0.0204	0.951 (0.914, 0.99)	0.015	0.104	3.89E-08	TRUE
CD8 NC	*FCRL3*	Wald ratio	1	0.0158	0.0195	1.016 (0.978, 1.055)	0.416	0.688	5.50E-10	TRUE
CD8 NC	*FGFR1*	Wald ratio	1	-0.0031	0.0221	0.997 (0.955, 1.041)	0.889	0.955	9.79E-09	TRUE
CD8 NC	*FGL2*	Wald ratio	1	-0.0145	0.0148	0.986 (0.957, 1.015)	0.325	0.62	7.28E-12	TRUE
CD8 NC	*FHIT*	Wald ratio	1	-0.0500	0.0184	0.951 (0.918, 0.986)	0.007	0.06	8.57E-09	TRUE
CD8 NC	*FHL3*	Wald ratio	1	-0.0082	0.0144	0.992 (0.964, 1.02)	0.57	0.783	1.98E-14	TRUE
CD8 NC	*FIGNL1*	Wald ratio	1	0.0035	0.0136	1.004 (0.977, 1.031)	0.795	0.911	7.34E-17	TRUE
CD8 NC	*FIS1*	Wald ratio	1	0.0073	0.0160	1.007 (0.976, 1.039)	0.647	0.834	1.72E-14	TRUE
CD8 NC	*FKBP11*	Wald ratio	1	0.0030	0.0109	1.003 (0.982, 1.025)	0.78	0.904	2.79E-25	TRUE
CD8 NC	*FLVCR1-AS1*	Wald ratio	1	-0.0118	0.0105	0.988 (0.968, 1.009)	0.264	0.558	4.11E-27	TRUE
CD8 NC	*FMN1*	Wald ratio	1	-0.0066	0.0054	0.993 (0.983, 1.004)	0.217	0.508	5.74E-88	TRUE
CD8 NC	*FN3KRP*	Wald ratio	1	0.0260	0.0140	1.026 (0.999, 1.055)	0.063	0.255	4.50E-16	TRUE
CD8 NC	*FNBP4*	Wald ratio	1	-0.0759	0.0162	0.927 (0.898, 0.957)	2.84E-06	2.39E-04	3.14E-12	TRUE
CD8 NC	*FNTB*	Wald ratio	1	0.0090	0.0193	1.009 (0.971, 1.048)	0.643	0.832	2.91E-08	TRUE
CD8 NC	*FOXN2*	Wald ratio	1	0.0994	0.0230	1.105 (1.056, 1.155)	1.48E-05	7.56E-04	1.64E-08	TRUE
CD8 NC	*FTCDNL1*	Wald ratio	1	-0.0012	0.0177	0.999 (0.965, 1.034)	0.945	0.979	1.22E-12	TRUE
CD8 NC	*FUT10*	Wald ratio	1	-0.0006	0.0195	0.999 (0.962, 1.038)	0.974	0.989	1.21E-08	TRUE
CD8 NC	*FUT2*	Wald ratio	1	0.0214	0.0088	1.022 (1.004, 1.039)	0.015	0.104	4.24E-34	TRUE
CD8 NC	*FXYD2*	IVW	2	0.0073	0.0126	1.007 (0.983, 1.032)	0.561	0.778	3.12E-19	TRUE
CD8 NC	*G3BP2*	Wald ratio	1	0.0021	0.0153	1.002 (0.973, 1.033)	0.891	0.956	2.82E-13	TRUE
CD8 NC	*GALNT2*	Wald ratio	1	-0.0157	0.0145	0.984 (0.957, 1.013)	0.278	0.571	1.65E-14	TRUE
CD8 NC	*GALT*	Wald ratio	1	0.0966	0.0195	1.101 (1.06, 1.144)	7.32E-07	9.75E-05	1.14E-08	TRUE
CD8 NC	*GATM*	Wald ratio	1	-0.0386	0.0126	0.962 (0.939, 0.986)	0.002	0.028	1.83E-17	TRUE
CD8 NC	*GBP3*	Wald ratio	1	0.0219	0.0201	1.022 (0.983, 1.063)	0.276	0.569	4.22E-08	TRUE
CD8 NC	*GBP5*	Wald ratio	1	-0.0289	0.0113	0.972 (0.95, 0.993)	0.011	0.085	9.57E-23	TRUE
CD8 NC	*GBP7*	Wald ratio	1	0.0005	0.0076	1.001 (0.986, 1.016)	0.946	0.979	5.63E-51	TRUE
CD8 NC	*GCC2*	Wald ratio	1	0.0073	0.0090	1.007 (0.99, 1.025)	0.414	0.686	3.72E-40	TRUE
CD8 NC	*GDI2*	Wald ratio	1	-0.0068	0.0105	0.993 (0.973, 1.014)	0.516	0.751	2.13E-27	TRUE
CD8 NC	*GIMAP2*	Wald ratio	1	-0.0237	0.0186	0.977 (0.942, 1.013)	0.202	0.486	2.41E-09	TRUE
CD8 NC	*GIMAP4*	Wald ratio	1	0.0146	0.0210	1.015 (0.974, 1.057)	0.488	0.738	1.19E-08	TRUE
CD8 NC	*GJB6*	Wald ratio	1	0.0242	0.0117	1.025 (1.001, 1.048)	0.039	0.19	7.27E-20	TRUE
CD8 NC	*GLIPR1*	Wald ratio	1	0.0120	0.0191	1.012 (0.975, 1.051)	0.531	0.758	1.06E-09	TRUE
CD8 NC	*GLIPR1L2*	Wald ratio	1	0.0253	0.0180	1.026 (0.99, 1.062)	0.158	0.43	4.55E-10	TRUE
CD8 NC	*GLIS3*	Wald ratio	1	-0.0267	0.0173	0.974 (0.941, 1.007)	0.122	0.375	8.06E-09	TRUE
CD8 NC	*GLRX5*	Wald ratio	1	0.0274	0.0157	1.028 (0.997, 1.06)	0.081	0.299	1.43E-12	TRUE
CD8 NC	*GLTP*	Wald ratio	1	-0.0318	0.0191	0.969 (0.933, 1.006)	0.096	0.329	6.46E-09	TRUE
CD8 NC	*GLTSCR2*	Wald ratio	1	-0.0079	0.0128	0.992 (0.968, 1.017)	0.537	0.762	3.63E-17	TRUE
CD8 NC	*GNG8*	Wald ratio	1	-0.0027	0.0148	0.997 (0.969, 1.027)	0.858	0.939	7.79E-15	TRUE
CD8 NC	*GNLY*	Wald ratio	1	-0.0235	0.0179	0.977 (0.943, 1.012)	0.189	0.469	7.36E-09	TRUE
CD8 NC	*GNPTG*	Wald ratio	1	-0.0162	0.0129	0.984 (0.959, 1.009)	0.208	0.494	9.84E-15	TRUE
CD8 NC	*GOLT1B*	Wald ratio	1	0.0537	0.0227	1.055 (1.009, 1.103)	0.018	0.118	6.69E-09	TRUE
CD8 NC	*GPR160*	Wald ratio	1	0.0306	0.0099	1.031 (1.011, 1.051)	0.002	0.026	2.14E-28	TRUE
CD8 NC	*GPR18*	Wald ratio	1	0.0309	0.0186	1.031 (0.994, 1.07)	0.097	0.331	4.43E-09	TRUE
CD8 NC	*GPX4*	Wald ratio	1	-0.0165	0.0178	0.984 (0.95, 1.019)	0.354	0.645	1.69E-11	TRUE
CD8 NC	*GRAMD1A*	Wald ratio	1	-0.0240	0.0181	0.976 (0.942, 1.011)	0.184	0.464	4.89E-10	TRUE
CD8 NC	*GRHPR*	Wald ratio	1	-0.0092	0.0076	0.991 (0.976, 1.006)	0.227	0.52	2.70E-46	TRUE
CD8 NC	*GS1-124K5.4*	Wald ratio	1	0.0038	0.0193	1.004 (0.966, 1.043)	0.845	0.935	3.14E-09	TRUE
CD8 NC	*GSTM3*	Wald ratio	1	-0.0097	0.0064	0.99 (0.978, 1.003)	0.125	0.38	1.66E-71	TRUE
CD8 NC	*GSTO1*	Wald ratio	1	0.0318	0.0141	1.032 (1.004, 1.061)	0.024	0.143	8.01E-16	TRUE
CD8 NC	*GSTP1*	Wald ratio	1	0.0248	0.0083	1.025 (1.009, 1.042)	0.003	0.034	1.41E-41	TRUE
CD8 NC	*GTF2H4*	Wald ratio	1	0.0274	0.0090	1.028 (1.01, 1.046)	0.002	0.03	1.28E-31	TRUE
CD8 NC	*GTPBP8*	Wald ratio	1	-0.0016	0.0187	0.998 (0.962, 1.036)	0.933	0.972	4.43E-08	TRUE
CD8 NC	*GTSCR1*	Wald ratio	1	0.0007	0.0165	1.001 (0.969, 1.034)	0.967	0.989	1.92E-10	TRUE
CD8 NC	*GYPC*	Wald ratio	1	-0.0094	0.0097	0.991 (0.972, 1.01)	0.334	0.627	3.68E-28	TRUE
CD8 NC	*GZMM*	Wald ratio	1	0.0150	0.0221	1.015 (0.972, 1.06)	0.498	0.741	2.12E-10	TRUE
CD8 NC	*H6PD*	Wald ratio	1	-0.0349	0.0199	0.966 (0.929, 1.004)	0.079	0.294	1.58E-09	TRUE
CD8 NC	*HAGH*	Wald ratio	1	0.0460	0.0099	1.047 (1.027, 1.068)	3.61E-06	2.70E-04	2.98E-24	TRUE
CD8 NC	*HAGHL*	Wald ratio	1	0.0290	0.0199	1.029 (0.99, 1.07)	0.145	0.415	5.11E-08	TRUE
CD8 NC	*HAX1*	Wald ratio	1	-0.0131	0.0146	0.987 (0.959, 1.016)	0.368	0.656	6.45E-13	TRUE
CD8 NC	*HCG25*	Wald ratio	1	0.0061	0.0111	1.006 (0.984, 1.028)	0.583	0.792	8.03E-24	TRUE
CD8 NC	*HDGFRP3*	Wald ratio	1	0.0048	0.0184	1.005 (0.969, 1.042)	0.792	0.909	1.05E-09	TRUE
CD8 NC	*HEBP1*	Wald ratio	1	-0.0269	0.0190	0.973 (0.938, 1.01)	0.157	0.428	6.46E-09	TRUE
CD8 NC	*HEBP2*	Wald ratio	1	0.0200	0.0177	1.02 (0.985, 1.056)	0.258	0.552	1.51E-12	TRUE
CD8 NC	*HELLS*	Wald ratio	1	-0.0215	0.0196	0.979 (0.942, 1.017)	0.274	0.568	8.13E-09	TRUE
CD8 NC	*HFE*	Wald ratio	1	0.0044	0.0255	1.004 (0.955, 1.056)	0.864	0.943	4.57E-08	TRUE
CD8 NC	*HIGD2A*	Wald ratio	1	-0.0083	0.0096	0.992 (0.973, 1.01)	0.385	0.668	2.81E-33	TRUE
CD8 NC	*HIST1H2AD*	Wald ratio	1	-0.0055	0.0233	0.995 (0.95, 1.041)	0.814	0.921	4.93E-09	TRUE
CD8 NC	*HIST1H2AI*	Wald ratio	1	-0.0316	0.0234	0.969 (0.925, 1.014)	0.177	0.456	1.34E-10	TRUE
CD8 NC	*HIST1H2BB*	Wald ratio	1	0.0255	0.0189	1.026 (0.989, 1.064)	0.178	0.457	2.96E-11	TRUE
CD8 NC	*HIST1H2BF*	Wald ratio	1	0.0332	0.0165	1.034 (1.001, 1.068)	0.045	0.208	3.52E-09	TRUE

CD8 NC	*HIST1H2BI*	Wald ratio	1	0.0203	0.0224	1.021 (0.977, 1.066)	0.364	0.653	1.16E-11	TRUE
CD8 NC	*HIST1H2BJ*	Wald ratio	1	0.0290	0.0253	1.029 (0.98, 1.082)	0.25	0.543	3.46E-08	TRUE
CD8 NC	*HIST1H2BL*	Wald ratio	1	-0.0061	0.0125	0.994 (0.97, 1.019)	0.626	0.822	1.28E-44	TRUE
CD8 NC	*HIST1H2BO*	Wald ratio	1	-0.0188	0.0090	0.981 (0.964, 0.999)	0.036	0.182	3.17E-46	TRUE
CD8 NC	*HIST1H3H*	Wald ratio	1	0.0134	0.0276	1.013 (0.96, 1.07)	0.628	0.822	4.15E-09	TRUE
CD8 NC	*HIST1H3I*	Wald ratio	1	0.0162	0.0175	1.016 (0.982, 1.052)	0.355	0.646	3.05E-17	TRUE
CD8 NC	*HIVEP3*	Wald ratio	1	0.0102	0.0142	1.01 (0.983, 1.039)	0.474	0.73	1.73E-15	TRUE
CD8 NC	*HLA-B*	IVW	3	0.0030	0.0206	1.003 (0.963, 1.044)	0.886	0.954	2.83E-108	TRUE
CD8 NC	*HLA-B*	MR Egger	3	0.2356	0.0443	1.266 (1.16, 1.381)	0.118	NA	NA	NA
CD8 NC	*HLA-B*	Weighted median	3	0.0029	0.0064	1.003 (0.99, 1.016)	0.651	NA	NA	NA
CD8 NC	*HLA-B*	Simple mode	3	-0.0346	0.0106	0.966 (0.946, 0.986)	0.083	NA	NA	NA
CD8 NC	*HLA-B*	Weighted mode	3	0.0260	0.0077	1.026 (1.011, 1.042)	0.077	NA	NA	NA
CD8 NC	*HLA-C*	Wald ratio	1	-0.0149	0.0102	0.985 (0.966, 1.005)	0.146	0.415	1.44E-24	TRUE
CD8 NC	*HLA-DQB2*	Wald ratio	1	-0.0703	0.0166	0.932 (0.902, 0.963)	2.33E-05	0.001	1.91E-12	TRUE
CD8 NC	*HLA-DRB5*	Wald ratio	1	0.0468	0.0095	1.048 (1.029, 1.068)	7.43E-07	9.75E-05	9.35E-39	TRUE
CD8 NC	*HLA-F*	Wald ratio	1	-0.0134	0.0152	0.987 (0.958, 1.017)	0.379	0.665	6.05E-14	TRUE
CD8 NC	*HMBOX1*	Wald ratio	1	-0.0135	0.0074	0.987 (0.972, 1.001)	0.066	0.263	3.35E-48	TRUE
CD8 NC	*HNRNPU*	Wald ratio	1	-0.0616	0.0176	0.94 (0.908, 0.973)	4.59E-04	0.01	7.20E-09	TRUE
CD8 NC	*HOXB-AS1*	Wald ratio	1	-0.0114	0.0110	0.989 (0.968, 1.01)	0.299	0.594	3.54E-24	TRUE
CD8 NC	*HOXB2*	Wald ratio	1	-0.0206	0.0086	0.98 (0.963, 0.996)	0.017	0.116	1.25E-37	TRUE
CD8 NC	*HOXB4*	Wald ratio	1	-0.0204	0.0145	0.98 (0.952, 1.008)	0.161	0.434	1.73E-14	TRUE
CD8 NC	*HPCAL4*	Wald ratio	1	-0.0187	0.0165	0.981 (0.95, 1.014)	0.255	0.549	1.67E-11	TRUE
CD8 NC	*HSD17B12*	Wald ratio	1	-0.0613	0.0186	0.941 (0.907, 0.976)	9.95E-04	0.017	8.51E-09	TRUE
CD8 NC	*HSD17B14*	Wald ratio	1	-0.0155	0.0132	0.985 (0.959, 1.01)	0.241	0.534	1.66E-16	TRUE
CD8 NC	*HSPBAP1*	Wald ratio	1	0.0072	0.0073	1.007 (0.993, 1.022)	0.327	0.62	4.47E-44	TRUE
CD8 NC	*IAH1*	Wald ratio	1	0.0211	0.0194	1.021 (0.983, 1.061)	0.278	0.571	1.52E-08	TRUE
CD8 NC	*ICA1L*	Wald ratio	1	-0.0353	0.0149	0.965 (0.938, 0.994)	0.018	0.118	5.74E-15	TRUE
CD8 NC	*ICAM3*	Wald ratio	1	0.0721	0.0152	1.075 (1.043, 1.107)	2.05E-06	1.89E-04	8.02E-10	TRUE
CD8 NC	*IFI27L2*	Wald ratio	1	0.0067	0.0118	1.007 (0.984, 1.03)	0.568	0.782	1.09E-20	TRUE
CD8 NC	*IFITM2*	Wald ratio	1	-0.0035	0.0095	0.997 (0.978, 1.015)	0.713	0.872	2.21E-31	TRUE
CD8 NC	*IFNG-AS1*	Wald ratio	1	-0.0059	0.0141	0.994 (0.967, 1.022)	0.673	0.851	2.20E-15	TRUE
CD8 NC	*IKZF1*	Wald ratio	1	-0.0022	0.0191	0.998 (0.961, 1.036)	0.907	0.961	3.22E-09	TRUE
CD8 NC	*IL24*	Wald ratio	1	0.0202	0.0172	1.02 (0.987, 1.055)	0.24	0.534	5.87E-11	TRUE
CD8 NC	*IL32*	IVW	2	-0.0110	0.0126	0.989 (0.965, 1.014)	0.381	0.666	1.99E-26	TRUE
CD8 NC	*IMMT*	Wald ratio	1	-0.0233	0.0198	0.977 (0.94, 1.016)	0.24	0.534	2.66E-08	TRUE
CD8 NC	*INF2*	Wald ratio	1	0.0153	0.0133	1.015 (0.989, 1.042)	0.248	0.541	2.93E-14	TRUE
CD8 NC	*INPP4B*	Wald ratio	1	-0.0690	0.0227	0.933 (0.893, 0.976)	0.002	0.029	1.18E-08	TRUE
CD8 NC	*INTS5*	Wald ratio	1	-0.0262	0.0201	0.974 (0.936, 1.013)	0.193	0.474	1.11E-09	TRUE
CD8 NC	*IQSEC3*	Wald ratio	1	-0.0386	0.0197	0.962 (0.926, 1)	0.05	0.222	4.29E-08	TRUE
CD8 NC	*IRF7*	Wald ratio	1	-0.0038	0.0191	0.996 (0.96, 1.034)	0.842	0.934	1.09E-09	TRUE
CD8 NC	*ISCA1*	Wald ratio	1	0.0258	0.0132	1.026 (1, 1.053)	0.051	0.224	6.64E-16	TRUE
CD8 NC	*ISCA2*	Wald ratio	1	0.0063	0.0098	1.006 (0.987, 1.026)	0.519	0.753	3.53E-30	TRUE
CD8 NC	*ISG15*	Wald ratio	1	-0.0375	0.0160	0.963 (0.933, 0.994)	0.019	0.123	1.71E-11	TRUE
CD8 NC	*ITGB2*	Wald ratio	1	-0.0007	0.0056	0.999 (0.988, 1.01)	0.897	0.957	2.43E-79	TRUE
CD8 NC	*ITGB2-AS1*	Wald ratio	1	-0.0122	0.0201	0.988 (0.95, 1.028)	0.544	0.766	7.26E-09	TRUE
CD8 NC	*ITGB3BP*	Wald ratio	1	0.0078	0.0128	1.008 (0.983, 1.034)	0.541	0.763	1.84E-17	TRUE
CD8 NC	*ITGB7*	Wald ratio	1	-0.0349	0.0122	0.966 (0.943, 0.989)	0.004	0.045	3.02E-13	TRUE
CD8 NC	*ITM2C*	Wald ratio	1	0.0029	0.0113	1.003 (0.981, 1.025)	0.795	0.911	3.58E-24	TRUE
CD8 NC	*JAKMIP2*	Wald ratio	1	0.0564	0.0181	1.058 (1.021, 1.096)	0.002	0.025	2.54E-10	TRUE
CD8 NC	*KANSL1-AS1*	Wald ratio	1	-0.0174	0.0071	0.983 (0.969, 0.997)	0.014	0.102	2.72E-152	TRUE
CD8 NC	*KCNAB2*	Wald ratio	1	0.0174	0.0145	1.018 (0.989, 1.047)	0.23	0.523	1.10E-17	TRUE
CD8 NC	*KCNN1*	Wald ratio	1	0.0212	0.0200	1.021 (0.982, 1.062)	0.288	0.581	1.34E-08	TRUE
CD8 NC	*KCTD21-AS1*	Wald ratio	1	0.0035	0.0155	1.004 (0.973, 1.035)	0.821	0.925	7.19E-13	TRUE
CD8 NC	*KDELR2*	Wald ratio	1	0.0336	0.0104	1.034 (1.013, 1.056)	0.001	0.02	3.61E-25	TRUE
CD8 NC	*KIAA1430*	Wald ratio	1	0.0064	0.0146	1.006 (0.978, 1.036)	0.663	0.845	1.25E-13	TRUE
CD8 NC	*KIF16B*	Wald ratio	1	0.0013	0.0157	1.001 (0.971, 1.033)	0.936	0.974	8.05E-12	TRUE
CD8 NC	*KISS1R*	Wald ratio	1	-0.0033	0.0179	0.997 (0.962, 1.032)	0.855	0.938	5.30E-12	TRUE
CD8 NC	*KLF13*	Wald ratio	1	0.0222	0.0201	1.022 (0.983, 1.064)	0.271	0.564	5.10E-08	TRUE
CD8 NC	*KLRC4*	Wald ratio	1	-0.0107	0.0096	0.989 (0.971, 1.008)	0.268	0.562	8.93E-31	TRUE
CD8 NC	*KLRD1*	Wald ratio	1	-0.0246	0.0146	0.976 (0.948, 1.004)	0.092	0.323	2.65E-13	TRUE
CD8 NC	*KRT1*	Wald ratio	1	-0.0031	0.0060	0.997 (0.985, 1.009)	0.604	0.806	2.47E-83	TRUE
CD8 NC	*KRT10*	Wald ratio	1	0.0169	0.0192	1.017 (0.98, 1.056)	0.379	0.665	1.18E-08	TRUE
CD8 NC	*KRT72*	Wald ratio	1	-0.0335	0.0122	0.967 (0.944, 0.991)	0.006	0.058	2.60E-18	TRUE
CD8 NC	*KRTCAP3*	Wald ratio	1	-0.0241	0.0097	0.976 (0.958, 0.995)	0.013	0.095	3.66E-30	TRUE
CD8 NC	*KTN1*	Wald ratio	1	0.0356	0.0114	1.036 (1.013, 1.06)	0.002	0.025	8.08E-22	TRUE
CD8 NC	*L3MBTL3*	Wald ratio	1	-0.0042	0.0120	0.996 (0.973, 1.02)	0.726	0.878	5.87E-23	TRUE
CD8 NC	*LA16c-358B7.3*	Wald ratio	1	0.0416	0.0104	1.043 (1.021, 1.064)	6.58E-05	0.002	1.41E-28	TRUE
CD8 NC	*LAIR2*	Wald ratio	1	0.0675	0.0231	1.07 (1.022, 1.119)	0.003	0.039	4.40E-08	TRUE
CD8 NC	*LAMB1*	Wald ratio	1	0.0074	0.0078	1.007 (0.992, 1.023)	0.343	0.635	1.91E-48	TRUE
CD8 NC	*LAMTOR4*	Wald ratio	1	-0.0109	0.0146	0.989 (0.961, 1.018)	0.456	0.718	3.12E-14	TRUE
CD8 NC	*LCLAT1*	Wald ratio	1	-0.0006	0.0089	0.999 (0.982, 1.017)	0.947	0.979	1.90E-40	TRUE
CD8 NC	*LCNL1*	Wald ratio	1	0.0093	0.0205	1.009 (0.97, 1.051)	0.651	0.836	3.92E-08	TRUE
CD8 NC	*LDHC*	Wald ratio	1	-0.0160	0.0112	0.984 (0.963, 1.006)	0.153	0.423	3.12E-27	TRUE
CD8 NC	*LDLRAP1*	Wald ratio	1	0.0075	0.0101	1.008 (0.988, 1.028)	0.458	0.719	6.66E-29	TRUE
CD8 NC	*LEPROT*	Wald ratio	1	0.0005	0.0128	1.001 (0.976, 1.026)	0.969	0.989	1.08E-19	TRUE
CD8 NC	*LGALS3*	Wald ratio	1	0.0121	0.0175	1.012 (0.978, 1.048)	0.489	0.738	9.29E-10	TRUE
CD8 NC	*LIMD1*	Wald ratio	1	0.0092	0.0102	1.009 (0.989, 1.03)	0.368	0.656	2.15E-28	TRUE
CD8 NC	*LINC00339*	Wald ratio	1	0.0145	0.0129	1.015 (0.989, 1.041)	0.261	0.556	8.26E-18	TRUE
CD8 NC	*LINC00649*	Wald ratio	1	-0.0217	0.0100	0.979 (0.959, 0.998)	0.031	0.166	1.98E-26	TRUE

CD8 NC	*LINC00884*	Wald ratio	1	0.0022	0.0094	1.002 (0.984, 1.021)	0.816	0.921	2.89E-33	TRUE
CD8 NC	*LINC00954*	Wald ratio	1	-0.0102	0.0118	0.99 (0.967, 1.013)	0.385	0.668	4.66E-26	TRUE
CD8 NC	*LINC00969*	Wald ratio	1	-0.0221	0.0102	0.978 (0.959, 0.998)	0.03	0.162	5.33E-27	TRUE
CD8 NC	*LIPC*	Wald ratio	1	-0.0645	0.0231	0.938 (0.896, 0.981)	0.005	0.051	3.73E-11	TRUE
CD8 NC	*LL22NC03-2H8.5*	Wald ratio	1	0.0056	0.0180	1.006 (0.971, 1.042)	0.758	0.889	3.50E-08	TRUE
CD8 NC	*LPIN1*	Wald ratio	1	-0.0064	0.0126	0.994 (0.969, 1.019)	0.612	0.812	4.52E-18	TRUE
CD8 NC	*LPIN2*	Wald ratio	1	-0.0226	0.0141	0.978 (0.951, 1.005)	0.108	0.349	1.52E-16	TRUE
CD8 NC	*LRRC14*	Wald ratio	1	0.0143	0.0164	1.014 (0.982, 1.048)	0.382	0.666	1.03E-11	TRUE
CD8 NC	*LRRC16A*	Wald ratio	1	-0.0191	0.0146	0.981 (0.953, 1.01)	0.192	0.473	6.53E-15	TRUE
CD8 NC	*LRRC37A*	Wald ratio	1	-0.0537	0.0117	0.948 (0.926, 0.97)	4.32E-06	3.02E-04	7.95E-18	TRUE
CD8 NC	*LRRC37A2*	Wald ratio	1	-0.0622	0.0257	0.94 (0.894, 0.988)	0.015	0.106	5.85E-11	TRUE
CD8 NC	*LRRC61*	Wald ratio	1	-0.0789	0.0169	0.924 (0.894, 0.955)	3.12E-06	2.60E-04	3.29E-09	TRUE
CD8 NC	*LRRIQ3*	Wald ratio	1	-0.0063	0.0183	0.994 (0.959, 1.03)	0.731	0.88	1.35E-09	TRUE
CD8 NC	*LSM7*	Wald ratio	1	0.0551	0.0172	1.057 (1.022, 1.093)	0.001	0.02	3.19E-10	TRUE
CD8 NC	*LYAR*	Wald ratio	1	-0.0261	0.0197	0.974 (0.937, 1.012)	0.184	0.463	2.57E-08	TRUE
CD8 NC	*LYRM4*	Wald ratio	1	0.0136	0.0105	1.014 (0.993, 1.035)	0.197	0.48	6.37E-22	TRUE
CD8 NC	*MACF1*	Wald ratio	1	0.0261	0.0252	1.026 (0.977, 1.078)	0.3	0.596	8.57E-09	TRUE
CD8 NC	*MANBA*	Wald ratio	1	0.0627	0.0174	1.065 (1.029, 1.102)	3.16E-04	0.007	9.46E-10	TRUE
CD8 NC	*MAST3*	Wald ratio	1	0.0543	0.0182	1.056 (1.019, 1.094)	0.003	0.033	1.71E-09	TRUE
CD8 NC	*MBP*	Wald ratio	1	-0.0184	0.0189	0.982 (0.946, 1.019)	0.331	0.625	4.68E-10	TRUE
CD8 NC	*MCC*	Wald ratio	1	0.0302	0.0139	1.031 (1.003, 1.059)	0.03	0.162	4.70E-14	TRUE
CD8 NC	*MDGA1*	Wald ratio	1	-0.0104	0.0133	0.99 (0.964, 1.016)	0.435	0.702	1.67E-17	TRUE
CD8 NC	*MDH1*	Wald ratio	1	0.0241	0.0220	1.024 (0.981, 1.069)	0.272	0.566	3.10E-08	TRUE
CD8 NC	*MDM4*	Wald ratio	1	-0.0503	0.0134	0.951 (0.926, 0.976)	1.71E-04	0.004	1.11E-20	TRUE
CD8 NC	*MDS2*	Wald ratio	1	-0.0154	0.0206	0.985 (0.946, 1.025)	0.454	0.717	5.43E-08	TRUE
CD8 NC	*MEAF6*	Wald ratio	1	-0.0276	0.0164	0.973 (0.942, 1.005)	0.092	0.323	3.35E-12	TRUE
CD8 NC	*MED16*	Wald ratio	1	0.0003	0.0098	1 (0.981, 1.02)	0.973	0.989	2.07E-36	TRUE
CD8 NC	*METTL10*	Wald ratio	1	0.0116	0.0171	1.012 (0.978, 1.046)	0.497	0.741	5.06E-11	TRUE
CD8 NC	*METTL21B*	Wald ratio	1	-0.0193	0.0169	0.981 (0.949, 1.014)	0.252	0.545	9.19E-11	TRUE
CD8 NC	*MFF*	Wald ratio	1	-0.0048	0.0211	0.995 (0.955, 1.037)	0.82	0.924	2.56E-09	TRUE
CD8 NC	*MGMT*	Wald ratio	1	-0.0103	0.0117	0.99 (0.967, 1.013)	0.381	0.666	3.96E-21	TRUE
CD8 NC	*MGST2*	Wald ratio	1	-0.0074	0.0153	0.993 (0.963, 1.023)	0.627	0.822	1.77E-12	TRUE
CD8 NC	*MGST3*	Wald ratio	1	-0.0129	0.0130	0.987 (0.962, 1.013)	0.319	0.614	3.50E-18	TRUE
CD8 NC	*MICU3*	Wald ratio	1	0.0252	0.0175	1.026 (0.991, 1.061)	0.15	0.42	4.61E-09	TRUE
CD8 NC	*MORN3*	Wald ratio	1	-0.0134	0.0138	0.987 (0.96, 1.014)	0.334	0.627	6.62E-16	TRUE
CD8 NC	*MPHOSPH9*	Wald ratio	1	-0.0510	0.0164	0.95 (0.92, 0.981)	0.002	0.025	1.24E-11	TRUE
CD8 NC	*MPZL3*	Wald ratio	1	0.0108	0.0176	1.011 (0.977, 1.046)	0.539	0.762	3.27E-10	TRUE
CD8 NC	*MRPL14*	Wald ratio	1	-0.0083	0.0179	0.992 (0.958, 1.027)	0.645	0.834	5.67E-10	TRUE
CD8 NC	*MRPL18*	Wald ratio	1	-0.0041	0.0182	0.996 (0.961, 1.032)	0.823	0.926	1.41E-11	TRUE
CD8 NC	*MRPL21*	Wald ratio	1	0.0102	0.0096	1.01 (0.992, 1.029)	0.284	0.577	2.71E-28	TRUE
CD8 NC	*MRPL32*	Wald ratio	1	-0.0115	0.0233	0.989 (0.944, 1.035)	0.623	0.822	4.25E-12	TRUE
CD8 NC	*MRPL42*	Wald ratio	1	0.0214	0.0149	1.022 (0.992, 1.052)	0.152	0.423	7.64E-13	TRUE
CD8 NC	*MRPL48*	Wald ratio	1	-0.0136	0.0189	0.986 (0.951, 1.024)	0.471	0.729	6.65E-09	TRUE
CD8 NC	*MRPL53*	Wald ratio	1	-0.0125	0.0161	0.988 (0.957, 1.019)	0.437	0.703	8.23E-11	TRUE
CD8 NC	*MRPL54*	Wald ratio	1	0.0085	0.0109	1.009 (0.987, 1.03)	0.434	0.702	8.30E-25	TRUE
CD8 NC	*MRPS18C*	Wald ratio	1	-0.0030	0.0167	0.997 (0.965, 1.03)	0.858	0.939	2.59E-11	TRUE
CD8 NC	*MSH5*	Wald ratio	1	-0.0138	0.0194	0.986 (0.949, 1.025)	0.477	0.732	6.62E-08	TRUE
CD8 NC	*MT1X*	Wald ratio	1	0.0038	0.0101	1.004 (0.984, 1.024)	0.702	0.866	7.49E-31	TRUE
CD8 NC	*MT2A*	Wald ratio	1	-0.0180	0.0098	0.982 (0.963, 1.001)	0.067	0.265	3.02E-35	TRUE
CD8 NC	*MTERFD2*	Wald ratio	1	-0.0073	0.0170	0.993 (0.96, 1.026)	0.669	0.848	2.74E-10	TRUE
CD8 NC	*MTG2*	Wald ratio	1	0.0333	0.0208	1.034 (0.992, 1.077)	0.111	0.354	5.91E-08	TRUE
CD8 NC	*MTSS1*	Wald ratio	1	0.0242	0.0205	1.024 (0.984, 1.066)	0.237	0.532	7.60E-09	TRUE
CD8 NC	*MTUS1*	Wald ratio	1	0.0064	0.0182	1.006 (0.971, 1.043)	0.726	0.878	2.57E-09	TRUE
CD8 NC	*MUC20*	Wald ratio	1	-0.0180	0.0154	0.982 (0.953, 1.012)	0.244	0.537	1.55E-12	TRUE
CD8 NC	*MX1*	Wald ratio	1	0.0234	0.0178	1.024 (0.989, 1.06)	0.189	0.47	7.11E-10	TRUE
CD8 NC	*MYCBPAP*	Wald ratio	1	-0.0045	0.0168	0.996 (0.963, 1.029)	0.789	0.909	3.16E-11	TRUE
CD8 NC	*MYH11*	Wald ratio	1	-0.0172	0.0127	0.983 (0.959, 1.008)	0.176	0.456	9.90E-18	TRUE
CD8 NC	*MYO15B*	Wald ratio	1	-0.0150	0.0204	0.985 (0.947, 1.025)	0.462	0.721	3.78E-11	TRUE
CD8 NC	*MYO16*	Wald ratio	1	0.0329	0.0099	1.033 (1.014, 1.054)	9.16E-04	0.016	2.17E-31	TRUE
CD8 NC	*MYOM2*	Wald ratio	1	-0.0063	0.0099	0.994 (0.975, 1.013)	0.524	0.754	1.01E-27	TRUE
CD8 NC	*MZT2A*	Wald ratio	1	-0.0106	0.0063	0.989 (0.977, 1.002)	0.092	0.321	3.65E-56	TRUE
CD8 NC	*NAA38*	Wald ratio	1	-0.0087	0.0119	0.991 (0.969, 1.015)	0.465	0.725	6.19E-28	TRUE
CD8 NC	*NAAA*	Wald ratio	1	0.0228	0.0149	1.023 (0.994, 1.053)	0.127	0.382	1.59E-13	TRUE
CD8 NC	*NABP1*	Wald ratio	1	-0.0189	0.0190	0.981 (0.945, 1.019)	0.321	0.616	3.19E-10	TRUE
CD8 NC	*NAP1L1*	Wald ratio	1	-0.0086	0.0110	0.991 (0.97, 1.013)	0.432	0.701	1.80E-24	TRUE
CD8 NC	*NAPRT1*	Wald ratio	1	-0.0126	0.0076	0.988 (0.973, 1.002)	0.099	0.333	1.81E-48	TRUE
CD8 NC	*NBL1*	IVW	2	0.0037	0.0095	1.004 (0.985, 1.023)	0.702	0.866	6.14E-31	TRUE
CD8 NC	*NBPF3*	Wald ratio	1	-0.0028	0.0195	0.997 (0.96, 1.036)	0.887	0.955	1.36E-08	TRUE
CD8 NC	*NCAPG2*	Wald ratio	1	-0.0201	0.0218	0.98 (0.939, 1.023)	0.357	0.648	2.07E-09	TRUE
CD8 NC	*NCF2*	Wald ratio	1	-0.0180	0.0124	0.982 (0.959, 1.006)	0.148	0.417	9.27E-20	TRUE
CD8 NC	*NCF4*	Wald ratio	1	-0.0314	0.0189	0.969 (0.934, 1.006)	0.096	0.329	1.16E-10	TRUE
CD8 NC	*NCL*	Wald ratio	1	-0.0437	0.0204	0.957 (0.92, 0.996)	0.032	0.169	1.65E-08	TRUE
CD8 NC	*NDUFA10*	Wald ratio	1	-0.0355	0.0181	0.965 (0.932, 1)	0.049	0.22	2.62E-10	TRUE
CD8 NC	*NDUFA12*	Wald ratio	1	0.0039	0.0105	1.004 (0.983, 1.025)	0.712	0.872	8.01E-32	TRUE
CD8 NC	*NDUFA3*	Wald ratio	1	0.0045	0.0127	1.004 (0.98, 1.03)	0.725	0.878	1.43E-19	TRUE
CD8 NC	*NDUFS5*	Wald ratio	1	-0.0062	0.0102	0.994 (0.974, 1.014)	0.54	0.763	2.39E-32	TRUE
CD8 NC	*NEXN*	Wald ratio	1	-0.0158	0.0155	0.984 (0.955, 1.015)	0.309	0.603	5.69E-11	TRUE
CD8 NC	*NINJ2*	Wald ratio	1	0.0235	0.0148	1.024 (0.994, 1.054)	0.113	0.359	2.68E-14	TRUE
CD8 NC	*NME1*	Wald ratio	1	0.0046	0.0136	1.005 (0.978, 1.032)	0.737	0.88	1.71E-16	TRUE

CD8 NC	*NMI*	Wald ratio	1	0.0239	0.0113	1.024 (1.002, 1.047)	0.034	0.175	8.22E-23	TRUE
CD8 NC	*NMRK1*	Wald ratio	1	-0.0064	0.0094	0.994 (0.975, 1.012)	0.495	0.74	7.65E-39	TRUE
CD8 NC	*NMT2*	Wald ratio	1	-0.0209	0.0112	0.979 (0.958, 1.001)	0.062	0.253	2.19E-23	TRUE
CD8 NC	*NOTCH4*	Wald ratio	1	-0.0326	0.0145	0.968 (0.941, 0.996)	0.024	0.144	3.14E-30	TRUE
CD8 NC	*NPC2*	Wald ratio	1	0.0056	0.0087	1.006 (0.989, 1.023)	0.519	0.753	3.88E-38	TRUE
CD8 NC	*NPDC1*	Wald ratio	1	0.0151	0.0165	1.015 (0.983, 1.049)	0.359	0.65	1.08E-11	TRUE
CD8 NC	*NQO2*	Wald ratio	1	-0.0221	0.0180	0.978 (0.944, 1.013)	0.22	0.511	6.39E-10	TRUE
CD8 NC	*NSA2*	Wald ratio	1	0.0174	0.0076	1.018 (1.003, 1.033)	0.022	0.134	2.93E-56	TRUE
CD8 NC	*NSG1*	Wald ratio	1	0.0098	0.0075	1.01 (0.995, 1.025)	0.191	0.472	4.27E-66	TRUE
CD8 NC	*NT5C3A*	Wald ratio	1	-0.0197	0.0131	0.981 (0.956, 1.006)	0.135	0.396	2.22E-18	TRUE
CD8 NC	*NT5C3B*	Wald ratio	1	0.0001	0.0090	1 (0.983, 1.018)	0.991	0.996	7.16E-31	TRUE
CD8 NC	*NTPCR*	Wald ratio	1	0.0125	0.0083	1.013 (0.996, 1.029)	0.13	0.391	4.51E-49	TRUE
CD8 NC	*NUCKS1*	Wald ratio	1	0.0246	0.0198	1.025 (0.986, 1.065)	0.213	0.502	3.63E-08	TRUE
CD8 NC	*NUDT18*	Wald ratio	1	-0.0277	0.0172	0.973 (0.94, 1.006)	0.108	0.35	3.41E-11	TRUE
CD8 NC	*NUDT2*	Wald ratio	1	0.0031	0.0181	1.003 (0.968, 1.039)	0.862	0.942	8.71E-10	TRUE
CD8 NC	*NUP107*	Wald ratio	1	-0.0314	0.0180	0.969 (0.935, 1.004)	0.081	0.298	4.35E-09	TRUE
CD8 NC	*NUTF2*	Wald ratio	1	0.0158	0.0229	1.016 (0.971, 1.063)	0.491	0.738	2.66E-11	TRUE
CD8 NC	*ORMDL3*	Wald ratio	1	-0.0029	0.0082	0.997 (0.981, 1.013)	0.724	0.878	2.71E-33	TRUE
CD8 NC	*OSBPL10*	Wald ratio	1	0.0081	0.0164	1.008 (0.976, 1.041)	0.621	0.82	5.45E-12	TRUE
CD8 NC	*OSGIN1*	Wald ratio	1	-0.0082	0.0161	0.992 (0.961, 1.024)	0.612	0.812	1.54E-10	TRUE
CD8 NC	*OSTF1*	Wald ratio	1	-0.0102	0.0144	0.99 (0.962, 1.018)	0.477	0.732	1.57E-16	TRUE
CD8 NC	*OXLD1*	Wald ratio	1	0.0037	0.0087	1.004 (0.987, 1.021)	0.668	0.848	1.65E-36	TRUE
CD8 NC	*OXNAD1*	Wald ratio	1	-0.0238	0.0203	0.976 (0.938, 1.016)	0.24	0.534	1.39E-09	TRUE
CD8 NC	*P4HA3*	Wald ratio	1	-0.0051	0.0079	0.995 (0.98, 1.011)	0.521	0.753	1.41E-47	TRUE
CD8 NC	*PADI4*	Wald ratio	1	0.0000	0.0216	1 (0.959, 1.043)	1	1	9.76E-09	TRUE
CD8 NC	*PARK7*	Wald ratio	1	-0.0093	0.0185	0.991 (0.955, 1.027)	0.617	0.817	3.54E-10	TRUE
CD8 NC	*PARVB*	Wald ratio	1	0.0085	0.0153	1.009 (0.979, 1.039)	0.578	0.789	1.17E-13	TRUE
CD8 NC	*PASK*	Wald ratio	1	-0.0034	0.0079	0.997 (0.981, 1.012)	0.669	0.848	1.01E-43	TRUE
CD8 NC	*PAX8*	Wald ratio	1	0.0199	0.0133	1.02 (0.994, 1.047)	0.135	0.396	2.52E-17	TRUE
CD8 NC	*PCBP2*	Wald ratio	1	-0.0831	0.0210	0.92 (0.883, 0.959)	7.45E-05	0.003	3.74E-08	TRUE
CD8 NC	*PCM1*	Wald ratio	1	-0.0151	0.0173	0.985 (0.952, 1.019)	0.383	0.667	1.91E-11	TRUE
CD8 NC	*PCSK7*	Wald ratio	1	-0.0260	0.0186	0.974 (0.94, 1.01)	0.162	0.435	3.30E-08	TRUE
CD8 NC	*PDE6G*	Wald ratio	1	0.0073	0.0170	1.007 (0.974, 1.041)	0.668	0.848	1.49E-10	TRUE
CD8 NC	*PDE9A*	Wald ratio	1	0.0065	0.0160	1.007 (0.976, 1.039)	0.682	0.855	4.49E-13	TRUE
CD8 NC	*PDIA6*	Wald ratio	1	-0.0120	0.0186	0.988 (0.953, 1.025)	0.519	0.753	2.11E-09	TRUE
CD8 NC	*PDXDC1*	Wald ratio	1	-0.0418	0.0198	0.959 (0.923, 0.997)	0.034	0.175	1.10E-09	TRUE
CD8 NC	*PDXK*	Wald ratio	1	0.0042	0.0128	1.004 (0.979, 1.03)	0.741	0.881	1.88E-16	TRUE
CD8 NC	*PGLS*	Wald ratio	1	-0.0087	0.0198	0.991 (0.954, 1.031)	0.66	0.843	1.18E-08	TRUE
CD8 NC	*PHACTR4*	Wald ratio	1	0.0644	0.0178	1.067 (1.03, 1.104)	2.97E-04	0.007	1.60E-09	TRUE
CD8 NC	*PHLDB3*	Wald ratio	1	-0.0018	0.0152	0.998 (0.969, 1.028)	0.905	0.96	2.88E-12	TRUE
CD8 NC	*PI16*	Wald ratio	1	-0.0080	0.0125	0.992 (0.968, 1.017)	0.521	0.753	1.73E-21	TRUE
CD8 NC	*PI4K2B*	Wald ratio	1	0.0057	0.0268	1.006 (0.954, 1.06)	0.833	0.93	3.41E-09	TRUE
CD8 NC	*PIGP*	Wald ratio	1	0.0006	0.0132	1.001 (0.975, 1.027)	0.963	0.988	1.01E-16	TRUE
CD8 NC	*PILRB*	Wald ratio	1	0.0053	0.0073	1.005 (0.991, 1.02)	0.467	0.725	3.29E-50	TRUE
CD8 NC	*PKIA*	Wald ratio	1	-0.0130	0.0121	0.987 (0.964, 1.011)	0.282	0.575	6.46E-19	TRUE
CD8 NC	*PLEC*	Wald ratio	1	0.0533	0.0181	1.055 (1.018, 1.093)	0.003	0.037	1.09E-09	TRUE
CD8 NC	*PLEK*	Wald ratio	1	0.0079	0.0182	1.008 (0.973, 1.044)	0.665	0.847	8.49E-10	TRUE
CD8 NC	*PLEKHA1*	Wald ratio	1	0.0081	0.0144	1.008 (0.98, 1.037)	0.577	0.788	7.56E-17	TRUE
CD8 NC	*PLEKHH2*	Wald ratio	1	0.0243	0.0146	1.025 (0.996, 1.054)	0.096	0.33	1.13E-13	TRUE
CD8 NC	*PLGRKT*	Wald ratio	1	-0.0105	0.0153	0.99 (0.96, 1.02)	0.492	0.738	6.64E-13	TRUE
CD8 NC	*POLM*	Wald ratio	1	0.0092	0.0146	1.009 (0.981, 1.038)	0.531	0.758	4.31E-12	TRUE
CD8 NC	*POLR1D*	Wald ratio	1	0.0012	0.0088	1.001 (0.984, 1.019)	0.888	0.955	9.29E-49	TRUE
CD8 NC	*POLR2J3*	Wald ratio	1	0.0406	0.0164	1.041 (1.009, 1.075)	0.013	0.097	6.24E-11	TRUE
CD8 NC	*POMC*	Wald ratio	1	-0.0252	0.0127	0.975 (0.951, 1)	0.047	0.213	3.62E-16	TRUE
CD8 NC	*POMZP3*	Wald ratio	1	0.0199	0.0146	1.02 (0.991, 1.05)	0.174	0.453	2.08E-17	TRUE
CD8 NC	*POU5F1*	Wald ratio	1	-0.0107	0.0440	0.989 (0.908, 1.078)	0.807	0.917	6.98E-13	TRUE
CD8 NC	*PPA1*	Wald ratio	1	0.0027	0.0051	1.003 (0.993, 1.013)	0.603	0.806	1.82E-108	TRUE
CD8 NC	*PPDPF*	Wald ratio	1	-0.0069	0.0065	0.993 (0.981, 1.006)	0.283	0.575	1.02E-62	TRUE
CD8 NC	*PPFIA3*	Wald ratio	1	-0.0090	0.0089	0.991 (0.974, 1.008)	0.311	0.605	1.50E-44	TRUE
CD8 NC	*PPFIBP2*	Wald ratio	1	-0.0062	0.0095	0.994 (0.975, 1.013)	0.517	0.752	1.74E-32	TRUE
CD8 NC	*PPIA*	Wald ratio	1	0.0121	0.0116	1.012 (0.989, 1.035)	0.298	0.593	3.71E-21	TRUE
CD8 NC	*PPIL3*	Wald ratio	1	0.0060	0.0070	1.006 (0.992, 1.02)	0.393	0.672	3.31E-57	TRUE
CD8 NC	*PPIL6*	Wald ratio	1	-0.0321	0.0156	0.968 (0.939, 0.998)	0.04	0.193	6.38E-09	TRUE
CD8 NC	*PPWD1*	Wald ratio	1	0.0210	0.0147	1.021 (0.992, 1.051)	0.155	0.426	6.34E-14	TRUE
CD8 NC	*PQLC3*	Wald ratio	1	0.0425	0.0188	1.043 (1.006, 1.083)	0.024	0.142	6.26E-08	TRUE
CD8 NC	*PRDX6*	Wald ratio	1	-0.0083	0.0066	0.992 (0.979, 1.005)	0.207	0.494	3.09E-62	TRUE
CD8 NC	*PRELID1*	Wald ratio	1	0.0702	0.0138	1.073 (1.044, 1.102)	3.52E-07	8.32E-05	8.54E-15	TRUE
CD8 NC	*PRKAR1B*	Wald ratio	1	-0.0468	0.0202	0.954 (0.917, 0.993)	0.021	0.13	5.86E-08	TRUE
CD8 NC	*PRKCB*	Wald ratio	1	0.0121	0.0099	1.012 (0.993, 1.032)	0.222	0.514	7.54E-38	TRUE
CD8 NC	*PRKCQ-AS1*	Wald ratio	1	-0.0043	0.0068	0.996 (0.983, 1.009)	0.53	0.758	1.28E-62	TRUE
CD8 NC	*PRMT2*	IVW	2	0.0034	0.0113	1.003 (0.982, 1.026)	0.76	0.892	2.02E-45	TRUE
CD8 NC	*PRPF6*	Wald ratio	1	0.0106	0.0129	1.011 (0.985, 1.037)	0.41	0.685	3.94E-17	TRUE
CD8 NC	*PRR4*	Wald ratio	1	-0.0079	0.0201	0.992 (0.954, 1.032)	0.694	0.863	3.23E-13	TRUE
CD8 NC	*PRR7-AS1*	Wald ratio	1	0.0078	0.0069	1.008 (0.994, 1.021)	0.258	0.552	2.72E-61	TRUE
CD8 NC	*PRSS16*	Wald ratio	1	0.0174	0.0268	1.018 (0.966, 1.072)	0.516	0.751	1.64E-08	TRUE
CD8 NC	*PSD4*	Wald ratio	1	-0.0140	0.0151	0.986 (0.957, 1.016)	0.353	0.644	1.98E-13	TRUE
CD8 NC	*PSMB9*	Wald ratio	1	-0.0088	0.0188	0.991 (0.955, 1.028)	0.638	0.829	3.16E-09	TRUE
CD8 NC	*PSMD5-AS1*	Wald ratio	1	0.0039	0.0043	1.004 (0.996, 1.012)	0.361	0.65	3.82E-183	TRUE
CD8 NC	*PSPH*	Wald ratio	1	-0.0052	0.0144	0.995 (0.967, 1.023)	0.716	0.874	4.61E-12	TRUE

CD8 NC	*PSTPIP1*	Wald ratio	1	0.0078	0.0135	1.008 (0.981, 1.035)	0.566	0.782	7.19E-18	TRUE
CD8 NC	*PTGER2*	Wald ratio	1	0.0012	0.0181	1.001 (0.966, 1.037)	0.946	0.979	8.04E-09	TRUE
CD8 NC	*PTGIR*	Wald ratio	1	0.0056	0.0144	1.006 (0.978, 1.034)	0.696	0.864	1.17E-16	TRUE
CD8 NC	*PTPLAD2*	Wald ratio	1	-0.0251	0.0182	0.975 (0.941, 1.011)	0.169	0.444	8.93E-10	TRUE
CD8 NC	*PTPN4*	Wald ratio	1	-0.0038	0.0114	0.996 (0.974, 1.019)	0.737	0.88	7.60E-21	TRUE
CD8 NC	*PVRIG*	Wald ratio	1	-0.0146	0.0156	0.986 (0.956, 1.016)	0.35	0.643	5.83E-14	TRUE
CD8 NC	*PWWP2A*	Wald ratio	1	-0.0024	0.0178	0.998 (0.963, 1.033)	0.894	0.957	8.12E-11	TRUE
CD8 NC	*PYHIN1*	Wald ratio	1	0.0057	0.0174	1.006 (0.972, 1.041)	0.742	0.881	1.18E-09	TRUE
CD8 NC	*RABAC1*	Wald ratio	1	0.0150	0.0099	1.015 (0.996, 1.035)	0.132	0.393	6.68E-33	TRUE
CD8 NC	*RAC1*	Wald ratio	1	-0.0237	0.0095	0.977 (0.959, 0.995)	0.012	0.094	2.49E-32	TRUE
CD8 NC	*RBAK-RBAKDN*	Wald ratio	1	-0.0104	0.0230	0.99 (0.946, 1.035)	0.652	0.836	1.83E-12	TRUE
CD8 NC	*RBL2*	Wald ratio	1	0.0021	0.0138	1.002 (0.975, 1.03)	0.882	0.952	4.72E-17	TRUE
CD8 NC	*RBM43*	Wald ratio	1	-0.0230	0.0098	0.977 (0.959, 0.996)	0.019	0.122	5.53E-30	TRUE
CD8 NC	*RBM6*	Wald ratio	1	-0.0775	0.0180	0.925 (0.893, 0.959)	1.75E-05	8.68E-04	1.69E-09	TRUE
CD8 NC	*RCSD1*	IVW	2	-0.0047	0.0097	0.995 (0.977, 1.014)	0.626	0.822	9.69E-35	TRUE
CD8 NC	*RDH13*	Wald ratio	1	-0.0243	0.0190	0.976 (0.94, 1.013)	0.201	0.484	2.10E-09	TRUE
CD8 NC	*RGS12*	Wald ratio	1	-0.0369	0.0192	0.964 (0.928, 1.001)	0.054	0.233	1.45E-08	TRUE
CD8 NC	*RGS14*	Wald ratio	1	-0.0200	0.0184	0.98 (0.945, 1.016)	0.277	0.57	1.47E-08	TRUE
CD8 NC	*RIC3*	Wald ratio	1	-0.0071	0.0203	0.993 (0.954, 1.033)	0.727	0.878	3.20E-08	TRUE
CD8 NC	*RNASEH2C*	Wald ratio	1	0.0038	0.0176	1.004 (0.97, 1.039)	0.831	0.929	2.04E-11	TRUE
CD8 NC	*RNASET2*	Wald ratio	1	-0.0171	0.0060	0.983 (0.971, 0.995)	0.005	0.047	1.36E-80	TRUE
CD8 NC	*RNF145*	Wald ratio	1	0.0532	0.0187	1.055 (1.017, 1.094)	0.004	0.046	1.32E-08	TRUE
CD8 NC	*RNF168*	Wald ratio	1	-0.0048	0.0158	0.995 (0.965, 1.026)	0.76	0.892	4.32E-12	TRUE
CD8 NC	*RNF175*	Wald ratio	1	0.0225	0.0207	1.023 (0.982, 1.065)	0.277	0.571	7.19E-08	TRUE
CD8 NC	*RNF24*	Wald ratio	1	-0.0487	0.0191	0.952 (0.917, 0.989)	0.011	0.086	5.94E-09	TRUE
CD8 NC	*RP1-257A7.5*	Wald ratio	1	-0.0352	0.0157	0.965 (0.936, 0.996)	0.025	0.145	2.52E-12	TRUE
CD8 NC	*RP1-265C24.8*	Wald ratio	1	-0.0107	0.0206	0.989 (0.95, 1.03)	0.604	0.806	1.37E-10	TRUE
CD8 NC	*RP1-29C18.10*	Wald ratio	1	-0.0160	0.0074	0.984 (0.97, 0.998)	0.03	0.164	7.05E-53	TRUE
CD8 NC	*RP1-30M3.5*	Wald ratio	1	-0.0165	0.0174	0.984 (0.951, 1.018)	0.343	0.635	1.36E-12	TRUE
CD8 NC	*RP1-3J17.3*	Wald ratio	1	0.0010	0.0075	1.001 (0.986, 1.016)	0.893	0.957	6.61E-48	TRUE
CD8 NC	*RP1-40E16.12*	Wald ratio	1	-0.0160	0.0148	0.984 (0.956, 1.013)	0.282	0.575	3.30E-14	TRUE
CD8 NC	*RP11-103G8.2*	Wald ratio	1	0.0229	0.0200	1.023 (0.984, 1.064)	0.251	0.544	3.02E-09	TRUE
CD8 NC	*RP11-1060J15.4*	Wald ratio	1	-0.0115	0.0115	0.989 (0.967, 1.011)	0.32	0.615	1.25E-36	TRUE
CD8 NC	*RP11-108M9.4*	Wald ratio	1	0.0186	0.0160	1.019 (0.987, 1.051)	0.246	0.539	8.59E-12	TRUE
CD8 NC	*RP11-1191J2.5*	Wald ratio	1	0.0248	0.0172	1.025 (0.991, 1.06)	0.149	0.418	1.08E-09	TRUE
CD8 NC	*RP11-137H2.4*	Wald ratio	1	0.0161	0.0187	1.016 (0.98, 1.054)	0.391	0.672	1.15E-09	TRUE
CD8 NC	*RP11-138A9.1*	Wald ratio	1	0.0053	0.0140	1.005 (0.978, 1.033)	0.706	0.868	1.98E-12	TRUE
CD8 NC	*RP11-138A9.2*	Wald ratio	1	0.0025	0.0169	1.002 (0.97, 1.036)	0.882	0.952	3.37E-14	TRUE
CD8 NC	*RP11-1398P2.1*	Wald ratio	1	-0.0299	0.0136	0.971 (0.945, 0.997)	0.028	0.157	8.28E-16	TRUE
CD8 NC	*RP11-158I9.7*	Wald ratio	1	-0.0021	0.0078	0.998 (0.983, 1.013)	0.784	0.907	1.70E-35	TRUE
CD8 NC	*RP11-166B2.1*	Wald ratio	1	-0.0170	0.0072	0.983 (0.969, 0.997)	0.017	0.116	1.21E-50	TRUE
CD8 NC	*RP11-166B2.5*	Wald ratio	1	-0.0050	0.0132	0.995 (0.969, 1.021)	0.705	0.867	3.12E-16	TRUE
CD8 NC	*RP11-186N15.3*	Wald ratio	1	-0.0233	0.0124	0.977 (0.953, 1.001)	0.06	0.248	5.22E-19	TRUE
CD8 NC	*RP11-18H21.1*	IVW	2	0.0058	0.0249	1.006 (0.958, 1.056)	0.816	0.921	1.19E-87	TRUE
CD8 NC	*RP11-245G13.2*	Wald ratio	1	-0.0106	0.0077	0.989 (0.975, 1.004)	0.168	0.444	2.94E-69	TRUE
CD8 NC	*RP11-273G15.2*	Wald ratio	1	-0.0942	0.0193	0.91 (0.876, 0.945)	1.01E-06	1.13E-04	4.11E-08	TRUE
CD8 NC	*RP11-294J22.6*	Wald ratio	1	-0.0170	0.0208	0.983 (0.944, 1.024)	0.413	0.686	3.24E-08	TRUE
CD8 NC	*RP11-298J20.3*	Wald ratio	1	0.0142	0.0054	1.014 (1.004, 1.025)	0.009	0.073	3.12E-96	TRUE
CD8 NC	*RP11-299J3.8*	Wald ratio	1	0.0012	0.0169	1.001 (0.969, 1.035)	0.944	0.978	2.59E-13	TRUE
CD8 NC	*RP11-326C3.11*	Wald ratio	1	-0.0101	0.0074	0.99 (0.976, 1.004)	0.169	0.444	3.46E-65	TRUE
CD8 NC	*RP11-326C3.12*	Wald ratio	1	-0.0069	0.0070	0.993 (0.979, 1.007)	0.325	0.62	6.97E-58	TRUE
CD8 NC	*RP11-326C3.15*	Wald ratio	1	0.0115	0.0130	1.012 (0.986, 1.038)	0.377	0.665	1.29E-17	TRUE
CD8 NC	*RP11-334C17.5*	Wald ratio	1	-0.0185	0.0196	0.982 (0.945, 1.02)	0.347	0.64	2.09E-08	TRUE
CD8 NC	*RP11-345J4.5*	Wald ratio	1	-0.0244	0.0110	0.976 (0.955, 0.997)	0.027	0.154	5.00E-25	TRUE
CD8 NC	*RP11-347P5.1*	Wald ratio	1	0.0016	0.0072	1.002 (0.988, 1.016)	0.826	0.927	2.43E-56	TRUE
CD8 NC	*RP11-395B7.4*	Wald ratio	1	0.0092	0.0218	1.009 (0.967, 1.053)	0.674	0.852	5.80E-09	TRUE
CD8 NC	*RP11-403A21.2*	Wald ratio	1	0.0058	0.0138	1.006 (0.979, 1.033)	0.678	0.853	4.44E-14	TRUE
CD8 NC	*RP11-415F23.2*	Wald ratio	1	0.0142	0.0090	1.014 (0.997, 1.032)	0.114	0.36	1.24E-33	TRUE
CD8 NC	*RP11-430H10.1*	Wald ratio	1	-0.0250	0.0126	0.975 (0.951, 1)	0.047	0.214	1.07E-19	TRUE
CD8 NC	*RP11-434C1.1*	Wald ratio	1	-0.0051	0.0200	0.995 (0.957, 1.035)	0.799	0.913	1.10E-08	TRUE
CD8 NC	*RP11-457M11.5*	Wald ratio	1	0.0329	0.0192	1.033 (0.995, 1.073)	0.087	0.312	6.35E-09	TRUE
CD8 NC	*RP11-473I1.5*	Wald ratio	1	-0.0253	0.0118	0.975 (0.953, 0.998)	0.032	0.168	3.32E-18	TRUE
CD8 NC	*RP11-473M20.9*	Wald ratio	1	-0.0117	0.0119	0.988 (0.965, 1.012)	0.326	0.62	4.15E-21	TRUE
CD8 NC	*RP11-526I2.5*	Wald ratio	1	0.0178	0.0142	1.018 (0.99, 1.047)	0.212	0.499	9.96E-15	TRUE
CD8 NC	*RP11-536K7.3*	Wald ratio	1	-0.0162	0.0174	0.984 (0.951, 1.018)	0.353	0.644	2.83E-11	TRUE
CD8 NC	*RP11-539L10.3*	Wald ratio	1	-0.0114	0.0110	0.989 (0.968, 1.01)	0.299	0.594	4.23E-23	TRUE
CD8 NC	*RP11-53O19.3*	Wald ratio	1	0.0069	0.0082	1.007 (0.991, 1.023)	0.401	0.679	4.36E-43	TRUE
CD8 NC	*RP11-542K23.7*	Wald ratio	1	0.0093	0.0124	1.009 (0.985, 1.034)	0.453	0.717	1.85E-36	TRUE
CD8 NC	*RP11-545E17.3*	Wald ratio	1	-0.0131	0.0090	0.987 (0.97, 1.005)	0.146	0.415	9.62E-35	TRUE
CD8 NC	*RP11-589P10.5*	Wald ratio	1	-0.0315	0.0209	0.969 (0.93, 1.009)	0.131	0.392	3.55E-08	TRUE
CD8 NC	*RP11-596D21.1*	Wald ratio	1	0.0316	0.0128	1.032 (1.007, 1.058)	0.013	0.098	1.98E-17	TRUE
CD8 NC	*RP11-641A6.2*	Wald ratio	1	-0.0107	0.0163	0.989 (0.958, 1.021)	0.51	0.748	2.27E-18	TRUE
CD8 NC	*RP11-660L16.2*	Wald ratio	1	-0.0067	0.0093	0.993 (0.975, 1.012)	0.47	0.727	3.75E-24	TRUE
CD8 NC	*RP11-664D1.1*	IVW	2	0.0077	0.0102	1.008 (0.988, 1.028)	0.453	0.717	8.17E-44	TRUE
CD8 NC	*RP11-669E14.6*	Wald ratio	1	0.0236	0.0176	1.024 (0.989, 1.06)	0.181	0.461	5.53E-22	TRUE
CD8 NC	*RP11-677M14.3*	Wald ratio	1	0.0058	0.0087	1.006 (0.989, 1.023)	0.501	0.743	2.09E-39	TRUE
CD8 NC	*RP11-69E11.4*	Wald ratio	1	0.0331	0.0156	1.034 (1.002, 1.066)	0.034	0.175	1.38E-11	TRUE
CD8 NC	*RP11-712L6.5*	Wald ratio	1	0.0228	0.0158	1.023 (0.992, 1.055)	0.15	0.419	5.34E-13	TRUE
CD8 NC	*RP11-774O3.3*	Wald ratio	1	0.0125	0.0201	1.013 (0.973, 1.053)	0.536	0.762	5.59E-08	TRUE

CD8 NC	*RP11-77P16.4*	Wald ratio	1	-0.0114	0.0139	0.989 (0.962, 1.016)	0.412	0.686	3.86E-16	TRUE
CD8 NC	*RP11-792A8.4*	Wald ratio	1	0.0047	0.0087	1.005 (0.988, 1.022)	0.587	0.794	1.06E-17	TRUE
CD8 NC	*RP11-841O20.2*	Wald ratio	1	0.0181	0.0157	1.018 (0.987, 1.05)	0.251	0.545	3.16E-11	TRUE
CD8 NC	*RP11-936I5.1*	Wald ratio	1	0.0124	0.0120	1.012 (0.989, 1.037)	0.302	0.598	2.63E-19	TRUE
CD8 NC	*RP11-950C14.3*	Wald ratio	1	0.0255	0.0152	1.026 (0.996, 1.057)	0.095	0.326	3.24E-13	TRUE
CD8 NC	*RP11-96H19.1*	Wald ratio	1	-0.0137	0.0175	0.986 (0.953, 1.021)	0.434	0.702	2.55E-10	TRUE
CD8 NC	*RP3-325F22.5*	Wald ratio	1	0.0133	0.0188	1.013 (0.977, 1.051)	0.481	0.734	4.00E-10	TRUE
CD8 NC	*RP4-539M6.22*	Wald ratio	1	-0.0049	0.0200	0.995 (0.957, 1.035)	0.808	0.918	2.48E-08	TRUE
CD8 NC	*RP4-594I10.3*	Wald ratio	1	-0.0101	0.0115	0.99 (0.968, 1.013)	0.381	0.666	5.12E-22	TRUE
CD8 NC	*RP4-798A10.2*	Wald ratio	1	-0.0038	0.0179	0.996 (0.962, 1.032)	0.833	0.93	4.47E-10	TRUE
CD8 NC	*RP5-1021I20.1*	Wald ratio	1	-0.0541	0.0173	0.947 (0.916, 0.98)	0.002	0.025	4.83E-10	TRUE
CD8 NC	*RP5-1042K10.10*	Wald ratio	1	-0.0160	0.0110	0.984 (0.963, 1.006)	0.146	0.415	1.06E-20	TRUE
CD8 NC	*RP5-827C21.4*	Wald ratio	1	-0.0062	0.0151	0.994 (0.965, 1.024)	0.68	0.853	5.96E-20	TRUE
CD8 NC	*RPL10A*	Wald ratio	1	-0.0094	0.0071	0.991 (0.977, 1.005)	0.187	0.466	6.43E-60	TRUE
CD8 NC	*RPL11*	Wald ratio	1	-0.0131	0.0084	0.987 (0.971, 1.003)	0.119	0.369	2.72E-40	TRUE
CD8 NC	*RPL12*	IVW	2	-0.0136	0.0198	0.986 (0.949, 1.026)	0.492	0.738	3.27E-122	TRUE
CD8 NC	*RPL13*	Wald ratio	1	-0.0230	0.0114	0.977 (0.956, 0.999)	0.043	0.202	1.02E-40	TRUE
CD8 NC	*RPL14*	IVW	2	0.0113	0.0100	1.011 (0.992, 1.031)	0.26	0.554	3.36E-42	TRUE
CD8 NC	*RPL22L1*	Wald ratio	1	0.0042	0.0199	1.004 (0.966, 1.044)	0.835	0.93	1.73E-10	TRUE
CD8 NC	*RPL23A*	Wald ratio	1	-0.0190	0.0103	0.981 (0.962, 1.001)	0.064	0.258	7.38E-29	TRUE
CD8 NC	*RPL27A*	Wald ratio	1	0.0104	0.0123	1.01 (0.986, 1.035)	0.4	0.678	5.83E-18	TRUE
CD8 NC	*RPL28*	Wald ratio	1	0.0036	0.0047	1.004 (0.994, 1.013)	0.438	0.703	1.19E-146	TRUE
CD8 NC	*RPL3*	Wald ratio	1	0.0168	0.0180	1.017 (0.982, 1.053)	0.35	0.643	4.93E-10	TRUE
CD8 NC	*RPL32*	Wald ratio	1	0.0217	0.0129	1.022 (0.996, 1.048)	0.093	0.323	3.87E-19	TRUE
CD8 NC	*RPL36AL*	Wald ratio	1	-0.0035	0.0055	0.996 (0.986, 1.007)	0.524	0.754	7.03E-103	TRUE
CD8 NC	*RPL8*	Wald ratio	1	-0.0033	0.0045	0.997 (0.988, 1.006)	0.467	0.725	2.88E-164	TRUE
CD8 NC	*RPLP2*	Wald ratio	1	-0.0179	0.0069	0.982 (0.969, 0.996)	0.009	0.076	2.49E-62	TRUE
CD8 NC	*RPS10*	Wald ratio	1	-0.0066	0.0064	0.993 (0.981, 1.006)	0.305	0.599	2.07E-60	TRUE
CD8 NC	*RPS11*	Wald ratio	1	0.0264	0.0111	1.027 (1.005, 1.049)	0.017	0.115	3.60E-26	TRUE
CD8 NC	*RPS12*	Wald ratio	1	0.0188	0.0121	1.019 (0.995, 1.043)	0.12	0.37	7.28E-13	TRUE
CD8 NC	*RPS14*	Wald ratio	1	-0.0195	0.0176	0.981 (0.947, 1.015)	0.267	0.561	6.76E-10	TRUE
CD8 NC	*RPS16*	Wald ratio	1	-0.0129	0.0126	0.987 (0.963, 1.012)	0.304	0.598	8.21E-18	TRUE
CD8 NC	*RPS18*	Wald ratio	1	-0.0120	0.0070	0.988 (0.975, 1.002)	0.087	0.312	7.27E-74	TRUE
CD8 NC	*RPS2*	Wald ratio	1	-0.0241	0.0090	0.976 (0.959, 0.994)	0.007	0.066	1.59E-37	TRUE
CD8 NC	*RPS23*	Wald ratio	1	-0.0216	0.0151	0.979 (0.95, 1.008)	0.151	0.422	5.26E-15	TRUE
CD8 NC	*RPS24*	Wald ratio	1	-0.0031	0.0168	0.997 (0.965, 1.03)	0.855	0.938	2.11E-11	TRUE
CD8 NC	*RPS25*	Wald ratio	1	-0.0008	0.0073	0.999 (0.985, 1.014)	0.908	0.961	2.21E-52	TRUE
CD8 NC	*RPS26*	Wald ratio	1	-0.0099	0.0043	0.99 (0.982, 0.999)	0.022	0.132	3.87E-190	TRUE
CD8 NC	*RPS3*	Wald ratio	1	-0.0149	0.0084	0.985 (0.969, 1.002)	0.075	0.287	9.20E-25	TRUE
CD8 NC	*RPS3A*	Wald ratio	1	-0.0040	0.0062	0.996 (0.984, 1.008)	0.514	0.75	2.66E-37	TRUE
CD8 NC	*RPS5*	Wald ratio	1	0.0073	0.0054	1.007 (0.997, 1.018)	0.177	0.456	1.25E-114	TRUE
CD8 NC	*RPS6*	Wald ratio	1	-0.0110	0.0094	0.989 (0.971, 1.007)	0.242	0.534	2.35E-44	TRUE
CD8 NC	*RPS6KA2*	Wald ratio	1	0.0279	0.0173	1.028 (0.994, 1.064)	0.108	0.348	4.99E-08	TRUE
CD8 NC	*RPS7*	Wald ratio	1	0.0030	0.0099	1.003 (0.984, 1.023)	0.761	0.892	2.00E-46	TRUE
CD8 NC	*RPS8*	Wald ratio	1	0.0024	0.0089	1.002 (0.985, 1.02)	0.791	0.909	3.48E-37	TRUE
CD8 NC	*RPS9*	Wald ratio	1	0.0225	0.0060	1.023 (1.011, 1.035)	1.64E-04	0.004	4.13E-85	TRUE
CD8 NC	*RPSA*	Wald ratio	1	-0.0223	0.0109	0.978 (0.957, 0.999)	0.04	0.195	7.83E-32	TRUE
CD8 NC	*RSRC2*	Wald ratio	1	-0.0077	0.0162	0.992 (0.961, 1.024)	0.635	0.827	2.04E-14	TRUE
CD8 NC	*RTKN2*	Wald ratio	1	0.0152	0.0196	1.015 (0.977, 1.055)	0.438	0.703	3.70E-12	TRUE
CD8 NC	*S100B*	IVW	2	0.0087	0.0133	1.009 (0.983, 1.035)	0.514	0.75	1.11E-22	TRUE
CD8 NC	*SAMD3*	Wald ratio	1	-0.0027	0.0081	0.997 (0.982, 1.013)	0.739	0.881	3.34E-45	TRUE
CD8 NC	*SAMM50*	Wald ratio	1	-0.0027	0.0159	0.997 (0.967, 1.029)	0.866	0.944	2.78E-08	TRUE
CD8 NC	*SAR1A*	Wald ratio	1	0.0093	0.0124	1.009 (0.985, 1.034)	0.453	0.717	8.68E-19	TRUE
CD8 NC	*SAT2*	Wald ratio	1	-0.0998	0.0190	0.905 (0.872, 0.939)	1.40E-07	6.60E-05	1.28E-08	TRUE
CD8 NC	*SCAMP3*	Wald ratio	1	0.0039	0.0141	1.004 (0.977, 1.032)	0.783	0.906	1.06E-15	TRUE
CD8 NC	*SCD5*	Wald ratio	1	-0.0913	0.0196	0.913 (0.878, 0.949)	3.19E-06	2.62E-04	2.27E-09	TRUE
CD8 NC	*SCGB3A1*	Wald ratio	1	0.0060	0.0055	1.006 (0.995, 1.017)	0.277	0.571	1.13E-104	TRUE
CD8 NC	*SDK2*	Wald ratio	1	0.0310	0.0188	1.031 (0.994, 1.07)	0.099	0.333	5.99E-09	TRUE
CD8 NC	*SELM*	Wald ratio	1	0.0392	0.0166	1.04 (1.007, 1.074)	0.018	0.12	3.38E-11	TRUE
CD8 NC	*SEPT2*	Wald ratio	1	0.0059	0.0090	1.006 (0.988, 1.024)	0.512	0.748	1.18E-39	TRUE
CD8 NC	*SERPINB6*	Wald ratio	1	0.0059	0.0079	1.006 (0.99, 1.022)	0.456	0.719	2.28E-51	TRUE
CD8 NC	*SERPINB9*	Wald ratio	1	-0.0152	0.0138	0.985 (0.959, 1.012)	0.27	0.563	8.51E-13	TRUE
CD8 NC	*SERPINE2*	Wald ratio	1	-0.0484	0.0203	0.953 (0.916, 0.991)	0.017	0.115	5.18E-08	TRUE
CD8 NC	*SERPINF1*	Wald ratio	1	0.0110	0.0132	1.011 (0.985, 1.038)	0.404	0.681	5.20E-16	TRUE
CD8 NC	*SESN1*	Wald ratio	1	-0.0251	0.0180	0.975 (0.941, 1.01)	0.164	0.438	9.05E-09	TRUE
CD8 NC	*SESN3*	Wald ratio	1	-0.0132	0.0166	0.987 (0.955, 1.019)	0.425	0.695	5.13E-12	TRUE
CD8 NC	*SESTD1*	Wald ratio	1	-0.0075	0.0183	0.993 (0.958, 1.029)	0.683	0.856	7.27E-10	TRUE
CD8 NC	*SETD5-AS1*	Wald ratio	1	-0.0052	0.0175	0.995 (0.961, 1.03)	0.767	0.897	3.32E-10	TRUE
CD8 NC	*SETD9*	Wald ratio	1	-0.0357	0.0132	0.965 (0.94, 0.99)	0.007	0.062	5.61E-15	TRUE
CD8 NC	*SF1*	Wald ratio	1	-0.0349	0.0163	0.966 (0.935, 0.997)	0.032	0.17	3.04E-09	TRUE
CD8 NC	*SFT2D1*	Wald ratio	1	0.0314	0.0144	1.032 (1.003, 1.061)	0.029	0.158	1.91E-13	TRUE
CD8 NC	*SH3YL1*	Wald ratio	1	0.0203	0.0050	1.021 (1.01, 1.031)	5.45E-05	0.002	6.04E-124	TRUE
CD8 NC	*SIDT1-AS1*	Wald ratio	1	-0.0279	0.0136	0.972 (0.947, 0.999)	0.04	0.195	2.09E-22	TRUE
CD8 NC	*SIRPG*	Wald ratio	1	0.0134	0.0180	1.014 (0.978, 1.05)	0.456	0.719	1.48E-09	TRUE
CD8 NC	*SKAP2*	Wald ratio	1	-0.0139	0.0169	0.986 (0.954, 1.019)	0.412	0.686	6.26E-13	TRUE
CD8 NC	*SLC12A5*	Wald ratio	1	-0.0260	0.0118	0.974 (0.952, 0.997)	0.028	0.157	8.27E-21	TRUE
CD8 NC	*SLC12A7*	Wald ratio	1	-0.0118	0.0108	0.988 (0.968, 1.009)	0.275	0.568	6.26E-26	TRUE
CD8 NC	*SLC22A18*	Wald ratio	1	-0.0099	0.0164	0.99 (0.959, 1.023)	0.546	0.767	2.50E-14	TRUE
CD8 NC	*SLC25A1*	Wald ratio	1	-0.0166	0.0171	0.984 (0.951, 1.017)	0.332	0.626	4.10E-11	TRUE

CD8 NC	*SLC25A26*	Wald ratio	1	-0.0145	0.0153	0.986 (0.957, 1.015)	0.341	0.634	4.73E-13	TRUE
CD8 NC	*SLC25A29*	Wald ratio	1	-0.0071	0.0100	0.993 (0.974, 1.012)	0.475	0.73	4.03E-29	TRUE
CD8 NC	*SLC25A3*	Wald ratio	1	0.0068	0.0164	1.007 (0.975, 1.04)	0.678	0.853	3.36E-15	TRUE
CD8 NC	*SLC25A51*	Wald ratio	1	-0.0414	0.0174	0.959 (0.927, 0.993)	0.018	0.117	1.31E-08	TRUE
CD8 NC	*SLC2A1-AS1*	Wald ratio	1	0.0197	0.0169	1.02 (0.987, 1.054)	0.244	0.537	1.63E-08	TRUE
CD8 NC	*SLC35F3*	Wald ratio	1	0.0010	0.0150	1.001 (0.972, 1.031)	0.947	0.979	1.62E-13	TRUE
CD8 NC	*SLC38A1*	Wald ratio	1	-0.0180	0.0200	0.982 (0.944, 1.021)	0.367	0.655	4.32E-08	TRUE
CD8 NC	*SLC40A1*	Wald ratio	1	0.0500	0.0204	1.051 (1.01, 1.094)	0.014	0.101	1.05E-07	TRUE
CD8 NC	*SLC7A8*	Wald ratio	1	-0.0047	0.0135	0.995 (0.969, 1.022)	0.73	0.88	6.63E-17	TRUE
CD8 NC	*SLFN5*	Wald ratio	1	-0.0083	0.0140	0.992 (0.965, 1.019)	0.55	0.769	9.45E-16	TRUE
CD8 NC	*SMAD5*	Wald ratio	1	0.0239	0.0176	1.024 (0.989, 1.06)	0.176	0.456	5.21E-10	TRUE
CD8 NC	*SMAGP*	Wald ratio	1	0.0171	0.0190	1.017 (0.98, 1.056)	0.37	0.658	2.36E-08	TRUE
CD8 NC	*SMDT1*	Wald ratio	1	0.0075	0.0039	1.008 (1, 1.015)	0.057	0.239	1.35E-211	TRUE
CD8 NC	*SMIM19*	Wald ratio	1	0.0129	0.0183	1.013 (0.977, 1.05)	0.481	0.734	4.67E-10	TRUE
CD8 NC	*SNAP29*	Wald ratio	1	0.0385	0.0167	1.039 (1.006, 1.074)	0.022	0.132	8.21E-14	TRUE
CD8 NC	*SNHG7*	Wald ratio	1	-0.0137	0.0085	0.986 (0.97, 1.003)	0.107	0.348	3.41E-61	TRUE
CD8 NC	*SNHG8*	Wald ratio	1	-0.0023	0.0050	0.998 (0.988, 1.007)	0.647	0.834	3.80E-151	TRUE
CD8 NC	*SNORD3B-2*	Wald ratio	1	-0.0150	0.0209	0.985 (0.946, 1.026)	0.473	0.73	5.90E-11	TRUE
CD8 NC	*SNRNP25*	Wald ratio	1	0.0384	0.0190	1.039 (1.001, 1.079)	0.043	0.205	3.50E-08	TRUE
CD8 NC	*SNRPC*	Wald ratio	1	-0.0576	0.0165	0.944 (0.914, 0.975)	4.98E-04	0.01	8.00E-11	TRUE
CD8 NC	*SNRPD2*	Wald ratio	1	-0.0247	0.0079	0.976 (0.961, 0.991)	0.002	0.025	4.22E-44	TRUE
CD8 NC	*SNTG2*	Wald ratio	1	-0.0055	0.0098	0.994 (0.975, 1.014)	0.573	0.786	1.74E-28	TRUE
CD8 NC	*SPATA20*	Wald ratio	1	0.0216	0.0132	1.022 (0.996, 1.049)	0.101	0.337	1.45E-17	TRUE
CD8 NC	*SPATC1L*	Wald ratio	1	0.0017	0.0097	1.002 (0.983, 1.021)	0.864	0.943	1.24E-42	TRUE
CD8 NC	*SPINK2*	Wald ratio	1	-0.0046	0.0131	0.995 (0.97, 1.021)	0.722	0.877	1.12E-17	TRUE
CD8 NC	*SPNS1*	Wald ratio	1	0.0358	0.0190	1.036 (0.999, 1.076)	0.06	0.247	2.95E-08	TRUE
CD8 NC	*SPRYD4*	Wald ratio	1	0.0339	0.0205	1.034 (0.994, 1.077)	0.098	0.333	2.82E-08	TRUE
CD8 NC	*SPSB2*	Wald ratio	1	0.0087	0.0168	1.009 (0.976, 1.043)	0.604	0.806	3.36E-12	TRUE
CD8 NC	*SRA1*	Wald ratio	1	-0.0036	0.0177	0.996 (0.962, 1.032)	0.84	0.932	2.34E-09	TRUE
CD8 NC	*SRD5A3*	Wald ratio	1	-0.0339	0.0149	0.967 (0.939, 0.995)	0.023	0.138	3.26E-12	TRUE
CD8 NC	*SRI*	Wald ratio	1	-0.0196	0.0166	0.981 (0.949, 1.013)	0.239	0.533	6.48E-11	TRUE
CD8 NC	*SRP14*	Wald ratio	1	0.0546	0.0146	1.056 (1.026, 1.087)	1.83E-04	0.005	3.25E-11	TRUE
CD8 NC	*SRR*	Wald ratio	1	0.0095	0.0192	1.01 (0.972, 1.048)	0.619	0.819	5.18E-09	TRUE
CD8 NC	*SSBP4*	Wald ratio	1	0.0162	0.0193	1.016 (0.979, 1.056)	0.403	0.68	1.42E-08	TRUE
CD8 NC	*ST6GALNAC6*	Wald ratio	1	0.0112	0.0165	1.011 (0.979, 1.044)	0.498	0.741	6.55E-11	TRUE
CD8 NC	*STK32C*	Wald ratio	1	0.0780	0.0269	1.081 (1.026, 1.14)	0.004	0.041	2.34E-08	TRUE
CD8 NC	*STK4*	Wald ratio	1	-0.0054	0.0197	0.995 (0.957, 1.034)	0.785	0.907	5.76E-08	TRUE
CD8 NC	*STMN3*	Wald ratio	1	0.0182	0.0093	1.018 (1, 1.037)	0.05	0.222	1.53E-33	TRUE
CD8 NC	*STOX1*	Wald ratio	1	-0.0175	0.0087	0.983 (0.966, 1)	0.045	0.209	8.91E-27	TRUE
CD8 NC	*STYX*	Wald ratio	1	0.0365	0.0192	1.037 (0.999, 1.077)	0.058	0.243	3.30E-08	TRUE
CD8 NC	*SULF2*	Wald ratio	1	0.0058	0.0059	1.006 (0.994, 1.017)	0.324	0.618	3.43E-85	TRUE
CD8 NC	*SUPT3H*	Wald ratio	1	-0.0222	0.0137	0.978 (0.952, 1.005)	0.106	0.345	5.24E-16	TRUE
CD8 NC	*SURF1*	Wald ratio	1	-0.0275	0.0137	0.973 (0.947, 0.999)	0.045	0.208	2.78E-16	TRUE
CD8 NC	*SVIP*	Wald ratio	1	-0.0026	0.0175	0.997 (0.964, 1.032)	0.88	0.951	3.81E-10	TRUE
CD8 NC	*SYNGR1*	Wald ratio	1	-0.0201	0.0105	0.98 (0.96, 1)	0.054	0.233	1.35E-24	TRUE
CD8 NC	*SYNJ2*	Wald ratio	1	-0.0147	0.0190	0.985 (0.949, 1.023)	0.44	0.705	5.53E-09	TRUE
CD8 NC	*TAGLN2*	Wald ratio	1	-0.0209	0.0205	0.979 (0.941, 1.02)	0.309	0.603	7.13E-08	TRUE
CD8 NC	*TAPBP*	Wald ratio	1	0.0085	0.0129	1.009 (0.983, 1.034)	0.511	0.748	2.18E-16	TRUE
CD8 NC	*TAPBPL*	Wald ratio	1	0.0054	0.0144	1.005 (0.977, 1.034)	0.706	0.868	8.49E-15	TRUE
CD8 NC	*TBCA*	Wald ratio	1	0.0081	0.0096	1.008 (0.989, 1.027)	0.399	0.678	4.14E-32	TRUE
CD8 NC	*TBCD*	Wald ratio	1	0.0184	0.0146	1.019 (0.99, 1.048)	0.21	0.497	2.52E-14	TRUE
CD8 NC	*TC2N*	Wald ratio	1	0.0030	0.0191	1.003 (0.966, 1.041)	0.877	0.949	5.87E-09	TRUE
CD8 NC	*TCTEX1D2*	Wald ratio	1	-0.0097	0.0190	0.99 (0.954, 1.028)	0.61	0.81	5.86E-09	TRUE
CD8 NC	*TEN1*	Wald ratio	1	-0.0141	0.0191	0.986 (0.95, 1.023)	0.458	0.719	3.05E-11	TRUE
CD8 NC	*THAP5*	Wald ratio	1	0.0352	0.0186	1.036 (0.999, 1.074)	0.058	0.243	6.61E-08	TRUE
CD8 NC	*THAP7-AS1*	Wald ratio	1	0.0305	0.0143	1.031 (1.003, 1.06)	0.032	0.17	2.30E-13	TRUE
CD8 NC	*THAP9-AS1*	Wald ratio	1	0.0777	0.0167	1.081 (1.046, 1.117)	3.33E-06	2.62E-04	1.52E-12	TRUE
CD8 NC	*THEM4*	Wald ratio	1	0.0391	0.0185	1.04 (1.003, 1.078)	0.035	0.176	1.34E-09	TRUE
CD8 NC	*THEMIS2*	Wald ratio	1	0.0075	0.0079	1.007 (0.992, 1.023)	0.345	0.637	7.03E-47	TRUE
CD8 NC	*TIMM10*	Wald ratio	1	-0.0010	0.0063	0.999 (0.987, 1.011)	0.875	0.948	1.43E-91	TRUE
CD8 NC	*TIPARP-AS1*	Wald ratio	1	0.0467	0.0183	1.048 (1.011, 1.086)	0.011	0.085	2.54E-10	TRUE
CD8 NC	*TIPRL*	Wald ratio	1	0.0194	0.0194	1.02 (0.982, 1.059)	0.317	0.613	1.24E-08	TRUE
CD8 NC	*TLE4*	Wald ratio	1	-0.0100	0.0124	0.99 (0.966, 1.014)	0.419	0.69	2.45E-20	TRUE
CD8 NC	*TLK1*	Wald ratio	1	0.0113	0.0153	1.011 (0.982, 1.042)	0.459	0.719	1.58E-14	TRUE
CD8 NC	*TMEM101*	Wald ratio	1	-0.0381	0.0262	0.963 (0.914, 1.013)	0.146	0.415	5.70E-12	TRUE
CD8 NC	*TMEM120B*	Wald ratio	1	-0.0173	0.0146	0.983 (0.955, 1.011)	0.235	0.529	4.87E-22	TRUE
CD8 NC	*TMEM121*	Wald ratio	1	0.0082	0.0122	1.008 (0.984, 1.033)	0.502	0.743	3.00E-18	TRUE
CD8 NC	*TMEM156*	Wald ratio	1	0.0038	0.0092	1.004 (0.986, 1.022)	0.684	0.856	1.31E-33	TRUE
CD8 NC	*TMEM18*	Wald ratio	1	0.0034	0.0153	1.003 (0.974, 1.034)	0.824	0.926	1.19E-13	TRUE
CD8 NC	*TMEM2*	Wald ratio	1	-0.0143	0.0199	0.986 (0.948, 1.025)	0.471	0.729	4.17E-08	TRUE
CD8 NC	*TMEM204*	Wald ratio	1	-0.0131	0.0169	0.987 (0.955, 1.02)	0.437	0.703	3.54E-15	TRUE
CD8 NC	*TMEM230*	Wald ratio	1	0.0060	0.0101	1.006 (0.986, 1.026)	0.551	0.769	4.52E-29	TRUE
CD8 NC	*TMEM244*	Wald ratio	1	0.0120	0.0119	1.012 (0.989, 1.036)	0.313	0.608	3.73E-22	TRUE
CD8 NC	*TMEM254-AS1*	Wald ratio	1	-0.0002	0.0154	1 (0.97, 1.031)	0.991	0.996	3.98E-10	TRUE
CD8 NC	*TMEM258*	Wald ratio	1	0.0001	0.0173	1 (0.967, 1.034)	0.996	0.999	6.33E-15	TRUE
CD8 NC	*TMEM41B*	Wald ratio	1	-0.0094	0.0172	0.991 (0.958, 1.025)	0.585	0.794	1.99E-10	TRUE
CD8 NC	*TMEM45B*	Wald ratio	1	-0.0140	0.0162	0.986 (0.955, 1.018)	0.386	0.669	1.52E-11	TRUE
CD8 NC	*TMEM50A*	Wald ratio	1	0.0302	0.0204	1.031 (0.99, 1.073)	0.138	0.401	5.01E-08	TRUE
CD8 NC	*TMEM50B*	Wald ratio	1	-0.0307	0.0096	0.97 (0.952, 0.988)	0.001	0.02	7.68E-31	TRUE

CD8 NC	*TMEM80*	Wald ratio	1	-0.0354	0.0119	0.965 (0.943, 0.988)	0.003	0.035	4.00E-20	TRUE
CD8 NC	*TNFRSF14*	Wald ratio	1	0.0269	0.0189	1.027 (0.99, 1.066)	0.155	0.426	4.55E-10	TRUE
CD8 NC	*TNFSF13B*	Wald ratio	1	0.0466	0.0210	1.048 (1.005, 1.092)	0.026	0.151	9.99E-08	TRUE
CD8 NC	*TNFSF8*	Wald ratio	1	-0.0128	0.0179	0.987 (0.953, 1.022)	0.472	0.729	1.46E-08	TRUE
CD8 NC	*TNNI2*	Wald ratio	1	-0.0149	0.0126	0.985 (0.961, 1.01)	0.235	0.53	2.56E-28	TRUE
CD8 NC	*TOMM7*	Wald ratio	1	0.0028	0.0043	1.003 (0.995, 1.011)	0.503	0.743	1.02E-166	TRUE
CD8 NC	*TP53TG1*	Wald ratio	1	-0.0031	0.0193	0.997 (0.96, 1.035)	0.871	0.947	9.06E-09	TRUE
CD8 NC	*TPD52*	Wald ratio	1	-0.0206	0.0122	0.98 (0.956, 1.003)	0.092	0.321	1.70E-19	TRUE
CD8 NC	*TPGS1*	Wald ratio	1	-0.0001	0.0190	1 (0.963, 1.038)	0.996	0.999	1.44E-11	TRUE
CD8 NC	*TPGS2*	Wald ratio	1	-0.0033	0.0114	0.997 (0.975, 1.019)	0.769	0.897	1.27E-24	TRUE
CD8 NC	*TPRA1*	Wald ratio	1	-0.0571	0.0165	0.945 (0.914, 0.976)	5.55E-04	0.011	4.32E-08	TRUE
CD8 NC	*TRAPPC12-AS1*	Wald ratio	1	0.0471	0.0152	1.048 (1.017, 1.08)	0.002	0.025	1.45E-12	TRUE
CD8 NC	*TRAPPC2L*	Wald ratio	1	0.0346	0.0130	1.035 (1.009, 1.062)	0.008	0.069	7.12E-17	TRUE
CD8 NC	*TRAPPC4*	Wald ratio	1	0.0269	0.0170	1.027 (0.994, 1.062)	0.113	0.359	8.96E-11	TRUE
CD8 NC	*TRIM23*	Wald ratio	1	0.0311	0.0197	1.032 (0.993, 1.072)	0.114	0.361	2.46E-08	TRUE
CD8 NC	*TRMT112*	Wald ratio	1	0.0167	0.0144	1.017 (0.988, 1.046)	0.247	0.54	1.67E-19	TRUE
CD8 NC	*TSGA10*	Wald ratio	1	-0.0026	0.0159	0.997 (0.967, 1.029)	0.871	0.947	8.36E-12	TRUE
CD8 NC	*TSHZ2*	Wald ratio	1	-0.0013	0.0169	0.999 (0.966, 1.032)	0.94	0.976	1.32E-10	TRUE
CD8 NC	*TSPO*	Wald ratio	1	0.0154	0.0138	1.015 (0.988, 1.043)	0.264	0.558	3.66E-16	TRUE
CD8 NC	*TSTD1*	IVW	2	0.0065	0.0074	1.007 (0.992, 1.021)	0.375	0.663	3.43E-46	TRUE
CD8 NC	*TSTD3*	Wald ratio	1	-0.0111	0.0134	0.989 (0.963, 1.015)	0.405	0.681	8.68E-15	TRUE
CD8 NC	*TTC16*	Wald ratio	1	-0.0195	0.0213	0.981 (0.941, 1.022)	0.359	0.65	3.59E-08	TRUE
CD8 NC	*TTC32*	Wald ratio	1	0.0263	0.0124	1.027 (1.002, 1.052)	0.034	0.175	2.17E-20	TRUE
CD8 NC	*TTC39B*	Wald ratio	1	0.0351	0.0193	1.036 (0.997, 1.076)	0.07	0.273	3.32E-08	TRUE
CD8 NC	*TUFM*	Wald ratio	1	0.0132	0.0066	1.013 (1, 1.026)	0.046	0.21	1.50E-64	TRUE
CD8 NC	*TXK*	Wald ratio	1	0.0057	0.0160	1.006 (0.975, 1.038)	0.722	0.877	4.14E-09	TRUE
CD8 NC	*TXLNB*	Wald ratio	1	0.0224	0.0174	1.023 (0.988, 1.058)	0.197	0.479	2.06E-13	TRUE
CD8 NC	*TXN2*	Wald ratio	1	0.0088	0.0168	1.009 (0.976, 1.043)	0.601	0.805	8.33E-11	TRUE
CD8 NC	*TYMP*	Wald ratio	1	-0.0043	0.0198	0.996 (0.958, 1.035)	0.829	0.929	7.00E-15	TRUE
CD8 NC	*UBA52*	Wald ratio	1	0.0090	0.0165	1.009 (0.977, 1.042)	0.586	0.794	7.79E-12	TRUE
CD8 NC	*UBA6*	Wald ratio	1	0.0135	0.0166	1.014 (0.981, 1.047)	0.415	0.687	1.68E-11	TRUE
CD8 NC	*UBASH3A*	Wald ratio	1	-0.0213	0.0166	0.979 (0.947, 1.011)	0.2	0.484	2.83E-11	TRUE
CD8 NC	*UBE2F*	Wald ratio	1	-0.0320	0.0246	0.969 (0.923, 1.016)	0.193	0.474	5.43E-08	TRUE
CD8 NC	*UBE2L3*	Wald ratio	1	-0.0130	0.0171	0.987 (0.955, 1.021)	0.446	0.711	4.57E-08	TRUE
CD8 NC	*UBXN6*	Wald ratio	1	-0.0315	0.0180	0.969 (0.935, 1.004)	0.08	0.297	5.37E-12	TRUE
CD8 NC	*UCP2*	Wald ratio	1	0.0042	0.0090	1.004 (0.987, 1.022)	0.64	0.83	3.09E-34	TRUE
CD8 NC	*ULK4*	Wald ratio	1	0.0166	0.0138	1.017 (0.99, 1.045)	0.228	0.521	3.43E-13	TRUE
CD8 NC	*UPK3BL*	Wald ratio	1	-0.0165	0.0094	0.984 (0.966, 1.002)	0.079	0.293	5.85E-31	TRUE
CD8 NC	*UQCRH*	Wald ratio	1	-0.0250	0.0119	0.975 (0.953, 0.998)	0.035	0.179	9.64E-26	TRUE
CD8 NC	*USF1*	Wald ratio	1	-0.0205	0.0192	0.98 (0.944, 1.017)	0.285	0.577	5.04E-10	TRUE
CD8 NC	*USP36*	Wald ratio	1	0.1024	0.0208	1.108 (1.064, 1.154)	8.57E-07	1.03E-04	1.53E-07	TRUE
CD8 NC	*USP53*	Wald ratio	1	-0.0036	0.0065	0.996 (0.984, 1.009)	0.584	0.793	1.36E-46	TRUE
CD8 NC	*UTS2*	Wald ratio	1	0.0373	0.0174	1.038 (1.003, 1.074)	0.032	0.168	2.26E-10	TRUE
CD8 NC	*VAMP2*	Wald ratio	1	0.0226	0.0132	1.023 (0.997, 1.05)	0.086	0.311	4.18E-20	TRUE
CD8 NC	*VAMP4*	Wald ratio	1	0.0222	0.0197	1.022 (0.984, 1.063)	0.26	0.554	6.00E-10	TRUE
CD8 NC	*VAMP5*	Wald ratio	1	0.0161	0.0123	1.016 (0.992, 1.041)	0.193	0.474	3.84E-27	TRUE
CD8 NC	*VCL*	Wald ratio	1	-0.0212	0.0137	0.979 (0.953, 1.006)	0.12	0.37	6.81E-16	TRUE
CD8 NC	*VIM*	Wald ratio	1	0.0825	0.0160	1.086 (1.052, 1.121)	2.49E-07	7.98E-05	9.13E-11	TRUE
CD8 NC	*VPS28*	Wald ratio	1	0.0203	0.0125	1.021 (0.996, 1.046)	0.102	0.338	1.53E-16	TRUE
CD8 NC	*VRK3*	Wald ratio	1	-0.0167	0.0209	0.983 (0.944, 1.024)	0.424	0.694	3.88E-08	TRUE
CD8 NC	*VWDE*	Wald ratio	1	-0.0109	0.0211	0.989 (0.949, 1.031)	0.604	0.806	1.84E-10	TRUE
CD8 NC	*WAC-AS1*	Wald ratio	1	0.0238	0.0146	1.024 (0.995, 1.054)	0.105	0.343	9.59E-16	TRUE
CD8 NC	*WBP1L*	Wald ratio	1	0.0059	0.0191	1.006 (0.969, 1.044)	0.759	0.89	5.38E-09	TRUE
CD8 NC	*WBSCR27*	Wald ratio	1	0.0130	0.0078	1.013 (0.998, 1.029)	0.097	0.331	2.84E-40	TRUE
CD8 NC	*WIPI2*	Wald ratio	1	-0.0198	0.0166	0.98 (0.949, 1.013)	0.234	0.527	1.56E-10	TRUE
CD8 NC	*XBP1*	Wald ratio	1	0.0082	0.0101	1.008 (0.988, 1.028)	0.418	0.689	8.35E-22	TRUE
CD8 NC	*XRCC6BP1*	Wald ratio	1	-0.0302	0.0153	0.97 (0.942, 1)	0.048	0.215	5.37E-13	TRUE
CD8 NC	*XRRA1*	Wald ratio	1	-0.0061	0.0050	0.994 (0.984, 1.004)	0.228	0.521	1.56E-114	TRUE
CD8 NC	*XXbac-BPG181B23.7*	Wald ratio	1	-0.0044	0.0204	0.996 (0.957, 1.036)	0.828	0.928	6.17E-08	TRUE
CD8 NC	*XXbac-BPG252P9.9*	IVW	2	-0.0348	0.0469	0.966 (0.881, 1.059)	0.457	0.719	1.67E-30	TRUE
CD8 NC	*XXbac-BPG299F13.17*	IVW	2	0.0065	0.0050	1.006 (0.997, 1.016)	0.198	0.48	5.64E-107	TRUE
CD8 NC	*YBEY*	Wald ratio	1	-0.0009	0.0072	0.999 (0.985, 1.013)	0.898	0.957	3.30E-48	TRUE
CD8 NC	*YBX3*	Wald ratio	1	0.0274	0.0164	1.028 (0.995, 1.061)	0.095	0.327	2.62E-12	TRUE
CD8 NC	*YEATS4*	Wald ratio	1	-0.0018	0.0159	0.998 (0.968, 1.03)	0.911	0.962	1.19E-12	TRUE
CD8 NC	*YME1L1*	Wald ratio	1	0.0119	0.0191	1.012 (0.975, 1.051)	0.532	0.76	6.21E-09	TRUE
CD8 NC	*YWHAH*	Wald ratio	1	-0.0621	0.0207	0.94 (0.902, 0.979)	0.003	0.032	9.22E-08	TRUE
CD8 NC	*ZC3HAV1*	Wald ratio	1	0.0063	0.0085	1.006 (0.99, 1.023)	0.459	0.72	7.64E-40	TRUE
CD8 NC	*ZFAS1*	Wald ratio	1	0.0151	0.0088	1.015 (0.998, 1.033)	0.088	0.313	4.31E-37	TRUE
CD8 NC	*ZFP57*	Wald ratio	1	-0.0165	0.0180	0.984 (0.95, 1.019)	0.359	0.65	6.65E-11	TRUE
CD8 NC	*ZFP90*	Wald ratio	1	0.0294	0.0205	1.03 (0.989, 1.072)	0.152	0.422	5.47E-09	TRUE
CD8 NC	*ZNF100*	Wald ratio	1	-0.0769	0.0144	0.926 (0.9, 0.952)	8.65E-08	6.60E-05	7.83E-14	TRUE
CD8 NC	*ZNF160*	Wald ratio	1	0.0456	0.0239	1.047 (0.999, 1.097)	0.056	0.239	5.63E-08	TRUE
CD8 NC	*ZNF266*	Wald ratio	1	0.0470	0.0187	1.048 (1.01, 1.087)	0.012	0.093	2.40E-09	TRUE
CD8 NC	*ZNF302*	Wald ratio	1	-0.0114	0.0112	0.989 (0.967, 1.011)	0.307	0.6	3.53E-22	TRUE
CD8 NC	*ZNF429*	Wald ratio	1	-0.0325	0.0202	0.968 (0.93, 1.007)	0.107	0.347	3.88E-08	TRUE
CD8 NC	*ZNF584*	Wald ratio	1	0.0221	0.0130	1.022 (0.997, 1.049)	0.089	0.317	6.05E-17	TRUE
CD8 NC	*ZNF593*	Wald ratio	1	0.0352	0.0179	1.036 (1, 1.073)	0.049	0.221	2.27E-10	TRUE
CD8 NC	*ZNF638*	Wald ratio	1	0.0474	0.0198	1.049 (1.009, 1.09)	0.017	0.113	2.03E-08	TRUE
CD8 NC	*ZNF670*	Wald ratio	1	0.0005	0.0128	1.001 (0.976, 1.026)	0.966	0.988	1.65E-16	TRUE

CD8 NC	*ZNF677*	Wald ratio	1	-0.0168	0.0126	0.983 (0.959, 1.008)	0.184	0.464	6.61E-20	TRUE
CD8 NC	*ZNF749*	Wald ratio	1	-0.0180	0.0135	0.982 (0.957, 1.008)	0.181	0.461	1.32E-16	TRUE
CD8 NC	*ZNF786*	Wald ratio	1	0.0147	0.0214	1.015 (0.973, 1.058)	0.492	0.738	5.05E-08	TRUE
CD8 NC	*ZNF83*	Wald ratio	1	-0.0173	0.0102	0.983 (0.963, 1.003)	0.09	0.318	1.43E-27	TRUE
CD8 NC	*ZNF85*	Wald ratio	1	0.0198	0.0119	1.02 (0.996, 1.044)	0.097	0.331	2.15E-22	TRUE
CD8 NC	*ZNF880*	Wald ratio	1	-0.0268	0.0201	0.974 (0.936, 1.013)	0.183	0.463	4.28E-08	TRUE
CD8 NC	*ZSCAN18*	Wald ratio	1	-0.0230	0.0180	0.977 (0.943, 1.012)	0.202	0.486	7.42E-09	TRUE
CD8 NC	*ZSCAN31*	Wald ratio	1	0.0047	0.0227	1.005 (0.961, 1.05)	0.835	0.93	1.13E-11	TRUE
CD8 NC	*ZSCAN9*	Wald ratio	1	0.0121	0.0177	1.012 (0.978, 1.048)	0.495	0.74	2.06E-18	TRUE
CD8 NC	*ZSWIM7*	Wald ratio	1	0.0612	0.0191	1.063 (1.024, 1.104)	0.001	0.02	9.56E-09	TRUE
CD8 S100B	*AC006369.2*	Wald ratio	1	0.0117	0.0104	1.012 (0.991, 1.033)	0.259	0.553	6.81E-29	TRUE
CD8 S100B	*AC013264.2*	Wald ratio	1	0.0124	0.0102	1.012 (0.992, 1.033)	0.223	0.516	1.36E-26	TRUE
CD8 S100B	*AC090186.1*	Wald ratio	1	-0.0284	0.0202	0.972 (0.934, 1.011)	0.16	0.433	1.67E-08	TRUE
CD8 S100B	*AHI1*	Wald ratio	1	-0.0285	0.0181	0.972 (0.938, 1.007)	0.116	0.362	7.95E-10	TRUE
CD8 S100B	*AKAP5*	Wald ratio	1	0.0308	0.0159	1.031 (1, 1.064)	0.053	0.231	9.61E-23	TRUE
CD8 S100B	*ANKDD1A*	Wald ratio	1	-0.0037	0.0094	0.996 (0.978, 1.015)	0.696	0.864	1.66E-31	TRUE
CD8 S100B	*ANXA6*	Wald ratio	1	0.0009	0.0165	1.001 (0.969, 1.034)	0.958	0.985	7.41E-10	TRUE
CD8 S100B	*AOAH*	IVW	2	-0.0409	0.0124	0.96 (0.937, 0.983)	9.32E-04	0.016	2.51E-21	TRUE
CD8 S100B	*AP003774.1*	Wald ratio	1	-0.0529	0.0154	0.948 (0.92, 0.978)	5.86E-04	0.011	3.05E-12	TRUE
CD8 S100B	*APOM*	Wald ratio	1	0.0125	0.0166	1.013 (0.98, 1.046)	0.449	0.714	2.19E-18	TRUE
CD8 S100B	*ARHGAP15*	Wald ratio	1	0.0101	0.0151	1.01 (0.981, 1.04)	0.505	0.745	8.83E-17	TRUE
CD8 S100B	*ARHGEF3*	Wald ratio	1	-0.0035	0.0187	0.996 (0.961, 1.034)	0.85	0.936	2.20E-08	TRUE
CD8 S100B	*ARL16*	Wald ratio	1	0.0200	0.0164	1.02 (0.988, 1.053)	0.222	0.514	4.39E-11	TRUE
CD8 S100B	*ATHL1*	Wald ratio	1	-0.0057	0.0181	0.994 (0.96, 1.03)	0.752	0.887	2.00E-11	TRUE
CD8 S100B	*ATP6V1G2*	Wald ratio	1	0.0014	0.0125	1.001 (0.977, 1.026)	0.911	0.962	8.51E-30	TRUE
CD8 S100B	*BIN1*	Wald ratio	1	0.0290	0.0153	1.029 (0.999, 1.061)	0.058	0.243	2.30E-12	TRUE
CD8 S100B	*BTF3*	Wald ratio	1	0.0463	0.0155	1.047 (1.016, 1.08)	0.003	0.034	3.47E-11	TRUE
CD8 S100B	*BTN3A2*	Wald ratio	1	0.0187	0.0131	1.019 (0.993, 1.045)	0.153	0.423	5.86E-24	TRUE
CD8 S100B	*C10orf128*	Wald ratio	1	-0.0071	0.0118	0.993 (0.97, 1.016)	0.549	0.769	1.04E-20	TRUE
CD8 S100B	*C10orf32*	Wald ratio	1	-0.0893	0.0182	0.915 (0.883, 0.948)	8.67E-07	1.03E-04	1.38E-09	TRUE
CD8 S100B	*C17orf97*	Wald ratio	1	0.0097	0.0080	1.01 (0.994, 1.026)	0.23	0.524	4.16E-44	TRUE
CD8 S100B	*C22orf34*	Wald ratio	1	0.0235	0.0104	1.024 (1.003, 1.045)	0.024	0.142	6.06E-26	TRUE
CD8 S100B	*C9orf135*	Wald ratio	1	-0.0185	0.0123	0.982 (0.958, 1.006)	0.132	0.394	7.53E-17	TRUE
CD8 S100B	*CAMLG*	Wald ratio	1	0.0000	0.0151	1 (0.971, 1.03)	0.999	0.999	2.63E-09	TRUE
CD8 S100B	*CARHSP1*	Wald ratio	1	0.0232	0.0164	1.023 (0.991, 1.057)	0.157	0.428	4.15E-11	TRUE
CD8 S100B	*CARS*	Wald ratio	1	0.0177	0.0119	1.018 (0.994, 1.042)	0.137	0.399	8.74E-20	TRUE
CD8 S100B	*CASP8*	Wald ratio	1	-0.0437	0.0156	0.957 (0.928, 0.987)	0.005	0.051	3.77E-12	TRUE
CD8 S100B	*CAST*	Wald ratio	1	-0.0071	0.0212	0.993 (0.953, 1.035)	0.737	0.88	2.13E-08	TRUE
CD8 S100B	*CCDC122*	Wald ratio	1	0.0077	0.0140	1.008 (0.98, 1.036)	0.584	0.793	1.76E-15	TRUE
CD8 S100B	*CCDC146*	Wald ratio	1	0.0141	0.0238	1.014 (0.968, 1.063)	0.555	0.774	3.94E-09	TRUE
CD8 S100B	*CCDC163P*	Wald ratio	1	0.0130	0.0115	1.013 (0.99, 1.036)	0.262	0.557	5.10E-22	TRUE
CD8 S100B	*CCDC167*	Wald ratio	1	-0.0011	0.0102	0.999 (0.979, 1.019)	0.913	0.962	2.21E-26	TRUE
CD8 S100B	*CCDC58*	Wald ratio	1	0.0272	0.0146	1.028 (0.999, 1.057)	0.063	0.256	2.10E-12	TRUE
CD8 S100B	*CCNG2*	Wald ratio	1	0.0040	0.0173	1.004 (0.971, 1.039)	0.816	0.921	7.85E-11	TRUE
CD8 S100B	*CCT3*	Wald ratio	1	0.0320	0.0187	1.032 (0.995, 1.071)	0.087	0.313	5.01E-09	TRUE
CD8 S100B	*CCT8*	Wald ratio	1	-0.0011	0.0115	0.999 (0.977, 1.022)	0.922	0.965	1.57E-35	TRUE
CD8 S100B	*CCZ1B*	Wald ratio	1	-0.0190	0.0123	0.981 (0.958, 1.005)	0.122	0.375	7.64E-21	TRUE
CD8 S100B	*CD151*	Wald ratio	1	0.0685	0.0204	1.071 (1.029, 1.115)	7.95E-04	0.014	2.63E-08	TRUE
CD8 S100B	*CD300A*	Wald ratio	1	-0.0116	0.0190	0.988 (0.952, 1.026)	0.541	0.763	5.41E-09	TRUE
CD8 S100B	*CDC42*	Wald ratio	1	0.0277	0.0168	1.028 (0.995, 1.063)	0.1	0.334	3.20E-10	TRUE
CD8 S100B	*CELA1*	Wald ratio	1	-0.0144	0.0209	0.986 (0.946, 1.027)	0.491	0.738	3.28E-09	TRUE
CD8 S100B	*CENPK*	Wald ratio	1	0.0100	0.0072	1.01 (0.996, 1.024)	0.165	0.439	5.10E-56	TRUE
CD8 S100B	*CFD*	Wald ratio	1	-0.0013	0.0183	0.999 (0.963, 1.035)	0.944	0.978	4.03E-11	TRUE
CD8 S100B	*CHCHD2*	Wald ratio	1	0.0083	0.0155	1.008 (0.978, 1.04)	0.591	0.797	6.11E-14	TRUE
CD8 S100B	*CHPT1*	Wald ratio	1	0.0005	0.0154	1 (0.971, 1.031)	0.974	0.99	1.69E-13	TRUE
CD8 S100B	*CLEC2B*	Wald ratio	1	0.0063	0.0203	1.006 (0.967, 1.047)	0.755	0.888	4.48E-08	TRUE
CD8 S100B	*CMC1*	Wald ratio	1	0.0350	0.0188	1.036 (0.998, 1.075)	0.062	0.255	3.50E-10	TRUE
CD8 S100B	*CMTM8*	Wald ratio	1	0.0026	0.0154	1.003 (0.973, 1.033)	0.865	0.943	5.19E-15	TRUE
CD8 S100B	*COG5*	Wald ratio	1	0.0725	0.0145	1.075 (1.045, 1.106)	5.96E-07	9.75E-05	4.50E-13	TRUE
CD8 S100B	*COL18A1*	Wald ratio	1	-0.0673	0.0235	0.935 (0.893, 0.979)	0.004	0.044	3.54E-14	TRUE
CD8 S100B	*COL5A3*	Wald ratio	1	-0.0321	0.0170	0.968 (0.937, 1.001)	0.059	0.244	2.56E-13	TRUE
CD8 S100B	*COMMD6*	Wald ratio	1	0.0250	0.0154	1.025 (0.995, 1.057)	0.104	0.343	4.69E-13	TRUE
CD8 S100B	*CPA5*	Wald ratio	1	-0.0070	0.0079	0.993 (0.978, 1.009)	0.378	0.665	2.09E-47	TRUE
CD8 S100B	*CPNE1*	Wald ratio	1	-0.0098	0.0208	0.99 (0.951, 1.032)	0.639	0.83	3.94E-09	TRUE
CD8 S100B	*CSTB*	Wald ratio	1	-0.0038	0.0148	0.996 (0.968, 1.026)	0.798	0.913	2.78E-13	TRUE
CD8 S100B	*CTA-29F11.1*	Wald ratio	1	-0.0088	0.0138	0.991 (0.965, 1.019)	0.527	0.756	3.57E-16	TRUE
CD8 S100B	*CTC-524C5.2*	Wald ratio	1	0.0219	0.0123	1.022 (0.998, 1.047)	0.076	0.288	1.03E-15	TRUE
CD8 S100B	*CTD-2368P22.1*	Wald ratio	1	-0.0034	0.0146	0.997 (0.969, 1.026)	0.818	0.922	1.18E-15	TRUE
CD8 S100B	*CTD-2371O3.2*	Wald ratio	1	0.0033	0.0086	1.003 (0.986, 1.02)	0.704	0.867	4.03E-41	TRUE
CD8 S100B	*CTLA4*	Wald ratio	1	-0.0080	0.0098	0.992 (0.973, 1.011)	0.418	0.689	2.32E-29	TRUE
CD8 S100B	*CTSW*	Wald ratio	1	-0.0307	0.0073	0.97 (0.956, 0.984)	2.34E-05	0.001	1.22E-63	TRUE
CD8 S100B	*DCXR*	Wald ratio	1	-0.0172	0.0190	0.983 (0.947, 1.02)	0.365	0.653	6.08E-08	TRUE
CD8 S100B	*DDX11*	Wald ratio	1	-0.0219	0.0165	0.978 (0.947, 1.01)	0.183	0.462	1.62E-11	TRUE
CD8 S100B	*DDX5*	Wald ratio	1	0.0579	0.0149	1.06 (1.029, 1.091)	1.06E-04	0.003	2.55E-12	TRUE
CD8 S100B	*DIP2A*	Wald ratio	1	0.0132	0.0111	1.013 (0.991, 1.036)	0.236	0.53	1.43E-24	TRUE
CD8 S100B	*DNAJC15*	Wald ratio	1	0.0100	0.0108	1.01 (0.989, 1.032)	0.355	0.646	1.05E-24	TRUE
CD8 S100B	*DTX2*	Wald ratio	1	-0.0124	0.0186	0.988 (0.952, 1.024)	0.506	0.745	3.36E-12	TRUE
CD8 S100B	*DYNLL2*	Wald ratio	1	-0.0419	0.0193	0.959 (0.923, 0.996)	0.03	0.161	2.54E-09	TRUE
CD8 S100B	*EBPL*	Wald ratio	1	0.0086	0.0146	1.009 (0.98, 1.038)	0.556	0.774	2.55E-14	TRUE

CD8 S100B	*EEF2*	Wald ratio	1	0.0861	0.0193	1.09 (1.049, 1.132)	8.31E-06	4.97E-04	2.11E-08	TRUE
CD8 S100B	*EIF2A*	Wald ratio	1	-0.0214	0.0154	0.979 (0.95, 1.009)	0.163	0.437	4.11E-12	TRUE
CD8 S100B	*EIF5A*	Wald ratio	1	-0.0086	0.0057	0.991 (0.98, 1.003)	0.134	0.394	8.27E-104	TRUE
CD8 S100B	*EPSTI1*	Wald ratio	1	0.0093	0.0169	1.009 (0.976, 1.043)	0.581	0.791	5.43E-12	TRUE
CD8 S100B	*ERAP2*	Wald ratio	1	0.0361	0.0080	1.037 (1.021, 1.053)	6.43E-06	4.08E-04	2.24E-45	TRUE
CD8 S100B	*ERN1*	Wald ratio	1	-0.0510	0.0190	0.95 (0.916, 0.986)	0.007	0.066	3.11E-09	TRUE
CD8 S100B	*ESR2*	Wald ratio	1	-0.0507	0.0234	0.951 (0.908, 0.995)	0.03	0.162	1.18E-09	TRUE
CD8 S100B	*EXOC3*	Wald ratio	1	-0.0186	0.0186	0.982 (0.946, 1.018)	0.318	0.613	2.23E-09	TRUE
CD8 S100B	*FADS1*	Wald ratio	1	-0.0358	0.0106	0.965 (0.945, 0.985)	7.62E-04	0.014	7.33E-28	TRUE
CD8 S100B	*FAM118A*	Wald ratio	1	0.0007	0.0050	1.001 (0.991, 1.011)	0.895	0.957	1.47E-88	TRUE
CD8 S100B	*FAM153B*	Wald ratio	1	0.0463	0.0173	1.047 (1.012, 1.083)	0.007	0.066	1.94E-10	TRUE
CD8 S100B	*FAM184B*	Wald ratio	1	0.0018	0.0093	1.002 (0.984, 1.02)	0.847	0.935	8.51E-37	TRUE
CD8 S100B	*FAM26F*	Wald ratio	1	-0.0470	0.0146	0.954 (0.927, 0.982)	0.001	0.02	4.03E-15	TRUE
CD8 S100B	*FAM85A*	Wald ratio	1	-0.0479	0.0097	0.953 (0.935, 0.971)	7.01E-07	9.75E-05	3.62E-29	TRUE
CD8 S100B	*FAM86B1*	Wald ratio	1	-0.0285	0.0102	0.972 (0.953, 0.992)	0.005	0.053	3.81E-32	TRUE
CD8 S100B	*FAU*	Wald ratio	1	-0.0007	0.0194	0.999 (0.962, 1.038)	0.973	0.989	1.06E-08	TRUE
CD8 S100B	*FBLN5*	Wald ratio	1	0.0038	0.0136	1.004 (0.977, 1.031)	0.779	0.904	2.48E-16	TRUE
CD8 S100B	*FDFT1*	Wald ratio	1	0.0664	0.0207	1.069 (1.026, 1.113)	0.001	0.02	5.01E-09	TRUE
CD8 S100B	*FHL3*	Wald ratio	1	0.0137	0.0202	1.014 (0.974, 1.055)	0.499	0.741	1.62E-08	TRUE
CD8 S100B	*FKBP11*	Wald ratio	1	0.0377	0.0192	1.038 (1, 1.078)	0.049	0.221	1.08E-08	TRUE
CD8 S100B	*FMN1*	Wald ratio	1	0.0098	0.0076	1.01 (0.995, 1.025)	0.195	0.477	2.88E-43	TRUE
CD8 S100B	*GBP5*	Wald ratio	1	-0.0571	0.0199	0.945 (0.908, 0.982)	0.004	0.043	6.05E-08	TRUE
CD8 S100B	*GBP7*	Wald ratio	1	-0.0024	0.0121	0.998 (0.974, 1.022)	0.844	0.935	1.01E-20	TRUE
CD8 S100B	*GCC2*	Wald ratio	1	0.0105	0.0140	1.011 (0.983, 1.039)	0.451	0.716	3.57E-17	TRUE
CD8 S100B	*GLTSCR2*	Wald ratio	1	-0.0085	0.0138	0.991 (0.965, 1.019)	0.537	0.762	7.30E-15	TRUE
CD8 S100B	*GNLY*	Wald ratio	1	-0.0139	0.0098	0.986 (0.967, 1.005)	0.157	0.428	7.70E-27	TRUE
CD8 S100B	*GPR160*	Wald ratio	1	-0.0398	0.0179	0.961 (0.928, 0.995)	0.026	0.149	1.14E-09	TRUE
CD8 S100B	*GRHPR*	Wald ratio	1	-0.0221	0.0183	0.978 (0.944, 1.014)	0.227	0.52	5.79E-09	TRUE
CD8 S100B	*GSTM3*	Wald ratio	1	-0.0090	0.0135	0.991 (0.965, 1.018)	0.506	0.745	2.62E-16	TRUE
CD8 S100B	*GSTP1*	Wald ratio	1	0.0264	0.0152	1.027 (0.997, 1.058)	0.082	0.301	4.46E-14	TRUE
CD8 S100B	*GYPC*	Wald ratio	1	-0.0162	0.0164	0.984 (0.953, 1.016)	0.324	0.618	1.12E-10	TRUE
CD8 S100B	*HCG27*	IVW	2	-0.0280	0.0228	0.972 (0.93, 1.017)	0.219	0.51	1.17E-62	TRUE
CD8 S100B	*HIGD2A*	Wald ratio	1	-0.0145	0.0184	0.986 (0.951, 1.022)	0.429	0.699	6.71E-10	TRUE
CD8 S100B	*HIST1H2AB*	Wald ratio	1	0.0138	0.0189	1.014 (0.977, 1.052)	0.467	0.725	7.81E-16	TRUE
CD8 S100B	*HIST1H2AG*	Wald ratio	1	0.0209	0.0243	1.021 (0.974, 1.071)	0.39	0.671	2.89E-10	TRUE
CD8 S100B	*HIST1H2AK*	Wald ratio	1	0.0039	0.0163	1.004 (0.972, 1.037)	0.812	0.92	2.02E-21	TRUE
CD8 S100B	*HIST1H2BF*	Wald ratio	1	0.0194	0.0226	1.02 (0.975, 1.066)	0.39	0.671	1.13E-11	TRUE
CD8 S100B	*HIST1H2BG*	Wald ratio	1	-0.0239	0.0270	0.976 (0.926, 1.029)	0.376	0.664	3.45E-08	TRUE
CD8 S100B	*HIST1H3D*	Wald ratio	1	0.0233	0.0184	1.024 (0.987, 1.061)	0.207	0.494	3.30E-16	TRUE
CD8 S100B	*HLA-A*	Wald ratio	1	0.0467	0.0200	1.048 (1.008, 1.09)	0.019	0.124	3.14E-08	TRUE
CD8 S100B	*HLA-B*	IVW	3	0.0030	0.0219	1.003 (0.961, 1.047)	0.891	0.956	2.89E-92	TRUE
CD8 S100B	*HLA-B*	MR Egger	3	0.2605	0.0489	1.298 (1.179, 1.428)	0.118	NA	NA	NA
CD8 S100B	*HLA-B*	Weighted median	3	0.0031	0.0068	1.003 (0.99, 1.017)	0.649	NA	NA	NA
CD8 S100B	*HLA-B*	Simple mode	3	-0.0364	0.0122	0.964 (0.942, 0.987)	0.096	NA	NA	NA
CD8 S100B	*HLA-B*	Weighted mode	3	0.0278	0.0088	1.028 (1.011, 1.046)	0.087	NA	NA	NA
CD8 S100B	*HLA-C*	Wald ratio	1	-0.0199	0.0137	0.98 (0.954, 1.007)	0.146	0.415	2.51E-14	TRUE
CD8 S100B	*HLA-DOA*	Wald ratio	1	-0.0221	0.0157	0.978 (0.949, 1.009)	0.158	0.429	3.99E-17	TRUE
CD8 S100B	*HLA-DOB*	Wald ratio	1	0.0197	0.0239	1.02 (0.973, 1.069)	0.411	0.686	1.36E-09	TRUE
CD8 S100B	*HLA-DQA2*	Wald ratio	1	0.0227	0.0130	1.023 (0.997, 1.049)	0.081	0.298	6.76E-18	TRUE
CD8 S100B	*HLA-DQB1*	Wald ratio	1	-0.0305	0.0181	0.97 (0.936, 1.005)	0.093	0.323	4.67E-11	TRUE
CD8 S100B	*HLA-DRB5*	Wald ratio	1	0.0213	0.0187	1.021 (0.985, 1.06)	0.257	0.551	1.63E-09	TRUE
CD8 S100B	*HMBOX1*	Wald ratio	1	-0.0233	0.0127	0.977 (0.953, 1.002)	0.066	0.263	1.05E-16	TRUE
CD8 S100B	*HOXB2*	Wald ratio	1	-0.0275	0.0116	0.973 (0.951, 0.995)	0.017	0.116	2.90E-21	TRUE
CD8 S100B	*HSPA1L*	Wald ratio	1	-0.0533	0.0135	0.948 (0.923, 0.973)	7.48E-05	0.003	3.82E-22	TRUE
CD8 S100B	*HSPBAP1*	Wald ratio	1	0.0216	0.0140	1.022 (0.994, 1.05)	0.124	0.379	1.61E-12	TRUE
CD8 S100B	*IFITM2*	Wald ratio	1	-0.0041	0.0111	0.996 (0.974, 1.018)	0.713	0.872	7.14E-23	TRUE
CD8 S100B	*IFITM3*	Wald ratio	1	0.0003	0.0093	1 (0.982, 1.019)	0.978	0.991	1.49E-34	TRUE
CD8 S100B	*IFNG-AS1*	Wald ratio	1	-0.0140	0.0194	0.986 (0.949, 1.024)	0.471	0.729	1.14E-08	TRUE
CD8 S100B	*IL32*	Wald ratio	1	-0.0276	0.0161	0.973 (0.943, 1.004)	0.087	0.312	2.71E-08	TRUE
CD8 S100B	*ISCA2*	Wald ratio	1	0.0153	0.0192	1.015 (0.978, 1.054)	0.424	0.694	9.84E-09	TRUE
CD8 S100B	*ITGB2*	Wald ratio	1	-0.0011	0.0085	0.999 (0.982, 1.016)	0.897	0.957	4.63E-34	TRUE
CD8 S100B	*KANSL1-AS1*	Wald ratio	1	-0.0236	0.0096	0.977 (0.958, 0.995)	0.014	0.103	3.39E-74	TRUE
CD8 S100B	*KCNAB2*	Wald ratio	1	0.0189	0.0208	1.019 (0.978, 1.061)	0.365	0.653	1.54E-08	TRUE
CD8 S100B	*KDELR2*	Wald ratio	1	0.0462	0.0143	1.047 (1.018, 1.077)	0.001	0.02	8.69E-14	TRUE
CD8 S100B	*KIAA0040*	Wald ratio	1	0.0936	0.0194	1.098 (1.057, 1.141)	1.40E-06	1.45E-04	2.88E-08	TRUE
CD8 S100B	*KIAA1430*	Wald ratio	1	0.0060	0.0193	1.006 (0.969, 1.045)	0.756	0.888	2.16E-08	TRUE
CD8 S100B	*KLRC4*	Wald ratio	1	-0.0191	0.0169	0.981 (0.949, 1.014)	0.257	0.551	8.31E-11	TRUE
CD8 S100B	*KRTCAP3*	Wald ratio	1	0.0350	0.0146	1.036 (1.006, 1.066)	0.016	0.112	9.24E-14	TRUE
CD8 S100B	*KTN1*	Wald ratio	1	0.0363	0.0191	1.037 (0.999, 1.076)	0.057	0.24	8.54E-09	TRUE
CD8 S100B	*LAMB1*	Wald ratio	1	-0.0087	0.0096	0.991 (0.973, 1.01)	0.365	0.653	6.26E-32	TRUE
CD8 S100B	*LDLRAP1*	Wald ratio	1	0.0134	0.0181	1.014 (0.978, 1.05)	0.458	0.719	7.97E-10	TRUE
CD8 S100B	*LINC00339*	Wald ratio	1	0.0174	0.0175	1.018 (0.983, 1.053)	0.319	0.615	2.92E-10	TRUE
CD8 S100B	*LINC00649*	Wald ratio	1	-0.0080	0.0194	0.992 (0.955, 1.03)	0.679	0.853	1.24E-08	TRUE
CD8 S100B	*LINC00969*	Wald ratio	1	0.0103	0.0171	1.01 (0.977, 1.045)	0.546	0.767	8.22E-11	TRUE
CD8 S100B	*LRRC16A*	Wald ratio	1	0.0347	0.0148	1.035 (1.006, 1.066)	0.019	0.124	1.30E-13	TRUE
CD8 S100B	*LYRM4*	Wald ratio	1	0.0217	0.0169	1.022 (0.989, 1.056)	0.197	0.48	2.59E-09	TRUE
CD8 S100B	*MDC1*	Wald ratio	1	0.0380	0.0225	1.039 (0.994, 1.085)	0.091	0.319	8.91E-08	TRUE
CD8 S100B	*MDM4*	Wald ratio	1	-0.0749	0.0199	0.928 (0.892, 0.965)	1.64E-04	0.004	5.70E-10	TRUE
CD8 S100B	*MED16*	Wald ratio	1	-0.0013	0.0195	0.999 (0.961, 1.038)	0.948	0.98	3.75E-10	TRUE

CD8 S100B	*MGST3*	Wald ratio	1	-0.0193	0.0193	0.981 (0.944, 1.019)	0.319	0.614	6.51E-09	TRUE
CD8 S100B	*MRPL21*	Wald ratio	1	0.0195	0.0183	1.02 (0.984, 1.057)	0.286	0.578	1.37E-08	TRUE
CD8 S100B	*MRPL34*	Wald ratio	1	0.0149	0.0182	1.015 (0.979, 1.052)	0.413	0.686	7.31E-10	TRUE
CD8 S100B	*MT1F*	Wald ratio	1	0.0167	0.0204	1.017 (0.977, 1.058)	0.411	0.686	4.56E-08	TRUE
CD8 S100B	*MT1X*	Wald ratio	1	0.0078	0.0204	1.008 (0.968, 1.049)	0.702	0.866	1.91E-08	TRUE
CD8 S100B	*MT2A*	Wald ratio	1	-0.0148	0.0093	0.985 (0.968, 1.003)	0.11	0.353	1.82E-39	TRUE
CD8 S100B	*MYOM2*	Wald ratio	1	0.0172	0.0122	1.017 (0.993, 1.042)	0.16	0.433	3.96E-18	TRUE
CD8 S100B	*MZT2A*	Wald ratio	1	-0.0172	0.0101	0.983 (0.964, 1.003)	0.088	0.315	8.74E-22	TRUE
CD8 S100B	*NAA38*	Wald ratio	1	-0.0126	0.0172	0.987 (0.955, 1.021)	0.465	0.725	6.60E-14	TRUE
CD8 S100B	*NAP1L1*	Wald ratio	1	-0.0179	0.0200	0.982 (0.945, 1.021)	0.37	0.658	2.76E-08	TRUE
CD8 S100B	*NAPRT1*	Wald ratio	1	-0.0127	0.0110	0.987 (0.966, 1.009)	0.245	0.539	2.51E-21	TRUE
CD8 S100B	*NCAPG2*	Wald ratio	1	-0.0189	0.0206	0.981 (0.942, 1.022)	0.36	0.65	2.59E-10	TRUE
CD8 S100B	*NDUFA12*	Wald ratio	1	-0.0071	0.0207	0.993 (0.953, 1.034)	0.733	0.88	9.11E-10	TRUE
CD8 S100B	*NDUFA3*	Wald ratio	1	0.0221	0.0186	1.022 (0.986, 1.06)	0.233	0.526	4.84E-09	TRUE
CD8 S100B	*NDUFS5*	Wald ratio	1	-0.0107	0.0174	0.989 (0.956, 1.024)	0.54	0.763	7.99E-12	TRUE
CD8 S100B	*NMI*	Wald ratio	1	0.0405	0.0193	1.041 (1.003, 1.081)	0.036	0.18	1.47E-08	TRUE
CD8 S100B	*NMRK1*	Wald ratio	1	-0.0035	0.0174	0.996 (0.963, 1.031)	0.84	0.932	1.08E-10	TRUE
CD8 S100B	*NPC2*	Wald ratio	1	0.0156	0.0195	1.016 (0.978, 1.055)	0.424	0.694	1.63E-08	TRUE
CD8 S100B	*NPDC1*	Wald ratio	1	0.0185	0.0201	1.019 (0.979, 1.06)	0.359	0.65	2.86E-08	TRUE
CD8 S100B	*NQO2*	Wald ratio	1	-0.0172	0.0221	0.983 (0.941, 1.026)	0.436	0.703	4.78E-08	TRUE
CD8 S100B	*NSA2*	Wald ratio	1	0.0332	0.0144	1.034 (1.005, 1.063)	0.021	0.132	6.07E-16	TRUE
CD8 S100B	*NSG1*	Wald ratio	1	0.0117	0.0089	1.012 (0.994, 1.03)	0.191	0.472	1.31E-45	TRUE
CD8 S100B	*NT5C3B*	Wald ratio	1	-0.0002	0.0133	1 (0.974, 1.026)	0.986	0.995	6.40E-15	TRUE
CD8 S100B	*NTPCR*	Wald ratio	1	0.0317	0.0194	1.032 (0.994, 1.072)	0.103	0.339	2.61E-09	TRUE
CD8 S100B	*NUDT2*	Wald ratio	1	-0.0050	0.0088	0.995 (0.978, 1.012)	0.569	0.782	1.12E-34	TRUE
CD8 S100B	*ORMDL3*	Wald ratio	1	-0.0013	0.0127	0.999 (0.974, 1.024)	0.916	0.963	6.33E-19	TRUE
CD8 S100B	*OXLD1*	Wald ratio	1	0.0082	0.0192	1.008 (0.971, 1.047)	0.668	0.848	1.39E-08	TRUE
CD8 S100B	*PASK*	Wald ratio	1	-0.0049	0.0150	0.995 (0.966, 1.025)	0.742	0.881	3.23E-13	TRUE
CD8 S100B	*PEX6*	Wald ratio	1	0.0132	0.0186	1.013 (0.977, 1.051)	0.478	0.733	1.68E-09	TRUE
CD8 S100B	*PIGP*	Wald ratio	1	0.0133	0.0177	1.013 (0.979, 1.049)	0.453	0.717	5.44E-10	TRUE
CD8 S100B	*PILRB*	Wald ratio	1	0.0075	0.0103	1.008 (0.987, 1.028)	0.465	0.725	6.64E-26	TRUE
CD8 S100B	*PLEK*	Wald ratio	1	0.0051	0.0197	1.005 (0.967, 1.045)	0.795	0.911	1.08E-08	TRUE
CD8 S100B	*PNRC2*	Wald ratio	1	-0.0208	0.0180	0.979 (0.946, 1.015)	0.248	0.541	7.13E-10	TRUE
CD8 S100B	*POLR1D*	Wald ratio	1	0.0019	0.0178	1.002 (0.968, 1.037)	0.915	0.963	4.36E-13	TRUE
CD8 S100B	*PPA1*	Wald ratio	1	0.0040	0.0078	1.004 (0.989, 1.019)	0.603	0.806	2.37E-44	TRUE
CD8 S100B	*PPAP2C*	Wald ratio	1	-0.0063	0.0109	0.994 (0.973, 1.015)	0.561	0.778	1.53E-25	TRUE
CD8 S100B	*PPDPF*	Wald ratio	1	-0.0091	0.0085	0.991 (0.975, 1.008)	0.283	0.575	6.16E-36	TRUE
CD8 S100B	*PPIA*	Wald ratio	1	0.0208	0.0150	1.021 (0.991, 1.052)	0.166	0.442	2.21E-13	TRUE
CD8 S100B	*PPIL3*	Wald ratio	1	0.0099	0.0137	1.01 (0.983, 1.037)	0.467	0.725	1.48E-15	TRUE
CD8 S100B	*PRDX6*	Wald ratio	1	-0.0184	0.0146	0.982 (0.954, 1.01)	0.207	0.494	3.44E-13	TRUE
CD8 S100B	*PRKCB*	Wald ratio	1	0.0218	0.0178	1.022 (0.987, 1.058)	0.221	0.513	1.31E-12	TRUE
CD8 S100B	*PRKCQ-AS1*	Wald ratio	1	-0.0081	0.0124	0.992 (0.968, 1.016)	0.511	0.748	2.70E-19	TRUE
CD8 S100B	*PRMT2*	IVW	2	0.0148	0.0091	1.015 (0.997, 1.033)	0.106	0.345	3.17E-34	TRUE
CD8 S100B	*PRR7-AS1*	Wald ratio	1	0.0287	0.0145	1.029 (1, 1.059)	0.048	0.215	1.41E-14	TRUE
CD8 S100B	*PRSS16*	Wald ratio	1	0.0158	0.0265	1.016 (0.965, 1.07)	0.55	0.769	1.19E-09	TRUE
CD8 S100B	*PSMD5-AS1*	Wald ratio	1	0.0047	0.0060	1.005 (0.993, 1.017)	0.434	0.702	7.41E-82	TRUE
CD8 S100B	*PSPH*	Wald ratio	1	0.0006	0.0090	1.001 (0.983, 1.018)	0.95	0.981	2.32E-27	TRUE
CD8 S100B	*RAB6B*	Wald ratio	1	0.0110	0.0104	1.011 (0.991, 1.032)	0.291	0.584	1.39E-23	TRUE
CD8 S100B	*RAC1*	Wald ratio	1	-0.0346	0.0178	0.966 (0.933, 1)	0.053	0.228	5.06E-10	TRUE
CD8 S100B	*RBM43*	Wald ratio	1	-0.0301	0.0135	0.97 (0.945, 0.996)	0.026	0.149	3.45E-16	TRUE
CD8 S100B	*RNASET2*	Wald ratio	1	-0.0256	0.0108	0.975 (0.954, 0.996)	0.018	0.117	3.06E-25	TRUE
CD8 S100B	*RP1-29C18.10*	Wald ratio	1	-0.0301	0.0151	0.97 (0.942, 0.999)	0.046	0.21	1.58E-14	TRUE
CD8 S100B	*RP1-3J17.3*	IVW	2	0.0027	0.0113	1.003 (0.981, 1.025)	0.813	0.921	4.63E-25	TRUE
CD8 S100B	*RP11-18H21.1*	Wald ratio	1	-0.0029	0.0107	0.997 (0.976, 1.018)	0.784	0.907	1.43E-29	TRUE
CD8 S100B	*RP11-291B21.2*	Wald ratio	1	-0.0282	0.0203	0.972 (0.934, 1.012)	0.166	0.441	7.88E-08	TRUE
CD8 S100B	*RP11-298J20.3*	Wald ratio	1	0.0313	0.0118	1.032 (1.008, 1.056)	0.008	0.069	1.17E-19	TRUE
CD8 S100B	*RP11-326C3.11*	Wald ratio	1	-0.0149	0.0108	0.985 (0.964, 1.006)	0.169	0.444	8.49E-30	TRUE
CD8 S100B	*RP11-326C3.12*	Wald ratio	1	-0.0136	0.0154	0.986 (0.957, 1.017)	0.377	0.665	7.47E-13	TRUE
CD8 S100B	*RP11-347P5.1*	Wald ratio	1	0.0016	0.0094	1.002 (0.983, 1.02)	0.861	0.942	2.15E-34	TRUE
CD8 S100B	*RP11-403A21.2*	Wald ratio	1	-0.0284	0.0196	0.972 (0.935, 1.01)	0.147	0.416	3.09E-08	TRUE
CD8 S100B	*RP11-457M11.5*	Wald ratio	1	0.0208	0.0188	1.021 (0.984, 1.059)	0.268	0.562	7.12E-16	TRUE
CD8 S100B	*RP11-473I1.5*	Wald ratio	1	-0.0334	0.0184	0.967 (0.933, 1.003)	0.069	0.271	4.01E-08	TRUE
CD8 S100B	*RP11-473M20.9*	Wald ratio	1	-0.0143	0.0146	0.986 (0.958, 1.014)	0.326	0.62	1.59E-14	TRUE
CD8 S100B	*RP11-664D1.1*	IVW	2	0.0179	0.0140	1.018 (0.99, 1.046)	0.202	0.486	2.91E-17	TRUE
CD8 S100B	*RP11-81H14.2*	Wald ratio	1	-0.0069	0.0182	0.993 (0.958, 1.029)	0.705	0.867	1.09E-10	TRUE
CD8 S100B	*RP5-1042K10.10*	Wald ratio	1	0.0126	0.0086	1.013 (0.996, 1.03)	0.147	0.415	1.42E-33	TRUE
CD8 S100B	*RPL10A*	Wald ratio	1	-0.0123	0.0094	0.988 (0.97, 1.006)	0.187	0.466	1.98E-34	TRUE
CD8 S100B	*RPL12*	Wald ratio	1	-0.0223	0.0067	0.978 (0.965, 0.991)	8.72E-04	0.015	1.74E-71	TRUE
CD8 S100B	*RPL13*	Wald ratio	1	-0.0289	0.0119	0.972 (0.949, 0.995)	0.016	0.107	9.35E-30	TRUE
CD8 S100B	*RPL14*	Wald ratio	1	0.0192	0.0172	1.019 (0.986, 1.054)	0.264	0.558	7.51E-13	TRUE
CD8 S100B	*RPL23A*	Wald ratio	1	-0.0265	0.0143	0.974 (0.947, 1.002)	0.064	0.258	2.44E-15	TRUE
CD8 S100B	*RPL28*	Wald ratio	1	0.0040	0.0051	1.004 (0.994, 1.014)	0.438	0.703	2.54E-117	TRUE
CD8 S100B	*RPL3*	Wald ratio	1	0.0060	0.0195	1.006 (0.968, 1.045)	0.758	0.89	4.89E-09	TRUE
CD8 S100B	*RPL32*	Wald ratio	1	0.0359	0.0214	1.037 (0.994, 1.081)	0.093	0.323	8.71E-08	TRUE
CD8 S100B	*RPL36AL*	Wald ratio	1	-0.0069	0.0083	0.993 (0.977, 1.009)	0.405	0.681	2.26E-44	TRUE
CD8 S100B	*RPL8*	Wald ratio	1	-0.0057	0.0055	0.994 (0.984, 1.005)	0.303	0.598	1.99E-100	TRUE
CD8 S100B	*RPLP2*	Wald ratio	1	-0.0289	0.0107	0.972 (0.951, 0.992)	0.007	0.062	5.09E-26	TRUE
CD8 S100B	*RPS10*	Wald ratio	1	-0.0068	0.0066	0.993 (0.981, 1.006)	0.305	0.599	4.62E-57	TRUE
CD8 S100B	*RPS11*	Wald ratio	1	0.0356	0.0149	1.036 (1.006, 1.067)	0.017	0.115	6.85E-15	TRUE

CD8 S100B	*RPS12*	Wald ratio	1	0.0692	0.0163	1.072 (1.038, 1.106)	2.21E-05	0.001	3.30E-10	TRUE
CD8 S100B	*RPS18*	Wald ratio	1	-0.0128	0.0075	0.987 (0.973, 1.002)	0.087	0.312	1.81E-64	TRUE
CD8 S100B	*RPS19*	Wald ratio	1	0.0247	0.0191	1.025 (0.987, 1.064)	0.197	0.48	6.15E-09	TRUE
CD8 S100B	*RPS2*	Wald ratio	1	-0.0324	0.0121	0.968 (0.946, 0.991)	0.007	0.066	1.71E-19	TRUE
CD8 S100B	*RPS23*	Wald ratio	1	-0.0193	0.0145	0.981 (0.953, 1.009)	0.184	0.464	1.21E-16	TRUE
CD8 S100B	*RPS24*	Wald ratio	1	-0.0035	0.0192	0.997 (0.96, 1.035)	0.855	0.938	4.89E-09	TRUE
CD8 S100B	*RPS25*	Wald ratio	1	-0.0013	0.0110	0.999 (0.977, 1.021)	0.908	0.961	2.01E-23	TRUE
CD8 S100B	*RPS26*	Wald ratio	1	-0.0100	0.0043	0.99 (0.982, 0.999)	0.022	0.132	9.58E-188	TRUE
CD8 S100B	*RPS3A*	Wald ratio	1	-0.0046	0.0068	0.995 (0.982, 1.009)	0.492	0.738	4.77E-31	TRUE
CD8 S100B	*RPS5*	Wald ratio	1	0.0094	0.0069	1.009 (0.996, 1.023)	0.177	0.456	8.20E-66	TRUE
CD8 S100B	*RPS6*	Wald ratio	1	-0.0156	0.0133	0.985 (0.959, 1.011)	0.242	0.534	1.83E-22	TRUE
CD8 S100B	*RPS7*	Wald ratio	1	0.0041	0.0132	1.004 (0.978, 1.03)	0.754	0.888	3.59E-26	TRUE
CD8 S100B	*RPS8*	Wald ratio	1	0.0027	0.0101	1.003 (0.983, 1.023)	0.791	0.909	3.60E-29	TRUE
CD8 S100B	*RPS9*	Wald ratio	1	0.0287	0.0075	1.029 (1.014, 1.044)	1.26E-04	0.003	6.40E-51	TRUE
CD8 S100B	*RPSA*	Wald ratio	1	-0.0059	0.0147	0.994 (0.966, 1.023)	0.688	0.859	1.37E-21	TRUE
CD8 S100B	*S100B*	IVW	2	0.0095	0.0058	1.01 (0.998, 1.021)	0.103	0.339	3.52E-95	TRUE
CD8 S100B	*SCD5*	Wald ratio	1	0.0613	0.0141	1.063 (1.034, 1.093)	1.30E-05	6.87E-04	3.80E-15	TRUE
CD8 S100B	*SCGB3A1*	Wald ratio	1	0.0077	0.0065	1.008 (0.995, 1.021)	0.234	0.527	1.18E-71	TRUE
CD8 S100B	*SEPT2*	Wald ratio	1	0.0130	0.0198	1.013 (0.975, 1.053)	0.511	0.748	2.96E-09	TRUE
CD8 S100B	*SERPINB6*	Wald ratio	1	0.0126	0.0162	1.013 (0.981, 1.045)	0.436	0.703	2.77E-13	TRUE
CD8 S100B	*SERPINF1*	Wald ratio	1	-0.0165	0.0172	0.984 (0.951, 1.017)	0.336	0.629	5.94E-10	TRUE
CD8 S100B	*SETD9*	Wald ratio	1	0.0565	0.0170	1.058 (1.023, 1.094)	9.16E-04	0.016	5.21E-10	TRUE
CD8 S100B	*SH3YL1*	Wald ratio	1	0.0378	0.0087	1.039 (1.021, 1.056)	1.23E-05	6.72E-04	2.52E-37	TRUE
CD8 S100B	*SLC25A29*	Wald ratio	1	-0.0042	0.0128	0.996 (0.971, 1.021)	0.743	0.881	9.75E-17	TRUE
CD8 S100B	*SLC25A3*	Wald ratio	1	0.0098	0.0237	1.01 (0.964, 1.058)	0.678	0.853	5.39E-08	TRUE
CD8 S100B	*SLFN5*	Wald ratio	1	-0.0032	0.0176	0.997 (0.963, 1.032)	0.856	0.939	2.14E-10	TRUE
CD8 S100B	*SMDT1*	Wald ratio	1	0.0087	0.0049	1.009 (0.999, 1.018)	0.076	0.289	1.37E-118	TRUE
CD8 S100B	*SMIM19*	Wald ratio	1	0.0143	0.0206	1.014 (0.974, 1.056)	0.487	0.738	4.59E-08	TRUE
CD8 S100B	*SNHG7*	Wald ratio	1	-0.0181	0.0112	0.982 (0.961, 1.004)	0.107	0.348	8.12E-35	TRUE
CD8 S100B	*SNHG8*	Wald ratio	1	-0.0029	0.0065	0.997 (0.984, 1.01)	0.663	0.845	2.74E-79	TRUE
CD8 S100B	*SNORD3B-1*	Wald ratio	1	0.0084	0.0121	1.008 (0.985, 1.033)	0.486	0.738	6.19E-16	TRUE
CD8 S100B	*SNORD3B-2*	Wald ratio	1	0.0134	0.0191	1.013 (0.976, 1.052)	0.482	0.735	1.04E-08	TRUE
CD8 S100B	*SNRPD2*	Wald ratio	1	-0.0480	0.0154	0.953 (0.925, 0.982)	0.002	0.025	2.62E-12	TRUE
CD8 S100B	*SPATC1L*	Wald ratio	1	0.0035	0.0205	1.004 (0.964, 1.045)	0.864	0.943	1.86E-10	TRUE
CD8 S100B	*SRP14*	Wald ratio	1	0.0618	0.0159	1.064 (1.031, 1.098)	1.05E-04	0.003	9.04E-10	TRUE
CD8 S100B	*SRSF10*	Wald ratio	1	-0.0233	0.0201	0.977 (0.939, 1.016)	0.248	0.541	4.01E-08	TRUE
CD8 S100B	*STMN3*	Wald ratio	1	0.0344	0.0176	1.035 (1, 1.071)	0.05	0.222	3.76E-10	TRUE
CD8 S100B	*STXBP5*	Wald ratio	1	0.0003	0.0198	1 (0.962, 1.04)	0.989	0.996	1.19E-08	TRUE
CD8 S100B	*SULF2*	Wald ratio	1	0.0133	0.0102	1.013 (0.993, 1.034)	0.191	0.471	6.43E-30	TRUE
CD8 S100B	*SURF1*	Wald ratio	1	-0.0244	0.0209	0.976 (0.937, 1.017)	0.242	0.535	4.99E-09	TRUE
CD8 S100B	*SYNGR1*	Wald ratio	1	-0.0186	0.0097	0.982 (0.963, 1)	0.054	0.233	1.04E-28	TRUE
CD8 S100B	*TBCA*	Wald ratio	1	0.0123	0.0142	1.012 (0.985, 1.041)	0.385	0.668	1.31E-15	TRUE
CD8 S100B	*THEMIS2*	Wald ratio	1	0.0099	0.0108	1.01 (0.989, 1.032)	0.362	0.651	3.25E-25	TRUE
CD8 S100B	*TIMM10*	Wald ratio	1	-0.0009	0.0107	0.999 (0.978, 1.02)	0.937	0.974	1.83E-30	TRUE
CD8 S100B	*TIMM13*	Wald ratio	1	0.0632	0.0193	1.065 (1.026, 1.106)	0.001	0.018	1.80E-08	TRUE
CD8 S100B	*TLE4*	Wald ratio	1	-0.0056	0.0149	0.994 (0.966, 1.024)	0.705	0.867	3.18E-14	TRUE
CD8 S100B	*TMEM120B*	Wald ratio	1	-0.0247	0.0226	0.976 (0.933, 1.02)	0.275	0.568	8.48E-09	TRUE
CD8 S100B	*TMEM156*	Wald ratio	1	0.0066	0.0163	1.007 (0.975, 1.039)	0.684	0.856	1.34E-11	TRUE
CD8 S100B	*TMEM204*	Wald ratio	1	-0.0024	0.0263	0.998 (0.947, 1.05)	0.928	0.969	4.77E-08	TRUE
CD8 S100B	*TMEM50B*	Wald ratio	1	-0.0536	0.0167	0.948 (0.917, 0.979)	0.001	0.02	6.89E-11	TRUE
CD8 S100B	*TNFSF13B*	Wald ratio	1	-0.0676	0.0205	0.935 (0.898, 0.973)	9.57E-04	0.016	6.61E-08	TRUE
CD8 S100B	*TOMM7*	Wald ratio	1	0.0035	0.0052	1.003 (0.993, 1.014)	0.503	0.743	2.07E-101	TRUE
CD8 S100B	*TPGS2*	Wald ratio	1	-0.0198	0.0198	0.98 (0.943, 1.019)	0.318	0.614	9.08E-09	TRUE
CD8 S100B	*TPP2*	Wald ratio	1	0.0094	0.0208	1.009 (0.969, 1.051)	0.651	0.836	4.93E-08	TRUE
CD8 S100B	*TRMT112*	Wald ratio	1	0.0190	0.0220	1.019 (0.976, 1.064)	0.387	0.669	1.91E-09	TRUE
CD8 S100B	*TTC12*	Wald ratio	1	-0.0338	0.0111	0.967 (0.946, 0.988)	0.002	0.029	7.76E-24	TRUE
CD8 S100B	*TUFM*	Wald ratio	1	0.0206	0.0107	1.021 (1, 1.042)	0.054	0.232	1.10E-24	TRUE
CD8 S100B	*U2AF1L4*	Wald ratio	1	0.0155	0.0276	1.016 (0.962, 1.072)	0.574	0.786	4.76E-08	TRUE
CD8 S100B	*UBA52*	Wald ratio	1	0.0082	0.0174	1.008 (0.975, 1.043)	0.635	0.827	8.33E-11	TRUE
CD8 S100B	*UCP2*	Wald ratio	1	-0.0140	0.0155	0.986 (0.957, 1.016)	0.366	0.654	1.68E-11	TRUE
CD8 S100B	*USP53*	Wald ratio	1	-0.0064	0.0117	0.994 (0.971, 1.017)	0.584	0.793	3.39E-15	TRUE
CD8 S100B	*VAMP2*	Wald ratio	1	0.0159	0.0199	1.016 (0.977, 1.056)	0.425	0.695	1.56E-08	TRUE
CD8 S100B	*VAMP5*	Wald ratio	1	0.0328	0.0226	1.033 (0.989, 1.08)	0.147	0.416	7.21E-09	TRUE
CD8 S100B	*VARS2*	Wald ratio	1	-0.0200	0.0189	0.98 (0.944, 1.017)	0.29	0.583	3.58E-15	TRUE
CD8 S100B	*XBP1*	Wald ratio	1	0.0913	0.0193	1.096 (1.055, 1.138)	2.34E-06	2.10E-04	9.84E-08	TRUE
CD8 S100B	*XCL1*	Wald ratio	1	0.0303	0.0165	1.031 (0.998, 1.065)	0.066	0.263	1.11E-10	TRUE
CD8 S100B	*XRRA1*	Wald ratio	1	-0.0151	0.0103	0.985 (0.965, 1.005)	0.143	0.41	5.23E-26	TRUE
CD8 S100B	*XXbac-BPG181B23.7*	IVW	2	0.0058	0.0110	1.006 (0.984, 1.028)	0.596	0.801	2.45E-28	TRUE
CD8 S100B	*XXbac-BPG252P9.10*	IVW	3	0.0237	0.0292	1.024 (0.967, 1.084)	0.417	0.689	4.38E-53	TRUE
CD8 S100B	*XXbac-BPG252P9.10*	MR Egger	3	0.2296	0.1211	1.258 (0.992, 1.595)	0.309			
CD8 S100B	*XXbac-BPG252P9.10*	Weighted median	3	0.0332	0.0104	1.034 (1.013, 1.055)	0.001			
CD8 S100B	*XXbac-BPG252P9.10*	Simple mode	3	0.0291	0.0201	1.03 (0.99, 1.071)	0.285			
CD8 S100B	*XXbac-BPG252P9.10*	Weighted mode	3	0.0427	0.0102	1.044 (1.023, 1.065)	0.053			
CD8 S100B	*XXbac-BPG299F13.17*	Wald ratio	1	0.0113	0.0081	1.011 (0.995, 1.027)	0.162	0.436	9.41E-37	TRUE
CD8 S100B	*YBEY*	Wald ratio	1	-0.0017	0.0131	0.998 (0.973, 1.024)	0.898	0.957	2.72E-15	TRUE
CD8 S100B	*ZBTB9*	IVW	2	-0.0220	0.0303	0.978 (0.922, 1.038)	0.468	0.726	2.64E-50	TRUE
CD8 S100B	*ZC3HAV1*	Wald ratio	1	0.0080	0.0134	1.008 (0.982, 1.035)	0.548	0.769	7.74E-17	TRUE
CD8 S100B	*ZFAS1*	Wald ratio	1	0.0098	0.0121	1.01 (0.986, 1.034)	0.421	0.691	2.52E-21	TRUE
CD8 S100B	*ZFP57*	IVW	2	-0.0011	0.0087	0.999 (0.982, 1.016)	0.898	0.957	8.92E-58	TRUE

CD8 S100B	*ZKSCAN3*	Wald ratio	1	-0.0055	0.0143	0.995 (0.967, 1.023)	0.701	0.866	1.08E-28	TRUE
CD8 S100B	*ZKSCAN4*	Wald ratio	1	0.0102	0.0211	1.01 (0.969, 1.053)	0.628	0.822	1.18E-14	TRUE
CD8 S100B	*ZNF677*	Wald ratio	1	-0.0296	0.0201	0.971 (0.933, 1.01)	0.141	0.408	1.18E-08	TRUE
CD8 S100B	*ZNF85*	Wald ratio	1	-0.0223	0.0167	0.978 (0.946, 1.01)	0.181	0.462	9.55E-13	TRUE
CD8 S100B	*ZP3*	Wald ratio	1	-0.0326	0.0131	0.968 (0.943, 0.993)	0.013	0.096	8.26E-21	TRUE
CD8 S100B	*ZSCAN12*	Wald ratio	1	0.0137	0.0202	1.014 (0.974, 1.055)	0.499	0.742	5.61E-14	TRUE
DC	*AC000003.2*	Wald ratio	1	0.0254	0.0169	1.026 (0.992, 1.06)	0.133	0.394	1.62E-10	TRUE
DC	*AGER*	IVW	2	0.0105	0.0178	1.011 (0.976, 1.046)	0.554	0.773	6.35E-36	TRUE
DC	*ANPEP*	Wald ratio	1	0.0134	0.0145	1.014 (0.985, 1.043)	0.354	0.646	1.76E-14	TRUE
DC	*ANXA1*	Wald ratio	1	0.0152	0.0181	1.015 (0.98, 1.052)	0.401	0.679	3.47E-08	TRUE
DC	*ANXA5*	Wald ratio	1	-0.0104	0.0213	0.99 (0.949, 1.032)	0.625	0.822	4.63E-09	TRUE
DC	*APOM*	Wald ratio	1	0.0014	0.0154	1.001 (0.972, 1.032)	0.927	0.968	4.68E-16	TRUE
DC	*ATAT1*	Wald ratio	1	0.0503	0.0139	1.052 (1.023, 1.081)	2.91E-04	0.007	1.54E-13	TRUE
DC	*B3GALT4*	Wald ratio	1	-0.0192	0.0187	0.981 (0.946, 1.018)	0.304	0.598	2.82E-11	TRUE
DC	*BAG2*	Wald ratio	1	-0.0205	0.0167	0.98 (0.948, 1.012)	0.22	0.511	7.69E-10	TRUE
DC	*BLOC1S1*	Wald ratio	1	0.0023	0.0203	1.002 (0.963, 1.043)	0.91	0.962	2.46E-08	TRUE
DC	*BRK1*	Wald ratio	1	-0.0183	0.0180	0.982 (0.948, 1.017)	0.31	0.604	5.43E-12	TRUE
DC	*C20orf27*	IVW	2	0.0187	0.0099	1.019 (0.999, 1.039)	0.059	0.244	3.28E-29	TRUE
DC	*C6orf136*	IVW	2	-0.0056	0.0147	0.994 (0.966, 1.023)	0.705	0.867	5.35E-26	TRUE
DC	*CCHCR1*	IVW	2	0.0190	0.0176	1.019 (0.985, 1.055)	0.28	0.574	7.00E-25	TRUE
DC	*CCT8*	Wald ratio	1	-0.0012	0.0126	0.999 (0.974, 1.024)	0.922	0.965	2.11E-29	TRUE
DC	*CD52*	Wald ratio	1	-0.0004	0.0097	1 (0.981, 1.019)	0.967	0.989	7.43E-28	TRUE
DC	*CFD*	Wald ratio	1	0.0002	0.0149	1 (0.971, 1.03)	0.99	0.996	4.14E-17	TRUE
DC	*CLEC12A*	Wald ratio	1	-0.0084	0.0101	0.992 (0.972, 1.011)	0.406	0.682	9.96E-30	TRUE
DC	*COMMD6*	Wald ratio	1	0.0200	0.0155	1.02 (0.99, 1.052)	0.197	0.479	2.32E-12	TRUE
DC	*CPNE1*	Wald ratio	1	-0.0105	0.0179	0.99 (0.955, 1.025)	0.557	0.775	1.38E-11	TRUE
DC	*CTC-524C5.2*	Wald ratio	1	-0.0167	0.0119	0.983 (0.961, 1.007)	0.159	0.43	8.48E-17	TRUE
DC	*CTSH*	Wald ratio	1	-0.0395	0.0158	0.961 (0.932, 0.992)	0.012	0.094	2.75E-11	TRUE
DC	*DBI*	Wald ratio	1	-0.0076	0.0118	0.992 (0.97, 1.016)	0.52	0.753	1.54E-22	TRUE
DC	*DCXR*	Wald ratio	1	-0.0166	0.0184	0.984 (0.949, 1.02)	0.365	0.653	2.39E-08	TRUE
DC	*DDT*	Wald ratio	1	-0.0221	0.0180	0.978 (0.944, 1.013)	0.219	0.511	7.97E-10	TRUE
DC	*DDX5*	Wald ratio	1	0.0741	0.0191	1.077 (1.037, 1.118)	1.06E-04	0.003	6.90E-08	TRUE
DC	*DNAJA1*	Wald ratio	1	-0.0607	0.0202	0.941 (0.905, 0.979)	0.003	0.032	5.13E-08	TRUE
DC	*DNAJC15*	Wald ratio	1	0.0108	0.0133	1.011 (0.985, 1.038)	0.418	0.689	9.38E-16	TRUE
DC	*DOPEY2*	Wald ratio	1	0.0167	0.0196	1.017 (0.979, 1.057)	0.394	0.673	3.43E-08	TRUE
DC	*DRAM2*	Wald ratio	1	-0.0127	0.0199	0.987 (0.95, 1.027)	0.524	0.754	2.29E-08	TRUE
DC	*EIF5A*	Wald ratio	1	-0.0096	0.0064	0.99 (0.978, 1.003)	0.134	0.394	2.98E-80	TRUE
DC	*ENPP4*	Wald ratio	1	-0.0039	0.0130	0.996 (0.971, 1.022)	0.762	0.893	4.59E-21	TRUE
DC	*ERAP2*	Wald ratio	1	0.0793	0.0148	1.082 (1.052, 1.114)	8.19E-08	6.60E-05	3.42E-13	TRUE
DC	*F13A1*	Wald ratio	1	-0.0358	0.0203	0.965 (0.927, 1.004)	0.077	0.291	5.22E-08	TRUE
DC	*FAM118A*	Wald ratio	1	0.0006	0.0057	1.001 (0.99, 1.012)	0.912	0.962	1.19E-67	TRUE
DC	*FAM184B*	Wald ratio	1	0.0115	0.0100	1.012 (0.992, 1.031)	0.249	0.542	8.02E-30	TRUE
DC	*FAM26F*	Wald ratio	1	-0.0153	0.0110	0.985 (0.964, 1.006)	0.162	0.436	1.29E-23	TRUE
DC	*FCER1G*	Wald ratio	1	-0.0053	0.0156	0.995 (0.965, 1.026)	0.734	0.88	7.73E-12	TRUE
DC	*FCGR2B*	Wald ratio	1	0.0111	0.0137	1.011 (0.984, 1.039)	0.415	0.687	1.81E-17	TRUE
DC	*FCN1*	Wald ratio	1	0.0032	0.0195	1.003 (0.966, 1.042)	0.868	0.945	1.42E-08	TRUE
DC	*FPR1*	Wald ratio	1	0.0098	0.0158	1.01 (0.979, 1.042)	0.535	0.761	7.99E-14	TRUE
DC	*GAA*	Wald ratio	1	0.0033	0.0175	1.003 (0.97, 1.038)	0.85	0.936	3.31E-10	TRUE
DC	*GDI2*	Wald ratio	1	-0.0099	0.0168	0.99 (0.958, 1.023)	0.555	0.774	1.16E-11	TRUE
DC	*GNPDA2*	Wald ratio	1	-0.0181	0.0125	0.982 (0.958, 1.006)	0.149	0.418	5.23E-25	TRUE
DC	*GRHPR*	Wald ratio	1	-0.0199	0.0155	0.98 (0.951, 1.01)	0.198	0.481	5.64E-12	TRUE
DC	*GSTM4*	Wald ratio	1	-0.0366	0.0195	0.964 (0.928, 1.002)	0.061	0.25	2.72E-08	TRUE
DC	*HCG11*	Wald ratio	1	-0.0208	0.0188	0.979 (0.944, 1.016)	0.268	0.562	1.09E-15	TRUE
DC	*HIST1H2BN*	Wald ratio	1	-0.0118	0.0243	0.988 (0.942, 1.036)	0.626	0.822	1.53E-12	TRUE
DC	*HIVEP2*	Wald ratio	1	0.0046	0.0124	1.005 (0.98, 1.029)	0.71	0.871	1.68E-19	TRUE
DC	*HLA-B*	IVW	2	0.0233	0.0168	1.024 (0.99, 1.058)	0.165	0.439	2.35E-47	TRUE
DC	*HLA-DPA1*	Wald ratio	1	0.0375	0.0177	1.038 (1.003, 1.075)	0.034	0.175	1.17E-09	TRUE
DC	*HLA-DQA1*	Wald ratio	1	-0.0038	0.0102	0.996 (0.976, 1.016)	0.709	0.87	3.32E-22	TRUE
DC	*HLA-DQA2*	Wald ratio	1	0.0624	0.0175	1.064 (1.028, 1.101)	3.61E-04	0.008	2.04E-12	TRUE
DC	*HLA-DQB2*	Wald ratio	1	0.0449	0.0144	1.046 (1.017, 1.076)	0.002	0.025	3.61E-14	TRUE
DC	*HLA-DRB1*	Wald ratio	1	-0.0230	0.0114	0.977 (0.956, 0.999)	0.043	0.202	8.09E-21	TRUE
DC	*HLA-DRB5*	IVW	2	0.0122	0.0143	1.012 (0.984, 1.041)	0.393	0.672	5.59E-117	TRUE
DC	*HYLS1*	Wald ratio	1	-0.0031	0.0091	0.997 (0.979, 1.015)	0.734	0.88	4.63E-25	TRUE
DC	*IFITM3*	Wald ratio	1	0.0063	0.0112	1.006 (0.984, 1.029)	0.573	0.786	1.92E-23	TRUE
DC	*IGFLR1*	Wald ratio	1	0.0137	0.0218	1.014 (0.971, 1.058)	0.531	0.759	2.95E-10	TRUE
DC	*IGLL1*	Wald ratio	1	0.0354	0.0192	1.036 (0.998, 1.076)	0.065	0.262	5.13E-08	TRUE
DC	*ITGB2*	Wald ratio	1	-0.0015	0.0113	0.999 (0.977, 1.021)	0.897	0.957	1.04E-19	TRUE
DC	*KANSL1-AS1*	Wald ratio	1	-0.0460	0.0193	0.955 (0.92, 0.992)	0.017	0.115	3.52E-18	TRUE
DC	*KCNK17*	Wald ratio	1	0.0109	0.0163	1.011 (0.979, 1.044)	0.503	0.744	7.44E-12	TRUE
DC	*LGALS1*	Wald ratio	1	-0.0398	0.0170	0.961 (0.93, 0.994)	0.019	0.123	7.71E-11	TRUE
DC	*LGALS2*	Wald ratio	1	0.0061	0.0084	1.006 (0.99, 1.023)	0.463	0.723	1.04E-43	TRUE
DC	*LY6G5C*	Wald ratio	1	-0.0045	0.0128	0.995 (0.971, 1.021)	0.725	0.878	1.04E-23	TRUE
DC	*LYZ*	Wald ratio	1	-0.0021	0.0078	0.998 (0.983, 1.013)	0.788	0.909	4.18E-49	TRUE
DC	*MDC1*	Wald ratio	1	-0.0077	0.0123	0.992 (0.969, 1.017)	0.532	0.76	1.76E-19	TRUE
DC	*MICA*	Wald ratio	1	-0.0262	0.0134	0.974 (0.949, 1)	0.05	0.222	2.10E-17	TRUE
DC	*MICB*	Wald ratio	1	0.0425	0.0132	1.043 (1.017, 1.071)	0.001	0.02	4.54E-16	TRUE
DC	*MZT2A*	Wald ratio	1	-0.0278	0.0177	0.973 (0.939, 1.007)	0.116	0.363	6.80E-08	TRUE
DC	*NAPRT1*	Wald ratio	1	-0.0230	0.0139	0.977 (0.951, 1.004)	0.099	0.333	7.33E-15	TRUE
DC	*NDUFS5*	Wald ratio	1	-0.0160	0.0184	0.984 (0.949, 1.02)	0.383	0.667	9.43E-11	TRUE

DC	*NQO2*	Wald ratio	1	-0.0041	0.0199	0.996 (0.958, 1.036)	0.836	0.93	1.91E-08	TRUE
DC	*NSA2*	Wald ratio	1	0.0438	0.0194	1.045 (1.006, 1.085)	0.024	0.143	2.97E-09	TRUE
DC	*NT5C3B*	Wald ratio	1	-0.0005	0.0169	0.999 (0.967, 1.033)	0.974	0.99	2.14E-09	TRUE
DC	*PPA1*	Wald ratio	1	0.0068	0.0120	1.007 (0.983, 1.031)	0.57	0.784	7.92E-19	TRUE
DC	*PPARA*	Wald ratio	1	0.0160	0.0106	1.016 (0.995, 1.038)	0.133	0.394	4.68E-25	TRUE
DC	*PPDPF*	Wald ratio	1	-0.0123	0.0114	0.988 (0.966, 1.01)	0.283	0.575	4.37E-20	TRUE
DC	*PPIL3*	Wald ratio	1	0.0158	0.0185	1.016 (0.98, 1.053)	0.393	0.672	5.42E-09	TRUE
DC	*PSMD5-AS1*	Wald ratio	1	0.0084	0.0111	1.008 (0.987, 1.031)	0.449	0.714	1.95E-23	TRUE
DC	*RAC1*	Wald ratio	1	-0.0160	0.0088	0.984 (0.967, 1.001)	0.067	0.265	2.43E-37	TRUE
DC	*RNASET2*	Wald ratio	1	-0.0507	0.0188	0.951 (0.916, 0.986)	0.007	0.064	8.67E-09	TRUE
DC	*ROGDI*	Wald ratio	1	-0.0623	0.0195	0.94 (0.904, 0.976)	0.001	0.021	3.20E-08	TRUE
DC	*RP1-3J17.3*	Wald ratio	1	-0.0053	0.0112	0.995 (0.973, 1.017)	0.636	0.827	5.87E-22	TRUE
DC	*RP11-1143G9.4*	Wald ratio	1	0.0033	0.0065	1.003 (0.991, 1.016)	0.608	0.809	2.29E-70	TRUE
DC	*RP11-347P5.1*	Wald ratio	1	0.0022	0.0122	1.002 (0.979, 1.026)	0.859	0.94	1.01E-20	TRUE
DC	*RP11-385F7.1*	Wald ratio	1	0.0074	0.0136	1.007 (0.981, 1.035)	0.586	0.794	2.41E-16	TRUE
DC	*RP11-47L3.1*	Wald ratio	1	-0.0289	0.0254	0.972 (0.924, 1.021)	0.255	0.549	3.61E-09	TRUE
DC	*RP6-91H8.3*	Wald ratio	1	-0.0366	0.0155	0.964 (0.935, 0.994)	0.018	0.119	6.63E-13	TRUE
DC	*RPL10A*	Wald ratio	1	-0.0151	0.0169	0.985 (0.953, 1.018)	0.372	0.661	3.99E-11	TRUE
DC	*RPL12*	Wald ratio	1	-0.0307	0.0091	0.97 (0.953, 0.987)	7.26E-04	0.013	1.09E-37	TRUE
DC	*RPL13*	Wald ratio	1	-0.0384	0.0190	0.962 (0.927, 0.999)	0.043	0.202	5.47E-15	TRUE
DC	*RPL22L1*	Wald ratio	1	0.0137	0.0202	1.014 (0.974, 1.055)	0.498	0.741	3.71E-08	TRUE
DC	*RPL28*	Wald ratio	1	0.0056	0.0072	1.006 (0.991, 1.02)	0.438	0.703	1.36E-54	TRUE
DC	*RPL36AL*	Wald ratio	1	-0.0001	0.0152	1 (0.971, 1.03)	0.997	0.999	5.83E-14	TRUE
DC	*RPL8*	Wald ratio	1	-0.0078	0.0076	0.992 (0.978, 1.007)	0.303	0.598	3.76E-50	TRUE
DC	*RPP21*	Wald ratio	1	-0.0151	0.0241	0.985 (0.94, 1.033)	0.531	0.758	6.83E-09	TRUE
DC	*RPPH1*	Wald ratio	1	-0.0009	0.0195	0.999 (0.962, 1.038)	0.965	0.988	4.80E-08	TRUE
DC	*RPS10*	Wald ratio	1	-0.0105	0.0108	0.99 (0.969, 1.011)	0.33	0.624	1.41E-22	TRUE
DC	*RPS11*	Wald ratio	1	0.0437	0.0183	1.045 (1.008, 1.083)	0.017	0.114	1.92E-10	TRUE
DC	*RPS16*	Wald ratio	1	-0.0155	0.0150	0.985 (0.956, 1.014)	0.304	0.598	1.01E-12	TRUE
DC	*RPS18*	Wald ratio	1	-0.0145	0.0085	0.986 (0.969, 1.002)	0.087	0.312	2.89E-49	TRUE
DC	*RPS19*	Wald ratio	1	0.0226	0.0204	1.023 (0.983, 1.065)	0.269	0.563	7.21E-08	TRUE
DC	*RPS2*	Wald ratio	1	-0.0331	0.0115	0.967 (0.946, 0.99)	0.004	0.044	2.28E-22	TRUE
DC	*RPS26*	Wald ratio	1	-0.0102	0.0044	0.99 (0.981, 0.998)	0.022	0.132	5.86E-173	TRUE
DC	*RPS3A*	Wald ratio	1	0.0192	0.0205	1.019 (0.979, 1.061)	0.35	0.643	1.43E-16	TRUE
DC	*RPS5*	Wald ratio	1	0.0142	0.0105	1.014 (0.994, 1.035)	0.177	0.456	3.98E-28	TRUE
DC	*RPS7*	Wald ratio	1	0.0071	0.0226	1.007 (0.963, 1.053)	0.754	0.888	1.06E-09	TRUE
DC	*RPS8*	Wald ratio	1	0.0041	0.0157	1.004 (0.974, 1.035)	0.791	0.909	1.17E-12	TRUE
DC	*RPS9*	Wald ratio	1	0.0343	0.0111	1.035 (1.013, 1.058)	0.002	0.026	7.33E-22	TRUE
DC	*SESN1*	Wald ratio	1	-0.0265	0.0139	0.974 (0.948, 1.001)	0.056	0.239	3.24E-17	TRUE
DC	*SH3BGRL3*	Wald ratio	1	0.0002	0.0168	1 (0.968, 1.034)	0.989	0.996	3.63E-10	TRUE
DC	*SIRPB1*	Wald ratio	1	-0.0175	0.0205	0.983 (0.944, 1.023)	0.394	0.673	1.16E-08	TRUE
DC	*SMDT1*	Wald ratio	1	0.0136	0.0070	1.014 (1, 1.028)	0.05	0.222	1.36E-54	TRUE
DC	*SNAP29*	Wald ratio	1	0.0531	0.0232	1.055 (1.008, 1.104)	0.022	0.134	1.00E-07	TRUE
DC	*SNHG7*	Wald ratio	1	-0.0278	0.0172	0.973 (0.94, 1.006)	0.107	0.348	3.38E-15	TRUE
DC	*SNHG8*	Wald ratio	1	-0.0048	0.0104	0.995 (0.975, 1.016)	0.647	0.834	3.66E-30	TRUE
DC	*SULF2*	Wald ratio	1	0.0547	0.0196	1.056 (1.016, 1.098)	0.005	0.053	9.33E-11	TRUE
DC	*SYNGAP1*	Wald ratio	1	0.0283	0.0093	1.029 (1.01, 1.048)	0.002	0.029	3.52E-35	TRUE
DC	*SYNGR1*	Wald ratio	1	-0.0353	0.0172	0.965 (0.933, 0.998)	0.04	0.193	6.72E-09	TRUE
DC	*THEMIS2*	Wald ratio	1	-0.0033	0.0127	0.997 (0.972, 1.022)	0.795	0.911	1.29E-17	TRUE
DC	*TIMM10*	Wald ratio	1	0.0001	0.0153	1 (0.971, 1.031)	0.993	0.997	4.41E-15	TRUE
DC	*TMEM176A*	Wald ratio	1	0.0275	0.0079	1.028 (1.012, 1.044)	5.05E-04	0.01	3.99E-42	TRUE
DC	*TMEM176B*	Wald ratio	1	0.0308	0.0112	1.031 (1.009, 1.054)	0.006	0.058	5.85E-20	TRUE
DC	*TNF*	Wald ratio	1	-0.0217	0.0160	0.979 (0.948, 1.01)	0.175	0.454	4.03E-15	TRUE
DC	*TOMM7*	Wald ratio	1	0.0057	0.0085	1.006 (0.989, 1.023)	0.503	0.743	4.62E-36	TRUE
DC	*TRAM2*	Wald ratio	1	0.0216	0.0110	1.022 (1, 1.044)	0.05	0.222	1.07E-19	TRUE
DC	*TRIM39*	IVW	2	-0.0123	0.0103	0.988 (0.968, 1.008)	0.234	0.527	7.49E-40	TRUE
DC	*TSTD1*	Wald ratio	1	0.0311	0.0169	1.032 (0.998, 1.066)	0.066	0.263	4.43E-10	TRUE
DC	*TUFM*	Wald ratio	1	0.0219	0.0111	1.022 (1, 1.045)	0.049	0.221	1.44E-22	TRUE
DC	*TYMP*	Wald ratio	1	0.0289	0.0142	1.029 (1.001, 1.058)	0.042	0.201	1.41E-14	TRUE
DC	*VARS2*	IVW	2	0.0230	0.0097	1.023 (1.004, 1.043)	0.018	0.117	3.68E-27	TRUE
DC	*VPS28*	Wald ratio	1	0.0289	0.0177	1.029 (0.994, 1.066)	0.102	0.338	1.05E-08	TRUE
DC	*WASF1*	Wald ratio	1	0.0324	0.0155	1.033 (1.002, 1.065)	0.036	0.182	1.17E-10	TRUE
DC	*XRRA1*	Wald ratio	1	0.0055	0.0142	1.006 (0.978, 1.034)	0.697	0.864	1.12E-14	TRUE
DC	*XXbac-BPG299F13.17*	Wald ratio	1	-0.0264	0.0189	0.974 (0.938, 1.011)	0.164	0.438	4.77E-09	TRUE
DC	*ZBTB22*	Wald ratio	1	-0.0116	0.0147	0.988 (0.96, 1.017)	0.431	0.7	1.85E-15	TRUE
DC	*ZNF322*	Wald ratio	1	0.0217	0.0199	1.022 (0.983, 1.063)	0.275	0.568	9.72E-14	TRUE
DC	*ZSCAN16*	Wald ratio	1	0.0399	0.0414	1.041 (0.96, 1.129)	0.335	0.628	1.40E-10	TRUE
Mono C	*ABCC3*	Wald ratio	1	-0.0035	0.0146	0.996 (0.968, 1.025)	0.809	0.918	6.77E-15	TRUE
Mono C	*ABHD16A*	IVW	3	0.0003	0.0117	1 (0.978, 1.024)	0.976	0.991	4.79E-47	TRUE
Mono C	*ABHD16A*	MR Egger	3	-0.0459	0.0250	0.955 (0.909, 1.003)	0.317	NA	NA	NA
Mono C	*ABHD16A*	Weighted median	3	-0.0064	0.0088	0.994 (0.977, 1.011)	0.469	NA	NA	NA
Mono C	*ABHD16A*	Simple mode	3	-0.0063	0.0137	0.994 (0.967, 1.021)	0.692	NA	NA	NA
Mono C	*ABHD16A*	Weighted mode	3	-0.0064	0.0094	0.994 (0.975, 1.012)	0.569	NA	NA	NA
Mono C	*ABRACL*	Wald ratio	1	0.0760	0.0190	1.079 (1.04, 1.12)	6.23E-05	0.002	3.11E-09	TRUE
Mono C	*ABTB1*	Wald ratio	1	-0.0072	0.0126	0.993 (0.969, 1.018)	0.568	0.782	8.14E-16	TRUE
Mono C	*AC000003.2*	Wald ratio	1	0.0231	0.0154	1.023 (0.993, 1.055)	0.133	0.394	2.00E-12	TRUE
Mono C	*AC016757.3*	Wald ratio	1	0.0082	0.0085	1.008 (0.992, 1.025)	0.333	0.626	1.37E-41	TRUE
Mono C	*ACP5*	Wald ratio	1	-0.0340	0.0160	0.967 (0.937, 0.997)	0.034	0.175	8.60E-13	TRUE
Mono C	*ACTA2*	Wald ratio	1	0.0105	0.0159	1.011 (0.98, 1.043)	0.508	0.747	2.53E-12	TRUE

Mono C	*ADK*	Wald ratio	1	0.0295	0.0101	1.03 (1.01, 1.051)	0.004	0.04	2.03E-21	TRUE
Mono C	*AFAP1*	Wald ratio	1	0.0012	0.0076	1.001 (0.986, 1.016)	0.877	0.949	5.92E-51	TRUE
Mono C	*ANAPC13*	Wald ratio	1	-0.0141	0.0170	0.986 (0.954, 1.019)	0.405	0.681	1.69E-11	TRUE
Mono C	*ANP32E*	Wald ratio	1	-0.0952	0.0182	0.909 (0.877, 0.942)	1.73E-07	6.60E-05	3.92E-08	TRUE
Mono C	*ANPEP*	Wald ratio	1	-0.0002	0.0090	1 (0.982, 1.018)	0.984	0.994	5.54E-37	TRUE
Mono C	*ANXA6*	Wald ratio	1	-0.0049	0.0177	0.995 (0.961, 1.03)	0.783	0.906	2.94E-10	TRUE
Mono C	*AOAH*	Wald ratio	1	-0.0664	0.0196	0.936 (0.9, 0.973)	7.29E-04	0.013	1.18E-08	TRUE
Mono C	*AP003774.1*	Wald ratio	1	0.0539	0.0157	1.055 (1.023, 1.088)	5.86E-04	0.011	1.03E-11	TRUE
Mono C	*APBB3*	Wald ratio	1	0.0020	0.0156	1.002 (0.972, 1.033)	0.9	0.958	1.41E-11	TRUE
Mono C	*APOBEC3A*	Wald ratio	1	-0.0299	0.0135	0.971 (0.945, 0.996)	0.026	0.151	2.00E-13	TRUE
Mono C	*APOBEC3B*	Wald ratio	1	0.0314	0.0141	1.032 (1.004, 1.061)	0.026	0.151	2.48E-12	TRUE
Mono C	*ARPC5*	Wald ratio	1	-0.0222	0.0156	0.978 (0.949, 1.008)	0.154	0.425	8.21E-13	TRUE
Mono C	*ARRDC4*	Wald ratio	1	-0.0004	0.0163	1 (0.968, 1.032)	0.981	0.993	2.27E-09	TRUE
Mono C	*B3GNT5*	Wald ratio	1	-0.0102	0.0191	0.99 (0.954, 1.028)	0.593	0.799	5.46E-09	TRUE
Mono C	*BCAT1*	Wald ratio	1	-0.0127	0.0173	0.987 (0.954, 1.021)	0.462	0.723	1.75E-11	TRUE
Mono C	*BRI3*	Wald ratio	1	0.0051	0.0203	1.005 (0.966, 1.046)	0.801	0.915	4.66E-08	TRUE
Mono C	*BRK1*	Wald ratio	1	-0.0225	0.0155	0.978 (0.948, 1.008)	0.147	0.416	4.24E-16	TRUE
Mono C	*BSG*	Wald ratio	1	0.0002	0.0158	1 (0.97, 1.032)	0.991	0.996	2.10E-10	TRUE
Mono C	*BTBD9*	Wald ratio	1	0.0075	0.0148	1.008 (0.979, 1.037)	0.613	0.813	8.19E-17	TRUE
Mono C	*C10orf32*	Wald ratio	1	-0.0835	0.0180	0.92 (0.888, 0.953)	3.45E-06	2.63E-04	1.33E-09	TRUE
Mono C	*C10orf88*	Wald ratio	1	0.0090	0.0160	1.009 (0.978, 1.041)	0.575	0.787	8.82E-17	TRUE
Mono C	*C11orf21*	Wald ratio	1	-0.0420	0.0194	0.959 (0.923, 0.996)	0.03	0.163	7.02E-09	TRUE
Mono C	*C17orf97*	Wald ratio	1	0.0118	0.0088	1.012 (0.995, 1.029)	0.179	0.459	1.37E-36	TRUE
Mono C	*C19orf60*	Wald ratio	1	0.0055	0.0162	1.006 (0.974, 1.038)	0.734	0.88	5.59E-12	TRUE
Mono C	*C1orf85*	Wald ratio	1	-0.0227	0.0114	0.978 (0.956, 1)	0.046	0.212	6.74E-23	TRUE
Mono C	*C20orf27*	Wald ratio	1	0.0197	0.0088	1.02 (1.003, 1.038)	0.025	0.145	2.51E-27	TRUE
Mono C	*CAPG*	Wald ratio	1	-0.0268	0.0175	0.974 (0.941, 1.008)	0.126	0.382	7.16E-14	TRUE
Mono C	*CASC8*	Wald ratio	1	0.0066	0.0074	1.007 (0.992, 1.021)	0.375	0.663	2.80E-36	TRUE
Mono C	*CAST*	Wald ratio	1	-0.0158	0.0163	0.984 (0.953, 1.016)	0.331	0.625	1.87E-13	TRUE
Mono C	*CAT*	Wald ratio	1	0.0333	0.0178	1.034 (0.998, 1.071)	0.062	0.253	1.36E-09	TRUE
Mono C	*CCHCR1*	Wald ratio	1	-0.0041	0.0149	0.996 (0.967, 1.025)	0.781	0.905	5.89E-14	TRUE
Mono C	*CCT8*	Wald ratio	1	-0.0016	0.0163	0.998 (0.967, 1.031)	0.922	0.965	4.14E-18	TRUE
Mono C	*CD151*	Wald ratio	1	0.0337	0.0122	1.034 (1.01, 1.059)	0.006	0.056	1.47E-20	TRUE
Mono C	*CD52*	IVW	2	-0.0023	0.0088	0.998 (0.981, 1.015)	0.791	0.909	3.94E-108	TRUE
Mono C	*CD55*	Wald ratio	1	0.0275	0.0110	1.028 (1.006, 1.05)	0.012	0.094	1.67E-26	TRUE
Mono C	*CD9*	Wald ratio	1	-0.0019	0.0144	0.998 (0.97, 1.027)	0.894	0.957	3.97E-14	TRUE
Mono C	*CDA*	Wald ratio	1	0.0091	0.0124	1.009 (0.985, 1.034)	0.465	0.725	6.29E-23	TRUE
Mono C	*CDC42EP1*	Wald ratio	1	0.0060	0.0162	1.006 (0.975, 1.039)	0.712	0.872	2.73E-12	TRUE
Mono C	*CEACAM21*	Wald ratio	1	0.0158	0.0117	1.016 (0.993, 1.039)	0.177	0.456	3.99E-19	TRUE
Mono C	*CES1*	Wald ratio	1	-0.0123	0.0120	0.988 (0.965, 1.011)	0.306	0.599	1.82E-21	TRUE
Mono C	*CFD*	Wald ratio	1	0.0001	0.0081	1 (0.984, 1.016)	0.99	0.996	8.39E-56	TRUE
Mono C	*CHCHD2*	Wald ratio	1	0.0075	0.0176	1.007 (0.973, 1.043)	0.671	0.849	4.77E-11	TRUE
Mono C	*CHN2*	Wald ratio	1	-0.0012	0.0139	0.999 (0.972, 1.026)	0.933	0.972	7.79E-16	TRUE
Mono C	*CHST13*	Wald ratio	1	0.0062	0.0140	1.006 (0.979, 1.034)	0.658	0.842	7.85E-15	TRUE
Mono C	*CLEC12A*	Wald ratio	1	-0.0034	0.0063	0.997 (0.984, 1.009)	0.588	0.795	1.25E-78	TRUE
Mono C	*CNN2*	Wald ratio	1	0.0001	0.0213	1 (0.959, 1.043)	0.996	0.999	1.52E-10	TRUE
Mono C	*COMMD6*	Wald ratio	1	0.0302	0.0186	1.031 (0.994, 1.069)	0.105	0.343	3.52E-09	TRUE
Mono C	*COX6B1*	Wald ratio	1	-0.0367	0.0165	0.964 (0.933, 0.996)	0.026	0.149	3.37E-11	TRUE
Mono C	*CRYZ*	Wald ratio	1	0.0210	0.0167	1.021 (0.988, 1.055)	0.21	0.497	2.51E-11	TRUE
Mono C	*CSGALNACT1*	Wald ratio	1	0.0129	0.0164	1.013 (0.981, 1.046)	0.431	0.7	1.50E-11	TRUE
Mono C	*CST3*	Wald ratio	1	-0.0059	0.0086	0.994 (0.978, 1.011)	0.492	0.738	8.18E-39	TRUE
Mono C	*CTC-524C5.2*	Wald ratio	1	-0.0241	0.0137	0.976 (0.95, 1.003)	0.078	0.292	6.27E-13	TRUE
Mono C	*CTSB*	Wald ratio	1	-0.0433	0.0159	0.958 (0.928, 0.988)	0.006	0.059	3.15E-14	TRUE
Mono C	*CTSH*	Wald ratio	1	-0.0322	0.0129	0.968 (0.944, 0.993)	0.012	0.094	2.54E-16	TRUE
Mono C	*CTSS*	Wald ratio	1	0.0299	0.0178	1.03 (0.995, 1.067)	0.094	0.326	2.57E-12	TRUE
Mono C	*DARS2*	Wald ratio	1	-0.0020	0.0111	0.998 (0.977, 1.02)	0.859	0.94	1.15E-23	TRUE
Mono C	*DBI*	Wald ratio	1	-0.0150	0.0147	0.985 (0.957, 1.014)	0.31	0.604	1.53E-15	TRUE
Mono C	*DEGS1*	Wald ratio	1	0.0108	0.0208	1.011 (0.97, 1.053)	0.603	0.806	4.40E-09	TRUE
Mono C	*DNAJC15*	Wald ratio	1	0.0087	0.0099	1.009 (0.989, 1.029)	0.382	0.666	1.36E-27	TRUE
Mono C	*DRAM2*	Wald ratio	1	-0.0038	0.0113	0.996 (0.974, 1.018)	0.734	0.88	2.57E-22	TRUE
Mono C	*DYSF*	Wald ratio	1	0.0362	0.0179	1.037 (1.001, 1.074)	0.043	0.204	3.30E-09	TRUE
Mono C	*EBPL*	Wald ratio	1	0.0302	0.0195	1.031 (0.992, 1.071)	0.122	0.375	1.26E-08	TRUE
Mono C	*EIF5A*	Wald ratio	1	-0.0139	0.0093	0.986 (0.968, 1.004)	0.134	0.394	1.76E-37	TRUE
Mono C	*EIF6*	Wald ratio	1	-0.0491	0.0175	0.952 (0.92, 0.985)	0.005	0.05	1.24E-09	TRUE
Mono C	*EPHA1-AS1*	Wald ratio	1	0.0010	0.0092	1.001 (0.983, 1.019)	0.914	0.962	1.74E-32	TRUE
Mono C	*F13A1*	Wald ratio	1	-0.0178	0.0145	0.982 (0.955, 1.011)	0.218	0.509	8.30E-16	TRUE
Mono C	*FADS2*	Wald ratio	1	-0.0268	0.0189	0.974 (0.938, 1.01)	0.156	0.427	2.84E-13	TRUE
Mono C	*FAM118A*	Wald ratio	1	0.0009	0.0066	1.001 (0.988, 1.014)	0.895	0.957	1.72E-48	TRUE
Mono C	*FAM184B*	Wald ratio	1	-0.0108	0.0110	0.989 (0.968, 1.011)	0.328	0.622	2.76E-28	TRUE
Mono C	*FAM26F*	Wald ratio	1	-0.0091	0.0095	0.991 (0.973, 1.01)	0.339	0.632	6.08E-32	TRUE
Mono C	*FAR1*	Wald ratio	1	-0.0045	0.0181	0.996 (0.961, 1.032)	0.806	0.916	3.32E-10	TRUE
Mono C	*FCER1A*	Wald ratio	1	0.0064	0.0113	1.006 (0.984, 1.029)	0.573	0.786	2.10E-22	TRUE
Mono C	*FCER1G*	Wald ratio	1	-0.0040	0.0084	0.996 (0.98, 1.012)	0.632	0.826	2.84E-38	TRUE
Mono C	*FCER2*	Wald ratio	1	-0.0043	0.0203	0.996 (0.957, 1.036)	0.833	0.93	1.83E-08	TRUE
Mono C	*FCGR1A*	Wald ratio	1	-0.0684	0.0198	0.934 (0.898, 0.971)	5.59E-04	0.011	4.81E-11	TRUE
Mono C	*FCN1*	Wald ratio	1	0.0010	0.0085	1.001 (0.984, 1.018)	0.908	0.961	2.26E-39	TRUE
Mono C	*FHL3*	Wald ratio	1	-0.0099	0.0193	0.99 (0.953, 1.028)	0.607	0.808	5.31E-10	TRUE
Mono C	*FKBPL*	Wald ratio	1	-0.0475	0.0119	0.954 (0.932, 0.976)	7.11E-05	0.002	2.21E-21	TRUE
Mono C	*FOLR3*	Wald ratio	1	0.0081	0.0069	1.008 (0.995, 1.022)	0.238	0.532	8.74E-44	TRUE

Mono C	*FPR1*	Wald ratio	1	0.0081	0.0133	1.008 (0.982, 1.035)	0.543	0.765	1.94E-18	TRUE
Mono C	*GAA*	Wald ratio	1	0.0069	0.0196	1.007 (0.969, 1.046)	0.724	0.878	1.33E-08	TRUE
Mono C	*GBP7*	Wald ratio	1	-0.0009	0.0078	0.999 (0.984, 1.014)	0.903	0.959	1.79E-48	TRUE
Mono C	*GPR27*	Wald ratio	1	0.0142	0.0154	1.014 (0.984, 1.045)	0.355	0.646	5.09E-11	TRUE
Mono C	*GPX4*	Wald ratio	1	-0.0106	0.0198	0.989 (0.952, 1.029)	0.59	0.797	3.71E-09	TRUE
Mono C	*GRHPR*	Wald ratio	1	-0.0160	0.0132	0.984 (0.959, 1.01)	0.227	0.52	8.51E-16	TRUE
Mono C	*GSTK1*	Wald ratio	1	0.0466	0.0217	1.048 (1.004, 1.093)	0.032	0.168	2.18E-08	TRUE
Mono C	*GSTM4*	Wald ratio	1	-0.0380	0.0201	0.963 (0.926, 1.001)	0.058	0.243	7.59E-08	TRUE
Mono C	*GSTP1*	Wald ratio	1	-0.0327	0.0158	0.968 (0.938, 0.998)	0.039	0.19	3.86E-13	TRUE
Mono C	*H1F0*	Wald ratio	1	0.0294	0.0176	1.03 (0.995, 1.066)	0.096	0.329	4.18E-10	TRUE
Mono C	*HCG27*	IVW	2	-0.0034	0.0095	0.997 (0.978, 1.015)	0.721	0.876	4.71E-61	TRUE
Mono C	*HIGD2A*	Wald ratio	1	-0.0140	0.0161	0.986 (0.955, 1.018)	0.385	0.668	2.38E-12	TRUE
Mono C	*HIST1H2AE*	Wald ratio	1	0.0354	0.0162	1.036 (1.004, 1.069)	0.029	0.159	7.81E-14	TRUE
Mono C	*HIST1H3D*	Wald ratio	1	-0.0158	0.0243	0.984 (0.938, 1.032)	0.516	0.751	6.39E-10	TRUE
Mono C	*HIST1H4A*	Wald ratio	1	0.0081	0.0158	1.008 (0.977, 1.04)	0.607	0.808	1.24E-21	TRUE
Mono C	*HIST1H4J*	Wald ratio	1	-0.0125	0.0240	0.988 (0.942, 1.035)	0.604	0.806	4.43E-08	TRUE
Mono C	*HLA-B*	IVW	3	0.0078	0.0213	1.008 (0.967, 1.051)	0.716	0.873	7.46E-80	TRUE
Mono C	*HLA-B*	MR Egger	3	0.0844	0.0718	1.088 (0.945, 1.253)	0.449	NA	NA	NA
Mono C	*HLA-B*	Weighted median	3	0.0094	0.0072	1.009 (0.995, 1.024)	0.194	NA	NA	NA
Mono C	*HLA-B*	Simple mode	3	-0.0353	0.0173	0.965 (0.933, 0.999)	0.178	NA	NA	NA
Mono C	*HLA-B*	Weighted mode	3	0.0271	0.0076	1.027 (1.012, 1.043)	0.071	NA	NA	NA
Mono C	*HLA-C*	Wald ratio	1	0.0140	0.0103	1.014 (0.994, 1.035)	0.173	0.451	4.71E-27	TRUE
Mono C	*HLA-DOB*	Wald ratio	1	0.0089	0.0144	1.009 (0.981, 1.038)	0.536	0.762	1.12E-19	TRUE
Mono C	*HLA-DPB1*	Wald ratio	1	0.0145	0.0202	1.015 (0.975, 1.056)	0.473	0.73	5.57E-08	TRUE
Mono C	*HLA-DQA2*	IVW	2	-0.0098	0.0150	0.99 (0.961, 1.02)	0.514	0.75	1.47E-16	TRUE
Mono C	*HLA-DQB1*	Wald ratio	1	0.0054	0.0181	1.005 (0.97, 1.042)	0.767	0.897	3.39E-09	TRUE
Mono C	*HLA-DQB2*	Wald ratio	1	0.0289	0.0126	1.029 (1.004, 1.055)	0.021	0.132	3.12E-15	TRUE
Mono C	*HLA-DRB5*	IVW	2	0.0128	0.0192	1.013 (0.976, 1.052)	0.503	0.744	2.16E-112	TRUE
Mono C	*HLA-G*	Wald ratio	1	0.0620	0.0372	1.064 (0.989, 1.144)	0.096	0.329	4.54E-12	TRUE
Mono C	*HMBOX1*	Wald ratio	1	-0.0353	0.0192	0.965 (0.93, 1.002)	0.066	0.263	6.55E-08	TRUE
Mono C	*HMGN1*	Wald ratio	1	0.0290	0.0208	1.029 (0.988, 1.072)	0.163	0.437	3.55E-08	TRUE
Mono C	*HP*	Wald ratio	1	-0.0322	0.0098	0.968 (0.95, 0.987)	0.001	0.018	2.32E-28	TRUE
Mono C	*HSBP1L1*	Wald ratio	1	-0.0761	0.0176	0.927 (0.895, 0.959)	1.56E-05	7.86E-04	2.14E-09	TRUE
Mono C	*HSD17B8*	Wald ratio	1	0.0129	0.0169	1.013 (0.98, 1.047)	0.445	0.709	1.05E-12	TRUE
Mono C	*ICAM4*	Wald ratio	1	0.0342	0.0130	1.035 (1.009, 1.062)	0.008	0.072	7.95E-20	TRUE
Mono C	*IFITM3*	Wald ratio	1	0.0052	0.0093	1.005 (0.987, 1.024)	0.573	0.786	9.42E-34	TRUE
Mono C	*IGFLR1*	Wald ratio	1	0.0033	0.0238	1.003 (0.958, 1.051)	0.889	0.955	3.51E-09	TRUE
Mono C	*ITGB2*	Wald ratio	1	-0.0006	0.0049	0.999 (0.99, 1.009)	0.897	0.957	3.05E-108	TRUE
Mono C	*ITPR3*	Wald ratio	1	-0.0259	0.0127	0.974 (0.95, 0.999)	0.041	0.197	4.49E-18	TRUE
Mono C	*JUP*	Wald ratio	1	-0.0487	0.0119	0.952 (0.93, 0.975)	4.57E-05	0.002	7.92E-17	TRUE
Mono C	*KANSL1-AS1*	Wald ratio	1	-0.0327	0.0139	0.968 (0.942, 0.994)	0.018	0.12	7.68E-35	TRUE
Mono C	*KCNMA1*	Wald ratio	1	-0.0150	0.0167	0.985 (0.953, 1.018)	0.369	0.657	3.57E-11	TRUE
Mono C	*KDELR2*	Wald ratio	1	0.0485	0.0150	1.05 (1.019, 1.081)	0.001	0.02	1.72E-12	TRUE
Mono C	*KIAA1598*	Wald ratio	1	0.0223	0.0118	1.023 (0.999, 1.047)	0.06	0.248	6.56E-24	TRUE
Mono C	*LAP3*	Wald ratio	1	0.0035	0.0121	1.004 (0.98, 1.028)	0.769	0.897	5.08E-22	TRUE
Mono C	*LGALS1*	Wald ratio	1	-0.0250	0.0134	0.975 (0.95, 1.001)	0.062	0.253	1.51E-22	TRUE
Mono C	*LGALS12*	Wald ratio	1	0.0081	0.0217	1.008 (0.966, 1.052)	0.709	0.87	2.07E-08	TRUE
Mono C	*LGALS2*	Wald ratio	1	0.0039	0.0049	1.004 (0.994, 1.014)	0.433	0.701	2.97E-138	TRUE
Mono C	*LGALS9*	Wald ratio	1	0.0406	0.0140	1.041 (1.013, 1.07)	0.004	0.042	1.36E-16	TRUE
Mono C	*LILRA3*	Wald ratio	1	0.0060	0.0073	1.006 (0.992, 1.021)	0.411	0.686	2.40E-44	TRUE
Mono C	*LILRA5*	Wald ratio	1	0.0181	0.0143	1.018 (0.99, 1.047)	0.206	0.493	9.90E-18	TRUE
Mono C	*LINC00969*	Wald ratio	1	-0.0341	0.0163	0.967 (0.936, 0.998)	0.036	0.182	1.00E-10	TRUE
Mono C	*LIPA*	Wald ratio	1	-0.0093	0.0097	0.991 (0.972, 1.01)	0.339	0.632	3.49E-28	TRUE
Mono C	*LITAF*	Wald ratio	1	0.0263	0.0111	1.027 (1.005, 1.049)	0.018	0.117	7.66E-30	TRUE
Mono C	*LMNA*	Wald ratio	1	-0.0818	0.0226	0.921 (0.882, 0.963)	2.97E-04	0.007	1.69E-11	TRUE
Mono C	*LPAR1*	Wald ratio	1	0.0238	0.0137	1.024 (0.997, 1.052)	0.083	0.304	2.46E-16	TRUE
Mono C	*LRRC8B*	Wald ratio	1	0.0005	0.0189	1 (0.964, 1.038)	0.981	0.993	4.61E-09	TRUE
Mono C	*LTA4H*	IVW	2	0.0051	0.0123	1.005 (0.981, 1.03)	0.679	0.853	1.36E-24	TRUE
Mono C	*LY96*	Wald ratio	1	0.0245	0.0236	1.025 (0.979, 1.073)	0.298	0.593	4.36E-08	TRUE
Mono C	*LYZ*	Wald ratio	1	-0.0015	0.0056	0.998 (0.988, 1.01)	0.788	0.909	3.42E-98	TRUE
Mono C	*MAK*	Wald ratio	1	0.0007	0.0104	1.001 (0.981, 1.021)	0.946	0.979	6.41E-39	TRUE
Mono C	*MANBA*	Wald ratio	1	0.0735	0.0173	1.076 (1.041, 1.113)	2.04E-05	9.61E-04	5.95E-10	TRUE
Mono C	*MANEA*	Wald ratio	1	0.0113	0.0120	1.011 (0.988, 1.035)	0.343	0.635	2.22E-19	TRUE
Mono C	*MARCO*	Wald ratio	1	-0.0067	0.0110	0.993 (0.972, 1.015)	0.546	0.767	2.69E-20	TRUE
Mono C	*MDC1*	IVW	2	0.0322	0.0392	1.033 (0.956, 1.115)	0.412	0.686	4.15E-22	TRUE
Mono C	*METTL22*	Wald ratio	1	-0.0277	0.0244	0.973 (0.927, 1.02)	0.256	0.551	6.75E-09	TRUE
Mono C	*MGMT*	Wald ratio	1	-0.0166	0.0154	0.984 (0.954, 1.014)	0.281	0.575	9.88E-10	TRUE
Mono C	*MGST3*	Wald ratio	1	0.0005	0.0121	1 (0.977, 1.024)	0.97	0.989	3.26E-20	TRUE
Mono C	*MICALL1*	Wald ratio	1	0.0017	0.0161	1.002 (0.971, 1.034)	0.918	0.965	2.47E-10	TRUE
Mono C	*MIF*	Wald ratio	1	0.0187	0.0152	1.019 (0.989, 1.05)	0.219	0.511	3.25E-13	TRUE
Mono C	*MRPL21*	Wald ratio	1	0.0148	0.0169	1.015 (0.982, 1.049)	0.382	0.667	7.38E-10	TRUE
Mono C	*MRPL54*	Wald ratio	1	0.0124	0.0141	1.012 (0.985, 1.041)	0.377	0.665	8.34E-15	TRUE
Mono C	*MX1*	Wald ratio	1	0.0307	0.0212	1.031 (0.989, 1.075)	0.147	0.416	1.79E-08	TRUE
Mono C	*NAAA*	IVW	2	0.0332	0.0124	1.034 (1.009, 1.059)	0.007	0.065	4.79E-18	TRUE
Mono C	*NAGK*	Wald ratio	1	0.0211	0.0166	1.021 (0.989, 1.055)	0.205	0.492	4.37E-12	TRUE
Mono C	*NAP1L1*	Wald ratio	1	-0.0135	0.0172	0.987 (0.954, 1.02)	0.432	0.701	1.29E-10	TRUE
Mono C	*NAPRT1*	Wald ratio	1	-0.0188	0.0114	0.981 (0.96, 1.004)	0.099	0.333	1.50E-21	TRUE
Mono C	*NDUFA12*	Wald ratio	1	0.0075	0.0153	1.007 (0.978, 1.038)	0.626	0.822	5.04E-15	TRUE
Mono C	*NDUFS5*	Wald ratio	1	-0.0061	0.0099	0.994 (0.975, 1.013)	0.536	0.762	9.49E-34	TRUE

Mono C	*NDUFV3*	Wald ratio	1	0.0223	0.0165	1.023 (0.99, 1.056)	0.178	0.457	3.14E-08	TRUE
Mono C	*NEU4*	Wald ratio	1	0.0180	0.0169	1.018 (0.985, 1.052)	0.286	0.577	4.83E-15	TRUE
Mono C	*NFXL1*	Wald ratio	1	0.0039	0.0135	1.004 (0.978, 1.031)	0.77	0.898	3.29E-15	TRUE
Mono C	*NKAPL*	Wald ratio	1	0.0047	0.0192	1.005 (0.968, 1.043)	0.805	0.916	8.96E-14	TRUE
Mono C	*NME1*	Wald ratio	1	0.0062	0.0186	1.006 (0.97, 1.044)	0.737	0.88	2.18E-09	TRUE
Mono C	*NOP10*	Wald ratio	1	-0.0045	0.0095	0.995 (0.977, 1.014)	0.635	0.827	2.08E-45	TRUE
Mono C	*NQO2*	Wald ratio	1	-0.0010	0.0091	0.999 (0.981, 1.017)	0.914	0.962	2.19E-36	TRUE
Mono C	*NRG1*	Wald ratio	1	-0.0175	0.0099	0.983 (0.964, 1.002)	0.077	0.289	9.41E-30	TRUE
Mono C	*NT5C3B*	Wald ratio	1	0.0000	0.0106	1 (0.979, 1.021)	0.997	0.999	3.30E-22	TRUE
Mono C	*NT5DC3*	Wald ratio	1	-0.0116	0.0123	0.988 (0.965, 1.012)	0.343	0.635	2.42E-24	TRUE
Mono C	*NUDT14*	Wald ratio	1	0.0049	0.0201	1.005 (0.966, 1.045)	0.808	0.918	3.46E-08	TRUE
Mono C	*NUDT2*	Wald ratio	1	-0.0193	0.0130	0.981 (0.956, 1.006)	0.137	0.399	1.61E-15	TRUE
Mono C	*OLIG1*	Wald ratio	1	-0.0068	0.0199	0.993 (0.955, 1.033)	0.733	0.88	6.45E-09	TRUE
Mono C	*OSCAR*	Wald ratio	1	-0.0055	0.0144	0.995 (0.967, 1.023)	0.704	0.867	1.74E-14	TRUE
Mono C	*OSTF1*	Wald ratio	1	-0.0094	0.0207	0.991 (0.951, 1.032)	0.65	0.835	2.14E-08	TRUE
Mono C	*PADI2*	Wald ratio	1	-0.0184	0.0157	0.982 (0.952, 1.012)	0.241	0.534	8.23E-12	TRUE
Mono C	*PLA2G16*	Wald ratio	1	-0.0114	0.0199	0.989 (0.951, 1.028)	0.567	0.782	4.66E-12	TRUE
Mono C	*PNRC2*	Wald ratio	1	-0.0211	0.0183	0.979 (0.945, 1.015)	0.248	0.541	1.80E-09	TRUE
Mono C	*POLR1D*	Wald ratio	1	0.0023	0.0162	1.002 (0.971, 1.035)	0.888	0.955	5.38E-15	TRUE
Mono C	*POLR2L*	Wald ratio	1	-0.0346	0.0132	0.966 (0.941, 0.991)	0.009	0.075	8.30E-18	TRUE
Mono C	*PPA1*	Wald ratio	1	0.0081	0.0178	1.008 (0.974, 1.044)	0.648	0.834	2.54E-09	TRUE
Mono C	*PPDPF*	Wald ratio	1	-0.0103	0.0096	0.99 (0.971, 1.008)	0.283	0.575	2.91E-28	TRUE
Mono C	*PPIL3*	Wald ratio	1	0.0115	0.0135	1.012 (0.985, 1.039)	0.393	0.672	8.37E-16	TRUE
Mono C	*PPT1*	Wald ratio	1	0.0350	0.0120	1.036 (1.012, 1.06)	0.003	0.039	6.11E-19	TRUE
Mono C	*PRELID1*	Wald ratio	1	0.0408	0.0111	1.042 (1.019, 1.065)	2.46E-04	0.006	5.24E-20	TRUE
Mono C	*PRKCB*	Wald ratio	1	0.0239	0.0117	1.024 (1.001, 1.048)	0.042	0.201	3.21E-22	TRUE
Mono C	*PROK2*	Wald ratio	1	0.0132	0.0143	1.013 (0.985, 1.042)	0.355	0.646	1.25E-12	TRUE
Mono C	*PRR7-AS1*	Wald ratio	1	-0.0167	0.0131	0.983 (0.959, 1.009)	0.2	0.484	4.17E-17	TRUE
Mono C	*PSAP*	Wald ratio	1	0.0075	0.0168	1.007 (0.975, 1.041)	0.656	0.84	3.46E-10	TRUE
Mono C	*PSMD5-AS1*	Wald ratio	1	0.0085	0.0106	1.009 (0.988, 1.03)	0.423	0.693	1.72E-25	TRUE
Mono C	*PVRL2*	Wald ratio	1	0.0528	0.0176	1.054 (1.018, 1.091)	0.003	0.033	1.85E-11	TRUE
Mono C	*RAD51C*	Wald ratio	1	0.0051	0.0189	1.005 (0.968, 1.043)	0.789	0.909	3.73E-08	TRUE
Mono C	*RGS1*	Wald ratio	1	-0.0119	0.0107	0.988 (0.968, 1.009)	0.267	0.561	1.99E-29	TRUE
Mono C	*RNASE2*	Wald ratio	1	-0.0015	0.0136	0.999 (0.972, 1.025)	0.913	0.962	2.89E-16	TRUE
Mono C	*RNF144B*	Wald ratio	1	-0.0241	0.0152	0.976 (0.948, 1.006)	0.113	0.359	6.87E-17	TRUE
Mono C	*RNF166*	Wald ratio	1	-0.0021	0.0204	0.998 (0.959, 1.039)	0.918	0.965	3.14E-08	TRUE
Mono C	*RNPEP*	Wald ratio	1	-0.0182	0.0132	0.982 (0.957, 1.008)	0.168	0.444	4.88E-17	TRUE
Mono C	*ROGDI*	Wald ratio	1	-0.0294	0.0092	0.971 (0.954, 0.989)	0.001	0.021	2.51E-33	TRUE
Mono C	*RP1-3J17.3*	Wald ratio	1	-0.0024	0.0085	0.998 (0.981, 1.014)	0.781	0.905	7.35E-37	TRUE
Mono C	*RP11-1143G9.4*	Wald ratio	1	0.0026	0.0051	1.003 (0.993, 1.013)	0.608	0.809	1.65E-120	TRUE
Mono C	*RP11-245G13.2*	Wald ratio	1	-0.0391	0.0197	0.962 (0.925, 0.999)	0.047	0.214	2.24E-10	TRUE
Mono C	*RP11-290F20.3*	Wald ratio	1	-0.0245	0.0219	0.976 (0.935, 1.019)	0.262	0.557	6.60E-08	TRUE
Mono C	*RP11-347P5.1*	Wald ratio	1	0.0044	0.0100	1.004 (0.985, 1.024)	0.658	0.842	4.27E-29	TRUE
Mono C	*RP11-362F19.1*	Wald ratio	1	0.0048	0.0147	1.005 (0.976, 1.034)	0.746	0.883	2.99E-14	TRUE
Mono C	*RP11-661A12.5*	Wald ratio	1	0.0090	0.0102	1.009 (0.989, 1.029)	0.38	0.666	2.53E-28	TRUE
Mono C	*RP3-460G2.2*	Wald ratio	1	0.0102	0.0121	1.01 (0.987, 1.034)	0.399	0.678	9.12E-20	TRUE
Mono C	*RPH3A*	Wald ratio	1	-0.0477	0.0135	0.953 (0.929, 0.979)	3.96E-04	0.009	4.85E-17	TRUE
Mono C	*RPL10A*	Wald ratio	1	-0.0211	0.0160	0.979 (0.949, 1.01)	0.187	0.466	2.22E-12	TRUE
Mono C	*RPL12*	Wald ratio	1	-0.0330	0.0098	0.968 (0.949, 0.986)	7.92E-04	0.014	3.62E-32	TRUE
Mono C	*RPL13*	Wald ratio	1	-0.0500	0.0250	0.951 (0.906, 0.999)	0.045	0.21	2.29E-09	TRUE
Mono C	*RPL14*	Wald ratio	1	0.0590	0.0194	1.061 (1.021, 1.102)	0.002	0.03	2.74E-08	TRUE
Mono C	*RPL28*	Wald ratio	1	0.0097	0.0124	1.01 (0.985, 1.035)	0.438	0.703	9.34E-19	TRUE
Mono C	*RPL36AL*	Wald ratio	1	-0.0077	0.0119	0.992 (0.969, 1.016)	0.517	0.752	1.50E-21	TRUE
Mono C	*RPL8*	Wald ratio	1	-0.0067	0.0065	0.993 (0.981, 1.006)	0.303	0.598	7.07E-69	TRUE
Mono C	*RPLP2*	Wald ratio	1	-0.0553	0.0205	0.946 (0.909, 0.985)	0.007	0.062	7.89E-08	TRUE
Mono C	*RPPH1*	Wald ratio	1	0.0079	0.0097	1.008 (0.989, 1.027)	0.411	0.686	7.82E-25	TRUE
Mono C	*RPS10*	Wald ratio	1	-0.0104	0.0106	0.99 (0.969, 1.011)	0.33	0.624	3.57E-23	TRUE
Mono C	*RPS16*	Wald ratio	1	-0.0080	0.0088	0.992 (0.975, 1.009)	0.363	0.651	4.61E-35	TRUE
Mono C	*RPS18*	Wald ratio	1	-0.0199	0.0117	0.98 (0.958, 1.003)	0.087	0.312	2.97E-26	TRUE
Mono C	*RPS19*	Wald ratio	1	0.0222	0.0201	1.022 (0.983, 1.064)	0.27	0.563	4.66E-08	TRUE
Mono C	*RPS2*	Wald ratio	1	-0.0338	0.0118	0.967 (0.945, 0.989)	0.004	0.044	1.60E-21	TRUE
Mono C	*RPS25*	Wald ratio	1	-0.0202	0.0150	0.98 (0.952, 1.009)	0.179	0.458	4.14E-10	TRUE
Mono C	*RPS26*	Wald ratio	1	-0.0106	0.0046	0.989 (0.981, 0.998)	0.022	0.132	1.66E-156	TRUE
Mono C	*RPS3*	Wald ratio	1	-0.0259	0.0146	0.974 (0.947, 1.003)	0.075	0.287	7.62E-09	TRUE
Mono C	*RPS3A*	Wald ratio	1	-0.0060	0.0092	0.994 (0.976, 1.012)	0.514	0.75	2.13E-17	TRUE
Mono C	*RPS5*	Wald ratio	1	0.0249	0.0185	1.025 (0.989, 1.063)	0.177	0.456	6.64E-10	TRUE
Mono C	*RPS8*	Wald ratio	1	0.0095	0.0192	1.01 (0.972, 1.048)	0.622	0.821	1.16E-08	TRUE
Mono C	*RPS9*	Wald ratio	1	0.0333	0.0087	1.034 (1.016, 1.052)	1.26E-04	0.003	4.76E-37	TRUE
Mono C	*S100A12*	IVW	2	0.0190	0.0415	1.019 (0.94, 1.106)	0.648	0.834	1.15E-24	TRUE
Mono C	*S100A8*	Wald ratio	1	-0.0604	0.0203	0.941 (0.905, 0.98)	0.003	0.034	3.31E-09	TRUE
Mono C	*S100A9*	Wald ratio	1	-0.0553	0.0185	0.946 (0.912, 0.981)	0.003	0.034	9.02E-11	TRUE
Mono C	*S100P*	Wald ratio	1	-0.0063	0.0160	0.994 (0.963, 1.025)	0.695	0.863	3.92E-13	TRUE
Mono C	*SCML4*	Wald ratio	1	-0.0034	0.0129	0.997 (0.972, 1.022)	0.793	0.91	4.13E-13	TRUE
Mono C	*SELL*	Wald ratio	1	0.0012	0.0105	1.001 (0.981, 1.022)	0.908	0.961	1.22E-28	TRUE
Mono C	*SERPINB10*	Wald ratio	1	-0.0027	0.0130	0.997 (0.972, 1.023)	0.837	0.932	3.28E-16	TRUE
Mono C	*SETD9*	Wald ratio	1	-0.0526	0.0189	0.949 (0.914, 0.985)	0.005	0.053	9.60E-08	TRUE
Mono C	*SH3BGRL3*	Wald ratio	1	0.0001	0.0090	1 (0.983, 1.018)	0.989	0.996	4.13E-32	TRUE
Mono C	*SH3BP5*	Wald ratio	1	0.0311	0.0179	1.032 (0.996, 1.068)	0.082	0.301	7.10E-10	TRUE
Mono C	*SIRPB1*	Wald ratio	1	-0.0100	0.0097	0.99 (0.971, 1.009)	0.299	0.594	7.62E-35	TRUE

Mono C	*SLC12A7*	Wald ratio	1	-0.0195	0.0179	0.981 (0.947, 1.016)	0.275	0.568	4.23E-10	TRUE
Mono C	*SLC22A18AS*	Wald ratio	1	-0.0201	0.0154	0.98 (0.951, 1.01)	0.19	0.471	7.15E-14	TRUE
Mono C	*SLC24A4*	Wald ratio	1	-0.0276	0.0196	0.973 (0.936, 1.011)	0.159	0.432	8.27E-08	TRUE
Mono C	*SMDT1*	Wald ratio	1	0.0128	0.0067	1.013 (1, 1.026)	0.057	0.239	6.38E-58	TRUE
Mono C	*SNHG7*	Wald ratio	1	-0.0304	0.0179	0.97 (0.937, 1.005)	0.091	0.318	3.46E-14	TRUE
Mono C	*SNHG8*	Wald ratio	1	-0.0042	0.0091	0.996 (0.978, 1.014)	0.647	0.834	7.10E-40	TRUE
Mono C	*SNORD3B-1*	Wald ratio	1	-0.0039	0.0116	0.996 (0.974, 1.019)	0.736	0.88	1.02E-16	TRUE
Mono C	*SNORD3B-2*	Wald ratio	1	-0.0121	0.0169	0.988 (0.956, 1.021)	0.473	0.73	7.25E-16	TRUE
Mono C	*SNORD3D*	Wald ratio	1	0.0114	0.0155	1.011 (0.981, 1.043)	0.461	0.721	4.65E-11	TRUE
Mono C	*SPATA20*	Wald ratio	1	0.0221	0.0137	1.022 (0.995, 1.05)	0.106	0.346	3.35E-16	TRUE
Mono C	*SRD5A3*	Wald ratio	1	-0.0425	0.0194	0.958 (0.923, 0.995)	0.028	0.157	4.01E-09	TRUE
Mono C	*STEAP4*	Wald ratio	1	-0.0017	0.0205	0.998 (0.959, 1.039)	0.933	0.972	6.88E-10	TRUE
Mono C	*STXBP2*	Wald ratio	1	0.0100	0.0091	1.01 (0.992, 1.028)	0.274	0.568	1.50E-39	TRUE
Mono C	*SULF2*	Wald ratio	1	-0.0199	0.0203	0.98 (0.942, 1.02)	0.327	0.62	3.43E-11	TRUE
Mono C	*SUSD3*	Wald ratio	1	-0.0053	0.0169	0.995 (0.962, 1.028)	0.752	0.887	5.75E-11	TRUE
Mono C	*TACSTD2*	Wald ratio	1	-0.0336	0.0135	0.967 (0.942, 0.993)	0.013	0.096	2.04E-20	TRUE
Mono C	*TBKBP1*	Wald ratio	1	0.0086	0.0123	1.009 (0.985, 1.033)	0.484	0.736	8.22E-20	TRUE
Mono C	*TCF19*	Wald ratio	1	0.0024	0.0150	1.002 (0.973, 1.032)	0.873	0.947	8.40E-12	TRUE
Mono C	*TEN1*	Wald ratio	1	-0.0157	0.0212	0.984 (0.944, 1.026)	0.458	0.719	2.94E-09	TRUE
Mono C	*THBS1*	Wald ratio	1	0.0097	0.0128	1.01 (0.985, 1.035)	0.45	0.714	4.36E-16	TRUE
Mono C	*THEMIS*	Wald ratio	1	-0.0067	0.0101	0.993 (0.974, 1.013)	0.507	0.745	4.73E-25	TRUE
Mono C	*THEMIS2*	Wald ratio	1	0.0103	0.0129	1.01 (0.985, 1.036)	0.422	0.692	1.52E-17	TRUE
Mono C	*TIMM10*	Wald ratio	1	-0.0018	0.0116	0.998 (0.976, 1.021)	0.875	0.948	3.98E-26	TRUE
Mono C	*TKT*	Wald ratio	1	-0.0026	0.0153	0.997 (0.968, 1.028)	0.864	0.943	3.18E-12	TRUE
Mono C	*TLE4*	Wald ratio	1	-0.0242	0.0153	0.976 (0.947, 1.006)	0.114	0.36	1.17E-13	TRUE
Mono C	*TMC4*	Wald ratio	1	-0.0180	0.0166	0.982 (0.951, 1.015)	0.278	0.571	4.66E-11	TRUE
Mono C	*TMEM150B*	Wald ratio	1	-0.0391	0.0191	0.962 (0.926, 0.998)	0.041	0.197	1.32E-08	TRUE
Mono C	*TMEM176A*	Wald ratio	1	-0.0158	0.0046	0.984 (0.975, 0.993)	6.36E-04	0.012	1.34E-137	TRUE
Mono C	*TMEM176B*	Wald ratio	1	-0.0152	0.0044	0.985 (0.977, 0.993)	5.05E-04	0.01	6.79E-159	TRUE
Mono C	*TMEM220*	Wald ratio	1	0.0108	0.0123	1.011 (0.987, 1.036)	0.381	0.666	4.81E-19	TRUE
Mono C	*TMEM50A*	Wald ratio	1	0.0229	0.0161	1.023 (0.991, 1.056)	0.156	0.427	6.63E-12	TRUE
Mono C	*TMEM91*	Wald ratio	1	0.0173	0.0263	1.017 (0.966, 1.071)	0.51	0.748	4.33E-08	TRUE
Mono C	*TMTC1*	Wald ratio	1	0.0002	0.0085	1 (0.984, 1.017)	0.98	0.992	1.20E-38	TRUE
Mono C	*TNNT1*	Wald ratio	1	-0.0147	0.0095	0.985 (0.967, 1.004)	0.123	0.377	1.55E-29	TRUE
Mono C	*TOMM7*	Wald ratio	1	0.0042	0.0062	1.004 (0.992, 1.017)	0.503	0.743	2.35E-67	TRUE
Mono C	*TPTEP1*	Wald ratio	1	-0.0103	0.0213	0.99 (0.949, 1.032)	0.629	0.824	6.07E-08	TRUE
Mono C	*TREML1*	Wald ratio	1	0.0264	0.0076	1.027 (1.012, 1.042)	5.34E-04	0.011	2.89E-40	TRUE
Mono C	*TSPAN33*	Wald ratio	1	-0.0124	0.0174	0.988 (0.955, 1.022)	0.478	0.733	1.82E-10	TRUE
Mono C	*TSPAN4*	Wald ratio	1	-0.0396	0.0158	0.961 (0.932, 0.991)	0.012	0.094	6.00E-13	TRUE
Mono C	*TSPO*	Wald ratio	1	-0.0109	0.0160	0.989 (0.959, 1.021)	0.497	0.741	2.51E-12	TRUE
Mono C	*TSTD1*	Wald ratio	1	0.0357	0.0194	1.036 (0.998, 1.077)	0.066	0.263	5.89E-08	TRUE
Mono C	*TTC39C*	Wald ratio	1	0.0322	0.0134	1.033 (1.006, 1.06)	0.016	0.111	5.39E-14	TRUE
Mono C	*TUFM*	Wald ratio	1	0.0238	0.0119	1.024 (1, 1.048)	0.046	0.21	1.11E-19	TRUE
Mono C	*TYMP*	Wald ratio	1	0.0277	0.0120	1.028 (1.004, 1.053)	0.022	0.132	4.93E-20	TRUE
Mono C	*UAP1L1*	Wald ratio	1	0.0154	0.0184	1.015 (0.98, 1.053)	0.404	0.681	1.92E-10	TRUE
Mono C	*UBE2D1*	Wald ratio	1	0.0356	0.0185	1.036 (0.999, 1.075)	0.055	0.235	1.68E-09	TRUE
Mono C	*UCP2*	Wald ratio	1	0.0047	0.0126	1.005 (0.98, 1.03)	0.711	0.871	4.09E-18	TRUE
Mono C	*UPK3A*	Wald ratio	1	-0.0043	0.0191	0.996 (0.959, 1.034)	0.823	0.926	1.12E-08	TRUE
Mono C	*UTP20*	Wald ratio	1	-0.0059	0.0094	0.994 (0.976, 1.013)	0.529	0.757	2.80E-25	TRUE
Mono C	*VARS2*	Wald ratio	1	-0.0031	0.0121	0.997 (0.974, 1.021)	0.798	0.913	2.21E-19	TRUE
Mono C	*VASH1*	Wald ratio	1	0.0045	0.0144	1.004 (0.976, 1.033)	0.757	0.889	1.37E-13	TRUE
Mono C	*VNN1*	Wald ratio	1	0.0285	0.0143	1.029 (1.001, 1.058)	0.046	0.21	1.06E-13	TRUE
Mono C	*VPS28*	Wald ratio	1	0.0182	0.0111	1.018 (0.996, 1.041)	0.102	0.338	4.10E-20	TRUE
Mono C	*VSTM1*	Wald ratio	1	-0.0046	0.0100	0.995 (0.976, 1.015)	0.645	0.834	9.42E-29	TRUE
Mono C	*WAC-AS1*	Wald ratio	1	0.0214	0.0192	1.022 (0.984, 1.061)	0.265	0.56	1.02E-09	TRUE
Mono C	*XRCC6BP1*	Wald ratio	1	-0.0374	0.0187	0.963 (0.929, 0.999)	0.046	0.21	5.74E-09	TRUE
Mono C	*XRRA1*	Wald ratio	1	-0.0096	0.0160	0.99 (0.96, 1.022)	0.55	0.769	1.04E-12	TRUE
Mono C	*XXbac-BPG181B23.7*	Wald ratio	1	-0.0694	0.0150	0.933 (0.906, 0.961)	3.77E-06	2.77E-04	1.30E-12	TRUE
Mono C	*XXbac-BPG252P9.10*	Wald ratio	1	0.0147	0.0256	1.015 (0.965, 1.067)	0.564	0.78	4.16E-08	TRUE
Mono C	*XXbac-BPG299F13.17*	Wald ratio	1	0.0170	0.0224	1.017 (0.973, 1.063)	0.448	0.713	2.05E-08	TRUE
Mono C	*ZFAS1*	Wald ratio	1	0.0093	0.0120	1.009 (0.986, 1.033)	0.437	0.703	1.07E-21	TRUE
Mono C	*ZKSCAN3*	Wald ratio	1	0.0136	0.0172	1.014 (0.98, 1.048)	0.43	0.7	1.75E-12	TRUE
Mono C	*ZKSCAN4*	Wald ratio	1	-0.0046	0.0155	0.995 (0.966, 1.026)	0.769	0.897	1.72E-20	TRUE
Mono C	*ZNF184*	Wald ratio	1	0.0140	0.0227	1.014 (0.97, 1.06)	0.538	0.762	2.85E-10	TRUE
Mono C	*ZNF506*	Wald ratio	1	0.0304	0.0184	1.031 (0.994, 1.069)	0.099	0.333	3.68E-08	TRUE
Mono C	*ZNF593*	Wald ratio	1	-0.0034	0.0178	0.997 (0.962, 1.032)	0.846	0.935	2.73E-09	TRUE
Mono C	*ZP3*	Wald ratio	1	-0.0050	0.0173	0.995 (0.962, 1.029)	0.774	0.901	2.82E-10	TRUE
Mono C	*ZSCAN12*	Wald ratio	1	-0.0062	0.0172	0.994 (0.961, 1.028)	0.717	0.874	1.22E-18	TRUE
Mono NC	*ABCC3*	Wald ratio	1	-0.0079	0.0204	0.992 (0.953, 1.033)	0.7	0.866	3.13E-08	TRUE
Mono NC	*ABHD16A*	Wald ratio	1	0.0317	0.0125	1.032 (1.007, 1.058)	0.011	0.088	1.22E-18	TRUE
Mono NC	*ABRACL*	Wald ratio	1	0.0773	0.0193	1.08 (1.04, 1.122)	6.23E-05	0.002	1.09E-08	TRUE
Mono NC	*AC000003.2*	Wald ratio	1	0.0177	0.0118	1.018 (0.995, 1.042)	0.133	0.394	1.06E-19	TRUE
Mono NC	*AC002451.3*	Wald ratio	1	0.0034	0.0078	1.003 (0.988, 1.019)	0.664	0.846	1.95E-48	TRUE
Mono NC	*ACP5*	Wald ratio	1	-0.0101	0.0087	0.99 (0.973, 1.007)	0.243	0.535	5.23E-32	TRUE
Mono NC	*ACTA2*	Wald ratio	1	-0.0085	0.0108	0.992 (0.971, 1.013)	0.433	0.701	4.04E-24	TRUE
Mono NC	*ADK*	Wald ratio	1	0.0275	0.0094	1.028 (1.009, 1.047)	0.004	0.04	1.10E-23	TRUE
Mono NC	*AGER*	Wald ratio	1	0.0189	0.0137	1.019 (0.992, 1.047)	0.167	0.442	5.10E-21	TRUE
Mono NC	*AHI1*	Wald ratio	1	0.0199	0.0187	1.02 (0.983, 1.058)	0.286	0.577	1.58E-08	TRUE
Mono NC	*ANPEP*	Wald ratio	1	0.0028	0.0126	1.003 (0.978, 1.028)	0.825	0.926	1.03E-18	TRUE

Mono NC	*ANXA1*	Wald ratio	1	0.0264	0.0168	1.027 (0.993, 1.061)	0.117	0.365	2.48E-09	TRUE
Mono NC	*ANXA4*	Wald ratio	1	-0.0292	0.0130	0.971 (0.947, 0.996)	0.025	0.145	2.93E-18	TRUE
Mono NC	*AP1AR*	Wald ratio	1	0.0247	0.0160	1.025 (0.993, 1.058)	0.123	0.378	6.46E-13	TRUE
Mono NC	*APOBEC3A*	Wald ratio	1	-0.0321	0.0145	0.968 (0.941, 0.996)	0.026	0.151	1.94E-11	TRUE
Mono NC	*BATF3*	Wald ratio	1	0.0092	0.0217	1.009 (0.967, 1.053)	0.673	0.85	9.93E-09	TRUE
Mono NC	*BCAT1*	Wald ratio	1	0.0116	0.0160	1.012 (0.98, 1.044)	0.467	0.725	8.77E-13	TRUE
Mono NC	*BCR*	Wald ratio	1	-0.0367	0.0150	0.964 (0.936, 0.993)	0.015	0.103	1.94E-11	TRUE
Mono NC	*BEGAIN*	Wald ratio	1	0.0104	0.0084	1.01 (0.994, 1.027)	0.212	0.5	1.91E-38	TRUE
Mono NC	*BIRC7*	Wald ratio	1	0.0059	0.0120	1.006 (0.982, 1.03)	0.627	0.822	3.33E-31	TRUE
Mono NC	*BLVRA*	Wald ratio	1	-0.0044	0.0133	0.996 (0.97, 1.022)	0.739	0.881	1.47E-16	TRUE
Mono NC	*BRK1*	Wald ratio	1	-0.0261	0.0184	0.974 (0.94, 1.01)	0.157	0.428	2.15E-11	TRUE
Mono NC	*C1QA*	Wald ratio	1	0.0015	0.0160	1.001 (0.971, 1.033)	0.926	0.968	4.16E-11	TRUE
Mono NC	*C1QB*	Wald ratio	1	0.0022	0.0136	1.002 (0.976, 1.029)	0.875	0.948	6.19E-15	TRUE
Mono NC	*C20orf27*	Wald ratio	1	0.0223	0.0099	1.023 (1.003, 1.043)	0.025	0.145	6.62E-21	TRUE
Mono NC	*CAT*	Wald ratio	1	0.0142	0.0204	1.014 (0.975, 1.056)	0.487	0.738	5.93E-08	TRUE
Mono NC	*CCDC170*	Wald ratio	1	-0.0196	0.0130	0.981 (0.956, 1.006)	0.133	0.394	2.20E-15	TRUE
Mono NC	*CCT8*	Wald ratio	1	-0.0109	0.0201	0.989 (0.951, 1.029)	0.586	0.794	5.24E-12	TRUE
Mono NC	*CD151*	Wald ratio	1	0.0220	0.0088	1.022 (1.005, 1.04)	0.013	0.095	2.35E-38	TRUE
Mono NC	*CD52*	Wald ratio	1	-0.0001	0.0049	1 (0.99, 1.01)	0.989	0.996	2.28E-109	TRUE
Mono NC	*CD55*	Wald ratio	1	0.0243	0.0097	1.025 (1.005, 1.044)	0.012	0.094	9.64E-33	TRUE
Mono NC	*CDA*	Wald ratio	1	-0.0105	0.0090	0.99 (0.972, 1.007)	0.243	0.536	1.57E-34	TRUE
Mono NC	*CEACAM3*	Wald ratio	1	0.0030	0.0058	1.003 (0.992, 1.014)	0.604	0.806	2.80E-85	TRUE
Mono NC	*CFD*	Wald ratio	1	0.0001	0.0077	1 (0.985, 1.015)	0.99	0.996	8.88E-61	TRUE
Mono NC	*CHMP4B*	Wald ratio	1	-0.0402	0.0177	0.961 (0.928, 0.994)	0.023	0.138	8.61E-10	TRUE
Mono NC	*CHST13*	Wald ratio	1	0.0035	0.0124	1.003 (0.979, 1.028)	0.778	0.904	2.13E-16	TRUE
Mono NC	*CHST2*	Wald ratio	1	0.0000	0.0199	1 (0.962, 1.04)	0.998	0.999	3.61E-08	TRUE
Mono NC	*CKB*	Wald ratio	1	-0.0216	0.0169	0.979 (0.947, 1.012)	0.201	0.484	1.12E-10	TRUE
Mono NC	*CLCN1*	Wald ratio	1	0.0117	0.0136	1.012 (0.985, 1.039)	0.391	0.672	2.12E-11	TRUE
Mono NC	*CLEC12A*	Wald ratio	1	-0.0049	0.0059	0.995 (0.984, 1.007)	0.404	0.681	1.15E-87	TRUE
Mono NC	*CLEC4F*	Wald ratio	1	0.0066	0.0138	1.007 (0.98, 1.034)	0.63	0.824	1.67E-17	TRUE
Mono NC	*CNN2*	Wald ratio	1	0.0001	0.0195	1 (0.963, 1.039)	0.996	0.999	5.62E-12	TRUE
Mono NC	*COPG2*	Wald ratio	1	-0.0214	0.0182	0.979 (0.944, 1.014)	0.24	0.534	3.41E-09	TRUE
Mono NC	*COTL1*	Wald ratio	1	-0.0132	0.0199	0.987 (0.949, 1.026)	0.508	0.747	3.61E-08	TRUE
Mono NC	*CRYZ*	Wald ratio	1	0.0143	0.0111	1.014 (0.993, 1.037)	0.198	0.481	3.10E-23	TRUE
Mono NC	*CST3*	Wald ratio	1	-0.0020	0.0082	0.998 (0.982, 1.014)	0.806	0.916	1.01E-41	TRUE
Mono NC	*CTB-61M7.2*	Wald ratio	1	-0.0121	0.0072	0.988 (0.974, 1.002)	0.092	0.322	1.45E-47	TRUE
Mono NC	*CTD-2006K23.1*	Wald ratio	1	-0.0105	0.0121	0.99 (0.966, 1.013)	0.388	0.669	2.36E-19	TRUE
Mono NC	*CTSH*	Wald ratio	1	-0.0331	0.0124	0.967 (0.944, 0.991)	0.007	0.066	5.04E-18	TRUE
Mono NC	*CYBRD1*	Wald ratio	1	0.0109	0.0124	1.011 (0.987, 1.036)	0.377	0.665	8.97E-15	TRUE
Mono NC	*CYP1B1*	Wald ratio	1	-0.0145	0.0206	0.986 (0.947, 1.026)	0.482	0.735	3.46E-08	TRUE
Mono NC	*DNAJC15*	Wald ratio	1	0.0101	0.0123	1.01 (0.986, 1.035)	0.413	0.686	9.21E-18	TRUE
Mono NC	*DOK2*	Wald ratio	1	0.0229	0.0125	1.023 (0.998, 1.049)	0.066	0.263	1.61E-19	TRUE
Mono NC	*EIF5A*	Wald ratio	1	-0.0105	0.0070	0.99 (0.976, 1.003)	0.134	0.394	1.33E-63	TRUE
Mono NC	*ELP2*	Wald ratio	1	0.0147	0.0188	1.015 (0.978, 1.053)	0.435	0.702	5.91E-09	TRUE
Mono NC	*EPHA1-AS1*	Wald ratio	1	0.0074	0.0127	1.007 (0.983, 1.033)	0.561	0.779	1.22E-17	TRUE
Mono NC	*FADS2*	Wald ratio	1	-0.0152	0.0121	0.985 (0.962, 1.009)	0.209	0.496	4.22E-30	TRUE
Mono NC	*FAM118A*	Wald ratio	1	-0.0017	0.0069	0.998 (0.985, 1.012)	0.81	0.919	2.25E-51	TRUE
Mono NC	*FAM184B*	Wald ratio	1	0.0029	0.0146	1.003 (0.975, 1.032)	0.843	0.935	4.17E-15	TRUE
Mono NC	*FAM26F*	Wald ratio	1	0.0026	0.0070	1.003 (0.989, 1.016)	0.706	0.868	4.10E-42	TRUE
Mono NC	*FBP1*	Wald ratio	1	-0.0207	0.0204	0.98 (0.941, 1.019)	0.311	0.605	7.22E-08	TRUE
Mono NC	*FCAR*	Wald ratio	1	0.0088	0.0111	1.009 (0.987, 1.031)	0.424	0.695	5.13E-21	TRUE
Mono NC	*FCER1G*	Wald ratio	1	-0.0077	0.0114	0.992 (0.97, 1.015)	0.501	0.743	6.21E-20	TRUE
Mono NC	*FCN1*	Wald ratio	1	0.0087	0.0087	1.009 (0.992, 1.026)	0.318	0.614	4.74E-31	TRUE
Mono NC	*FHL3*	Wald ratio	1	-0.0080	0.0156	0.992 (0.962, 1.023)	0.607	0.808	4.56E-14	TRUE
Mono NC	*FIBCD1*	Wald ratio	1	0.0105	0.0163	1.011 (0.979, 1.043)	0.52	0.753	8.57E-11	TRUE
Mono NC	*FKBPL*	Wald ratio	1	-0.0311	0.0147	0.969 (0.942, 0.998)	0.034	0.175	6.95E-15	TRUE
Mono NC	*FLVCR1-AS1*	Wald ratio	1	0.0194	0.0144	1.02 (0.991, 1.049)	0.178	0.458	2.07E-14	TRUE
Mono NC	*FPR1*	Wald ratio	1	0.0069	0.0098	1.007 (0.988, 1.026)	0.482	0.735	8.41E-32	TRUE
Mono NC	*GAPDH*	Wald ratio	1	0.0291	0.0133	1.03 (1.003, 1.057)	0.029	0.158	5.33E-14	TRUE
Mono NC	*GAPT*	Wald ratio	1	-0.0072	0.0190	0.993 (0.957, 1.03)	0.703	0.866	9.38E-10	TRUE
Mono NC	*GBP7*	Wald ratio	1	0.0010	0.0150	1.001 (0.972, 1.031)	0.946	0.979	2.65E-13	TRUE
Mono NC	*GDI2*	Wald ratio	1	0.0224	0.0196	1.023 (0.984, 1.063)	0.252	0.545	2.36E-08	TRUE
Mono NC	*GFI1B*	Wald ratio	1	0.0009	0.0128	1.001 (0.976, 1.026)	0.943	0.978	3.13E-20	TRUE
Mono NC	*GLIPR1*	Wald ratio	1	0.0368	0.0174	1.037 (1.003, 1.074)	0.035	0.177	2.69E-09	TRUE
Mono NC	*GLUL*	Wald ratio	1	-0.0221	0.0167	0.978 (0.947, 1.011)	0.185	0.465	1.83E-10	TRUE
Mono NC	*GM2A*	Wald ratio	1	0.0204	0.0118	1.021 (0.997, 1.045)	0.085	0.308	1.78E-19	TRUE
Mono NC	*GMPR*	Wald ratio	1	0.0154	0.0114	1.016 (0.993, 1.038)	0.176	0.456	9.57E-22	TRUE
Mono NC	*GPRC5C*	Wald ratio	1	-0.0075	0.0118	0.993 (0.97, 1.016)	0.525	0.755	8.07E-26	TRUE
Mono NC	*GRAMD1A*	Wald ratio	1	-0.0093	0.0133	0.991 (0.965, 1.017)	0.485	0.736	1.05E-15	TRUE
Mono NC	*GSTP1*	Wald ratio	1	0.0115	0.0068	1.012 (0.998, 1.025)	0.09	0.317	2.64E-65	TRUE
Mono NC	*GTF2H2*	Wald ratio	1	-0.0043	0.0200	0.996 (0.958, 1.035)	0.831	0.929	1.58E-08	TRUE
Mono NC	*HCAR2*	Wald ratio	1	-0.0111	0.0072	0.989 (0.975, 1.003)	0.123	0.378	1.21E-52	TRUE
Mono NC	*HCG18*	Wald ratio	1	-0.0090	0.0221	0.991 (0.949, 1.035)	0.684	0.856	1.81E-08	TRUE
Mono NC	*HIGD2A*	Wald ratio	1	-0.0119	0.0151	0.988 (0.959, 1.018)	0.429	0.699	1.82E-13	TRUE
Mono NC	*HIST1H2BN*	Wald ratio	1	-0.0231	0.0185	0.977 (0.942, 1.013)	0.211	0.498	2.38E-10	TRUE
Mono NC	*HIST1H3D*	Wald ratio	1	0.0300	0.0244	1.03 (0.982, 1.081)	0.219	0.51	5.64E-10	TRUE
Mono NC	*HLA-B*	IVW	2	0.0156	0.0227	1.016 (0.972, 1.062)	0.491	0.738	9.48E-83	TRUE
Mono NC	*HLA-C*	Wald ratio	1	0.0157	0.0115	1.016 (0.993, 1.039)	0.173	0.451	5.68E-21	TRUE
Mono NC	*HLA-DOA*	Wald ratio	1	0.0350	0.0576	1.036 (0.925, 1.159)	0.543	0.765	9.61E-11	TRUE

Mono NC	*HLA-DOB*	IVW	2	0.0226	0.0086	1.023 (1.006, 1.04)	0.008	0.072	2.71E-36	TRUE
Mono NC	*HLA-DPA1*	Wald ratio	1	0.0487	0.0164	1.05 (1.017, 1.084)	0.003	0.036	2.40E-09	TRUE
Mono NC	*HLA-DQA2*	Wald ratio	1	0.0495	0.0194	1.051 (1.012, 1.091)	0.011	0.085	4.36E-11	TRUE
Mono NC	*HLA-DRB5*	Wald ratio	1	0.0063	0.0056	1.006 (0.995, 1.017)	0.257	0.551	1.28E-96	TRUE
Mono NC	*HLA-G*	Wald ratio	1	0.0078	0.0158	1.008 (0.977, 1.04)	0.619	0.819	2.62E-15	TRUE
Mono NC	*HMGN1*	Wald ratio	1	0.0375	0.0128	1.038 (1.012, 1.065)	0.003	0.038	9.35E-19	TRUE
Mono NC	*HSPA1B*	IVW	2	-0.0419	0.0109	0.959 (0.939, 0.98)	1.15E-04	0.003	2.52E-33	TRUE
Mono NC	*HSPBAP1*	Wald ratio	1	0.0129	0.0131	1.013 (0.987, 1.039)	0.327	0.62	8.92E-14	TRUE
Mono NC	*ICAM3*	Wald ratio	1	0.0401	0.0174	1.041 (1.006, 1.077)	0.021	0.132	1.81E-10	TRUE
Mono NC	*ICAM4*	Wald ratio	1	0.0478	0.0177	1.049 (1.013, 1.086)	0.007	0.064	8.01E-11	TRUE
Mono NC	*IFI30*	Wald ratio	1	-0.0402	0.0181	0.961 (0.927, 0.995)	0.026	0.15	7.90E-09	TRUE
Mono NC	*IFITM2*	Wald ratio	1	-0.0148	0.0119	0.985 (0.963, 1.009)	0.214	0.503	6.16E-19	TRUE
Mono NC	*IFITM3*	Wald ratio	1	0.0045	0.0080	1.005 (0.989, 1.02)	0.573	0.786	5.83E-44	TRUE
Mono NC	*IQGAP2*	Wald ratio	1	0.0264	0.0155	1.027 (0.996, 1.058)	0.089	0.317	2.09E-12	TRUE
Mono NC	*ITGB2*	Wald ratio	1	-0.0010	0.0081	0.999 (0.983, 1.015)	0.897	0.957	4.68E-36	TRUE
Mono NC	*JUP*	Wald ratio	1	-0.0360	0.0088	0.965 (0.948, 0.981)	4.30E-05	0.002	3.95E-29	TRUE
Mono NC	*KANSL1-AS1*	Wald ratio	1	-0.0300	0.0120	0.97 (0.948, 0.994)	0.013	0.094	2.21E-44	TRUE
Mono NC	*KCNMA1*	Wald ratio	1	0.0067	0.0083	1.007 (0.99, 1.023)	0.419	0.69	5.21E-40	TRUE
Mono NC	*KCNN4*	Wald ratio	1	0.0134	0.0142	1.013 (0.986, 1.042)	0.349	0.641	8.08E-15	TRUE
Mono NC	*KIAA1586*	Wald ratio	1	0.0407	0.0150	1.042 (1.011, 1.073)	0.007	0.062	9.55E-13	TRUE
Mono NC	*KNDC1*	Wald ratio	1	-0.0019	0.0176	0.998 (0.964, 1.033)	0.914	0.962	5.43E-09	TRUE
Mono NC	*L1TD1*	Wald ratio	1	0.0285	0.0163	1.029 (0.997, 1.062)	0.08	0.296	4.24E-08	TRUE
Mono NC	*L3MBTL3*	Wald ratio	1	-0.0073	0.0210	0.993 (0.953, 1.034)	0.726	0.878	4.12E-08	TRUE
Mono NC	*LAIR2*	Wald ratio	1	0.0065	0.0185	1.007 (0.971, 1.044)	0.725	0.878	1.56E-13	TRUE
Mono NC	*LAP3*	Wald ratio	1	0.0060	0.0133	1.006 (0.98, 1.033)	0.654	0.838	8.27E-18	TRUE
Mono NC	*LGALS2*	Wald ratio	1	-0.0131	0.0145	0.987 (0.959, 1.015)	0.367	0.656	6.52E-15	TRUE
Mono NC	*LGALS9*	Wald ratio	1	0.0327	0.0112	1.033 (1.011, 1.056)	0.003	0.039	2.61E-25	TRUE
Mono NC	*LILRA2*	Wald ratio	1	-0.0028	0.0139	0.997 (0.97, 1.025)	0.839	0.932	1.89E-15	TRUE
Mono NC	*LILRA3*	Wald ratio	1	0.0072	0.0074	1.007 (0.993, 1.022)	0.332	0.626	3.69E-42	TRUE
Mono NC	*LILRA5*	Wald ratio	1	0.0172	0.0143	1.017 (0.989, 1.046)	0.226	0.52	1.68E-17	TRUE
Mono NC	*LIMD1*	Wald ratio	1	0.0187	0.0178	1.019 (0.984, 1.055)	0.295	0.589	3.46E-09	TRUE
Mono NC	*LINC00969*	Wald ratio	1	-0.0411	0.0189	0.96 (0.925, 0.996)	0.03	0.162	2.65E-08	TRUE
Mono NC	*LIPA*	Wald ratio	1	-0.0126	0.0127	0.988 (0.963, 1.012)	0.322	0.617	7.90E-18	TRUE
Mono NC	*LITAF*	Wald ratio	1	0.0393	0.0166	1.04 (1.007, 1.074)	0.018	0.117	1.68E-13	TRUE
Mono NC	*LPL*	Wald ratio	1	-0.0366	0.0168	0.964 (0.933, 0.996)	0.029	0.159	3.50E-14	TRUE
Mono NC	*LST1*	Wald ratio	1	0.0646	0.0135	1.067 (1.039, 1.095)	1.75E-06	1.71E-04	6.26E-14	TRUE
Mono NC	*LTA*	Wald ratio	1	-0.0012	0.0302	0.999 (0.941, 1.06)	0.969	0.989	5.05E-09	TRUE
Mono NC	*LTA4H*	Wald ratio	1	0.0115	0.0098	1.012 (0.992, 1.031)	0.239	0.533	1.95E-14	TRUE
Mono NC	*LY6G5C*	Wald ratio	1	-0.0154	0.0106	0.985 (0.964, 1.006)	0.149	0.418	1.45E-22	TRUE
Mono NC	*LY86*	Wald ratio	1	-0.0078	0.0129	0.992 (0.968, 1.018)	0.546	0.767	1.77E-17	TRUE
Mono NC	*LY96*	Wald ratio	1	0.0156	0.0172	1.016 (0.982, 1.051)	0.365	0.654	5.79E-14	TRUE
Mono NC	*LYPD2*	Wald ratio	1	-0.0005	0.0157	0.999 (0.969, 1.031)	0.973	0.989	4.48E-13	TRUE
Mono NC	*LYZ*	Wald ratio	1	-0.0016	0.0058	0.998 (0.987, 1.01)	0.788	0.909	1.02E-88	TRUE
Mono NC	*MAD1L1*	Wald ratio	1	0.0352	0.0175	1.036 (1.001, 1.072)	0.044	0.208	6.01E-10	TRUE
Mono NC	*MAP3K7CL*	Wald ratio	1	0.0086	0.0141	1.009 (0.981, 1.037)	0.541	0.763	5.97E-14	TRUE
Mono NC	*MDC1*	IVW	3	-0.0135	0.0280	0.987 (0.934, 1.042)	0.629	0.824	1.22E-39	TRUE
Mono NC	*MDC1*	MR Egger	3	-0.0185	0.1612	0.982 (0.716, 1.346)	0.927	NA	NA	NA
Mono NC	*MDC1*	Weighted median	3	-0.0116	0.0152	0.988 (0.96, 1.018)	0.445	NA	NA	NA
Mono NC	*MDC1*	Simple mode	3	-0.0032	0.0278	0.997 (0.944, 1.053)	0.919	NA	NA	NA
Mono NC	*MDC1*	Weighted mode	3	-0.0116	0.0258	0.988 (0.94, 1.04)	0.696	NA	NA	NA
Mono NC	*MGLL*	Wald ratio	1	-0.0069	0.0171	0.993 (0.96, 1.027)	0.687	0.858	1.52E-12	TRUE
Mono NC	*MGME1*	Wald ratio	1	0.0160	0.0244	1.016 (0.969, 1.066)	0.511	0.748	2.29E-08	TRUE
Mono NC	*MGST3*	Wald ratio	1	0.0005	0.0141	1.001 (0.973, 1.028)	0.97	0.989	9.95E-15	TRUE
Mono NC	*MIF*	Wald ratio	1	0.0083	0.0200	1.008 (0.969, 1.049)	0.68	0.853	2.66E-08	TRUE
Mono NC	*MLLT4*	Wald ratio	1	-0.0017	0.0103	0.998 (0.978, 1.019)	0.866	0.944	1.50E-25	TRUE
Mono NC	*MMEL1*	Wald ratio	1	0.0147	0.0185	1.015 (0.979, 1.052)	0.426	0.696	5.59E-09	TRUE
Mono NC	*MROH6*	Wald ratio	1	0.0192	0.0156	1.019 (0.989, 1.051)	0.22	0.512	1.69E-09	TRUE
Mono NC	*MRPL54*	Wald ratio	1	0.0138	0.0176	1.014 (0.979, 1.05)	0.434	0.702	8.03E-10	TRUE
Mono NC	*MX1*	Wald ratio	1	0.0263	0.0181	1.027 (0.991, 1.064)	0.147	0.416	1.03E-10	TRUE
Mono NC	*NAAA*	Wald ratio	1	0.0264	0.0131	1.027 (1.001, 1.054)	0.045	0.208	1.62E-16	TRUE
Mono NC	*NAGK*	Wald ratio	1	0.0228	0.0145	1.023 (0.994, 1.053)	0.115	0.362	6.19E-14	TRUE
Mono NC	*NAP1L1*	Wald ratio	1	-0.0193	0.0194	0.981 (0.944, 1.019)	0.32	0.615	3.41E-08	TRUE
Mono NC	*NAPRT1*	Wald ratio	1	-0.0198	0.0120	0.98 (0.958, 1.004)	0.099	0.333	6.41E-19	TRUE
Mono NC	*NCR3*	Wald ratio	1	-0.0578	0.0129	0.944 (0.92, 0.968)	7.06E-06	4.45E-04	1.69E-18	TRUE
Mono NC	*NDUFA3*	Wald ratio	1	0.0078	0.0187	1.008 (0.971, 1.045)	0.679	0.853	5.32E-09	TRUE
Mono NC	*NDUFS5*	Wald ratio	1	-0.0093	0.0151	0.991 (0.962, 1.02)	0.536	0.762	1.07E-14	TRUE
Mono NC	*NOP10*	Wald ratio	1	-0.0023	0.0104	0.998 (0.977, 1.018)	0.823	0.926	2.21E-37	TRUE
Mono NC	*NQO2*	Wald ratio	1	-0.0072	0.0174	0.993 (0.96, 1.027)	0.677	0.853	9.01E-14	TRUE
Mono NC	*NRM*	IVW	2	-0.0224	0.0325	0.978 (0.918, 1.042)	0.492	0.738	1.25E-21	TRUE
Mono NC	*NUDT14*	Wald ratio	1	0.0011	0.0199	1.001 (0.963, 1.041)	0.956	0.984	3.84E-08	TRUE
Mono NC	*NUDT2*	Wald ratio	1	-0.0175	0.0133	0.983 (0.957, 1.009)	0.19	0.47	1.15E-14	TRUE
Mono NC	*OAS1*	Wald ratio	1	-0.0041	0.0122	0.996 (0.972, 1.02)	0.738	0.88	2.80E-21	TRUE
Mono NC	*OLIG1*	Wald ratio	1	-0.0079	0.0190	0.992 (0.956, 1.03)	0.676	0.853	8.81E-09	TRUE
Mono NC	*OSBPL8*	Wald ratio	1	-0.0060	0.0185	0.994 (0.959, 1.031)	0.744	0.882	1.77E-09	TRUE
Mono NC	*OSCAR*	Wald ratio	1	-0.0064	0.0138	0.994 (0.967, 1.021)	0.645	0.834	4.51E-15	TRUE
Mono NC	*OSTF1*	Wald ratio	1	0.0015	0.0191	1.001 (0.965, 1.04)	0.938	0.975	1.87E-10	TRUE
Mono NC	*P2RX1*	Wald ratio	1	-0.0139	0.0129	0.986 (0.962, 1.011)	0.281	0.575	9.81E-18	TRUE
Mono NC	*PDGFC*	Wald ratio	1	-0.0418	0.0185	0.959 (0.925, 0.995)	0.024	0.143	3.50E-10	TRUE
Mono NC	*PHF11*	Wald ratio	1	0.0523	0.0197	1.054 (1.014, 1.095)	0.008	0.069	6.29E-08	TRUE

Mono NC	*PLEK*	Wald ratio	1	0.0038	0.0176	1.004 (0.97, 1.039)	0.829	0.929	1.02E-09	TRUE
Mono NC	*POLR1D*	Wald ratio	1	0.0021	0.0192	1.002 (0.965, 1.041)	0.915	0.963	7.14E-11	TRUE
Mono NC	*POLR2L*	Wald ratio	1	-0.0517	0.0184	0.95 (0.916, 0.985)	0.005	0.051	2.31E-09	TRUE
Mono NC	*POMZP3*	Wald ratio	1	-0.0011	0.0192	0.999 (0.962, 1.037)	0.953	0.983	9.05E-09	TRUE
Mono NC	*PPA1*	Wald ratio	1	-0.0022	0.0163	0.998 (0.966, 1.03)	0.891	0.957	1.16E-10	TRUE
Mono NC	*PPDPF*	Wald ratio	1	-0.0082	0.0077	0.992 (0.977, 1.007)	0.283	0.575	2.92E-42	TRUE
Mono NC	*PPP1R17*	Wald ratio	1	0.0175	0.0112	1.018 (0.996, 1.04)	0.117	0.365	8.14E-20	TRUE
Mono NC	*PPT1*	Wald ratio	1	-0.0031	0.0173	0.997 (0.964, 1.031)	0.857	0.939	8.18E-13	TRUE
Mono NC	*PRELID1*	Wald ratio	1	0.0588	0.0120	1.061 (1.036, 1.086)	8.60E-07	1.03E-04	1.23E-14	TRUE
Mono NC	*PRR7-AS1*	Wald ratio	1	-0.0120	0.0080	0.988 (0.973, 1.004)	0.136	0.397	6.49E-43	TRUE
Mono NC	*PSAP*	Wald ratio	1	0.0082	0.0164	1.008 (0.976, 1.041)	0.616	0.816	2.88E-10	TRUE
Mono NC	*PSMB9*	Wald ratio	1	-0.0281	0.0185	0.972 (0.938, 1.008)	0.129	0.389	7.25E-10	TRUE
Mono NC	*PSMD5-AS1*	Wald ratio	1	0.0087	0.0107	1.009 (0.988, 1.03)	0.418	0.689	5.23E-24	TRUE
Mono NC	*RAC1*	Wald ratio	1	-0.0315	0.0105	0.969 (0.949, 0.989)	0.003	0.032	3.62E-24	TRUE
Mono NC	*RIN1*	Wald ratio	1	0.0317	0.0191	1.032 (0.994, 1.072)	0.097	0.331	2.75E-12	TRUE
Mono NC	*RIN3*	Wald ratio	1	-0.0310	0.0193	0.969 (0.933, 1.007)	0.109	0.352	2.95E-08	TRUE
Mono NC	*RNF13*	Wald ratio	1	0.0136	0.0163	1.014 (0.982, 1.047)	0.402	0.68	2.39E-12	TRUE
Mono NC	*RNF144B*	Wald ratio	1	-0.0194	0.0122	0.981 (0.958, 1.005)	0.113	0.359	1.74E-24	TRUE
Mono NC	*RNPEP*	Wald ratio	1	-0.0191	0.0131	0.981 (0.956, 1.007)	0.145	0.415	1.47E-16	TRUE
Mono NC	*ROGDI*	Wald ratio	1	-0.0415	0.0130	0.959 (0.935, 0.984)	0.001	0.021	1.42E-16	TRUE
Mono NC	*RP1-102H19.8*	Wald ratio	1	-0.0071	0.0196	0.993 (0.956, 1.032)	0.717	0.874	5.67E-09	TRUE
Mono NC	*RP1-197B17.3*	Wald ratio	1	-0.0378	0.0170	0.963 (0.931, 0.996)	0.026	0.15	7.94E-08	TRUE
Mono NC	*RP1-29C18.10*	Wald ratio	1	0.0019	0.0126	1.002 (0.978, 1.027)	0.878	0.95	3.37E-19	TRUE
Mono NC	*RP1-3J17.3*	IVW	2	0.0027	0.0109	1.003 (0.981, 1.024)	0.803	0.915	3.45E-25	TRUE
Mono NC	*RP11-1143G9.4*	Wald ratio	1	0.0044	0.0085	1.004 (0.988, 1.021)	0.608	0.809	7.79E-39	TRUE
Mono NC	*RP11-298J20.3*	Wald ratio	1	-0.0205	0.0096	0.98 (0.961, 0.998)	0.032	0.168	1.63E-31	TRUE
Mono NC	*RP11-347P5.1*	Wald ratio	1	0.0064	0.0088	1.006 (0.989, 1.024)	0.466	0.725	2.18E-36	TRUE
Mono NC	*RP11-362F19.1*	Wald ratio	1	0.0052	0.0164	1.005 (0.973, 1.038)	0.749	0.886	2.39E-11	TRUE
Mono NC	*RP11-367G6.3*	Wald ratio	1	-0.0152	0.0184	0.985 (0.95, 1.021)	0.408	0.683	1.21E-15	TRUE
Mono NC	*RP11-386I14.4*	Wald ratio	1	-0.0208	0.0190	0.979 (0.944, 1.017)	0.273	0.567	6.49E-08	TRUE
Mono NC	*RP11-514O12.4*	Wald ratio	1	0.0052	0.0167	1.005 (0.973, 1.039)	0.755	0.888	9.45E-10	TRUE
Mono NC	*RP3-460G2.2*	Wald ratio	1	-0.0111	0.0083	0.989 (0.973, 1.005)	0.183	0.462	2.08E-39	TRUE
Mono NC	*RP3-525N10.2*	Wald ratio	1	-0.0324	0.0117	0.968 (0.946, 0.991)	0.006	0.056	2.47E-21	TRUE
Mono NC	*RPH3A*	Wald ratio	1	-0.0566	0.0155	0.945 (0.917, 0.974)	2.54E-04	0.006	1.14E-12	TRUE
Mono NC	*RPL10A*	Wald ratio	1	-0.0211	0.0160	0.979 (0.949, 1.01)	0.187	0.466	5.12E-12	TRUE
Mono NC	*RPL12*	Wald ratio	1	-0.0424	0.0119	0.958 (0.936, 0.981)	3.66E-04	0.008	2.49E-21	TRUE
Mono NC	*RPL28*	Wald ratio	1	0.0077	0.0099	1.008 (0.988, 1.027)	0.438	0.703	5.04E-28	TRUE
Mono NC	*RPL36AL*	Wald ratio	1	-0.0018	0.0123	0.998 (0.975, 1.023)	0.886	0.954	2.55E-19	TRUE
Mono NC	*RPL8*	Wald ratio	1	-0.0069	0.0069	0.993 (0.98, 1.007)	0.317	0.612	1.37E-59	TRUE
Mono NC	*RPS10*	Wald ratio	1	-0.0104	0.0102	0.99 (0.97, 1.01)	0.305	0.599	4.05E-23	TRUE
Mono NC	*RPS16*	Wald ratio	1	-0.0056	0.0062	0.994 (0.982, 1.007)	0.363	0.651	1.34E-69	TRUE
Mono NC	*RPS18*	Wald ratio	1	-0.0159	0.0122	0.984 (0.961, 1.008)	0.193	0.474	1.42E-24	TRUE
Mono NC	*RPS19*	Wald ratio	1	0.0088	0.0080	1.009 (0.993, 1.025)	0.269	0.563	1.27E-43	TRUE
Mono NC	*RPS2*	Wald ratio	1	-0.0562	0.0196	0.945 (0.91, 0.982)	0.004	0.044	3.24E-08	TRUE
Mono NC	*RPS23*	Wald ratio	1	-0.0262	0.0216	0.974 (0.934, 1.016)	0.225	0.519	7.19E-08	TRUE
Mono NC	*RPS25*	Wald ratio	1	-0.0158	0.0121	0.984 (0.961, 1.008)	0.192	0.473	1.51E-14	TRUE
Mono NC	*RPS26*	Wald ratio	1	-0.0124	0.0042	0.988 (0.979, 0.996)	0.003	0.038	5.60E-183	TRUE
Mono NC	*RPS3*	Wald ratio	1	-0.0238	0.0136	0.976 (0.951, 1.003)	0.079	0.295	6.30E-10	TRUE
Mono NC	*RPS3A*	Wald ratio	1	-0.0055	0.0082	0.994 (0.979, 1.011)	0.502	0.743	8.33E-21	TRUE
Mono NC	*RPS5*	Wald ratio	1	0.0199	0.0147	1.02 (0.991, 1.05)	0.177	0.456	2.38E-14	TRUE
Mono NC	*RPS7*	Wald ratio	1	0.0068	0.0216	1.007 (0.965, 1.05)	0.754	0.888	3.41E-10	TRUE
Mono NC	*RPS8*	Wald ratio	1	0.0051	0.0192	1.005 (0.968, 1.044)	0.791	0.909	1.35E-08	TRUE
Mono NC	*RPS9*	Wald ratio	1	0.0490	0.0128	1.05 (1.024, 1.077)	1.26E-04	0.003	6.09E-17	TRUE
Mono NC	*S100A10*	Wald ratio	1	-0.0523	0.0187	0.949 (0.915, 0.985)	0.005	0.052	9.63E-08	TRUE
Mono NC	*SECTM1*	Wald ratio	1	0.0100	0.0194	1.01 (0.972, 1.049)	0.607	0.808	1.95E-08	TRUE
Mono NC	*SH3BGRL3*	Wald ratio	1	0.0001	0.0084	1 (0.984, 1.017)	0.989	0.996	8.66E-36	TRUE
Mono NC	*SIDT2*	Wald ratio	1	-0.0450	0.0119	0.956 (0.934, 0.979)	1.53E-04	0.004	3.78E-17	TRUE
Mono NC	*SIRPB1*	Wald ratio	1	-0.0100	0.0096	0.99 (0.972, 1.009)	0.299	0.594	4.45E-34	TRUE
Mono NC	*SLC11A1*	Wald ratio	1	-0.0046	0.0197	0.995 (0.958, 1.034)	0.814	0.921	4.07E-08	TRUE
Mono NC	*SLC17A5*	Wald ratio	1	-0.0358	0.0144	0.965 (0.938, 0.992)	0.013	0.095	5.78E-16	TRUE
Mono NC	*SLC22A18*	IVW	2	0.0200	0.0120	1.02 (0.997, 1.044)	0.095	0.326	2.09E-19	TRUE
Mono NC	*SLC24A4*	Wald ratio	1	-0.0247	0.0176	0.976 (0.943, 1.01)	0.159	0.432	4.13E-09	TRUE
Mono NC	*SMDT1*	Wald ratio	1	0.0209	0.0107	1.021 (1, 1.043)	0.05	0.222	1.77E-22	TRUE
Mono NC	*SNAP29*	Wald ratio	1	0.0459	0.0211	1.047 (1.005, 1.091)	0.03	0.161	5.20E-09	TRUE
Mono NC	*SNHG8*	Wald ratio	1	-0.0039	0.0084	0.996 (0.98, 1.013)	0.647	0.834	8.62E-45	TRUE
Mono NC	*SOD1*	Wald ratio	1	0.0122	0.0173	1.012 (0.979, 1.047)	0.478	0.733	7.57E-10	TRUE
Mono NC	*SOD2*	Wald ratio	1	0.0084	0.0197	1.008 (0.97, 1.048)	0.67	0.848	4.51E-09	TRUE
Mono NC	*SQRDL*	Wald ratio	1	-0.0476	0.0219	0.953 (0.913, 0.995)	0.03	0.161	1.09E-08	TRUE
Mono NC	*SSBP3*	Wald ratio	1	0.0480	0.0197	1.049 (1.009, 1.09)	0.015	0.104	6.86E-08	TRUE
Mono NC	*ST3GAL3*	Wald ratio	1	-0.0066	0.0116	0.993 (0.971, 1.016)	0.572	0.786	3.02E-20	TRUE
Mono NC	*SULF2*	IVW	2	0.0168	0.0392	1.017 (0.942, 1.098)	0.668	0.848	1.61E-16	TRUE
Mono NC	*TCF19*	Wald ratio	1	0.0010	0.0082	1.001 (0.985, 1.017)	0.903	0.959	2.02E-35	TRUE
Mono NC	*THEMIS2*	Wald ratio	1	-0.0045	0.0127	0.996 (0.971, 1.021)	0.724	0.878	8.85E-17	TRUE
Mono NC	*TIMM10*	Wald ratio	1	-0.0116	0.0184	0.988 (0.953, 1.025)	0.53	0.758	1.15E-09	TRUE
Mono NC	*TLE4*	Wald ratio	1	-0.0003	0.0166	1 (0.968, 1.033)	0.984	0.994	4.89E-11	TRUE
Mono NC	*TMEM176A*	Wald ratio	1	-0.0192	0.0055	0.981 (0.97, 0.992)	5.05E-04	0.01	8.44E-87	TRUE
Mono NC	*TMEM176B*	Wald ratio	1	-0.0158	0.0048	0.984 (0.975, 0.994)	9.61E-04	0.016	7.41E-122	TRUE
Mono NC	*TMEM50A*	Wald ratio	1	0.0386	0.0148	1.039 (1.01, 1.07)	0.009	0.076	7.76E-14	TRUE
Mono NC	*TMPRSS3*	Wald ratio	1	-0.0102	0.0083	0.99 (0.974, 1.006)	0.218	0.51	1.12E-44	TRUE

Mono NC	*TMTC1*	Wald ratio	1	0.0117	0.0188	1.012 (0.975, 1.05)	0.535	0.761	1.25E-08	TRUE
Mono NC	*TNNI2*	Wald ratio	1	-0.0733	0.0173	0.929 (0.898, 0.961)	2.27E-05	0.001	1.11E-09	TRUE
Mono NC	*TNNT1*	Wald ratio	1	-0.0246	0.0159	0.976 (0.946, 1.007)	0.123	0.377	6.43E-11	TRUE
Mono NC	*TOMM7*	Wald ratio	1	0.0045	0.0067	1.005 (0.991, 1.018)	0.503	0.743	1.86E-55	TRUE
Mono NC	*TTC39C*	Wald ratio	1	0.0374	0.0153	1.038 (1.007, 1.07)	0.015	0.104	9.94E-11	TRUE
Mono NC	*TUFM*	Wald ratio	1	0.0221	0.0115	1.022 (1, 1.046)	0.054	0.232	1.14E-20	TRUE
Mono NC	*TYMP*	Wald ratio	1	0.0350	0.0152	1.036 (1.005, 1.067)	0.022	0.132	1.69E-12	TRUE
Mono NC	*UCP2*	Wald ratio	1	0.0044	0.0133	1.004 (0.979, 1.031)	0.739	0.881	7.12E-16	TRUE
Mono NC	*USP49*	Wald ratio	1	-0.0585	0.0173	0.943 (0.912, 0.976)	6.96E-04	0.013	1.93E-08	TRUE
Mono NC	*UTF1*	Wald ratio	1	-0.0016	0.0144	0.998 (0.971, 1.027)	0.914	0.962	7.88E-13	TRUE
Mono NC	*VARS2*	Wald ratio	1	0.0027	0.0113	1.003 (0.981, 1.025)	0.811	0.919	4.90E-16	TRUE
Mono NC	*VASH1*	Wald ratio	1	0.0034	0.0164	1.003 (0.972, 1.036)	0.838	0.932	2.08E-10	TRUE
Mono NC	*VMO1*	Wald ratio	1	-0.0004	0.0131	1 (0.974, 1.026)	0.975	0.99	2.56E-17	TRUE
Mono NC	*VNN1*	Wald ratio	1	0.0279	0.0143	1.028 (1, 1.058)	0.051	0.224	2.37E-13	TRUE
Mono NC	*VPS52*	Wald ratio	1	-0.0457	0.0154	0.955 (0.927, 0.985)	0.003	0.035	1.16E-11	TRUE
Mono NC	*VPS53*	Wald ratio	1	-0.0409	0.0117	0.96 (0.938, 0.982)	4.75E-04	0.01	6.46E-21	TRUE
Mono NC	*VSTM1*	Wald ratio	1	-0.0166	0.0085	0.984 (0.967, 1)	0.05	0.222	7.47E-38	TRUE
Mono NC	*WAC-AS1*	Wald ratio	1	0.0255	0.0168	1.026 (0.993, 1.06)	0.128	0.385	8.13E-12	TRUE
Mono NC	*XRRA1*	Wald ratio	1	-0.0101	0.0170	0.99 (0.957, 1.023)	0.55	0.769	4.17E-11	TRUE
Mono NC	*XXbac-BPG181B23.7*	IVW	2	-0.0262	0.0344	0.974 (0.911, 1.042)	0.446	0.71	1.34E-20	TRUE
Mono NC	*XXbac-BPG299F13.17*	Wald ratio	1	-0.0502	0.0189	0.951 (0.916, 0.987)	0.008	0.069	1.47E-08	TRUE
Mono NC	*YBEY*	Wald ratio	1	-0.0085	0.0162	0.992 (0.961, 1.023)	0.599	0.803	1.07E-08	TRUE
Mono NC	*ZFAS1*	Wald ratio	1	0.0083	0.0112	1.008 (0.986, 1.031)	0.458	0.719	9.99E-24	TRUE
Mono NC	*ZFP36L1*	Wald ratio	1	0.0528	0.0155	1.054 (1.023, 1.087)	6.62E-04	0.012	5.22E-12	TRUE
Mono NC	*ZKSCAN8*	Wald ratio	1	-0.0131	0.0269	0.987 (0.936, 1.04)	0.626	0.822	4.01E-10	TRUE
Mono NC	*ZNF593*	Wald ratio	1	0.0288	0.0113	1.029 (1.007, 1.052)	0.011	0.086	1.74E-21	TRUE
Mono NC	*ZNRD1-AS1*	Wald ratio	1	-0.0281	0.0143	0.972 (0.945, 1)	0.05	0.222	4.59E-13	TRUE
Mono NC	*ZP3*	Wald ratio	1	-0.0194	0.0095	0.981 (0.963, 0.999)	0.041	0.197	4.46E-36	TRUE
NK R	*AC000003.2*	Wald ratio	1	0.0056	0.0144	1.006 (0.978, 1.034)	0.696	0.864	3.05E-13	TRUE
NK R	*AC006369.2*	Wald ratio	1	-0.0057	0.0103	0.994 (0.974, 1.015)	0.579	0.789	1.12E-28	TRUE
NK R	*AC016831.7*	Wald ratio	1	-0.0336	0.0111	0.967 (0.946, 0.988)	0.003	0.031	4.69E-33	TRUE
NK R	*APOM*	Wald ratio	1	-0.0552	0.0169	0.946 (0.915, 0.978)	0.001	0.018	2.11E-11	TRUE
NK R	*ATXN1*	Wald ratio	1	-0.0176	0.0162	0.983 (0.952, 1.014)	0.277	0.571	1.69E-12	TRUE
NK R	*BIN1*	Wald ratio	1	0.0277	0.0146	1.028 (0.999, 1.058)	0.058	0.243	3.23E-13	TRUE
NK R	*BTN3A2*	Wald ratio	1	0.0288	0.0197	1.029 (0.99, 1.07)	0.143	0.411	1.89E-10	TRUE
NK R	*C11orf21*	Wald ratio	1	-0.0485	0.0207	0.953 (0.915, 0.992)	0.019	0.123	3.23E-09	TRUE
NK R	*C19orf83*	Wald ratio	1	-0.0407	0.0186	0.96 (0.926, 0.996)	0.029	0.159	4.63E-09	TRUE
NK R	*C6orf136*	Wald ratio	1	0.0187	0.0161	1.019 (0.987, 1.051)	0.245	0.538	1.57E-11	TRUE
NK R	*CAPG*	Wald ratio	1	-0.0188	0.0195	0.981 (0.945, 1.02)	0.336	0.629	5.33E-11	TRUE
NK R	*CCHCR1*	Wald ratio	1	0.0228	0.0118	1.023 (1, 1.047)	0.052	0.227	1.31E-25	TRUE
NK R	*CCT8*	Wald ratio	1	-0.0020	0.0203	0.998 (0.959, 1.038)	0.922	0.965	3.42E-12	TRUE
NK R	*CD3E*	Wald ratio	1	-0.0139	0.0062	0.986 (0.974, 0.998)	0.024	0.143	4.27E-75	TRUE
NK R	*CD52*	Wald ratio	1	-0.0007	0.0173	0.999 (0.966, 1.034)	0.969	0.989	1.02E-09	TRUE
NK R	*CDC42*	Wald ratio	1	0.0306	0.0198	1.031 (0.992, 1.072)	0.123	0.377	3.66E-08	TRUE
NK R	*CHI3L2*	Wald ratio	1	-0.0119	0.0155	0.988 (0.959, 1.019)	0.444	0.709	6.54E-13	TRUE
NK R	*CLIC3*	Wald ratio	1	0.0098	0.0188	1.01 (0.973, 1.048)	0.601	0.805	2.48E-09	TRUE
NK R	*COMMD6*	Wald ratio	1	-0.0019	0.0165	0.998 (0.966, 1.031)	0.907	0.961	5.18E-12	TRUE
NK R	*CST3*	Wald ratio	1	-0.0028	0.0201	0.997 (0.959, 1.037)	0.887	0.955	5.08E-08	TRUE
NK R	*CSTB*	Wald ratio	1	0.0052	0.0150	1.005 (0.976, 1.035)	0.73	0.88	2.86E-12	TRUE
NK R	*CTD-2368P22.1*	Wald ratio	1	0.0012	0.0145	1.001 (0.973, 1.03)	0.936	0.974	1.40E-14	TRUE
NK R	*CTSW*	Wald ratio	1	-0.0728	0.0186	0.93 (0.897, 0.964)	8.96E-05	0.003	1.91E-10	TRUE
NK R	*CYTL1*	Wald ratio	1	-0.0290	0.0142	0.971 (0.945, 0.999)	0.042	0.199	1.92E-19	TRUE
NK R	*DCBLD1*	Wald ratio	1	-0.0102	0.0104	0.99 (0.97, 1.01)	0.327	0.62	7.81E-23	TRUE
NK R	*DCXR*	Wald ratio	1	0.0101	0.0205	1.01 (0.97, 1.052)	0.62	0.819	1.54E-09	TRUE
NK R	*DHX16*	Wald ratio	1	-0.0320	0.0177	0.968 (0.935, 1.003)	0.071	0.275	3.32E-09	TRUE
NK R	*DIP2A*	Wald ratio	1	0.0132	0.0112	1.013 (0.991, 1.036)	0.239	0.533	9.19E-24	TRUE
NK R	*DNAJC15*	Wald ratio	1	0.0200	0.0194	1.02 (0.982, 1.06)	0.303	0.598	2.11E-08	TRUE
NK R	*EIF3G*	Wald ratio	1	-0.0062	0.0117	0.994 (0.971, 1.017)	0.595	0.801	5.14E-22	TRUE
NK R	*EIF5A*	Wald ratio	1	-0.0097	0.0065	0.99 (0.978, 1.003)	0.134	0.394	7.12E-79	TRUE
NK R	*ERAP2*	Wald ratio	1	0.0504	0.0113	1.052 (1.029, 1.075)	8.07E-06	4.89E-04	8.09E-23	TRUE
NK R	*FAM118A*	Wald ratio	1	0.0011	0.0093	1.001 (0.983, 1.02)	0.902	0.959	6.31E-25	TRUE
NK R	*FAM184B*	Wald ratio	1	0.0092	0.0111	1.009 (0.988, 1.031)	0.404	0.681	6.84E-24	TRUE
NK R	*FAM26F*	Wald ratio	1	-0.0127	0.0200	0.987 (0.949, 1.027)	0.525	0.755	3.52E-08	TRUE
NK R	*FCER1G*	Wald ratio	1	0.0156	0.0188	1.016 (0.979, 1.054)	0.405	0.681	8.51E-10	TRUE
NK R	*FCRL3*	Wald ratio	1	-0.0184	0.0174	0.982 (0.949, 1.016)	0.29	0.583	3.09E-10	TRUE
NK R	*FGFR1OP*	Wald ratio	1	0.0164	0.0113	1.017 (0.994, 1.039)	0.148	0.417	1.61E-20	TRUE
NK R	*GCC2*	Wald ratio	1	0.0129	0.0167	1.013 (0.98, 1.047)	0.44	0.704	3.22E-12	TRUE
NK R	*GNLY*	Wald ratio	1	-0.0109	0.0095	0.989 (0.971, 1.008)	0.25	0.543	2.53E-34	TRUE
NK R	*HIST1H2BC*	Wald ratio	1	-0.0153	0.0178	0.985 (0.951, 1.02)	0.39	0.671	9.27E-18	TRUE
NK R	*HIST1H4E*	Wald ratio	1	0.0002	0.0120	1 (0.977, 1.024)	0.988	0.996	4.79E-25	TRUE
NK R	*HLA-B*	IVW	2	0.0192	0.0167	1.019 (0.987, 1.053)	0.25	0.543	3.95E-59	TRUE
NK R	*HLA-C*	Wald ratio	1	-0.0363	0.0125	0.964 (0.941, 0.988)	0.004	0.039	2.13E-12	TRUE
NK R	*HLA-DQA2*	IVW	2	0.0488	0.0134	1.05 (1.023, 1.078)	2.83E-04	0.007	2.15E-19	TRUE
NK R	*HLA-DRB1*	Wald ratio	1	-0.0257	0.0196	0.975 (0.938, 1.013)	0.189	0.47	3.18E-08	TRUE
NK R	*HLA-G*	IVW	2	-0.0071	0.0105	0.993 (0.973, 1.014)	0.499	0.741	7.08E-42	TRUE
NK R	*HMGN4*	Wald ratio	1	0.0154	0.0146	1.016 (0.987, 1.045)	0.292	0.586	3.47E-25	TRUE
NK R	*HSF2*	Wald ratio	1	0.0076	0.0091	1.008 (0.99, 1.026)	0.403	0.681	5.56E-32	TRUE
NK R	*HSP90AA1*	Wald ratio	1	-0.0218	0.0212	0.978 (0.939, 1.02)	0.302	0.597	3.06E-08	TRUE
NK R	*HSPA1B*	Wald ratio	1	0.0154	0.0111	1.016 (0.994, 1.038)	0.164	0.438	3.08E-32	TRUE

NK R	*IFITM2*	Wald ratio	1	-0.0056	0.0152	0.994 (0.965, 1.025)	0.713	0.872	9.90E-13	TRUE
NK R	*IFITM3*	Wald ratio	1	0.0049	0.0088	1.005 (0.988, 1.022)	0.573	0.786	5.60E-38	TRUE
NK R	*IL32*	Wald ratio	1	-0.0208	0.0202	0.979 (0.941, 1.019)	0.303	0.598	3.74E-14	TRUE
NK R	*ITGB2*	Wald ratio	1	-0.0010	0.0077	0.999 (0.984, 1.014)	0.897	0.957	1.94E-41	TRUE
NK R	*ITPR3*	Wald ratio	1	-0.0143	0.0191	0.986 (0.95, 1.024)	0.456	0.719	3.29E-10	TRUE
NK R	*KANSL1-AS1*	Wald ratio	1	-0.0402	0.0170	0.961 (0.929, 0.993)	0.018	0.12	2.51E-23	TRUE
NK R	*KLRC2*	Wald ratio	1	-0.0075	0.0125	0.993 (0.968, 1.017)	0.548	0.769	2.58E-19	TRUE
NK R	*KLRD1*	Wald ratio	1	-0.0253	0.0186	0.975 (0.94, 1.011)	0.174	0.452	9.22E-09	TRUE
NK R	*LY6G5C*	Wald ratio	1	0.0256	0.0162	1.026 (0.994, 1.059)	0.114	0.361	1.77E-15	TRUE
NK R	*LYAR*	Wald ratio	1	-0.0148	0.0111	0.985 (0.964, 1.007)	0.182	0.462	6.25E-24	TRUE
NK R	*MAP3K5*	Wald ratio	1	-0.0293	0.0088	0.971 (0.955, 0.988)	8.91E-04	0.016	1.25E-36	TRUE
NK R	*MBOAT1*	Wald ratio	1	-0.0132	0.0102	0.987 (0.967, 1.007)	0.199	0.482	4.68E-28	TRUE
NK R	*MICA*	IVW	2	0.0231	0.0660	1.023 (0.899, 1.165)	0.727	0.878	1.15E-25	TRUE
NK R	*MORC2*	Wald ratio	1	-0.0199	0.0063	0.98 (0.968, 0.992)	0.001	0.021	4.14E-74	TRUE
NK R	*MYOM2*	Wald ratio	1	-0.0177	0.0126	0.982 (0.959, 1.007)	0.16	0.433	5.32E-17	TRUE
NK R	*NSA2*	Wald ratio	1	0.0459	0.0200	1.047 (1.007, 1.089)	0.022	0.134	8.60E-09	TRUE
NK R	*NT5C3B*	Wald ratio	1	-0.0126	0.0150	0.987 (0.959, 1.017)	0.4	0.679	2.70E-11	TRUE
NK R	*OSTF1*	Wald ratio	1	-0.0099	0.0223	0.99 (0.948, 1.034)	0.655	0.839	1.52E-08	TRUE
NK R	*PABPC1*	Wald ratio	1	-0.0255	0.0182	0.975 (0.941, 1.01)	0.161	0.434	1.93E-10	TRUE
NK R	*PILRB*	Wald ratio	1	0.0114	0.0158	1.012 (0.981, 1.043)	0.468	0.726	1.19E-11	TRUE
NK R	*PPDPF*	Wald ratio	1	-0.0129	0.0120	0.987 (0.964, 1.011)	0.283	0.575	3.18E-18	TRUE
NK R	*PRMT2*	Wald ratio	1	0.0145	0.0123	1.015 (0.991, 1.039)	0.236	0.53	4.51E-20	TRUE
NK R	*PRR7-AS1*	Wald ratio	1	-0.0187	0.0094	0.982 (0.964, 1)	0.048	0.215	1.06E-32	TRUE
NK R	*PSMD5-AS1*	Wald ratio	1	0.0074	0.0094	1.007 (0.989, 1.026)	0.434	0.702	2.86E-32	TRUE
NK R	*PTGDR*	Wald ratio	1	0.0302	0.0150	1.031 (1.001, 1.061)	0.044	0.206	6.95E-13	TRUE
NK R	*QRSL1*	Wald ratio	1	-0.0074	0.0184	0.993 (0.958, 1.029)	0.687	0.858	6.90E-10	TRUE
NK R	*RAC1*	Wald ratio	1	-0.0697	0.0191	0.933 (0.898, 0.968)	2.66E-04	0.006	3.27E-08	TRUE
NK R	*RAMP1*	IVW	2	-0.0007	0.0207	0.999 (0.96, 1.041)	0.973	0.989	4.43E-21	TRUE
NK R	*RP1-3J17.3*	Wald ratio	1	0.0310	0.0187	1.031 (0.994, 1.07)	0.098	0.333	9.65E-12	TRUE
NK R	*RP11-291B21.2*	Wald ratio	1	-0.0145	0.0109	0.986 (0.965, 1.007)	0.185	0.465	1.85E-23	TRUE
NK R	*RP11-326C3.11*	Wald ratio	1	-0.0123	0.0172	0.988 (0.955, 1.022)	0.474	0.73	2.95E-11	TRUE
NK R	*RP11-347P5.1*	Wald ratio	1	0.0033	0.0151	1.003 (0.974, 1.034)	0.826	0.927	2.47E-13	TRUE
NK R	*RP11-81H14.2*	Wald ratio	1	0.0171	0.0154	1.017 (0.987, 1.048)	0.266	0.561	1.28E-13	TRUE
NK R	*RPL10A*	Wald ratio	1	-0.0169	0.0190	0.983 (0.947, 1.02)	0.372	0.661	3.94E-09	TRUE
NK R	*RPL12*	Wald ratio	1	-0.0390	0.0114	0.962 (0.94, 0.983)	6.21E-04	0.012	4.78E-24	TRUE
NK R	*RPL13*	Wald ratio	1	-0.0501	0.0244	0.951 (0.907, 0.998)	0.04	0.195	1.16E-09	TRUE
NK R	*RPL28*	Wald ratio	1	0.0050	0.0064	1.005 (0.992, 1.018)	0.438	0.703	8.55E-70	TRUE
NK R	*RPL36AL*	Wald ratio	1	-0.0088	0.0137	0.991 (0.965, 1.018)	0.524	0.754	2.25E-16	TRUE
NK R	*RPL8*	Wald ratio	1	-0.0083	0.0080	0.992 (0.976, 1.008)	0.303	0.598	1.50E-44	TRUE
NK R	*RPLP2*	Wald ratio	1	-0.0275	0.0199	0.973 (0.936, 1.012)	0.166	0.442	7.17E-08	TRUE
NK R	*RPS10*	Wald ratio	1	-0.0096	0.0087	0.99 (0.974, 1.007)	0.271	0.565	1.12E-32	TRUE
NK R	*RPS18*	Wald ratio	1	-0.0142	0.0083	0.986 (0.97, 1.002)	0.087	0.312	2.48E-51	TRUE
NK R	*RPS2*	Wald ratio	1	-0.0495	0.0185	0.952 (0.918, 0.987)	0.007	0.066	6.88E-09	TRUE
NK R	*RPS26*	Wald ratio	1	-0.0101	0.0044	0.99 (0.981, 0.999)	0.022	0.132	3.02E-178	TRUE
NK R	*RPS3A*	Wald ratio	1	-0.0059	0.0085	0.994 (0.978, 1.011)	0.492	0.738	1.53E-19	TRUE
NK R	*RPS5*	Wald ratio	1	0.0142	0.0105	1.014 (0.994, 1.036)	0.177	0.456	6.70E-28	TRUE
NK R	*RPS7*	Wald ratio	1	0.0003	0.0224	1 (0.957, 1.045)	0.99	0.996	1.27E-10	TRUE
NK R	*RPS8*	Wald ratio	1	0.0038	0.0147	1.004 (0.975, 1.033)	0.794	0.911	3.61E-14	TRUE
NK R	*RPS9*	Wald ratio	1	0.0345	0.0111	1.035 (1.013, 1.058)	0.002	0.025	1.38E-21	TRUE
NK R	*RPSA*	Wald ratio	1	-0.0478	0.0234	0.953 (0.911, 0.998)	0.041	0.197	9.25E-08	TRUE
NK R	*S100A13*	Wald ratio	1	-0.0328	0.0147	0.968 (0.94, 0.996)	0.026	0.15	1.35E-13	TRUE
NK R	*S100A4*	Wald ratio	1	0.0009	0.0198	1.001 (0.963, 1.04)	0.963	0.988	4.47E-10	TRUE
NK R	*S100B*	Wald ratio	1	-0.0040	0.0132	0.996 (0.97, 1.022)	0.762	0.893	4.66E-18	TRUE
NK R	*SMDT1*	Wald ratio	1	0.0197	0.0098	1.02 (1, 1.04)	0.045	0.208	9.70E-28	TRUE
NK R	*SNHG8*	Wald ratio	1	-0.0050	0.0110	0.995 (0.974, 1.017)	0.647	0.834	3.17E-27	TRUE
NK R	*SPINT2*	Wald ratio	1	-0.0212	0.0212	0.979 (0.939, 1.021)	0.318	0.614	4.87E-08	TRUE
NK R	*SYNGAP1*	Wald ratio	1	-0.0601	0.0132	0.942 (0.918, 0.966)	5.62E-06	3.66E-04	3.04E-16	TRUE
NK R	*SYNGR1*	Wald ratio	1	0.0191	0.0104	1.019 (0.999, 1.04)	0.067	0.265	1.06E-18	TRUE
NK R	*TCF19*	IVW	2	0.0183	0.0196	1.018 (0.98, 1.058)	0.349	0.642	3.87E-31	TRUE
NK R	*TCIRG1*	Wald ratio	1	-0.0460	0.0172	0.955 (0.923, 0.988)	0.007	0.066	5.62E-09	TRUE
NK R	*THEMIS2*	Wald ratio	1	0.0116	0.0124	1.012 (0.988, 1.037)	0.346	0.639	2.67E-19	TRUE
NK R	*TIMM10*	Wald ratio	1	-0.0031	0.0196	0.997 (0.959, 1.036)	0.875	0.948	5.42E-10	TRUE
NK R	*TJAP1*	Wald ratio	1	-0.0246	0.0201	0.976 (0.938, 1.015)	0.221	0.513	4.27E-08	TRUE
NK R	*TOMM7*	Wald ratio	1	0.0056	0.0083	1.006 (0.989, 1.022)	0.503	0.743	1.76E-37	TRUE
NK R	*TSPO*	Wald ratio	1	-0.0008	0.0199	0.999 (0.961, 1.039)	0.968	0.989	2.89E-08	TRUE
NK R	*UCP2*	Wald ratio	1	0.0075	0.0161	1.008 (0.976, 1.04)	0.64	0.83	1.74E-11	TRUE
NK R	*VPS52*	Wald ratio	1	0.0093	0.0118	1.009 (0.986, 1.033)	0.431	0.7	2.82E-23	TRUE
NK R	*WRNIP1*	Wald ratio	1	0.0315	0.0204	1.032 (0.992, 1.074)	0.123	0.377	5.42E-08	TRUE
NK R	*XRRA1*	Wald ratio	1	-0.0198	0.0171	0.98 (0.948, 1.014)	0.247	0.541	5.38E-10	TRUE
NK R	*XXbac-BPG299F13.17*	Wald ratio	1	-0.0718	0.0176	0.931 (0.899, 0.963)	4.59E-05	0.002	1.02E-09	TRUE
NK R	*YBEY*	Wald ratio	1	0.0025	0.0186	1.003 (0.967, 1.04)	0.893	0.957	2.02E-09	TRUE
NK R	*ZBTB22*	Wald ratio	1	-0.0333	0.0102	0.967 (0.948, 0.987)	0.001	0.017	8.35E-18	TRUE
NK R	*ZKSCAN8*	Wald ratio	1	-0.0597	0.0171	0.942 (0.911, 0.974)	4.61E-04	0.01	6.51E-11	TRUE
NK R	*ZNF667-AS1*	Wald ratio	1	0.0002	0.0203	1 (0.961, 1.041)	0.994	0.998	5.84E-09	TRUE
NK R	*ZNRD1-AS1*	IVW	2	0.0189	0.0094	1.019 (1, 1.038)	0.045	0.209	6.61E-61	TRUE
NK R	*ZSCAN16*	Wald ratio	1	0.0228	0.0274	1.023 (0.97, 1.079)	0.404	0.681	3.04E-08	TRUE
NK	*ABR*	Wald ratio	1	-0.0616	0.0200	0.94 (0.904, 0.978)	0.002	0.026	1.06E-07	TRUE
NK	*ABRACL*	Wald ratio	1	-0.0211	0.0210	0.979 (0.94, 1.02)	0.314	0.608	8.77E-09	TRUE
NK	*AC000003.2*	Wald ratio	1	0.0154	0.0102	1.015 (0.995, 1.036)	0.133	0.394	5.66E-27	TRUE

NK	*AC005037.3*	Wald ratio	1	-0.0142	0.0168	0.986 (0.954, 1.019)	0.398	0.678	2.99E-12	TRUE
NK	*AC005082.12*	Wald ratio	1	0.0021	0.0089	1.002 (0.985, 1.02)	0.816	0.921	1.63E-36	TRUE
NK	*AC005253.2*	Wald ratio	1	0.0062	0.0131	1.006 (0.981, 1.032)	0.635	0.827	4.20E-18	TRUE
NK	*AC006369.2*	Wald ratio	1	0.0064	0.0060	1.006 (0.995, 1.018)	0.283	0.575	1.52E-88	TRUE
NK	*AC007383.4*	Wald ratio	1	-0.0017	0.0078	0.998 (0.983, 1.014)	0.822	0.926	2.51E-47	TRUE
NK	*AC009120.10*	Wald ratio	1	0.0235	0.0092	1.024 (1.006, 1.042)	0.01	0.084	4.64E-31	TRUE
NK	*AC010642.1*	Wald ratio	1	0.0199	0.0207	1.02 (0.98, 1.062)	0.336	0.628	6.12E-12	TRUE
NK	*AC016757.3*	Wald ratio	1	-0.0221	0.0129	0.978 (0.954, 1.003)	0.087	0.312	3.89E-19	TRUE
NK	*AC021860.1*	Wald ratio	1	0.0330	0.0147	1.034 (1.004, 1.064)	0.025	0.145	2.03E-16	TRUE
NK	*AC022498.1*	Wald ratio	1	-0.0050	0.0187	0.995 (0.959, 1.032)	0.789	0.909	2.82E-13	TRUE
NK	*AC023590.1*	Wald ratio	1	-0.0082	0.0172	0.992 (0.959, 1.026)	0.633	0.826	2.40E-15	TRUE
NK	*AC026202.1*	Wald ratio	1	-0.0072	0.0069	0.993 (0.979, 1.006)	0.297	0.591	2.30E-73	TRUE
NK	*AC067959.1*	Wald ratio	1	-0.0073	0.0081	0.993 (0.977, 1.009)	0.364	0.653	6.20E-34	TRUE
NK	*AC069277.2*	Wald ratio	1	0.0218	0.0108	1.022 (1.001, 1.044)	0.044	0.207	6.88E-43	TRUE
NK	*AC090186.1*	Wald ratio	1	-0.0318	0.0125	0.969 (0.945, 0.993)	0.011	0.088	6.06E-16	TRUE
NK	*AC091729.9*	Wald ratio	1	-0.0366	0.0177	0.964 (0.931, 0.998)	0.038	0.189	5.87E-10	TRUE
NK	*AC093157.1*	Wald ratio	1	0.0208	0.0166	1.021 (0.988, 1.055)	0.21	0.497	2.44E-11	TRUE
NK	*AC100830.3*	Wald ratio	1	0.0378	0.0178	1.039 (1.003, 1.075)	0.034	0.175	1.60E-12	TRUE
NK	*AC104653.1*	Wald ratio	1	0.0122	0.0099	1.012 (0.993, 1.032)	0.218	0.51	2.35E-30	TRUE
NK	*AC132186.1*	Wald ratio	1	-0.0059	0.0095	0.994 (0.976, 1.013)	0.538	0.762	1.14E-28	TRUE
NK	*ACAA2*	Wald ratio	1	-0.0166	0.0143	0.983 (0.956, 1.011)	0.245	0.538	1.83E-15	TRUE
NK	*ACAP1*	Wald ratio	1	0.0148	0.0212	1.015 (0.974, 1.058)	0.485	0.736	2.81E-08	TRUE
NK	*ACCS*	Wald ratio	1	-0.0035	0.0183	0.996 (0.961, 1.033)	0.847	0.935	4.07E-11	TRUE
NK	*ACOT4*	Wald ratio	1	0.0248	0.0158	1.025 (0.994, 1.057)	0.115	0.362	1.83E-12	TRUE
NK	*ACTA2*	Wald ratio	1	0.0091	0.0116	1.009 (0.986, 1.032)	0.433	0.701	3.98E-22	TRUE
NK	*ACTN4*	Wald ratio	1	0.0567	0.0277	1.058 (1.002, 1.117)	0.041	0.196	1.95E-08	TRUE
NK	*ACYP2*	Wald ratio	1	0.0061	0.0073	1.006 (0.992, 1.021)	0.4	0.679	4.55E-53	TRUE
NK	*ADAM10*	Wald ratio	1	-0.0516	0.0184	0.95 (0.916, 0.985)	0.005	0.051	9.20E-17	TRUE
NK	*ADARB2*	Wald ratio	1	-0.0089	0.0084	0.991 (0.975, 1.008)	0.291	0.583	7.44E-42	TRUE
NK	*ADCY7*	Wald ratio	1	-0.0385	0.0158	0.962 (0.933, 0.992)	0.015	0.104	5.44E-13	TRUE
NK	*ADCY9*	Wald ratio	1	-0.0153	0.0112	0.985 (0.963, 1.007)	0.172	0.45	7.93E-22	TRUE
NK	*ADHFE1*	Wald ratio	1	-0.0399	0.0153	0.961 (0.933, 0.99)	0.009	0.076	5.84E-13	TRUE
NK	*ADRB1*	Wald ratio	1	-0.0448	0.0115	0.956 (0.935, 0.978)	9.15E-05	0.003	4.18E-17	TRUE
NK	*AE000662.92*	Wald ratio	1	-0.0470	0.0198	0.954 (0.918, 0.992)	0.017	0.116	5.70E-08	TRUE
NK	*AGA*	Wald ratio	1	-0.0281	0.0174	0.972 (0.94, 1.006)	0.106	0.346	4.78E-10	TRUE
NK	*AGPAT2*	Wald ratio	1	-0.0187	0.0166	0.981 (0.95, 1.014)	0.26	0.554	3.07E-19	TRUE
NK	*AHI1*	Wald ratio	1	-0.0143	0.0091	0.986 (0.968, 1.004)	0.116	0.362	8.21E-36	TRUE
NK	*AHNAK*	Wald ratio	1	0.0352	0.0240	1.036 (0.988, 1.086)	0.143	0.41	7.08E-08	TRUE
NK	*AK5*	Wald ratio	1	0.0019	0.0052	1.002 (0.992, 1.012)	0.718	0.874	6.64E-121	TRUE
NK	*AKAP8*	Wald ratio	1	0.0225	0.0195	1.023 (0.984, 1.063)	0.248	0.541	2.87E-09	TRUE
NK	*AKR1C3*	Wald ratio	1	-0.0236	0.0137	0.977 (0.951, 1.003)	0.086	0.311	1.78E-15	TRUE
NK	*AL359091.2*	Wald ratio	1	0.0376	0.0205	1.038 (0.997, 1.081)	0.066	0.263	4.61E-08	TRUE
NK	*ALDH16A1*	Wald ratio	1	0.0238	0.0178	1.024 (0.989, 1.06)	0.182	0.462	1.43E-08	TRUE
NK	*ALG1L*	Wald ratio	1	-0.0099	0.0114	0.99 (0.968, 1.012)	0.383	0.667	7.02E-20	TRUE
NK	*AMICA1*	Wald ratio	1	0.0036	0.0135	1.004 (0.977, 1.031)	0.787	0.909	3.66E-15	TRUE
NK	*ANAPC13*	Wald ratio	1	-0.0133	0.0159	0.987 (0.957, 1.018)	0.403	0.681	4.16E-13	TRUE
NK	*ANKDD1A*	Wald ratio	1	0.0066	0.0168	1.007 (0.974, 1.04)	0.696	0.864	8.61E-11	TRUE
NK	*ANXA11*	Wald ratio	1	-0.0124	0.0150	0.988 (0.959, 1.017)	0.407	0.682	1.21E-16	TRUE
NK	*ANXA2*	Wald ratio	1	-0.0415	0.0169	0.959 (0.928, 0.992)	0.014	0.101	2.61E-14	TRUE
NK	*ANXA2R*	Wald ratio	1	0.0040	0.0178	1.004 (0.97, 1.04)	0.823	0.926	1.60E-12	TRUE
NK	*ANXA5*	Wald ratio	1	-0.0051	0.0139	0.995 (0.968, 1.022)	0.714	0.872	1.78E-25	TRUE
NK	*ANXA6*	Wald ratio	1	0.0006	0.0109	1.001 (0.979, 1.022)	0.958	0.985	1.43E-20	TRUE
NK	*AOAH*	IVW	2	-0.0233	0.0154	0.977 (0.948, 1.007)	0.131	0.392	2.35E-44	TRUE
NK	*AP001055.6*	Wald ratio	1	0.0432	0.0212	1.044 (1.002, 1.089)	0.042	0.2	1.02E-08	TRUE
NK	*AP003774.1*	Wald ratio	1	-0.0220	0.0067	0.978 (0.966, 0.991)	9.48E-04	0.016	5.04E-63	TRUE
NK	*AP2A1*	Wald ratio	1	0.0017	0.0116	1.002 (0.979, 1.025)	0.884	0.953	2.71E-12	TRUE
NK	*AP2A2*	Wald ratio	1	0.0441	0.0113	1.045 (1.022, 1.068)	9.29E-05	0.003	2.87E-22	TRUE
NK	*APC2*	Wald ratio	1	0.0018	0.0096	1.002 (0.983, 1.021)	0.848	0.935	1.57E-37	TRUE
NK	*APMAP*	Wald ratio	1	0.0153	0.0229	1.015 (0.971, 1.062)	0.503	0.744	1.60E-12	TRUE
NK	*APOBEC3G*	Wald ratio	1	0.0098	0.0118	1.01 (0.987, 1.033)	0.407	0.682	8.80E-22	TRUE
NK	*APOBEC3H*	Wald ratio	1	0.0138	0.0155	1.014 (0.984, 1.045)	0.372	0.661	2.87E-12	TRUE
NK	*ARHGAP15*	Wald ratio	1	0.0124	0.0219	1.012 (0.97, 1.057)	0.573	0.786	2.75E-09	TRUE
NK	*ARHGEF10*	Wald ratio	1	0.0067	0.0133	1.007 (0.981, 1.033)	0.616	0.816	4.05E-11	TRUE
NK	*ARHGEF3*	Wald ratio	1	-0.0020	0.0109	0.998 (0.977, 1.019)	0.85	0.936	3.58E-22	TRUE
NK	*ARL16*	Wald ratio	1	0.0113	0.0093	1.011 (0.993, 1.03)	0.222	0.514	5.32E-32	TRUE
NK	*ARPC4*	Wald ratio	1	0.0213	0.0137	1.021 (0.994, 1.049)	0.12	0.37	1.72E-18	TRUE
NK	*ARPC5*	Wald ratio	1	-0.0064	0.0147	0.994 (0.965, 1.023)	0.663	0.845	2.14E-13	TRUE
NK	*ARSA*	Wald ratio	1	0.0170	0.0169	1.017 (0.984, 1.051)	0.314	0.608	2.50E-19	TRUE
NK	*ASCL2*	Wald ratio	1	-0.0150	0.0115	0.985 (0.963, 1.008)	0.193	0.474	3.44E-22	TRUE
NK	*ASNSD1*	Wald ratio	1	-0.0120	0.0160	0.988 (0.958, 1.02)	0.454	0.717	4.58E-11	TRUE
NK	*ATG10*	Wald ratio	1	0.0307	0.0166	1.031 (0.998, 1.065)	0.064	0.258	1.46E-12	TRUE
NK	*ATHL1*	Wald ratio	1	-0.0029	0.0093	0.997 (0.979, 1.015)	0.756	0.888	4.87E-40	TRUE
NK	*ATIC*	Wald ratio	1	-0.0112	0.0174	0.989 (0.956, 1.023)	0.52	0.753	3.46E-09	TRUE
NK	*ATP5D*	Wald ratio	1	-0.0536	0.0193	0.948 (0.913, 0.984)	0.005	0.053	2.70E-08	TRUE
NK	*ATP5G1*	Wald ratio	1	-0.0764	0.0148	0.926 (0.9, 0.954)	2.64E-07	8.12E-05	2.87E-13	TRUE
NK	*ATP5G3*	Wald ratio	1	-0.0052	0.0210	0.995 (0.955, 1.037)	0.804	0.916	1.20E-09	TRUE
NK	*ATP6AP1L*	Wald ratio	1	0.0243	0.0161	1.025 (0.993, 1.058)	0.131	0.392	4.63E-12	TRUE
NK	*ATP6V1G2*	Wald ratio	1	-0.0497	0.0108	0.952 (0.932, 0.972)	3.87E-06	2.79E-04	2.12E-27	TRUE
NK	*ATP9A*	Wald ratio	1	0.0080	0.0097	1.008 (0.989, 1.027)	0.41	0.685	6.62E-31	TRUE

NK	*ATP9B*	Wald ratio	1	-0.0302	0.0188	0.97 (0.935, 1.007)	0.107	0.348	5.04E-09	TRUE
NK	*B3GAT1*	Wald ratio	1	-0.0216	0.0192	0.979 (0.942, 1.016)	0.26	0.554	2.09E-10	TRUE
NK	*B3GAT2*	Wald ratio	1	0.0309	0.0236	1.031 (0.985, 1.08)	0.191	0.471	5.62E-09	TRUE
NK	*B3GNT7*	Wald ratio	1	-0.0157	0.0147	0.984 (0.957, 1.013)	0.285	0.577	1.75E-11	TRUE
NK	*B4GALNT3*	Wald ratio	1	0.0067	0.0085	1.007 (0.99, 1.024)	0.432	0.701	1.62E-42	TRUE
NK	*B4GALT1*	Wald ratio	1	-0.0241	0.0146	0.976 (0.949, 1.005)	0.1	0.334	3.65E-17	TRUE
NK	*B4GALT4*	Wald ratio	1	-0.0296	0.0182	0.971 (0.937, 1.006)	0.104	0.342	7.89E-12	TRUE
NK	*BIN1*	Wald ratio	1	0.0110	0.0057	1.011 (1, 1.022)	0.052	0.226	4.38E-86	TRUE
NK	*BOK*	Wald ratio	1	-0.0281	0.0133	0.972 (0.947, 0.998)	0.035	0.176	5.49E-28	TRUE
NK	*BRD7*	Wald ratio	1	-0.0342	0.0140	0.966 (0.94, 0.993)	0.015	0.104	3.98E-16	TRUE
NK	*BRK1*	Wald ratio	1	-0.0255	0.0177	0.975 (0.942, 1.009)	0.148	0.417	9.79E-13	TRUE
NK	*BSPRY*	Wald ratio	1	-0.0037	0.0189	0.996 (0.96, 1.034)	0.846	0.935	1.12E-12	TRUE
NK	*BST1*	Wald ratio	1	-0.0228	0.0157	0.977 (0.948, 1.008)	0.146	0.415	1.74E-13	TRUE
NK	*BTBD9*	Wald ratio	1	0.0086	0.0126	1.009 (0.984, 1.034)	0.493	0.739	5.24E-16	TRUE
NK	*BTN2A1*	Wald ratio	1	0.0406	0.0157	1.041 (1.01, 1.074)	0.01	0.079	4.72E-13	TRUE
NK	*BTN3A1*	Wald ratio	1	0.0833	0.0188	1.087 (1.048, 1.128)	9.36E-06	5.39E-04	6.25E-08	TRUE
NK	*BTN3A2*	Wald ratio	1	0.0125	0.0087	1.013 (0.995, 1.03)	0.153	0.423	4.59E-53	TRUE
NK	*C10orf128*	IVW	2	0.0006	0.0075	1.001 (0.986, 1.015)	0.933	0.972	2.44E-68	TRUE
NK	*C10orf32*	Wald ratio	1	-0.0582	0.0134	0.943 (0.919, 0.969)	1.40E-05	7.21E-04	4.52E-17	TRUE
NK	*C10orf54*	Wald ratio	1	0.0121	0.0130	1.012 (0.987, 1.038)	0.353	0.644	1.54E-18	TRUE
NK	*C10orf95*	Wald ratio	1	-0.0482	0.0233	0.953 (0.91, 0.997)	0.039	0.19	6.27E-08	TRUE
NK	*C11orf21*	Wald ratio	1	-0.0314	0.0134	0.969 (0.944, 0.995)	0.019	0.123	1.56E-20	TRUE
NK	*C11orf24*	Wald ratio	1	0.0000	0.0113	1 (0.978, 1.022)	0.997	0.999	5.72E-23	TRUE
NK	*C14orf2*	Wald ratio	1	0.0322	0.0181	1.033 (0.997, 1.07)	0.075	0.287	8.40E-10	TRUE
NK	*C16orf13*	Wald ratio	1	0.0000	0.0118	1 (0.977, 1.023)	0.997	0.999	4.09E-15	TRUE
NK	*C17orf49*	Wald ratio	1	-0.0074	0.0101	0.993 (0.973, 1.012)	0.461	0.721	8.68E-14	TRUE
NK	*C17orf89*	Wald ratio	1	0.0340	0.0117	1.035 (1.011, 1.059)	0.004	0.041	3.27E-21	TRUE
NK	*C17orf97*	Wald ratio	1	-0.0114	0.0095	0.989 (0.97, 1.007)	0.23	0.524	1.18E-31	TRUE
NK	*C19orf24*	Wald ratio	1	0.0439	0.0201	1.045 (1.004, 1.087)	0.029	0.16	2.27E-09	TRUE
NK	*C19orf66*	Wald ratio	1	-0.0126	0.0203	0.988 (0.949, 1.028)	0.537	0.762	3.33E-08	TRUE
NK	*C19orf83*	Wald ratio	1	-0.0172	0.0079	0.983 (0.968, 0.998)	0.029	0.159	3.22E-46	TRUE
NK	*C1orf173*	Wald ratio	1	-0.0202	0.0155	0.98 (0.951, 1.01)	0.192	0.473	3.80E-12	TRUE
NK	*C1orf86*	Wald ratio	1	0.0032	0.0157	1.003 (0.973, 1.034)	0.84	0.932	1.47E-12	TRUE
NK	*C2*	Wald ratio	1	0.0125	0.0106	1.013 (0.992, 1.034)	0.238	0.533	1.58E-40	TRUE
NK	*C22orf34*	Wald ratio	1	-0.0209	0.0117	0.979 (0.957, 1.002)	0.075	0.287	1.54E-21	TRUE
NK	*C2ORF15*	Wald ratio	1	0.0018	0.0187	1.002 (0.966, 1.039)	0.924	0.967	9.48E-09	TRUE
NK	*C2orf27A*	Wald ratio	1	0.0171	0.0115	1.017 (0.995, 1.04)	0.137	0.399	2.93E-24	TRUE
NK	*C4orf33*	Wald ratio	1	0.0092	0.0203	1.009 (0.97, 1.05)	0.65	0.835	2.91E-09	TRUE
NK	*C5orf17*	Wald ratio	1	-0.0065	0.0104	0.993 (0.973, 1.014)	0.529	0.757	1.42E-26	TRUE
NK	*C5orf34*	Wald ratio	1	0.0138	0.0154	1.014 (0.984, 1.045)	0.369	0.657	6.56E-13	TRUE
NK	*C5orf56*	Wald ratio	1	0.0427	0.0157	1.044 (1.012, 1.076)	0.007	0.062	1.86E-12	TRUE
NK	*C6orf62*	Wald ratio	1	0.0666	0.0194	1.069 (1.029, 1.11)	5.87E-04	0.011	1.33E-08	TRUE
NK	*C9orf156*	Wald ratio	1	0.0073	0.0138	1.007 (0.981, 1.035)	0.594	0.799	4.90E-17	TRUE
NK	*C9orf78*	Wald ratio	1	-0.0014	0.0122	0.999 (0.975, 1.023)	0.906	0.96	2.81E-20	TRUE
NK	*CACNA1A*	Wald ratio	1	0.0080	0.0176	1.008 (0.974, 1.043)	0.648	0.834	2.84E-10	TRUE
NK	*CALR*	Wald ratio	1	0.0019	0.0211	1.002 (0.961, 1.044)	0.929	0.97	3.17E-08	TRUE
NK	*CAMLG*	Wald ratio	1	-0.0008	0.0129	0.999 (0.974, 1.025)	0.948	0.98	9.41E-13	TRUE
NK	*CAP1*	Wald ratio	1	-0.0839	0.0219	0.919 (0.881, 0.96)	1.24E-04	0.003	9.36E-08	TRUE
NK	*CAPN12*	Wald ratio	1	-0.0109	0.0114	0.989 (0.967, 1.012)	0.34	0.634	1.31E-28	TRUE
NK	*CARD16*	Wald ratio	1	0.0342	0.0146	1.035 (1.006, 1.065)	0.019	0.124	2.00E-11	TRUE
NK	*CARHSP1*	Wald ratio	1	0.0156	0.0095	1.016 (0.997, 1.035)	0.098	0.333	5.43E-31	TRUE
NK	*CASP8*	Wald ratio	1	-0.0385	0.0126	0.962 (0.939, 0.986)	0.002	0.029	5.13E-18	TRUE
NK	*CAST*	Wald ratio	1	-0.0030	0.0090	0.997 (0.979, 1.015)	0.737	0.88	1.55E-40	TRUE
NK	*CAT*	Wald ratio	1	0.0273	0.0177	1.028 (0.993, 1.064)	0.123	0.378	1.56E-10	TRUE
NK	*CCDC101*	Wald ratio	1	-0.0183	0.0203	0.982 (0.944, 1.022)	0.365	0.654	3.78E-08	TRUE
NK	*CCDC122*	Wald ratio	1	0.0029	0.0191	1.003 (0.966, 1.041)	0.879	0.951	4.31E-09	TRUE
NK	*CCDC146*	Wald ratio	1	0.0080	0.0139	1.008 (0.981, 1.036)	0.567	0.782	4.61E-24	TRUE
NK	*CCDC152*	Wald ratio	1	0.0195	0.0141	1.02 (0.992, 1.048)	0.167	0.442	5.05E-14	TRUE
NK	*CCDC163P*	Wald ratio	1	-0.0097	0.0116	0.99 (0.968, 1.013)	0.402	0.68	3.52E-22	TRUE
NK	*CCDC25*	Wald ratio	1	-0.0281	0.0186	0.972 (0.938, 1.008)	0.131	0.392	3.39E-09	TRUE
NK	*CCDC50*	Wald ratio	1	0.0161	0.0108	1.016 (0.995, 1.038)	0.137	0.399	1.92E-23	TRUE
NK	*CCDC66*	Wald ratio	1	0.0148	0.0126	1.015 (0.99, 1.04)	0.241	0.534	2.30E-20	TRUE
NK	*CCDC69*	Wald ratio	1	0.0057	0.0204	1.006 (0.966, 1.047)	0.781	0.905	5.10E-08	TRUE
NK	*CCDC74A*	Wald ratio	1	-0.0110	0.0200	0.989 (0.951, 1.029)	0.582	0.792	7.40E-12	TRUE
NK	*CCDC88B*	Wald ratio	1	0.0523	0.0178	1.054 (1.018, 1.091)	0.003	0.037	1.47E-09	TRUE
NK	*CCS*	Wald ratio	1	0.0115	0.0139	1.012 (0.984, 1.04)	0.411	0.686	1.13E-15	TRUE
NK	*CCSAP*	Wald ratio	1	-0.0456	0.0182	0.955 (0.922, 0.99)	0.012	0.094	4.18E-09	TRUE
NK	*CCSER2*	Wald ratio	1	-0.0289	0.0151	0.972 (0.943, 1.001)	0.056	0.238	3.49E-16	TRUE
NK	*CCT8*	Wald ratio	1	-0.0005	0.0127	0.999 (0.975, 1.025)	0.967	0.989	1.50E-31	TRUE
NK	*CCZ1*	IVW	2	0.0122	0.0072	1.012 (0.998, 1.027)	0.089	0.317	1.11E-60	TRUE
NK	*CD151*	Wald ratio	1	0.0321	0.0102	1.033 (1.012, 1.053)	0.002	0.022	1.46E-30	TRUE
NK	*CD27-AS1*	Wald ratio	1	0.0060	0.0203	1.006 (0.967, 1.047)	0.768	0.897	3.53E-08	TRUE
NK	*CD300A*	IVW	2	0.0121	0.0098	1.012 (0.993, 1.032)	0.215	0.504	1.01E-40	TRUE
NK	*CD300C*	Wald ratio	1	-0.0361	0.0202	0.965 (0.927, 1.003)	0.073	0.284	4.89E-08	TRUE
NK	*CD300LB*	Wald ratio	1	0.0065	0.0108	1.007 (0.985, 1.028)	0.545	0.767	5.52E-25	TRUE
NK	*CD37*	Wald ratio	1	0.0014	0.0146	1.001 (0.973, 1.03)	0.925	0.967	6.15E-20	TRUE
NK	*CD3E*	Wald ratio	1	-0.0106	0.0047	0.989 (0.98, 0.999)	0.024	0.143	2.81E-142	TRUE
NK	*CD47*	Wald ratio	1	-0.0119	0.0121	0.988 (0.965, 1.012)	0.324	0.618	1.18E-20	TRUE
NK	*CD48*	Wald ratio	1	0.0159	0.0141	1.016 (0.988, 1.045)	0.259	0.553	1.14E-15	TRUE

NK	*CD52*	Wald ratio	1	0.0018	0.0092	1.002 (0.984, 1.02)	0.846	0.935	1.99E-31	TRUE
NK	*CD55*	Wald ratio	1	-0.0437	0.0194	0.957 (0.922, 0.994)	0.024	0.142	1.46E-09	TRUE
NK	*CD79B*	Wald ratio	1	0.0063	0.0154	1.006 (0.976, 1.037)	0.682	0.856	2.22E-10	TRUE
NK	*CD81*	Wald ratio	1	-0.0432	0.0148	0.958 (0.93, 0.986)	0.004	0.04	9.13E-10	TRUE
NK	*CD8A*	Wald ratio	1	-0.0135	0.0097	0.987 (0.968, 1.006)	0.167	0.442	2.87E-26	TRUE
NK	*CDC14A*	Wald ratio	1	-0.0107	0.0156	0.989 (0.96, 1.02)	0.491	0.738	1.62E-12	TRUE
NK	*CDC42*	Wald ratio	1	0.0253	0.0158	1.026 (0.994, 1.058)	0.108	0.35	9.04E-11	TRUE
NK	*CDC42SE2*	Wald ratio	1	0.0126	0.0124	1.013 (0.988, 1.038)	0.311	0.605	7.85E-20	TRUE
NK	*CDK10*	Wald ratio	1	-0.0426	0.0129	0.958 (0.934, 0.983)	9.77E-04	0.017	9.55E-17	TRUE
NK	*CEACAM21*	Wald ratio	1	-0.0127	0.0105	0.987 (0.967, 1.008)	0.227	0.52	2.92E-23	TRUE
NK	*CEBPZ-AS1*	Wald ratio	1	0.0186	0.0146	1.019 (0.99, 1.048)	0.205	0.492	3.22E-20	TRUE
NK	*CELA1*	Wald ratio	1	-0.0014	0.0190	0.999 (0.962, 1.036)	0.943	0.978	6.08E-11	TRUE
NK	*CENPK*	Wald ratio	1	0.0139	0.0091	1.014 (0.996, 1.032)	0.127	0.382	7.45E-35	TRUE
NK	*CENPU*	Wald ratio	1	0.0261	0.0189	1.026 (0.989, 1.065)	0.167	0.442	2.47E-09	TRUE
NK	*CEP63*	Wald ratio	1	-0.0180	0.0212	0.982 (0.942, 1.024)	0.394	0.673	6.06E-08	TRUE
NK	*CEP78*	Wald ratio	1	-0.0012	0.0078	0.999 (0.984, 1.014)	0.882	0.952	2.41E-28	TRUE
NK	*CES1*	Wald ratio	1	-0.0210	0.0200	0.979 (0.942, 1.018)	0.293	0.587	1.08E-08	TRUE
NK	*CFD*	Wald ratio	1	0.0005	0.0114	1.001 (0.978, 1.023)	0.963	0.988	1.05E-26	TRUE
NK	*CFLAR*	Wald ratio	1	0.0336	0.0159	1.034 (1.003, 1.067)	0.034	0.175	3.01E-14	TRUE
NK	*CHCHD2*	Wald ratio	1	0.0047	0.0090	1.005 (0.987, 1.023)	0.599	0.803	2.27E-38	TRUE
NK	*CHI3L2*	Wald ratio	1	-0.0044	0.0057	0.996 (0.984, 1.007)	0.444	0.709	7.19E-93	TRUE
NK	*CHMP4A*	Wald ratio	1	0.0180	0.0166	1.018 (0.986, 1.052)	0.278	0.571	1.19E-09	TRUE
NK	*CHMP4B*	Wald ratio	1	-0.0305	0.0156	0.97 (0.941, 1)	0.05	0.222	1.18E-12	TRUE
NK	*CHPT1*	Wald ratio	1	0.0200	0.0104	1.02 (1, 1.041)	0.054	0.233	3.35E-27	TRUE
NK	*CHURC1*	Wald ratio	1	0.0142	0.0169	1.014 (0.981, 1.049)	0.401	0.679	1.34E-08	TRUE
NK	*CIAO1*	Wald ratio	1	0.0195	0.0209	1.02 (0.979, 1.062)	0.35	0.643	5.99E-09	TRUE
NK	*CIB1*	Wald ratio	1	-0.0207	0.0143	0.979 (0.952, 1.007)	0.148	0.417	2.42E-15	TRUE
NK	*CISD1*	Wald ratio	1	-0.0032	0.0177	0.997 (0.963, 1.032)	0.857	0.939	4.26E-09	TRUE
NK	*CLEC11A*	Wald ratio	1	0.0069	0.0195	1.007 (0.969, 1.046)	0.723	0.877	4.92E-08	TRUE
NK	*CLEC12A*	Wald ratio	1	-0.0192	0.0150	0.981 (0.953, 1.01)	0.199	0.482	2.05E-13	TRUE
NK	*CLEC2B*	Wald ratio	1	0.0035	0.0107	1.004 (0.983, 1.025)	0.741	0.881	1.94E-25	TRUE
NK	*CLHC1*	Wald ratio	1	-0.0964	0.0233	0.908 (0.868, 0.95)	3.38E-05	0.001	1.56E-09	TRUE
NK	*CLIC3*	Wald ratio	1	0.0066	0.0126	1.007 (0.982, 1.032)	0.601	0.805	2.87E-19	TRUE
NK	*CLK4*	Wald ratio	1	0.0112	0.0178	1.011 (0.977, 1.047)	0.529	0.758	6.00E-11	TRUE
NK	*CMC1*	Wald ratio	1	0.0248	0.0155	1.025 (0.994, 1.057)	0.11	0.353	4.31E-13	TRUE
NK	*CMTM3*	Wald ratio	1	0.0146	0.0145	1.015 (0.986, 1.044)	0.315	0.609	2.08E-14	TRUE
NK	*CNN2*	Wald ratio	1	0.0126	0.0130	1.013 (0.987, 1.039)	0.333	0.626	6.94E-17	TRUE
NK	*CNOT6L*	Wald ratio	1	0.0291	0.0142	1.03 (1.001, 1.059)	0.041	0.196	9.93E-16	TRUE
NK	*COA1*	Wald ratio	1	0.0042	0.0145	1.004 (0.976, 1.033)	0.775	0.901	4.02E-16	TRUE
NK	*COG5*	Wald ratio	1	0.0471	0.0095	1.048 (1.029, 1.068)	6.43E-07	9.75E-05	1.18E-29	TRUE
NK	*COL18A1*	Wald ratio	1	0.0444	0.0186	1.045 (1.008, 1.084)	0.017	0.115	1.98E-10	TRUE
NK	*COL5A3*	Wald ratio	1	-0.0181	0.0103	0.982 (0.962, 1.002)	0.077	0.291	1.37E-34	TRUE
NK	*COL6A1*	Wald ratio	1	-0.0045	0.0079	0.996 (0.98, 1.011)	0.574	0.786	1.80E-47	TRUE
NK	*COLGALT2*	Wald ratio	1	0.0005	0.0080	1 (0.985, 1.016)	0.951	0.981	9.08E-46	TRUE
NK	*COMMD5*	Wald ratio	1	-0.0018	0.0142	0.998 (0.971, 1.026)	0.902	0.959	1.10E-15	TRUE
NK	*COMMD6*	Wald ratio	1	0.0166	0.0102	1.017 (0.997, 1.037)	0.105	0.343	6.89E-28	TRUE
NK	*CORO1B*	Wald ratio	1	0.0019	0.0087	1.002 (0.985, 1.019)	0.828	0.928	6.92E-40	TRUE
NK	*COX20*	Wald ratio	1	0.0063	0.0201	1.006 (0.967, 1.047)	0.755	0.888	5.78E-11	TRUE
NK	*COX6B1*	Wald ratio	1	-0.0358	0.0162	0.965 (0.935, 0.996)	0.027	0.155	1.15E-11	TRUE
NK	*COX7A2L*	Wald ratio	1	-0.0562	0.0192	0.945 (0.911, 0.982)	0.003	0.038	6.53E-09	TRUE
NK	*CPNE1*	Wald ratio	1	-0.0060	0.0102	0.994 (0.974, 1.014)	0.557	0.775	5.37E-34	TRUE
NK	*CPSF1*	Wald ratio	1	-0.0016	0.0192	0.998 (0.962, 1.037)	0.934	0.972	7.46E-09	TRUE
NK	*CRIP1*	Wald ratio	1	-0.0101	0.0150	0.99 (0.961, 1.02)	0.502	0.743	6.33E-13	TRUE
NK	*CRIPAK*	Wald ratio	1	0.0399	0.0233	1.041 (0.994, 1.089)	0.087	0.312	8.62E-08	TRUE
NK	*CRLF3*	Wald ratio	1	0.0367	0.0150	1.037 (1.007, 1.068)	0.015	0.104	1.24E-16	TRUE
NK	*CROCC*	Wald ratio	1	-0.0560	0.0272	0.946 (0.897, 0.997)	0.039	0.192	1.58E-08	TRUE
NK	*CRTAM*	Wald ratio	1	-0.0082	0.0112	0.992 (0.97, 1.014)	0.467	0.725	6.45E-22	TRUE
NK	*CRYBB2*	Wald ratio	1	0.0085	0.0068	1.009 (0.995, 1.022)	0.21	0.497	6.14E-63	TRUE
NK	*CRYZ*	Wald ratio	1	-0.0223	0.0187	0.978 (0.943, 1.014)	0.231	0.524	3.61E-09	TRUE
NK	*CST3*	Wald ratio	1	-0.0036	0.0146	0.996 (0.968, 1.025)	0.806	0.916	2.10E-14	TRUE
NK	*CST7*	Wald ratio	1	-0.0120	0.0225	0.988 (0.945, 1.033)	0.594	0.799	1.28E-13	TRUE
NK	*CSTB*	Wald ratio	1	0.0029	0.0084	1.003 (0.986, 1.02)	0.73	0.88	9.31E-37	TRUE
NK	*CTA-29F11.1*	Wald ratio	1	-0.0068	0.0108	0.993 (0.972, 1.014)	0.527	0.756	7.86E-26	TRUE
NK	*CTA-445C9.15*	Wald ratio	1	0.0117	0.0093	1.012 (0.994, 1.03)	0.207	0.494	4.52E-33	TRUE
NK	*CTBP1-AS2*	Wald ratio	1	-0.0204	0.0155	0.98 (0.951, 1.01)	0.187	0.466	1.25E-12	TRUE
NK	*CTC-425F1.4*	Wald ratio	1	-0.0025	0.0181	0.997 (0.963, 1.034)	0.889	0.955	1.06E-10	TRUE
NK	*CTC-503J8.4*	Wald ratio	1	-0.0168	0.0154	0.983 (0.954, 1.014)	0.277	0.571	3.06E-17	TRUE
NK	*CTC-524C5.2*	Wald ratio	1	0.0170	0.0096	1.017 (0.998, 1.037)	0.076	0.289	7.32E-25	TRUE
NK	*CTD-2007H13.3*	Wald ratio	1	-0.0229	0.0252	0.977 (0.93, 1.027)	0.364	0.653	5.96E-08	TRUE
NK	*CTD-2325M2.1*	Wald ratio	1	-0.0046	0.0149	0.995 (0.967, 1.025)	0.755	0.888	6.56E-14	TRUE
NK	*CTD-2353F22.1*	Wald ratio	1	0.0091	0.0115	1.009 (0.987, 1.032)	0.427	0.697	2.75E-22	TRUE
NK	*CTD-2357A8.3*	Wald ratio	1	0.0170	0.0129	1.017 (0.992, 1.043)	0.19	0.47	1.22E-17	TRUE
NK	*CTD-2368P22.1*	Wald ratio	1	0.0025	0.0072	1.002 (0.988, 1.017)	0.734	0.88	7.87E-61	TRUE
NK	*CTD-2371O3.2*	Wald ratio	1	-0.0012	0.0073	0.999 (0.985, 1.013)	0.874	0.948	1.43E-57	TRUE
NK	*CTD-3138B18.5*	Wald ratio	1	0.0153	0.0179	1.015 (0.98, 1.052)	0.394	0.673	4.37E-10	TRUE
NK	*CTD-3162L10.1*	Wald ratio	1	0.0206	0.0101	1.021 (1.001, 1.041)	0.04	0.195	3.75E-28	TRUE
NK	*CTSF*	Wald ratio	1	-0.0163	0.0143	0.984 (0.957, 1.012)	0.254	0.548	4.54E-16	TRUE
NK	*CTSG*	Wald ratio	1	0.0080	0.0095	1.008 (0.989, 1.027)	0.4	0.679	3.32E-26	TRUE
NK	*CTSS*	Wald ratio	1	-0.0328	0.0174	0.968 (0.935, 1.001)	0.06	0.248	7.23E-10	TRUE

NK	*CTSW*	Wald ratio	1	-0.0269	0.0064	0.974 (0.961, 0.986)	3.13E-05	0.001	5.22E-81	TRUE
NK	*CXCR4*	Wald ratio	1	0.0324	0.0179	1.033 (0.997, 1.07)	0.069	0.272	4.22E-08	TRUE
NK	*CYTL1*	Wald ratio	1	-0.0120	0.0083	0.988 (0.972, 1.004)	0.149	0.418	6.32E-55	TRUE
NK	*DAD1*	Wald ratio	1	-0.0015	0.0187	0.998 (0.963, 1.036)	0.934	0.972	7.31E-09	TRUE
NK	*DAP*	Wald ratio	1	-0.0203	0.0162	0.98 (0.949, 1.011)	0.21	0.497	1.94E-11	TRUE
NK	*DAPK1*	Wald ratio	1	0.0000	0.0135	1 (0.974, 1.027)	0.998	0.999	9.35E-16	TRUE
NK	*DAZAP1*	Wald ratio	1	0.0597	0.0196	1.062 (1.021, 1.103)	0.002	0.03	1.68E-08	TRUE
NK	*DBI*	Wald ratio	1	0.0237	0.0164	1.024 (0.992, 1.057)	0.149	0.418	7.97E-13	TRUE
NK	*DBNL*	Wald ratio	1	0.0423	0.0222	1.043 (0.999, 1.09)	0.056	0.239	2.17E-08	TRUE
NK	*DCTN5*	Wald ratio	1	0.0209	0.0179	1.021 (0.986, 1.058)	0.242	0.535	3.79E-09	TRUE
NK	*DCXR*	Wald ratio	1	-0.0060	0.0066	0.994 (0.981, 1.007)	0.365	0.653	1.49E-57	TRUE
NK	*DDR1*	Wald ratio	1	-0.0137	0.0160	0.986 (0.956, 1.018)	0.391	0.672	1.59E-10	TRUE
NK	*DDRGK1*	Wald ratio	1	-0.0233	0.0227	0.977 (0.934, 1.022)	0.305	0.599	1.32E-08	TRUE
NK	*DDTL*	Wald ratio	1	-0.0108	0.0104	0.989 (0.969, 1.01)	0.298	0.593	4.60E-27	TRUE
NK	*DDX11*	Wald ratio	1	-0.0116	0.0087	0.988 (0.972, 1.005)	0.183	0.462	1.54E-38	TRUE
NK	*DDX42*	Wald ratio	1	-0.0758	0.0195	0.927 (0.892, 0.963)	1.01E-04	0.003	2.01E-09	TRUE
NK	*DDX5*	Wald ratio	1	0.0380	0.0098	1.039 (1.019, 1.059)	1.06E-04	0.003	2.69E-27	TRUE
NK	*DDX6*	Wald ratio	1	-0.0294	0.0188	0.971 (0.936, 1.008)	0.119	0.369	7.63E-09	TRUE
NK	*DFNB31*	Wald ratio	1	0.0424	0.0222	1.043 (0.999, 1.09)	0.056	0.238	9.58E-08	TRUE
NK	*DGKQ*	Wald ratio	1	-0.0165	0.0202	0.984 (0.946, 1.023)	0.414	0.686	3.63E-08	TRUE
NK	*DHRS4*	Wald ratio	1	0.0285	0.0139	1.029 (1.001, 1.057)	0.04	0.193	8.33E-19	TRUE
NK	*DHRS7*	Wald ratio	1	-0.0168	0.0152	0.983 (0.954, 1.013)	0.27	0.563	1.20E-08	TRUE
NK	*DHX32*	Wald ratio	1	0.0112	0.0193	1.011 (0.974, 1.05)	0.562	0.779	8.35E-09	TRUE
NK	*DLG5*	Wald ratio	1	-0.0546	0.0195	0.947 (0.911, 0.984)	0.005	0.051	3.51E-08	TRUE
NK	*DLL1*	Wald ratio	1	0.0305	0.0203	1.031 (0.991, 1.073)	0.134	0.394	1.23E-08	TRUE
NK	*DMKN*	Wald ratio	1	-0.0118	0.0142	0.988 (0.961, 1.016)	0.405	0.681	1.95E-22	TRUE
NK	*DNAJA1*	Wald ratio	1	-0.0596	0.0166	0.942 (0.912, 0.973)	3.34E-04	0.008	1.53E-11	TRUE
NK	*DNAJC15*	Wald ratio	1	0.0066	0.0068	1.007 (0.993, 1.02)	0.334	0.627	9.58E-62	TRUE
NK	*DOCK8*	Wald ratio	1	0.0337	0.0170	1.034 (1, 1.069)	0.047	0.214	6.04E-15	TRUE
NK	*DPYSL4*	Wald ratio	1	0.0269	0.0196	1.027 (0.989, 1.068)	0.169	0.446	4.24E-10	TRUE
NK	*DRAM2*	Wald ratio	1	0.0064	0.0154	1.006 (0.977, 1.037)	0.676	0.853	5.36E-13	TRUE
NK	*DRAXIN*	Wald ratio	1	-0.0299	0.0140	0.971 (0.944, 0.998)	0.033	0.173	4.63E-12	TRUE
NK	*DST*	Wald ratio	1	0.0129	0.0184	1.013 (0.977, 1.05)	0.482	0.735	5.72E-08	TRUE
NK	*DSTN*	IVW	2	-0.0386	0.0118	0.962 (0.94, 0.985)	0.001	0.018	6.62E-21	TRUE
NK	*DTD1*	Wald ratio	1	-0.0321	0.0172	0.968 (0.936, 1.002)	0.063	0.255	3.03E-09	TRUE
NK	*DUOX1*	Wald ratio	1	0.0420	0.0147	1.043 (1.013, 1.073)	0.004	0.045	3.07E-12	TRUE
NK	*DYNLL2*	Wald ratio	1	-0.0428	0.0151	0.958 (0.93, 0.987)	0.004	0.046	1.13E-15	TRUE
NK	*DYNLRB1*	Wald ratio	1	0.0118	0.0162	1.012 (0.98, 1.044)	0.466	0.725	3.41E-12	TRUE
NK	*DZIP3*	Wald ratio	1	0.0155	0.0100	1.016 (0.996, 1.036)	0.121	0.373	2.92E-28	TRUE
NK	*EBPL*	IVW	2	0.0142	0.0166	1.014 (0.982, 1.048)	0.393	0.672	2.67E-47	TRUE
NK	*ECHDC2*	Wald ratio	1	-0.0194	0.0167	0.981 (0.949, 1.013)	0.245	0.538	1.02E-10	TRUE
NK	*EFCAB4B*	Wald ratio	1	-0.0206	0.0180	0.98 (0.946, 1.015)	0.254	0.548	1.19E-11	TRUE
NK	*EFHB*	Wald ratio	1	0.0054	0.0170	1.005 (0.973, 1.039)	0.751	0.886	2.26E-11	TRUE
NK	*EGFL8*	Wald ratio	1	0.0466	0.0117	1.048 (1.024, 1.072)	7.11E-05	0.002	1.96E-22	TRUE
NK	*EIF2A*	Wald ratio	1	0.0003	0.0192	1 (0.963, 1.039)	0.986	0.994	1.57E-09	TRUE
NK	*EIF3G*	Wald ratio	1	-0.0033	0.0062	0.997 (0.985, 1.009)	0.595	0.801	5.34E-78	TRUE
NK	*EIF3K*	Wald ratio	1	0.0161	0.0198	1.016 (0.978, 1.056)	0.415	0.687	1.89E-08	TRUE
NK	*EIF4G3*	Wald ratio	1	-0.0335	0.0119	0.967 (0.945, 0.99)	0.005	0.051	3.30E-20	TRUE
NK	*EIF5A*	Wald ratio	1	-0.0066	0.0044	0.993 (0.985, 1.002)	0.134	0.394	1.78E-207	TRUE
NK	*EMILIN1*	Wald ratio	1	0.0431	0.0191	1.044 (1.006, 1.084)	0.024	0.142	9.69E-09	TRUE
NK	*EML4*	Wald ratio	1	0.0145	0.0159	1.015 (0.983, 1.047)	0.362	0.651	2.15E-15	TRUE
NK	*ENC1*	Wald ratio	1	-0.0006	0.0150	0.999 (0.971, 1.029)	0.969	0.989	1.01E-18	TRUE
NK	*ENDOG*	Wald ratio	1	0.0368	0.0204	1.038 (0.997, 1.08)	0.072	0.278	6.55E-09	TRUE
NK	*ENO1*	Wald ratio	1	-0.0057	0.0116	0.994 (0.972, 1.017)	0.624	0.822	4.22E-22	TRUE
NK	*ENPP4*	Wald ratio	1	-0.0022	0.0143	0.998 (0.97, 1.026)	0.88	0.951	6.16E-15	TRUE
NK	*ENPP5*	Wald ratio	1	0.0041	0.0157	1.004 (0.974, 1.036)	0.793	0.909	1.57E-12	TRUE
NK	*ENTPD3-AS1*	Wald ratio	1	-0.0512	0.0158	0.95 (0.921, 0.98)	0.001	0.02	2.40E-11	TRUE
NK	*EPDR1*	Wald ratio	1	0.0119	0.0180	1.012 (0.977, 1.048)	0.509	0.747	4.56E-10	TRUE
NK	*EPHX4*	Wald ratio	1	0.0681	0.0189	1.07 (1.032, 1.111)	3.16E-04	0.007	3.38E-09	TRUE
NK	*EPSTI1*	Wald ratio	1	0.0060	0.0098	1.006 (0.987, 1.026)	0.54	0.762	1.02E-32	TRUE
NK	*ERAP1*	Wald ratio	1	-0.0962	0.0195	0.908 (0.874, 0.944)	8.09E-07	1.03E-04	2.77E-08	TRUE
NK	*ERMP1*	Wald ratio	1	0.0286	0.0209	1.029 (0.988, 1.072)	0.172	0.45	7.38E-11	TRUE
NK	*ERN1*	Wald ratio	1	-0.0261	0.0099	0.974 (0.956, 0.993)	0.008	0.07	5.12E-31	TRUE
NK	*ESR2*	IVW	2	0.0064	0.0395	1.006 (0.931, 1.087)	0.871	0.947	5.05E-49	TRUE
NK	*ETV7*	Wald ratio	1	-0.0137	0.0117	0.986 (0.964, 1.009)	0.241	0.534	2.63E-21	TRUE
NK	*EVA1C*	Wald ratio	1	-0.0329	0.0128	0.968 (0.944, 0.992)	0.01	0.081	4.83E-18	TRUE
NK	*EVI2A*	Wald ratio	1	-0.0147	0.0109	0.985 (0.965, 1.007)	0.177	0.456	8.36E-24	TRUE
NK	*EXOC3*	Wald ratio	1	-0.0039	0.0125	0.996 (0.972, 1.021)	0.755	0.888	2.35E-19	TRUE
NK	*F2R*	Wald ratio	1	-0.0241	0.0102	0.976 (0.957, 0.996)	0.018	0.117	1.29E-28	TRUE
NK	*FADS1*	Wald ratio	1	0.0352	0.0182	1.036 (1, 1.073)	0.052	0.228	1.72E-08	TRUE
NK	*FADS2*	Wald ratio	1	0.0127	0.0089	1.013 (0.995, 1.031)	0.153	0.423	1.39E-57	TRUE
NK	*FAH*	Wald ratio	1	-0.0002	0.0088	1 (0.983, 1.017)	0.978	0.991	6.78E-36	TRUE
NK	*FAM101B*	Wald ratio	1	0.0096	0.0217	1.01 (0.968, 1.053)	0.659	0.842	2.35E-09	TRUE
NK	*FAM118A*	Wald ratio	1	0.0004	0.0048	1 (0.991, 1.01)	0.928	0.969	1.98E-97	TRUE
NK	*FAM129A*	Wald ratio	1	-0.0113	0.0162	0.989 (0.958, 1.021)	0.484	0.736	2.46E-12	TRUE
NK	*FAM132B*	Wald ratio	1	0.0336	0.0191	1.034 (0.996, 1.074)	0.079	0.293	1.22E-08	TRUE
NK	*FAM154B*	Wald ratio	1	-0.0083	0.0135	0.992 (0.966, 1.018)	0.537	0.762	1.63E-14	TRUE
NK	*FAM26F*	IVW	2	-0.0146	0.0157	0.986 (0.956, 1.016)	0.353	0.644	2.51E-84	TRUE
NK	*FAM3C*	Wald ratio	1	-0.0012	0.0208	0.999 (0.959, 1.04)	0.954	0.983	4.52E-08	TRUE

NK	*FAM43A*	Wald ratio	1	-0.0181	0.0168	0.982 (0.95, 1.015)	0.282	0.575	3.54E-12	TRUE
NK	*FAM53B*	Wald ratio	1	-0.0074	0.0210	0.993 (0.953, 1.034)	0.723	0.877	4.98E-10	TRUE
NK	*FAM63B*	Wald ratio	1	-0.0725	0.0259	0.93 (0.884, 0.979)	0.005	0.051	4.32E-09	TRUE
NK	*FAM64A*	Wald ratio	1	-0.0131	0.0128	0.987 (0.963, 1.012)	0.303	0.598	1.39E-16	TRUE
NK	*FAR1*	Wald ratio	1	-0.0033	0.0108	0.997 (0.976, 1.018)	0.759	0.89	7.38E-27	TRUE
NK	*FAU*	Wald ratio	1	-0.0020	0.0163	0.998 (0.967, 1.03)	0.901	0.958	8.95E-12	TRUE
NK	*FBLN5*	Wald ratio	1	-0.0073	0.0160	0.993 (0.962, 1.024)	0.648	0.835	3.20E-12	TRUE
NK	*FBP1*	Wald ratio	1	0.0086	0.0186	1.009 (0.973, 1.046)	0.643	0.832	1.74E-09	TRUE
NK	*FBXO2*	Wald ratio	1	-0.0355	0.0145	0.965 (0.938, 0.993)	0.015	0.104	7.47E-15	TRUE
NK	*FBXO6*	Wald ratio	1	0.0064	0.0150	1.006 (0.977, 1.037)	0.672	0.85	2.04E-14	TRUE
NK	*FCER1G*	Wald ratio	1	-0.0205	0.0233	0.98 (0.936, 1.025)	0.379	0.666	2.58E-08	TRUE
NK	*FCGR2A*	Wald ratio	1	0.0087	0.0128	1.009 (0.984, 1.034)	0.497	0.741	8.28E-19	TRUE
NK	*FCRL3*	Wald ratio	1	0.0045	0.0127	1.004 (0.98, 1.03)	0.725	0.878	1.02E-18	TRUE
NK	*FGFBP2*	Wald ratio	1	0.0162	0.0104	1.016 (0.996, 1.037)	0.121	0.372	1.95E-27	TRUE
NK	*FGFR1*	Wald ratio	1	0.0261	0.0087	1.026 (1.009, 1.044)	0.003	0.032	7.74E-38	TRUE
NK	*FGL2*	Wald ratio	1	-0.0097	0.0099	0.99 (0.971, 1.01)	0.325	0.62	8.52E-25	TRUE
NK	*FHAD1*	Wald ratio	1	0.0127	0.0096	1.013 (0.994, 1.032)	0.187	0.466	6.68E-32	TRUE
NK	*FIBP*	Wald ratio	1	0.0535	0.0126	1.055 (1.029, 1.081)	2.34E-05	0.001	6.88E-21	TRUE
NK	*FIG4*	Wald ratio	1	0.0358	0.0183	1.036 (1, 1.074)	0.051	0.224	2.19E-09	TRUE
NK	*FIS1*	Wald ratio	1	0.0066	0.0145	1.007 (0.978, 1.036)	0.649	0.835	2.19E-15	TRUE
NK	*FKBP11*	Wald ratio	1	0.0025	0.0095	1.003 (0.984, 1.021)	0.792	0.909	1.59E-31	TRUE
NK	*FKRP*	Wald ratio	1	0.0000	0.0211	1 (0.959, 1.042)	0.999	0.999	1.41E-08	TRUE
NK	*FLVCR1-AS1*	Wald ratio	1	-0.0137	0.0117	0.986 (0.964, 1.009)	0.243	0.535	4.10E-22	TRUE
NK	*FMN1*	Wald ratio	1	0.0020	0.0151	1.002 (0.973, 1.032)	0.896	0.957	3.19E-14	TRUE
NK	*FN3KRP*	Wald ratio	1	0.0173	0.0098	1.017 (0.998, 1.037)	0.077	0.291	1.26E-31	TRUE
NK	*FOXN2*	Wald ratio	1	0.0488	0.0196	1.05 (1.01, 1.091)	0.013	0.095	4.81E-12	TRUE
NK	*FOXRED1*	Wald ratio	1	-0.0309	0.0207	0.97 (0.931, 1.01)	0.135	0.396	5.95E-08	TRUE
NK	*FRG1B*	Wald ratio	1	-0.0790	0.0188	0.924 (0.891, 0.959)	2.70E-05	0.001	8.31E-09	TRUE
NK	*FUT7*	Wald ratio	1	-0.0062	0.0108	0.994 (0.973, 1.015)	0.562	0.779	3.07E-25	TRUE
NK	*FXYD5*	Wald ratio	1	-0.0118	0.0165	0.988 (0.957, 1.021)	0.477	0.732	1.26E-14	TRUE
NK	*G3BP2*	Wald ratio	1	0.0019	0.0136	1.002 (0.976, 1.029)	0.891	0.956	2.31E-16	TRUE
NK	*GADD45G*	Wald ratio	1	0.0212	0.0160	1.021 (0.99, 1.054)	0.185	0.464	5.08E-12	TRUE
NK	*GALNT2*	Wald ratio	1	0.0305	0.0175	1.031 (0.996, 1.067)	0.08	0.297	6.95E-09	TRUE
NK	*GALT*	Wald ratio	1	0.0891	0.0180	1.093 (1.055, 1.132)	7.32E-07	9.75E-05	4.97E-10	TRUE
NK	*GAPDH*	Wald ratio	1	-0.0376	0.0152	0.963 (0.935, 0.992)	0.013	0.098	2.07E-12	TRUE
NK	*GBP3*	Wald ratio	1	0.0168	0.0179	1.017 (0.982, 1.053)	0.348	0.64	7.60E-10	TRUE
NK	*GBP5*	Wald ratio	1	-0.0350	0.0137	0.966 (0.94, 0.992)	0.011	0.085	8.45E-16	TRUE
NK	*GBP7*	Wald ratio	1	0.0004	0.0062	1 (0.988, 1.013)	0.946	0.979	1.34E-78	TRUE
NK	*GCC2*	Wald ratio	1	0.0087	0.0107	1.009 (0.988, 1.03)	0.414	0.686	2.11E-28	TRUE
NK	*GCHFR*	Wald ratio	1	0.0229	0.0108	1.023 (1.002, 1.045)	0.034	0.175	2.10E-42	TRUE
NK	*GDE1*	Wald ratio	1	-0.0016	0.0229	0.998 (0.955, 1.044)	0.944	0.978	3.45E-08	TRUE
NK	*GDI2*	Wald ratio	1	-0.0078	0.0132	0.992 (0.967, 1.018)	0.555	0.774	3.90E-18	TRUE
NK	*GEMIN7*	Wald ratio	1	-0.0198	0.0157	0.98 (0.951, 1.011)	0.208	0.495	1.52E-13	TRUE
NK	*GGA1*	Wald ratio	1	0.0260	0.0206	1.026 (0.986, 1.069)	0.207	0.494	4.61E-08	TRUE
NK	*GIMAP2*	Wald ratio	1	-0.0287	0.0163	0.972 (0.941, 1.003)	0.078	0.292	1.61E-11	TRUE
NK	*GIPC1*	Wald ratio	1	-0.0389	0.0146	0.962 (0.935, 0.99)	0.008	0.067	8.29E-15	TRUE
NK	*GLRX*	Wald ratio	1	-0.0320	0.0203	0.969 (0.931, 1.008)	0.115	0.361	4.24E-08	TRUE
NK	*GLTP*	Wald ratio	1	0.0309	0.0187	1.031 (0.994, 1.07)	0.098	0.333	1.03E-09	TRUE
NK	*GLTPD1*	Wald ratio	1	-0.0353	0.0140	0.965 (0.939, 0.992)	0.012	0.092	3.14E-09	TRUE
NK	*GLTSCR2*	Wald ratio	1	-0.0147	0.0137	0.985 (0.959, 1.012)	0.282	0.575	5.49E-16	TRUE
NK	*GMIP*	Wald ratio	1	-0.0004	0.0181	1 (0.965, 1.036)	0.981	0.993	2.35E-09	TRUE
NK	*GNB5*	Wald ratio	1	0.0548	0.0262	1.056 (1.004, 1.112)	0.036	0.181	2.60E-08	TRUE
NK	*GNG5*	Wald ratio	1	0.0117	0.0238	1.012 (0.966, 1.06)	0.622	0.821	3.51E-08	TRUE
NK	*GNGT2*	Wald ratio	1	0.0847	0.0170	1.088 (1.053, 1.125)	6.47E-07	9.75E-05	1.19E-09	TRUE
NK	*GNLY*	Wald ratio	1	-0.0067	0.0059	0.993 (0.982, 1.005)	0.25	0.543	2.30E-94	TRUE
NK	*GNMT*	Wald ratio	1	0.0231	0.0140	1.023 (0.996, 1.052)	0.099	0.333	7.66E-15	TRUE
NK	*GNPTG*	Wald ratio	1	-0.0100	0.0101	0.99 (0.971, 1.01)	0.32	0.615	9.23E-25	TRUE
NK	*GOLGA8Q*	Wald ratio	1	-0.0052	0.0260	0.995 (0.945, 1.047)	0.843	0.934	2.24E-08	TRUE
NK	*GOLM1*	Wald ratio	1	-0.0008	0.0188	0.999 (0.963, 1.037)	0.966	0.988	1.23E-12	TRUE
NK	*GPLD1*	Wald ratio	1	0.0502	0.0194	1.051 (1.012, 1.092)	0.01	0.079	1.79E-20	TRUE
NK	*GPR56*	Wald ratio	1	-0.0282	0.0162	0.972 (0.942, 1.004)	0.083	0.302	1.65E-12	TRUE
NK	*GRAMD1C*	Wald ratio	1	-0.0165	0.0107	0.984 (0.963, 1.004)	0.122	0.375	1.17E-26	TRUE
NK	*GRHPR*	Wald ratio	1	-0.0119	0.0099	0.988 (0.969, 1.007)	0.227	0.52	8.75E-28	TRUE
NK	*GRIK4*	Wald ratio	1	0.0061	0.0054	1.006 (0.996, 1.017)	0.257	0.551	7.30E-108	TRUE
NK	*GS1-124K5.4*	Wald ratio	1	0.0009	0.0158	1.001 (0.97, 1.032)	0.954	0.983	4.13E-13	TRUE
NK	*GS1-251I9.4*	Wald ratio	1	-0.0045	0.0198	0.996 (0.958, 1.035)	0.82	0.925	2.92E-08	TRUE
NK	*GSAP*	Wald ratio	1	0.0008	0.0079	1.001 (0.985, 1.016)	0.916	0.963	2.97E-46	TRUE
NK	*GSC*	Wald ratio	1	0.0074	0.0166	1.007 (0.975, 1.041)	0.658	0.842	1.05E-12	TRUE
NK	*GSDMA*	Wald ratio	1	-0.0009	0.0158	0.999 (0.969, 1.03)	0.954	0.983	1.42E-09	TRUE
NK	*GSTM1*	Wald ratio	1	0.0750	0.0199	1.078 (1.037, 1.121)	1.65E-04	0.004	5.24E-08	TRUE
NK	*GSTM3*	Wald ratio	1	-0.0182	0.0119	0.982 (0.959, 1.005)	0.125	0.38	1.97E-20	TRUE
NK	*GSTO1*	Wald ratio	1	0.0353	0.0176	1.036 (1.001, 1.072)	0.045	0.209	1.10E-10	TRUE
NK	*GSTP1*	Wald ratio	1	0.0340	0.0114	1.035 (1.012, 1.058)	0.003	0.034	3.08E-22	TRUE
NK	*GTF2H4*	Wald ratio	1	-0.0085	0.0151	0.992 (0.963, 1.021)	0.572	0.786	3.86E-21	TRUE
NK	*GTPBP4*	Wald ratio	1	-0.0276	0.0259	0.973 (0.925, 1.023)	0.287	0.579	1.22E-21	TRUE
NK	*GYPC*	Wald ratio	1	-0.0229	0.0248	0.977 (0.931, 1.026)	0.357	0.648	5.72E-08	TRUE
NK	*GZMH*	Wald ratio	1	-0.0171	0.0150	0.983 (0.955, 1.012)	0.252	0.546	1.19E-13	TRUE
NK	*H2AFV*	Wald ratio	1	-0.0075	0.0192	0.993 (0.956, 1.031)	0.697	0.864	7.83E-09	TRUE
NK	*H6PD*	Wald ratio	1	-0.0329	0.0194	0.968 (0.932, 1.005)	0.09	0.318	5.68E-10	TRUE

NK	*HAGH*	Wald ratio	1	0.0464	0.0112	1.048 (1.025, 1.071)	3.48E-05	0.001	2.46E-19	TRUE
NK	*HBA1*	Wald ratio	1	-0.0078	0.0053	0.992 (0.982, 1.002)	0.137	0.399	7.47E-63	TRUE
NK	*HBA2*	Wald ratio	1	-0.0083	0.0056	0.992 (0.981, 1.003)	0.137	0.399	4.16E-55	TRUE
NK	*HBQ1*	Wald ratio	1	-0.0078	0.0082	0.992 (0.976, 1.008)	0.342	0.635	4.45E-25	TRUE
NK	*HCG17*	Wald ratio	1	0.0005	0.0088	1.001 (0.983, 1.018)	0.951	0.981	6.02E-36	TRUE
NK	*HCG27*	Wald ratio	1	0.0004	0.0133	1 (0.975, 1.027)	0.977	0.991	8.93E-15	TRUE
NK	*HDHD3*	Wald ratio	1	-0.0170	0.0143	0.983 (0.956, 1.011)	0.232	0.525	8.29E-14	TRUE
NK	*HEBP2*	Wald ratio	1	0.0133	0.0121	1.013 (0.99, 1.038)	0.269	0.563	1.32E-27	TRUE
NK	*HEG1*	Wald ratio	1	-0.0217	0.0120	0.979 (0.956, 1.002)	0.07	0.273	2.96E-20	TRUE
NK	*HELLS*	Wald ratio	1	-0.0154	0.0162	0.985 (0.954, 1.017)	0.342	0.635	2.65E-12	TRUE
NK	*HELZ2*	Wald ratio	1	0.0268	0.0247	1.027 (0.979, 1.078)	0.279	0.573	1.54E-09	TRUE
NK	*HES6*	Wald ratio	1	0.0009	0.0116	1.001 (0.978, 1.024)	0.935	0.973	3.90E-23	TRUE
NK	*HEXDC*	Wald ratio	1	0.0069	0.0134	1.007 (0.981, 1.034)	0.608	0.809	2.99E-19	TRUE
NK	*HHEX*	Wald ratio	1	0.0090	0.0118	1.009 (0.986, 1.033)	0.444	0.709	2.65E-21	TRUE
NK	*HIF1A-AS2*	Wald ratio	1	-0.0107	0.0135	0.989 (0.964, 1.016)	0.425	0.695	1.67E-15	TRUE
NK	*HIGD2A*	Wald ratio	1	-0.0111	0.0134	0.989 (0.963, 1.015)	0.404	0.681	6.28E-17	TRUE
NK	*HIP1*	Wald ratio	1	-0.0130	0.0138	0.987 (0.961, 1.014)	0.347	0.64	1.28E-18	TRUE
NK	*HIST1H1B*	Wald ratio	1	0.0460	0.0477	1.047 (0.954, 1.15)	0.335	0.628	2.14E-08	TRUE
NK	*HIST1H2AD*	IVW	2	0.0237	0.0396	1.024 (0.947, 1.107)	0.55	0.769	4.63E-34	TRUE
NK	*HIST1H2AJ*	Wald ratio	1	-0.0092	0.0190	0.991 (0.955, 1.028)	0.628	0.822	1.16E-17	TRUE
NK	*HIST1H2BB*	Wald ratio	1	-0.0167	0.0274	0.983 (0.932, 1.038)	0.543	0.765	4.88E-08	TRUE
NK	*HIST1H2BI*	Wald ratio	1	-0.0188	0.0219	0.981 (0.94, 1.024)	0.39	0.671	2.52E-12	TRUE
NK	*HIST1H2BL*	Wald ratio	1	0.0200	0.0177	1.02 (0.985, 1.056)	0.259	0.553	1.03E-17	TRUE
NK	*HIST1H2BO*	Wald ratio	1	0.0002	0.0141	1 (0.973, 1.028)	0.987	0.995	1.43E-24	TRUE
NK	*HIST1H3H*	Wald ratio	1	0.0304	0.0130	1.031 (1.005, 1.058)	0.019	0.124	1.70E-20	TRUE
NK	*HIST1H3I*	Wald ratio	1	-0.0268	0.0279	0.974 (0.922, 1.028)	0.335	0.628	3.86E-22	TRUE
NK	*HIST1H4B*	Wald ratio	1	-0.0165	0.0205	0.984 (0.945, 1.024)	0.423	0.693	1.72E-12	TRUE
NK	*HIST1H4I*	Wald ratio	1	0.0125	0.0197	1.013 (0.974, 1.052)	0.527	0.756	2.87E-12	TRUE
NK	*HIST1H4K*	Wald ratio	1	-0.0182	0.0270	0.982 (0.931, 1.035)	0.499	0.742	1.99E-08	TRUE
NK	*HLA-B*	IVW	2	0.0133	0.0257	1.013 (0.964, 1.066)	0.606	0.807	1.43E-86	TRUE
NK	*HLA-C*	Wald ratio	1	-0.0271	0.0097	0.973 (0.955, 0.992)	0.005	0.051	1.98E-20	TRUE
NK	*HLA-DOA*	Wald ratio	1	0.0739	0.0216	1.077 (1.032, 1.123)	6.10E-04	0.012	9.54E-09	TRUE
NK	*HLA-DPA1*	Wald ratio	1	0.0385	0.0137	1.039 (1.012, 1.067)	0.005	0.049	6.09E-10	TRUE
NK	*HLA-F*	Wald ratio	1	-0.0099	0.0109	0.99 (0.969, 1.011)	0.362	0.651	5.23E-27	TRUE
NK	*HLA-G*	IVW	2	0.0259	0.0180	1.026 (0.991, 1.063)	0.15	0.419	7.43E-15	TRUE
NK	*HMBOX1*	Wald ratio	1	-0.0123	0.0067	0.988 (0.975, 1.001)	0.066	0.263	6.30E-59	TRUE
NK	*HMGN2*	Wald ratio	1	0.0305	0.0186	1.031 (0.994, 1.069)	0.102	0.338	3.15E-09	TRUE
NK	*HNRNPU*	Wald ratio	1	-0.0562	0.0180	0.945 (0.912, 0.979)	0.002	0.025	3.42E-08	TRUE
NK	*HOPX*	Wald ratio	1	-0.0336	0.0172	0.967 (0.935, 1)	0.051	0.223	1.02E-10	TRUE
NK	*HOXB-AS1*	Wald ratio	1	-0.0094	0.0156	0.991 (0.961, 1.021)	0.546	0.767	1.02E-12	TRUE
NK	*HOXB2*	Wald ratio	1	-0.0240	0.0101	0.976 (0.957, 0.996)	0.017	0.116	1.06E-27	TRUE
NK	*HPGD*	IVW	2	-0.0056	0.0089	0.994 (0.977, 1.012)	0.529	0.757	1.61E-38	TRUE
NK	*HSBP1L1*	Wald ratio	1	-0.0767	0.0200	0.926 (0.891, 0.963)	1.23E-04	0.003	5.16E-10	TRUE
NK	*HSD17B11*	Wald ratio	1	0.0100	0.0204	1.01 (0.97, 1.051)	0.623	0.822	5.81E-09	TRUE
NK	*HSD17B12*	Wald ratio	1	-0.0650	0.0197	0.937 (0.902, 0.974)	9.95E-04	0.017	5.84E-08	TRUE
NK	*HSP90B1*	Wald ratio	1	-0.0263	0.0203	0.974 (0.936, 1.014)	0.195	0.477	4.86E-08	TRUE
NK	*HSPBAP1*	Wald ratio	1	0.0081	0.0087	1.008 (0.991, 1.026)	0.352	0.644	2.83E-31	TRUE
NK	*IAH1*	Wald ratio	1	0.0274	0.0181	1.028 (0.992, 1.065)	0.129	0.389	7.36E-09	TRUE
NK	*ICA1L*	Wald ratio	1	-0.0086	0.0182	0.991 (0.957, 1.028)	0.636	0.827	2.64E-09	TRUE
NK	*IDI1*	Wald ratio	1	-0.0304	0.0283	0.97 (0.918, 1.025)	0.282	0.575	2.37E-18	TRUE
NK	*IFI27L1*	Wald ratio	1	-0.0321	0.0152	0.968 (0.94, 0.998)	0.035	0.176	4.25E-12	TRUE
NK	*IFITM1*	Wald ratio	1	-0.0202	0.0170	0.98 (0.948, 1.013)	0.234	0.528	4.30E-13	TRUE
NK	*IFITM2*	IVW	2	-0.0069	0.0063	0.993 (0.981, 1.005)	0.272	0.566	2.96E-92	TRUE
NK	*IFITM3*	IVW	3	0.0056	0.0051	1.006 (0.996, 1.016)	0.272	0.565	4.03E-137	TRUE
NK	*IFITM3*	MR Egger	3	-0.0049	0.0120	0.995 (0.972, 1.019)	0.753	NA	NA	NA
NK	*IFITM3*	Weighted median	3	0.0049	0.0052	1.005 (0.995, 1.015)	0.339	NA	NA	NA
NK	*IFITM3*	Simple mode	3	0.0117	0.0105	1.012 (0.991, 1.033)	0.382	NA	NA	NA
NK	*IFITM3*	Weighted mode	3	0.0039	0.0055	1.004 (0.993, 1.015)	0.552	NA	NA	NA
NK	*IGF2BP2*	Wald ratio	1	0.0666	0.0183	1.069 (1.031, 1.108)	2.73E-04	0.007	4.05E-08	TRUE
NK	*IGFBP7*	IVW	2	-0.0106	0.0126	0.989 (0.965, 1.014)	0.401	0.679	3.17E-18	TRUE
NK	*IGLL1*	Wald ratio	1	-0.0103	0.0071	0.99 (0.976, 1.004)	0.146	0.415	4.41E-56	TRUE
NK	*IKBKE*	Wald ratio	1	-0.0039	0.0160	0.996 (0.965, 1.028)	0.807	0.917	3.77E-11	TRUE
NK	*IL10RB*	Wald ratio	1	0.0322	0.0190	1.033 (0.995, 1.072)	0.089	0.316	4.00E-08	TRUE
NK	*IL12RB1*	Wald ratio	1	-0.0473	0.0183	0.954 (0.92, 0.989)	0.01	0.081	3.48E-09	TRUE
NK	*IL12RB2*	Wald ratio	1	0.0233	0.0215	1.024 (0.981, 1.068)	0.28	0.574	6.72E-12	TRUE
NK	*IL18R1*	Wald ratio	1	0.0016	0.0161	1.002 (0.97, 1.034)	0.919	0.965	1.24E-11	TRUE
NK	*IL18RAP*	Wald ratio	1	0.0155	0.0176	1.016 (0.981, 1.051)	0.378	0.665	3.02E-10	TRUE
NK	*IL27RA*	Wald ratio	1	-0.0250	0.0168	0.975 (0.944, 1.008)	0.138	0.401	8.32E-11	TRUE
NK	*IL32*	IVW	2	-0.0168	0.0103	0.983 (0.964, 1.003)	0.105	0.343	2.87E-27	TRUE
NK	*ILKAP*	Wald ratio	1	0.0409	0.0192	1.042 (1.003, 1.082)	0.033	0.172	7.06E-09	TRUE
NK	*IMPDH1*	Wald ratio	1	0.0154	0.0148	1.016 (0.987, 1.045)	0.297	0.592	5.75E-10	TRUE
NK	*IP6K2*	Wald ratio	1	-0.0067	0.0185	0.993 (0.958, 1.03)	0.719	0.874	1.55E-09	TRUE
NK	*IQCB1*	Wald ratio	1	-0.0143	0.0201	0.986 (0.948, 1.025)	0.477	0.733	1.96E-08	TRUE
NK	*IQSEC3*	Wald ratio	1	0.0043	0.0103	1.004 (0.984, 1.025)	0.675	0.852	9.45E-28	TRUE
NK	*IRF1*	Wald ratio	1	0.0117	0.0187	1.012 (0.975, 1.049)	0.531	0.758	8.62E-11	TRUE
NK	*IRF6*	Wald ratio	1	0.0179	0.0125	1.018 (0.993, 1.043)	0.152	0.422	8.72E-19	TRUE
NK	*IRF7*	Wald ratio	1	-0.0059	0.0211	0.994 (0.954, 1.036)	0.778	0.904	5.40E-08	TRUE
NK	*ISCA1*	Wald ratio	1	0.0326	0.0184	1.033 (0.997, 1.071)	0.076	0.288	7.22E-09	TRUE
NK	*ITCH*	Wald ratio	1	-0.0121	0.0171	0.988 (0.955, 1.022)	0.481	0.735	4.53E-11	TRUE

NK	*ITCH-AS1*	Wald ratio	1	-0.0112	0.0172	0.989 (0.956, 1.023)	0.514	0.75	1.72E-10	TRUE
NK	*ITGB2*	Wald ratio	1	-0.0006	0.0046	0.999 (0.991, 1.008)	0.897	0.957	8.42E-128	TRUE
NK	*ITGB2-AS1*	Wald ratio	1	-0.0091	0.0198	0.991 (0.953, 1.03)	0.645	0.834	9.98E-09	TRUE
NK	*ITPA*	Wald ratio	1	0.0198	0.0137	1.02 (0.993, 1.048)	0.148	0.417	2.49E-20	TRUE
NK	*ITPR3*	Wald ratio	1	-0.0180	0.0213	0.982 (0.942, 1.024)	0.399	0.678	2.71E-08	TRUE
NK	*IVNS1ABP*	Wald ratio	1	0.0069	0.0071	1.007 (0.993, 1.021)	0.331	0.624	1.22E-60	TRUE
NK	*JAKMIP1*	Wald ratio	1	0.0057	0.0094	1.006 (0.987, 1.024)	0.546	0.767	4.61E-33	TRUE
NK	*JAKMIP2*	Wald ratio	1	0.0666	0.0214	1.069 (1.025, 1.115)	0.002	0.025	9.58E-08	TRUE
NK	*KANSL1-AS1*	Wald ratio	1	-0.0161	0.0069	0.984 (0.971, 0.997)	0.02	0.125	4.74E-149	TRUE
NK	*KCNAB2*	Wald ratio	1	0.0120	0.0135	1.012 (0.986, 1.039)	0.374	0.662	2.80E-20	TRUE
NK	*KCTD10*	Wald ratio	1	0.0059	0.0081	1.006 (0.99, 1.022)	0.466	0.725	3.74E-39	TRUE
NK	*KDELR2*	Wald ratio	1	0.0239	0.0074	1.024 (1.009, 1.039)	0.001	0.02	1.34E-49	TRUE
NK	*KDSR*	Wald ratio	1	-0.0029	0.0186	0.997 (0.961, 1.034)	0.875	0.948	1.09E-09	TRUE
NK	*KHK*	Wald ratio	1	-0.0754	0.0206	0.927 (0.891, 0.966)	2.50E-04	0.006	1.05E-07	TRUE
NK	*KIAA1377*	Wald ratio	1	0.0102	0.0206	1.01 (0.97, 1.052)	0.62	0.819	6.64E-08	TRUE
NK	*KIAA1671*	Wald ratio	1	-0.0064	0.0181	0.994 (0.959, 1.029)	0.723	0.877	1.25E-10	TRUE
NK	*KIAA1683*	Wald ratio	1	-0.0086	0.0187	0.991 (0.956, 1.029)	0.646	0.834	9.68E-09	TRUE
NK	*KIR2DL3*	Wald ratio	1	0.0158	0.0144	1.016 (0.988, 1.045)	0.274	0.567	1.14E-09	TRUE
NK	*KIR3DL1*	Wald ratio	1	0.0055	0.0101	1.006 (0.986, 1.026)	0.586	0.794	1.59E-26	TRUE
NK	*KIR3DL2*	IVW	2	-0.0067	0.0110	0.993 (0.972, 1.015)	0.541	0.763	6.95E-25	TRUE
NK	*KIR3DX1*	Wald ratio	1	0.0194	0.0203	1.02 (0.98, 1.061)	0.339	0.632	6.60E-08	TRUE
NK	*KLB*	Wald ratio	1	0.0131	0.0116	1.013 (0.99, 1.036)	0.261	0.555	2.54E-21	TRUE
NK	*KLF7*	Wald ratio	1	-0.0280	0.0196	0.972 (0.936, 1.011)	0.154	0.425	1.15E-08	TRUE
NK	*KLHDC4*	Wald ratio	1	-0.0024	0.0101	0.998 (0.978, 1.018)	0.816	0.921	1.10E-21	TRUE
NK	*KLHL25*	Wald ratio	1	-0.0369	0.0177	0.964 (0.931, 0.998)	0.038	0.187	3.10E-10	TRUE
NK	*KLRC2*	Wald ratio	1	-0.0112	0.0092	0.989 (0.971, 1.007)	0.223	0.515	3.40E-35	TRUE
NK	*KLRC3*	Wald ratio	1	0.0164	0.0148	1.016 (0.988, 1.046)	0.268	0.562	9.94E-14	TRUE
NK	*KLRC4*	Wald ratio	1	0.0221	0.0160	1.022 (0.991, 1.055)	0.166	0.442	4.27E-12	TRUE
NK	*KLRD1*	Wald ratio	1	-0.0098	0.0099	0.99 (0.971, 1.01)	0.323	0.618	2.58E-28	TRUE
NK	*KLRK1*	Wald ratio	1	-0.0238	0.0181	0.977 (0.942, 1.012)	0.19	0.471	3.71E-10	TRUE
NK	*KRT10*	Wald ratio	1	-0.0332	0.0162	0.967 (0.937, 0.999)	0.04	0.195	1.01E-09	TRUE
NK	*KRT72*	Wald ratio	1	-0.0375	0.0136	0.963 (0.938, 0.989)	0.006	0.056	5.06E-15	TRUE
NK	*KRT73*	Wald ratio	1	-0.0060	0.0241	0.994 (0.948, 1.042)	0.803	0.915	3.60E-08	TRUE
NK	*KTN1*	Wald ratio	1	0.0433	0.0141	1.044 (1.016, 1.073)	0.002	0.027	9.81E-15	TRUE
NK	*KYNU*	Wald ratio	1	-0.0061	0.0193	0.994 (0.957, 1.032)	0.754	0.888	1.61E-10	TRUE
NK	*L3MBTL3*	Wald ratio	1	-0.0129	0.0151	0.987 (0.958, 1.017)	0.393	0.672	1.02E-13	TRUE
NK	*LA16c-358B7.3*	Wald ratio	1	0.0358	0.0090	1.036 (1.018, 1.055)	6.58E-05	0.002	1.60E-38	TRUE
NK	*LAIR2*	IVW	2	0.0279	0.0093	1.028 (1.01, 1.047)	0.003	0.032	7.19E-43	TRUE
NK	*LAMB1*	Wald ratio	1	0.0088	0.0090	1.009 (0.991, 1.027)	0.332	0.625	7.17E-36	TRUE
NK	*LAPTM5*	Wald ratio	1	-0.0652	0.0200	0.937 (0.901, 0.974)	0.001	0.018	1.18E-07	TRUE
NK	*LAT*	Wald ratio	1	0.0120	0.0133	1.012 (0.986, 1.039)	0.368	0.656	2.53E-13	TRUE
NK	*LBX2*	Wald ratio	1	-0.0258	0.0203	0.975 (0.937, 1.014)	0.203	0.488	3.06E-10	TRUE
NK	*LCNL1*	Wald ratio	1	0.0102	0.0196	1.01 (0.972, 1.05)	0.601	0.805	9.10E-09	TRUE
NK	*LCP1*	Wald ratio	1	-0.0220	0.0218	0.978 (0.937, 1.021)	0.313	0.607	1.98E-09	TRUE
NK	*LDHC*	Wald ratio	1	-0.0167	0.0118	0.983 (0.961, 1.006)	0.157	0.428	8.95E-25	TRUE
NK	*LEPROT*	Wald ratio	1	0.0198	0.0125	1.02 (0.995, 1.045)	0.112	0.358	4.72E-19	TRUE
NK	*LGR6*	Wald ratio	1	0.0396	0.0190	1.04 (1.002, 1.08)	0.037	0.186	1.49E-08	TRUE
NK	*LILRB4*	Wald ratio	1	0.0005	0.0154	1.001 (0.971, 1.031)	0.973	0.989	2.08E-11	TRUE
NK	*LIMD1*	Wald ratio	1	0.0116	0.0129	1.012 (0.986, 1.038)	0.368	0.656	2.73E-18	TRUE
NK	*LIN52*	Wald ratio	1	-0.0023	0.0209	0.998 (0.958, 1.039)	0.912	0.962	4.25E-08	TRUE
NK	*LINC00299*	Wald ratio	1	0.0024	0.0171	1.002 (0.969, 1.037)	0.888	0.955	5.25E-10	TRUE
NK	*LINC00339*	Wald ratio	1	-0.0159	0.0097	0.984 (0.966, 1.003)	0.1	0.334	1.29E-28	TRUE
NK	*LINC00565*	Wald ratio	1	-0.0287	0.0114	0.972 (0.95, 0.994)	0.012	0.093	3.79E-22	TRUE
NK	*LINC00665*	Wald ratio	1	-0.0488	0.0191	0.952 (0.917, 0.989)	0.011	0.085	1.26E-08	TRUE
NK	*LINC00861*	Wald ratio	1	-0.0306	0.0200	0.97 (0.933, 1.009)	0.125	0.38	6.50E-08	TRUE
NK	*LINC00886*	Wald ratio	1	-0.0147	0.0149	0.985 (0.957, 1.015)	0.323	0.618	1.70E-13	TRUE
NK	*LINC00920*	Wald ratio	1	0.0035	0.0075	1.004 (0.989, 1.018)	0.636	0.827	3.28E-52	TRUE
NK	*LINC00944*	Wald ratio	1	0.0084	0.0155	1.008 (0.978, 1.04)	0.59	0.797	3.45E-08	TRUE
NK	*LINC00954*	Wald ratio	1	-0.0147	0.0169	0.985 (0.953, 1.019)	0.385	0.668	2.69E-13	TRUE
NK	*LINC00969*	Wald ratio	1	-0.0082	0.0094	0.992 (0.974, 1.01)	0.384	0.668	1.85E-32	TRUE
NK	*LINC01137*	Wald ratio	1	-0.0024	0.0187	0.998 (0.962, 1.035)	0.897	0.957	1.88E-08	TRUE
NK	*LINGO2*	Wald ratio	1	0.0314	0.0177	1.032 (0.997, 1.068)	0.076	0.289	1.64E-09	TRUE
NK	*LIPC*	Wald ratio	1	-0.0610	0.0218	0.941 (0.902, 0.982)	0.005	0.051	2.40E-12	TRUE
NK	*LL22NC03-2H8.5*	Wald ratio	1	0.0017	0.0166	1.002 (0.97, 1.035)	0.916	0.963	1.01E-11	TRUE
NK	*LLGL1*	Wald ratio	1	0.0945	0.0194	1.099 (1.058, 1.142)	1.07E-06	1.17E-04	3.12E-08	TRUE
NK	*LPIN1*	Wald ratio	1	0.0254	0.0106	1.026 (1.005, 1.047)	0.016	0.111	9.95E-22	TRUE
NK	*LRRC37A2*	Wald ratio	1	-0.0637	0.0269	0.938 (0.89, 0.989)	0.018	0.119	4.98E-10	TRUE
NK	*LRRC6*	Wald ratio	1	0.0067	0.0094	1.007 (0.988, 1.026)	0.476	0.732	4.43E-33	TRUE
NK	*LRRC61*	Wald ratio	1	-0.0542	0.0181	0.947 (0.914, 0.981)	0.003	0.033	3.74E-09	TRUE
NK	*LRRIQ3*	Wald ratio	1	-0.0015	0.0152	0.999 (0.969, 1.029)	0.922	0.965	2.65E-13	TRUE
NK	*LSM7*	Wald ratio	1	0.0413	0.0127	1.042 (1.017, 1.068)	0.001	0.018	5.83E-18	TRUE
NK	*LSS*	Wald ratio	1	0.0085	0.0205	1.009 (0.969, 1.05)	0.678	0.853	1.89E-08	TRUE
NK	*LTA4H*	Wald ratio	1	-0.0172	0.0141	0.983 (0.956, 1.011)	0.224	0.517	5.53E-08	TRUE
NK	*LTBP1*	Wald ratio	1	-0.0219	0.0097	0.978 (0.96, 0.997)	0.024	0.144	3.38E-31	TRUE
NK	*LY6G5B*	Wald ratio	1	-0.1099	0.0214	0.896 (0.859, 0.934)	2.99E-07	8.32E-05	7.14E-09	TRUE
NK	*LY6G6F*	Wald ratio	1	-0.0243	0.0156	0.976 (0.947, 1.006)	0.118	0.367	3.47E-20	TRUE
NK	*LYAR*	Wald ratio	1	-0.0100	0.0075	0.99 (0.976, 1.005)	0.182	0.462	1.07E-52	TRUE
NK	*LYRM4*	Wald ratio	1	0.0169	0.0131	1.017 (0.991, 1.043)	0.197	0.48	1.40E-14	TRUE
NK	*LYST*	Wald ratio	1	-0.0228	0.0218	0.977 (0.936, 1.02)	0.296	0.591	3.09E-08	TRUE

NK	*MAD1L1*	Wald ratio	1	0.0157	0.0151	1.016 (0.986, 1.046)	0.299	0.594	5.02E-14	TRUE
NK	*MAF1*	Wald ratio	1	0.0479	0.0295	1.049 (0.99, 1.112)	0.105	0.343	3.56E-08	TRUE
NK	*MAFF*	Wald ratio	1	0.0002	0.0203	1 (0.961, 1.041)	0.994	0.998	4.42E-08	TRUE
NK	*MAGEF1*	Wald ratio	1	0.0152	0.0185	1.015 (0.979, 1.053)	0.414	0.686	5.40E-09	TRUE
NK	*MAP6D1*	Wald ratio	1	-0.0122	0.0173	0.988 (0.955, 1.022)	0.481	0.735	1.53E-11	TRUE
NK	*MARCH10*	Wald ratio	1	0.0299	0.0175	1.03 (0.996, 1.066)	0.088	0.313	2.60E-09	TRUE
NK	*MARCH2*	Wald ratio	1	0.0379	0.0200	1.039 (0.999, 1.08)	0.059	0.244	3.80E-09	TRUE
NK	*MAST3*	Wald ratio	1	0.0311	0.0114	1.032 (1.009, 1.055)	0.007	0.06	2.92E-22	TRUE
NK	*MAST4*	Wald ratio	1	0.0257	0.0169	1.026 (0.992, 1.061)	0.13	0.39	4.53E-11	TRUE
NK	*MBP*	IVW	2	0.0118	0.0308	1.012 (0.953, 1.075)	0.702	0.866	2.11E-40	TRUE
NK	*MC1R*	Wald ratio	1	0.0124	0.0100	1.013 (0.993, 1.033)	0.215	0.504	1.56E-35	TRUE
NK	*MDGA1*	Wald ratio	1	-0.0063	0.0081	0.994 (0.978, 1.01)	0.435	0.702	1.76E-45	TRUE
NK	*MDM4*	Wald ratio	1	-0.0797	0.0205	0.923 (0.887, 0.961)	1.02E-04	0.003	2.18E-09	TRUE
NK	*MEAF6*	Wald ratio	1	-0.0382	0.0163	0.963 (0.932, 0.994)	0.019	0.124	2.47E-10	TRUE
NK	*MED16*	Wald ratio	1	-0.0005	0.0077	1 (0.984, 1.015)	0.948	0.98	6.41E-59	TRUE
NK	*MEI1*	Wald ratio	1	0.0572	0.0186	1.059 (1.021, 1.098)	0.002	0.027	5.66E-08	TRUE
NK	*MESP1*	Wald ratio	1	0.0200	0.0197	1.02 (0.982, 1.06)	0.31	0.604	6.29E-11	TRUE
NK	*METTL18*	Wald ratio	1	0.0000	0.0218	1 (0.958, 1.044)	1	1	1.14E-08	TRUE
NK	*MFF*	Wald ratio	1	-0.0029	0.0170	0.997 (0.964, 1.031)	0.866	0.944	7.36E-09	TRUE
NK	*MGME1*	Wald ratio	1	0.0130	0.0188	1.013 (0.976, 1.051)	0.489	0.738	7.92E-14	TRUE
NK	*MGST2*	Wald ratio	1	-0.0100	0.0156	0.99 (0.96, 1.021)	0.522	0.754	3.43E-12	TRUE
NK	*MGST3*	Wald ratio	1	0.0003	0.0088	1 (0.983, 1.018)	0.97	0.989	8.19E-38	TRUE
NK	*MIAT*	Wald ratio	1	-0.0025	0.0083	0.998 (0.981, 1.014)	0.768	0.897	5.84E-36	TRUE
NK	*MICAL3*	Wald ratio	1	-0.0180	0.0177	0.982 (0.949, 1.017)	0.307	0.601	3.09E-10	TRUE
NK	*MICB*	Wald ratio	1	0.0478	0.0227	1.049 (1.003, 1.097)	0.035	0.179	1.57E-08	TRUE
NK	*MIF*	IVW	2	0.0088	0.0083	1.009 (0.993, 1.025)	0.291	0.584	2.71E-43	TRUE
NK	*MIR181A2HG*	Wald ratio	1	0.0093	0.0158	1.009 (0.978, 1.041)	0.558	0.776	2.05E-12	TRUE
NK	*MKRN1*	Wald ratio	1	-0.0229	0.0192	0.977 (0.941, 1.015)	0.233	0.527	3.30E-09	TRUE
NK	*MLC1*	Wald ratio	1	0.0411	0.0198	1.042 (1.002, 1.083)	0.038	0.189	3.47E-09	TRUE
NK	*MLF1*	Wald ratio	1	0.0715	0.0132	1.074 (1.047, 1.102)	6.83E-08	6.60E-05	2.11E-13	TRUE
NK	*MLF2*	Wald ratio	1	-0.0173	0.0177	0.983 (0.949, 1.018)	0.329	0.623	4.17E-10	TRUE
NK	*MLKL*	Wald ratio	1	-0.0723	0.0200	0.93 (0.895, 0.967)	2.98E-04	0.007	6.52E-08	TRUE
NK	*MMAB*	Wald ratio	1	0.0193	0.0232	1.019 (0.974, 1.067)	0.405	0.681	8.98E-09	TRUE
NK	*MMEL1*	Wald ratio	1	0.0336	0.0154	1.034 (1.003, 1.066)	0.029	0.16	7.19E-13	TRUE
NK	*MPHOSPH9*	Wald ratio	1	-0.0461	0.0147	0.955 (0.928, 0.983)	0.002	0.024	1.15E-12	TRUE
NK	*MRPL10*	Wald ratio	1	-0.0168	0.0090	0.983 (0.966, 1.001)	0.062	0.253	6.06E-35	TRUE
NK	*MRPL14*	Wald ratio	1	-0.0089	0.0194	0.991 (0.954, 1.03)	0.645	0.834	1.06E-08	TRUE
NK	*MRPL18*	Wald ratio	1	-0.0022	0.0223	0.998 (0.955, 1.042)	0.92	0.965	3.58E-08	TRUE
NK	*MRPL21*	Wald ratio	1	0.0118	0.0126	1.012 (0.987, 1.037)	0.349	0.643	1.16E-16	TRUE
NK	*MRPL32*	Wald ratio	1	0.0262	0.0185	1.027 (0.99, 1.065)	0.158	0.429	3.87E-16	TRUE
NK	*MRPL41*	Wald ratio	1	-0.0110	0.0095	0.989 (0.971, 1.008)	0.248	0.541	3.91E-34	TRUE
NK	*MRPL54*	Wald ratio	1	0.0085	0.0096	1.009 (0.99, 1.028)	0.377	0.665	1.31E-30	TRUE
NK	*MRPS18C*	Wald ratio	1	-0.0022	0.0176	0.998 (0.964, 1.033)	0.899	0.958	2.27E-10	TRUE
NK	*MRPS6*	Wald ratio	1	-0.0454	0.0185	0.956 (0.922, 0.991)	0.014	0.101	1.18E-09	TRUE
NK	*MSH3*	Wald ratio	1	0.0347	0.0187	1.035 (0.998, 1.074)	0.063	0.256	5.68E-10	TRUE
NK	*MT1E*	Wald ratio	1	-0.0315	0.0172	0.969 (0.937, 1.002)	0.067	0.265	3.76E-12	TRUE
NK	*MT1X*	Wald ratio	1	0.0079	0.0206	1.008 (0.968, 1.049)	0.702	0.866	2.55E-08	TRUE
NK	*MT2A*	Wald ratio	1	-0.0099	0.0062	0.99 (0.978, 1.002)	0.11	0.353	5.93E-91	TRUE
NK	*MTSS1*	IVW	2	0.0173	0.0152	1.017 (0.988, 1.048)	0.254	0.548	3.17E-23	TRUE
NK	*MTUS1*	Wald ratio	1	-0.0058	0.0154	0.994 (0.965, 1.025)	0.705	0.867	9.82E-15	TRUE
NK	*MVP*	Wald ratio	1	-0.0435	0.0211	0.957 (0.919, 0.998)	0.039	0.192	1.37E-08	TRUE
NK	*MX1*	Wald ratio	1	0.0171	0.0153	1.017 (0.987, 1.048)	0.263	0.558	5.87E-13	TRUE
NK	*MX2*	Wald ratio	1	0.0095	0.0145	1.01 (0.981, 1.039)	0.514	0.75	2.70E-14	TRUE
NK	*MXRA7*	Wald ratio	1	0.0057	0.0157	1.006 (0.975, 1.037)	0.715	0.873	1.42E-09	TRUE
NK	*MYCBPAP*	Wald ratio	1	0.0038	0.0141	1.004 (0.976, 1.032)	0.789	0.909	2.10E-15	TRUE
NK	*MYH11*	Wald ratio	1	-0.0225	0.0140	0.978 (0.951, 1.005)	0.109	0.35	9.04E-16	TRUE
NK	*MYL12A*	Wald ratio	1	0.0049	0.0076	1.005 (0.99, 1.02)	0.521	0.754	2.74E-31	TRUE
NK	*MYO15B*	Wald ratio	1	-0.0167	0.0227	0.983 (0.941, 1.028)	0.462	0.721	3.38E-09	TRUE
NK	*MYO1F*	Wald ratio	1	-0.0214	0.0129	0.979 (0.954, 1.004)	0.097	0.331	1.01E-18	TRUE
NK	*MYO1G*	Wald ratio	1	-0.0299	0.0173	0.971 (0.938, 1.004)	0.084	0.308	1.34E-09	TRUE
NK	*MYOM2*	Wald ratio	1	-0.0038	0.0044	0.996 (0.988, 1.005)	0.392	0.672	3.50E-172	TRUE
NK	*MYRF*	Wald ratio	1	-0.0315	0.0153	0.969 (0.94, 0.999)	0.04	0.193	4.98E-13	TRUE
NK	*MZT2A*	Wald ratio	1	-0.0117	0.0062	0.988 (0.977, 1)	0.058	0.244	2.78E-70	TRUE
NK	*NAA38*	Wald ratio	1	-0.0086	0.0125	0.991 (0.967, 1.016)	0.494	0.739	4.97E-25	TRUE
NK	*NAA40*	Wald ratio	1	-0.0669	0.0205	0.935 (0.898, 0.974)	0.001	0.018	1.08E-07	TRUE
NK	*NAAA*	IVW	2	0.0193	0.0121	1.019 (0.996, 1.044)	0.11	0.353	2.00E-34	TRUE
NK	*NAPRT1*	Wald ratio	1	-0.0119	0.0072	0.988 (0.974, 1.002)	0.099	0.333	5.41E-54	TRUE
NK	*NBEAL2*	Wald ratio	1	0.0104	0.0118	1.01 (0.987, 1.034)	0.379	0.666	2.54E-21	TRUE
NK	*NBPF1*	Wald ratio	1	-0.0431	0.0218	0.958 (0.918, 1)	0.048	0.216	4.64E-08	TRUE
NK	*NBPF3*	Wald ratio	1	-0.0001	0.0132	1 (0.974, 1.026)	0.997	0.999	1.03E-17	TRUE
NK	*NCL*	Wald ratio	1	-0.0172	0.0113	0.983 (0.961, 1.005)	0.129	0.388	6.09E-19	TRUE
NK	*NCR3*	Wald ratio	1	-0.0042	0.0122	0.996 (0.972, 1.02)	0.731	0.88	6.68E-24	TRUE
NK	*NDUFA10*	Wald ratio	1	-0.0361	0.0183	0.965 (0.931, 1)	0.049	0.22	4.73E-10	TRUE
NK	*NDUFA12*	Wald ratio	1	0.0036	0.0075	1.004 (0.989, 1.018)	0.626	0.822	3.37E-61	TRUE
NK	*NDUFA3*	Wald ratio	1	0.0014	0.0115	1.001 (0.979, 1.024)	0.9	0.958	4.58E-22	TRUE
NK	*NDUFA6*	Wald ratio	1	0.0016	0.0248	1.002 (0.954, 1.051)	0.948	0.98	4.62E-09	TRUE
NK	*NDUFB2*	Wald ratio	1	-0.0236	0.0153	0.977 (0.948, 1.006)	0.122	0.376	5.26E-11	TRUE
NK	*NDUFC2*	Wald ratio	1	0.0396	0.0207	1.04 (0.999, 1.083)	0.056	0.237	7.79E-09	TRUE
NK	*NDUFS5*	Wald ratio	1	-0.0033	0.0075	0.997 (0.982, 1.012)	0.663	0.845	6.69E-53	TRUE

NK	*NDUFS8*	Wald ratio	1	-0.0285	0.0105	0.972 (0.952, 0.992)	0.007	0.063	1.49E-21	TRUE
NK	*NEAT1*	Wald ratio	1	-0.0209	0.0204	0.979 (0.941, 1.019)	0.306	0.599	7.32E-11	TRUE
NK	*NEXN*	Wald ratio	1	-0.0223	0.0179	0.978 (0.944, 1.013)	0.213	0.501	2.55E-11	TRUE
NK	*NFE2L3*	Wald ratio	1	0.0077	0.0094	1.008 (0.989, 1.026)	0.409	0.685	6.20E-34	TRUE
NK	*NGRN*	Wald ratio	1	-0.0633	0.0213	0.939 (0.9, 0.979)	0.003	0.034	7.65E-08	TRUE
NK	*NINJ1*	Wald ratio	1	0.0142	0.0118	1.014 (0.991, 1.038)	0.228	0.521	4.40E-18	TRUE
NK	*NIPAL2*	Wald ratio	1	0.0013	0.0191	1.001 (0.964, 1.04)	0.947	0.979	5.59E-08	TRUE
NK	*NKAPL*	Wald ratio	1	-0.0168	0.0248	0.983 (0.937, 1.032)	0.497	0.741	7.88E-09	TRUE
NK	*NLRP2*	Wald ratio	1	-0.0094	0.0151	0.991 (0.962, 1.02)	0.533	0.76	1.30E-13	TRUE
NK	*NME1*	Wald ratio	1	0.0049	0.0133	1.005 (0.979, 1.031)	0.71	0.871	3.20E-17	TRUE
NK	*NMI*	Wald ratio	1	0.0261	0.0124	1.026 (1.002, 1.052)	0.036	0.18	7.60E-19	TRUE
NK	*NMRAL1*	IVW	2	0.0207	0.0141	1.021 (0.993, 1.05)	0.142	0.41	2.32E-16	TRUE
NK	*NMRK1*	Wald ratio	1	-0.0060	0.0088	0.994 (0.977, 1.011)	0.495	0.74	1.76E-44	TRUE
NK	*NMUR1*	Wald ratio	1	-0.0173	0.0136	0.983 (0.957, 1.009)	0.202	0.487	9.32E-15	TRUE
NK	*NOP10*	Wald ratio	1	-0.0110	0.0232	0.989 (0.945, 1.035)	0.635	0.827	1.00E-08	TRUE
NK	*NPRL3*	Wald ratio	1	-0.0102	0.0108	0.99 (0.969, 1.011)	0.342	0.635	3.72E-15	TRUE
NK	*NQO2*	Wald ratio	1	-0.0220	0.0188	0.978 (0.943, 1.015)	0.24	0.534	3.54E-09	TRUE
NK	*NRROS*	Wald ratio	1	-0.0096	0.0121	0.99 (0.967, 1.014)	0.426	0.696	3.17E-19	TRUE
NK	*NSA2*	Wald ratio	1	0.0217	0.0092	1.022 (1.004, 1.04)	0.018	0.119	1.41E-38	TRUE
NK	*NSUN4*	Wald ratio	1	-0.0002	0.0153	1 (0.97, 1.03)	0.991	0.996	7.51E-13	TRUE
NK	*NSUN5*	Wald ratio	1	-0.0409	0.0187	0.96 (0.925, 0.996)	0.029	0.158	3.02E-09	TRUE
NK	*NT5C*	Wald ratio	1	0.0037	0.0184	1.004 (0.968, 1.041)	0.841	0.933	2.01E-09	TRUE
NK	*NT5C3B*	Wald ratio	1	-0.0001	0.0059	1 (0.988, 1.011)	0.986	0.995	1.39E-73	TRUE
NK	*NTPCR*	Wald ratio	1	0.0186	0.0135	1.019 (0.992, 1.046)	0.17	0.446	6.22E-19	TRUE
NK	*NUB1*	Wald ratio	1	0.0000	0.0208	1 (0.96, 1.042)	1	1	1.19E-08	TRUE
NK	*NUCB2*	Wald ratio	1	-0.0370	0.0074	0.964 (0.95, 0.978)	6.26E-07	9.75E-05	5.17E-51	TRUE
NK	*NUDT14*	Wald ratio	1	0.0025	0.0112	1.002 (0.981, 1.025)	0.826	0.927	1.21E-23	TRUE
NK	*NUDT2*	Wald ratio	1	-0.0054	0.0094	0.995 (0.976, 1.013)	0.567	0.782	2.63E-30	TRUE
NK	*NUP107*	Wald ratio	1	-0.0252	0.0171	0.975 (0.943, 1.008)	0.14	0.405	3.22E-10	TRUE
NK	*NUTF2*	Wald ratio	1	0.0149	0.0200	1.015 (0.976, 1.056)	0.457	0.719	1.23E-13	TRUE
NK	*NXPE3*	Wald ratio	1	-0.0135	0.0166	0.987 (0.955, 1.019)	0.416	0.688	3.07E-10	TRUE
NK	*OAT*	Wald ratio	1	-0.0053	0.0240	0.995 (0.949, 1.043)	0.827	0.928	3.87E-09	TRUE
NK	*ORAI1*	Wald ratio	1	-0.0350	0.0228	0.966 (0.923, 1.01)	0.125	0.38	3.64E-08	TRUE
NK	*ORAI2*	Wald ratio	1	-0.0036	0.0129	0.996 (0.972, 1.022)	0.783	0.906	3.68E-18	TRUE
NK	*ORMDL3*	Wald ratio	1	-0.0025	0.0063	0.997 (0.985, 1.01)	0.692	0.861	6.76E-75	TRUE
NK	*OSBPL5*	Wald ratio	1	0.0076	0.0140	1.008 (0.98, 1.036)	0.584	0.793	1.56E-17	TRUE
NK	*OSTF1*	IVW	2	-0.0008	0.0091	0.999 (0.982, 1.017)	0.934	0.972	2.35E-36	TRUE
NK	*OVCH1-AS1*	Wald ratio	1	0.0215	0.0143	1.022 (0.994, 1.051)	0.132	0.393	2.24E-16	TRUE
NK	*OXNAD1*	Wald ratio	1	-0.0519	0.0112	0.949 (0.929, 0.97)	3.24E-06	2.62E-04	1.73E-22	TRUE
NK	*P2RY11*	Wald ratio	1	-0.0353	0.0199	0.965 (0.928, 1.004)	0.076	0.288	2.84E-10	TRUE
NK	*P4HA3*	Wald ratio	1	-0.0039	0.0069	0.996 (0.983, 1.01)	0.573	0.786	2.38E-63	TRUE
NK	*PABPC1*	Wald ratio	1	-0.0174	0.0124	0.983 (0.959, 1.007)	0.161	0.434	2.54E-21	TRUE
NK	*PADI4*	Wald ratio	1	-0.0305	0.0115	0.97 (0.948, 0.992)	0.008	0.07	2.98E-20	TRUE
NK	*PAK1*	Wald ratio	1	-0.0166	0.0198	0.984 (0.946, 1.023)	0.403	0.681	3.87E-08	TRUE
NK	*PAPD4*	Wald ratio	1	0.0201	0.0202	1.02 (0.981, 1.062)	0.319	0.614	5.28E-08	TRUE
NK	*PARK7*	Wald ratio	1	-0.0055	0.0143	0.994 (0.967, 1.023)	0.699	0.865	1.36E-15	TRUE
NK	*PARP14*	Wald ratio	1	0.0013	0.0107	1.001 (0.98, 1.023)	0.906	0.96	4.33E-11	TRUE
NK	*PARP8*	Wald ratio	1	0.0325	0.0176	1.033 (0.998, 1.069)	0.065	0.262	6.93E-10	TRUE
NK	*PARVB*	Wald ratio	1	0.0120	0.0137	1.012 (0.985, 1.04)	0.38	0.666	1.12E-16	TRUE
NK	*PAX8*	Wald ratio	1	0.0220	0.0138	1.022 (0.995, 1.05)	0.111	0.355	5.80E-14	TRUE
NK	*PCBP2*	Wald ratio	1	-0.0906	0.0200	0.913 (0.878, 0.95)	5.80E-06	3.74E-04	1.04E-08	TRUE
NK	*PCNT*	Wald ratio	1	0.0217	0.0185	1.022 (0.986, 1.06)	0.239	0.533	1.08E-09	TRUE
NK	*PCNX*	Wald ratio	1	0.0412	0.0155	1.042 (1.011, 1.074)	0.008	0.068	1.17E-12	TRUE
NK	*PCSK5*	Wald ratio	1	-0.0039	0.0120	0.996 (0.973, 1.02)	0.744	0.882	1.82E-20	TRUE
NK	*PCSK6*	Wald ratio	1	-0.0320	0.0183	0.968 (0.934, 1.004)	0.08	0.296	5.27E-09	TRUE
NK	*PCSK7*	Wald ratio	1	-0.0191	0.0158	0.981 (0.951, 1.012)	0.227	0.52	8.42E-11	TRUE
NK	*PDCD5*	Wald ratio	1	-0.0162	0.0167	0.984 (0.952, 1.017)	0.33	0.624	7.72E-11	TRUE
NK	*PDE4B*	Wald ratio	1	-0.0292	0.0116	0.971 (0.949, 0.994)	0.012	0.09	3.47E-22	TRUE
NK	*PDLIM1*	Wald ratio	1	0.0510	0.0320	1.052 (0.988, 1.12)	0.111	0.354	2.64E-11	TRUE
NK	*PDS5B*	Wald ratio	1	0.0034	0.0194	1.003 (0.966, 1.042)	0.862	0.942	9.89E-09	TRUE
NK	*PDXDC1*	Wald ratio	1	-0.0124	0.0175	0.988 (0.954, 1.022)	0.479	0.733	3.50E-10	TRUE
NK	*PDXK*	Wald ratio	1	0.0043	0.0130	1.004 (0.979, 1.03)	0.741	0.881	5.01E-16	TRUE
NK	*PEX6*	Wald ratio	1	0.0136	0.0151	1.014 (0.984, 1.044)	0.367	0.655	2.83E-14	TRUE
NK	*PGLS*	Wald ratio	1	0.0070	0.0127	1.007 (0.982, 1.032)	0.58	0.789	6.90E-19	TRUE
NK	*PHPT1*	Wald ratio	1	-0.0376	0.0147	0.963 (0.936, 0.991)	0.01	0.084	1.32E-13	TRUE
NK	*PI4K2B*	Wald ratio	1	0.0049	0.0231	1.005 (0.96, 1.051)	0.833	0.93	5.31E-12	TRUE
NK	*PIF1*	Wald ratio	1	0.0295	0.0143	1.03 (1.001, 1.059)	0.039	0.192	4.53E-15	TRUE
NK	*PIGC*	Wald ratio	1	-0.0794	0.0193	0.924 (0.889, 0.959)	3.95E-05	0.002	1.53E-07	TRUE
NK	*PIGP*	Wald ratio	1	0.0064	0.0161	1.006 (0.975, 1.039)	0.692	0.861	5.96E-12	TRUE
NK	*PILRB*	Wald ratio	1	0.0037	0.0051	1.004 (0.994, 1.014)	0.468	0.726	4.95E-108	TRUE
NK	*PIP4K2A*	Wald ratio	1	-0.0232	0.0183	0.977 (0.943, 1.013)	0.206	0.494	1.34E-09	TRUE
NK	*PLEK*	Wald ratio	1	0.0127	0.0137	1.013 (0.986, 1.04)	0.351	0.644	6.53E-16	TRUE
NK	*PLGRKT*	Wald ratio	1	-0.0115	0.0167	0.989 (0.957, 1.022)	0.492	0.738	5.44E-11	TRUE
NK	*PLOD1*	Wald ratio	1	-0.0169	0.0079	0.983 (0.968, 0.998)	0.031	0.167	4.99E-42	TRUE
NK	*PLXDC1*	Wald ratio	1	0.0034	0.0071	1.003 (0.989, 1.018)	0.637	0.828	6.93E-73	TRUE
NK	*PLXND1*	Wald ratio	1	0.0201	0.0191	1.02 (0.983, 1.059)	0.293	0.587	1.99E-09	TRUE
NK	*PNPLA2*	Wald ratio	1	0.0246	0.0208	1.025 (0.984, 1.068)	0.237	0.53	4.46E-08	TRUE
NK	*PNRC2*	Wald ratio	1	-0.0102	0.0088	0.99 (0.973, 1.007)	0.248	0.541	3.71E-37	TRUE
NK	*POLM*	Wald ratio	1	0.0117	0.0183	1.012 (0.976, 1.049)	0.523	0.754	3.05E-08	TRUE

NK	*POLR1D*	Wald ratio	1	0.0047	0.0166	1.005 (0.973, 1.038)	0.776	0.902	2.36E-14	TRUE
NK	*POLR2I*	Wald ratio	1	0.0186	0.0143	1.019 (0.991, 1.048)	0.195	0.477	3.33E-15	TRUE
NK	*POLR2J3*	Wald ratio	1	0.0112	0.0176	1.011 (0.977, 1.047)	0.524	0.754	3.80E-11	TRUE
NK	*POMC*	Wald ratio	1	-0.0284	0.0143	0.972 (0.945, 1)	0.047	0.213	5.41E-13	TRUE
NK	*POU5F1*	IVW	3	-0.0138	0.0361	0.986 (0.919, 1.059)	0.703	0.866	3.87E-62	TRUE
NK	*POU5F1*	MR Egger	3	-0.0635	0.1326	0.938 (0.724, 1.217)	0.716	NA	NA	NA
NK	*POU5F1*	Weighted median	3	-0.0411	0.0104	0.96 (0.94, 0.979)	7.18E-05	NA	NA	NA
NK	*POU5F1*	Simple mode	3	-0.0409	0.0130	0.96 (0.936, 0.985)	0.088	NA	NA	NA
NK	*POU5F1*	Weighted mode	3	-0.0426	0.0093	0.958 (0.941, 0.976)	0.044	NA	NA	NA
NK	*PPA1*	Wald ratio	1	0.0041	0.0080	1.004 (0.989, 1.02)	0.603	0.806	3.69E-42	TRUE
NK	*PPDPF*	Wald ratio	1	-0.0071	0.0066	0.993 (0.98, 1.006)	0.283	0.575	5.32E-60	TRUE
NK	*PPFIBP2*	Wald ratio	1	-0.0084	0.0154	0.992 (0.962, 1.022)	0.585	0.794	2.90E-13	TRUE
NK	*PPIA*	Wald ratio	1	0.0159	0.0163	1.016 (0.984, 1.049)	0.33	0.624	8.38E-12	TRUE
NK	*PPIE*	Wald ratio	1	0.0215	0.0128	1.022 (0.996, 1.048)	0.094	0.326	8.19E-18	TRUE
NK	*PPIL3*	Wald ratio	1	0.0078	0.0091	1.008 (0.99, 1.026)	0.393	0.672	1.30E-33	TRUE
NK	*PPM1M*	Wald ratio	1	-0.0415	0.0164	0.959 (0.929, 0.991)	0.012	0.09	2.12E-11	TRUE
NK	*PPP1R9A*	Wald ratio	1	0.0241	0.0110	1.024 (1.003, 1.047)	0.028	0.156	1.17E-24	TRUE
NK	*PPP5C*	Wald ratio	1	0.0096	0.0103	1.01 (0.99, 1.03)	0.35	0.643	1.94E-32	TRUE
NK	*PQLC3*	Wald ratio	1	0.0563	0.0205	1.058 (1.016, 1.101)	0.006	0.057	2.94E-16	TRUE
NK	*PRDM11*	Wald ratio	1	0.0047	0.0085	1.005 (0.988, 1.022)	0.579	0.789	4.32E-40	TRUE
NK	*PRDX3*	Wald ratio	1	-0.0304	0.0199	0.97 (0.933, 1.009)	0.126	0.381	2.42E-08	TRUE
NK	*PRDX5*	Wald ratio	1	-0.0065	0.0078	0.994 (0.978, 1.009)	0.407	0.682	8.41E-54	TRUE
NK	*PRELID1*	Wald ratio	1	0.0535	0.0119	1.055 (1.031, 1.08)	7.56E-06	4.73E-04	1.29E-17	TRUE
NK	*PRKCB*	IVW	2	0.0085	0.0146	1.009 (0.98, 1.038)	0.561	0.778	6.49E-23	TRUE
NK	*PRMT2*	Wald ratio	1	0.0129	0.0113	1.013 (0.991, 1.036)	0.254	0.548	2.35E-18	TRUE
NK	*PRR7-AS1*	Wald ratio	1	0.0096	0.0076	1.01 (0.995, 1.025)	0.209	0.496	2.92E-49	TRUE
NK	*PRSS57*	Wald ratio	1	0.0017	0.0076	1.002 (0.987, 1.017)	0.823	0.926	6.53E-49	TRUE
NK	*PRX*	Wald ratio	1	-0.0243	0.0114	0.976 (0.954, 0.998)	0.034	0.175	6.06E-18	TRUE
NK	*PSD4*	Wald ratio	1	-0.0137	0.0147	0.986 (0.958, 1.015)	0.353	0.644	5.17E-14	TRUE
NK	*PSMB9*	Wald ratio	1	-0.0234	0.0157	0.977 (0.947, 1.007)	0.137	0.399	7.84E-14	TRUE
NK	*PSMD5-AS1*	Wald ratio	1	0.0041	0.0045	1.004 (0.995, 1.013)	0.361	0.65	2.51E-164	TRUE
NK	*PSPH*	Wald ratio	1	-0.0008	0.0141	0.999 (0.972, 1.027)	0.952	0.982	1.14E-11	TRUE
NK	*PSTPIP1*	Wald ratio	1	-0.0306	0.0130	0.97 (0.946, 0.995)	0.018	0.119	6.23E-18	TRUE
NK	*PTGDR*	Wald ratio	1	0.0187	0.0093	1.019 (1, 1.038)	0.044	0.208	4.43E-32	TRUE
NK	*PTGDS*	Wald ratio	1	0.0074	0.0090	1.007 (0.99, 1.025)	0.413	0.686	3.51E-37	TRUE
NK	*PTGER4*	Wald ratio	1	0.0185	0.0157	1.019 (0.988, 1.051)	0.239	0.533	3.24E-13	TRUE
NK	*PWWP2A*	Wald ratio	1	0.0038	0.0170	1.004 (0.971, 1.038)	0.825	0.926	5.96E-12	TRUE
NK	*PXK*	Wald ratio	1	-0.0111	0.0196	0.989 (0.952, 1.028)	0.572	0.786	3.48E-09	TRUE
NK	*PYCR2*	Wald ratio	1	-0.0086	0.0193	0.991 (0.955, 1.03)	0.655	0.839	3.49E-09	TRUE
NK	*PYHIN1*	Wald ratio	1	0.0041	0.0091	1.004 (0.986, 1.022)	0.65	0.835	1.06E-31	TRUE
NK	*PYROXD2*	Wald ratio	1	-0.0436	0.0202	0.957 (0.92, 0.996)	0.031	0.165	1.10E-08	TRUE
NK	*QDPR*	Wald ratio	1	0.0294	0.0139	1.03 (1.002, 1.058)	0.034	0.175	1.85E-17	TRUE
NK	*R3HDM4*	Wald ratio	1	0.0012	0.0108	1.001 (0.98, 1.023)	0.914	0.962	7.56E-30	TRUE
NK	*RAB11A*	Wald ratio	1	-0.0098	0.0168	0.99 (0.958, 1.023)	0.558	0.776	9.81E-11	TRUE
NK	*RAB24*	Wald ratio	1	-0.0376	0.0200	0.963 (0.926, 1.002)	0.06	0.247	1.46E-09	TRUE
NK	*RAB38*	Wald ratio	1	-0.0421	0.0158	0.959 (0.93, 0.989)	0.008	0.067	3.56E-11	TRUE
NK	*RAC1*	Wald ratio	1	-0.0217	0.0076	0.979 (0.964, 0.993)	0.004	0.044	2.11E-50	TRUE
NK	*RAD51C*	Wald ratio	1	0.0025	0.0105	1.002 (0.982, 1.023)	0.815	0.921	1.14E-23	TRUE
NK	*RAI14*	Wald ratio	1	0.0045	0.0135	1.005 (0.978, 1.031)	0.739	0.881	8.90E-17	TRUE
NK	*RAMP1*	IVW	2	0.0044	0.0075	1.004 (0.99, 1.019)	0.557	0.775	1.03E-49	TRUE
NK	*RAP1GAP2*	Wald ratio	1	-0.0064	0.0176	0.994 (0.96, 1.029)	0.715	0.873	5.33E-12	TRUE
NK	*RASA3*	Wald ratio	1	-0.0359	0.0128	0.965 (0.941, 0.989)	0.005	0.051	1.65E-17	TRUE
NK	*RBAK-RBAKDN*	Wald ratio	1	-0.0204	0.0255	0.98 (0.932, 1.03)	0.424	0.694	1.00E-08	TRUE
NK	*RBL2*	Wald ratio	1	0.0004	0.0164	1 (0.969, 1.033)	0.982	0.993	1.37E-12	TRUE
NK	*RBM43*	Wald ratio	1	-0.0206	0.0088	0.98 (0.963, 0.997)	0.019	0.122	3.69E-37	TRUE
NK	*RBM6*	Wald ratio	1	-0.0653	0.0143	0.937 (0.911, 0.963)	5.24E-06	3.48E-04	2.41E-14	TRUE
NK	*RBPMS2*	Wald ratio	1	0.0252	0.0118	1.025 (1.002, 1.05)	0.034	0.175	5.12E-26	TRUE
NK	*RCSD1*	Wald ratio	1	0.0051	0.0153	1.005 (0.975, 1.036)	0.74	0.881	8.62E-14	TRUE
NK	*REEP4*	Wald ratio	1	0.0223	0.0141	1.023 (0.995, 1.051)	0.113	0.359	3.27E-18	TRUE
NK	*RFC4*	Wald ratio	1	0.0163	0.0166	1.016 (0.984, 1.05)	0.325	0.62	3.91E-09	TRUE
NK	*RFTN1*	Wald ratio	1	-0.0074	0.0175	0.993 (0.959, 1.027)	0.674	0.851	3.85E-09	TRUE
NK	*RGS10*	Wald ratio	1	0.0171	0.0128	1.017 (0.992, 1.043)	0.183	0.462	5.30E-17	TRUE
NK	*RGS17*	Wald ratio	1	0.0034	0.0102	1.003 (0.984, 1.024)	0.736	0.88	2.37E-28	TRUE
NK	*RHOC*	Wald ratio	1	-0.0404	0.0126	0.96 (0.937, 0.984)	0.001	0.02	9.84E-19	TRUE
NK	*RMDN3*	Wald ratio	1	0.0315	0.0137	1.032 (1.005, 1.06)	0.021	0.132	7.15E-15	TRUE
NK	*RNASEH2C*	Wald ratio	1	0.0025	0.0117	1.002 (0.98, 1.026)	0.831	0.929	2.32E-24	TRUE
NK	*RNASET2*	Wald ratio	1	-0.0244	0.0086	0.976 (0.96, 0.992)	0.004	0.046	1.07E-38	TRUE
NK	*RNF167*	Wald ratio	1	-0.0068	0.0184	0.993 (0.958, 1.03)	0.71	0.871	1.07E-08	TRUE
NK	*RNF168*	Wald ratio	1	-0.0221	0.0183	0.978 (0.944, 1.014)	0.229	0.522	8.08E-10	TRUE
NK	*RNF19A*	Wald ratio	1	0.0088	0.0191	1.009 (0.972, 1.047)	0.644	0.834	1.83E-08	TRUE
NK	*RORB*	Wald ratio	1	-0.0037	0.0152	0.996 (0.967, 1.026)	0.807	0.917	2.58E-14	TRUE
NK	*RP1-168L15.5*	Wald ratio	1	-0.0219	0.0202	0.978 (0.94, 1.018)	0.28	0.574	3.78E-08	TRUE
NK	*RP1-265C24.8*	Wald ratio	1	-0.0272	0.0220	0.973 (0.932, 1.016)	0.217	0.509	8.87E-10	TRUE
NK	*RP1-29C18.10*	Wald ratio	1	-0.0132	0.0066	0.987 (0.974, 1)	0.046	0.21	4.32E-73	TRUE
NK	*RP1-30M3.5*	Wald ratio	1	0.0131	0.0202	1.013 (0.974, 1.054)	0.516	0.751	6.02E-15	TRUE
NK	*RP1-3J17.3*	IVW	2	0.0023	0.0062	1.002 (0.99, 1.015)	0.712	0.872	5.59E-80	TRUE
NK	*RP1-40E16.12*	Wald ratio	1	-0.0195	0.0196	0.981 (0.944, 1.019)	0.319	0.614	1.34E-08	TRUE
NK	*RP1-8B1.4*	Wald ratio	1	0.0011	0.0064	1.001 (0.989, 1.014)	0.87	0.946	2.89E-71	TRUE
NK	*RP11-1000B6.3*	Wald ratio	1	-0.0041	0.0187	0.996 (0.96, 1.033)	0.825	0.926	3.85E-15	TRUE

NK	*RP11-1055B8.4*	Wald ratio	1	-0.0154	0.0164	0.985 (0.954, 1.017)	0.347	0.64	2.57E-11	TRUE
NK	*RP11-1055B8.7*	Wald ratio	1	-0.0227	0.0094	0.978 (0.96, 0.996)	0.016	0.108	5.34E-28	TRUE
NK	*RP11-108M9.4*	Wald ratio	1	0.0238	0.0146	1.024 (0.995, 1.054)	0.105	0.343	2.23E-13	TRUE
NK	*RP11-119F19.2*	Wald ratio	1	-0.0322	0.0158	0.968 (0.939, 0.999)	0.041	0.196	6.72E-12	TRUE
NK	*RP11-121A8.1*	Wald ratio	1	-0.0149	0.0154	0.985 (0.956, 1.015)	0.334	0.627	1.04E-11	TRUE
NK	*RP11-137H2.4*	Wald ratio	1	-0.0150	0.0075	0.985 (0.971, 1)	0.047	0.214	9.71E-53	TRUE
NK	*RP11-138A9.2*	Wald ratio	1	0.0032	0.0219	1.003 (0.961, 1.047)	0.882	0.952	6.15E-09	TRUE
NK	*RP11-157J24.2*	Wald ratio	1	-0.0040	0.0156	0.996 (0.966, 1.027)	0.797	0.913	3.67E-13	TRUE
NK	*RP11-166B2.5*	Wald ratio	1	0.0008	0.0167	1.001 (0.969, 1.034)	0.963	0.988	4.16E-11	TRUE
NK	*RP11-245G13.2*	Wald ratio	1	-0.0102	0.0074	0.99 (0.976, 1.004)	0.167	0.442	2.20E-76	TRUE
NK	*RP11-254F7.2*	Wald ratio	1	0.0191	0.0246	1.019 (0.971, 1.07)	0.437	0.703	8.49E-09	TRUE
NK	*RP11-266L9.2*	Wald ratio	1	-0.0004	0.0148	1 (0.971, 1.029)	0.98	0.992	5.46E-18	TRUE
NK	*RP11-273G15.2*	Wald ratio	1	-0.0499	0.0092	0.951 (0.934, 0.969)	6.44E-08	6.60E-05	5.87E-34	TRUE
NK	*RP11-294J22.6*	Wald ratio	1	-0.0445	0.0282	0.956 (0.905, 1.011)	0.114	0.361	1.49E-09	TRUE
NK	*RP11-298J20.3*	Wald ratio	1	0.0142	0.0054	1.014 (1.004, 1.025)	0.008	0.071	4.79E-97	TRUE
NK	*RP11-299J3.8*	Wald ratio	1	-0.0287	0.0250	0.972 (0.925, 1.021)	0.252	0.545	1.80E-08	TRUE
NK	*RP11-305L7.1*	Wald ratio	1	-0.0193	0.0137	0.981 (0.955, 1.008)	0.16	0.433	7.01E-18	TRUE
NK	*RP11-305L7.3*	Wald ratio	1	-0.0179	0.0128	0.982 (0.958, 1.007)	0.162	0.435	2.89E-20	TRUE
NK	*RP11-306I1.2*	Wald ratio	1	-0.0224	0.0151	0.978 (0.949, 1.007)	0.137	0.399	4.13E-14	TRUE
NK	*RP11-308N19.1*	Wald ratio	1	-0.0096	0.0133	0.99 (0.965, 1.017)	0.472	0.729	7.30E-15	TRUE
NK	*RP11-324I22.4*	Wald ratio	1	0.0093	0.0198	1.009 (0.971, 1.049)	0.638	0.829	1.03E-08	TRUE
NK	*RP11-326C3.11*	IVW	2	-0.0042	0.0135	0.996 (0.97, 1.023)	0.757	0.889	5.30E-94	TRUE
NK	*RP11-326C3.15*	IVW	2	-0.0013	0.0106	0.999 (0.978, 1.02)	0.904	0.959	3.16E-27	TRUE
NK	*RP11-345J4.5*	Wald ratio	1	-0.0208	0.0094	0.979 (0.961, 0.998)	0.027	0.154	5.00E-34	TRUE
NK	*RP11-347P5.1*	Wald ratio	1	0.0015	0.0077	1.001 (0.986, 1.017)	0.85	0.936	1.45E-50	TRUE
NK	*RP11-369E15.3*	Wald ratio	1	0.0039	0.0124	1.004 (0.98, 1.029)	0.755	0.888	5.57E-17	TRUE
NK	*RP11-395B7.4*	Wald ratio	1	0.0065	0.0185	1.007 (0.971, 1.044)	0.725	0.878	2.01E-09	TRUE
NK	*RP11-403P17.3*	Wald ratio	1	0.0608	0.0180	1.063 (1.026, 1.101)	7.05E-04	0.013	9.24E-08	TRUE
NK	*RP11-413H22.2*	Wald ratio	1	0.0078	0.0168	1.008 (0.975, 1.042)	0.645	0.834	2.37E-11	TRUE
NK	*RP11-426C22.5*	Wald ratio	1	0.0288	0.0205	1.029 (0.989, 1.071)	0.159	0.432	1.96E-08	TRUE
NK	*RP11-430H10.1*	Wald ratio	1	-0.0344	0.0174	0.966 (0.934, 1)	0.047	0.214	5.57E-11	TRUE
NK	*RP11-434C1.1*	Wald ratio	1	-0.0005	0.0141	0.999 (0.972, 1.028)	0.971	0.989	2.05E-16	TRUE
NK	*RP11-448A19.1*	Wald ratio	1	-0.0023	0.0141	0.998 (0.971, 1.026)	0.871	0.947	2.87E-15	TRUE
NK	*RP11-473I1.5*	Wald ratio	1	-0.0183	0.0085	0.982 (0.966, 0.998)	0.032	0.168	6.74E-34	TRUE
NK	*RP11-473M20.7*	Wald ratio	1	-0.0466	0.0190	0.954 (0.92, 0.991)	0.014	0.101	1.04E-09	TRUE
NK	*RP11-473M20.9*	Wald ratio	1	-0.0097	0.0099	0.99 (0.971, 1.01)	0.326	0.62	2.07E-30	TRUE
NK	*RP11-48B3.3*	Wald ratio	1	0.0181	0.0130	1.018 (0.993, 1.045)	0.162	0.436	4.74E-16	TRUE
NK	*RP11-493L12.4*	Wald ratio	1	-0.0192	0.0175	0.981 (0.948, 1.015)	0.273	0.567	5.47E-12	TRUE
NK	*RP11-509J21.2*	Wald ratio	1	-0.0118	0.0153	0.988 (0.959, 1.018)	0.438	0.703	2.01E-14	TRUE
NK	*RP11-514O12.4*	Wald ratio	1	0.0418	0.0148	1.043 (1.013, 1.073)	0.005	0.047	1.09E-13	TRUE
NK	*RP11-539G18.2*	Wald ratio	1	0.0035	0.0073	1.004 (0.989, 1.018)	0.626	0.822	2.10E-57	TRUE
NK	*RP11-539L10.2*	Wald ratio	1	0.0044	0.0132	1.004 (0.979, 1.031)	0.739	0.881	6.76E-19	TRUE
NK	*RP11-539L10.3*	Wald ratio	1	-0.0152	0.0140	0.985 (0.958, 1.012)	0.278	0.571	1.03E-14	TRUE
NK	*RP11-596D21.1*	Wald ratio	1	0.0250	0.0099	1.025 (1.006, 1.045)	0.011	0.086	2.69E-28	TRUE
NK	*RP11-611D20.2*	Wald ratio	1	0.0006	0.0165	1.001 (0.969, 1.034)	0.972	0.989	6.70E-11	TRUE
NK	*RP11-63P12.6*	Wald ratio	1	-0.0342	0.0228	0.966 (0.924, 1.01)	0.133	0.394	4.14E-08	TRUE
NK	*RP11-656D10.3*	Wald ratio	1	0.0298	0.0145	1.03 (1.001, 1.06)	0.04	0.195	1.41E-14	TRUE
NK	*RP11-69E11.4*	Wald ratio	1	0.0198	0.0094	1.02 (1.001, 1.039)	0.034	0.175	3.54E-30	TRUE
NK	*RP11-791G15.2*	Wald ratio	1	0.0110	0.0171	1.011 (0.978, 1.045)	0.519	0.753	1.31E-10	TRUE
NK	*RP11-792A8.4*	Wald ratio	1	0.0081	0.0115	1.008 (0.986, 1.031)	0.483	0.736	3.95E-16	TRUE
NK	*RP11-81H14.2*	Wald ratio	1	-0.0018	0.0048	0.998 (0.989, 1.008)	0.705	0.867	1.00E-153	TRUE
NK	*RP11-841O20.2*	Wald ratio	1	-0.0359	0.0227	0.965 (0.923, 1.009)	0.113	0.359	3.26E-10	TRUE
NK	*RP11-894P9.1*	Wald ratio	1	-0.0307	0.0183	0.97 (0.936, 1.005)	0.093	0.323	3.34E-09	TRUE
NK	*RP11-932O9.9*	Wald ratio	1	0.0145	0.0187	1.015 (0.978, 1.053)	0.438	0.703	2.97E-09	TRUE
NK	*RP11-950C14.3*	Wald ratio	1	-0.0177	0.0106	0.982 (0.962, 1.003)	0.095	0.326	4.62E-26	TRUE
NK	*RP11-973H7.1*	Wald ratio	1	0.0193	0.0110	1.02 (0.998, 1.042)	0.08	0.296	2.19E-23	TRUE
NK	*RP11-973H7.4*	Wald ratio	1	0.0243	0.0137	1.025 (0.997, 1.053)	0.076	0.289	1.60E-15	TRUE
NK	*RP13-582O9.5*	Wald ratio	1	0.0023	0.0196	1.002 (0.964, 1.042)	0.905	0.96	1.18E-08	TRUE
NK	*RP3-325F22.5*	Wald ratio	1	0.0090	0.0172	1.009 (0.976, 1.044)	0.601	0.805	9.98E-13	TRUE
NK	*RP5-1021I20.1*	Wald ratio	1	-0.0400	0.0129	0.961 (0.937, 0.985)	0.002	0.025	2.43E-17	TRUE
NK	*RP5-821D11.7*	Wald ratio	1	-0.0239	0.0174	0.976 (0.944, 1.01)	0.171	0.449	2.74E-10	TRUE
NK	*RP5-827C21.4*	Wald ratio	1	0.0063	0.0146	1.006 (0.978, 1.036)	0.664	0.846	5.65E-15	TRUE
NK	*RP6-109B7.3*	Wald ratio	1	-0.0003	0.0217	1 (0.958, 1.043)	0.988	0.995	4.60E-08	TRUE
NK	*RPA2*	Wald ratio	1	0.0136	0.0147	1.014 (0.985, 1.043)	0.353	0.644	4.50E-14	TRUE
NK	*RPL10A*	Wald ratio	1	-0.0112	0.0085	0.989 (0.973, 1.005)	0.187	0.466	6.56E-42	TRUE
NK	*RPL11*	Wald ratio	1	-0.0133	0.0085	0.987 (0.97, 1.003)	0.119	0.369	4.24E-39	TRUE
NK	*RPL12*	Wald ratio	1	-0.0199	0.0059	0.98 (0.969, 0.992)	7.26E-04	0.013	2.69E-94	TRUE
NK	*RPL13*	Wald ratio	1	-0.0282	0.0139	0.972 (0.946, 0.999)	0.043	0.202	2.85E-27	TRUE
NK	*RPL13A*	Wald ratio	1	0.0311	0.0143	1.032 (1.003, 1.061)	0.03	0.161	1.15E-15	TRUE
NK	*RPL14*	Wald ratio	1	0.0391	0.0129	1.04 (1.014, 1.067)	0.003	0.031	1.07E-16	TRUE
NK	*RPL22L1*	Wald ratio	1	0.0028	0.0130	1.003 (0.978, 1.029)	0.83	0.929	9.69E-23	TRUE
NK	*RPL23A*	Wald ratio	1	-0.0280	0.0146	0.972 (0.945, 1.001)	0.055	0.236	8.23E-15	TRUE
NK	*RPL27A*	Wald ratio	1	0.0149	0.0177	1.015 (0.98, 1.051)	0.4	0.678	2.32E-09	TRUE
NK	*RPL28*	Wald ratio	1	0.0033	0.0043	1.003 (0.995, 1.012)	0.438	0.703	5.11E-184	TRUE
NK	*RPL3*	Wald ratio	1	-0.0203	0.0176	0.98 (0.947, 1.014)	0.249	0.542	6.00E-10	TRUE
NK	*RPL32*	Wald ratio	1	0.0286	0.0170	1.029 (0.995, 1.064)	0.093	0.323	1.48E-11	TRUE
NK	*RPL36AL*	Wald ratio	1	-0.0041	0.0064	0.996 (0.983, 1.009)	0.524	0.754	1.49E-73	TRUE
NK	*RPL8*	Wald ratio	1	-0.0058	0.0057	0.994 (0.983, 1.005)	0.303	0.598	1.27E-93	TRUE
NK	*RPL9*	Wald ratio	1	-0.0196	0.0125	0.981 (0.957, 1.005)	0.117	0.365	8.27E-18	TRUE

NK	*RPPH1*	Wald ratio	1	0.0147	0.0160	1.015 (0.983, 1.047)	0.361	0.65	5.34E-10	TRUE
NK	*RPS10*	Wald ratio	1	-0.0061	0.0059	0.994 (0.982, 1.006)	0.305	0.599	1.12E-70	TRUE
NK	*RPS11*	Wald ratio	1	0.0470	0.0197	1.048 (1.008, 1.089)	0.017	0.115	4.82E-09	TRUE
NK	*RPS12*	Wald ratio	1	0.0724	0.0175	1.075 (1.039, 1.113)	3.46E-05	0.001	1.87E-08	TRUE
NK	*RPS14*	Wald ratio	1	-0.0205	0.0185	0.98 (0.945, 1.016)	0.267	0.561	4.33E-09	TRUE
NK	*RPS16*	Wald ratio	1	-0.0095	0.0093	0.99 (0.973, 1.009)	0.304	0.598	7.58E-32	TRUE
NK	*RPS18*	Wald ratio	1	-0.0123	0.0072	0.988 (0.974, 1.002)	0.087	0.312	1.96E-70	TRUE
NK	*RPS2*	Wald ratio	1	-0.0333	0.0125	0.967 (0.944, 0.991)	0.007	0.066	6.76E-20	TRUE
NK	*RPS23*	Wald ratio	1	-0.0207	0.0144	0.98 (0.952, 1.008)	0.151	0.422	2.88E-16	TRUE
NK	*RPS24*	Wald ratio	1	-0.0017	0.0140	0.998 (0.971, 1.026)	0.906	0.96	1.06E-15	TRUE
NK	*RPS25*	Wald ratio	1	-0.0102	0.0076	0.99 (0.975, 1.005)	0.179	0.458	1.32E-36	TRUE
NK	*RPS26*	Wald ratio	1	-0.0099	0.0043	0.99 (0.982, 0.999)	0.022	0.132	3.19E-191	TRUE
NK	*RPS3*	Wald ratio	1	-0.0166	0.0093	0.984 (0.966, 1.002)	0.074	0.286	3.67E-20	TRUE
NK	*RPS3A*	Wald ratio	1	0.0139	0.0149	1.014 (0.985, 1.044)	0.348	0.641	2.96E-31	TRUE
NK	*RPS5*	Wald ratio	1	0.0078	0.0058	1.008 (0.996, 1.019)	0.177	0.456	3.17E-97	TRUE
NK	*RPS6KL1*	Wald ratio	1	0.0562	0.0168	1.058 (1.023, 1.093)	8.35E-04	0.015	3.27E-12	TRUE
NK	*RPS7*	Wald ratio	1	0.0045	0.0143	1.004 (0.977, 1.033)	0.754	0.888	1.25E-22	TRUE
NK	*RPS8*	Wald ratio	1	0.0020	0.0075	1.002 (0.987, 1.017)	0.791	0.909	1.04E-52	TRUE
NK	*RPS9*	Wald ratio	1	0.0217	0.0058	1.022 (1.01, 1.034)	1.64E-04	0.004	8.38E-92	TRUE
NK	*RPSA*	Wald ratio	1	-0.0242	0.0121	0.976 (0.953, 0.999)	0.045	0.209	5.40E-26	TRUE
NK	*RPTOR*	Wald ratio	1	0.0131	0.0176	1.013 (0.979, 1.049)	0.456	0.718	3.32E-10	TRUE
NK	*RRAS2*	Wald ratio	1	-0.0141	0.0128	0.986 (0.962, 1.011)	0.27	0.563	9.44E-15	TRUE
NK	*RSRC2*	Wald ratio	1	-0.0071	0.0126	0.993 (0.969, 1.018)	0.573	0.786	6.82E-23	TRUE
NK	*RTDR1*	Wald ratio	1	0.0011	0.0072	1.001 (0.987, 1.015)	0.877	0.949	6.89E-54	TRUE
NK	*RTN4*	Wald ratio	1	-0.0374	0.0185	0.963 (0.929, 0.999)	0.044	0.206	6.33E-10	TRUE
NK	*RXRA*	Wald ratio	1	0.0074	0.0181	1.007 (0.972, 1.044)	0.683	0.856	1.63E-10	TRUE
NK	*S100A10*	Wald ratio	1	-0.0256	0.0145	0.975 (0.947, 1.003)	0.078	0.292	5.02E-15	TRUE
NK	*S100A13*	Wald ratio	1	-0.0280	0.0179	0.972 (0.939, 1.007)	0.117	0.364	5.38E-10	TRUE
NK	*S100B*	Wald ratio	1	0.0054	0.0046	1.005 (0.996, 1.015)	0.239	0.533	1.31E-157	TRUE
NK	*S1PR5*	Wald ratio	1	-0.0110	0.0169	0.989 (0.957, 1.022)	0.515	0.75	2.78E-11	TRUE
NK	*SACM1L*	Wald ratio	1	-0.0067	0.0066	0.993 (0.981, 1.006)	0.313	0.608	4.36E-61	TRUE
NK	*SAMD12*	Wald ratio	1	-0.0094	0.0152	0.991 (0.962, 1.021)	0.537	0.762	1.83E-18	TRUE
NK	*SAMD3*	Wald ratio	1	-0.0065	0.0196	0.993 (0.956, 1.032)	0.739	0.881	9.83E-09	TRUE
NK	*SAMHD1*	Wald ratio	1	-0.0134	0.0121	0.987 (0.964, 1.01)	0.267	0.561	2.99E-18	TRUE
NK	*SAMSN1*	Wald ratio	1	0.0076	0.0157	1.008 (0.977, 1.039)	0.628	0.822	1.17E-12	TRUE
NK	*SAR1A*	Wald ratio	1	0.0070	0.0093	1.007 (0.989, 1.026)	0.453	0.717	2.43E-32	TRUE
NK	*SAT2*	Wald ratio	1	-0.0820	0.0155	0.921 (0.894, 0.95)	1.14E-07	6.60E-05	1.65E-12	TRUE
NK	*SBF1*	Wald ratio	1	0.0189	0.0190	1.019 (0.982, 1.058)	0.319	0.614	1.20E-08	TRUE
NK	*SCAMP3*	Wald ratio	1	-0.0099	0.0192	0.99 (0.954, 1.028)	0.607	0.808	9.06E-09	TRUE
NK	*SCAND1*	Wald ratio	1	-0.0110	0.0196	0.989 (0.952, 1.028)	0.575	0.787	3.29E-10	TRUE
NK	*SCD5*	Wald ratio	1	-0.0533	0.0122	0.948 (0.926, 0.971)	1.30E-05	6.87E-04	1.24E-19	TRUE
NK	*SCRN2*	Wald ratio	1	-0.0449	0.0146	0.956 (0.929, 0.984)	0.002	0.028	6.43E-14	TRUE
NK	*SDSL*	Wald ratio	1	-0.0285	0.0122	0.972 (0.949, 0.995)	0.019	0.123	8.34E-18	TRUE
NK	*SEC61B*	Wald ratio	1	-0.0153	0.0127	0.985 (0.961, 1.01)	0.228	0.521	9.71E-27	TRUE
NK	*SELL*	Wald ratio	1	-0.0096	0.0207	0.99 (0.951, 1.031)	0.641	0.831	3.64E-08	TRUE
NK	*SEMA4D*	Wald ratio	1	-0.0263	0.0180	0.974 (0.94, 1.009)	0.144	0.411	4.01E-10	TRUE
NK	*SEPT2*	Wald ratio	1	0.0103	0.0125	1.01 (0.986, 1.035)	0.409	0.685	3.98E-23	TRUE
NK	*SERAC1*	Wald ratio	1	-0.0491	0.0173	0.952 (0.92, 0.985)	0.005	0.048	1.44E-10	TRUE
NK	*SERPINB6*	IVW	2	0.0185	0.0096	1.019 (1, 1.038)	0.054	0.233	5.38E-36	TRUE
NK	*SERPINB9*	Wald ratio	1	0.0072	0.0159	1.007 (0.976, 1.039)	0.652	0.836	1.36E-11	TRUE
NK	*SERPINE2*	Wald ratio	1	-0.0269	0.0117	0.973 (0.951, 0.996)	0.022	0.134	1.47E-21	TRUE
NK	*SETD5-AS1*	Wald ratio	1	-0.0060	0.0154	0.994 (0.964, 1.024)	0.696	0.864	9.29E-13	TRUE
NK	*SETD9*	Wald ratio	1	-0.0381	0.0152	0.963 (0.934, 0.992)	0.012	0.093	1.33E-12	TRUE
NK	*SF1*	Wald ratio	1	0.0042	0.0152	1.004 (0.975, 1.035)	0.785	0.907	1.10E-10	TRUE
NK	*SH3BP5*	Wald ratio	1	-0.0108	0.0178	0.989 (0.955, 1.024)	0.543	0.765	3.00E-10	TRUE
NK	*SH3YL1*	Wald ratio	1	0.0682	0.0169	1.071 (1.036, 1.107)	5.45E-05	0.002	1.01E-10	TRUE
NK	*SHFM1*	Wald ratio	1	0.0007	0.0173	1.001 (0.967, 1.035)	0.969	0.989	1.38E-08	TRUE
NK	*SHMT1*	Wald ratio	1	0.0369	0.0185	1.038 (1.001, 1.076)	0.046	0.21	1.56E-09	TRUE
NK	*SIDT1-AS1*	Wald ratio	1	-0.0086	0.0093	0.991 (0.974, 1.01)	0.356	0.647	4.64E-40	TRUE
NK	*SKAP2*	Wald ratio	1	-0.0093	0.0122	0.991 (0.967, 1.015)	0.447	0.712	7.14E-24	TRUE
NK	*SLC12A5*	Wald ratio	1	-0.0328	0.0151	0.968 (0.94, 0.997)	0.03	0.162	3.54E-13	TRUE
NK	*SLC12A7*	Wald ratio	1	-0.0080	0.0073	0.992 (0.978, 1.006)	0.275	0.568	1.52E-55	TRUE
NK	*SLC14A1*	Wald ratio	1	-0.0130	0.0165	0.987 (0.956, 1.019)	0.429	0.699	5.87E-15	TRUE
NK	*SLC15A4*	IVW	2	0.0174	0.0105	1.018 (0.997, 1.039)	0.098	0.333	1.83E-27	TRUE
NK	*SLC16A4*	Wald ratio	1	0.0292	0.0151	1.03 (1, 1.061)	0.052	0.227	2.70E-13	TRUE
NK	*SLC1A7*	IVW	2	-0.0253	0.0145	0.975 (0.948, 1.003)	0.082	0.301	3.51E-17	TRUE
NK	*SLC22A18*	Wald ratio	1	-0.0094	0.0189	0.991 (0.955, 1.028)	0.617	0.817	8.64E-11	TRUE
NK	*SLC22A5*	Wald ratio	1	-0.0256	0.0116	0.975 (0.953, 0.997)	0.026	0.151	3.70E-24	TRUE
NK	*SLC25A1*	Wald ratio	1	-0.0096	0.0099	0.99 (0.971, 1.01)	0.332	0.626	1.41E-30	TRUE
NK	*SLC25A24*	Wald ratio	1	-0.0252	0.0151	0.975 (0.947, 1.004)	0.096	0.329	1.87E-12	TRUE
NK	*SLC25A26*	Wald ratio	1	-0.0403	0.0152	0.961 (0.932, 0.99)	0.008	0.069	2.72E-11	TRUE
NK	*SLC25A29*	Wald ratio	1	-0.0039	0.0055	0.996 (0.985, 1.007)	0.475	0.73	2.04E-99	TRUE
NK	*SLC25A3*	Wald ratio	1	0.0082	0.0198	1.008 (0.97, 1.048)	0.678	0.853	6.67E-11	TRUE
NK	*SLC25A51*	Wald ratio	1	-0.0419	0.0171	0.959 (0.927, 0.992)	0.014	0.101	5.25E-09	TRUE
NK	*SLC27A1*	Wald ratio	1	0.0042	0.0132	1.004 (0.979, 1.031)	0.751	0.886	4.76E-13	TRUE
NK	*SLC44A2*	Wald ratio	1	0.0177	0.0196	1.018 (0.979, 1.058)	0.368	0.656	2.38E-10	TRUE
NK	*SLC44A4*	Wald ratio	1	-0.0106	0.0184	0.989 (0.954, 1.026)	0.564	0.78	8.71E-10	TRUE
NK	*SLC7A7*	Wald ratio	1	-0.0267	0.0190	0.974 (0.938, 1.011)	0.159	0.432	1.18E-09	TRUE
NK	*SLFN12*	Wald ratio	1	0.0450	0.0265	1.046 (0.993, 1.102)	0.09	0.318	1.10E-08	TRUE

NK	*SLFN5*	Wald ratio	1	-0.0073	0.0123	0.993 (0.969, 1.017)	0.55	0.769	5.24E-20	TRUE
NK	*SMAP1*	Wald ratio	1	0.0043	0.0177	1.004 (0.97, 1.04)	0.81	0.919	3.02E-10	TRUE
NK	*SMDT1*	Wald ratio	1	0.0109	0.0056	1.011 (1, 1.022)	0.05	0.222	4.15E-88	TRUE
NK	*SMIM19*	Wald ratio	1	0.0085	0.0124	1.009 (0.984, 1.033)	0.493	0.738	4.84E-20	TRUE
NK	*SMIM20*	Wald ratio	1	0.0288	0.0187	1.029 (0.992, 1.068)	0.123	0.377	1.18E-08	TRUE
NK	*SMOX*	Wald ratio	1	-0.0156	0.0264	0.985 (0.935, 1.037)	0.554	0.773	6.33E-09	TRUE
NK	*SMPD3*	Wald ratio	1	-0.0582	0.0193	0.943 (0.908, 0.98)	0.003	0.032	1.90E-08	TRUE
NK	*SMPDL3B*	Wald ratio	1	-0.0084	0.0088	0.992 (0.975, 1.009)	0.343	0.635	2.23E-37	TRUE
NK	*SNAI3*	Wald ratio	1	-0.0343	0.0195	0.966 (0.93, 1.004)	0.078	0.292	9.44E-10	TRUE
NK	*SNAP29*	Wald ratio	1	0.0449	0.0097	1.046 (1.026, 1.066)	3.40E-06	2.63E-04	2.00E-35	TRUE
NK	*SNHG7*	Wald ratio	1	0.0150	0.0104	1.015 (0.995, 1.036)	0.149	0.419	2.86E-28	TRUE
NK	*SNHG8*	Wald ratio	1	-0.0022	0.0067	0.998 (0.985, 1.011)	0.737	0.88	1.34E-75	TRUE
NK	*SNORD3B-1*	Wald ratio	1	-0.0092	0.0120	0.991 (0.968, 1.015)	0.446	0.71	4.23E-16	TRUE
NK	*SNORD3B-2*	Wald ratio	1	0.0096	0.0136	1.01 (0.983, 1.037)	0.482	0.735	8.13E-16	TRUE
NK	*SNRNP25*	Wald ratio	1	0.0404	0.0185	1.041 (1.004, 1.08)	0.029	0.159	8.21E-09	TRUE
NK	*SNRPD2*	Wald ratio	1	-0.0425	0.0132	0.958 (0.934, 0.983)	0.001	0.02	1.82E-16	TRUE
NK	*SNX18*	Wald ratio	1	0.0001	0.0162	1 (0.969, 1.032)	0.993	0.998	1.41E-12	TRUE
NK	*SNX9*	Wald ratio	1	0.0075	0.0199	1.008 (0.969, 1.048)	0.707	0.869	9.03E-09	TRUE
NK	*SPATA20*	Wald ratio	1	0.0182	0.0113	1.018 (0.996, 1.041)	0.106	0.346	1.41E-23	TRUE
NK	*SPATC1L*	IVW	2	0.0006	0.0094	1.001 (0.982, 1.019)	0.952	0.982	2.27E-40	TRUE
NK	*SPECC1*	Wald ratio	1	0.0023	0.0131	1.002 (0.977, 1.028)	0.858	0.94	7.84E-19	TRUE
NK	*SPNS1*	Wald ratio	1	0.0305	0.0167	1.031 (0.998, 1.065)	0.068	0.266	2.38E-10	TRUE
NK	*SPOCK2*	Wald ratio	1	0.0279	0.0160	1.028 (0.996, 1.061)	0.082	0.301	2.43E-12	TRUE
NK	*SPON2*	Wald ratio	1	-0.0037	0.0136	0.996 (0.97, 1.023)	0.784	0.906	1.74E-15	TRUE
NK	*SPRYD4*	Wald ratio	1	0.0320	0.0203	1.033 (0.992, 1.074)	0.115	0.361	1.89E-09	TRUE
NK	*SPSB1*	Wald ratio	1	0.0203	0.0127	1.02 (0.995, 1.046)	0.111	0.354	3.58E-18	TRUE
NK	*SPSB2*	Wald ratio	1	-0.0003	0.0102	1 (0.98, 1.02)	0.973	0.989	3.45E-31	TRUE
NK	*SPTBN1*	Wald ratio	1	0.0013	0.0126	1.001 (0.977, 1.026)	0.918	0.965	6.69E-24	TRUE
NK	*SPTLC3*	Wald ratio	1	-0.0008	0.0173	0.999 (0.966, 1.034)	0.961	0.987	1.34E-10	TRUE
NK	*SRD5A3*	Wald ratio	1	-0.0271	0.0119	0.973 (0.951, 0.996)	0.023	0.138	1.89E-18	TRUE
NK	*SRI*	Wald ratio	1	-0.0230	0.0196	0.977 (0.94, 1.015)	0.239	0.533	3.00E-08	TRUE
NK	*SRP14*	Wald ratio	1	0.0591	0.0139	1.061 (1.032, 1.09)	2.14E-05	9.86E-04	1.96E-12	TRUE
NK	*SRR*	Wald ratio	1	0.0236	0.0185	1.024 (0.987, 1.062)	0.202	0.486	3.54E-09	TRUE
NK	*ST3GAL5*	Wald ratio	1	-0.0496	0.0144	0.952 (0.925, 0.979)	5.65E-04	0.011	6.42E-12	TRUE
NK	*ST6GAL1*	Wald ratio	1	0.0257	0.0213	1.026 (0.984, 1.07)	0.227	0.52	4.23E-08	TRUE
NK	*STK32C*	Wald ratio	1	-0.0068	0.0193	0.993 (0.956, 1.031)	0.723	0.877	4.79E-08	TRUE
NK	*STYX*	Wald ratio	1	0.0437	0.0164	1.045 (1.012, 1.079)	0.008	0.068	5.50E-12	TRUE
NK	*SULF2*	Wald ratio	1	0.0068	0.0070	1.007 (0.993, 1.021)	0.33	0.624	7.60E-59	TRUE
NK	*SURF1*	Wald ratio	1	-0.0236	0.0118	0.977 (0.954, 0.999)	0.045	0.208	9.83E-22	TRUE
NK	*SVIP*	Wald ratio	1	-0.0021	0.0138	0.998 (0.971, 1.025)	0.88	0.951	1.96E-15	TRUE
NK	*SYK*	Wald ratio	1	-0.0292	0.0129	0.971 (0.947, 0.996)	0.024	0.141	2.16E-13	TRUE
NK	*SYNC*	Wald ratio	1	-0.0078	0.0161	0.992 (0.961, 1.024)	0.628	0.822	3.62E-11	TRUE
NK	*SYNGR1*	Wald ratio	1	-0.0098	0.0051	0.99 (0.98, 1)	0.054	0.233	2.77E-108	TRUE
NK	*SYTL2*	Wald ratio	1	-0.0325	0.0211	0.968 (0.929, 1.009)	0.123	0.377	3.70E-08	TRUE
NK	*TAGLN2*	Wald ratio	1	0.0036	0.0143	1.004 (0.976, 1.032)	0.804	0.915	6.44E-15	TRUE
NK	*TAMM41*	Wald ratio	1	-0.0066	0.0203	0.993 (0.955, 1.034)	0.746	0.883	5.05E-08	TRUE
NK	*TAPBP*	Wald ratio	1	0.0646	0.0211	1.067 (1.023, 1.112)	0.002	0.028	1.71E-08	TRUE
NK	*TAPBPL*	Wald ratio	1	0.0023	0.0088	1.002 (0.985, 1.02)	0.791	0.909	1.36E-37	TRUE
NK	*TBC1D12*	Wald ratio	1	0.0611	0.0155	1.063 (1.031, 1.096)	7.75E-05	0.003	5.98E-12	TRUE
NK	*TBCA*	Wald ratio	1	0.0241	0.0185	1.024 (0.988, 1.062)	0.193	0.474	1.03E-09	TRUE
NK	*TC2N*	Wald ratio	1	0.0026	0.0092	1.003 (0.985, 1.021)	0.779	0.904	3.24E-34	TRUE
NK	*TCF25*	Wald ratio	1	0.0087	0.0070	1.009 (0.995, 1.023)	0.211	0.498	6.26E-76	TRUE
NK	*TCIRG1*	Wald ratio	1	-0.0326	0.0122	0.968 (0.945, 0.991)	0.007	0.066	7.61E-17	TRUE
NK	*TECR*	Wald ratio	1	0.0084	0.0083	1.008 (0.992, 1.025)	0.312	0.606	3.76E-42	TRUE
NK	*TEN1*	Wald ratio	1	-0.0125	0.0158	0.988 (0.957, 1.019)	0.43	0.7	1.10E-15	TRUE
NK	*TESC*	Wald ratio	1	-0.0245	0.0252	0.976 (0.929, 1.025)	0.331	0.624	3.87E-11	TRUE
NK	*TFPT*	Wald ratio	1	-0.0070	0.0134	0.993 (0.967, 1.02)	0.604	0.806	7.13E-19	TRUE
NK	*THAP7-AS1*	Wald ratio	1	0.0291	0.0136	1.03 (1.002, 1.057)	0.032	0.17	1.35E-14	TRUE
NK	*THAP9-AS1*	Wald ratio	1	0.0612	0.0132	1.063 (1.036, 1.091)	3.76E-06	2.77E-04	2.12E-19	TRUE
NK	*THEM6*	Wald ratio	1	0.0271	0.0209	1.027 (0.986, 1.07)	0.194	0.474	8.04E-08	TRUE
NK	*THEMIS2*	Wald ratio	1	0.0050	0.0053	1.005 (0.995, 1.015)	0.343	0.635	2.24E-109	TRUE
NK	*TIAM1*	Wald ratio	1	0.0025	0.0165	1.003 (0.971, 1.035)	0.879	0.951	2.19E-10	TRUE
NK	*TIGIT*	Wald ratio	1	-0.0300	0.0210	0.97 (0.931, 1.011)	0.152	0.423	4.57E-08	TRUE
NK	*TIMM10*	Wald ratio	1	-0.0014	0.0090	0.999 (0.981, 1.016)	0.875	0.948	8.88E-44	TRUE
NK	*TIPARP-AS1*	Wald ratio	1	0.0412	0.0170	1.042 (1.008, 1.077)	0.015	0.105	5.55E-11	TRUE
NK	*TLE4*	Wald ratio	1	-0.0082	0.0101	0.992 (0.972, 1.012)	0.419	0.69	4.86E-30	TRUE
NK	*TLK1*	Wald ratio	1	0.0121	0.0153	1.012 (0.982, 1.043)	0.431	0.701	1.91E-14	TRUE
NK	*TLR6*	Wald ratio	1	0.0389	0.0150	1.04 (1.009, 1.071)	0.01	0.079	4.72E-12	TRUE
NK	*TM7SF3*	Wald ratio	1	-0.0296	0.0158	0.971 (0.941, 1.001)	0.062	0.253	1.01E-09	TRUE
NK	*TMA7*	Wald ratio	1	-0.0066	0.0198	0.993 (0.956, 1.033)	0.741	0.881	1.28E-08	TRUE
NK	*TMC4*	Wald ratio	1	-0.0118	0.0100	0.988 (0.969, 1.008)	0.238	0.532	1.24E-31	TRUE
NK	*TMC6*	Wald ratio	1	0.0217	0.0227	1.022 (0.977, 1.068)	0.34	0.634	2.76E-08	TRUE
NK	*TMEM101*	Wald ratio	1	-0.0459	0.0316	0.955 (0.898, 1.016)	0.146	0.415	1.15E-08	TRUE
NK	*TMEM120B*	Wald ratio	1	-0.0149	0.0108	0.985 (0.965, 1.006)	0.168	0.444	4.43E-40	TRUE
NK	*TMEM156*	Wald ratio	1	0.0030	0.0073	1.003 (0.989, 1.017)	0.684	0.856	7.52E-54	TRUE
NK	*TMEM163*	Wald ratio	1	0.0362	0.0166	1.037 (1.004, 1.071)	0.029	0.16	2.41E-11	TRUE
NK	*TMEM171*	Wald ratio	1	-0.0139	0.0163	0.986 (0.955, 1.018)	0.394	0.673	1.15E-09	TRUE
NK	*TMEM2*	Wald ratio	1	-0.0117	0.0130	0.988 (0.963, 1.014)	0.369	0.657	4.81E-15	TRUE
NK	*TMEM204*	Wald ratio	1	0.0074	0.0105	1.007 (0.987, 1.028)	0.481	0.734	3.46E-49	TRUE

NK	*TMEM220*	Wald ratio	1	-0.0213	0.0182	0.979 (0.945, 1.014)	0.242	0.534	1.63E-09	TRUE
NK	*TMEM230*	Wald ratio	1	0.0064	0.0115	1.006 (0.984, 1.029)	0.575	0.786	1.03E-22	TRUE
NK	*TMEM241*	Wald ratio	1	0.0676	0.0188	1.07 (1.031, 1.11)	3.25E-04	0.007	7.40E-09	TRUE
NK	*TMEM50B*	Wald ratio	1	-0.0304	0.0095	0.97 (0.952, 0.988)	0.001	0.021	2.65E-31	TRUE
NK	*TMEM55A*	Wald ratio	1	-0.0004	0.0157	1 (0.969, 1.031)	0.982	0.993	1.68E-11	TRUE
NK	*TMEM71*	Wald ratio	1	-0.0039	0.0166	0.996 (0.964, 1.029)	0.814	0.921	1.36E-11	TRUE
NK	*TMEM80*	Wald ratio	1	-0.0361	0.0132	0.965 (0.94, 0.99)	0.006	0.058	7.99E-17	TRUE
NK	*TMEM8A*	Wald ratio	1	-0.0083	0.0173	0.992 (0.959, 1.026)	0.63	0.824	9.89E-11	TRUE
NK	*TMOD1*	Wald ratio	1	0.0089	0.0156	1.009 (0.979, 1.04)	0.567	0.782	6.28E-13	TRUE
NK	*TMPPE*	Wald ratio	1	0.0182	0.0109	1.018 (0.997, 1.04)	0.095	0.326	9.09E-21	TRUE
NK	*TMSB10*	Wald ratio	1	0.0042	0.0175	1.004 (0.97, 1.039)	0.81	0.919	3.41E-08	TRUE
NK	*TMTC4*	Wald ratio	1	-0.0225	0.0166	0.978 (0.947, 1.01)	0.174	0.452	3.22E-10	TRUE
NK	*TNFRSF14*	Wald ratio	1	0.0231	0.0163	1.023 (0.991, 1.057)	0.155	0.427	9.46E-12	TRUE
NK	*TNFRSF18*	Wald ratio	1	0.0169	0.0105	1.017 (0.996, 1.038)	0.109	0.351	1.14E-22	TRUE
NK	*TNFRSF4*	Wald ratio	1	0.0187	0.0137	1.019 (0.992, 1.047)	0.172	0.451	1.80E-14	TRUE
NK	*TNIP1*	Wald ratio	1	0.0263	0.0197	1.027 (0.988, 1.067)	0.183	0.462	2.18E-08	TRUE
NK	*TNRC18*	Wald ratio	1	0.0152	0.0183	1.015 (0.98, 1.052)	0.407	0.682	2.81E-10	TRUE
NK	*TOMM34*	Wald ratio	1	-0.0094	0.0212	0.991 (0.95, 1.033)	0.656	0.84	5.66E-08	TRUE
NK	*TOMM7*	Wald ratio	1	0.0030	0.0045	1.003 (0.994, 1.012)	0.503	0.743	1.32E-143	TRUE
NK	*TOX*	Wald ratio	1	-0.0291	0.0191	0.971 (0.936, 1.008)	0.126	0.382	1.03E-11	TRUE
NK	*TP53I11*	Wald ratio	1	-0.0462	0.0183	0.955 (0.921, 0.99)	0.011	0.089	6.42E-10	TRUE
NK	*TP53TG1*	Wald ratio	1	-0.0028	0.0171	0.997 (0.964, 1.031)	0.871	0.947	9.55E-11	TRUE
NK	*TPD52*	Wald ratio	1	-0.0124	0.0079	0.988 (0.973, 1.003)	0.117	0.365	6.79E-46	TRUE
NK	*TPGS1*	Wald ratio	1	-0.0052	0.0162	0.995 (0.964, 1.027)	0.749	0.886	1.92E-12	TRUE
NK	*TPGS2*	Wald ratio	1	-0.0038	0.0128	0.996 (0.972, 1.022)	0.769	0.897	1.23E-19	TRUE
NK	*TPM1*	Wald ratio	1	-0.0267	0.0184	0.974 (0.939, 1.009)	0.146	0.415	7.81E-09	TRUE
NK	*TPRA1*	Wald ratio	1	0.0050	0.0155	1.005 (0.975, 1.036)	0.746	0.883	4.66E-12	TRUE
NK	*TPST2*	Wald ratio	1	-0.0199	0.0158	0.98 (0.951, 1.011)	0.207	0.494	2.77E-12	TRUE
NK	*TRAM1*	Wald ratio	1	0.0006	0.0201	1.001 (0.962, 1.041)	0.977	0.991	3.27E-08	TRUE
NK	*TRAPPC12-AS1*	Wald ratio	1	0.0442	0.0143	1.045 (1.016, 1.075)	0.002	0.026	4.47E-14	TRUE
NK	*TRAPPC2L*	Wald ratio	1	0.0221	0.0178	1.022 (0.987, 1.059)	0.216	0.506	1.79E-10	TRUE
NK	*TRAPPC4*	Wald ratio	1	0.0164	0.0123	1.017 (0.992, 1.041)	0.182	0.462	1.28E-19	TRUE
NK	*TRIM47*	Wald ratio	1	0.0541	0.0239	1.056 (1.007, 1.106)	0.023	0.14	3.75E-09	TRUE
NK	*TRMT112*	Wald ratio	1	-0.0087	0.0105	0.991 (0.971, 1.012)	0.407	0.682	3.53E-30	TRUE
NK	*TRMT12*	Wald ratio	1	-0.0154	0.0188	0.985 (0.949, 1.022)	0.412	0.686	2.40E-09	TRUE
NK	*TRPC3*	Wald ratio	1	0.0250	0.0071	1.025 (1.011, 1.04)	4.64E-04	0.01	2.64E-61	TRUE
NK	*TSGA10*	Wald ratio	1	0.0016	0.0139	1.002 (0.975, 1.029)	0.91	0.962	2.97E-15	TRUE
NK	*TSPAN15*	Wald ratio	1	-0.0082	0.0131	0.992 (0.967, 1.018)	0.535	0.761	9.35E-20	TRUE
NK	*TSPO*	Wald ratio	1	0.0055	0.0107	1.006 (0.985, 1.027)	0.606	0.807	6.85E-27	TRUE
NK	*TSTD1*	Wald ratio	1	0.0029	0.0081	1.003 (0.987, 1.019)	0.718	0.874	1.82E-39	TRUE
NK	*TSTD3*	Wald ratio	1	-0.0047	0.0111	0.995 (0.974, 1.017)	0.675	0.852	7.48E-24	TRUE
NK	*TTC16*	Wald ratio	1	-0.0126	0.0176	0.987 (0.954, 1.022)	0.474	0.73	6.10E-12	TRUE
NK	*TTC32*	Wald ratio	1	0.0302	0.0142	1.031 (1.002, 1.06)	0.034	0.175	1.14E-15	TRUE
NK	*TTC38*	Wald ratio	1	0.0003	0.0084	1 (0.984, 1.017)	0.97	0.989	5.02E-44	TRUE
NK	*TUBB2A*	Wald ratio	1	0.0204	0.0214	1.021 (0.979, 1.064)	0.341	0.634	5.58E-08	TRUE
NK	*TUFM*	Wald ratio	1	0.0162	0.0081	1.016 (1, 1.033)	0.046	0.21	2.90E-42	TRUE
NK	*TXK*	Wald ratio	1	0.0031	0.0094	1.003 (0.985, 1.022)	0.741	0.881	2.24E-23	TRUE
NK	*TYROBP*	Wald ratio	1	0.0285	0.0122	1.029 (1.005, 1.054)	0.019	0.123	1.36E-21	TRUE
NK	*U2AF1L4*	Wald ratio	1	-0.0091	0.0142	0.991 (0.964, 1.019)	0.522	0.754	7.53E-24	TRUE
NK	*UBA52*	Wald ratio	1	0.0071	0.0131	1.007 (0.982, 1.033)	0.585	0.794	3.63E-18	TRUE
NK	*UBA6*	Wald ratio	1	0.0106	0.0127	1.011 (0.986, 1.036)	0.404	0.681	1.06E-18	TRUE
NK	*UBA6-AS1*	Wald ratio	1	-0.0313	0.0193	0.969 (0.933, 1.007)	0.105	0.343	3.80E-09	TRUE
NK	*UBE2L3*	Wald ratio	1	-0.0014	0.0123	0.999 (0.975, 1.023)	0.91	0.962	3.03E-14	TRUE
NK	*UBE3B*	Wald ratio	1	0.0782	0.0222	1.081 (1.035, 1.129)	4.20E-04	0.009	1.15E-07	TRUE
NK	*UFL1*	Wald ratio	1	0.0171	0.0213	1.017 (0.976, 1.061)	0.423	0.693	2.74E-08	TRUE
NK	*UFSP2*	Wald ratio	1	-0.0191	0.0209	0.981 (0.942, 1.022)	0.36	0.65	5.88E-08	TRUE
NK	*ULK4*	Wald ratio	1	0.0219	0.0104	1.022 (1.001, 1.043)	0.036	0.181	4.41E-24	TRUE
NK	*UNC5B-AS1*	Wald ratio	1	0.0057	0.0171	1.006 (0.973, 1.04)	0.737	0.88	1.44E-09	TRUE
NK	*UPF3A*	Wald ratio	1	0.0458	0.0179	1.047 (1.011, 1.084)	0.011	0.085	1.60E-11	TRUE
NK	*UPK3BL*	Wald ratio	1	0.0126	0.0168	1.013 (0.98, 1.047)	0.454	0.717	1.32E-12	TRUE
NK	*UQCRH*	Wald ratio	1	-0.0336	0.0160	0.967 (0.937, 0.998)	0.035	0.179	9.48E-15	TRUE
NK	*USF1*	Wald ratio	1	-0.0162	0.0152	0.984 (0.955, 1.014)	0.285	0.577	3.08E-15	TRUE
NK	*UVSSA*	Wald ratio	1	-0.0586	0.0225	0.943 (0.902, 0.986)	0.009	0.076	9.62E-08	TRUE
NK	*VAMP2*	Wald ratio	1	0.0377	0.0189	1.038 (1.001, 1.078)	0.046	0.211	1.02E-09	TRUE
NK	*VAMP4*	Wald ratio	1	0.0111	0.0203	1.011 (0.972, 1.052)	0.582	0.792	3.39E-09	TRUE
NK	*VIM*	Wald ratio	1	0.0831	0.0214	1.087 (1.042, 1.133)	1.04E-04	0.003	7.38E-08	TRUE
NK	*VIPR2*	Wald ratio	1	0.0322	0.0166	1.033 (1, 1.067)	0.052	0.227	1.19E-11	TRUE
NK	*VIT*	Wald ratio	1	-0.0060	0.0150	0.994 (0.965, 1.024)	0.689	0.859	1.52E-12	TRUE
NK	*VOPP1*	Wald ratio	1	-0.0037	0.0204	0.996 (0.957, 1.037)	0.857	0.939	3.48E-08	TRUE
NK	*VPS28*	Wald ratio	1	0.0149	0.0091	1.015 (0.997, 1.033)	0.102	0.338	1.10E-29	TRUE
NK	*WAC-AS1*	IVW	2	0.0220	0.0095	1.022 (1.003, 1.041)	0.021	0.131	3.72E-36	TRUE
NK	*WARS*	Wald ratio	1	-0.0694	0.0168	0.933 (0.903, 0.964)	3.52E-05	0.001	4.35E-12	TRUE
NK	*WDR1*	Wald ratio	1	0.0016	0.0182	1.002 (0.967, 1.038)	0.932	0.971	8.51E-10	TRUE
NK	*WDR37*	Wald ratio	1	-0.0409	0.0411	0.96 (0.886, 1.041)	0.32	0.615	1.77E-09	TRUE
NK	*WDR60*	Wald ratio	1	-0.0259	0.0195	0.974 (0.938, 1.012)	0.185	0.464	8.03E-08	TRUE
NK	*WDR93*	Wald ratio	1	0.0090	0.0076	1.009 (0.994, 1.024)	0.231	0.524	1.36E-64	TRUE
NK	*WIPI1*	Wald ratio	1	-0.0099	0.0210	0.99 (0.95, 1.032)	0.636	0.827	4.84E-11	TRUE
NK	*XCL1*	Wald ratio	1	0.0115	0.0063	1.012 (0.999, 1.024)	0.066	0.263	4.34E-69	TRUE
NK	*XRCC6BP1*	Wald ratio	1	-0.0328	0.0163	0.968 (0.937, 0.999)	0.044	0.208	1.62E-11	TRUE

NK	*XRRA1*	Wald ratio	1	-0.0064	0.0053	0.994 (0.983, 1.004)	0.232	0.525	1.23E-98	TRUE
NK	*XXbac-BPG252P9.9*	Wald ratio	1	0.0144	0.0124	1.014 (0.99, 1.04)	0.249	0.542	5.08E-17	TRUE
NK	*XXbac-BPG299F13.17*	IVW	3	0.0014	0.0142	1.001 (0.974, 1.03)	0.919	0.965	1.27E-139	TRUE
NK	*XXbac-BPG299F13.17*	MR Egger	3	0.0348	0.0118	1.035 (1.012, 1.06)	0.208	NA	NA	NA
NK	*XXbac-BPG299F13.17*	Weighted median	3	0.0069	0.0050	1.007 (0.997, 1.017)	0.169	NA	NA	NA
NK	*XXbac-BPG299F13.17*	Simple mode	3	0.0059	0.0084	1.006 (0.989, 1.023)	0.557	NA	NA	NA
NK	*XXbac-BPG299F13.17*	Weighted mode	3	0.0072	0.0048	1.007 (0.998, 1.017)	0.275	NA	NA	NA
NK	*XXYLT1-AS2*	Wald ratio	1	-0.0301	0.0136	0.97 (0.945, 0.997)	0.027	0.155	7.20E-19	TRUE
NK	*YBEY*	Wald ratio	1	-0.0011	0.0084	0.999 (0.983, 1.016)	0.898	0.957	5.71E-35	TRUE
NK	*YEATS4*	Wald ratio	1	-0.0011	0.0148	0.999 (0.97, 1.028)	0.942	0.977	7.21E-15	TRUE
NK	*YWHAB*	Wald ratio	1	0.0227	0.0130	1.023 (0.997, 1.049)	0.081	0.3	3.35E-19	TRUE
NK	*ZAP70*	Wald ratio	1	-0.0243	0.0207	0.976 (0.937, 1.016)	0.24	0.534	6.25E-08	TRUE
NK	*ZC3H7A*	Wald ratio	1	-0.0121	0.0199	0.988 (0.95, 1.027)	0.544	0.765	4.72E-09	TRUE
NK	*ZC3HAV1*	Wald ratio	1	0.0058	0.0095	1.006 (0.987, 1.025)	0.538	0.762	1.42E-32	TRUE
NK	*ZDHHC4*	Wald ratio	1	-0.0713	0.0170	0.931 (0.901, 0.963)	2.72E-05	0.001	3.65E-09	TRUE
NK	*ZFAND3*	Wald ratio	1	-0.0297	0.0202	0.971 (0.933, 1.01)	0.142	0.409	3.62E-08	TRUE
NK	*ZFAS1*	Wald ratio	1	0.0101	0.0130	1.01 (0.985, 1.036)	0.436	0.703	9.54E-19	TRUE
NK	*ZFP57*	IVW	2	0.0118	0.0189	1.012 (0.975, 1.05)	0.532	0.76	3.47E-23	TRUE
NK	*ZFP90*	Wald ratio	1	0.0185	0.0124	1.019 (0.994, 1.044)	0.137	0.399	1.96E-22	TRUE
NK	*ZMAT4*	Wald ratio	1	-0.0058	0.0067	0.994 (0.981, 1.007)	0.385	0.668	5.92E-31	TRUE
NK	*ZNF100*	Wald ratio	1	-0.0574	0.0126	0.944 (0.921, 0.968)	5.52E-06	3.62E-04	1.20E-17	TRUE
NK	*ZNF165*	Wald ratio	1	0.0003	0.0194	1 (0.963, 1.039)	0.987	0.995	1.64E-13	TRUE
NK	*ZNF264*	Wald ratio	1	0.0072	0.0173	1.007 (0.974, 1.042)	0.678	0.853	8.69E-10	TRUE
NK	*ZNF266*	Wald ratio	1	0.0334	0.0132	1.034 (1.008, 1.061)	0.011	0.088	7.49E-17	TRUE
NK	*ZNF302*	Wald ratio	1	-0.0110	0.0108	0.989 (0.968, 1.01)	0.307	0.6	7.65E-24	TRUE
NK	*ZNF324*	Wald ratio	1	0.0367	0.0184	1.037 (1.001, 1.075)	0.046	0.211	4.61E-09	TRUE
NK	*ZNF391*	Wald ratio	1	-0.0130	0.0093	0.987 (0.969, 1.005)	0.162	0.436	2.58E-36	TRUE
NK	*ZNF429*	Wald ratio	1	0.0188	0.0204	1.019 (0.979, 1.061)	0.357	0.648	2.89E-08	TRUE
NK	*ZNF44*	Wald ratio	1	-0.0227	0.0124	0.978 (0.954, 1.002)	0.068	0.266	1.14E-19	TRUE
NK	*ZNF492*	Wald ratio	1	0.0086	0.0087	1.009 (0.992, 1.026)	0.323	0.618	8.42E-26	TRUE
NK	*ZNF506*	Wald ratio	1	0.0236	0.0143	1.024 (0.996, 1.053)	0.099	0.333	8.58E-13	TRUE
NK	*ZNF584*	Wald ratio	1	0.0081	0.0132	1.008 (0.982, 1.035)	0.538	0.762	5.30E-14	TRUE
NK	*ZNF587*	Wald ratio	1	-0.0037	0.0190	0.996 (0.96, 1.034)	0.847	0.935	1.49E-08	TRUE
NK	*ZNF593*	Wald ratio	1	0.0453	0.0176	1.046 (1.011, 1.083)	0.01	0.083	1.64E-10	TRUE
NK	*ZNF677*	Wald ratio	1	-0.0231	0.0174	0.977 (0.944, 1.011)	0.184	0.464	5.38E-10	TRUE
NK	*ZNF718*	Wald ratio	1	-0.0066	0.0155	0.993 (0.964, 1.024)	0.669	0.848	7.75E-13	TRUE
NK	*ZNF749*	Wald ratio	1	-0.0227	0.0126	0.978 (0.954, 1.002)	0.071	0.276	1.28E-18	TRUE
NK	*ZNF791*	Wald ratio	1	-0.0819	0.0187	0.921 (0.888, 0.956)	1.14E-05	6.36E-04	1.08E-08	TRUE
NK	*ZNF823*	Wald ratio	1	-0.0072	0.0143	0.993 (0.965, 1.021)	0.617	0.817	3.12E-14	TRUE
NK	*ZNF83*	Wald ratio	1	-0.0240	0.0129	0.976 (0.952, 1.001)	0.063	0.255	1.20E-17	TRUE
NK	*ZNF85*	Wald ratio	1	0.0141	0.0093	1.014 (0.996, 1.033)	0.13	0.39	4.11E-38	TRUE
NK	*ZNF880*	Wald ratio	1	-0.0306	0.0189	0.97 (0.934, 1.007)	0.106	0.346	7.28E-09	TRUE
NK	*ZNHIT1*	Wald ratio	1	0.0199	0.0177	1.02 (0.985, 1.056)	0.261	0.555	2.91E-12	TRUE
NK	*ZNHIT6*	Wald ratio	1	-0.0268	0.0202	0.974 (0.936, 1.013)	0.184	0.464	3.79E-08	TRUE
NK	*ZNRD1*	Wald ratio	1	-0.0043	0.0223	0.996 (0.953, 1.04)	0.848	0.935	2.06E-12	TRUE
NK	*ZP3*	Wald ratio	1	0.0185	0.0074	1.019 (1.004, 1.034)	0.013	0.096	1.35E-64	TRUE
NK	*ZSWIM7*	Wald ratio	1	0.0437	0.0125	1.045 (1.019, 1.07)	4.62E-04	0.01	2.91E-19	TRUE
Plasma	*AIF1*	Wald ratio	1	-0.0128	0.0148	0.987 (0.959, 1.016)	0.389	0.671	3.61E-10	TRUE
Plasma	*ANXA6*	Wald ratio	1	-0.0188	0.0165	0.981 (0.95, 1.014)	0.255	0.549	2.09E-10	TRUE
Plasma	*APOBEC3G*	Wald ratio	1	-0.0178	0.0188	0.982 (0.947, 1.019)	0.342	0.635	7.77E-08	TRUE
Plasma	*APOM*	Wald ratio	1	0.0184	0.0134	1.019 (0.992, 1.046)	0.168	0.444	1.66E-18	TRUE
Plasma	*BIK*	Wald ratio	1	0.0171	0.0189	1.017 (0.98, 1.056)	0.366	0.654	6.05E-08	TRUE
Plasma	*C11orf24*	Wald ratio	1	-0.0036	0.0132	0.996 (0.971, 1.023)	0.787	0.909	3.33E-14	TRUE
Plasma	*C6orf136*	Wald ratio	1	-0.0449	0.0163	0.956 (0.926, 0.987)	0.006	0.055	9.82E-10	TRUE
Plasma	*CCHCR1*	Wald ratio	1	-0.0011	0.0124	0.999 (0.975, 1.023)	0.929	0.97	2.72E-16	TRUE
Plasma	*CCT8*	Wald ratio	1	-0.0020	0.0208	0.998 (0.958, 1.039)	0.922	0.965	8.46E-10	TRUE
Plasma	*CDKN1A*	Wald ratio	1	0.0043	0.0162	1.004 (0.973, 1.037)	0.791	0.909	5.65E-10	TRUE
Plasma	*CSTB*	Wald ratio	1	0.0049	0.0142	1.005 (0.977, 1.033)	0.73	0.88	1.99E-11	TRUE
Plasma	*CTC-524C5.2*	Wald ratio	1	-0.0057	0.0096	0.994 (0.976, 1.013)	0.556	0.774	1.74E-22	TRUE
Plasma	*CTSH*	Wald ratio	1	-0.0079	0.0124	0.992 (0.968, 1.017)	0.524	0.754	1.03E-14	TRUE
Plasma	*CTSS*	Wald ratio	1	-0.0307	0.0171	0.97 (0.938, 1.003)	0.072	0.279	6.83E-09	TRUE
Plasma	*DDX39B*	Wald ratio	1	0.0056	0.0133	1.006 (0.98, 1.032)	0.671	0.849	8.52E-13	TRUE
Plasma	*DEK*	Wald ratio	1	-0.0099	0.0264	0.99 (0.94, 1.043)	0.706	0.868	5.35E-08	TRUE
Plasma	*DXO*	Wald ratio	1	0.0302	0.0197	1.031 (0.992, 1.071)	0.125	0.38	3.16E-09	TRUE
Plasma	*EHMT2*	Wald ratio	1	-0.0467	0.0184	0.954 (0.921, 0.989)	0.011	0.087	9.24E-09	TRUE
Plasma	*EIF5A*	Wald ratio	1	-0.0094	0.0062	0.991 (0.979, 1.003)	0.134	0.394	1.42E-68	TRUE
Plasma	*ELL2*	Wald ratio	1	0.0056	0.0170	1.006 (0.973, 1.04)	0.74	0.881	1.01E-08	TRUE
Plasma	*FAM118A*	Wald ratio	1	0.0016	0.0119	1.002 (0.978, 1.025)	0.895	0.957	4.85E-13	TRUE
Plasma	*HIST1H2BN*	Wald ratio	1	0.0140	0.0219	1.014 (0.971, 1.059)	0.523	0.754	3.18E-10	TRUE
Plasma	*HLA-A*	Wald ratio	1	0.0487	0.0181	1.05 (1.013, 1.088)	0.007	0.066	2.95E-08	TRUE
Plasma	*HLA-B*	IVW	2	0.0035	0.0521	1.003 (0.906, 1.111)	0.947	0.979	7.72E-29	TRUE
Plasma	*HLA-C*	Wald ratio	1	0.0210	0.0154	1.021 (0.991, 1.053)	0.173	0.451	1.19E-10	TRUE
Plasma	*HLA-DQB1*	Wald ratio	1	-0.0708	0.0148	0.932 (0.905, 0.959)	1.77E-06	1.71E-04	1.72E-10	TRUE
Plasma	*HLA-G*	Wald ratio	1	0.0534	0.0343	1.055 (0.986, 1.128)	0.119	0.369	9.91E-12	TRUE
Plasma	*IFNG-AS1*	Wald ratio	1	-0.0059	0.0106	0.994 (0.974, 1.015)	0.576	0.787	2.92E-20	TRUE
Plasma	*ITPR3*	Wald ratio	1	-0.0780	0.0184	0.925 (0.892, 0.959)	2.33E-05	0.001	1.23E-08	TRUE
Plasma	*KANSL1-AS1*	Wald ratio	1	0.0517	0.0207	1.053 (1.011, 1.097)	0.013	0.094	2.61E-13	TRUE
Plasma	*LCN8*	Wald ratio	1	-0.0210	0.0135	0.979 (0.954, 1.005)	0.119	0.369	2.12E-18	TRUE
Plasma	*LEMD2*	Wald ratio	1	-0.0384	0.0381	0.962 (0.893, 1.037)	0.313	0.608	4.96E-14	TRUE

Plasma	*LIMD1*	Wald ratio	1	0.0141	0.0155	1.014 (0.984, 1.045)	0.362	0.651	7.63E-11	TRUE
Plasma	*LRRC16A*	Wald ratio	1	0.0180	0.0073	1.018 (1.004, 1.033)	0.014	0.101	5.35E-43	TRUE
Plasma	*LST1*	Wald ratio	1	-0.0473	0.0170	0.954 (0.923, 0.986)	0.005	0.053	4.77E-10	TRUE
Plasma	*MED23*	Wald ratio	1	-0.0091	0.0166	0.991 (0.959, 1.024)	0.583	0.792	8.47E-10	TRUE
Plasma	*MICB*	IVW	2	-0.0178	0.0092	0.982 (0.965, 1)	0.053	0.231	1.36E-34	TRUE
Plasma	*MTHFD1L*	Wald ratio	1	0.0109	0.0141	1.011 (0.983, 1.039)	0.441	0.706	1.73E-12	TRUE
Plasma	*NFKBIL1*	Wald ratio	1	0.0537	0.0137	1.055 (1.027, 1.084)	8.51E-05	0.003	3.71E-14	TRUE
Plasma	*NT5C3B*	Wald ratio	1	-0.0401	0.0127	0.961 (0.937, 0.985)	0.002	0.022	9.75E-12	TRUE
Plasma	*NUCB2*	Wald ratio	1	-0.0703	0.0141	0.932 (0.907, 0.958)	6.26E-07	9.75E-05	5.23E-12	TRUE
Plasma	*PAQR6*	Wald ratio	1	0.0246	0.0111	1.025 (1.003, 1.048)	0.027	0.153	1.14E-19	TRUE
Plasma	*PKHD1L1*	Wald ratio	1	0.0078	0.0168	1.008 (0.975, 1.042)	0.641	0.83	2.34E-09	TRUE
Plasma	*PPDPF*	Wald ratio	1	-0.0171	0.0159	0.983 (0.953, 1.014)	0.283	0.575	3.38E-09	TRUE
Plasma	*PRR3*	IVW	2	0.0090	0.0151	1.009 (0.98, 1.039)	0.55	0.769	5.40E-23	TRUE
Plasma	*PSMD5-AS1*	Wald ratio	1	0.0127	0.0139	1.013 (0.986, 1.041)	0.361	0.65	9.67E-13	TRUE
Plasma	*RMND1*	Wald ratio	1	0.0159	0.0127	1.016 (0.991, 1.042)	0.21	0.497	1.50E-12	TRUE
Plasma	*RNF146*	Wald ratio	1	-0.0262	0.0076	0.974 (0.96, 0.989)	5.57E-04	0.011	5.25E-37	TRUE
Plasma	*RP1-3J17.3*	Wald ratio	1	-0.0014	0.0073	0.999 (0.985, 1.013)	0.85	0.936	2.04E-42	TRUE
Plasma	*RP11-356I2.4*	Wald ratio	1	-0.0247	0.0193	0.976 (0.939, 1.013)	0.201	0.485	1.15E-08	TRUE
Plasma	*RPL12*	Wald ratio	1	-0.0461	0.0128	0.955 (0.931, 0.979)	3.09E-04	0.007	3.89E-16	TRUE
Plasma	*RPL28*	Wald ratio	1	0.0100	0.0130	1.01 (0.985, 1.036)	0.438	0.703	1.73E-14	TRUE
Plasma	*RPL36AL*	Wald ratio	1	-0.0041	0.0165	0.996 (0.964, 1.029)	0.802	0.915	1.65E-09	TRUE
Plasma	*RPL8*	Wald ratio	1	-0.0103	0.0100	0.99 (0.97, 1.009)	0.303	0.598	1.17E-23	TRUE
Plasma	*RPP21*	IVW	2	-0.0034	0.0091	0.997 (0.979, 1.014)	0.705	0.868	2.10E-40	TRUE
Plasma	*RPS10*	Wald ratio	1	-0.0148	0.0152	0.985 (0.956, 1.015)	0.33	0.624	5.07E-10	TRUE
Plasma	*RPS18*	Wald ratio	1	-0.0327	0.0191	0.968 (0.932, 1.005)	0.087	0.312	7.16E-09	TRUE
Plasma	*RXRB*	Wald ratio	1	0.0730	0.0187	1.076 (1.037, 1.116)	9.79E-05	0.003	8.17E-08	TRUE
Plasma	*SIRT5*	Wald ratio	1	0.0511	0.0139	1.052 (1.024, 1.081)	2.31E-04	0.006	7.35E-17	TRUE
Plasma	*STK19*	IVW	2	-0.0503	0.0386	0.951 (0.882, 1.026)	0.192	0.473	6.18E-20	TRUE
Plasma	*SULF2*	Wald ratio	1	0.0103	0.0106	1.01 (0.99, 1.032)	0.33	0.624	6.76E-21	TRUE
Plasma	*TIMM10*	Wald ratio	1	-0.0022	0.0137	0.998 (0.971, 1.025)	0.875	0.948	8.87E-16	TRUE
Plasma	*TMEM156*	Wald ratio	1	0.0106	0.0137	1.011 (0.984, 1.038)	0.438	0.703	1.39E-12	TRUE
Plasma	*TSPO*	Wald ratio	1	0.0014	0.0165	1.001 (0.97, 1.034)	0.93	0.97	1.64E-09	TRUE
Plasma	*TXNDC11*	Wald ratio	1	-0.0379	0.0164	0.963 (0.932, 0.994)	0.021	0.131	1.83E-09	TRUE
Plasma	*XXbac-BPG299F13.17*	Wald ratio	1	-0.0084	0.0172	0.992 (0.959, 1.026)	0.627	0.822	5.03E-09	TRUE
Plasma	*YBEY*	Wald ratio	1	0.0011	0.0125	1.001 (0.977, 1.026)	0.928	0.969	1.05E-15	TRUE
Plasma	*ZBTB22*	Wald ratio	1	-0.0361	0.0116	0.965 (0.943, 0.987)	0.002	0.025	1.74E-17	TRUE
Plasma	*ZP3*	Wald ratio	1	-0.0057	0.0113	0.994 (0.973, 1.016)	0.612	0.812	7.06E-20	TRUE

Table S3. Replication MR analyses on the associations between cell type-specific eGenes and hypertension.

Cell type	eGene	Method	nSNP	β	SE	OR (95% CI)	*P*-value	Steiger pval	Steiger direction
B IN	*ABCA1*	Wald ratio	1	0.0058	0.0038	1.006 (0.998, 1.013)	0.129	1.27E-08	TRUE
B IN	*ACAT1*	Wald ratio	1	-0.0094	0.0039	0.991 (0.983, 0.998)	0.015	2.10E-08	TRUE
B IN	*ANXA4*	Wald ratio	1	-0.0007	0.0023	0.999 (0.995, 1.004)	0.769	5.62E-22	TRUE
B IN	*AP003774.1*	Wald ratio	1	-0.0085	0.0023	0.992 (0.987, 0.996)	2.75E-04	1.25E-20	TRUE
B IN	*BCL2A1*	Wald ratio	1	0.0002	0.0012	1 (0.998, 1.003)	0.875	3.31E-78	TRUE
B IN	*BIRC5*	Wald ratio	1	-0.0024	0.0019	0.998 (0.994, 1.001)	0.201	5.67E-36	TRUE
B IN	*BLK*	Wald ratio	1	-0.0106	0.0022	0.989 (0.985, 0.994)	2.24E-06	8.66E-23	TRUE
B IN	*C10orf32*	Wald ratio	1	-0.0114	0.0034	0.989 (0.982, 0.995)	7.34E-04	2.73E-10	TRUE
B IN	*C6orf48*	Wald ratio	1	0.0066	0.0030	1.007 (1.001, 1.012)	0.025	3.20E-13	TRUE
B IN	*C6orf62*	Wald ratio	1	0.0111	0.0040	1.011 (1.003, 1.019)	0.006	1.01E-07	TRUE
B IN	*CD164*	Wald ratio	1	-0.0087	0.0041	0.991 (0.983, 0.999)	0.033	8.85E-08	TRUE
B IN	*CENPV*	Wald ratio	1	0.0126	0.0032	1.013 (1.006, 1.019)	9.78E-05	2.27E-11	TRUE
B IN	*CLLU1*	Wald ratio	1	0.0048	0.0029	1.005 (0.999, 1.011)	0.105	7.29E-14	TRUE
B IN	*CLLU1OS*	Wald ratio	1	0.0019	0.0019	1.002 (0.998, 1.006)	0.319	8.65E-30	TRUE
B IN	*CLN8*	Wald ratio	1	-0.0099	0.0031	0.99 (0.984, 0.996)	0.001	9.55E-13	TRUE
B IN	*CNPPD1*	Wald ratio	1	-0.0037	0.0036	0.996 (0.989, 1.003)	0.309	1.23E-09	TRUE
B IN	*CTD-3110H11.1*	Wald ratio	1	-0.0036	0.0024	0.996 (0.992, 1.001)	0.137	9.56E-20	TRUE
B IN	*DDX5*	Wald ratio	1	0.0127	0.0032	1.013 (1.006, 1.019)	7.78E-05	1.73E-11	TRUE
B IN	*DYNLL2*	Wald ratio	1	0.0050	0.0038	1.005 (0.998, 1.013)	0.19	5.99E-09	TRUE
B IN	*ERAP2*	Wald ratio	1	0.0027	0.0017	1.003 (0.999, 1.006)	0.103	1.28E-41	TRUE
B IN	*FAM167A*	Wald ratio	1	-0.0113	0.0024	0.989 (0.984, 0.993)	2.07E-06	5.13E-20	TRUE
B IN	*FAM21A*	Wald ratio	1	0.0003	0.0033	1 (0.994, 1.007)	0.932	8.01E-12	TRUE
B IN	*FANCA*	Wald ratio	1	-0.0090	0.0040	0.991 (0.983, 0.999)	0.024	5.60E-08	TRUE
B IN	*FNBP4*	Wald ratio	1	-0.0137	0.0034	0.986 (0.98, 0.993)	5.29E-05	1.91E-10	TRUE
B IN	*GALT*	Wald ratio	1	0.0071	0.0041	1.007 (0.999, 1.015)	0.082	8.15E-08	TRUE
B IN	*GNG7*	Wald ratio	1	-0.0034	0.0040	0.997 (0.989, 1.004)	0.387	2.40E-08	TRUE
B IN	*GTF2H4*	IVW	4	-0.0049	0.0023	0.995 (0.991, 0.999)	0.029	3.02E-61	TRUE
B IN	*GTF2H4*	MR Egger	4	0.0101	0.0079	1.01 (0.995, 1.026)	0.329	NA	NA
B IN	*GTF2H4*	Weighted median	4	-0.0038	0.0019	0.996 (0.993, 1)	0.04	NA	NA
B IN	*GTF2H4*	Simple mode	4	-0.0039	0.0022	0.996 (0.992, 1)	0.179	NA	NA
B IN	*GTF2H4*	Weighted mode	4	-0.0031	0.0019	0.997 (0.993, 1.001)	0.195	NA	NA
B IN	*HAGHL*	Wald ratio	1	0.0085	0.0038	1.009 (1.001, 1.016)	0.026	9.06E-09	TRUE
B IN	*HLA-DQA1*	Wald ratio	1	0.0035	0.0020	1.003 (1, 1.007)	0.079	4.99E-29	TRUE
B IN	*HSBP1L1*	Wald ratio	1	-0.0078	0.0040	0.992 (0.984, 1)	0.053	4.79E-08	TRUE
B IN	*HSD17B11*	Wald ratio	1	0.0098	0.0040	1.01 (1.002, 1.018)	0.013	3.23E-08	TRUE
B IN	*KDELR2*	Wald ratio	1	0.0027	0.0027	1.003 (0.997, 1.008)	0.328	1.55E-16	TRUE
B IN	*KTN1*	Wald ratio	1	0.0050	0.0035	1.005 (0.998, 1.012)	0.146	3.97E-10	TRUE
B IN	*LA16c-358B7.3*	Wald ratio	1	0.0123	0.0029	1.012 (1.007, 1.018)	1.88E-05	4.49E-14	TRUE
B IN	*LSM7*	Wald ratio	1	0.0039	0.0023	1.004 (0.999, 1.008)	0.084	3.18E-22	TRUE
B IN	*LY6G5C*	IVW	2	0.0026	0.0021	1.003 (0.999, 1.007)	0.212	3.56E-29	TRUE
B IN	*MDM4*	Wald ratio	1	0.0075	0.0021	1.008 (1.003, 1.012)	2.88E-04	5.18E-27	TRUE
B IN	*MSMP*	Wald ratio	1	-0.0072	0.0035	0.993 (0.986, 1)	0.04	5.55E-10	TRUE
B IN	*NOMO1*	Wald ratio	1	-0.0065	0.0030	0.994 (0.988, 0.999)	0.033	1.41E-12	TRUE
B IN	*PCBP2*	Wald ratio	1	-0.0053	0.0039	0.995 (0.987, 1.002)	0.168	2.19E-08	TRUE
B IN	*PRKD3*	Wald ratio	1	0.0102	0.0035	1.01 (1.003, 1.017)	0.004	8.27E-10	TRUE
B IN	*PTPLAD2*	Wald ratio	1	-0.0025	0.0020	0.998 (0.994, 1.001)	0.219	2.24E-30	TRUE
B IN	*RAC1*	Wald ratio	1	-0.0077	0.0026	0.992 (0.987, 0.998)	0.004	9.49E-17	TRUE
B IN	*RBM6*	Wald ratio	1	-0.0046	0.0034	0.995 (0.989, 1.002)	0.179	9.09E-11	TRUE
B IN	*RP11-69E11.4*	Wald ratio	1	0.0018	0.0041	1.002 (0.994, 1.01)	0.665	3.18E-08	TRUE
B IN	*RP13-514E23.2*	Wald ratio	1	-0.0039	0.0018	0.996 (0.993, 1)	0.032	1.45E-34	TRUE
B IN	*RPS9*	Wald ratio	1	0.0041	0.0014	1.004 (1.001, 1.007)	0.003	1.82E-60	TRUE
B IN	*SAG*	Wald ratio	1	0.0037	0.0019	1.004 (1, 1.008)	0.056	5.24E-32	TRUE
B IN	*SH3YL1*	Wald ratio	1	0.0003	0.0020	1 (0.996, 1.004)	0.889	3.70E-29	TRUE
B IN	*SIDT2*	Wald ratio	1	0.0110	0.0027	1.011 (1.006, 1.017)	5.86E-05	3.41E-16	TRUE
B IN	*SNRPD2*	Wald ratio	1	-0.0054	0.0026	0.995 (0.99, 1)	0.036	3.43E-18	TRUE
B IN	*THAP9-AS1*	Wald ratio	1	-0.0136	0.0038	0.986 (0.979, 0.994)	3.88E-04	3.10E-08	TRUE
B IN	*WARS*	Wald ratio	1	-0.0053	0.0015	0.995 (0.992, 0.998)	3.54E-04	3.16E-51	TRUE
B Mem	*ADAMTS6*	Wald ratio	1	0.0018	0.0039	1.002 (0.994, 1.01)	0.637	1.45E-08	TRUE
B Mem	*ANXA4*	Wald ratio	1	-0.0006	0.0022	0.999 (0.995, 1.004)	0.769	1.84E-25	TRUE
B Mem	*AP003774.1*	Wald ratio	1	-0.0099	0.0028	0.99 (0.985, 0.996)	3.44E-04	6.42E-15	TRUE
B Mem	*BCL2A1*	Wald ratio	1	0.0003	0.0023	1 (0.996, 1.005)	0.904	1.44E-22	TRUE
B Mem	*BLK*	Wald ratio	1	-0.0081	0.0018	0.992 (0.988, 0.995)	6.08E-06	5.92E-36	TRUE
B Mem	*BTF3*	Wald ratio	1	-0.0017	0.0041	0.998 (0.99, 1.006)	0.674	2.77E-08	TRUE
B Mem	*C6orf48*	Wald ratio	1	0.0091	0.0040	1.009 (1.001, 1.017)	0.023	3.06E-08	TRUE
B Mem	*CCDC163P*	Wald ratio	1	0.0048	0.0024	1.005 (1, 1.01)	0.041	6.53E-21	TRUE
B Mem	*CLLU1OS*	Wald ratio	1	-0.0017	0.0015	0.998 (0.995, 1.001)	0.233	7.02E-52	TRUE
B Mem	*COL9A3*	Wald ratio	1	0.0039	0.0038	1.004 (0.996, 1.011)	0.305	1.66E-08	TRUE
B Mem	*DDX5*	Wald ratio	1	0.0148	0.0038	1.015 (1.007, 1.022)	7.78E-05	1.08E-08	TRUE
B Mem	*ERAP2*	Wald ratio	1	0.0026	0.0015	1.003 (1, 1.006)	0.089	1.17E-47	TRUE
B Mem	*FAM167A*	Wald ratio	1	0.0126	0.0028	1.013 (1.007, 1.018)	7.04E-06	7.74E-15	TRUE
B Mem	*FNBP4*	Wald ratio	1	-0.0157	0.0032	0.984 (0.978, 0.991)	8.36E-07	6.33E-12	TRUE
B Mem	*HAGH*	Wald ratio	1	0.0113	0.0033	1.011 (1.005, 1.018)	6.71E-04	1.08E-10	TRUE
B Mem	*HAGHL*	Wald ratio	1	0.0103	0.0036	1.01 (1.003, 1.017)	0.004	1.16E-09	TRUE
B Mem	*HIST1H2AG*	Wald ratio	1	0.0109	0.0029	1.011 (1.005, 1.017)	2.22E-04	4.11E-13	TRUE
B Mem	*HIST1H2BJ*	Wald ratio	1	-0.0091	0.0041	0.991 (0.983, 0.999)	0.028	5.62E-08	TRUE
B Mem	*HIST1H3D*	Wald ratio	1	-0.0067	0.0023	0.993 (0.989, 0.998)	0.004	7.62E-20	TRUE

B Mem	*HOXB-AS1*	Wald ratio	1	-0.0028	0.0031	0.997 (0.991, 1.003)	0.356	4.33E-13	TRUE
B Mem	*HSBP1L1*	Wald ratio	1	-0.0051	0.0035	0.995 (0.988, 1.002)	0.141	1.65E-10	TRUE
B Mem	*KDELR2*	Wald ratio	1	0.0028	0.0028	1.003 (0.997, 1.008)	0.328	3.51E-15	TRUE
B Mem	*KRTAP5-AS1*	Wald ratio	1	-0.0052	0.0018	0.995 (0.991, 0.998)	0.005	1.70E-33	TRUE
B Mem	*LA16c-358B7.3*	Wald ratio	1	0.0092	0.0025	1.009 (1.004, 1.014)	2.83E-04	3.47E-18	TRUE
B Mem	*LGALS9*	Wald ratio	1	0.0063	0.0037	1.006 (0.999, 1.014)	0.085	2.63E-09	TRUE
B Mem	*LSM7*	Wald ratio	1	0.0049	0.0028	1.005 (0.999, 1.01)	0.082	5.21E-15	TRUE
B Mem	*MDM4*	Wald ratio	1	-0.0112	0.0030	0.989 (0.983, 0.995)	1.69E-04	1.88E-13	TRUE
B Mem	*PIM3*	Wald ratio	1	0.0068	0.0038	1.007 (0.999, 1.014)	0.077	9.27E-09	TRUE
B Mem	*PRKD3*	Wald ratio	1	0.0110	0.0035	1.011 (1.004, 1.018)	0.002	8.26E-10	TRUE
B Mem	*PTPLAD2*	Wald ratio	1	-0.0055	0.0036	0.995 (0.988, 1.002)	0.133	1.17E-09	TRUE
B Mem	*RP13-514E23.2*	Wald ratio	1	-0.0033	0.0016	0.997 (0.994, 1)	0.04	1.22E-44	TRUE
B Mem	*RPL12*	Wald ratio	1	0.0004	0.0011	1 (0.998, 1.003)	0.687	1.42E-101	TRUE
B Mem	*RPL14*	Wald ratio	1	-0.0041	0.0039	0.996 (0.988, 1.004)	0.298	1.15E-08	TRUE
B Mem	*RPS9*	Wald ratio	1	0.0043	0.0015	1.004 (1.001, 1.007)	0.003	2.38E-53	TRUE
B Mem	*SERAC1*	Wald ratio	1	0.0039	0.0021	1.004 (1, 1.008)	0.069	1.59E-26	TRUE
B Mem	*SH3YL1*	Wald ratio	1	0.0003	0.0018	1 (0.997, 1.004)	0.856	1.54E-36	TRUE
B Mem	*SNRPD2*	Wald ratio	1	-0.0054	0.0026	0.995 (0.99, 1)	0.035	3.40E-18	TRUE
B Mem	*TESC*	Wald ratio	1	0.0067	0.0038	1.007 (0.999, 1.014)	0.076	1.01E-08	TRUE
B Mem	*TIMM13*	Wald ratio	1	0.0024	0.0041	1.002 (0.994, 1.01)	0.551	5.78E-08	TRUE
B Mem	*TMEM50B*	Wald ratio	1	-0.0007	0.0035	0.999 (0.993, 1.006)	0.846	1.24E-10	TRUE
B Mem	*WARS*	Wald ratio	1	-0.0062	0.0017	0.994 (0.99, 0.997)	3.54E-04	3.30E-37	TRUE
B Mem	*ZNF100*	Wald ratio	1	0.0101	0.0032	1.01 (1.004, 1.017)	0.002	1.32E-11	TRUE
CD4 ET	*ABRACL*	Wald ratio	1	0.0018	0.0034	1.002 (0.995, 1.008)	0.592	6.12E-11	TRUE
CD4 ET	*AIF1*	Wald ratio	1	0.0084	0.0030	1.008 (1.003, 1.014)	0.005	2.16E-13	TRUE
CD4 ET	*AP003774.1*	Wald ratio	1	-0.0081	0.0024	0.992 (0.987, 0.997)	8.00E-04	1.06E-19	TRUE
CD4 ET	*BST2*	Wald ratio	1	0.0011	0.0038	1.001 (0.994, 1.009)	0.772	2.73E-09	TRUE
CD4 ET	*BTF3*	Wald ratio	1	-0.0014	0.0033	0.999 (0.992, 1.005)	0.674	3.46E-12	TRUE
CD4 ET	*C10orf32*	Wald ratio	1	-0.0089	0.0030	0.991 (0.985, 0.997)	0.003	1.33E-12	TRUE
CD4 ET	*C17orf89*	Wald ratio	1	0.0120	0.0033	1.012 (1.006, 1.019)	2.44E-04	3.59E-11	TRUE
CD4 ET	*CD151*	Wald ratio	1	0.0061	0.0026	1.006 (1.001, 1.011)	0.021	2.60E-16	TRUE
CD4 ET	*CDK10*	Wald ratio	1	-0.0108	0.0033	0.989 (0.983, 0.996)	0.001	3.26E-09	TRUE
CD4 ET	*COG5*	Wald ratio	1	0.0064	0.0034	1.006 (1, 1.013)	0.06	2.08E-10	TRUE
CD4 ET	*COL5A3*	Wald ratio	1	-0.0014	0.0023	0.999 (0.994, 1.003)	0.53	5.97E-22	TRUE
CD4 ET	*COX14*	Wald ratio	1	-0.0075	0.0041	0.993 (0.985, 1.001)	0.066	9.81E-08	TRUE
CD4 ET	*CTD-2336O2.1*	Wald ratio	1	-0.0082	0.0028	0.992 (0.986, 0.997)	0.003	1.64E-15	TRUE
CD4 ET	*CTSW*	Wald ratio	1	-0.0054	0.0017	0.995 (0.991, 0.998)	0.001	5.87E-39	TRUE
CD4 ET	*DAZAP2*	Wald ratio	1	-0.0021	0.0037	0.998 (0.991, 1.005)	0.562	1.50E-09	TRUE
CD4 ET	*DDX5*	Wald ratio	1	0.0099	0.0025	1.01 (1.005, 1.015)	7.78E-05	3.63E-18	TRUE
CD4 ET	*FAM21A*	Wald ratio	1	-0.0001	0.0040	1 (0.992, 1.008)	0.974	3.41E-08	TRUE
CD4 ET	*FDFT1*	Wald ratio	1	0.0074	0.0025	1.007 (1.002, 1.012)	0.003	6.84E-18	TRUE
CD4 ET	*FGF9*	Wald ratio	1	-0.0073	0.0036	0.993 (0.986, 1)	0.041	8.49E-10	TRUE
CD4 ET	*FKBPL*	Wald ratio	1	0.0006	0.0034	1.001 (0.994, 1.007)	0.868	6.62E-11	TRUE
CD4 ET	*FNBP4*	Wald ratio	1	-0.0155	0.0038	0.985 (0.977, 0.992)	5.29E-05	1.89E-08	TRUE
CD4 ET	*HAGH*	Wald ratio	1	0.0105	0.0031	1.011 (1.004, 1.017)	7.25E-04	4.23E-12	TRUE
CD4 ET	*JAKMIP2*	Wald ratio	1	0.0040	0.0036	1.004 (0.997, 1.011)	0.259	3.95E-10	TRUE
CD4 ET	*KDELR2*	Wald ratio	1	0.0024	0.0025	1.002 (0.998, 1.007)	0.328	1.99E-19	TRUE
CD4 ET	*LA16c-358B7.3*	Wald ratio	1	0.0121	0.0028	1.012 (1.007, 1.018)	1.52E-05	8.74E-15	TRUE
CD4 ET	*LSM7*	Wald ratio	1	0.0063	0.0036	1.006 (0.999, 1.013)	0.082	1.45E-09	TRUE
CD4 ET	*MANBA*	Wald ratio	1	0.0176	0.0034	1.018 (1.011, 1.025)	2.91E-07	4.53E-10	TRUE
CD4 ET	*MDM4*	Wald ratio	1	-0.0161	0.0037	0.984 (0.977, 0.991)	1.21E-05	5.27E-09	TRUE
CD4 ET	*PLEKHA1*	Wald ratio	1	0.0123	0.0036	1.012 (1.005, 1.019)	5.53E-04	1.22E-09	TRUE
CD4 ET	*PPT2*	Wald ratio	1	0.0069	0.0033	1.007 (1, 1.014)	0.037	6.63E-11	TRUE
CD4 ET	*PRELID1*	Wald ratio	1	-0.0145	0.0034	0.986 (0.979, 0.992)	2.12E-05	1.81E-10	TRUE
CD4 ET	*RNASET2*	Wald ratio	1	-0.0015	0.0014	0.999 (0.996, 1.001)	0.307	8.35E-57	TRUE
CD4 ET	*RNF166*	Wald ratio	1	-0.0073	0.0036	0.993 (0.986, 1)	0.045	1.70E-09	TRUE
CD4 ET	*RP11-499P20.2*	Wald ratio	1	-0.0131	0.0041	0.987 (0.979, 0.995)	0.001	1.45E-07	TRUE
CD4 ET	*RP5-1021I20.1*	Wald ratio	1	0.0050	0.0027	1.005 (1, 1.01)	0.066	7.34E-17	TRUE
CD4 ET	*RPL12*	Wald ratio	1	0.0004	0.0010	1 (0.998, 1.002)	0.666	3.37E-120	TRUE
CD4 ET	*RPS9*	Wald ratio	1	0.0038	0.0013	1.004 (1.001, 1.006)	0.003	3.41E-69	TRUE
CD4 ET	*SH3YL1*	Wald ratio	1	0.0002	0.0011	1 (0.998, 1.002)	0.889	9.88E-100	TRUE
CD4 ET	*SNRPD2*	Wald ratio	1	-0.0054	0.0025	0.995 (0.99, 1)	0.035	1.05E-18	TRUE
CD4 ET	*SRP14*	Wald ratio	1	0.0038	0.0035	1.004 (0.997, 1.011)	0.277	4.17E-10	TRUE
CD4 ET	*THEM4*	Wald ratio	1	0.0038	0.0031	1.004 (0.998, 1.01)	0.214	1.32E-12	TRUE
CD4 ET	*TIMM13*	Wald ratio	1	0.0020	0.0034	1.002 (0.995, 1.009)	0.551	9.91E-11	TRUE
CD4 ET	*TMEM50B*	Wald ratio	1	-0.0004	0.0025	1 (0.995, 1.005)	0.884	5.09E-19	TRUE
CD4 ET	*TNFSF13B*	Wald ratio	1	-0.0077	0.0032	0.992 (0.986, 0.999)	0.016	1.66E-11	TRUE
CD4 NC	*ABRACL*	Wald ratio	1	0.0018	0.0034	1.002 (0.995, 1.009)	0.588	8.42E-11	TRUE
CD4 NC	*AC010982.1*	Wald ratio	1	-0.0177	0.0040	0.982 (0.975, 0.99)	1.01E-05	7.44E-08	TRUE
CD4 NC	*AC068282.3*	Wald ratio	1	-0.0002	0.0023	1 (0.995, 1.004)	0.926	1.39E-22	TRUE
CD4 NC	*AC090186.1*	IVW	2	-0.0011	0.0024	0.999 (0.994, 1.004)	0.641	2.60E-37	TRUE
CD4 NC	*AC137932.1*	Wald ratio	1	0.0002	0.0025	1 (0.995, 1.005)	0.93	1.35E-18	TRUE
CD4 NC	*ACAT1*	Wald ratio	1	-0.0054	0.0020	0.995 (0.991, 0.998)	0.006	2.53E-29	TRUE
CD4 NC	*ACOT13*	Wald ratio	1	-0.0109	0.0040	0.989 (0.981, 0.997)	0.007	4.68E-08	TRUE
CD4 NC	*ACP1*	Wald ratio	1	-0.0022	0.0037	0.998 (0.991, 1.005)	0.554	1.45E-09	TRUE
CD4 NC	*ACY1*	Wald ratio	1	0.0005	0.0038	1.001 (0.993, 1.008)	0.889	2.98E-09	TRUE
CD4 NC	*ADTRP*	Wald ratio	1	-0.0008	0.0027	0.999 (0.994, 1.005)	0.773	3.43E-15	TRUE
CD4 NC	*AGTRAP*	Wald ratio	1	-0.0007	0.0035	0.999 (0.992, 1.006)	0.847	1.72E-10	TRUE
CD4 NC	*AIF1*	Wald ratio	1	0.0059	0.0019	1.006 (1.002, 1.01)	0.002	1.93E-33	TRUE

CD4 NC	*ANXA2*	Wald ratio	1	-0.0103	0.0037	0.99 (0.983, 0.997)	0.005	3.04E-09	TRUE
CD4 NC	*AOAH*	Wald ratio	1	-0.0020	0.0020	0.998 (0.994, 1.002)	0.33	2.01E-27	TRUE
CD4 NC	*AP003774.1*	Wald ratio	1	-0.0044	0.0012	0.996 (0.993, 0.998)	2.75E-04	1.07E-79	TRUE
CD4 NC	*AP2A2*	Wald ratio	1	0.0081	0.0038	1.008 (1.001, 1.016)	0.034	1.15E-08	TRUE
CD4 NC	*ATF5*	Wald ratio	1	-0.0014	0.0034	0.999 (0.992, 1.005)	0.688	1.37E-10	TRUE
CD4 NC	*B3GALNT1*	Wald ratio	1	-0.0028	0.0035	0.997 (0.99, 1.004)	0.42	1.62E-10	TRUE
CD4 NC	*BIK*	Wald ratio	1	-0.0088	0.0033	0.991 (0.985, 0.998)	0.008	1.22E-11	TRUE
CD4 NC	*BIN2*	Wald ratio	1	0.0020	0.0036	1.002 (0.995, 1.009)	0.574	6.65E-10	TRUE
CD4 NC	*BLK*	Wald ratio	1	-0.0112	0.0023	0.989 (0.984, 0.993)	1.86E-06	8.84E-21	TRUE
CD4 NC	*BLMH*	Wald ratio	1	-0.0116	0.0039	0.988 (0.981, 0.996)	0.003	3.78E-08	TRUE
CD4 NC	*BST2*	Wald ratio	1	0.0008	0.0019	1.001 (0.997, 1.005)	0.679	5.23E-34	TRUE
CD4 NC	*BTF3*	Wald ratio	1	-0.0010	0.0023	0.999 (0.995, 1.004)	0.674	2.12E-24	TRUE
CD4 NC	*C10orf32*	Wald ratio	1	-0.0058	0.0017	0.994 (0.991, 0.998)	7.10E-04	8.34E-38	TRUE
CD4 NC	*C17orf89*	Wald ratio	1	0.0032	0.0019	1.003 (1, 1.007)	0.087	2.17E-32	TRUE
CD4 NC	*C2orf74*	Wald ratio	1	-0.0114	0.0031	0.989 (0.983, 0.995)	2.82E-04	1.21E-11	TRUE
CD4 NC	*C6orf132*	Wald ratio	1	0.0038	0.0040	1.004 (0.996, 1.012)	0.349	5.55E-08	TRUE
CD4 NC	*C6orf48*	Wald ratio	1	0.0055	0.0023	1.006 (1.001, 1.01)	0.017	6.47E-22	TRUE
CD4 NC	*C6orf57*	Wald ratio	1	-0.0021	0.0039	0.998 (0.99, 1.006)	0.594	1.54E-08	TRUE
CD4 NC	*C6orf62*	Wald ratio	1	0.0105	0.0038	1.011 (1.003, 1.018)	0.006	1.73E-08	TRUE
CD4 NC	*CASP8*	Wald ratio	1	0.0003	0.0021	1 (0.996, 1.004)	0.882	1.56E-26	TRUE
CD4 NC	*CCDC104*	Wald ratio	1	0.0067	0.0028	1.007 (1.001, 1.012)	0.017	7.74E-15	TRUE
CD4 NC	*CCDC88B*	Wald ratio	1	0.0088	0.0024	1.009 (1.004, 1.014)	3.42E-04	5.92E-19	TRUE
CD4 NC	*CCT4*	Wald ratio	1	0.0012	0.0029	1.001 (0.996, 1.007)	0.683	1.33E-13	TRUE
CD4 NC	*CD151*	Wald ratio	1	0.0057	0.0024	1.006 (1.001, 1.01)	0.018	2.31E-19	TRUE
CD4 NC	*CD53*	IVW	2	-0.0028	0.0020	0.997 (0.993, 1.001)	0.161	3.77E-30	TRUE
CD4 NC	*CDK10*	Wald ratio	1	-0.0067	0.0021	0.993 (0.989, 0.997)	0.001	2.88E-22	TRUE
CD4 NC	*CHRNA1*	Wald ratio	1	0.0025	0.0037	1.002 (0.995, 1.01)	0.504	3.11E-09	TRUE
CD4 NC	*CKB*	Wald ratio	1	-0.0027	0.0037	0.997 (0.99, 1.005)	0.473	1.84E-09	TRUE
CD4 NC	*CLHC1*	Wald ratio	1	0.0028	0.0029	1.003 (0.997, 1.008)	0.34	1.97E-14	TRUE
CD4 NC	*CLN8*	Wald ratio	1	-0.0131	0.0040	0.987 (0.979, 0.995)	0.001	7.80E-08	TRUE
CD4 NC	*CLNS1A*	Wald ratio	1	0.0085	0.0024	1.009 (1.004, 1.013)	4.93E-04	9.54E-18	TRUE
CD4 NC	*CLUAP1*	Wald ratio	1	0.0094	0.0025	1.009 (1.005, 1.014)	1.67E-04	1.73E-18	TRUE
CD4 NC	*COG5*	Wald ratio	1	0.0030	0.0015	1.003 (1, 1.006)	0.051	1.36E-48	TRUE
CD4 NC	*COMMD7*	Wald ratio	1	0.0100	0.0034	1.01 (1.003, 1.017)	0.004	1.09E-10	TRUE
CD4 NC	*COX14*	Wald ratio	1	-0.0049	0.0026	0.995 (0.99, 1)	0.061	5.09E-17	TRUE
CD4 NC	*COX7A2L*	Wald ratio	1	0.0006	0.0019	1.001 (0.997, 1.004)	0.759	8.97E-33	TRUE
CD4 NC	*CRHR1*	Wald ratio	1	-0.0092	0.0028	0.991 (0.985, 0.996)	9.39E-04	6.75E-16	TRUE
CD4 NC	*CTBP1-AS2*	Wald ratio	1	-0.0006	0.0020	0.999 (0.995, 1.003)	0.746	1.42E-29	TRUE
CD4 NC	*CTD-2336O2.1*	Wald ratio	1	0.0041	0.0013	1.004 (1.001, 1.007)	0.002	6.01E-65	TRUE
CD4 NC	*CTSW*	Wald ratio	1	-0.0033	0.0010	0.997 (0.995, 0.999)	0.001	9.84E-114	TRUE
CD4 NC	*CUBN*	Wald ratio	1	0.0074	0.0028	1.007 (1.002, 1.013)	0.008	8.07E-15	TRUE
CD4 NC	*CYP2D6*	Wald ratio	1	-0.0111	0.0039	0.989 (0.981, 0.997)	0.005	3.99E-08	TRUE
CD4 NC	*DAZAP2*	Wald ratio	1	-0.0016	0.0027	0.998 (0.993, 1.004)	0.562	6.22E-17	TRUE
CD4 NC	*DDX5*	Wald ratio	1	0.0083	0.0021	1.008 (1.004, 1.012)	7.78E-05	1.31E-25	TRUE
CD4 NC	*DDX59*	Wald ratio	1	-0.0032	0.0037	0.997 (0.99, 1.004)	0.391	2.92E-09	TRUE
CD4 NC	*DECR2*	Wald ratio	1	0.0039	0.0037	1.004 (0.997, 1.011)	0.293	2.71E-09	TRUE
CD4 NC	*DNAJA1*	Wald ratio	1	-0.0026	0.0025	0.997 (0.993, 1.002)	0.302	2.69E-18	TRUE
CD4 NC	*DNAJA3*	Wald ratio	1	-0.0015	0.0039	0.999 (0.991, 1.006)	0.701	5.93E-10	TRUE
CD4 NC	*DSTN*	IVW	2	-0.0024	0.0024	0.998 (0.993, 1.002)	0.334	3.10E-20	TRUE
CD4 NC	*EEF2*	Wald ratio	1	0.0110	0.0038	1.011 (1.003, 1.019)	0.004	6.13E-09	TRUE
CD4 NC	*EGFL8*	Wald ratio	1	0.0166	0.0033	1.017 (1.01, 1.023)	3.86E-07	1.92E-10	TRUE
CD4 NC	*ENTPD3-AS1*	Wald ratio	1	-0.0040	0.0033	0.996 (0.99, 1.002)	0.22	1.09E-11	TRUE
CD4 NC	*EPC1*	Wald ratio	1	-0.0073	0.0038	0.993 (0.985, 1)	0.051	4.71E-09	TRUE
CD4 NC	*ERAP2*	Wald ratio	1	0.0010	0.0008	1.001 (0.999, 1.003)	0.247	3.50E-199	TRUE
CD4 NC	*ERP29*	Wald ratio	1	0.0164	0.0031	1.017 (1.01, 1.023)	9.30E-08	1.26E-12	TRUE
CD4 NC	*FAM134A*	Wald ratio	1	-0.0028	0.0037	0.997 (0.99, 1.004)	0.445	2.47E-09	TRUE
CD4 NC	*FAM153B*	Wald ratio	1	0.0023	0.0012	1.002 (1, 1.005)	0.045	2.57E-92	TRUE
CD4 NC	*FAM21A*	Wald ratio	1	-0.0002	0.0022	1 (0.995, 1.004)	0.93	8.21E-24	TRUE
CD4 NC	*FAM92A1*	Wald ratio	1	0.0005	0.0038	1 (0.993, 1.008)	0.899	4.09E-09	TRUE
CD4 NC	*FANCA*	Wald ratio	1	-0.0109	0.0033	0.989 (0.983, 0.996)	9.94E-04	3.00E-11	TRUE
CD4 NC	*FDFT1*	Wald ratio	1	0.0035	0.0012	1.003 (1.001, 1.006)	0.003	7.49E-82	TRUE
CD4 NC	*FNBP4*	Wald ratio	1	-0.0074	0.0018	0.993 (0.989, 0.996)	2.81E-05	8.84E-36	TRUE
CD4 NC	*FOXN2*	Wald ratio	1	0.0066	0.0024	1.007 (1.002, 1.011)	0.005	4.52E-20	TRUE
CD4 NC	*FRG1B*	Wald ratio	1	-0.0036	0.0030	0.996 (0.99, 1.002)	0.231	4.12E-13	TRUE
CD4 NC	*GALT*	Wald ratio	1	0.0035	0.0020	1.003 (1, 1.007)	0.082	3.96E-29	TRUE
CD4 NC	*GOSR2*	Wald ratio	1	0.0108	0.0025	1.011 (1.006, 1.016)	1.92E-05	9.53E-18	TRUE
CD4 NC	*GPR160*	Wald ratio	1	-0.0027	0.0015	0.997 (0.994, 1)	0.067	4.77E-53	TRUE
CD4 NC	*HAGH*	Wald ratio	1	0.0047	0.0014	1.005 (1.002, 1.007)	7.25E-04	9.22E-59	TRUE
CD4 NC	*HAGHL*	Wald ratio	1	0.0054	0.0030	1.005 (1, 1.011)	0.069	8.40E-14	TRUE
CD4 NC	*HEATR6*	Wald ratio	1	-0.0012	0.0035	0.999 (0.992, 1.006)	0.739	5.79E-11	TRUE
CD4 NC	*HINT1*	Wald ratio	1	-0.0027	0.0039	0.997 (0.99, 1.005)	0.492	6.90E-09	TRUE
CD4 NC	*HLA-DQA1*	Wald ratio	1	0.0120	0.0034	1.012 (1.005, 1.019)	4.32E-04	1.75E-10	TRUE
CD4 NC	*HLA-DRB5*	Wald ratio	1	0.0050	0.0017	1.005 (1.002, 1.008)	0.003	9.81E-40	TRUE
CD4 NC	*HMBS*	Wald ratio	1	0.0055	0.0023	1.006 (1.001, 1.01)	0.016	2.14E-21	TRUE
CD4 NC	*HNRNPU*	Wald ratio	1	-0.0015	0.0031	0.998 (0.992, 1.005)	0.622	1.33E-12	TRUE
CD4 NC	*HOTAIRM1*	Wald ratio	1	0.0001	0.0033	1 (0.994, 1.006)	0.978	6.41E-12	TRUE
CD4 NC	*HSBP1L1*	Wald ratio	1	-0.0058	0.0030	0.994 (0.988, 1)	0.053	1.19E-13	TRUE
CD4 NC	*HSD17B11*	Wald ratio	1	0.0074	0.0030	1.007 (1.002, 1.013)	0.013	1.07E-13	TRUE
CD4 NC	*HSD17B12*	Wald ratio	1	-0.0085	0.0020	0.992 (0.988, 0.995)	2.83E-05	1.39E-26	TRUE

CD4 NC	*ICAM3*	Wald ratio	1	0.0037	0.0032	1.004 (0.998, 1.01)	0.239	1.11E-11	TRUE
CD4 NC	*INPP4B*	Wald ratio	1	-0.0024	0.0024	0.998 (0.993, 1.002)	0.3	5.09E-22	TRUE
CD4 NC	*IQCH-AS1*	Wald ratio	1	0.0021	0.0035	1.002 (0.995, 1.009)	0.55	1.34E-10	TRUE
CD4 NC	*KB-1507C5.2*	Wald ratio	1	0.0103	0.0032	1.01 (1.004, 1.017)	0.001	2.09E-11	TRUE
CD4 NC	*KCNQ1OT1*	Wald ratio	1	0.0001	0.0037	1 (0.993, 1.007)	0.973	1.37E-09	TRUE
CD4 NC	*KDELR2*	Wald ratio	1	0.0013	0.0013	1.001 (0.999, 1.004)	0.328	3.30E-72	TRUE
CD4 NC	*KRTAP5-2*	Wald ratio	1	-0.0078	0.0019	0.992 (0.989, 0.996)	3.25E-05	1.13E-31	TRUE
CD4 NC	*KTN1*	Wald ratio	1	0.0031	0.0018	1.003 (0.999, 1.007)	0.093	3.55E-33	TRUE
CD4 NC	*LA16c-358B7.3*	Wald ratio	1	0.0056	0.0013	1.006 (1.003, 1.008)	1.88E-05	3.42E-67	TRUE
CD4 NC	*LAIR2*	Wald ratio	1	0.0047	0.0023	1.005 (1, 1.009)	0.043	7.85E-22	TRUE
CD4 NC	*LAPTM5*	Wald ratio	1	0.0057	0.0042	1.006 (0.997, 1.014)	0.177	2.40E-08	TRUE
CD4 NC	*LILRB3*	Wald ratio	1	0.0017	0.0040	1.002 (0.994, 1.009)	0.676	1.77E-08	TRUE
CD4 NC	*LIMD2*	Wald ratio	1	-0.0127	0.0039	0.987 (0.98, 0.995)	0.001	2.46E-08	TRUE
CD4 NC	*LLGL1*	Wald ratio	1	0.0127	0.0031	1.013 (1.007, 1.019)	3.67E-05	1.98E-12	TRUE
CD4 NC	*LRRC61*	Wald ratio	1	-0.0041	0.0019	0.996 (0.992, 1)	0.029	1.33E-31	TRUE
CD4 NC	*LSM4*	Wald ratio	1	-0.0071	0.0032	0.993 (0.987, 0.999)	0.029	4.04E-12	TRUE
CD4 NC	*LSM6*	Wald ratio	1	0.0006	0.0030	1.001 (0.995, 1.006)	0.834	6.13E-14	TRUE
CD4 NC	*LSM7*	Wald ratio	1	0.0031	0.0018	1.003 (1, 1.007)	0.082	1.10E-36	TRUE
CD4 NC	*LST1*	Wald ratio	1	0.0005	0.0027	1 (0.995, 1.006)	0.864	5.28E-16	TRUE
CD4 NC	*MACF1*	Wald ratio	1	0.0081	0.0025	1.008 (1.003, 1.013)	0.001	2.15E-17	TRUE
CD4 NC	*MAP2K1*	Wald ratio	1	-0.0049	0.0033	0.995 (0.989, 1.002)	0.138	2.07E-11	TRUE
CD4 NC	*MARK3*	Wald ratio	1	-0.0154	0.0032	0.985 (0.979, 0.991)	1.61E-06	2.54E-11	TRUE
CD4 NC	*MAST3*	Wald ratio	1	0.0019	0.0022	1.002 (0.998, 1.006)	0.39	2.52E-24	TRUE
CD4 NC	*MDM4*	Wald ratio	1	0.0074	0.0020	1.007 (1.003, 1.011)	2.88E-04	9.83E-28	TRUE
CD4 NC	*MEI1*	Wald ratio	1	0.0062	0.0031	1.006 (1, 1.012)	0.044	5.00E-13	TRUE
CD4 NC	*MKRN1*	Wald ratio	1	0.0014	0.0025	1.001 (0.997, 1.006)	0.575	5.76E-20	TRUE
CD4 NC	*MLKL*	Wald ratio	1	0.0029	0.0039	1.003 (0.995, 1.011)	0.458	1.23E-08	TRUE
CD4 NC	*MRPL41*	Wald ratio	1	0.0057	0.0031	1.006 (1, 1.012)	0.063	8.67E-13	TRUE
CD4 NC	*MRPS21*	Wald ratio	1	-0.0007	0.0024	0.999 (0.995, 1.004)	0.77	5.86E-20	TRUE
CD4 NC	*MRPS34*	Wald ratio	1	0.0116	0.0040	1.012 (1.004, 1.02)	0.004	7.05E-08	TRUE
CD4 NC	*MRPS36*	Wald ratio	1	0.0027	0.0032	1.003 (0.996, 1.009)	0.396	3.56E-12	TRUE
CD4 NC	*MYO16*	Wald ratio	1	-0.0045	0.0019	0.996 (0.992, 0.999)	0.016	6.57E-32	TRUE
CD4 NC	*NARFL*	Wald ratio	1	0.0075	0.0034	1.008 (1.001, 1.014)	0.027	9.76E-11	TRUE
CD4 NC	*NEIL2*	Wald ratio	1	0.0101	0.0034	1.01 (1.003, 1.017)	0.003	4.77E-10	TRUE
CD4 NC	*NMB*	Wald ratio	1	-0.0067	0.0037	0.993 (0.986, 1.001)	0.071	7.62E-09	TRUE
CD4 NC	*NME3*	Wald ratio	1	-0.0082	0.0032	0.992 (0.985, 0.998)	0.011	3.61E-11	TRUE
CD4 NC	*OSER1-AS1*	Wald ratio	1	0.0090	0.0029	1.009 (1.003, 1.015)	0.002	7.99E-14	TRUE
CD4 NC	*PABPC4*	Wald ratio	1	-0.0136	0.0039	0.986 (0.979, 0.994)	4.56E-04	7.27E-08	TRUE
CD4 NC	*PARP15*	Wald ratio	1	0.0042	0.0039	1.004 (0.997, 1.012)	0.281	1.84E-08	TRUE
CD4 NC	*PCBP2*	Wald ratio	1	-0.0047	0.0029	0.995 (0.99, 1.001)	0.11	2.96E-14	TRUE
CD4 NC	*PCNX*	Wald ratio	1	0.0081	0.0023	1.008 (1.004, 1.013)	4.47E-04	3.51E-21	TRUE
CD4 NC	*PDGFB*	Wald ratio	1	0.0010	0.0035	1.001 (0.994, 1.008)	0.775	4.62E-10	TRUE
CD4 NC	*PGPEP1*	Wald ratio	1	0.0122	0.0031	1.012 (1.006, 1.018)	8.50E-05	2.77E-12	TRUE
CD4 NC	*PHACTR4*	Wald ratio	1	0.0065	0.0025	1.007 (1.002, 1.011)	0.009	3.79E-19	TRUE
CD4 NC	*PKMYT1*	Wald ratio	1	-0.0063	0.0021	0.994 (0.99, 0.998)	0.003	2.20E-25	TRUE
CD4 NC	*PLEKHG1*	Wald ratio	1	0.0045	0.0033	1.005 (0.998, 1.011)	0.169	2.52E-11	TRUE
CD4 NC	*PLEKHO1*	Wald ratio	1	-0.0044	0.0030	0.996 (0.99, 1.001)	0.135	1.76E-13	TRUE
CD4 NC	*PPT1*	Wald ratio	1	-0.0015	0.0031	0.999 (0.992, 1.005)	0.629	3.02E-13	TRUE
CD4 NC	*PRELID1*	Wald ratio	1	-0.0080	0.0019	0.992 (0.988, 0.996)	2.12E-05	3.96E-32	TRUE
CD4 NC	*PRKAG2-AS1*	Wald ratio	1	0.0047	0.0031	1.005 (0.999, 1.011)	0.129	1.10E-12	TRUE
CD4 NC	*PRKD3*	Wald ratio	1	0.0088	0.0030	1.009 (1.003, 1.015)	0.004	6.43E-13	TRUE
CD4 NC	*PYROXD1*	Wald ratio	1	0.0010	0.0024	1.001 (0.996, 1.006)	0.679	4.33E-20	TRUE
CD4 NC	*RAC1*	Wald ratio	1	-0.0041	0.0014	0.996 (0.993, 0.999)	0.004	4.10E-56	TRUE
CD4 NC	*RASA4*	Wald ratio	1	0.0008	0.0039	1.001 (0.993, 1.008)	0.836	2.48E-09	TRUE
CD4 NC	*RBM6*	Wald ratio	1	-0.0030	0.0023	0.997 (0.993, 1.001)	0.18	1.68E-23	TRUE
CD4 NC	*RBMS1*	Wald ratio	1	0.0025	0.0018	1.003 (0.999, 1.006)	0.17	1.50E-32	TRUE
CD4 NC	*REEP3*	Wald ratio	1	0.0134	0.0041	1.014 (1.005, 1.022)	0.001	1.12E-07	TRUE
CD4 NC	*RNASET2*	Wald ratio	1	-0.0010	0.0009	0.999 (0.997, 1.001)	0.291	9.18E-158	TRUE
CD4 NC	*RNF145*	Wald ratio	1	0.0060	0.0033	1.006 (1, 1.013)	0.066	1.00E-11	TRUE
CD4 NC	*RNF24*	Wald ratio	1	-0.0031	0.0023	0.997 (0.992, 1.001)	0.173	4.78E-22	TRUE
CD4 NC	*RNF8*	Wald ratio	1	-0.0110	0.0039	0.989 (0.981, 0.997)	0.005	4.20E-08	TRUE
CD4 NC	*RP1-39G22.7*	Wald ratio	1	0.0029	0.0035	1.003 (0.996, 1.01)	0.407	2.34E-10	TRUE
CD4 NC	*RP11-179B2.2*	Wald ratio	1	-0.0056	0.0037	0.994 (0.987, 1.002)	0.126	2.36E-09	TRUE
CD4 NC	*RP11-223C24.1*	Wald ratio	1	-0.0028	0.0027	0.997 (0.992, 1.002)	0.3	1.92E-17	TRUE
CD4 NC	*RP11-273G15.2*	Wald ratio	1	0.0097	0.0025	1.01 (1.005, 1.015)	1.10E-04	4.57E-18	TRUE
CD4 NC	*RP11-293M10.2*	Wald ratio	1	-0.0042	0.0024	0.996 (0.991, 1)	0.078	2.56E-20	TRUE
CD4 NC	*RP11-298J20.3*	Wald ratio	1	-0.0007	0.0009	0.999 (0.998, 1.001)	0.455	8.33E-161	TRUE
CD4 NC	*RP11-379F4.9*	Wald ratio	1	0.0022	0.0029	1.002 (0.997, 1.008)	0.445	1.66E-14	TRUE
CD4 NC	*RP11-47L3.1*	Wald ratio	1	-0.0003	0.0030	1 (0.994, 1.006)	0.93	1.00E-14	TRUE
CD4 NC	*RP11-502I4.3*	Wald ratio	1	-0.0023	0.0041	0.998 (0.99, 1.006)	0.576	6.00E-08	TRUE
CD4 NC	*RP11-796G6.2*	Wald ratio	1	0.0158	0.0038	1.016 (1.008, 1.024)	3.84E-05	1.38E-08	TRUE
CD4 NC	*RP11-797A18.4*	Wald ratio	1	-0.0020	0.0027	0.998 (0.993, 1.003)	0.446	4.09E-16	TRUE
CD4 NC	*RP11-817O13.8*	Wald ratio	1	0.0023	0.0043	1.002 (0.994, 1.011)	0.593	3.23E-08	TRUE
CD4 NC	*RP11-92G12.3*	Wald ratio	1	-0.0012	0.0016	0.999 (0.996, 1.002)	0.465	2.37E-42	TRUE
CD4 NC	*RP5-1021I20.1*	Wald ratio	1	-0.0034	0.0019	0.997 (0.993, 1)	0.066	4.11E-34	TRUE
CD4 NC	*RP5-1092A3.4*	Wald ratio	1	0.0004	0.0028	1 (0.995, 1.006)	0.888	6.75E-15	TRUE
CD4 NC	*RPRD2*	Wald ratio	1	-0.0052	0.0037	0.995 (0.988, 1.002)	0.153	2.73E-09	TRUE
CD4 NC	*RPS27L*	Wald ratio	1	-0.0075	0.0040	0.993 (0.985, 1)	0.063	6.50E-08	TRUE
CD4 NC	*RPS6KL1*	Wald ratio	1	0.0069	0.0039	1.007 (0.999, 1.015)	0.081	3.55E-08	TRUE

CD4 NC	*RPS9*	Wald ratio	1	0.0033	0.0011	1.003 (1.001, 1.005)	0.003	5.96E-97	TRUE
CD4 NC	*RSU1*	Wald ratio	1	-0.0003	0.0036	1 (0.993, 1.007)	0.944	4.44E-10	TRUE
CD4 NC	*SARDH*	Wald ratio	1	0.0023	0.0034	1.002 (0.996, 1.009)	0.483	1.92E-11	TRUE
CD4 NC	*SAT2*	Wald ratio	1	-0.0055	0.0022	0.994 (0.99, 0.999)	0.013	3.03E-23	TRUE
CD4 NC	*SH3YL1*	Wald ratio	1	-0.0003	0.0009	1 (0.998, 1.001)	0.77	2.70E-171	TRUE
CD4 NC	*SLC22A5*	Wald ratio	1	0.0202	0.0039	1.02 (1.013, 1.028)	1.82E-07	3.80E-08	TRUE
CD4 NC	*SNRPC*	Wald ratio	1	-0.0072	0.0021	0.993 (0.989, 0.997)	5.47E-04	1.89E-25	TRUE
CD4 NC	*SNRPD2*	Wald ratio	1	-0.0027	0.0013	0.997 (0.995, 1)	0.035	1.02E-73	TRUE
CD4 NC	*SNX16*	Wald ratio	1	0.0151	0.0031	1.015 (1.009, 1.021)	1.71E-06	9.26E-12	TRUE
CD4 NC	*SPCS1*	Wald ratio	1	0.0085	0.0030	1.009 (1.003, 1.015)	0.005	7.22E-13	TRUE
CD4 NC	*TESC*	Wald ratio	1	0.0027	0.0033	1.003 (0.996, 1.009)	0.424	3.23E-11	TRUE
CD4 NC	*THAP9-AS1*	Wald ratio	1	-0.0074	0.0021	0.993 (0.989, 0.997)	3.88E-04	2.18E-25	TRUE
CD4 NC	*TMEM199*	Wald ratio	1	-0.0100	0.0035	0.99 (0.983, 0.997)	0.004	4.45E-10	TRUE
CD4 NC	*TMEM50B*	Wald ratio	1	-0.0001	0.0012	1 (0.998, 1.002)	0.904	4.00E-89	TRUE
CD4 NC	*TMEM87B*	Wald ratio	1	0.0096	0.0033	1.01 (1.003, 1.016)	0.004	5.03E-11	TRUE
CD4 NC	*TMEM97*	Wald ratio	1	-0.0083	0.0033	0.992 (0.985, 0.998)	0.013	5.95E-11	TRUE
CD4 NC	*TNFRSF18*	Wald ratio	1	0.0091	0.0042	1.009 (1.001, 1.018)	0.03	9.45E-08	TRUE
CD4 NC	*TNRC6A*	Wald ratio	1	-0.0023	0.0038	0.998 (0.99, 1.005)	0.547	4.49E-09	TRUE
CD4 NC	*TRAPPC12-AS1*	Wald ratio	1	0.0030	0.0018	1.003 (0.999, 1.007)	0.1	7.62E-35	TRUE
CD4 NC	*TRAT1*	Wald ratio	1	0.0014	0.0039	1.001 (0.994, 1.009)	0.724	1.51E-08	TRUE
CD4 NC	*TSPAN32*	Wald ratio	1	-0.0003	0.0030	1 (0.994, 1.006)	0.917	2.49E-13	TRUE
CD4 NC	*TTC12*	Wald ratio	1	0.0123	0.0034	1.012 (1.006, 1.019)	3.10E-04	3.41E-10	TRUE
CD4 NC	*TXNL4A*	Wald ratio	1	-0.0074	0.0038	0.993 (0.985, 1)	0.053	8.44E-09	TRUE
CD4 NC	*UACA*	Wald ratio	1	0.0077	0.0037	1.008 (1, 1.015)	0.04	2.65E-09	TRUE
CD4 NC	*UBE2I*	Wald ratio	1	0.0087	0.0034	1.009 (1.002, 1.016)	0.011	1.17E-10	TRUE
CD4 NC	*UBE2Q2*	Wald ratio	1	0.0023	0.0028	1.002 (0.997, 1.008)	0.412	1.82E-14	TRUE
CD4 NC	*UBE3B*	Wald ratio	1	0.0094	0.0034	1.009 (1.003, 1.016)	0.006	5.66E-10	TRUE
CD4 NC	*USP36*	Wald ratio	1	0.0129	0.0033	1.013 (1.006, 1.02)	9.78E-05	6.40E-11	TRUE
CD4 NC	*VIM*	Wald ratio	1	0.0154	0.0039	1.015 (1.008, 1.023)	9.39E-05	5.98E-08	TRUE
CD4 NC	*VPS53*	Wald ratio	1	-0.0073	0.0033	0.993 (0.986, 0.999)	0.027	2.83E-11	TRUE
CD4 NC	*WBSCR16*	Wald ratio	1	-0.0116	0.0037	0.988 (0.981, 0.996)	0.002	7.72E-09	TRUE
CD4 NC	*WRB*	Wald ratio	1	-0.0049	0.0041	0.995 (0.987, 1.003)	0.234	6.11E-08	TRUE
CD4 NC	*ZDHHC4*	Wald ratio	1	0.0024	0.0023	1.002 (0.998, 1.007)	0.302	3.82E-22	TRUE
CD4 NC	*ZNF100*	Wald ratio	1	-0.0052	0.0016	0.995 (0.992, 0.998)	0.001	7.78E-43	TRUE
CD4 NC	*ZNF502*	Wald ratio	1	-0.0051	0.0018	0.995 (0.991, 0.999)	0.006	2.51E-34	TRUE
CD4 NC	*ZSWIM7*	Wald ratio	1	0.0057	0.0026	1.006 (1.001, 1.011)	0.028	2.67E-17	TRUE
CD4 SOX4	*DAXX*	Wald ratio	1	0.0176	0.0036	1.018 (1.011, 1.025)	1.06E-06	5.51E-08	TRUE
CD4 SOX4	*DXO*	Wald ratio	1	-0.0127	0.0026	0.987 (0.982, 0.992)	1.17E-06	1.47E-14	TRUE
CD4 SOX4	*ERAP2*	Wald ratio	1	0.0057	0.0035	1.006 (0.999, 1.013)	0.104	7.37E-09	TRUE
CD4 SOX4	*HLA-B*	IVW	2	0.0041	0.0035	1.004 (0.997, 1.011)	0.24	2.86E-31	TRUE
CD4 SOX4	*HLA-DRB5*	Wald ratio	1	-0.0062	0.0037	0.994 (0.987, 1.001)	0.096	3.25E-08	TRUE
CD4 SOX4	*RPL12*	Wald ratio	1	-0.0008	0.0028	0.999 (0.994, 1.005)	0.767	8.12E-14	TRUE
CD4 SOX4	*RPS26*	Wald ratio	1	-0.0039	0.0008	0.996 (0.994, 0.998)	3.51E-06	5.71E-164	TRUE
CD4 SOX4	*RPS9*	Wald ratio	1	0.0094	0.0031	1.009 (1.003, 1.016)	0.003	1.18E-11	TRUE
CD4 SOX4	*RXRB*	Wald ratio	1	0.0124	0.0025	1.013 (1.008, 1.017)	5.60E-07	3.73E-16	TRUE
CD4 SOX4	*SH3YL1*	Wald ratio	1	0.0007	0.0033	1.001 (0.994, 1.007)	0.825	4.52E-10	TRUE
CD4 SOX4	*SYNCRIP*	Wald ratio	1	0.0043	0.0029	1.004 (0.999, 1.01)	0.141	9.04E-13	TRUE
CD4 SOX4	*TRIM39*	Wald ratio	1	-0.0012	0.0038	0.999 (0.991, 1.006)	0.754	1.74E-08	TRUE
CD8 ET	*ABRACL*	Wald ratio	1	0.0020	0.0036	1.002 (0.995, 1.009)	0.587	9.43E-10	TRUE
CD8 ET	*ADRB1*	Wald ratio	1	-0.0097	0.0027	0.99 (0.985, 0.996)	3.93E-04	7.97E-15	TRUE
CD8 ET	*AP003774.1*	Wald ratio	1	-0.0049	0.0014	0.995 (0.992, 0.998)	8.00E-04	2.58E-54	TRUE
CD8 ET	*AP2A2*	Wald ratio	1	0.0067	0.0034	1.007 (1, 1.013)	0.048	8.57E-11	TRUE
CD8 ET	*BTN3A1*	Wald ratio	1	0.0083	0.0035	1.008 (1.001, 1.015)	0.017	7.65E-10	TRUE
CD8 ET	*C10orf32*	Wald ratio	1	-0.0082	0.0024	0.992 (0.987, 0.997)	7.10E-04	5.01E-19	TRUE
CD8 ET	*C17orf89*	Wald ratio	1	0.0039	0.0023	1.004 (0.999, 1.008)	0.09	2.84E-22	TRUE
CD8 ET	*C2*	Wald ratio	1	-0.0056	0.0034	0.994 (0.988, 1.001)	0.098	1.49E-10	TRUE
CD8 ET	*C2orf74*	Wald ratio	1	0.0101	0.0033	1.01 (1.004, 1.017)	0.002	8.02E-11	TRUE
CD8 ET	*C6orf62*	Wald ratio	1	0.0112	0.0040	1.011 (1.003, 1.019)	0.006	1.09E-07	TRUE
CD8 ET	*CASP8*	Wald ratio	1	-0.0004	0.0026	1 (0.994, 1.005)	0.886	1.71E-17	TRUE
CD8 ET	*CCDC88B*	Wald ratio	1	0.0114	0.0038	1.011 (1.004, 1.019)	0.003	1.64E-08	TRUE
CD8 ET	*CDK10*	Wald ratio	1	-0.0086	0.0026	0.991 (0.986, 0.997)	0.001	7.04E-14	TRUE
CD8 ET	*CIB1*	Wald ratio	1	-0.0004	0.0020	1 (0.996, 1.004)	0.859	1.07E-28	TRUE
CD8 ET	*CKB*	Wald ratio	1	-0.0026	0.0033	0.997 (0.991, 1.004)	0.43	1.39E-11	TRUE
CD8 ET	*CLHC1*	Wald ratio	1	0.0006	0.0039	1.001 (0.993, 1.008)	0.886	7.78E-09	TRUE
CD8 ET	*CLIC3*	Wald ratio	1	-0.0061	0.0036	0.994 (0.987, 1.001)	0.095	1.56E-09	TRUE
CD8 ET	*COG5*	Wald ratio	1	0.0033	0.0017	1.003 (1, 1.007)	0.051	1.41E-38	TRUE
CD8 ET	*COL18A1*	Wald ratio	1	-0.0023	0.0020	0.998 (0.994, 1.002)	0.251	1.77E-29	TRUE
CD8 ET	*COX7A2L*	Wald ratio	1	0.0011	0.0031	1.001 (0.995, 1.007)	0.73	8.01E-13	TRUE
CD8 ET	*CTA-14H9.5*	Wald ratio	1	0.0068	0.0039	1.007 (0.999, 1.014)	0.079	3.19E-08	TRUE
CD8 ET	*CTD-2515A14.1*	Wald ratio	1	-0.0111	0.0039	0.989 (0.981, 0.997)	0.005	2.91E-08	TRUE
CD8 ET	*CTSW*	Wald ratio	1	-0.0033	0.0010	0.997 (0.995, 0.999)	0.001	9.28E-108	TRUE
CD8 ET	*DDX5*	Wald ratio	1	0.0084	0.0021	1.008 (1.004, 1.013)	7.78E-05	5.58E-25	TRUE
CD8 ET	*DGKD*	Wald ratio	1	0.0127	0.0040	1.013 (1.005, 1.021)	0.001	9.24E-08	TRUE
CD8 ET	*DNAJA1*	Wald ratio	1	-0.0049	0.0039	0.995 (0.987, 1.003)	0.211	3.36E-08	TRUE
CD8 ET	*ENTPD3-AS1*	Wald ratio	1	0.0002	0.0040	1 (0.992, 1.008)	0.967	1.98E-08	TRUE
CD8 ET	*FAM179A*	Wald ratio	1	-0.0044	0.0037	0.996 (0.988, 1.003)	0.234	2.60E-09	TRUE
CD8 ET	*FAM21A*	Wald ratio	1	0.0002	0.0030	1 (0.994, 1.006)	0.956	1.61E-13	TRUE
CD8 ET	*FDFT1*	Wald ratio	1	0.0087	0.0030	1.009 (1.003, 1.015)	0.003	3.48E-13	TRUE
CD8 ET	*FIBP*	Wald ratio	1	0.0106	0.0036	1.011 (1.004, 1.018)	0.003	1.43E-09	TRUE

CD8 ET	*FNBP4*	Wald ratio	1	-0.0115	0.0027	0.989 (0.983, 0.994)	2.66E-05	2.14E-15	TRUE
CD8 ET	*FOXN2*	Wald ratio	1	0.0091	0.0033	1.009 (1.003, 1.016)	0.006	5.94E-11	TRUE
CD8 ET	*FRG1B*	Wald ratio	1	-0.0055	0.0038	0.994 (0.987, 1.002)	0.149	1.28E-08	TRUE
CD8 ET	*GALT*	Wald ratio	1	0.0063	0.0036	1.006 (0.999, 1.013)	0.082	9.35E-10	TRUE
CD8 ET	*HAGH*	Wald ratio	1	0.0082	0.0023	1.008 (1.004, 1.013)	3.32E-04	2.54E-21	TRUE
CD8 ET	*HCG25*	Wald ratio	1	0.0044	0.0026	1.004 (0.999, 1.01)	0.093	3.02E-17	TRUE
CD8 ET	*HIST1H4K*	Wald ratio	1	-0.0068	0.0040	0.993 (0.985, 1.001)	0.09	1.99E-08	TRUE
CD8 ET	*HLA-DQB2*	Wald ratio	1	-0.0120	0.0025	0.988 (0.983, 0.993)	1.47E-06	1.67E-17	TRUE
CD8 ET	*HMGN1*	Wald ratio	1	-0.0031	0.0036	0.997 (0.99, 1.004)	0.383	4.15E-10	TRUE
CD8 ET	*HNRNPU*	Wald ratio	1	-0.0019	0.0038	0.998 (0.991, 1.006)	0.622	5.12E-09	TRUE
CD8 ET	*HSBP1L1*	Wald ratio	1	-0.0055	0.0032	0.995 (0.988, 1.001)	0.086	3.77E-12	TRUE
CD8 ET	*IGHMBP2*	Wald ratio	1	-0.0010	0.0039	0.999 (0.992, 1.007)	0.804	9.53E-09	TRUE
CD8 ET	*KDELR2*	Wald ratio	1	0.0017	0.0017	1.002 (0.998, 1.005)	0.328	2.84E-40	TRUE
CD8 ET	*KRT73*	Wald ratio	1	-0.0005	0.0029	0.999 (0.994, 1.005)	0.851	1.83E-15	TRUE
CD8 ET	*KTN1*	Wald ratio	1	0.0047	0.0029	1.005 (0.999, 1.01)	0.099	3.39E-14	TRUE
CD8 ET	*LA16c-358B7.3*	Wald ratio	1	0.0085	0.0020	1.008 (1.005, 1.012)	1.88E-05	4.53E-29	TRUE
CD8 ET	*LAIR2*	Wald ratio	1	0.0042	0.0020	1.004 (1, 1.008)	0.036	6.63E-30	TRUE
CD8 ET	*LAPTM5*	Wald ratio	1	0.0056	0.0042	1.006 (0.997, 1.014)	0.177	2.20E-08	TRUE
CD8 ET	*LATS2*	Wald ratio	1	0.0028	0.0035	1.003 (0.996, 1.01)	0.417	5.65E-10	TRUE
CD8 ET	*LLGL1*	Wald ratio	1	0.0137	0.0033	1.014 (1.007, 1.02)	3.75E-05	8.25E-11	TRUE
CD8 ET	*LRRC37A*	Wald ratio	1	-0.0068	0.0021	0.993 (0.989, 0.997)	0.001	6.18E-24	TRUE
CD8 ET	*LRRC61*	Wald ratio	1	-0.0081	0.0038	0.992 (0.985, 0.999)	0.033	1.30E-08	TRUE
CD8 ET	*LSM7*	Wald ratio	1	0.0046	0.0026	1.005 (0.999, 1.01)	0.082	7.92E-17	TRUE
CD8 ET	*MAD1L1*	Wald ratio	1	0.0017	0.0034	1.002 (0.995, 1.008)	0.619	8.36E-11	TRUE
CD8 ET	*MDM4*	Wald ratio	1	-0.0121	0.0031	0.988 (0.982, 0.994)	8.72E-05	1.36E-12	TRUE
CD8 ET	*MINK1*	Wald ratio	1	0.0050	0.0035	1.005 (0.998, 1.012)	0.154	1.91E-10	TRUE
CD8 ET	*MKRN1*	Wald ratio	1	0.0019	0.0034	1.002 (0.995, 1.009)	0.575	3.57E-11	TRUE
CD8 ET	*MLF1*	Wald ratio	1	0.0151	0.0029	1.015 (1.009, 1.021)	2.26E-07	7.86E-13	TRUE
CD8 ET	*MPHOSPH9*	Wald ratio	1	-0.0124	0.0039	0.988 (0.98, 0.995)	0.001	7.18E-08	TRUE
CD8 ET	*MRPS21*	Wald ratio	1	0.0030	0.0038	1.003 (0.995, 1.011)	0.438	6.76E-09	TRUE
CD8 ET	*MSH5*	Wald ratio	1	0.0180	0.0034	1.018 (1.011, 1.025)	1.22E-07	6.29E-10	TRUE
CD8 ET	*NCOA7*	Wald ratio	1	0.0062	0.0037	1.006 (0.999, 1.014)	0.098	4.30E-09	TRUE
CD8 ET	*NOTCH4*	Wald ratio	1	-0.0136	0.0037	0.986 (0.979, 0.994)	2.29E-04	9.36E-09	TRUE
CD8 ET	*NUCB2*	Wald ratio	1	-0.0058	0.0014	0.994 (0.992, 0.997)	1.58E-05	4.96E-62	TRUE
CD8 ET	*OXNAD1*	Wald ratio	1	-0.0083	0.0028	0.992 (0.986, 0.997)	0.002	1.89E-15	TRUE
CD8 ET	*PCBP2*	Wald ratio	1	-0.0060	0.0037	0.994 (0.987, 1.001)	0.11	3.32E-09	TRUE
CD8 ET	*PDCD6*	Wald ratio	1	0.0079	0.0037	1.008 (1.001, 1.015)	0.034	2.83E-09	TRUE
CD8 ET	*PHACTR4*	Wald ratio	1	0.0100	0.0037	1.01 (1.003, 1.018)	0.007	4.06E-09	TRUE
CD8 ET	*PHPT1*	Wald ratio	1	0.0053	0.0034	1.005 (0.999, 1.012)	0.119	1.38E-10	TRUE
CD8 ET	*PIWIL4*	Wald ratio	1	-0.0051	0.0025	0.995 (0.99, 1)	0.044	1.05E-17	TRUE
CD8 ET	*PLCB2*	Wald ratio	1	-0.0040	0.0037	0.996 (0.989, 1.003)	0.277	2.86E-09	TRUE
CD8 ET	*PPT1*	Wald ratio	1	-0.0017	0.0038	0.998 (0.991, 1.006)	0.659	4.33E-09	TRUE
CD8 ET	*PRELID1*	Wald ratio	1	-0.0124	0.0029	0.988 (0.982, 0.993)	2.12E-05	5.51E-14	TRUE
CD8 ET	*REEP5*	Wald ratio	1	0.0098	0.0036	1.01 (1.003, 1.017)	0.007	1.94E-09	TRUE
CD8 ET	*RP4-799D16.1*	Wald ratio	1	0.0176	0.0035	1.018 (1.011, 1.025)	5.44E-07	3.87E-09	TRUE
CD8 ET	*RP5-1021I20.1*	Wald ratio	1	-0.0037	0.0026	0.996 (0.991, 1.001)	0.154	2.76E-19	TRUE
CD8 ET	*RPL12*	Wald ratio	1	0.0004	0.0010	1 (0.998, 1.002)	0.687	1.28E-121	TRUE
CD8 ET	*RPS9*	Wald ratio	1	0.0033	0.0011	1.003 (1.001, 1.005)	0.003	2.19E-96	TRUE
CD8 ET	*SAT2*	Wald ratio	1	-0.0070	0.0030	0.993 (0.987, 0.999)	0.02	2.63E-13	TRUE
CD8 ET	*SCD5*	Wald ratio	1	-0.0084	0.0024	0.992 (0.987, 0.996)	5.69E-04	3.33E-19	TRUE
CD8 ET	*SCRN2*	Wald ratio	1	-0.0006	0.0028	0.999 (0.994, 1.005)	0.842	3.06E-15	TRUE
CD8 ET	*SH3YL1*	Wald ratio	1	0.0006	0.0026	1.001 (0.995, 1.006)	0.825	3.38E-17	TRUE
CD8 ET	*SNRPC*	Wald ratio	1	-0.0136	0.0040	0.986 (0.979, 0.994)	6.33E-04	1.01E-07	TRUE
CD8 ET	*SNRPD2*	Wald ratio	1	-0.0037	0.0018	0.996 (0.993, 1)	0.035	3.14E-38	TRUE
CD8 ET	*SRA1*	Wald ratio	1	0.0024	0.0041	1.002 (0.994, 1.01)	0.556	5.52E-08	TRUE
CD8 ET	*TESC*	Wald ratio	1	0.0030	0.0038	1.003 (0.996, 1.011)	0.424	5.11E-09	TRUE
CD8 ET	*THAP9-AS1*	Wald ratio	1	0.0085	0.0024	1.009 (1.004, 1.013)	4.60E-04	2.71E-19	TRUE
CD8 ET	*THEM4*	Wald ratio	1	0.0051	0.0034	1.005 (0.998, 1.012)	0.131	1.14E-10	TRUE
CD8 ET	*TRAPPC12-AS1*	Wald ratio	1	0.0043	0.0027	1.004 (0.999, 1.01)	0.119	8.98E-16	TRUE
CD8 ET	*UAP1*	Wald ratio	1	-0.0017	0.0040	0.998 (0.991, 1.006)	0.679	3.70E-08	TRUE
CD8 ET	*VIM*	Wald ratio	1	0.0122	0.0033	1.012 (1.006, 1.019)	2.26E-04	6.35E-11	TRUE
CD8 ET	*WARS*	Wald ratio	1	-0.0153	0.0038	0.985 (0.977, 0.992)	5.83E-05	3.01E-08	TRUE
CD8 ET	*XBP1*	Wald ratio	1	0.0086	0.0036	1.009 (1.002, 1.016)	0.016	5.64E-10	TRUE
CD8 ET	*XXbac-BPG252P9.9*	Wald ratio	1	-0.0051	0.0036	0.995 (0.988, 1.002)	0.157	3.16E-10	TRUE
CD8 ET	*ZDHHC4*	Wald ratio	1	0.0033	0.0032	1.003 (0.997, 1.01)	0.302	3.87E-12	TRUE
CD8 ET	*ZNF100*	Wald ratio	1	-0.0084	0.0027	0.992 (0.986, 0.997)	0.002	1.11E-15	TRUE
CD8 ET	*ZNF257*	Wald ratio	1	0.0070	0.0037	1.007 (1, 1.014)	0.058	3.13E-09	TRUE
CD8 ET	*ZNF266*	Wald ratio	1	0.0036	0.0036	1.004 (0.997, 1.011)	0.312	9.04E-10	TRUE
CD8 ET	*ZNF391*	Wald ratio	1	0.0038	0.0020	1.004 (1, 1.008)	0.06	2.65E-28	TRUE
CD8 ET	*ZNF502*	Wald ratio	1	-0.0103	0.0037	0.99 (0.983, 0.997)	0.006	3.08E-09	TRUE
CD8 ET	*ZSWIM7*	Wald ratio	1	0.0065	0.0030	1.007 (1.001, 1.012)	0.028	1.52E-13	TRUE
CD8 NC	*ABCG2*	Wald ratio	1	0.0004	0.0017	1 (0.997, 1.004)	0.795	5.66E-41	TRUE
CD8 NC	*AC064834.2*	Wald ratio	1	0.0035	0.0033	1.004 (0.997, 1.01)	0.282	2.58E-11	TRUE
CD8 NC	*AC092301.3*	Wald ratio	1	0.0003	0.0023	1 (0.996, 1.005)	0.884	2.57E-22	TRUE
CD8 NC	*ACAT1*	Wald ratio	1	0.0001	0.0031	1 (0.994, 1.006)	0.967	7.20E-13	TRUE
CD8 NC	*AOAH*	Wald ratio	1	-0.0032	0.0033	0.997 (0.99, 1.003)	0.33	1.63E-11	TRUE
CD8 NC	*AP003774.1*	Wald ratio	1	-0.0064	0.0018	0.994 (0.99, 0.997)	2.75E-04	4.62E-36	TRUE
CD8 NC	*ATP5G2*	Wald ratio	1	0.0069	0.0036	1.007 (1, 1.014)	0.054	1.39E-09	TRUE
CD8 NC	*B3GALNT1*	Wald ratio	1	-0.0014	0.0014	0.999 (0.996, 1.001)	0.324	2.98E-57	TRUE

CD8 NC	*BST2*	Wald ratio	1	0.0014	0.0033	1.001 (0.995, 1.008)	0.679	9.97E-12	TRUE
CD8 NC	*BTF3*	Wald ratio	1	-0.0011	0.0026	0.999 (0.994, 1.004)	0.674	4.39E-19	TRUE
CD8 NC	*BTN3A1*	Wald ratio	1	0.0082	0.0033	1.008 (1.002, 1.015)	0.013	9.00E-11	TRUE
CD8 NC	*C17orf89*	Wald ratio	1	0.0045	0.0026	1.004 (0.999, 1.01)	0.087	2.73E-17	TRUE
CD8 NC	*C2*	Wald ratio	1	-0.0044	0.0024	0.996 (0.991, 1)	0.069	5.21E-22	TRUE
CD8 NC	*C6orf48*	Wald ratio	1	0.0067	0.0029	1.007 (1.001, 1.012)	0.023	2.29E-14	TRUE
CD8 NC	*C6orf57*	Wald ratio	1	-0.0008	0.0036	0.999 (0.992, 1.006)	0.825	1.89E-09	TRUE
CD8 NC	*CASP8*	Wald ratio	1	0.0005	0.0032	1 (0.994, 1.007)	0.882	1.89E-12	TRUE
CD8 NC	*CEBPZ*	Wald ratio	1	0.0120	0.0040	1.012 (1.004, 1.02)	0.003	6.30E-08	TRUE
CD8 NC	*CIB1*	Wald ratio	1	-0.0005	0.0027	1 (0.994, 1.005)	0.859	8.14E-17	TRUE
CD8 NC	*CLIC3*	Wald ratio	1	-0.0066	0.0040	0.993 (0.986, 1.001)	0.095	3.29E-08	TRUE
CD8 NC	*CLNS1A*	Wald ratio	1	0.0111	0.0036	1.011 (1.004, 1.018)	0.002	1.11E-08	TRUE
CD8 NC	*CLUAP1*	Wald ratio	1	0.0139	0.0037	1.014 (1.007, 1.021)	2.00E-04	8.69E-09	TRUE
CD8 NC	*COG5*	Wald ratio	1	0.0039	0.0021	1.004 (1, 1.008)	0.063	1.76E-25	TRUE
CD8 NC	*COX14*	Wald ratio	1	-0.0076	0.0036	0.992 (0.985, 0.999)	0.036	1.86E-09	TRUE
CD8 NC	*COX7A2L*	Wald ratio	1	0.0009	0.0026	1.001 (0.996, 1.006)	0.734	6.80E-18	TRUE
CD8 NC	*CRHR1*	Wald ratio	1	0.0074	0.0030	1.007 (1.001, 1.013)	0.014	5.92E-13	TRUE
CD8 NC	*CTBP1-AS2*	Wald ratio	1	-0.0025	0.0033	0.997 (0.991, 1.004)	0.452	2.71E-11	TRUE
CD8 NC	*CTD-2336O2.1*	Wald ratio	1	0.0060	0.0020	1.006 (1.002, 1.01)	0.002	1.23E-29	TRUE
CD8 NC	*CTD-3110H11.1*	Wald ratio	1	-0.0048	0.0031	0.995 (0.989, 1.001)	0.119	8.99E-13	TRUE
CD8 NC	*CTSW*	Wald ratio	1	-0.0033	0.0010	0.997 (0.995, 0.999)	0.001	8.73E-108	TRUE
CD8 NC	*DAZAP2*	Wald ratio	1	-0.0023	0.0039	0.998 (0.99, 1.005)	0.562	9.92E-09	TRUE
CD8 NC	*DCP2*	Wald ratio	1	0.0093	0.0036	1.009 (1.002, 1.016)	0.009	1.28E-09	TRUE
CD8 NC	*DDR1*	IVW	2	-0.0088	0.0026	0.991 (0.986, 0.996)	6.71E-04	7.45E-28	TRUE
CD8 NC	*DDX5*	Wald ratio	1	0.0099	0.0025	1.01 (1.005, 1.015)	7.78E-05	2.93E-18	TRUE
CD8 NC	*DNAJA1*	Wald ratio	1	-0.0045	0.0036	0.995 (0.989, 1.002)	0.206	1.26E-09	TRUE
CD8 NC	*FAM21A*	Wald ratio	1	-0.0003	0.0036	1 (0.993, 1.007)	0.932	6.18E-10	TRUE
CD8 NC	*FNBP4*	Wald ratio	1	-0.0130	0.0031	0.987 (0.981, 0.993)	2.81E-05	3.56E-12	TRUE
CD8 NC	*FOXN2*	Wald ratio	1	0.0106	0.0038	1.011 (1.003, 1.018)	0.005	1.31E-08	TRUE
CD8 NC	*GALT*	Wald ratio	1	0.0066	0.0038	1.007 (0.999, 1.014)	0.082	5.53E-09	TRUE
CD8 NC	*GATM*	Wald ratio	1	0.0011	0.0026	1.001 (0.996, 1.006)	0.655	7.27E-18	TRUE
CD8 NC	*GPR160*	Wald ratio	1	-0.0037	0.0020	0.996 (0.992, 1)	0.067	1.45E-28	TRUE
CD8 NC	*GSTP1*	Wald ratio	1	-0.0013	0.0017	0.999 (0.996, 1.002)	0.446	4.47E-42	TRUE
CD8 NC	*GTF2H4*	Wald ratio	1	0.0030	0.0021	1.003 (0.999, 1.007)	0.15	6.97E-32	TRUE
CD8 NC	*HAGH*	Wald ratio	1	0.0076	0.0021	1.008 (1.003, 1.012)	3.32E-04	2.68E-24	TRUE
CD8 NC	*HLA-DQB2*	Wald ratio	1	-0.0047	0.0031	0.995 (0.989, 1.001)	0.123	9.23E-13	TRUE
CD8 NC	*HLA-DRB5*	Wald ratio	1	0.0045	0.0017	1.004 (1.001, 1.008)	0.009	3.57E-39	TRUE
CD8 NC	*HNRNPU*	Wald ratio	1	-0.0029	0.0038	0.997 (0.99, 1.005)	0.447	3.68E-09	TRUE
CD8 NC	*HSD17B12*	Wald ratio	1	-0.0161	0.0037	0.984 (0.977, 0.991)	1.78E-05	1.39E-08	TRUE
CD8 NC	*ICAM3*	Wald ratio	1	0.0040	0.0034	1.004 (0.997, 1.011)	0.239	3.23E-10	TRUE
CD8 NC	*INPP4B*	Wald ratio	1	-0.0040	0.0039	0.996 (0.988, 1.004)	0.3	7.54E-09	TRUE
CD8 NC	*ITGB7*	Wald ratio	1	-0.0020	0.0031	0.998 (0.992, 1.004)	0.529	1.56E-13	TRUE
CD8 NC	*JAKMIP2*	Wald ratio	1	0.0039	0.0035	1.004 (0.997, 1.011)	0.259	1.56E-10	TRUE
CD8 NC	*KDELR2*	Wald ratio	1	0.0021	0.0022	1.002 (0.998, 1.006)	0.328	1.47E-25	TRUE
CD8 NC	*KTN1*	Wald ratio	1	0.0034	0.0023	1.003 (0.999, 1.008)	0.136	4.77E-22	TRUE
CD8 NC	*LA16c-358B7.3*	Wald ratio	1	0.0086	0.0020	1.009 (1.005, 1.013)	1.88E-05	2.57E-28	TRUE
CD8 NC	*LAIR2*	Wald ratio	1	0.0085	0.0040	1.008 (1.001, 1.016)	0.036	3.99E-08	TRUE
CD8 NC	*LRRC37A*	Wald ratio	1	-0.0091	0.0025	0.991 (0.986, 0.996)	2.54E-04	7.52E-18	TRUE
CD8 NC	*LRRC61*	Wald ratio	1	-0.0079	0.0036	0.992 (0.985, 0.999)	0.028	1.95E-09	TRUE
CD8 NC	*LSM7*	Wald ratio	1	0.0060	0.0034	1.006 (0.999, 1.013)	0.082	2.37E-10	TRUE
CD8 NC	*MAST3*	Wald ratio	1	0.0028	0.0036	1.003 (0.996, 1.01)	0.442	9.89E-10	TRUE
CD8 NC	*MDM4*	Wald ratio	1	0.0086	0.0024	1.009 (1.004, 1.013)	2.88E-04	1.45E-20	TRUE
CD8 NC	*MPHOSPH9*	Wald ratio	1	-0.0091	0.0031	0.991 (0.985, 0.997)	0.003	1.44E-11	TRUE
CD8 NC	*MYO16*	Wald ratio	1	0.0046	0.0019	1.005 (1.001, 1.008)	0.016	1.84E-31	TRUE
CD8 NC	*PCBP2*	Wald ratio	1	-0.0031	0.0039	0.997 (0.989, 1.005)	0.426	1.79E-08	TRUE
CD8 NC	*PHACTR4*	Wald ratio	1	0.0092	0.0036	1.009 (1.002, 1.016)	0.011	1.39E-09	TRUE
CD8 NC	*PLEC*	Wald ratio	1	0.0033	0.0036	1.003 (0.996, 1.01)	0.354	6.71E-10	TRUE
CD8 NC	*PRELID1*	Wald ratio	1	-0.0120	0.0028	0.988 (0.983, 0.994)	2.12E-05	8.78E-15	TRUE
CD8 NC	*RBM6*	Wald ratio	1	-0.0059	0.0036	0.994 (0.987, 1.001)	0.101	9.11E-10	TRUE
CD8 NC	*RNASET2*	Wald ratio	1	-0.0012	0.0012	0.999 (0.996, 1.001)	0.335	3.87E-81	TRUE
CD8 NC	*RNF145*	Wald ratio	1	0.0072	0.0039	1.007 (1, 1.015)	0.066	1.13E-08	TRUE
CD8 NC	*RP11-273G15.2*	Wald ratio	1	-0.0136	0.0039	0.987 (0.979, 0.994)	5.26E-04	3.48E-08	TRUE
CD8 NC	*RP5-1021I20.1*	Wald ratio	1	-0.0069	0.0036	0.993 (0.986, 1)	0.054	3.92E-10	TRUE
CD8 NC	*RPS9*	Wald ratio	1	0.0037	0.0012	1.004 (1.001, 1.006)	0.003	3.90E-85	TRUE
CD8 NC	*SAT2*	Wald ratio	1	-0.0096	0.0038	0.99 (0.983, 0.998)	0.011	7.34E-09	TRUE
CD8 NC	*SCD5*	Wald ratio	1	-0.0132	0.0036	0.987 (0.98, 0.994)	2.13E-04	2.17E-09	TRUE
CD8 NC	*SH3YL1*	Wald ratio	1	0.0001	0.0010	1 (0.998, 1.002)	0.889	1.39E-125	TRUE
CD8 NC	*SNRPC*	Wald ratio	1	-0.0113	0.0033	0.989 (0.982, 0.995)	6.22E-04	9.74E-11	TRUE
CD8 NC	*SNRPD2*	Wald ratio	1	-0.0034	0.0016	0.997 (0.993, 1)	0.036	3.16E-44	TRUE
CD8 NC	*SRP14*	Wald ratio	1	0.0023	0.0033	1.002 (0.996, 1.009)	0.473	1.41E-11	TRUE
CD8 NC	*STK32C*	Wald ratio	1	0.0128	0.0039	1.013 (1.005, 1.021)	0.001	3.05E-08	TRUE
CD8 NC	*THAP9-AS1*	Wald ratio	1	0.0112	0.0030	1.011 (1.005, 1.017)	2.29E-04	1.44E-12	TRUE
CD8 NC	*TMEM50B*	Wald ratio	1	-0.0003	0.0019	1 (0.996, 1.004)	0.889	1.74E-31	TRUE
CD8 NC	*TMEM80*	Wald ratio	1	-0.0013	0.0024	0.999 (0.994, 1.003)	0.593	1.61E-20	TRUE
CD8 NC	*TPRA1*	Wald ratio	1	-0.0067	0.0038	0.993 (0.986, 1.001)	0.076	3.17E-08	TRUE
CD8 NC	*TRAPPC12-AS1*	Wald ratio	1	0.0050	0.0031	1.005 (0.999, 1.011)	0.1	1.03E-12	TRUE
CD8 NC	*USP36*	Wald ratio	1	0.0159	0.0041	1.016 (1.008, 1.024)	9.37E-05	1.47E-07	TRUE
CD8 NC	*VIM*	Wald ratio	1	0.0131	0.0033	1.013 (1.007, 1.02)	7.79E-05	8.21E-11	TRUE
CD8 NC	*YWHAH*	Wald ratio	1	-0.0071	0.0040	0.993 (0.985, 1.001)	0.078	7.53E-08	TRUE

CD8 NC	*ZNF100*	Wald ratio	1	-0.0087	0.0029	0.991 (0.986, 0.997)	0.003	4.46E-14	TRUE
CD8 NC	*ZSWIM7*	Wald ratio	1	0.0083	0.0038	1.008 (1.001, 1.016)	0.028	8.38E-09	TRUE
CD8 S100B	*AOAH*	IVW	2	-0.0025	0.0024	0.998 (0.993, 1.002)	0.299	1.18E-21	TRUE
CD8 S100B	*AP003774.1*	Wald ratio	1	-0.0113	0.0031	0.989 (0.983, 0.995)	2.75E-04	4.12E-12	TRUE
CD8 S100B	*BTF3*	Wald ratio	1	-0.0014	0.0034	0.999 (0.992, 1.005)	0.674	1.70E-11	TRUE
CD8 S100B	*C10orf32*	Wald ratio	1	-0.0103	0.0035	0.99 (0.983, 0.997)	0.003	9.64E-10	TRUE
CD8 S100B	*CD151*	Wald ratio	1	0.0089	0.0038	1.009 (1.001, 1.017)	0.019	2.31E-08	TRUE
CD8 S100B	*COG5*	Wald ratio	1	0.0055	0.0029	1.005 (1, 1.011)	0.063	1.88E-13	TRUE
CD8 S100B	*COL18A1*	Wald ratio	1	-0.0039	0.0030	0.996 (0.99, 1.002)	0.195	2.32E-14	TRUE
CD8 S100B	*CTSW*	Wald ratio	1	-0.0042	0.0013	0.996 (0.993, 0.998)	0.001	1.02E-63	TRUE
CD8 S100B	*DDX5*	Wald ratio	1	0.0123	0.0031	1.012 (1.006, 1.019)	7.78E-05	3.37E-12	TRUE
CD8 S100B	*EEF2*	Wald ratio	1	0.0113	0.0039	1.011 (1.004, 1.019)	0.004	1.65E-08	TRUE
CD8 S100B	*ERAP2*	Wald ratio	1	0.0026	0.0016	1.003 (0.999, 1.006)	0.104	4.96E-46	TRUE
CD8 S100B	*FADS1*	Wald ratio	1	-0.0035	0.0020	0.996 (0.993, 1)	0.077	4.22E-28	TRUE
CD8 S100B	*FAM26F*	Wald ratio	1	0.0032	0.0028	1.003 (0.998, 1.009)	0.244	2.19E-15	TRUE
CD8 S100B	*FAM85A*	Wald ratio	1	-0.0091	0.0020	0.991 (0.987, 0.995)	3.53E-06	5.06E-29	TRUE
CD8 S100B	*FDFT1*	Wald ratio	1	0.0108	0.0037	1.011 (1.004, 1.018)	0.003	5.50E-09	TRUE
CD8 S100B	*GBP5*	Wald ratio	1	0.0021	0.0040	1.002 (0.994, 1.01)	0.598	3.54E-08	TRUE
CD8 S100B	*HSPA1L*	Wald ratio	1	-0.0115	0.0022	0.989 (0.984, 0.993)	2.72E-07	1.00E-21	TRUE
CD8 S100B	*KDELR2*	Wald ratio	1	0.0029	0.0030	1.003 (0.997, 1.009)	0.328	4.48E-14	TRUE
CD8 S100B	*KIAA0040*	Wald ratio	1	0.0132	0.0039	1.013 (1.006, 1.021)	6.05E-04	2.45E-08	TRUE
CD8 S100B	*MDM4*	Wald ratio	1	-0.0129	0.0035	0.987 (0.98, 0.994)	2.45E-04	6.87E-10	TRUE
CD8 S100B	*RPL12*	Wald ratio	1	0.0005	0.0013	1.001 (0.998, 1.003)	0.666	2.21E-72	TRUE
CD8 S100B	*RPS12*	Wald ratio	1	0.0128	0.0034	1.013 (1.006, 1.02)	1.67E-04	3.63E-10	TRUE
CD8 S100B	*RPS9*	Wald ratio	1	0.0045	0.0015	1.004 (1.002, 1.007)	0.003	5.90E-51	TRUE
CD8 S100B	*SCD5*	Wald ratio	1	0.0095	0.0027	1.01 (1.004, 1.015)	5.69E-04	3.58E-15	TRUE
CD8 S100B	*SETD9*	Wald ratio	1	0.0031	0.0034	1.003 (0.996, 1.01)	0.362	2.86E-10	TRUE
CD8 S100B	*SH3YL1*	Wald ratio	1	0.0003	0.0017	1 (0.997, 1.004)	0.878	2.59E-38	TRUE
CD8 S100B	*SNRPD2*	Wald ratio	1	-0.0067	0.0032	0.993 (0.987, 1)	0.036	2.22E-12	TRUE
CD8 S100B	*SRP14*	Wald ratio	1	-0.0028	0.0035	0.997 (0.99, 1.004)	0.429	4.10E-10	TRUE
CD8 S100B	*TIMM13*	Wald ratio	1	0.0053	0.0039	1.005 (0.998, 1.013)	0.168	1.21E-08	TRUE
CD8 S100B	*TMEM50B*	Wald ratio	1	-0.0005	0.0033	1 (0.993, 1.006)	0.89	2.89E-11	TRUE
CD8 S100B	*TNFSF13B*	Wald ratio	1	-0.0085	0.0040	0.992 (0.984, 0.999)	0.032	5.59E-08	TRUE
CD8 S100B	*TTC12*	Wald ratio	1	-0.0076	0.0022	0.992 (0.988, 0.997)	6.12E-04	1.28E-23	TRUE
CD8 S100B	*XBP1*	Wald ratio	1	0.0097	0.0041	1.01 (1.002, 1.018)	0.017	6.36E-08	TRUE
DC	*ATAT1*	Wald ratio	1	0.0059	0.0029	1.006 (1, 1.012)	0.044	1.06E-13	TRUE
DC	*DDX5*	Wald ratio	1	0.0157	0.0040	1.016 (1.008, 1.024)	7.78E-05	8.54E-08	TRUE
DC	*DNAJA1*	Wald ratio	1	-0.0041	0.0039	0.996 (0.988, 1.004)	0.297	3.39E-08	TRUE
DC	*ERAP2*	Wald ratio	1	0.0027	0.0030	1.003 (0.997, 1.009)	0.358	8.85E-14	TRUE
DC	*HLA-DQA2*	IVW	2	0.0026	0.0016	1.003 (0.999, 1.006)	0.115	1.19E-166	TRUE
DC	*HLA-DQB2*	Wald ratio	1	-0.0055	0.0029	0.995 (0.989, 1)	0.056	2.82E-14	TRUE
DC	*MICB*	Wald ratio	1	0.0080	0.0027	1.008 (1.003, 1.013)	0.003	5.21E-16	TRUE
DC	*ROGDI*	Wald ratio	1	0.0005	0.0040	1 (0.993, 1.008)	0.907	1.51E-08	TRUE
DC	*RPL12*	Wald ratio	1	0.0007	0.0017	1.001 (0.997, 1.004)	0.687	2.33E-38	TRUE
DC	*RPS2*	Wald ratio	1	-0.0016	0.0022	0.998 (0.994, 1.003)	0.477	1.00E-22	TRUE
DC	*RPS9*	Wald ratio	1	-0.0006	0.0023	0.999 (0.995, 1.004)	0.803	2.34E-22	TRUE
DC	*SYNGAP1*	IVW	2	0.0039	0.0031	1.004 (0.998, 1.01)	0.219	4.82E-49	TRUE
DC	*TMEM176A*	Wald ratio	1	0.0003	0.0016	1 (0.997, 1.004)	0.839	6.31E-43	TRUE
Mono C	*ABRACL*	Wald ratio	1	-0.0020	0.0036	0.998 (0.991, 1.005)	0.583	1.29E-09	TRUE
Mono C	*ADK*	Wald ratio	1	-0.0005	0.0023	1 (0.995, 1.004)	0.832	6.95E-22	TRUE
Mono C	*ANP32E*	Wald ratio	1	-0.0057	0.0039	0.994 (0.987, 1.002)	0.143	1.68E-08	TRUE
Mono C	*AOAH*	Wald ratio	1	-0.0068	0.0038	0.993 (0.986, 1.001)	0.072	8.74E-09	TRUE
Mono C	*AP003774.1*	Wald ratio	1	0.0115	0.0032	1.012 (1.005, 1.018)	2.75E-04	1.38E-11	TRUE
Mono C	*C10orf32*	Wald ratio	1	-0.0118	0.0035	0.988 (0.982, 0.995)	7.34E-04	1.15E-09	TRUE
Mono C	*FCGR1A*	Wald ratio	1	-0.0043	0.0034	0.996 (0.989, 1.002)	0.2	2.78E-11	TRUE
Mono C	*FKBPL*	Wald ratio	1	0.0120	0.0023	1.012 (1.008, 1.017)	1.22E-07	6.06E-21	TRUE
Mono C	*HP*	Wald ratio	1	-0.0066	0.0020	0.993 (0.99, 0.997)	8.04E-04	3.49E-28	TRUE
Mono C	*HSBP1L1*	Wald ratio	1	-0.0017	0.0036	0.998 (0.991, 1.005)	0.625	8.00E-10	TRUE
Mono C	*JUP*	Wald ratio	1	-0.0032	0.0025	0.997 (0.992, 1.002)	0.203	3.17E-17	TRUE
Mono C	*KDELR2*	Wald ratio	1	0.0030	0.0031	1.003 (0.997, 1.009)	0.328	9.23E-13	TRUE
Mono C	*LGALS9*	Wald ratio	1	0.0037	0.0026	1.004 (0.998, 1.009)	0.166	8.89E-17	TRUE
Mono C	*LMNA*	Wald ratio	1	-0.0046	0.0034	0.995 (0.989, 1.002)	0.178	9.40E-12	TRUE
Mono C	*PPT1*	Wald ratio	1	0.0011	0.0025	1.001 (0.996, 1.006)	0.659	2.47E-19	TRUE
Mono C	*PRELID1*	Wald ratio	1	0.0101	0.0024	1.01 (1.005, 1.015)	3.67E-05	9.01E-20	TRUE
Mono C	*PVRL2*	Wald ratio	1	0.0063	0.0033	1.006 (1, 1.013)	0.057	1.52E-11	TRUE
Mono C	*ROGDI*	Wald ratio	1	0.0002	0.0019	1 (0.997, 1.004)	0.907	5.32E-34	TRUE
Mono C	*RPH3A*	Wald ratio	1	-0.0049	0.0026	0.995 (0.99, 1)	0.06	3.10E-17	TRUE
Mono C	*RPL12*	Wald ratio	1	0.0008	0.0019	1.001 (0.997, 1.004)	0.673	8.85E-33	TRUE
Mono C	*RPL14*	Wald ratio	1	0.0009	0.0039	1.001 (0.993, 1.009)	0.815	1.40E-08	TRUE
Mono C	*RPS2*	Wald ratio	1	-0.0016	0.0023	0.998 (0.994, 1.003)	0.477	7.14E-22	TRUE
Mono C	*RPS9*	Wald ratio	1	0.0052	0.0017	1.005 (1.002, 1.009)	0.003	4.40E-37	TRUE
Mono C	*S100A8*	Wald ratio	1	0.0015	0.0038	1.002 (0.994, 1.009)	0.69	1.73E-09	TRUE
Mono C	*S100A9*	Wald ratio	1	0.0014	0.0035	1.001 (0.995, 1.008)	0.69	4.45E-11	TRUE
Mono C	*TMEM176A*	Wald ratio	1	-0.0002	0.0010	1 (0.998, 1.002)	0.837	5.53E-139	TRUE
Mono C	*TMEM176B*	Wald ratio	1	-0.0002	0.0009	1 (0.998, 1.002)	0.839	2.08E-160	TRUE
Mono C	*TREML1*	Wald ratio	1	0.0032	0.0016	1.003 (1, 1.006)	0.047	1.66E-40	TRUE
Mono NC	*ABRACL*	Wald ratio	1	-0.0020	0.0037	0.998 (0.991, 1.005)	0.583	4.75E-09	TRUE
Mono NC	*ADK*	Wald ratio	1	-0.0005	0.0022	1 (0.995, 1.004)	0.832	3.63E-24	TRUE
Mono NC	*HLA-DPA1*	Wald ratio	1	0.0018	0.0035	1.002 (0.995, 1.009)	0.603	1.32E-09	TRUE

Mono NC	*HMGN1*	Wald ratio	1	-0.0010	0.0024	0.999 (0.994, 1.004)	0.685	3.80E-19	TRUE
Mono NC	*HSPA1B*	IVW	2	-0.0018	0.0043	0.998 (0.99, 1.007)	0.678	1.73E-33	TRUE
Mono NC	*JUP*	Wald ratio	1	-0.0024	0.0019	0.998 (0.994, 1.001)	0.19	1.23E-29	TRUE
Mono NC	*LGALS9*	Wald ratio	1	0.0038	0.0021	1.004 (1, 1.008)	0.067	1.96E-25	TRUE
Mono NC	*LST1*	Wald ratio	1	0.0005	0.0028	1 (0.995, 1.006)	0.864	1.45E-14	TRUE
Mono NC	*NCR3*	Wald ratio	1	-0.0130	0.0024	0.987 (0.983, 0.992)	5.05E-08	3.73E-18	TRUE
Mono NC	*PRELID1*	Wald ratio	1	0.0154	0.0029	1.015 (1.01, 1.021)	1.19E-07	2.01E-14	TRUE
Mono NC	*RAC1*	Wald ratio	1	-0.0040	0.0021	0.996 (0.992, 1)	0.062	2.71E-24	TRUE
Mono NC	*ROGDI*	Wald ratio	1	0.0003	0.0026	1 (0.995, 1.006)	0.907	4.89E-17	TRUE
Mono NC	*RPH3A*	Wald ratio	1	-0.0060	0.0030	0.994 (0.988, 1)	0.045	7.93E-13	TRUE
Mono NC	*RPL12*	Wald ratio	1	-0.0014	0.0023	0.999 (0.994, 1.003)	0.54	8.18E-22	TRUE
Mono NC	*RPS2*	Wald ratio	1	-0.0028	0.0038	0.997 (0.99, 1.005)	0.453	2.03E-08	TRUE
Mono NC	*RPS26*	Wald ratio	1	-0.0039	0.0008	0.996 (0.994, 0.998)	3.51E-06	1.86E-181	TRUE
Mono NC	*RPS9*	Wald ratio	1	0.0076	0.0025	1.008 (1.003, 1.013)	0.003	5.71E-17	TRUE
Mono NC	*SIDT2*	Wald ratio	1	0.0105	0.0026	1.011 (1.005, 1.016)	5.86E-05	5.63E-17	TRUE
Mono NC	*TMEM176A*	Wald ratio	1	-0.0002	0.0011	1 (0.998, 1.002)	0.839	6.45E-88	TRUE
Mono NC	*TMEM176B*	Wald ratio	1	-0.0004	0.0010	1 (0.998, 1.002)	0.72	5.14E-123	TRUE
Mono NC	*USP49*	Wald ratio	1	-0.0068	0.0037	0.993 (0.986, 1.001)	0.07	1.45E-08	TRUE
Mono NC	*VPS52*	Wald ratio	1	-0.0155	0.0031	0.985 (0.979, 0.991)	5.28E-07	2.86E-11	TRUE
Mono NC	*VPS53*	Wald ratio	1	-0.0038	0.0023	0.996 (0.992, 1.001)	0.097	3.65E-21	TRUE
Mono NC	*ZFP36L1*	Wald ratio	1	-0.0015	0.0031	0.998 (0.992, 1.005)	0.62	2.29E-12	TRUE
NK R	*AC016831.7*	Wald ratio	1	-0.0039	0.0019	0.996 (0.992, 1)	0.045	3.60E-33	TRUE
NK R	*APOM*	Wald ratio	1	-0.0152	0.0032	0.985 (0.979, 0.991)	2.22E-06	4.33E-11	TRUE
NK R	*CTSW*	Wald ratio	1	-0.0101	0.0034	0.99 (0.983, 0.997)	0.003	1.78E-10	TRUE
NK R	*ERAP2*	Wald ratio	1	0.0029	0.0022	1.003 (0.999, 1.007)	0.196	2.40E-23	TRUE
NK R	*HLA-C*	Wald ratio	1	0.0021	0.0033	1.002 (0.996, 1.009)	0.535	1.11E-12	TRUE
NK R	*HLA-DQA2*	IVW	3	0.0033	0.0039	1.003 (0.996, 1.011)	0.389	7.02E-43	TRUE
NK R	*HLA-DQA2*	MR Egger	3	0.0196	0.0077	1.02 (1.004, 1.035)	0.24	NA	NA
NK R	*HLA-DQA2*	Weighted median	3	0.0007	0.0022	1.001 (0.996, 1.005)	0.757	NA	NA
NK R	*HLA-DQA2*	Simple mode	3	-0.0021	0.0035	0.998 (0.991, 1.005)	0.606	NA	NA
NK R	*HLA-DQA2*	Weighted mode	3	0.0086	0.0027	1.009 (1.003, 1.014)	0.085	NA	NA
NK R	*MAP3K5*	Wald ratio	1	-0.0015	0.0018	0.999 (0.995, 1.002)	0.395	3.74E-37	TRUE
NK R	*MORC2*	Wald ratio	1	-0.0001	0.0012	1 (0.998, 1.002)	0.964	3.97E-75	TRUE
NK R	*RAC1*	Wald ratio	1	-0.0090	0.0039	0.991 (0.983, 0.999)	0.021	2.68E-08	TRUE
NK R	*RPL12*	Wald ratio	1	0.0011	0.0022	1.001 (0.997, 1.005)	0.616	1.43E-24	TRUE
NK R	*RPS9*	Wald ratio	1	-0.0005	0.0023	0.999 (0.995, 1.004)	0.815	4.38E-22	TRUE
NK R	*SYNGAP1*	Wald ratio	1	-0.0119	0.0026	0.988 (0.983, 0.993)	4.72E-06	4.37E-16	TRUE
NK R	*XXbac-BPG299F13.17*	Wald ratio	1	-0.0157	0.0035	0.984 (0.978, 0.991)	9.10E-06	1.45E-09	TRUE
NK R	*ZBTB22*	Wald ratio	1	0.0042	0.0025	1.004 (0.999, 1.009)	0.088	5.41E-18	TRUE
NK R	*ZKSCAN8*	Wald ratio	1	0.0073	0.0033	1.007 (1.001, 1.014)	0.029	5.15E-11	TRUE
NK	*ABR*	Wald ratio	1	-0.0017	0.0042	0.998 (0.99, 1.007)	0.691	5.80E-08	TRUE
NK	*ADRB1*	Wald ratio	1	-0.0090	0.0025	0.991 (0.986, 0.996)	3.93E-04	4.83E-17	TRUE
NK	*AP003774.1*	Wald ratio	1	-0.0047	0.0013	0.995 (0.993, 0.998)	4.87E-04	1.03E-62	TRUE
NK	*AP2A2*	Wald ratio	1	0.0049	0.0023	1.005 (1, 1.009)	0.032	1.72E-22	TRUE
NK	*ATP5G1*	Wald ratio	1	-0.0141	0.0030	0.986 (0.98, 0.992)	1.89E-06	3.50E-13	TRUE
NK	*BTN3A1*	Wald ratio	1	0.0093	0.0039	1.009 (1.002, 1.017)	0.017	4.38E-08	TRUE
NK	*C10orf32*	Wald ratio	1	-0.0087	0.0026	0.991 (0.986, 0.996)	7.10E-04	4.20E-17	TRUE
NK	*C17orf89*	Wald ratio	1	0.0040	0.0023	1.004 (0.999, 1.009)	0.087	2.39E-21	TRUE
NK	*C6orf62*	Wald ratio	1	0.0105	0.0038	1.011 (1.003, 1.018)	0.006	1.31E-08	TRUE
NK	*CAP1*	Wald ratio	1	-0.0041	0.0042	0.996 (0.988, 1.004)	0.329	5.05E-08	TRUE
NK	*CASP8*	Wald ratio	1	-0.0004	0.0026	1 (0.995, 1.005)	0.864	1.83E-18	TRUE
NK	*CCDC88B*	Wald ratio	1	0.0104	0.0036	1.01 (1.003, 1.018)	0.003	1.72E-09	TRUE
NK	*CD151*	Wald ratio	1	0.0045	0.0019	1.004 (1.001, 1.008)	0.018	1.30E-30	TRUE
NK	*CD81*	Wald ratio	1	0.0009	0.0036	1.001 (0.994, 1.008)	0.804	4.61E-10	TRUE
NK	*CDK10*	Wald ratio	1	-0.0078	0.0024	0.992 (0.988, 0.997)	0.001	1.23E-16	TRUE
NK	*CLHC1*	Wald ratio	1	0.0034	0.0036	1.003 (0.996, 1.01)	0.34	7.13E-10	TRUE
NK	*COG5*	Wald ratio	1	0.0038	0.0019	1.004 (1, 1.008)	0.051	3.50E-30	TRUE
NK	*COX7A2L*	Wald ratio	1	0.0013	0.0038	1.001 (0.994, 1.009)	0.73	3.50E-09	TRUE
NK	*CTSW*	Wald ratio	1	-0.0036	0.0012	0.996 (0.994, 0.999)	0.002	3.99E-81	TRUE
NK	*DAZAP1*	Wald ratio	1	-0.0001	0.0038	1 (0.992, 1.007)	0.984	8.07E-09	TRUE
NK	*DDX42*	Wald ratio	1	-0.0131	0.0036	0.987 (0.98, 0.994)	2.88E-04	2.29E-09	TRUE
NK	*DDX5*	Wald ratio	1	0.0081	0.0020	1.008 (1.004, 1.012)	7.78E-05	4.13E-27	TRUE
NK	*DNAJA1*	Wald ratio	1	-0.0040	0.0032	0.996 (0.99, 1.002)	0.209	8.48E-12	TRUE
NK	*DSTN*	IVW	2	-0.0020	0.0023	0.998 (0.993, 1.003)	0.392	2.78E-21	TRUE
NK	*DUOX1*	Wald ratio	1	0.0002	0.0032	1 (0.994, 1.006)	0.96	1.34E-12	TRUE
NK	*DYNLL2*	Wald ratio	1	0.0036	0.0028	1.004 (0.998, 1.009)	0.19	7.45E-16	TRUE
NK	*EGFL8*	Wald ratio	1	-0.0118	0.0022	0.988 (0.984, 0.993)	1.22E-07	5.40E-22	TRUE
NK	*ENTPD3-AS1*	Wald ratio	1	-0.0051	0.0033	0.995 (0.989, 1.001)	0.117	1.65E-11	TRUE
NK	*EPHX4*	Wald ratio	1	0.0081	0.0037	1.008 (1.001, 1.015)	0.03	2.65E-09	TRUE
NK	*ERAP1*	Wald ratio	1	-0.0128	0.0038	0.987 (0.98, 0.995)	8.43E-04	2.24E-08	TRUE
NK	*FGFR1*	Wald ratio	1	0.0019	0.0017	1.002 (0.998, 1.005)	0.276	3.26E-38	TRUE
NK	*FIBP*	Wald ratio	1	0.0074	0.0023	1.007 (1.003, 1.012)	0.001	6.08E-21	TRUE
NK	*FRG1B*	Wald ratio	1	-0.0071	0.0038	0.993 (0.986, 1)	0.057	5.18E-09	TRUE
NK	*GALT*	Wald ratio	1	0.0060	0.0035	1.006 (0.999, 1.013)	0.082	2.32E-10	TRUE
NK	*GNGT2*	Wald ratio	1	0.0177	0.0035	1.018 (1.011, 1.025)	5.81E-07	1.58E-09	TRUE
NK	*GSTM1*	Wald ratio	1	0.0016	0.0039	1.002 (0.994, 1.009)	0.69	2.41E-08	TRUE
NK	*GSTP1*	Wald ratio	1	-0.0017	0.0023	0.998 (0.994, 1.003)	0.446	1.35E-22	TRUE
NK	*HAGH*	Wald ratio	1	0.0081	0.0024	1.008 (1.003, 1.013)	7.25E-04	2.46E-19	TRUE
NK	*HLA-DOA*	Wald ratio	1	0.0183	0.0037	1.018 (1.011, 1.026)	8.89E-07	1.78E-08	TRUE

NK	*HLA-DPA1*	Wald ratio	1	-0.0023	0.0035	0.998 (0.991, 1.004)	0.506	3.59E-10	TRUE
NK	*HNRNPU*	Wald ratio	1	-0.0019	0.0039	0.998 (0.99, 1.006)	0.622	1.87E-08	TRUE
NK	*HSBP1L1*	Wald ratio	1	-0.0065	0.0035	0.994 (0.987, 1)	0.062	3.39E-10	TRUE
NK	*HSD17B12*	Wald ratio	1	0.0172	0.0040	1.017 (1.009, 1.025)	1.47E-05	9.24E-08	TRUE
NK	*IGF2BP2*	Wald ratio	1	0.0109	0.0039	1.011 (1.003, 1.019)	0.006	3.79E-08	TRUE
NK	*JAKMIP2*	Wald ratio	1	0.0047	0.0041	1.005 (0.997, 1.013)	0.259	6.39E-08	TRUE
NK	*KDELR2*	Wald ratio	1	0.0015	0.0015	1.001 (0.998, 1.005)	0.328	3.95E-50	TRUE
NK	*KHK*	Wald ratio	1	-0.0081	0.0041	0.992 (0.984, 1)	0.047	7.89E-08	TRUE
NK	*KTN1*	Wald ratio	1	0.0044	0.0028	1.004 (0.999, 1.01)	0.114	6.76E-15	TRUE
NK	*LA16c-358B7.3*	Wald ratio	1	0.0074	0.0017	1.007 (1.004, 1.011)	1.88E-05	3.20E-38	TRUE
NK	*LAIR2*	IVW	2	0.0025	0.0028	1.003 (0.997, 1.008)	0.369	6.06E-43	TRUE
NK	*LAPTM5*	Wald ratio	1	0.0058	0.0040	1.006 (0.998, 1.014)	0.15	8.37E-08	TRUE
NK	*LLGL1*	Wald ratio	1	0.0160	0.0039	1.016 (1.008, 1.024)	3.75E-05	3.21E-08	TRUE
NK	*LRRC61*	Wald ratio	1	-0.0076	0.0036	0.992 (0.985, 1)	0.037	3.35E-09	TRUE
NK	*LSM7*	Wald ratio	1	0.0043	0.0025	1.004 (0.999, 1.009)	0.087	3.85E-18	TRUE
NK	*MDM4*	Wald ratio	1	-0.0143	0.0036	0.986 (0.979, 0.993)	8.72E-05	2.72E-09	TRUE
NK	*MEI1*	Wald ratio	1	0.0066	0.0041	1.007 (0.999, 1.015)	0.105	4.37E-08	TRUE
NK	*MLF1*	Wald ratio	1	0.0151	0.0029	1.015 (1.01, 1.021)	1.49E-07	2.87E-13	TRUE
NK	*MLKL*	Wald ratio	1	0.0030	0.0040	1.003 (0.995, 1.011)	0.458	3.44E-08	TRUE
NK	*MPHOSPH9*	Wald ratio	1	-0.0080	0.0030	0.992 (0.986, 0.998)	0.007	1.21E-12	TRUE
NK	*NAA40*	Wald ratio	1	-0.0098	0.0041	0.99 (0.982, 0.998)	0.016	1.00E-07	TRUE
NK	*NGRN*	Wald ratio	1	0.0039	0.0040	1.004 (0.996, 1.012)	0.334	5.04E-08	TRUE
NK	*NUCB2*	Wald ratio	1	-0.0065	0.0015	0.994 (0.991, 0.996)	1.25E-05	6.54E-51	TRUE
NK	*OXNAD1*	Wald ratio	1	-0.0090	0.0022	0.991 (0.987, 0.995)	5.61E-05	1.93E-22	TRUE
NK	*PCBP2*	Wald ratio	1	-0.0062	0.0037	0.994 (0.987, 1.001)	0.096	5.58E-09	TRUE
NK	*PIGC*	Wald ratio	1	-0.0051	0.0042	0.995 (0.987, 1.003)	0.218	8.41E-08	TRUE
NK	*PRELID1*	Wald ratio	1	0.0120	0.0027	1.012 (1.007, 1.017)	6.67E-06	1.86E-17	TRUE
NK	*RAC1*	Wald ratio	1	-0.0044	0.0015	0.996 (0.993, 0.999)	0.004	3.37E-50	TRUE
NK	*RBM6*	Wald ratio	1	-0.0048	0.0028	0.995 (0.99, 1.001)	0.092	1.07E-14	TRUE
NK	*RHOC*	Wald ratio	1	-0.0004	0.0026	1 (0.995, 1.005)	0.89	3.18E-19	TRUE
NK	*RNASET2*	Wald ratio	1	-0.0017	0.0017	0.998 (0.995, 1.002)	0.307	4.64E-39	TRUE
NK	*RP11-273G15.2*	Wald ratio	1	-0.0041	0.0019	0.996 (0.992, 1)	0.029	1.44E-34	TRUE
NK	*RP11-403P17.3*	Wald ratio	1	0.0056	0.0039	1.006 (0.998, 1.013)	0.152	6.31E-08	TRUE
NK	*RP11-514O12.4*	Wald ratio	1	0.0031	0.0029	1.003 (0.997, 1.009)	0.291	6.71E-14	TRUE
NK	*RP5-1021I20.1*	Wald ratio	1	-0.0052	0.0027	0.995 (0.99, 1)	0.052	1.88E-17	TRUE
NK	*RPL12*	Wald ratio	1	0.0005	0.0011	1 (0.998, 1.003)	0.687	2.52E-95	TRUE
NK	*RPL14*	Wald ratio	1	0.0022	0.0027	1.002 (0.997, 1.007)	0.414	5.30E-17	TRUE
NK	*RPS12*	Wald ratio	1	0.0128	0.0038	1.013 (1.005, 1.02)	6.71E-04	1.87E-08	TRUE
NK	*RPS6KL1*	Wald ratio	1	0.0051	0.0031	1.005 (0.999, 1.011)	0.099	2.21E-12	TRUE
NK	*RPS9*	Wald ratio	1	0.0035	0.0012	1.004 (1.001, 1.006)	0.003	7.94E-92	TRUE
NK	*SAT2*	Wald ratio	1	-0.0071	0.0031	0.993 (0.987, 0.999)	0.021	7.72E-13	TRUE
NK	*SCD5*	Wald ratio	1	-0.0084	0.0024	0.992 (0.987, 0.996)	4.60E-04	1.19E-19	TRUE
NK	*SCRN2*	Wald ratio	1	-0.0006	0.0029	0.999 (0.994, 1.005)	0.842	2.63E-14	TRUE
NK	*SERAC1*	Wald ratio	1	-0.0068	0.0034	0.993 (0.987, 1)	0.046	1.30E-10	TRUE
NK	*SH3YL1*	Wald ratio	1	0.0005	0.0034	1 (0.994, 1.007)	0.889	3.45E-11	TRUE
NK	*SMPD3*	Wald ratio	1	-0.0049	0.0039	0.995 (0.988, 1.003)	0.206	1.33E-08	TRUE
NK	*SNAP29*	Wald ratio	1	0.0035	0.0018	1.004 (1, 1.007)	0.049	6.33E-36	TRUE
NK	*SNRPD2*	Wald ratio	1	-0.0057	0.0027	0.994 (0.989, 1)	0.035	1.46E-16	TRUE
NK	*SRP14*	Wald ratio	1	0.0033	0.0030	1.003 (0.997, 1.009)	0.277	8.15E-13	TRUE
NK	*ST3GAL5*	Wald ratio	1	-0.0099	0.0032	0.99 (0.984, 0.996)	0.002	7.18E-12	TRUE
NK	*TAPBP*	Wald ratio	1	0.0054	0.0038	1.005 (0.998, 1.013)	0.161	1.24E-08	TRUE
NK	*TBC1D12*	Wald ratio	1	0.0157	0.0032	1.016 (1.01, 1.022)	6.98E-07	1.10E-11	TRUE
NK	*THAP9-AS1*	Wald ratio	1	0.0082	0.0024	1.008 (1.004, 1.013)	6.05E-04	1.78E-19	TRUE
NK	*TMEM241*	Wald ratio	1	0.0092	0.0038	1.009 (1.002, 1.017)	0.015	6.34E-09	TRUE
NK	*TMEM50B*	Wald ratio	1	-0.0003	0.0019	1 (0.996, 1.003)	0.893	6.22E-32	TRUE
NK	*TRAPPC12-AS1*	Wald ratio	1	0.0045	0.0029	1.005 (0.999, 1.01)	0.119	3.06E-14	TRUE
NK	*TRPC3*	Wald ratio	1	0.0002	0.0014	1 (0.998, 1.003)	0.871	2.82E-62	TRUE
NK	*UBE3B*	Wald ratio	1	0.0107	0.0039	1.011 (1.003, 1.019)	0.006	1.10E-07	TRUE
NK	*VIM*	Wald ratio	1	0.0111	0.0040	1.011 (1.003, 1.019)	0.005	6.55E-08	TRUE
NK	*WARS*	Wald ratio	1	-0.0123	0.0031	0.988 (0.982, 0.994)	6.32E-05	5.37E-12	TRUE
NK	*ZDHHC4*	Wald ratio	1	0.0038	0.0037	1.004 (0.997, 1.011)	0.302	1.72E-09	TRUE
NK	*ZNF100*	Wald ratio	1	-0.0080	0.0025	0.992 (0.987, 0.997)	0.002	9.18E-18	TRUE
NK	*ZNF791*	Wald ratio	1	0.0016	0.0038	1.002 (0.994, 1.009)	0.672	4.12E-09	TRUE
Plasma	*HLA-DQB1*	Wald ratio	1	-0.0126	0.0031	0.988 (0.982, 0.993)	3.92E-05	1.80E-10	TRUE
Plasma	*ITPR3*	Wald ratio	1	-0.0053	0.0034	0.995 (0.988, 1.001)	0.123	7.59E-09	TRUE
Plasma	*NFKBIL1*	Wald ratio	1	0.0123	0.0026	1.012 (1.007, 1.018)	1.59E-06	6.16E-14	TRUE
Plasma	*NT5C3B*	Wald ratio	1	0.0015	0.0029	1.001 (0.996, 1.007)	0.609	5.28E-12	TRUE
Plasma	*NUCB2*	Wald ratio	1	-0.0123	0.0028	0.988 (0.982, 0.993)	1.25E-05	5.61E-12	TRUE
Plasma	*RNF146*	Wald ratio	1	-0.0070	0.0016	0.993 (0.99, 0.996)	7.64E-06	1.29E-36	TRUE
Plasma	*RPL12*	Wald ratio	1	-0.0002	0.0024	1 (0.995, 1.005)	0.92	1.43E-16	TRUE
Plasma	*RXRB*	Wald ratio	1	0.0171	0.0036	1.017 (1.01, 1.024)	1.64E-06	1.17E-07	TRUE
Plasma	*SIRT5*	Wald ratio	1	0.0037	0.0025	1.004 (0.999, 1.009)	0.137	4.24E-17	TRUE
Plasma	*ZBTB22*	Wald ratio	1	-0.0003	0.0023	1 (0.995, 1.004)	0.886	7.24E-18	TRUE

Table S4. Colocalization evidence for the associations between cell type-specific eGenes and hypertension (COLOC method).

Cell type	eGene	PP.H0.abf	PP.H1.abf	PP.H2.abf	PP.H3.abf	PP.H4.abf
B IN	*ACAT1*	2.33E-04	0.45	2.08E-04	0.402	0.148
B IN	*AP003774.1*	5.78E-16	0.222	1.53E-15	0.589	0.189
B IN	*BLK*	3.01E-27	1.27E-10	2.37E-17	1	2.45E-08
B IN	*C10orf32*	2.89E-26	1.06E-21	2.73E-05	1	2.76E-06
B IN	*C6orf48*	1.34E-17	7.18E-12	1.86E-06	1	6.95E-08
B IN	*C6orf62*	3.54E-04	0.012	0.027	0.948	0.012
B IN	*CD164*	0.002	0.929	6.81E-05	0.027	0.042
B IN	*CENPV*	1.62E-07	0.482	1.10E-07	0.328	0.191
B IN	*DDX5*	2.21E-08	0.003	7.19E-06	0.983	0.014
B IN	*FAM167A*	6.59E-13	3.54E-11	0.018	0.981	8.65E-04
B IN	*FANCA*	1.83E-08	3.05E-06	0.006	0.991	0.003
B IN	*FNBP4*	1.23E-11	3.73E-06	3.32E-07	0.1	0.9
B IN	*GTF2H4*	7.41E-22	1.33E-10	5.59E-12	1	7.68E-11
B IN	*HAGHL*	9.03E-09	9.22E-06	9.79E-04	0.999	3.86E-05
B IN	*HSD17B11*	5.53E-04	0.106	0.005	0.872	0.017
B IN	*LA16c-358B7.3*	5.47E-14	9.20E-06	5.94E-09	1	5.68E-05
B IN	*MSMP*	3.79E-11	7.50E-07	5.05E-05	1	4.05E-06
B IN	*NOMO1*	0.1	0.466	0.013	0.061	0.36
B IN	*PRKD3*	1.66E-08	2.01E-04	8.28E-05	0.999	2.43E-04
B IN	*RAC1*	1.20E-12	0.137	7.15E-12	0.811	0.052
B IN	*RP13-514E23.2*	2.05E-11	3.74E-07	5.48E-05	1	3.74E-06
B IN	*RPS9*	4.59E-59	0.18	2.18E-59	0.084	0.736
B IN	*SNRPD2*	1.04E-12	0.114	7.60E-12	0.832	0.054
B IN	*WARS*	7.47E-49	0.001	3.77E-47	0.057	0.942
B Mem	*AP003774.1*	1.25E-10	0.227	3.30E-10	0.602	0.171
B Mem	*BLK*	9.85E-41	1.27E-10	7.75E-31	1	2.69E-08
B Mem	*C6orf48*	2.53E-14	7.15E-12	0.004	0.996	1.20E-04
B Mem	*CCDC163P*	6.50E-10	0.268	1.77E-09	0.73	0.001
B Mem	*DDX5*	8.52E-06	0.003	0.003	0.981	0.013
B Mem	*FAM167A*	2.46E-20	3.61E-11	6.83E-10	1	7.39E-09
B Mem	*FNBP4*	1.31E-13	7.68E-07	3.55E-09	0.02	0.98
B Mem	*HAGH*	1.35E-08	8.62E-04	1.45E-05	0.929	0.07
B Mem	*HAGHL*	1.89E-09	9.22E-06	2.05E-04	1	8.44E-06
B Mem	*HIST1H2AG*	0.037	0.017	0.631	0.28	0.035
B Mem	*HIST1H2BJ*	0.033	0.02	0.556	0.333	0.059
B Mem	*HIST1H3D*	3.18E-11	5.25E-11	0.371	0.614	0.015
B Mem	*KRTAP5-AS1*	4.76E-25	1.13E-24	0.29	0.686	0.025
B Mem	*LA16c-358B7.3*	5.23E-18	9.20E-06	5.68E-13	1	5.50E-05
B Mem	*MDM4*	3.71E-10	0.089	3.18E-10	0.075	0.836
B Mem	*PRKD3*	2.18E-08	2.01E-04	1.08E-04	1	5.91E-05
B Mem	*RP13-514E23.2*	3.49E-09	3.71E-07	0.009	0.99	5.36E-04
B Mem	*RPS9*	6.29E-51	0.18	2.98E-51	0.085	0.735
B Mem	*SNRPD2*	9.45E-13	0.113	6.90E-12	0.824	0.063
B Mem	*WARS*	7.56E-35	0.001	3.82E-33	0.07	0.929
B Mem	*ZNF100*	9.58E-07	1.03E-04	0.004	0.455	0.54
CD4 ET	*AIF1*	1.19E-18	7.12E-12	1.67E-07	0.999	0.001
CD4 ET	*AP003774.1*	1.18E-14	0.24	3.13E-14	0.636	0.125
CD4 ET	*C10orf32*	6.00E-29	1.06E-21	5.66E-08	1	5.02E-09
CD4 ET	*C17orf89*	4.64E-06	0.646	1.49E-06	0.207	0.147
CD4 ET	*CD151*	2.99E-11	0.159	8.42E-11	0.445	0.396

CD4 ET	*CDK10*	2.65E-10	4.54E-07	8.62E-05	0.147	0.853
CD4 ET	*CTSW*	7.36E-41	7.34E-09	1.00E-32	1	1.84E-07
CD4 ET	*DDX5*	8.40E-15	0.003	2.73E-12	0.983	0.014
CD4 ET	*FDFT1*	2.37E-21	1.38E-10	1.71E-11	1	1.05E-10
CD4 ET	*FGF9*	4.97E-10	1.12E-10	0.781	0.177	0.042
CD4 ET	*FNBP4*	3.23E-09	2.02E-05	8.75E-05	0.547	0.453
CD4 ET	*HAGH*	3.68E-10	7.48E-04	3.97E-07	0.806	0.193
CD4 ET	*LA16c-358B7.3*	5.37E-15	9.20E-06	5.84E-10	1	7.16E-05
CD4 ET	*MANBA*	2.15E-06	0.182	4.96E-06	0.421	0.397
CD4 ET	*MDM4*	5.94E-06	0.081	5.09E-06	0.069	0.85
CD4 ET	*PLEKHA1*	8.40E-07	0.067	1.15E-05	0.915	0.018
CD4 ET	*PPT2*	1.74E-09	1.32E-08	0.113	0.861	0.026
CD4 ET	*RNF166*	4.24E-06	0.024	1.64E-04	0.925	0.051
CD4 ET	*RP11-499P20.2*	4.64E-08	1.10E-07	0.292	0.693	0.015
CD4 ET	*RPS9*	4.60E-69	0.179	2.18E-69	0.084	0.736
CD4 ET	*SNRPD2*	3.17E-13	0.113	2.32E-12	0.828	0.059
CD4 ET	*TNFSF13B*	8.02E-07	0.399	3.04E-07	0.15	0.451
CD4 NC	*AC010982.1*	0.584	0.129	0.213	0.047	0.026
CD4 NC	*ACAT1*	7.64E-23	0.453	6.83E-23	0.405	0.141
CD4 NC	*ACOT13*	3.85E-05	0.003	0.011	0.954	0.031
CD4 NC	*AIF1*	2.52E-37	7.13E-12	3.54E-26	1	2.21E-06
CD4 NC	*AP003774.1*	1.78E-80	0.217	4.73E-80	0.576	0.207
CD4 NC	*AP2A2*	5.34E-06	0.078	3.51E-05	0.514	0.408
CD4 NC	*BIK*	2.45E-07	0.052	3.85E-06	0.82	0.128
CD4 NC	*BLK*	3.92E-25	1.27E-10	3.09E-15	1	3.31E-08
CD4 NC	*BLMH*	5.07E-06	0.023	3.93E-05	0.179	0.798
CD4 NC	*C10orf32*	1.38E-52	1.06E-21	1.30E-31	1	6.18E-20
CD4 NC	*C2orf74*	8.75E-09	0.025	3.24E-07	0.929	0.046
CD4 NC	*C6orf48*	7.31E-26	7.18E-12	1.02E-14	1	8.46E-12
CD4 NC	*C6orf62*	7.34E-05	0.013	0.006	0.97	0.012
CD4 NC	*CCDC104*	5.43E-12	0.002	1.03E-09	0.405	0.593
CD4 NC	*CCDC88B*	1.48E-14	0.245	3.74E-14	0.618	0.137
CD4 NC	*CD151*	2.14E-13	0.225	6.01E-13	0.632	0.143
CD4 NC	*CDK10*	1.19E-22	1.51E-06	3.86E-17	0.489	0.511
CD4 NC	*CLNS1A*	1.27E-13	0.066	4.52E-13	0.235	0.699
CD4 NC	*CLUAP1*	6.45E-15	0.011	2.99E-13	0.501	0.488
CD4 NC	*CRHR1*	3.87E-13	0.077	4.62E-12	0.917	0.007
CD4 NC	*CTD-2336O2.1*	2.84E-62	0.491	2.31E-62	0.4	0.109
CD4 NC	*CTSW*	4.97E-127	7.34E-09	6.77E-119	1	1.88E-07
CD4 NC	*CUBN*	1.33E-11	0.02	4.80E-10	0.737	0.243
CD4 NC	*CYP2D6*	2.09E-04	0.399	9.98E-05	0.19	0.411
CD4 NC	*DDX5*	3.78E-22	0.003	1.23E-19	0.983	0.014
CD4 NC	*EEF2*	7.80E-06	0.013	2.61E-05	0.044	0.942
CD4 NC	*EGFL8*	4.46E-14	1.59E-08	2.74E-06	0.978	0.022
CD4 NC	*ERP29*	7.28E-40	5.69E-33	1.28E-07	1	4.48E-07
CD4 NC	*FAM153B*	1.32E-94	0.771	2.03E-95	0.118	0.111
CD4 NC	*FANCA*	1.58E-11	3.08E-06	5.12E-06	1	1.34E-05
CD4 NC	*FDFT1*	4.50E-89	1.38E-10	3.25E-79	1	7.91E-11
CD4 NC	*FNBP4*	2.71E-36	3.63E-05	7.35E-32	0.983	0.017
CD4 NC	*FOXN2*	1.42E-14	0.023	4.55E-14	0.072	0.905
CD4 NC	*GOSR2*	3.56E-28	1.87E-15	1.90E-13	1	1.75E-12
CD4 NC	*HAGH*	1.92E-57	9.01E-04	2.07E-54	0.972	0.028
CD4 NC	*HLA-DQA1*	2.39E-13	8.10E-09	1.81E-05	0.613	0.387
CD4 NC	*HLA-DRB5*	1.11E-42	1.32E-08	8.45E-35	1	1.27E-05

CD4 NC	*HMBS*	7.98E-17	0.667	1.48E-17	0.123	0.209
CD4 NC	*HSD17B11*	3.66E-09	0.107	3.00E-08	0.88	0.013
CD4 NC	*HSD17B12*	2.25E-21	0.268	3.54E-21	0.421	0.311
CD4 NC	*KB-1507C5.2*	2.65E-09	0.003	8.21E-07	0.922	0.075
CD4 NC	*KRTAP5-2*	9.15E-29	1.64E-24	5.57E-05	1	2.98E-06
CD4 NC	*LA16c-358B7.3*	3.44E-70	9.20E-06	3.74E-65	1	6.99E-05
CD4 NC	*LAIR2*	2.88E-14	0.658	6.12E-15	0.14	0.203
CD4 NC	*LIMD2*	5.45E-11	9.46E-08	5.75E-04	0.999	5.08E-05
CD4 NC	*LLGL1*	3.68E-10	0.002	2.38E-09	0.014	0.983
CD4 NC	*LRRC61*	2.96E-30	4.65E-05	6.31E-26	0.993	0.007
CD4 NC	*LSM4*	6.67E-08	0.006	1.08E-05	0.976	0.017
CD4 NC	*MACF1*	1.04E-14	0.004	3.55E-13	0.144	0.852
CD4 NC	*MARK3*	2.87E-12	1.24E-05	2.32E-07	0.999	7.46E-04
CD4 NC	*MEI1*	8.34E-09	0.597	2.84E-09	0.203	0.2
CD4 NC	*MRPS34*	3.35E-07	3.32E-04	3.62E-04	0.357	0.642
CD4 NC	*MYO16*	1.35E-25	0.374	9.00E-26	0.25	0.376
CD4 NC	*NARFL*	4.10E-10	9.23E-06	4.44E-05	1	9.98E-06
CD4 NC	*NEIL2*	1.73E-14	1.10E-10	1.25E-04	0.794	0.206
CD4 NC	*OSER1-AS1*	9.86E-12	3.14E-05	3.14E-07	0.999	7.47E-04
CD4 NC	*PABPC4*	2.08E-05	0.005	7.10E-04	0.174	0.821
CD4 NC	*PCNX*	1.13E-16	0.445	7.22E-17	0.284	0.271
CD4 NC	*PGPEP1*	2.73E-09	0.006	4.43E-07	0.988	0.006
CD4 NC	*PHACTR4*	5.50E-16	0.036	1.26E-14	0.835	0.128
CD4 NC	*PKMYT1*	0.08	0.096	0.142	0.17	0.511
CD4 NC	*PRKD3*	1.53E-11	2.01E-04	7.63E-08	1	1.77E-04
CD4 NC	*RAC1*	2.16E-53	0.142	1.28E-52	0.844	0.013
CD4 NC	*REEP3*	1.59E-07	4.48E-05	0.003	0.748	0.249
CD4 NC	*RNF8*	0.004	0.619	0.002	0.265	0.11
CD4 NC	*RP11-273G15.2*	1.46E-18	3.58E-06	4.06E-13	1	3.83E-04
CD4 NC	*RP11-796G6.2*	2.42E-04	0.454	9.91E-05	0.185	0.36
CD4 NC	*RPS9*	9.04E-103	0.179	4.29E-103	0.084	0.736
CD4 NC	*SAT2*	9.84E-30	8.30E-13	1.18E-17	1	2.26E-09
CD4 NC	*SLC22A5*	4.09E-05	0.104	2.90E-04	0.736	0.16
CD4 NC	*SNRPC*	1.62E-22	0.017	9.45E-21	0.967	0.016
CD4 NC	*SNRPD2*	2.94E-70	0.114	2.15E-69	0.829	0.058
CD4 NC	*SNX16*	1.96E-08	0.207	2.63E-08	0.277	0.517
CD4 NC	*SPCS1*	2.76E-10	0.024	1.12E-08	0.961	0.016
CD4 NC	*TMEM199*	1.37E-06	0.099	4.79E-06	0.345	0.555
CD4 NC	*TMEM87B*	3.08E-10	1.64E-04	1.87E-06	0.999	4.00E-04
CD4 NC	*TMEM97*	1.00E-06	0.176	3.43E-06	0.601	0.223
CD4 NC	*TNFRSF18*	0.008	0.551	0.005	0.329	0.107
CD4 NC	*TTC12*	2.40E-06	0.288	4.44E-06	0.533	0.18
CD4 NC	*UACA*	4.88E-04	0.46	4.91E-05	0.046	0.494
CD4 NC	*UBE2I*	8.25E-11	9.20E-06	8.96E-06	1	5.62E-05
CD4 NC	*UBE3B*	2.28E-08	1.87E-04	1.22E-04	0.999	3.94E-04
CD4 NC	*USP36*	2.78E-09	0.002	8.29E-07	0.665	0.333
CD4 NC	*VIM*	9.73E-07	5.86E-04	3.96E-05	0.023	0.976
CD4 NC	*VPS53*	3.13E-07	0.055	3.66E-07	0.064	0.881
CD4 NC	*WBSCR16*	1.11E-05	0.016	3.63E-05	0.05	0.934
CD4 NC	*ZNF100*	2.86E-42	1.08E-04	1.26E-38	0.474	0.526
CD4 NC	*ZNF502*	1.12E-27	0.566	3.55E-28	0.18	0.254
CD4 NC	*ZSWIM7*	5.79E-13	0.468	3.96E-13	0.32	0.212
CD4 SOX4	*DAXX*	5.05E-08	1.73E-08	0.701	0.24	0.059
CD4 SOX4	*DXO*	4.88E-13	1.01E-11	0.046	0.946	0.009

CD4 SOX4	*RPS26*	9.32E-215	0.708	1.60E-215	0.121	0.171
CD4 SOX4	*RPS9*	1.68E-07	0.196	7.96E-08	0.092	0.711
CD8 ET	*ADRB1*	5.17E-31	6.32E-23	8.18E-09	1	5.76E-10
CD8 ET	*AP003774.1*	1.68E-50	0.246	4.44E-50	0.653	0.101
CD8 ET	*AP2A2*	1.51E-07	0.079	9.94E-07	0.516	0.406
CD8 ET	*BTN3A1*	2.13E-15	9.02E-11	2.36E-05	1	1.31E-06
CD8 ET	*C10orf32*	2.66E-34	1.06E-21	2.51E-13	1	1.42E-14
CD8 ET	*C2orf74*	4.93E-05	0.026	0.002	0.962	0.01
CD8 ET	*C6orf62*	3.58E-04	0.012	0.027	0.944	0.016
CD8 ET	*CCDC88B*	1.68E-04	0.252	4.23E-04	0.635	0.112
CD8 ET	*CDK10*	1.09E-12	9.69E-07	3.55E-07	0.314	0.686
CD8 ET	*CTD-2515A14.1*	0.001	0.611	1.02E-04	0.042	0.345
CD8 ET	*CTSW*	2.30E-119	7.34E-09	3.14E-111	1	1.88E-07
CD8 ET	*DDX5*	1.62E-21	0.003	5.25E-19	0.983	0.014
CD8 ET	*DGKD*	0.005	0.409	0.002	0.177	0.406
CD8 ET	*FDFT1*	5.96E-17	1.38E-10	4.31E-07	1	1.91E-08
CD8 ET	*FIBP*	5.93E-13	7.34E-09	8.08E-05	1	6.93E-06
CD8 ET	*FNBP4*	7.04E-16	3.45E-05	1.91E-11	0.935	0.065
CD8 ET	*FOXN2*	1.99E-06	0.043	6.34E-06	0.136	0.821
CD8 ET	*HAGH*	4.13E-19	9.10E-04	4.46E-16	0.981	0.018
CD8 ET	*HLA-DQB2*	1.10E-14	5.65E-08	1.95E-07	1	2.54E-04
CD8 ET	*LA16c-358B7.3*	3.46E-29	9.20E-06	3.76E-24	1	6.30E-05
CD8 ET	*LAIR2*	1.15E-21	0.656	2.45E-22	0.139	0.205
CD8 ET	*LLGL1*	1.07E-08	0.002	6.89E-08	0.013	0.985
CD8 ET	*LRRC37A*	6.55E-04	0.004	0.046	0.277	0.672
CD8 ET	*LRRC61*	2.33E-08	4.62E-05	4.97E-04	0.986	0.013
CD8 ET	*MDM4*	3.13E-09	0.147	2.68E-09	0.125	0.728
CD8 ET	*MLF1*	2.45E-12	8.35E-05	2.15E-08	0.731	0.269
CD8 ET	*MPHOSPH9*	5.05E-04	0.7	5.63E-05	0.078	0.221
CD8 ET	*NOTCH4*	9.74E-12	1.61E-08	6.03E-04	0.999	2.33E-04
CD8 ET	*NUCB2*	2.32E-63	5.73E-04	2.38E-60	0.59	0.41
CD8 ET	*OXNAD1*	3.02E-12	0.031	1.15E-11	0.116	0.854
CD8 ET	*PDCD6*	1.81E-05	0.07	1.92E-04	0.739	0.191
CD8 ET	*PHACTR4*	5.36E-07	0.036	1.23E-05	0.813	0.152
CD8 ET	*REEP5*	1.60E-05	0.225	5.13E-05	0.724	0.051
CD8 ET	*RP4-799D16.1*	1.47E-08	3.75E-05	3.93E-04	0.999	1.27E-04
CD8 ET	*RPS9*	4.58E-102	0.18	2.17E-102	0.084	0.736
CD8 ET	*SAT2*	7.53E-20	8.30E-13	9.07E-08	1	6.29E-09
CD8 ET	*SCD5*	8.84E-16	0.015	3.72E-15	0.064	0.92
CD8 ET	*SNRPC*	6.23E-06	0.016	3.64E-04	0.938	0.045
CD8 ET	*SNRPD2*	4.56E-32	0.113	3.33E-31	0.826	0.061
CD8 ET	*THAP9-AS1*	3.39E-16	0.01	1.42E-15	0.041	0.948
CD8 ET	*VIM*	3.69E-09	2.82E-04	1.50E-07	0.01	0.989
CD8 ET	*WARS*	1.45E-06	0.003	7.33E-05	0.149	0.848
CD8 ET	*XBP1*	1.99E-15	1.56E-10	1.27E-05	1	4.44E-05
CD8 ET	*ZNF100*	1.82E-14	1.08E-04	8.04E-11	0.477	0.523
CD8 ET	*ZNF502*	9.70E-04	0.583	3.08E-04	0.185	0.231
CD8 ET	*ZSWIM7*	4.25E-09	0.469	2.90E-09	0.321	0.21
CD8 NC	*AP003774.1*	7.49E-32	0.224	1.99E-31	0.593	0.183
CD8 NC	*BTN3A1*	2.11E-16	9.02E-11	2.34E-06	1	1.37E-07
CD8 NC	*C6orf48*	4.43E-19	7.18E-12	6.17E-08	1	2.51E-09
CD8 NC	*CEBPZ*	1.04E-06	2.47E-04	0.004	0.995	8.01E-04
CD8 NC	*CLNS1A*	7.06E-06	0.065	2.50E-05	0.23	0.705
CD8 NC	*CLUAP1*	5.67E-06	0.009	2.63E-04	0.433	0.557

CD8 NC	*COX14*	4.39E-22	1.08E-17	4.07E-05	1	8.91E-06
CD8 NC	*CRHR1*	5.19E-05	0.076	6.19E-04	0.906	0.017
CD8 NC	*CTD-2336O2.1*	2.02E-24	0.373	1.65E-24	0.303	0.324
CD8 NC	*CTSW*	2.15E-119	7.34E-09	2.94E-111	1	1.87E-07
CD8 NC	*DCP2*	8.07E-05	0.228	2.61E-04	0.739	0.032
CD8 NC	*DDR1*	5.70E-25	1.33E-10	4.30E-15	1	3.31E-08
CD8 NC	*DDX5*	6.82E-15	0.003	2.21E-12	0.983	0.014
CD8 NC	*FNBP4*	8.30E-13	3.06E-05	2.25E-08	0.83	0.17
CD8 NC	*FOXN2*	1.64E-04	0.02	5.23E-04	0.064	0.915
CD8 NC	*HAGH*	1.24E-21	8.85E-04	1.33E-18	0.954	0.045
CD8 NC	*HLA-DRB5*	1.70E-42	1.32E-08	1.29E-34	1	9.66E-06
CD8 NC	*HSD17B12*	5.82E-05	0.291	9.16E-05	0.458	0.251
CD8 NC	*LA16c-358B7.3*	6.30E-28	9.20E-06	6.84E-23	1	6.89E-05
CD8 NC	*LAIR2*	0.022	0.649	0.005	0.138	0.186
CD8 NC	*LRRC37A*	1.90E-04	0.006	0.013	0.428	0.552
CD8 NC	*LRRC61*	3.24E-09	4.66E-05	6.91E-05	0.994	0.005
CD8 NC	*MPHOSPH9*	1.77E-07	0.7	1.97E-08	0.078	0.222
CD8 NC	*MYO16*	0.024	0.388	0.016	0.259	0.314
CD8 NC	*PHACTR4*	3.01E-07	0.036	6.90E-06	0.82	0.144
CD8 NC	*RP11-273G15.2*	2.80E-07	3.26E-06	0.078	0.91	0.012
CD8 NC	*RPS9*	4.87E-88	0.18	2.31E-88	0.085	0.735
CD8 NC	*SAT2*	7.06E-16	8.30E-13	8.50E-04	0.999	3.09E-05
CD8 NC	*SCD5*	4.59E-07	0.008	1.93E-06	0.033	0.959
CD8 NC	*SNRPC*	1.04E-08	0.016	6.10E-07	0.958	0.026
CD8 NC	*SNRPD2*	3.46E-38	0.115	2.53E-37	0.836	0.049
CD8 NC	*STK32C*	0.001	0.341	0.002	0.626	0.03
CD8 NC	*THAP9-AS1*	6.87E-10	0.009	2.88E-09	0.037	0.954
CD8 NC	*USP36*	7.67E-06	0.002	0.002	0.593	0.403
CD8 NC	*VIM*	4.81E-09	6.19E-04	1.96E-07	0.024	0.975
CD8 NC	*ZNF100*	4.17E-13	1.07E-04	1.84E-09	0.472	0.528
CD8 NC	*ZSWIM7*	1.43E-04	0.444	9.76E-05	0.303	0.253
CD8 S100B	*AP003774.1*	5.81E-08	0.222	1.54E-07	0.589	0.189
CD8 S100B	*C10orf32*	1.33E-25	1.06E-21	1.25E-04	1	6.33E-06
CD8 S100B	*CD151*	7.03E-04	0.203	0.002	0.569	0.225
CD8 S100B	*CTSW*	3.17E-67	7.34E-09	4.32E-59	1	1.69E-07
CD8 S100B	*DDX5*	4.58E-09	0.003	1.49E-06	0.983	0.014
CD8 S100B	*EEF2*	1.10E-05	0.01	3.69E-05	0.032	0.958
CD8 S100B	*FAM85A*	3.15E-09	2.61E-08	0.033	0.276	0.691
CD8 S100B	*FDFT1*	3.71E-13	1.38E-10	0.003	0.997	1.37E-04
CD8 S100B	*HSPA1L*	2.65E-27	7.18E-12	3.70E-16	1	5.45E-05
CD8 S100B	*KIAA0040*	1.26E-08	2.04E-05	3.98E-04	0.645	0.355
CD8 S100B	*MDM4*	1.80E-06	0.186	1.55E-06	0.159	0.656
CD8 S100B	*RPS12*	2.23E-05	0.142	1.31E-05	0.082	0.776
CD8 S100B	*RPS9*	1.70E-48	0.18	8.05E-49	0.084	0.736
CD8 S100B	*SCD5*	7.56E-04	0.015	0.003	0.064	0.917
CD8 S100B	*SNRPD2*	1.09E-07	0.112	7.99E-07	0.82	0.068
CD8 S100B	*TNFSF13B*	0.002	0.39	7.25E-04	0.147	0.46
CD8 S100B	*TTC12*	0.074	0.217	0.137	0.401	0.171
CD8 S100B	*XBP1*	2.40E-13	1.56E-10	0.002	0.998	2.08E-04
DC	*ATAT1*	5.85E-12	5.19E-10	0.006	0.504	0.491
DC	*DDX5*	5.99E-05	0.003	0.019	0.952	0.026
DC	*MICB*	6.44E-22	7.13E-12	9.03E-11	1	2.45E-11
Mono C	*AP003774.1*	0.01	0.225	0.028	0.597	0.14
Mono C	*C10orf32*	4.02E-26	1.06E-21	3.79E-05	1	4.69E-06

Mono C	*HP*	1.02E-23	0.325	1.71E-23	0.545	0.13
Mono C	*PRELID1*	3.09E-15	0.016	1.84E-13	0.942	0.042
Mono C	*RPS9*	1.18E-33	0.18	5.57E-34	0.085	0.735
Mono C	*TREML1*	0.03	0.009	0.218	0.063	0.681
Mono NC	*NCR3*	1.95E-24	7.13E-12	2.73E-13	1	2.71E-08
Mono NC	*PRELID1*	1.44E-11	0.002	8.57E-10	0.101	0.898
Mono NC	*RPH3A*	5.62E-31	3.92E-24	1.43E-07	1	4.41E-09
Mono NC	*RPS26*	3.05E-236	0.708	5.22E-237	0.121	0.171
Mono NC	*RPS9*	2.37E-13	0.18	1.12E-13	0.085	0.735
Mono NC	*VPS52*	3.82E-08	3.01E-08	0.53	0.418	0.051
NK R	*AC016831.7*	0.013	0.002	0.814	0.123	0.047
NK R	*APOM*	2.63E-16	7.16E-12	3.66E-05	0.997	0.003
NK R	*CTSW*	1.28E-13	7.34E-09	1.75E-05	1	9.43E-06
NK R	*RAC1*	7.43E-05	0.043	4.41E-04	0.252	0.705
NK R	*SYNGAP1*	2.85E-10	3.37E-07	8.45E-04	0.999	1.04E-04
NK R	*XXbac-BPG299F13.17*	1.23E-15	7.13E-12	1.73E-04	1	2.08E-05
NK	*ADRB1*	1.04E-32	6.32E-23	1.64E-10	1	7.76E-12
NK	*AP003774.1*	1.12E-60	0.222	2.96E-60	0.589	0.188
NK	*AP2A2*	4.15E-19	0.071	2.73E-18	0.466	0.463
NK	*ATP5G1*	4.52E-16	1.38E-07	3.27E-09	0.997	0.003
NK	*BTN3A1*	6.92E-14	9.01E-11	7.68E-04	0.999	3.72E-05
NK	*C10orf32*	3.24E-32	1.06E-21	3.06E-11	1	2.97E-12
NK	*C6orf62*	6.51E-05	0.013	0.005	0.97	0.012
NK	*CCDC88B*	1.66E-05	0.266	4.17E-05	0.67	0.064
NK	*CD151*	3.83E-24	0.226	1.08E-23	0.635	0.138
NK	*CDK10*	1.92E-13	3.08E-06	6.23E-08	1	2.19E-04
NK	*CTSW*	4.39E-87	7.34E-09	5.98E-79	1	1.74E-07
NK	*DDX42*	3.04E-12	9.47E-08	3.21E-05	1	2.55E-05
NK	*DDX5*	1.46E-23	0.003	4.74E-21	0.983	0.014
NK	*EPHX4*	7.60E-05	0.182	7.88E-05	0.188	0.629
NK	*ERAP1*	1.84E-15	2.58E-12	6.94E-04	0.976	0.023
NK	*FIBP*	2.82E-23	7.34E-09	3.84E-15	1	1.52E-07
NK	*GNGT2*	7.74E-12	5.09E-08	5.91E-05	0.388	0.612
NK	*HAGH*	1.77E-16	9.02E-04	1.91E-13	0.973	0.026
NK	*HLA-DOA*	1.34E-09	5.52E-08	0.024	0.975	0.001
NK	*IGF2BP2*	1.87E-04	0.073	0.002	0.755	0.17
NK	*KHK*	7.14E-52	2.08E-49	0.003	0.996	1.06E-04
NK	*LA16c-358B7.3*	3.42E-38	9.20E-06	3.72E-33	1	6.85E-05
NK	*LLGL1*	2.89E-06	0.003	1.87E-05	0.015	0.982
NK	*LRRC61*	6.29E-09	4.65E-05	1.34E-04	0.992	0.008
NK	*MDM4*	5.20E-06	0.148	4.46E-06	0.126	0.726
NK	*MLF1*	1.71E-12	8.27E-05	1.50E-08	0.725	0.275
NK	*MPHOSPH9*	3.89E-08	0.671	4.34E-09	0.075	0.254
NK	*NAA40*	2.52E-04	0.185	6.49E-04	0.477	0.337
NK	*NUCB2*	3.56E-50	5.97E-04	3.67E-47	0.614	0.385
NK	*OXNAD1*	3.85E-19	0.011	1.47E-18	0.04	0.949
NK	*PRELID1*	1.68E-13	0.01	1.00E-11	0.621	0.369
NK	*RAC1*	1.04E-46	0.142	6.18E-46	0.844	0.014
NK	*RP11-273G15.2*	1.45E-06	1.35E-06	0.406	0.377	0.217
NK	*RPS12*	6.63E-04	0.169	3.88E-04	0.098	0.731
NK	*RPS9*	8.88E-96	0.187	4.21E-96	0.088	0.726
NK	*SAT2*	9.32E-20	8.30E-13	1.12E-07	1	8.06E-09
NK	*SCD5*	4.58E-16	0.014	1.93E-15	0.057	0.93
NK	*SERAC1*	1.44E-05	0.86	9.00E-07	0.054	0.086

NK	*SNAP29*	2.01E-33	0.006	3.09E-32	0.097	0.897
NK	*SNRPD2*	1.24E-11	0.111	9.07E-11	0.814	0.075
NK	*ST3GAL5*	3.13E-07	0.488	1.74E-08	0.027	0.485
NK	*TBC1D12*	4.65E-14	4.99E-12	0.009	0.961	0.03
NK	*THAP9-AS1*	4.83E-16	0.006	2.03E-15	0.025	0.969
NK	*TMEM241*	5.48E-08	1.08E-04	5.08E-04	0.999	3.61E-04
NK	*UBE3B*	9.22E-07	1.86E-04	0.005	0.994	7.73E-04
NK	*VIM*	1.41E-05	0.013	5.73E-04	0.549	0.437
NK	*WARS*	3.53E-09	0.015	1.78E-07	0.733	0.252
NK	*ZNF100*	5.72E-16	2.21E-04	2.52E-12	0.974	0.026
Plasma	*HLA-DQB1*	2.21E-13	1.38E-08	1.60E-05	1	3.88E-05
Plasma	*NFKBIL1*	1.18E-19	7.13E-12	1.66E-08	1	3.72E-08
Plasma	*NUCB2*	4.52E-10	5.93E-04	4.65E-07	0.61	0.389
Plasma	*RNF146*	7.19E-28	2.63E-28	0.7	0.256	0.043
Plasma	*RXRB*	2.08E-08	4.74E-08	0.299	0.681	0.02

Table S5. Colocalization evidence for the associations between cell type-specific eGenes and hypertension (SuSiE method).

Cell type	eGene	hit1	hit2	PP.H0.abf	PP.H1.abf	PP.H2.abf	PP.H3.abf	PP.H4.abf
B IN	*FNBP4*	NA	NA	NA	NA	NA	NA	NA
B IN	*WARS*	rs8015259	rs10149438	6.68E-36	0.002	2.16E-34	0.076	0.921
B Mem	*FNBP4*	NA	NA	NA	NA	NA	NA	NA
B Mem	*MDM4*	NA	NA	NA	NA	NA	NA	NA
B Mem	*WARS*	rs8015259	rs10149438	6.87E-31	0.003	2.22E-29	0.083	0.915
CD4 ET	*CDK10*	NA	NA	NA	NA	NA	NA	NA
CD4 ET	*MDM4*	NA	NA	NA	NA	NA	NA	NA
CD4 NC	*EEF2*	NA	NA	NA	NA	NA	NA	NA
CD4 NC	*FOXN2*	NA	NA	NA	NA	NA	NA	NA
CD4 NC	*LLGL1*	rs8066387	rs1975821	2.13E-11	1.33E-04	4.87E-09	0.028	0.971
CD4 NC	*MACF1*	NA	NA	NA	NA	NA	NA	NA
CD4 NC	*PABPC4*	NA	NA	NA	NA	NA	NA	NA
CD4 NC	*VIM*	rs1980662	rs3758411	4.28E-06	4.04E-04	3.27E-04	0.029	0.97
CD4 NC	*VPS53*	NA	NA	NA	NA	NA	NA	NA
CD4 NC	*WBSCR16*	rs6955671	rs10276861	1.30E-04	0.162	6.53E-04	0.81	0.027
CD8 ET	*FOXN2*	NA	NA	NA	NA	NA	NA	NA
CD8 ET	*LLGL1*	rs8076336	rs1975821	7.96E-10	1.21E-04	1.82E-07	0.026	0.974
CD8 ET	*OXNAD1*	NA	NA	NA	NA	NA	NA	NA
CD8 ET	*SCD5*	rs4615165	rs3811792	2.22E-16	0.008	3.22E-15	0.108	0.885
CD8 ET	*THAP9-AS1*	rs4484290	rs3811792	3.76E-16	0.005	5.65E-15	0.073	0.923
CD8 ET	*VIM*	rs3758411	rs3758411	1.82E-09	2.47E-04	1.39E-07	0.017	0.983
CD8 ET	*WARS*	rs1570305	rs10149438	2.49E-06	0.006	8.02E-05	0.187	0.807
CD8 NC	*FOXN2*	NA	NA	NA	NA	NA	NA	NA
CD8 NC	*SCD5*	rs4461514	rs3811792	1.77E-07	0.004	2.56E-06	0.058	0.937
CD8 NC	*THAP9-AS1*	rs4560402	rs3811792	2.37E-10	0.004	3.56E-09	0.066	0.93
CD8 NC	*VIM*	rs4747300	rs3758411	1.23E-09	4.08E-04	9.38E-08	0.029	0.97
CD8 S100B	*EEF2*	NA	NA	NA	NA	NA	NA	NA
CD8 S100B	*SCD5*	NA	NA	NA	NA	NA	NA	NA
Mono NC	*PRELID1*	rs17078781	rs28419182	2.46E-12	0.002	2.78E-10	0.169	0.829
NK	*LLGL1*	rs8076336	rs1975821	1.90E-07	1.44E-04	4.35E-05	0.031	0.969
NK	*OXNAD1*	NA	NA	NA	NA	NA	NA	NA
NK	*SCD5*	rs4484290	rs3811792	6.96E-17	0.007	1.01E-15	0.094	0.899
NK	*SNAP29*	rs165866	rs178046	9.80E-20	0.002	1.03E-17	0.18	0.818
NK	*THAP9-AS1*	rs12506410	rs3811792	6.82E-14	0.003	1.02E-12	0.048	0.949

Table S6. MR and colocalization analyses on the cell type-specific associations between hypertension-related eGenes and hypertensive heart disease.

Cell type	eGene	Method	nSNP	OR (95% CI)	*P*-value	*FDR*	Steiger pval	Steiger direction	PP.H4.abf
B IN	*ACAT1*	Wald ratio	1	0.992 (0.907, 1.086)	0.862	0.878	1.13E-08	TRUE	0.009
B IN	*AP003774.1*	Wald ratio	1	0.94 (0.882, 1.002)	0.056	0.159	1.88E-20	TRUE	0.037
B IN	*BLK*	Wald ratio	1	0.936 (0.88, 0.995)	0.034	0.136	9.82E-23	TRUE	6.16E-04
B IN	*C10orf32*	Wald ratio	1	0.968 (0.879, 1.066)	0.51	0.618	1.47E-10	TRUE	0.009
B IN	*C6orf48*	Wald ratio	1	1.115 (1.052, 1.181)	2.54E-04	0.012	3.58E-12	TRUE	0.181
B IN	*C6orf62*	Wald ratio	1	1.083 (0.967, 1.212)	0.167	0.286	1.15E-07	TRUE	0.016
B IN	*CD164*	Wald ratio	1	0.91 (0.813, 1.02)	0.105	0.223	1.43E-07	TRUE	0.012
B IN	*CENPV*	Wald ratio	1	0.955 (0.874, 1.044)	0.309	0.452	1.32E-11	TRUE	0.009
B IN	*DDX5*	Wald ratio	1	1.078 (0.991, 1.174)	0.08	0.19	1.83E-11	TRUE	0.032
B IN	*FAM167A*	Wald ratio	1	0.931 (0.872, 0.994)	0.033	0.136	5.77E-20	TRUE	0.002
B IN	*FANCA*	Wald ratio	1	0.878 (0.777, 0.992)	0.036	0.137	1.20E-07	TRUE	0.044
B IN	*FNBP4*	Wald ratio	1	0.809 (0.735, 0.889)	1.20E-05	0.002	1.46E-09	TRUE	0.87
B IN	*GTF2H4*	IVW	3	0.95 (0.903, 0.998)	0.044	0.147	1.46E-33	TRUE	0.006
B IN	*GTF2H4*	MR Egger	3	1.159 (0.847, 1.585)	0.526	NA	NA	NA	NA
B IN	*GTF2H4*	Weighted median	3	0.951 (0.899, 1.005)	0.076	NA	NA	NA	NA
B IN	*GTF2H4*	Simple mode	3	0.921 (0.844, 1.006)	0.208	NA	NA	NA	NA
B IN	*GTF2H4*	Weighted mode	3	0.973 (0.914, 1.036)	0.481	NA	NA	NA	NA
B IN	*HAGHL*	Wald ratio	1	1.08 (0.986, 1.183)	0.098	0.216	1.50E-08	TRUE	0.036
B IN	*HSD17B11*	Wald ratio	1	1.142 (1.031, 1.265)	0.011	0.084	8.90E-08	TRUE	0.107
B IN	*LA16c-358B7.3*	Wald ratio	1	1.066 (0.982, 1.158)	0.129	0.254	3.35E-14	TRUE	0.022
B IN	*MSMP*	Wald ratio	1	0.875 (0.795, 0.962)	0.006	0.07	2.32E-09	TRUE	0.136
B IN	*NOMO1*	Wald ratio	1	0.94 (0.858, 1.031)	0.192	0.308	2.00E-12	TRUE	0.022
B IN	*PRKD3*	Wald ratio	1	1.148 (1.04, 1.268)	0.006	0.07	2.54E-09	TRUE	0.144
B IN	*RAC1*	Wald ratio	1	0.989 (0.92, 1.064)	0.767	0.821	3.58E-17	TRUE	0.008
B IN	*RP13-514E23.2*	Wald ratio	1	0.976 (0.929, 1.026)	0.339	0.476	1.55E-34	TRUE	0.014
B IN	*RPS9*	Wald ratio	1	1.029 (0.991, 1.068)	0.142	0.265	2.69E-60	TRUE	0.02
B IN	*SNRPD2*	Wald ratio	1	0.918 (0.858, 0.984)	0.015	0.087	1.72E-17	TRUE	0.133
B IN	*WARS*	Wald ratio	1	1.013 (0.969, 1.059)	0.562	0.665	5.49E-52	TRUE	0.009
B Mem	*AP003774.1*	Wald ratio	1	0.929 (0.862, 1.002)	0.057	0.161	9.18E-15	TRUE	0.035
B Mem	*BLK*	Wald ratio	1	0.949 (0.904, 0.996)	0.035	0.137	7.75E-36	TRUE	6.10E-04
B Mem	*C6orf48*	Wald ratio	1	1.105 (1.022, 1.193)	0.012	0.084	8.84E-08	TRUE	0.022
B Mem	*CCDC163P*	Wald ratio	1	1.096 (1.03, 1.167)	0.004	0.057	6.40E-20	TRUE	0.013
B Mem	*DDX5*	Wald ratio	1	1.092 (0.989, 1.206)	0.08	0.19	1.13E-08	TRUE	0.03
B Mem	*FAM167A*	Wald ratio	1	1.092 (1.01, 1.181)	0.027	0.119	1.03E-14	TRUE	5.68E-04
B Mem	*FNBP4*	Wald ratio	1	0.88 (0.799, 0.969)	0.009	0.084	9.43E-12	TRUE	0.856
B Mem	*HAGH*	Wald ratio	1	0.976 (0.897, 1.063)	0.581	0.685	5.19E-11	TRUE	0.013
B Mem	*HAGHL*	Wald ratio	1	1.077 (0.986, 1.175)	0.099	0.216	1.59E-09	TRUE	0.026
B Mem	*HIST1H2AG*	Wald ratio	1	1.03 (0.955, 1.111)	0.445	0.572	1.97E-13	TRUE	0.02
B Mem	*HIST1H2BJ*	Wald ratio	1	1.051 (0.92, 1.201)	0.466	0.593	4.90E-08	TRUE	0.019
B Mem	*HIST1H3D*	Wald ratio	1	0.966 (0.909, 1.026)	0.261	0.401	6.81E-20	TRUE	0.046
B Mem	*KRTAP5-AS1*	Wald ratio	1	0.934 (0.888, 0.982)	0.008	0.077	1.41E-32	TRUE	0.045
B Mem	*LA16c-358B7.3*	Wald ratio	1	1.061 (0.987, 1.141)	0.109	0.226	3.67E-18	TRUE	0.022
B Mem	*MDM4*	Wald ratio	1	0.953 (0.868, 1.046)	0.308	0.452	1.10E-13	TRUE	0.014
B Mem	*PRKD3*	Wald ratio	1	1.148 (1.04, 1.268)	0.006	0.07	2.37E-09	TRUE	0.12
B Mem	*RP13-514E23.2*	Wald ratio	1	0.976 (0.934, 1.019)	0.263	0.403	1.84E-44	TRUE	0.013
B Mem	*RPS9*	Wald ratio	1	1.03 (0.99, 1.073)	0.143	0.267	3.56E-53	TRUE	0.02
B Mem	*SNRPD2*	Wald ratio	1	0.916 (0.855, 0.981)	0.012	0.084	1.84E-17	TRUE	0.139
B Mem	*WARS*	Wald ratio	1	1.015 (0.964, 1.069)	0.562	0.665	7.47E-38	TRUE	0.011
B Mem	*ZNF100*	Wald ratio	1	1.135 (1.041, 1.239)	0.004	0.057	4.63E-11	TRUE	0.21
CD4 ET	*AIF1*	Wald ratio	1	1.077 (1.007, 1.152)	0.031	0.132	5.13E-13	TRUE	0.029
CD4 ET	*AP003774.1*	Wald ratio	1	0.935 (0.876, 0.997)	0.04	0.141	2.11E-19	TRUE	0.043
CD4 ET	*C10orf32*	Wald ratio	1	0.972 (0.891, 1.06)	0.522	0.624	7.75E-13	TRUE	0.007
CD4 ET	*C17orf89*	Wald ratio	1	1.09 (0.997, 1.192)	0.059	0.164	4.67E-11	TRUE	0.029
CD4 ET	*CD151*	Wald ratio	1	1.09 (1.007, 1.179)	0.032	0.136	8.22E-16	TRUE	0.065
CD4 ET	*CDK10*	Wald ratio	1	0.967 (0.877, 1.067)	0.505	0.618	1.88E-09	TRUE	0.806
CD4 ET	*CTSW*	Wald ratio	1	0.95 (0.903, 0.999)	0.047	0.148	1.55E-38	TRUE	0.041
CD4 ET	*DDX5*	Wald ratio	1	1.061 (0.993, 1.133)	0.08	0.19	3.88E-18	TRUE	0.032
CD4 ET	*FDFT1*	Wald ratio	1	1.056 (0.977, 1.142)	0.168	0.286	7.83E-18	TRUE	2.23E-04
CD4 ET	*FGF9*	Wald ratio	1	0.923 (0.836, 1.019)	0.113	0.233	1.44E-09	TRUE	0.007
CD4 ET	*FNBP4*	Wald ratio	1	0.787 (0.707, 0.876)	1.20E-05	0.002	1.15E-07	TRUE	0.844
CD4 ET	*HAGH*	Wald ratio	1	0.96 (0.887, 1.039)	0.316	0.452	2.89E-12	TRUE	0.015
CD4 ET	*LA16c-358B7.3*	Wald ratio	1	1.063 (0.981, 1.153)	0.134	0.263	6.23E-15	TRUE	0.021
CD4 ET	*MANBA*	Wald ratio	1	0.934 (0.851, 1.025)	0.152	0.279	2.50E-10	TRUE	0.02
CD4 ET	*MDM4*	Wald ratio	1	0.91 (0.807, 1.026)	0.123	0.252	4.15E-09	TRUE	0.014
CD4 ET	*PLEKHA1*	Wald ratio	1	1.089 (0.984, 1.205)	0.1	0.216	1.39E-09	TRUE	0.007
CD4 ET	*PPT2*	Wald ratio	1	1.064 (0.978, 1.158)	0.15	0.278	1.03E-10	TRUE	0.052
CD4 ET	*RNF166*	Wald ratio	1	0.873 (0.789, 0.966)	0.008	0.077	6.33E-09	TRUE	0.143
CD4 ET	*RP11-499P20.2*	Wald ratio	1	0.939 (0.839, 1.052)	0.276	0.415	1.21E-07	TRUE	0.007
CD4 ET	*RPS9*	Wald ratio	1	1.027 (0.991, 1.064)	0.142	0.265	5.19E-69	TRUE	0.02
CD4 ET	*SNRPD2*	Wald ratio	1	0.917 (0.857, 0.981)	0.012	0.084	5.85E-18	TRUE	0.139
CD4 ET	*TNFSF13B*	Wald ratio	1	0.926 (0.844, 1.015)	0.099	0.216	2.78E-11	TRUE	0.036
CD4 NC	*AC010982.1*	Wald ratio	1	0.888 (0.796, 0.991)	0.034	0.136	8.69E-08	TRUE	0.005
CD4 NC	*ACAT1*	Wald ratio	1	0.986 (0.943, 1.032)	0.543	0.647	1.15E-29	TRUE	0.009
CD4 NC	*ACOT13*	Wald ratio	1	0.942 (0.835, 1.062)	0.327	0.465	4.14E-08	TRUE	0.016

CD4 NC	*AIF1*	Wald ratio	1	1.047 (1.008, 1.088)	0.017	0.096	9.01E-33	TRUE	0.027
CD4 NC	*AP003774.1*	Wald ratio	1	0.969 (0.937, 1.001)	0.056	0.159	2.44E-79	TRUE	0.038
CD4 NC	*AP2A2*	Wald ratio	1	1.132 (1.02, 1.256)	0.02	0.101	3.09E-08	TRUE	0.084
CD4 NC	*BIK*	Wald ratio	1	0.892 (0.805, 0.99)	0.031	0.132	2.85E-11	TRUE	0.126
CD4 NC	*BLK*	Wald ratio	1	0.947 (0.888, 1.01)	0.099	0.216	5.65E-21	TRUE	2.47E-04
CD4 NC	*BLMH*	Wald ratio	1	0.885 (0.794, 0.986)	0.027	0.119	7.25E-08	TRUE	0.073
CD4 NC	*C10orf32*	Wald ratio	1	0.988 (0.941, 1.038)	0.635	0.717	1.80E-38	TRUE	0.008
CD4 NC	*C2orf74*	Wald ratio	1	0.939 (0.861, 1.024)	0.154	0.283	1.09E-11	TRUE	0.021
CD4 NC	*C6orf48*	Wald ratio	1	1.062 (1.015, 1.111)	0.01	0.084	4.14E-21	TRUE	0.018
CD4 NC	*C6orf62*	Wald ratio	1	1.078 (0.969, 1.199)	0.167	0.286	1.97E-08	TRUE	0.017
CD4 NC	*CCDC104*	Wald ratio	1	1.153 (1.061, 1.254)	8.27E-04	0.018	7.04E-14	TRUE	0.213
CD4 NC	*CCDC88B*	Wald ratio	1	1.056 (0.988, 1.129)	0.107	0.226	6.47E-19	TRUE	0.019
CD4 NC	*CD151*	Wald ratio	1	1.069 (0.997, 1.147)	0.061	0.164	5.90E-19	TRUE	0.046
CD4 NC	*CDK10*	Wald ratio	1	0.98 (0.922, 1.041)	0.505	0.618	1.19E-22	TRUE	0.825
CD4 NC	*CLNS1A*	Wald ratio	1	1.093 (1.037, 1.151)	8.95E-04	0.018	6.55E-17	TRUE	0.548
CD4 NC	*CLUAP1*	Wald ratio	1	1.084 (1.012, 1.161)	0.022	0.103	3.58E-18	TRUE	0.057
CD4 NC	*CRHR1*	Wald ratio	1	1.073 (0.945, 1.218)	0.275	0.415	4.91E-16	TRUE	0.015
CD4 NC	*CTD-2336O2.1*	Wald ratio	1	1.025 (0.988, 1.065)	0.191	0.308	5.90E-65	TRUE	0.017
CD4 NC	*CTSW*	Wald ratio	1	0.969 (0.94, 1)	0.047	0.148	5.34E-113	TRUE	0.041
CD4 NC	*CUBN*	Wald ratio	1	1.071 (0.986, 1.163)	0.103	0.22	1.31E-14	TRUE	0.03
CD4 NC	*CYP2D6*	Wald ratio	1	0.925 (0.831, 1.031)	0.158	0.283	4.57E-08	TRUE	0.019
CD4 NC	*DDX5*	Wald ratio	1	1.051 (0.994, 1.111)	0.08	0.19	1.42E-25	TRUE	0.032
CD4 NC	*EEF2*	Wald ratio	1	1.137 (1.027, 1.26)	0.013	0.087	1.48E-08	TRUE	0.127
CD4 NC	*EGFL8*	Wald ratio	1	1.025 (0.935, 1.124)	0.598	0.693	5.12E-11	TRUE	0.001
CD4 NC	*ERP29*	Wald ratio	1	1 (0.927, 1.078)	0.995	0.995	1.64E-13	TRUE	0.01
CD4 NC	*FAM153B*	Wald ratio	1	1.034 (1.002, 1.067)	0.035	0.137	5.69E-91	TRUE	0.046
CD4 NC	*FANCA*	Wald ratio	1	0.919 (0.832, 1.013)	0.09	0.209	3.80E-11	TRUE	0.034
CD4 NC	*FDFT1*	Wald ratio	1	1.026 (0.989, 1.064)	0.168	0.286	1.01E-81	TRUE	2.23E-04
CD4 NC	*FNBP4*	Wald ratio	1	0.907 (0.862, 0.954)	1.46E-04	0.011	1.78E-34	TRUE	0.809
CD4 NC	*FOXN2*	Wald ratio	1	1.033 (0.954, 1.119)	0.42	0.547	2.91E-20	TRUE	0.016
CD4 NC	*GOSR2*	Wald ratio	1	1.066 (0.994, 1.143)	0.073	0.185	9.16E-18	TRUE	0.004
CD4 NC	*HAGH*	Wald ratio	1	0.982 (0.948, 1.017)	0.316	0.452	3.85E-59	TRUE	0.01
CD4 NC	*HLA-DQA1*	Wald ratio	1	1.138 (1.036, 1.249)	0.007	0.071	4.40E-10	TRUE	0.014
CD4 NC	*HLA-DRB5*	Wald ratio	1	1.079 (1.024, 1.137)	0.004	0.057	1.21E-38	TRUE	0.079
CD4 NC	*HMBS*	Wald ratio	1	1.062 (0.992, 1.137)	0.084	0.196	4.80E-21	TRUE	0.031
CD4 NC	*HSD17B11*	Wald ratio	1	1.105 (1.023, 1.193)	0.011	0.084	4.10E-13	TRUE	0.09
CD4 NC	*HSD17B12*	Wald ratio	1	0.934 (0.883, 0.987)	0.015	0.089	3.15E-26	TRUE	0.109
CD4 NC	*KB-1507C5.2*	Wald ratio	1	1.097 (1.006, 1.195)	0.036	0.137	3.84E-11	TRUE	0.041
CD4 NC	*KRTAP5-2*	Wald ratio	1	0.946 (0.897, 0.997)	0.04	0.141	1.69E-31	TRUE	2.07E-06
CD4 NC	*LA16c-358B7.3*	Wald ratio	1	1.03 (0.992, 1.069)	0.129	0.254	1.77E-67	TRUE	0.021
CD4 NC	*LAIR2*	Wald ratio	1	0.959 (0.89, 1.033)	0.273	0.414	1.03E-21	TRUE	0.021
CD4 NC	*LIMD2*	Wald ratio	1	1.015 (0.905, 1.137)	0.801	0.836	1.09E-08	TRUE	0.008
CD4 NC	*LLGL1*	Wald ratio	1	1.038 (0.954, 1.129)	0.39	0.524	8.91E-13	TRUE	0.01
CD4 NC	*LRRC61*	Wald ratio	1	0.959 (0.913, 1.007)	0.094	0.214	3.80E-31	TRUE	0.026
CD4 NC	*LSM4*	Wald ratio	1	0.892 (0.831, 0.957)	0.002	0.027	2.56E-11	TRUE	0.246
CD4 NC	*MACF1*	Wald ratio	1	1.025 (0.957, 1.097)	0.483	0.612	1.06E-17	TRUE	0.013
CD4 NC	*MARK3*	Wald ratio	1	0.929 (0.85, 1.016)	0.106	0.224	1.78E-11	TRUE	0.025
CD4 NC	*MEI1*	Wald ratio	1	1.085 (1.004, 1.173)	0.039	0.141	1.45E-12	TRUE	0.085
CD4 NC	*MRPS34*	Wald ratio	1	0.964 (0.864, 1.075)	0.512	0.618	4.79E-08	TRUE	0.01
CD4 NC	*MYO16*	Wald ratio	1	0.956 (0.906, 1.009)	0.099	0.216	1.61E-31	TRUE	0.022
CD4 NC	*NARFL*	Wald ratio	1	1.083 (1.001, 1.172)	0.048	0.148	2.24E-10	TRUE	0.049
CD4 NC	*NEIL2*	Wald ratio	1	1.078 (0.969, 1.199)	0.168	0.286	5.26E-10	TRUE	0.176
CD4 NC	*OSER1-AS1*	Wald ratio	1	1.056 (0.979, 1.14)	0.16	0.285	8.53E-14	TRUE	0.024
CD4 NC	*PABPC4*	Wald ratio	1	0.946 (0.843, 1.062)	0.347	0.485	4.98E-08	TRUE	0.011
CD4 NC	*PCNX*	Wald ratio	1	1.012 (0.95, 1.078)	0.711	0.783	9.38E-22	TRUE	0.009
CD4 NC	*PGPEP1*	Wald ratio	1	1.041 (0.952, 1.139)	0.376	0.512	1.38E-12	TRUE	0.009
CD4 NC	*PHACTR4*	Wald ratio	1	1.011 (0.947, 1.08)	0.733	0.803	1.60E-19	TRUE	0.008
CD4 NC	*PKMYT1*	Wald ratio	1	0.952 (0.901, 1.005)	0.073	0.185	4.22E-25	TRUE	0.025
CD4 NC	*PRKD3*	Wald ratio	1	1.126 (1.034, 1.226)	0.006	0.07	2.38E-12	TRUE	0.12
CD4 NC	*RAC1*	Wald ratio	1	0.994 (0.956, 1.034)	0.767	0.821	6.60E-57	TRUE	0.007
CD4 NC	*REEP3*	Wald ratio	1	1.12 (0.994, 1.263)	0.062	0.164	1.52E-07	TRUE	0.044
CD4 NC	*RNF8*	Wald ratio	1	1.013 (0.906, 1.133)	0.815	0.837	2.15E-08	TRUE	0.014
CD4 NC	*RP11-273G15.2*	Wald ratio	1	1.084 (1.012, 1.161)	0.021	0.101	9.14E-18	TRUE	0.15
CD4 NC	*RP11-796G6.2*	Wald ratio	1	1.294 (1.103, 1.516)	0.002	0.027	4.00E-08	TRUE	0.6
CD4 NC	*RPS9*	Wald ratio	1	1.023 (0.992, 1.055)	0.142	0.265	9.82E-97	TRUE	0.02
CD4 NC	*SAT2*	Wald ratio	1	0.936 (0.88, 0.996)	0.036	0.137	1.06E-22	TRUE	0.045
CD4 NC	*SLC22A5*	Wald ratio	1	1.045 (0.941, 1.16)	0.414	0.543	1.42E-08	TRUE	0.008
CD4 NC	*SNRPC*	Wald ratio	1	0.947 (0.894, 1.004)	0.066	0.172	3.02E-25	TRUE	0.03
CD4 NC	*SNRPD2*	Wald ratio	1	0.957 (0.925, 0.99)	0.012	0.084	3.44E-72	TRUE	0.14
CD4 NC	*SNX16*	Wald ratio	1	0.996 (0.907, 1.093)	0.933	0.942	1.71E-12	TRUE	0.008
CD4 NC	*SPCS1*	Wald ratio	1	1.037 (0.955, 1.127)	0.385	0.522	5.35E-13	TRUE	0.009
CD4 NC	*TMEM199*	Wald ratio	1	1.007 (0.914, 1.109)	0.892	0.906	1.88E-10	TRUE	0.007
CD4 NC	*TMEM87B*	Wald ratio	1	1.068 (0.971, 1.175)	0.176	0.294	5.50E-11	TRUE	0.015
CD4 NC	*TMEM97*	Wald ratio	1	1.003 (0.915, 1.099)	0.955	0.957	2.60E-11	TRUE	0.008
CD4 NC	*TNFRSF18*	Wald ratio	1	1.109 (0.998, 1.232)	0.053	0.159	1.85E-07	TRUE	0.038
CD4 NC	*TTC12*	Wald ratio	1	1.026 (0.933, 1.128)	0.592	0.688	1.55E-10	TRUE	0.007
CD4 NC	*UACA*	Wald ratio	1	1.155 (1.052, 1.267)	0.002	0.039	1.31E-08	TRUE	0.616
CD4 NC	*UBE2I*	Wald ratio	1	1.039 (0.931, 1.159)	0.491	0.614	8.44E-11	TRUE	0.014
CD4 NC	*UBE3B*	Wald ratio	1	1.074 (0.966, 1.194)	0.184	0.303	6.34E-10	TRUE	0.038

CD4 NC	*USP36*	Wald ratio	1	1.066 (0.971, 1.17)	0.18	0.299	4.92E-11	TRUE	0.012
CD4 NC	*VIM*	Wald ratio	1	1.074 (0.967, 1.193)	0.181	0.301	4.78E-08	TRUE	0.02
CD4 NC	*VPS53*	Wald ratio	1	0.888 (0.81, 0.975)	0.012	0.084	1.01E-10	TRUE	0.152
CD4 NC	*WBSCR16*	Wald ratio	1	0.896 (0.805, 0.996)	0.042	0.144	1.27E-08	TRUE	0.034
CD4 NC	*ZNF100*	Wald ratio	1	0.924 (0.884, 0.966)	4.90E-04	0.013	2.57E-41	TRUE	0.265
CD4 NC	*ZNF502*	Wald ratio	1	0.949 (0.903, 0.997)	0.037	0.138	9.44E-34	TRUE	0.064
CD4 NC	*ZSWIM7*	Wald ratio	1	1.024 (0.953, 1.101)	0.513	0.618	1.98E-17	TRUE	0.008
CD4 SOX4	*DAXX*	Wald ratio	1	0.976 (0.87, 1.094)	0.671	0.745	1.88E-08	TRUE	0.015
CD4 SOX4	*DXO*	Wald ratio	1	1.01 (0.938, 1.088)	0.785	0.83	2.90E-15	TRUE	0.009
CD4 SOX4	*RPS26*	Wald ratio	1	0.997 (0.974, 1.021)	0.802	0.836	2.60E-166	TRUE	0.006
CD4 SOX4	*RPS9*	Wald ratio	1	1.062 (0.977, 1.155)	0.158	0.283	1.33E-11	TRUE	0.021
CD8 ET	*ADRB1*	Wald ratio	1	0.966 (0.903, 1.033)	0.312	0.452	4.89E-15	TRUE	0.01
CD8 ET	*AP003774.1*	Wald ratio	1	0.96 (0.924, 0.998)	0.04	0.141	8.26E-54	TRUE	0.046
CD8 ET	*AP2A2*	Wald ratio	1	1.116 (1.017, 1.224)	0.02	0.101	2.75E-10	TRUE	0.084
CD8 ET	*BTN3A1*	Wald ratio	1	1.034 (0.942, 1.134)	0.487	0.612	5.98E-10	TRUE	0.007
CD8 ET	*C10orf32*	Wald ratio	1	0.983 (0.917, 1.054)	0.635	0.717	1.72E-19	TRUE	0.01
CD8 ET	*C2orf74*	Wald ratio	1	1.074 (0.982, 1.174)	0.12	0.246	9.78E-11	TRUE	0.027
CD8 ET	*C6orf62*	Wald ratio	1	1.083 (0.967, 1.212)	0.167	0.286	1.24E-07	TRUE	0.016
CD8 ET	*CCDC88B*	Wald ratio	1	1.043 (0.94, 1.157)	0.428	0.556	1.16E-08	TRUE	0.013
CD8 ET	*CDK10*	Wald ratio	1	0.974 (0.9, 1.053)	0.505	0.618	3.55E-14	TRUE	0.831
CD8 ET	*CTD-2515A14.1*	Wald ratio	1	0.896 (0.805, 0.996)	0.042	0.144	5.12E-08	TRUE	0.049
CD8 ET	*CTSW*	Wald ratio	1	0.969 (0.939, 1)	0.047	0.148	4.80E-107	TRUE	0.041
CD8 ET	*DDX5*	Wald ratio	1	1.051 (0.994, 1.112)	0.08	0.19	6.05E-25	TRUE	0.032
CD8 ET	*DGKD*	Wald ratio	1	1.107 (0.991, 1.236)	0.072	0.185	1.24E-07	TRUE	0.023
CD8 ET	*FDFT1*	Wald ratio	1	1.067 (0.973, 1.169)	0.168	0.286	3.90E-13	TRUE	2.23E-04
CD8 ET	*FIBP*	Wald ratio	1	1.146 (1.028, 1.277)	0.014	0.087	3.49E-09	TRUE	0.059
CD8 ET	*FNBP4*	Wald ratio	1	0.861 (0.796, 0.931)	1.71E-04	0.011	1.40E-14	TRUE	0.851
CD8 ET	*FOXN2*	Wald ratio	1	1.058 (0.952, 1.176)	0.297	0.439	5.29E-11	TRUE	0.016
CD8 ET	*HAGH*	Wald ratio	1	0.986 (0.93, 1.046)	0.646	0.722	7.20E-22	TRUE	0.016
CD8 ET	*HLA-DQB2*	Wald ratio	1	1.035 (0.962, 1.113)	0.356	0.495	5.01E-18	TRUE	0.001
CD8 ET	*LA16c-358B7.3*	Wald ratio	1	1.045 (0.987, 1.106)	0.129	0.254	2.95E-29	TRUE	0.021
CD8 ET	*LAIR2*	Wald ratio	1	0.942 (0.886, 1.003)	0.061	0.164	2.61E-29	TRUE	0.061
CD8 ET	*LLGL1*	Wald ratio	1	1.042 (0.951, 1.142)	0.374	0.512	4.04E-11	TRUE	0.011
CD8 ET	*LRRC37A*	Wald ratio	1	0.934 (0.884, 0.987)	0.015	0.087	2.33E-23	TRUE	0.308
CD8 ET	*LRRC61*	Wald ratio	1	0.975 (0.879, 1.081)	0.627	0.716	9.58E-09	TRUE	0.018
CD8 ET	*MDM4*	Wald ratio	1	0.959 (0.872, 1.055)	0.391	0.524	6.58E-13	TRUE	0.01
CD8 ET	*MLF1*	Wald ratio	1	1.142 (1.062, 1.228)	3.52E-04	0.012	2.53E-12	TRUE	0.681
CD8 ET	*MPHOSPH9*	Wald ratio	1	1.019 (0.911, 1.141)	0.737	0.804	3.53E-08	TRUE	0.01
CD8 ET	*NOTCH4*	Wald ratio	1	1.02 (0.907, 1.146)	0.744	0.809	3.77E-09	TRUE	0.005
CD8 ET	*NUCB2*	Wald ratio	1	0.957 (0.922, 0.993)	0.021	0.101	1.28E-61	TRUE	0.069
CD8 ET	*OXNAD1*	Wald ratio	1	0.928 (0.859, 1.002)	0.056	0.159	3.44E-15	TRUE	0.045
CD8 ET	*PDCD6*	Wald ratio	1	1.012 (0.915, 1.118)	0.82	0.84	1.70E-09	TRUE	0.008
CD8 ET	*PHACTR4*	Wald ratio	1	1.026 (0.93, 1.132)	0.608	0.702	2.54E-09	TRUE	0.009
CD8 ET	*REEP5*	Wald ratio	1	1.047 (0.947, 1.158)	0.368	0.507	1.61E-09	TRUE	0.013
CD8 ET	*RP4-799D16.1*	Wald ratio	1	1.067 (0.968, 1.175)	0.192	0.308	2.08E-09	TRUE	0.016
CD8 ET	*RPS9*	Wald ratio	1	1.023 (0.992, 1.055)	0.142	0.265	3.60E-96	TRUE	0.02
CD8 ET	*SAT2*	Wald ratio	1	0.916 (0.843, 0.996)	0.04	0.141	6.82E-13	TRUE	0.041
CD8 ET	*SCD5*	Wald ratio	1	0.992 (0.926, 1.062)	0.811	0.837	8.47E-20	TRUE	0.008
CD8 ET	*SNRPC*	Wald ratio	1	0.9 (0.806, 1.005)	0.062	0.164	1.33E-07	TRUE	0.032
CD8 ET	*SNRPD2*	Wald ratio	1	0.942 (0.899, 0.987)	0.012	0.084	3.84E-37	TRUE	0.143
CD8 ET	*THAP9-AS1*	Wald ratio	1	1.009 (0.943, 1.079)	0.803	0.836	6.76E-20	TRUE	0.009
CD8 ET	*VIM*	Wald ratio	1	1.069 (0.978, 1.167)	0.14	0.265	5.86E-11	TRUE	0.018
CD8 ET	*WARS*	Wald ratio	1	1.057 (0.944, 1.184)	0.336	0.474	1.77E-08	TRUE	0.012
CD8 ET	*XBP1*	Wald ratio	1	1.118 (1.019, 1.227)	0.018	0.096	1.56E-09	TRUE	0.084
CD8 ET	*ZNF100*	Wald ratio	1	0.876 (0.813, 0.944)	4.72E-04	0.013	9.17E-15	TRUE	0.262
CD8 ET	*ZNF502*	Wald ratio	1	0.9 (0.815, 0.994)	0.037	0.138	5.92E-09	TRUE	0.059
CD8 ET	*ZSWIM7*	Wald ratio	1	1.028 (0.947, 1.116)	0.513	0.618	1.17E-13	TRUE	0.008
CD8 NC	*AP003774.1*	Wald ratio	1	0.954 (0.91, 1.001)	0.056	0.159	7.99E-36	TRUE	0.037
CD8 NC	*BTN3A1*	Wald ratio	1	1.032 (0.945, 1.126)	0.486	0.612	6.71E-11	TRUE	0.007
CD8 NC	*C6orf48*	Wald ratio	1	1.075 (1.016, 1.138)	0.012	0.084	9.71E-14	TRUE	0.018
CD8 NC	*CEBPZ*	Wald ratio	1	1.174 (1.051, 1.311)	0.004	0.057	1.79E-07	TRUE	0.25
CD8 NC	*CLNS1A*	Wald ratio	1	1.143 (1.056, 1.236)	8.85E-04	0.018	4.60E-08	TRUE	0.543
CD8 NC	*CLUAP1*	Wald ratio	1	1.062 (0.958, 1.178)	0.25	0.386	6.32E-09	TRUE	0.028
CD8 NC	*COX14*	Wald ratio	1	0.939 (0.846, 1.043)	0.241	0.378	2.28E-09	TRUE	0.007
CD8 NC	*CRHR1*	Wald ratio	1	0.94 (0.814, 1.085)	0.395	0.528	4.98E-13	TRUE	0.016
CD8 NC	*CTD-2336O2.1*	Wald ratio	1	1.051 (0.992, 1.113)	0.092	0.211	2.04E-29	TRUE	0.025
CD8 NC	*CTSW*	Wald ratio	1	0.969 (0.939, 1)	0.047	0.148	4.52E-107	TRUE	0.041
CD8 NC	*DCP2*	Wald ratio	1	1.059 (0.967, 1.16)	0.216	0.34	1.41E-09	TRUE	0.014
CD8 NC	*DDR1*	IVW	2	1.003 (0.924, 1.089)	0.939	0.945	3.66E-28	TRUE	0.004
CD8 NC	*DDX5*	Wald ratio	1	1.061 (0.993, 1.133)	0.08	0.19	3.13E-18	TRUE	0.032
CD8 NC	*FNBP4*	Wald ratio	1	0.842 (0.771, 0.92)	1.46E-04	0.011	1.91E-11	TRUE	0.812
CD8 NC	*FOXN2*	Wald ratio	1	1.054 (0.929, 1.196)	0.416	0.545	9.92E-09	TRUE	0.016
CD8 NC	*HAGH*	Wald ratio	1	0.987 (0.935, 1.043)	0.646	0.722	6.93E-25	TRUE	0.01
CD8 NC	*HLA-DRB5*	Wald ratio	1	1.086 (1.032, 1.144)	0.002	0.028	8.56E-38	TRUE	0.096
CD8 NC	*HSD17B12*	Wald ratio	1	0.886 (0.8, 0.981)	0.02	0.101	1.94E-08	TRUE	0.094
CD8 NC	*LA16c-358B7.3*	Wald ratio	1	1.045 (0.987, 1.107)	0.129	0.254	1.68E-28	TRUE	0.021
CD8 NC	*LAIR2*	Wald ratio	1	0.887 (0.782, 1.005)	0.061	0.164	7.83E-08	TRUE	0.05
CD8 NC	*LRRC37A*	Wald ratio	1	0.921 (0.863, 0.982)	0.012	0.084	2.07E-17	TRUE	0.119
CD8 NC	*LRRC61*	Wald ratio	1	0.921 (0.839, 1.011)	0.082	0.193	3.52E-09	TRUE	0.021

CD8 NC	*MPHOSPH9*	Wald ratio	1	1.009 (0.923, 1.105)	0.838	0.856	5.89E-12	TRUE	0.01
CD8 NC	*MYO16*	Wald ratio	1	1.056 (1, 1.115)	0.051	0.156	6.82E-31	TRUE	0.029
CD8 NC	*PHACTR4*	Wald ratio	1	1.016 (0.922, 1.12)	0.75	0.812	7.76E-10	TRUE	0.009
CD8 NC	*RP11-273G15.2*	Wald ratio	1	0.879 (0.791, 0.977)	0.017	0.096	6.44E-08	TRUE	0.25
CD8 NC	*RPS9*	Wald ratio	1	1.024 (0.991, 1.058)	0.158	0.283	5.65E-85	TRUE	0.02
CD8 NC	*SAT2*	Wald ratio	1	0.892 (0.804, 0.989)	0.031	0.132	1.58E-08	TRUE	0.053
CD8 NC	*SCD5*	Wald ratio	1	0.971 (0.872, 1.081)	0.587	0.688	9.91E-10	TRUE	0.009
CD8 NC	*SNRPC*	Wald ratio	1	0.916 (0.836, 1.004)	0.06	0.164	1.37E-10	TRUE	0.031
CD8 NC	*SNRPD2*	Wald ratio	1	0.947 (0.907, 0.99)	0.015	0.087	4.19E-43	TRUE	0.13
CD8 NC	*STK32C*	Wald ratio	1	1.106 (0.953, 1.283)	0.186	0.303	2.92E-08	TRUE	0.015
CD8 NC	*THAP9-AS1*	Wald ratio	1	1.026 (0.936, 1.124)	0.59	0.688	5.77E-13	TRUE	0.009
CD8 NC	*USP36*	Wald ratio	1	1.083 (0.965, 1.214)	0.175	0.294	1.19E-07	TRUE	0.013
CD8 NC	*VIM*	Wald ratio	1	1.062 (0.972, 1.16)	0.183	0.302	6.17E-11	TRUE	0.017
CD8 NC	*ZNF100*	Wald ratio	1	0.868 (0.802, 0.939)	4.34E-04	0.013	3.46E-13	TRUE	0.267
CD8 NC	*ZSWIM7*	Wald ratio	1	1.036 (0.932, 1.15)	0.513	0.618	6.81E-09	TRUE	0.008
CD8 S100B	*AP003774.1*	Wald ratio	1	0.921 (0.846, 1.002)	0.056	0.159	5.60E-12	TRUE	0.036
CD8 S100B	*C10orf32*	Wald ratio	1	0.968 (0.875, 1.07)	0.522	0.624	6.03E-10	TRUE	0.008
CD8 S100B	*CD151*	Wald ratio	1	1.12 (1.002, 1.253)	0.046	0.148	4.60E-08	TRUE	0.059
CD8 S100B	*CTSW*	Wald ratio	1	0.96 (0.923, 1)	0.047	0.148	3.60E-63	TRUE	0.047
CD8 S100B	*DDX5*	Wald ratio	1	1.076 (0.991, 1.168)	0.08	0.19	3.55E-12	TRUE	0.032
CD8 S100B	*EEF2*	Wald ratio	1	1.142 (1.028, 1.268)	0.013	0.087	3.89E-08	TRUE	0.11
CD8 S100B	*FAM85A*	Wald ratio	1	0.953 (0.904, 1.005)	0.075	0.189	3.88E-29	TRUE	0.317
CD8 S100B	*FDFT1*	Wald ratio	1	1.083 (0.967, 1.213)	0.168	0.286	6.03E-09	TRUE	3.73E-04
CD8 S100B	*HSPA1L*	Wald ratio	1	0.989 (0.919, 1.065)	0.778	0.825	1.03E-22	TRUE	0.001
CD8 S100B	*KIAA0040*	Wald ratio	1	1.025 (0.922, 1.14)	0.647	0.722	1.20E-08	TRUE	0.009
CD8 S100B	*MDM4*	Wald ratio	1	0.954 (0.856, 1.064)	0.402	0.534	3.90E-10	TRUE	0.01
CD8 S100B	*RPS12*	Wald ratio	1	1.095 (1.001, 1.199)	0.048	0.148	4.82E-10	TRUE	0.029
CD8 S100B	*RPS9*	Wald ratio	1	1.031 (0.99, 1.075)	0.142	0.265	8.45E-51	TRUE	0.02
CD8 S100B	*SCD5*	Wald ratio	1	1.009 (0.934, 1.091)	0.811	0.837	1.07E-15	TRUE	0.009
CD8 S100B	*SNRPD2*	Wald ratio	1	0.901 (0.828, 0.98)	0.015	0.087	8.21E-12	TRUE	0.136
CD8 S100B	*TNFSF13B*	Wald ratio	1	0.908 (0.812, 1.016)	0.094	0.213	9.36E-08	TRUE	0.033
CD8 S100B	*TTC12*	Wald ratio	1	1.009 (0.95, 1.072)	0.771	0.822	2.99E-24	TRUE	0.007
CD8 S100B	*XBP1*	Wald ratio	1	1.132 (1.018, 1.259)	0.023	0.105	1.48E-07	TRUE	0.076
DC	*ATAT1*	Wald ratio	1	1.029 (0.954, 1.11)	0.462	0.591	9.48E-14	TRUE	0.006
DC	*DDX5*	Wald ratio	1	1.098 (0.989, 1.219)	0.08	0.19	8.90E-08	TRUE	0.032
DC	*MICB*	Wald ratio	1	1.029 (0.957, 1.106)	0.442	0.57	3.18E-16	TRUE	0.004
Mono C	*AP003774.1*	Wald ratio	1	1.088 (0.998, 1.185)	0.056	0.159	1.86E-11	TRUE	0.028
Mono C	*C10orf32*	Wald ratio	1	0.967 (0.876, 1.068)	0.51	0.618	6.36E-10	TRUE	0.007
Mono C	*HP*	Wald ratio	1	0.929 (0.88, 0.98)	0.007	0.071	1.83E-27	TRUE	0.219
Mono C	*PRELID1*	Wald ratio	1	1.119 (1.053, 1.19)	3.19E-04	0.012	6.91E-19	TRUE	0.808
Mono C	*RPS9*	Wald ratio	1	1.036 (0.988, 1.087)	0.142	0.265	5.95E-37	TRUE	0.02
Mono C	*TREML1*	Wald ratio	1	1.018 (0.976, 1.061)	0.403	0.534	1.61E-40	TRUE	0.014
Mono NC	*NCR3*	Wald ratio	1	0.982 (0.915, 1.054)	0.612	0.705	5.45E-19	TRUE	0.003
Mono NC	*PRELID1*	Wald ratio	1	1.119 (1.047, 1.195)	8.86E-04	0.018	5.45E-14	TRUE	0.642
Mono NC	*RPH3A*	Wald ratio	1	0.956 (0.878, 1.04)	0.294	0.436	9.33E-13	TRUE	0.014
Mono NC	*RPS26*	Wald ratio	1	0.997 (0.974, 1.02)	0.802	0.836	5.01E-184	TRUE	0.006
Mono NC	*RPS9*	Wald ratio	1	1.054 (0.983, 1.131)	0.142	0.265	6.94E-17	TRUE	0.02
Mono NC	*VPS52*	Wald ratio	1	1.034 (0.95, 1.125)	0.44	0.569	9.36E-12	TRUE	0.014
NK R	*AC016831.7*	Wald ratio	1	0.972 (0.914, 1.033)	0.362	0.501	3.92E-33	TRUE	0.004
NK R	*APOM*	Wald ratio	1	1.056 (0.963, 1.159)	0.246	0.384	2.13E-11	TRUE	0.003
NK R	*CTSW*	Wald ratio	1	0.88 (0.794, 0.974)	0.014	0.087	4.43E-10	TRUE	0.075
NK R	*RAC1*	Wald ratio	1	0.934 (0.841, 1.037)	0.2	0.319	3.27E-08	TRUE	0.011
NK R	*SYNGAP1*	Wald ratio	1	0.988 (0.919, 1.063)	0.751	0.812	8.38E-17	TRUE	0.067
NK R	*XXbac-BPG299F13.17*	Wald ratio	1	0.91 (0.825, 1.002)	0.055	0.159	1.47E-09	TRUE	0.008
NK	*ADRB1*	Wald ratio	1	0.968 (0.91, 1.031)	0.312	0.452	2.90E-17	TRUE	0.01
NK	*AP003774.1*	Wald ratio	1	0.966 (0.932, 1.002)	0.065	0.17	2.09E-62	TRUE	0.036
NK	*AP2A2*	Wald ratio	1	1.075 (1.011, 1.144)	0.021	0.101	8.43E-22	TRUE	0.089
NK	*ATP5G1*	Wald ratio	1	0.956 (0.881, 1.037)	0.279	0.416	1.51E-13	TRUE	0.033
NK	*BTN3A1*	Wald ratio	1	1.038 (0.935, 1.152)	0.487	0.612	3.53E-08	TRUE	0.008
NK	*C10orf32*	Wald ratio	1	0.982 (0.912, 1.058)	0.635	0.717	1.57E-17	TRUE	0.008
NK	*C6orf62*	Wald ratio	1	1.077 (0.969, 1.198)	0.167	0.286	1.49E-08	TRUE	0.017
NK	*CCDC88B*	Wald ratio	1	1.059 (0.961, 1.168)	0.248	0.385	1.61E-09	TRUE	0.012
NK	*CD151*	Wald ratio	1	1.054 (0.998, 1.114)	0.061	0.164	4.16E-30	TRUE	0.042
NK	*CDK10*	Wald ratio	1	0.976 (0.909, 1.048)	0.505	0.618	5.88E-17	TRUE	0.012
NK	*CTSW*	Wald ratio	1	0.965 (0.931, 1)	0.047	0.148	1.83E-80	TRUE	0.041
NK	*DDX42*	Wald ratio	1	1.02 (0.916, 1.136)	0.715	0.785	9.62E-10	TRUE	0.008
NK	*DDX5*	Wald ratio	1	1.049 (0.994, 1.107)	0.08	0.19	4.48E-27	TRUE	0.032
NK	*EPHX4*	Wald ratio	1	1.071 (0.964, 1.19)	0.201	0.32	3.45E-09	TRUE	0.032
NK	*ERAP1*	Wald ratio	1	0.863 (0.775, 0.961)	0.007	0.071	5.23E-08	TRUE	0.185
NK	*FIBP*	Wald ratio	1	1.073 (1.001, 1.15)	0.047	0.148	1.20E-20	TRUE	0.04
NK	*GNGT2*	Wald ratio	1	1.014 (0.923, 1.113)	0.778	0.825	3.96E-10	TRUE	0.008
NK	*HAGH*	Wald ratio	1	0.969 (0.911, 1.031)	0.316	0.452	1.52E-19	TRUE	0.011
NK	*HLA-DOA*	Wald ratio	1	0.975 (0.866, 1.097)	0.671	0.745	5.52E-09	TRUE	0.017
NK	*IGF2BP2*	Wald ratio	1	1.123 (1.015, 1.242)	0.024	0.111	7.75E-08	TRUE	0.076
NK	*KHK*	Wald ratio	1	0.872 (0.779, 0.977)	0.018	0.096	2.11E-07	TRUE	1.05E-04
NK	*LA16c-358B7.3*	Wald ratio	1	1.039 (0.989, 1.091)	0.129	0.254	1.97E-38	TRUE	0.021
NK	*LLGL1*	Wald ratio	1	1.049 (0.943, 1.167)	0.374	0.512	1.77E-08	TRUE	0.012
NK	*LRRC61*	Wald ratio	1	0.976 (0.883, 1.078)	0.629	0.716	2.49E-09	TRUE	0.021
NK	*MDM4*	Wald ratio	1	0.952 (0.851, 1.065)	0.391	0.524	1.50E-09	TRUE	0.01

NK	*MLF1*	Wald ratio	1	1.144 (1.063, 1.23)	2.92E-04	0.012	9.30E-13	TRUE	0.69
NK	*MPHOSPH9*	Wald ratio	1	1.01 (0.931, 1.095)	0.814	0.837	5.47E-13	TRUE	0.009
NK	*NAA40*	Wald ratio	1	0.954 (0.852, 1.068)	0.411	0.542	8.76E-08	TRUE	0.016
NK	*NUCB2*	Wald ratio	1	0.952 (0.914, 0.992)	0.018	0.096	1.58E-50	TRUE	0.068
NK	*OXNAD1*	Wald ratio	1	0.958 (0.901, 1.019)	0.171	0.29	1.27E-22	TRUE	0.021
NK	*PRELID1*	Wald ratio	1	1.133 (1.06, 1.21)	2.14E-04	0.012	1.13E-16	TRUE	0.828
NK	*RAC1*	Wald ratio	1	0.994 (0.953, 1.036)	0.767	0.821	6.29E-51	TRUE	0.008
NK	*RP11-273G15.2*	Wald ratio	1	0.958 (0.91, 1.008)	0.096	0.216	4.23E-34	TRUE	0.039
NK	*RPS12*	Wald ratio	1	1.113 (1.01, 1.226)	0.03	0.132	3.04E-08	TRUE	0.032
NK	*RPS9*	Wald ratio	1	1.023 (0.991, 1.056)	0.158	0.283	1.15E-91	TRUE	0.019
NK	*SAT2*	Wald ratio	1	0.915 (0.84, 0.996)	0.04	0.141	1.93E-12	TRUE	0.045
NK	*SCD5*	Wald ratio	1	0.992 (0.927, 1.06)	0.803	0.836	3.05E-20	TRUE	0.008
NK	*SERAC1*	Wald ratio	1	0.938 (0.853, 1.031)	0.186	0.303	1.86E-10	TRUE	0.014
NK	*SNAP29*	Wald ratio	1	1.045 (0.99, 1.102)	0.108	0.226	1.97E-35	TRUE	0.027
NK	*SNRPD2*	Wald ratio	1	0.912 (0.848, 0.98)	0.012	0.084	6.97E-16	TRUE	0.137
NK	*ST3GAL5*	Wald ratio	1	0.962 (0.889, 1.041)	0.339	0.476	5.21E-12	TRUE	0.013
NK	*TBC1D12*	Wald ratio	1	1.057 (0.971, 1.15)	0.203	0.321	5.43E-12	TRUE	0.016
NK	*THAP9-AS1*	Wald ratio	1	1.019 (0.947, 1.095)	0.615	0.706	6.29E-20	TRUE	0.01
NK	*TMEM241*	Wald ratio	1	1.153 (1.04, 1.278)	0.007	0.071	2.00E-08	TRUE	0.121
NK	*UBE3B*	Wald ratio	1	1.101 (0.974, 1.244)	0.125	0.254	1.39E-07	TRUE	0.04
NK	*VIM*	Wald ratio	1	1.069 (0.95, 1.203)	0.268	0.408	6.23E-08	TRUE	0.026
NK	*WARS*	Wald ratio	1	1.046 (0.955, 1.146)	0.333	0.472	2.88E-12	TRUE	0.013
NK	*ZNF100*	Wald ratio	1	0.903 (0.843, 0.968)	0.004	0.057	4.41E-17	TRUE	0.051
Plasma	*HLA-DQB1*	Wald ratio	1	1.017 (0.938, 1.104)	0.682	0.754	7.52E-11	TRUE	9.89E-04
Plasma	*NFKBIL1*	Wald ratio	1	1.042 (0.967, 1.123)	0.279	0.416	2.96E-14	TRUE	0.003
Plasma	*NUCB2*	Wald ratio	1	0.911 (0.843, 0.984)	0.018	0.096	7.90E-12	TRUE	0.069
Plasma	*RNF146*	Wald ratio	1	0.988 (0.948, 1.031)	0.583	0.686	2.33E-37	TRUE	0.023
Plasma	*RXRB*	Wald ratio	1	1.005 (0.907, 1.114)	0.92	0.931	4.04E-08	TRUE	0.024

Table S7. MR and colocalization analyses on the cell type-specific associations between hypertension-related eGenes and hypertensive renal disease.

Cell type	eGene	Method	nSNP	OR (95% CI)	*P*-value	*FDR*	Steiger pval	Steiger direction	PP.H4.abf
B IN	*ACAT1*	Wald ratio	1	0.878 (0.684, 1.128)	0.308	0.807	7.14E-08	TRUE	0.036
B IN	*AP003774.1*	Wald ratio	1	0.939 (0.788, 1.119)	0.482	0.807	1.94E-20	TRUE	0.021
B IN	*BLK*	Wald ratio	1	0.958 (0.808, 1.136)	0.621	0.848	3.97E-23	TRUE	0.021
B IN	*C10orf32*	Wald ratio	1	1.096 (0.841, 1.429)	0.498	0.822	3.81E-10	TRUE	0.024
B IN	*C6orf48*	Wald ratio	1	1.139 (0.972, 1.336)	0.108	0.664	6.82E-12	TRUE	0.097
B IN	*C6orf62*	Wald ratio	1	1.238 (0.907, 1.69)	0.179	0.778	5.17E-07	TRUE	0.034
B IN	*CD164*	Wald ratio	1	0.859 (0.628, 1.174)	0.341	0.807	2.75E-07	TRUE	0.044
B IN	*CENPV*	Wald ratio	1	0.893 (0.699, 1.141)	0.366	0.807	4.47E-11	TRUE	0.026
B IN	*DDX5*	Wald ratio	1	1.043 (0.826, 1.317)	0.723	0.848	9.56E-12	TRUE	0.02
B IN	*FAM167A*	Wald ratio	1	0.957 (0.798, 1.148)	0.638	0.848	2.27E-20	TRUE	0.02
B IN	*FANCA*	Wald ratio	1	0.867 (0.619, 1.214)	0.407	0.807	1.36E-07	TRUE	0.027
B IN	*FNBP4*	Wald ratio	1	0.665 (0.513, 0.862)	0.002	0.285	2.68E-08	TRUE	0.35
B IN	*GTF2H4*	IVW	3	0.899 (0.786, 1.029)	0.122	0.692	9.96E-32	TRUE	0.025
B IN	*GTF2H4*	MR Egger	3	0.525 (0.221, 1.246)	0.382	NA	NA	NA	NA
B IN	*GTF2H4*	Weighted median	3	0.904 (0.776, 1.053)	0.194	NA	NA	NA	NA
B IN	*GTF2H4*	Simple mode	3	0.963 (0.764, 1.214)	0.781	NA	NA	NA	NA
B IN	*GTF2H4*	Weighted mode	3	0.851 (0.718, 1.01)	0.206	NA	NA	NA	NA
B IN	*HAGHL*	Wald ratio	1	0.949 (0.738, 1.22)	0.682	0.848	1.03E-08	TRUE	0.02
B IN	*HSD17B11*	Wald ratio	1	1.012 (0.762, 1.344)	0.933	0.964	1.82E-08	TRUE	0.028
B IN	*LA16c-358B7.3*	Wald ratio	1	1.239 (0.986, 1.556)	0.066	0.564	8.56E-13	TRUE	0.079
B IN	*MSMP*	Wald ratio	1	1.025 (0.788, 1.332)	0.855	0.93	4.16E-10	TRUE	0.019
B IN	*NOMO1*	Wald ratio	1	0.99 (0.768, 1.277)	0.939	0.965	7.66E-13	TRUE	0.025
B IN	*PRKD3*	Wald ratio	1	0.776 (0.59, 1.021)	0.07	0.564	1.32E-08	TRUE	0.067
B IN	*RAC1*	Wald ratio	1	1.067 (0.873, 1.304)	0.528	0.824	1.58E-16	TRUE	0.022
B IN	*RP13-514E23.2*	Wald ratio	1	0.932 (0.813, 1.07)	0.317	0.807	2.28E-33	TRUE	0.05
B IN	*RPS9*	Wald ratio	1	1.041 (0.938, 1.156)	0.45	0.807	9.22E-60	TRUE	0.02
B IN	*SNRPD2*	Wald ratio	1	0.896 (0.742, 1.081)	0.252	0.778	3.64E-17	TRUE	0.037
B IN	*WARS*	Wald ratio	1	0.897 (0.795, 1.013)	0.08	0.571	1.19E-48	TRUE	0.075
B Mem	*AP003774.1*	Wald ratio	1	0.927 (0.753, 1.142)	0.477	0.807	9.75E-15	TRUE	0.022
B Mem	*BLK*	Wald ratio	1	0.974 (0.851, 1.115)	0.702	0.848	1.56E-36	TRUE	0.02
B Mem	*C6orf48*	Wald ratio	1	1.262 (1.02, 1.562)	0.033	0.564	7.94E-07	TRUE	0.088
B Mem	*CCDC163P*	Wald ratio	1	1.086 (0.915, 1.289)	0.345	0.807	4.65E-20	TRUE	0.047
B Mem	*DDX5*	Wald ratio	1	1.051 (0.8, 1.38)	0.723	0.848	6.48E-09	TRUE	0.02
B Mem	*FAM167A*	Wald ratio	1	1.059 (0.853, 1.315)	0.602	0.848	4.92E-15	TRUE	0.02
B Mem	*FNBP4*	Wald ratio	1	0.729 (0.558, 0.951)	0.02	0.515	2.13E-10	TRUE	0.25
B Mem	*HAGH*	Wald ratio	1	0.892 (0.705, 1.127)	0.337	0.807	2.77E-10	TRUE	0.025
B Mem	*HAGHL*	Wald ratio	1	0.927 (0.727, 1.181)	0.538	0.824	1.66E-09	TRUE	0.021
B Mem	*HIST1H2AG*	Wald ratio	1	0.967 (0.784, 1.192)	0.753	0.867	2.18E-13	TRUE	0.009
B Mem	*HIST1H2BJ*	Wald ratio	1	0.961 (0.666, 1.385)	0.83	0.92	4.46E-08	TRUE	0.025
B Mem	*HIST1H3D*	Wald ratio	1	0.917 (0.776, 1.085)	0.313	0.807	4.26E-19	TRUE	0.022
B Mem	*KRTAP5-AS1*	Wald ratio	1	1.076 (0.936, 1.237)	0.302	0.807	1.89E-32	TRUE	0.019
B Mem	*LA16c-358B7.3*	Wald ratio	1	1.229 (1.007, 1.501)	0.043	0.564	2.31E-16	TRUE	0.077
B Mem	*MDM4*	Wald ratio	1	1.014 (0.785, 1.31)	0.913	0.95	5.67E-14	TRUE	0.023
B Mem	*PRKD3*	Wald ratio	1	0.778 (0.592, 1.022)	0.071	0.564	1.22E-08	TRUE	0.071
B Mem	*RP13-514E23.2*	Wald ratio	1	0.919 (0.815, 1.036)	0.165	0.778	1.82E-42	TRUE	0.045
B Mem	*RPS9*	Wald ratio	1	1.043 (0.934, 1.166)	0.452	0.807	1.13E-52	TRUE	0.02
B Mem	*SNRPD2*	Wald ratio	1	0.921 (0.762, 1.112)	0.391	0.807	1.58E-17	TRUE	0.04
B Mem	*WARS*	Wald ratio	1	0.881 (0.764, 1.015)	0.08	0.571	4.98E-35	TRUE	0.1
B Mem	*ZNF100*	Wald ratio	1	1.036 (0.815, 1.316)	0.772	0.883	8.31E-12	TRUE	0.06
CD4 ET	*AIF1*	Wald ratio	1	1.242 (1.031, 1.497)	0.022	0.535	1.88E-11	TRUE	0.143
CD4 ET	*AP003774.1*	Wald ratio	1	0.949 (0.794, 1.134)	0.564	0.824	1.27E-19	TRUE	0.02
CD4 ET	*C10orf32*	Wald ratio	1	1.078 (0.849, 1.37)	0.536	0.824	1.98E-12	TRUE	0.038
CD4 ET	*C17orf89*	Wald ratio	1	1.171 (0.914, 1.5)	0.213	0.778	1.65E-10	TRUE	0.032
CD4 ET	*CD151*	Wald ratio	1	1.39 (1.115, 1.733)	0.003	0.285	2.59E-13	TRUE	0.482
CD4 ET	*CDK10*	Wald ratio	1	1.201 (0.917, 1.574)	0.184	0.778	1.62E-08	TRUE	0.036
CD4 ET	*CTSW*	Wald ratio	1	0.946 (0.823, 1.088)	0.44	0.807	1.96E-38	TRUE	0.03
CD4 ET	*DDX5*	Wald ratio	1	1.034 (0.861, 1.24)	0.723	0.848	1.70E-18	TRUE	0.02
CD4 ET	*FDFT1*	Wald ratio	1	0.841 (0.68, 1.041)	0.112	0.664	1.75E-16	TRUE	0.069
CD4 ET	*FGF9*	Wald ratio	1	1.062 (0.808, 1.396)	0.665	0.848	1.08E-09	TRUE	0.01
CD4 ET	*FNBP4*	Wald ratio	1	0.631 (0.472, 0.845)	0.002	0.285	1.47E-06	TRUE	0.328
CD4 ET	*HAGH*	Wald ratio	1	0.917 (0.737, 1.141)	0.436	0.807	7.71E-12	TRUE	0.026
CD4 ET	*LA16c-358B7.3*	Wald ratio	1	1.197 (0.958, 1.495)	0.114	0.664	9.36E-14	TRUE	0.06
CD4 ET	*MANBA*	Wald ratio	1	0.924 (0.715, 1.194)	0.548	0.824	2.98E-10	TRUE	0.019
CD4 ET	*MDM4*	Wald ratio	1	1.047 (0.754, 1.453)	0.785	0.884	2.30E-09	TRUE	0.022
CD4 ET	*PLEKHA1*	Wald ratio	1	1.017 (0.77, 1.343)	0.907	0.95	5.04E-10	TRUE	0.018
CD4 ET	*PPT2*	Wald ratio	1	1.265 (1.003, 1.596)	0.047	0.564	2.28E-09	TRUE	0.027
CD4 ET	*RNF166*	Wald ratio	1	0.789 (0.597, 1.043)	0.096	0.641	2.56E-08	TRUE	0.059
CD4 ET	*RP11-499P20.2*	Wald ratio	1	0.723 (0.529, 0.987)	0.041	0.564	2.15E-06	TRUE	0.037
CD4 ET	*RPS9*	Wald ratio	1	1.038 (0.942, 1.145)	0.45	0.807	1.95E-68	TRUE	0.02
CD4 ET	*SNRPD2*	Wald ratio	1	0.922 (0.765, 1.11)	0.391	0.807	5.01E-18	TRUE	0.036
CD4 ET	*TNFSF13B*	Wald ratio	1	0.867 (0.673, 1.117)	0.269	0.778	8.73E-11	TRUE	0.029
CD4 NC	*AC010982.1*	Wald ratio	1	0.806 (0.597, 1.09)	0.162	0.778	2.71E-07	TRUE	0.007
CD4 NC	*ACAT1*	Wald ratio	1	0.934 (0.824, 1.058)	0.28	0.795	2.99E-28	TRUE	0.036
CD4 NC	*ACOT13*	Wald ratio	1	1.006 (0.723, 1.401)	0.971	0.977	2.26E-08	TRUE	0.025

CD4 NC	*AIF1*	Wald ratio	1	1.149 (1.035, 1.277)	0.009	0.371	8.89E-30	TRUE	0.288
CD4 NC	*AP003774.1*	Wald ratio	1	0.968 (0.885, 1.06)	0.482	0.807	2.64E-79	TRUE	0.021
CD4 NC	*AP2A2*	Wald ratio	1	1.137 (0.853, 1.515)	0.382	0.807	3.28E-08	TRUE	0.021
CD4 NC	*BIK*	Wald ratio	1	0.936 (0.702, 1.248)	0.653	0.848	1.34E-11	TRUE	0.029
CD4 NC	*BLK*	Wald ratio	1	0.975 (0.816, 1.166)	0.785	0.884	2.02E-21	TRUE	0.019
CD4 NC	*BLMH*	Wald ratio	1	1.177 (0.873, 1.587)	0.285	0.795	1.18E-07	TRUE	0.028
CD4 NC	*C10orf32*	Wald ratio	1	1.041 (0.909, 1.192)	0.56	0.824	1.08E-37	TRUE	0.023
CD4 NC	*C2orf74*	Wald ratio	1	0.961 (0.757, 1.221)	0.745	0.864	6.98E-12	TRUE	0.02
CD4 NC	*C6orf48*	Wald ratio	1	1.143 (1.009, 1.294)	0.035	0.564	1.61E-19	TRUE	0.125
CD4 NC	*C6orf62*	Wald ratio	1	1.224 (0.912, 1.643)	0.179	0.778	9.67E-08	TRUE	0.035
CD4 NC	*CCDC104*	Wald ratio	1	1.148 (0.914, 1.442)	0.234	0.778	6.43E-14	TRUE	0.035
CD4 NC	*CCDC88B*	Wald ratio	1	1.059 (0.881, 1.272)	0.541	0.824	7.05E-19	TRUE	0.026
CD4 NC	*CD151*	Wald ratio	1	1.266 (1.043, 1.536)	0.017	0.471	8.53E-17	TRUE	0.266
CD4 NC	*CDK10*	Wald ratio	1	1.12 (0.947, 1.325)	0.184	0.778	3.91E-21	TRUE	0.039
CD4 NC	*CLNS1A*	Wald ratio	1	1.116 (0.964, 1.292)	0.141	0.772	1.49E-16	TRUE	0.06
CD4 NC	*CLUAP1*	Wald ratio	1	1.115 (0.922, 1.348)	0.261	0.778	8.49E-18	TRUE	0.025
CD4 NC	*CRHR1*	Wald ratio	1	0.81 (0.574, 1.144)	0.231	0.778	4.16E-15	TRUE	0.052
CD4 NC	*CTD-2336O2.1*	Wald ratio	1	1.034 (0.932, 1.148)	0.525	0.824	1.51E-64	TRUE	0.024
CD4 NC	*CTSW*	Wald ratio	1	0.967 (0.889, 1.053)	0.44	0.807	8.02E-113	TRUE	0.03
CD4 NC	*CUBN*	Wald ratio	1	1.114 (0.888, 1.397)	0.35	0.807	3.18E-14	TRUE	0.029
CD4 NC	*CYP2D6*	Wald ratio	1	0.871 (0.647, 1.173)	0.363	0.807	9.55E-08	TRUE	0.029
CD4 NC	*DDX5*	Wald ratio	1	1.028 (0.883, 1.197)	0.723	0.848	5.26E-26	TRUE	0.02
CD4 NC	*EEF2*	Wald ratio	1	1.113 (0.839, 1.477)	0.458	0.807	1.10E-08	TRUE	0.028
CD4 NC	*EGFL8*	Wald ratio	1	1.149 (0.891, 1.481)	0.284	0.795	3.52E-10	TRUE	0.027
CD4 NC	*ERP29*	Wald ratio	1	1.159 (0.944, 1.424)	0.159	0.778	4.93E-12	TRUE	0.11
CD4 NC	*FAM153B*	Wald ratio	1	1.12 (1.027, 1.22)	0.01	0.371	9.63E-86	TRUE	0.274
CD4 NC	*FANCA*	Wald ratio	1	0.888 (0.677, 1.165)	0.391	0.807	6.56E-11	TRUE	0.028
CD4 NC	*FDFT1*	Wald ratio	1	0.922 (0.834, 1.019)	0.112	0.664	1.04E-78	TRUE	0.069
CD4 NC	*FNBP4*	Wald ratio	1	0.829 (0.723, 0.952)	0.008	0.365	2.85E-32	TRUE	0.312
CD4 NC	*FOXN2*	Wald ratio	1	0.979 (0.787, 1.219)	0.849	0.93	2.12E-20	TRUE	0.03
CD4 NC	*GOSR2*	Wald ratio	1	1.148 (0.947, 1.391)	0.161	0.778	7.93E-17	TRUE	0.037
CD4 NC	*HAGH*	Wald ratio	1	0.962 (0.873, 1.06)	0.436	0.807	3.70E-58	TRUE	0.022
CD4 NC	*HLA-DQA1*	Wald ratio	1	0.982 (0.757, 1.274)	0.891	0.949	7.12E-11	TRUE	0.029
CD4 NC	*HLA-DRB5*	Wald ratio	1	0.992 (0.858, 1.147)	0.91	0.95	2.09E-40	TRUE	0.015
CD4 NC	*HMBS*	Wald ratio	1	1.196 (0.993, 1.442)	0.06	0.564	2.33E-19	TRUE	0.092
CD4 NC	*HSD17B11*	Wald ratio	1	1.009 (0.816, 1.248)	0.933	0.964	4.98E-14	TRUE	0.028
CD4 NC	*HSD17B12*	Wald ratio	1	1.06 (0.91, 1.235)	0.451	0.807	2.01E-26	TRUE	0.023
CD4 NC	*KB-1507C5.2*	Wald ratio	1	1.21 (0.955, 1.534)	0.115	0.664	2.34E-10	TRUE	0.049
CD4 NC	*KRTAP5-2*	Wald ratio	1	1.031 (0.891, 1.193)	0.683	0.848	4.46E-32	TRUE	0.022
CD4 NC	*LA16c-358B7.3*	Wald ratio	1	1.102 (0.994, 1.223)	0.066	0.564	3.00E-64	TRUE	0.07
CD4 NC	*LAIR2*	Wald ratio	1	1.052 (0.858, 1.29)	0.626	0.848	1.37E-21	TRUE	0.029
CD4 NC	*LIMD2*	Wald ratio	1	1.12 (0.82, 1.53)	0.476	0.807	3.63E-08	TRUE	0.025
CD4 NC	*LLGL1*	Wald ratio	1	1.102 (0.874, 1.389)	0.411	0.807	3.04E-12	TRUE	0.028
CD4 NC	*LRRC61*	Wald ratio	1	0.89 (0.777, 1.018)	0.089	0.61	2.58E-29	TRUE	0.066
CD4 NC	*LSM4*	Wald ratio	1	1.046 (0.855, 1.28)	0.661	0.848	5.22E-12	TRUE	0.024
CD4 NC	*MACF1*	Wald ratio	1	0.974 (0.804, 1.179)	0.786	0.884	1.13E-17	TRUE	0.021
CD4 NC	*MARK3*	Wald ratio	1	1.01 (0.79, 1.291)	0.938	0.965	5.44E-12	TRUE	0.018
CD4 NC	*MEI1*	Wald ratio	1	1.035 (0.836, 1.281)	0.752	0.867	5.04E-13	TRUE	0.021
CD4 NC	*MRPS34*	Wald ratio	1	0.931 (0.689, 1.258)	0.641	0.848	7.36E-08	TRUE	0.021
CD4 NC	*MYO16*	Wald ratio	1	0.92 (0.794, 1.066)	0.269	0.778	1.18E-30	TRUE	0.032
CD4 NC	*NARFL*	Wald ratio	1	0.948 (0.763, 1.178)	0.629	0.848	1.34E-10	TRUE	0.021
CD4 NC	*NEIL2*	Wald ratio	1	0.789 (0.59, 1.056)	0.112	0.664	5.00E-09	TRUE	0.044
CD4 NC	*OSER1-AS1*	Wald ratio	1	1.048 (0.851, 1.292)	0.658	0.848	7.17E-14	TRUE	0.025
CD4 NC	*PABPC4*	Wald ratio	1	0.819 (0.594, 1.13)	0.225	0.778	2.53E-07	TRUE	0.03
CD4 NC	*PCNX*	Wald ratio	1	0.96 (0.806, 1.143)	0.646	0.848	2.68E-21	TRUE	0.02
CD4 NC	*PGPEP1*	Wald ratio	1	0.861 (0.672, 1.102)	0.235	0.778	1.08E-11	TRUE	0.034
CD4 NC	*PHACTR4*	Wald ratio	1	1.084 (0.905, 1.299)	0.38	0.807	1.57E-18	TRUE	0.032
CD4 NC	*PKMYT1*	Wald ratio	1	0.971 (0.836, 1.129)	0.704	0.848	1.65E-25	TRUE	0.012
CD4 NC	*PRKD3*	Wald ratio	1	0.805 (0.636, 1.018)	0.07	0.564	1.64E-11	TRUE	0.064
CD4 NC	*RAC1*	Wald ratio	1	1.036 (0.929, 1.155)	0.528	0.824	1.07E-55	TRUE	0.02
CD4 NC	*REEP3*	Wald ratio	1	1.219 (0.875, 1.698)	0.242	0.778	3.74E-07	TRUE	0.035
CD4 NC	*RNF8*	Wald ratio	1	1 (0.734, 1.36)	0.998	0.998	1.84E-08	TRUE	0.022
CD4 NC	*RP11-273G15.2*	Wald ratio	1	1.035 (0.857, 1.251)	0.718	0.848	2.27E-18	TRUE	0.019
CD4 NC	*RP11-796G6.2*	Wald ratio	1	0.975 (0.646, 1.472)	0.905	0.95	4.91E-09	TRUE	0.043
CD4 NC	*RPS9*	Wald ratio	1	1.033 (0.95, 1.123)	0.45	0.807	4.74E-96	TRUE	0.02
CD4 NC	*SAT2*	Wald ratio	1	0.854 (0.72, 1.012)	0.068	0.564	3.47E-21	TRUE	0.081
CD4 NC	*SLC22A5*	Wald ratio	1	0.979 (0.733, 1.307)	0.886	0.949	1.05E-08	TRUE	0.021
CD4 NC	*SNRPC*	Wald ratio	1	0.959 (0.818, 1.124)	0.603	0.848	1.80E-25	TRUE	0.021
CD4 NC	*SNRPD2*	Wald ratio	1	0.96 (0.874, 1.054)	0.391	0.807	2.51E-72	TRUE	0.032
CD4 NC	*SNX16*	Wald ratio	1	0.929 (0.719, 1.199)	0.569	0.825	6.17E-12	TRUE	0.022
CD4 NC	*SPCS1*	Wald ratio	1	1.168 (0.928, 1.469)	0.185	0.778	6.15E-12	TRUE	0.039
CD4 NC	*TMEM199*	Wald ratio	1	0.907 (0.696, 1.183)	0.473	0.807	7.88E-10	TRUE	0.022
CD4 NC	*TMEM87B*	Wald ratio	1	1.036 (0.796, 1.348)	0.794	0.889	3.28E-11	TRUE	0.021
CD4 NC	*TMEM97*	Wald ratio	1	0.937 (0.729, 1.205)	0.613	0.848	7.75E-11	TRUE	0.02
CD4 NC	*TNFRSF18*	Wald ratio	1	1.01 (0.759, 1.343)	0.948	0.965	5.71E-08	TRUE	0.032
CD4 NC	*TTC12*	Wald ratio	1	0.922 (0.71, 1.198)	0.543	0.824	3.81E-10	TRUE	0.021
CD4 NC	*UACA*	Wald ratio	1	1.061 (0.824, 1.366)	0.646	0.848	3.55E-09	TRUE	0.028
CD4 NC	*UBE2I*	Wald ratio	1	1.505 (1.129, 2.004)	0.005	0.352	1.22E-08	TRUE	0.44
CD4 NC	*UBE3B*	Wald ratio	1	1.021 (0.76, 1.371)	0.892	0.949	3.03E-10	TRUE	0.032

CD4 NC	*USP36*	Wald ratio	1	0.993 (0.769, 1.283)	0.959	0.968	1.83E-11	TRUE	0.012
CD4 NC	*VIM*	Wald ratio	1	1.342 (1.004, 1.793)	0.047	0.564	7.29E-07	TRUE	0.089
CD4 NC	*VPS53*	Wald ratio	1	0.751 (0.581, 0.97)	0.028	0.564	1.61E-09	TRUE	0.127
CD4 NC	*WBSCR16*	Wald ratio	1	1.135 (0.847, 1.52)	0.396	0.807	1.58E-08	TRUE	0.035
CD4 NC	*ZNF100*	Wald ratio	1	0.96 (0.85, 1.084)	0.506	0.824	1.66E-42	TRUE	0.019
CD4 NC	*ZNF502*	Wald ratio	1	1.005 (0.878, 1.149)	0.948	0.965	5.57E-35	TRUE	0.02
CD4 NC	*ZSWIM7*	Wald ratio	1	0.965 (0.791, 1.177)	0.724	0.848	2.76E-17	TRUE	0.019
CD4 SOX4	*DAXX*	Wald ratio	1	1.073 (0.783, 1.471)	0.661	0.848	3.13E-08	TRUE	0.015
CD4 SOX4	*DXO*	Wald ratio	1	0.908 (0.742, 1.112)	0.351	0.807	2.30E-14	TRUE	0.027
CD4 SOX4	*RPS26*	Wald ratio	1	1.016 (0.952, 1.083)	0.635	0.848	7.16E-165	TRUE	0.019
CD4 SOX4	*RPS9*	Wald ratio	1	1.064 (0.844, 1.341)	0.6	0.848	1.38E-11	TRUE	0.02
CD8 ET	*ADRB1*	Wald ratio	1	0.822 (0.682, 0.989)	0.038	0.564	3.88E-13	TRUE	0.121
CD8 ET	*AP003774.1*	Wald ratio	1	0.969 (0.871, 1.078)	0.564	0.824	3.52E-54	TRUE	0.02
CD8 ET	*AP2A2*	Wald ratio	1	1.129 (0.875, 1.457)	0.352	0.807	3.34E-10	TRUE	0.021
CD8 ET	*BTN3A1*	Wald ratio	1	1.118 (0.865, 1.444)	0.395	0.807	2.10E-09	TRUE	0.032
CD8 ET	*C10orf32*	Wald ratio	1	1.059 (0.874, 1.284)	0.56	0.824	5.99E-19	TRUE	0.025
CD8 ET	*C2orf74*	Wald ratio	1	1.05 (0.821, 1.343)	0.7	0.848	6.57E-11	TRUE	0.025
CD8 ET	*C6orf62*	Wald ratio	1	1.239 (0.907, 1.692)	0.179	0.778	5.56E-07	TRUE	0.034
CD8 ET	*CCDC88B*	Wald ratio	1	1.193 (0.896, 1.59)	0.227	0.778	6.80E-08	TRUE	0.032
CD8 ET	*CDK10*	Wald ratio	1	1.157 (0.933, 1.435)	0.184	0.778	5.29E-13	TRUE	0.042
CD8 ET	*CTD-2515A14.1*	Wald ratio	1	1.089 (0.813, 1.46)	0.567	0.825	3.75E-08	TRUE	0.018
CD8 ET	*CTSW*	Wald ratio	1	0.966 (0.886, 1.054)	0.44	0.807	7.14E-107	TRUE	0.03
CD8 ET	*DDX5*	Wald ratio	1	1.028 (0.881, 1.2)	0.723	0.848	2.27E-25	TRUE	0.02
CD8 ET	*DGKD*	Wald ratio	1	1.148 (0.845, 1.558)	0.377	0.807	1.90E-07	TRUE	0.029
CD8 ET	*FDFT1*	Wald ratio	1	0.816 (0.635, 1.048)	0.112	0.664	5.39E-12	TRUE	0.069
CD8 ET	*FIBP*	Wald ratio	1	1.06 (0.787, 1.428)	0.702	0.848	1.21E-09	TRUE	0.029
CD8 ET	*FNBP4*	Wald ratio	1	0.758 (0.611, 0.941)	0.012	0.405	2.67E-13	TRUE	0.327
CD8 ET	*FOXN2*	Wald ratio	1	1.186 (0.886, 1.588)	0.252	0.778	2.87E-10	TRUE	0.038
CD8 ET	*HAGH*	Wald ratio	1	0.905 (0.771, 1.064)	0.227	0.778	2.02E-20	TRUE	0.028
CD8 ET	*HLA-DQB2*	Wald ratio	1	0.936 (0.765, 1.144)	0.516	0.824	1.26E-17	TRUE	0.03
CD8 ET	*LA16c-358B7.3*	Wald ratio	1	1.158 (0.99, 1.354)	0.066	0.564	3.57E-27	TRUE	0.071
CD8 ET	*LAIR2*	Wald ratio	1	1.104 (0.931, 1.308)	0.257	0.778	1.44E-28	TRUE	0.045
CD8 ET	*LLGL1*	Wald ratio	1	1.152 (0.896, 1.479)	0.269	0.778	2.27E-10	TRUE	0.029
CD8 ET	*LRRC37A*	Wald ratio	1	0.992 (0.852, 1.154)	0.913	0.95	1.63E-24	TRUE	0.022
CD8 ET	*LRRC61*	Wald ratio	1	0.862 (0.648, 1.145)	0.304	0.807	4.96E-08	TRUE	0.052
CD8 ET	*MDM4*	Wald ratio	1	1.064 (0.82, 1.381)	0.64	0.848	9.63E-13	TRUE	0.022
CD8 ET	*MLF1*	Wald ratio	1	1.035 (0.845, 1.269)	0.737	0.858	2.50E-13	TRUE	0.022
CD8 ET	*MPHOSPH9*	Wald ratio	1	0.901 (0.661, 1.23)	0.513	0.824	9.65E-08	TRUE	0.029
CD8 ET	*NOTCH4*	Wald ratio	1	0.731 (0.53, 1.01)	0.057	0.564	1.16E-07	TRUE	0.037
CD8 ET	*NUCB2*	Wald ratio	1	0.94 (0.848, 1.041)	0.234	0.778	9.24E-61	TRUE	0.033
CD8 ET	*OXNAD1*	Wald ratio	1	0.901 (0.729, 1.114)	0.337	0.807	7.03E-15	TRUE	0.025
CD8 ET	*PDCD6*	Wald ratio	1	0.979 (0.742, 1.292)	0.882	0.949	1.96E-09	TRUE	0.019
CD8 ET	*PHACTR4*	Wald ratio	1	1.167 (0.889, 1.532)	0.265	0.778	1.63E-08	TRUE	0.031
CD8 ET	*REEP5*	Wald ratio	1	1.1 (0.835, 1.45)	0.497	0.822	3.31E-09	TRUE	0.029
CD8 ET	*RP4-799D16.1*	Wald ratio	1	1.05 (0.804, 1.372)	0.719	0.848	1.65E-09	TRUE	0.02
CD8 ET	*RPS9*	Wald ratio	1	1.033 (0.95, 1.124)	0.45	0.807	1.73E-95	TRUE	0.02
CD8 ET	*SAT2*	Wald ratio	1	0.804 (0.639, 1.011)	0.062	0.564	9.93E-12	TRUE	0.086
CD8 ET	*SCD5*	Wald ratio	1	0.928 (0.769, 1.12)	0.437	0.807	7.08E-19	TRUE	0.026
CD8 ET	*SNRPC*	Wald ratio	1	0.911 (0.672, 1.235)	0.549	0.824	1.15E-07	TRUE	0.022
CD8 ET	*SNRPD2*	Wald ratio	1	0.945 (0.831, 1.075)	0.391	0.807	3.06E-37	TRUE	0.031
CD8 ET	*THAP9-AS1*	Wald ratio	1	1.073 (0.89, 1.293)	0.462	0.807	4.95E-19	TRUE	0.028
CD8 ET	*VIM*	Wald ratio	1	1.257 (0.986, 1.604)	0.065	0.564	1.02E-09	TRUE	0.084
CD8 ET	*WARS*	Wald ratio	1	0.754 (0.552, 1.029)	0.075	0.564	2.49E-07	TRUE	0.081
CD8 ET	*XBP1*	Wald ratio	1	0.962 (0.745, 1.243)	0.768	0.882	4.78E-10	TRUE	0.019
CD8 ET	*ZNF100*	Wald ratio	1	0.94 (0.767, 1.153)	0.555	0.824	1.49E-15	TRUE	0.019
CD8 ET	*ZNF502*	Wald ratio	1	1.009 (0.769, 1.323)	0.948	0.965	1.46E-09	TRUE	0.021
CD8 ET	*ZSWIM7*	Wald ratio	1	0.96 (0.765, 1.205)	0.724	0.848	1.57E-13	TRUE	0.019
CD8 NC	*AP003774.1*	Wald ratio	1	0.954 (0.836, 1.088)	0.482	0.807	8.38E-36	TRUE	0.021
CD8 NC	*BTN3A1*	Wald ratio	1	1.118 (0.878, 1.424)	0.364	0.807	2.85E-10	TRUE	0.032
CD8 NC	*C6orf48*	Wald ratio	1	1.186 (1.014, 1.386)	0.033	0.564	2.03E-12	TRUE	0.122
CD8 NC	*CEBPZ*	Wald ratio	1	0.821 (0.605, 1.114)	0.206	0.778	2.73E-07	TRUE	0.049
CD8 NC	*CLNS1A*	Wald ratio	1	1.169 (0.938, 1.456)	0.164	0.778	6.78E-08	TRUE	0.061
CD8 NC	*CLUAP1*	Wald ratio	1	1.157 (0.871, 1.537)	0.315	0.807	2.01E-08	TRUE	0.024
CD8 NC	*COX14*	Wald ratio	1	1.068 (0.802, 1.423)	0.651	0.848	2.39E-09	TRUE	0.021
CD8 NC	*CRHR1*	Wald ratio	1	1.277 (0.864, 1.888)	0.22	0.778	4.46E-12	TRUE	0.052
CD8 NC	*CTD-2336O2.1*	Wald ratio	1	1.027 (0.877, 1.204)	0.737	0.858	7.21E-30	TRUE	0.022
CD8 NC	*CTSW*	Wald ratio	1	0.966 (0.886, 1.054)	0.44	0.807	6.72E-107	TRUE	0.03
CD8 NC	*DCP2*	Wald ratio	1	1.024 (0.797, 1.317)	0.851	0.93	8.19E-10	TRUE	0.018
CD8 NC	*DDR1*	IVW	2	1.009 (0.86, 1.184)	0.909	0.95	2.07E-28	TRUE	0.015
CD8 NC	*DDX5*	Wald ratio	1	1.033 (0.862, 1.239)	0.723	0.848	1.37E-18	TRUE	0.02
CD8 NC	*FNBP4*	Wald ratio	1	0.72 (0.566, 0.917)	0.008	0.365	3.09E-10	TRUE	0.319
CD8 NC	*FOXN2*	Wald ratio	1	0.966 (0.681, 1.369)	0.845	0.93	8.16E-09	TRUE	0.031
CD8 NC	*HAGH*	Wald ratio	1	0.911 (0.784, 1.059)	0.227	0.778	2.47E-23	TRUE	0.024
CD8 NC	*HLA-DRB5*	Wald ratio	1	0.99 (0.857, 1.143)	0.89	0.949	1.10E-39	TRUE	0.015
CD8 NC	*HSD17B12*	Wald ratio	1	1.063 (0.802, 1.409)	0.672	0.848	8.58E-09	TRUE	0.019
CD8 NC	*LA16c-358B7.3*	Wald ratio	1	1.16 (0.99, 1.36)	0.066	0.564	1.91E-26	TRUE	0.069
CD8 NC	*LAIR2*	Wald ratio	1	1.221 (0.864, 1.725)	0.257	0.778	1.79E-07	TRUE	0.04
CD8 NC	*LRRC37A*	Wald ratio	1	1.003 (0.838, 1.199)	0.977	0.98	1.54E-18	TRUE	0.02
CD8 NC	*LRRC61*	Wald ratio	1	0.799 (0.619, 1.033)	0.087	0.606	2.99E-08	TRUE	0.063

CD8 NC	*MPHOSPH9*	Wald ratio	1	0.911 (0.71, 1.169)	0.465	0.807	2.73E-11	TRUE	0.03
CD8 NC	*MYO16*	Wald ratio	1	1.088 (0.937, 1.265)	0.269	0.778	3.22E-30	TRUE	0.029
CD8 NC	*PHACTR4*	Wald ratio	1	1.102 (0.841, 1.443)	0.482	0.807	2.64E-09	TRUE	0.031
CD8 NC	*RP11-273G15.2*	Wald ratio	1	1.173 (0.877, 1.57)	0.282	0.795	9.48E-08	TRUE	0.024
CD8 NC	*RPS9*	Wald ratio	1	1.025 (0.936, 1.122)	0.6	0.848	6.29E-85	TRUE	0.02
CD8 NC	*SAT2*	Wald ratio	1	0.77 (0.577, 1.026)	0.074	0.564	1.07E-07	TRUE	0.084
CD8 NC	*SCD5*	Wald ratio	1	0.885 (0.659, 1.189)	0.418	0.807	3.52E-09	TRUE	0.027
CD8 NC	*SNRPC*	Wald ratio	1	0.927 (0.72, 1.192)	0.553	0.824	1.13E-10	TRUE	0.022
CD8 NC	*SNRPD2*	Wald ratio	1	0.933 (0.828, 1.051)	0.252	0.778	1.39E-42	TRUE	0.036
CD8 NC	*STK32C*	Wald ratio	1	1.11 (0.738, 1.67)	0.617	0.848	3.02E-08	TRUE	0.065
CD8 NC	*THAP9-AS1*	Wald ratio	1	1.109 (0.862, 1.427)	0.42	0.807	2.54E-12	TRUE	0.028
CD8 NC	*USP36*	Wald ratio	1	0.991 (0.723, 1.359)	0.956	0.968	5.29E-08	TRUE	0.012
CD8 NC	*VIM*	Wald ratio	1	1.268 (0.995, 1.617)	0.055	0.564	1.38E-09	TRUE	0.106
CD8 NC	*ZNF100*	Wald ratio	1	0.936 (0.754, 1.162)	0.546	0.824	6.34E-14	TRUE	0.019
CD8 NC	*ZSWIM7*	Wald ratio	1	0.949 (0.711, 1.268)	0.724	0.848	8.57E-09	TRUE	0.019
CD8 S100B	*AP003774.1*	Wald ratio	1	0.92 (0.729, 1.161)	0.482	0.807	5.74E-12	TRUE	0.022
CD8 S100B	*C10orf32*	Wald ratio	1	1.091 (0.828, 1.438)	0.536	0.824	1.36E-09	TRUE	0.029
CD8 S100B	*CD151*	Wald ratio	1	1.598 (1.169, 2.183)	0.003	0.285	2.48E-06	TRUE	0.511
CD8 S100B	*CTSW*	Wald ratio	1	0.958 (0.858, 1.069)	0.44	0.807	4.85E-63	TRUE	0.03
CD8 S100B	*DDX5*	Wald ratio	1	1.042 (0.831, 1.305)	0.723	0.848	1.82E-12	TRUE	0.02
CD8 S100B	*EEF2*	Wald ratio	1	1.116 (0.835, 1.493)	0.458	0.807	2.92E-08	TRUE	0.026
CD8 S100B	*FAM85A*	Wald ratio	1	0.989 (0.854, 1.145)	0.882	0.949	5.82E-30	TRUE	0.02
CD8 S100B	*FDFT1*	Wald ratio	1	0.777 (0.57, 1.06)	0.112	0.664	4.97E-08	TRUE	0.064
CD8 S100B	*HSPA1L*	Wald ratio	1	0.963 (0.787, 1.18)	0.719	0.848	2.40E-22	TRUE	0.022
CD8 S100B	*KIAA0040*	Wald ratio	1	1.181 (0.881, 1.583)	0.266	0.778	7.30E-08	TRUE	0.026
CD8 S100B	*MDM4*	Wald ratio	1	1.085 (0.805, 1.463)	0.593	0.848	6.36E-10	TRUE	0.023
CD8 S100B	*RPS12*	Wald ratio	1	1.111 (0.867, 1.423)	0.406	0.807	6.11E-10	TRUE	0.035
CD8 S100B	*RPS9*	Wald ratio	1	1.045 (0.933, 1.17)	0.45	0.807	2.61E-50	TRUE	0.02
CD8 S100B	*SCD5*	Wald ratio	1	1.088 (0.879, 1.347)	0.437	0.807	6.95E-15	TRUE	0.025
CD8 S100B	*SNRPD2*	Wald ratio	1	0.873 (0.693, 1.101)	0.252	0.778	1.50E-11	TRUE	0.055
CD8 S100B	*TNFSF13B*	Wald ratio	1	0.911 (0.669, 1.241)	0.555	0.824	9.05E-08	TRUE	0.025
CD8 S100B	*TTC12*	Wald ratio	1	1.1 (0.931, 1.301)	0.263	0.778	9.33E-23	TRUE	0.017
CD8 S100B	*XBP1*	Wald ratio	1	0.966 (0.721, 1.296)	0.82	0.912	4.88E-08	TRUE	0.026
DC	*ATAT1*	Wald ratio	1	1.216 (0.986, 1.5)	0.067	0.564	4.20E-12	TRUE	0.026
DC	*DDX5*	Wald ratio	1	1.054 (0.79, 1.405)	0.723	0.848	5.30E-08	TRUE	0.02
DC	*MICB*	Wald ratio	1	1.026 (0.841, 1.252)	0.801	0.895	2.96E-16	TRUE	0.017
Mono C	*AP003774.1*	Wald ratio	1	1.089 (0.859, 1.381)	0.482	0.807	1.90E-11	TRUE	0.024
Mono C	*C10orf32*	Wald ratio	1	1.099 (0.836, 1.444)	0.498	0.822	1.59E-09	TRUE	0.036
Mono C	*HP*	Wald ratio	1	0.93 (0.802, 1.079)	0.341	0.807	1.69E-27	TRUE	0.027
Mono C	*PRELID1*	Wald ratio	1	0.996 (0.842, 1.177)	0.959	0.968	1.46E-20	TRUE	0.021
Mono C	*RPS9*	Wald ratio	1	1.052 (0.922, 1.2)	0.45	0.807	1.54E-36	TRUE	0.02
Mono C	*TREML1*	Wald ratio	1	0.943 (0.841, 1.058)	0.321	0.807	3.40E-39	TRUE	0.014
Mono NC	*NCR3*	Wald ratio	1	1.088 (0.897, 1.321)	0.391	0.807	3.82E-18	TRUE	0.027
Mono NC	*PRELID1*	Wald ratio	1	1.045 (0.872, 1.251)	0.635	0.848	8.30E-15	TRUE	0.025
Mono NC	*RPH3A*	Wald ratio	1	0.946 (0.749, 1.194)	0.638	0.848	1.16E-12	TRUE	0.02
Mono NC	*RPS26*	Wald ratio	1	1.016 (0.953, 1.083)	0.635	0.848	1.91E-182	TRUE	0.019
Mono NC	*RPS9*	Wald ratio	1	1.077 (0.888, 1.307)	0.45	0.807	1.28E-16	TRUE	0.02
Mono NC	*VPS52*	Wald ratio	1	0.859 (0.681, 1.083)	0.198	0.778	8.61E-11	TRUE	0.019
NK R	*AC016831.7*	Wald ratio	1	0.91 (0.771, 1.075)	0.269	0.778	8.20E-32	TRUE	0.007
NK R	*APOM*	Wald ratio	1	0.864 (0.671, 1.114)	0.26	0.778	1.02E-10	TRUE	0.019
NK R	*CTSW*	Wald ratio	1	0.945 (0.713, 1.252)	0.695	0.848	1.51E-10	TRUE	0.028
NK R	*RAC1*	Wald ratio	1	0.868 (0.651, 1.157)	0.334	0.807	8.26E-08	TRUE	0.026
NK R	*SYNGAP1*	Wald ratio	1	0.911 (0.746, 1.113)	0.363	0.807	7.52E-16	TRUE	0.023
NK R	*XXbac-BPG299F13.17*	Wald ratio	1	1.075 (0.822, 1.405)	0.597	0.848	1.04E-09	TRUE	0.021
NK	*ADRB1*	Wald ratio	1	0.833 (0.701, 0.99)	0.038	0.564	2.87E-15	TRUE	0.129
NK	*AP003774.1*	Wald ratio	1	0.962 (0.87, 1.064)	0.452	0.807	3.32E-62	TRUE	0.021
NK	*AP2A2*	Wald ratio	1	1.071 (0.903, 1.27)	0.428	0.807	7.35E-22	TRUE	0.021
NK	*ATP5G1*	Wald ratio	1	0.863 (0.689, 1.081)	0.201	0.778	1.27E-12	TRUE	0.037
NK	*BTN3A1*	Wald ratio	1	1.133 (0.85, 1.51)	0.395	0.807	1.05E-07	TRUE	0.032
NK	*C10orf32*	Wald ratio	1	1.062 (0.867, 1.302)	0.56	0.824	4.95E-17	TRUE	0.023
NK	*C6orf62*	Wald ratio	1	1.222 (0.912, 1.636)	0.179	0.778	7.09E-08	TRUE	0.036
NK	*CCDC88B*	Wald ratio	1	1.164 (0.89, 1.523)	0.268	0.778	6.30E-09	TRUE	0.031
NK	*CD151*	Wald ratio	1	1.204 (1.034, 1.403)	0.017	0.471	2.08E-27	TRUE	0.224
NK	*CDK10*	Wald ratio	1	1.142 (0.939, 1.388)	0.184	0.778	1.07E-15	TRUE	0.064
NK	*CTSW*	Wald ratio	1	0.963 (0.873, 1.062)	0.447	0.807	2.37E-80	TRUE	0.03
NK	*DDX42*	Wald ratio	1	1.096 (0.817, 1.47)	0.541	0.824	2.53E-09	TRUE	0.024
NK	*DDX5*	Wald ratio	1	1.027 (0.886, 1.191)	0.723	0.848	1.66E-27	TRUE	0.02
NK	*EPHX4*	Wald ratio	1	1.209 (0.908, 1.611)	0.194	0.778	1.70E-08	TRUE	0.063
NK	*ERAP1*	Wald ratio	1	0.806 (0.601, 1.082)	0.152	0.778	1.19E-07	TRUE	0.053
NK	*FIBP*	Wald ratio	1	1.078 (0.89, 1.306)	0.44	0.807	1.41E-20	TRUE	0.029
NK	*GNGT2*	Wald ratio	1	1.17 (0.905, 1.513)	0.231	0.778	3.66E-09	TRUE	0.068
NK	*HAGH*	Wald ratio	1	0.935 (0.789, 1.108)	0.436	0.807	5.21E-19	TRUE	0.022
NK	*HLA-DOA*	Wald ratio	1	1.076 (0.777, 1.489)	0.661	0.848	9.56E-09	TRUE	0.065
NK	*IGF2BP2*	Wald ratio	1	0.978 (0.74, 1.293)	0.878	0.949	2.27E-08	TRUE	0.03
NK	*KHK*	Wald ratio	1	0.797 (0.584, 1.089)	0.154	0.778	5.56E-07	TRUE	0.039
NK	*LA16c-358B7.3*	Wald ratio	1	1.136 (0.992, 1.302)	0.066	0.564	4.33E-36	TRUE	0.068
NK	*LLGL1*	Wald ratio	1	1.179 (0.88, 1.58)	0.269	0.778	7.47E-08	TRUE	0.033
NK	*LRRC61*	Wald ratio	1	0.812 (0.617, 1.068)	0.136	0.758	3.13E-08	TRUE	0.06
NK	*MDM4*	Wald ratio	1	1.076 (0.791, 1.466)	0.64	0.848	2.05E-09	TRUE	0.023

NK	*MLF1*	Wald ratio	1	1.047 (0.855, 1.282)	0.657	0.848	1.17E-13	TRUE	0.022
NK	*MPHOSPH9*	Wald ratio	1	0.929 (0.743, 1.16)	0.514	0.824	2.11E-12	TRUE	0.027
NK	*NAA40*	Wald ratio	1	1.121 (0.822, 1.53)	0.469	0.807	1.86E-07	TRUE	0.023
NK	*NUCB2*	Wald ratio	1	0.935 (0.835, 1.046)	0.238	0.778	7.77E-50	TRUE	0.032
NK	*OXNAD1*	Wald ratio	1	0.905 (0.764, 1.071)	0.245	0.778	1.06E-21	TRUE	0.029
NK	*PRELID1*	Wald ratio	1	1.01 (0.844, 1.209)	0.912	0.95	3.51E-18	TRUE	0.022
NK	*RAC1*	Wald ratio	1	1.038 (0.925, 1.163)	0.528	0.824	8.13E-50	TRUE	0.019
NK	*RP11-273G15.2*	Wald ratio	1	1.052 (0.915, 1.211)	0.473	0.807	6.67E-34	TRUE	0.013
NK	*RPS12*	Wald ratio	1	1.086 (0.833, 1.416)	0.542	0.824	2.17E-08	TRUE	0.032
NK	*RPS9*	Wald ratio	1	1.024 (0.938, 1.117)	0.6	0.848	1.29E-91	TRUE	0.02
NK	*SAT2*	Wald ratio	1	0.8 (0.633, 1.012)	0.062	0.564	2.48E-11	TRUE	0.086
NK	*SCD5*	Wald ratio	1	0.933 (0.775, 1.123)	0.462	0.807	2.15E-19	TRUE	0.026
NK	*SERAC1*	Wald ratio	1	0.78 (0.6, 1.014)	0.063	0.564	3.10E-09	TRUE	0.14
NK	*SNAP29*	Wald ratio	1	0.93 (0.803, 1.076)	0.33	0.807	9.53E-35	TRUE	0.028
NK	*SNRPD2*	Wald ratio	1	0.917 (0.752, 1.118)	0.391	0.807	6.05E-16	TRUE	0.063
NK	*ST3GAL5*	Wald ratio	1	0.98 (0.788, 1.219)	0.854	0.93	3.60E-12	TRUE	0.019
NK	*TBC1D12*	Wald ratio	1	1.047 (0.828, 1.324)	0.7	0.848	4.57E-12	TRUE	0.018
NK	*THAP9-AS1*	Wald ratio	1	1.074 (0.88, 1.312)	0.48	0.807	3.01E-19	TRUE	0.028
NK	*TMEM241*	Wald ratio	1	1.068 (0.803, 1.419)	0.652	0.848	7.30E-09	TRUE	0.019
NK	*UBE3B*	Wald ratio	1	1.037 (0.737, 1.459)	0.834	0.922	7.50E-08	TRUE	0.032
NK	*VIM*	Wald ratio	1	1.201 (0.868, 1.663)	0.268	0.778	2.20E-07	TRUE	0.03
NK	*WARS*	Wald ratio	1	0.796 (0.619, 1.024)	0.076	0.564	6.87E-11	TRUE	0.078
NK	*ZNF100*	Wald ratio	1	0.973 (0.804, 1.177)	0.775	0.883	5.22E-18	TRUE	0.017
Plasma	*HLA-DQB1*	Wald ratio	1	0.799 (0.639, 0.999)	0.049	0.564	1.76E-09	TRUE	0.07
Plasma	*NFKBIL1*	Wald ratio	1	0.971 (0.79, 1.193)	0.778	0.884	2.34E-14	TRUE	0.021
Plasma	*NUCB2*	Wald ratio	1	0.879 (0.71, 1.089)	0.238	0.778	1.46E-11	TRUE	0.032
Plasma	*RNF146*	Wald ratio	1	0.921 (0.82, 1.033)	0.16	0.778	1.52E-35	TRUE	0.017
Plasma	*RXRB*	Wald ratio	1	1.146 (0.864, 1.52)	0.346	0.807	1.58E-07	TRUE	0.029

Table S8. MR and colocalization analyses on the cell type-specific associations between hypertension-related eGenes and hypertensive encephalopathy.

Cell type	eGene	Method	nSNP	OR (95% CI)	*P* -value	*FDR*	Steiger pval	Steiger direction	PP.H4.abf
B IN	*ACAT1*	Wald ratio	1	0.558 (0.245, 1.272)	0.165	0.661	2.74E-05	TRUE	0.099
B IN	*AP003774.1*	Wald ratio	1	0.518 (0.292, 0.92)	0.025	0.278	1.84E-12	TRUE	0.225
B IN	*BLK*	Wald ratio	1	2.014 (1.174, 3.455)	0.011	0.208	1.90E-13	TRUE	0.293
B IN	*C10orf32*	Wald ratio	1	1.409 (0.596, 3.334)	0.435	0.818	1.69E-08	TRUE	0.07
B IN	*C6orf48*	Wald ratio	1	1.297 (0.77, 2.187)	0.328	0.78	2.48E-10	TRUE	0.048
B IN	*C6orf62*	Wald ratio	1	0.634 (0.229, 1.759)	0.381	0.799	6.37E-06	TRUE	0.055
B IN	*CD164*	Wald ratio	1	0.872 (0.313, 2.428)	0.793	0.91	2.30E-07	TRUE	0.054
B IN	*CENPV*	Wald ratio	1	1.311 (0.587, 2.928)	0.509	0.829	6.96E-10	TRUE	0.05
B IN	*DDX5*	Wald ratio	1	0.715 (0.333, 1.534)	0.389	0.799	2.12E-09	TRUE	0.062
B IN	*FAM167A*	Wald ratio	1	2.115 (1.189, 3.762)	0.011	0.208	2.32E-11	TRUE	0.297
B IN	*FANCA*	Wald ratio	1	0.907 (0.302, 2.728)	0.862	0.941	8.33E-08	TRUE	0.06
B IN	*FNBP4*	Wald ratio	1	1.338 (0.566, 3.162)	0.506	0.829	4.83E-09	TRUE	0.048
B IN	*GTF2H4*	IVW	3	0.714 (0.46, 1.109)	0.134	0.65	1.02E-26	TRUE	0.037
B IN	*GTF2H4*	MR Egger	3	0.632 (0.037, 10.642)	0.804	NA	NA	NA	NA
B IN	*GTF2H4*	Weighted median	3	0.689 (0.417, 1.138)	0.146	NA	NA	NA	NA
B IN	*GTF2H4*	Simple mode	3	0.68 (0.374, 1.237)	0.334	NA	NA	NA	NA
B IN	*GTF2H4*	Weighted mode	3	0.684 (0.375, 1.246)	0.34	NA	NA	NA	NA
B IN	*HAGHL*	Wald ratio	1	0.972 (0.426, 2.214)	0.946	0.971	7.05E-09	TRUE	0.046
B IN	*HSD17B11*	Wald ratio	1	1.376 (0.545, 3.48)	0.499	0.829	8.93E-07	TRUE	0.055
B IN	*LA16c-358B7.3*	Wald ratio	1	1.891 (0.896, 3.99)	0.094	0.608	2.93E-09	TRUE	0.11
B IN	*MSMP*	Wald ratio	1	0.877 (0.372, 2.067)	0.764	0.897	2.20E-09	TRUE	0.047
B IN	*NOMO1*	Wald ratio	1	0.796 (0.348, 1.822)	0.59	0.874	3.99E-11	TRUE	0.056
B IN	*PRKD3*	Wald ratio	1	1.406 (0.574, 3.442)	0.456	0.826	4.36E-08	TRUE	0.049
B IN	*RAC1*	Wald ratio	1	0.695 (0.36, 1.34)	0.277	0.725	3.47E-13	TRUE	0.066
B IN	*RP13-514E23.2*	Wald ratio	1	1.084 (0.692, 1.697)	0.725	0.897	3.99E-33	TRUE	0.054
B IN	*RPS9*	Wald ratio	1	1.117 (0.794, 1.572)	0.525	0.829	1.13E-56	TRUE	0.049
B IN	*SNRPD2*	Wald ratio	1	2.55 (1.375, 4.729)	0.003	0.124	2.60E-08	TRUE	0.444
B IN	*WARS*	Wald ratio	1	1.349 (0.908, 2.004)	0.139	0.65	2.34E-42	TRUE	0.093
B Mem	*AP003774.1*	Wald ratio	1	0.457 (0.231, 0.904)	0.024	0.278	3.44E-08	TRUE	0.224
B Mem	*BLK*	Wald ratio	1	1.758 (1.144, 2.7)	0.01	0.208	1.48E-23	TRUE	0.309
B Mem	*C6orf48*	Wald ratio	1	0.857 (0.425, 1.726)	0.665	0.886	2.22E-07	TRUE	0.043
B Mem	*CCDC163P*	Wald ratio	1	1.435 (0.819, 2.515)	0.207	0.674	5.01E-16	TRUE	0.055
B Mem	*DDX5*	Wald ratio	1	0.675 (0.277, 1.649)	0.389	0.799	6.40E-07	TRUE	0.061
B Mem	*FAM167A*	Wald ratio	1	0.413 (0.208, 0.818)	0.011	0.208	1.15E-07	TRUE	0.289
B Mem	*FNBP4*	Wald ratio	1	0.778 (0.326, 1.859)	0.573	0.856	7.30E-11	TRUE	0.053
B Mem	*HAGH*	Wald ratio	1	0.59 (0.274, 1.271)	0.178	0.661	2.53E-07	TRUE	0.063
B Mem	*HAGHL*	Wald ratio	1	0.974 (0.44, 2.153)	0.947	0.971	7.07E-10	TRUE	0.045
B Mem	*HIST1H2AG*	Wald ratio	1	2.393 (1.207, 4.744)	0.012	0.208	2.77E-06	TRUE	0.018
B Mem	*HIST1H2BJ*	Wald ratio	1	0.608 (0.181, 2.035)	0.419	0.818	3.45E-06	TRUE	0.033
B Mem	*HIST1H3D*	Wald ratio	1	0.88 (0.508, 1.524)	0.649	0.886	1.79E-18	TRUE	0.038
B Mem	*KRTAP5-AS1*	Wald ratio	1	1.054 (0.667, 1.663)	0.823	0.916	5.44E-33	TRUE	0.058
B Mem	*LA16c-358B7.3*	Wald ratio	1	1.858 (0.968, 3.567)	0.063	0.512	7.51E-12	TRUE	0.112
B Mem	*MDM4*	Wald ratio	1	0.542 (0.235, 1.249)	0.15	0.661	2.11E-09	TRUE	0.072
B Mem	*PRKD3*	Wald ratio	1	1.405 (0.574, 3.434)	0.456	0.826	4.08E-08	TRUE	0.046
B Mem	*RP13-514E23.2*	Wald ratio	1	1.106 (0.749, 1.633)	0.612	0.875	5.85E-42	TRUE	0.054
B Mem	*RPS9*	Wald ratio	1	1.125 (0.783, 1.617)	0.525	0.829	8.96E-50	TRUE	0.049
B Mem	*SNRPD2*	Wald ratio	1	2.618 (1.407, 4.874)	0.002	0.124	4.06E-08	TRUE	0.43
B Mem	*WARS*	Wald ratio	1	1.419 (0.893, 2.256)	0.139	0.65	9.49E-30	TRUE	0.087
B Mem	*ZNF100*	Wald ratio	1	0.601 (0.274, 1.317)	0.203	0.674	3.07E-08	TRUE	0.081
CD4 ET	*AIF1*	Wald ratio	1	1.026 (0.557, 1.89)	0.935	0.971	1.40E-13	TRUE	0.038
CD4 ET	*AP003774.1*	Wald ratio	1	0.49 (0.283, 0.85)	0.011	0.208	3.12E-11	TRUE	0.292
CD4 ET	*C10orf32*	Wald ratio	1	1.795 (0.826, 3.9)	0.14	0.65	1.71E-08	TRUE	0.062
CD4 ET	*C17orf89*	Wald ratio	1	0.919 (0.409, 2.066)	0.839	0.93	4.44E-11	TRUE	0.045
CD4 ET	*CD151*	Wald ratio	1	1.445 (0.713, 2.928)	0.307	0.758	6.01E-13	TRUE	0.061
CD4 ET	*CDK10*	Wald ratio	1	0.862 (0.357, 2.083)	0.742	0.897	9.96E-09	TRUE	0.052
CD4 ET	*CTSW*	Wald ratio	1	1.375 (0.871, 2.168)	0.171	0.661	8.41E-32	TRUE	0.09
CD4 ET	*DDX5*	Wald ratio	1	0.769 (0.423, 1.397)	0.389	0.799	1.84E-15	TRUE	0.063
CD4 ET	*FDFT1*	Wald ratio	1	0.801 (0.398, 1.613)	0.534	0.829	5.98E-16	TRUE	0.058
CD4 ET	*FGF9*	Wald ratio	1	0.975 (0.396, 2.404)	0.957	0.971	6.25E-10	TRUE	0.015
CD4 ET	*FNBP4*	Wald ratio	1	1.389 (0.527, 3.66)	0.506	0.829	3.29E-07	TRUE	0.048
CD4 ET	*HAGH*	Wald ratio	1	0.629 (0.307, 1.289)	0.206	0.674	1.09E-08	TRUE	0.059
CD4 ET	*LA16c-358B7.3*	Wald ratio	1	1.82 (0.879, 3.768)	0.107	0.617	5.25E-10	TRUE	0.102
CD4 ET	*MANBA*	Wald ratio	1	0.973 (0.418, 2.263)	0.949	0.971	1.25E-10	TRUE	0.049
CD4 ET	*MDM4*	Wald ratio	1	0.75 (0.256, 2.193)	0.599	0.875	3.90E-08	TRUE	0.071
CD4 ET	*PLEKHA1*	Wald ratio	1	1.739 (0.701, 4.315)	0.233	0.705	7.22E-07	TRUE	0.078
CD4 ET	*PPT2*	Wald ratio	1	1.707 (0.796, 3.661)	0.17	0.661	2.80E-07	TRUE	0.054
CD4 ET	*RNF166*	Wald ratio	1	1.016 (0.408, 2.534)	0.972	0.981	1.11E-09	TRUE	0.044
CD4 ET	*RP11-499P20.2*	Wald ratio	1	1.521 (0.548, 4.223)	0.421	0.818	5.61E-06	TRUE	0.057
CD4 ET	*RPS9*	Wald ratio	1	1.11 (0.805, 1.529)	0.525	0.829	3.98E-65	TRUE	0.049
CD4 ET	*SNRPD2*	Wald ratio	1	2.582 (1.4, 4.763)	0.002	0.124	1.91E-08	TRUE	0.446
CD4 ET	*TNFSF13B*	Wald ratio	1	1.571 (0.684, 3.608)	0.287	0.725	1.27E-08	TRUE	0.076
CD4 NC	*AC010982.1*	Wald ratio	1	0.887 (0.33, 2.385)	0.812	0.916	8.73E-08	TRUE	0.012
CD4 NC	*ACAT1*	Wald ratio	1	0.713 (0.472, 1.075)	0.106	0.617	7.61E-22	TRUE	0.107
CD4 NC	*ACOT13*	Wald ratio	1	1.227 (0.406, 3.705)	0.717	0.897	1.95E-07	TRUE	0.057

CD4 NC	*AIF1*	Wald ratio	1	0.986 (0.701, 1.388)	0.936	0.971	7.41E-34	TRUE	0.037
CD4 NC	*AP003774.1*	Wald ratio	1	0.713 (0.531, 0.958)	0.025	0.278	3.58E-62	TRUE	0.216
CD4 NC	*AP2A2*	Wald ratio	1	3.147 (1.317, 7.522)	0.01	0.208	0.001	TRUE	0.314
CD4 NC	*BIK*	Wald ratio	1	0.323 (0.127, 0.818)	0.017	0.25	1.61E-05	TRUE	0.186
CD4 NC	*BLK*	Wald ratio	1	2.062 (1.174, 3.621)	0.012	0.208	4.39E-12	TRUE	0.28
CD4 NC	*BLMH*	Wald ratio	1	1.439 (0.542, 3.822)	0.465	0.829	1.20E-06	TRUE	0.057
CD4 NC	*C10orf32*	Wald ratio	1	1.295 (0.835, 2.01)	0.248	0.705	5.20E-32	TRUE	0.075
CD4 NC	*C2orf74*	Wald ratio	1	0.44 (0.201, 0.961)	0.039	0.377	2.52E-06	TRUE	0.172
CD4 NC	*C6orf48*	Wald ratio	1	0.915 (0.608, 1.375)	0.669	0.886	1.75E-20	TRUE	0.043
CD4 NC	*C6orf62*	Wald ratio	1	0.65 (0.248, 1.706)	0.381	0.799	1.39E-06	TRUE	0.056
CD4 NC	*CCDC104*	Wald ratio	1	0.896 (0.424, 1.891)	0.772	0.897	3.40E-14	TRUE	0.048
CD4 NC	*CCDC88B*	Wald ratio	1	1.951 (1.07, 3.557)	0.029	0.316	2.23E-11	TRUE	0.172
CD4 NC	*CD151*	Wald ratio	1	1.272 (0.675, 2.397)	0.456	0.826	9.55E-17	TRUE	0.056
CD4 NC	*CDK10*	Wald ratio	1	0.912 (0.528, 1.576)	0.742	0.897	1.75E-21	TRUE	0.046
CD4 NC	*CLNS1A*	Wald ratio	1	0.897 (0.555, 1.451)	0.658	0.886	1.38E-16	TRUE	0.053
CD4 NC	*CLUAP1*	Wald ratio	1	1.159 (0.624, 2.152)	0.64	0.881	2.65E-17	TRUE	0.048
CD4 NC	*CRHR1*	Wald ratio	1	0.553 (0.177, 1.728)	0.308	0.758	9.64E-13	TRUE	0.078
CD4 NC	*CTD-2336O2.1*	Wald ratio	1	1.295 (0.92, 1.822)	0.138	0.65	1.40E-54	TRUE	0.085
CD4 NC	*CTSW*	Wald ratio	1	1.213 (0.92, 1.599)	0.171	0.661	4.14E-101	TRUE	0.09
CD4 NC	*CUBN*	Wald ratio	1	1.28 (0.612, 2.676)	0.512	0.829	6.51E-13	TRUE	0.045
CD4 NC	*CYP2D6*	Wald ratio	1	0.418 (0.158, 1.103)	0.078	0.589	1.97E-04	TRUE	0.116
CD4 NC	*DDX5*	Wald ratio	1	0.803 (0.487, 1.323)	0.389	0.799	2.40E-22	TRUE	0.063
CD4 NC	*EEF2*	Wald ratio	1	1.738 (0.688, 4.387)	0.242	0.705	2.81E-06	TRUE	0.078
CD4 NC	*EGFL8*	Wald ratio	1	1.057 (0.46, 2.428)	0.896	0.958	8.53E-11	TRUE	0.05
CD4 NC	*ERP29*	Wald ratio	1	1.547 (0.788, 3.038)	0.205	0.674	1.95E-09	TRUE	0.089
CD4 NC	*FAM153B*	Wald ratio	1	1.18 (0.888, 1.568)	0.255	0.717	2.01E-82	TRUE	0.072
CD4 NC	*FANCA*	Wald ratio	1	0.925 (0.382, 2.24)	0.863	0.941	3.35E-11	TRUE	0.054
CD4 NC	*FDFT1*	Wald ratio	1	0.901 (0.648, 1.252)	0.534	0.829	1.57E-77	TRUE	0.058
CD4 NC	*FNBP4*	Wald ratio	1	1.028 (0.65, 1.625)	0.907	0.961	2.75E-36	TRUE	0.048
CD4 NC	*FOXN2*	Wald ratio	1	1.154 (0.564, 2.36)	0.695	0.897	5.90E-19	TRUE	0.059
CD4 NC	*GOSR2*	Wald ratio	1	1.467 (0.781, 2.756)	0.234	0.705	6.43E-14	TRUE	0.074
CD4 NC	*HAGH*	Wald ratio	1	0.814 (0.591, 1.12)	0.206	0.674	1.49E-50	TRUE	0.07
CD4 NC	*HLA-DQA1*	Wald ratio	1	0.872 (0.372, 2.044)	0.752	0.897	4.98E-10	TRUE	0.042
CD4 NC	*HLA-DRB5*	Wald ratio	1	1.121 (0.698, 1.801)	0.637	0.881	1.12E-37	TRUE	0.042
CD4 NC	*HMBS*	Wald ratio	1	1.421 (0.771, 2.619)	0.26	0.721	4.53E-17	TRUE	0.07
CD4 NC	*HSD17B11*	Wald ratio	1	1.271 (0.634, 2.547)	0.499	0.829	9.19E-12	TRUE	0.055
CD4 NC	*HSD17B12*	Wald ratio	1	1.283 (0.779, 2.112)	0.328	0.78	8.91E-23	TRUE	0.056
CD4 NC	*KB-1507C5.2*	Wald ratio	1	0.698 (0.32, 1.52)	0.365	0.799	4.34E-09	TRUE	0.054
CD4 NC	*KRTAP5-2*	Wald ratio	1	1.59 (0.989, 2.556)	0.056	0.49	7.28E-23	TRUE	0.097
CD4 NC	*LA16c-358B7.3*	Wald ratio	1	1.336 (0.951, 1.877)	0.094	0.608	1.28E-55	TRUE	0.102
CD4 NC	*LAIR2*	Wald ratio	1	1.126 (0.572, 2.215)	0.732	0.897	1.04E-20	TRUE	0.06
CD4 NC	*LIMD2*	Wald ratio	1	1.929 (0.698, 5.332)	0.205	0.674	1.25E-05	TRUE	0.084
CD4 NC	*LLGL1*	Wald ratio	1	1.162 (0.544, 2.482)	0.698	0.897	8.62E-12	TRUE	0.051
CD4 NC	*LRRC61*	Wald ratio	1	0.835 (0.537, 1.3)	0.426	0.818	7.44E-28	TRUE	0.056
CD4 NC	*LSM4*	Wald ratio	1	0.783 (0.408, 1.502)	0.462	0.827	4.43E-10	TRUE	0.061
CD4 NC	*MACF1*	Wald ratio	1	0.623 (0.334, 1.16)	0.135	0.65	2.54E-12	TRUE	0.074
CD4 NC	*MARK3*	Wald ratio	1	0.806 (0.36, 1.805)	0.601	0.875	2.24E-10	TRUE	0.05
CD4 NC	*MEI1*	Wald ratio	1	1.895 (0.939, 3.823)	0.074	0.58	7.80E-08	TRUE	0.127
CD4 NC	*MRPS34*	Wald ratio	1	0.44 (0.164, 1.181)	0.103	0.617	1.78E-04	TRUE	0.051
CD4 NC	*MYO16*	Wald ratio	1	1.301 (0.801, 2.112)	0.287	0.725	8.78E-27	TRUE	0.064
CD4 NC	*NARFL*	Wald ratio	1	1.032 (0.506, 2.104)	0.93	0.971	8.54E-11	TRUE	0.045
CD4 NC	*NEIL2*	Wald ratio	1	0.738 (0.283, 1.925)	0.534	0.829	1.21E-08	TRUE	0.06
CD4 NC	*OSER1-AS1*	Wald ratio	1	0.569 (0.289, 1.119)	0.102	0.617	4.82E-09	TRUE	0.107
CD4 NC	*PABPC4*	Wald ratio	1	1.021 (0.358, 2.915)	0.968	0.98	3.31E-08	TRUE	0.057
CD4 NC	*PCNX*	Wald ratio	1	1.276 (0.721, 2.261)	0.403	0.813	2.92E-18	TRUE	0.053
CD4 NC	*PGPEP1*	Wald ratio	1	1.266 (0.564, 2.84)	0.567	0.852	5.05E-11	TRUE	0.052
CD4 NC	*PHACTR4*	Wald ratio	1	0.837 (0.462, 1.514)	0.555	0.849	3.36E-17	TRUE	0.058
CD4 NC	*PKMYT1*	Wald ratio	1	0.952 (0.581, 1.561)	0.846	0.931	3.98E-25	TRUE	0.028
CD4 NC	*PRKD3*	Wald ratio	1	1.339 (0.621, 2.888)	0.456	0.826	6.70E-11	TRUE	0.043
CD4 NC	*RAC1*	Wald ratio	1	0.821 (0.575, 1.172)	0.277	0.725	3.15E-49	TRUE	0.072
CD4 NC	*REEP3*	Wald ratio	1	0.638 (0.217, 1.88)	0.415	0.818	4.63E-06	TRUE	0.059
CD4 NC	*RNF8*	Wald ratio	1	1.736 (0.636, 4.738)	0.281	0.725	8.56E-06	TRUE	0.054
CD4 NC	*RP11-273G15.2*	Wald ratio	1	1.615 (0.87, 2.998)	0.129	0.65	5.91E-13	TRUE	0.094
CD4 NC	*RP11-796G6.2*	Wald ratio	1	2.809 (0.71, 11.11)	0.141	0.65	1.74E-05	TRUE	0.084
CD4 NC	*RPS9*	Wald ratio	1	1.093 (0.831, 1.439)	0.525	0.829	4.05E-92	TRUE	0.049
CD4 NC	*SAT2*	Wald ratio	1	1.408 (0.809, 2.452)	0.226	0.705	2.36E-18	TRUE	0.075
CD4 NC	*SLC22A5*	Wald ratio	1	0.911 (0.352, 2.354)	0.847	0.931	2.70E-08	TRUE	0.048
CD4 NC	*SNRPC*	Wald ratio	1	0.674 (0.401, 1.134)	0.138	0.65	2.84E-19	TRUE	0.089
CD4 NC	*SNRPD2*	Wald ratio	1	1.612 (1.185, 2.195)	0.002	0.124	1.43E-50	TRUE	0.459
CD4 NC	*SNX16*	Wald ratio	1	0.827 (0.36, 1.901)	0.654	0.886	4.70E-11	TRUE	0.049
CD4 NC	*SPCS1*	Wald ratio	1	0.759 (0.359, 1.607)	0.472	0.829	6.45E-11	TRUE	0.056
CD4 NC	*TMEM199*	Wald ratio	1	0.972 (0.407, 2.321)	0.949	0.971	2.62E-10	TRUE	0.048
CD4 NC	*TMEM87B*	Wald ratio	1	2.742 (1.288, 5.836)	0.009	0.208	2.53E-05	TRUE	0.337
CD4 NC	*TMEM97*	Wald ratio	1	0.9 (0.394, 2.056)	0.802	0.916	1.54E-10	TRUE	0.051
CD4 NC	*TNFRSF18*	Wald ratio	1	0.474 (0.182, 1.237)	0.127	0.65	1.73E-04	TRUE	0.087
CD4 NC	*TTC12*	Wald ratio	1	1.002 (0.427, 2.349)	0.997	0.997	1.02E-10	TRUE	0.045
CD4 NC	*UACA*	Wald ratio	1	1.181 (0.519, 2.69)	0.691	0.897	1.83E-08	TRUE	0.063
CD4 NC	*UBE2I*	Wald ratio	1	1.95 (0.732, 5.193)	0.181	0.661	2.82E-07	TRUE	0.078
CD4 NC	*UBE3B*	Wald ratio	1	0.829 (0.318, 2.164)	0.702	0.897	3.20E-09	TRUE	0.065

CD4 NC	*USP36*	Wald ratio	1	1.612 (0.699, 3.715)	0.263	0.721	3.47E-08	TRUE	0.057
CD4 NC	*VIM*	Wald ratio	1	0.864 (0.334, 2.235)	0.764	0.897	1.21E-07	TRUE	0.043
CD4 NC	*VPS53*	Wald ratio	1	0.672 (0.292, 1.548)	0.351	0.799	8.94E-09	TRUE	0.054
CD4 NC	*WBSCR16*	Wald ratio	1	1.122 (0.428, 2.942)	0.815	0.916	1.35E-08	TRUE	0.054
CD4 NC	*ZNF100*	Wald ratio	1	1.306 (0.877, 1.945)	0.189	0.674	1.35E-35	TRUE	0.074
CD4 NC	*ZNF502*	Wald ratio	1	0.807 (0.518, 1.258)	0.344	0.799	8.82E-30	TRUE	0.066
CD4 NC	*ZSWIM7*	Wald ratio	1	0.704 (0.367, 1.349)	0.29	0.725	1.18E-13	TRUE	0.069
CD4 SOX4	*DAXX*	Wald ratio	1	1.127 (0.402, 3.163)	0.82	0.916	5.29E-08	TRUE	0.018
CD4 SOX4	*DXO*	Wald ratio	1	0.542 (0.279, 1.054)	0.071	0.567	1.33E-09	TRUE	0.048
CD4 SOX4	*RPS26*	Wald ratio	1	1.025 (0.83, 1.266)	0.818	0.916	6.31E-164	TRUE	0.049
CD4 SOX4	*RPS9*	Wald ratio	1	1.158 (0.543, 2.471)	0.704	0.897	6.46E-11	TRUE	0.051
CD8 ET	*ADRB1*	Wald ratio	1	1.041 (0.563, 1.924)	0.898	0.958	5.55E-15	TRUE	0.049
CD8 ET	*AP003774.1*	Wald ratio	1	0.652 (0.468, 0.907)	0.011	0.208	4.32E-39	TRUE	0.314
CD8 ET	*AP2A2*	Wald ratio	1	2.604 (1.202, 5.642)	0.015	0.243	3.45E-05	TRUE	0.313
CD8 ET	*BTN3A1*	Wald ratio	1	1.325 (0.573, 3.062)	0.511	0.829	2.79E-08	TRUE	0.059
CD8 ET	*C10orf32*	Wald ratio	1	1.444 (0.774, 2.695)	0.248	0.705	4.71E-15	TRUE	0.069
CD8 ET	*C2orf74*	Wald ratio	1	2.375 (1.061, 5.318)	0.035	0.37	1.35E-05	TRUE	0.146
CD8 ET	*C6orf62*	Wald ratio	1	0.633 (0.228, 1.761)	0.381	0.799	6.80E-06	TRUE	0.057
CD8 ET	*CCDC88B*	Wald ratio	1	2.465 (0.964, 6.302)	0.06	0.499	1.89E-04	TRUE	0.247
CD8 ET	*CDK10*	Wald ratio	1	0.888 (0.44, 1.794)	0.742	0.897	2.86E-13	TRUE	0.049
CD8 ET	*CTD-2515A14.1*	Wald ratio	1	0.868 (0.332, 2.268)	0.773	0.897	7.49E-08	TRUE	0.044
CD8 ET	*CTSW*	Wald ratio	1	1.218 (0.918, 1.617)	0.171	0.661	1.75E-95	TRUE	0.09
CD8 ET	*DDX5*	Wald ratio	1	0.801 (0.483, 1.328)	0.389	0.799	9.28E-22	TRUE	0.063
CD8 ET	*DGKD*	Wald ratio	1	2.373 (0.874, 6.446)	0.09	0.608	2.79E-04	TRUE	0.075
CD8 ET	*FDFT1*	Wald ratio	1	0.77 (0.338, 1.756)	0.534	0.829	1.52E-11	TRUE	0.058
CD8 ET	*FIBP*	Wald ratio	1	0.512 (0.194, 1.351)	0.177	0.661	2.20E-06	TRUE	0.09
CD8 ET	*FNBP4*	Wald ratio	1	1.123 (0.554, 2.275)	0.748	0.897	6.08E-15	TRUE	0.048
CD8 ET	*FOXN2*	Wald ratio	1	1.05 (0.404, 2.725)	0.921	0.971	4.66E-11	TRUE	0.058
CD8 ET	*HAGH*	Wald ratio	1	0.72 (0.425, 1.22)	0.222	0.701	8.13E-17	TRUE	0.053
CD8 ET	*HLA-DQB2*	Wald ratio	1	1.221 (0.633, 2.353)	0.551	0.847	4.70E-16	TRUE	0.04
CD8 ET	*LA16c-358B7.3*	Wald ratio	1	1.548 (0.928, 2.581)	0.094	0.608	9.57E-22	TRUE	0.107
CD8 ET	*LAIR2*	Wald ratio	1	1.026 (0.582, 1.809)	0.929	0.971	5.73E-30	TRUE	0.058
CD8 ET	*LLGL1*	Wald ratio	1	1.274 (0.559, 2.902)	0.564	0.852	1.20E-09	TRUE	0.052
CD8 ET	*LRRC37A*	Wald ratio	1	0.798 (0.487, 1.307)	0.37	0.799	1.83E-20	TRUE	0.066
CD8 ET	*LRRC61*	Wald ratio	1	0.7 (0.276, 1.778)	0.454	0.826	6.46E-07	TRUE	0.055
CD8 ET	*MDM4*	Wald ratio	1	0.574 (0.246, 1.34)	0.199	0.674	3.60E-09	TRUE	0.076
CD8 ET	*MLF1*	Wald ratio	1	0.889 (0.459, 1.722)	0.726	0.897	1.79E-12	TRUE	0.048
CD8 ET	*MPHOSPH9*	Wald ratio	1	0.741 (0.266, 2.066)	0.567	0.852	8.65E-07	TRUE	0.056
CD8 ET	*NOTCH4*	Wald ratio	1	1.202 (0.417, 3.468)	0.733	0.897	2.66E-08	TRUE	0.047
CD8 ET	*NUCB2*	Wald ratio	1	0.917 (0.655, 1.283)	0.613	0.875	1.13E-59	TRUE	0.047
CD8 ET	*OXNAD1*	Wald ratio	1	2.457 (1.281, 4.71)	0.007	0.208	9.99E-08	TRUE	0.346
CD8 ET	*PDCD6*	Wald ratio	1	0.804 (0.325, 1.986)	0.636	0.881	3.17E-08	TRUE	0.048
CD8 ET	*PHACTR4*	Wald ratio	1	0.702 (0.288, 1.713)	0.437	0.818	2.34E-07	TRUE	0.058
CD8 ET	*REEP5*	Wald ratio	1	0.898 (0.363, 2.222)	0.817	0.916	3.86E-09	TRUE	0.047
CD8 ET	*RP4-799D16.1*	Wald ratio	1	2.418 (1.007, 5.808)	0.048	0.449	7.02E-05	TRUE	0.158
CD8 ET	*RPS9*	Wald ratio	1	1.094 (0.83, 1.441)	0.525	0.829	1.44E-91	TRUE	0.049
CD8 ET	*SAT2*	Wald ratio	1	1.566 (0.74, 3.313)	0.241	0.705	7.55E-10	TRUE	0.074
CD8 ET	*SCD5*	Wald ratio	1	0.863 (0.464, 1.605)	0.641	0.881	6.63E-18	TRUE	0.053
CD8 ET	*SNRPC*	Wald ratio	1	0.478 (0.177, 1.291)	0.145	0.65	9.51E-05	TRUE	0.087
CD8 ET	*SNRPD2*	Wald ratio	1	1.928 (1.262, 2.945)	0.002	0.124	2.49E-22	TRUE	0.463
CD8 ET	*THAP9-AS1*	Wald ratio	1	1.158 (0.625, 2.146)	0.641	0.881	5.28E-18	TRUE	0.055
CD8 ET	*VIM*	Wald ratio	1	1.022 (0.46, 2.27)	0.957	0.971	2.57E-11	TRUE	0.045
CD8 ET	*WARS*	Wald ratio	1	1.66 (0.601, 4.585)	0.328	0.78	2.70E-06	TRUE	0.075
CD8 ET	*XBP1*	Wald ratio	1	0.848 (0.368, 1.953)	0.699	0.897	3.55E-09	TRUE	0.054
CD8 ET	*ZNF100*	Wald ratio	1	1.577 (0.808, 3.077)	0.181	0.661	1.89E-11	TRUE	0.076
CD8 ET	*ZNF502*	Wald ratio	1	0.65 (0.266, 1.587)	0.344	0.799	4.59E-07	TRUE	0.065
CD8 ET	*ZSWIM7*	Wald ratio	1	0.67 (0.318, 1.408)	0.29	0.725	2.24E-10	TRUE	0.066
CD8 NC	*AP003774.1*	Wald ratio	1	0.61 (0.396, 0.94)	0.025	0.278	9.34E-25	TRUE	0.231
CD8 NC	*BTN3A1*	Wald ratio	1	1.304 (0.592, 2.872)	0.51	0.829	3.88E-09	TRUE	0.059
CD8 NC	*C6orf48*	Wald ratio	1	0.893 (0.535, 1.491)	0.665	0.886	3.44E-13	TRUE	0.043
CD8 NC	*CEBPZ*	Wald ratio	1	2.875 (1.06, 7.797)	0.038	0.374	9.99E-04	TRUE	0.078
CD8 NC	*CLNS1A*	Wald ratio	1	0.85 (0.412, 1.751)	0.659	0.886	7.52E-08	TRUE	0.053
CD8 NC	*CLUAP1*	Wald ratio	1	1.375 (0.544, 3.478)	0.501	0.829	1.85E-07	TRUE	0.049
CD8 NC	*COX14*	Wald ratio	1	0.864 (0.335, 2.226)	0.762	0.897	7.05E-09	TRUE	0.051
CD8 NC	*CRHR1*	Wald ratio	1	2.02 (0.562, 7.267)	0.282	0.725	7.02E-10	TRUE	0.078
CD8 NC	*CTD-2336O2.1*	Wald ratio	1	1.652 (0.983, 2.777)	0.058	0.499	5.56E-21	TRUE	0.107
CD8 NC	*CTSW*	Wald ratio	1	1.218 (0.918, 1.616)	0.171	0.661	1.65E-95	TRUE	0.09
CD8 NC	*DCP2*	Wald ratio	1	0.811 (0.356, 1.844)	0.617	0.875	1.55E-08	TRUE	0.053
CD8 NC	*DDR1*	IVW	2	0.996 (0.43, 2.305)	0.993	0.996	4.21E-21	TRUE	0.043
CD8 NC	*DDX5*	Wald ratio	1	0.77 (0.424, 1.396)	0.389	0.799	1.51E-15	TRUE	0.063
CD8 NC	*FNBP4*	Wald ratio	1	1.049 (0.47, 2.342)	0.907	0.961	1.86E-12	TRUE	0.048
CD8 NC	*FOXN2*	Wald ratio	1	1.251 (0.4, 3.916)	0.7	0.897	6.26E-08	TRUE	0.06
CD8 NC	*HAGH*	Wald ratio	1	0.736 (0.449, 1.204)	0.222	0.701	1.88E-19	TRUE	0.068
CD8 NC	*HLA-DRB5*	Wald ratio	1	1.194 (0.746, 1.911)	0.459	0.827	2.03E-35	TRUE	0.043
CD8 NC	*HSD17B12*	Wald ratio	1	1.165 (0.464, 2.927)	0.745	0.897	2.93E-08	TRUE	0.048
CD8 NC	*LA16c-358B7.3*	Wald ratio	1	1.557 (0.927, 2.614)	0.094	0.608	4.25E-21	TRUE	0.103
CD8 NC	*LAIR2*	Wald ratio	1	1.054 (0.334, 3.327)	0.929	0.971	3.75E-08	TRUE	0.057
CD8 NC	*LRRC37A*	Wald ratio	1	0.792 (0.442, 1.418)	0.432	0.818	2.17E-15	TRUE	0.058
CD8 NC	*LRRC61*	Wald ratio	1	0.714 (0.308, 1.654)	0.432	0.818	1.47E-07	TRUE	0.056

CD8 NC	*MPHOSPH9*	Wald ratio	1	0.94 (0.413, 2.139)	0.883	0.958	1.52E-11	TRUE	0.056
CD8 NC	*MYO16*	Wald ratio	1	0.805 (0.492, 1.318)	0.389	0.799	2.02E-27	TRUE	0.055
CD8 NC	*PHACTR4*	Wald ratio	1	0.637 (0.263, 1.542)	0.317	0.776	3.52E-07	TRUE	0.058
CD8 NC	*RP11-273G15.2*	Wald ratio	1	0.395 (0.153, 1.02)	0.055	0.49	3.05E-04	TRUE	0.114
CD8 NC	*RPS9*	Wald ratio	1	1.059 (0.788, 1.425)	0.704	0.897	6.64E-83	TRUE	0.049
CD8 NC	*SAT2*	Wald ratio	1	1.809 (0.708, 4.621)	0.215	0.693	5.10E-06	TRUE	0.075
CD8 NC	*SCD5*	Wald ratio	1	1.286 (0.484, 3.416)	0.614	0.875	1.91E-08	TRUE	0.054
CD8 NC	*SNRPC*	Wald ratio	1	0.542 (0.238, 1.236)	0.146	0.65	4.43E-07	TRUE	0.087
CD8 NC	*SNRPD2*	Wald ratio	1	1.811 (1.224, 2.679)	0.003	0.124	2.73E-27	TRUE	0.446
CD8 NC	*STK32C*	Wald ratio	1	0.745 (0.197, 2.82)	0.664	0.886	1.65E-07	TRUE	0.057
CD8 NC	*THAP9-AS1*	Wald ratio	1	0.807 (0.351, 1.858)	0.614	0.875	1.86E-11	TRUE	0.055
CD8 NC	*USP36*	Wald ratio	1	1.801 (0.644, 5.039)	0.263	0.721	2.25E-05	TRUE	0.058
CD8 NC	*VIM*	Wald ratio	1	0.89 (0.401, 1.976)	0.775	0.897	1.68E-10	TRUE	0.042
CD8 NC	*ZNF100*	Wald ratio	1	1.623 (0.799, 3.298)	0.181	0.661	4.41E-10	TRUE	0.074
CD8 NC	*ZSWIM7*	Wald ratio	1	0.6 (0.233, 1.546)	0.29	0.725	2.37E-06	TRUE	0.067
CD8 S100B	*AP003774.1*	Wald ratio	1	0.418 (0.195, 0.896)	0.025	0.278	3.05E-06	TRUE	0.213
CD8 S100B	*C10orf32*	Wald ratio	1	1.963 (0.802, 4.807)	0.14	0.65	3.11E-06	TRUE	0.076
CD8 S100B	*CD151*	Wald ratio	1	1.386 (0.51, 3.767)	0.523	0.829	5.31E-07	TRUE	0.052
CD8 S100B	*CTSW*	Wald ratio	1	1.285 (0.897, 1.839)	0.171	0.661	2.10E-54	TRUE	0.09
CD8 S100B	*DDX5*	Wald ratio	1	0.723 (0.345, 1.512)	0.389	0.799	4.87E-10	TRUE	0.063
CD8 S100B	*EEF2*	Wald ratio	1	1.765 (0.681, 4.575)	0.242	0.705	6.28E-06	TRUE	0.086
CD8 S100B	*FAM85A*	Wald ratio	1	0.919 (0.57, 1.483)	0.73	0.897	2.29E-28	TRUE	0.049
CD8 S100B	*FDFT1*	Wald ratio	1	0.723 (0.261, 2.008)	0.534	0.829	1.14E-07	TRUE	0.057
CD8 S100B	*HSPA1L*	Wald ratio	1	1.114 (0.575, 2.16)	0.749	0.897	2.14E-21	TRUE	0.108
CD8 S100B	*KIAA0040*	Wald ratio	1	2.085 (0.797, 5.451)	0.134	0.65	3.86E-05	TRUE	0.123
CD8 S100B	*MDM4*	Wald ratio	1	0.551 (0.208, 1.46)	0.23	0.705	4.21E-07	TRUE	0.075
CD8 S100B	*RPS12*	Wald ratio	1	1.126 (0.496, 2.558)	0.777	0.897	7.64E-10	TRUE	0.067
CD8 S100B	*RPS9*	Wald ratio	1	1.128 (0.778, 1.634)	0.525	0.829	1.75E-47	TRUE	0.049
CD8 S100B	*SCD5*	Wald ratio	1	1.182 (0.585, 2.388)	0.641	0.881	4.97E-14	TRUE	0.052
CD8 S100B	*SNRPD2*	Wald ratio	1	3.167 (1.481, 6.776)	0.003	0.124	9.97E-05	TRUE	0.372
CD8 S100B	*TNFSF13B*	Wald ratio	1	2.06 (0.746, 5.689)	0.163	0.661	6.09E-05	TRUE	0.083
CD8 S100B	*TTC12*	Wald ratio	1	1.247 (0.722, 2.154)	0.429	0.818	1.05E-20	TRUE	0.037
CD8 S100B	*XBP1*	Wald ratio	1	0.726 (0.279, 1.893)	0.513	0.829	1.45E-06	TRUE	0.057
DC	*ATAT1*	Wald ratio	1	1.357 (0.683, 2.693)	0.383	0.799	4.30E-11	TRUE	0.047
DC	*DDX5*	Wald ratio	1	0.66 (0.257, 1.697)	0.389	0.799	3.81E-06	TRUE	0.062
DC	*MICB*	Wald ratio	1	1.356 (0.706, 2.605)	0.361	0.799	3.69E-13	TRUE	0.044
Mono C	*AP003774.1*	Wald ratio	1	2.432 (1.119, 5.288)	0.025	0.278	6.54E-06	TRUE	0.195
Mono C	*C10orf32*	Wald ratio	1	1.423 (0.587, 3.453)	0.435	0.818	6.01E-08	TRUE	0.082
Mono C	*HP*	Wald ratio	1	0.888 (0.545, 1.447)	0.632	0.881	1.51E-26	TRUE	0.054
Mono C	*PRELID1*	Wald ratio	1	1.27 (0.733, 2.2)	0.393	0.799	4.86E-17	TRUE	0.065
Mono C	*RPS9*	Wald ratio	1	1.15 (0.747, 1.77)	0.525	0.829	3.69E-34	TRUE	0.049
Mono C	*TREML1*	Wald ratio	1	0.974 (0.667, 1.424)	0.894	0.958	2.83E-40	TRUE	0.017
Mono NC	*NCR3*	Wald ratio	1	0.766 (0.405, 1.448)	0.412	0.818	5.83E-16	TRUE	0.084
Mono NC	*PRELID1*	Wald ratio	1	1.204 (0.668, 2.172)	0.537	0.829	3.78E-13	TRUE	0.062
Mono NC	*RPH3A*	Wald ratio	1	0.776 (0.361, 1.668)	0.517	0.829	4.84E-11	TRUE	0.053
Mono NC	*RPS26*	Wald ratio	1	1.025 (0.831, 1.264)	0.818	0.916	2.03E-181	TRUE	0.049
Mono NC	*RPS9*	Wald ratio	1	1.228 (0.652, 2.314)	0.525	0.829	4.39E-15	TRUE	0.049
Mono NC	*VPS52*	Wald ratio	1	0.52 (0.242, 1.115)	0.093	0.608	3.15E-07	TRUE	0.044
NK R	*AC016831.7*	Wald ratio	1	0.56 (0.325, 0.966)	0.037	0.374	6.04E-23	TRUE	0.01
NK R	*APOM*	Wald ratio	1	0.628 (0.272, 1.447)	0.274	0.725	1.60E-08	TRUE	0.042
NK R	*CTSW*	Wald ratio	1	1.88 (0.752, 4.698)	0.177	0.661	3.89E-07	TRUE	0.09
NK R	*RAC1*	Wald ratio	1	0.598 (0.231, 1.546)	0.289	0.725	6.05E-06	TRUE	0.063
NK R	*SYNGAP1*	Wald ratio	1	0.903 (0.47, 1.737)	0.761	0.897	9.26E-16	TRUE	0.039
NK R	*XXbac-BPG299F13.17*	Wald ratio	1	1.169 (0.487, 2.81)	0.727	0.897	3.65E-09	TRUE	0.043
NK	*ADRB1*	Wald ratio	1	1.038 (0.587, 1.835)	0.898	0.958	3.31E-17	TRUE	0.049
NK	*AP003774.1*	Wald ratio	1	0.683 (0.491, 0.95)	0.023	0.278	1.69E-47	TRUE	0.215
NK	*AP2A2*	Wald ratio	1	1.971 (1.176, 3.302)	0.01	0.208	2.12E-13	TRUE	0.312
NK	*ATP5G1*	Wald ratio	1	1.734 (0.829, 3.628)	0.144	0.65	2.62E-09	TRUE	0.116
NK	*BTN3A1*	Wald ratio	1	1.37 (0.536, 3.505)	0.511	0.829	9.90E-07	TRUE	0.058
NK	*C10orf32*	Wald ratio	1	1.475 (0.762, 2.854)	0.248	0.705	1.85E-13	TRUE	0.072
NK	*C6orf62*	Wald ratio	1	0.652 (0.25, 1.699)	0.381	0.799	9.73E-07	TRUE	0.056
NK	*CCDC88B*	Wald ratio	1	2.081 (0.862, 5.023)	0.103	0.617	9.37E-06	TRUE	0.117
NK	*CD151*	Wald ratio	1	1.209 (0.734, 1.992)	0.456	0.826	2.39E-27	TRUE	0.056
NK	*CDK10*	Wald ratio	1	0.898 (0.475, 1.7)	0.742	0.897	5.51E-16	TRUE	0.063
NK	*CTSW*	Wald ratio	1	1.25 (0.909, 1.719)	0.17	0.661	9.68E-71	TRUE	0.09
NK	*DDX42*	Wald ratio	1	1.772 (0.682, 4.608)	0.24	0.705	8.69E-07	TRUE	0.08
NK	*DDX5*	Wald ratio	1	0.808 (0.498, 1.312)	0.389	0.799	7.80E-24	TRUE	0.063
NK	*EPHX4*	Wald ratio	1	1.167 (0.451, 3.022)	0.75	0.897	1.07E-08	TRUE	0.066
NK	*ERAP1*	Wald ratio	1	0.419 (0.159, 1.106)	0.079	0.589	1.03E-04	TRUE	0.053
NK	*FIBP*	Wald ratio	1	0.646 (0.346, 1.208)	0.171	0.661	5.33E-16	TRUE	0.086
NK	*GNGT2*	Wald ratio	1	1.006 (0.432, 2.345)	0.988	0.994	3.50E-10	TRUE	0.046
NK	*HAGH*	Wald ratio	1	0.698 (0.4, 1.218)	0.206	0.674	5.61E-15	TRUE	0.068
NK	*HLA-DOA*	Wald ratio	1	1.131 (0.39, 3.28)	0.82	0.916	1.68E-08	TRUE	0.049
NK	*IGF2BP2*	Wald ratio	1	0.67 (0.268, 1.675)	0.392	0.799	2.36E-06	TRUE	0.059
NK	*KHK*	Wald ratio	1	1.205 (0.435, 3.336)	0.72	0.897	3.58E-07	TRUE	0.051
NK	*LA16c-358B7.3*	Wald ratio	1	1.463 (0.937, 2.284)	0.094	0.608	6.44E-30	TRUE	0.103
NK	*LLGL1*	Wald ratio	1	1.327 (0.507, 3.47)	0.564	0.852	2.98E-07	TRUE	0.053
NK	*LRRC61*	Wald ratio	1	0.728 (0.296, 1.789)	0.488	0.829	1.27E-07	TRUE	0.055
NK	*MDM4*	Wald ratio	1	0.518 (0.189, 1.415)	0.199	0.674	1.64E-06	TRUE	0.077

NK	*MLF1*	Wald ratio	1	0.892 (0.462, 1.723)	0.734	0.897	5.82E-13	TRUE	0.048
NK	*MPHOSPH9*	Wald ratio	1	0.969 (0.465, 2.02)	0.934	0.971	8.47E-13	TRUE	0.053
NK	*NAA40*	Wald ratio	1	0.93 (0.337, 2.571)	0.889	0.958	1.15E-07	TRUE	0.046
NK	*NUCB2*	Wald ratio	1	0.909 (0.629, 1.315)	0.614	0.875	7.28E-49	TRUE	0.047
NK	*OXNAD1*	Wald ratio	1	1.86 (1.118, 3.093)	0.017	0.25	2.72E-14	TRUE	0.231
NK	*PRELID1*	Wald ratio	1	1.043 (0.578, 1.879)	0.889	0.958	9.10E-18	TRUE	0.058
NK	*RAC1*	Wald ratio	1	0.812 (0.558, 1.182)	0.277	0.725	6.98E-44	TRUE	0.071
NK	*RP11-273G15.2*	Wald ratio	1	0.809 (0.513, 1.277)	0.363	0.799	3.92E-30	TRUE	0.046
NK	*RPS12*	Wald ratio	1	1.091 (0.452, 2.632)	0.846	0.931	2.30E-08	TRUE	0.06
NK	*RPS9*	Wald ratio	1	1.057 (0.794, 1.406)	0.704	0.897	1.42E-89	TRUE	0.048
NK	*SAT2*	Wald ratio	1	1.578 (0.735, 3.388)	0.242	0.705	1.53E-09	TRUE	0.073
NK	*SCD5*	Wald ratio	1	0.865 (0.469, 1.593)	0.641	0.881	2.21E-18	TRUE	0.053
NK	*SERAC1*	Wald ratio	1	0.973 (0.412, 2.298)	0.95	0.971	1.02E-10	TRUE	0.043
NK	*SNAP29*	Wald ratio	1	0.937 (0.582, 1.51)	0.791	0.91	5.87E-35	TRUE	0.048
NK	*SNRPD2*	Wald ratio	1	2.751 (1.432, 5.286)	0.002	0.124	2.75E-07	TRUE	0.327
NK	*ST3GAL5*	Wald ratio	1	1.349 (0.657, 2.771)	0.415	0.818	8.30E-10	TRUE	0.06
NK	*TBC1D12*	Wald ratio	1	0.554 (0.257, 1.192)	0.131	0.65	5.44E-08	TRUE	0.082
NK	*THAP9-AS1*	Wald ratio	1	0.838 (0.433, 1.621)	0.6	0.875	5.77E-18	TRUE	0.056
NK	*TMEM241*	Wald ratio	1	2.027 (0.801, 5.131)	0.136	0.65	1.37E-05	TRUE	0.105
NK	*UBE3B*	Wald ratio	1	0.785 (0.259, 2.383)	0.669	0.886	6.04E-07	TRUE	0.064
NK	*VIM*	Wald ratio	1	1.177 (0.409, 3.389)	0.763	0.897	1.75E-07	TRUE	0.046
NK	*WARS*	Wald ratio	1	1.506 (0.663, 3.42)	0.328	0.78	1.29E-09	TRUE	0.066
NK	*ZNF100*	Wald ratio	1	1.513 (0.811, 2.822)	0.193	0.674	1.89E-13	TRUE	0.073
Plasma	*HLA-DQB1*	Wald ratio	1	0.734 (0.352, 1.53)	0.409	0.818	5.90E-09	TRUE	0.039
Plasma	*NFKBIL1*	Wald ratio	1	0.902 (0.458, 1.778)	0.766	0.897	9.80E-14	TRUE	0.039
Plasma	*NUCB2*	Wald ratio	1	0.835 (0.414, 1.684)	0.614	0.875	3.49E-11	TRUE	0.047
Plasma	*RNF146*	Wald ratio	1	1.374 (0.944, 2)	0.097	0.612	6.58E-30	TRUE	0.03
Plasma	*RXRB*	Wald ratio	1	0.774 (0.308, 1.947)	0.586	0.872	5.21E-07	TRUE	0.035

Table S9. Cell type-specific DEGs between PBMCs of patients with and without hypertension.

Cell type	Gene	Pct (Hypertension)	Pct (Control)	avg_logFC	*P* -value	*P* .adjust	Change
T IN	*RPS26*	1	0.898	1.1185	0	0	UP
T IN	*MT-ND3*	0.994	0.996	-0.6531	2.29E-292	4.00E-288	DOWN
T IN	*HLA-B*	0.994	0.999	-0.5338	2.59E-286	4.52E-282	DOWN
T IN	*MT-CYB*	0.999	0.999	-0.3921	6.10E-140	1.06E-135	DOWN
T IN	*MT-ND2*	0.879	0.928	-0.5296	1.33E-116	2.32E-112	DOWN
T IN	*MT-ND1*	0.973	0.988	-0.3859	5.01E-98	8.74E-94	DOWN
T IN	*MT-ATP8*	0.96	0.975	-0.3917	2.46E-93	4.30E-89	DOWN
T IN	*MT-ATP6*	0.993	0.996	-0.3298	1.18E-81	2.06E-77	DOWN
T IN	*MT-ND4*	0.889	0.923	-0.3841	2.43E-70	4.24E-66	DOWN
T IN	*S100A4*	0.589	0.469	0.3562	1.08E-25	1.89E-21	UP
T IN	*GIMAP7*	0.669	0.575	0.2587	9.46E-16	1.65E-11	UP
T IN	*CRIP1*	0.622	0.517	0.2669	1.33E-14	2.32E-10	UP
CD4 ET	*RPS26*	1	0.96	0.7574	4.30E-213	7.50E-209	UP
CD4 ET	*HLA-B*	0.998	1	-0.4636	1.22E-144	2.13E-140	DOWN
CD4 ET	*MT-ND3*	0.994	0.994	-0.5208	3.73E-109	6.51E-105	DOWN
CD4 ET	*MT-CYB*	0.999	0.999	-0.3086	7.98E-51	1.39E-46	DOWN
CD4 ET	*MT-ATP6*	0.996	0.995	-0.2830	3.19E-40	5.57E-36	DOWN
CD4 ET	*MT-ATP8*	0.976	0.977	-0.3010	1.31E-34	2.29E-30	DOWN
CD4 ET	*MT-ND2*	0.907	0.917	-0.3842	1.97E-34	3.43E-30	DOWN
CD4 ET	*MT-ND1*	0.985	0.985	-0.2905	2.41E-33	4.21E-29	DOWN
CD4 ET	*MT-ND4*	0.907	0.91	-0.2960	6.45E-25	1.13E-20	DOWN
NK	*KLRB1*	0.575	0.219	1.0335	8.11E-29	1.42E-24	UP
NK	*MT-ND3*	0.986	0.999	-0.4800	1.46E-25	2.55E-21	DOWN
NK	*MT-CYB*	1	1	-0.4078	2.48E-25	4.32E-21	DOWN
NK	*GZMB*	0.854	0.596	0.8352	1.29E-23	2.26E-19	UP
NK	*GNLY*	0.934	0.767	0.8038	1.38E-23	2.40E-19	UP
NK	*MT-CO3*	1	1	-0.3683	4.97E-23	8.67E-19	DOWN
NK	*PRF1*	0.856	0.689	0.7394	9.36E-19	1.63E-14	UP
NK	*FCGR3A*	0.627	0.361	0.7786	1.72E-17	3.00E-13	UP
NK	*MT-ATP6*	0.992	0.997	-0.3713	3.99E-17	6.96E-13	DOWN
NK	*HLA-B*	1	1	-0.3126	1.53E-16	2.66E-12	DOWN
NK	*CMC1*	0.373	0.59	-0.7452	1.29E-13	2.25E-09	DOWN
NK	*PRMT2*	0.646	0.416	0.5195	2.53E-12	4.43E-08	UP
NK	*HOPX*	0.655	0.41	0.5421	5.83E-12	1.02E-07	UP
NK	*MT-ATP8*	0.978	0.991	-0.3303	1.63E-11	2.85E-07	DOWN
NK	*CTSW*	0.928	0.852	0.3934	9.37E-11	1.64E-06	UP
NK	*CD7*	0.68	0.508	0.5927	6.69E-10	1.17E-05	UP
NK	*CD74*	0.812	0.862	-0.4437	4.08E-09	7.12E-05	DOWN
NK	*CD247*	0.691	0.517	0.4690	4.46E-09	7.79E-05	UP
NK	*KLRD1*	0.707	0.524	0.4585	9.13E-09	1.60E-04	UP
NK	*MT-ND1*	0.972	0.985	-0.3000	1.48E-08	2.58E-04	DOWN
NK	*MT-ND2*	0.895	0.934	-0.3723	3.87E-08	6.75E-04	DOWN
NK	*GIMAP7*	0.63	0.457	0.4194	1.18E-06	0.021	UP
T Un	*RPS26*	0.994	0.868	0.9363	4.47E-38	7.81E-34	UP
T Un	*MT-ND3*	1	1	-0.3996	8.89E-18	1.55E-13	DOWN
T Un	*HLA-B*	0.978	0.983	-0.3565	2.16E-12	3.77E-08	DOWN
T Un	*RPS21*	0.994	0.983	0.2586	3.32E-12	5.79E-08	UP
T Un	*RPS29*	0.992	0.96	0.3296	1.01E-11	1.76E-07	UP
T Un	*EEF1A1*	1	0.998	-0.2577	1.10E-08	1.92E-04	DOWN

T Un	*TMA7*	0.916	0.833	0.3714	7.81E-07	0.014	UP
CD8 ET	*MT-ND3*	0.998	0.999	-0.4742	1.77E-33	3.09E-29	DOWN
CD8 ET	*MT-CYB*	1	1	-0.4267	4.32E-33	7.54E-29	DOWN
CD8 ET	*RPS26*	1	0.982	0.4655	4.66E-25	8.14E-21	UP
CD8 ET	*HLA-B*	1	1	-0.3347	2.89E-24	5.04E-20	DOWN
CD8 ET	*MT-CO3*	0.998	1	-0.2770	1.15E-19	2.01E-15	DOWN
CD8 ET	*MT-ND2*	0.936	0.961	-0.4791	6.54E-19	1.14E-14	DOWN
CD8 ET	*GZMK*	0.625	0.847	-0.6054	7.28E-19	1.27E-14	DOWN
CD8 ET	*JUNB*	0.692	0.854	-0.4521	3.29E-18	5.75E-14	DOWN
CD8 ET	*MT-ND1*	0.996	0.987	-0.3526	1.60E-17	2.80E-13	DOWN
CD8 ET	*CD74*	0.809	0.877	-0.7294	3.38E-16	5.90E-12	DOWN
CD8 ET	*CXCR4*	0.565	0.758	-0.4966	4.29E-15	7.49E-11	DOWN
CD8 ET	*GIMAP7*	0.637	0.43	0.5921	2.58E-14	4.50E-10	UP
CD8 ET	*GIMAP4*	0.533	0.343	0.6497	1.91E-13	3.33E-09	UP
CD8 ET	*CST7*	0.566	0.735	-0.5126	2.38E-13	4.15E-09	DOWN
CD8 ET	*DUSP2*	0.549	0.696	-0.5171	2.91E-11	5.08E-07	DOWN
CD8 ET	*DNAJB1*	0.481	0.605	-0.4913	6.07E-11	1.06E-06	DOWN
CD8 ET	*H3F3B*	0.94	0.957	-0.3254	3.00E-10	5.24E-06	DOWN
CD8 ET	*UBC*	0.935	0.954	-0.2611	1.11E-09	1.94E-05	DOWN
CD8 ET	*HSPA8*	0.83	0.734	0.4075	1.95E-09	3.40E-05	UP
CD8 ET	*CIRBP*	0.789	0.848	-0.3012	8.66E-09	1.51E-04	DOWN
CD8 ET	*GZMA*	0.607	0.75	-0.3928	1.30E-08	2.27E-04	DOWN
CD8 ET	*RBM3*	0.676	0.714	-0.3626	7.57E-08	0.001	DOWN
CD8 ET	*VIM*	0.897	0.818	0.3216	2.05E-07	0.004	UP
CD8 ET	*S100A4*	0.97	0.915	0.2814	2.32E-07	0.004	UP
CD4 NC	*RPS26*	1	0.997	0.8851	3.97E-266	6.93E-262	UP
CD4 NC	*MT-ATP8*	0.93	0.98	-0.8203	2.85E-105	4.97E-101	DOWN
CD4 NC	*MT-ATP6*	0.99	0.998	-0.6507	7.79E-97	1.36E-92	DOWN
CD4 NC	*HLA-B*	0.998	0.999	-0.4894	3.66E-84	6.38E-80	DOWN
CD4 NC	*MT-CO3*	1	1	-0.2973	3.38E-38	5.89E-34	DOWN
CD4 NC	*PPDPF*	0.884	0.937	-0.4240	4.86E-28	8.48E-24	DOWN
CD4 NC	*MT-ND1*	0.981	0.985	-0.3820	1.46E-27	2.55E-23	DOWN
CD4 NC	*MT-CYB*	1	0.999	-0.2597	1.20E-26	2.09E-22	DOWN
CD4 NC	*GIMAP4*	0.708	0.505	0.5741	1.93E-26	3.37E-22	UP
CD4 NC	*BTG1*	0.945	0.85	0.4138	9.25E-26	1.61E-21	UP
CD4 NC	*DDX5*	0.896	0.781	0.3746	6.82E-20	1.19E-15	UP
CD4 NC	*IL32*	0.836	0.648	0.3909	2.36E-19	4.13E-15	UP
CD4 NC	*HSPA8*	0.906	0.844	0.3339	1.92E-15	3.35E-11	UP
CD4 NC	*GIMAP1*	0.588	0.423	0.4151	1.06E-13	1.85E-09	UP
CD4 NC	*MT-ND2*	0.925	0.944	-0.2893	6.97E-13	1.22E-08	DOWN
CD4 NC	*CD3G*	0.707	0.544	0.3595	1.05E-12	1.83E-08	UP
CD4 NC	*H3F3B*	0.893	0.906	-0.2826	2.54E-10	4.44E-06	DOWN
CD4 NC	*KLF2*	0.547	0.613	-0.2977	8.09E-08	0.001	DOWN
CD4 NC	*HSP90AA1*	0.668	0.566	0.2811	3.51E-07	0.006	UP
Mono C	*LYZ*	1	1	-1.2230	3.59E-27	6.26E-23	DOWN
Mono C	*HLA-C*	1	0.99	0.5908	1.02E-09	1.78E-05	UP
Mono C	*IFITM3*	0.623	0.263	1.0683	3.37E-08	5.89E-04	UP
Mono C	*DYNLL1*	0.702	0.414	1.1107	3.58E-08	6.25E-04	UP
Mono C	*NOP10*	0.763	0.485	0.9027	5.05E-08	8.82E-04	UP
Mono C	*ITGB2*	0.93	0.869	0.6931	1.43E-07	0.003	UP
CD8 NC	*RPS26*	1	0.992	0.5242	2.74E-135	4.79E-131	UP
CD8 NC	*RPS4Y1*	0.534	0.151	1.3646	7.43E-86	1.30E-81	UP
CD8 NC	*HLA-B*	0.993	0.998	-0.3613	2.38E-47	4.15E-43	DOWN
CD8 NC	*MT-ATP8*	0.978	0.993	-0.4324	1.78E-42	3.11E-38	DOWN

CD8 NC	*MT-ATP6*	0.997	0.999	-0.3379	7.01E-33	1.22E-28	DOWN
CD8 NC	*MT-ND1*	0.989	0.996	-0.2784	6.70E-23	1.17E-18	DOWN
CD8 NC	*RPL36AL*	0.948	0.894	0.3001	4.13E-19	7.21E-15	UP
CD8 NC	*MT-ND2*	0.978	0.974	-0.2686	2.47E-13	4.30E-09	DOWN
CD8 NC	*SNHG8*	0.821	0.731	0.2829	4.62E-13	8.07E-09	UP
CD8 NC	*EIF2S3*	0.519	0.615	-0.3184	3.17E-12	5.53E-08	DOWN
CD8 NC	*NME2*	0.646	0.706	-0.3058	6.53E-11	1.14E-06	DOWN
CD8 NC	*MT-ND6*	0.58	0.455	0.3851	1.30E-10	2.28E-06	UP
CD8 NC	*DDX5*	0.853	0.783	0.2588	1.81E-10	3.15E-06	UP
CD8 NC	*SRSF5*	0.761	0.687	0.2872	5.80E-09	1.01E-04	UP
CD8 NC	*GIMAP7*	0.653	0.568	0.2871	2.11E-07	0.004	UP
B cell	*HLA-DRB5*	0.925	0.603	1.2304	1.31E-10	2.28E-06	UP

Table S10. Druggability assessment of Tier 1 and 2 eGene targets with the Therapeutic Target Database.

eGene	Target name	Target type	Drug name	Drug type	Indication
MDM4	p53-binding protein Mdm4	Clinical trial Target	ALRN-6924	Inhibitor	Haematological malignancy; Acute myeloid leukaemia
DDX5	ATP-dependent RNA helicase DDX5	Clinical trial Target	RX-5902	Modulator	Triple negative breast cancer; Solid tumour/cancer
FNBP4	NA	NA	NA	NA	NA
FOXN2	NA	NA	NA	NA	NA
LLGL1	NA	NA	NA	NA	NA
MACF1	NA	NA	NA	NA	NA
OXNAD1	NA	NA	NA	NA	NA
PABPC4	NA	NA	NA	NA	NA
PRELID1	NA	NA	NA	NA	NA
SCD5	NA	NA	NA	NA	NA
SNAP29	NA	NA	NA	NA	NA
THAP9-AS1	NA	NA	NA	NA	NA
VIM	NA	NA	NA	NA	NA
VPS53	NA	NA	NA	NA	NA
WARS	NA	NA	NA	NA	NA
WBSCR16	NA	NA	NA	NA	NA
CTSW	NA	NA	NA	NA	NA
CDK10	NA	NA	NA	NA	NA
EEF2	NA	NA	NA	NA	NA

Table S11. Druggability assessment of Tier 1 and 2 eGene targets with the DrugBank.

eGene	Drug name	Drug type	Stage	Indication
WARS	Tryptophan	Small Molecule (Inhibitor)	Approved (investigational)	Acute renal failure (arf);<br>Chronic renal failure (crf);<br>Depression
	Tryptophanamide	Small Molecule	Experimental	NA
	Tryptophanyl-5'amp	Small Molecule	Experimental	NA
	Tryptophan	Small Molecule (Substrate, Inhibitor)	Approved (investigational)	Acute renal failure (arf);<br>Chronic renal failure (crf);<br>Depression
EEF2	Adenosine-5-Diphosphoribose	Small Molecule	Experimental	NA
	Diphthamide	Small Molecule	Experimental	NA
	Esketamine	Small Molecule (Inhibitor)	Approved (investigational)	Major depressive disorder (mdd);<br>Major depressive disorder, recurrent episode;<br>Pain;<br>Treatment resistant depression (trd)
	Guanosine-5'-Diphosphate	Small Molecule	Experimental	NA
	Moxetumomab pasudotox	Small Molecule (Inactivator)	Approved (investigational)	Relapsed/refractory hairy cell leukemia
VIM	Artenimol	Small Molecule (Ligand)	Approved (investigational)	Malaria caused by plasmodium falciparum
	Phenethyl Isothiocyanate	Small Molecule	Investigational	NA
DDX5	Artenimol	Small Molecule (Ligand)	Approved (investigational)	Malaria caused by plasmodium falciparum
	Supinoxin	Small Molecule (Modulator)	Investigational	NA
FNBP4	NA	NA	NA	NA
FOXN2	NA	NA	NA	NA
LLGL1	NA	NA	NA	NA
MACF1	NA	NA	NA	NA
MDM4	NA	NA	NA	NA
OXNAD1	NA	NA	NA	NA
PABPC4	NA	NA	NA	NA
PRELID1	NA	NA	NA	NA
SCD5	NA	NA	NA	NA
SNAP29	NA	NA	NA	NA
THAP9-AS1	NA	NA	NA	NA
VPS53	NA	NA	NA	NA
WBSCR16	NA	NA	NA	NA
CTSW	NA	NA	NA	NA
CDK10	NA	NA	NA	NA

Table S12. Druggability assessment of Tier 1 and 2 eGene targets with the Open Targets Database.

eGene	Drug name	Drug type	Mechanism of action	Action type	Disease	Phase	Status
CDK10	At-7519	Small molecule	Cyclin-dependent kinase inhibitor	Inhibitor	Chronic lymphocytic leukemia	II	Completed
	At-7519	Small molecule	Cyclin-dependent kinase inhibitor	Inhibitor	Mantle cell lymphoma	II	Completed
	At-7519	Small molecule	Cyclin-dependent kinase inhibitor	Inhibitor	Lymphoma	I	Completed
	At-7519	Small molecule	Cyclin-dependent kinase inhibitor	Inhibitor	Neoplasm	I	Active, not recruiting
	At-7519	Small molecule	Cyclin-dependent kinase inhibitor	Inhibitor	Neoplasm	I	Completed
	At-7519	Small molecule	Cyclin-dependent kinase inhibitor	Inhibitor	Multiple myeloma	I	Completed
	Azd-5438	Small molecule	Cyclin-dependent kinase inhibitor	Inhibitor	Neoplasm	I	Completed
	Pha-793887	Small molecule	Cyclin-dependent kinase inhibitor	Inhibitor	Neoplasm	I	Terminated
	Roniciclib	Small molecule	Cyclin-dependent kinase inhibitor	Inhibitor	Neoplasm	II	Withdrawn
	Roniciclib	Small molecule	Cyclin-dependent kinase inhibitor	Inhibitor	Small cell lung carcinoma	II	Terminated
	Roniciclib	Small molecule	Cyclin-dependent kinase inhibitor	Inhibitor	Neoplasm	I	Completed
	Roniciclib	Small molecule	Cyclin-dependent kinase inhibitor	Inhibitor	Small cell lung carcinoma	I	Terminated
	Roniciclib	Small molecule	Cyclin-dependent kinase inhibitor	Inhibitor	Non-small cell lung carcinoma	I	Withdrawn
	Denileukin diftitox	Protein	Elongation factor 2 inhibitor	Binding agent (inhibitor)	Neoplasm	IV	NA
	Denileukin diftitox	Protein	Elongation factor 2 inhibitor	Binding agent (inhibitor)	Cutaneous t-cell lymphoma	IV	NA
	Denileukin diftitox	Protein	Elongation factor 2 inhibitor	Binding agent (inhibitor)	Cutaneous t-cell lymphoma	IV	Completed
	Denileukin diftitox	Protein	Elongation factor 2 inhibitor	Binding agent (inhibitor)	Unspecified peripheral t-cell lymphoma	III	Withdrawn
	Denileukin diftitox	Protein	Elongation factor 2 inhibitor	Binding agent (inhibitor)	Lymphoma	III	Completed
	Denileukin diftitox	Protein	Elongation factor 2 inhibitor	Binding agent (inhibitor)	Chronic lymphocytic leukemia	II	Completed
	Denileukin diftitox	Protein	Elongation factor 2 inhibitor	Binding agent (inhibitor)	Unspecified peripheral t-cell lymphoma	II	Terminated
	Denileukin diftitox	Protein	Elongation factor 2 inhibitor	Binding agent (inhibitor)	Unspecified peripheral t-cell lymphoma	II	Completed
	Denileukin diftitox	Protein	Elongation factor 2 inhibitor	Binding agent (inhibitor)	Acute myeloid leukemia	II	Terminated
	Denileukin diftitox	Protein	Elongation factor 2 inhibitor	Binding agent (inhibitor)	T-cell acute lymphoblastic leukemia	II	Terminated
	Denileukin diftitox	Protein	Elongation factor 2 inhibitor	Binding agent (inhibitor)	Leukemia	II	Completed
	Denileukin diftitox	Protein	Elongation factor 2 inhibitor	Binding agent (inhibitor)	Lymphoma	II	Unknown status
	Denileukin diftitox	Protein	Elongation factor 2 inhibitor	Binding agent (inhibitor)	Leukemia	II	Unknown status
	Denileukin diftitox	Protein	Elongation factor 2 inhibitor	Binding agent (inhibitor)	Lymphoma	II	Completed
	Denileukin diftitox	Protein	Elongation factor 2 inhibitor	Binding agent (inhibitor)	Lymphoma	II	Withdrawn
	Denileukin diftitox	Protein	Elongation factor 2 inhibitor	Binding agent (inhibitor)	Melanoma	II	Completed
	Denileukin diftitox	Protein	Elongation factor 2 inhibitor	Binding agent (inhibitor)	Melanoma	II	Terminated
	Denileukin diftitox	Protein	Elongation factor 2 inhibitor	Binding agent (inhibitor)	Pancreatic carcinoma	II	Terminated
	Denileukin diftitox	Protein	Elongation factor 2 inhibitor	Binding agent (inhibitor)	Non-hodgkins lymphoma	II	Completed
	Denileukin diftitox	Protein	Elongation factor 2 inhibitor	Binding agent (inhibitor)	Toxicity	II	Completed
	Denileukin diftitox	Protein	Elongation factor 2 inhibitor	Binding agent (inhibitor)	Fallopian tube carcinoma	II	Completed
	Denileukin diftitox	Protein	Elongation factor 2 inhibitor	Binding agent (inhibitor)	Fallopian tube carcinoma	II	Terminated
	Denileukin diftitox	Protein	Elongation factor 2 inhibitor	Binding agent (inhibitor)	Peritoneum cancer	II	Completed
	Denileukin diftitox	Protein	Elongation factor 2 inhibitor	Binding agent (inhibitor)	Peritoneum cancer	II	Terminated
	Denileukin diftitox	Protein	Elongation factor 2 inhibitor	Binding agent (inhibitor)	Ovarian cancer	II	Completed
	Denileukin diftitox	Protein	Elongation factor 2 inhibitor	Binding agent (inhibitor)	Ovarian cancer	II	Terminated

	Denileukin diftitox	Protein	Elongation factor 2 inhibitor	Binding agent (inhibitor)	Graft versus host disease	II	Completed
	Denileukin diftitox	Protein	Elongation factor 2 inhibitor	Binding agent (inhibitor)	Unspecified peripheral t-cell lymphoma	I	Completed
	Denileukin diftitox	Protein	Elongation factor 2 inhibitor	Binding agent (inhibitor)	Cutaneous melanoma	I	Completed
	Denileukin diftitox	Protein	Elongation factor 2 inhibitor	Binding agent (inhibitor)	Endometrioid carcinoma	I	Completed
	Denileukin diftitox	Protein	Elongation factor 2 inhibitor	Binding agent (inhibitor)	Leukemia	I	Withdrawn
	Denileukin diftitox	Protein	Elongation factor 2 inhibitor	Binding agent (inhibitor)	Melanoma	I	Terminated
	Denileukin diftitox	Protein	Elongation factor 2 inhibitor	Binding agent (inhibitor)	Ovarian carcinoma	I	Completed
	Denileukin diftitox	Protein	Elongation factor 2 inhibitor	Binding agent (inhibitor)	Multiple myeloma	I	Terminated
	Denileukin diftitox	Protein	Elongation factor 2 inhibitor	Binding agent (inhibitor)	Male breast carcinoma	I	Completed
	Denileukin diftitox	Protein	Elongation factor 2 inhibitor	Binding agent (inhibitor)	Ovarian serous cystadenocarcinoma	I	Completed
	Denileukin diftitox	Protein	Elongation factor 2 inhibitor	Binding agent (inhibitor)	Ovarian mucinous cystadenocarcinoma	I	Completed
	Denileukin diftitox	Protein	Elongation factor 2 inhibitor	Binding agent (inhibitor)	Peritoneum cancer	I	Completed
	Denileukin diftitox	Protein	Elongation factor 2 inhibitor	Binding agent (inhibitor)	Fallopian tube cancer	I	Completed
	Denileukin diftitox	Protein	Elongation factor 2 inhibitor	Binding agent (inhibitor)	Ovarian clear cell cystadenocarcinoma	I	Completed
	Denileukin diftitox	Protein	Elongation factor 2 inhibitor	Binding agent (inhibitor)	Ovarian cancer	I	Recruiting
	Denileukin diftitox	Protein	Elongation factor 2 inhibitor	Binding agent (inhibitor)	Kidney cancer	NA	Completed
EEF2	Lmb-100	Unknown	Elongation factor 2 disrupting agent	Binding agent (inhibitor)	Lung adenocarcinoma	II	Terminated
	Lmb-100	Unknown	Elongation factor 2 disrupting agent	Binding agent (inhibitor)	Mesothelioma	II	Terminated
	Lmb-100	Unknown	Elongation factor 2 disrupting agent	Binding agent (inhibitor)	Mesothelioma	I	Terminated
	Lmb-100	Unknown	Elongation factor 2 disrupting agent	Binding agent (inhibitor)	Mesothelioma	I	Completed
	Lmb-100	Unknown	Elongation factor 2 disrupting agent	Binding agent (inhibitor)	Pancreatic neoplasm	I	Completed
	Lmb-100	Unknown	Elongation factor 2 disrupting agent	Binding agent (inhibitor)	Cancer	I	Terminated
	Moxetumomab pasudotox	Antibody drug conjugate	Elongation factor 2 inhibitor	Binding agent (inhibitor)	Neoplasm	IV	NA
	Moxetumomab pasudotox	Antibody drug conjugate	Elongation factor 2 inhibitor	Binding agent (inhibitor)	Hairy cell leukemia	IV	NA
	Moxetumomab pasudotox	Antibody drug conjugate	Elongation factor 2 inhibitor	Binding agent (inhibitor)	Hairy cell leukemia	III	Completed
	Moxetumomab pasudotox	Antibody drug conjugate	Elongation factor 2 inhibitor	Binding agent (inhibitor)	Acute lymphoblastic leukemia	II	Terminated
	Moxetumomab pasudotox	Antibody drug conjugate	Elongation factor 2 inhibitor	Binding agent (inhibitor)	Childhood acute lymphoblastic leukemia	II	Terminated
	Moxetumomab pasudotox	Antibody drug conjugate	Elongation factor 2 inhibitor	Binding agent (inhibitor)	Chronic lymphocytic leukemia	I	Terminated
	Moxetumomab pasudotox	Antibody drug conjugate	Elongation factor 2 inhibitor	Binding agent (inhibitor)	Chronic lymphocytic leukemia	I	Completed
	Moxetumomab pasudotox	Antibody drug conjugate	Elongation factor 2 inhibitor	Binding agent (inhibitor)	Acute lymphoblastic leukemia	I	Completed
	Moxetumomab pasudotox	Antibody drug conjugate	Elongation factor 2 inhibitor	Binding agent (inhibitor)	Leukemia	I	Unknown status
	Moxetumomab pasudotox	Antibody drug conjugate	Elongation factor 2 inhibitor	Binding agent (inhibitor)	Leukemia	I	Terminated
	Moxetumomab pasudotox	Antibody drug conjugate	Elongation factor 2 inhibitor	Binding agent (inhibitor)	Lymphoma	I	Terminated
	Moxetumomab pasudotox	Antibody drug conjugate	Elongation factor 2 inhibitor	Binding agent (inhibitor)	Non-hodgkins lymphoma	I	Unknown status
	Moxetumomab pasudotox	Antibody drug conjugate	Elongation factor 2 inhibitor	Binding agent (inhibitor)	Non-hodgkins lymphoma	I	Completed
	Moxetumomab pasudotox	Antibody drug conjugate	Elongation factor 2 inhibitor	Binding agent (inhibitor)	Hairy cell leukemia	I	Unknown status
	Moxetumomab pasudotox	Antibody drug conjugate	Elongation factor 2 inhibitor	Binding agent (inhibitor)	Hairy cell leukemia	I	Active, not recruiting
	Moxetumomab pasudotox	Antibody drug conjugate	Elongation factor 2 inhibitor	Binding agent (inhibitor)	Hairy cell leukemia	I	Completed
	Resimmune	Unknown	Elongation factor 2 inhibitor	Binding agent (inhibitor)	Cutaneous t-cell lymphoma	II	Completed
	Resimmune	Unknown	Elongation factor 2 inhibitor	Binding agent (inhibitor)	T-cell leukemia	II	Completed
	Resimmune	Unknown	Elongation factor 2 inhibitor	Binding agent (inhibitor)	Sezary's disease	II	Completed

	Resimmune	Unknown	Elongation factor 2 inhibitor	Binding agent (inhibitor)	Mycosis fungoides	II	Completed
	Resimmune	Unknown	Elongation factor 2 inhibitor	Binding agent (inhibitor)	Mycosis fungoides	II	Unknown status
	Resimmune	Unknown	Elongation factor 2 inhibitor	Binding agent (inhibitor)	Melanoma	I	Unknown status
	Resimmune	Unknown	Elongation factor 2 inhibitor	Binding agent (inhibitor)	Melanoma	I	Terminated
	Tagraxofusp	Protein	Elongation factor 2 inhibitor	Binding agent (inhibitor)	Acute myeloid leukemia	IV	NA
	Tagraxofusp	Protein	Elongation factor 2 inhibitor	Binding agent (inhibitor)	Lymphoma	IV	NA
	Tagraxofusp	Protein	Elongation factor 2 inhibitor	Binding agent (inhibitor)	Neoplasm	IV	NA
	Tagraxofusp	Protein	Elongation factor 2 inhibitor	Binding agent (inhibitor)	Acute myeloid leukemia	II	Not yet recruiting
	Tagraxofusp	Protein	Elongation factor 2 inhibitor	Binding agent (inhibitor)	Acute myeloid leukemia	II	Recruiting
	Tagraxofusp	Protein	Elongation factor 2 inhibitor	Binding agent (inhibitor)	Blastic plasmacytoid dendritic cell neoplasm	II	Recruiting
	Tagraxofusp	Protein	Elongation factor 2 inhibitor	Binding agent (inhibitor)	Blastic plasmacytoid dendritic cell neoplasm	II	Completed
	Tagraxofusp	Protein	Elongation factor 2 inhibitor	Binding agent (inhibitor)	Myelodysplastic syndrome	I	Recruiting
	Tagraxofusp	Protein	Elongation factor 2 inhibitor	Binding agent (inhibitor)	Acute myeloid leukemia	I	Withdrawn
	Tagraxofusp	Protein	Elongation factor 2 inhibitor	Binding agent (inhibitor)	Acute myeloid leukemia	I	Recruiting
	Tagraxofusp	Protein	Elongation factor 2 inhibitor	Binding agent (inhibitor)	Acute myeloid leukemia	I	Terminated
	Tagraxofusp	Protein	Elongation factor 2 inhibitor	Binding agent (inhibitor)	Blastic plasmacytoid dendritic cell neoplasm	I	Recruiting
	Tagraxofusp	Protein	Elongation factor 2 inhibitor	Binding agent (inhibitor)	Chronic myelomonocytic leukemia	I	Recruiting
	Tagraxofusp	Protein	Elongation factor 2 inhibitor	Binding agent (inhibitor)	Myelofibrosis	NA	Not yet recruiting
FNBP4	NA	NA	NA	NA	NA	NA	NA
FOXN2	NA	NA	NA	NA	NA	NA	NA
LLGL1	NA	NA	NA	NA	NA	NA	NA
MACF1	NA	NA	NA	NA	NA	NA	NA
MDM4	NA	NA	NA	NA	NA	NA	NA
OXNAD1	NA	NA	NA	NA	NA	NA	NA
PABPC4	NA	NA	NA	NA	NA	NA	NA
PRELID1	NA	NA	NA	NA	NA	NA	NA
SCD5	NA	NA	NA	NA	NA	NA	NA
SNAP29	NA	NA	NA	NA	NA	NA	NA
THAP9-AS1	NA	NA	NA	NA	NA	NA	NA
VIM	NA	NA	NA	NA	NA	NA	NA
VPS53	NA	NA	NA	NA	NA	NA	NA
WARS	NA	NA	NA	NA	NA	NA	NA
WBSCR16	NA	NA	NA	NA	NA	NA	NA
CTSW	NA	NA	NA	NA	NA	NA	NA
DDX5	NA	NA	NA	NA	NA	NA	NA

Table S13. Phenome-wide MR analyses on genetically mimicked targeting of CD8 ET-specific *VIM* .

Phenotype	Category	Method	nSNP	OR (95% CI)	*P* -value	*FDR*
Rheumatic disease of the heart valves	Circulatory system	Wald ratio	1	0.928 (0.81, 1.062)	0.277	0.995
Mitral valve disease	Circulatory system	Wald ratio	1	0.874 (0.737, 1.036)	0.121	0.958
Aortic valve disease	Circulatory system	Wald ratio	1	0.933 (0.72, 1.209)	0.601	0.995
Disease of tricuspid valve	Circulatory system	Wald ratio	1	1.034 (0.779, 1.371)	0.819	0.995
Heart valve disorders	Circulatory system	Wald ratio	1	0.879 (0.763, 1.012)	0.073	0.881
Nonrheumatic mitral valve disorders	Circulatory system	Wald ratio	1	0.898 (0.757, 1.065)	0.214	0.975
Nonrheumatic aortic valve disorders	Circulatory system	Wald ratio	1	0.95 (0.531, 1.701)	0.864	0.995
Nonrheumatic tricuspid valve disorders	Circulatory system	Wald ratio	1	0.583 (0.241, 1.408)	0.23	0.995
Nonrheumatic pulmonary valve disorders	Circulatory system	Wald ratio	1	0.583 (0.227, 1.493)	0.261	0.995
Heart valve replaced	Circulatory system	Wald ratio	1	0.85 (0.668, 1.082)	0.188	0.958
Abnormal heart sounds	Circulatory system	Wald ratio	1	1.148 (0.866, 1.523)	0.338	0.995
Hypertensive heart and or or renal disease	Circulatory system	Wald ratio	1	1.138 (0.91, 1.423)	0.258	0.995
Hypertensive heart disease	Circulatory system	Wald ratio	1	1.183 (0.584, 2.396)	0.641	0.995
Hypertensive chronic kidney disease	Circulatory system	Wald ratio	1	1.138 (0.899, 1.44)	0.282	0.995
Other hypertensive complications	Circulatory system	Wald ratio	1	1.215 (0.566, 2.611)	0.617	0.995
Elevated blood pressure reading without diagnosis of hypertension	Circulatory system	Wald ratio	1	0.903 (0.71, 1.149)	0.407	0.995
Ischemic Heart Disease	Circulatory system	Wald ratio	1	1.088 (1.026, 1.153)	0.005	0.464
Unstable angina (intermediate coronary syndrome)	Circulatory system	Wald ratio	1	0.977 (0.858, 1.112)	0.723	0.995
Myocardial infarction	Circulatory system	Wald ratio	1	1.02 (0.934, 1.114)	0.655	0.995
Angina pectoris	Circulatory system	Wald ratio	1	1.071 (0.993, 1.157)	0.077	0.889
Coronary atherosclerosis	Circulatory system	Wald ratio	1	1.121 (1.044, 1.203)	0.002	0.383
Aneurysm and dissection of heart	Circulatory system	Wald ratio	1	0.936 (0.662, 1.324)	0.709	0.995
Other chronic ischemic heart disease, unspecified	Circulatory system	Wald ratio	1	1.028 (0.946, 1.116)	0.516	0.995
Other acute and subacute forms of ischemic heart disease	Circulatory system	Wald ratio	1	0.908 (0.693, 1.191)	0.487	0.995
Other forms of chronic heart disease	Circulatory system	Wald ratio	1	1.009 (0.812, 1.255)	0.933	0.995
ASCVD	Circulatory system	Wald ratio	1	0.445 (0.145, 1.36)	0.155	0.958
Pulmonary heart disease	Circulatory system	Wald ratio	1	0.992 (0.862, 1.143)	0.914	0.995
Acute pulmonary heart disease	Circulatory system	Wald ratio	1	1.306 (0.453, 3.765)	0.621	0.995
Pulmonary embolism and infarction, acute	Circulatory system	Wald ratio	1	1.306 (0.453, 3.765)	0.621	0.995
Chronic pulmonary heart disease	Circulatory system	Wald ratio	1	0.719 (0.493, 1.047)	0.086	0.9
Primary pulmonary hypertension	Circulatory system	Wald ratio	1	0.677 (0.438, 1.046)	0.079	0.899
Cardiomegaly	Circulatory system	Wald ratio	1	1.037 (0.864, 1.244)	0.699	0.995
Nonspecific chest pain	Circulatory system	Wald ratio	1	0.995 (0.942, 1.052)	0.865	0.995
Precordial pain	Circulatory system	Wald ratio	1	1.078 (0.925, 1.256)	0.336	0.995
Carditis	Circulatory system	Wald ratio	1	0.997 (0.812, 1.225)	0.98	0.995
Myocarditis	Circulatory system	Wald ratio	1	1.616 (0.669, 3.906)	0.286	0.995
Pericarditis	Circulatory system	Wald ratio	1	0.947 (0.731, 1.227)	0.682	0.995
Acute pericarditis	Circulatory system	Wald ratio	1	0.768 (0.426, 1.383)	0.379	0.995
Endocarditis	Circulatory system	Wald ratio	1	1.037 (0.728, 1.475)	0.841	0.995
Cardiomyopathy	Circulatory system	Wald ratio	1	0.911 (0.703, 1.18)	0.481	0.995
Primary or intrinsic cardiomyopathies	Circulatory system	Wald ratio	1	0.908 (0.697, 1.184)	0.477	0.995
Hypertrophic obstructive cardiomyopathy	Circulatory system	Wald ratio	1	0.754 (0.331, 1.718)	0.502	0.995
Other hypertrophic cardiomyopathy	Circulatory system	Wald ratio	1	1.131 (0.558, 2.291)	0.733	0.995
Other cardiomyopathy	Circulatory system	Wald ratio	1	0.777 (0.24, 2.52)	0.674	0.995
Cardiac conduction disorders	Circulatory system	Wald ratio	1	1.121 (1.002, 1.253)	0.046	0.869
Atrioventricular [AV] block	Circulatory system	Wald ratio	1	1.056 (0.864, 1.289)	0.597	0.995
First degree AV block	Circulatory system	Wald ratio	1	1.138 (0.848, 1.527)	0.39	0.995
Second degree AV block	Circulatory system	Wald ratio	1	1.091 (0.71, 1.676)	0.691	0.995
Atrioventricular block, complete	Circulatory system	Wald ratio	1	0.942 (0.643, 1.38)	0.758	0.995
Other heart block	Circulatory system	Wald ratio	1	0.903 (0.396, 2.057)	0.808	0.995
Bundle branch block	Circulatory system	Wald ratio	1	1.158 (0.988, 1.358)	0.07	0.879
Right bundle branch block	Circulatory system	Wald ratio	1	1.201 (0.944, 1.528)	0.137	0.958
Left bundle branch block	Circulatory system	Wald ratio	1	1.138 (0.915, 1.414)	0.245	0.995
Anomalous atrioventricular excitation	Circulatory system	Wald ratio	1	0.931 (0.517, 1.676)	0.81	0.995
Other cardiac conduction disorders	Circulatory system	Wald ratio	1	1.326 (0.409, 4.299)	0.638	0.995
Cardiac pacemaker or device in situ	Circulatory system	Wald ratio	1	1.148 (0.951, 1.386)	0.151	0.958
Cardiac pacemaker in situ	Circulatory system	Wald ratio	1	1.134 (0.934, 1.377)	0.203	0.958
Cardiac dysrhythmias	Circulatory system	Wald ratio	1	1.03 (0.966, 1.099)	0.363	0.995
Paroxysmal tachycardia, unspecified	Circulatory system	Wald ratio	1	1.121 (0.951, 1.321)	0.175	0.958
Paroxysmal supraventricular tachycardia	Circulatory system	Wald ratio	1	1.148 (0.951, 1.386)	0.151	0.958
Paroxysmal ventricular tachycardia	Circulatory system	Wald ratio	1	0.965 (0.715, 1.302)	0.814	0.995
Atrial fibrillation and flutter	Circulatory system	Wald ratio	1	1.029 (0.948, 1.117)	0.497	0.995
Other specified cardiac dysrhythmias	Circulatory system	Wald ratio	1	1.097 (0.931, 1.294)	0.268	0.995
Cardiac arrest and ventricular fibrillation	Circulatory system	Wald ratio	1	1.075 (0.82, 1.409)	0.602	0.995
Ventricular fibrillation and flutter	Circulatory system	Wald ratio	1	1.078 (0.65, 1.788)	0.771	0.995
Cardiac arrest	Circulatory system	Wald ratio	1	1.065 (0.789, 1.438)	0.681	0.995
Arrhythmia (cardiac) NOS	Circulatory system	Wald ratio	1	0.89 (0.655, 1.208)	0.453	0.995
Premature beats	Circulatory system	Wald ratio	1	1.025 (0.691, 1.52)	0.903	0.995
Supraventricular premature beats	Circulatory system	Wald ratio	1	0.868 (0.381, 1.979)	0.737	0.995
Tachycardia NOS	Circulatory system	Wald ratio	1	0.933 (0.764, 1.14)	0.499	0.995
Sinoatrial node dysfunction (Bradycardia)	Circulatory system	Wald ratio	1	0.974 (0.615, 1.54)	0.909	0.995
Palpitations	Circulatory system	Wald ratio	1	0.974 (0.841, 1.129)	0.728	0.995
Congestive heart failure; nonhypertensive	Circulatory system	Wald ratio	1	0.98 (0.861, 1.115)	0.754	0.995
Congestive heart failure (CHF) NOS	Circulatory system	Wald ratio	1	1.071 (0.872, 1.316)	0.511	0.995
Heart failure NOS	Circulatory system	Wald ratio	1	0.919 (0.798, 1.059)	0.243	0.995
Ill-defined descriptions and complications of heart disease	Circulatory system	Wald ratio	1	0.992 (0.718, 1.371)	0.962	0.995
Heart transplant or surgery	Circulatory system	Wald ratio	1	1.024 (0.335, 3.132)	0.966	0.995

Abnormal function study of cardiovascular system	Circulatory system	Wald ratio	1	0.928 (0.622, 1.384)	0.713	0.995
Symptoms involving cardiovascular system	Circulatory system	Wald ratio	1	1.013 (0.531, 1.935)	0.968	0.995
Intracranial hemorrhage	Circulatory system	Wald ratio	1	0.898 (0.722, 1.116)	0.331	0.995
Subarachnoid hemorrhage	Circulatory system	Wald ratio	1	0.947 (0.686, 1.309)	0.743	0.995
Intracerebral hemorrhage	Circulatory system	Wald ratio	1	0.843 (0.596, 1.192)	0.334	0.995
Subdural hemorrhage	Circulatory system	Wald ratio	1	1.005 (0.568, 1.777)	0.988	0.995
Cerebrovascular disease	Circulatory system	Wald ratio	1	1.012 (0.916, 1.119)	0.814	0.995
Occlusion and stenosis of precerebral arteries	Circulatory system	Wald ratio	1	1.33 (1.021, 1.733)	0.035	0.838
Cerebral atherosclerosis	Circulatory system	Wald ratio	1	1.03 (0.572, 1.854)	0.923	0.995
Occlusion of cerebral arteries	Circulatory system	Wald ratio	1	1.037 (0.895, 1.201)	0.631	0.995
Cerebral artery occlusion, with cerebral infarction	Circulatory system	Wald ratio	1	0.833 (0.654, 1.06)	0.137	0.958
Cerebral ischemia	Circulatory system	Wald ratio	1	0.928 (0.782, 1.1)	0.389	0.995
Transient cerebral ischemia	Circulatory system	Wald ratio	1	0.98 (0.802, 1.197)	0.844	0.995
Cerebral aneurysm	Circulatory system	Wald ratio	1	1.124 (0.702, 1.8)	0.626	0.995
Late effects of cerebrovascular disease	Circulatory system	Wald ratio	1	1.201 (0.927, 1.556)	0.166	0.958
Atherosclerosis	Circulatory system	Wald ratio	1	1.052 (0.817, 1.355)	0.693	0.995
Atherosclerosis of renal artery	Circulatory system	Wald ratio	1	1.769 (0.732, 4.274)	0.205	0.958
Atherosclerosis of the extremities	Circulatory system	Wald ratio	1	1.013 (0.733, 1.399)	0.939	0.995
Atherosclerosis of aorta	Circulatory system	Wald ratio	1	1.194 (0.691, 2.063)	0.526	0.995
Vascular insufficiency of intestine	Circulatory system	Wald ratio	1	0.947 (0.646, 1.389)	0.782	0.995
Acute vascular insufficiency of intestine	Circulatory system	Wald ratio	1	1.075 (0.611, 1.89)	0.803	0.995
Other aneurysm	Circulatory system	Wald ratio	1	1.155 (0.929, 1.436)	0.195	0.958
Aortic aneurysm	Circulatory system	Wald ratio	1	1.176 (0.919, 1.505)	0.199	0.958
Abdominal aortic aneurysm	Circulatory system	Wald ratio	1	1.138 (0.838, 1.545)	0.408	0.995
Aneurysm of iliac artery	Circulatory system	Wald ratio	1	1.522 (0.594, 3.901)	0.382	0.995
Aneurysm of artery of lower extremity	Circulatory system	Wald ratio	1	1.134 (0.56, 2.298)	0.726	0.995
Arterial dissection	Circulatory system	Wald ratio	1	2.182 (0.634, 7.504)	0.216	0.975
Aneurysm of other specified artery	Circulatory system	Wald ratio	1	0.853 (0.447, 1.629)	0.63	0.995
Peripheral vascular disease	Circulatory system	Wald ratio	1	1.023 (0.883, 1.185)	0.764	0.995
Raynaud's syndrome	Circulatory system	Wald ratio	1	0.887 (0.677, 1.162)	0.385	0.995
Peripheral angiopathy in diseases classified elsewhere	Circulatory system	Wald ratio	1	0.965 (0.603, 1.544)	0.881	0.995
Peripheral vascular disease, unspecified	Circulatory system	Wald ratio	1	1.114 (0.928, 1.337)	0.246	0.995
Arterial embolism and thrombosis	Circulatory system	Wald ratio	1	1.391 (1.025, 1.889)	0.034	0.838
Arterial embolism and thrombosis of lower extremity artery	Circulatory system	Wald ratio	1	1.433 (0.972, 2.113)	0.069	0.879
Polyarteritis nodosa and allied conditions	Circulatory system	Wald ratio	1	1.433 (1.043, 1.969)	0.026	0.793
Wegener's granulomatosis	Circulatory system	Wald ratio	1	1.878 (0.983, 3.587)	0.056	0.869
Giant cell arteritis	Circulatory system	Wald ratio	1	1.194 (0.75, 1.9)	0.455	0.995
Arteritis NOS	Circulatory system	Wald ratio	1	1.275 (0.708, 2.296)	0.418	0.995
Other disorders of arteries and arterioles	Circulatory system	Wald ratio	1	0.953 (0.74, 1.227)	0.71	0.995
Stricture of artery	Circulatory system	Wald ratio	1	1.071 (0.784, 1.463)	0.664	0.995
Disease of capillaries	Circulatory system	Wald ratio	1	1.015 (0.472, 2.18)	0.97	0.995
Noninfectious disorders of lymphatic channels	Circulatory system	Wald ratio	1	1.071 (0.757, 1.516)	0.697	0.995
Phlebitis and thrombophlebitis	Circulatory system	Wald ratio	1	0.968 (0.835, 1.121)	0.66	0.995
Phlebitis and thrombophlebitis of lower extremities	Circulatory system	Wald ratio	1	0.939 (0.806, 1.094)	0.419	0.995
Other venous embolism and thrombosis	Circulatory system	Wald ratio	1	0.84 (0.57, 1.239)	0.38	0.995
Varicose veins	Circulatory system	Wald ratio	1	0.965 (0.883, 1.054)	0.424	0.995
Varicose veins of lower extremity	Circulatory system	Wald ratio	1	0.968 (0.886, 1.057)	0.463	0.995
Varicose veins of lower extremity, symptomtic	Circulatory system	Wald ratio	1	0.818 (0.568, 1.178)	0.28	0.995
Hemorrhoids	Circulatory system	Wald ratio	1	0.968 (0.907, 1.032)	0.317	0.995
Chronic venous insufficiency [CVI]	Circulatory system	Wald ratio	1	0.794 (0.416, 1.516)	0.484	0.995
Encounter for long-term (current) use of anticoagulants, antithrombotics, aspirin	Circulatory system	Wald ratio	1	1.314 (0.544, 3.175)	0.544	0.995
Hypotension	Circulatory system	Wald ratio	1	0.933 (0.825, 1.056)	0.273	0.995
Orthostatic hypotension	Circulatory system	Wald ratio	1	0.777 (0.604, 1.001)	0.051	0.869
Iatrogenic hypotension	Circulatory system	Wald ratio	1	0.925 (0.514, 1.666)	0.795	0.995
Hypotension NOS	Circulatory system	Wald ratio	1	0.95 (0.816, 1.107)	0.513	0.995
Other disorders of circulatory system	Circulatory system	Wald ratio	1	1.006 (0.932, 1.086)	0.884	0.995
Hemorrhage NOS	Circulatory system	Wald ratio	1	1.569 (0.612, 4.02)	0.349	0.995
Circulatory disease NEC	Circulatory system	Wald ratio	1	0.999 (0.926, 1.079)	0.987	0.995
Nonspecific abnormal findings on radiological and other examination of other intrathoracic organs (echocardiogram, etc)	Circulatory system	Wald ratio	1	1.068 (0.725, 1.575)	0.739	0.995
Cardiac and circulatory congenital anomalies	Congenital anomalies	Wald ratio	1	0.998 (0.836, 1.19)	0.978	0.995
Cardiac congenital anomalies	Congenital anomalies	Wald ratio	1	1.002 (0.835, 1.202)	0.986	0.995
Cardiac shunt or heart septal defect	Congenital anomalies	Wald ratio	1	1.03 (0.703, 1.509)	0.88	0.995
Valvular heart disease or heart chambers	Congenital anomalies	Wald ratio	1	0.619 (0.344, 1.114)	0.11	0.948
Congenital anomalies of great vessels	Congenital anomalies	Wald ratio	1	0.962 (0.774, 1.196)	0.725	0.995
Congenital anomalies of peripheral vascular system	Congenital anomalies	Wald ratio	1	0.959 (0.473, 1.942)	0.907	0.995
Anomalies of respiratory system, congenital	Congenital anomalies	Wald ratio	1	1.256 (0.344, 4.582)	0.73	0.995
Congenital anomalies of face and neck	Congenital anomalies	Wald ratio	1	0.752 (0.451, 1.254)	0.275	0.995
Digestive congenital anomalies	Congenital anomalies	Wald ratio	1	1.291 (0.912, 1.826)	0.15	0.958
Upper gastrointestinal congenital anomalies	Congenital anomalies	Wald ratio	1	1.165 (0.707, 1.921)	0.549	0.995
Congenital anomalies of mouth or tongue	Congenital anomalies	Wald ratio	1	1.172 (0.614, 2.239)	0.63	0.995
Lower gastrointestinal congenital anomalies	Congenital anomalies	Wald ratio	1	1.391 (0.859, 2.253)	0.18	0.958
Congenital anomalies of intestine	Congenital anomalies	Wald ratio	1	1.19 (0.661, 2.143)	0.562	0.995
Congenital anomaly of gallbladder, bile ducts, liver, pancreas	Congenital anomalies	Wald ratio	1	1.569 (0.688, 3.574)	0.284	0.995
Genitourinary congenital anomalies	Congenital anomalies	Wald ratio	1	0.9 (0.716, 1.132)	0.369	0.995
Congenital anomalies of genital organs	Congenital anomalies	Wald ratio	1	0.825 (0.587, 1.161)	0.27	0.995
Congenital anomalies of female genital organs	Congenital anomalies	Wald ratio	1	1.145 (0.745, 1.758)	0.538	0.995
Congenital anomalies of urinary system	Congenital anomalies	Wald ratio	1	0.968 (0.708, 1.321)	0.836	0.995

Cystic kidney disease	Congenital anomalies	Wald ratio	1	0.981 (0.631, 1.525)	0.932	0.995
Other specified congenital anomalies of kidney	Congenital anomalies	Wald ratio	1	0.983 (0.596, 1.621)	0.947	0.995
Nervous system congenital anomalies	Congenital anomalies	Wald ratio	1	1.138 (0.69, 1.876)	0.613	0.995
Neural tube defects	Congenital anomalies	Wald ratio	1	0.919 (0.517, 1.636)	0.775	0.995
Spina bifida	Congenital anomalies	Wald ratio	1	1.201 (0.629, 2.293)	0.579	0.995
Other specified congenital anomalies of nervous system	Congenital anomalies	Wald ratio	1	1.19 (0.438, 3.235)	0.733	0.995
Congenital anomalies of the eye	Congenital anomalies	Wald ratio	1	1.158 (0.508, 2.639)	0.726	0.995
Congenital cataract and lens anomalies	Congenital anomalies	Wald ratio	1	1.179 (0.409, 3.4)	0.76	0.995
Congenital musculoskeletal deformities of spine	Congenital anomalies	Wald ratio	1	1.35 (0.559, 3.262)	0.505	0.995
Congenital anomalies of limbs	Congenital anomalies	Wald ratio	1	0.871 (0.564, 1.346)	0.534	0.995
Congenital deformities of feet	Congenital anomalies	Wald ratio	1	0.922 (0.483, 1.761)	0.806	0.995
Other congenital anomalies of lower limb, including pelvic girdle	Congenital anomalies	Wald ratio	1	0.992 (0.49, 2.01)	0.983	0.995
Congenital hip dysplasia and deformity	Congenital anomalies	Wald ratio	1	1.151 (0.477, 2.782)	0.754	0.995
Other congenital musculoskeletal anomalies	Congenital anomalies	Wald ratio	1	0.815 (0.56, 1.188)	0.288	0.995
Congenital anomalies of muscle, tendon, fascia, and connective tissue	Congenital anomalies	Wald ratio	1	0.914 (0.336, 2.484)	0.86	0.995
Congenital osteodystrophies	Congenital anomalies	Wald ratio	1	0.956 (0.615, 1.486)	0.841	0.995
Chromosomal anomalies and genetic disorders	Congenital anomalies	Wald ratio	1	1.569 (0.821, 2.996)	0.173	0.958
Chromosomal anomalies	Congenital anomalies	Wald ratio	1	2.055 (0.802, 5.266)	0.134	0.958
Other and unspecified congenital anomalies	Congenital anomalies	Wald ratio	1	0.933 (0.518, 1.681)	0.818	0.995
Anomalies of endocrine glands, congenital	Congenital anomalies	Wald ratio	1	0.501 (0.208, 1.212)	0.125	0.958
Superficial cellulitis and abscess	Dermatologic	Wald ratio	1	0.931 (0.837, 1.034)	0.182	0.958
Cellulitis and abscess of fingers or toes	Dermatologic	Wald ratio	1	0.745 (0.505, 1.099)	0.138	0.958
Cellulitis and abscess of face or neck	Dermatologic	Wald ratio	1	1.052 (0.705, 1.57)	0.803	0.995
Cellulitis and abscess of arm or hand	Dermatologic	Wald ratio	1	0.968 (0.855, 1.095)	0.6	0.995
Cellulitis and abscess of leg, except foot	Dermatologic	Wald ratio	1	0.965 (0.853, 1.091)	0.568	0.995
Cellulitis and abscess of foot, toe	Dermatologic	Wald ratio	1	0.972 (0.859, 1.099)	0.648	0.995
Cellulitis and abscess of trunk	Dermatologic	Wald ratio	1	0.759 (0.521, 1.106)	0.151	0.958
Other local infections of skin and subcutaneous tissue	Dermatologic	Wald ratio	1	1.007 (0.88, 1.153)	0.917	0.995
Carbuncle and furuncle	Dermatologic	Wald ratio	1	0.908 (0.748, 1.103)	0.332	0.995
Pilonidal cyst	Dermatologic	Wald ratio	1	1.011 (0.69, 1.482)	0.956	0.995
Pyogenic granuloma	Dermatologic	Wald ratio	1	1.23 (0.759, 1.992)	0.4	0.995
Symptoms affecting skin	Dermatologic	Wald ratio	1	1.018 (0.9, 1.152)	0.775	0.995
Rash and other nonspecific skin eruption	Dermatologic	Wald ratio	1	1.145 (0.937, 1.398)	0.186	0.958
Changes in skin texture	Dermatologic	Wald ratio	1	1.822 (0.754, 4.404)	0.182	0.958
Disturbance of skin sensation	Dermatologic	Wald ratio	1	0.984 (0.83, 1.167)	0.852	0.995
Disorder of skin and subcutaneous tissue NOS	Dermatologic	Wald ratio	1	1.151 (1.018, 1.303)	0.025	0.793
Erythematosquamous dermatosis	Dermatologic	Wald ratio	1	1.935 (0.671, 5.579)	0.222	0.986
Seborrheic dermatitis	Dermatologic	Wald ratio	1	2.249 (0.654, 7.733)	0.199	0.958
Congenital anomalies of skin	Dermatologic	Wald ratio	1	0.853 (0.374, 1.943)	0.705	0.995
Dyschromia and Vitiligo	Dermatologic	Wald ratio	1	1.084 (0.808, 1.455)	0.589	0.995
Vitiligo	Dermatologic	Wald ratio	1	1.569 (0.484, 5.086)	0.453	0.995
Other dyschromia	Dermatologic	Wald ratio	1	1.049 (0.755, 1.458)	0.775	0.995
Vascular disorders of skin	Dermatologic	Wald ratio	1	1.101 (0.483, 2.508)	0.819	0.995
Erythematous conditions	Dermatologic	Wald ratio	1	0.947 (0.785, 1.144)	0.574	0.995
Toxic erythema	Dermatologic	Wald ratio	1	0.85 (0.352, 2.055)	0.719	0.995
Bullous dermatoses	Dermatologic	Wald ratio	1	0.501 (0.233, 1.077)	0.077	0.889
Dermatitis herpetiformis	Dermatologic	Wald ratio	1	1.23 (0.358, 4.23)	0.742	0.995
Pemphigus and pemphigoid	Dermatologic	Wald ratio	1	0.267 (0.093, 0.77)	0.015	0.709
Rosacea	Dermatologic	Wald ratio	1	0.911 (0.54, 1.538)	0.728	0.995
Lupus (localized and systemic)	Dermatologic	Wald ratio	1	0.75 (0.479, 1.172)	0.207	0.958
Cutaneous lupus erythematosus	Dermatologic	Wald ratio	1	0.787 (0.345, 1.792)	0.568	0.995
Systemic lupus erythematosus	Dermatologic	Wald ratio	1	0.719 (0.436, 1.185)	0.196	0.958
Prurigo and Lichen	Dermatologic	Wald ratio	1	1.04 (0.748, 1.445)	0.816	0.995
Other specified erythematous conditions	Dermatologic	Wald ratio	1	1.391 (0.773, 2.505)	0.271	0.995
Erythema nodosum	Dermatologic	Wald ratio	1	1.769 (0.614, 5.098)	0.291	0.995
Unspecified erythematous condition	Dermatologic	Wald ratio	1	1.131 (0.74, 1.727)	0.569	0.995
Psoriasis and related disorders	Dermatologic	Wald ratio	1	0.845 (0.696, 1.026)	0.09	0.913
Psoriasis	Dermatologic	Wald ratio	1	0.868 (0.715, 1.054)	0.154	0.958
Psoriasis vulgaris	Dermatologic	Wald ratio	1	0.945 (0.755, 1.181)	0.617	0.995
Psoriatic arthropathy	Dermatologic	Wald ratio	1	0.741 (0.524, 1.048)	0.09	0.913
Sarcoidosis	Dermatologic	Wald ratio	1	0.815 (0.55, 1.209)	0.31	0.995
Pruritus and related conditions	Dermatologic	Wald ratio	1	0.974 (0.701, 1.354)	0.875	0.995
Corns and callosities	Dermatologic	Wald ratio	1	1.052 (0.616, 1.797)	0.852	0.995
Other hypertrophic and atrophic conditions of skin	Dermatologic	Wald ratio	1	0.848 (0.741, 0.971)	0.017	0.709
Keratoderma, acquired	Dermatologic	Wald ratio	1	0.458 (0.169, 1.246)	0.126	0.958
Scar conditions and fibrosis of skin	Dermatologic	Wald ratio	1	0.892 (0.739, 1.077)	0.235	0.995
Circumscribed scleroderma	Dermatologic	Wald ratio	1	0.565 (0.174, 1.833)	0.342	0.995
Keloid scar	Dermatologic	Wald ratio	1	0.6 (0.314, 1.147)	0.122	0.958
Abnormal granulation tissue	Dermatologic	Wald ratio	1	1.013 (0.66, 1.557)	0.952	0.995
Acquired acanthosis nigricans	Dermatologic	Wald ratio	1	0.895 (0.37, 2.162)	0.805	0.995
Degenerative skin conditions and other dermatoses	Dermatologic	Wald ratio	1	1.043 (0.922, 1.18)	0.505	0.995
Actinic keratosis	Dermatologic	Wald ratio	1	1.056 (0.88, 1.267)	0.561	0.995
Seborrheic keratosis	Dermatologic	Wald ratio	1	1.046 (0.887, 1.233)	0.592	0.995
Diseases of nail, NOS	Dermatologic	Wald ratio	1	0.936 (0.723, 1.213)	0.617	0.995
Ingrowing nail	Dermatologic	Wald ratio	1	0.828 (0.617, 1.111)	0.208	0.958
Diseases of hair and hair follicles	Dermatologic	Wald ratio	1	0.925 (0.813, 1.053)	0.237	0.995
Alopecia	Dermatologic	Wald ratio	1	0.741 (0.325, 1.688)	0.475	0.995
Other specified diseases of hair and hair follicles	Dermatologic	Wald ratio	1	1.294 (0.449, 3.732)	0.633	0.995
Disorders of sweat glands	Dermatologic	Wald ratio	1	0.892 (0.646, 1.233)	0.49	0.995

Hidradenitis	Dermatologic	Wald ratio	1	0.89 (0.466, 1.699)	0.723	0.995
Hyperhidrosis	Dermatologic	Wald ratio	1	0.917 (0.629, 1.336)	0.65	0.995
Diseases of sebaceous glands	Dermatologic	Wald ratio	1	0.988 (0.894, 1.092)	0.809	0.995
Acne	Dermatologic	Wald ratio	1	1.208 (0.419, 3.483)	0.726	0.995
Sebaceous cyst	Dermatologic	Wald ratio	1	0.993 (0.898, 1.097)	0.888	0.995
Chronic ulcer of skin	Dermatologic	Wald ratio	1	0.861 (0.701, 1.057)	0.153	0.958
Decubitus ulcer	Dermatologic	Wald ratio	1	0.863 (0.636, 1.172)	0.346	0.995
Diffuse diseases of connective tissue	Dermatologic	Wald ratio	1	0.823 (0.702, 0.964)	0.016	0.709
Sicca syndrome	Dermatologic	Wald ratio	1	0.887 (0.591, 1.331)	0.562	0.995
Systemic sclerosis	Dermatologic	Wald ratio	1	0.698 (0.344, 1.413)	0.317	0.995
Other specified diffuse diseases of connective tissue	Dermatologic	Wald ratio	1	0.828 (0.255, 2.684)	0.753	0.995
Unspecified diffuse connective tissue disease	Dermatologic	Wald ratio	1	0.798 (0.669, 0.953)	0.012	0.709
Atopic or contact dermatitis due to other or unspecified	Dermatologic	Wald ratio	1	0.919 (0.753, 1.123)	0.41	0.995
Disorders of tooth development	Digestive	Wald ratio	1	0.984 (0.815, 1.187)	0.864	0.995
Hereditary disturbances in tooth structure	Digestive	Wald ratio	1	1.275 (0.371, 4.385)	0.7	0.995
Disturbances in tooth eruption	Digestive	Wald ratio	1	0.975 (0.808, 1.177)	0.793	0.995
Diseases of hard tissues of teeth	Digestive	Wald ratio	1	0.971 (0.824, 1.145)	0.729	0.995
Dental caries	Digestive	Wald ratio	1	0.979 (0.83, 1.154)	0.797	0.995
Diseases of pulp and periapical tissues	Digestive	Wald ratio	1	1.111 (0.894, 1.381)	0.344	0.995
Periapical abscess	Digestive	Wald ratio	1	1.101 (0.84, 1.443)	0.487	0.995
Gingival and periodontal diseases	Digestive	Wald ratio	1	1.179 (0.949, 1.466)	0.137	0.958
Gingivitis	Digestive	Wald ratio	1	1.097 (0.454, 2.652)	0.836	0.995
Periodontitis (acute or chronic)	Digestive	Wald ratio	1	1.169 (0.897, 1.523)	0.248	0.995
Acute periodontitis	Digestive	Wald ratio	1	1.145 (0.804, 1.629)	0.453	0.995
Chronic periodontitis	Digestive	Wald ratio	1	1.223 (0.829, 1.803)	0.31	0.995
Dentofacial anomalies, including malocclusion	Digestive	Wald ratio	1	1.334 (0.814, 2.186)	0.253	0.995
Anomalies of tooth position or malocclusion	Digestive	Wald ratio	1	1.522 (0.902, 2.569)	0.116	0.958
Other diseases of the teeth and supporting structures	Digestive	Wald ratio	1	1.059 (0.887, 1.263)	0.527	0.995
Atrophy of edentulous alveolar ridge	Digestive	Wald ratio	1	0.532 (0.185, 1.535)	0.243	0.995
Diseases of the jaws	Digestive	Wald ratio	1	1.128 (0.84, 1.513)	0.424	0.995
Cysts of the jaws	Digestive	Wald ratio	1	1.346 (0.812, 2.232)	0.25	0.995
Anomalies of jaw size or symmetry	Digestive	Wald ratio	1	0.95 (0.293, 3.081)	0.932	0.995
Temporomandibular joint disorders	Digestive	Wald ratio	1	1.104 (0.613, 1.988)	0.741	0.995
Temporomandibular joint disorder, unspecified	Digestive	Wald ratio	1	1.187 (0.659, 2.137)	0.569	0.995
Inflammatory conditions of jaw	Digestive	Wald ratio	1	1.201 (0.497, 2.902)	0.684	0.995
Jaw disease NOS	Digestive	Wald ratio	1	0.756 (0.42, 1.362)	0.352	0.995
Diseases of the salivary glands	Digestive	Wald ratio	1	1.172 (0.829, 1.659)	0.369	0.995
Hypertrophy of salivary gland	Digestive	Wald ratio	1	0.487 (0.179, 1.323)	0.158	0.958
Sialoadenitis	Digestive	Wald ratio	1	1.666 (1.028, 2.698)	0.038	0.838
Disturbance of salivary secretion	Digestive	Wald ratio	1	2.249 (0.693, 7.291)	0.177	0.958
Other specified diseases of the salivary glands	Digestive	Wald ratio	1	0.85 (0.373, 1.938)	0.7	0.995
Diseases of the oral soft tissues, excluding lesions specific for gingiva and tongue	Digestive	Wald ratio	1	0.992 (0.856, 1.149)	0.911	0.995
Stomatitis and mucositis	Digestive	Wald ratio	1	0.517 (0.338, 0.789)	0.002	0.383
Stomatitis and mucositis (ulcerative)	Digestive	Wald ratio	1	0.394 (0.248, 0.628)	8.71E-05	0.111
Oral aphthae	Digestive	Wald ratio	1	1.716 (0.631, 4.665)	0.29	0.995
Cellulitis and abscess of oral soft tissues	Digestive	Wald ratio	1	1.334 (0.659, 2.702)	0.424	0.995
Cysts of oral soft tissues	Digestive	Wald ratio	1	0.719 (0.316, 1.638)	0.432	0.995
Cyst of the salivary gland	Digestive	Wald ratio	1	1.049 (0.409, 2.689)	0.92	0.995
Diseases of lips	Digestive	Wald ratio	1	1.212 (0.851, 1.725)	0.286	0.995
Leukoplakia of oral mucosa	Digestive	Wald ratio	1	1.049 (0.622, 1.771)	0.857	0.995
Sialolithiasis	Digestive	Wald ratio	1	0.845 (0.504, 1.418)	0.525	0.995
Diseases and other conditions of the tongue	Digestive	Wald ratio	1	0.741 (0.572, 0.96)	0.023	0.771
Glossitis	Digestive	Wald ratio	1	0.741 (0.436, 1.258)	0.267	0.995
Diseases of esophagus	Digestive	Wald ratio	1	1.021 (0.969, 1.077)	0.437	0.995
Esophagitis, GERD and related diseases	Digestive	Wald ratio	1	1.025 (0.97, 1.083)	0.383	0.995
GERD	Digestive	Wald ratio	1	1.003 (0.929, 1.083)	0.939	0.995
Ulcer of esophagus	Digestive	Wald ratio	1	1.158 (1.018, 1.318)	0.026	0.793
Reflux esophagitis	Digestive	Wald ratio	1	0.99 (0.901, 1.088)	0.837	0.995
Esophageal bleeding (varices or hemorrhage)	Digestive	Wald ratio	1	0.956 (0.765, 1.195)	0.693	0.995
Stricture and stenosis of esophagus	Digestive	Wald ratio	1	0.99 (0.801, 1.224)	0.929	0.995
Disorders of esophageal motility	Digestive	Wald ratio	1	1.223 (0.849, 1.761)	0.28	0.995
Diverticulum of esophagus, acquired	Digestive	Wald ratio	1	1.024 (0.477, 2.199)	0.952	0.995
Gastroesophageal laceration-hemorrhage syndrome	Digestive	Wald ratio	1	1.275 (0.778, 2.09)	0.335	0.995
Heartburn	Digestive	Wald ratio	1	1.04 (0.851, 1.27)	0.702	0.995
Peptic ulcer (excl. esophageal)	Digestive	Wald ratio	1	1.075 (0.967, 1.195)	0.182	0.958
Hemorrhage from gastrointestinal ulcer	Digestive	Wald ratio	1	0.903 (0.623, 1.308)	0.589	0.995
Gastric ulcer	Digestive	Wald ratio	1	1.046 (0.903, 1.212)	0.549	0.995
Duodenal ulcer	Digestive	Wald ratio	1	1.111 (0.937, 1.317)	0.227	0.995
Peptic ulcer, site unspecified	Digestive	Wald ratio	1	1.35 (0.863, 2.111)	0.188	0.958
Dysphagia	Digestive	Wald ratio	1	0.98 (0.871, 1.102)	0.73	0.995
Gastritis and duodenitis	Digestive	Wald ratio	1	1.01 (0.953, 1.07)	0.736	0.995
Acute gastritis	Digestive	Wald ratio	1	0.968 (0.743, 1.261)	0.807	0.995
Atrophic gastritis	Digestive	Wald ratio	1	0.9 (0.559, 1.45)	0.666	0.995
Duodenitis	Digestive	Wald ratio	1	1.018 (0.915, 1.131)	0.747	0.995
Other specified gastritis	Digestive	Wald ratio	1	0.983 (0.884, 1.093)	0.751	0.995
Gastritis and duodenitis, NOS	Digestive	Wald ratio	1	0.657 (0.288, 1.497)	0.317	0.995
Disorders of function of stomach	Digestive	Wald ratio	1	0.754 (0.434, 1.311)	0.317	0.995
Dyspepsia and other specified disorders of function of stomach	Digestive	Wald ratio	1	0.759 (0.431, 1.335)	0.338	0.995

Other disorders of stomach and duodenum	Digestive	Wald ratio	1	0.976 (0.833, 1.144)	0.764	0.995
Appendiceal conditions	Digestive	Wald ratio	1	1.043 (0.89, 1.222)	0.604	0.995
Appendicitis	Digestive	Wald ratio	1	1.043 (0.885, 1.23)	0.617	0.995
Acute appendicitis	Digestive	Wald ratio	1	1.056 (0.88, 1.267)	0.561	0.995
Abdominal hernia	Digestive	Wald ratio	1	1.005 (0.961, 1.052)	0.825	0.995
Inguinal hernia	Digestive	Wald ratio	1	1.034 (0.957, 1.116)	0.397	0.995
Diaphragmatic hernia	Digestive	Wald ratio	1	1.016 (0.958, 1.078)	0.589	0.995
Femoral hernia	Digestive	Wald ratio	1	0.898 (0.627, 1.285)	0.555	0.995
Umbilical hernia	Digestive	Wald ratio	1	1.01 (0.867, 1.177)	0.899	0.995
Ventral hernia	Digestive	Wald ratio	1	0.853 (0.728, 1)	0.05	0.869
Inflammatory bowel disease and other gastroenteritis and colitis	Digestive	Wald ratio	1	0.874 (0.759, 1.006)	0.061	0.869
Regional enteritis	Digestive	Wald ratio	1	0.868 (0.695, 1.086)	0.216	0.975
Ulcerative colitis	Digestive	Wald ratio	1	0.884 (0.75, 1.043)	0.143	0.958
Ulcerative colitis (chronic)	Digestive	Wald ratio	1	1.391 (0.938, 2.063)	0.101	0.914
Ulceration of the lower GI tract	Digestive	Wald ratio	1	0.787 (0.593, 1.043)	0.096	0.913
Ulceration of intestine	Digestive	Wald ratio	1	0.818 (0.575, 1.164)	0.264	0.995
Intestinal malabsorption (non-celiac)	Digestive	Wald ratio	1	1.094 (0.896, 1.336)	0.378	0.995
Celiac disease	Digestive	Wald ratio	1	1.052 (0.852, 1.301)	0.637	0.995
Noninfectious gastroenteritis	Digestive	Wald ratio	1	0.992 (0.919, 1.071)	0.835	0.995
Ileostomy status	Digestive	Wald ratio	1	0.892 (0.709, 1.122)	0.33	0.995
Intestinal obstruction without mention of hernia	Digestive	Wald ratio	1	0.982 (0.847, 1.137)	0.804	0.995
Paralytic ileus	Digestive	Wald ratio	1	1.194 (0.8, 1.781)	0.386	0.995
Impaction of intestine	Digestive	Wald ratio	1	0.83 (0.472, 1.46)	0.518	0.995
Peritoneal or intestinal adhesions	Digestive	Wald ratio	1	1.052 (0.766, 1.446)	0.753	0.995
Other intestinal obstruction	Digestive	Wald ratio	1	0.962 (0.821, 1.127)	0.63	0.995
Symptoms involving digestive system	Digestive	Wald ratio	1	1.034 (0.957, 1.116)	0.397	0.995
Diarrhea	Digestive	Wald ratio	1	1.019 (0.667, 1.557)	0.929	0.995
Flatulence	Digestive	Wald ratio	1	0.95 (0.76, 1.188)	0.655	0.995
Diverticulosis and diverticulitis	Digestive	Wald ratio	1	0.977 (0.922, 1.037)	0.447	0.995
Diverticulosis	Digestive	Wald ratio	1	0.977 (0.921, 1.036)	0.435	0.995
Constipation	Digestive	Wald ratio	1	0.882 (0.802, 0.969)	0.009	0.68
Functional digestive disorders	Digestive	Wald ratio	1	1.043 (0.978, 1.113)	0.203	0.958
Irritable Bowel Syndrome	Digestive	Wald ratio	1	1.091 (0.964, 1.234)	0.167	0.958
Abnormal findings on exam of gastrointestinal tract or abdominal area	Digestive	Wald ratio	1	1.024 (0.819, 1.28)	0.837	0.995
Personal history of diseases of digestive system	Digestive	Wald ratio	1	1.056 (0.978, 1.139)	0.166	0.958
Anal and rectal conditions	Digestive	Wald ratio	1	0.97 (0.899, 1.048)	0.442	0.995
Anal and rectal polyp	Digestive	Wald ratio	1	0.991 (0.891, 1.101)	0.863	0.995
Peritonitis and retroperitoneal infections	Digestive	Wald ratio	1	0.999 (0.736, 1.357)	0.996	0.998
Other disorders of peritoneum	Digestive	Wald ratio	1	1.056 (0.901, 1.237)	0.505	0.995
Peritoneal adhesions (postoperative) (postinfection)	Digestive	Wald ratio	1	1.015 (0.861, 1.196)	0.861	0.995
Other disorders of intestine	Digestive	Wald ratio	1	0.887 (0.77, 1.021)	0.096	0.913
Toxic gastroenteritis and colitis	Digestive	Wald ratio	1	0.914 (0.425, 1.963)	0.817	0.995
Gastrointestinal complications	Digestive	Wald ratio	1	1.059 (0.697, 1.607)	0.789	0.995
Chronic liver disease and cirrhosis	Digestive	Wald ratio	1	1.101 (0.928, 1.306)	0.27	0.995
Other chronic nonalcoholic liver disease	Digestive	Wald ratio	1	1.194 (0.955, 1.493)	0.121	0.958
Cirrhosis of liver without mention of alcohol	Digestive	Wald ratio	1	1.037 (0.429, 2.505)	0.936	0.995
Primary biliary cirrhosis	Digestive	Wald ratio	1	1.145 (0.643, 2.037)	0.646	0.995
Liver abscess and sequelae of chronic liver disease	Digestive	Wald ratio	1	0.876 (0.649, 1.183)	0.388	0.995
Portal hypertension	Digestive	Wald ratio	1	0.942 (0.631, 1.405)	0.769	0.995
Malposition and malpresentation of fetus or obstruction	Digestive	Wald ratio	1	0.953 (0.758, 1.199)	0.682	0.995
Simple and unspecified goiter	Endocrine/metabolic	Wald ratio	1	0.977 (0.671, 1.424)	0.905	0.995
Nontoxic nodular goiter	Endocrine/metabolic	Wald ratio	1	1.075 (0.82, 1.409)	0.602	0.995
Nontoxic uninodular goiter	Endocrine/metabolic	Wald ratio	1	0.942 (0.617, 1.438)	0.781	0.995
Nontoxic multinodular goiter	Endocrine/metabolic	Wald ratio	1	1.197 (0.841, 1.704)	0.317	0.995
Thyrotoxicosis with or without goiter	Endocrine/metabolic	Wald ratio	1	0.78 (0.631, 0.963)	0.021	0.758
Graves' disease	Endocrine/metabolic	Wald ratio	1	0.808 (0.526, 1.241)	0.331	0.995
Toxic multinodular goiter	Endocrine/metabolic	Wald ratio	1	1.204 (0.47, 3.087)	0.698	0.995
Exophthalmos	Endocrine/metabolic	Wald ratio	1	0.931 (0.433, 1.999)	0.854	0.995
Hypothyroidism	Endocrine/metabolic	Wald ratio	1	1.059 (0.975, 1.15)	0.175	0.958
Secondary hypothyroidism	Endocrine/metabolic	Wald ratio	1	1.037 (0.786, 1.367)	0.798	0.995
Acquired hypothyroidism	Endocrine/metabolic	Wald ratio	1	0.931 (0.385, 2.249)	0.873	0.995
Hypothyroidism NOS	Endocrine/metabolic	Wald ratio	1	1.071 (0.987, 1.163)	0.1	0.914
Thyroiditis	Endocrine/metabolic	Wald ratio	1	0.973 (0.57, 1.662)	0.92	0.995
Chronic thyroiditis	Endocrine/metabolic	Wald ratio	1	0.754 (0.419, 1.358)	0.347	0.995
Chronic lymphocytic thyroiditis	Endocrine/metabolic	Wald ratio	1	0.756 (0.396, 1.445)	0.398	0.995
Other disorders of thyroid	Endocrine/metabolic	Wald ratio	1	1.716 (1.104, 2.668)	0.016	0.709
Diabetes mellitus	Endocrine/metabolic	Wald ratio	1	1.005 (0.936, 1.078)	0.901	0.995
Type 1 diabetes	Endocrine/metabolic	Wald ratio	1	0.993 (0.827, 1.191)	0.938	0.995
Type 1 diabetes with ketoacidosis	Endocrine/metabolic	Wald ratio	1	0.501 (0.278, 0.903)	0.021	0.758
Type 1 diabetes with renal manifestations	Endocrine/metabolic	Wald ratio	1	2.055 (0.713, 5.924)	0.182	0.958
Type 2 diabetes	Endocrine/metabolic	Wald ratio	1	1.009 (0.94, 1.083)	0.803	0.995
Type 2 diabetes with ketoacidosis	Endocrine/metabolic	Wald ratio	1	1.091 (0.451, 2.636)	0.847	0.995
Type 2 diabetes with renal manifestations	Endocrine/metabolic	Wald ratio	1	0.989 (0.518, 1.889)	0.973	0.995
Type 2 diabetes with ophthalmic manifestations	Endocrine/metabolic	Wald ratio	1	1.052 (0.812, 1.363)	0.699	0.995
Type 2 diabetes with neurological manifestations	Endocrine/metabolic	Wald ratio	1	0.917 (0.625, 1.344)	0.655	0.995
Abnormal glucose	Endocrine/metabolic	Wald ratio	1	0.922 (0.648, 1.312)	0.653	0.995
Impaired fasting glucose	Endocrine/metabolic	Wald ratio	1	0.9 (0.512, 1.583)	0.715	0.995
Other abnormal glucose	Endocrine/metabolic	Wald ratio	1	0.956 (0.611, 1.495)	0.844	0.995
Glycosuria or Acetonuria	Endocrine/metabolic	Wald ratio	1	1.212 (0.532, 2.761)	0.648	0.995

Polyneuropathy in diabetes	Endocrine/metabolic	Wald ratio	1	0.719 (0.441, 1.171)	0.185	0.958
Diabetic retinopathy	Endocrine/metabolic	Wald ratio	1	1.037 (0.805, 1.335)	0.78	0.995
Other disorders of pancreatic internal secretion	Endocrine/metabolic	Wald ratio	1	0.879 (0.651, 1.186)	0.399	0.995
Hypoglycemia	Endocrine/metabolic	Wald ratio	1	0.861 (0.638, 1.162)	0.327	0.995
Disorders of parathyroid gland	Endocrine/metabolic	Wald ratio	1	0.798 (0.585, 1.091)	0.157	0.958
Hyperparathyroidism	Endocrine/metabolic	Wald ratio	1	0.823 (0.592, 1.144)	0.246	0.995
Hypoparathyroidism	Endocrine/metabolic	Wald ratio	1	0.719 (0.335, 1.544)	0.397	0.995
Disorders of the pituitary gland and its hypothalamic control	Endocrine/metabolic	Wald ratio	1	0.903 (0.638, 1.278)	0.564	0.995
Pituitary hyperfunction	Endocrine/metabolic	Wald ratio	1	0.914 (0.479, 1.745)	0.785	0.995
Acromegaly and gigantism	Endocrine/metabolic	Wald ratio	1	0.876 (0.342, 2.246)	0.783	0.995
Pituitary hypofunction	Endocrine/metabolic	Wald ratio	1	0.75 (0.426, 1.319)	0.317	0.995
Diabetes insipidus	Endocrine/metabolic	Wald ratio	1	2.535 (0.989, 6.497)	0.053	0.869
Other disorders of neurohypophysis	Endocrine/metabolic	Wald ratio	1	0.698 (0.344, 1.413)	0.317	0.995
Disorders of adrenal glands	Endocrine/metabolic	Wald ratio	1	0.898 (0.623, 1.293)	0.561	0.995
Adrenal hyperfunction	Endocrine/metabolic	Wald ratio	1	0.884 (0.437, 1.791)	0.733	0.995
Cushing's syndrome	Endocrine/metabolic	Wald ratio	1	0.719 (0.297, 1.737)	0.463	0.995
Hyperaldosteronism	Endocrine/metabolic	Wald ratio	1	1.391 (0.483, 4.01)	0.541	0.995
Adrenal hypofunction	Endocrine/metabolic	Wald ratio	1	0.796 (0.491, 1.289)	0.354	0.995
Glucocorticoid deficiency	Endocrine/metabolic	Wald ratio	1	0.796 (0.491, 1.289)	0.354	0.995
Ovarian dysfunction	Endocrine/metabolic	Wald ratio	1	0.565 (0.314, 1.018)	0.057	0.869
Polycystic ovaries	Endocrine/metabolic	Wald ratio	1	0.532 (0.279, 1.017)	0.056	0.869
Testicular dysfunction	Endocrine/metabolic	Wald ratio	1	1.215 (0.6, 2.462)	0.588	0.995
Testicular hypofunction	Endocrine/metabolic	Wald ratio	1	1.065 (0.526, 2.157)	0.861	0.995
Iatrogenic endocrine disorders	Endocrine/metabolic	Wald ratio	1	0.759 (0.333, 1.729)	0.511	0.995
Other endocrine disorders	Endocrine/metabolic	Wald ratio	1	0.677 (0.354, 1.293)	0.237	0.995
Protein-calorie malnutrition	Endocrine/metabolic	Wald ratio	1	0.982 (0.74, 1.302)	0.899	0.995
Anorexia	Endocrine/metabolic	Wald ratio	1	1.128 (0.821, 1.549)	0.459	0.995
Vitamin deficiency	Endocrine/metabolic	Wald ratio	1	1.138 (0.873, 1.482)	0.339	0.995
Vitamin B-complex deficiencies	Endocrine/metabolic	Wald ratio	1	1.03 (0.737, 1.441)	0.861	0.995
Vitamin D deficiency	Endocrine/metabolic	Wald ratio	1	1.264 (0.818, 1.953)	0.292	0.995
Rickets or osteomalacia	Endocrine/metabolic	Wald ratio	1	0.756 (0.262, 2.181)	0.605	0.995
Mineral deficiency NEC	Endocrine/metabolic	Wald ratio	1	0.825 (0.458, 1.486)	0.522	0.995
Proteinuria	Endocrine/metabolic	Wald ratio	1	0.619 (0.375, 1.02)	0.06	0.869
Disorders of protein plasma or amino-acid transport and metabolism	Endocrine/metabolic	Wald ratio	1	0.956 (0.608, 1.504)	0.846	0.995
Disorders of plasma protein metabolism	Endocrine/metabolic	Wald ratio	1	0.936 (0.578, 1.516)	0.788	0.995
Paraproteinemia	Endocrine/metabolic	Wald ratio	1	0.75 (0.393, 1.432)	0.383	0.995
Amyloidosis	Endocrine/metabolic	Wald ratio	1	1.391 (0.576, 3.362)	0.463	0.995
Macroglobulinemia	Endocrine/metabolic	Wald ratio	1	1.433 (0.469, 4.383)	0.528	0.995
Disorders of carbohydrate transport and metabolism	Endocrine/metabolic	Wald ratio	1	0.787 (0.388, 1.593)	0.505	0.995
Intestinal disaccharidase deficiencies and disaccharide malabsorption	Endocrine/metabolic	Wald ratio	1	0.884 (0.412, 1.9)	0.752	0.995
Disorders of lipoid metabolism	Endocrine/metabolic	Wald ratio	1	1.004 (0.951, 1.06)	0.888	0.995
Hyperlipidemia	Endocrine/metabolic	Wald ratio	1	1.005 (0.952, 1.061)	0.862	0.995
Hypercholesterolemia	Endocrine/metabolic	Wald ratio	1	0.994 (0.94, 1.051)	0.841	0.995
Hyperglyceridemia	Endocrine/metabolic	Wald ratio	1	0.275 (0.085, 0.892)	0.032	0.838
Mixed hyperlipidemia	Endocrine/metabolic	Wald ratio	1	0.936 (0.462, 1.896)	0.855	0.995
Unspecified disorder of lipoid metabolism	Endocrine/metabolic	Wald ratio	1	0.863 (0.452, 1.649)	0.656	0.995
Gout and other crystal arthropathies	Endocrine/metabolic	Wald ratio	1	0.992 (0.852, 1.156)	0.92	0.995
Gout	Endocrine/metabolic	Wald ratio	1	1.075 (0.911, 1.267)	0.391	0.995
Gouty arthropathy	Endocrine/metabolic	Wald ratio	1	0.794 (0.328, 1.918)	0.608	0.995
Crystal arthropathies	Endocrine/metabolic	Wald ratio	1	0.638 (0.44, 0.923)	0.017	0.709
Chondrocalcinosis	Endocrine/metabolic	Wald ratio	1	0.619 (0.42, 0.912)	0.015	0.709
Disorders of mineral metabolism	Endocrine/metabolic	Wald ratio	1	0.89 (0.728, 1.086)	0.251	0.995
Disorders of magnesium metabolism	Endocrine/metabolic	Wald ratio	1	0.845 (0.525, 1.361)	0.489	0.995
Disorders of calcium or phosphorus metabolism	Endocrine/metabolic	Wald ratio	1	0.898 (0.689, 1.17)	0.424	0.995
Disorders of phosphorus metabolism	Endocrine/metabolic	Wald ratio	1	1.201 (0.593, 2.432)	0.611	0.995
Disorders of fluid, electrolyte, and acid-base balance	Endocrine/metabolic	Wald ratio	1	1.046 (0.941, 1.163)	0.405	0.995
Electrolyte imbalance	Endocrine/metabolic	Wald ratio	1	1.151 (0.994, 1.334)	0.06	0.869
Hyperosmolality and or or hypernatremia	Endocrine/metabolic	Wald ratio	1	2.317 (1.287, 4.172)	0.005	0.464
Hyposmolality and or or hyponatremia	Endocrine/metabolic	Wald ratio	1	1.006 (0.809, 1.25)	0.959	0.995
Hyperpotassemia	Endocrine/metabolic	Wald ratio	1	1.162 (0.866, 1.559)	0.317	0.995
Hypopotassemia	Endocrine/metabolic	Wald ratio	1	1.19 (0.935, 1.515)	0.157	0.958
Acid-base balance disorder	Endocrine/metabolic	Wald ratio	1	1.071 (0.817, 1.404)	0.617	0.995
Acidosis	Endocrine/metabolic	Wald ratio	1	1.04 (0.784, 1.379)	0.787	0.995
Alkalosis	Endocrine/metabolic	Wald ratio	1	1.616 (0.669, 3.906)	0.286	0.995
Hypovolemia	Endocrine/metabolic	Wald ratio	1	0.917 (0.773, 1.087)	0.317	0.995
Fluid overload	Endocrine/metabolic	Wald ratio	1	0.928 (0.604, 1.425)	0.732	0.995
Polydipsia	Endocrine/metabolic	Wald ratio	1	1.31 (0.575, 2.985)	0.52	0.995
Other disorders of metabolism	Endocrine/metabolic	Wald ratio	1	1.02 (0.796, 1.305)	0.877	0.995
Disorders of bilirubin excretion	Endocrine/metabolic	Wald ratio	1	1.201 (0.741, 1.945)	0.457	0.995
Other disorders of lipoid metabolism	Endocrine/metabolic	Wald ratio	1	0.638 (0.354, 1.148)	0.134	0.958
Lipoprotein disorders	Endocrine/metabolic	Wald ratio	1	1.326 (0.364, 4.836)	0.669	0.995
Overweight, obesity and other hyperalimentation	Endocrine/metabolic	Wald ratio	1	0.968 (0.886, 1.057)	0.463	0.995
Obesity	Endocrine/metabolic	Wald ratio	1	0.968 (0.881, 1.063)	0.492	0.995
Localized adiposity	Endocrine/metabolic	Wald ratio	1	1.391 (0.512, 3.781)	0.518	0.995
Abnormal weight gain	Endocrine/metabolic	Wald ratio	1	0.406 (0.133, 1.243)	0.114	0.958
Disorders involving the immune mechanism	Endocrine/metabolic	Wald ratio	1	0.97 (0.639, 1.473)	0.888	0.995
Immunity deficiency	Endocrine/metabolic	Wald ratio	1	0.85 (0.445, 1.624)	0.623	0.995
Deficiency of humoral immunity	Endocrine/metabolic	Wald ratio	1	0.565 (0.263, 1.215)	0.144	0.958
Other immunological findings	Endocrine/metabolic	Wald ratio	1	1.097 (0.613, 1.965)	0.754	0.995

Noninflammatory female genital disorders	Genitourinary	Wald ratio	1	1.071 (0.975, 1.177)	0.151	0.958
Noninflammatory disorders of vulva and perineum	Genitourinary	Wald ratio	1	0.895 (0.695, 1.152)	0.39	0.995
Dysplasia of female genital organs	Genitourinary	Wald ratio	1	0.741 (0.257, 2.135)	0.579	0.995
Endometrial hyperplasia	Genitourinary	Wald ratio	1	1.114 (0.85, 1.46)	0.434	0.995
Polyp of female genital organs	Genitourinary	Wald ratio	1	0.956 (0.875, 1.044)	0.317	0.995
Polyp of corpus uteri	Genitourinary	Wald ratio	1	0.959 (0.863, 1.066)	0.437	0.995
Mucous polyp of cervix	Genitourinary	Wald ratio	1	0.895 (0.763, 1.049)	0.171	0.958
Hypertrophy of female genital organs	Genitourinary	Wald ratio	1	0.95 (0.734, 1.231)	0.699	0.995
Symptoms involving female genital tract	Genitourinary	Wald ratio	1	1.03 (0.916, 1.159)	0.617	0.995
Dystrophy of female genital tract	Genitourinary	Wald ratio	1	1.124 (0.624, 2.024)	0.697	0.995
Atrophy of female genital tract	Genitourinary	Wald ratio	1	0.719 (0.465, 1.111)	0.137	0.958
stress incontinence, female	Genitourinary	Wald ratio	1	1.065 (0.941, 1.205)	0.317	0.995
Pain and other symptoms associated with female genital organs	Genitourinary	Wald ratio	1	0.768 (0.647, 0.911)	0.002	0.383
Dyspareunia	Genitourinary	Wald ratio	1	0.811 (0.622, 1.056)	0.12	0.958
Disorders of menstruation and other abnormal bleeding from female genital tract	Genitourinary	Wald ratio	1	1.03 (0.955, 1.112)	0.442	0.995
Irregular menstrual cycle or bleeding	Genitourinary	Wald ratio	1	1.043 (0.966, 1.126)	0.282	0.995
Absent or infrequent menstruation	Genitourinary	Wald ratio	1	2.055 (0.957, 4.414)	0.065	0.879
Excessive or frequent menstruation	Genitourinary	Wald ratio	1	1.034 (0.935, 1.142)	0.518	0.995
Irregular menstrual cycle	Genitourinary	Wald ratio	1	1.052 (0.857, 1.293)	0.627	0.995
Irregular menstrual bleeding	Genitourinary	Wald ratio	1	1.037 (0.89, 1.208)	0.644	0.995
Dysmenorrhea	Genitourinary	Wald ratio	1	1.179 (0.949, 1.466)	0.137	0.958
Premenstrual tension syndromes	Genitourinary	Wald ratio	1	2.774 (1.37, 5.619)	0.005	0.464
Infertility, female	Genitourinary	Wald ratio	1	1.002 (0.778, 1.29)	0.99	0.995
Menopausal and postmenopausal disorders	Genitourinary	Wald ratio	1	1.134 (1.039, 1.239)	0.005	0.464
Postmenopausal bleeding	Genitourinary	Wald ratio	1	1.121 (1.014, 1.239)	0.025	0.793
Symptomatic menopause	Genitourinary	Wald ratio	1	1.052 (0.52, 2.132)	0.887	0.995
Postmenopausal atrophic vaginitis	Genitourinary	Wald ratio	1	1.223 (0.927, 1.612)	0.154	0.958
Premenopausal menorrhagia	Genitourinary	Wald ratio	1	1.306 (0.774, 2.205)	0.317	0.995
Ovarian cyst	Genitourinary	Wald ratio	1	0.982 (0.858, 1.124)	0.791	0.995
Disorders of iron metabolism	Hematopoietic	Wald ratio	1	0.898 (0.631, 1.277)	0.549	0.995
Iron deficiency anemias	Hematopoietic	Wald ratio	1	1.03 (0.927, 1.145)	0.582	0.995
Iron deficiency anemias, unspecified or not due to blood loss	Hematopoietic	Wald ratio	1	1.034 (0.93, 1.149)	0.541	0.995
Iron deficiency anemia secondary to blood loss (chronic)	Hematopoietic	Wald ratio	1	0.933 (0.607, 1.434)	0.753	0.995
Other deficiency anemia	Hematopoietic	Wald ratio	1	0.962 (0.734, 1.261)	0.777	0.995
Megaloblastic anemia	Hematopoietic	Wald ratio	1	0.987 (0.745, 1.31)	0.93	0.995
Pernicious anemia	Hematopoietic	Wald ratio	1	1.006 (0.719, 1.406)	0.973	0.995
Other vitamin B12 deficiency anemia	Hematopoietic	Wald ratio	1	0.501 (0.248, 1.016)	0.055	0.869
Folate-deficiency anemia	Hematopoietic	Wald ratio	1	1.162 (0.608, 2.219)	0.649	0.995
Deficiency anemias	Hematopoietic	Wald ratio	1	0.472 (0.137, 1.624)	0.234	0.995
Hereditary hemolytic anemias	Hematopoietic	Wald ratio	1	1.049 (0.518, 2.125)	0.894	0.995
Other hemoglobinopathies	Hematopoietic	Wald ratio	1	1.037 (0.32, 3.362)	0.952	0.995
Other hereditary hemolytic anemias	Hematopoietic	Wald ratio	1	1.391 (0.512, 3.781)	0.518	0.995
Acquired hemolytic anemias	Hematopoietic	Wald ratio	1	0.698 (0.325, 1.499)	0.356	0.995
Autoimmune hemolytic anemias	Hematopoietic	Wald ratio	1	0.383 (0.158, 0.925)	0.033	0.838
Aplastic anemia	Hematopoietic	Wald ratio	1	1.059 (0.681, 1.646)	0.8	0.995
Other anemias	Hematopoietic	Wald ratio	1	0.994 (0.915, 1.079)	0.886	0.995
Acute posthemorrhagic anemia	Hematopoietic	Wald ratio	1	0.922 (0.527, 1.612)	0.776	0.995
Anemia of chronic disease	Hematopoietic	Wald ratio	1	1.028 (0.726, 1.454)	0.877	0.995
Anemia in neoplastic disease	Hematopoietic	Wald ratio	1	1.005 (0.643, 1.571)	0.983	0.995
Coagulation defects	Hematopoietic	Wald ratio	1	1.219 (0.903, 1.645)	0.196	0.958
Congenital coagulation defects	Hematopoietic	Wald ratio	1	0.968 (0.633, 1.478)	0.879	0.995
Von willebrand's disease	Hematopoietic	Wald ratio	1	1.322 (0.516, 3.388)	0.561	0.995
Congenital deficiency of other clotting factors (including factor VII)	Hematopoietic	Wald ratio	1	0.775 (0.459, 1.308)	0.34	0.995
Congenital factor VIII disorder	Hematopoietic	Wald ratio	1	1.935 (0.597, 6.275)	0.271	0.995
Hemorrhagic disorder due to intrinsic circulating anticoagulants	Hematopoietic	Wald ratio	1	0.583 (0.214, 1.584)	0.29	0.995
Other and unspecified coagulation defects	Hematopoietic	Wald ratio	1	1.522 (0.956, 2.423)	0.076	0.889
Purpura and other hemorrhagic conditions	Hematopoietic	Wald ratio	1	1.391 (1.119, 1.729)	0.003	0.408
Spontaneous ecchymoses	Hematopoietic	Wald ratio	1	0.874 (0.383, 1.991)	0.748	0.995
Allergic purpura	Hematopoietic	Wald ratio	1	0.565 (0.155, 2.062)	0.388	0.995
Thrombocytopenia	Hematopoietic	Wald ratio	1	1.477 (1.167, 1.869)	0.001	0.383
Primary thrombocytopenia	Hematopoietic	Wald ratio	1	1.249 (0.799, 1.952)	0.33	0.995
Secondary thrombocytopenia	Hematopoietic	Wald ratio	1	2.859 (0.992, 8.241)	0.052	0.869
Diseases of white blood cells	Hematopoietic	Wald ratio	1	1.059 (0.914, 1.226)	0.447	0.995
Decreased white blood cell count	Hematopoietic	Wald ratio	1	1.03 (0.874, 1.215)	0.721	0.995
Neutropenia	Hematopoietic	Wald ratio	1	1.03 (0.874, 1.215)	0.721	0.995
Eosinophilia	Hematopoietic	Wald ratio	1	1.35 (0.468, 3.892)	0.579	0.995
Other diseases of blood and blood-forming organs	Hematopoietic	Wald ratio	1	1.008 (0.875, 1.161)	0.91	0.995
Personal history of diseases of blood and blood-forming organs	Hematopoietic	Wald ratio	1	0.843 (0.493, 1.439)	0.531	0.995
Lymphadenitis	Hematopoietic	Wald ratio	1	1.071 (0.898, 1.278)	0.443	0.995
Diseases of spleen	Hematopoietic	Wald ratio	1	0.968 (0.645, 1.452)	0.873	0.995
Polycythemia, secondary	Hematopoietic	Wald ratio	1	0.823 (0.482, 1.405)	0.475	0.995
Abnormality of red blood cells	Hematopoietic	Wald ratio	1	0.868 (0.339, 2.226)	0.769	0.995
Intestinal infection	Infectious diseases	Wald ratio	1	0.9 (0.815, 0.995)	0.04	0.838
Bacterial enteritis	Infectious diseases	Wald ratio	1	0.936 (0.785, 1.117)	0.463	0.995
Intestinal infection due to C. difficile	Infectious diseases	Wald ratio	1	0.871 (0.608, 1.247)	0.451	0.995
Viral Enteritis	Infectious diseases	Wald ratio	1	0.768 (0.562, 1.049)	0.097	0.913
Intestinal infection due to protozoa	Infectious diseases	Wald ratio	1	0.719 (0.209, 2.472)	0.6	0.995
Tuberculosis	Infectious diseases	Wald ratio	1	0.719 (0.355, 1.456)	0.359	0.995

Septicemia	Infectious diseases	Wald ratio	1	1.138 (0.982, 1.318)	0.085	0.9
Gram negative septicemia	Infectious diseases	Wald ratio	1	1.287 (0.937, 1.768)	0.12	0.958
Gram positive septicemia	Infectious diseases	Wald ratio	1	1.078 (0.714, 1.627)	0.721	0.995
Bacterial infection NOS	Infectious diseases	Wald ratio	1	1.012 (0.932, 1.099)	0.77	0.995
Staphylococcus infections	Infectious diseases	Wald ratio	1	0.983 (0.834, 1.159)	0.836	0.995
Streptococcus infection	Infectious diseases	Wald ratio	1	0.942 (0.753, 1.178)	0.599	0.995
E. coli	Infectious diseases	Wald ratio	1	1.084 (0.909, 1.294)	0.368	0.995
Herpes zoster	Infectious diseases	Wald ratio	1	1.245 (0.796, 1.947)	0.337	0.995
Herpes zoster with nervous system complications	Infectious diseases	Wald ratio	1	0.973 (0.38, 2.495)	0.955	0.995
Herpes simplex	Infectious diseases	Wald ratio	1	1.078 (0.564, 2.059)	0.82	0.995
Viral hepatitis	Infectious diseases	Wald ratio	1	0.997 (0.765, 1.299)	0.98	0.995
Viral hepatitis A	Infectious diseases	Wald ratio	1	2.249 (0.735, 6.875)	0.155	0.958
Viral hepatitis B	Infectious diseases	Wald ratio	1	1.569 (0.688, 3.574)	0.284	0.995
Viral hepatitis C	Infectious diseases	Wald ratio	1	1.666 (0.936, 2.964)	0.083	0.899
Chronic hepatitis	Infectious diseases	Wald ratio	1	0.6 (0.345, 1.044)	0.071	0.879
Hepatitis NOS	Infectious diseases	Wald ratio	1	0.971 (0.625, 1.509)	0.896	0.995
Human immunodeficiency virus [HIV] disease	Infectious diseases	Wald ratio	1	0.619 (0.288, 1.329)	0.218	0.982
HIV infection, symptomatic	Infectious diseases	Wald ratio	1	0.619 (0.256, 1.495)	0.286	0.995
Viral warts & HPV	Infectious diseases	Wald ratio	1	0.815 (0.611, 1.088)	0.165	0.958
Viral infection	Infectious diseases	Wald ratio	1	0.97 (0.818, 1.151)	0.73	0.995
Varicella infection	Infectious diseases	Wald ratio	1	0.848 (0.277, 2.592)	0.772	0.995
Infectious mononucleosis	Infectious diseases	Wald ratio	1	0.638 (0.209, 1.949)	0.43	0.995
Postoperative infection	Infectious diseases	Wald ratio	1	0.989 (0.859, 1.139)	0.881	0.995
Infection or inflammation of internal prosthetic device; implant; and graft	Infectious diseases	Wald ratio	1	0.882 (0.735, 1.058)	0.175	0.958
Sexually transmitted infections (not HIV or hepatitis)	Infectious diseases	Wald ratio	1	1.822 (0.632, 5.254)	0.267	0.995
Dermatophytosis or Dermatomycosis	Infectious diseases	Wald ratio	1	0.977 (0.569, 1.679)	0.934	0.995
Dermatophytosis	Infectious diseases	Wald ratio	1	1.017 (0.588, 1.757)	0.952	0.995
Dermatophytosis of nail	Infectious diseases	Wald ratio	1	0.565 (0.196, 1.63)	0.291	0.995
Althete's foot	Infectious diseases	Wald ratio	1	1.391 (0.648, 2.988)	0.397	0.995
Candidiasis	Infectious diseases	Wald ratio	1	0.986 (0.808, 1.205)	0.892	0.995
Mycoses	Infectious diseases	Wald ratio	1	2.317 (1.325, 4.051)	0.003	0.408
Aspergillosis	Infectious diseases	Wald ratio	1	3.035 (1.499, 6.148)	0.002	0.383
Spirochetal infection	Infectious diseases	Wald ratio	1	0.35 (0.129, 0.951)	0.04	0.838
Arthropod-borne diseases	Infectious diseases	Wald ratio	1	1.666 (0.545, 5.092)	0.371	0.995
Helminthiases	Infectious diseases	Wald ratio	1	0.698 (0.306, 1.589)	0.391	0.995
Intestinal helminthiases	Infectious diseases	Wald ratio	1	0.777 (0.322, 1.878)	0.575	0.995
Other infectious and parasitic diseases	Infectious diseases	Wald ratio	1	0.811 (0.424, 1.548)	0.525	0.995
Sepsis and SIRS	Infectious diseases	Wald ratio	1	1.141 (0.962, 1.353)	0.129	0.958
Sepsis	Infectious diseases	Wald ratio	1	1.141 (0.962, 1.353)	0.129	0.958
Burns	Injuries & poisonings	Wald ratio	1	0.984 (0.671, 1.442)	0.933	0.995
Foreign body injury	Injuries & poisonings	Wald ratio	1	0.879 (0.675, 1.145)	0.339	0.995
Crushing injury	Injuries & poisonings	Wald ratio	1	0.83 (0.469, 1.469)	0.523	0.995
Injury to blood vessels	Injuries & poisonings	Wald ratio	1	1.716 (0.959, 3.072)	0.069	0.879
Crushing or internal injury to organs	Injuries & poisonings	Wald ratio	1	1.131 (0.848, 1.509)	0.403	0.995
Injury, NOS	Injuries & poisonings	Wald ratio	1	1.008 (0.912, 1.113)	0.883	0.995
Complications of surgical and medical procedures	Injuries & poisonings	Wald ratio	1	0.917 (0.834, 1.007)	0.07	0.879
Late effect	Injuries & poisonings	Wald ratio	1	0.565 (0.234, 1.366)	0.205	0.958
Effects of heat, cold and air pressure	Injuries & poisonings	Wald ratio	1	0.825 (0.27, 2.523)	0.736	0.995
Complications of in utero procedures	Injuries & poisonings	Wald ratio	1	0.445 (0.122, 1.622)	0.22	0.984
Nonspecific abnormal findings on radiological and other examination of musculoskeletal system	Injuries & poisonings	Wald ratio	1	0.971 (0.686, 1.374)	0.868	0.995
Fracture of lower limb	Injuries & poisonings	Wald ratio	1	0.942 (0.842, 1.053)	0.293	0.995
Fracture of neck of femur	Injuries & poisonings	Wald ratio	1	0.868 (0.699, 1.08)	0.204	0.958
Fracture of unspecified part of femur	Injuries & poisonings	Wald ratio	1	0.768 (0.379, 1.555)	0.463	0.995
Fracture of tibia and fibula	Injuries & poisonings	Wald ratio	1	0.887 (0.726, 1.083)	0.239	0.995
Fracture of patella	Injuries & poisonings	Wald ratio	1	0.959 (0.69, 1.333)	0.803	0.995
Fracture of ankle and foot	Injuries & poisonings	Wald ratio	1	0.95 (0.783, 1.154)	0.606	0.995
Fracture of foot	Injuries & poisonings	Wald ratio	1	0.976 (0.785, 1.213)	0.825	0.995
Fracture of pelvis	Injuries & poisonings	Wald ratio	1	1.075 (0.764, 1.512)	0.679	0.995
Fracture of upper limb	Injuries & poisonings	Wald ratio	1	0.945 (0.855, 1.044)	0.264	0.995
Fracture of humerus	Injuries & poisonings	Wald ratio	1	0.845 (0.676, 1.057)	0.141	0.958
Fracture of radius and ulna	Injuries & poisonings	Wald ratio	1	0.953 (0.837, 1.085)	0.467	0.995
Fracture of clavicle or scapula	Injuries & poisonings	Wald ratio	1	0.945 (0.747, 1.195)	0.635	0.995
Fracture of hand or wrist	Injuries & poisonings	Wald ratio	1	0.892 (0.761, 1.046)	0.159	0.958
Fracture of vertebral column without mention of spinal cord injury	Injuries & poisonings	Wald ratio	1	0.911 (0.712, 1.167)	0.46	0.995
Fracture of ribs	Injuries & poisonings	Wald ratio	1	0.906 (0.652, 1.259)	0.556	0.995
Fracture of unspecified bones	Injuries & poisonings	Wald ratio	1	0.986 (0.766, 1.27)	0.913	0.995
Concussion	Injuries & poisonings	Wald ratio	1	0.657 (0.365, 1.183)	0.162	0.958
Intracranial hemorrhage (injury)	Injuries & poisonings	Wald ratio	1	0.919 (0.613, 1.38)	0.685	0.995
Skull and face fracture and other intercranial injury	Injuries & poisonings	Wald ratio	1	0.942 (0.794, 1.117)	0.49	0.995
Torus fracture	Injuries & poisonings	Wald ratio	1	0.882 (0.705, 1.102)	0.269	0.995
Dislocation	Injuries & poisonings	Wald ratio	1	0.858 (0.703, 1.048)	0.134	0.958
Internal derangement of knee	Injuries & poisonings	Wald ratio	1	0.998 (0.925, 1.078)	0.966	0.995
Traumatic arthropathy	Injuries & poisonings	Wald ratio	1	1.201 (0.805, 1.791)	0.37	0.995
Sprains and strains	Injuries & poisonings	Wald ratio	1	1.234 (0.817, 1.862)	0.317	0.995
Hemorrhage or hematoma complicating a procedure	Injuries & poisonings	Wald ratio	1	1.019 (0.895, 1.16)	0.778	0.995
Complications of transplants and reattached limbs	Injuries & poisonings	Wald ratio	1	0.939 (0.464, 1.902)	0.861	0.995
Complication of colostomy or enterostomy	Injuries & poisonings	Wald ratio	1	0.863 (0.572, 1.303)	0.484	0.995
Complications of cardiac or vascular device, implant, and graft	Injuries & poisonings	Wald ratio	1	1.165 (0.937, 1.449)	0.168	0.958

Mechanical complication of unspecified genitourinary device, implant, and graft	Injuries & poisonings	Wald ratio	1	0.975 (0.752, 1.262)	0.845	0.995
Complication of internal orthopedic device	Injuries & poisonings	Wald ratio	1	0.884 (0.75, 1.043)	0.143	0.958
Complication due to other implant and internal device	Injuries & poisonings	Wald ratio	1	1.062 (0.901, 1.252)	0.475	0.995
Open wounds of head; neck; and trunk	Injuries & poisonings	Wald ratio	1	1.094 (0.945, 1.268)	0.23	0.995
Open wound or laceration of eye or eyelid	Injuries & poisonings	Wald ratio	1	1.477 (0.85, 2.568)	0.167	0.958
Open wound of ear	Injuries & poisonings	Wald ratio	1	1.111 (0.582, 2.121)	0.75	0.995
Other open wound of head and face	Injuries & poisonings	Wald ratio	1	1.101 (0.928, 1.306)	0.27	0.995
Open wound of nose and sinus	Injuries & poisonings	Wald ratio	1	0.741 (0.411, 1.334)	0.317	0.995
Open wound of neck	Injuries & poisonings	Wald ratio	1	0.818 (0.485, 1.38)	0.452	0.995
Open wounds of extremities	Injuries & poisonings	Wald ratio	1	1.002 (0.87, 1.153)	0.983	0.995
Open wound of hand except finger(s)	Injuries & poisonings	Wald ratio	1	1.298 (0.378, 4.465)	0.679	0.995
Open wound of finger(s)	Injuries & poisonings	Wald ratio	1	0.843 (0.329, 2.16)	0.722	0.995
Open wound of foot except toe(s) alone	Injuries & poisonings	Wald ratio	1	0.657 (0.324, 1.331)	0.243	0.995
Traumatic amputation	Injuries & poisonings	Wald ratio	1	1.172 (0.768, 1.791)	0.462	0.995
Broken tooth	Injuries & poisonings	Wald ratio	1	0.719 (0.399, 1.294)	0.271	0.995
Complication of amputation stump	Injuries & poisonings	Wald ratio	1	1.994 (0.985, 4.039)	0.055	0.869
Late effects of musculoskeletal and connective tissue injuries	Injuries & poisonings	Wald ratio	1	1.011 (0.499, 2.048)	0.975	0.995
Injuries to the nervous system	Injuries & poisonings	Wald ratio	1	1.002 (0.778, 1.29)	0.988	0.995
Superficial injury without mention of infection	Injuries & poisonings	Wald ratio	1	1.004 (0.872, 1.156)	0.957	0.995
Contusion	Injuries & poisonings	Wald ratio	1	0.895 (0.703, 1.139)	0.367	0.995
Allergic reaction to food	Injuries & poisonings	Wald ratio	1	1.935 (0.955, 3.92)	0.067	0.879
Dermatitis due to solar radiation	Injuries & poisonings	Wald ratio	1	0.754 (0.474, 1.2)	0.234	0.995
Chronic dermatitis due to solar radiation	Injuries & poisonings	Wald ratio	1	0.657 (0.369, 1.169)	0.153	0.958
Adverse reaction to serum or vaccine	Injuries & poisonings	Wald ratio	1	1.131 (0.628, 2.037)	0.682	0.995
Infusion and transfusion reaction	Injuries & poisonings	Wald ratio	1	1.433 (0.593, 3.464)	0.424	0.995
Anaphylactic shock NOS	Injuries & poisonings	Wald ratio	1	1.298 (0.875, 1.925)	0.194	0.958
Urticaria	Injuries & poisonings	Wald ratio	1	0.823 (0.511, 1.325)	0.422	0.995
Allergies, other	Injuries & poisonings	Wald ratio	1	1.215 (0.768, 1.923)	0.405	0.995
Spinal cord injury without evidence of spinal bone injury	Injuries & poisonings	Wald ratio	1	0.962 (0.615, 1.504)	0.864	0.995
Injury to other and unspecified nerves	Injuries & poisonings	Wald ratio	1	1.068 (0.611, 1.868)	0.817	0.995
Certain early complications of trauma or procedure	Injuries & poisonings	Wald ratio	1	0.752 (0.498, 1.135)	0.175	0.958
Postoperative shock	Injuries & poisonings	Wald ratio	1	0.936 (0.272, 3.219)	0.917	0.995
Traumatic and surgical subcutaneous emphysema	Injuries & poisonings	Wald ratio	1	0.292 (0.101, 0.842)	0.023	0.771
Poisoning by antibiotics	Injuries & poisonings	Wald ratio	1	1.015 (0.946, 1.089)	0.677	0.995
Allergy or adverse effect of penicillin	Injuries & poisonings	Wald ratio	1	1.008 (0.934, 1.088)	0.835	0.995
Poisoning by other anti-infectives	Injuries & poisonings	Wald ratio	1	1.124 (0.868, 1.456)	0.375	0.995
Poisoning or allergy of sulfonamides	Injuries & poisonings	Wald ratio	1	1.35 (0.988, 1.844)	0.059	0.869
Poisoning by hormones and synthetic substitutes	Injuries & poisonings	Wald ratio	1	1.23 (0.768, 1.969)	0.388	0.995
Hormones and synthetic substitutes causing adverse effects in therapeutic use	Injuries & poisonings	Wald ratio	1	1.23 (0.759, 1.992)	0.4	0.995
Poisoning by primarily systemic agents	Injuries & poisonings	Wald ratio	1	1.291 (0.717, 2.324)	0.395	0.995
Poisoning by agents primarily affecting blood constituents	Injuries & poisonings	Wald ratio	1	1.241 (0.706, 2.183)	0.453	0.995
Anticoagulants causing adverse effects	Injuries & poisonings	Wald ratio	1	1.226 (0.689, 2.183)	0.488	0.995
Poisoning by analgesics, antipyretics, and antirheumatics	Injuries & poisonings	Wald ratio	1	0.999 (0.893, 1.117)	0.986	0.995
Opiates and related narcotics causing adverse effects in therapeutic use	Injuries & poisonings	Wald ratio	1	0.866 (0.661, 1.135)	0.297	0.995
Antirheumatics causing adverse effects in therapeutic use	Injuries & poisonings	Wald ratio	1	1.433 (0.944, 2.177)	0.091	0.913
Salicylates causing adverse effects in therapeutic use	Injuries & poisonings	Wald ratio	1	1.01 (0.642, 1.588)	0.967	0.995
Poisoning by anticonvulsants and anti-Parkinsonism drugs	Injuries & poisonings	Wald ratio	1	0.583 (0.388, 0.874)	0.009	0.68
Adverse effects of sedatives or other central nervous system depressants and anesthetics	Injuries & poisonings	Wald ratio	1	0.882 (0.605, 1.285)	0.512	0.995
Poisoning by psychotropic agents	Injuries & poisonings	Wald ratio	1	0.993 (0.804, 1.227)	0.949	0.995
Poisoning by drugs primarily affecting the autonomic nervous system	Injuries & poisonings	Wald ratio	1	0.942 (0.493, 1.799)	0.856	0.995
Poisoning by agents primarily affecting the cardiovascular system	Injuries & poisonings	Wald ratio	1	1.059 (0.613, 1.829)	0.838	0.995
Antihypertensive agents causing adverse effects	Injuries & poisonings	Wald ratio	1	1.097 (0.404, 2.983)	0.855	0.995
Poisoning by agents primarily affecting the gastrointestinal system	Injuries & poisonings	Wald ratio	1	0.947 (0.392, 2.289)	0.904	0.995
Poisoning by water, mineral, and uric acid metabolism drugs	Injuries & poisonings	Wald ratio	1	0.472 (0.195, 1.141)	0.096	0.913
Poisoning by agents primarily acting on the smooth and skeletal muscles and respiratory system	Injuries & poisonings	Wald ratio	1	0.903 (0.278, 2.928)	0.865	0.995
Poisoning by agents primarily affecting skin & mucous membrane, ophthalmological, otorhinolaryngological, & dental drugs	Injuries & poisonings	Wald ratio	1	0.284 (0.087, 0.919)	0.036	0.838
Personal history of allergy to medicinal agents	Injuries & poisonings	Wald ratio	1	1.878 (1.043, 3.382)	0.036	0.838
Adverse drug events and drug allergies	Injuries & poisonings	Wald ratio	1	1.264 (0.893, 1.788)	0.186	0.958
Toxic effect of (non-ethyl) alcohol and petroleum and other solvents	Injuries & poisonings	Wald ratio	1	1.522 (0.797, 2.907)	0.203	0.958
Toxic effect of carbon monoxide	Injuries & poisonings	Wald ratio	1	2.118 (0.693, 6.474)	0.188	0.958
Toxic effect of other gases, fumes, or vapors	Injuries & poisonings	Wald ratio	1	0.458 (0.19, 1.107)	0.083	0.899
Toxic effect of noxious substances eaten as food	Injuries & poisonings	Wald ratio	1	1.878 (0.777, 4.538)	0.162	0.958
Toxic effect of other substances, chiefly nonmedicinal as to source	Injuries & poisonings	Wald ratio	1	0.801 (0.247, 2.597)	0.711	0.995
Effects radiation NOS	Injuries & poisonings	Wald ratio	1	0.965 (0.818, 1.137)	0.668	0.995
Delirium dementia and amnestic and other cognitive disorders	Mental disorders	Wald ratio	1	1.012 (0.824, 1.243)	0.909	0.995
Dementias	Mental disorders	Wald ratio	1	0.884 (0.655, 1.194)	0.421	0.995
Alzheimer's disease	Mental disorders	Wald ratio	1	0.6 (0.379, 0.95)	0.029	0.83
Vascular dementia	Mental disorders	Wald ratio	1	1.194 (0.625, 2.28)	0.592	0.995
Delirium due to conditions classified elsewhere	Mental disorders	Wald ratio	1	1.027 (0.718, 1.471)	0.883	0.995
Other persistent mental disorders due to conditions classified elsewhere	Mental disorders	Wald ratio	1	0.818 (0.284, 2.358)	0.71	0.995
Other specified nonpsychotic and or or transient mental disorders	Mental disorders	Wald ratio	1	1.078 (0.749, 1.552)	0.687	0.995
Transient mental disorders due to conditions classified elsewhere	Mental disorders	Wald ratio	1	0.835 (0.367, 1.903)	0.668	0.995
Specific nonpsychotic mental disorders due to brain damage	Mental disorders	Wald ratio	1	0.833 (0.306, 2.263)	0.72	0.995
Alteration of consciousness	Mental disorders	Wald ratio	1	1.234 (0.798, 1.907)	0.344	0.995
Neurological disorders	Mental disorders	Wald ratio	1	0.962 (0.84, 1.101)	0.572	0.995

Aphasia or speech disturbance	Mental disorders	Wald ratio	1	1.002 (0.792, 1.268)	0.988	0.995
Mild cognitive impairment	Mental disorders	Wald ratio	1	1.148 (0.601, 2.193)	0.676	0.995
Memory loss	Mental disorders	Wald ratio	1	0.984 (0.695, 1.392)	0.927	0.995
Altered mental status	Mental disorders	Wald ratio	1	0.962 (0.792, 1.168)	0.694	0.995
Hallucinations	Mental disorders	Wald ratio	1	0.794 (0.47, 1.34)	0.387	0.995
Symptoms involving head and neck	Mental disorders	Wald ratio	1	1.003 (0.846, 1.19)	0.972	0.995
Swelling, mass, or lump in head and neck [Space-occupying lesion, intracranial NOS]	Mental disorders	Wald ratio	1	1.322 (0.974, 1.795)	0.074	0.881
Schizophrenia and other psychotic disorders	Mental disorders	Wald ratio	1	1.201 (0.874, 1.65)	0.259	0.995
Schizophrenia	Mental disorders	Wald ratio	1	1.145 (0.781, 1.678)	0.489	0.995
Paranoid disorders	Mental disorders	Wald ratio	1	1.716 (0.847, 3.476)	0.134	0.958
Psychosis	Mental disorders	Wald ratio	1	1.046 (0.516, 2.119)	0.901	0.995
Mood disorders	Mental disorders	Wald ratio	1	1.091 (1.005, 1.185)	0.038	0.838
Bipolar	Mental disorders	Wald ratio	1	0.987 (0.744, 1.309)	0.927	0.995
Depression	Mental disorders	Wald ratio	1	1.094 (1.002, 1.195)	0.046	0.869
Major depressive disorder	Mental disorders	Wald ratio	1	0.719 (0.444, 1.164)	0.18	0.958
Suicidal ideation or attempt	Mental disorders	Wald ratio	1	0.968 (0.59, 1.586)	0.896	0.995
Suicide or self-inflicted injury	Mental disorders	Wald ratio	1	0.986 (0.591, 1.645)	0.958	0.995
Anxiety disorders	Mental disorders	Wald ratio	1	1.081 (0.967, 1.209)	0.171	0.958
Anxiety disorder	Mental disorders	Wald ratio	1	1.052 (0.936, 1.184)	0.395	0.995
Generalized anxiety disorder	Mental disorders	Wald ratio	1	0.741 (0.345, 1.591)	0.442	0.995
Agorophobia, social phobia, and panic disorder	Mental disorders	Wald ratio	1	0.868 (0.614, 1.229)	0.426	0.995
Phobia	Mental disorders	Wald ratio	1	0.719 (0.476, 1.085)	0.116	0.958
Obsessive-compulsive disorders	Mental disorders	Wald ratio	1	1.769 (0.823, 3.799)	0.144	0.958
Dysthymic disorder	Mental disorders	Wald ratio	1	0.583 (0.169, 2.004)	0.391	0.995
Acute reaction to stress	Mental disorders	Wald ratio	1	0.976 (0.428, 2.224)	0.954	0.995
Posttraumatic stress disorder	Mental disorders	Wald ratio	1	0.743 (0.307, 1.795)	0.509	0.995
Personality disorders	Mental disorders	Wald ratio	1	0.848 (0.514, 1.398)	0.518	0.995
Antisocial or borderline personality disorder	Mental disorders	Wald ratio	1	0.823 (0.361, 1.875)	0.642	0.995
Sexual and gender identity disorders	Mental disorders	Wald ratio	1	1.271 (0.762, 2.121)	0.358	0.995
Psychogenic and somatoform disorders	Mental disorders	Wald ratio	1	1.128 (0.756, 1.682)	0.556	0.995
Psychogenic disorder	Mental disorders	Wald ratio	1	1.23 (0.755, 2.004)	0.406	0.995
Somatoform disorder	Mental disorders	Wald ratio	1	0.823 (0.361, 1.875)	0.642	0.995
Adjustment reaction	Mental disorders	Wald ratio	1	0.962 (0.563, 1.643)	0.886	0.995
Eating disorder (no controls excluded)	Mental disorders	Wald ratio	1	1.131 (0.441, 2.898)	0.798	0.995
Eating disorder	Mental disorders	Wald ratio	1	1.134 (0.417, 3.083)	0.805	0.995
Other mental disorder	Mental disorders	Wald ratio	1	1.027 (0.969, 1.09)	0.368	0.995
Tension headache	Mental disorders	Wald ratio	1	0.914 (0.545, 1.534)	0.733	0.995
Pervasive developmental disorders	Mental disorders	Wald ratio	1	0.898 (0.311, 2.588)	0.841	0.995
Autism	Mental disorders	Wald ratio	1	0.698 (0.203, 2.399)	0.568	0.995
Develomental delays and disorders	Mental disorders	Wald ratio	1	1.091 (0.723, 1.647)	0.679	0.995
Learning disorder	Mental disorders	Wald ratio	1	1.059 (0.523, 2.144)	0.874	0.995
Speech and language disorder	Mental disorders	Wald ratio	1	6.053 (2.227, 16.453)	4.16E-04	0.265
Substance addiction and disorders	Mental disorders	Wald ratio	1	0.947 (0.595, 1.508)	0.82	0.995
Polyneuropathy due to drugs	Mental disorders	Wald ratio	1	0.517 (0.159, 1.676)	0.271	0.995
Alcohol-related disorders	Mental disorders	Wald ratio	1	1.084 (0.999, 1.177)	0.054	0.869
Alcoholism	Mental disorders	Wald ratio	1	1.021 (0.924, 1.128)	0.685	0.995
Alcoholic liver damage	Mental disorders	Wald ratio	1	0.887 (0.642, 1.226)	0.467	0.995
Tobacco use disorder	Mental disorders	Wald ratio	1	1.04 (0.969, 1.116)	0.279	0.995
Osteomyelitis, periostitis, and other infections involving bone	Musculoskeletal	Wald ratio	1	0.798 (0.551, 1.157)	0.234	0.995
Osteomyelitis	Musculoskeletal	Wald ratio	1	0.789 (0.535, 1.163)	0.231	0.995
Chronic osteomyelitis	Musculoskeletal	Wald ratio	1	0.887 (0.464, 1.694)	0.716	0.995
Unspecified osteomyelitis	Musculoskeletal	Wald ratio	1	0.741 (0.468, 1.172)	0.2	0.958
Arthropathy associated with infections	Musculoskeletal	Wald ratio	1	0.999 (0.643, 1.554)	0.998	0.999
Pyogenic arthritis	Musculoskeletal	Wald ratio	1	0.898 (0.541, 1.489)	0.676	0.995
Infective connective tissue disorders	Musculoskeletal	Wald ratio	1	0.698 (0.257, 1.896)	0.48	0.995
Arthropathy associated with other disorders classified elsewhere	Musculoskeletal	Wald ratio	1	0.97 (0.452, 2.085)	0.939	0.995
Arthropathy associated with neurological disorders	Musculoskeletal	Wald ratio	1	0.925 (0.303, 2.828)	0.891	0.995
Rheumatoid arthritis and other inflammatory polyarthropathies	Musculoskeletal	Wald ratio	1	0.994 (0.868, 1.138)	0.931	0.995
Rheumatoid arthritis	Musculoskeletal	Wald ratio	1	1.017 (0.883, 1.171)	0.816	0.995
Other inflammatory spondylopathies	Musculoskeletal	Wald ratio	1	0.953 (0.762, 1.192)	0.674	0.995
Sacroiliitis NEC	Musculoskeletal	Wald ratio	1	1.477 (0.611, 3.569)	0.386	0.995
Ankylosing spondylitis	Musculoskeletal	Wald ratio	1	0.908 (0.627, 1.316)	0.611	0.995
Other arthropathies	Musculoskeletal	Wald ratio	1	1.001 (0.951, 1.054)	0.961	0.995
Unspecified polyarthropathy or polyarthritis	Musculoskeletal	Wald ratio	1	1.043 (0.89, 1.222)	0.604	0.995
Unspecified monoarthritis	Musculoskeletal	Wald ratio	1	0.987 (0.915, 1.066)	0.747	0.995
Arthropathy NOS	Musculoskeletal	Wald ratio	1	0.993 (0.942, 1.047)	0.79	0.995
Polymyalgia Rheumatica	Musculoskeletal	Wald ratio	1	0.887 (0.677, 1.162)	0.385	0.995
Spinal stenosis	Musculoskeletal	Wald ratio	1	1.027 (0.881, 1.197)	0.732	0.995
Spondylosis and allied disorders	Musculoskeletal	Wald ratio	1	1.02 (0.918, 1.134)	0.71	0.995
Spondylosis without myelopathy	Musculoskeletal	Wald ratio	1	1.014 (0.891, 1.154)	0.834	0.995
Spondylosis with myelopathy	Musculoskeletal	Wald ratio	1	0.747 (0.391, 1.427)	0.378	0.995
Other allied disorders of spine	Musculoskeletal	Wald ratio	1	1.034 (0.634, 1.684)	0.895	0.995
Intervertebral disc disorders	Musculoskeletal	Wald ratio	1	0.989 (0.895, 1.093)	0.832	0.995
Displacement of intervertebral disc	Musculoskeletal	Wald ratio	1	0.959 (0.639, 1.439)	0.839	0.995
Degeneration of intervertebral disc	Musculoskeletal	Wald ratio	1	0.968 (0.811, 1.154)	0.714	0.995
Postlaminectomy syndrome	Musculoskeletal	Wald ratio	1	1.078 (0.353, 3.296)	0.895	0.995
Other and unspecified disc disorder	Musculoskeletal	Wald ratio	1	0.985 (0.856, 1.135)	0.838	0.995
Other disorders of cervical region	Musculoskeletal	Wald ratio	1	0.828 (0.552, 1.242)	0.361	0.995

Torticollis	Musculoskeletal	Wald ratio	1	0.988 (0.409, 2.388)	0.979	0.995
Other and unspecified disorders of back	Musculoskeletal	Wald ratio	1	0.947 (0.776, 1.157)	0.597	0.995
Disorders of sacrum	Musculoskeletal	Wald ratio	1	0.968 (0.612, 1.531)	0.888	0.995
Disorders of coccyx	Musculoskeletal	Wald ratio	1	0.986 (0.627, 1.551)	0.951	0.995
Other symptoms referable to back	Musculoskeletal	Wald ratio	1	1.433 (0.442, 4.648)	0.549	0.995
Other unspecified back disorders	Musculoskeletal	Wald ratio	1	0.917 (0.729, 1.153)	0.457	0.995
Peripheral enthesopathies and allied syndromes	Musculoskeletal	Wald ratio	1	0.985 (0.912, 1.063)	0.689	0.995
Enthesopathy	Musculoskeletal	Wald ratio	1	0.972 (0.885, 1.068)	0.557	0.995
Synoviopathy	Musculoskeletal	Wald ratio	1	1.283 (0.778, 2.115)	0.329	0.995
Bursitis	Musculoskeletal	Wald ratio	1	0.808 (0.592, 1.104)	0.18	0.958
Other disorders of synovium, tendon, and bursa	Musculoskeletal	Wald ratio	1	0.974 (0.876, 1.082)	0.621	0.995
Synovitis and tenosynovitis	Musculoskeletal	Wald ratio	1	1.155 (0.968, 1.378)	0.11	0.948
Bursitis disorders	Musculoskeletal	Wald ratio	1	1.065 (0.441, 2.574)	0.889	0.995
Ganglion and cyst of synovium, tendon, and bursa	Musculoskeletal	Wald ratio	1	0.833 (0.706, 0.982)	0.029	0.83
Rupture of synovium	Musculoskeletal	Wald ratio	1	1.091 (0.749, 1.59)	0.65	0.995
Rupture of tendon, nontraumatic	Musculoskeletal	Wald ratio	1	1.121 (0.688, 1.826)	0.647	0.995
Contracture of tendon (sheath)	Musculoskeletal	Wald ratio	1	0.6 (0.279, 1.29)	0.191	0.958
Disorders of muscle, ligament, and fascia	Musculoskeletal	Wald ratio	1	0.95 (0.825, 1.094)	0.479	0.995
Laxity of ligament or hypermobility syndrome	Musculoskeletal	Wald ratio	1	0.806 (0.515, 1.26)	0.343	0.995
Fasciitis	Musculoskeletal	Wald ratio	1	0.939 (0.806, 1.094)	0.419	0.995
Contracture of palmar fascia [Dupuytren's disease]	Musculoskeletal	Wald ratio	1	0.959 (0.818, 1.124)	0.604	0.995
Other disorders of soft tissues	Musculoskeletal	Wald ratio	1	0.996 (0.886, 1.12)	0.948	0.995
Rheumatism, unspecified and fibrositis	Musculoskeletal	Wald ratio	1	1.128 (0.845, 1.504)	0.414	0.995
Panniculitis	Musculoskeletal	Wald ratio	1	1.02 (0.422, 2.466)	0.964	0.995
Osteitis deformans and osteopathies associated with other disorders classified elsewhere	Musculoskeletal	Wald ratio	1	0.657 (0.38, 1.135)	0.132	0.958
Osteitis deformans [Paget's disease of bone]	Musculoskeletal	Wald ratio	1	0.677 (0.385, 1.191)	0.176	0.958
Osteochondropathies	Musculoskeletal	Wald ratio	1	0.657 (0.43, 1.003)	0.052	0.869
Juvenile osteochondrosis	Musculoskeletal	Wald ratio	1	0.657 (0.344, 1.255)	0.203	0.958
Osteochondritis dissecans	Musculoskeletal	Wald ratio	1	0.863 (0.266, 2.799)	0.806	0.995
Other disorders of bone and cartilage	Musculoskeletal	Wald ratio	1	1.056 (0.911, 1.223)	0.472	0.995
Cyst of bone	Musculoskeletal	Wald ratio	1	1.084 (0.65, 1.809)	0.756	0.995
Aseptic necrosis of bone	Musculoskeletal	Wald ratio	1	1.043 (0.703, 1.547)	0.834	0.995
Costochondritis	Musculoskeletal	Wald ratio	1	1.088 (0.477, 2.478)	0.841	0.995
Malunion and nonunion of fracture	Musculoskeletal	Wald ratio	1	1.194 (0.922, 1.546)	0.18	0.958
Chondromalacia	Musculoskeletal	Wald ratio	1	0.848 (0.372, 1.932)	0.694	0.995
Acquired foot deformities	Musculoskeletal	Wald ratio	1	0.965 (0.878, 1.06)	0.453	0.995
Flat foot	Musculoskeletal	Wald ratio	1	1.134 (0.594, 2.166)	0.703	0.995
Acquired toe deformities	Musculoskeletal	Wald ratio	1	1.022 (0.898, 1.163)	0.747	0.995
Hammer toe (acquired)	Musculoskeletal	Wald ratio	1	1.075 (0.87, 1.328)	0.505	0.995
Hallux rigidus	Musculoskeletal	Wald ratio	1	1.056 (0.834, 1.335)	0.653	0.995
Hallux valgus (Bunion)	Musculoskeletal	Wald ratio	1	0.928 (0.825, 1.044)	0.211	0.97
Other acquired deformities of limbs	Musculoskeletal	Wald ratio	1	1.037 (0.829, 1.296)	0.752	0.995
Acquired deformities of finger	Musculoskeletal	Wald ratio	1	0.741 (0.414, 1.326)	0.312	0.995
Curvature of spine	Musculoskeletal	Wald ratio	1	0.914 (0.693, 1.205)	0.523	0.995
Kyphoscoliosis and scoliosis	Musculoskeletal	Wald ratio	1	0.956 (0.721, 1.268)	0.755	0.995
Other acquired musculoskeletal deformity	Musculoskeletal	Wald ratio	1	1.023 (0.828, 1.264)	0.833	0.995
Acquired spondylolisthesis	Musculoskeletal	Wald ratio	1	0.884 (0.699, 1.119)	0.305	0.995
Contracture of joint	Musculoskeletal	Wald ratio	1	1.04 (0.689, 1.569)	0.853	0.995
Osteoarthrosis	Musculoskeletal	Wald ratio	1	0.947 (0.893, 1.005)	0.072	0.879
Osteoarthritis; localized	Musculoskeletal	Wald ratio	1	0.906 (0.844, 0.972)	0.006	0.505
Osteoarthrosis, localized, primary	Musculoskeletal	Wald ratio	1	0.903 (0.817, 0.998)	0.046	0.869
Osteoarthrosis, localized, secondary	Musculoskeletal	Wald ratio	1	0.698 (0.426, 1.143)	0.153	0.958
Osteoarthrosis, generalized	Musculoskeletal	Wald ratio	1	0.698 (0.479, 1.016)	0.061	0.869
Osteoarthrosis NOS	Musculoskeletal	Wald ratio	1	0.988 (0.904, 1.079)	0.785	0.995
Symptoms and disorders of the joints	Musculoskeletal	Wald ratio	1	1.02 (0.875, 1.188)	0.803	0.995
Ankylosis of joint	Musculoskeletal	Wald ratio	1	0.84 (0.348, 2.03)	0.699	0.995
Stiffness of joint	Musculoskeletal	Wald ratio	1	0.761 (0.525, 1.102)	0.149	0.958
Difficulty in walking	Musculoskeletal	Wald ratio	1	1.477 (0.729, 2.992)	0.279	0.995
Joint effusions	Musculoskeletal	Wald ratio	1	0.985 (0.774, 1.254)	0.905	0.995
Hemarthrosis	Musculoskeletal	Wald ratio	1	1.569 (0.649, 3.79)	0.317	0.995
Villonodular synovitis	Musculoskeletal	Wald ratio	1	0.756 (0.22, 2.602)	0.658	0.995
Derangement of joint, non-traumatic	Musculoskeletal	Wald ratio	1	0.898 (0.757, 1.065)	0.214	0.975
Loose body in joint	Musculoskeletal	Wald ratio	1	0.823 (0.496, 1.364)	0.45	0.995
Pathological, developmental or recurrent dislocation	Musculoskeletal	Wald ratio	1	0.903 (0.352, 2.314)	0.832	0.995
Articular cartilage disorder	Musculoskeletal	Wald ratio	1	0.6 (0.398, 0.906)	0.015	0.709
Other derangement of joint	Musculoskeletal	Wald ratio	1	0.992 (0.807, 1.219)	0.939	0.995
Osteoporosis, osteopenia and pathological fracture	Musculoskeletal	Wald ratio	1	0.989 (0.89, 1.1)	0.841	0.995
Osteoporosis	Musculoskeletal	Wald ratio	1	1.014 (0.901, 1.14)	0.822	0.995
Osteoporosis NOS	Musculoskeletal	Wald ratio	1	0.983 (0.869, 1.113)	0.79	0.995
Other specified osteoporosis	Musculoskeletal	Wald ratio	1	1.155 (0.774, 1.723)	0.48	0.995
Pathologic fracture	Musculoskeletal	Wald ratio	1	0.768 (0.512, 1.152)	0.202	0.958
Osteopenia or other disorder of bone and cartilage	Musculoskeletal	Wald ratio	1	0.947 (0.686, 1.309)	0.743	0.995
Pain in joint	Musculoskeletal	Wald ratio	1	1.107 (0.996, 1.231)	0.059	0.869
Cancer of mouth	Neoplasms	Wald ratio	1	1.322 (0.923, 1.892)	0.127	0.958
Cancer of tongue	Neoplasms	Wald ratio	1	1.35 (0.8, 2.279)	0.261	0.995
Cancer of major salivary glands	Neoplasms	Wald ratio	1	0.914 (0.378, 2.208)	0.841	0.995
Cancer of the mouth floor	Neoplasms	Wald ratio	1	0.619 (0.18, 2.128)	0.446	0.995
Cancer of larynx, pharynx, nasal cavities	Neoplasms	Wald ratio	1	0.698 (0.484, 1.005)	0.053	0.869

Cancer of oropharynx	Neoplasms	Wald ratio	1	0.838 (0.465, 1.509)	0.555	0.995
Cancer of larynx	Neoplasms	Wald ratio	1	0.487 (0.275, 0.861)	0.013	0.709
Cancer of of nasal cavities	Neoplasms	Wald ratio	1	3.128 (0.91, 10.757)	0.07	0.879
Cancer of esophagus	Neoplasms	Wald ratio	1	1.111 (0.79, 1.562)	0.546	0.995
Cancer of stomach	Neoplasms	Wald ratio	1	0.9 (0.611, 1.327)	0.596	0.995
Colorectal cancer	Neoplasms	Wald ratio	1	1.046 (0.908, 1.205)	0.532	0.995
Colon cancer	Neoplasms	Wald ratio	1	1.084 (0.914, 1.286)	0.352	0.995
Malignant neoplasm of rectum, rectosigmoid junction, and anus	Neoplasms	Wald ratio	1	1.002 (0.821, 1.224)	0.981	0.995
Cancer of liver and intrahepatic bile duct	Neoplasms	Wald ratio	1	1.094 (0.668, 1.793)	0.721	0.995
Malignant neoplasm of liver, primary	Neoplasms	Wald ratio	1	1.716 (0.799, 3.687)	0.166	0.958
Pancreatic cancer	Neoplasms	Wald ratio	1	1.117 (0.767, 1.628)	0.563	0.995
Neoplasm of unspecified nature of digestive system	Neoplasms	Wald ratio	1	0.976 (0.736, 1.294)	0.864	0.995
Malignant neoplasm of other and ill-defined sites within the digestive organs and peritoneum	Neoplasms	Wald ratio	1	1.075 (0.95, 1.216)	0.253	0.995
Malignant neoplasm of small intestine, including duodenum	Neoplasms	Wald ratio	1	0.843 (0.416, 1.707)	0.635	0.995
Malignant neoplasm of gallbladder and extrahepatic bile ducts	Neoplasms	Wald ratio	1	2.317 (1.213, 4.425)	0.011	0.709
Malignant neoplasm of retroperitoneum and peritoneum	Neoplasms	Wald ratio	1	0.989 (0.488, 2.003)	0.975	0.995
Cancer of intrathoracic organs	Neoplasms	Wald ratio	1	0.85 (0.247, 2.925)	0.797	0.995
Cancer within the respiratory system	Neoplasms	Wald ratio	1	0.945 (0.792, 1.127)	0.527	0.995
Cancer of bronchus; lung	Neoplasms	Wald ratio	1	0.945 (0.773, 1.154)	0.576	0.995
Cancer of bone and connective tissue	Neoplasms	Wald ratio	1	1.279 (0.818, 2)	0.281	0.995
Bone cancer	Neoplasms	Wald ratio	1	1.616 (0.709, 3.683)	0.253	0.995
Cancer of connective tissue	Neoplasms	Wald ratio	1	1.134 (0.676, 1.903)	0.633	0.995
Skin cancer	Neoplasms	Wald ratio	1	1.028 (0.947, 1.116)	0.511	0.995
Melanomas of skin, dx or hx	Neoplasms	Wald ratio	1	1.049 (0.879, 1.252)	0.594	0.995
Melanomas of skin	Neoplasms	Wald ratio	1	1.049 (0.879, 1.252)	0.594	0.995
Other non-epithelial cancer of skin	Neoplasms	Wald ratio	1	1.015 (0.929, 1.108)	0.749	0.995
Carcinoma in situ of skin	Neoplasms	Wald ratio	1	1.338 (0.935, 1.915)	0.112	0.958
Neoplasm of uncertain behavior of skin	Neoplasms	Wald ratio	1	0.756 (0.373, 1.532)	0.438	0.995
Breast cancer	Neoplasms	Wald ratio	1	0.922 (0.844, 1.007)	0.072	0.879
Breast cancer [female]	Neoplasms	Wald ratio	1	0.931 (0.852, 1.016)	0.11	0.948
Malignant neoplasm of female breast	Neoplasms	Wald ratio	1	0.917 (0.839, 1.001)	0.053	0.869
Neoplasm of uncertain behavior of breast	Neoplasms	Wald ratio	1	0.853 (0.374, 1.943)	0.705	0.995
Acquired absence of breast	Neoplasms	Wald ratio	1	0.884 (0.733, 1.067)	0.2	0.958
Cervical cancer and dysplasia	Neoplasms	Wald ratio	1	1 (0.859, 1.166)	0.995	0.998
Cervical cancer	Neoplasms	Wald ratio	1	1.014 (0.806, 1.275)	0.908	0.995
Cervical intraepithelial neoplasia [CIN] [Cervical dysplasia]	Neoplasms	Wald ratio	1	0.975 (0.799, 1.191)	0.807	0.995
Malignant neoplasm of uterus	Neoplasms	Wald ratio	1	1.104 (0.852, 1.43)	0.453	0.995
Cancer of other female genital organs	Neoplasms	Wald ratio	1	0.965 (0.799, 1.164)	0.708	0.995
Malignant neoplasm of ovary and other uterine adnexa	Neoplasms	Wald ratio	1	1.008 (0.826, 1.232)	0.934	0.995
Malignant neoplasm of ovary	Neoplasms	Wald ratio	1	0.991 (0.812, 1.211)	0.932	0.995
Cancer of other female genital organs (excluding uterus and ovary)	Neoplasms	Wald ratio	1	1.018 (0.599, 1.728)	0.948	0.995
Malignant neoplasm of testis	Neoplasms	Wald ratio	1	1.034 (0.892, 1.197)	0.66	0.995
Neoplasm of uncertain behavior of male genital organs	Neoplasms	Wald ratio	1	0.458 (0.19, 1.107)	0.083	0.899
Cancer of urinary organs (incl. kidney and bladder)	Neoplasms	Wald ratio	1	0.992 (0.857, 1.149)	0.917	0.995
Cancer of kidney and renal pelvis	Neoplasms	Wald ratio	1	1.094 (0.825, 1.451)	0.532	0.995
Malignant neoplasm of kidney, except pelvis	Neoplasms	Wald ratio	1	1.141 (0.855, 1.522)	0.369	0.995
Malignant neoplasm of renal pelvis	Neoplasms	Wald ratio	1	0.638 (0.209, 1.949)	0.43	0.995
Cancer of bladder	Neoplasms	Wald ratio	1	0.993 (0.823, 1.199)	0.945	0.995
Malignant neoplasm of bladder	Neoplasms	Wald ratio	1	0.993 (0.813, 1.213)	0.948	0.995
Malignant neoplasm of other urinary organs	Neoplasms	Wald ratio	1	1.391 (0.611, 3.17)	0.432	0.995
Cancer of eye	Neoplasms	Wald ratio	1	0.835 (0.412, 1.692)	0.617	0.995
Manlignant and unknown neoplasms of brain and nervous system	Neoplasms	Wald ratio	1	1.111 (0.776, 1.59)	0.566	0.995
Cancer of brain and nervous system	Neoplasms	Wald ratio	1	1.169 (0.784, 1.744)	0.444	0.995
Cancer of brain	Neoplasms	Wald ratio	1	1.138 (0.749, 1.727)	0.545	0.995
Thyroid cancer	Neoplasms	Wald ratio	1	1.252 (0.769, 2.041)	0.366	0.995
Cancer of other endocrine glands	Neoplasms	Wald ratio	1	0.419 (0.163, 1.073)	0.07	0.879
Cancer, suspected or other	Neoplasms	Wald ratio	1	0.99 (0.922, 1.062)	0.777	0.995
Malignant neoplasm, other	Neoplasms	Wald ratio	1	0.997 (0.924, 1.077)	0.948	0.995
Chemotherapy	Neoplasms	Wald ratio	1	1.008 (0.944, 1.075)	0.82	0.995
Secondary malignant neoplasm	Neoplasms	Wald ratio	1	0.95 (0.865, 1.044)	0.288	0.995
Secondary malignancy of lymph nodes	Neoplasms	Wald ratio	1	0.914 (0.808, 1.034)	0.153	0.958
Secondary malignancy of respiratory organs	Neoplasms	Wald ratio	1	0.863 (0.711, 1.048)	0.138	0.958
Secondary malignant neoplasm of digestive systems	Neoplasms	Wald ratio	1	1.052 (0.832, 1.331)	0.671	0.995
Secondary malignant neoplasm of liver	Neoplasms	Wald ratio	1	0.953 (0.794, 1.144)	0.606	0.995
Secondary malignancy of brain or spine	Neoplasms	Wald ratio	1	0.996 (0.721, 1.376)	0.98	0.995
Secondary malignancy of bone	Neoplasms	Wald ratio	1	0.956 (0.783, 1.168)	0.659	0.995
Secondary malignant neoplasm of skin	Neoplasms	Wald ratio	1	1.111 (0.582, 2.121)	0.75	0.995
Neoplasm of uncertain behavior	Neoplasms	Wald ratio	1	1.012 (0.791, 1.296)	0.922	0.995
Neurofibromatosis	Neoplasms	Wald ratio	1	1.31 (0.511, 3.357)	0.574	0.995
Myeloproliferative disease	Neoplasms	Wald ratio	1	1.012 (0.754, 1.358)	0.936	0.995
Polycythemia vera	Neoplasms	Wald ratio	1	0.992 (0.623, 1.579)	0.973	0.995
Hodgkin's disease	Neoplasms	Wald ratio	1	1.037 (0.586, 1.834)	0.902	0.995
Cancer of other lymphoid, histiocytic tissue	Neoplasms	Wald ratio	1	0.962 (0.792, 1.168)	0.694	0.995
Non-Hodgkins lymphoma	Neoplasms	Wald ratio	1	1.056 (0.849, 1.312)	0.627	0.995
Nodular lymphoma	Neoplasms	Wald ratio	1	1.306 (0.811, 2.103)	0.272	0.995
Large cell lymphoma	Neoplasms	Wald ratio	1	0.719 (0.49, 1.054)	0.091	0.913
Leukemia	Neoplasms	Wald ratio	1	0.974 (0.779, 1.218)	0.817	0.995
Lymphoid leukemia	Neoplasms	Wald ratio	1	1.094 (0.747, 1.604)	0.644	0.995

Lymphoid leukemia, acute	Neoplasms	Wald ratio	1	1.35 (0.416, 4.378)	0.617	0.995
Lymphoid leukemia, chronic	Neoplasms	Wald ratio	1	1.018 (0.675, 1.537)	0.932	0.995
Myeloid leukemia	Neoplasms	Wald ratio	1	0.947 (0.62, 1.447)	0.803	0.995
Myeloid leukemia, acute	Neoplasms	Wald ratio	1	1.091 (0.654, 1.82)	0.739	0.995
Myeloid leukemia, chronic	Neoplasms	Wald ratio	1	0.83 (0.386, 1.784)	0.633	0.995
Multiple myeloma	Neoplasms	Wald ratio	1	1.059 (0.718, 1.561)	0.773	0.995
Benign neoplasm of colon	Neoplasms	Wald ratio	1	0.942 (0.878, 1.011)	0.096	0.913
Benign neoplasm of lip, oral cavity, and pharynx	Neoplasms	Wald ratio	1	1.097 (0.818, 1.473)	0.535	0.995
Benign neoplasm of other parts of digestive system	Neoplasms	Wald ratio	1	0.965 (0.848, 1.098)	0.585	0.995
Benign neoplasm of respiratory and intrathoracic organs	Neoplasms	Wald ratio	1	0.9 (0.549, 1.476)	0.677	0.995
Benign neoplasm of bone and articular cartilage	Neoplasms	Wald ratio	1	0.843 (0.505, 1.406)	0.512	0.995
Lipoma	Neoplasms	Wald ratio	1	1.101 (0.979, 1.238)	0.11	0.948
Lipoma of skin and subcutaneous tissue	Neoplasms	Wald ratio	1	1.071 (0.936, 1.227)	0.317	0.995
Other benign neoplasm of connective and other soft tissue	Neoplasms	Wald ratio	1	0.782 (0.593, 1.031)	0.081	0.899
Benign neoplasm of skin	Neoplasms	Wald ratio	1	1.022 (0.919, 1.136)	0.689	0.995
Vascular hamartomas and non-neoplastic nevi	Neoplasms	Wald ratio	1	1.204 (0.841, 1.724)	0.309	0.995
Nevus, non-neoplastic	Neoplasms	Wald ratio	1	1.326 (0.91, 1.932)	0.142	0.958
Benign neoplasm of uterus	Neoplasms	Wald ratio	1	1.124 (1.023, 1.235)	0.015	0.709
Uterine leiomyoma	Neoplasms	Wald ratio	1	1.117 (1.017, 1.228)	0.021	0.758
Other benign neoplasm of uterus	Neoplasms	Wald ratio	1	1.433 (0.88, 2.336)	0.148	0.958
Benign neoplasm of ovary	Neoplasms	Wald ratio	1	0.866 (0.68, 1.102)	0.242	0.995
Benign neoplasm of other female genital organs	Neoplasms	Wald ratio	1	1.249 (0.693, 2.249)	0.459	0.995
Benign neoplasm of kidney and other urinary organs	Neoplasms	Wald ratio	1	0.956 (0.472, 1.936)	0.901	0.995
Benign neoplasm of eye	Neoplasms	Wald ratio	1	0.89 (0.39, 2.027)	0.781	0.995
Benign neoplasm of eye, uveal	Neoplasms	Wald ratio	1	0.815 (0.267, 2.493)	0.72	0.995
Benign neoplasm of brain and other parts of nervous system	Neoplasms	Wald ratio	1	1.124 (0.818, 1.544)	0.47	0.995
Benign neoplasm of brain, cranial nerves, meninges	Neoplasms	Wald ratio	1	1.059 (0.762, 1.472)	0.734	0.995
Benign neoplasm of spinal cord, meninges	Neoplasms	Wald ratio	1	2.182 (0.673, 7.076)	0.194	0.958
Benign neoplasm of thyroid glands	Neoplasms	Wald ratio	1	1.176 (0.684, 2.02)	0.557	0.995
Benign neoplasm of other endocrine glands and related structures	Neoplasms	Wald ratio	1	0.863 (0.632, 1.179)	0.355	0.995
Benign neoplasm of adrenal gland	Neoplasms	Wald ratio	1	0.719 (0.355, 1.456)	0.359	0.995
Benign neoplasm of parathyroid gland	Neoplasms	Wald ratio	1	0.85 (0.528, 1.369)	0.505	0.995
Benign neoplasm of pituitary gland and craniopharyngeal duct (pouch)	Neoplasms	Wald ratio	1	0.994 (0.589, 1.677)	0.981	0.995
Hemangioma and lymphangioma, any site	Neoplasms	Wald ratio	1	0.853 (0.678, 1.073)	0.174	0.958
Benign neoplasm of unspecified sites	Neoplasms	Wald ratio	1	0.97 (0.804, 1.171)	0.755	0.995
Bone marrow or stem cell transplant	Neoplasms	Wald ratio	1	0.898 (0.33, 2.44)	0.832	0.995
Meningitis	Neurological	Wald ratio	1	0.887 (0.574, 1.371)	0.589	0.995
Encephalitis	Neurological	Wald ratio	1	1.35 (0.824, 2.213)	0.234	0.995
Acute (transverse) myelitis	Neurological	Wald ratio	1	1.878 (0.651, 5.414)	0.243	0.995
Other CNS infection and poliomyelitis	Neurological	Wald ratio	1	1.35 (0.786, 2.319)	0.277	0.995
Sleep disorders	Neurological	Wald ratio	1	0.996 (0.875, 1.133)	0.949	0.995
Sleep apnea	Neurological	Wald ratio	1	0.98 (0.851, 1.129)	0.783	0.995
Insomnia	Neurological	Wald ratio	1	1.134 (0.56, 2.298)	0.726	0.995
Organic or persistent insomnia	Neurological	Wald ratio	1	1.35 (0.628, 2.9)	0.442	0.995
Other cerebral degenerations	Neurological	Wald ratio	1	0.813 (0.617, 1.072)	0.142	0.958
Hydrocephalus	Neurological	Wald ratio	1	0.657 (0.435, 0.992)	0.046	0.869
Cerebral degeneration, unspecified	Neurological	Wald ratio	1	0.791 (0.506, 1.237)	0.305	0.995
Parkinson's disease	Neurological	Wald ratio	1	0.945 (0.716, 1.245)	0.686	0.995
Extrapyramidal disease and abnormal movement disorders	Neurological	Wald ratio	1	1.049 (0.773, 1.425)	0.758	0.995
Essential tremor	Neurological	Wald ratio	1	0.565 (0.296, 1.08)	0.084	0.899
Myoclonus	Neurological	Wald ratio	1	1.124 (0.439, 2.881)	0.807	0.995
Torsion dystonia	Neurological	Wald ratio	1	1.027 (0.577, 1.828)	0.928	0.995
Degenerative disease of the spinal cord	Neurological	Wald ratio	1	0.879 (0.707, 1.093)	0.245	0.995
Spinocerebellar disease	Neurological	Wald ratio	1	0.97 (0.426, 2.211)	0.943	0.995
Anterior horn cell disease	Neurological	Wald ratio	1	0.657 (0.365, 1.183)	0.162	0.958
Multiple sclerosis	Neurological	Wald ratio	1	0.782 (0.607, 1.007)	0.057	0.869
Disorders of the autonomic nervous system	Neurological	Wald ratio	1	0.806 (0.458, 1.417)	0.453	0.995
Peripheral autonomic neuropathy	Neurological	Wald ratio	1	0.956 (0.332, 2.756)	0.934	0.995
Acute pain	Neurological	Wald ratio	1	1.241 (0.78, 1.975)	0.362	0.995
Chronic pain	Neurological	Wald ratio	1	1.043 (0.651, 1.67)	0.861	0.995
Other headache syndromes	Neurological	Wald ratio	1	0.998 (0.898, 1.11)	0.976	0.995
Migraine	Neurological	Wald ratio	1	0.925 (0.78, 1.097)	0.37	0.995
Migrain with aura	Neurological	Wald ratio	1	1.35 (0.707, 2.578)	0.363	0.995
Other demyelinating diseases of central nervous system	Neurological	Wald ratio	1	0.782 (0.439, 1.391)	0.403	0.995
Hemiplegia	Neurological	Wald ratio	1	1.187 (0.932, 1.51)	0.164	0.958
Infantile cerebral palsy	Neurological	Wald ratio	1	1.197 (0.557, 2.572)	0.644	0.995
Other paralytic syndromes	Neurological	Wald ratio	1	0.919 (0.642, 1.316)	0.646	0.995
Epilepsy, recurrent seizures, convulsions	Neurological	Wald ratio	1	0.974 (0.855, 1.108)	0.686	0.995
Epilepsy	Neurological	Wald ratio	1	1.145 (0.843, 1.554)	0.387	0.995
Generalized convulsive epilepsy	Neurological	Wald ratio	1	1.011 (0.654, 1.563)	0.96	0.995
Partial epilepsy	Neurological	Wald ratio	1	1.935 (1.12, 3.344)	0.018	0.71
Convulsions	Neurological	Wald ratio	1	0.985 (0.811, 1.196)	0.88	0.995
Abnormal findings on study of brain and or or nervous system	Neurological	Wald ratio	1	0.993 (0.52, 1.896)	0.983	0.995
Nonspecific abnormal findings on radiological and other examination of skull and head	Neurological	Wald ratio	1	1.569 (0.73, 3.37)	0.249	0.995
Other conditions of brain	Neurological	Wald ratio	1	0.892 (0.701, 1.136)	0.354	0.995
Cerebral edema and compression of brain	Neurological	Wald ratio	1	1.037 (0.586, 1.834)	0.902	0.995
Cerebral cysts	Neurological	Wald ratio	1	0.89 (0.414, 1.911)	0.764	0.995
Coma	Neurological	Wald ratio	1	0.871 (0.538, 1.411)	0.575	0.995

Encephalopathy, not elsewhere classified	Neurological	Wald ratio	1	1.094 (0.608, 1.97)	0.764	0.995
Other conditions of brain, NOS	Neurological	Wald ratio	1	0.977 (0.614, 1.556)	0.923	0.995
Other and unspecified disorders of the nervous system	Neurological	Wald ratio	1	0.931 (0.564, 1.534)	0.778	0.995
Abnormal movement	Neurological	Wald ratio	1	0.922 (0.773, 1.1)	0.368	0.995
Abnormal involuntary movements	Neurological	Wald ratio	1	1.141 (0.84, 1.549)	0.397	0.995
Abnormality of gait	Neurological	Wald ratio	1	0.876 (0.697, 1.102)	0.259	0.995
Lack of coordination	Neurological	Wald ratio	1	0.997 (0.616, 1.615)	0.991	0.995
Disturbances of sensation of smell and taste	Neurological	Wald ratio	1	0.677 (0.197, 2.328)	0.536	0.995
Other peripheral nerve disorders	Neurological	Wald ratio	1	1.012 (0.932, 1.099)	0.77	0.995
Disorders of other cranial nerves	Neurological	Wald ratio	1	0.953 (0.744, 1.22)	0.703	0.995
Trigeminal nerve disorders [CN5]	Neurological	Wald ratio	1	0.833 (0.545, 1.272)	0.397	0.995
Facial nerve disorders [CN7]	Neurological	Wald ratio	1	1.03 (0.754, 1.407)	0.85	0.995
Nerve root and plexus disorders	Neurological	Wald ratio	1	0.906 (0.691, 1.187)	0.473	0.995
Nerve plexus lesions	Neurological	Wald ratio	1	0.657 (0.272, 1.587)	0.351	0.995
Nerve root lesions	Neurological	Wald ratio	1	0.657 (0.191, 2.259)	0.505	0.995
Hereditary and idiopathic peripheral neuropathy	Neurological	Wald ratio	1	1.249 (0.581, 2.682)	0.569	0.995
Inflammatory and toxic neuropathy	Neurological	Wald ratio	1	0.796 (0.622, 1.019)	0.07	0.879
Myoneural disorders	Neurological	Wald ratio	1	0.745 (0.39, 1.423)	0.373	0.995
Myasthenia gravis	Neurological	Wald ratio	1	0.754 (0.395, 1.44)	0.393	0.995
Muscular dystrophies and other myopathies	Neurological	Wald ratio	1	0.698 (0.457, 1.065)	0.096	0.913
Muscular dystrophies	Neurological	Wald ratio	1	1.046 (0.487, 2.247)	0.908	0.995
Myopathy	Neurological	Wald ratio	1	0.638 (0.394, 1.033)	0.067	0.879
Miscarriage; stillbirth	Pregnancy complications	Wald ratio	1	1.059 (0.925, 1.212)	0.409	0.995
Missed abortion or Hydatidiform mole	Pregnancy complications	Wald ratio	1	1.194 (0.916, 1.555)	0.19	0.958
Ectopic pregnancy	Pregnancy complications	Wald ratio	1	0.638 (0.384, 1.057)	0.081	0.899
Hemorrhage during pregnancy; childbirth and postpartum	Pregnancy complications	Wald ratio	1	0.898 (0.726, 1.109)	0.317	0.995
Antepartum hemorrhage, abruptio placentae, and placenta previa	Pregnancy complications	Wald ratio	1	0.777 (0.566, 1.068)	0.12	0.958
Placenta previa and abruptio placenta	Pregnancy complications	Wald ratio	1	0.97 (0.745, 1.265)	0.824	0.995
Early or threatened labor; hemorrhage in early pregnancy	Pregnancy complications	Wald ratio	1	1.023 (0.848, 1.235)	0.812	0.995
Early onset of delivery	Pregnancy complications	Wald ratio	1	1.094 (0.751, 1.594)	0.639	0.995
Hemorrhage in early pregnancy	Pregnancy complications	Wald ratio	1	0.99 (0.747, 1.314)	0.947	0.995
Other high-risk pregnancy	Pregnancy complications	Wald ratio	1	0.868 (0.284, 2.655)	0.805	0.995
Hypertension complicating pregnancy, childbirth, and the puerperium	Pregnancy complications	Wald ratio	1	0.919 (0.693, 1.219)	0.56	0.995
Preeclampsia and eclampsia	Pregnancy complications	Wald ratio	1	0.85 (0.504, 1.435)	0.544	0.995
Excessive vomiting in pregnancy	Pregnancy complications	Wald ratio	1	1.569 (0.871, 2.825)	0.134	0.958
Hyperemesis gravidarum	Pregnancy complications	Wald ratio	1	2.055 (0.957, 4.414)	0.065	0.879
Late pregnancy and failed induction	Pregnancy complications	Wald ratio	1	0.895 (0.687, 1.166)	0.411	0.995
Other complications of pregnancy NEC	Pregnancy complications	Wald ratio	1	1.005 (0.822, 1.227)	0.965	0.995
Infectious and parasitic complications affecting pregnancy	Pregnancy complications	Wald ratio	1	0.798 (0.496, 1.286)	0.354	0.995
Infections of genitourinary tract during pregnancy	Pregnancy complications	Wald ratio	1	0.811 (0.464, 1.417)	0.461	0.995
Other conditions or status of the mother complicating pregnancy, childbirth, or the puerperium	Pregnancy complications	Wald ratio	1	1.35 (0.79, 2.306)	0.272	0.995
Diabetes or abnormal glucose tolerance complicating pregnancy	Pregnancy complications	Wald ratio	1	1.279 (0.67, 2.443)	0.456	0.995
Normal delivery	Pregnancy complications	Wald ratio	1	0.988 (0.795, 1.228)	0.912	0.995
Multiple gestation	Pregnancy complications	Wald ratio	1	1 (0.607, 1.649)	1	1
Problems associated with amniotic cavity and membranes	Pregnancy complications	Wald ratio	1	0.942 (0.731, 1.213)	0.642	0.995
Other and unspecified complications of birth; puerperium affecting management of mother	Pregnancy complications	Wald ratio	1	1.037 (0.483, 2.227)	0.926	0.995
Known or suspected fetal abnormality affecting management of mother	Pregnancy complications	Wald ratio	1	1.025 (0.88, 1.194)	0.752	0.995
Other perinatal conditions of fetus or newborn	Pregnancy complications	Wald ratio	1	3.035 (1.256, 7.335)	0.014	0.709
Fetal distress and abnormal forces of labor	Pregnancy complications	Wald ratio	1	0.887 (0.722, 1.09)	0.253	0.995
Umbilical cord complications during labor and delivery	Pregnancy complications	Wald ratio	1	1.226 (0.822, 1.829)	0.317	0.995
Obstetrical or birth trauma	Pregnancy complications	Wald ratio	1	1.046 (0.908, 1.205)	0.532	0.995
Complications of labor and delivery NEC	Pregnancy complications	Wald ratio	1	1.034 (0.851, 1.255)	0.739	0.995
Venous or cerebrovascular complications embolism in pregnancy and the puerperium	Pregnancy complications	Wald ratio	1	1.104 (0.545, 2.236)	0.783	0.995
Other complications of the puerperium NEC	Pregnancy complications	Wald ratio	1	1.071 (0.606, 1.896)	0.813	0.995
Other disorders of the breast associated with childbirth and disorders of lactation	Pregnancy complications	Wald ratio	1	2.388 (0.736, 7.742)	0.147	0.958
Asphyxia and hypoxemia	Respiratory	Wald ratio	1	0.759 (0.421, 1.366)	0.358	0.995
Acute sinusitis	Respiratory	Wald ratio	1	0.936 (0.49, 1.788)	0.841	0.995
Acute upper respiratory infections of multiple or unspecified sites	Respiratory	Wald ratio	1	1.049 (0.869, 1.266)	0.617	0.995
Acute pharyngitis	Respiratory	Wald ratio	1	1.197 (0.877, 1.635)	0.258	0.995
Acute laryngitis and tracheitis	Respiratory	Wald ratio	1	0.741 (0.366, 1.5)	0.405	0.995
Septal Deviations or Turbinate Hypertrophy	Respiratory	Wald ratio	1	0.933 (0.82, 1.062)	0.296	0.995
Nasal polyps	Respiratory	Wald ratio	1	0.871 (0.739, 1.027)	0.1	0.914
Chronic pharyngitis and nasopharyngitis	Respiratory	Wald ratio	1	0.78 (0.581, 1.046)	0.097	0.913
Diseases of the larynx and vocal cords	Respiratory	Wald ratio	1	0.798 (0.669, 0.953)	0.012	0.709
Chronic laryngitis	Respiratory	Wald ratio	1	1.068 (0.442, 2.581)	0.883	0.995
Paralysis or spasm of vocal cords or larynx	Respiratory	Wald ratio	1	1.183 (0.673, 2.081)	0.56	0.995
Voice disturbance	Respiratory	Wald ratio	1	0.747 (0.567, 0.985)	0.039	0.838
Acute and chronic tonsillitis	Respiratory	Wald ratio	1	0.9 (0.733, 1.106)	0.317	0.995
Acute tonsillitis	Respiratory	Wald ratio	1	0.968 (0.668, 1.402)	0.861	0.995
Chronic tonsillitis and adenoiditis	Respiratory	Wald ratio	1	0.887 (0.677, 1.162)	0.385	0.995
Chronic sinusitis	Respiratory	Wald ratio	1	0.791 (0.659, 0.95)	0.012	0.709
Allergic rhinitis	Respiratory	Wald ratio	1	1.046 (0.789, 1.387)	0.755	0.995
Epistaxis or throat hemorrhage	Respiratory	Wald ratio	1	1.059 (0.877, 1.278)	0.553	0.995
Throat pain	Respiratory	Wald ratio	1	1.04 (0.65, 1.665)	0.871	0.995
Other upper respiratory disease	Respiratory	Wald ratio	1	0.9 (0.782, 1.037)	0.145	0.958
Pneumonia	Respiratory	Wald ratio	1	1.002 (0.912, 1.101)	0.966	0.995

Bacterial pneumonia	Respiratory	Wald ratio	1	1.043 (0.933, 1.166)	0.461	0.995
Pneumococcal pneumonia	Respiratory	Wald ratio	1	1.049 (0.927, 1.187)	0.446	0.995
Pseudomonal pneumonia	Respiratory	Wald ratio	1	1.769 (0.69, 4.533)	0.235	0.995
Viral pneumonia	Respiratory	Wald ratio	1	0.698 (0.242, 2.011)	0.505	0.995
Pneumonia due to fungus (mycoses)	Respiratory	Wald ratio	1	2.535 (0.989, 6.497)	0.053	0.869
Bronchopneumonia and lung abscess	Respiratory	Wald ratio	1	1.318 (0.818, 2.122)	0.256	0.995
Influenza	Respiratory	Wald ratio	1	1.151 (0.694, 1.91)	0.585	0.995
Acute bronchitis and bronchiolitis	Respiratory	Wald ratio	1	1.878 (0.983, 3.587)	0.056	0.869
Asthma	Respiratory	Wald ratio	1	0.985 (0.929, 1.045)	0.624	0.995
Asthma with exacerbation	Respiratory	Wald ratio	1	1.245 (0.7, 2.216)	0.456	0.995
Chronic airway obstruction	Respiratory	Wald ratio	1	0.953 (0.868, 1.047)	0.317	0.995
Emphysema	Respiratory	Wald ratio	1	0.825 (0.66, 1.032)	0.092	0.913
Chronic bronchitis	Respiratory	Wald ratio	1	0.911 (0.768, 1.081)	0.285	0.995
Obstructive chronic bronchitis	Respiratory	Wald ratio	1	0.919 (0.771, 1.097)	0.351	0.995
Bronchiectasis	Respiratory	Wald ratio	1	1.028 (0.832, 1.271)	0.796	0.995
Bronchitis	Respiratory	Wald ratio	1	1.021 (0.709, 1.47)	0.913	0.995
Lung disease due to external agents	Respiratory	Wald ratio	1	0.975 (0.616, 1.542)	0.913	0.995
Extrinsic allergic alveolitis	Respiratory	Wald ratio	1	0.419 (0.154, 1.138)	0.088	0.913
Pneumoconiosis	Respiratory	Wald ratio	1	1.477 (0.82, 2.66)	0.194	0.958
Pneumonitis due to inhalation of food or vomitus	Respiratory	Wald ratio	1	1.279 (0.873, 1.875)	0.207	0.958
Postinflammatory pulmonary fibrosis	Respiratory	Wald ratio	1	0.761 (0.56, 1.033)	0.08	0.899
Pulmonary congestion and hypostasis	Respiratory	Wald ratio	1	1.062 (0.691, 1.631)	0.784	0.995
Other alveolar and parietoalveolar pneumonopathy	Respiratory	Wald ratio	1	0.756 (0.446, 1.284)	0.301	0.995
Other pulmonary inflamation or edema	Respiratory	Wald ratio	1	1.616 (0.498, 5.241)	0.424	0.995
Empyema and pneumothorax	Respiratory	Wald ratio	1	1.326 (1.018, 1.728)	0.037	0.838
Pleurisy; pleural effusion	Respiratory	Wald ratio	1	1.081 (0.967, 1.209)	0.171	0.958
Pulmonary collapse; interstitial and compensatory emphysema	Respiratory	Wald ratio	1	0.866 (0.705, 1.064)	0.17	0.958
Respiratory failure, insufficiency, arrest	Respiratory	Wald ratio	1	0.959 (0.799, 1.151)	0.652	0.995
Respiratory failure	Respiratory	Wald ratio	1	0.942 (0.767, 1.157)	0.568	0.995
Respiratory insufficiency	Respiratory	Wald ratio	1	1.003 (0.807, 1.247)	0.976	0.995
Respiratory arrest	Respiratory	Wald ratio	1	0.843 (0.37, 1.92)	0.684	0.995
Dependence on respirator [Ventilator] or supplemental oxygen	Respiratory	Wald ratio	1	1.33 (0.836, 2.117)	0.229	0.995
Other diseases of lung	Respiratory	Wald ratio	1	1.141 (0.821, 1.586)	0.432	0.995
Other symptoms of respiratory system	Respiratory	Wald ratio	1	0.936 (0.847, 1.035)	0.196	0.958
Wheezing	Respiratory	Wald ratio	1	0.825 (0.458, 1.486)	0.522	0.995
Painful respiration	Respiratory	Wald ratio	1	0.953 (0.499, 1.82)	0.884	0.995
Shortness of breath	Respiratory	Wald ratio	1	0.947 (0.837, 1.072)	0.391	0.995
Cough	Respiratory	Wald ratio	1	0.987 (0.832, 1.17)	0.879	0.995
Other dyspnea	Respiratory	Wald ratio	1	0.782 (0.576, 1.062)	0.115	0.958
Respiratory abnormalities	Respiratory	Wald ratio	1	0.82 (0.566, 1.188)	0.295	0.995
Hypoventilation	Respiratory	Wald ratio	1	0.698 (0.257, 1.896)	0.48	0.995
Hyperventilation	Respiratory	Wald ratio	1	0.997 (0.574, 1.734)	0.992	0.996
Disorders of diaphragm	Respiratory	Wald ratio	1	0.917 (0.427, 1.969)	0.823	0.995
Abnormal findings examination of lungs	Respiratory	Wald ratio	1	1.002 (0.835, 1.202)	0.986	0.995
Abnormal sputum	Respiratory	Wald ratio	1	0.942 (0.771, 1.15)	0.556	0.995
Hemoptysis	Respiratory	Wald ratio	1	0.956 (0.783, 1.168)	0.659	0.995
Other diseases of respiratory system, not elsewhere classified	Respiratory	Wald ratio	1	0.98 (0.892, 1.076)	0.671	0.995
Respiratory complications	Respiratory	Wald ratio	1	0.657 (0.385, 1.122)	0.124	0.958
Other diseases of respiratory system, NEC	Respiratory	Wald ratio	1	0.989 (0.895, 1.093)	0.823	0.995
Symptoms involving respiratory system and other chest symptoms	Respiratory	Wald ratio	1	0.82 (0.486, 1.385)	0.458	0.995
Disorders of the globe	Sense organs	Wald ratio	1	0.657 (0.398, 1.083)	0.1	0.914
Progressive myopia	Sense organs	Wald ratio	1	0.698 (0.392, 1.241)	0.221	0.985
Retinal detachments and defects	Sense organs	Wald ratio	1	0.917 (0.777, 1.081)	0.3	0.995
Retinal detachment with retinal defect	Sense organs	Wald ratio	1	0.959 (0.749, 1.228)	0.739	0.995
Retinoschisis and retinal cysts	Sense organs	Wald ratio	1	1.35 (0.442, 4.127)	0.599	0.995
Other retinal disorders	Sense organs	Wald ratio	1	0.99 (0.855, 1.147)	0.895	0.995
Degeneration of macula and posterior pole of retina	Sense organs	Wald ratio	1	0.868 (0.711, 1.061)	0.167	0.958
Macular degeneration (senile) of retina NOS	Sense organs	Wald ratio	1	0.871 (0.713, 1.064)	0.176	0.958
Other nondiabetic retinopathy	Sense organs	Wald ratio	1	1.204 (0.595, 2.44)	0.605	0.995
Separation of retinal layers	Sense organs	Wald ratio	1	1.071 (0.418, 2.746)	0.886	0.995
Retinal vascular changes and abnomalities	Sense organs	Wald ratio	1	0.887 (0.646, 1.218)	0.459	0.995
Peripheral retinal degenerations	Sense organs	Wald ratio	1	1.346 (0.664, 2.726)	0.409	0.995
Hereditary retinal dystrophies	Sense organs	Wald ratio	1	0.741 (0.273, 2.013)	0.556	0.995
Retinal hemorrhage or ischemia	Sense organs	Wald ratio	1	2.46 (1.08, 5.606)	0.032	0.838
Chorioretinal inflammations, scars, and other disorders of choroid	Sense organs	Wald ratio	1	1.065 (0.526, 2.157)	0.861	0.995
Chorioretinal scars	Sense organs	Wald ratio	1	0.823 (0.269, 2.516)	0.732	0.995
Corneal opacity and other disorders of cornea	Sense organs	Wald ratio	1	1.037 (0.737, 1.458)	0.836	0.995
Corneal opacity	Sense organs	Wald ratio	1	0.931 (0.363, 2.385)	0.881	0.995
Corneal edema	Sense organs	Wald ratio	1	0.657 (0.242, 1.786)	0.41	0.995
Corneal degenerations	Sense organs	Wald ratio	1	1.769 (0.926, 3.378)	0.084	0.899
Keratoconus	Sense organs	Wald ratio	1	2.535 (0.933, 6.891)	0.068	0.879
Corneal dystrophy	Sense organs	Wald ratio	1	1.078 (0.564, 2.059)	0.82	0.995
Cornea replaced by transplant	Sense organs	Wald ratio	1	2.182 (0.851, 5.592)	0.104	0.933
Glaucoma	Sense organs	Wald ratio	1	1.013 (0.88, 1.167)	0.858	0.995
Open-angle glaucoma	Sense organs	Wald ratio	1	1.148 (0.861, 1.532)	0.348	0.995
Primary open angle glaucoma	Sense organs	Wald ratio	1	1.169 (0.876, 1.559)	0.289	0.995
Primary angle-closure glaucoma	Sense organs	Wald ratio	1	1.111 (0.785, 1.572)	0.553	0.995
Cataract	Sense organs	Wald ratio	1	0.994 (0.927, 1.067)	0.874	0.995
Nonsenile Cataract	Sense organs	Wald ratio	1	1.028 (0.336, 3.142)	0.962	0.995

Senile cataract	Sense organs	Wald ratio	1	0.953 (0.857, 1.06)	0.374	0.995
Traumatic cataract	Sense organs	Wald ratio	1	1.35 (0.559, 3.262)	0.505	0.995
Disorders of refraction and accommodation; blindness and low vision	Sense organs	Wald ratio	1	1.124 (0.931, 1.357)	0.223	0.988
Myopia	Sense organs	Wald ratio	1	1.056 (0.815, 1.367)	0.682	0.995
Astigmatism	Sense organs	Wald ratio	1	1.037 (0.543, 1.98)	0.913	0.995
Hypermetropia	Sense organs	Wald ratio	1	1.121 (0.63, 1.995)	0.698	0.995
Blindness and low vision	Sense organs	Wald ratio	1	1.326 (0.943, 1.865)	0.105	0.935
Visual disturbances	Sense organs	Wald ratio	1	0.988 (0.843, 1.158)	0.885	0.995
Amblyopia	Sense organs	Wald ratio	1	0.863 (0.582, 1.28)	0.465	0.995
Diplopia and disorders of binocular vision	Sense organs	Wald ratio	1	1.391 (0.989, 1.957)	0.058	0.869
Anisometropia	Sense organs	Wald ratio	1	0.741 (0.366, 1.5)	0.405	0.995
Visual field defects	Sense organs	Wald ratio	1	0.78 (0.473, 1.285)	0.329	0.995
Subjective visual disturbances	Sense organs	Wald ratio	1	1.03 (0.711, 1.493)	0.874	0.995
Infection of the eye	Sense organs	Wald ratio	1	0.914 (0.62, 1.347)	0.649	0.995
Eye infection, viral	Sense organs	Wald ratio	1	0.933 (0.41, 2.126)	0.87	0.995
Conjunctivitis, infectious	Sense organs	Wald ratio	1	1.001 (0.524, 1.912)	0.997	0.999
Keratitis	Sense organs	Wald ratio	1	0.853 (0.502, 1.448)	0.556	0.995
Corneal ulcer	Sense organs	Wald ratio	1	0.835 (0.389, 1.794)	0.644	0.995
Inflammation of the eye	Sense organs	Wald ratio	1	1.052 (0.893, 1.241)	0.544	0.995
Uveitis, noninfectious or NOS	Sense organs	Wald ratio	1	0.989 (0.651, 1.501)	0.957	0.995
Inflammation of eyelids	Sense organs	Wald ratio	1	1.046 (0.867, 1.263)	0.639	0.995
Disorders of conjunctiva	Sense organs	Wald ratio	1	1.716 (1.227, 2.4)	0.002	0.383
Other disorders of eyelids	Sense organs	Wald ratio	1	1.014 (0.896, 1.147)	0.83	0.995
Ectropion or entropion	Sense organs	Wald ratio	1	1.091 (0.823, 1.447)	0.546	0.995
Ptosis of eyelid	Sense organs	Wald ratio	1	1.019 (0.82, 1.267)	0.863	0.995
Disorders of lacrimal system	Sense organs	Wald ratio	1	0.911 (0.75, 1.106)	0.348	0.995
Epiphora	Sense organs	Wald ratio	1	1.101 (0.811, 1.495)	0.538	0.995
Disorders of the orbit	Sense organs	Wald ratio	1	0.828 (0.305, 2.25)	0.711	0.995
Disorders of optic nerve and visual pathways	Sense organs	Wald ratio	1	1.029 (0.639, 1.657)	0.907	0.995
Optic atrophy	Sense organs	Wald ratio	1	0.775 (0.239, 2.513)	0.671	0.995
Optic neuritis or neuropathy	Sense organs	Wald ratio	1	1.237 (0.543, 2.819)	0.612	0.995
Strabismus and other disorders of binocular eye movements	Sense organs	Wald ratio	1	1.101 (0.865, 1.401)	0.435	0.995
Strabismus (not specified as paralytic)	Sense organs	Wald ratio	1	0.979 (0.721, 1.33)	0.893	0.995
Nystagmus and other irregular eye movements	Sense organs	Wald ratio	1	1.522 (0.797, 2.907)	0.203	0.958
Paralytic strabismus	Sense organs	Wald ratio	1	1.279 (0.736, 2.223)	0.383	0.995
Other disorders of eye	Sense organs	Wald ratio	1	0.868 (0.75, 1.006)	0.06	0.869
Disorders of vitreous body	Sense organs	Wald ratio	1	0.811 (0.633, 1.038)	0.096	0.913
Aphakia and other disorders of lens	Sense organs	Wald ratio	1	0.898 (0.722, 1.116)	0.331	0.995
Anomalies of pupillary function	Sense organs	Wald ratio	1	0.698 (0.242, 2.011)	0.505	0.995
Disorders of iris and ciliary body	Sense organs	Wald ratio	1	0.906 (0.576, 1.425)	0.668	0.995
Pain, swelling or discharge of eye	Sense organs	Wald ratio	1	1.008 (0.498, 2.043)	0.981	0.995
Disorders of external ear	Sense organs	Wald ratio	1	0.939 (0.738, 1.195)	0.609	0.995
Otitis externa	Sense organs	Wald ratio	1	0.983 (0.659, 1.467)	0.934	0.995
Impacted cerumen	Sense organs	Wald ratio	1	0.9 (0.556, 1.458)	0.67	0.995
Otitis media and Eustachian tube disorders	Sense organs	Wald ratio	1	1.046 (0.861, 1.27)	0.649	0.995
Otitis media	Sense organs	Wald ratio	1	1.059 (0.852, 1.316)	0.608	0.995
Suppurative and unspecified otitis media	Sense organs	Wald ratio	1	1.107 (0.811, 1.513)	0.521	0.995
Eustachian tube disorders	Sense organs	Wald ratio	1	0.619 (0.324, 1.182)	0.146	0.958
Mastoiditis & related conditions	Sense organs	Wald ratio	1	0.903 (0.446, 1.829)	0.777	0.995
Otorrhea	Sense organs	Wald ratio	1	1.716 (0.993, 2.966)	0.053	0.869
Otalgia	Sense organs	Wald ratio	1	0.801 (0.474, 1.352)	0.406	0.995
Otosclerosis	Sense organs	Wald ratio	1	1.194 (0.707, 2.015)	0.507	0.995
Other disorders of tympanic membrane	Sense organs	Wald ratio	1	1.322 (1.033, 1.692)	0.027	0.793
Perforation of tympanic membrane	Sense organs	Wald ratio	1	1.208 (0.911, 1.602)	0.189	0.958
Other disorders of middle ear and mastoid	Sense organs	Wald ratio	1	1.084 (0.789, 1.49)	0.617	0.995
Cholesteatoma	Sense organs	Wald ratio	1	1.226 (0.847, 1.776)	0.28	0.995
Tympanosclerosis and middle ear disease related to otitis media	Sense organs	Wald ratio	1	0.677 (0.376, 1.219)	0.194	0.958
Vertiginous syndromes and other disorders of vestibular system	Sense organs	Wald ratio	1	1.034 (0.919, 1.163)	0.582	0.995
Meniere's disease	Sense organs	Wald ratio	1	1.056 (0.72, 1.547)	0.782	0.995
Peripheral or central vertigo	Sense organs	Wald ratio	1	1.391 (0.869, 2.227)	0.169	0.958
Labyrinthitis	Sense organs	Wald ratio	1	1.165 (0.833, 1.63)	0.371	0.995
Dizziness and giddiness (Light-headedness and vertigo)	Sense organs	Wald ratio	1	1.008 (0.881, 1.155)	0.903	0.995
Other disorders of ear	Sense organs	Wald ratio	1	1.716 (0.899, 3.278)	0.102	0.918
Hearing loss	Sense organs	Wald ratio	1	1.046 (0.908, 1.205)	0.532	0.995
Sensorineural hearing loss	Sense organs	Wald ratio	1	1.433 (0.933, 2.202)	0.1	0.914
Conductive hearing loss	Sense organs	Wald ratio	1	1.04 (0.673, 1.607)	0.861	0.995
Tinnitus	Sense organs	Wald ratio	1	0.89 (0.593, 1.335)	0.572	0.995
Symptoms concerning nutrition, metabolism, and development	Symptoms	Wald ratio	1	0.989 (0.874, 1.119)	0.86	0.995
Other signs and symptoms involving emotional state	Symptoms	Wald ratio	1	0.677 (0.399, 1.149)	0.149	0.958
Other symptoms	Symptoms	Wald ratio	1	0.99 (0.759, 1.289)	0.938	0.995
Other tests	Symptoms	Wald ratio	1	0.928 (0.82, 1.05)	0.234	0.995
Effects of other external causes	Symptoms	Wald ratio	1	1.088 (0.944, 1.253)	0.243	0.995
Other ill-defined and unknown causes of morbidity and mortality	Symptoms	Wald ratio	1	0.962 (0.891, 1.038)	0.317	0.995
Pain	Symptoms	Wald ratio	1	1.138 (0.818, 1.582)	0.443	0.995
Localized superficial swelling, mass, or lump	Symptoms	Wald ratio	1	0.698 (0.404, 1.206)	0.197	0.958
Back pain	Symptoms	Wald ratio	1	0.983 (0.9, 1.073)	0.699	0.995
Cervicalgia	Symptoms	Wald ratio	1	1.011 (0.813, 1.257)	0.922	0.995
Sciatica	Symptoms	Wald ratio	1	1.03 (0.854, 1.244)	0.755	0.995
Neuralgia, neuritis, and radiculitis NOS	Symptoms	Wald ratio	1	0.887 (0.677, 1.162)	0.385	0.995

Myalgia and myositis unspecified	Symptoms	Wald ratio	1	0.879 (0.61, 1.266)	0.488	0.995
Musculoskeletal symptoms referable to limbs	Symptoms	Wald ratio	1	0.925 (0.817, 1.047)	0.216	0.975
Swelling of limb	Symptoms	Wald ratio	1	0.962 (0.84, 1.101)	0.572	0.995
Symptoms of the muscles	Symptoms	Wald ratio	1	0.975 (0.689, 1.379)	0.884	0.995
Pain in limb	Symptoms	Wald ratio	1	1.017 (0.904, 1.144)	0.779	0.995
Hypothermia or Chills	Symptoms	Wald ratio	1	1.31 (0.647, 2.654)	0.453	0.995
Symptoms involving nervous and musculoskeletal systems	Symptoms	Wald ratio	1	0.995 (0.805, 1.23)	0.962	0.995
Symptoms involving skin and other integumentary tissue	Symptoms	Wald ratio	1	1.007 (0.81, 1.252)	0.95	0.995
Edema	Symptoms	Wald ratio	1	0.962 (0.765, 1.21)	0.739	0.995
Pallor and flushing	Symptoms	Wald ratio	1	1.477 (0.729, 2.992)	0.279	0.995
Fever of unknown origin	Symptoms	Wald ratio	1	1.016 (0.877, 1.177)	0.832	0.995
Abdominal pain	Symptoms	Wald ratio	1	0.998 (0.952, 1.046)	0.925	0.995
Syncope and collapse	Symptoms	Wald ratio	1	1.012 (0.916, 1.119)	0.814	0.995
Nausea and vomiting	Symptoms	Wald ratio	1	1.007 (0.922, 1.099)	0.883	0.995
Nonspecific findings on examination of blood	Symptoms	Wald ratio	1	1.065 (0.947, 1.198)	0.294	0.995
Elevated sedimentation rate	Symptoms	Wald ratio	1	0.768 (0.283, 2.087)	0.605	0.995
Other abnormal blood chemistry	Symptoms	Wald ratio	1	1.059 (0.941, 1.191)	0.342	0.995
Gangrene	Symptoms	Wald ratio	1	0.882 (0.594, 1.307)	0.531	0.995
Abnormal Papanicolaou smear of cervix and cervical HPV	Symptoms	Wald ratio	1	1.433 (0.593, 3.464)	0.424	0.995
Abnormal results of other function studies (bladder, pancreas, placenta, spleen, etc)	Symptoms	Wald ratio	1	2.692 (1.182, 6.134)	0.018	0.71
Shock	Symptoms	Wald ratio	1	0.472 (0.207, 1.076)	0.074	0.881
Cardiogenic shock	Symptoms	Wald ratio	1	0.549 (0.202, 1.491)	0.239	0.995
Malaise and fatigue	Symptoms	Wald ratio	1	0.903 (0.77, 1.058)	0.208	0.958
Chronic fatigue syndrome	Symptoms	Wald ratio	1	0.85 (0.584, 1.239)	0.399	0.995

Table S14. Phenome-wide MR analyses on genetically mimicked targeting of CD8 NC-specific *DDX5* .

Phenotype	Category	Method	nSNP	OR (95% CI)	*P* -value	*FDR*
Rheumatic disease of the heart valves	Circulatory system	Wald ratio	1	0.964 (0.873, 1.065)	0.467	0.974
Mitral valve disease	Circulatory system	Wald ratio	1	0.981 (0.864, 1.113)	0.761	0.981
Aortic valve disease	Circulatory system	Wald ratio	1	0.839 (0.691, 1.019)	0.077	0.874
Disease of tricuspid valve	Circulatory system	Wald ratio	1	1.03 (0.833, 1.274)	0.782	0.981
Heart valve disorders	Circulatory system	Wald ratio	1	1.006 (0.903, 1.121)	0.914	0.983
Nonrheumatic mitral valve disorders	Circulatory system	Wald ratio	1	0.985 (0.864, 1.123)	0.817	0.981
Nonrheumatic aortic valve disorders	Circulatory system	Wald ratio	1	1.102 (0.708, 1.715)	0.668	0.981
Nonrheumatic tricuspid valve disorders	Circulatory system	Wald ratio	1	0.853 (0.433, 1.68)	0.646	0.981
Nonrheumatic pulmonary valve disorders	Circulatory system	Wald ratio	1	1.55 (0.787, 3.051)	0.205	0.917
Heart valve replaced	Circulatory system	Wald ratio	1	1.038 (0.866, 1.243)	0.689	0.981
Abnormal heart sounds	Circulatory system	Wald ratio	1	0.849 (0.684, 1.055)	0.139	0.916
Hypertensive heart and or or renal disease	Circulatory system	Wald ratio	1	1.062 (0.898, 1.255)	0.482	0.974
Hypertensive heart disease	Circulatory system	Wald ratio	1	0.776 (0.451, 1.335)	0.359	0.963
Hypertensive chronic kidney disease	Circulatory system	Wald ratio	1	1.094 (0.917, 1.305)	0.317	0.923
Other hypertensive complications	Circulatory system	Wald ratio	1	0.861 (0.478, 1.549)	0.617	0.981
Elevated blood pressure reading without diagnosis of hypertension	Circulatory system	Wald ratio	1	1.003 (0.837, 1.201)	0.976	0.991
Ischemic Heart Disease	Circulatory system	Wald ratio	1	1.016 (0.972, 1.061)	0.483	0.974
Unstable angina (intermediate coronary syndrome)	Circulatory system	Wald ratio	1	1.005 (0.91, 1.11)	0.92	0.984
Myocardial infarction	Circulatory system	Wald ratio	1	0.968 (0.905, 1.036)	0.351	0.962
Angina pectoris	Circulatory system	Wald ratio	1	1.016 (0.958, 1.077)	0.601	0.981
Coronary atherosclerosis	Circulatory system	Wald ratio	1	0.988 (0.936, 1.043)	0.665	0.981
Aneurysm and dissection of heart	Circulatory system	Wald ratio	1	1.156 (0.89, 1.503)	0.277	0.923
Other chronic ischemic heart disease, unspecified	Circulatory system	Wald ratio	1	1.018 (0.96, 1.079)	0.554	0.981
Other acute and subacute forms of ischemic heart disease	Circulatory system	Wald ratio	1	0.929 (0.758, 1.138)	0.477	0.974
Other forms of chronic heart disease	Circulatory system	Wald ratio	1	1.05 (0.892, 1.235)	0.56	0.981
ASCVD	Circulatory system	Wald ratio	1	0.794 (0.337, 1.874)	0.599	0.981
Pulmonary heart disease	Circulatory system	Wald ratio	1	0.984 (0.883, 1.097)	0.777	0.981
Acute pulmonary heart disease	Circulatory system	Wald ratio	1	0.524 (0.233, 1.183)	0.12	0.916
Pulmonary embolism and infarction, acute	Circulatory system	Wald ratio	1	0.524 (0.233, 1.183)	0.12	0.916
Chronic pulmonary heart disease	Circulatory system	Wald ratio	1	1.087 (0.817, 1.444)	0.568	0.981
Primary pulmonary hypertension	Circulatory system	Wald ratio	1	0.99 (0.712, 1.377)	0.954	0.989
Cardiomegaly	Circulatory system	Wald ratio	1	1.026 (0.892, 1.18)	0.723	0.981
Nonspecific chest pain	Circulatory system	Wald ratio	1	1.006 (0.965, 1.049)	0.772	0.981
Precordial pain	Circulatory system	Wald ratio	1	1.035 (0.92, 1.164)	0.564	0.981
Carditis	Circulatory system	Wald ratio	1	1.042 (0.894, 1.215)	0.597	0.981
Myocarditis	Circulatory system	Wald ratio	1	1.319 (0.67, 2.597)	0.424	0.974
Pericarditis	Circulatory system	Wald ratio	1	1.074 (0.884, 1.304)	0.471	0.974
Acute pericarditis	Circulatory system	Wald ratio	1	1.381 (0.879, 2.169)	0.162	0.916
Endocarditis	Circulatory system	Wald ratio	1	0.929 (0.712, 1.213)	0.588	0.981
Cardiomyopathy	Circulatory system	Wald ratio	1	1.004 (0.823, 1.225)	0.967	0.991
Primary or intrinsic cardiomyopathies	Circulatory system	Wald ratio	1	1.018 (0.834, 1.241)	0.863	0.981
Hypertrophic obstructive cardiomyopathy	Circulatory system	Wald ratio	1	1.907 (1.06, 3.431)	0.031	0.749
Other hypertrophic cardiomyopathy	Circulatory system	Wald ratio	1	1.623 (0.944, 2.79)	0.08	0.874
Other cardiomyopathy	Circulatory system	Wald ratio	1	0.524 (0.212, 1.295)	0.162	0.916
Cardiac conduction disorders	Circulatory system	Wald ratio	1	0.973 (0.893, 1.06)	0.528	0.981
Atrioventricular [AV] block	Circulatory system	Wald ratio	1	0.97 (0.836, 1.127)	0.694	0.981
First degree AV block	Circulatory system	Wald ratio	1	1.045 (0.837, 1.304)	0.698	0.981
Second degree AV block	Circulatory system	Wald ratio	1	0.813 (0.587, 1.125)	0.211	0.917
Atrioventricular block, complete	Circulatory system	Wald ratio	1	0.982 (0.739, 1.306)	0.901	0.983
Other heart block	Circulatory system	Wald ratio	1	1.514 (0.842, 2.724)	0.166	0.916
Bundle branch block	Circulatory system	Wald ratio	1	0.922 (0.817, 1.042)	0.195	0.917
Right bundle branch block	Circulatory system	Wald ratio	1	1.072 (0.894, 1.284)	0.453	0.974
Left bundle branch block	Circulatory system	Wald ratio	1	0.758 (0.642, 0.896)	0.001	0.125
Anomalous atrioventricular excitation	Circulatory system	Wald ratio	1	1.072 (0.682, 1.684)	0.764	0.981
Other cardiac conduction disorders	Circulatory system	Wald ratio	1	0.489 (0.198, 1.208)	0.121	0.916
Cardiac pacemaker or device in situ	Circulatory system	Wald ratio	1	0.99 (0.86, 1.138)	0.885	0.983
Cardiac pacemaker in situ	Circulatory system	Wald ratio	1	1.038 (0.898, 1.199)	0.617	0.981
Cardiac dysrhythmias	Circulatory system	Wald ratio	1	1.022 (0.977, 1.069)	0.347	0.959
Paroxysmal tachycardia, unspecified	Circulatory system	Wald ratio	1	0.982 (0.869, 1.11)	0.773	0.981
Paroxysmal supraventricular tachycardia	Circulatory system	Wald ratio	1	0.975 (0.844, 1.127)	0.731	0.981
Paroxysmal ventricular tachycardia	Circulatory system	Wald ratio	1	0.975 (0.778, 1.222)	0.826	0.981
Atrial fibrillation and flutter	Circulatory system	Wald ratio	1	1.033 (0.974, 1.095)	0.282	0.923
Other specified cardiac dysrhythmias	Circulatory system	Wald ratio	1	1.214 (1.074, 1.371)	0.002	0.148
Cardiac arrest and ventricular fibrillation	Circulatory system	Wald ratio	1	1.122 (0.916, 1.375)	0.267	0.923
Ventricular fibrillation and flutter	Circulatory system	Wald ratio	1	1.072 (0.733, 1.566)	0.721	0.981
Cardiac arrest	Circulatory system	Wald ratio	1	1.175 (0.937, 1.473)	0.162	0.916
Arrhythmia (cardiac) NOS	Circulatory system	Wald ratio	1	0.995 (0.79, 1.253)	0.967	0.991
Premature beats	Circulatory system	Wald ratio	1	0.933 (0.693, 1.257)	0.649	0.981
Supraventricular premature beats	Circulatory system	Wald ratio	1	1.55 (0.823, 2.917)	0.175	0.916
Tachycardia NOS	Circulatory system	Wald ratio	1	0.935 (0.806, 1.086)	0.38	0.974
Sinoatrial node dysfunction (Bradycardia)	Circulatory system	Wald ratio	1	1.13 (0.802, 1.593)	0.486	0.974
Palpitations	Circulatory system	Wald ratio	1	0.953 (0.851, 1.067)	0.401	0.974
Congestive heart failure; nonhypertensive	Circulatory system	Wald ratio	1	0.942 (0.857, 1.036)	0.216	0.917
Congestive heart failure (CHF) NOS	Circulatory system	Wald ratio	1	1.018 (0.873, 1.187)	0.819	0.981
Heart failure NOS	Circulatory system	Wald ratio	1	0.922 (0.828, 1.028)	0.145	0.916
Ill-defined descriptions and complications of heart disease	Circulatory system	Wald ratio	1	0.944 (0.74, 1.205)	0.643	0.981
Heart transplant or surgery	Circulatory system	Wald ratio	1	2.14 (0.907, 5.048)	0.082	0.874

Abnormal function study of cardiovascular system	Circulatory system	Wald ratio	1	0.8 (0.591, 1.082)	0.148	0.916
Symptoms involving cardiovascular system	Circulatory system	Wald ratio	1	1.17 (0.712, 1.923)	0.536	0.981
Intracranial hemorrhage	Circulatory system	Wald ratio	1	1.009 (0.858, 1.187)	0.914	0.983
Subarachnoid hemorrhage	Circulatory system	Wald ratio	1	1.162 (0.91, 1.483)	0.229	0.917
Intracerebral hemorrhage	Circulatory system	Wald ratio	1	0.997 (0.767, 1.296)	0.982	0.991
Subdural hemorrhage	Circulatory system	Wald ratio	1	0.708 (0.461, 1.087)	0.114	0.916
Cerebrovascular disease	Circulatory system	Wald ratio	1	1.001 (0.927, 1.081)	0.976	0.991
Occlusion and stenosis of precerebral arteries	Circulatory system	Wald ratio	1	1.02 (0.833, 1.251)	0.845	0.981
Cerebral atherosclerosis	Circulatory system	Wald ratio	1	1.48 (0.942, 2.325)	0.089	0.874
Occlusion of cerebral arteries	Circulatory system	Wald ratio	1	1.072 (0.961, 1.194)	0.211	0.917
Cerebral artery occlusion, with cerebral infarction	Circulatory system	Wald ratio	1	0.996 (0.832, 1.194)	0.968	0.991
Cerebral ischemia	Circulatory system	Wald ratio	1	1.057 (0.927, 1.205)	0.408	0.974
Transient cerebral ischemia	Circulatory system	Wald ratio	1	1.054 (0.908, 1.224)	0.486	0.974
Cerebral aneurysm	Circulatory system	Wald ratio	1	0.912 (0.638, 1.303)	0.613	0.981
Late effects of cerebrovascular disease	Circulatory system	Wald ratio	1	0.893 (0.735, 1.085)	0.254	0.919
Atherosclerosis	Circulatory system	Wald ratio	1	1.107 (0.915, 1.338)	0.295	0.923
Atherosclerosis of renal artery	Circulatory system	Wald ratio	1	0.933 (0.496, 1.757)	0.83	0.981
Atherosclerosis of the extremities	Circulatory system	Wald ratio	1	1.062 (0.832, 1.355)	0.63	0.981
Atherosclerosis of aorta	Circulatory system	Wald ratio	1	1.117 (0.74, 1.685)	0.598	0.981
Vascular insufficiency of intestine	Circulatory system	Wald ratio	1	1.079 (0.808, 1.441)	0.606	0.981
Acute vascular insufficiency of intestine	Circulatory system	Wald ratio	1	1.013 (0.659, 1.556)	0.953	0.989
Other aneurysm	Circulatory system	Wald ratio	1	1.104 (0.938, 1.299)	0.232	0.917
Aortic aneurysm	Circulatory system	Wald ratio	1	1.092 (0.903, 1.32)	0.366	0.97
Abdominal aortic aneurysm	Circulatory system	Wald ratio	1	1.084 (0.861, 1.365)	0.493	0.974
Aneurysm of iliac artery	Circulatory system	Wald ratio	1	0.912 (0.463, 1.796)	0.79	0.981
Aneurysm of artery of lower extremity	Circulatory system	Wald ratio	1	0.955 (0.555, 1.642)	0.868	0.981
Arterial dissection	Circulatory system	Wald ratio	1	1.779 (0.689, 4.595)	0.234	0.917
Aneurysm of other specified artery	Circulatory system	Wald ratio	1	0.953 (0.58, 1.566)	0.849	0.981
Peripheral vascular disease	Circulatory system	Wald ratio	1	0.999 (0.892, 1.118)	0.98	0.991
Raynaud's syndrome	Circulatory system	Wald ratio	1	0.845 (0.69, 1.036)	0.105	0.902
Peripheral angiopathy in diseases classified elsewhere	Circulatory system	Wald ratio	1	0.995 (0.7, 1.416)	0.98	0.991
Peripheral vascular disease, unspecified	Circulatory system	Wald ratio	1	1.094 (0.951, 1.259)	0.208	0.917
Arterial embolism and thrombosis	Circulatory system	Wald ratio	1	1.289 (1.023, 1.622)	0.031	0.749
Arterial embolism and thrombosis of lower extremity artery	Circulatory system	Wald ratio	1	1.202 (0.897, 1.613)	0.218	0.917
Polyarteritis nodosa and allied conditions	Circulatory system	Wald ratio	1	1.023 (0.805, 1.3)	0.853	0.981
Wegener's granulomatosis	Circulatory system	Wald ratio	1	0.899 (0.547, 1.478)	0.676	0.981
Giant cell arteritis	Circulatory system	Wald ratio	1	1.064 (0.748, 1.514)	0.729	0.981
Arteritis NOS	Circulatory system	Wald ratio	1	0.918 (0.584, 1.443)	0.711	0.981
Other disorders of arteries and arterioles	Circulatory system	Wald ratio	1	1.143 (0.945, 1.382)	0.167	0.916
Stricture of artery	Circulatory system	Wald ratio	1	1.057 (0.836, 1.337)	0.644	0.981
Disease of capillaries	Circulatory system	Wald ratio	1	0.871 (0.484, 1.567)	0.644	0.981
Noninfectious disorders of lymphatic channels	Circulatory system	Wald ratio	1	0.966 (0.743, 1.255)	0.796	0.981
Phlebitis and thrombophlebitis	Circulatory system	Wald ratio	1	0.859 (0.767, 0.962)	0.008	0.502
Phlebitis and thrombophlebitis of lower extremities	Circulatory system	Wald ratio	1	0.871 (0.774, 0.979)	0.021	0.688
Other venous embolism and thrombosis	Circulatory system	Wald ratio	1	1.259 (0.939, 1.689)	0.124	0.916
Varicose veins	Circulatory system	Wald ratio	1	0.983 (0.919, 1.052)	0.626	0.981
Varicose veins of lower extremity	Circulatory system	Wald ratio	1	0.989 (0.925, 1.059)	0.759	0.981
Varicose veins of lower extremity, symptomtic	Circulatory system	Wald ratio	1	0.816 (0.62, 1.075)	0.149	0.916
Hemorrhoids	Circulatory system	Wald ratio	1	0.964 (0.917, 1.013)	0.146	0.916
Chronic venous insufficiency [CVI]	Circulatory system	Wald ratio	1	1.349 (0.821, 2.218)	0.237	0.919
Encounter for long-term (current) use of anticoagulants, antithrombotics, aspirin	Circulatory system	Wald ratio	1	1.55 (0.787, 3.051)	0.205	0.917
Hypotension	Circulatory system	Wald ratio	1	1.038 (0.948, 1.136)	0.424	0.974
Orthostatic hypotension	Circulatory system	Wald ratio	1	0.996 (0.824, 1.204)	0.964	0.99
Iatrogenic hypotension	Circulatory system	Wald ratio	1	0.906 (0.576, 1.423)	0.667	0.981
Hypotension NOS	Circulatory system	Wald ratio	1	1.05 (0.933, 1.18)	0.419	0.974
Other disorders of circulatory system	Circulatory system	Wald ratio	1	0.975 (0.924, 1.029)	0.359	0.963
Hemorrhage NOS	Circulatory system	Wald ratio	1	0.602 (0.306, 1.186)	0.142	0.916
Circulatory disease NEC	Circulatory system	Wald ratio	1	0.975 (0.919, 1.034)	0.397	0.974
Nonspecific abnormal findings on radiological and other examination of other intrathoracic organs (echocardiogram, etc)	Circulatory system	Wald ratio	1	0.975 (0.727, 1.308)	0.866	0.981
Cardiac and circulatory congenital anomalies	Congenital anomalies	Wald ratio	1	1.009 (0.885, 1.151)	0.89	0.983
Cardiac congenital anomalies	Congenital anomalies	Wald ratio	1	0.995 (0.869, 1.139)	0.942	0.986
Cardiac shunt or heart septal defect	Congenital anomalies	Wald ratio	1	0.933 (0.702, 1.24)	0.634	0.981
Valvular heart disease or heart chambers	Congenital anomalies	Wald ratio	1	1.514 (0.964, 2.379)	0.072	0.874
Congenital anomalies of great vessels	Congenital anomalies	Wald ratio	1	0.994 (0.845, 1.17)	0.945	0.987
Congenital anomalies of peripheral vascular system	Congenital anomalies	Wald ratio	1	1.167 (0.679, 2.007)	0.577	0.981
Anomalies of respiratory system, congenital	Congenital anomalies	Wald ratio	1	1.349 (0.523, 3.485)	0.536	0.981
Congenital anomalies of face and neck	Congenital anomalies	Wald ratio	1	0.942 (0.642, 1.383)	0.76	0.981
Digestive congenital anomalies	Congenital anomalies	Wald ratio	1	0.887 (0.683, 1.153)	0.37	0.972
Upper gastrointestinal congenital anomalies	Congenital anomalies	Wald ratio	1	0.807 (0.552, 1.18)	0.268	0.923
Congenital anomalies of mouth or tongue	Congenital anomalies	Wald ratio	1	0.879 (0.535, 1.445)	0.611	0.981
Lower gastrointestinal congenital anomalies	Congenital anomalies	Wald ratio	1	1.016 (0.705, 1.465)	0.932	0.986
Congenital anomalies of intestine	Congenital anomalies	Wald ratio	1	0.859 (0.547, 1.349)	0.509	0.978
Congenital anomaly of gallbladder, bile ducts, liver, pancreas	Congenital anomalies	Wald ratio	1	1.319 (0.701, 2.482)	0.391	0.974
Genitourinary congenital anomalies	Congenital anomalies	Wald ratio	1	1.035 (0.872, 1.229)	0.693	0.981
Congenital anomalies of genital organs	Congenital anomalies	Wald ratio	1	1.038 (0.802, 1.342)	0.779	0.981
Congenital anomalies of female genital organs	Congenital anomalies	Wald ratio	1	0.966 (0.698, 1.337)	0.835	0.981
Congenital anomalies of urinary system	Congenital anomalies	Wald ratio	1	1.05 (0.826, 1.334)	0.692	0.981

Cystic kidney disease	Congenital anomalies	Wald ratio	1	0.942 (0.674, 1.316)	0.725	0.981
Other specified congenital anomalies of kidney	Congenital anomalies	Wald ratio	1	1.228 (0.844, 1.786)	0.284	0.923
Nervous system congenital anomalies	Congenital anomalies	Wald ratio	1	0.776 (0.531, 1.134)	0.19	0.917
Neural tube defects	Congenital anomalies	Wald ratio	1	0.758 (0.491, 1.17)	0.211	0.917
Spina bifida	Congenital anomalies	Wald ratio	1	0.893 (0.543, 1.468)	0.656	0.981
Other specified congenital anomalies of nervous system	Congenital anomalies	Wald ratio	1	0.676 (0.314, 1.457)	0.317	0.923
Congenital anomalies of the eye	Congenital anomalies	Wald ratio	1	0.692 (0.384, 1.244)	0.218	0.917
Congenital cataract and lens anomalies	Congenital anomalies	Wald ratio	1	0.549 (0.244, 1.239)	0.149	0.916
Congenital musculoskeletal deformities of spine	Congenital anomalies	Wald ratio	1	0.816 (0.415, 1.608)	0.557	0.981
Congenital anomalies of limbs	Congenital anomalies	Wald ratio	1	0.951 (0.687, 1.316)	0.76	0.981
Congenital deformities of feet	Congenital anomalies	Wald ratio	1	0.912 (0.555, 1.499)	0.716	0.981
Other congenital anomalies of lower limb, including pelvic girdle	Congenital anomalies	Wald ratio	1	0.995 (0.578, 1.711)	0.985	0.991
Congenital hip dysplasia and deformity	Congenital anomalies	Wald ratio	1	1.125 (0.571, 2.215)	0.734	0.981
Other congenital musculoskeletal anomalies	Congenital anomalies	Wald ratio	1	0.841 (0.633, 1.118)	0.234	0.917
Congenital anomalies of muscle, tendon, fascia, and connective tissue	Congenital anomalies	Wald ratio	1	0.758 (0.352, 1.635)	0.48	0.974
Congenital osteodystrophies	Congenital anomalies	Wald ratio	1	0.883 (0.632, 1.233)	0.466	0.974
Chromosomal anomalies and genetic disorders	Congenital anomalies	Wald ratio	1	0.887 (0.54, 1.458)	0.636	0.981
Chromosomal anomalies	Congenital anomalies	Wald ratio	1	0.867 (0.44, 1.707)	0.679	0.981
Other and unspecified congenital anomalies	Congenital anomalies	Wald ratio	1	0.877 (0.558, 1.378)	0.569	0.981
Anomalies of endocrine glands, congenital	Congenital anomalies	Wald ratio	1	0.885 (0.449, 1.743)	0.724	0.981
Superficial cellulitis and abscess	Dermatologic	Wald ratio	1	0.981 (0.904, 1.064)	0.641	0.981
Cellulitis and abscess of fingers or toes	Dermatologic	Wald ratio	1	0.798 (0.595, 1.07)	0.132	0.916
Cellulitis and abscess of face or neck	Dermatologic	Wald ratio	1	1.164 (0.86, 1.576)	0.325	0.934
Cellulitis and abscess of arm or hand	Dermatologic	Wald ratio	1	0.996 (0.906, 1.095)	0.935	0.986
Cellulitis and abscess of leg, except foot	Dermatologic	Wald ratio	1	1.003 (0.912, 1.103)	0.947	0.988
Cellulitis and abscess of foot, toe	Dermatologic	Wald ratio	1	0.998 (0.908, 1.097)	0.97	0.991
Cellulitis and abscess of trunk	Dermatologic	Wald ratio	1	0.968 (0.728, 1.287)	0.824	0.981
Other local infections of skin and subcutaneous tissue	Dermatologic	Wald ratio	1	0.98 (0.884, 1.088)	0.708	0.981
Carbuncle and furuncle	Dermatologic	Wald ratio	1	0.944 (0.817, 1.091)	0.435	0.974
Pilonidal cyst	Dermatologic	Wald ratio	1	0.776 (0.581, 1.036)	0.086	0.874
Pyogenic granuloma	Dermatologic	Wald ratio	1	1.015 (0.704, 1.464)	0.935	0.986
Symptoms affecting skin	Dermatologic	Wald ratio	1	1.04 (0.946, 1.143)	0.418	0.974
Rash and other nonspecific skin eruption	Dermatologic	Wald ratio	1	1.011 (0.871, 1.173)	0.889	0.983
Changes in skin texture	Dermatologic	Wald ratio	1	0.758 (0.385, 1.493)	0.424	0.974
Disturbance of skin sensation	Dermatologic	Wald ratio	1	1.082 (0.949, 1.233)	0.241	0.919
Disorder of skin and subcutaneous tissue NOS	Dermatologic	Wald ratio	1	0.951 (0.868, 1.04)	0.271	0.923
Erythematosquamous dermatosis	Dermatologic	Wald ratio	1	0.849 (0.376, 1.915)	0.693	0.981
Seborrheic dermatitis	Dermatologic	Wald ratio	1	0.758 (0.294, 1.958)	0.568	0.981
Congenital anomalies of skin	Dermatologic	Wald ratio	1	0.724 (0.385, 1.363)	0.317	0.923
Dyschromia and Vitiligo	Dermatologic	Wald ratio	1	1.008 (0.808, 1.258)	0.941	0.986
Vitiligo	Dermatologic	Wald ratio	1	0.66 (0.268, 1.63)	0.368	0.972
Other dyschromia	Dermatologic	Wald ratio	1	1.069 (0.834, 1.371)	0.598	0.981
Vascular disorders of skin	Dermatologic	Wald ratio	1	0.935 (0.497, 1.761)	0.836	0.981
Erythematous conditions	Dermatologic	Wald ratio	1	1.159 (1.007, 1.333)	0.039	0.792
Toxic erythema	Dermatologic	Wald ratio	1	1.514 (0.769, 2.982)	0.23	0.917
Bullous dermatoses	Dermatologic	Wald ratio	1	1.446 (0.804, 2.602)	0.218	0.917
Dermatitis herpetiformis	Dermatologic	Wald ratio	1	1.586 (0.642, 3.914)	0.317	0.923
Pemphigus and pemphigoid	Dermatologic	Wald ratio	1	1.349 (0.598, 3.043)	0.47	0.974
Rosacea	Dermatologic	Wald ratio	1	1.319 (0.89, 1.954)	0.168	0.916
Lupus (localized and systemic)	Dermatologic	Wald ratio	1	0.887 (0.632, 1.245)	0.488	0.974
Cutaneous lupus erythematosus	Dermatologic	Wald ratio	1	1.007 (0.535, 1.896)	0.982	0.991
Systemic lupus erythematosus	Dermatologic	Wald ratio	1	0.879 (0.604, 1.279)	0.5	0.974
Prurigo and Lichen	Dermatologic	Wald ratio	1	1.233 (0.962, 1.581)	0.098	0.877
Other specified erythematous conditions	Dermatologic	Wald ratio	1	1.251 (0.803, 1.947)	0.322	0.932
Erythema nodosum	Dermatologic	Wald ratio	1	0.98 (0.435, 2.21)	0.961	0.99
Unspecified erythematous condition	Dermatologic	Wald ratio	1	1.018 (0.739, 1.403)	0.913	0.983
Psoriasis and related disorders	Dermatologic	Wald ratio	1	0.906 (0.784, 1.047)	0.179	0.916
Psoriasis	Dermatologic	Wald ratio	1	0.912 (0.786, 1.059)	0.225	0.917
Psoriasis vulgaris	Dermatologic	Wald ratio	1	0.891 (0.754, 1.053)	0.177	0.916
Psoriatic arthropathy	Dermatologic	Wald ratio	1	0.997 (0.767, 1.296)	0.982	0.991
Sarcoidosis	Dermatologic	Wald ratio	1	0.899 (0.668, 1.212)	0.486	0.974
Pruritus and related conditions	Dermatologic	Wald ratio	1	1.289 (1.005, 1.652)	0.046	0.792
Corns and callosities	Dermatologic	Wald ratio	1	1.349 (0.899, 2.026)	0.149	0.916
Other hypertrophic and atrophic conditions of skin	Dermatologic	Wald ratio	1	1.052 (0.952, 1.162)	0.317	0.923
Keratoderma, acquired	Dermatologic	Wald ratio	1	0.602 (0.292, 1.241)	0.169	0.916
Scar conditions and fibrosis of skin	Dermatologic	Wald ratio	1	1.138 (0.985, 1.315)	0.08	0.874
Circumscribed scleroderma	Dermatologic	Wald ratio	1	0.826 (0.335, 2.039)	0.678	0.981
Keloid scar	Dermatologic	Wald ratio	1	0.805 (0.513, 1.265)	0.347	0.959
Abnormal granulation tissue	Dermatologic	Wald ratio	1	1.143 (0.826, 1.582)	0.42	0.974
Acquired acanthosis nigricans	Dermatologic	Wald ratio	1	0.501 (0.254, 0.986)	0.046	0.792
Degenerative skin conditions and other dermatoses	Dermatologic	Wald ratio	1	0.988 (0.898, 1.086)	0.797	0.981
Actinic keratosis	Dermatologic	Wald ratio	1	0.973 (0.849, 1.114)	0.689	0.981
Seborrheic keratosis	Dermatologic	Wald ratio	1	0.997 (0.878, 1.131)	0.957	0.989
Diseases of nail, NOS	Dermatologic	Wald ratio	1	1.143 (0.941, 1.388)	0.177	0.916
Ingrowing nail	Dermatologic	Wald ratio	1	1.214 (0.968, 1.521)	0.093	0.874
Diseases of hair and hair follicles	Dermatologic	Wald ratio	1	0.914 (0.831, 1.005)	0.063	0.874
Alopecia	Dermatologic	Wald ratio	1	0.776 (0.412, 1.461)	0.432	0.974
Other specified diseases of hair and hair follicles	Dermatologic	Wald ratio	1	1.514 (0.671, 3.415)	0.317	0.923
Disorders of sweat glands	Dermatologic	Wald ratio	1	1.067 (0.836, 1.361)	0.604	0.981

Hidradenitis	Dermatologic	Wald ratio	1	0.616 (0.375, 1.013)	0.056	0.87
Hyperhidrosis	Dermatologic	Wald ratio	1	1.208 (0.909, 1.606)	0.193	0.917
Diseases of sebaceous glands	Dermatologic	Wald ratio	1	0.977 (0.905, 1.055)	0.556	0.981
Acne	Dermatologic	Wald ratio	1	1.289 (0.598, 2.778)	0.518	0.981
Sebaceous cyst	Dermatologic	Wald ratio	1	0.975 (0.903, 1.053)	0.518	0.981
Chronic ulcer of skin	Dermatologic	Wald ratio	1	1.038 (0.886, 1.215)	0.648	0.981
Decubitus ulcer	Dermatologic	Wald ratio	1	1.17 (0.929, 1.473)	0.182	0.916
Diffuse diseases of connective tissue	Dermatologic	Wald ratio	1	0.985 (0.876, 1.107)	0.797	0.981
Sicca syndrome	Dermatologic	Wald ratio	1	1.092 (0.803, 1.484)	0.576	0.981
Systemic sclerosis	Dermatologic	Wald ratio	1	1.48 (0.86, 2.545)	0.157	0.916
Other specified diffuse diseases of connective tissue	Dermatologic	Wald ratio	1	1.55 (0.657, 3.656)	0.317	0.923
Unspecified diffuse connective tissue disease	Dermatologic	Wald ratio	1	0.948 (0.828, 1.086)	0.443	0.974
Atopic or contact dermatitis due to other or unspecified	Dermatologic	Wald ratio	1	1.094 (0.938, 1.276)	0.251	0.919
Disorders of tooth development	Digestive	Wald ratio	1	0.925 (0.804, 1.064)	0.273	0.923
Hereditary disturbances in tooth structure	Digestive	Wald ratio	1	0.908 (0.352, 2.344)	0.841	0.981
Disturbances in tooth eruption	Digestive	Wald ratio	1	0.929 (0.804, 1.073)	0.317	0.923
Diseases of hard tissues of teeth	Digestive	Wald ratio	1	1.008 (0.888, 1.144)	0.901	0.983
Dental caries	Digestive	Wald ratio	1	1.008 (0.888, 1.144)	0.901	0.983
Diseases of pulp and periapical tissues	Digestive	Wald ratio	1	0.986 (0.838, 1.16)	0.865	0.981
Periapical abscess	Digestive	Wald ratio	1	1.028 (0.839, 1.26)	0.79	0.981
Gingival and periodontal diseases	Digestive	Wald ratio	1	0.944 (0.799, 1.116)	0.499	0.974
Gingivitis	Digestive	Wald ratio	1	0.776 (0.412, 1.461)	0.432	0.974
Periodontitis (acute or chronic)	Digestive	Wald ratio	1	0.895 (0.734, 1.092)	0.275	0.923
Acute periodontitis	Digestive	Wald ratio	1	0.798 (0.611, 1.041)	0.097	0.876
Chronic periodontitis	Digestive	Wald ratio	1	0.977 (0.729, 1.311)	0.879	0.983
Dentofacial anomalies, including malocclusion	Digestive	Wald ratio	1	0.758 (0.521, 1.103)	0.148	0.916
Anomalies of tooth position or malocclusion	Digestive	Wald ratio	1	0.758 (0.51, 1.129)	0.173	0.916
Other diseases of the teeth and supporting structures	Digestive	Wald ratio	1	1.028 (0.898, 1.177)	0.689	0.981
Atrophy of edentulous alveolar ridge	Digestive	Wald ratio	1	0.859 (0.381, 1.937)	0.714	0.981
Diseases of the jaws	Digestive	Wald ratio	1	0.993 (0.792, 1.244)	0.949	0.988
Cysts of the jaws	Digestive	Wald ratio	1	0.942 (0.642, 1.383)	0.76	0.981
Anomalies of jaw size or symmetry	Digestive	Wald ratio	1	0.616 (0.25, 1.521)	0.294	0.923
Temporomandibular joint disorders	Digestive	Wald ratio	1	1.033 (0.66, 1.615)	0.888	0.983
Temporomandibular joint disorder, unspecified	Digestive	Wald ratio	1	1.026 (0.653, 1.611)	0.912	0.983
Inflammatory conditions of jaw	Digestive	Wald ratio	1	2.241 (1.138, 4.412)	0.02	0.688
Jaw disease NOS	Digestive	Wald ratio	1	0.883 (0.562, 1.387)	0.589	0.981
Diseases of the salivary glands	Digestive	Wald ratio	1	0.834 (0.641, 1.083)	0.173	0.916
Hypertrophy of salivary gland	Digestive	Wald ratio	1	1.028 (0.477, 2.216)	0.944	0.986
Sialoadenitis	Digestive	Wald ratio	1	0.839 (0.585, 1.205)	0.342	0.954
Disturbance of salivary secretion	Digestive	Wald ratio	1	0.955 (0.405, 2.253)	0.916	0.983
Other specified diseases of the salivary glands	Digestive	Wald ratio	1	0.631 (0.335, 1.187)	0.153	0.916
Diseases of the oral soft tissues, excluding lesions specific for gingiva and tongue	Digestive	Wald ratio	1	1.018 (0.909, 1.14)	0.758	0.981
Stomatitis and mucositis	Digestive	Wald ratio	1	0.901 (0.654, 1.242)	0.526	0.981
Stomatitis and mucositis (ulcerative)	Digestive	Wald ratio	1	1.04 (0.731, 1.479)	0.827	0.981
Oral aphthae	Digestive	Wald ratio	1	0.416 (0.193, 0.898)	0.025	0.717
Cellulitis and abscess of oral soft tissues	Digestive	Wald ratio	1	0.959 (0.584, 1.577)	0.87	0.981
Cysts of oral soft tissues	Digestive	Wald ratio	1	1.117 (0.593, 2.103)	0.732	0.981
Cyst of the salivary gland	Digestive	Wald ratio	1	1.202 (0.584, 2.477)	0.617	0.981
Diseases of lips	Digestive	Wald ratio	1	1.054 (0.808, 1.377)	0.697	0.981
Leukoplakia of oral mucosa	Digestive	Wald ratio	1	1.079 (0.728, 1.599)	0.704	0.981
Sialolithiasis	Digestive	Wald ratio	1	0.966 (0.652, 1.431)	0.863	0.981
Diseases and other conditions of the tongue	Digestive	Wald ratio	1	0.984 (0.807, 1.201)	0.875	0.983
Glossitis	Digestive	Wald ratio	1	0.897 (0.6, 1.341)	0.597	0.981
Diseases of esophagus	Digestive	Wald ratio	1	1.016 (0.976, 1.057)	0.446	0.974
Esophagitis, GERD and related diseases	Digestive	Wald ratio	1	1.021 (0.979, 1.065)	0.323	0.932
GERD	Digestive	Wald ratio	1	0.975 (0.919, 1.034)	0.397	0.974
Ulcer of esophagus	Digestive	Wald ratio	1	1.057 (0.957, 1.167)	0.275	0.923
Reflux esophagitis	Digestive	Wald ratio	1	1.01 (0.944, 1.081)	0.769	0.981
Esophageal bleeding (varices or hemorrhage)	Digestive	Wald ratio	1	1.069 (0.9, 1.269)	0.445	0.974
Stricture and stenosis of esophagus	Digestive	Wald ratio	1	0.97 (0.829, 1.137)	0.71	0.981
Disorders of esophageal motility	Digestive	Wald ratio	1	0.853 (0.648, 1.124)	0.258	0.921
Diverticulum of esophagus, acquired	Digestive	Wald ratio	1	0.631 (0.367, 1.085)	0.096	0.874
Gastroesophageal laceration-hemorrhage syndrome	Digestive	Wald ratio	1	0.822 (0.565, 1.196)	0.306	0.923
Heartburn	Digestive	Wald ratio	1	0.937 (0.804, 1.093)	0.41	0.974
Peptic ulcer (excl. esophageal)	Digestive	Wald ratio	1	0.955 (0.88, 1.036)	0.267	0.923
Hemorrhage from gastrointestinal ulcer	Digestive	Wald ratio	1	0.826 (0.624, 1.093)	0.181	0.916
Gastric ulcer	Digestive	Wald ratio	1	0.968 (0.869, 1.079)	0.56	0.981
Duodenal ulcer	Digestive	Wald ratio	1	0.916 (0.807, 1.04)	0.175	0.916
Peptic ulcer, site unspecified	Digestive	Wald ratio	1	1.059 (0.755, 1.487)	0.739	0.981
Dysphagia	Digestive	Wald ratio	1	0.993 (0.911, 1.082)	0.875	0.983
Gastritis and duodenitis	Digestive	Wald ratio	1	1.006 (0.964, 1.051)	0.771	0.981
Acute gastritis	Digestive	Wald ratio	1	0.879 (0.717, 1.077)	0.213	0.917
Atrophic gastritis	Digestive	Wald ratio	1	1.079 (0.752, 1.549)	0.68	0.981
Duodenitis	Digestive	Wald ratio	1	0.966 (0.891, 1.048)	0.405	0.974
Other specified gastritis	Digestive	Wald ratio	1	1.038 (0.961, 1.12)	0.347	0.959
Gastritis and duodenitis, NOS	Digestive	Wald ratio	1	0.692 (0.367, 1.302)	0.253	0.919
Disorders of function of stomach	Digestive	Wald ratio	1	1.236 (0.812, 1.882)	0.323	0.932
Dyspepsia and other specified disorders of function of stomach	Digestive	Wald ratio	1	1.259 (0.82, 1.934)	0.293	0.923

Other disorders of stomach and duodenum	Digestive	Wald ratio	1	0.981 (0.868, 1.108)	0.759	0.981
Appendiceal conditions	Digestive	Wald ratio	1	0.807 (0.714, 0.912)	5.72E-04	0.109
Appendicitis	Digestive	Wald ratio	1	0.794 (0.703, 0.897)	2.12E-04	0.109
Acute appendicitis	Digestive	Wald ratio	1	0.803 (0.702, 0.92)	0.002	0.131
Abdominal hernia	Digestive	Wald ratio	1	0.989 (0.955, 1.023)	0.519	0.981
Inguinal hernia	Digestive	Wald ratio	1	0.975 (0.919, 1.034)	0.397	0.974
Diaphragmatic hernia	Digestive	Wald ratio	1	0.981 (0.937, 1.026)	0.395	0.974
Femoral hernia	Digestive	Wald ratio	1	1.028 (0.784, 1.348)	0.841	0.981
Umbilical hernia	Digestive	Wald ratio	1	1.057 (0.944, 1.183)	0.337	0.952
Ventral hernia	Digestive	Wald ratio	1	1.052 (0.935, 1.183)	0.397	0.974
Inflammatory bowel disease and other gastroenteritis and colitis	Digestive	Wald ratio	1	0.99 (0.893, 1.099)	0.855	0.981
Regional enteritis	Digestive	Wald ratio	1	1.089 (0.921, 1.287)	0.317	0.923
Ulcerative colitis	Digestive	Wald ratio	1	0.997 (0.878, 1.131)	0.96	0.99
Ulcerative colitis (chronic)	Digestive	Wald ratio	1	1.057 (0.784, 1.424)	0.716	0.981
Ulceration of the lower GI tract	Digestive	Wald ratio	1	1.2 (0.97, 1.484)	0.093	0.874
Ulceration of intestine	Digestive	Wald ratio	1	1.413 (1.082, 1.845)	0.011	0.564
Intestinal malabsorption (non-celiac)	Digestive	Wald ratio	1	1.03 (0.884, 1.201)	0.702	0.981
Celiac disease	Digestive	Wald ratio	1	1.007 (0.856, 1.185)	0.929	0.986
Noninfectious gastroenteritis	Digestive	Wald ratio	1	1.035 (0.976, 1.098)	0.249	0.919
Ileostomy status	Digestive	Wald ratio	1	1.102 (0.928, 1.308)	0.269	0.923
Intestinal obstruction without mention of hernia	Digestive	Wald ratio	1	0.951 (0.853, 1.059)	0.359	0.963
Paralytic ileus	Digestive	Wald ratio	1	1.225 (0.905, 1.658)	0.189	0.917
Impaction of intestine	Digestive	Wald ratio	1	1.446 (0.946, 2.211)	0.089	0.874
Peritoneal or intestinal adhesions	Digestive	Wald ratio	1	0.94 (0.74, 1.194)	0.61	0.981
Other intestinal obstruction	Digestive	Wald ratio	1	0.897 (0.794, 1.014)	0.082	0.874
Symptoms involving digestive system	Digestive	Wald ratio	1	1.033 (0.978, 1.09)	0.243	0.919
Diarrhea	Digestive	Wald ratio	1	1.045 (0.758, 1.44)	0.789	0.981
Flatulence	Digestive	Wald ratio	1	1.112 (0.941, 1.314)	0.214	0.917
Diverticulosis and diverticulitis	Digestive	Wald ratio	1	1.012 (0.967, 1.058)	0.617	0.981
Diverticulosis	Digestive	Wald ratio	1	1.012 (0.968, 1.059)	0.596	0.981
Constipation	Digestive	Wald ratio	1	1.026 (0.958, 1.098)	0.463	0.974
Functional digestive disorders	Digestive	Wald ratio	1	0.997 (0.948, 1.047)	0.892	0.983
Irritable Bowel Syndrome	Digestive	Wald ratio	1	1.077 (0.979, 1.184)	0.128	0.916
Abnormal findings on exam of gastrointestinal tract or abdominal area	Digestive	Wald ratio	1	0.996 (0.839, 1.182)	0.962	0.99
Personal history of diseases of digestive system	Digestive	Wald ratio	1	0.979 (0.923, 1.038)	0.474	0.974
Anal and rectal conditions	Digestive	Wald ratio	1	1.052 (0.992, 1.116)	0.091	0.874
Anal and rectal polyp	Digestive	Wald ratio	1	1.054 (0.972, 1.144)	0.201	0.917
Peritonitis and retroperitoneal infections	Digestive	Wald ratio	1	0.822 (0.653, 1.035)	0.096	0.874
Other disorders of peritoneum	Digestive	Wald ratio	1	0.995 (0.881, 1.125)	0.941	0.986
Peritoneal adhesions (postoperative) (postinfection)	Digestive	Wald ratio	1	1.011 (0.891, 1.147)	0.867	0.981
Other disorders of intestine	Digestive	Wald ratio	1	1.135 (1.019, 1.265)	0.022	0.688
Toxic gastroenteritis and colitis	Digestive	Wald ratio	1	1.054 (0.586, 1.897)	0.86	0.981
Gastrointestinal complications	Digestive	Wald ratio	1	1.178 (0.858, 1.616)	0.31	0.923
Other chronic nonalcoholic liver disease	Digestive	Wald ratio	1	0.984 (0.829, 1.168)	0.854	0.981
Cirrhosis of liver without mention of alcohol	Digestive	Wald ratio	1	0.849 (0.451, 1.598)	0.612	0.981
Primary biliary cirrhosis	Digestive	Wald ratio	1	1.623 (1.047, 2.515)	0.03	0.749
Liver abscess and sequelae of chronic liver disease	Digestive	Wald ratio	1	1.057 (0.843, 1.325)	0.631	0.981
Portal hypertension	Digestive	Wald ratio	1	1.191 (0.88, 1.613)	0.257	0.919
Malposition and malpresentation of fetus or obstruction	Digestive	Wald ratio	1	1.023 (0.858, 1.22)	0.802	0.981
Simple and unspecified goiter	Endocrine/metabolic	Wald ratio	1	0.857 (0.645, 1.139)	0.288	0.923
Nontoxic nodular goiter	Endocrine/metabolic	Wald ratio	1	1.079 (0.877, 1.328)	0.473	0.974
Nontoxic uninodular goiter	Endocrine/metabolic	Wald ratio	1	1.289 (0.935, 1.776)	0.121	0.916
Nontoxic multinodular goiter	Endocrine/metabolic	Wald ratio	1	0.899 (0.689, 1.174)	0.436	0.974
Thyrotoxicosis with or without goiter	Endocrine/metabolic	Wald ratio	1	0.98 (0.833, 1.153)	0.805	0.981
Graves' disease	Endocrine/metabolic	Wald ratio	1	0.724 (0.523, 1.003)	0.052	0.856
Toxic multinodular goiter	Endocrine/metabolic	Wald ratio	1	0.805 (0.391, 1.659)	0.557	0.981
Exophthalmos	Endocrine/metabolic	Wald ratio	1	0.66 (0.367, 1.188)	0.166	0.916
Hypothyroidism	Endocrine/metabolic	Wald ratio	1	1.033 (0.974, 1.095)	0.282	0.923
Secondary hypothyroidism	Endocrine/metabolic	Wald ratio	1	0.889 (0.722, 1.094)	0.268	0.923
Acquired hypothyroidism	Endocrine/metabolic	Wald ratio	1	0.975 (0.495, 1.92)	0.942	0.986
Hypothyroidism NOS	Endocrine/metabolic	Wald ratio	1	1.042 (0.978, 1.11)	0.199	0.917
Thyroiditis	Endocrine/metabolic	Wald ratio	1	0.977 (0.651, 1.467)	0.912	0.983
Chronic thyroiditis	Endocrine/metabolic	Wald ratio	1	0.83 (0.528, 1.304)	0.418	0.974
Chronic lymphocytic thyroiditis	Endocrine/metabolic	Wald ratio	1	0.869 (0.529, 1.428)	0.579	0.981
Other disorders of thyroid	Endocrine/metabolic	Wald ratio	1	1.082 (0.778, 1.504)	0.641	0.981
Diabetes mellitus	Endocrine/metabolic	Wald ratio	1	1.052 (0.997, 1.111)	0.067	0.874
Type 1 diabetes	Endocrine/metabolic	Wald ratio	1	0.991 (0.865, 1.134)	0.891	0.983
Type 1 diabetes with ketoacidosis	Endocrine/metabolic	Wald ratio	1	1.514 (0.964, 2.379)	0.072	0.874
Type 1 diabetes with renal manifestations	Endocrine/metabolic	Wald ratio	1	0.724 (0.321, 1.633)	0.437	0.974
Type 2 diabetes	Endocrine/metabolic	Wald ratio	1	1.05 (0.994, 1.108)	0.08	0.874
Type 2 diabetes with ketoacidosis	Endocrine/metabolic	Wald ratio	1	0.816 (0.415, 1.608)	0.557	0.981
Type 2 diabetes with renal manifestations	Endocrine/metabolic	Wald ratio	1	0.959 (0.584, 1.577)	0.87	0.981
Type 2 diabetes with ophthalmic manifestations	Endocrine/metabolic	Wald ratio	1	0.873 (0.719, 1.06)	0.17	0.916
Type 2 diabetes with neurological manifestations	Endocrine/metabolic	Wald ratio	1	0.724 (0.542, 0.967)	0.029	0.749
Abnormal glucose	Endocrine/metabolic	Wald ratio	1	1.381 (1.058, 1.803)	0.018	0.68
Impaired fasting glucose	Endocrine/metabolic	Wald ratio	1	1.143 (0.748, 1.748)	0.537	0.981
Other abnormal glucose	Endocrine/metabolic	Wald ratio	1	1.514 (1.079, 2.125)	0.016	0.68
Glycosuria or Acetonuria	Endocrine/metabolic	Wald ratio	1	1.143 (0.635, 2.056)	0.655	0.981
Polyneuropathy in diabetes	Endocrine/metabolic	Wald ratio	1	1.02 (0.704, 1.478)	0.916	0.983

Diabetic retinopathy	Endocrine/metabolic	Wald ratio	1	0.962 (0.795, 1.162)	0.686	0.981
Other disorders of pancreatic internal secretion	Endocrine/metabolic	Wald ratio	1	0.994 (0.793, 1.245)	0.955	0.989
Hypoglycemia	Endocrine/metabolic	Wald ratio	1	0.994 (0.793, 1.245)	0.955	0.989
Disorders of parathyroid gland	Endocrine/metabolic	Wald ratio	1	0.741 (0.586, 0.937)	0.012	0.564
Hyperparathyroidism	Endocrine/metabolic	Wald ratio	1	0.708 (0.552, 0.907)	0.006	0.406
Hypoparathyroidism	Endocrine/metabolic	Wald ratio	1	1.779 (0.989, 3.201)	0.054	0.87
Disorders of the pituitary gland and its hypothalamic control	Endocrine/metabolic	Wald ratio	1	0.805 (0.62, 1.046)	0.105	0.902
Pituitary hyperfunction	Endocrine/metabolic	Wald ratio	1	0.708 (0.431, 1.163)	0.173	0.916
Acromegaly and gigantism	Endocrine/metabolic	Wald ratio	1	0.524 (0.255, 1.081)	0.08	0.874
Pituitary hypofunction	Endocrine/metabolic	Wald ratio	1	0.807 (0.525, 1.24)	0.328	0.94
Diabetes insipidus	Endocrine/metabolic	Wald ratio	1	0.776 (0.377, 1.599)	0.492	0.974
Other disorders of neurohypophysis	Endocrine/metabolic	Wald ratio	1	1.164 (0.677, 2.002)	0.582	0.981
Disorders of adrenal glands	Endocrine/metabolic	Wald ratio	1	1.02 (0.774, 1.343)	0.89	0.983
Adrenal hyperfunction	Endocrine/metabolic	Wald ratio	1	0.953 (0.58, 1.566)	0.849	0.981
Cushing's syndrome	Endocrine/metabolic	Wald ratio	1	1.064 (0.54, 2.096)	0.857	0.981
Hyperaldosteronism	Endocrine/metabolic	Wald ratio	1	0.887 (0.393, 2)	0.773	0.981
Adrenal hypofunction	Endocrine/metabolic	Wald ratio	1	1.151 (0.798, 1.66)	0.451	0.974
Glucocorticoid deficiency	Endocrine/metabolic	Wald ratio	1	1.154 (0.8, 1.663)	0.444	0.974
Ovarian dysfunction	Endocrine/metabolic	Wald ratio	1	1.008 (0.644, 1.576)	0.973	0.991
Polycystic ovaries	Endocrine/metabolic	Wald ratio	1	1.064 (0.647, 1.749)	0.806	0.981
Testicular dysfunction	Endocrine/metabolic	Wald ratio	1	1.104 (0.642, 1.899)	0.72	0.981
Testicular hypofunction	Endocrine/metabolic	Wald ratio	1	1.099 (0.639, 1.89)	0.733	0.981
Iatrogenic endocrine disorders	Endocrine/metabolic	Wald ratio	1	0.904 (0.502, 1.626)	0.735	0.981
Other endocrine disorders	Endocrine/metabolic	Wald ratio	1	0.944 (0.574, 1.552)	0.82	0.981
Protein-calorie malnutrition	Endocrine/metabolic	Wald ratio	1	0.937 (0.758, 1.159)	0.551	0.981
Anorexia	Endocrine/metabolic	Wald ratio	1	0.957 (0.753, 1.216)	0.72	0.981
Vitamin deficiency	Endocrine/metabolic	Wald ratio	1	1.022 (0.838, 1.247)	0.827	0.981
Vitamin B-complex deficiencies	Endocrine/metabolic	Wald ratio	1	1.109 (0.861, 1.429)	0.422	0.974
Vitamin D deficiency	Endocrine/metabolic	Wald ratio	1	0.863 (0.623, 1.195)	0.374	0.974
Rickets or osteomalacia	Endocrine/metabolic	Wald ratio	1	1.162 (0.539, 2.504)	0.702	0.981
Mineral deficiency NEC	Endocrine/metabolic	Wald ratio	1	1.413 (0.903, 2.21)	0.13	0.916
Proteinuria	Endocrine/metabolic	Wald ratio	1	1.089 (0.748, 1.584)	0.656	0.981
Disorders of protein plasma or amino-acid transport and metabolism	Endocrine/metabolic	Wald ratio	1	0.964 (0.684, 1.359)	0.833	0.981
Disorders of plasma protein metabolism	Endocrine/metabolic	Wald ratio	1	0.981 (0.68, 1.414)	0.916	0.983
Paraproteinemia	Endocrine/metabolic	Wald ratio	1	0.839 (0.511, 1.38)	0.49	0.974
Amyloidosis	Endocrine/metabolic	Wald ratio	1	1.319 (0.67, 2.597)	0.424	0.974
Macroglobulinemia	Endocrine/metabolic	Wald ratio	1	1.004 (0.445, 2.264)	0.992	0.995
Disorders of carbohydrate transport and metabolism	Endocrine/metabolic	Wald ratio	1	1.181 (0.686, 2.03)	0.549	0.981
Intestinal disaccharidase deficiencies and disaccharide malabsorption	Endocrine/metabolic	Wald ratio	1	1.057 (0.587, 1.901)	0.854	0.981
Disorders of lipoid metabolism	Endocrine/metabolic	Wald ratio	1	1.035 (0.994, 1.078)	0.096	0.874
Hyperlipidemia	Endocrine/metabolic	Wald ratio	1	1.033 (0.992, 1.076)	0.12	0.916
Hypercholesterolemia	Endocrine/metabolic	Wald ratio	1	1.035 (0.993, 1.08)	0.107	0.905
Hyperglyceridemia	Endocrine/metabolic	Wald ratio	1	1.779 (0.721, 4.392)	0.211	0.917
Mixed hyperlipidemia	Endocrine/metabolic	Wald ratio	1	1.097 (0.638, 1.886)	0.739	0.981
Unspecified disorder of lipoid metabolism	Endocrine/metabolic	Wald ratio	1	1.381 (0.84, 2.27)	0.203	0.917
Gout and other crystal arthropathies	Endocrine/metabolic	Wald ratio	1	1.026 (0.916, 1.148)	0.66	0.981
Gout	Endocrine/metabolic	Wald ratio	1	1.015 (0.895, 1.152)	0.814	0.981
Gouty arthropathy	Endocrine/metabolic	Wald ratio	1	1.319 (0.701, 2.482)	0.391	0.974
Crystal arthropathies	Endocrine/metabolic	Wald ratio	1	1.104 (0.834, 1.461)	0.488	0.974
Chondrocalcinosis	Endocrine/metabolic	Wald ratio	1	1.104 (0.823, 1.481)	0.508	0.977
Disorders of mineral metabolism	Endocrine/metabolic	Wald ratio	1	1.099 (0.943, 1.282)	0.228	0.917
Disorders of magnesium metabolism	Endocrine/metabolic	Wald ratio	1	1.151 (0.805, 1.645)	0.44	0.974
Disorders of calcium or phosphorus metabolism	Endocrine/metabolic	Wald ratio	1	1.248 (1.023, 1.522)	0.029	0.749
Disorders of phosphorus metabolism	Endocrine/metabolic	Wald ratio	1	1.077 (0.626, 1.851)	0.79	0.981
Disorders of fluid, electrolyte, and acid-base balance	Endocrine/metabolic	Wald ratio	1	0.992 (0.915, 1.076)	0.85	0.981
Electrolyte imbalance	Endocrine/metabolic	Wald ratio	1	0.942 (0.845, 1.05)	0.279	0.923
Hyperosmolality and or or hypernatremia	Endocrine/metabolic	Wald ratio	1	0.927 (0.593, 1.449)	0.739	0.981
Hyposmolality and or or hyponatremia	Endocrine/metabolic	Wald ratio	1	0.959 (0.815, 1.129)	0.617	0.981
Hyperpotassemia	Endocrine/metabolic	Wald ratio	1	0.881 (0.706, 1.099)	0.262	0.923
Hypopotassemia	Endocrine/metabolic	Wald ratio	1	0.908 (0.754, 1.092)	0.306	0.923
Acid-base balance disorder	Endocrine/metabolic	Wald ratio	1	0.805 (0.657, 0.987)	0.037	0.792
Acidosis	Endocrine/metabolic	Wald ratio	1	0.794 (0.642, 0.982)	0.033	0.771
Alkalosis	Endocrine/metabolic	Wald ratio	1	1.062 (0.564, 1.999)	0.853	0.981
Hypovolemia	Endocrine/metabolic	Wald ratio	1	1.099 (0.964, 1.253)	0.157	0.916
Fluid overload	Endocrine/metabolic	Wald ratio	1	1.011 (0.73, 1.399)	0.948	0.988
Polydipsia	Endocrine/metabolic	Wald ratio	1	0.758 (0.403, 1.427)	0.391	0.974
Other disorders of metabolism	Endocrine/metabolic	Wald ratio	1	1.107 (0.92, 1.332)	0.283	0.923
Disorders of bilirubin excretion	Endocrine/metabolic	Wald ratio	1	1.14 (0.795, 1.637)	0.476	0.974
Other disorders of lipoid metabolism	Endocrine/metabolic	Wald ratio	1	1.072 (0.682, 1.684)	0.764	0.981
Lipoprotein disorders	Endocrine/metabolic	Wald ratio	1	1.197 (0.464, 3.091)	0.71	0.981
Overweight, obesity and other hyperalimentation	Endocrine/metabolic	Wald ratio	1	0.975 (0.911, 1.043)	0.463	0.974
Obesity	Endocrine/metabolic	Wald ratio	1	0.981 (0.917, 1.05)	0.575	0.981
Localized adiposity	Endocrine/metabolic	Wald ratio	1	0.855 (0.397, 1.843)	0.689	0.981
Abnormal weight gain	Endocrine/metabolic	Wald ratio	1	1.13 (0.479, 2.666)	0.78	0.981
Disorders involving the immune mechanism	Endocrine/metabolic	Wald ratio	1	0.841 (0.613, 1.154)	0.284	0.923
Immunity deficiency	Endocrine/metabolic	Wald ratio	1	0.692 (0.421, 1.137)	0.146	0.916
Deficiency of humoral immunity	Endocrine/metabolic	Wald ratio	1	0.631 (0.351, 1.135)	0.124	0.916
Other immunological findings	Endocrine/metabolic	Wald ratio	1	0.916 (0.591, 1.42)	0.695	0.981
Noninflammatory female genital disorders	Genitourinary	Wald ratio	1	0.994 (0.925, 1.069)	0.881	0.983

Noninflammatory disorders of vulva and perineum	Genitourinary	Wald ratio	1	1.014 (0.839, 1.226)	0.885	0.983
Dysplasia of female genital organs	Genitourinary	Wald ratio	1	1.047 (0.464, 2.362)	0.912	0.983
Endometrial hyperplasia	Genitourinary	Wald ratio	1	1.175 (0.955, 1.447)	0.128	0.916
Polyp of female genital organs	Genitourinary	Wald ratio	1	1.004 (0.939, 1.075)	0.899	0.983
Polyp of corpus uteri	Genitourinary	Wald ratio	1	1.014 (0.935, 1.1)	0.731	0.981
Mucous polyp of cervix	Genitourinary	Wald ratio	1	0.944 (0.839, 1.062)	0.336	0.952
Hypertrophy of female genital organs	Genitourinary	Wald ratio	1	1.127 (0.928, 1.369)	0.227	0.917
Symptoms involving female genital tract	Genitourinary	Wald ratio	1	1.022 (0.938, 1.114)	0.617	0.981
Dystrophy of female genital tract	Genitourinary	Wald ratio	1	0.979 (0.623, 1.538)	0.926	0.986
Atrophy of female genital tract	Genitourinary	Wald ratio	1	1.289 (0.927, 1.792)	0.132	0.916
stress incontinence, female	Genitourinary	Wald ratio	1	1.02 (0.928, 1.122)	0.682	0.981
Pain and other symptoms associated with female genital organs	Genitourinary	Wald ratio	1	1.042 (0.919, 1.183)	0.52	0.981
Dyspareunia	Genitourinary	Wald ratio	1	0.994 (0.811, 1.218)	0.956	0.989
Disorders of menstruation and other abnormal bleeding from female genital tract	Genitourinary	Wald ratio	1	1.003 (0.951, 1.059)	0.901	0.983
Irregular menstrual cycle or bleeding	Genitourinary	Wald ratio	1	1.01 (0.952, 1.071)	0.747	0.981
Absent or infrequent menstruation	Genitourinary	Wald ratio	1	1.079 (0.6, 1.941)	0.8	0.981
Excessive or frequent menstruation	Genitourinary	Wald ratio	1	1.02 (0.949, 1.097)	0.587	0.981
Irregular menstrual cycle	Genitourinary	Wald ratio	1	1.04 (0.888, 1.218)	0.627	0.981
Irregular menstrual bleeding	Genitourinary	Wald ratio	1	1.018 (0.909, 1.14)	0.755	0.981
Dysmenorrhea	Genitourinary	Wald ratio	1	0.985 (0.834, 1.165)	0.863	0.981
Premenstrual tension syndromes	Genitourinary	Wald ratio	1	1.739 (1.011, 2.99)	0.046	0.792
Infertility, female	Genitourinary	Wald ratio	1	0.944 (0.777, 1.146)	0.561	0.981
Menopausal and postmenopausal disorders	Genitourinary	Wald ratio	1	1.004 (0.938, 1.075)	0.904	0.983
Postmenopausal bleeding	Genitourinary	Wald ratio	1	0.999 (0.925, 1.079)	0.982	0.991
Symptomatic menopause	Genitourinary	Wald ratio	1	0.724 (0.441, 1.19)	0.203	0.917
Postmenopausal atrophic vaginitis	Genitourinary	Wald ratio	1	1.164 (0.946, 1.433)	0.151	0.916
Premenopausal menorrhagia	Genitourinary	Wald ratio	1	0.899 (0.607, 1.332)	0.597	0.981
Ovarian cyst	Genitourinary	Wald ratio	1	0.957 (0.863, 1.062)	0.409	0.974
Disorders of iron metabolism	Hematopoietic	Wald ratio	1	0.92 (0.705, 1.201)	0.542	0.981
Iron deficiency anemias	Hematopoietic	Wald ratio	1	0.992 (0.914, 1.076)	0.846	0.981
Iron deficiency anemias, unspecified or not due to blood loss	Hematopoietic	Wald ratio	1	0.986 (0.909, 1.069)	0.731	0.981
Iron deficiency anemia secondary to blood loss (chronic)	Hematopoietic	Wald ratio	1	1.133 (0.818, 1.568)	0.453	0.974
Other deficiency anemia	Hematopoietic	Wald ratio	1	0.832 (0.676, 1.024)	0.082	0.874
Megaloblastic anemia	Hematopoietic	Wald ratio	1	0.826 (0.668, 1.021)	0.077	0.874
Pernicious anemia	Hematopoietic	Wald ratio	1	0.776 (0.603, 0.999)	0.049	0.839
Other vitamin B12 deficiency anemia	Hematopoietic	Wald ratio	1	0.741 (0.431, 1.274)	0.279	0.923
Folate-deficiency anemia	Hematopoietic	Wald ratio	1	1.074 (0.653, 1.765)	0.778	0.981
Deficiency anemias	Hematopoietic	Wald ratio	1	1.062 (0.411, 2.742)	0.901	0.983
Hereditary hemolytic anemias	Hematopoietic	Wald ratio	1	1.259 (0.732, 2.165)	0.405	0.974
Other hemoglobinopathies	Hematopoietic	Wald ratio	1	0.987 (0.418, 2.329)	0.976	0.991
Other hereditary hemolytic anemias	Hematopoietic	Wald ratio	1	1.319 (0.612, 2.842)	0.48	0.974
Acquired hemolytic anemias	Hematopoietic	Wald ratio	1	1.821 (1.012, 3.276)	0.046	0.792
Autoimmune hemolytic anemias	Hematopoietic	Wald ratio	1	1.586 (0.805, 3.123)	0.182	0.916
Aplastic anemia	Hematopoietic	Wald ratio	1	0.978 (0.7, 1.366)	0.897	0.983
Other anemias	Hematopoietic	Wald ratio	1	0.968 (0.909, 1.031)	0.317	0.923
Acute posthemorrhagic anemia	Hematopoietic	Wald ratio	1	1.087 (0.714, 1.654)	0.699	0.981
Anemia of chronic disease	Hematopoietic	Wald ratio	1	0.839 (0.646, 1.091)	0.19	0.917
Anemia in neoplastic disease	Hematopoietic	Wald ratio	1	1.127 (0.803, 1.582)	0.488	0.974
Coagulation defects	Hematopoietic	Wald ratio	1	1.072 (0.855, 1.343)	0.549	0.981
Congenital coagulation defects	Hematopoietic	Wald ratio	1	0.933 (0.677, 1.286)	0.673	0.981
Von willebrand's disease	Hematopoietic	Wald ratio	1	0.631 (0.32, 1.242)	0.182	0.916
Congenital deficiency of other clotting factors (including factor VII)	Hematopoietic	Wald ratio	1	0.92 (0.621, 1.364)	0.679	0.981
Congenital factor VIII disorder	Hematopoietic	Wald ratio	1	1.236 (0.524, 2.917)	0.628	0.981
Hemorrhagic disorder due to intrinsic circulating anticoagulants	Hematopoietic	Wald ratio	1	1.821 (0.845, 3.925)	0.126	0.916
Other and unspecified coagulation defects	Hematopoietic	Wald ratio	1	1.13 (0.794, 1.607)	0.497	0.974
Purpura and other hemorrhagic conditions	Hematopoietic	Wald ratio	1	1.002 (0.852, 1.179)	0.978	0.991
Spontaneous ecchymoses	Hematopoietic	Wald ratio	1	0.891 (0.473, 1.677)	0.721	0.981
Allergic purpura	Hematopoietic	Wald ratio	1	2.293 (0.888, 5.921)	0.086	0.874
Thrombocytopenia	Hematopoietic	Wald ratio	1	0.981 (0.823, 1.17)	0.831	0.981
Primary thrombocytopenia	Hematopoietic	Wald ratio	1	0.818 (0.583, 1.148)	0.246	0.919
Secondary thrombocytopenia	Hematopoietic	Wald ratio	1	1.319 (0.585, 2.974)	0.505	0.974
Diseases of white blood cells	Hematopoietic	Wald ratio	1	0.906 (0.809, 1.014)	0.085	0.874
Decreased white blood cell count	Hematopoietic	Wald ratio	1	0.881 (0.78, 0.995)	0.042	0.792
Neutropenia	Hematopoietic	Wald ratio	1	0.881 (0.78, 0.995)	0.042	0.792
Eosinophilia	Hematopoietic	Wald ratio	1	0.631 (0.28, 1.422)	0.267	0.923
Other diseases of blood and blood-forming organs	Hematopoietic	Wald ratio	1	1.014 (0.91, 1.13)	0.799	0.981
Personal history of diseases of blood and blood-forming organs	Hematopoietic	Wald ratio	1	0.944 (0.629, 1.418)	0.781	0.981
Lymphadenitis	Hematopoietic	Wald ratio	1	1.04 (0.908, 1.191)	0.571	0.981
Diseases of spleen	Hematopoietic	Wald ratio	1	1.002 (0.737, 1.362)	0.991	0.995
Polycythemia, secondary	Hematopoietic	Wald ratio	1	1.064 (0.712, 1.591)	0.762	0.981
Abnormality of red blood cells	Hematopoietic	Wald ratio	1	1.167 (0.566, 2.404)	0.675	0.981
Intestinal infection	Infectious diseases	Wald ratio	1	1.022 (0.951, 1.099)	0.553	0.981
Bacterial enteritis	Infectious diseases	Wald ratio	1	1.099 (0.964, 1.253)	0.157	0.916
Intestinal infection due to C. difficile	Infectious diseases	Wald ratio	1	0.942 (0.718, 1.235)	0.665	0.981
Viral Enteritis	Infectious diseases	Wald ratio	1	0.885 (0.7, 1.119)	0.308	0.923
Intestinal infection due to protozoa	Infectious diseases	Wald ratio	1	0.631 (0.244, 1.629)	0.341	0.954
Tuberculosis	Infectious diseases	Wald ratio	1	1.413 (0.822, 2.43)	0.211	0.917
Septicemia	Infectious diseases	Wald ratio	1	0.959 (0.861, 1.069)	0.453	0.974

Gram negative septicemia	Infectious diseases	Wald ratio	1	1.045 (0.822, 1.327)	0.72	0.981
Gram positive septicemia	Infectious diseases	Wald ratio	1	0.881 (0.645, 1.203)	0.425	0.974
Bacterial infection NOS	Infectious diseases	Wald ratio	1	0.966 (0.907, 1.029)	0.284	0.923
Staphylococcus infections	Infectious diseases	Wald ratio	1	0.987 (0.87, 1.121)	0.844	0.981
Streptococcus infection	Infectious diseases	Wald ratio	1	0.929 (0.782, 1.103)	0.4	0.974
E. coli	Infectious diseases	Wald ratio	1	0.935 (0.82, 1.066)	0.317	0.923
Herpes zoster	Infectious diseases	Wald ratio	1	1.054 (0.751, 1.48)	0.759	0.981
Herpes zoster with nervous system complications	Infectious diseases	Wald ratio	1	0.602 (0.292, 1.241)	0.169	0.916
Herpes simplex	Infectious diseases	Wald ratio	1	1.259 (0.801, 1.978)	0.317	0.923
Viral hepatitis	Infectious diseases	Wald ratio	1	0.851 (0.698, 1.038)	0.112	0.916
Viral hepatitis A	Infectious diseases	Wald ratio	1	0.904 (0.401, 2.038)	0.807	0.981
Viral hepatitis B	Infectious diseases	Wald ratio	1	0.676 (0.376, 1.216)	0.191	0.917
Viral hepatitis C	Infectious diseases	Wald ratio	1	1.052 (0.682, 1.623)	0.819	0.981
Chronic hepatitis	Infectious diseases	Wald ratio	1	0.794 (0.524, 1.203)	0.277	0.923
Hepatitis NOS	Infectious diseases	Wald ratio	1	0.815 (0.583, 1.138)	0.229	0.917
Human immunodeficiency virus [HIV] disease	Infectious diseases	Wald ratio	1	1.349 (0.75, 2.428)	0.317	0.923
HIV infection, symptomatic	Infectious diseases	Wald ratio	1	1.242 (0.66, 2.338)	0.502	0.974
Viral warts & HPV	Infectious diseases	Wald ratio	1	0.832 (0.669, 1.033)	0.096	0.874
Viral infection	Infectious diseases	Wald ratio	1	0.973 (0.853, 1.109)	0.679	0.981
Varicella infection	Infectious diseases	Wald ratio	1	0.676 (0.286, 1.594)	0.371	0.972
Infectious mononucleosis	Infectious diseases	Wald ratio	1	1.259 (0.534, 2.971)	0.599	0.981
Postoperative infection	Infectious diseases	Wald ratio	1	1.005 (0.906, 1.115)	0.927	0.986
Infection or inflammation of internal prosthetic device; implant; and graft	Infectious diseases	Wald ratio	1	1.05 (0.912, 1.207)	0.498	0.974
Sexually transmitted infections (not HIV or hepatitis)	Infectious diseases	Wald ratio	1	0.537 (0.238, 1.21)	0.134	0.916
Dermatophytosis or Dermatomycosis	Infectious diseases	Wald ratio	1	0.758 (0.503, 1.144)	0.187	0.917
Dermatophytosis	Infectious diseases	Wald ratio	1	0.758 (0.5, 1.149)	0.192	0.917
Dermatophytosis of nail	Infectious diseases	Wald ratio	1	0.575 (0.255, 1.297)	0.182	0.916
Althete's foot	Infectious diseases	Wald ratio	1	0.708 (0.393, 1.273)	0.249	0.919
Candidiasis	Infectious diseases	Wald ratio	1	1.05 (0.904, 1.218)	0.525	0.981
Mycoses	Infectious diseases	Wald ratio	1	1.008 (0.659, 1.541)	0.97	0.991
Aspergillosis	Infectious diseases	Wald ratio	1	0.861 (0.501, 1.48)	0.588	0.981
Spirochetal infection	Infectious diseases	Wald ratio	1	1.997 (0.926, 4.304)	0.078	0.874
Arthropod-borne diseases	Infectious diseases	Wald ratio	1	0.436 (0.193, 0.984)	0.046	0.792
Helminthiases	Infectious diseases	Wald ratio	1	0.933 (0.496, 1.757)	0.83	0.981
Intestinal helminthiases	Infectious diseases	Wald ratio	1	0.918 (0.466, 1.808)	0.805	0.981
Other infectious and parasitic diseases	Infectious diseases	Wald ratio	1	0.927 (0.564, 1.523)	0.764	0.981
Sepsis and SIRS	Infectious diseases	Wald ratio	1	0.94 (0.824, 1.071)	0.352	0.962
Sepsis	Infectious diseases	Wald ratio	1	0.94 (0.824, 1.071)	0.352	0.962
Burns	Injuries & poisonings	Wald ratio	1	1.047 (0.784, 1.398)	0.755	0.981
Foreign body injury	Injuries & poisonings	Wald ratio	1	1.125 (0.922, 1.372)	0.246	0.919
Crushing injury	Injuries & poisonings	Wald ratio	1	0.942 (0.61, 1.453)	0.787	0.981
Injury to blood vessels	Injuries & poisonings	Wald ratio	1	1.04 (0.671, 1.612)	0.861	0.981
Crushing or internal injury to organs	Injuries & poisonings	Wald ratio	1	1.319 (1.062, 1.638)	0.012	0.564
Injury, NOS	Injuries & poisonings	Wald ratio	1	1.097 (1.016, 1.184)	0.019	0.688
Complications of surgical and medical procedures	Injuries & poisonings	Wald ratio	1	0.998 (0.928, 1.072)	0.95	0.988
Late effect	Injuries & poisonings	Wald ratio	1	0.794 (0.403, 1.564)	0.505	0.974
Effects of heat, cold and air pressure	Injuries & poisonings	Wald ratio	1	0.676 (0.286, 1.594)	0.371	0.972
Complications of in utero procedures	Injuries & poisonings	Wald ratio	1	1.054 (0.408, 2.723)	0.913	0.983
Nonspecific abnormal findings on radiological and other examination of musculoskeletal system	Injuries & poisonings	Wald ratio	1	1.04 (0.8, 1.352)	0.769	0.981
Fracture of lower limb	Injuries & poisonings	Wald ratio	1	0.91 (0.839, 0.987)	0.023	0.688
Fracture of neck of femur	Injuries & poisonings	Wald ratio	1	0.871 (0.74, 1.025)	0.096	0.874
Fracture of unspecified part of femur	Injuries & poisonings	Wald ratio	1	1.289 (0.749, 2.216)	0.359	0.963
Fracture of tibia and fibula	Injuries & poisonings	Wald ratio	1	0.962 (0.828, 1.116)	0.606	0.981
Fracture of patella	Injuries & poisonings	Wald ratio	1	0.879 (0.682, 1.132)	0.317	0.923
Fracture of ankle and foot	Injuries & poisonings	Wald ratio	1	1.052 (0.91, 1.216)	0.492	0.974
Fracture of foot	Injuries & poisonings	Wald ratio	1	1.138 (0.967, 1.339)	0.12	0.916
Fracture of pelvis	Injuries & poisonings	Wald ratio	1	1.045 (0.808, 1.352)	0.739	0.981
Fracture of upper limb	Injuries & poisonings	Wald ratio	1	0.97 (0.899, 1.048)	0.444	0.974
Fracture of humerus	Injuries & poisonings	Wald ratio	1	0.908 (0.768, 1.073)	0.256	0.919
Fracture of radius and ulna	Injuries & poisonings	Wald ratio	1	0.977 (0.885, 1.079)	0.649	0.981
Fracture of clavicle or scapula	Injuries & poisonings	Wald ratio	1	0.927 (0.777, 1.105)	0.397	0.974
Fracture of hand or wrist	Injuries & poisonings	Wald ratio	1	0.989 (0.88, 1.113)	0.86	0.981
Fracture of vertebral column without mention of spinal cord injury	Injuries & poisonings	Wald ratio	1	1.154 (0.954, 1.395)	0.14	0.916
Fracture of ribs	Injuries & poisonings	Wald ratio	1	1.12 (0.873, 1.435)	0.373	0.973
Fracture of unspecified bones	Injuries & poisonings	Wald ratio	1	1.349 (1.111, 1.639)	0.003	0.177
Concussion	Injuries & poisonings	Wald ratio	1	0.931 (0.598, 1.45)	0.752	0.981
Intracranial hemorrhage (injury)	Injuries & poisonings	Wald ratio	1	0.776 (0.571, 1.055)	0.106	0.902
Skull and face fracture and other intercranial injury	Injuries & poisonings	Wald ratio	1	0.94 (0.828, 1.066)	0.335	0.952
Torus fracture	Injuries & poisonings	Wald ratio	1	1 (0.842, 1.187)	0.997	0.997
Dislocation	Injuries & poisonings	Wald ratio	1	1.045 (0.896, 1.218)	0.576	0.981
Internal derangement of knee	Injuries & poisonings	Wald ratio	1	0.984 (0.928, 1.044)	0.59	0.981
Traumatic arthropathy	Injuries & poisonings	Wald ratio	1	0.818 (0.605, 1.108)	0.194	0.917
Sprains and strains	Injuries & poisonings	Wald ratio	1	1.259 (0.922, 1.72)	0.147	0.916
Hemorrhage or hematoma complicating a procedure	Injuries & poisonings	Wald ratio	1	1.069 (0.972, 1.176)	0.167	0.916
Complications of transplants and reattached limbs	Injuries & poisonings	Wald ratio	1	0.929 (0.54, 1.597)	0.79	0.981
Complication of colostomy or enterostomy	Injuries & poisonings	Wald ratio	1	1.159 (0.849, 1.583)	0.354	0.962
Complications of cardiac or vascular device, implant, and graft	Injuries & poisonings	Wald ratio	1	1.107 (0.941, 1.302)	0.222	0.917

Mechanical complication of unspecified genitourinary device, implant, and graft	Injuries & poisonings	Wald ratio	1	1.064 (0.872, 1.298)	0.539	0.981
Complication of internal orthopedic device	Injuries & poisonings	Wald ratio	1	0.962 (0.847, 1.091)	0.544	0.981
Complication due to other implant and internal device	Injuries & poisonings	Wald ratio	1	1.038 (0.918, 1.172)	0.553	0.981
Open wounds of head; neck; and trunk	Injuries & poisonings	Wald ratio	1	0.94 (0.839, 1.052)	0.28	0.923
Open wound or laceration of eye or eyelid	Injuries & poisonings	Wald ratio	1	1.054 (0.693, 1.605)	0.805	0.981
Open wound of ear	Injuries & poisonings	Wald ratio	1	0.935 (0.569, 1.537)	0.792	0.981
Other open wound of head and face	Injuries & poisonings	Wald ratio	1	0.906 (0.794, 1.032)	0.138	0.916
Open wound of nose and sinus	Injuries & poisonings	Wald ratio	1	0.879 (0.559, 1.381)	0.575	0.981
Open wound of neck	Injuries & poisonings	Wald ratio	1	0.977 (0.657, 1.454)	0.91	0.983
Open wounds of extremities	Injuries & poisonings	Wald ratio	1	1.104 (0.991, 1.231)	0.073	0.874
Open wound of hand except finger(s)	Injuries & poisonings	Wald ratio	1	1.14 (0.442, 2.945)	0.786	0.981
Open wound of finger(s)	Injuries & poisonings	Wald ratio	1	1.197 (0.581, 2.466)	0.626	0.981
Open wound of foot except toe(s) alone	Injuries & poisonings	Wald ratio	1	0.631 (0.367, 1.085)	0.096	0.874
Traumatic amputation	Injuries & poisonings	Wald ratio	1	1.197 (0.872, 1.642)	0.265	0.923
Broken tooth	Injuries & poisonings	Wald ratio	1	1.319 (0.839, 2.072)	0.23	0.917
Complication of amputation stump	Injuries & poisonings	Wald ratio	1	0.922 (0.536, 1.586)	0.771	0.981
Late effects of musculoskeletal and connective tissue injuries	Injuries & poisonings	Wald ratio	1	0.692 (0.402, 1.189)	0.182	0.916
Injuries to the nervous system	Injuries & poisonings	Wald ratio	1	1.143 (0.945, 1.382)	0.167	0.916
Superficial injury without mention of infection	Injuries & poisonings	Wald ratio	1	0.975 (0.875, 1.087)	0.647	0.981
Contusion	Injuries & poisonings	Wald ratio	1	0.979 (0.818, 1.173)	0.822	0.981
Allergic reaction to food	Injuries & poisonings	Wald ratio	1	1.143 (0.695, 1.879)	0.598	0.981
Dermatitis due to solar radiation	Injuries & poisonings	Wald ratio	1	1.062 (0.746, 1.51)	0.739	0.981
Chronic dermatitis due to solar radiation	Injuries & poisonings	Wald ratio	1	1.028 (0.663, 1.593)	0.902	0.983
Adverse reaction to serum or vaccine	Injuries & poisonings	Wald ratio	1	0.822 (0.523, 1.292)	0.395	0.974
Infusion and transfusion reaction	Injuries & poisonings	Wald ratio	1	0.914 (0.464, 1.8)	0.795	0.981
Anaphylactic shock NOS	Injuries & poisonings	Wald ratio	1	1.003 (0.745, 1.352)	0.982	0.991
Urticaria	Injuries & poisonings	Wald ratio	1	1.381 (0.962, 1.982)	0.08	0.874
Allergies, other	Injuries & poisonings	Wald ratio	1	1.019 (0.719, 1.442)	0.917	0.983
Spinal cord injury without evidence of spinal bone injury	Injuries & poisonings	Wald ratio	1	1.125 (0.801, 1.578)	0.497	0.974
Injury to other and unspecified nerves	Injuries & poisonings	Wald ratio	1	0.889 (0.584, 1.353)	0.583	0.981
Certain early complications of trauma or procedure	Injuries & poisonings	Wald ratio	1	0.962 (0.704, 1.313)	0.805	0.981
Postoperative shock	Injuries & poisonings	Wald ratio	1	0.389 (0.151, 1.004)	0.051	0.851
Traumatic and surgical subcutaneous emphysema	Injuries & poisonings	Wald ratio	1	1.863 (0.826, 4.202)	0.134	0.916
Poisoning by antibiotics	Injuries & poisonings	Wald ratio	1	0.982 (0.93, 1.036)	0.5	0.974
Allergy or adverse effect of penicillin	Injuries & poisonings	Wald ratio	1	0.977 (0.921, 1.036)	0.442	0.974
Poisoning by other anti-infectives	Injuries & poisonings	Wald ratio	1	1.122 (0.92, 1.369)	0.256	0.919
Poisoning or allergy of sulfonamides	Injuries & poisonings	Wald ratio	1	1.146 (0.906, 1.449)	0.257	0.919
Poisoning by hormones and synthetic substitutes	Injuries & poisonings	Wald ratio	1	1.172 (0.82, 1.675)	0.382	0.974
Hormones and synthetic substitutes causing adverse effects in therapeutic use	Injuries & poisonings	Wald ratio	1	1.125 (0.78, 1.622)	0.529	0.981
Poisoning by primarily systemic agents	Injuries & poisonings	Wald ratio	1	0.847 (0.539, 1.331)	0.472	0.974
Poisoning by agents primarily affecting blood constituents	Injuries & poisonings	Wald ratio	1	2.091 (1.361, 3.212)	7.56E-04	0.109
Anticoagulants causing adverse effects	Injuries & poisonings	Wald ratio	1	2.14 (1.387, 3.301)	5.87E-04	0.109
Poisoning by analgesics, antipyretics, and antirheumatics	Injuries & poisonings	Wald ratio	1	0.97 (0.891, 1.057)	0.494	0.974
Opiates and related narcotics causing adverse effects in therapeutic use	Injuries & poisonings	Wald ratio	1	0.92 (0.748, 1.133)	0.434	0.974
Antirheumatics causing adverse effects in therapeutic use	Injuries & poisonings	Wald ratio	1	0.776 (0.566, 1.065)	0.116	0.916
Salicylates causing adverse effects in therapeutic use	Injuries & poisonings	Wald ratio	1	0.885 (0.628, 1.248)	0.486	0.974
Poisoning by anticonvulsants and anti-Parkinsonism drugs	Injuries & poisonings	Wald ratio	1	0.741 (0.545, 1.008)	0.056	0.87
Adverse effects of sedatives or other central nervous system depressants and anesthetics	Injuries & poisonings	Wald ratio	1	1.319 (0.988, 1.761)	0.061	0.874
Poisoning by psychotropic agents	Injuries & poisonings	Wald ratio	1	0.906 (0.773, 1.061)	0.219	0.917
Poisoning by drugs primarily affecting the autonomic nervous system	Injuries & poisonings	Wald ratio	1	1.191 (0.725, 1.958)	0.49	0.974
Poisoning by agents primarily affecting the cardiovascular system	Injuries & poisonings	Wald ratio	1	0.887 (0.585, 1.344)	0.572	0.981
Antihypertensive agents causing adverse effects	Injuries & poisonings	Wald ratio	1	0.904 (0.419, 1.948)	0.796	0.981
Poisoning by agents primarily affecting the gastrointestinal system	Injuries & poisonings	Wald ratio	1	0.457 (0.232, 0.899)	0.023	0.692
Poisoning by water, mineral, and uric acid metabolism drugs	Injuries & poisonings	Wald ratio	1	0.616 (0.313, 1.214)	0.162	0.916
Poisoning by agents primarily acting on the smooth and skeletal muscles and respiratory system	Injuries & poisonings	Wald ratio	1	0.724 (0.307, 1.709)	0.461	0.974
Poisoning by agents primarily affecting skin & mucous membrane, ophthalmological, otorhinolaryngological, & dental drugs	Injuries & poisonings	Wald ratio	1	1.381 (0.559, 3.408)	0.484	0.974
Personal history of allergy to medicinal agents	Injuries & poisonings	Wald ratio	1	1.02 (0.649, 1.603)	0.931	0.986
Adverse drug events and drug allergies	Injuries & poisonings	Wald ratio	1	0.982 (0.756, 1.276)	0.893	0.983
Toxic effect of (non-ethyl) alcohol and petroleum and other solvents	Injuries & poisonings	Wald ratio	1	0.901 (0.548, 1.482)	0.682	0.981
Toxic effect of carbon monoxide	Injuries & poisonings	Wald ratio	1	0.776 (0.329, 1.831)	0.563	0.981
Toxic effect of other gases, fumes, or vapors	Injuries & poisonings	Wald ratio	1	1.186 (0.602, 2.336)	0.622	0.981
Toxic effect of noxious substances eaten as food	Injuries & poisonings	Wald ratio	1	1.289 (0.685, 2.426)	0.432	0.974
Toxic effect of other substances, chiefly nonmedicinal as to source	Injuries & poisonings	Wald ratio	1	0.724 (0.293, 1.788)	0.484	0.974
Effects radiation NOS	Injuries & poisonings	Wald ratio	1	0.97 (0.855, 1.101)	0.642	0.981
Delirium dementia and amnestic and other cognitive disorders	Mental disorders	Wald ratio	1	0.959 (0.819, 1.124)	0.607	0.981
Dementias	Mental disorders	Wald ratio	1	0.946 (0.755, 1.186)	0.631	0.981
Alzheimer's disease	Mental disorders	Wald ratio	1	1.045 (0.741, 1.473)	0.803	0.981
Vascular dementia	Mental disorders	Wald ratio	1	0.794 (0.483, 1.305)	0.363	0.97
Delirium due to conditions classified elsewhere	Mental disorders	Wald ratio	1	0.922 (0.703, 1.21)	0.56	0.981
Other persistent mental disorders due to conditions classified elsewhere	Mental disorders	Wald ratio	1	0.871 (0.386, 1.964)	0.739	0.981
Other specified nonpsychotic and or or transient mental disorders	Mental disorders	Wald ratio	1	1.112 (0.844, 1.465)	0.451	0.974
Transient mental disorders due to conditions classified elsewhere	Mental disorders	Wald ratio	1	1.446 (0.768, 2.722)	0.253	0.919
Specific nonpsychotic mental disorders due to brain damage	Mental disorders	Wald ratio	1	0.741 (0.344, 1.597)	0.444	0.974
Alteration of consciousness	Mental disorders	Wald ratio	1	1.094 (0.787, 1.521)	0.593	0.981
Neurological disorders	Mental disorders	Wald ratio	1	0.998 (0.9, 1.107)	0.971	0.991

Aphasia or speech disturbance	Mental disorders	Wald ratio	1	0.982 (0.819, 1.176)	0.841	0.981
Mild cognitive impairment	Mental disorders	Wald ratio	1	1.104 (0.672, 1.815)	0.696	0.981
Memory loss	Mental disorders	Wald ratio	1	1.021 (0.785, 1.327)	0.878	0.983
Altered mental status	Mental disorders	Wald ratio	1	0.986 (0.849, 1.144)	0.851	0.981
Hallucinations	Mental disorders	Wald ratio	1	1.059 (0.712, 1.576)	0.776	0.981
Symptoms involving head and neck	Mental disorders	Wald ratio	1	0.989 (0.871, 1.122)	0.858	0.981
Swelling, mass, or lump in head and neck [Space-occupying lesion, intracranial NOS]	Mental disorders	Wald ratio	1	0.959 (0.762, 1.208)	0.724	0.981
Schizophrenia and other psychotic disorders	Mental disorders	Wald ratio	1	0.881 (0.693, 1.119)	0.299	0.923
Schizophrenia	Mental disorders	Wald ratio	1	0.875 (0.655, 1.168)	0.365	0.97
Paranoid disorders	Mental disorders	Wald ratio	1	0.832 (0.484, 1.43)	0.505	0.974
Psychosis	Mental disorders	Wald ratio	1	1.127 (0.656, 1.939)	0.665	0.981
Mood disorders	Mental disorders	Wald ratio	1	1.006 (0.945, 1.072)	0.841	0.981
Bipolar	Mental disorders	Wald ratio	1	1.102 (0.891, 1.362)	0.372	0.972
Depression	Mental disorders	Wald ratio	1	0.992 (0.927, 1.062)	0.826	0.981
Major depressive disorder	Mental disorders	Wald ratio	1	0.855 (0.593, 1.233)	0.401	0.974
Suicidal ideation or attempt	Mental disorders	Wald ratio	1	1.094 (0.752, 1.592)	0.638	0.981
Suicide or self-inflicted injury	Mental disorders	Wald ratio	1	1.102 (0.747, 1.625)	0.625	0.981
Anxiety disorders	Mental disorders	Wald ratio	1	0.962 (0.882, 1.048)	0.371	0.972
Anxiety disorder	Mental disorders	Wald ratio	1	0.951 (0.868, 1.04)	0.271	0.923
Generalized anxiety disorder	Mental disorders	Wald ratio	1	1.082 (0.629, 1.86)	0.777	0.981
Agorophobia, social phobia, and panic disorder	Mental disorders	Wald ratio	1	0.815 (0.627, 1.059)	0.125	0.916
Phobia	Mental disorders	Wald ratio	1	0.927 (0.679, 1.266)	0.632	0.981
Obsessive-compulsive disorders	Mental disorders	Wald ratio	1	1.413 (0.785, 2.542)	0.249	0.919
Dysthymic disorder	Mental disorders	Wald ratio	1	0.676 (0.262, 1.745)	0.418	0.974
Acute reaction to stress	Mental disorders	Wald ratio	1	0.758 (0.403, 1.427)	0.391	0.974
Posttraumatic stress disorder	Mental disorders	Wald ratio	1	0.524 (0.279, 0.987)	0.046	0.792
Personality disorders	Mental disorders	Wald ratio	1	0.94 (0.643, 1.373)	0.748	0.981
Antisocial or borderline personality disorder	Mental disorders	Wald ratio	1	0.975 (0.518, 1.835)	0.937	0.986
Sexual and gender identity disorders	Mental disorders	Wald ratio	1	0.835 (0.566, 1.232)	0.364	0.97
Psychogenic and somatoform disorders	Mental disorders	Wald ratio	1	0.931 (0.688, 1.26)	0.644	0.981
Psychogenic disorder	Mental disorders	Wald ratio	1	0.957 (0.661, 1.386)	0.817	0.981
Somatoform disorder	Mental disorders	Wald ratio	1	0.805 (0.428, 1.516)	0.502	0.974
Adjustment reaction	Mental disorders	Wald ratio	1	0.758 (0.507, 1.134)	0.178	0.916
Eating disorder (no controls excluded)	Mental disorders	Wald ratio	1	0.631 (0.306, 1.299)	0.211	0.917
Eating disorder	Mental disorders	Wald ratio	1	0.631 (0.306, 1.299)	0.211	0.917
Other mental disorder	Mental disorders	Wald ratio	1	0.996 (0.953, 1.041)	0.846	0.981
Tension headache	Mental disorders	Wald ratio	1	0.853 (0.576, 1.264)	0.428	0.974
Pervasive developmental disorders	Mental disorders	Wald ratio	1	0.478 (0.222, 1.031)	0.06	0.874
Autism	Mental disorders	Wald ratio	1	0.457 (0.177, 1.179)	0.105	0.902
Develomental delays and disorders	Mental disorders	Wald ratio	1	1.023 (0.749, 1.398)	0.885	0.983
Learning disorder	Mental disorders	Wald ratio	1	1.079 (0.627, 1.856)	0.783	0.981
Speech and language disorder	Mental disorders	Wald ratio	1	0.589 (0.286, 1.213)	0.151	0.916
Substance addiction and disorders	Mental disorders	Wald ratio	1	1.349 (0.949, 1.919)	0.096	0.874
Polyneuropathy due to drugs	Mental disorders	Wald ratio	1	2.19 (0.887, 5.405)	0.089	0.874
Alcohol-related disorders	Mental disorders	Wald ratio	1	1.03 (0.967, 1.098)	0.353	0.962
Alcoholism	Mental disorders	Wald ratio	1	0.993 (0.92, 1.072)	0.86	0.981
Alcoholic liver damage	Mental disorders	Wald ratio	1	1.052 (0.824, 1.343)	0.684	0.981
Tobacco use disorder	Mental disorders	Wald ratio	1	0.983 (0.931, 1.038)	0.537	0.981
Osteomyelitis, periostitis, and other infections involving bone	Musculoskeletal	Wald ratio	1	1.074 (0.812, 1.421)	0.617	0.981
Osteomyelitis	Musculoskeletal	Wald ratio	1	1.084 (0.808, 1.454)	0.59	0.981
Chronic osteomyelitis	Musculoskeletal	Wald ratio	1	1.208 (0.735, 1.986)	0.456	0.974
Unspecified osteomyelitis	Musculoskeletal	Wald ratio	1	0.957 (0.676, 1.355)	0.805	0.981
Arthropathy associated with infections	Musculoskeletal	Wald ratio	1	0.922 (0.66, 1.289)	0.636	0.981
Pyogenic arthritis	Musculoskeletal	Wald ratio	1	0.931 (0.634, 1.367)	0.715	0.981
Infective connective tissue disorders	Musculoskeletal	Wald ratio	1	2.19 (1.016, 4.72)	0.046	0.792
Arthropathy associated with other disorders classified elsewhere	Musculoskeletal	Wald ratio	1	1.03 (0.599, 1.772)	0.914	0.983
Arthropathy associated with neurological disorders	Musculoskeletal	Wald ratio	1	1.069 (0.474, 2.411)	0.872	0.983
Rheumatoid arthritis and other inflammatory polyarthropathies	Musculoskeletal	Wald ratio	1	0.998 (0.903, 1.102)	0.967	0.991
Rheumatoid arthritis	Musculoskeletal	Wald ratio	1	1.026 (0.92, 1.143)	0.647	0.981
Other inflammatory spondylopathies	Musculoskeletal	Wald ratio	1	1.225 (1.032, 1.454)	0.021	0.688
Sacroiliitis NEC	Musculoskeletal	Wald ratio	1	0.851 (0.452, 1.602)	0.617	0.981
Ankylosing spondylitis	Musculoskeletal	Wald ratio	1	1.125 (0.85, 1.488)	0.411	0.974
Other arthropathies	Musculoskeletal	Wald ratio	1	0.987 (0.949, 1.027)	0.527	0.981
Unspecified polyarthropathy or polyarthritis	Musculoskeletal	Wald ratio	1	0.931 (0.828, 1.047)	0.233	0.917
Unspecified monoarthritis	Musculoskeletal	Wald ratio	1	0.962 (0.907, 1.02)	0.191	0.917
Arthropathy NOS	Musculoskeletal	Wald ratio	1	0.989 (0.95, 1.029)	0.585	0.981
Polymyalgia Rheumatica	Musculoskeletal	Wald ratio	1	1.013 (0.826, 1.241)	0.903	0.983
Spinal stenosis	Musculoskeletal	Wald ratio	1	1.014 (0.906, 1.136)	0.804	0.981
Spondylosis and allied disorders	Musculoskeletal	Wald ratio	1	1.045 (0.963, 1.133)	0.291	0.923
Spondylosis without myelopathy	Musculoskeletal	Wald ratio	1	1.008 (0.913, 1.114)	0.87	0.981
Spondylosis with myelopathy	Musculoskeletal	Wald ratio	1	0.708 (0.431, 1.163)	0.173	0.916
Other allied disorders of spine	Musculoskeletal	Wald ratio	1	1.248 (0.861, 1.807)	0.242	0.919
Intervertebral disc disorders	Musculoskeletal	Wald ratio	1	0.959 (0.892, 1.031)	0.261	0.923
Displacement of intervertebral disc	Musculoskeletal	Wald ratio	1	0.575 (0.423, 0.782)	4.16E-04	0.109
Degeneration of intervertebral disc	Musculoskeletal	Wald ratio	1	1.087 (0.953, 1.239)	0.214	0.917
Postlaminectomy syndrome	Musculoskeletal	Wald ratio	1	1.094 (0.485, 2.467)	0.828	0.981
Other and unspecified disc disorder	Musculoskeletal	Wald ratio	1	0.927 (0.835, 1.028)	0.151	0.916
Other disorders of cervical region	Musculoskeletal	Wald ratio	1	0.931 (0.688, 1.26)	0.644	0.981

Torticollis	Musculoskeletal	Wald ratio	1	0.66 (0.335, 1.301)	0.23	0.917
Other and unspecified disorders of back	Musculoskeletal	Wald ratio	1	1.097 (0.94, 1.279)	0.239	0.919
Disorders of sacrum	Musculoskeletal	Wald ratio	1	0.964 (0.684, 1.359)	0.833	0.981
Disorders of coccyx	Musculoskeletal	Wald ratio	1	0.933 (0.662, 1.315)	0.693	0.981
Other symptoms referable to back	Musculoskeletal	Wald ratio	1	1.699 (0.72, 4.009)	0.226	0.917
Other unspecified back disorders	Musculoskeletal	Wald ratio	1	1.114 (0.939, 1.323)	0.216	0.917
Peripheral enthesopathies and allied syndromes	Musculoskeletal	Wald ratio	1	1.003 (0.946, 1.064)	0.914	0.983
Enthesopathy	Musculoskeletal	Wald ratio	1	1.023 (0.951, 1.099)	0.544	0.981
Synoviopathy	Musculoskeletal	Wald ratio	1	1.233 (0.844, 1.803)	0.279	0.923
Bursitis	Musculoskeletal	Wald ratio	1	1.03 (0.815, 1.303)	0.803	0.981
Other disorders of synovium, tendon, and bursa	Musculoskeletal	Wald ratio	1	0.957 (0.882, 1.038)	0.291	0.923
Synovitis and tenosynovitis	Musculoskeletal	Wald ratio	1	0.853 (0.748, 0.972)	0.017	0.68
Bursitis disorders	Musculoskeletal	Wald ratio	1	0.807 (0.41, 1.589)	0.535	0.981
Ganglion and cyst of synovium, tendon, and bursa	Musculoskeletal	Wald ratio	1	1.064 (0.942, 1.202)	0.317	0.923
Rupture of synovium	Musculoskeletal	Wald ratio	1	1.003 (0.754, 1.333)	0.985	0.991
Rupture of tendon, nontraumatic	Musculoskeletal	Wald ratio	1	0.776 (0.536, 1.124)	0.18	0.916
Contracture of tendon (sheath)	Musculoskeletal	Wald ratio	1	1.052 (0.585, 1.893)	0.866	0.981
Disorders of muscle, ligament, and fascia	Musculoskeletal	Wald ratio	1	1.002 (0.903, 1.112)	0.968	0.991
Laxity of ligament or hypermobility syndrome	Musculoskeletal	Wald ratio	1	0.929 (0.662, 1.304)	0.67	0.981
Fasciitis	Musculoskeletal	Wald ratio	1	1 (0.894, 1.12)	0.993	0.996
Contracture of palmar fascia [Dupuytren's disease]	Musculoskeletal	Wald ratio	1	1.006 (0.895, 1.132)	0.914	0.983
Other disorders of soft tissues	Musculoskeletal	Wald ratio	1	0.998 (0.912, 1.092)	0.962	0.99
Rheumatism, unspecified and fibrositis	Musculoskeletal	Wald ratio	1	1.087 (0.871, 1.356)	0.463	0.974
Panniculitis	Musculoskeletal	Wald ratio	1	0.951 (0.483, 1.872)	0.883	0.983
Osteitis deformans and osteopathies associated with other disorders classified elsewhere	Musculoskeletal	Wald ratio	1	1.042 (0.688, 1.58)	0.845	0.981
Osteitis deformans [Paget's disease of bone]	Musculoskeletal	Wald ratio	1	0.973 (0.636, 1.487)	0.898	0.983
Osteochondropathies	Musculoskeletal	Wald ratio	1	1.191 (0.865, 1.642)	0.284	0.923
Juvenile osteochondrosis	Musculoskeletal	Wald ratio	1	1.319 (0.802, 2.167)	0.275	0.923
Osteochondritis dissecans	Musculoskeletal	Wald ratio	1	0.575 (0.233, 1.42)	0.23	0.917
Other disorders of bone and cartilage	Musculoskeletal	Wald ratio	1	0.978 (0.873, 1.095)	0.695	0.981
Cyst of bone	Musculoskeletal	Wald ratio	1	0.834 (0.565, 1.229)	0.358	0.963
Aseptic necrosis of bone	Musculoskeletal	Wald ratio	1	0.985 (0.731, 1.327)	0.919	0.984
Costochondritis	Musculoskeletal	Wald ratio	1	1.084 (0.576, 2.04)	0.803	0.981
Malunion and nonunion of fracture	Musculoskeletal	Wald ratio	1	0.966 (0.795, 1.173)	0.727	0.981
Chondromalacia	Musculoskeletal	Wald ratio	1	0.927 (0.492, 1.744)	0.814	0.981
Acquired foot deformities	Musculoskeletal	Wald ratio	1	1.018 (0.947, 1.094)	0.635	0.981
Flat foot	Musculoskeletal	Wald ratio	1	0.816 (0.497, 1.342)	0.424	0.974
Acquired toe deformities	Musculoskeletal	Wald ratio	1	1.084 (0.981, 1.197)	0.112	0.916
Hammer toe (acquired)	Musculoskeletal	Wald ratio	1	1.02 (0.871, 1.195)	0.801	0.981
Hallux rigidus	Musculoskeletal	Wald ratio	1	1.092 (0.915, 1.302)	0.33	0.944
Hallux valgus (Bunion)	Musculoskeletal	Wald ratio	1	0.942 (0.864, 1.026)	0.171	0.916
Other acquired deformities of limbs	Musculoskeletal	Wald ratio	1	0.834 (0.702, 0.99)	0.038	0.792
Acquired deformities of finger	Musculoskeletal	Wald ratio	1	0.813 (0.524, 1.26)	0.353	0.962
Curvature of spine	Musculoskeletal	Wald ratio	1	1.125 (0.914, 1.385)	0.268	0.923
Kyphoscoliosis and scoliosis	Musculoskeletal	Wald ratio	1	1.107 (0.895, 1.369)	0.349	0.962
Other acquired musculoskeletal deformity	Musculoskeletal	Wald ratio	1	1.191 (1.017, 1.396)	0.03	0.749
Acquired spondylolisthesis	Musculoskeletal	Wald ratio	1	1.194 (1.001, 1.424)	0.048	0.83
Contracture of joint	Musculoskeletal	Wald ratio	1	0.994 (0.728, 1.358)	0.971	0.991
Osteoarthrosis	Musculoskeletal	Wald ratio	1	1.004 (0.961, 1.05)	0.854	0.981
Osteoarthritis; localized	Musculoskeletal	Wald ratio	1	0.977 (0.926, 1.032)	0.405	0.974
Osteoarthrosis, localized, primary	Musculoskeletal	Wald ratio	1	1.009 (0.935, 1.09)	0.814	0.981
Osteoarthrosis, localized, secondary	Musculoskeletal	Wald ratio	1	0.883 (0.61, 1.279)	0.51	0.978
Osteoarthrosis, generalized	Musculoskeletal	Wald ratio	1	1.211 (0.911, 1.61)	0.188	0.917
Osteoarthrosis NOS	Musculoskeletal	Wald ratio	1	1.033 (0.969, 1.1)	0.317	0.923
Symptoms and disorders of the joints	Musculoskeletal	Wald ratio	1	0.922 (0.824, 1.033)	0.162	0.916
Ankylosis of joint	Musculoskeletal	Wald ratio	1	0.575 (0.292, 1.133)	0.11	0.916
Stiffness of joint	Musculoskeletal	Wald ratio	1	0.822 (0.621, 1.088)	0.17	0.916
Difficulty in walking	Musculoskeletal	Wald ratio	1	1.17 (0.68, 2.011)	0.571	0.981
Joint effusions	Musculoskeletal	Wald ratio	1	1.04 (0.864, 1.252)	0.678	0.981
Hemarthrosis	Musculoskeletal	Wald ratio	1	1.094 (0.556, 2.155)	0.795	0.981
Villonodular synovitis	Musculoskeletal	Wald ratio	1	0.776 (0.301, 2.004)	0.6	0.981
Derangement of joint, non-traumatic	Musculoskeletal	Wald ratio	1	0.968 (0.849, 1.104)	0.629	0.981
Loose body in joint	Musculoskeletal	Wald ratio	1	0.794 (0.543, 1.161)	0.234	0.917
Pathological, developmental or recurrent dislocation	Musculoskeletal	Wald ratio	1	1.349 (0.655, 2.78)	0.417	0.974
Articular cartilage disorder	Musculoskeletal	Wald ratio	1	1.2 (0.878, 1.639)	0.252	0.919
Other derangement of joint	Musculoskeletal	Wald ratio	1	0.937 (0.8, 1.098)	0.424	0.974
Osteoporosis, osteopenia and pathological fracture	Musculoskeletal	Wald ratio	1	0.94 (0.866, 1.019)	0.134	0.916
Osteoporosis	Musculoskeletal	Wald ratio	1	0.946 (0.864, 1.036)	0.23	0.917
Osteoporosis NOS	Musculoskeletal	Wald ratio	1	0.975 (0.887, 1.072)	0.6	0.981
Other specified osteoporosis	Musculoskeletal	Wald ratio	1	0.776 (0.573, 1.05)	0.101	0.894
Pathologic fracture	Musculoskeletal	Wald ratio	1	0.834 (0.613, 1.133)	0.245	0.919
Osteopenia or other disorder of bone and cartilage	Musculoskeletal	Wald ratio	1	0.948 (0.743, 1.21)	0.67	0.981
Pain in joint	Musculoskeletal	Wald ratio	1	1.006 (0.932, 1.087)	0.869	0.981
Cancer of mouth	Neoplasms	Wald ratio	1	1.259 (0.96, 1.651)	0.096	0.874
Cancer of tongue	Neoplasms	Wald ratio	1	1.127 (0.761, 1.67)	0.55	0.981
Cancer of major salivary glands	Neoplasms	Wald ratio	1	0.741 (0.376, 1.459)	0.386	0.974
Cancer of the mouth floor	Neoplasms	Wald ratio	1	0.758 (0.294, 1.958)	0.568	0.981
Cancer of larynx, pharynx, nasal cavities	Neoplasms	Wald ratio	1	1.104 (0.838, 1.455)	0.481	0.974

Cancer of oropharynx	Neoplasms	Wald ratio	1	0.955 (0.613, 1.487)	0.838	0.981
Cancer of larynx	Neoplasms	Wald ratio	1	1.259 (0.816, 1.943)	0.298	0.923
Cancer of of nasal cavities	Neoplasms	Wald ratio	1	0.893 (0.362, 2.205)	0.806	0.981
Cancer of esophagus	Neoplasms	Wald ratio	1	1.026 (0.793, 1.327)	0.847	0.981
Cancer of stomach	Neoplasms	Wald ratio	1	1.514 (1.129, 2.031)	0.006	0.376
Colorectal cancer	Neoplasms	Wald ratio	1	0.994 (0.896, 1.102)	0.903	0.983
Colon cancer	Neoplasms	Wald ratio	1	1.015 (0.894, 1.152)	0.819	0.981
Malignant neoplasm of rectum, rectosigmoid junction, and anus	Neoplasms	Wald ratio	1	0.97 (0.832, 1.132)	0.702	0.981
Cancer of liver and intrahepatic bile duct	Neoplasms	Wald ratio	1	0.853 (0.586, 1.241)	0.406	0.974
Malignant neoplasm of liver, primary	Neoplasms	Wald ratio	1	0.489 (0.272, 0.881)	0.017	0.68
Pancreatic cancer	Neoplasms	Wald ratio	1	0.841 (0.633, 1.118)	0.234	0.917
Neoplasm of unspecified nature of digestive system	Neoplasms	Wald ratio	1	0.955 (0.772, 1.181)	0.67	0.981
Malignant neoplasm of other and ill-defined sites within the digestive organs and peritoneum	Neoplasms	Wald ratio	1	1.047 (0.952, 1.151)	0.341	0.954
Malignant neoplasm of small intestine, including duodenum	Neoplasms	Wald ratio	1	0.708 (0.431, 1.163)	0.173	0.916
Malignant neoplasm of gallbladder and extrahepatic bile ducts	Neoplasms	Wald ratio	1	1.026 (0.624, 1.686)	0.92	0.984
Malignant neoplasm of retroperitoneum and peritoneum	Neoplasms	Wald ratio	1	1.03 (0.627, 1.694)	0.906	0.983
Cancer of intrathoracic organs	Neoplasms	Wald ratio	1	1.699 (0.688, 4.194)	0.25	0.919
Cancer within the respiratory system	Neoplasms	Wald ratio	1	1.054 (0.921, 1.207)	0.443	0.974
Cancer of bronchus; lung	Neoplasms	Wald ratio	1	1.087 (0.932, 1.267)	0.29	0.923
Cancer of bone and connective tissue	Neoplasms	Wald ratio	1	1.349 (0.966, 1.885)	0.079	0.874
Bone cancer	Neoplasms	Wald ratio	1	1.446 (0.768, 2.722)	0.253	0.919
Cancer of connective tissue	Neoplasms	Wald ratio	1	1.319 (0.89, 1.954)	0.168	0.916
Skin cancer	Neoplasms	Wald ratio	1	0.999 (0.938, 1.065)	0.987	0.992
Melanomas of skin, dx or hx	Neoplasms	Wald ratio	1	1.05 (0.917, 1.202)	0.484	0.974
Melanomas of skin	Neoplasms	Wald ratio	1	1.05 (0.917, 1.202)	0.484	0.974
Other non-epithelial cancer of skin	Neoplasms	Wald ratio	1	0.984 (0.92, 1.053)	0.646	0.981
Carcinoma in situ of skin	Neoplasms	Wald ratio	1	0.853 (0.65, 1.119)	0.25	0.919
Neoplasm of uncertain behavior of skin	Neoplasms	Wald ratio	1	0.865 (0.526, 1.422)	0.567	0.981
Breast cancer	Neoplasms	Wald ratio	1	0.989 (0.929, 1.054)	0.737	0.981
Breast cancer [female]	Neoplasms	Wald ratio	1	0.982 (0.922, 1.047)	0.582	0.981
Malignant neoplasm of female breast	Neoplasms	Wald ratio	1	0.975 (0.911, 1.043)	0.463	0.974
Neoplasm of uncertain behavior of breast	Neoplasms	Wald ratio	1	1.66 (0.882, 3.125)	0.116	0.916
Acquired absence of breast	Neoplasms	Wald ratio	1	0.973 (0.842, 1.124)	0.708	0.981
Cervical cancer and dysplasia	Neoplasms	Wald ratio	1	0.959 (0.853, 1.079)	0.489	0.974
Cervical cancer	Neoplasms	Wald ratio	1	0.948 (0.799, 1.126)	0.545	0.981
Cervical intraepithelial neoplasia [CIN] [Cervical dysplasia]	Neoplasms	Wald ratio	1	0.975 (0.836, 1.137)	0.746	0.981
Malignant neoplasm of uterus	Neoplasms	Wald ratio	1	1.089 (0.897, 1.323)	0.39	0.974
Cancer of other female genital organs	Neoplasms	Wald ratio	1	0.929 (0.808, 1.069)	0.302	0.923
Malignant neoplasm of ovary and other uterine adnexa	Neoplasms	Wald ratio	1	0.908 (0.778, 1.058)	0.217	0.917
Malignant neoplasm of ovary	Neoplasms	Wald ratio	1	0.908 (0.778, 1.058)	0.217	0.917
Cancer of other female genital organs (excluding uterus and ovary)	Neoplasms	Wald ratio	1	0.984 (0.658, 1.471)	0.937	0.986
Malignant neoplasm of testis	Neoplasms	Wald ratio	1	1.007 (0.899, 1.127)	0.908	0.983
Neoplasm of uncertain behavior of male genital organs	Neoplasms	Wald ratio	1	1.259 (0.639, 2.48)	0.505	0.974
Cancer of urinary organs (incl. kidney and bladder)	Neoplasms	Wald ratio	1	0.944 (0.847, 1.052)	0.298	0.923
Cancer of kidney and renal pelvis	Neoplasms	Wald ratio	1	0.964 (0.776, 1.197)	0.739	0.981
Malignant neoplasm of kidney, except pelvis	Neoplasms	Wald ratio	1	0.955 (0.765, 1.192)	0.683	0.981
Malignant neoplasm of renal pelvis	Neoplasms	Wald ratio	1	1.319 (0.559, 3.111)	0.528	0.981
Cancer of bladder	Neoplasms	Wald ratio	1	0.946 (0.819, 1.093)	0.453	0.974
Malignant neoplasm of bladder	Neoplasms	Wald ratio	1	0.94 (0.806, 1.096)	0.427	0.974
Malignant neoplasm of other urinary organs	Neoplasms	Wald ratio	1	0.964 (0.536, 1.734)	0.902	0.983
Cancer of eye	Neoplasms	Wald ratio	1	0.794 (0.462, 1.366)	0.405	0.974
Manlignant and unknown neoplasms of brain and nervous system	Neoplasms	Wald ratio	1	1.084 (0.827, 1.422)	0.56	0.981
Cancer of brain and nervous system	Neoplasms	Wald ratio	1	1.092 (0.806, 1.477)	0.571	0.981
Cancer of brain	Neoplasms	Wald ratio	1	1.064 (0.776, 1.46)	0.7	0.981
Thyroid cancer	Neoplasms	Wald ratio	1	0.877 (0.608, 1.264)	0.482	0.974
Cancer of other endocrine glands	Neoplasms	Wald ratio	1	0.489 (0.238, 1.008)	0.053	0.858
Cancer, suspected or other	Neoplasms	Wald ratio	1	0.989 (0.937, 1.045)	0.701	0.981
Malignant neoplasm, other	Neoplasms	Wald ratio	1	0.989 (0.937, 1.044)	0.683	0.981
Chemotherapy	Neoplasms	Wald ratio	1	1.005 (0.956, 1.056)	0.856	0.981
Secondary malignant neoplasm	Neoplasms	Wald ratio	1	0.99 (0.921, 1.064)	0.788	0.981
Secondary malignancy of lymph nodes	Neoplasms	Wald ratio	1	1.01 (0.919, 1.111)	0.834	0.981
Secondary malignancy of respiratory organs	Neoplasms	Wald ratio	1	0.962 (0.828, 1.116)	0.606	0.981
Secondary malignant neoplasm of digestive systems	Neoplasms	Wald ratio	1	0.977 (0.819, 1.165)	0.798	0.981
Secondary malignant neoplasm of liver	Neoplasms	Wald ratio	1	0.916 (0.8, 1.049)	0.205	0.917
Secondary malignancy of brain or spine	Neoplasms	Wald ratio	1	1.077 (0.843, 1.374)	0.553	0.981
Secondary malignancy of bone	Neoplasms	Wald ratio	1	1.164 (1.003, 1.352)	0.046	0.792
Secondary malignant neoplasm of skin	Neoplasms	Wald ratio	1	0.998 (0.607, 1.641)	0.994	0.996
Neoplasm of uncertain behavior	Neoplasms	Wald ratio	1	1.17 (0.972, 1.408)	0.097	0.876
Neurofibromatosis	Neoplasms	Wald ratio	1	1.48 (0.718, 3.049)	0.288	0.923
Myeloproliferative disease	Neoplasms	Wald ratio	1	0.891 (0.714, 1.112)	0.308	0.923
Polycythemia vera	Neoplasms	Wald ratio	1	0.776 (0.546, 1.104)	0.158	0.916
Hodgkin's disease	Neoplasms	Wald ratio	1	1.251 (0.81, 1.93)	0.312	0.923
Cancer of other lymphoid, histiocytic tissue	Neoplasms	Wald ratio	1	1.045 (0.904, 1.207)	0.553	0.981
Non-Hodgkins lymphoma	Neoplasms	Wald ratio	1	1.033 (0.878, 1.215)	0.697	0.981
Nodular lymphoma	Neoplasms	Wald ratio	1	1.01 (0.704, 1.45)	0.957	0.989
Large cell lymphoma	Neoplasms	Wald ratio	1	1.033 (0.773, 1.379)	0.827	0.981
Leukemia	Neoplasms	Wald ratio	1	1.178 (0.992, 1.398)	0.062	0.874
Lymphoid leukemia	Neoplasms	Wald ratio	1	0.962 (0.72, 1.284)	0.791	0.981

Lymphoid leukemia, acute	Neoplasms	Wald ratio	1	0.631 (0.255, 1.557)	0.317	0.923
Lymphoid leukemia, chronic	Neoplasms	Wald ratio	1	0.989 (0.727, 1.344)	0.941	0.986
Myeloid leukemia	Neoplasms	Wald ratio	1	1.211 (0.879, 1.669)	0.242	0.919
Myeloid leukemia, acute	Neoplasms	Wald ratio	1	1.035 (0.702, 1.527)	0.862	0.981
Myeloid leukemia, chronic	Neoplasms	Wald ratio	1	1.623 (0.944, 2.79)	0.08	0.874
Multiple myeloma	Neoplasms	Wald ratio	1	1.319 (0.983, 1.769)	0.065	0.874
Benign neoplasm of colon	Neoplasms	Wald ratio	1	1.033 (0.983, 1.085)	0.203	0.917
Benign neoplasm of lip, oral cavity, and pharynx	Neoplasms	Wald ratio	1	1.016 (0.814, 1.267)	0.89	0.983
Benign neoplasm of other parts of digestive system	Neoplasms	Wald ratio	1	0.99 (0.901, 1.089)	0.841	0.981
Benign neoplasm of respiratory and intrathoracic organs	Neoplasms	Wald ratio	1	1.154 (0.796, 1.671)	0.45	0.974
Benign neoplasm of bone and articular cartilage	Neoplasms	Wald ratio	1	0.794 (0.538, 1.171)	0.245	0.919
Lipoma	Neoplasms	Wald ratio	1	0.991 (0.909, 1.08)	0.833	0.981
Lipoma of skin and subcutaneous tissue	Neoplasms	Wald ratio	1	1.008 (0.908, 1.118)	0.882	0.983
Other benign neoplasm of connective and other soft tissue	Neoplasms	Wald ratio	1	1.586 (1.288, 1.952)	1.37E-05	0.017
Benign neoplasm of skin	Neoplasms	Wald ratio	1	1.022 (0.942, 1.108)	0.605	0.981
Vascular hamartomas and non-neoplastic nevi	Neoplasms	Wald ratio	1	1.045 (0.797, 1.37)	0.751	0.981
Nevus, non-neoplastic	Neoplasms	Wald ratio	1	1.092 (0.821, 1.451)	0.546	0.981
Benign neoplasm of uterus	Neoplasms	Wald ratio	1	1.026 (0.958, 1.098)	0.463	0.974
Uterine leiomyoma	Neoplasms	Wald ratio	1	1.028 (0.956, 1.105)	0.453	0.974
Other benign neoplasm of uterus	Neoplasms	Wald ratio	1	0.879 (0.607, 1.273)	0.495	0.974
Benign neoplasm of ovary	Neoplasms	Wald ratio	1	0.99 (0.827, 1.187)	0.916	0.983
Benign neoplasm of other female genital organs	Neoplasms	Wald ratio	1	0.875 (0.557, 1.374)	0.562	0.981
Benign neoplasm of kidney and other urinary organs	Neoplasms	Wald ratio	1	1.208 (0.702, 2.077)	0.494	0.974
Benign neoplasm of eye	Neoplasms	Wald ratio	1	1.381 (0.734, 2.599)	0.317	0.923
Benign neoplasm of eye, uveal	Neoplasms	Wald ratio	1	2.757 (1.169, 6.505)	0.021	0.688
Benign neoplasm of brain and other parts of nervous system	Neoplasms	Wald ratio	1	0.966 (0.76, 1.227)	0.777	0.981
Benign neoplasm of brain, cranial nerves, meninges	Neoplasms	Wald ratio	1	0.99 (0.772, 1.269)	0.938	0.986
Benign neoplasm of spinal cord, meninges	Neoplasms	Wald ratio	1	0.645 (0.261, 1.593)	0.342	0.954
Benign neoplasm of thyroid glands	Neoplasms	Wald ratio	1	0.692 (0.458, 1.043)	0.079	0.874
Benign neoplasm of other endocrine glands and related structures	Neoplasms	Wald ratio	1	0.741 (0.586, 0.937)	0.012	0.564
Benign neoplasm of adrenal gland	Neoplasms	Wald ratio	1	0.776 (0.472, 1.276)	0.317	0.923
Benign neoplasm of parathyroid gland	Neoplasms	Wald ratio	1	0.724 (0.505, 1.039)	0.08	0.874
Benign neoplasm of pituitary gland and craniopharyngeal duct (pouch)	Neoplasms	Wald ratio	1	0.708 (0.478, 1.048)	0.085	0.874
Hemangioma and lymphangioma, any site	Neoplasms	Wald ratio	1	1.052 (0.886, 1.249)	0.563	0.981
Benign neoplasm of unspecified sites	Neoplasms	Wald ratio	1	0.962 (0.836, 1.106)	0.583	0.981
Bone marrow or stem cell transplant	Neoplasms	Wald ratio	1	1.117 (0.518, 2.408)	0.778	0.981
Meningitis	Neurological	Wald ratio	1	0.867 (0.623, 1.205)	0.396	0.974
Encephalitis	Neurological	Wald ratio	1	0.676 (0.464, 0.983)	0.041	0.792
Acute (transverse) myelitis	Neurological	Wald ratio	1	0.257 (0.114, 0.579)	0.001	0.121
Other CNS infection and poliomyelitis	Neurological	Wald ratio	1	0.951 (0.633, 1.427)	0.807	0.981
Sleep disorders	Neurological	Wald ratio	1	1.045 (0.946, 1.154)	0.388	0.974
Sleep apnea	Neurological	Wald ratio	1	1.064 (0.959, 1.181)	0.24	0.919
Insomnia	Neurological	Wald ratio	1	0.692 (0.402, 1.189)	0.182	0.916
Organic or persistent insomnia	Neurological	Wald ratio	1	0.92 (0.512, 1.656)	0.782	0.981
Other cerebral degenerations	Neurological	Wald ratio	1	1.005 (0.817, 1.238)	0.96	0.99
Hydrocephalus	Neurological	Wald ratio	1	1.194 (0.874, 1.631)	0.264	0.923
Cerebral degeneration, unspecified	Neurological	Wald ratio	1	0.837 (0.597, 1.175)	0.305	0.923
Parkinson's disease	Neurological	Wald ratio	1	0.724 (0.588, 0.891)	0.002	0.175
Extrapyramidal disease and abnormal movement disorders	Neurological	Wald ratio	1	0.893 (0.709, 1.125)	0.337	0.952
Essential tremor	Neurological	Wald ratio	1	0.875 (0.557, 1.374)	0.562	0.981
Myoclonus	Neurological	Wald ratio	1	1.446 (0.702, 2.979)	0.317	0.923
Torsion dystonia	Neurological	Wald ratio	1	1.062 (0.685, 1.646)	0.789	0.981
Degenerative disease of the spinal cord	Neurological	Wald ratio	1	0.964 (0.819, 1.134)	0.657	0.981
Spinocerebellar disease	Neurological	Wald ratio	1	0.881 (0.468, 1.658)	0.694	0.981
Anterior horn cell disease	Neurological	Wald ratio	1	1.259 (0.801, 1.978)	0.317	0.923
Multiple sclerosis	Neurological	Wald ratio	1	0.794 (0.657, 0.96)	0.017	0.68
Disorders of the autonomic nervous system	Neurological	Wald ratio	1	1.04 (0.68, 1.59)	0.856	0.981
Peripheral autonomic neuropathy	Neurological	Wald ratio	1	1.349 (0.598, 3.043)	0.47	0.974
Acute pain	Neurological	Wald ratio	1	0.929 (0.656, 1.315)	0.678	0.981
Chronic pain	Neurological	Wald ratio	1	1.514 (1.06, 2.164)	0.023	0.688
Other headache syndromes	Neurological	Wald ratio	1	0.97 (0.895, 1.053)	0.47	0.974
Migraine	Neurological	Wald ratio	1	0.964 (0.845, 1.099)	0.581	0.981
Migrain with aura	Neurological	Wald ratio	1	0.822 (0.523, 1.292)	0.395	0.974
Other demyelinating diseases of central nervous system	Neurological	Wald ratio	1	0.809 (0.524, 1.248)	0.338	0.952
Hemiplegia	Neurological	Wald ratio	1	0.997 (0.832, 1.194)	0.972	0.991
Infantile cerebral palsy	Neurological	Wald ratio	1	1.189 (0.661, 2.139)	0.564	0.981
Other paralytic syndromes	Neurological	Wald ratio	1	1.035 (0.789, 1.357)	0.803	0.981
Epilepsy, recurrent seizures, convulsions	Neurological	Wald ratio	1	1.067 (0.966, 1.178)	0.203	0.917
Epilepsy	Neurological	Wald ratio	1	1.089 (0.865, 1.371)	0.468	0.974
Generalized convulsive epilepsy	Neurological	Wald ratio	1	1.159 (0.833, 1.612)	0.381	0.974
Partial epilepsy	Neurological	Wald ratio	1	1.289 (0.85, 1.953)	0.232	0.917
Convulsions	Neurological	Wald ratio	1	1.04 (0.896, 1.207)	0.606	0.981
Abnormal findings on study of brain and or or nervous system	Neurological	Wald ratio	1	1.062 (0.646, 1.745)	0.813	0.981
Nonspecific abnormal findings on radiological and other examination of skull and head	Neurological	Wald ratio	1	0.776 (0.431, 1.396)	0.397	0.974
Other conditions of brain	Neurological	Wald ratio	1	1.012 (0.841, 1.218)	0.897	0.983
Cerebral edema and compression of brain	Neurological	Wald ratio	1	0.692 (0.448, 1.067)	0.096	0.874
Cerebral cysts	Neurological	Wald ratio	1	1.259 (0.7, 2.266)	0.442	0.974
Coma	Neurological	Wald ratio	1	0.92 (0.638, 1.327)	0.657	0.981

Encephalopathy, not elsewhere classified	Neurological	Wald ratio	1	1.214 (0.772, 1.907)	0.401	0.974
Other conditions of brain, NOS	Neurological	Wald ratio	1	1.069 (0.752, 1.521)	0.71	0.981
Other and unspecified disorders of the nervous system	Neurological	Wald ratio	1	0.91 (0.623, 1.33)	0.625	0.981
Abnormal movement	Neurological	Wald ratio	1	1.052 (0.923, 1.199)	0.448	0.974
Abnormal involuntary movements	Neurological	Wald ratio	1	1.094 (0.869, 1.378)	0.444	0.974
Abnormality of gait	Neurological	Wald ratio	1	1.03 (0.868, 1.223)	0.732	0.981
Lack of coordination	Neurological	Wald ratio	1	1.069 (0.741, 1.542)	0.72	0.981
Disturbances of sensation of smell and taste	Neurological	Wald ratio	1	0.602 (0.233, 1.555)	0.295	0.923
Other peripheral nerve disorders	Neurological	Wald ratio	1	1.069 (1.004, 1.139)	0.038	0.792
Disorders of other cranial nerves	Neurological	Wald ratio	1	1.117 (0.928, 1.344)	0.242	0.919
Trigeminal nerve disorders [CN5]	Neurological	Wald ratio	1	1.514 (1.099, 2.087)	0.011	0.564
Facial nerve disorders [CN7]	Neurological	Wald ratio	1	0.951 (0.752, 1.202)	0.672	0.981
Nerve root and plexus disorders	Neurological	Wald ratio	1	0.875 (0.711, 1.077)	0.207	0.917
Nerve plexus lesions	Neurological	Wald ratio	1	0.501 (0.266, 0.943)	0.032	0.756
Nerve root lesions	Neurological	Wald ratio	1	0.195 (0.075, 0.503)	7.22E-04	0.109
Hereditary and idiopathic peripheral neuropathy	Neurological	Wald ratio	1	0.631 (0.367, 1.085)	0.096	0.874
Inflammatory and toxic neuropathy	Neurological	Wald ratio	1	1.007 (0.833, 1.218)	0.941	0.986
Myoneural disorders	Neurological	Wald ratio	1	0.959 (0.611, 1.507)	0.857	0.981
Myasthenia gravis	Neurological	Wald ratio	1	0.916 (0.557, 1.506)	0.73	0.981
Muscular dystrophies and other myopathies	Neurological	Wald ratio	1	0.931 (0.676, 1.283)	0.662	0.981
Muscular dystrophies	Neurological	Wald ratio	1	1.077 (0.598, 1.937)	0.806	0.981
Myopathy	Neurological	Wald ratio	1	0.946 (0.656, 1.364)	0.767	0.981
Miscarriage; stillbirth	Pregnancy complications	Wald ratio	1	0.957 (0.867, 1.057)	0.388	0.974
Missed abortion or Hydatidiform mole	Pregnancy complications	Wald ratio	1	1.028 (0.843, 1.254)	0.785	0.981
Ectopic pregnancy	Pregnancy complications	Wald ratio	1	1.109 (0.756, 1.629)	0.597	0.981
Hemorrhage during pregnancy; childbirth and postpartum	Pregnancy complications	Wald ratio	1	0.891 (0.757, 1.049)	0.165	0.916
Antepartum hemorrhage, abruptio placentae, and placenta previa	Pregnancy complications	Wald ratio	1	1.042 (0.82, 1.324)	0.734	0.981
Placenta previa and abruptio placenta	Pregnancy complications	Wald ratio	1	0.828 (0.675, 1.014)	0.068	0.874
Early or threatened labor; hemorrhage in early pregnancy	Pregnancy complications	Wald ratio	1	0.847 (0.733, 0.979)	0.024	0.706
Early onset of delivery	Pregnancy complications	Wald ratio	1	0.758 (0.571, 1.008)	0.057	0.87
Hemorrhage in early pregnancy	Pregnancy complications	Wald ratio	1	0.758 (0.611, 0.942)	0.012	0.564
Other high-risk pregnancy	Pregnancy complications	Wald ratio	1	1.289 (0.571, 2.906)	0.541	0.981
Hypertension complicating pregnancy, childbirth, and the puerperium	Pregnancy complications	Wald ratio	1	0.998 (0.807, 1.234)	0.983	0.991
Preeclampsia and eclampsia	Pregnancy complications	Wald ratio	1	0.997 (0.67, 1.484)	0.99	0.995
Excessive vomiting in pregnancy	Pregnancy complications	Wald ratio	1	0.901 (0.574, 1.416)	0.653	0.981
Hyperemesis gravidarum	Pregnancy complications	Wald ratio	1	0.865 (0.503, 1.487)	0.6	0.981
Late pregnancy and failed induction	Pregnancy complications	Wald ratio	1	0.873 (0.712, 1.07)	0.19	0.917
Other complications of pregnancy NEC	Pregnancy complications	Wald ratio	1	0.925 (0.793, 1.078)	0.317	0.923
Infectious and parasitic complications affecting pregnancy	Pregnancy complications	Wald ratio	1	0.867 (0.607, 1.239)	0.433	0.974
Infections of genitourinary tract during pregnancy	Pregnancy complications	Wald ratio	1	0.914 (0.6, 1.391)	0.675	0.981
Other conditions or status of the mother complicating pregnancy, childbirth, or the puerperium	Pregnancy complications	Wald ratio	1	1.04 (0.696, 1.555)	0.849	0.981
Diabetes or abnormal glucose tolerance complicating pregnancy	Pregnancy complications	Wald ratio	1	0.692 (0.421, 1.137)	0.146	0.916
Normal delivery	Pregnancy complications	Wald ratio	1	0.933 (0.793, 1.098)	0.405	0.974
Multiple gestation	Pregnancy complications	Wald ratio	1	0.97 (0.667, 1.412)	0.876	0.983
Problems associated with amniotic cavity and membranes	Pregnancy complications	Wald ratio	1	0.975 (0.803, 1.184)	0.798	0.981
Other and unspecified complications of birth; puerperium affecting management of mother	Pregnancy complications	Wald ratio	1	0.589 (0.327, 1.059)	0.077	0.874
Known or suspected fetal abnormality affecting management of mother	Pregnancy complications	Wald ratio	1	0.927 (0.828, 1.038)	0.187	0.917
Other perinatal conditions of fetus or newborn	Pregnancy complications	Wald ratio	1	1.117 (0.567, 2.2)	0.749	0.981
Fetal distress and abnormal forces of labor	Pregnancy complications	Wald ratio	1	0.931 (0.798, 1.086)	0.362	0.968
Umbilical cord complications during labor and delivery	Pregnancy complications	Wald ratio	1	0.935 (0.688, 1.272)	0.67	0.981
Obstetrical or birth trauma	Pregnancy complications	Wald ratio	1	1.01 (0.906, 1.126)	0.855	0.981
Complications of labor and delivery NEC	Pregnancy complications	Wald ratio	1	0.94 (0.81, 1.091)	0.413	0.974
Venous or cerebrovascular complications embolism in pregnancy and the puerperium	Pregnancy complications	Wald ratio	1	1.205 (0.701, 2.073)	0.5	0.974
Other complications of the puerperium NEC	Pregnancy complications	Wald ratio	1	0.708 (0.459, 1.092)	0.118	0.916
Other disorders of the breast associated with childbirth and disorders of lactation	Pregnancy complications	Wald ratio	1	0.19 (0.077, 0.47)	3.18E-04	0.109
Asphyxia and hypoxemia	Respiratory	Wald ratio	1	0.83 (0.528, 1.304)	0.418	0.974
Acute sinusitis	Respiratory	Wald ratio	1	1.054 (0.642, 1.733)	0.834	0.981
Acute upper respiratory infections of multiple or unspecified sites	Respiratory	Wald ratio	1	1.109 (0.96, 1.282)	0.16	0.916
Acute pharyngitis	Respiratory	Wald ratio	1	1.194 (0.944, 1.51)	0.139	0.916
Acute laryngitis and tracheitis	Respiratory	Wald ratio	1	0.758 (0.441, 1.304)	0.317	0.923
Septal Deviations or Turbinate Hypertrophy	Respiratory	Wald ratio	1	1.05 (0.95, 1.159)	0.34	0.954
Nasal polyps	Respiratory	Wald ratio	1	1.028 (0.91, 1.161)	0.657	0.981
Chronic pharyngitis and nasopharyngitis	Respiratory	Wald ratio	1	1.248 (0.995, 1.564)	0.055	0.87
Diseases of the larynx and vocal cords	Respiratory	Wald ratio	1	1.069 (0.934, 1.224)	0.334	0.952
Chronic laryngitis	Respiratory	Wald ratio	1	1.03 (0.523, 2.029)	0.931	0.986
Paralysis or spasm of vocal cords or larynx	Respiratory	Wald ratio	1	0.933 (0.61, 1.427)	0.75	0.981
Voice disturbance	Respiratory	Wald ratio	1	1.164 (0.942, 1.44)	0.16	0.916
Acute and chronic tonsillitis	Respiratory	Wald ratio	1	1.104 (0.947, 1.288)	0.206	0.917
Acute tonsillitis	Respiratory	Wald ratio	1	1.233 (0.932, 1.632)	0.142	0.916
Chronic tonsillitis and adenoiditis	Respiratory	Wald ratio	1	1.04 (0.845, 1.28)	0.712	0.981
Chronic sinusitis	Respiratory	Wald ratio	1	0.889 (0.776, 1.018)	0.089	0.874
Allergic rhinitis	Respiratory	Wald ratio	1	1.211 (0.979, 1.497)	0.077	0.874
Epistaxis or throat hemorrhage	Respiratory	Wald ratio	1	1.087 (0.945, 1.25)	0.246	0.919
Throat pain	Respiratory	Wald ratio	1	1.319 (0.923, 1.884)	0.129	0.916
Other upper respiratory disease	Respiratory	Wald ratio	1	0.957 (0.859, 1.067)	0.429	0.974
Pneumonia	Respiratory	Wald ratio	1	1.006 (0.936, 1.082)	0.861	0.981

Bacterial pneumonia	Respiratory	Wald ratio	1	1.062 (0.974, 1.157)	0.171	0.916
Pneumococcal pneumonia	Respiratory	Wald ratio	1	1.074 (0.981, 1.176)	0.121	0.916
Pseudomonal pneumonia	Respiratory	Wald ratio	1	0.741 (0.36, 1.527)	0.417	0.974
Viral pneumonia	Respiratory	Wald ratio	1	0.389 (0.172, 0.876)	0.023	0.688
Pneumonia due to fungus (mycoses)	Respiratory	Wald ratio	1	0.776 (0.394, 1.528)	0.463	0.974
Bronchopneumonia and lung abscess	Respiratory	Wald ratio	1	0.855 (0.598, 1.222)	0.389	0.974
Influenza	Respiratory	Wald ratio	1	1.057 (0.72, 1.552)	0.778	0.981
Acute bronchitis and bronchiolitis	Respiratory	Wald ratio	1	1.164 (0.708, 1.914)	0.549	0.981
Asthma	Respiratory	Wald ratio	1	1.079 (1.031, 1.129)	9.67E-04	0.121
Asthma with exacerbation	Respiratory	Wald ratio	1	1.413 (0.912, 2.19)	0.122	0.916
Chronic airway obstruction	Respiratory	Wald ratio	1	1.045 (0.972, 1.123)	0.235	0.919
Emphysema	Respiratory	Wald ratio	1	0.962 (0.814, 1.137)	0.646	0.981
Chronic bronchitis	Respiratory	Wald ratio	1	1.114 (0.978, 1.27)	0.105	0.902
Obstructive chronic bronchitis	Respiratory	Wald ratio	1	1.109 (0.969, 1.27)	0.134	0.916
Bronchiectasis	Respiratory	Wald ratio	1	0.935 (0.795, 1.101)	0.42	0.974
Bronchitis	Respiratory	Wald ratio	1	1.122 (0.852, 1.478)	0.412	0.974
Lung disease due to external agents	Respiratory	Wald ratio	1	0.964 (0.681, 1.365)	0.835	0.981
Extrinsic allergic alveolitis	Respiratory	Wald ratio	1	0.853 (0.396, 1.839)	0.685	0.981
Pneumoconiosis	Respiratory	Wald ratio	1	1.052 (0.67, 1.653)	0.826	0.981
Pneumonitis due to inhalation of food or vomitus	Respiratory	Wald ratio	1	1.03 (0.772, 1.376)	0.839	0.981
Postinflammatory pulmonary fibrosis	Respiratory	Wald ratio	1	1.097 (0.867, 1.387)	0.442	0.974
Pulmonary congestion and hypostasis	Respiratory	Wald ratio	1	1.17 (0.845, 1.619)	0.345	0.959
Other alveolar and parietoalveolar pneumonopathy	Respiratory	Wald ratio	1	1.159 (0.775, 1.733)	0.472	0.974
Other pulmonary inflamation or edema	Respiratory	Wald ratio	1	2.091 (0.847, 5.161)	0.11	0.916
Empyema and pneumothorax	Respiratory	Wald ratio	1	0.984 (0.803, 1.206)	0.878	0.983
Pleurisy; pleural effusion	Respiratory	Wald ratio	1	0.989 (0.907, 1.077)	0.796	0.981
Pulmonary collapse; interstitial and compensatory emphysema	Respiratory	Wald ratio	1	0.973 (0.834, 1.134)	0.724	0.981
Respiratory failure, insufficiency, arrest	Respiratory	Wald ratio	1	0.994 (0.864, 1.143)	0.928	0.986
Respiratory failure	Respiratory	Wald ratio	1	1.008 (0.865, 1.176)	0.916	0.983
Respiratory insufficiency	Respiratory	Wald ratio	1	1.033 (0.874, 1.221)	0.705	0.981
Respiratory arrest	Respiratory	Wald ratio	1	1.48 (0.786, 2.785)	0.225	0.917
Dependence on respirator [Ventilator] or supplemental oxygen	Respiratory	Wald ratio	1	0.724 (0.509, 1.03)	0.073	0.874
Other diseases of lung	Respiratory	Wald ratio	1	1.012 (0.789, 1.297)	0.926	0.986
Other symptoms of respiratory system	Respiratory	Wald ratio	1	1.069 (0.995, 1.149)	0.07	0.874
Wheezing	Respiratory	Wald ratio	1	1.211 (0.771, 1.902)	0.407	0.974
Painful respiration	Respiratory	Wald ratio	1	1.586 (0.965, 2.606)	0.069	0.874
Shortness of breath	Respiratory	Wald ratio	1	1.042 (0.952, 1.141)	0.368	0.972
Cough	Respiratory	Wald ratio	1	1.067 (0.936, 1.216)	0.334	0.952
Other dyspnea	Respiratory	Wald ratio	1	1.349 (1.072, 1.699)	0.011	0.564
Respiratory abnormalities	Respiratory	Wald ratio	1	1.062 (0.802, 1.405)	0.675	0.981
Hypoventilation	Respiratory	Wald ratio	1	1.289 (0.598, 2.778)	0.518	0.981
Hyperventilation	Respiratory	Wald ratio	1	0.869 (0.573, 1.317)	0.507	0.977
Disorders of diaphragm	Respiratory	Wald ratio	1	1.259 (0.732, 2.165)	0.405	0.974
Abnormal findings examination of lungs	Respiratory	Wald ratio	1	1.033 (0.898, 1.188)	0.652	0.981
Abnormal sputum	Respiratory	Wald ratio	1	1.122 (0.967, 1.303)	0.13	0.916
Hemoptysis	Respiratory	Wald ratio	1	1.133 (0.971, 1.321)	0.112	0.916
Other diseases of respiratory system, not elsewhere classified	Respiratory	Wald ratio	1	0.994 (0.925, 1.069)	0.876	0.983
Respiratory complications	Respiratory	Wald ratio	1	1.017 (0.68, 1.52)	0.936	0.986
Other diseases of respiratory system, NEC	Respiratory	Wald ratio	1	0.998 (0.928, 1.073)	0.952	0.989
Symptoms involving respiratory system and other chest symptoms	Respiratory	Wald ratio	1	1.017 (0.686, 1.507)	0.933	0.986
Disorders of the globe	Sense organs	Wald ratio	1	0.901 (0.617, 1.318)	0.592	0.981
Progressive myopia	Sense organs	Wald ratio	1	0.94 (0.606, 1.456)	0.781	0.981
Retinal detachments and defects	Sense organs	Wald ratio	1	1.107 (0.98, 1.25)	0.103	0.902
Retinal detachment with retinal defect	Sense organs	Wald ratio	1	1.102 (0.915, 1.326)	0.306	0.923
Retinoschisis and retinal cysts	Sense organs	Wald ratio	1	1.047 (0.444, 2.471)	0.916	0.983
Other retinal disorders	Sense organs	Wald ratio	1	1.102 (0.984, 1.233)	0.093	0.874
Degeneration of macula and posterior pole of retina	Sense organs	Wald ratio	1	1.022 (0.88, 1.186)	0.776	0.981
Macular degeneration (senile) of retina NOS	Sense organs	Wald ratio	1	1.02 (0.878, 1.183)	0.799	0.981
Other nondiabetic retinopathy	Sense organs	Wald ratio	1	1.189 (0.691, 2.044)	0.532	0.981
Separation of retinal layers	Sense organs	Wald ratio	1	0.82 (0.416, 1.615)	0.566	0.981
Retinal vascular changes and abnomalities	Sense organs	Wald ratio	1	1.112 (0.875, 1.413)	0.385	0.974
Peripheral retinal degenerations	Sense organs	Wald ratio	1	1.12 (0.681, 1.84)	0.656	0.981
Hereditary retinal dystrophies	Sense organs	Wald ratio	1	0.758 (0.352, 1.635)	0.48	0.974
Retinal hemorrhage or ischemia	Sense organs	Wald ratio	1	0.951 (0.505, 1.789)	0.875	0.983
Chorioretinal inflammations, scars, and other disorders of choroid	Sense organs	Wald ratio	1	1.162 (0.675, 1.998)	0.588	0.981
Chorioretinal scars	Sense organs	Wald ratio	1	1.319 (0.585, 2.974)	0.505	0.974
Corneal opacity and other disorders of cornea	Sense organs	Wald ratio	1	1.109 (0.857, 1.435)	0.43	0.974
Corneal opacity	Sense organs	Wald ratio	1	1.289 (0.625, 2.655)	0.492	0.974
Corneal edema	Sense organs	Wald ratio	1	0.944 (0.438, 2.035)	0.883	0.983
Corneal degenerations	Sense organs	Wald ratio	1	1.17 (0.712, 1.923)	0.536	0.981
Keratoconus	Sense organs	Wald ratio	1	1.623 (0.788, 3.343)	0.189	0.917
Corneal dystrophy	Sense organs	Wald ratio	1	1.047 (0.667, 1.645)	0.841	0.981
Cornea replaced by transplant	Sense organs	Wald ratio	1	1.222 (0.621, 2.407)	0.562	0.981
Glaucoma	Sense organs	Wald ratio	1	1.033 (0.931, 1.146)	0.543	0.981
Open-angle glaucoma	Sense organs	Wald ratio	1	1.015 (0.817, 1.261)	0.892	0.983
Primary open angle glaucoma	Sense organs	Wald ratio	1	1 (0.805, 1.243)	0.997	0.997
Primary angle-closure glaucoma	Sense organs	Wald ratio	1	0.91 (0.7, 1.182)	0.48	0.974
Cataract	Sense organs	Wald ratio	1	1.089 (1.036, 1.145)	7.69E-04	0.109
Nonsenile Cataract	Sense organs	Wald ratio	1	0.828 (0.367, 1.867)	0.649	0.981

Senile cataract	Sense organs	Wald ratio	1	1.077 (0.997, 1.162)	0.06	0.874
Traumatic cataract	Sense organs	Wald ratio	1	0.741 (0.376, 1.459)	0.386	0.974
Disorders of refraction and accommodation; blindness and low vision	Sense organs	Wald ratio	1	1.042 (0.906, 1.199)	0.561	0.981
Myopia	Sense organs	Wald ratio	1	1.159 (0.954, 1.407)	0.137	0.916
Astigmatism	Sense organs	Wald ratio	1	1.082 (0.658, 1.778)	0.757	0.981
Hypermetropia	Sense organs	Wald ratio	1	1.259 (0.812, 1.952)	0.303	0.923
Blindness and low vision	Sense organs	Wald ratio	1	0.944 (0.73, 1.221)	0.661	0.981
Visual disturbances	Sense organs	Wald ratio	1	0.968 (0.857, 1.094)	0.604	0.981
Amblyopia	Sense organs	Wald ratio	1	0.988 (0.733, 1.331)	0.935	0.986
Diplopia and disorders of binocular vision	Sense organs	Wald ratio	1	0.992 (0.766, 1.283)	0.948	0.988
Anisometropia	Sense organs	Wald ratio	1	0.776 (0.451, 1.335)	0.359	0.963
Visual field defects	Sense organs	Wald ratio	1	1.092 (0.747, 1.595)	0.651	0.981
Subjective visual disturbances	Sense organs	Wald ratio	1	0.994 (0.751, 1.315)	0.964	0.99
Infection of the eye	Sense organs	Wald ratio	1	0.881 (0.657, 1.182)	0.397	0.974
Eye infection, viral	Sense organs	Wald ratio	1	0.575 (0.306, 1.083)	0.086	0.874
Conjunctivitis, infectious	Sense organs	Wald ratio	1	0.758 (0.483, 1.191)	0.23	0.917
Keratitis	Sense organs	Wald ratio	1	0.94 (0.629, 1.405)	0.762	0.981
Corneal ulcer	Sense organs	Wald ratio	1	0.724 (0.403, 1.303)	0.282	0.923
Inflammation of the eye	Sense organs	Wald ratio	1	0.98 (0.868, 1.107)	0.747	0.981
Uveitis, noninfectious or NOS	Sense organs	Wald ratio	1	1.012 (0.737, 1.388)	0.943	0.986
Inflammation of eyelids	Sense organs	Wald ratio	1	0.964 (0.838, 1.109)	0.606	0.981
Disorders of conjunctiva	Sense organs	Wald ratio	1	1.167 (0.906, 1.503)	0.232	0.917
Other disorders of eyelids	Sense organs	Wald ratio	1	0.966 (0.879, 1.062)	0.475	0.974
Ectropion or entropion	Sense organs	Wald ratio	1	0.937 (0.758, 1.159)	0.551	0.981
Ptosis of eyelid	Sense organs	Wald ratio	1	0.937 (0.797, 1.103)	0.437	0.974
Disorders of lacrimal system	Sense organs	Wald ratio	1	0.962 (0.828, 1.116)	0.606	0.981
Epiphora	Sense organs	Wald ratio	1	1.094 (0.869, 1.378)	0.444	0.974
Disorders of the orbit	Sense organs	Wald ratio	1	0.66 (0.306, 1.424)	0.29	0.923
Disorders of optic nerve and visual pathways	Sense organs	Wald ratio	1	1.12 (0.78, 1.607)	0.54	0.981
Optic atrophy	Sense organs	Wald ratio	1	0.524 (0.212, 1.295)	0.162	0.916
Optic neuritis or neuropathy	Sense organs	Wald ratio	1	1.289 (0.685, 2.426)	0.432	0.974
Strabismus and other disorders of binocular eye movements	Sense organs	Wald ratio	1	1.067 (0.89, 1.278)	0.484	0.974
Strabismus (not specified as paralytic)	Sense organs	Wald ratio	1	1.05 (0.834, 1.322)	0.681	0.981
Nystagmus and other irregular eye movements	Sense organs	Wald ratio	1	1.042 (0.663, 1.638)	0.857	0.981
Paralytic strabismus	Sense organs	Wald ratio	1	1.319 (0.87, 1.998)	0.192	0.917
Other disorders of eye	Sense organs	Wald ratio	1	1.133 (1.012, 1.268)	0.031	0.749
Disorders of vitreous body	Sense organs	Wald ratio	1	1.054 (0.872, 1.275)	0.584	0.981
Aphakia and other disorders of lens	Sense organs	Wald ratio	1	1.107 (0.941, 1.302)	0.222	0.917
Anomalies of pupillary function	Sense organs	Wald ratio	1	0.914 (0.405, 2.061)	0.828	0.981
Disorders of iris and ciliary body	Sense organs	Wald ratio	1	1.289 (0.914, 1.816)	0.148	0.916
Pain, swelling or discharge of eye	Sense organs	Wald ratio	1	0.776 (0.451, 1.335)	0.359	0.963
Disorders of external ear	Sense organs	Wald ratio	1	0.966 (0.803, 1.163)	0.714	0.981
Otitis externa	Sense organs	Wald ratio	1	1.026 (0.758, 1.388)	0.87	0.981
Impacted cerumen	Sense organs	Wald ratio	1	0.935 (0.649, 1.349)	0.72	0.981
Otitis media and Eustachian tube disorders	Sense organs	Wald ratio	1	0.979 (0.844, 1.137)	0.785	0.981
Otitis media	Sense organs	Wald ratio	1	1.019 (0.866, 1.199)	0.824	0.981
Suppurative and unspecified otitis media	Sense organs	Wald ratio	1	1.052 (0.828, 1.337)	0.678	0.981
Eustachian tube disorders	Sense organs	Wald ratio	1	0.953 (0.58, 1.566)	0.849	0.981
Mastoiditis & related conditions	Sense organs	Wald ratio	1	0.835 (0.486, 1.437)	0.516	0.981
Otorrhea	Sense organs	Wald ratio	1	0.859 (0.567, 1.301)	0.473	0.974
Otalgia	Sense organs	Wald ratio	1	0.794 (0.536, 1.177)	0.25	0.919
Otosclerosis	Sense organs	Wald ratio	1	1.019 (0.688, 1.51)	0.925	0.986
Other disorders of tympanic membrane	Sense organs	Wald ratio	1	1.05 (0.868, 1.269)	0.617	0.981
Perforation of tympanic membrane	Sense organs	Wald ratio	1	1.127 (0.908, 1.4)	0.279	0.923
Other disorders of middle ear and mastoid	Sense organs	Wald ratio	1	0.968 (0.762, 1.23)	0.792	0.981
Cholesteatoma	Sense organs	Wald ratio	1	0.931 (0.704, 1.232)	0.617	0.981
Tympanosclerosis and middle ear disease related to otitis media	Sense organs	Wald ratio	1	1.175 (0.751, 1.838)	0.48	0.974
Vertiginous syndromes and other disorders of vestibular system	Sense organs	Wald ratio	1	1.026 (0.937, 1.123)	0.582	0.981
Meniere's disease	Sense organs	Wald ratio	1	1.107 (0.829, 1.478)	0.492	0.974
Peripheral or central vertigo	Sense organs	Wald ratio	1	1.172 (0.82, 1.675)	0.382	0.974
Labyrinthitis	Sense organs	Wald ratio	1	1.082 (0.84, 1.393)	0.544	0.981
Dizziness and giddiness (Light-headedness and vertigo)	Sense organs	Wald ratio	1	0.985 (0.888, 1.093)	0.777	0.981
Other disorders of ear	Sense organs	Wald ratio	1	0.741 (0.451, 1.218)	0.237	0.919
Hearing loss	Sense organs	Wald ratio	1	0.978 (0.877, 1.09)	0.686	0.981
Sensorineural hearing loss	Sense organs	Wald ratio	1	1.259 (0.91, 1.743)	0.165	0.916
Conductive hearing loss	Sense organs	Wald ratio	1	1.349 (0.975, 1.868)	0.071	0.874
Tinnitus	Sense organs	Wald ratio	1	0.97 (0.714, 1.319)	0.848	0.981
Symptoms concerning nutrition, metabolism, and development	Symptoms	Wald ratio	1	1.011 (0.919, 1.111)	0.823	0.981
Other signs and symptoms involving emotional state	Symptoms	Wald ratio	1	0.887 (0.596, 1.32)	0.555	0.981
Other symptoms	Symptoms	Wald ratio	1	1.072 (0.878, 1.307)	0.495	0.974
Other tests	Symptoms	Wald ratio	1	0.944 (0.862, 1.033)	0.211	0.917
Effects of other external causes	Symptoms	Wald ratio	1	0.968 (0.869, 1.079)	0.56	0.981
Other ill-defined and unknown causes of morbidity and mortality	Symptoms	Wald ratio	1	0.987 (0.931, 1.047)	0.667	0.981
Pain	Symptoms	Wald ratio	1	1.164 (0.908, 1.493)	0.23	0.917
Localized superficial swelling, mass, or lump	Symptoms	Wald ratio	1	1.156 (0.767, 1.744)	0.489	0.974
Back pain	Symptoms	Wald ratio	1	1.009 (0.942, 1.079)	0.805	0.981
Cervicalgia	Symptoms	Wald ratio	1	0.979 (0.832, 1.151)	0.794	0.981
Sciatica	Symptoms	Wald ratio	1	0.929 (0.804, 1.073)	0.317	0.923
Neuralgia, neuritis, and radiculitis NOS	Symptoms	Wald ratio	1	1.12 (0.914, 1.372)	0.276	0.923

Myalgia and myositis unspecified	Symptoms	Wald ratio	1	1.033 (0.784, 1.361)	0.818	0.981
Musculoskeletal symptoms referable to limbs	Symptoms	Wald ratio	1	1.001 (0.911, 1.101)	0.978	0.991
Swelling of limb	Symptoms	Wald ratio	1	0.983 (0.886, 1.09)	0.744	0.981
Symptoms of the muscles	Symptoms	Wald ratio	1	1.011 (0.778, 1.314)	0.933	0.986
Pain in limb	Symptoms	Wald ratio	1	0.951 (0.868, 1.04)	0.271	0.923
Hypothermia or Chills	Symptoms	Wald ratio	1	0.692 (0.402, 1.189)	0.182	0.916
Symptoms involving nervous and musculoskeletal systems	Symptoms	Wald ratio	1	0.897 (0.763, 1.056)	0.192	0.917
Symptoms involving skin and other integumentary tissue	Symptoms	Wald ratio	1	0.955 (0.812, 1.124)	0.579	0.981
Edema	Symptoms	Wald ratio	1	0.98 (0.825, 1.163)	0.817	0.981
Pallor and flushing	Symptoms	Wald ratio	1	0.692 (0.402, 1.189)	0.182	0.916
Fever of unknown origin	Symptoms	Wald ratio	1	0.982 (0.877, 1.1)	0.758	0.981
Abdominal pain	Symptoms	Wald ratio	1	1.026 (0.99, 1.063)	0.164	0.916
Syncope and collapse	Symptoms	Wald ratio	1	1.077 (1.001, 1.157)	0.046	0.792
Nausea and vomiting	Symptoms	Wald ratio	1	1.03 (0.963, 1.103)	0.386	0.974
Nonspecific findings on examination of blood	Symptoms	Wald ratio	1	1.102 (1.011, 1.2)	0.027	0.748
Elevated sedimentation rate	Symptoms	Wald ratio	1	2.241 (1.039, 4.83)	0.04	0.792
Other abnormal blood chemistry	Symptoms	Wald ratio	1	1.097 (1.002, 1.2)	0.046	0.792
Gangrene	Symptoms	Wald ratio	1	1.159 (0.864, 1.555)	0.325	0.934
Abnormal Papanicolaou smear of cervix and cervical HPV	Symptoms	Wald ratio	1	1.064 (0.54, 2.096)	0.857	0.981
Abnormal results of other function studies (bladder, pancreas, placenta, spleen, etc)	Symptoms	Wald ratio	1	0.575 (0.306, 1.083)	0.086	0.874
Shock	Symptoms	Wald ratio	1	0.828 (0.46, 1.489)	0.528	0.981
Cardiogenic shock	Symptoms	Wald ratio	1	0.724 (0.336, 1.561)	0.41	0.974
Malaise and fatigue	Symptoms	Wald ratio	1	1.004 (0.893, 1.13)	0.942	0.986
Chronic fatigue syndrome	Symptoms	Wald ratio	1	1.135 (0.854, 1.509)	0.383	0.974

Table S15. Phenome-wide MR analyses on genetically mimicked targeting of CD4 NC-specific *DDX5* .

Phenotype	Category	Method	nSNP	OR (95% CI)	*P* -value	*FDR*
Rheumatic disease of the heart valves	Circulatory system	Wald ratio	1	0.97 (0.892, 1.054)	0.467	0.974
Mitral valve disease	Circulatory system	Wald ratio	1	0.984 (0.885, 1.094)	0.761	0.981
Aortic valve disease	Circulatory system	Wald ratio	1	0.863 (0.733, 1.016)	0.077	0.874
Disease of tricuspid valve	Circulatory system	Wald ratio	1	1.025 (0.858, 1.225)	0.782	0.981
Heart valve disorders	Circulatory system	Wald ratio	1	1.005 (0.918, 1.101)	0.914	0.983
Nonrheumatic mitral valve disorders	Circulatory system	Wald ratio	1	0.987 (0.884, 1.102)	0.817	0.981
Nonrheumatic aortic valve disorders	Circulatory system	Wald ratio	1	1.085 (0.748, 1.573)	0.668	0.981
Nonrheumatic tricuspid valve disorders	Circulatory system	Wald ratio	1	0.875 (0.496, 1.545)	0.646	0.981
Nonrheumatic pulmonary valve disorders	Circulatory system	Wald ratio	1	1.444 (0.818, 2.55)	0.205	0.917
Heart valve replaced	Circulatory system	Wald ratio	1	1.031 (0.886, 1.2)	0.689	0.981
Abnormal heart sounds	Circulatory system	Wald ratio	1	0.872 (0.727, 1.046)	0.139	0.916
Hypertensive heart and or or renal disease	Circulatory system	Wald ratio	1	1.052 (0.914, 1.21)	0.482	0.974
Hypertensive heart disease	Circulatory system	Wald ratio	1	0.808 (0.513, 1.274)	0.359	0.963
Hypertensive chronic kidney disease	Circulatory system	Wald ratio	1	1.078 (0.93, 1.25)	0.317	0.923
Other hypertensive complications	Circulatory system	Wald ratio	1	0.882 (0.539, 1.444)	0.617	0.981
Elevated blood pressure reading without diagnosis of hypertension	Circulatory system	Wald ratio	1	1.002 (0.861, 1.166)	0.976	0.991
Ischemic Heart Disease	Circulatory system	Wald ratio	1	1.013 (0.977, 1.051)	0.483	0.974
Unstable angina (intermediate coronary syndrome)	Circulatory system	Wald ratio	1	1.004 (0.924, 1.092)	0.92	0.984
Myocardial infarction	Circulatory system	Wald ratio	1	0.973 (0.919, 1.03)	0.351	0.962
Angina pectoris	Circulatory system	Wald ratio	1	1.013 (0.965, 1.064)	0.601	0.981
Coronary atherosclerosis	Circulatory system	Wald ratio	1	0.99 (0.946, 1.036)	0.665	0.981
Aneurysm and dissection of heart	Circulatory system	Wald ratio	1	1.13 (0.907, 1.407)	0.277	0.923
Other chronic ischemic heart disease, unspecified	Circulatory system	Wald ratio	1	1.015 (0.966, 1.066)	0.554	0.981
Other acute and subacute forms of ischemic heart disease	Circulatory system	Wald ratio	1	0.94 (0.793, 1.115)	0.477	0.974
Other forms of chronic heart disease	Circulatory system	Wald ratio	1	1.041 (0.909, 1.194)	0.56	0.981
ASCVD	Circulatory system	Wald ratio	1	0.824 (0.401, 1.694)	0.599	0.981
Pulmonary heart disease	Circulatory system	Wald ratio	1	0.987 (0.901, 1.081)	0.777	0.981
Acute pulmonary heart disease	Circulatory system	Wald ratio	1	0.582 (0.294, 1.151)	0.12	0.916
Pulmonary embolism and infarction, acute	Circulatory system	Wald ratio	1	0.582 (0.294, 1.151)	0.12	0.916
Chronic pulmonary heart disease	Circulatory system	Wald ratio	1	1.072 (0.844, 1.361)	0.568	0.981
Primary pulmonary hypertension	Circulatory system	Wald ratio	1	0.992 (0.752, 1.308)	0.954	0.989
Cardiomegaly	Circulatory system	Wald ratio	1	1.022 (0.908, 1.149)	0.723	0.981
Nonspecific chest pain	Circulatory system	Wald ratio	1	1.005 (0.97, 1.041)	0.772	0.981
Precordial pain	Circulatory system	Wald ratio	1	1.029 (0.933, 1.136)	0.564	0.981
Carditis	Circulatory system	Wald ratio	1	1.035 (0.91, 1.178)	0.597	0.981
Myocarditis	Circulatory system	Wald ratio	1	1.261 (0.714, 2.227)	0.424	0.974
Pericarditis	Circulatory system	Wald ratio	1	1.062 (0.902, 1.25)	0.471	0.974
Acute pericarditis	Circulatory system	Wald ratio	1	1.311 (0.897, 1.915)	0.162	0.916
Endocarditis	Circulatory system	Wald ratio	1	0.94 (0.752, 1.176)	0.588	0.981
Cardiomyopathy	Circulatory system	Wald ratio	1	1.003 (0.849, 1.186)	0.967	0.991
Primary or intrinsic cardiomyopathies	Circulatory system	Wald ratio	1	1.015 (0.859, 1.199)	0.863	0.981
Hypertrophic obstructive cardiomyopathy	Circulatory system	Wald ratio	1	1.719 (1.05, 2.813)	0.031	0.749
Other hypertrophic cardiomyopathy	Circulatory system	Wald ratio	1	1.501 (0.952, 2.366)	0.08	0.874
Other cardiomyopathy	Circulatory system	Wald ratio	1	0.582 (0.273, 1.242)	0.162	0.916
Cardiac conduction disorders	Circulatory system	Wald ratio	1	0.977 (0.909, 1.05)	0.528	0.981
Atrioventricular [AV] block	Circulatory system	Wald ratio	1	0.975 (0.861, 1.105)	0.694	0.981
First degree AV block	Circulatory system	Wald ratio	1	1.037 (0.862, 1.249)	0.698	0.981
Second degree AV block	Circulatory system	Wald ratio	1	0.84 (0.64, 1.104)	0.211	0.917
Atrioventricular block, complete	Circulatory system	Wald ratio	1	0.985 (0.776, 1.251)	0.901	0.983
Other heart block	Circulatory system	Wald ratio	1	1.416 (0.865, 2.319)	0.166	0.916
Bundle branch block	Circulatory system	Wald ratio	1	0.935 (0.844, 1.035)	0.195	0.917
Right bundle branch block	Circulatory system	Wald ratio	1	1.06 (0.911, 1.233)	0.453	0.974
Left bundle branch block	Circulatory system	Wald ratio	1	0.793 (0.689, 0.912)	0.001	0.125
Anomalous atrioventricular excitation	Circulatory system	Wald ratio	1	1.06 (0.725, 1.548)	0.764	0.981
Other cardiac conduction disorders	Circulatory system	Wald ratio	1	0.549 (0.257, 1.172)	0.121	0.916
Cardiac pacemaker or device in situ	Circulatory system	Wald ratio	1	0.991 (0.881, 1.115)	0.885	0.983
Cardiac pacemaker in situ	Circulatory system	Wald ratio	1	1.031 (0.914, 1.164)	0.617	0.981
Cardiac dysrhythmias	Circulatory system	Wald ratio	1	1.018 (0.98, 1.058)	0.347	0.959
Paroxysmal tachycardia, unspecified	Circulatory system	Wald ratio	1	0.985 (0.889, 1.091)	0.773	0.981
Paroxysmal supraventricular tachycardia	Circulatory system	Wald ratio	1	0.979 (0.867, 1.105)	0.731	0.981
Paroxysmal ventricular tachycardia	Circulatory system	Wald ratio	1	0.979 (0.81, 1.183)	0.826	0.981
Atrial fibrillation and flutter	Circulatory system	Wald ratio	1	1.027 (0.978, 1.079)	0.282	0.923
Other specified cardiac dysrhythmias	Circulatory system	Wald ratio	1	1.176 (1.062, 1.303)	0.002	0.148
Cardiac arrest and ventricular fibrillation	Circulatory system	Wald ratio	1	1.102 (0.929, 1.306)	0.267	0.923
Ventricular fibrillation and flutter	Circulatory system	Wald ratio	1	1.06 (0.771, 1.457)	0.721	0.981
Cardiac arrest	Circulatory system	Wald ratio	1	1.145 (0.947, 1.384)	0.162	0.916
Arrhythmia (cardiac) NOS	Circulatory system	Wald ratio	1	0.996 (0.821, 1.208)	0.967	0.991
Premature beats	Circulatory system	Wald ratio	1	0.944 (0.735, 1.212)	0.649	0.981
Supraventricular premature beats	Circulatory system	Wald ratio	1	1.444 (0.849, 2.455)	0.175	0.916
Tachycardia NOS	Circulatory system	Wald ratio	1	0.945 (0.834, 1.071)	0.38	0.974
Sinoatrial node dysfunction (Bradycardia)	Circulatory system	Wald ratio	1	1.108 (0.831, 1.478)	0.486	0.974
Palpitations	Circulatory system	Wald ratio	1	0.96 (0.873, 1.056)	0.401	0.974
Congestive heart failure; nonhypertensive	Circulatory system	Wald ratio	1	0.951 (0.878, 1.03)	0.216	0.917
Congestive heart failure (CHF) NOS	Circulatory system	Wald ratio	1	1.015 (0.892, 1.155)	0.819	0.981
Heart failure NOS	Circulatory system	Wald ratio	1	0.935 (0.853, 1.024)	0.145	0.916
Ill-defined descriptions and complications of heart disease	Circulatory system	Wald ratio	1	0.953 (0.776, 1.169)	0.643	0.981
Heart transplant or surgery	Circulatory system	Wald ratio	1	1.893 (0.921, 3.89)	0.082	0.874

Abnormal function study of cardiovascular system	Circulatory system	Wald ratio	1	0.829 (0.643, 1.069)	0.148	0.916
Symptoms involving cardiovascular system	Circulatory system	Wald ratio	1	1.141 (0.752, 1.731)	0.536	0.981
Intracranial hemorrhage	Circulatory system	Wald ratio	1	1.008 (0.879, 1.155)	0.914	0.983
Subarachnoid hemorrhage	Circulatory system	Wald ratio	1	1.134 (0.924, 1.392)	0.229	0.917
Intracerebral hemorrhage	Circulatory system	Wald ratio	1	0.997 (0.801, 1.243)	0.982	0.991
Subdural hemorrhage	Circulatory system	Wald ratio	1	0.748 (0.522, 1.073)	0.114	0.916
Cerebrovascular disease	Circulatory system	Wald ratio	1	1.001 (0.939, 1.068)	0.976	0.991
Occlusion and stenosis of precerebral arteries	Circulatory system	Wald ratio	1	1.017 (0.858, 1.206)	0.845	0.981
Cerebral atherosclerosis	Circulatory system	Wald ratio	1	1.389 (0.951, 2.03)	0.089	0.874
Occlusion of cerebral arteries	Circulatory system	Wald ratio	1	1.06 (0.968, 1.161)	0.211	0.917
Cerebral artery occlusion, with cerebral infarction	Circulatory system	Wald ratio	1	0.997 (0.857, 1.16)	0.968	0.991
Cerebral ischemia	Circulatory system	Wald ratio	1	1.048 (0.938, 1.169)	0.408	0.974
Transient cerebral ischemia	Circulatory system	Wald ratio	1	1.045 (0.923, 1.185)	0.486	0.974
Cerebral aneurysm	Circulatory system	Wald ratio	1	0.926 (0.686, 1.249)	0.613	0.981
Late effects of cerebrovascular disease	Circulatory system	Wald ratio	1	0.91 (0.773, 1.071)	0.254	0.919
Atherosclerosis	Circulatory system	Wald ratio	1	1.089 (0.929, 1.277)	0.295	0.923
Atherosclerosis of renal artery	Circulatory system	Wald ratio	1	0.944 (0.555, 1.604)	0.83	0.981
Atherosclerosis of the extremities	Circulatory system	Wald ratio	1	1.052 (0.857, 1.29)	0.63	0.981
Atherosclerosis of aorta	Circulatory system	Wald ratio	1	1.097 (0.777, 1.549)	0.598	0.981
Vascular insufficiency of intestine	Circulatory system	Wald ratio	1	1.066 (0.836, 1.359)	0.606	0.981
Acute vascular insufficiency of intestine	Circulatory system	Wald ratio	1	1.011 (0.705, 1.449)	0.953	0.989
Other aneurysm	Circulatory system	Wald ratio	1	1.087 (0.948, 1.246)	0.232	0.917
Aortic aneurysm	Circulatory system	Wald ratio	1	1.076 (0.918, 1.262)	0.366	0.97
Abdominal aortic aneurysm	Circulatory system	Wald ratio	1	1.07 (0.882, 1.298)	0.493	0.974
Aneurysm of iliac artery	Circulatory system	Wald ratio	1	0.926 (0.524, 1.634)	0.79	0.981
Aneurysm of artery of lower extremity	Circulatory system	Wald ratio	1	0.962 (0.61, 1.516)	0.868	0.981
Arterial dissection	Circulatory system	Wald ratio	1	1.622 (0.732, 3.595)	0.234	0.917
Aneurysm of other specified artery	Circulatory system	Wald ratio	1	0.96 (0.633, 1.457)	0.849	0.981
Peripheral vascular disease	Circulatory system	Wald ratio	1	0.999 (0.908, 1.098)	0.98	0.991
Raynaud's syndrome	Circulatory system	Wald ratio	1	0.868 (0.732, 1.03)	0.105	0.902
Peripheral angiopathy in diseases classified elsewhere	Circulatory system	Wald ratio	1	0.996 (0.741, 1.339)	0.98	0.991
Peripheral vascular disease, unspecified	Circulatory system	Wald ratio	1	1.078 (0.959, 1.213)	0.208	0.917
Arterial embolism and thrombosis	Circulatory system	Wald ratio	1	1.237 (1.02, 1.501)	0.031	0.749
Arterial embolism and thrombosis of lower extremity artery	Circulatory system	Wald ratio	1	1.167 (0.912, 1.494)	0.218	0.917
Polyarteritis nodosa and allied conditions	Circulatory system	Wald ratio	1	1.019 (0.834, 1.246)	0.853	0.981
Wegener's granulomatosis	Circulatory system	Wald ratio	1	0.915 (0.603, 1.388)	0.676	0.981
Giant cell arteritis	Circulatory system	Wald ratio	1	1.054 (0.784, 1.416)	0.729	0.981
Arteritis NOS	Circulatory system	Wald ratio	1	0.931 (0.637, 1.36)	0.711	0.981
Other disorders of arteries and arterioles	Circulatory system	Wald ratio	1	1.119 (0.954, 1.312)	0.167	0.916
Stricture of artery	Circulatory system	Wald ratio	1	1.048 (0.86, 1.276)	0.644	0.981
Disease of capillaries	Circulatory system	Wald ratio	1	0.89 (0.544, 1.458)	0.644	0.981
Noninfectious disorders of lymphatic channels	Circulatory system	Wald ratio	1	0.971 (0.78, 1.21)	0.796	0.981
Phlebitis and thrombophlebitis	Circulatory system	Wald ratio	1	0.88 (0.801, 0.968)	0.008	0.502
Phlebitis and thrombophlebitis of lower extremities	Circulatory system	Wald ratio	1	0.89 (0.807, 0.983)	0.021	0.688
Other venous embolism and thrombosis	Circulatory system	Wald ratio	1	1.213 (0.948, 1.552)	0.124	0.916
Varicose veins	Circulatory system	Wald ratio	1	0.986 (0.931, 1.044)	0.626	0.981
Varicose veins of lower extremity	Circulatory system	Wald ratio	1	0.991 (0.936, 1.049)	0.759	0.981
Varicose veins of lower extremity, symptomtic	Circulatory system	Wald ratio	1	0.843 (0.669, 1.063)	0.149	0.916
Hemorrhoids	Circulatory system	Wald ratio	1	0.97 (0.93, 1.011)	0.146	0.916
Chronic venous insufficiency [CVI]	Circulatory system	Wald ratio	1	1.286 (0.847, 1.951)	0.237	0.919
Encounter for long-term (current) use of anticoagulants, antithrombotics, aspirin	Circulatory system	Wald ratio	1	1.444 (0.818, 2.55)	0.205	0.917
Hypotension	Circulatory system	Wald ratio	1	1.031 (0.956, 1.113)	0.424	0.974
Orthostatic hypotension	Circulatory system	Wald ratio	1	0.996 (0.85, 1.168)	0.964	0.99
Iatrogenic hypotension	Circulatory system	Wald ratio	1	0.92 (0.63, 1.344)	0.667	0.981
Hypotension NOS	Circulatory system	Wald ratio	1	1.041 (0.944, 1.149)	0.419	0.974
Other disorders of circulatory system	Circulatory system	Wald ratio	1	0.979 (0.935, 1.025)	0.359	0.963
Hemorrhage NOS	Circulatory system	Wald ratio	1	0.653 (0.37, 1.154)	0.142	0.916
Circulatory disease NEC	Circulatory system	Wald ratio	1	0.979 (0.932, 1.028)	0.397	0.974
Nonspecific abnormal findings on radiological and other examination of other intrathoracic organs (echocardiogram, etc)	Circulatory system	Wald ratio	1	0.979 (0.765, 1.252)	0.866	0.981
Cardiac and circulatory congenital anomalies	Congenital anomalies	Wald ratio	1	1.008 (0.903, 1.125)	0.89	0.983
Cardiac congenital anomalies	Congenital anomalies	Wald ratio	1	0.996 (0.889, 1.116)	0.942	0.986
Cardiac shunt or heart septal defect	Congenital anomalies	Wald ratio	1	0.944 (0.743, 1.198)	0.634	0.981
Valvular heart disease or heart chambers	Congenital anomalies	Wald ratio	1	1.416 (0.97, 2.069)	0.072	0.874
Congenital anomalies of great vessels	Congenital anomalies	Wald ratio	1	0.995 (0.868, 1.141)	0.945	0.987
Congenital anomalies of peripheral vascular system	Congenital anomalies	Wald ratio	1	1.138 (0.722, 1.794)	0.577	0.981
Anomalies of respiratory system, congenital	Congenital anomalies	Wald ratio	1	1.286 (0.58, 2.851)	0.536	0.981
Congenital anomalies of face and neck	Congenital anomalies	Wald ratio	1	0.951 (0.689, 1.312)	0.76	0.981
Digestive congenital anomalies	Congenital anomalies	Wald ratio	1	0.904 (0.726, 1.127)	0.37	0.972
Upper gastrointestinal congenital anomalies	Congenital anomalies	Wald ratio	1	0.835 (0.608, 1.149)	0.268	0.923
Congenital anomalies of mouth or tongue	Congenital anomalies	Wald ratio	1	0.897 (0.591, 1.362)	0.611	0.981
Lower gastrointestinal congenital anomalies	Congenital anomalies	Wald ratio	1	1.013 (0.745, 1.378)	0.932	0.986
Congenital anomalies of intestine	Congenital anomalies	Wald ratio	1	0.88 (0.602, 1.286)	0.509	0.978
Congenital anomaly of gallbladder, bile ducts, liver, pancreas	Congenital anomalies	Wald ratio	1	1.261 (0.742, 2.144)	0.391	0.974
Genitourinary congenital anomalies	Congenital anomalies	Wald ratio	1	1.029 (0.891, 1.189)	0.693	0.981
Congenital anomalies of genital organs	Congenital anomalies	Wald ratio	1	1.031 (0.831, 1.28)	0.779	0.981
Congenital anomalies of female genital organs	Congenital anomalies	Wald ratio	1	0.971 (0.739, 1.276)	0.835	0.981
Congenital anomalies of urinary system	Congenital anomalies	Wald ratio	1	1.041 (0.852, 1.273)	0.692	0.981

Cystic kidney disease	Congenital anomalies	Wald ratio	1	0.951 (0.718, 1.259)	0.725	0.981
Other specified congenital anomalies of kidney	Congenital anomalies	Wald ratio	1	1.188 (0.867, 1.627)	0.284	0.923
Nervous system congenital anomalies	Congenital anomalies	Wald ratio	1	0.808 (0.588, 1.111)	0.19	0.917
Neural tube defects	Congenital anomalies	Wald ratio	1	0.793 (0.551, 1.141)	0.211	0.917
Spina bifida	Congenital anomalies	Wald ratio	1	0.91 (0.599, 1.38)	0.656	0.981
Other specified congenital anomalies of nervous system	Congenital anomalies	Wald ratio	1	0.72 (0.378, 1.371)	0.317	0.923
Congenital anomalies of the eye	Congenital anomalies	Wald ratio	1	0.734 (0.448, 1.201)	0.218	0.917
Congenital cataract and lens anomalies	Congenital anomalies	Wald ratio	1	0.605 (0.306, 1.197)	0.149	0.916
Congenital musculoskeletal deformities of spine	Congenital anomalies	Wald ratio	1	0.843 (0.478, 1.489)	0.557	0.981
Congenital anomalies of limbs	Congenital anomalies	Wald ratio	1	0.958 (0.729, 1.259)	0.76	0.981
Congenital deformities of feet	Congenital anomalies	Wald ratio	1	0.926 (0.61, 1.404)	0.716	0.981
Other congenital anomalies of lower limb, including pelvic girdle	Congenital anomalies	Wald ratio	1	0.996 (0.632, 1.569)	0.985	0.991
Congenital hip dysplasia and deformity	Congenital anomalies	Wald ratio	1	1.104 (0.625, 1.949)	0.734	0.981
Other congenital musculoskeletal anomalies	Congenital anomalies	Wald ratio	1	0.865 (0.681, 1.098)	0.234	0.917
Congenital anomalies of muscle, tendon, fascia, and connective tissue	Congenital anomalies	Wald ratio	1	0.793 (0.416, 1.51)	0.48	0.974
Congenital osteodystrophies	Congenital anomalies	Wald ratio	1	0.901 (0.68, 1.193)	0.466	0.974
Chromosomal anomalies and genetic disorders	Congenital anomalies	Wald ratio	1	0.904 (0.596, 1.372)	0.636	0.981
Chromosomal anomalies	Congenital anomalies	Wald ratio	1	0.887 (0.502, 1.566)	0.679	0.981
Other and unspecified congenital anomalies	Congenital anomalies	Wald ratio	1	0.896 (0.613, 1.308)	0.569	0.981
Anomalies of endocrine glands, congenital	Congenital anomalies	Wald ratio	1	0.903 (0.511, 1.594)	0.724	0.981
Superficial cellulitis and abscess	Dermatologic	Wald ratio	1	0.984 (0.919, 1.053)	0.641	0.981
Cellulitis and abscess of fingers or toes	Dermatologic	Wald ratio	1	0.827 (0.647, 1.059)	0.132	0.916
Cellulitis and abscess of face or neck	Dermatologic	Wald ratio	1	1.136 (0.881, 1.465)	0.325	0.934
Cellulitis and abscess of arm or hand	Dermatologic	Wald ratio	1	0.997 (0.92, 1.079)	0.935	0.986
Cellulitis and abscess of leg, except foot	Dermatologic	Wald ratio	1	1.003 (0.926, 1.086)	0.947	0.988
Cellulitis and abscess of foot, toe	Dermatologic	Wald ratio	1	0.998 (0.922, 1.081)	0.97	0.991
Cellulitis and abscess of trunk	Dermatologic	Wald ratio	1	0.973 (0.767, 1.236)	0.824	0.981
Other local infections of skin and subcutaneous tissue	Dermatologic	Wald ratio	1	0.984 (0.901, 1.073)	0.708	0.981
Carbuncle and furuncle	Dermatologic	Wald ratio	1	0.953 (0.844, 1.076)	0.435	0.974
Pilonidal cyst	Dermatologic	Wald ratio	1	0.808 (0.634, 1.03)	0.086	0.874
Pyogenic granuloma	Dermatologic	Wald ratio	1	1.013 (0.745, 1.377)	0.935	0.986
Symptoms affecting skin	Dermatologic	Wald ratio	1	1.033 (0.954, 1.119)	0.418	0.974
Rash and other nonspecific skin eruption	Dermatologic	Wald ratio	1	1.009 (0.89, 1.143)	0.889	0.983
Changes in skin texture	Dermatologic	Wald ratio	1	0.793 (0.449, 1.4)	0.424	0.974
Disturbance of skin sensation	Dermatologic	Wald ratio	1	1.068 (0.957, 1.192)	0.241	0.919
Disorder of skin and subcutaneous tissue NOS	Dermatologic	Wald ratio	1	0.958 (0.888, 1.034)	0.271	0.923
Erythematosquamous dermatosis	Dermatologic	Wald ratio	1	0.872 (0.441, 1.725)	0.693	0.981
Seborrheic dermatitis	Dermatologic	Wald ratio	1	0.793 (0.358, 1.758)	0.568	0.981
Congenital anomalies of skin	Dermatologic	Wald ratio	1	0.763 (0.449, 1.297)	0.317	0.923
Dyschromia and Vitiligo	Dermatologic	Wald ratio	1	1.007 (0.836, 1.213)	0.941	0.986
Vitiligo	Dermatologic	Wald ratio	1	0.706 (0.331, 1.507)	0.368	0.972
Other dyschromia	Dermatologic	Wald ratio	1	1.058 (0.859, 1.303)	0.598	0.981
Vascular disorders of skin	Dermatologic	Wald ratio	1	0.945 (0.556, 1.607)	0.836	0.981
Erythematous conditions	Dermatologic	Wald ratio	1	1.132 (1.006, 1.273)	0.039	0.792
Toxic erythema	Dermatologic	Wald ratio	1	1.416 (0.802, 2.501)	0.23	0.917
Bullous dermatoses	Dermatologic	Wald ratio	1	1.363 (0.832, 2.231)	0.218	0.917
Dermatitis herpetiformis	Dermatologic	Wald ratio	1	1.472 (0.69, 3.142)	0.317	0.923
Pemphigus and pemphigoid	Dermatologic	Wald ratio	1	1.286 (0.65, 2.544)	0.47	0.974
Rosacea	Dermatologic	Wald ratio	1	1.261 (0.907, 1.754)	0.168	0.916
Lupus (localized and systemic)	Dermatologic	Wald ratio	1	0.904 (0.681, 1.202)	0.488	0.974
Cutaneous lupus erythematosus	Dermatologic	Wald ratio	1	1.006 (0.592, 1.711)	0.982	0.991
Systemic lupus erythematosus	Dermatologic	Wald ratio	1	0.897 (0.655, 1.229)	0.5	0.974
Prurigo and Lichen	Dermatologic	Wald ratio	1	1.192 (0.968, 1.469)	0.098	0.877
Other specified erythematous conditions	Dermatologic	Wald ratio	1	1.206 (0.832, 1.749)	0.322	0.932
Erythema nodosum	Dermatologic	Wald ratio	1	0.983 (0.497, 1.945)	0.961	0.99
Unspecified erythematous condition	Dermatologic	Wald ratio	1	1.015 (0.776, 1.329)	0.913	0.983
Psoriasis and related disorders	Dermatologic	Wald ratio	1	0.92 (0.815, 1.039)	0.179	0.916
Psoriasis	Dermatologic	Wald ratio	1	0.926 (0.817, 1.049)	0.225	0.917
Psoriasis vulgaris	Dermatologic	Wald ratio	1	0.908 (0.789, 1.045)	0.177	0.916
Psoriatic arthropathy	Dermatologic	Wald ratio	1	0.997 (0.801, 1.243)	0.982	0.991
Sarcoidosis	Dermatologic	Wald ratio	1	0.915 (0.712, 1.175)	0.486	0.974
Pruritus and related conditions	Dermatologic	Wald ratio	1	1.237 (1.004, 1.524)	0.046	0.792
Corns and callosities	Dermatologic	Wald ratio	1	1.286 (0.914, 1.809)	0.149	0.916
Other hypertrophic and atrophic conditions of skin	Dermatologic	Wald ratio	1	1.043 (0.96, 1.134)	0.317	0.923
Keratoderma, acquired	Dermatologic	Wald ratio	1	0.653 (0.356, 1.198)	0.169	0.916
Scar conditions and fibrosis of skin	Dermatologic	Wald ratio	1	1.114 (0.987, 1.258)	0.08	0.874
Circumscribed scleroderma	Dermatologic	Wald ratio	1	0.852 (0.399, 1.818)	0.678	0.981
Keloid scar	Dermatologic	Wald ratio	1	0.834 (0.571, 1.218)	0.347	0.959
Abnormal granulation tissue	Dermatologic	Wald ratio	1	1.119 (0.851, 1.47)	0.42	0.974
Acquired acanthosis nigricans	Dermatologic	Wald ratio	1	0.56 (0.317, 0.988)	0.046	0.792
Degenerative skin conditions and other dermatoses	Dermatologic	Wald ratio	1	0.99 (0.914, 1.072)	0.797	0.981
Actinic keratosis	Dermatologic	Wald ratio	1	0.977 (0.872, 1.095)	0.689	0.981
Seborrheic keratosis	Dermatologic	Wald ratio	1	0.997 (0.897, 1.109)	0.957	0.989
Diseases of nail, NOS	Dermatologic	Wald ratio	1	1.119 (0.95, 1.317)	0.177	0.916
Ingrowing nail	Dermatologic	Wald ratio	1	1.176 (0.973, 1.422)	0.093	0.874
Diseases of hair and hair follicles	Dermatologic	Wald ratio	1	0.927 (0.856, 1.004)	0.063	0.874
Alopecia	Dermatologic	Wald ratio	1	0.808 (0.475, 1.374)	0.432	0.974
Other specified diseases of hair and hair follicles	Dermatologic	Wald ratio	1	1.416 (0.716, 2.802)	0.317	0.923
Disorders of sweat glands	Dermatologic	Wald ratio	1	1.056 (0.86, 1.295)	0.604	0.981

Hidradenitis	Dermatologic	Wald ratio	1	0.666 (0.439, 1.011)	0.056	0.87
Hyperhidrosis	Dermatologic	Wald ratio	1	1.172 (0.923, 1.488)	0.193	0.917
Diseases of sebaceous glands	Dermatologic	Wald ratio	1	0.981 (0.92, 1.046)	0.556	0.981
Acne	Dermatologic	Wald ratio	1	1.237 (0.649, 2.357)	0.518	0.981
Sebaceous cyst	Dermatologic	Wald ratio	1	0.979 (0.918, 1.044)	0.518	0.981
Chronic ulcer of skin	Dermatologic	Wald ratio	1	1.031 (0.903, 1.178)	0.648	0.981
Decubitus ulcer	Dermatologic	Wald ratio	1	1.141 (0.94, 1.384)	0.182	0.916
Diffuse diseases of connective tissue	Dermatologic	Wald ratio	1	0.987 (0.894, 1.089)	0.797	0.981
Sicca syndrome	Dermatologic	Wald ratio	1	1.076 (0.832, 1.393)	0.576	0.981
Systemic sclerosis	Dermatologic	Wald ratio	1	1.389 (0.882, 2.19)	0.157	0.916
Other specified diffuse diseases of connective tissue	Dermatologic	Wald ratio	1	1.444 (0.703, 2.968)	0.317	0.923
Unspecified diffuse connective tissue disease	Dermatologic	Wald ratio	1	0.956 (0.854, 1.072)	0.443	0.974
Atopic or contact dermatitis due to other or unspecified	Dermatologic	Wald ratio	1	1.078 (0.948, 1.227)	0.251	0.919
Disorders of tooth development	Digestive	Wald ratio	1	0.936 (0.833, 1.053)	0.273	0.923
Hereditary disturbances in tooth structure	Digestive	Wald ratio	1	0.922 (0.416, 2.044)	0.841	0.981
Disturbances in tooth eruption	Digestive	Wald ratio	1	0.94 (0.833, 1.061)	0.317	0.923
Diseases of hard tissues of teeth	Digestive	Wald ratio	1	1.007 (0.905, 1.12)	0.901	0.983
Dental caries	Digestive	Wald ratio	1	1.007 (0.905, 1.12)	0.901	0.983
Diseases of pulp and periapical tissues	Digestive	Wald ratio	1	0.988 (0.862, 1.133)	0.865	0.981
Periapical abscess	Digestive	Wald ratio	1	1.023 (0.863, 1.214)	0.79	0.981
Gingival and periodontal diseases	Digestive	Wald ratio	1	0.953 (0.828, 1.096)	0.499	0.974
Gingivitis	Digestive	Wald ratio	1	0.808 (0.475, 1.374)	0.432	0.974
Periodontitis (acute or chronic)	Digestive	Wald ratio	1	0.911 (0.771, 1.077)	0.275	0.923
Acute periodontitis	Digestive	Wald ratio	1	0.827 (0.662, 1.035)	0.097	0.876
Chronic periodontitis	Digestive	Wald ratio	1	0.981 (0.767, 1.255)	0.879	0.983
Dentofacial anomalies, including malocclusion	Digestive	Wald ratio	1	0.793 (0.579, 1.086)	0.148	0.916
Anomalies of tooth position or malocclusion	Digestive	Wald ratio	1	0.793 (0.568, 1.107)	0.173	0.916
Other diseases of the teeth and supporting structures	Digestive	Wald ratio	1	1.023 (0.913, 1.147)	0.689	0.981
Atrophy of edentulous alveolar ridge	Digestive	Wald ratio	1	0.88 (0.445, 1.741)	0.714	0.981
Diseases of the jaws	Digestive	Wald ratio	1	0.994 (0.822, 1.201)	0.949	0.988
Cysts of the jaws	Digestive	Wald ratio	1	0.951 (0.689, 1.312)	0.76	0.981
Anomalies of jaw size or symmetry	Digestive	Wald ratio	1	0.666 (0.312, 1.422)	0.294	0.923
Temporomandibular joint disorders	Digestive	Wald ratio	1	1.027 (0.706, 1.495)	0.888	0.983
Temporomandibular joint disorder, unspecified	Digestive	Wald ratio	1	1.022 (0.699, 1.492)	0.912	0.983
Inflammatory conditions of jaw	Digestive	Wald ratio	1	1.968 (1.114, 3.475)	0.02	0.688
Jaw disease NOS	Digestive	Wald ratio	1	0.901 (0.617, 1.316)	0.589	0.981
Diseases of the salivary glands	Digestive	Wald ratio	1	0.858 (0.689, 1.069)	0.173	0.916
Hypertrophy of salivary gland	Digestive	Wald ratio	1	1.023 (0.537, 1.95)	0.944	0.986
Sialoadenitis	Digestive	Wald ratio	1	0.863 (0.637, 1.169)	0.342	0.954
Disturbance of salivary secretion	Digestive	Wald ratio	1	0.962 (0.468, 1.977)	0.916	0.983
Other specified diseases of the salivary glands	Digestive	Wald ratio	1	0.679 (0.4, 1.155)	0.153	0.916
Diseases of the oral soft tissues, excluding lesions specific for gingiva and tongue	Digestive	Wald ratio	1	1.015 (0.923, 1.116)	0.758	0.981
Stomatitis and mucositis	Digestive	Wald ratio	1	0.917 (0.7, 1.2)	0.526	0.981
Stomatitis and mucositis (ulcerative)	Digestive	Wald ratio	1	1.033 (0.769, 1.389)	0.827	0.981
Oral aphthae	Digestive	Wald ratio	1	0.48 (0.252, 0.913)	0.025	0.717
Cellulitis and abscess of oral soft tissues	Digestive	Wald ratio	1	0.966 (0.636, 1.465)	0.87	0.981
Cysts of oral soft tissues	Digestive	Wald ratio	1	1.097 (0.645, 1.866)	0.732	0.981
Cyst of the salivary gland	Digestive	Wald ratio	1	1.167 (0.636, 2.141)	0.617	0.981
Diseases of lips	Digestive	Wald ratio	1	1.045 (0.836, 1.308)	0.697	0.981
Leukoplakia of oral mucosa	Digestive	Wald ratio	1	1.066 (0.766, 1.482)	0.704	0.981
Sialolithiasis	Digestive	Wald ratio	1	0.971 (0.699, 1.351)	0.863	0.981
Diseases and other conditions of the tongue	Digestive	Wald ratio	1	0.987 (0.835, 1.166)	0.875	0.983
Glossitis	Digestive	Wald ratio	1	0.913 (0.652, 1.28)	0.597	0.981
Diseases of esophagus	Digestive	Wald ratio	1	1.013 (0.98, 1.047)	0.446	0.974
Esophagitis, GERD and related diseases	Digestive	Wald ratio	1	1.018 (0.983, 1.054)	0.323	0.932
GERD	Digestive	Wald ratio	1	0.979 (0.932, 1.028)	0.397	0.974
Ulcer of esophagus	Digestive	Wald ratio	1	1.048 (0.964, 1.139)	0.275	0.923
Reflux esophagitis	Digestive	Wald ratio	1	1.009 (0.953, 1.068)	0.769	0.981
Esophageal bleeding (varices or hemorrhage)	Digestive	Wald ratio	1	1.058 (0.916, 1.222)	0.445	0.974
Stricture and stenosis of esophagus	Digestive	Wald ratio	1	0.975 (0.854, 1.114)	0.71	0.981
Disorders of esophageal motility	Digestive	Wald ratio	1	0.875 (0.694, 1.103)	0.258	0.921
Diverticulum of esophagus, acquired	Digestive	Wald ratio	1	0.679 (0.431, 1.07)	0.096	0.874
Gastroesophageal laceration-hemorrhage syndrome	Digestive	Wald ratio	1	0.848 (0.619, 1.162)	0.306	0.923
Heartburn	Digestive	Wald ratio	1	0.947 (0.833, 1.078)	0.41	0.974
Peptic ulcer (excl. esophageal)	Digestive	Wald ratio	1	0.962 (0.899, 1.03)	0.267	0.923
Hemorrhage from gastrointestinal ulcer	Digestive	Wald ratio	1	0.852 (0.673, 1.077)	0.181	0.916
Gastric ulcer	Digestive	Wald ratio	1	0.973 (0.889, 1.066)	0.56	0.981
Duodenal ulcer	Digestive	Wald ratio	1	0.929 (0.836, 1.033)	0.175	0.916
Peptic ulcer, site unspecified	Digestive	Wald ratio	1	1.05 (0.79, 1.395)	0.739	0.981
Dysphagia	Digestive	Wald ratio	1	0.994 (0.925, 1.068)	0.875	0.983
Gastritis and duodenitis	Digestive	Wald ratio	1	1.005 (0.969, 1.043)	0.771	0.981
Acute gastritis	Digestive	Wald ratio	1	0.897 (0.757, 1.064)	0.213	0.917
Atrophic gastritis	Digestive	Wald ratio	1	1.066 (0.787, 1.444)	0.68	0.981
Duodenitis	Digestive	Wald ratio	1	0.971 (0.907, 1.04)	0.405	0.974
Other specified gastritis	Digestive	Wald ratio	1	1.031 (0.967, 1.1)	0.347	0.959
Gastritis and duodenitis, NOS	Digestive	Wald ratio	1	0.734 (0.432, 1.248)	0.253	0.919
Disorders of function of stomach	Digestive	Wald ratio	1	1.195 (0.84, 1.7)	0.323	0.932
Dyspepsia and other specified disorders of function of stomach	Digestive	Wald ratio	1	1.213 (0.846, 1.739)	0.293	0.923

Other disorders of stomach and duodenum	Digestive	Wald ratio	1	0.984 (0.888, 1.09)	0.759	0.981
Appendiceal conditions	Digestive	Wald ratio	1	0.835 (0.754, 0.925)	5.72E-04	0.109
Appendicitis	Digestive	Wald ratio	1	0.824 (0.744, 0.913)	2.12E-04	0.109
Acute appendicitis	Digestive	Wald ratio	1	0.832 (0.743, 0.932)	0.002	0.131
Abdominal hernia	Digestive	Wald ratio	1	0.991 (0.962, 1.02)	0.519	0.981
Inguinal hernia	Digestive	Wald ratio	1	0.979 (0.932, 1.028)	0.397	0.974
Diaphragmatic hernia	Digestive	Wald ratio	1	0.984 (0.947, 1.022)	0.395	0.974
Femoral hernia	Digestive	Wald ratio	1	1.023 (0.815, 1.285)	0.841	0.981
Umbilical hernia	Digestive	Wald ratio	1	1.048 (0.953, 1.152)	0.337	0.952
Ventral hernia	Digestive	Wald ratio	1	1.043 (0.946, 1.152)	0.397	0.974
Inflammatory bowel disease and other gastroenteritis and colitis	Digestive	Wald ratio	1	0.992 (0.909, 1.082)	0.855	0.981
Regional enteritis	Digestive	Wald ratio	1	1.074 (0.934, 1.236)	0.317	0.923
Ulcerative colitis	Digestive	Wald ratio	1	0.997 (0.897, 1.109)	0.96	0.99
Ulcerative colitis (chronic)	Digestive	Wald ratio	1	1.048 (0.816, 1.345)	0.716	0.981
Ulceration of the lower GI tract	Digestive	Wald ratio	1	1.165 (0.975, 1.392)	0.093	0.874
Ulceration of intestine	Digestive	Wald ratio	1	1.337 (1.069, 1.672)	0.011	0.564
Intestinal malabsorption (non-celiac)	Digestive	Wald ratio	1	1.025 (0.901, 1.167)	0.702	0.981
Celiac disease	Digestive	Wald ratio	1	1.006 (0.878, 1.153)	0.929	0.986
Noninfectious gastroenteritis	Digestive	Wald ratio	1	1.029 (0.98, 1.081)	0.249	0.919
Ileostomy status	Digestive	Wald ratio	1	1.085 (0.939, 1.253)	0.269	0.923
Intestinal obstruction without mention of hernia	Digestive	Wald ratio	1	0.958 (0.875, 1.05)	0.359	0.963
Paralytic ileus	Digestive	Wald ratio	1	1.186 (0.92, 1.528)	0.189	0.917
Impaction of intestine	Digestive	Wald ratio	1	1.363 (0.954, 1.946)	0.089	0.874
Peritoneal or intestinal adhesions	Digestive	Wald ratio	1	0.949 (0.776, 1.16)	0.61	0.981
Other intestinal obstruction	Digestive	Wald ratio	1	0.913 (0.824, 1.012)	0.082	0.874
Symptoms involving digestive system	Digestive	Wald ratio	1	1.027 (0.982, 1.075)	0.243	0.919
Diarrhea	Digestive	Wald ratio	1	1.037 (0.793, 1.358)	0.789	0.981
Flatulence	Digestive	Wald ratio	1	1.093 (0.95, 1.258)	0.214	0.917
Diverticulosis and diverticulitis	Digestive	Wald ratio	1	1.01 (0.972, 1.049)	0.617	0.981
Diverticulosis	Digestive	Wald ratio	1	1.01 (0.973, 1.049)	0.596	0.981
Constipation	Digestive	Wald ratio	1	1.022 (0.965, 1.081)	0.463	0.974
Functional digestive disorders	Digestive	Wald ratio	1	0.997 (0.956, 1.04)	0.892	0.983
Irritable Bowel Syndrome	Digestive	Wald ratio	1	1.064 (0.982, 1.152)	0.128	0.916
Abnormal findings on exam of gastrointestinal tract or abdominal area	Digestive	Wald ratio	1	0.997 (0.863, 1.151)	0.962	0.99
Personal history of diseases of digestive system	Digestive	Wald ratio	1	0.982 (0.935, 1.032)	0.474	0.974
Anal and rectal conditions	Digestive	Wald ratio	1	1.043 (0.993, 1.096)	0.091	0.874
Anal and rectal polyp	Digestive	Wald ratio	1	1.045 (0.977, 1.119)	0.201	0.917
Peritonitis and retroperitoneal infections	Digestive	Wald ratio	1	0.848 (0.699, 1.029)	0.096	0.874
Other disorders of peritoneum	Digestive	Wald ratio	1	0.996 (0.899, 1.103)	0.941	0.986
Peritoneal adhesions (postoperative) (postinfection)	Digestive	Wald ratio	1	1.009 (0.908, 1.122)	0.867	0.981
Other disorders of intestine	Digestive	Wald ratio	1	1.112 (1.016, 1.218)	0.022	0.688
Toxic gastroenteritis and colitis	Digestive	Wald ratio	1	1.045 (0.639, 1.711)	0.86	0.981
Gastrointestinal complications	Digestive	Wald ratio	1	1.147 (0.88, 1.496)	0.31	0.923
Other chronic nonalcoholic liver disease	Digestive	Wald ratio	1	0.987 (0.854, 1.139)	0.854	0.981
Cirrhosis of liver without mention of alcohol	Digestive	Wald ratio	1	0.872 (0.513, 1.482)	0.612	0.981
Primary biliary cirrhosis	Digestive	Wald ratio	1	1.501 (1.039, 2.168)	0.03	0.749
Liver abscess and sequelae of chronic liver disease	Digestive	Wald ratio	1	1.048 (0.867, 1.266)	0.631	0.981
Portal hypertension	Digestive	Wald ratio	1	1.158 (0.899, 1.493)	0.257	0.919
Malposition and malpresentation of fetus or obstruction	Digestive	Wald ratio	1	1.019 (0.879, 1.182)	0.802	0.981
Simple and unspecified goiter	Endocrine/metabolic	Wald ratio	1	0.878 (0.692, 1.115)	0.288	0.923
Nontoxic nodular goiter	Endocrine/metabolic	Wald ratio	1	1.066 (0.895, 1.269)	0.473	0.974
Nontoxic uninodular goiter	Endocrine/metabolic	Wald ratio	1	1.237 (0.945, 1.619)	0.121	0.916
Nontoxic multinodular goiter	Endocrine/metabolic	Wald ratio	1	0.915 (0.732, 1.144)	0.436	0.974
Thyrotoxicosis with or without goiter	Endocrine/metabolic	Wald ratio	1	0.983 (0.858, 1.127)	0.805	0.981
Graves' disease	Endocrine/metabolic	Wald ratio	1	0.763 (0.581, 1.002)	0.052	0.856
Toxic multinodular goiter	Endocrine/metabolic	Wald ratio	1	0.834 (0.455, 1.529)	0.557	0.981
Exophthalmos	Endocrine/metabolic	Wald ratio	1	0.706 (0.431, 1.156)	0.166	0.916
Hypothyroidism	Endocrine/metabolic	Wald ratio	1	1.027 (0.978, 1.079)	0.282	0.923
Secondary hypothyroidism	Endocrine/metabolic	Wald ratio	1	0.906 (0.761, 1.079)	0.268	0.923
Acquired hypothyroidism	Endocrine/metabolic	Wald ratio	1	0.979 (0.554, 1.729)	0.942	0.986
Hypothyroidism NOS	Endocrine/metabolic	Wald ratio	1	1.035 (0.982, 1.092)	0.199	0.917
Thyroiditis	Endocrine/metabolic	Wald ratio	1	0.981 (0.697, 1.38)	0.912	0.983
Chronic thyroiditis	Endocrine/metabolic	Wald ratio	1	0.855 (0.585, 1.249)	0.418	0.974
Chronic lymphocytic thyroiditis	Endocrine/metabolic	Wald ratio	1	0.889 (0.586, 1.349)	0.579	0.981
Other disorders of thyroid	Endocrine/metabolic	Wald ratio	1	1.068 (0.81, 1.408)	0.641	0.981
Diabetes mellitus	Endocrine/metabolic	Wald ratio	1	1.043 (0.997, 1.092)	0.067	0.874
Type 1 diabetes	Endocrine/metabolic	Wald ratio	1	0.992 (0.885, 1.112)	0.891	0.983
Type 1 diabetes with ketoacidosis	Endocrine/metabolic	Wald ratio	1	1.416 (0.97, 2.069)	0.072	0.874
Type 1 diabetes with renal manifestations	Endocrine/metabolic	Wald ratio	1	0.763 (0.386, 1.509)	0.437	0.974
Type 2 diabetes	Endocrine/metabolic	Wald ratio	1	1.041 (0.995, 1.09)	0.08	0.874
Type 2 diabetes with ketoacidosis	Endocrine/metabolic	Wald ratio	1	0.843 (0.478, 1.489)	0.557	0.981
Type 2 diabetes with renal manifestations	Endocrine/metabolic	Wald ratio	1	0.966 (0.636, 1.465)	0.87	0.981
Type 2 diabetes with ophthalmic manifestations	Endocrine/metabolic	Wald ratio	1	0.892 (0.758, 1.05)	0.17	0.916
Type 2 diabetes with neurological manifestations	Endocrine/metabolic	Wald ratio	1	0.763 (0.598, 0.972)	0.029	0.749
Abnormal glucose	Endocrine/metabolic	Wald ratio	1	1.311 (1.048, 1.64)	0.018	0.68
Impaired fasting glucose	Endocrine/metabolic	Wald ratio	1	1.119 (0.783, 1.598)	0.537	0.981
Other abnormal glucose	Endocrine/metabolic	Wald ratio	1	1.416 (1.066, 1.882)	0.016	0.68
Glycosuria or Acetonuria	Endocrine/metabolic	Wald ratio	1	1.119 (0.683, 1.831)	0.655	0.981
Polyneuropathy in diabetes	Endocrine/metabolic	Wald ratio	1	1.017 (0.745, 1.388)	0.916	0.983

Diabetic retinopathy	Endocrine/metabolic	Wald ratio	1	0.968 (0.825, 1.135)	0.686	0.981
Other disorders of pancreatic internal secretion	Endocrine/metabolic	Wald ratio	1	0.995 (0.823, 1.202)	0.955	0.989
Hypoglycemia	Endocrine/metabolic	Wald ratio	1	0.995 (0.823, 1.202)	0.955	0.989
Disorders of parathyroid gland	Endocrine/metabolic	Wald ratio	1	0.778 (0.639, 0.947)	0.012	0.564
Hyperparathyroidism	Endocrine/metabolic	Wald ratio	1	0.748 (0.607, 0.922)	0.006	0.406
Hypoparathyroidism	Endocrine/metabolic	Wald ratio	1	1.622 (0.991, 2.655)	0.054	0.87
Disorders of the pituitary gland and its hypothalamic control	Endocrine/metabolic	Wald ratio	1	0.834 (0.669, 1.039)	0.105	0.902
Pituitary hyperfunction	Endocrine/metabolic	Wald ratio	1	0.748 (0.493, 1.135)	0.173	0.916
Acromegaly and gigantism	Endocrine/metabolic	Wald ratio	1	0.582 (0.317, 1.067)	0.08	0.874
Pituitary hypofunction	Endocrine/metabolic	Wald ratio	1	0.835 (0.583, 1.198)	0.328	0.94
Diabetes insipidus	Endocrine/metabolic	Wald ratio	1	0.808 (0.441, 1.483)	0.492	0.974
Other disorders of neurohypophysis	Endocrine/metabolic	Wald ratio	1	1.136 (0.721, 1.791)	0.582	0.981
Disorders of adrenal glands	Endocrine/metabolic	Wald ratio	1	1.016 (0.807, 1.281)	0.89	0.983
Adrenal hyperfunction	Endocrine/metabolic	Wald ratio	1	0.96 (0.633, 1.457)	0.849	0.981
Cushing's syndrome	Endocrine/metabolic	Wald ratio	1	1.054 (0.597, 1.86)	0.857	0.981
Hyperaldosteronism	Endocrine/metabolic	Wald ratio	1	0.904 (0.457, 1.789)	0.773	0.981
Adrenal hypofunction	Endocrine/metabolic	Wald ratio	1	1.125 (0.828, 1.53)	0.451	0.974
Glucocorticoid deficiency	Endocrine/metabolic	Wald ratio	1	1.127 (0.829, 1.533)	0.444	0.974
Ovarian dysfunction	Endocrine/metabolic	Wald ratio	1	1.007 (0.692, 1.465)	0.973	0.991
Polycystic ovaries	Endocrine/metabolic	Wald ratio	1	1.054 (0.694, 1.599)	0.806	0.981
Testicular dysfunction	Endocrine/metabolic	Wald ratio	1	1.087 (0.69, 1.713)	0.72	0.981
Testicular hypofunction	Endocrine/metabolic	Wald ratio	1	1.083 (0.687, 1.706)	0.733	0.981
Iatrogenic endocrine disorders	Endocrine/metabolic	Wald ratio	1	0.918 (0.561, 1.503)	0.735	0.981
Other endocrine disorders	Endocrine/metabolic	Wald ratio	1	0.953 (0.628, 1.446)	0.82	0.981
Protein-calorie malnutrition	Endocrine/metabolic	Wald ratio	1	0.947 (0.793, 1.132)	0.551	0.981
Anorexia	Endocrine/metabolic	Wald ratio	1	0.964 (0.788, 1.178)	0.72	0.981
Vitamin deficiency	Endocrine/metabolic	Wald ratio	1	1.019 (0.862, 1.204)	0.827	0.981
Vitamin B-complex deficiencies	Endocrine/metabolic	Wald ratio	1	1.091 (0.882, 1.349)	0.422	0.974
Vitamin D deficiency	Endocrine/metabolic	Wald ratio	1	0.884 (0.673, 1.161)	0.374	0.974
Rickets or osteomalacia	Endocrine/metabolic	Wald ratio	1	1.134 (0.595, 2.16)	0.702	0.981
Mineral deficiency NEC	Endocrine/metabolic	Wald ratio	1	1.337 (0.918, 1.945)	0.13	0.916
Proteinuria	Endocrine/metabolic	Wald ratio	1	1.074 (0.784, 1.471)	0.656	0.981
Disorders of protein plasma or amino-acid transport and metabolism	Endocrine/metabolic	Wald ratio	1	0.97 (0.727, 1.293)	0.833	0.981
Disorders of plasma protein metabolism	Endocrine/metabolic	Wald ratio	1	0.984 (0.724, 1.337)	0.916	0.983
Paraproteinemia	Endocrine/metabolic	Wald ratio	1	0.863 (0.569, 1.31)	0.49	0.974
Amyloidosis	Endocrine/metabolic	Wald ratio	1	1.261 (0.714, 2.227)	0.424	0.974
Macroglobulinemia	Endocrine/metabolic	Wald ratio	1	1.003 (0.507, 1.985)	0.992	0.995
Disorders of carbohydrate transport and metabolism	Endocrine/metabolic	Wald ratio	1	1.149 (0.729, 1.811)	0.549	0.981
Intestinal disaccharidase deficiencies and disaccharide malabsorption	Endocrine/metabolic	Wald ratio	1	1.048 (0.64, 1.715)	0.854	0.981
Disorders of lipoid metabolism	Endocrine/metabolic	Wald ratio	1	1.029 (0.995, 1.065)	0.096	0.874
Hyperlipidemia	Endocrine/metabolic	Wald ratio	1	1.027 (0.993, 1.063)	0.12	0.916
Hypercholesterolemia	Endocrine/metabolic	Wald ratio	1	1.029 (0.994, 1.066)	0.107	0.905
Hyperglyceridemia	Endocrine/metabolic	Wald ratio	1	1.622 (0.76, 3.461)	0.211	0.917
Mixed hyperlipidemia	Endocrine/metabolic	Wald ratio	1	1.08 (0.686, 1.703)	0.739	0.981
Unspecified disorder of lipoid metabolism	Endocrine/metabolic	Wald ratio	1	1.311 (0.864, 1.989)	0.203	0.917
Gout and other crystal arthropathies	Endocrine/metabolic	Wald ratio	1	1.022 (0.929, 1.123)	0.66	0.981
Gout	Endocrine/metabolic	Wald ratio	1	1.013 (0.911, 1.126)	0.814	0.981
Gouty arthropathy	Endocrine/metabolic	Wald ratio	1	1.261 (0.742, 2.144)	0.391	0.974
Crystal arthropathies	Endocrine/metabolic	Wald ratio	1	1.087 (0.859, 1.375)	0.488	0.974
Chondrocalcinosis	Endocrine/metabolic	Wald ratio	1	1.087 (0.849, 1.39)	0.508	0.977
Disorders of mineral metabolism	Endocrine/metabolic	Wald ratio	1	1.083 (0.952, 1.231)	0.228	0.917
Disorders of magnesium metabolism	Endocrine/metabolic	Wald ratio	1	1.125 (0.834, 1.518)	0.44	0.974
Disorders of calcium or phosphorus metabolism	Endocrine/metabolic	Wald ratio	1	1.204 (1.019, 1.423)	0.029	0.749
Disorders of phosphorus metabolism	Endocrine/metabolic	Wald ratio	1	1.064 (0.675, 1.677)	0.79	0.981
Disorders of fluid, electrolyte, and acid-base balance	Endocrine/metabolic	Wald ratio	1	0.993 (0.928, 1.064)	0.85	0.981
Electrolyte imbalance	Endocrine/metabolic	Wald ratio	1	0.951 (0.868, 1.042)	0.279	0.923
Hyperosmolality and or or hypernatremia	Endocrine/metabolic	Wald ratio	1	0.938 (0.645, 1.365)	0.739	0.981
Hyposmolality and or or hyponatremia	Endocrine/metabolic	Wald ratio	1	0.966 (0.843, 1.107)	0.617	0.981
Hyperpotassemia	Endocrine/metabolic	Wald ratio	1	0.899 (0.747, 1.083)	0.262	0.923
Hypopotassemia	Endocrine/metabolic	Wald ratio	1	0.922 (0.789, 1.077)	0.306	0.923
Acid-base balance disorder	Endocrine/metabolic	Wald ratio	1	0.834 (0.703, 0.989)	0.037	0.792
Acidosis	Endocrine/metabolic	Wald ratio	1	0.824 (0.69, 0.985)	0.033	0.771
Alkalosis	Endocrine/metabolic	Wald ratio	1	1.052 (0.619, 1.788)	0.853	0.981
Hypovolemia	Endocrine/metabolic	Wald ratio	1	1.083 (0.97, 1.208)	0.157	0.916
Fluid overload	Endocrine/metabolic	Wald ratio	1	1.009 (0.768, 1.326)	0.948	0.988
Polydipsia	Endocrine/metabolic	Wald ratio	1	0.793 (0.466, 1.348)	0.391	0.974
Other disorders of metabolism	Endocrine/metabolic	Wald ratio	1	1.089 (0.932, 1.272)	0.283	0.923
Disorders of bilirubin excretion	Endocrine/metabolic	Wald ratio	1	1.117 (0.824, 1.512)	0.476	0.974
Other disorders of lipoid metabolism	Endocrine/metabolic	Wald ratio	1	1.06 (0.725, 1.548)	0.764	0.981
Lipoprotein disorders	Endocrine/metabolic	Wald ratio	1	1.163 (0.525, 2.578)	0.71	0.981
Overweight, obesity and other hyperalimentation	Endocrine/metabolic	Wald ratio	1	0.979 (0.925, 1.036)	0.463	0.974
Obesity	Endocrine/metabolic	Wald ratio	1	0.984 (0.93, 1.041)	0.575	0.981
Localized adiposity	Endocrine/metabolic	Wald ratio	1	0.877 (0.46, 1.67)	0.689	0.981
Abnormal weight gain	Endocrine/metabolic	Wald ratio	1	1.108 (0.539, 2.277)	0.78	0.981
Disorders involving the immune mechanism	Endocrine/metabolic	Wald ratio	1	0.865 (0.663, 1.128)	0.284	0.923
Immunity deficiency	Endocrine/metabolic	Wald ratio	1	0.734 (0.484, 1.114)	0.146	0.916
Deficiency of humoral immunity	Endocrine/metabolic	Wald ratio	1	0.679 (0.415, 1.112)	0.124	0.916
Other immunological findings	Endocrine/metabolic	Wald ratio	1	0.929 (0.643, 1.342)	0.695	0.981
Noninflammatory female genital disorders	Genitourinary	Wald ratio	1	0.995 (0.937, 1.058)	0.881	0.983

Noninflammatory disorders of vulva and perineum	Genitourinary	Wald ratio	1	1.012 (0.863, 1.187)	0.885	0.983
Dysplasia of female genital organs	Genitourinary	Wald ratio	1	1.039 (0.525, 2.057)	0.912	0.983
Endometrial hyperplasia	Genitourinary	Wald ratio	1	1.145 (0.962, 1.363)	0.128	0.916
Polyp of female genital organs	Genitourinary	Wald ratio	1	1.004 (0.948, 1.062)	0.899	0.983
Polyp of corpus uteri	Genitourinary	Wald ratio	1	1.012 (0.945, 1.084)	0.731	0.981
Mucous polyp of cervix	Genitourinary	Wald ratio	1	0.953 (0.863, 1.051)	0.336	0.952
Hypertrophy of female genital organs	Genitourinary	Wald ratio	1	1.106 (0.939, 1.302)	0.227	0.917
Symptoms involving female genital tract	Genitourinary	Wald ratio	1	1.019 (0.948, 1.095)	0.617	0.981
Dystrophy of female genital tract	Genitourinary	Wald ratio	1	0.982 (0.672, 1.435)	0.926	0.986
Atrophy of female genital tract	Genitourinary	Wald ratio	1	1.237 (0.938, 1.631)	0.132	0.916
stress incontinence, female	Genitourinary	Wald ratio	1	1.017 (0.939, 1.101)	0.682	0.981
Pain and other symptoms associated with female genital organs	Genitourinary	Wald ratio	1	1.035 (0.931, 1.151)	0.52	0.981
Dyspareunia	Genitourinary	Wald ratio	1	0.995 (0.839, 1.18)	0.956	0.989
Disorders of menstruation and other abnormal bleeding from female genital tract	Genitourinary	Wald ratio	1	1.003 (0.958, 1.05)	0.901	0.983
Irregular menstrual cycle or bleeding	Genitourinary	Wald ratio	1	1.008 (0.96, 1.059)	0.747	0.981
Absent or infrequent menstruation	Genitourinary	Wald ratio	1	1.066 (0.651, 1.745)	0.8	0.981
Excessive or frequent menstruation	Genitourinary	Wald ratio	1	1.017 (0.957, 1.081)	0.587	0.981
Irregular menstrual cycle	Genitourinary	Wald ratio	1	1.033 (0.905, 1.18)	0.627	0.981
Irregular menstrual bleeding	Genitourinary	Wald ratio	1	1.015 (0.923, 1.116)	0.755	0.981
Dysmenorrhea	Genitourinary	Wald ratio	1	0.988 (0.858, 1.136)	0.863	0.981
Premenstrual tension syndromes	Genitourinary	Wald ratio	1	1.591 (1.009, 2.507)	0.046	0.792
Infertility, female	Genitourinary	Wald ratio	1	0.953 (0.809, 1.121)	0.561	0.981
Menopausal and postmenopausal disorders	Genitourinary	Wald ratio	1	1.003 (0.948, 1.062)	0.904	0.983
Postmenopausal bleeding	Genitourinary	Wald ratio	1	0.999 (0.937, 1.066)	0.982	0.991
Symptomatic menopause	Genitourinary	Wald ratio	1	0.763 (0.503, 1.157)	0.203	0.917
Postmenopausal atrophic vaginitis	Genitourinary	Wald ratio	1	1.136 (0.954, 1.353)	0.151	0.916
Premenopausal menorrhagia	Genitourinary	Wald ratio	1	0.915 (0.658, 1.272)	0.597	0.981
Ovarian cyst	Genitourinary	Wald ratio	1	0.964 (0.883, 1.052)	0.409	0.974
Disorders of iron metabolism	Hematopoietic	Wald ratio	1	0.933 (0.746, 1.167)	0.542	0.981
Iron deficiency anemias	Hematopoietic	Wald ratio	1	0.993 (0.928, 1.063)	0.846	0.981
Iron deficiency anemias, unspecified or not due to blood loss	Hematopoietic	Wald ratio	1	0.988 (0.923, 1.058)	0.731	0.981
Iron deficiency anemia secondary to blood loss (chronic)	Hematopoietic	Wald ratio	1	1.11 (0.845, 1.458)	0.453	0.974
Other deficiency anemia	Hematopoietic	Wald ratio	1	0.857 (0.72, 1.02)	0.082	0.874
Megaloblastic anemia	Hematopoietic	Wald ratio	1	0.852 (0.713, 1.018)	0.077	0.874
Pernicious anemia	Hematopoietic	Wald ratio	1	0.808 (0.654, 1)	0.049	0.839
Other vitamin B12 deficiency anemia	Hematopoietic	Wald ratio	1	0.778 (0.493, 1.226)	0.279	0.923
Folate-deficiency anemia	Hematopoietic	Wald ratio	1	1.062 (0.7, 1.611)	0.778	0.981
Deficiency anemias	Hematopoietic	Wald ratio	1	1.052 (0.474, 2.331)	0.901	0.983
Hereditary hemolytic anemias	Hematopoietic	Wald ratio	1	1.213 (0.77, 1.912)	0.405	0.974
Other hemoglobinopathies	Hematopoietic	Wald ratio	1	0.989 (0.481, 2.032)	0.976	0.991
Other hereditary hemolytic anemias	Hematopoietic	Wald ratio	1	1.261 (0.662, 2.403)	0.48	0.974
Acquired hemolytic anemias	Hematopoietic	Wald ratio	1	1.653 (1.01, 2.707)	0.046	0.792
Autoimmune hemolytic anemias	Hematopoietic	Wald ratio	1	1.472 (0.834, 2.6)	0.182	0.916
Aplastic anemia	Hematopoietic	Wald ratio	1	0.982 (0.741, 1.299)	0.897	0.983
Other anemias	Hematopoietic	Wald ratio	1	0.973 (0.923, 1.026)	0.317	0.923
Acute posthemorrhagic anemia	Hematopoietic	Wald ratio	1	1.072 (0.754, 1.525)	0.699	0.981
Anemia of chronic disease	Hematopoietic	Wald ratio	1	0.863 (0.693, 1.076)	0.19	0.917
Anemia in neoplastic disease	Hematopoietic	Wald ratio	1	1.106 (0.832, 1.469)	0.488	0.974
Coagulation defects	Hematopoietic	Wald ratio	1	1.06 (0.877, 1.281)	0.549	0.981
Congenital coagulation defects	Hematopoietic	Wald ratio	1	0.944 (0.721, 1.235)	0.673	0.981
Von willebrand's disease	Hematopoietic	Wald ratio	1	0.679 (0.385, 1.199)	0.182	0.916
Congenital deficiency of other clotting factors (including factor VII)	Hematopoietic	Wald ratio	1	0.933 (0.671, 1.297)	0.679	0.981
Congenital factor VIII disorder	Hematopoietic	Wald ratio	1	1.195 (0.581, 2.455)	0.628	0.981
Hemorrhagic disorder due to intrinsic circulating anticoagulants	Hematopoietic	Wald ratio	1	1.653 (0.868, 3.15)	0.126	0.916
Other and unspecified coagulation defects	Hematopoietic	Wald ratio	1	1.108 (0.824, 1.489)	0.497	0.974
Purpura and other hemorrhagic conditions	Hematopoietic	Wald ratio	1	1.002 (0.874, 1.148)	0.978	0.991
Spontaneous ecchymoses	Hematopoietic	Wald ratio	1	0.908 (0.534, 1.543)	0.721	0.981
Allergic purpura	Hematopoietic	Wald ratio	1	2.006 (0.905, 4.447)	0.086	0.874
Thrombocytopenia	Hematopoietic	Wald ratio	1	0.984 (0.849, 1.141)	0.831	0.981
Primary thrombocytopenia	Hematopoietic	Wald ratio	1	0.845 (0.636, 1.123)	0.246	0.919
Secondary thrombocytopenia	Hematopoietic	Wald ratio	1	1.261 (0.637, 2.495)	0.505	0.974
Diseases of white blood cells	Hematopoietic	Wald ratio	1	0.92 (0.837, 1.012)	0.085	0.874
Decreased white blood cell count	Hematopoietic	Wald ratio	1	0.899 (0.812, 0.996)	0.042	0.792
Neutropenia	Hematopoietic	Wald ratio	1	0.899 (0.812, 0.996)	0.042	0.792
Eosinophilia	Hematopoietic	Wald ratio	1	0.679 (0.343, 1.344)	0.267	0.923
Other diseases of blood and blood-forming organs	Hematopoietic	Wald ratio	1	1.012 (0.924, 1.108)	0.799	0.981
Personal history of diseases of blood and blood-forming organs	Hematopoietic	Wald ratio	1	0.953 (0.677, 1.34)	0.781	0.981
Lymphadenitis	Hematopoietic	Wald ratio	1	1.033 (0.922, 1.158)	0.571	0.981
Diseases of spleen	Hematopoietic	Wald ratio	1	1.002 (0.774, 1.296)	0.991	0.995
Polycythemia, secondary	Hematopoietic	Wald ratio	1	1.054 (0.752, 1.476)	0.762	0.981
Abnormality of red blood cells	Hematopoietic	Wald ratio	1	1.138 (0.621, 2.088)	0.675	0.981
Intestinal infection	Infectious diseases	Wald ratio	1	1.019 (0.959, 1.082)	0.553	0.981
Bacterial enteritis	Infectious diseases	Wald ratio	1	1.083 (0.97, 1.208)	0.157	0.916
Intestinal infection due to C. difficile	Infectious diseases	Wald ratio	1	0.951 (0.757, 1.194)	0.665	0.981
Viral Enteritis	Infectious diseases	Wald ratio	1	0.903 (0.741, 1.099)	0.308	0.923
Intestinal infection due to protozoa	Infectious diseases	Wald ratio	1	0.679 (0.306, 1.506)	0.341	0.954
Tuberculosis	Infectious diseases	Wald ratio	1	1.337 (0.848, 2.106)	0.211	0.917
Septicemia	Infectious diseases	Wald ratio	1	0.966 (0.882, 1.058)	0.453	0.974

Gram negative septicemia	Infectious diseases	Wald ratio	1	1.037 (0.849, 1.268)	0.72	0.981
Gram positive septicemia	Infectious diseases	Wald ratio	1	0.899 (0.692, 1.168)	0.425	0.974
Bacterial infection NOS	Infectious diseases	Wald ratio	1	0.971 (0.921, 1.024)	0.284	0.923
Staphylococcus infections	Infectious diseases	Wald ratio	1	0.989 (0.89, 1.1)	0.844	0.981
Streptococcus infection	Infectious diseases	Wald ratio	1	0.94 (0.814, 1.086)	0.4	0.974
E. coli	Infectious diseases	Wald ratio	1	0.945 (0.847, 1.055)	0.317	0.923
Herpes zoster	Infectious diseases	Wald ratio	1	1.045 (0.787, 1.389)	0.759	0.981
Herpes zoster with nervous system complications	Infectious diseases	Wald ratio	1	0.653 (0.356, 1.198)	0.169	0.916
Herpes simplex	Infectious diseases	Wald ratio	1	1.213 (0.831, 1.773)	0.317	0.923
Viral hepatitis	Infectious diseases	Wald ratio	1	0.873 (0.739, 1.032)	0.112	0.916
Viral hepatitis A	Infectious diseases	Wald ratio	1	0.918 (0.464, 1.817)	0.807	0.981
Viral hepatitis B	Infectious diseases	Wald ratio	1	0.72 (0.44, 1.178)	0.191	0.917
Viral hepatitis C	Infectious diseases	Wald ratio	1	1.043 (0.725, 1.502)	0.819	0.981
Chronic hepatitis	Infectious diseases	Wald ratio	1	0.824 (0.581, 1.168)	0.277	0.923
Hepatitis NOS	Infectious diseases	Wald ratio	1	0.842 (0.636, 1.114)	0.229	0.917
Human immunodeficiency virus [HIV] disease	Infectious diseases	Wald ratio	1	1.286 (0.786, 2.105)	0.317	0.923
HIV infection, symptomatic	Infectious diseases	Wald ratio	1	1.199 (0.705, 2.039)	0.502	0.974
Viral warts & HPV	Infectious diseases	Wald ratio	1	0.857 (0.714, 1.028)	0.096	0.874
Viral infection	Infectious diseases	Wald ratio	1	0.977 (0.875, 1.091)	0.679	0.981
Varicella infection	Infectious diseases	Wald ratio	1	0.72 (0.35, 1.479)	0.371	0.972
Infectious mononucleosis	Infectious diseases	Wald ratio	1	1.213 (0.59, 2.493)	0.599	0.981
Postoperative infection	Infectious diseases	Wald ratio	1	1.004 (0.92, 1.096)	0.927	0.986
Infection or inflammation of internal prosthetic device; implant; and graft	Infectious diseases	Wald ratio	1	1.041 (0.926, 1.171)	0.498	0.974
Sexually transmitted infections (not HIV or hepatitis)	Infectious diseases	Wald ratio	1	0.593 (0.3, 1.174)	0.134	0.916
Dermatophytosis or Dermatomycosis	Infectious diseases	Wald ratio	1	0.793 (0.562, 1.119)	0.187	0.917
Dermatophytosis	Infectious diseases	Wald ratio	1	0.793 (0.559, 1.124)	0.192	0.917
Dermatophytosis of nail	Infectious diseases	Wald ratio	1	0.629 (0.318, 1.244)	0.182	0.916
Althete's foot	Infectious diseases	Wald ratio	1	0.748 (0.457, 1.225)	0.249	0.919
Candidiasis	Infectious diseases	Wald ratio	1	1.041 (0.919, 1.18)	0.525	0.981
Mycoses	Infectious diseases	Wald ratio	1	1.007 (0.705, 1.438)	0.97	0.991
Aspergillosis	Infectious diseases	Wald ratio	1	0.882 (0.56, 1.39)	0.588	0.981
Spirochetal infection	Infectious diseases	Wald ratio	1	1.786 (0.938, 3.403)	0.078	0.874
Arthropod-borne diseases	Infectious diseases	Wald ratio	1	0.498 (0.252, 0.986)	0.046	0.792
Helminthiases	Infectious diseases	Wald ratio	1	0.944 (0.555, 1.604)	0.83	0.981
Intestinal helminthiases	Infectious diseases	Wald ratio	1	0.931 (0.527, 1.644)	0.805	0.981
Other infectious and parasitic diseases	Infectious diseases	Wald ratio	1	0.938 (0.618, 1.424)	0.764	0.981
Sepsis and SIRS	Infectious diseases	Wald ratio	1	0.949 (0.85, 1.059)	0.352	0.962
Sepsis	Infectious diseases	Wald ratio	1	0.949 (0.85, 1.059)	0.352	0.962
Burns	Injuries & poisonings	Wald ratio	1	1.039 (0.816, 1.325)	0.755	0.981
Foreign body injury	Injuries & poisonings	Wald ratio	1	1.104 (0.934, 1.304)	0.246	0.919
Crushing injury	Injuries & poisonings	Wald ratio	1	0.951 (0.661, 1.368)	0.787	0.981
Injury to blood vessels	Injuries & poisonings	Wald ratio	1	1.033 (0.715, 1.493)	0.861	0.981
Crushing or internal injury to organs	Injuries & poisonings	Wald ratio	1	1.261 (1.051, 1.513)	0.012	0.564
Injury, NOS	Injuries & poisonings	Wald ratio	1	1.08 (1.013, 1.152)	0.019	0.688
Complications of surgical and medical procedures	Injuries & poisonings	Wald ratio	1	0.998 (0.939, 1.06)	0.95	0.988
Late effect	Injuries & poisonings	Wald ratio	1	0.824 (0.467, 1.455)	0.505	0.974
Effects of heat, cold and air pressure	Injuries & poisonings	Wald ratio	1	0.72 (0.35, 1.479)	0.371	0.972
Complications of in utero procedures	Injuries & poisonings	Wald ratio	1	1.045 (0.472, 2.318)	0.913	0.983
Nonspecific abnormal findings on radiological and other examination of musculoskeletal system	Injuries & poisonings	Wald ratio	1	1.033 (0.829, 1.288)	0.769	0.981
Fracture of lower limb	Injuries & poisonings	Wald ratio	1	0.924 (0.863, 0.989)	0.023	0.688
Fracture of neck of femur	Injuries & poisonings	Wald ratio	1	0.89 (0.777, 1.021)	0.096	0.874
Fracture of unspecified part of femur	Injuries & poisonings	Wald ratio	1	1.237 (0.785, 1.95)	0.359	0.963
Fracture of tibia and fibula	Injuries & poisonings	Wald ratio	1	0.968 (0.854, 1.097)	0.606	0.981
Fracture of patella	Injuries & poisonings	Wald ratio	1	0.897 (0.726, 1.11)	0.317	0.923
Fracture of ankle and foot	Injuries & poisonings	Wald ratio	1	1.043 (0.924, 1.178)	0.492	0.974
Fracture of foot	Injuries & poisonings	Wald ratio	1	1.114 (0.972, 1.277)	0.12	0.916
Fracture of pelvis	Injuries & poisonings	Wald ratio	1	1.037 (0.836, 1.288)	0.739	0.981
Fracture of upper limb	Injuries & poisonings	Wald ratio	1	0.975 (0.914, 1.04)	0.444	0.974
Fracture of humerus	Injuries & poisonings	Wald ratio	1	0.922 (0.801, 1.061)	0.256	0.919
Fracture of radius and ulna	Injuries & poisonings	Wald ratio	1	0.981 (0.902, 1.066)	0.649	0.981
Fracture of clavicle or scapula	Injuries & poisonings	Wald ratio	1	0.938 (0.809, 1.088)	0.397	0.974
Fracture of hand or wrist	Injuries & poisonings	Wald ratio	1	0.991 (0.898, 1.094)	0.86	0.981
Fracture of vertebral column without mention of spinal cord injury	Injuries & poisonings	Wald ratio	1	1.127 (0.961, 1.322)	0.14	0.916
Fracture of ribs	Injuries & poisonings	Wald ratio	1	1.099 (0.893, 1.354)	0.373	0.973
Fracture of unspecified bones	Injuries & poisonings	Wald ratio	1	1.286 (1.092, 1.514)	0.003	0.177
Concussion	Injuries & poisonings	Wald ratio	1	0.942 (0.65, 1.366)	0.752	0.981
Intracranial hemorrhage (injury)	Injuries & poisonings	Wald ratio	1	0.808 (0.625, 1.046)	0.106	0.902
Skull and face fracture and other intercranial injury	Injuries & poisonings	Wald ratio	1	0.949 (0.854, 1.055)	0.335	0.952
Torus fracture	Injuries & poisonings	Wald ratio	1	1 (0.866, 1.155)	0.997	0.997
Dislocation	Injuries & poisonings	Wald ratio	1	1.037 (0.912, 1.18)	0.576	0.981
Internal derangement of knee	Injuries & poisonings	Wald ratio	1	0.987 (0.939, 1.036)	0.59	0.981
Traumatic arthropathy	Injuries & poisonings	Wald ratio	1	0.845 (0.656, 1.089)	0.194	0.917
Sprains and strains	Injuries & poisonings	Wald ratio	1	1.213 (0.934, 1.576)	0.147	0.916
Hemorrhage or hematoma complicating a procedure	Injuries & poisonings	Wald ratio	1	1.058 (0.977, 1.145)	0.167	0.916
Complications of transplants and reattached limbs	Injuries & poisonings	Wald ratio	1	0.94 (0.596, 1.481)	0.79	0.981
Complication of colostomy or enterostomy	Injuries & poisonings	Wald ratio	1	1.132 (0.871, 1.47)	0.354	0.962
Complications of cardiac or vascular device, implant, and graft	Injuries & poisonings	Wald ratio	1	1.089 (0.95, 1.248)	0.222	0.917

Mechanical complication of unspecified genitourinary device, implant, and graft	Injuries & poisonings	Wald ratio	1	1.054 (0.892, 1.245)	0.539	0.981
Complication of internal orthopedic device	Injuries & poisonings	Wald ratio	1	0.968 (0.87, 1.076)	0.544	0.981
Complication due to other implant and internal device	Injuries & poisonings	Wald ratio	1	1.031 (0.931, 1.143)	0.553	0.981
Open wounds of head; neck; and trunk	Injuries & poisonings	Wald ratio	1	0.949 (0.863, 1.043)	0.28	0.923
Open wound or laceration of eye or eyelid	Injuries & poisonings	Wald ratio	1	1.045 (0.735, 1.487)	0.805	0.981
Open wound of ear	Injuries & poisonings	Wald ratio	1	0.945 (0.623, 1.435)	0.792	0.981
Other open wound of head and face	Injuries & poisonings	Wald ratio	1	0.92 (0.824, 1.027)	0.138	0.916
Open wound of nose and sinus	Injuries & poisonings	Wald ratio	1	0.897 (0.614, 1.311)	0.575	0.981
Open wound of neck	Injuries & poisonings	Wald ratio	1	0.981 (0.703, 1.369)	0.91	0.983
Open wounds of extremities	Injuries & poisonings	Wald ratio	1	1.087 (0.992, 1.19)	0.073	0.874
Open wound of hand except finger(s)	Injuries & poisonings	Wald ratio	1	1.117 (0.504, 2.475)	0.786	0.981
Open wound of finger(s)	Injuries & poisonings	Wald ratio	1	1.163 (0.634, 2.133)	0.626	0.981
Open wound of foot except toe(s) alone	Injuries & poisonings	Wald ratio	1	0.679 (0.431, 1.07)	0.096	0.874
Traumatic amputation	Injuries & poisonings	Wald ratio	1	1.163 (0.892, 1.516)	0.265	0.923
Broken tooth	Injuries & poisonings	Wald ratio	1	1.261 (0.863, 1.843)	0.23	0.917
Complication of amputation stump	Injuries & poisonings	Wald ratio	1	0.935 (0.593, 1.473)	0.771	0.981
Late effects of musculoskeletal and connective tissue injuries	Injuries & poisonings	Wald ratio	1	0.734 (0.466, 1.157)	0.182	0.916
Injuries to the nervous system	Injuries & poisonings	Wald ratio	1	1.119 (0.954, 1.312)	0.167	0.916
Superficial injury without mention of infection	Injuries & poisonings	Wald ratio	1	0.979 (0.894, 1.072)	0.647	0.981
Contusion	Injuries & poisonings	Wald ratio	1	0.983 (0.844, 1.144)	0.822	0.981
Allergic reaction to food	Injuries & poisonings	Wald ratio	1	1.119 (0.737, 1.698)	0.598	0.981
Dermatitis due to solar radiation	Injuries & poisonings	Wald ratio	1	1.052 (0.782, 1.413)	0.739	0.981
Chronic dermatitis due to solar radiation	Injuries & poisonings	Wald ratio	1	1.023 (0.709, 1.478)	0.902	0.983
Adverse reaction to serum or vaccine	Injuries & poisonings	Wald ratio	1	0.848 (0.581, 1.239)	0.395	0.974
Infusion and transfusion reaction	Injuries & poisonings	Wald ratio	1	0.927 (0.525, 1.638)	0.795	0.981
Anaphylactic shock NOS	Injuries & poisonings	Wald ratio	1	1.003 (0.781, 1.288)	0.982	0.991
Urticaria	Injuries & poisonings	Wald ratio	1	1.311 (0.968, 1.775)	0.08	0.874
Allergies, other	Injuries & poisonings	Wald ratio	1	1.016 (0.758, 1.36)	0.917	0.983
Spinal cord injury without evidence of spinal bone injury	Injuries & poisonings	Wald ratio	1	1.104 (0.831, 1.467)	0.497	0.974
Injury to other and unspecified nerves	Injuries & poisonings	Wald ratio	1	0.906 (0.637, 1.289)	0.583	0.981
Certain early complications of trauma or procedure	Injuries & poisonings	Wald ratio	1	0.968 (0.745, 1.257)	0.805	0.981
Postoperative shock	Injuries & poisonings	Wald ratio	1	0.452 (0.204, 1.003)	0.051	0.851
Traumatic and surgical subcutaneous emphysema	Injuries & poisonings	Wald ratio	1	1.686 (0.852, 3.335)	0.134	0.916
Poisoning by antibiotics	Injuries & poisonings	Wald ratio	1	0.984 (0.941, 1.03)	0.5	0.974
Allergy or adverse effect of penicillin	Injuries & poisonings	Wald ratio	1	0.981 (0.934, 1.03)	0.442	0.974
Poisoning by other anti-infectives	Injuries & poisonings	Wald ratio	1	1.102 (0.932, 1.301)	0.256	0.919
Poisoning or allergy of sulfonamides	Injuries & poisonings	Wald ratio	1	1.121 (0.92, 1.365)	0.257	0.919
Poisoning by hormones and synthetic substitutes	Injuries & poisonings	Wald ratio	1	1.143 (0.847, 1.542)	0.382	0.974
Hormones and synthetic substitutes causing adverse effects in therapeutic use	Injuries & poisonings	Wald ratio	1	1.104 (0.812, 1.5)	0.529	0.981
Poisoning by primarily systemic agents	Injuries & poisonings	Wald ratio	1	0.87 (0.596, 1.271)	0.472	0.974
Poisoning by agents primarily affecting blood constituents	Injuries & poisonings	Wald ratio	1	1.857 (1.295, 2.662)	7.56E-04	0.109
Anticoagulants causing adverse effects	Injuries & poisonings	Wald ratio	1	1.893 (1.316, 2.724)	5.87E-04	0.109
Poisoning by analgesics, antipyretics, and antirheumatics	Injuries & poisonings	Wald ratio	1	0.975 (0.907, 1.048)	0.494	0.974
Opiates and related narcotics causing adverse effects in therapeutic use	Injuries & poisonings	Wald ratio	1	0.933 (0.783, 1.11)	0.434	0.974
Antirheumatics causing adverse effects in therapeutic use	Injuries & poisonings	Wald ratio	1	0.808 (0.62, 1.054)	0.116	0.916
Salicylates causing adverse effects in therapeutic use	Injuries & poisonings	Wald ratio	1	0.903 (0.677, 1.204)	0.486	0.974
Poisoning by anticonvulsants and anti-Parkinsonism drugs	Injuries & poisonings	Wald ratio	1	0.778 (0.601, 1.006)	0.056	0.87
Adverse effects of sedatives or other central nervous system depressants and anesthetics	Injuries & poisonings	Wald ratio	1	1.261 (0.99, 1.608)	0.061	0.874
Poisoning by psychotropic agents	Injuries & poisonings	Wald ratio	1	0.92 (0.806, 1.051)	0.219	0.917
Poisoning by drugs primarily affecting the autonomic nervous system	Injuries & poisonings	Wald ratio	1	1.158 (0.763, 1.758)	0.49	0.974
Poisoning by agents primarily affecting the cardiovascular system	Injuries & poisonings	Wald ratio	1	0.904 (0.638, 1.282)	0.572	0.981
Antihypertensive agents causing adverse effects	Injuries & poisonings	Wald ratio	1	0.918 (0.482, 1.75)	0.796	0.981
Poisoning by agents primarily affecting the gastrointestinal system	Injuries & poisonings	Wald ratio	1	0.518 (0.293, 0.915)	0.023	0.692
Poisoning by water, mineral, and uric acid metabolism drugs	Injuries & poisonings	Wald ratio	1	0.666 (0.377, 1.176)	0.162	0.916
Poisoning by agents primarily acting on the smooth and skeletal muscles and respiratory system	Injuries & poisonings	Wald ratio	1	0.763 (0.371, 1.567)	0.461	0.974
Poisoning by agents primarily affecting skin & mucous membrane, ophthalmological, otorhinolaryngological, & dental drugs	Injuries & poisonings	Wald ratio	1	1.311 (0.614, 2.798)	0.484	0.974
Personal history of allergy to medicinal agents	Injuries & poisonings	Wald ratio	1	1.017 (0.696, 1.485)	0.931	0.986
Adverse drug events and drug allergies	Injuries & poisonings	Wald ratio	1	0.985 (0.791, 1.227)	0.893	0.983
Toxic effect of (non-ethyl) alcohol and petroleum and other solvents	Injuries & poisonings	Wald ratio	1	0.917 (0.604, 1.391)	0.682	0.981
Toxic effect of carbon monoxide	Injuries & poisonings	Wald ratio	1	0.808 (0.393, 1.661)	0.563	0.981
Toxic effect of other gases, fumes, or vapors	Injuries & poisonings	Wald ratio	1	1.154 (0.653, 2.038)	0.622	0.981
Toxic effect of noxious substances eaten as food	Injuries & poisonings	Wald ratio	1	1.237 (0.728, 2.103)	0.432	0.974
Toxic effect of other substances, chiefly nonmedicinal as to source	Injuries & poisonings	Wald ratio	1	0.763 (0.357, 1.628)	0.484	0.974
Effects radiation NOS	Injuries & poisonings	Wald ratio	1	0.975 (0.877, 1.084)	0.642	0.981
Delirium dementia and amnestic and other cognitive disorders	Mental disorders	Wald ratio	1	0.966 (0.846, 1.103)	0.607	0.981
Dementias	Mental disorders	Wald ratio	1	0.955 (0.79, 1.154)	0.631	0.981
Alzheimer's disease	Mental disorders	Wald ratio	1	1.037 (0.778, 1.384)	0.803	0.981
Vascular dementia	Mental disorders	Wald ratio	1	0.824 (0.543, 1.251)	0.363	0.97
Delirium due to conditions classified elsewhere	Mental disorders	Wald ratio	1	0.935 (0.744, 1.173)	0.56	0.981
Other persistent mental disorders due to conditions classified elsewhere	Mental disorders	Wald ratio	1	0.89 (0.45, 1.762)	0.739	0.981
Other specified nonpsychotic and or or transient mental disorders	Mental disorders	Wald ratio	1	1.093 (0.867, 1.377)	0.451	0.974
Transient mental disorders due to conditions classified elsewhere	Mental disorders	Wald ratio	1	1.363 (0.802, 2.317)	0.253	0.919
Specific nonpsychotic mental disorders due to brain damage	Mental disorders	Wald ratio	1	0.778 (0.408, 1.481)	0.444	0.974
Alteration of consciousness	Mental disorders	Wald ratio	1	1.078 (0.818, 1.422)	0.593	0.981
Neurological disorders	Mental disorders	Wald ratio	1	0.998 (0.915, 1.089)	0.971	0.991

Aphasia or speech disturbance	Mental disorders	Wald ratio	1	0.985 (0.846, 1.146)	0.841	0.981
Mild cognitive impairment	Mental disorders	Wald ratio	1	1.087 (0.716, 1.649)	0.696	0.981
Memory loss	Mental disorders	Wald ratio	1	1.017 (0.817, 1.268)	0.878	0.983
Altered mental status	Mental disorders	Wald ratio	1	0.988 (0.872, 1.12)	0.851	0.981
Hallucinations	Mental disorders	Wald ratio	1	1.05 (0.752, 1.465)	0.776	0.981
Symptoms involving head and neck	Mental disorders	Wald ratio	1	0.99 (0.891, 1.101)	0.858	0.981
Swelling, mass, or lump in head and neck [Space-occupying lesion, intracranial NOS]	Mental disorders	Wald ratio	1	0.966 (0.796, 1.172)	0.724	0.981
Schizophrenia and other psychotic disorders	Mental disorders	Wald ratio	1	0.899 (0.735, 1.099)	0.299	0.923
Schizophrenia	Mental disorders	Wald ratio	1	0.894 (0.701, 1.139)	0.365	0.97
Paranoid disorders	Mental disorders	Wald ratio	1	0.857 (0.544, 1.35)	0.505	0.974
Psychosis	Mental disorders	Wald ratio	1	1.106 (0.702, 1.743)	0.665	0.981
Mood disorders	Mental disorders	Wald ratio	1	1.005 (0.953, 1.06)	0.841	0.981
Bipolar	Mental disorders	Wald ratio	1	1.085 (0.908, 1.296)	0.372	0.972
Depression	Mental disorders	Wald ratio	1	0.994 (0.939, 1.052)	0.826	0.981
Major depressive disorder	Mental disorders	Wald ratio	1	0.877 (0.645, 1.192)	0.401	0.974
Suicidal ideation or attempt	Mental disorders	Wald ratio	1	1.078 (0.787, 1.477)	0.638	0.981
Suicide or self-inflicted injury	Mental disorders	Wald ratio	1	1.085 (0.783, 1.503)	0.625	0.981
Anxiety disorders	Mental disorders	Wald ratio	1	0.968 (0.9, 1.04)	0.371	0.972
Anxiety disorder	Mental disorders	Wald ratio	1	0.958 (0.888, 1.034)	0.271	0.923
Generalized anxiety disorder	Mental disorders	Wald ratio	1	1.068 (0.678, 1.683)	0.777	0.981
Agorophobia, social phobia, and panic disorder	Mental disorders	Wald ratio	1	0.842 (0.676, 1.049)	0.125	0.916
Phobia	Mental disorders	Wald ratio	1	0.938 (0.722, 1.219)	0.632	0.981
Obsessive-compulsive disorders	Mental disorders	Wald ratio	1	1.337 (0.817, 2.188)	0.249	0.919
Dysthymic disorder	Mental disorders	Wald ratio	1	0.72 (0.325, 1.596)	0.418	0.974
Acute reaction to stress	Mental disorders	Wald ratio	1	0.793 (0.466, 1.348)	0.391	0.974
Posttraumatic stress disorder	Mental disorders	Wald ratio	1	0.582 (0.342, 0.989)	0.046	0.792
Personality disorders	Mental disorders	Wald ratio	1	0.949 (0.69, 1.305)	0.748	0.981
Antisocial or borderline personality disorder	Mental disorders	Wald ratio	1	0.979 (0.576, 1.664)	0.937	0.986
Sexual and gender identity disorders	Mental disorders	Wald ratio	1	0.86 (0.621, 1.191)	0.364	0.97
Psychogenic and somatoform disorders	Mental disorders	Wald ratio	1	0.942 (0.731, 1.214)	0.644	0.981
Psychogenic disorder	Mental disorders	Wald ratio	1	0.964 (0.706, 1.315)	0.817	0.981
Somatoform disorder	Mental disorders	Wald ratio	1	0.834 (0.49, 1.418)	0.502	0.974
Adjustment reaction	Mental disorders	Wald ratio	1	0.793 (0.566, 1.111)	0.178	0.916
Eating disorder (no controls excluded)	Mental disorders	Wald ratio	1	0.679 (0.37, 1.246)	0.211	0.917
Eating disorder	Mental disorders	Wald ratio	1	0.679 (0.37, 1.246)	0.211	0.917
Other mental disorder	Mental disorders	Wald ratio	1	0.996 (0.96, 1.034)	0.846	0.981
Tension headache	Mental disorders	Wald ratio	1	0.875 (0.629, 1.217)	0.428	0.974
Pervasive developmental disorders	Mental disorders	Wald ratio	1	0.539 (0.283, 1.026)	0.06	0.874
Autism	Mental disorders	Wald ratio	1	0.518 (0.234, 1.149)	0.105	0.902
Develomental delays and disorders	Mental disorders	Wald ratio	1	1.02 (0.785, 1.324)	0.885	0.983
Learning disorder	Mental disorders	Wald ratio	1	1.066 (0.676, 1.68)	0.783	0.981
Speech and language disorder	Mental disorders	Wald ratio	1	0.641 (0.349, 1.175)	0.151	0.916
Substance addiction and disorders	Mental disorders	Wald ratio	1	1.286 (0.957, 1.728)	0.096	0.874
Polyneuropathy due to drugs	Mental disorders	Wald ratio	1	1.93 (0.904, 4.12)	0.089	0.874
Alcohol-related disorders	Mental disorders	Wald ratio	1	1.025 (0.972, 1.081)	0.353	0.962
Alcoholism	Mental disorders	Wald ratio	1	0.994 (0.932, 1.06)	0.86	0.981
Alcoholic liver damage	Mental disorders	Wald ratio	1	1.043 (0.85, 1.281)	0.684	0.981
Tobacco use disorder	Mental disorders	Wald ratio	1	0.986 (0.942, 1.032)	0.537	0.981
Osteomyelitis, periostitis, and other infections involving bone	Musculoskeletal	Wald ratio	1	1.062 (0.839, 1.343)	0.617	0.981
Osteomyelitis	Musculoskeletal	Wald ratio	1	1.07 (0.836, 1.369)	0.59	0.981
Chronic osteomyelitis	Musculoskeletal	Wald ratio	1	1.172 (0.772, 1.778)	0.456	0.974
Unspecified osteomyelitis	Musculoskeletal	Wald ratio	1	0.964 (0.72, 1.291)	0.805	0.981
Arthropathy associated with infections	Musculoskeletal	Wald ratio	1	0.935 (0.706, 1.237)	0.636	0.981
Pyogenic arthritis	Musculoskeletal	Wald ratio	1	0.942 (0.682, 1.3)	0.715	0.981
Infective connective tissue disorders	Musculoskeletal	Wald ratio	1	1.93 (1.013, 3.677)	0.046	0.792
Arthropathy associated with other disorders classified elsewhere	Musculoskeletal	Wald ratio	1	1.025 (0.651, 1.616)	0.914	0.983
Arthropathy associated with neurological disorders	Musculoskeletal	Wald ratio	1	1.058 (0.535, 2.093)	0.872	0.983
Rheumatoid arthritis and other inflammatory polyarthropathies	Musculoskeletal	Wald ratio	1	0.998 (0.918, 1.085)	0.967	0.991
Rheumatoid arthritis	Musculoskeletal	Wald ratio	1	1.022 (0.933, 1.119)	0.647	0.981
Other inflammatory spondylopathies	Musculoskeletal	Wald ratio	1	1.186 (1.026, 1.369)	0.021	0.688
Sacroiliitis NEC	Musculoskeletal	Wald ratio	1	0.873 (0.514, 1.485)	0.617	0.981
Ankylosing spondylitis	Musculoskeletal	Wald ratio	1	1.104 (0.873, 1.396)	0.411	0.974
Other arthropathies	Musculoskeletal	Wald ratio	1	0.989 (0.957, 1.023)	0.527	0.981
Unspecified polyarthropathy or polyarthritis	Musculoskeletal	Wald ratio	1	0.942 (0.853, 1.039)	0.233	0.917
Unspecified monoarthritis	Musculoskeletal	Wald ratio	1	0.968 (0.921, 1.017)	0.191	0.917
Arthropathy NOS	Musculoskeletal	Wald ratio	1	0.991 (0.958, 1.024)	0.585	0.981
Polymyalgia Rheumatica	Musculoskeletal	Wald ratio	1	1.011 (0.852, 1.199)	0.903	0.983
Spinal stenosis	Musculoskeletal	Wald ratio	1	1.012 (0.921, 1.113)	0.804	0.981
Spondylosis and allied disorders	Musculoskeletal	Wald ratio	1	1.037 (0.969, 1.111)	0.291	0.923
Spondylosis without myelopathy	Musculoskeletal	Wald ratio	1	1.007 (0.926, 1.095)	0.87	0.981
Spondylosis with myelopathy	Musculoskeletal	Wald ratio	1	0.748 (0.493, 1.135)	0.173	0.916
Other allied disorders of spine	Musculoskeletal	Wald ratio	1	1.204 (0.882, 1.643)	0.242	0.919
Intervertebral disc disorders	Musculoskeletal	Wald ratio	1	0.966 (0.909, 1.026)	0.261	0.923
Displacement of intervertebral disc	Musculoskeletal	Wald ratio	1	0.629 (0.486, 0.814)	4.16E-04	0.109
Degeneration of intervertebral disc	Musculoskeletal	Wald ratio	1	1.072 (0.96, 1.197)	0.214	0.917
Postlaminectomy syndrome	Musculoskeletal	Wald ratio	1	1.078 (0.545, 2.134)	0.828	0.981
Other and unspecified disc disorder	Musculoskeletal	Wald ratio	1	0.938 (0.86, 1.024)	0.151	0.916
Other disorders of cervical region	Musculoskeletal	Wald ratio	1	0.942 (0.731, 1.214)	0.644	0.981

Torticollis	Musculoskeletal	Wald ratio	1	0.706 (0.4, 1.247)	0.23	0.917
Other and unspecified disorders of back	Musculoskeletal	Wald ratio	1	1.08 (0.95, 1.229)	0.239	0.919
Disorders of sacrum	Musculoskeletal	Wald ratio	1	0.97 (0.727, 1.293)	0.833	0.981
Disorders of coccyx	Musculoskeletal	Wald ratio	1	0.944 (0.707, 1.259)	0.693	0.981
Other symptoms referable to back	Musculoskeletal	Wald ratio	1	1.56 (0.759, 3.206)	0.226	0.917
Other unspecified back disorders	Musculoskeletal	Wald ratio	1	1.095 (0.948, 1.265)	0.216	0.917
Peripheral enthesopathies and allied syndromes	Musculoskeletal	Wald ratio	1	1.003 (0.954, 1.053)	0.914	0.983
Enthesopathy	Musculoskeletal	Wald ratio	1	1.019 (0.959, 1.083)	0.544	0.981
Synoviopathy	Musculoskeletal	Wald ratio	1	1.192 (0.867, 1.64)	0.279	0.923
Bursitis	Musculoskeletal	Wald ratio	1	1.025 (0.842, 1.249)	0.803	0.981
Other disorders of synovium, tendon, and bursa	Musculoskeletal	Wald ratio	1	0.964 (0.9, 1.032)	0.291	0.923
Synovitis and tenosynovitis	Musculoskeletal	Wald ratio	1	0.875 (0.784, 0.977)	0.017	0.68
Bursitis disorders	Musculoskeletal	Wald ratio	1	0.835 (0.473, 1.475)	0.535	0.981
Ganglion and cyst of synovium, tendon, and bursa	Musculoskeletal	Wald ratio	1	1.054 (0.951, 1.167)	0.317	0.923
Rupture of synovium	Musculoskeletal	Wald ratio	1	1.002 (0.789, 1.273)	0.985	0.991
Rupture of tendon, nontraumatic	Musculoskeletal	Wald ratio	1	0.808 (0.592, 1.103)	0.18	0.916
Contracture of tendon (sheath)	Musculoskeletal	Wald ratio	1	1.043 (0.637, 1.708)	0.866	0.981
Disorders of muscle, ligament, and fascia	Musculoskeletal	Wald ratio	1	1.002 (0.918, 1.093)	0.968	0.991
Laxity of ligament or hypermobility syndrome	Musculoskeletal	Wald ratio	1	0.94 (0.707, 1.249)	0.67	0.981
Fasciitis	Musculoskeletal	Wald ratio	1	1 (0.91, 1.1)	0.993	0.996
Contracture of palmar fascia [Dupuytren's disease]	Musculoskeletal	Wald ratio	1	1.005 (0.911, 1.11)	0.914	0.983
Other disorders of soft tissues	Musculoskeletal	Wald ratio	1	0.998 (0.925, 1.077)	0.962	0.99
Rheumatism, unspecified and fibrositis	Musculoskeletal	Wald ratio	1	1.072 (0.89, 1.291)	0.463	0.974
Panniculitis	Musculoskeletal	Wald ratio	1	0.958 (0.543, 1.692)	0.883	0.983
Osteitis deformans and osteopathies associated with other disorders classified elsewhere	Musculoskeletal	Wald ratio	1	1.035 (0.731, 1.468)	0.845	0.981
Osteitis deformans [Paget's disease of bone]	Musculoskeletal	Wald ratio	1	0.977 (0.684, 1.395)	0.898	0.983
Osteochondropathies	Musculoskeletal	Wald ratio	1	1.158 (0.885, 1.516)	0.284	0.923
Juvenile osteochondrosis	Musculoskeletal	Wald ratio	1	1.261 (0.831, 1.914)	0.275	0.923
Osteochondritis dissecans	Musculoskeletal	Wald ratio	1	0.629 (0.295, 1.342)	0.23	0.917
Other disorders of bone and cartilage	Musculoskeletal	Wald ratio	1	0.981 (0.893, 1.079)	0.695	0.981
Cyst of bone	Musculoskeletal	Wald ratio	1	0.858 (0.62, 1.189)	0.358	0.963
Aseptic necrosis of bone	Musculoskeletal	Wald ratio	1	0.987 (0.769, 1.268)	0.919	0.984
Costochondritis	Musculoskeletal	Wald ratio	1	1.07 (0.629, 1.819)	0.803	0.981
Malunion and nonunion of fracture	Musculoskeletal	Wald ratio	1	0.971 (0.825, 1.143)	0.727	0.981
Chondromalacia	Musculoskeletal	Wald ratio	1	0.938 (0.552, 1.595)	0.814	0.981
Acquired foot deformities	Musculoskeletal	Wald ratio	1	1.015 (0.955, 1.078)	0.635	0.981
Flat foot	Musculoskeletal	Wald ratio	1	0.843 (0.556, 1.28)	0.424	0.974
Acquired toe deformities	Musculoskeletal	Wald ratio	1	1.07 (0.984, 1.163)	0.112	0.916
Hammer toe (acquired)	Musculoskeletal	Wald ratio	1	1.017 (0.891, 1.161)	0.801	0.981
Hallux rigidus	Musculoskeletal	Wald ratio	1	1.076 (0.928, 1.248)	0.33	0.944
Hallux valgus (Bunion)	Musculoskeletal	Wald ratio	1	0.951 (0.885, 1.022)	0.171	0.916
Other acquired deformities of limbs	Musculoskeletal	Wald ratio	1	0.858 (0.743, 0.991)	0.038	0.792
Acquired deformities of finger	Musculoskeletal	Wald ratio	1	0.84 (0.582, 1.214)	0.353	0.962
Curvature of spine	Musculoskeletal	Wald ratio	1	1.104 (0.927, 1.314)	0.268	0.923
Kyphoscoliosis and scoliosis	Musculoskeletal	Wald ratio	1	1.089 (0.911, 1.301)	0.349	0.962
Other acquired musculoskeletal deformity	Musculoskeletal	Wald ratio	1	1.158 (1.014, 1.323)	0.03	0.749
Acquired spondylolisthesis	Musculoskeletal	Wald ratio	1	1.161 (1.001, 1.345)	0.048	0.83
Contracture of joint	Musculoskeletal	Wald ratio	1	0.995 (0.766, 1.293)	0.971	0.991
Osteoarthrosis	Musculoskeletal	Wald ratio	1	1.003 (0.967, 1.041)	0.854	0.981
Osteoarthritis; localized	Musculoskeletal	Wald ratio	1	0.981 (0.937, 1.026)	0.405	0.974
Osteoarthrosis, localized, primary	Musculoskeletal	Wald ratio	1	1.008 (0.945, 1.075)	0.814	0.981
Osteoarthrosis, localized, secondary	Musculoskeletal	Wald ratio	1	0.901 (0.66, 1.229)	0.51	0.978
Osteoarthrosis, generalized	Musculoskeletal	Wald ratio	1	1.174 (0.925, 1.491)	0.188	0.917
Osteoarthrosis NOS	Musculoskeletal	Wald ratio	1	1.027 (0.974, 1.083)	0.317	0.923
Symptoms and disorders of the joints	Musculoskeletal	Wald ratio	1	0.935 (0.85, 1.027)	0.162	0.916
Ankylosis of joint	Musculoskeletal	Wald ratio	1	0.629 (0.356, 1.11)	0.11	0.916
Stiffness of joint	Musculoskeletal	Wald ratio	1	0.848 (0.671, 1.073)	0.17	0.916
Difficulty in walking	Musculoskeletal	Wald ratio	1	1.141 (0.724, 1.798)	0.571	0.981
Joint effusions	Musculoskeletal	Wald ratio	1	1.033 (0.885, 1.207)	0.678	0.981
Hemarthrosis	Musculoskeletal	Wald ratio	1	1.078 (0.611, 1.904)	0.795	0.981
Villonodular synovitis	Musculoskeletal	Wald ratio	1	0.808 (0.365, 1.792)	0.6	0.981
Derangement of joint, non-traumatic	Musculoskeletal	Wald ratio	1	0.973 (0.872, 1.086)	0.629	0.981
Loose body in joint	Musculoskeletal	Wald ratio	1	0.824 (0.599, 1.133)	0.234	0.917
Pathological, developmental or recurrent dislocation	Musculoskeletal	Wald ratio	1	1.286 (0.701, 2.358)	0.417	0.974
Articular cartilage disorder	Musculoskeletal	Wald ratio	1	1.165 (0.897, 1.513)	0.252	0.919
Other derangement of joint	Musculoskeletal	Wald ratio	1	0.947 (0.83, 1.082)	0.424	0.974
Osteoporosis, osteopenia and pathological fracture	Musculoskeletal	Wald ratio	1	0.949 (0.887, 1.016)	0.134	0.916
Osteoporosis	Musculoskeletal	Wald ratio	1	0.955 (0.885, 1.03)	0.23	0.917
Osteoporosis NOS	Musculoskeletal	Wald ratio	1	0.979 (0.904, 1.06)	0.6	0.981
Other specified osteoporosis	Musculoskeletal	Wald ratio	1	0.808 (0.627, 1.042)	0.101	0.894
Pathologic fracture	Musculoskeletal	Wald ratio	1	0.858 (0.663, 1.111)	0.245	0.919
Osteopenia or other disorder of bone and cartilage	Musculoskeletal	Wald ratio	1	0.956 (0.779, 1.174)	0.67	0.981
Pain in joint	Musculoskeletal	Wald ratio	1	1.005 (0.943, 1.072)	0.869	0.981
Cancer of mouth	Neoplasms	Wald ratio	1	1.213 (0.967, 1.523)	0.096	0.874
Cancer of tongue	Neoplasms	Wald ratio	1	1.106 (0.795, 1.538)	0.55	0.981
Cancer of major salivary glands	Neoplasms	Wald ratio	1	0.778 (0.44, 1.373)	0.386	0.974
Cancer of the mouth floor	Neoplasms	Wald ratio	1	0.793 (0.358, 1.758)	0.568	0.981
Cancer of larynx, pharynx, nasal cavities	Neoplasms	Wald ratio	1	1.087 (0.862, 1.369)	0.481	0.974

Cancer of oropharynx	Neoplasms	Wald ratio	1	0.962 (0.664, 1.395)	0.838	0.981
Cancer of larynx	Neoplasms	Wald ratio	1	1.213 (0.843, 1.746)	0.298	0.923
Cancer of of nasal cavities	Neoplasms	Wald ratio	1	0.91 (0.426, 1.941)	0.806	0.981
Cancer of esophagus	Neoplasms	Wald ratio	1	1.022 (0.823, 1.268)	0.847	0.981
Cancer of stomach	Neoplasms	Wald ratio	1	1.416 (1.107, 1.812)	0.006	0.376
Colorectal cancer	Neoplasms	Wald ratio	1	0.995 (0.912, 1.085)	0.903	0.983
Colon cancer	Neoplasms	Wald ratio	1	1.012 (0.91, 1.126)	0.819	0.981
Malignant neoplasm of rectum, rectosigmoid junction, and anus	Neoplasms	Wald ratio	1	0.975 (0.857, 1.109)	0.702	0.981
Cancer of liver and intrahepatic bile duct	Neoplasms	Wald ratio	1	0.875 (0.639, 1.199)	0.406	0.974
Malignant neoplasm of liver, primary	Neoplasms	Wald ratio	1	0.549 (0.335, 0.899)	0.017	0.68
Pancreatic cancer	Neoplasms	Wald ratio	1	0.865 (0.681, 1.098)	0.234	0.917
Neoplasm of unspecified nature of digestive system	Neoplasms	Wald ratio	1	0.962 (0.805, 1.15)	0.67	0.981
Malignant neoplasm of other and ill-defined sites within the digestive organs and peritoneum	Neoplasms	Wald ratio	1	1.039 (0.96, 1.126)	0.341	0.954
Malignant neoplasm of small intestine, including duodenum	Neoplasms	Wald ratio	1	0.748 (0.493, 1.135)	0.173	0.916
Malignant neoplasm of gallbladder and extrahepatic bile ducts	Neoplasms	Wald ratio	1	1.022 (0.673, 1.55)	0.92	0.984
Malignant neoplasm of retroperitoneum and peritoneum	Neoplasms	Wald ratio	1	1.025 (0.676, 1.556)	0.906	0.983
Cancer of intrathoracic organs	Neoplasms	Wald ratio	1	1.56 (0.731, 3.33)	0.25	0.919
Cancer within the respiratory system	Neoplasms	Wald ratio	1	1.045 (0.933, 1.171)	0.443	0.974
Cancer of bronchus; lung	Neoplasms	Wald ratio	1	1.072 (0.942, 1.22)	0.29	0.923
Cancer of bone and connective tissue	Neoplasms	Wald ratio	1	1.286 (0.971, 1.702)	0.079	0.874
Bone cancer	Neoplasms	Wald ratio	1	1.363 (0.802, 2.317)	0.253	0.919
Cancer of connective tissue	Neoplasms	Wald ratio	1	1.261 (0.907, 1.754)	0.168	0.916
Skin cancer	Neoplasms	Wald ratio	1	1 (0.948, 1.054)	0.987	0.992
Melanomas of skin, dx or hx	Neoplasms	Wald ratio	1	1.041 (0.93, 1.167)	0.484	0.974
Melanomas of skin	Neoplasms	Wald ratio	1	1.041 (0.93, 1.167)	0.484	0.974
Other non-epithelial cancer of skin	Neoplasms	Wald ratio	1	0.987 (0.932, 1.044)	0.646	0.981
Carcinoma in situ of skin	Neoplasms	Wald ratio	1	0.875 (0.697, 1.099)	0.25	0.919
Neoplasm of uncertain behavior of skin	Neoplasms	Wald ratio	1	0.885 (0.583, 1.343)	0.567	0.981
Breast cancer	Neoplasms	Wald ratio	1	0.991 (0.94, 1.045)	0.737	0.981
Breast cancer [female]	Neoplasms	Wald ratio	1	0.985 (0.934, 1.039)	0.582	0.981
Malignant neoplasm of female breast	Neoplasms	Wald ratio	1	0.979 (0.925, 1.036)	0.463	0.974
Neoplasm of uncertain behavior of breast	Neoplasms	Wald ratio	1	1.53 (0.9, 2.602)	0.116	0.916
Acquired absence of breast	Neoplasms	Wald ratio	1	0.977 (0.865, 1.103)	0.708	0.981
Cervical cancer and dysplasia	Neoplasms	Wald ratio	1	0.966 (0.875, 1.066)	0.489	0.974
Cervical cancer	Neoplasms	Wald ratio	1	0.956 (0.828, 1.105)	0.545	0.981
Cervical intraepithelial neoplasia [CIN] [Cervical dysplasia]	Neoplasms	Wald ratio	1	0.979 (0.861, 1.114)	0.746	0.981
Malignant neoplasm of uterus	Neoplasms	Wald ratio	1	1.074 (0.913, 1.264)	0.39	0.974
Cancer of other female genital organs	Neoplasms	Wald ratio	1	0.94 (0.836, 1.057)	0.302	0.923
Malignant neoplasm of ovary and other uterine adnexa	Neoplasms	Wald ratio	1	0.922 (0.81, 1.049)	0.217	0.917
Malignant neoplasm of ovary	Neoplasms	Wald ratio	1	0.922 (0.81, 1.049)	0.217	0.917
Cancer of other female genital organs (excluding uterus and ovary)	Neoplasms	Wald ratio	1	0.987 (0.704, 1.382)	0.937	0.986
Malignant neoplasm of testis	Neoplasms	Wald ratio	1	1.006 (0.915, 1.106)	0.908	0.983
Neoplasm of uncertain behavior of male genital organs	Neoplasms	Wald ratio	1	1.213 (0.687, 2.143)	0.505	0.974
Cancer of urinary organs (incl. kidney and bladder)	Neoplasms	Wald ratio	1	0.953 (0.87, 1.044)	0.298	0.923
Cancer of kidney and renal pelvis	Neoplasms	Wald ratio	1	0.97 (0.808, 1.163)	0.739	0.981
Malignant neoplasm of kidney, except pelvis	Neoplasms	Wald ratio	1	0.962 (0.799, 1.158)	0.683	0.981
Malignant neoplasm of renal pelvis	Neoplasms	Wald ratio	1	1.261 (0.614, 2.592)	0.528	0.981
Cancer of bladder	Neoplasms	Wald ratio	1	0.955 (0.846, 1.078)	0.453	0.974
Malignant neoplasm of bladder	Neoplasms	Wald ratio	1	0.949 (0.834, 1.08)	0.427	0.974
Malignant neoplasm of other urinary organs	Neoplasms	Wald ratio	1	0.97 (0.592, 1.587)	0.902	0.983
Cancer of eye	Neoplasms	Wald ratio	1	0.824 (0.523, 1.299)	0.405	0.974
Manlignant and unknown neoplasms of brain and nervous system	Neoplasms	Wald ratio	1	1.07 (0.852, 1.343)	0.56	0.981
Cancer of brain and nervous system	Neoplasms	Wald ratio	1	1.076 (0.835, 1.387)	0.571	0.981
Cancer of brain	Neoplasms	Wald ratio	1	1.054 (0.808, 1.374)	0.7	0.981
Thyroid cancer	Neoplasms	Wald ratio	1	0.896 (0.659, 1.218)	0.482	0.974
Cancer of other endocrine glands	Neoplasms	Wald ratio	1	0.549 (0.299, 1.007)	0.053	0.858
Cancer, suspected or other	Neoplasms	Wald ratio	1	0.991 (0.947, 1.037)	0.701	0.981
Malignant neoplasm, other	Neoplasms	Wald ratio	1	0.991 (0.947, 1.037)	0.683	0.981
Chemotherapy	Neoplasms	Wald ratio	1	1.004 (0.963, 1.047)	0.856	0.981
Secondary malignant neoplasm	Neoplasms	Wald ratio	1	0.992 (0.933, 1.054)	0.788	0.981
Secondary malignancy of lymph nodes	Neoplasms	Wald ratio	1	1.009 (0.931, 1.092)	0.834	0.981
Secondary malignancy of respiratory organs	Neoplasms	Wald ratio	1	0.968 (0.854, 1.097)	0.606	0.981
Secondary malignant neoplasm of digestive systems	Neoplasms	Wald ratio	1	0.981 (0.846, 1.137)	0.798	0.981
Secondary malignant neoplasm of liver	Neoplasms	Wald ratio	1	0.929 (0.829, 1.041)	0.205	0.917
Secondary malignancy of brain or spine	Neoplasms	Wald ratio	1	1.064 (0.867, 1.306)	0.553	0.981
Secondary malignancy of bone	Neoplasms	Wald ratio	1	1.136 (1.003, 1.288)	0.046	0.792
Secondary malignant neoplasm of skin	Neoplasms	Wald ratio	1	0.998 (0.658, 1.515)	0.994	0.996
Neoplasm of uncertain behavior	Neoplasms	Wald ratio	1	1.141 (0.976, 1.332)	0.097	0.876
Neurofibromatosis	Neoplasms	Wald ratio	1	1.389 (0.757, 2.548)	0.288	0.923
Myeloproliferative disease	Neoplasms	Wald ratio	1	0.908 (0.754, 1.093)	0.308	0.923
Polycythemia vera	Neoplasms	Wald ratio	1	0.808 (0.601, 1.086)	0.158	0.916
Hodgkin's disease	Neoplasms	Wald ratio	1	1.206 (0.838, 1.736)	0.312	0.923
Cancer of other lymphoid, histiocytic tissue	Neoplasms	Wald ratio	1	1.037 (0.919, 1.171)	0.553	0.981
Non-Hodgkins lymphoma	Neoplasms	Wald ratio	1	1.027 (0.896, 1.178)	0.697	0.981
Nodular lymphoma	Neoplasms	Wald ratio	1	1.008 (0.745, 1.366)	0.957	0.989
Large cell lymphoma	Neoplasms	Wald ratio	1	1.027 (0.806, 1.31)	0.827	0.981
Leukemia	Neoplasms	Wald ratio	1	1.147 (0.993, 1.325)	0.062	0.874
Lymphoid leukemia	Neoplasms	Wald ratio	1	0.968 (0.759, 1.233)	0.791	0.981

Lymphoid leukemia, acute	Neoplasms	Wald ratio	1	0.679 (0.318, 1.45)	0.317	0.923
Lymphoid leukemia, chronic	Neoplasms	Wald ratio	1	0.99 (0.765, 1.282)	0.941	0.986
Myeloid leukemia	Neoplasms	Wald ratio	1	1.174 (0.897, 1.537)	0.242	0.919
Myeloid leukemia, acute	Neoplasms	Wald ratio	1	1.029 (0.743, 1.426)	0.862	0.981
Myeloid leukemia, chronic	Neoplasms	Wald ratio	1	1.501 (0.952, 2.366)	0.08	0.874
Multiple myeloma	Neoplasms	Wald ratio	1	1.261 (0.986, 1.614)	0.065	0.874
Benign neoplasm of colon	Neoplasms	Wald ratio	1	1.027 (0.985, 1.071)	0.203	0.917
Benign neoplasm of lip, oral cavity, and pharynx	Neoplasms	Wald ratio	1	1.013 (0.841, 1.22)	0.89	0.983
Benign neoplasm of other parts of digestive system	Neoplasms	Wald ratio	1	0.992 (0.916, 1.074)	0.841	0.981
Benign neoplasm of respiratory and intrathoracic organs	Neoplasms	Wald ratio	1	1.127 (0.826, 1.538)	0.45	0.974
Benign neoplasm of bone and articular cartilage	Neoplasms	Wald ratio	1	0.824 (0.595, 1.142)	0.245	0.919
Lipoma	Neoplasms	Wald ratio	1	0.992 (0.923, 1.066)	0.833	0.981
Lipoma of skin and subcutaneous tissue	Neoplasms	Wald ratio	1	1.007 (0.923, 1.098)	0.882	0.983
Other benign neoplasm of connective and other soft tissue	Neoplasms	Wald ratio	1	1.472 (1.237, 1.753)	1.37E-05	0.017
Benign neoplasm of skin	Neoplasms	Wald ratio	1	1.018 (0.951, 1.09)	0.605	0.981
Vascular hamartomas and non-neoplastic nevi	Neoplasms	Wald ratio	1	1.037 (0.826, 1.302)	0.751	0.981
Nevus, non-neoplastic	Neoplasms	Wald ratio	1	1.076 (0.848, 1.367)	0.546	0.981
Benign neoplasm of uterus	Neoplasms	Wald ratio	1	1.022 (0.965, 1.081)	0.463	0.974
Uterine leiomyoma	Neoplasms	Wald ratio	1	1.023 (0.963, 1.087)	0.453	0.974
Other benign neoplasm of uterus	Neoplasms	Wald ratio	1	0.897 (0.658, 1.225)	0.495	0.974
Benign neoplasm of ovary	Neoplasms	Wald ratio	1	0.992 (0.852, 1.154)	0.916	0.983
Benign neoplasm of other female genital organs	Neoplasms	Wald ratio	1	0.894 (0.612, 1.306)	0.562	0.981
Benign neoplasm of kidney and other urinary organs	Neoplasms	Wald ratio	1	1.172 (0.744, 1.847)	0.494	0.974
Benign neoplasm of eye	Neoplasms	Wald ratio	1	1.311 (0.771, 2.229)	0.317	0.923
Benign neoplasm of eye, uveal	Neoplasms	Wald ratio	1	2.342 (1.14, 4.813)	0.021	0.688
Benign neoplasm of brain and other parts of nervous system	Neoplasms	Wald ratio	1	0.971 (0.795, 1.188)	0.777	0.981
Benign neoplasm of brain, cranial nerves, meninges	Neoplasms	Wald ratio	1	0.992 (0.805, 1.222)	0.938	0.986
Benign neoplasm of spinal cord, meninges	Neoplasms	Wald ratio	1	0.692 (0.324, 1.478)	0.342	0.954
Benign neoplasm of thyroid glands	Neoplasms	Wald ratio	1	0.734 (0.52, 1.036)	0.079	0.874
Benign neoplasm of other endocrine glands and related structures	Neoplasms	Wald ratio	1	0.778 (0.639, 0.947)	0.012	0.564
Benign neoplasm of adrenal gland	Neoplasms	Wald ratio	1	0.808 (0.533, 1.227)	0.317	0.923
Benign neoplasm of parathyroid gland	Neoplasms	Wald ratio	1	0.763 (0.563, 1.033)	0.08	0.874
Benign neoplasm of pituitary gland and craniopharyngeal duct (pouch)	Neoplasms	Wald ratio	1	0.748 (0.538, 1.04)	0.085	0.874
Hemangioma and lymphangioma, any site	Neoplasms	Wald ratio	1	1.043 (0.903, 1.205)	0.563	0.981
Benign neoplasm of unspecified sites	Neoplasms	Wald ratio	1	0.968 (0.86, 1.088)	0.583	0.981
Bone marrow or stem cell transplant	Neoplasms	Wald ratio	1	1.097 (0.576, 2.09)	0.778	0.981
Meningitis	Neurological	Wald ratio	1	0.887 (0.673, 1.17)	0.396	0.974
Encephalitis	Neurological	Wald ratio	1	0.72 (0.525, 0.986)	0.041	0.792
Acute (transverse) myelitis	Neurological	Wald ratio	1	0.319 (0.161, 0.632)	0.001	0.121
Other CNS infection and poliomyelitis	Neurological	Wald ratio	1	0.958 (0.681, 1.348)	0.807	0.981
Sleep disorders	Neurological	Wald ratio	1	1.037 (0.954, 1.128)	0.388	0.974
Sleep apnea	Neurological	Wald ratio	1	1.054 (0.966, 1.15)	0.24	0.919
Insomnia	Neurological	Wald ratio	1	0.734 (0.466, 1.157)	0.182	0.916
Organic or persistent insomnia	Neurological	Wald ratio	1	0.933 (0.57, 1.527)	0.782	0.981
Other cerebral degenerations	Neurological	Wald ratio	1	1.004 (0.844, 1.196)	0.96	0.99
Hydrocephalus	Neurological	Wald ratio	1	1.161 (0.893, 1.508)	0.264	0.923
Cerebral degeneration, unspecified	Neurological	Wald ratio	1	0.862 (0.648, 1.145)	0.305	0.923
Parkinson's disease	Neurological	Wald ratio	1	0.763 (0.641, 0.908)	0.002	0.175
Extrapyramidal disease and abnormal movement disorders	Neurological	Wald ratio	1	0.91 (0.75, 1.104)	0.337	0.952
Essential tremor	Neurological	Wald ratio	1	0.894 (0.612, 1.306)	0.562	0.981
Myoclonus	Neurological	Wald ratio	1	1.363 (0.743, 2.499)	0.317	0.923
Torsion dystonia	Neurological	Wald ratio	1	1.052 (0.728, 1.519)	0.789	0.981
Degenerative disease of the spinal cord	Neurological	Wald ratio	1	0.97 (0.846, 1.111)	0.657	0.981
Spinocerebellar disease	Neurological	Wald ratio	1	0.899 (0.529, 1.529)	0.694	0.981
Anterior horn cell disease	Neurological	Wald ratio	1	1.213 (0.831, 1.773)	0.317	0.923
Multiple sclerosis	Neurological	Wald ratio	1	0.824 (0.703, 0.966)	0.017	0.68
Disorders of the autonomic nervous system	Neurological	Wald ratio	1	1.033 (0.724, 1.476)	0.856	0.981
Peripheral autonomic neuropathy	Neurological	Wald ratio	1	1.286 (0.65, 2.544)	0.47	0.974
Acute pain	Neurological	Wald ratio	1	0.94 (0.702, 1.259)	0.678	0.981
Chronic pain	Neurological	Wald ratio	1	1.416 (1.05, 1.911)	0.023	0.688
Other headache syndromes	Neurological	Wald ratio	1	0.975 (0.911, 1.044)	0.47	0.974
Migraine	Neurological	Wald ratio	1	0.97 (0.869, 1.082)	0.581	0.981
Migrain with aura	Neurological	Wald ratio	1	0.848 (0.581, 1.239)	0.395	0.974
Other demyelinating diseases of central nervous system	Neurological	Wald ratio	1	0.837 (0.582, 1.204)	0.338	0.952
Hemiplegia	Neurological	Wald ratio	1	0.997 (0.857, 1.161)	0.972	0.991
Infantile cerebral palsy	Neurological	Wald ratio	1	1.156 (0.706, 1.892)	0.564	0.981
Other paralytic syndromes	Neurological	Wald ratio	1	1.029 (0.82, 1.292)	0.803	0.981
Epilepsy, recurrent seizures, convulsions	Neurological	Wald ratio	1	1.056 (0.971, 1.147)	0.203	0.917
Epilepsy	Neurological	Wald ratio	1	1.074 (0.885, 1.303)	0.468	0.974
Generalized convulsive epilepsy	Neurological	Wald ratio	1	1.132 (0.858, 1.493)	0.381	0.974
Partial epilepsy	Neurological	Wald ratio	1	1.237 (0.873, 1.753)	0.232	0.917
Convulsions	Neurological	Wald ratio	1	1.033 (0.912, 1.171)	0.606	0.981
Abnormal findings on study of brain and or or nervous system	Neurological	Wald ratio	1	1.052 (0.693, 1.596)	0.813	0.981
Nonspecific abnormal findings on radiological and other examination of skull and head	Neurological	Wald ratio	1	0.808 (0.494, 1.323)	0.397	0.974
Other conditions of brain	Neurological	Wald ratio	1	1.01 (0.865, 1.18)	0.897	0.983
Cerebral edema and compression of brain	Neurological	Wald ratio	1	0.734 (0.51, 1.056)	0.096	0.874
Cerebral cysts	Neurological	Wald ratio	1	1.213 (0.741, 1.986)	0.442	0.974
Coma	Neurological	Wald ratio	1	0.933 (0.686, 1.268)	0.657	0.981

Encephalopathy, not elsewhere classified	Neurological	Wald ratio	1	1.176 (0.805, 1.719)	0.401	0.974
Other conditions of brain, NOS	Neurological	Wald ratio	1	1.058 (0.787, 1.422)	0.71	0.981
Other and unspecified disorders of the nervous system	Neurological	Wald ratio	1	0.924 (0.672, 1.27)	0.625	0.981
Abnormal movement	Neurological	Wald ratio	1	1.043 (0.935, 1.165)	0.448	0.974
Abnormal involuntary movements	Neurological	Wald ratio	1	1.078 (0.889, 1.308)	0.444	0.974
Abnormality of gait	Neurological	Wald ratio	1	1.025 (0.888, 1.184)	0.732	0.981
Lack of coordination	Neurological	Wald ratio	1	1.058 (0.778, 1.438)	0.72	0.981
Disturbances of sensation of smell and taste	Neurological	Wald ratio	1	0.653 (0.295, 1.449)	0.295	0.923
Other peripheral nerve disorders	Neurological	Wald ratio	1	1.058 (1.003, 1.115)	0.038	0.792
Disorders of other cranial nerves	Neurological	Wald ratio	1	1.097 (0.939, 1.282)	0.242	0.919
Trigeminal nerve disorders [CN5]	Neurological	Wald ratio	1	1.416 (1.082, 1.854)	0.011	0.564
Facial nerve disorders [CN7]	Neurological	Wald ratio	1	0.958 (0.787, 1.167)	0.672	0.981
Nerve root and plexus disorders	Neurological	Wald ratio	1	0.894 (0.751, 1.064)	0.207	0.917
Nerve plexus lesions	Neurological	Wald ratio	1	0.56 (0.329, 0.952)	0.032	0.756
Nerve root lesions	Neurological	Wald ratio	1	0.253 (0.114, 0.562)	7.22E-04	0.109
Hereditary and idiopathic peripheral neuropathy	Neurological	Wald ratio	1	0.679 (0.431, 1.07)	0.096	0.874
Inflammatory and toxic neuropathy	Neurological	Wald ratio	1	1.006 (0.858, 1.18)	0.941	0.986
Myoneural disorders	Neurological	Wald ratio	1	0.966 (0.661, 1.411)	0.857	0.981
Myasthenia gravis	Neurological	Wald ratio	1	0.929 (0.612, 1.41)	0.73	0.981
Muscular dystrophies and other myopathies	Neurological	Wald ratio	1	0.942 (0.72, 1.233)	0.662	0.981
Muscular dystrophies	Neurological	Wald ratio	1	1.064 (0.65, 1.741)	0.806	0.981
Myopathy	Neurological	Wald ratio	1	0.955 (0.702, 1.298)	0.767	0.981
Miscarriage; stillbirth	Pregnancy complications	Wald ratio	1	0.964 (0.887, 1.048)	0.388	0.974
Missed abortion or Hydatidiform mole	Pregnancy complications	Wald ratio	1	1.023 (0.866, 1.209)	0.785	0.981
Ectopic pregnancy	Pregnancy complications	Wald ratio	1	1.091 (0.79, 1.506)	0.597	0.981
Hemorrhage during pregnancy; childbirth and postpartum	Pregnancy complications	Wald ratio	1	0.908 (0.792, 1.041)	0.165	0.916
Antepartum hemorrhage, abruptio placentae, and placenta previa	Pregnancy complications	Wald ratio	1	1.035 (0.847, 1.266)	0.734	0.981
Placenta previa and abruptio placenta	Pregnancy complications	Wald ratio	1	0.853 (0.72, 1.012)	0.068	0.874
Early or threatened labor; hemorrhage in early pregnancy	Pregnancy complications	Wald ratio	1	0.87 (0.771, 0.982)	0.024	0.706
Early onset of delivery	Pregnancy complications	Wald ratio	1	0.793 (0.624, 1.007)	0.057	0.87
Hemorrhage in early pregnancy	Pregnancy complications	Wald ratio	1	0.793 (0.661, 0.951)	0.012	0.564
Other high-risk pregnancy	Pregnancy complications	Wald ratio	1	1.237 (0.625, 2.448)	0.541	0.981
Hypertension complicating pregnancy, childbirth, and the puerperium	Pregnancy complications	Wald ratio	1	0.998 (0.835, 1.193)	0.983	0.991
Preeclampsia and eclampsia	Pregnancy complications	Wald ratio	1	0.998 (0.715, 1.393)	0.99	0.995
Excessive vomiting in pregnancy	Pregnancy complications	Wald ratio	1	0.917 (0.627, 1.339)	0.653	0.981
Hyperemesis gravidarum	Pregnancy complications	Wald ratio	1	0.885 (0.562, 1.395)	0.6	0.981
Late pregnancy and failed induction	Pregnancy complications	Wald ratio	1	0.892 (0.752, 1.058)	0.19	0.917
Other complications of pregnancy NEC	Pregnancy complications	Wald ratio	1	0.936 (0.823, 1.065)	0.317	0.923
Infectious and parasitic complications affecting pregnancy	Pregnancy complications	Wald ratio	1	0.887 (0.657, 1.197)	0.433	0.974
Infections of genitourinary tract during pregnancy	Pregnancy complications	Wald ratio	1	0.927 (0.652, 1.319)	0.675	0.981
Other conditions or status of the mother complicating pregnancy, childbirth, or the puerperium	Pregnancy complications	Wald ratio	1	1.033 (0.737, 1.448)	0.849	0.981
Diabetes or abnormal glucose tolerance complicating pregnancy	Pregnancy complications	Wald ratio	1	0.734 (0.484, 1.114)	0.146	0.916
Normal delivery	Pregnancy complications	Wald ratio	1	0.944 (0.823, 1.082)	0.405	0.974
Multiple gestation	Pregnancy complications	Wald ratio	1	0.975 (0.712, 1.336)	0.876	0.983
Problems associated with amniotic cavity and membranes	Pregnancy complications	Wald ratio	1	0.979 (0.832, 1.152)	0.798	0.981
Other and unspecified complications of birth; puerperium affecting management of mother	Pregnancy complications	Wald ratio	1	0.641 (0.392, 1.049)	0.077	0.874
Known or suspected fetal abnormality affecting management of mother	Pregnancy complications	Wald ratio	1	0.938 (0.853, 1.031)	0.187	0.917
Other perinatal conditions of fetus or newborn	Pregnancy complications	Wald ratio	1	1.097 (0.621, 1.938)	0.749	0.981
Fetal distress and abnormal forces of labor	Pregnancy complications	Wald ratio	1	0.942 (0.828, 1.071)	0.362	0.968
Umbilical cord complications during labor and delivery	Pregnancy complications	Wald ratio	1	0.945 (0.731, 1.223)	0.67	0.981
Obstetrical or birth trauma	Pregnancy complications	Wald ratio	1	1.009 (0.921, 1.105)	0.855	0.981
Complications of labor and delivery NEC	Pregnancy complications	Wald ratio	1	0.949 (0.838, 1.076)	0.413	0.974
Venous or cerebrovascular complications embolism in pregnancy and the puerperium	Pregnancy complications	Wald ratio	1	1.17 (0.742, 1.843)	0.5	0.974
Other complications of the puerperium NEC	Pregnancy complications	Wald ratio	1	0.748 (0.52, 1.077)	0.118	0.916
Other disorders of the breast associated with childbirth and disorders of lactation	Pregnancy complications	Wald ratio	1	0.248 (0.116, 0.53)	3.18E-04	0.109
Asphyxia and hypoxemia	Respiratory	Wald ratio	1	0.855 (0.585, 1.249)	0.418	0.974
Acute sinusitis	Respiratory	Wald ratio	1	1.045 (0.689, 1.586)	0.834	0.981
Acute upper respiratory infections of multiple or unspecified sites	Respiratory	Wald ratio	1	1.091 (0.966, 1.232)	0.16	0.916
Acute pharyngitis	Respiratory	Wald ratio	1	1.161 (0.953, 1.413)	0.139	0.916
Acute laryngitis and tracheitis	Respiratory	Wald ratio	1	0.793 (0.503, 1.25)	0.317	0.923
Septal Deviations or Turbinate Hypertrophy	Respiratory	Wald ratio	1	1.041 (0.958, 1.132)	0.34	0.954
Nasal polyps	Respiratory	Wald ratio	1	1.023 (0.924, 1.134)	0.657	0.981
Chronic pharyngitis and nasopharyngitis	Respiratory	Wald ratio	1	1.204 (0.996, 1.455)	0.055	0.87
Diseases of the larynx and vocal cords	Respiratory	Wald ratio	1	1.058 (0.944, 1.185)	0.334	0.952
Chronic laryngitis	Respiratory	Wald ratio	1	1.025 (0.581, 1.811)	0.931	0.986
Paralysis or spasm of vocal cords or larynx	Respiratory	Wald ratio	1	0.944 (0.661, 1.348)	0.75	0.981
Voice disturbance	Respiratory	Wald ratio	1	1.136 (0.951, 1.358)	0.16	0.916
Acute and chronic tonsillitis	Respiratory	Wald ratio	1	1.087 (0.955, 1.236)	0.206	0.917
Acute tonsillitis	Respiratory	Wald ratio	1	1.192 (0.943, 1.508)	0.142	0.916
Chronic tonsillitis and adenoiditis	Respiratory	Wald ratio	1	1.033 (0.868, 1.23)	0.712	0.981
Chronic sinusitis	Respiratory	Wald ratio	1	0.906 (0.809, 1.015)	0.089	0.874
Allergic rhinitis	Respiratory	Wald ratio	1	1.174 (0.983, 1.403)	0.077	0.874
Epistaxis or throat hemorrhage	Respiratory	Wald ratio	1	1.072 (0.953, 1.206)	0.246	0.919
Throat pain	Respiratory	Wald ratio	1	1.261 (0.935, 1.702)	0.129	0.916
Other upper respiratory disease	Respiratory	Wald ratio	1	0.964 (0.88, 1.056)	0.429	0.974
Pneumonia	Respiratory	Wald ratio	1	1.005 (0.946, 1.068)	0.861	0.981

Bacterial pneumonia	Respiratory	Wald ratio	1	1.052 (0.978, 1.13)	0.171	0.916
Pneumococcal pneumonia	Respiratory	Wald ratio	1	1.062 (0.984, 1.145)	0.121	0.916
Pseudomonal pneumonia	Respiratory	Wald ratio	1	0.778 (0.424, 1.426)	0.417	0.974
Viral pneumonia	Respiratory	Wald ratio	1	0.452 (0.229, 0.895)	0.023	0.688
Pneumonia due to fungus (mycoses)	Respiratory	Wald ratio	1	0.808 (0.458, 1.427)	0.463	0.974
Bronchopneumonia and lung abscess	Respiratory	Wald ratio	1	0.877 (0.65, 1.183)	0.389	0.974
Influenza	Respiratory	Wald ratio	1	1.048 (0.759, 1.446)	0.778	0.981
Acute bronchitis and bronchiolitis	Respiratory	Wald ratio	1	1.136 (0.749, 1.724)	0.549	0.981
Asthma	Respiratory	Wald ratio	1	1.066 (1.026, 1.107)	9.67E-04	0.121
Asthma with exacerbation	Respiratory	Wald ratio	1	1.337 (0.925, 1.931)	0.122	0.916
Chronic airway obstruction	Respiratory	Wald ratio	1	1.037 (0.976, 1.102)	0.235	0.919
Emphysema	Respiratory	Wald ratio	1	0.968 (0.841, 1.113)	0.646	0.981
Chronic bronchitis	Respiratory	Wald ratio	1	1.095 (0.981, 1.222)	0.105	0.902
Obstructive chronic bronchitis	Respiratory	Wald ratio	1	1.091 (0.974, 1.222)	0.134	0.916
Bronchiectasis	Respiratory	Wald ratio	1	0.945 (0.825, 1.084)	0.42	0.974
Bronchitis	Respiratory	Wald ratio	1	1.102 (0.874, 1.388)	0.412	0.974
Lung disease due to external agents	Respiratory	Wald ratio	1	0.97 (0.724, 1.298)	0.835	0.981
Extrinsic allergic alveolitis	Respiratory	Wald ratio	1	0.875 (0.459, 1.667)	0.685	0.981
Pneumoconiosis	Respiratory	Wald ratio	1	1.043 (0.714, 1.524)	0.826	0.981
Pneumonitis due to inhalation of food or vomitus	Respiratory	Wald ratio	1	1.025 (0.805, 1.307)	0.839	0.981
Postinflammatory pulmonary fibrosis	Respiratory	Wald ratio	1	1.08 (0.887, 1.316)	0.442	0.974
Pulmonary congestion and hypostasis	Respiratory	Wald ratio	1	1.141 (0.868, 1.498)	0.345	0.959
Other alveolar and parietoalveolar pneumonopathy	Respiratory	Wald ratio	1	1.132 (0.808, 1.586)	0.472	0.974
Other pulmonary inflamation or edema	Respiratory	Wald ratio	1	1.857 (0.87, 3.963)	0.11	0.916
Empyema and pneumothorax	Respiratory	Wald ratio	1	0.987 (0.832, 1.17)	0.878	0.983
Pleurisy; pleural effusion	Respiratory	Wald ratio	1	0.991 (0.922, 1.065)	0.796	0.981
Pulmonary collapse; interstitial and compensatory emphysema	Respiratory	Wald ratio	1	0.977 (0.859, 1.111)	0.724	0.981
Respiratory failure, insufficiency, arrest	Respiratory	Wald ratio	1	0.995 (0.884, 1.119)	0.928	0.986
Respiratory failure	Respiratory	Wald ratio	1	1.007 (0.885, 1.146)	0.916	0.983
Respiratory insufficiency	Respiratory	Wald ratio	1	1.027 (0.893, 1.182)	0.705	0.981
Respiratory arrest	Respiratory	Wald ratio	1	1.389 (0.817, 2.362)	0.225	0.917
Dependence on respirator [Ventilator] or supplemental oxygen	Respiratory	Wald ratio	1	0.763 (0.568, 1.025)	0.073	0.874
Other diseases of lung	Respiratory	Wald ratio	1	1.01 (0.82, 1.244)	0.926	0.986
Other symptoms of respiratory system	Respiratory	Wald ratio	1	1.058 (0.995, 1.124)	0.07	0.874
Wheezing	Respiratory	Wald ratio	1	1.174 (0.804, 1.715)	0.407	0.974
Painful respiration	Respiratory	Wald ratio	1	1.472 (0.97, 2.234)	0.069	0.874
Shortness of breath	Respiratory	Wald ratio	1	1.035 (0.96, 1.117)	0.368	0.972
Cough	Respiratory	Wald ratio	1	1.056 (0.946, 1.178)	0.334	0.952
Other dyspnea	Respiratory	Wald ratio	1	1.286 (1.06, 1.56)	0.011	0.564
Respiratory abnormalities	Respiratory	Wald ratio	1	1.052 (0.831, 1.33)	0.675	0.981
Hypoventilation	Respiratory	Wald ratio	1	1.237 (0.649, 2.357)	0.518	0.981
Hyperventilation	Respiratory	Wald ratio	1	0.889 (0.627, 1.26)	0.507	0.977
Disorders of diaphragm	Respiratory	Wald ratio	1	1.213 (0.77, 1.912)	0.405	0.974
Abnormal findings examination of lungs	Respiratory	Wald ratio	1	1.027 (0.914, 1.156)	0.652	0.981
Abnormal sputum	Respiratory	Wald ratio	1	1.102 (0.972, 1.248)	0.13	0.916
Hemoptysis	Respiratory	Wald ratio	1	1.11 (0.976, 1.263)	0.112	0.916
Other diseases of respiratory system, not elsewhere classified	Respiratory	Wald ratio	1	0.995 (0.937, 1.057)	0.876	0.983
Respiratory complications	Respiratory	Wald ratio	1	1.014 (0.724, 1.421)	0.936	0.986
Other diseases of respiratory system, NEC	Respiratory	Wald ratio	1	0.998 (0.939, 1.061)	0.952	0.989
Symptoms involving respiratory system and other chest symptoms	Respiratory	Wald ratio	1	1.014 (0.729, 1.41)	0.933	0.986
Disorders of the globe	Sense organs	Wald ratio	1	0.917 (0.667, 1.26)	0.592	0.981
Progressive myopia	Sense organs	Wald ratio	1	0.949 (0.657, 1.371)	0.781	0.981
Retinal detachments and defects	Sense organs	Wald ratio	1	1.089 (0.983, 1.206)	0.103	0.902
Retinal detachment with retinal defect	Sense organs	Wald ratio	1	1.085 (0.928, 1.267)	0.306	0.923
Retinoschisis and retinal cysts	Sense organs	Wald ratio	1	1.039 (0.506, 2.136)	0.916	0.983
Other retinal disorders	Sense organs	Wald ratio	1	1.085 (0.987, 1.192)	0.093	0.874
Degeneration of macula and posterior pole of retina	Sense organs	Wald ratio	1	1.018 (0.899, 1.154)	0.776	0.981
Macular degeneration (senile) of retina NOS	Sense organs	Wald ratio	1	1.016 (0.897, 1.152)	0.799	0.981
Other nondiabetic retinopathy	Sense organs	Wald ratio	1	1.156 (0.734, 1.822)	0.532	0.981
Separation of retinal layers	Sense organs	Wald ratio	1	0.847 (0.48, 1.495)	0.566	0.981
Retinal vascular changes and abnomalities	Sense organs	Wald ratio	1	1.093 (0.894, 1.336)	0.385	0.974
Peripheral retinal degenerations	Sense organs	Wald ratio	1	1.099 (0.725, 1.668)	0.656	0.981
Hereditary retinal dystrophies	Sense organs	Wald ratio	1	0.793 (0.416, 1.51)	0.48	0.974
Retinal hemorrhage or ischemia	Sense organs	Wald ratio	1	0.958 (0.564, 1.629)	0.875	0.983
Chorioretinal inflammations, scars, and other disorders of choroid	Sense organs	Wald ratio	1	1.134 (0.72, 1.787)	0.588	0.981
Chorioretinal scars	Sense organs	Wald ratio	1	1.261 (0.637, 2.495)	0.505	0.974
Corneal opacity and other disorders of cornea	Sense organs	Wald ratio	1	1.091 (0.879, 1.354)	0.43	0.974
Corneal opacity	Sense organs	Wald ratio	1	1.237 (0.675, 2.269)	0.492	0.974
Corneal edema	Sense organs	Wald ratio	1	0.953 (0.5, 1.815)	0.883	0.983
Corneal degenerations	Sense organs	Wald ratio	1	1.141 (0.752, 1.731)	0.536	0.981
Keratoconus	Sense organs	Wald ratio	1	1.501 (0.818, 2.753)	0.189	0.917
Corneal dystrophy	Sense organs	Wald ratio	1	1.039 (0.711, 1.519)	0.841	0.981
Cornea replaced by transplant	Sense organs	Wald ratio	1	1.183 (0.67, 2.089)	0.562	0.981
Glaucoma	Sense organs	Wald ratio	1	1.027 (0.942, 1.121)	0.543	0.981
Open-angle glaucoma	Sense organs	Wald ratio	1	1.013 (0.844, 1.215)	0.892	0.983
Primary open angle glaucoma	Sense organs	Wald ratio	1	1 (0.834, 1.2)	0.997	0.997
Primary angle-closure glaucoma	Sense organs	Wald ratio	1	0.924 (0.741, 1.151)	0.48	0.974
Cataract	Sense organs	Wald ratio	1	1.074 (1.03, 1.12)	7.69E-04	0.109
Nonsenile Cataract	Sense organs	Wald ratio	1	0.853 (0.431, 1.688)	0.649	0.981

Senile cataract	Sense organs	Wald ratio	1	1.064 (0.997, 1.135)	0.06	0.874
Traumatic cataract	Sense organs	Wald ratio	1	0.778 (0.44, 1.373)	0.386	0.974
Disorders of refraction and accommodation; blindness and low vision	Sense organs	Wald ratio	1	1.035 (0.921, 1.165)	0.561	0.981
Myopia	Sense organs	Wald ratio	1	1.132 (0.962, 1.332)	0.137	0.916
Astigmatism	Sense organs	Wald ratio	1	1.068 (0.704, 1.621)	0.757	0.981
Hypermetropia	Sense organs	Wald ratio	1	1.213 (0.84, 1.753)	0.303	0.923
Blindness and low vision	Sense organs	Wald ratio	1	0.953 (0.768, 1.183)	0.661	0.981
Visual disturbances	Sense organs	Wald ratio	1	0.973 (0.879, 1.078)	0.604	0.981
Amblyopia	Sense organs	Wald ratio	1	0.99 (0.771, 1.271)	0.935	0.986
Diplopia and disorders of binocular vision	Sense organs	Wald ratio	1	0.993 (0.8, 1.232)	0.948	0.988
Anisometropia	Sense organs	Wald ratio	1	0.808 (0.513, 1.274)	0.359	0.963
Visual field defects	Sense organs	Wald ratio	1	1.076 (0.783, 1.48)	0.651	0.981
Subjective visual disturbances	Sense organs	Wald ratio	1	0.995 (0.786, 1.258)	0.964	0.99
Infection of the eye	Sense organs	Wald ratio	1	0.899 (0.703, 1.15)	0.397	0.974
Eye infection, viral	Sense organs	Wald ratio	1	0.629 (0.37, 1.069)	0.086	0.874
Conjunctivitis, infectious	Sense organs	Wald ratio	1	0.793 (0.543, 1.158)	0.23	0.917
Keratitis	Sense organs	Wald ratio	1	0.949 (0.677, 1.33)	0.762	0.981
Corneal ulcer	Sense organs	Wald ratio	1	0.763 (0.466, 1.249)	0.282	0.923
Inflammation of the eye	Sense organs	Wald ratio	1	0.983 (0.888, 1.089)	0.747	0.981
Uveitis, noninfectious or NOS	Sense organs	Wald ratio	1	1.01 (0.774, 1.317)	0.943	0.986
Inflammation of eyelids	Sense organs	Wald ratio	1	0.97 (0.862, 1.09)	0.606	0.981
Disorders of conjunctiva	Sense organs	Wald ratio	1	1.138 (0.921, 1.408)	0.232	0.917
Other disorders of eyelids	Sense organs	Wald ratio	1	0.971 (0.897, 1.052)	0.475	0.974
Ectropion or entropion	Sense organs	Wald ratio	1	0.947 (0.793, 1.132)	0.551	0.981
Ptosis of eyelid	Sense organs	Wald ratio	1	0.947 (0.826, 1.086)	0.437	0.974
Disorders of lacrimal system	Sense organs	Wald ratio	1	0.968 (0.854, 1.097)	0.606	0.981
Epiphora	Sense organs	Wald ratio	1	1.078 (0.889, 1.308)	0.444	0.974
Disorders of the orbit	Sense organs	Wald ratio	1	0.706 (0.371, 1.345)	0.29	0.923
Disorders of optic nerve and visual pathways	Sense organs	Wald ratio	1	1.099 (0.812, 1.489)	0.54	0.981
Optic atrophy	Sense organs	Wald ratio	1	0.582 (0.273, 1.242)	0.162	0.916
Optic neuritis or neuropathy	Sense organs	Wald ratio	1	1.237 (0.728, 2.103)	0.432	0.974
Strabismus and other disorders of binocular eye movements	Sense organs	Wald ratio	1	1.056 (0.907, 1.228)	0.484	0.974
Strabismus (not specified as paralytic)	Sense organs	Wald ratio	1	1.041 (0.858, 1.264)	0.681	0.981
Nystagmus and other irregular eye movements	Sense organs	Wald ratio	1	1.035 (0.709, 1.513)	0.857	0.981
Paralytic strabismus	Sense organs	Wald ratio	1	1.261 (0.89, 1.788)	0.192	0.917
Other disorders of eye	Sense organs	Wald ratio	1	1.11 (1.01, 1.22)	0.031	0.749
Disorders of vitreous body	Sense organs	Wald ratio	1	1.045 (0.892, 1.226)	0.584	0.981
Aphakia and other disorders of lens	Sense organs	Wald ratio	1	1.089 (0.95, 1.248)	0.222	0.917
Anomalies of pupillary function	Sense organs	Wald ratio	1	0.927 (0.469, 1.835)	0.828	0.981
Disorders of iris and ciliary body	Sense organs	Wald ratio	1	1.237 (0.927, 1.65)	0.148	0.916
Pain, swelling or discharge of eye	Sense organs	Wald ratio	1	0.808 (0.513, 1.274)	0.359	0.963
Disorders of external ear	Sense organs	Wald ratio	1	0.971 (0.832, 1.135)	0.714	0.981
Otitis externa	Sense organs	Wald ratio	1	1.022 (0.792, 1.317)	0.87	0.981
Impacted cerumen	Sense organs	Wald ratio	1	0.945 (0.695, 1.285)	0.72	0.981
Otitis media and Eustachian tube disorders	Sense organs	Wald ratio	1	0.983 (0.867, 1.114)	0.785	0.981
Otitis media	Sense organs	Wald ratio	1	1.016 (0.886, 1.164)	0.824	0.981
Suppurative and unspecified otitis media	Sense organs	Wald ratio	1	1.043 (0.854, 1.276)	0.678	0.981
Eustachian tube disorders	Sense organs	Wald ratio	1	0.96 (0.633, 1.457)	0.849	0.981
Mastoiditis & related conditions	Sense organs	Wald ratio	1	0.86 (0.546, 1.355)	0.516	0.981
Otorrhea	Sense organs	Wald ratio	1	0.88 (0.621, 1.247)	0.473	0.974
Otalgia	Sense organs	Wald ratio	1	0.824 (0.593, 1.146)	0.25	0.919
Otosclerosis	Sense organs	Wald ratio	1	1.016 (0.731, 1.413)	0.925	0.986
Other disorders of tympanic membrane	Sense organs	Wald ratio	1	1.041 (0.888, 1.221)	0.617	0.981
Perforation of tympanic membrane	Sense organs	Wald ratio	1	1.106 (0.922, 1.326)	0.279	0.923
Other disorders of middle ear and mastoid	Sense organs	Wald ratio	1	0.973 (0.796, 1.19)	0.792	0.981
Cholesteatoma	Sense organs	Wald ratio	1	0.942 (0.745, 1.191)	0.617	0.981
Tympanosclerosis and middle ear disease related to otitis media	Sense organs	Wald ratio	1	1.145 (0.787, 1.666)	0.48	0.974
Vertiginous syndromes and other disorders of vestibular system	Sense organs	Wald ratio	1	1.022 (0.947, 1.102)	0.582	0.981
Meniere's disease	Sense organs	Wald ratio	1	1.089 (0.854, 1.388)	0.492	0.974
Peripheral or central vertigo	Sense organs	Wald ratio	1	1.143 (0.847, 1.542)	0.382	0.974
Labyrinthitis	Sense organs	Wald ratio	1	1.068 (0.864, 1.321)	0.544	0.981
Dizziness and giddiness (Light-headedness and vertigo)	Sense organs	Wald ratio	1	0.988 (0.905, 1.077)	0.777	0.981
Other disorders of ear	Sense organs	Wald ratio	1	0.778 (0.513, 1.18)	0.237	0.919
Hearing loss	Sense organs	Wald ratio	1	0.981 (0.896, 1.075)	0.686	0.981
Sensorineural hearing loss	Sense organs	Wald ratio	1	1.213 (0.924, 1.594)	0.165	0.916
Conductive hearing loss	Sense organs	Wald ratio	1	1.286 (0.979, 1.689)	0.071	0.874
Tinnitus	Sense organs	Wald ratio	1	0.975 (0.754, 1.262)	0.848	0.981
Symptoms concerning nutrition, metabolism, and development	Symptoms	Wald ratio	1	1.009 (0.932, 1.093)	0.823	0.981
Other signs and symptoms involving emotional state	Symptoms	Wald ratio	1	0.904 (0.648, 1.262)	0.555	0.981
Other symptoms	Symptoms	Wald ratio	1	1.06 (0.897, 1.252)	0.495	0.974
Other tests	Symptoms	Wald ratio	1	0.953 (0.883, 1.028)	0.211	0.917
Effects of other external causes	Symptoms	Wald ratio	1	0.973 (0.889, 1.066)	0.56	0.981
Other ill-defined and unknown causes of morbidity and mortality	Symptoms	Wald ratio	1	0.989 (0.942, 1.039)	0.667	0.981
Pain	Symptoms	Wald ratio	1	1.136 (0.922, 1.4)	0.23	0.917
Localized superficial swelling, mass, or lump	Symptoms	Wald ratio	1	1.13 (0.8, 1.595)	0.489	0.974
Back pain	Symptoms	Wald ratio	1	1.007 (0.952, 1.066)	0.805	0.981
Cervicalgia	Symptoms	Wald ratio	1	0.982 (0.857, 1.126)	0.794	0.981
Sciatica	Symptoms	Wald ratio	1	0.94 (0.833, 1.061)	0.317	0.923
Neuralgia, neuritis, and radiculitis NOS	Symptoms	Wald ratio	1	1.099 (0.927, 1.304)	0.276	0.923

Myalgia and myositis unspecified	Symptoms	Wald ratio	1	1.027 (0.815, 1.295)	0.818	0.981
Musculoskeletal symptoms referable to limbs	Symptoms	Wald ratio	1	1.001 (0.925, 1.084)	0.978	0.991
Swelling of limb	Symptoms	Wald ratio	1	0.986 (0.903, 1.075)	0.744	0.981
Symptoms of the muscles	Symptoms	Wald ratio	1	1.01 (0.81, 1.258)	0.933	0.986
Pain in limb	Symptoms	Wald ratio	1	0.958 (0.888, 1.034)	0.271	0.923
Hypothermia or Chills	Symptoms	Wald ratio	1	0.734 (0.466, 1.157)	0.182	0.916
Symptoms involving nervous and musculoskeletal systems	Symptoms	Wald ratio	1	0.913 (0.797, 1.047)	0.192	0.917
Symptoms involving skin and other integumentary tissue	Symptoms	Wald ratio	1	0.962 (0.839, 1.103)	0.579	0.981
Edema	Symptoms	Wald ratio	1	0.983 (0.851, 1.135)	0.817	0.981
Pallor and flushing	Symptoms	Wald ratio	1	0.734 (0.466, 1.157)	0.182	0.916
Fever of unknown origin	Symptoms	Wald ratio	1	0.985 (0.896, 1.083)	0.758	0.981
Abdominal pain	Symptoms	Wald ratio	1	1.022 (0.991, 1.053)	0.164	0.916
Syncope and collapse	Symptoms	Wald ratio	1	1.064 (1.001, 1.13)	0.046	0.792
Nausea and vomiting	Symptoms	Wald ratio	1	1.025 (0.969, 1.085)	0.386	0.974
Nonspecific findings on examination of blood	Symptoms	Wald ratio	1	1.085 (1.009, 1.166)	0.027	0.748
Elevated sedimentation rate	Symptoms	Wald ratio	1	1.968 (1.033, 3.749)	0.04	0.792
Other abnormal blood chemistry	Symptoms	Wald ratio	1	1.08 (1.002, 1.166)	0.046	0.792
Gangrene	Symptoms	Wald ratio	1	1.132 (0.885, 1.448)	0.325	0.934
Abnormal Papanicolaou smear of cervix and cervical HPV	Symptoms	Wald ratio	1	1.054 (0.597, 1.86)	0.857	0.981
Abnormal results of other function studies (bladder, pancreas, placenta, spleen, etc)	Symptoms	Wald ratio	1	0.629 (0.37, 1.069)	0.086	0.874
Shock	Symptoms	Wald ratio	1	0.853 (0.521, 1.397)	0.528	0.981
Cardiogenic shock	Symptoms	Wald ratio	1	0.763 (0.4, 1.453)	0.41	0.974
Malaise and fatigue	Symptoms	Wald ratio	1	1.004 (0.909, 1.108)	0.942	0.986
Chronic fatigue syndrome	Symptoms	Wald ratio	1	1.112 (0.876, 1.412)	0.383	0.974

Table S16. Phenome-wide MR analyses on genetically mimicked targeting of NK-specific *CTSW*.

Phenotype	Category	Method	nSNP	OR (95% CI)	*P*-value	*FDR*
Rheumatic disease of the heart valves	Circulatory system	Wald ratio	1	0.965 (0.92, 1.012)	0.144	0.859
Mitral valve disease	Circulatory system	Wald ratio	1	0.984 (0.926, 1.046)	0.606	0.956
Aortic valve disease	Circulatory system	Wald ratio	1	0.893 (0.814, 0.979)	0.016	0.594
Disease of tricuspid valve	Circulatory system	Wald ratio	1	0.991 (0.897, 1.095)	0.857	0.984
Heart valve disorders	Circulatory system	Wald ratio	1	0.969 (0.92, 1.021)	0.239	0.879
Nonrheumatic mitral valve disorders	Circulatory system	Wald ratio	1	0.996 (0.937, 1.058)	0.892	0.984
Nonrheumatic aortic valve disorders	Circulatory system	Wald ratio	1	1.009 (0.823, 1.236)	0.933	0.984
Nonrheumatic tricuspid valve disorders	Circulatory system	Wald ratio	1	0.868 (0.634, 1.189)	0.378	0.899
Nonrheumatic pulmonary valve disorders	Circulatory system	Wald ratio	1	1.028 (0.737, 1.433)	0.872	0.984
Heart valve replaced	Circulatory system	Wald ratio	1	0.931 (0.855, 1.013)	0.098	0.835
Abnormal heart sounds	Circulatory system	Wald ratio	1	1.032 (0.932, 1.142)	0.549	0.949
Hypertensive heart and or or renal disease	Circulatory system	Wald ratio	1	1.048 (0.968, 1.135)	0.245	0.879
Hypertensive heart disease	Circulatory system	Wald ratio	1	1.174 (0.906, 1.52)	0.225	0.879
Hypertensive chronic kidney disease	Circulatory system	Wald ratio	1	1.032 (0.949, 1.121)	0.463	0.919
Other hypertensive complications	Circulatory system	Wald ratio	1	0.97 (0.735, 1.28)	0.831	0.984
Elevated blood pressure reading without diagnosis of hypertension	Circulatory system	Wald ratio	1	0.996 (0.915, 1.084)	0.927	0.984
Ischemic Heart Disease	Circulatory system	Wald ratio	1	0.988 (0.968, 1.008)	0.237	0.879
Unstable angina (intermediate coronary syndrome)	Circulatory system	Wald ratio	1	0.956 (0.913, 1.001)	0.055	0.778
Myocardial infarction	Circulatory system	Wald ratio	1	0.979 (0.949, 1.011)	0.196	0.879
Angina pectoris	Circulatory system	Wald ratio	1	1.004 (0.977, 1.032)	0.769	0.982
Coronary atherosclerosis	Circulatory system	Wald ratio	1	1.003 (0.977, 1.029)	0.83	0.984
Aneurysm and dissection of heart	Circulatory system	Wald ratio	1	0.918 (0.811, 1.039)	0.174	0.879
Other chronic ischemic heart disease, unspecified	Circulatory system	Wald ratio	1	0.993 (0.966, 1.021)	0.617	0.956
Other acute and subacute forms of ischemic heart disease	Circulatory system	Wald ratio	1	1.042 (0.947, 1.147)	0.397	0.908
Other forms of chronic heart disease	Circulatory system	Wald ratio	1	0.97 (0.898, 1.049)	0.446	0.917
ASCVD	Circulatory system	Wald ratio	1	0.783 (0.521, 1.175)	0.237	0.879
Pulmonary heart disease	Circulatory system	Wald ratio	1	0.967 (0.92, 1.016)	0.182	0.879
Acute pulmonary heart disease	Circulatory system	Wald ratio	1	0.828 (0.572, 1.198)	0.317	0.879
Pulmonary embolism and infarction, acute	Circulatory system	Wald ratio	1	0.828 (0.572, 1.198)	0.317	0.879
Chronic pulmonary heart disease	Circulatory system	Wald ratio	1	0.97 (0.848, 1.11)	0.661	0.974
Primary pulmonary hypertension	Circulatory system	Wald ratio	1	0.967 (0.828, 1.129)	0.668	0.974
Cardiomegaly	Circulatory system	Wald ratio	1	0.959 (0.899, 1.023)	0.209	0.879
Nonspecific chest pain	Circulatory system	Wald ratio	1	1.005 (0.985, 1.026)	0.636	0.967
Precordial pain	Circulatory system	Wald ratio	1	0.992 (0.939, 1.049)	0.79	0.984
Carditis	Circulatory system	Wald ratio	1	1.009 (0.937, 1.086)	0.816	0.984
Myocarditis	Circulatory system	Wald ratio	1	1.23 (0.899, 1.685)	0.196	0.879
Pericarditis	Circulatory system	Wald ratio	1	0.995 (0.908, 1.092)	0.922	0.984
Acute pericarditis	Circulatory system	Wald ratio	1	0.992 (0.795, 1.239)	0.947	0.986
Endocarditis	Circulatory system	Wald ratio	1	1.015 (0.895, 1.151)	0.814	0.984
Cardiomyopathy	Circulatory system	Wald ratio	1	1.012 (0.923, 1.11)	0.795	0.984
Primary or intrinsic cardiomyopathies	Circulatory system	Wald ratio	1	1.011 (0.92, 1.111)	0.814	0.984
Hypertrophic obstructive cardiomyopathy	Circulatory system	Wald ratio	1	0.79 (0.599, 1.042)	0.096	0.835
Other hypertrophic cardiomyopathy	Circulatory system	Wald ratio	1	0.893 (0.689, 1.157)	0.391	0.903
Other cardiomyopathy	Circulatory system	Wald ratio	1	1.185 (0.775, 1.813)	0.434	0.917
Cardiac conduction disorders	Circulatory system	Wald ratio	1	1.03 (0.989, 1.072)	0.159	0.879
Atrioventricular [AV] block	Circulatory system	Wald ratio	1	1.019 (0.95, 1.093)	0.599	0.955
First degree AV block	Circulatory system	Wald ratio	1	1.021 (0.919, 1.134)	0.7	0.979
Second degree AV block	Circulatory system	Wald ratio	1	1.109 (0.952, 1.293)	0.185	0.879
Atrioventricular block, complete	Circulatory system	Wald ratio	1	0.976 (0.853, 1.117)	0.722	0.982
Other heart block	Circulatory system	Wald ratio	1	1.099 (0.833, 1.45)	0.505	0.943
Bundle branch block	Circulatory system	Wald ratio	1	1.023 (0.966, 1.083)	0.439	0.917
Right bundle branch block	Circulatory system	Wald ratio	1	1.048 (0.963, 1.141)	0.277	0.879
Left bundle branch block	Circulatory system	Wald ratio	1	1.001 (0.924, 1.084)	0.985	0.991
Anomalous atrioventricular excitation	Circulatory system	Wald ratio	1	1.02 (0.817, 1.273)	0.861	0.984
Other cardiac conduction disorders	Circulatory system	Wald ratio	1	0.573 (0.375, 0.877)	0.01	0.55
Cardiac pacemaker or device in situ	Circulatory system	Wald ratio	1	1.064 (0.996, 1.137)	0.067	0.794
Cardiac pacemaker in situ	Circulatory system	Wald ratio	1	1.067 (0.997, 1.143)	0.062	0.789
Cardiac dysrhythmias	Circulatory system	Wald ratio	1	0.998 (0.976, 1.02)	0.835	0.984
Paroxysmal tachycardia, unspecified	Circulatory system	Wald ratio	1	1.011 (0.953, 1.073)	0.708	0.982
Paroxysmal supraventricular tachycardia	Circulatory system	Wald ratio	1	0.991 (0.926, 1.061)	0.797	0.984
Paroxysmal ventricular tachycardia	Circulatory system	Wald ratio	1	1.028 (0.923, 1.144)	0.617	0.956
Atrial fibrillation and flutter	Circulatory system	Wald ratio	1	0.996 (0.969, 1.024)	0.769	0.982
Other specified cardiac dysrhythmias	Circulatory system	Wald ratio	1	0.997 (0.942, 1.056)	0.931	0.984
Cardiac arrest and ventricular fibrillation	Circulatory system	Wald ratio	1	0.932 (0.846, 1.026)	0.149	0.879
Ventricular fibrillation and flutter	Circulatory system	Wald ratio	1	0.941 (0.786, 1.125)	0.503	0.943
Cardiac arrest	Circulatory system	Wald ratio	1	0.919 (0.825, 1.023)	0.121	0.847
Arrhythmia (cardiac) NOS	Circulatory system	Wald ratio	1	0.973 (0.874, 1.083)	0.617	0.956
Premature beats	Circulatory system	Wald ratio	1	1.185 (1.03, 1.364)	0.018	0.594
Supraventricular premature beats	Circulatory system	Wald ratio	1	1.109 (0.825, 1.491)	0.492	0.94
Tachycardia NOS	Circulatory system	Wald ratio	1	0.975 (0.909, 1.046)	0.477	0.93
Sinoatrial node dysfunction (Bradycardia)	Circulatory system	Wald ratio	1	1.109 (0.943, 1.305)	0.211	0.879
Palpitations	Circulatory system	Wald ratio	1	1.046 (0.992, 1.104)	0.098	0.835
Congestive heart failure; nonhypertensive	Circulatory system	Wald ratio	1	0.949 (0.906, 0.993)	0.025	0.665
Congestive heart failure (CHF) NOS	Circulatory system	Wald ratio	1	0.947 (0.879, 1.019)	0.147	0.87
Heart failure NOS	Circulatory system	Wald ratio	1	0.959 (0.911, 1.01)	0.116	0.847
Ill-defined descriptions and complications of heart disease	Circulatory system	Wald ratio	1	1.033 (0.919, 1.16)	0.589	0.955
Heart transplant or surgery	Circulatory system	Wald ratio	1	1.365 (0.909, 2.05)	0.134	0.85

Abnormal function study of cardiovascular system	Circulatory system	Wald ratio	1	0.989 (0.858, 1.14)	0.876	0.984
Symptoms involving cardiovascular system	Circulatory system	Wald ratio	1	1.076 (0.846, 1.369)	0.549	0.949
Intracranial hemorrhage	Circulatory system	Wald ratio	1	1.002 (0.927, 1.083)	0.954	0.99
Subarachnoid hemorrhage	Circulatory system	Wald ratio	1	1.024 (0.913, 1.148)	0.687	0.979
Intracerebral hemorrhage	Circulatory system	Wald ratio	1	1.076 (0.951, 1.218)	0.244	0.879
Subdural hemorrhage	Circulatory system	Wald ratio	1	0.956 (0.78, 1.171)	0.663	0.974
Cerebrovascular disease	Circulatory system	Wald ratio	1	1 (0.965, 1.035)	0.987	0.991
Occlusion and stenosis of precerebral arteries	Circulatory system	Wald ratio	1	1.008 (0.918, 1.108)	0.863	0.984
Cerebral atherosclerosis	Circulatory system	Wald ratio	1	1.04 (0.833, 1.299)	0.726	0.982
Occlusion of cerebral arteries	Circulatory system	Wald ratio	1	0.984 (0.934, 1.036)	0.544	0.949
Cerebral artery occlusion, with cerebral infarction	Circulatory system	Wald ratio	1	0.967 (0.888, 1.052)	0.434	0.917
Cerebral ischemia	Circulatory system	Wald ratio	1	0.999 (0.94, 1.062)	0.979	0.991
Transient cerebral ischemia	Circulatory system	Wald ratio	1	1.005 (0.937, 1.078)	0.895	0.984
Cerebral aneurysm	Circulatory system	Wald ratio	1	1.018 (0.862, 1.202)	0.833	0.984
Late effects of cerebrovascular disease	Circulatory system	Wald ratio	1	0.969 (0.884, 1.063)	0.509	0.944
Atherosclerosis	Circulatory system	Wald ratio	1	0.957 (0.874, 1.047)	0.337	0.898
Atherosclerosis of renal artery	Circulatory system	Wald ratio	1	0.956 (0.698, 1.309)	0.778	0.984
Atherosclerosis of the extremities	Circulatory system	Wald ratio	1	0.998 (0.89, 1.119)	0.974	0.991
Atherosclerosis of aorta	Circulatory system	Wald ratio	1	0.91 (0.756, 1.095)	0.317	0.879
Vascular insufficiency of intestine	Circulatory system	Wald ratio	1	0.934 (0.815, 1.071)	0.331	0.898
Acute vascular insufficiency of intestine	Circulatory system	Wald ratio	1	0.955 (0.779, 1.17)	0.656	0.974
Other aneurysm	Circulatory system	Wald ratio	1	0.983 (0.91, 1.063)	0.668	0.974
Aortic aneurysm	Circulatory system	Wald ratio	1	0.979 (0.895, 1.069)	0.632	0.967
Abdominal aortic aneurysm	Circulatory system	Wald ratio	1	1.045 (0.937, 1.166)	0.426	0.917
Aneurysm of iliac artery	Circulatory system	Wald ratio	1	1.208 (0.866, 1.684)	0.267	0.879
Aneurysm of artery of lower extremity	Circulatory system	Wald ratio	1	0.82 (0.633, 1.063)	0.134	0.85
Arterial dissection	Circulatory system	Wald ratio	1	1.242 (0.797, 1.935)	0.338	0.898
Aneurysm of other specified artery	Circulatory system	Wald ratio	1	1.196 (0.958, 1.493)	0.113	0.847
Peripheral vascular disease	Circulatory system	Wald ratio	1	0.976 (0.925, 1.03)	0.37	0.899
Raynaud's syndrome	Circulatory system	Wald ratio	1	1.01 (0.918, 1.112)	0.832	0.984
Peripheral angiopathy in diseases classified elsewhere	Circulatory system	Wald ratio	1	1.077 (0.912, 1.272)	0.38	0.899
Peripheral vascular disease, unspecified	Circulatory system	Wald ratio	1	0.951 (0.892, 1.015)	0.13	0.85
Arterial embolism and thrombosis	Circulatory system	Wald ratio	1	0.934 (0.839, 1.04)	0.214	0.879
Arterial embolism and thrombosis of lower extremity artery	Circulatory system	Wald ratio	1	0.868 (0.756, 0.997)	0.046	0.752
Polyarteritis nodosa and allied conditions	Circulatory system	Wald ratio	1	1.032 (0.92, 1.157)	0.595	0.955
Wegener's granulomatosis	Circulatory system	Wald ratio	1	0.931 (0.732, 1.184)	0.559	0.951
Giant cell arteritis	Circulatory system	Wald ratio	1	1.098 (0.93, 1.296)	0.271	0.879
Arteritis NOS	Circulatory system	Wald ratio	1	0.93 (0.745, 1.161)	0.521	0.949
Other disorders of arteries and arterioles	Circulatory system	Wald ratio	1	1.001 (0.914, 1.096)	0.985	0.991
Stricture of artery	Circulatory system	Wald ratio	1	0.988 (0.884, 1.104)	0.828	0.984
Disease of capillaries	Circulatory system	Wald ratio	1	0.994 (0.753, 1.311)	0.965	0.991
Noninfectious disorders of lymphatic channels	Circulatory system	Wald ratio	1	0.885 (0.782, 1.001)	0.052	0.778
Phlebitis and thrombophlebitis	Circulatory system	Wald ratio	1	0.995 (0.943, 1.05)	0.863	0.984
Phlebitis and thrombophlebitis of lower extremities	Circulatory system	Wald ratio	1	0.995 (0.942, 1.052)	0.865	0.984
Other venous embolism and thrombosis	Circulatory system	Wald ratio	1	1.13 (0.984, 1.298)	0.083	0.825
Varicose veins	Circulatory system	Wald ratio	1	0.982 (0.952, 1.014)	0.264	0.879
Varicose veins of lower extremity	Circulatory system	Wald ratio	1	0.98 (0.95, 1.012)	0.217	0.879
Varicose veins of lower extremity, symptomtic	Circulatory system	Wald ratio	1	1.043 (0.917, 1.187)	0.52	0.949
Hemorrhoids	Circulatory system	Wald ratio	1	0.981 (0.96, 1.003)	0.096	0.835
Chronic venous insufficiency [CVI]	Circulatory system	Wald ratio	1	1.055 (0.83, 1.342)	0.661	0.974
Encounter for long-term (current) use of anticoagulants, antithrombotics, aspirin	Circulatory system	Wald ratio	1	1.035 (0.742, 1.443)	0.841	0.984
Hypotension	Circulatory system	Wald ratio	1	1.032 (0.989, 1.076)	0.151	0.879
Orthostatic hypotension	Circulatory system	Wald ratio	1	0.999 (0.914, 1.092)	0.982	0.991
Iatrogenic hypotension	Circulatory system	Wald ratio	1	1.196 (0.958, 1.493)	0.113	0.847
Hypotension NOS	Circulatory system	Wald ratio	1	1.035 (0.98, 1.095)	0.217	0.879
Other disorders of circulatory system	Circulatory system	Wald ratio	1	1.008 (0.983, 1.035)	0.525	0.949
Hemorrhage NOS	Circulatory system	Wald ratio	1	0.933 (0.669, 1.302)	0.685	0.979
Circulatory disease NEC	Circulatory system	Wald ratio	1	1.007 (0.982, 1.034)	0.582	0.955
Nonspecific abnormal findings on radiological and other examination of other intrathoracic organs (echocardiogram, etc)	Circulatory system	Wald ratio	1	1.035 (0.903, 1.187)	0.617	0.956
Cardiac and circulatory congenital anomalies	Congenital anomalies	Wald ratio	1	0.988 (0.928, 1.052)	0.702	0.979
Cardiac congenital anomalies	Congenital anomalies	Wald ratio	1	0.995 (0.932, 1.061)	0.873	0.984
Cardiac shunt or heart septal defect	Congenital anomalies	Wald ratio	1	1.109 (0.969, 1.269)	0.132	0.85
Valvular heart disease or heart chambers	Congenital anomalies	Wald ratio	1	1.095 (0.877, 1.367)	0.424	0.917
Congenital anomalies of great vessels	Congenital anomalies	Wald ratio	1	0.952 (0.881, 1.029)	0.216	0.879
Congenital anomalies of peripheral vascular system	Congenital anomalies	Wald ratio	1	0.86 (0.664, 1.114)	0.253	0.879
Anomalies of respiratory system, congenital	Congenital anomalies	Wald ratio	1	1.302 (0.82, 2.067)	0.263	0.879
Congenital anomalies of face and neck	Congenital anomalies	Wald ratio	1	0.893 (0.745, 1.07)	0.221	0.879
Digestive congenital anomalies	Congenital anomalies	Wald ratio	1	0.983 (0.869, 1.113)	0.788	0.984
Upper gastrointestinal congenital anomalies	Congenital anomalies	Wald ratio	1	1.041 (0.87, 1.246)	0.658	0.974
Congenital anomalies of mouth or tongue	Congenital anomalies	Wald ratio	1	1.196 (0.941, 1.521)	0.144	0.859
Lower gastrointestinal congenital anomalies	Congenital anomalies	Wald ratio	1	0.937 (0.789, 1.113)	0.458	0.919
Congenital anomalies of intestine	Congenital anomalies	Wald ratio	1	0.868 (0.695, 1.084)	0.211	0.879
Congenital anomaly of gallbladder, bile ducts, liver, pancreas	Congenital anomalies	Wald ratio	1	1.035 (0.77, 1.39)	0.822	0.984
Genitourinary congenital anomalies	Congenital anomalies	Wald ratio	1	0.949 (0.874, 1.029)	0.203	0.879
Congenital anomalies of genital organs	Congenital anomalies	Wald ratio	1	0.918 (0.812, 1.037)	0.168	0.879
Congenital anomalies of female genital organs	Congenital anomalies	Wald ratio	1	0.933 (0.801, 1.088)	0.379	0.899
Congenital anomalies of urinary system	Congenital anomalies	Wald ratio	1	0.969 (0.868, 1.083)	0.582	0.955

Cystic kidney disease	Congenital anomalies	Wald ratio	1	1.013 (0.866, 1.186)	0.869	0.984
Other specified congenital anomalies of kidney	Congenital anomalies	Wald ratio	1	0.964 (0.807, 1.151)	0.685	0.979
Nervous system congenital anomalies	Congenital anomalies	Wald ratio	1	0.955 (0.8, 1.14)	0.61	0.956
Neural tube defects	Congenital anomalies	Wald ratio	1	1.007 (0.822, 1.234)	0.947	0.986
Spina bifida	Congenital anomalies	Wald ratio	1	0.989 (0.792, 1.234)	0.92	0.984
Other specified congenital anomalies of nervous system	Congenital anomalies	Wald ratio	1	0.805 (0.556, 1.165)	0.25	0.879
Congenital anomalies of the eye	Congenital anomalies	Wald ratio	1	1.13 (0.857, 1.491)	0.386	0.901
Congenital cataract and lens anomalies	Congenital anomalies	Wald ratio	1	1.095 (0.756, 1.584)	0.631	0.967
Congenital musculoskeletal deformities of spine	Congenital anomalies	Wald ratio	1	0.726 (0.53, 0.994)	0.046	0.752
Congenital anomalies of limbs	Congenital anomalies	Wald ratio	1	1.163 (0.997, 1.356)	0.054	0.778
Congenital deformities of feet	Congenital anomalies	Wald ratio	1	1.13 (0.906, 1.411)	0.279	0.879
Other congenital anomalies of lower limb, including pelvic girdle	Congenital anomalies	Wald ratio	1	1.052 (0.812, 1.363)	0.7	0.979
Congenital hip dysplasia and deformity	Congenital anomalies	Wald ratio	1	0.798 (0.583, 1.092)	0.158	0.879
Other congenital musculoskeletal anomalies	Congenital anomalies	Wald ratio	1	1.019 (0.89, 1.166)	0.784	0.984
Congenital anomalies of muscle, tendon, fascia, and connective tissue	Congenital anomalies	Wald ratio	1	1.015 (0.702, 1.469)	0.936	0.984
Congenital osteodystrophies	Congenital anomalies	Wald ratio	1	1.027 (0.878, 1.201)	0.742	0.982
Chromosomal anomalies and genetic disorders	Congenital anomalies	Wald ratio	1	0.893 (0.702, 1.136)	0.356	0.898
Chromosomal anomalies	Congenital anomalies	Wald ratio	1	0.876 (0.628, 1.222)	0.437	0.917
Other and unspecified congenital anomalies	Congenital anomalies	Wald ratio	1	0.79 (0.633, 0.986)	0.037	0.752
Anomalies of endocrine glands, congenital	Congenital anomalies	Wald ratio	1	0.917 (0.67, 1.255)	0.588	0.955
Superficial cellulitis and abscess	Dermatologic	Wald ratio	1	0.964 (0.927, 1.002)	0.063	0.789
Cellulitis and abscess of fingers or toes	Dermatologic	Wald ratio	1	1.06 (0.925, 1.216)	0.402	0.911
Cellulitis and abscess of face or neck	Dermatologic	Wald ratio	1	0.946 (0.82, 1.091)	0.444	0.917
Cellulitis and abscess of arm or hand	Dermatologic	Wald ratio	1	0.949 (0.908, 0.993)	0.022	0.649
Cellulitis and abscess of leg, except foot	Dermatologic	Wald ratio	1	0.948 (0.907, 0.991)	0.018	0.594
Cellulitis and abscess of foot, toe	Dermatologic	Wald ratio	1	0.95 (0.909, 0.993)	0.024	0.665
Cellulitis and abscess of trunk	Dermatologic	Wald ratio	1	1.079 (0.945, 1.233)	0.261	0.879
Other local infections of skin and subcutaneous tissue	Dermatologic	Wald ratio	1	1.012 (0.965, 1.062)	0.617	0.956
Carbuncle and furuncle	Dermatologic	Wald ratio	1	1.01 (0.944, 1.082)	0.766	0.982
Pilonidal cyst	Dermatologic	Wald ratio	1	0.983 (0.859, 1.125)	0.805	0.984
Pyogenic granuloma	Dermatologic	Wald ratio	1	1.152 (0.97, 1.368)	0.107	0.847
Symptoms affecting skin	Dermatologic	Wald ratio	1	1.002 (0.959, 1.048)	0.92	0.984
Rash and other nonspecific skin eruption	Dermatologic	Wald ratio	1	1.028 (0.958, 1.102)	0.445	0.917
Changes in skin texture	Dermatologic	Wald ratio	1	0.79 (0.577, 1.082)	0.141	0.859
Disturbance of skin sensation	Dermatologic	Wald ratio	1	1.005 (0.945, 1.068)	0.88	0.984
Disorder of skin and subcutaneous tissue NOS	Dermatologic	Wald ratio	1	1.002 (0.961, 1.046)	0.917	0.984
Erythematosquamous dermatosis	Dermatologic	Wald ratio	1	1.278 (0.867, 1.884)	0.216	0.879
Seborrheic dermatitis	Dermatologic	Wald ratio	1	1.587 (1.019, 2.473)	0.041	0.752
Congenital anomalies of skin	Dermatologic	Wald ratio	1	0.82 (0.61, 1.103)	0.189	0.879
Dyschromia and Vitiligo	Dermatologic	Wald ratio	1	0.988 (0.891, 1.096)	0.816	0.984
Vitiligo	Dermatologic	Wald ratio	1	1.219 (0.797, 1.865)	0.361	0.899
Other dyschromia	Dermatologic	Wald ratio	1	0.956 (0.851, 1.074)	0.446	0.917
Vascular disorders of skin	Dermatologic	Wald ratio	1	0.971 (0.723, 1.305)	0.846	0.984
Erythematous conditions	Dermatologic	Wald ratio	1	0.985 (0.922, 1.053)	0.657	0.974
Toxic erythema	Dermatologic	Wald ratio	1	1.048 (0.766, 1.435)	0.769	0.982
Bullous dermatoses	Dermatologic	Wald ratio	1	0.836 (0.634, 1.103)	0.205	0.879
Dermatitis herpetiformis	Dermatologic	Wald ratio	1	0.692 (0.453, 1.059)	0.09	0.835
Pemphigus and pemphigoid	Dermatologic	Wald ratio	1	0.86 (0.594, 1.244)	0.424	0.917
Rosacea	Dermatologic	Wald ratio	1	1.096 (0.911, 1.318)	0.332	0.898
Lupus (localized and systemic)	Dermatologic	Wald ratio	1	0.925 (0.789, 1.084)	0.334	0.898
Cutaneous lupus erythematosus	Dermatologic	Wald ratio	1	0.975 (0.739, 1.286)	0.857	0.984
Systemic lupus erythematosus	Dermatologic	Wald ratio	1	0.926 (0.776, 1.106)	0.399	0.908
Prurigo and Lichen	Dermatologic	Wald ratio	1	1.059 (0.941, 1.192)	0.341	0.898
Other specified erythematous conditions	Dermatologic	Wald ratio	1	0.893 (0.729, 1.094)	0.275	0.879
Erythema nodosum	Dermatologic	Wald ratio	1	0.775 (0.536, 1.122)	0.177	0.879
Unspecified erythematous condition	Dermatologic	Wald ratio	1	0.913 (0.785, 1.063)	0.242	0.879
Psoriasis and related disorders	Dermatologic	Wald ratio	1	0.951 (0.888, 1.019)	0.152	0.879
Psoriasis	Dermatologic	Wald ratio	1	0.95 (0.886, 1.019)	0.155	0.879
Psoriasis vulgaris	Dermatologic	Wald ratio	1	1.009 (0.932, 1.093)	0.816	0.984
Psoriatic arthropathy	Dermatologic	Wald ratio	1	0.868 (0.768, 0.981)	0.023	0.665
Sarcoidosis	Dermatologic	Wald ratio	1	1.01 (0.878, 1.163)	0.885	0.984
Pruritus and related conditions	Dermatologic	Wald ratio	1	1.021 (0.909, 1.147)	0.727	0.982
Corns and callosities	Dermatologic	Wald ratio	1	0.876 (0.728, 1.054)	0.162	0.879
Other hypertrophic and atrophic conditions of skin	Dermatologic	Wald ratio	1	1.025 (0.977, 1.075)	0.317	0.879
Keratoderma, acquired	Dermatologic	Wald ratio	1	0.692 (0.487, 0.984)	0.04	0.752
Scar conditions and fibrosis of skin	Dermatologic	Wald ratio	1	1.021 (0.955, 1.091)	0.541	0.949
Circumscribed scleroderma	Dermatologic	Wald ratio	1	1.431 (0.935, 2.189)	0.098	0.835
Keloid scar	Dermatologic	Wald ratio	1	1.12 (0.897, 1.398)	0.317	0.879
Abnormal granulation tissue	Dermatologic	Wald ratio	1	1.099 (0.943, 1.281)	0.228	0.879
Acquired acanthosis nigricans	Dermatologic	Wald ratio	1	1.054 (0.77, 1.443)	0.742	0.982
Degenerative skin conditions and other dermatoses	Dermatologic	Wald ratio	1	0.996 (0.952, 1.041)	0.845	0.984
Actinic keratosis	Dermatologic	Wald ratio	1	0.989 (0.927, 1.055)	0.732	0.982
Seborrheic keratosis	Dermatologic	Wald ratio	1	1.001 (0.944, 1.062)	0.968	0.991
Diseases of nail, NOS	Dermatologic	Wald ratio	1	0.988 (0.902, 1.081)	0.791	0.984
Ingrowing nail	Dermatologic	Wald ratio	1	1.001 (0.901, 1.113)	0.979	0.991
Diseases of hair and hair follicles	Dermatologic	Wald ratio	1	0.991 (0.946, 1.038)	0.698	0.979
Alopecia	Dermatologic	Wald ratio	1	1.045 (0.778, 1.405)	0.769	0.982
Other specified diseases of hair and hair follicles	Dermatologic	Wald ratio	1	1.141 (0.789, 1.651)	0.484	0.934
Disorders of sweat glands	Dermatologic	Wald ratio	1	1.043 (0.93, 1.17)	0.468	0.923

Hidradenitis	Dermatologic	Wald ratio	1	1.12 (0.881, 1.424)	0.356	0.898
Hyperhidrosis	Dermatologic	Wald ratio	1	1.017 (0.889, 1.164)	0.805	0.984
Diseases of sebaceous glands	Dermatologic	Wald ratio	1	1.014 (0.979, 1.05)	0.43	0.917
Acne	Dermatologic	Wald ratio	1	0.719 (0.497, 1.04)	0.08	0.825
Sebaceous cyst	Dermatologic	Wald ratio	1	1.017 (0.982, 1.053)	0.343	0.898
Chronic ulcer of skin	Dermatologic	Wald ratio	1	0.965 (0.896, 1.039)	0.342	0.898
Decubitus ulcer	Dermatologic	Wald ratio	1	0.981 (0.88, 1.094)	0.735	0.982
Diffuse diseases of connective tissue	Dermatologic	Wald ratio	1	0.926 (0.876, 0.978)	0.006	0.532
Sicca syndrome	Dermatologic	Wald ratio	1	0.937 (0.811, 1.082)	0.376	0.899
Systemic sclerosis	Dermatologic	Wald ratio	1	1.163 (0.915, 1.479)	0.218	0.879
Other specified diffuse diseases of connective tissue	Dermatologic	Wald ratio	1	0.932 (0.62, 1.399)	0.733	0.982
Unspecified diffuse connective tissue disease	Dermatologic	Wald ratio	1	0.901 (0.847, 0.96)	0.001	0.258
Atopic or contact dermatitis due to other or unspecified	Dermatologic	Wald ratio	1	0.999 (0.93, 1.074)	0.98	0.991
Disorders of tooth development	Digestive	Wald ratio	1	0.988 (0.924, 1.056)	0.718	0.982
Hereditary disturbances in tooth structure	Digestive	Wald ratio	1	0.82 (0.527, 1.278)	0.382	0.899
Disturbances in tooth eruption	Digestive	Wald ratio	1	0.991 (0.928, 1.06)	0.8	0.984
Diseases of hard tissues of teeth	Digestive	Wald ratio	1	0.974 (0.918, 1.033)	0.382	0.899
Dental caries	Digestive	Wald ratio	1	0.973 (0.917, 1.032)	0.365	0.899
Diseases of pulp and periapical tissues	Digestive	Wald ratio	1	0.995 (0.92, 1.075)	0.89	0.984
Periapical abscess	Digestive	Wald ratio	1	0.975 (0.886, 1.073)	0.604	0.956
Gingival and periodontal diseases	Digestive	Wald ratio	1	1.008 (0.933, 1.089)	0.843	0.984
Gingivitis	Digestive	Wald ratio	1	0.868 (0.634, 1.189)	0.378	0.899
Periodontitis (acute or chronic)	Digestive	Wald ratio	1	0.974 (0.886, 1.07)	0.583	0.955
Acute periodontitis	Digestive	Wald ratio	1	1.032 (0.908, 1.172)	0.632	0.967
Chronic periodontitis	Digestive	Wald ratio	1	0.92 (0.802, 1.054)	0.229	0.879
Dentofacial anomalies, including malocclusion	Digestive	Wald ratio	1	0.936 (0.785, 1.116)	0.461	0.919
Anomalies of tooth position or malocclusion	Digestive	Wald ratio	1	0.91 (0.756, 1.095)	0.317	0.879
Other diseases of the teeth and supporting structures	Digestive	Wald ratio	1	1.015 (0.953, 1.081)	0.638	0.967
Atrophy of edentulous alveolar ridge	Digestive	Wald ratio	1	0.733 (0.497, 1.08)	0.116	0.847
Diseases of the jaws	Digestive	Wald ratio	1	1.067 (0.961, 1.186)	0.226	0.879
Cysts of the jaws	Digestive	Wald ratio	1	1.035 (0.864, 1.241)	0.706	0.981
Anomalies of jaw size or symmetry	Digestive	Wald ratio	1	0.844 (0.552, 1.291)	0.434	0.917
Temporomandibular joint disorders	Digestive	Wald ratio	1	1.085 (0.886, 1.33)	0.429	0.917
Temporomandibular joint disorder, unspecified	Digestive	Wald ratio	1	1.12 (0.897, 1.398)	0.317	0.879
Inflammatory conditions of jaw	Digestive	Wald ratio	1	1.065 (0.778, 1.458)	0.693	0.979
Jaw disease NOS	Digestive	Wald ratio	1	1.152 (0.923, 1.438)	0.211	0.879
Diseases of the salivary glands	Digestive	Wald ratio	1	0.912 (0.807, 1.03)	0.138	0.859
Hypertrophy of salivary gland	Digestive	Wald ratio	1	0.957 (0.673, 1.359)	0.805	0.984
Sialoadenitis	Digestive	Wald ratio	1	0.973 (0.821, 1.153)	0.753	0.982
Disturbance of salivary secretion	Digestive	Wald ratio	1	0.579 (0.385, 0.869)	0.008	0.55
Other specified diseases of the salivary glands	Digestive	Wald ratio	1	0.912 (0.678, 1.225)	0.54	0.949
Diseases of the oral soft tissues, excluding lesions specific for gingiva and tongue	Digestive	Wald ratio	1	0.974 (0.925, 1.026)	0.317	0.879
Stomatitis and mucositis	Digestive	Wald ratio	1	0.86 (0.74, 0.999)	0.048	0.778
Stomatitis and mucositis (ulcerative)	Digestive	Wald ratio	1	0.852 (0.723, 1.004)	0.056	0.778
Oral aphthae	Digestive	Wald ratio	1	0.901 (0.635, 1.281)	0.563	0.951
Cellulitis and abscess of oral soft tissues	Digestive	Wald ratio	1	0.964 (0.758, 1.226)	0.764	0.982
Cysts of oral soft tissues	Digestive	Wald ratio	1	0.912 (0.678, 1.225)	0.54	0.949
Cyst of the salivary gland	Digestive	Wald ratio	1	0.93 (0.667, 1.297)	0.669	0.974
Diseases of lips	Digestive	Wald ratio	1	0.961 (0.848, 1.09)	0.537	0.949
Leukoplakia of oral mucosa	Digestive	Wald ratio	1	0.998 (0.829, 1.2)	0.981	0.991
Sialolithiasis	Digestive	Wald ratio	1	0.918 (0.763, 1.104)	0.363	0.899
Diseases and other conditions of the tongue	Digestive	Wald ratio	1	0.954 (0.87, 1.046)	0.317	0.879
Glossitis	Digestive	Wald ratio	1	0.975 (0.81, 1.173)	0.787	0.984
Diseases of esophagus	Digestive	Wald ratio	1	1.001 (0.983, 1.02)	0.881	0.984
Esophagitis, GERD and related diseases	Digestive	Wald ratio	1	1.001 (0.981, 1.022)	0.892	0.984
GERD	Digestive	Wald ratio	1	0.975 (0.948, 1.002)	0.072	0.809
Ulcer of esophagus	Digestive	Wald ratio	1	1.03 (0.983, 1.078)	0.215	0.879
Reflux esophagitis	Digestive	Wald ratio	1	0.992 (0.96, 1.026)	0.645	0.967
Esophageal bleeding (varices or hemorrhage)	Digestive	Wald ratio	1	1.035 (0.956, 1.121)	0.39	0.903
Stricture and stenosis of esophagus	Digestive	Wald ratio	1	0.989 (0.917, 1.067)	0.77	0.982
Disorders of esophageal motility	Digestive	Wald ratio	1	0.932 (0.819, 1.06)	0.284	0.879
Diverticulum of esophagus, acquired	Digestive	Wald ratio	1	1.254 (0.968, 1.624)	0.086	0.835
Gastroesophageal laceration-hemorrhage syndrome	Digestive	Wald ratio	1	0.92 (0.771, 1.096)	0.349	0.898
Heartburn	Digestive	Wald ratio	1	1.033 (0.961, 1.11)	0.383	0.9
Peptic ulcer (excl. esophageal)	Digestive	Wald ratio	1	1.006 (0.968, 1.046)	0.764	0.982
Hemorrhage from gastrointestinal ulcer	Digestive	Wald ratio	1	1.052 (0.923, 1.2)	0.447	0.917
Gastric ulcer	Digestive	Wald ratio	1	1.01 (0.959, 1.064)	0.694	0.979
Duodenal ulcer	Digestive	Wald ratio	1	0.979 (0.922, 1.041)	0.505	0.943
Peptic ulcer, site unspecified	Digestive	Wald ratio	1	1.078 (0.92, 1.264)	0.352	0.898
Dysphagia	Digestive	Wald ratio	1	0.998 (0.958, 1.039)	0.91	0.984
Gastritis and duodenitis	Digestive	Wald ratio	1	1.003 (0.983, 1.024)	0.771	0.982
Acute gastritis	Digestive	Wald ratio	1	1.039 (0.944, 1.144)	0.43	0.917
Atrophic gastritis	Digestive	Wald ratio	1	1.002 (0.845, 1.188)	0.98	0.991
Duodenitis	Digestive	Wald ratio	1	0.984 (0.947, 1.023)	0.418	0.917
Other specified gastritis	Digestive	Wald ratio	1	0.998 (0.962, 1.036)	0.928	0.984
Gastritis and duodenitis, NOS	Digestive	Wald ratio	1	1.185 (0.882, 1.593)	0.261	0.879
Disorders of function of stomach	Digestive	Wald ratio	1	0.926 (0.755, 1.134)	0.456	0.919
Dyspepsia and other specified disorders of function of stomach	Digestive	Wald ratio	1	0.938 (0.765, 1.149)	0.536	0.949

Other disorders of stomach and duodenum	Digestive	Wald ratio	1	0.996 (0.94, 1.054)	0.885	0.984
Appendiceal conditions	Digestive	Wald ratio	1	1.002 (0.946, 1.061)	0.949	0.987
Appendicitis	Digestive	Wald ratio	1	1.009 (0.951, 1.07)	0.779	0.984
Acute appendicitis	Digestive	Wald ratio	1	1.01 (0.947, 1.078)	0.753	0.982
Abdominal hernia	Digestive	Wald ratio	1	1.006 (0.99, 1.023)	0.448	0.917
Inguinal hernia	Digestive	Wald ratio	1	1.029 (1.001, 1.058)	0.046	0.752
Diaphragmatic hernia	Digestive	Wald ratio	1	0.996 (0.976, 1.017)	0.723	0.982
Femoral hernia	Digestive	Wald ratio	1	0.989 (0.87, 1.123)	0.862	0.984
Umbilical hernia	Digestive	Wald ratio	1	0.982 (0.931, 1.036)	0.512	0.945
Ventral hernia	Digestive	Wald ratio	1	1.021 (0.966, 1.079)	0.463	0.919
Inflammatory bowel disease and other gastroenteritis and colitis	Digestive	Wald ratio	1	0.961 (0.914, 1.01)	0.12	0.847
Regional enteritis	Digestive	Wald ratio	1	0.955 (0.882, 1.034)	0.254	0.879
Ulcerative colitis	Digestive	Wald ratio	1	0.973 (0.917, 1.032)	0.365	0.899
Ulcerative colitis (chronic)	Digestive	Wald ratio	1	0.995 (0.865, 1.145)	0.943	0.984
Ulceration of the lower GI tract	Digestive	Wald ratio	1	0.933 (0.844, 1.03)	0.171	0.879
Ulceration of intestine	Digestive	Wald ratio	1	0.914 (0.806, 1.037)	0.162	0.879
Intestinal malabsorption (non-celiac)	Digestive	Wald ratio	1	0.968 (0.901, 1.041)	0.383	0.9
Celiac disease	Digestive	Wald ratio	1	0.949 (0.878, 1.025)	0.182	0.879
Noninfectious gastroenteritis	Digestive	Wald ratio	1	0.966 (0.939, 0.993)	0.014	0.55
Ileostomy status	Digestive	Wald ratio	1	0.99 (0.912, 1.074)	0.803	0.984
Intestinal obstruction without mention of hernia	Digestive	Wald ratio	1	0.994 (0.944, 1.047)	0.819	0.984
Paralytic ileus	Digestive	Wald ratio	1	0.941 (0.817, 1.085)	0.406	0.911
Impaction of intestine	Digestive	Wald ratio	1	0.965 (0.787, 1.182)	0.73	0.982
Peritoneal or intestinal adhesions	Digestive	Wald ratio	1	1.016 (0.908, 1.137)	0.78	0.984
Other intestinal obstruction	Digestive	Wald ratio	1	0.997 (0.942, 1.056)	0.92	0.984
Symptoms involving digestive system	Digestive	Wald ratio	1	0.988 (0.963, 1.014)	0.353	0.898
Diarrhea	Digestive	Wald ratio	1	0.925 (0.795, 1.076)	0.311	0.879
Flatulence	Digestive	Wald ratio	1	1.053 (0.973, 1.14)	0.201	0.879
Diverticulosis and diverticulitis	Digestive	Wald ratio	1	0.959 (0.938, 0.981)	2.46E-04	0.104
Diverticulosis	Digestive	Wald ratio	1	0.96 (0.939, 0.982)	3.39E-04	0.108
Constipation	Digestive	Wald ratio	1	0.975 (0.943, 1.008)	0.134	0.85
Functional digestive disorders	Digestive	Wald ratio	1	0.983 (0.96, 1.007)	0.166	0.879
Irritable Bowel Syndrome	Digestive	Wald ratio	1	0.958 (0.916, 1.001)	0.055	0.778
Abnormal findings on exam of gastrointestinal tract or abdominal area	Digestive	Wald ratio	1	0.959 (0.884, 1.041)	0.317	0.879
Personal history of diseases of digestive system	Digestive	Wald ratio	1	1.002 (0.974, 1.03)	0.91	0.984
Anal and rectal conditions	Digestive	Wald ratio	1	1.007 (0.98, 1.036)	0.598	0.955
Anal and rectal polyp	Digestive	Wald ratio	1	1.009 (0.971, 1.049)	0.634	0.967
Peritonitis and retroperitoneal infections	Digestive	Wald ratio	1	1.109 (0.995, 1.237)	0.062	0.789
Other disorders of peritoneum	Digestive	Wald ratio	1	0.958 (0.905, 1.015)	0.147	0.87
Peritoneal adhesions (postoperative) (postinfection)	Digestive	Wald ratio	1	0.961 (0.906, 1.02)	0.189	0.879
Other disorders of intestine	Digestive	Wald ratio	1	1.052 (0.999, 1.108)	0.054	0.778
Toxic gastroenteritis and colitis	Digestive	Wald ratio	1	1.254 (0.968, 1.624)	0.086	0.835
Gastrointestinal complications	Digestive	Wald ratio	1	0.998 (0.861, 1.157)	0.983	0.991
Chronic liver disease and cirrhosis	Digestive	Wald ratio	1	1.012 (0.952, 1.076)	0.694	0.979
Other chronic nonalcoholic liver disease	Digestive	Wald ratio	1	0.993 (0.917, 1.075)	0.856	0.984
Cirrhosis of liver without mention of alcohol	Digestive	Wald ratio	1	0.968 (0.707, 1.326)	0.841	0.984
Primary biliary cirrhosis	Digestive	Wald ratio	1	1.067 (0.871, 1.308)	0.53	0.949
Liver abscess and sequelae of chronic liver disease	Digestive	Wald ratio	1	1.006 (0.904, 1.12)	0.907	0.984
Portal hypertension	Digestive	Wald ratio	1	1.08 (0.937, 1.246)	0.287	0.879
Malposition and malpresentation of fetus or obstruction	Digestive	Wald ratio	1	0.968 (0.893, 1.05)	0.44	0.917
Simple and unspecified goiter	Endocrine/metabolic	Wald ratio	1	0.852 (0.746, 0.973)	0.018	0.594
Nontoxic nodular goiter	Endocrine/metabolic	Wald ratio	1	0.983 (0.893, 1.082)	0.729	0.982
Nontoxic uninodular goiter	Endocrine/metabolic	Wald ratio	1	0.962 (0.828, 1.117)	0.613	0.956
Nontoxic multinodular goiter	Endocrine/metabolic	Wald ratio	1	0.984 (0.868, 1.116)	0.803	0.984
Thyrotoxicosis with or without goiter	Endocrine/metabolic	Wald ratio	1	0.966 (0.895, 1.042)	0.367	0.899
Graves' disease	Endocrine/metabolic	Wald ratio	1	0.949 (0.814, 1.106)	0.5	0.943
Toxic multinodular goiter	Endocrine/metabolic	Wald ratio	1	0.893 (0.64, 1.245)	0.505	0.943
Exophthalmos	Endocrine/metabolic	Wald ratio	1	1.12 (0.849, 1.477)	0.424	0.917
Hypothyroidism	Endocrine/metabolic	Wald ratio	1	1.022 (0.994, 1.051)	0.125	0.847
Secondary hypothyroidism	Endocrine/metabolic	Wald ratio	1	0.99 (0.897, 1.092)	0.836	0.984
Acquired hypothyroidism	Endocrine/metabolic	Wald ratio	1	1.038 (0.745, 1.448)	0.824	0.984
Hypothyroidism NOS	Endocrine/metabolic	Wald ratio	1	1.024 (0.994, 1.055)	0.118	0.847
Thyroiditis	Endocrine/metabolic	Wald ratio	1	0.916 (0.761, 1.102)	0.352	0.898
Chronic thyroiditis	Endocrine/metabolic	Wald ratio	1	0.901 (0.722, 1.125)	0.359	0.899
Chronic lymphocytic thyroiditis	Endocrine/metabolic	Wald ratio	1	0.92 (0.737, 1.148)	0.458	0.919
Other disorders of thyroid	Endocrine/metabolic	Wald ratio	1	0.893 (0.763, 1.045)	0.158	0.879
Diabetes mellitus	Endocrine/metabolic	Wald ratio	1	0.97 (0.947, 0.994)	0.014	0.55
Type 1 diabetes	Endocrine/metabolic	Wald ratio	1	1.057 (0.991, 1.128)	0.092	0.835
Type 1 diabetes with ketoacidosis	Endocrine/metabolic	Wald ratio	1	1.067 (0.855, 1.332)	0.565	0.951
Type 1 diabetes with renal manifestations	Endocrine/metabolic	Wald ratio	1	1.23 (0.85, 1.781)	0.271	0.879
Type 2 diabetes	Endocrine/metabolic	Wald ratio	1	0.966 (0.941, 0.991)	0.008	0.55
Type 2 diabetes with ketoacidosis	Endocrine/metabolic	Wald ratio	1	0.876 (0.64, 1.2)	0.41	0.911
Type 2 diabetes with renal manifestations	Endocrine/metabolic	Wald ratio	1	1.099 (0.88, 1.372)	0.405	0.911
Type 2 diabetes with ophthalmic manifestations	Endocrine/metabolic	Wald ratio	1	0.999 (0.912, 1.093)	0.979	0.991
Type 2 diabetes with neurological manifestations	Endocrine/metabolic	Wald ratio	1	1.077 (0.94, 1.235)	0.286	0.879
Abnormal glucose	Endocrine/metabolic	Wald ratio	1	0.935 (0.826, 1.059)	0.289	0.879
Impaired fasting glucose	Endocrine/metabolic	Wald ratio	1	1.073 (0.876, 1.315)	0.495	0.942
Other abnormal glucose	Endocrine/metabolic	Wald ratio	1	0.86 (0.734, 1.008)	0.063	0.789
Glycosuria or Acetonuria	Endocrine/metabolic	Wald ratio	1	1.066 (0.808, 1.407)	0.65	0.97

Polyneuropathy in diabetes	Endocrine/metabolic	Wald ratio	1	1.085 (0.912, 1.291)	0.355	0.898
Diabetic retinopathy	Endocrine/metabolic	Wald ratio	1	1.003 (0.916, 1.098)	0.943	0.984
Other disorders of pancreatic internal secretion	Endocrine/metabolic	Wald ratio	1	0.945 (0.849, 1.052)	0.301	0.879
Hypoglycemia	Endocrine/metabolic	Wald ratio	1	0.945 (0.849, 1.052)	0.301	0.879
Disorders of parathyroid gland	Endocrine/metabolic	Wald ratio	1	0.901 (0.807, 1.007)	0.067	0.794
Hyperparathyroidism	Endocrine/metabolic	Wald ratio	1	0.91 (0.809, 1.024)	0.118	0.847
Hypoparathyroidism	Endocrine/metabolic	Wald ratio	1	0.914 (0.693, 1.206)	0.527	0.949
Disorders of the pituitary gland and its hypothalamic control	Endocrine/metabolic	Wald ratio	1	1.03 (0.91, 1.165)	0.644	0.967
Pituitary hyperfunction	Endocrine/metabolic	Wald ratio	1	1.059 (0.833, 1.347)	0.639	0.967
Acromegaly and gigantism	Endocrine/metabolic	Wald ratio	1	1.097 (0.786, 1.53)	0.586	0.955
Pituitary hypofunction	Endocrine/metabolic	Wald ratio	1	0.928 (0.757, 1.137)	0.473	0.927
Diabetes insipidus	Endocrine/metabolic	Wald ratio	1	1.042 (0.747, 1.454)	0.807	0.984
Other disorders of neurohypophysis	Endocrine/metabolic	Wald ratio	1	1.302 (1.005, 1.687)	0.046	0.752
Disorders of adrenal glands	Endocrine/metabolic	Wald ratio	1	0.92 (0.808, 1.046)	0.204	0.879
Adrenal hyperfunction	Endocrine/metabolic	Wald ratio	1	0.86 (0.676, 1.093)	0.218	0.879
Cushing's syndrome	Endocrine/metabolic	Wald ratio	1	0.868 (0.634, 1.189)	0.378	0.899
Hyperaldosteronism	Endocrine/metabolic	Wald ratio	1	0.836 (0.578, 1.21)	0.342	0.898
Adrenal hypofunction	Endocrine/metabolic	Wald ratio	1	1.09 (0.918, 1.294)	0.328	0.898
Glucocorticoid deficiency	Endocrine/metabolic	Wald ratio	1	1.088 (0.916, 1.291)	0.339	0.898
Ovarian dysfunction	Endocrine/metabolic	Wald ratio	1	0.876 (0.715, 1.074)	0.203	0.879
Polycystic ovaries	Endocrine/metabolic	Wald ratio	1	0.844 (0.676, 1.053)	0.134	0.85
Testicular dysfunction	Endocrine/metabolic	Wald ratio	1	0.885 (0.683, 1.146)	0.353	0.898
Testicular hypofunction	Endocrine/metabolic	Wald ratio	1	0.876 (0.677, 1.135)	0.317	0.879
Iatrogenic endocrine disorders	Endocrine/metabolic	Wald ratio	1	1.13 (0.857, 1.491)	0.386	0.901
Other endocrine disorders	Endocrine/metabolic	Wald ratio	1	1.242 (0.977, 1.579)	0.077	0.825
Protein-calorie malnutrition	Endocrine/metabolic	Wald ratio	1	0.936 (0.847, 1.034)	0.195	0.879
Anorexia	Endocrine/metabolic	Wald ratio	1	0.939 (0.839, 1.051)	0.272	0.879
Vitamin deficiency	Endocrine/metabolic	Wald ratio	1	0.993 (0.903, 1.091)	0.878	0.984
Vitamin B-complex deficiencies	Endocrine/metabolic	Wald ratio	1	1.004 (0.891, 1.133)	0.944	0.984
Vitamin D deficiency	Endocrine/metabolic	Wald ratio	1	1.023 (0.876, 1.195)	0.775	0.984
Rickets or osteomalacia	Endocrine/metabolic	Wald ratio	1	0.893 (0.617, 1.292)	0.549	0.949
Mineral deficiency NEC	Endocrine/metabolic	Wald ratio	1	0.993 (0.81, 1.216)	0.943	0.984
Proteinuria	Endocrine/metabolic	Wald ratio	1	1.003 (0.84, 1.198)	0.974	0.991
Disorders of protein plasma or amino-acid transport and metabolism	Endocrine/metabolic	Wald ratio	1	0.852 (0.724, 1.002)	0.053	0.778
Disorders of plasma protein metabolism	Endocrine/metabolic	Wald ratio	1	0.868 (0.731, 1.031)	0.107	0.847
Paraproteinemia	Endocrine/metabolic	Wald ratio	1	0.868 (0.683, 1.104)	0.249	0.879
Amyloidosis	Endocrine/metabolic	Wald ratio	1	0.852 (0.622, 1.166)	0.317	0.879
Macroglobulinemia	Endocrine/metabolic	Wald ratio	1	1.025 (0.695, 1.511)	0.901	0.984
Disorders of carbohydrate transport and metabolism	Endocrine/metabolic	Wald ratio	1	1.208 (0.932, 1.564)	0.153	0.879
Intestinal disaccharidase deficiencies and disaccharide malabsorption	Endocrine/metabolic	Wald ratio	1	1.242 (0.941, 1.639)	0.125	0.847
Disorders of lipoid metabolism	Endocrine/metabolic	Wald ratio	1	0.991 (0.972, 1.009)	0.317	0.879
Hyperlipidemia	Endocrine/metabolic	Wald ratio	1	0.991 (0.973, 1.009)	0.332	0.898
Hypercholesterolemia	Endocrine/metabolic	Wald ratio	1	0.99 (0.97, 1.01)	0.317	0.879
Hyperglyceridemia	Endocrine/metabolic	Wald ratio	1	0.828 (0.541, 1.267)	0.385	0.901
Mixed hyperlipidemia	Endocrine/metabolic	Wald ratio	1	0.885 (0.683, 1.146)	0.353	0.898
Unspecified disorder of lipoid metabolism	Endocrine/metabolic	Wald ratio	1	0.936 (0.75, 1.169)	0.56	0.951
Gout and other crystal arthropathies	Endocrine/metabolic	Wald ratio	1	0.996 (0.944, 1.051)	0.893	0.984
Gout	Endocrine/metabolic	Wald ratio	1	1.005 (0.948, 1.067)	0.861	0.984
Gouty arthropathy	Endocrine/metabolic	Wald ratio	1	0.885 (0.646, 1.211)	0.444	0.917
Crystal arthropathies	Endocrine/metabolic	Wald ratio	1	0.95 (0.834, 1.084)	0.447	0.917
Chondrocalcinosis	Endocrine/metabolic	Wald ratio	1	0.943 (0.821, 1.083)	0.408	0.911
Disorders of mineral metabolism	Endocrine/metabolic	Wald ratio	1	1.008 (0.938, 1.083)	0.835	0.984
Disorders of magnesium metabolism	Endocrine/metabolic	Wald ratio	1	0.951 (0.804, 1.125)	0.56	0.951
Disorders of calcium or phosphorus metabolism	Endocrine/metabolic	Wald ratio	1	0.989 (0.9, 1.086)	0.814	0.984
Disorders of phosphorus metabolism	Endocrine/metabolic	Wald ratio	1	0.893 (0.689, 1.157)	0.391	0.903
Disorders of fluid, electrolyte, and acid-base balance	Endocrine/metabolic	Wald ratio	1	1.006 (0.968, 1.046)	0.75	0.982
Electrolyte imbalance	Endocrine/metabolic	Wald ratio	1	1.015 (0.964, 1.069)	0.568	0.951
Hyperosmolality and or or hypernatremia	Endocrine/metabolic	Wald ratio	1	0.933 (0.761, 1.143)	0.501	0.943
Hyposmolality and or or hyponatremia	Endocrine/metabolic	Wald ratio	1	1.045 (0.967, 1.13)	0.263	0.879
Hyperpotassemia	Endocrine/metabolic	Wald ratio	1	0.976 (0.878, 1.084)	0.648	0.97
Hypopotassemia	Endocrine/metabolic	Wald ratio	1	1.023 (0.938, 1.116)	0.61	0.956
Acid-base balance disorder	Endocrine/metabolic	Wald ratio	1	0.983 (0.893, 1.082)	0.729	0.982
Acidosis	Endocrine/metabolic	Wald ratio	1	0.956 (0.865, 1.056)	0.374	0.899
Alkalosis	Endocrine/metabolic	Wald ratio	1	1.254 (0.916, 1.717)	0.158	0.879
Hypovolemia	Endocrine/metabolic	Wald ratio	1	1.003 (0.944, 1.066)	0.916	0.984
Fluid overload	Endocrine/metabolic	Wald ratio	1	0.93 (0.798, 1.084)	0.354	0.898
Polydipsia	Endocrine/metabolic	Wald ratio	1	0.988 (0.735, 1.328)	0.935	0.984
Other disorders of metabolism	Endocrine/metabolic	Wald ratio	1	0.983 (0.901, 1.072)	0.702	0.979
Disorders of bilirubin excretion	Endocrine/metabolic	Wald ratio	1	1.004 (0.845, 1.192)	0.965	0.991
Other disorders of lipoid metabolism	Endocrine/metabolic	Wald ratio	1	0.893 (0.715, 1.115)	0.317	0.879
Lipoprotein disorders	Endocrine/metabolic	Wald ratio	1	1.152 (0.726, 1.828)	0.549	0.949
Overweight, obesity and other hyperalimentation	Endocrine/metabolic	Wald ratio	1	0.963 (0.931, 0.996)	0.026	0.667
Obesity	Endocrine/metabolic	Wald ratio	1	0.965 (0.933, 0.997)	0.035	0.749
Localized adiposity	Endocrine/metabolic	Wald ratio	1	0.836 (0.588, 1.188)	0.317	0.879
Abnormal weight gain	Endocrine/metabolic	Wald ratio	1	1.039 (0.692, 1.561)	0.852	0.984
Disorders involving the immune mechanism	Endocrine/metabolic	Wald ratio	1	0.942 (0.813, 1.092)	0.431	0.917
Immunity deficiency	Endocrine/metabolic	Wald ratio	1	0.976 (0.782, 1.218)	0.828	0.984
Deficiency of humoral immunity	Endocrine/metabolic	Wald ratio	1	0.922 (0.699, 1.217)	0.566	0.951
Other immunological findings	Endocrine/metabolic	Wald ratio	1	0.944 (0.77, 1.157)	0.579	0.955

Noninflammatory female genital disorders	Genitourinary	Wald ratio	1	0.982 (0.948, 1.017)	0.317	0.879
Noninflammatory disorders of vulva and perineum	Genitourinary	Wald ratio	1	0.982 (0.899, 1.073)	0.692	0.979
Dysplasia of female genital organs	Genitourinary	Wald ratio	1	1.219 (0.827, 1.797)	0.317	0.879
Endometrial hyperplasia	Genitourinary	Wald ratio	1	0.943 (0.855, 1.04)	0.242	0.879
Polyp of female genital organs	Genitourinary	Wald ratio	1	0.977 (0.945, 1.01)	0.165	0.879
Polyp of corpus uteri	Genitourinary	Wald ratio	1	0.967 (0.932, 1.003)	0.072	0.809
Mucous polyp of cervix	Genitourinary	Wald ratio	1	0.983 (0.93, 1.039)	0.549	0.949
Hypertrophy of female genital organs	Genitourinary	Wald ratio	1	0.93 (0.848, 1.02)	0.124	0.847
Symptoms involving female genital tract	Genitourinary	Wald ratio	1	1.037 (0.996, 1.08)	0.076	0.825
Dystrophy of female genital tract	Genitourinary	Wald ratio	1	1.014 (0.813, 1.266)	0.901	0.984
Atrophy of female genital tract	Genitourinary	Wald ratio	1	0.972 (0.832, 1.135)	0.721	0.982
stress incontinence, female	Genitourinary	Wald ratio	1	1.044 (0.999, 1.092)	0.055	0.778
Pain and other symptoms associated with female genital organs	Genitourinary	Wald ratio	1	1.037 (0.976, 1.103)	0.237	0.879
Dyspareunia	Genitourinary	Wald ratio	1	1.023 (0.931, 1.124)	0.638	0.967
Disorders of menstruation and other abnormal bleeding from female genital tract	Genitourinary	Wald ratio	1	0.985 (0.96, 1.011)	0.253	0.879
Irregular menstrual cycle or bleeding	Genitourinary	Wald ratio	1	0.982 (0.955, 1.01)	0.205	0.879
Absent or infrequent menstruation	Genitourinary	Wald ratio	1	0.981 (0.744, 1.295)	0.894	0.984
Excessive or frequent menstruation	Genitourinary	Wald ratio	1	0.985 (0.951, 1.02)	0.4	0.908
Irregular menstrual cycle	Genitourinary	Wald ratio	1	0.988 (0.917, 1.064)	0.745	0.982
Irregular menstrual bleeding	Genitourinary	Wald ratio	1	0.98 (0.929, 1.034)	0.469	0.923
Dysmenorrhea	Genitourinary	Wald ratio	1	0.893 (0.826, 0.965)	0.004	0.418
Premenstrual tension syndromes	Genitourinary	Wald ratio	1	0.885 (0.683, 1.146)	0.353	0.898
Infertility, female	Genitourinary	Wald ratio	1	1.055 (0.964, 1.155)	0.245	0.879
Menopausal and postmenopausal disorders	Genitourinary	Wald ratio	1	0.987 (0.955, 1.02)	0.437	0.917
Postmenopausal bleeding	Genitourinary	Wald ratio	1	0.991 (0.957, 1.027)	0.621	0.961
Symptomatic menopause	Genitourinary	Wald ratio	1	0.928 (0.73, 1.18)	0.543	0.949
Postmenopausal atrophic vaginitis	Genitourinary	Wald ratio	1	0.917 (0.831, 1.011)	0.083	0.825
Premenopausal menorrhagia	Genitourinary	Wald ratio	1	1.08 (0.898, 1.3)	0.412	0.913
Ovarian cyst	Genitourinary	Wald ratio	1	1.005 (0.958, 1.054)	0.841	0.984
Disorders of iron metabolism	Hematopoietic	Wald ratio	1	1.019 (0.899, 1.155)	0.769	0.982
Iron deficiency anemias	Hematopoietic	Wald ratio	1	0.986 (0.95, 1.023)	0.453	0.917
Iron deficiency anemias, unspecified or not due to blood loss	Hematopoietic	Wald ratio	1	0.978 (0.94, 1.016)	0.253	0.879
Iron deficiency anemia secondary to blood loss (chronic)	Hematopoietic	Wald ratio	1	1.141 (0.979, 1.33)	0.092	0.835
Other deficiency anemia	Hematopoietic	Wald ratio	1	1.015 (0.92, 1.12)	0.763	0.982
Megaloblastic anemia	Hematopoietic	Wald ratio	1	1.019 (0.922, 1.126)	0.711	0.982
Pernicious anemia	Hematopoietic	Wald ratio	1	1.007 (0.893, 1.135)	0.913	0.984
Other vitamin B12 deficiency anemia	Hematopoietic	Wald ratio	1	1.141 (0.897, 1.451)	0.282	0.879
Folate-deficiency anemia	Hematopoietic	Wald ratio	1	1.029 (0.809, 1.308)	0.817	0.984
Deficiency anemias	Hematopoietic	Wald ratio	1	0.885 (0.568, 1.378)	0.588	0.955
Hereditary hemolytic anemias	Hematopoietic	Wald ratio	1	0.852 (0.658, 1.103)	0.225	0.879
Other hemoglobinopathies	Hematopoietic	Wald ratio	1	1.029 (0.685, 1.545)	0.892	0.984
Other hereditary hemolytic anemias	Hematopoietic	Wald ratio	1	0.813 (0.572, 1.155)	0.247	0.879
Acquired hemolytic anemias	Hematopoietic	Wald ratio	1	0.836 (0.634, 1.103)	0.205	0.879
Autoimmune hemolytic anemias	Hematopoietic	Wald ratio	1	0.754 (0.55, 1.032)	0.078	0.825
Aplastic anemia	Hematopoietic	Wald ratio	1	0.934 (0.799, 1.093)	0.397	0.908
Other anemias	Hematopoietic	Wald ratio	1	1 (0.971, 1.03)	0.983	0.991
Acute posthemorrhagic anemia	Hematopoietic	Wald ratio	1	1.036 (0.846, 1.27)	0.73	0.982
Anemia of chronic disease	Hematopoietic	Wald ratio	1	1.025 (0.905, 1.16)	0.698	0.979
Anemia in neoplastic disease	Hematopoietic	Wald ratio	1	1.061 (0.904, 1.246)	0.469	0.923
Coagulation defects	Hematopoietic	Wald ratio	1	1.098 (0.986, 1.222)	0.088	0.835
Congenital coagulation defects	Hematopoietic	Wald ratio	1	1.052 (0.906, 1.222)	0.505	0.943
Von willebrand's disease	Hematopoietic	Wald ratio	1	1.068 (0.766, 1.49)	0.697	0.979
Congenital deficiency of other clotting factors (including factor VII)	Hematopoietic	Wald ratio	1	1.12 (0.931, 1.347)	0.23	0.879
Congenital factor VIII disorder	Hematopoietic	Wald ratio	1	0.844 (0.562, 1.267)	0.413	0.914
Hemorrhagic disorder due to intrinsic circulating anticoagulants	Hematopoietic	Wald ratio	1	1.051 (0.74, 1.493)	0.78	0.984
Other and unspecified coagulation defects	Hematopoietic	Wald ratio	1	1.152 (0.975, 1.36)	0.096	0.835
Purpura and other hemorrhagic conditions	Hematopoietic	Wald ratio	1	0.922 (0.853, 0.997)	0.041	0.752
Spontaneous ecchymoses	Hematopoietic	Wald ratio	1	1.185 (0.882, 1.593)	0.261	0.879
Allergic purpura	Hematopoietic	Wald ratio	1	0.974 (0.614, 1.546)	0.911	0.984
Thrombocytopenia	Hematopoietic	Wald ratio	1	0.901 (0.83, 0.98)	0.015	0.559
Primary thrombocytopenia	Hematopoietic	Wald ratio	1	0.966 (0.824, 1.132)	0.667	0.974
Secondary thrombocytopenia	Hematopoietic	Wald ratio	1	1.009 (0.685, 1.488)	0.963	0.991
Diseases of white blood cells	Hematopoietic	Wald ratio	1	0.974 (0.923, 1.028)	0.334	0.898
Decreased white blood cell count	Hematopoietic	Wald ratio	1	0.971 (0.917, 1.028)	0.317	0.879
Neutropenia	Hematopoietic	Wald ratio	1	0.971 (0.917, 1.028)	0.317	0.879
Eosinophilia	Hematopoietic	Wald ratio	1	1.152 (0.781, 1.698)	0.475	0.927
Other diseases of blood and blood-forming organs	Hematopoietic	Wald ratio	1	1.012 (0.961, 1.066)	0.642	0.967
Personal history of diseases of blood and blood-forming organs	Hematopoietic	Wald ratio	1	1.23 (1.023, 1.48)	0.028	0.667
Lymphadenitis	Hematopoietic	Wald ratio	1	1.016 (0.953, 1.084)	0.627	0.967
Diseases of spleen	Hematopoietic	Wald ratio	1	0.925 (0.801, 1.068)	0.287	0.879
Polycythemia, secondary	Hematopoietic	Wald ratio	1	1.045 (0.869, 1.257)	0.638	0.967
Abnormality of red blood cells	Hematopoietic	Wald ratio	1	1.302 (0.917, 1.85)	0.141	0.859
Intestinal infection	Infectious diseases	Wald ratio	1	1.02 (0.985, 1.056)	0.269	0.879
Bacterial enteritis	Infectious diseases	Wald ratio	1	0.995 (0.934, 1.059)	0.865	0.984
Intestinal infection due to C. difficile	Infectious diseases	Wald ratio	1	0.893 (0.786, 1.014)	0.082	0.825
Viral Enteritis	Infectious diseases	Wald ratio	1	1.093 (0.978, 1.221)	0.117	0.847
Intestinal infection due to protozoa	Infectious diseases	Wald ratio	1	0.733 (0.47, 1.142)	0.169	0.879
Tuberculosis	Infectious diseases	Wald ratio	1	1.045 (0.807, 1.354)	0.737	0.982

Septicemia	Infectious diseases	Wald ratio	1	0.974 (0.925, 1.026)	0.317	0.879
Gram negative septicemia	Infectious diseases	Wald ratio	1	0.98 (0.876, 1.097)	0.731	0.982
Gram positive septicemia	Infectious diseases	Wald ratio	1	0.916 (0.79, 1.062)	0.245	0.879
Bacterial infection NOS	Infectious diseases	Wald ratio	1	0.988 (0.959, 1.017)	0.417	0.917
Staphylococcus infections	Infectious diseases	Wald ratio	1	0.998 (0.941, 1.059)	0.958	0.99
Streptococcus infection	Infectious diseases	Wald ratio	1	0.995 (0.918, 1.08)	0.911	0.984
E. coli	Infectious diseases	Wald ratio	1	0.985 (0.925, 1.049)	0.638	0.967
Herpes zoster	Infectious diseases	Wald ratio	1	1.088 (0.928, 1.275)	0.301	0.879
Herpes zoster with nervous system complications	Infectious diseases	Wald ratio	1	1.152 (0.826, 1.606)	0.405	0.911
Herpes simplex	Infectious diseases	Wald ratio	1	0.844 (0.676, 1.053)	0.134	0.85
Viral hepatitis	Infectious diseases	Wald ratio	1	1.005 (0.914, 1.104)	0.923	0.984
Viral hepatitis A	Infectious diseases	Wald ratio	1	1.444 (0.98, 2.129)	0.063	0.789
Viral hepatitis B	Infectious diseases	Wald ratio	1	1.005 (0.762, 1.327)	0.969	0.991
Viral hepatitis C	Infectious diseases	Wald ratio	1	0.975 (0.796, 1.195)	0.806	0.984
Chronic hepatitis	Infectious diseases	Wald ratio	1	0.893 (0.729, 1.094)	0.275	0.879
Hepatitis NOS	Infectious diseases	Wald ratio	1	1.092 (0.933, 1.277)	0.274	0.879
Human immunodeficiency virus [HIV] disease	Infectious diseases	Wald ratio	1	1.031 (0.781, 1.36)	0.831	0.984
HIV infection, symptomatic	Infectious diseases	Wald ratio	1	1.092 (0.797, 1.495)	0.584	0.955
Viral warts & HPV	Infectious diseases	Wald ratio	1	0.852 (0.768, 0.945)	0.002	0.409
Viral infection	Infectious diseases	Wald ratio	1	1.019 (0.959, 1.083)	0.544	0.949
Varicella infection	Infectious diseases	Wald ratio	1	1.444 (0.962, 2.169)	0.076	0.825
Infectious mononucleosis	Infectious diseases	Wald ratio	1	0.783 (0.531, 1.154)	0.216	0.879
Postoperative infection	Infectious diseases	Wald ratio	1	0.979 (0.932, 1.03)	0.415	0.917
Infection or inflammation of internal prosthetic device; implant; and graft	Infectious diseases	Wald ratio	1	1.015 (0.952, 1.083)	0.648	0.97
Sexually transmitted infections (not HIV or hepatitis)	Infectious diseases	Wald ratio	1	1.141 (0.774, 1.682)	0.505	0.943
Dermatophytosis or Dermatomycosis	Infectious diseases	Wald ratio	1	0.93 (0.773, 1.119)	0.441	0.917
Dermatophytosis	Infectious diseases	Wald ratio	1	0.934 (0.763, 1.145)	0.513	0.945
Dermatophytosis of nail	Infectious diseases	Wald ratio	1	1.242 (0.858, 1.798)	0.25	0.879
Althete's foot	Infectious diseases	Wald ratio	1	0.86 (0.652, 1.135)	0.286	0.879
Candidiasis	Infectious diseases	Wald ratio	1	0.935 (0.872, 1.003)	0.062	0.789
Mycoses	Infectious diseases	Wald ratio	1	1.071 (0.874, 1.313)	0.507	0.943
Aspergillosis	Infectious diseases	Wald ratio	1	1.163 (0.898, 1.506)	0.253	0.879
Spirochetal infection	Infectious diseases	Wald ratio	1	1.254 (0.866, 1.815)	0.23	0.879
Arthropod-borne diseases	Infectious diseases	Wald ratio	1	0.999 (0.678, 1.473)	0.997	0.997
Helminthiases	Infectious diseases	Wald ratio	1	0.798 (0.604, 1.052)	0.11	0.847
Intestinal helminthiases	Infectious diseases	Wald ratio	1	0.876 (0.64, 1.2)	0.41	0.911
Other infectious and parasitic diseases	Infectious diseases	Wald ratio	1	0.868 (0.695, 1.084)	0.211	0.879
Sepsis and SIRS	Infectious diseases	Wald ratio	1	0.996 (0.937, 1.059)	0.901	0.984
Sepsis	Infectious diseases	Wald ratio	1	0.996 (0.937, 1.059)	0.901	0.984
Burns	Injuries & poisonings	Wald ratio	1	0.946 (0.827, 1.082)	0.419	0.917
Foreign body injury	Injuries & poisonings	Wald ratio	1	1.04 (0.947, 1.143)	0.41	0.911
Crushing injury	Injuries & poisonings	Wald ratio	1	0.978 (0.798, 1.198)	0.827	0.984
Injury to blood vessels	Injuries & poisonings	Wald ratio	1	0.868 (0.708, 1.064)	0.173	0.879
Crushing or internal injury to organs	Injuries & poisonings	Wald ratio	1	1.054 (0.951, 1.169)	0.317	0.879
Injury, NOS	Injuries & poisonings	Wald ratio	1	0.986 (0.95, 1.023)	0.453	0.917
Complications of surgical and medical procedures	Injuries & poisonings	Wald ratio	1	1.003 (0.969, 1.039)	0.854	0.984
Late effect	Injuries & poisonings	Wald ratio	1	0.885 (0.646, 1.211)	0.444	0.917
Effects of heat, cold and air pressure	Injuries & poisonings	Wald ratio	1	0.82 (0.546, 1.232)	0.34	0.898
Complications of in utero procedures	Injuries & poisonings	Wald ratio	1	1.033 (0.663, 1.609)	0.887	0.984
Nonspecific abnormal findings on radiological and other examination of musculoskeletal system	Injuries & poisonings	Wald ratio	1	1.001 (0.884, 1.133)	0.989	0.992
Fracture of lower limb	Injuries & poisonings	Wald ratio	1	1.007 (0.969, 1.047)	0.728	0.982
Fracture of neck of femur	Injuries & poisonings	Wald ratio	1	1.026 (0.949, 1.109)	0.52	0.949
Fracture of unspecified part of femur	Injuries & poisonings	Wald ratio	1	0.977 (0.754, 1.265)	0.858	0.984
Fracture of tibia and fibula	Injuries & poisonings	Wald ratio	1	0.988 (0.921, 1.06)	0.732	0.982
Fracture of patella	Injuries & poisonings	Wald ratio	1	0.981 (0.872, 1.105)	0.755	0.982
Fracture of ankle and foot	Injuries & poisonings	Wald ratio	1	0.986 (0.921, 1.056)	0.685	0.979
Fracture of foot	Injuries & poisonings	Wald ratio	1	0.987 (0.915, 1.065)	0.733	0.982
Fracture of pelvis	Injuries & poisonings	Wald ratio	1	1.098 (0.972, 1.24)	0.134	0.85
Fracture of upper limb	Injuries & poisonings	Wald ratio	1	1.002 (0.966, 1.04)	0.904	0.984
Fracture of humerus	Injuries & poisonings	Wald ratio	1	1.042 (0.963, 1.129)	0.306	0.879
Fracture of radius and ulna	Injuries & poisonings	Wald ratio	1	1.006 (0.96, 1.053)	0.807	0.984
Fracture of clavicle or scapula	Injuries & poisonings	Wald ratio	1	0.983 (0.905, 1.068)	0.689	0.979
Fracture of hand or wrist	Injuries & poisonings	Wald ratio	1	1.033 (0.977, 1.091)	0.257	0.879
Fracture of vertebral column without mention of spinal cord injury	Injuries & poisonings	Wald ratio	1	1.051 (0.962, 1.149)	0.27	0.879
Fracture of ribs	Injuries & poisonings	Wald ratio	1	1.152 (1.023, 1.297)	0.019	0.607
Fracture of unspecified bones	Injuries & poisonings	Wald ratio	1	1.074 (0.981, 1.176)	0.121	0.847
Concussion	Injuries & poisonings	Wald ratio	1	1.071 (0.874, 1.313)	0.507	0.943
Intracranial hemorrhage (injury)	Injuries & poisonings	Wald ratio	1	0.924 (0.798, 1.069)	0.288	0.879
Skull and face fracture and other intercranial injury	Injuries & poisonings	Wald ratio	1	0.995 (0.936, 1.057)	0.865	0.984
Torus fracture	Injuries & poisonings	Wald ratio	1	1.005 (0.928, 1.088)	0.911	0.984
Dislocation	Injuries & poisonings	Wald ratio	1	0.928 (0.864, 0.998)	0.043	0.752
Internal derangement of knee	Injuries & poisonings	Wald ratio	1	0.976 (0.949, 1.003)	0.083	0.825
Traumatic arthropathy	Injuries & poisonings	Wald ratio	1	0.981 (0.851, 1.131)	0.795	0.984
Sprains and strains	Injuries & poisonings	Wald ratio	1	0.933 (0.805, 1.082)	0.362	0.899
Hemorrhage or hematoma complicating a procedure	Injuries & poisonings	Wald ratio	1	0.979 (0.935, 1.026)	0.379	0.899
Complications of transplants and reattached limbs	Injuries & poisonings	Wald ratio	1	1.391 (1.074, 1.802)	0.012	0.55
Complication of colostomy or enterostomy	Injuries & poisonings	Wald ratio	1	0.91 (0.786, 1.053)	0.206	0.879
Complications of cardiac or vascular device, implant, and graft	Injuries & poisonings	Wald ratio	1	0.98 (0.907, 1.06)	0.617	0.956

Mechanical complication of unspecified genitourinary device, implant, and graft	Injuries & poisonings	Wald ratio	1	1.011 (0.92, 1.111)	0.814	0.984
Complication of internal orthopedic device	Injuries & poisonings	Wald ratio	1	0.996 (0.939, 1.057)	0.901	0.984
Complication due to other implant and internal device	Injuries & poisonings	Wald ratio	1	0.987 (0.932, 1.045)	0.652	0.97
Open wounds of head; neck; and trunk	Injuries & poisonings	Wald ratio	1	0.983 (0.932, 1.037)	0.535	0.949
Open wound or laceration of eye or eyelid	Injuries & poisonings	Wald ratio	1	0.988 (0.806, 1.21)	0.906	0.984
Open wound of ear	Injuries & poisonings	Wald ratio	1	1.061 (0.835, 1.349)	0.628	0.967
Other open wound of head and face	Injuries & poisonings	Wald ratio	1	0.996 (0.937, 1.058)	0.887	0.984
Open wound of nose and sinus	Injuries & poisonings	Wald ratio	1	0.868 (0.695, 1.084)	0.211	0.879
Open wound of neck	Injuries & poisonings	Wald ratio	1	0.97 (0.807, 1.167)	0.749	0.982
Open wounds of extremities	Injuries & poisonings	Wald ratio	1	0.977 (0.929, 1.027)	0.354	0.898
Open wound of hand except finger(s)	Injuries & poisonings	Wald ratio	1	0.82 (0.527, 1.278)	0.382	0.899
Open wound of finger(s)	Injuries & poisonings	Wald ratio	1	1.208 (0.866, 1.684)	0.267	0.879
Open wound of foot except toe(s) alone	Injuries & poisonings	Wald ratio	1	0.934 (0.735, 1.188)	0.58	0.955
Traumatic amputation	Injuries & poisonings	Wald ratio	1	1.061 (0.914, 1.233)	0.437	0.917
Broken tooth	Injuries & poisonings	Wald ratio	1	1.03 (0.825, 1.285)	0.796	0.984
Complication of amputation stump	Injuries & poisonings	Wald ratio	1	1.029 (0.794, 1.332)	0.83	0.984
Late effects of musculoskeletal and connective tissue injuries	Injuries & poisonings	Wald ratio	1	1.208 (0.932, 1.564)	0.153	0.879
Injuries to the nervous system	Injuries & poisonings	Wald ratio	1	0.988 (0.902, 1.081)	0.791	0.984
Superficial injury without mention of infection	Injuries & poisonings	Wald ratio	1	0.975 (0.927, 1.025)	0.317	0.879
Contusion	Injuries & poisonings	Wald ratio	1	0.981 (0.901, 1.068)	0.664	0.974
Allergic reaction to food	Injuries & poisonings	Wald ratio	1	1.043 (0.821, 1.327)	0.729	0.982
Dermatitis due to solar radiation	Injuries & poisonings	Wald ratio	1	1.028 (0.872, 1.211)	0.745	0.982
Chronic dermatitis due to solar radiation	Injuries & poisonings	Wald ratio	1	1.185 (0.967, 1.452)	0.102	0.835
Adverse reaction to serum or vaccine	Injuries & poisonings	Wald ratio	1	0.936 (0.75, 1.169)	0.56	0.951
Infusion and transfusion reaction	Injuries & poisonings	Wald ratio	1	0.726 (0.53, 0.994)	0.046	0.752
Anaphylactic shock NOS	Injuries & poisonings	Wald ratio	1	0.876 (0.762, 1.008)	0.065	0.794
Urticaria	Injuries & poisonings	Wald ratio	1	0.922 (0.779, 1.091)	0.345	0.898
Allergies, other	Injuries & poisonings	Wald ratio	1	1.024 (0.87, 1.205)	0.776	0.984
Spinal cord injury without evidence of spinal bone injury	Injuries & poisonings	Wald ratio	1	1.047 (0.893, 1.228)	0.569	0.951
Injury to other and unspecified nerves	Injuries & poisonings	Wald ratio	1	1.001 (0.817, 1.226)	0.995	0.996
Certain early complications of trauma or procedure	Injuries & poisonings	Wald ratio	1	0.963 (0.831, 1.116)	0.617	0.956
Postoperative shock	Injuries & poisonings	Wald ratio	1	0.552 (0.354, 0.86)	0.009	0.55
Traumatic and surgical subcutaneous emphysema	Injuries & poisonings	Wald ratio	1	1.266 (0.875, 1.832)	0.211	0.879
Poisoning by antibiotics	Injuries & poisonings	Wald ratio	1	0.997 (0.972, 1.024)	0.841	0.984
Allergy or adverse effect of penicillin	Injuries & poisonings	Wald ratio	1	0.999 (0.972, 1.027)	0.957	0.99
Poisoning by other anti-infectives	Injuries & poisonings	Wald ratio	1	1.02 (0.93, 1.119)	0.674	0.979
Poisoning or allergy of sulfonamides	Injuries & poisonings	Wald ratio	1	1.044 (0.935, 1.167)	0.443	0.917
Poisoning by hormones and synthetic substitutes	Injuries & poisonings	Wald ratio	1	0.948 (0.801, 1.121)	0.531	0.949
Hormones and synthetic substitutes causing adverse effects in therapeutic use	Injuries & poisonings	Wald ratio	1	0.967 (0.814, 1.148)	0.699	0.979
Poisoning by primarily systemic agents	Injuries & poisonings	Wald ratio	1	1.039 (0.833, 1.297)	0.733	0.982
Poisoning by agents primarily affecting blood constituents	Injuries & poisonings	Wald ratio	1	0.798 (0.651, 0.977)	0.029	0.674
Anticoagulants causing adverse effects	Injuries & poisonings	Wald ratio	1	0.798 (0.651, 0.977)	0.029	0.674
Poisoning by analgesics, antipyretics, and antirheumatics	Injuries & poisonings	Wald ratio	1	1.019 (0.978, 1.061)	0.363	0.899
Opiates and related narcotics causing adverse effects in therapeutic use	Injuries & poisonings	Wald ratio	1	1.055 (0.959, 1.162)	0.273	0.879
Antirheumatics causing adverse effects in therapeutic use	Injuries & poisonings	Wald ratio	1	0.96 (0.827, 1.115)	0.596	0.955
Salicylates causing adverse effects in therapeutic use	Injuries & poisonings	Wald ratio	1	1.13 (0.961, 1.33)	0.14	0.859
Poisoning by anticonvulsants and anti-Parkinsonism drugs	Injuries & poisonings	Wald ratio	1	0.985 (0.853, 1.138)	0.837	0.984
Adverse effects of sedatives or other central nervous system depressants and anesthetics	Injuries & poisonings	Wald ratio	1	0.997 (0.871, 1.141)	0.966	0.991
Poisoning by psychotropic agents	Injuries & poisonings	Wald ratio	1	0.964 (0.895, 1.038)	0.33	0.898
Poisoning by drugs primarily affecting the autonomic nervous system	Injuries & poisonings	Wald ratio	1	0.79 (0.621, 1.005)	0.054	0.778
Poisoning by agents primarily affecting the cardiovascular system	Injuries & poisonings	Wald ratio	1	1.018 (0.846, 1.225)	0.849	0.984
Antihypertensive agents causing adverse effects	Injuries & poisonings	Wald ratio	1	1.208 (0.85, 1.715)	0.293	0.879
Poisoning by agents primarily affecting the gastrointestinal system	Injuries & poisonings	Wald ratio	1	0.933 (0.682, 1.278)	0.668	0.974
Poisoning by water, mineral, and uric acid metabolism drugs	Injuries & poisonings	Wald ratio	1	1.094 (0.799, 1.497)	0.576	0.955
Poisoning by agents primarily acting on the smooth and skeletal muscles and respiratory system	Injuries & poisonings	Wald ratio	1	1.082 (0.721, 1.625)	0.703	0.979
Poisoning by agents primarily affecting skin & mucous membrane, ophthalmological, otorhinolaryngological, & dental drugs	Injuries & poisonings	Wald ratio	1	1.13 (0.739, 1.729)	0.572	0.953
Personal history of allergy to medicinal agents	Injuries & poisonings	Wald ratio	1	1.038 (0.832, 1.296)	0.739	0.982
Adverse drug events and drug allergies	Injuries & poisonings	Wald ratio	1	1.046 (0.924, 1.184)	0.474	0.927
Toxic effect of (non-ethyl) alcohol and petroleum and other solvents	Injuries & poisonings	Wald ratio	1	1.185 (0.932, 1.507)	0.166	0.879
Toxic effect of carbon monoxide	Injuries & poisonings	Wald ratio	1	0.917 (0.622, 1.352)	0.661	0.974
Toxic effect of other gases, fumes, or vapors	Injuries & poisonings	Wald ratio	1	0.868 (0.634, 1.189)	0.378	0.899
Toxic effect of noxious substances eaten as food	Injuries & poisonings	Wald ratio	1	1.12 (0.818, 1.533)	0.48	0.931
Toxic effect of other substances, chiefly nonmedicinal as to source	Injuries & poisonings	Wald ratio	1	0.965 (0.631, 1.476)	0.869	0.984
Effects radiation NOS	Injuries & poisonings	Wald ratio	1	1.034 (0.974, 1.097)	0.274	0.879
Delirium dementia and amnestic and other cognitive disorders	Mental disorders	Wald ratio	1	1.021 (0.948, 1.099)	0.582	0.955
Dementias	Mental disorders	Wald ratio	1	1.096 (0.986, 1.217)	0.089	0.835
Alzheimer's disease	Mental disorders	Wald ratio	1	1.163 (0.988, 1.368)	0.069	0.806
Vascular dementia	Mental disorders	Wald ratio	1	0.901 (0.709, 1.146)	0.397	0.908
Delirium due to conditions classified elsewhere	Mental disorders	Wald ratio	1	0.979 (0.862, 1.113)	0.75	0.982
Other persistent mental disorders due to conditions classified elsewhere	Mental disorders	Wald ratio	1	1.071 (0.727, 1.579)	0.728	0.982
Other specified nonpsychotic and or or transient mental disorders	Mental disorders	Wald ratio	1	0.993 (0.872, 1.13)	0.914	0.984
Transient mental disorders due to conditions classified elsewhere	Mental disorders	Wald ratio	1	1.09 (0.811, 1.464)	0.57	0.951
Specific nonpsychotic mental disorders due to brain damage	Mental disorders	Wald ratio	1	1.196 (0.842, 1.699)	0.317	0.879
Alteration of consciousness	Mental disorders	Wald ratio	1	0.94 (0.805, 1.097)	0.432	0.917
Neurological disorders	Mental disorders	Wald ratio	1	1.007 (0.96, 1.057)	0.77	0.982

Aphasia or speech disturbance	Mental disorders	Wald ratio	1	0.986 (0.906, 1.073)	0.744	0.982
Mild cognitive impairment	Mental disorders	Wald ratio	1	0.935 (0.736, 1.189)	0.585	0.955
Memory loss	Mental disorders	Wald ratio	1	1.088 (0.961, 1.231)	0.184	0.879
Altered mental status	Mental disorders	Wald ratio	1	1.013 (0.945, 1.087)	0.713	0.982
Hallucinations	Mental disorders	Wald ratio	1	0.971 (0.807, 1.168)	0.757	0.982
Symptoms involving head and neck	Mental disorders	Wald ratio	1	0.974 (0.918, 1.033)	0.382	0.899
Swelling, mass, or lump in head and neck [Space-occupying lesion, intracranial NOS]	Mental disorders	Wald ratio	1	0.926 (0.83, 1.032)	0.165	0.879
Schizophrenia and other psychotic disorders	Mental disorders	Wald ratio	1	1.093 (0.976, 1.223)	0.123	0.847
Schizophrenia	Mental disorders	Wald ratio	1	1.047 (0.913, 1.201)	0.508	0.943
Paranoid disorders	Mental disorders	Wald ratio	1	1.109 (0.872, 1.41)	0.397	0.908
Psychosis	Mental disorders	Wald ratio	1	1.219 (0.941, 1.579)	0.134	0.85
Mood disorders	Mental disorders	Wald ratio	1	1.017 (0.987, 1.048)	0.261	0.879
Bipolar	Mental disorders	Wald ratio	1	1.12 (1.013, 1.237)	0.026	0.667
Depression	Mental disorders	Wald ratio	1	1.013 (0.982, 1.046)	0.41	0.911
Major depressive disorder	Mental disorders	Wald ratio	1	1.208 (1.017, 1.434)	0.032	0.716
Suicidal ideation or attempt	Mental disorders	Wald ratio	1	1.07 (0.898, 1.276)	0.449	0.917
Suicide or self-inflicted injury	Mental disorders	Wald ratio	1	1.097 (0.913, 1.317)	0.322	0.89
Anxiety disorders	Mental disorders	Wald ratio	1	0.988 (0.948, 1.029)	0.555	0.951
Anxiety disorder	Mental disorders	Wald ratio	1	0.975 (0.936, 1.015)	0.22	0.879
Generalized anxiety disorder	Mental disorders	Wald ratio	1	1.003 (0.774, 1.299)	0.985	0.991
Agorophobia, social phobia, and panic disorder	Mental disorders	Wald ratio	1	0.931 (0.822, 1.054)	0.257	0.879
Phobia	Mental disorders	Wald ratio	1	1.012 (0.875, 1.171)	0.869	0.984
Obsessive-compulsive disorders	Mental disorders	Wald ratio	1	1.078 (0.817, 1.423)	0.594	0.955
Dysthymic disorder	Mental disorders	Wald ratio	1	1.242 (0.797, 1.935)	0.338	0.898
Acute reaction to stress	Mental disorders	Wald ratio	1	0.935 (0.696, 1.257)	0.657	0.974
Posttraumatic stress disorder	Mental disorders	Wald ratio	1	0.913 (0.667, 1.249)	0.568	0.951
Personality disorders	Mental disorders	Wald ratio	1	1.009 (0.845, 1.205)	0.922	0.984
Antisocial or borderline personality disorder	Mental disorders	Wald ratio	1	0.836 (0.622, 1.124)	0.235	0.879
Sexual and gender identity disorders	Mental disorders	Wald ratio	1	1.098 (0.916, 1.316)	0.312	0.879
Psychogenic and somatoform disorders	Mental disorders	Wald ratio	1	1.13 (0.98, 1.303)	0.091	0.835
Psychogenic disorder	Mental disorders	Wald ratio	1	1.109 (0.932, 1.32)	0.242	0.879
Somatoform disorder	Mental disorders	Wald ratio	1	1.094 (0.814, 1.47)	0.553	0.951
Adjustment reaction	Mental disorders	Wald ratio	1	1.266 (1.052, 1.523)	0.012	0.55
Eating disorder (no controls excluded)	Mental disorders	Wald ratio	1	0.59 (0.415, 0.838)	0.003	0.409
Eating disorder	Mental disorders	Wald ratio	1	0.59 (0.415, 0.838)	0.003	0.409
Other mental disorder	Mental disorders	Wald ratio	1	0.986 (0.966, 1.006)	0.173	0.879
Tension headache	Mental disorders	Wald ratio	1	0.992 (0.825, 1.194)	0.936	0.984
Pervasive developmental disorders	Mental disorders	Wald ratio	1	0.959 (0.663, 1.388)	0.826	0.984
Autism	Mental disorders	Wald ratio	1	0.836 (0.537, 1.303)	0.429	0.917
Develomental delays and disorders	Mental disorders	Wald ratio	1	1.006 (0.87, 1.164)	0.933	0.984
Learning disorder	Mental disorders	Wald ratio	1	1.07 (0.826, 1.386)	0.607	0.956
Speech and language disorder	Mental disorders	Wald ratio	1	1.174 (0.826, 1.668)	0.371	0.899
Substance addiction and disorders	Mental disorders	Wald ratio	1	0.992 (0.842, 1.17)	0.927	0.984
Polyneuropathy due to drugs	Mental disorders	Wald ratio	1	1.152 (0.753, 1.762)	0.514	0.947
Alcohol-related disorders	Mental disorders	Wald ratio	1	1.006 (0.977, 1.036)	0.68	0.979
Alcoholism	Mental disorders	Wald ratio	1	1.027 (0.991, 1.063)	0.141	0.859
Alcoholic liver damage	Mental disorders	Wald ratio	1	1.009 (0.9, 1.131)	0.878	0.984
Tobacco use disorder	Mental disorders	Wald ratio	1	1.01 (0.986, 1.035)	0.397	0.908
Osteomyelitis, periostitis, and other infections involving bone	Musculoskeletal	Wald ratio	1	0.94 (0.824, 1.071)	0.353	0.898
Osteomyelitis	Musculoskeletal	Wald ratio	1	0.955 (0.833, 1.095)	0.508	0.943
Chronic osteomyelitis	Musculoskeletal	Wald ratio	1	1.12 (0.881, 1.424)	0.356	0.898
Unspecified osteomyelitis	Musculoskeletal	Wald ratio	1	0.901 (0.766, 1.061)	0.211	0.879
Arthropathy associated with infections	Musculoskeletal	Wald ratio	1	1.007 (0.859, 1.18)	0.935	0.984
Pyogenic arthritis	Musculoskeletal	Wald ratio	1	1.022 (0.853, 1.225)	0.814	0.984
Infective connective tissue disorders	Musculoskeletal	Wald ratio	1	1.152 (0.796, 1.667)	0.453	0.917
Arthropathy associated with other disorders classified elsewhere	Musculoskeletal	Wald ratio	1	1.009 (0.779, 1.308)	0.943	0.984
Arthropathy associated with neurological disorders	Musculoskeletal	Wald ratio	1	1.314 (0.892, 1.938)	0.167	0.879
Rheumatoid arthritis and other inflammatory polyarthropathies	Musculoskeletal	Wald ratio	1	0.968 (0.923, 1.016)	0.191	0.879
Rheumatoid arthritis	Musculoskeletal	Wald ratio	1	0.966 (0.919, 1.015)	0.171	0.879
Other inflammatory spondylopathies	Musculoskeletal	Wald ratio	1	1.079 (0.997, 1.169)	0.06	0.789
Sacroiliitis NEC	Musculoskeletal	Wald ratio	1	1.009 (0.737, 1.382)	0.955	0.99
Ankylosing spondylitis	Musculoskeletal	Wald ratio	1	1.065 (0.934, 1.215)	0.345	0.898
Other arthropathies	Musculoskeletal	Wald ratio	1	0.992 (0.974, 1.011)	0.424	0.917
Unspecified polyarthropathy or polyarthritis	Musculoskeletal	Wald ratio	1	1 (0.946, 1.057)	0.991	0.993
Unspecified monoarthritis	Musculoskeletal	Wald ratio	1	0.979 (0.953, 1.007)	0.142	0.859
Arthropathy NOS	Musculoskeletal	Wald ratio	1	0.991 (0.972, 1.009)	0.317	0.879
Polymyalgia Rheumatica	Musculoskeletal	Wald ratio	1	0.993 (0.902, 1.094)	0.893	0.984
Spinal stenosis	Musculoskeletal	Wald ratio	1	0.997 (0.945, 1.052)	0.909	0.984
Spondylosis and allied disorders	Musculoskeletal	Wald ratio	1	0.978 (0.942, 1.014)	0.23	0.879
Spondylosis without myelopathy	Musculoskeletal	Wald ratio	1	0.989 (0.944, 1.035)	0.631	0.967
Spondylosis with myelopathy	Musculoskeletal	Wald ratio	1	0.876 (0.689, 1.114)	0.282	0.879
Other allied disorders of spine	Musculoskeletal	Wald ratio	1	0.925 (0.777, 1.1)	0.377	0.899
Intervertebral disc disorders	Musculoskeletal	Wald ratio	1	0.981 (0.947, 1.016)	0.293	0.879
Displacement of intervertebral disc	Musculoskeletal	Wald ratio	1	0.951 (0.824, 1.099)	0.497	0.943
Degeneration of intervertebral disc	Musculoskeletal	Wald ratio	1	0.938 (0.881, 0.999)	0.046	0.752
Postlaminectomy syndrome	Musculoskeletal	Wald ratio	1	1.12 (0.76, 1.651)	0.568	0.951
Other and unspecified disc disorder	Musculoskeletal	Wald ratio	1	0.974 (0.927, 1.024)	0.3	0.879
Other disorders of cervical region	Musculoskeletal	Wald ratio	1	0.91 (0.788, 1.051)	0.2	0.879

Torticollis	Musculoskeletal	Wald ratio	1	0.82 (0.599, 1.123)	0.217	0.879
Other and unspecified disorders of back	Musculoskeletal	Wald ratio	1	1.016 (0.946, 1.092)	0.663	0.974
Disorders of sacrum	Musculoskeletal	Wald ratio	1	0.955 (0.812, 1.123)	0.578	0.955
Disorders of coccyx	Musculoskeletal	Wald ratio	1	0.944 (0.802, 1.111)	0.488	0.937
Other symptoms referable to back	Musculoskeletal	Wald ratio	1	1.278 (0.851, 1.919)	0.237	0.879
Other unspecified back disorders	Musculoskeletal	Wald ratio	1	1.032 (0.951, 1.119)	0.453	0.917
Peripheral enthesopathies and allied syndromes	Musculoskeletal	Wald ratio	1	1.023 (0.995, 1.052)	0.11	0.847
Enthesopathy	Musculoskeletal	Wald ratio	1	1.045 (1.011, 1.081)	0.009	0.55
Synoviopathy	Musculoskeletal	Wald ratio	1	0.991 (0.828, 1.185)	0.918	0.984
Bursitis	Musculoskeletal	Wald ratio	1	1.06 (0.949, 1.185)	0.301	0.879
Other disorders of synovium, tendon, and bursa	Musculoskeletal	Wald ratio	1	1.005 (0.966, 1.044)	0.819	0.984
Synovitis and tenosynovitis	Musculoskeletal	Wald ratio	1	0.995 (0.934, 1.059)	0.865	0.984
Bursitis disorders	Musculoskeletal	Wald ratio	1	1.208 (0.882, 1.653)	0.239	0.879
Ganglion and cyst of synovium, tendon, and bursa	Musculoskeletal	Wald ratio	1	1.021 (0.962, 1.083)	0.492	0.94
Rupture of synovium	Musculoskeletal	Wald ratio	1	1.044 (0.913, 1.195)	0.529	0.949
Rupture of tendon, nontraumatic	Musculoskeletal	Wald ratio	1	1.055 (0.887, 1.255)	0.544	0.949
Contracture of tendon (sheath)	Musculoskeletal	Wald ratio	1	0.852 (0.646, 1.124)	0.257	0.879
Disorders of muscle, ligament, and fascia	Musculoskeletal	Wald ratio	1	1.06 (1.009, 1.114)	0.022	0.649
Laxity of ligament or hypermobility syndrome	Musculoskeletal	Wald ratio	1	0.994 (0.848, 1.165)	0.936	0.984
Fasciitis	Musculoskeletal	Wald ratio	1	1.062 (1.007, 1.121)	0.027	0.667
Contracture of palmar fascia [Dupuytren's disease]	Musculoskeletal	Wald ratio	1	1.06 (1.003, 1.121)	0.039	0.752
Other disorders of soft tissues	Musculoskeletal	Wald ratio	1	0.966 (0.926, 1.008)	0.108	0.847
Rheumatism, unspecified and fibrositis	Musculoskeletal	Wald ratio	1	0.941 (0.849, 1.044)	0.253	0.879
Panniculitis	Musculoskeletal	Wald ratio	1	0.82 (0.599, 1.123)	0.217	0.879
Osteitis deformans and osteopathies associated with other disorders classified elsewhere	Musculoskeletal	Wald ratio	1	1.098 (0.896, 1.345)	0.368	0.899
Osteitis deformans [Paget's disease of bone]	Musculoskeletal	Wald ratio	1	1.099 (0.897, 1.347)	0.363	0.899
Osteochondropathies	Musculoskeletal	Wald ratio	1	1.057 (0.91, 1.228)	0.466	0.923
Juvenile osteochondrosis	Musculoskeletal	Wald ratio	1	1.12 (0.897, 1.398)	0.317	0.879
Osteochondritis dissecans	Musculoskeletal	Wald ratio	1	0.805 (0.526, 1.231)	0.317	0.879
Other disorders of bone and cartilage	Musculoskeletal	Wald ratio	1	1.029 (0.975, 1.085)	0.301	0.879
Cyst of bone	Musculoskeletal	Wald ratio	1	1.036 (0.863, 1.245)	0.701	0.979
Aseptic necrosis of bone	Musculoskeletal	Wald ratio	1	0.987 (0.858, 1.136)	0.854	0.984
Costochondritis	Musculoskeletal	Wald ratio	1	1.219 (0.907, 1.638)	0.189	0.879
Malunion and nonunion of fracture	Musculoskeletal	Wald ratio	1	1.019 (0.929, 1.118)	0.689	0.979
Chondromalacia	Musculoskeletal	Wald ratio	1	1.12 (0.833, 1.505)	0.453	0.917
Acquired foot deformities	Musculoskeletal	Wald ratio	1	1.017 (0.984, 1.052)	0.317	0.879
Flat foot	Musculoskeletal	Wald ratio	1	1.099 (0.864, 1.397)	0.442	0.917
Acquired toe deformities	Musculoskeletal	Wald ratio	1	1.001 (0.955, 1.048)	0.979	0.991
Hammer toe (acquired)	Musculoskeletal	Wald ratio	1	0.994 (0.922, 1.073)	0.882	0.984
Hallux rigidus	Musculoskeletal	Wald ratio	1	0.987 (0.908, 1.072)	0.756	0.982
Hallux valgus (Bunion)	Musculoskeletal	Wald ratio	1	1.037 (0.996, 1.08)	0.076	0.825
Other acquired deformities of limbs	Musculoskeletal	Wald ratio	1	0.926 (0.853, 1.004)	0.062	0.789
Acquired deformities of finger	Musculoskeletal	Wald ratio	1	0.844 (0.689, 1.034)	0.102	0.835
Curvature of spine	Musculoskeletal	Wald ratio	1	1.023 (0.927, 1.128)	0.651	0.97
Kyphoscoliosis and scoliosis	Musculoskeletal	Wald ratio	1	1.012 (0.916, 1.119)	0.81	0.984
Other acquired musculoskeletal deformity	Musculoskeletal	Wald ratio	1	1.003 (0.931, 1.08)	0.94	0.984
Acquired spondylolisthesis	Musculoskeletal	Wald ratio	1	1.02 (0.939, 1.108)	0.641	0.967
Contracture of joint	Musculoskeletal	Wald ratio	1	1.013 (0.874, 1.175)	0.861	0.984
Osteoarthrosis	Musculoskeletal	Wald ratio	1	0.988 (0.968, 1.008)	0.237	0.879
Osteoarthritis; localized	Musculoskeletal	Wald ratio	1	0.998 (0.973, 1.025)	0.903	0.984
Osteoarthrosis, localized, primary	Musculoskeletal	Wald ratio	1	0.982 (0.948, 1.017)	0.317	0.879
Osteoarthrosis, localized, secondary	Musculoskeletal	Wald ratio	1	0.901 (0.756, 1.074)	0.247	0.879
Osteoarthrosis, generalized	Musculoskeletal	Wald ratio	1	0.948 (0.83, 1.083)	0.429	0.917
Osteoarthrosis NOS	Musculoskeletal	Wald ratio	1	0.974 (0.944, 1.005)	0.1	0.835
Symptoms and disorders of the joints	Musculoskeletal	Wald ratio	1	1 (0.948, 1.056)	0.987	0.991
Ankylosis of joint	Musculoskeletal	Wald ratio	1	0.885 (0.646, 1.211)	0.444	0.917
Stiffness of joint	Musculoskeletal	Wald ratio	1	1.007 (0.883, 1.149)	0.914	0.984
Difficulty in walking	Musculoskeletal	Wald ratio	1	1.099 (0.848, 1.423)	0.475	0.927
Joint effusions	Musculoskeletal	Wald ratio	1	0.978 (0.896, 1.066)	0.61	0.956
Hemarthrosis	Musculoskeletal	Wald ratio	1	0.86 (0.628, 1.177)	0.347	0.898
Villonodular synovitis	Musculoskeletal	Wald ratio	1	0.79 (0.507, 1.231)	0.298	0.879
Derangement of joint, non-traumatic	Musculoskeletal	Wald ratio	1	1.042 (0.981, 1.108)	0.182	0.879
Loose body in joint	Musculoskeletal	Wald ratio	1	1.163 (0.972, 1.391)	0.099	0.835
Pathological, developmental or recurrent dislocation	Musculoskeletal	Wald ratio	1	1.185 (0.85, 1.653)	0.317	0.879
Articular cartilage disorder	Musculoskeletal	Wald ratio	1	0.979 (0.846, 1.133)	0.781	0.984
Other derangement of joint	Musculoskeletal	Wald ratio	1	1.023 (0.95, 1.101)	0.549	0.949
Osteoporosis, osteopenia and pathological fracture	Musculoskeletal	Wald ratio	1	0.98 (0.943, 1.019)	0.317	0.879
Osteoporosis	Musculoskeletal	Wald ratio	1	0.976 (0.937, 1.016)	0.237	0.879
Osteoporosis NOS	Musculoskeletal	Wald ratio	1	0.97 (0.928, 1.014)	0.182	0.879
Other specified osteoporosis	Musculoskeletal	Wald ratio	1	1.039 (0.902, 1.198)	0.594	0.955
Pathologic fracture	Musculoskeletal	Wald ratio	1	0.928 (0.804, 1.072)	0.311	0.879
Osteopenia or other disorder of bone and cartilage	Musculoskeletal	Wald ratio	1	1.027 (0.916, 1.151)	0.652	0.97
Pain in joint	Musculoskeletal	Wald ratio	1	0.97 (0.935, 1.007)	0.11	0.847
Cancer of mouth	Neoplasms	Wald ratio	1	1.089 (0.956, 1.239)	0.199	0.879
Cancer of tongue	Neoplasms	Wald ratio	1	1.208 (1.004, 1.453)	0.046	0.752
Cancer of major salivary glands	Neoplasms	Wald ratio	1	0.91 (0.665, 1.246)	0.556	0.951
Cancer of the mouth floor	Neoplasms	Wald ratio	1	0.844 (0.542, 1.315)	0.453	0.917
Cancer of larynx, pharynx, nasal cavities	Neoplasms	Wald ratio	1	1.13 (0.993, 1.286)	0.063	0.789

Cancer of oropharynx	Neoplasms	Wald ratio	1	1.23 (1.004, 1.508)	0.046	0.752
Cancer of larynx	Neoplasms	Wald ratio	1	1.13 (0.922, 1.385)	0.237	0.879
Cancer of of nasal cavities	Neoplasms	Wald ratio	1	0.993 (0.649, 1.52)	0.976	0.991
Cancer of esophagus	Neoplasms	Wald ratio	1	1.109 (0.982, 1.253)	0.096	0.835
Cancer of stomach	Neoplasms	Wald ratio	1	0.986 (0.858, 1.133)	0.841	0.984
Colorectal cancer	Neoplasms	Wald ratio	1	0.998 (0.949, 1.049)	0.926	0.984
Colon cancer	Neoplasms	Wald ratio	1	1.007 (0.949, 1.069)	0.81	0.984
Malignant neoplasm of rectum, rectosigmoid junction, and anus	Neoplasms	Wald ratio	1	1.017 (0.946, 1.093)	0.644	0.967
Cancer of liver and intrahepatic bile duct	Neoplasms	Wald ratio	1	1.13 (0.948, 1.347)	0.171	0.879
Malignant neoplasm of liver, primary	Neoplasms	Wald ratio	1	1.196 (0.907, 1.578)	0.205	0.879
Pancreatic cancer	Neoplasms	Wald ratio	1	1.001 (0.875, 1.146)	0.985	0.991
Neoplasm of unspecified nature of digestive system	Neoplasms	Wald ratio	1	0.978 (0.885, 1.08)	0.657	0.974
Malignant neoplasm of other and ill-defined sites within the digestive organs and peritoneum	Neoplasms	Wald ratio	1	1.008 (0.965, 1.054)	0.714	0.982
Malignant neoplasm of small intestine, including duodenum	Neoplasms	Wald ratio	1	0.92 (0.723, 1.169)	0.494	0.94
Malignant neoplasm of gallbladder and extrahepatic bile ducts	Neoplasms	Wald ratio	1	0.91 (0.716, 1.157)	0.442	0.917
Malignant neoplasm of retroperitoneum and peritoneum	Neoplasms	Wald ratio	1	1.042 (0.82, 1.325)	0.735	0.982
Cancer of intrathoracic organs	Neoplasms	Wald ratio	1	0.66 (0.432, 1.01)	0.056	0.778
Cancer within the respiratory system	Neoplasms	Wald ratio	1	1.019 (0.957, 1.085)	0.556	0.951
Cancer of bronchus; lung	Neoplasms	Wald ratio	1	1.015 (0.945, 1.091)	0.682	0.979
Cancer of bone and connective tissue	Neoplasms	Wald ratio	1	0.945 (0.806, 1.108)	0.485	0.934
Bone cancer	Neoplasms	Wald ratio	1	1.185 (0.882, 1.593)	0.261	0.879
Cancer of connective tissue	Neoplasms	Wald ratio	1	0.868 (0.722, 1.044)	0.134	0.85
Skin cancer	Neoplasms	Wald ratio	1	0.965 (0.937, 0.994)	0.018	0.594
Melanomas of skin, dx or hx	Neoplasms	Wald ratio	1	0.99 (0.928, 1.056)	0.753	0.982
Melanomas of skin	Neoplasms	Wald ratio	1	0.99 (0.928, 1.056)	0.753	0.982
Other non-epithelial cancer of skin	Neoplasms	Wald ratio	1	0.954 (0.924, 0.984)	0.003	0.409
Carcinoma in situ of skin	Neoplasms	Wald ratio	1	0.959 (0.845, 1.09)	0.524	0.949
Neoplasm of uncertain behavior of skin	Neoplasms	Wald ratio	1	1.097 (0.863, 1.395)	0.451	0.917
Breast cancer	Neoplasms	Wald ratio	1	0.994 (0.963, 1.026)	0.715	0.982
Breast cancer [female]	Neoplasms	Wald ratio	1	0.993 (0.962, 1.025)	0.659	0.974
Malignant neoplasm of female breast	Neoplasms	Wald ratio	1	0.99 (0.959, 1.021)	0.518	0.949
Neoplasm of uncertain behavior of breast	Neoplasms	Wald ratio	1	0.947 (0.692, 1.296)	0.733	0.982
Acquired absence of breast	Neoplasms	Wald ratio	1	0.981 (0.918, 1.049)	0.579	0.955
Cervical cancer and dysplasia	Neoplasms	Wald ratio	1	0.963 (0.911, 1.018)	0.182	0.879
Cervical cancer	Neoplasms	Wald ratio	1	0.973 (0.897, 1.055)	0.51	0.944
Cervical intraepithelial neoplasia [CIN] [Cervical dysplasia]	Neoplasms	Wald ratio	1	0.949 (0.883, 1.019)	0.151	0.879
Malignant neoplasm of uterus	Neoplasms	Wald ratio	1	0.947 (0.863, 1.038)	0.246	0.879
Cancer of other female genital organs	Neoplasms	Wald ratio	1	0.984 (0.921, 1.052)	0.637	0.967
Malignant neoplasm of ovary and other uterine adnexa	Neoplasms	Wald ratio	1	0.979 (0.911, 1.052)	0.555	0.951
Malignant neoplasm of ovary	Neoplasms	Wald ratio	1	0.973 (0.905, 1.046)	0.457	0.919
Cancer of other female genital organs (excluding uterus and ovary)	Neoplasms	Wald ratio	1	0.992 (0.824, 1.193)	0.929	0.984
Malignant neoplasm of testis	Neoplasms	Wald ratio	1	1.026 (0.972, 1.082)	0.352	0.898
Neoplasm of uncertain behavior of male genital organs	Neoplasms	Wald ratio	1	0.828 (0.605, 1.134)	0.239	0.879
Cancer of urinary organs (incl. kidney and bladder)	Neoplasms	Wald ratio	1	1.002 (0.951, 1.055)	0.943	0.984
Cancer of kidney and renal pelvis	Neoplasms	Wald ratio	1	0.971 (0.877, 1.075)	0.573	0.954
Malignant neoplasm of kidney, except pelvis	Neoplasms	Wald ratio	1	0.975 (0.879, 1.081)	0.63	0.967
Malignant neoplasm of renal pelvis	Neoplasms	Wald ratio	1	1.006 (0.67, 1.51)	0.978	0.991
Cancer of bladder	Neoplasms	Wald ratio	1	1.042 (0.975, 1.114)	0.222	0.879
Malignant neoplasm of bladder	Neoplasms	Wald ratio	1	1.052 (0.979, 1.131)	0.166	0.879
Malignant neoplasm of other urinary organs	Neoplasms	Wald ratio	1	0.679 (0.515, 0.896)	0.006	0.532
Cancer of eye	Neoplasms	Wald ratio	1	0.828 (0.651, 1.053)	0.124	0.847
Manlignant and unknown neoplasms of brain and nervous system	Neoplasms	Wald ratio	1	1.067 (0.939, 1.212)	0.317	0.879
Cancer of brain and nervous system	Neoplasms	Wald ratio	1	1.13 (0.98, 1.303)	0.091	0.835
Cancer of brain	Neoplasms	Wald ratio	1	1.185 (1.022, 1.374)	0.024	0.665
Thyroid cancer	Neoplasms	Wald ratio	1	1.063 (0.895, 1.263)	0.485	0.934
Cancer of other endocrine glands	Neoplasms	Wald ratio	1	0.911 (0.641, 1.294)	0.602	0.956
Cancer, suspected or other	Neoplasms	Wald ratio	1	0.994 (0.969, 1.02)	0.668	0.974
Malignant neoplasm, other	Neoplasms	Wald ratio	1	0.995 (0.969, 1.021)	0.679	0.979
Chemotherapy	Neoplasms	Wald ratio	1	1.004 (0.982, 1.027)	0.708	0.982
Secondary malignant neoplasm	Neoplasms	Wald ratio	1	1.008 (0.974, 1.044)	0.639	0.967
Secondary malignancy of lymph nodes	Neoplasms	Wald ratio	1	1.006 (0.963, 1.052)	0.787	0.984
Secondary malignancy of respiratory organs	Neoplasms	Wald ratio	1	1.024 (0.954, 1.098)	0.511	0.944
Secondary malignant neoplasm of digestive systems	Neoplasms	Wald ratio	1	1.001 (0.921, 1.088)	0.98	0.991
Secondary malignant neoplasm of liver	Neoplasms	Wald ratio	1	1.016 (0.953, 1.084)	0.627	0.967
Secondary malignancy of brain or spine	Neoplasms	Wald ratio	1	0.943 (0.841, 1.058)	0.317	0.879
Secondary malignancy of bone	Neoplasms	Wald ratio	1	0.993 (0.925, 1.065)	0.835	0.984
Secondary malignant neoplasm of skin	Neoplasms	Wald ratio	1	0.893 (0.702, 1.136)	0.356	0.898
Neoplasm of uncertain behavior	Neoplasms	Wald ratio	1	1.028 (0.942, 1.121)	0.537	0.949
Neurofibromatosis	Neoplasms	Wald ratio	1	1.074 (0.77, 1.498)	0.673	0.979
Myeloproliferative disease	Neoplasms	Wald ratio	1	1.074 (0.969, 1.191)	0.175	0.879
Polycythemia vera	Neoplasms	Wald ratio	1	1.099 (0.931, 1.298)	0.267	0.879
Hodgkin's disease	Neoplasms	Wald ratio	1	1.055 (0.861, 1.293)	0.604	0.956
Cancer of other lymphoid, histiocytic tissue	Neoplasms	Wald ratio	1	1.081 (1.01, 1.158)	0.025	0.665
Non-Hodgkins lymphoma	Neoplasms	Wald ratio	1	1.072 (0.992, 1.159)	0.078	0.825
Nodular lymphoma	Neoplasms	Wald ratio	1	0.933 (0.788, 1.106)	0.427	0.917
Large cell lymphoma	Neoplasms	Wald ratio	1	1.12 (0.977, 1.284)	0.105	0.847
Leukemia	Neoplasms	Wald ratio	1	0.954 (0.879, 1.035)	0.256	0.879
Lymphoid leukemia	Neoplasms	Wald ratio	1	0.972 (0.848, 1.115)	0.685	0.979

Lymphoid leukemia, acute	Neoplasms	Wald ratio	1	0.844 (0.552, 1.291)	0.434	0.917
Lymphoid leukemia, chronic	Neoplasms	Wald ratio	1	0.958 (0.827, 1.108)	0.56	0.951
Myeloid leukemia	Neoplasms	Wald ratio	1	0.885 (0.76, 1.029)	0.113	0.847
Myeloid leukemia, acute	Neoplasms	Wald ratio	1	0.885 (0.737, 1.062)	0.189	0.879
Myeloid leukemia, chronic	Neoplasms	Wald ratio	1	0.912 (0.704, 1.181)	0.484	0.934
Multiple myeloma	Neoplasms	Wald ratio	1	0.931 (0.81, 1.069)	0.311	0.879
Benign neoplasm of colon	Neoplasms	Wald ratio	1	1.001 (0.978, 1.026)	0.908	0.984
Benign neoplasm of lip, oral cavity, and pharynx	Neoplasms	Wald ratio	1	1.049 (0.946, 1.164)	0.362	0.899
Benign neoplasm of other parts of digestive system	Neoplasms	Wald ratio	1	0.994 (0.949, 1.041)	0.786	0.984
Benign neoplasm of respiratory and intrathoracic organs	Neoplasms	Wald ratio	1	0.965 (0.809, 1.15)	0.689	0.979
Benign neoplasm of bone and articular cartilage	Neoplasms	Wald ratio	1	0.973 (0.81, 1.168)	0.77	0.982
Lipoma	Neoplasms	Wald ratio	1	1.011 (0.971, 1.053)	0.585	0.955
Lipoma of skin and subcutaneous tissue	Neoplasms	Wald ratio	1	1.003 (0.956, 1.052)	0.911	0.984
Other benign neoplasm of connective and other soft tissue	Neoplasms	Wald ratio	1	0.984 (0.892, 1.085)	0.748	0.982
Benign neoplasm of skin	Neoplasms	Wald ratio	1	0.989 (0.951, 1.028)	0.568	0.951
Vascular hamartomas and non-neoplastic nevi	Neoplasms	Wald ratio	1	1.12 (0.984, 1.274)	0.086	0.835
Nevus, non-neoplastic	Neoplasms	Wald ratio	1	1.109 (0.971, 1.267)	0.127	0.85
Benign neoplasm of uterus	Neoplasms	Wald ratio	1	0.961 (0.93, 0.994)	0.02	0.609
Uterine leiomyoma	Neoplasms	Wald ratio	1	0.958 (0.927, 0.991)	0.012	0.55
Other benign neoplasm of uterus	Neoplasms	Wald ratio	1	1.056 (0.888, 1.257)	0.537	0.949
Benign neoplasm of ovary	Neoplasms	Wald ratio	1	0.926 (0.85, 1.008)	0.075	0.825
Benign neoplasm of other female genital organs	Neoplasms	Wald ratio	1	0.957 (0.766, 1.194)	0.695	0.979
Benign neoplasm of kidney and other urinary organs	Neoplasms	Wald ratio	1	0.844 (0.664, 1.073)	0.166	0.879
Benign neoplasm of eye	Neoplasms	Wald ratio	1	0.932 (0.706, 1.229)	0.617	0.956
Benign neoplasm of eye, uveal	Neoplasms	Wald ratio	1	1.052 (0.714, 1.551)	0.797	0.984
Benign neoplasm of brain and other parts of nervous system	Neoplasms	Wald ratio	1	1.035 (0.923, 1.16)	0.561	0.951
Benign neoplasm of brain, cranial nerves, meninges	Neoplasms	Wald ratio	1	1.038 (0.923, 1.169)	0.532	0.949
Benign neoplasm of spinal cord, meninges	Neoplasms	Wald ratio	1	1.079 (0.706, 1.651)	0.725	0.982
Benign neoplasm of thyroid glands	Neoplasms	Wald ratio	1	0.813 (0.676, 0.978)	0.028	0.667
Benign neoplasm of other endocrine glands and related structures	Neoplasms	Wald ratio	1	0.998 (0.893, 1.115)	0.972	0.991
Benign neoplasm of adrenal gland	Neoplasms	Wald ratio	1	0.987 (0.776, 1.255)	0.914	0.984
Benign neoplasm of parathyroid gland	Neoplasms	Wald ratio	1	1.017 (0.858, 1.206)	0.845	0.984
Benign neoplasm of pituitary gland and craniopharyngeal duct (pouch)	Neoplasms	Wald ratio	1	1.01 (0.84, 1.216)	0.912	0.984
Hemangioma and lymphangioma, any site	Neoplasms	Wald ratio	1	0.998 (0.92, 1.083)	0.966	0.991
Benign neoplasm of unspecified sites	Neoplasms	Wald ratio	1	0.99 (0.926, 1.058)	0.76	0.982
Bone marrow or stem cell transplant	Neoplasms	Wald ratio	1	1.028 (0.71, 1.487)	0.885	0.984
Meningitis	Neurological	Wald ratio	1	1.13 (0.968, 1.32)	0.122	0.847
Encephalitis	Neurological	Wald ratio	1	1.037 (0.87, 1.237)	0.681	0.979
Acute (transverse) myelitis	Neurological	Wald ratio	1	0.844 (0.583, 1.221)	0.368	0.899
Other CNS infection and poliomyelitis	Neurological	Wald ratio	1	1.008 (0.838, 1.213)	0.933	0.984
Sleep disorders	Neurological	Wald ratio	1	1.023 (0.977, 1.071)	0.337	0.898
Sleep apnea	Neurological	Wald ratio	1	1.009 (0.96, 1.061)	0.717	0.982
Insomnia	Neurological	Wald ratio	1	1.064 (0.822, 1.378)	0.637	0.967
Organic or persistent insomnia	Neurological	Wald ratio	1	1.152 (0.873, 1.52)	0.317	0.879
Other cerebral degenerations	Neurological	Wald ratio	1	1.045 (0.948, 1.153)	0.375	0.899
Hydrocephalus	Neurological	Wald ratio	1	1.013 (0.876, 1.173)	0.859	0.984
Cerebral degeneration, unspecified	Neurological	Wald ratio	1	1.13 (0.963, 1.328)	0.135	0.854
Parkinson's disease	Neurological	Wald ratio	1	1.008 (0.914, 1.111)	0.879	0.984
Extrapyramidal disease and abnormal movement disorders	Neurological	Wald ratio	1	1.025 (0.919, 1.143)	0.659	0.974
Essential tremor	Neurological	Wald ratio	1	1.087 (0.87, 1.356)	0.463	0.919
Myoclonus	Neurological	Wald ratio	1	0.836 (0.599, 1.166)	0.291	0.879
Torsion dystonia	Neurological	Wald ratio	1	0.893 (0.729, 1.094)	0.275	0.879
Degenerative disease of the spinal cord	Neurological	Wald ratio	1	0.978 (0.905, 1.057)	0.568	0.951
Spinocerebellar disease	Neurological	Wald ratio	1	1.095 (0.815, 1.471)	0.549	0.949
Anterior horn cell disease	Neurological	Wald ratio	1	1.008 (0.807, 1.258)	0.944	0.984
Multiple sclerosis	Neurological	Wald ratio	1	0.928 (0.849, 1.014)	0.1	0.835
Disorders of the autonomic nervous system	Neurological	Wald ratio	1	0.82 (0.669, 1.005)	0.056	0.778
Peripheral autonomic neuropathy	Neurological	Wald ratio	1	0.885 (0.6, 1.304)	0.536	0.949
Acute pain	Neurological	Wald ratio	1	0.901 (0.765, 1.063)	0.216	0.879
Chronic pain	Neurological	Wald ratio	1	1.04 (0.881, 1.229)	0.641	0.967
Other headache syndromes	Neurological	Wald ratio	1	0.995 (0.959, 1.032)	0.787	0.984
Migraine	Neurological	Wald ratio	1	1.031 (0.97, 1.095)	0.332	0.898
Migrain with aura	Neurological	Wald ratio	1	0.968 (0.776, 1.209)	0.777	0.984
Other demyelinating diseases of central nervous system	Neurological	Wald ratio	1	0.959 (0.783, 1.176)	0.689	0.979
Hemiplegia	Neurological	Wald ratio	1	0.987 (0.906, 1.074)	0.761	0.982
Infantile cerebral palsy	Neurological	Wald ratio	1	0.964 (0.731, 1.272)	0.795	0.984
Other paralytic syndromes	Neurological	Wald ratio	1	0.996 (0.877, 1.132)	0.956	0.99
Epilepsy, recurrent seizures, convulsions	Neurological	Wald ratio	1	1.006 (0.961, 1.054)	0.795	0.984
Epilepsy	Neurological	Wald ratio	1	1.018 (0.913, 1.135)	0.747	0.982
Generalized convulsive epilepsy	Neurological	Wald ratio	1	1.013 (0.868, 1.183)	0.868	0.984
Partial epilepsy	Neurological	Wald ratio	1	1.057 (0.863, 1.295)	0.592	0.955
Convulsions	Neurological	Wald ratio	1	0.982 (0.916, 1.054)	0.617	0.956
Abnormal findings on study of brain and or or nervous system	Neurological	Wald ratio	1	0.975 (0.767, 1.24)	0.835	0.984
Nonspecific abnormal findings on radiological and other examination of skull and head	Neurological	Wald ratio	1	0.913 (0.692, 1.205)	0.522	0.949
Other conditions of brain	Neurological	Wald ratio	1	1.014 (0.93, 1.106)	0.75	0.982
Cerebral edema and compression of brain	Neurological	Wald ratio	1	0.969 (0.791, 1.188)	0.764	0.982
Cerebral cysts	Neurological	Wald ratio	1	1.013 (0.768, 1.337)	0.926	0.984
Coma	Neurological	Wald ratio	1	1.027 (0.865, 1.219)	0.763	0.982

Encephalopathy, not elsewhere classified	Neurological	Wald ratio	1	0.868 (0.695, 1.084)	0.211	0.879
Other conditions of brain, NOS	Neurological	Wald ratio	1	1.054 (0.893, 1.245)	0.534	0.949
Other and unspecified disorders of the nervous system	Neurological	Wald ratio	1	1.057 (0.884, 1.265)	0.543	0.949
Abnormal movement	Neurological	Wald ratio	1	1.012 (0.951, 1.078)	0.702	0.979
Abnormal involuntary movements	Neurological	Wald ratio	1	0.994 (0.891, 1.108)	0.908	0.984
Abnormality of gait	Neurological	Wald ratio	1	1.027 (0.947, 1.114)	0.525	0.949
Lack of coordination	Neurological	Wald ratio	1	0.999 (0.841, 1.186)	0.99	0.992
Disturbances of sensation of smell and taste	Neurological	Wald ratio	1	0.979 (0.628, 1.525)	0.924	0.984
Other peripheral nerve disorders	Neurological	Wald ratio	1	0.991 (0.962, 1.02)	0.532	0.949
Disorders of other cranial nerves	Neurological	Wald ratio	1	1.031 (0.943, 1.126)	0.505	0.943
Trigeminal nerve disorders [CN5]	Neurological	Wald ratio	1	1.08 (0.928, 1.257)	0.317	0.879
Facial nerve disorders [CN7]	Neurological	Wald ratio	1	0.996 (0.891, 1.113)	0.942	0.984
Nerve root and plexus disorders	Neurological	Wald ratio	1	1.041 (0.944, 1.149)	0.417	0.917
Nerve plexus lesions	Neurological	Wald ratio	1	1.017 (0.743, 1.393)	0.916	0.984
Nerve root lesions	Neurological	Wald ratio	1	1.219 (0.797, 1.865)	0.361	0.899
Hereditary and idiopathic peripheral neuropathy	Neurological	Wald ratio	1	0.921 (0.711, 1.193)	0.534	0.949
Inflammatory and toxic neuropathy	Neurological	Wald ratio	1	1.035 (0.947, 1.13)	0.453	0.917
Myoneural disorders	Neurological	Wald ratio	1	0.954 (0.764, 1.191)	0.677	0.979
Myasthenia gravis	Neurological	Wald ratio	1	1.017 (0.8, 1.293)	0.89	0.984
Muscular dystrophies and other myopathies	Neurological	Wald ratio	1	0.975 (0.838, 1.134)	0.742	0.982
Muscular dystrophies	Neurological	Wald ratio	1	1.004 (0.761, 1.325)	0.976	0.991
Myopathy	Neurological	Wald ratio	1	1.003 (0.844, 1.191)	0.974	0.991
Miscarriage; stillbirth	Pregnancy complications	Wald ratio	1	1.026 (0.978, 1.076)	0.299	0.879
Missed abortion or Hydatidiform mole	Pregnancy complications	Wald ratio	1	1.099 (1, 1.207)	0.05	0.778
Ectopic pregnancy	Pregnancy complications	Wald ratio	1	1.12 (0.936, 1.34)	0.216	0.879
Hemorrhage during pregnancy; childbirth and postpartum	Pregnancy complications	Wald ratio	1	1.005 (0.932, 1.084)	0.895	0.984
Antepartum hemorrhage, abruptio placentae, and placenta previa	Pregnancy complications	Wald ratio	1	0.958 (0.855, 1.072)	0.451	0.917
Placenta previa and abruptio placenta	Pregnancy complications	Wald ratio	1	1.036 (0.943, 1.139)	0.456	0.919
Early or threatened labor; hemorrhage in early pregnancy	Pregnancy complications	Wald ratio	1	0.994 (0.93, 1.062)	0.855	0.984
Early onset of delivery	Pregnancy complications	Wald ratio	1	1.005 (0.88, 1.148)	0.938	0.984
Hemorrhage in early pregnancy	Pregnancy complications	Wald ratio	1	1.018 (0.921, 1.125)	0.725	0.982
Other high-risk pregnancy	Pregnancy complications	Wald ratio	1	0.885 (0.6, 1.304)	0.536	0.949
Hypertension complicating pregnancy, childbirth, and the puerperium	Pregnancy complications	Wald ratio	1	0.952 (0.862, 1.052)	0.336	0.898
Preeclampsia and eclampsia	Pregnancy complications	Wald ratio	1	0.986 (0.82, 1.186)	0.881	0.984
Excessive vomiting in pregnancy	Pregnancy complications	Wald ratio	1	1.021 (0.818, 1.274)	0.855	0.984
Hyperemesis gravidarum	Pregnancy complications	Wald ratio	1	1.095 (0.845, 1.418)	0.493	0.94
Late pregnancy and failed induction	Pregnancy complications	Wald ratio	1	0.976 (0.888, 1.072)	0.61	0.956
Other complications of pregnancy NEC	Pregnancy complications	Wald ratio	1	1.007 (0.937, 1.082)	0.848	0.984
Infectious and parasitic complications affecting pregnancy	Pregnancy complications	Wald ratio	1	0.998 (0.843, 1.181)	0.98	0.991
Infections of genitourinary tract during pregnancy	Pregnancy complications	Wald ratio	1	1.035 (0.845, 1.269)	0.737	0.982
Other conditions or status of the mother complicating pregnancy, childbirth, or the puerperium	Pregnancy complications	Wald ratio	1	1.365 (1.135, 1.642)	9.67E-04	0.246
Diabetes or abnormal glucose tolerance complicating pregnancy	Pregnancy complications	Wald ratio	1	1.254 (0.986, 1.594)	0.065	0.794
Normal delivery	Pregnancy complications	Wald ratio	1	1.013 (0.938, 1.095)	0.739	0.982
Multiple gestation	Pregnancy complications	Wald ratio	1	1.082 (0.906, 1.293)	0.382	0.899
Problems associated with amniotic cavity and membranes	Pregnancy complications	Wald ratio	1	0.961 (0.878, 1.052)	0.391	0.903
Other and unspecified complications of birth; puerperium affecting management of mother	Pregnancy complications	Wald ratio	1	0.82 (0.622, 1.082)	0.162	0.879
Known or suspected fetal abnormality affecting management of mother	Pregnancy complications	Wald ratio	1	1.003 (0.951, 1.059)	0.898	0.984
Other perinatal conditions of fetus or newborn	Pregnancy complications	Wald ratio	1	0.91 (0.665, 1.246)	0.556	0.951
Fetal distress and abnormal forces of labor	Pregnancy complications	Wald ratio	1	1.047 (0.974, 1.126)	0.209	0.879
Umbilical cord complications during labor and delivery	Pregnancy complications	Wald ratio	1	1.014 (0.88, 1.169)	0.846	0.984
Obstetrical or birth trauma	Pregnancy complications	Wald ratio	1	1.045 (0.994, 1.099)	0.082	0.825
Complications of labor and delivery NEC	Pregnancy complications	Wald ratio	1	1.057 (0.987, 1.132)	0.111	0.847
Venous or cerebrovascular complications embolism in pregnancy and the puerperium	Pregnancy complications	Wald ratio	1	1.073 (0.829, 1.39)	0.592	0.955
Other complications of the puerperium NEC	Pregnancy complications	Wald ratio	1	0.916 (0.748, 1.123)	0.398	0.908
Other disorders of the breast associated with childbirth and disorders of lactation	Pregnancy complications	Wald ratio	1	1.23 (0.819, 1.848)	0.317	0.879
Asphyxia and hypoxemia	Respiratory	Wald ratio	1	0.997 (0.814, 1.222)	0.978	0.991
Acute sinusitis	Respiratory	Wald ratio	1	1.022 (0.804, 1.299)	0.86	0.984
Acute upper respiratory infections of multiple or unspecified sites	Respiratory	Wald ratio	1	1.013 (0.946, 1.085)	0.705	0.981
Acute pharyngitis	Respiratory	Wald ratio	1	0.981 (0.878, 1.096)	0.739	0.982
Acute laryngitis and tracheitis	Respiratory	Wald ratio	1	1.152 (0.889, 1.492)	0.284	0.879
Septal Deviations or Turbinate Hypertrophy	Respiratory	Wald ratio	1	1.033 (0.986, 1.081)	0.174	0.879
Nasal polyps	Respiratory	Wald ratio	1	1.045 (0.987, 1.107)	0.129	0.85
Chronic pharyngitis and nasopharyngitis	Respiratory	Wald ratio	1	1.006 (0.906, 1.118)	0.909	0.984
Diseases of the larynx and vocal cords	Respiratory	Wald ratio	1	1.037 (0.972, 1.107)	0.265	0.879
Chronic laryngitis	Respiratory	Wald ratio	1	0.893 (0.652, 1.223)	0.48	0.931
Paralysis or spasm of vocal cords or larynx	Respiratory	Wald ratio	1	1.032 (0.842, 1.264)	0.764	0.982
Voice disturbance	Respiratory	Wald ratio	1	0.977 (0.884, 1.079)	0.643	0.967
Acute and chronic tonsillitis	Respiratory	Wald ratio	1	0.951 (0.885, 1.022)	0.174	0.879
Acute tonsillitis	Respiratory	Wald ratio	1	0.92 (0.807, 1.049)	0.215	0.879
Chronic tonsillitis and adenoiditis	Respiratory	Wald ratio	1	0.985 (0.893, 1.086)	0.763	0.982
Chronic sinusitis	Respiratory	Wald ratio	1	0.997 (0.935, 1.064)	0.932	0.984
Allergic rhinitis	Respiratory	Wald ratio	1	0.991 (0.897, 1.095)	0.866	0.984
Epistaxis or throat hemorrhage	Respiratory	Wald ratio	1	0.92 (0.86, 0.983)	0.013	0.55
Throat pain	Respiratory	Wald ratio	1	0.876 (0.741, 1.037)	0.124	0.847
Other upper respiratory disease	Respiratory	Wald ratio	1	1.039 (0.987, 1.095)	0.143	0.859
Pneumonia	Respiratory	Wald ratio	1	0.981 (0.949, 1.015)	0.267	0.879

Bacterial pneumonia	Respiratory	Wald ratio	1	0.968 (0.929, 1.008)	0.112	0.847
Pneumococcal pneumonia	Respiratory	Wald ratio	1	0.955 (0.915, 0.996)	0.033	0.739
Pseudomonal pneumonia	Respiratory	Wald ratio	1	0.893 (0.629, 1.269)	0.528	0.949
Viral pneumonia	Respiratory	Wald ratio	1	1.152 (0.781, 1.698)	0.475	0.927
Pneumonia due to fungus (mycoses)	Respiratory	Wald ratio	1	0.876 (0.628, 1.222)	0.437	0.917
Bronchopneumonia and lung abscess	Respiratory	Wald ratio	1	0.876 (0.741, 1.037)	0.124	0.847
Influenza	Respiratory	Wald ratio	1	1.093 (0.912, 1.31)	0.337	0.898
Acute bronchitis and bronchiolitis	Respiratory	Wald ratio	1	0.92 (0.723, 1.169)	0.494	0.94
Asthma	Respiratory	Wald ratio	1	0.986 (0.964, 1.008)	0.211	0.879
Asthma with exacerbation	Respiratory	Wald ratio	1	1.174 (0.958, 1.438)	0.122	0.847
Chronic airway obstruction	Respiratory	Wald ratio	1	0.978 (0.946, 1.011)	0.182	0.879
Emphysema	Respiratory	Wald ratio	1	0.926 (0.856, 1.003)	0.06	0.789
Chronic bronchitis	Respiratory	Wald ratio	1	1.008 (0.948, 1.071)	0.806	0.984
Obstructive chronic bronchitis	Respiratory	Wald ratio	1	1.002 (0.939, 1.069)	0.95	0.987
Bronchiectasis	Respiratory	Wald ratio	1	0.94 (0.871, 1.014)	0.107	0.847
Bronchitis	Respiratory	Wald ratio	1	0.876 (0.77, 0.997)	0.046	0.752
Lung disease due to external agents	Respiratory	Wald ratio	1	1.053 (0.895, 1.239)	0.532	0.949
Extrinsic allergic alveolitis	Respiratory	Wald ratio	1	1.141 (0.789, 1.651)	0.484	0.934
Pneumoconiosis	Respiratory	Wald ratio	1	1.01 (0.809, 1.261)	0.927	0.984
Pneumonitis due to inhalation of food or vomitus	Respiratory	Wald ratio	1	0.893 (0.78, 1.022)	0.1	0.835
Postinflammatory pulmonary fibrosis	Respiratory	Wald ratio	1	0.868 (0.778, 0.968)	0.011	0.55
Pulmonary congestion and hypostasis	Respiratory	Wald ratio	1	0.933 (0.801, 1.088)	0.379	0.899
Other alveolar and parietoalveolar pneumonopathy	Respiratory	Wald ratio	1	0.893 (0.742, 1.074)	0.23	0.879
Other pulmonary inflamation or edema	Respiratory	Wald ratio	1	1.431 (0.935, 2.189)	0.098	0.835
Empyema and pneumothorax	Respiratory	Wald ratio	1	0.98 (0.891, 1.079)	0.686	0.979
Pleurisy; pleural effusion	Respiratory	Wald ratio	1	1.008 (0.968, 1.05)	0.696	0.979
Pulmonary collapse; interstitial and compensatory emphysema	Respiratory	Wald ratio	1	1.007 (0.936, 1.085)	0.845	0.984
Respiratory failure, insufficiency, arrest	Respiratory	Wald ratio	1	0.967 (0.906, 1.031)	0.304	0.879
Respiratory failure	Respiratory	Wald ratio	1	0.954 (0.886, 1.027)	0.211	0.879
Respiratory insufficiency	Respiratory	Wald ratio	1	0.988 (0.914, 1.068)	0.757	0.982
Respiratory arrest	Respiratory	Wald ratio	1	1.09 (0.811, 1.464)	0.57	0.951
Dependence on respirator [Ventilator] or supplemental oxygen	Respiratory	Wald ratio	1	0.976 (0.828, 1.15)	0.77	0.982
Other diseases of lung	Respiratory	Wald ratio	1	0.915 (0.815, 1.028)	0.136	0.854
Other symptoms of respiratory system	Respiratory	Wald ratio	1	1.013 (0.978, 1.049)	0.461	0.919
Wheezing	Respiratory	Wald ratio	1	0.893 (0.715, 1.115)	0.317	0.879
Painful respiration	Respiratory	Wald ratio	1	1.027 (0.823, 1.282)	0.816	0.984
Shortness of breath	Respiratory	Wald ratio	1	1.01 (0.968, 1.054)	0.632	0.967
Cough	Respiratory	Wald ratio	1	1.005 (0.946, 1.069)	0.86	0.984
Other dyspnea	Respiratory	Wald ratio	1	1.004 (0.9, 1.12)	0.941	0.984
Respiratory abnormalities	Respiratory	Wald ratio	1	0.949 (0.83, 1.084)	0.437	0.917
Hypoventilation	Respiratory	Wald ratio	1	0.928 (0.641, 1.343)	0.693	0.979
Hyperventilation	Respiratory	Wald ratio	1	0.969 (0.791, 1.188)	0.764	0.982
Disorders of diaphragm	Respiratory	Wald ratio	1	0.955 (0.737, 1.237)	0.726	0.982
Abnormal findings examination of lungs	Respiratory	Wald ratio	1	0.99 (0.928, 1.056)	0.753	0.982
Abnormal sputum	Respiratory	Wald ratio	1	0.94 (0.876, 1.008)	0.082	0.825
Hemoptysis	Respiratory	Wald ratio	1	0.943 (0.878, 1.014)	0.112	0.847
Other diseases of respiratory system, not elsewhere classified	Respiratory	Wald ratio	1	1.009 (0.976, 1.044)	0.579	0.955
Respiratory complications	Respiratory	Wald ratio	1	1.093 (0.908, 1.314)	0.347	0.898
Other diseases of respiratory system, NEC	Respiratory	Wald ratio	1	1.005 (0.97, 1.041)	0.792	0.984
Symptoms involving respiratory system and other chest symptoms	Respiratory	Wald ratio	1	1.002 (0.833, 1.205)	0.985	0.991
Disorders of the globe	Sense organs	Wald ratio	1	0.965 (0.806, 1.154)	0.695	0.979
Progressive myopia	Sense organs	Wald ratio	1	0.914 (0.746, 1.12)	0.388	0.903
Retinal detachments and defects	Sense organs	Wald ratio	1	1.141 (1.076, 1.211)	1.21E-05	0.015
Retinal detachment with retinal defect	Sense organs	Wald ratio	1	1.141 (1.044, 1.247)	0.004	0.409
Retinoschisis and retinal cysts	Sense organs	Wald ratio	1	1.486 (1.008, 2.19)	0.046	0.752
Other retinal disorders	Sense organs	Wald ratio	1	1.02 (0.967, 1.076)	0.469	0.923
Degeneration of macula and posterior pole of retina	Sense organs	Wald ratio	1	1.036 (0.966, 1.112)	0.317	0.879
Macular degeneration (senile) of retina NOS	Sense organs	Wald ratio	1	1.035 (0.964, 1.11)	0.343	0.898
Other nondiabetic retinopathy	Sense organs	Wald ratio	1	1.12 (0.865, 1.45)	0.391	0.903
Separation of retinal layers	Sense organs	Wald ratio	1	1.12 (0.803, 1.562)	0.505	0.943
Retinal vascular changes and abnomalities	Sense organs	Wald ratio	1	1.011 (0.904, 1.132)	0.844	0.984
Peripheral retinal degenerations	Sense organs	Wald ratio	1	1.005 (0.79, 1.278)	0.969	0.991
Hereditary retinal dystrophies	Sense organs	Wald ratio	1	1.045 (0.736, 1.485)	0.805	0.984
Retinal hemorrhage or ischemia	Sense organs	Wald ratio	1	1.12 (0.833, 1.505)	0.453	0.917
Chorioretinal inflammations, scars, and other disorders of choroid	Sense organs	Wald ratio	1	0.926 (0.715, 1.199)	0.558	0.951
Chorioretinal scars	Sense organs	Wald ratio	1	1.029 (0.698, 1.516)	0.886	0.984
Corneal opacity and other disorders of cornea	Sense organs	Wald ratio	1	1.109 (0.984, 1.251)	0.091	0.835
Corneal opacity	Sense organs	Wald ratio	1	0.951 (0.682, 1.327)	0.768	0.982
Corneal edema	Sense organs	Wald ratio	1	1.009 (0.697, 1.46)	0.961	0.991
Corneal degenerations	Sense organs	Wald ratio	1	1.208 (0.967, 1.507)	0.096	0.835
Keratoconus	Sense organs	Wald ratio	1	1.23 (0.866, 1.748)	0.247	0.879
Corneal dystrophy	Sense organs	Wald ratio	1	1.099 (0.88, 1.372)	0.405	0.911
Cornea replaced by transplant	Sense organs	Wald ratio	1	1.064 (0.763, 1.484)	0.714	0.982
Glaucoma	Sense organs	Wald ratio	1	1.076 (1.024, 1.131)	0.004	0.41
Open-angle glaucoma	Sense organs	Wald ratio	1	1.059 (0.957, 1.173)	0.267	0.879
Primary open angle glaucoma	Sense organs	Wald ratio	1	1.064 (0.961, 1.178)	0.23	0.879
Primary angle-closure glaucoma	Sense organs	Wald ratio	1	0.993 (0.877, 1.124)	0.909	0.984
Cataract	Sense organs	Wald ratio	1	1.013 (0.989, 1.038)	0.282	0.879
Nonsenile Cataract	Sense organs	Wald ratio	1	0.775 (0.526, 1.143)	0.199	0.879

Senile cataract	Sense organs	Wald ratio	1	1.004 (0.967, 1.042)	0.838	0.984
Traumatic cataract	Sense organs	Wald ratio	1	0.79 (0.577, 1.082)	0.141	0.859
Disorders of refraction and accommodation; blindness and low vision	Sense organs	Wald ratio	1	1.057 (0.989, 1.13)	0.101	0.835
Myopia	Sense organs	Wald ratio	1	1.042 (0.95, 1.143)	0.379	0.899
Astigmatism	Sense organs	Wald ratio	1	1.068 (0.84, 1.358)	0.59	0.955
Hypermetropia	Sense organs	Wald ratio	1	0.876 (0.715, 1.074)	0.203	0.879
Blindness and low vision	Sense organs	Wald ratio	1	1.141 (1.01, 1.289)	0.034	0.744
Visual disturbances	Sense organs	Wald ratio	1	1.015 (0.959, 1.075)	0.606	0.956
Amblyopia	Sense organs	Wald ratio	1	0.987 (0.858, 1.136)	0.854	0.984
Diplopia and disorders of binocular vision	Sense organs	Wald ratio	1	0.976 (0.864, 1.102)	0.694	0.979
Anisometropia	Sense organs	Wald ratio	1	1.093 (0.844, 1.415)	0.502	0.943
Visual field defects	Sense organs	Wald ratio	1	1.174 (0.981, 1.404)	0.08	0.825
Subjective visual disturbances	Sense organs	Wald ratio	1	1.081 (0.948, 1.233)	0.242	0.879
Infection of the eye	Sense organs	Wald ratio	1	0.987 (0.859, 1.134)	0.852	0.984
Eye infection, viral	Sense organs	Wald ratio	1	1.035 (0.77, 1.39)	0.822	0.984
Conjunctivitis, infectious	Sense organs	Wald ratio	1	0.876 (0.702, 1.094)	0.243	0.879
Keratitis	Sense organs	Wald ratio	1	0.925 (0.769, 1.112)	0.407	0.911
Corneal ulcer	Sense organs	Wald ratio	1	1.08 (0.819, 1.425)	0.585	0.955
Inflammation of the eye	Sense organs	Wald ratio	1	0.992 (0.936, 1.05)	0.774	0.984
Uveitis, noninfectious or NOS	Sense organs	Wald ratio	1	0.971 (0.838, 1.126)	0.698	0.979
Inflammation of eyelids	Sense organs	Wald ratio	1	1.009 (0.944, 1.079)	0.788	0.984
Disorders of conjunctiva	Sense organs	Wald ratio	1	0.968 (0.86, 1.09)	0.595	0.955
Other disorders of eyelids	Sense organs	Wald ratio	1	1.007 (0.963, 1.052)	0.774	0.984
Ectropion or entropion	Sense organs	Wald ratio	1	1.057 (0.957, 1.168)	0.275	0.879
Ptosis of eyelid	Sense organs	Wald ratio	1	1.005 (0.93, 1.086)	0.907	0.984
Disorders of lacrimal system	Sense organs	Wald ratio	1	0.933 (0.87, 1.001)	0.055	0.778
Epiphora	Sense organs	Wald ratio	1	0.972 (0.872, 1.084)	0.611	0.956
Disorders of the orbit	Sense organs	Wald ratio	1	1.163 (0.819, 1.652)	0.4	0.908
Disorders of optic nerve and visual pathways	Sense organs	Wald ratio	1	1.09 (0.919, 1.291)	0.323	0.89
Optic atrophy	Sense organs	Wald ratio	1	1.174 (0.767, 1.796)	0.46	0.919
Optic neuritis or neuropathy	Sense organs	Wald ratio	1	1.174 (0.873, 1.578)	0.288	0.879
Strabismus and other disorders of binocular eye movements	Sense organs	Wald ratio	1	1.018 (0.935, 1.108)	0.68	0.979
Strabismus (not specified as paralytic)	Sense organs	Wald ratio	1	1.017 (0.914, 1.132)	0.756	0.982
Nystagmus and other irregular eye movements	Sense organs	Wald ratio	1	0.991 (0.794, 1.237)	0.935	0.984
Paralytic strabismus	Sense organs	Wald ratio	1	1.076 (0.878, 1.319)	0.478	0.93
Other disorders of eye	Sense organs	Wald ratio	1	1.002 (0.95, 1.058)	0.929	0.984
Disorders of vitreous body	Sense organs	Wald ratio	1	1.005 (0.92, 1.098)	0.915	0.984
Aphakia and other disorders of lens	Sense organs	Wald ratio	1	1.029 (0.952, 1.112)	0.475	0.927
Anomalies of pupillary function	Sense organs	Wald ratio	1	0.726 (0.492, 1.07)	0.105	0.847
Disorders of iris and ciliary body	Sense organs	Wald ratio	1	0.982 (0.835, 1.156)	0.829	0.984
Pain, swelling or discharge of eye	Sense organs	Wald ratio	1	0.966 (0.746, 1.251)	0.792	0.984
Disorders of external ear	Sense organs	Wald ratio	1	1.028 (0.942, 1.121)	0.537	0.949
Otitis externa	Sense organs	Wald ratio	1	0.964 (0.836, 1.111)	0.613	0.956
Impacted cerumen	Sense organs	Wald ratio	1	1.01 (0.851, 1.2)	0.906	0.984
Otitis media and Eustachian tube disorders	Sense organs	Wald ratio	1	1.099 (1.026, 1.177)	0.007	0.547
Otitis media	Sense organs	Wald ratio	1	1.08 (1, 1.168)	0.051	0.778
Suppurative and unspecified otitis media	Sense organs	Wald ratio	1	1.063 (0.952, 1.188)	0.279	0.879
Eustachian tube disorders	Sense organs	Wald ratio	1	1.208 (0.95, 1.535)	0.124	0.847
Mastoiditis & related conditions	Sense organs	Wald ratio	1	1.302 (1.005, 1.687)	0.046	0.752
Otorrhea	Sense organs	Wald ratio	1	0.962 (0.8, 1.157)	0.682	0.979
Otalgia	Sense organs	Wald ratio	1	1.185 (0.985, 1.425)	0.072	0.809
Otosclerosis	Sense organs	Wald ratio	1	1.431 (1.189, 1.721)	1.45E-04	0.092
Other disorders of tympanic membrane	Sense organs	Wald ratio	1	0.974 (0.891, 1.064)	0.56	0.951
Perforation of tympanic membrane	Sense organs	Wald ratio	1	0.973 (0.879, 1.077)	0.598	0.955
Other disorders of middle ear and mastoid	Sense organs	Wald ratio	1	1.003 (0.896, 1.123)	0.958	0.99
Cholesteatoma	Sense organs	Wald ratio	1	1.051 (0.92, 1.201)	0.462	0.919
Tympanosclerosis and middle ear disease related to otitis media	Sense organs	Wald ratio	1	0.91 (0.743, 1.115)	0.363	0.899
Vertiginous syndromes and other disorders of vestibular system	Sense organs	Wald ratio	1	1.018 (0.976, 1.062)	0.409	0.911
Meniere's disease	Sense organs	Wald ratio	1	0.868 (0.757, 0.995)	0.043	0.752
Peripheral or central vertigo	Sense organs	Wald ratio	1	1.23 (1.04, 1.456)	0.016	0.585
Labyrinthitis	Sense organs	Wald ratio	1	0.968 (0.86, 1.09)	0.595	0.955
Dizziness and giddiness (Light-headedness and vertigo)	Sense organs	Wald ratio	1	1.034 (0.985, 1.084)	0.178	0.879
Other disorders of ear	Sense organs	Wald ratio	1	1.23 (0.986, 1.536)	0.067	0.794
Hearing loss	Sense organs	Wald ratio	1	1.065 (1.013, 1.12)	0.013	0.55
Sensorineural hearing loss	Sense organs	Wald ratio	1	1.079 (0.926, 1.258)	0.329	0.898
Conductive hearing loss	Sense organs	Wald ratio	1	1.044 (0.896, 1.217)	0.579	0.955
Tinnitus	Sense organs	Wald ratio	1	1.01 (0.875, 1.167)	0.888	0.984
Symptoms concerning nutrition, metabolism, and development	Symptoms	Wald ratio	1	1.011 (0.968, 1.057)	0.617	0.956
Other signs and symptoms involving emotional state	Symptoms	Wald ratio	1	1.052 (0.875, 1.266)	0.589	0.955
Other symptoms	Symptoms	Wald ratio	1	0.988 (0.899, 1.085)	0.799	0.984
Other tests	Symptoms	Wald ratio	1	0.945 (0.906, 0.986)	0.009	0.55
Effects of other external causes	Symptoms	Wald ratio	1	0.963 (0.916, 1.012)	0.138	0.859
Other ill-defined and unknown causes of morbidity and mortality	Symptoms	Wald ratio	1	1.002 (0.974, 1.03)	0.894	0.984
Pain	Symptoms	Wald ratio	1	0.966 (0.858, 1.087)	0.563	0.951
Localized superficial swelling, mass, or lump	Symptoms	Wald ratio	1	0.951 (0.791, 1.144)	0.596	0.955
Back pain	Symptoms	Wald ratio	1	1.001 (0.97, 1.033)	0.939	0.984
Cervicalgia	Symptoms	Wald ratio	1	0.946 (0.875, 1.022)	0.16	0.879
Sciatica	Symptoms	Wald ratio	1	1.024 (0.958, 1.094)	0.487	0.937
Neuralgia, neuritis, and radiculitis NOS	Symptoms	Wald ratio	1	0.95 (0.863, 1.046)	0.299	0.879

Myalgia and myositis unspecified	Symptoms	Wald ratio	1	1.013 (0.89, 1.153)	0.841	0.984
Musculoskeletal symptoms referable to limbs	Symptoms	Wald ratio	1	0.98 (0.938, 1.025)	0.382	0.899
Swelling of limb	Symptoms	Wald ratio	1	0.977 (0.931, 1.025)	0.336	0.898
Symptoms of the muscles	Symptoms	Wald ratio	1	0.933 (0.824, 1.056)	0.269	0.879
Pain in limb	Symptoms	Wald ratio	1	0.961 (0.921, 1.003)	0.068	0.799
Hypothermia or Chills	Symptoms	Wald ratio	1	1.069 (0.826, 1.385)	0.612	0.956
Symptoms involving nervous and musculoskeletal systems	Symptoms	Wald ratio	1	0.992 (0.92, 1.071)	0.843	0.984
Symptoms involving skin and other integumentary tissue	Symptoms	Wald ratio	1	0.976 (0.903, 1.055)	0.536	0.949
Edema	Symptoms	Wald ratio	1	0.974 (0.898, 1.056)	0.525	0.949
Pallor and flushing	Symptoms	Wald ratio	1	0.993 (0.781, 1.262)	0.952	0.989
Fever of unknown origin	Symptoms	Wald ratio	1	0.97 (0.921, 1.022)	0.253	0.879
Abdominal pain	Symptoms	Wald ratio	1	0.998 (0.981, 1.015)	0.809	0.984
Syncope and collapse	Symptoms	Wald ratio	1	0.994 (0.96, 1.03)	0.748	0.982
Nausea and vomiting	Symptoms	Wald ratio	1	0.981 (0.951, 1.013)	0.239	0.879
Nonspecific findings on examination of blood	Symptoms	Wald ratio	1	1.024 (0.983, 1.066)	0.256	0.879
Elevated sedimentation rate	Symptoms	Wald ratio	1	0.828 (0.583, 1.176)	0.293	0.879
Other abnormal blood chemistry	Symptoms	Wald ratio	1	1.029 (0.986, 1.073)	0.192	0.879
Gangrene	Symptoms	Wald ratio	1	0.943 (0.821, 1.083)	0.408	0.911
Abnormal Papanicolaou smear of cervix and cervical HPV	Symptoms	Wald ratio	1	1.152 (0.841, 1.577)	0.378	0.899
Abnormal results of other function studies (bladder, pancreas, placenta, spleen, etc)	Symptoms	Wald ratio	1	1.314 (0.978, 1.767)	0.07	0.808
Shock	Symptoms	Wald ratio	1	0.957 (0.725, 1.262)	0.754	0.982
Cardiogenic shock	Symptoms	Wald ratio	1	0.901 (0.635, 1.281)	0.563	0.951
Malaise and fatigue	Symptoms	Wald ratio	1	1.035 (0.98, 1.095)	0.217	0.879
Chronic fatigue syndrome	Symptoms	Wald ratio	1	0.957 (0.836, 1.095)	0.52	0.949
