# Supplementary material for: Unveiling the Immune Mechanisms of Hypertension With Single‐Cell Transcriptome‐Wide Mendelian Randomization
Source: FASEB J. 2026 Aug 20;40(16):e72206. doi: 10.1096/fj.202600899R (PMC13492368; doi:10.1096/fj.202600899R)

Unveiling the immune mechanisms of hypertension with single-cell transcriptome-wide Mendelian randomization

Yukang Mao[1†], Peng Li[1†], Chen Cheng[1†], Xiangqing Kong[1], Wei Sun[1*], Tingting Wu[1*]

[1]Department of Cardiology, The First Affiliated Hospital with Nanjing Medical University, Nanjing 210029, China

***Correspondence**

Tingting Wu, M.D., Department of Cardiology, The First Affiliated Hospital with Nanjing Medical University, Nanjing, 210029, China; Email: wutingting@jsph.org.cn

Wei Sun, M.D., Ph.D., Department of Cardiology, The First Affiliated Hospital with Nanjing Medical University, Nanjing, 210029, China; Email: weisun7919@njmu.edu.cn

SUPPLEMENTARY FIGURES

Figure S1. Functional characterization of eGenes causally associated with hypertension.

Figure S2. Regional genomic plots showing the colocalization results for the associations between eGenes and hypertension.

Figure S3. Regional genomic plots showing the colocalization results for the associations between hypertension-related eGenes and hypertensive heart disease.

Figure S1. Functional characterization of eGenes causally associated with hypertension.

(A) Functional annotations of causal eGenes based on the Gene Ontology (GO) terms.

(B) Functional annotations of causal eGenes based on the Kyoto Encyclopedia of Genes and Genomes (KEGG) terms.

(C) Functional annotations of causal eGenes based on the Reactome terms.

(D) The protein–protein interaction (PPI) network of causal eGenes generated with GeneMANIA.

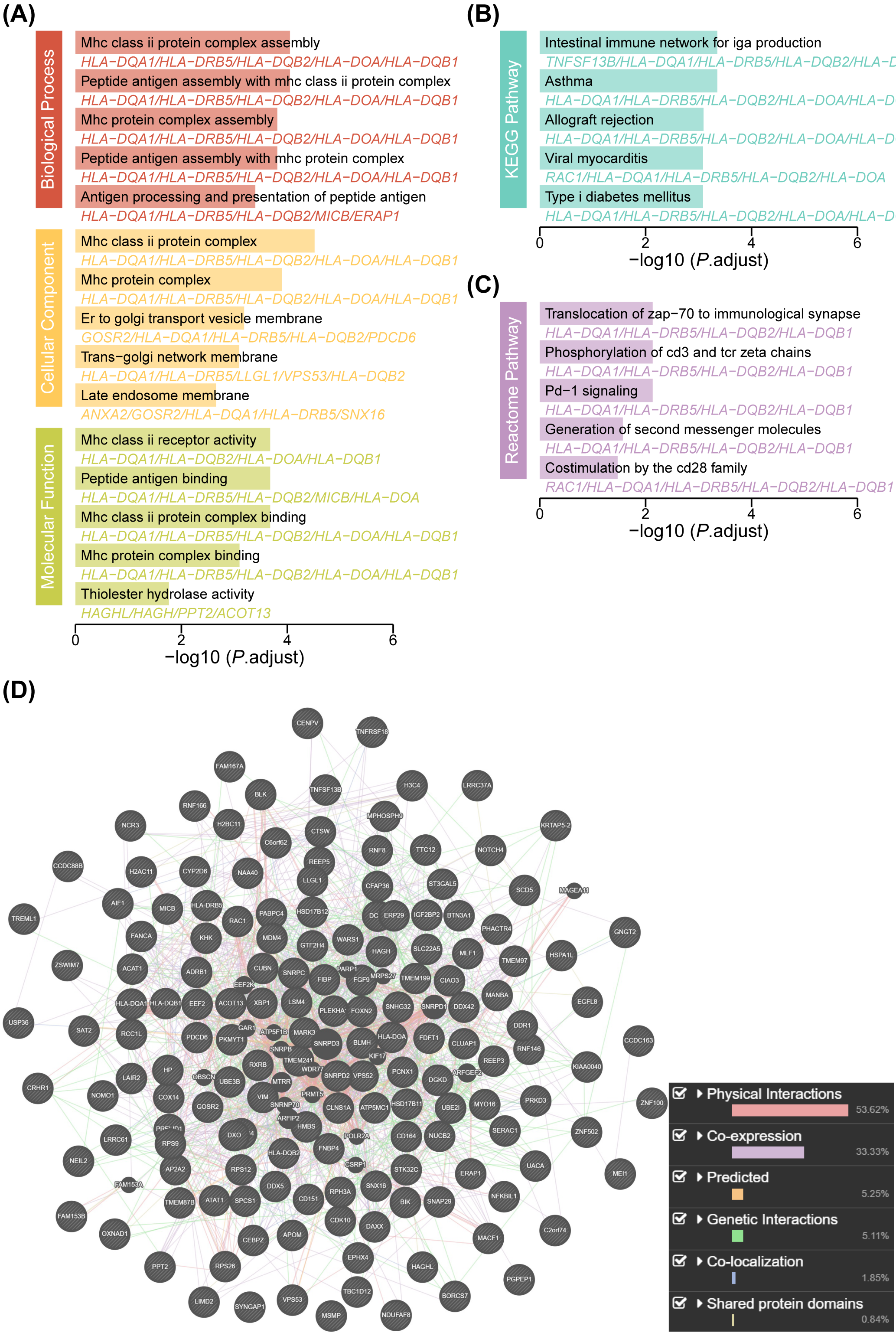

Figure S2. Regional genomic plots showing the colocalization results for the associations between eGenes and hypertension.

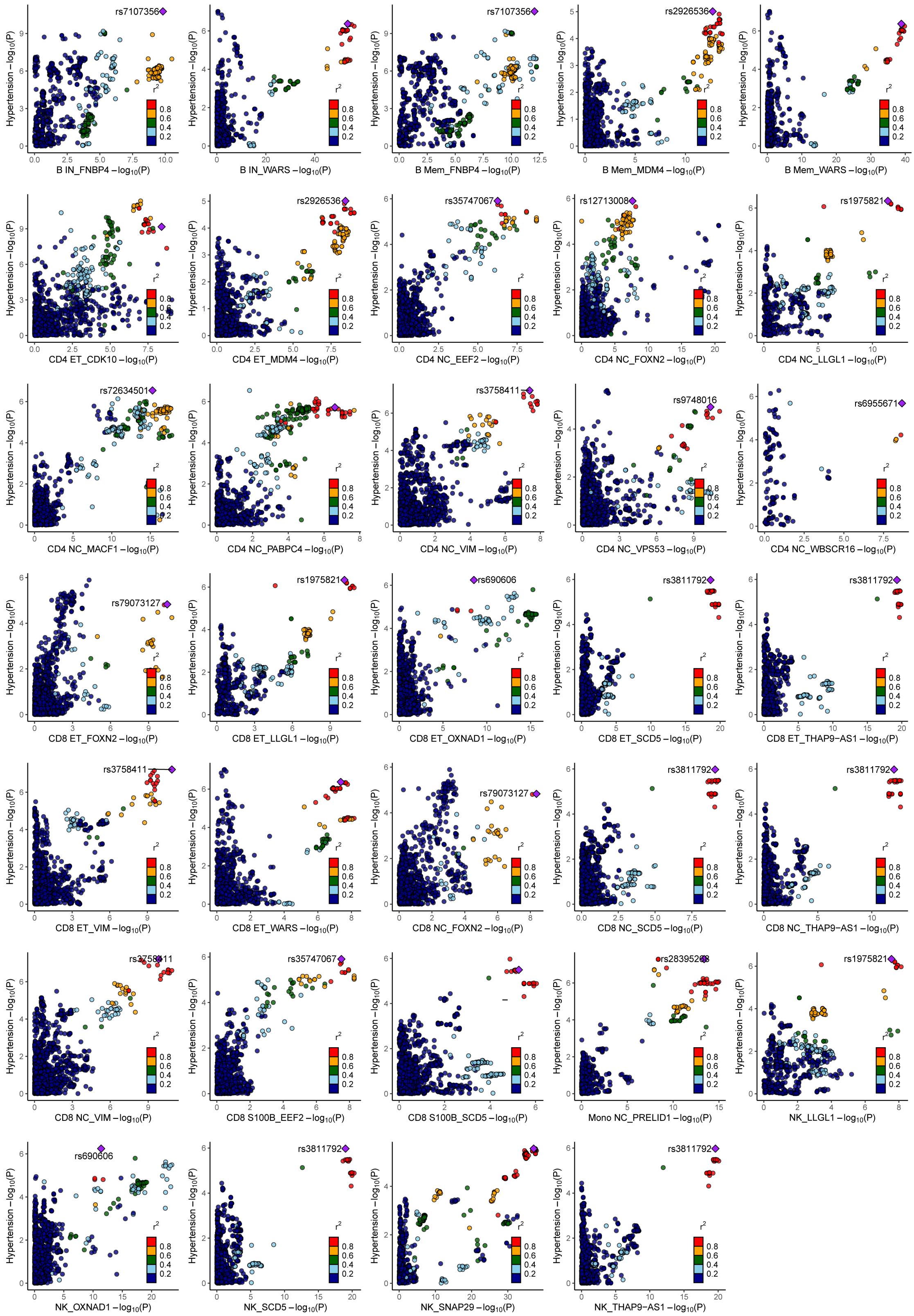

Figure S3. Regional genomic plots showing the colocalization results for the associations between hypertension-related eGenes and hypertensive heart disease.

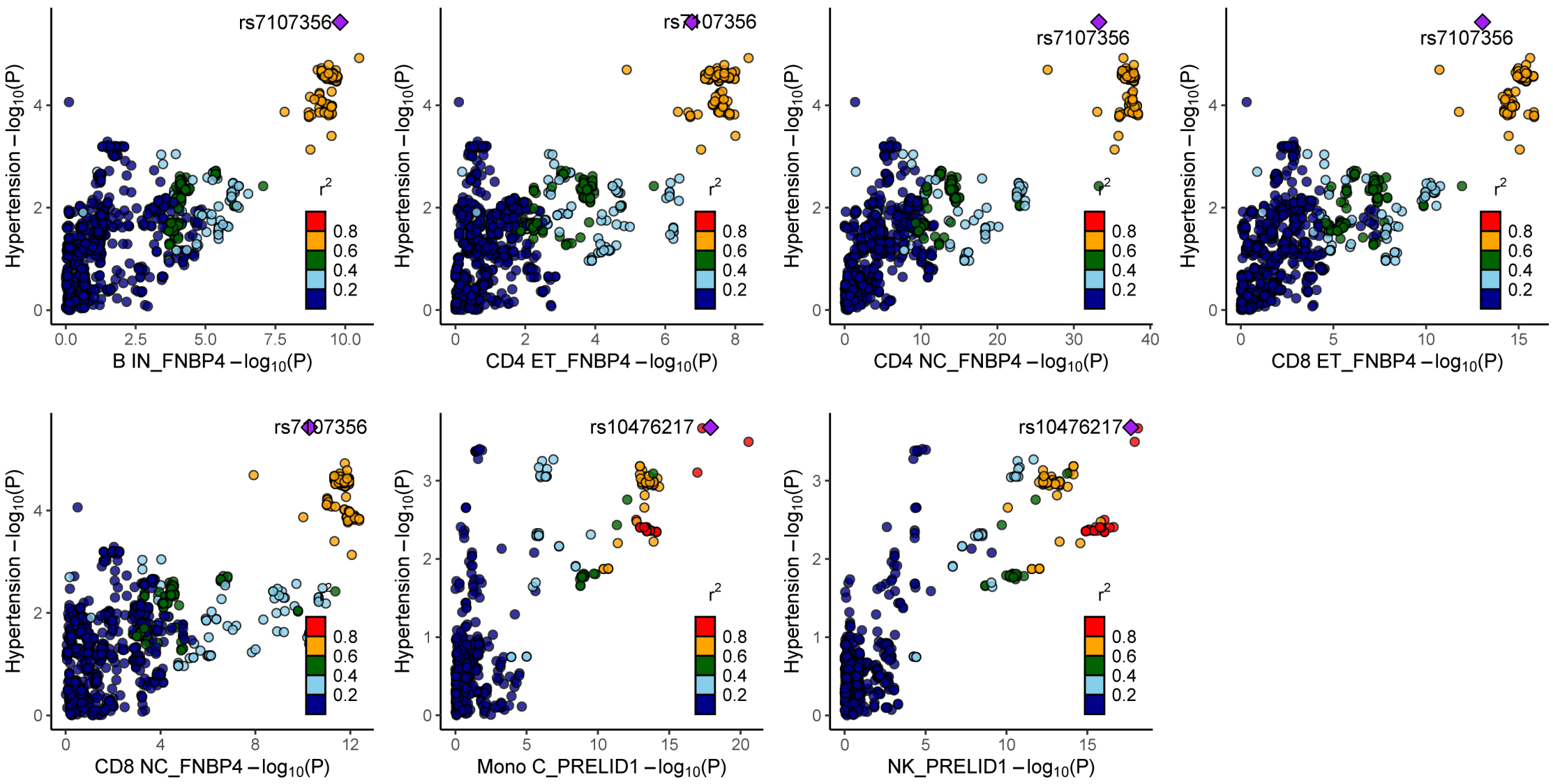

Supplement: Supplementary file 2 [file FSB2-40-e72206-s002.pdf]
